# KOPI: Kinase inhibitOr Proteome Impact analysis

**DOI:** 10.1038/s41598-022-16557-w

**Published:** 2022-07-29

**Authors:** Ginny Xiaohe Li, Tianyun Zhao, Loo Chien Wang, Hyungwon Choi, Yan Ting Lim, Radoslaw M. Sobota

**Affiliations:** 1 Department of Medicine, Yong Loo Lin School of Medicine, National University of Singapore, Singapore, Singapore; 2 Functional Proteomics Laboratory, Institute of Molecular and Cell Biology, Agency for Science, Technology and Research, Singapore, Singapore; 3 School of Biological Sciences, Nanyang Technological University, Singapore, Singapore; 4 Department of Pathology, University of Michigan, Ann Arbor, Michigan, USA

**Keywords:** Drug screening, Target identification, Post-translational modifications, Proteomics

## Abstract

Kinase inhibitors often exert on/off-target effects, and efficient data analysis is essential for assessing these effects on the proteome. We developed a workflow for rapidly performing such a proteomic assessment, termed as kinase inhibitor proteome impact analysis (KOPI). We demonstrate KOPI’s utility with staurosporine (STS) on the leukemic K562 cell proteome. We identified systematically staurosporine’s non-kinome interactors, and showed for the first time that it caused paradoxical hyper- and biphasic phosphorylation.

## Introduction

Kinases catalyse phosphorylation of serine, threonine or tyrosine residues on target substrate. Their dysregulation underlies many disorders, making them an important protein target class^1^. Kinase-targeted drug discovery has generated several classes of kinase inhibitors (KIs), of which more than 70 are used clinically^2,3^. The current strategy seeks to inhibit the aberrant kinase and the hyperactivated pathway, and refine its specificity within the kinome^4–8^. The assumption that an overactive kinase or hyper-phosphorylation drives oncogenesis has nonetheless been repeatedly challenged. KIs can behave as agonists in vitro^9,10^*.* Kinases also show non-catalytic functions such as competition for protein interactions or exert allosteric effects on partner proteins that regulate critical biochemical pathways^11,12^. KIs, though designed to target specifically within the kinome, can also interact with non-kinase targets^13,14^. Hence, a systems-wide understanding of the mode of action for KIs gives the first cue to its potential as a viable therapeutic agent. An ensemble of experimental and computational workflows that complements existing kinase-centric compound refinement processes can facilitate the selection of the lead compound and contribute to its success.

**Figure 1:**
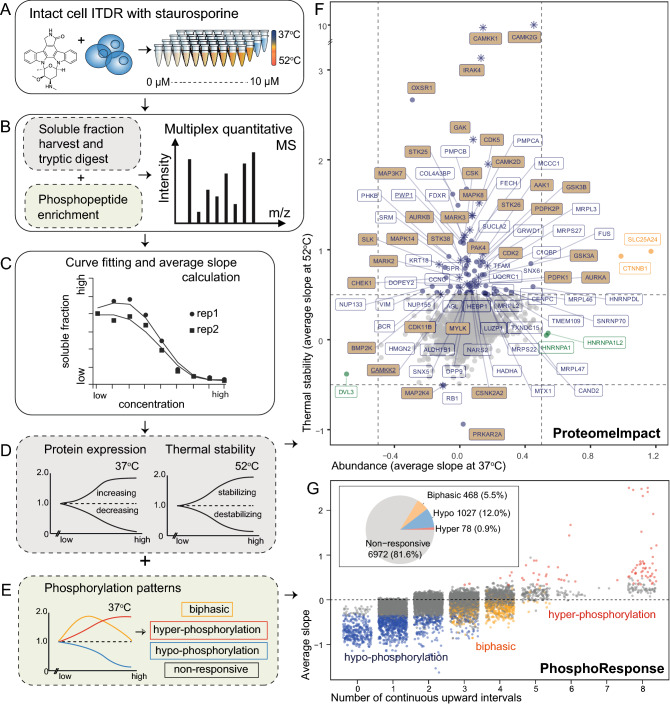
Kinase inhibitor proteome impact analysis (KOPI) workflow and outputs. (**A**) The cell line is treated with kinase inhibitor/s at different concentrations under normal (37 °C) temperature and a heat challenge (52 °C). (**B**) The soluble fraction is harvested for tryptic digestion and multiplexed for quantitative mass spectrometry. (**C**) The fold changes relative to the untreated are fitted with a smoothing curve. (**D**) The average slope per curve is calculated to indicate the proteins’ response to drug treatment. The same analysis is performed on the enriched phospho-peptides to quantify the phosphoproteome’s response. (**E**) Each phosphosite was sorted into 4 possible responses based on the trend of their curves. The results are represented as the (**F**) ProteomeImpact and the (**G**) Phospho-Response. (**F**) ProteomeImpact: the average slope of each protein’s dose–response at 37 °C and 52 °C. Proteins with the average slope value on either axis larger than 0.5 are annotated, with kinases in beige. Stars represent common hits between this publication and Savitski et al. (**G**) PhosphoResponse: phosphopeptides are plotted against their number of continuous upward intervals (x-axis) and average slope under 37 °C (y-axis). Response categories: hypo-(blue), hyper-phosphorylation (red), biphasic (yellow) and non-responsive (grey). The pie chart indicates global proportions.

Proteomics coupled with thermal shift assays can provide an unbiased view of a compound’s effect on the proteome by quantifying its effects on protein expression and turnover, phosphorylation and the interactome^15–17^. A critical bottleneck to the analysis is the lack of an open-source tool for performing user-friendly analytics on such data. Although a few solutions are freely available for analysing thermal stability data on Bioconductor^18–20^, phosphoproteomics for target tracking is not a part of the process. KOPI fills this gap by providing seamless analysis of drug-induced proteome dynamics based on both thermal stability and phosphoproteomics experiments (Fig. 1A–E).

## Results

To demonstrate KOPI, we generated a training data set with K526 cells treated with staurosporine, a potent KI chosen for its polypharmacology. The incubation duration was 30 min to emphasize transient events such as protein turnover and post-translational modification over protein transcription and translation. We performed thermal proteome profiling using the isothermal dose-response^20^. The cells were treated with nine different drug doses up to 10 µM and heat pulsed at 37 °C and 52 °C (Fig. 1A), and the soluble proteome was prepared for quantitative proteomic and phosphoproteomic analysis in each experiment (Fig. 1B, Supplementary Information).

KOPI is implemented as a web-based Rshiny application, accounting for three levels of proteomic data concurrently; constitutive protein abundance, thermal stability (Fig. 1B,D) and phosphopeptide abundance (Fig. 1B,E). A critical departure from the existing methods is that we did not limit the dose–response analysis to sigmoidal curves. The existing methods select hits with a coefficient of determination above a set threshold, and use the compound’s saturating concentrations for hit identification^18,20,21^. Instead, we generated a metric, the average slope, to describe the protein/peptide’s dose–response (Fig. 1C). Each dose–response curve was smoothened by a Gaussian kernel with a user-defined bandwidth parameter to summarize the overall trend. Its average slope was calculated by taking the average of the tangent of every pair of adjacent temperature points. Peptide intensities from the same protein were summed prior to curve fitting to achieve a global abundance measurement for constitutive protein and thermal stability changes.

The KOPI app generates two key diagrams, namely “ProteomeImpact” and “PhosphoResponse”. ProteomeImpact summarizes constitutive abundance changes at 37 °C and thermal stability changes at 52 °C (Fig. 1F). Hits that meet the user-defined average slope criteria are annotated. PhosphoResponse summarizes the dose-phosphorylation patterns at 37 °C as a function of the average slope and number of successive upward trends (Fig. 1G).

We observed four possible phosphorylation patterns in the phosphoproteome with the KOPI app; hyper-phosphorylation (hyper, Supplementary Data S1), hypo-phosphorylation (hypo, Supplementary Data S2), biphasic (Supplementary Data S3) and non-responsive (NR, Supplementary Data S4). There are four user-defined parameters *T*, *C*, *B* and *E* that determine this classification. *T* defines the range of the curve’s fold changes for a responsive phosphopeptide. *B* and *C* determine the number of consecutive upward or downward trends for assigning a hyper-phosphorylation or hypo-phosphorylation respectively. A biphasic curve requires both consecutive upward and downward trends in at least *B* or *C* adjacent dose pairs, and the difference between the ends of the curve should not be larger than *E* times the range. Phosphopeptides are assigned as responders when the response patterns in both replicates are consistent, summarized by the mean average slope between replicates.

**Figure 2:**
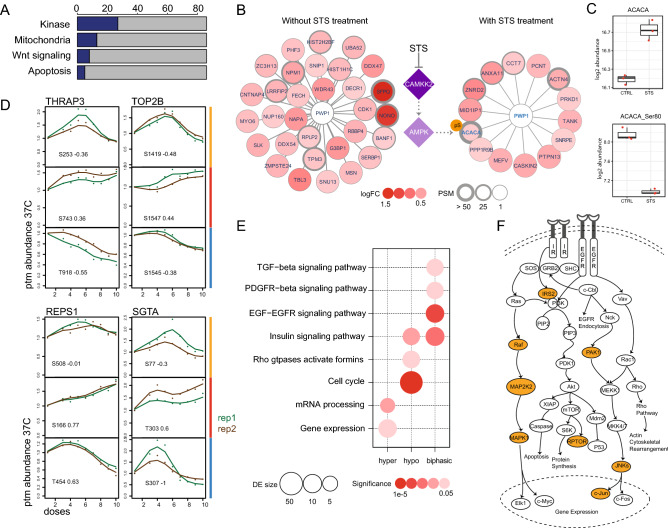
Impact of STS on the non-kinome and phosphoproteome. (**A**) The number of hits identified as kinases, and/or localized to the mitochondria, related to Wnt signaling and/or apoptosis. (**B**) Changes in PWP1 protein complex upon STS treatment as analysed by MS-immunoprecipitation. Hit proteins are defined by average log2 fold change (logFC) < − 0.5 or > 0.5, adjusted p-value < 0.05, from three replicates. PSM (number of peptide-spectra match per protein). CAMKK2 and AMPK were identified in the ProteomeImpact experiment (Fig. 1F), with CAMKK2 identified as a direct interactor of STS (solid purple fill). Known regulation of AMPK by CAMKK2^27–30^ (as indicated by the faint purple fill and dotted lines). (**C**) Log 2 abundance intensity of the total protein (ACACA) and its phosphosite ACACA-Ser80 with (STS) and without (CTRL) treatment. (**D**) Phosphopeptide response curves for four proteins with all three response patterns: thyroid hormone receptor-associated protein 3 (THRAP3), DNA topoisomerase 2-beta (TOP2B), RalBP1-associated Eps domain-containing protein 1 (REPS1), small glutamine-rich tetratricopeptide repeat-containing protein alpha (SGTA). The doses were represented as ordinal numbers, with the highest dose 10.0 µM at position number 10 (x-axis). The ptm (post-translation modification) abundance 37C (y-axis) represents the abundance fold change relative to the untreated condition. Coloured bars indicate biphasic (yellow), hyper-phosphorylation (red) and hypo-phosphorylation (blue), green and brown lines represent two biological replicates. (**E**) Differential phosphosites are clustered by biological processes involved. DE size (differential expression size), significance (enrichment significance). (**F**) Signaling network of epidermal growth factor receptor (EGFR) and insulin receptor (IR). Proteins with biphasic phosphorylation response in this pathway (shaded yellow).

We applied the training STS dataset to the KOPI pipeline to demonstrate how it visualizes STS’s effect on the proteome. We quantified abundance and thermal stability of 4000 proteins. STS’s ProteomeImpact diagram showed 86 protein hits with changes in either thermal stability and/or abundance (Fig. 1F, Supplementary Table S1), of which 34 were kinases. Gene ontology (GO) analysis showed enrichment for kinases, Wnt signalling pathway, apoptosis and localization to the mitochondria (Fig. 2A, Supplementary Table S2).

We compared these hits with the list of hits reported in the literature as the kinase targets of STS based on thermal profiling^21^, which confirmed 18 kinases. The common hits have larger abundance changes upon treatment (Supplementary Fig. S1A, Supplementary Table S1)^21^. More than half of our hits were non-kinases. We hypothesize that it was because the experiment was performed in live intact cells. We observed the dynamic effect of STS on Wnt signalling (Fig. 1F), where STS induced thermal stability changes to AP2-associated kinase (AAK1), glycogen synthase alpha and beta (GSK3a, GSKb), reduced and increased the abundance of dishevelled protein 3 (DVL3) and beta-catenin (CTNNB1) respectively^22,23^. Due to the short incubation period in this experiment, we inferred that STS downregulated Wnt signalling by altering DVL3’s cytoplasmic localization and reducing CTNNB1 degradation. To evaluate the downstream consequence of CTNNB1 in K562, we pulsed the cells with STS followed by two days of recovery. We observed a transient increase in CTNNB1 and a sustained increase in CD44 (Supplementary Fig. S1B,C), a cancer stem cell marker downstream of CTNNB1^24,25^.

We also identified a novel non-kinase protein that showed thermal stability changes with STS, the periodic tryptophan protein 1 (PWP1). No thermal stability changes were observed in experiments with K562 lysates, which indicated that the interaction of PWP1 with STS was indirect (Supplementary Fig. S1D). We hypothesized that PWP1’s response to STS is due to a change in its protein complex. We performed mass spectrometry-immunoprecipitation (MS-IP) on cells treated with STS and observed a significant change in its complex composition (Fig. 2B, Supplementary Table S3), whereby STS induced PWP1’s association with acetyl-CoA carboxylase 1 (ACACA), the rate-limiting enzyme in fatty acid biosynthesis^26^. Although there was an increase in total ACACA associated with the PWP1-protein complex from STS-treated cells, there was a corresponding dephosphorylation of ACACA-Ser80 (Fig. 2C, Supplementary Table S3). This phosphosite modulates its own enzymatic activity and is phosphorylated by AMP-activated protein kinase (AMPK)^27^. This implied a reduction in AMPK’s activity and its upstream kinases, LKB1 or CAMKK, of which CAMKK2 is a more potent activator than CAMKK1^28–30^. We quantified both CAMKKs and AMPK in the thermal proteome profile, and detected interaction of STS with both CAMKK1 and CAMKK2 only (Fig. 1F, Supplementary Table S1). The interaction of STS with CAMKK2 may have inhibited AMPK and resulted in dephosphorylation of ACACA-Ser80. We postulate that PWP1’s thermal stability change was caused by changes to its partner proteins, triggered by STS’s interaction with CAMKK (Fig. 2B).

We quantified 8545 phosphosites, of which 6005 were annotated in PhosphositePlus. STS’s PhosphoResponse diagram showed 6972 non-responsive phosphosites, 1027 with hypo-phosphorylation, 468 with biphasic phosphorylation and 78 with hyper-phosphorylation (Fig. 1G, Supplementary Table S4). These 1573 responsive sites were identified on 600 proteins (Supplementary Table S5). The majority showed homogenous phosphorylation. Several proteins showed heterogeneous (two or more possible phosphorylation trends) changes (53 proteins with both biphasic and hypo-phosphorylation, 23 with hyper-and hypo-phosphorylation, and nine with biphasic and hyper-phosphorylation). Four proteins had sites in all three categories, SGTA, TOP2B, REPS1 and THRAP3 (Fig. 2D), which did not show significant thermal stability or abundance changes in response to STS (Supplementary Fig. S1E).

We observed paradoxical hyper-phosphorylation and biphasic phospho-response, of which the former is more described^9,10^. We observed an enrichment in biphasic phosphorylation in the epidermal growth factor receptor (EGFR) signalling pathway (Fig. 2E,F, Supplementary Table S6). The biphasic response was previously shown for the RAF inhibitor PLX 4720 on phospho-ERK1/2, but it was described as an activation of the RAF–MEK–ERK pathway^10^. We showed here for the first time a systematic biphasic response to a kinase inhibitor. We propose that this biphasic phenomenon is a signature of kinases with kinase-independent roles. This postulate is only made possible by performing a detailed drug dilution series and not penalizing curves with a poor goodness-of-fit.

## Discussion

Unbiased proteomic approaches have shown that KI interactions with unintended kinases and non-kinase targets^31,32^. Advances in the proteomic approaches extend the coverage of the proteome or kinome^33,34^, but they have been limited to integrating the chemoproteome, the phosphotyrosine response and the transcriptome^35,36^. To the best of our knowledge, KOPI is the first workflow that integrates the responses from the proteome, phosphoproteome and interactome to a KI.

STS is a potent compound that is routinely used to trigger apoptosis. It causes cell death in K562 cells by inducing caspase-3 activity^37^. It also causes cell death in anticancer-drug resistant Jurkat T cells by inducing caspase-9 activity, but this induction can be independent of an apoptosome^38^. Our KOPI workflow showed for the first time a comprehensive impact of STS on the proteome by measuring and integrating the dose–response changes at three sublevels, the constitutive abundance, the interactome by thermal profiling, and the phosphoproteome. We showed that STS has a larger footprint on the kinome and beyond than previously described, and described candidate protein interactors that have not been considered thus far.

The strongest responses were observed in the interactome and the phosphoproteome in our data. Only three proteins showed significant changes in constitutive abundance, which is likely due to the changes in protein localization rather than the changes in protein expression. Thermal profiling identified a greater number of proteins with increased abundance with increasing STS concentration, implying ligand-induced stabilization. Interestingly, only half of the stabilized proteins were kinases. Most non-kinase interactors were either enriched for mitochondrial localization or involved in the Wnt/β-catenin pathway. We also showed that PWP1 was interacting indirectly with STS, through changes in its protein complex. In the significantly perturbed phosphoproteome, we discovered hypo- and hyper-phosphorylation, and more interestingly, systemic biphasic phosphorylation patterns, where phosphorylation levels rise and fall with the increase in the drug concentration. This unexpected quantitative variation adds a new layer of complexity to a KI’s impact on the proteome.

There are limitations and areas for improvement for the KOPI workflow. In order to take full advantage of the capabilities offered by the tool, a complete data set consisting of the proteome, the interactome and phosphoproteome is required, which is labour intensive. Without the full data set, it is still possible to visualise the curves with the KOPI app, but there are other existing tools that can visualise either one or two levels of the subproteome^20,39–41^.

Recent advances in thermal proteome profiling also simplified the workflow for studying ligand-proteome interaction, such as reducing the number of conditions and having higher replication^42^. However, a reduction in dose conditions will not capture the biphasic trends we report in this work. The detectable thermal proteome has also been expanded to membrane proteins such as G-coupled protein receptors through the use of mild detergent conditions during protein extraction^43^. Advances in isobaric labelling expanded the number of conditions that can be simultaneously acquired, increasing the throughput^44^. These modifications could be considered for the next iteration of KOPI.

In summary, the KOPI workflow seamlessly delineated the proteomic impact and phosphorylation responses induced by STS, providing a road map to understanding its complex mechanism of action. While the current implementation of KOPI was focused on phosphorylation, it can be readily applied to other post-translational modifications. Importantly, we generated the web-based KOPI tool for self-service analytics, allowing researchers to gain systems-wide insight into the complex proteomic interactions caused by specific KIs of interest.

## Methods

### Reagent and cell culture

Reagents and media were purchased from either Merck or Thermo unless stated otherwise. K562 were maintained as previously described^16^. Cells were washed with 1 × PBS unless stated otherwise.

### STS treatment

The cells were resuspended in media and treated with 9 doses of staurosporine (0, 0.0024, 0.0098, 0.0390, 0.1563, 0.6250, 2.5, 5.0 and 10.0 µM) for 30 min at 37 °C, thereafter washed and resuspended in PBS. They were heat pulsed at 37 °C or 52 °C for 3 min, followed by 3 min at 4 °C. They were harvested, washed and re-suspended in 1 × kinase lysis buffer (50 mM HEPES, pH 7.5, 5 mM beta-glycerophosphate, 0.1 mM sodium orthovanadate (Na_3_VO_4_), 10 mM MgCl_2_, 2 mM TCEP, 1 × protease inhibitor cocktail). The soluble proteome was extracted for MS analysis (Supplementary Methods).

### Immunoprecipitation

The cells were treated with 1 µM staurosporine or DMSO for 30 min at 37 °C, washed, harvested and lysed with 1 × kinase lysis buffer. The cleared supernatant was incubated with anti-PWP1 antibody (Novus Biologicals) for an hour, followed by Protein A Dynabeads at 4 °C overnight. The protein complexes were extracted with 30% 2,2,2-trifluoroethanol (TFE) with 100 mM glycine for digestion.

### Protein digestion and processing for MS

The supernatants were quantified using the BCA assay. Samples were reduced with 20 mM TCEP and 0.1% (w/v) RapiGest (Waters) at 55 °C for 20 min and alkylated with 55 mM CAA at RT for 30 min. Proteins were digested with LysC for 3–4 h followed by trypsin overnight at 37 °C. After digestion, the RapiGest was hydrolyzed with 1% TFA at 37 °C for 45 min. The samples were then centrifuged at 20,000*g* for 10 min and the supernatants were collected and dried. The samples were solubilized in 100 mM TEAB at a final concentration of 1 μg/μl. 25 μg of the digested peptides was labelled with Tandem Mass Tags-10plexTMT. The 127N channel (the 2nd position in the multiplex) is a common channel between multiplexed peptides, which was generated by pooling samples from the 37 °C condition series. The labelling was performed in 100 mM TEAB buffer for at least 1 h before quenching with 1 M Tris, pH 7.4. The samples were desalted using C18 Sep-Pak cartridge (Waters) and pre-fractionated into 96 fractions using a high pH reverse phase Zorbax 300 Extend C-18 4.6 mm × 250 mm (Agilent) column and liquid chromatography AKTA Micro (GE) system. The fractions were pooled into 20 fractions, and every alternate fraction was acquired for MS analysis.

The immunoprecipitated samples were reduced, alkylated, digested, desalted using 10 mg HLB cartridge (Waters), labelled with TMT10 and quenched with 1 M ammonium formate, pH 10. The samples were step fractionated with ReproSil-Pur Basic 10 µM C18 resin (Dr Maisch) with 14%, 24%, 60% acetonitrile in 10 mM ammonium formate.

### Phosphopeptide enrichment

Phosphopeptides were enriched from the remaining pooled labelled samples with TiO_2_ beads. The samples were resuspended in 1.5% TFA in 30% acetonitrile and incubated with the beads at room temperature for 10 min. This incubation was repeated once with fresh beads. The beads were loaded over a C8 mesh, washed once with 2% TFA in 80% acetonitrile, and eluted with 2% ammonia water in 40% acetonitrile. The phosphopeptides were desalted and fractionated with 9%, 12% 15% 17% 25% and 50% of acetonitrile in 10 mM ammonium formate.

### LC–MS analysis

Samples were analysed using reverse phase liquid chromatography with the Proxeon 1000 UHPLC system coupled with a Orbitrap Fusion Tribrid mass spectrometer (Thermo Scientific) and separated on a 50 cm × 75 µm Easy Spray column. The samples were separated with a 70 min gradient for proteomics and a 100 min gradient for phosphoproteomics, using mobile phase A (0.1% formic acid in water) and mobile phase B (0.1% formic acid in 99% acetonitrile).

For proteomics, peptides were eluted at a constant flow rate of 300 nl/min using 2–21% acetonitrile over 58 min, ramped to 60% over 2 min, then to 90% over 5 min and held for 5 min. Acquisition parameters: data dependent acquisition (DDA) with survey scan of 60,000 resolution, AGC target of 4e5, and maximum injection time (IT) of 100 ms; MS/MS at 45,000 resolution, AGC target of 8e4, and maximum IT of 105 ms; HCD collision energy 42%, isolation window 1.0 m/z.

For phosphoproteomics, peptides were eluted at a constant flow rate of 300 nl/min using 2—23% acetonitrile over 84 min and ramped to 60% over 5 min. The flow rate was increased to 350 nl/min and acetonitrile was ramped to 90% over 5 min, and held for 6 min. Acquisition parameters: DDA with survey scan of 60,000 resolution, AGC target of 4e5, and maximum IT of 100 ms; MS/MS at 50,000 resolution, AGC target of 8e4, and maximum IT of 150 ms; HCD collision energy 43%, isolation window 1.2 m/z.

For the MS-IP, peptides were eluted at a constant flow rate of 300 nl/min using 2—33% acetonitrile over 56 min, was ramped to 45% over 5 min, then to 95% over 5 min, and held for 4 min. Acquisition parameters: DDA with survey scan of 60,000 resolution, AGC target of 8e5, and maximum IT of 50 ms; MS/MS at 50,000 resolution, AGC target of 1e5, and maximum IT of 80 ms; HCD collision energy 42%, isolation window 1.0 m/z.

### Peptide quantification and processing

Raw files were analysed by MaxQuant version 1.6.5. Peptides in MS/MS spectra were identified by the Andromeda search engine, against the forward/decoy/Human Uniprot database (42,412 entries, retrieved 20,180,410). Search parameters as per default settings for Orbitrap: MS precursor mass tolerance 4.5 ppm, reporter mass tolerance 20 ppm, 3 missed cleavages; fixed modifications: Carbamidomethyl (C); variable modifications: Oxidation (M), Deamidated (NQ), Acetyl (protein N-term), Phospho (STY). Peptide-spectrum matches false discovery rate (PSM FDR) at 0.01.

Peak lists from the MS-IP were generated in Proteome Discoverer 2.4 (Thermo Scientific) using Mascot 2.6.1 (Matrix Science) and concatenated forward/decoy Human Uniprot database. Search parameters: MS precursor mass tolerance 30 ppm, MS/MS fragment mass tolerance 0.06 Da, 3 missed cleavages; static modifications: Carbamidomethyl (C); variable modifications: Oxidation (M), Deamidated (NQ), Acetyl N-terminal protein, TMT10plex (N-term), TMT10plex (K), Phospho (STY). False discovery rate estimation with 2 levels: Strict = FDR 1%, Medium = FDR 5%. Differential analysis was performed with the *limma* package in R and Cytoscape.

### Data analysis

#### Dose curve fitting with Gaussian kernel

Phosphosites quantified in the phosphoproteome dataset with localization probability lower than 50% from the MaxQuant output were filtered. Peptides and phosphopeptides with non-zero values in both replicates were retained. Peptides of the same protein were aggregated to achieve a summarised abundance measurement.

Each dose was normalized by dividing the abundances with the value of the first dose. Curves were fitted with a Gaussian kernel function estimator, while the bandwidth parameter *b* is left a user-defined parameter. For each point $(x_{i} ,\;y_{i} )$, $i \in \left\{ {1,\;2, \ldots ,\;n} \right\}$ a bandwidth-scaled distance *d* was calculated to represent the closeness between *x*_*i*_ and $x_{k} , \;k \in \left\{ {1,\;2, \ldots ,\,n} \right\}$, on which the impact of the other points on point $(x_{i} ,\;y_{i} )$ was evaluated. The impact of all the points on *y*_*i*_ is evaluated as the weight coefficients {*wt*}, which is the probability density of *d* under a standard Gaussian distribution. Using these weights, the fitted $\widehat{{y_{i} }}$ is calculated as a weighted average of all $y_{k} , k \in \left\{ {1,\;2, \ldots ,\;n} \right\}$.

$$d_{k} = (x_{k} - x_{i} )/b$$

$$wt_{k} = \frac{1}{{\sqrt {2\pi } }}exp\left( { - \frac{1}{2}d_{k}^{2} } \right)$$

$$\widehat{{y_{i} }} = \left( {\mathop \sum \limits_{k = 1}^{n} wt_{k} *y_{k} } \right)/\mathop \sum \limits_{k = 1}^{n} wt_{k}$$

After smoothing, an average slope for each curve was calculated by taking the average of the tangent of every pair of adjacent fitted points. The software accounts for potential technical bias resulting from experimental handling by excluding user-specified outlier data points before fitting curves.

#### Response pattern classification

The response patterns for phosphopeptides were categorised into four groups: hyper-phosphorylation (hyper), hypo-phosphorylation (hypo), biphasic and non-responsive (NR). A phosphopeptide curve with a range larger than the value *T* is identified as responsive to STS. Curves can be further categorized as “hyper” or “hypo” if it has a positive or negative average slope, at least *B* or *C* consecutive upward or downward trends respectively, between adjacent points. A biphasic curve has both consecutive upward and downward trends in at least *B* adjacent dose pairs, and the difference in abundance of the right and left ends of the curve are *E* times smaller than the range value *T*. *T*, *C*, *B* and *E* are all user-defined parameters. For the current dataset, the abundances were normalized with the firstdose and *T*, *C*, *B* and *E* were set at 0.3, 5, 3, 0.45 respectively. Each replicate was individually fitted, categorized and assigned an average slope value. Each phosphopeptide was assigned the mean value of their replicates’ average slopes. They are assigned a final response category as a responding phosphopeptide (not “NR”) only when the response pattern is concordant in the two replicates.

#### Gene set enrichment analysis

The list of genes encoding the hit proteins and phosphopeptides of interest was compared against the background list of all identified proteins using the ConsensusPathDB database^45^ as the reference. Significance was determined with the threshold of p < 0.05, calculated using hypergeometric probability.

## Supplementary Information

Supplementary Information.

Supplementary Table S1.

Supplementary Table S2.

Supplementary Table S3.

Supplementary Table S4.

Supplementary Table S5.

Supplementary Table S6.

## Data Availability

The raw spectra and search data were uploaded to the Jpost repository with the following accession numbers: JPST001389 (jPOST) and PXD029924 (ProteomeXchange). The data is available at https://repository.jpostdb.org/entry/JPST001389. The KOPI software is available on https://ginnyintifa.shinyapps.io/ProteomeNodesShiny/.
