# Supplementary material for: KOPI: Kinase inhibitOr Proteome Impact analysis
Source: Sci Rep. 2022 Jul 29;12:13015. doi: 10.1038/s41598-022-16557-w (PMC9338059; doi:10.1038/s41598-022-16557-w)
Supplement: Supplementary file 1 — Supplementary Information. [file 41598_2022_16557_MOESM1_ESM.pdf]

For Nature Scientific Reports

Supplementary information

KOPI – Kinase inhibitOr Proteome Impact analysis

Ginny Xiaohe Li, Tianyun Zhao, Loo Chien Wang, Hyungwon Choi, Yan Ting Lim,

Radoslaw M Sobota

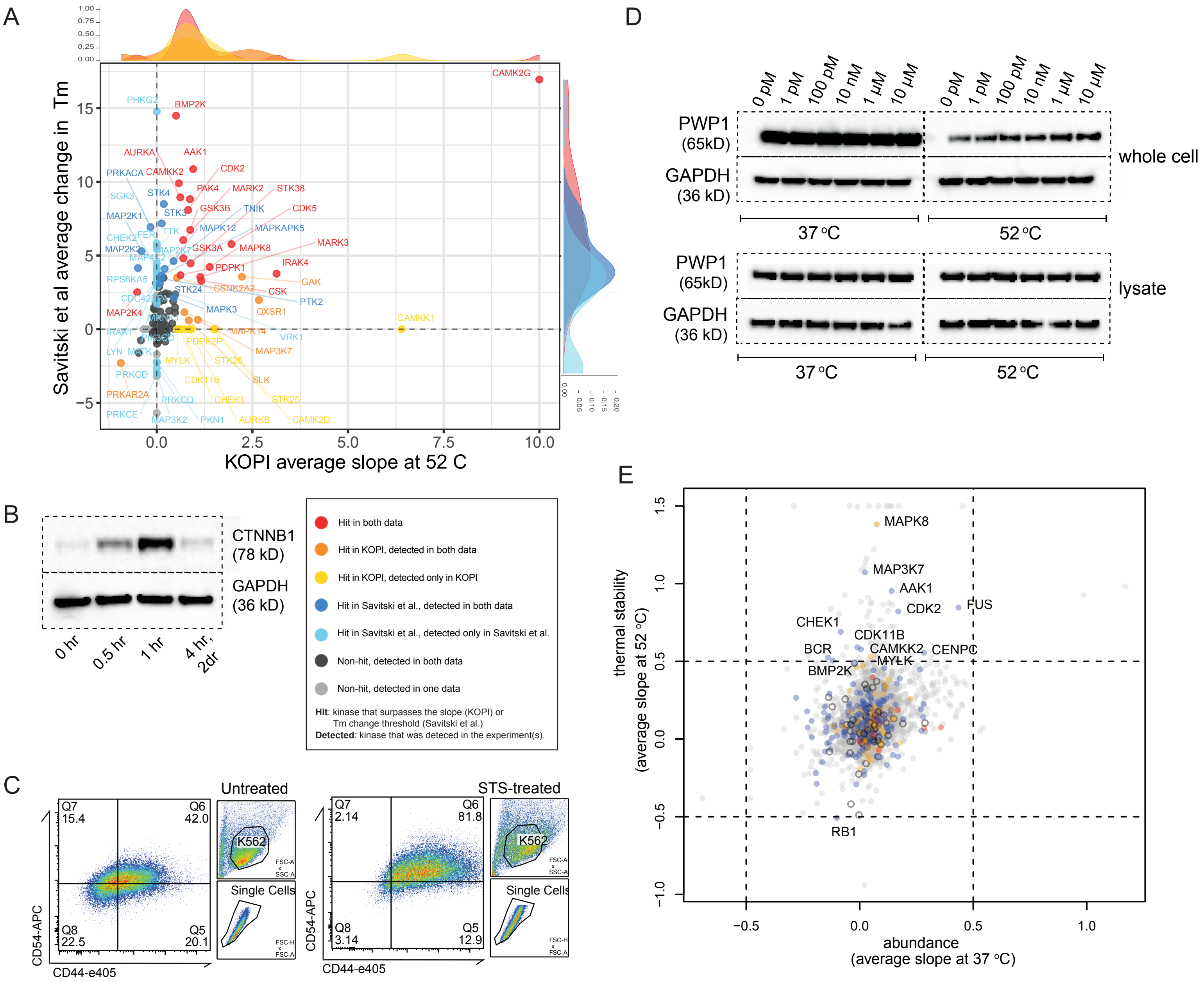

Supplementary Figure 1. (A) Average slope calculated by KOPI (x axis) and ΔTm calculated in Savitski et al. (y axis) of kinases profiled in at least one of the experiments. Kinases only profiled in one experiment and not reported as hit kinases (grey); Kinases profiled in both experiments and not reported as hit kinases (black); Kinases only profiled in Savitski et al. and reported to be hits (sky blue); Kinases profiled in both experiments and reported to be hits in Savitski et al. only (dark blue). Kinases only profiled in KOPI and reported to be hits (yellow); Kinases profiled in both experiments and reported to be hits in KOPI only (orange); Kinases profiled in both experiments and reported to be hits in both experiments (red). Density plots of kinase hits in at least one experiment are on the right and top margin of the scatter plot. (B) B-catenin levels after STS treatment at 1 μM for 0 hr, 0.5 hr, 1 hr or 4 hr with 2 days of recovery (2dr). GAPDH as loading control. (C) CD44 and CD54 surface expression after STS treatment by flow cytometry. Result is representative of 2 independent experiments, gating for single cells shown. (D) Whole cell and lysate ITDR with STS. Cells or lysates were treated with 0-10 μM of STS, heat pulsed at 37 oC and 52 oC, and probed for PWP1 and GAPDH (loading control) in the soluble fraction. (E) Comparison of phosphosites and protein thermal stability changes. Average slopes of each protein dose response curves at 37 oC and 52 oC. Thermal stabi- lized kinases with responsive phosphopeptides are labelled with names. Red, blue, yellow and grey colored proteins with hyper-, hypo-, biphasic, and non-responsive phosphopeptides respectively. Hollow circles represent proteins with more than one type of responsive phosphopeptides.

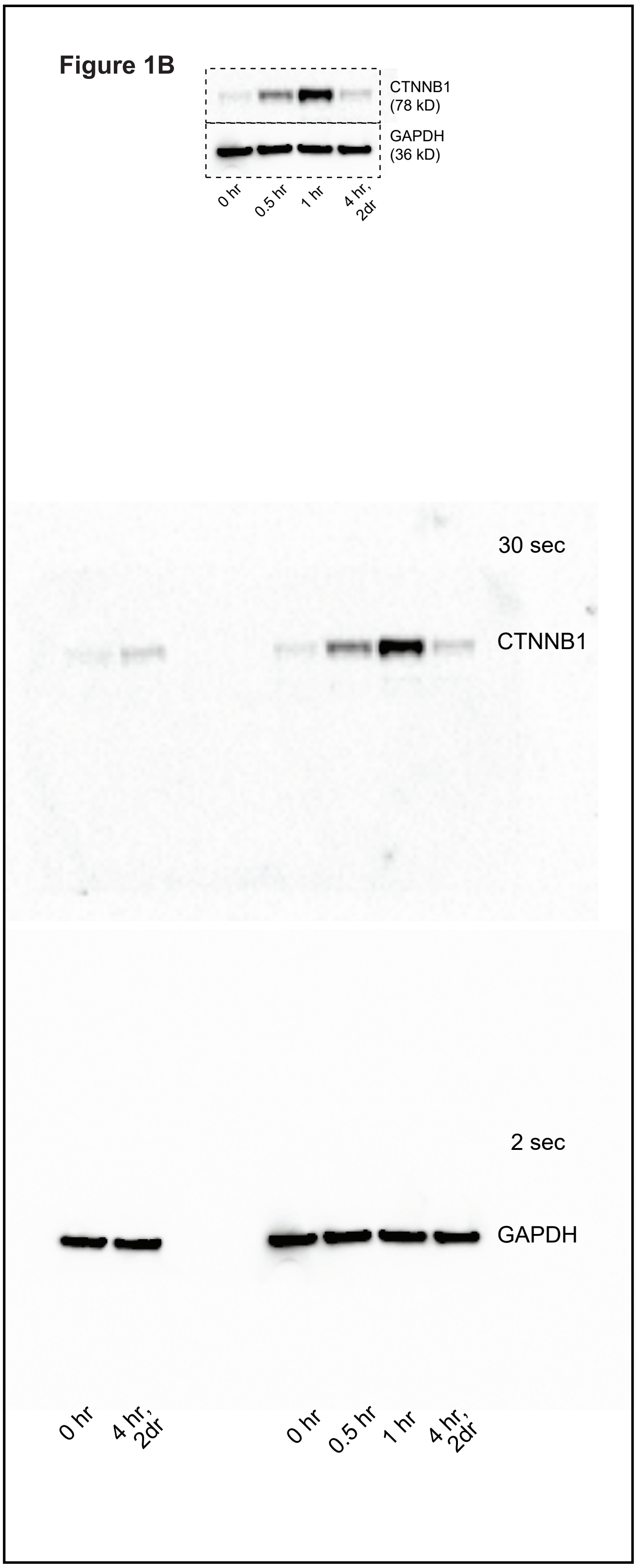

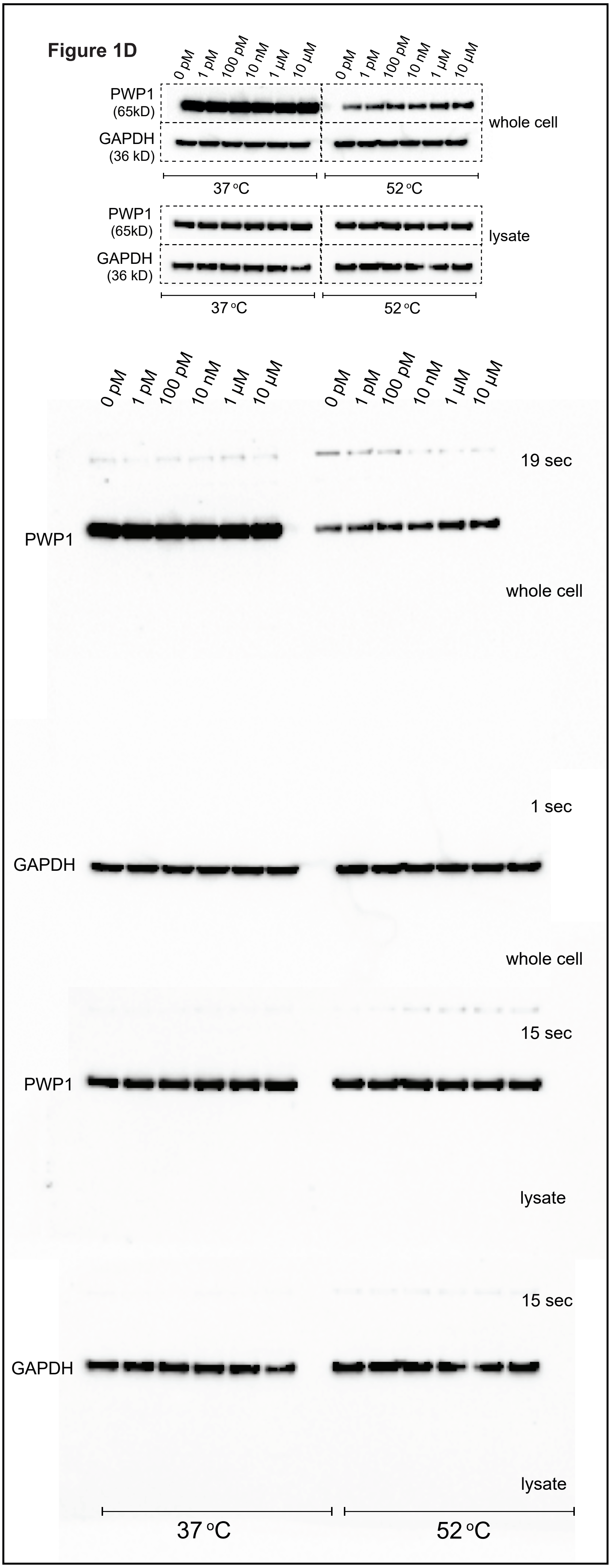

Supplementary Figure 2A. Original full length blots of Supplementary Figure 1B and 1D. 2dr: 2 days recovery. The exposure times used for Supplementary Figure 1B and 1D were indicated in this figure.

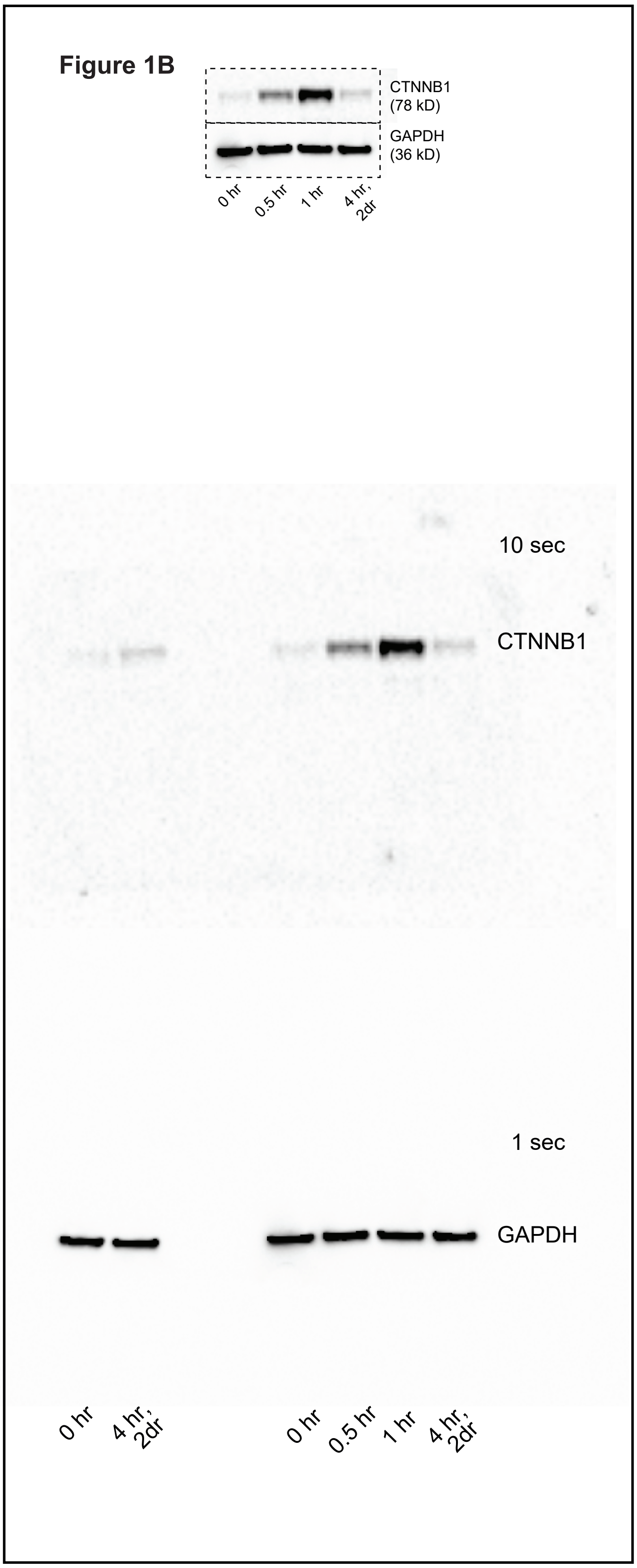

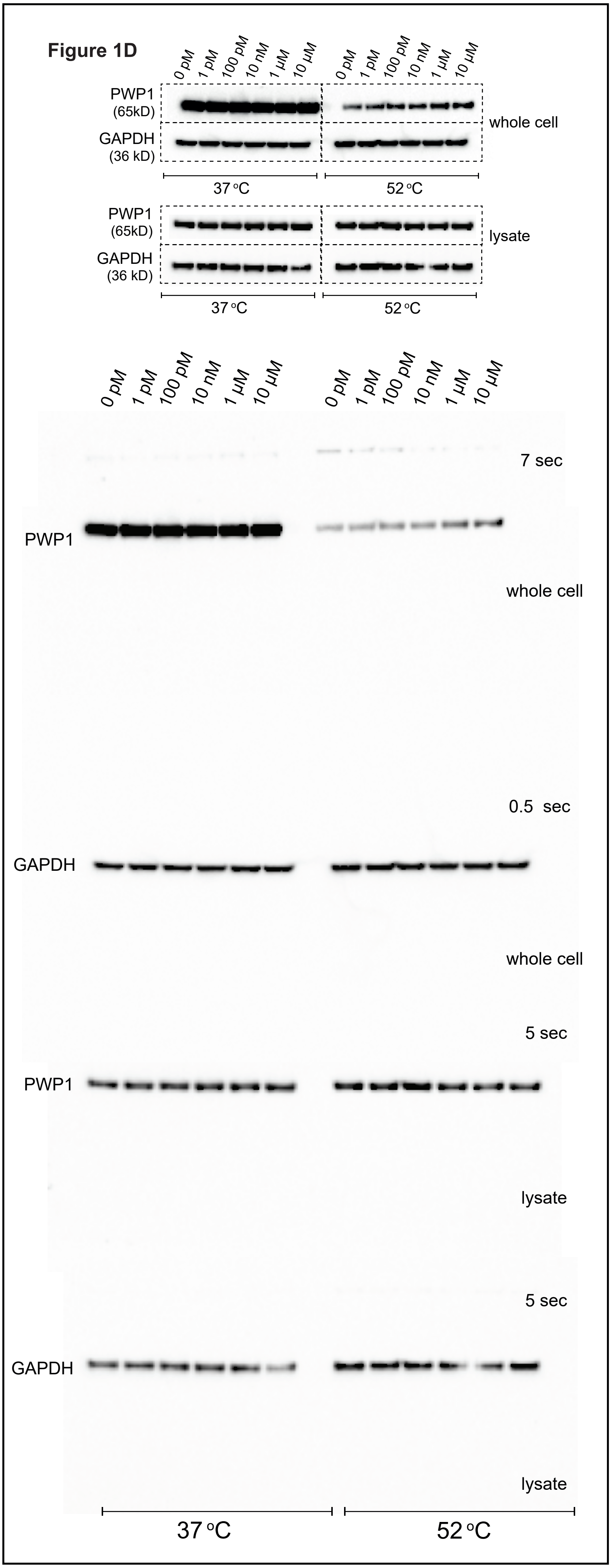

Supplementary Figure 2B. Original full length blots of Supplementary Figure 1B and 1D. 2dr: 2 days recovery. The exposure times indicated here were less than the duration used for Supplementary Figure 1B and 1D.

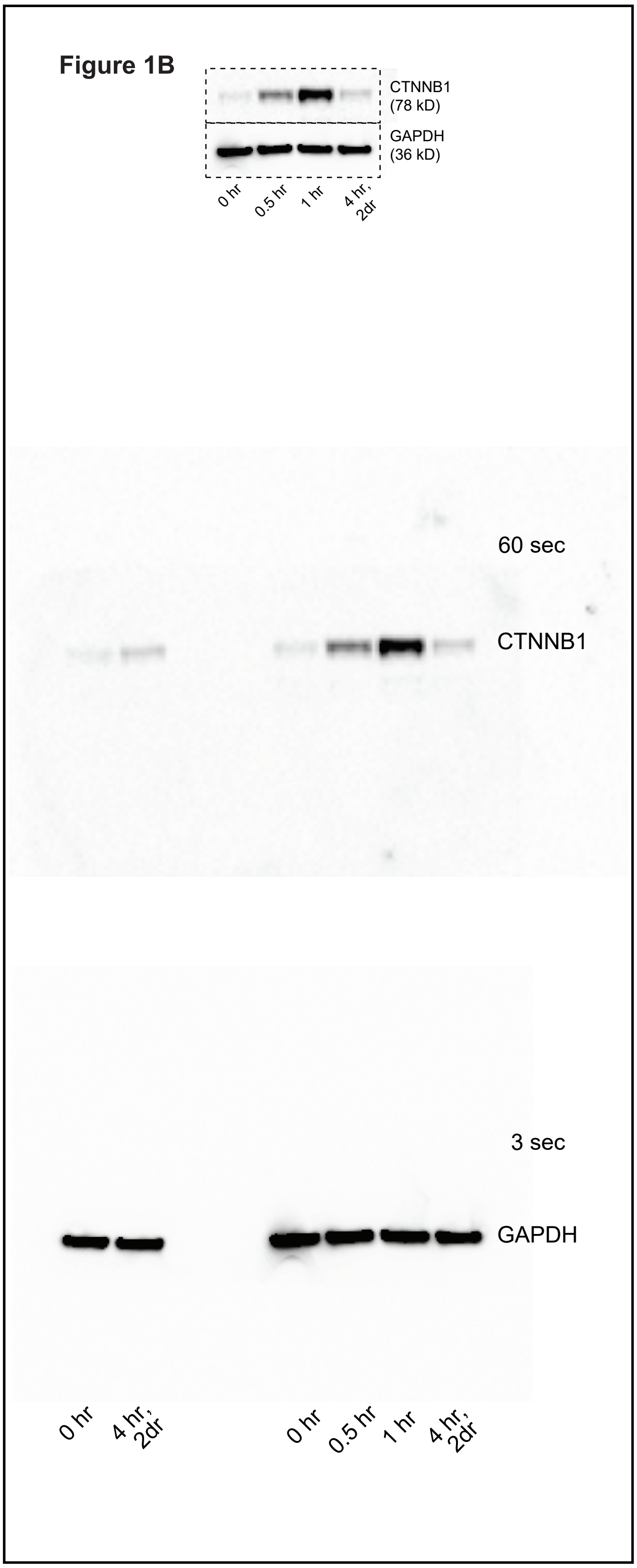

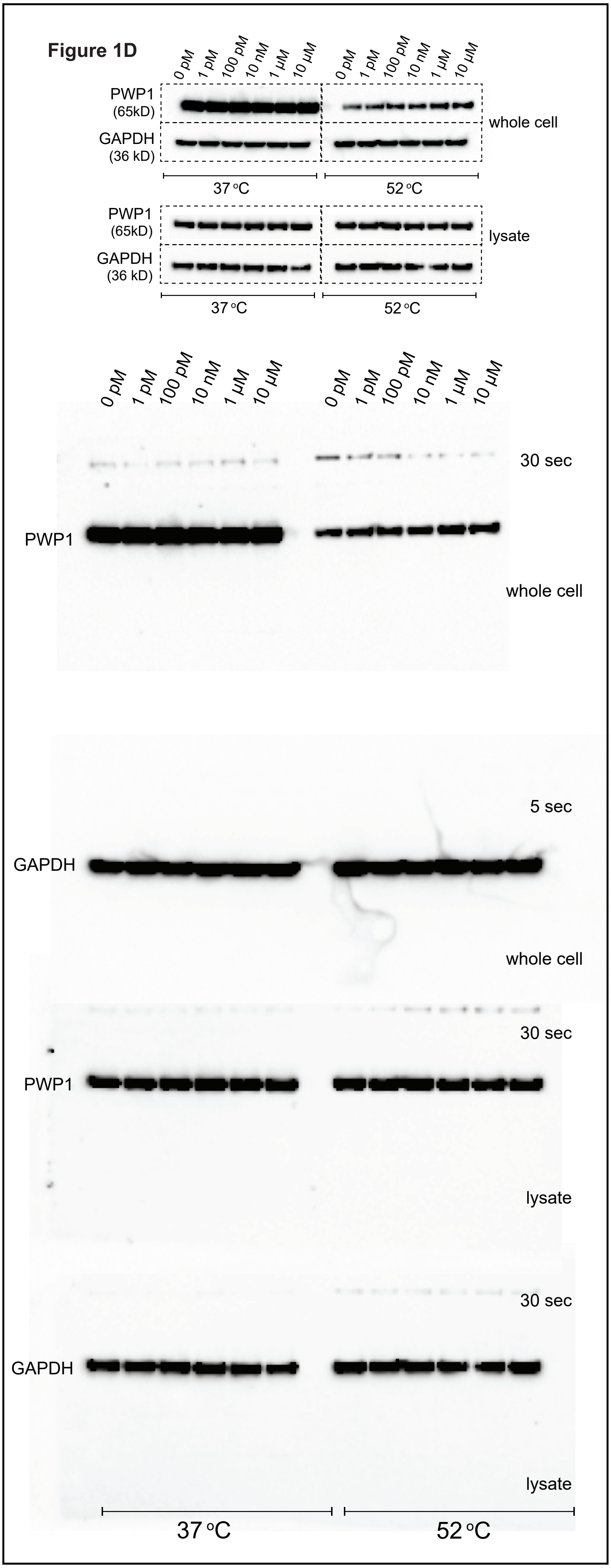

Supplementary Figure 2C. Original full length blots of Supplementary Figure 1B and 1D. 2dr: 2 days recovery. The exposure times indicated here were more than the duration used for Supplementary Figure 1B and 1D.

Supplementary Methods

Flow cytometry and Western blot

Cells were treated with 1 μM staurosporine for 30 min, 1 hr, or 4 hr with 2 days of recovery in fresh culture medium. Cells were washed, lysed and were resolved on a 4-12% gradient NuPAGE gel for western blotting with 1:2000 anti-β-Catenin (Cell Signaling) and 1:5000 anti-GAPDH (Abcam), with appropriate secondary antibodies. Cells that were treated for 4 hr with 2 days of recovery were washed and co-stained with 1:250 APC anti-CD44 antibody (BD Pharmingen) and Pacific Blue anti-CD54 (Biolegend) antibody in the staining buffer (PBS with 0.1% FBS) for 30 min on ice. Isotype control was also performed as background control. Cells were washed and fixed with 4% paraformaldehyde overnight at 4 °C before flow cytometry analysis.

Western blot-CETSA

Untreated K562 cell lysate was prepared by freeze-thaw using the 1X kinase lysis buffer. The cleared supernatant was used for lysate-based experiments. Lysate concentration was determined by BCA. 50 μg of the lysate was taken and treated with 6 different doses of STS as 0, 0.001 nM, 0.1 nM, 10 nM, 1 μM and 10 μM for 3 min at room temperature, followed by a heat pulse at 37 °C or 52 °C for 3 min. The samples were centrifuged for 20 mins again to remove aggregates. The supernatant was loaded on a 4-12% gradient gel. Membranes were incubated with the primary antibodies against the PWP1 (Novus Biologicals) or GAPDH and the appropriate secondary antibodies.

Preparation of the soluble proteome

The cells were lysed by two freeze-thaw cycles with liquid nitrogen, followed by mechanical shearing with a syringe and a final freeze-thaw cycle. The cell debris was removed by centrifugation at 20,000 g for 20 min at 4 °C and the supernatant was transferred into new microtubes for MS sample preparation

Supplementary Data 1: Phosphosites assigned as hyper-phosphorylated. Doses are represented as ordinal numbers (highest dose 10 μM as the 10th position; x-axis), abundance fold change relative to the untreated condition (y-axis).

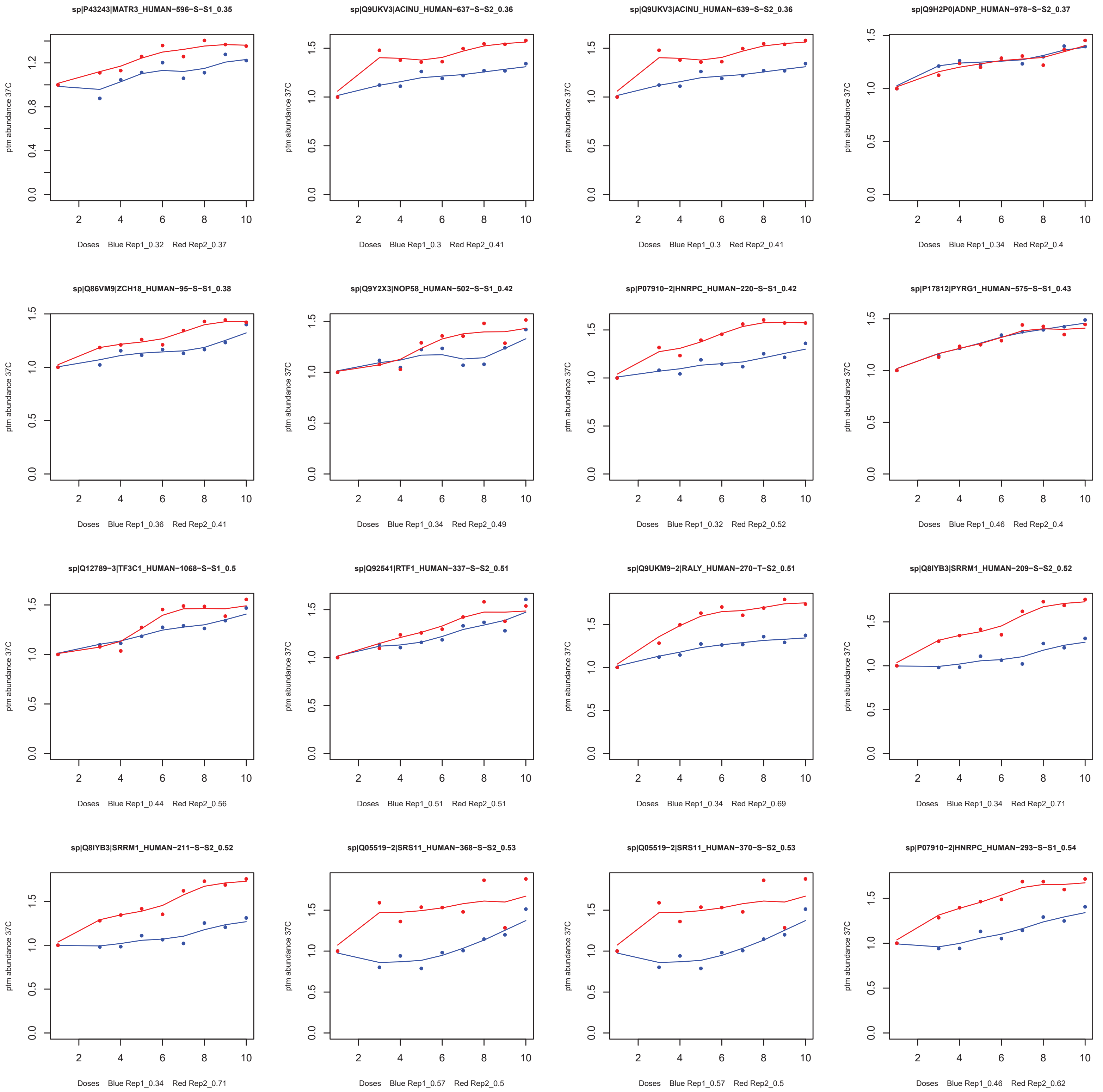

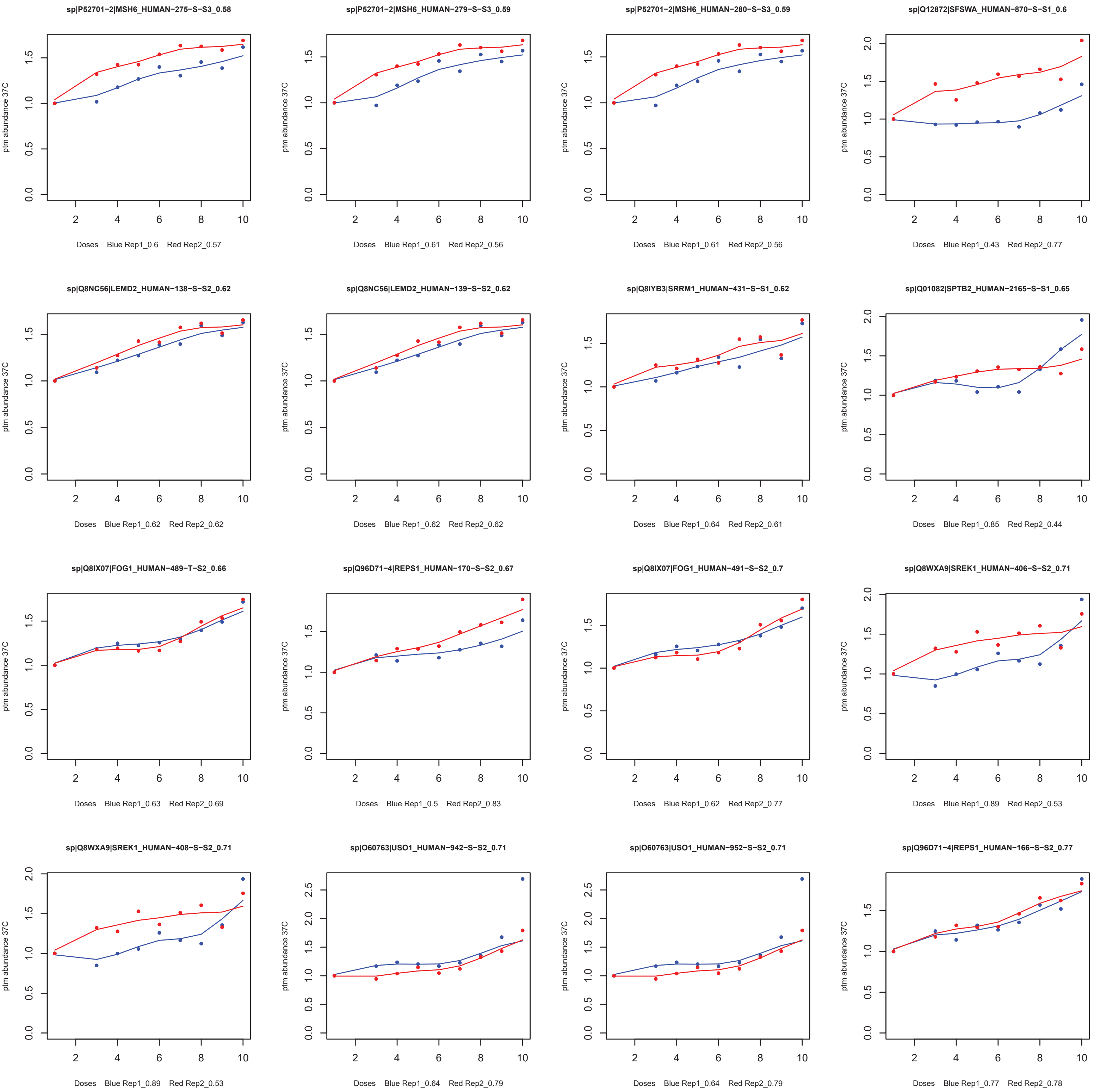
sp|P52701−2|MSH6_HUMAN−275−S−S3_0.58
ptm abundance 37C
Doses Blue Rep1_0.6 Red Rep2_0.57
sp|P52701−2|MSH6_HUMAN−279−S−S3_0.59
ptm abundance 37C
Doses Blue Rep1_0.61 Red Rep2_0.56
sp|P52701−2|MSH6_HUMAN−280−S−S3_0.59
ptm abundance 37C
Doses Blue Rep1_0.61 Red Rep2_0.56
sp|Q12872|SFSWA_HUMAN−870−S−S1_0.6
ptm abundance 37C
Doses Blue Rep1_0.43 Red Rep2_0.77
sp|Q8NC56|LEMD2_HUMAN−138−S−S2_0.62
ptm abundance 37C
Doses Blue Rep1_0.62 Red Rep2_0.62
sp|Q8NC56|LEMD2_HUMAN−139−S−S2_0.62
ptm abundance 37C
Doses Blue Rep1_0.62 Red Rep2_0.62
sp|Q8IYB3|SRRM1_HUMAN−431−S−S1_0.62
ptm abundance 37C
Doses Blue Rep1_0.64 Red Rep2_0.61
sp|Q01082|SPTB2_HUMAN−2165−S−S1_0.65
ptm abundance 37C
Doses Blue Rep1_0.85 Red Rep2_0.44
sp|Q8IX07|FOG1_HUMAN−489−T−S2_0.66
ptm abundance 37C
Doses Blue Rep1_0.63 Red Rep2_0.69
sp|Q96D71−4|REPS1_HUMAN−170−S−S2_0.67
ptm abundance 37C
Doses Blue Rep1_0.5 Red Rep2_0.83
sp|Q8IX07|FOG1_HUMAN−491−S−S2_0.7
ptm abundance 37C
Doses Blue Rep1_0.62 Red Rep2_0.77
sp|Q8WXA9|SREK1_HUMAN−406−S−S2_0.71
ptm abundance 37C
Doses Blue Rep1_0.89 Red Rep2_0.53
sp|Q8WXA9|SREK1_HUMAN−408−S−S2_0.71
ptm abundance 37C
Doses Blue Rep1_0.89 Red Rep2_0.53
sp|O60763|USO1_HUMAN−942−S−S2_0.71
ptm abundance 37C
Doses Blue Rep1_0.64 Red Rep2_0.79
sp|O60763|USO1_HUMAN−952−S−S2_0.71
ptm abundance 37C
Doses Blue Rep1_0.64 Red Rep2_0.79
sp|Q96D71−4|REPS1_HUMAN−166−S−S2_0.77
ptm abundance 37C
Doses Blue Rep1_0.77 Red Rep2_0.78

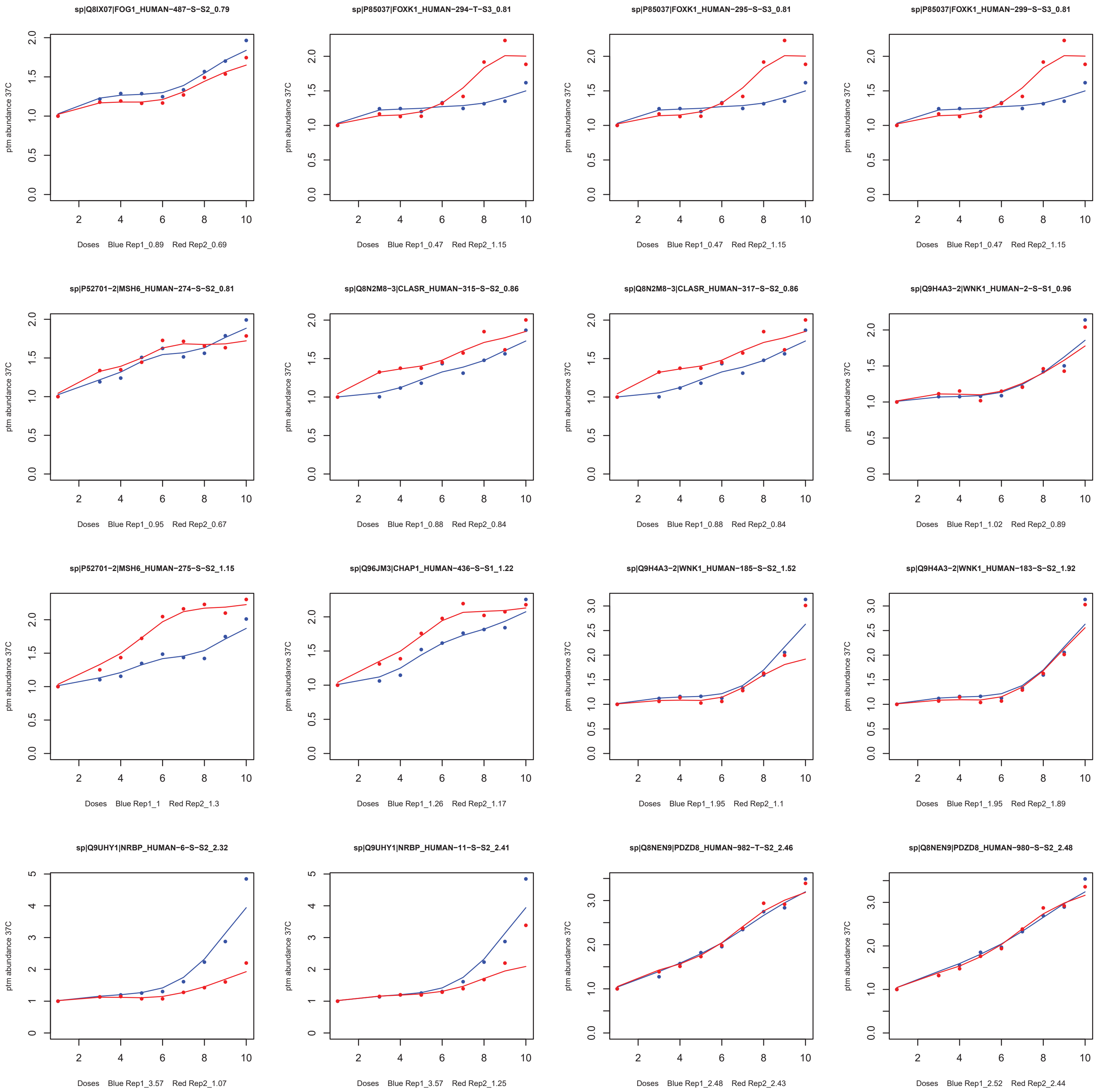

sp|Q8IX07|FOG1_HUMAN−487−S−S2_0.79
Doses Blue Rep1_0.89 Red Rep2_0.69
sp|P85037|FOXK1_HUMAN−294−T−S3_0.81
Doses Blue Rep1_0.47 Red Rep2_1.15
sp|P85037|FOXK1_HUMAN−295−S−S3_0.81
Doses Blue Rep1_0.47 Red Rep2_1.15
sp|P85037|FOXK1_HUMAN−299−S−S3_0.81
Doses Blue Rep1_0.47 Red Rep2_1.15
sp|P52701−2|MSH6_HUMAN−274−S−S2_0.81
Doses Blue Rep1_0.95 Red Rep2_0.67
sp|Q8N2M8−3|CLASR_HUMAN−315−S−S2_0.86
Doses Blue Rep1_0.88 Red Rep2_0.84
sp|Q8N2M8−3|CLASR_HUMAN−317−S−S2_0.86
Doses Blue Rep1_0.88 Red Rep2_0.84
sp|Q9H4A3−2|WNK1_HUMAN−2−S−S1_0.96
Doses Blue Rep1_1.02 Red Rep2_0.89
sp|P52701−2|MSH6_HUMAN−275−S−S2_1.15
Doses Blue Rep1_1 Red Rep2_1.3
sp|Q96JM3|CHAP1_HUMAN−436−S−S1_1.22
Doses Blue Rep1_1.26 Red Rep2_1.17
sp|Q9H4A3−2|WNK1_HUMAN−185−S−S2_1.52
Doses Blue Rep1_1.95 Red Rep2_1.1
sp|Q9H4A3−2|WNK1_HUMAN−183−S−S2_1.92
Doses Blue Rep1_1.95 Red Rep2_1.89
sp|Q9UHY1|NRBP_HUMAN−6−S−S2_2.32
Doses Blue Rep1_3.57 Red Rep2_1.07
sp|Q9UHY1|NRBP_HUMAN−11−S−S2_2.41
Doses Blue Rep1_3.57 Red Rep2_1.25
sp|Q8NEN9|PDZD8_HUMAN−982−T−S2_2.46
Doses Blue Rep1_2.48 Red Rep2_2.43
sp|Q8NEN9|PDZD8_HUMAN−980−S−S2_2.48
Doses Blue Rep1_2.52 Red Rep2_2.44
ptm abundance 37C

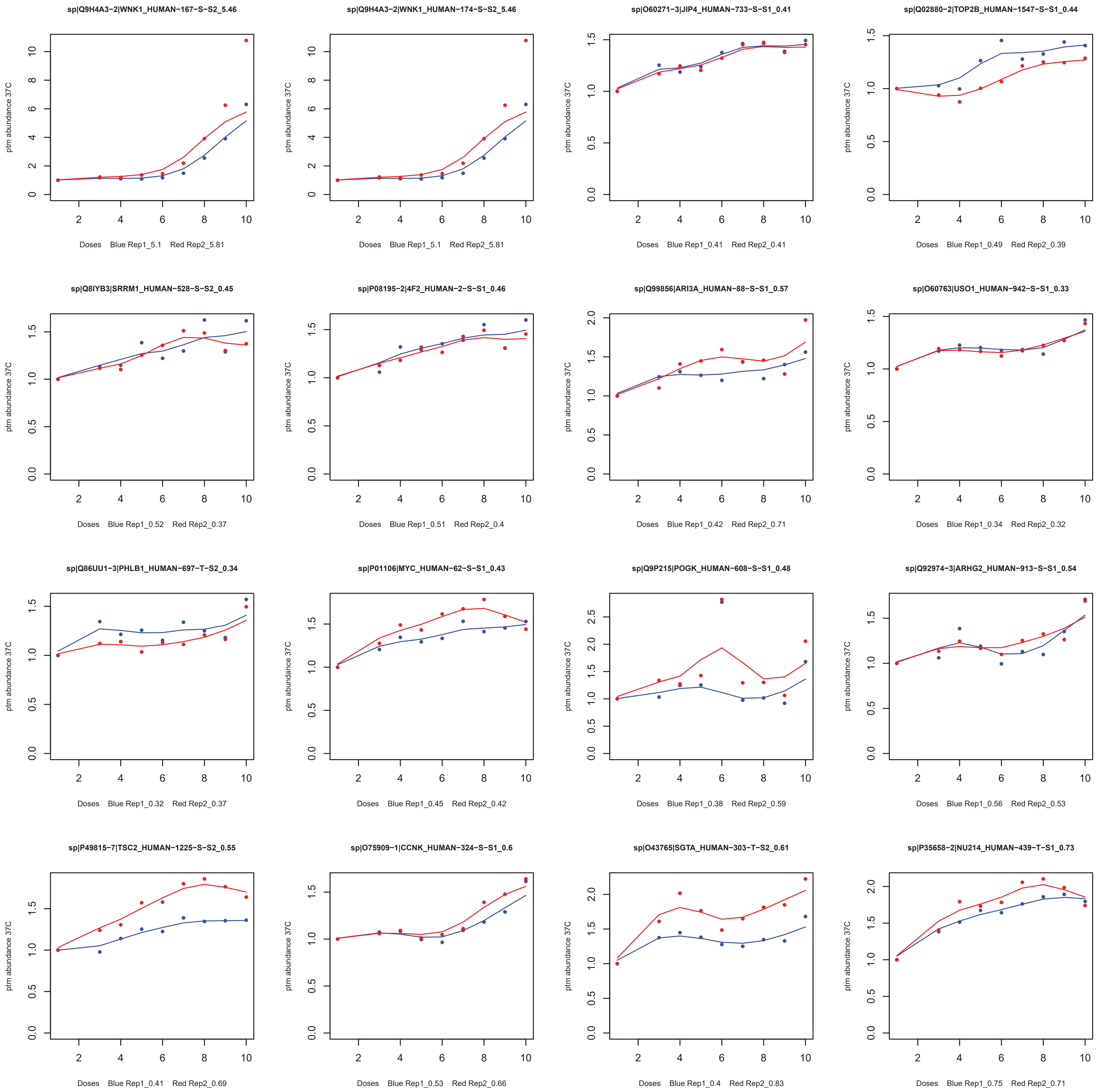
sp|Q9H4A3−2|WNK1_HUMAN−167−S−S2_5.46
Doses Blue Rep1_5.1 Red Rep2_5.81
sp|Q9H4A3−2|WNK1_HUMAN−174−S−S2_5.46
Doses Blue Rep1_5.1 Red Rep2_5.81
sp|O60271−3|JIP4_HUMAN−733−S−S1_0.41
Doses Blue Rep1_0.41 Red Rep2_0.41
sp|Q02880−2|TOP2B_HUMAN−1547−S−S1_0.44
Doses Blue Rep1_0.49 Red Rep2_0.39
sp|Q8IYB3|SRRM1_HUMAN−528−S−S2_0.45
Doses Blue Rep1_0.52 Red Rep2_0.37
sp|P08195−2|4F2_HUMAN−2−S−S1_0.46
Doses Blue Rep1_0.51 Red Rep2_0.4
sp|Q99856|ARI3A_HUMAN−88−S−S1_0.57
Doses Blue Rep1_0.42 Red Rep2_0.71
sp|O60763|USO1_HUMAN−942−S−S1_0.33
Doses Blue Rep1_0.34 Red Rep2_0.32
sp|Q86UU1−3|PHLB1_HUMAN−697−T−S2_0.34
Doses Blue Rep1_0.32 Red Rep2_0.37
sp|P01106|MYC_HUMAN−62−S−S1_0.43
Doses Blue Rep1_0.45 Red Rep2_0.42
sp|Q9P215|POGK_HUMAN−608−S−S1_0.48
Doses Blue Rep1_0.38 Red Rep2_0.59
sp|Q92974−3|ARHG2_HUMAN−913−S−S1_0.54
Doses Blue Rep1_0.56 Red Rep2_0.53
sp|P49815−7|TSC2_HUMAN−1225−S−S2_0.55
Doses Blue Rep1_0.41 Red Rep2_0.69
sp|O75909−1|CCNK_HUMAN−324−S−S1_0.6
Doses Blue Rep1_0.53 Red Rep2_0.66
sp|O43765|SGTA_HUMAN−303−T−S2_0.61
Doses Blue Rep1_0.4 Red Rep2_0.83
sp|P35658−2|NU214_HUMAN−439−T−S1_0.73
Doses Blue Rep1_0.75 Red Rep2_0.71
ptm abundance 37C

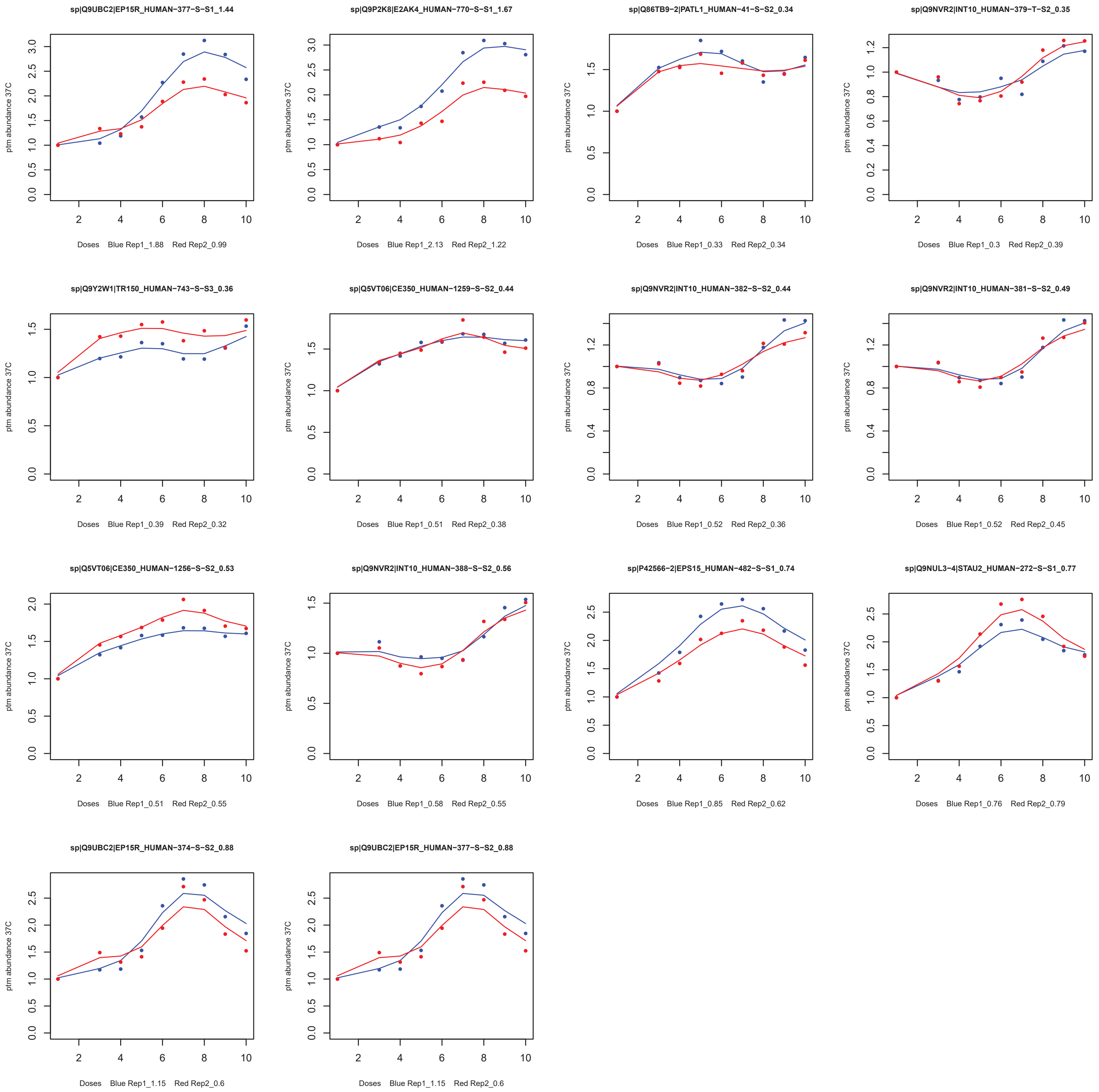
sp|Q9UBC2|EP15R_HUMAN−377−S−S1_1.44
ptm abundance 37C
Doses Blue Rep1_1.88 Red Rep2_0.99
sp|Q9P2K8|E2AK4_HUMAN−770−S−S1_1.67
ptm abundance 37C
Doses Blue Rep1_2.13 Red Rep2_1.22
sp|Q86TB9−2|PATL1_HUMAN−41−S−S2_0.34
ptm abundance 37C
Doses Blue Rep1_0.33 Red Rep2_0.34
sp|Q9NVR2|INT10_HUMAN−379−T−S2_0.35
ptm abundance 37C
Doses Blue Rep1_0.3 Red Rep2_0.39
sp|Q9Y2W1|TR150_HUMAN−743−S−S3_0.36
ptm abundance 37C
Doses Blue Rep1_0.39 Red Rep2_0.32
sp|Q5VT06|CE350_HUMAN−1259−S−S2_0.44
ptm abundance 37C
Doses Blue Rep1_0.51 Red Rep2_0.38
sp|Q9NVR2|INT10_HUMAN−382−S−S2_0.44
ptm abundance 37C
Doses Blue Rep1_0.52 Red Rep2_0.36
sp|Q9NVR2|INT10_HUMAN−381−S−S2_0.49
ptm abundance 37C
Doses Blue Rep1_0.52 Red Rep2_0.45
sp|Q5VT06|CE350_HUMAN−1256−S−S2_0.53
ptm abundance 37C
Doses Blue Rep1_0.51 Red Rep2_0.55
sp|Q9NVR2|INT10_HUMAN−388−S−S2_0.56
ptm abundance 37C
Doses Blue Rep1_0.58 Red Rep2_0.55
sp|P42566−2|EPS15_HUMAN−482−S−S1_0.74
ptm abundance 37C
Doses Blue Rep1_0.85 Red Rep2_0.62
sp|Q9NUL3−4|STAU2_HUMAN−272−S−S1_0.77
ptm abundance 37C
Doses Blue Rep1_0.76 Red Rep2_0.79
sp|Q9UBC2|EP15R_HUMAN−374−S−S2_0.88
ptm abundance 37C
Doses Blue Rep1_1.15 Red Rep2_0.6
sp|Q9UBC2|EP15R_HUMAN−377−S−S2_0.88
ptm abundance 37C
Doses Blue Rep1_1.15 Red Rep2_0.6

Supplementary Data 2: Phosphosites assigned as hypo-phosphorylated. Doses are represented as ordinal numbers (highest dose 10 µM as the 10th position; x-axis), abundance fold change relative to the untreated condition (y-axis).

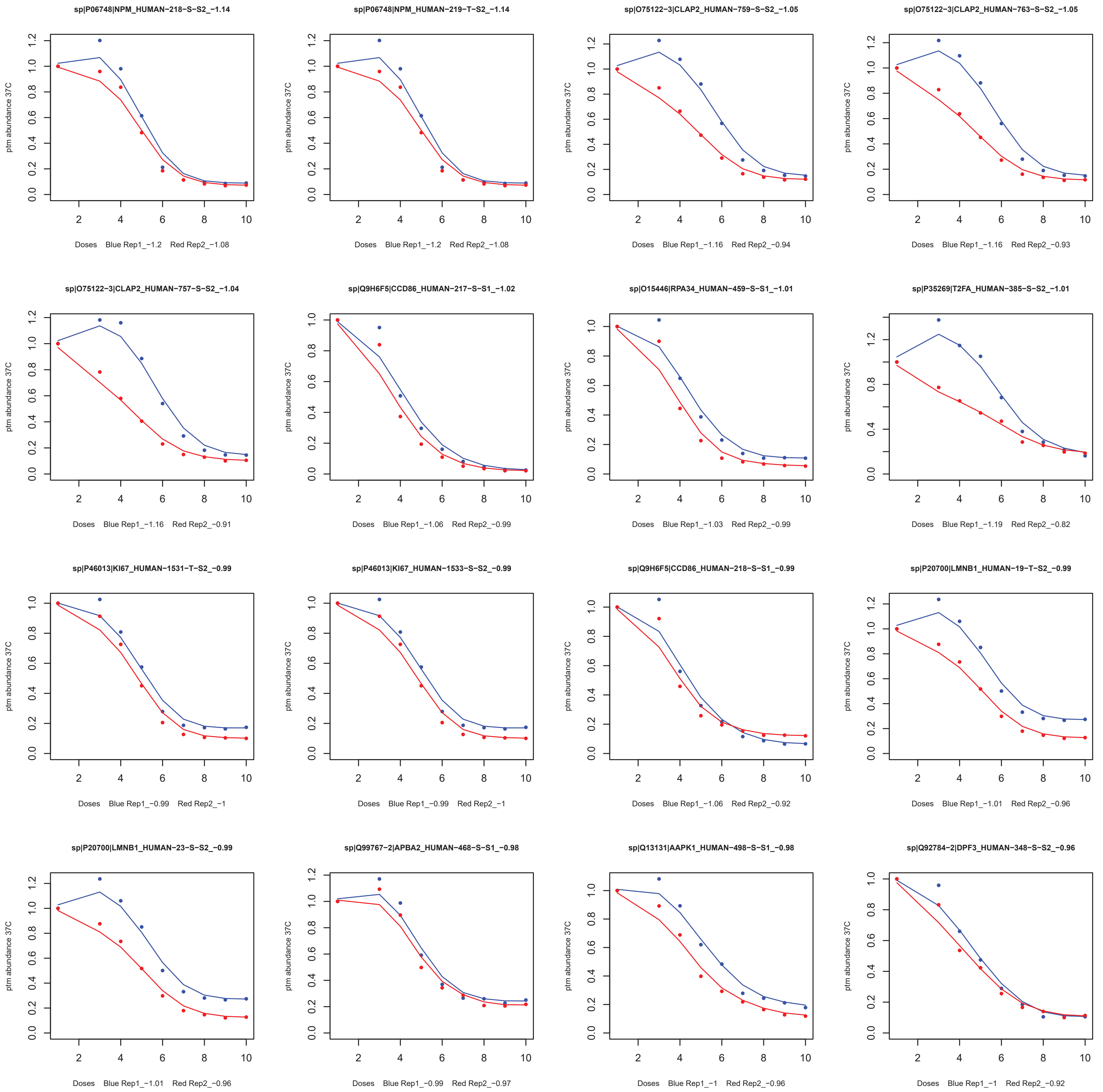

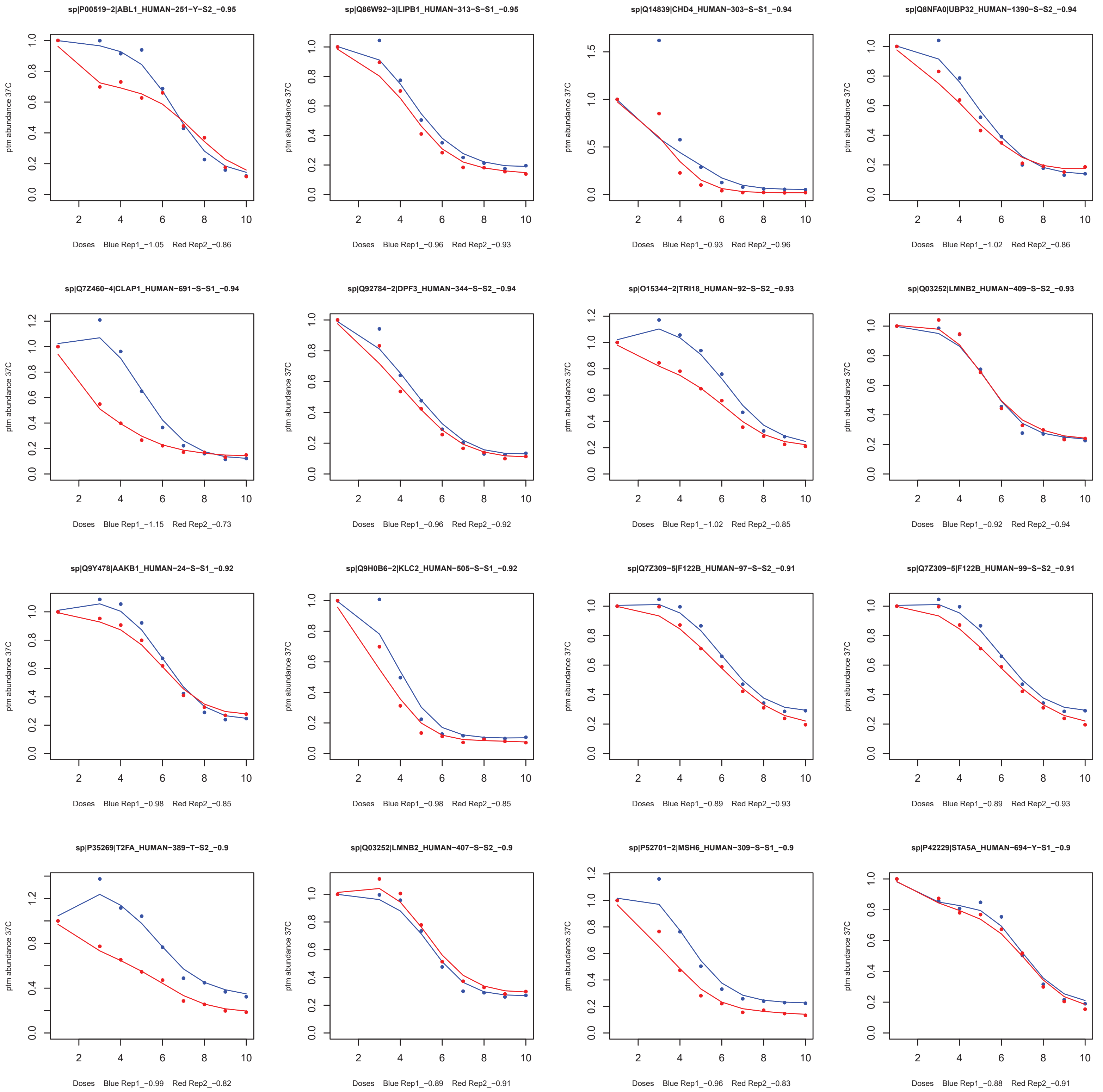
sp|P00519−2|ABL1_HUMAN−251−Y−S2_−0.95
ptm abundance 37C
Doses Blue Rep1_−1.05 Red Rep2_−0.86
sp|Q86W92−3|LIPB1_HUMAN−313−S−S1_−0.95
ptm abundance 37C
Doses Blue Rep1_−0.96 Red Rep2_−0.93
sp|Q14839|CHD4_HUMAN−303−S−S1_−0.94
ptm abundance 37C
Doses Blue Rep1_−0.93 Red Rep2_−0.96
sp|Q8NFA0|UBP32_HUMAN−1390−S−S2_−0.94
ptm abundance 37C
Doses Blue Rep1_−1.02 Red Rep2_−0.86
sp|Q7Z460−4|CLAP1_HUMAN−691−S−S1_−0.94
ptm abundance 37C
Doses Blue Rep1_−1.15 Red Rep2_−0.73
sp|Q92784−2|DPF3_HUMAN−344−S−S2_−0.94
ptm abundance 37C
Doses Blue Rep1_−0.96 Red Rep2_−0.92
sp|O15344−2|TRI18_HUMAN−92−S−S2_−0.93
ptm abundance 37C
Doses Blue Rep1_−1.02 Red Rep2_−0.85
sp|Q03252|LMNB2_HUMAN−409−S−S2_−0.93
ptm abundance 37C
Doses Blue Rep1_−0.92 Red Rep2_−0.94
sp|Q9Y478|AAKB1_HUMAN−24−S−S1_−0.92
ptm abundance 37C
Doses Blue Rep1_−0.98 Red Rep2_−0.85
sp|Q9H0B6−2|KLC2_HUMAN−505−S−S1_−0.92
ptm abundance 37C
Doses Blue Rep1_−0.98 Red Rep2_−0.85
sp|Q7Z309−5|F122B_HUMAN−97−S−S2_−0.91
ptm abundance 37C
Doses Blue Rep1_−0.89 Red Rep2_−0.93
sp|Q7Z309−5|F122B_HUMAN−99−S−S2_−0.91
ptm abundance 37C
Doses Blue Rep1_−0.89 Red Rep2_−0.93
sp|P35269|T2FA_HUMAN−389−T−S2_−0.9
ptm abundance 37C
Doses Blue Rep1_−0.99 Red Rep2_−0.82
sp|Q03252|LMNB2_HUMAN−407−S−S2_−0.9
ptm abundance 37C
Doses Blue Rep1_−0.89 Red Rep2_−0.91
sp|P52701−2|MSH6_HUMAN−309−S−S1_−0.9
ptm abundance 37C
Doses Blue Rep1_−0.96 Red Rep2_−0.83
sp|P42229|STA5A_HUMAN−694−Y−S1_−0.9
ptm abundance 37C
Doses Blue Rep1_−0.88 Red Rep2_−0.91

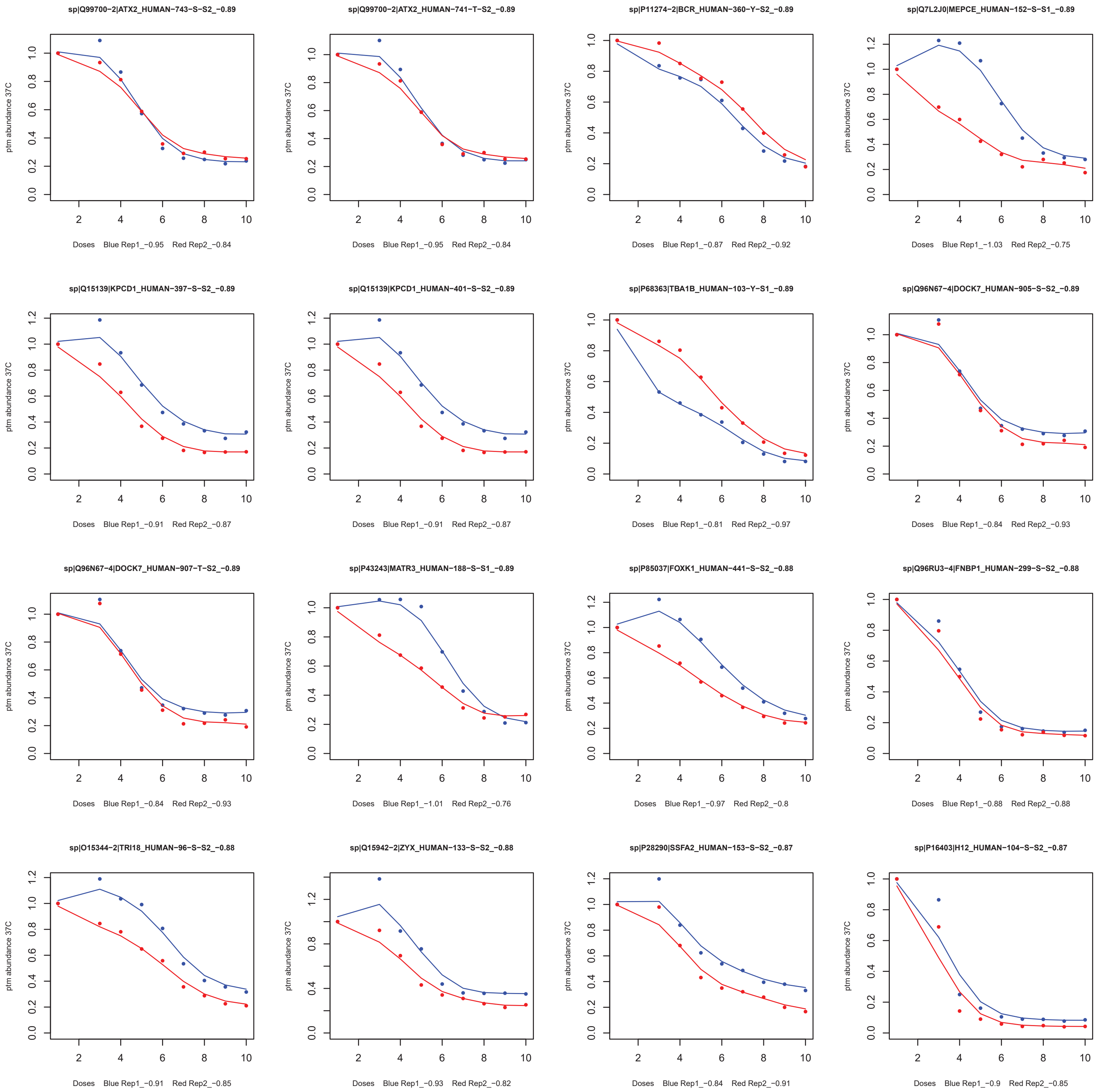

sp|Q99700−2|ATX2_HUMAN−743−S−S2_−0.89
ptm abundance 37C
Doses Blue Rep1_−0.95 Red Rep2_−0.84
sp|Q99700−2|ATX2_HUMAN−741−T−S2_−0.89
ptm abundance 37C
Doses Blue Rep1_−0.95 Red Rep2_−0.84
sp|P11274−2|BCR_HUMAN−360−Y−S2_−0.89
ptm abundance 37C
Doses Blue Rep1_−0.87 Red Rep2_−0.92
sp|Q7L2J0|MEPCE_HUMAN−152−S−S1_−0.89
ptm abundance 37C
Doses Blue Rep1_−1.03 Red Rep2_−0.75
sp|Q15139|KPCD1_HUMAN−397−S−S2_−0.89
ptm abundance 37C
Doses Blue Rep1_−0.91 Red Rep2_−0.87
sp|Q15139|KPCD1_HUMAN−401−S−S2_−0.89
ptm abundance 37C
Doses Blue Rep1_−0.91 Red Rep2_−0.87
sp|P68363|TBA1B_HUMAN−103−Y−S1_−0.89
ptm abundance 37C
Doses Blue Rep1_−0.81 Red Rep2_−0.97
sp|Q96N67−4|DOCK7_HUMAN−905−S−S2_−0.89
ptm abundance 37C
Doses Blue Rep1_−0.84 Red Rep2_−0.93
sp|Q96N67−4|DOCK7_HUMAN−907−T−S2_−0.89
ptm abundance 37C
Doses Blue Rep1_−0.84 Red Rep2_−0.93
sp|P43243|MATR3_HUMAN−188−S−S1_−0.89
ptm abundance 37C
Doses Blue Rep1_−1.01 Red Rep2_−0.76
sp|P85037|FOXK1_HUMAN−441−S−S2_−0.88
ptm abundance 37C
Doses Blue Rep1_−0.97 Red Rep2_−0.8
sp|Q96RU3−4|FNBP1_HUMAN−299−S−S2_−0.88
ptm abundance 37C
Doses Blue Rep1_−0.88 Red Rep2_−0.88
sp|O15344−2|TRI18_HUMAN−96−S−S2_−0.88
ptm abundance 37C
Doses Blue Rep1_−0.91 Red Rep2_−0.85
sp|Q15942−2|ZYX_HUMAN−133−S−S2_−0.88
ptm abundance 37C
Doses Blue Rep1_−0.93 Red Rep2_−0.82
sp|P28290|SSFA2_HUMAN−153−S−S2_−0.87
ptm abundance 37C
Doses Blue Rep1_−0.84 Red Rep2_−0.91
sp|P16403|H12_HUMAN−104−S−S2_−0.87
ptm abundance 37C
Doses Blue Rep1_−0.9 Red Rep2_−0.85

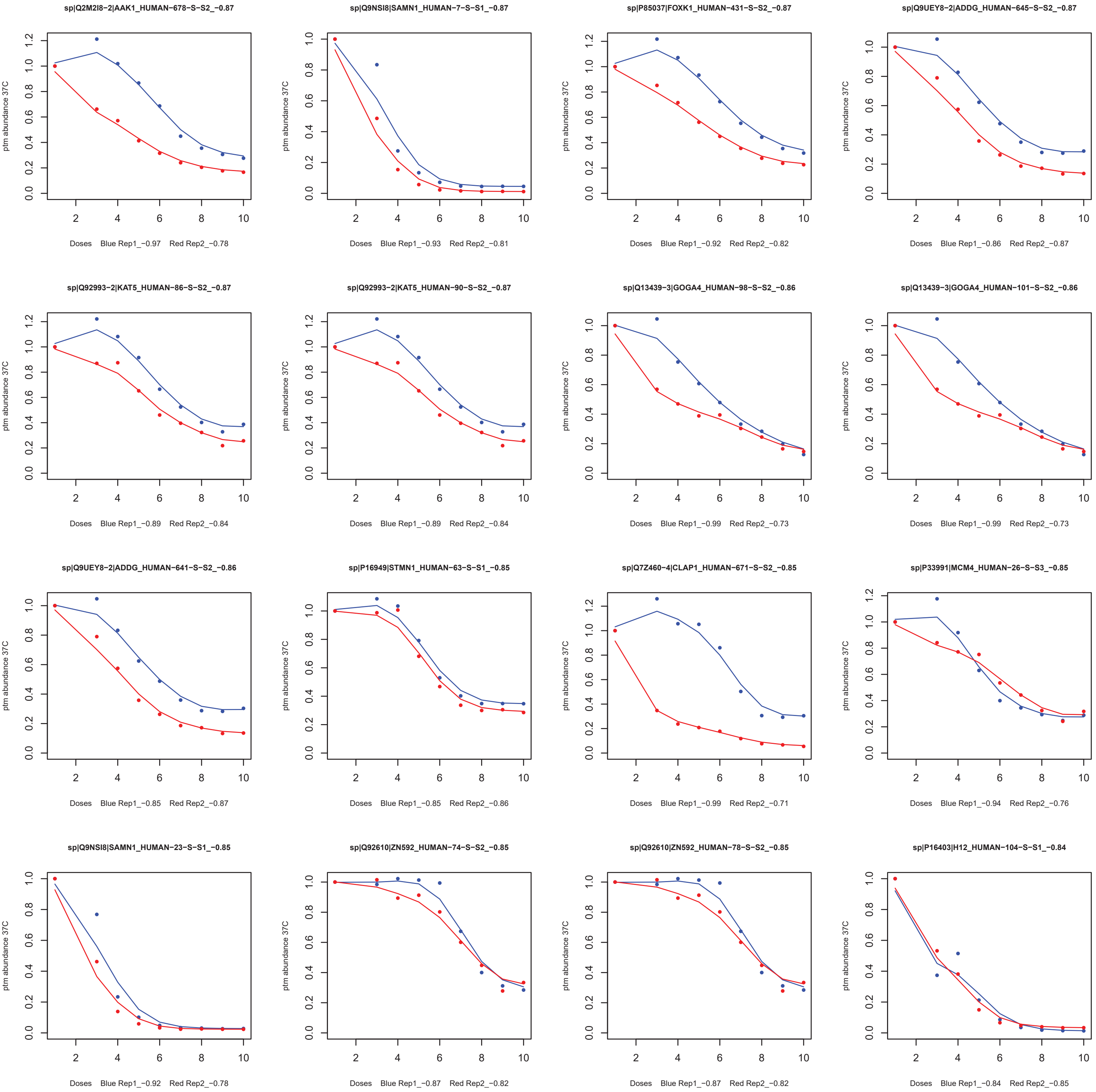
sp|Q2M2I8−2|AAK1_HUMAN−678−S−S2_−0.87
ptm abundance 37C
Doses Blue Rep1_−0.97 Red Rep2_−0.78
sp|Q9NSI8|SAMN1_HUMAN−7−S−S1_−0.87
ptm abundance 37C
Doses Blue Rep1_−0.93 Red Rep2_−0.81
sp|P85037|FOXK1_HUMAN−431−S−S2_−0.87
ptm abundance 37C
Doses Blue Rep1_−0.92 Red Rep2_−0.82
sp|Q9UEY8−2|ADDG_HUMAN−645−S−S2_−0.87
ptm abundance 37C
Doses Blue Rep1_−0.86 Red Rep2_−0.87
sp|Q92993−2|KAT5_HUMAN−86−S−S2_−0.87
ptm abundance 37C
Doses Blue Rep1_−0.89 Red Rep2_−0.84
sp|Q92993−2|KAT5_HUMAN−90−S−S2_−0.87
ptm abundance 37C
Doses Blue Rep1_−0.89 Red Rep2_−0.84
sp|Q13439−3|GOGA4_HUMAN−98−S−S2_−0.86
ptm abundance 37C
Doses Blue Rep1_−0.99 Red Rep2_−0.73
sp|Q13439−3|GOGA4_HUMAN−101−S−S2_−0.86
ptm abundance 37C
Doses Blue Rep1_−0.99 Red Rep2_−0.73
sp|Q9UEY8−2|ADDG_HUMAN−641−S−S2_−0.86
ptm abundance 37C
Doses Blue Rep1_−0.85 Red Rep2_−0.87
sp|P16949|STMN1_HUMAN−63−S−S1_−0.85
ptm abundance 37C
Doses Blue Rep1_−0.85 Red Rep2_−0.86
sp|Q7Z460−4|CLAP1_HUMAN−671−S−S2_−0.85
ptm abundance 37C
Doses Blue Rep1_−0.99 Red Rep2_−0.71
sp|P33991|MCM4_HUMAN−26−S−S3_−0.85
ptm abundance 37C
Doses Blue Rep1_−0.94 Red Rep2_−0.76
sp|Q9NSI8|SAMN1_HUMAN−23−S−S1_−0.85
ptm abundance 37C
Doses Blue Rep1_−0.92 Red Rep2_−0.78
sp|Q92610|ZN592_HUMAN−74−S−S2_−0.85
ptm abundance 37C
Doses Blue Rep1_−0.87 Red Rep2_−0.82
sp|Q92610|ZN592_HUMAN−78−S−S2_−0.85
ptm abundance 37C
Doses Blue Rep1_−0.87 Red Rep2_−0.82
sp|P16403|H12_HUMAN−104−S−S1_−0.84
ptm abundance 37C
Doses Blue Rep1_−0.84 Red Rep2_−0.85

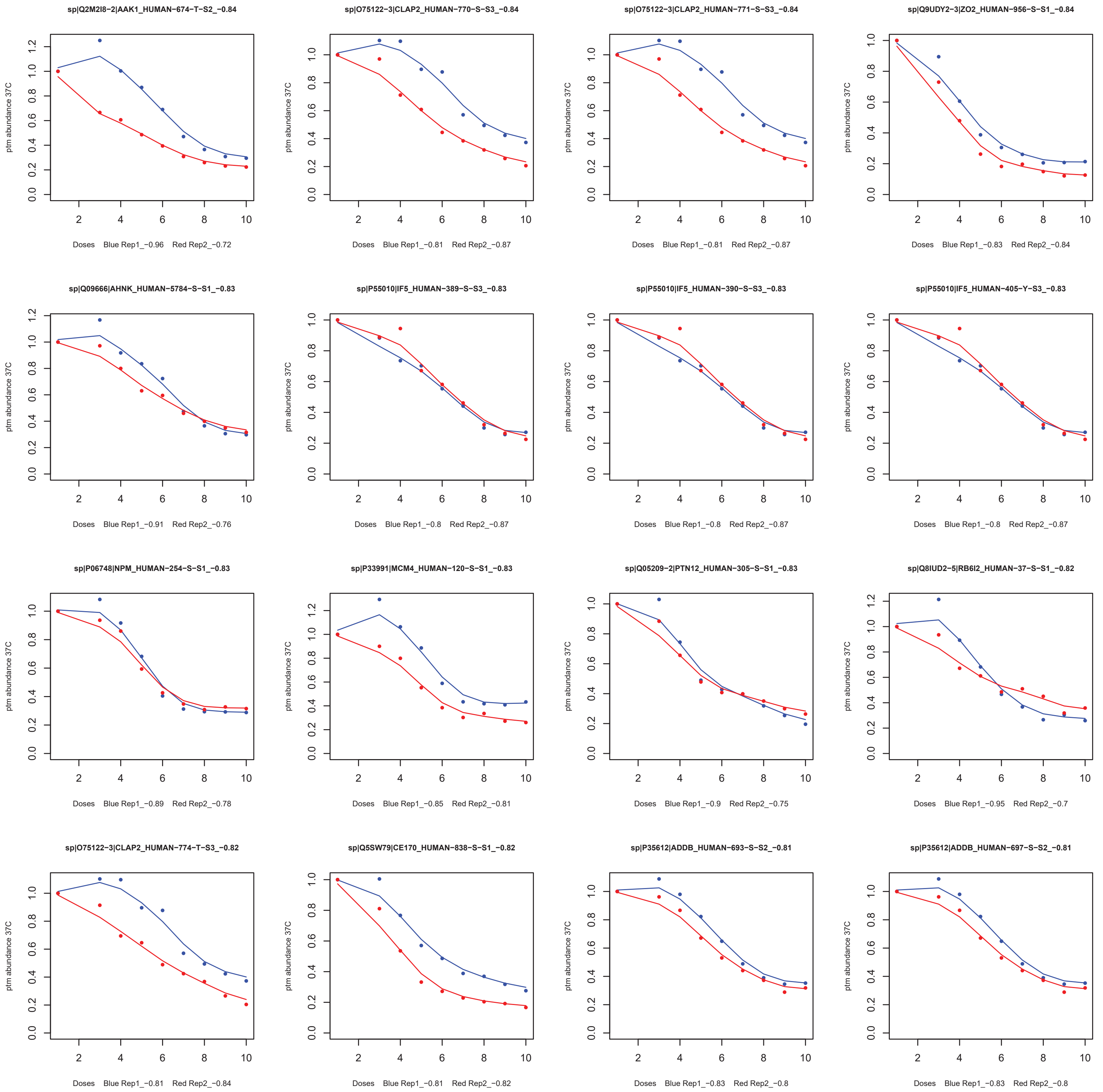
sp|Q2M2I8−2|AAK1_HUMAN−674−T−S2_−0.84
ptm abundance 37C
Doses Blue Rep1_−0.96 Red Rep2_−0.72
sp|O75122−3|CLAP2_HUMAN−770−S−S3_−0.84
ptm abundance 37C
Doses Blue Rep1_−0.81 Red Rep2_−0.87
sp|O75122−3|CLAP2_HUMAN−771−S−S3_−0.84
ptm abundance 37C
Doses Blue Rep1_−0.81 Red Rep2_−0.87
sp|Q9UDY2−3|ZO2_HUMAN−956−S−S1_−0.84
ptm abundance 37C
Doses Blue Rep1_−0.83 Red Rep2_−0.84
sp|Q09666|AHNK_HUMAN−5784−S−S1_−0.83
ptm abundance 37C
Doses Blue Rep1_−0.91 Red Rep2_−0.76
sp|P55010|IF5_HUMAN−389−S−S3_−0.83
ptm abundance 37C
Doses Blue Rep1_−0.8 Red Rep2_−0.87
sp|P55010|IF5_HUMAN−390−S−S3_−0.83
ptm abundance 37C
Doses Blue Rep1_−0.8 Red Rep2_−0.87
sp|P55010|IF5_HUMAN−405−Y−S3_−0.83
ptm abundance 37C
Doses Blue Rep1_−0.8 Red Rep2_−0.87
sp|P06748|NPM_HUMAN−254−S−S1_−0.83
ptm abundance 37C
Doses Blue Rep1_−0.89 Red Rep2_−0.78
sp|P33991|MCM4_HUMAN−120−S−S1_−0.83
ptm abundance 37C
Doses Blue Rep1_−0.85 Red Rep2_−0.81
sp|Q05209−2|PTN12_HUMAN−305−S−S1_−0.83
ptm abundance 37C
Doses Blue Rep1_−0.9 Red Rep2_−0.75
sp|Q8IUD2−5|RB6I2_HUMAN−37−S−S1_−0.82
ptm abundance 37C
Doses Blue Rep1_−0.95 Red Rep2_−0.7
sp|O75122−3|CLAP2_HUMAN−774−T−S3_−0.82
ptm abundance 37C
Doses Blue Rep1_−0.81 Red Rep2_−0.84
sp|Q5SW79|CE170_HUMAN−838−S−S1_−0.82
ptm abundance 37C
Doses Blue Rep1_−0.81 Red Rep2_−0.82
sp|P35612|ADDB_HUMAN−693−S−S2_−0.81
ptm abundance 37C
Doses Blue Rep1_−0.83 Red Rep2_−0.8
sp|P35612|ADDB_HUMAN−697−S−S2_−0.81
ptm abundance 37C
Doses Blue Rep1_−0.83 Red Rep2_−0.8

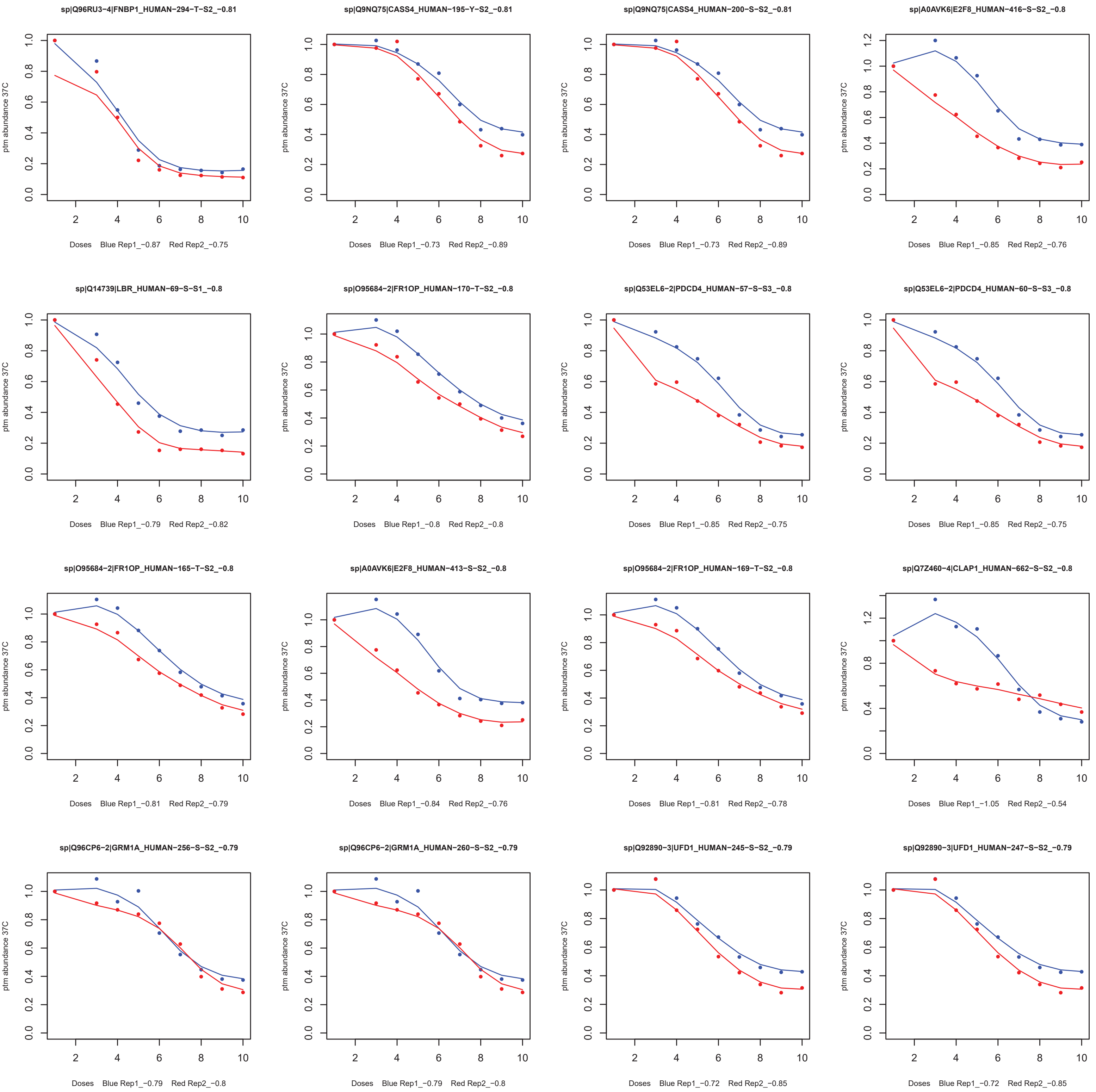
sp|Q96RU3−4|FNBP1_HUMAN−294−T−S2_−0.81
Doses Blue Rep1_−0.87 Red Rep2_−0.75
sp|Q9NQ75|CASS4_HUMAN−195−Y−S2_−0.81
Doses Blue Rep1_−0.73 Red Rep2_−0.89
sp|Q9NQ75|CASS4_HUMAN−200−S−S2_−0.81
Doses Blue Rep1_−0.73 Red Rep2_−0.89
sp|A0AVK6|E2F8_HUMAN−416−S−S2_−0.8
Doses Blue Rep1_−0.85 Red Rep2_−0.76
sp|Q14739|LBR_HUMAN−69−S−S1_−0.8
Doses Blue Rep1_−0.79 Red Rep2_−0.82
sp|O95684−2|FR1OP_HUMAN−170−T−S2_−0.8
Doses Blue Rep1_−0.8 Red Rep2_−0.8
sp|Q53EL6−2|PDCD4_HUMAN−57−S−S3_−0.8
Doses Blue Rep1_−0.85 Red Rep2_−0.75
sp|Q53EL6−2|PDCD4_HUMAN−60−S−S3_−0.8
Doses Blue Rep1_−0.85 Red Rep2_−0.75
sp|O95684−2|FR1OP_HUMAN−165−T−S2_−0.8
Doses Blue Rep1_−0.81 Red Rep2_−0.79
sp|A0AVK6|E2F8_HUMAN−413−S−S2_−0.8
Doses Blue Rep1_−0.84 Red Rep2_−0.76
sp|O95684−2|FR1OP_HUMAN−169−T−S2_−0.8
Doses Blue Rep1_−0.81 Red Rep2_−0.78
sp|Q7Z460−4|CLAP1_HUMAN−662−S−S2_−0.8
Doses Blue Rep1_−1.05 Red Rep2_−0.54
sp|Q96CP6−2|GRM1A_HUMAN−256−S−S2_−0.79
Doses Blue Rep1_−0.79 Red Rep2_−0.8
sp|Q96CP6−2|GRM1A_HUMAN−260−S−S2_−0.79
Doses Blue Rep1_−0.79 Red Rep2_−0.8
sp|Q92890−3|UFD1_HUMAN−245−S−S2_−0.79
Doses Blue Rep1_−0.72 Red Rep2_−0.85
sp|Q92890−3|UFD1_HUMAN−247−S−S2_−0.79
Doses Blue Rep1_−0.72 Red Rep2_−0.85
ptm abundance 37C

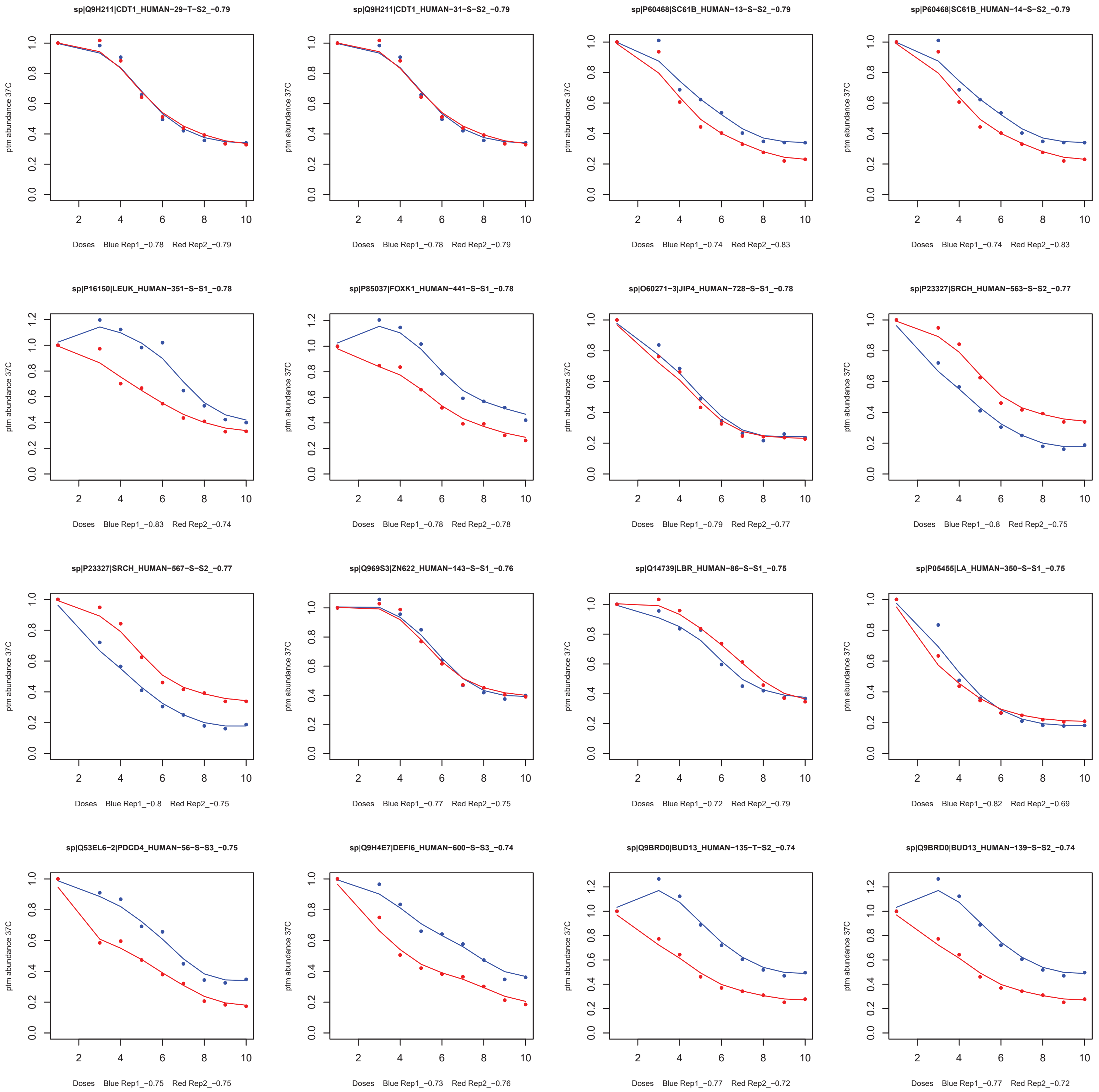

sp|Q9H211|CDT1_HUMAN−29−T−S2_−0.79
ptm abundance 37C
Doses Blue Rep1_−0.78 Red Rep2_−0.79
sp|Q9H211|CDT1_HUMAN−31−S−S2_−0.79
ptm abundance 37C
Doses Blue Rep1_−0.78 Red Rep2_−0.79
sp|P60468|SC61B_HUMAN−13−S−S2_−0.79
ptm abundance 37C
Doses Blue Rep1_−0.74 Red Rep2_−0.83
sp|P60468|SC61B_HUMAN−14−S−S2_−0.79
ptm abundance 37C
Doses Blue Rep1_−0.74 Red Rep2_−0.83
sp|P16150|LEUK_HUMAN−351−S−S1_−0.78
ptm abundance 37C
Doses Blue Rep1_−0.83 Red Rep2_−0.74
sp|P85037|FOXK1_HUMAN−441−S−S1_−0.78
ptm abundance 37C
Doses Blue Rep1_−0.78 Red Rep2_−0.78
sp|O60271−3|JIP4_HUMAN−728−S−S1_−0.78
ptm abundance 37C
Doses Blue Rep1_−0.79 Red Rep2_−0.77
sp|P23327|SRCH_HUMAN−563−S−S2_−0.77
ptm abundance 37C
Doses Blue Rep1_−0.8 Red Rep2_−0.75
sp|P23327|SRCH_HUMAN−567−S−S2_−0.77
ptm abundance 37C
Doses Blue Rep1_−0.8 Red Rep2_−0.75
sp|Q969S3|ZN622_HUMAN−143−S−S1_−0.76
ptm abundance 37C
Doses Blue Rep1_−0.77 Red Rep2_−0.75
sp|Q14739|LBR_HUMAN−86−S−S1_−0.75
ptm abundance 37C
Doses Blue Rep1_−0.72 Red Rep2_−0.79
sp|P05455|LA_HUMAN−350−S−S1_−0.75
ptm abundance 37C
Doses Blue Rep1_−0.82 Red Rep2_−0.69
sp|Q53EL6−2|PDCD4_HUMAN−56−S−S3_−0.75
ptm abundance 37C
Doses Blue Rep1_−0.75 Red Rep2_−0.75
sp|Q9H4E7|DEFI6_HUMAN−600−S−S3_−0.74
ptm abundance 37C
Doses Blue Rep1_−0.73 Red Rep2_−0.76
sp|Q9BRD0|BUD13_HUMAN−135−T−S2_−0.74
ptm abundance 37C
Doses Blue Rep1_−0.77 Red Rep2_−0.72
sp|Q9BRD0|BUD13_HUMAN−139−S−S2_−0.74
ptm abundance 37C
Doses Blue Rep1_−0.77 Red Rep2_−0.72

sp|Q14004−2|CDK13_HUMAN−437−S−S2_−0.74

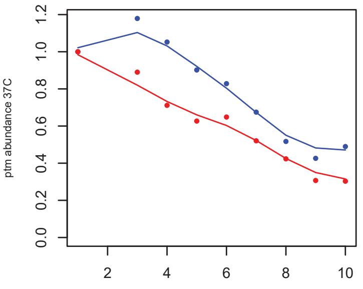

Doses Blue Rep1_−0.74 Red Rep2_−0.73

sp|Q14004−2|CDK13_HUMAN−439−S−S2_−0.74

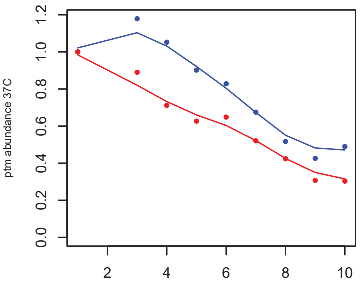

Doses Blue Rep1_−0.74 Red Rep2_−0.73

sp|P22681|CBL_HUMAN−674−Y−S2_−0.73

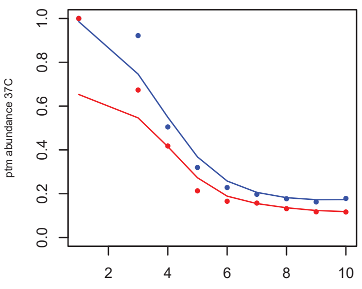

Doses Blue Rep1_−0.87 Red Rep2_−0.6

sp|Q8WWM7−6|ATX2L_HUMAN−339−S−S2_−0.73

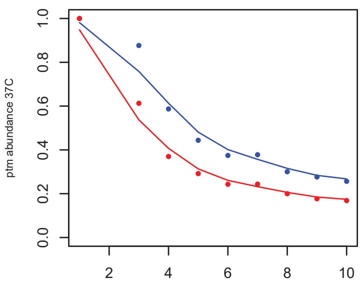

Doses Blue Rep1_−0.75 Red Rep2_−0.71

sp|Q5TEJ8−6|THMS2_HUMAN−371−S−S2_−0.73

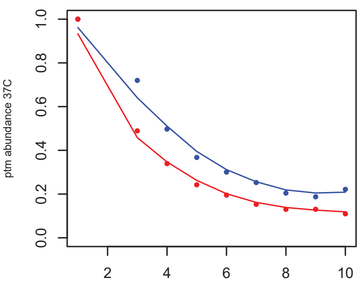

Doses Blue Rep1_−0.74 Red Rep2_−0.72

sp|Q5TEJ8−6|THMS2_HUMAN−372−S−S2_−0.73

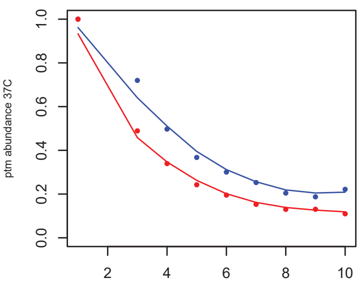

Doses Blue Rep1_−0.74 Red Rep2_−0.72

sp|Q12982|BNIP2_HUMAN−119−T−S1_−0.73

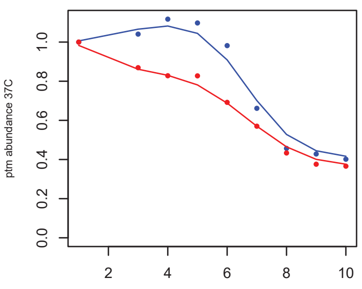

Doses Blue Rep1_−0.77 Red Rep2_−0.68

sp|Q8IV63−2|VRK3_HUMAN−58−T−S2_−0.73

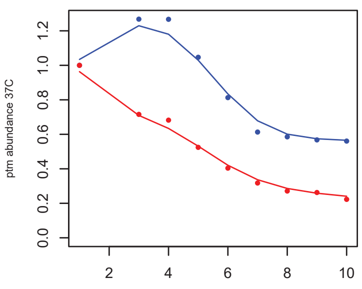

Doses Blue Rep1_−0.71 Red Rep2_−0.74

sp|Q9UIG0−2|BAZ1B_HUMAN−1338−S−S1_−0.72

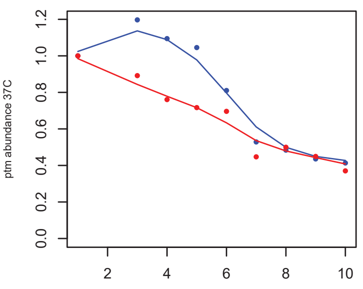

Doses Blue Rep1_−0.82 Red Rep2_−0.63

sp|Q9UKY7|CDV3_HUMAN−244−Y−S1_−0.72

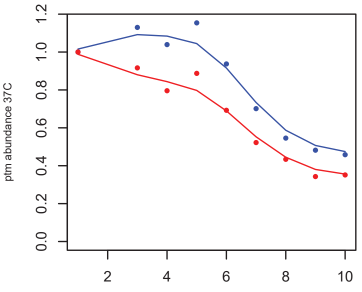

Doses Blue Rep1_−0.72 Red Rep2_−0.72

sp|Q8WWM7−6|ATX2L_HUMAN−335−S−S2_−0.72

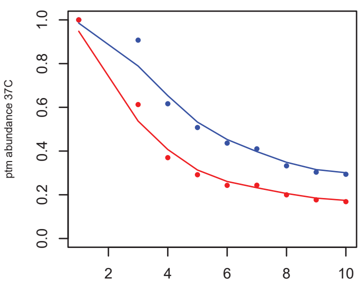

Doses Blue Rep1_−0.73 Red Rep2_−0.71

sp|Q5UIP0−2|RIF1_HUMAN−1688−S−S1_−0.72

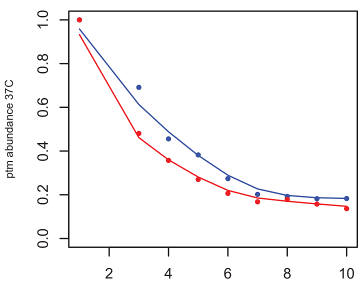

Doses Blue Rep1_−0.75 Red Rep2_−0.69

sp|P22059|OSBP1_HUMAN−385−S−S3_−0.72

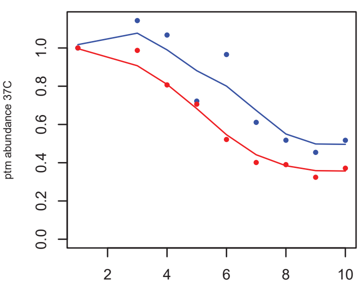

Doses Blue Rep1_−0.69 Red Rep2_−0.74

sp|P22059|OSBP1_HUMAN−386−S−S3_−0.72

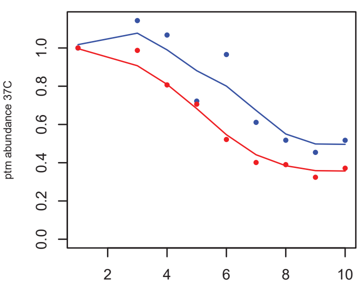

Doses Blue Rep1_−0.69 Red Rep2_−0.74

sp|Q9NV70−2|EXOC1_HUMAN−456−T−S1_−0.72

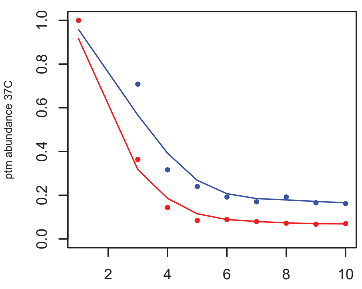

Doses Blue Rep1_−0.75 Red Rep2_−0.68

sp|Q9H4E7|DEFI6_HUMAN−595−T−S3_−0.72

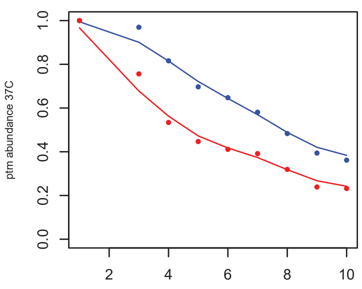

Doses Blue Rep1_−0.71 Red Rep2_−0.73

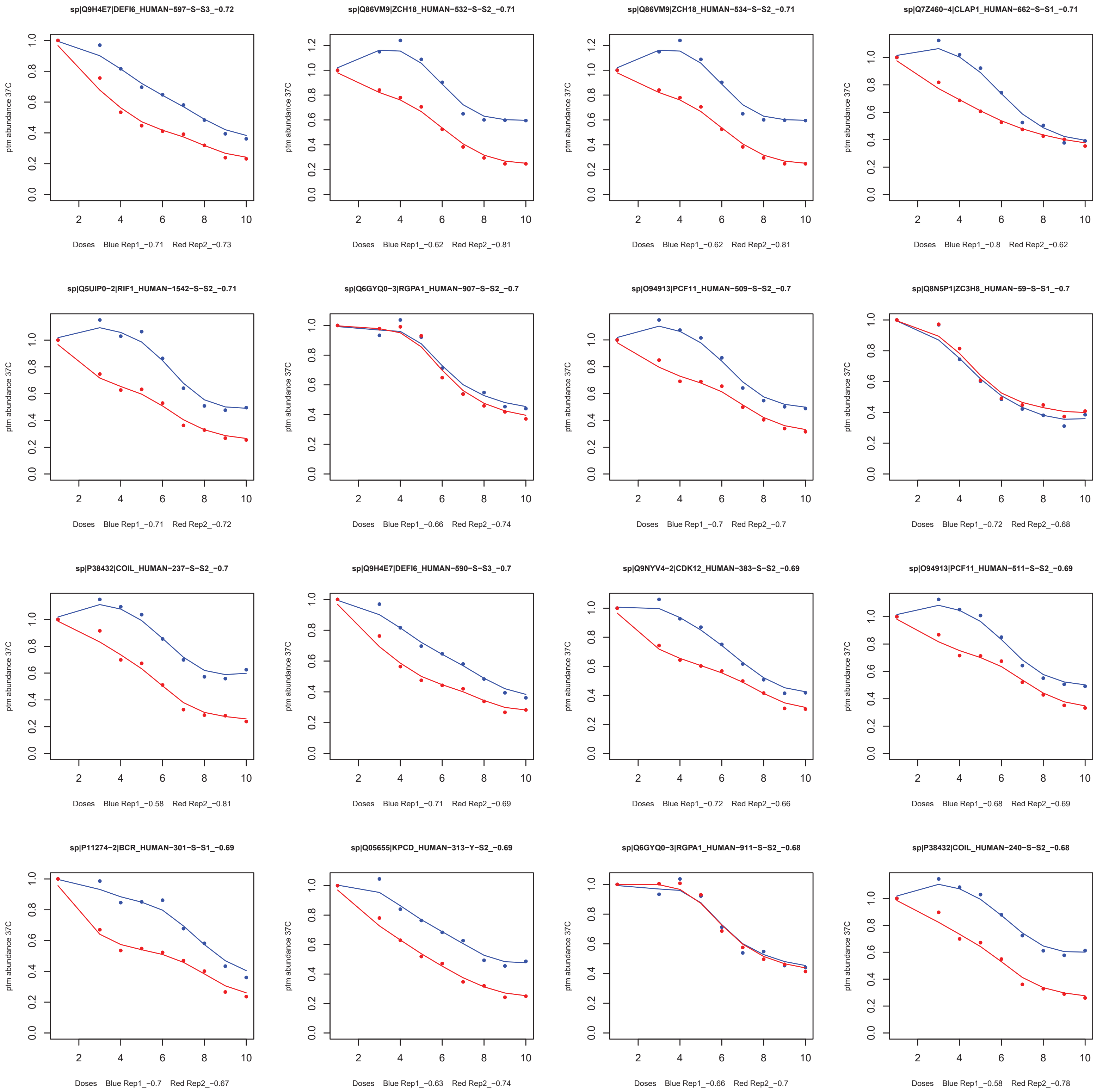
sp|Q9H4E7|DEFI6_HUMAN−597−S−S3_−0.72
Doses Blue Rep1_−0.71 Red Rep2_−0.73
sp|Q86VM9|ZCH18_HUMAN−532−S−S2_−0.71
Doses Blue Rep1_−0.62 Red Rep2_−0.81
sp|Q86VM9|ZCH18_HUMAN−534−S−S2_−0.71
Doses Blue Rep1_−0.62 Red Rep2_−0.81
sp|Q7Z460−4|CLAP1_HUMAN−662−S−S1_−0.71
Doses Blue Rep1_−0.8 Red Rep2_−0.62
sp|Q5UIP0−2|RIF1_HUMAN−1542−S−S2_−0.71
Doses Blue Rep1_−0.71 Red Rep2_−0.72
sp|Q6GYQ0−3|RGPA1_HUMAN−907−S−S2_−0.7
Doses Blue Rep1_−0.66 Red Rep2_−0.74
sp|O94913|PCF11_HUMAN−509−S−S2_−0.7
Doses Blue Rep1_−0.7 Red Rep2_−0.7
sp|Q8N5P1|ZC3H8_HUMAN−59−S−S1_−0.7
Doses Blue Rep1_−0.72 Red Rep2_−0.68
sp|P38432|COIL_HUMAN−237−S−S2_−0.7
Doses Blue Rep1_−0.58 Red Rep2_−0.81
sp|Q9H4E7|DEFI6_HUMAN−590−S−S3_−0.7
Doses Blue Rep1_−0.71 Red Rep2_−0.69
sp|Q9NYV4−2|CDK12_HUMAN−383−S−S2_−0.69
Doses Blue Rep1_−0.72 Red Rep2_−0.66
sp|O94913|PCF11_HUMAN−511−S−S2_−0.69
Doses Blue Rep1_−0.68 Red Rep2_−0.69
sp|P11274−2|BCR_HUMAN−301−S−S1_−0.69
Doses Blue Rep1_−0.7 Red Rep2_−0.67
sp|Q05655|KPCD_HUMAN−313−Y−S2_−0.69
Doses Blue Rep1_−0.63 Red Rep2_−0.74
sp|Q6GYQ0−3|RGPA1_HUMAN−911−S−S2_−0.68
Doses Blue Rep1_−0.66 Red Rep2_−0.7
sp|P38432|COIL_HUMAN−240−S−S2_−0.68
Doses Blue Rep1_−0.58 Red Rep2_−0.78
ptm abundance 37C

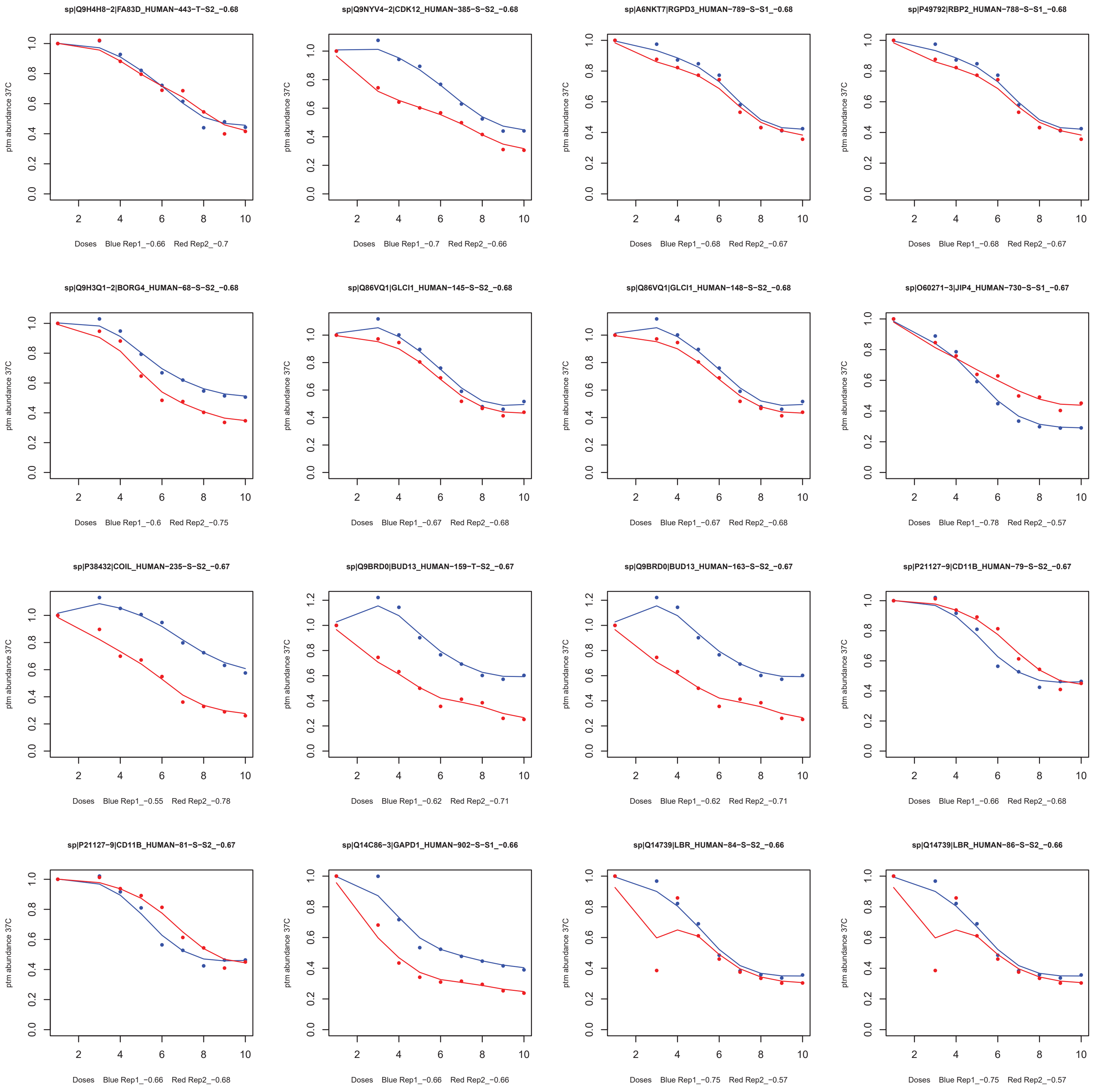
sp|Q9H4H8−2|FA83D_HUMAN−443−T−S2_−0.68
Doses Blue Rep1_−0.66 Red Rep2_−0.7
sp|Q9NYV4−2|CDK12_HUMAN−385−S−S2_−0.68
Doses Blue Rep1_−0.7 Red Rep2_−0.66
sp|A6NKT7|RGPD3_HUMAN−789−S−S1_−0.68
Doses Blue Rep1_−0.68 Red Rep2_−0.67
sp|P49792|RBP2_HUMAN−788−S−S1_−0.68
Doses Blue Rep1_−0.68 Red Rep2_−0.67
sp|Q9H3Q1−2|BORG4_HUMAN−68−S−S2_−0.68
Doses Blue Rep1_−0.6 Red Rep2_−0.75
sp|Q86VQ1|GLCI1_HUMAN−145−S−S2_−0.68
Doses Blue Rep1_−0.67 Red Rep2_−0.68
sp|Q86VQ1|GLCI1_HUMAN−148−S−S2_−0.68
Doses Blue Rep1_−0.67 Red Rep2_−0.68
sp|O60271−3|JIP4_HUMAN−730−S−S1_−0.67
Doses Blue Rep1_−0.78 Red Rep2_−0.57
sp|P38432|COIL_HUMAN−235−S−S2_−0.67
Doses Blue Rep1_−0.55 Red Rep2_−0.78
sp|Q9BRD0|BUD13_HUMAN−159−T−S2_−0.67
Doses Blue Rep1_−0.62 Red Rep2_−0.71
sp|Q9BRD0|BUD13_HUMAN−163−S−S2_−0.67
Doses Blue Rep1_−0.62 Red Rep2_−0.71
sp|P21127−9|CD11B_HUMAN−79−S−S2_−0.67
Doses Blue Rep1_−0.66 Red Rep2_−0.68
sp|P21127−9|CD11B_HUMAN−81−S−S2_−0.67
Doses Blue Rep1_−0.66 Red Rep2_−0.68
sp|Q14C86−3|GAPD1_HUMAN−902−S−S1_−0.66
Doses Blue Rep1_−0.66 Red Rep2_−0.66
sp|Q14739|LBR_HUMAN−84−S−S2_−0.66
Doses Blue Rep1_−0.75 Red Rep2_−0.57
sp|Q14739|LBR_HUMAN−86−S−S2_−0.66
Doses Blue Rep1_−0.75 Red Rep2_−0.57
ptm abundance 37C

sp|Q9Y3T9|NOC2L_HUMAN−60−S−S2_−0.66

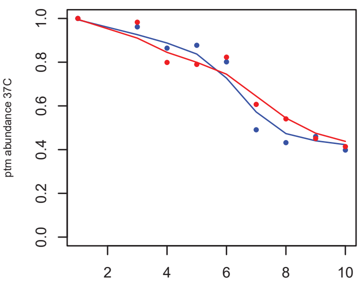

Doses Blue Rep1_−0.67 Red Rep2_−0.64

sp|P22059|OSBP1_HUMAN−240−S−S1_−0.65

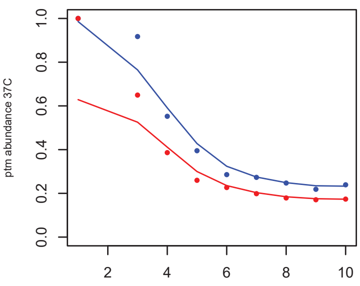

Doses Blue Rep1_−0.8 Red Rep2_−0.5

sp|Q5T4S7−3|UBR4_HUMAN−1762−S−S2_−0.65

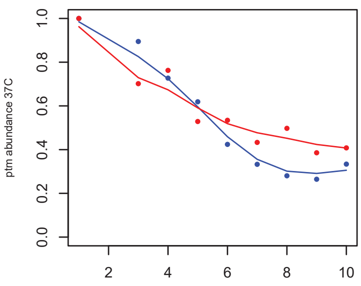

Doses Blue Rep1_−0.75 Red Rep2_−0.55

sp|Q9H4H8−2|FA83D_HUMAN−446−S−S2_−0.65

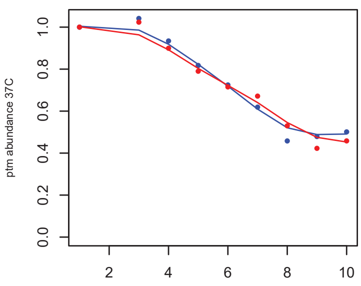

Doses Blue Rep1_−0.63 Red Rep2_−0.66

sp|Q9Y3T9|NOC2L_HUMAN−49−S−S2_−0.64

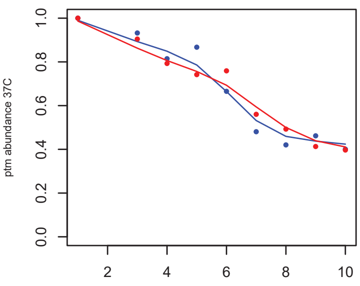

Doses Blue Rep1_−0.65 Red Rep2_−0.64

sp|Q9Y3T9|NOC2L_HUMAN−56−S−S2_−0.64

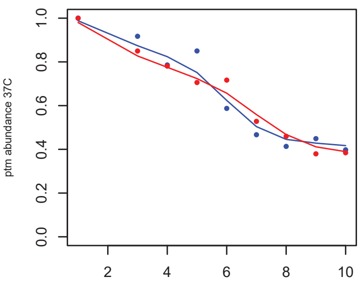

Doses Blue Rep1_−0.64 Red Rep2_−0.64

sp|Q7Z460−4|CLAP1_HUMAN−663−S−S2_−0.63

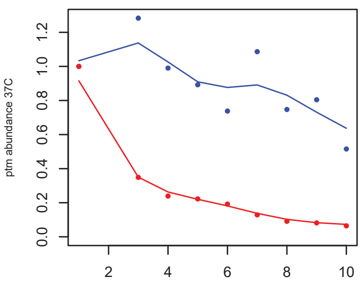

Doses Blue Rep1_−0.56 Red Rep2_−0.7

sp|Q9NYV4−2|CDK12_HUMAN−325−S−S2_−0.63

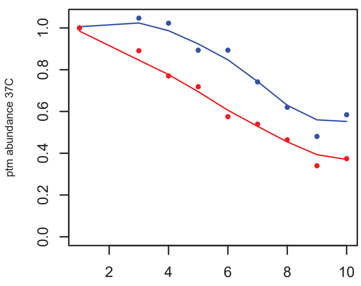

Doses Blue Rep1_−0.58 Red Rep2_−0.68

sp|Q9NYV4−2|CDK12_HUMAN−291−S−S2_−0.63

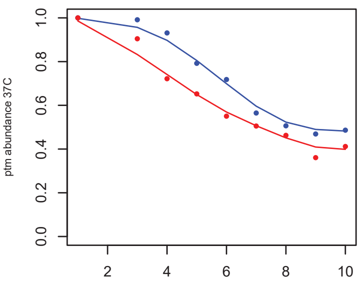

Doses Blue Rep1_−0.62 Red Rep2_−0.64

sp|Q9UPU5|UBP24_HUMAN−1143−S−S2_−0.63

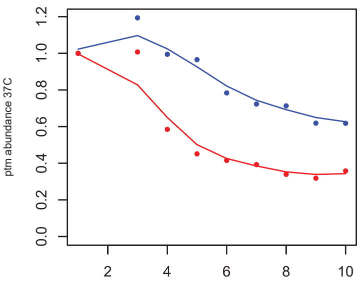

Doses Blue Rep1_−0.54 Red Rep2_−0.71

sp|Q9NYV4−2|CDK12_HUMAN−293−S−S2_−0.63

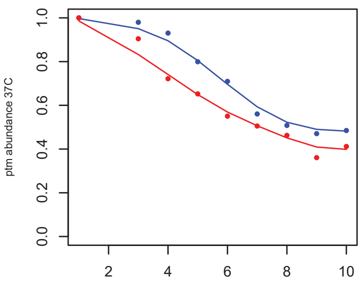

Doses Blue Rep1_−0.61 Red Rep2_−0.64

sp|Q9NYV4−2|CDK12_HUMAN−323−S−S2_−0.63

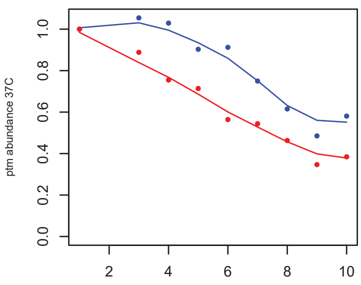

Doses Blue Rep1_−0.58 Red Rep2_−0.67

sp|P23528|COF1_HUMAN−3−S−S1_−0.62

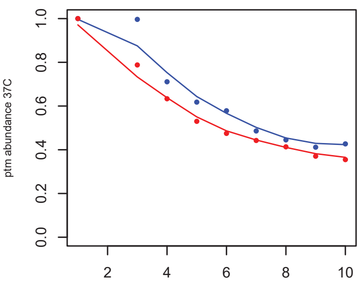

Doses Blue Rep1_−0.64 Red Rep2_−0.61

sp|P11274−2|BCR_HUMAN−354−S−S2_−0.61

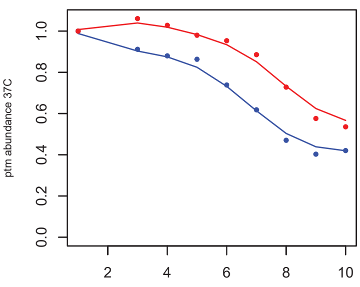

Doses Blue Rep1_−0.66 Red Rep2_−0.57

sp|Q9H3Q1−2|BORG4_HUMAN−72−S−S2_−0.61

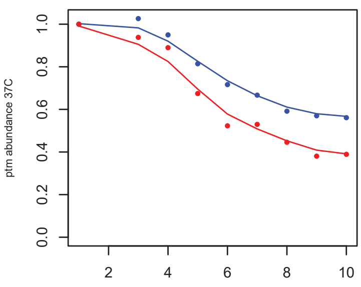

Doses Blue Rep1_−0.53 Red Rep2_−0.7

sp|Q15276|RABE1_HUMAN−413−T−S2_−0.61

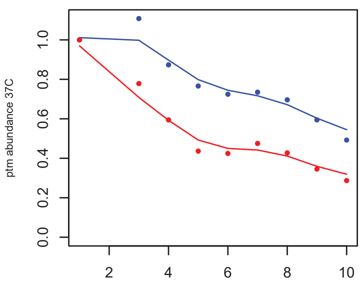

Doses Blue Rep1_−0.57 Red Rep2_−0.65

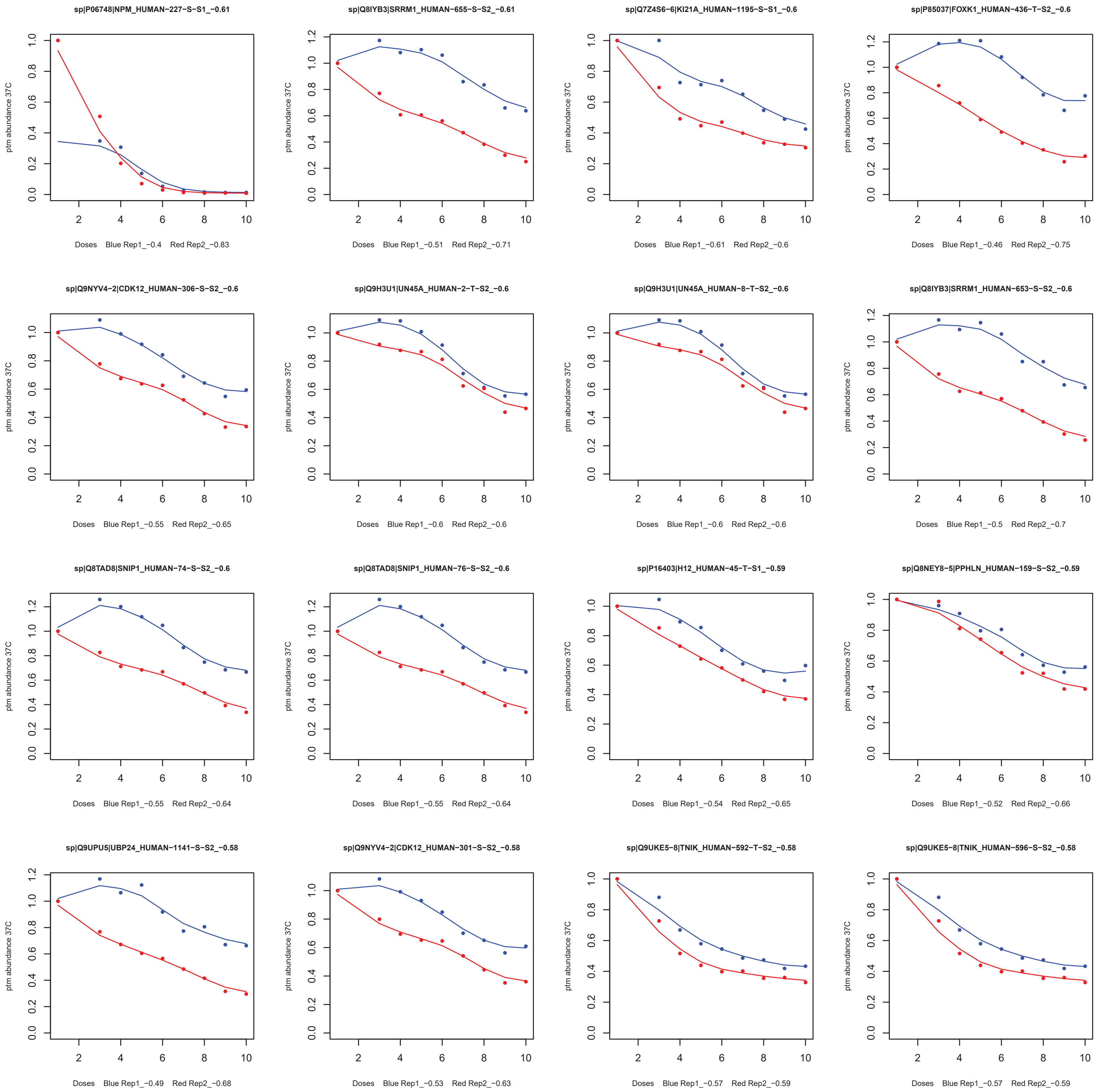
sp|P06748|NPM_HUMAN−227−S−S1_−0.61
Doses Blue Rep1_−0.4 Red Rep2_−0.83
sp|Q8IYB3|SRRM1_HUMAN−655−S−S2_−0.61
Doses Blue Rep1_−0.51 Red Rep2_−0.71
sp|Q7Z4S6−6|KI21A_HUMAN−1195−S−S1_−0.6
Doses Blue Rep1_−0.61 Red Rep2_−0.6
sp|P85037|FOXK1_HUMAN−436−T−S2_−0.6
Doses Blue Rep1_−0.46 Red Rep2_−0.75
sp|Q9NYV4−2|CDK12_HUMAN−306−S−S2_−0.6
Doses Blue Rep1_−0.55 Red Rep2_−0.65
sp|Q9H3U1|UN45A_HUMAN−2−T−S2_−0.6
Doses Blue Rep1_−0.6 Red Rep2_−0.6
sp|Q9H3U1|UN45A_HUMAN−8−T−S2_−0.6
Doses Blue Rep1_−0.6 Red Rep2_−0.6
sp|Q8IYB3|SRRM1_HUMAN−653−S−S2_−0.6
Doses Blue Rep1_−0.5 Red Rep2_−0.7
sp|Q8TAD8|SNIP1_HUMAN−74−S−S2_−0.6
Doses Blue Rep1_−0.55 Red Rep2_−0.64
sp|Q8TAD8|SNIP1_HUMAN−76−S−S2_−0.6
Doses Blue Rep1_−0.55 Red Rep2_−0.64
sp|P16403|H12_HUMAN−45−T−S1_−0.59
Doses Blue Rep1_−0.54 Red Rep2_−0.65
sp|Q8NEY8−5|PPHLN_HUMAN−159−S−S2_−0.59
Doses Blue Rep1_−0.52 Red Rep2_−0.66
sp|Q9UPU5|UBP24_HUMAN−1141−S−S2_−0.58
Doses Blue Rep1_−0.49 Red Rep2_−0.68
sp|Q9NYV4−2|CDK12_HUMAN−301−S−S2_−0.58
Doses Blue Rep1_−0.53 Red Rep2_−0.63
sp|Q9UKE5−8|TNIK_HUMAN−592−T−S2_−0.58
Doses Blue Rep1_−0.57 Red Rep2_−0.59
sp|Q9UKE5−8|TNIK_HUMAN−596−S−S2_−0.58
Doses Blue Rep1_−0.57 Red Rep2_−0.59
ptm abundance 37C

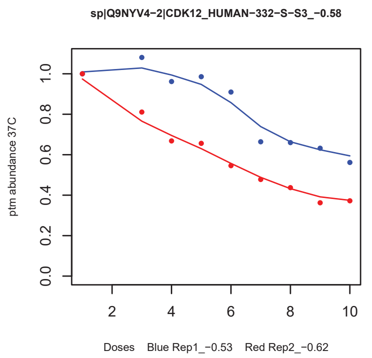
sp|Q9NYV4-2|CDK12_HUMAN-332-S-S3_-0.58
ptm abundance 37C
Doses Blue Rep1_-0.53 Red Rep2_-0.62

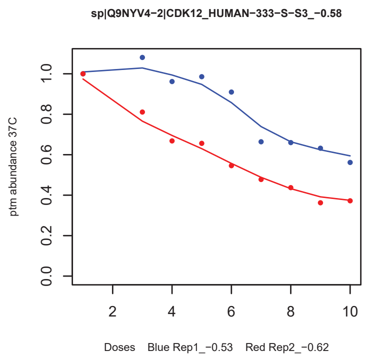
sp|Q9NYV4-2|CDK12_HUMAN-333-S-S3_-0.58
ptm abundance 37C
Doses Blue Rep1_-0.53 Red Rep2_-0.62

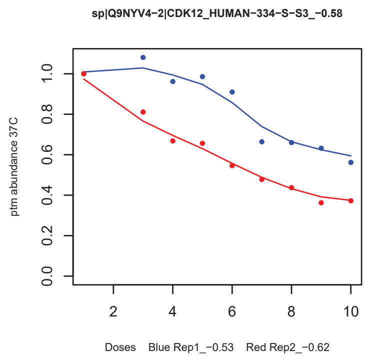
sp|Q9NYV4-2|CDK12_HUMAN-334-S-S3_-0.58
ptm abundance 37C
Doses Blue Rep1_-0.53 Red Rep2_-0.62

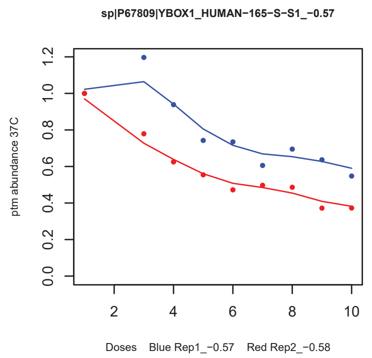
sp|P67809|YBOX1_HUMAN-165-S-S1_-0.57
ptm abundance 37C
Doses Blue Rep1_-0.57 Red Rep2_-0.58

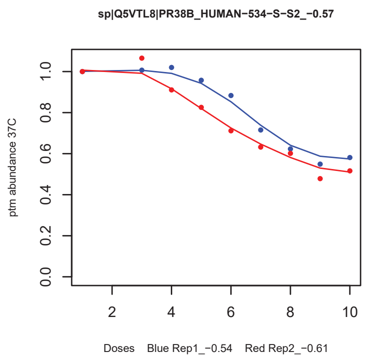
sp|Q5VTL8|PR38B_HUMAN-534-S-S2_-0.57
ptm abundance 37C
Doses Blue Rep1_-0.54 Red Rep2_-0.61

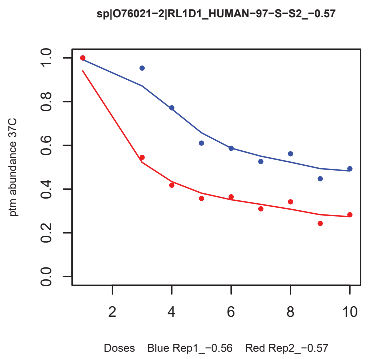
sp|O76021-2|RL1D1_HUMAN-97-S-S2_-0.57
ptm abundance 37C
Doses Blue Rep1_-0.56 Red Rep2_-0.57

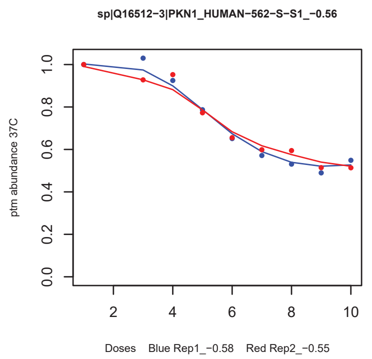
sp|Q16512-3|PKN1_HUMAN-562-S-S1_-0.56
ptm abundance 37C
Doses Blue Rep1_-0.58 Red Rep2_-0.55

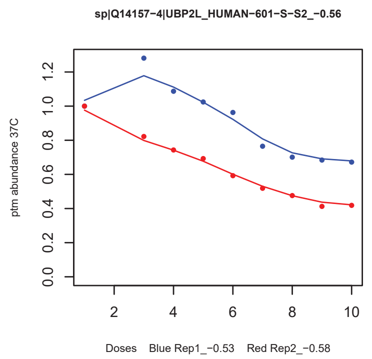
sp|Q14157-4|UBP2L_HUMAN-601-S-S2_-0.56
ptm abundance 37C
Doses Blue Rep1_-0.53 Red Rep2_-0.58

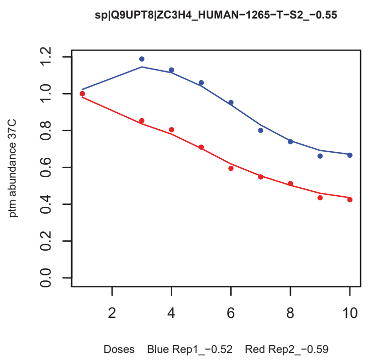
sp|Q9UPT8|ZC3H4_HUMAN-1265-T-S2_-0.55
ptm abundance 37C
Doses Blue Rep1_-0.52 Red Rep2_-0.59

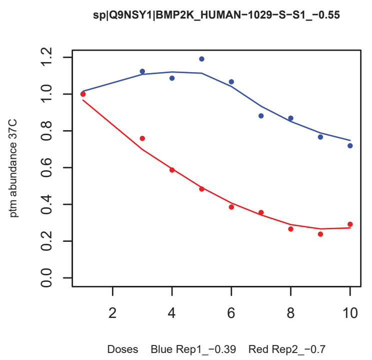
sp|Q9NSY1|BMP2K_HUMAN-1029-S-S1_-0.55
ptm abundance 37C
Doses Blue Rep1_-0.39 Red Rep2_-0.7

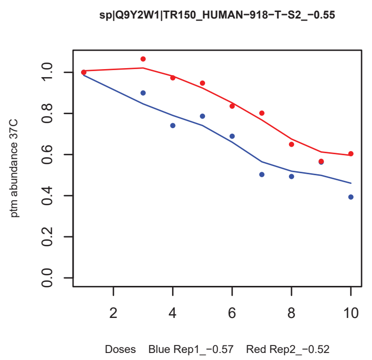
sp|Q9Y2W1|TR150_HUMAN-918-T-S2_-0.55
ptm abundance 37C
Doses Blue Rep1_-0.57 Red Rep2_-0.52

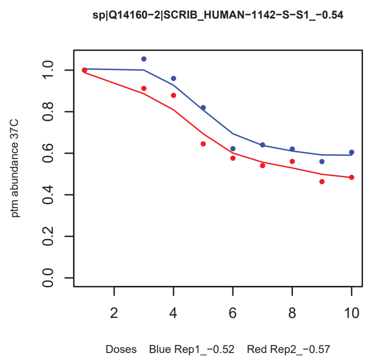
sp|Q14160-2|SCRIB_HUMAN-1142-S-S1_-0.54
ptm abundance 37C
Doses Blue Rep1_-0.52 Red Rep2_-0.57

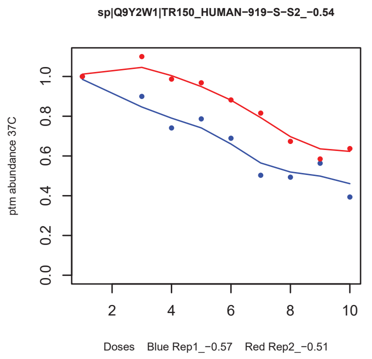
sp|Q9Y2W1|TR150_HUMAN-919-S-S2_-0.54
ptm abundance 37C
Doses Blue Rep1_-0.57 Red Rep2_-0.51

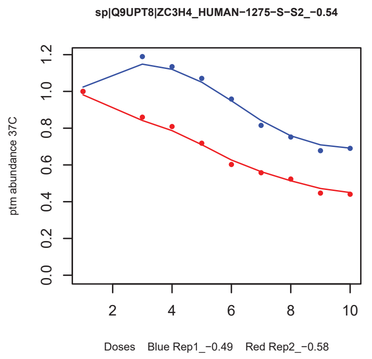
sp|Q9UPT8|ZC3H4_HUMAN-1275-S-S2_-0.54
ptm abundance 37C
Doses Blue Rep1_-0.49 Red Rep2_-0.58

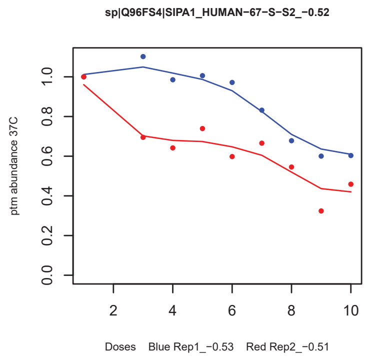
sp|Q96FS4|SIPA1_HUMAN-67-S-S2_-0.52
ptm abundance 37C
Doses Blue Rep1_-0.53 Red Rep2_-0.51

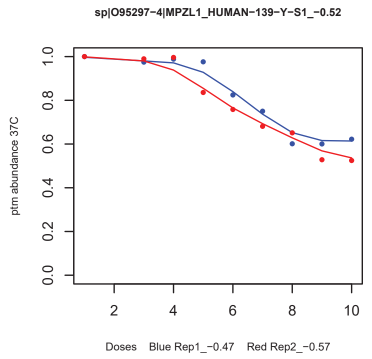
sp|O95297-4|MPZL1_HUMAN-139-Y-S1_-0.52
ptm abundance 37C
Doses Blue Rep1_-0.47 Red Rep2_-0.57

sp|Q5TC79|ZBT37_HUMAN-326-S-S2_-0.51

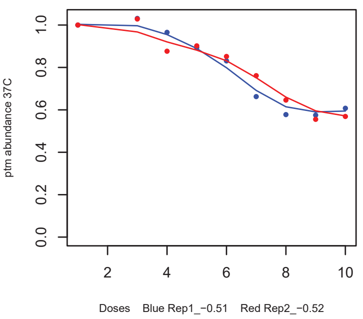

sp|Q5TC79|ZBT37_HUMAN-328-S-S2_-0.51

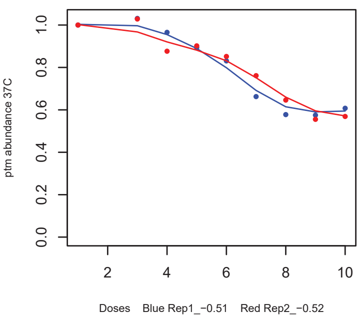

sp|P55198|AF17_HUMAN-224-S-S3_-0.49

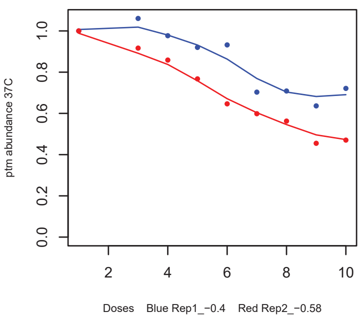

sp|Q9Y281|COF2_HUMAN-3-S-S1_-0.49

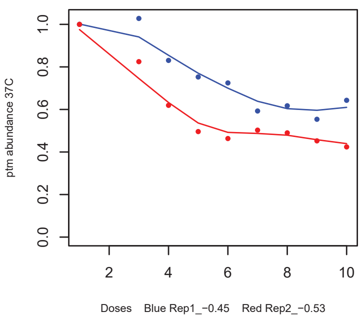

sp|O60271-3|JIP4_HUMAN-728-S-S2_-0.48

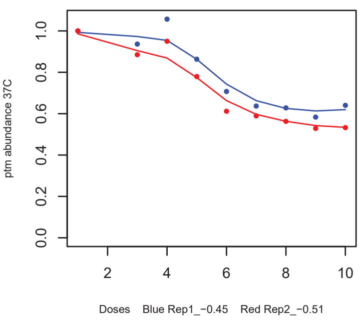

sp|O95819-4|M4K4_HUMAN-544-S-S2_-0.48

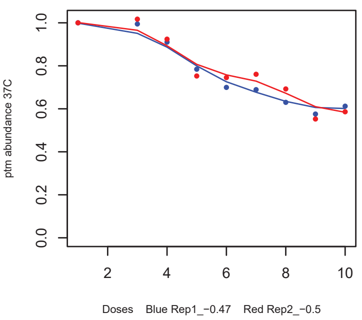

sp|O95819-4|M4K4_HUMAN-546-S-S2_-0.48

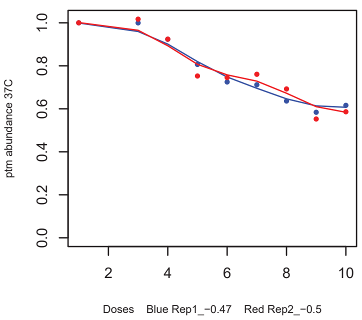

sp|Q15276|RABE1_HUMAN-408-T-S2_-0.48

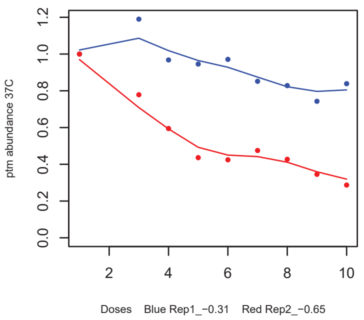

sp|O60271-3|JIP4_HUMAN-733-S-S2_-0.48

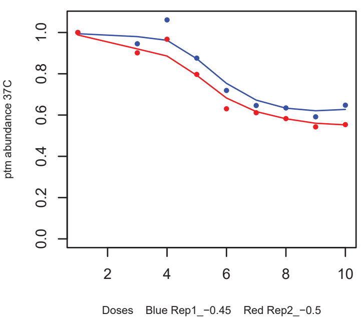

sp|Q9NYV4-2|CDK12_HUMAN-333-S-S2_-0.47

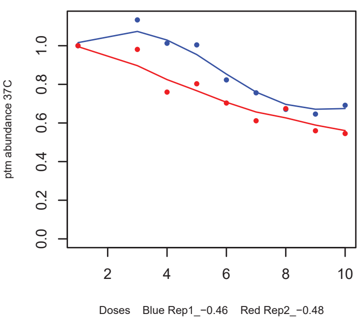

sp|P53396|ACLY_HUMAN-455-S-S1_-0.47

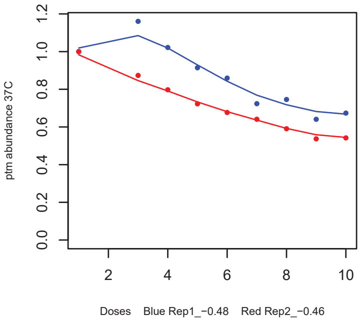

sp|Q16630-3|CPSF6_HUMAN-74-Y-S1_-0.47

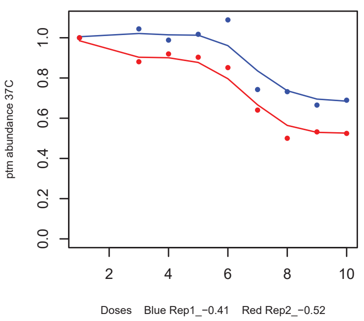

sp|Q9NYV4-2|CDK12_HUMAN-303-S-S2_-0.46

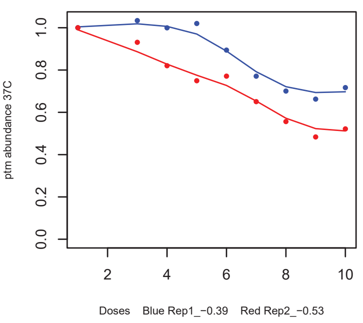

sp|Q9NYV4-2|CDK12_HUMAN-265-S-S1_-0.46

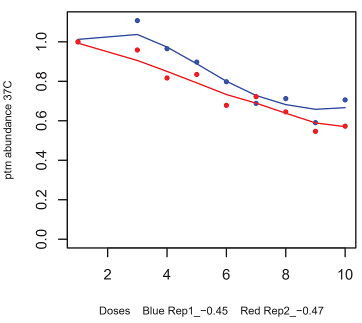

sp|P42167-3|LAP2B_HUMAN-168-S-S2_-0.46

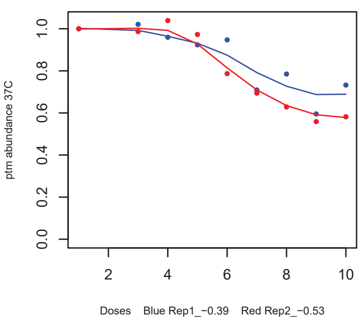

sp|Q14004-2|CDK13_HUMAN-315-S-S2_-0.46

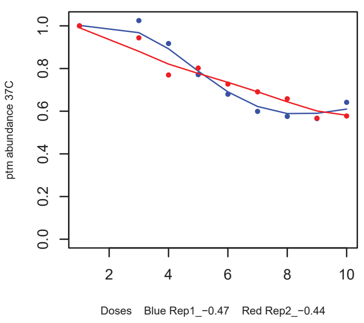

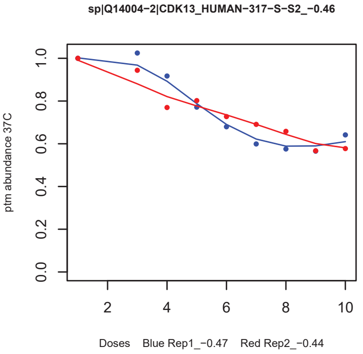
sp|Q14004−2|CDK13_HUMAN−317−S−S2_−0.46
ptm abundance 37C
Doses Blue Rep1_−0.47 Red Rep2_−0.44

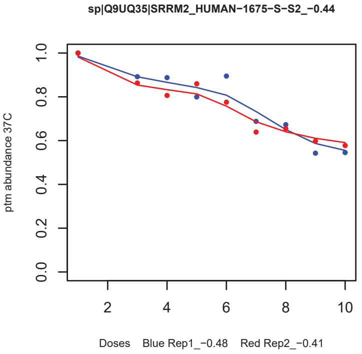
sp|Q9UQ35|SRRM2_HUMAN−1675−S−S2_−0.44
ptm abundance 37C
Doses Blue Rep1_−0.48 Red Rep2_−0.41

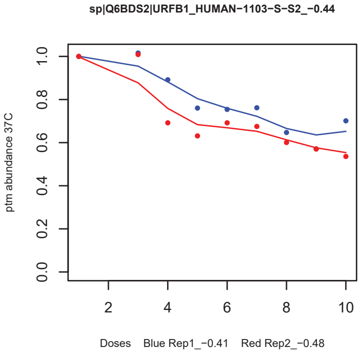
sp|Q6BDS2|URFB1_HUMAN−1103−S−S2_−0.44
ptm abundance 37C
Doses Blue Rep1_−0.41 Red Rep2_−0.48

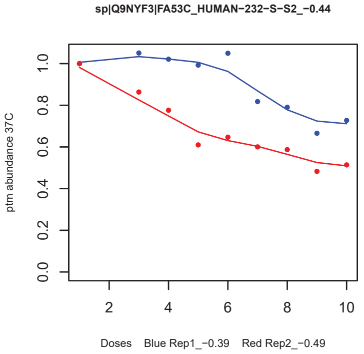
sp|Q9NYF3|FA53C_HUMAN−232−S−S2_−0.44
ptm abundance 37C
Doses Blue Rep1_−0.39 Red Rep2_−0.49

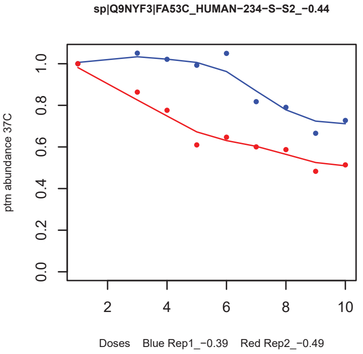
sp|Q9NYF3|FA53C_HUMAN−234−S−S2_−0.44
ptm abundance 37C
Doses Blue Rep1_−0.39 Red Rep2_−0.49

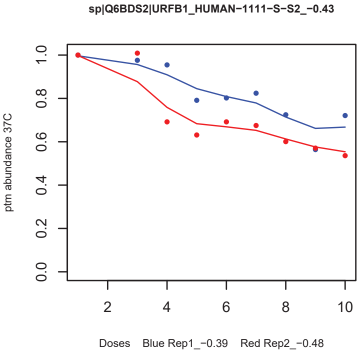
sp|Q6BDS2|URFB1_HUMAN−1111−S−S2_−0.43
ptm abundance 37C
Doses Blue Rep1_−0.39 Red Rep2_−0.48

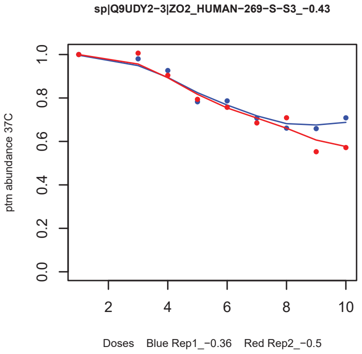
sp|Q9UDY2−3|ZO2_HUMAN−269−S−S3_−0.43
ptm abundance 37C
Doses Blue Rep1_−0.36 Red Rep2_−0.5

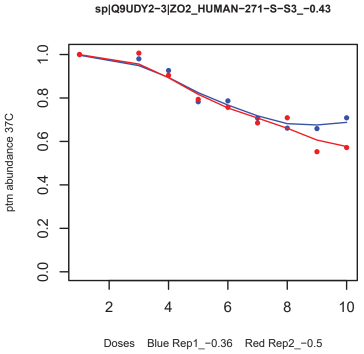
sp|Q9UDY2−3|ZO2_HUMAN−271−S−S3_−0.43
ptm abundance 37C
Doses Blue Rep1_−0.36 Red Rep2_−0.5

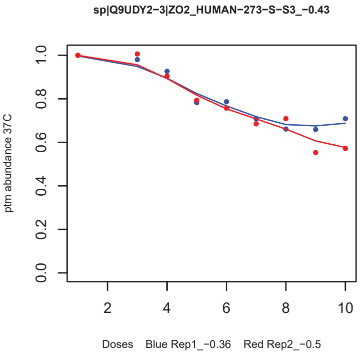
sp|Q9UDY2−3|ZO2_HUMAN−273−S−S3_−0.43
ptm abundance 37C
Doses Blue Rep1_−0.36 Red Rep2_−0.5

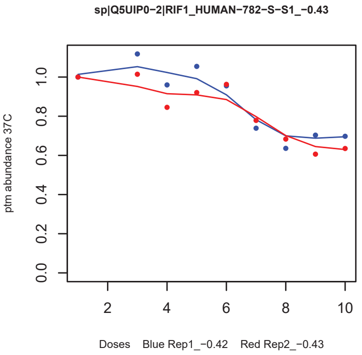
sp|Q5UIP0−2|RIF1_HUMAN−782−S−S1_−0.43
ptm abundance 37C
Doses Blue Rep1_−0.42 Red Rep2_−0.43

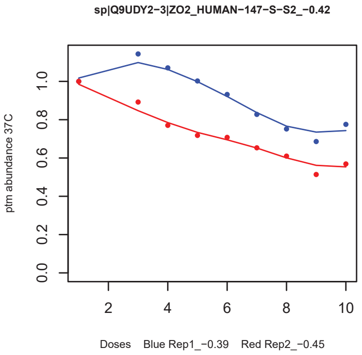
sp|Q9UDY2−3|ZO2_HUMAN−147−S−S2_−0.42
ptm abundance 37C
Doses Blue Rep1_−0.39 Red Rep2_−0.45

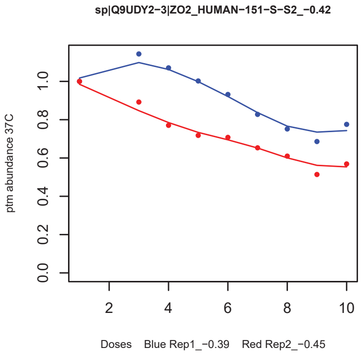
sp|Q9UDY2−3|ZO2_HUMAN−151−S−S2_−0.42
ptm abundance 37C
Doses Blue Rep1_−0.39 Red Rep2_−0.45

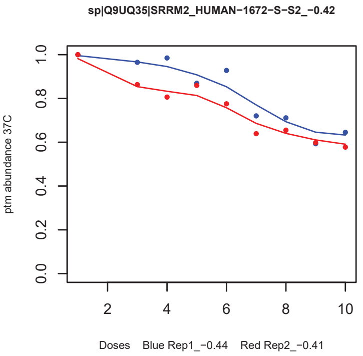
sp|Q9UQ35|SRRM2_HUMAN−1672−S−S2_−0.42
ptm abundance 37C
Doses Blue Rep1_−0.44 Red Rep2_−0.41

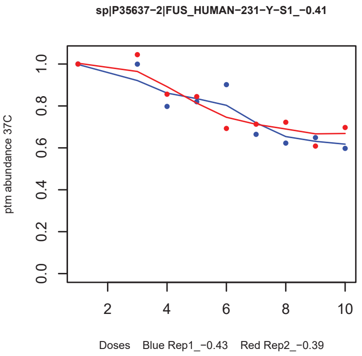
sp|P35637−2|FUS_HUMAN−231−Y−S1_−0.41
ptm abundance 37C
Doses Blue Rep1_−0.43 Red Rep2_−0.39

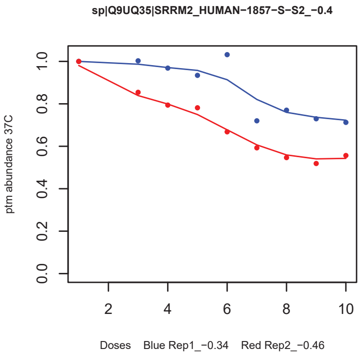
sp|Q9UQ35|SRRM2_HUMAN−1857−S−S2_−0.4
ptm abundance 37C
Doses Blue Rep1_−0.34 Red Rep2_−0.46

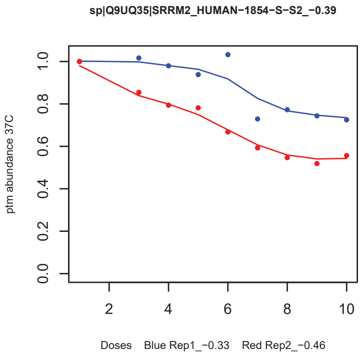
sp|Q9UQ35|SRRM2_HUMAN−1854−S−S2_−0.39
ptm abundance 37C
Doses Blue Rep1_−0.33 Red Rep2_−0.46

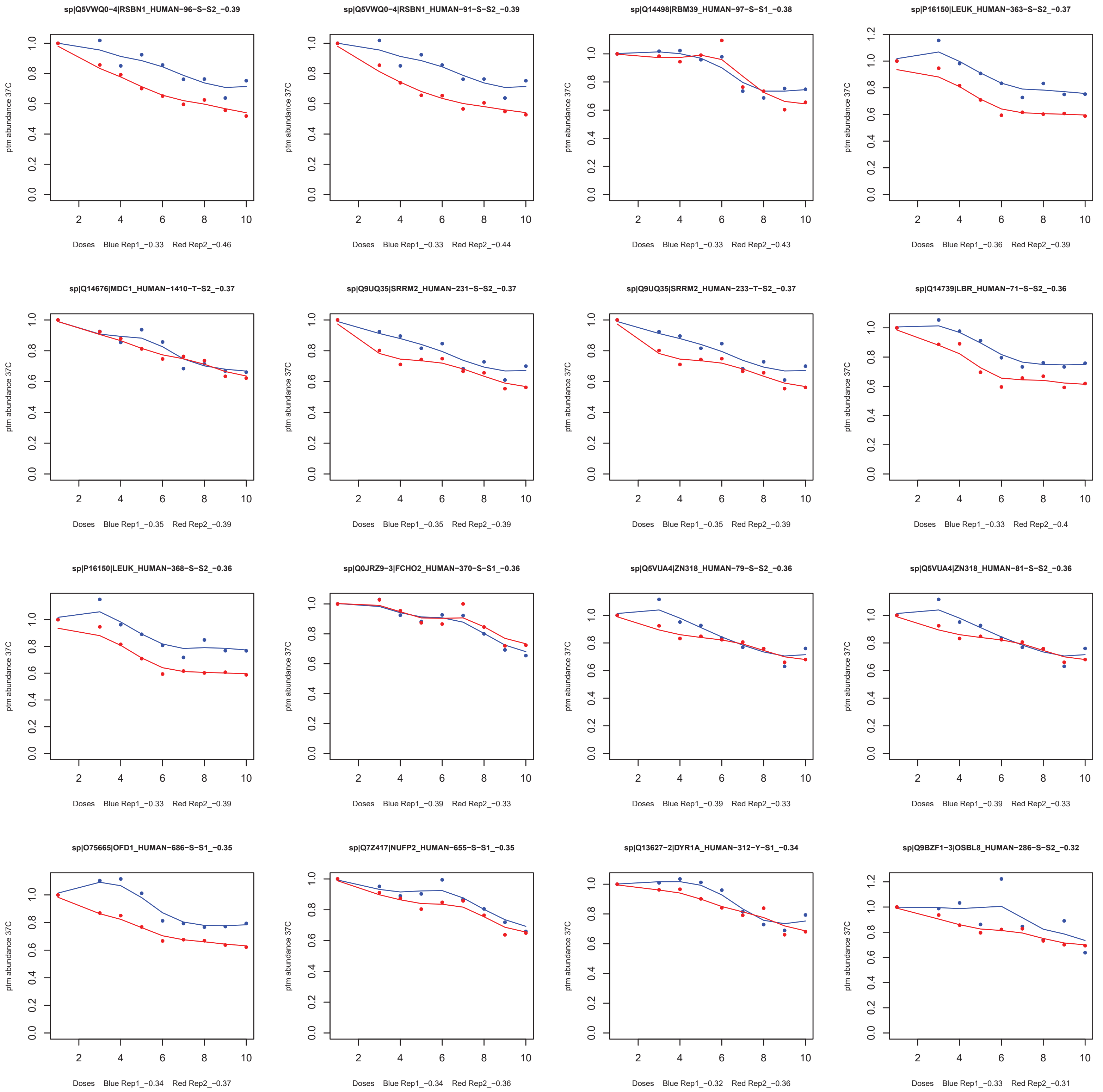
sp|Q5VWQ0−4|RSBN1_HUMAN−96−S−S2_−0.39
Doses Blue Rep1_−0.33 Red Rep2_−0.46
sp|Q5VWQ0−4|RSBN1_HUMAN−91−S−S2_−0.39
Doses Blue Rep1_−0.33 Red Rep2_−0.44
sp|Q14498|RBM39_HUMAN−97−S−S1_−0.38
Doses Blue Rep1_−0.33 Red Rep2_−0.43
sp|P16150|LEUK_HUMAN−363−S−S2_−0.37
Doses Blue Rep1_−0.36 Red Rep2_−0.39
sp|Q14676|MDC1_HUMAN−1410−T−S2_−0.37
Doses Blue Rep1_−0.35 Red Rep2_−0.39
sp|Q9UQ35|SRRM2_HUMAN−231−S−S2_−0.37
Doses Blue Rep1_−0.35 Red Rep2_−0.39
sp|Q9UQ35|SRRM2_HUMAN−233−T−S2_−0.37
Doses Blue Rep1_−0.35 Red Rep2_−0.39
sp|Q14739|LBR_HUMAN−71−S−S2_−0.36
Doses Blue Rep1_−0.33 Red Rep2_−0.4
sp|P16150|LEUK_HUMAN−368−S−S2_−0.36
Doses Blue Rep1_−0.33 Red Rep2_−0.39
sp|Q0JRZ9−3|FCHO2_HUMAN−370−S−S1_−0.36
Doses Blue Rep1_−0.39 Red Rep2_−0.33
sp|Q5VUA4|ZN318_HUMAN−79−S−S2_−0.36
Doses Blue Rep1_−0.39 Red Rep2_−0.33
sp|Q5VUA4|ZN318_HUMAN−81−S−S2_−0.36
Doses Blue Rep1_−0.39 Red Rep2_−0.33
sp|O75665|OFD1_HUMAN−686−S−S1_−0.35
Doses Blue Rep1_−0.34 Red Rep2_−0.37
sp|Q7Z417|NUFP2_HUMAN−655−S−S1_−0.35
Doses Blue Rep1_−0.34 Red Rep2_−0.36
sp|Q13627−2|DYR1A_HUMAN−312−Y−S1_−0.34
Doses Blue Rep1_−0.32 Red Rep2_−0.36
sp|Q9BZF1−3|OSBL8_HUMAN−286−S−S2_−0.32
Doses Blue Rep1_−0.33 Red Rep2_−0.31
ptm abundance 37C

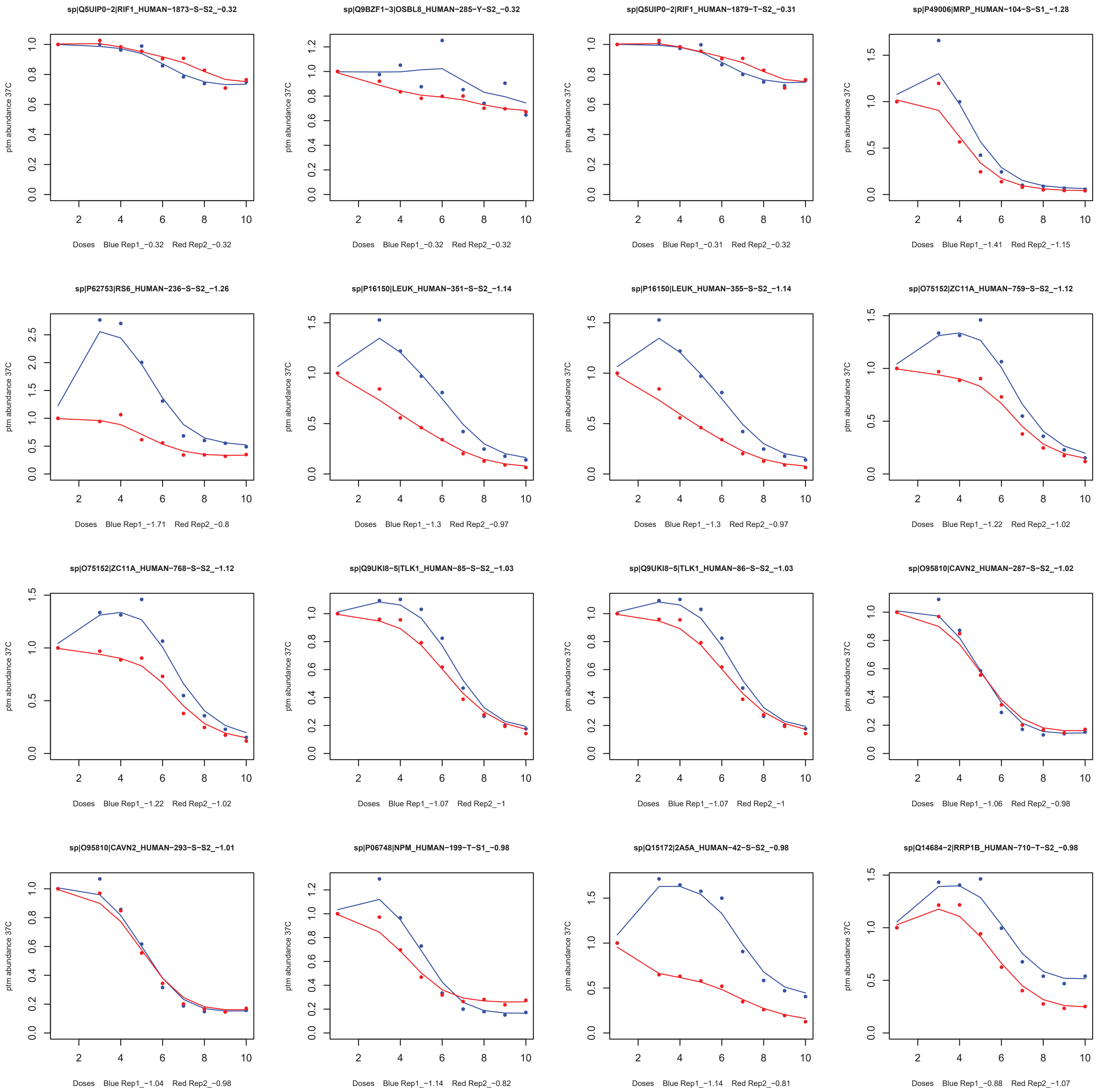
sp|Q5UIP0−2|RIF1_HUMAN−1873−S−S2_−0.32
Doses Blue Rep1_−0.32 Red Rep2_−0.32
sp|Q9BZF1−3|OSBL8_HUMAN−285−Y−S2_−0.32
Doses Blue Rep1_−0.32 Red Rep2_−0.32
sp|Q5UIP0−2|RIF1_HUMAN−1879−T−S2_−0.31
Doses Blue Rep1_−0.31 Red Rep2_−0.32
sp|P49006|MRP_HUMAN−104−S−S1_−1.28
Doses Blue Rep1_−1.41 Red Rep2_−1.15
sp|P62753|RS6_HUMAN−236−S−S2_−1.26
Doses Blue Rep1_−1.71 Red Rep2_−0.8
sp|P16150|LEUK_HUMAN−351−S−S2_−1.14
Doses Blue Rep1_−1.3 Red Rep2_−0.97
sp|P16150|LEUK_HUMAN−355−S−S2_−1.14
Doses Blue Rep1_−1.3 Red Rep2_−0.97
sp|O75152|ZC11A_HUMAN−759−S−S2_−1.12
Doses Blue Rep1_−1.22 Red Rep2_−1.02
sp|O75152|ZC11A_HUMAN−768−S−S2_−1.12
Doses Blue Rep1_−1.22 Red Rep2_−1.02
sp|Q9UKI8−5|TLK1_HUMAN−85−S−S2_−1.03
Doses Blue Rep1_−1.07 Red Rep2_−1
sp|Q9UKI8−5|TLK1_HUMAN−86−S−S2_−1.03
Doses Blue Rep1_−1.07 Red Rep2_−1
sp|O95810|CAVN2_HUMAN−287−S−S2_−1.02
Doses Blue Rep1_−1.06 Red Rep2_−0.98
sp|O95810|CAVN2_HUMAN−293−S−S2_−1.01
Doses Blue Rep1_−1.04 Red Rep2_−0.98
sp|P06748|NPM_HUMAN−199−T−S1_−0.98
Doses Blue Rep1_−1.14 Red Rep2_−0.82
sp|Q15172|2A5A_HUMAN−42−S−S2_−0.98
Doses Blue Rep1_−1.14 Red Rep2_−0.81
sp|Q14684−2|RRP1B_HUMAN−710−T−S2_−0.98
Doses Blue Rep1_−0.88 Red Rep2_−1.07
ptm abundance 37C

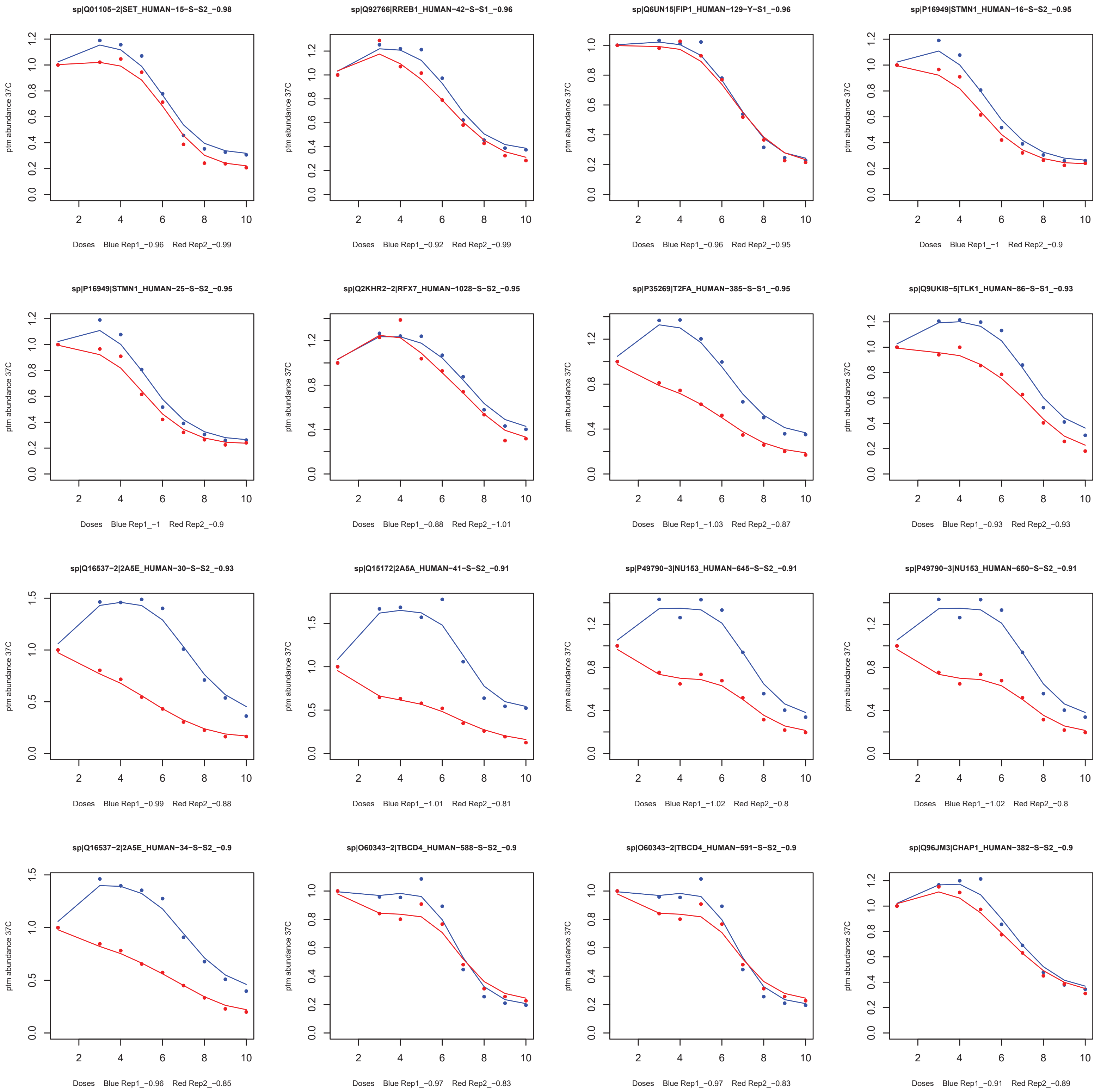

sp|Q01105−2|SET_HUMAN−15−S−S2_−0.98
ptm abundance 37C
Doses Blue Rep1_−0.96 Red Rep2_−0.99
sp|Q92766|RREB1_HUMAN−42−S−S1_−0.96
ptm abundance 37C
Doses Blue Rep1_−0.92 Red Rep2_−0.99
sp|Q6UN15|FIP1_HUMAN−129−Y−S1_−0.96
ptm abundance 37C
Doses Blue Rep1_−0.96 Red Rep2_−0.95
sp|P16949|STMN1_HUMAN−16−S−S2_−0.95
ptm abundance 37C
Doses Blue Rep1_−1 Red Rep2_−0.9
sp|P16949|STMN1_HUMAN−25−S−S2_−0.95
ptm abundance 37C
Doses Blue Rep1_−1 Red Rep2_−0.9
sp|Q2KHR2−2|RFX7_HUMAN−1028−S−S2_−0.95
ptm abundance 37C
Doses Blue Rep1_−0.88 Red Rep2_−1.01
sp|P35269|T2FA_HUMAN−385−S−S1_−0.95
ptm abundance 37C
Doses Blue Rep1_−1.03 Red Rep2_−0.87
sp|Q9UKI8−5|TLK1_HUMAN−86−S−S1_−0.93
ptm abundance 37C
Doses Blue Rep1_−0.93 Red Rep2_−0.93
sp|Q16537−2|2A5E_HUMAN−30−S−S2_−0.93
ptm abundance 37C
Doses Blue Rep1_−0.99 Red Rep2_−0.88
sp|Q15172|2A5A_HUMAN−41−S−S2_−0.91
ptm abundance 37C
Doses Blue Rep1_−1.01 Red Rep2_−0.81
sp|P49790−3|NU153_HUMAN−645−S−S2_−0.91
ptm abundance 37C
Doses Blue Rep1_−1.02 Red Rep2_−0.8
sp|P49790−3|NU153_HUMAN−650−S−S2_−0.91
ptm abundance 37C
Doses Blue Rep1_−1.02 Red Rep2_−0.8
sp|Q16537−2|2A5E_HUMAN−34−S−S2_−0.9
ptm abundance 37C
Doses Blue Rep1_−0.96 Red Rep2_−0.85
sp|O60343−2|TBCD4_HUMAN−588−S−S2_−0.9
ptm abundance 37C
Doses Blue Rep1_−0.97 Red Rep2_−0.83
sp|O60343−2|TBCD4_HUMAN−591−S−S2_−0.9
ptm abundance 37C
Doses Blue Rep1_−0.97 Red Rep2_−0.83
sp|Q96JM3|CHAP1_HUMAN−382−S−S2_−0.9
ptm abundance 37C
Doses Blue Rep1_−0.91 Red Rep2_−0.89

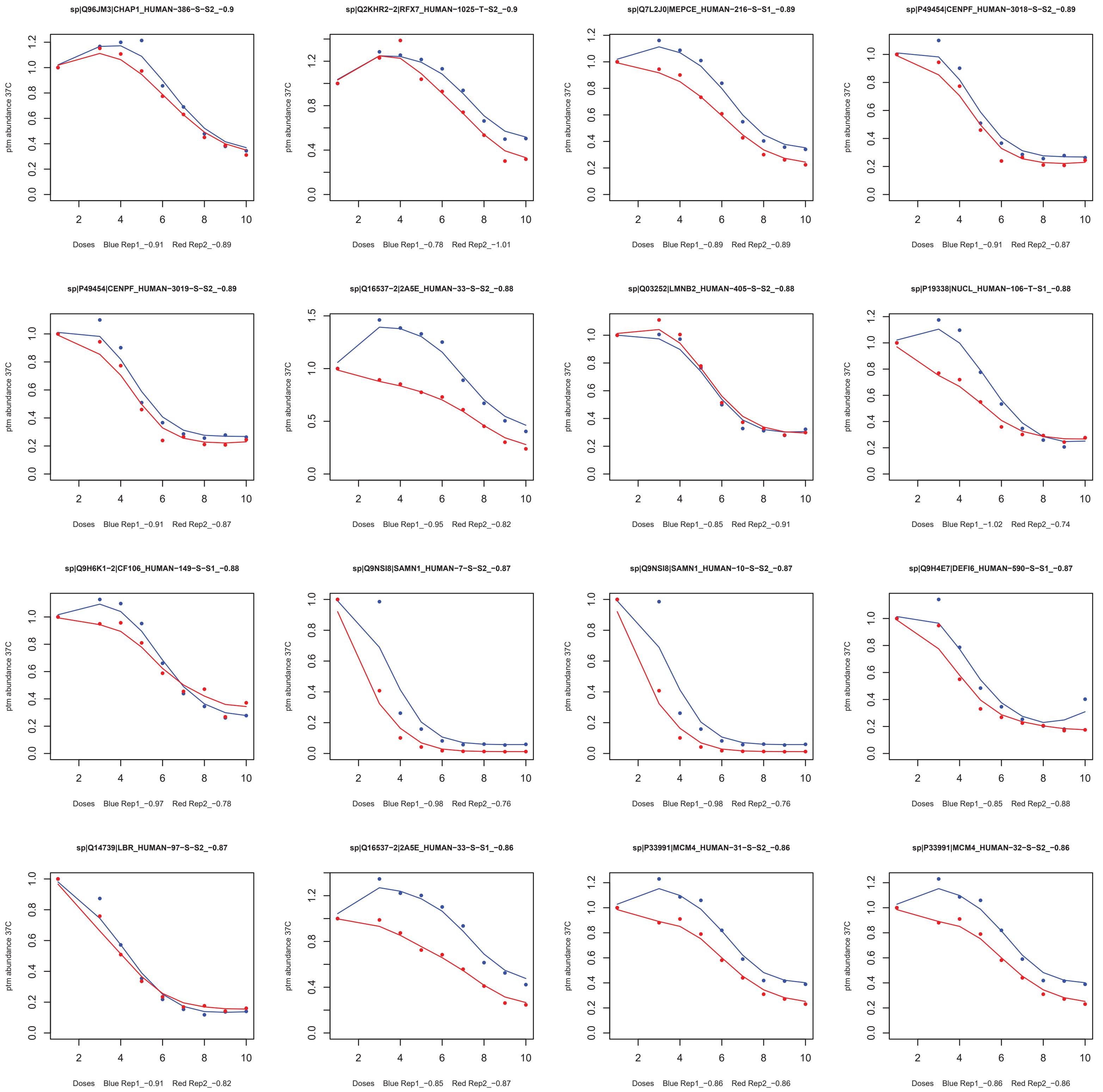
sp|Q96JM3|CHAP1_HUMAN−386−S−S2_−0.9
ptm abundance 37C
Doses Blue Rep1_−0.91 Red Rep2_−0.89
sp|Q2KHR2−2|RFX7_HUMAN−1025−T−S2_−0.9
ptm abundance 37C
Doses Blue Rep1_−0.78 Red Rep2_−1.01
sp|Q7L2J0|MEPCE_HUMAN−216−S−S1_−0.89
ptm abundance 37C
Doses Blue Rep1_−0.89 Red Rep2_−0.89
sp|P49454|CENPF_HUMAN−3018−S−S2_−0.89
ptm abundance 37C
Doses Blue Rep1_−0.91 Red Rep2_−0.87
sp|P49454|CENPF_HUMAN−3019−S−S2_−0.89
ptm abundance 37C
Doses Blue Rep1_−0.91 Red Rep2_−0.87
sp|Q16537−2|2A5E_HUMAN−33−S−S2_−0.88
ptm abundance 37C
Doses Blue Rep1_−0.95 Red Rep2_−0.82
sp|Q03252|LMNB2_HUMAN−405−S−S2_−0.88
ptm abundance 37C
Doses Blue Rep1_−0.85 Red Rep2_−0.91
sp|P19338|NUCL_HUMAN−106−T−S1_−0.88
ptm abundance 37C
Doses Blue Rep1_−1.02 Red Rep2_−0.74
sp|Q9H6K1−2|CF106_HUMAN−149−S−S1_−0.88
ptm abundance 37C
Doses Blue Rep1_−0.97 Red Rep2_−0.78
sp|Q9NSI8|SAMN1_HUMAN−7−S−S2_−0.87
ptm abundance 37C
Doses Blue Rep1_−0.98 Red Rep2_−0.76
sp|Q9NSI8|SAMN1_HUMAN−10−S−S2_−0.87
ptm abundance 37C
Doses Blue Rep1_−0.98 Red Rep2_−0.76
sp|Q9H4E7|DEFI6_HUMAN−590−S−S1_−0.87
ptm abundance 37C
Doses Blue Rep1_−0.85 Red Rep2_−0.88
sp|Q14739|LBR_HUMAN−97−S−S2_−0.87
ptm abundance 37C
Doses Blue Rep1_−0.91 Red Rep2_−0.82
sp|Q16537−2|2A5E_HUMAN−33−S−S1_−0.86
ptm abundance 37C
Doses Blue Rep1_−0.85 Red Rep2_−0.87
sp|P33991|MCM4_HUMAN−31−S−S2_−0.86
ptm abundance 37C
Doses Blue Rep1_−0.86 Red Rep2_−0.86
sp|P33991|MCM4_HUMAN−32−S−S2_−0.86
ptm abundance 37C
Doses Blue Rep1_−0.86 Red Rep2_−0.86

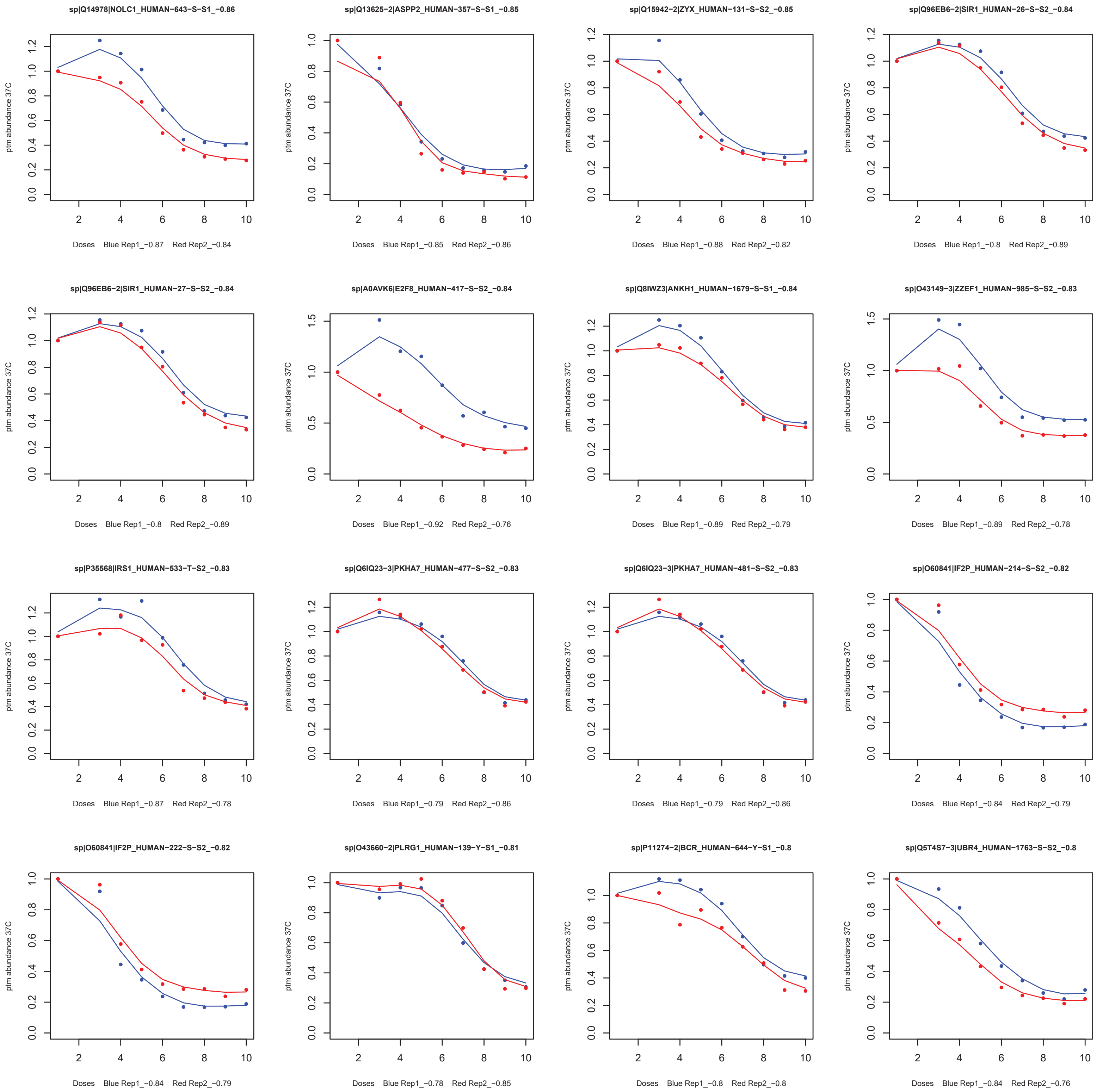
sp|Q14978|NOLC1_HUMAN−643−S−S1_−0.86
ptm abundance 37C
Doses Blue Rep1_−0.87 Red Rep2_−0.84
sp|Q13625−2|ASPP2_HUMAN−357−S−S1_−0.85
ptm abundance 37C
Doses Blue Rep1_−0.85 Red Rep2_−0.86
sp|Q15942−2|ZYX_HUMAN−131−S−S2_−0.85
ptm abundance 37C
Doses Blue Rep1_−0.88 Red Rep2_−0.82
sp|Q96EB6−2|SIR1_HUMAN−26−S−S2_−0.84
ptm abundance 37C
Doses Blue Rep1_−0.8 Red Rep2_−0.89
sp|Q96EB6−2|SIR1_HUMAN−27−S−S2_−0.84
ptm abundance 37C
Doses Blue Rep1_−0.8 Red Rep2_−0.89
sp|A0AVK6|E2F8_HUMAN−417−S−S2_−0.84
ptm abundance 37C
Doses Blue Rep1_−0.92 Red Rep2_−0.76
sp|Q8IWZ3|ANKH1_HUMAN−1679−S−S1_−0.84
ptm abundance 37C
Doses Blue Rep1_−0.89 Red Rep2_−0.79
sp|O43149−3|ZZEF1_HUMAN−985−S−S2_−0.83
ptm abundance 37C
Doses Blue Rep1_−0.89 Red Rep2_−0.78
sp|P35568|IRS1_HUMAN−533−T−S2_−0.83
ptm abundance 37C
Doses Blue Rep1_−0.87 Red Rep2_−0.78
sp|Q6IQ23−3|PKHA7_HUMAN−477−S−S2_−0.83
ptm abundance 37C
Doses Blue Rep1_−0.79 Red Rep2_−0.86
sp|Q6IQ23−3|PKHA7_HUMAN−481−S−S2_−0.83
ptm abundance 37C
Doses Blue Rep1_−0.79 Red Rep2_−0.86
sp|O60841|IF2P_HUMAN−214−S−S2_−0.82
ptm abundance 37C
Doses Blue Rep1_−0.84 Red Rep2_−0.79
sp|O60841|IF2P_HUMAN−222−S−S2_−0.82
ptm abundance 37C
Doses Blue Rep1_−0.84 Red Rep2_−0.79
sp|O43660−2|PLRG1_HUMAN−139−Y−S1_−0.81
ptm abundance 37C
Doses Blue Rep1_−0.78 Red Rep2_−0.85
sp|P11274−2|BCR_HUMAN−644−Y−S1_−0.8
ptm abundance 37C
Doses Blue Rep1_−0.8 Red Rep2_−0.8
sp|Q5T4S7−3|UBR4_HUMAN−1763−S−S2_−0.8
ptm abundance 37C
Doses Blue Rep1_−0.84 Red Rep2_−0.76

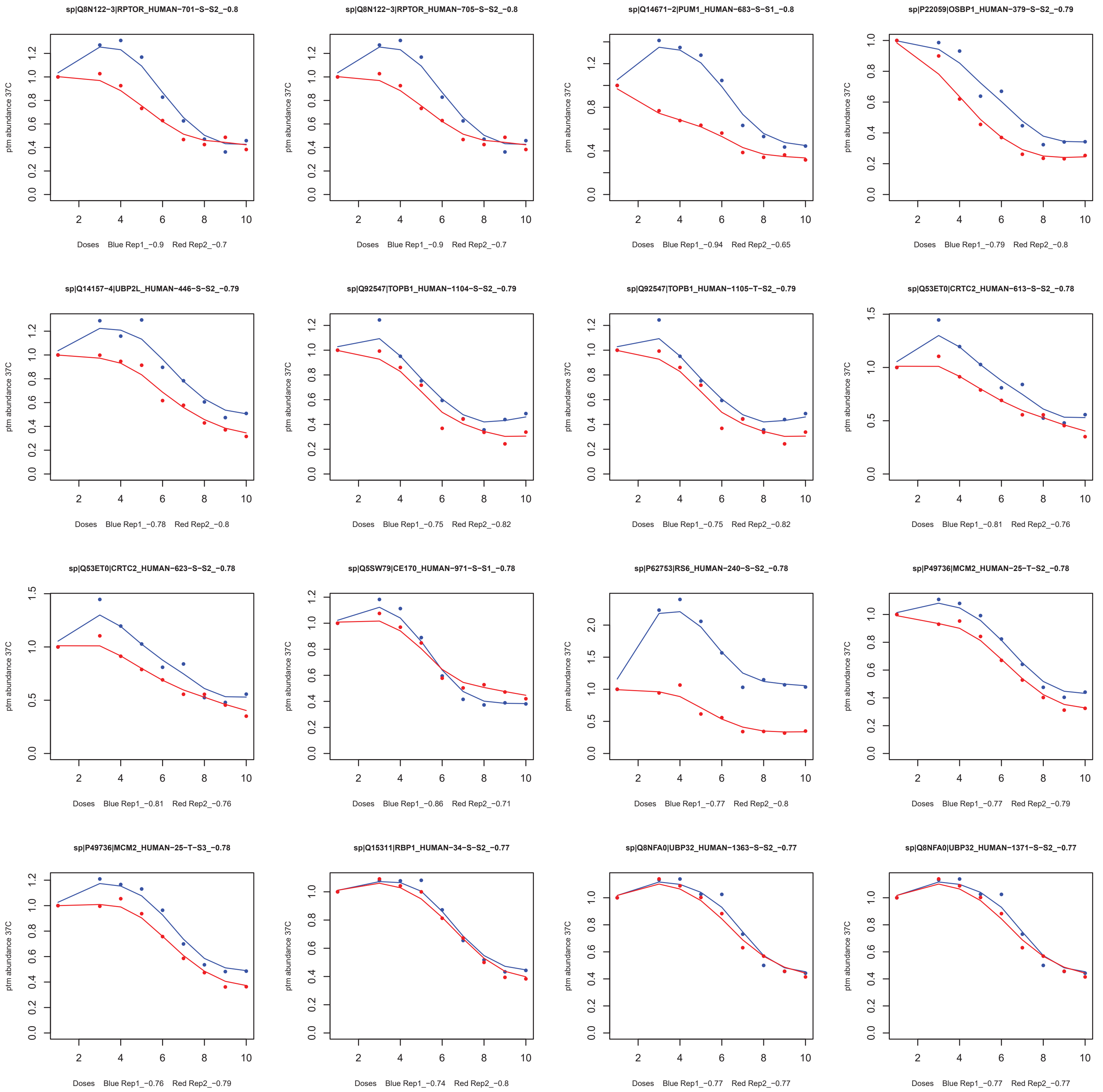
sp|Q8N122−3|RPTOR_HUMAN−701−S−S2_−0.8
ptm abundance 37C
Doses Blue Rep1_−0.9 Red Rep2_−0.7
sp|Q8N122−3|RPTOR_HUMAN−705−S−S2_−0.8
ptm abundance 37C
Doses Blue Rep1_−0.9 Red Rep2_−0.7
sp|Q14671−2|PUM1_HUMAN−683−S−S1_−0.8
ptm abundance 37C
Doses Blue Rep1_−0.94 Red Rep2_−0.65
sp|P22059|OSBP1_HUMAN−379−S−S2_−0.79
ptm abundance 37C
Doses Blue Rep1_−0.79 Red Rep2_−0.8
sp|Q14157−4|UBP2L_HUMAN−446−S−S2_−0.79
ptm abundance 37C
Doses Blue Rep1_−0.78 Red Rep2_−0.8
sp|Q92547|TOPB1_HUMAN−1104−S−S2_−0.79
ptm abundance 37C
Doses Blue Rep1_−0.75 Red Rep2_−0.82
sp|Q92547|TOPB1_HUMAN−1105−T−S2_−0.79
ptm abundance 37C
Doses Blue Rep1_−0.75 Red Rep2_−0.82
sp|Q53ET0|CRTC2_HUMAN−613−S−S2_−0.78
ptm abundance 37C
Doses Blue Rep1_−0.81 Red Rep2_−0.76
sp|Q53ET0|CRTC2_HUMAN−623−S−S2_−0.78
ptm abundance 37C
Doses Blue Rep1_−0.81 Red Rep2_−0.76
sp|Q5SW79|CE170_HUMAN−971−S−S1_−0.78
ptm abundance 37C
Doses Blue Rep1_−0.86 Red Rep2_−0.71
sp|P62753|RS6_HUMAN−240−S−S2_−0.78
ptm abundance 37C
Doses Blue Rep1_−0.77 Red Rep2_−0.8
sp|P49736|MCM2_HUMAN−25−T−S2_−0.78
ptm abundance 37C
Doses Blue Rep1_−0.77 Red Rep2_−0.79
sp|P49736|MCM2_HUMAN−25−T−S3_−0.78
ptm abundance 37C
Doses Blue Rep1_−0.76 Red Rep2_−0.79
sp|Q15311|RBP1_HUMAN−34−S−S2_−0.77
ptm abundance 37C
Doses Blue Rep1_−0.74 Red Rep2_−0.8
sp|Q8NFA0|UBP32_HUMAN−1363−S−S2_−0.77
ptm abundance 37C
Doses Blue Rep1_−0.77 Red Rep2_−0.77
sp|Q8NFA0|UBP32_HUMAN−1371−S−S2_−0.77
ptm abundance 37C
Doses Blue Rep1_−0.77 Red Rep2_−0.77

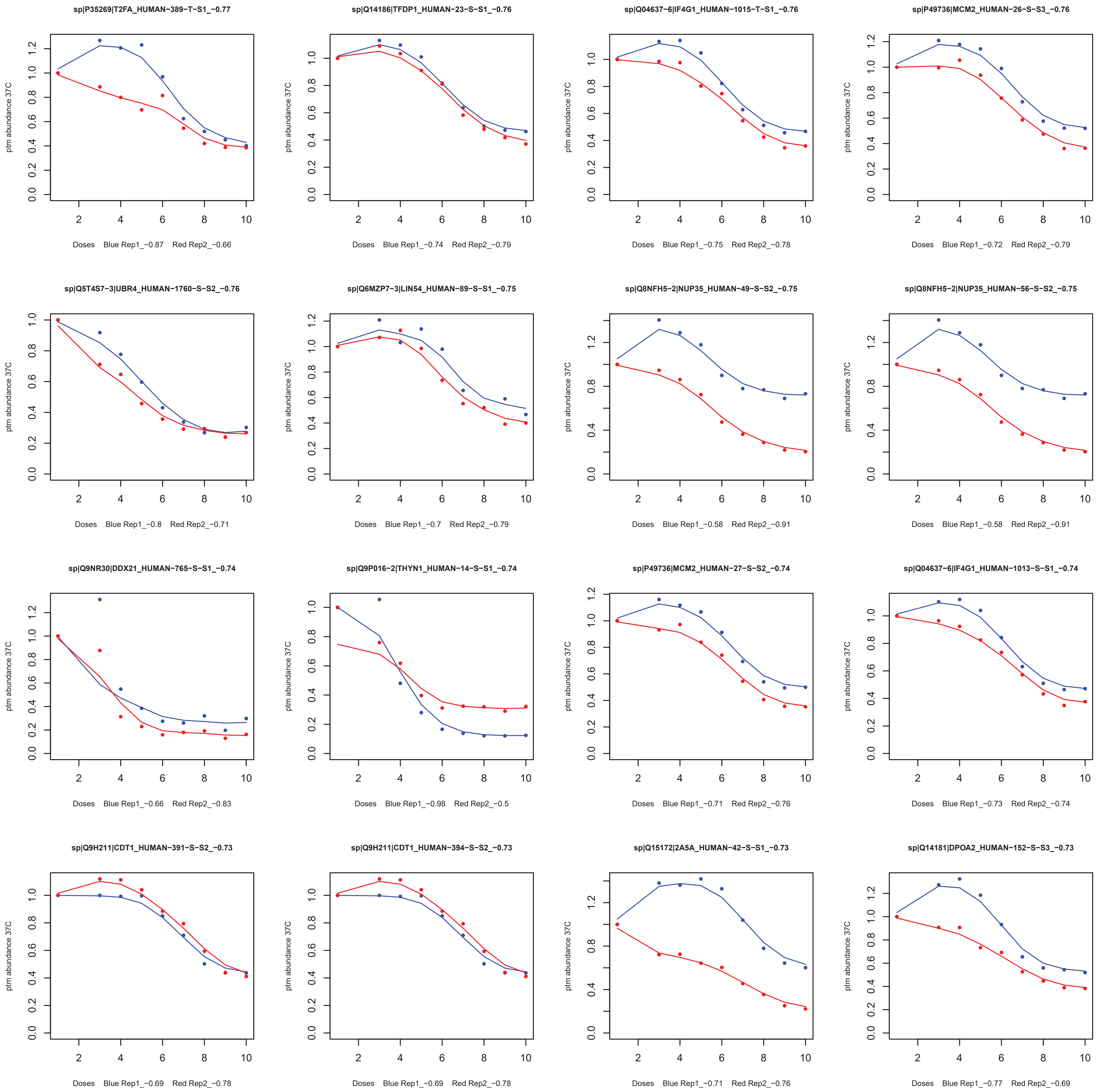

sp|P35269|T2FA_HUMAN−389−T−S1_−0.77
Doses Blue Rep1_−0.87 Red Rep2_−0.66
sp|Q14186|TFDP1_HUMAN−23−S−S1_−0.76
Doses Blue Rep1_−0.74 Red Rep2_−0.79
sp|Q04637−6|IF4G1_HUMAN−1015−T−S1_−0.76
Doses Blue Rep1_−0.75 Red Rep2_−0.78
sp|P49736|MCM2_HUMAN−26−S−S3_−0.76
Doses Blue Rep1_−0.72 Red Rep2_−0.79
sp|Q5T4S7−3|UBR4_HUMAN−1760−S−S2_−0.76
Doses Blue Rep1_−0.8 Red Rep2_−0.71
sp|Q6MZP7−3|LIN54_HUMAN−89−S−S1_−0.75
Doses Blue Rep1_−0.7 Red Rep2_−0.79
sp|Q8NFH5−2|NUP35_HUMAN−49−S−S2_−0.75
Doses Blue Rep1_−0.58 Red Rep2_−0.91
sp|Q8NFH5−2|NUP35_HUMAN−56−S−S2_−0.75
Doses Blue Rep1_−0.58 Red Rep2_−0.91
sp|Q9NR30|DDX21_HUMAN−765−S−S1_−0.74
Doses Blue Rep1_−0.66 Red Rep2_−0.83
sp|Q9P016−2|THYN1_HUMAN−14−S−S1_−0.74
Doses Blue Rep1_−0.98 Red Rep2_−0.5
sp|P49736|MCM2_HUMAN−27−S−S2_−0.74
Doses Blue Rep1_−0.71 Red Rep2_−0.76
sp|Q04637−6|IF4G1_HUMAN−1013−S−S1_−0.74
Doses Blue Rep1_−0.73 Red Rep2_−0.74
sp|Q9H211|CDT1_HUMAN−391−S−S2_−0.73
Doses Blue Rep1_−0.69 Red Rep2_−0.78
sp|Q9H211|CDT1_HUMAN−394−S−S2_−0.73
Doses Blue Rep1_−0.69 Red Rep2_−0.78
sp|Q15172|2A5A_HUMAN−42−S−S1_−0.73
Doses Blue Rep1_−0.71 Red Rep2_−0.76
sp|Q14181|DPOA2_HUMAN−152−S−S3_−0.73
Doses Blue Rep1_−0.77 Red Rep2_−0.69
ptm abundance 37C

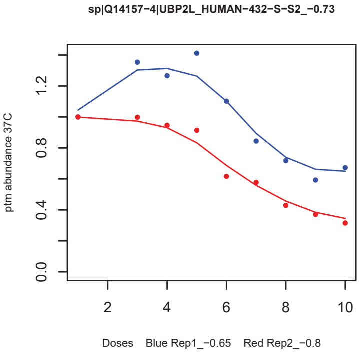
sp|Q14157−4|UBP2L_HUMAN−432−S−S2_−0.73
ptm abundance 37C
Doses Blue Rep1_−0.65 Red Rep2_−0.8

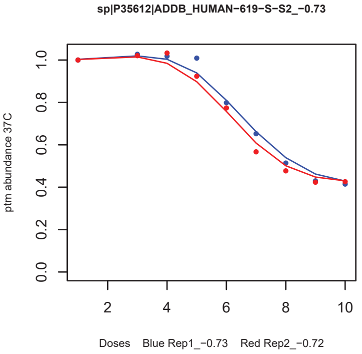
sp|P35612|ADDB_HUMAN−619−S−S2_−0.73
ptm abundance 37C
Doses Blue Rep1_−0.73 Red Rep2_−0.72

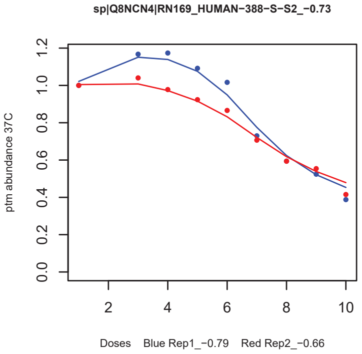
sp|Q8NCN4|RN169_HUMAN−388−S−S2_−0.73
ptm abundance 37C
Doses Blue Rep1_−0.79 Red Rep2_−0.66

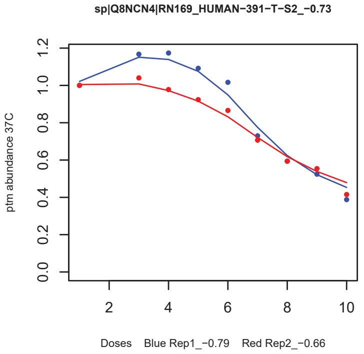
sp|Q8NCN4|RN169_HUMAN−391−T−S2_−0.73
ptm abundance 37C
Doses Blue Rep1_−0.79 Red Rep2_−0.66

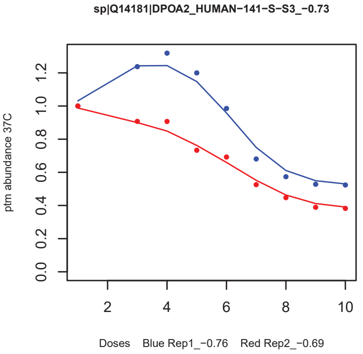
sp|Q14181|DPOA2_HUMAN−141−S−S3_−0.73
ptm abundance 37C
Doses Blue Rep1_−0.76 Red Rep2_−0.69

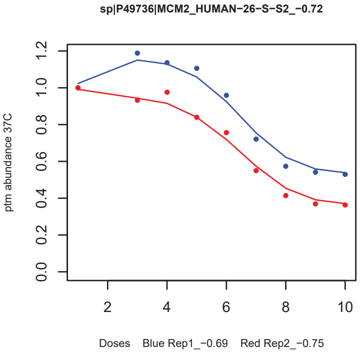
sp|P49736|MCM2_HUMAN−26−S−S2_−0.72
ptm abundance 37C
Doses Blue Rep1_−0.69 Red Rep2_−0.75

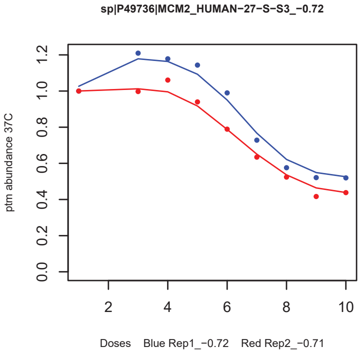
sp|P49736|MCM2_HUMAN−27−S−S3_−0.72
ptm abundance 37C
Doses Blue Rep1_−0.72 Red Rep2_−0.71

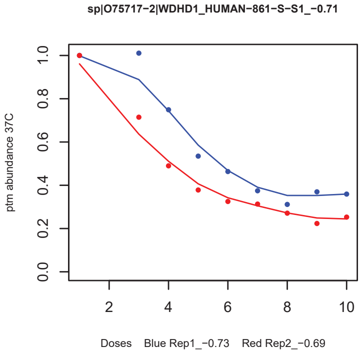
sp|O75717−2|WDHD1_HUMAN−861−S−S1_−0.71
ptm abundance 37C
Doses Blue Rep1_−0.73 Red Rep2_−0.69

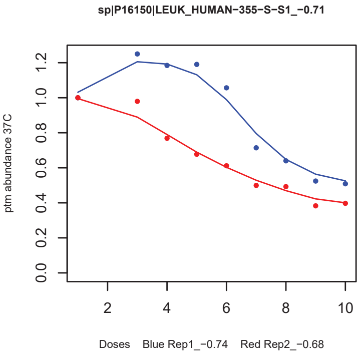
sp|P16150|LEUK_HUMAN−355−S−S1_−0.71
ptm abundance 37C
Doses Blue Rep1_−0.74 Red Rep2_−0.68

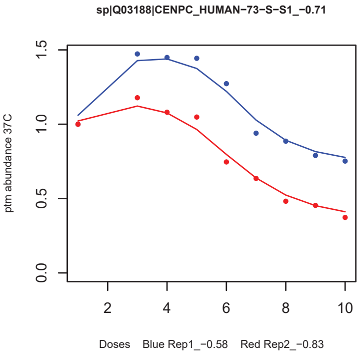
sp|Q03188|CENPC_HUMAN−73−S−S1_−0.71
ptm abundance 37C
Doses Blue Rep1_−0.58 Red Rep2_−0.83

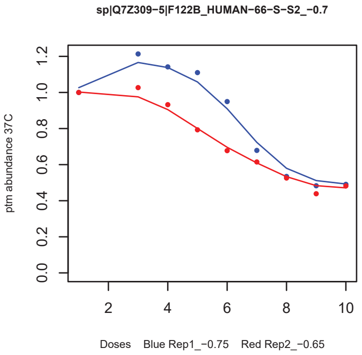
sp|Q7Z309−5|F122B_HUMAN−66−S−S2_−0.7
ptm abundance 37C
Doses Blue Rep1_−0.75 Red Rep2_−0.65

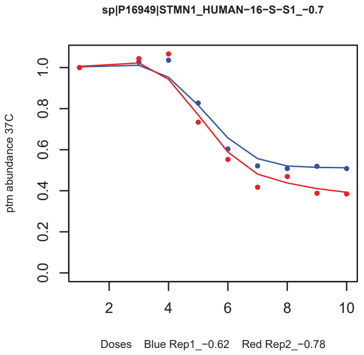
sp|P16949|STMN1_HUMAN−16−S−S1_−0.7
ptm abundance 37C
Doses Blue Rep1_−0.62 Red Rep2_−0.78

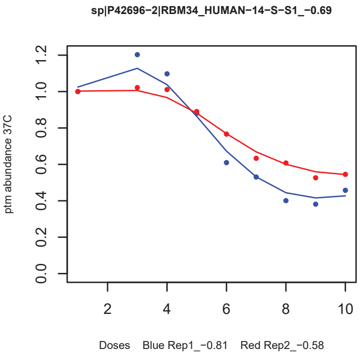
sp|P42696−2|RBM34_HUMAN−14−S−S1_−0.69
ptm abundance 37C
Doses Blue Rep1_−0.81 Red Rep2_−0.58

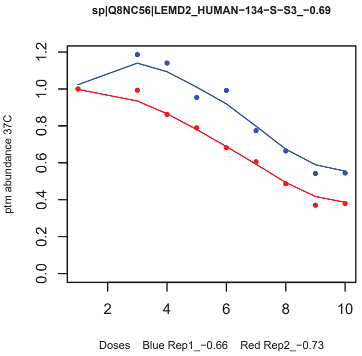
sp|Q8NC56|LEMD2_HUMAN−134−S−S3_−0.69
ptm abundance 37C
Doses Blue Rep1_−0.66 Red Rep2_−0.73

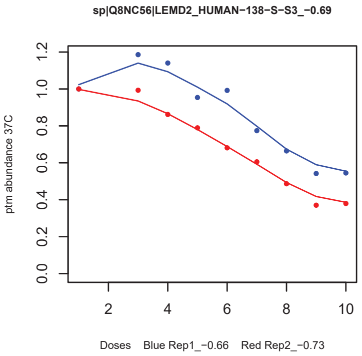
sp|Q8NC56|LEMD2_HUMAN−138−S−S3_−0.69
ptm abundance 37C
Doses Blue Rep1_−0.66 Red Rep2_−0.73

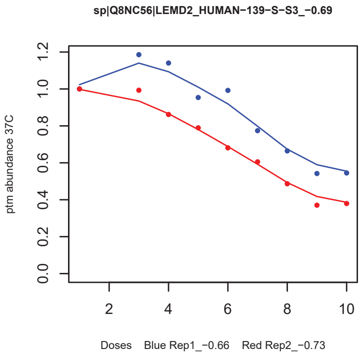
sp|Q8NC56|LEMD2_HUMAN−139−S−S3_−0.69
ptm abundance 37C
Doses Blue Rep1_−0.66 Red Rep2_−0.73

sp|Q9Y520−6|PRC2C_HUMAN−2617−T−S2_−0.69

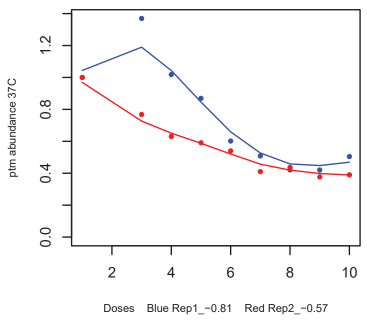

sp|Q9Y520−6|PRC2C_HUMAN−2621−S−S2_−0.69

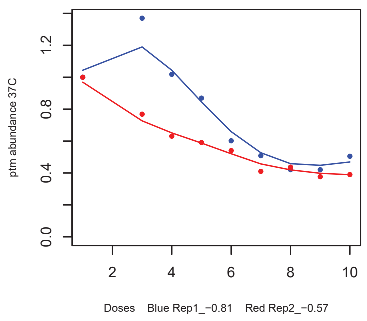

sp|Q9H1E3|NUCKS_HUMAN−179−T−S2_−0.69

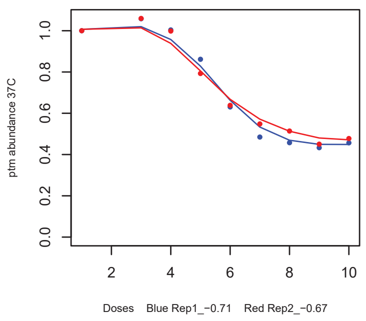

sp|Q9H1E3|NUCKS_HUMAN−181−S−S2_−0.69

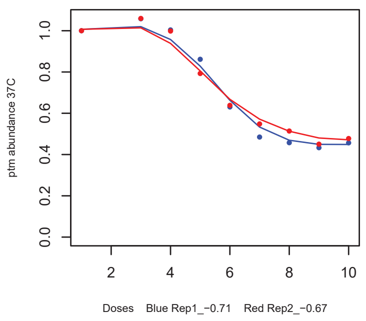

sp|Q14C86−3|GAPD1_HUMAN−758−S−S2_−0.69

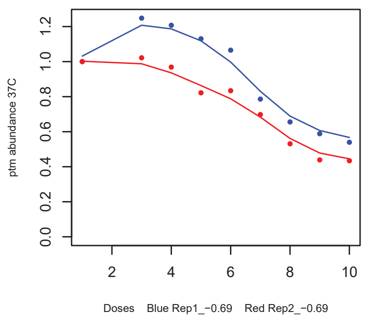

sp|O94913|PCF11_HUMAN−515−T−S2_−0.69

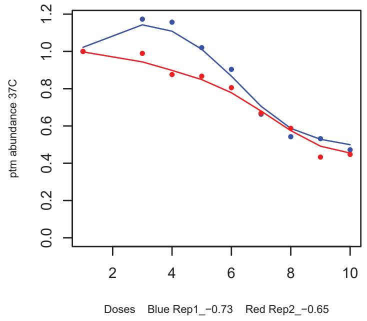

sp|P06400|RB_HUMAN−807−S−S1_−0.69

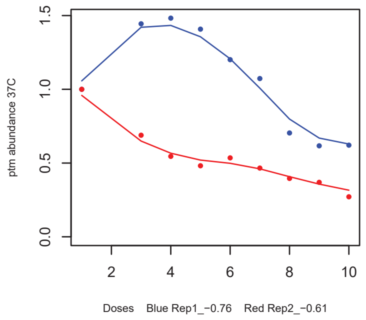

sp|Q14181|DPOA2_HUMAN−149−S−S3_−0.69

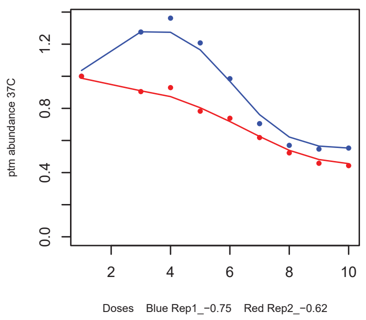

sp|Q9UQC2−2|GAB2_HUMAN−367−S−S2_−0.68

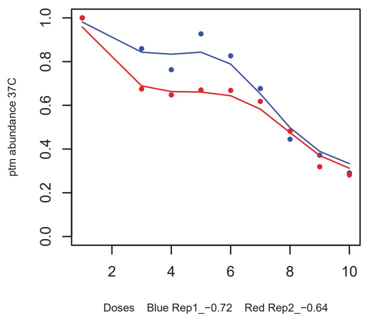

sp|P52594−2|AGFG1_HUMAN−181−S−S1_−0.68

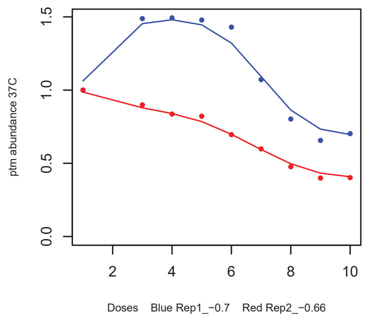

sp|Q14739|LBR_HUMAN−71−S−S1_−0.68

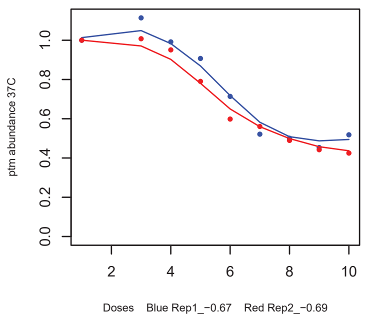

sp|Q6PKG0|LARP1_HUMAN−517−S−S2_−0.68

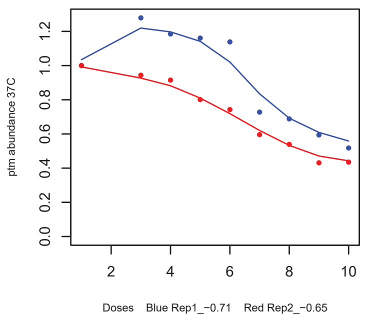

sp|Q6PKG0|LARP1_HUMAN−521−S−S2_−0.68

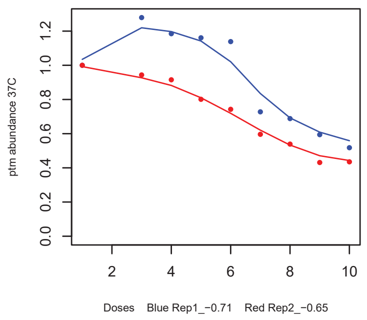

sp|Q9UHR4|BI2L1_HUMAN−257−T−S2_−0.68

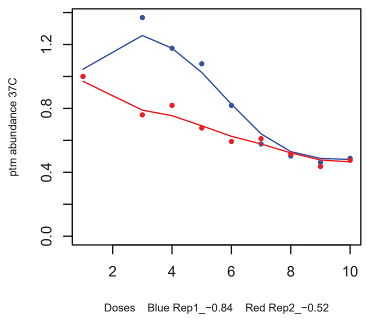

sp|Q14C86−3|GAPD1_HUMAN−772−S−S2_−0.68

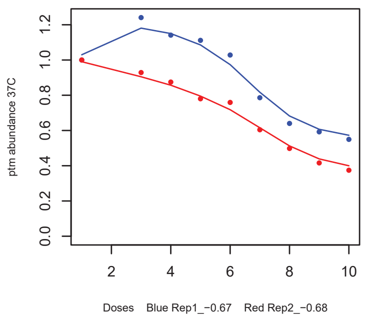

sp|O75122−3|CLAP2_HUMAN−771−S−S2_−0.67

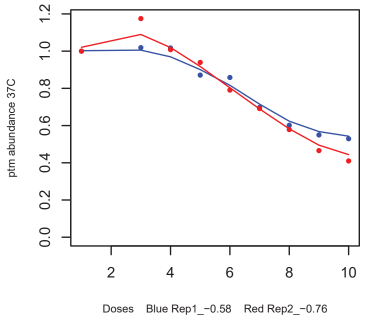

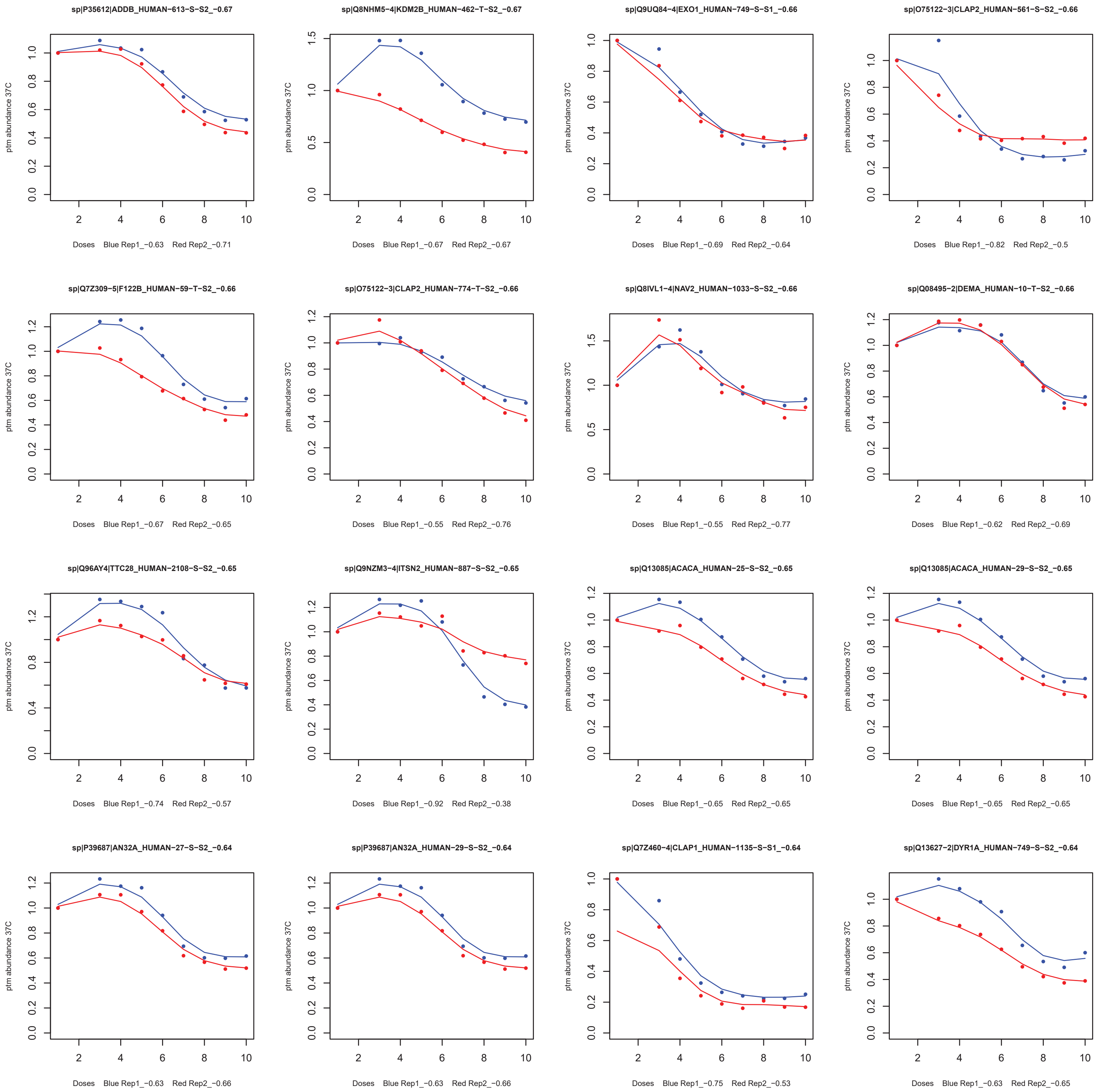
sp|P35612|ADDB_HUMAN−613−S−S2_−0.67
Doses Blue Rep1_−0.63 Red Rep2_−0.71
sp|Q8NHM5−4|KDM2B_HUMAN−462−T−S2_−0.67
Doses Blue Rep1_−0.67 Red Rep2_−0.67
sp|Q9UQ84−4|EXO1_HUMAN−749−S−S1_−0.66
Doses Blue Rep1_−0.69 Red Rep2_−0.64
sp|O75122−3|CLAP2_HUMAN−561−S−S2_−0.66
Doses Blue Rep1_−0.82 Red Rep2_−0.5
sp|Q7Z309−5|F122B_HUMAN−59−T−S2_−0.66
Doses Blue Rep1_−0.67 Red Rep2_−0.65
sp|O75122−3|CLAP2_HUMAN−774−T−S2_−0.66
Doses Blue Rep1_−0.55 Red Rep2_−0.76
sp|Q8IVL1−4|NAV2_HUMAN−1033−S−S2_−0.66
Doses Blue Rep1_−0.55 Red Rep2_−0.77
sp|Q08495−2|DEMA_HUMAN−10−T−S2_−0.66
Doses Blue Rep1_−0.62 Red Rep2_−0.69
sp|Q96AY4|TTC28_HUMAN−2108−S−S2_−0.65
Doses Blue Rep1_−0.74 Red Rep2_−0.57
sp|Q9NZM3−4|ITSN2_HUMAN−887−S−S2_−0.65
Doses Blue Rep1_−0.92 Red Rep2_−0.38
sp|Q13085|ACACA_HUMAN−25−S−S2_−0.65
Doses Blue Rep1_−0.65 Red Rep2_−0.65
sp|Q13085|ACACA_HUMAN−29−S−S2_−0.65
Doses Blue Rep1_−0.65 Red Rep2_−0.65
sp|P39687|AN32A_HUMAN−27−S−S2_−0.64
Doses Blue Rep1_−0.63 Red Rep2_−0.66
sp|P39687|AN32A_HUMAN−29−S−S2_−0.64
Doses Blue Rep1_−0.63 Red Rep2_−0.66
sp|Q7Z460−4|CLAP1_HUMAN−1135−S−S1_−0.64
Doses Blue Rep1_−0.75 Red Rep2_−0.53
sp|Q13627−2|DYR1A_HUMAN−749−S−S2_−0.64
Doses Blue Rep1_−0.63 Red Rep2_−0.65
ptm abundance 37C

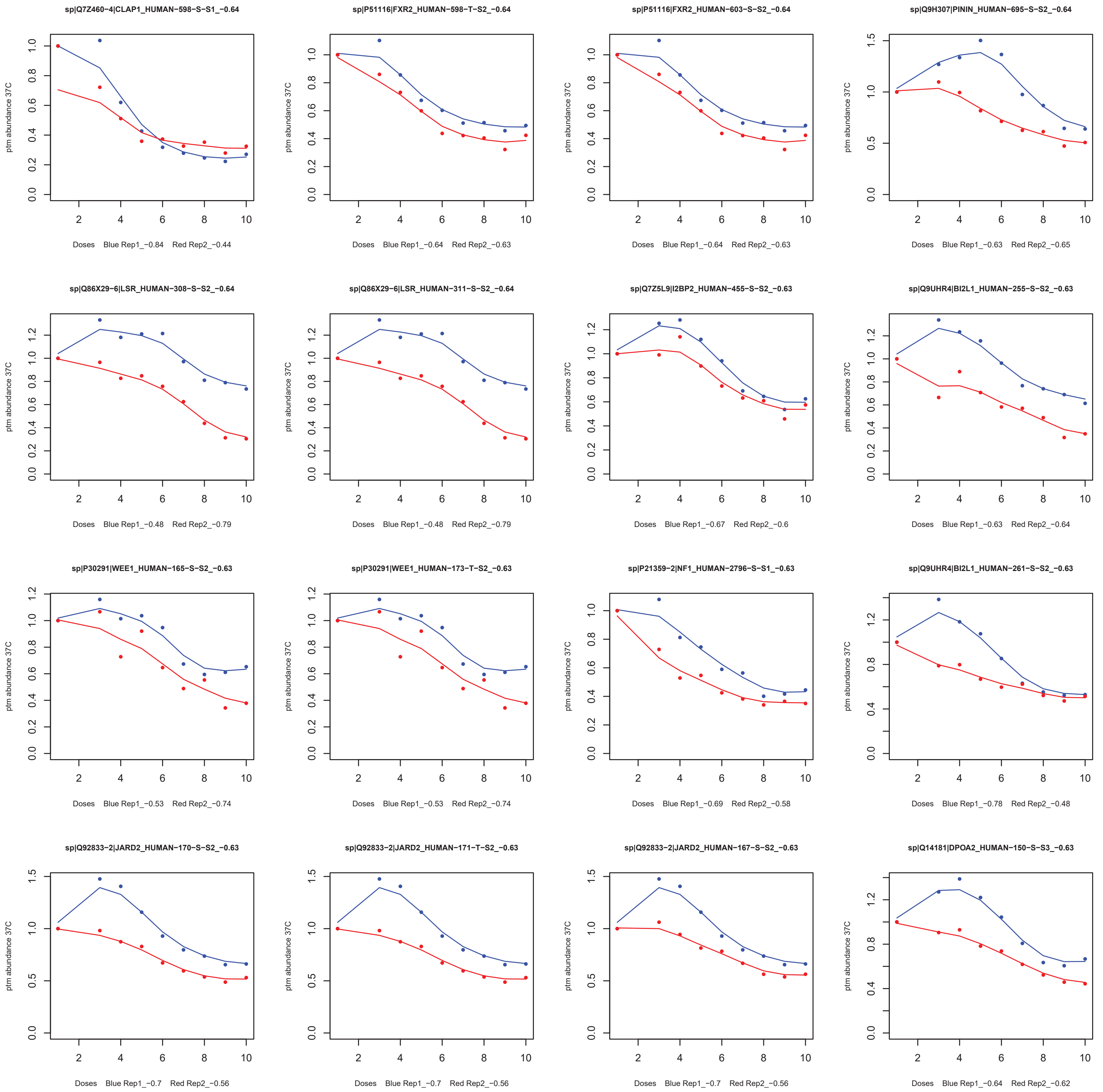
sp|Q7Z460-4|CLAP1_HUMAN-598-S-S1_-0.64
ptm abundance 37C
Doses Blue Rep1_-0.84 Red Rep2_-0.44
sp|P51116|FXR2_HUMAN-598-T-S2_-0.64
ptm abundance 37C
Doses Blue Rep1_-0.64 Red Rep2_-0.63
sp|P51116|FXR2_HUMAN-603-S-S2_-0.64
ptm abundance 37C
Doses Blue Rep1_-0.64 Red Rep2_-0.63
sp|Q9H307|PININ_HUMAN-695-S-S2_-0.64
ptm abundance 37C
Doses Blue Rep1_-0.63 Red Rep2_-0.65
sp|Q86X29-6|LSR_HUMAN-308-S-S2_-0.64
ptm abundance 37C
Doses Blue Rep1_-0.48 Red Rep2_-0.79
sp|Q86X29-6|LSR_HUMAN-311-S-S2_-0.64
ptm abundance 37C
Doses Blue Rep1_-0.48 Red Rep2_-0.79
sp|Q7Z5L9|I2BP2_HUMAN-455-S-S2_-0.63
ptm abundance 37C
Doses Blue Rep1_-0.67 Red Rep2_-0.6
sp|Q9UHR4|BI2L1_HUMAN-255-S-S2_-0.63
ptm abundance 37C
Doses Blue Rep1_-0.63 Red Rep2_-0.64
sp|P30291|WEE1_HUMAN-165-S-S2_-0.63
ptm abundance 37C
Doses Blue Rep1_-0.53 Red Rep2_-0.74
sp|P30291|WEE1_HUMAN-173-T-S2_-0.63
ptm abundance 37C
Doses Blue Rep1_-0.53 Red Rep2_-0.74
sp|P21359-2|NF1_HUMAN-2796-S-S1_-0.63
ptm abundance 37C
Doses Blue Rep1_-0.69 Red Rep2_-0.58
sp|Q9UHR4|BI2L1_HUMAN-261-S-S2_-0.63
ptm abundance 37C
Doses Blue Rep1_-0.78 Red Rep2_-0.48
sp|Q92833-2|JARD2_HUMAN-170-S-S2_-0.63
ptm abundance 37C
Doses Blue Rep1_-0.7 Red Rep2_-0.56
sp|Q92833-2|JARD2_HUMAN-171-T-S2_-0.63
ptm abundance 37C
Doses Blue Rep1_-0.7 Red Rep2_-0.56
sp|Q92833-2|JARD2_HUMAN-167-S-S2_-0.63
ptm abundance 37C
Doses Blue Rep1_-0.7 Red Rep2_-0.56
sp|Q14181|DPOA2_HUMAN-150-S-S3_-0.63
ptm abundance 37C
Doses Blue Rep1_-0.64 Red Rep2_-0.62

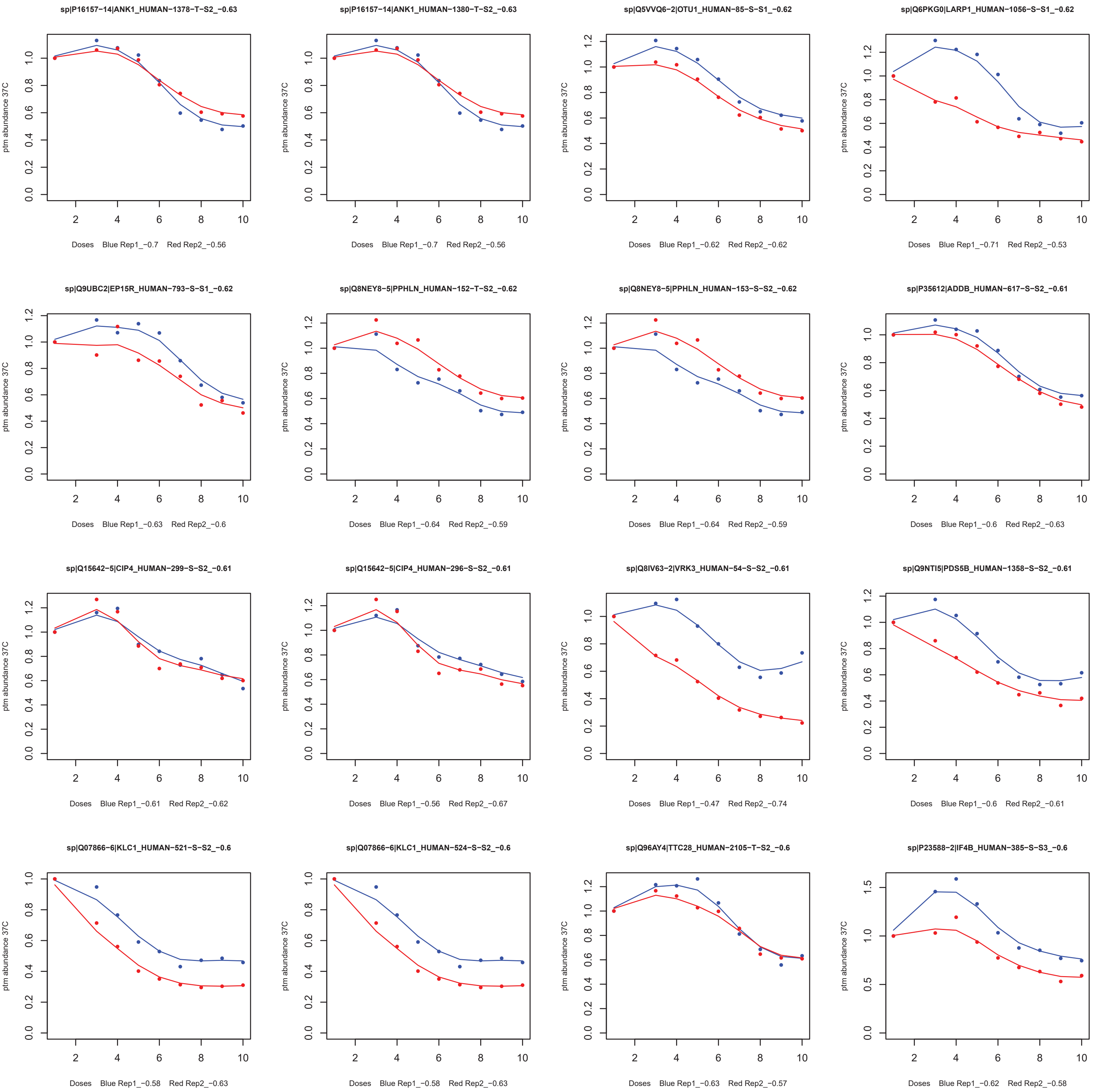
sp|P16157−14|ANK1_HUMAN−1378−T−S2_−0.63
ptm abundance 37C
Doses Blue Rep1_−0.7 Red Rep2_−0.56
sp|P16157−14|ANK1_HUMAN−1380−T−S2_−0.63
ptm abundance 37C
Doses Blue Rep1_−0.7 Red Rep2_−0.56
sp|Q5VVQ6−2|OTU1_HUMAN−85−S−S1_−0.62
ptm abundance 37C
Doses Blue Rep1_−0.62 Red Rep2_−0.62
sp|Q6PKG0|LARP1_HUMAN−1056−S−S1_−0.62
ptm abundance 37C
Doses Blue Rep1_−0.71 Red Rep2_−0.53
sp|Q9UBC2|EP15R_HUMAN−793−S−S1_−0.62
ptm abundance 37C
Doses Blue Rep1_−0.63 Red Rep2_−0.6
sp|Q8NEY8−5|PPHLN_HUMAN−152−T−S2_−0.62
ptm abundance 37C
Doses Blue Rep1_−0.64 Red Rep2_−0.59
sp|Q8NEY8−5|PPHLN_HUMAN−153−S−S2_−0.62
ptm abundance 37C
Doses Blue Rep1_−0.64 Red Rep2_−0.59
sp|P35612|ADDB_HUMAN−617−S−S2_−0.61
ptm abundance 37C
Doses Blue Rep1_−0.6 Red Rep2_−0.63
sp|Q15642−5|CIP4_HUMAN−299−S−S2_−0.61
ptm abundance 37C
Doses Blue Rep1_−0.61 Red Rep2_−0.62
sp|Q15642−5|CIP4_HUMAN−296−S−S2_−0.61
ptm abundance 37C
Doses Blue Rep1_−0.56 Red Rep2_−0.67
sp|Q8IV63−2|VRK3_HUMAN−54−S−S2_−0.61
ptm abundance 37C
Doses Blue Rep1_−0.47 Red Rep2_−0.74
sp|Q9NTI5|PDS5B_HUMAN−1358−S−S2_−0.61
ptm abundance 37C
Doses Blue Rep1_−0.6 Red Rep2_−0.61
sp|Q07866−6|KLC1_HUMAN−521−S−S2_−0.6
ptm abundance 37C
Doses Blue Rep1_−0.58 Red Rep2_−0.63
sp|Q07866−6|KLC1_HUMAN−524−S−S2_−0.6
ptm abundance 37C
Doses Blue Rep1_−0.58 Red Rep2_−0.63
sp|Q96AY4|TTC28_HUMAN−2105−T−S2_−0.6
ptm abundance 37C
Doses Blue Rep1_−0.63 Red Rep2_−0.57
sp|P23588−2|IF4B_HUMAN−385−S−S3_−0.6
ptm abundance 37C
Doses Blue Rep1_−0.62 Red Rep2_−0.58

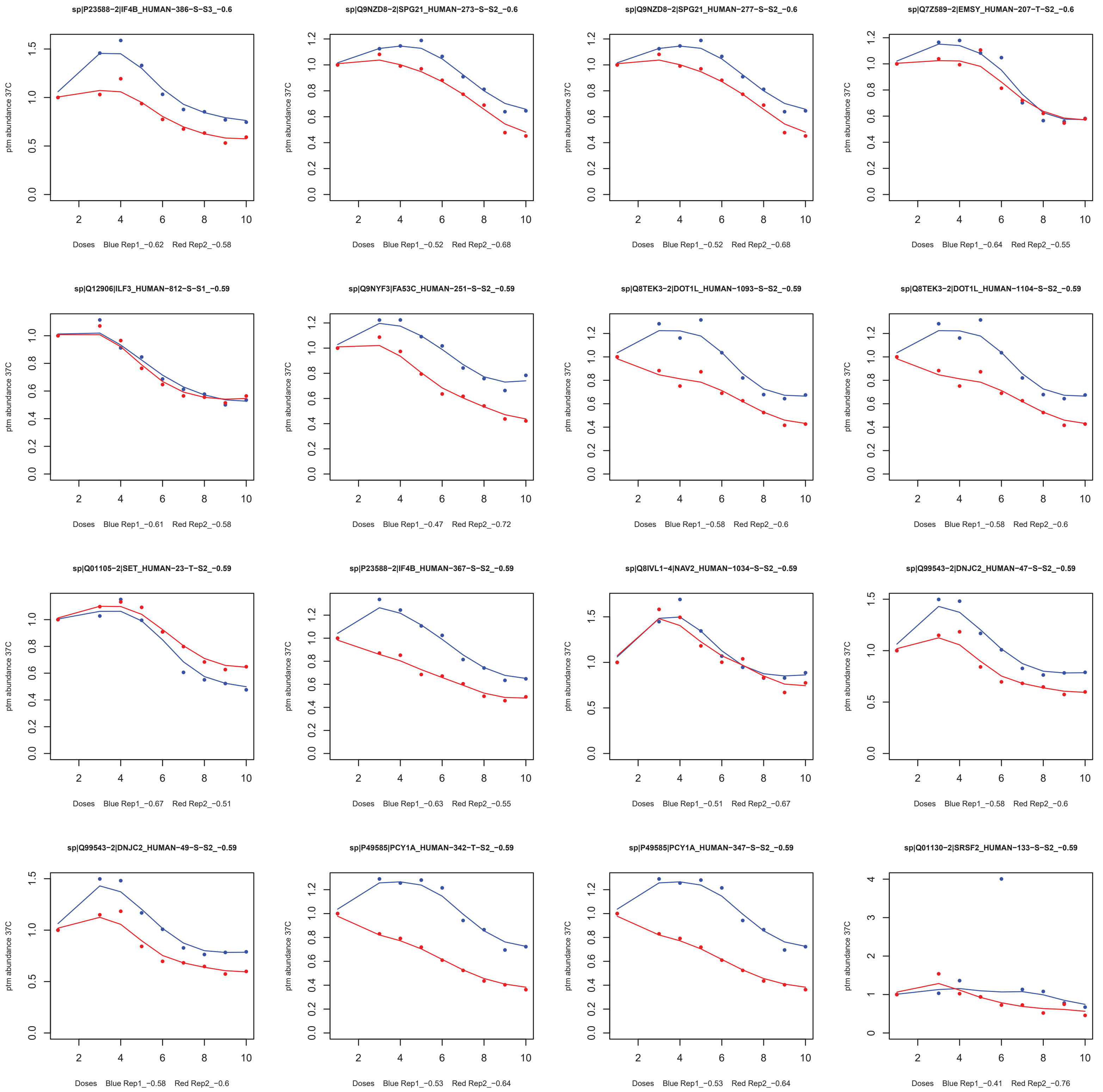

sp|P23588−2|IF4B_HUMAN−386−S−S3_−0.6
Doses Blue Rep1_−0.62 Red Rep2_−0.58
sp|Q9NZD8−2|SPG21_HUMAN−273−S−S2_−0.6
Doses Blue Rep1_−0.52 Red Rep2_−0.68
sp|Q9NZD8−2|SPG21_HUMAN−277−S−S2_−0.6
Doses Blue Rep1_−0.52 Red Rep2_−0.68
sp|Q7Z589−2|EMSY_HUMAN−207−T−S2_−0.6
Doses Blue Rep1_−0.64 Red Rep2_−0.55
sp|Q12906|ILF3_HUMAN−812−S−S1_−0.59
Doses Blue Rep1_−0.61 Red Rep2_−0.58
sp|Q9NYF3|FA53C_HUMAN−251−S−S2_−0.59
Doses Blue Rep1_−0.47 Red Rep2_−0.72
sp|Q8TEK3−2|DOT1L_HUMAN−1093−S−S2_−0.59
Doses Blue Rep1_−0.58 Red Rep2_−0.6
sp|Q8TEK3−2|DOT1L_HUMAN−1104−S−S2_−0.59
Doses Blue Rep1_−0.58 Red Rep2_−0.6
sp|Q01105−2|SET_HUMAN−23−T−S2_−0.59
Doses Blue Rep1_−0.67 Red Rep2_−0.51
sp|P23588−2|IF4B_HUMAN−367−S−S2_−0.59
Doses Blue Rep1_−0.63 Red Rep2_−0.55
sp|Q8IVL1−4|NAV2_HUMAN−1034−S−S2_−0.59
Doses Blue Rep1_−0.51 Red Rep2_−0.67
sp|Q99543−2|DNJC2_HUMAN−47−S−S2_−0.59
Doses Blue Rep1_−0.58 Red Rep2_−0.6
sp|Q99543−2|DNJC2_HUMAN−49−S−S2_−0.59
Doses Blue Rep1_−0.58 Red Rep2_−0.6
sp|P49585|PCY1A_HUMAN−342−T−S2_−0.59
Doses Blue Rep1_−0.53 Red Rep2_−0.64
sp|P49585|PCY1A_HUMAN−347−S−S2_−0.59
Doses Blue Rep1_−0.53 Red Rep2_−0.64
sp|Q01130−2|SRSF2_HUMAN−133−S−S2_−0.59
Doses Blue Rep1_−0.41 Red Rep2_−0.76
ptm abundance 37C

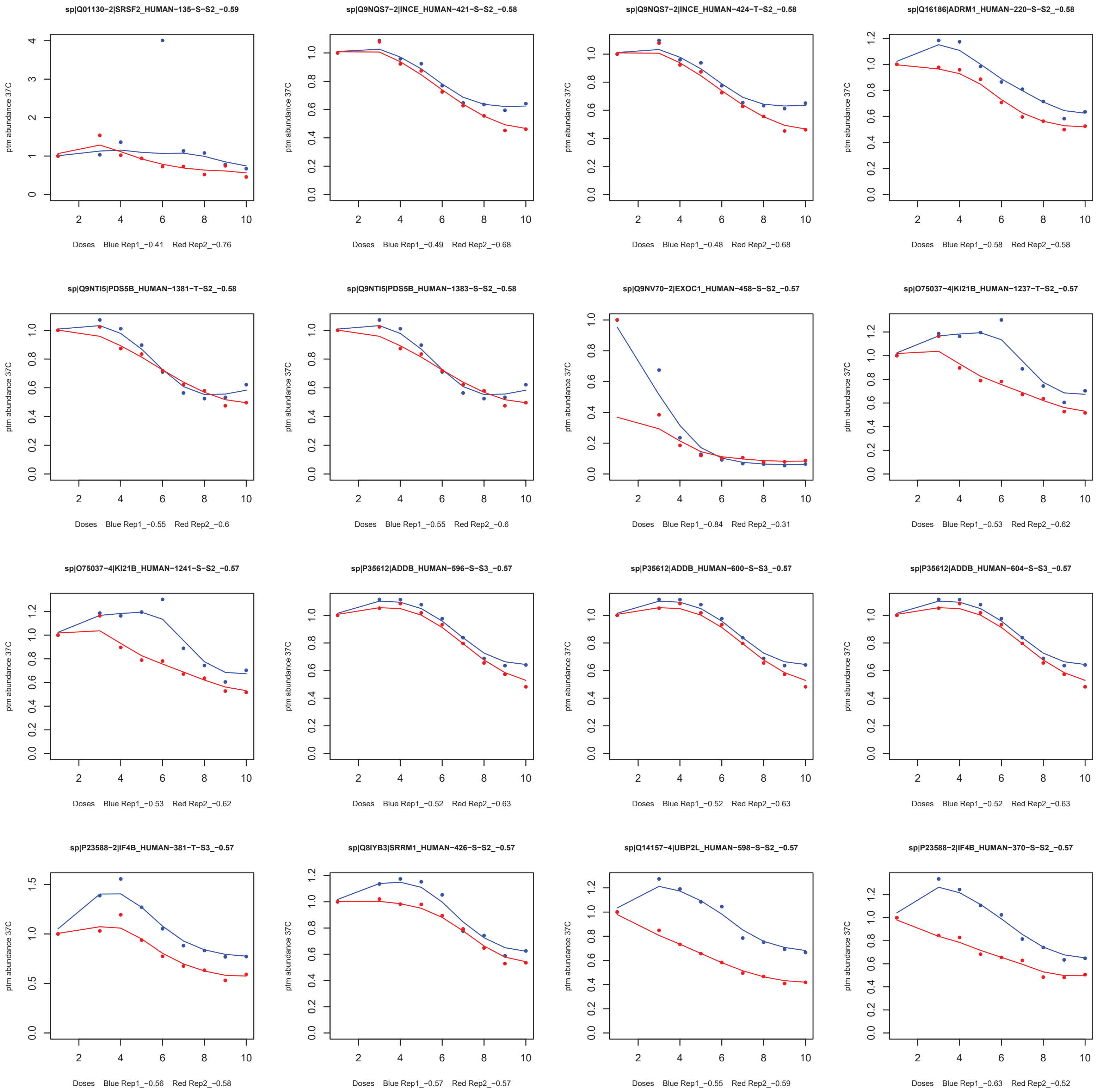

sp|Q01130−2|SRSF2_HUMAN−135−S−S2_−0.59
ptm abundance 37C
Doses Blue Rep1_−0.41 Red Rep2_−0.76
sp|Q9NQS7−2|INCE_HUMAN−421−S−S2_−0.58
ptm abundance 37C
Doses Blue Rep1_−0.49 Red Rep2_−0.68
sp|Q9NQS7−2|INCE_HUMAN−424−T−S2_−0.58
ptm abundance 37C
Doses Blue Rep1_−0.48 Red Rep2_−0.68
sp|Q16186|ADRM1_HUMAN−220−S−S2_−0.58
ptm abundance 37C
Doses Blue Rep1_−0.58 Red Rep2_−0.58
sp|Q9NTI5|PDS5B_HUMAN−1381−T−S2_−0.58
ptm abundance 37C
Doses Blue Rep1_−0.55 Red Rep2_−0.6
sp|Q9NTI5|PDS5B_HUMAN−1383−S−S2_−0.58
ptm abundance 37C
Doses Blue Rep1_−0.55 Red Rep2_−0.6
sp|Q9NV70−2|EXOC1_HUMAN−458−S−S2_−0.57
ptm abundance 37C
Doses Blue Rep1_−0.84 Red Rep2_−0.31
sp|O75037−4|KI21B_HUMAN−1237−T−S2_−0.57
ptm abundance 37C
Doses Blue Rep1_−0.53 Red Rep2_−0.62
sp|O75037−4|KI21B_HUMAN−1241−S−S2_−0.57
ptm abundance 37C
Doses Blue Rep1_−0.53 Red Rep2_−0.62
sp|P35612|ADDB_HUMAN−596−S−S3_−0.57
ptm abundance 37C
Doses Blue Rep1_−0.52 Red Rep2_−0.63
sp|P35612|ADDB_HUMAN−600−S−S3_−0.57
ptm abundance 37C
Doses Blue Rep1_−0.52 Red Rep2_−0.63
sp|P35612|ADDB_HUMAN−604−S−S3_−0.57
ptm abundance 37C
Doses Blue Rep1_−0.52 Red Rep2_−0.63
sp|P23588−2|IF4B_HUMAN−381−T−S3_−0.57
ptm abundance 37C
Doses Blue Rep1_−0.56 Red Rep2_−0.58
sp|Q8IYB3|SRRM1_HUMAN−426−S−S2_−0.57
ptm abundance 37C
Doses Blue Rep1_−0.57 Red Rep2_−0.57
sp|Q14157−4|UBP2L_HUMAN−598−S−S2_−0.57
ptm abundance 37C
Doses Blue Rep1_−0.55 Red Rep2_−0.59
sp|P23588−2|IF4B_HUMAN−370−S−S2_−0.57
ptm abundance 37C
Doses Blue Rep1_−0.63 Red Rep2_−0.52

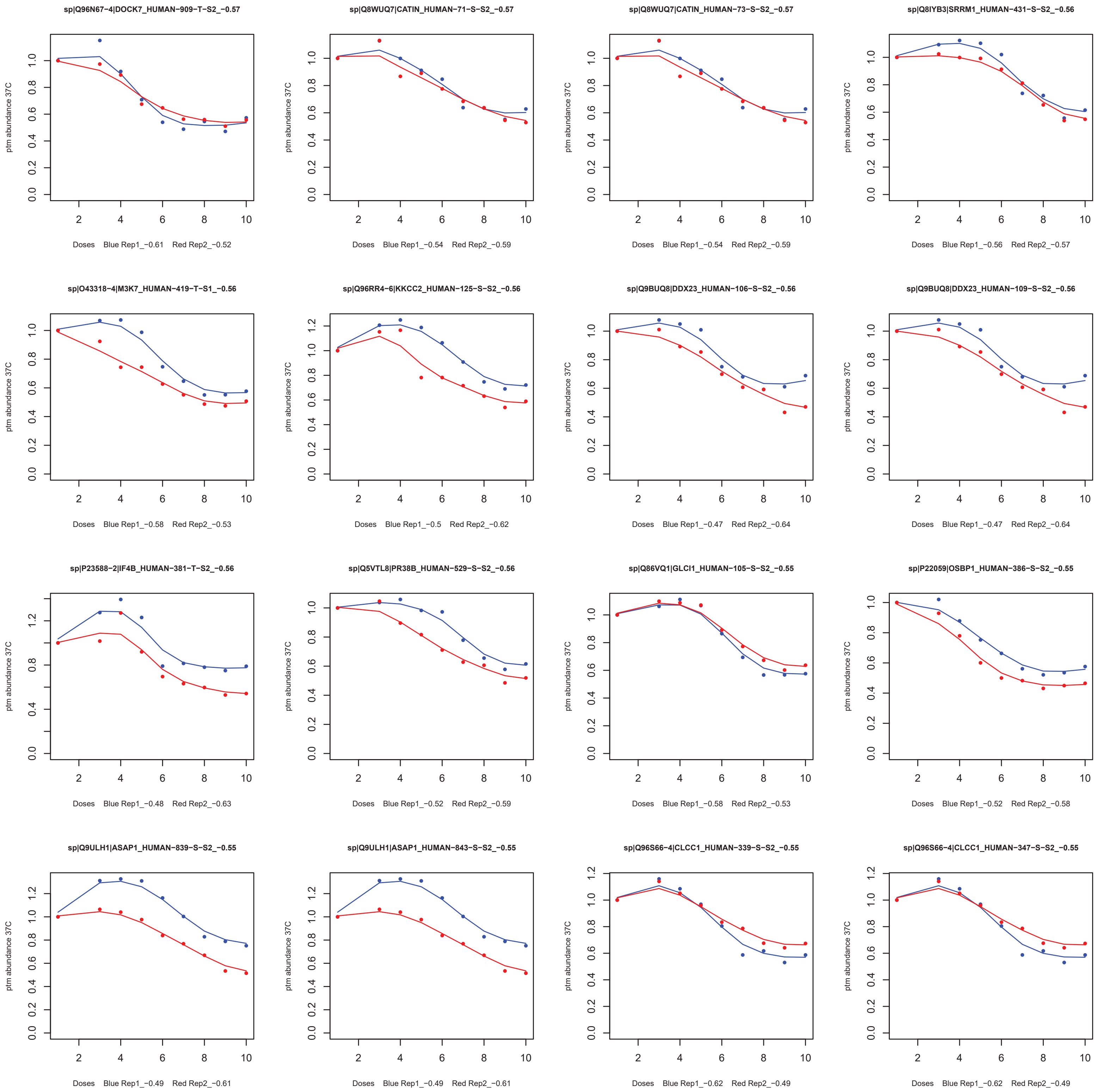
sp|Q96N67−4|DOCK7_HUMAN−909−T−S2_−0.57
ptm abundance 37C
Doses Blue Rep1_−0.61 Red Rep2_−0.52
sp|Q8WUQ7|CATIN_HUMAN−71−S−S2_−0.57
ptm abundance 37C
Doses Blue Rep1_−0.54 Red Rep2_−0.59
sp|Q8WUQ7|CATIN_HUMAN−73−S−S2_−0.57
ptm abundance 37C
Doses Blue Rep1_−0.54 Red Rep2_−0.59
sp|Q8IYB3|SRRM1_HUMAN−431−S−S2_−0.56
ptm abundance 37C
Doses Blue Rep1_−0.56 Red Rep2_−0.57
sp|O43318−4|M3K7_HUMAN−419−T−S1_−0.56
ptm abundance 37C
Doses Blue Rep1_−0.58 Red Rep2_−0.53
sp|Q96RR4−6|KKCC2_HUMAN−125−S−S2_−0.56
ptm abundance 37C
Doses Blue Rep1_−0.5 Red Rep2_−0.62
sp|Q9BUQ8|DDX23_HUMAN−106−S−S2_−0.56
ptm abundance 37C
Doses Blue Rep1_−0.47 Red Rep2_−0.64
sp|Q9BUQ8|DDX23_HUMAN−109−S−S2_−0.56
ptm abundance 37C
Doses Blue Rep1_−0.47 Red Rep2_−0.64
sp|P23588−2|IF4B_HUMAN−381−T−S2_−0.56
ptm abundance 37C
Doses Blue Rep1_−0.48 Red Rep2_−0.63
sp|Q5VTL8|PR38B_HUMAN−529−S−S2_−0.56
ptm abundance 37C
Doses Blue Rep1_−0.52 Red Rep2_−0.59
sp|Q86VQ1|GLCI1_HUMAN−105−S−S2_−0.55
ptm abundance 37C
Doses Blue Rep1_−0.58 Red Rep2_−0.53
sp|P22059|OSBP1_HUMAN−386−S−S2_−0.55
ptm abundance 37C
Doses Blue Rep1_−0.52 Red Rep2_−0.58
sp|Q9ULH1|ASAP1_HUMAN−839−S−S2_−0.55
ptm abundance 37C
Doses Blue Rep1_−0.49 Red Rep2_−0.61
sp|Q9ULH1|ASAP1_HUMAN−843−S−S2_−0.55
ptm abundance 37C
Doses Blue Rep1_−0.49 Red Rep2_−0.61
sp|Q96S66−4|CLCC1_HUMAN−339−S−S2_−0.55
ptm abundance 37C
Doses Blue Rep1_−0.62 Red Rep2_−0.49
sp|Q96S66−4|CLCC1_HUMAN−347−S−S2_−0.55
ptm abundance 37C
Doses Blue Rep1_−0.62 Red Rep2_−0.49

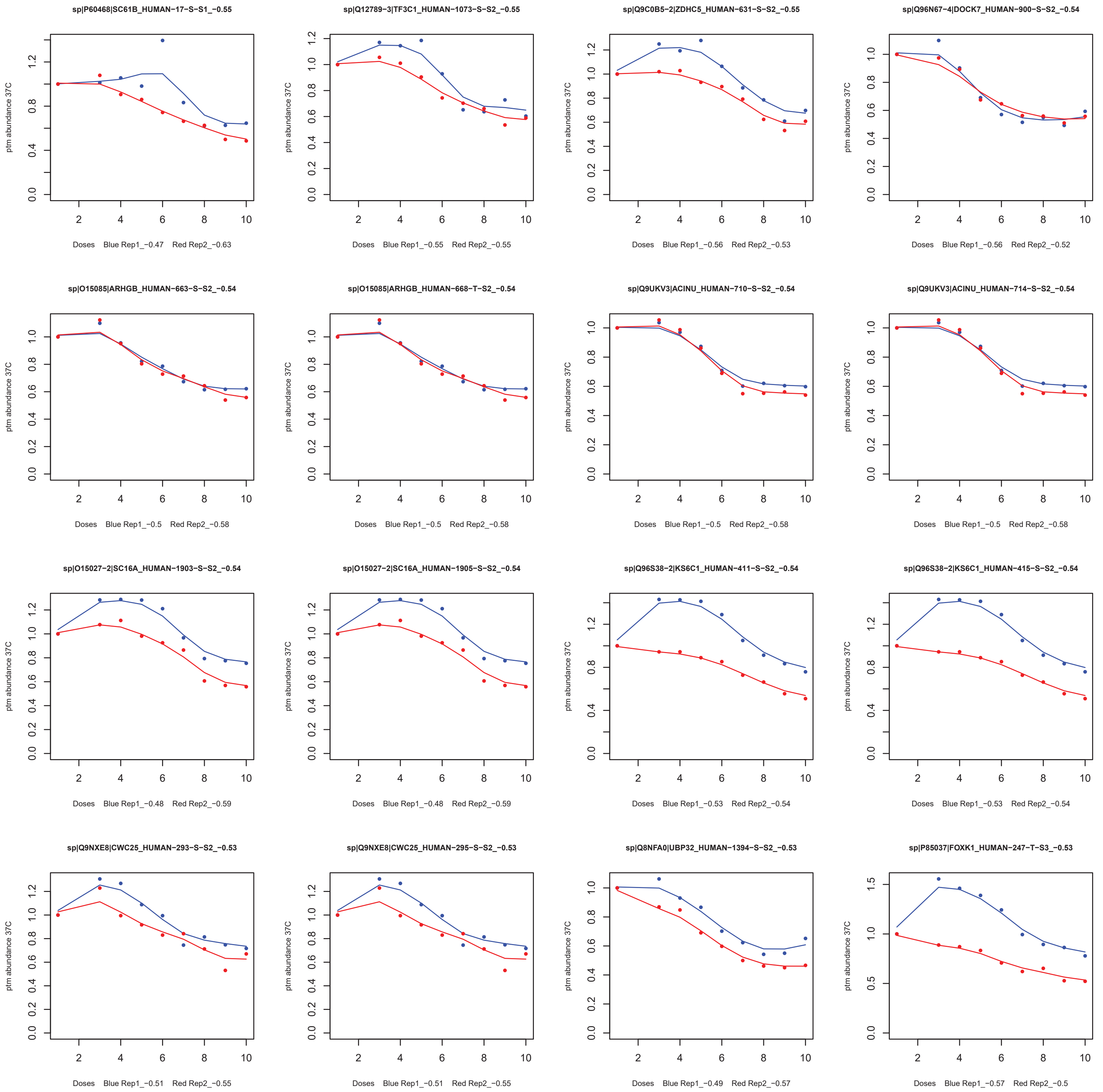

sp|P60468|SC61B_HUMAN−17−S−S1_−0.55
ptm abundance 37C
Doses Blue Rep1_−0.47 Red Rep2_−0.63
sp|Q12789−3|TF3C1_HUMAN−1073−S−S2_−0.55
ptm abundance 37C
Doses Blue Rep1_−0.55 Red Rep2_−0.55
sp|Q9C0B5−2|ZDHC5_HUMAN−631−S−S2_−0.55
ptm abundance 37C
Doses Blue Rep1_−0.56 Red Rep2_−0.53
sp|Q96N67−4|DOCK7_HUMAN−900−S−S2_−0.54
ptm abundance 37C
Doses Blue Rep1_−0.56 Red Rep2_−0.52
sp|O15085|ARHGB_HUMAN−663−S−S2_−0.54
ptm abundance 37C
Doses Blue Rep1_−0.5 Red Rep2_−0.58
sp|O15085|ARHGB_HUMAN−668−T−S2_−0.54
ptm abundance 37C
Doses Blue Rep1_−0.5 Red Rep2_−0.58
sp|Q9UKV3|ACINU_HUMAN−710−S−S2_−0.54
ptm abundance 37C
Doses Blue Rep1_−0.5 Red Rep2_−0.58
sp|Q9UKV3|ACINU_HUMAN−714−S−S2_−0.54
ptm abundance 37C
Doses Blue Rep1_−0.5 Red Rep2_−0.58
sp|O15027−2|SC16A_HUMAN−1903−S−S2_−0.54
ptm abundance 37C
Doses Blue Rep1_−0.48 Red Rep2_−0.59
sp|O15027−2|SC16A_HUMAN−1905−S−S2_−0.54
ptm abundance 37C
Doses Blue Rep1_−0.48 Red Rep2_−0.59
sp|Q96S38−2|KS6C1_HUMAN−411−S−S2_−0.54
ptm abundance 37C
Doses Blue Rep1_−0.53 Red Rep2_−0.54
sp|Q96S38−2|KS6C1_HUMAN−415−S−S2_−0.54
ptm abundance 37C
Doses Blue Rep1_−0.53 Red Rep2_−0.54
sp|Q9NXE8|CWC25_HUMAN−293−S−S2_−0.53
ptm abundance 37C
Doses Blue Rep1_−0.51 Red Rep2_−0.55
sp|Q9NXE8|CWC25_HUMAN−295−S−S2_−0.53
ptm abundance 37C
Doses Blue Rep1_−0.51 Red Rep2_−0.55
sp|Q8NFA0|UBP32_HUMAN−1394−S−S2_−0.53
ptm abundance 37C
Doses Blue Rep1_−0.49 Red Rep2_−0.57
sp|P85037|FOXK1_HUMAN−247−T−S3_−0.53
ptm abundance 37C
Doses Blue Rep1_−0.57 Red Rep2_−0.5

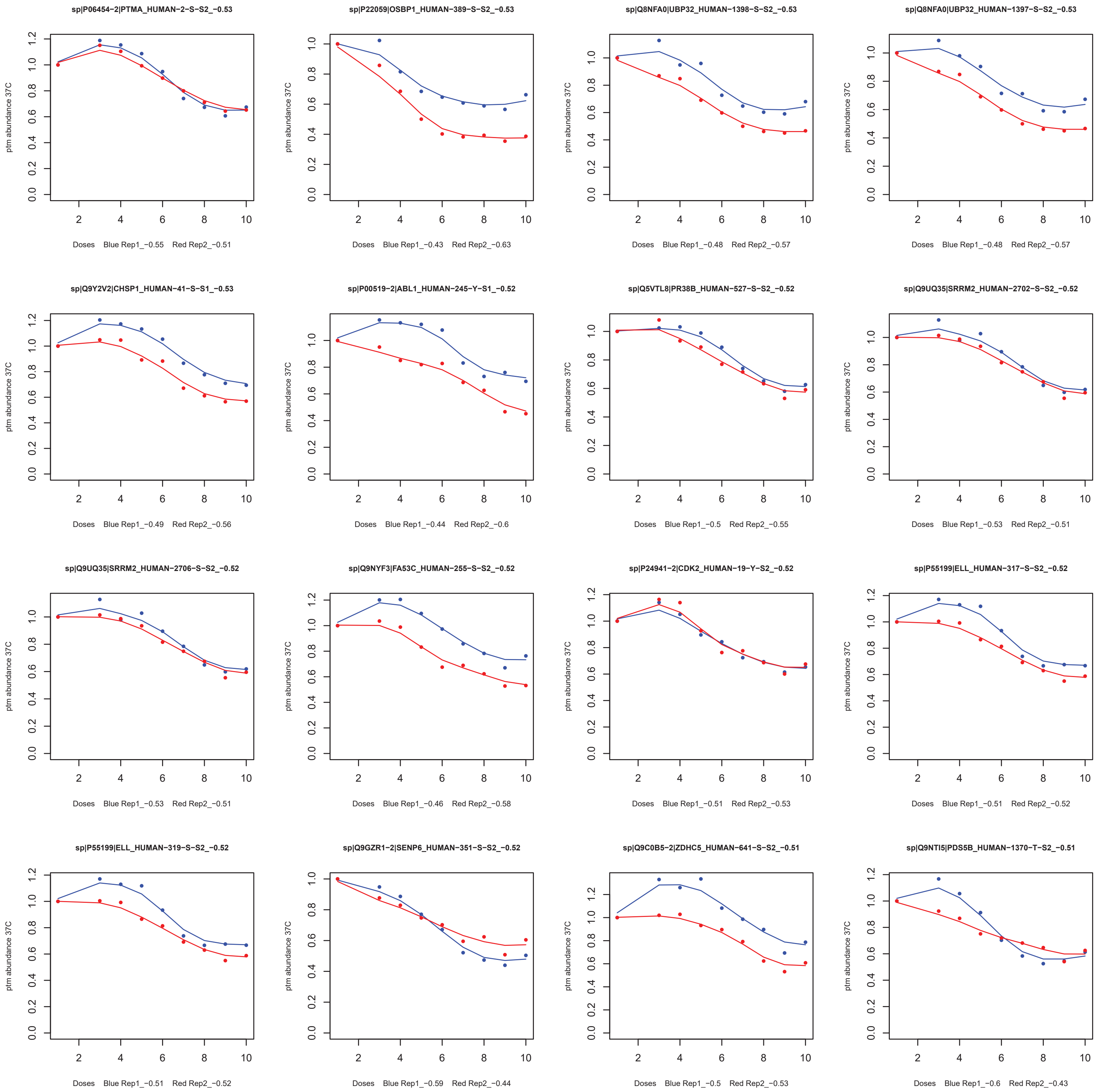

sp|P06454−2|PTMA_HUMAN−2−S−S2_−0.53
Doses Blue Rep1_−0.55 Red Rep2_−0.51
sp|P22059|OSBP1_HUMAN−389−S−S2_−0.53
Doses Blue Rep1_−0.43 Red Rep2_−0.63
sp|Q8NFA0|UBP32_HUMAN−1398−S−S2_−0.53
Doses Blue Rep1_−0.48 Red Rep2_−0.57
sp|Q8NFA0|UBP32_HUMAN−1397−S−S2_−0.53
Doses Blue Rep1_−0.48 Red Rep2_−0.57
sp|Q9Y2V2|CHSP1_HUMAN−41−S−S1_−0.53
Doses Blue Rep1_−0.49 Red Rep2_−0.56
sp|P00519−2|ABL1_HUMAN−245−Y−S1_−0.52
Doses Blue Rep1_−0.44 Red Rep2_−0.6
sp|Q5VTL8|PR38B_HUMAN−527−S−S2_−0.52
Doses Blue Rep1_−0.5 Red Rep2_−0.55
sp|Q9UQ35|SRRM2_HUMAN−2702−S−S2_−0.52
Doses Blue Rep1_−0.53 Red Rep2_−0.51
sp|Q9UQ35|SRRM2_HUMAN−2706−S−S2_−0.52
Doses Blue Rep1_−0.53 Red Rep2_−0.51
sp|Q9NYF3|FA53C_HUMAN−255−S−S2_−0.52
Doses Blue Rep1_−0.46 Red Rep2_−0.58
sp|P24941−2|CDK2_HUMAN−19−Y−S2_−0.52
Doses Blue Rep1_−0.51 Red Rep2_−0.53
sp|P55199|ELL_HUMAN−317−S−S2_−0.52
Doses Blue Rep1_−0.51 Red Rep2_−0.52
sp|P55199|ELL_HUMAN−319−S−S2_−0.52
Doses Blue Rep1_−0.51 Red Rep2_−0.52
sp|Q9GZR1−2|SENP6_HUMAN−351−S−S2_−0.52
Doses Blue Rep1_−0.59 Red Rep2_−0.44
sp|Q9C0B5−2|ZDHC5_HUMAN−641−S−S2_−0.51
Doses Blue Rep1_−0.5 Red Rep2_−0.53
sp|Q9NTI5|PDS5B_HUMAN−1370−T−S2_−0.51
Doses Blue Rep1_−0.6 Red Rep2_−0.43
ptm abundance 37C

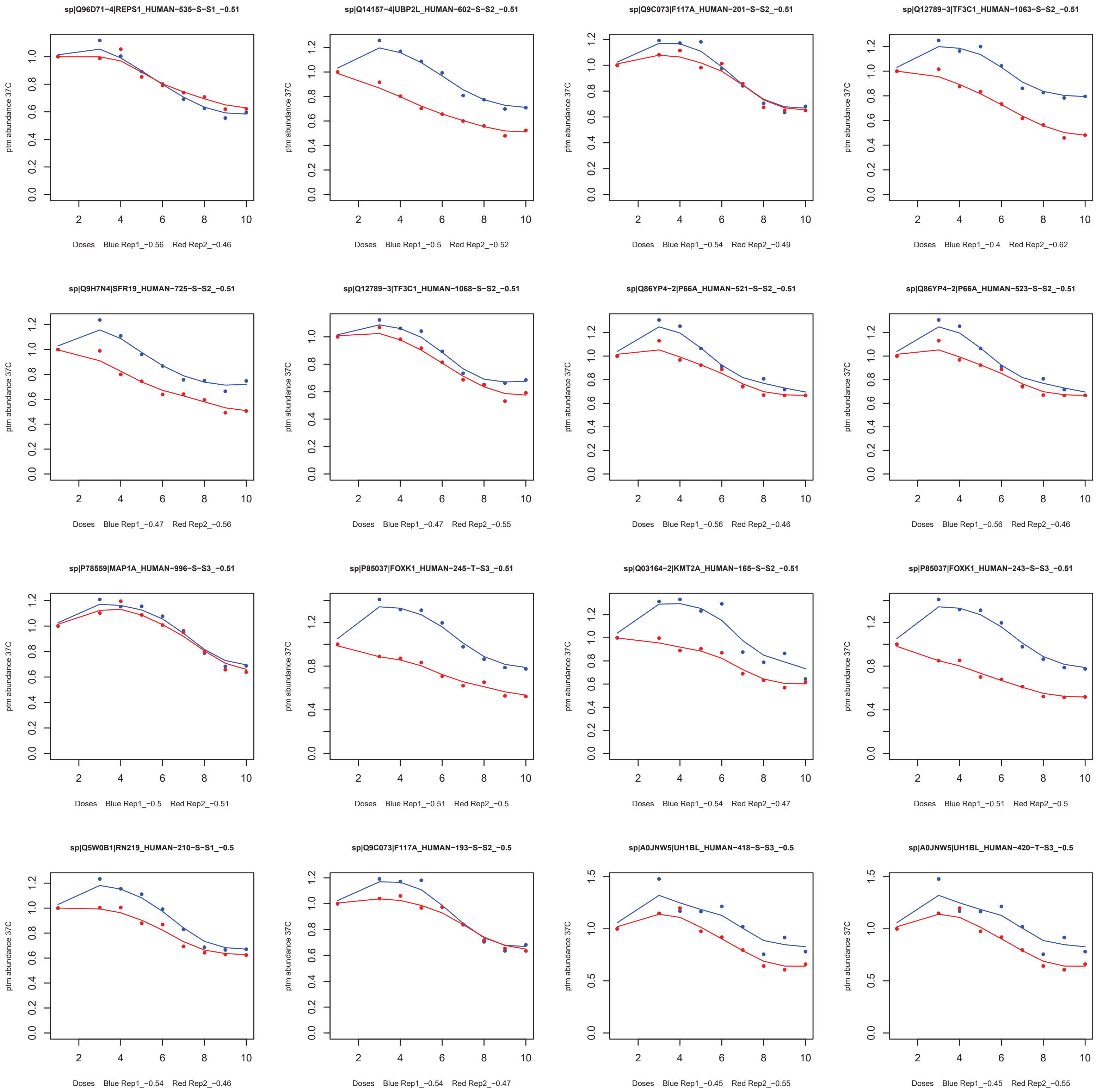
sp|Q96D71−4|REPS1_HUMAN−535−S−S1_−0.51
Doses Blue Rep1_−0.56 Red Rep2_−0.46
sp|Q14157−4|UBP2L_HUMAN−602−S−S2_−0.51
Doses Blue Rep1_−0.5 Red Rep2_−0.52
sp|Q9C073|F117A_HUMAN−201−S−S2_−0.51
Doses Blue Rep1_−0.54 Red Rep2_−0.49
sp|Q12789−3|TF3C1_HUMAN−1063−S−S2_−0.51
Doses Blue Rep1_−0.4 Red Rep2_−0.62
sp|Q9H7N4|SFR19_HUMAN−725−S−S2_−0.51
Doses Blue Rep1_−0.47 Red Rep2_−0.56
sp|Q12789−3|TF3C1_HUMAN−1068−S−S2_−0.51
Doses Blue Rep1_−0.47 Red Rep2_−0.55
sp|Q86YP4−2|P66A_HUMAN−521−S−S2_−0.51
Doses Blue Rep1_−0.56 Red Rep2_−0.46
sp|Q86YP4−2|P66A_HUMAN−523−S−S2_−0.51
Doses Blue Rep1_−0.56 Red Rep2_−0.46
sp|P78559|MAP1A_HUMAN−996−S−S3_−0.51
Doses Blue Rep1_−0.5 Red Rep2_−0.51
sp|P85037|FOXK1_HUMAN−245−T−S3_−0.51
Doses Blue Rep1_−0.51 Red Rep2_−0.5
sp|Q03164−2|KMT2A_HUMAN−165−S−S2_−0.51
Doses Blue Rep1_−0.54 Red Rep2_−0.47
sp|P85037|FOXK1_HUMAN−243−S−S3_−0.51
Doses Blue Rep1_−0.51 Red Rep2_−0.5
sp|Q5W0B1|RN219_HUMAN−210−S−S1_−0.5
Doses Blue Rep1_−0.54 Red Rep2_−0.46
sp|Q9C073|F117A_HUMAN−193−S−S2_−0.5
Doses Blue Rep1_−0.54 Red Rep2_−0.47
sp|A0JNW5|UH1BL_HUMAN−418−S−S3_−0.5
Doses Blue Rep1_−0.45 Red Rep2_−0.55
sp|A0JNW5|UH1BL_HUMAN−420−T−S3_−0.5
Doses Blue Rep1_−0.45 Red Rep2_−0.55
ptm abundance 37C

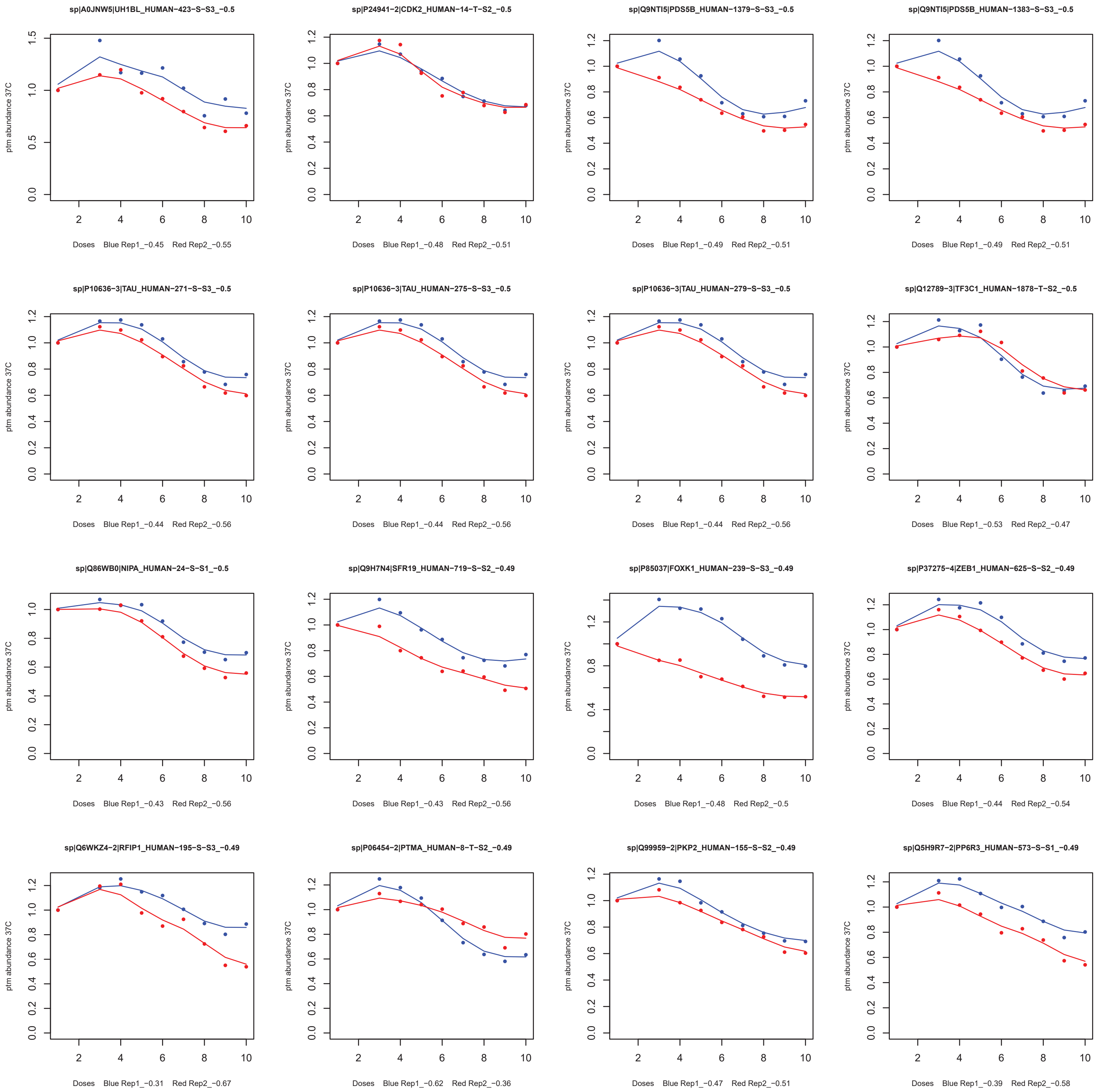
sp|A0JNW5|UH1BL_HUMAN−423−S−S3_−0.5
ptm abundance 37C
Doses Blue Rep1_−0.45 Red Rep2_−0.55
sp|P24941−2|CDK2_HUMAN−14−T−S2_−0.5
ptm abundance 37C
Doses Blue Rep1_−0.48 Red Rep2_−0.51
sp|Q9NTI5|PDS5B_HUMAN−1379−S−S3_−0.5
ptm abundance 37C
Doses Blue Rep1_−0.49 Red Rep2_−0.51
sp|Q9NTI5|PDS5B_HUMAN−1383−S−S3_−0.5
ptm abundance 37C
Doses Blue Rep1_−0.49 Red Rep2_−0.51
sp|P10636−3|TAU_HUMAN−271−S−S3_−0.5
ptm abundance 37C
Doses Blue Rep1_−0.44 Red Rep2_−0.56
sp|P10636−3|TAU_HUMAN−275−S−S3_−0.5
ptm abundance 37C
Doses Blue Rep1_−0.44 Red Rep2_−0.56
sp|P10636−3|TAU_HUMAN−279−S−S3_−0.5
ptm abundance 37C
Doses Blue Rep1_−0.44 Red Rep2_−0.56
sp|Q12789−3|TF3C1_HUMAN−1878−T−S2_−0.5
ptm abundance 37C
Doses Blue Rep1_−0.53 Red Rep2_−0.47
sp|Q86WB0|NIPA_HUMAN−24−S−S1_−0.5
ptm abundance 37C
Doses Blue Rep1_−0.43 Red Rep2_−0.56
sp|Q9H7N4|SFR19_HUMAN−719−S−S2_−0.49
ptm abundance 37C
Doses Blue Rep1_−0.43 Red Rep2_−0.56
sp|P85037|FOXK1_HUMAN−239−S−S3_−0.49
ptm abundance 37C
Doses Blue Rep1_−0.48 Red Rep2_−0.5
sp|P37275−4|ZEB1_HUMAN−625−S−S2_−0.49
ptm abundance 37C
Doses Blue Rep1_−0.44 Red Rep2_−0.54
sp|Q6WKZ4−2|RFIP1_HUMAN−195−S−S3_−0.49
ptm abundance 37C
Doses Blue Rep1_−0.31 Red Rep2_−0.67
sp|P06454−2|PTMA_HUMAN−8−T−S2_−0.49
ptm abundance 37C
Doses Blue Rep1_−0.62 Red Rep2_−0.36
sp|Q99959−2|PKP2_HUMAN−155−S−S2_−0.49
ptm abundance 37C
Doses Blue Rep1_−0.47 Red Rep2_−0.51
sp|Q5H9R7−2|PP6R3_HUMAN−573−S−S1_−0.49
ptm abundance 37C
Doses Blue Rep1_−0.39 Red Rep2_−0.58

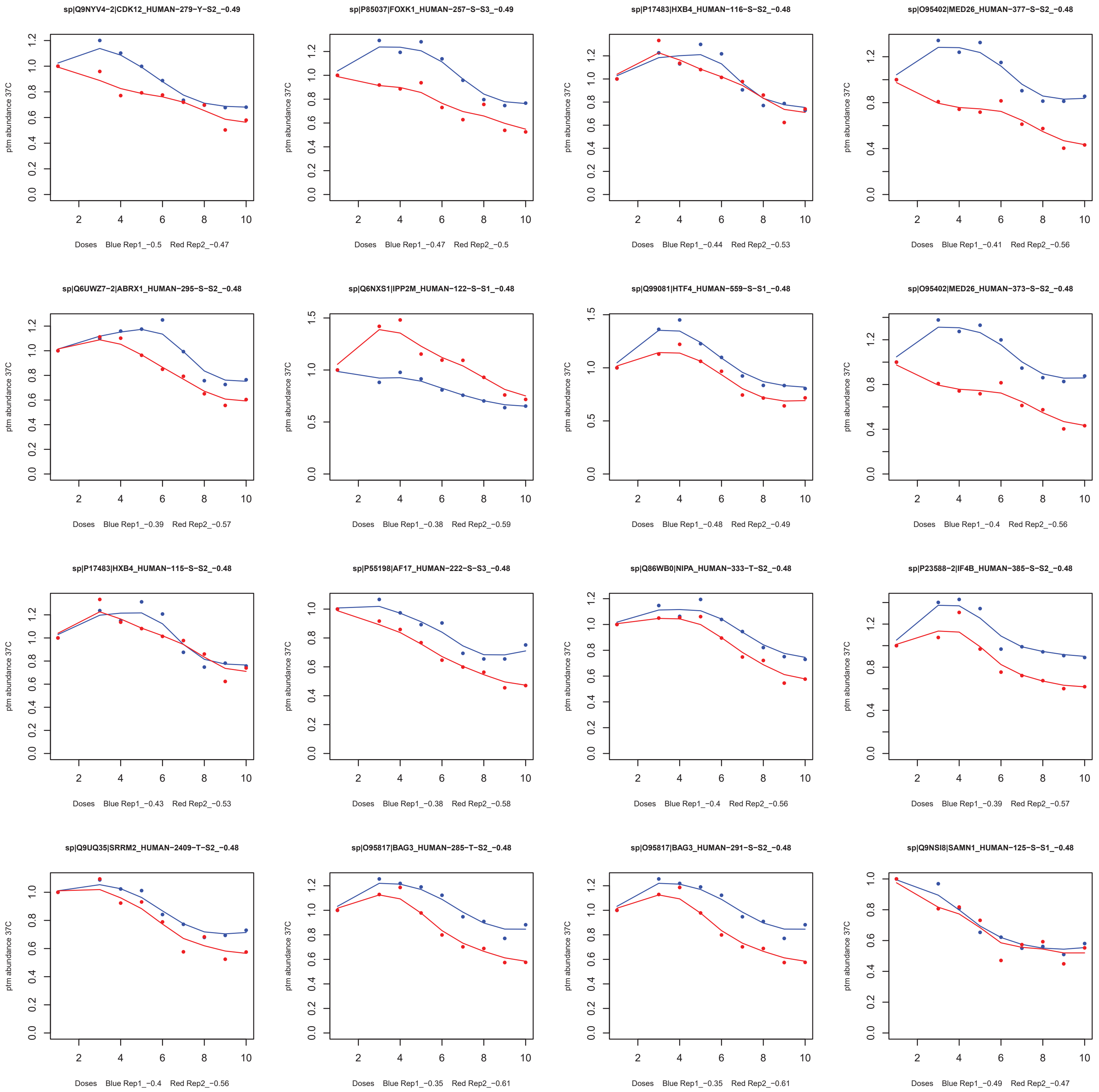

sp|Q9NYV4−2|CDK12_HUMAN−279−Y−S2_−0.49
Doses Blue Rep1_−0.5 Red Rep2_−0.47
sp|P85037|FOXK1_HUMAN−257−S−S3_−0.49
Doses Blue Rep1_−0.47 Red Rep2_−0.5
sp|P17483|HXB4_HUMAN−116−S−S2_−0.48
Doses Blue Rep1_−0.44 Red Rep2_−0.53
sp|O95402|MED26_HUMAN−377−S−S2_−0.48
Doses Blue Rep1_−0.41 Red Rep2_−0.56
sp|Q6UWZ7−2|ABRX1_HUMAN−295−S−S2_−0.48
Doses Blue Rep1_−0.39 Red Rep2_−0.57
sp|Q6NXS1|IPP2M_HUMAN−122−S−S1_−0.48
Doses Blue Rep1_−0.38 Red Rep2_−0.59
sp|Q99081|HTF4_HUMAN−559−S−S1_−0.48
Doses Blue Rep1_−0.48 Red Rep2_−0.49
sp|O95402|MED26_HUMAN−373−S−S2_−0.48
Doses Blue Rep1_−0.4 Red Rep2_−0.56
sp|P17483|HXB4_HUMAN−115−S−S2_−0.48
Doses Blue Rep1_−0.43 Red Rep2_−0.53
sp|P55198|AF17_HUMAN−222−S−S3_−0.48
Doses Blue Rep1_−0.38 Red Rep2_−0.58
sp|Q86WB0|NIPA_HUMAN−333−T−S2_−0.48
Doses Blue Rep1_−0.4 Red Rep2_−0.56
sp|P23588−2|IF4B_HUMAN−385−S−S2_−0.48
Doses Blue Rep1_−0.39 Red Rep2_−0.57
sp|Q9UQ35|SRRM2_HUMAN−2409−T−S2_−0.48
Doses Blue Rep1_−0.4 Red Rep2_−0.56
sp|O95817|BAG3_HUMAN−285−T−S2_−0.48
Doses Blue Rep1_−0.35 Red Rep2_−0.61
sp|O95817|BAG3_HUMAN−291−S−S2_−0.48
Doses Blue Rep1_−0.35 Red Rep2_−0.61
sp|Q9NSI8|SAMN1_HUMAN−125−S−S1_−0.48
Doses Blue Rep1_−0.49 Red Rep2_−0.47
ptm abundance 37C

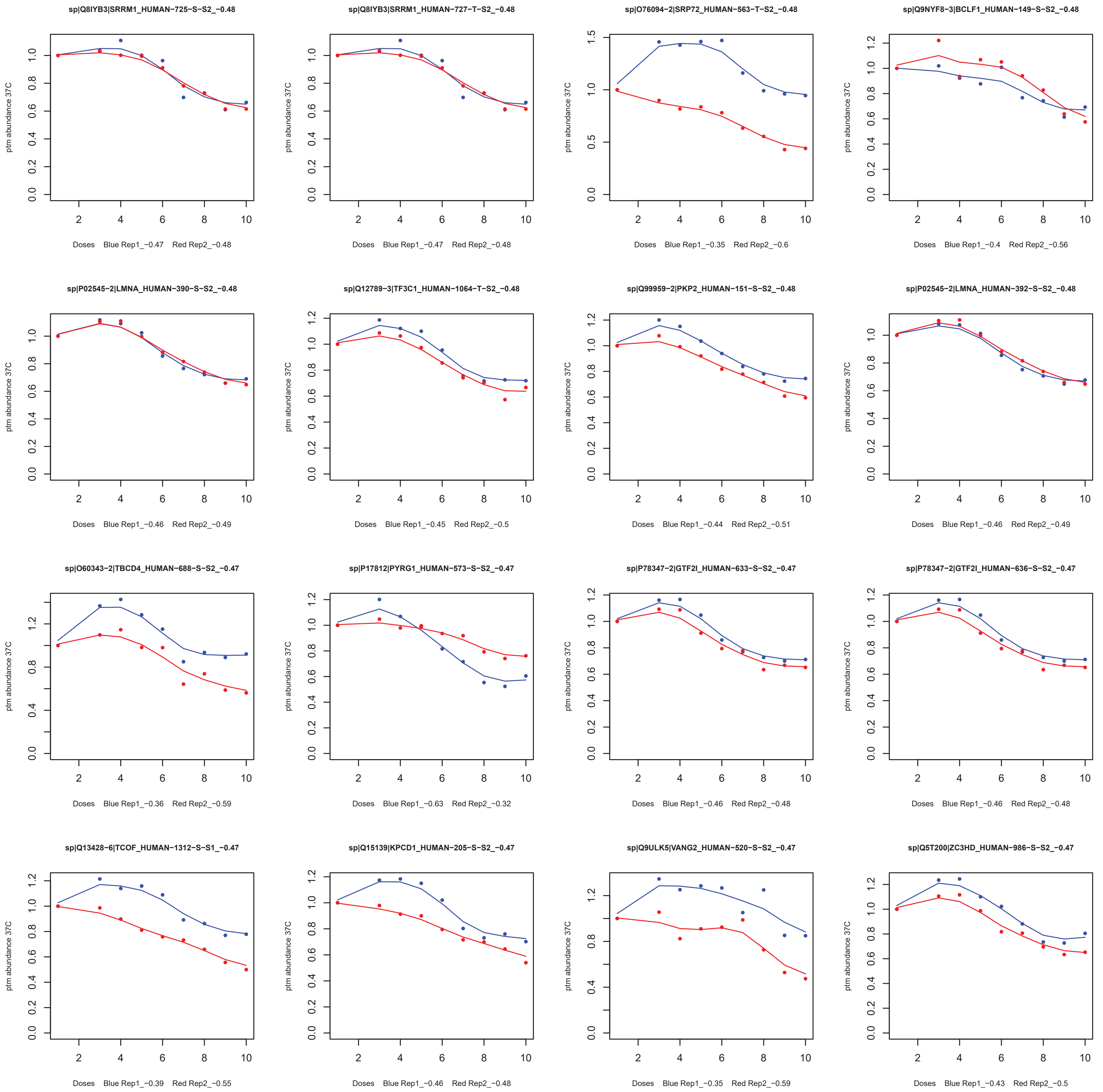
sp|Q8IYB3|SRRM1_HUMAN−725−S−S2_−0.48
ptm abundance 37C
Doses Blue Rep1_−0.47 Red Rep2_−0.48
sp|Q8IYB3|SRRM1_HUMAN−727−T−S2_−0.48
ptm abundance 37C
Doses Blue Rep1_−0.47 Red Rep2_−0.48
sp|O76094−2|SRP72_HUMAN−563−T−S2_−0.48
ptm abundance 37C
Doses Blue Rep1_−0.35 Red Rep2_−0.6
sp|Q9NYF8−3|BCLF1_HUMAN−149−S−S2_−0.48
ptm abundance 37C
Doses Blue Rep1_−0.4 Red Rep2_−0.56
sp|P02545−2|LMNA_HUMAN−390−S−S2_−0.48
ptm abundance 37C
Doses Blue Rep1_−0.46 Red Rep2_−0.49
sp|Q12789−3|TF3C1_HUMAN−1064−T−S2_−0.48
ptm abundance 37C
Doses Blue Rep1_−0.45 Red Rep2_−0.5
sp|Q99959−2|PKP2_HUMAN−151−S−S2_−0.48
ptm abundance 37C
Doses Blue Rep1_−0.44 Red Rep2_−0.51
sp|P02545−2|LMNA_HUMAN−392−S−S2_−0.48
ptm abundance 37C
Doses Blue Rep1_−0.46 Red Rep2_−0.49
sp|O60343−2|TBCD4_HUMAN−688−S−S2_−0.47
ptm abundance 37C
Doses Blue Rep1_−0.36 Red Rep2_−0.59
sp|P17812|PYRG1_HUMAN−573−S−S2_−0.47
ptm abundance 37C
Doses Blue Rep1_−0.63 Red Rep2_−0.32
sp|P78347−2|GTF2I_HUMAN−633−S−S2_−0.47
ptm abundance 37C
Doses Blue Rep1_−0.46 Red Rep2_−0.48
sp|P78347−2|GTF2I_HUMAN−636−S−S2_−0.47
ptm abundance 37C
Doses Blue Rep1_−0.46 Red Rep2_−0.48
sp|Q13428−6|TCOF_HUMAN−1312−S−S1_−0.47
ptm abundance 37C
Doses Blue Rep1_−0.39 Red Rep2_−0.55
sp|Q15139|KPCD1_HUMAN−205−S−S2_−0.47
ptm abundance 37C
Doses Blue Rep1_−0.46 Red Rep2_−0.48
sp|Q9ULK5|VANG2_HUMAN−520−S−S2_−0.47
ptm abundance 37C
Doses Blue Rep1_−0.35 Red Rep2_−0.59
sp|Q5T200|ZC3HD_HUMAN−986−S−S2_−0.47
ptm abundance 37C
Doses Blue Rep1_−0.43 Red Rep2_−0.5

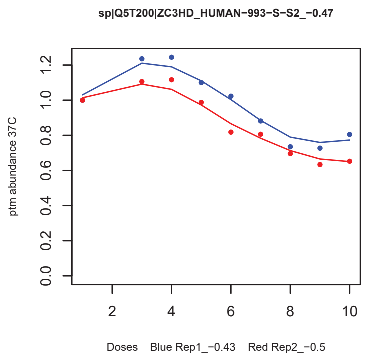
sp|Q5T200|ZC3HD_HUMAN−993−S−S2_−0.47
ptm abundance 37C
Doses Blue Rep1_−0.43 Red Rep2_−0.5

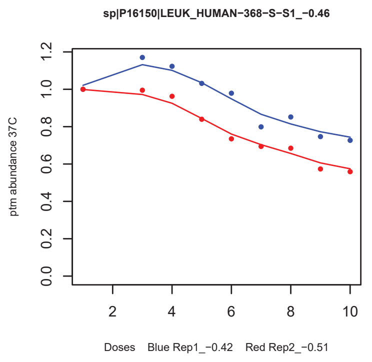
sp|P16150|LEUK_HUMAN−368−S−S1_−0.46
ptm abundance 37C
Doses Blue Rep1_−0.42 Red Rep2_−0.51

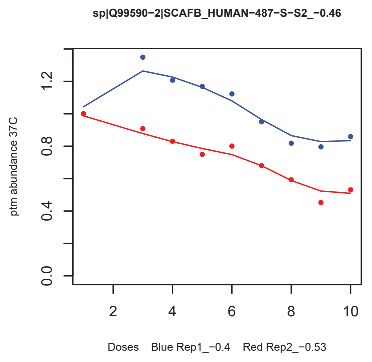
sp|Q99590−2|SCAFB_HUMAN−487−S−S2_−0.46
ptm abundance 37C
Doses Blue Rep1_−0.4 Red Rep2_−0.53

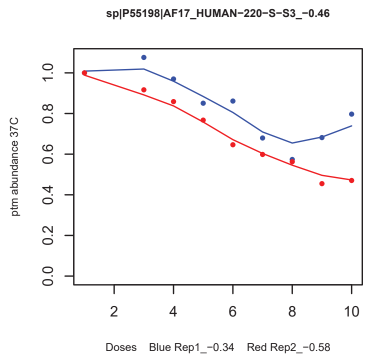
sp|P55198|AF17_HUMAN−220−S−S3_−0.46
ptm abundance 37C
Doses Blue Rep1_−0.34 Red Rep2_−0.58

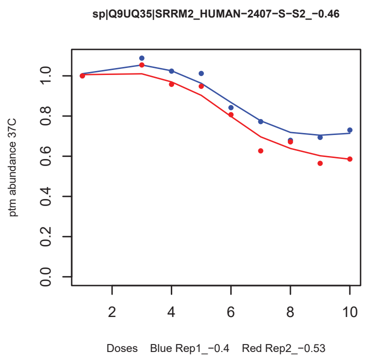
sp|Q9UQ35|SRRM2_HUMAN−2407−S−S2_−0.46
ptm abundance 37C
Doses Blue Rep1_−0.4 Red Rep2_−0.53

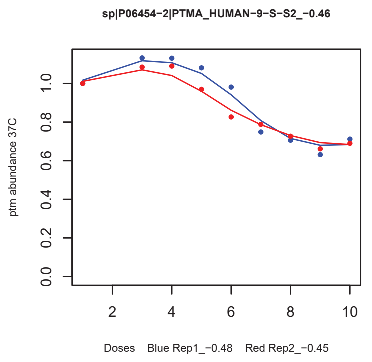
sp|P06454−2|PTMA_HUMAN−9−S−S2_−0.46
ptm abundance 37C
Doses Blue Rep1_−0.48 Red Rep2_−0.45

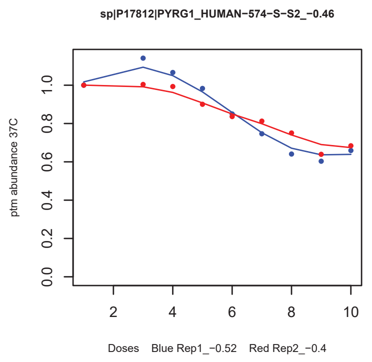
sp|P17812|PYRG1_HUMAN−574−S−S2_−0.46
ptm abundance 37C
Doses Blue Rep1_−0.52 Red Rep2_−0.4

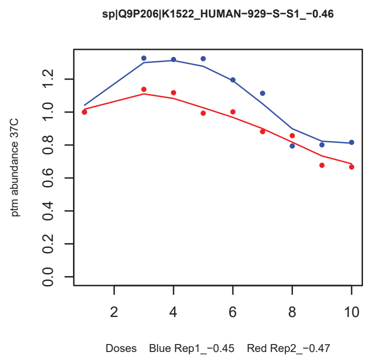
sp|Q9P206|K1522_HUMAN−929−S−S1_−0.46
ptm abundance 37C
Doses Blue Rep1_−0.45 Red Rep2_−0.47

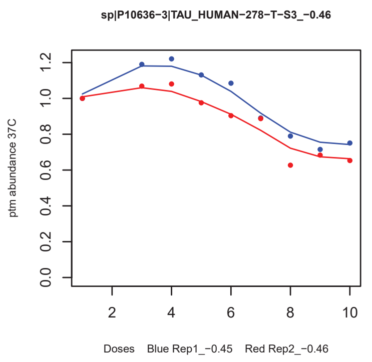
sp|P10636−3|TAU_HUMAN−278−T−S3_−0.46
ptm abundance 37C
Doses Blue Rep1_−0.45 Red Rep2_−0.46

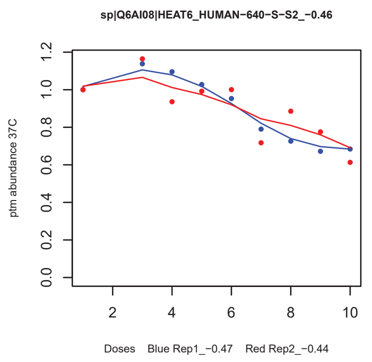
sp|Q6AI08|HEAT6_HUMAN−640−S−S2_−0.46
ptm abundance 37C
Doses Blue Rep1_−0.47 Red Rep2_−0.44

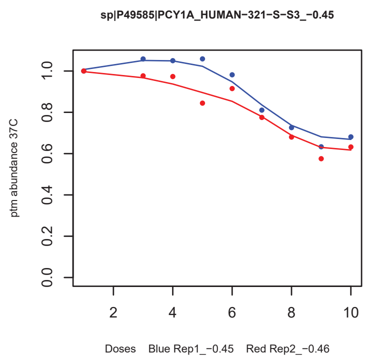
sp|P49585|PCY1A_HUMAN−321−S−S3_−0.45
ptm abundance 37C
Doses Blue Rep1_−0.45 Red Rep2_−0.46

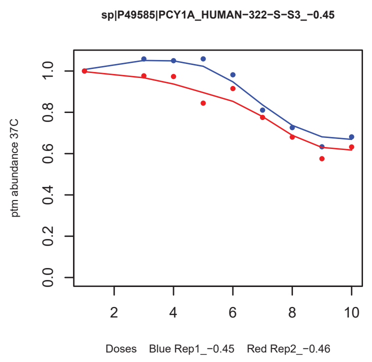
sp|P49585|PCY1A_HUMAN−322−S−S3_−0.45
ptm abundance 37C
Doses Blue Rep1_−0.45 Red Rep2_−0.46

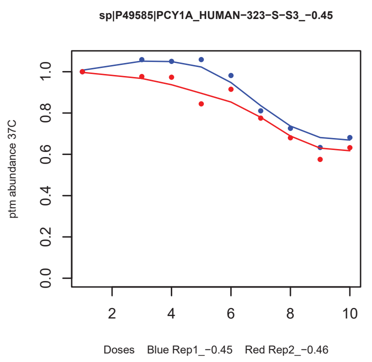
sp|P49585|PCY1A_HUMAN−323−S−S3_−0.45
ptm abundance 37C
Doses Blue Rep1_−0.45 Red Rep2_−0.46

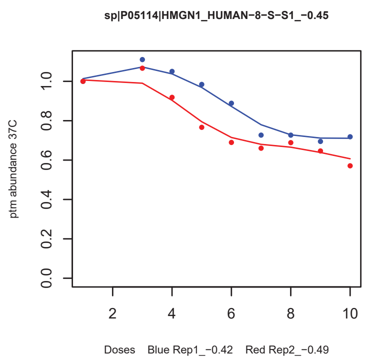
sp|P05114|HMGN1_HUMAN−8−S−S1_−0.45
ptm abundance 37C
Doses Blue Rep1_−0.42 Red Rep2_−0.49

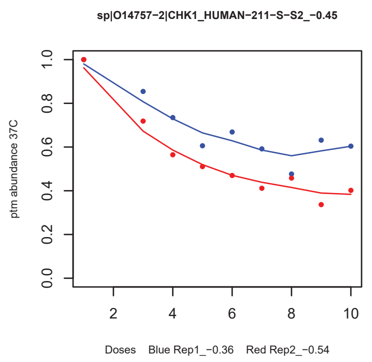
sp|O14757−2|CHK1_HUMAN−211−S−S2_−0.45
ptm abundance 37C
Doses Blue Rep1_−0.36 Red Rep2_−0.54

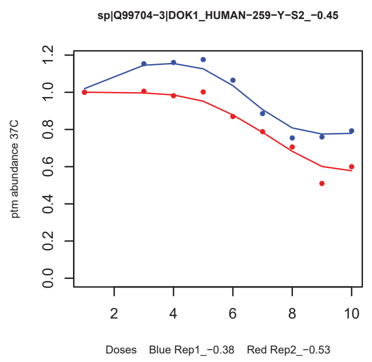
sp|Q99704−3|DOK1_HUMAN−259−Y−S2_−0.45
ptm abundance 37C
Doses Blue Rep1_−0.38 Red Rep2_−0.53

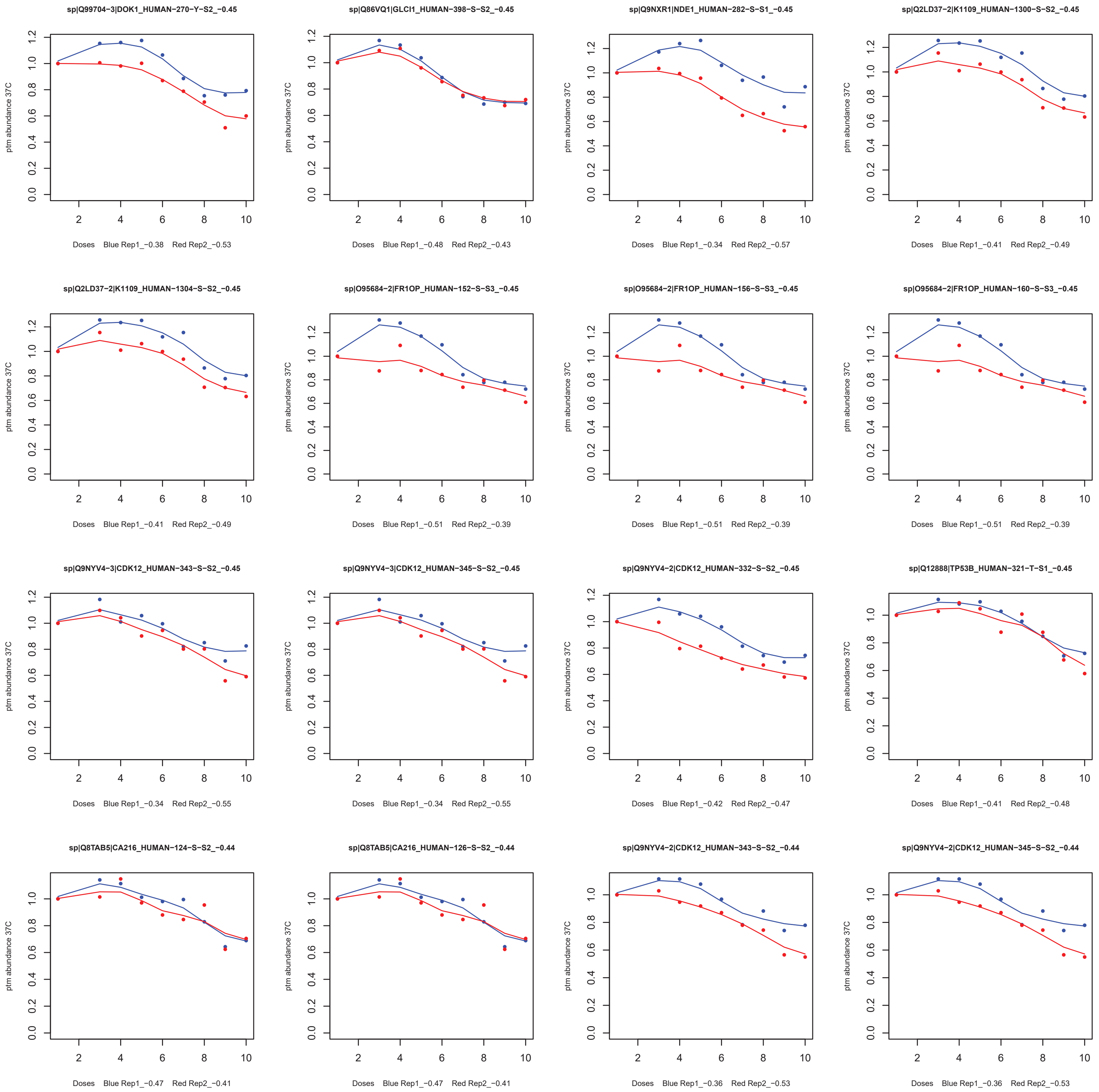
sp|Q99704-3|DOK1_HUMAN-270-Y-S2_-0.45
ptm abundance 37C
Doses Blue Rep1_-0.38 Red Rep2_-0.53
sp|Q86VQ1|GLCI1_HUMAN-398-S-S2_-0.45
ptm abundance 37C
Doses Blue Rep1_-0.48 Red Rep2_-0.43
sp|Q9NXR1|NDE1_HUMAN-282-S-S1_-0.45
ptm abundance 37C
Doses Blue Rep1_-0.34 Red Rep2_-0.57
sp|Q2LD37-2|K1109_HUMAN-1300-S-S2_-0.45
ptm abundance 37C
Doses Blue Rep1_-0.41 Red Rep2_-0.49
sp|Q2LD37-2|K1109_HUMAN-1304-S-S2_-0.45
ptm abundance 37C
Doses Blue Rep1_-0.41 Red Rep2_-0.49
sp|O95684-2|FR1OP_HUMAN-152-S-S3_-0.45
ptm abundance 37C
Doses Blue Rep1_-0.51 Red Rep2_-0.39
sp|O95684-2|FR1OP_HUMAN-156-S-S3_-0.45
ptm abundance 37C
Doses Blue Rep1_-0.51 Red Rep2_-0.39
sp|O95684-2|FR1OP_HUMAN-160-S-S3_-0.45
ptm abundance 37C
Doses Blue Rep1_-0.51 Red Rep2_-0.39
sp|Q9NYV4-3|CDK12_HUMAN-343-S-S2_-0.45
ptm abundance 37C
Doses Blue Rep1_-0.34 Red Rep2_-0.55
sp|Q9NYV4-3|CDK12_HUMAN-345-S-S2_-0.45
ptm abundance 37C
Doses Blue Rep1_-0.34 Red Rep2_-0.55
sp|Q9NYV4-2|CDK12_HUMAN-332-S-S2_-0.45
ptm abundance 37C
Doses Blue Rep1_-0.42 Red Rep2_-0.47
sp|Q12888|TP53B_HUMAN-321-T-S1_-0.45
ptm abundance 37C
Doses Blue Rep1_-0.41 Red Rep2_-0.48
sp|Q8TAB5|CA216_HUMAN-124-S-S2_-0.44
ptm abundance 37C
Doses Blue Rep1_-0.47 Red Rep2_-0.41
sp|Q8TAB5|CA216_HUMAN-126-S-S2_-0.44
ptm abundance 37C
Doses Blue Rep1_-0.47 Red Rep2_-0.41
sp|Q9NYV4-2|CDK12_HUMAN-343-S-S2_-0.44
ptm abundance 37C
Doses Blue Rep1_-0.36 Red Rep2_-0.53
sp|Q9NYV4-2|CDK12_HUMAN-345-S-S2_-0.44
ptm abundance 37C
Doses Blue Rep1_-0.36 Red Rep2_-0.53

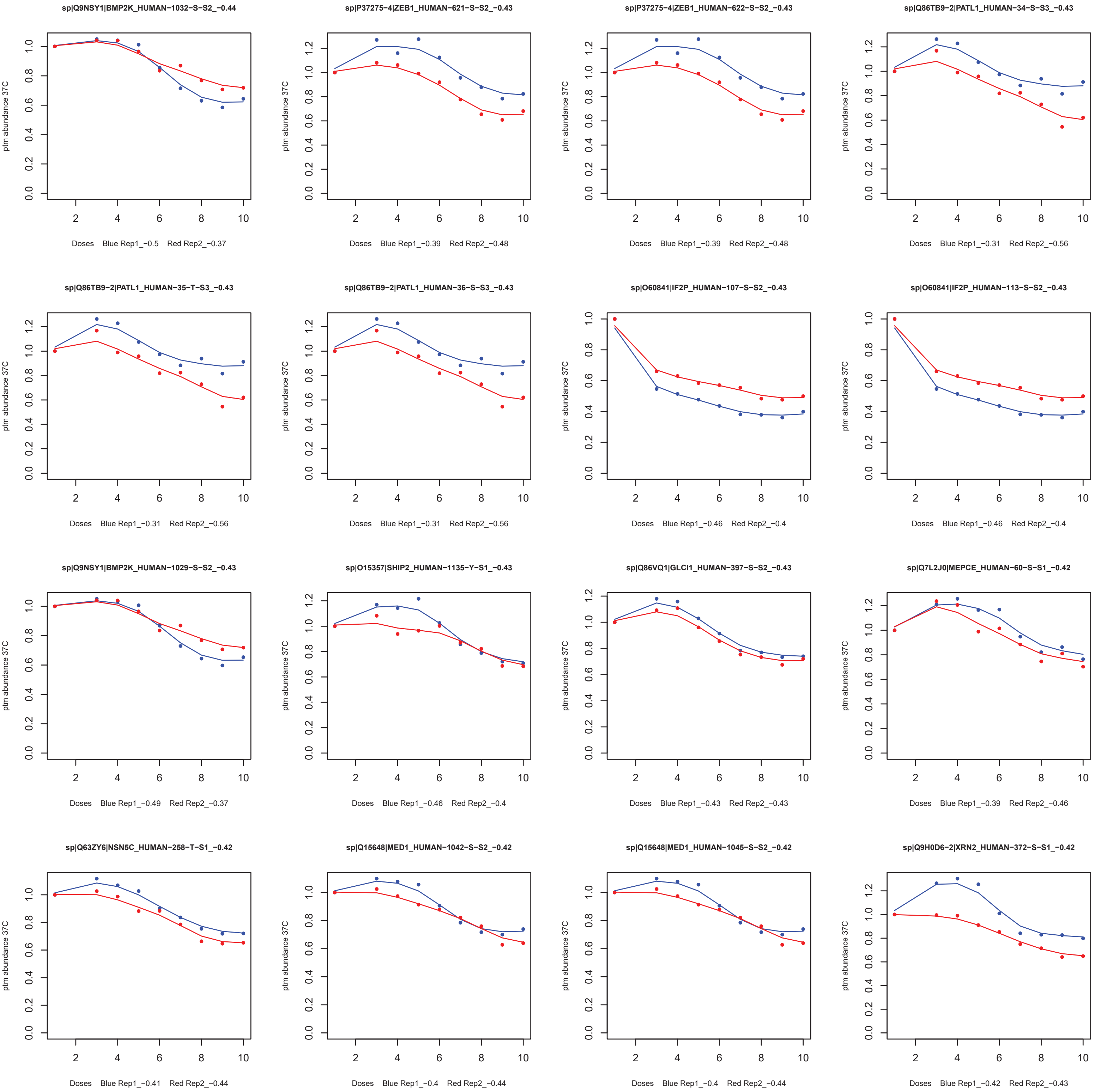
sp|Q9NSY1|BMP2K_HUMAN−1032−S−S2_−0.44
ptm abundance 37C
Doses Blue Rep1_−0.5 Red Rep2_−0.37
sp|P37275−4|ZEB1_HUMAN−621−S−S2_−0.43
ptm abundance 37C
Doses Blue Rep1_−0.39 Red Rep2_−0.48
sp|P37275−4|ZEB1_HUMAN−622−S−S2_−0.43
ptm abundance 37C
Doses Blue Rep1_−0.39 Red Rep2_−0.48
sp|Q86TB9−2|PATL1_HUMAN−34−S−S3_−0.43
ptm abundance 37C
Doses Blue Rep1_−0.31 Red Rep2_−0.56
sp|Q86TB9−2|PATL1_HUMAN−35−T−S3_−0.43
ptm abundance 37C
Doses Blue Rep1_−0.31 Red Rep2_−0.56
sp|Q86TB9−2|PATL1_HUMAN−36−S−S3_−0.43
ptm abundance 37C
Doses Blue Rep1_−0.31 Red Rep2_−0.56
sp|O60841|IF2P_HUMAN−107−S−S2_−0.43
ptm abundance 37C
Doses Blue Rep1_−0.46 Red Rep2_−0.4
sp|O60841|IF2P_HUMAN−113−S−S2_−0.43
ptm abundance 37C
Doses Blue Rep1_−0.46 Red Rep2_−0.4
sp|Q9NSY1|BMP2K_HUMAN−1029−S−S2_−0.43
ptm abundance 37C
Doses Blue Rep1_−0.49 Red Rep2_−0.37
sp|O15357|SHIP2_HUMAN−1135−Y−S1_−0.43
ptm abundance 37C
Doses Blue Rep1_−0.46 Red Rep2_−0.4
sp|Q86VQ1|GLCI1_HUMAN−397−S−S2_−0.43
ptm abundance 37C
Doses Blue Rep1_−0.43 Red Rep2_−0.43
sp|Q7L2J0|MEPCE_HUMAN−60−S−S1_−0.42
ptm abundance 37C
Doses Blue Rep1_−0.39 Red Rep2_−0.46
sp|Q63ZY6|NSN5C_HUMAN−258−T−S1_−0.42
ptm abundance 37C
Doses Blue Rep1_−0.41 Red Rep2_−0.44
sp|Q15648|MED1_HUMAN−1042−S−S2_−0.42
ptm abundance 37C
Doses Blue Rep1_−0.4 Red Rep2_−0.44
sp|Q15648|MED1_HUMAN−1045−S−S2_−0.42
ptm abundance 37C
Doses Blue Rep1_−0.4 Red Rep2_−0.44
sp|Q9H0D6−2|XRN2_HUMAN−372−S−S1_−0.42
ptm abundance 37C
Doses Blue Rep1_−0.42 Red Rep2_−0.43

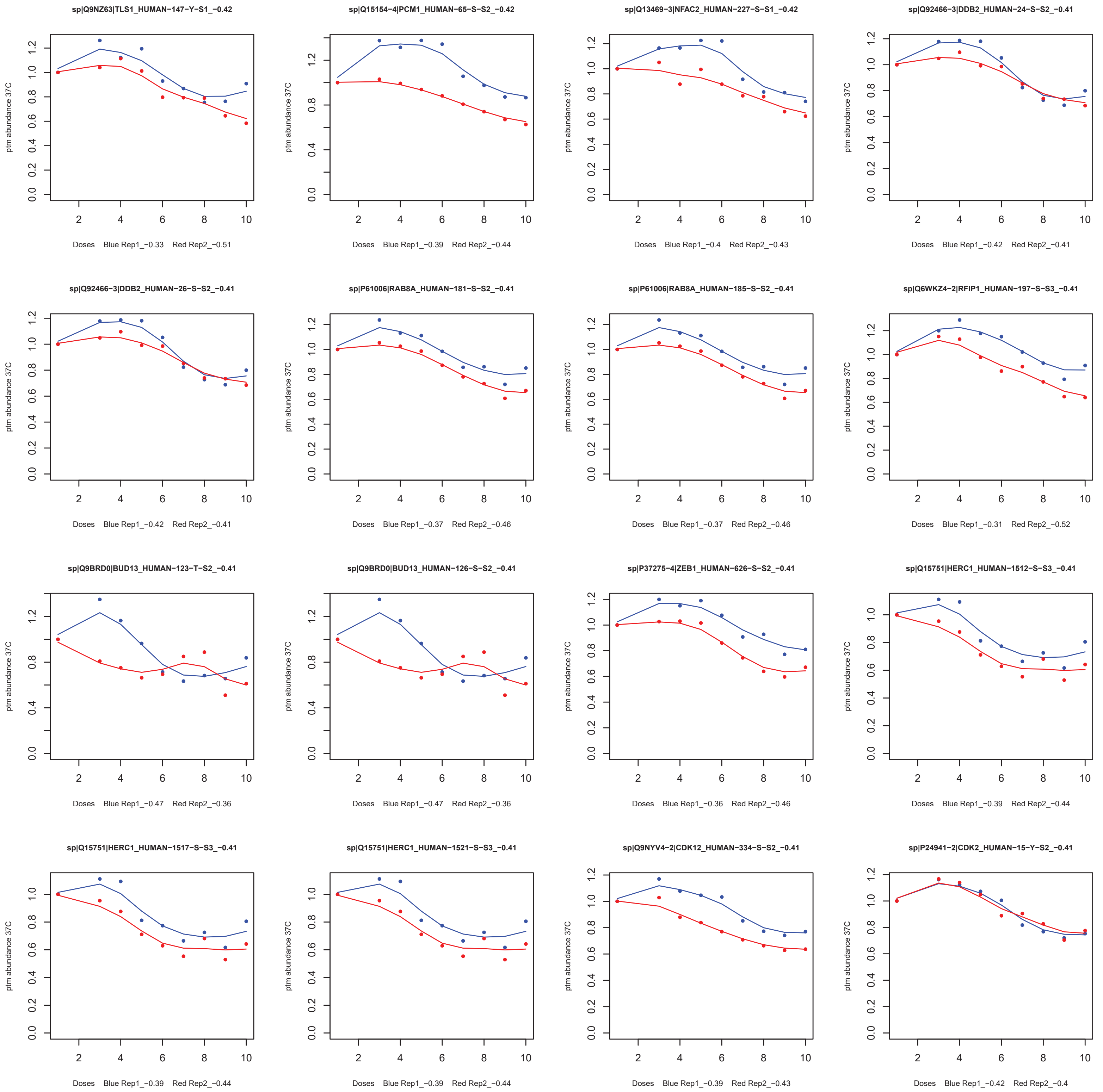
sp|Q9NZ63|TLS1_HUMAN−147−Y−S1_−0.42
Doses Blue Rep1_−0.33 Red Rep2_−0.51
sp|Q15154−4|PCM1_HUMAN−65−S−S2_−0.42
Doses Blue Rep1_−0.39 Red Rep2_−0.44
sp|Q13469−3|NFAC2_HUMAN−227−S−S1_−0.42
Doses Blue Rep1_−0.4 Red Rep2_−0.43
sp|Q92466−3|DDB2_HUMAN−24−S−S2_−0.41
Doses Blue Rep1_−0.42 Red Rep2_−0.41
sp|Q92466−3|DDB2_HUMAN−26−S−S2_−0.41
Doses Blue Rep1_−0.42 Red Rep2_−0.41
sp|P61006|RAB8A_HUMAN−181−S−S2_−0.41
Doses Blue Rep1_−0.37 Red Rep2_−0.46
sp|P61006|RAB8A_HUMAN−185−S−S2_−0.41
Doses Blue Rep1_−0.37 Red Rep2_−0.46
sp|Q6WKZ4−2|RFIP1_HUMAN−197−S−S3_−0.41
Doses Blue Rep1_−0.31 Red Rep2_−0.52
sp|Q9BRD0|BUD13_HUMAN−123−T−S2_−0.41
Doses Blue Rep1_−0.47 Red Rep2_−0.36
sp|Q9BRD0|BUD13_HUMAN−126−S−S2_−0.41
Doses Blue Rep1_−0.47 Red Rep2_−0.36
sp|P37275−4|ZEB1_HUMAN−626−S−S2_−0.41
Doses Blue Rep1_−0.36 Red Rep2_−0.46
sp|Q15751|HERC1_HUMAN−1512−S−S3_−0.41
Doses Blue Rep1_−0.39 Red Rep2_−0.44
sp|Q15751|HERC1_HUMAN−1517−S−S3_−0.41
Doses Blue Rep1_−0.39 Red Rep2_−0.44
sp|Q15751|HERC1_HUMAN−1521−S−S3_−0.41
Doses Blue Rep1_−0.39 Red Rep2_−0.44
sp|Q9NYV4−2|CDK12_HUMAN−334−S−S2_−0.41
Doses Blue Rep1_−0.39 Red Rep2_−0.43
sp|P24941−2|CDK2_HUMAN−15−Y−S2_−0.41
Doses Blue Rep1_−0.42 Red Rep2_−0.4
ptm abundance 37C

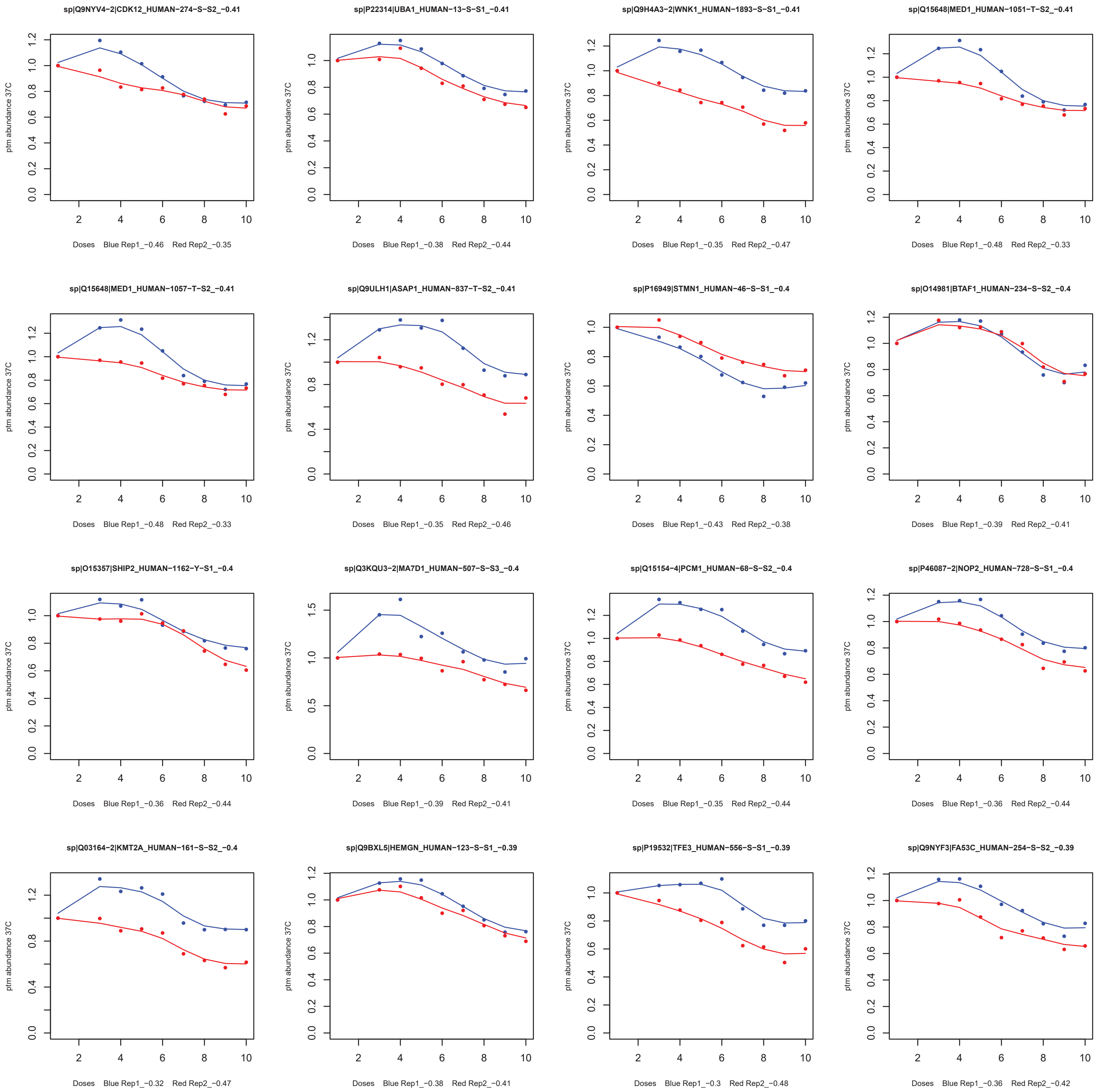
sp|Q9NYV4−2|CDK12_HUMAN−274−S−S2_−0.41
Doses Blue Rep1_−0.46 Red Rep2_−0.35
sp|P22314|UBA1_HUMAN−13−S−S1_−0.41
Doses Blue Rep1_−0.38 Red Rep2_−0.44
sp|Q9H4A3−2|WNK1_HUMAN−1893−S−S1_−0.41
Doses Blue Rep1_−0.35 Red Rep2_−0.47
sp|Q15648|MED1_HUMAN−1051−T−S2_−0.41
Doses Blue Rep1_−0.48 Red Rep2_−0.33
sp|Q15648|MED1_HUMAN−1057−T−S2_−0.41
Doses Blue Rep1_−0.48 Red Rep2_−0.33
sp|Q9ULH1|ASAP1_HUMAN−837−T−S2_−0.41
Doses Blue Rep1_−0.35 Red Rep2_−0.46
sp|P16949|STMN1_HUMAN−46−S−S1_−0.4
Doses Blue Rep1_−0.43 Red Rep2_−0.38
sp|O14981|BTAF1_HUMAN−234−S−S2_−0.4
Doses Blue Rep1_−0.39 Red Rep2_−0.41
sp|O15357|SHIP2_HUMAN−1162−Y−S1_−0.4
Doses Blue Rep1_−0.36 Red Rep2_−0.44
sp|Q3KQU3−2|MA7D1_HUMAN−507−S−S3_−0.4
Doses Blue Rep1_−0.39 Red Rep2_−0.41
sp|Q15154−4|PCM1_HUMAN−68−S−S2_−0.4
Doses Blue Rep1_−0.35 Red Rep2_−0.44
sp|P46087−2|NOP2_HUMAN−728−S−S1_−0.4
Doses Blue Rep1_−0.36 Red Rep2_−0.44
sp|Q03164−2|KMT2A_HUMAN−161−S−S2_−0.4
Doses Blue Rep1_−0.32 Red Rep2_−0.47
sp|Q9BXL5|HEMGN_HUMAN−123−S−S1_−0.39
Doses Blue Rep1_−0.38 Red Rep2_−0.41
sp|P19532|TFE3_HUMAN−556−S−S1_−0.39
Doses Blue Rep1_−0.3 Red Rep2_−0.48
sp|Q9NYF3|FA53C_HUMAN−254−S−S2_−0.39
Doses Blue Rep1_−0.36 Red Rep2_−0.42
ptm abundance 37C

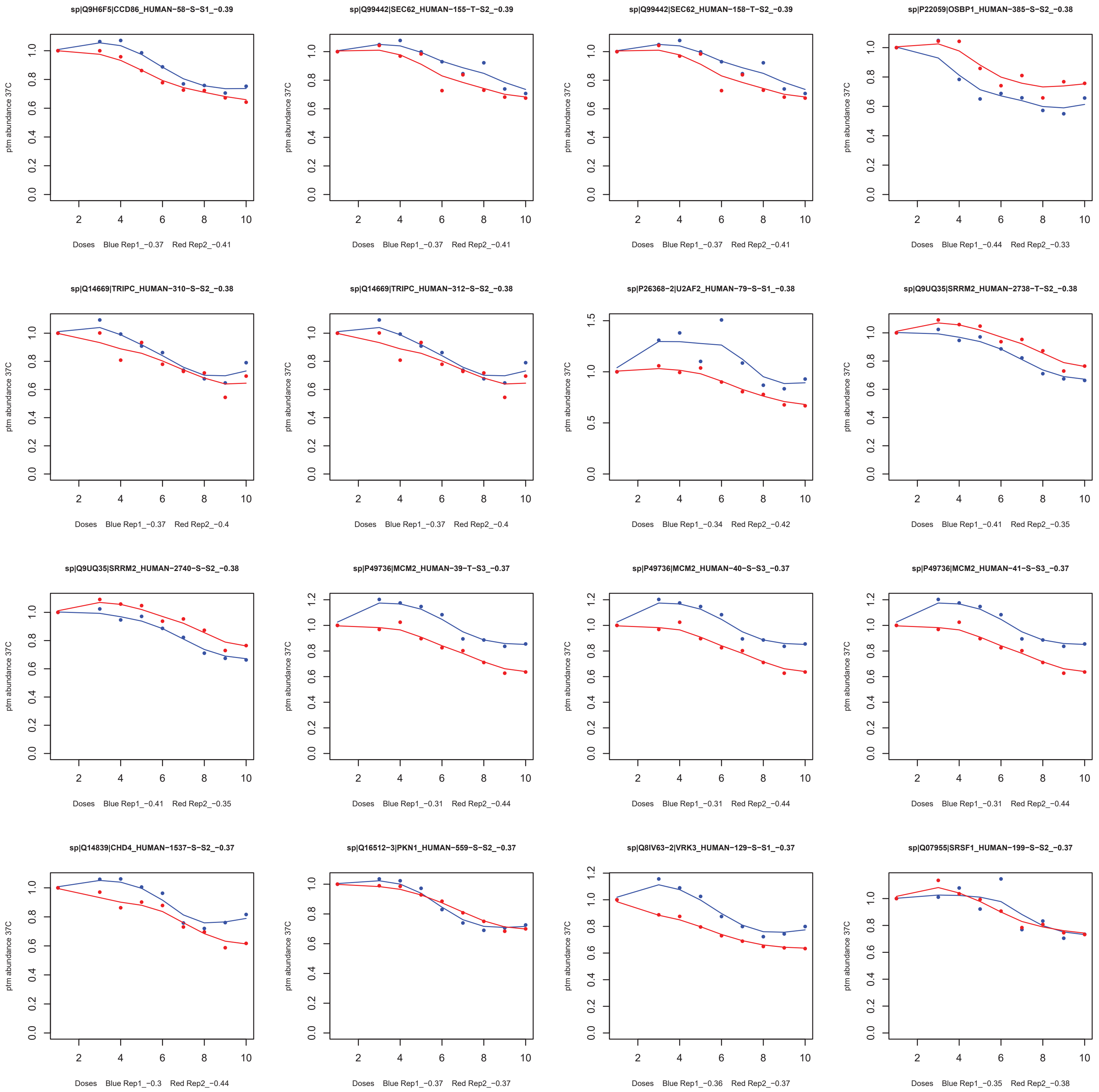
sp|Q9H6F5|CCD86_HUMAN−58−S−S1_−0.39
ptm abundance 37C
Doses Blue Rep1_−0.37 Red Rep2_−0.41
sp|Q99442|SEC62_HUMAN−155−T−S2_−0.39
ptm abundance 37C
Doses Blue Rep1_−0.37 Red Rep2_−0.41
sp|Q99442|SEC62_HUMAN−158−T−S2_−0.39
ptm abundance 37C
Doses Blue Rep1_−0.37 Red Rep2_−0.41
sp|P22059|OSBP1_HUMAN−385−S−S2_−0.38
ptm abundance 37C
Doses Blue Rep1_−0.44 Red Rep2_−0.33
sp|Q14669|TRIPC_HUMAN−310−S−S2_−0.38
ptm abundance 37C
Doses Blue Rep1_−0.37 Red Rep2_−0.4
sp|Q14669|TRIPC_HUMAN−312−S−S2_−0.38
ptm abundance 37C
Doses Blue Rep1_−0.37 Red Rep2_−0.4
sp|P26368−2|U2AF2_HUMAN−79−S−S1_−0.38
ptm abundance 37C
Doses Blue Rep1_−0.34 Red Rep2_−0.42
sp|Q9UQ35|SRRM2_HUMAN−2738−T−S2_−0.38
ptm abundance 37C
Doses Blue Rep1_−0.41 Red Rep2_−0.35
sp|Q9UQ35|SRRM2_HUMAN−2740−S−S2_−0.38
ptm abundance 37C
Doses Blue Rep1_−0.41 Red Rep2_−0.35
sp|P49736|MCM2_HUMAN−39−T−S3_−0.37
ptm abundance 37C
Doses Blue Rep1_−0.31 Red Rep2_−0.44
sp|P49736|MCM2_HUMAN−40−S−S3_−0.37
ptm abundance 37C
Doses Blue Rep1_−0.31 Red Rep2_−0.44
sp|P49736|MCM2_HUMAN−41−S−S3_−0.37
ptm abundance 37C
Doses Blue Rep1_−0.31 Red Rep2_−0.44
sp|Q14839|CHD4_HUMAN−1537−S−S2_−0.37
ptm abundance 37C
Doses Blue Rep1_−0.3 Red Rep2_−0.44
sp|Q16512−3|PKN1_HUMAN−559−S−S2_−0.37
ptm abundance 37C
Doses Blue Rep1_−0.37 Red Rep2_−0.37
sp|Q8IV63−2|VRK3_HUMAN−129−S−S1_−0.37
ptm abundance 37C
Doses Blue Rep1_−0.36 Red Rep2_−0.37
sp|Q07955|SRSF1_HUMAN−199−S−S2_−0.37
ptm abundance 37C
Doses Blue Rep1_−0.35 Red Rep2_−0.38

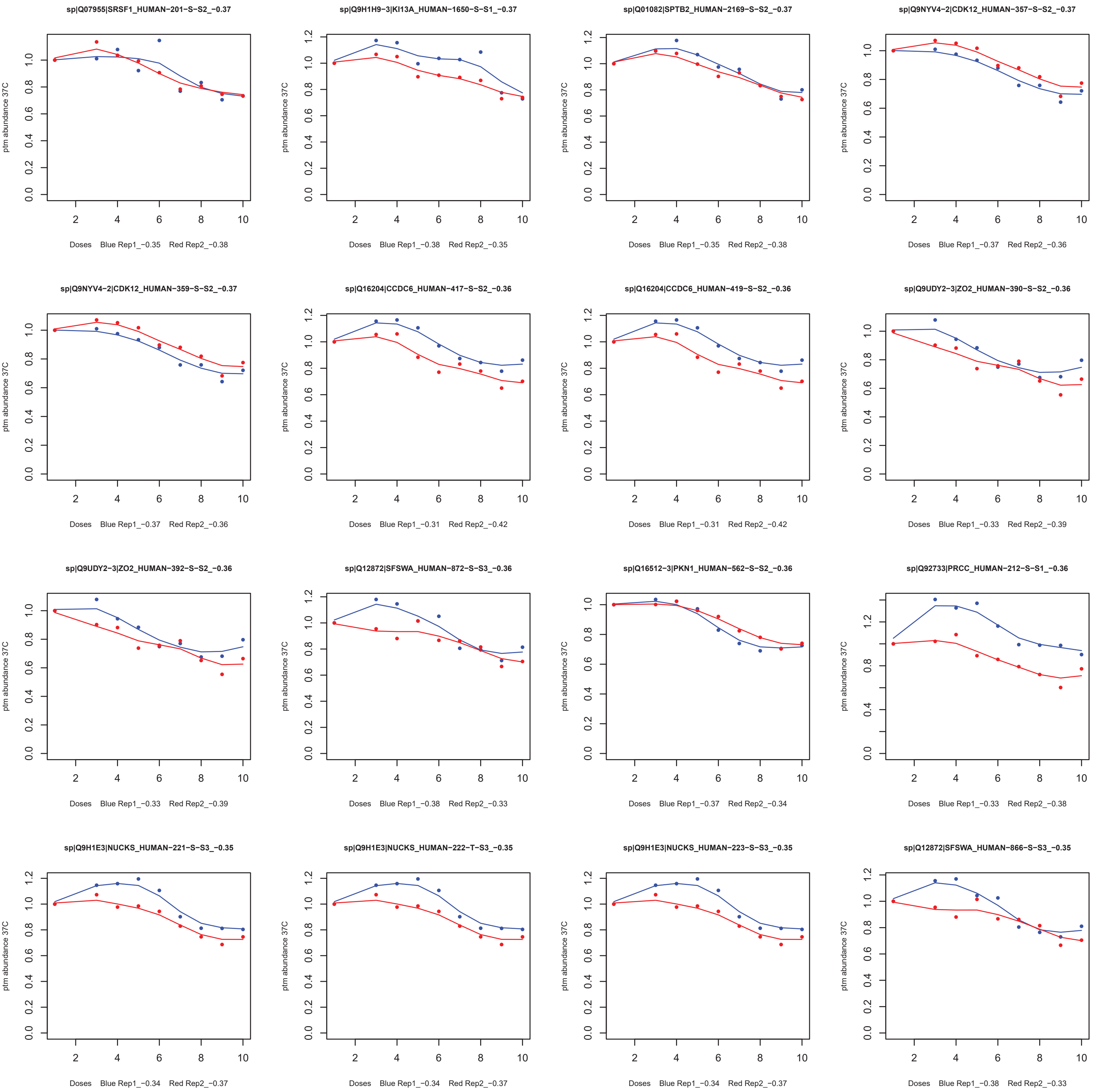
sp|Q07955|SRSF1_HUMAN−201−S−S2_−0.37
ptm abundance 37C
Doses Blue Rep1_−0.35 Red Rep2_−0.38
sp|Q9H1H9−3|KI13A_HUMAN−1650−S−S1_−0.37
ptm abundance 37C
Doses Blue Rep1_−0.38 Red Rep2_−0.35
sp|Q01082|SPTB2_HUMAN−2169−S−S2_−0.37
ptm abundance 37C
Doses Blue Rep1_−0.35 Red Rep2_−0.38
sp|Q9NYV4−2|CDK12_HUMAN−357−S−S2_−0.37
ptm abundance 37C
Doses Blue Rep1_−0.37 Red Rep2_−0.36
sp|Q9NYV4−2|CDK12_HUMAN−359−S−S2_−0.37
ptm abundance 37C
Doses Blue Rep1_−0.37 Red Rep2_−0.36
sp|Q16204|CCDC6_HUMAN−417−S−S2_−0.36
ptm abundance 37C
Doses Blue Rep1_−0.31 Red Rep2_−0.42
sp|Q16204|CCDC6_HUMAN−419−S−S2_−0.36
ptm abundance 37C
Doses Blue Rep1_−0.31 Red Rep2_−0.42
sp|Q9UDY2−3|ZO2_HUMAN−390−S−S2_−0.36
ptm abundance 37C
Doses Blue Rep1_−0.33 Red Rep2_−0.39
sp|Q9UDY2−3|ZO2_HUMAN−392−S−S2_−0.36
ptm abundance 37C
Doses Blue Rep1_−0.33 Red Rep2_−0.39
sp|Q12872|SFSWA_HUMAN−872−S−S3_−0.36
ptm abundance 37C
Doses Blue Rep1_−0.38 Red Rep2_−0.33
sp|Q16512−3|PKN1_HUMAN−562−S−S2_−0.36
ptm abundance 37C
Doses Blue Rep1_−0.37 Red Rep2_−0.34
sp|Q92733|PRCC_HUMAN−212−S−S1_−0.36
ptm abundance 37C
Doses Blue Rep1_−0.33 Red Rep2_−0.38
sp|Q9H1E3|NUCKS_HUMAN−221−S−S3_−0.35
ptm abundance 37C
Doses Blue Rep1_−0.34 Red Rep2_−0.37
sp|Q9H1E3|NUCKS_HUMAN−222−T−S3_−0.35
ptm abundance 37C
Doses Blue Rep1_−0.34 Red Rep2_−0.37
sp|Q9H1E3|NUCKS_HUMAN−223−S−S3_−0.35
ptm abundance 37C
Doses Blue Rep1_−0.34 Red Rep2_−0.37
sp|Q12872|SFSWA_HUMAN−866−S−S3_−0.35
ptm abundance 37C
Doses Blue Rep1_−0.38 Red Rep2_−0.33

sp|Q12872|SFSWA_HUMAN−868−S−S3_−0.35

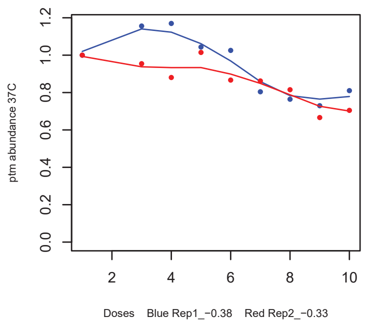

sp|Q12872|SFSWA_HUMAN−870−S−S3_−0.35

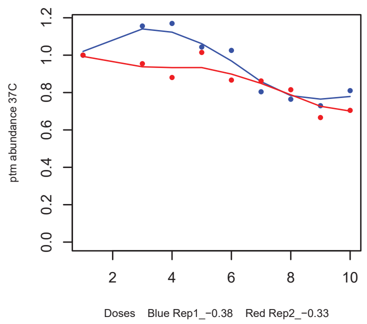

sp|Q8NI27−2|THOC2_HUMAN−397−S−S1_−0.35

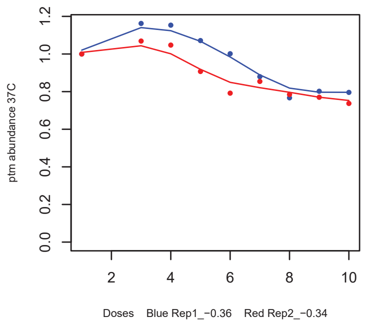

sp|Q01082|SPTB2_HUMAN−2165−S−S2_−0.35

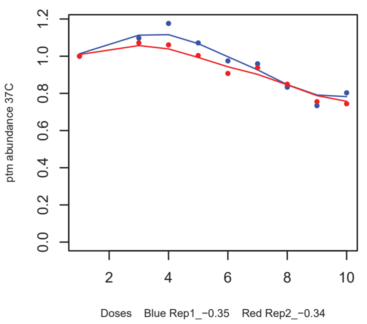

sp|P49736|MCM2_HUMAN−39−T−S2_−0.35

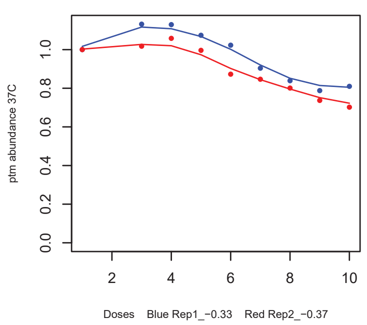

sp|Q969F1|TF3C6_HUMAN−160−S−S3_−0.34

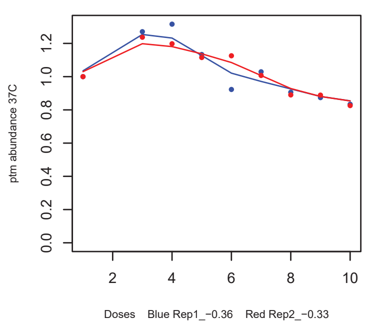

sp|Q969F1|TF3C6_HUMAN−173−S−S3_−0.34

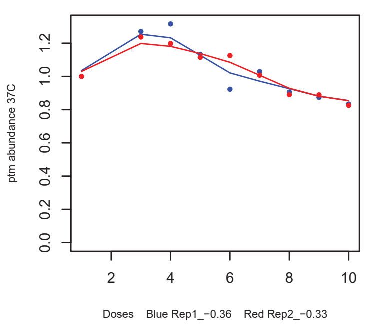

sp|Q969F1|TF3C6_HUMAN−174−S−S3_−0.34

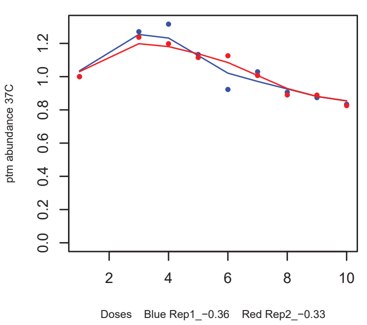

sp|Q969F1|TF3C6_HUMAN−177−S−S3_−0.34

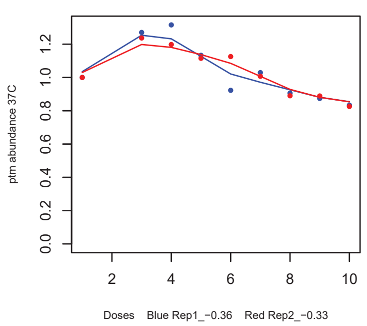

sp|P49736|MCM2_HUMAN−41−S−S2_−0.34

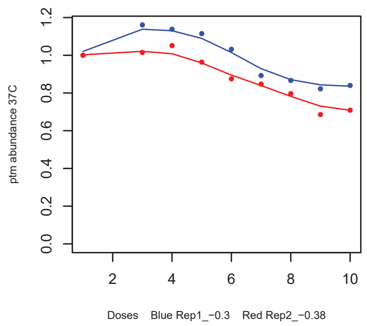

sp|Q96T37−4|RBM15_HUMAN−250−S−S1_−0.34

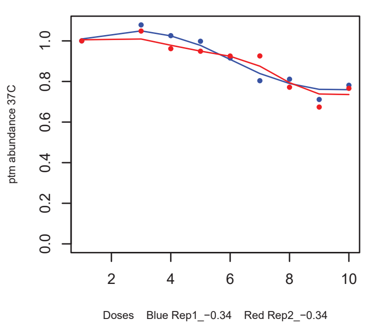

sp|Q96T37−4|RBM15_HUMAN−213−S−S2_−0.33

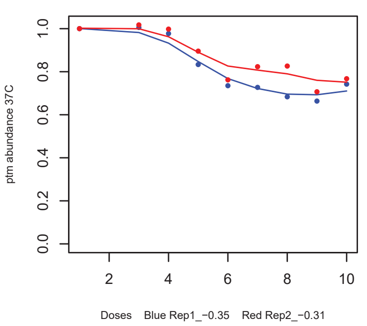

sp|Q96T37−4|RBM15_HUMAN−215−S−S2_−0.33

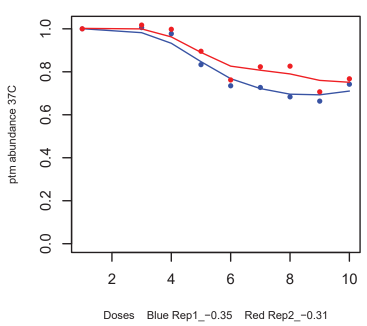

sp|Q9NYV4−2|CDK12_HUMAN−276−S−S2_−0.33

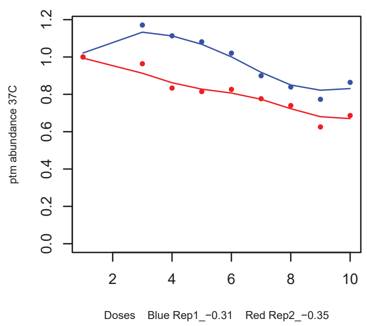

sp|Q9UID3|VPS51_HUMAN−644−S−S2_−0.33

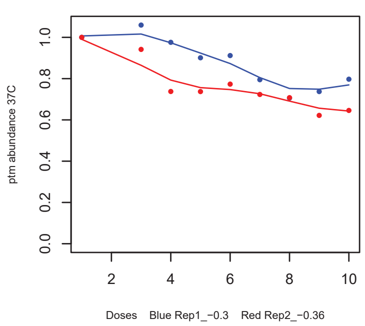

sp|O43491−4|E41L2_HUMAN−648−S−S1_−0.33

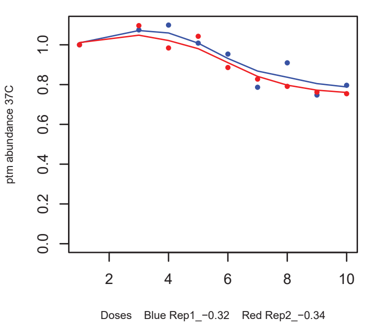

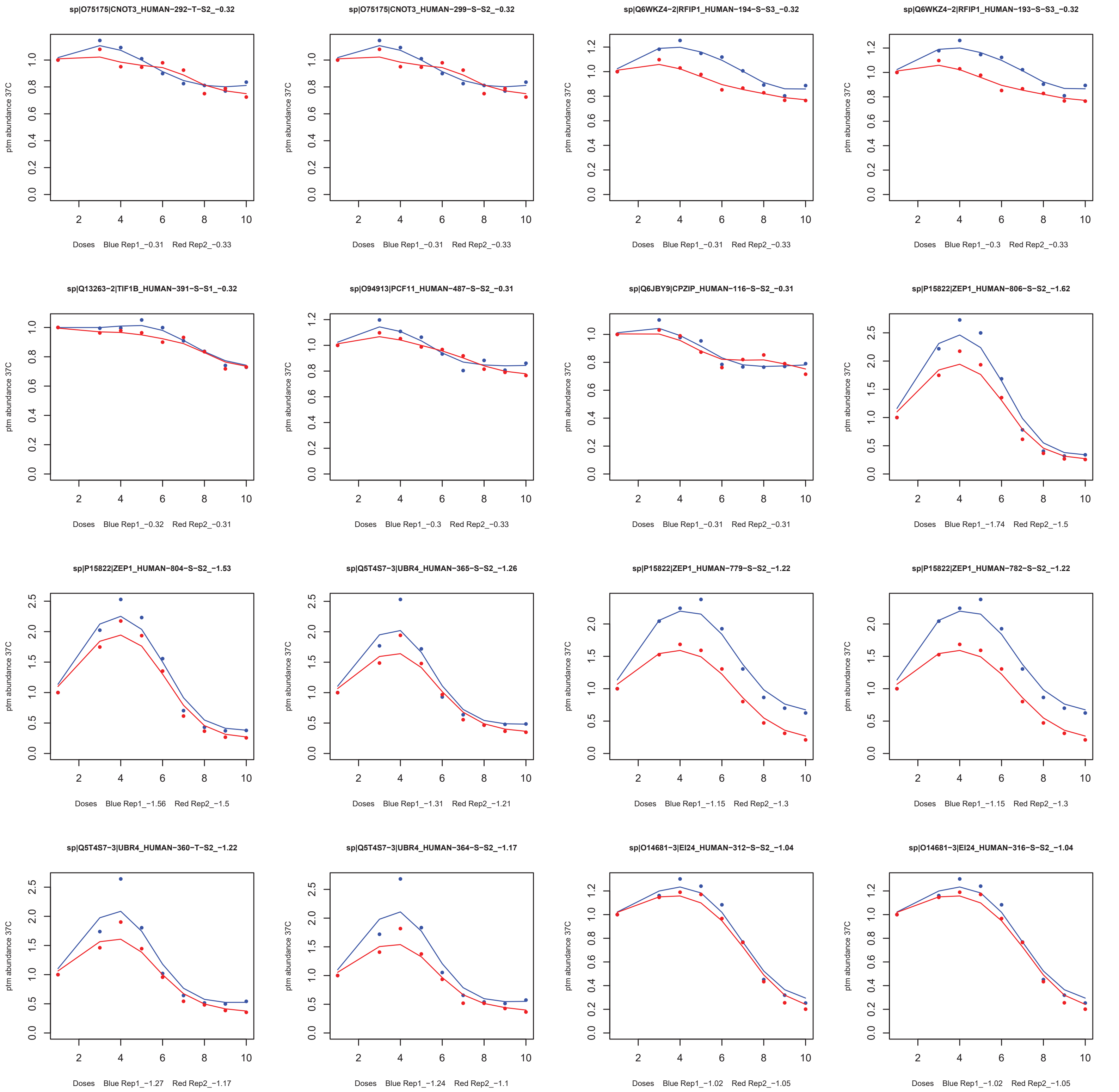

sp|O75175|CNOT3_HUMAN−292−T−S2_−0.32
ptm abundance 37C
Doses Blue Rep1_−0.31 Red Rep2_−0.33
sp|O75175|CNOT3_HUMAN−299−S−S2_−0.32
ptm abundance 37C
Doses Blue Rep1_−0.31 Red Rep2_−0.33
sp|Q6WKZ4−2|RFIP1_HUMAN−194−S−S3_−0.32
ptm abundance 37C
Doses Blue Rep1_−0.31 Red Rep2_−0.33
sp|Q6WKZ4−2|RFIP1_HUMAN−193−S−S3_−0.32
ptm abundance 37C
Doses Blue Rep1_−0.3 Red Rep2_−0.33
sp|Q13263−2|TIF1B_HUMAN−391−S−S1_−0.32
ptm abundance 37C
Doses Blue Rep1_−0.32 Red Rep2_−0.31
sp|O94913|PCF11_HUMAN−487−S−S2_−0.31
ptm abundance 37C
Doses Blue Rep1_−0.3 Red Rep2_−0.33
sp|Q6JBY9|CPZIP_HUMAN−116−S−S2_−0.31
ptm abundance 37C
Doses Blue Rep1_−0.31 Red Rep2_−0.31
sp|P15822|ZEP1_HUMAN−806−S−S2_−1.62
ptm abundance 37C
Doses Blue Rep1_−1.74 Red Rep2_−1.5
sp|P15822|ZEP1_HUMAN−804−S−S2_−1.53
ptm abundance 37C
Doses Blue Rep1_−1.56 Red Rep2_−1.5
sp|Q5T4S7−3|UBR4_HUMAN−365−S−S2_−1.26
ptm abundance 37C
Doses Blue Rep1_−1.31 Red Rep2_−1.21
sp|P15822|ZEP1_HUMAN−779−S−S2_−1.22
ptm abundance 37C
Doses Blue Rep1_−1.15 Red Rep2_−1.3
sp|P15822|ZEP1_HUMAN−782−S−S2_−1.22
ptm abundance 37C
Doses Blue Rep1_−1.15 Red Rep2_−1.3
sp|Q5T4S7−3|UBR4_HUMAN−360−T−S2_−1.22
ptm abundance 37C
Doses Blue Rep1_−1.27 Red Rep2_−1.17
sp|Q5T4S7−3|UBR4_HUMAN−364−S−S2_−1.17
ptm abundance 37C
Doses Blue Rep1_−1.24 Red Rep2_−1.1
sp|O14681−3|EI24_HUMAN−312−S−S2_−1.04
ptm abundance 37C
Doses Blue Rep1_−1.02 Red Rep2_−1.05
sp|O14681−3|EI24_HUMAN−316−S−S2_−1.04
ptm abundance 37C
Doses Blue Rep1_−1.02 Red Rep2_−1.05

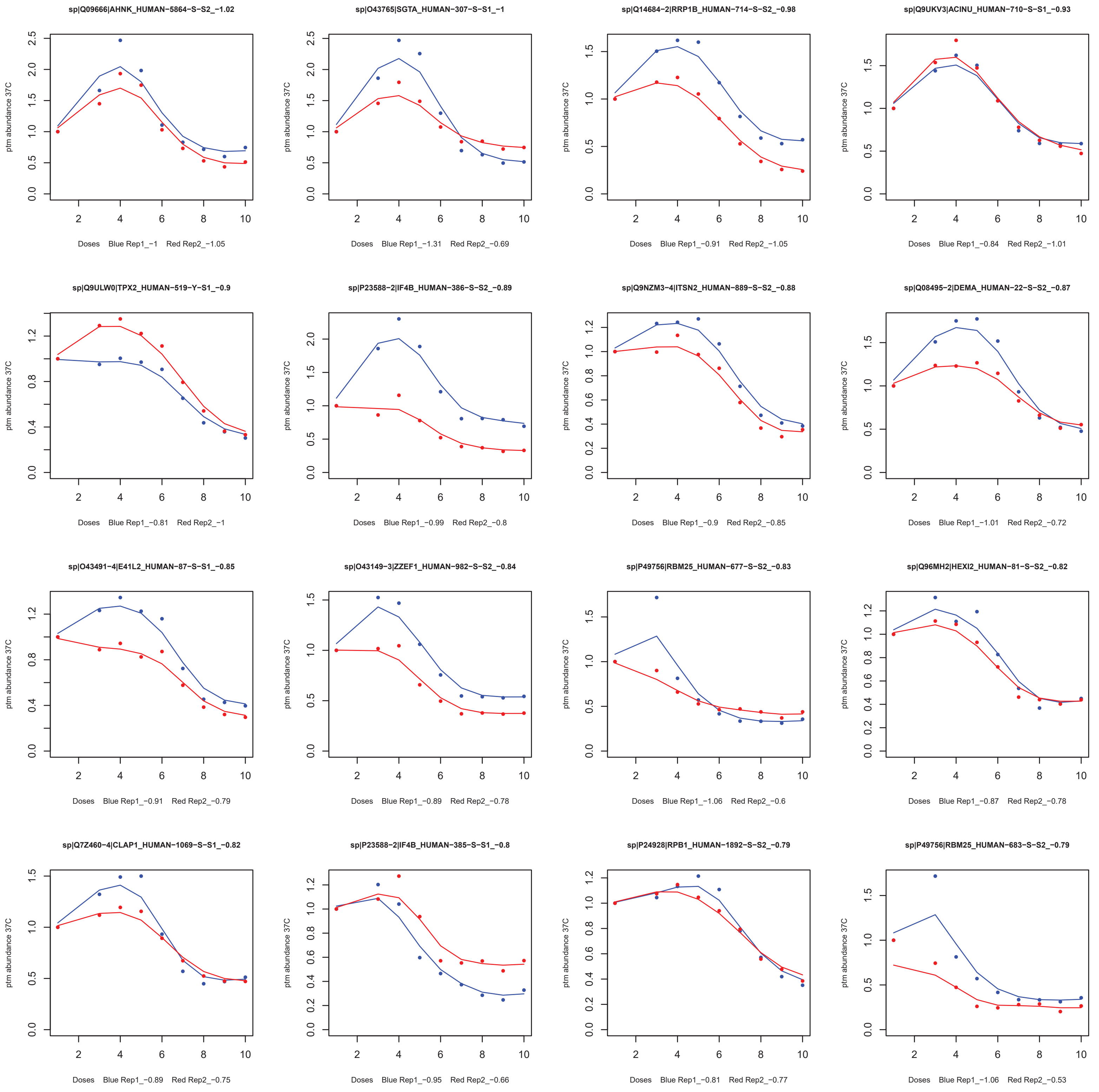

sp|Q09666|AHNK_HUMAN−5864−S−S2_−1.02
Doses Blue Rep1_−1 Red Rep2_−1.05
sp|O43765|SGTA_HUMAN−307−S−S1_−1
Doses Blue Rep1_−1.31 Red Rep2_−0.69
sp|Q14684−2|RRP1B_HUMAN−714−S−S2_−0.98
Doses Blue Rep1_−0.91 Red Rep2_−1.05
sp|Q9UKV3|ACINU_HUMAN−710−S−S1_−0.93
Doses Blue Rep1_−0.84 Red Rep2_−1.01
sp|Q9ULW0|TPX2_HUMAN−519−Y−S1_−0.9
Doses Blue Rep1_−0.81 Red Rep2_−1
sp|P23588−2|IF4B_HUMAN−386−S−S2_−0.89
Doses Blue Rep1_−0.99 Red Rep2_−0.8
sp|Q9NZM3−4|ITSN2_HUMAN−889−S−S2_−0.88
Doses Blue Rep1_−0.9 Red Rep2_−0.85
sp|Q08495−2|DEMA_HUMAN−22−S−S2_−0.87
Doses Blue Rep1_−1.01 Red Rep2_−0.72
sp|O43491−4|E41L2_HUMAN−87−S−S1_−0.85
Doses Blue Rep1_−0.91 Red Rep2_−0.79
sp|O43149−3|ZZEF1_HUMAN−982−S−S2_−0.84
Doses Blue Rep1_−0.89 Red Rep2_−0.78
sp|P49756|RBM25_HUMAN−677−S−S2_−0.83
Doses Blue Rep1_−1.06 Red Rep2_−0.6
sp|Q96MH2|HEXI2_HUMAN−81−S−S2_−0.82
Doses Blue Rep1_−0.87 Red Rep2_−0.78
sp|Q7Z460−4|CLAP1_HUMAN−1069−S−S1_−0.82
Doses Blue Rep1_−0.89 Red Rep2_−0.75
sp|P23588−2|IF4B_HUMAN−385−S−S1_−0.8
Doses Blue Rep1_−0.95 Red Rep2_−0.66
sp|P24928|RPB1_HUMAN−1892−S−S2_−0.79
Doses Blue Rep1_−0.81 Red Rep2_−0.77
sp|P49756|RBM25_HUMAN−683−S−S2_−0.79
Doses Blue Rep1_−1.06 Red Rep2_−0.53
ptm abundance 37C

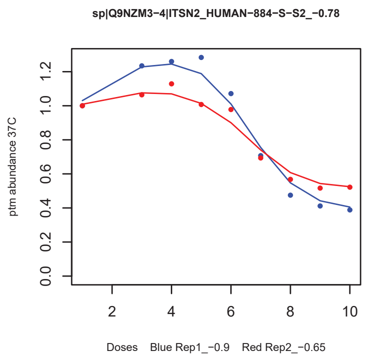
sp|Q9NZM3−4|ITSN2_HUMAN−884−S−S2_−0.78
ptm abundance 37C
Doses Blue Rep1_−0.9 Red Rep2_−0.65

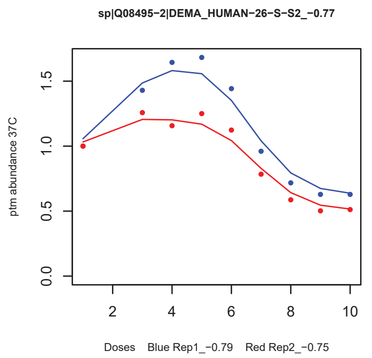
sp|Q08495−2|DEMA_HUMAN−26−S−S2_−0.77
ptm abundance 37C
Doses Blue Rep1_−0.79 Red Rep2_−0.75

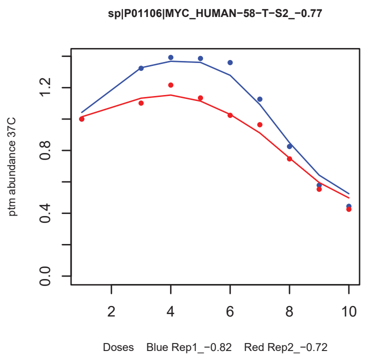
sp|P01106|MYC_HUMAN−58−T−S2_−0.77
ptm abundance 37C
Doses Blue Rep1_−0.82 Red Rep2_−0.72

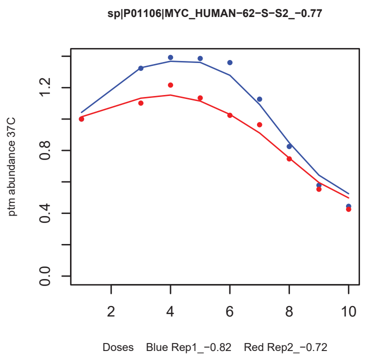
sp|P01106|MYC_HUMAN−62−S−S2_−0.77
ptm abundance 37C
Doses Blue Rep1_−0.82 Red Rep2_−0.72

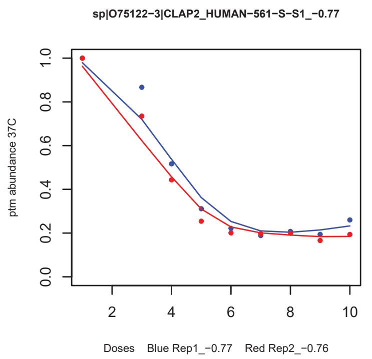
sp|O75122−3|CLAP2_HUMAN−561−S−S1_−0.77
ptm abundance 37C
Doses Blue Rep1_−0.77 Red Rep2_−0.76

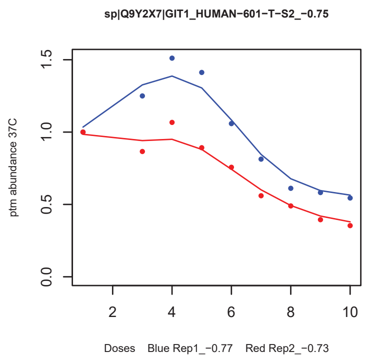
sp|Q9Y2X7|GIT1_HUMAN−601−T−S2_−0.75
ptm abundance 37C
Doses Blue Rep1_−0.77 Red Rep2_−0.73

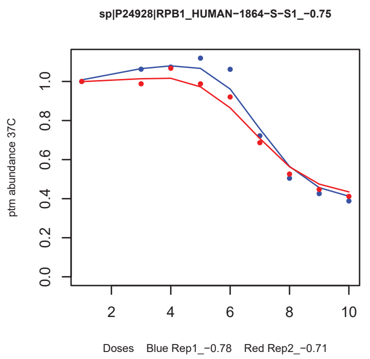
sp|P24928|RPB1_HUMAN−1864−S−S1_−0.75
ptm abundance 37C
Doses Blue Rep1_−0.78 Red Rep2_−0.71

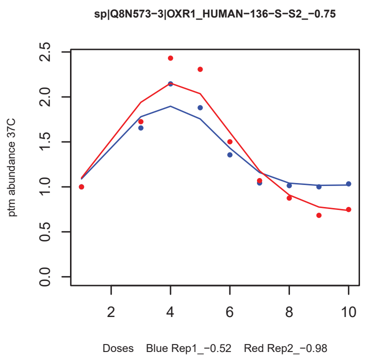
sp|Q8N573−3|OXR1_HUMAN−136−S−S2_−0.75
ptm abundance 37C
Doses Blue Rep1_−0.52 Red Rep2_−0.98

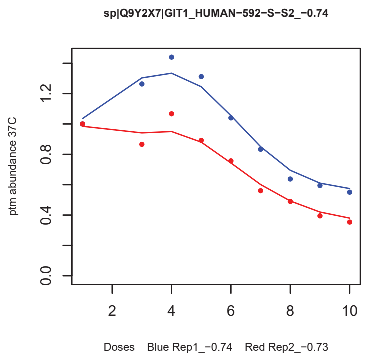
sp|Q9Y2X7|GIT1_HUMAN−592−S−S2_−0.74
ptm abundance 37C
Doses Blue Rep1_−0.74 Red Rep2_−0.73

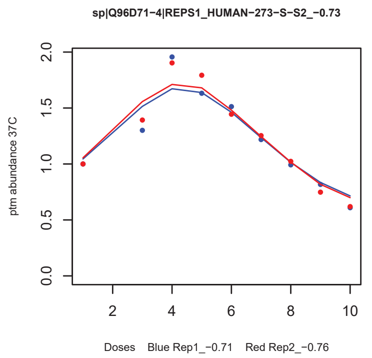
sp|Q96D71−4|REPS1_HUMAN−273−S−S2_−0.73
ptm abundance 37C
Doses Blue Rep1_−0.71 Red Rep2_−0.76

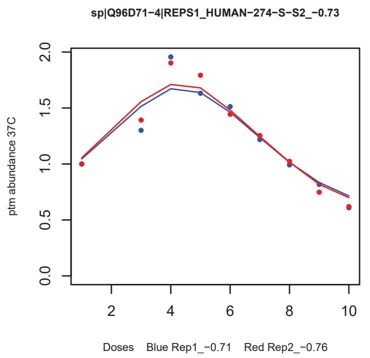
sp|Q96D71−4|REPS1_HUMAN−274−S−S2_−0.73
ptm abundance 37C
Doses Blue Rep1_−0.71 Red Rep2_−0.76

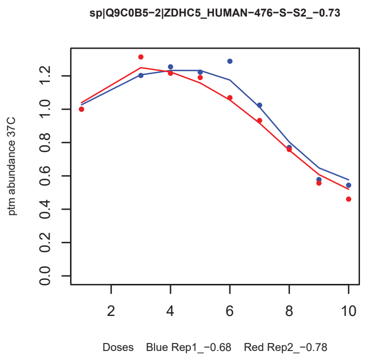
sp|Q9C0B5−2|ZDHC5_HUMAN−476−S−S2_−0.73
ptm abundance 37C
Doses Blue Rep1_−0.68 Red Rep2_−0.78

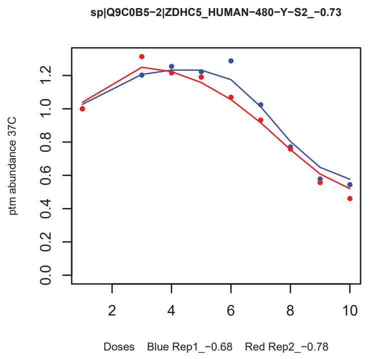
sp|Q9C0B5−2|ZDHC5_HUMAN−480−Y−S2_−0.73
ptm abundance 37C
Doses Blue Rep1_−0.68 Red Rep2_−0.78

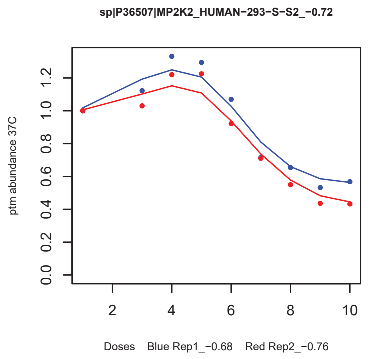
sp|P36507|MP2K2_HUMAN−293−S−S2_−0.72
ptm abundance 37C
Doses Blue Rep1_−0.68 Red Rep2_−0.76

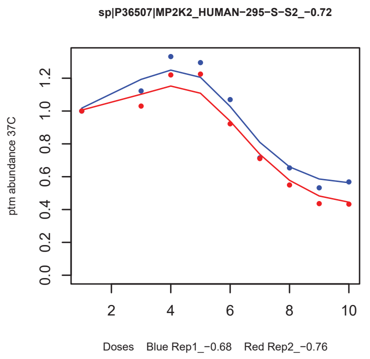
sp|P36507|MP2K2_HUMAN−295−S−S2_−0.72
ptm abundance 37C
Doses Blue Rep1_−0.68 Red Rep2_−0.76

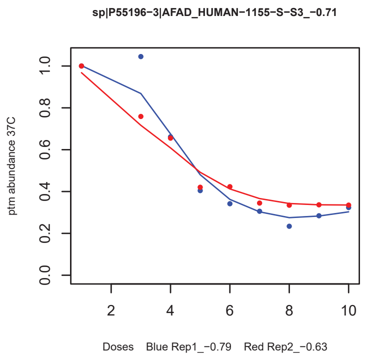
sp|P55196−3|AFAD_HUMAN−1155−S−S3_−0.71
ptm abundance 37C
Doses Blue Rep1_−0.79 Red Rep2_−0.63

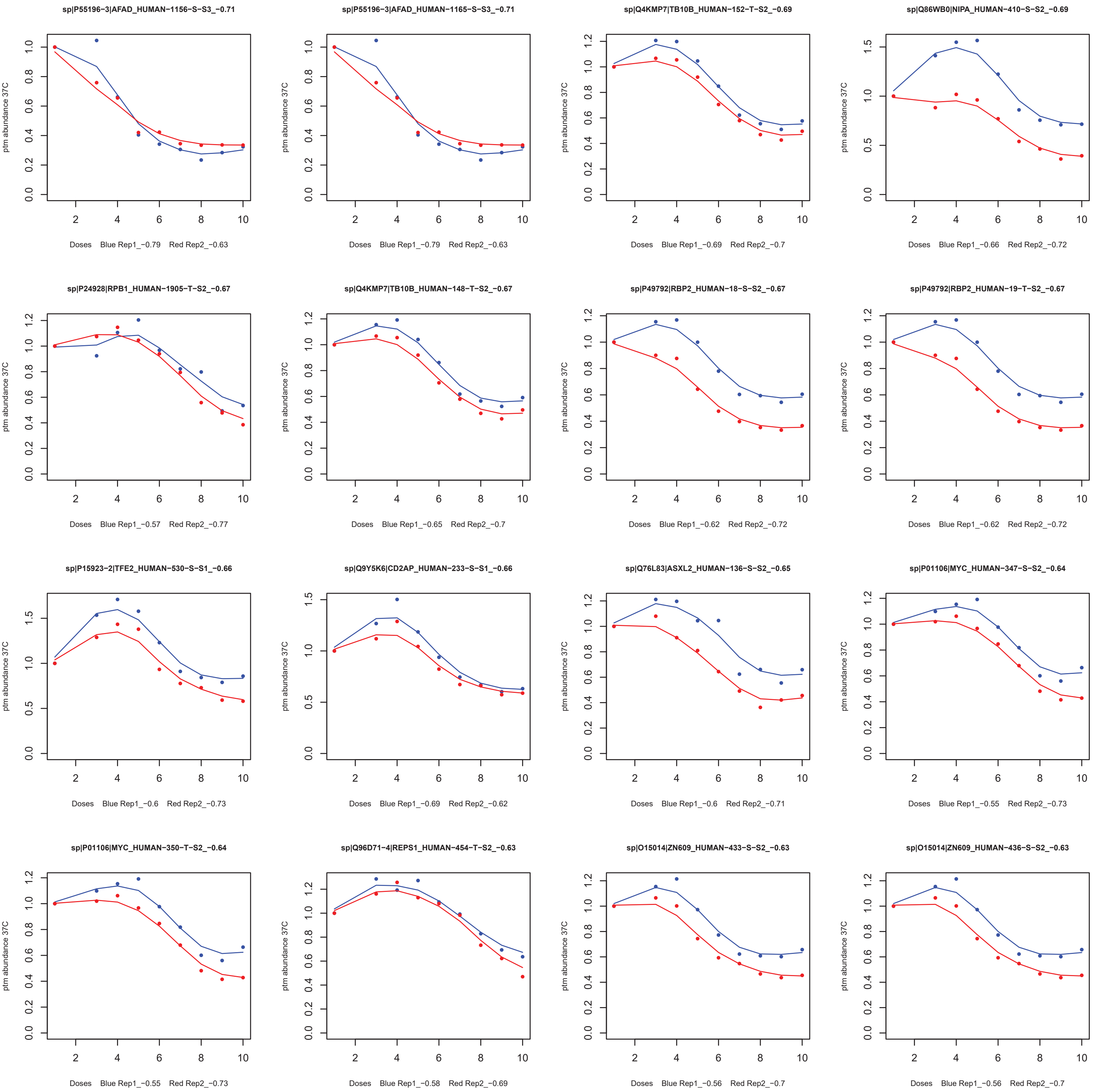
sp|P55196−3|AFAD_HUMAN−1156−S−S3_−0.71
ptm abundance 37C
Doses Blue Rep1_−0.79 Red Rep2_−0.63
sp|P55196−3|AFAD_HUMAN−1165−S−S3_−0.71
ptm abundance 37C
Doses Blue Rep1_−0.79 Red Rep2_−0.63
sp|Q4KMP7|TB10B_HUMAN−152−T−S2_−0.69
ptm abundance 37C
Doses Blue Rep1_−0.69 Red Rep2_−0.7
sp|Q86WB0|NIPA_HUMAN−410−S−S2_−0.69
ptm abundance 37C
Doses Blue Rep1_−0.66 Red Rep2_−0.72
sp|P24928|RPB1_HUMAN−1905−T−S2_−0.67
ptm abundance 37C
Doses Blue Rep1_−0.57 Red Rep2_−0.77
sp|Q4KMP7|TB10B_HUMAN−148−T−S2_−0.67
ptm abundance 37C
Doses Blue Rep1_−0.65 Red Rep2_−0.7
sp|P49792|RBP2_HUMAN−18−S−S2_−0.67
ptm abundance 37C
Doses Blue Rep1_−0.62 Red Rep2_−0.72
sp|P49792|RBP2_HUMAN−19−T−S2_−0.67
ptm abundance 37C
Doses Blue Rep1_−0.62 Red Rep2_−0.72
sp|P15923−2|TFE2_HUMAN−530−S−S1_−0.66
ptm abundance 37C
Doses Blue Rep1_−0.6 Red Rep2_−0.73
sp|Q9Y5K6|CD2AP_HUMAN−233−S−S1_−0.66
ptm abundance 37C
Doses Blue Rep1_−0.69 Red Rep2_−0.62
sp|Q76L83|ASXL2_HUMAN−136−S−S2_−0.65
ptm abundance 37C
Doses Blue Rep1_−0.6 Red Rep2_−0.71
sp|P01106|MYC_HUMAN−347−S−S2_−0.64
ptm abundance 37C
Doses Blue Rep1_−0.55 Red Rep2_−0.73
sp|P01106|MYC_HUMAN−350−T−S2_−0.64
ptm abundance 37C
Doses Blue Rep1_−0.55 Red Rep2_−0.73
sp|Q96D71−4|REPS1_HUMAN−454−T−S2_−0.63
ptm abundance 37C
Doses Blue Rep1_−0.58 Red Rep2_−0.69
sp|O15014|ZN609_HUMAN−433−S−S2_−0.63
ptm abundance 37C
Doses Blue Rep1_−0.56 Red Rep2_−0.7
sp|O15014|ZN609_HUMAN−436−S−S2_−0.63
ptm abundance 37C
Doses Blue Rep1_−0.56 Red Rep2_−0.7

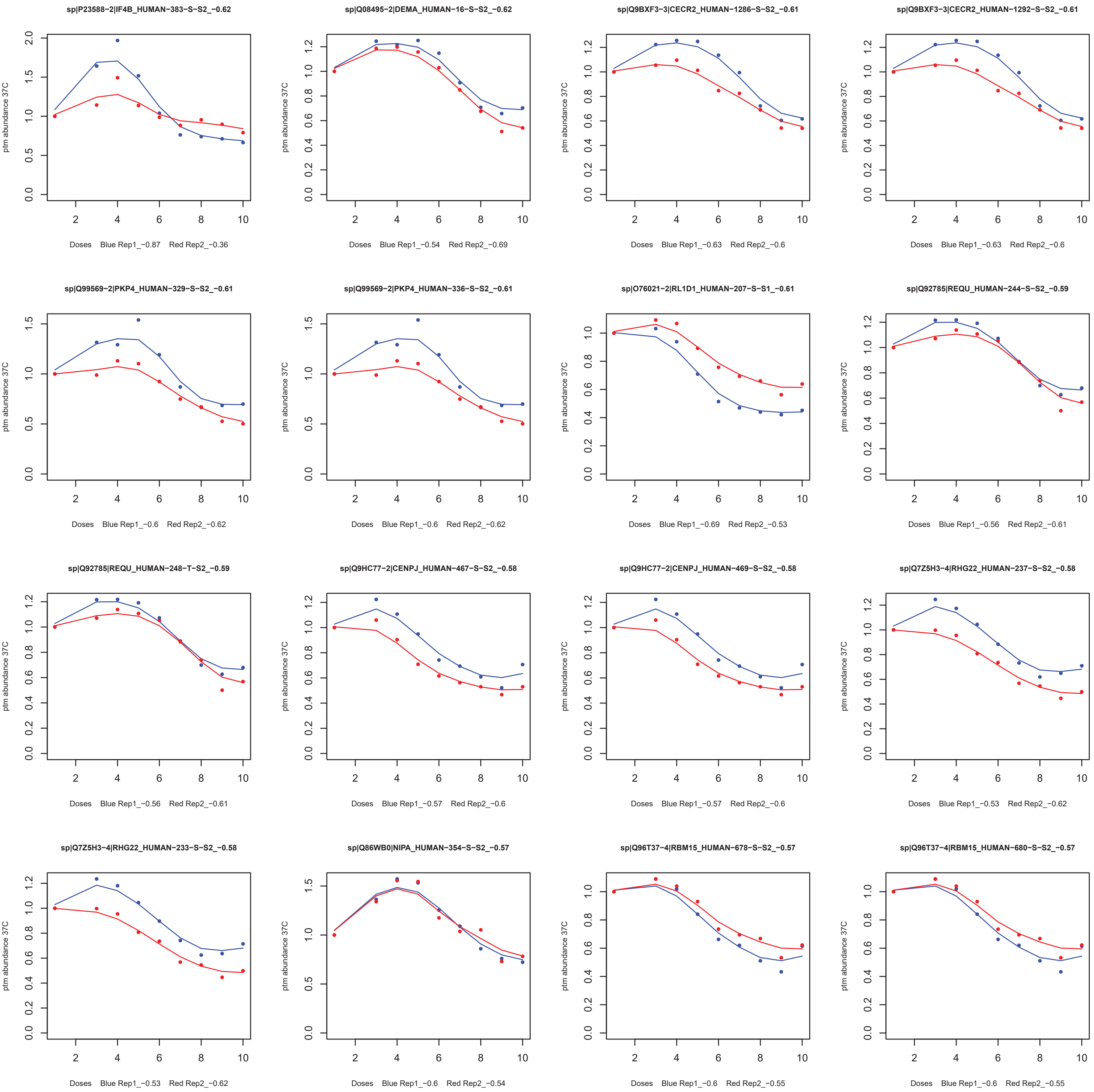

sp|P23588−2|IF4B_HUMAN−383−S−S2_−0.62
Doses Blue Rep1_−0.87 Red Rep2_−0.36
sp|Q08495−2|DEMA_HUMAN−16−S−S2_−0.62
Doses Blue Rep1_−0.54 Red Rep2_−0.69
sp|Q9BXF3−3|CECR2_HUMAN−1286−S−S2_−0.61
Doses Blue Rep1_−0.63 Red Rep2_−0.6
sp|Q9BXF3−3|CECR2_HUMAN−1292−S−S2_−0.61
Doses Blue Rep1_−0.63 Red Rep2_−0.6
sp|Q99569−2|PKP4_HUMAN−329−S−S2_−0.61
Doses Blue Rep1_−0.6 Red Rep2_−0.62
sp|Q99569−2|PKP4_HUMAN−336−S−S2_−0.61
Doses Blue Rep1_−0.6 Red Rep2_−0.62
sp|O76021−2|RL1D1_HUMAN−207−S−S1_−0.61
Doses Blue Rep1_−0.69 Red Rep2_−0.53
sp|Q92785|REQU_HUMAN−244−S−S2_−0.59
Doses Blue Rep1_−0.56 Red Rep2_−0.61
sp|Q92785|REQU_HUMAN−248−T−S2_−0.59
Doses Blue Rep1_−0.56 Red Rep2_−0.61
sp|Q9HC77−2|CENPJ_HUMAN−467−S−S2_−0.58
Doses Blue Rep1_−0.57 Red Rep2_−0.6
sp|Q9HC77−2|CENPJ_HUMAN−469−S−S2_−0.58
Doses Blue Rep1_−0.57 Red Rep2_−0.6
sp|Q7Z5H3−4|RHG22_HUMAN−237−S−S2_−0.58
Doses Blue Rep1_−0.53 Red Rep2_−0.62
sp|Q7Z5H3−4|RHG22_HUMAN−233−S−S2_−0.58
Doses Blue Rep1_−0.53 Red Rep2_−0.62
sp|Q86WB0|NIPA_HUMAN−354−S−S2_−0.57
Doses Blue Rep1_−0.6 Red Rep2_−0.54
sp|Q96T37−4|RBM15_HUMAN−678−S−S2_−0.57
Doses Blue Rep1_−0.6 Red Rep2_−0.55
sp|Q96T37−4|RBM15_HUMAN−680−S−S2_−0.57
Doses Blue Rep1_−0.6 Red Rep2_−0.55
ptm abundance 37C

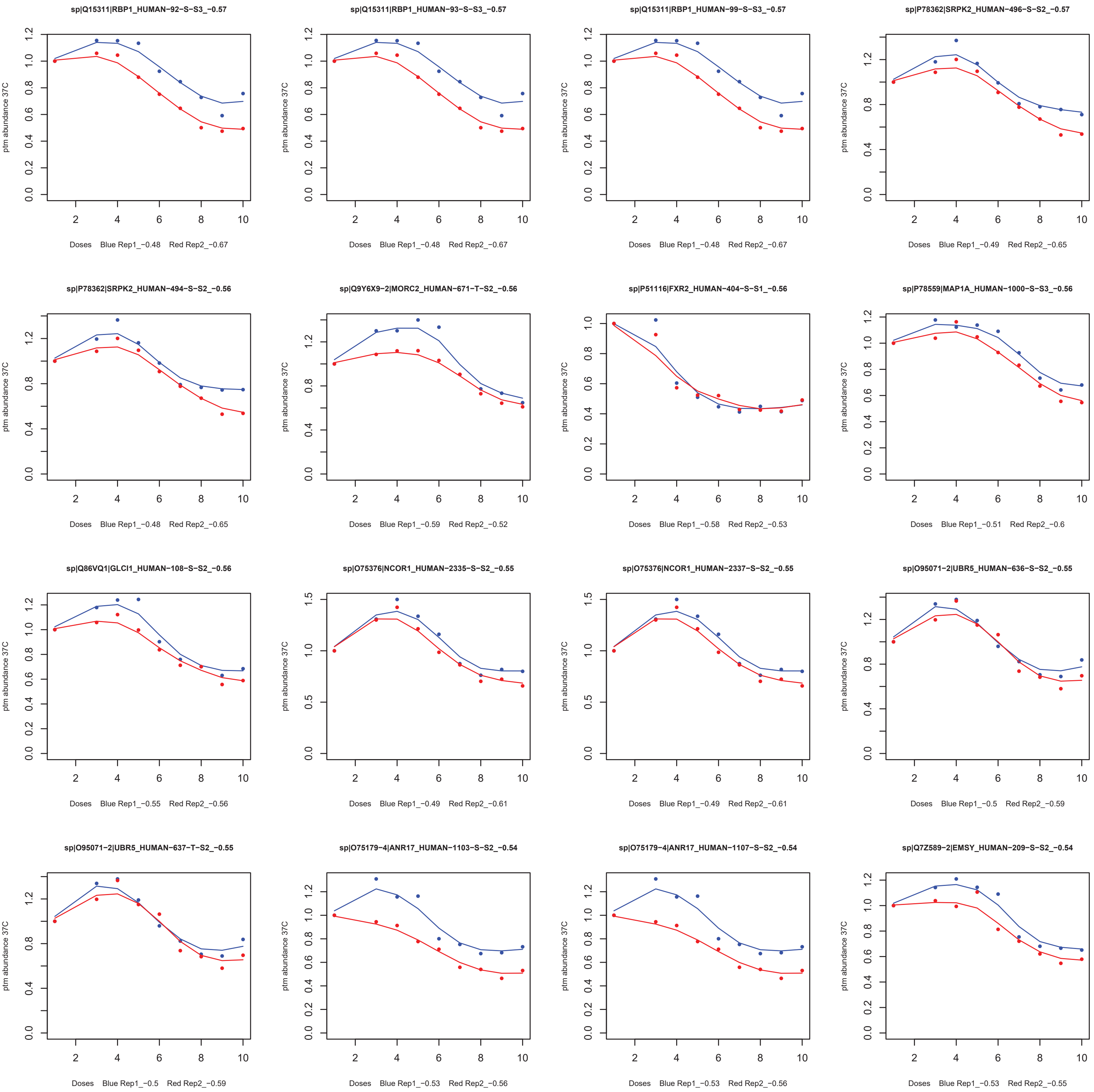
sp|Q15311|RBP1_HUMAN−92−S−S3_−0.57
ptm abundance 37C
Doses Blue Rep1_−0.48 Red Rep2_−0.67
sp|Q15311|RBP1_HUMAN−93−S−S3_−0.57
ptm abundance 37C
Doses Blue Rep1_−0.48 Red Rep2_−0.67
sp|Q15311|RBP1_HUMAN−99−S−S3_−0.57
ptm abundance 37C
Doses Blue Rep1_−0.48 Red Rep2_−0.67
sp|P78362|SRPK2_HUMAN−496−S−S2_−0.57
ptm abundance 37C
Doses Blue Rep1_−0.49 Red Rep2_−0.65
sp|P78362|SRPK2_HUMAN−494−S−S2_−0.56
ptm abundance 37C
Doses Blue Rep1_−0.48 Red Rep2_−0.65
sp|Q9Y6X9−2|MORC2_HUMAN−671−T−S2_−0.56
ptm abundance 37C
Doses Blue Rep1_−0.59 Red Rep2_−0.52
sp|P51116|FXR2_HUMAN−404−S−S1_−0.56
ptm abundance 37C
Doses Blue Rep1_−0.58 Red Rep2_−0.53
sp|P78559|MAP1A_HUMAN−1000−S−S3_−0.56
ptm abundance 37C
Doses Blue Rep1_−0.51 Red Rep2_−0.6
sp|Q86VQ1|GLCI1_HUMAN−108−S−S2_−0.56
ptm abundance 37C
Doses Blue Rep1_−0.55 Red Rep2_−0.56
sp|O75376|NCOR1_HUMAN−2335−S−S2_−0.55
ptm abundance 37C
Doses Blue Rep1_−0.49 Red Rep2_−0.61
sp|O75376|NCOR1_HUMAN−2337−S−S2_−0.55
ptm abundance 37C
Doses Blue Rep1_−0.49 Red Rep2_−0.61
sp|O95071−2|UBR5_HUMAN−636−S−S2_−0.55
ptm abundance 37C
Doses Blue Rep1_−0.5 Red Rep2_−0.59
sp|O95071−2|UBR5_HUMAN−637−T−S2_−0.55
ptm abundance 37C
Doses Blue Rep1_−0.5 Red Rep2_−0.59
sp|O75179−4|ANR17_HUMAN−1103−S−S2_−0.54
ptm abundance 37C
Doses Blue Rep1_−0.53 Red Rep2_−0.56
sp|O75179−4|ANR17_HUMAN−1107−S−S2_−0.54
ptm abundance 37C
Doses Blue Rep1_−0.53 Red Rep2_−0.56
sp|Q7Z589−2|EMSY_HUMAN−209−S−S2_−0.54
ptm abundance 37C
Doses Blue Rep1_−0.53 Red Rep2_−0.55

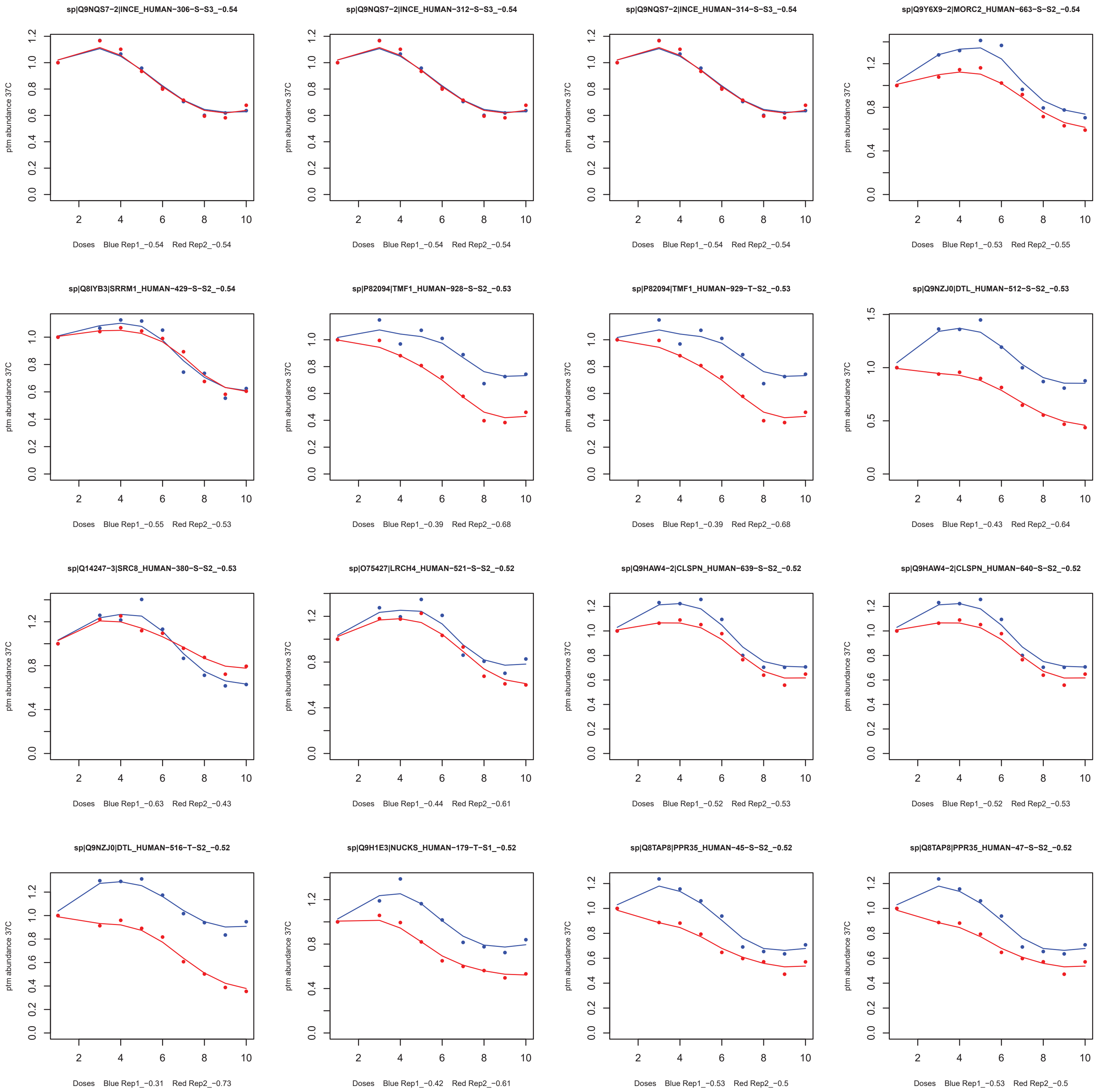

sp|Q9NQS7−2|INCE_HUMAN−306−S−S3_−0.54
ptm abundance 37C
Doses Blue Rep1_−0.54 Red Rep2_−0.54
sp|Q9NQS7−2|INCE_HUMAN−312−S−S3_−0.54
ptm abundance 37C
Doses Blue Rep1_−0.54 Red Rep2_−0.54
sp|Q9NQS7−2|INCE_HUMAN−314−S−S3_−0.54
ptm abundance 37C
Doses Blue Rep1_−0.54 Red Rep2_−0.54
sp|Q9Y6X9−2|MORC2_HUMAN−663−S−S2_−0.54
ptm abundance 37C
Doses Blue Rep1_−0.53 Red Rep2_−0.55
sp|Q8IYB3|SRRM1_HUMAN−429−S−S2_−0.54
ptm abundance 37C
Doses Blue Rep1_−0.55 Red Rep2_−0.53
sp|P82094|TMF1_HUMAN−928−S−S2_−0.53
ptm abundance 37C
Doses Blue Rep1_−0.39 Red Rep2_−0.68
sp|P82094|TMF1_HUMAN−929−T−S2_−0.53
ptm abundance 37C
Doses Blue Rep1_−0.39 Red Rep2_−0.68
sp|Q9NZJ0|DTL_HUMAN−512−S−S2_−0.53
ptm abundance 37C
Doses Blue Rep1_−0.43 Red Rep2_−0.64
sp|Q14247−3|SRC8_HUMAN−380−S−S2_−0.53
ptm abundance 37C
Doses Blue Rep1_−0.63 Red Rep2_−0.43
sp|O75427|LRCH4_HUMAN−521−S−S2_−0.52
ptm abundance 37C
Doses Blue Rep1_−0.44 Red Rep2_−0.61
sp|Q9HAW4−2|CLSPN_HUMAN−639−S−S2_−0.52
ptm abundance 37C
Doses Blue Rep1_−0.52 Red Rep2_−0.53
sp|Q9HAW4−2|CLSPN_HUMAN−640−S−S2_−0.52
ptm abundance 37C
Doses Blue Rep1_−0.52 Red Rep2_−0.53
sp|Q9NZJ0|DTL_HUMAN−516−T−S2_−0.52
ptm abundance 37C
Doses Blue Rep1_−0.31 Red Rep2_−0.73
sp|Q9H1E3|NUCKS_HUMAN−179−T−S1_−0.52
ptm abundance 37C
Doses Blue Rep1_−0.42 Red Rep2_−0.61
sp|Q8TAP8|PPR35_HUMAN−45−S−S2_−0.52
ptm abundance 37C
Doses Blue Rep1_−0.53 Red Rep2_−0.5
sp|Q8TAP8|PPR35_HUMAN−47−S−S2_−0.52
ptm abundance 37C
Doses Blue Rep1_−0.53 Red Rep2_−0.5

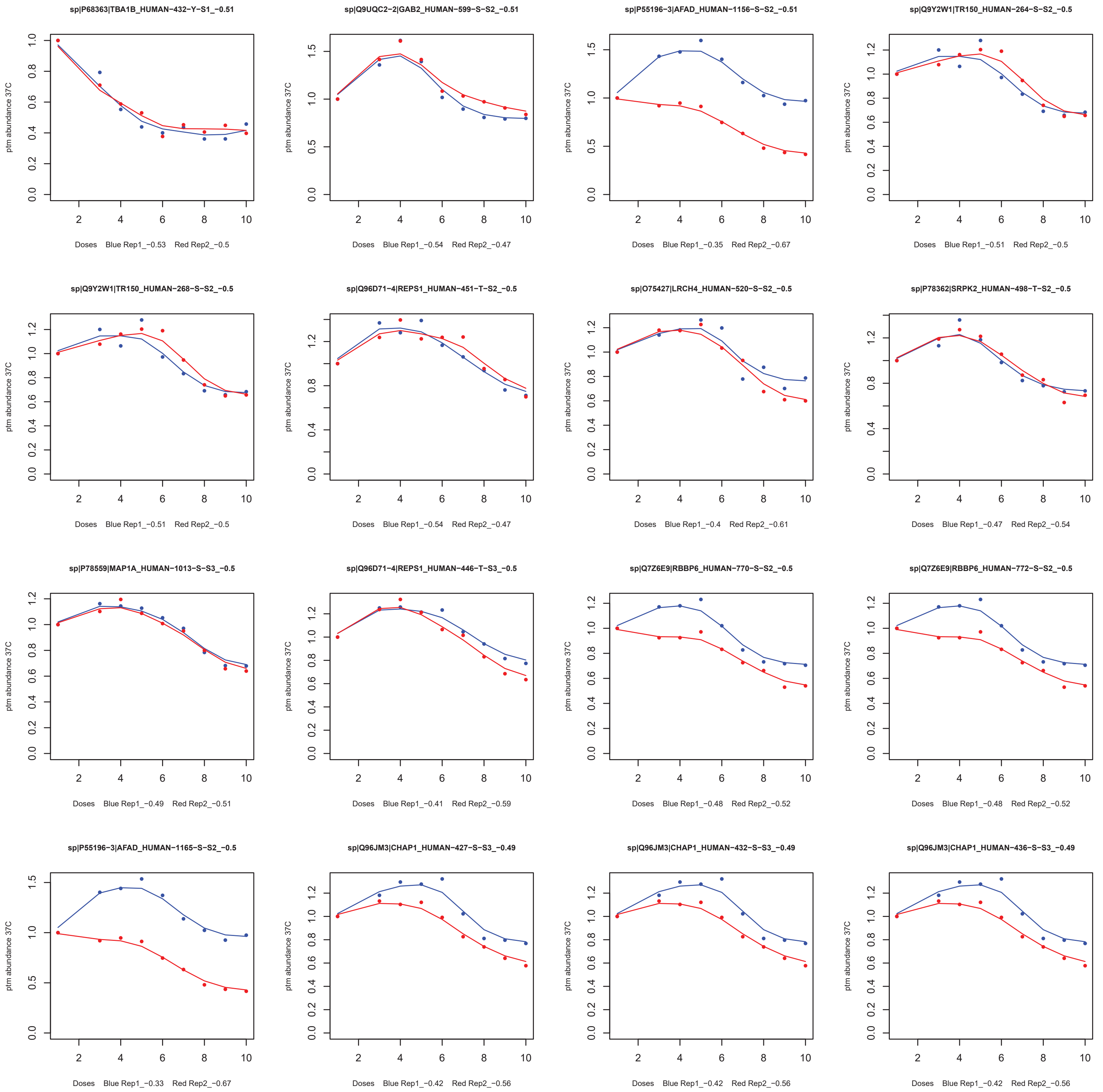

sp|P68363|TBA1B_HUMAN−432−Y−S1_−0.51
ptm abundance 37C
Doses Blue Rep1_−0.53 Red Rep2_−0.5
sp|Q9UQC2−2|GAB2_HUMAN−599−S−S2_−0.51
ptm abundance 37C
Doses Blue Rep1_−0.54 Red Rep2_−0.47
sp|P55196−3|AFAD_HUMAN−1156−S−S2_−0.51
ptm abundance 37C
Doses Blue Rep1_−0.35 Red Rep2_−0.67
sp|Q9Y2W1|TR150_HUMAN−264−S−S2_−0.5
ptm abundance 37C
Doses Blue Rep1_−0.51 Red Rep2_−0.5
sp|Q9Y2W1|TR150_HUMAN−268−S−S2_−0.5
ptm abundance 37C
Doses Blue Rep1_−0.51 Red Rep2_−0.5
sp|Q96D71−4|REPS1_HUMAN−451−T−S2_−0.5
ptm abundance 37C
Doses Blue Rep1_−0.54 Red Rep2_−0.47
sp|O75427|LRCH4_HUMAN−520−S−S2_−0.5
ptm abundance 37C
Doses Blue Rep1_−0.4 Red Rep2_−0.61
sp|P78362|SRPK2_HUMAN−498−T−S2_−0.5
ptm abundance 37C
Doses Blue Rep1_−0.47 Red Rep2_−0.54
sp|P78559|MAP1A_HUMAN−1013−S−S3_−0.5
ptm abundance 37C
Doses Blue Rep1_−0.49 Red Rep2_−0.51
sp|Q96D71−4|REPS1_HUMAN−446−T−S3_−0.5
ptm abundance 37C
Doses Blue Rep1_−0.41 Red Rep2_−0.59
sp|Q7Z6E9|RBBP6_HUMAN−770−S−S2_−0.5
ptm abundance 37C
Doses Blue Rep1_−0.48 Red Rep2_−0.52
sp|Q7Z6E9|RBBP6_HUMAN−772−S−S2_−0.5
ptm abundance 37C
Doses Blue Rep1_−0.48 Red Rep2_−0.52
sp|P55196−3|AFAD_HUMAN−1165−S−S2_−0.5
ptm abundance 37C
Doses Blue Rep1_−0.33 Red Rep2_−0.67
sp|Q96JM3|CHAP1_HUMAN−427−S−S3_−0.49
ptm abundance 37C
Doses Blue Rep1_−0.42 Red Rep2_−0.56
sp|Q96JM3|CHAP1_HUMAN−432−S−S3_−0.49
ptm abundance 37C
Doses Blue Rep1_−0.42 Red Rep2_−0.56
sp|Q96JM3|CHAP1_HUMAN−436−S−S3_−0.49
ptm abundance 37C
Doses Blue Rep1_−0.42 Red Rep2_−0.56

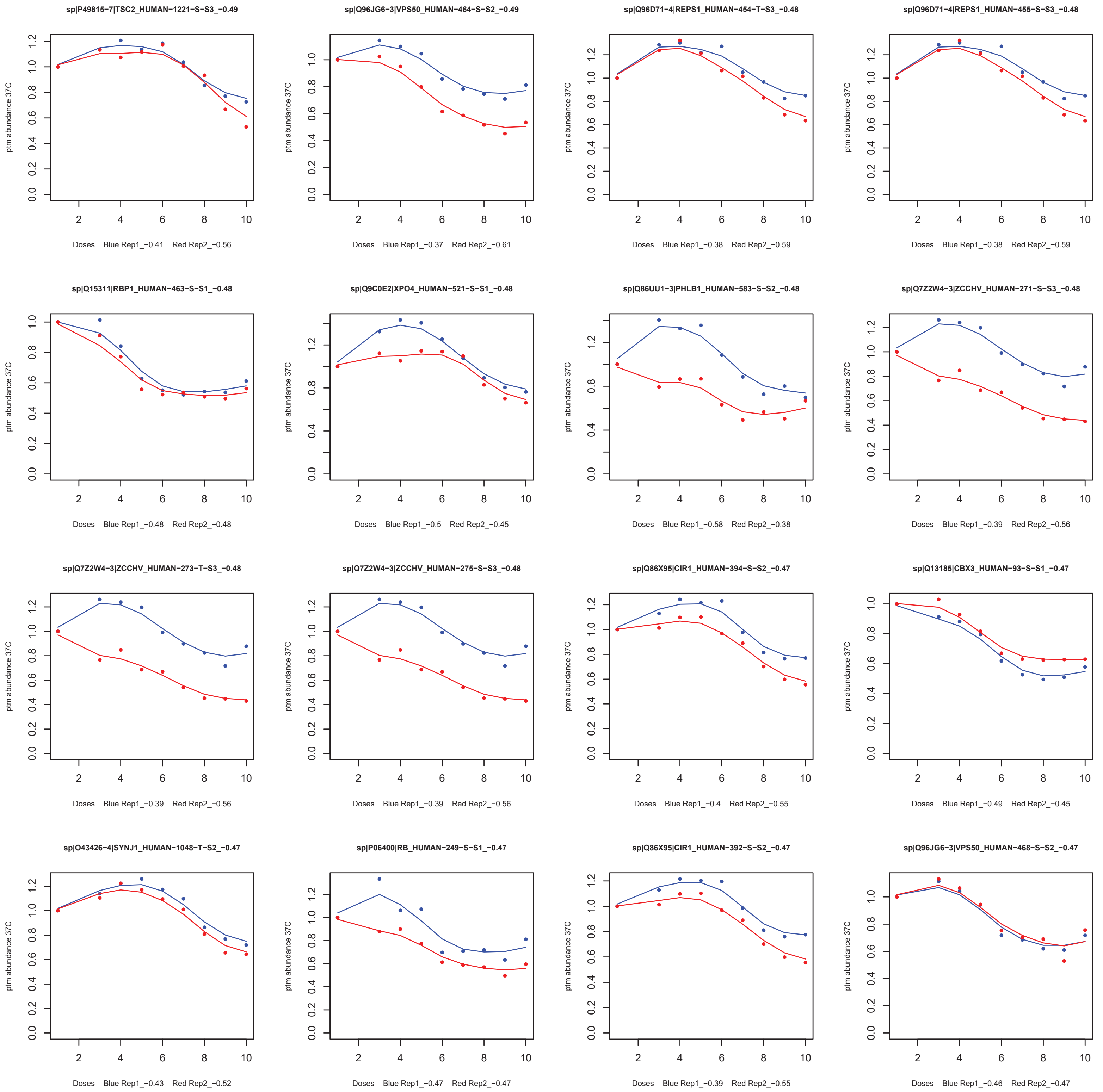
sp|P49815−7|TSC2_HUMAN−1221−S−S3_−0.49
Doses Blue Rep1_−0.41 Red Rep2_−0.56
sp|Q96JG6−3|VPS50_HUMAN−464−S−S2_−0.49
Doses Blue Rep1_−0.37 Red Rep2_−0.61
sp|Q96D71−4|REPS1_HUMAN−454−T−S3_−0.48
Doses Blue Rep1_−0.38 Red Rep2_−0.59
sp|Q96D71−4|REPS1_HUMAN−455−S−S3_−0.48
Doses Blue Rep1_−0.38 Red Rep2_−0.59
sp|Q15311|RBP1_HUMAN−463−S−S1_−0.48
Doses Blue Rep1_−0.48 Red Rep2_−0.48
sp|Q9C0E2|XPO4_HUMAN−521−S−S1_−0.48
Doses Blue Rep1_−0.5 Red Rep2_−0.45
sp|Q86UU1−3|PHLB1_HUMAN−583−S−S2_−0.48
Doses Blue Rep1_−0.58 Red Rep2_−0.38
sp|Q7Z2W4−3|ZCCHV_HUMAN−271−S−S3_−0.48
Doses Blue Rep1_−0.39 Red Rep2_−0.56
sp|Q7Z2W4−3|ZCCHV_HUMAN−273−T−S3_−0.48
Doses Blue Rep1_−0.39 Red Rep2_−0.56
sp|Q7Z2W4−3|ZCCHV_HUMAN−275−S−S3_−0.48
Doses Blue Rep1_−0.39 Red Rep2_−0.56
sp|Q86X95|CIR1_HUMAN−394−S−S2_−0.47
Doses Blue Rep1_−0.4 Red Rep2_−0.55
sp|Q13185|CBX3_HUMAN−93−S−S1_−0.47
Doses Blue Rep1_−0.49 Red Rep2_−0.45
sp|O43426−4|SYNJ1_HUMAN−1048−T−S2_−0.47
Doses Blue Rep1_−0.43 Red Rep2_−0.52
sp|P06400|RB_HUMAN−249−S−S1_−0.47
Doses Blue Rep1_−0.47 Red Rep2_−0.47
sp|Q86X95|CIR1_HUMAN−392−S−S2_−0.47
Doses Blue Rep1_−0.39 Red Rep2_−0.55
sp|Q96JG6−3|VPS50_HUMAN−468−S−S2_−0.47
Doses Blue Rep1_−0.46 Red Rep2_−0.47
ptm abundance 37C

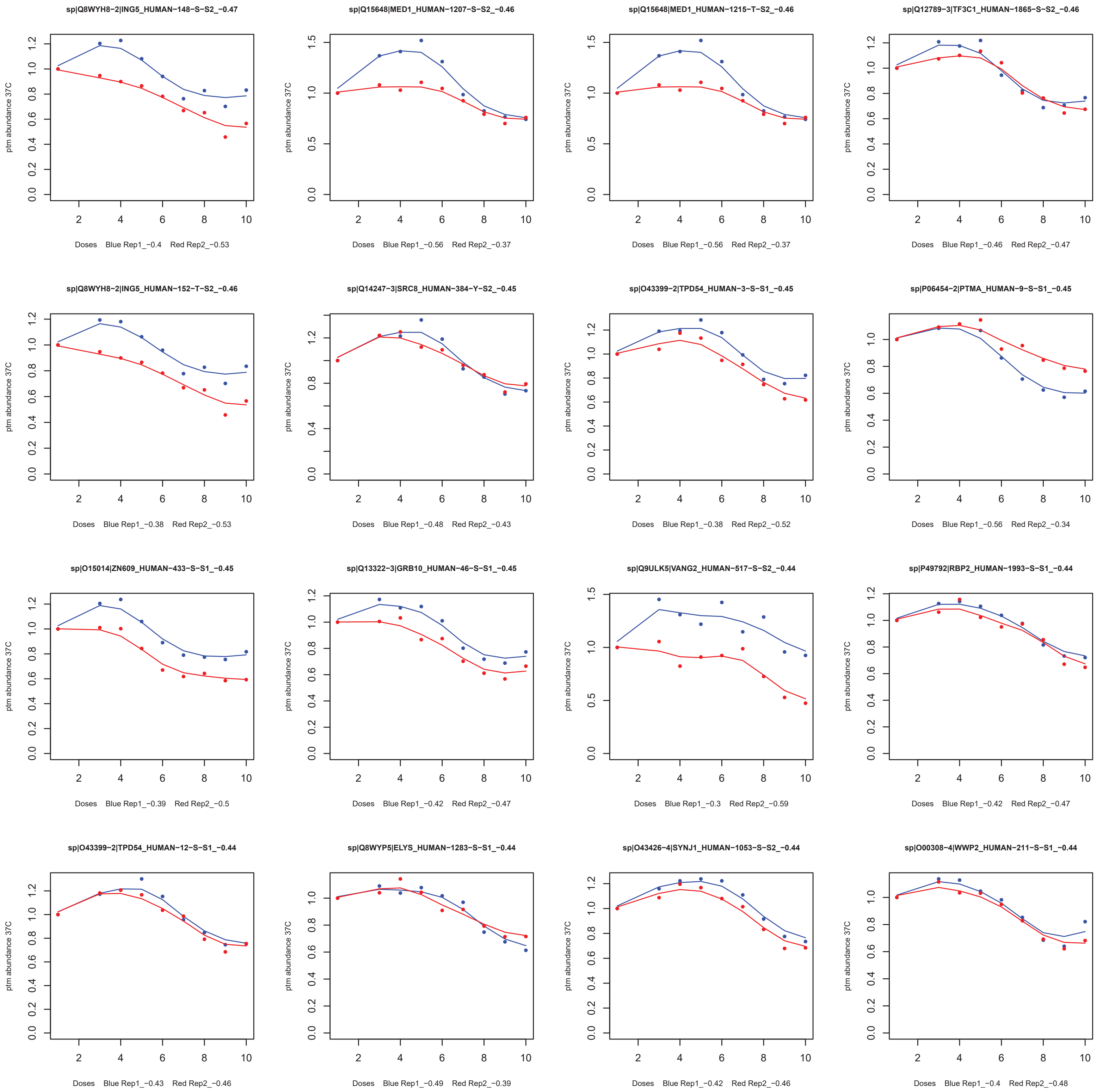

sp|Q8WYH8−2|ING5_HUMAN−148−S−S2_−0.47
ptm abundance 37C
Doses Blue Rep1_−0.4 Red Rep2_−0.53
sp|Q15648|MED1_HUMAN−1207−S−S2_−0.46
ptm abundance 37C
Doses Blue Rep1_−0.56 Red Rep2_−0.37
sp|Q15648|MED1_HUMAN−1215−T−S2_−0.46
ptm abundance 37C
Doses Blue Rep1_−0.56 Red Rep2_−0.37
sp|Q12789−3|TF3C1_HUMAN−1865−S−S2_−0.46
ptm abundance 37C
Doses Blue Rep1_−0.46 Red Rep2_−0.47
sp|Q8WYH8−2|ING5_HUMAN−152−T−S2_−0.46
ptm abundance 37C
Doses Blue Rep1_−0.38 Red Rep2_−0.53
sp|Q14247−3|SRC8_HUMAN−384−Y−S2_−0.45
ptm abundance 37C
Doses Blue Rep1_−0.48 Red Rep2_−0.43
sp|O43399−2|TPD54_HUMAN−3−S−S1_−0.45
ptm abundance 37C
Doses Blue Rep1_−0.38 Red Rep2_−0.52
sp|P06454−2|PTMA_HUMAN−9−S−S1_−0.45
ptm abundance 37C
Doses Blue Rep1_−0.56 Red Rep2_−0.34
sp|O15014|ZN609_HUMAN−433−S−S1_−0.45
ptm abundance 37C
Doses Blue Rep1_−0.39 Red Rep2_−0.5
sp|Q13322−3|GRB10_HUMAN−46−S−S1_−0.45
ptm abundance 37C
Doses Blue Rep1_−0.42 Red Rep2_−0.47
sp|Q9ULK5|VANG2_HUMAN−517−S−S2_−0.44
ptm abundance 37C
Doses Blue Rep1_−0.3 Red Rep2_−0.59
sp|P49792|RBP2_HUMAN−1993−S−S1_−0.44
ptm abundance 37C
Doses Blue Rep1_−0.42 Red Rep2_−0.47
sp|O43399−2|TPD54_HUMAN−12−S−S1_−0.44
ptm abundance 37C
Doses Blue Rep1_−0.43 Red Rep2_−0.46
sp|Q8WYP5|ELYS_HUMAN−1283−S−S1_−0.44
ptm abundance 37C
Doses Blue Rep1_−0.49 Red Rep2_−0.39
sp|O43426−4|SYNJ1_HUMAN−1053−S−S2_−0.44
ptm abundance 37C
Doses Blue Rep1_−0.42 Red Rep2_−0.46
sp|O00308−4|WWP2_HUMAN−211−S−S1_−0.44
ptm abundance 37C
Doses Blue Rep1_−0.4 Red Rep2_−0.48

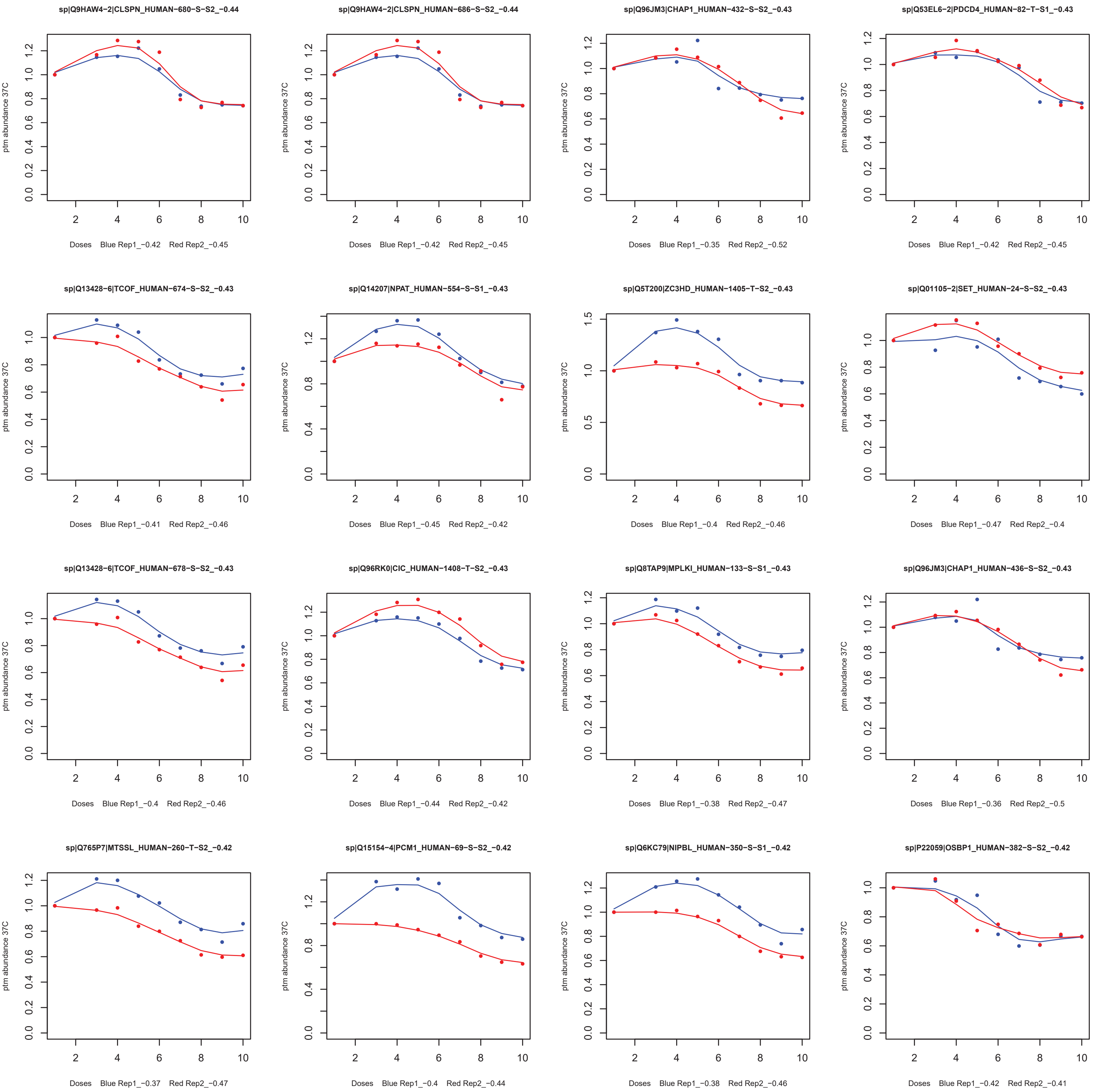
sp|Q9HAW4−2|CLSPN_HUMAN−680−S−S2_−0.44
ptm abundance 37C
Doses Blue Rep1_−0.42 Red Rep2_−0.45
sp|Q9HAW4−2|CLSPN_HUMAN−686−S−S2_−0.44
ptm abundance 37C
Doses Blue Rep1_−0.42 Red Rep2_−0.45
sp|Q96JM3|CHAP1_HUMAN−432−S−S2_−0.43
ptm abundance 37C
Doses Blue Rep1_−0.35 Red Rep2_−0.52
sp|Q53EL6−2|PDCD4_HUMAN−82−T−S1_−0.43
ptm abundance 37C
Doses Blue Rep1_−0.42 Red Rep2_−0.45
sp|Q13428−6|TCOF_HUMAN−674−S−S2_−0.43
ptm abundance 37C
Doses Blue Rep1_−0.41 Red Rep2_−0.46
sp|Q14207|NPAT_HUMAN−554−S−S1_−0.43
ptm abundance 37C
Doses Blue Rep1_−0.45 Red Rep2_−0.42
sp|Q5T200|ZC3HD_HUMAN−1405−T−S2_−0.43
ptm abundance 37C
Doses Blue Rep1_−0.4 Red Rep2_−0.46
sp|Q01105−2|SET_HUMAN−24−S−S2_−0.43
ptm abundance 37C
Doses Blue Rep1_−0.47 Red Rep2_−0.4
sp|Q13428−6|TCOF_HUMAN−678−S−S2_−0.43
ptm abundance 37C
Doses Blue Rep1_−0.4 Red Rep2_−0.46
sp|Q96RK0|CIC_HUMAN−1408−T−S2_−0.43
ptm abundance 37C
Doses Blue Rep1_−0.44 Red Rep2_−0.42
sp|Q8TAP9|MPLKI_HUMAN−133−S−S1_−0.43
ptm abundance 37C
Doses Blue Rep1_−0.38 Red Rep2_−0.47
sp|Q96JM3|CHAP1_HUMAN−436−S−S2_−0.43
ptm abundance 37C
Doses Blue Rep1_−0.36 Red Rep2_−0.5
sp|Q765P7|MTSSL_HUMAN−260−T−S2_−0.42
ptm abundance 37C
Doses Blue Rep1_−0.37 Red Rep2_−0.47
sp|Q15154−4|PCM1_HUMAN−69−S−S2_−0.42
ptm abundance 37C
Doses Blue Rep1_−0.4 Red Rep2_−0.44
sp|Q6KC79|NIPBL_HUMAN−350−S−S1_−0.42
ptm abundance 37C
Doses Blue Rep1_−0.38 Red Rep2_−0.46
sp|P22059|OSBP1_HUMAN−382−S−S2_−0.42
ptm abundance 37C
Doses Blue Rep1_−0.42 Red Rep2_−0.41

sp|Q15139|KPCD1_HUMAN−208−S−S2_−0.42

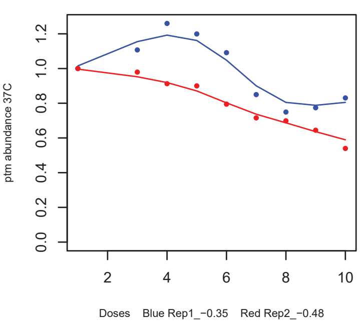

sp|Q86WB0|NIPA_HUMAN−58−S−S2_−0.41

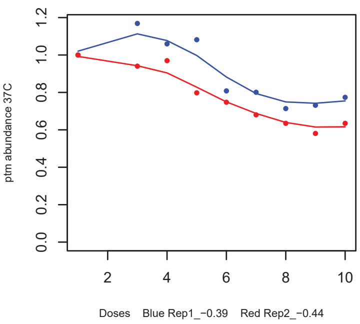

sp|Q86WB0|NIPA_HUMAN−62−S−S2_−0.41

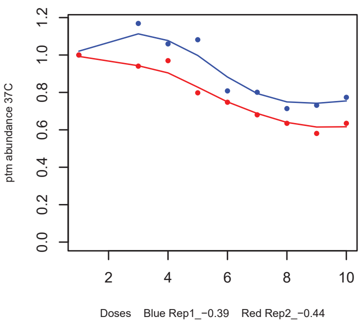

sp|Q63ZY6|NSN5C_HUMAN−258−T−S2_−0.41

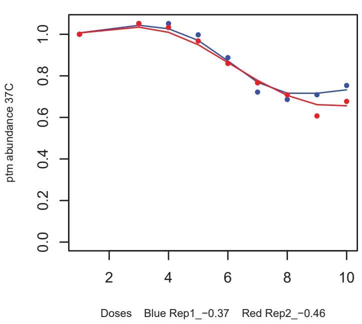

sp|Q63ZY6|NSN5C_HUMAN−260−S−S2_−0.41

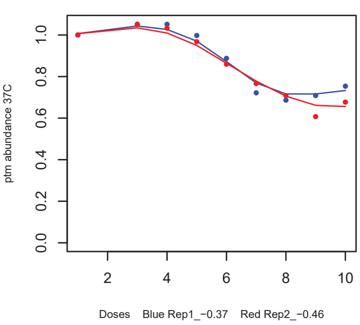

sp|Q5T200|ZC3HD_HUMAN−1406−S−S2_−0.41

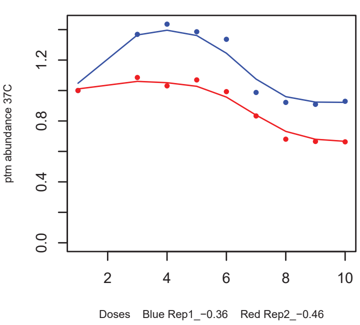

sp|O75179−4|ANR17_HUMAN−1116−S−S1_−0.41

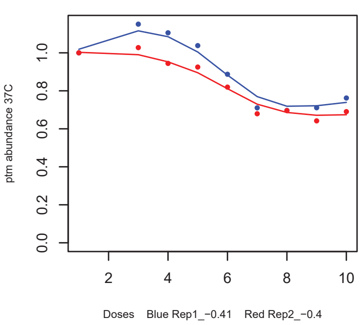

sp|P46013|KI67_HUMAN−357−S−S1_−0.41

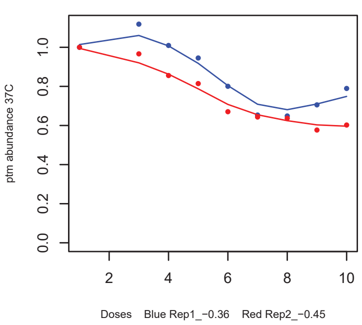

sp|Q04726−2|TLE3_HUMAN−286−S−S2_−0.41

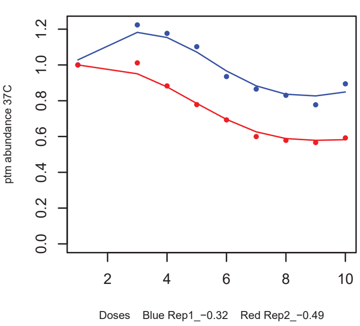

sp|P00519−2|ABL1_HUMAN−412−Y−S1_−0.4

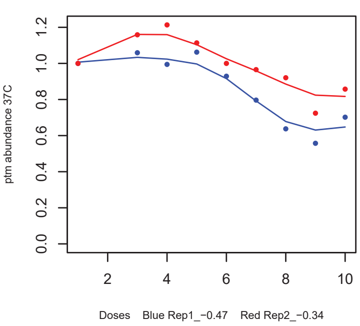

sp|Q14247−3|SRC8_HUMAN−381−S−S2_−0.4

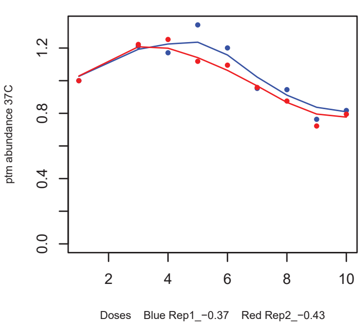

sp|O60271−5|JIP4_HUMAN−254−S−S2_−0.4

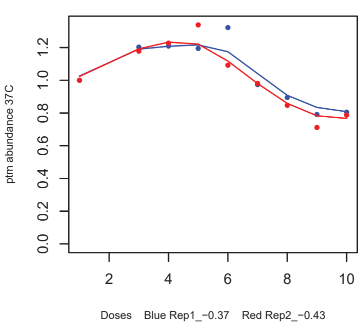

sp|Q96RK0|CIC_HUMAN−1409−S−S2_−0.4

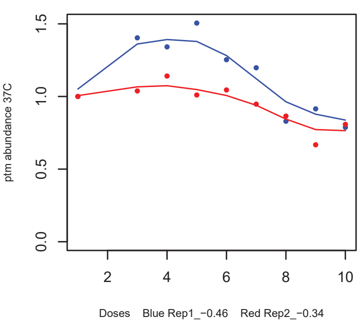

sp|Q8N5C8−2|TAB3_HUMAN−644−S−S1_−0.4

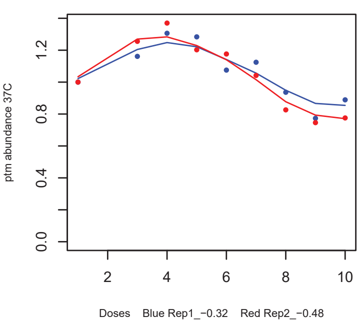

sp|Q15648|MED1_HUMAN−1447−S−S1_−0.4

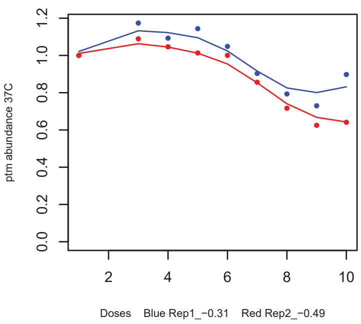

sp|Q99590−2|SCAFB_HUMAN−483−S−S2_−0.4

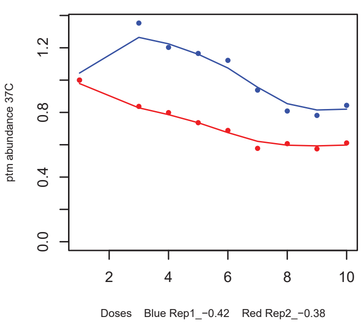

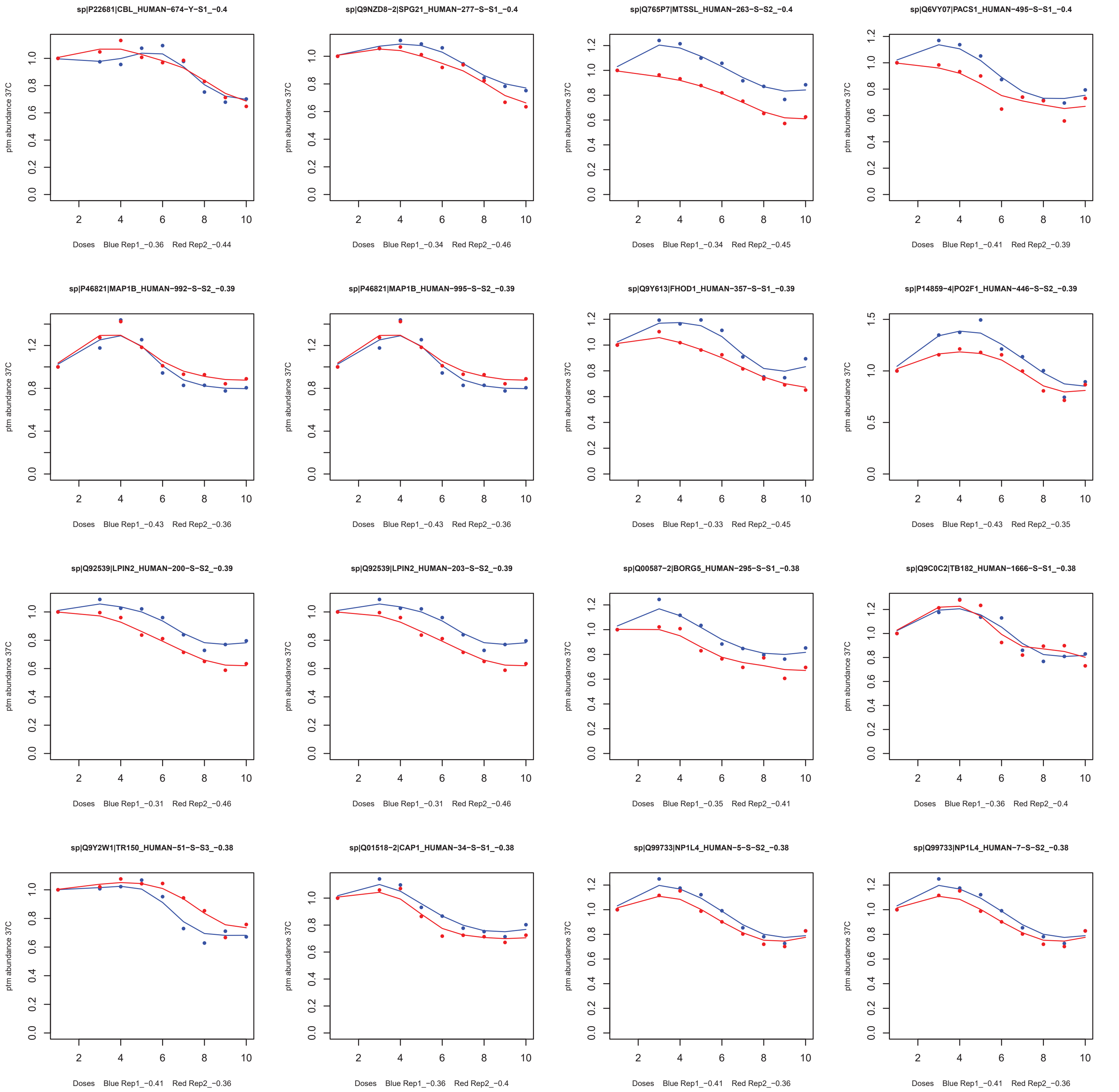
sp|P22681|CBL_HUMAN−674−Y−S1_−0.4
Doses Blue Rep1_−0.36 Red Rep2_−0.44
sp|Q9NZD8−2|SPG21_HUMAN−277−S−S1_−0.4
Doses Blue Rep1_−0.34 Red Rep2_−0.46
sp|Q765P7|MTSSL_HUMAN−263−S−S2_−0.4
Doses Blue Rep1_−0.34 Red Rep2_−0.45
sp|Q6VY07|PACS1_HUMAN−495−S−S1_−0.4
Doses Blue Rep1_−0.41 Red Rep2_−0.39
sp|P46821|MAP1B_HUMAN−992−S−S2_−0.39
Doses Blue Rep1_−0.43 Red Rep2_−0.36
sp|P46821|MAP1B_HUMAN−995−S−S2_−0.39
Doses Blue Rep1_−0.43 Red Rep2_−0.36
sp|Q9Y613|FHOD1_HUMAN−357−S−S1_−0.39
Doses Blue Rep1_−0.33 Red Rep2_−0.45
sp|P14859−4|PO2F1_HUMAN−446−S−S2_−0.39
Doses Blue Rep1_−0.43 Red Rep2_−0.35
sp|Q92539|LPIN2_HUMAN−200−S−S2_−0.39
Doses Blue Rep1_−0.31 Red Rep2_−0.46
sp|Q92539|LPIN2_HUMAN−203−S−S2_−0.39
Doses Blue Rep1_−0.31 Red Rep2_−0.46
sp|Q00587−2|BORG5_HUMAN−295−S−S1_−0.38
Doses Blue Rep1_−0.35 Red Rep2_−0.41
sp|Q9C0C2|TB182_HUMAN−1666−S−S1_−0.38
Doses Blue Rep1_−0.36 Red Rep2_−0.4
sp|Q9Y2W1|TR150_HUMAN−51−S−S3_−0.38
Doses Blue Rep1_−0.41 Red Rep2_−0.36
sp|Q01518−2|CAP1_HUMAN−34−S−S1_−0.38
Doses Blue Rep1_−0.36 Red Rep2_−0.4
sp|Q99733|NP1L4_HUMAN−5−S−S2_−0.38
Doses Blue Rep1_−0.41 Red Rep2_−0.36
sp|Q99733|NP1L4_HUMAN−7−S−S2_−0.38
Doses Blue Rep1_−0.41 Red Rep2_−0.36
ptm abundance 37C

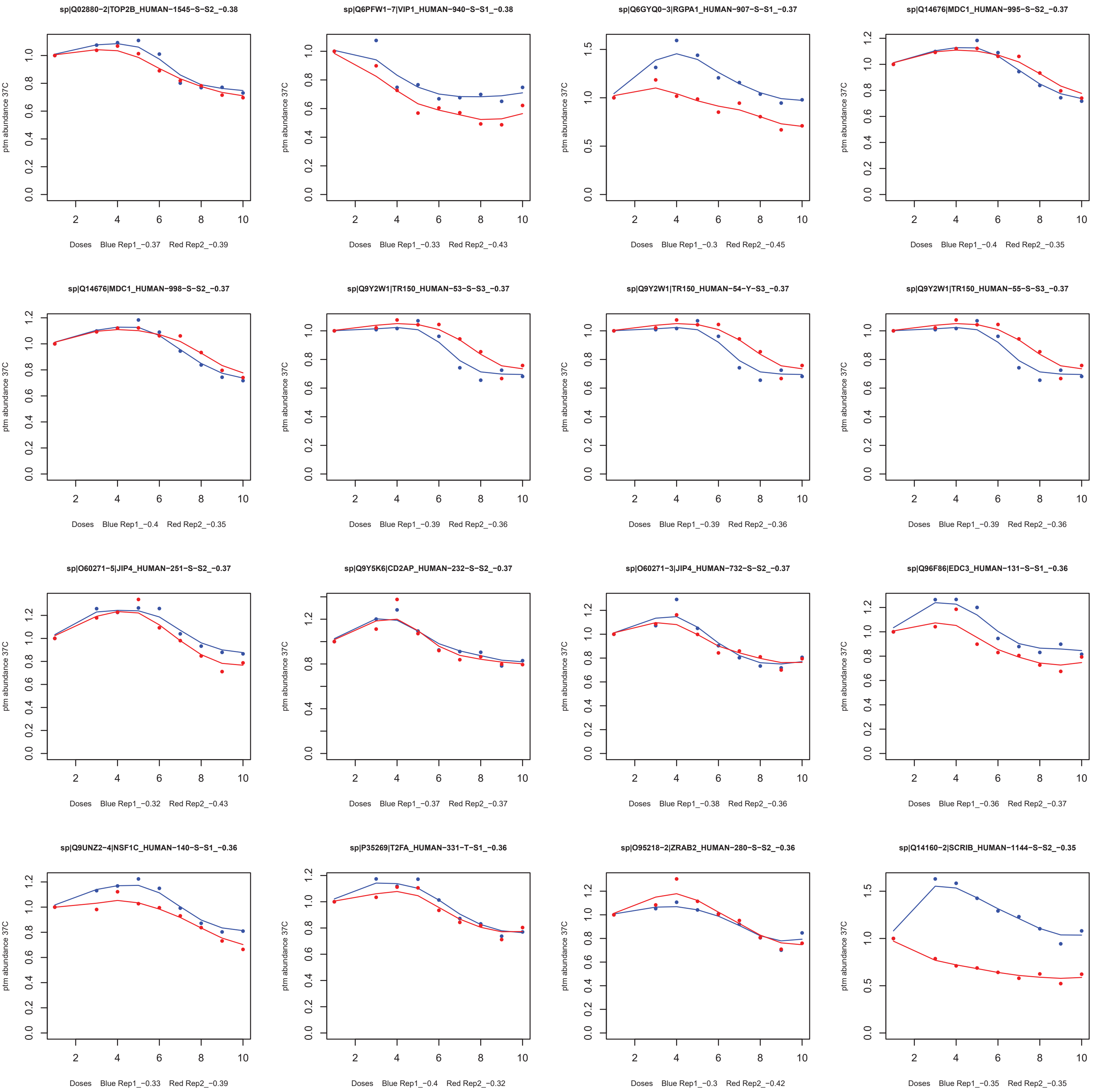
sp|Q02880−2|TOP2B_HUMAN−1545−S−S2_−0.38
Doses Blue Rep1_−0.37 Red Rep2_−0.39
sp|Q6PFW1−7|VIP1_HUMAN−940−S−S1_−0.38
Doses Blue Rep1_−0.33 Red Rep2_−0.43
sp|Q6GYQ0−3|RGPA1_HUMAN−907−S−S1_−0.37
Doses Blue Rep1_−0.3 Red Rep2_−0.45
sp|Q14676|MDC1_HUMAN−995−S−S2_−0.37
Doses Blue Rep1_−0.4 Red Rep2_−0.35
sp|Q14676|MDC1_HUMAN−998−S−S2_−0.37
Doses Blue Rep1_−0.4 Red Rep2_−0.35
sp|Q9Y2W1|TR150_HUMAN−53−S−S3_−0.37
Doses Blue Rep1_−0.39 Red Rep2_−0.36
sp|Q9Y2W1|TR150_HUMAN−54−Y−S3_−0.37
Doses Blue Rep1_−0.39 Red Rep2_−0.36
sp|Q9Y2W1|TR150_HUMAN−55−S−S3_−0.37
Doses Blue Rep1_−0.39 Red Rep2_−0.36
sp|O60271−5|JIP4_HUMAN−251−S−S2_−0.37
Doses Blue Rep1_−0.32 Red Rep2_−0.43
sp|Q9Y5K6|CD2AP_HUMAN−232−S−S2_−0.37
Doses Blue Rep1_−0.37 Red Rep2_−0.37
sp|O60271−3|JIP4_HUMAN−732−S−S2_−0.37
Doses Blue Rep1_−0.38 Red Rep2_−0.36
sp|Q96F86|EDC3_HUMAN−131−S−S1_−0.36
Doses Blue Rep1_−0.36 Red Rep2_−0.37
sp|Q9UNZ2−4|NSF1C_HUMAN−140−S−S1_−0.36
Doses Blue Rep1_−0.33 Red Rep2_−0.39
sp|P35269|T2FA_HUMAN−331−T−S1_−0.36
Doses Blue Rep1_−0.4 Red Rep2_−0.32
sp|O95218−2|ZRAB2_HUMAN−280−S−S2_−0.36
Doses Blue Rep1_−0.3 Red Rep2_−0.42
sp|Q14160−2|SCRIB_HUMAN−1144−S−S2_−0.35
Doses Blue Rep1_−0.35 Red Rep2_−0.35
ptm abundance 37C

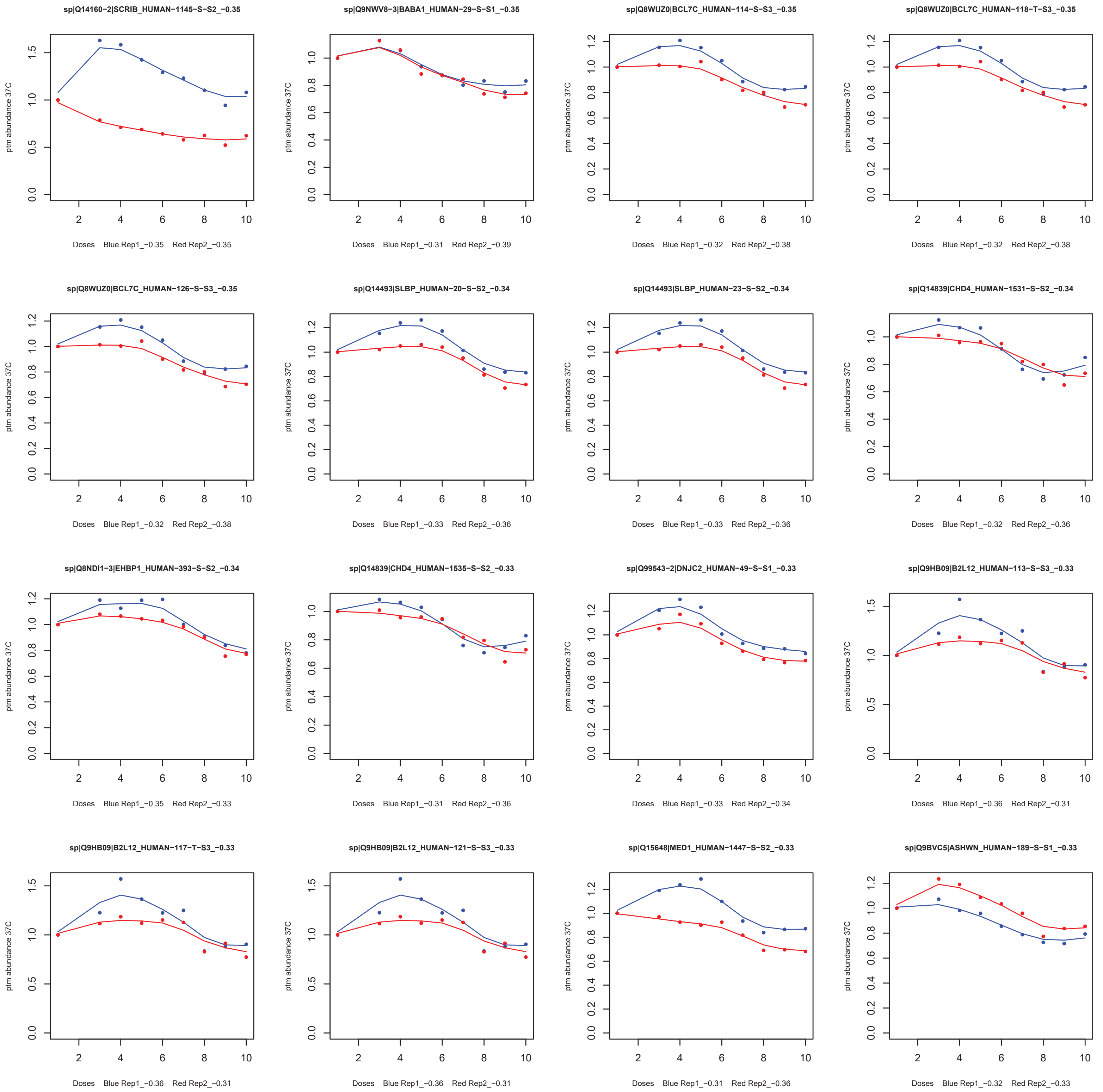

sp|Q14160−2|SCRIB_HUMAN−1145−S−S2_−0.35
ptm abundance 37C
Doses Blue Rep1_−0.35 Red Rep2_−0.35
sp|Q9NWV8−3|BABA1_HUMAN−29−S−S1_−0.35
ptm abundance 37C
Doses Blue Rep1_−0.31 Red Rep2_−0.39
sp|Q8WUZ0|BCL7C_HUMAN−114−S−S3_−0.35
ptm abundance 37C
Doses Blue Rep1_−0.32 Red Rep2_−0.38
sp|Q8WUZ0|BCL7C_HUMAN−118−T−S3_−0.35
ptm abundance 37C
Doses Blue Rep1_−0.32 Red Rep2_−0.38
sp|Q8WUZ0|BCL7C_HUMAN−126−S−S3_−0.35
ptm abundance 37C
Doses Blue Rep1_−0.32 Red Rep2_−0.38
sp|Q14493|SLBP_HUMAN−20−S−S2_−0.34
ptm abundance 37C
Doses Blue Rep1_−0.33 Red Rep2_−0.36
sp|Q14493|SLBP_HUMAN−23−S−S2_−0.34
ptm abundance 37C
Doses Blue Rep1_−0.33 Red Rep2_−0.36
sp|Q14839|CHD4_HUMAN−1531−S−S2_−0.34
ptm abundance 37C
Doses Blue Rep1_−0.32 Red Rep2_−0.36
sp|Q8NDI1−3|EHBP1_HUMAN−393−S−S2_−0.34
ptm abundance 37C
Doses Blue Rep1_−0.35 Red Rep2_−0.33
sp|Q14839|CHD4_HUMAN−1535−S−S2_−0.33
ptm abundance 37C
Doses Blue Rep1_−0.31 Red Rep2_−0.36
sp|Q99543−2|DNJC2_HUMAN−49−S−S1_−0.33
ptm abundance 37C
Doses Blue Rep1_−0.33 Red Rep2_−0.34
sp|Q9HB09|B2L12_HUMAN−113−S−S3_−0.33
ptm abundance 37C
Doses Blue Rep1_−0.36 Red Rep2_−0.31
sp|Q9HB09|B2L12_HUMAN−117−T−S3_−0.33
ptm abundance 37C
Doses Blue Rep1_−0.36 Red Rep2_−0.31
sp|Q9HB09|B2L12_HUMAN−121−S−S3_−0.33
ptm abundance 37C
Doses Blue Rep1_−0.36 Red Rep2_−0.31
sp|Q15648|MED1_HUMAN−1447−S−S2_−0.33
ptm abundance 37C
Doses Blue Rep1_−0.31 Red Rep2_−0.36
sp|Q9BVC5|ASHWN_HUMAN−189−S−S1_−0.33
ptm abundance 37C
Doses Blue Rep1_−0.32 Red Rep2_−0.33

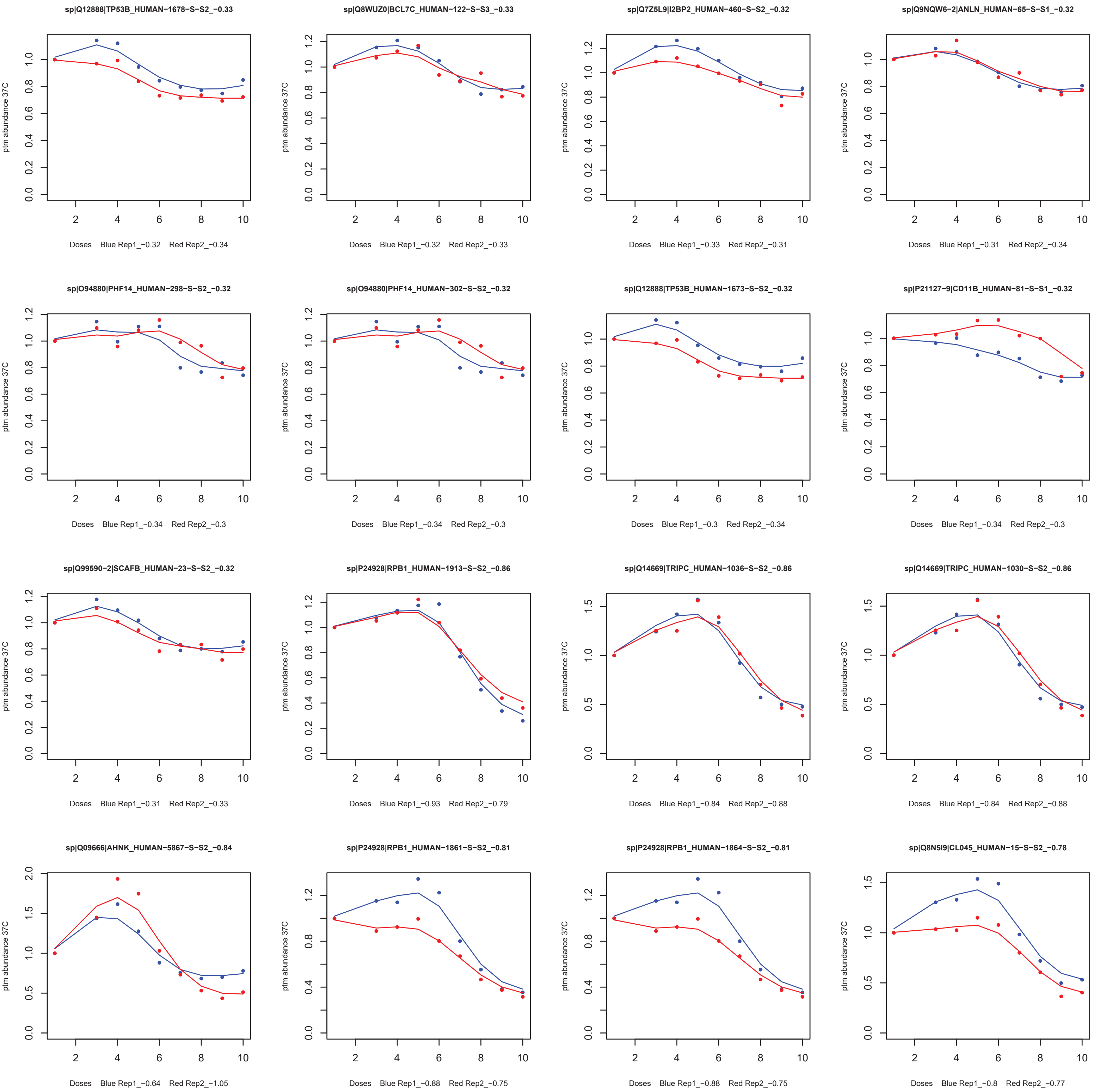

sp|Q12888|TP53B_HUMAN−1678−S−S2_−0.33
Doses Blue Rep1_−0.32 Red Rep2_−0.34
sp|Q8WUZ0|BCL7C_HUMAN−122−S−S3_−0.33
Doses Blue Rep1_−0.32 Red Rep2_−0.33
sp|Q7Z5L9|I2BP2_HUMAN−460−S−S2_−0.32
Doses Blue Rep1_−0.33 Red Rep2_−0.31
sp|Q9NQW6−2|ANLN_HUMAN−65−S−S1_−0.32
Doses Blue Rep1_−0.31 Red Rep2_−0.34
sp|O94880|PHF14_HUMAN−298−S−S2_−0.32
Doses Blue Rep1_−0.34 Red Rep2_−0.3
sp|O94880|PHF14_HUMAN−302−S−S2_−0.32
Doses Blue Rep1_−0.34 Red Rep2_−0.3
sp|Q12888|TP53B_HUMAN−1673−S−S2_−0.32
Doses Blue Rep1_−0.3 Red Rep2_−0.34
sp|P21127−9|CD11B_HUMAN−81−S−S1_−0.32
Doses Blue Rep1_−0.34 Red Rep2_−0.3
sp|Q99590−2|SCAFB_HUMAN−23−S−S2_−0.32
Doses Blue Rep1_−0.31 Red Rep2_−0.33
sp|P24928|RPB1_HUMAN−1913−S−S2_−0.86
Doses Blue Rep1_−0.93 Red Rep2_−0.79
sp|Q14669|TRIPC_HUMAN−1036−S−S2_−0.86
Doses Blue Rep1_−0.84 Red Rep2_−0.88
sp|Q14669|TRIPC_HUMAN−1030−S−S2_−0.86
Doses Blue Rep1_−0.84 Red Rep2_−0.88
sp|Q09666|AHNK_HUMAN−5867−S−S2_−0.84
Doses Blue Rep1_−0.64 Red Rep2_−1.05
sp|P24928|RPB1_HUMAN−1861−S−S2_−0.81
Doses Blue Rep1_−0.88 Red Rep2_−0.75
sp|P24928|RPB1_HUMAN−1864−S−S2_−0.81
Doses Blue Rep1_−0.88 Red Rep2_−0.75
sp|Q8N5I9|CL045_HUMAN−15−S−S2_−0.78
Doses Blue Rep1_−0.8 Red Rep2_−0.77
ptm abundance 37C

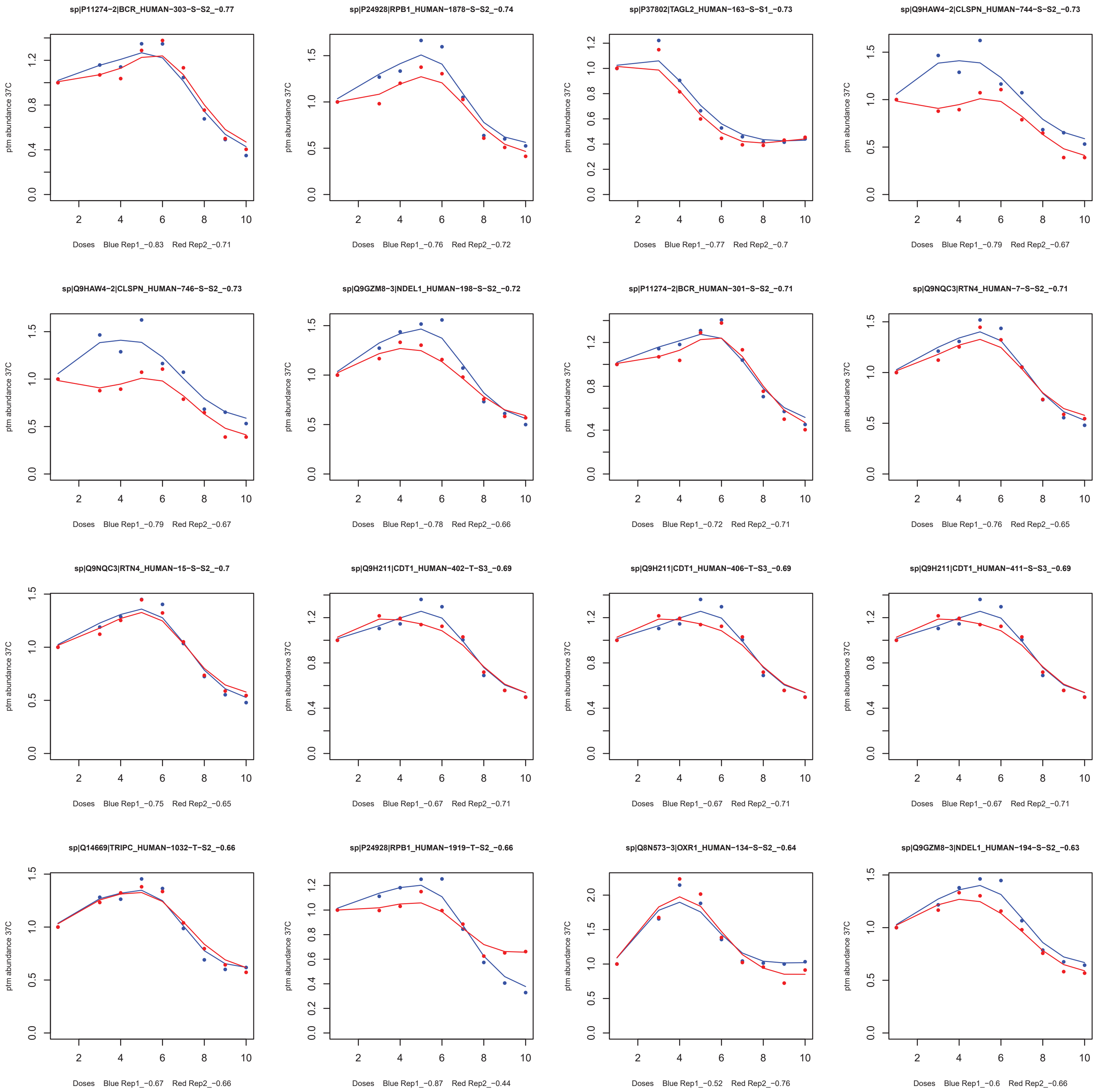

sp|P11274−2|BCR_HUMAN−303−S−S2_−0.77
ptm abundance 37C
Doses Blue Rep1_−0.83 Red Rep2_−0.71
sp|P24928|RPB1_HUMAN−1878−S−S2_−0.74
ptm abundance 37C
Doses Blue Rep1_−0.76 Red Rep2_−0.72
sp|P37802|TAGL2_HUMAN−163−S−S1_−0.73
ptm abundance 37C
Doses Blue Rep1_−0.77 Red Rep2_−0.7
sp|Q9HAW4−2|CLSPN_HUMAN−744−S−S2_−0.73
ptm abundance 37C
Doses Blue Rep1_−0.79 Red Rep2_−0.67
sp|Q9HAW4−2|CLSPN_HUMAN−746−S−S2_−0.73
ptm abundance 37C
Doses Blue Rep1_−0.79 Red Rep2_−0.67
sp|Q9GZM8−3|NDEL1_HUMAN−198−S−S2_−0.72
ptm abundance 37C
Doses Blue Rep1_−0.78 Red Rep2_−0.66
sp|P11274−2|BCR_HUMAN−301−S−S2_−0.71
ptm abundance 37C
Doses Blue Rep1_−0.72 Red Rep2_−0.71
sp|Q9NQC3|RTN4_HUMAN−7−S−S2_−0.71
ptm abundance 37C
Doses Blue Rep1_−0.76 Red Rep2_−0.65
sp|Q9NQC3|RTN4_HUMAN−15−S−S2_−0.7
ptm abundance 37C
Doses Blue Rep1_−0.75 Red Rep2_−0.65
sp|Q9H211|CDT1_HUMAN−402−T−S3_−0.69
ptm abundance 37C
Doses Blue Rep1_−0.67 Red Rep2_−0.71
sp|Q9H211|CDT1_HUMAN−406−T−S3_−0.69
ptm abundance 37C
Doses Blue Rep1_−0.67 Red Rep2_−0.71
sp|Q9H211|CDT1_HUMAN−411−S−S3_−0.69
ptm abundance 37C
Doses Blue Rep1_−0.67 Red Rep2_−0.71
sp|Q14669|TRIPC_HUMAN−1032−T−S2_−0.66
ptm abundance 37C
Doses Blue Rep1_−0.67 Red Rep2_−0.66
sp|P24928|RPB1_HUMAN−1919−T−S2_−0.66
ptm abundance 37C
Doses Blue Rep1_−0.87 Red Rep2_−0.44
sp|Q8N573−3|OXR1_HUMAN−134−S−S2_−0.64
ptm abundance 37C
Doses Blue Rep1_−0.52 Red Rep2_−0.76
sp|Q9GZM8−3|NDEL1_HUMAN−194−S−S2_−0.63
ptm abundance 37C
Doses Blue Rep1_−0.6 Red Rep2_−0.66

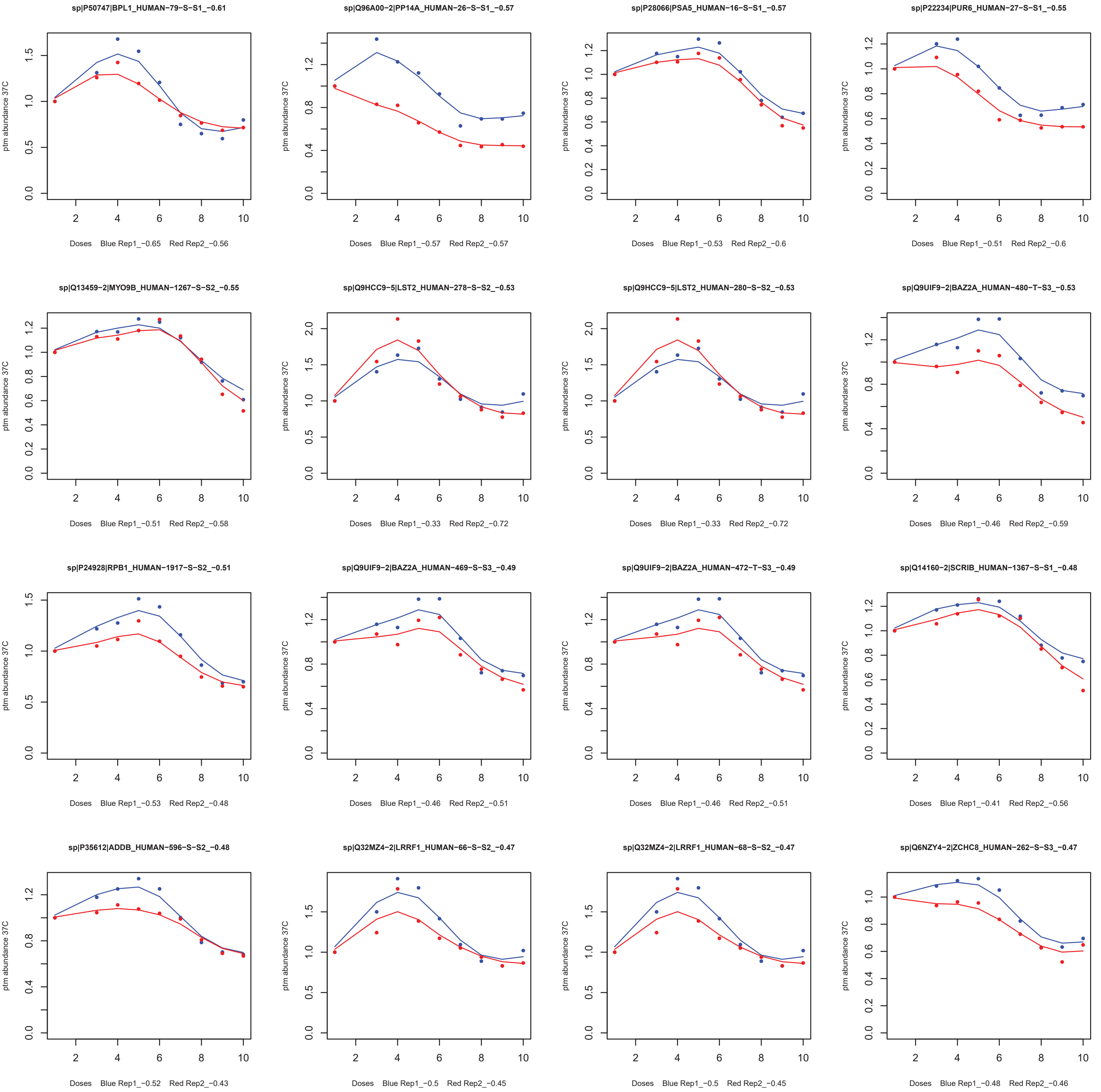

sp|P50747|BPL1_HUMAN−79−S−S1_−0.61
Doses Blue Rep1_−0.65 Red Rep2_−0.56
sp|Q96A00−2|PP14A_HUMAN−26−S−S1_−0.57
Doses Blue Rep1_−0.57 Red Rep2_−0.57
sp|P28066|PSA5_HUMAN−16−S−S1_−0.57
Doses Blue Rep1_−0.53 Red Rep2_−0.6
sp|P22234|PUR6_HUMAN−27−S−S1_−0.55
Doses Blue Rep1_−0.51 Red Rep2_−0.6
sp|Q13459−2|MYO9B_HUMAN−1267−S−S2_−0.55
Doses Blue Rep1_−0.51 Red Rep2_−0.58
sp|Q9HCC9−5|LST2_HUMAN−278−S−S2_−0.53
Doses Blue Rep1_−0.33 Red Rep2_−0.72
sp|Q9HCC9−5|LST2_HUMAN−280−S−S2_−0.53
Doses Blue Rep1_−0.33 Red Rep2_−0.72
sp|Q9UIF9−2|BAZ2A_HUMAN−480−T−S3_−0.53
Doses Blue Rep1_−0.46 Red Rep2_−0.59
sp|P24928|RPB1_HUMAN−1917−S−S2_−0.51
Doses Blue Rep1_−0.53 Red Rep2_−0.48
sp|Q9UIF9−2|BAZ2A_HUMAN−469−S−S3_−0.49
Doses Blue Rep1_−0.46 Red Rep2_−0.51
sp|Q9UIF9−2|BAZ2A_HUMAN−472−T−S3_−0.49
Doses Blue Rep1_−0.46 Red Rep2_−0.51
sp|Q14160−2|SCRIB_HUMAN−1367−S−S1_−0.48
Doses Blue Rep1_−0.41 Red Rep2_−0.56
sp|P35612|ADDB_HUMAN−596−S−S2_−0.48
Doses Blue Rep1_−0.52 Red Rep2_−0.43
sp|Q32MZ4−2|LRRF1_HUMAN−66−S−S2_−0.47
Doses Blue Rep1_−0.5 Red Rep2_−0.45
sp|Q32MZ4−2|LRRF1_HUMAN−68−S−S2_−0.47
Doses Blue Rep1_−0.5 Red Rep2_−0.45
sp|Q6NZY4−2|ZCHC8_HUMAN−262−S−S3_−0.47
Doses Blue Rep1_−0.48 Red Rep2_−0.46
ptm abundance 37C

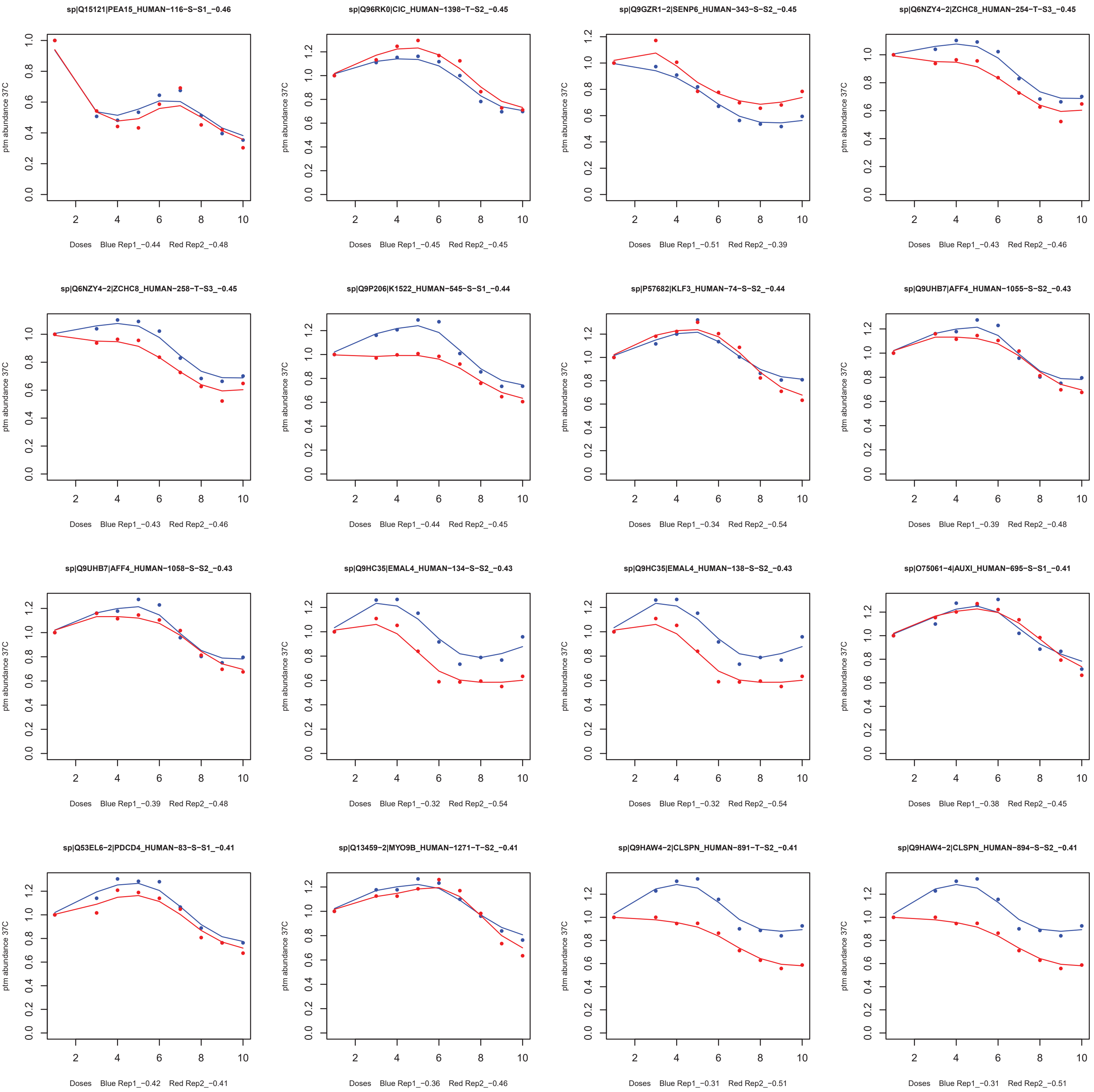
sp|Q15121|PEA15_HUMAN−116−S−S1_−0.46
ptm abundance 37C
Doses Blue Rep1_−0.44 Red Rep2_−0.48
sp|Q96RK0|CIC_HUMAN−1398−T−S2_−0.45
ptm abundance 37C
Doses Blue Rep1_−0.45 Red Rep2_−0.45
sp|Q9GZR1−2|SENP6_HUMAN−343−S−S2_−0.45
ptm abundance 37C
Doses Blue Rep1_−0.51 Red Rep2_−0.39
sp|Q6NZY4−2|ZCHC8_HUMAN−254−T−S3_−0.45
ptm abundance 37C
Doses Blue Rep1_−0.43 Red Rep2_−0.46
sp|Q6NZY4−2|ZCHC8_HUMAN−258−T−S3_−0.45
ptm abundance 37C
Doses Blue Rep1_−0.43 Red Rep2_−0.46
sp|Q9P206|K1522_HUMAN−545−S−S1_−0.44
ptm abundance 37C
Doses Blue Rep1_−0.44 Red Rep2_−0.45
sp|P57682|KLF3_HUMAN−74−S−S2_−0.44
ptm abundance 37C
Doses Blue Rep1_−0.34 Red Rep2_−0.54
sp|Q9UHB7|AFF4_HUMAN−1055−S−S2_−0.43
ptm abundance 37C
Doses Blue Rep1_−0.39 Red Rep2_−0.48
sp|Q9UHB7|AFF4_HUMAN−1058−S−S2_−0.43
ptm abundance 37C
Doses Blue Rep1_−0.39 Red Rep2_−0.48
sp|Q9HC35|EMAL4_HUMAN−134−S−S2_−0.43
ptm abundance 37C
Doses Blue Rep1_−0.32 Red Rep2_−0.54
sp|Q9HC35|EMAL4_HUMAN−138−S−S2_−0.43
ptm abundance 37C
Doses Blue Rep1_−0.32 Red Rep2_−0.54
sp|O75061−4|AUXI_HUMAN−695−S−S1_−0.41
ptm abundance 37C
Doses Blue Rep1_−0.38 Red Rep2_−0.45
sp|Q53EL6−2|PDCD4_HUMAN−83−S−S1_−0.41
ptm abundance 37C
Doses Blue Rep1_−0.42 Red Rep2_−0.41
sp|Q13459−2|MYO9B_HUMAN−1271−T−S2_−0.41
ptm abundance 37C
Doses Blue Rep1_−0.36 Red Rep2_−0.46
sp|Q9HAW4−2|CLSPN_HUMAN−891−T−S2_−0.41
ptm abundance 37C
Doses Blue Rep1_−0.31 Red Rep2_−0.51
sp|Q9HAW4−2|CLSPN_HUMAN−894−S−S2_−0.41
ptm abundance 37C
Doses Blue Rep1_−0.31 Red Rep2_−0.51

sp|O14497|ARI1A_HUMAN−696−S−S3_−0.41

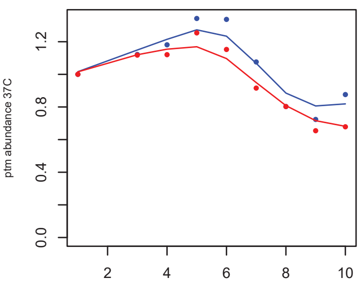

Doses Blue Rep1_−0.33 Red Rep2_−0.48

sp|O14497|ARI1A_HUMAN−698−S−S3_−0.41

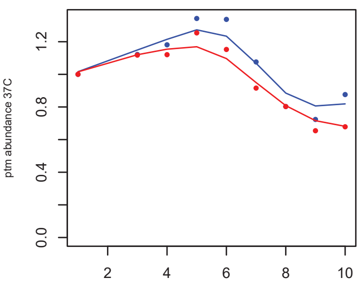

Doses Blue Rep1_−0.33 Red Rep2_−0.48

sp|O14497|ARI1A_HUMAN−702−S−S3_−0.41

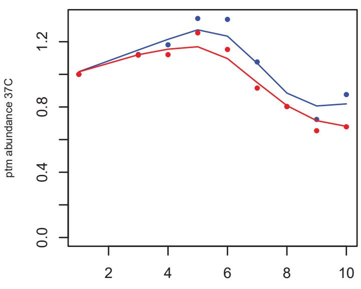

Doses Blue Rep1_−0.33 Red Rep2_−0.48

sp|Q5VTL8|PR38B_HUMAN−529−S−S1_−0.39

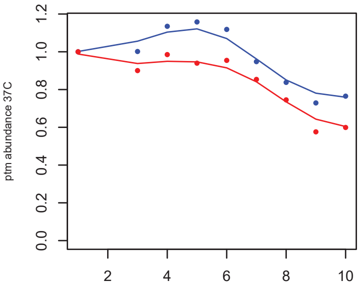

Doses Blue Rep1_−0.34 Red Rep2_−0.45

sp|Q12789−3|TF3C1_HUMAN−1868−S−S2_−0.38

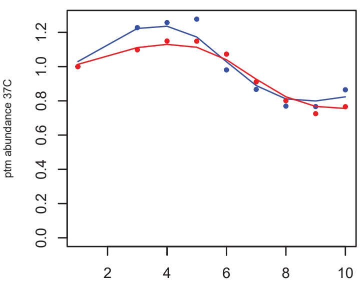

Doses Blue Rep1_−0.38 Red Rep2_−0.38

sp|O43426−4|SYNJ1_HUMAN−1049−S−S2_−0.38

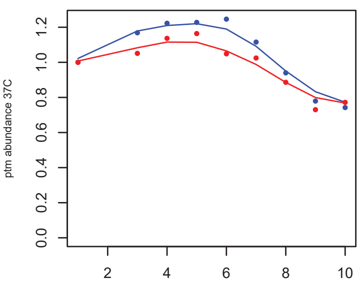

Doses Blue Rep1_−0.41 Red Rep2_−0.35

sp|Q9Y5U2−2|TSSC4_HUMAN−79−S−S2_−0.38

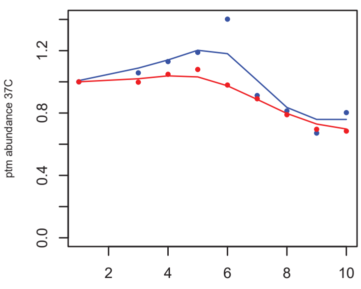

Doses Blue Rep1_−0.36 Red Rep2_−0.39

sp|Q9Y5U2−2|TSSC4_HUMAN−82−S−S2_−0.38

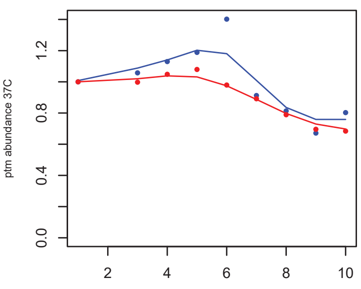

Doses Blue Rep1_−0.36 Red Rep2_−0.39

sp|Q13136−2|LIPA1_HUMAN−741−S−S2_−0.36

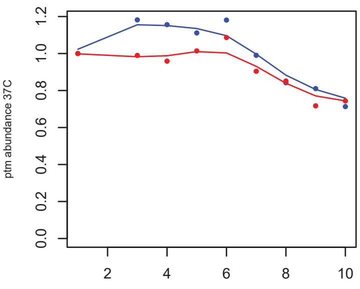

Doses Blue Rep1_−0.41 Red Rep2_−0.31

sp|Q13136−2|LIPA1_HUMAN−745−S−S2_−0.36

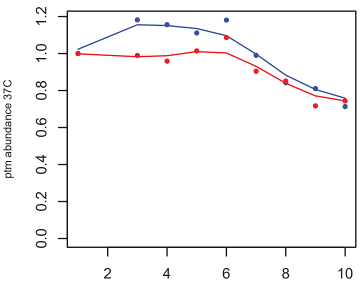

Doses Blue Rep1_−0.41 Red Rep2_−0.31

sp|Q96JM3|CHAP1_HUMAN−459−S−S2_−0.36

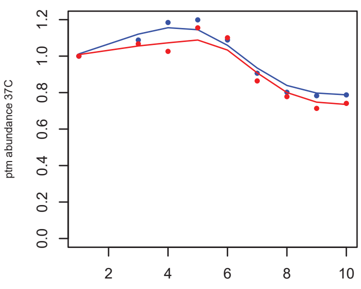

Doses Blue Rep1_−0.35 Red Rep2_−0.37

sp|Q96JM3|CHAP1_HUMAN−462−S−S2_−0.36

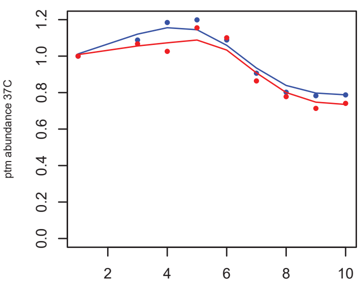

Doses Blue Rep1_−0.35 Red Rep2_−0.37

sp|Q9UIG0−2|BAZ1B_HUMAN−349−S−S1_−0.35

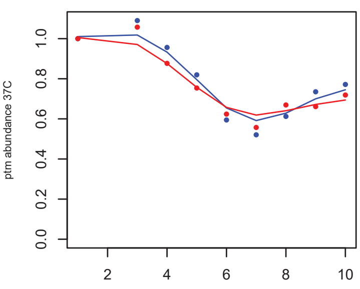

Doses Blue Rep1_−0.34 Red Rep2_−0.37

sp|Q96CP6−2|GRM1A_HUMAN−277−S−S2_−0.35

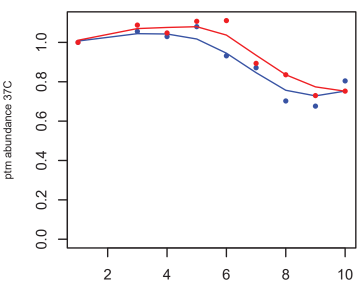

Doses Blue Rep1_−0.34 Red Rep2_−0.36

sp|Q96CP6−2|GRM1A_HUMAN−278−S−S2_−0.35

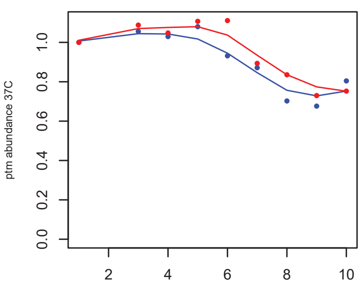

Doses Blue Rep1_−0.34 Red Rep2_−0.36

sp|Q13045−2|FLII_HUMAN−381−S−S1_−0.34

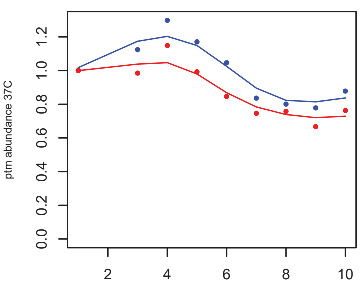

Doses Blue Rep1_−0.32 Red Rep2_−0.36

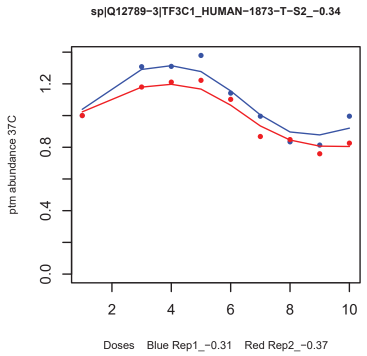
sp|Q12789−3|TF3C1_HUMAN−1873−T−S2_−0.34
ptm abundance 37C
0.0
0.4
0.8
1.2
2
4
6
8
10
Doses Blue Rep1_−0.31 Red Rep2_−0.37

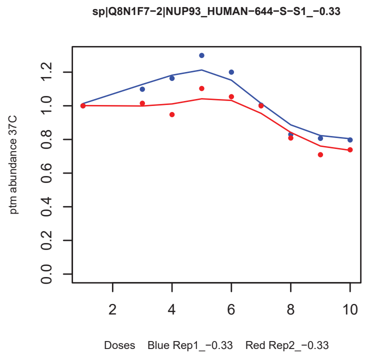
sp|Q8N1F7−2|NUP93_HUMAN−644−S−S1_−0.33
ptm abundance 37C
0.0
0.2
0.4
0.6
0.8
1.0
1.2
2
4
6
8
10
Doses Blue Rep1_−0.33 Red Rep2_−0.33

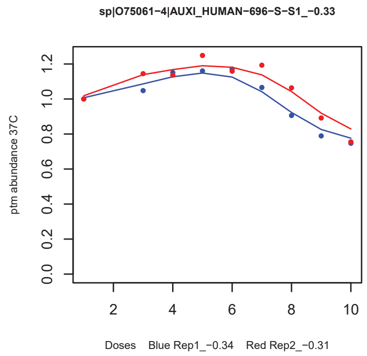
sp|O75061−4|AUXI_HUMAN−696−S−S1_−0.33
ptm abundance 37C
0.0
0.2
0.4
0.6
0.8
1.0
1.2
2
4
6
8
10
Doses Blue Rep1_−0.34 Red Rep2_−0.31

Supplementary Data 3: Phosphosites assigned as biphasically phosphorylated. Doses are represented as ordinal numbers (highest dose 10 µM as the 10th position; x-axis), abundance fold change relative to the untreated condition (y-axis).

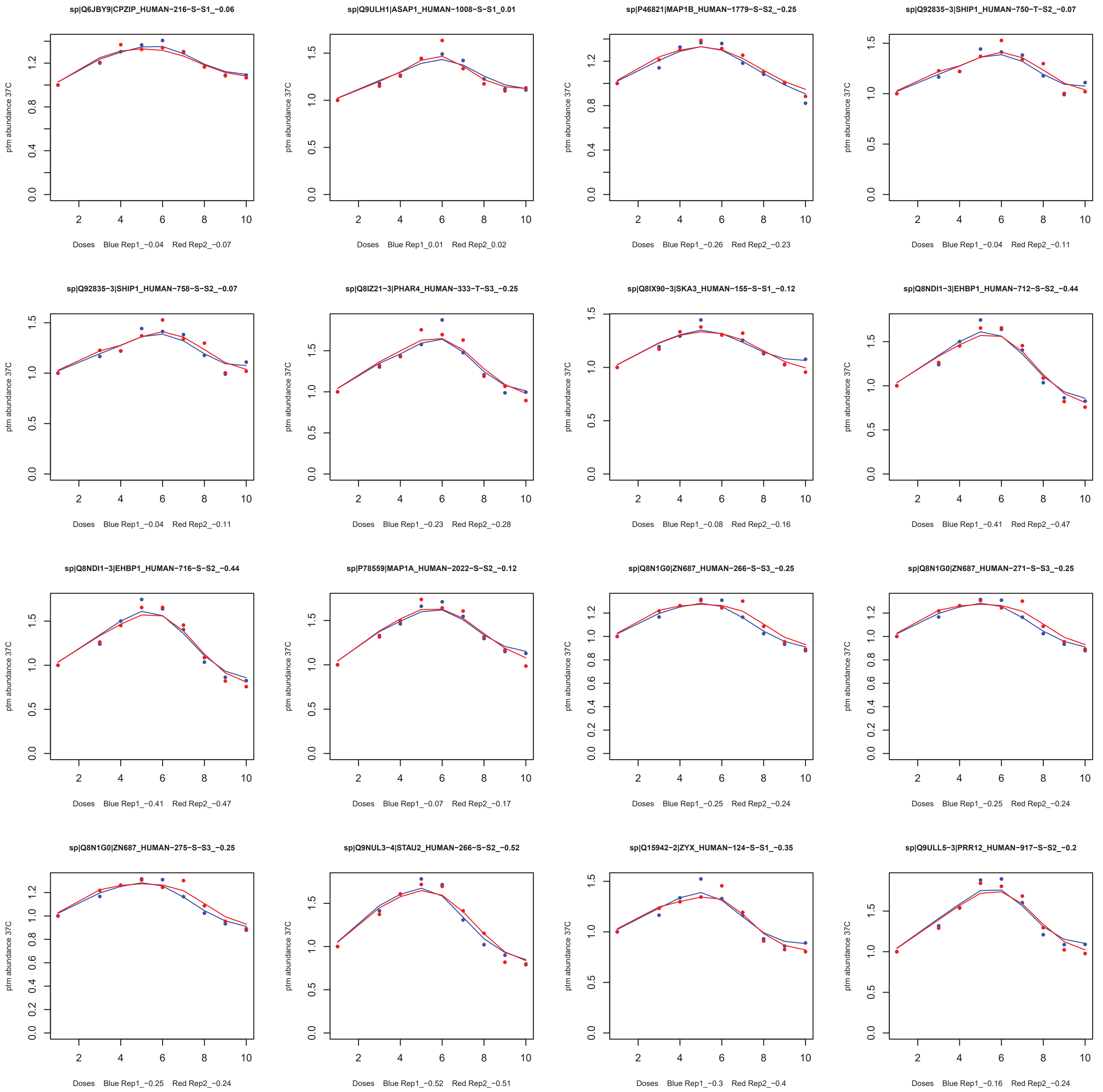

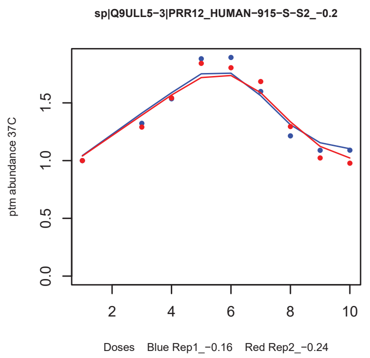
sp|Q9ULL5−3|PRR12_HUMAN−915−S−S2_−0.2
ptm abundance 37C
Doses Blue Rep1_−0.16 Red Rep2_−0.24

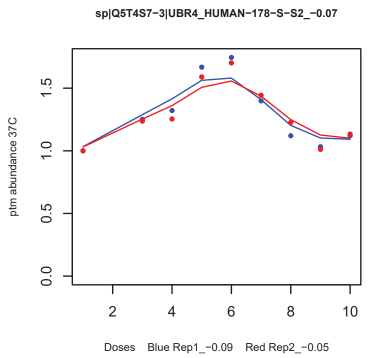
sp|Q5T4S7−3|UBR4_HUMAN−178−S−S2_−0.07
ptm abundance 37C
Doses Blue Rep1_−0.09 Red Rep2_−0.05

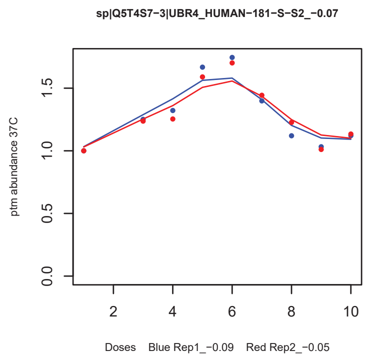
sp|Q5T4S7−3|UBR4_HUMAN−181−S−S2_−0.07
ptm abundance 37C
Doses Blue Rep1_−0.09 Red Rep2_−0.05

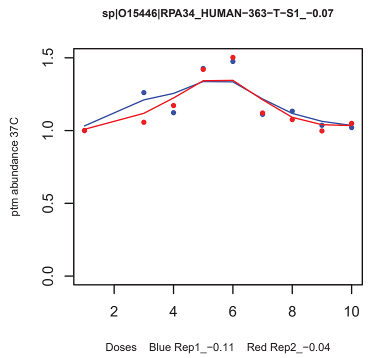
sp|O15446|RPA34_HUMAN−363−T−S1_−0.07
ptm abundance 37C
Doses Blue Rep1_−0.11 Red Rep2_−0.04

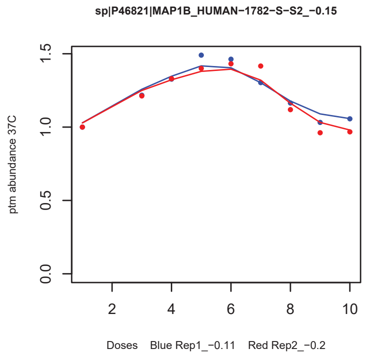
sp|P46821|MAP1B_HUMAN−1782−S−S2_−0.15
ptm abundance 37C
Doses Blue Rep1_−0.11 Red Rep2_−0.2

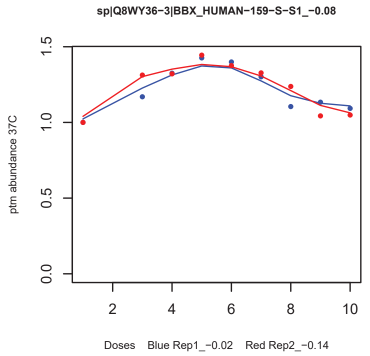
sp|Q8WY36−3|BBX_HUMAN−159−S−S1_−0.08
ptm abundance 37C
Doses Blue Rep1_−0.02 Red Rep2_−0.14

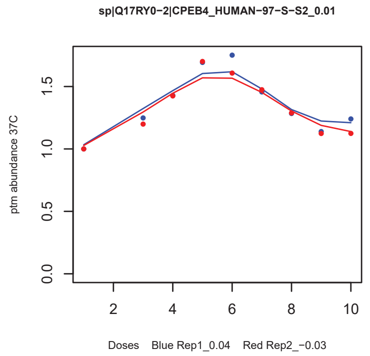
sp|Q17RY0−2|CPEB4_HUMAN−97−S−S2_0.01
ptm abundance 37C
Doses Blue Rep1_0.04 Red Rep2_−0.03

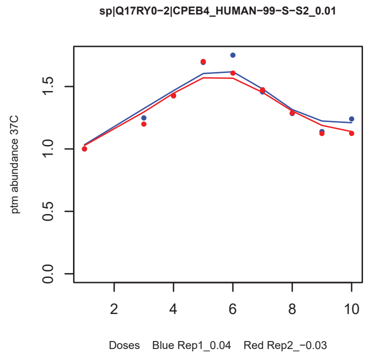
sp|Q17RY0−2|CPEB4_HUMAN−99−S−S2_0.01
ptm abundance 37C
Doses Blue Rep1_0.04 Red Rep2_−0.03

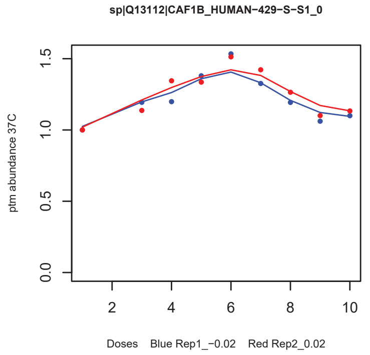
sp|Q13112|CAF1B_HUMAN−429−S−S1_0
ptm abundance 37C
Doses Blue Rep1_−0.02 Red Rep2_0.02

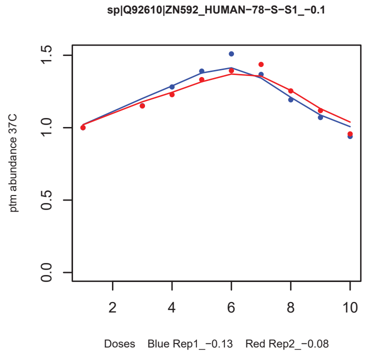
sp|Q92610|ZN592_HUMAN−78−S−S1_−0.1
ptm abundance 37C
Doses Blue Rep1_−0.13 Red Rep2_−0.08

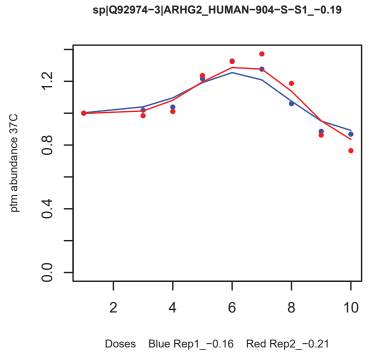
sp|Q92974−3|ARHG2_HUMAN−904−S−S1_−0.19
ptm abundance 37C
Doses Blue Rep1_−0.16 Red Rep2_−0.21

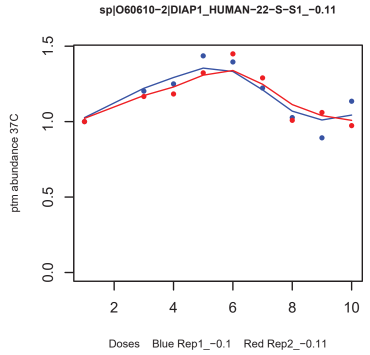
sp|O60610−2|DIAP1_HUMAN−22−S−S1_−0.11
ptm abundance 37C
Doses Blue Rep1_−0.1 Red Rep2_−0.11

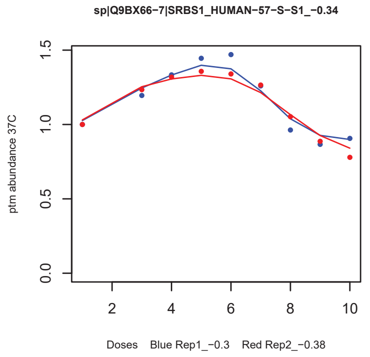
sp|Q9BX66−7|SRBS1_HUMAN−57−S−S1_−0.34
ptm abundance 37C
Doses Blue Rep1_−0.3 Red Rep2_−0.38

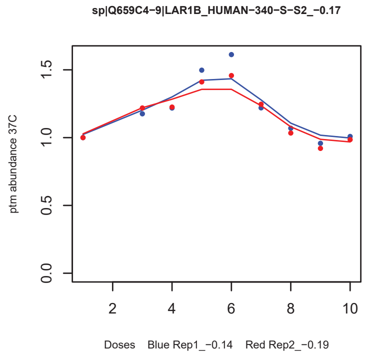
sp|Q659C4−9|LAR1B_HUMAN−340−S−S2_−0.17
ptm abundance 37C
Doses Blue Rep1_−0.14 Red Rep2_−0.19

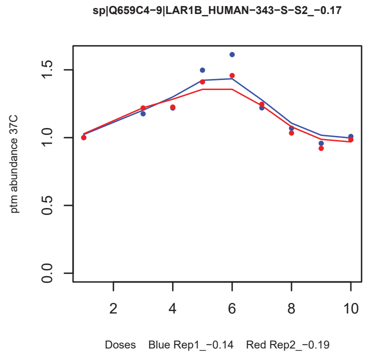
sp|Q659C4−9|LAR1B_HUMAN−343−S−S2_−0.17
ptm abundance 37C
Doses Blue Rep1_−0.14 Red Rep2_−0.19

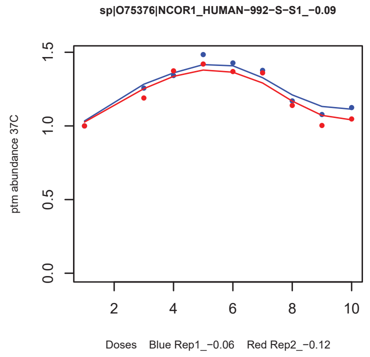
sp|O75376|NCOR1_HUMAN−992−S−S1_−0.09
ptm abundance 37C
Doses Blue Rep1_−0.06 Red Rep2_−0.12

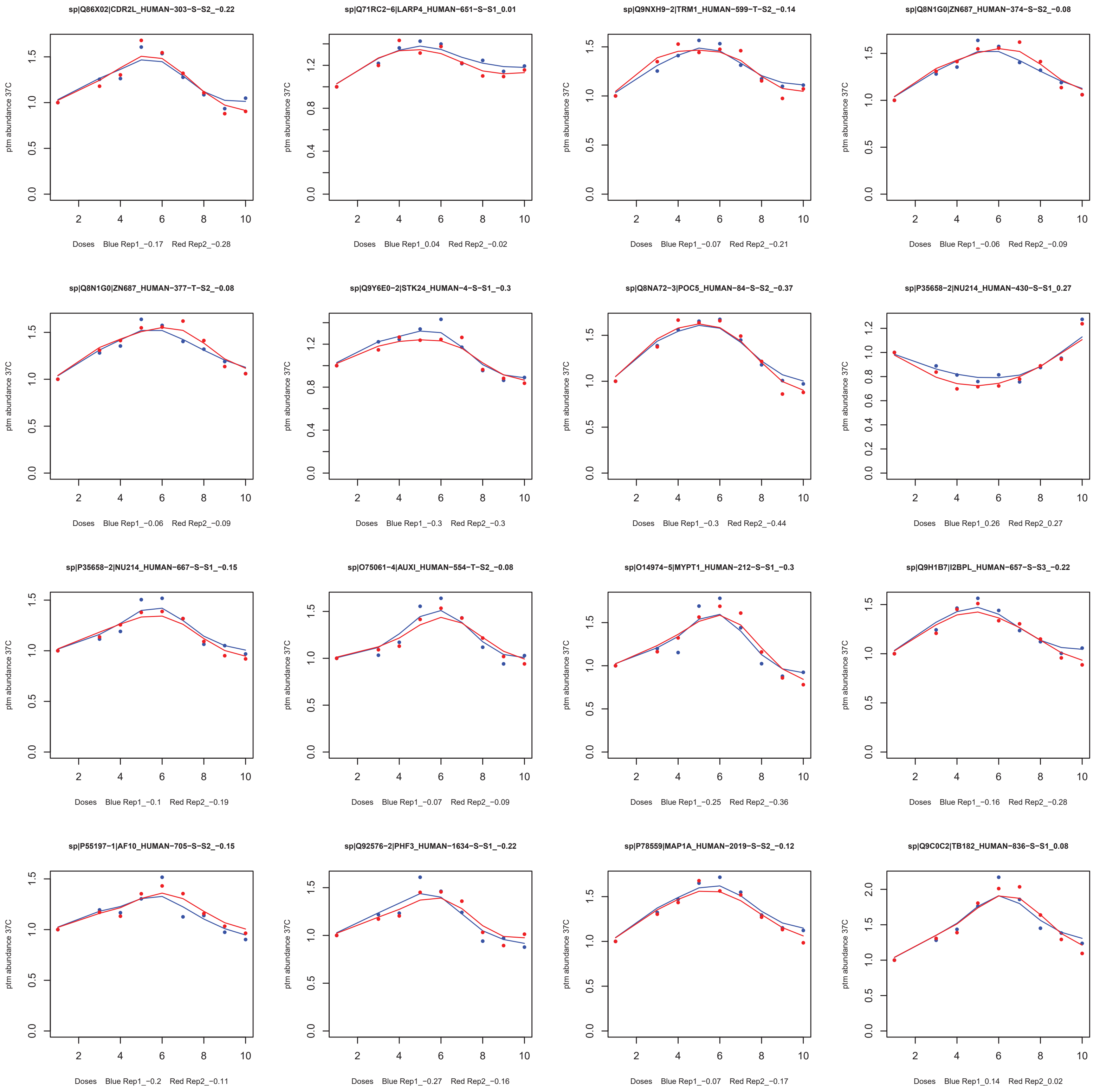
sp|Q86X02|CDR2L_HUMAN−303−S−S2_−0.22
Doses Blue Rep1_−0.17 Red Rep2_−0.28
sp|Q71RC2−6|LARP4_HUMAN−651−S−S1_0.01
Doses Blue Rep1_0.04 Red Rep2_−0.02
sp|Q9NXH9−2|TRM1_HUMAN−599−T−S2_−0.14
Doses Blue Rep1_−0.07 Red Rep2_−0.21
sp|Q8N1G0|ZN687_HUMAN−374−S−S2_−0.08
Doses Blue Rep1_−0.06 Red Rep2_−0.09
sp|Q8N1G0|ZN687_HUMAN−377−T−S2_−0.08
Doses Blue Rep1_−0.06 Red Rep2_−0.09
sp|Q9Y6E0−2|STK24_HUMAN−4−S−S1_−0.3
Doses Blue Rep1_−0.3 Red Rep2_−0.3
sp|Q8NA72−3|POC5_HUMAN−84−S−S2_−0.37
Doses Blue Rep1_−0.3 Red Rep2_−0.44
sp|P35658−2|NU214_HUMAN−430−S−S1_0.27
Doses Blue Rep1_0.26 Red Rep2_0.27
sp|P35658−2|NU214_HUMAN−667−S−S1_−0.15
Doses Blue Rep1_−0.1 Red Rep2_−0.19
sp|O75061−4|AUXI_HUMAN−554−T−S2_−0.08
Doses Blue Rep1_−0.07 Red Rep2_−0.09
sp|O14974−5|MYPT1_HUMAN−212−S−S1_−0.3
Doses Blue Rep1_−0.25 Red Rep2_−0.36
sp|Q9H1B7|I2BPL_HUMAN−657−S−S3_−0.22
Doses Blue Rep1_−0.16 Red Rep2_−0.28
sp|P55197−1|AF10_HUMAN−705−S−S2_−0.15
Doses Blue Rep1_−0.2 Red Rep2_−0.11
sp|Q92576−2|PHF3_HUMAN−1634−S−S1_−0.22
Doses Blue Rep1_−0.27 Red Rep2_−0.16
sp|P78559|MAP1A_HUMAN−2019−S−S2_−0.12
Doses Blue Rep1_−0.07 Red Rep2_−0.17
sp|Q9C0C2|TB182_HUMAN−836−S−S1_0.08
Doses Blue Rep1_0.14 Red Rep2_0.02
ptm abundance 37C

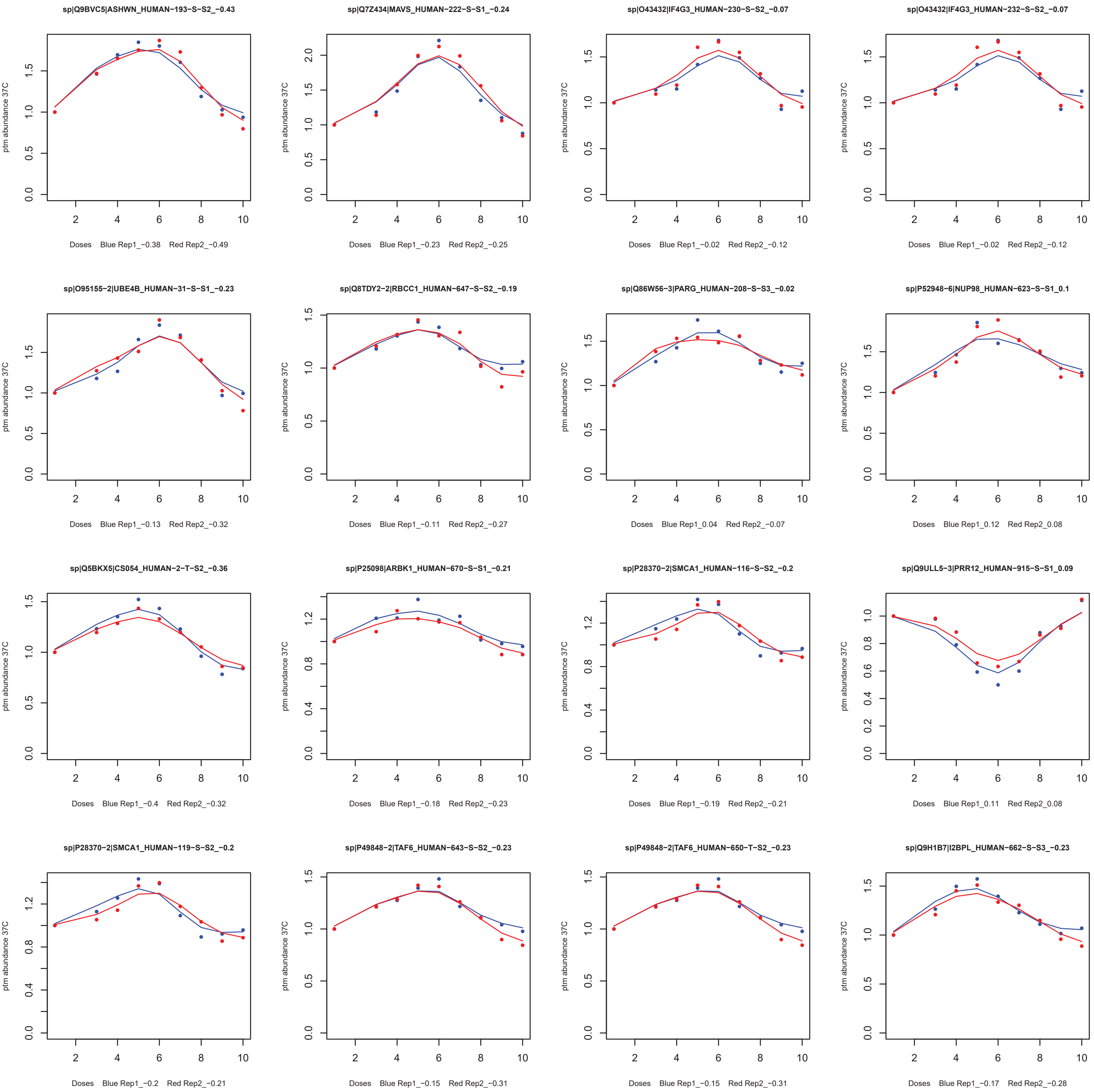

sp|Q9BVC5|ASHWN_HUMAN−193−S−S2_−0.43
ptm abundance 37C
Doses Blue Rep1_−0.38 Red Rep2_−0.49
sp|Q7Z434|MAVS_HUMAN−222−S−S1_−0.24
ptm abundance 37C
Doses Blue Rep1_−0.23 Red Rep2_−0.25
sp|O43432|IF4G3_HUMAN−230−S−S2_−0.07
ptm abundance 37C
Doses Blue Rep1_−0.02 Red Rep2_−0.12
sp|O43432|IF4G3_HUMAN−232−S−S2_−0.07
ptm abundance 37C
Doses Blue Rep1_−0.02 Red Rep2_−0.12
sp|O95155−2|UBE4B_HUMAN−31−S−S1_−0.23
ptm abundance 37C
Doses Blue Rep1_−0.13 Red Rep2_−0.32
sp|Q8TDY2−2|RBCC1_HUMAN−647−S−S2_−0.19
ptm abundance 37C
Doses Blue Rep1_−0.11 Red Rep2_−0.27
sp|Q86W56−3|PARG_HUMAN−208−S−S3_−0.02
ptm abundance 37C
Doses Blue Rep1_0.04 Red Rep2_−0.07
sp|P52948−6|NUP98_HUMAN−623−S−S1_0.1
ptm abundance 37C
Doses Blue Rep1_0.12 Red Rep2_0.08
sp|Q5BKX5|CS054_HUMAN−2−T−S2_−0.36
ptm abundance 37C
Doses Blue Rep1_−0.4 Red Rep2_−0.32
sp|P25098|ARBK1_HUMAN−670−S−S1_−0.21
ptm abundance 37C
Doses Blue Rep1_−0.18 Red Rep2_−0.23
sp|P28370−2|SMCA1_HUMAN−116−S−S2_−0.2
ptm abundance 37C
Doses Blue Rep1_−0.19 Red Rep2_−0.21
sp|Q9ULL5−3|PRR12_HUMAN−915−S−S1_0.09
ptm abundance 37C
Doses Blue Rep1_0.11 Red Rep2_0.08
sp|P28370−2|SMCA1_HUMAN−119−S−S2_−0.2
ptm abundance 37C
Doses Blue Rep1_−0.2 Red Rep2_−0.21
sp|P49848−2|TAF6_HUMAN−643−S−S2_−0.23
ptm abundance 37C
Doses Blue Rep1_−0.15 Red Rep2_−0.31
sp|P49848−2|TAF6_HUMAN−650−T−S2_−0.23
ptm abundance 37C
Doses Blue Rep1_−0.15 Red Rep2_−0.31
sp|Q9H1B7|I2BPL_HUMAN−662−S−S3_−0.23
ptm abundance 37C
Doses Blue Rep1_−0.17 Red Rep2_−0.28

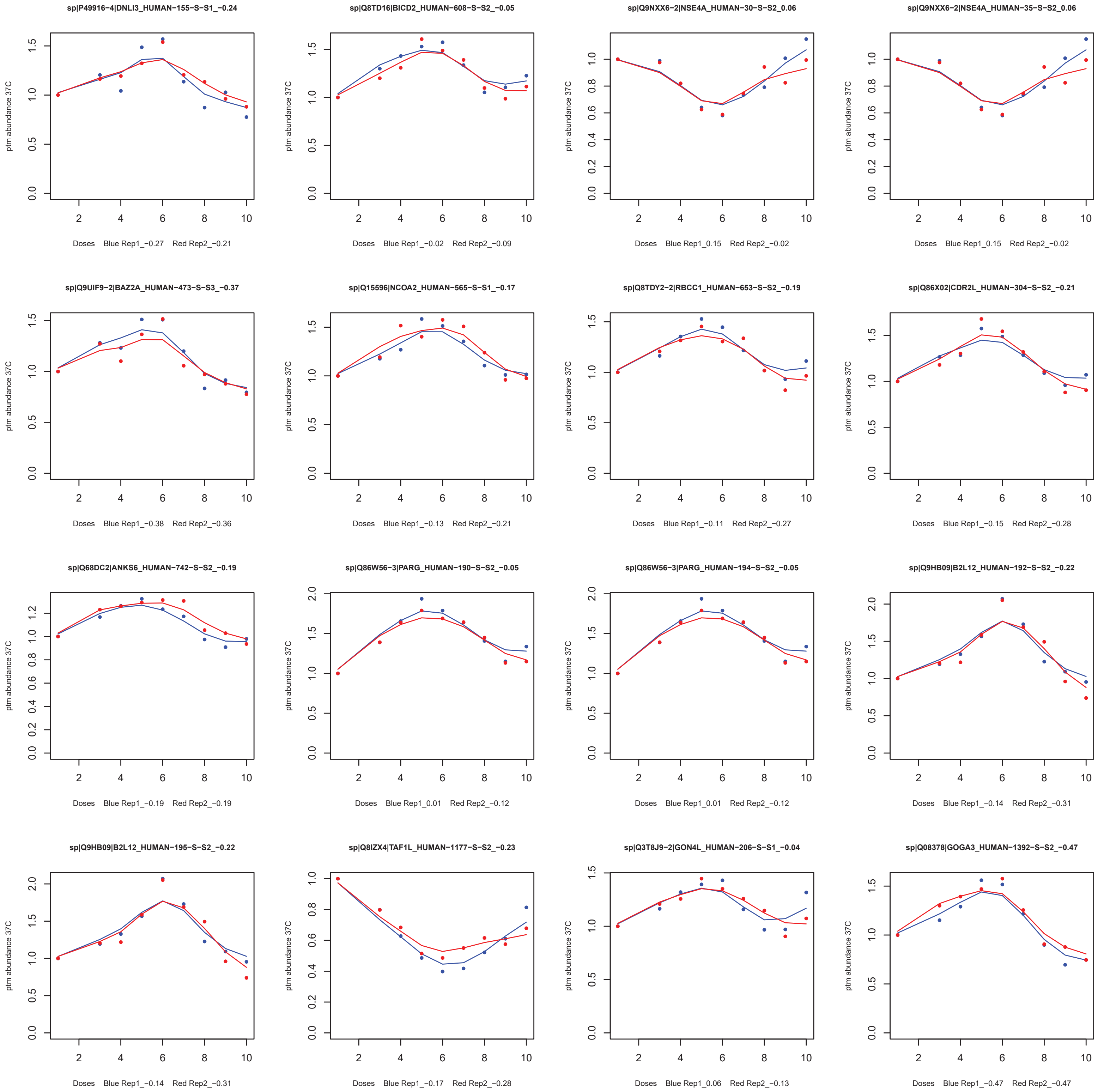
sp|P49916−4|DNLI3_HUMAN−155−S−S1_−0.24
ptm abundance 37C
Doses Blue Rep1_−0.27 Red Rep2_−0.21
sp|Q8TD16|BICD2_HUMAN−608−S−S2_−0.05
ptm abundance 37C
Doses Blue Rep1_−0.02 Red Rep2_−0.09
sp|Q9NXX6−2|NSE4A_HUMAN−30−S−S2_0.06
ptm abundance 37C
Doses Blue Rep1_0.15 Red Rep2_−0.02
sp|Q9NXX6−2|NSE4A_HUMAN−35−S−S2_0.06
ptm abundance 37C
Doses Blue Rep1_0.15 Red Rep2_−0.02
sp|Q9UIF9−2|BAZ2A_HUMAN−473−S−S3_−0.37
ptm abundance 37C
Doses Blue Rep1_−0.38 Red Rep2_−0.36
sp|Q15596|NCOA2_HUMAN−565−S−S1_−0.17
ptm abundance 37C
Doses Blue Rep1_−0.13 Red Rep2_−0.21
sp|Q8TDY2−2|RBCC1_HUMAN−653−S−S2_−0.19
ptm abundance 37C
Doses Blue Rep1_−0.11 Red Rep2_−0.27
sp|Q86X02|CDR2L_HUMAN−304−S−S2_−0.21
ptm abundance 37C
Doses Blue Rep1_−0.15 Red Rep2_−0.28
sp|Q68DC2|ANKS6_HUMAN−742−S−S2_−0.19
ptm abundance 37C
Doses Blue Rep1_−0.19 Red Rep2_−0.19
sp|Q86W56−3|PARG_HUMAN−190−S−S2_−0.05
ptm abundance 37C
Doses Blue Rep1_0.01 Red Rep2_−0.12
sp|Q86W56−3|PARG_HUMAN−194−S−S2_−0.05
ptm abundance 37C
Doses Blue Rep1_0.01 Red Rep2_−0.12
sp|Q9HB09|B2L12_HUMAN−192−S−S2_−0.22
ptm abundance 37C
Doses Blue Rep1_−0.14 Red Rep2_−0.31
sp|Q9HB09|B2L12_HUMAN−195−S−S2_−0.22
ptm abundance 37C
Doses Blue Rep1_−0.14 Red Rep2_−0.31
sp|Q8IZX4|TAF1L_HUMAN−1177−S−S2_−0.23
ptm abundance 37C
Doses Blue Rep1_−0.17 Red Rep2_−0.28
sp|Q3T8J9−2|GON4L_HUMAN−206−S−S1_−0.04
ptm abundance 37C
Doses Blue Rep1_0.06 Red Rep2_−0.13
sp|Q08378|GOGA3_HUMAN−1392−S−S2_−0.47
ptm abundance 37C
Doses Blue Rep1_−0.47 Red Rep2_−0.47

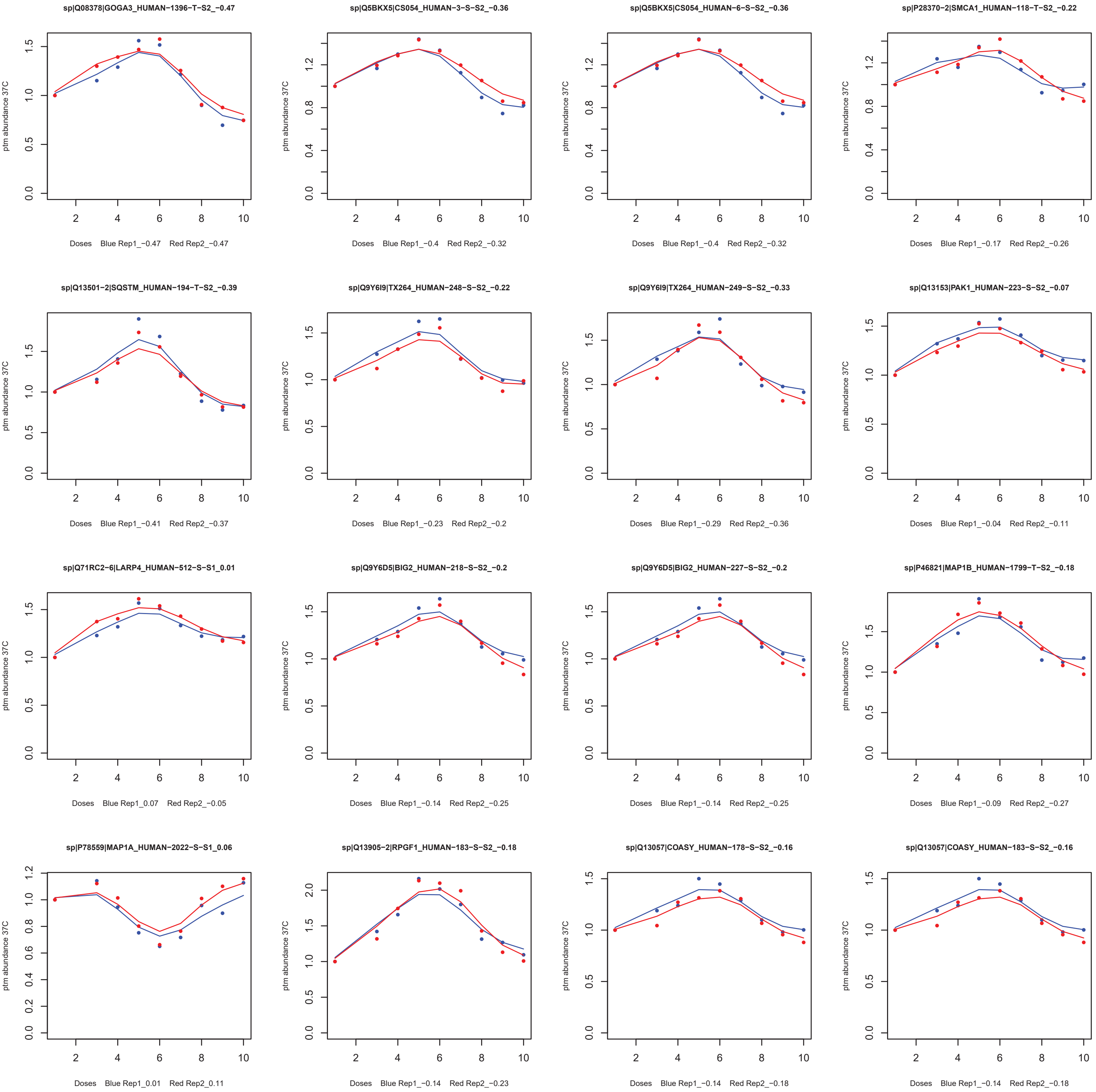
sp|Q08378|GOGA3_HUMAN−1396−T−S2_−0.47
Doses Blue Rep1_−0.47 Red Rep2_−0.47
sp|Q5BKX5|CS054_HUMAN−3−S−S2_−0.36
Doses Blue Rep1_−0.4 Red Rep2_−0.32
sp|Q5BKX5|CS054_HUMAN−6−S−S2_−0.36
Doses Blue Rep1_−0.4 Red Rep2_−0.32
sp|P28370−2|SMCA1_HUMAN−118−T−S2_−0.22
Doses Blue Rep1_−0.17 Red Rep2_−0.26
sp|Q13501−2|SQSTM_HUMAN−194−T−S2_−0.39
Doses Blue Rep1_−0.41 Red Rep2_−0.37
sp|Q9Y6I9|TX264_HUMAN−248−S−S2_−0.22
Doses Blue Rep1_−0.23 Red Rep2_−0.2
sp|Q9Y6I9|TX264_HUMAN−249−S−S2_−0.33
Doses Blue Rep1_−0.29 Red Rep2_−0.36
sp|Q13153|PAK1_HUMAN−223−S−S2_−0.07
Doses Blue Rep1_−0.04 Red Rep2_−0.11
sp|Q71RC2−6|LARP4_HUMAN−512−S−S1_0.01
Doses Blue Rep1_0.07 Red Rep2_−0.05
sp|Q9Y6D5|BIG2_HUMAN−218−S−S2_−0.2
Doses Blue Rep1_−0.14 Red Rep2_−0.25
sp|Q9Y6D5|BIG2_HUMAN−227−S−S2_−0.2
Doses Blue Rep1_−0.14 Red Rep2_−0.25
sp|P46821|MAP1B_HUMAN−1799−T−S2_−0.18
Doses Blue Rep1_−0.09 Red Rep2_−0.27
sp|P78559|MAP1A_HUMAN−2022−S−S1_0.06
Doses Blue Rep1_0.01 Red Rep2_0.11
sp|Q13905−2|RPGF1_HUMAN−183−S−S2_−0.18
Doses Blue Rep1_−0.14 Red Rep2_−0.23
sp|Q13057|COASY_HUMAN−178−S−S2_−0.16
Doses Blue Rep1_−0.14 Red Rep2_−0.18
sp|Q13057|COASY_HUMAN−183−S−S2_−0.16
Doses Blue Rep1_−0.14 Red Rep2_−0.18
ptm abundance 37C

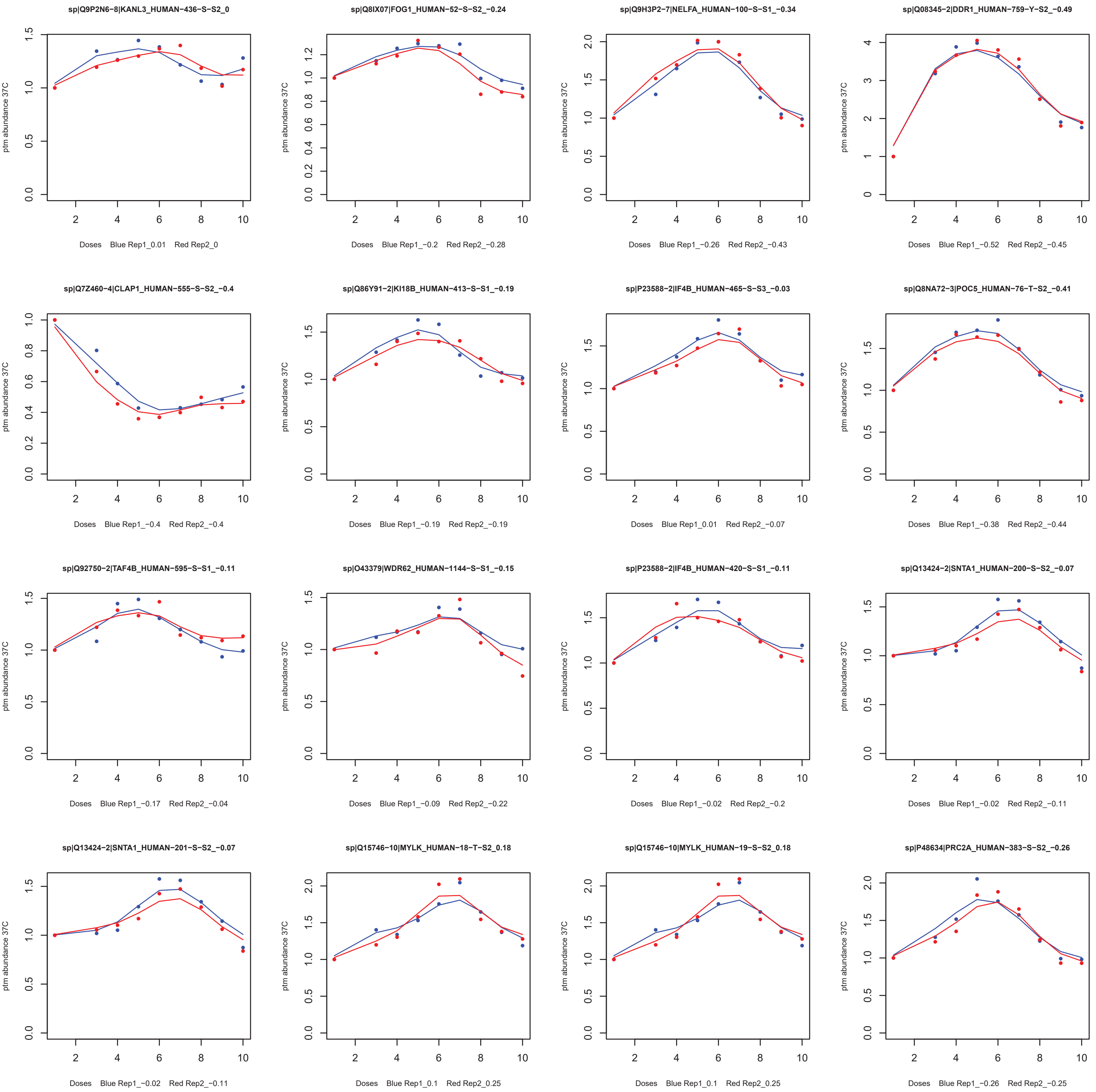

sp|Q9P2N6−8|KANL3_HUMAN−436−S−S2_0
ptm abundance 37C
Doses Blue Rep1_0.01 Red Rep2_0
sp|Q8IX07|FOG1_HUMAN−52−S−S2_−0.24
ptm abundance 37C
Doses Blue Rep1_−0.2 Red Rep2_−0.28
sp|Q9H3P2−7|NELFA_HUMAN−100−S−S1_−0.34
ptm abundance 37C
Doses Blue Rep1_−0.26 Red Rep2_−0.43
sp|Q08345−2|DDR1_HUMAN−759−Y−S2_−0.49
ptm abundance 37C
Doses Blue Rep1_−0.52 Red Rep2_−0.45
sp|Q7Z460−4|CLAP1_HUMAN−555−S−S2_−0.4
ptm abundance 37C
Doses Blue Rep1_−0.4 Red Rep2_−0.4
sp|Q86Y91−2|KI18B_HUMAN−413−S−S1_−0.19
ptm abundance 37C
Doses Blue Rep1_−0.19 Red Rep2_−0.19
sp|P23588−2|IF4B_HUMAN−465−S−S3_−0.03
ptm abundance 37C
Doses Blue Rep1_0.01 Red Rep2_−0.07
sp|Q8NA72−3|POC5_HUMAN−76−T−S2_−0.41
ptm abundance 37C
Doses Blue Rep1_−0.38 Red Rep2_−0.44
sp|Q92750−2|TAF4B_HUMAN−595−S−S1_−0.11
ptm abundance 37C
Doses Blue Rep1_−0.17 Red Rep2_−0.04
sp|O43379|WDR62_HUMAN−1144−S−S1_−0.15
ptm abundance 37C
Doses Blue Rep1_−0.09 Red Rep2_−0.22
sp|P23588−2|IF4B_HUMAN−420−S−S1_−0.11
ptm abundance 37C
Doses Blue Rep1_−0.02 Red Rep2_−0.2
sp|Q13424−2|SNTA1_HUMAN−200−S−S2_−0.07
ptm abundance 37C
Doses Blue Rep1_−0.02 Red Rep2_−0.11
sp|Q13424−2|SNTA1_HUMAN−201−S−S2_−0.07
ptm abundance 37C
Doses Blue Rep1_−0.02 Red Rep2_−0.11
sp|Q15746−10|MYLK_HUMAN−18−T−S2_0.18
ptm abundance 37C
Doses Blue Rep1_0.1 Red Rep2_0.25
sp|Q15746−10|MYLK_HUMAN−19−S−S2_0.18
ptm abundance 37C
Doses Blue Rep1_0.1 Red Rep2_0.25
sp|P48634|PRC2A_HUMAN−383−S−S2_−0.26
ptm abundance 37C
Doses Blue Rep1_−0.26 Red Rep2_−0.25

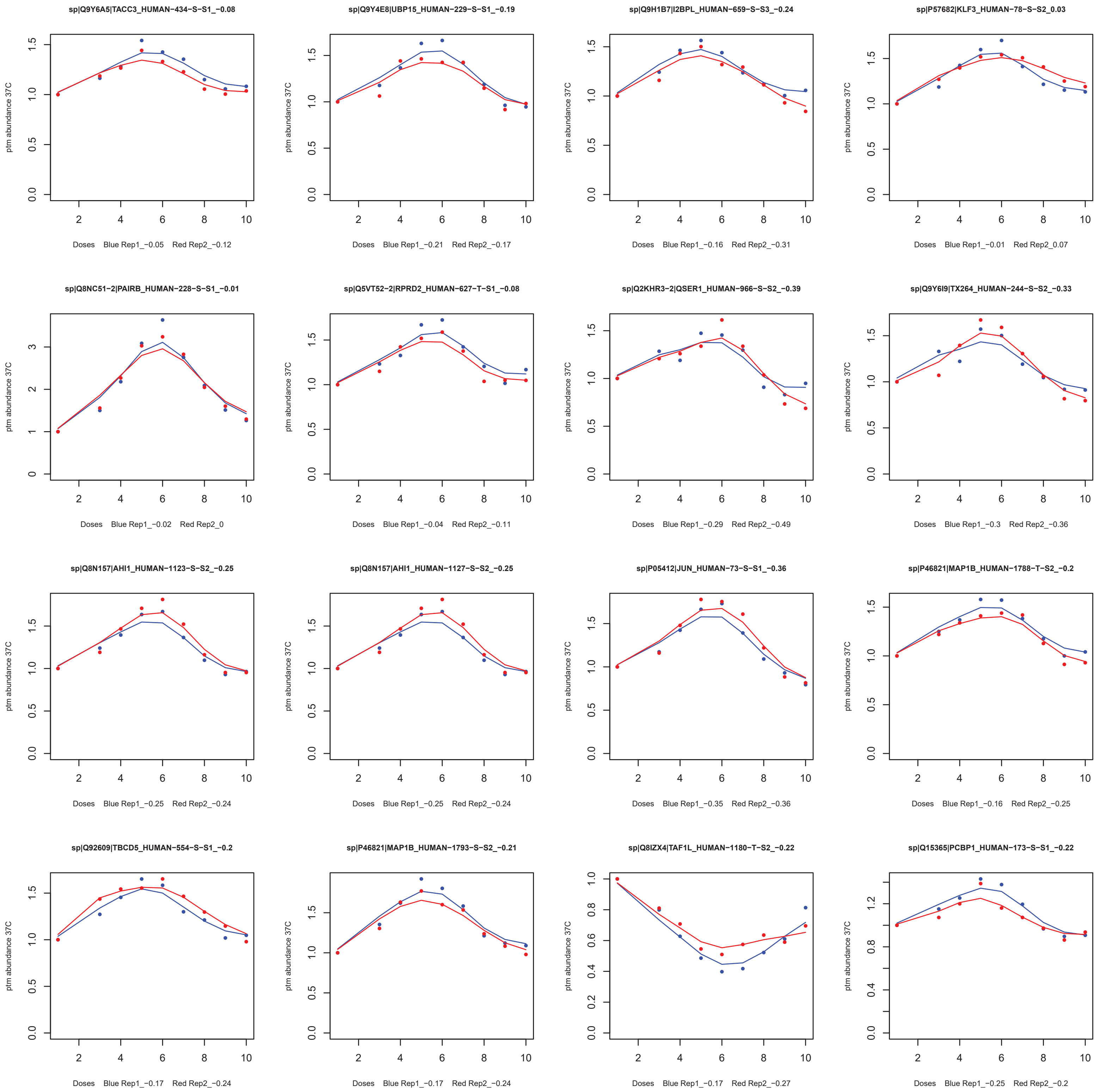
sp|Q9Y6A5|TACC3_HUMAN−434−S−S1_−0.08
Doses Blue Rep1_−0.05 Red Rep2_−0.12
sp|Q9Y4E8|UBP15_HUMAN−229−S−S1_−0.19
Doses Blue Rep1_−0.21 Red Rep2_−0.17
sp|Q9H1B7|I2BPL_HUMAN−659−S−S3_−0.24
Doses Blue Rep1_−0.16 Red Rep2_−0.31
sp|P57682|KLF3_HUMAN−78−S−S2_0.03
Doses Blue Rep1_−0.01 Red Rep2_0.07
sp|Q8NC51−2|PAIRB_HUMAN−228−S−S1_−0.01
Doses Blue Rep1_−0.02 Red Rep2_0
sp|Q5VT52−2|RPRD2_HUMAN−627−T−S1_−0.08
Doses Blue Rep1_−0.04 Red Rep2_−0.11
sp|Q2KHR3−2|QSER1_HUMAN−966−S−S2_−0.39
Doses Blue Rep1_−0.29 Red Rep2_−0.49
sp|Q9Y6I9|TX264_HUMAN−244−S−S2_−0.33
Doses Blue Rep1_−0.3 Red Rep2_−0.36
sp|Q8N157|AHI1_HUMAN−1123−S−S2_−0.25
Doses Blue Rep1_−0.25 Red Rep2_−0.24
sp|Q8N157|AHI1_HUMAN−1127−S−S2_−0.25
Doses Blue Rep1_−0.25 Red Rep2_−0.24
sp|P05412|JUN_HUMAN−73−S−S1_−0.36
Doses Blue Rep1_−0.35 Red Rep2_−0.36
sp|P46821|MAP1B_HUMAN−1788−T−S2_−0.2
Doses Blue Rep1_−0.16 Red Rep2_−0.25
sp|Q92609|TBCD5_HUMAN−554−S−S1_−0.2
Doses Blue Rep1_−0.17 Red Rep2_−0.24
sp|P46821|MAP1B_HUMAN−1793−S−S2_−0.21
Doses Blue Rep1_−0.17 Red Rep2_−0.24
sp|Q8IZX4|TAF1L_HUMAN−1180−T−S2_−0.22
Doses Blue Rep1_−0.17 Red Rep2_−0.27
sp|Q15365|PCBP1_HUMAN−173−S−S1_−0.22
Doses Blue Rep1_−0.25 Red Rep2_−0.2
ptm abundance 37C

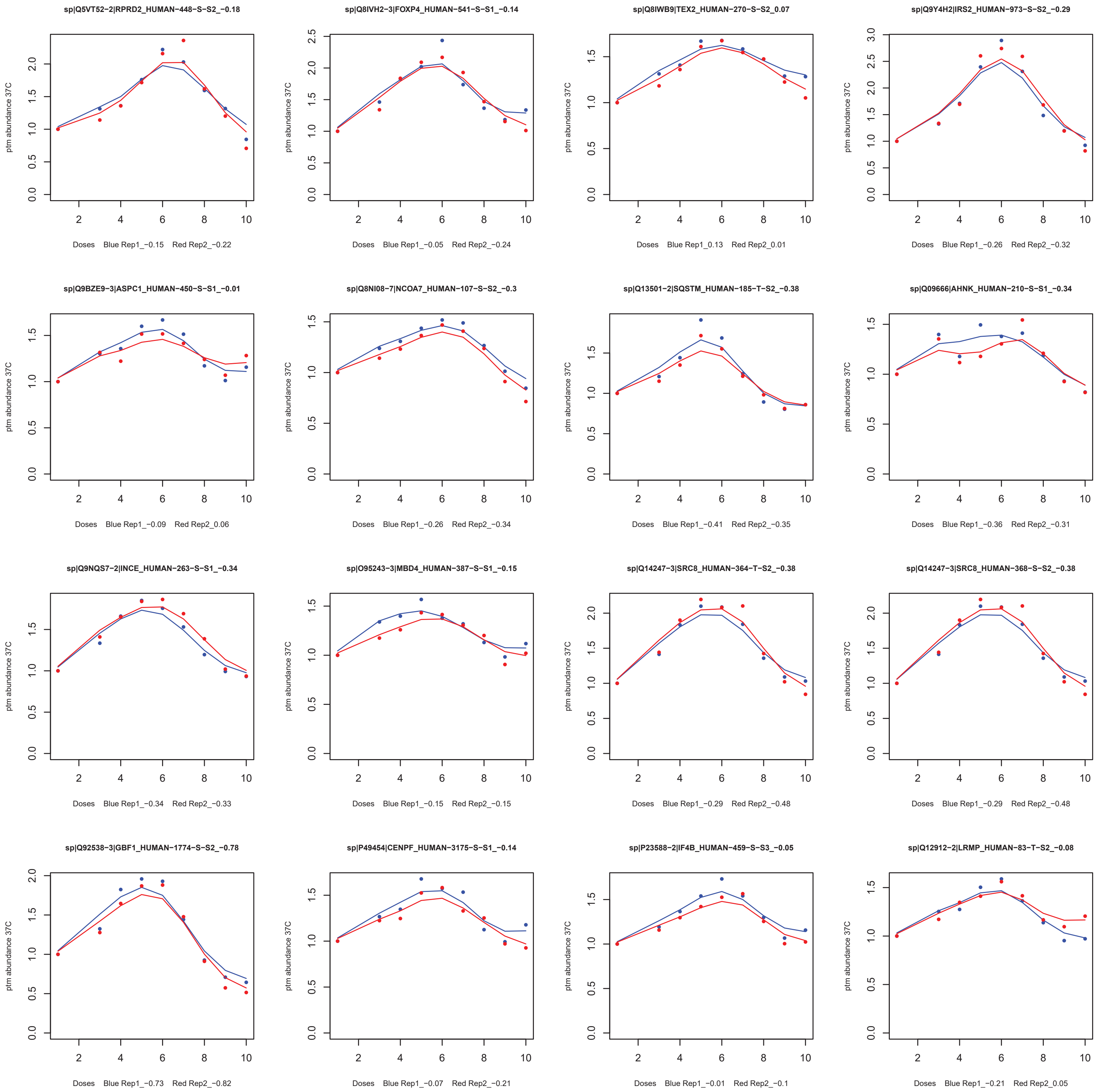
sp|Q5VT52−2|RPRD2_HUMAN−448−S−S2_−0.18
ptm abundance 37C
Doses Blue Rep1_−0.15 Red Rep2_−0.22
sp|Q8IVH2−3|FOXP4_HUMAN−541−S−S1_−0.14
ptm abundance 37C
Doses Blue Rep1_−0.05 Red Rep2_−0.24
sp|Q8IWB9|TEX2_HUMAN−270−S−S2_0.07
ptm abundance 37C
Doses Blue Rep1_0.13 Red Rep2_0.01
sp|Q9Y4H2|IRS2_HUMAN−973−S−S2_−0.29
ptm abundance 37C
Doses Blue Rep1_−0.26 Red Rep2_−0.32
sp|Q9BZE9−3|ASPC1_HUMAN−450−S−S1_−0.01
ptm abundance 37C
Doses Blue Rep1_−0.09 Red Rep2_0.06
sp|Q8NI08−7|NCOA7_HUMAN−107−S−S2_−0.3
ptm abundance 37C
Doses Blue Rep1_−0.26 Red Rep2_−0.34
sp|Q13501−2|SQSTM_HUMAN−185−T−S2_−0.38
ptm abundance 37C
Doses Blue Rep1_−0.41 Red Rep2_−0.35
sp|Q09666|AHNK_HUMAN−210−S−S1_−0.34
ptm abundance 37C
Doses Blue Rep1_−0.36 Red Rep2_−0.31
sp|Q9NQS7−2|INCE_HUMAN−263−S−S1_−0.34
ptm abundance 37C
Doses Blue Rep1_−0.34 Red Rep2_−0.33
sp|O95243−3|MBD4_HUMAN−387−S−S1_−0.15
ptm abundance 37C
Doses Blue Rep1_−0.15 Red Rep2_−0.15
sp|Q14247−3|SRC8_HUMAN−364−T−S2_−0.38
ptm abundance 37C
Doses Blue Rep1_−0.29 Red Rep2_−0.48
sp|Q14247−3|SRC8_HUMAN−368−S−S2_−0.38
ptm abundance 37C
Doses Blue Rep1_−0.29 Red Rep2_−0.48
sp|Q92538−3|GBF1_HUMAN−1774−S−S2_−0.78
ptm abundance 37C
Doses Blue Rep1_−0.73 Red Rep2_−0.82
sp|P49454|CENPF_HUMAN−3175−S−S1_−0.14
ptm abundance 37C
Doses Blue Rep1_−0.07 Red Rep2_−0.21
sp|P23588−2|IF4B_HUMAN−459−S−S3_−0.05
ptm abundance 37C
Doses Blue Rep1_−0.01 Red Rep2_−0.1
sp|Q12912−2|LRMP_HUMAN−83−T−S2_−0.08
ptm abundance 37C
Doses Blue Rep1_−0.21 Red Rep2_0.05

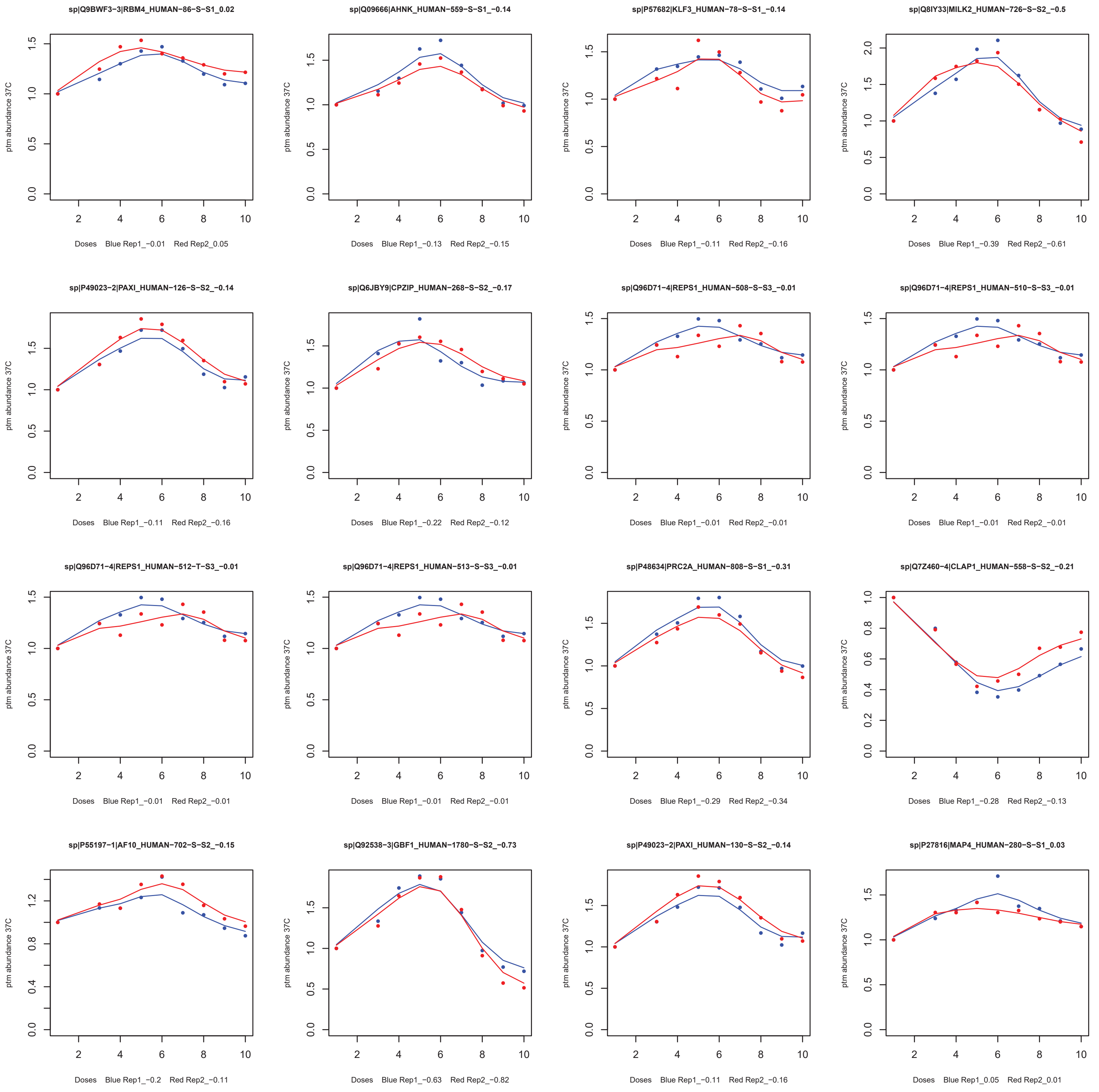

sp|Q9BWF3−3|RBM4_HUMAN−86−S−S1_0.02
ptm abundance 37C
Doses Blue Rep1_−0.01 Red Rep2_0.05
sp|Q09666|AHNK_HUMAN−559−S−S1_−0.14
ptm abundance 37C
Doses Blue Rep1_−0.13 Red Rep2_−0.15
sp|P57682|KLF3_HUMAN−78−S−S1_−0.14
ptm abundance 37C
Doses Blue Rep1_−0.11 Red Rep2_−0.16
sp|Q8IY33|MILK2_HUMAN−726−S−S2_−0.5
ptm abundance 37C
Doses Blue Rep1_−0.39 Red Rep2_−0.61
sp|P49023−2|PAXI_HUMAN−126−S−S2_−0.14
ptm abundance 37C
Doses Blue Rep1_−0.11 Red Rep2_−0.16
sp|Q6JBY9|CPZIP_HUMAN−268−S−S2_−0.17
ptm abundance 37C
Doses Blue Rep1_−0.22 Red Rep2_−0.12
sp|Q96D71−4|REPS1_HUMAN−508−S−S3_−0.01
ptm abundance 37C
Doses Blue Rep1_−0.01 Red Rep2_−0.01
sp|Q96D71−4|REPS1_HUMAN−510−S−S3_−0.01
ptm abundance 37C
Doses Blue Rep1_−0.01 Red Rep2_−0.01
sp|Q96D71−4|REPS1_HUMAN−512−T−S3_−0.01
ptm abundance 37C
Doses Blue Rep1_−0.01 Red Rep2_−0.01
sp|Q96D71−4|REPS1_HUMAN−513−S−S3_−0.01
ptm abundance 37C
Doses Blue Rep1_−0.01 Red Rep2_−0.01
sp|P48634|PRC2A_HUMAN−808−S−S1_−0.31
ptm abundance 37C
Doses Blue Rep1_−0.29 Red Rep2_−0.34
sp|Q7Z460−4|CLAP1_HUMAN−558−S−S2_−0.21
ptm abundance 37C
Doses Blue Rep1_−0.28 Red Rep2_−0.13
sp|P55197−1|AF10_HUMAN−702−S−S2_−0.15
ptm abundance 37C
Doses Blue Rep1_−0.2 Red Rep2_−0.11
sp|Q92538−3|GBF1_HUMAN−1780−S−S2_−0.73
ptm abundance 37C
Doses Blue Rep1_−0.63 Red Rep2_−0.82
sp|P49023−2|PAXI_HUMAN−130−S−S2_−0.14
ptm abundance 37C
Doses Blue Rep1_−0.11 Red Rep2_−0.16
sp|P27816|MAP4_HUMAN−280−S−S1_0.03
ptm abundance 37C
Doses Blue Rep1_0.05 Red Rep2_0.01

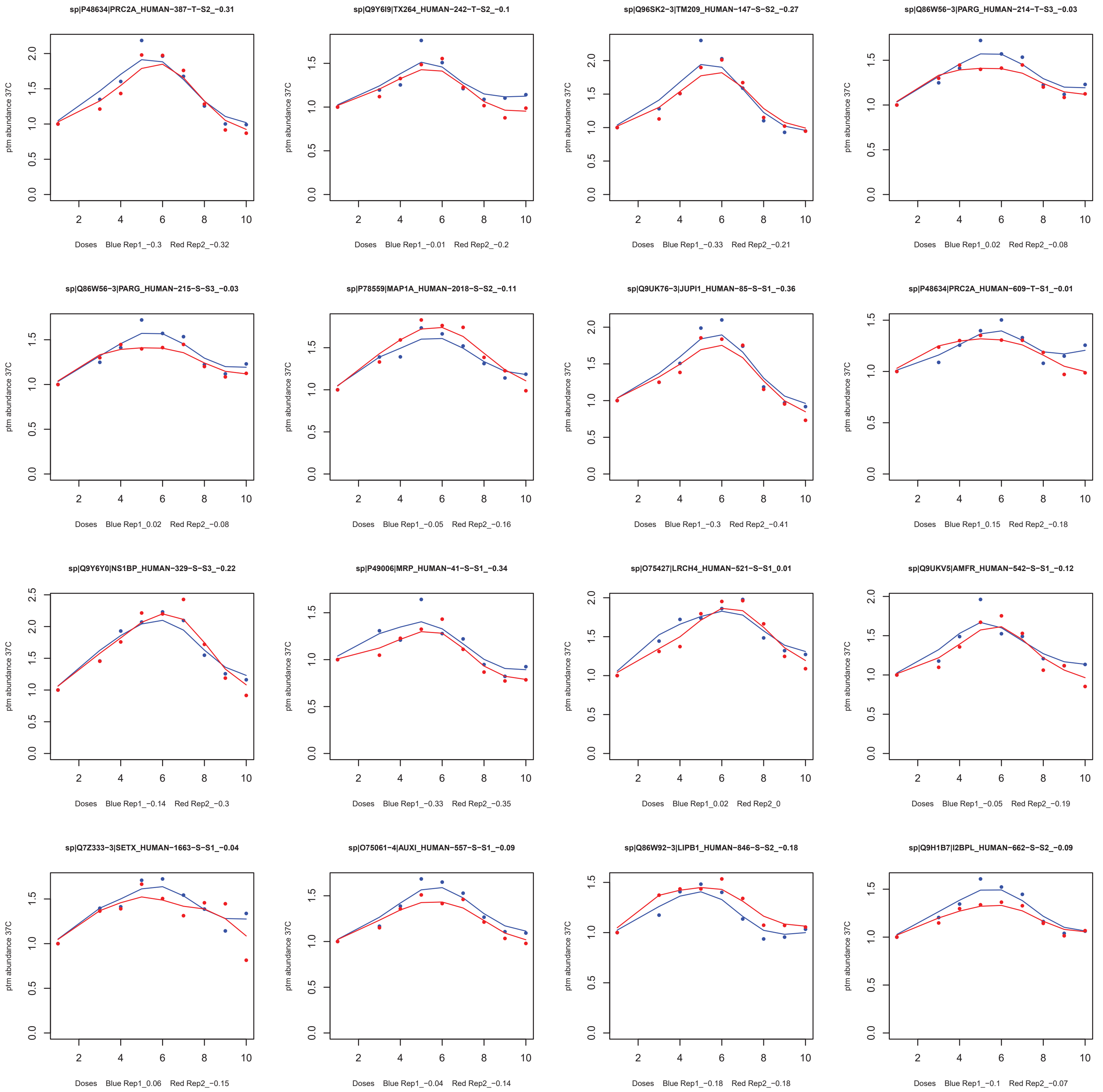

sp|P48634|PRC2A_HUMAN−387−T−S2_−0.31
ptm abundance 37C
Doses Blue Rep1_−0.3 Red Rep2_−0.32
sp|Q9Y6I9|TX264_HUMAN−242−T−S2_−0.1
ptm abundance 37C
Doses Blue Rep1_−0.01 Red Rep2_−0.2
sp|Q96SK2−3|TM209_HUMAN−147−S−S2_−0.27
ptm abundance 37C
Doses Blue Rep1_−0.33 Red Rep2_−0.21
sp|Q86W56−3|PARG_HUMAN−214−T−S3_−0.03
ptm abundance 37C
Doses Blue Rep1_0.02 Red Rep2_−0.08
sp|Q86W56−3|PARG_HUMAN−215−S−S3_−0.03
ptm abundance 37C
Doses Blue Rep1_0.02 Red Rep2_−0.08
sp|P78559|MAP1A_HUMAN−2018−S−S2_−0.11
ptm abundance 37C
Doses Blue Rep1_−0.05 Red Rep2_−0.16
sp|Q9UK76−3|JUPI1_HUMAN−85−S−S1_−0.36
ptm abundance 37C
Doses Blue Rep1_−0.3 Red Rep2_−0.41
sp|P48634|PRC2A_HUMAN−609−T−S1_−0.01
ptm abundance 37C
Doses Blue Rep1_0.15 Red Rep2_−0.18
sp|Q9Y6Y0|NS1BP_HUMAN−329−S−S3_−0.22
ptm abundance 37C
Doses Blue Rep1_−0.14 Red Rep2_−0.3
sp|P49006|MRP_HUMAN−41−S−S1_−0.34
ptm abundance 37C
Doses Blue Rep1_−0.33 Red Rep2_−0.35
sp|O75427|LRCH4_HUMAN−521−S−S1_0.01
ptm abundance 37C
Doses Blue Rep1_0.02 Red Rep2_0
sp|Q9UKV5|AMFR_HUMAN−542−S−S1_−0.12
ptm abundance 37C
Doses Blue Rep1_−0.05 Red Rep2_−0.19
sp|Q7Z333−3|SETX_HUMAN−1663−S−S1_−0.04
ptm abundance 37C
Doses Blue Rep1_0.06 Red Rep2_−0.15
sp|O75061−4|AUXI_HUMAN−557−S−S1_−0.09
ptm abundance 37C
Doses Blue Rep1_−0.04 Red Rep2_−0.14
sp|Q86W92−3|LIPB1_HUMAN−846−S−S2_−0.18
ptm abundance 37C
Doses Blue Rep1_−0.18 Red Rep2_−0.18
sp|Q9H1B7|I2BPL_HUMAN−662−S−S2_−0.09
ptm abundance 37C
Doses Blue Rep1_−0.1 Red Rep2_−0.07

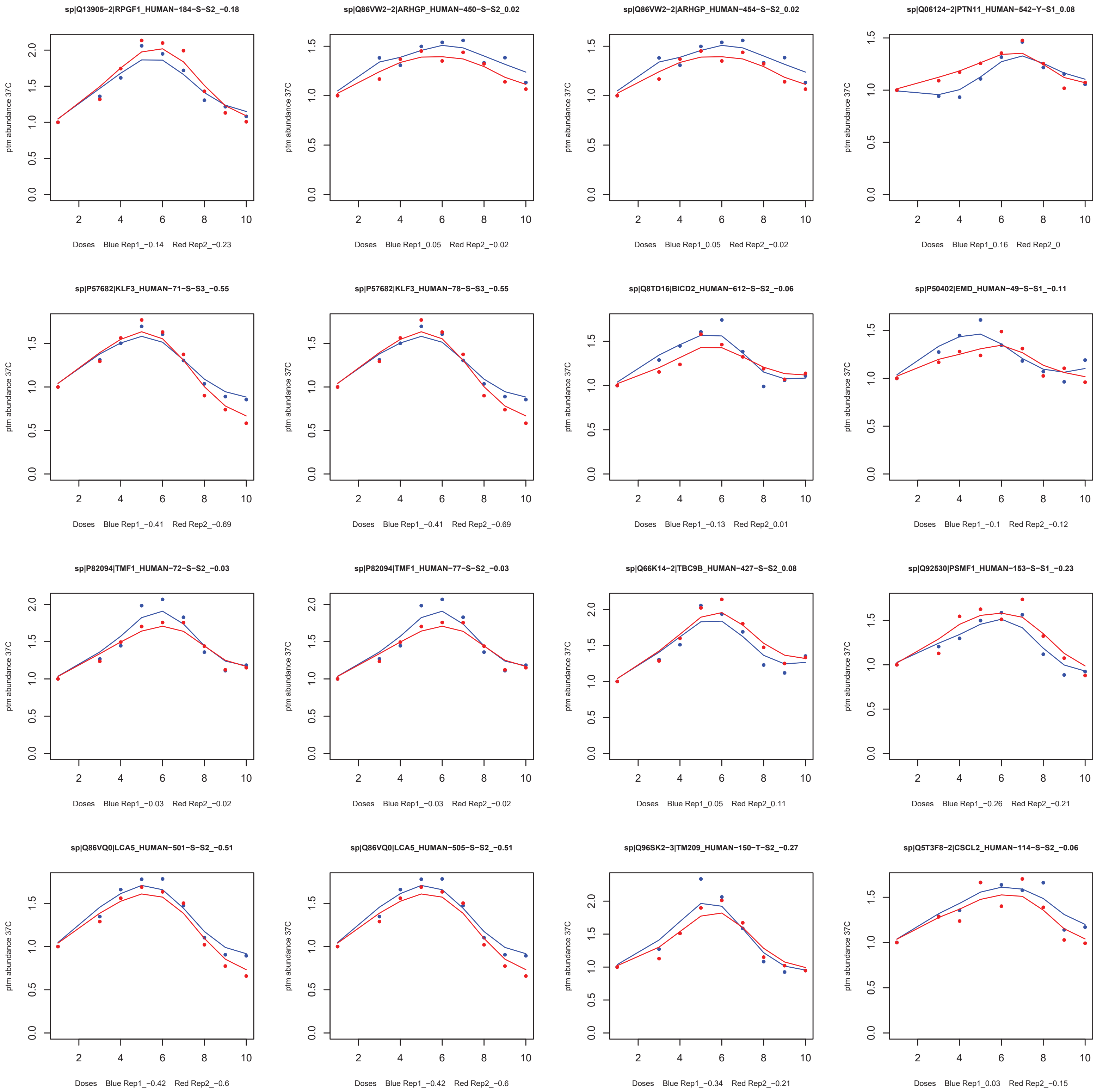
sp|Q13905−2|RPGF1_HUMAN−184−S−S2_−0.18
ptm abundance 37C
Doses Blue Rep1_−0.14 Red Rep2_−0.23
sp|Q86VW2−2|ARHGP_HUMAN−450−S−S2_0.02
ptm abundance 37C
Doses Blue Rep1_0.05 Red Rep2_−0.02
sp|Q86VW2−2|ARHGP_HUMAN−454−S−S2_0.02
ptm abundance 37C
Doses Blue Rep1_0.05 Red Rep2_−0.02
sp|Q06124−2|PTN11_HUMAN−542−Y−S1_0.08
ptm abundance 37C
Doses Blue Rep1_0.16 Red Rep2_0
sp|P57682|KLF3_HUMAN−71−S−S3_−0.55
ptm abundance 37C
Doses Blue Rep1_−0.41 Red Rep2_−0.69
sp|P57682|KLF3_HUMAN−78−S−S3_−0.55
ptm abundance 37C
Doses Blue Rep1_−0.41 Red Rep2_−0.69
sp|Q8TD16|BICD2_HUMAN−612−S−S2_−0.06
ptm abundance 37C
Doses Blue Rep1_−0.13 Red Rep2_0.01
sp|P50402|EMD_HUMAN−49−S−S1_−0.11
ptm abundance 37C
Doses Blue Rep1_−0.1 Red Rep2_−0.12
sp|P82094|TMF1_HUMAN−72−S−S2_−0.03
ptm abundance 37C
Doses Blue Rep1_−0.03 Red Rep2_−0.02
sp|P82094|TMF1_HUMAN−77−S−S2_−0.03
ptm abundance 37C
Doses Blue Rep1_−0.03 Red Rep2_−0.02
sp|Q66K14−2|TBC9B_HUMAN−427−S−S2_0.08
ptm abundance 37C
Doses Blue Rep1_0.05 Red Rep2_0.11
sp|Q92530|PSMF1_HUMAN−153−S−S1_−0.23
ptm abundance 37C
Doses Blue Rep1_−0.26 Red Rep2_−0.21
sp|Q86VQ0|LCA5_HUMAN−501−S−S2_−0.51
ptm abundance 37C
Doses Blue Rep1_−0.42 Red Rep2_−0.6
sp|Q86VQ0|LCA5_HUMAN−505−S−S2_−0.51
ptm abundance 37C
Doses Blue Rep1_−0.42 Red Rep2_−0.6
sp|Q96SK2−3|TM209_HUMAN−150−T−S2_−0.27
ptm abundance 37C
Doses Blue Rep1_−0.34 Red Rep2_−0.21
sp|Q5T3F8−2|CSCL2_HUMAN−114−S−S2_−0.06
ptm abundance 37C
Doses Blue Rep1_0.03 Red Rep2_−0.15

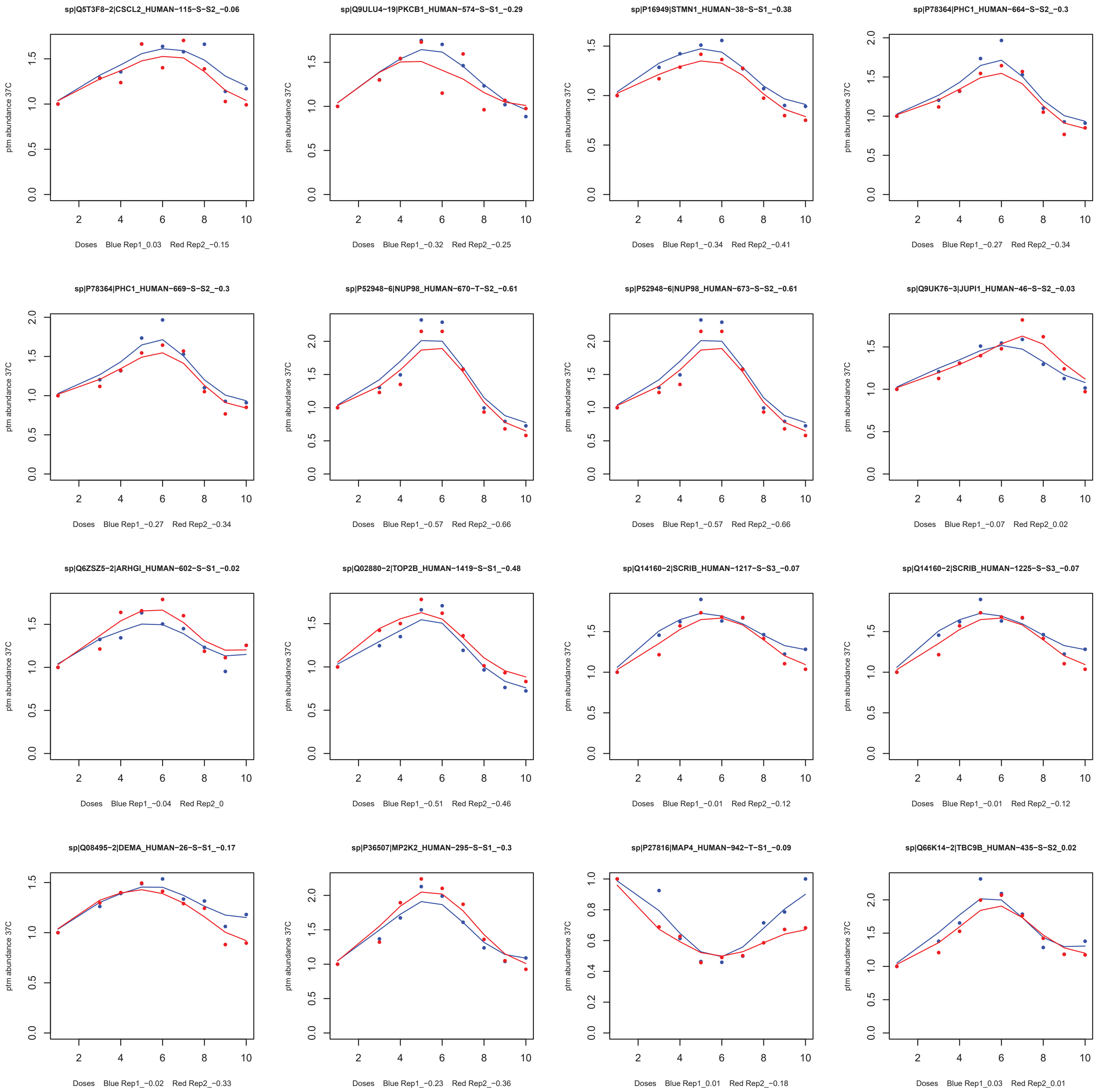
sp|Q5T3F8−2|CSCL2_HUMAN−115−S−S2_−0.06
ptm abundance 37C
Doses Blue Rep1_0.03 Red Rep2_−0.15
sp|Q9ULU4−19|PKCB1_HUMAN−574−S−S1_−0.29
ptm abundance 37C
Doses Blue Rep1_−0.32 Red Rep2_−0.25
sp|P16949|STMN1_HUMAN−38−S−S1_−0.38
ptm abundance 37C
Doses Blue Rep1_−0.34 Red Rep2_−0.41
sp|P78364|PHC1_HUMAN−664−S−S2_−0.3
ptm abundance 37C
Doses Blue Rep1_−0.27 Red Rep2_−0.34
sp|P78364|PHC1_HUMAN−669−S−S2_−0.3
ptm abundance 37C
Doses Blue Rep1_−0.27 Red Rep2_−0.34
sp|P52948−6|NUP98_HUMAN−670−T−S2_−0.61
ptm abundance 37C
Doses Blue Rep1_−0.57 Red Rep2_−0.66
sp|P52948−6|NUP98_HUMAN−673−S−S2_−0.61
ptm abundance 37C
Doses Blue Rep1_−0.57 Red Rep2_−0.66
sp|Q9UK76−3|JUPI1_HUMAN−46−S−S2_−0.03
ptm abundance 37C
Doses Blue Rep1_−0.07 Red Rep2_0.02
sp|Q6ZSZ5−2|ARHGI_HUMAN−602−S−S1_−0.02
ptm abundance 37C
Doses Blue Rep1_−0.04 Red Rep2_0
sp|Q02880−2|TOP2B_HUMAN−1419−S−S1_−0.48
ptm abundance 37C
Doses Blue Rep1_−0.51 Red Rep2_−0.46
sp|Q14160−2|SCRIB_HUMAN−1217−S−S3_−0.07
ptm abundance 37C
Doses Blue Rep1_−0.01 Red Rep2_−0.12
sp|Q14160−2|SCRIB_HUMAN−1225−S−S3_−0.07
ptm abundance 37C
Doses Blue Rep1_−0.01 Red Rep2_−0.12
sp|Q08495−2|DEMA_HUMAN−26−S−S1_−0.17
ptm abundance 37C
Doses Blue Rep1_−0.02 Red Rep2_−0.33
sp|P36507|MP2K2_HUMAN−295−S−S1_−0.3
ptm abundance 37C
Doses Blue Rep1_−0.23 Red Rep2_−0.36
sp|P27816|MAP4_HUMAN−942−T−S1_−0.09
ptm abundance 37C
Doses Blue Rep1_0.01 Red Rep2_−0.18
sp|Q66K14−2|TBC9B_HUMAN−435−S−S2_0.02
ptm abundance 37C
Doses Blue Rep1_0.03 Red Rep2_0.01

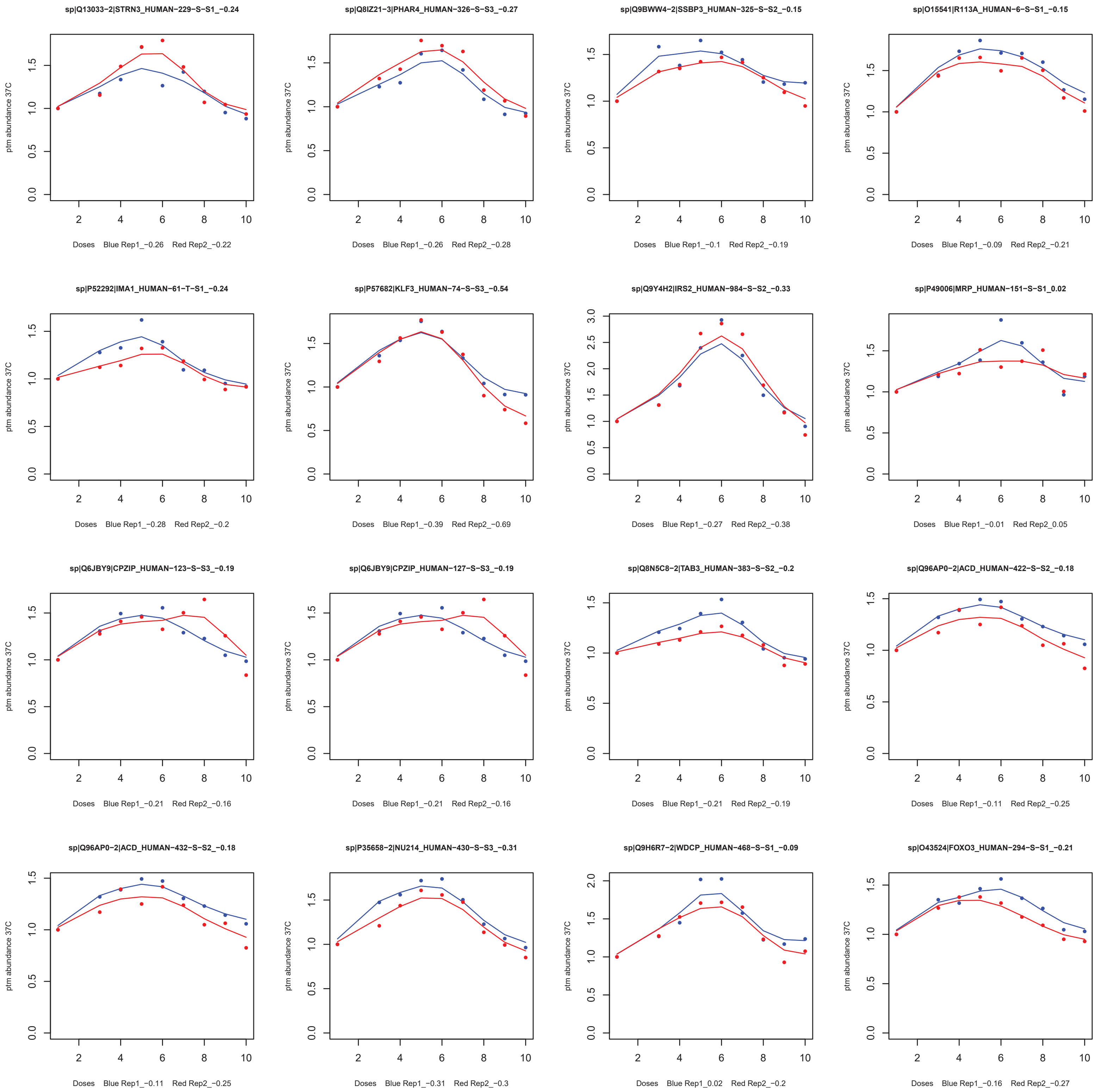
sp|Q13033−2|STRN3_HUMAN−229−S−S1_−0.24
Doses Blue Rep1_−0.26 Red Rep2_−0.22
sp|Q8IZ21−3|PHAR4_HUMAN−326−S−S3_−0.27
Doses Blue Rep1_−0.26 Red Rep2_−0.28
sp|Q9BWW4−2|SSBP3_HUMAN−325−S−S2_−0.15
Doses Blue Rep1_−0.1 Red Rep2_−0.19
sp|O15541|R113A_HUMAN−6−S−S1_−0.15
Doses Blue Rep1_−0.09 Red Rep2_−0.21
sp|P52292|IMA1_HUMAN−61−T−S1_−0.24
Doses Blue Rep1_−0.28 Red Rep2_−0.2
sp|P57682|KLF3_HUMAN−74−S−S3_−0.54
Doses Blue Rep1_−0.39 Red Rep2_−0.69
sp|Q9Y4H2|IRS2_HUMAN−984−S−S2_−0.33
Doses Blue Rep1_−0.27 Red Rep2_−0.38
sp|P49006|MRP_HUMAN−151−S−S1_0.02
Doses Blue Rep1_−0.01 Red Rep2_0.05
sp|Q6JBY9|CPZIP_HUMAN−123−S−S3_−0.19
Doses Blue Rep1_−0.21 Red Rep2_−0.16
sp|Q6JBY9|CPZIP_HUMAN−127−S−S3_−0.19
Doses Blue Rep1_−0.21 Red Rep2_−0.16
sp|Q8N5C8−2|TAB3_HUMAN−383−S−S2_−0.2
Doses Blue Rep1_−0.21 Red Rep2_−0.19
sp|Q96AP0−2|ACD_HUMAN−422−S−S2_−0.18
Doses Blue Rep1_−0.11 Red Rep2_−0.25
sp|Q96AP0−2|ACD_HUMAN−432−S−S2_−0.18
Doses Blue Rep1_−0.11 Red Rep2_−0.25
sp|P35658−2|NU214_HUMAN−430−S−S3_−0.31
Doses Blue Rep1_−0.31 Red Rep2_−0.3
sp|Q9H6R7−2|WDCP_HUMAN−468−S−S1_−0.09
Doses Blue Rep1_0.02 Red Rep2_−0.2
sp|O43524|FOXO3_HUMAN−294−S−S1_−0.21
Doses Blue Rep1_−0.16 Red Rep2_−0.27
ptm abundance 37C

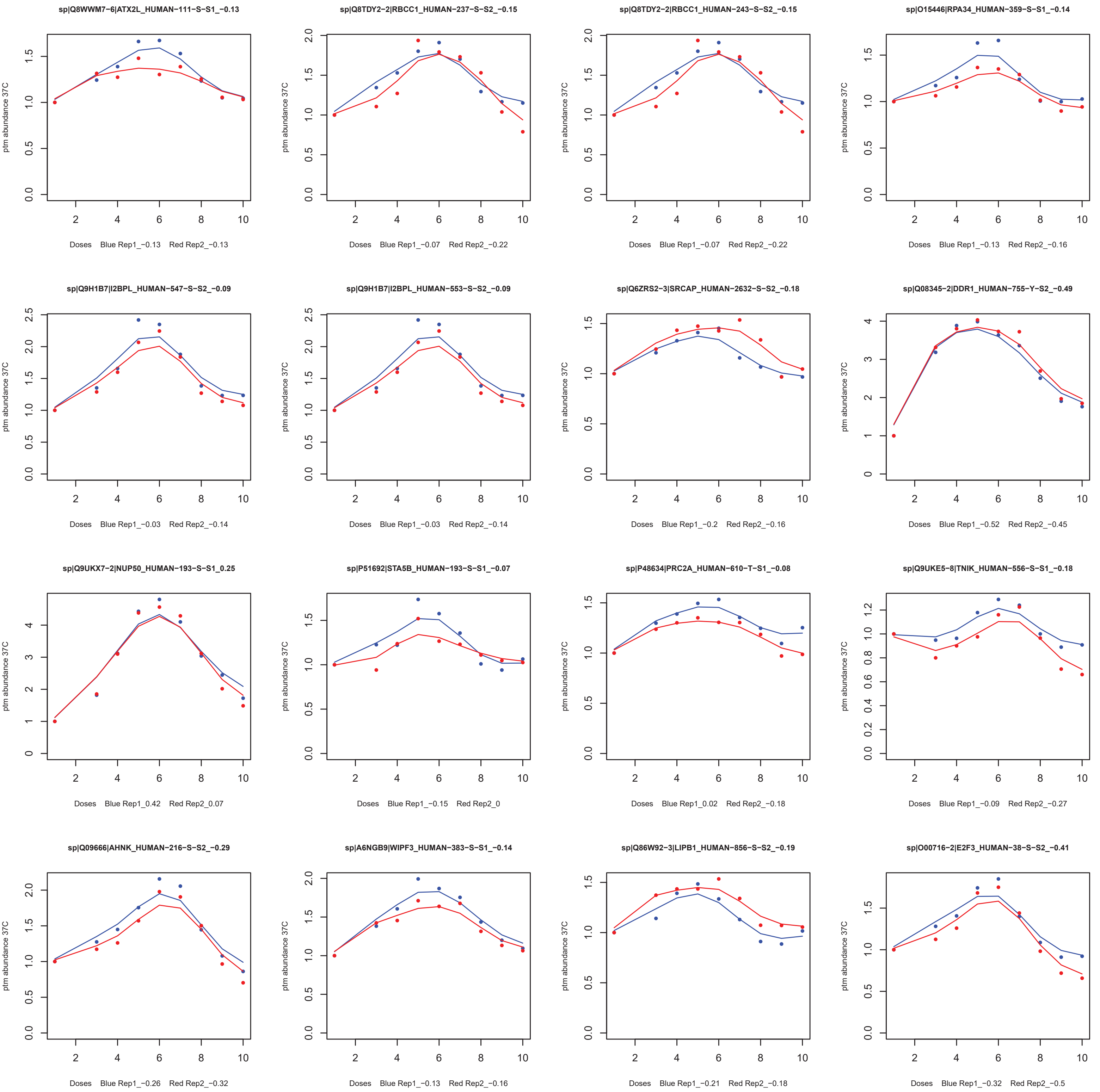
sp|Q8WWM7−6|ATX2L_HUMAN−111−S−S1_−0.13
Doses Blue Rep1_−0.13 Red Rep2_−0.13
sp|Q8TDY2−2|RBCC1_HUMAN−237−S−S2_−0.15
Doses Blue Rep1_−0.07 Red Rep2_−0.22
sp|Q8TDY2−2|RBCC1_HUMAN−243−S−S2_−0.15
Doses Blue Rep1_−0.07 Red Rep2_−0.22
sp|O15446|RPA34_HUMAN−359−S−S1_−0.14
Doses Blue Rep1_−0.13 Red Rep2_−0.16
sp|Q9H1B7|I2BPL_HUMAN−547−S−S2_−0.09
Doses Blue Rep1_−0.03 Red Rep2_−0.14
sp|Q9H1B7|I2BPL_HUMAN−553−S−S2_−0.09
Doses Blue Rep1_−0.03 Red Rep2_−0.14
sp|Q6ZRS2−3|SRCAP_HUMAN−2632−S−S2_−0.18
Doses Blue Rep1_−0.2 Red Rep2_−0.16
sp|Q08345−2|DDR1_HUMAN−755−Y−S2_−0.49
Doses Blue Rep1_−0.52 Red Rep2_−0.45
sp|Q9UKX7−2|NUP50_HUMAN−193−S−S1_0.25
Doses Blue Rep1_0.42 Red Rep2_0.07
sp|P51692|STA5B_HUMAN−193−S−S1_−0.07
Doses Blue Rep1_−0.15 Red Rep2_0
sp|P48634|PRC2A_HUMAN−610−T−S1_−0.08
Doses Blue Rep1_0.02 Red Rep2_−0.18
sp|Q9UKE5−8|TNIK_HUMAN−556−S−S1_−0.18
Doses Blue Rep1_−0.09 Red Rep2_−0.27
sp|Q09666|AHNK_HUMAN−216−S−S2_−0.29
Doses Blue Rep1_−0.26 Red Rep2_−0.32
sp|A6NGB9|WIPF3_HUMAN−383−S−S1_−0.14
Doses Blue Rep1_−0.13 Red Rep2_−0.16
sp|Q86W92−3|LIPB1_HUMAN−856−S−S2_−0.19
Doses Blue Rep1_−0.21 Red Rep2_−0.18
sp|O00716−2|E2F3_HUMAN−38−S−S2_−0.41
Doses Blue Rep1_−0.32 Red Rep2_−0.5
ptm abundance 37C

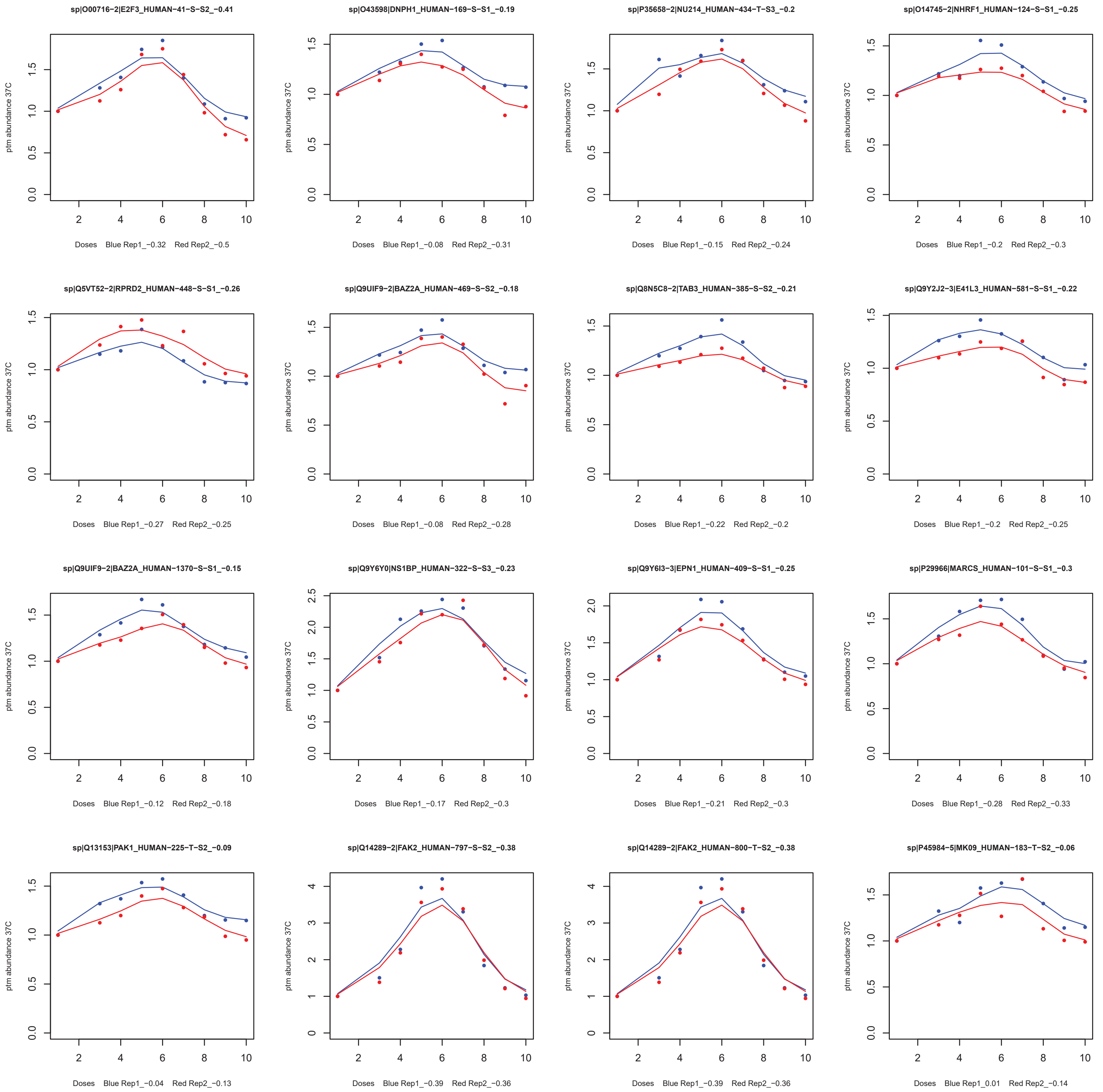
sp|O00716−2|E2F3_HUMAN−41−S−S2_−0.41
ptm abundance 37C
Doses Blue Rep1_−0.32 Red Rep2_−0.5
sp|O43598|DNPH1_HUMAN−169−S−S1_−0.19
ptm abundance 37C
Doses Blue Rep1_−0.08 Red Rep2_−0.31
sp|P35658−2|NU214_HUMAN−434−T−S3_−0.2
ptm abundance 37C
Doses Blue Rep1_−0.15 Red Rep2_−0.24
sp|O14745−2|NHRF1_HUMAN−124−S−S1_−0.25
ptm abundance 37C
Doses Blue Rep1_−0.2 Red Rep2_−0.3
sp|Q5VT52−2|RPRD2_HUMAN−448−S−S1_−0.26
ptm abundance 37C
Doses Blue Rep1_−0.27 Red Rep2_−0.25
sp|Q9UIF9−2|BAZ2A_HUMAN−469−S−S2_−0.18
ptm abundance 37C
Doses Blue Rep1_−0.08 Red Rep2_−0.28
sp|Q8N5C8−2|TAB3_HUMAN−385−S−S2_−0.21
ptm abundance 37C
Doses Blue Rep1_−0.22 Red Rep2_−0.2
sp|Q9Y2J2−3|E41L3_HUMAN−581−S−S1_−0.22
ptm abundance 37C
Doses Blue Rep1_−0.2 Red Rep2_−0.25
sp|Q9UIF9−2|BAZ2A_HUMAN−1370−S−S1_−0.15
ptm abundance 37C
Doses Blue Rep1_−0.12 Red Rep2_−0.18
sp|Q9Y6Y0|NS1BP_HUMAN−322−S−S3_−0.23
ptm abundance 37C
Doses Blue Rep1_−0.17 Red Rep2_−0.3
sp|Q9Y6I3−3|EPN1_HUMAN−409−S−S1_−0.25
ptm abundance 37C
Doses Blue Rep1_−0.21 Red Rep2_−0.3
sp|P29966|MARCS_HUMAN−101−S−S1_−0.3
ptm abundance 37C
Doses Blue Rep1_−0.28 Red Rep2_−0.33
sp|Q13153|PAK1_HUMAN−225−T−S2_−0.09
ptm abundance 37C
Doses Blue Rep1_−0.04 Red Rep2_−0.13
sp|Q14289−2|FAK2_HUMAN−797−S−S2_−0.38
ptm abundance 37C
Doses Blue Rep1_−0.39 Red Rep2_−0.36
sp|Q14289−2|FAK2_HUMAN−800−T−S2_−0.38
ptm abundance 37C
Doses Blue Rep1_−0.39 Red Rep2_−0.36
sp|P45984−5|MK09_HUMAN−183−T−S2_−0.06
ptm abundance 37C
Doses Blue Rep1_0.01 Red Rep2_−0.14

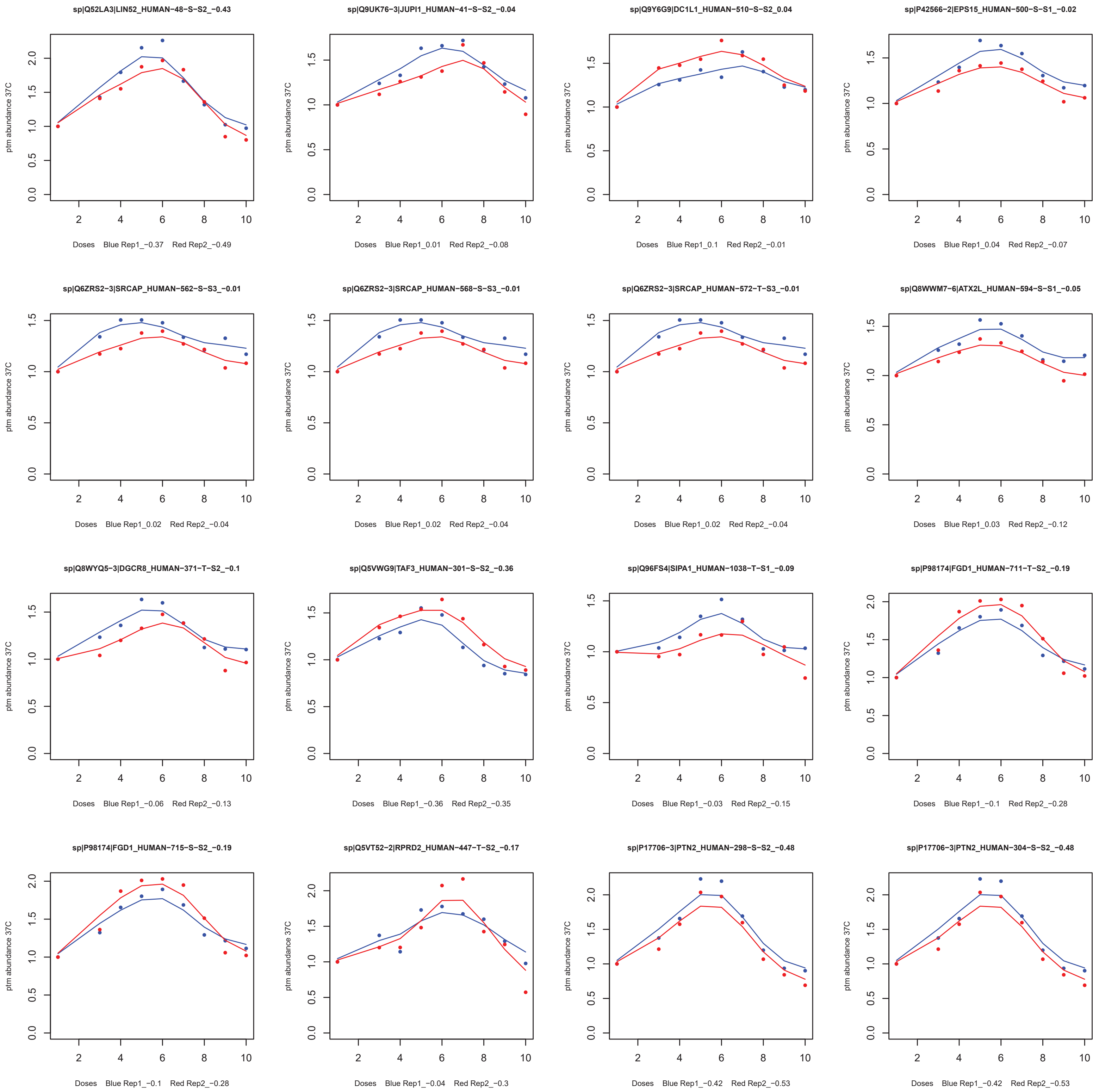

sp|Q52LA3|LIN52_HUMAN−48−S−S2_−0.43
ptm abundance 37C
Doses Blue Rep1_−0.37 Red Rep2_−0.49
sp|Q9UK76−3|JUPI1_HUMAN−41−S−S2_−0.04
ptm abundance 37C
Doses Blue Rep1_0.01 Red Rep2_−0.08
sp|Q9Y6G9|DC1L1_HUMAN−510−S−S2_0.04
ptm abundance 37C
Doses Blue Rep1_0.1 Red Rep2_−0.01
sp|P42566−2|EPS15_HUMAN−500−S−S1_−0.02
ptm abundance 37C
Doses Blue Rep1_0.04 Red Rep2_−0.07
sp|Q6ZRS2−3|SRCAP_HUMAN−562−S−S3_−0.01
ptm abundance 37C
Doses Blue Rep1_0.02 Red Rep2_−0.04
sp|Q6ZRS2−3|SRCAP_HUMAN−568−S−S3_−0.01
ptm abundance 37C
Doses Blue Rep1_0.02 Red Rep2_−0.04
sp|Q6ZRS2−3|SRCAP_HUMAN−572−T−S3_−0.01
ptm abundance 37C
Doses Blue Rep1_0.02 Red Rep2_−0.04
sp|Q8WWM7−6|ATX2L_HUMAN−594−S−S1_−0.05
ptm abundance 37C
Doses Blue Rep1_0.03 Red Rep2_−0.12
sp|Q8WYQ5−3|DGCR8_HUMAN−371−T−S2_−0.1
ptm abundance 37C
Doses Blue Rep1_−0.06 Red Rep2_−0.13
sp|Q5VWG9|TAF3_HUMAN−301−S−S2_−0.36
ptm abundance 37C
Doses Blue Rep1_−0.36 Red Rep2_−0.35
sp|Q96FS4|SIPA1_HUMAN−1038−T−S1_−0.09
ptm abundance 37C
Doses Blue Rep1_−0.03 Red Rep2_−0.15
sp|P98174|FGD1_HUMAN−711−T−S2_−0.19
ptm abundance 37C
Doses Blue Rep1_−0.1 Red Rep2_−0.28
sp|P98174|FGD1_HUMAN−715−S−S2_−0.19
ptm abundance 37C
Doses Blue Rep1_−0.1 Red Rep2_−0.28
sp|Q5VT52−2|RPRD2_HUMAN−447−T−S2_−0.17
ptm abundance 37C
Doses Blue Rep1_−0.04 Red Rep2_−0.3
sp|P17706−3|PTN2_HUMAN−298−S−S2_−0.48
ptm abundance 37C
Doses Blue Rep1_−0.42 Red Rep2_−0.53
sp|P17706−3|PTN2_HUMAN−304−S−S2_−0.48
ptm abundance 37C
Doses Blue Rep1_−0.42 Red Rep2_−0.53

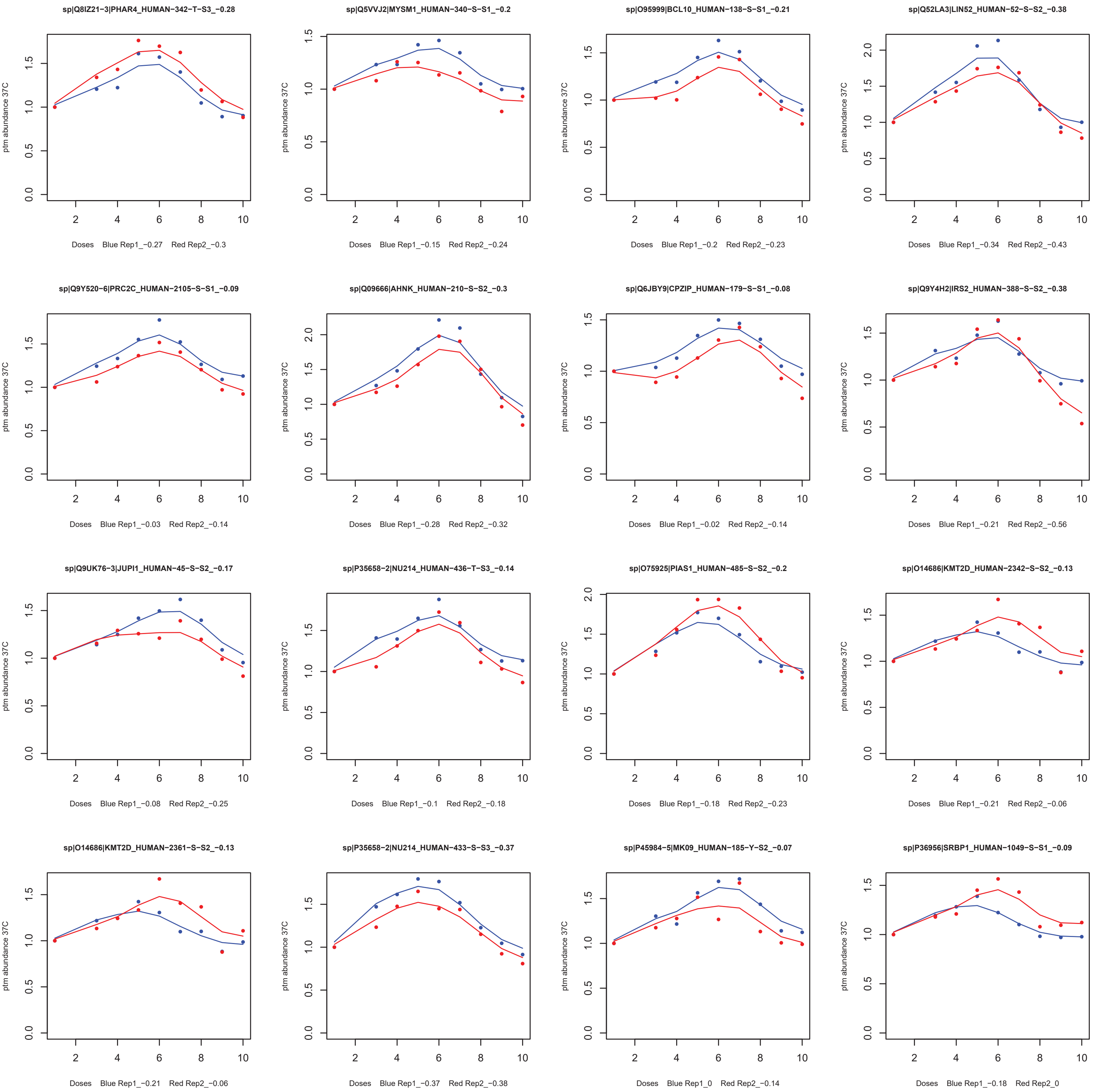

sp|Q8IZ21−3|PHAR4_HUMAN−342−T−S3_−0.28
Doses Blue Rep1_−0.27 Red Rep2_−0.3
sp|Q5VVJ2|MYSM1_HUMAN−340−S−S1_−0.2
Doses Blue Rep1_−0.15 Red Rep2_−0.24
sp|O95999|BCL10_HUMAN−138−S−S1_−0.21
Doses Blue Rep1_−0.2 Red Rep2_−0.23
sp|Q52LA3|LIN52_HUMAN−52−S−S2_−0.38
Doses Blue Rep1_−0.34 Red Rep2_−0.43
sp|Q9Y520−6|PRC2C_HUMAN−2105−S−S1_−0.09
Doses Blue Rep1_−0.03 Red Rep2_−0.14
sp|Q09666|AHNK_HUMAN−210−S−S2_−0.3
Doses Blue Rep1_−0.28 Red Rep2_−0.32
sp|Q6JBY9|CPZIP_HUMAN−179−S−S1_−0.08
Doses Blue Rep1_−0.02 Red Rep2_−0.14
sp|Q9Y4H2|IRS2_HUMAN−388−S−S2_−0.38
Doses Blue Rep1_−0.21 Red Rep2_−0.56
sp|Q9UK76−3|JUPI1_HUMAN−45−S−S2_−0.17
Doses Blue Rep1_−0.08 Red Rep2_−0.25
sp|P35658−2|NU214_HUMAN−436−T−S3_−0.14
Doses Blue Rep1_−0.1 Red Rep2_−0.18
sp|O75925|PIAS1_HUMAN−485−S−S2_−0.2
Doses Blue Rep1_−0.18 Red Rep2_−0.23
sp|O14686|KMT2D_HUMAN−2342−S−S2_−0.13
Doses Blue Rep1_−0.21 Red Rep2_−0.06
sp|O14686|KMT2D_HUMAN−2361−S−S2_−0.13
Doses Blue Rep1_−0.21 Red Rep2_−0.06
sp|P35658−2|NU214_HUMAN−433−S−S3_−0.37
Doses Blue Rep1_−0.37 Red Rep2_−0.38
sp|P45984−5|MK09_HUMAN−185−Y−S2_−0.07
Doses Blue Rep1_0 Red Rep2_−0.14
sp|P36956|SRBP1_HUMAN−1049−S−S1_−0.09
Doses Blue Rep1_−0.18 Red Rep2_0
ptm abundance 37C

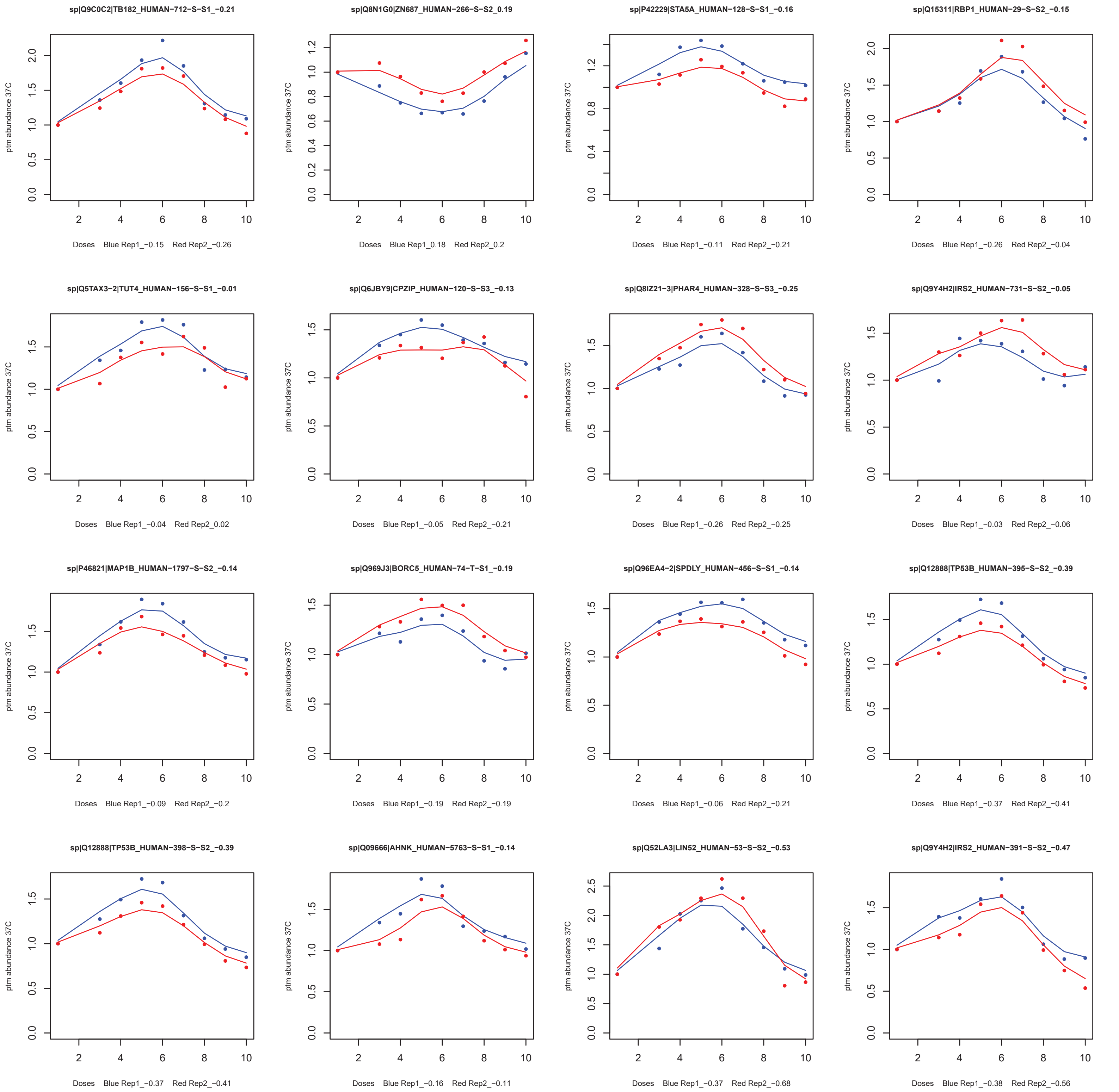
sp|Q9C0C2|TB182_HUMAN−712−S−S1_−0.21
ptm abundance 37C
Doses Blue Rep1_−0.15 Red Rep2_−0.26
sp|Q8N1G0|ZN687_HUMAN−266−S−S2_0.19
ptm abundance 37C
Doses Blue Rep1_0.18 Red Rep2_0.2
sp|P42229|STA5A_HUMAN−128−S−S1_−0.16
ptm abundance 37C
Doses Blue Rep1_−0.11 Red Rep2_−0.21
sp|Q15311|RBP1_HUMAN−29−S−S2_−0.15
ptm abundance 37C
Doses Blue Rep1_−0.26 Red Rep2_−0.04
sp|Q5TAX3−2|TUT4_HUMAN−156−S−S1_−0.01
ptm abundance 37C
Doses Blue Rep1_−0.04 Red Rep2_0.02
sp|Q6JBY9|CPZIP_HUMAN−120−S−S3_−0.13
ptm abundance 37C
Doses Blue Rep1_−0.05 Red Rep2_−0.21
sp|Q8IZ21−3|PHAR4_HUMAN−328−S−S3_−0.25
ptm abundance 37C
Doses Blue Rep1_−0.26 Red Rep2_−0.25
sp|Q9Y4H2|IRS2_HUMAN−731−S−S2_−0.05
ptm abundance 37C
Doses Blue Rep1_−0.03 Red Rep2_−0.06
sp|P46821|MAP1B_HUMAN−1797−S−S2_−0.14
ptm abundance 37C
Doses Blue Rep1_−0.09 Red Rep2_−0.2
sp|Q969J3|BORC5_HUMAN−74−T−S1_−0.19
ptm abundance 37C
Doses Blue Rep1_−0.19 Red Rep2_−0.19
sp|Q96EA4−2|SPDLY_HUMAN−456−S−S1_−0.14
ptm abundance 37C
Doses Blue Rep1_−0.06 Red Rep2_−0.21
sp|Q12888|TP53B_HUMAN−395−S−S2_−0.39
ptm abundance 37C
Doses Blue Rep1_−0.37 Red Rep2_−0.41
sp|Q12888|TP53B_HUMAN−398−S−S2_−0.39
ptm abundance 37C
Doses Blue Rep1_−0.37 Red Rep2_−0.41
sp|Q09666|AHNK_HUMAN−5763−S−S1_−0.14
ptm abundance 37C
Doses Blue Rep1_−0.16 Red Rep2_−0.11
sp|Q52LA3|LIN52_HUMAN−53−S−S2_−0.53
ptm abundance 37C
Doses Blue Rep1_−0.37 Red Rep2_−0.68
sp|Q9Y4H2|IRS2_HUMAN−391−S−S2_−0.47
ptm abundance 37C
Doses Blue Rep1_−0.38 Red Rep2_−0.56

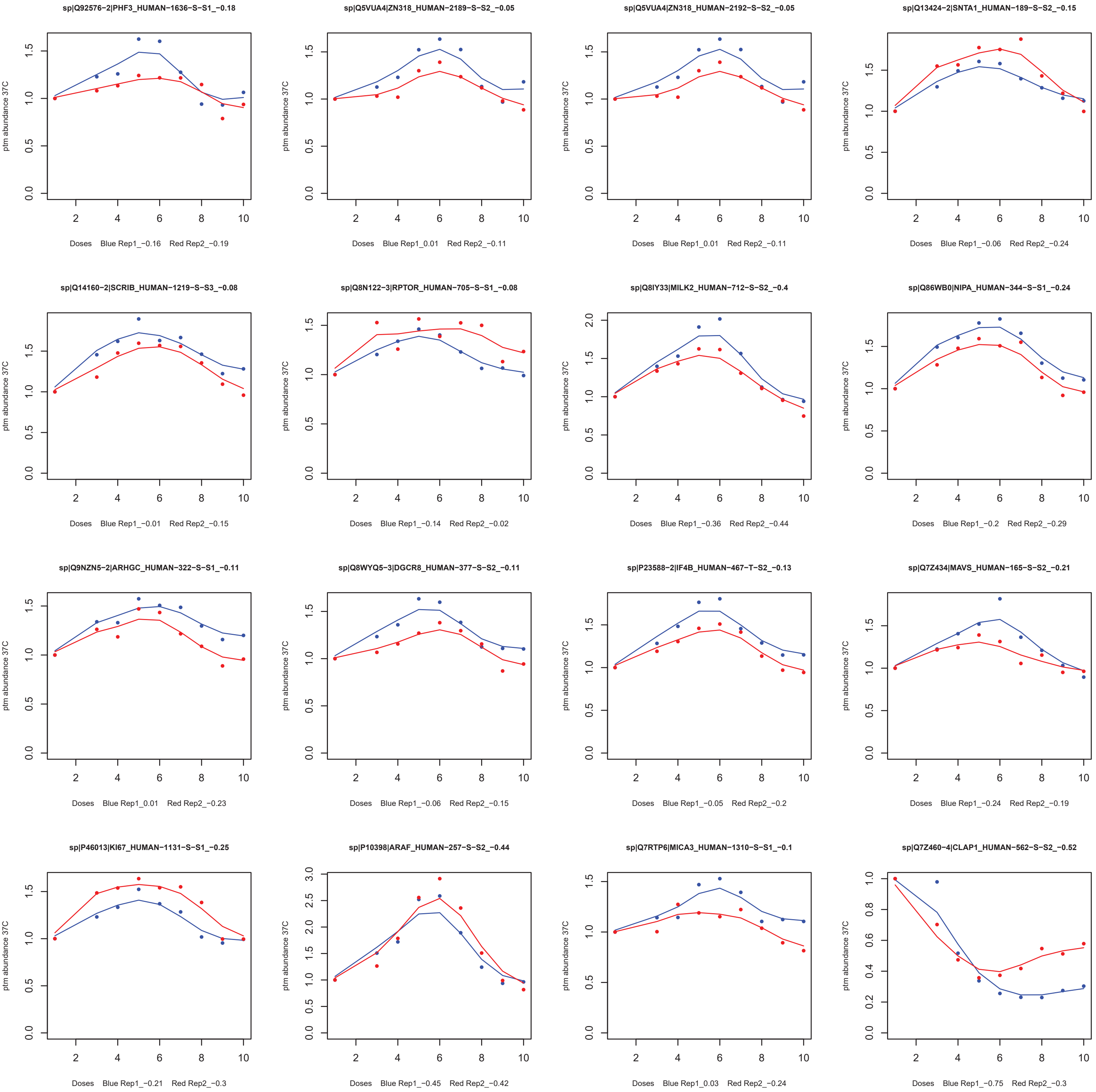
sp|Q92576−2|PHF3_HUMAN−1636−S−S1_−0.18
Doses Blue Rep1_−0.16 Red Rep2_−0.19
sp|Q5VUA4|ZN318_HUMAN−2189−S−S2_−0.05
Doses Blue Rep1_0.01 Red Rep2_−0.11
sp|Q5VUA4|ZN318_HUMAN−2192−S−S2_−0.05
Doses Blue Rep1_0.01 Red Rep2_−0.11
sp|Q13424−2|SNTA1_HUMAN−189−S−S2_−0.15
Doses Blue Rep1_−0.06 Red Rep2_−0.24
sp|Q14160−2|SCRIB_HUMAN−1219−S−S3_−0.08
Doses Blue Rep1_−0.01 Red Rep2_−0.15
sp|Q8N122−3|RPTOR_HUMAN−705−S−S1_−0.08
Doses Blue Rep1_−0.14 Red Rep2_−0.02
sp|Q8IY33|MILK2_HUMAN−712−S−S2_−0.4
Doses Blue Rep1_−0.36 Red Rep2_−0.44
sp|Q86WB0|NIPA_HUMAN−344−S−S1_−0.24
Doses Blue Rep1_−0.2 Red Rep2_−0.29
sp|Q9NZN5−2|ARHGC_HUMAN−322−S−S1_−0.11
Doses Blue Rep1_0.01 Red Rep2_−0.23
sp|Q8WYQ5−3|DGCR8_HUMAN−377−S−S2_−0.11
Doses Blue Rep1_−0.06 Red Rep2_−0.15
sp|P23588−2|IF4B_HUMAN−467−T−S2_−0.13
Doses Blue Rep1_−0.05 Red Rep2_−0.2
sp|Q7Z434|MAVS_HUMAN−165−S−S2_−0.21
Doses Blue Rep1_−0.24 Red Rep2_−0.19
sp|P46013|KI67_HUMAN−1131−S−S1_−0.25
Doses Blue Rep1_−0.21 Red Rep2_−0.3
sp|P10398|ARAF_HUMAN−257−S−S2_−0.44
Doses Blue Rep1_−0.45 Red Rep2_−0.42
sp|Q7RTP6|MICA3_HUMAN−1310−S−S1_−0.1
Doses Blue Rep1_0.03 Red Rep2_−0.24
sp|Q7Z460−4|CLAP1_HUMAN−562−S−S2_−0.52
Doses Blue Rep1_−0.75 Red Rep2_−0.3
ptm abundance 37C

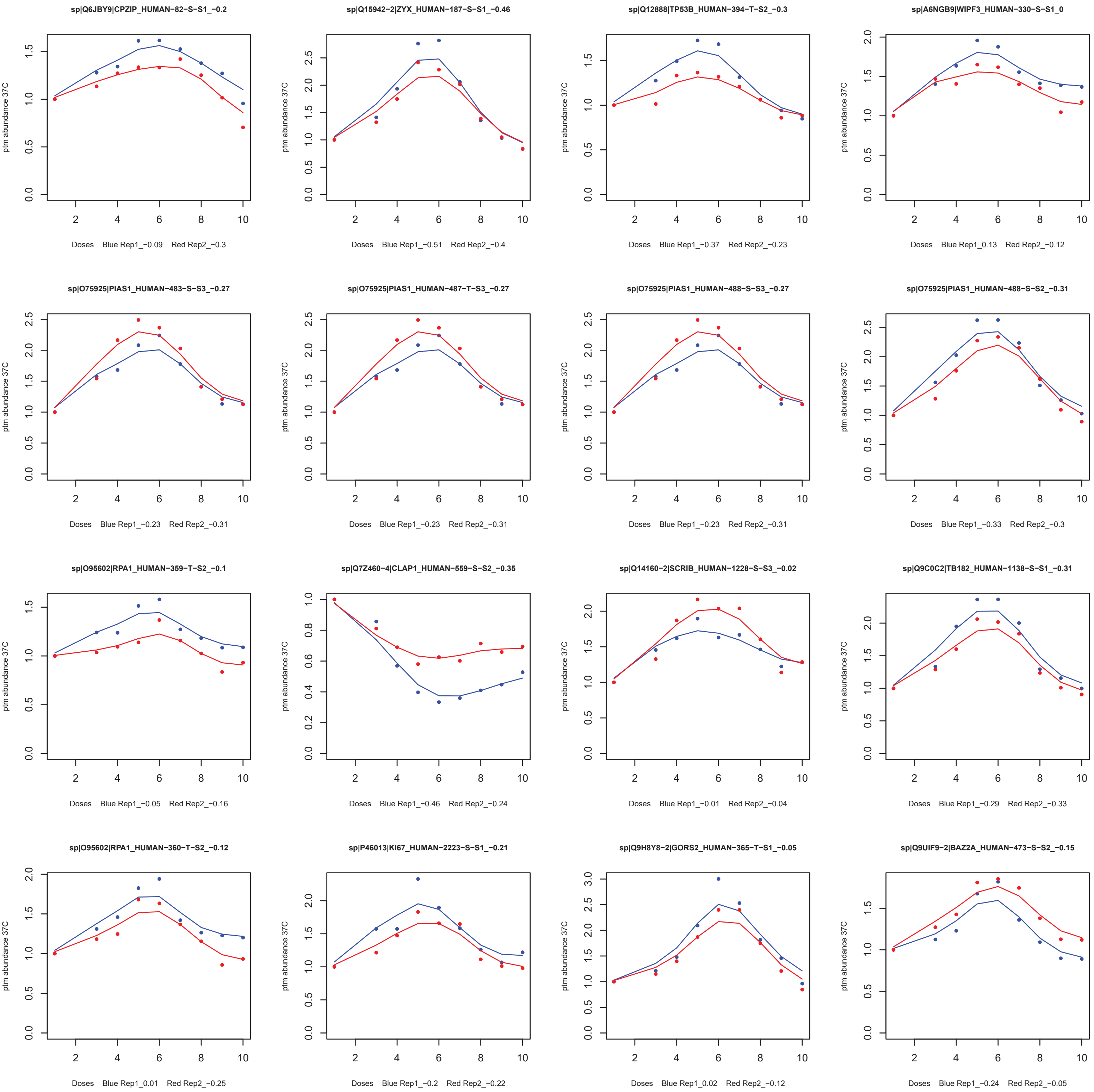
sp|Q6JBY9|CPZIP_HUMAN−82−S−S1_−0.2
ptm abundance 37C
Doses Blue Rep1_−0.09 Red Rep2_−0.3
sp|Q15942−2|ZYX_HUMAN−187−S−S1_−0.46
ptm abundance 37C
Doses Blue Rep1_−0.51 Red Rep2_−0.4
sp|Q12888|TP53B_HUMAN−394−T−S2_−0.3
ptm abundance 37C
Doses Blue Rep1_−0.37 Red Rep2_−0.23
sp|A6NGB9|WIPF3_HUMAN−330−S−S1_0
ptm abundance 37C
Doses Blue Rep1_0.13 Red Rep2_−0.12
sp|O75925|PIAS1_HUMAN−483−S−S3_−0.27
ptm abundance 37C
Doses Blue Rep1_−0.23 Red Rep2_−0.31
sp|O75925|PIAS1_HUMAN−487−T−S3_−0.27
ptm abundance 37C
Doses Blue Rep1_−0.23 Red Rep2_−0.31
sp|O75925|PIAS1_HUMAN−488−S−S3_−0.27
ptm abundance 37C
Doses Blue Rep1_−0.23 Red Rep2_−0.31
sp|O75925|PIAS1_HUMAN−488−S−S2_−0.31
ptm abundance 37C
Doses Blue Rep1_−0.33 Red Rep2_−0.3
sp|O95602|RPA1_HUMAN−359−T−S2_−0.1
ptm abundance 37C
Doses Blue Rep1_−0.05 Red Rep2_−0.16
sp|Q7Z460−4|CLAP1_HUMAN−559−S−S2_−0.35
ptm abundance 37C
Doses Blue Rep1_−0.46 Red Rep2_−0.24
sp|Q14160−2|SCRIB_HUMAN−1228−S−S3_−0.02
ptm abundance 37C
Doses Blue Rep1_−0.01 Red Rep2_−0.04
sp|Q9C0C2|TB182_HUMAN−1138−S−S1_−0.31
ptm abundance 37C
Doses Blue Rep1_−0.29 Red Rep2_−0.33
sp|O95602|RPA1_HUMAN−360−T−S2_−0.12
ptm abundance 37C
Doses Blue Rep1_0.01 Red Rep2_−0.25
sp|P46013|KI67_HUMAN−2223−S−S1_−0.21
ptm abundance 37C
Doses Blue Rep1_−0.2 Red Rep2_−0.22
sp|Q9H8Y8−2|GORS2_HUMAN−365−T−S1_−0.05
ptm abundance 37C
Doses Blue Rep1_0.02 Red Rep2_−0.12
sp|Q9UIF9−2|BAZ2A_HUMAN−473−S−S2_−0.15
ptm abundance 37C
Doses Blue Rep1_−0.24 Red Rep2_−0.05

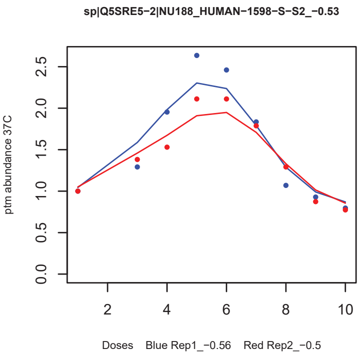
sp|Q5SRE5-2|NU188_HUMAN-1598-S-S2_-0.53
ptm abundance 37C
Doses Blue Rep1_-0.56 Red Rep2_-0.5

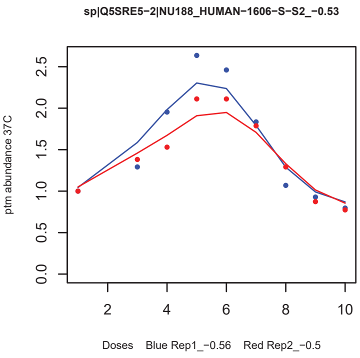
sp|Q5SRE5-2|NU188_HUMAN-1606-S-S2_-0.53
ptm abundance 37C
Doses Blue Rep1_-0.56 Red Rep2_-0.5

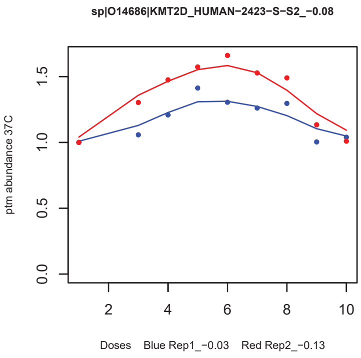
sp|O14686|KMT2D_HUMAN-2423-S-S2_-0.08
ptm abundance 37C
Doses Blue Rep1_-0.03 Red Rep2_-0.13

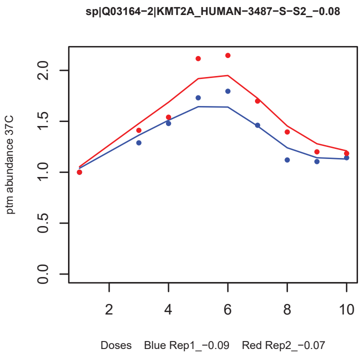
sp|Q03164-2|KMT2A_HUMAN-3487-S-S2_-0.08
ptm abundance 37C
Doses Blue Rep1_-0.09 Red Rep2_-0.07

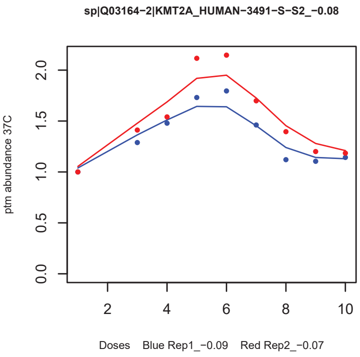
sp|Q03164-2|KMT2A_HUMAN-3491-S-S2_-0.08
ptm abundance 37C
Doses Blue Rep1_-0.09 Red Rep2_-0.07

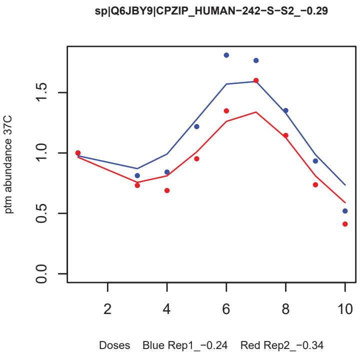
sp|Q6JBY9|CPZIP_HUMAN-242-S-S2_-0.29
ptm abundance 37C
Doses Blue Rep1_-0.24 Red Rep2_-0.34

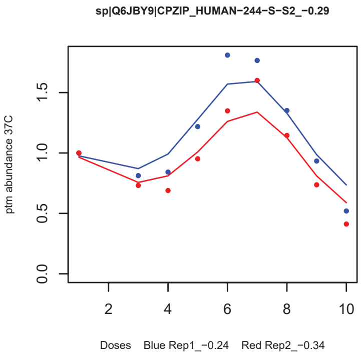
sp|Q6JBY9|CPZIP_HUMAN-244-S-S2_-0.29
ptm abundance 37C
Doses Blue Rep1_-0.24 Red Rep2_-0.34

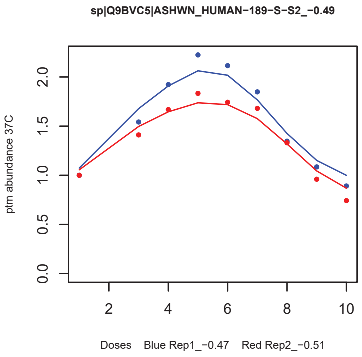
sp|Q9BVC5|ASHWN_HUMAN-189-S-S2_-0.49
ptm abundance 37C
Doses Blue Rep1_-0.47 Red Rep2_-0.51

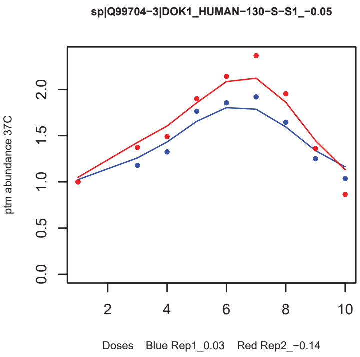
sp|Q99704-3|DOK1_HUMAN-130-S-S1_-0.05
ptm abundance 37C
Doses Blue Rep1_0.03 Red Rep2_-0.14

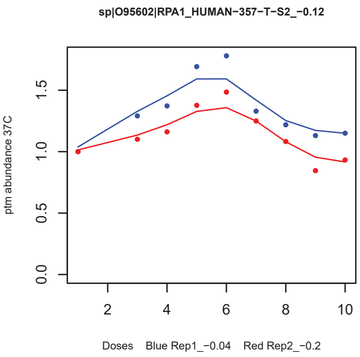
sp|O95602|RPA1_HUMAN-357-T-S2_-0.12
ptm abundance 37C
Doses Blue Rep1_-0.04 Red Rep2_-0.2

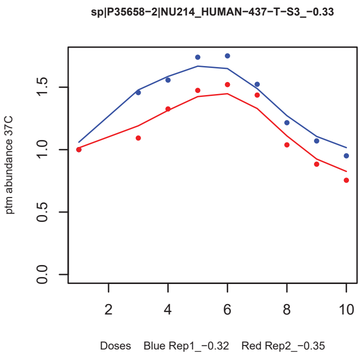
sp|P35658-2|NU214_HUMAN-437-T-S3_-0.33
ptm abundance 37C
Doses Blue Rep1_-0.32 Red Rep2_-0.35

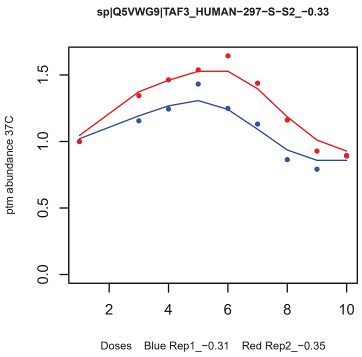
sp|Q5VWG9|TAF3_HUMAN-297-S-S2_-0.33
ptm abundance 37C
Doses Blue Rep1_-0.31 Red Rep2_-0.35

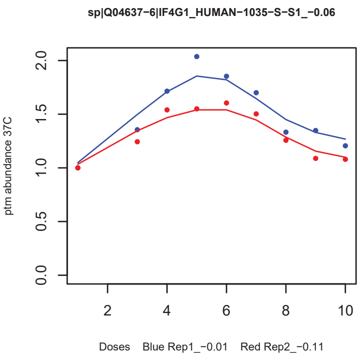
sp|Q04637-6|IF4G1_HUMAN-1035-S-S1_-0.06
ptm abundance 37C
Doses Blue Rep1_-0.01 Red Rep2_-0.11

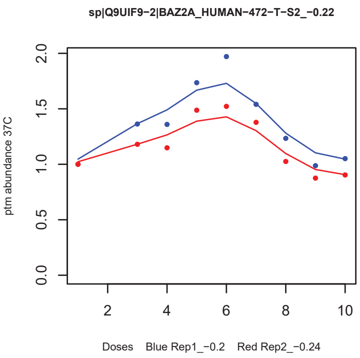
sp|Q9UIF9-2|BAZ2A_HUMAN-472-T-S2_-0.22
ptm abundance 37C
Doses Blue Rep1_-0.2 Red Rep2_-0.24

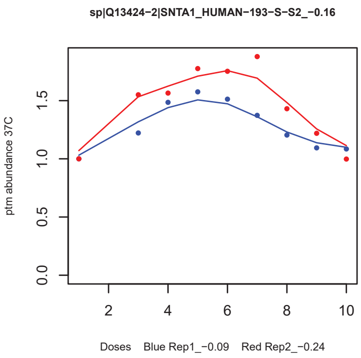
sp|Q13424-2|SNTA1_HUMAN-193-S-S2_-0.16
ptm abundance 37C
Doses Blue Rep1_-0.09 Red Rep2_-0.24

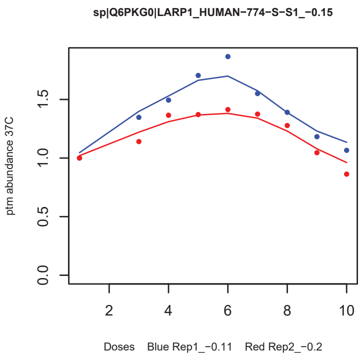
sp|Q6PKG0|LARP1_HUMAN-774-S-S1_-0.15
ptm abundance 37C
Doses Blue Rep1_-0.11 Red Rep2_-0.2

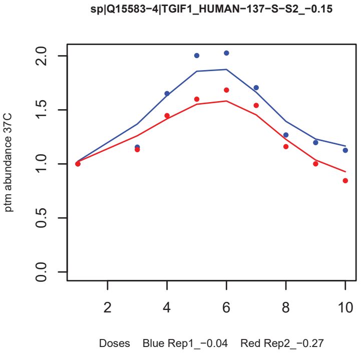
sp|Q15583−4|TGIF1_HUMAN−137−S−S2_−0.15
ptm abundance 37C
Doses Blue Rep1_−0.04 Red Rep2_−0.27

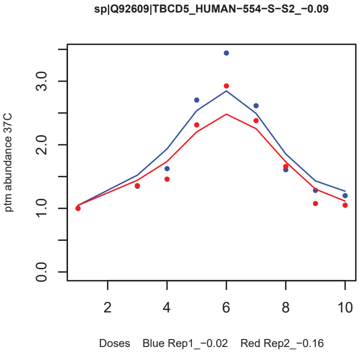
sp|Q92609|TBCD5_HUMAN−554−S−S2_−0.09
ptm abundance 37C
Doses Blue Rep1_−0.02 Red Rep2_−0.16

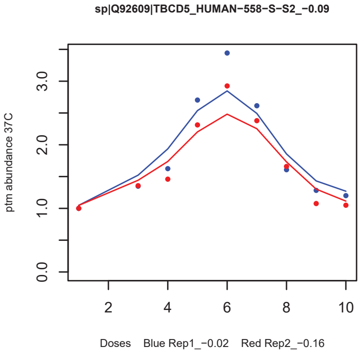
sp|Q92609|TBCD5_HUMAN−558−S−S2_−0.09
ptm abundance 37C
Doses Blue Rep1_−0.02 Red Rep2_−0.16

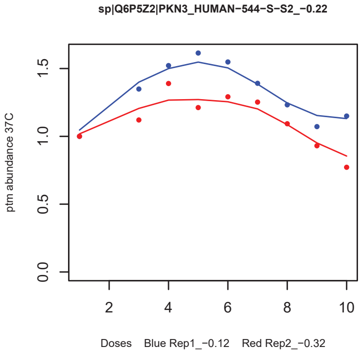
sp|Q6P5Z2|PKN3_HUMAN−544−S−S2_−0.22
ptm abundance 37C
Doses Blue Rep1_−0.12 Red Rep2_−0.32

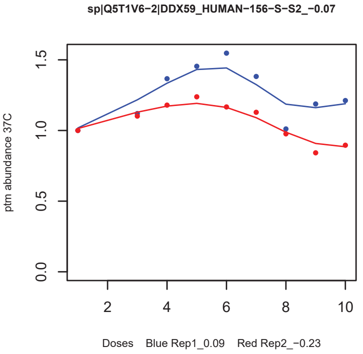
sp|Q5T1V6−2|DDX59_HUMAN−156−S−S2_−0.07
ptm abundance 37C
Doses Blue Rep1_0.09 Red Rep2_−0.23

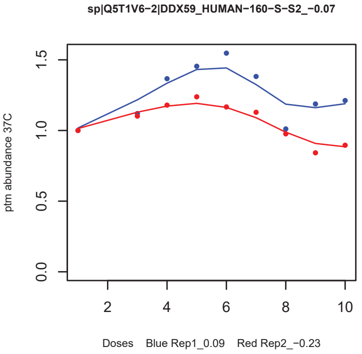
sp|Q5T1V6−2|DDX59_HUMAN−160−S−S2_−0.07
ptm abundance 37C
Doses Blue Rep1_0.09 Red Rep2_−0.23

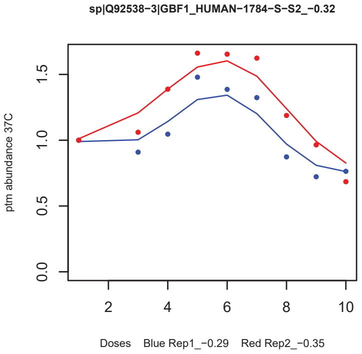
sp|Q92538−3|GBF1_HUMAN−1784−S−S2_−0.32
ptm abundance 37C
Doses Blue Rep1_−0.29 Red Rep2_−0.35

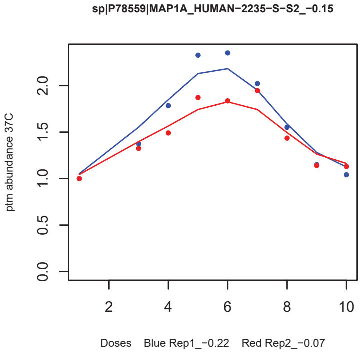
sp|P78559|MAP1A_HUMAN−2235−S−S2_−0.15
ptm abundance 37C
Doses Blue Rep1_−0.22 Red Rep2_−0.07

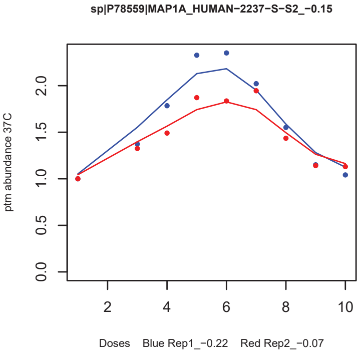
sp|P78559|MAP1A_HUMAN−2237−S−S2_−0.15
ptm abundance 37C
Doses Blue Rep1_−0.22 Red Rep2_−0.07

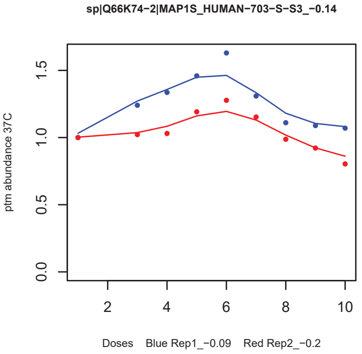
sp|Q66K74−2|MAP1S_HUMAN−703−S−S3_−0.14
ptm abundance 37C
Doses Blue Rep1_−0.09 Red Rep2_−0.2

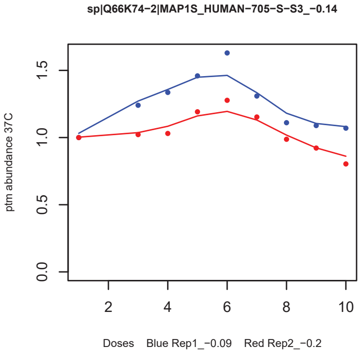
sp|Q66K74−2|MAP1S_HUMAN−705−S−S3_−0.14
ptm abundance 37C
Doses Blue Rep1_−0.09 Red Rep2_−0.2

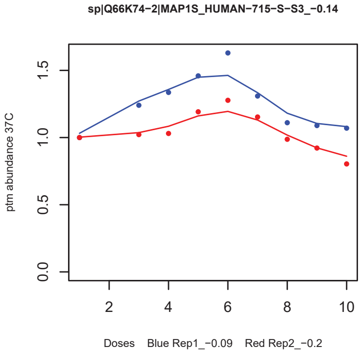
sp|Q66K74−2|MAP1S_HUMAN−715−S−S3_−0.14
ptm abundance 37C
Doses Blue Rep1_−0.09 Red Rep2_−0.2

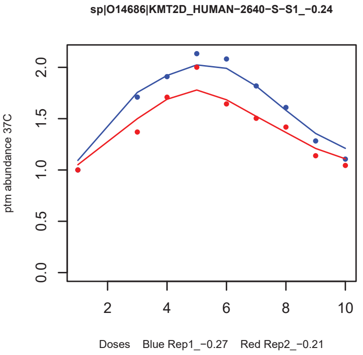
sp|O14686|KMT2D_HUMAN−2640−S−S1_−0.24
ptm abundance 37C
Doses Blue Rep1_−0.27 Red Rep2_−0.21

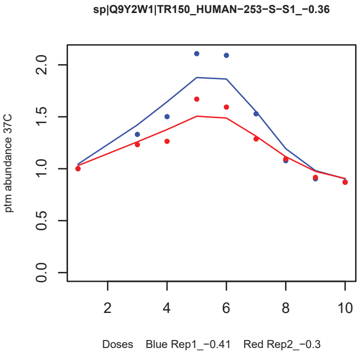
sp|Q9Y2W1|TR150_HUMAN−253−S−S1_−0.36
ptm abundance 37C
Doses Blue Rep1_−0.41 Red Rep2_−0.3

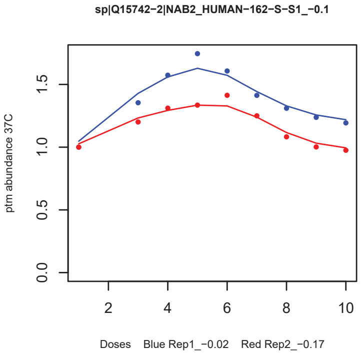
sp|Q15742−2|NAB2_HUMAN−162−S−S1_−0.1
ptm abundance 37C
Doses Blue Rep1_−0.02 Red Rep2_−0.17

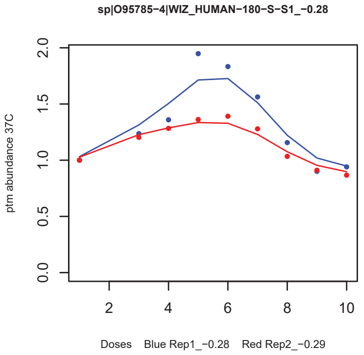
sp|O95785−4|WIZ_HUMAN−180−S−S1_−0.28
ptm abundance 37C
Doses Blue Rep1_−0.28 Red Rep2_−0.29

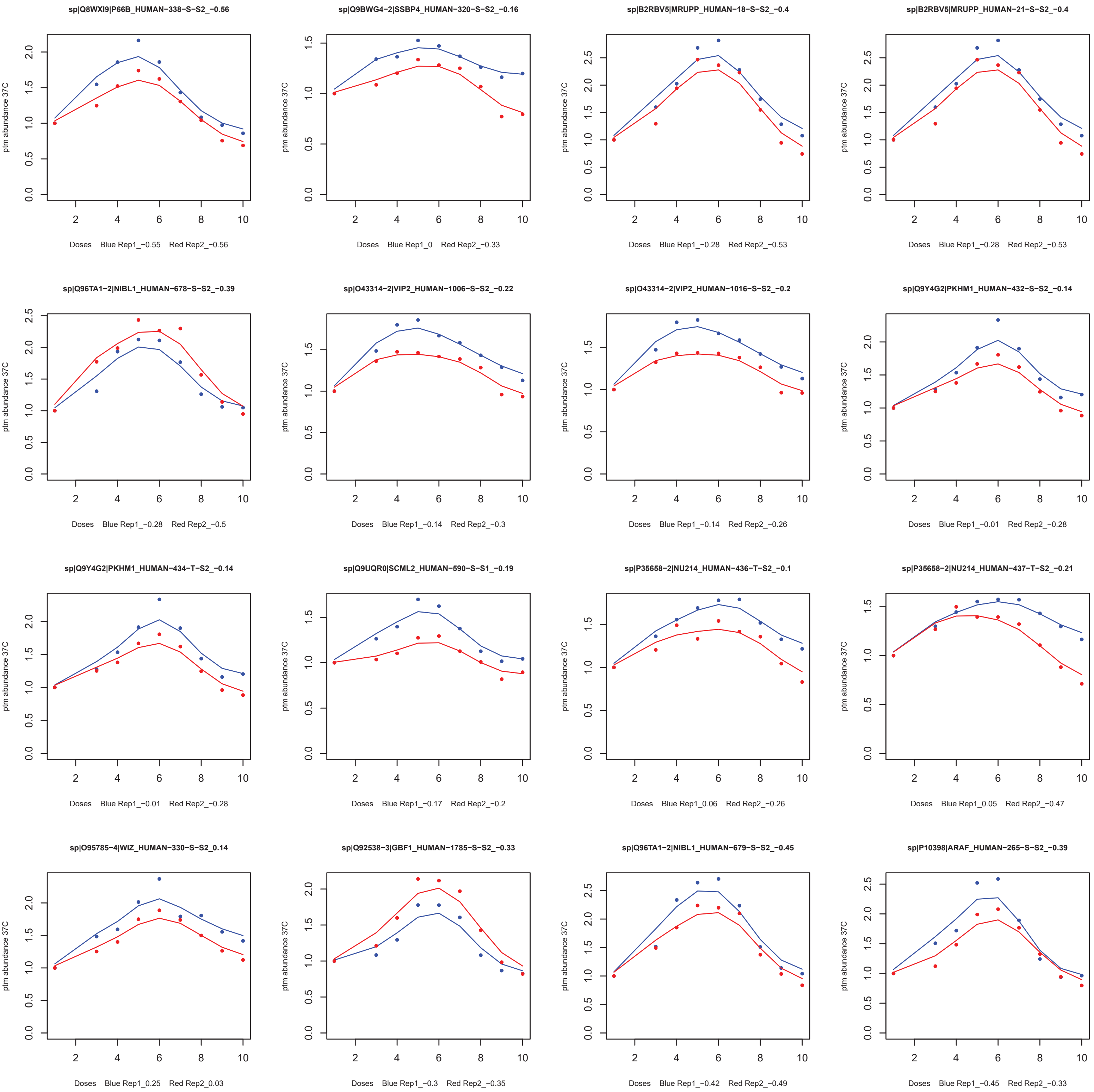
sp|Q8WXI9|P66B_HUMAN−338−S−S2_−0.56
Doses Blue Rep1_−0.55 Red Rep2_−0.56
sp|Q9BWG4−2|SSBP4_HUMAN−320−S−S2_−0.16
Doses Blue Rep1_0 Red Rep2_−0.33
sp|B2RBV5|MRUPP_HUMAN−18−S−S2_−0.4
Doses Blue Rep1_−0.28 Red Rep2_−0.53
sp|B2RBV5|MRUPP_HUMAN−21−S−S2_−0.4
Doses Blue Rep1_−0.28 Red Rep2_−0.53
sp|Q96TA1−2|NIBL1_HUMAN−678−S−S2_−0.39
Doses Blue Rep1_−0.28 Red Rep2_−0.5
sp|O43314−2|VIP2_HUMAN−1006−S−S2_−0.22
Doses Blue Rep1_−0.14 Red Rep2_−0.3
sp|O43314−2|VIP2_HUMAN−1016−S−S2_−0.2
Doses Blue Rep1_−0.14 Red Rep2_−0.26
sp|Q9Y4G2|PKHM1_HUMAN−432−S−S2_−0.14
Doses Blue Rep1_−0.01 Red Rep2_−0.28
sp|Q9Y4G2|PKHM1_HUMAN−434−T−S2_−0.14
Doses Blue Rep1_−0.01 Red Rep2_−0.28
sp|Q9UQR0|SCML2_HUMAN−590−S−S1_−0.19
Doses Blue Rep1_−0.17 Red Rep2_−0.2
sp|P35658−2|NU214_HUMAN−436−T−S2_−0.1
Doses Blue Rep1_0.06 Red Rep2_−0.26
sp|P35658−2|NU214_HUMAN−437−T−S2_−0.21
Doses Blue Rep1_0.05 Red Rep2_−0.47
sp|O95785−4|WIZ_HUMAN−330−S−S2_0.14
Doses Blue Rep1_0.25 Red Rep2_0.03
sp|Q92538−3|GBF1_HUMAN−1785−S−S2_−0.33
Doses Blue Rep1_−0.3 Red Rep2_−0.35
sp|Q96TA1−2|NIBL1_HUMAN−679−S−S2_−0.45
Doses Blue Rep1_−0.42 Red Rep2_−0.49
sp|P10398|ARAF_HUMAN−265−S−S2_−0.39
Doses Blue Rep1_−0.45 Red Rep2_−0.33
ptm abundance 37C

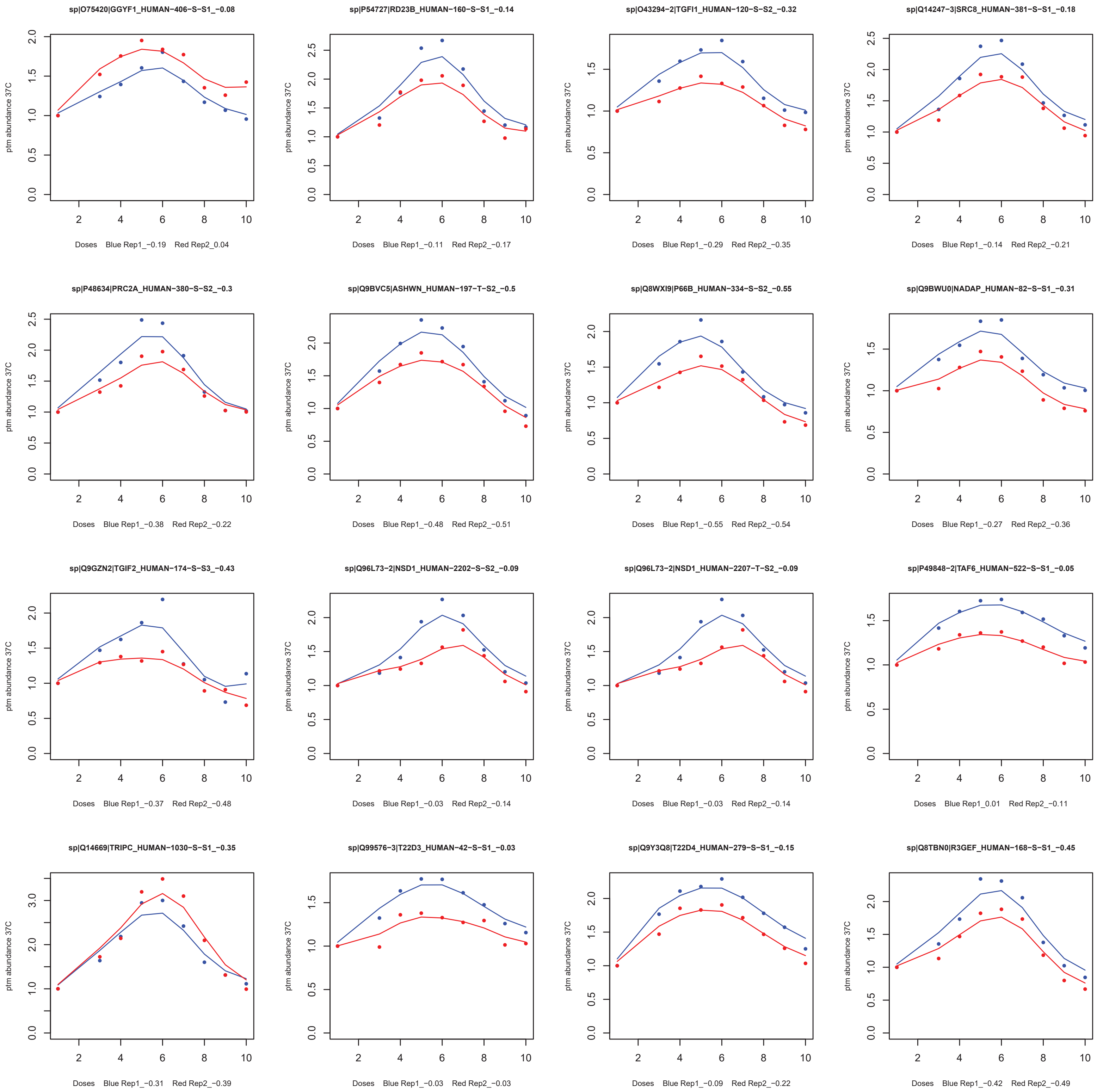
sp|O75420|GGYF1_HUMAN−406−S−S1_−0.08
Doses Blue Rep1_−0.19 Red Rep2_0.04
sp|P54727|RD23B_HUMAN−160−S−S1_−0.14
Doses Blue Rep1_−0.11 Red Rep2_−0.17
sp|O43294−2|TGFI1_HUMAN−120−S−S2_−0.32
Doses Blue Rep1_−0.29 Red Rep2_−0.35
sp|Q14247−3|SRC8_HUMAN−381−S−S1_−0.18
Doses Blue Rep1_−0.14 Red Rep2_−0.21
sp|P48634|PRC2A_HUMAN−380−S−S2_−0.3
Doses Blue Rep1_−0.38 Red Rep2_−0.22
sp|Q9BVC5|ASHWN_HUMAN−197−T−S2_−0.5
Doses Blue Rep1_−0.48 Red Rep2_−0.51
sp|Q8WXI9|P66B_HUMAN−334−S−S2_−0.55
Doses Blue Rep1_−0.55 Red Rep2_−0.54
sp|Q9BWU0|NADAP_HUMAN−82−S−S1_−0.31
Doses Blue Rep1_−0.27 Red Rep2_−0.36
sp|Q9GZN2|TGIF2_HUMAN−174−S−S3_−0.43
Doses Blue Rep1_−0.37 Red Rep2_−0.48
sp|Q96L73−2|NSD1_HUMAN−2202−S−S2_−0.09
Doses Blue Rep1_−0.03 Red Rep2_−0.14
sp|Q96L73−2|NSD1_HUMAN−2207−T−S2_−0.09
Doses Blue Rep1_−0.03 Red Rep2_−0.14
sp|P49848−2|TAF6_HUMAN−522−S−S1_−0.05
Doses Blue Rep1_0.01 Red Rep2_−0.11
sp|Q14669|TRIPC_HUMAN−1030−S−S1_−0.35
Doses Blue Rep1_−0.31 Red Rep2_−0.39
sp|Q99576−3|T22D3_HUMAN−42−S−S1_−0.03
Doses Blue Rep1_−0.03 Red Rep2_−0.03
sp|Q9Y3Q8|T22D4_HUMAN−279−S−S1_−0.15
Doses Blue Rep1_−0.09 Red Rep2_−0.22
sp|Q8TBN0|R3GEF_HUMAN−168−S−S1_−0.45
Doses Blue Rep1_−0.42 Red Rep2_−0.49
ptm abundance 37C

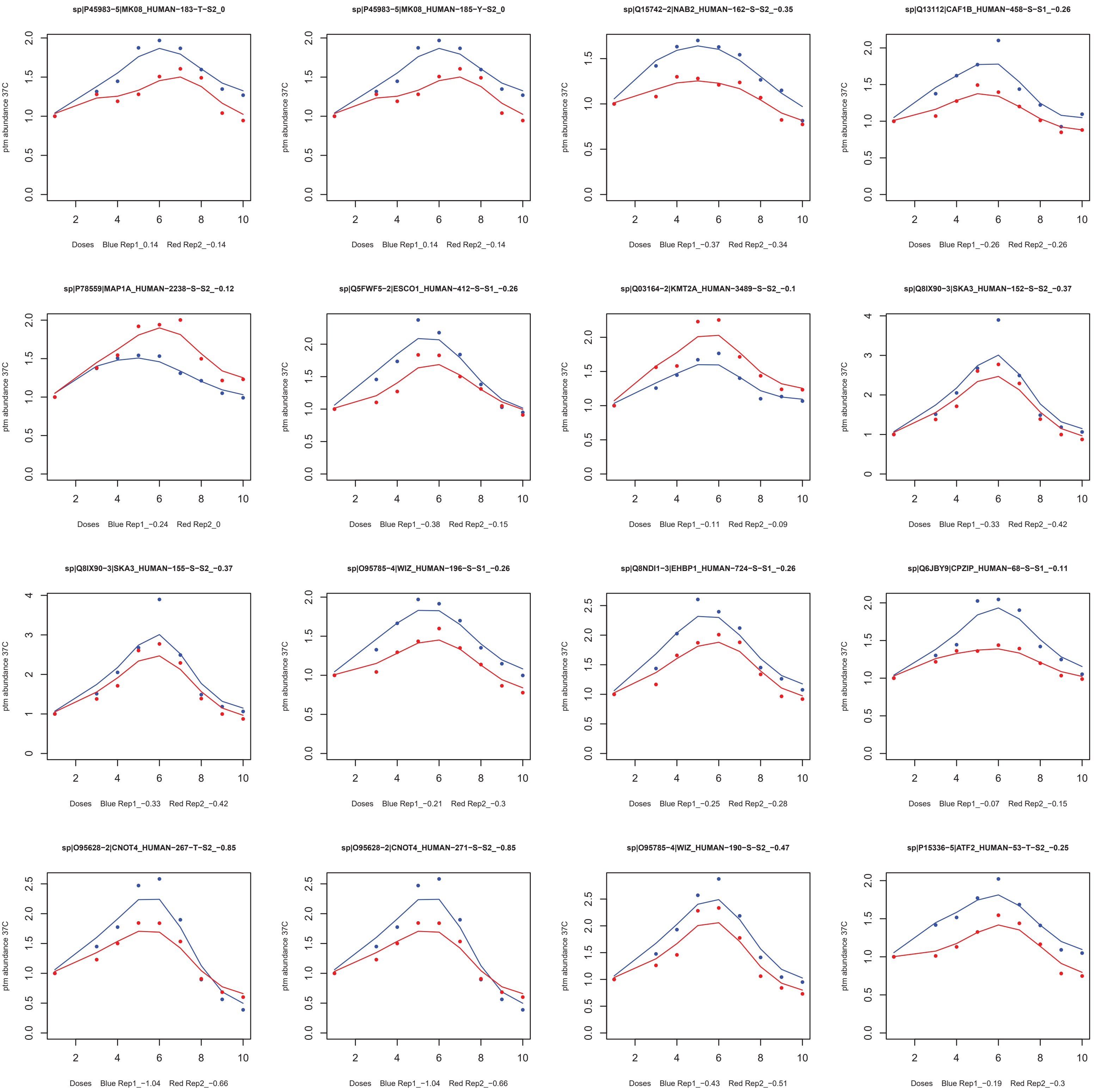
sp|P45983−5|MK08_HUMAN−183−T−S2_0
ptm abundance 37C
Doses Blue Rep1_0.14 Red Rep2_−0.14
sp|P45983−5|MK08_HUMAN−185−Y−S2_0
Doses Blue Rep1_0.14 Red Rep2_−0.14
sp|Q15742−2|NAB2_HUMAN−162−S−S2_−0.35
Doses Blue Rep1_−0.37 Red Rep2_−0.34
sp|Q13112|CAF1B_HUMAN−458−S−S1_−0.26
Doses Blue Rep1_−0.26 Red Rep2_−0.26
sp|P78559|MAP1A_HUMAN−2238−S−S2_−0.12
Doses Blue Rep1_−0.24 Red Rep2_0
sp|Q5FWF5−2|ESCO1_HUMAN−412−S−S1_−0.26
Doses Blue Rep1_−0.38 Red Rep2_−0.15
sp|Q03164−2|KMT2A_HUMAN−3489−S−S2_−0.1
Doses Blue Rep1_−0.11 Red Rep2_−0.09
sp|Q8IX90−3|SKA3_HUMAN−152−S−S2_−0.37
Doses Blue Rep1_−0.33 Red Rep2_−0.42
sp|Q8IX90−3|SKA3_HUMAN−155−S−S2_−0.37
Doses Blue Rep1_−0.33 Red Rep2_−0.42
sp|O95785−4|WIZ_HUMAN−196−S−S1_−0.26
Doses Blue Rep1_−0.21 Red Rep2_−0.3
sp|Q8NDI1−3|EHBP1_HUMAN−724−S−S1_−0.26
Doses Blue Rep1_−0.25 Red Rep2_−0.28
sp|Q6JBY9|CPZIP_HUMAN−68−S−S1_−0.11
Doses Blue Rep1_−0.07 Red Rep2_−0.15
sp|O95628−2|CNOT4_HUMAN−267−T−S2_−0.85
Doses Blue Rep1_−1.04 Red Rep2_−0.66
sp|O95628−2|CNOT4_HUMAN−271−S−S2_−0.85
Doses Blue Rep1_−1.04 Red Rep2_−0.66
sp|O95785−4|WIZ_HUMAN−190−S−S2_−0.47
Doses Blue Rep1_−0.43 Red Rep2_−0.51
sp|P15336−5|ATF2_HUMAN−53−T−S2_−0.25
Doses Blue Rep1_−0.19 Red Rep2_−0.3

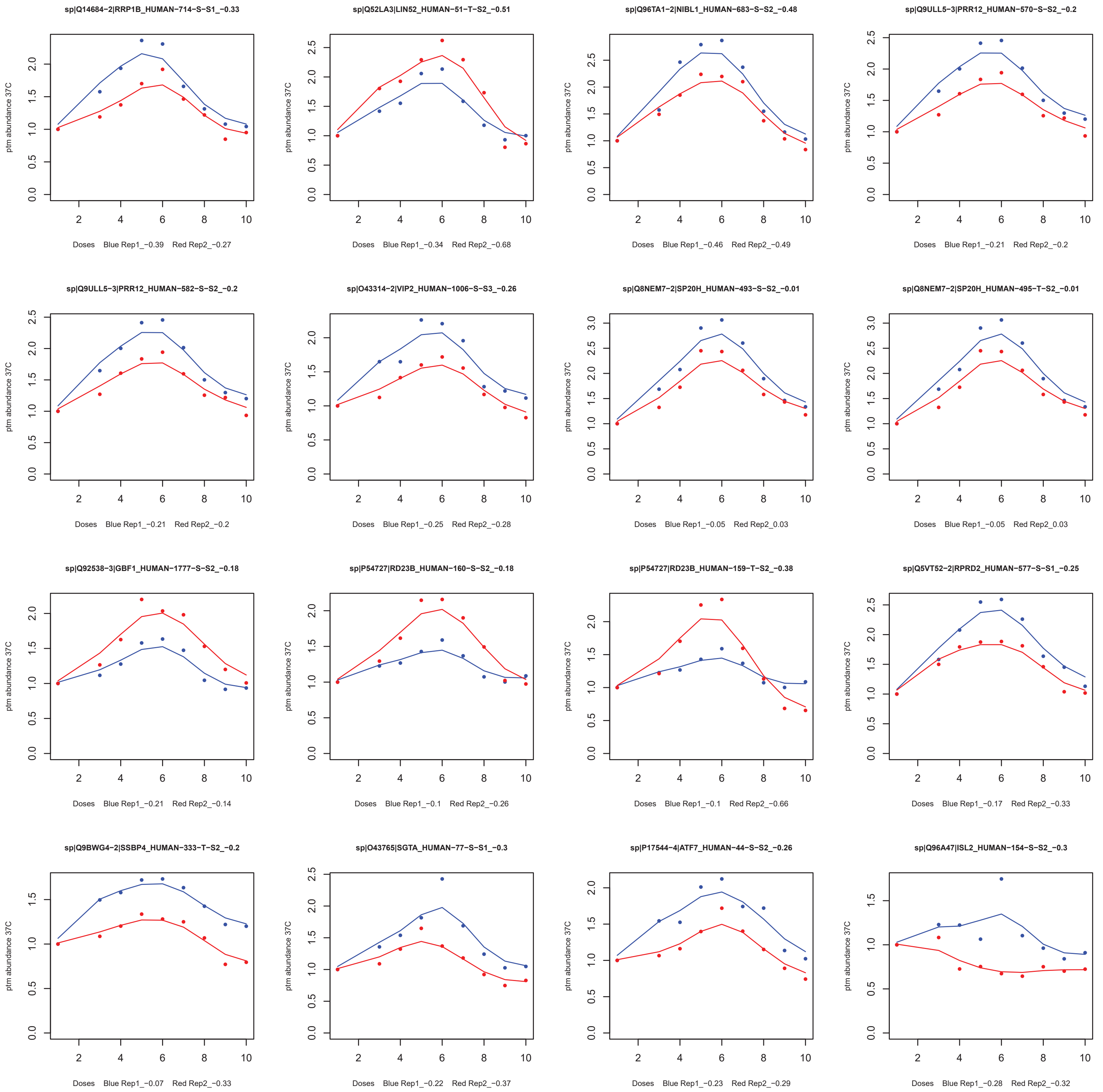
sp|Q14684−2|RRP1B_HUMAN−714−S−S1_−0.33
Doses Blue Rep1_−0.39 Red Rep2_−0.27
sp|Q52LA3|LIN52_HUMAN−51−T−S2_−0.51
Doses Blue Rep1_−0.34 Red Rep2_−0.68
sp|Q96TA1−2|NIBL1_HUMAN−683−S−S2_−0.48
Doses Blue Rep1_−0.46 Red Rep2_−0.49
sp|Q9ULL5−3|PRR12_HUMAN−570−S−S2_−0.2
Doses Blue Rep1_−0.21 Red Rep2_−0.2
sp|Q9ULL5−3|PRR12_HUMAN−582−S−S2_−0.2
Doses Blue Rep1_−0.21 Red Rep2_−0.2
sp|O43314−2|VIP2_HUMAN−1006−S−S3_−0.26
Doses Blue Rep1_−0.25 Red Rep2_−0.28
sp|Q8NEM7−2|SP20H_HUMAN−493−S−S2_−0.01
Doses Blue Rep1_−0.05 Red Rep2_0.03
sp|Q8NEM7−2|SP20H_HUMAN−495−T−S2_−0.01
Doses Blue Rep1_−0.05 Red Rep2_0.03
sp|Q92538−3|GBF1_HUMAN−1777−S−S2_−0.18
Doses Blue Rep1_−0.21 Red Rep2_−0.14
sp|P54727|RD23B_HUMAN−160−S−S2_−0.18
Doses Blue Rep1_−0.1 Red Rep2_−0.26
sp|P54727|RD23B_HUMAN−159−T−S2_−0.38
Doses Blue Rep1_−0.1 Red Rep2_−0.66
sp|Q5VT52−2|RPRD2_HUMAN−577−S−S1_−0.25
Doses Blue Rep1_−0.17 Red Rep2_−0.33
sp|Q9BWG4−2|SSBP4_HUMAN−333−T−S2_−0.2
Doses Blue Rep1_−0.07 Red Rep2_−0.33
sp|O43765|SGTA_HUMAN−77−S−S1_−0.3
Doses Blue Rep1_−0.22 Red Rep2_−0.37
sp|P17544−4|ATF7_HUMAN−44−S−S2_−0.26
Doses Blue Rep1_−0.23 Red Rep2_−0.29
sp|Q96A47|ISL2_HUMAN−154−S−S2_−0.3
Doses Blue Rep1_−0.28 Red Rep2_−0.32
ptm abundance 37C

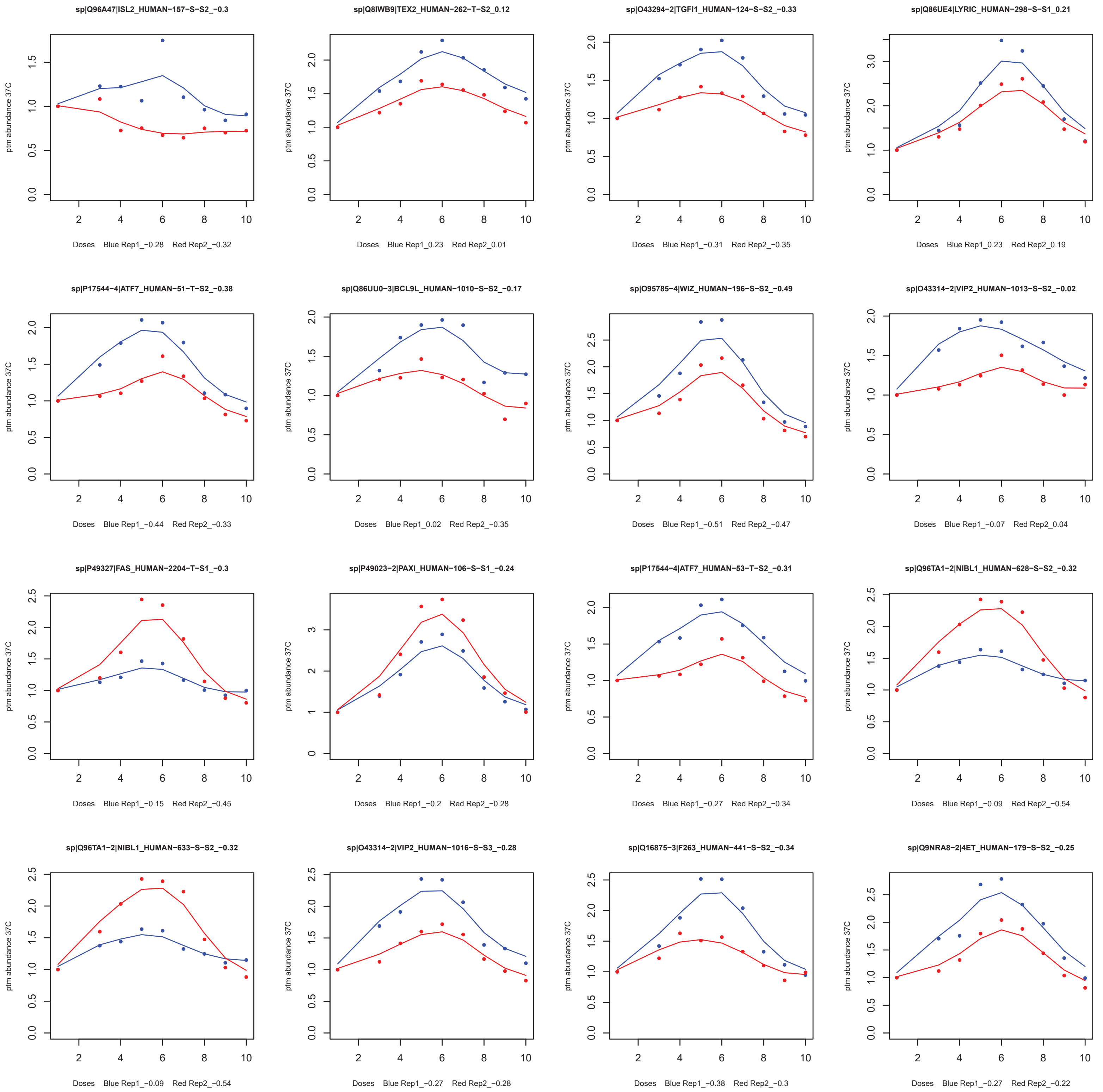
sp|Q96A47|ISL2_HUMAN−157−S−S2_−0.3
ptm abundance 37C
Doses Blue Rep1_−0.28 Red Rep2_−0.32
sp|Q8IWB9|TEX2_HUMAN−262−T−S2_0.12
ptm abundance 37C
Doses Blue Rep1_0.23 Red Rep2_0.01
sp|O43294−2|TGFI1_HUMAN−124−S−S2_−0.33
ptm abundance 37C
Doses Blue Rep1_−0.31 Red Rep2_−0.35
sp|Q86UE4|LYRIC_HUMAN−298−S−S1_0.21
ptm abundance 37C
Doses Blue Rep1_0.23 Red Rep2_0.19
sp|P17544−4|ATF7_HUMAN−51−T−S2_−0.38
ptm abundance 37C
Doses Blue Rep1_−0.44 Red Rep2_−0.33
sp|Q86UU0−3|BCL9L_HUMAN−1010−S−S2_−0.17
ptm abundance 37C
Doses Blue Rep1_0.02 Red Rep2_−0.35
sp|O95785−4|WIZ_HUMAN−196−S−S2_−0.49
ptm abundance 37C
Doses Blue Rep1_−0.51 Red Rep2_−0.47
sp|O43314−2|VIP2_HUMAN−1013−S−S2_−0.02
ptm abundance 37C
Doses Blue Rep1_−0.07 Red Rep2_0.04
sp|P49327|FAS_HUMAN−2204−T−S1_−0.3
ptm abundance 37C
Doses Blue Rep1_−0.15 Red Rep2_−0.45
sp|P49023−2|PAXI_HUMAN−106−S−S1_−0.24
ptm abundance 37C
Doses Blue Rep1_−0.2 Red Rep2_−0.28
sp|P17544−4|ATF7_HUMAN−53−T−S2_−0.31
ptm abundance 37C
Doses Blue Rep1_−0.27 Red Rep2_−0.34
sp|Q96TA1−2|NIBL1_HUMAN−628−S−S2_−0.32
ptm abundance 37C
Doses Blue Rep1_−0.09 Red Rep2_−0.54
sp|Q96TA1−2|NIBL1_HUMAN−633−S−S2_−0.32
ptm abundance 37C
Doses Blue Rep1_−0.09 Red Rep2_−0.54
sp|O43314−2|VIP2_HUMAN−1016−S−S3_−0.28
ptm abundance 37C
Doses Blue Rep1_−0.27 Red Rep2_−0.28
sp|Q16875−3|F263_HUMAN−441−S−S2_−0.34
ptm abundance 37C
Doses Blue Rep1_−0.38 Red Rep2_−0.3
sp|Q9NRA8−2|4ET_HUMAN−179−S−S2_−0.25
ptm abundance 37C
Doses Blue Rep1_−0.27 Red Rep2_−0.22

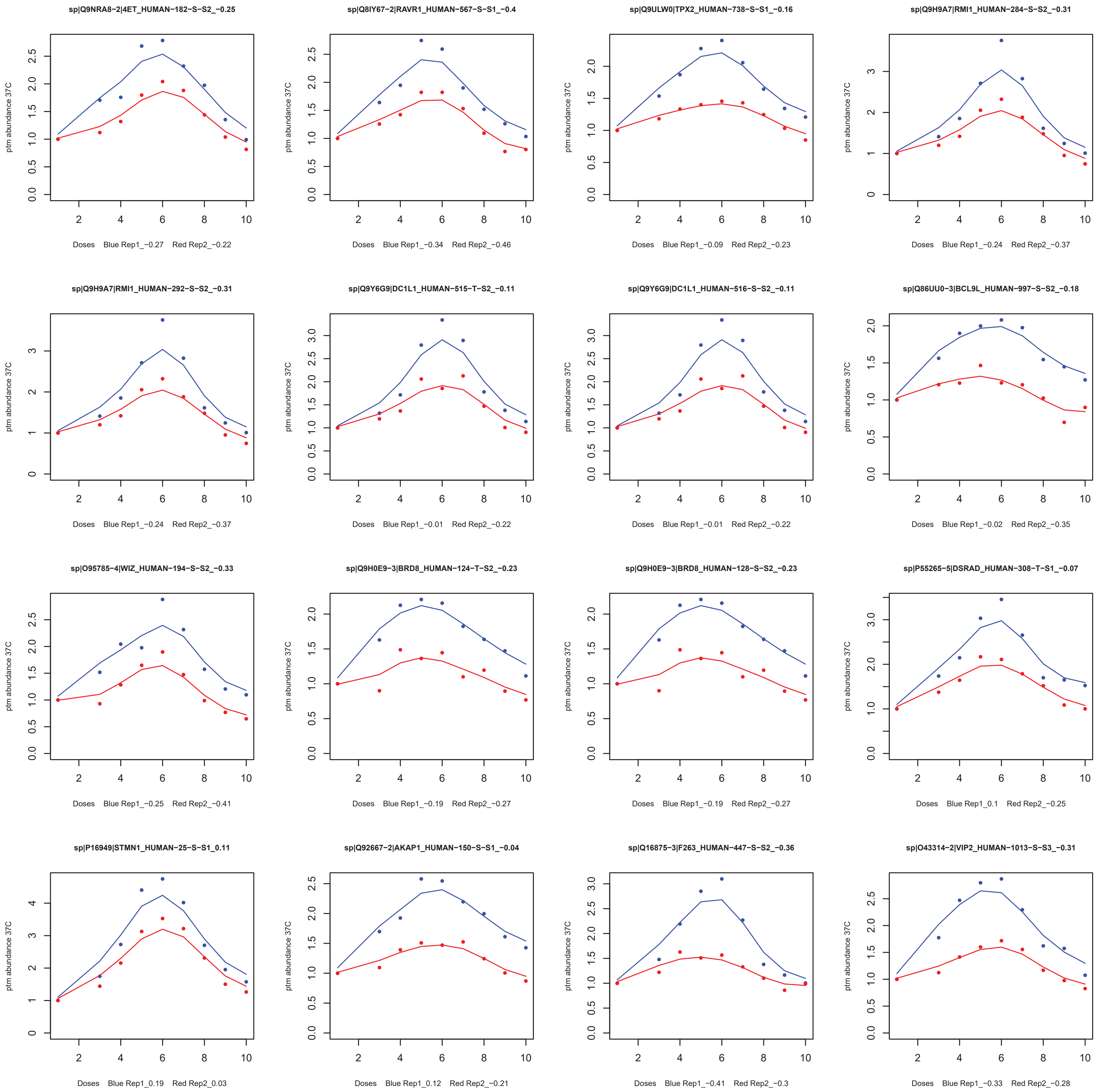

sp|Q9NRA8−2|4ET_HUMAN−182−S−S2_−0.25
Doses Blue Rep1_−0.27 Red Rep2_−0.22
sp|Q8IY67−2|RAVR1_HUMAN−567−S−S1_−0.4
Doses Blue Rep1_−0.34 Red Rep2_−0.46
sp|Q9ULW0|TPX2_HUMAN−738−S−S1_−0.16
Doses Blue Rep1_−0.09 Red Rep2_−0.23
sp|Q9H9A7|RMI1_HUMAN−284−S−S2_−0.31
Doses Blue Rep1_−0.24 Red Rep2_−0.37
sp|Q9H9A7|RMI1_HUMAN−292−S−S2_−0.31
Doses Blue Rep1_−0.24 Red Rep2_−0.37
sp|Q9Y6G9|DC1L1_HUMAN−515−T−S2_−0.11
Doses Blue Rep1_−0.01 Red Rep2_−0.22
sp|Q9Y6G9|DC1L1_HUMAN−516−S−S2_−0.11
Doses Blue Rep1_−0.01 Red Rep2_−0.22
sp|Q86UU0−3|BCL9L_HUMAN−997−S−S2_−0.18
Doses Blue Rep1_−0.02 Red Rep2_−0.35
sp|O95785−4|WIZ_HUMAN−194−S−S2_−0.33
Doses Blue Rep1_−0.25 Red Rep2_−0.41
sp|Q9H0E9−3|BRD8_HUMAN−124−T−S2_−0.23
Doses Blue Rep1_−0.19 Red Rep2_−0.27
sp|Q9H0E9−3|BRD8_HUMAN−128−S−S2_−0.23
Doses Blue Rep1_−0.19 Red Rep2_−0.27
sp|P55265−5|DSRAD_HUMAN−308−T−S1_−0.07
Doses Blue Rep1_0.1 Red Rep2_−0.25
sp|P16949|STMN1_HUMAN−25−S−S1_0.11
Doses Blue Rep1_0.19 Red Rep2_0.03
sp|Q92667−2|AKAP1_HUMAN−150−S−S1_−0.04
Doses Blue Rep1_0.12 Red Rep2_−0.21
sp|Q16875−3|F263_HUMAN−447−S−S2_−0.36
Doses Blue Rep1_−0.41 Red Rep2_−0.3
sp|O43314−2|VIP2_HUMAN−1013−S−S3_−0.31
Doses Blue Rep1_−0.33 Red Rep2_−0.28
ptm abundance 37C

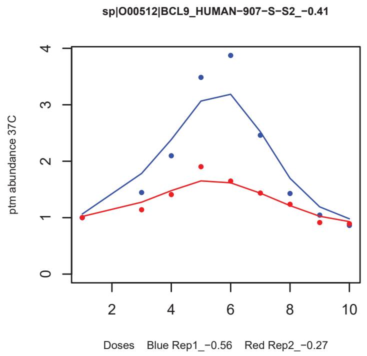
sp|O00512|BCL9_HUMAN−907−S−S2_−0.41
ptm abundance 37C
Doses Blue Rep1_−0.56 Red Rep2_−0.27

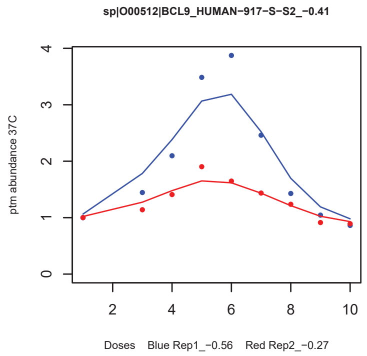
sp|O00512|BCL9_HUMAN−917−S−S2_−0.41
ptm abundance 37C
Doses Blue Rep1_−0.56 Red Rep2_−0.27

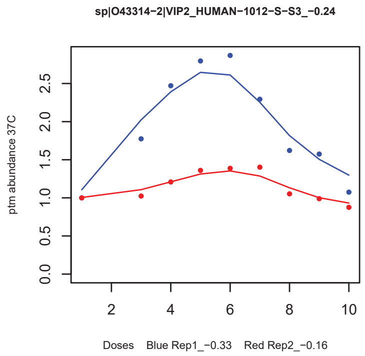
sp|O43314−2|VIP2_HUMAN−1012−S−S3_−0.24
ptm abundance 37C
Doses Blue Rep1_−0.33 Red Rep2_−0.16

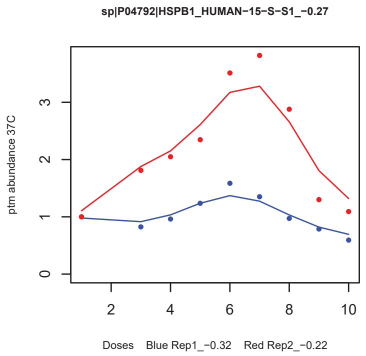
sp|P04792|HSPB1_HUMAN−15−S−S1_−0.27
ptm abundance 37C
Doses Blue Rep1_−0.32 Red Rep2_−0.22

Supplementary Data 4: Non-responsive phosphosites. Doses are represented as ordinal numbers (highest dose 10 µM as the 10th position; x-axis), abundance fold change relative to the untreated condition (y-axis).

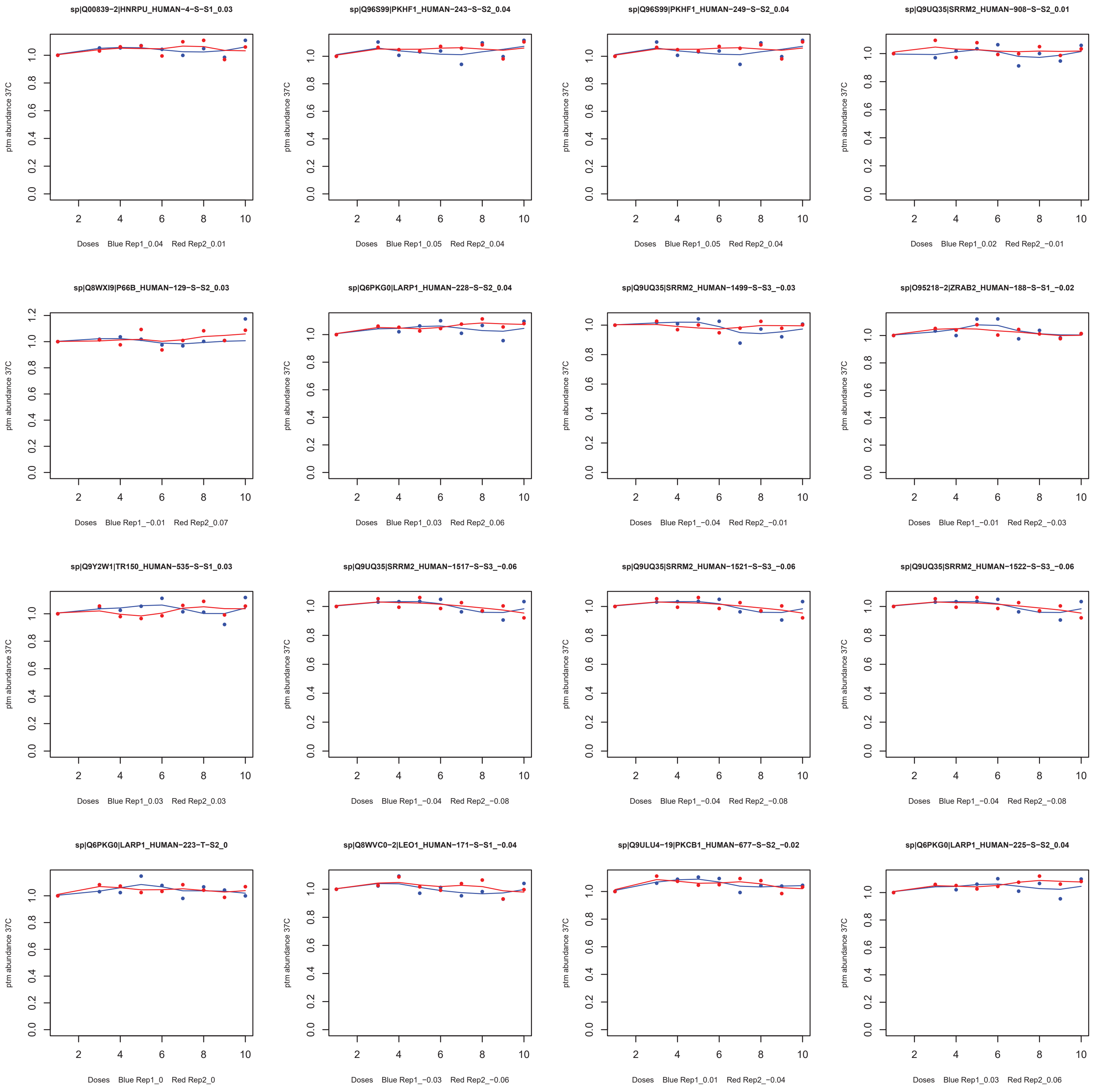

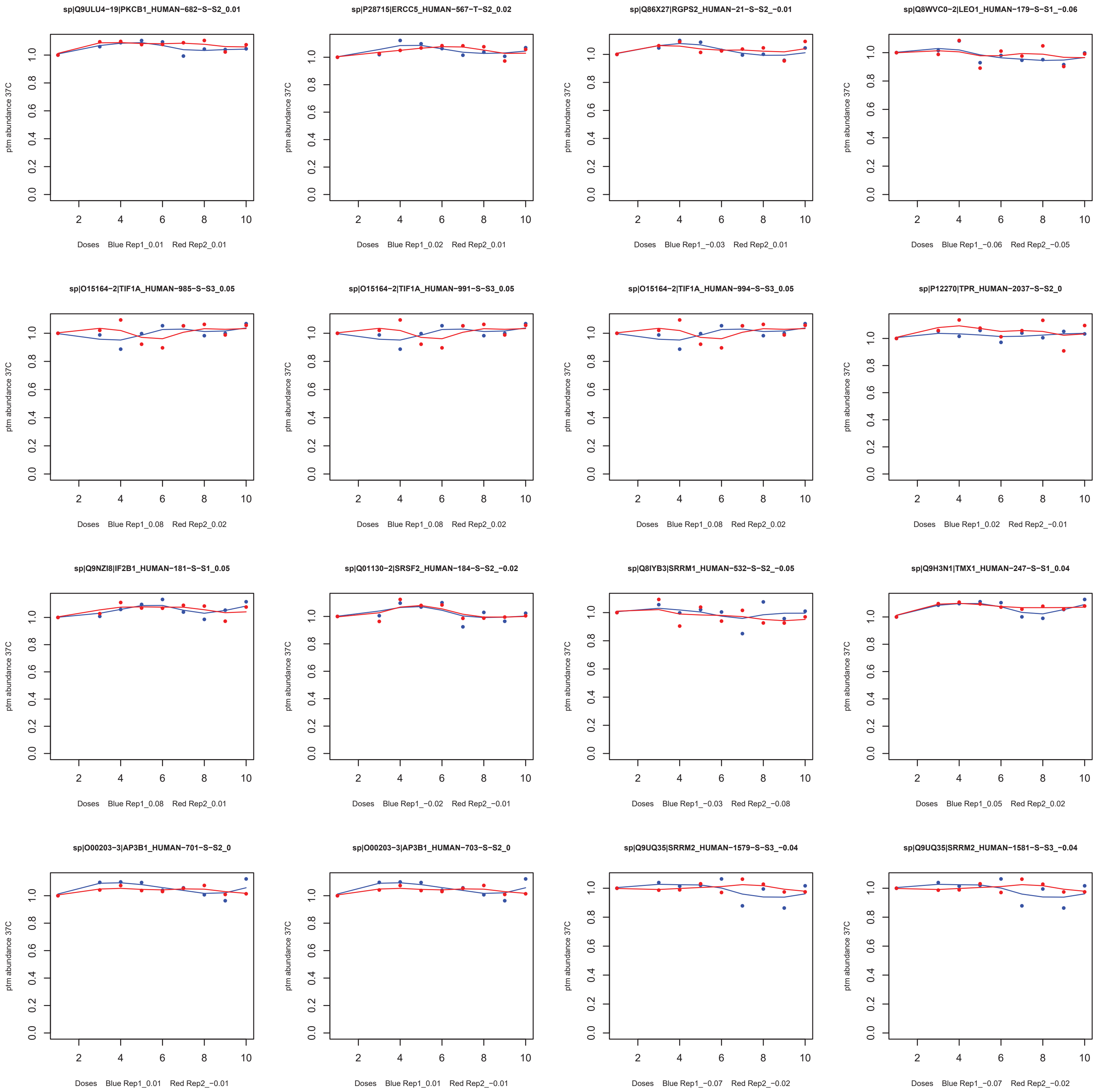
ptm abundance 37C
0.0 0.2 0.4 0.6 0.8 1.0
2 4 6 8 10
sp|Q9ULU4−19|PKCB1_HUMAN−682−S−S2_0.01
Doses Blue Rep1_0.01 Red Rep2_0.01
sp|P28715|ERCC5_HUMAN−567−T−S2_0.02
Doses Blue Rep1_0.02 Red Rep2_0.01
sp|Q86X27|RGPS2_HUMAN−21−S−S2_−0.01
Doses Blue Rep1_−0.03 Red Rep2_0.01
sp|Q8WVC0−2|LEO1_HUMAN−179−S−S1_−0.06
Doses Blue Rep1_−0.06 Red Rep2_−0.05
sp|O15164−2|TIF1A_HUMAN−985−S−S3_0.05
Doses Blue Rep1_0.08 Red Rep2_0.02
sp|O15164−2|TIF1A_HUMAN−991−S−S3_0.05
Doses Blue Rep1_0.08 Red Rep2_0.02
sp|O15164−2|TIF1A_HUMAN−994−S−S3_0.05
Doses Blue Rep1_0.08 Red Rep2_0.02
sp|P12270|TPR_HUMAN−2037−S−S2_0
Doses Blue Rep1_0.02 Red Rep2_−0.01
sp|Q9NZI8|IF2B1_HUMAN−181−S−S1_0.05
Doses Blue Rep1_0.08 Red Rep2_0.01
sp|Q01130−2|SRSF2_HUMAN−184−S−S2_−0.02
Doses Blue Rep1_−0.02 Red Rep2_−0.01
sp|Q8IYB3|SRRM1_HUMAN−532−S−S2_−0.05
Doses Blue Rep1_−0.03 Red Rep2_−0.08
sp|Q9H3N1|TMX1_HUMAN−247−S−S1_0.04
Doses Blue Rep1_0.05 Red Rep2_0.02
sp|O00203−3|AP3B1_HUMAN−701−S−S2_0
Doses Blue Rep1_0.01 Red Rep2_−0.01
sp|O00203−3|AP3B1_HUMAN−703−S−S2_0
Doses Blue Rep1_0.01 Red Rep2_−0.01
sp|Q9UQ35|SRRM2_HUMAN−1579−S−S3_−0.04
Doses Blue Rep1_−0.07 Red Rep2_−0.02
sp|Q9UQ35|SRRM2_HUMAN−1581−S−S3_−0.04
Doses Blue Rep1_−0.07 Red Rep2_−0.02

sp|Q9UQ35|SRRM2_HUMAN−1582−S−S3_−0.04

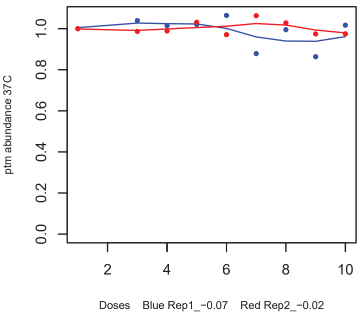

sp|Q9NRF8|PYRG2_HUMAN−576−S−S3_0

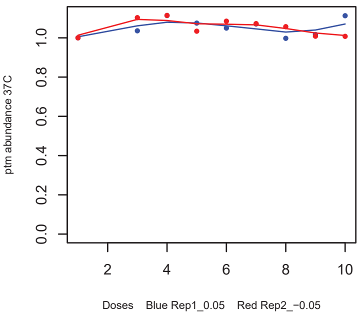

sp|O00418|EF2K_HUMAN−477−S−S2_0.01

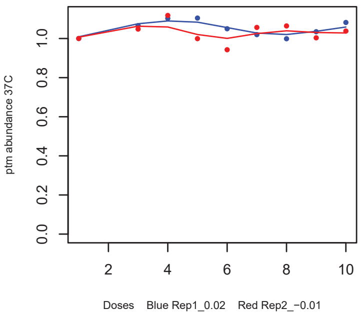

sp|Q01130−2|SRSF2_HUMAN−177−S−S2_−0.03

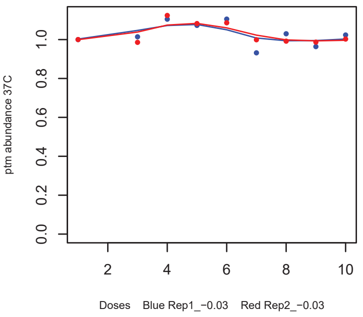

sp|Q13185|CBX3_HUMAN−95−S−S3_0.01

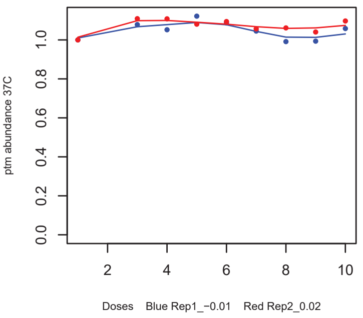

sp|Q13185|CBX3_HUMAN−97−S−S3_0.01

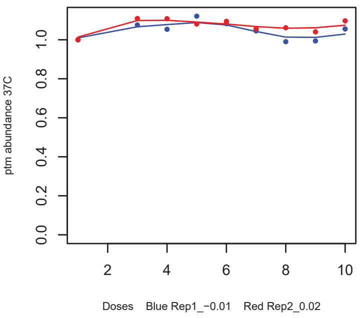

sp|Q3L8U1−2|CHD9_HUMAN−2059−S−S2_−0.02

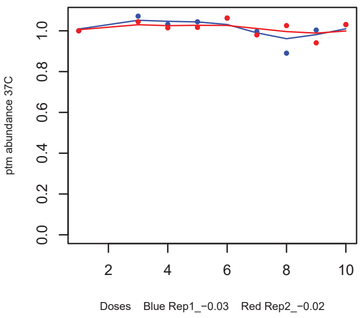

sp|P41227−2|NAA10_HUMAN−171−S−S1_0.01

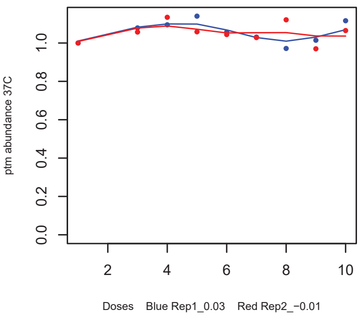

sp|Q9H5H4|ZN768_HUMAN−125−S−S1_0

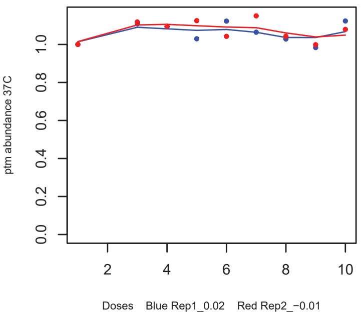

sp|Q9H4A6|GOLP3_HUMAN−35−S−S2_0.04

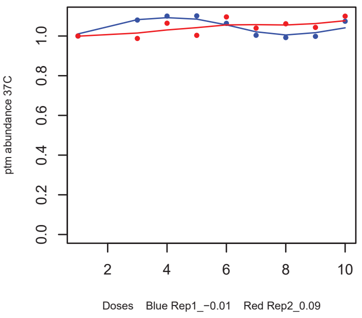

sp|Q9H4A6|GOLP3_HUMAN−36−S−S2_0.04

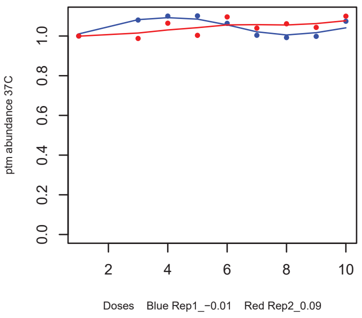

sp|Q9UIG0−2|BAZ1B_HUMAN−160−S−S3_−0.04

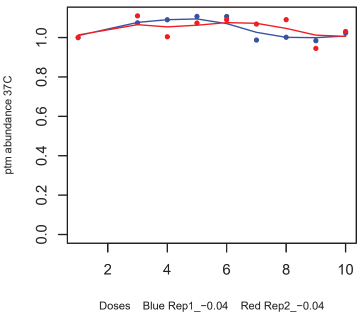

sp|Q9UQ35|SRRM2_HUMAN−353−S−S2_−0.06

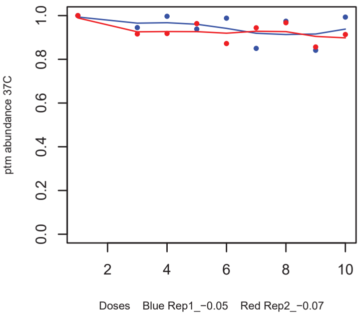

sp|P25205|MCM3_HUMAN−672−S−S2_0.03

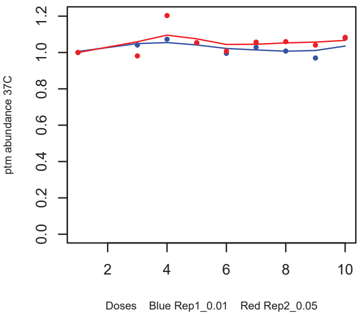

sp|Q13185|CBX3_HUMAN−93−S−S3_0

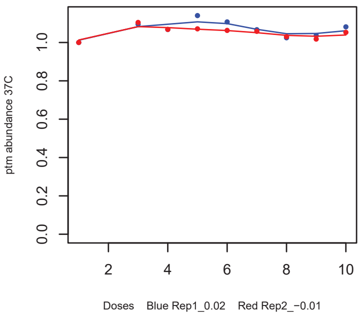

sp|Q12996|CSTF3_HUMAN−691−S−S1_−0.03

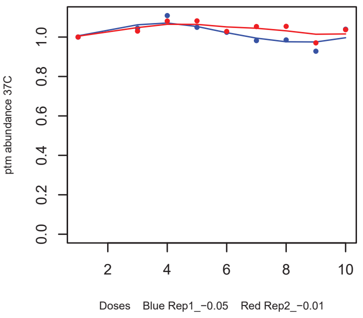

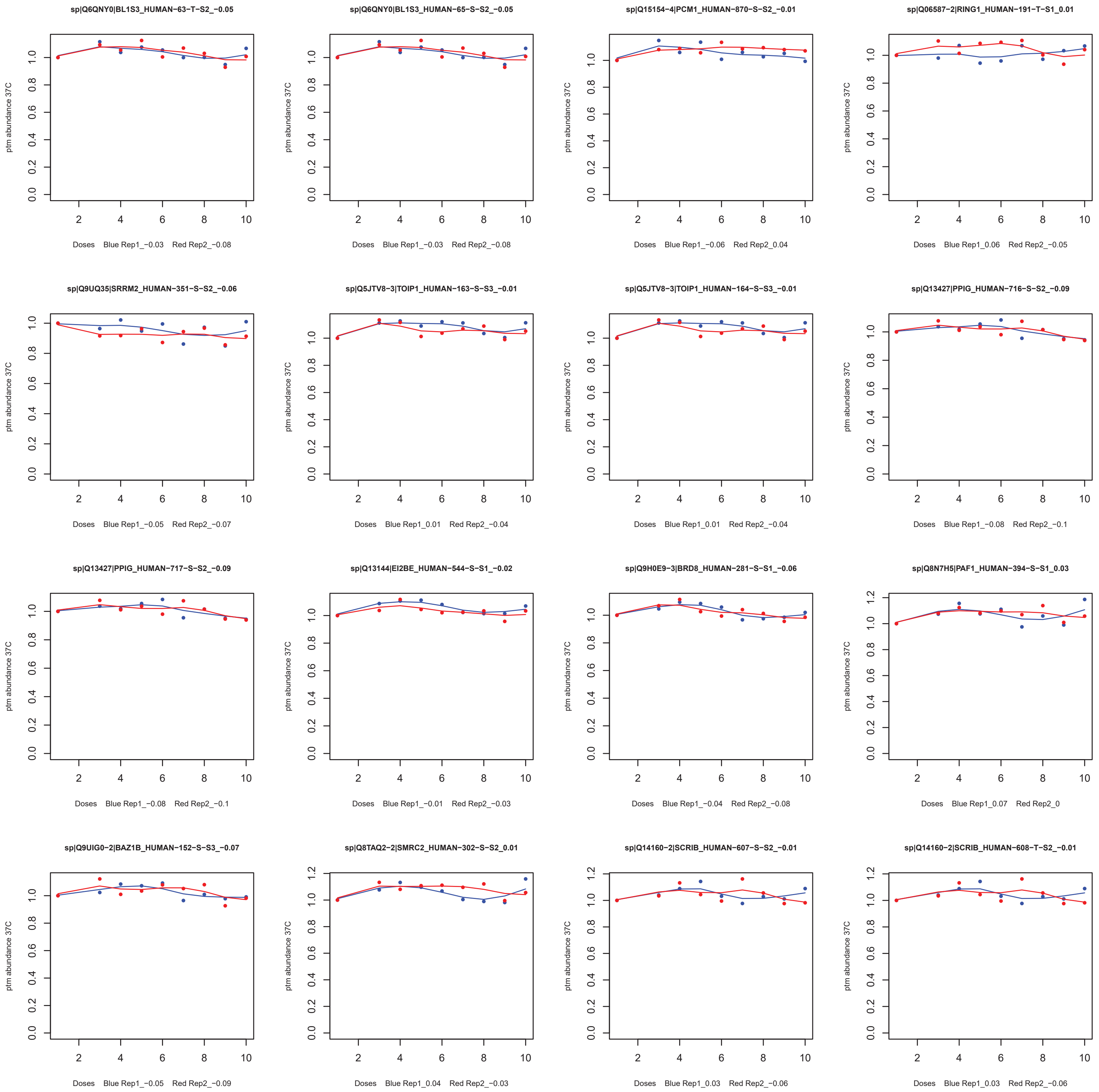

sp|Q6QNY0|BL1S3_HUMAN−63−T−S2_−0.05
ptm abundance 37C
Doses Blue Rep1_−0.03 Red Rep2_−0.08
sp|Q6QNY0|BL1S3_HUMAN−65−S−S2_−0.05
ptm abundance 37C
Doses Blue Rep1_−0.03 Red Rep2_−0.08
sp|Q15154−4|PCM1_HUMAN−870−S−S2_−0.01
ptm abundance 37C
Doses Blue Rep1_−0.06 Red Rep2_0.04
sp|Q06587−2|RING1_HUMAN−191−T−S1_0.01
ptm abundance 37C
Doses Blue Rep1_0.06 Red Rep2_−0.05
sp|Q9UQ35|SRRM2_HUMAN−351−S−S2_−0.06
ptm abundance 37C
Doses Blue Rep1_−0.05 Red Rep2_−0.07
sp|Q5JTV8−3|TOIP1_HUMAN−163−S−S3_−0.01
ptm abundance 37C
Doses Blue Rep1_0.01 Red Rep2_−0.04
sp|Q5JTV8−3|TOIP1_HUMAN−164−S−S3_−0.01
ptm abundance 37C
Doses Blue Rep1_0.01 Red Rep2_−0.04
sp|Q13427|PPIG_HUMAN−716−S−S2_−0.09
ptm abundance 37C
Doses Blue Rep1_−0.08 Red Rep2_−0.1
sp|Q13427|PPIG_HUMAN−717−S−S2_−0.09
ptm abundance 37C
Doses Blue Rep1_−0.08 Red Rep2_−0.1
sp|Q13144|EI2BE_HUMAN−544−S−S1_−0.02
ptm abundance 37C
Doses Blue Rep1_−0.01 Red Rep2_−0.03
sp|Q9H0E9−3|BRD8_HUMAN−281−S−S1_−0.06
ptm abundance 37C
Doses Blue Rep1_−0.04 Red Rep2_−0.08
sp|Q8N7H5|PAF1_HUMAN−394−S−S1_0.03
ptm abundance 37C
Doses Blue Rep1_0.07 Red Rep2_0
sp|Q9UIG0−2|BAZ1B_HUMAN−152−S−S3_−0.07
ptm abundance 37C
Doses Blue Rep1_−0.05 Red Rep2_−0.09
sp|Q8TAQ2−2|SMRC2_HUMAN−302−S−S2_0.01
ptm abundance 37C
Doses Blue Rep1_0.04 Red Rep2_−0.03
sp|Q14160−2|SCRIB_HUMAN−607−S−S2_−0.01
ptm abundance 37C
Doses Blue Rep1_0.03 Red Rep2_−0.06
sp|Q14160−2|SCRIB_HUMAN−608−T−S2_−0.01
ptm abundance 37C
Doses Blue Rep1_0.03 Red Rep2_−0.06

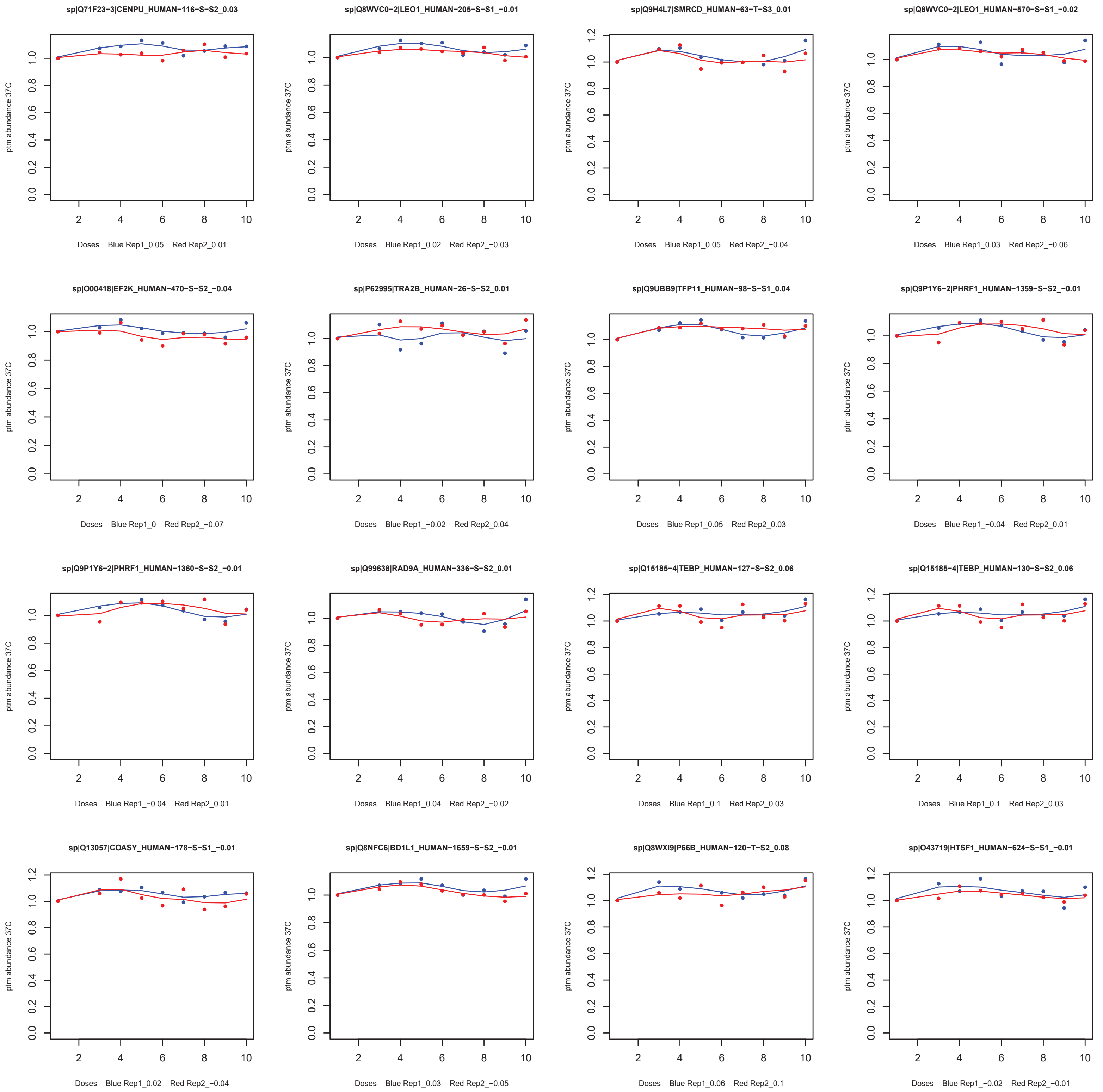
sp|Q71F23−3|CENPU_HUMAN−116−S−S2_0.03
Doses Blue Rep1_0.05 Red Rep2_0.01
sp|Q8WVC0−2|LEO1_HUMAN−205−S−S1_−0.01
Doses Blue Rep1_0.02 Red Rep2_−0.03
sp|Q9H4L7|SMRCD_HUMAN−63−T−S3_0.01
Doses Blue Rep1_0.05 Red Rep2_−0.04
sp|Q8WVC0−2|LEO1_HUMAN−570−S−S1_−0.02
Doses Blue Rep1_0.03 Red Rep2_−0.06
sp|O00418|EF2K_HUMAN−470−S−S2_−0.04
Doses Blue Rep1_0 Red Rep2_−0.07
sp|P62995|TRA2B_HUMAN−26−S−S2_0.01
Doses Blue Rep1_−0.02 Red Rep2_0.04
sp|Q9UBB9|TFP11_HUMAN−98−S−S1_0.04
Doses Blue Rep1_0.05 Red Rep2_0.03
sp|Q9P1Y6−2|PHRF1_HUMAN−1359−S−S2_−0.01
Doses Blue Rep1_−0.04 Red Rep2_0.01
sp|Q9P1Y6−2|PHRF1_HUMAN−1360−S−S2_−0.01
Doses Blue Rep1_−0.04 Red Rep2_0.01
sp|Q99638|RAD9A_HUMAN−336−S−S2_0.01
Doses Blue Rep1_0.04 Red Rep2_−0.02
sp|Q15185−4|TEBP_HUMAN−127−S−S2_0.06
Doses Blue Rep1_0.1 Red Rep2_0.03
sp|Q15185−4|TEBP_HUMAN−130−S−S2_0.06
Doses Blue Rep1_0.1 Red Rep2_0.03
sp|Q13057|COASY_HUMAN−178−S−S1_−0.01
Doses Blue Rep1_0.02 Red Rep2_−0.04
sp|Q8NFC6|BD1L1_HUMAN−1659−S−S2_−0.01
Doses Blue Rep1_0.03 Red Rep2_−0.05
sp|Q8WXI9|P66B_HUMAN−120−T−S2_0.08
Doses Blue Rep1_0.06 Red Rep2_0.1
sp|O43719|HTSF1_HUMAN−624−S−S1_−0.01
Doses Blue Rep1_−0.02 Red Rep2_−0.01
ptm abundance 37C

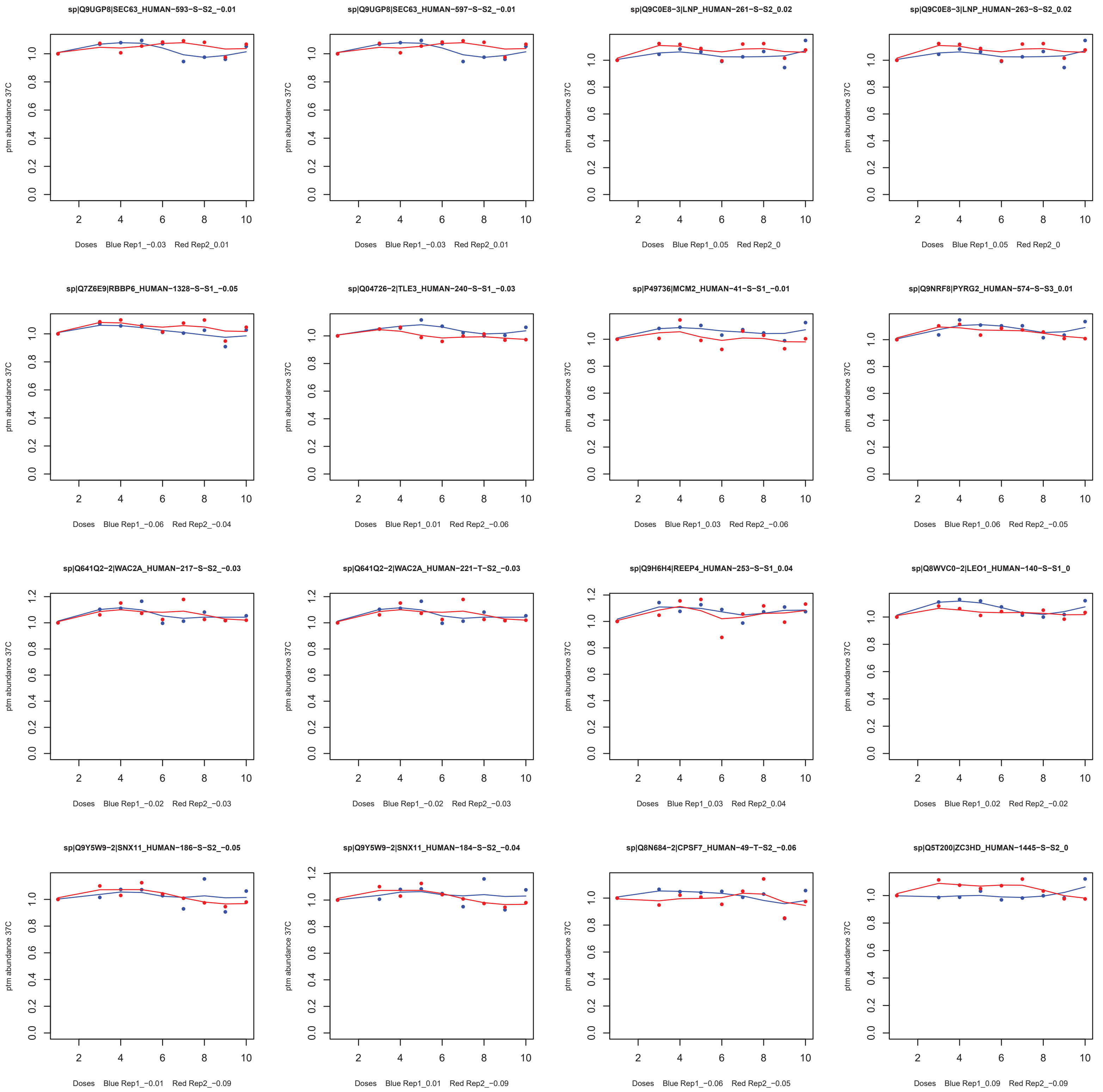

sp|Q9UGP8|SEC63_HUMAN−593−S−S2_−0.01
ptm abundance 37C
Doses Blue Rep1_−0.03 Red Rep2_0.01
sp|Q9UGP8|SEC63_HUMAN−597−S−S2_−0.01
ptm abundance 37C
Doses Blue Rep1_−0.03 Red Rep2_0.01
sp|Q9C0E8−3|LNP_HUMAN−261−S−S2_0.02
ptm abundance 37C
Doses Blue Rep1_0.05 Red Rep2_0
sp|Q9C0E8−3|LNP_HUMAN−263−S−S2_0.02
ptm abundance 37C
Doses Blue Rep1_0.05 Red Rep2_0
sp|Q7Z6E9|RBBP6_HUMAN−1328−S−S1_−0.05
ptm abundance 37C
Doses Blue Rep1_−0.06 Red Rep2_−0.04
sp|Q04726−2|TLE3_HUMAN−240−S−S1_−0.03
ptm abundance 37C
Doses Blue Rep1_0.01 Red Rep2_−0.06
sp|P49736|MCM2_HUMAN−41−S−S1_−0.01
ptm abundance 37C
Doses Blue Rep1_0.03 Red Rep2_−0.06
sp|Q9NRF8|PYRG2_HUMAN−574−S−S3_0.01
ptm abundance 37C
Doses Blue Rep1_0.06 Red Rep2_−0.05
sp|Q641Q2−2|WAC2A_HUMAN−217−S−S2_−0.03
ptm abundance 37C
Doses Blue Rep1_−0.02 Red Rep2_−0.03
sp|Q641Q2−2|WAC2A_HUMAN−221−T−S2_−0.03
ptm abundance 37C
Doses Blue Rep1_−0.02 Red Rep2_−0.03
sp|Q9H6H4|REEP4_HUMAN−253−S−S1_0.04
ptm abundance 37C
Doses Blue Rep1_0.03 Red Rep2_0.04
sp|Q8WVC0−2|LEO1_HUMAN−140−S−S1_0
ptm abundance 37C
Doses Blue Rep1_0.02 Red Rep2_−0.02
sp|Q9Y5W9−2|SNX11_HUMAN−186−S−S2_−0.05
ptm abundance 37C
Doses Blue Rep1_−0.01 Red Rep2_−0.09
sp|Q9Y5W9−2|SNX11_HUMAN−184−S−S2_−0.04
ptm abundance 37C
Doses Blue Rep1_0.01 Red Rep2_−0.09
sp|Q8N684−2|CPSF7_HUMAN−49−T−S2_−0.06
ptm abundance 37C
Doses Blue Rep1_−0.06 Red Rep2_−0.05
sp|Q5T200|ZC3HD_HUMAN−1445−S−S2_0
ptm abundance 37C
Doses Blue Rep1_0.09 Red Rep2_−0.09

sp|Q9P2E9|RRBP1_HUMAN−583−S−S1_−0.07

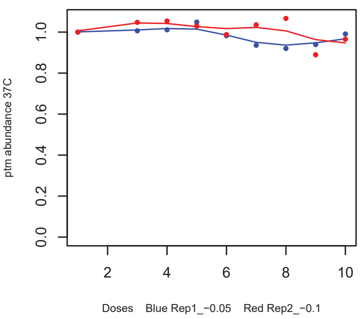

sp|Q9H1E3|NUCKS_HUMAN−214−S−S2_−0.05

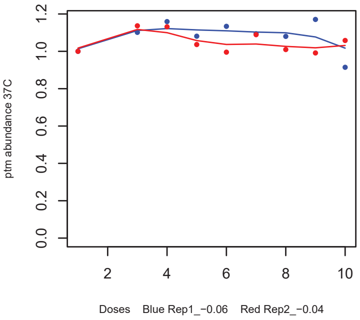

sp|O95218−2|ZRAB2_HUMAN−120−S−S1_0.03

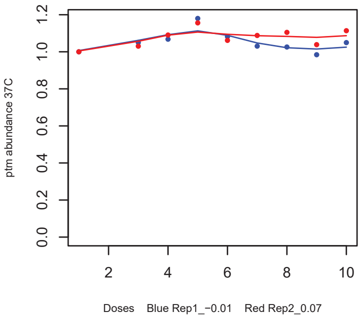

sp|Q86VM9|ZCH18_HUMAN−893−S−S1_−0.02

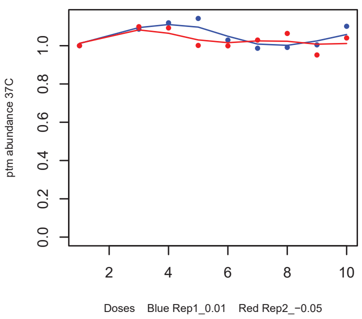

sp|Q06587−2|RING1_HUMAN−219−S−S2_−0.02

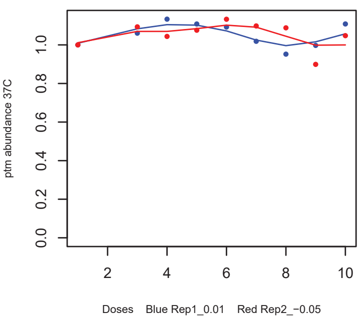

sp|Q06587−2|RING1_HUMAN−225−S−S2_−0.02

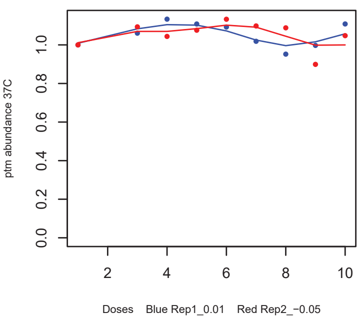

sp|Q5JTV8−3|TOIP1_HUMAN−166−T−S3_−0.03

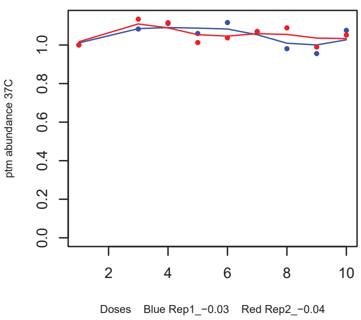

sp|P62995|TRA2B_HUMAN−20−S−S2_0.07

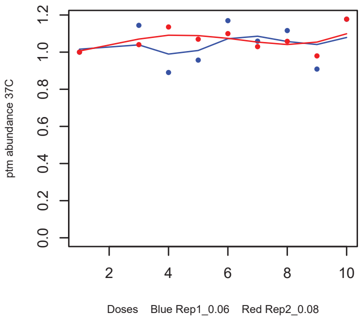

sp|Q13185|CBX3_HUMAN−93−S−S2_−0.06

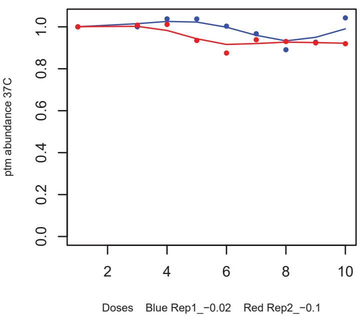

sp|P42167−3|LAP2B_HUMAN−66−S−S1_−0.05

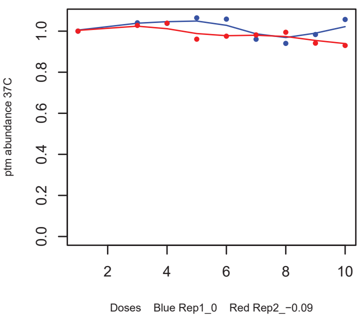

sp|Q9UQ35|SRRM2_HUMAN−1443−S−S2_−0.05

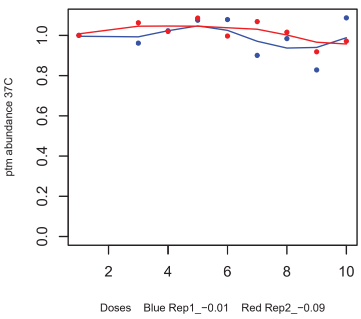

sp|Q8N684−2|CPSF7_HUMAN−46−S−S2_−0.06

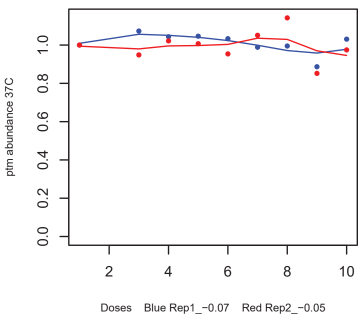

sp|Q8IWB9|TEX2_HUMAN−802−S−S3_0.03

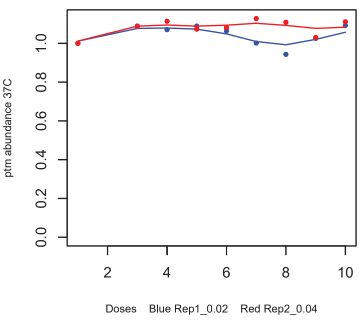

sp|Q9BVG4|PBDC1_HUMAN−197−S−S1_−0.04

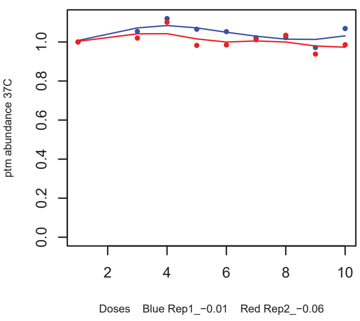

sp|Q8IWB9|TEX2_HUMAN−801−S−S3_0.05

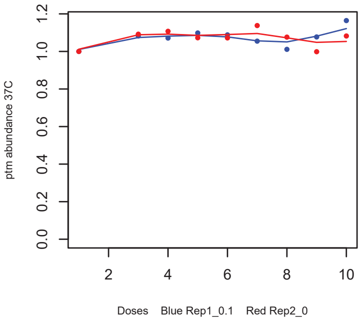

sp|Q12906|ILF3_HUMAN−477−S−S3_0

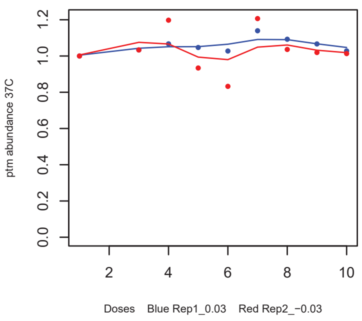

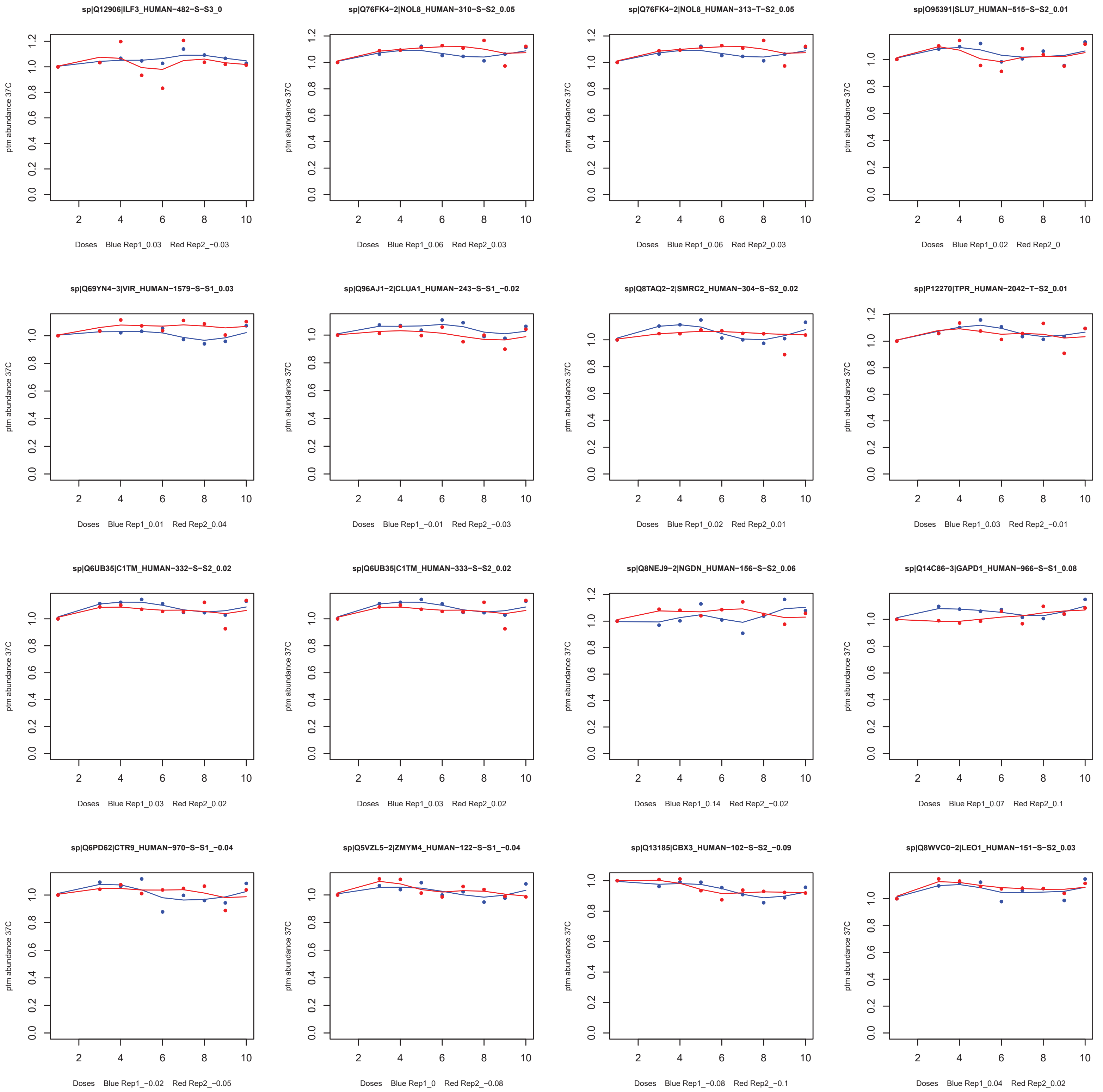
sp|Q12906|ILF3_HUMAN−482−S−S3_0
ptm abundance 37C
Doses Blue Rep1_0.03 Red Rep2_−0.03
sp|Q76FK4−2|NOL8_HUMAN−310−S−S2_0.05
ptm abundance 37C
Doses Blue Rep1_0.06 Red Rep2_0.03
sp|Q76FK4−2|NOL8_HUMAN−313−T−S2_0.05
ptm abundance 37C
Doses Blue Rep1_0.06 Red Rep2_0.03
sp|O95391|SLU7_HUMAN−515−S−S2_0.01
ptm abundance 37C
Doses Blue Rep1_0.02 Red Rep2_0
sp|Q69YN4−3|VIR_HUMAN−1579−S−S1_0.03
ptm abundance 37C
Doses Blue Rep1_0.01 Red Rep2_0.04
sp|Q96AJ1−2|CLUA1_HUMAN−243−S−S1_−0.02
ptm abundance 37C
Doses Blue Rep1_−0.01 Red Rep2_−0.03
sp|Q8TAQ2−2|SMRC2_HUMAN−304−S−S2_0.02
ptm abundance 37C
Doses Blue Rep1_0.02 Red Rep2_0.01
sp|P12270|TPR_HUMAN−2042−T−S2_0.01
ptm abundance 37C
Doses Blue Rep1_0.03 Red Rep2_−0.01
sp|Q6UB35|C1TM_HUMAN−332−S−S2_0.02
ptm abundance 37C
Doses Blue Rep1_0.03 Red Rep2_0.02
sp|Q6UB35|C1TM_HUMAN−333−S−S2_0.02
ptm abundance 37C
Doses Blue Rep1_0.03 Red Rep2_0.02
sp|Q8NEJ9−2|NGDN_HUMAN−156−S−S2_0.06
ptm abundance 37C
Doses Blue Rep1_0.14 Red Rep2_−0.02
sp|Q14C86−3|GAPD1_HUMAN−966−S−S1_0.08
ptm abundance 37C
Doses Blue Rep1_0.07 Red Rep2_0.1
sp|Q6PD62|CTR9_HUMAN−970−S−S1_−0.04
ptm abundance 37C
Doses Blue Rep1_−0.02 Red Rep2_−0.05
sp|Q5VZL5−2|ZMYM4_HUMAN−122−S−S1_−0.04
ptm abundance 37C
Doses Blue Rep1_0 Red Rep2_−0.08
sp|Q13185|CBX3_HUMAN−102−S−S2_−0.09
ptm abundance 37C
Doses Blue Rep1_−0.08 Red Rep2_−0.1
sp|Q8WVC0−2|LEO1_HUMAN−151−S−S2_0.03
ptm abundance 37C
Doses Blue Rep1_0.04 Red Rep2_0.02

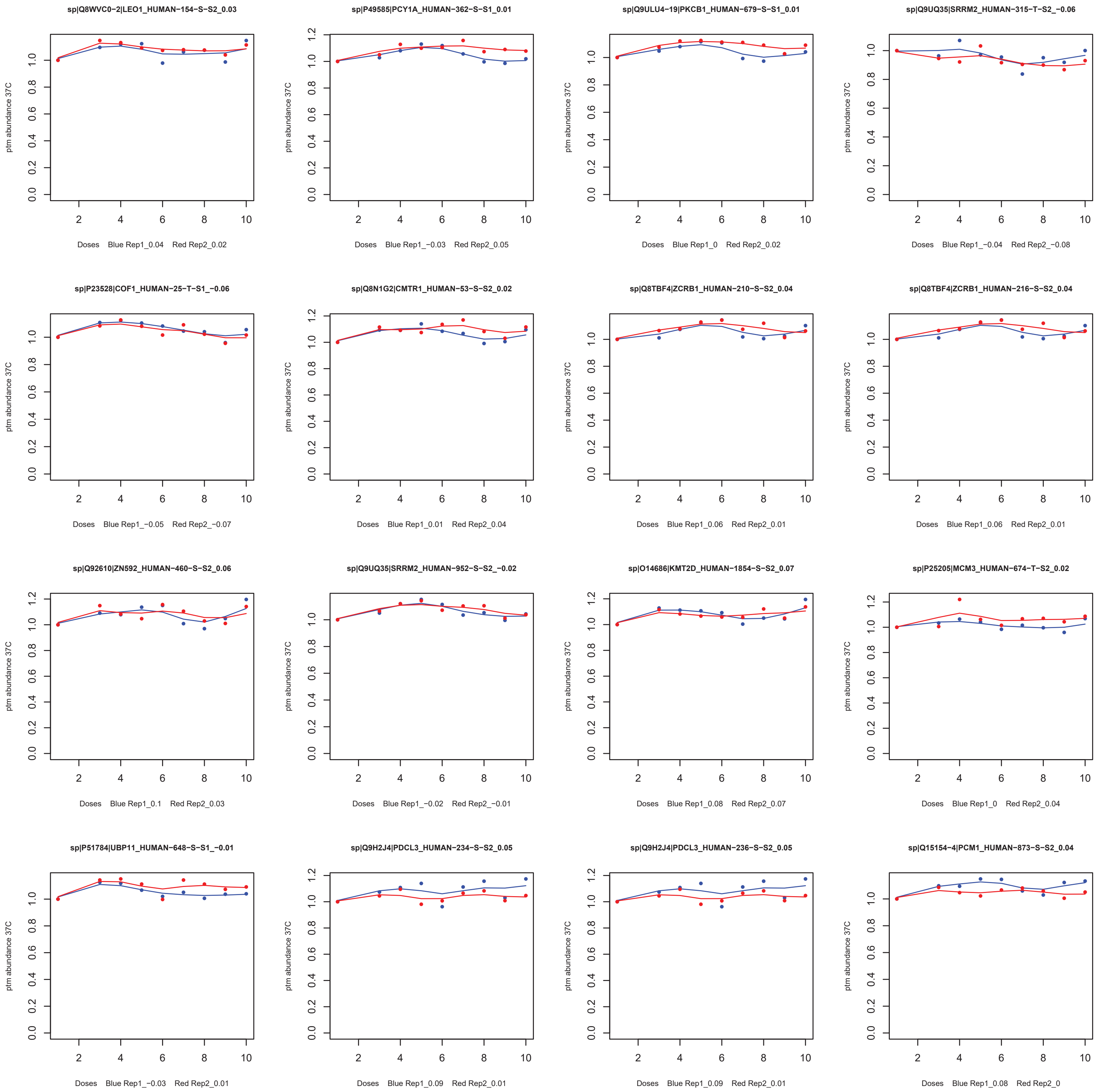
sp|Q8WVC0−2|LEO1_HUMAN−154−S−S2_0.03
ptm abundance 37C
Doses Blue Rep1_0.04 Red Rep2_0.02
sp|P49585|PCY1A_HUMAN−362−S−S1_0.01
ptm abundance 37C
Doses Blue Rep1_−0.03 Red Rep2_0.05
sp|Q9ULU4−19|PKCB1_HUMAN−679−S−S1_0.01
ptm abundance 37C
Doses Blue Rep1_0 Red Rep2_0.02
sp|Q9UQ35|SRRM2_HUMAN−315−T−S2_−0.06
ptm abundance 37C
Doses Blue Rep1_−0.04 Red Rep2_−0.08
sp|P23528|COF1_HUMAN−25−T−S1_−0.06
ptm abundance 37C
Doses Blue Rep1_−0.05 Red Rep2_−0.07
sp|Q8N1G2|CMTR1_HUMAN−53−S−S2_0.02
ptm abundance 37C
Doses Blue Rep1_0.01 Red Rep2_0.04
sp|Q8TBF4|ZCRB1_HUMAN−210−S−S2_0.04
ptm abundance 37C
Doses Blue Rep1_0.06 Red Rep2_0.01
sp|Q8TBF4|ZCRB1_HUMAN−216−S−S2_0.04
ptm abundance 37C
Doses Blue Rep1_0.06 Red Rep2_0.01
sp|Q92610|ZN592_HUMAN−460−S−S2_0.06
ptm abundance 37C
Doses Blue Rep1_0.1 Red Rep2_0.03
sp|Q9UQ35|SRRM2_HUMAN−952−S−S2_−0.02
ptm abundance 37C
Doses Blue Rep1_−0.02 Red Rep2_−0.01
sp|O14686|KMT2D_HUMAN−1854−S−S2_0.07
ptm abundance 37C
Doses Blue Rep1_0.08 Red Rep2_0.07
sp|P25205|MCM3_HUMAN−674−T−S2_0.02
ptm abundance 37C
Doses Blue Rep1_0 Red Rep2_0.04
sp|P51784|UBP11_HUMAN−648−S−S1_−0.01
ptm abundance 37C
Doses Blue Rep1_−0.03 Red Rep2_0.01
sp|Q9H2J4|PDCL3_HUMAN−234−S−S2_0.05
ptm abundance 37C
Doses Blue Rep1_0.09 Red Rep2_0.01
sp|Q9H2J4|PDCL3_HUMAN−236−S−S2_0.05
ptm abundance 37C
Doses Blue Rep1_0.09 Red Rep2_0.01
sp|Q15154−4|PCM1_HUMAN−873−S−S2_0.04
ptm abundance 37C
Doses Blue Rep1_0.08 Red Rep2_0

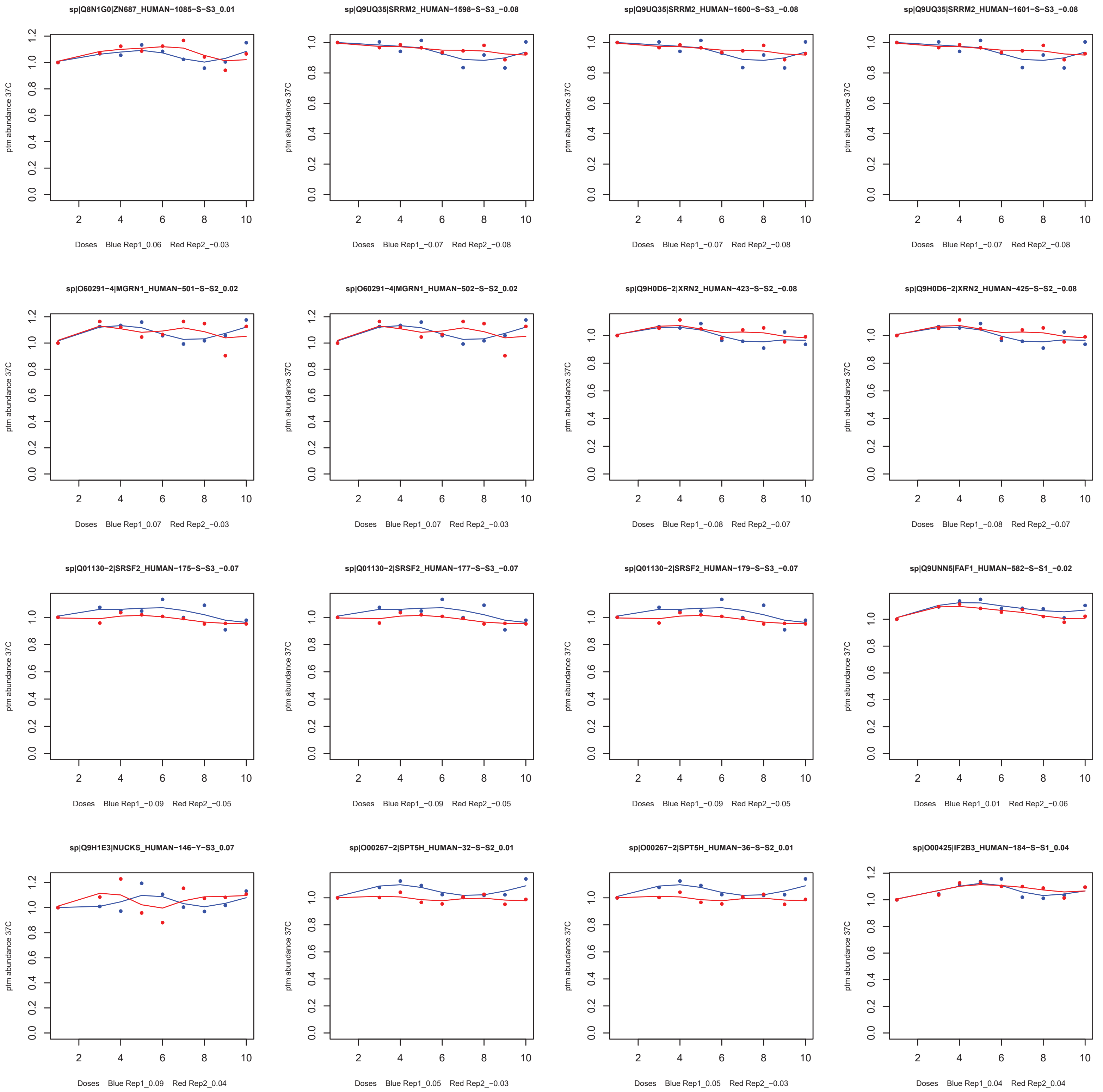

sp|Q8N1G0|ZN687_HUMAN−1085−S−S3_0.01
ptm abundance 37C
Doses Blue Rep1_0.06 Red Rep2_−0.03
sp|Q9UQ35|SRRM2_HUMAN−1598−S−S3_−0.08
ptm abundance 37C
Doses Blue Rep1_−0.07 Red Rep2_−0.08
sp|Q9UQ35|SRRM2_HUMAN−1600−S−S3_−0.08
ptm abundance 37C
Doses Blue Rep1_−0.07 Red Rep2_−0.08
sp|Q9UQ35|SRRM2_HUMAN−1601−S−S3_−0.08
ptm abundance 37C
Doses Blue Rep1_−0.07 Red Rep2_−0.08
sp|O60291−4|MGRN1_HUMAN−501−S−S2_0.02
ptm abundance 37C
Doses Blue Rep1_0.07 Red Rep2_−0.03
sp|O60291−4|MGRN1_HUMAN−502−S−S2_0.02
ptm abundance 37C
Doses Blue Rep1_0.07 Red Rep2_−0.03
sp|Q9H0D6−2|XRN2_HUMAN−423−S−S2_−0.08
ptm abundance 37C
Doses Blue Rep1_−0.08 Red Rep2_−0.07
sp|Q9H0D6−2|XRN2_HUMAN−425−S−S2_−0.08
ptm abundance 37C
Doses Blue Rep1_−0.08 Red Rep2_−0.07
sp|Q01130−2|SRSF2_HUMAN−175−S−S3_−0.07
ptm abundance 37C
Doses Blue Rep1_−0.09 Red Rep2_−0.05
sp|Q01130−2|SRSF2_HUMAN−177−S−S3_−0.07
ptm abundance 37C
Doses Blue Rep1_−0.09 Red Rep2_−0.05
sp|Q01130−2|SRSF2_HUMAN−179−S−S3_−0.07
ptm abundance 37C
Doses Blue Rep1_−0.09 Red Rep2_−0.05
sp|Q9UNN5|FAF1_HUMAN−582−S−S1_−0.02
ptm abundance 37C
Doses Blue Rep1_0.01 Red Rep2_−0.06
sp|Q9H1E3|NUCKS_HUMAN−146−Y−S3_0.07
ptm abundance 37C
Doses Blue Rep1_0.09 Red Rep2_0.04
sp|O00267−2|SPT5H_HUMAN−32−S−S2_0.01
ptm abundance 37C
Doses Blue Rep1_0.05 Red Rep2_−0.03
sp|O00267−2|SPT5H_HUMAN−36−S−S2_0.01
ptm abundance 37C
Doses Blue Rep1_0.05 Red Rep2_−0.03
sp|O00425|IF2B3_HUMAN−184−S−S1_0.04
ptm abundance 37C
Doses Blue Rep1_0.04 Red Rep2_0.04

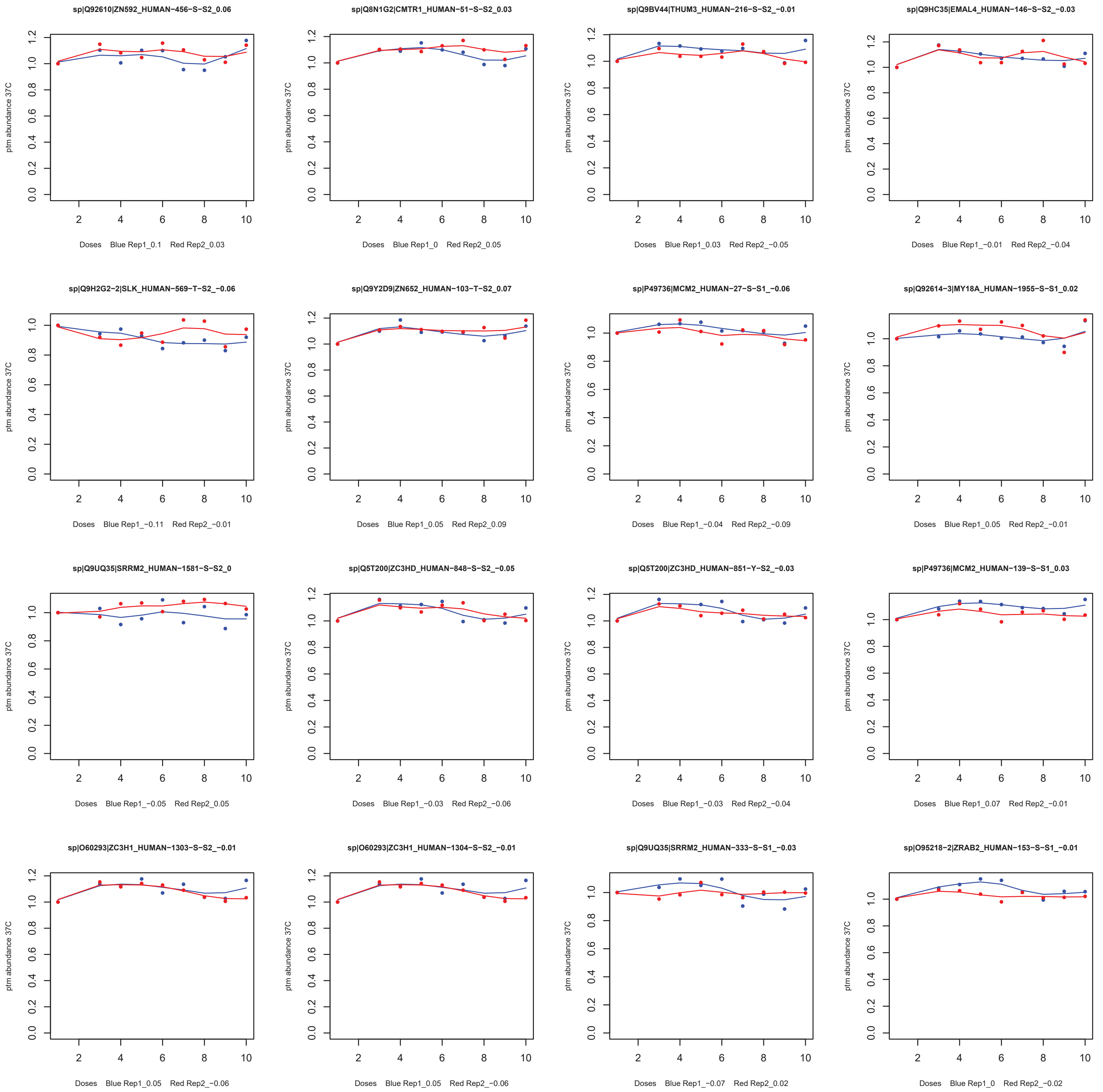
sp|Q92610|ZN592_HUMAN−456−S−S2_0.06
Doses Blue Rep1_0.1 Red Rep2_0.03
sp|Q8N1G2|CMTR1_HUMAN−51−S−S2_0.03
Doses Blue Rep1_0 Red Rep2_0.05
sp|Q9BV44|THUM3_HUMAN−216−S−S2_−0.01
Doses Blue Rep1_0.03 Red Rep2_−0.05
sp|Q9HC35|EMAL4_HUMAN−146−S−S2_−0.03
Doses Blue Rep1_−0.01 Red Rep2_−0.04
sp|Q9H2G2−2|SLK_HUMAN−569−T−S2_−0.06
Doses Blue Rep1_−0.11 Red Rep2_−0.01
sp|Q9Y2D9|ZN652_HUMAN−103−T−S2_0.07
Doses Blue Rep1_0.05 Red Rep2_0.09
sp|P49736|MCM2_HUMAN−27−S−S1_−0.06
Doses Blue Rep1_−0.04 Red Rep2_−0.09
sp|Q92614−3|MY18A_HUMAN−1955−S−S1_0.02
Doses Blue Rep1_0.05 Red Rep2_−0.01
sp|Q9UQ35|SRRM2_HUMAN−1581−S−S2_0
Doses Blue Rep1_−0.05 Red Rep2_0.05
sp|Q5T200|ZC3HD_HUMAN−848−S−S2_−0.05
Doses Blue Rep1_−0.03 Red Rep2_−0.06
sp|Q5T200|ZC3HD_HUMAN−851−Y−S2_−0.03
Doses Blue Rep1_−0.03 Red Rep2_−0.04
sp|P49736|MCM2_HUMAN−139−S−S1_0.03
Doses Blue Rep1_0.07 Red Rep2_−0.01
sp|O60293|ZC3H1_HUMAN−1303−S−S2_−0.01
Doses Blue Rep1_0.05 Red Rep2_−0.06
sp|O60293|ZC3H1_HUMAN−1304−S−S2_−0.01
Doses Blue Rep1_0.05 Red Rep2_−0.06
sp|Q9UQ35|SRRM2_HUMAN−333−S−S1_−0.03
Doses Blue Rep1_−0.07 Red Rep2_0.02
sp|O95218−2|ZRAB2_HUMAN−153−S−S1_−0.01
Doses Blue Rep1_0 Red Rep2_−0.02
ptm abundance 37C

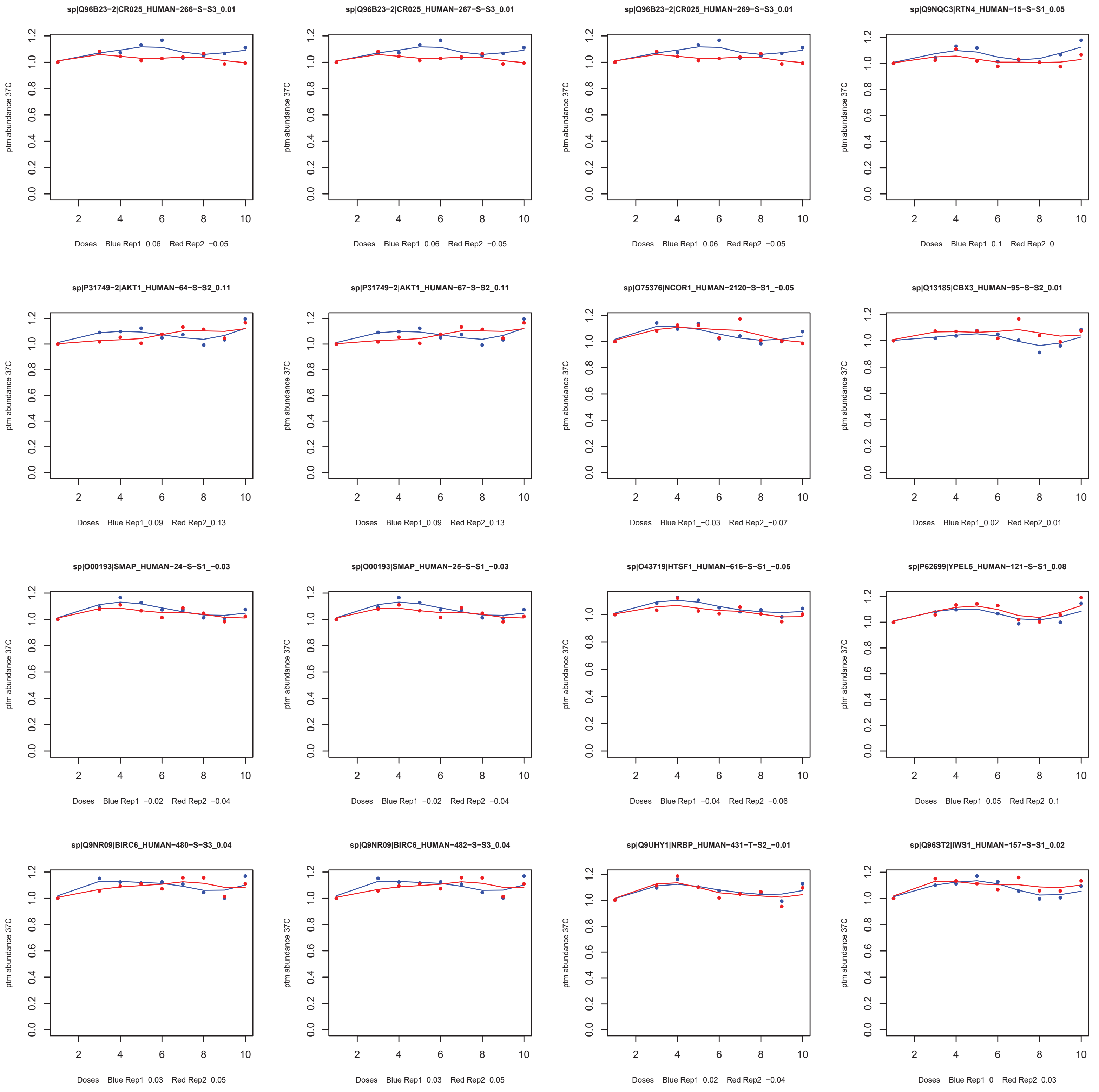
sp|Q96B23−2|CR025_HUMAN−266−S−S3_0.01
Doses Blue Rep1_0.06 Red Rep2_−0.05
sp|Q96B23−2|CR025_HUMAN−267−S−S3_0.01
Doses Blue Rep1_0.06 Red Rep2_−0.05
sp|Q96B23−2|CR025_HUMAN−269−S−S3_0.01
Doses Blue Rep1_0.06 Red Rep2_−0.05
sp|Q9NQC3|RTN4_HUMAN−15−S−S1_0.05
Doses Blue Rep1_0.1 Red Rep2_0
sp|P31749−2|AKT1_HUMAN−64−S−S2_0.11
Doses Blue Rep1_0.09 Red Rep2_0.13
sp|P31749−2|AKT1_HUMAN−67−S−S2_0.11
Doses Blue Rep1_0.09 Red Rep2_0.13
sp|O75376|NCOR1_HUMAN−2120−S−S1_−0.05
Doses Blue Rep1_−0.03 Red Rep2_−0.07
sp|Q13185|CBX3_HUMAN−95−S−S2_0.01
Doses Blue Rep1_0.02 Red Rep2_0.01
sp|O00193|SMAP_HUMAN−24−S−S1_−0.03
Doses Blue Rep1_−0.02 Red Rep2_−0.04
sp|O00193|SMAP_HUMAN−25−S−S1_−0.03
Doses Blue Rep1_−0.02 Red Rep2_−0.04
sp|O43719|HTSF1_HUMAN−616−S−S1_−0.05
Doses Blue Rep1_−0.04 Red Rep2_−0.06
sp|P62699|YPEL5_HUMAN−121−S−S1_0.08
Doses Blue Rep1_0.05 Red Rep2_0.1
sp|Q9NR09|BIRC6_HUMAN−480−S−S3_0.04
Doses Blue Rep1_0.03 Red Rep2_0.05
sp|Q9NR09|BIRC6_HUMAN−482−S−S3_0.04
Doses Blue Rep1_0.03 Red Rep2_0.05
sp|Q9UHY1|NRBP_HUMAN−431−T−S2_−0.01
Doses Blue Rep1_0.02 Red Rep2_−0.04
sp|Q96ST2|IWS1_HUMAN−157−S−S1_0.02
Doses Blue Rep1_0 Red Rep2_0.03
ptm abundance 37C

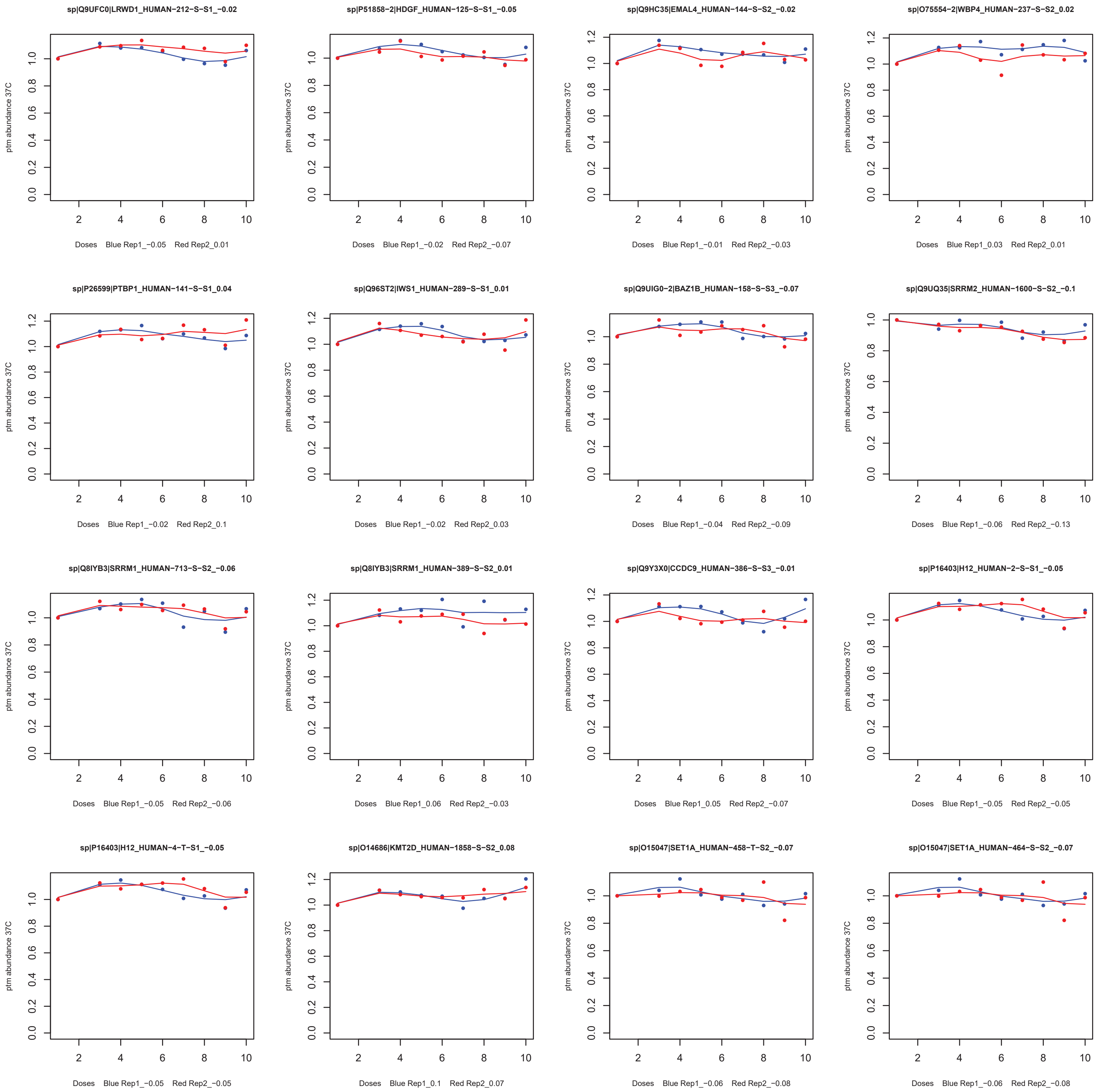
sp|Q9UFC0|LRWD1_HUMAN−212−S−S1_−0.02
ptm abundance 37C
Doses Blue Rep1_−0.05 Red Rep2_0.01
sp|P51858−2|HDGF_HUMAN−125−S−S1_−0.05
ptm abundance 37C
Doses Blue Rep1_−0.02 Red Rep2_−0.07
sp|Q9HC35|EMAL4_HUMAN−144−S−S2_−0.02
ptm abundance 37C
Doses Blue Rep1_−0.01 Red Rep2_−0.03
sp|O75554−2|WBP4_HUMAN−237−S−S2_0.02
ptm abundance 37C
Doses Blue Rep1_0.03 Red Rep2_0.01
sp|P26599|PTBP1_HUMAN−141−S−S1_0.04
ptm abundance 37C
Doses Blue Rep1_−0.02 Red Rep2_0.1
sp|Q96ST2|IWS1_HUMAN−289−S−S1_0.01
ptm abundance 37C
Doses Blue Rep1_−0.02 Red Rep2_0.03
sp|Q9UIG0−2|BAZ1B_HUMAN−158−S−S3_−0.07
ptm abundance 37C
Doses Blue Rep1_−0.04 Red Rep2_−0.09
sp|Q9UQ35|SRRM2_HUMAN−1600−S−S2_−0.1
ptm abundance 37C
Doses Blue Rep1_−0.06 Red Rep2_−0.13
sp|Q8IYB3|SRRM1_HUMAN−713−S−S2_−0.06
ptm abundance 37C
Doses Blue Rep1_−0.05 Red Rep2_−0.06
sp|Q8IYB3|SRRM1_HUMAN−389−S−S2_0.01
ptm abundance 37C
Doses Blue Rep1_0.06 Red Rep2_−0.03
sp|Q9Y3X0|CCDC9_HUMAN−386−S−S3_−0.01
ptm abundance 37C
Doses Blue Rep1_0.05 Red Rep2_−0.07
sp|P16403|H12_HUMAN−2−S−S1_−0.05
ptm abundance 37C
Doses Blue Rep1_−0.05 Red Rep2_−0.05
sp|P16403|H12_HUMAN−4−T−S1_−0.05
ptm abundance 37C
Doses Blue Rep1_−0.05 Red Rep2_−0.05
sp|O14686|KMT2D_HUMAN−1858−S−S2_0.08
ptm abundance 37C
Doses Blue Rep1_0.1 Red Rep2_0.07
sp|O15047|SET1A_HUMAN−458−T−S2_−0.07
ptm abundance 37C
Doses Blue Rep1_−0.06 Red Rep2_−0.08
sp|O15047|SET1A_HUMAN−464−S−S2_−0.07
ptm abundance 37C
Doses Blue Rep1_−0.06 Red Rep2_−0.08

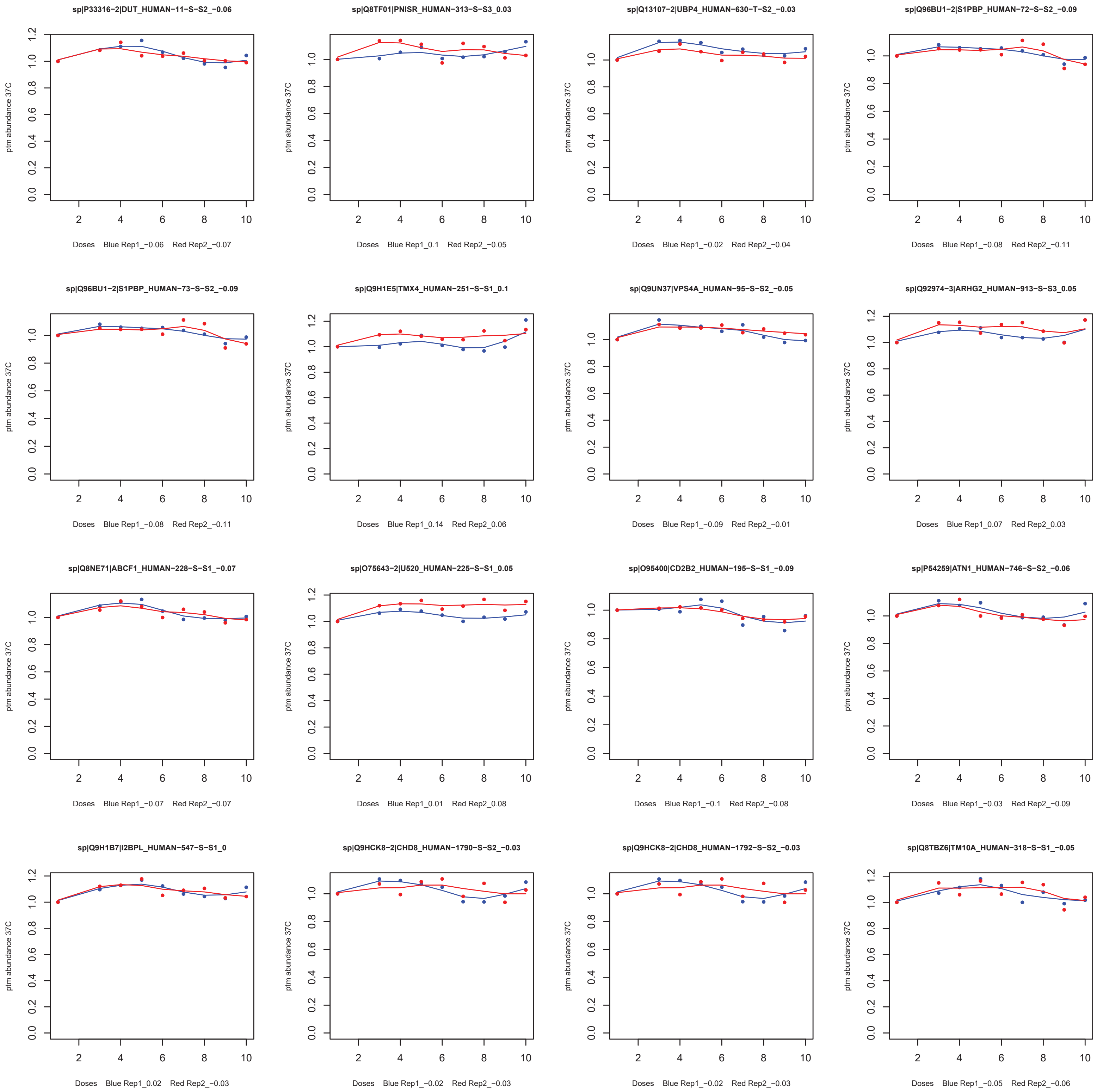
sp|P33316−2|DUT_HUMAN−11−S−S2_−0.06
ptm abundance 37C
Doses Blue Rep1_−0.06 Red Rep2_−0.07
sp|Q8TF01|PNISR_HUMAN−313−S−S3_0.03
ptm abundance 37C
Doses Blue Rep1_0.1 Red Rep2_−0.05
sp|Q13107−2|UBP4_HUMAN−630−T−S2_−0.03
ptm abundance 37C
Doses Blue Rep1_−0.02 Red Rep2_−0.04
sp|Q96BU1−2|S1PBP_HUMAN−72−S−S2_−0.09
ptm abundance 37C
Doses Blue Rep1_−0.08 Red Rep2_−0.11
sp|Q96BU1−2|S1PBP_HUMAN−73−S−S2_−0.09
ptm abundance 37C
Doses Blue Rep1_−0.08 Red Rep2_−0.11
sp|Q9H1E5|TMX4_HUMAN−251−S−S1_0.1
ptm abundance 37C
Doses Blue Rep1_0.14 Red Rep2_0.06
sp|Q9UN37|VPS4A_HUMAN−95−S−S2_−0.05
ptm abundance 37C
Doses Blue Rep1_−0.09 Red Rep2_−0.01
sp|Q92974−3|ARHG2_HUMAN−913−S−S3_0.05
ptm abundance 37C
Doses Blue Rep1_0.07 Red Rep2_0.03
sp|Q8NE71|ABCF1_HUMAN−228−S−S1_−0.07
ptm abundance 37C
Doses Blue Rep1_−0.07 Red Rep2_−0.07
sp|O75643−2|U520_HUMAN−225−S−S1_0.05
ptm abundance 37C
Doses Blue Rep1_0.01 Red Rep2_0.08
sp|O95400|CD2B2_HUMAN−195−S−S1_−0.09
ptm abundance 37C
Doses Blue Rep1_−0.1 Red Rep2_−0.08
sp|P54259|ATN1_HUMAN−746−S−S2_−0.06
ptm abundance 37C
Doses Blue Rep1_−0.03 Red Rep2_−0.09
sp|Q9H1B7|I2BPL_HUMAN−547−S−S1_0
ptm abundance 37C
Doses Blue Rep1_0.02 Red Rep2_−0.03
sp|Q9HCK8−2|CHD8_HUMAN−1790−S−S2_−0.03
ptm abundance 37C
Doses Blue Rep1_−0.02 Red Rep2_−0.03
sp|Q9HCK8−2|CHD8_HUMAN−1792−S−S2_−0.03
ptm abundance 37C
Doses Blue Rep1_−0.02 Red Rep2_−0.03
sp|Q8TBZ6|TM10A_HUMAN−318−S−S1_−0.05
ptm abundance 37C
Doses Blue Rep1_−0.05 Red Rep2_−0.06

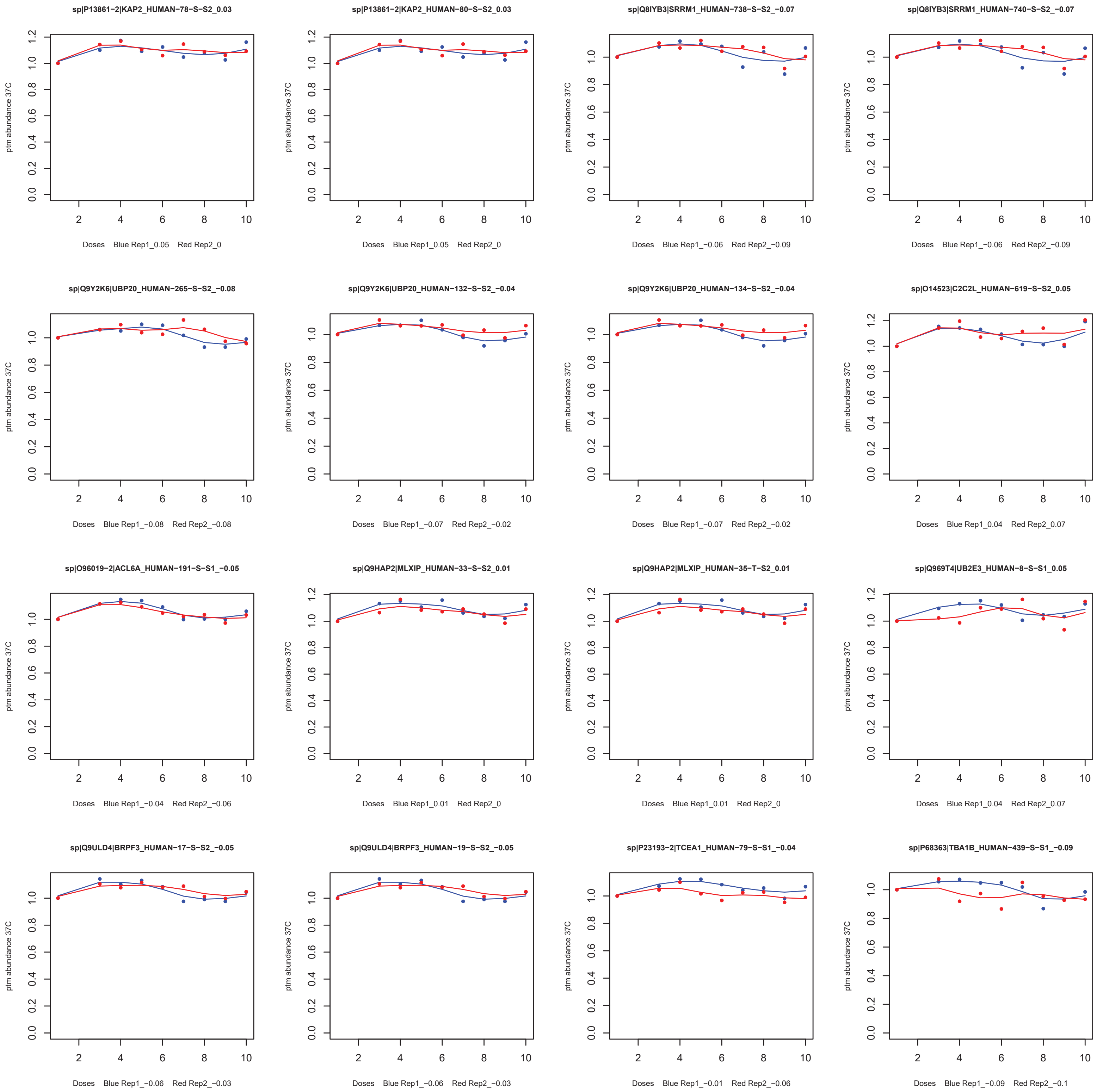

sp|P13861−2|KAP2_HUMAN−78−S−S2_0.03
Doses Blue Rep1_0.05 Red Rep2_0
sp|P13861−2|KAP2_HUMAN−80−S−S2_0.03
Doses Blue Rep1_0.05 Red Rep2_0
sp|Q8IYB3|SRRM1_HUMAN−738−S−S2_−0.07
Doses Blue Rep1_−0.06 Red Rep2_−0.09
sp|Q8IYB3|SRRM1_HUMAN−740−S−S2_−0.07
Doses Blue Rep1_−0.06 Red Rep2_−0.09
sp|Q9Y2K6|UBP20_HUMAN−265−S−S2_−0.08
Doses Blue Rep1_−0.08 Red Rep2_−0.08
sp|Q9Y2K6|UBP20_HUMAN−132−S−S2_−0.04
Doses Blue Rep1_−0.07 Red Rep2_−0.02
sp|Q9Y2K6|UBP20_HUMAN−134−S−S2_−0.04
Doses Blue Rep1_−0.07 Red Rep2_−0.02
sp|O14523|C2C2L_HUMAN−619−S−S2_0.05
Doses Blue Rep1_0.04 Red Rep2_0.07
sp|O96019−2|ACL6A_HUMAN−191−S−S1_−0.05
Doses Blue Rep1_−0.04 Red Rep2_−0.06
sp|Q9HAP2|MLXIP_HUMAN−33−S−S2_0.01
Doses Blue Rep1_0.01 Red Rep2_0
sp|Q9HAP2|MLXIP_HUMAN−35−T−S2_0.01
Doses Blue Rep1_0.01 Red Rep2_0
sp|Q969T4|UB2E3_HUMAN−8−S−S1_0.05
Doses Blue Rep1_0.04 Red Rep2_0.07
sp|Q9ULD4|BRPF3_HUMAN−17−S−S2_−0.05
Doses Blue Rep1_−0.06 Red Rep2_−0.03
sp|Q9ULD4|BRPF3_HUMAN−19−S−S2_−0.05
Doses Blue Rep1_−0.06 Red Rep2_−0.03
sp|P23193−2|TCEA1_HUMAN−79−S−S1_−0.04
Doses Blue Rep1_−0.01 Red Rep2_−0.06
sp|P68363|TBA1B_HUMAN−439−S−S1_−0.09
Doses Blue Rep1_−0.09 Red Rep2_−0.1
ptm abundance 37C

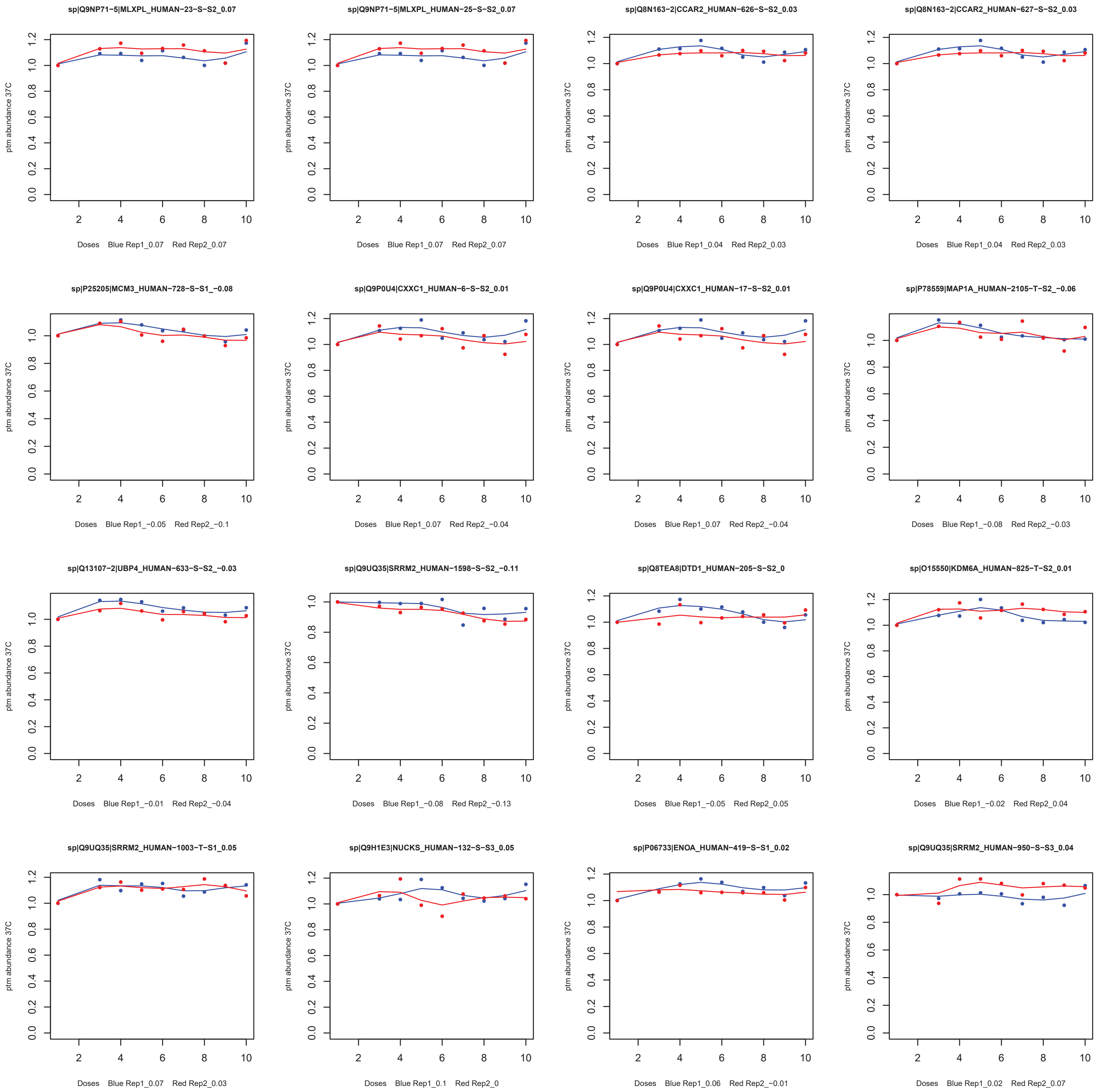

sp|Q9NP71−5|MLXPL_HUMAN−23−S−S2_0.07
Doses Blue Rep1_0.07 Red Rep2_0.07
sp|Q9NP71−5|MLXPL_HUMAN−25−S−S2_0.07
Doses Blue Rep1_0.07 Red Rep2_0.07
sp|Q8N163−2|CCAR2_HUMAN−626−S−S2_0.03
Doses Blue Rep1_0.04 Red Rep2_0.03
sp|Q8N163−2|CCAR2_HUMAN−627−S−S2_0.03
Doses Blue Rep1_0.04 Red Rep2_0.03
sp|P25205|MCM3_HUMAN−728−S−S1_−0.08
Doses Blue Rep1_−0.05 Red Rep2_−0.1
sp|Q9P0U4|CXXC1_HUMAN−6−S−S2_0.01
Doses Blue Rep1_0.07 Red Rep2_−0.04
sp|Q9P0U4|CXXC1_HUMAN−17−S−S2_0.01
Doses Blue Rep1_0.07 Red Rep2_−0.04
sp|P78559|MAP1A_HUMAN−2105−T−S2_−0.06
Doses Blue Rep1_−0.08 Red Rep2_−0.03
sp|Q13107−2|UBP4_HUMAN−633−S−S2_−0.03
Doses Blue Rep1_−0.01 Red Rep2_−0.04
sp|Q9UQ35|SRRM2_HUMAN−1598−S−S2_−0.11
Doses Blue Rep1_−0.08 Red Rep2_−0.13
sp|Q8TEA8|DTD1_HUMAN−205−S−S2_0
Doses Blue Rep1_−0.05 Red Rep2_0.05
sp|O15550|KDM6A_HUMAN−825−T−S2_0.01
Doses Blue Rep1_−0.02 Red Rep2_0.04
sp|Q9UQ35|SRRM2_HUMAN−1003−T−S1_0.05
Doses Blue Rep1_0.07 Red Rep2_0.03
sp|Q9H1E3|NUCKS_HUMAN−132−S−S3_0.05
Doses Blue Rep1_0.1 Red Rep2_0
sp|P06733|ENOA_HUMAN−419−S−S1_0.02
Doses Blue Rep1_0.06 Red Rep2_−0.01
sp|Q9UQ35|SRRM2_HUMAN−950−S−S3_0.04
Doses Blue Rep1_0.02 Red Rep2_0.07
ptm abundance 37C

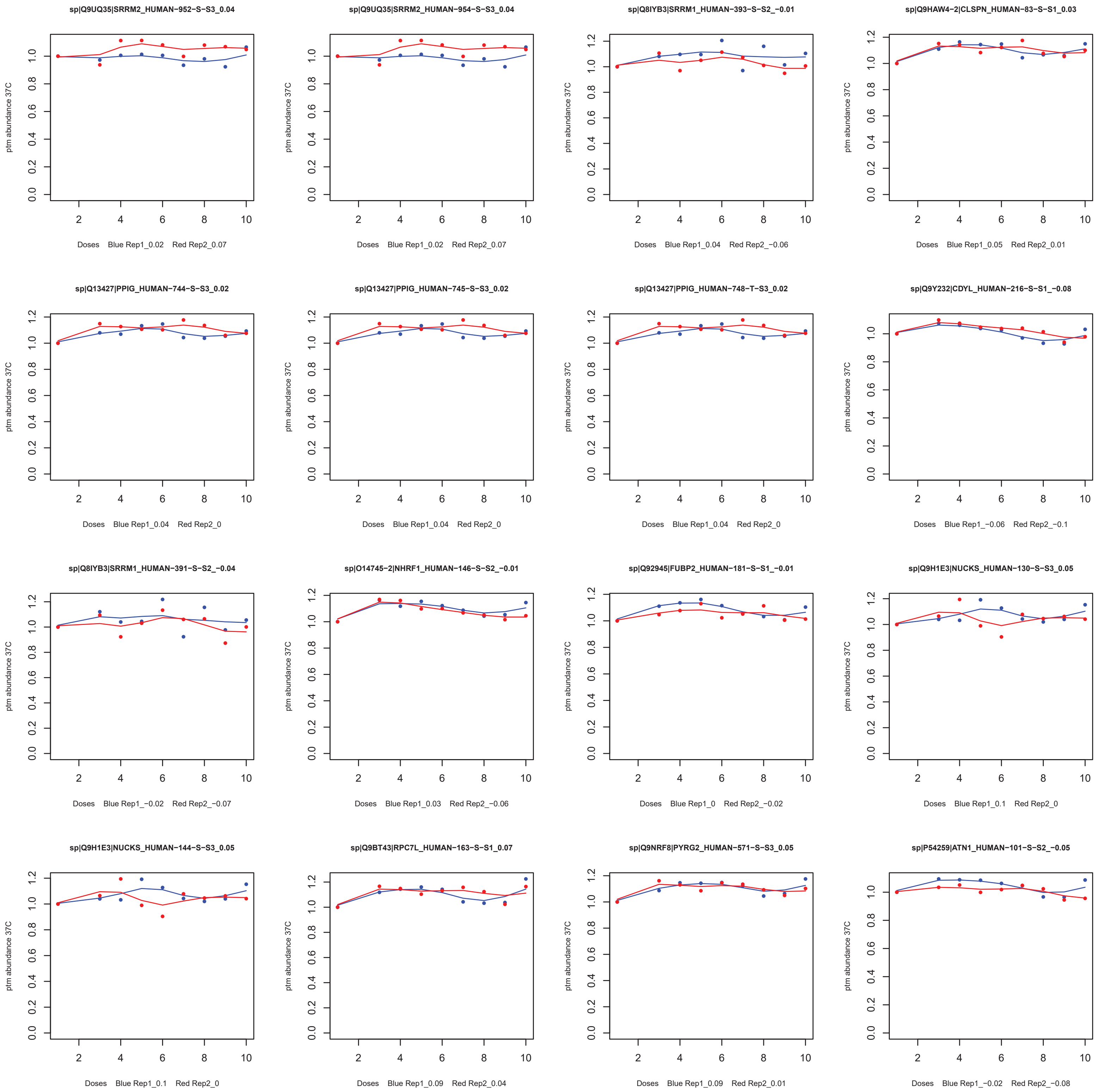
sp|Q9UQ35|SRRM2_HUMAN−952−S−S3_0.04
Doses Blue Rep1_0.02 Red Rep2_0.07
sp|Q9UQ35|SRRM2_HUMAN−954−S−S3_0.04
Doses Blue Rep1_0.02 Red Rep2_0.07
sp|Q8IYB3|SRRM1_HUMAN−393−S−S2_−0.01
Doses Blue Rep1_0.04 Red Rep2_−0.06
sp|Q9HAW4−2|CLSPN_HUMAN−83−S−S1_0.03
Doses Blue Rep1_0.05 Red Rep2_0.01
sp|Q13427|PPIG_HUMAN−744−S−S3_0.02
Doses Blue Rep1_0.04 Red Rep2_0
sp|Q13427|PPIG_HUMAN−745−S−S3_0.02
Doses Blue Rep1_0.04 Red Rep2_0
sp|Q13427|PPIG_HUMAN−748−T−S3_0.02
Doses Blue Rep1_0.04 Red Rep2_0
sp|Q9Y232|CDYL_HUMAN−216−S−S1_−0.08
Doses Blue Rep1_−0.06 Red Rep2_−0.1
sp|Q8IYB3|SRRM1_HUMAN−391−S−S2_−0.04
Doses Blue Rep1_−0.02 Red Rep2_−0.07
sp|O14745−2|NHRF1_HUMAN−146−S−S2_−0.01
Doses Blue Rep1_0.03 Red Rep2_−0.06
sp|Q92945|FUBP2_HUMAN−181−S−S1_−0.01
Doses Blue Rep1_0 Red Rep2_−0.02
sp|Q9H1E3|NUCKS_HUMAN−130−S−S3_0.05
Doses Blue Rep1_0.1 Red Rep2_0
sp|Q9H1E3|NUCKS_HUMAN−144−S−S3_0.05
Doses Blue Rep1_0.1 Red Rep2_0
sp|Q9BT43|RPC7L_HUMAN−163−S−S1_0.07
Doses Blue Rep1_0.09 Red Rep2_0.04
sp|Q9NRF8|PYRG2_HUMAN−571−S−S3_0.05
Doses Blue Rep1_0.09 Red Rep2_0.01
sp|P54259|ATN1_HUMAN−101−S−S2_−0.05
Doses Blue Rep1_−0.02 Red Rep2_−0.08
ptm abundance 37C

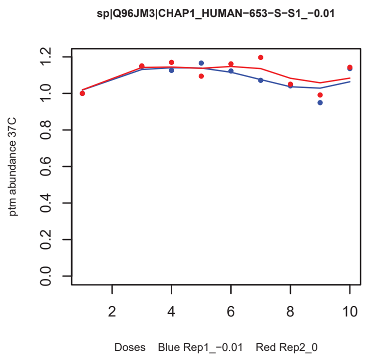
sp|Q96JM3|CHAP1_HUMAN−653−S−S1_−0.01
ptm abundance 37C
Doses Blue Rep1_−0.01 Red Rep2_0
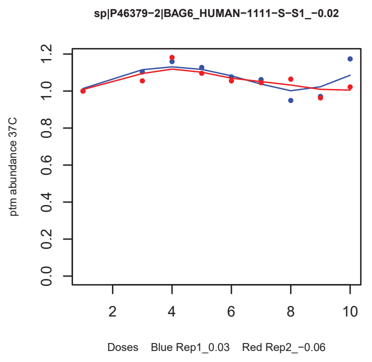
sp|P46379−2|BAG6_HUMAN−1111−S−S1_−0.02
ptm abundance 37C
Doses Blue Rep1_0.03 Red Rep2_−0.06
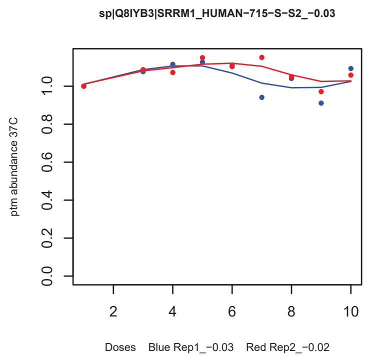
sp|Q8IYB3|SRRM1_HUMAN−715−S−S2_−0.03
ptm abundance 37C
Doses Blue Rep1_−0.03 Red Rep2_−0.02
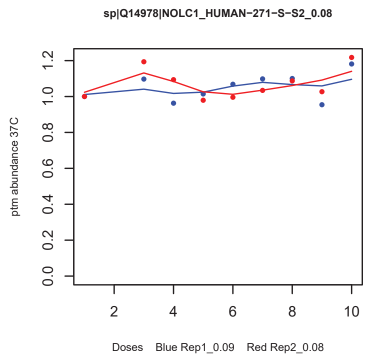
sp|Q14978|NOLC1_HUMAN−271−S−S2_0.08
ptm abundance 37C
Doses Blue Rep1_0.09 Red Rep2_0.08
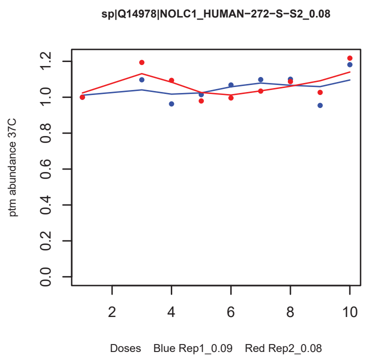
sp|Q14978|NOLC1_HUMAN−272−S−S2_0.08
ptm abundance 37C
Doses Blue Rep1_0.09 Red Rep2_0.08
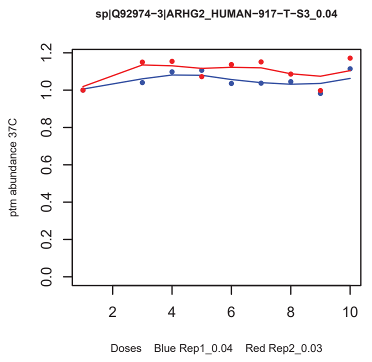
sp|Q92974−3|ARHG2_HUMAN−917−T−S3_0.04
ptm abundance 37C
Doses Blue Rep1_0.04 Red Rep2_0.03
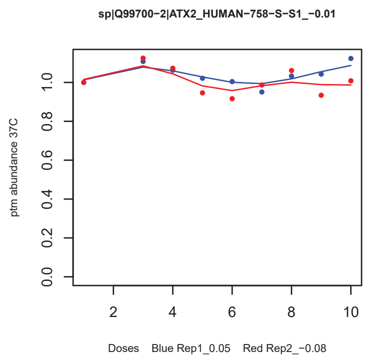
sp|Q99700−2|ATX2_HUMAN−758−S−S1_−0.01
ptm abundance 37C
Doses Blue Rep1_0.05 Red Rep2_−0.08
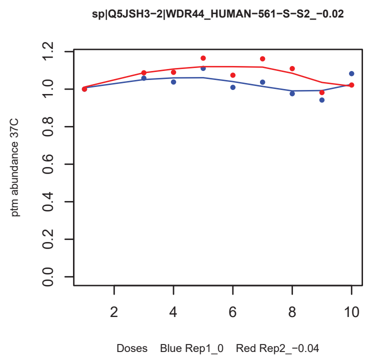
sp|Q5JSH3−2|WDR44_HUMAN−561−S−S2_−0.02
ptm abundance 37C
Doses Blue Rep1_0 Red Rep2_−0.04
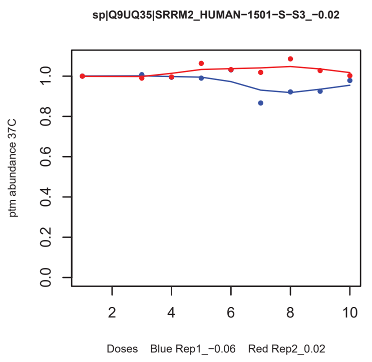
sp|Q9UQ35|SRRM2_HUMAN−1501−S−S3_−0.02
ptm abundance 37C
Doses Blue Rep1_−0.06 Red Rep2_0.02
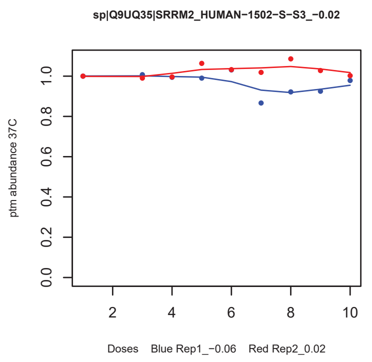
sp|Q9UQ35|SRRM2_HUMAN−1502−S−S3_−0.02
ptm abundance 37C
Doses Blue Rep1_−0.06 Red Rep2_0.02
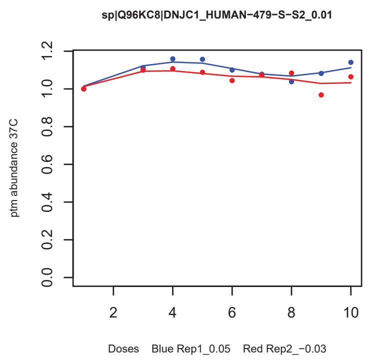
sp|Q96KC8|DNJC1_HUMAN−479−S−S2_0.01
ptm abundance 37C
Doses Blue Rep1_0.05 Red Rep2_−0.03
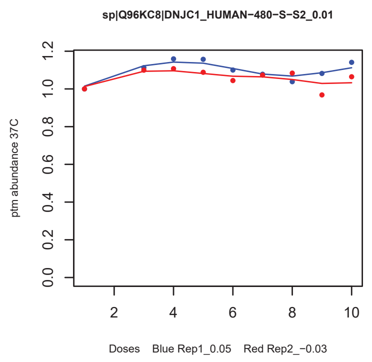
sp|Q96KC8|DNJC1_HUMAN−480−S−S2_0.01
ptm abundance 37C
Doses Blue Rep1_0.05 Red Rep2_−0.03
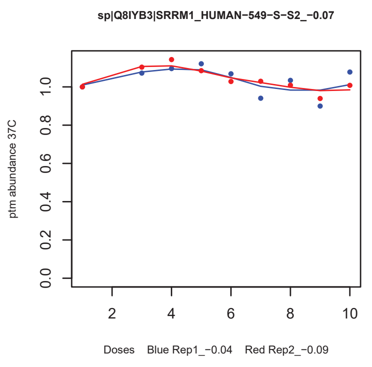
sp|Q8IYB3|SRRM1_HUMAN−549−S−S2_−0.07
ptm abundance 37C
Doses Blue Rep1_−0.04 Red Rep2_−0.09
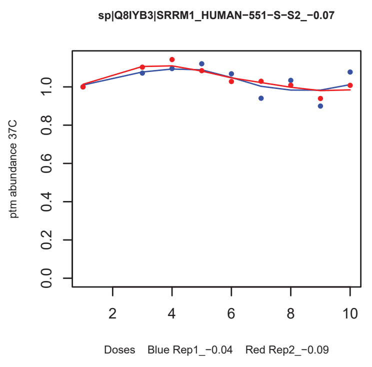
sp|Q8IYB3|SRRM1_HUMAN−551−S−S2_−0.07
ptm abundance 37C
Doses Blue Rep1_−0.04 Red Rep2_−0.09
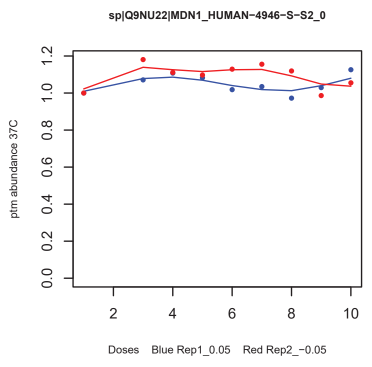
sp|Q9NU22|MDN1_HUMAN−4946−S−S2_0
ptm abundance 37C
Doses Blue Rep1_0.05 Red Rep2_−0.05
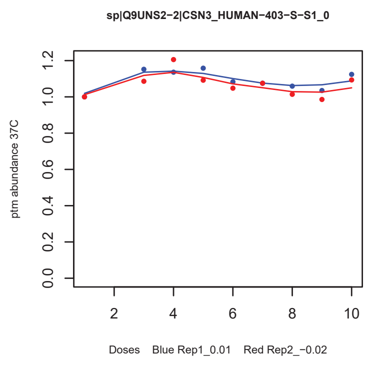
sp|Q9UNS2−2|CSN3_HUMAN−403−S−S1_0
ptm abundance 37C
Doses Blue Rep1_0.01 Red Rep2_−0.02

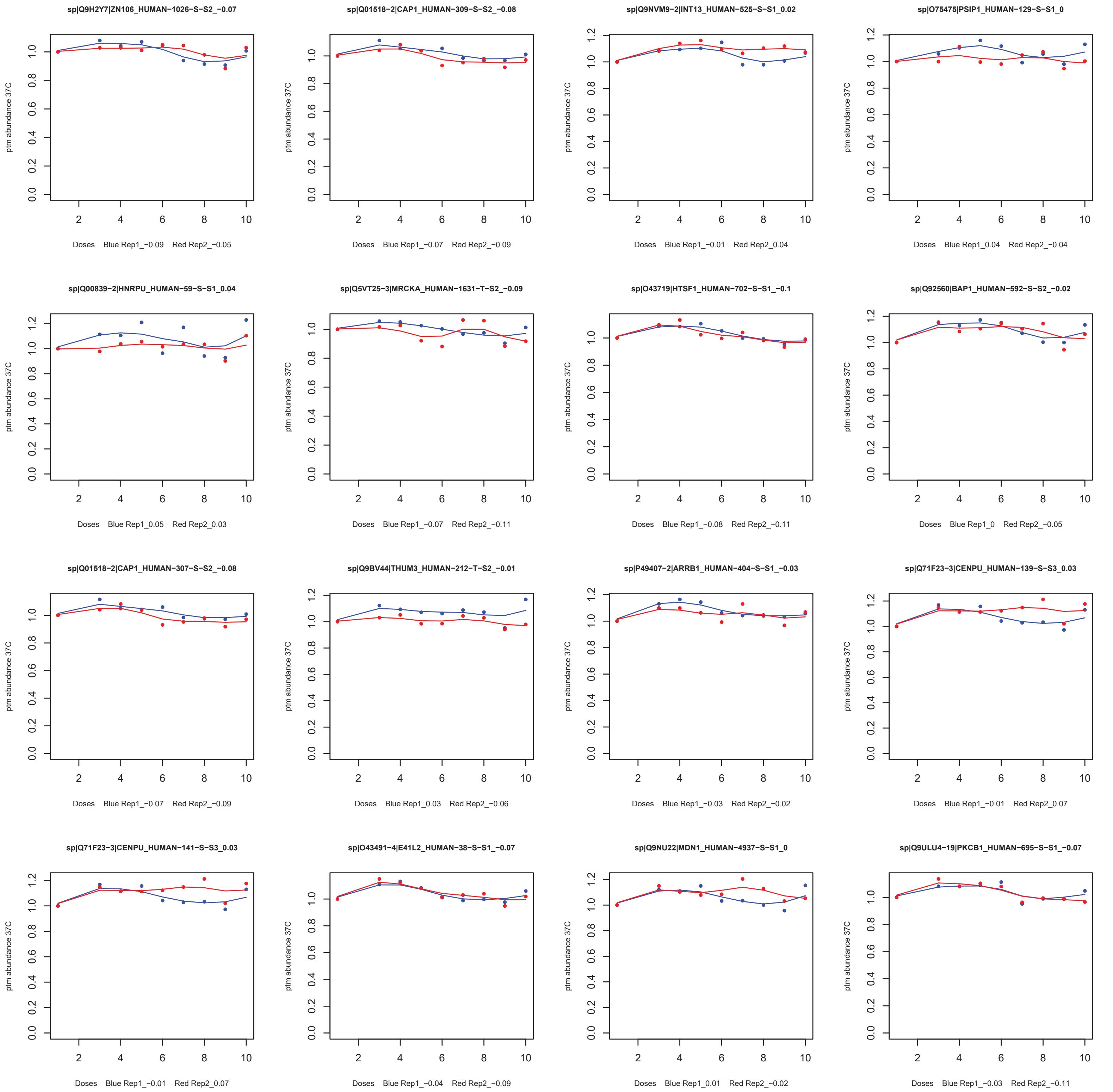

sp|Q9H2Y7|ZN106_HUMAN−1026−S−S2_−0.07
Doses Blue Rep1_−0.09 Red Rep2_−0.05
sp|Q01518−2|CAP1_HUMAN−309−S−S2_−0.08
Doses Blue Rep1_−0.07 Red Rep2_−0.09
sp|Q9NVM9−2|INT13_HUMAN−525−S−S1_0.02
Doses Blue Rep1_−0.01 Red Rep2_0.04
sp|O75475|PSIP1_HUMAN−129−S−S1_0
Doses Blue Rep1_0.04 Red Rep2_−0.04
sp|Q00839−2|HNRPU_HUMAN−59−S−S1_0.04
Doses Blue Rep1_0.05 Red Rep2_0.03
sp|Q5VT25−3|MRCKA_HUMAN−1631−T−S2_−0.09
Doses Blue Rep1_−0.07 Red Rep2_−0.11
sp|O43719|HTSF1_HUMAN−702−S−S1_−0.1
Doses Blue Rep1_−0.08 Red Rep2_−0.11
sp|Q92560|BAP1_HUMAN−592−S−S2_−0.02
Doses Blue Rep1_0 Red Rep2_−0.05
sp|Q01518−2|CAP1_HUMAN−307−S−S2_−0.08
Doses Blue Rep1_−0.07 Red Rep2_−0.09
sp|Q9BV44|THUM3_HUMAN−212−T−S2_−0.01
Doses Blue Rep1_0.03 Red Rep2_−0.06
sp|P49407−2|ARRB1_HUMAN−404−S−S1_−0.03
Doses Blue Rep1_−0.03 Red Rep2_−0.02
sp|Q71F23−3|CENPU_HUMAN−139−S−S3_0.03
Doses Blue Rep1_−0.01 Red Rep2_0.07
sp|Q71F23−3|CENPU_HUMAN−141−S−S3_0.03
Doses Blue Rep1_−0.01 Red Rep2_0.07
sp|O43491−4|E41L2_HUMAN−38−S−S1_−0.07
Doses Blue Rep1_−0.04 Red Rep2_−0.09
sp|Q9NU22|MDN1_HUMAN−4937−S−S1_0
Doses Blue Rep1_0.01 Red Rep2_−0.02
sp|Q9ULU4−19|PKCB1_HUMAN−695−S−S1_−0.07
Doses Blue Rep1_−0.03 Red Rep2_−0.11
ptm abundance 37C

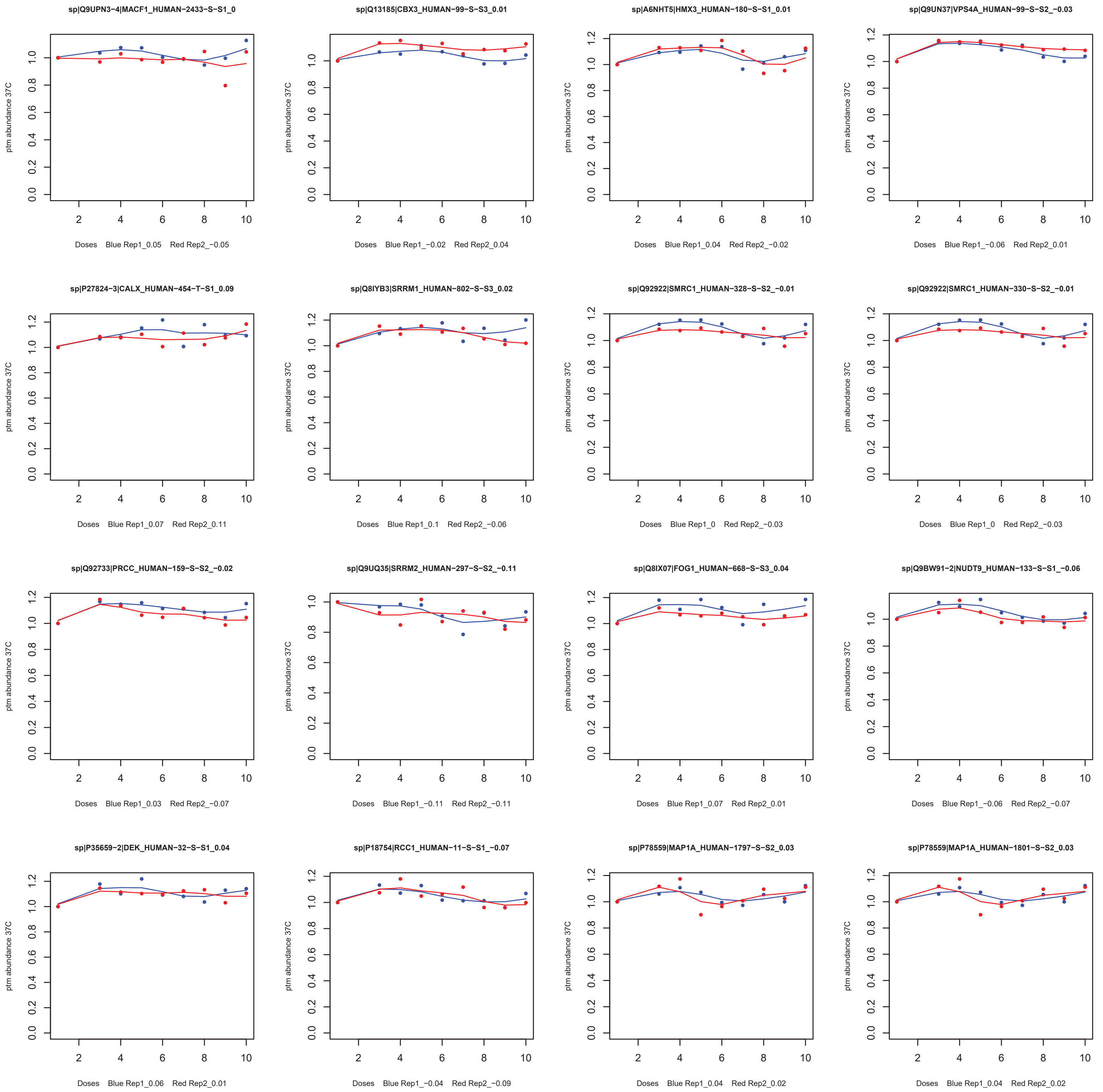

sp|Q9UPN3−4|MACF1_HUMAN−2433−S−S1_0
Doses Blue Rep1_0.05 Red Rep2_−0.05
sp|Q13185|CBX3_HUMAN−99−S−S3_0.01
Doses Blue Rep1_−0.02 Red Rep2_0.04
sp|A6NHT5|HMX3_HUMAN−180−S−S1_0.01
Doses Blue Rep1_0.04 Red Rep2_−0.02
sp|Q9UN37|VPS4A_HUMAN−99−S−S2_−0.03
Doses Blue Rep1_−0.06 Red Rep2_0.01
sp|P27824−3|CALX_HUMAN−454−T−S1_0.09
Doses Blue Rep1_0.07 Red Rep2_0.11
sp|Q8IYB3|SRRM1_HUMAN−802−S−S3_0.02
Doses Blue Rep1_0.1 Red Rep2_−0.06
sp|Q92922|SMRC1_HUMAN−328−S−S2_−0.01
Doses Blue Rep1_0 Red Rep2_−0.03
sp|Q92922|SMRC1_HUMAN−330−S−S2_−0.01
Doses Blue Rep1_0 Red Rep2_−0.03
sp|Q92733|PRCC_HUMAN−159−S−S2_−0.02
Doses Blue Rep1_0.03 Red Rep2_−0.07
sp|Q9UQ35|SRRM2_HUMAN−297−S−S2_−0.11
Doses Blue Rep1_−0.11 Red Rep2_−0.11
sp|Q8IX07|FOG1_HUMAN−668−S−S3_0.04
Doses Blue Rep1_0.07 Red Rep2_0.01
sp|Q9BW91−2|NUDT9_HUMAN−133−S−S1_−0.06
Doses Blue Rep1_−0.06 Red Rep2_−0.07
sp|P35659−2|DEK_HUMAN−32−S−S1_0.04
Doses Blue Rep1_0.06 Red Rep2_0.01
sp|P18754|RCC1_HUMAN−11−S−S1_−0.07
Doses Blue Rep1_−0.04 Red Rep2_−0.09
sp|P78559|MAP1A_HUMAN−1797−S−S2_0.03
Doses Blue Rep1_0.04 Red Rep2_0.02
sp|P78559|MAP1A_HUMAN−1801−S−S2_0.03
Doses Blue Rep1_0.04 Red Rep2_0.02
ptm abundance 37C

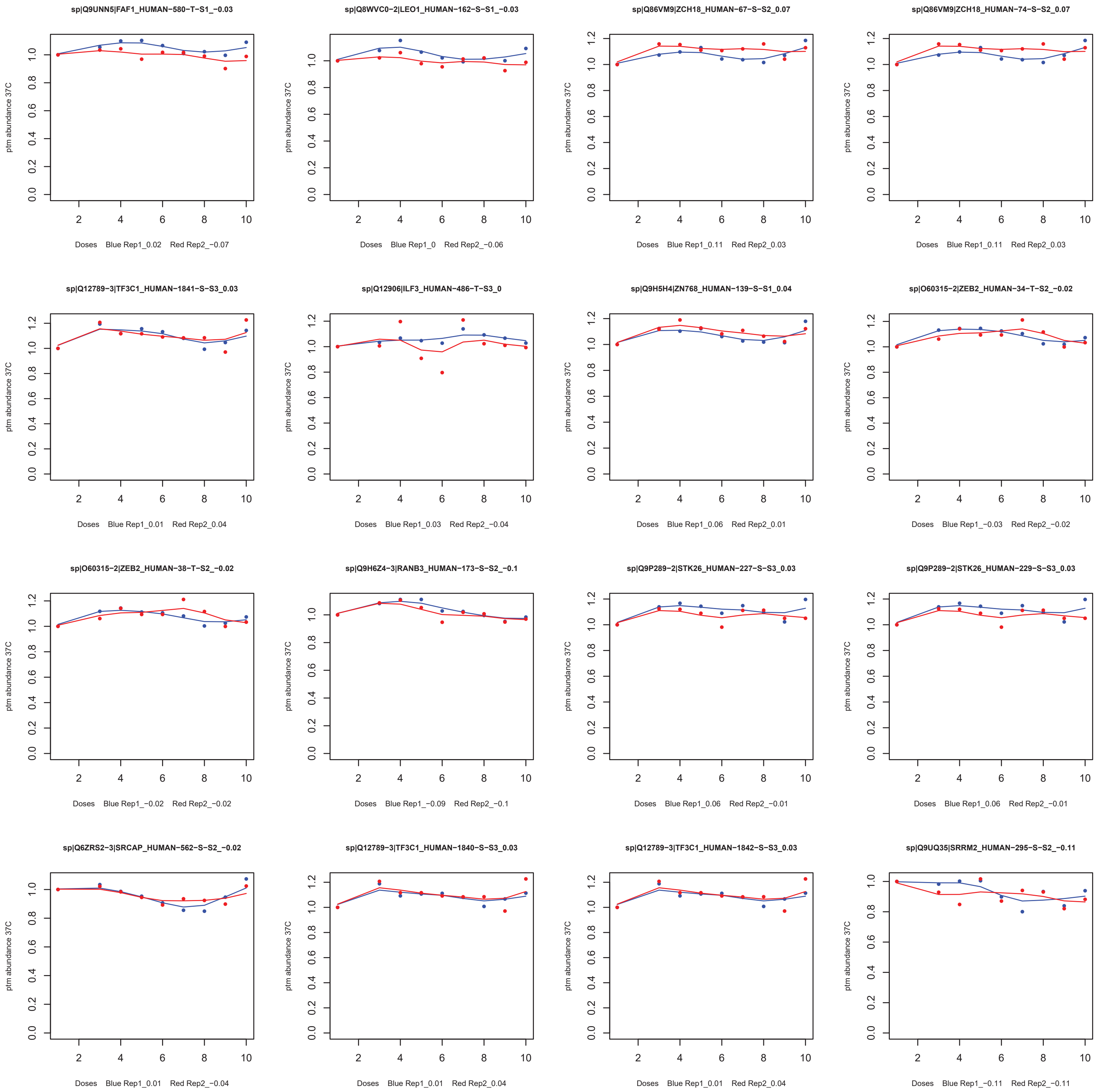
sp|Q9UNN5|FAF1_HUMAN−580−T−S1_−0.03
ptm abundance 37C
Doses Blue Rep1_0.02 Red Rep2_−0.07
sp|Q8WVC0−2|LEO1_HUMAN−162−S−S1_−0.03
ptm abundance 37C
Doses Blue Rep1_0 Red Rep2_−0.06
sp|Q86VM9|ZCH18_HUMAN−67−S−S2_0.07
ptm abundance 37C
Doses Blue Rep1_0.11 Red Rep2_0.03
sp|Q86VM9|ZCH18_HUMAN−74−S−S2_0.07
ptm abundance 37C
Doses Blue Rep1_0.11 Red Rep2_0.03
sp|Q12789−3|TF3C1_HUMAN−1841−S−S3_0.03
ptm abundance 37C
Doses Blue Rep1_0.01 Red Rep2_0.04
sp|Q12906|ILF3_HUMAN−486−T−S3_0
ptm abundance 37C
Doses Blue Rep1_0.03 Red Rep2_−0.04
sp|Q9H5H4|ZN768_HUMAN−139−S−S1_0.04
ptm abundance 37C
Doses Blue Rep1_0.06 Red Rep2_0.01
sp|O60315−2|ZEB2_HUMAN−34−T−S2_−0.02
ptm abundance 37C
Doses Blue Rep1_−0.03 Red Rep2_−0.02
sp|O60315−2|ZEB2_HUMAN−38−T−S2_−0.02
ptm abundance 37C
Doses Blue Rep1_−0.02 Red Rep2_−0.02
sp|Q9H6Z4−3|RANB3_HUMAN−173−S−S2_−0.1
ptm abundance 37C
Doses Blue Rep1_−0.09 Red Rep2_−0.1
sp|Q9P289−2|STK26_HUMAN−227−S−S3_0.03
ptm abundance 37C
Doses Blue Rep1_0.06 Red Rep2_−0.01
sp|Q9P289−2|STK26_HUMAN−229−S−S3_0.03
ptm abundance 37C
Doses Blue Rep1_0.06 Red Rep2_−0.01
sp|Q6ZRS2−3|SRCAP_HUMAN−562−S−S2_−0.02
ptm abundance 37C
Doses Blue Rep1_0.01 Red Rep2_−0.04
sp|Q12789−3|TF3C1_HUMAN−1840−S−S3_0.03
ptm abundance 37C
Doses Blue Rep1_0.01 Red Rep2_0.04
sp|Q12789−3|TF3C1_HUMAN−1842−S−S3_0.03
ptm abundance 37C
Doses Blue Rep1_0.01 Red Rep2_0.04
sp|Q9UQ35|SRRM2_HUMAN−295−S−S2_−0.11
ptm abundance 37C
Doses Blue Rep1_−0.11 Red Rep2_−0.11

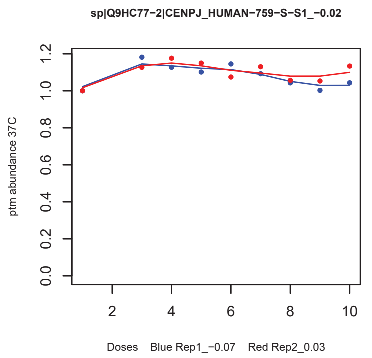
sp|Q9HC77−2|CENPJ_HUMAN−759−S−S1_−0.02
ptm abundance 37C
Doses Blue Rep1_−0.07 Red Rep2_0.03

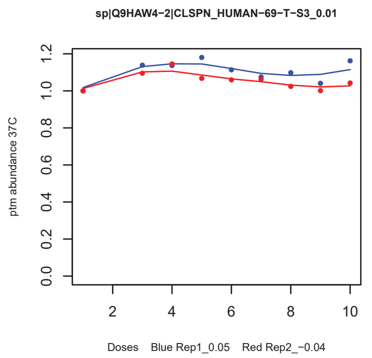
sp|Q9HAW4−2|CLSPN_HUMAN−69−T−S3_0.01
ptm abundance 37C
Doses Blue Rep1_0.05 Red Rep2_−0.04

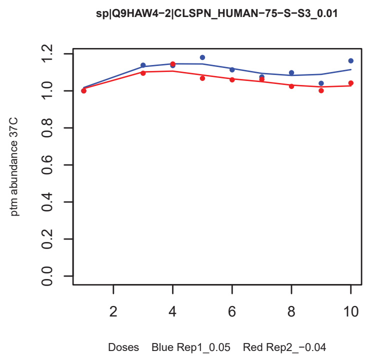
sp|Q9HAW4−2|CLSPN_HUMAN−75−S−S3_0.01
ptm abundance 37C
Doses Blue Rep1_0.05 Red Rep2_−0.04

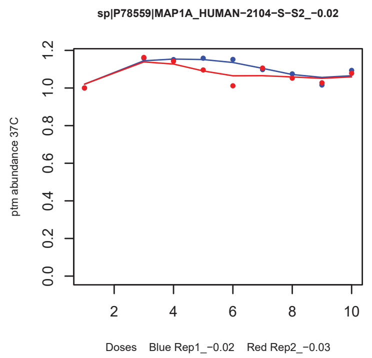
sp|P78559|MAP1A_HUMAN−2104−S−S2_−0.02
ptm abundance 37C
Doses Blue Rep1_−0.02 Red Rep2_−0.03

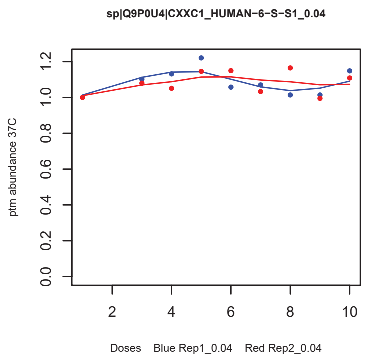
sp|Q9P0U4|CXXC1_HUMAN−6−S−S1_0.04
ptm abundance 37C
Doses Blue Rep1_0.04 Red Rep2_0.04

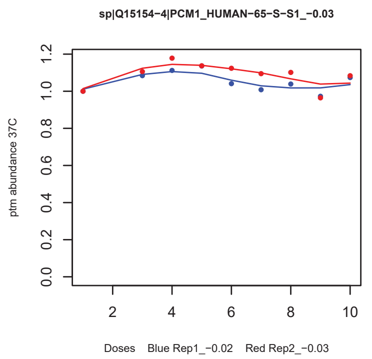
sp|Q15154−4|PCM1_HUMAN−65−S−S1_−0.03
ptm abundance 37C
Doses Blue Rep1_−0.02 Red Rep2_−0.03

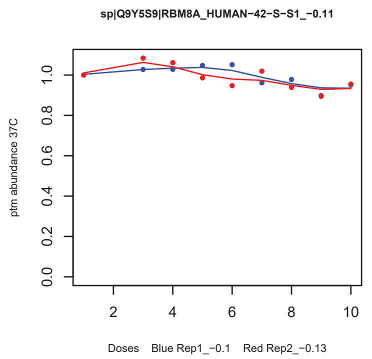
sp|Q9Y5S9|RBM8A_HUMAN−42−S−S1_−0.11
ptm abundance 37C
Doses Blue Rep1_−0.1 Red Rep2_−0.13

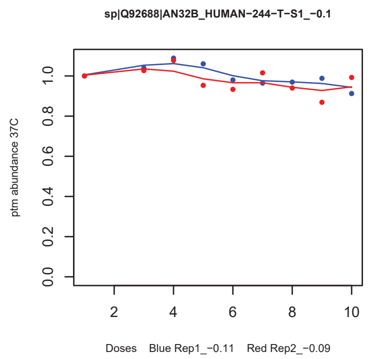
sp|Q92688|AN32B_HUMAN−244−T−S1_−0.1
ptm abundance 37C
Doses Blue Rep1_−0.11 Red Rep2_−0.09

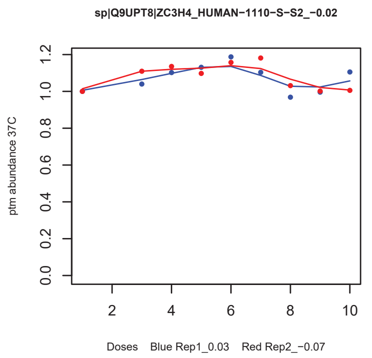
sp|Q9UPT8|ZC3H4_HUMAN−1110−S−S2_−0.02
ptm abundance 37C
Doses Blue Rep1_0.03 Red Rep2_−0.07

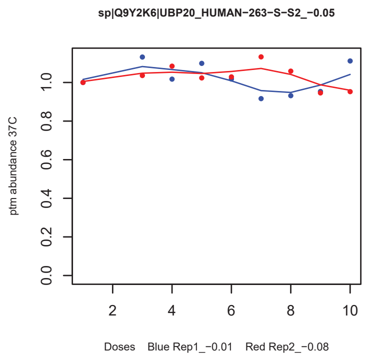
sp|Q9Y2K6|UBP20_HUMAN−263−S−S2_−0.05
ptm abundance 37C
Doses Blue Rep1_−0.01 Red Rep2_−0.08

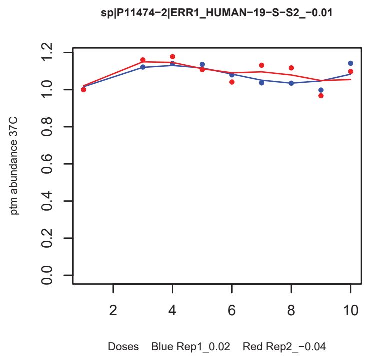
sp|P11474−2|ERR1_HUMAN−19−S−S2_−0.01
ptm abundance 37C
Doses Blue Rep1_0.02 Red Rep2_−0.04

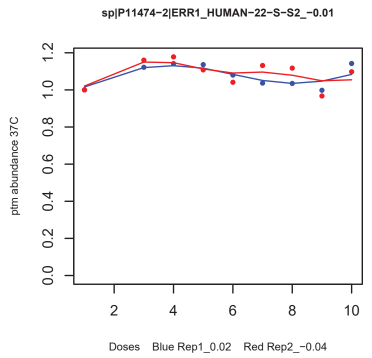
sp|P11474−2|ERR1_HUMAN−22−S−S2_−0.01
ptm abundance 37C
Doses Blue Rep1_0.02 Red Rep2_−0.04

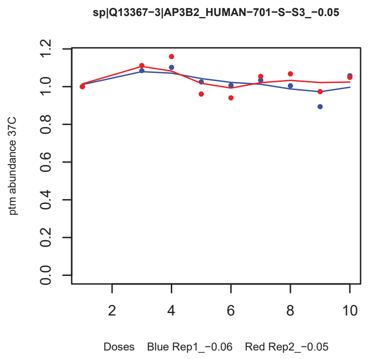
sp|Q13367−3|AP3B2_HUMAN−701−S−S3_−0.05
ptm abundance 37C
Doses Blue Rep1_−0.06 Red Rep2_−0.05

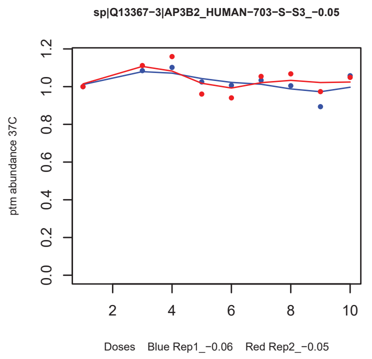
sp|Q13367−3|AP3B2_HUMAN−703−S−S3_−0.05
ptm abundance 37C
Doses Blue Rep1_−0.06 Red Rep2_−0.05

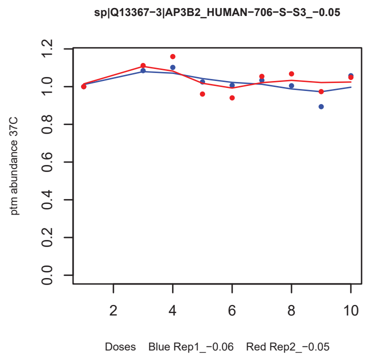
sp|Q13367−3|AP3B2_HUMAN−706−S−S3_−0.05
ptm abundance 37C
Doses Blue Rep1_−0.06 Red Rep2_−0.05

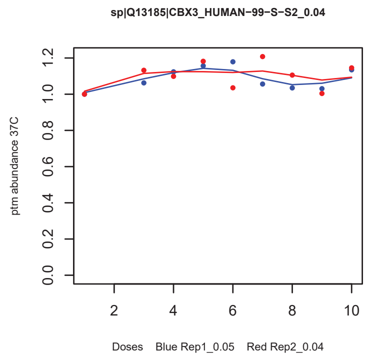
sp|Q13185|CBX3_HUMAN−99−S−S2_0.04
ptm abundance 37C
Doses Blue Rep1_0.05 Red Rep2_0.04

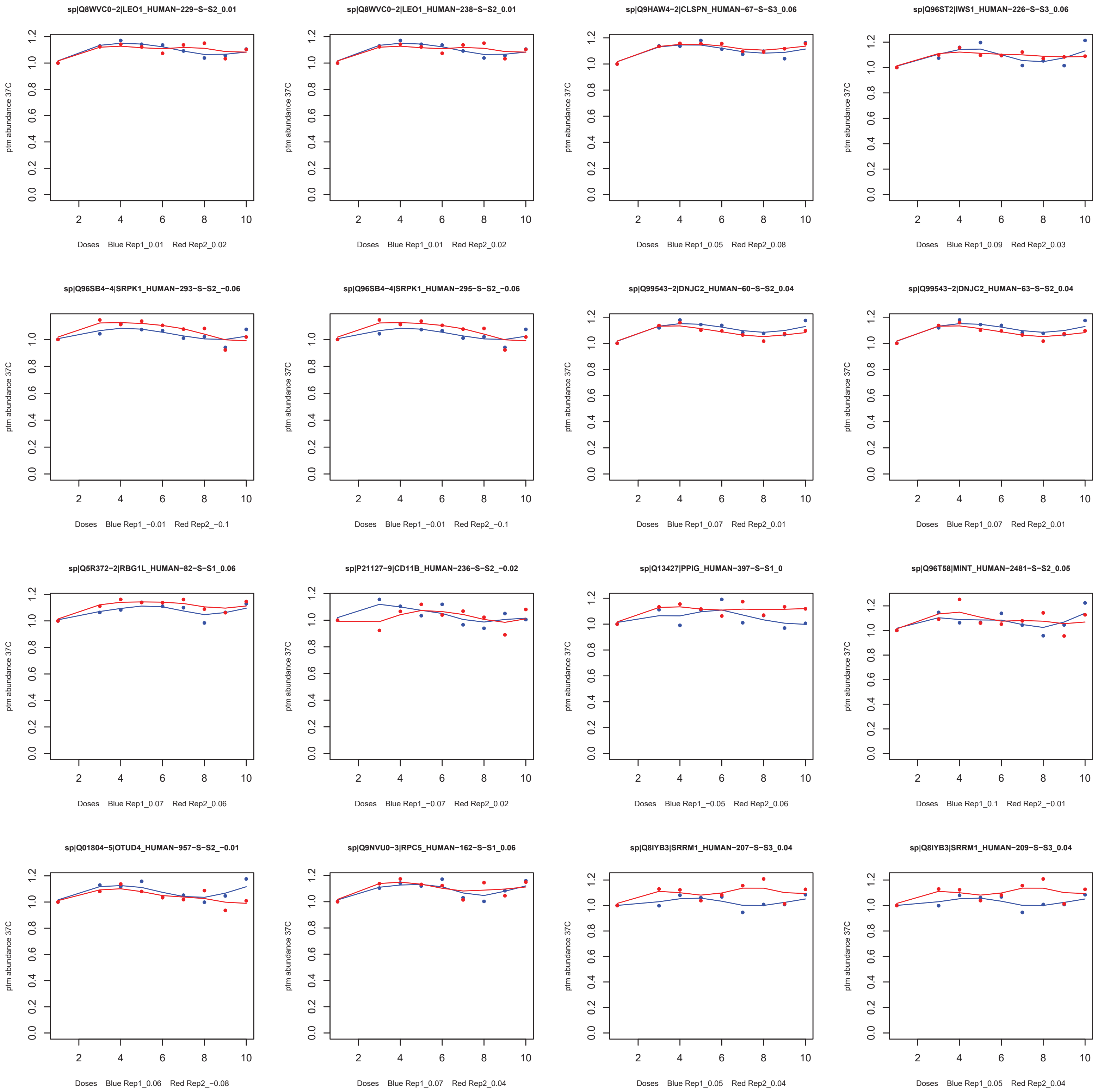
sp|Q8WVC0−2|LEO1_HUMAN−229−S−S2_0.01
Doses Blue Rep1_0.01 Red Rep2_0.02
sp|Q8WVC0−2|LEO1_HUMAN−238−S−S2_0.01
Doses Blue Rep1_0.01 Red Rep2_0.02
sp|Q9HAW4−2|CLSPN_HUMAN−67−S−S3_0.06
Doses Blue Rep1_0.05 Red Rep2_0.08
sp|Q96ST2|IWS1_HUMAN−226−S−S3_0.06
Doses Blue Rep1_0.09 Red Rep2_0.03
sp|Q96SB4−4|SRPK1_HUMAN−293−S−S2_−0.06
Doses Blue Rep1_−0.01 Red Rep2_−0.1
sp|Q96SB4−4|SRPK1_HUMAN−295−S−S2_−0.06
Doses Blue Rep1_−0.01 Red Rep2_−0.1
sp|Q99543−2|DNJC2_HUMAN−60−S−S2_0.04
Doses Blue Rep1_0.07 Red Rep2_0.01
sp|Q99543−2|DNJC2_HUMAN−63−S−S2_0.04
Doses Blue Rep1_0.07 Red Rep2_0.01
sp|Q5R372−2|RBG1L_HUMAN−82−S−S1_0.06
Doses Blue Rep1_0.07 Red Rep2_0.06
sp|P21127−9|CD11B_HUMAN−236−S−S2_−0.02
Doses Blue Rep1_−0.07 Red Rep2_0.02
sp|Q13427|PPIG_HUMAN−397−S−S1_0
Doses Blue Rep1_−0.05 Red Rep2_0.06
sp|Q96T58|MINT_HUMAN−2481−S−S2_0.05
Doses Blue Rep1_0.1 Red Rep2_−0.01
sp|Q01804−5|OTUD4_HUMAN−957−S−S2_−0.01
Doses Blue Rep1_0.06 Red Rep2_−0.08
sp|Q9NVU0−3|RPC5_HUMAN−162−S−S1_0.06
Doses Blue Rep1_0.07 Red Rep2_0.04
sp|Q8IYB3|SRRM1_HUMAN−207−S−S3_0.04
Doses Blue Rep1_0.05 Red Rep2_0.04
sp|Q8IYB3|SRRM1_HUMAN−209−S−S3_0.04
Doses Blue Rep1_0.05 Red Rep2_0.04
ptm abundance 37C

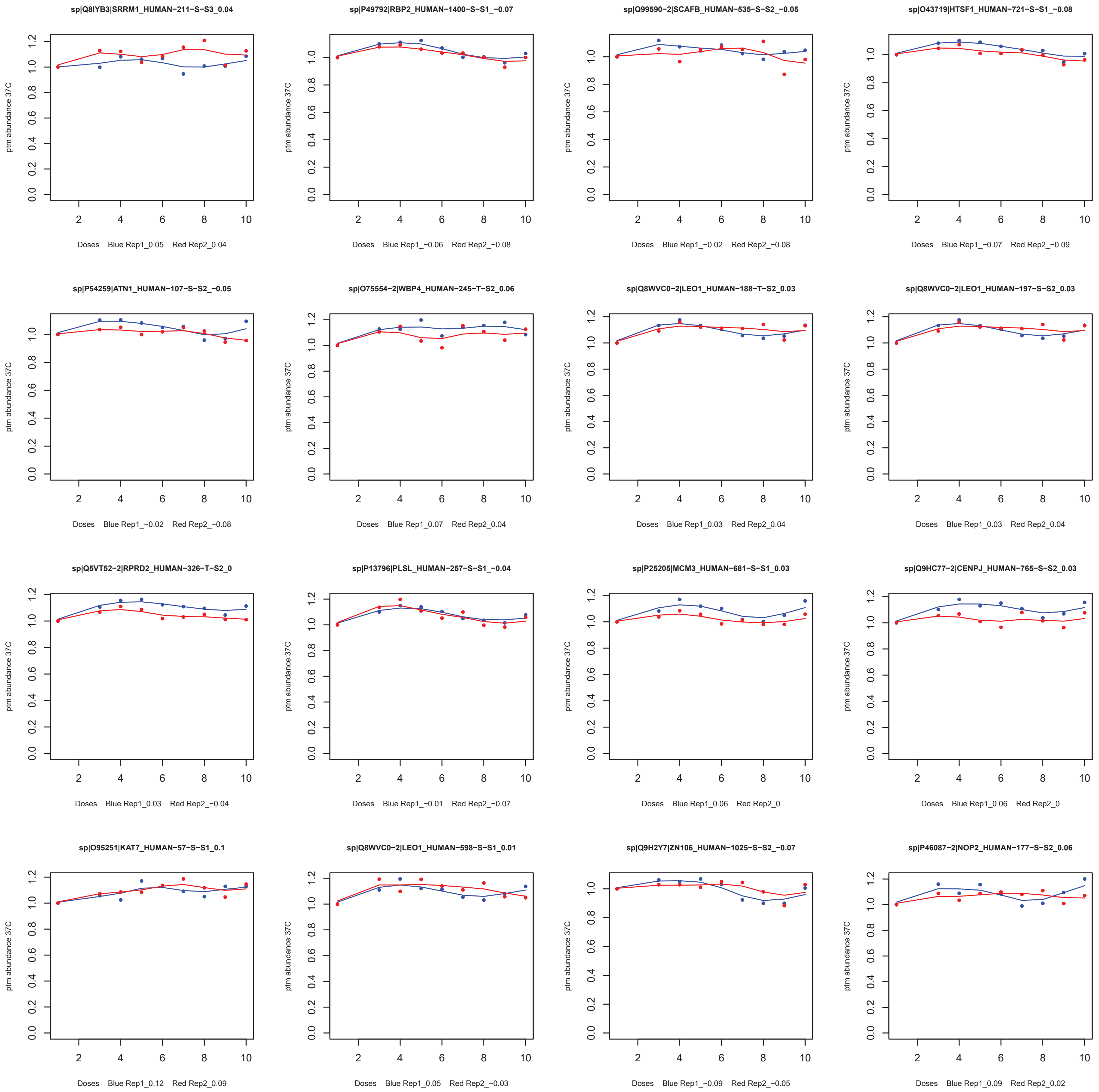
sp|Q8IYB3|SRRM1_HUMAN−211−S−S3_0.04
ptm abundance 37C
Doses Blue Rep1_0.05 Red Rep2_0.04
sp|P49792|RBP2_HUMAN−1400−S−S1_−0.07
ptm abundance 37C
Doses Blue Rep1_−0.06 Red Rep2_−0.08
sp|Q99590−2|SCAFB_HUMAN−535−S−S2_−0.05
ptm abundance 37C
Doses Blue Rep1_−0.02 Red Rep2_−0.08
sp|O43719|HTSF1_HUMAN−721−S−S1_−0.08
ptm abundance 37C
Doses Blue Rep1_−0.07 Red Rep2_−0.09
sp|P54259|ATN1_HUMAN−107−S−S2_−0.05
ptm abundance 37C
Doses Blue Rep1_−0.02 Red Rep2_−0.08
sp|O75554−2|WBP4_HUMAN−245−T−S2_0.06
ptm abundance 37C
Doses Blue Rep1_0.07 Red Rep2_0.04
sp|Q8WVC0−2|LEO1_HUMAN−188−T−S2_0.03
ptm abundance 37C
Doses Blue Rep1_0.03 Red Rep2_0.04
sp|Q8WVC0−2|LEO1_HUMAN−197−S−S2_0.03
ptm abundance 37C
Doses Blue Rep1_0.03 Red Rep2_0.04
sp|Q5VT52−2|RPRD2_HUMAN−326−T−S2_0
ptm abundance 37C
Doses Blue Rep1_0.03 Red Rep2_−0.04
sp|P13796|PLSL_HUMAN−257−S−S1_−0.04
ptm abundance 37C
Doses Blue Rep1_−0.01 Red Rep2_−0.07
sp|P25205|MCM3_HUMAN−681−S−S1_0.03
ptm abundance 37C
Doses Blue Rep1_0.06 Red Rep2_0
sp|Q9HC77−2|CENPJ_HUMAN−765−S−S2_0.03
ptm abundance 37C
Doses Blue Rep1_0.06 Red Rep2_0
sp|O95251|KAT7_HUMAN−57−S−S1_0.1
ptm abundance 37C
Doses Blue Rep1_0.12 Red Rep2_0.09
sp|Q8WVC0−2|LEO1_HUMAN−598−S−S1_0.01
ptm abundance 37C
Doses Blue Rep1_0.05 Red Rep2_−0.03
sp|Q9H2Y7|ZN106_HUMAN−1025−S−S2_−0.07
ptm abundance 37C
Doses Blue Rep1_−0.09 Red Rep2_−0.05
sp|P46087−2|NOP2_HUMAN−177−S−S2_0.06
ptm abundance 37C
Doses Blue Rep1_0.09 Red Rep2_0.02

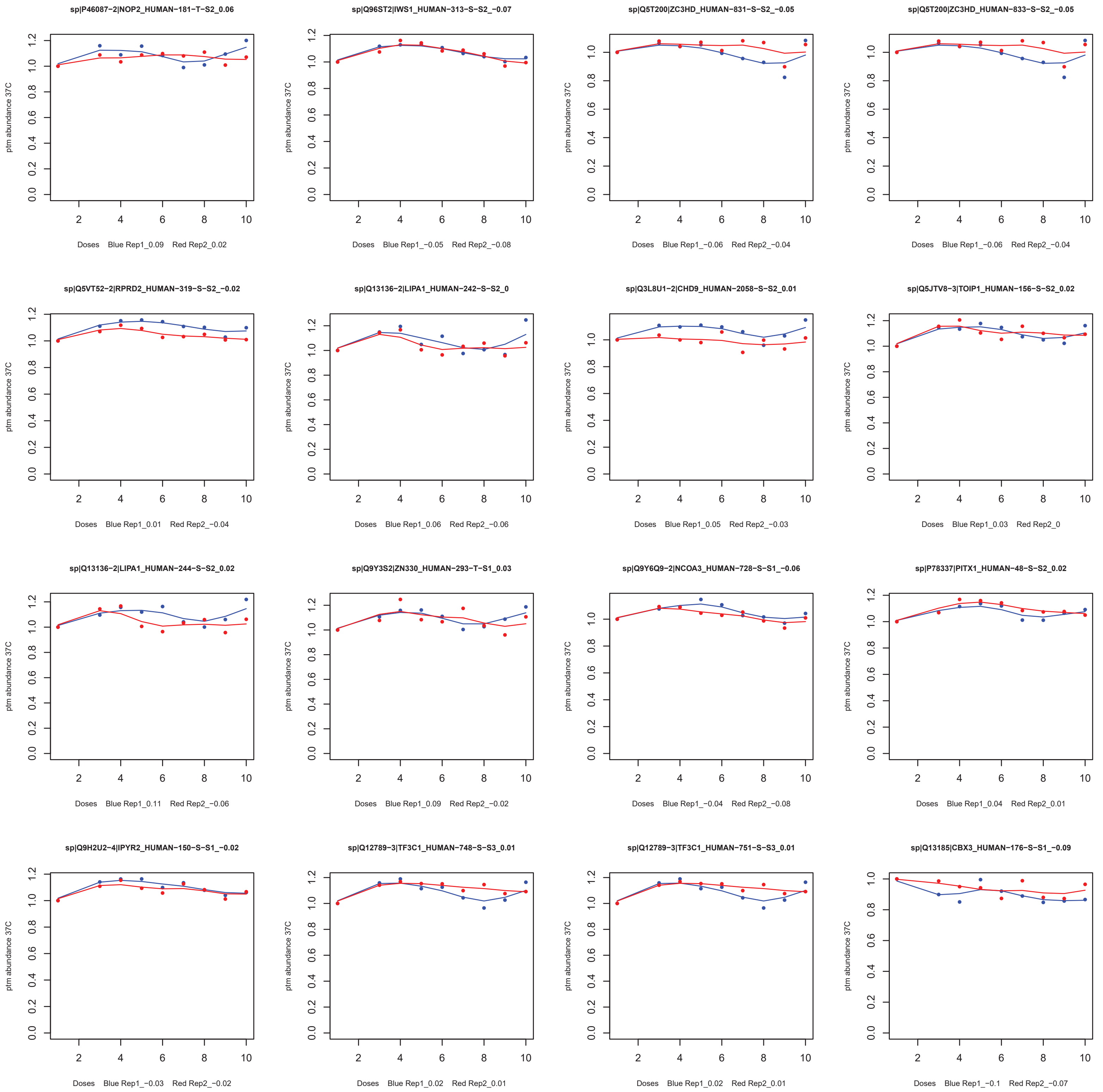
sp|P46087−2|NOP2_HUMAN−181−T−S2_0.06
Doses Blue Rep1_0.09 Red Rep2_0.02
sp|Q96ST2|IWS1_HUMAN−313−S−S2_−0.07
Doses Blue Rep1_−0.05 Red Rep2_−0.08
sp|Q5T200|ZC3HD_HUMAN−831−S−S2_−0.05
Doses Blue Rep1_−0.06 Red Rep2_−0.04
sp|Q5T200|ZC3HD_HUMAN−833−S−S2_−0.05
Doses Blue Rep1_−0.06 Red Rep2_−0.04
sp|Q5VT52−2|RPRD2_HUMAN−319−S−S2_−0.02
Doses Blue Rep1_0.01 Red Rep2_−0.04
sp|Q13136−2|LIPA1_HUMAN−242−S−S2_0
Doses Blue Rep1_0.06 Red Rep2_−0.06
sp|Q3L8U1−2|CHD9_HUMAN−2058−S−S2_0.01
Doses Blue Rep1_0.05 Red Rep2_−0.03
sp|Q5JTV8−3|TOIP1_HUMAN−156−S−S2_0.02
Doses Blue Rep1_0.03 Red Rep2_0
sp|Q13136−2|LIPA1_HUMAN−244−S−S2_0.02
Doses Blue Rep1_0.11 Red Rep2_−0.06
sp|Q9Y3S2|ZN330_HUMAN−293−T−S1_0.03
Doses Blue Rep1_0.09 Red Rep2_−0.02
sp|Q9Y6Q9−2|NCOA3_HUMAN−728−S−S1_−0.06
Doses Blue Rep1_−0.04 Red Rep2_−0.08
sp|P78337|PITX1_HUMAN−48−S−S2_0.02
Doses Blue Rep1_0.04 Red Rep2_0.01
sp|Q9H2U2−4|IPYR2_HUMAN−150−S−S1_−0.02
Doses Blue Rep1_−0.03 Red Rep2_−0.02
sp|Q12789−3|TF3C1_HUMAN−748−S−S3_0.01
Doses Blue Rep1_0.02 Red Rep2_0.01
sp|Q12789−3|TF3C1_HUMAN−751−S−S3_0.01
Doses Blue Rep1_0.02 Red Rep2_0.01
sp|Q13185|CBX3_HUMAN−176−S−S1_−0.09
Doses Blue Rep1_−0.1 Red Rep2_−0.07
ptm abundance 37C

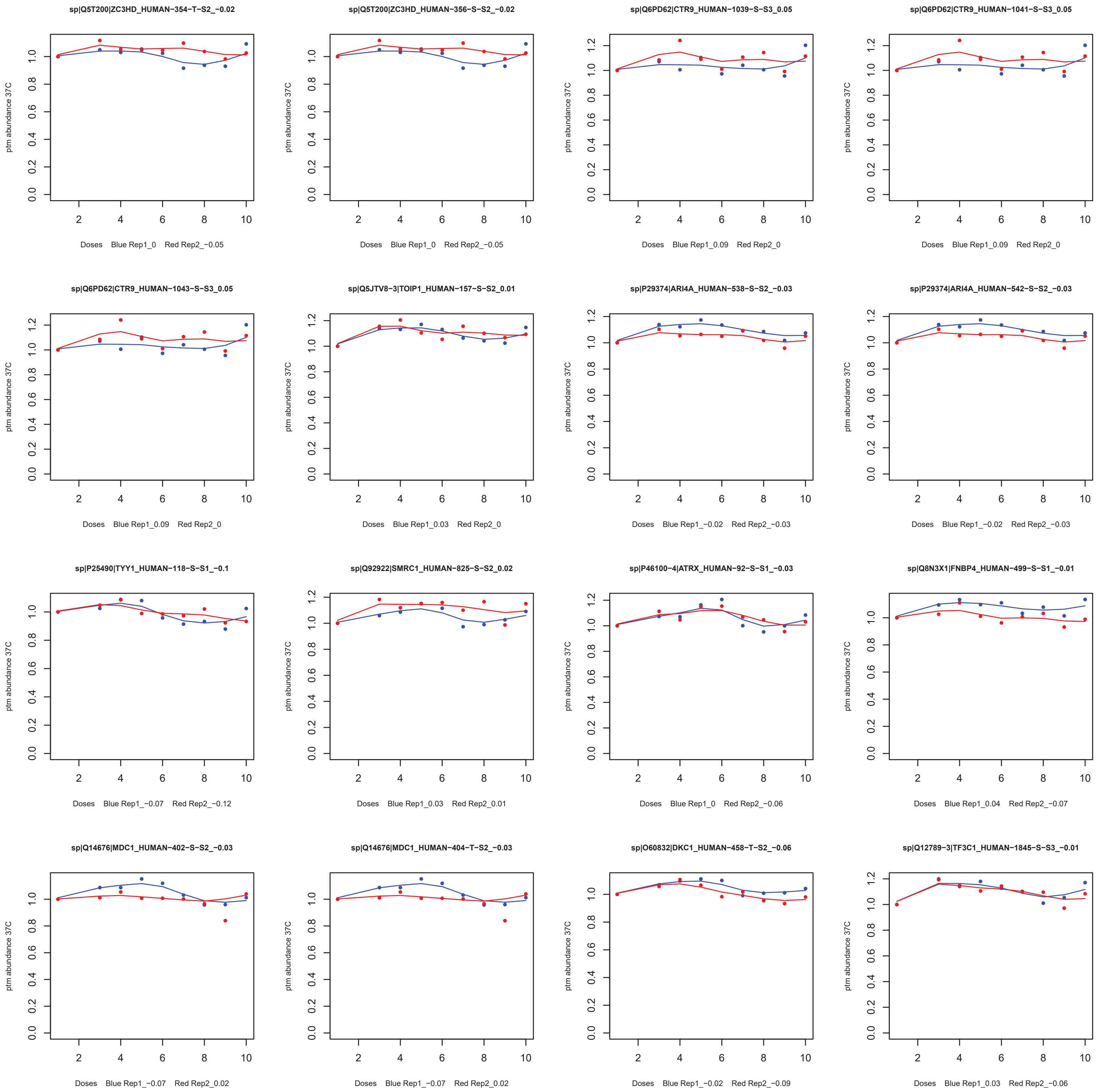

sp|Q5T200|ZC3HD_HUMAN−354−T−S2_−0.02
Doses Blue Rep1_0 Red Rep2_−0.05
sp|Q5T200|ZC3HD_HUMAN−356−S−S2_−0.02
Doses Blue Rep1_0 Red Rep2_−0.05
sp|Q6PD62|CTR9_HUMAN−1039−S−S3_0.05
Doses Blue Rep1_0.09 Red Rep2_0
sp|Q6PD62|CTR9_HUMAN−1041−S−S3_0.05
Doses Blue Rep1_0.09 Red Rep2_0
sp|Q6PD62|CTR9_HUMAN−1043−S−S3_0.05
Doses Blue Rep1_0.09 Red Rep2_0
sp|Q5JTV8−3|TOIP1_HUMAN−157−S−S2_0.01
Doses Blue Rep1_0.03 Red Rep2_0
sp|P29374|ARI4A_HUMAN−538−S−S2_−0.03
Doses Blue Rep1_−0.02 Red Rep2_−0.03
sp|P29374|ARI4A_HUMAN−542−S−S2_−0.03
Doses Blue Rep1_−0.02 Red Rep2_−0.03
sp|P25490|TYY1_HUMAN−118−S−S1_−0.1
Doses Blue Rep1_−0.07 Red Rep2_−0.12
sp|Q92922|SMRC1_HUMAN−825−S−S2_0.02
Doses Blue Rep1_0.03 Red Rep2_0.01
sp|P46100−4|ATRX_HUMAN−92−S−S1_−0.03
Doses Blue Rep1_0 Red Rep2_−0.06
sp|Q8N3X1|FNBP4_HUMAN−499−S−S1_−0.01
Doses Blue Rep1_0.04 Red Rep2_−0.07
sp|Q14676|MDC1_HUMAN−402−S−S2_−0.03
Doses Blue Rep1_−0.07 Red Rep2_0.02
sp|Q14676|MDC1_HUMAN−404−T−S2_−0.03
Doses Blue Rep1_−0.07 Red Rep2_0.02
sp|O60832|DKC1_HUMAN−458−T−S2_−0.06
Doses Blue Rep1_−0.02 Red Rep2_−0.09
sp|Q12789−3|TF3C1_HUMAN−1845−S−S3_−0.01
Doses Blue Rep1_0.03 Red Rep2_−0.06
ptm abundance 37C

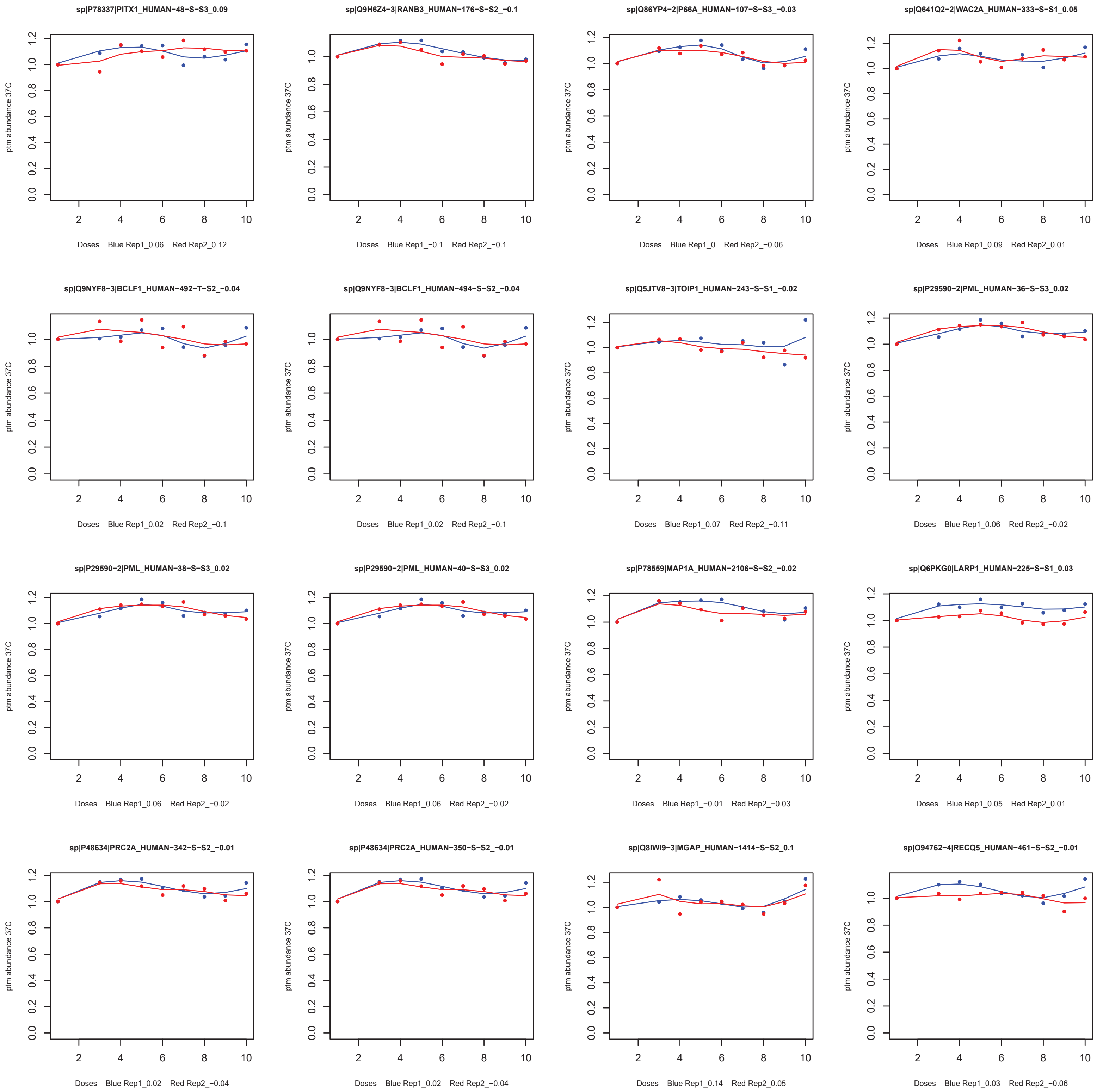
sp|P78337|PITX1_HUMAN−48−S−S3_0.09
Doses Blue Rep1_0.06 Red Rep2_0.12
sp|Q9H6Z4−3|RANB3_HUMAN−176−S−S2_−0.1
Doses Blue Rep1_−0.1 Red Rep2_−0.1
sp|Q86YP4−2|P66A_HUMAN−107−S−S3_−0.03
Doses Blue Rep1_0 Red Rep2_−0.06
sp|Q641Q2−2|WAC2A_HUMAN−333−S−S1_0.05
Doses Blue Rep1_0.09 Red Rep2_0.01
sp|Q9NYF8−3|BCLF1_HUMAN−492−T−S2_−0.04
Doses Blue Rep1_0.02 Red Rep2_−0.1
sp|Q9NYF8−3|BCLF1_HUMAN−494−S−S2_−0.04
Doses Blue Rep1_0.02 Red Rep2_−0.1
sp|Q5JTV8−3|TOIP1_HUMAN−243−S−S1_−0.02
Doses Blue Rep1_0.07 Red Rep2_−0.11
sp|P29590−2|PML_HUMAN−36−S−S3_0.02
Doses Blue Rep1_0.06 Red Rep2_−0.02
sp|P29590−2|PML_HUMAN−38−S−S3_0.02
Doses Blue Rep1_0.06 Red Rep2_−0.02
sp|P29590−2|PML_HUMAN−40−S−S3_0.02
Doses Blue Rep1_0.06 Red Rep2_−0.02
sp|P78559|MAP1A_HUMAN−2106−S−S2_−0.02
Doses Blue Rep1_−0.01 Red Rep2_−0.03
sp|Q6PKG0|LARP1_HUMAN−225−S−S1_0.03
Doses Blue Rep1_0.05 Red Rep2_0.01
sp|P48634|PRC2A_HUMAN−342−S−S2_−0.01
Doses Blue Rep1_0.02 Red Rep2_−0.04
sp|P48634|PRC2A_HUMAN−350−S−S2_−0.01
Doses Blue Rep1_0.02 Red Rep2_−0.04
sp|Q8IWI9−3|MGAP_HUMAN−1414−S−S2_0.1
Doses Blue Rep1_0.14 Red Rep2_0.05
sp|O94762−4|RECQ5_HUMAN−461−S−S2_−0.01
Doses Blue Rep1_0.03 Red Rep2_−0.06
ptm abundance 37C

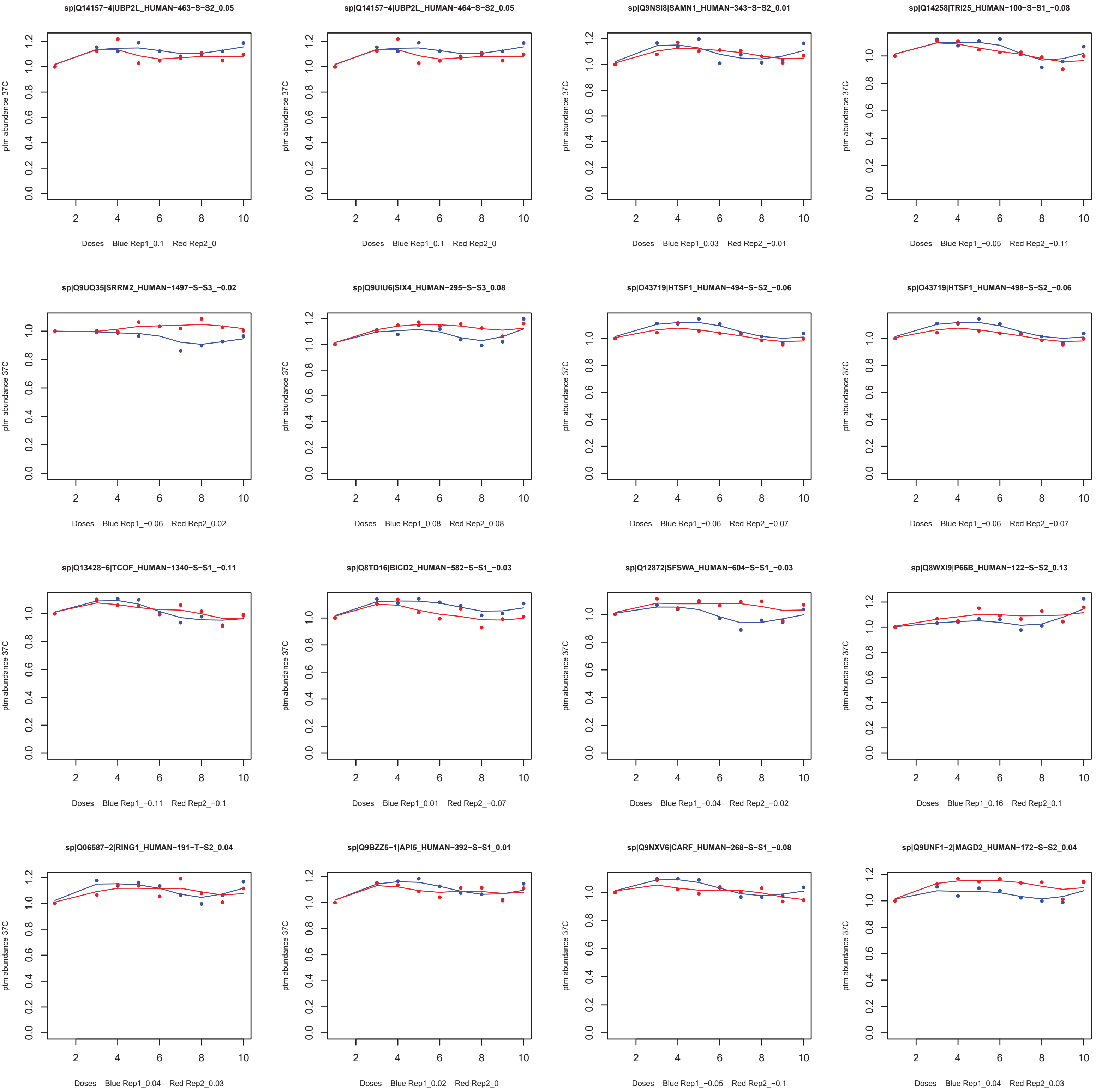
sp|Q14157−4|UBP2L_HUMAN−463−S−S2_0.05
ptm abundance 37C
Doses Blue Rep1_0.1 Red Rep2_0
sp|Q14157−4|UBP2L_HUMAN−464−S−S2_0.05
ptm abundance 37C
Doses Blue Rep1_0.1 Red Rep2_0
sp|Q9NSI8|SAMN1_HUMAN−343−S−S2_0.01
ptm abundance 37C
Doses Blue Rep1_0.03 Red Rep2_−0.01
sp|Q14258|TRI25_HUMAN−100−S−S1_−0.08
ptm abundance 37C
Doses Blue Rep1_−0.05 Red Rep2_−0.11
sp|Q9UQ35|SRRM2_HUMAN−1497−S−S3_−0.02
ptm abundance 37C
Doses Blue Rep1_−0.06 Red Rep2_0.02
sp|Q9UIU6|SIX4_HUMAN−295−S−S3_0.08
ptm abundance 37C
Doses Blue Rep1_0.08 Red Rep2_0.08
sp|O43719|HTSF1_HUMAN−494−S−S2_−0.06
ptm abundance 37C
Doses Blue Rep1_−0.06 Red Rep2_−0.07
sp|O43719|HTSF1_HUMAN−498−S−S2_−0.06
ptm abundance 37C
Doses Blue Rep1_−0.06 Red Rep2_−0.07
sp|Q13428−6|TCOF_HUMAN−1340−S−S1_−0.11
ptm abundance 37C
Doses Blue Rep1_−0.11 Red Rep2_−0.1
sp|Q8TD16|BICD2_HUMAN−582−S−S1_−0.03
ptm abundance 37C
Doses Blue Rep1_0.01 Red Rep2_−0.07
sp|Q12872|SFSWA_HUMAN−604−S−S1_−0.03
ptm abundance 37C
Doses Blue Rep1_−0.04 Red Rep2_−0.02
sp|Q8WXI9|P66B_HUMAN−122−S−S2_0.13
ptm abundance 37C
Doses Blue Rep1_0.16 Red Rep2_0.1
sp|Q06587−2|RING1_HUMAN−191−T−S2_0.04
ptm abundance 37C
Doses Blue Rep1_0.04 Red Rep2_0.03
sp|Q9BZZ5−1|API5_HUMAN−392−S−S1_0.01
ptm abundance 37C
Doses Blue Rep1_0.02 Red Rep2_0
sp|Q9NXV6|CARF_HUMAN−268−S−S1_−0.08
ptm abundance 37C
Doses Blue Rep1_−0.05 Red Rep2_−0.1
sp|Q9UNF1−2|MAGD2_HUMAN−172−S−S2_0.04
ptm abundance 37C
Doses Blue Rep1_0.04 Red Rep2_0.03

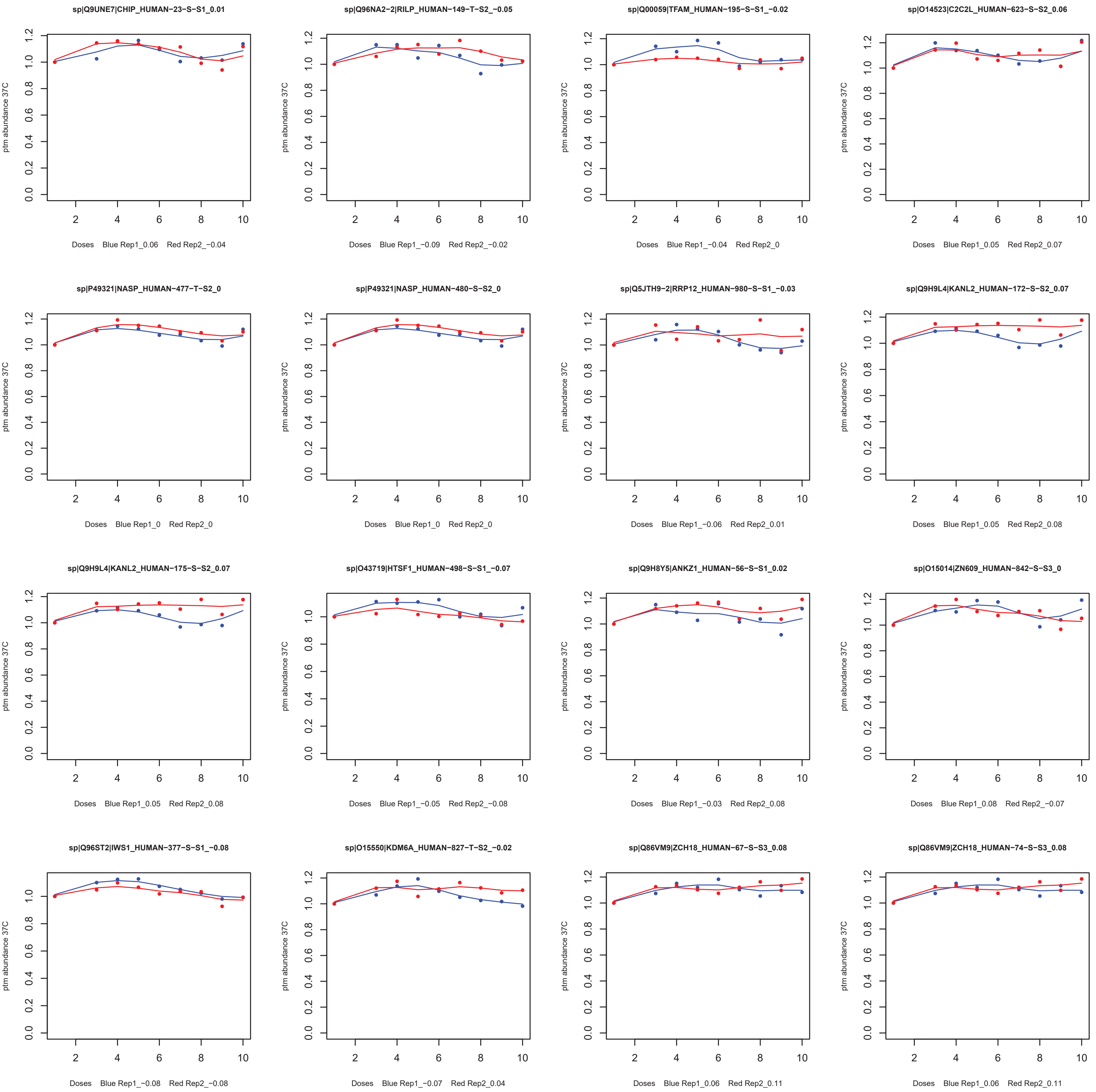
sp|Q9UNE7|CHIP_HUMAN−23−S−S1_0.01
Doses Blue Rep1_0.06 Red Rep2_−0.04
sp|Q96NA2−2|RILP_HUMAN−149−T−S2_−0.05
Doses Blue Rep1_−0.09 Red Rep2_−0.02
sp|Q00059|TFAM_HUMAN−195−S−S1_−0.02
Doses Blue Rep1_−0.04 Red Rep2_0
sp|O14523|C2C2L_HUMAN−623−S−S2_0.06
Doses Blue Rep1_0.05 Red Rep2_0.07
sp|P49321|NASP_HUMAN−477−T−S2_0
Doses Blue Rep1_0 Red Rep2_0
sp|P49321|NASP_HUMAN−480−S−S2_0
Doses Blue Rep1_0 Red Rep2_0
sp|Q5JTH9−2|RRP12_HUMAN−980−S−S1_−0.03
Doses Blue Rep1_−0.06 Red Rep2_0.01
sp|Q9H9L4|KANL2_HUMAN−172−S−S2_0.07
Doses Blue Rep1_0.05 Red Rep2_0.08
sp|Q9H9L4|KANL2_HUMAN−175−S−S2_0.07
Doses Blue Rep1_0.05 Red Rep2_0.08
sp|O43719|HTSF1_HUMAN−498−S−S1_−0.07
Doses Blue Rep1_−0.05 Red Rep2_−0.08
sp|Q9H8Y5|ANKZ1_HUMAN−56−S−S1_0.02
Doses Blue Rep1_−0.03 Red Rep2_0.08
sp|O15014|ZN609_HUMAN−842−S−S3_0
Doses Blue Rep1_0.08 Red Rep2_−0.07
sp|Q96ST2|IWS1_HUMAN−377−S−S1_−0.08
Doses Blue Rep1_−0.08 Red Rep2_−0.08
sp|O15550|KDM6A_HUMAN−827−T−S2_−0.02
Doses Blue Rep1_−0.07 Red Rep2_0.04
sp|Q86VM9|ZCH18_HUMAN−67−S−S3_0.08
Doses Blue Rep1_0.06 Red Rep2_0.11
sp|Q86VM9|ZCH18_HUMAN−74−S−S3_0.08
Doses Blue Rep1_0.06 Red Rep2_0.11
ptm abundance 37C

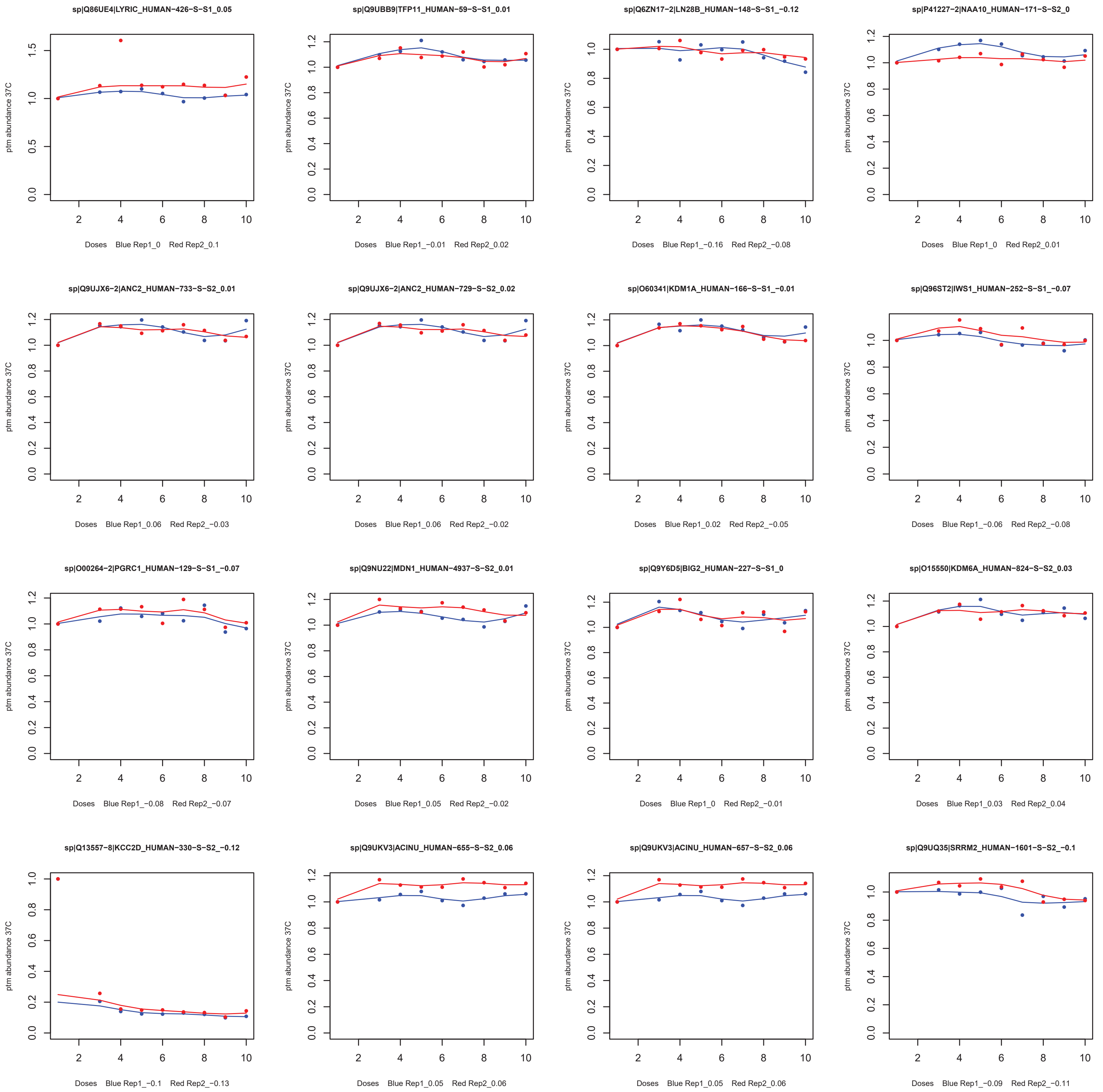

sp|Q86UE4|LYRIC_HUMAN−426−S−S1_0.05
Doses Blue Rep1_0 Red Rep2_0.1
sp|Q9UBB9|TFP11_HUMAN−59−S−S1_0.01
Doses Blue Rep1_−0.01 Red Rep2_0.02
sp|Q6ZN17−2|LN28B_HUMAN−148−S−S1_−0.12
Doses Blue Rep1_−0.16 Red Rep2_−0.08
sp|P41227−2|NAA10_HUMAN−171−S−S2_0
Doses Blue Rep1_0 Red Rep2_0.01
sp|Q9UJX6−2|ANC2_HUMAN−733−S−S2_0.01
Doses Blue Rep1_0.06 Red Rep2_−0.03
sp|Q9UJX6−2|ANC2_HUMAN−729−S−S2_0.02
Doses Blue Rep1_0.06 Red Rep2_−0.02
sp|O60341|KDM1A_HUMAN−166−S−S1_−0.01
Doses Blue Rep1_0.02 Red Rep2_−0.05
sp|Q96ST2|IWS1_HUMAN−252−S−S1_−0.07
Doses Blue Rep1_−0.06 Red Rep2_−0.08
sp|O00264−2|PGRC1_HUMAN−129−S−S1_−0.07
Doses Blue Rep1_−0.08 Red Rep2_−0.07
sp|Q9NU22|MDN1_HUMAN−4937−S−S2_0.01
Doses Blue Rep1_0.05 Red Rep2_−0.02
sp|Q9Y6D5|BIG2_HUMAN−227−S−S1_0
Doses Blue Rep1_0 Red Rep2_−0.01
sp|O15550|KDM6A_HUMAN−824−S−S2_0.03
Doses Blue Rep1_0.03 Red Rep2_0.04
sp|Q13557−8|KCC2D_HUMAN−330−S−S2_−0.12
Doses Blue Rep1_−0.1 Red Rep2_−0.13
sp|Q9UKV3|ACINU_HUMAN−655−S−S2_0.06
Doses Blue Rep1_0.05 Red Rep2_0.06
sp|Q9UKV3|ACINU_HUMAN−657−S−S2_0.06
Doses Blue Rep1_0.05 Red Rep2_0.06
sp|Q9UQ35|SRRM2_HUMAN−1601−S−S2_−0.1
Doses Blue Rep1_−0.09 Red Rep2_−0.11
ptm abundance 37C

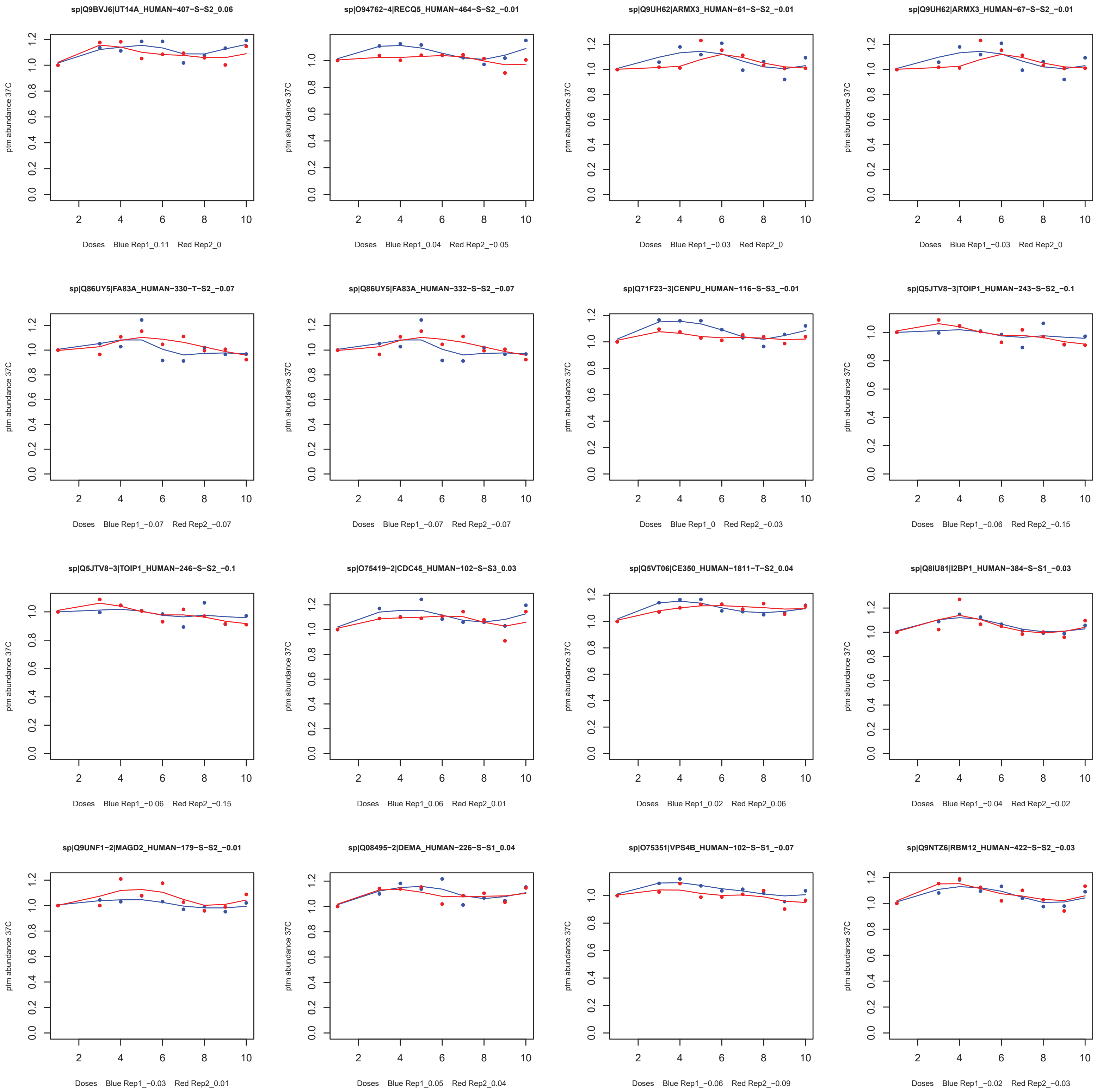
sp|Q9BVJ6|UT14A_HUMAN−407−S−S2_0.06
ptm abundance 37C
Doses Blue Rep1_0.11 Red Rep2_0
sp|O94762−4|RECQ5_HUMAN−464−S−S2_−0.01
ptm abundance 37C
Doses Blue Rep1_0.04 Red Rep2_−0.05
sp|Q9UH62|ARMX3_HUMAN−61−S−S2_−0.01
ptm abundance 37C
Doses Blue Rep1_−0.03 Red Rep2_0
sp|Q9UH62|ARMX3_HUMAN−67−S−S2_−0.01
ptm abundance 37C
Doses Blue Rep1_−0.03 Red Rep2_0
sp|Q86UY5|FA83A_HUMAN−330−T−S2_−0.07
ptm abundance 37C
Doses Blue Rep1_−0.07 Red Rep2_−0.07
sp|Q86UY5|FA83A_HUMAN−332−S−S2_−0.07
ptm abundance 37C
Doses Blue Rep1_−0.07 Red Rep2_−0.07
sp|Q71F23−3|CENPU_HUMAN−116−S−S3_−0.01
ptm abundance 37C
Doses Blue Rep1_0 Red Rep2_−0.03
sp|Q5JTV8−3|TOIP1_HUMAN−243−S−S2_−0.1
ptm abundance 37C
Doses Blue Rep1_−0.06 Red Rep2_−0.15
sp|Q5JTV8−3|TOIP1_HUMAN−246−S−S2_−0.1
ptm abundance 37C
Doses Blue Rep1_−0.06 Red Rep2_−0.15
sp|O75419−2|CDC45_HUMAN−102−S−S3_0.03
ptm abundance 37C
Doses Blue Rep1_0.06 Red Rep2_0.01
sp|Q5VT06|CE350_HUMAN−1811−T−S2_0.04
ptm abundance 37C
Doses Blue Rep1_0.02 Red Rep2_0.06
sp|Q8IU81|I2BP1_HUMAN−384−S−S1_−0.03
ptm abundance 37C
Doses Blue Rep1_−0.04 Red Rep2_−0.02
sp|Q9UNF1−2|MAGD2_HUMAN−179−S−S2_−0.01
ptm abundance 37C
Doses Blue Rep1_−0.03 Red Rep2_0.01
sp|Q08495−2|DEMA_HUMAN−226−S−S1_0.04
ptm abundance 37C
Doses Blue Rep1_0.05 Red Rep2_0.04
sp|O75351|VPS4B_HUMAN−102−S−S1_−0.07
ptm abundance 37C
Doses Blue Rep1_−0.06 Red Rep2_−0.09
sp|Q9NTZ6|RBM12_HUMAN−422−S−S2_−0.03
ptm abundance 37C
Doses Blue Rep1_−0.02 Red Rep2_−0.03

sp|O15164-2|TIF1A_HUMAN-991-S-S2_0.02

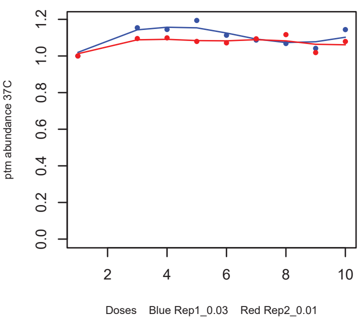

sp|O15164-2|TIF1A_HUMAN-994-S-S2_0.02

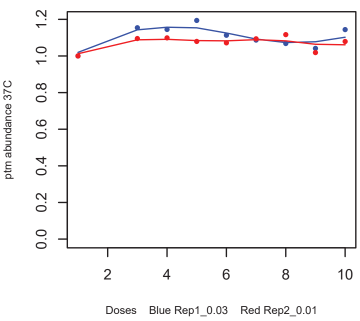

sp|Q92610|ZN592_HUMAN-356-S-S3_0.03

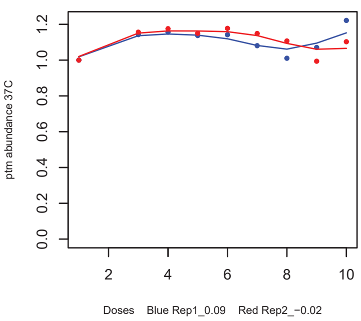

sp|Q92610|ZN592_HUMAN-357-S-S3_0.03

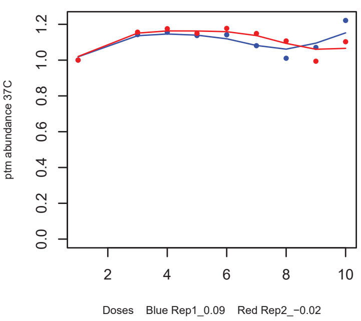

sp|O75153|CLU_HUMAN-664-S-S1_0

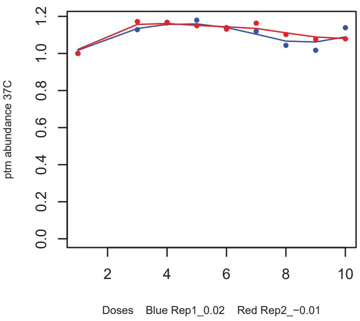

sp|Q9HC77-2|CENPJ_HUMAN-768-S-S2_0

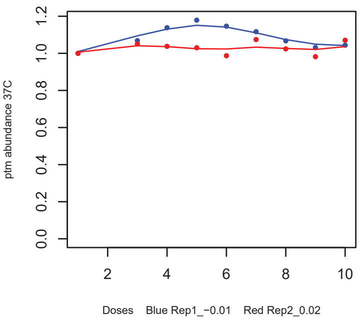

sp|Q9P2D1|CHD7_HUMAN-2251-S-S2_0.09

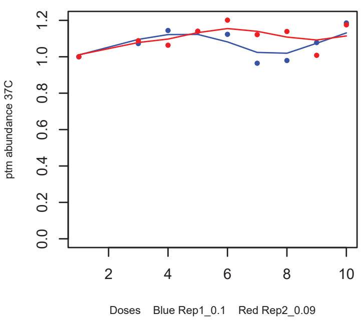

sp|Q9P2D1|CHD7_HUMAN-2254-S-S2_0.09

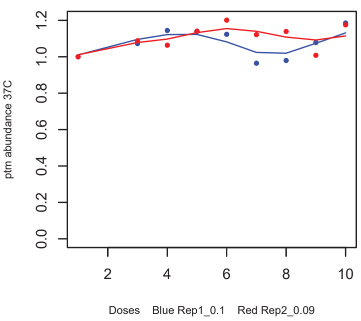

sp|Q8TAQ2-2|SMRC2_HUMAN-306-S-S2_0.01

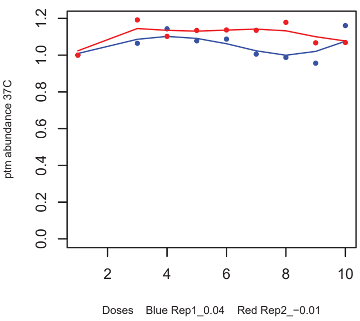

sp|Q99590-2|SCAFB_HUMAN-293-S-S1_-0.04

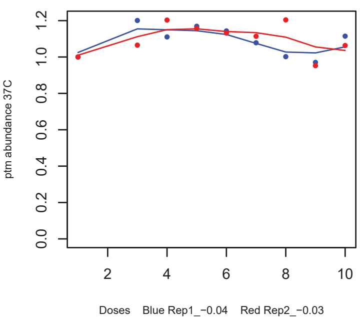

sp|Q01130-2|SRSF2_HUMAN-179-S-S2_-0.09

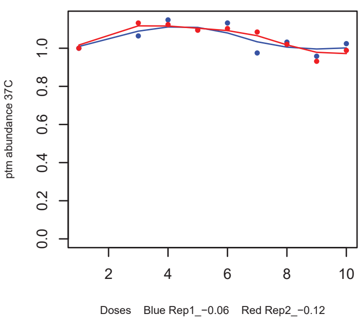

sp|Q68E01-2|INT3_HUMAN-536-S-S1_0.02

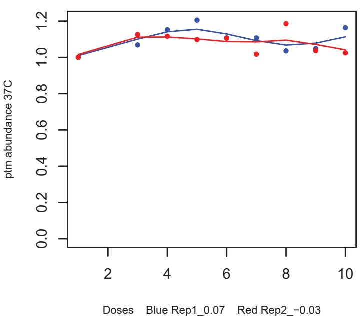

sp|Q92610|ZN592_HUMAN-358-S-S3_0.03

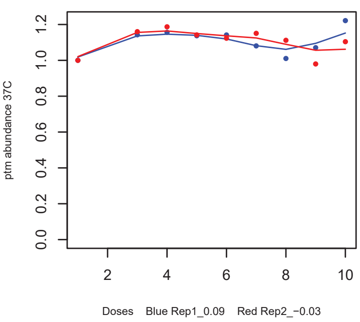

sp|Q9UQ84-4|EXO1_HUMAN-659-S-S2_0.11

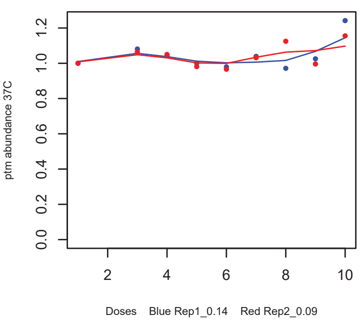

sp|Q96JM3|CHAP1_HUMAN-476-S-S1_-0.07

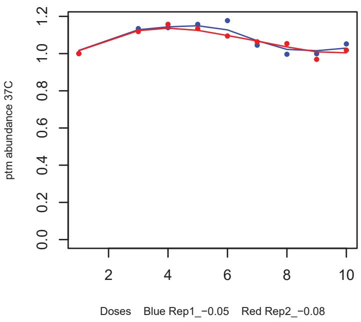

sp|P20929-4|NEBU_HUMAN-646-S-S1_0.01

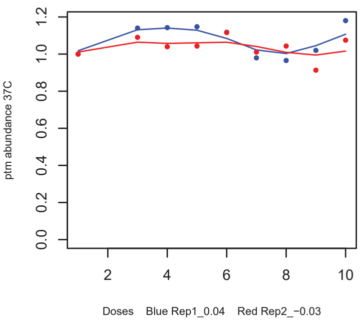

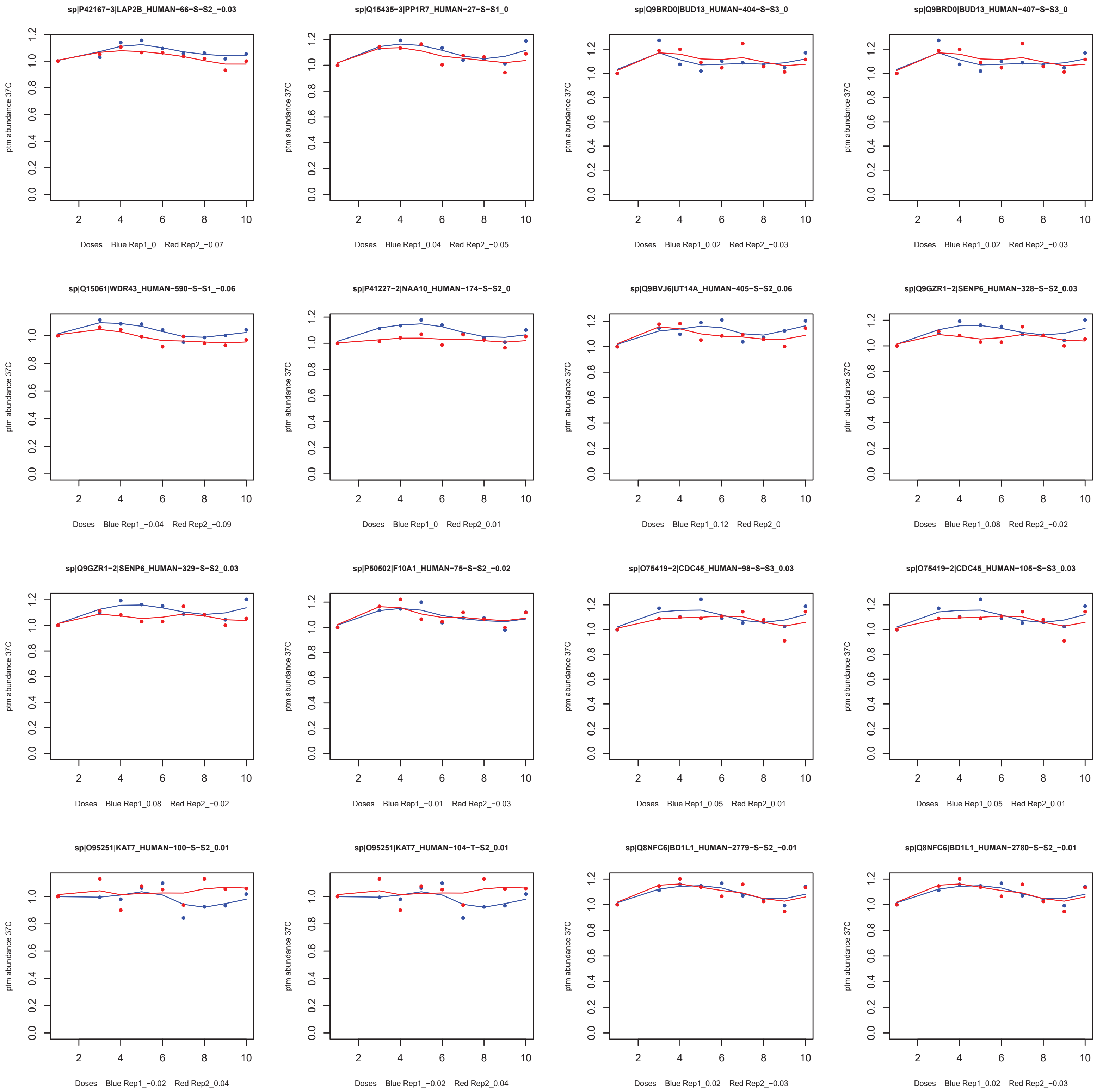
sp|P42167−3|LAP2B_HUMAN−66−S−S2_−0.03
Doses Blue Rep1_0 Red Rep2_−0.07
sp|Q15435−3|PP1R7_HUMAN−27−S−S1_0
Doses Blue Rep1_0.04 Red Rep2_−0.05
sp|Q9BRD0|BUD13_HUMAN−404−S−S3_0
Doses Blue Rep1_0.02 Red Rep2_−0.03
sp|Q9BRD0|BUD13_HUMAN−407−S−S3_0
Doses Blue Rep1_0.02 Red Rep2_−0.03
sp|Q15061|WDR43_HUMAN−590−S−S1_−0.06
Doses Blue Rep1_−0.04 Red Rep2_−0.09
sp|P41227−2|NAA10_HUMAN−174−S−S2_0
Doses Blue Rep1_0 Red Rep2_0.01
sp|Q9BVJ6|UT14A_HUMAN−405−S−S2_0.06
Doses Blue Rep1_0.12 Red Rep2_0
sp|Q9GZR1−2|SENP6_HUMAN−328−S−S2_0.03
Doses Blue Rep1_0.08 Red Rep2_−0.02
sp|Q9GZR1−2|SENP6_HUMAN−329−S−S2_0.03
Doses Blue Rep1_0.08 Red Rep2_−0.02
sp|P50502|F10A1_HUMAN−75−S−S2_−0.02
Doses Blue Rep1_−0.01 Red Rep2_−0.03
sp|O75419−2|CDC45_HUMAN−98−S−S3_0.03
Doses Blue Rep1_0.05 Red Rep2_0.01
sp|O75419−2|CDC45_HUMAN−105−S−S3_0.03
Doses Blue Rep1_0.05 Red Rep2_0.01
sp|O95251|KAT7_HUMAN−100−S−S2_0.01
Doses Blue Rep1_−0.02 Red Rep2_0.04
sp|O95251|KAT7_HUMAN−104−T−S2_0.01
Doses Blue Rep1_−0.02 Red Rep2_0.04
sp|Q8NFC6|BD1L1_HUMAN−2779−S−S2_−0.01
Doses Blue Rep1_0.02 Red Rep2_−0.03
sp|Q8NFC6|BD1L1_HUMAN−2780−S−S2_−0.01
Doses Blue Rep1_0.02 Red Rep2_−0.03
ptm abundance 37C

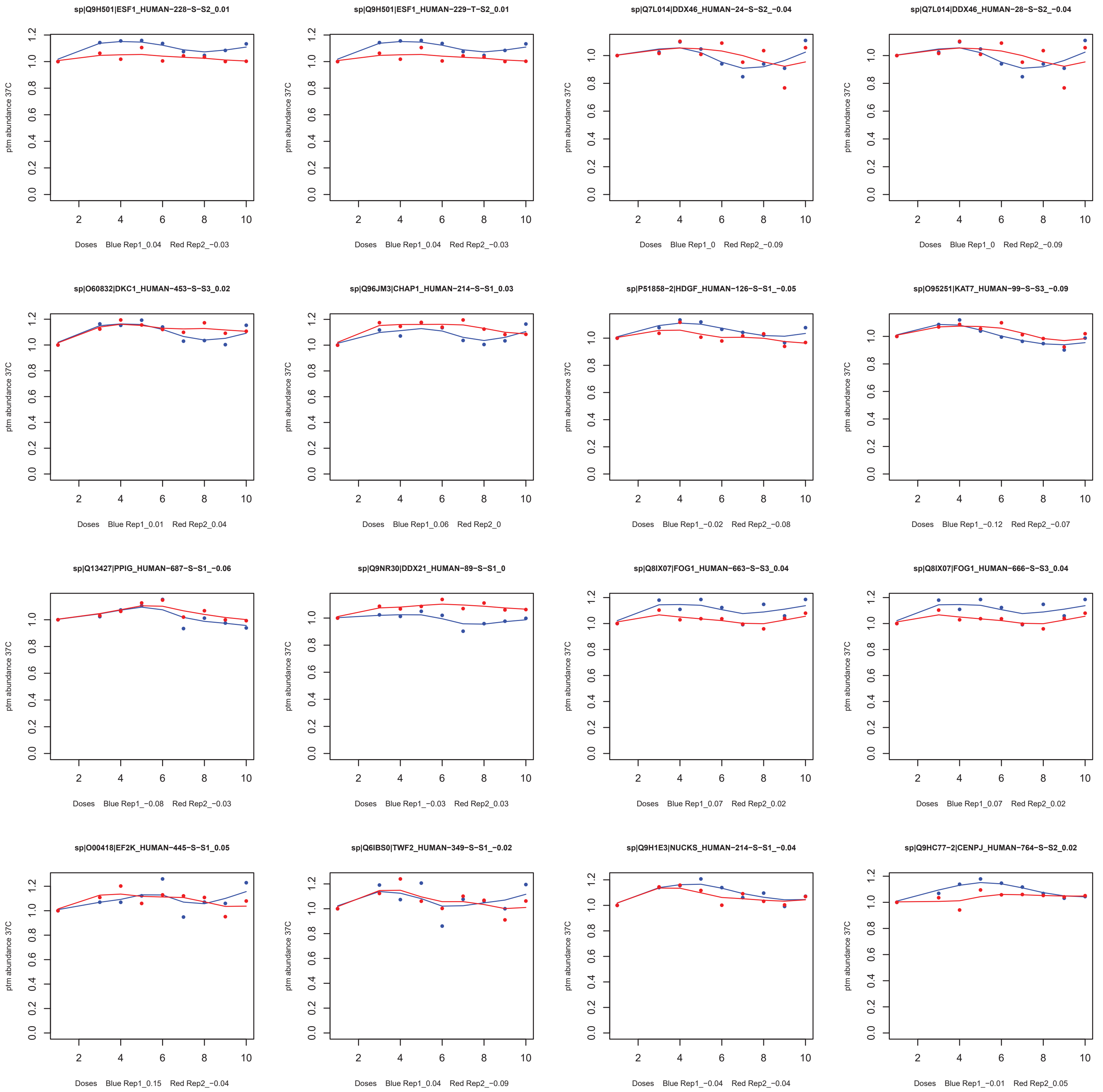

sp|Q9H501|ESF1_HUMAN−228−S−S2_0.01
Doses Blue Rep1_0.04 Red Rep2_−0.03
sp|Q9H501|ESF1_HUMAN−229−T−S2_0.01
Doses Blue Rep1_0.04 Red Rep2_−0.03
sp|Q7L014|DDX46_HUMAN−24−S−S2_−0.04
Doses Blue Rep1_0 Red Rep2_−0.09
sp|Q7L014|DDX46_HUMAN−28−S−S2_−0.04
Doses Blue Rep1_0 Red Rep2_−0.09
sp|O60832|DKC1_HUMAN−453−S−S3_0.02
Doses Blue Rep1_0.01 Red Rep2_0.04
sp|Q96JM3|CHAP1_HUMAN−214−S−S1_0.03
Doses Blue Rep1_0.06 Red Rep2_0
sp|P51858−2|HDGF_HUMAN−126−S−S1_−0.05
Doses Blue Rep1_−0.02 Red Rep2_−0.08
sp|O95251|KAT7_HUMAN−99−S−S3_−0.09
Doses Blue Rep1_−0.12 Red Rep2_−0.07
sp|Q13427|PPIG_HUMAN−687−S−S1_−0.06
Doses Blue Rep1_−0.08 Red Rep2_−0.03
sp|Q9NR30|DDX21_HUMAN−89−S−S1_0
Doses Blue Rep1_−0.03 Red Rep2_0.03
sp|Q8IX07|FOG1_HUMAN−663−S−S3_0.04
Doses Blue Rep1_0.07 Red Rep2_0.02
sp|Q8IX07|FOG1_HUMAN−666−S−S3_0.04
Doses Blue Rep1_0.07 Red Rep2_0.02
sp|O00418|EF2K_HUMAN−445−S−S1_0.05
Doses Blue Rep1_0.15 Red Rep2_−0.04
sp|Q6IBS0|TWF2_HUMAN−349−S−S1_−0.02
Doses Blue Rep1_0.04 Red Rep2_−0.09
sp|Q9H1E3|NUCKS_HUMAN−214−S−S1_−0.04
Doses Blue Rep1_−0.04 Red Rep2_−0.04
sp|Q9HC77−2|CENPJ_HUMAN−764−S−S2_0.02
Doses Blue Rep1_−0.01 Red Rep2_0.05
ptm abundance 37C

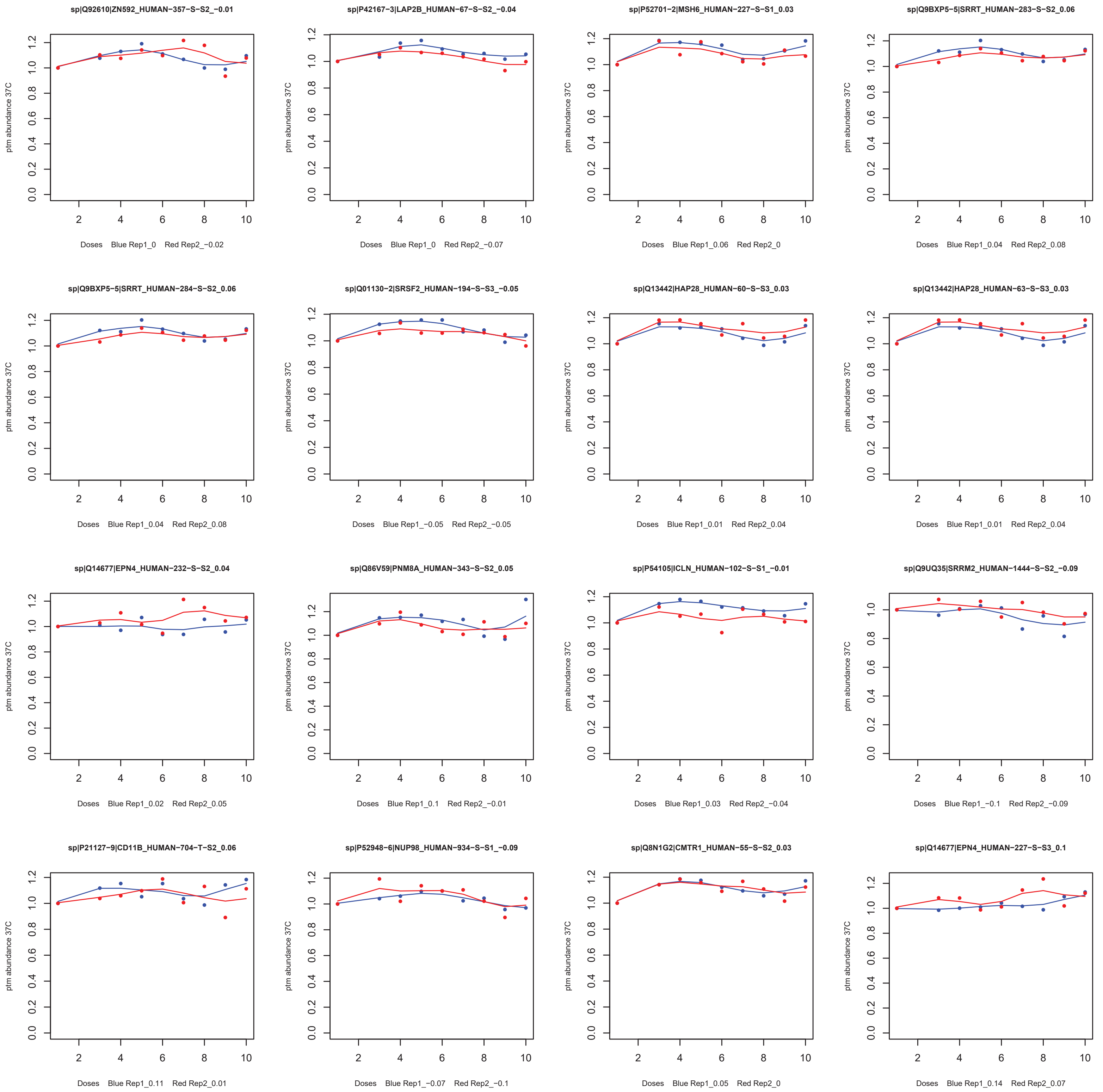
sp|Q92610|ZN592_HUMAN−357−S−S2_−0.01
Doses Blue Rep1_0 Red Rep2_−0.02
sp|P42167−3|LAP2B_HUMAN−67−S−S2_−0.04
Doses Blue Rep1_0 Red Rep2_−0.07
sp|P52701−2|MSH6_HUMAN−227−S−S1_0.03
Doses Blue Rep1_0.06 Red Rep2_0
sp|Q9BXP5−5|SRRT_HUMAN−283−S−S2_0.06
Doses Blue Rep1_0.04 Red Rep2_0.08
sp|Q9BXP5−5|SRRT_HUMAN−284−S−S2_0.06
Doses Blue Rep1_0.04 Red Rep2_0.08
sp|Q01130−2|SRSF2_HUMAN−194−S−S3_−0.05
Doses Blue Rep1_−0.05 Red Rep2_−0.05
sp|Q13442|HAP28_HUMAN−60−S−S3_0.03
Doses Blue Rep1_0.01 Red Rep2_0.04
sp|Q13442|HAP28_HUMAN−63−S−S3_0.03
Doses Blue Rep1_0.01 Red Rep2_0.04
sp|Q14677|EPN4_HUMAN−232−S−S2_0.04
Doses Blue Rep1_0.02 Red Rep2_0.05
sp|Q86V59|PNM8A_HUMAN−343−S−S2_0.05
Doses Blue Rep1_0.1 Red Rep2_−0.01
sp|P54105|ICLN_HUMAN−102−S−S1_−0.01
Doses Blue Rep1_0.03 Red Rep2_−0.04
sp|Q9UQ35|SRRM2_HUMAN−1444−S−S2_−0.09
Doses Blue Rep1_−0.1 Red Rep2_−0.09
sp|P21127−9|CD11B_HUMAN−704−T−S2_0.06
Doses Blue Rep1_0.11 Red Rep2_0.01
sp|P52948−6|NUP98_HUMAN−934−S−S1_−0.09
Doses Blue Rep1_−0.07 Red Rep2_−0.1
sp|Q8N1G2|CMTR1_HUMAN−55−S−S2_0.03
Doses Blue Rep1_0.05 Red Rep2_0
sp|Q14677|EPN4_HUMAN−227−S−S3_0.1
Doses Blue Rep1_0.14 Red Rep2_0.07
ptm abundance 37C

sp|Q14677|EPN4_HUMAN−232−S−S3_0.1

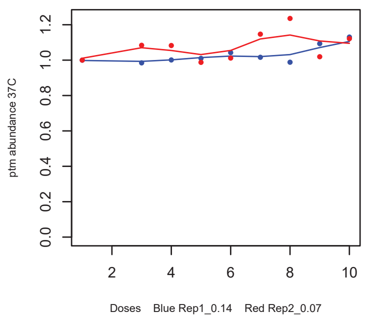

sp|Q14677|EPN4_HUMAN−234−S−S3_0.1

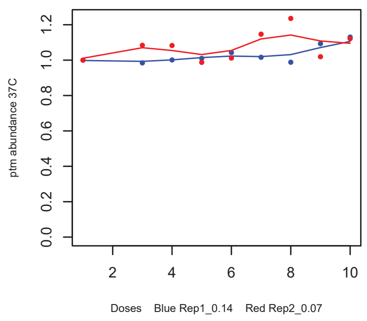

sp|B2RUZ4|SMIM1_HUMAN−28−S−S2_−0.03

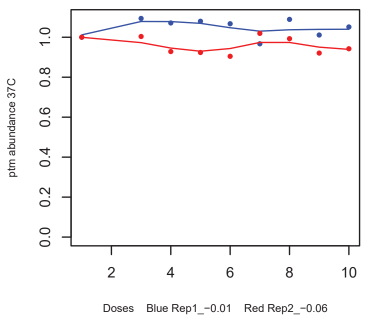

sp|Q9UQ35|SRRM2_HUMAN−1014−S−S1_−0.09

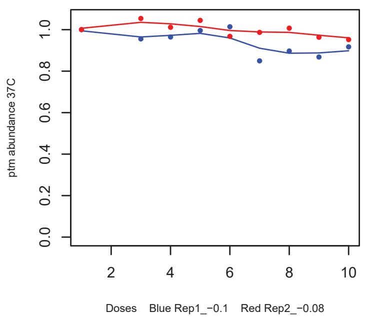

sp|Q9UJX5|APC4_HUMAN−757−S−S2_−0.01

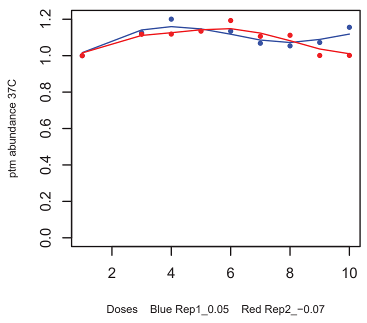

sp|Q9UJX5|APC4_HUMAN−758−S−S2_−0.01

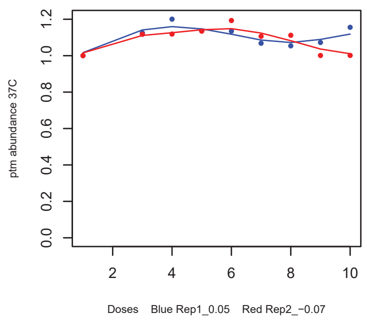

sp|Q9Y618−3|NCOR2_HUMAN−745−S−S3_0.07

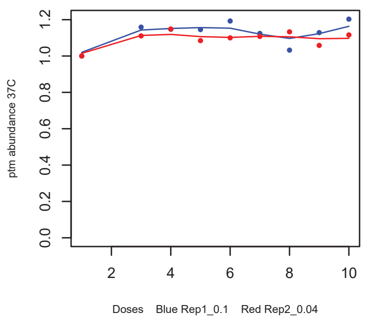

sp|Q9Y618−3|NCOR2_HUMAN−746−S−S3_0.07

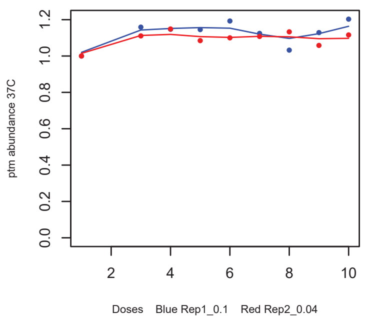

sp|Q9Y618−3|NCOR2_HUMAN−748−T−S3_0.07

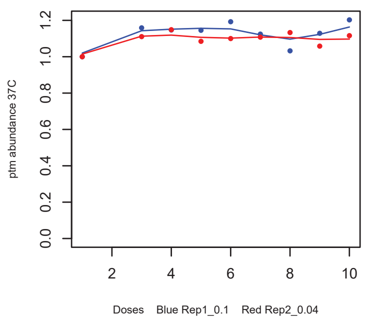

sp|Q9Y618−3|NCOR2_HUMAN−750−S−S3_0.07

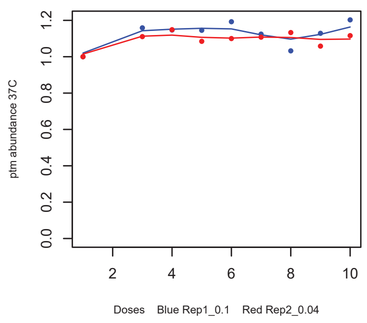

sp|O43719|HTSF1_HUMAN−676−S−S1_−0.01

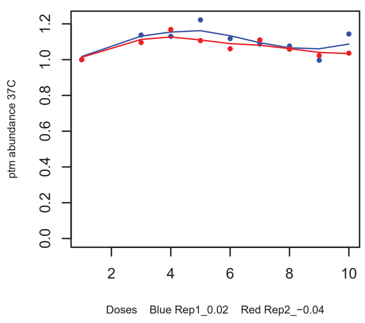

sp|Q15637−5|SF01_HUMAN−205−S−S2_0.1

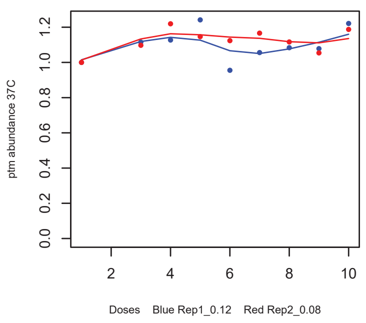

sp|Q15637−5|SF01_HUMAN−207−S−S2_0.1

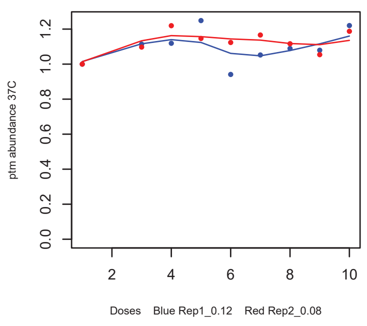

sp|Q96ST2|IWS1_HUMAN−224−S−S3_0.05

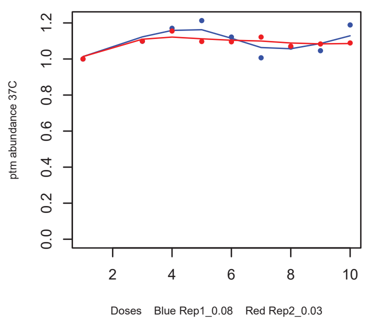

sp|Q8WUB8|PHF10_HUMAN−50−S−S1_0.1

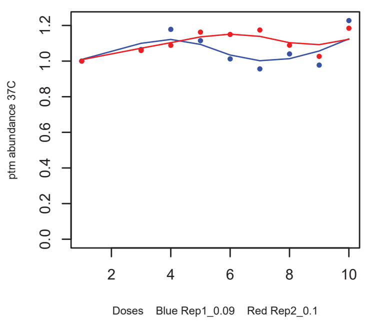

sp|Q13451|FKBP5_HUMAN−13−S−S1_−0.03

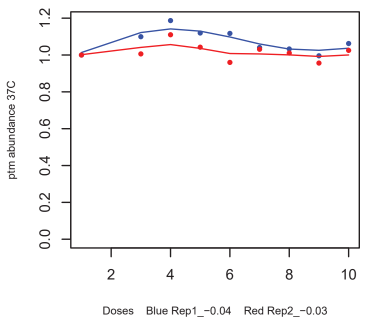

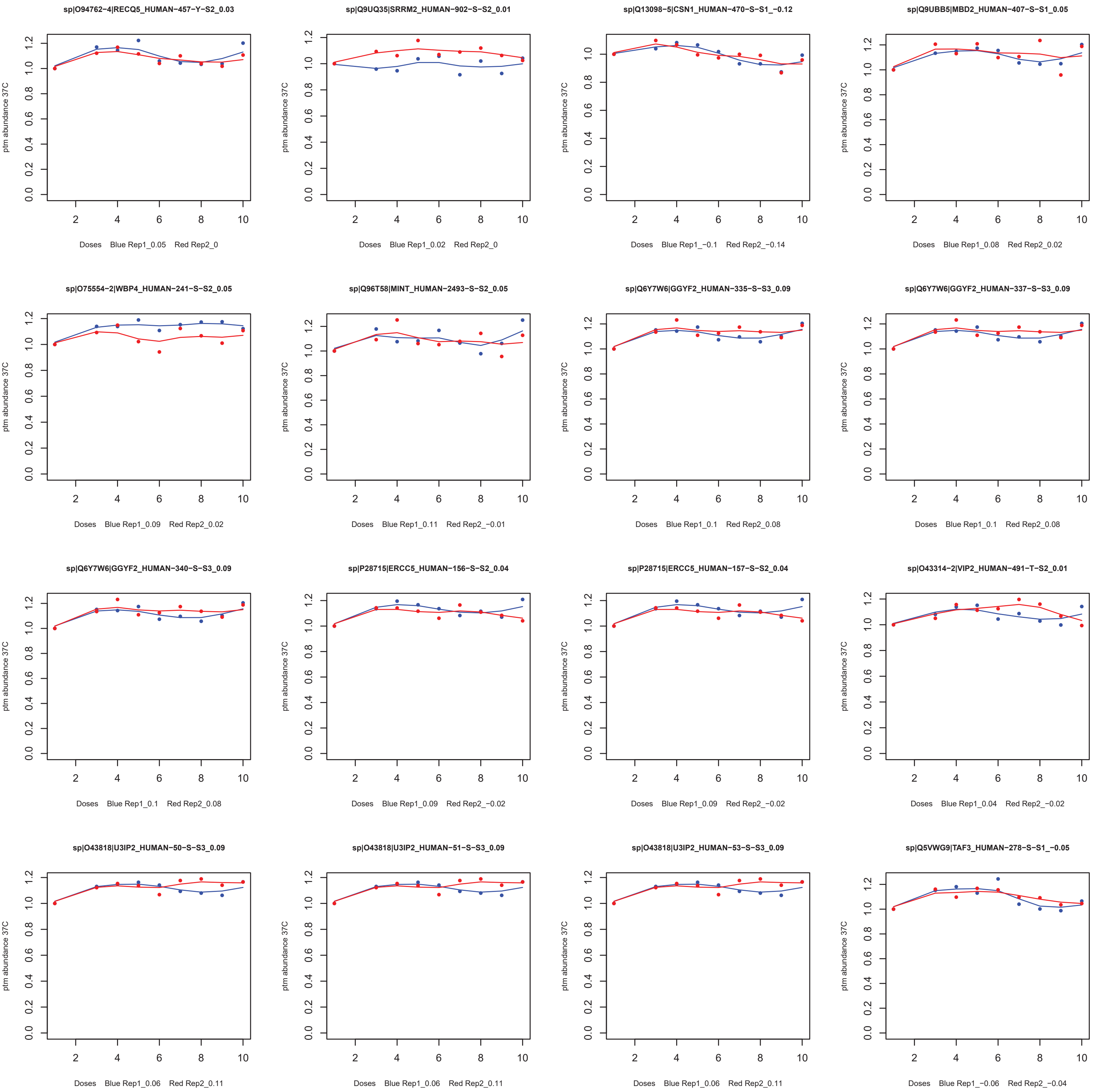
sp|O94762−4|RECQ5_HUMAN−457−Y−S2_0.03
Doses Blue Rep1_0.05 Red Rep2_0
sp|Q9UQ35|SRRM2_HUMAN−902−S−S2_0.01
Doses Blue Rep1_0.02 Red Rep2_0
sp|Q13098−5|CSN1_HUMAN−470−S−S1_−0.12
Doses Blue Rep1_−0.1 Red Rep2_−0.14
sp|Q9UBB5|MBD2_HUMAN−407−S−S1_0.05
Doses Blue Rep1_0.08 Red Rep2_0.02
sp|O75554−2|WBP4_HUMAN−241−S−S2_0.05
Doses Blue Rep1_0.09 Red Rep2_0.02
sp|Q96T58|MINT_HUMAN−2493−S−S2_0.05
Doses Blue Rep1_0.11 Red Rep2_−0.01
sp|Q6Y7W6|GGYF2_HUMAN−335−S−S3_0.09
Doses Blue Rep1_0.1 Red Rep2_0.08
sp|Q6Y7W6|GGYF2_HUMAN−337−S−S3_0.09
Doses Blue Rep1_0.1 Red Rep2_0.08
sp|Q6Y7W6|GGYF2_HUMAN−340−S−S3_0.09
Doses Blue Rep1_0.1 Red Rep2_0.08
sp|P28715|ERCC5_HUMAN−156−S−S2_0.04
Doses Blue Rep1_0.09 Red Rep2_−0.02
sp|P28715|ERCC5_HUMAN−157−S−S2_0.04
Doses Blue Rep1_0.09 Red Rep2_−0.02
sp|O43314−2|VIP2_HUMAN−491−T−S2_0.01
Doses Blue Rep1_0.04 Red Rep2_−0.02
sp|O43818|U3IP2_HUMAN−50−S−S3_0.09
Doses Blue Rep1_0.06 Red Rep2_0.11
sp|O43818|U3IP2_HUMAN−51−S−S3_0.09
Doses Blue Rep1_0.06 Red Rep2_0.11
sp|O43818|U3IP2_HUMAN−53−S−S3_0.09
Doses Blue Rep1_0.06 Red Rep2_0.11
sp|Q5VWG9|TAF3_HUMAN−278−S−S1_−0.05
Doses Blue Rep1_−0.06 Red Rep2_−0.04
ptm abundance 37C

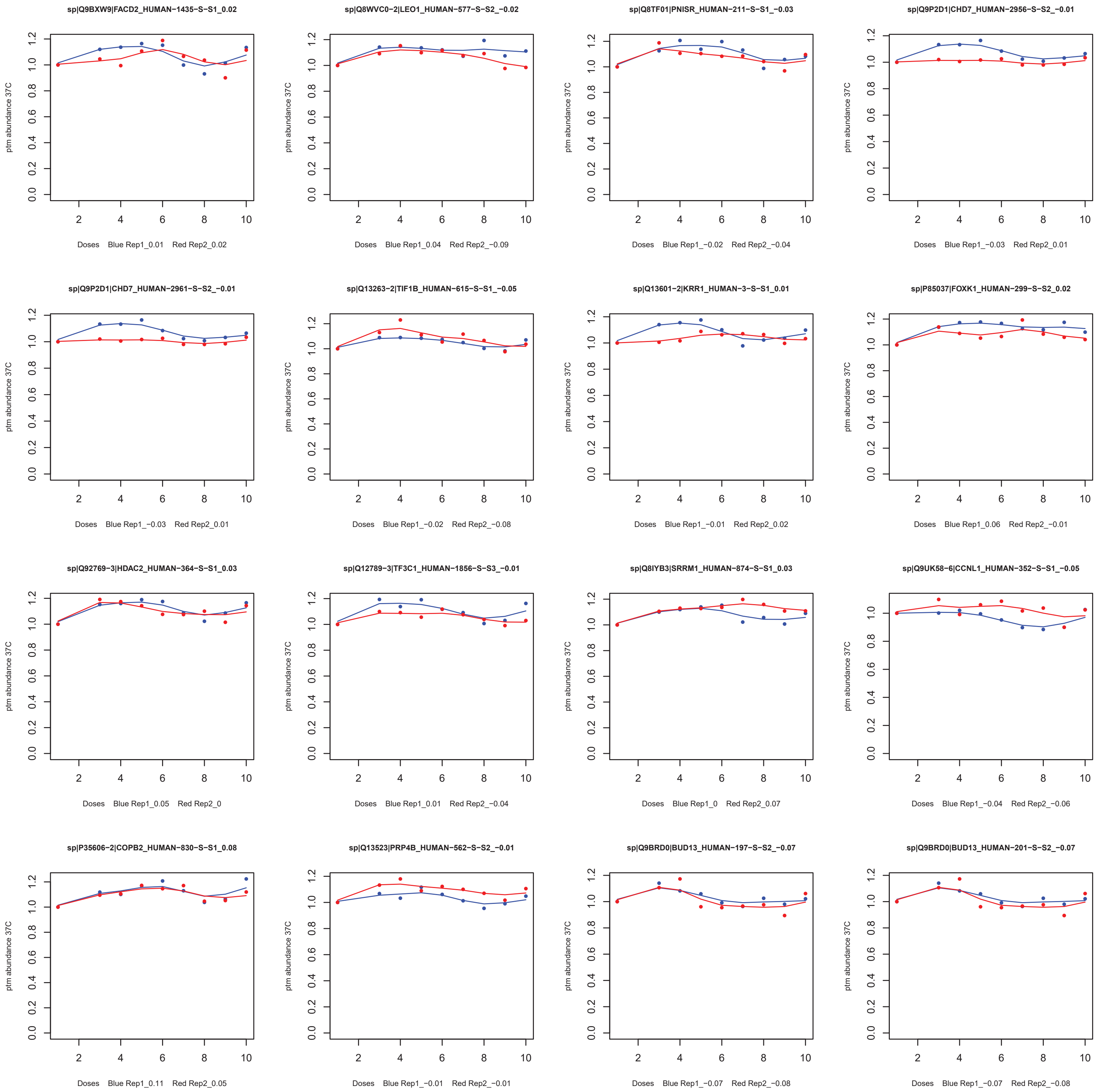
sp|Q9BXW9|FACD2_HUMAN−1435−S−S1_0.02
Doses Blue Rep1_0.01 Red Rep2_0.02
sp|Q8WVC0−2|LEO1_HUMAN−577−S−S2_−0.02
Doses Blue Rep1_0.04 Red Rep2_−0.09
sp|Q8TF01|PNISR_HUMAN−211−S−S1_−0.03
Doses Blue Rep1_−0.02 Red Rep2_−0.04
sp|Q9P2D1|CHD7_HUMAN−2956−S−S2_−0.01
Doses Blue Rep1_−0.03 Red Rep2_0.01
sp|Q9P2D1|CHD7_HUMAN−2961−S−S2_−0.01
Doses Blue Rep1_−0.03 Red Rep2_0.01
sp|Q13263−2|TIF1B_HUMAN−615−S−S1_−0.05
Doses Blue Rep1_−0.02 Red Rep2_−0.08
sp|Q13601−2|KRR1_HUMAN−3−S−S1_0.01
Doses Blue Rep1_−0.01 Red Rep2_0.02
sp|P85037|FOXK1_HUMAN−299−S−S2_0.02
Doses Blue Rep1_0.06 Red Rep2_−0.01
sp|Q92769−3|HDAC2_HUMAN−364−S−S1_0.03
Doses Blue Rep1_0.05 Red Rep2_0
sp|Q12789−3|TF3C1_HUMAN−1856−S−S3_−0.01
Doses Blue Rep1_0.01 Red Rep2_−0.04
sp|Q8IYB3|SRRM1_HUMAN−874−S−S1_0.03
Doses Blue Rep1_0 Red Rep2_0.07
sp|Q9UK58−6|CCNL1_HUMAN−352−S−S1_−0.05
Doses Blue Rep1_−0.04 Red Rep2_−0.06
sp|P35606−2|COPB2_HUMAN−830−S−S1_0.08
Doses Blue Rep1_0.11 Red Rep2_0.05
sp|Q13523|PRP4B_HUMAN−562−S−S2_−0.01
Doses Blue Rep1_−0.01 Red Rep2_−0.01
sp|Q9BRD0|BUD13_HUMAN−197−S−S2_−0.07
Doses Blue Rep1_−0.07 Red Rep2_−0.08
sp|Q9BRD0|BUD13_HUMAN−201−S−S2_−0.07
Doses Blue Rep1_−0.07 Red Rep2_−0.08
ptm abundance 37C

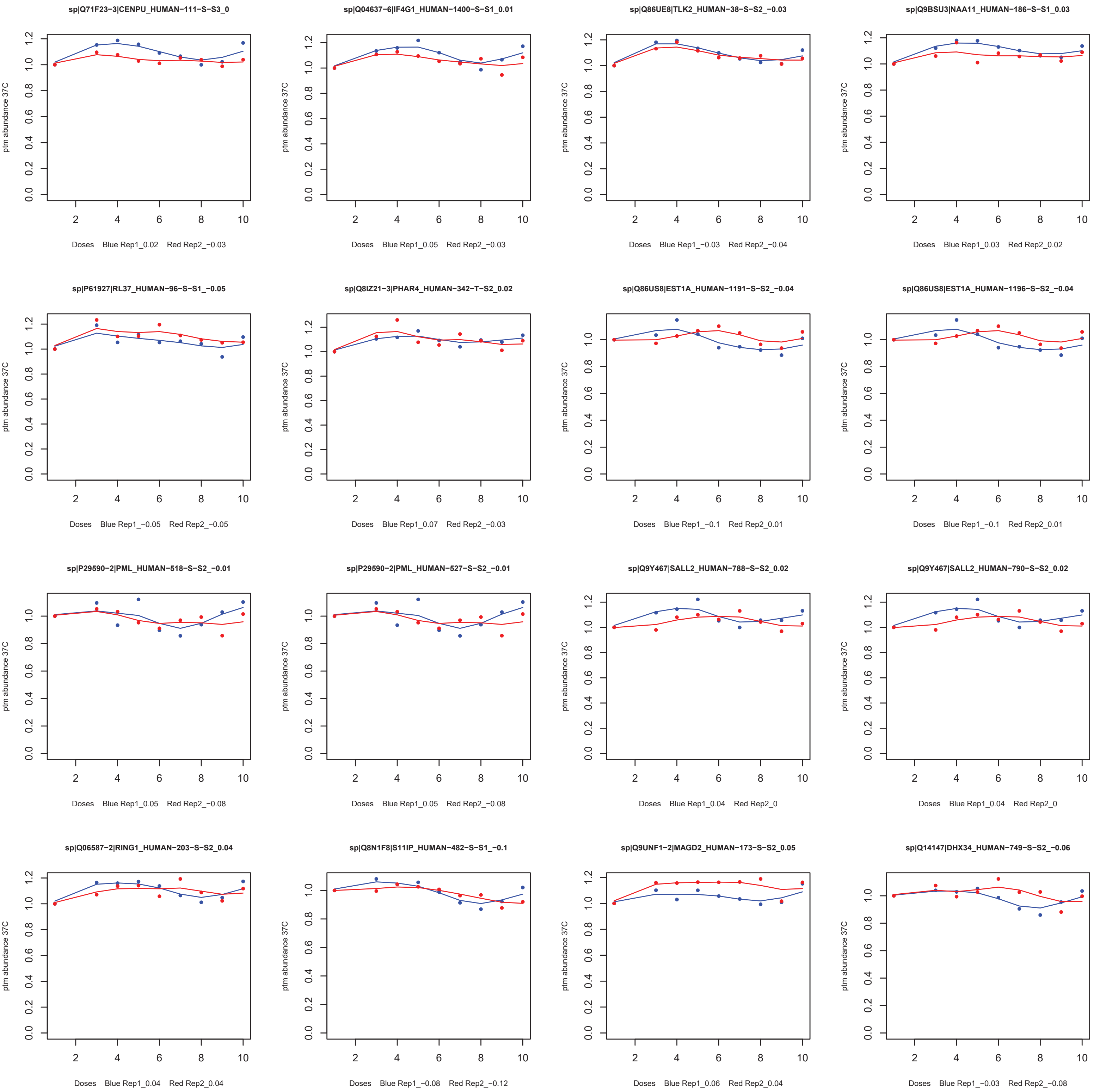

sp|Q71F23−3|CENPU_HUMAN−111−S−S3_0
ptm abundance 37C
Doses Blue Rep1_0.02 Red Rep2_−0.03
sp|Q04637−6|IF4G1_HUMAN−1400−S−S1_0.01
ptm abundance 37C
Doses Blue Rep1_0.05 Red Rep2_−0.03
sp|Q86UE8|TLK2_HUMAN−38−S−S2_−0.03
ptm abundance 37C
Doses Blue Rep1_−0.03 Red Rep2_−0.04
sp|Q9BSU3|NAA11_HUMAN−186−S−S1_0.03
ptm abundance 37C
Doses Blue Rep1_0.03 Red Rep2_0.02
sp|P61927|RL37_HUMAN−96−S−S1_−0.05
ptm abundance 37C
Doses Blue Rep1_−0.05 Red Rep2_−0.05
sp|Q8IZ21−3|PHAR4_HUMAN−342−T−S2_0.02
ptm abundance 37C
Doses Blue Rep1_0.07 Red Rep2_−0.03
sp|Q86US8|EST1A_HUMAN−1191−S−S2_−0.04
ptm abundance 37C
Doses Blue Rep1_−0.1 Red Rep2_0.01
sp|Q86US8|EST1A_HUMAN−1196−S−S2_−0.04
ptm abundance 37C
Doses Blue Rep1_−0.1 Red Rep2_0.01
sp|P29590−2|PML_HUMAN−518−S−S2_−0.01
ptm abundance 37C
Doses Blue Rep1_0.05 Red Rep2_−0.08
sp|P29590−2|PML_HUMAN−527−S−S2_−0.01
ptm abundance 37C
Doses Blue Rep1_0.05 Red Rep2_−0.08
sp|Q9Y467|SALL2_HUMAN−788−S−S2_0.02
ptm abundance 37C
Doses Blue Rep1_0.04 Red Rep2_0
sp|Q9Y467|SALL2_HUMAN−790−S−S2_0.02
ptm abundance 37C
Doses Blue Rep1_0.04 Red Rep2_0
sp|Q06587−2|RING1_HUMAN−203−S−S2_0.04
ptm abundance 37C
Doses Blue Rep1_0.04 Red Rep2_0.04
sp|Q8N1F8|S11IP_HUMAN−482−S−S1_−0.1
ptm abundance 37C
Doses Blue Rep1_−0.08 Red Rep2_−0.12
sp|Q9UNF1−2|MAGD2_HUMAN−173−S−S2_0.05
ptm abundance 37C
Doses Blue Rep1_0.06 Red Rep2_0.04
sp|Q14147|DHX34_HUMAN−749−S−S2_−0.06
ptm abundance 37C
Doses Blue Rep1_−0.03 Red Rep2_−0.08

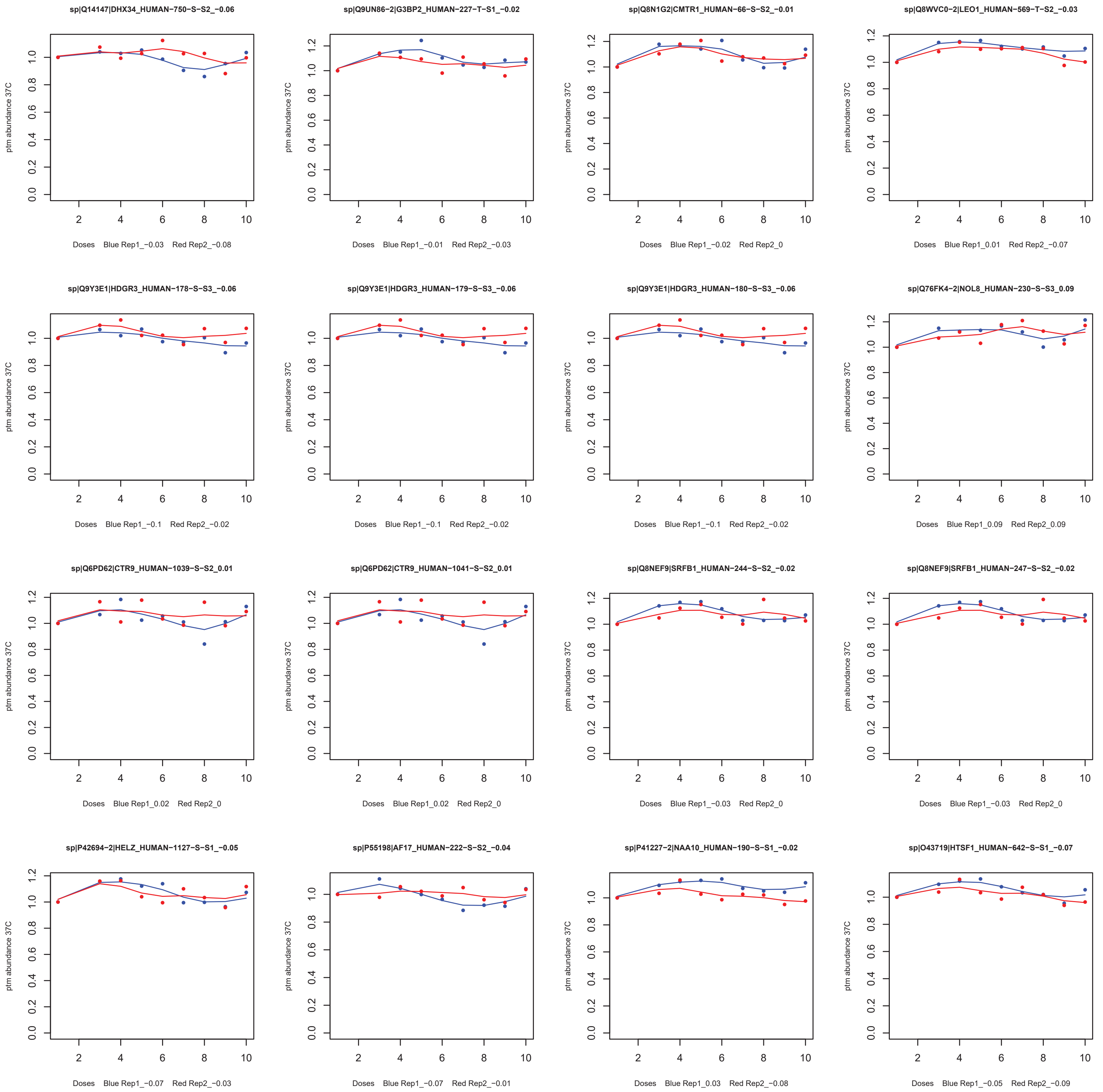
sp|Q14147|DHX34_HUMAN−750−S−S2_−0.06
Doses Blue Rep1_−0.03 Red Rep2_−0.08
sp|Q9UN86−2|G3BP2_HUMAN−227−T−S1_−0.02
Doses Blue Rep1_−0.01 Red Rep2_−0.03
sp|Q8N1G2|CMTR1_HUMAN−66−S−S2_−0.01
Doses Blue Rep1_−0.02 Red Rep2_0
sp|Q8WVC0−2|LEO1_HUMAN−569−T−S2_−0.03
Doses Blue Rep1_0.01 Red Rep2_−0.07
sp|Q9Y3E1|HDGR3_HUMAN−178−S−S3_−0.06
Doses Blue Rep1_−0.1 Red Rep2_−0.02
sp|Q9Y3E1|HDGR3_HUMAN−179−S−S3_−0.06
Doses Blue Rep1_−0.1 Red Rep2_−0.02
sp|Q9Y3E1|HDGR3_HUMAN−180−S−S3_−0.06
Doses Blue Rep1_−0.1 Red Rep2_−0.02
sp|Q76FK4−2|NOL8_HUMAN−230−S−S3_0.09
Doses Blue Rep1_0.09 Red Rep2_0.09
sp|Q6PD62|CTR9_HUMAN−1039−S−S2_0.01
Doses Blue Rep1_0.02 Red Rep2_0
sp|Q6PD62|CTR9_HUMAN−1041−S−S2_0.01
Doses Blue Rep1_0.02 Red Rep2_0
sp|Q8NEF9|SRFB1_HUMAN−244−S−S2_−0.02
Doses Blue Rep1_−0.03 Red Rep2_0
sp|Q8NEF9|SRFB1_HUMAN−247−S−S2_−0.02
Doses Blue Rep1_−0.03 Red Rep2_0
sp|P42694−2|HELZ_HUMAN−1127−S−S1_−0.05
Doses Blue Rep1_−0.07 Red Rep2_−0.03
sp|P55198|AF17_HUMAN−222−S−S2_−0.04
Doses Blue Rep1_−0.07 Red Rep2_−0.01
sp|P41227−2|NAA10_HUMAN−190−S−S1_−0.02
Doses Blue Rep1_0.03 Red Rep2_−0.08
sp|O43719|HTSF1_HUMAN−642−S−S1_−0.07
Doses Blue Rep1_−0.05 Red Rep2_−0.09
ptm abundance 37C

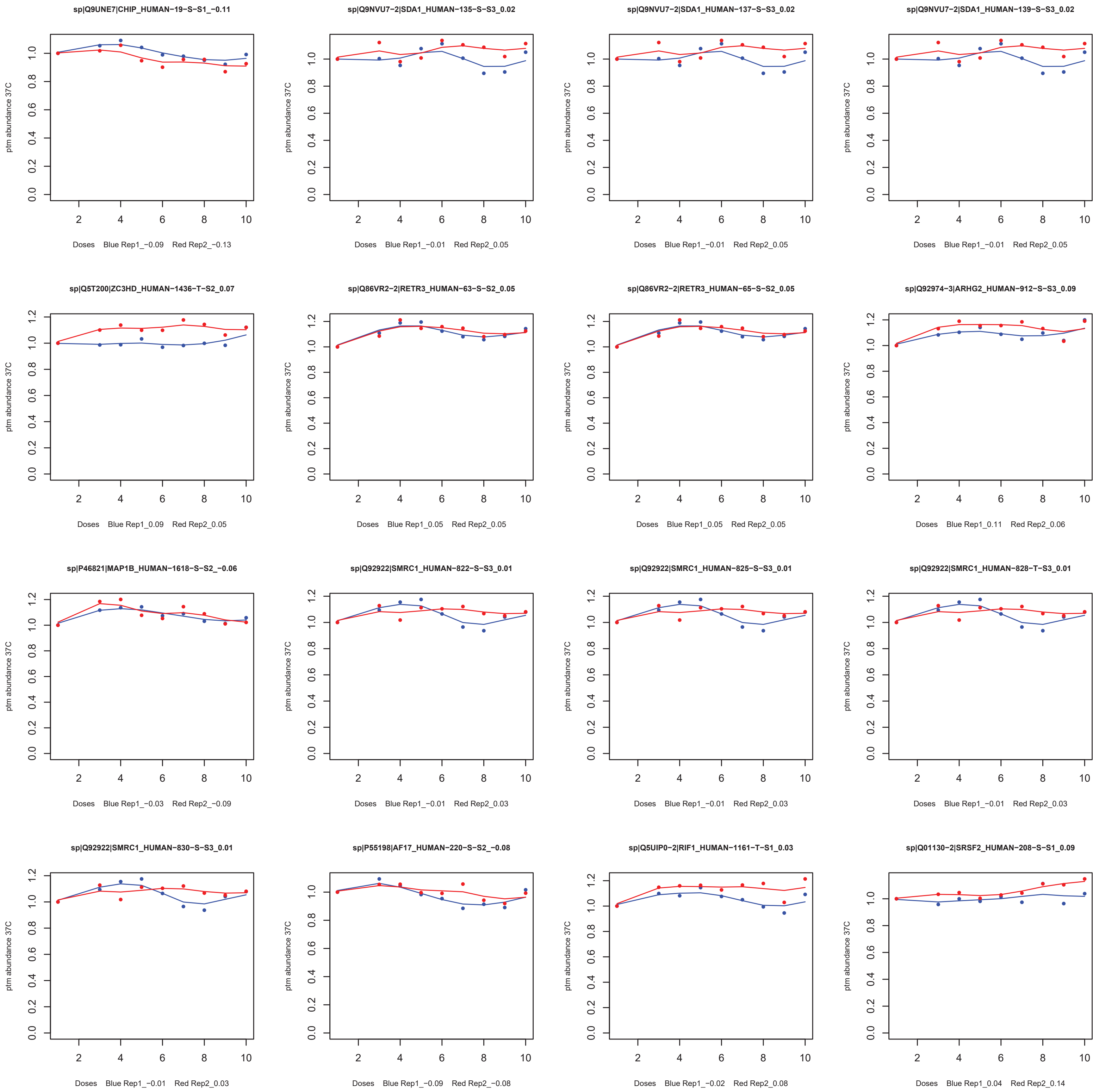
sp|Q9UNE7|CHIP_HUMAN−19−S−S1_−0.11
ptm abundance 37C
Doses Blue Rep1_−0.09 Red Rep2_−0.13
sp|Q9NVU7−2|SDA1_HUMAN−135−S−S3_0.02
ptm abundance 37C
Doses Blue Rep1_−0.01 Red Rep2_0.05
sp|Q9NVU7−2|SDA1_HUMAN−137−S−S3_0.02
ptm abundance 37C
Doses Blue Rep1_−0.01 Red Rep2_0.05
sp|Q9NVU7−2|SDA1_HUMAN−139−S−S3_0.02
ptm abundance 37C
Doses Blue Rep1_−0.01 Red Rep2_0.05
sp|Q5T200|ZC3HD_HUMAN−1436−T−S2_0.07
ptm abundance 37C
Doses Blue Rep1_0.09 Red Rep2_0.05
sp|Q86VR2−2|RETR3_HUMAN−63−S−S2_0.05
ptm abundance 37C
Doses Blue Rep1_0.05 Red Rep2_0.05
sp|Q86VR2−2|RETR3_HUMAN−65−S−S2_0.05
ptm abundance 37C
Doses Blue Rep1_0.05 Red Rep2_0.05
sp|Q92974−3|ARHG2_HUMAN−912−S−S3_0.09
ptm abundance 37C
Doses Blue Rep1_0.11 Red Rep2_0.06
sp|P46821|MAP1B_HUMAN−1618−S−S2_−0.06
ptm abundance 37C
Doses Blue Rep1_−0.03 Red Rep2_−0.09
sp|Q92922|SMRC1_HUMAN−822−S−S3_0.01
ptm abundance 37C
Doses Blue Rep1_−0.01 Red Rep2_0.03
sp|Q92922|SMRC1_HUMAN−825−S−S3_0.01
ptm abundance 37C
Doses Blue Rep1_−0.01 Red Rep2_0.03
sp|Q92922|SMRC1_HUMAN−828−T−S3_0.01
ptm abundance 37C
Doses Blue Rep1_−0.01 Red Rep2_0.03
sp|Q92922|SMRC1_HUMAN−830−S−S3_0.01
ptm abundance 37C
Doses Blue Rep1_−0.01 Red Rep2_0.03
sp|P55198|AF17_HUMAN−220−S−S2_−0.08
ptm abundance 37C
Doses Blue Rep1_−0.09 Red Rep2_−0.08
sp|Q5UIP0−2|RIF1_HUMAN−1161−T−S1_0.03
ptm abundance 37C
Doses Blue Rep1_−0.02 Red Rep2_0.08
sp|Q01130−2|SRSF2_HUMAN−208−S−S1_0.09
ptm abundance 37C
Doses Blue Rep1_0.04 Red Rep2_0.14

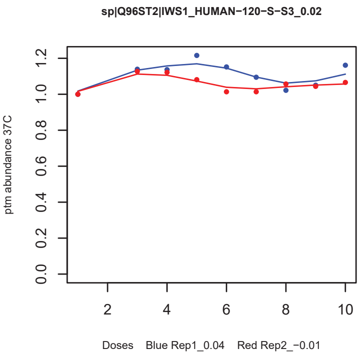
sp|Q96ST2|IWS1_HUMAN−120−S−S3_0.02
ptm abundance 37C
Doses Blue Rep1_0.04 Red Rep2_−0.01

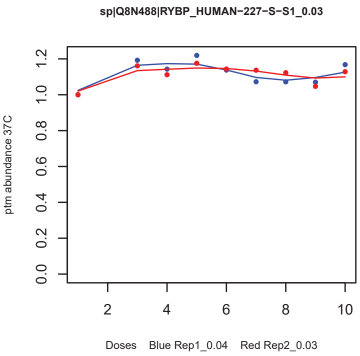
sp|Q8N488|RYBP_HUMAN−227−S−S1_0.03
ptm abundance 37C
Doses Blue Rep1_0.04 Red Rep2_0.03

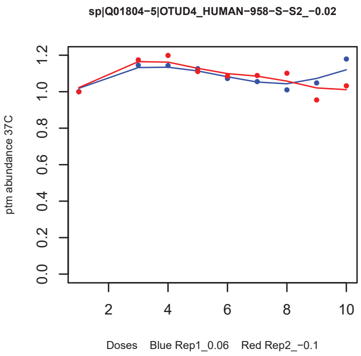
sp|Q01804−5|OTUD4_HUMAN−958−S−S2_−0.02
ptm abundance 37C
Doses Blue Rep1_0.06 Red Rep2_−0.1

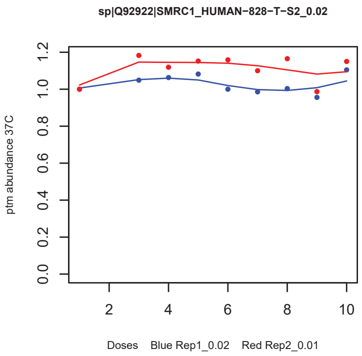
sp|Q92922|SMRC1_HUMAN−828−T−S2_0.02
ptm abundance 37C
Doses Blue Rep1_0.02 Red Rep2_0.01

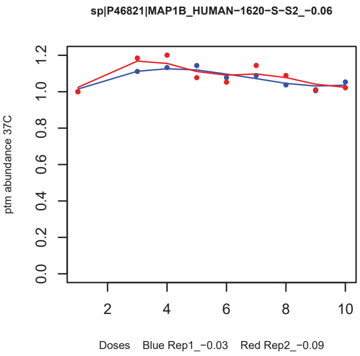
sp|P46821|MAP1B_HUMAN−1620−S−S2_−0.06
ptm abundance 37C
Doses Blue Rep1_−0.03 Red Rep2_−0.09

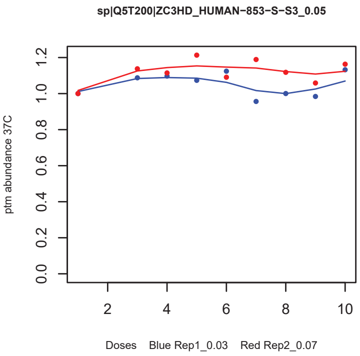
sp|Q5T200|ZC3HD_HUMAN−853−S−S3_0.05
ptm abundance 37C
Doses Blue Rep1_0.03 Red Rep2_0.07

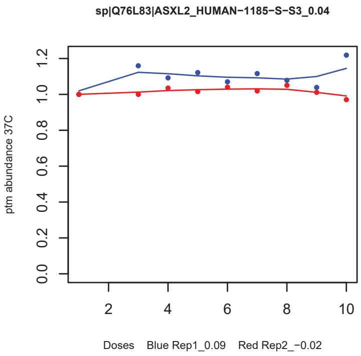
sp|Q76L83|ASXL2_HUMAN−1185−S−S3_0.04
ptm abundance 37C
Doses Blue Rep1_0.09 Red Rep2_−0.02

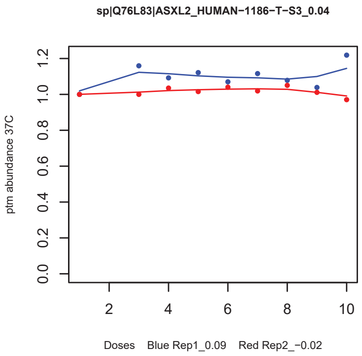
sp|Q76L83|ASXL2_HUMAN−1186−T−S3_0.04
ptm abundance 37C
Doses Blue Rep1_0.09 Red Rep2_−0.02

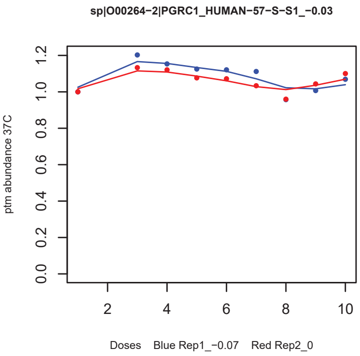
sp|O00264−2|PGRC1_HUMAN−57−S−S1_−0.03
ptm abundance 37C
Doses Blue Rep1_−0.07 Red Rep2_0

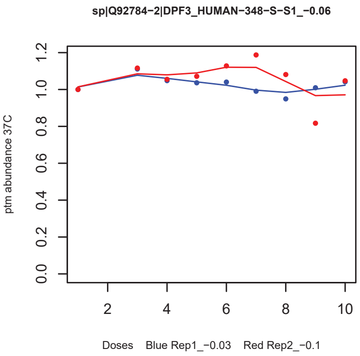
sp|Q92784−2|DPF3_HUMAN−348−S−S1_−0.06
ptm abundance 37C
Doses Blue Rep1_−0.03 Red Rep2_−0.1

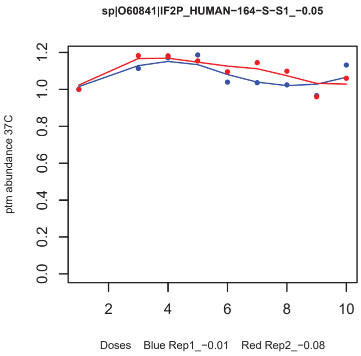
sp|O60841|IF2P_HUMAN−164−S−S1_−0.05
ptm abundance 37C
Doses Blue Rep1_−0.01 Red Rep2_−0.08

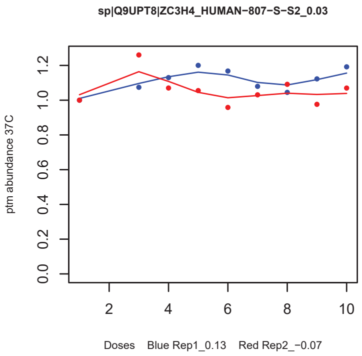
sp|Q9UPT8|ZC3H4_HUMAN−807−S−S2_0.03
ptm abundance 37C
Doses Blue Rep1_0.13 Red Rep2_−0.07

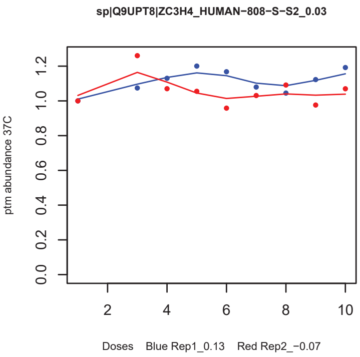
sp|Q9UPT8|ZC3H4_HUMAN−808−S−S2_0.03
ptm abundance 37C
Doses Blue Rep1_0.13 Red Rep2_−0.07

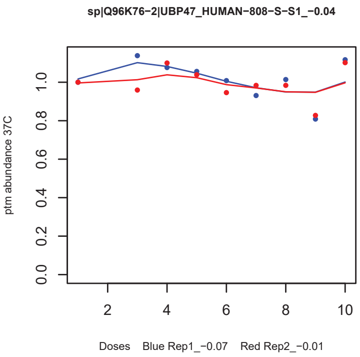
sp|Q96K76−2|UBP47_HUMAN−808−S−S1_−0.04
ptm abundance 37C
Doses Blue Rep1_−0.07 Red Rep2_−0.01

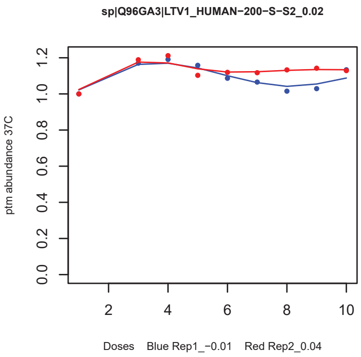
sp|Q96GA3|LTV1_HUMAN−200−S−S2_0.02
ptm abundance 37C
Doses Blue Rep1_−0.01 Red Rep2_0.04

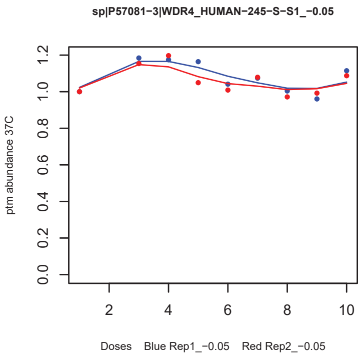
sp|P57081−3|WDR4_HUMAN−245−S−S1_−0.05
ptm abundance 37C
Doses Blue Rep1_−0.05 Red Rep2_−0.05

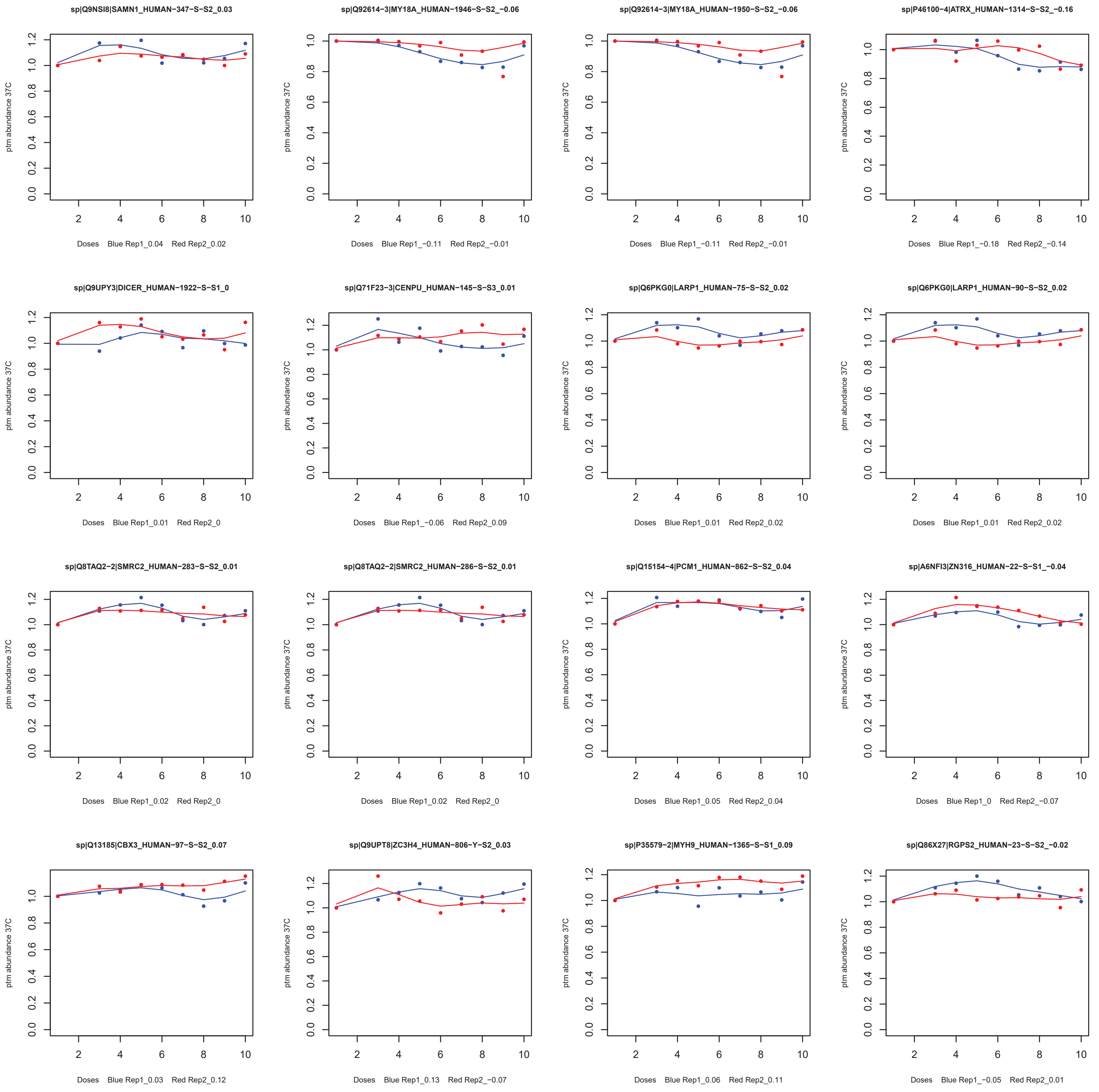

sp|Q9NSI8|SAMN1_HUMAN−347−S−S2_0.03
ptm abundance 37C
Doses Blue Rep1_0.04 Red Rep2_0.02
sp|Q92614−3|MY18A_HUMAN−1946−S−S2_−0.06
ptm abundance 37C
Doses Blue Rep1_−0.11 Red Rep2_−0.01
sp|Q92614−3|MY18A_HUMAN−1950−S−S2_−0.06
ptm abundance 37C
Doses Blue Rep1_−0.11 Red Rep2_−0.01
sp|P46100−4|ATRX_HUMAN−1314−S−S2_−0.16
ptm abundance 37C
Doses Blue Rep1_−0.18 Red Rep2_−0.14
sp|Q9UPY3|DICER_HUMAN−1922−S−S1_0
ptm abundance 37C
Doses Blue Rep1_0.01 Red Rep2_0
sp|Q71F23−3|CENPU_HUMAN−145−S−S3_0.01
ptm abundance 37C
Doses Blue Rep1_−0.06 Red Rep2_0.09
sp|Q6PKG0|LARP1_HUMAN−75−S−S2_0.02
ptm abundance 37C
Doses Blue Rep1_0.01 Red Rep2_0.02
sp|Q6PKG0|LARP1_HUMAN−90−S−S2_0.02
ptm abundance 37C
Doses Blue Rep1_0.01 Red Rep2_0.02
sp|Q8TAQ2−2|SMRC2_HUMAN−283−S−S2_0.01
ptm abundance 37C
Doses Blue Rep1_0.02 Red Rep2_0
sp|Q8TAQ2−2|SMRC2_HUMAN−286−S−S2_0.01
ptm abundance 37C
Doses Blue Rep1_0.02 Red Rep2_0
sp|Q15154−4|PCM1_HUMAN−862−S−S2_0.04
ptm abundance 37C
Doses Blue Rep1_0.05 Red Rep2_0.04
sp|A6NFI3|ZN316_HUMAN−22−S−S1_−0.04
ptm abundance 37C
Doses Blue Rep1_0 Red Rep2_−0.07
sp|Q13185|CBX3_HUMAN−97−S−S2_0.07
ptm abundance 37C
Doses Blue Rep1_0.03 Red Rep2_0.12
sp|Q9UPT8|ZC3H4_HUMAN−806−Y−S2_0.03
ptm abundance 37C
Doses Blue Rep1_0.13 Red Rep2_−0.07
sp|P35579−2|MYH9_HUMAN−1365−S−S1_0.09
ptm abundance 37C
Doses Blue Rep1_0.06 Red Rep2_0.11
sp|Q86X27|RGPS2_HUMAN−23−S−S2_−0.02
ptm abundance 37C
Doses Blue Rep1_−0.05 Red Rep2_0.01

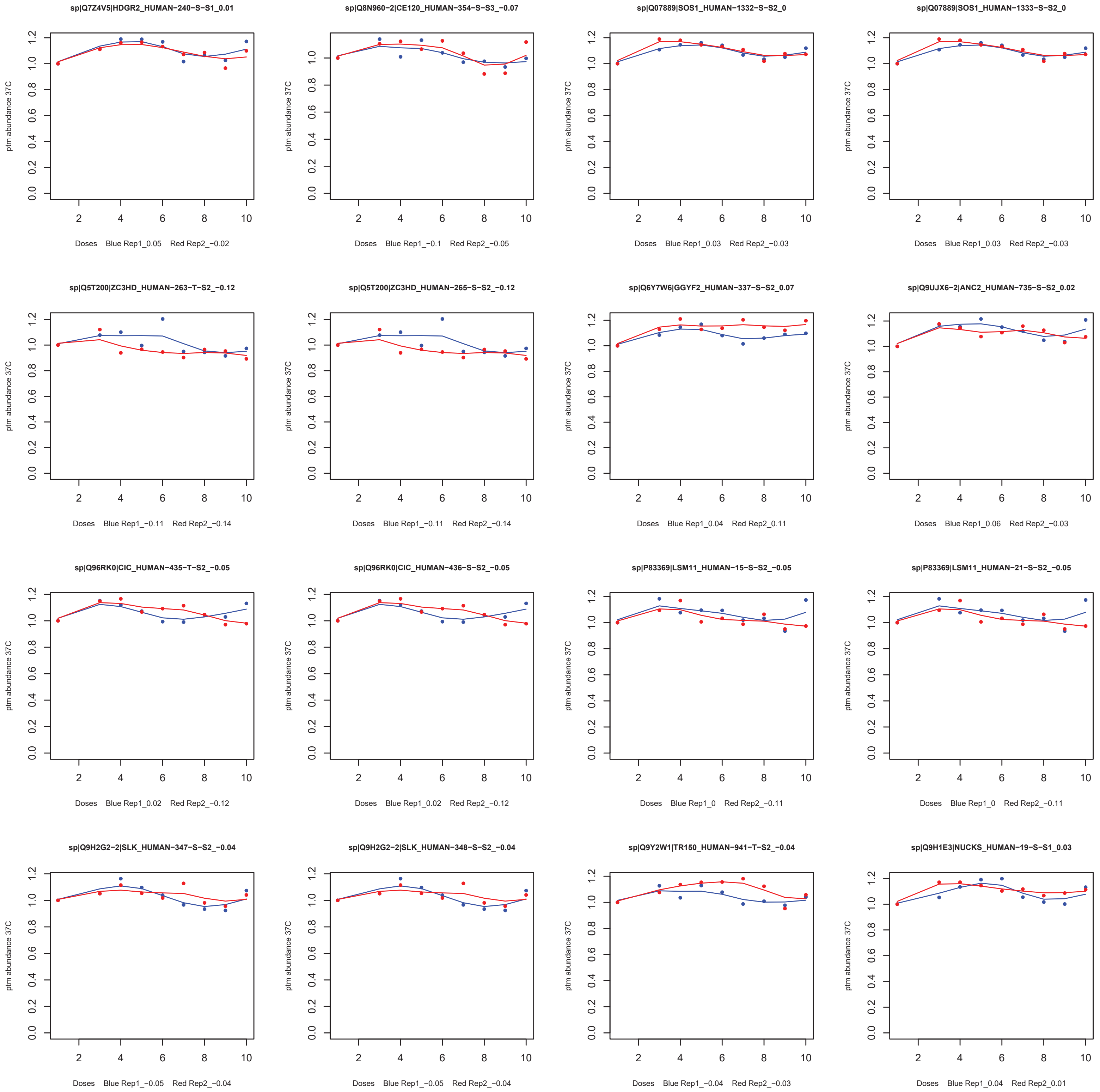
sp|Q7Z4V5|HDGR2_HUMAN−240−S−S1_0.01
Doses Blue Rep1_0.05 Red Rep2_−0.02
sp|Q8N960−2|CE120_HUMAN−354−S−S3_−0.07
Doses Blue Rep1_−0.1 Red Rep2_−0.05
sp|Q07889|SOS1_HUMAN−1332−S−S2_0
Doses Blue Rep1_0.03 Red Rep2_−0.03
sp|Q07889|SOS1_HUMAN−1333−S−S2_0
Doses Blue Rep1_0.03 Red Rep2_−0.03
sp|Q5T200|ZC3HD_HUMAN−263−T−S2_−0.12
Doses Blue Rep1_−0.11 Red Rep2_−0.14
sp|Q5T200|ZC3HD_HUMAN−265−S−S2_−0.12
Doses Blue Rep1_−0.11 Red Rep2_−0.14
sp|Q6Y7W6|GGYF2_HUMAN−337−S−S2_0.07
Doses Blue Rep1_0.04 Red Rep2_0.11
sp|Q9UJX6−2|ANC2_HUMAN−735−S−S2_0.02
Doses Blue Rep1_0.06 Red Rep2_−0.03
sp|Q96RK0|CIC_HUMAN−435−T−S2_−0.05
Doses Blue Rep1_0.02 Red Rep2_−0.12
sp|Q96RK0|CIC_HUMAN−436−S−S2_−0.05
Doses Blue Rep1_0.02 Red Rep2_−0.12
sp|P83369|LSM11_HUMAN−15−S−S2_−0.05
Doses Blue Rep1_0 Red Rep2_−0.11
sp|P83369|LSM11_HUMAN−21−S−S2_−0.05
Doses Blue Rep1_0 Red Rep2_−0.11
sp|Q9H2G2−2|SLK_HUMAN−347−S−S2_−0.04
Doses Blue Rep1_−0.05 Red Rep2_−0.04
sp|Q9H2G2−2|SLK_HUMAN−348−S−S2_−0.04
Doses Blue Rep1_−0.05 Red Rep2_−0.04
sp|Q9Y2W1|TR150_HUMAN−941−T−S2_−0.04
Doses Blue Rep1_−0.04 Red Rep2_−0.03
sp|Q9H1E3|NUCKS_HUMAN−19−S−S1_0.03
Doses Blue Rep1_0.04 Red Rep2_0.01
ptm abundance 37C

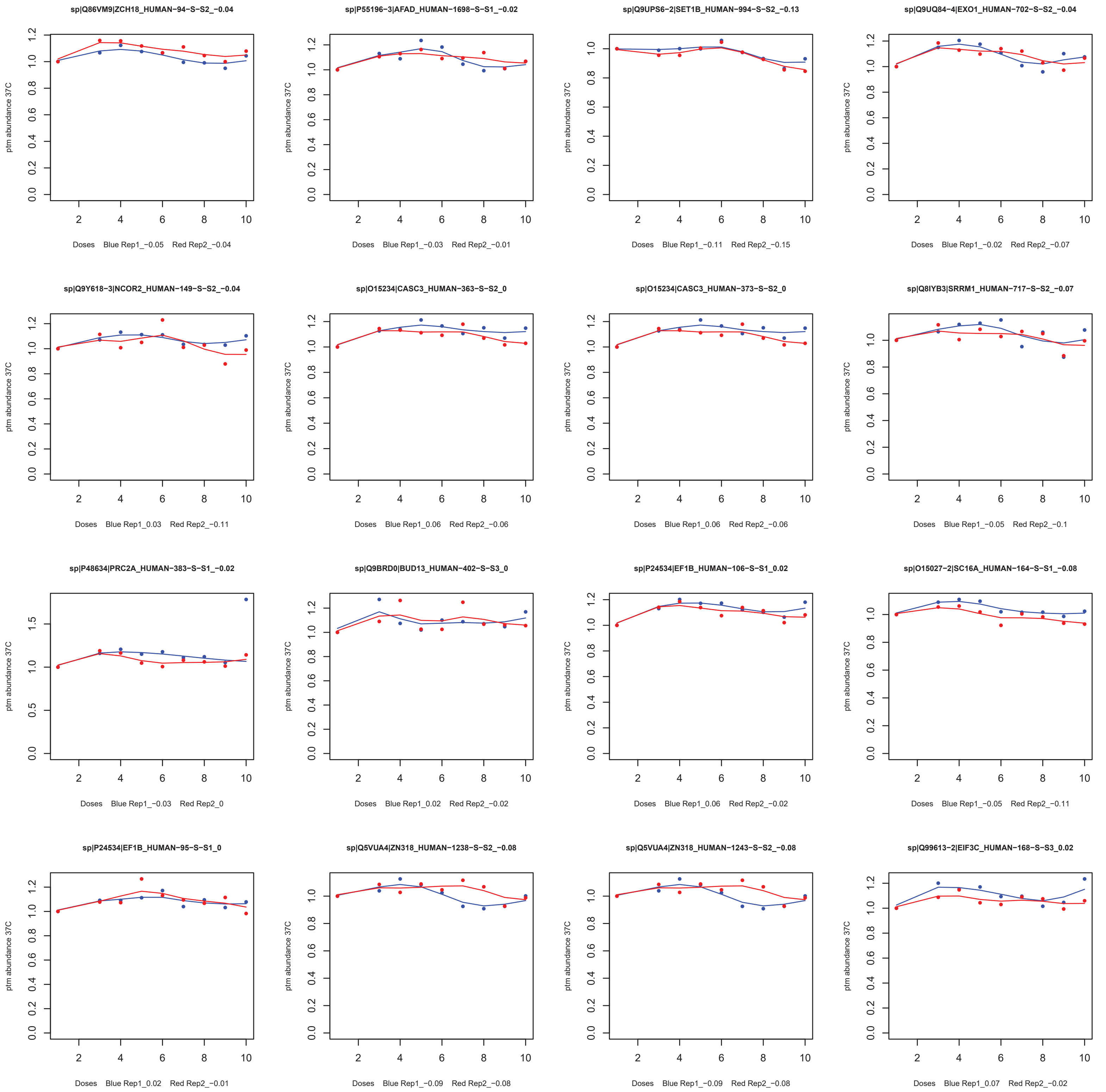
sp|Q86VM9|ZCH18_HUMAN−94−S−S2_−0.04
Doses Blue Rep1_−0.05 Red Rep2_−0.04
sp|P55196−3|AFAD_HUMAN−1698−S−S1_−0.02
Doses Blue Rep1_−0.03 Red Rep2_−0.01
sp|Q9UPS6−2|SET1B_HUMAN−994−S−S2_−0.13
Doses Blue Rep1_−0.11 Red Rep2_−0.15
sp|Q9UQ84−4|EXO1_HUMAN−702−S−S2_−0.04
Doses Blue Rep1_−0.02 Red Rep2_−0.07
sp|Q9Y618−3|NCOR2_HUMAN−149−S−S2_−0.04
Doses Blue Rep1_0.03 Red Rep2_−0.11
sp|O15234|CASC3_HUMAN−363−S−S2_0
Doses Blue Rep1_0.06 Red Rep2_−0.06
sp|O15234|CASC3_HUMAN−373−S−S2_0
Doses Blue Rep1_0.06 Red Rep2_−0.06
sp|Q8IYB3|SRRM1_HUMAN−717−S−S2_−0.07
Doses Blue Rep1_−0.05 Red Rep2_−0.1
sp|P48634|PRC2A_HUMAN−383−S−S1_−0.02
Doses Blue Rep1_−0.03 Red Rep2_0
sp|Q9BRD0|BUD13_HUMAN−402−S−S3_0
Doses Blue Rep1_0.02 Red Rep2_−0.02
sp|P24534|EF1B_HUMAN−106−S−S1_0.02
Doses Blue Rep1_0.06 Red Rep2_−0.02
sp|O15027−2|SC16A_HUMAN−164−S−S1_−0.08
Doses Blue Rep1_−0.05 Red Rep2_−0.11
sp|P24534|EF1B_HUMAN−95−S−S1_0
Doses Blue Rep1_0.02 Red Rep2_−0.01
sp|Q5VUA4|ZN318_HUMAN−1238−S−S2_−0.08
Doses Blue Rep1_−0.09 Red Rep2_−0.08
sp|Q5VUA4|ZN318_HUMAN−1243−S−S2_−0.08
Doses Blue Rep1_−0.09 Red Rep2_−0.08
sp|Q99613−2|EIF3C_HUMAN−168−S−S3_0.02
Doses Blue Rep1_0.07 Red Rep2_−0.02
ptm abundance 37C

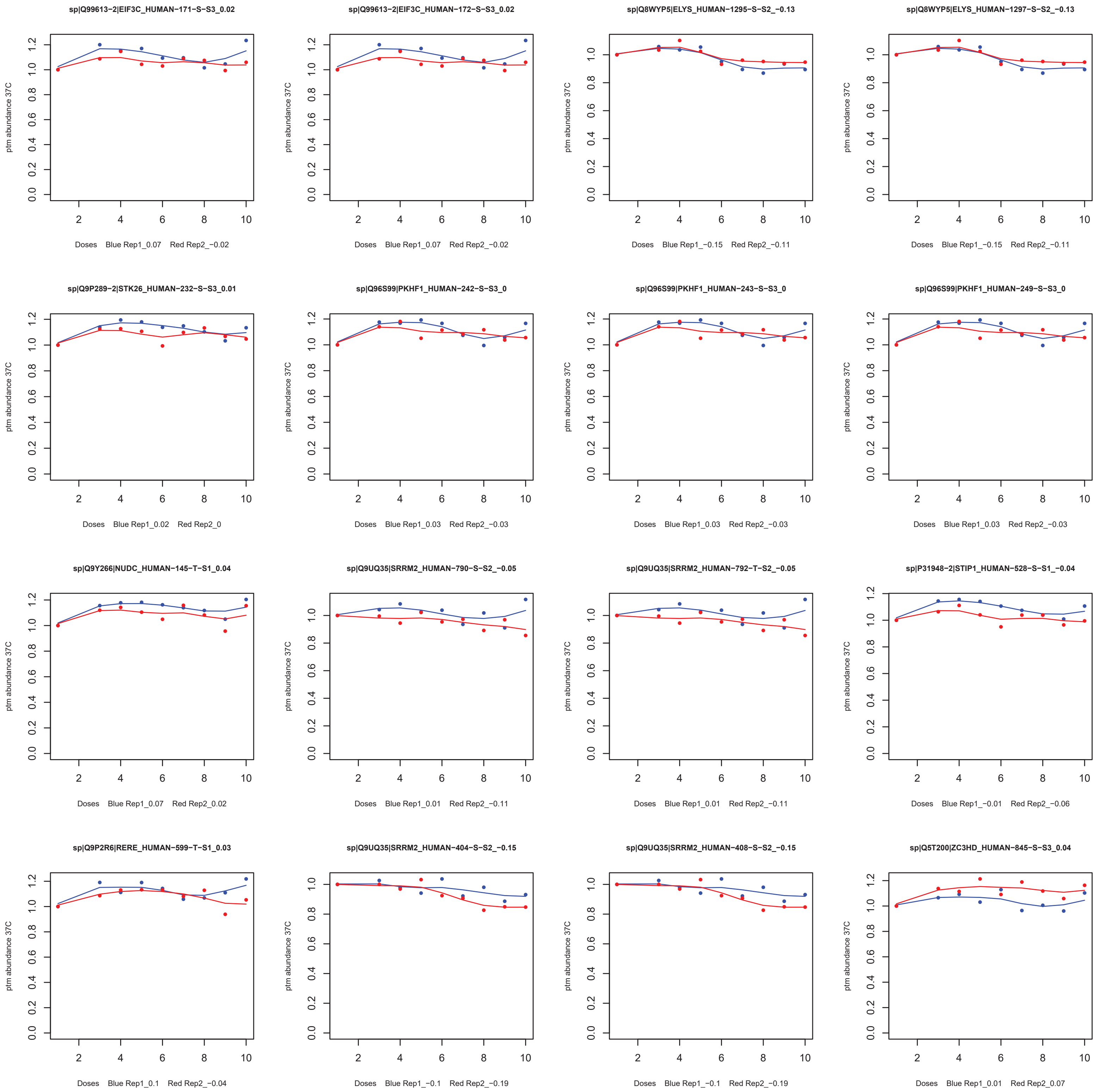
sp|Q99613-2|EIF3C_HUMAN-171-S-S3_0.02
ptm abundance 37C
Doses Blue Rep1_0.07 Red Rep2_-0.02
sp|Q99613-2|EIF3C_HUMAN-172-S-S3_0.02
ptm abundance 37C
Doses Blue Rep1_0.07 Red Rep2_-0.02
sp|Q8WYP5|ELYS_HUMAN-1295-S-S2_-0.13
ptm abundance 37C
Doses Blue Rep1_-0.15 Red Rep2_-0.11
sp|Q8WYP5|ELYS_HUMAN-1297-S-S2_-0.13
ptm abundance 37C
Doses Blue Rep1_-0.15 Red Rep2_-0.11
sp|Q9P289-2|STK26_HUMAN-232-S-S3_0.01
ptm abundance 37C
Doses Blue Rep1_0.02 Red Rep2_0
sp|Q96S99|PKHF1_HUMAN-242-S-S3_0
ptm abundance 37C
Doses Blue Rep1_0.03 Red Rep2_-0.03
sp|Q96S99|PKHF1_HUMAN-243-S-S3_0
ptm abundance 37C
Doses Blue Rep1_0.03 Red Rep2_-0.03
sp|Q96S99|PKHF1_HUMAN-249-S-S3_0
ptm abundance 37C
Doses Blue Rep1_0.03 Red Rep2_-0.03
sp|Q9Y266|NUDC_HUMAN-145-T-S1_0.04
ptm abundance 37C
Doses Blue Rep1_0.07 Red Rep2_0.02
sp|Q9UQ35|SRRM2_HUMAN-790-S-S2_-0.05
ptm abundance 37C
Doses Blue Rep1_0.01 Red Rep2_-0.11
sp|Q9UQ35|SRRM2_HUMAN-792-T-S2_-0.05
ptm abundance 37C
Doses Blue Rep1_0.01 Red Rep2_-0.11
sp|P31948-2|STIP1_HUMAN-528-S-S1_-0.04
ptm abundance 37C
Doses Blue Rep1_-0.01 Red Rep2_-0.06
sp|Q9P2R6|RERE_HUMAN-599-T-S1_0.03
ptm abundance 37C
Doses Blue Rep1_0.1 Red Rep2_-0.04
sp|Q9UQ35|SRRM2_HUMAN-404-S-S2_-0.15
ptm abundance 37C
Doses Blue Rep1_-0.1 Red Rep2_-0.19
sp|Q9UQ35|SRRM2_HUMAN-408-S-S2_-0.15
ptm abundance 37C
Doses Blue Rep1_-0.1 Red Rep2_-0.19
sp|Q5T200|ZC3HD_HUMAN-845-S-S3_0.04
ptm abundance 37C
Doses Blue Rep1_0.01 Red Rep2_0.07

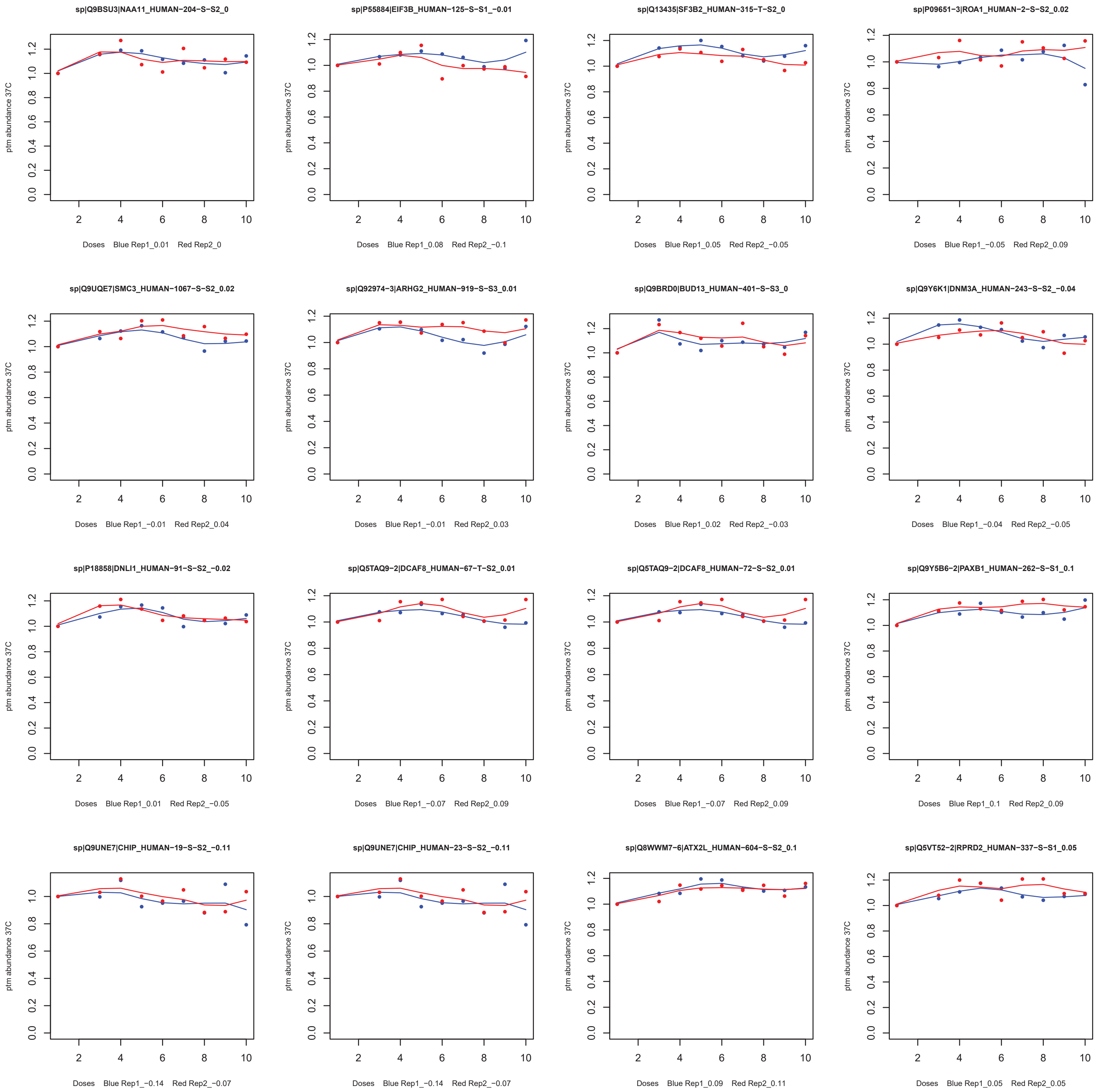
sp|Q9BSU3|NAA11_HUMAN−204−S−S2_0
Doses Blue Rep1_0.01 Red Rep2_0
sp|P55884|EIF3B_HUMAN−125−S−S1_−0.01
Doses Blue Rep1_0.08 Red Rep2_−0.1
sp|Q13435|SF3B2_HUMAN−315−T−S2_0
Doses Blue Rep1_0.05 Red Rep2_−0.05
sp|P09651−3|ROA1_HUMAN−2−S−S2_0.02
Doses Blue Rep1_−0.05 Red Rep2_0.09
sp|Q9UQE7|SMC3_HUMAN−1067−S−S2_0.02
Doses Blue Rep1_−0.01 Red Rep2_0.04
sp|Q92974−3|ARHG2_HUMAN−919−S−S3_0.01
Doses Blue Rep1_−0.01 Red Rep2_0.03
sp|Q9BRD0|BUD13_HUMAN−401−S−S3_0
Doses Blue Rep1_0.02 Red Rep2_−0.03
sp|Q9Y6K1|DNM3A_HUMAN−243−S−S2_−0.04
Doses Blue Rep1_−0.04 Red Rep2_−0.05
sp|P18858|DNLI1_HUMAN−91−S−S2_−0.02
Doses Blue Rep1_0.01 Red Rep2_−0.05
sp|Q5TAQ9−2|DCAF8_HUMAN−67−T−S2_0.01
Doses Blue Rep1_−0.07 Red Rep2_0.09
sp|Q5TAQ9−2|DCAF8_HUMAN−72−S−S2_0.01
Doses Blue Rep1_−0.07 Red Rep2_0.09
sp|Q9Y5B6−2|PAXB1_HUMAN−262−S−S1_0.1
Doses Blue Rep1_0.1 Red Rep2_0.09
sp|Q9UNE7|CHIP_HUMAN−19−S−S2_−0.11
Doses Blue Rep1_−0.14 Red Rep2_−0.07
sp|Q9UNE7|CHIP_HUMAN−23−S−S2_−0.11
Doses Blue Rep1_−0.14 Red Rep2_−0.07
sp|Q8WWM7−6|ATX2L_HUMAN−604−S−S2_0.1
Doses Blue Rep1_0.09 Red Rep2_0.11
sp|Q5VT52−2|RPRD2_HUMAN−337−S−S1_0.05
Doses Blue Rep1_0.05 Red Rep2_0.05
ptm abundance 37C

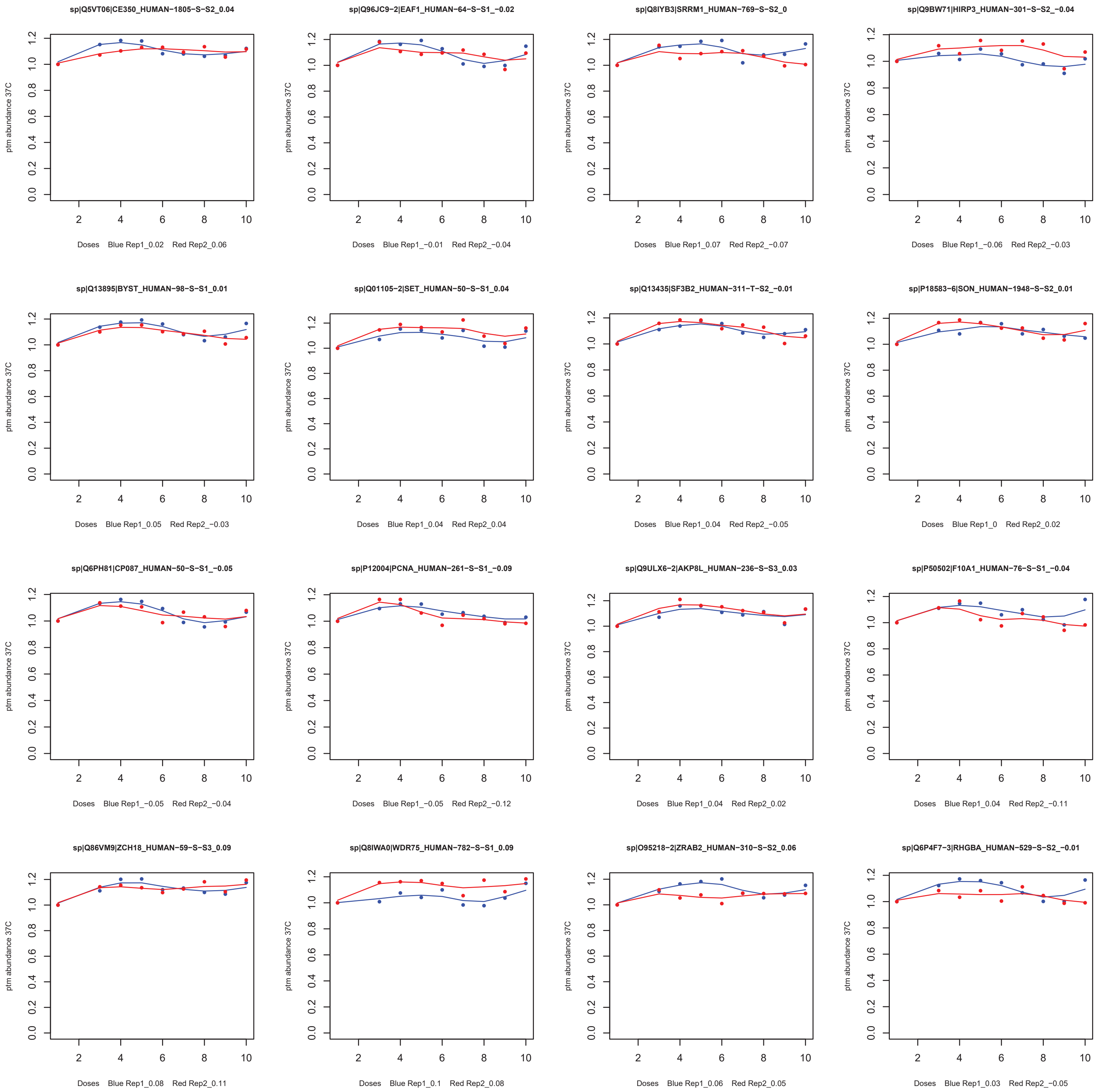
sp|Q5VT06|CE350_HUMAN−1805−S−S2_0.04
Doses Blue Rep1_0.02 Red Rep2_0.06
sp|Q96JC9−2|EAF1_HUMAN−64−S−S1_−0.02
Doses Blue Rep1_−0.01 Red Rep2_−0.04
sp|Q8IYB3|SRRM1_HUMAN−769−S−S2_0
Doses Blue Rep1_0.07 Red Rep2_−0.07
sp|Q9BW71|HIRP3_HUMAN−301−S−S2_−0.04
Doses Blue Rep1_−0.06 Red Rep2_−0.03
sp|Q13895|BYST_HUMAN−98−S−S1_0.01
Doses Blue Rep1_0.05 Red Rep2_−0.03
sp|Q01105−2|SET_HUMAN−50−S−S1_0.04
Doses Blue Rep1_0.04 Red Rep2_0.04
sp|Q13435|SF3B2_HUMAN−311−T−S2_−0.01
Doses Blue Rep1_0.04 Red Rep2_−0.05
sp|P18583−6|SON_HUMAN−1948−S−S2_0.01
Doses Blue Rep1_0 Red Rep2_0.02
sp|Q6PH81|CP087_HUMAN−50−S−S1_−0.05
Doses Blue Rep1_−0.05 Red Rep2_−0.04
sp|P12004|PCNA_HUMAN−261−S−S1_−0.09
Doses Blue Rep1_−0.05 Red Rep2_−0.12
sp|Q9ULX6−2|AKP8L_HUMAN−236−S−S3_0.03
Doses Blue Rep1_0.04 Red Rep2_0.02
sp|P50502|F10A1_HUMAN−76−S−S1_−0.04
Doses Blue Rep1_0.04 Red Rep2_−0.11
sp|Q86VM9|ZCH18_HUMAN−59−S−S3_0.09
Doses Blue Rep1_0.08 Red Rep2_0.11
sp|Q8IWA0|WDR75_HUMAN−782−S−S1_0.09
Doses Blue Rep1_0.1 Red Rep2_0.08
sp|O95218−2|ZRAB2_HUMAN−310−S−S2_0.06
Doses Blue Rep1_0.06 Red Rep2_0.05
sp|Q6P4F7−3|RHGBA_HUMAN−529−S−S2_−0.01
Doses Blue Rep1_0.03 Red Rep2_−0.05
ptm abundance 37C

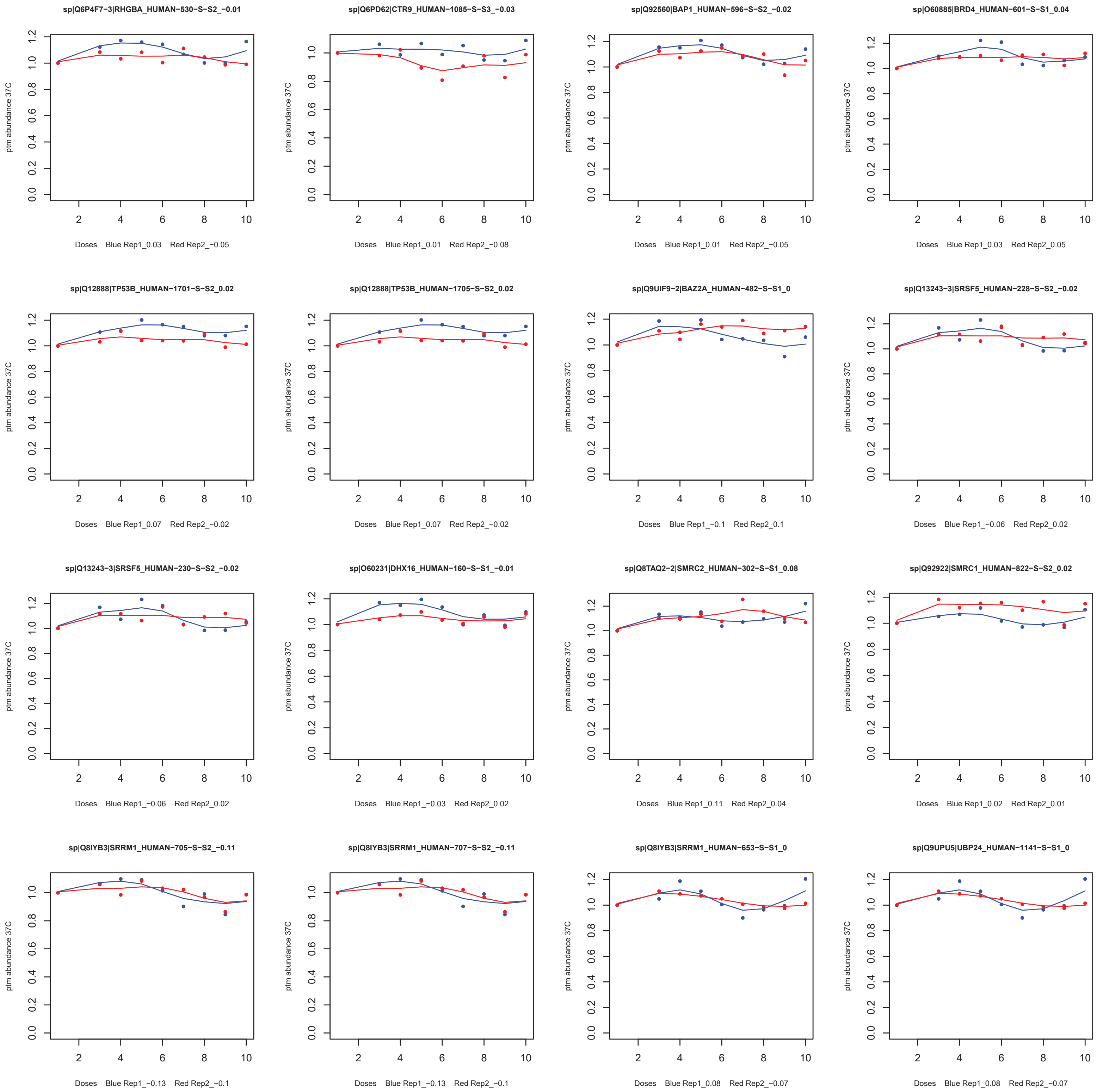
sp|Q6P4F7−3|RHGBA_HUMAN−530−S−S2_−0.01
ptm abundance 37C
Doses Blue Rep1_0.03 Red Rep2_−0.05
sp|Q6PD62|CTR9_HUMAN−1085−S−S3_−0.03
ptm abundance 37C
Doses Blue Rep1_0.01 Red Rep2_−0.08
sp|Q92560|BAP1_HUMAN−596−S−S2_−0.02
ptm abundance 37C
Doses Blue Rep1_0.01 Red Rep2_−0.05
sp|O60885|BRD4_HUMAN−601−S−S1_0.04
ptm abundance 37C
Doses Blue Rep1_0.03 Red Rep2_0.05
sp|Q12888|TP53B_HUMAN−1701−S−S2_0.02
ptm abundance 37C
Doses Blue Rep1_0.07 Red Rep2_−0.02
sp|Q12888|TP53B_HUMAN−1705−S−S2_0.02
ptm abundance 37C
Doses Blue Rep1_0.07 Red Rep2_−0.02
sp|Q9UIF9−2|BAZ2A_HUMAN−482−S−S1_0
ptm abundance 37C
Doses Blue Rep1_−0.1 Red Rep2_0.1
sp|Q13243−3|SRSF5_HUMAN−228−S−S2_−0.02
ptm abundance 37C
Doses Blue Rep1_−0.06 Red Rep2_0.02
sp|Q13243−3|SRSF5_HUMAN−230−S−S2_−0.02
ptm abundance 37C
Doses Blue Rep1_−0.06 Red Rep2_0.02
sp|O60231|DHX16_HUMAN−160−S−S1_−0.01
ptm abundance 37C
Doses Blue Rep1_−0.03 Red Rep2_0.02
sp|Q8TAQ2−2|SMRC2_HUMAN−302−S−S1_0.08
ptm abundance 37C
Doses Blue Rep1_0.11 Red Rep2_0.04
sp|Q92922|SMRC1_HUMAN−822−S−S2_0.02
ptm abundance 37C
Doses Blue Rep1_0.02 Red Rep2_0.01
sp|Q8IYB3|SRRM1_HUMAN−705−S−S2_−0.11
ptm abundance 37C
Doses Blue Rep1_−0.13 Red Rep2_−0.1
sp|Q8IYB3|SRRM1_HUMAN−707−S−S2_−0.11
ptm abundance 37C
Doses Blue Rep1_−0.13 Red Rep2_−0.1
sp|Q8IYB3|SRRM1_HUMAN−653−S−S1_0
ptm abundance 37C
Doses Blue Rep1_0.08 Red Rep2_−0.07
sp|Q9UPU5|UBP24_HUMAN−1141−S−S1_0
ptm abundance 37C
Doses Blue Rep1_0.08 Red Rep2_−0.07

sp|P78317|RNF4_HUMAN−94−S−S2_0.01

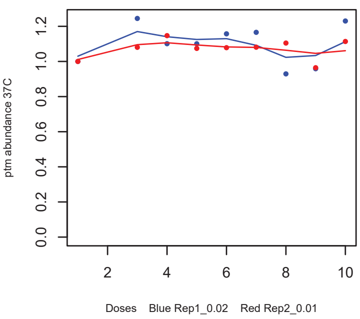

sp|Q13105−2|ZBT17_HUMAN−120−S−S1_0.03

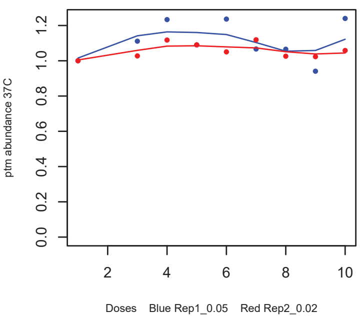

sp|Q12888|TP53B_HUMAN−1673−S−S1_0

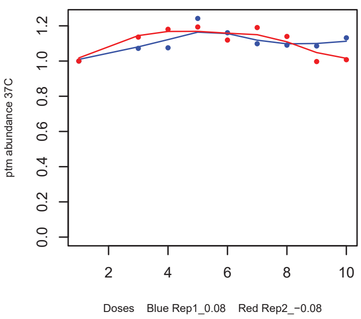

sp|Q93075|TATD2_HUMAN−51−S−S1_−0.15

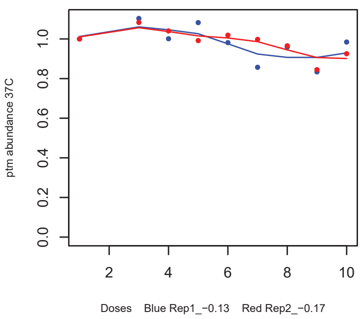

sp|O15417|TNC18_HUMAN−991−S−S2_−0.1

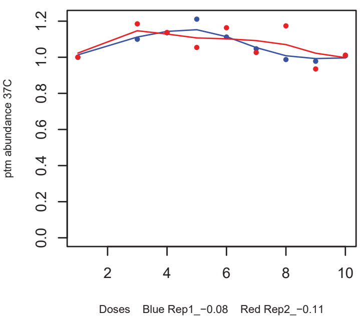

sp|O15417|TNC18_HUMAN−995−S−S2_−0.1

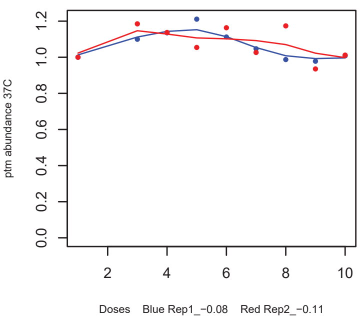

sp|Q9BSU3|NAA11_HUMAN−199−T−S2_−0.03

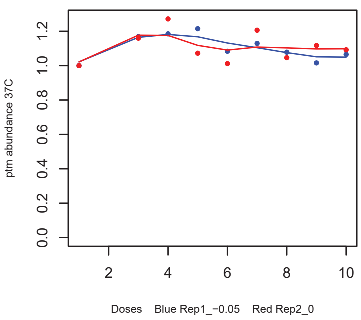

sp|Q9UGU0−2|TCF20_HUMAN−538−S−S2_−0.06

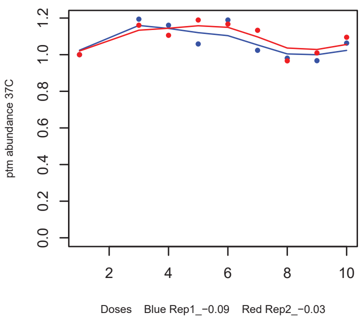

sp|Q8IYB3|SRRM1_HUMAN−775−S−S2_0

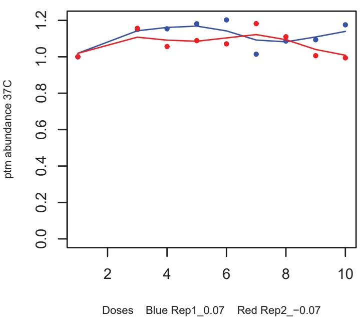

sp|Q9BVS4|RIOK2_HUMAN−382−S−S2_−0.04

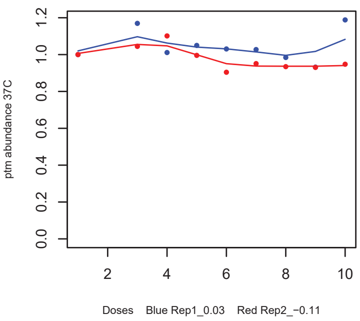

sp|Q9BVS4|RIOK2_HUMAN−385−S−S2_−0.04

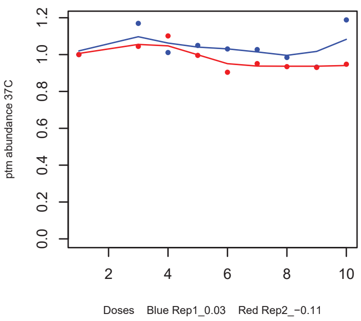

sp|O14976−2|GAK_HUMAN−747−S−S2_0.12

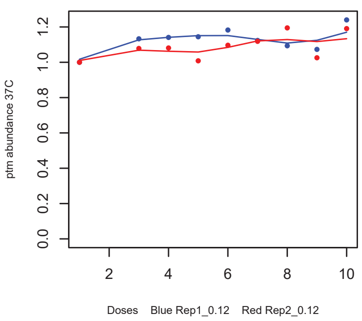

sp|O60583−2|CCNT2_HUMAN−536−S−S3_−0.03

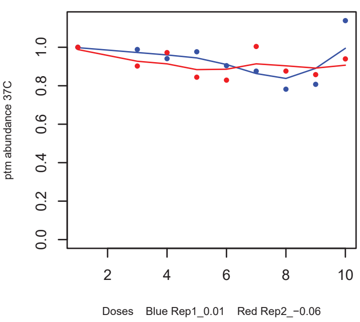

sp|O60583−2|CCNT2_HUMAN−537−S−S3_−0.03

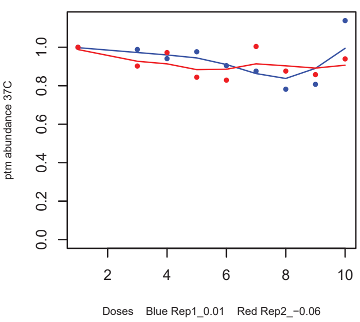

sp|O60583−2|CCNT2_HUMAN−538−S−S3_−0.03

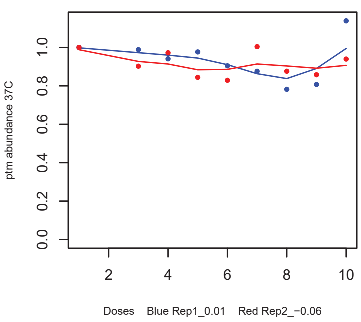

sp|Q8WWM7−6|ATX2L_HUMAN−600−T−S2_0.11

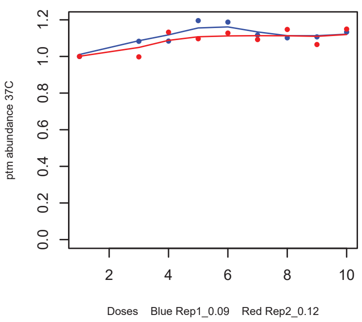

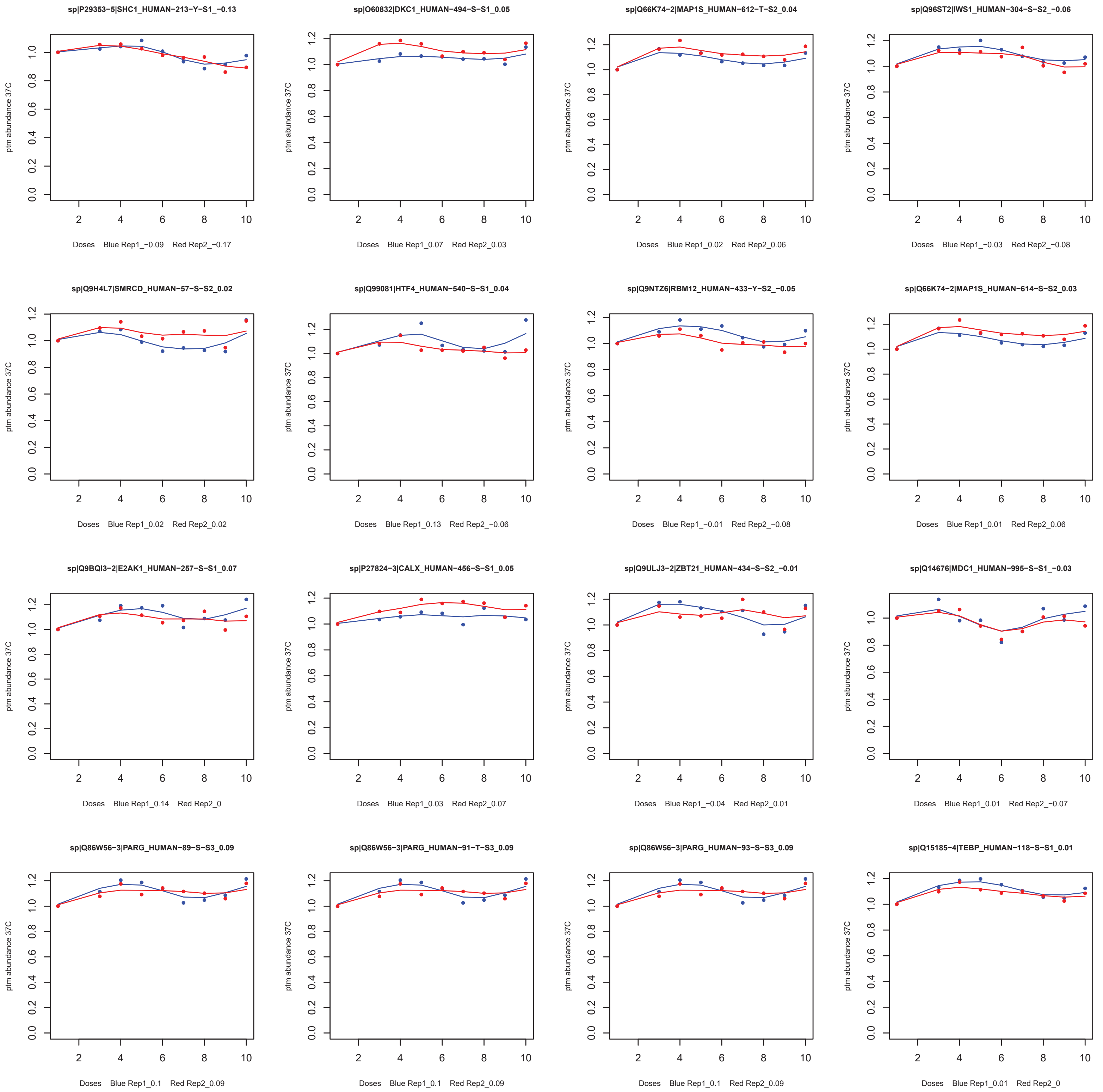
sp|P29353−5|SHC1_HUMAN−213−Y−S1_−0.13
Doses Blue Rep1_−0.09 Red Rep2_−0.17
sp|O60832|DKC1_HUMAN−494−S−S1_0.05
Doses Blue Rep1_0.07 Red Rep2_0.03
sp|Q66K74−2|MAP1S_HUMAN−612−T−S2_0.04
Doses Blue Rep1_0.02 Red Rep2_0.06
sp|Q96ST2|IWS1_HUMAN−304−S−S2_−0.06
Doses Blue Rep1_−0.03 Red Rep2_−0.08
sp|Q9H4L7|SMRCD_HUMAN−57−S−S2_0.02
Doses Blue Rep1_0.02 Red Rep2_0.02
sp|Q99081|HTF4_HUMAN−540−S−S1_0.04
Doses Blue Rep1_0.13 Red Rep2_−0.06
sp|Q9NTZ6|RBM12_HUMAN−433−Y−S2_−0.05
Doses Blue Rep1_−0.01 Red Rep2_−0.08
sp|Q66K74−2|MAP1S_HUMAN−614−S−S2_0.03
Doses Blue Rep1_0.01 Red Rep2_0.06
sp|Q9BQI3−2|E2AK1_HUMAN−257−S−S1_0.07
Doses Blue Rep1_0.14 Red Rep2_0
sp|P27824−3|CALX_HUMAN−456−S−S1_0.05
Doses Blue Rep1_0.03 Red Rep2_0.07
sp|Q9ULJ3−2|ZBT21_HUMAN−434−S−S2_−0.01
Doses Blue Rep1_−0.04 Red Rep2_0.01
sp|Q14676|MDC1_HUMAN−995−S−S1_−0.03
Doses Blue Rep1_0.01 Red Rep2_−0.07
sp|Q86W56−3|PARG_HUMAN−89−S−S3_0.09
Doses Blue Rep1_0.1 Red Rep2_0.09
sp|Q86W56−3|PARG_HUMAN−91−T−S3_0.09
Doses Blue Rep1_0.1 Red Rep2_0.09
sp|Q86W56−3|PARG_HUMAN−93−S−S3_0.09
Doses Blue Rep1_0.1 Red Rep2_0.09
sp|Q15185−4|TEBP_HUMAN−118−S−S1_0.01
Doses Blue Rep1_0.01 Red Rep2_0
ptm abundance 37C

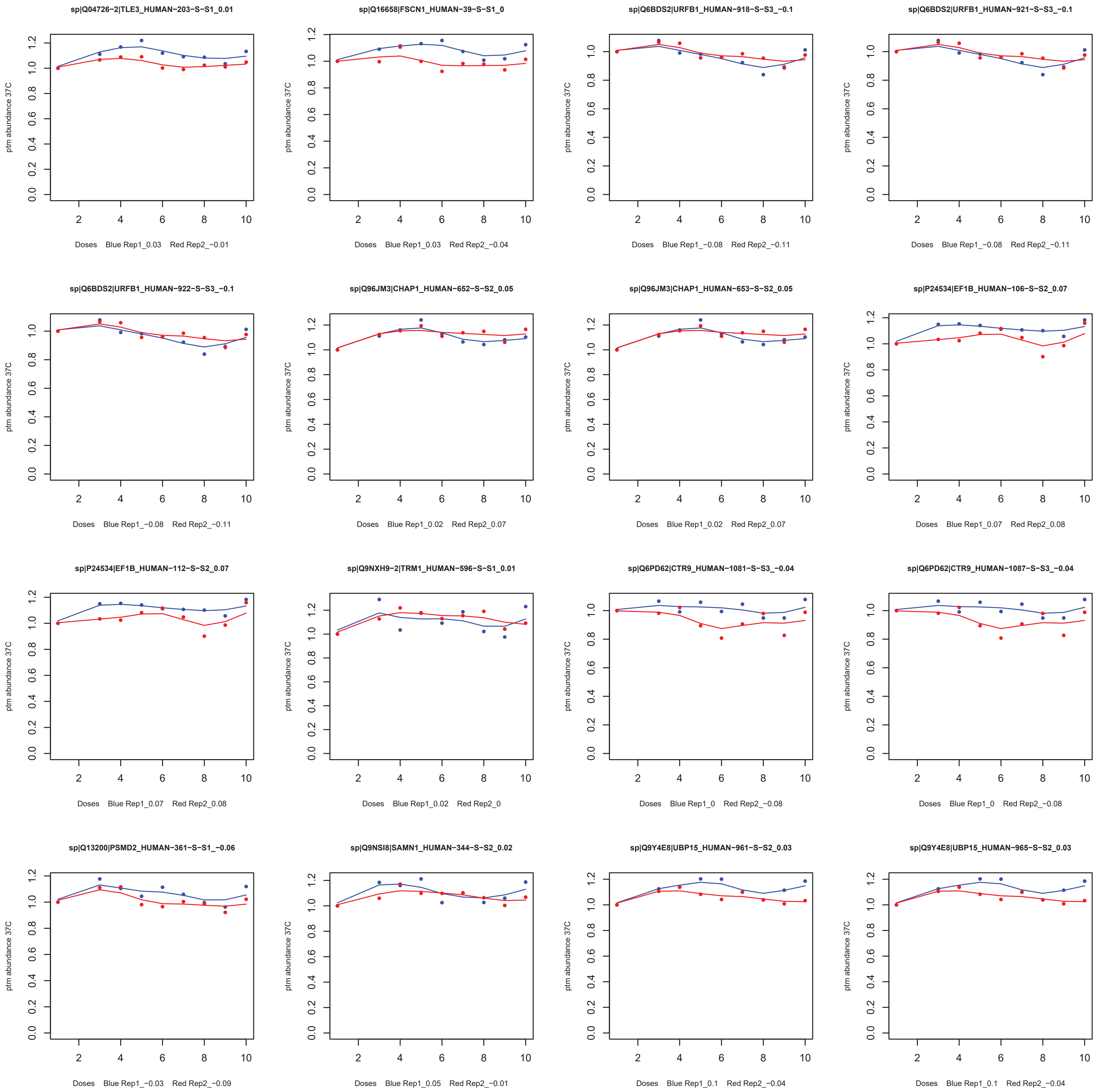
sp|Q04726−2|TLE3_HUMAN−203−S−S1_0.01
Doses Blue Rep1_0.03 Red Rep2_−0.01
sp|Q16658|FSCN1_HUMAN−39−S−S1_0
Doses Blue Rep1_0.03 Red Rep2_−0.04
sp|Q6BDS2|URFB1_HUMAN−918−S−S3_−0.1
Doses Blue Rep1_−0.08 Red Rep2_−0.11
sp|Q6BDS2|URFB1_HUMAN−921−S−S3_−0.1
Doses Blue Rep1_−0.08 Red Rep2_−0.11
sp|Q6BDS2|URFB1_HUMAN−922−S−S3_−0.1
Doses Blue Rep1_−0.08 Red Rep2_−0.11
sp|Q96JM3|CHAP1_HUMAN−652−S−S2_0.05
Doses Blue Rep1_0.02 Red Rep2_0.07
sp|Q96JM3|CHAP1_HUMAN−653−S−S2_0.05
Doses Blue Rep1_0.02 Red Rep2_0.07
sp|P24534|EF1B_HUMAN−106−S−S2_0.07
Doses Blue Rep1_0.07 Red Rep2_0.08
sp|P24534|EF1B_HUMAN−112−S−S2_0.07
Doses Blue Rep1_0.07 Red Rep2_0.08
sp|Q9NXH9−2|TRM1_HUMAN−596−S−S1_0.01
Doses Blue Rep1_0.02 Red Rep2_0
sp|Q6PD62|CTR9_HUMAN−1081−S−S3_−0.04
Doses Blue Rep1_0 Red Rep2_−0.08
sp|Q6PD62|CTR9_HUMAN−1087−S−S3_−0.04
Doses Blue Rep1_0 Red Rep2_−0.08
sp|Q13200|PSMD2_HUMAN−361−S−S1_−0.06
Doses Blue Rep1_−0.03 Red Rep2_−0.09
sp|Q9NSI8|SAMN1_HUMAN−344−S−S2_0.02
Doses Blue Rep1_0.05 Red Rep2_−0.01
sp|Q9Y4E8|UBP15_HUMAN−961−S−S2_0.03
Doses Blue Rep1_0.1 Red Rep2_−0.04
sp|Q9Y4E8|UBP15_HUMAN−965−S−S2_0.03
Doses Blue Rep1_0.1 Red Rep2_−0.04
ptm abundance 37C

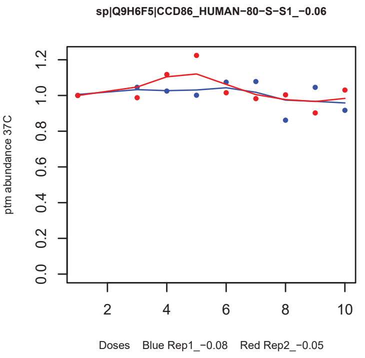
sp|Q9H6F5|CCD86_HUMAN−80−S−S1_−0.06
ptm abundance 37C
Doses Blue Rep1_−0.08 Red Rep2_−0.05

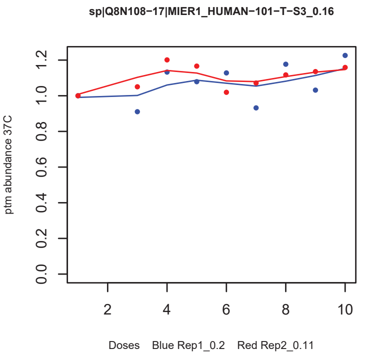
sp|Q8N108−17|MIER1_HUMAN−101−T−S3_0.16
ptm abundance 37C
Doses Blue Rep1_0.2 Red Rep2_0.11

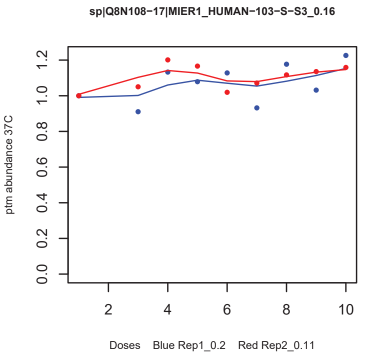
sp|Q8N108−17|MIER1_HUMAN−103−S−S3_0.16
ptm abundance 37C
Doses Blue Rep1_0.2 Red Rep2_0.11

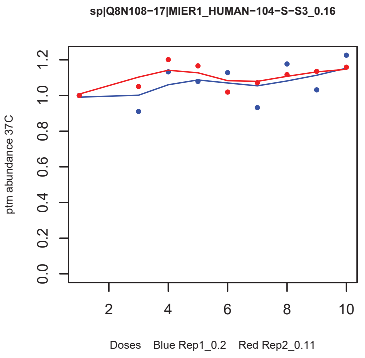
sp|Q8N108−17|MIER1_HUMAN−104−S−S3_0.16
ptm abundance 37C
Doses Blue Rep1_0.2 Red Rep2_0.11

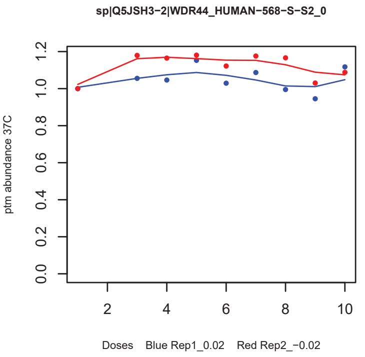
sp|Q5JSH3−2|WDR44_HUMAN−568−S−S2_0
ptm abundance 37C
Doses Blue Rep1_0.02 Red Rep2_−0.02

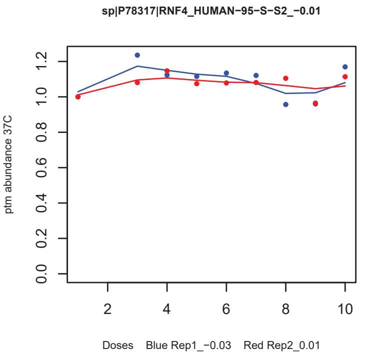
sp|P78317|RNF4_HUMAN−95−S−S2_−0.01
ptm abundance 37C
Doses Blue Rep1_−0.03 Red Rep2_0.01

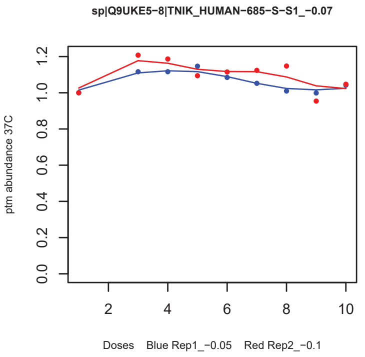
sp|Q9UKE5−8|TNIK_HUMAN−685−S−S1_−0.07
ptm abundance 37C
Doses Blue Rep1_−0.05 Red Rep2_−0.1

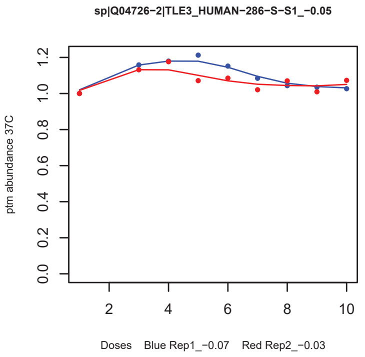
sp|Q04726−2|TLE3_HUMAN−286−S−S1_−0.05
ptm abundance 37C
Doses Blue Rep1_−0.07 Red Rep2_−0.03

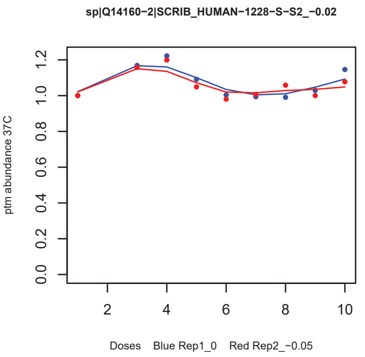
sp|Q14160−2|SCRIB_HUMAN−1228−S−S2_−0.02
ptm abundance 37C
Doses Blue Rep1_0 Red Rep2_−0.05

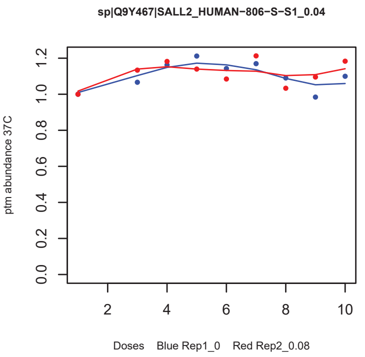
sp|Q9Y467|SALL2_HUMAN−806−S−S1_0.04
ptm abundance 37C
Doses Blue Rep1_0 Red Rep2_0.08

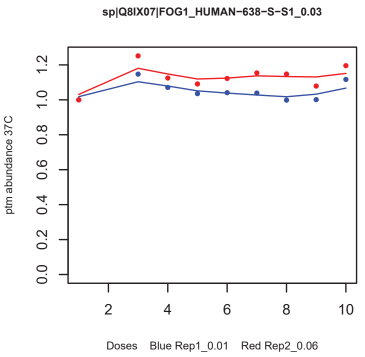
sp|Q8IX07|FOG1_HUMAN−638−S−S1_0.03
ptm abundance 37C
Doses Blue Rep1_0.01 Red Rep2_0.06

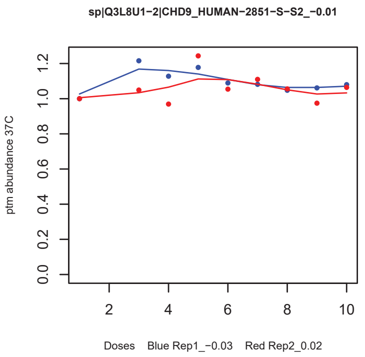
sp|Q3L8U1−2|CHD9_HUMAN−2851−S−S2_−0.01
ptm abundance 37C
Doses Blue Rep1_−0.03 Red Rep2_0.02

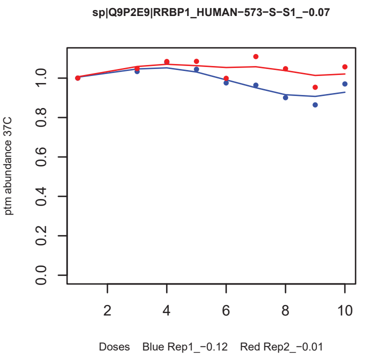
sp|Q9P2E9|RRBP1_HUMAN−573−S−S1_−0.07
ptm abundance 37C
Doses Blue Rep1_−0.12 Red Rep2_−0.01

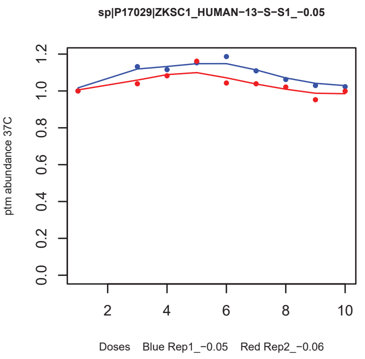
sp|P17029|ZKSC1_HUMAN−13−S−S1_−0.05
ptm abundance 37C
Doses Blue Rep1_−0.05 Red Rep2_−0.06

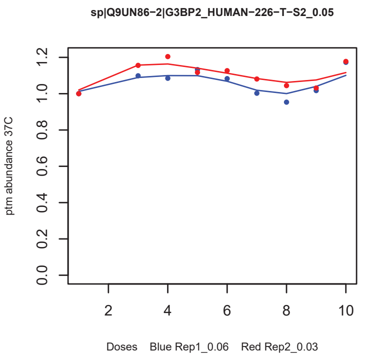
sp|Q9UN86−2|G3BP2_HUMAN−226−T−S2_0.05
ptm abundance 37C
Doses Blue Rep1_0.06 Red Rep2_0.03

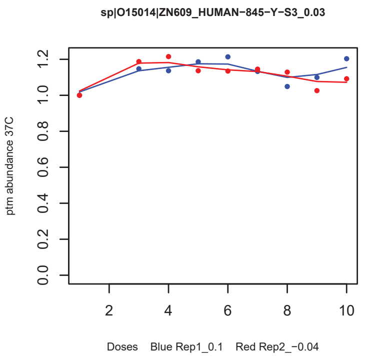
sp|O15014|ZN609_HUMAN−845−Y−S3_0.03
ptm abundance 37C
Doses Blue Rep1_0.1 Red Rep2_−0.04

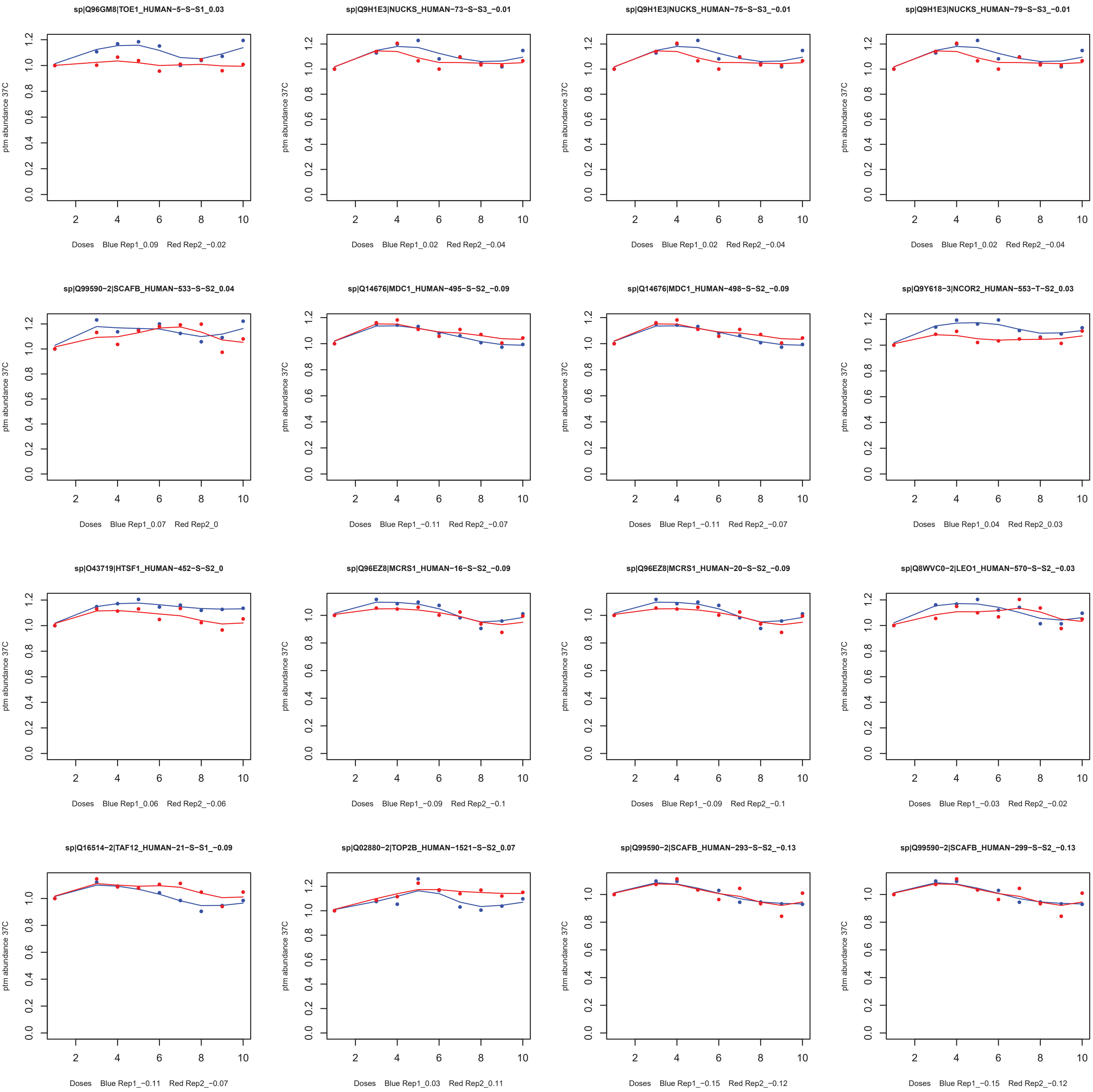
sp|Q96GM8|TOE1_HUMAN−5−S−S1_0.03
Doses Blue Rep1_0.09 Red Rep2_−0.02
sp|Q9H1E3|NUCKS_HUMAN−73−S−S3_−0.01
Doses Blue Rep1_0.02 Red Rep2_−0.04
sp|Q9H1E3|NUCKS_HUMAN−75−S−S3_−0.01
Doses Blue Rep1_0.02 Red Rep2_−0.04
sp|Q9H1E3|NUCKS_HUMAN−79−S−S3_−0.01
Doses Blue Rep1_0.02 Red Rep2_−0.04
sp|Q99590−2|SCAFB_HUMAN−533−S−S2_0.04
Doses Blue Rep1_0.07 Red Rep2_0
sp|Q14676|MDC1_HUMAN−495−S−S2_−0.09
Doses Blue Rep1_−0.11 Red Rep2_−0.07
sp|Q14676|MDC1_HUMAN−498−S−S2_−0.09
Doses Blue Rep1_−0.11 Red Rep2_−0.07
sp|Q9Y618−3|NCOR2_HUMAN−553−T−S2_0.03
Doses Blue Rep1_0.04 Red Rep2_0.03
sp|O43719|HTSF1_HUMAN−452−S−S2_0
Doses Blue Rep1_0.06 Red Rep2_−0.06
sp|Q96EZ8|MCRS1_HUMAN−16−S−S2_−0.09
Doses Blue Rep1_−0.09 Red Rep2_−0.1
sp|Q96EZ8|MCRS1_HUMAN−20−S−S2_−0.09
Doses Blue Rep1_−0.09 Red Rep2_−0.1
sp|Q8WVC0−2|LEO1_HUMAN−570−S−S2_−0.03
Doses Blue Rep1_−0.03 Red Rep2_−0.02
sp|Q16514−2|TAF12_HUMAN−21−S−S1_−0.09
Doses Blue Rep1_−0.11 Red Rep2_−0.07
sp|Q02880−2|TOP2B_HUMAN−1521−S−S2_0.07
Doses Blue Rep1_0.03 Red Rep2_0.11
sp|Q99590−2|SCAFB_HUMAN−293−S−S2_−0.13
Doses Blue Rep1_−0.15 Red Rep2_−0.12
sp|Q99590−2|SCAFB_HUMAN−299−S−S2_−0.13
Doses Blue Rep1_−0.15 Red Rep2_−0.12
ptm abundance 37C

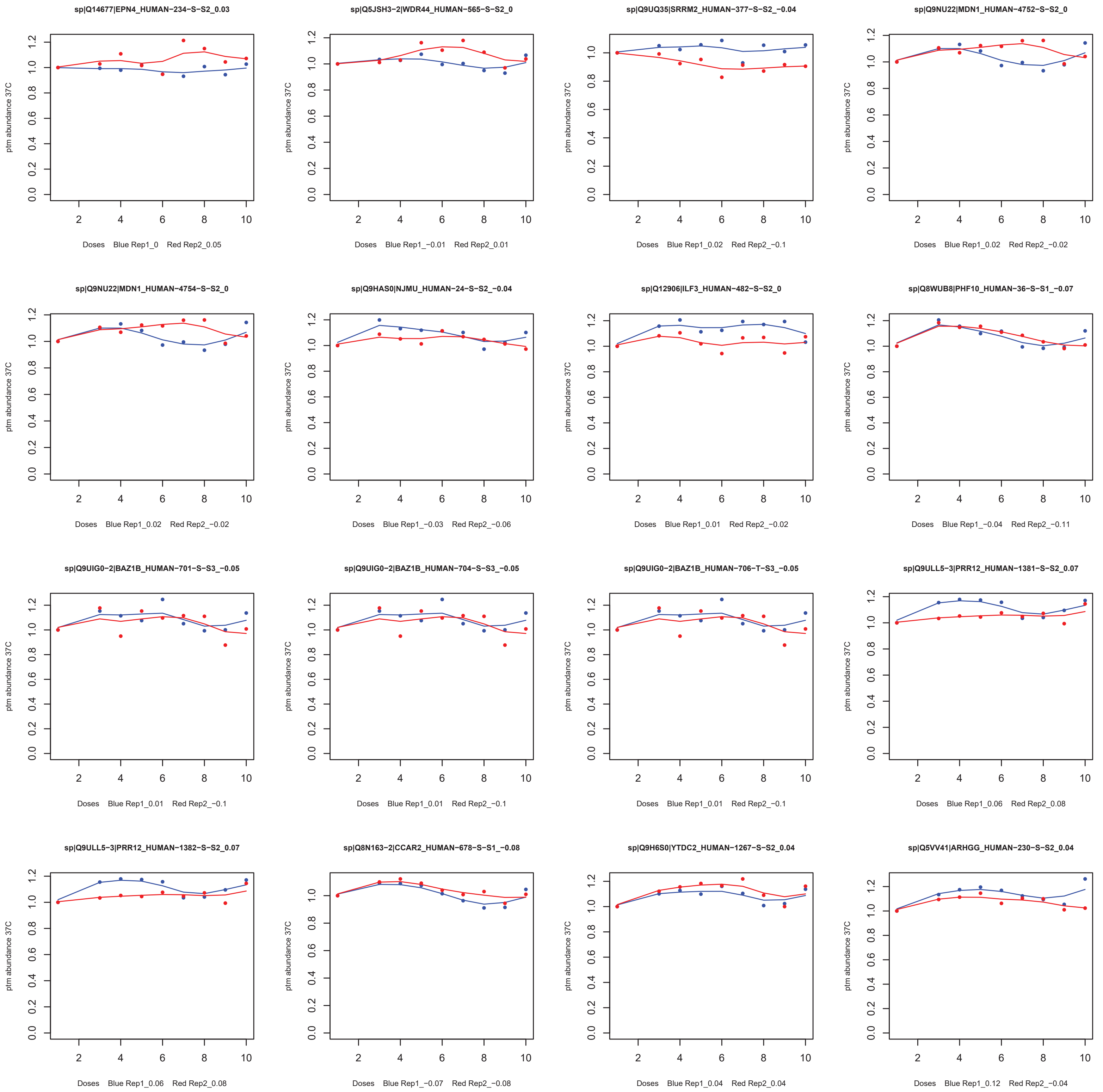
sp|Q14677|EPN4_HUMAN−234−S−S2_0.03
Doses Blue Rep1_0 Red Rep2_0.05
sp|Q5JSH3−2|WDR44_HUMAN−565−S−S2_0
Doses Blue Rep1_−0.01 Red Rep2_0.01
sp|Q9UQ35|SRRM2_HUMAN−377−S−S2_−0.04
Doses Blue Rep1_0.02 Red Rep2_−0.1
sp|Q9NU22|MDN1_HUMAN−4752−S−S2_0
Doses Blue Rep1_0.02 Red Rep2_−0.02
sp|Q9NU22|MDN1_HUMAN−4754−S−S2_0
Doses Blue Rep1_0.02 Red Rep2_−0.02
sp|Q9HAS0|NJMU_HUMAN−24−S−S2_−0.04
Doses Blue Rep1_−0.03 Red Rep2_−0.06
sp|Q12906|ILF3_HUMAN−482−S−S2_0
Doses Blue Rep1_0.01 Red Rep2_−0.02
sp|Q8WUB8|PHF10_HUMAN−36−S−S1_−0.07
Doses Blue Rep1_−0.04 Red Rep2_−0.11
sp|Q9UIG0−2|BAZ1B_HUMAN−701−S−S3_−0.05
Doses Blue Rep1_0.01 Red Rep2_−0.1
sp|Q9UIG0−2|BAZ1B_HUMAN−704−S−S3_−0.05
Doses Blue Rep1_0.01 Red Rep2_−0.1
sp|Q9UIG0−2|BAZ1B_HUMAN−706−T−S3_−0.05
Doses Blue Rep1_0.01 Red Rep2_−0.1
sp|Q9ULL5−3|PRR12_HUMAN−1381−S−S2_0.07
Doses Blue Rep1_0.06 Red Rep2_0.08
sp|Q9ULL5−3|PRR12_HUMAN−1382−S−S2_0.07
Doses Blue Rep1_0.06 Red Rep2_0.08
sp|Q8N163−2|CCAR2_HUMAN−678−S−S1_−0.08
Doses Blue Rep1_−0.07 Red Rep2_−0.08
sp|Q9H6S0|YTDC2_HUMAN−1267−S−S2_0.04
Doses Blue Rep1_0.04 Red Rep2_0.04
sp|Q5VV41|ARHGG_HUMAN−230−S−S2_0.04
Doses Blue Rep1_0.12 Red Rep2_−0.04
ptm abundance 37C

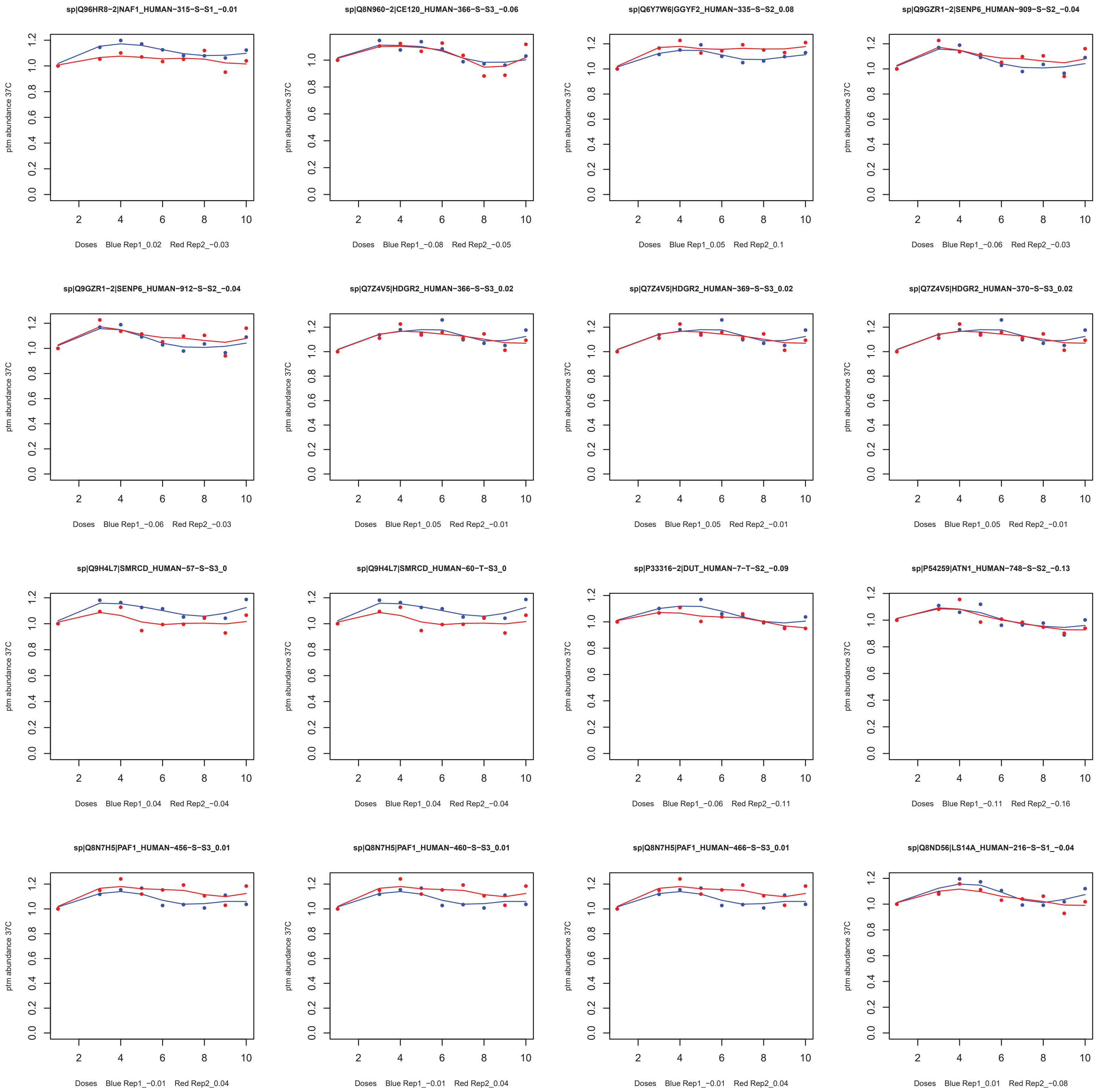

sp|Q96HR8−2|NAF1_HUMAN−315−S−S1_−0.01
Doses Blue Rep1_0.02 Red Rep2_−0.03
sp|Q8N960−2|CE120_HUMAN−366−S−S3_−0.06
Doses Blue Rep1_−0.08 Red Rep2_−0.05
sp|Q6Y7W6|GGYF2_HUMAN−335−S−S2_0.08
Doses Blue Rep1_0.05 Red Rep2_0.1
sp|Q9GZR1−2|SENP6_HUMAN−909−S−S2_−0.04
Doses Blue Rep1_−0.06 Red Rep2_−0.03
sp|Q9GZR1−2|SENP6_HUMAN−912−S−S2_−0.04
Doses Blue Rep1_−0.06 Red Rep2_−0.03
sp|Q7Z4V5|HDGR2_HUMAN−366−S−S3_0.02
Doses Blue Rep1_0.05 Red Rep2_−0.01
sp|Q7Z4V5|HDGR2_HUMAN−369−S−S3_0.02
Doses Blue Rep1_0.05 Red Rep2_−0.01
sp|Q7Z4V5|HDGR2_HUMAN−370−S−S3_0.02
Doses Blue Rep1_0.05 Red Rep2_−0.01
sp|Q9H4L7|SMRCD_HUMAN−57−S−S3_0
Doses Blue Rep1_0.04 Red Rep2_−0.04
sp|Q9H4L7|SMRCD_HUMAN−60−T−S3_0
Doses Blue Rep1_0.04 Red Rep2_−0.04
sp|P33316−2|DUT_HUMAN−7−T−S2_−0.09
Doses Blue Rep1_−0.06 Red Rep2_−0.11
sp|P54259|ATN1_HUMAN−748−S−S2_−0.13
Doses Blue Rep1_−0.11 Red Rep2_−0.16
sp|Q8N7H5|PAF1_HUMAN−456−S−S3_0.01
Doses Blue Rep1_−0.01 Red Rep2_0.04
sp|Q8N7H5|PAF1_HUMAN−460−S−S3_0.01
Doses Blue Rep1_−0.01 Red Rep2_0.04
sp|Q8N7H5|PAF1_HUMAN−466−S−S3_0.01
Doses Blue Rep1_−0.01 Red Rep2_0.04
sp|Q8ND56|LS14A_HUMAN−216−S−S1_−0.04
Doses Blue Rep1_0.01 Red Rep2_−0.08
ptm abundance 37C

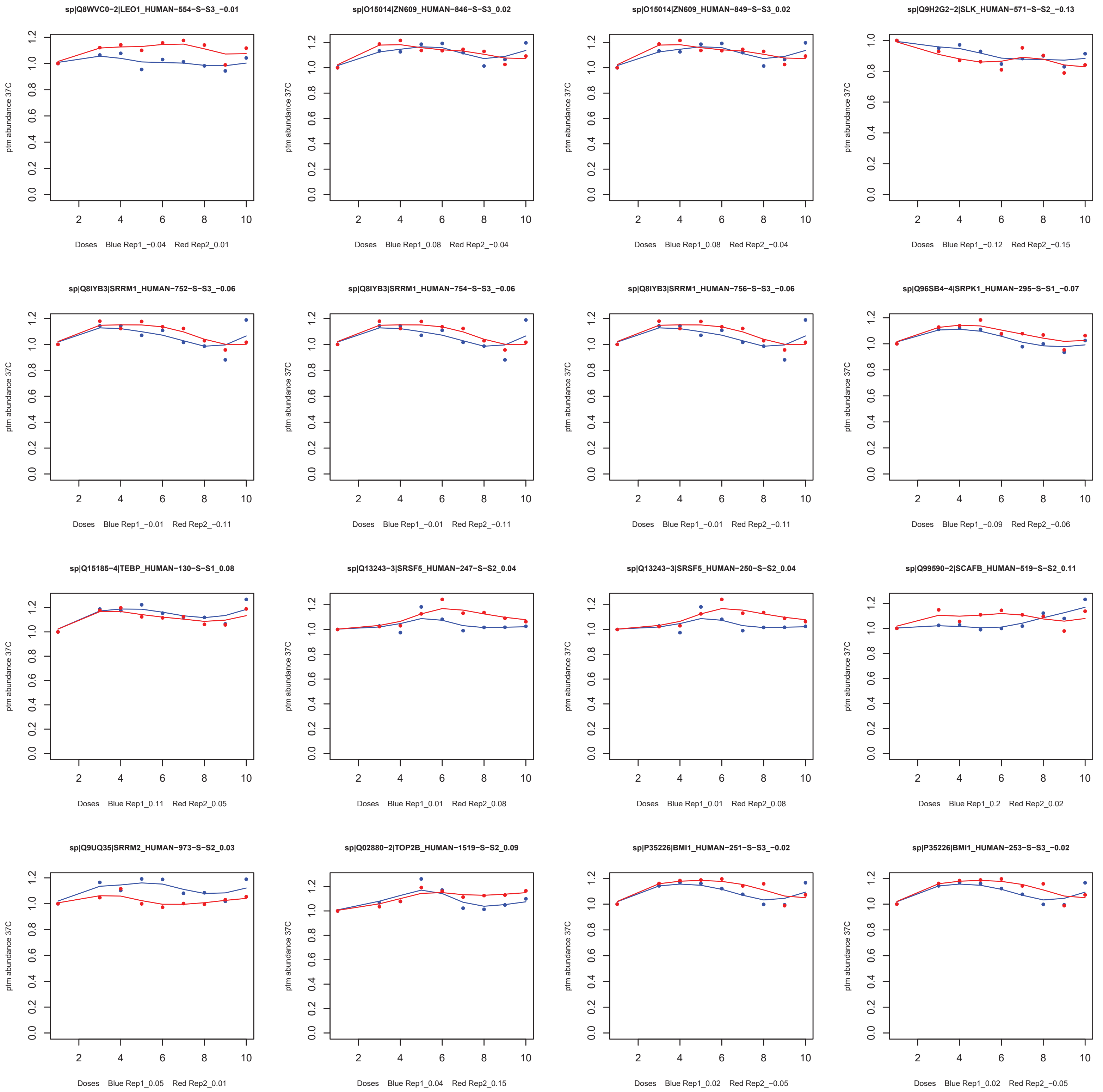
sp|Q8WVC0−2|LEO1_HUMAN−554−S−S3_−0.01
Doses Blue Rep1_−0.04 Red Rep2_0.01
sp|O15014|ZN609_HUMAN−846−S−S3_0.02
Doses Blue Rep1_0.08 Red Rep2_−0.04
sp|O15014|ZN609_HUMAN−849−S−S3_0.02
Doses Blue Rep1_0.08 Red Rep2_−0.04
sp|Q9H2G2−2|SLK_HUMAN−571−S−S2_−0.13
Doses Blue Rep1_−0.12 Red Rep2_−0.15
sp|Q8IYB3|SRRM1_HUMAN−752−S−S3_−0.06
Doses Blue Rep1_−0.01 Red Rep2_−0.11
sp|Q8IYB3|SRRM1_HUMAN−754−S−S3_−0.06
Doses Blue Rep1_−0.01 Red Rep2_−0.11
sp|Q8IYB3|SRRM1_HUMAN−756−S−S3_−0.06
Doses Blue Rep1_−0.01 Red Rep2_−0.11
sp|Q96SB4−4|SRPK1_HUMAN−295−S−S1_−0.07
Doses Blue Rep1_−0.09 Red Rep2_−0.06
sp|Q15185−4|TEBP_HUMAN−130−S−S1_0.08
Doses Blue Rep1_0.11 Red Rep2_0.05
sp|Q13243−3|SRSF5_HUMAN−247−S−S2_0.04
Doses Blue Rep1_0.01 Red Rep2_0.08
sp|Q13243−3|SRSF5_HUMAN−250−S−S2_0.04
Doses Blue Rep1_0.01 Red Rep2_0.08
sp|Q99590−2|SCAFB_HUMAN−519−S−S2_0.11
Doses Blue Rep1_0.2 Red Rep2_0.02
sp|Q9UQ35|SRRM2_HUMAN−973−S−S2_0.03
Doses Blue Rep1_0.05 Red Rep2_0.01
sp|Q02880−2|TOP2B_HUMAN−1519−S−S2_0.09
Doses Blue Rep1_0.04 Red Rep2_0.15
sp|P35226|BMI1_HUMAN−251−S−S3_−0.02
Doses Blue Rep1_0.02 Red Rep2_−0.05
sp|P35226|BMI1_HUMAN−253−S−S3_−0.02
Doses Blue Rep1_0.02 Red Rep2_−0.05
ptm abundance 37C

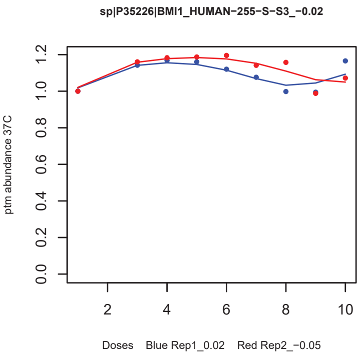
sp|P35226|BMI1_HUMAN−255−S−S3_−0.02
ptm abundance 37C
Doses Blue Rep1_0.02 Red Rep2_−0.05

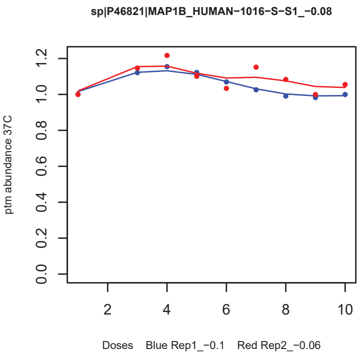
sp|P46821|MAP1B_HUMAN−1016−S−S1_−0.08
ptm abundance 37C
Doses Blue Rep1_−0.1 Red Rep2_−0.06

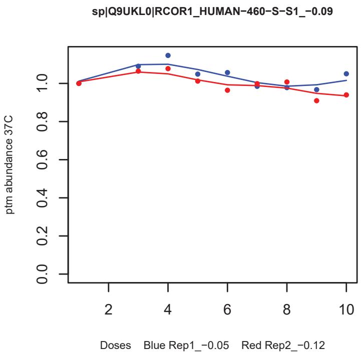
sp|Q9UKL0|RCOR1_HUMAN−460−S−S1_−0.09
ptm abundance 37C
Doses Blue Rep1_−0.05 Red Rep2_−0.12

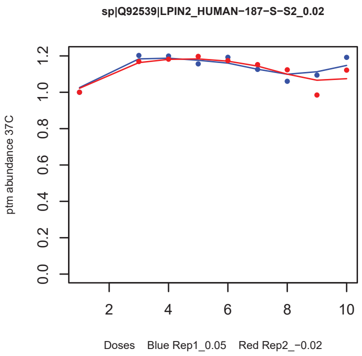
sp|Q92539|LPIN2_HUMAN−187−S−S2_0.02
ptm abundance 37C
Doses Blue Rep1_0.05 Red Rep2_−0.02

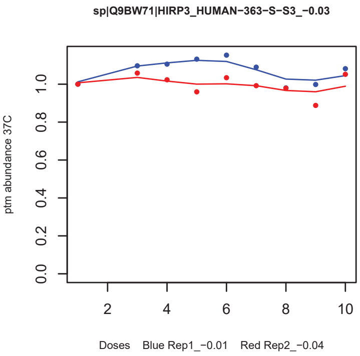
sp|Q9BW71|HIRP3_HUMAN−363−S−S3_−0.03
ptm abundance 37C
Doses Blue Rep1_−0.01 Red Rep2_−0.04

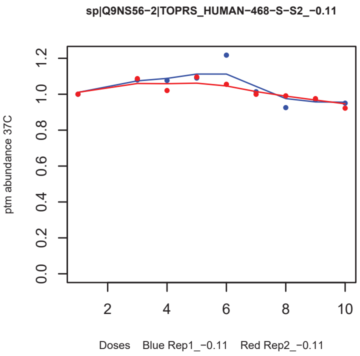
sp|Q9NS56−2|TOPRS_HUMAN−468−S−S2_−0.11
ptm abundance 37C
Doses Blue Rep1_−0.11 Red Rep2_−0.11

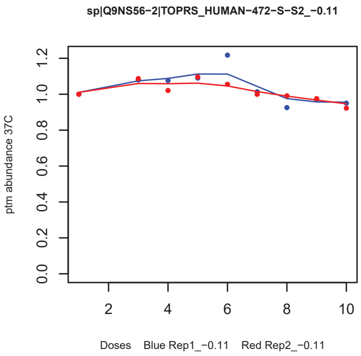
sp|Q9NS56−2|TOPRS_HUMAN−472−S−S2_−0.11
ptm abundance 37C
Doses Blue Rep1_−0.11 Red Rep2_−0.11

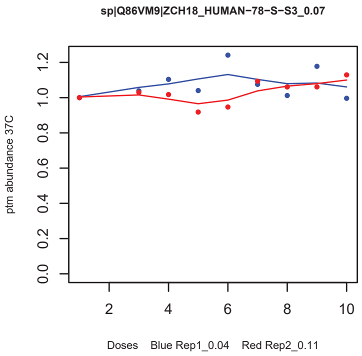
sp|Q86VM9|ZCH18_HUMAN−78−S−S3_0.07
ptm abundance 37C
Doses Blue Rep1_0.04 Red Rep2_0.11

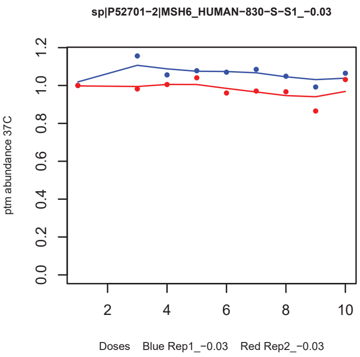
sp|P52701−2|MSH6_HUMAN−830−S−S1_−0.03
ptm abundance 37C
Doses Blue Rep1_−0.03 Red Rep2_−0.03

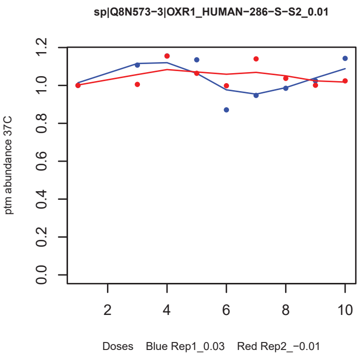
sp|Q8N573−3|OXR1_HUMAN−286−S−S2_0.01
ptm abundance 37C
Doses Blue Rep1_0.03 Red Rep2_−0.01

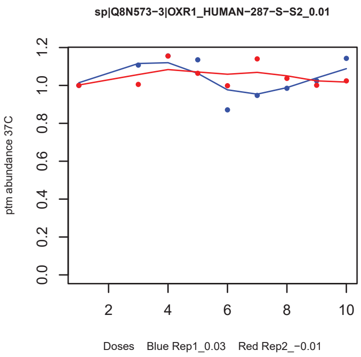
sp|Q8N573−3|OXR1_HUMAN−287−S−S2_0.01
ptm abundance 37C
Doses Blue Rep1_0.03 Red Rep2_−0.01

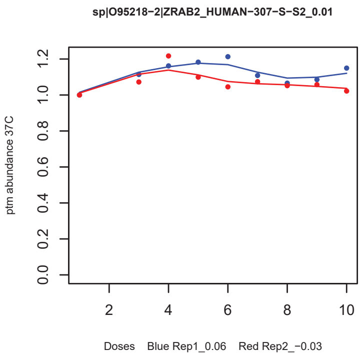
sp|O95218−2|ZRAB2_HUMAN−307−S−S2_0.01
ptm abundance 37C
Doses Blue Rep1_0.06 Red Rep2_−0.03

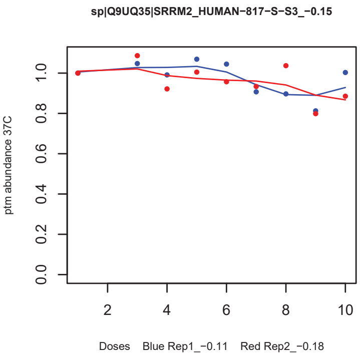
sp|Q9UQ35|SRRM2_HUMAN−817−S−S3_−0.15
ptm abundance 37C
Doses Blue Rep1_−0.11 Red Rep2_−0.18

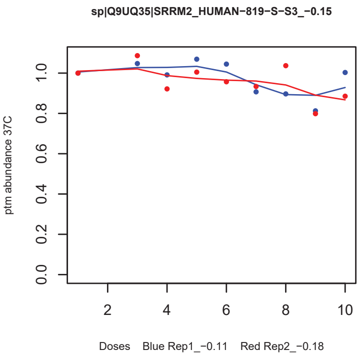
sp|Q9UQ35|SRRM2_HUMAN−819−S−S3_−0.15
ptm abundance 37C
Doses Blue Rep1_−0.11 Red Rep2_−0.18

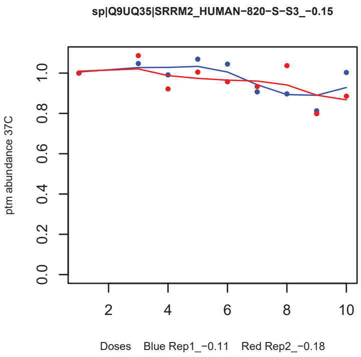
sp|Q9UQ35|SRRM2_HUMAN−820−S−S3_−0.15
ptm abundance 37C
Doses Blue Rep1_−0.11 Red Rep2_−0.18

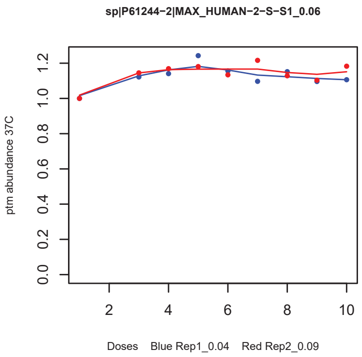
sp|P61244−2|MAX_HUMAN−2−S−S1_0.06
ptm abundance 37C
Doses Blue Rep1_0.04 Red Rep2_0.09

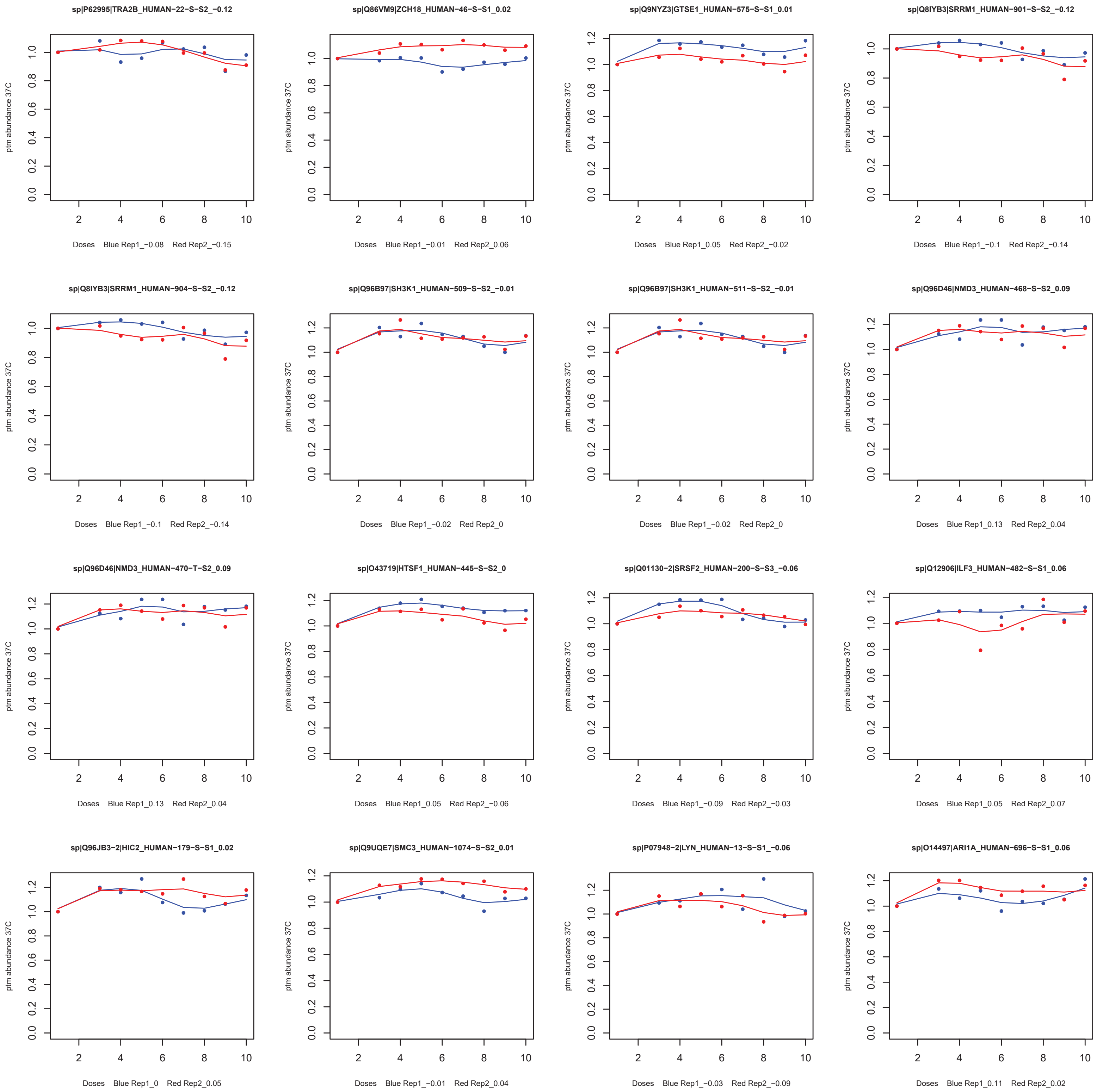
sp|P62995|TRA2B_HUMAN−22−S−S2_−0.12
ptm abundance 37C
Doses Blue Rep1_−0.08 Red Rep2_−0.15
sp|Q86VM9|ZCH18_HUMAN−46−S−S1_0.02
ptm abundance 37C
Doses Blue Rep1_−0.01 Red Rep2_0.06
sp|Q9NYZ3|GTSE1_HUMAN−575−S−S1_0.01
ptm abundance 37C
Doses Blue Rep1_0.05 Red Rep2_−0.02
sp|Q8IYB3|SRRM1_HUMAN−901−S−S2_−0.12
ptm abundance 37C
Doses Blue Rep1_−0.1 Red Rep2_−0.14
sp|Q8IYB3|SRRM1_HUMAN−904−S−S2_−0.12
ptm abundance 37C
Doses Blue Rep1_−0.1 Red Rep2_−0.14
sp|Q96B97|SH3K1_HUMAN−509−S−S2_−0.01
ptm abundance 37C
Doses Blue Rep1_−0.02 Red Rep2_0
sp|Q96B97|SH3K1_HUMAN−511−S−S2_−0.01
ptm abundance 37C
Doses Blue Rep1_−0.02 Red Rep2_0
sp|Q96D46|NMD3_HUMAN−468−S−S2_0.09
ptm abundance 37C
Doses Blue Rep1_0.13 Red Rep2_0.04
sp|Q96D46|NMD3_HUMAN−470−T−S2_0.09
ptm abundance 37C
Doses Blue Rep1_0.13 Red Rep2_0.04
sp|O43719|HTSF1_HUMAN−445−S−S2_0
ptm abundance 37C
Doses Blue Rep1_0.05 Red Rep2_−0.06
sp|Q01130−2|SRSF2_HUMAN−200−S−S3_−0.06
ptm abundance 37C
Doses Blue Rep1_−0.09 Red Rep2_−0.03
sp|Q12906|ILF3_HUMAN−482−S−S1_0.06
ptm abundance 37C
Doses Blue Rep1_0.05 Red Rep2_0.07
sp|Q96JB3−2|HIC2_HUMAN−179−S−S1_0.02
ptm abundance 37C
Doses Blue Rep1_0 Red Rep2_0.05
sp|Q9UQE7|SMC3_HUMAN−1074−S−S2_0.01
ptm abundance 37C
Doses Blue Rep1_−0.01 Red Rep2_0.04
sp|P07948−2|LYN_HUMAN−13−S−S1_−0.06
ptm abundance 37C
Doses Blue Rep1_−0.03 Red Rep2_−0.09
sp|O14497|ARI1A_HUMAN−696−S−S1_0.06
ptm abundance 37C
Doses Blue Rep1_0.11 Red Rep2_0.02

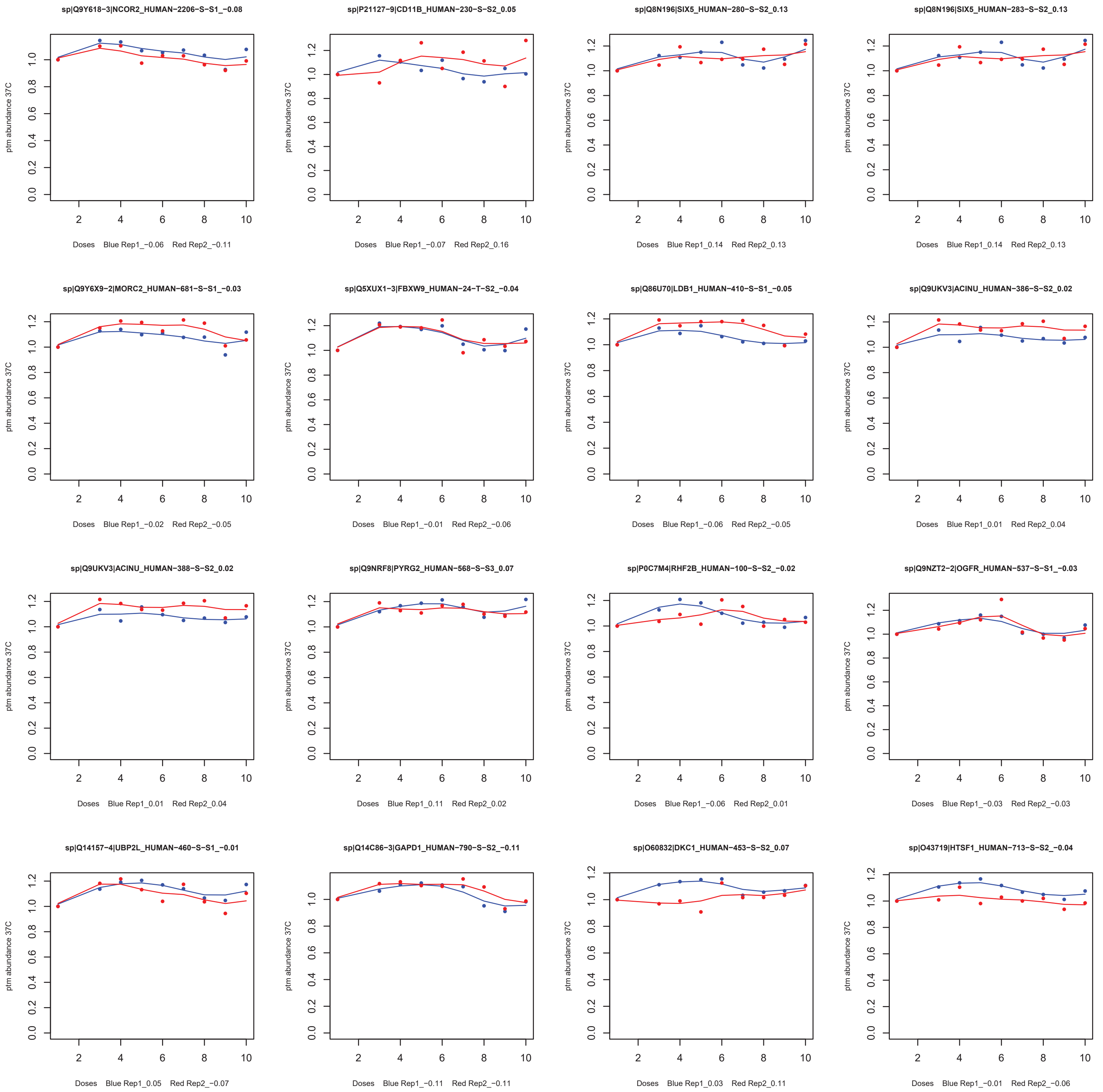
sp|Q9Y618-3|NCOR2_HUMAN-2206-S-S1_-0.08
ptm abundance 37C
Doses Blue Rep1_-0.06 Red Rep2_-0.11
sp|P21127-9|CD11B_HUMAN-230-S-S2_0.05
ptm abundance 37C
Doses Blue Rep1_-0.07 Red Rep2_0.16
sp|Q8N196|SIX5_HUMAN-280-S-S2_0.13
ptm abundance 37C
Doses Blue Rep1_0.14 Red Rep2_0.13
sp|Q8N196|SIX5_HUMAN-283-S-S2_0.13
ptm abundance 37C
Doses Blue Rep1_0.14 Red Rep2_0.13
sp|Q9Y6X9-2|MORC2_HUMAN-681-S-S1_-0.03
ptm abundance 37C
Doses Blue Rep1_-0.02 Red Rep2_-0.05
sp|Q5XUX1-3|FBXW9_HUMAN-24-T-S2_-0.04
ptm abundance 37C
Doses Blue Rep1_-0.01 Red Rep2_-0.06
sp|Q86U70|LDB1_HUMAN-410-S-S1_-0.05
ptm abundance 37C
Doses Blue Rep1_-0.06 Red Rep2_-0.05
sp|Q9UKV3|ACINU_HUMAN-386-S-S2_0.02
ptm abundance 37C
Doses Blue Rep1_0.01 Red Rep2_0.04
sp|Q9UKV3|ACINU_HUMAN-388-S-S2_0.02
ptm abundance 37C
Doses Blue Rep1_0.01 Red Rep2_0.04
sp|Q9NRF8|PYRG2_HUMAN-568-S-S3_0.07
ptm abundance 37C
Doses Blue Rep1_0.11 Red Rep2_0.02
sp|P0C7M4|RHF2B_HUMAN-100-S-S2_-0.02
ptm abundance 37C
Doses Blue Rep1_-0.06 Red Rep2_0.01
sp|Q9NZT2-2|OGFR_HUMAN-537-S-S1_-0.03
ptm abundance 37C
Doses Blue Rep1_-0.03 Red Rep2_-0.03
sp|Q14157-4|UBP2L_HUMAN-460-S-S1_-0.01
ptm abundance 37C
Doses Blue Rep1_0.05 Red Rep2_-0.07
sp|Q14C86-3|GAPD1_HUMAN-790-S-S2_-0.11
ptm abundance 37C
Doses Blue Rep1_-0.11 Red Rep2_-0.11
sp|O60832|DKC1_HUMAN-453-S-S2_0.07
ptm abundance 37C
Doses Blue Rep1_0.03 Red Rep2_0.11
sp|O43719|HTSF1_HUMAN-713-S-S2_-0.04
ptm abundance 37C
Doses Blue Rep1_-0.01 Red Rep2_-0.06

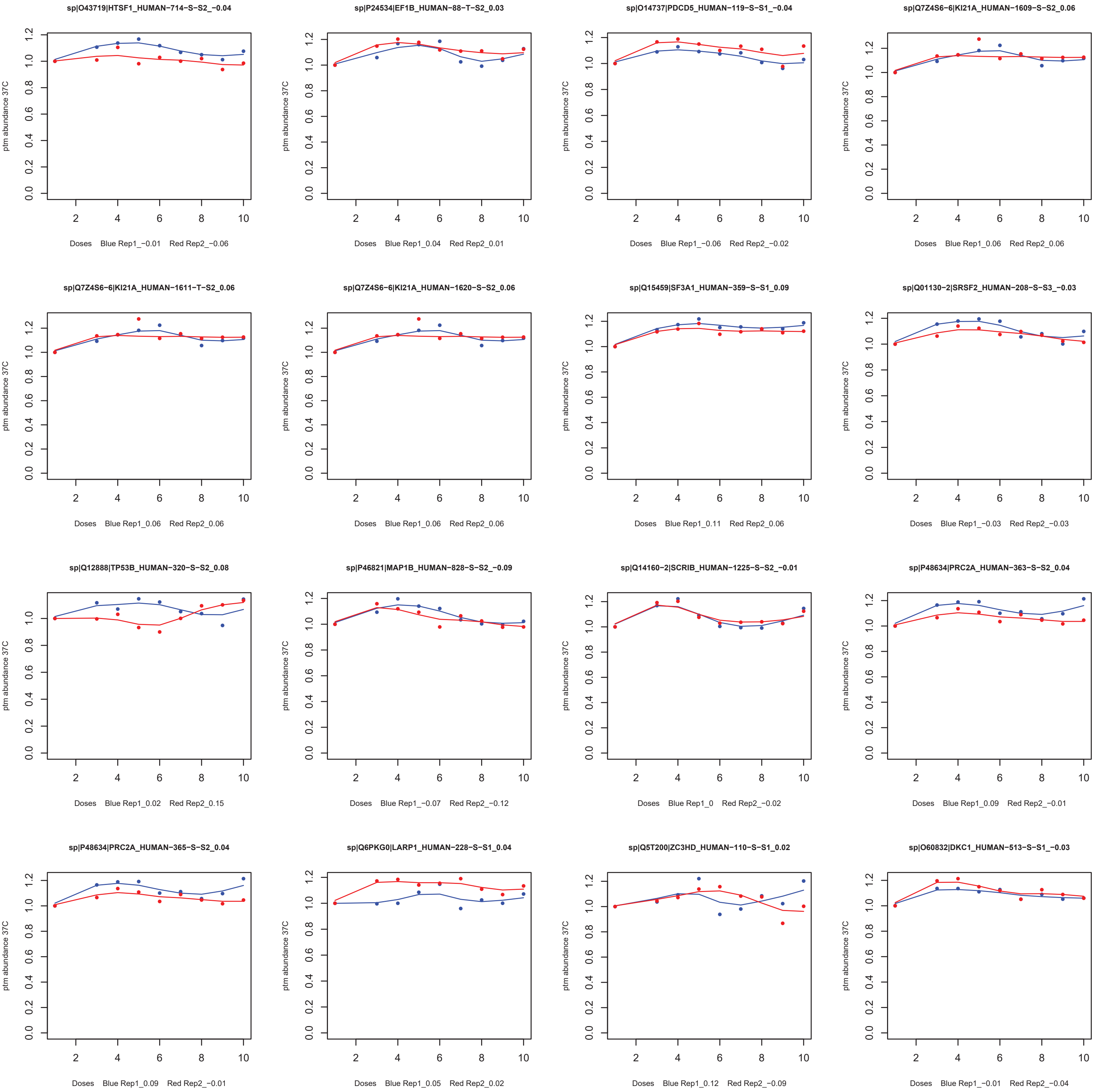
sp|O43719|HTSF1_HUMAN−714−S−S2_−0.04
Doses Blue Rep1_−0.01 Red Rep2_−0.06
sp|P24534|EF1B_HUMAN−88−T−S2_0.03
Doses Blue Rep1_0.04 Red Rep2_0.01
sp|O14737|PDCD5_HUMAN−119−S−S1_−0.04
Doses Blue Rep1_−0.06 Red Rep2_−0.02
sp|Q7Z4S6−6|KI21A_HUMAN−1609−S−S2_0.06
Doses Blue Rep1_0.06 Red Rep2_0.06
sp|Q7Z4S6−6|KI21A_HUMAN−1611−T−S2_0.06
Doses Blue Rep1_0.06 Red Rep2_0.06
sp|Q7Z4S6−6|KI21A_HUMAN−1620−S−S2_0.06
Doses Blue Rep1_0.06 Red Rep2_0.06
sp|Q15459|SF3A1_HUMAN−359−S−S1_0.09
Doses Blue Rep1_0.11 Red Rep2_0.06
sp|Q01130−2|SRSF2_HUMAN−208−S−S3_−0.03
Doses Blue Rep1_−0.03 Red Rep2_−0.03
sp|Q12888|TP53B_HUMAN−320−S−S2_0.08
Doses Blue Rep1_0.02 Red Rep2_0.15
sp|P46821|MAP1B_HUMAN−828−S−S2_−0.09
Doses Blue Rep1_−0.07 Red Rep2_−0.12
sp|Q14160−2|SCRIB_HUMAN−1225−S−S2_−0.01
Doses Blue Rep1_0 Red Rep2_−0.02
sp|P48634|PRC2A_HUMAN−363−S−S2_0.04
Doses Blue Rep1_0.09 Red Rep2_−0.01
sp|P48634|PRC2A_HUMAN−365−S−S2_0.04
Doses Blue Rep1_0.09 Red Rep2_−0.01
sp|Q6PKG0|LARP1_HUMAN−228−S−S1_0.04
Doses Blue Rep1_0.05 Red Rep2_0.02
sp|Q5T200|ZC3HD_HUMAN−110−S−S1_0.02
Doses Blue Rep1_0.12 Red Rep2_−0.09
sp|O60832|DKC1_HUMAN−513−S−S1_−0.03
Doses Blue Rep1_−0.01 Red Rep2_−0.04
ptm abundance 37C

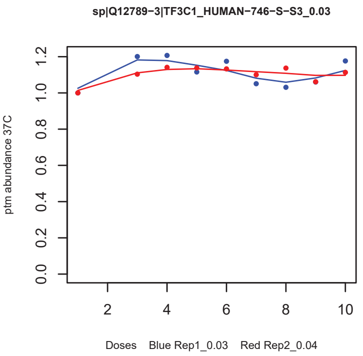
sp|Q12789−3|TF3C1_HUMAN−746−S−S3_0.03
ptm abundance 37C
Doses Blue Rep1_0.03 Red Rep2_0.04

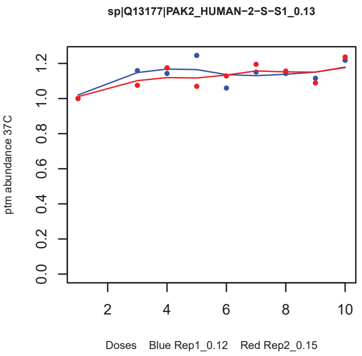
sp|Q13177|PAK2_HUMAN−2−S−S1_0.13
ptm abundance 37C
Doses Blue Rep1_0.12 Red Rep2_0.15

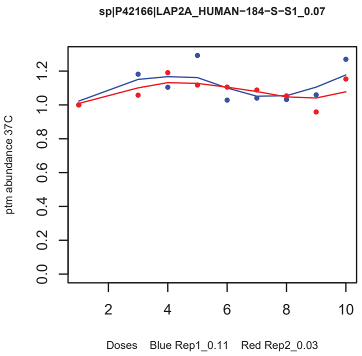
sp|P42166|LAP2A_HUMAN−184−S−S1_0.07
ptm abundance 37C
Doses Blue Rep1_0.11 Red Rep2_0.03

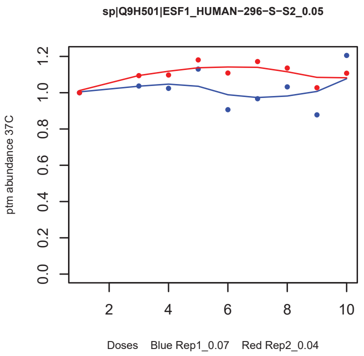
sp|Q9H501|ESF1_HUMAN−296−S−S2_0.05
ptm abundance 37C
Doses Blue Rep1_0.07 Red Rep2_0.04

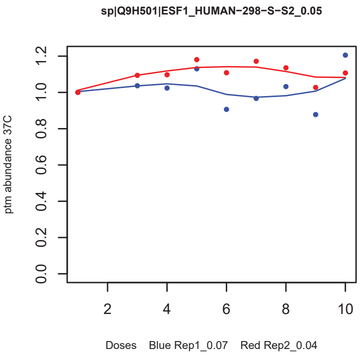
sp|Q9H501|ESF1_HUMAN−298−S−S2_0.05
ptm abundance 37C
Doses Blue Rep1_0.07 Red Rep2_0.04

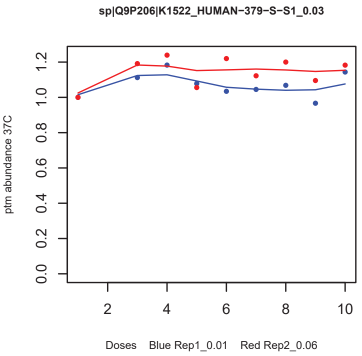
sp|Q9P206|K1522_HUMAN−379−S−S1_0.03
ptm abundance 37C
Doses Blue Rep1_0.01 Red Rep2_0.06

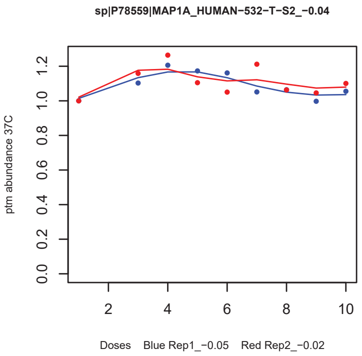
sp|P78559|MAP1A_HUMAN−532−T−S2_−0.04
ptm abundance 37C
Doses Blue Rep1_−0.05 Red Rep2_−0.02

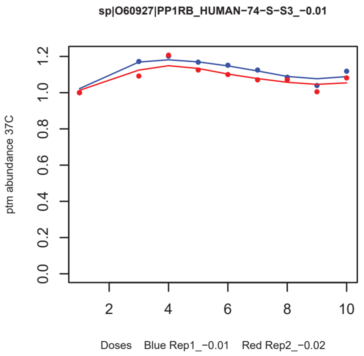
sp|O60927|PP1RB_HUMAN−74−S−S3_−0.01
ptm abundance 37C
Doses Blue Rep1_−0.01 Red Rep2_−0.02

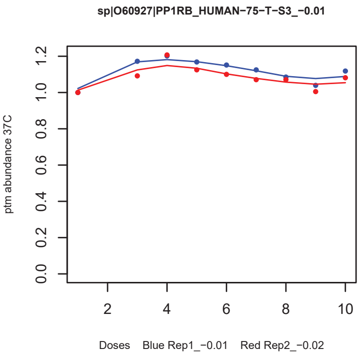
sp|O60927|PP1RB_HUMAN−75−T−S3_−0.01
ptm abundance 37C
Doses Blue Rep1_−0.01 Red Rep2_−0.02

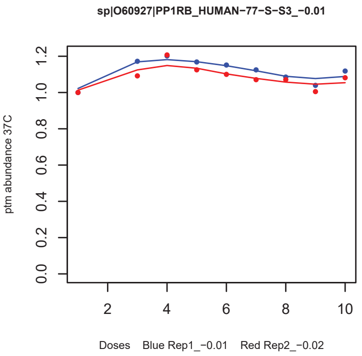
sp|O60927|PP1RB_HUMAN−77−S−S3_−0.01
ptm abundance 37C
Doses Blue Rep1_−0.01 Red Rep2_−0.02

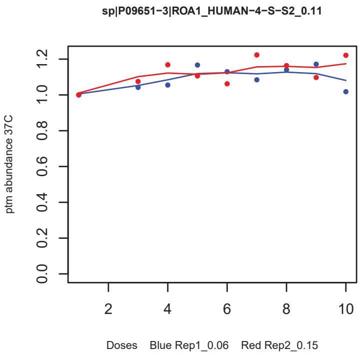
sp|P09651−3|ROA1_HUMAN−4−S−S2_0.11
ptm abundance 37C
Doses Blue Rep1_0.06 Red Rep2_0.15

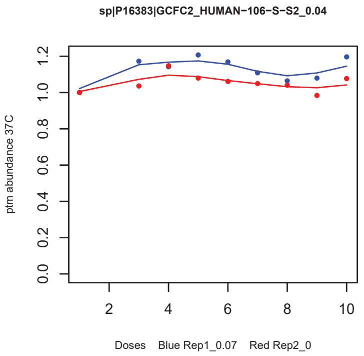
sp|P16383|GCFC2_HUMAN−106−S−S2_0.04
ptm abundance 37C
Doses Blue Rep1_0.07 Red Rep2_0

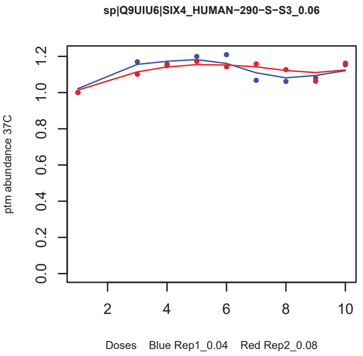
sp|Q9UIU6|SIX4_HUMAN−290−S−S3_0.06
ptm abundance 37C
Doses Blue Rep1_0.04 Red Rep2_0.08

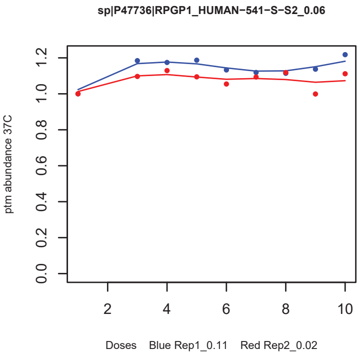
sp|P47736|RPGP1_HUMAN−541−S−S2_0.06
ptm abundance 37C
Doses Blue Rep1_0.11 Red Rep2_0.02

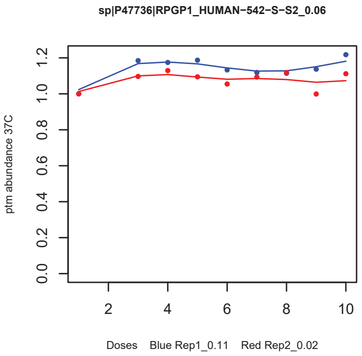
sp|P47736|RPGP1_HUMAN−542−S−S2_0.06
ptm abundance 37C
Doses Blue Rep1_0.11 Red Rep2_0.02

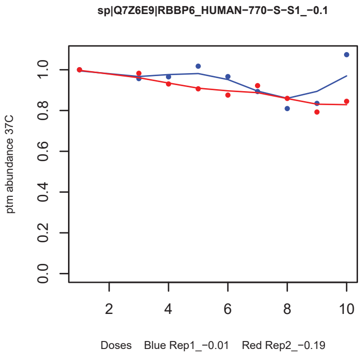
sp|Q7Z6E9|RBBP6_HUMAN−770−S−S1_−0.1
ptm abundance 37C
Doses Blue Rep1_−0.01 Red Rep2_−0.19

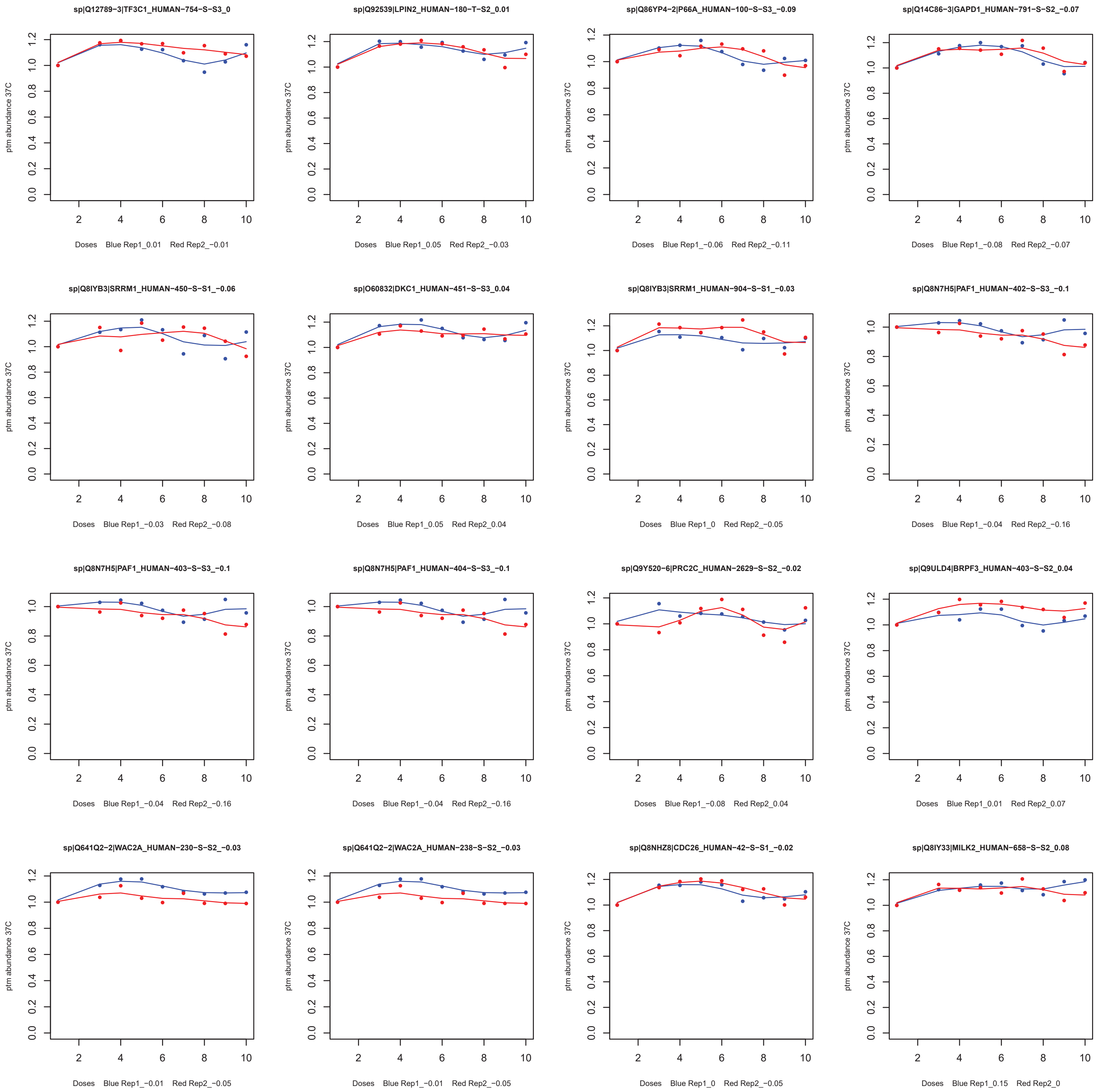

sp|Q12789−3|TF3C1_HUMAN−754−S−S3_0
ptm abundance 37C
Doses Blue Rep1_0.01 Red Rep2_−0.01
sp|Q92539|LPIN2_HUMAN−180−T−S2_0.01
ptm abundance 37C
Doses Blue Rep1_0.05 Red Rep2_−0.03
sp|Q86YP4−2|P66A_HUMAN−100−S−S3_−0.09
ptm abundance 37C
Doses Blue Rep1_−0.06 Red Rep2_−0.11
sp|Q14C86−3|GAPD1_HUMAN−791−S−S2_−0.07
ptm abundance 37C
Doses Blue Rep1_−0.08 Red Rep2_−0.07
sp|Q8IYB3|SRRM1_HUMAN−450−S−S1_−0.06
ptm abundance 37C
Doses Blue Rep1_−0.03 Red Rep2_−0.08
sp|O60832|DKC1_HUMAN−451−S−S3_0.04
ptm abundance 37C
Doses Blue Rep1_0.05 Red Rep2_0.04
sp|Q8IYB3|SRRM1_HUMAN−904−S−S1_−0.03
ptm abundance 37C
Doses Blue Rep1_0 Red Rep2_−0.05
sp|Q8N7H5|PAF1_HUMAN−402−S−S3_−0.1
ptm abundance 37C
Doses Blue Rep1_−0.04 Red Rep2_−0.16
sp|Q8N7H5|PAF1_HUMAN−403−S−S3_−0.1
ptm abundance 37C
Doses Blue Rep1_−0.04 Red Rep2_−0.16
sp|Q8N7H5|PAF1_HUMAN−404−S−S3_−0.1
ptm abundance 37C
Doses Blue Rep1_−0.04 Red Rep2_−0.16
sp|Q9Y520−6|PRC2C_HUMAN−2629−S−S2_−0.02
ptm abundance 37C
Doses Blue Rep1_−0.08 Red Rep2_0.04
sp|Q9ULD4|BRPF3_HUMAN−403−S−S2_0.04
ptm abundance 37C
Doses Blue Rep1_0.01 Red Rep2_0.07
sp|Q641Q2−2|WAC2A_HUMAN−230−S−S2_−0.03
ptm abundance 37C
Doses Blue Rep1_−0.01 Red Rep2_−0.05
sp|Q641Q2−2|WAC2A_HUMAN−238−S−S2_−0.03
ptm abundance 37C
Doses Blue Rep1_−0.01 Red Rep2_−0.05
sp|Q8NHZ8|CDC26_HUMAN−42−S−S1_−0.02
ptm abundance 37C
Doses Blue Rep1_0 Red Rep2_−0.05
sp|Q8IY33|MILK2_HUMAN−658−S−S2_0.08
ptm abundance 37C
Doses Blue Rep1_0.15 Red Rep2_0

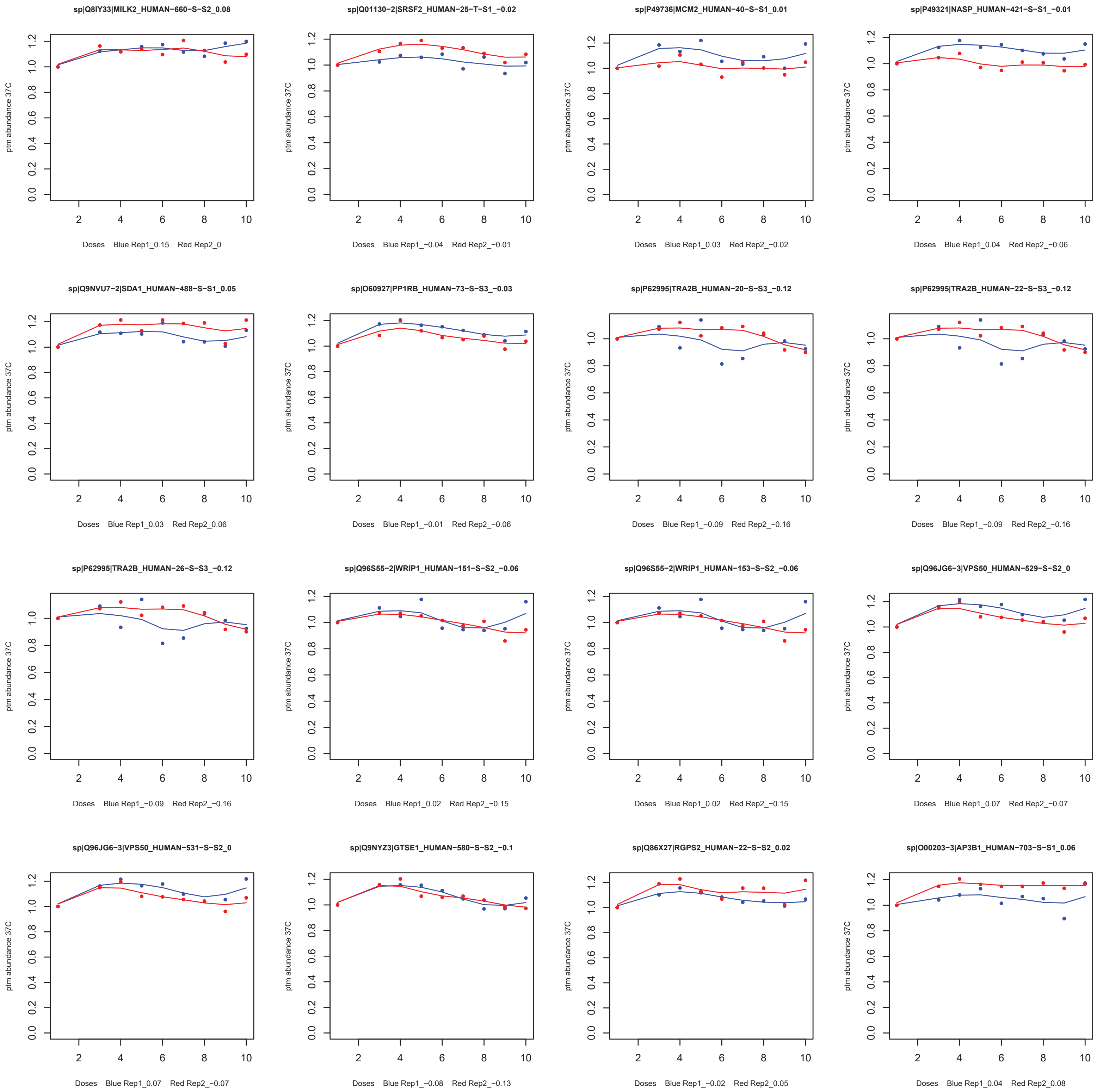

sp|Q8IY33|MILK2_HUMAN−660−S−S2_0.08
ptm abundance 37C
Doses Blue Rep1_0.15 Red Rep2_0
sp|Q01130−2|SRSF2_HUMAN−25−T−S1_−0.02
ptm abundance 37C
Doses Blue Rep1_−0.04 Red Rep2_−0.01
sp|P49736|MCM2_HUMAN−40−S−S1_0.01
ptm abundance 37C
Doses Blue Rep1_0.03 Red Rep2_−0.02
sp|P49321|NASP_HUMAN−421−S−S1_−0.01
ptm abundance 37C
Doses Blue Rep1_0.04 Red Rep2_−0.06
sp|Q9NVU7−2|SDA1_HUMAN−488−S−S1_0.05
ptm abundance 37C
Doses Blue Rep1_0.03 Red Rep2_0.06
sp|O60927|PP1RB_HUMAN−73−S−S3_−0.03
ptm abundance 37C
Doses Blue Rep1_−0.01 Red Rep2_−0.06
sp|P62995|TRA2B_HUMAN−20−S−S3_−0.12
ptm abundance 37C
Doses Blue Rep1_−0.09 Red Rep2_−0.16
sp|P62995|TRA2B_HUMAN−22−S−S3_−0.12
ptm abundance 37C
Doses Blue Rep1_−0.09 Red Rep2_−0.16
sp|P62995|TRA2B_HUMAN−26−S−S3_−0.12
ptm abundance 37C
Doses Blue Rep1_−0.09 Red Rep2_−0.16
sp|Q96S55−2|WRIP1_HUMAN−151−S−S2_−0.06
ptm abundance 37C
Doses Blue Rep1_0.02 Red Rep2_−0.15
sp|Q96S55−2|WRIP1_HUMAN−153−S−S2_−0.06
ptm abundance 37C
Doses Blue Rep1_0.02 Red Rep2_−0.15
sp|Q96JG6−3|VPS50_HUMAN−529−S−S2_0
ptm abundance 37C
Doses Blue Rep1_0.07 Red Rep2_−0.07
sp|Q96JG6−3|VPS50_HUMAN−531−S−S2_0
ptm abundance 37C
Doses Blue Rep1_0.07 Red Rep2_−0.07
sp|Q9NYZ3|GTSE1_HUMAN−580−S−S2_−0.1
ptm abundance 37C
Doses Blue Rep1_−0.08 Red Rep2_−0.13
sp|Q86X27|RGPS2_HUMAN−22−S−S2_0.02
ptm abundance 37C
Doses Blue Rep1_−0.02 Red Rep2_0.05
sp|O00203−3|AP3B1_HUMAN−703−S−S1_0.06
ptm abundance 37C
Doses Blue Rep1_0.04 Red Rep2_0.08

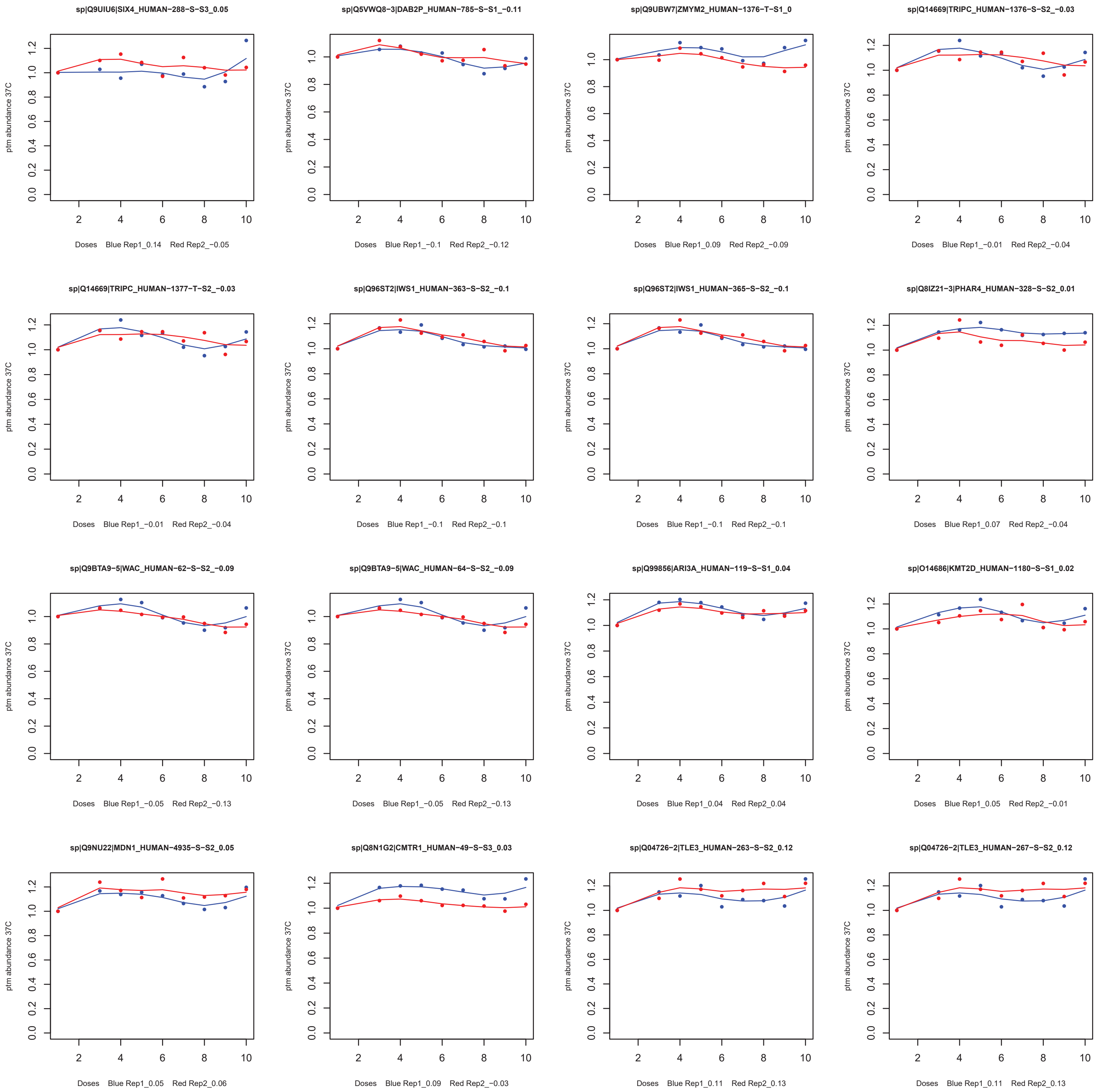
sp|Q9UIU6|SIX4_HUMAN−288−S−S3_0.05
Doses Blue Rep1_0.14 Red Rep2_−0.05
sp|Q5VWQ8−3|DAB2P_HUMAN−785−S−S1_−0.11
Doses Blue Rep1_−0.1 Red Rep2_−0.12
sp|Q9UBW7|ZMYM2_HUMAN−1376−T−S1_0
Doses Blue Rep1_0.09 Red Rep2_−0.09
sp|Q14669|TRIPC_HUMAN−1376−S−S2_−0.03
Doses Blue Rep1_−0.01 Red Rep2_−0.04
sp|Q14669|TRIPC_HUMAN−1377−T−S2_−0.03
Doses Blue Rep1_−0.01 Red Rep2_−0.04
sp|Q96ST2|IWS1_HUMAN−363−S−S2_−0.1
Doses Blue Rep1_−0.1 Red Rep2_−0.1
sp|Q96ST2|IWS1_HUMAN−365−S−S2_−0.1
Doses Blue Rep1_−0.1 Red Rep2_−0.1
sp|Q8IZ21−3|PHAR4_HUMAN−328−S−S2_0.01
Doses Blue Rep1_0.07 Red Rep2_−0.04
sp|Q9BTA9−5|WAC_HUMAN−62−S−S2_−0.09
Doses Blue Rep1_−0.05 Red Rep2_−0.13
sp|Q9BTA9−5|WAC_HUMAN−64−S−S2_−0.09
Doses Blue Rep1_−0.05 Red Rep2_−0.13
sp|Q99856|ARI3A_HUMAN−119−S−S1_0.04
Doses Blue Rep1_0.04 Red Rep2_0.04
sp|O14686|KMT2D_HUMAN−1180−S−S1_0.02
Doses Blue Rep1_0.05 Red Rep2_−0.01
sp|Q9NU22|MDN1_HUMAN−4935−S−S2_0.05
Doses Blue Rep1_0.05 Red Rep2_0.06
sp|Q8N1G2|CMTR1_HUMAN−49−S−S3_0.03
Doses Blue Rep1_0.09 Red Rep2_−0.03
sp|Q04726−2|TLE3_HUMAN−263−S−S2_0.12
Doses Blue Rep1_0.11 Red Rep2_0.13
sp|Q04726−2|TLE3_HUMAN−267−S−S2_0.12
Doses Blue Rep1_0.11 Red Rep2_0.13
ptm abundance 37C

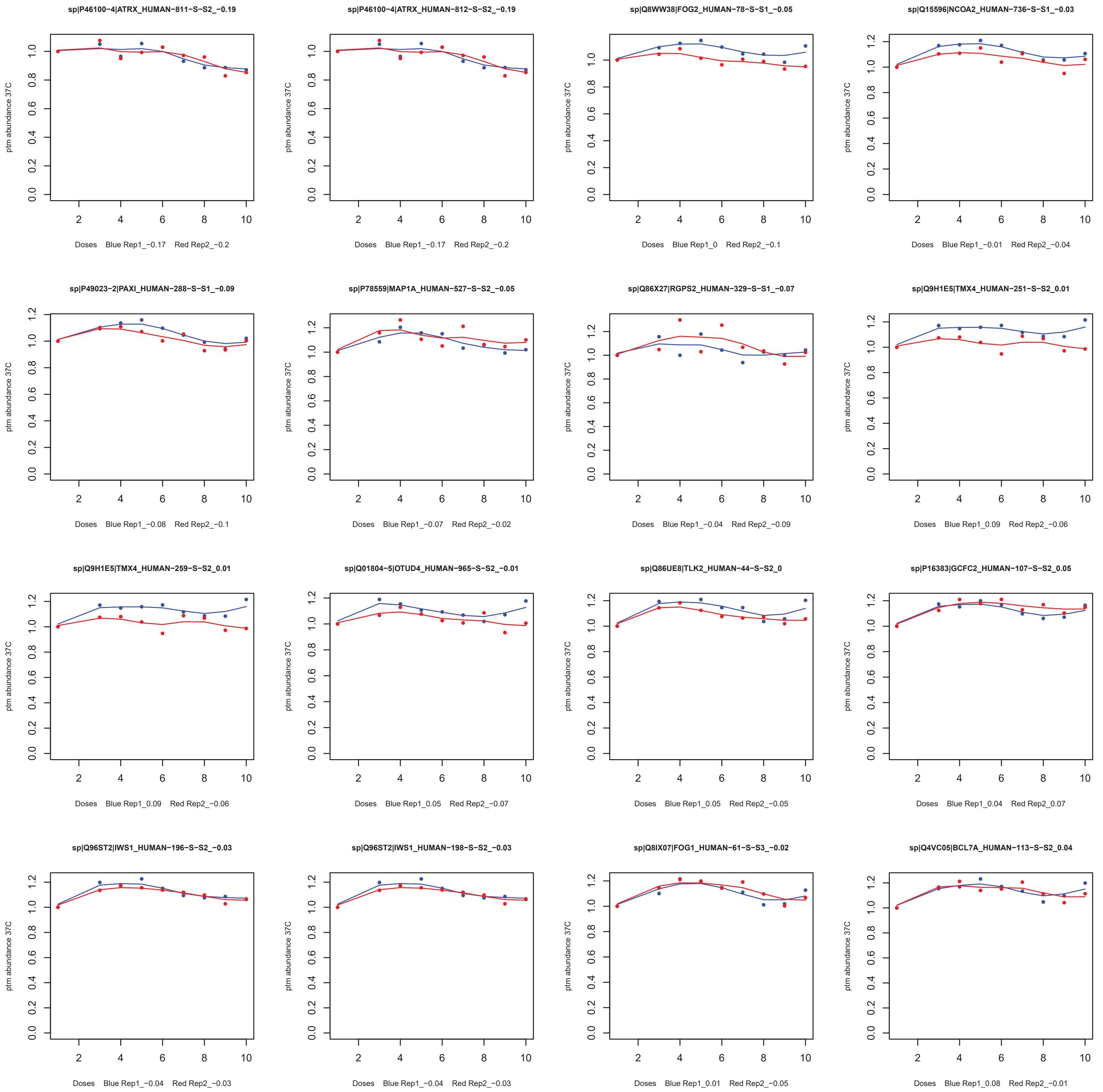
sp|P46100−4|ATRX_HUMAN−811−S−S2_−0.19
Doses Blue Rep1_−0.17 Red Rep2_−0.2
sp|P46100−4|ATRX_HUMAN−812−S−S2_−0.19
Doses Blue Rep1_−0.17 Red Rep2_−0.2
sp|Q8WW38|FOG2_HUMAN−78−S−S1_−0.05
Doses Blue Rep1_0 Red Rep2_−0.1
sp|Q15596|NCOA2_HUMAN−736−S−S1_−0.03
Doses Blue Rep1_−0.01 Red Rep2_−0.04
sp|P49023−2|PAXI_HUMAN−288−S−S1_−0.09
Doses Blue Rep1_−0.08 Red Rep2_−0.1
sp|P78559|MAP1A_HUMAN−527−S−S2_−0.05
Doses Blue Rep1_−0.07 Red Rep2_−0.02
sp|Q86X27|RGPS2_HUMAN−329−S−S1_−0.07
Doses Blue Rep1_−0.04 Red Rep2_−0.09
sp|Q9H1E5|TMX4_HUMAN−251−S−S2_0.01
Doses Blue Rep1_0.09 Red Rep2_−0.06
sp|Q9H1E5|TMX4_HUMAN−259−S−S2_0.01
Doses Blue Rep1_0.09 Red Rep2_−0.06
sp|Q01804−5|OTUD4_HUMAN−965−S−S2_−0.01
Doses Blue Rep1_0.05 Red Rep2_−0.07
sp|Q86UE8|TLK2_HUMAN−44−S−S2_0
Doses Blue Rep1_0.05 Red Rep2_−0.05
sp|P16383|GCFC2_HUMAN−107−S−S2_0.05
Doses Blue Rep1_0.04 Red Rep2_0.07
sp|Q96ST2|IWS1_HUMAN−196−S−S2_−0.03
Doses Blue Rep1_−0.04 Red Rep2_−0.03
sp|Q96ST2|IWS1_HUMAN−198−S−S2_−0.03
Doses Blue Rep1_−0.04 Red Rep2_−0.03
sp|Q8IX07|FOG1_HUMAN−61−S−S3_−0.02
Doses Blue Rep1_0.01 Red Rep2_−0.05
sp|Q4VC05|BCL7A_HUMAN−113−S−S2_0.04
Doses Blue Rep1_0.08 Red Rep2_−0.01
ptm abundance 37C

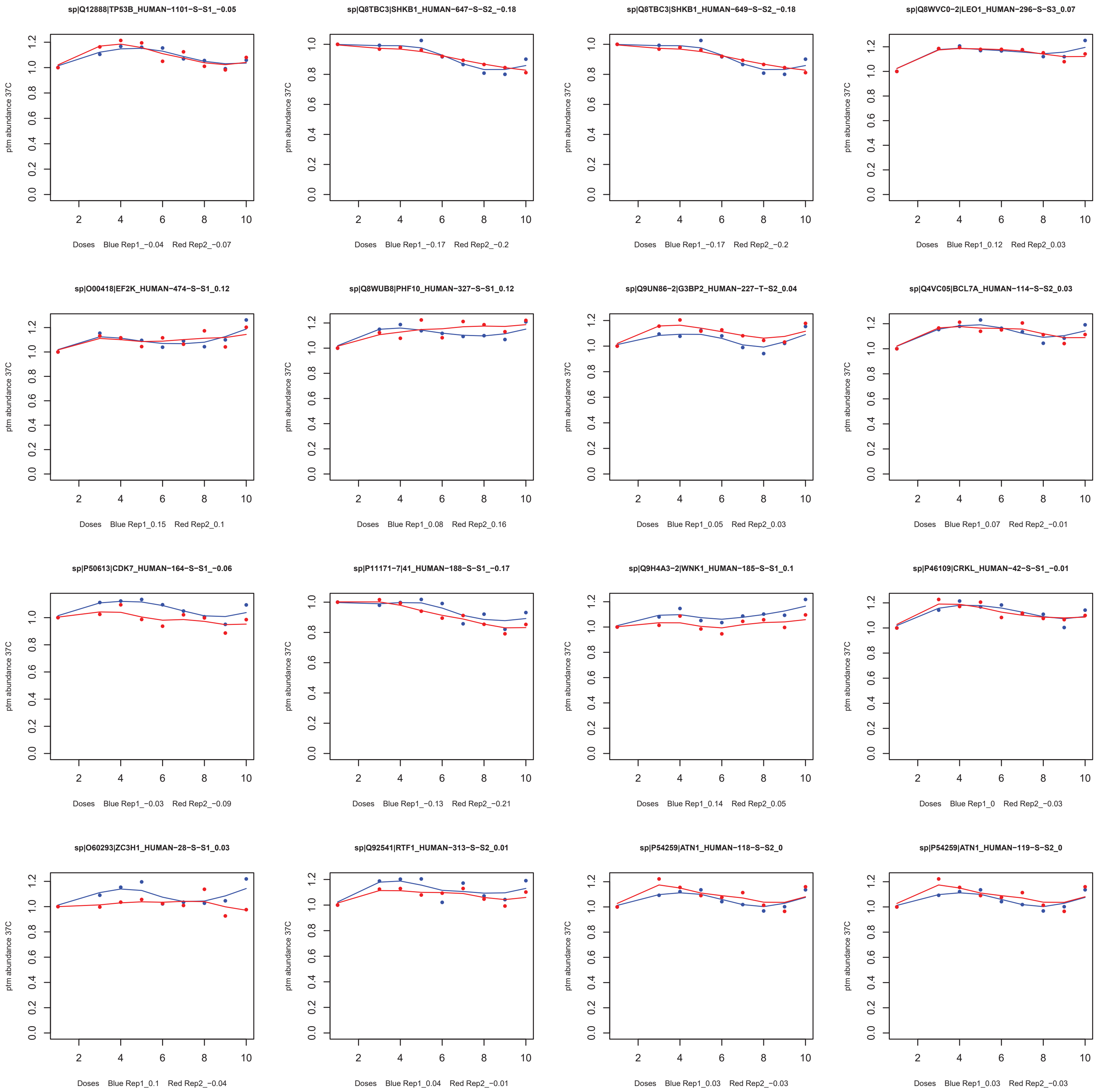
sp|Q12888|TP53B_HUMAN−1101−S−S1_−0.05
Doses Blue Rep1_−0.04 Red Rep2_−0.07
sp|Q8TBC3|SHKB1_HUMAN−647−S−S2_−0.18
Doses Blue Rep1_−0.17 Red Rep2_−0.2
sp|Q8TBC3|SHKB1_HUMAN−649−S−S2_−0.18
Doses Blue Rep1_−0.17 Red Rep2_−0.2
sp|Q8WVC0−2|LEO1_HUMAN−296−S−S3_0.07
Doses Blue Rep1_0.12 Red Rep2_0.03
sp|O00418|EF2K_HUMAN−474−S−S1_0.12
Doses Blue Rep1_0.15 Red Rep2_0.1
sp|Q8WUB8|PHF10_HUMAN−327−S−S1_0.12
Doses Blue Rep1_0.08 Red Rep2_0.16
sp|Q9UN86−2|G3BP2_HUMAN−227−T−S2_0.04
Doses Blue Rep1_0.05 Red Rep2_0.03
sp|Q4VC05|BCL7A_HUMAN−114−S−S2_0.03
Doses Blue Rep1_0.07 Red Rep2_−0.01
sp|P50613|CDK7_HUMAN−164−S−S1_−0.06
Doses Blue Rep1_−0.03 Red Rep2_−0.09
sp|P11171−7|41_HUMAN−188−S−S1_−0.17
Doses Blue Rep1_−0.13 Red Rep2_−0.21
sp|Q9H4A3−2|WNK1_HUMAN−185−S−S1_0.1
Doses Blue Rep1_0.14 Red Rep2_0.05
sp|P46109|CRKL_HUMAN−42−S−S1_−0.01
Doses Blue Rep1_0 Red Rep2_−0.03
sp|O60293|ZC3H1_HUMAN−28−S−S1_0.03
Doses Blue Rep1_0.1 Red Rep2_−0.04
sp|Q92541|RTF1_HUMAN−313−S−S2_0.01
Doses Blue Rep1_0.04 Red Rep2_−0.01
sp|P54259|ATN1_HUMAN−118−S−S2_0
Doses Blue Rep1_0.03 Red Rep2_−0.03
sp|P54259|ATN1_HUMAN−119−S−S2_0
Doses Blue Rep1_0.03 Red Rep2_−0.03
ptm abundance 37C

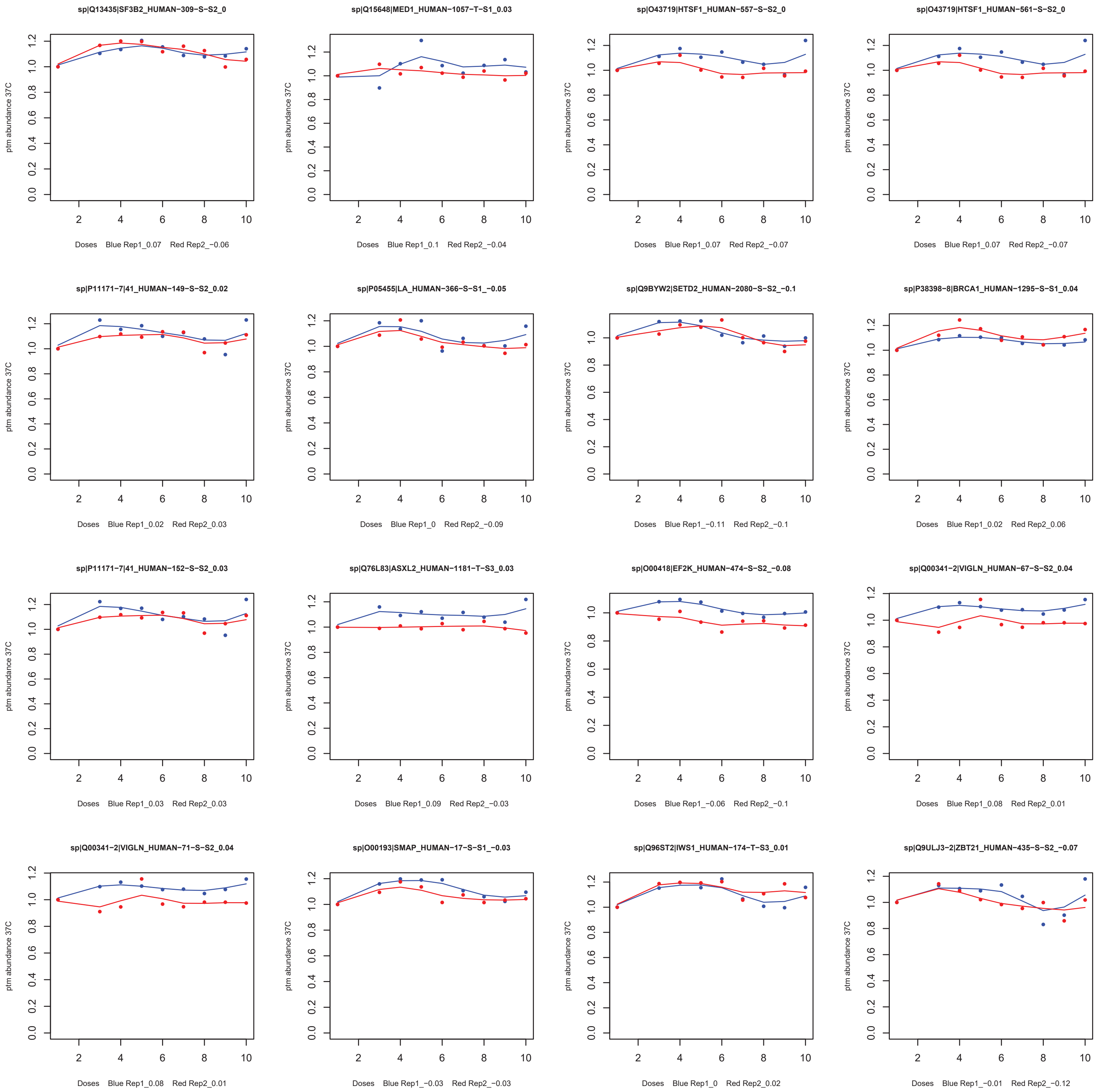

sp|Q13435|SF3B2_HUMAN−309−S−S2_0
Doses Blue Rep1_0.07 Red Rep2_−0.06
sp|Q15648|MED1_HUMAN−1057−T−S1_0.03
Doses Blue Rep1_0.1 Red Rep2_−0.04
sp|O43719|HTSF1_HUMAN−557−S−S2_0
Doses Blue Rep1_0.07 Red Rep2_−0.07
sp|O43719|HTSF1_HUMAN−561−S−S2_0
Doses Blue Rep1_0.07 Red Rep2_−0.07
sp|P11171−7|41_HUMAN−149−S−S2_0.02
Doses Blue Rep1_0.02 Red Rep2_0.03
sp|P05455|LA_HUMAN−366−S−S1_−0.05
Doses Blue Rep1_0 Red Rep2_−0.09
sp|Q9BYW2|SETD2_HUMAN−2080−S−S2_−0.1
Doses Blue Rep1_−0.11 Red Rep2_−0.1
sp|P38398−8|BRCA1_HUMAN−1295−S−S1_0.04
Doses Blue Rep1_0.02 Red Rep2_0.06
sp|P11171−7|41_HUMAN−152−S−S2_0.03
Doses Blue Rep1_0.03 Red Rep2_0.03
sp|Q76L83|ASXL2_HUMAN−1181−T−S3_0.03
Doses Blue Rep1_0.09 Red Rep2_−0.03
sp|O00418|EF2K_HUMAN−474−S−S2_−0.08
Doses Blue Rep1_−0.06 Red Rep2_−0.1
sp|Q00341−2|VIGLN_HUMAN−67−S−S2_0.04
Doses Blue Rep1_0.08 Red Rep2_0.01
sp|Q00341−2|VIGLN_HUMAN−71−S−S2_0.04
Doses Blue Rep1_0.08 Red Rep2_0.01
sp|O00193|SMAP_HUMAN−17−S−S1_−0.03
Doses Blue Rep1_−0.03 Red Rep2_−0.03
sp|Q96ST2|IWS1_HUMAN−174−T−S3_0.01
Doses Blue Rep1_0 Red Rep2_0.02
sp|Q9ULJ3−2|ZBT21_HUMAN−435−S−S2_−0.07
Doses Blue Rep1_−0.01 Red Rep2_−0.12
ptm abundance 37C

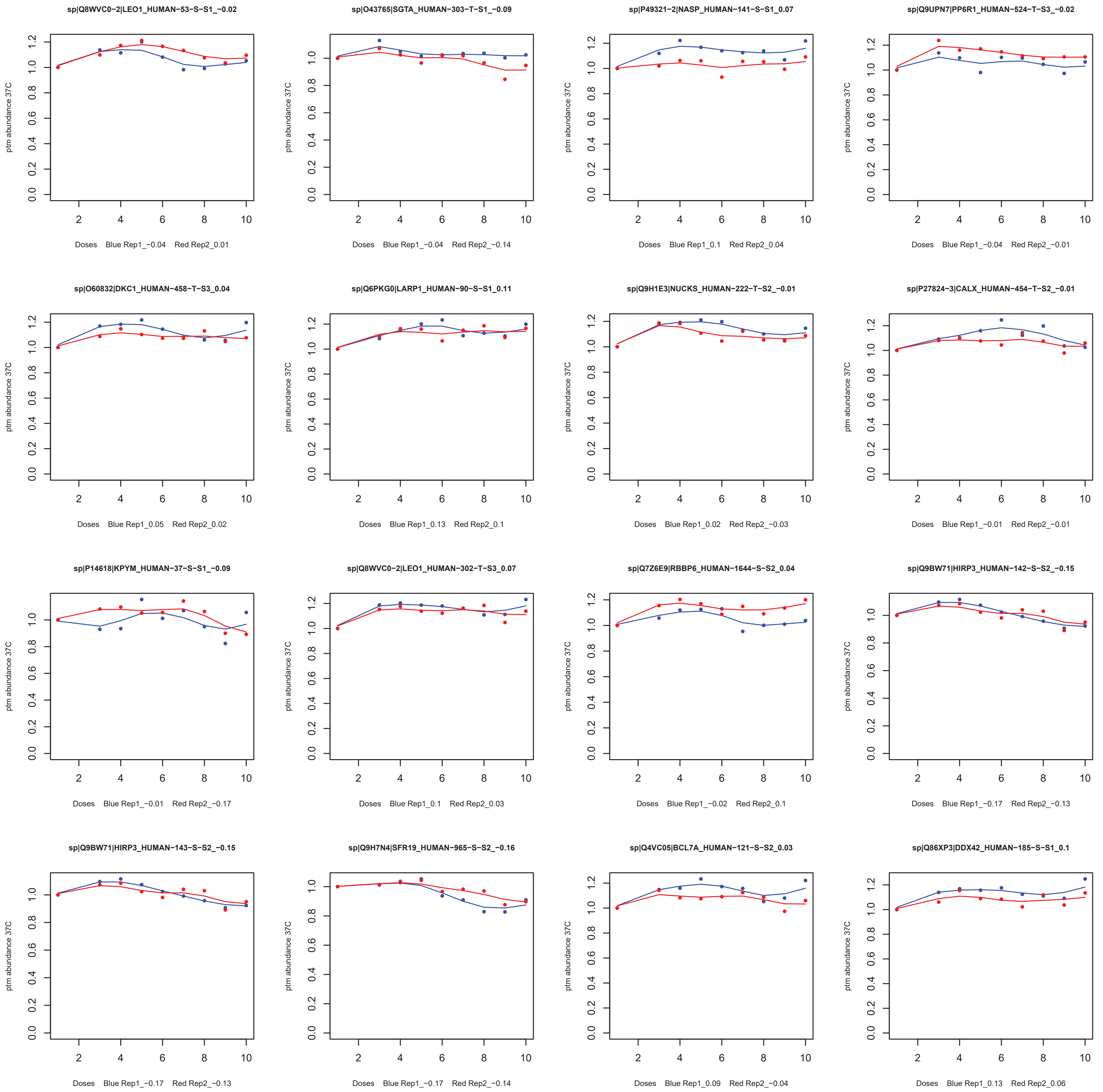
sp|Q8WVC0−2|LEO1_HUMAN−53−S−S1_−0.02
Doses Blue Rep1_−0.04 Red Rep2_0.01
sp|O43765|SGTA_HUMAN−303−T−S1_−0.09
Doses Blue Rep1_−0.04 Red Rep2_−0.14
sp|P49321−2|NASP_HUMAN−141−S−S1_0.07
Doses Blue Rep1_0.1 Red Rep2_0.04
sp|Q9UPN7|PP6R1_HUMAN−524−T−S3_−0.02
Doses Blue Rep1_−0.04 Red Rep2_−0.01
sp|O60832|DKC1_HUMAN−458−T−S3_0.04
Doses Blue Rep1_0.05 Red Rep2_0.02
sp|Q6PKG0|LARP1_HUMAN−90−S−S1_0.11
Doses Blue Rep1_0.13 Red Rep2_0.1
sp|Q9H1E3|NUCKS_HUMAN−222−T−S2_−0.01
Doses Blue Rep1_0.02 Red Rep2_−0.03
sp|P27824−3|CALX_HUMAN−454−T−S2_−0.01
Doses Blue Rep1_−0.01 Red Rep2_−0.01
sp|P14618|KPYM_HUMAN−37−S−S1_−0.09
Doses Blue Rep1_−0.01 Red Rep2_−0.17
sp|Q8WVC0−2|LEO1_HUMAN−302−T−S3_0.07
Doses Blue Rep1_0.1 Red Rep2_0.03
sp|Q7Z6E9|RBBP6_HUMAN−1644−S−S2_0.04
Doses Blue Rep1_−0.02 Red Rep2_0.1
sp|Q9BW71|HIRP3_HUMAN−142−S−S2_−0.15
Doses Blue Rep1_−0.17 Red Rep2_−0.13
sp|Q9BW71|HIRP3_HUMAN−143−S−S2_−0.15
Doses Blue Rep1_−0.17 Red Rep2_−0.13
sp|Q9H7N4|SFR19_HUMAN−965−S−S2_−0.16
Doses Blue Rep1_−0.17 Red Rep2_−0.14
sp|Q4VC05|BCL7A_HUMAN−121−S−S2_0.03
Doses Blue Rep1_0.09 Red Rep2_−0.04
sp|Q86XP3|DDX42_HUMAN−185−S−S1_0.1
Doses Blue Rep1_0.13 Red Rep2_0.06
ptm abundance 37C

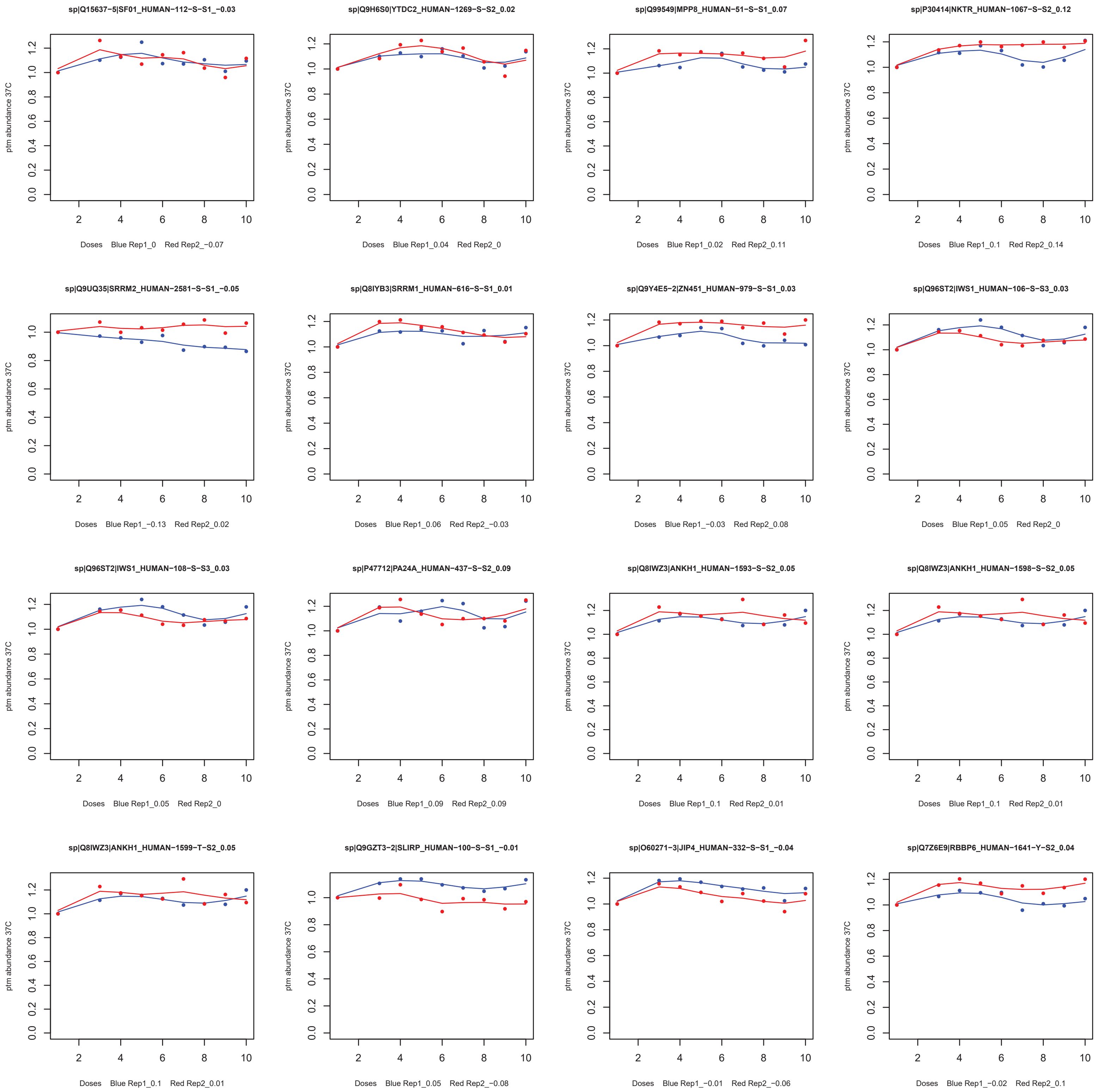
sp|Q15637−5|SF01_HUMAN−112−S−S1_−0.03
ptm abundance 37C
Doses Blue Rep1_0 Red Rep2_−0.07
sp|Q9H6S0|YTDC2_HUMAN−1269−S−S2_0.02
ptm abundance 37C
Doses Blue Rep1_0.04 Red Rep2_0
sp|Q99549|MPP8_HUMAN−51−S−S1_0.07
ptm abundance 37C
Doses Blue Rep1_0.02 Red Rep2_0.11
sp|P30414|NKTR_HUMAN−1067−S−S2_0.12
ptm abundance 37C
Doses Blue Rep1_0.1 Red Rep2_0.14
sp|Q9UQ35|SRRM2_HUMAN−2581−S−S1_−0.05
ptm abundance 37C
Doses Blue Rep1_−0.13 Red Rep2_0.02
sp|Q8IYB3|SRRM1_HUMAN−616−S−S1_0.01
ptm abundance 37C
Doses Blue Rep1_0.06 Red Rep2_−0.03
sp|Q9Y4E5−2|ZN451_HUMAN−979−S−S1_0.03
ptm abundance 37C
Doses Blue Rep1_−0.03 Red Rep2_0.08
sp|Q96ST2|IWS1_HUMAN−106−S−S3_0.03
ptm abundance 37C
Doses Blue Rep1_0.05 Red Rep2_0
sp|Q96ST2|IWS1_HUMAN−108−S−S3_0.03
ptm abundance 37C
Doses Blue Rep1_0.05 Red Rep2_0
sp|P47712|PA24A_HUMAN−437−S−S2_0.09
ptm abundance 37C
Doses Blue Rep1_0.09 Red Rep2_0.09
sp|Q8IWZ3|ANKH1_HUMAN−1593−S−S2_0.05
ptm abundance 37C
Doses Blue Rep1_0.1 Red Rep2_0.01
sp|Q8IWZ3|ANKH1_HUMAN−1598−S−S2_0.05
ptm abundance 37C
Doses Blue Rep1_0.1 Red Rep2_0.01
sp|Q8IWZ3|ANKH1_HUMAN−1599−T−S2_0.05
ptm abundance 37C
Doses Blue Rep1_0.1 Red Rep2_0.01
sp|Q9GZT3−2|SLIRP_HUMAN−100−S−S1_−0.01
ptm abundance 37C
Doses Blue Rep1_0.05 Red Rep2_−0.08
sp|O60271−3|JIP4_HUMAN−332−S−S1_−0.04
ptm abundance 37C
Doses Blue Rep1_−0.01 Red Rep2_−0.06
sp|Q7Z6E9|RBBP6_HUMAN−1641−Y−S2_0.04
ptm abundance 37C
Doses Blue Rep1_−0.02 Red Rep2_0.1

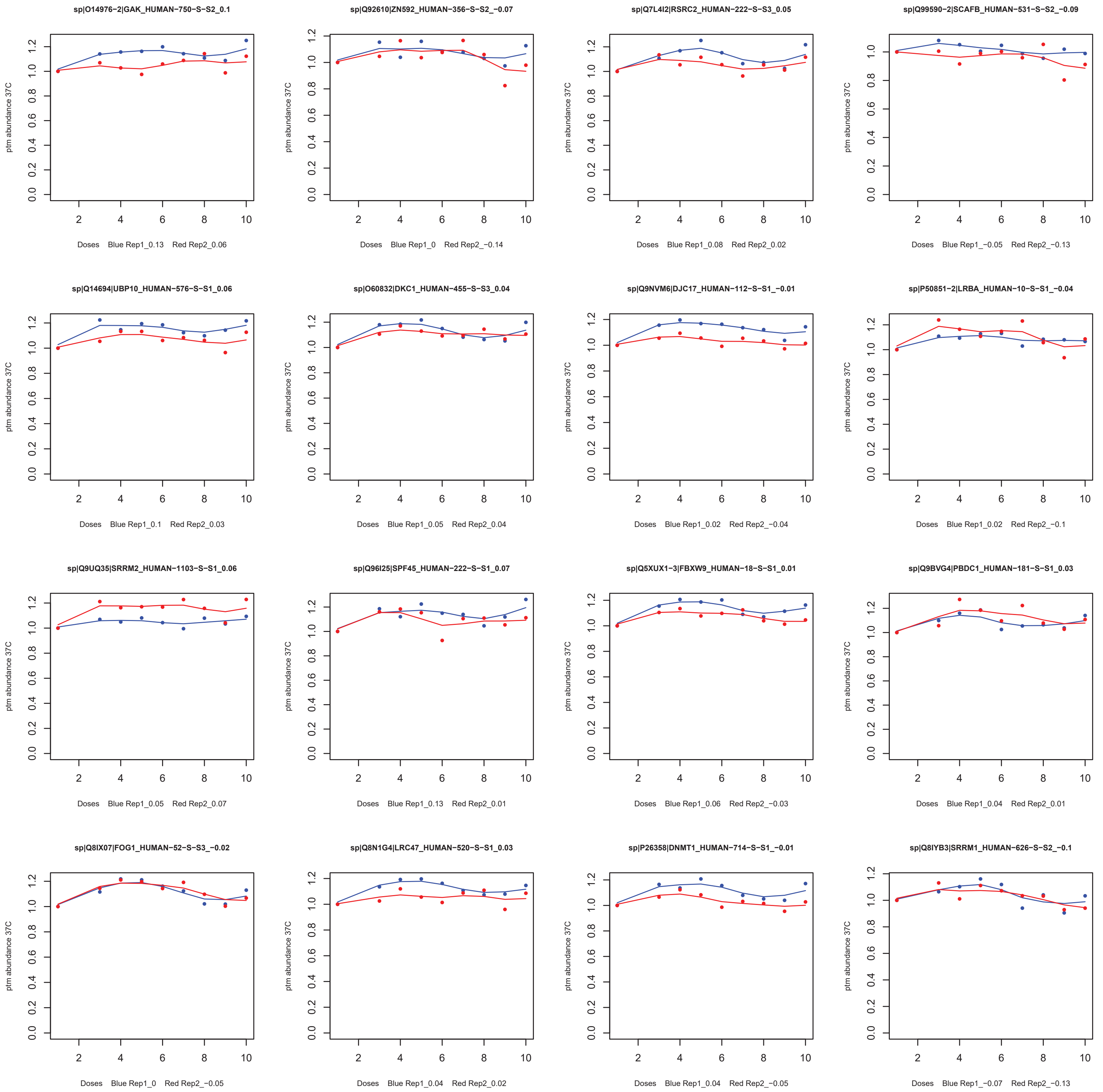

sp|O14976−2|GAK_HUMAN−750−S−S2_0.1
Doses Blue Rep1_0.13 Red Rep2_0.06
sp|Q92610|ZN592_HUMAN−356−S−S2_−0.07
Doses Blue Rep1_0 Red Rep2_−0.14
sp|Q7L4I2|RSRC2_HUMAN−222−S−S3_0.05
Doses Blue Rep1_0.08 Red Rep2_0.02
sp|Q99590−2|SCAFB_HUMAN−531−S−S2_−0.09
Doses Blue Rep1_−0.05 Red Rep2_−0.13
sp|Q14694|UBP10_HUMAN−576−S−S1_0.06
Doses Blue Rep1_0.1 Red Rep2_0.03
sp|O60832|DKC1_HUMAN−455−S−S3_0.04
Doses Blue Rep1_0.05 Red Rep2_0.04
sp|Q9NVM6|DJC17_HUMAN−112−S−S1_−0.01
Doses Blue Rep1_0.02 Red Rep2_−0.04
sp|P50851−2|LRBA_HUMAN−10−S−S1_−0.04
Doses Blue Rep1_0.02 Red Rep2_−0.1
sp|Q9UQ35|SRRM2_HUMAN−1103−S−S1_0.06
Doses Blue Rep1_0.05 Red Rep2_0.07
sp|Q96I25|SPF45_HUMAN−222−S−S1_0.07
Doses Blue Rep1_0.13 Red Rep2_0.01
sp|Q5XUX1−3|FBXW9_HUMAN−18−S−S1_0.01
Doses Blue Rep1_0.06 Red Rep2_−0.03
sp|Q9BVG4|PBDC1_HUMAN−181−S−S1_0.03
Doses Blue Rep1_0.04 Red Rep2_0.01
sp|Q8IX07|FOG1_HUMAN−52−S−S3_−0.02
Doses Blue Rep1_0 Red Rep2_−0.05
sp|Q8N1G4|LRC47_HUMAN−520−S−S1_0.03
Doses Blue Rep1_0.04 Red Rep2_0.02
sp|P26358|DNMT1_HUMAN−714−S−S1_−0.01
Doses Blue Rep1_0.04 Red Rep2_−0.05
sp|Q8IYB3|SRRM1_HUMAN−626−S−S2_−0.1
Doses Blue Rep1_−0.07 Red Rep2_−0.13
ptm abundance 37C

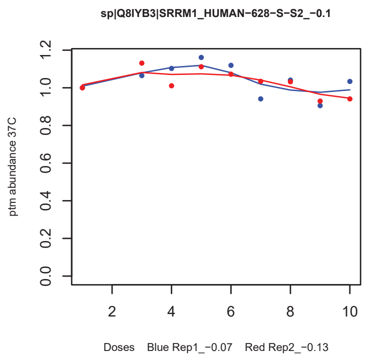
sp|Q8IYB3|SRRM1_HUMAN−628−S−S2_−0.1
ptm abundance 37C
Doses Blue Rep1_−0.07 Red Rep2_−0.13
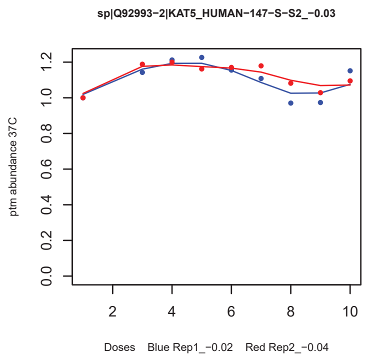
sp|Q92993−2|KAT5_HUMAN−147−S−S2_−0.03
ptm abundance 37C
Doses Blue Rep1_−0.02 Red Rep2_−0.04
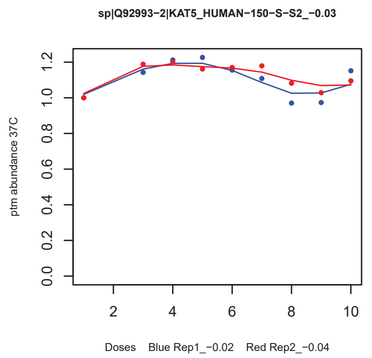
sp|Q92993−2|KAT5_HUMAN−150−S−S2_−0.03
ptm abundance 37C
Doses Blue Rep1_−0.02 Red Rep2_−0.04
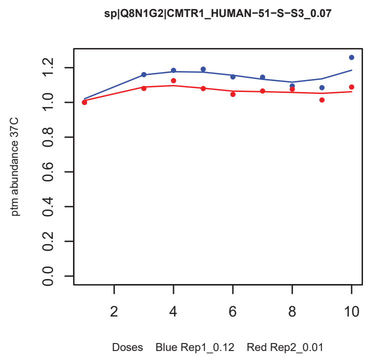
sp|Q8N1G2|CMTR1_HUMAN−51−S−S3_0.07
ptm abundance 37C
Doses Blue Rep1_0.12 Red Rep2_0.01
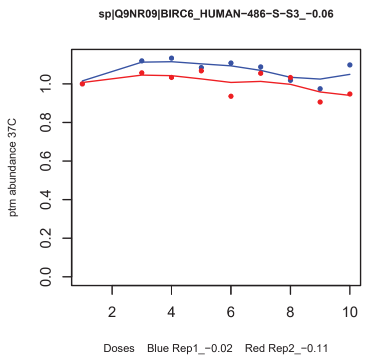
sp|Q9NR09|BIRC6_HUMAN−486−S−S3_−0.06
ptm abundance 37C
Doses Blue Rep1_−0.02 Red Rep2_−0.11
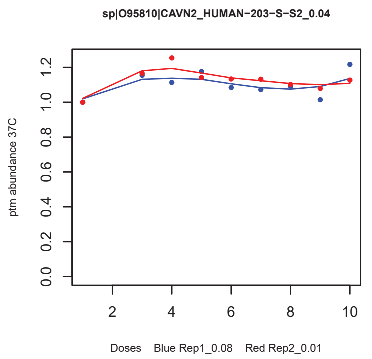
sp|O95810|CAVN2_HUMAN−203−S−S2_0.04
ptm abundance 37C
Doses Blue Rep1_0.08 Red Rep2_0.01
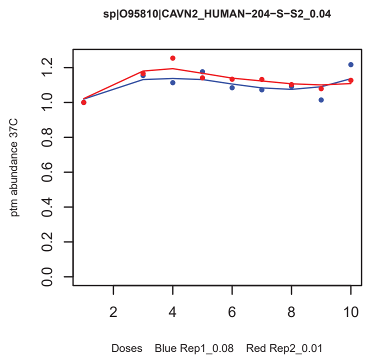
sp|O95810|CAVN2_HUMAN−204−S−S2_0.04
ptm abundance 37C
Doses Blue Rep1_0.08 Red Rep2_0.01
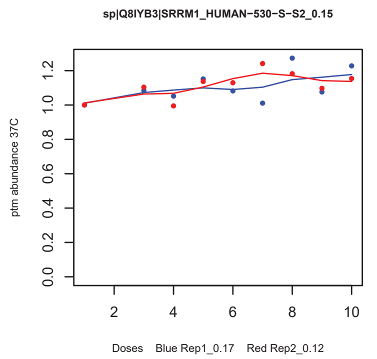
sp|Q8IYB3|SRRM1_HUMAN−530−S−S2_0.15
ptm abundance 37C
Doses Blue Rep1_0.17 Red Rep2_0.12
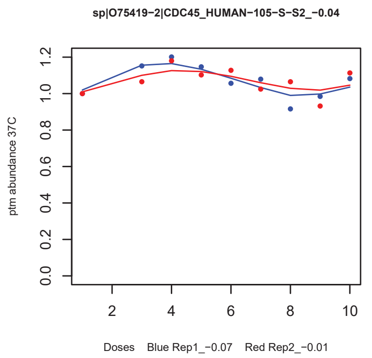
sp|O75419−2|CDC45_HUMAN−105−S−S2_−0.04
ptm abundance 37C
Doses Blue Rep1_−0.07 Red Rep2_−0.01
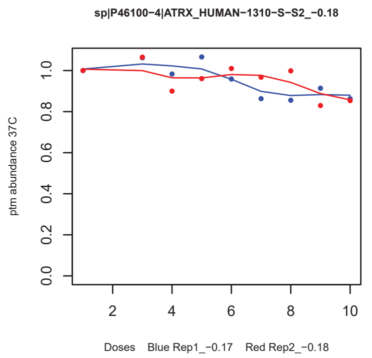
sp|P46100−4|ATRX_HUMAN−1310−S−S2_−0.18
ptm abundance 37C
Doses Blue Rep1_−0.17 Red Rep2_−0.18
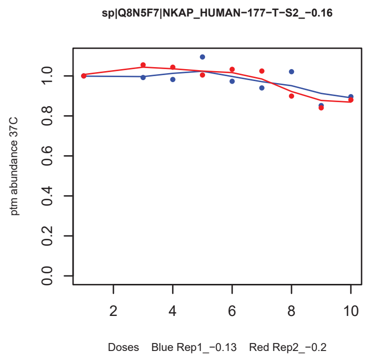
sp|Q8N5F7|NKAP_HUMAN−177−T−S2_−0.16
ptm abundance 37C
Doses Blue Rep1_−0.13 Red Rep2_−0.2
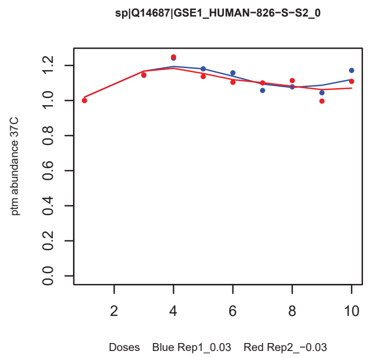
sp|Q14687|GSE1_HUMAN−826−S−S2_0
ptm abundance 37C
Doses Blue Rep1_0.03 Red Rep2_−0.03
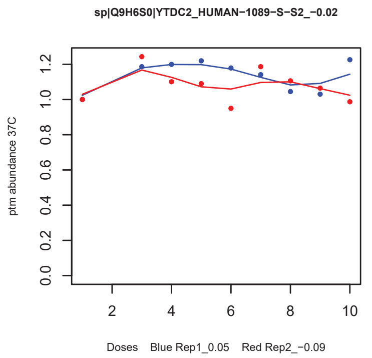
sp|Q9H6S0|YTDC2_HUMAN−1089−S−S2_−0.02
ptm abundance 37C
Doses Blue Rep1_0.05 Red Rep2_−0.09
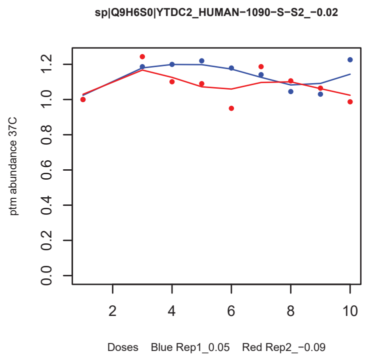
sp|Q9H6S0|YTDC2_HUMAN−1090−S−S2_−0.02
ptm abundance 37C
Doses Blue Rep1_0.05 Red Rep2_−0.09
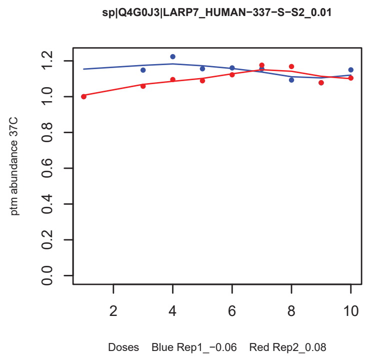
sp|Q4G0J3|LARP7_HUMAN−337−S−S2_0.01
ptm abundance 37C
Doses Blue Rep1_−0.06 Red Rep2_0.08
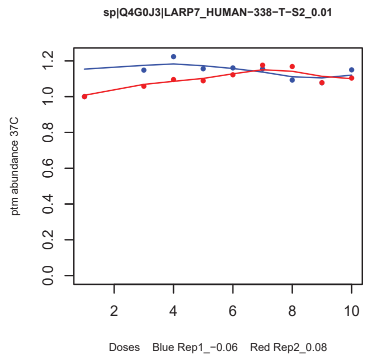
sp|Q4G0J3|LARP7_HUMAN−338−T−S2_0.01
ptm abundance 37C
Doses Blue Rep1_−0.06 Red Rep2_0.08

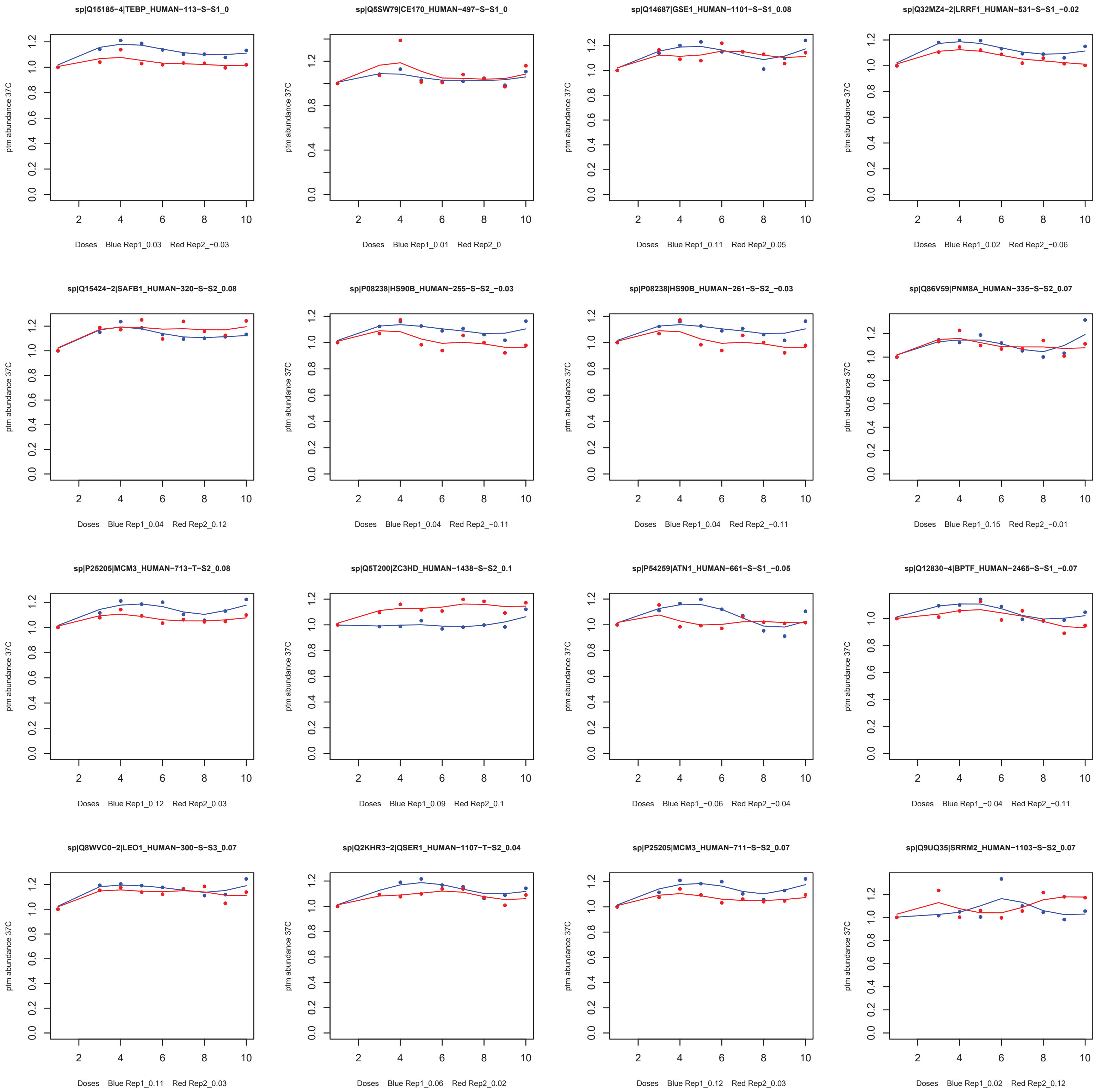
sp|Q15185−4|TEBP_HUMAN−113−S−S1_0
Doses Blue Rep1_0.03 Red Rep2_−0.03
sp|Q5SW79|CE170_HUMAN−497−S−S1_0
Doses Blue Rep1_0.01 Red Rep2_0
sp|Q14687|GSE1_HUMAN−1101−S−S1_0.08
Doses Blue Rep1_0.11 Red Rep2_0.05
sp|Q32MZ4−2|LRRF1_HUMAN−531−S−S1_−0.02
Doses Blue Rep1_0.02 Red Rep2_−0.06
sp|Q15424−2|SAFB1_HUMAN−320−S−S2_0.08
Doses Blue Rep1_0.04 Red Rep2_0.12
sp|P08238|HS90B_HUMAN−255−S−S2_−0.03
Doses Blue Rep1_0.04 Red Rep2_−0.11
sp|P08238|HS90B_HUMAN−261−S−S2_−0.03
Doses Blue Rep1_0.04 Red Rep2_−0.11
sp|Q86V59|PNM8A_HUMAN−335−S−S2_0.07
Doses Blue Rep1_0.15 Red Rep2_−0.01
sp|P25205|MCM3_HUMAN−713−T−S2_0.08
Doses Blue Rep1_0.12 Red Rep2_0.03
sp|Q5T200|ZC3HD_HUMAN−1438−S−S2_0.1
Doses Blue Rep1_0.09 Red Rep2_0.1
sp|P54259|ATN1_HUMAN−661−S−S1_−0.05
Doses Blue Rep1_−0.06 Red Rep2_−0.04
sp|Q12830−4|BPTF_HUMAN−2465−S−S1_−0.07
Doses Blue Rep1_−0.04 Red Rep2_−0.11
sp|Q8WVC0−2|LEO1_HUMAN−300−S−S3_0.07
Doses Blue Rep1_0.11 Red Rep2_0.03
sp|Q2KHR3−2|QSER1_HUMAN−1107−T−S2_0.04
Doses Blue Rep1_0.06 Red Rep2_0.02
sp|P25205|MCM3_HUMAN−711−S−S2_0.07
Doses Blue Rep1_0.12 Red Rep2_0.03
sp|Q9UQ35|SRRM2_HUMAN−1103−S−S2_0.07
Doses Blue Rep1_0.02 Red Rep2_0.12
ptm abundance 37C

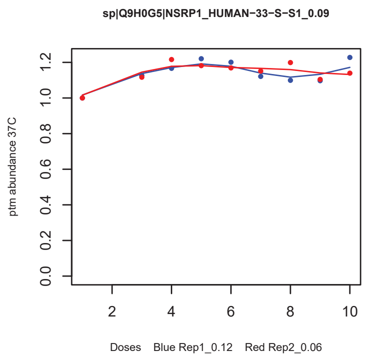
sp|Q9H0G5|NSRP1_HUMAN−33−S−S1_0.09
ptm abundance 37C
Doses Blue Rep1_0.12 Red Rep2_0.06

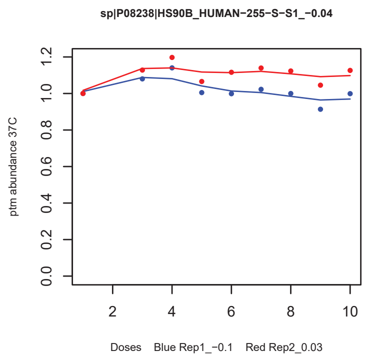
sp|P08238|HS90B_HUMAN−255−S−S1_−0.04
ptm abundance 37C
Doses Blue Rep1_−0.1 Red Rep2_0.03

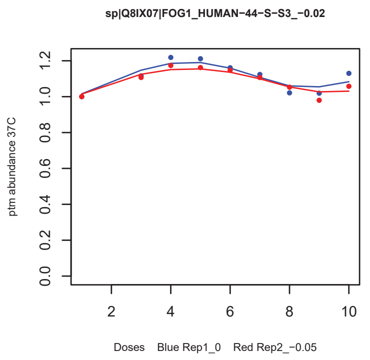
sp|Q8IX07|FOG1_HUMAN−44−S−S3_−0.02
ptm abundance 37C
Doses Blue Rep1_0 Red Rep2_−0.05

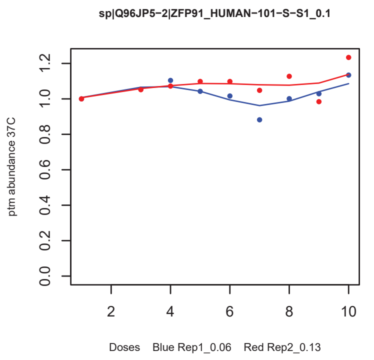
sp|Q96JP5−2|ZFP91_HUMAN−101−S−S1_0.1
ptm abundance 37C
Doses Blue Rep1_0.06 Red Rep2_0.13

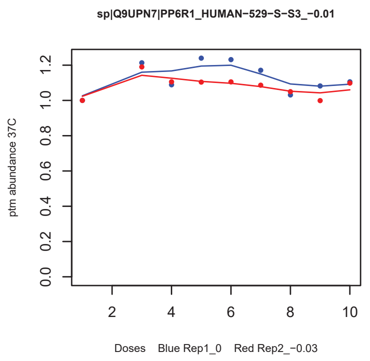
sp|Q9UPN7|PP6R1_HUMAN−529−S−S3_−0.01
ptm abundance 37C
Doses Blue Rep1_0 Red Rep2_−0.03

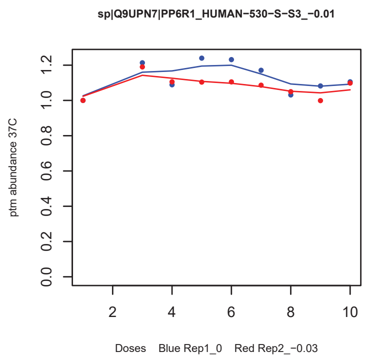
sp|Q9UPN7|PP6R1_HUMAN−530−S−S3_−0.01
ptm abundance 37C
Doses Blue Rep1_0 Red Rep2_−0.03

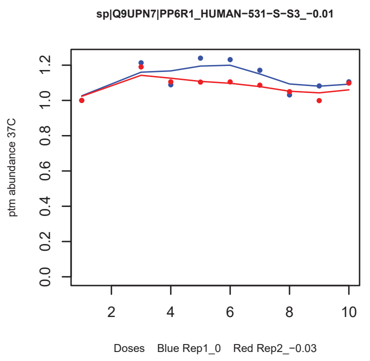
sp|Q9UPN7|PP6R1_HUMAN−531−S−S3_−0.01
ptm abundance 37C
Doses Blue Rep1_0 Red Rep2_−0.03

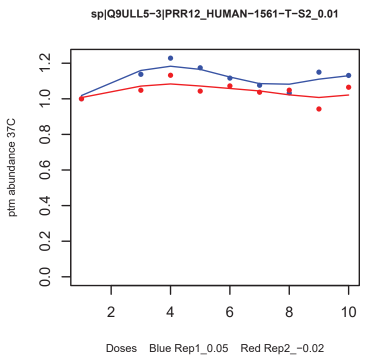
sp|Q9ULL5−3|PRR12_HUMAN−1561−T−S2_0.01
ptm abundance 37C
Doses Blue Rep1_0.05 Red Rep2_−0.02

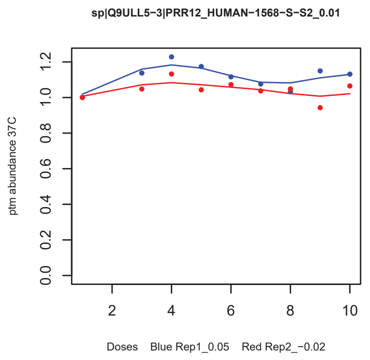
sp|Q9ULL5−3|PRR12_HUMAN−1568−S−S2_0.01
ptm abundance 37C
Doses Blue Rep1_0.05 Red Rep2_−0.02

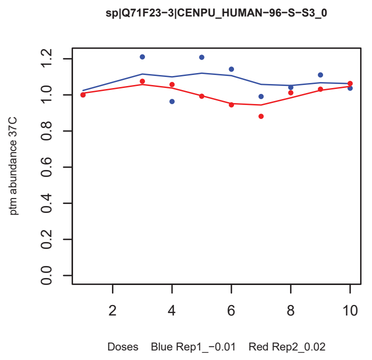
sp|Q71F23−3|CENPU_HUMAN−96−S−S3_0
ptm abundance 37C
Doses Blue Rep1_−0.01 Red Rep2_0.02

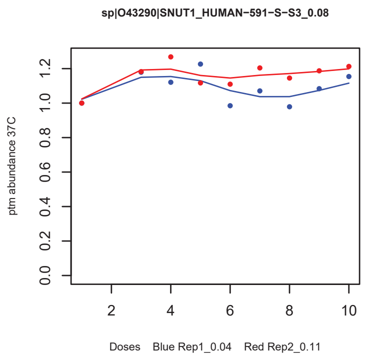
sp|O43290|SNUT1_HUMAN−591−S−S3_0.08
ptm abundance 37C
Doses Blue Rep1_0.04 Red Rep2_0.11

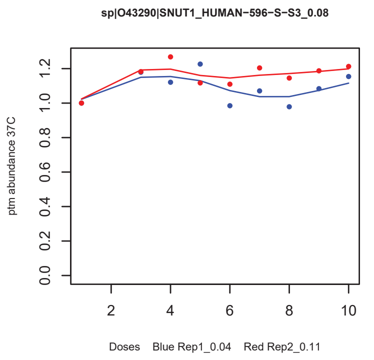
sp|O43290|SNUT1_HUMAN−596−S−S3_0.08
ptm abundance 37C
Doses Blue Rep1_0.04 Red Rep2_0.11

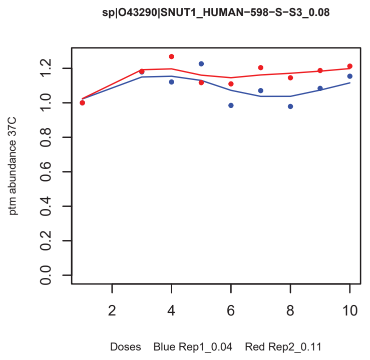
sp|O43290|SNUT1_HUMAN−598−S−S3_0.08
ptm abundance 37C
Doses Blue Rep1_0.04 Red Rep2_0.11

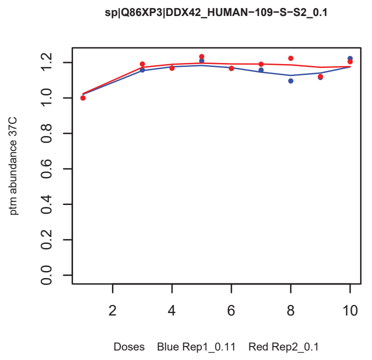
sp|Q86XP3|DDX42_HUMAN−109−S−S2_0.1
ptm abundance 37C
Doses Blue Rep1_0.11 Red Rep2_0.1

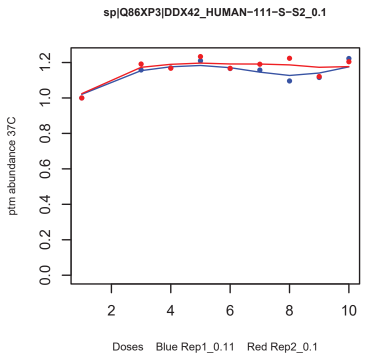
sp|Q86XP3|DDX42_HUMAN−111−S−S2_0.1
ptm abundance 37C
Doses Blue Rep1_0.11 Red Rep2_0.1

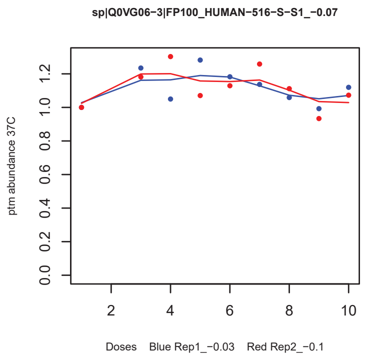
sp|Q0VG06−3|FP100_HUMAN−516−S−S1_−0.07
ptm abundance 37C
Doses Blue Rep1_−0.03 Red Rep2_−0.1

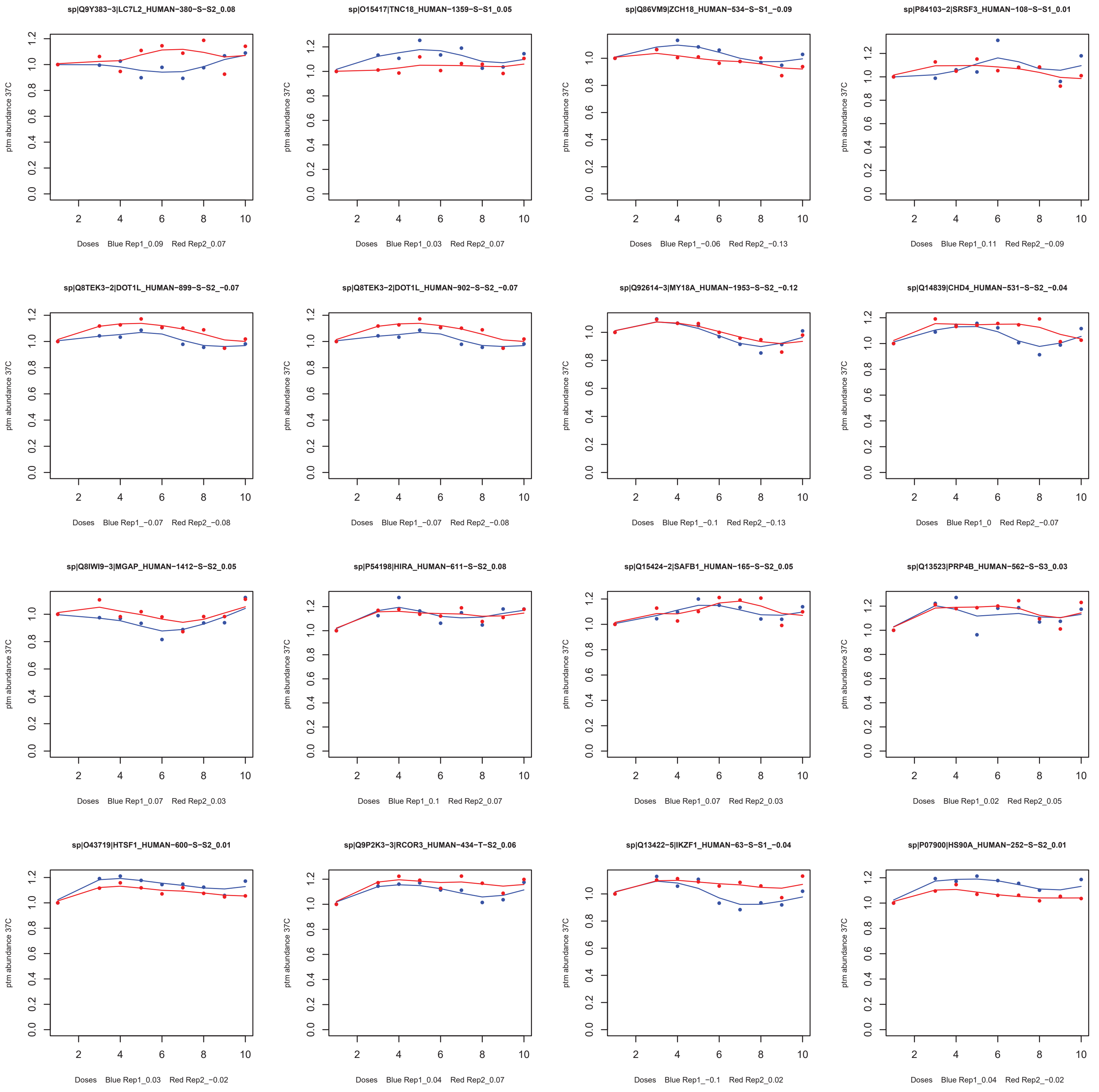
sp|Q9Y383−3|LC7L2_HUMAN−380−S−S2_0.08
Doses Blue Rep1_0.09 Red Rep2_0.07
sp|O15417|TNC18_HUMAN−1359−S−S1_0.05
Doses Blue Rep1_0.03 Red Rep2_0.07
sp|Q86VM9|ZCH18_HUMAN−534−S−S1_−0.09
Doses Blue Rep1_−0.06 Red Rep2_−0.13
sp|P84103−2|SRSF3_HUMAN−108−S−S1_0.01
Doses Blue Rep1_0.11 Red Rep2_−0.09
sp|Q8TEK3−2|DOT1L_HUMAN−899−S−S2_−0.07
Doses Blue Rep1_−0.07 Red Rep2_−0.08
sp|Q8TEK3−2|DOT1L_HUMAN−902−S−S2_−0.07
Doses Blue Rep1_−0.07 Red Rep2_−0.08
sp|Q92614−3|MY18A_HUMAN−1953−S−S2_−0.12
Doses Blue Rep1_−0.1 Red Rep2_−0.13
sp|Q14839|CHD4_HUMAN−531−S−S2_−0.04
Doses Blue Rep1_0 Red Rep2_−0.07
sp|Q8IWI9−3|MGAP_HUMAN−1412−S−S2_0.05
Doses Blue Rep1_0.07 Red Rep2_0.03
sp|P54198|HIRA_HUMAN−611−S−S2_0.08
Doses Blue Rep1_0.1 Red Rep2_0.07
sp|Q15424−2|SAFB1_HUMAN−165−S−S2_0.05
Doses Blue Rep1_0.07 Red Rep2_0.03
sp|Q13523|PRP4B_HUMAN−562−S−S3_0.03
Doses Blue Rep1_0.02 Red Rep2_0.05
sp|O43719|HTSF1_HUMAN−600−S−S2_0.01
Doses Blue Rep1_0.03 Red Rep2_−0.02
sp|Q9P2K3−3|RCOR3_HUMAN−434−T−S2_0.06
Doses Blue Rep1_0.04 Red Rep2_0.07
sp|Q13422−5|IKZF1_HUMAN−63−S−S1_−0.04
Doses Blue Rep1_−0.1 Red Rep2_0.02
sp|P07900|HS90A_HUMAN−252−S−S2_0.01
Doses Blue Rep1_0.04 Red Rep2_−0.02
ptm abundance 37C

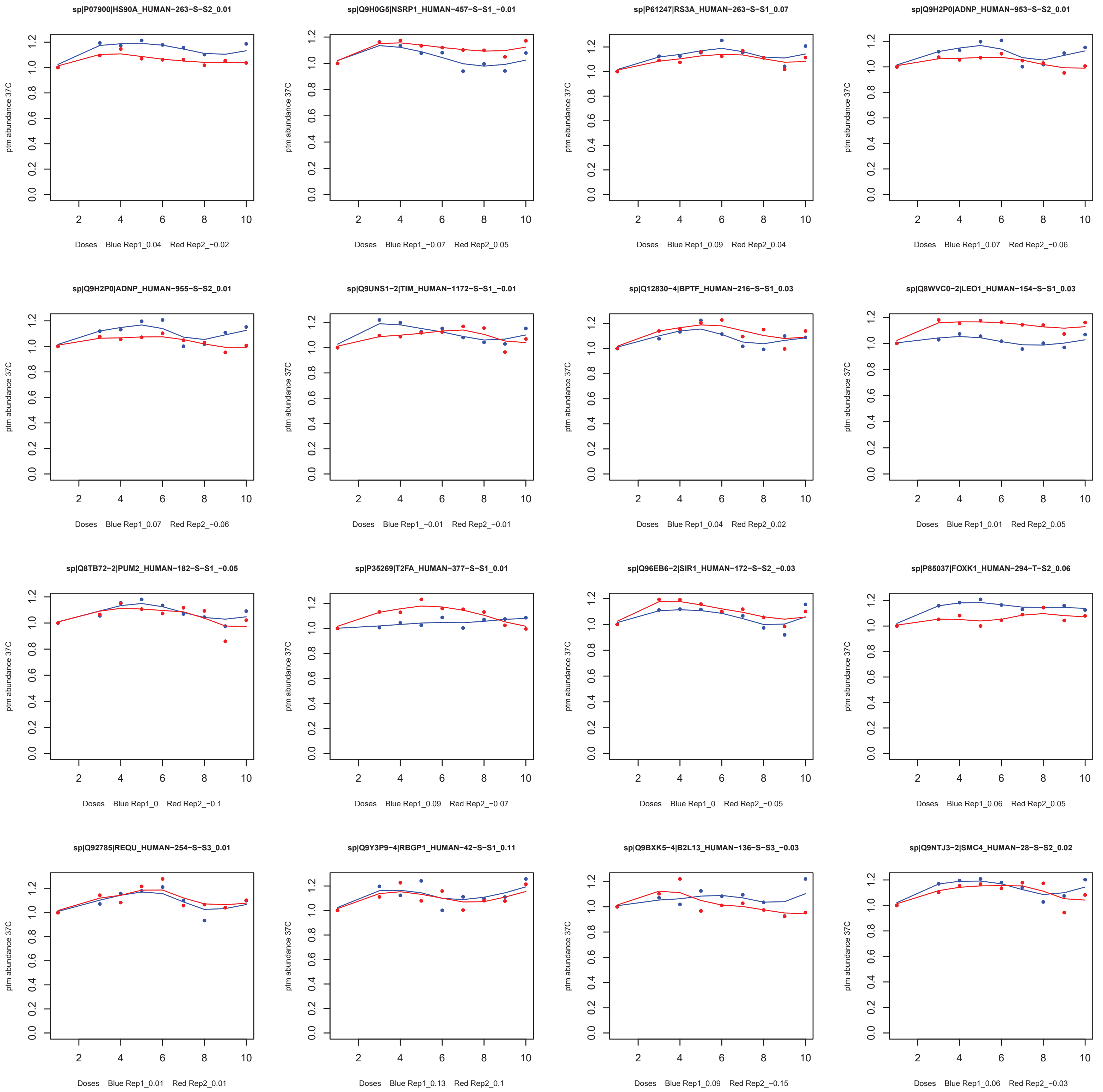

sp|P07900|HS90A_HUMAN−263−S−S2_0.01
Doses Blue Rep1_0.04 Red Rep2_−0.02
sp|Q9H0G5|NSRP1_HUMAN−457−S−S1_−0.01
Doses Blue Rep1_−0.07 Red Rep2_0.05
sp|P61247|RS3A_HUMAN−263−S−S1_0.07
Doses Blue Rep1_0.09 Red Rep2_0.04
sp|Q9H2P0|ADNP_HUMAN−953−S−S2_0.01
Doses Blue Rep1_0.07 Red Rep2_−0.06
sp|Q9H2P0|ADNP_HUMAN−955−S−S2_0.01
Doses Blue Rep1_0.07 Red Rep2_−0.06
sp|Q9UNS1−2|TIM_HUMAN−1172−S−S1_−0.01
Doses Blue Rep1_−0.01 Red Rep2_−0.01
sp|Q12830−4|BPTF_HUMAN−216−S−S1_0.03
Doses Blue Rep1_0.04 Red Rep2_0.02
sp|Q8WVC0−2|LEO1_HUMAN−154−S−S1_0.03
Doses Blue Rep1_0.01 Red Rep2_0.05
sp|Q8TB72−2|PUM2_HUMAN−182−S−S1_−0.05
Doses Blue Rep1_0 Red Rep2_−0.1
sp|P35269|T2FA_HUMAN−377−S−S1_0.01
Doses Blue Rep1_0.09 Red Rep2_−0.07
sp|Q96EB6−2|SIR1_HUMAN−172−S−S2_−0.03
Doses Blue Rep1_0 Red Rep2_−0.05
sp|P85037|FOXK1_HUMAN−294−T−S2_0.06
Doses Blue Rep1_0.06 Red Rep2_0.05
sp|Q92785|REQU_HUMAN−254−S−S3_0.01
Doses Blue Rep1_0.01 Red Rep2_0.01
sp|Q9Y3P9−4|RBGP1_HUMAN−42−S−S1_0.11
Doses Blue Rep1_0.13 Red Rep2_0.1
sp|Q9BXK5−4|B2L13_HUMAN−136−S−S3_−0.03
Doses Blue Rep1_0.09 Red Rep2_−0.15
sp|Q9NTJ3−2|SMC4_HUMAN−28−S−S2_0.02
Doses Blue Rep1_0.06 Red Rep2_−0.03
ptm abundance 37C
0.0 0.2 0.4 0.6 0.8 1.0 1.2
2 4 6 8 10

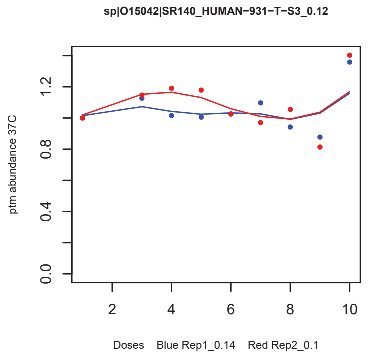
sp|O15042|SR140_HUMAN-931-T-S3_0.12
ptm abundance 37C
Doses Blue Rep1_0.14 Red Rep2_0.1

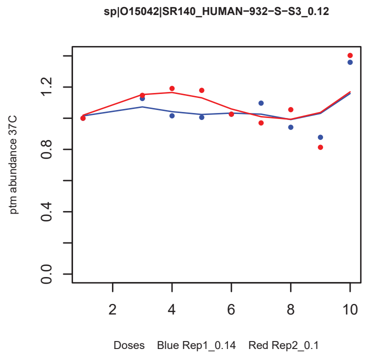
sp|O15042|SR140_HUMAN-932-S-S3_0.12
ptm abundance 37C
Doses Blue Rep1_0.14 Red Rep2_0.1

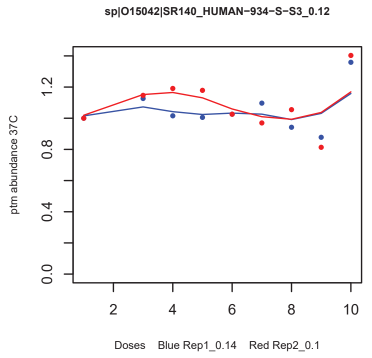
sp|O15042|SR140_HUMAN-934-S-S3_0.12
ptm abundance 37C
Doses Blue Rep1_0.14 Red Rep2_0.1

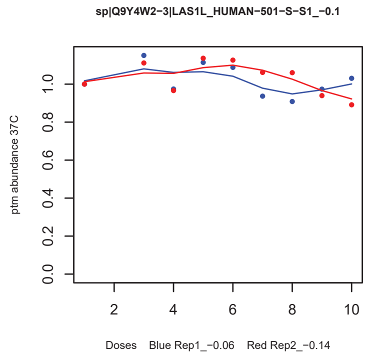
sp|Q9Y4W2-3|LAS1L_HUMAN-501-S-S1_-0.1
ptm abundance 37C
Doses Blue Rep1_-0.06 Red Rep2_-0.14

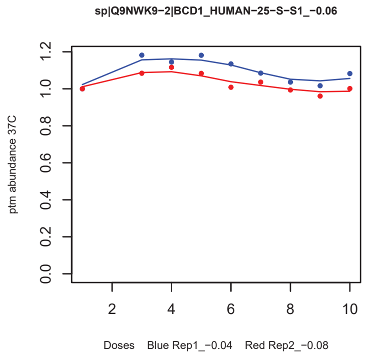
sp|Q9NWK9-2|BCD1_HUMAN-25-S-S1_-0.06
ptm abundance 37C
Doses Blue Rep1_-0.04 Red Rep2_-0.08

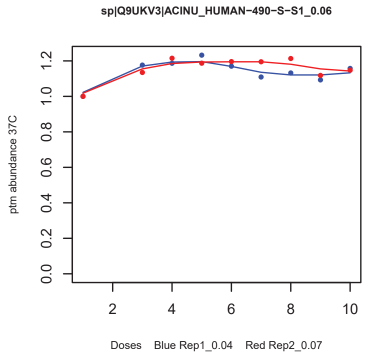
sp|Q9UKV3|ACINU_HUMAN-490-S-S1_0.06
ptm abundance 37C
Doses Blue Rep1_0.04 Red Rep2_0.07

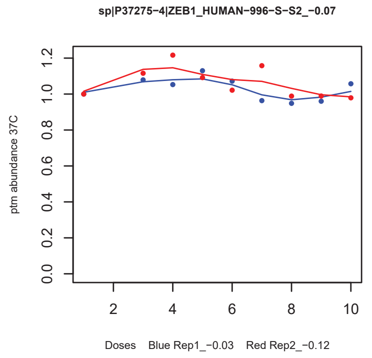
sp|P37275-4|ZEB1_HUMAN-996-S-S2_-0.07
ptm abundance 37C
Doses Blue Rep1_-0.03 Red Rep2_-0.12

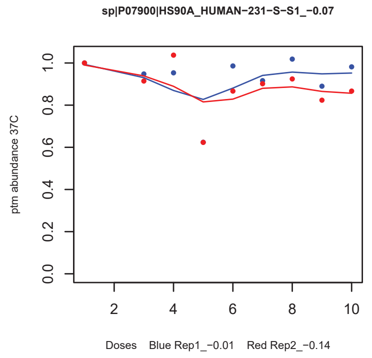
sp|P07900|HS90A_HUMAN-231-S-S1_-0.07
ptm abundance 37C
Doses Blue Rep1_-0.01 Red Rep2_-0.14

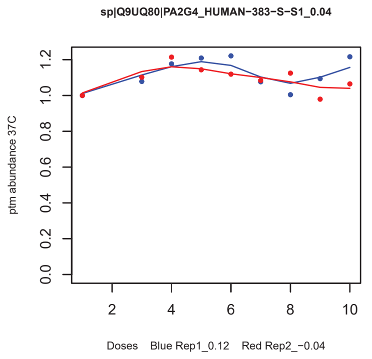
sp|Q9UQ80|PA2G4_HUMAN-383-S-S1_0.04
ptm abundance 37C
Doses Blue Rep1_0.12 Red Rep2_-0.04

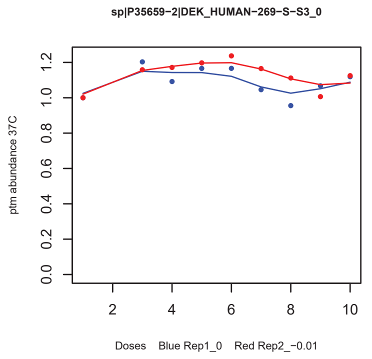
sp|P35659-2|DEK_HUMAN-269-S-S3_0
ptm abundance 37C
Doses Blue Rep1_0 Red Rep2_-0.01

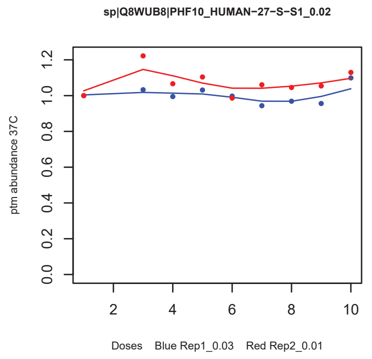
sp|Q8WUB8|PHF10_HUMAN-27-S-S1_0.02
ptm abundance 37C
Doses Blue Rep1_0.03 Red Rep2_0.01

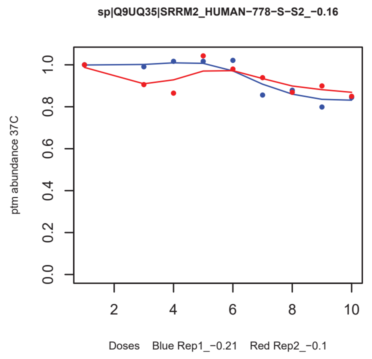
sp|Q9UQ35|SRRM2_HUMAN-778-S-S2_-0.16
ptm abundance 37C
Doses Blue Rep1_-0.21 Red Rep2_-0.1

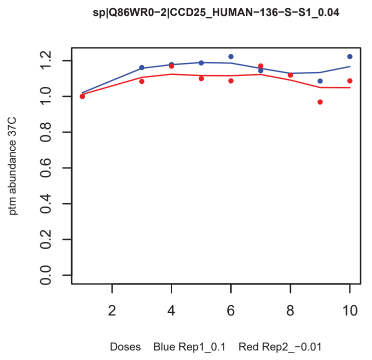
sp|Q86WR0-2|CCD25_HUMAN-136-S-S1_0.04
ptm abundance 37C
Doses Blue Rep1_0.1 Red Rep2_-0.01

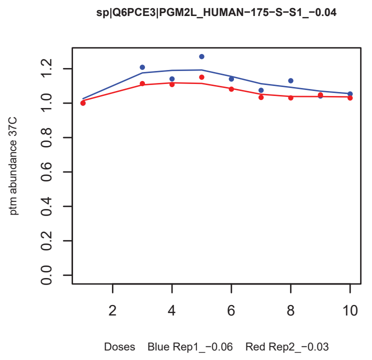
sp|Q6PCE3|PGM2L_HUMAN-175-S-S1_-0.04
ptm abundance 37C
Doses Blue Rep1_-0.06 Red Rep2_-0.03

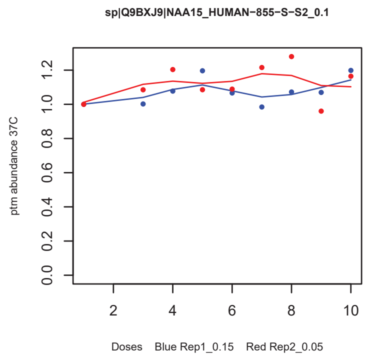
sp|Q9BXJ9|NAA15_HUMAN-855-S-S2_0.1
ptm abundance 37C
Doses Blue Rep1_0.15 Red Rep2_0.05

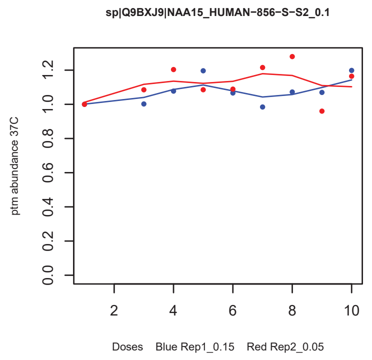
sp|Q9BXJ9|NAA15_HUMAN-856-S-S2_0.1
ptm abundance 37C
Doses Blue Rep1_0.15 Red Rep2_0.05

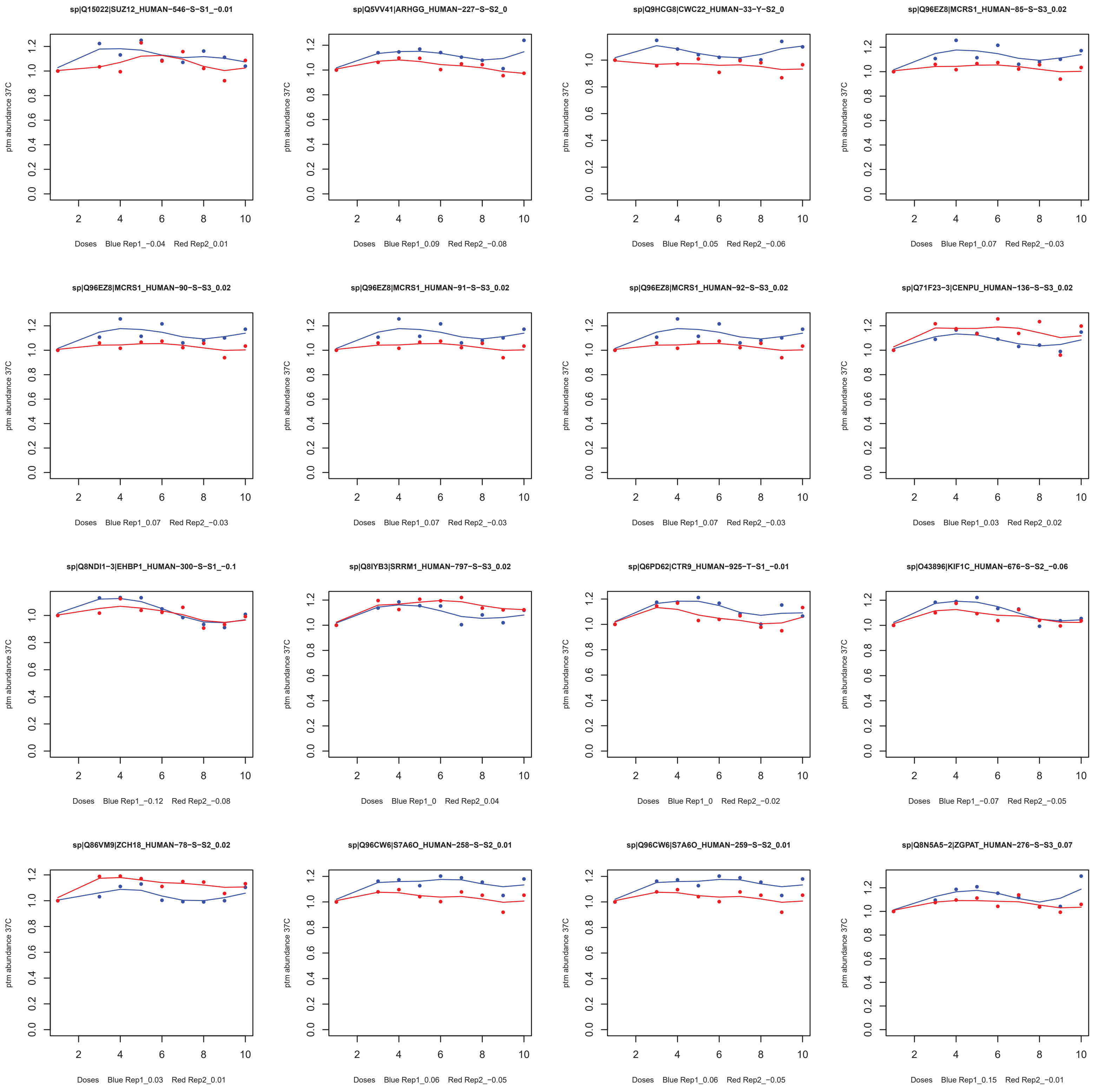
sp|Q15022|SUZ12_HUMAN−546−S−S1_−0.01
Doses Blue Rep1_−0.04 Red Rep2_0.01
sp|Q5VV41|ARHGG_HUMAN−227−S−S2_0
Doses Blue Rep1_0.09 Red Rep2_−0.08
sp|Q9HCG8|CWC22_HUMAN−33−Y−S2_0
Doses Blue Rep1_0.05 Red Rep2_−0.06
sp|Q96EZ8|MCRS1_HUMAN−85−S−S3_0.02
Doses Blue Rep1_0.07 Red Rep2_−0.03
sp|Q96EZ8|MCRS1_HUMAN−90−S−S3_0.02
Doses Blue Rep1_0.07 Red Rep2_−0.03
sp|Q96EZ8|MCRS1_HUMAN−91−S−S3_0.02
Doses Blue Rep1_0.07 Red Rep2_−0.03
sp|Q96EZ8|MCRS1_HUMAN−92−S−S3_0.02
Doses Blue Rep1_0.07 Red Rep2_−0.03
sp|Q71F23−3|CENPU_HUMAN−136−S−S3_0.02
Doses Blue Rep1_0.03 Red Rep2_0.02
sp|Q8NDI1−3|EHBP1_HUMAN−300−S−S1_−0.1
Doses Blue Rep1_−0.12 Red Rep2_−0.08
sp|Q8IYB3|SRRM1_HUMAN−797−S−S3_0.02
Doses Blue Rep1_0 Red Rep2_0.04
sp|Q6PD62|CTR9_HUMAN−925−T−S1_−0.01
Doses Blue Rep1_0 Red Rep2_−0.02
sp|O43896|KIF1C_HUMAN−676−S−S2_−0.06
Doses Blue Rep1_−0.07 Red Rep2_−0.05
sp|Q86VM9|ZCH18_HUMAN−78−S−S2_0.02
Doses Blue Rep1_0.03 Red Rep2_0.01
sp|Q96CW6|S7A6O_HUMAN−258−S−S2_0.01
Doses Blue Rep1_0.06 Red Rep2_−0.05
sp|Q96CW6|S7A6O_HUMAN−259−S−S2_0.01
Doses Blue Rep1_0.06 Red Rep2_−0.05
sp|Q8N5A5−2|ZGPAT_HUMAN−276−S−S3_0.07
Doses Blue Rep1_0.15 Red Rep2_−0.01
ptm abundance 37C

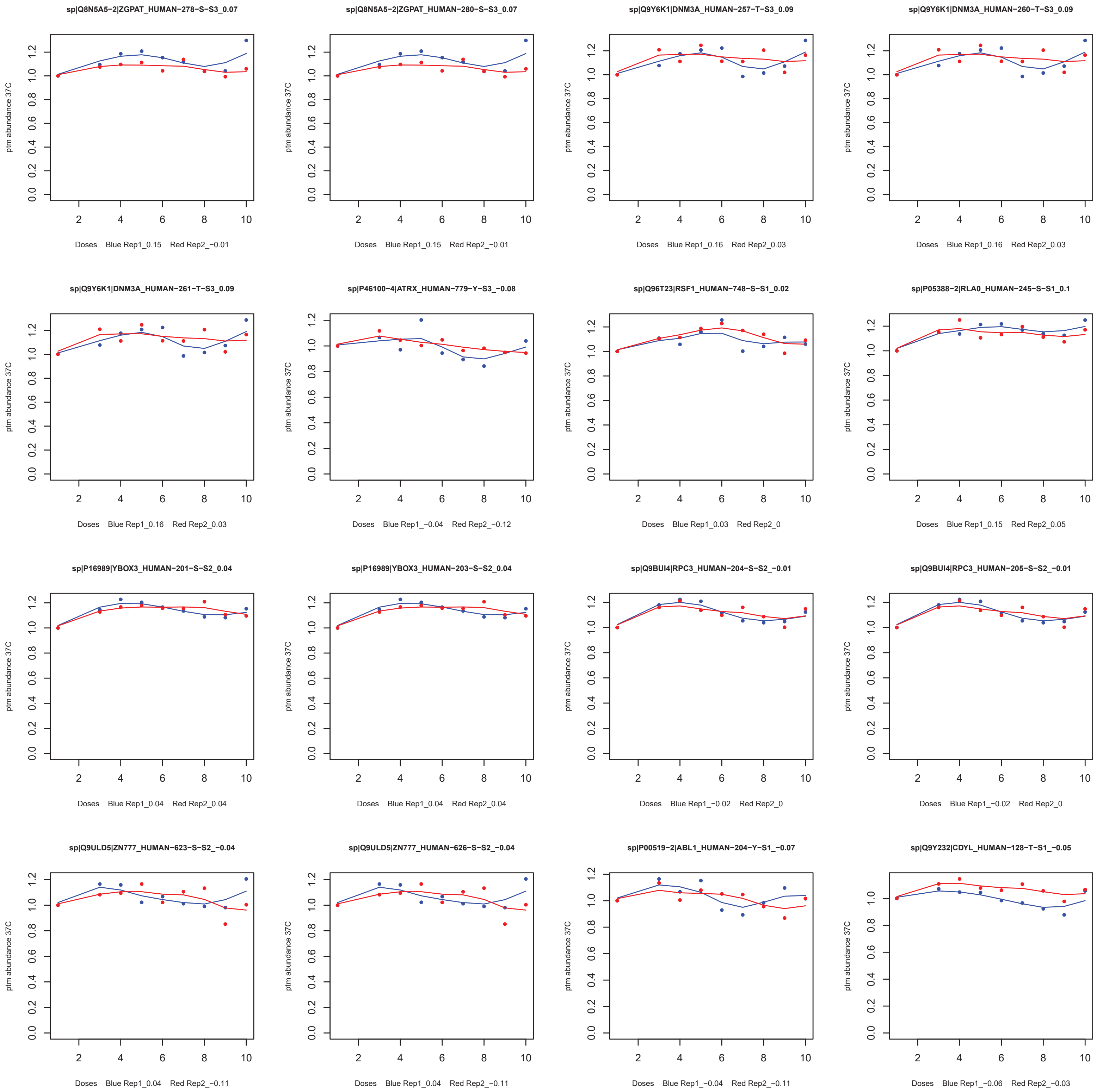
sp|Q8N5A5−2|ZGPAT_HUMAN−278−S−S3_0.07
ptm abundance 37C
Doses Blue Rep1_0.15 Red Rep2_−0.01
sp|Q8N5A5−2|ZGPAT_HUMAN−280−S−S3_0.07
ptm abundance 37C
Doses Blue Rep1_0.15 Red Rep2_−0.01
sp|Q9Y6K1|DNM3A_HUMAN−257−T−S3_0.09
ptm abundance 37C
Doses Blue Rep1_0.16 Red Rep2_0.03
sp|Q9Y6K1|DNM3A_HUMAN−260−T−S3_0.09
ptm abundance 37C
Doses Blue Rep1_0.16 Red Rep2_0.03
sp|Q9Y6K1|DNM3A_HUMAN−261−T−S3_0.09
ptm abundance 37C
Doses Blue Rep1_0.16 Red Rep2_0.03
sp|P46100−4|ATRX_HUMAN−779−Y−S3_−0.08
ptm abundance 37C
Doses Blue Rep1_−0.04 Red Rep2_−0.12
sp|Q96T23|RSF1_HUMAN−748−S−S1_0.02
ptm abundance 37C
Doses Blue Rep1_0.03 Red Rep2_0
sp|P05388−2|RLA0_HUMAN−245−S−S1_0.1
ptm abundance 37C
Doses Blue Rep1_0.15 Red Rep2_0.05
sp|P16989|YBOX3_HUMAN−201−S−S2_0.04
ptm abundance 37C
Doses Blue Rep1_0.04 Red Rep2_0.04
sp|P16989|YBOX3_HUMAN−203−S−S2_0.04
ptm abundance 37C
Doses Blue Rep1_0.04 Red Rep2_0.04
sp|Q9BUI4|RPC3_HUMAN−204−S−S2_−0.01
ptm abundance 37C
Doses Blue Rep1_−0.02 Red Rep2_0
sp|Q9BUI4|RPC3_HUMAN−205−S−S2_−0.01
ptm abundance 37C
Doses Blue Rep1_−0.02 Red Rep2_0
sp|Q9ULD5|ZN777_HUMAN−623−S−S2_−0.04
ptm abundance 37C
Doses Blue Rep1_0.04 Red Rep2_−0.11
sp|Q9ULD5|ZN777_HUMAN−626−S−S2_−0.04
ptm abundance 37C
Doses Blue Rep1_0.04 Red Rep2_−0.11
sp|P00519−2|ABL1_HUMAN−204−Y−S1_−0.07
ptm abundance 37C
Doses Blue Rep1_−0.04 Red Rep2_−0.11
sp|Q9Y232|CDYL_HUMAN−128−T−S1_−0.05
ptm abundance 37C
Doses Blue Rep1_−0.06 Red Rep2_−0.03

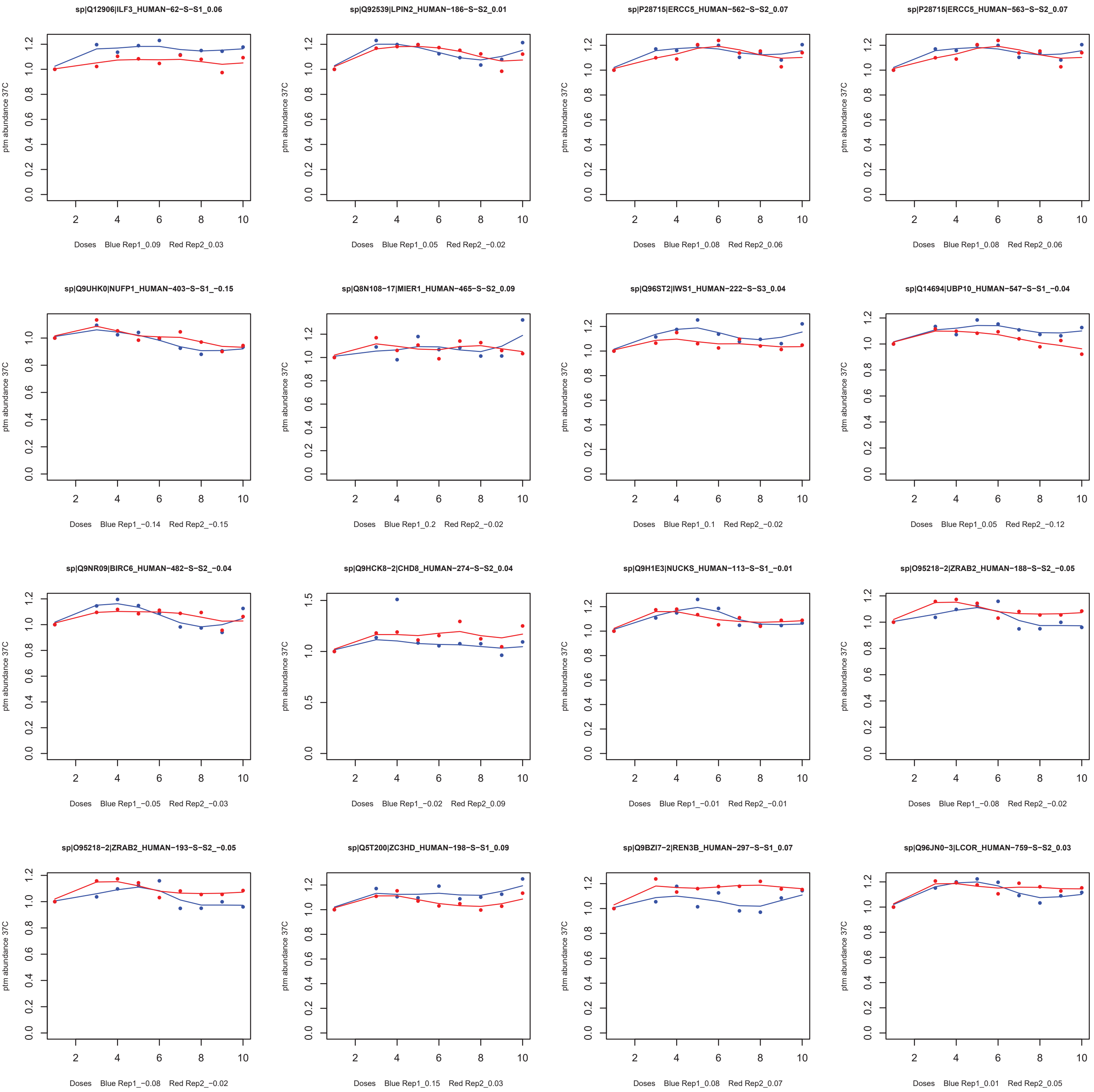
sp|Q12906|ILF3_HUMAN−62−S−S1_0.06
ptm abundance 37C
Doses Blue Rep1_0.09 Red Rep2_0.03
sp|Q92539|LPIN2_HUMAN−186−S−S2_0.01
Doses Blue Rep1_0.05 Red Rep2_−0.02
sp|P28715|ERCC5_HUMAN−562−S−S2_0.07
Doses Blue Rep1_0.08 Red Rep2_0.06
sp|P28715|ERCC5_HUMAN−563−S−S2_0.07
Doses Blue Rep1_0.08 Red Rep2_0.06
sp|Q9UHK0|NUFP1_HUMAN−403−S−S1_−0.15
Doses Blue Rep1_−0.14 Red Rep2_−0.15
sp|Q8N108−17|MIER1_HUMAN−465−S−S2_0.09
Doses Blue Rep1_0.2 Red Rep2_−0.02
sp|Q96ST2|IWS1_HUMAN−222−S−S3_0.04
Doses Blue Rep1_0.1 Red Rep2_−0.02
sp|Q14694|UBP10_HUMAN−547−S−S1_−0.04
Doses Blue Rep1_0.05 Red Rep2_−0.12
sp|Q9NR09|BIRC6_HUMAN−482−S−S2_−0.04
Doses Blue Rep1_−0.05 Red Rep2_−0.03
sp|Q9HCK8−2|CHD8_HUMAN−274−S−S2_0.04
Doses Blue Rep1_−0.02 Red Rep2_0.09
sp|Q9H1E3|NUCKS_HUMAN−113−S−S1_−0.01
Doses Blue Rep1_−0.01 Red Rep2_−0.01
sp|O95218−2|ZRAB2_HUMAN−188−S−S2_−0.05
Doses Blue Rep1_−0.08 Red Rep2_−0.02
sp|O95218−2|ZRAB2_HUMAN−193−S−S2_−0.05
Doses Blue Rep1_−0.08 Red Rep2_−0.02
sp|Q5T200|ZC3HD_HUMAN−198−S−S1_0.09
Doses Blue Rep1_0.15 Red Rep2_0.03
sp|Q9BZI7−2|REN3B_HUMAN−297−S−S1_0.07
Doses Blue Rep1_0.08 Red Rep2_0.07
sp|Q96JN0−3|LCOR_HUMAN−759−S−S2_0.03
Doses Blue Rep1_0.01 Red Rep2_0.05

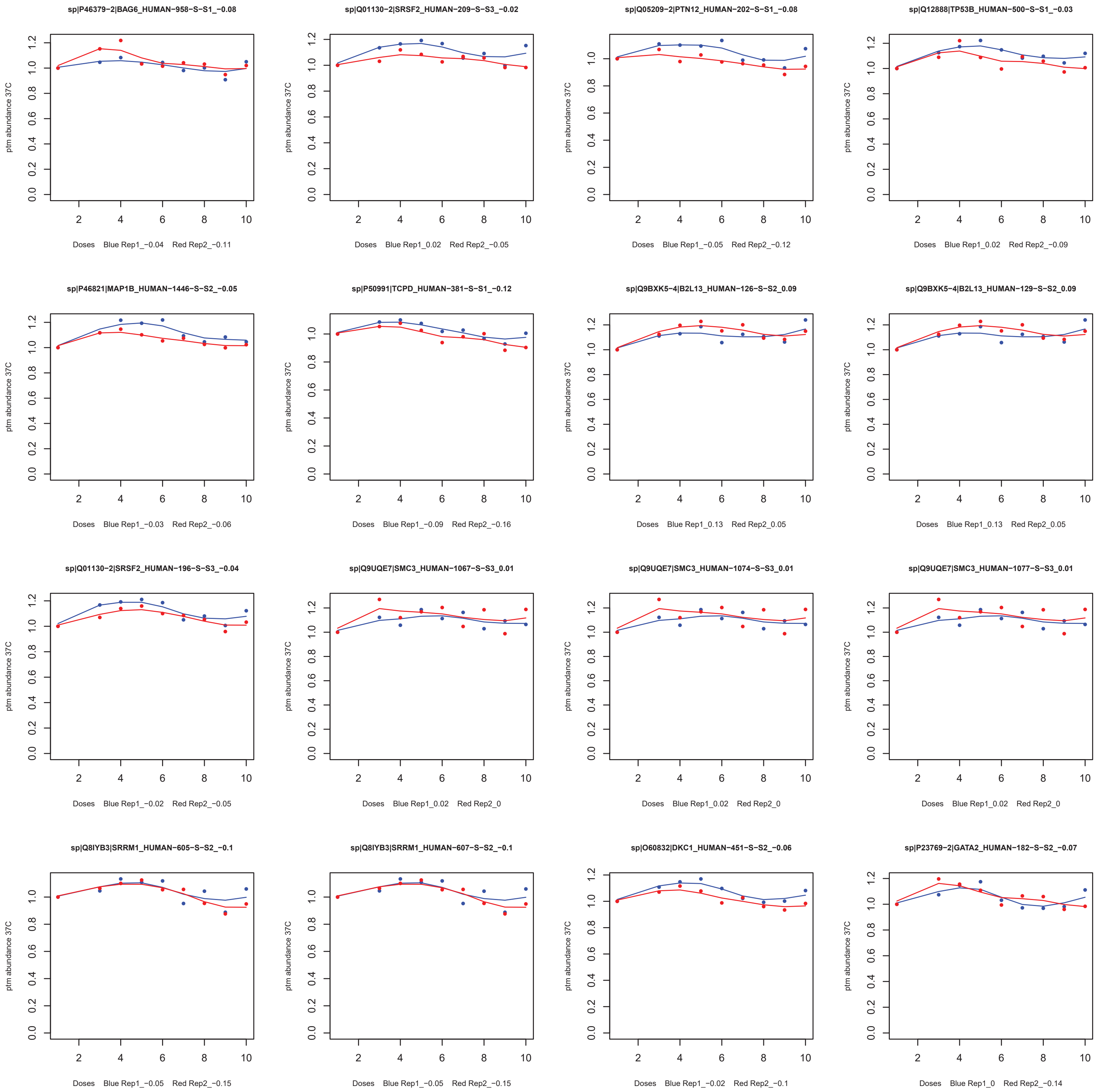

sp|P46379−2|BAG6_HUMAN−958−S−S1_−0.08
ptm abundance 37C
Doses Blue Rep1_−0.04 Red Rep2_−0.11
sp|Q01130−2|SRSF2_HUMAN−209−S−S3_−0.02
ptm abundance 37C
Doses Blue Rep1_0.02 Red Rep2_−0.05
sp|Q05209−2|PTN12_HUMAN−202−S−S1_−0.08
ptm abundance 37C
Doses Blue Rep1_−0.05 Red Rep2_−0.12
sp|Q12888|TP53B_HUMAN−500−S−S1_−0.03
ptm abundance 37C
Doses Blue Rep1_0.02 Red Rep2_−0.09
sp|P46821|MAP1B_HUMAN−1446−S−S2_−0.05
ptm abundance 37C
Doses Blue Rep1_−0.03 Red Rep2_−0.06
sp|P50991|TCPD_HUMAN−381−S−S1_−0.12
ptm abundance 37C
Doses Blue Rep1_−0.09 Red Rep2_−0.16
sp|Q9BXK5−4|B2L13_HUMAN−126−S−S2_0.09
ptm abundance 37C
Doses Blue Rep1_0.13 Red Rep2_0.05
sp|Q9BXK5−4|B2L13_HUMAN−129−S−S2_0.09
ptm abundance 37C
Doses Blue Rep1_0.13 Red Rep2_0.05
sp|Q01130−2|SRSF2_HUMAN−196−S−S3_−0.04
ptm abundance 37C
Doses Blue Rep1_−0.02 Red Rep2_−0.05
sp|Q9UQE7|SMC3_HUMAN−1067−S−S3_0.01
ptm abundance 37C
Doses Blue Rep1_0.02 Red Rep2_0
sp|Q9UQE7|SMC3_HUMAN−1074−S−S3_0.01
ptm abundance 37C
Doses Blue Rep1_0.02 Red Rep2_0
sp|Q9UQE7|SMC3_HUMAN−1077−S−S3_0.01
ptm abundance 37C
Doses Blue Rep1_0.02 Red Rep2_0
sp|Q8IYB3|SRRM1_HUMAN−605−S−S2_−0.1
ptm abundance 37C
Doses Blue Rep1_−0.05 Red Rep2_−0.15
sp|Q8IYB3|SRRM1_HUMAN−607−S−S2_−0.1
ptm abundance 37C
Doses Blue Rep1_−0.05 Red Rep2_−0.15
sp|O60832|DKC1_HUMAN−451−S−S2_−0.06
ptm abundance 37C
Doses Blue Rep1_−0.02 Red Rep2_−0.1
sp|P23769−2|GATA2_HUMAN−182−S−S2_−0.07
ptm abundance 37C
Doses Blue Rep1_0 Red Rep2_−0.14

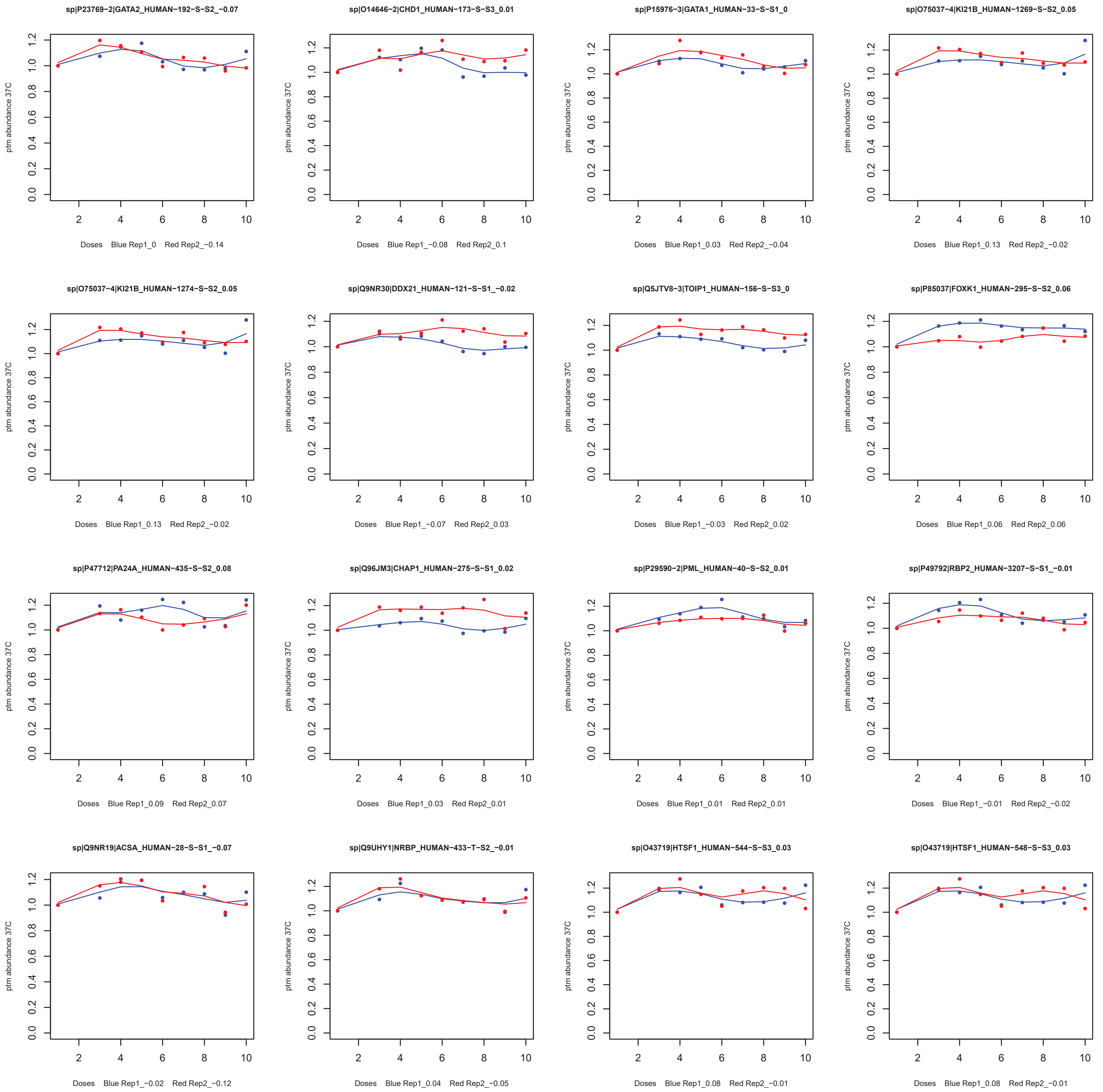

sp|P23769−2|GATA2_HUMAN−192−S−S2_−0.07
Doses Blue Rep1_0 Red Rep2_−0.14
sp|O14646−2|CHD1_HUMAN−173−S−S3_0.01
Doses Blue Rep1_−0.08 Red Rep2_0.1
sp|P15976−3|GATA1_HUMAN−33−S−S1_0
Doses Blue Rep1_0.03 Red Rep2_−0.04
sp|O75037−4|KI21B_HUMAN−1269−S−S2_0.05
Doses Blue Rep1_0.13 Red Rep2_−0.02
sp|O75037−4|KI21B_HUMAN−1274−S−S2_0.05
Doses Blue Rep1_0.13 Red Rep2_−0.02
sp|Q9NR30|DDX21_HUMAN−121−S−S1_−0.02
Doses Blue Rep1_−0.07 Red Rep2_0.03
sp|Q5JTV8−3|TOIP1_HUMAN−156−S−S3_0
Doses Blue Rep1_−0.03 Red Rep2_0.02
sp|P85037|FOXK1_HUMAN−295−S−S2_0.06
Doses Blue Rep1_0.06 Red Rep2_0.06
sp|P47712|PA24A_HUMAN−435−S−S2_0.08
Doses Blue Rep1_0.09 Red Rep2_0.07
sp|Q96JM3|CHAP1_HUMAN−275−S−S1_0.02
Doses Blue Rep1_0.03 Red Rep2_0.01
sp|P29590−2|PML_HUMAN−40−S−S2_0.01
Doses Blue Rep1_0.01 Red Rep2_0.01
sp|P49792|RBP2_HUMAN−3207−S−S1_−0.01
Doses Blue Rep1_−0.01 Red Rep2_−0.02
sp|Q9NR19|ACSA_HUMAN−28−S−S1_−0.07
Doses Blue Rep1_−0.02 Red Rep2_−0.12
sp|Q9UHY1|NRBP_HUMAN−433−T−S2_−0.01
Doses Blue Rep1_0.04 Red Rep2_−0.05
sp|O43719|HTSF1_HUMAN−544−S−S3_0.03
Doses Blue Rep1_0.08 Red Rep2_−0.01
sp|O43719|HTSF1_HUMAN−548−S−S3_0.03
Doses Blue Rep1_0.08 Red Rep2_−0.01
ptm abundance 37C

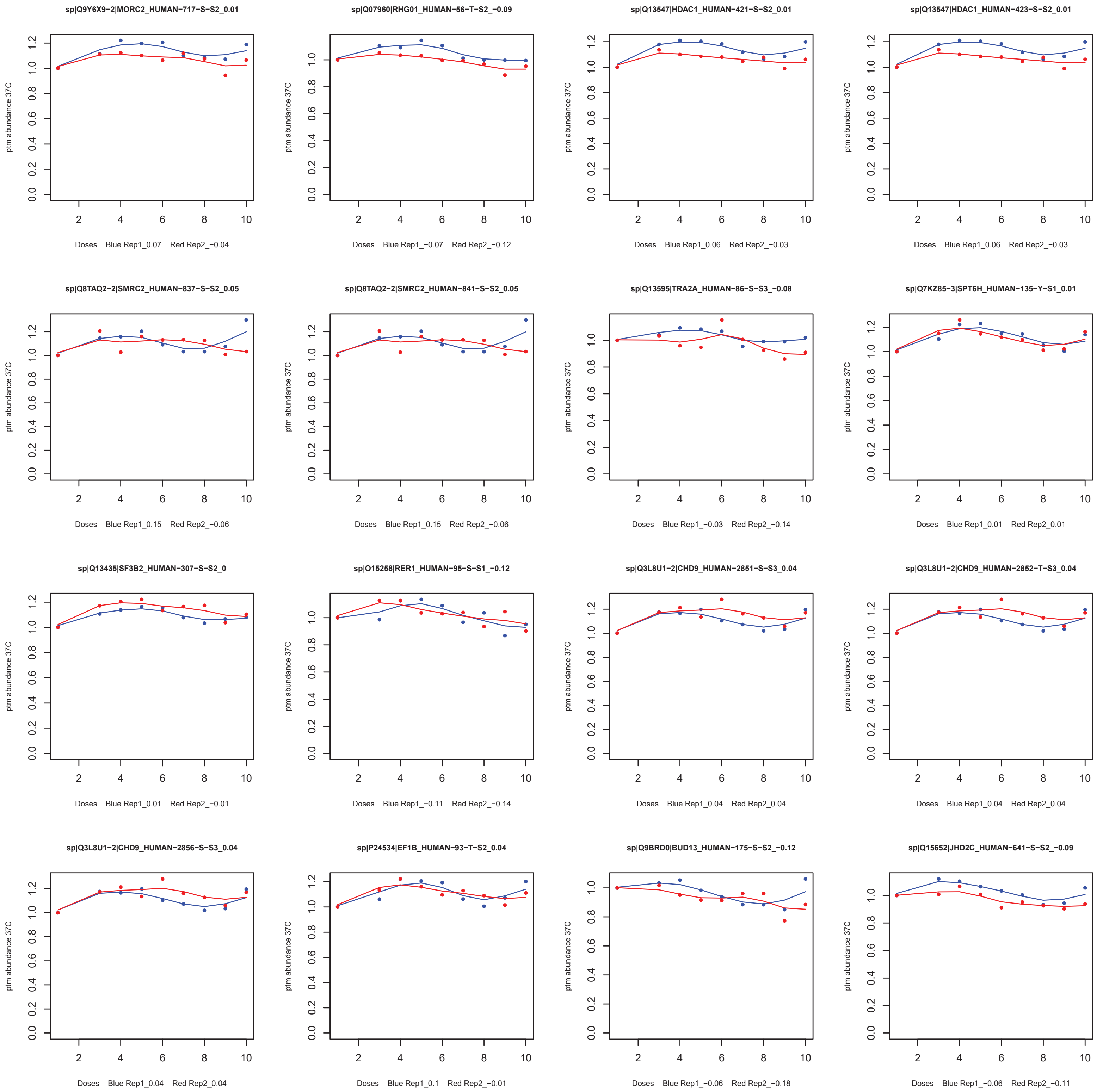
sp|Q9Y6X9−2|MORC2_HUMAN−717−S−S2_0.01
Doses Blue Rep1_0.07 Red Rep2_−0.04
sp|Q07960|RHG01_HUMAN−56−T−S2_−0.09
Doses Blue Rep1_−0.07 Red Rep2_−0.12
sp|Q13547|HDAC1_HUMAN−421−S−S2_0.01
Doses Blue Rep1_0.06 Red Rep2_−0.03
sp|Q13547|HDAC1_HUMAN−423−S−S2_0.01
Doses Blue Rep1_0.06 Red Rep2_−0.03
sp|Q8TAQ2−2|SMRC2_HUMAN−837−S−S2_0.05
Doses Blue Rep1_0.15 Red Rep2_−0.06
sp|Q8TAQ2−2|SMRC2_HUMAN−841−S−S2_0.05
Doses Blue Rep1_0.15 Red Rep2_−0.06
sp|Q13595|TRA2A_HUMAN−86−S−S3_−0.08
Doses Blue Rep1_−0.03 Red Rep2_−0.14
sp|Q7KZ85−3|SPT6H_HUMAN−135−Y−S1_0.01
Doses Blue Rep1_0.01 Red Rep2_0.01
sp|Q13435|SF3B2_HUMAN−307−S−S2_0
Doses Blue Rep1_0.01 Red Rep2_−0.01
sp|O15258|RER1_HUMAN−95−S−S1_−0.12
Doses Blue Rep1_−0.11 Red Rep2_−0.14
sp|Q3L8U1−2|CHD9_HUMAN−2851−S−S3_0.04
Doses Blue Rep1_0.04 Red Rep2_0.04
sp|Q3L8U1−2|CHD9_HUMAN−2852−T−S3_0.04
Doses Blue Rep1_0.04 Red Rep2_0.04
sp|Q3L8U1−2|CHD9_HUMAN−2856−S−S3_0.04
Doses Blue Rep1_0.04 Red Rep2_0.04
sp|P24534|EF1B_HUMAN−93−T−S2_0.04
Doses Blue Rep1_0.1 Red Rep2_−0.01
sp|Q9BRD0|BUD13_HUMAN−175−S−S2_−0.12
Doses Blue Rep1_−0.06 Red Rep2_−0.18
sp|Q15652|JHD2C_HUMAN−641−S−S2_−0.09
Doses Blue Rep1_−0.06 Red Rep2_−0.11
ptm abundance 37C

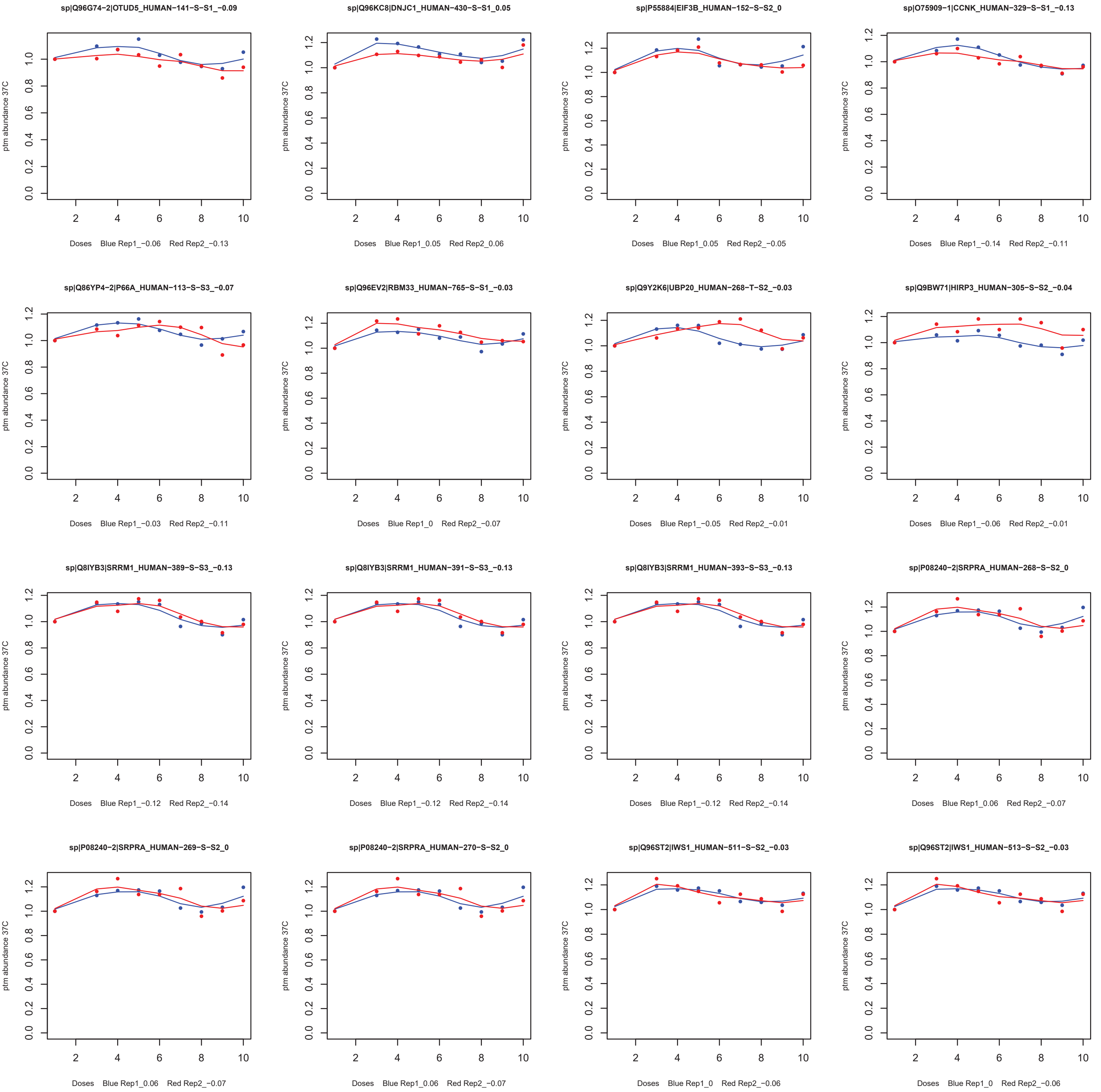

sp|Q96G74−2|OTUD5_HUMAN−141−S−S1_−0.09
Doses Blue Rep1_−0.06 Red Rep2_−0.13
sp|Q96KC8|DNJC1_HUMAN−430−S−S1_0.05
Doses Blue Rep1_0.05 Red Rep2_0.06
sp|P55884|EIF3B_HUMAN−152−S−S2_0
Doses Blue Rep1_0.05 Red Rep2_−0.05
sp|O75909−1|CCNK_HUMAN−329−S−S1_−0.13
Doses Blue Rep1_−0.14 Red Rep2_−0.11
sp|Q86YP4−2|P66A_HUMAN−113−S−S3_−0.07
Doses Blue Rep1_−0.03 Red Rep2_−0.11
sp|Q96EV2|RBM33_HUMAN−765−S−S1_−0.03
Doses Blue Rep1_0 Red Rep2_−0.07
sp|Q9Y2K6|UBP20_HUMAN−268−T−S2_−0.03
Doses Blue Rep1_−0.05 Red Rep2_−0.01
sp|Q9BW71|HIRP3_HUMAN−305−S−S2_−0.04
Doses Blue Rep1_−0.06 Red Rep2_−0.01
sp|Q8IYB3|SRRM1_HUMAN−389−S−S3_−0.13
Doses Blue Rep1_−0.12 Red Rep2_−0.14
sp|Q8IYB3|SRRM1_HUMAN−391−S−S3_−0.13
Doses Blue Rep1_−0.12 Red Rep2_−0.14
sp|Q8IYB3|SRRM1_HUMAN−393−S−S3_−0.13
Doses Blue Rep1_−0.12 Red Rep2_−0.14
sp|P08240−2|SRPRA_HUMAN−268−S−S2_0
Doses Blue Rep1_0.06 Red Rep2_−0.07
sp|P08240−2|SRPRA_HUMAN−269−S−S2_0
Doses Blue Rep1_0.06 Red Rep2_−0.07
sp|P08240−2|SRPRA_HUMAN−270−S−S2_0
Doses Blue Rep1_0.06 Red Rep2_−0.07
sp|Q96ST2|IWS1_HUMAN−511−S−S2_−0.03
Doses Blue Rep1_0 Red Rep2_−0.06
sp|Q96ST2|IWS1_HUMAN−513−S−S2_−0.03
Doses Blue Rep1_0 Red Rep2_−0.06
ptm abundance 37C

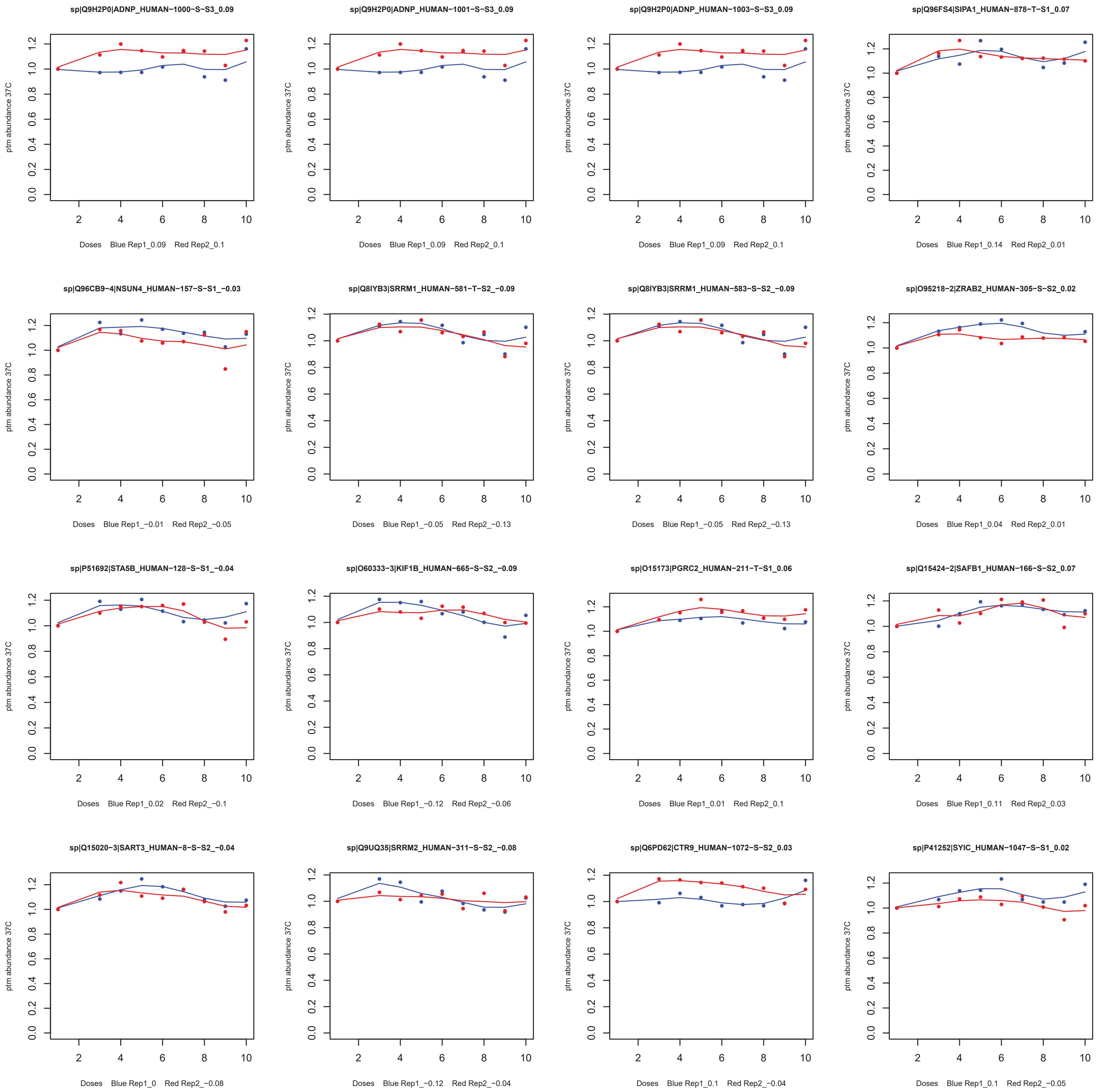

sp|Q9H2P0|ADNP_HUMAN−1000−S−S3_0.09
Doses Blue Rep1_0.09 Red Rep2_0.1
sp|Q9H2P0|ADNP_HUMAN−1001−S−S3_0.09
Doses Blue Rep1_0.09 Red Rep2_0.1
sp|Q9H2P0|ADNP_HUMAN−1003−S−S3_0.09
Doses Blue Rep1_0.09 Red Rep2_0.1
sp|Q96FS4|SIPA1_HUMAN−878−T−S1_0.07
Doses Blue Rep1_0.14 Red Rep2_0.01
sp|Q96CB9−4|NSUN4_HUMAN−157−S−S1_−0.03
Doses Blue Rep1_−0.01 Red Rep2_−0.05
sp|Q8IYB3|SRRM1_HUMAN−581−T−S2_−0.09
Doses Blue Rep1_−0.05 Red Rep2_−0.13
sp|Q8IYB3|SRRM1_HUMAN−583−S−S2_−0.09
Doses Blue Rep1_−0.05 Red Rep2_−0.13
sp|O95218−2|ZRAB2_HUMAN−305−S−S2_0.02
Doses Blue Rep1_0.04 Red Rep2_0.01
sp|P51692|STA5B_HUMAN−128−S−S1_−0.04
Doses Blue Rep1_0.02 Red Rep2_−0.1
sp|O60333−3|KIF1B_HUMAN−665−S−S2_−0.09
Doses Blue Rep1_−0.12 Red Rep2_−0.06
sp|O15173|PGRC2_HUMAN−211−T−S1_0.06
Doses Blue Rep1_0.01 Red Rep2_0.1
sp|Q15424−2|SAFB1_HUMAN−166−S−S2_0.07
Doses Blue Rep1_0.11 Red Rep2_0.03
sp|Q15020−3|SART3_HUMAN−8−S−S2_−0.04
Doses Blue Rep1_0 Red Rep2_−0.08
sp|Q9UQ35|SRRM2_HUMAN−311−S−S2_−0.08
Doses Blue Rep1_−0.12 Red Rep2_−0.04
sp|Q6PD62|CTR9_HUMAN−1072−S−S2_0.03
Doses Blue Rep1_0.1 Red Rep2_−0.04
sp|P41252|SYIC_HUMAN−1047−S−S1_0.02
Doses Blue Rep1_0.1 Red Rep2_−0.05
ptm abundance 37C

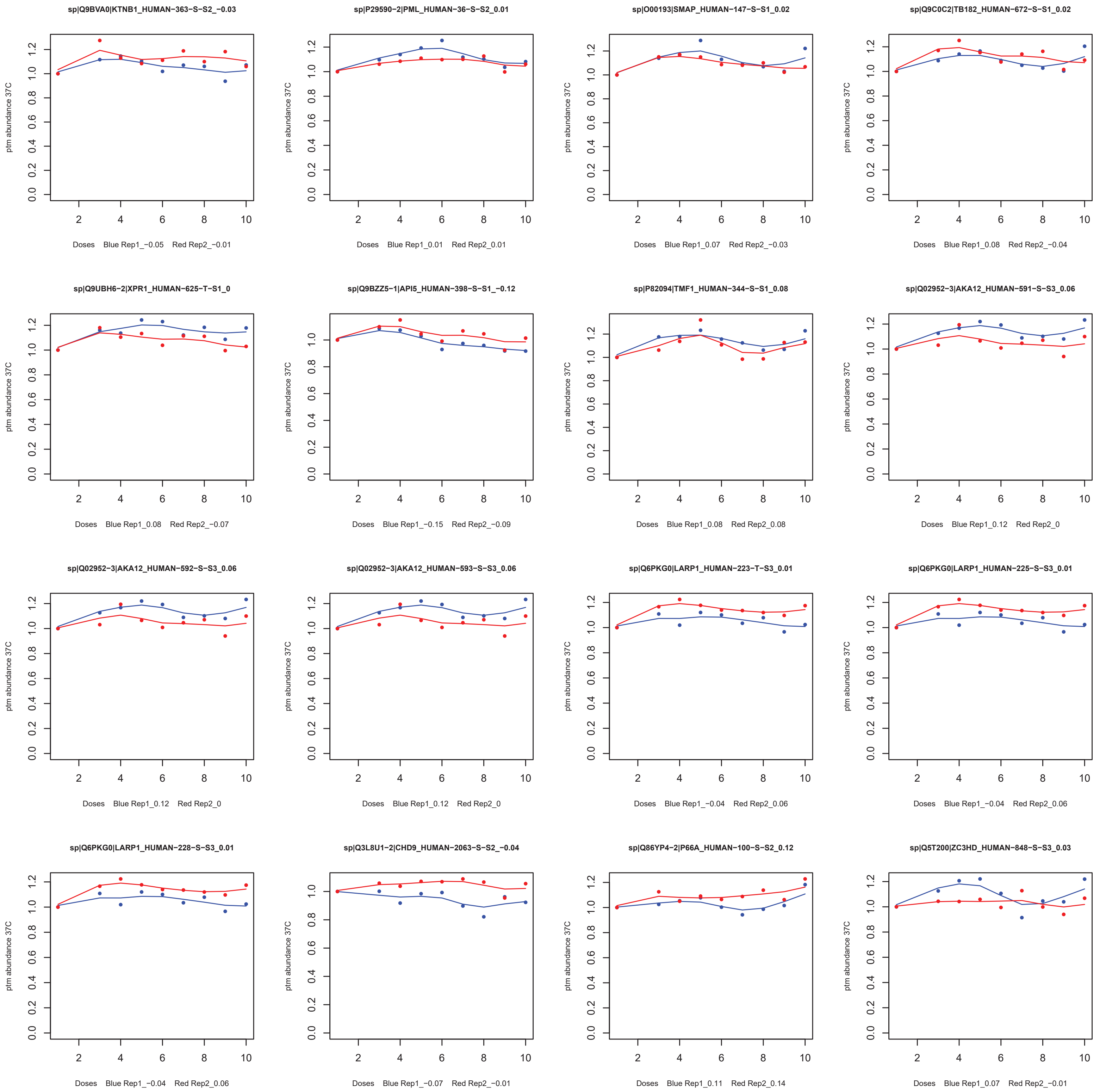
sp|Q9BVA0|KTNB1_HUMAN−363−S−S2_−0.03
Doses Blue Rep1_−0.05 Red Rep2_−0.01
sp|P29590−2|PML_HUMAN−36−S−S2_0.01
Doses Blue Rep1_0.01 Red Rep2_0.01
sp|O00193|SMAP_HUMAN−147−S−S1_0.02
Doses Blue Rep1_0.07 Red Rep2_−0.03
sp|Q9C0C2|TB182_HUMAN−672−S−S1_0.02
Doses Blue Rep1_0.08 Red Rep2_−0.04
sp|Q9UBH6−2|XPR1_HUMAN−625−T−S1_0
Doses Blue Rep1_0.08 Red Rep2_−0.07
sp|Q9BZZ5−1|API5_HUMAN−398−S−S1_−0.12
Doses Blue Rep1_−0.15 Red Rep2_−0.09
sp|P82094|TMF1_HUMAN−344−S−S1_0.08
Doses Blue Rep1_0.08 Red Rep2_0.08
sp|Q02952−3|AKA12_HUMAN−591−S−S3_0.06
Doses Blue Rep1_0.12 Red Rep2_0
sp|Q02952−3|AKA12_HUMAN−592−S−S3_0.06
Doses Blue Rep1_0.12 Red Rep2_0
sp|Q02952−3|AKA12_HUMAN−593−S−S3_0.06
Doses Blue Rep1_0.12 Red Rep2_0
sp|Q6PKG0|LARP1_HUMAN−223−T−S3_0.01
Doses Blue Rep1_−0.04 Red Rep2_0.06
sp|Q6PKG0|LARP1_HUMAN−225−S−S3_0.01
Doses Blue Rep1_−0.04 Red Rep2_0.06
sp|Q6PKG0|LARP1_HUMAN−228−S−S3_0.01
Doses Blue Rep1_−0.04 Red Rep2_0.06
sp|Q3L8U1−2|CHD9_HUMAN−2063−S−S2_−0.04
Doses Blue Rep1_−0.07 Red Rep2_−0.01
sp|Q86YP4−2|P66A_HUMAN−100−S−S2_0.12
Doses Blue Rep1_0.11 Red Rep2_0.14
sp|Q5T200|ZC3HD_HUMAN−848−S−S3_0.03
Doses Blue Rep1_0.07 Red Rep2_−0.01
ptm abundance 37C

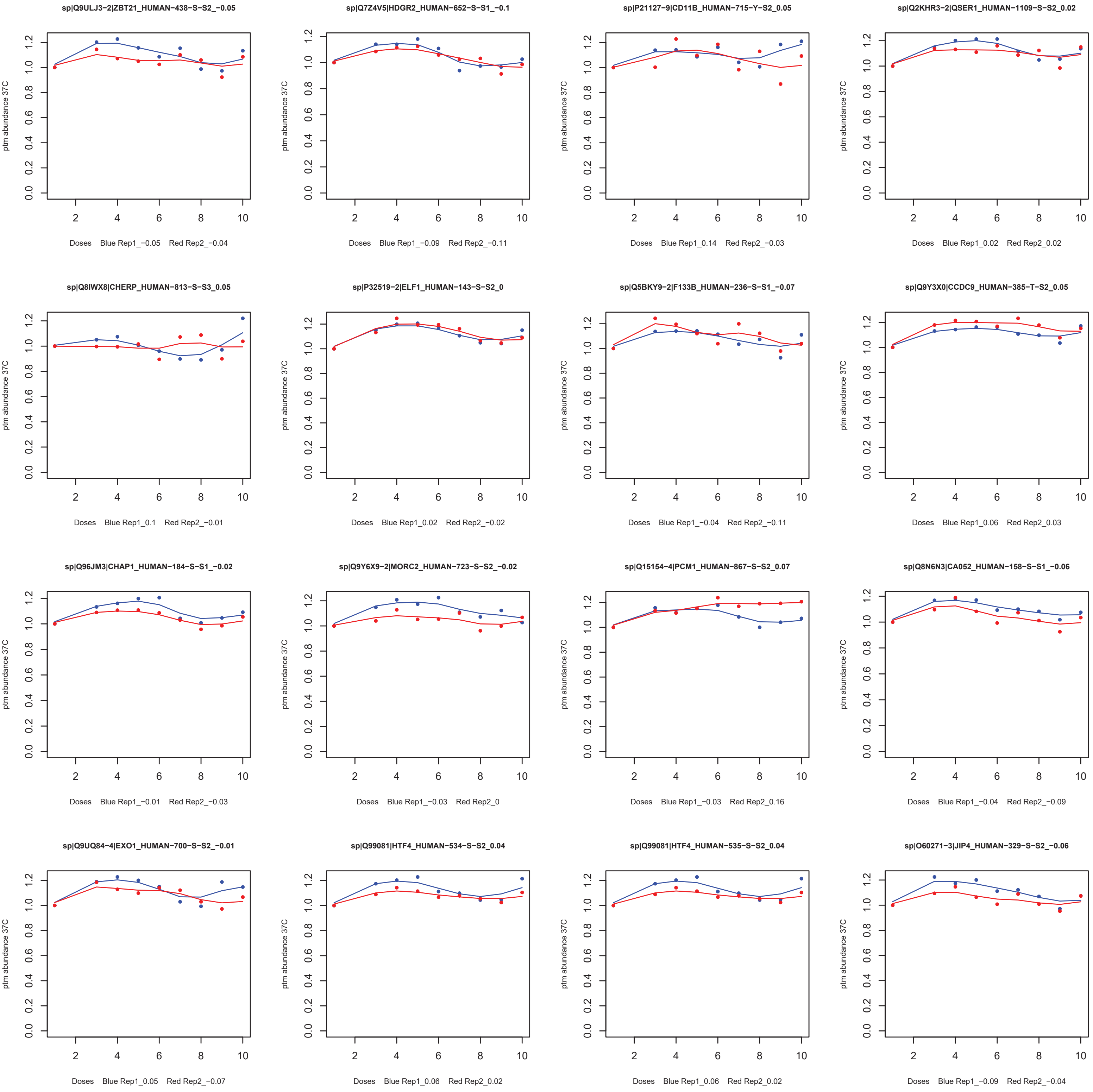
sp|Q9ULJ3−2|ZBT21_HUMAN−438−S−S2_−0.05
Doses Blue Rep1_−0.05 Red Rep2_−0.04
sp|Q7Z4V5|HDGR2_HUMAN−652−S−S1_−0.1
Doses Blue Rep1_−0.09 Red Rep2_−0.11
sp|P21127−9|CD11B_HUMAN−715−Y−S2_0.05
Doses Blue Rep1_0.14 Red Rep2_−0.03
sp|Q2KHR3−2|QSER1_HUMAN−1109−S−S2_0.02
Doses Blue Rep1_0.02 Red Rep2_0.02
sp|Q8IWX8|CHERP_HUMAN−813−S−S3_0.05
Doses Blue Rep1_0.1 Red Rep2_−0.01
sp|P32519−2|ELF1_HUMAN−143−S−S2_0
Doses Blue Rep1_0.02 Red Rep2_−0.02
sp|Q5BKY9−2|F133B_HUMAN−236−S−S1_−0.07
Doses Blue Rep1_−0.04 Red Rep2_−0.11
sp|Q9Y3X0|CCDC9_HUMAN−385−T−S2_0.05
Doses Blue Rep1_0.06 Red Rep2_0.03
sp|Q96JM3|CHAP1_HUMAN−184−S−S1_−0.02
Doses Blue Rep1_−0.01 Red Rep2_−0.03
sp|Q9Y6X9−2|MORC2_HUMAN−723−S−S2_−0.02
Doses Blue Rep1_−0.03 Red Rep2_0
sp|Q15154−4|PCM1_HUMAN−867−S−S2_0.07
Doses Blue Rep1_−0.03 Red Rep2_0.16
sp|Q8N6N3|CA052_HUMAN−158−S−S1_−0.06
Doses Blue Rep1_−0.04 Red Rep2_−0.09
sp|Q9UQ84−4|EXO1_HUMAN−700−S−S2_−0.01
Doses Blue Rep1_0.05 Red Rep2_−0.07
sp|Q99081|HTF4_HUMAN−534−S−S2_0.04
Doses Blue Rep1_0.06 Red Rep2_0.02
sp|Q99081|HTF4_HUMAN−535−S−S2_0.04
Doses Blue Rep1_0.06 Red Rep2_0.02
sp|O60271−3|JIP4_HUMAN−329−S−S2_−0.06
Doses Blue Rep1_−0.09 Red Rep2_−0.04
ptm abundance 37C

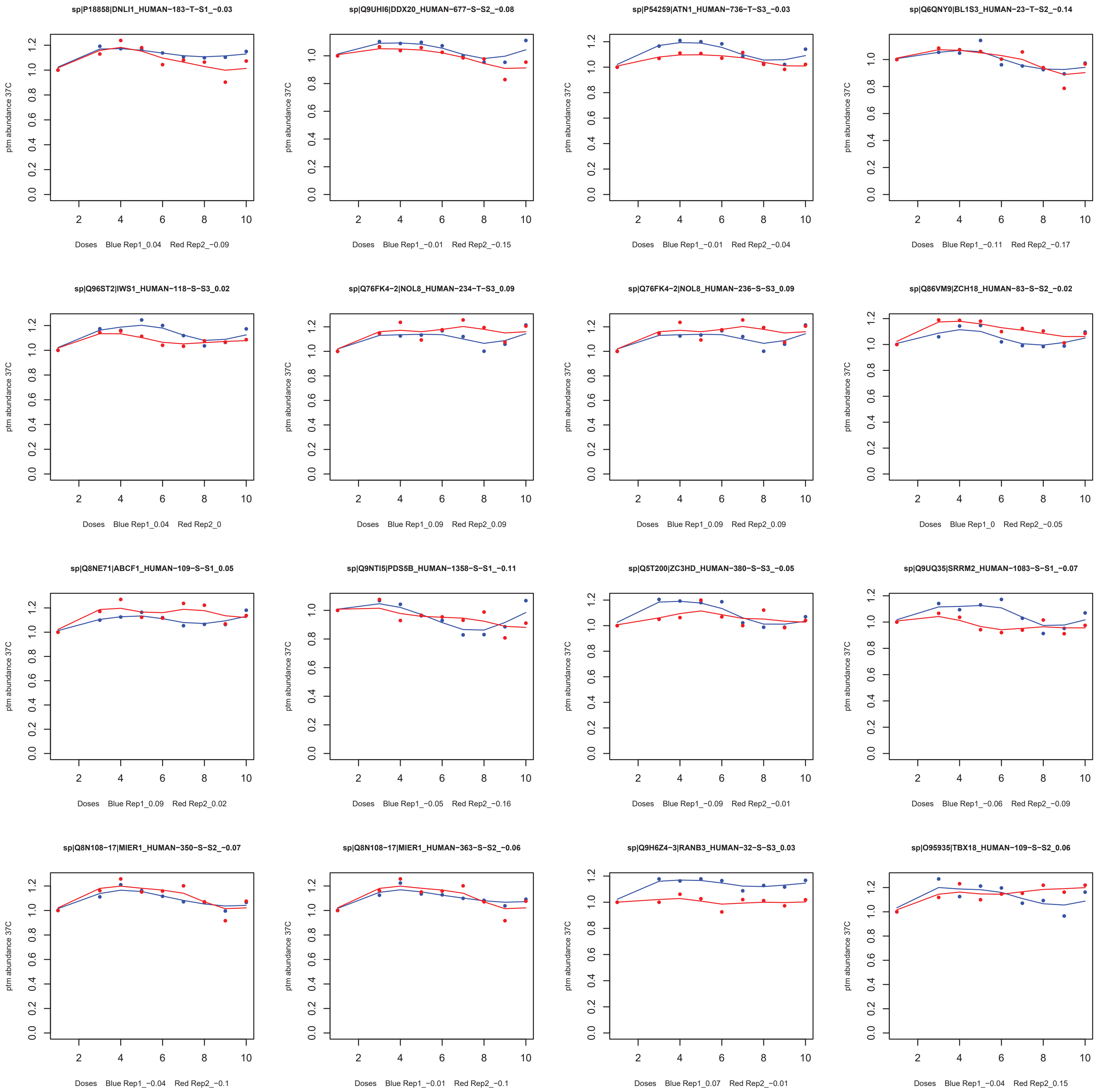

sp|P18858|DNLI1_HUMAN−183−T−S1_−0.03
Doses Blue Rep1_0.04 Red Rep2_−0.09
sp|Q9UHI6|DDX20_HUMAN−677−S−S2_−0.08
Doses Blue Rep1_−0.01 Red Rep2_−0.15
sp|P54259|ATN1_HUMAN−736−T−S3_−0.03
Doses Blue Rep1_−0.01 Red Rep2_−0.04
sp|Q6QNY0|BL1S3_HUMAN−23−T−S2_−0.14
Doses Blue Rep1_−0.11 Red Rep2_−0.17
sp|Q96ST2|IWS1_HUMAN−118−S−S3_0.02
Doses Blue Rep1_0.04 Red Rep2_0
sp|Q76FK4−2|NOL8_HUMAN−234−T−S3_0.09
Doses Blue Rep1_0.09 Red Rep2_0.09
sp|Q76FK4−2|NOL8_HUMAN−236−S−S3_0.09
Doses Blue Rep1_0.09 Red Rep2_0.09
sp|Q86VM9|ZCH18_HUMAN−83−S−S2_−0.02
Doses Blue Rep1_0 Red Rep2_−0.05
sp|Q8NE71|ABCF1_HUMAN−109−S−S1_0.05
Doses Blue Rep1_0.09 Red Rep2_0.02
sp|Q9NTI5|PDS5B_HUMAN−1358−S−S1_−0.11
Doses Blue Rep1_−0.05 Red Rep2_−0.16
sp|Q5T200|ZC3HD_HUMAN−380−S−S3_−0.05
Doses Blue Rep1_−0.09 Red Rep2_−0.01
sp|Q9UQ35|SRRM2_HUMAN−1083−S−S1_−0.07
Doses Blue Rep1_−0.06 Red Rep2_−0.09
sp|Q8N108−17|MIER1_HUMAN−350−S−S2_−0.07
Doses Blue Rep1_−0.04 Red Rep2_−0.1
sp|Q8N108−17|MIER1_HUMAN−363−S−S2_−0.06
Doses Blue Rep1_−0.01 Red Rep2_−0.1
sp|Q9H6Z4−3|RANB3_HUMAN−32−S−S3_0.03
Doses Blue Rep1_0.07 Red Rep2_−0.01
sp|O95935|TBX18_HUMAN−109−S−S2_0.06
Doses Blue Rep1_−0.04 Red Rep2_0.15
ptm abundance 37C

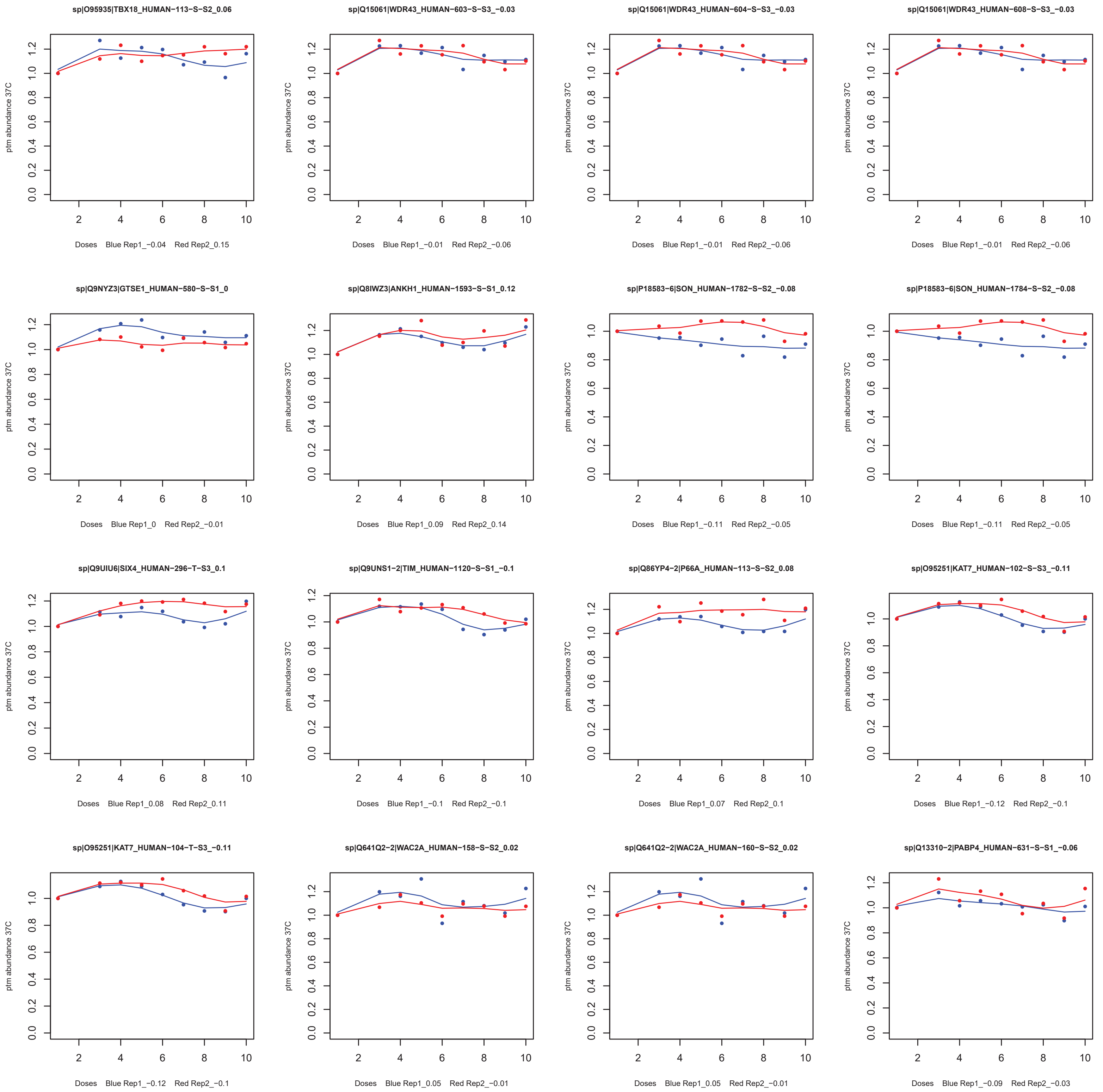

sp|O95935|TBX18_HUMAN−113−S−S2_0.06
Doses Blue Rep1_−0.04 Red Rep2_0.15
sp|Q15061|WDR43_HUMAN−603−S−S3_−0.03
Doses Blue Rep1_−0.01 Red Rep2_−0.06
sp|Q15061|WDR43_HUMAN−604−S−S3_−0.03
Doses Blue Rep1_−0.01 Red Rep2_−0.06
sp|Q15061|WDR43_HUMAN−608−S−S3_−0.03
Doses Blue Rep1_−0.01 Red Rep2_−0.06
sp|Q9NYZ3|GTSE1_HUMAN−580−S−S1_0
Doses Blue Rep1_0 Red Rep2_−0.01
sp|Q8IWZ3|ANKH1_HUMAN−1593−S−S1_0.12
Doses Blue Rep1_0.09 Red Rep2_0.14
sp|P18583−6|SON_HUMAN−1782−S−S2_−0.08
Doses Blue Rep1_−0.11 Red Rep2_−0.05
sp|P18583−6|SON_HUMAN−1784−S−S2_−0.08
Doses Blue Rep1_−0.11 Red Rep2_−0.05
sp|Q9UIU6|SIX4_HUMAN−296−T−S3_0.1
Doses Blue Rep1_0.08 Red Rep2_0.11
sp|Q9UNS1−2|TIM_HUMAN−1120−S−S1_−0.1
Doses Blue Rep1_−0.1 Red Rep2_−0.1
sp|Q86YP4−2|P66A_HUMAN−113−S−S2_0.08
Doses Blue Rep1_0.07 Red Rep2_0.1
sp|O95251|KAT7_HUMAN−102−S−S3_−0.11
Doses Blue Rep1_−0.12 Red Rep2_−0.1
sp|O95251|KAT7_HUMAN−104−T−S3_−0.11
Doses Blue Rep1_−0.12 Red Rep2_−0.1
sp|Q641Q2−2|WAC2A_HUMAN−158−S−S2_0.02
Doses Blue Rep1_0.05 Red Rep2_−0.01
sp|Q641Q2−2|WAC2A_HUMAN−160−S−S2_0.02
Doses Blue Rep1_0.05 Red Rep2_−0.01
sp|Q13310−2|PABP4_HUMAN−631−S−S1_−0.06
Doses Blue Rep1_−0.09 Red Rep2_−0.03
ptm abundance 37C

sp|P04075|ALDOA_HUMAN−36−S−S1_−0.02

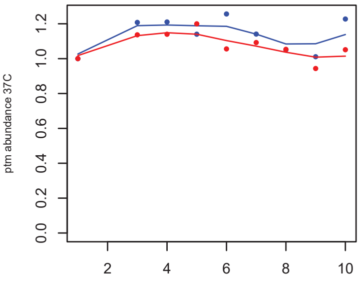

Doses Blue Rep1_0.04 Red Rep2_−0.08

sp|Q8IYH5−4|ZZZ3_HUMAN−130−S−S3_−0.04

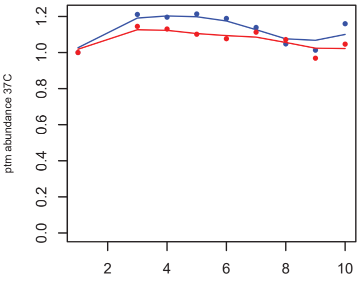

Doses Blue Rep1_−0.01 Red Rep2_−0.06

sp|Q8IYH5−4|ZZZ3_HUMAN−131−S−S3_−0.04

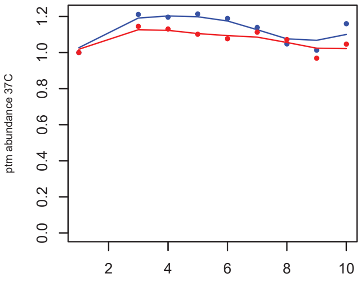

Doses Blue Rep1_−0.01 Red Rep2_−0.06

sp|Q8IYH5−4|ZZZ3_HUMAN−135−S−S3_−0.04

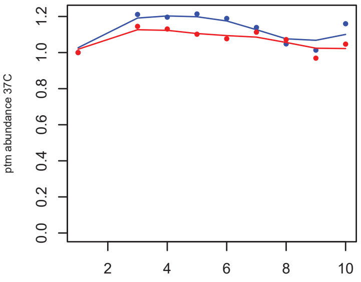

Doses Blue Rep1_−0.01 Red Rep2_−0.06

sp|Q9NRF8|PYRG2_HUMAN−567−Y−S3_0.11

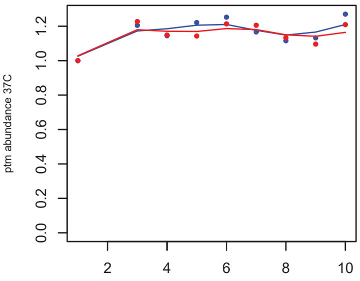

Doses Blue Rep1_0.14 Red Rep2_0.08

sp|P08238|HS90B_HUMAN−226−S−S1_−0.02

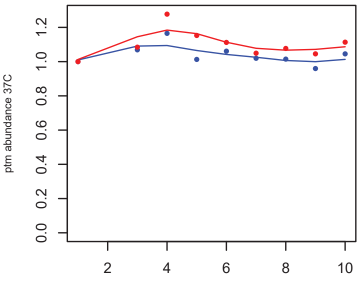

Doses Blue Rep1_−0.05 Red Rep2_0.01

sp|P41227−2|NAA10_HUMAN−220−S−S3_0.08

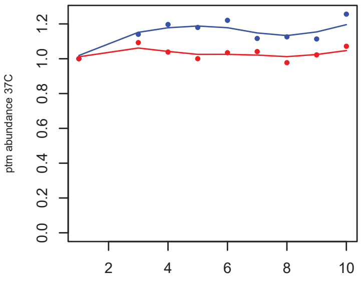

Doses Blue Rep1_0.14 Red Rep2_0.01

sp|P49321|NASP_HUMAN−191−S−S1_−0.01

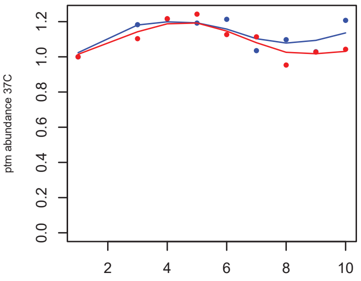

Doses Blue Rep1_0.04 Red Rep2_−0.06

sp|Q9Y6X9−2|MORC2_HUMAN−715−S−S2_0.01

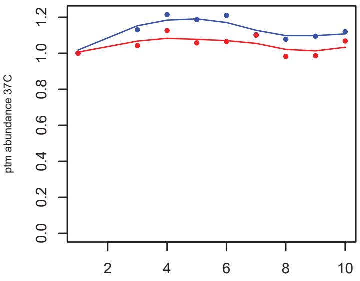

Doses Blue Rep1_0.03 Red Rep2_0

sp|Q9BVS4|RIOK2_HUMAN−337−S−S1_0.04

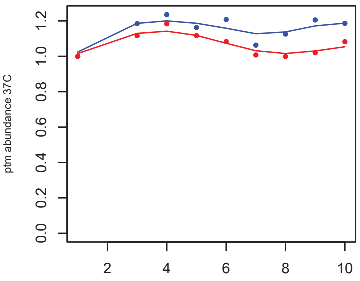

Doses Blue Rep1_0.1 Red Rep2_−0.02

sp|Q00839−2|HNRPU_HUMAN−2−S−S2_−0.09

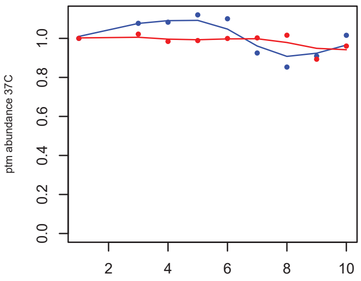

Doses Blue Rep1_−0.1 Red Rep2_−0.08

sp|Q00839−2|HNRPU_HUMAN−3−S−S2_−0.08

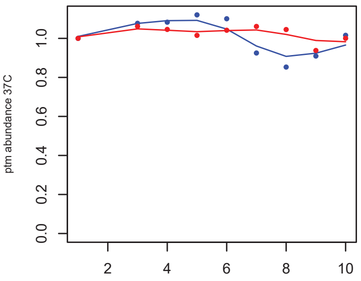

Doses Blue Rep1_−0.1 Red Rep2_−0.06

sp|Q8TDD1|DDX54_HUMAN−39−S−S2_0.1

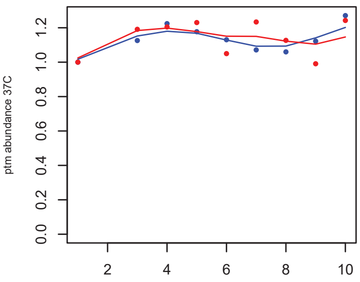

Doses Blue Rep1_0.15 Red Rep2_0.05

sp|Q8TDD1|DDX54_HUMAN−41−S−S2_0.1

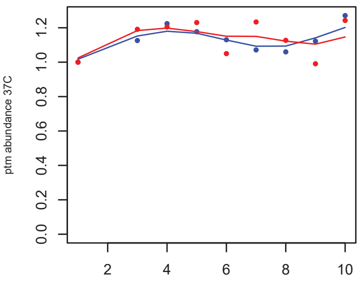

Doses Blue Rep1_0.15 Red Rep2_0.05

sp|Q9NYZ3|GTSE1_HUMAN−575−S−S2_−0.1

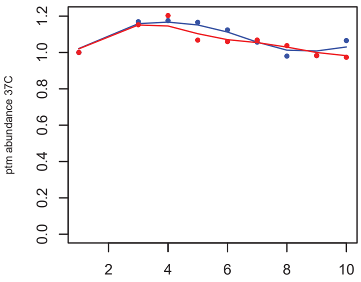

Doses Blue Rep1_−0.07 Red Rep2_−0.13

sp|Q96JM3|CHAP1_HUMAN−627−S−S1_0.04

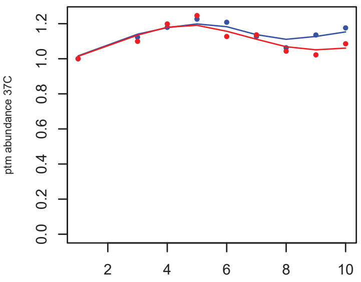

Doses Blue Rep1_0.09 Red Rep2_−0.02

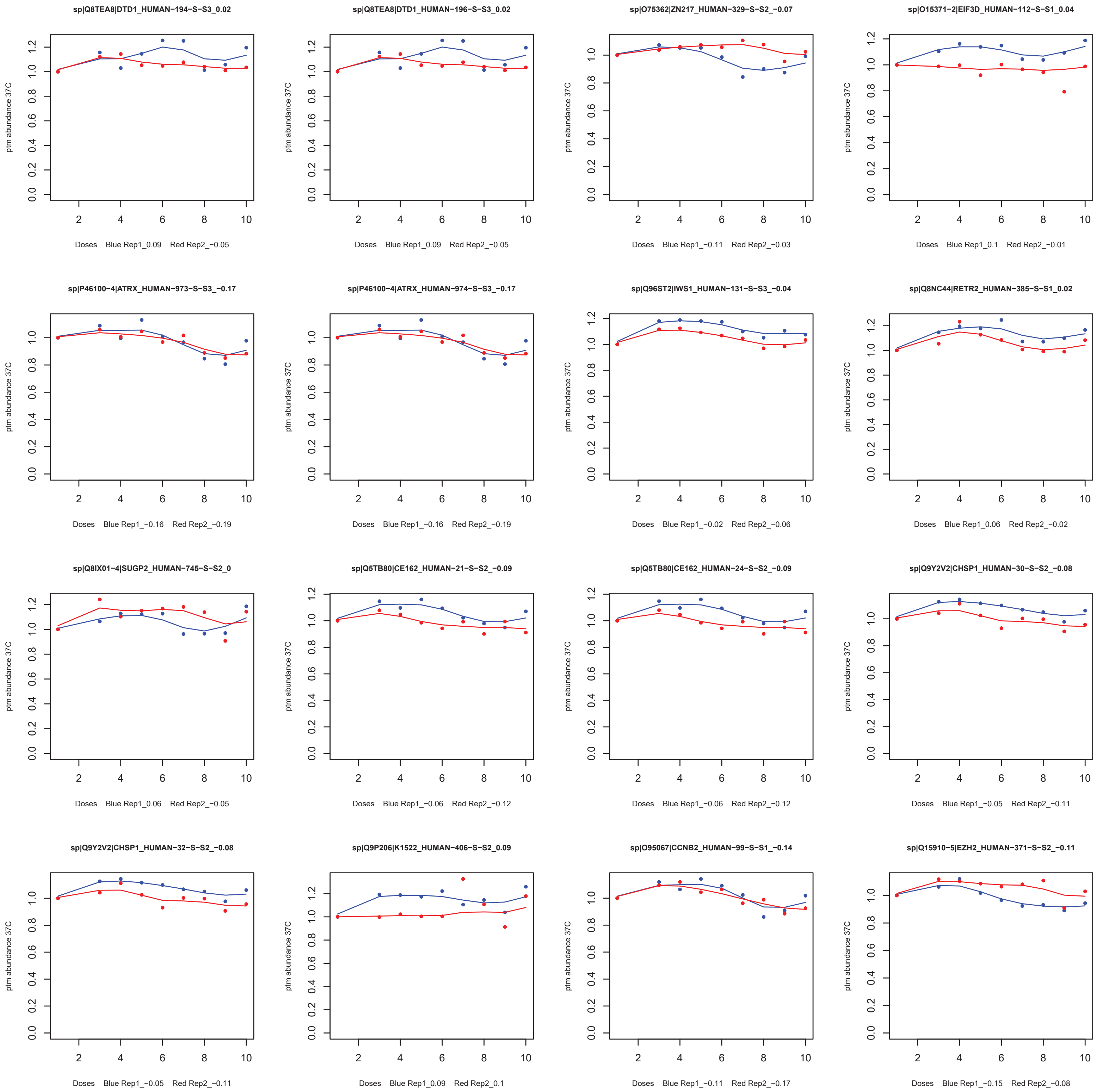

sp|Q8TEA8|DTD1_HUMAN−194−S−S3_0.02
ptm abundance 37C
Doses Blue Rep1_0.09 Red Rep2_−0.05
sp|Q8TEA8|DTD1_HUMAN−196−S−S3_0.02
ptm abundance 37C
Doses Blue Rep1_0.09 Red Rep2_−0.05
sp|O75362|ZN217_HUMAN−329−S−S2_−0.07
ptm abundance 37C
Doses Blue Rep1_−0.11 Red Rep2_−0.03
sp|O15371−2|EIF3D_HUMAN−112−S−S1_0.04
ptm abundance 37C
Doses Blue Rep1_0.1 Red Rep2_−0.01
sp|P46100−4|ATRX_HUMAN−973−S−S3_−0.17
ptm abundance 37C
Doses Blue Rep1_−0.16 Red Rep2_−0.19
sp|P46100−4|ATRX_HUMAN−974−S−S3_−0.17
ptm abundance 37C
Doses Blue Rep1_−0.16 Red Rep2_−0.19
sp|Q96ST2|IWS1_HUMAN−131−S−S3_−0.04
ptm abundance 37C
Doses Blue Rep1_−0.02 Red Rep2_−0.06
sp|Q8NC44|RETR2_HUMAN−385−S−S1_0.02
ptm abundance 37C
Doses Blue Rep1_0.06 Red Rep2_−0.02
sp|Q8IX01−4|SUGP2_HUMAN−745−S−S2_0
ptm abundance 37C
Doses Blue Rep1_0.06 Red Rep2_−0.05
sp|Q5TB80|CE162_HUMAN−21−S−S2_−0.09
ptm abundance 37C
Doses Blue Rep1_−0.06 Red Rep2_−0.12
sp|Q5TB80|CE162_HUMAN−24−S−S2_−0.09
ptm abundance 37C
Doses Blue Rep1_−0.06 Red Rep2_−0.12
sp|Q9Y2V2|CHSP1_HUMAN−30−S−S2_−0.08
ptm abundance 37C
Doses Blue Rep1_−0.05 Red Rep2_−0.11
sp|Q9Y2V2|CHSP1_HUMAN−32−S−S2_−0.08
ptm abundance 37C
Doses Blue Rep1_−0.05 Red Rep2_−0.11
sp|Q9P206|K1522_HUMAN−406−S−S2_0.09
ptm abundance 37C
Doses Blue Rep1_0.09 Red Rep2_0.1
sp|O95067|CCNB2_HUMAN−99−S−S1_−0.14
ptm abundance 37C
Doses Blue Rep1_−0.11 Red Rep2_−0.17
sp|Q15910−5|EZH2_HUMAN−371−S−S2_−0.11
ptm abundance 37C
Doses Blue Rep1_−0.15 Red Rep2_−0.08

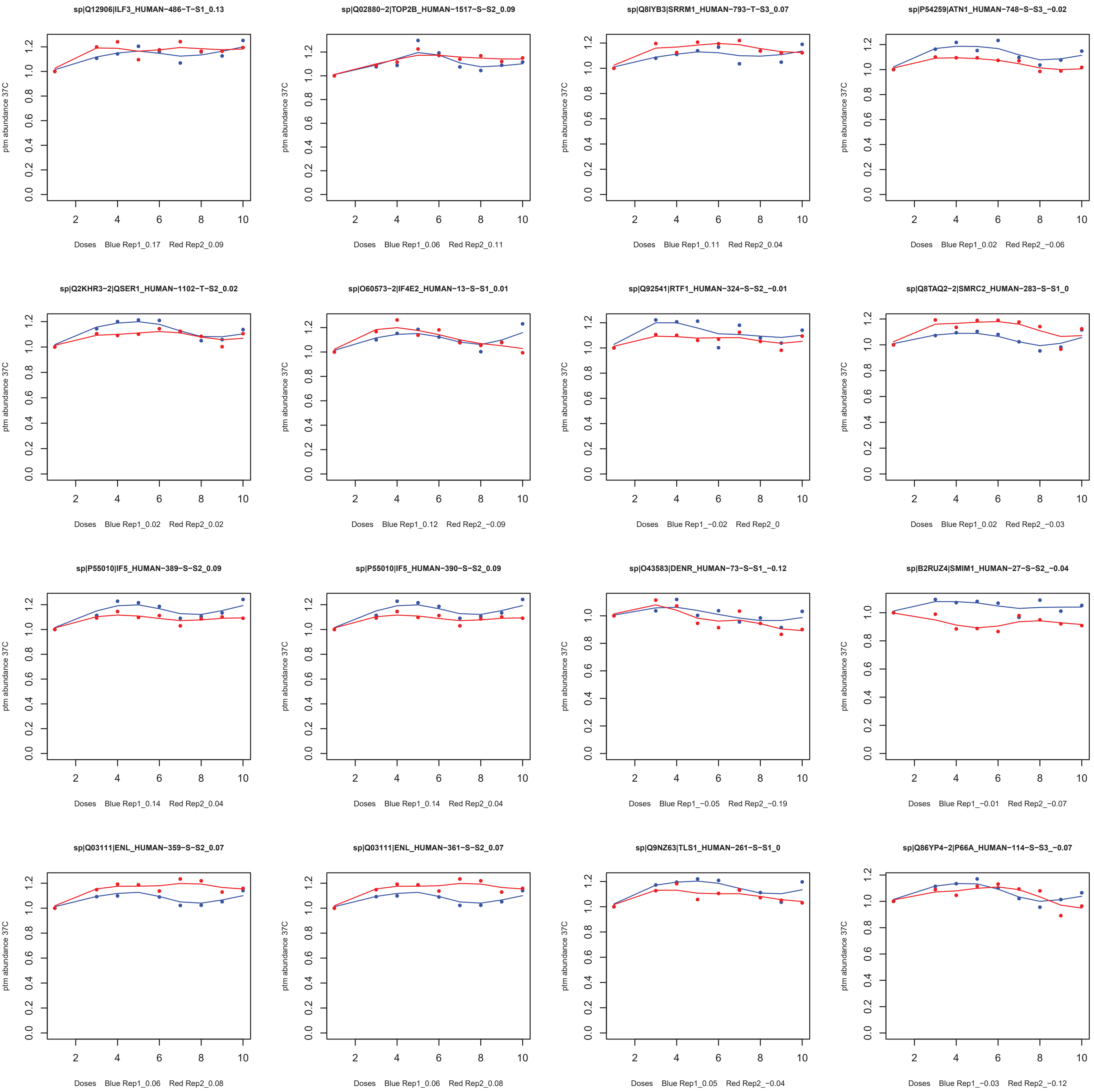

sp|Q12906|ILF3_HUMAN−486−T−S1_0.13
ptm abundance 37C
Doses Blue Rep1_0.17 Red Rep2_0.09
sp|Q02880−2|TOP2B_HUMAN−1517−S−S2_0.09
ptm abundance 37C
Doses Blue Rep1_0.06 Red Rep2_0.11
sp|Q8IYB3|SRRM1_HUMAN−793−T−S3_0.07
ptm abundance 37C
Doses Blue Rep1_0.11 Red Rep2_0.04
sp|P54259|ATN1_HUMAN−748−S−S3_−0.02
ptm abundance 37C
Doses Blue Rep1_0.02 Red Rep2_−0.06
sp|Q2KHR3−2|QSER1_HUMAN−1102−T−S2_0.02
ptm abundance 37C
Doses Blue Rep1_0.02 Red Rep2_0.02
sp|O60573−2|IF4E2_HUMAN−13−S−S1_0.01
ptm abundance 37C
Doses Blue Rep1_0.12 Red Rep2_−0.09
sp|Q92541|RTF1_HUMAN−324−S−S2_−0.01
ptm abundance 37C
Doses Blue Rep1_−0.02 Red Rep2_0
sp|Q8TAQ2−2|SMRC2_HUMAN−283−S−S1_0
ptm abundance 37C
Doses Blue Rep1_0.02 Red Rep2_−0.03
sp|P55010|IF5_HUMAN−389−S−S2_0.09
ptm abundance 37C
Doses Blue Rep1_0.14 Red Rep2_0.04
sp|P55010|IF5_HUMAN−390−S−S2_0.09
ptm abundance 37C
Doses Blue Rep1_0.14 Red Rep2_0.04
sp|O43583|DENR_HUMAN−73−S−S1_−0.12
ptm abundance 37C
Doses Blue Rep1_−0.05 Red Rep2_−0.19
sp|B2RUZ4|SMIM1_HUMAN−27−S−S2_−0.04
ptm abundance 37C
Doses Blue Rep1_−0.01 Red Rep2_−0.07
sp|Q03111|ENL_HUMAN−359−S−S2_0.07
ptm abundance 37C
Doses Blue Rep1_0.06 Red Rep2_0.08
sp|Q03111|ENL_HUMAN−361−S−S2_0.07
ptm abundance 37C
Doses Blue Rep1_0.06 Red Rep2_0.08
sp|Q9NZ63|TLS1_HUMAN−261−S−S1_0
ptm abundance 37C
Doses Blue Rep1_0.05 Red Rep2_−0.04
sp|Q86YP4−2|P66A_HUMAN−114−S−S3_−0.07
ptm abundance 37C
Doses Blue Rep1_−0.03 Red Rep2_−0.12

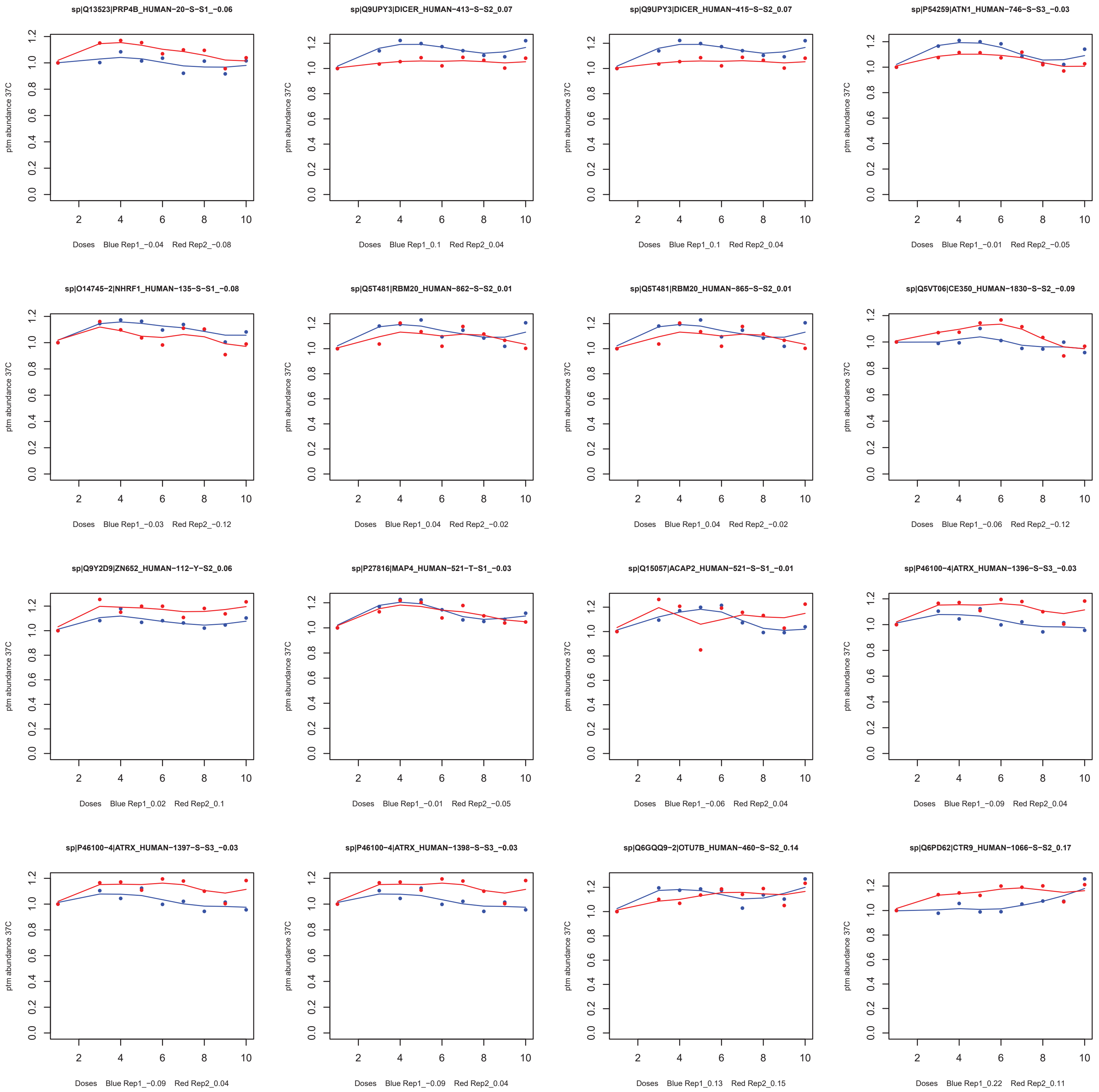

sp|Q13523|PRP4B_HUMAN−20−S−S1_−0.06
ptm abundance 37C
Doses Blue Rep1_−0.04 Red Rep2_−0.08
sp|Q9UPY3|DICER_HUMAN−413−S−S2_0.07
ptm abundance 37C
Doses Blue Rep1_0.1 Red Rep2_0.04
sp|Q9UPY3|DICER_HUMAN−415−S−S2_0.07
ptm abundance 37C
Doses Blue Rep1_0.1 Red Rep2_0.04
sp|P54259|ATN1_HUMAN−746−S−S3_−0.03
ptm abundance 37C
Doses Blue Rep1_−0.01 Red Rep2_−0.05
sp|O14745−2|NHRF1_HUMAN−135−S−S1_−0.08
ptm abundance 37C
Doses Blue Rep1_−0.03 Red Rep2_−0.12
sp|Q5T481|RBM20_HUMAN−862−S−S2_0.01
ptm abundance 37C
Doses Blue Rep1_0.04 Red Rep2_−0.02
sp|Q5T481|RBM20_HUMAN−865−S−S2_0.01
ptm abundance 37C
Doses Blue Rep1_0.04 Red Rep2_−0.02
sp|Q5VT06|CE350_HUMAN−1830−S−S2_−0.09
ptm abundance 37C
Doses Blue Rep1_−0.06 Red Rep2_−0.12
sp|Q9Y2D9|ZN652_HUMAN−112−Y−S2_0.06
ptm abundance 37C
Doses Blue Rep1_0.02 Red Rep2_0.1
sp|P27816|MAP4_HUMAN−521−T−S1_−0.03
ptm abundance 37C
Doses Blue Rep1_−0.01 Red Rep2_−0.05
sp|Q15057|ACAP2_HUMAN−521−S−S1_−0.01
ptm abundance 37C
Doses Blue Rep1_−0.06 Red Rep2_0.04
sp|P46100−4|ATRX_HUMAN−1396−S−S3_−0.03
ptm abundance 37C
Doses Blue Rep1_−0.09 Red Rep2_0.04
sp|P46100−4|ATRX_HUMAN−1397−S−S3_−0.03
ptm abundance 37C
Doses Blue Rep1_−0.09 Red Rep2_0.04
sp|P46100−4|ATRX_HUMAN−1398−S−S3_−0.03
ptm abundance 37C
Doses Blue Rep1_−0.09 Red Rep2_0.04
sp|Q6GQQ9−2|OTU7B_HUMAN−460−S−S2_0.14
ptm abundance 37C
Doses Blue Rep1_0.13 Red Rep2_0.15
sp|Q6PD62|CTR9_HUMAN−1066−S−S2_0.17
ptm abundance 37C
Doses Blue Rep1_0.22 Red Rep2_0.11

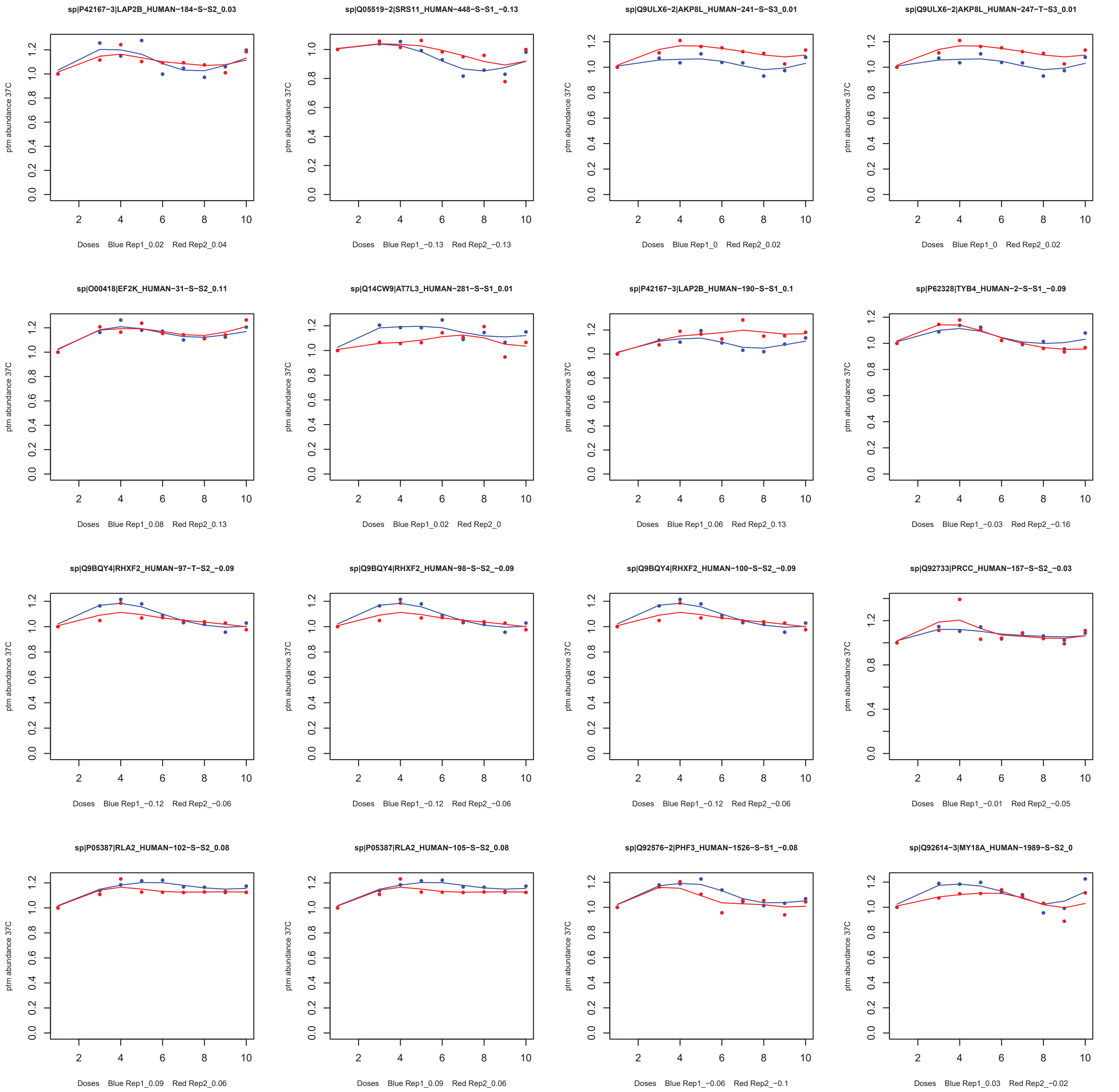
sp|P42167−3|LAP2B_HUMAN−184−S−S2_0.03
ptm abundance 37C
Doses Blue Rep1_0.02 Red Rep2_0.04
sp|Q05519−2|SRS11_HUMAN−448−S−S1_−0.13
ptm abundance 37C
Doses Blue Rep1_−0.13 Red Rep2_−0.13
sp|Q9ULX6−2|AKP8L_HUMAN−241−S−S3_0.01
ptm abundance 37C
Doses Blue Rep1_0 Red Rep2_0.02
sp|Q9ULX6−2|AKP8L_HUMAN−247−T−S3_0.01
ptm abundance 37C
Doses Blue Rep1_0 Red Rep2_0.02
sp|O00418|EF2K_HUMAN−31−S−S2_0.11
ptm abundance 37C
Doses Blue Rep1_0.08 Red Rep2_0.13
sp|Q14CW9|AT7L3_HUMAN−281−S−S1_0.01
ptm abundance 37C
Doses Blue Rep1_0.02 Red Rep2_0
sp|P42167−3|LAP2B_HUMAN−190−S−S1_0.1
ptm abundance 37C
Doses Blue Rep1_0.06 Red Rep2_0.13
sp|P62328|TYB4_HUMAN−2−S−S1_−0.09
ptm abundance 37C
Doses Blue Rep1_−0.03 Red Rep2_−0.16
sp|Q9BQY4|RHXF2_HUMAN−97−T−S2_−0.09
ptm abundance 37C
Doses Blue Rep1_−0.12 Red Rep2_−0.06
sp|Q9BQY4|RHXF2_HUMAN−98−S−S2_−0.09
ptm abundance 37C
Doses Blue Rep1_−0.12 Red Rep2_−0.06
sp|Q9BQY4|RHXF2_HUMAN−100−S−S2_−0.09
ptm abundance 37C
Doses Blue Rep1_−0.12 Red Rep2_−0.06
sp|Q92733|PRCC_HUMAN−157−S−S2_−0.03
ptm abundance 37C
Doses Blue Rep1_−0.01 Red Rep2_−0.05
sp|P05387|RLA2_HUMAN−102−S−S2_0.08
ptm abundance 37C
Doses Blue Rep1_0.09 Red Rep2_0.06
sp|P05387|RLA2_HUMAN−105−S−S2_0.08
ptm abundance 37C
Doses Blue Rep1_0.09 Red Rep2_0.06
sp|Q92576−2|PHF3_HUMAN−1526−S−S1_−0.08
ptm abundance 37C
Doses Blue Rep1_−0.06 Red Rep2_−0.1
sp|Q92614−3|MY18A_HUMAN−1989−S−S2_0
ptm abundance 37C
Doses Blue Rep1_0.03 Red Rep2_−0.02

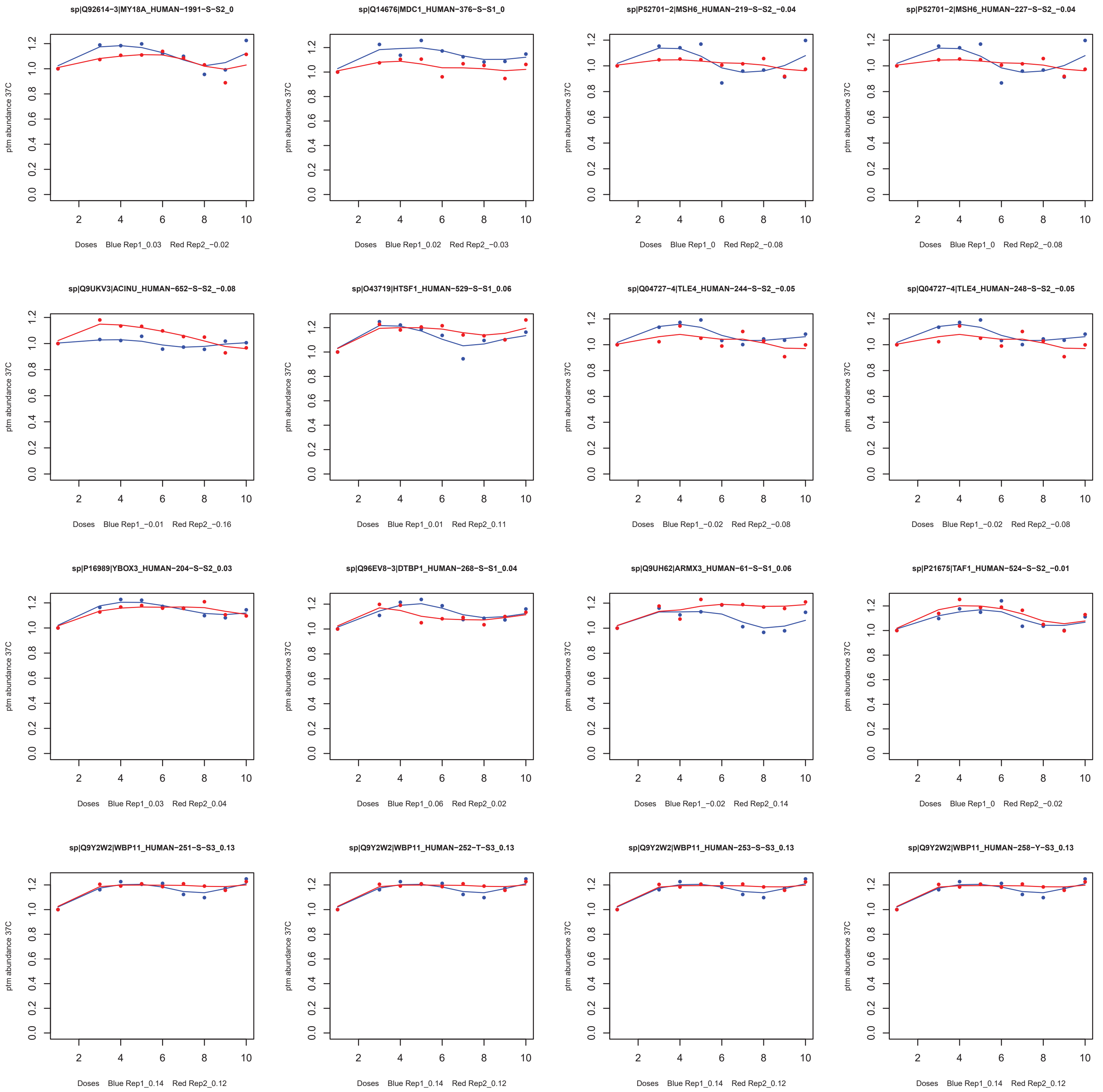

sp|Q92614−3|MY18A_HUMAN−1991−S−S2_0
Doses Blue Rep1_0.03 Red Rep2_−0.02
sp|Q14676|MDC1_HUMAN−376−S−S1_0
Doses Blue Rep1_0.02 Red Rep2_−0.03
sp|P52701−2|MSH6_HUMAN−219−S−S2_−0.04
Doses Blue Rep1_0 Red Rep2_−0.08
sp|P52701−2|MSH6_HUMAN−227−S−S2_−0.04
Doses Blue Rep1_0 Red Rep2_−0.08
sp|Q9UKV3|ACINU_HUMAN−652−S−S2_−0.08
Doses Blue Rep1_−0.01 Red Rep2_−0.16
sp|O43719|HTSF1_HUMAN−529−S−S1_0.06
Doses Blue Rep1_0.01 Red Rep2_0.11
sp|Q04727−4|TLE4_HUMAN−244−S−S2_−0.05
Doses Blue Rep1_−0.02 Red Rep2_−0.08
sp|Q04727−4|TLE4_HUMAN−248−S−S2_−0.05
Doses Blue Rep1_−0.02 Red Rep2_−0.08
sp|P16989|YBOX3_HUMAN−204−S−S2_0.03
Doses Blue Rep1_0.03 Red Rep2_0.04
sp|Q96EV8−3|DTBP1_HUMAN−268−S−S1_0.04
Doses Blue Rep1_0.06 Red Rep2_0.02
sp|Q9UH62|ARMX3_HUMAN−61−S−S1_0.06
Doses Blue Rep1_−0.02 Red Rep2_0.14
sp|P21675|TAF1_HUMAN−524−S−S2_−0.01
Doses Blue Rep1_0 Red Rep2_−0.02
sp|Q9Y2W2|WBP11_HUMAN−251−S−S3_0.13
Doses Blue Rep1_0.14 Red Rep2_0.12
sp|Q9Y2W2|WBP11_HUMAN−252−T−S3_0.13
Doses Blue Rep1_0.14 Red Rep2_0.12
sp|Q9Y2W2|WBP11_HUMAN−253−S−S3_0.13
Doses Blue Rep1_0.14 Red Rep2_0.12
sp|Q9Y2W2|WBP11_HUMAN−258−Y−S3_0.13
Doses Blue Rep1_0.14 Red Rep2_0.12
ptm abundance 37C

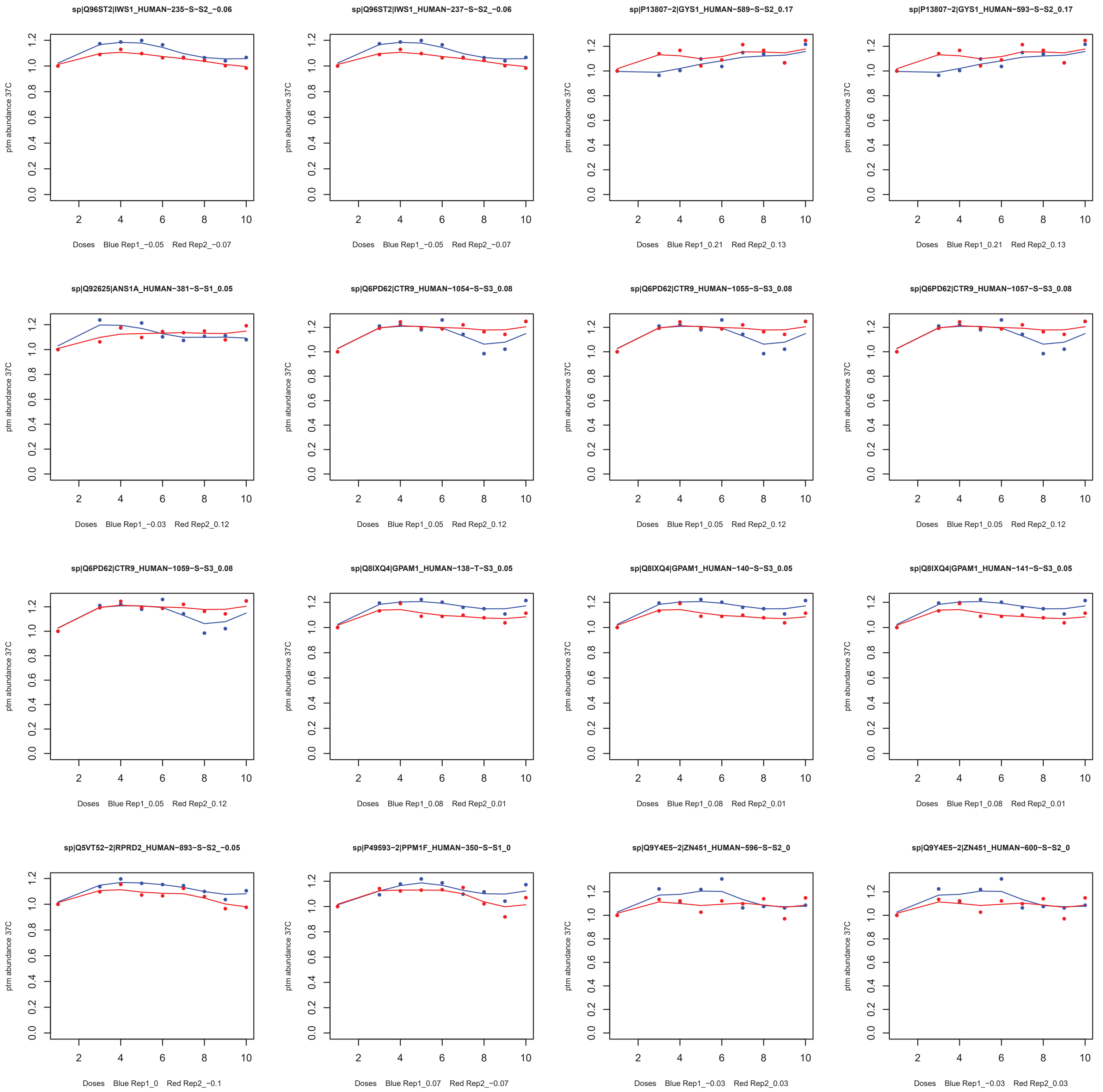
sp|Q96ST2|IWS1_HUMAN−235−S−S2_−0.06
ptm abundance 37C
Doses Blue Rep1_−0.05 Red Rep2_−0.07
sp|Q96ST2|IWS1_HUMAN−237−S−S2_−0.06
ptm abundance 37C
Doses Blue Rep1_−0.05 Red Rep2_−0.07
sp|P13807−2|GYS1_HUMAN−589−S−S2_0.17
ptm abundance 37C
Doses Blue Rep1_0.21 Red Rep2_0.13
sp|P13807−2|GYS1_HUMAN−593−S−S2_0.17
ptm abundance 37C
Doses Blue Rep1_0.21 Red Rep2_0.13
sp|Q92625|ANS1A_HUMAN−381−S−S1_0.05
ptm abundance 37C
Doses Blue Rep1_−0.03 Red Rep2_0.12
sp|Q6PD62|CTR9_HUMAN−1054−S−S3_0.08
ptm abundance 37C
Doses Blue Rep1_0.05 Red Rep2_0.12
sp|Q6PD62|CTR9_HUMAN−1055−S−S3_0.08
ptm abundance 37C
Doses Blue Rep1_0.05 Red Rep2_0.12
sp|Q6PD62|CTR9_HUMAN−1057−S−S3_0.08
ptm abundance 37C
Doses Blue Rep1_0.05 Red Rep2_0.12
sp|Q6PD62|CTR9_HUMAN−1059−S−S3_0.08
ptm abundance 37C
Doses Blue Rep1_0.05 Red Rep2_0.12
sp|Q8IXQ4|GPAM1_HUMAN−138−T−S3_0.05
ptm abundance 37C
Doses Blue Rep1_0.08 Red Rep2_0.01
sp|Q8IXQ4|GPAM1_HUMAN−140−S−S3_0.05
ptm abundance 37C
Doses Blue Rep1_0.08 Red Rep2_0.01
sp|Q8IXQ4|GPAM1_HUMAN−141−S−S3_0.05
ptm abundance 37C
Doses Blue Rep1_0.08 Red Rep2_0.01
sp|Q5VT52−2|RPRD2_HUMAN−893−S−S2_−0.05
ptm abundance 37C
Doses Blue Rep1_0 Red Rep2_−0.1
sp|P49593−2|PPM1F_HUMAN−350−S−S1_0
ptm abundance 37C
Doses Blue Rep1_0.07 Red Rep2_−0.07
sp|Q9Y4E5−2|ZN451_HUMAN−596−S−S2_0
ptm abundance 37C
Doses Blue Rep1_−0.03 Red Rep2_0.03
sp|Q9Y4E5−2|ZN451_HUMAN−600−S−S2_0
ptm abundance 37C
Doses Blue Rep1_−0.03 Red Rep2_0.03

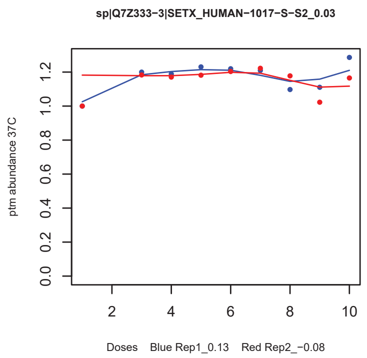
sp|Q7Z333−3|SETX_HUMAN−1017−S−S2_0.03
ptm abundance 37C
Doses Blue Rep1_0.13 Red Rep2_−0.08

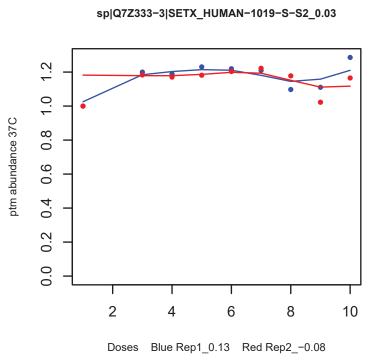
sp|Q7Z333−3|SETX_HUMAN−1019−S−S2_0.03
ptm abundance 37C
Doses Blue Rep1_0.13 Red Rep2_−0.08

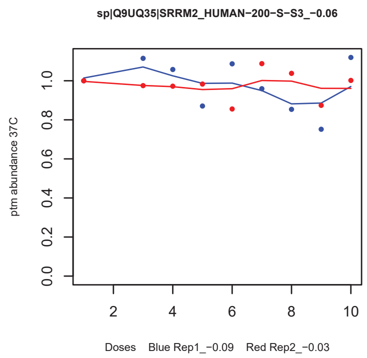
sp|Q9UQ35|SRRM2_HUMAN−200−S−S3_−0.06
ptm abundance 37C
Doses Blue Rep1_−0.09 Red Rep2_−0.03

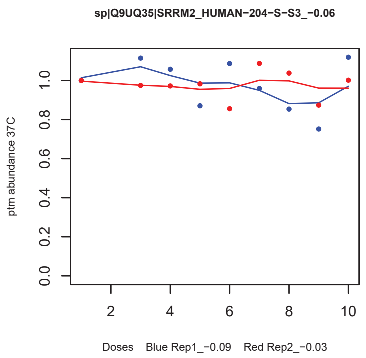
sp|Q9UQ35|SRRM2_HUMAN−204−S−S3_−0.06
ptm abundance 37C
Doses Blue Rep1_−0.09 Red Rep2_−0.03

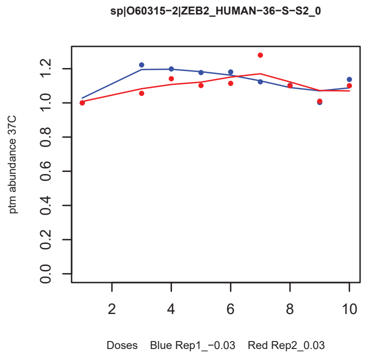
sp|O60315−2|ZEB2_HUMAN−36−S−S2_0
ptm abundance 37C
Doses Blue Rep1_−0.03 Red Rep2_0.03

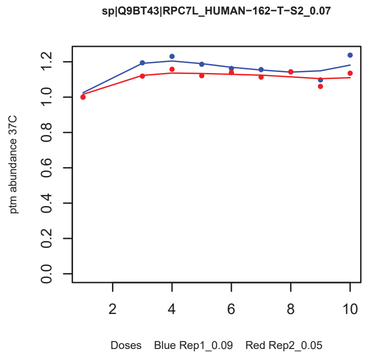
sp|Q9BT43|RPC7L_HUMAN−162−T−S2_0.07
ptm abundance 37C
Doses Blue Rep1_0.09 Red Rep2_0.05

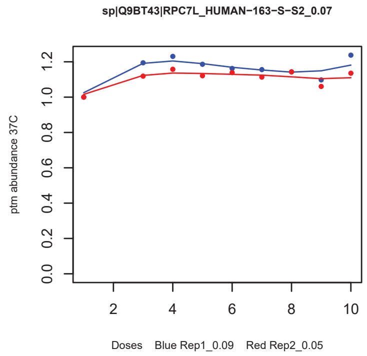
sp|Q9BT43|RPC7L_HUMAN−163−S−S2_0.07
ptm abundance 37C
Doses Blue Rep1_0.09 Red Rep2_0.05

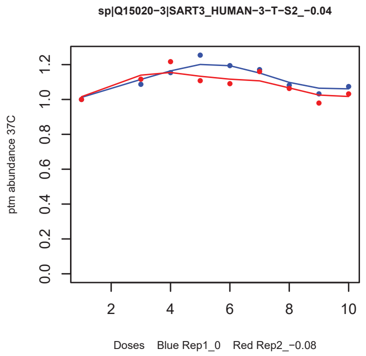
sp|Q15020−3|SART3_HUMAN−3−T−S2_−0.04
ptm abundance 37C
Doses Blue Rep1_0 Red Rep2_−0.08

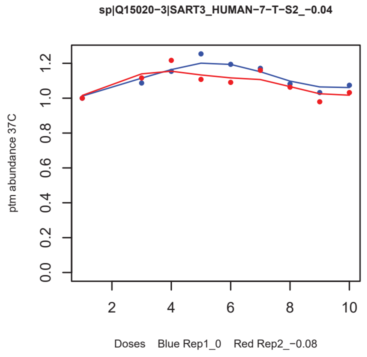
sp|Q15020−3|SART3_HUMAN−7−T−S2_−0.04
ptm abundance 37C
Doses Blue Rep1_0 Red Rep2_−0.08

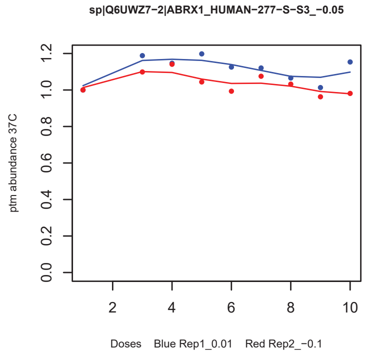
sp|Q6UWZ7−2|ABRX1_HUMAN−277−S−S3_−0.05
ptm abundance 37C
Doses Blue Rep1_0.01 Red Rep2_−0.1

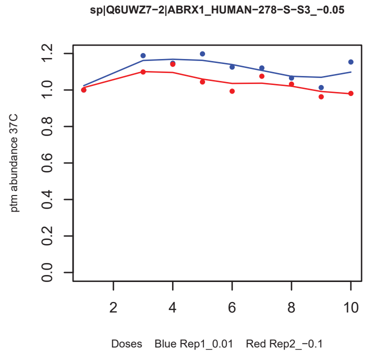
sp|Q6UWZ7−2|ABRX1_HUMAN−278−S−S3_−0.05
ptm abundance 37C
Doses Blue Rep1_0.01 Red Rep2_−0.1

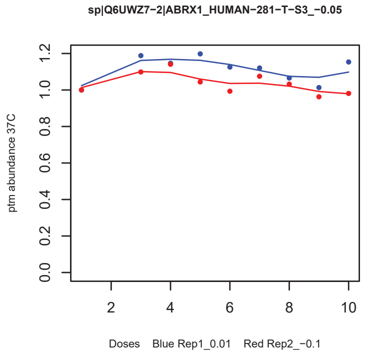
sp|Q6UWZ7−2|ABRX1_HUMAN−281−T−S3_−0.05
ptm abundance 37C
Doses Blue Rep1_0.01 Red Rep2_−0.1

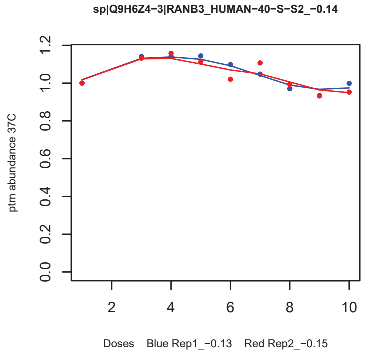
sp|Q9H6Z4−3|RANB3_HUMAN−40−S−S2_−0.14
ptm abundance 37C
Doses Blue Rep1_−0.13 Red Rep2_−0.15

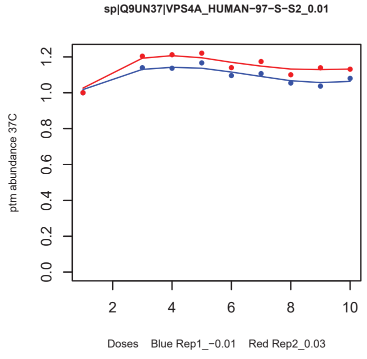
sp|Q9UN37|VPS4A_HUMAN−97−S−S2_0.01
ptm abundance 37C
Doses Blue Rep1_−0.01 Red Rep2_0.03

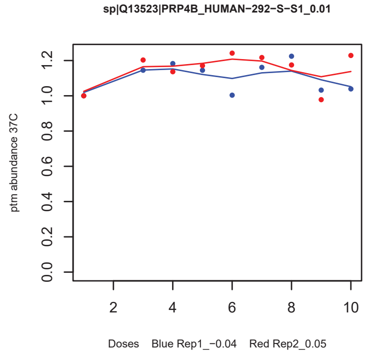
sp|Q13523|PRP4B_HUMAN−292−S−S1_0.01
ptm abundance 37C
Doses Blue Rep1_−0.04 Red Rep2_0.05

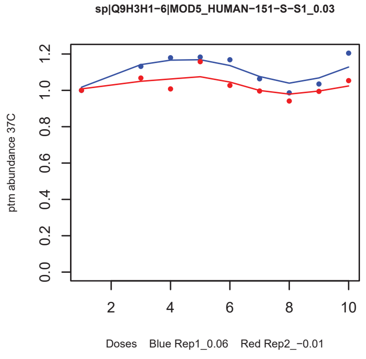
sp|Q9H3H1−6|MOD5_HUMAN−151−S−S1_0.03
ptm abundance 37C
Doses Blue Rep1_0.06 Red Rep2_−0.01

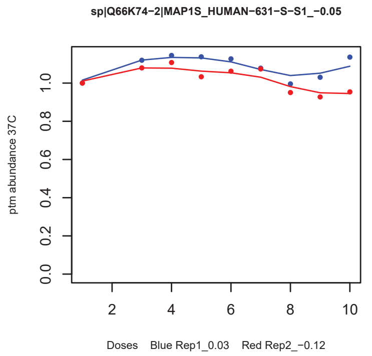
sp|Q66K74−2|MAP1S_HUMAN−631−S−S1_−0.05
ptm abundance 37C
Doses Blue Rep1_0.03 Red Rep2_−0.12

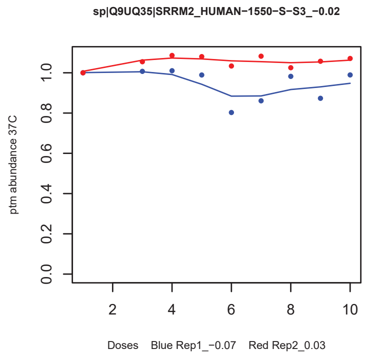
sp|Q9UQ35|SRRM2_HUMAN−1550−S−S3_−0.02
ptm abundance 37C
Doses Blue Rep1_−0.07 Red Rep2_0.03

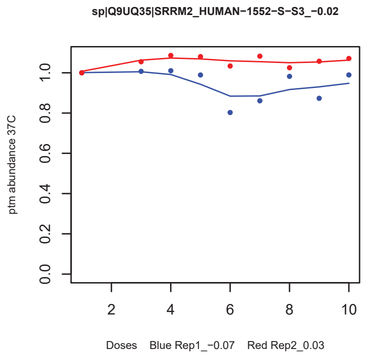
sp|Q9UQ35|SRRM2_HUMAN−1552−S−S3_−0.02
ptm abundance 37C
Doses Blue Rep1_−0.07 Red Rep2_0.03

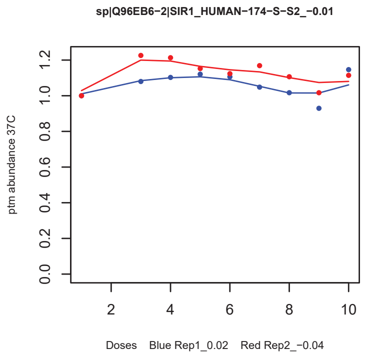
sp|Q96EB6−2|SIR1_HUMAN−174−S−S2_−0.01
ptm abundance 37C
Doses Blue Rep1_0.02 Red Rep2_−0.04

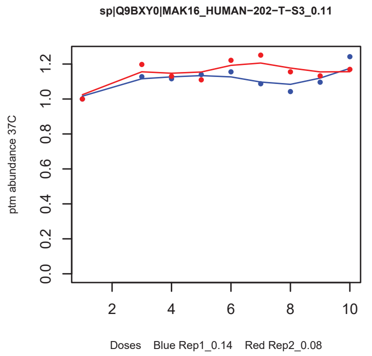
sp|Q9BXY0|MAK16_HUMAN−202−T−S3_0.11
ptm abundance 37C
Doses Blue Rep1_0.14 Red Rep2_0.08

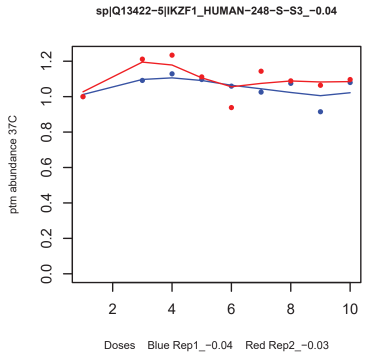
sp|Q13422−5|IKZF1_HUMAN−248−S−S3_−0.04
ptm abundance 37C
Doses Blue Rep1_−0.04 Red Rep2_−0.03

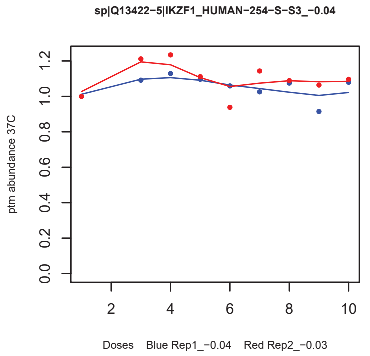
sp|Q13422−5|IKZF1_HUMAN−254−S−S3_−0.04
ptm abundance 37C
Doses Blue Rep1_−0.04 Red Rep2_−0.03

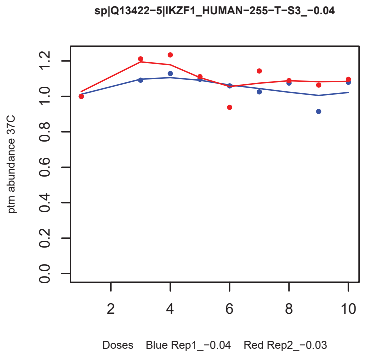
sp|Q13422−5|IKZF1_HUMAN−255−T−S3_−0.04
ptm abundance 37C
Doses Blue Rep1_−0.04 Red Rep2_−0.03

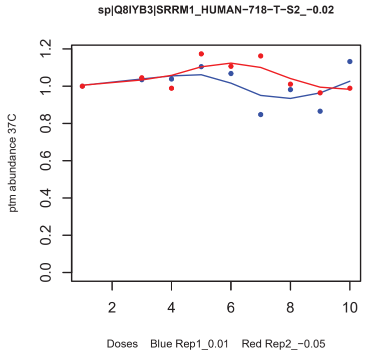
sp|Q8IYB3|SRRM1_HUMAN−718−T−S2_−0.02
ptm abundance 37C
Doses Blue Rep1_0.01 Red Rep2_−0.05

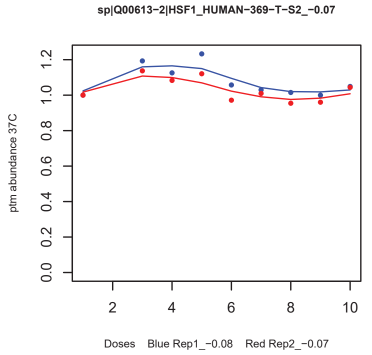
sp|Q00613−2|HSF1_HUMAN−369−T−S2_−0.07
ptm abundance 37C
Doses Blue Rep1_−0.08 Red Rep2_−0.07

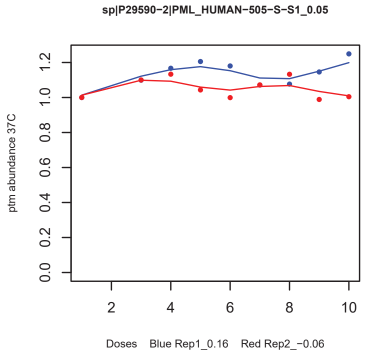
sp|P29590−2|PML_HUMAN−505−S−S1_0.05
ptm abundance 37C
Doses Blue Rep1_0.16 Red Rep2_−0.06

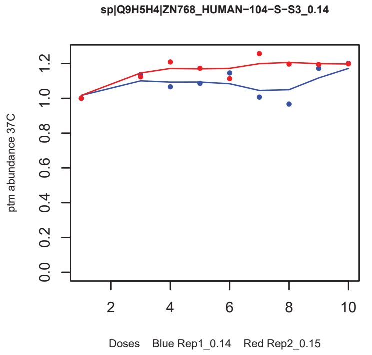
sp|Q9H5H4|ZN768_HUMAN−104−S−S3_0.14
ptm abundance 37C
Doses Blue Rep1_0.14 Red Rep2_0.15

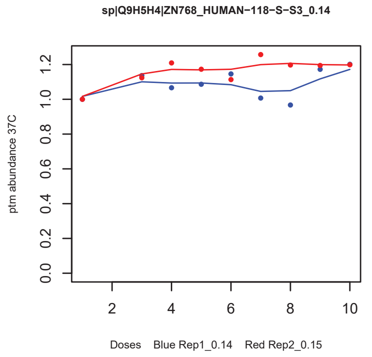
sp|Q9H5H4|ZN768_HUMAN−118−S−S3_0.14
ptm abundance 37C
Doses Blue Rep1_0.14 Red Rep2_0.15

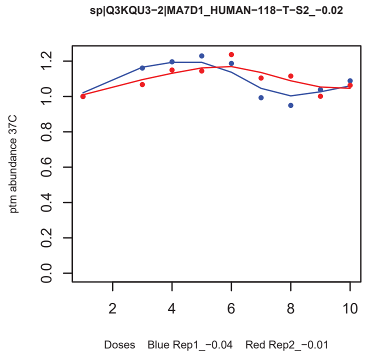
sp|Q3KQU3−2|MA7D1_HUMAN−118−T−S2_−0.02
ptm abundance 37C
Doses Blue Rep1_−0.04 Red Rep2_−0.01

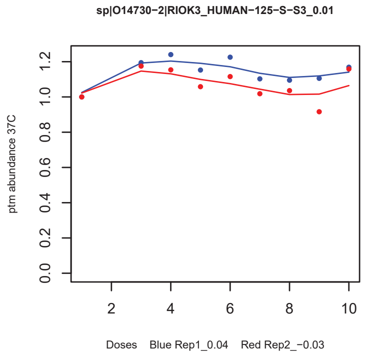
sp|O14730−2|RIOK3_HUMAN−125−S−S3_0.01
ptm abundance 37C
Doses Blue Rep1_0.04 Red Rep2_−0.03

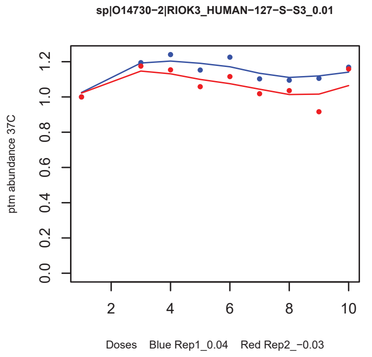
sp|O14730−2|RIOK3_HUMAN−127−S−S3_0.01
ptm abundance 37C
Doses Blue Rep1_0.04 Red Rep2_−0.03

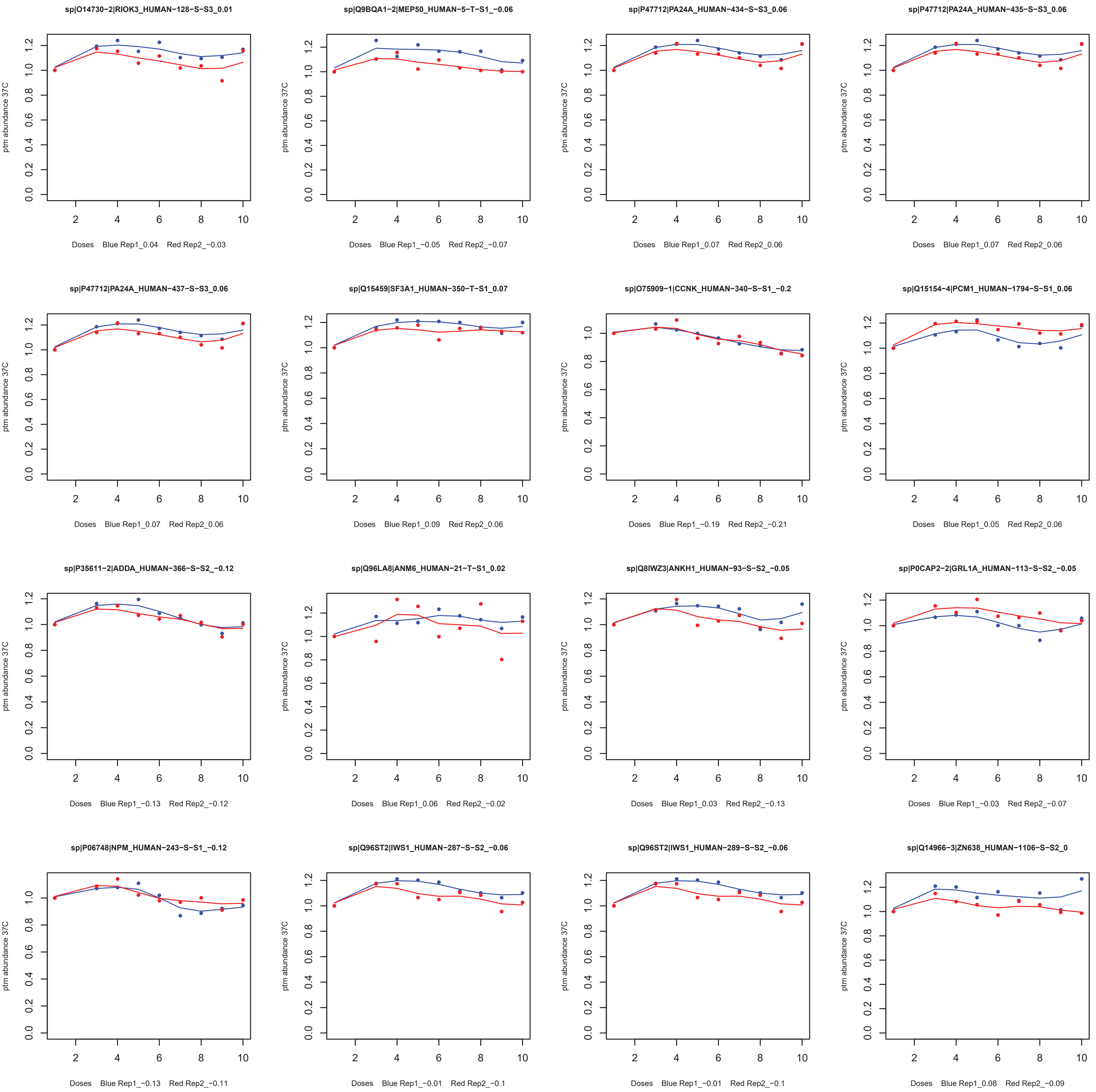
sp|O14730−2|RIOK3_HUMAN−128−S−S3_0.01
Doses Blue Rep1_0.04 Red Rep2_−0.03
sp|Q9BQA1−2|MEP50_HUMAN−5−T−S1_−0.06
Doses Blue Rep1_−0.05 Red Rep2_−0.07
sp|P47712|PA24A_HUMAN−434−S−S3_0.06
Doses Blue Rep1_0.07 Red Rep2_0.06
sp|P47712|PA24A_HUMAN−435−S−S3_0.06
Doses Blue Rep1_0.07 Red Rep2_0.06
sp|P47712|PA24A_HUMAN−437−S−S3_0.06
Doses Blue Rep1_0.07 Red Rep2_0.06
sp|Q15459|SF3A1_HUMAN−350−T−S1_0.07
Doses Blue Rep1_0.09 Red Rep2_0.06
sp|O75909−1|CCNK_HUMAN−340−S−S1_−0.2
Doses Blue Rep1_−0.19 Red Rep2_−0.21
sp|Q15154−4|PCM1_HUMAN−1794−S−S1_0.06
Doses Blue Rep1_0.05 Red Rep2_0.06
sp|P35611−2|ADDA_HUMAN−366−S−S2_−0.12
Doses Blue Rep1_−0.13 Red Rep2_−0.12
sp|Q96LA8|ANM6_HUMAN−21−T−S1_0.02
Doses Blue Rep1_0.06 Red Rep2_−0.02
sp|Q8IWZ3|ANKH1_HUMAN−93−S−S2_−0.05
Doses Blue Rep1_0.03 Red Rep2_−0.13
sp|P0CAP2−2|GRL1A_HUMAN−113−S−S2_−0.05
Doses Blue Rep1_−0.03 Red Rep2_−0.07
sp|P06748|NPM_HUMAN−243−S−S1_−0.12
Doses Blue Rep1_−0.13 Red Rep2_−0.11
sp|Q96ST2|IWS1_HUMAN−287−S−S2_−0.06
Doses Blue Rep1_−0.01 Red Rep2_−0.1
sp|Q96ST2|IWS1_HUMAN−289−S−S2_−0.06
Doses Blue Rep1_−0.01 Red Rep2_−0.1
sp|Q14966−3|ZN638_HUMAN−1106−S−S2_0
Doses Blue Rep1_0.08 Red Rep2_−0.09
ptm abundance 37C

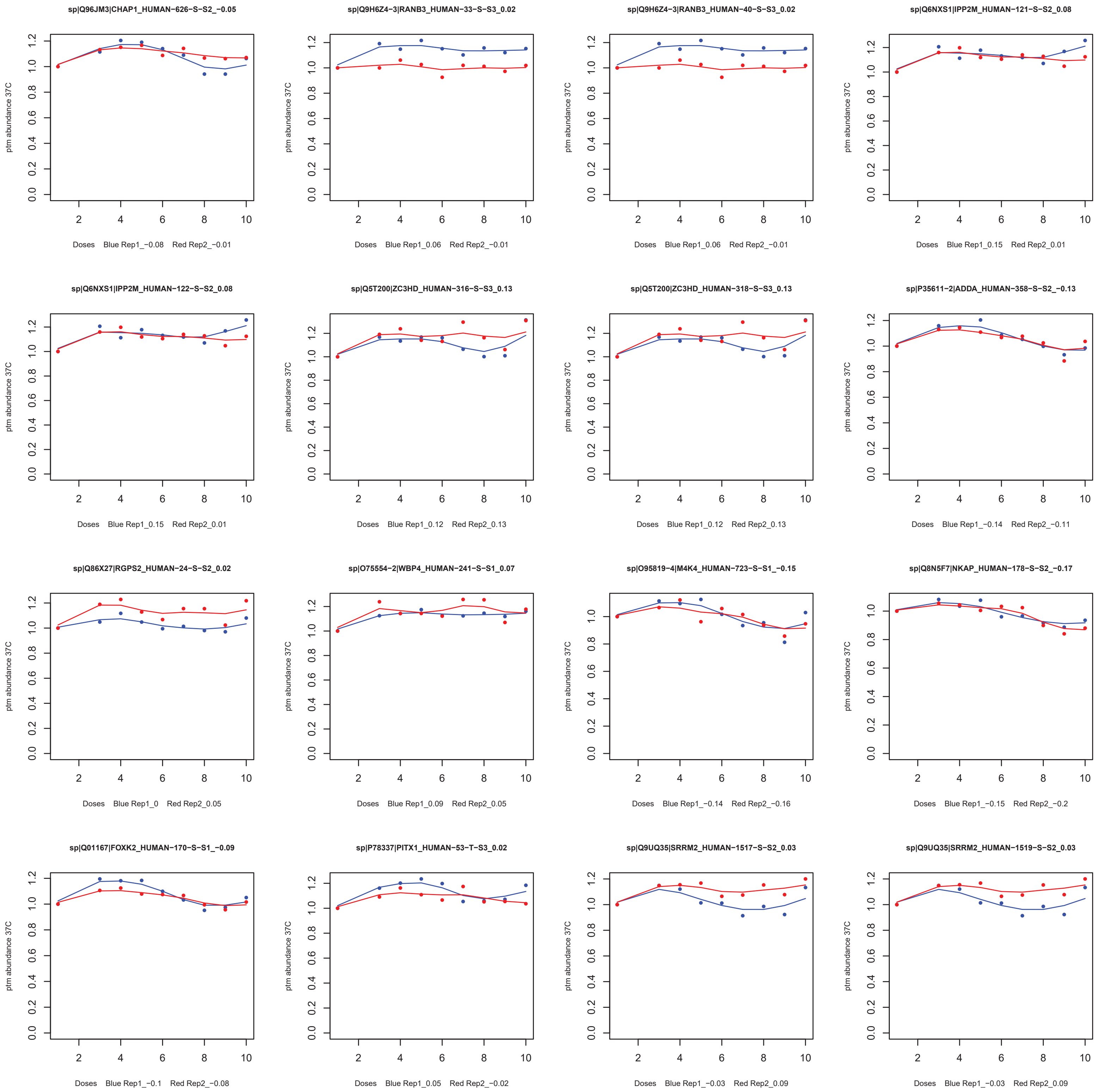
sp|Q96JM3|CHAP1_HUMAN−626−S−S2_−0.05
ptm abundance 37C
Doses Blue Rep1_−0.08 Red Rep2_−0.01
sp|Q9H6Z4−3|RANB3_HUMAN−33−S−S3_0.02
ptm abundance 37C
Doses Blue Rep1_0.06 Red Rep2_−0.01
sp|Q9H6Z4−3|RANB3_HUMAN−40−S−S3_0.02
ptm abundance 37C
Doses Blue Rep1_0.06 Red Rep2_−0.01
sp|Q6NXS1|IPP2M_HUMAN−121−S−S2_0.08
ptm abundance 37C
Doses Blue Rep1_0.15 Red Rep2_0.01
sp|Q6NXS1|IPP2M_HUMAN−122−S−S2_0.08
ptm abundance 37C
Doses Blue Rep1_0.15 Red Rep2_0.01
sp|Q5T200|ZC3HD_HUMAN−316−S−S3_0.13
ptm abundance 37C
Doses Blue Rep1_0.12 Red Rep2_0.13
sp|Q5T200|ZC3HD_HUMAN−318−S−S3_0.13
ptm abundance 37C
Doses Blue Rep1_0.12 Red Rep2_0.13
sp|P35611−2|ADDA_HUMAN−358−S−S2_−0.13
ptm abundance 37C
Doses Blue Rep1_−0.14 Red Rep2_−0.11
sp|Q86X27|RGPS2_HUMAN−24−S−S2_0.02
ptm abundance 37C
Doses Blue Rep1_0 Red Rep2_0.05
sp|O75554−2|WBP4_HUMAN−241−S−S1_0.07
ptm abundance 37C
Doses Blue Rep1_0.09 Red Rep2_0.05
sp|O95819−4|M4K4_HUMAN−723−S−S1_−0.15
ptm abundance 37C
Doses Blue Rep1_−0.14 Red Rep2_−0.16
sp|Q8N5F7|NKAP_HUMAN−178−S−S2_−0.17
ptm abundance 37C
Doses Blue Rep1_−0.15 Red Rep2_−0.2
sp|Q01167|FOXK2_HUMAN−170−S−S1_−0.09
ptm abundance 37C
Doses Blue Rep1_−0.1 Red Rep2_−0.08
sp|P78337|PITX1_HUMAN−53−T−S3_0.02
ptm abundance 37C
Doses Blue Rep1_0.05 Red Rep2_−0.02
sp|Q9UQ35|SRRM2_HUMAN−1517−S−S2_0.03
ptm abundance 37C
Doses Blue Rep1_−0.03 Red Rep2_0.09
sp|Q9UQ35|SRRM2_HUMAN−1519−S−S2_0.03
ptm abundance 37C
Doses Blue Rep1_−0.03 Red Rep2_0.09

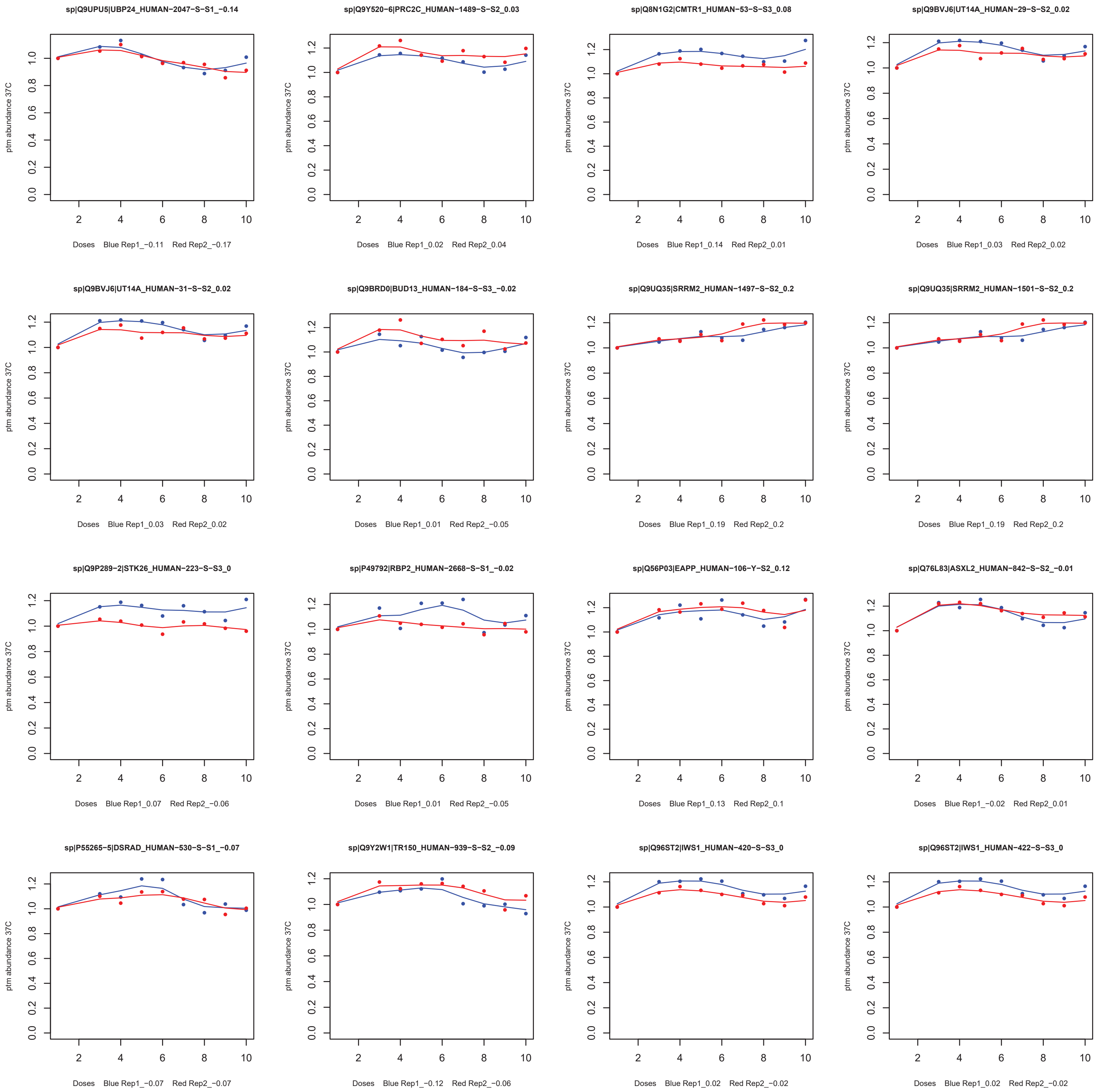
sp|Q9UPU5|UBP24_HUMAN−2047−S−S1_−0.14
Doses Blue Rep1_−0.11 Red Rep2_−0.17
sp|Q9Y520−6|PRC2C_HUMAN−1489−S−S2_0.03
Doses Blue Rep1_0.02 Red Rep2_0.04
sp|Q8N1G2|CMTR1_HUMAN−53−S−S3_0.08
Doses Blue Rep1_0.14 Red Rep2_0.01
sp|Q9BVJ6|UT14A_HUMAN−29−S−S2_0.02
Doses Blue Rep1_0.03 Red Rep2_0.02
sp|Q9BVJ6|UT14A_HUMAN−31−S−S2_0.02
Doses Blue Rep1_0.03 Red Rep2_0.02
sp|Q9BRD0|BUD13_HUMAN−184−S−S3_−0.02
Doses Blue Rep1_0.01 Red Rep2_−0.05
sp|Q9UQ35|SRRM2_HUMAN−1497−S−S2_0.2
Doses Blue Rep1_0.19 Red Rep2_0.2
sp|Q9UQ35|SRRM2_HUMAN−1501−S−S2_0.2
Doses Blue Rep1_0.19 Red Rep2_0.2
sp|Q9P289−2|STK26_HUMAN−223−S−S3_0
Doses Blue Rep1_0.07 Red Rep2_−0.06
sp|P49792|RBP2_HUMAN−2668−S−S1_−0.02
Doses Blue Rep1_0.01 Red Rep2_−0.05
sp|Q56P03|EAPP_HUMAN−106−Y−S2_0.12
Doses Blue Rep1_0.13 Red Rep2_0.1
sp|Q76L83|ASXL2_HUMAN−842−S−S2_−0.01
Doses Blue Rep1_−0.02 Red Rep2_0.01
sp|P55265−5|DSRAD_HUMAN−530−S−S1_−0.07
Doses Blue Rep1_−0.07 Red Rep2_−0.07
sp|Q9Y2W1|TR150_HUMAN−939−S−S2_−0.09
Doses Blue Rep1_−0.12 Red Rep2_−0.06
sp|Q96ST2|IWS1_HUMAN−420−S−S3_0
Doses Blue Rep1_0.02 Red Rep2_−0.02
sp|Q96ST2|IWS1_HUMAN−422−S−S3_0
Doses Blue Rep1_0.02 Red Rep2_−0.02
ptm abundance 37C

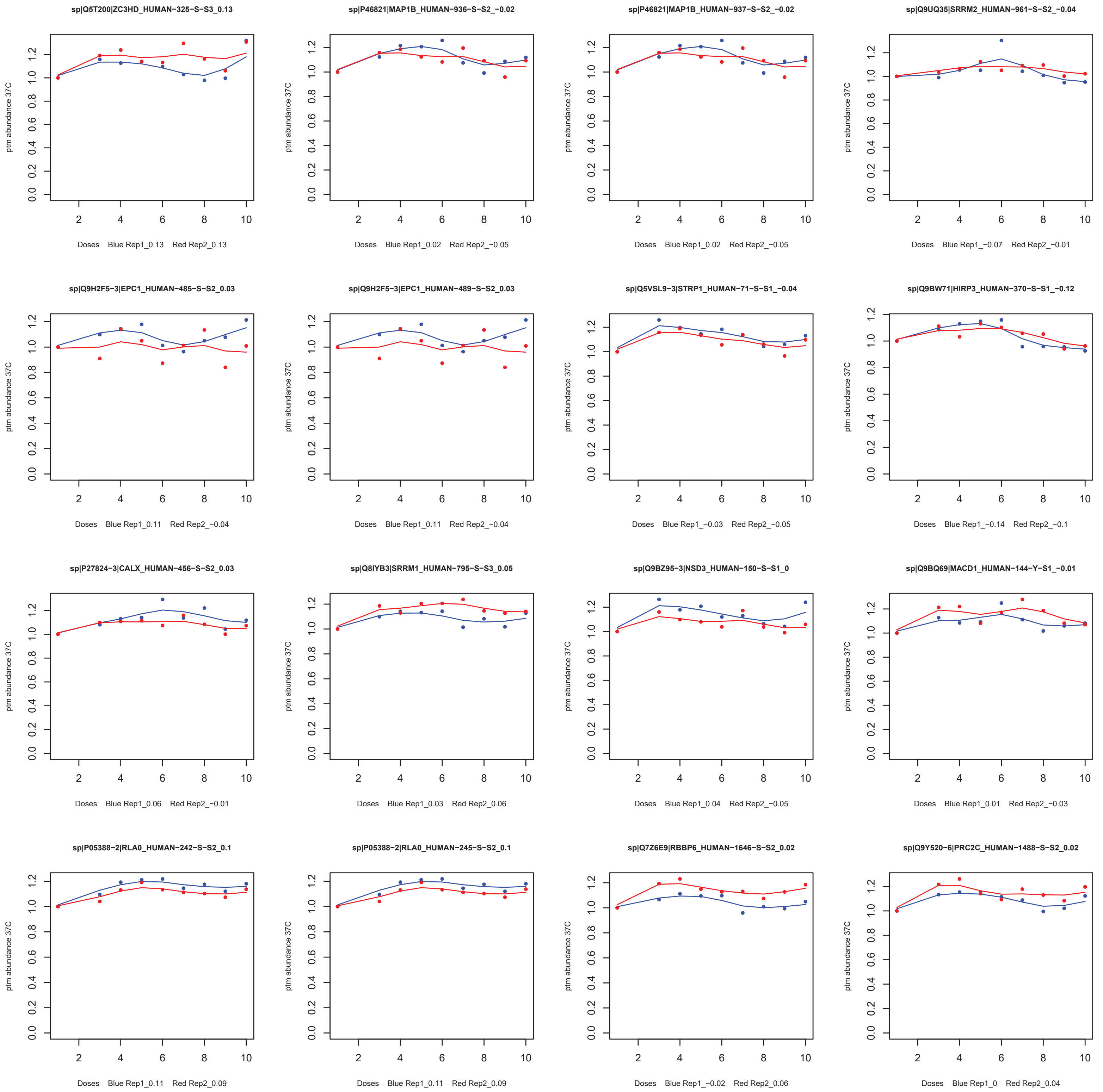

sp|Q5T200|ZC3HD_HUMAN−325−S−S3_0.13
Doses Blue Rep1_0.13 Red Rep2_0.13
sp|P46821|MAP1B_HUMAN−936−S−S2_−0.02
Doses Blue Rep1_0.02 Red Rep2_−0.05
sp|P46821|MAP1B_HUMAN−937−S−S2_−0.02
Doses Blue Rep1_0.02 Red Rep2_−0.05
sp|Q9UQ35|SRRM2_HUMAN−961−S−S2_−0.04
Doses Blue Rep1_−0.07 Red Rep2_−0.01
sp|Q9H2F5−3|EPC1_HUMAN−485−S−S2_0.03
Doses Blue Rep1_0.11 Red Rep2_−0.04
sp|Q9H2F5−3|EPC1_HUMAN−489−S−S2_0.03
Doses Blue Rep1_0.11 Red Rep2_−0.04
sp|Q5VSL9−3|STRP1_HUMAN−71−S−S1_−0.04
Doses Blue Rep1_−0.03 Red Rep2_−0.05
sp|Q9BW71|HIRP3_HUMAN−370−S−S1_−0.12
Doses Blue Rep1_−0.14 Red Rep2_−0.1
sp|P27824−3|CALX_HUMAN−456−S−S2_0.03
Doses Blue Rep1_0.06 Red Rep2_−0.01
sp|Q8IYB3|SRRM1_HUMAN−795−S−S3_0.05
Doses Blue Rep1_0.03 Red Rep2_0.06
sp|Q9BZ95−3|NSD3_HUMAN−150−S−S1_0
Doses Blue Rep1_0.04 Red Rep2_−0.05
sp|Q9BQ69|MACD1_HUMAN−144−Y−S1_−0.01
Doses Blue Rep1_0.01 Red Rep2_−0.03
sp|P05388−2|RLA0_HUMAN−242−S−S2_0.1
Doses Blue Rep1_0.11 Red Rep2_0.09
sp|P05388−2|RLA0_HUMAN−245−S−S2_0.1
Doses Blue Rep1_0.11 Red Rep2_0.09
sp|Q7Z6E9|RBBP6_HUMAN−1646−S−S2_0.02
Doses Blue Rep1_−0.02 Red Rep2_0.06
sp|Q9Y520−6|PRC2C_HUMAN−1488−S−S2_0.02
Doses Blue Rep1_0 Red Rep2_0.04
ptm abundance 37C

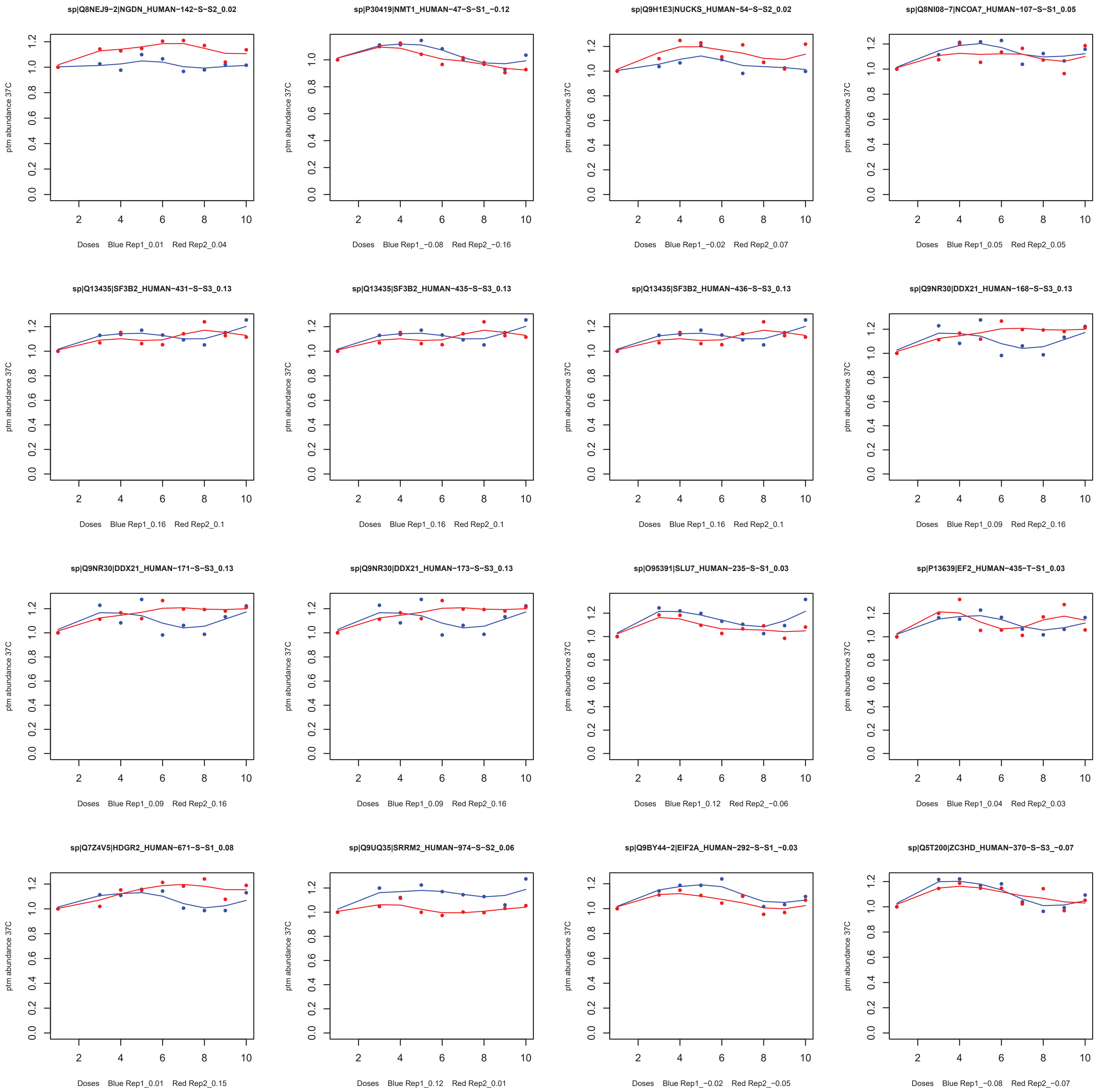

sp|Q8NEJ9−2|NGDN_HUMAN−142−S−S2_0.02
sp|P30419|NMT1_HUMAN−47−S−S1_−0.12
sp|Q9H1E3|NUCKS_HUMAN−54−S−S2_0.02
sp|Q8NI08−7|NCOA7_HUMAN−107−S−S1_0.05
Doses Blue Rep1_0.01 Red Rep2_0.04
Doses Blue Rep1_−0.08 Red Rep2_−0.16
Doses Blue Rep1_−0.02 Red Rep2_0.07
Doses Blue Rep1_0.05 Red Rep2_0.05
sp|Q13435|SF3B2_HUMAN−431−S−S3_0.13
sp|Q13435|SF3B2_HUMAN−435−S−S3_0.13
sp|Q13435|SF3B2_HUMAN−436−S−S3_0.13
sp|Q9NR30|DDX21_HUMAN−168−S−S3_0.13
Doses Blue Rep1_0.16 Red Rep2_0.1
Doses Blue Rep1_0.16 Red Rep2_0.1
Doses Blue Rep1_0.16 Red Rep2_0.1
Doses Blue Rep1_0.09 Red Rep2_0.16
sp|Q9NR30|DDX21_HUMAN−171−S−S3_0.13
sp|Q9NR30|DDX21_HUMAN−173−S−S3_0.13
sp|O95391|SLU7_HUMAN−235−S−S1_0.03
sp|P13639|EF2_HUMAN−435−T−S1_0.03
Doses Blue Rep1_0.09 Red Rep2_0.16
Doses Blue Rep1_0.09 Red Rep2_0.16
Doses Blue Rep1_0.12 Red Rep2_−0.06
Doses Blue Rep1_0.04 Red Rep2_0.03
sp|Q7Z4V5|HDGR2_HUMAN−671−S−S1_0.08
sp|Q9UQ35|SRRM2_HUMAN−974−S−S2_0.06
sp|Q9BY44−2|EIF2A_HUMAN−292−S−S1_−0.03
sp|Q5T200|ZC3HD_HUMAN−370−S−S3_−0.07
Doses Blue Rep1_0.01 Red Rep2_0.15
Doses Blue Rep1_0.12 Red Rep2_0.01
Doses Blue Rep1_−0.02 Red Rep2_−0.05
Doses Blue Rep1_−0.08 Red Rep2_−0.07
ptm abundance 37C

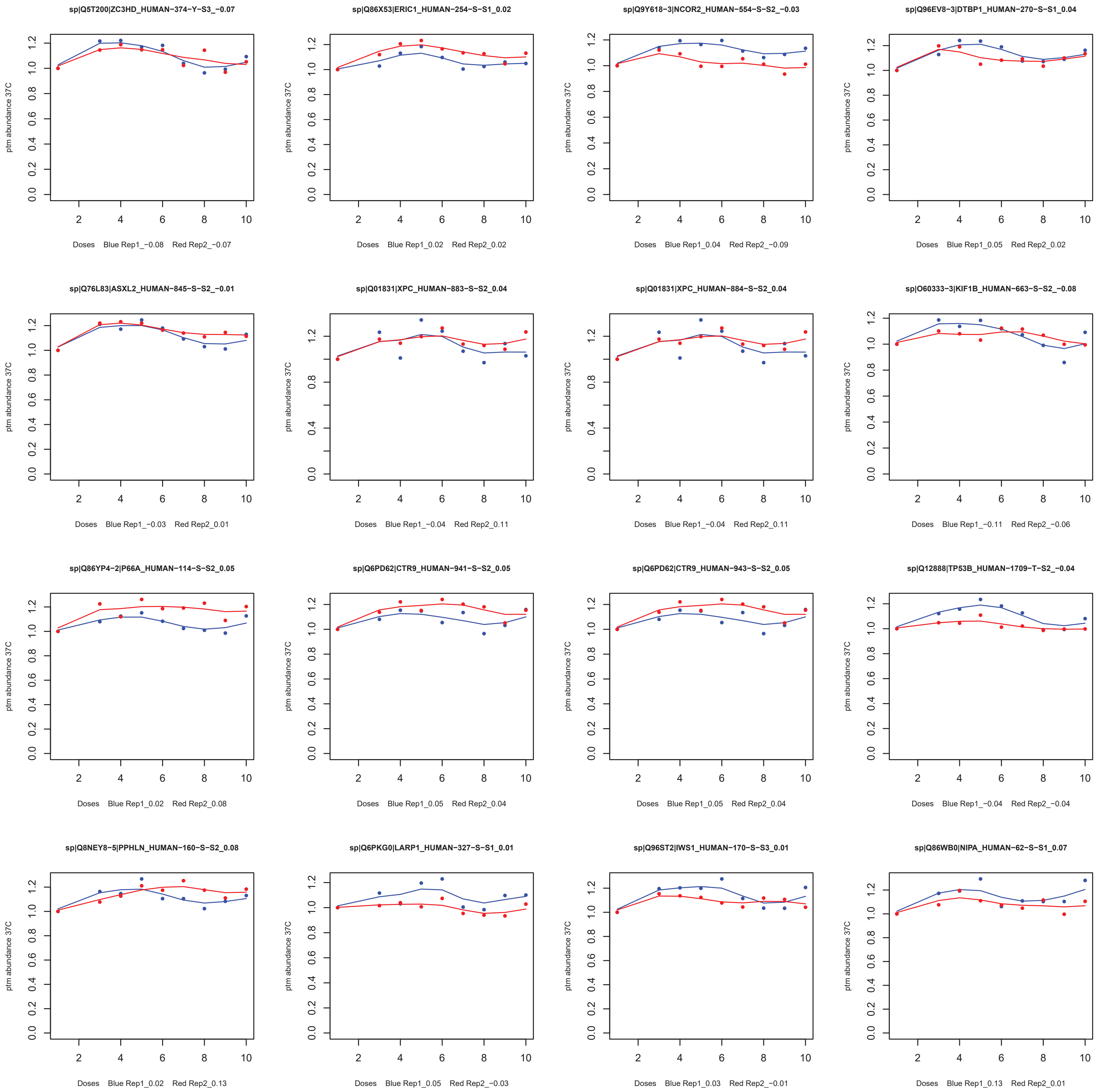
sp|Q5T200|ZC3HD_HUMAN−374−Y−S3_−0.07
ptm abundance 37C
Doses Blue Rep1_−0.08 Red Rep2_−0.07
sp|Q86X53|ERIC1_HUMAN−254−S−S1_0.02
ptm abundance 37C
Doses Blue Rep1_0.02 Red Rep2_0.02
sp|Q9Y618−3|NCOR2_HUMAN−554−S−S2_−0.03
ptm abundance 37C
Doses Blue Rep1_0.04 Red Rep2_−0.09
sp|Q96EV8−3|DTBP1_HUMAN−270−S−S1_0.04
ptm abundance 37C
Doses Blue Rep1_0.05 Red Rep2_0.02
sp|Q76L83|ASXL2_HUMAN−845−S−S2_−0.01
ptm abundance 37C
Doses Blue Rep1_−0.03 Red Rep2_0.01
sp|Q01831|XPC_HUMAN−883−S−S2_0.04
ptm abundance 37C
Doses Blue Rep1_−0.04 Red Rep2_0.11
sp|Q01831|XPC_HUMAN−884−S−S2_0.04
ptm abundance 37C
Doses Blue Rep1_−0.04 Red Rep2_0.11
sp|O60333−3|KIF1B_HUMAN−663−S−S2_−0.08
ptm abundance 37C
Doses Blue Rep1_−0.11 Red Rep2_−0.06
sp|Q86YP4−2|P66A_HUMAN−114−S−S2_0.05
ptm abundance 37C
Doses Blue Rep1_0.02 Red Rep2_0.08
sp|Q6PD62|CTR9_HUMAN−941−S−S2_0.05
ptm abundance 37C
Doses Blue Rep1_0.05 Red Rep2_0.04
sp|Q6PD62|CTR9_HUMAN−943−S−S2_0.05
ptm abundance 37C
Doses Blue Rep1_0.05 Red Rep2_0.04
sp|Q12888|TP53B_HUMAN−1709−T−S2_−0.04
ptm abundance 37C
Doses Blue Rep1_−0.04 Red Rep2_−0.04
sp|Q8NEY8−5|PPHLN_HUMAN−160−S−S2_0.08
ptm abundance 37C
Doses Blue Rep1_0.02 Red Rep2_0.13
sp|Q6PKG0|LARP1_HUMAN−327−S−S1_0.01
ptm abundance 37C
Doses Blue Rep1_0.05 Red Rep2_−0.03
sp|Q96ST2|IWS1_HUMAN−170−S−S3_0.01
ptm abundance 37C
Doses Blue Rep1_0.03 Red Rep2_−0.01
sp|Q86WB0|NIPA_HUMAN−62−S−S1_0.07
ptm abundance 37C
Doses Blue Rep1_0.13 Red Rep2_0.01

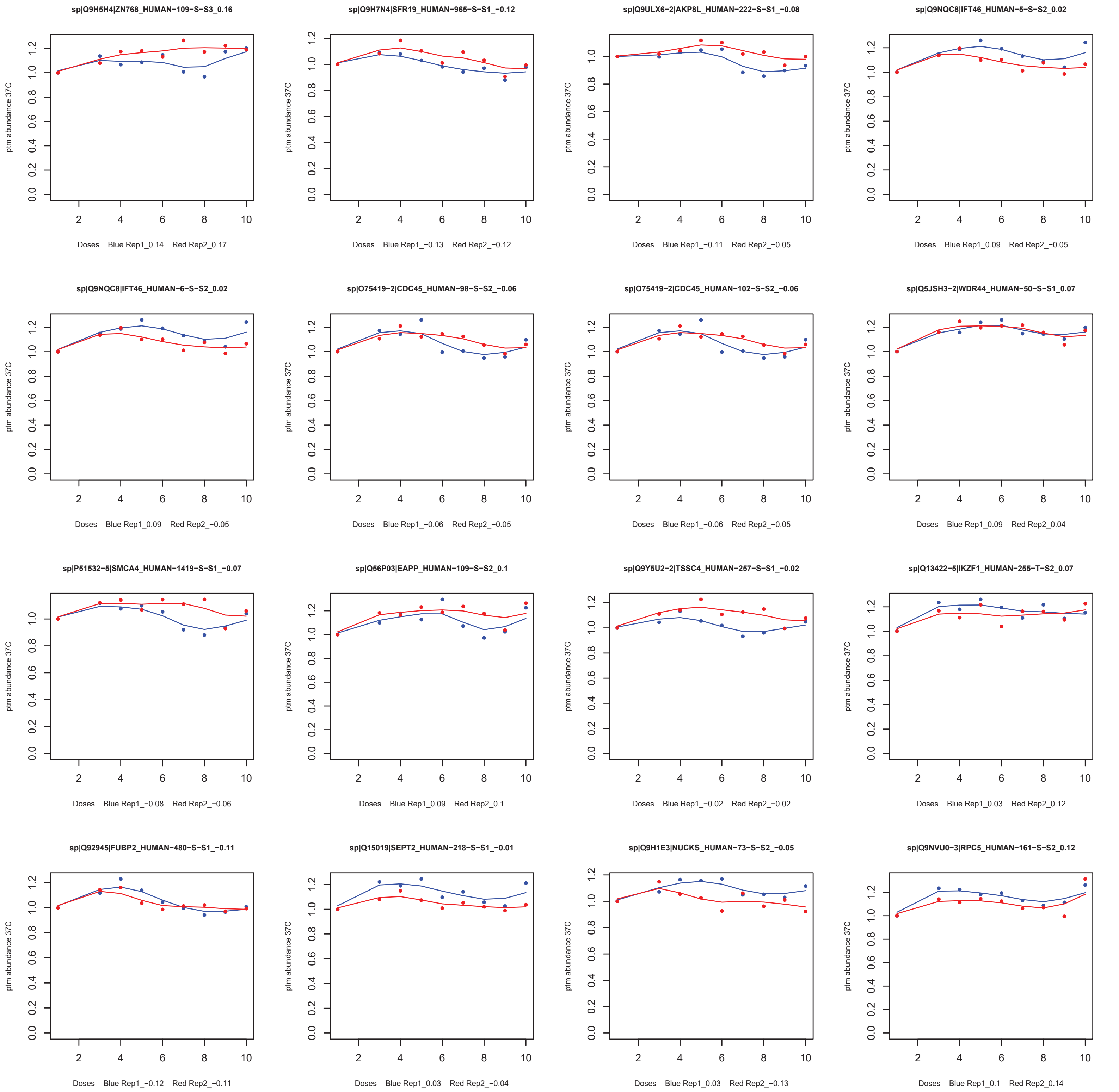
sp|Q9H5H4|ZN768_HUMAN−109−S−S3_0.16
ptm abundance 37C
Doses Blue Rep1_0.14 Red Rep2_0.17
sp|Q9H7N4|SFR19_HUMAN−965−S−S1_−0.12
ptm abundance 37C
Doses Blue Rep1_−0.13 Red Rep2_−0.12
sp|Q9ULX6−2|AKP8L_HUMAN−222−S−S1_−0.08
ptm abundance 37C
Doses Blue Rep1_−0.11 Red Rep2_−0.05
sp|Q9NQC8|IFT46_HUMAN−5−S−S2_0.02
ptm abundance 37C
Doses Blue Rep1_0.09 Red Rep2_−0.05
sp|Q9NQC8|IFT46_HUMAN−6−S−S2_0.02
ptm abundance 37C
Doses Blue Rep1_0.09 Red Rep2_−0.05
sp|O75419−2|CDC45_HUMAN−98−S−S2_−0.06
ptm abundance 37C
Doses Blue Rep1_−0.06 Red Rep2_−0.05
sp|O75419−2|CDC45_HUMAN−102−S−S2_−0.06
ptm abundance 37C
Doses Blue Rep1_−0.06 Red Rep2_−0.05
sp|Q5JSH3−2|WDR44_HUMAN−50−S−S1_0.07
ptm abundance 37C
Doses Blue Rep1_0.09 Red Rep2_0.04
sp|P51532−5|SMCA4_HUMAN−1419−S−S1_−0.07
ptm abundance 37C
Doses Blue Rep1_−0.08 Red Rep2_−0.06
sp|Q56P03|EAPP_HUMAN−109−S−S2_0.1
ptm abundance 37C
Doses Blue Rep1_0.09 Red Rep2_0.1
sp|Q9Y5U2−2|TSSC4_HUMAN−257−S−S1_−0.02
ptm abundance 37C
Doses Blue Rep1_−0.02 Red Rep2_−0.02
sp|Q13422−5|IKZF1_HUMAN−255−T−S2_0.07
ptm abundance 37C
Doses Blue Rep1_0.03 Red Rep2_0.12
sp|Q92945|FUBP2_HUMAN−480−S−S1_−0.11
ptm abundance 37C
Doses Blue Rep1_−0.12 Red Rep2_−0.11
sp|Q15019|SEPT2_HUMAN−218−S−S1_−0.01
ptm abundance 37C
Doses Blue Rep1_0.03 Red Rep2_−0.04
sp|Q9H1E3|NUCKS_HUMAN−73−S−S2_−0.05
ptm abundance 37C
Doses Blue Rep1_0.03 Red Rep2_−0.13
sp|Q9NVU0−3|RPC5_HUMAN−161−S−S2_0.12
ptm abundance 37C
Doses Blue Rep1_0.1 Red Rep2_0.14

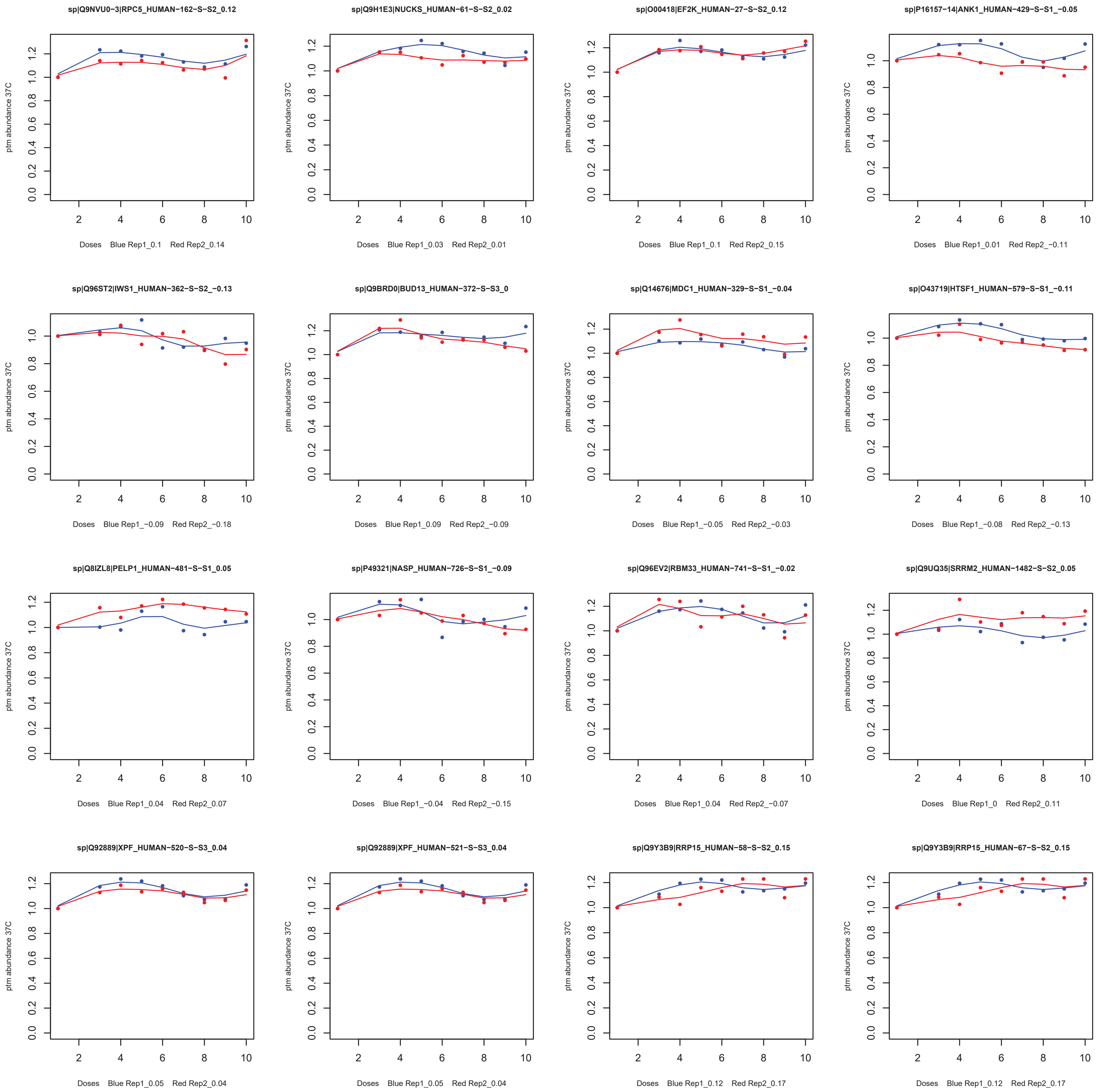

sp|Q9NVU0−3|RPC5_HUMAN−162−S−S2_0.12
ptm abundance 37C
Doses Blue Rep1_0.1 Red Rep2_0.14
sp|Q9H1E3|NUCKS_HUMAN−61−S−S2_0.02
ptm abundance 37C
Doses Blue Rep1_0.03 Red Rep2_0.01
sp|O00418|EF2K_HUMAN−27−S−S2_0.12
ptm abundance 37C
Doses Blue Rep1_0.1 Red Rep2_0.15
sp|P16157−14|ANK1_HUMAN−429−S−S1_−0.05
ptm abundance 37C
Doses Blue Rep1_0.01 Red Rep2_−0.11
sp|Q96ST2|IWS1_HUMAN−362−S−S2_−0.13
ptm abundance 37C
Doses Blue Rep1_−0.09 Red Rep2_−0.18
sp|Q9BRD0|BUD13_HUMAN−372−S−S3_0
ptm abundance 37C
Doses Blue Rep1_0.09 Red Rep2_−0.09
sp|Q14676|MDC1_HUMAN−329−S−S1_−0.04
ptm abundance 37C
Doses Blue Rep1_−0.05 Red Rep2_−0.03
sp|O43719|HTSF1_HUMAN−579−S−S1_−0.11
ptm abundance 37C
Doses Blue Rep1_−0.08 Red Rep2_−0.13
sp|Q8IZL8|PELP1_HUMAN−481−S−S1_0.05
ptm abundance 37C
Doses Blue Rep1_0.04 Red Rep2_0.07
sp|P49321|NASP_HUMAN−726−S−S1_−0.09
ptm abundance 37C
Doses Blue Rep1_−0.04 Red Rep2_−0.15
sp|Q96EV2|RBM33_HUMAN−741−S−S1_−0.02
ptm abundance 37C
Doses Blue Rep1_0.04 Red Rep2_−0.07
sp|Q9UQ35|SRRM2_HUMAN−1482−S−S2_0.05
ptm abundance 37C
Doses Blue Rep1_0 Red Rep2_0.11
sp|Q92889|XPF_HUMAN−520−S−S3_0.04
ptm abundance 37C
Doses Blue Rep1_0.05 Red Rep2_0.04
sp|Q92889|XPF_HUMAN−521−S−S3_0.04
ptm abundance 37C
Doses Blue Rep1_0.05 Red Rep2_0.04
sp|Q9Y3B9|RRP15_HUMAN−58−S−S2_0.15
ptm abundance 37C
Doses Blue Rep1_0.12 Red Rep2_0.17
sp|Q9Y3B9|RRP15_HUMAN−67−S−S2_0.15
ptm abundance 37C
Doses Blue Rep1_0.12 Red Rep2_0.17

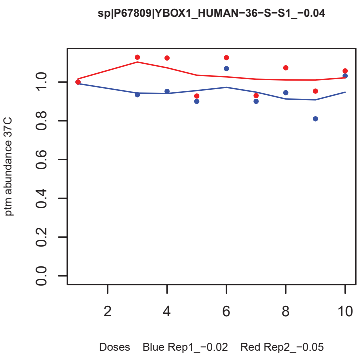
sp|P67809|YBOX1_HUMAN−36−S−S1_−0.04
ptm abundance 37C
Doses Blue Rep1_−0.02 Red Rep2_−0.05

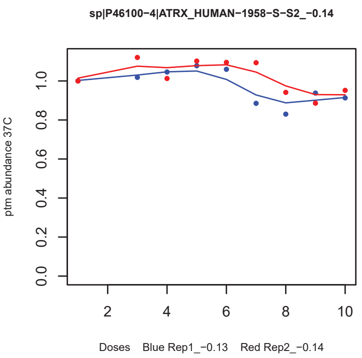
sp|P46100−4|ATRX_HUMAN−1958−S−S2_−0.14
ptm abundance 37C
Doses Blue Rep1_−0.13 Red Rep2_−0.14

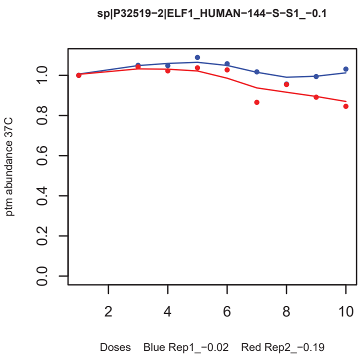
sp|P32519−2|ELF1_HUMAN−144−S−S1_−0.1
ptm abundance 37C
Doses Blue Rep1_−0.02 Red Rep2_−0.19

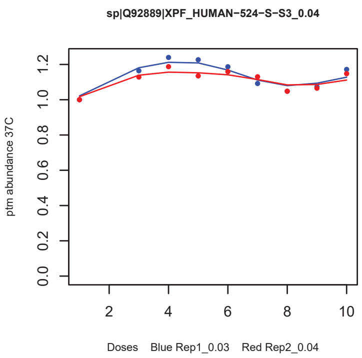
sp|Q92889|XPF_HUMAN−524−S−S3_0.04
ptm abundance 37C
Doses Blue Rep1_0.03 Red Rep2_0.04

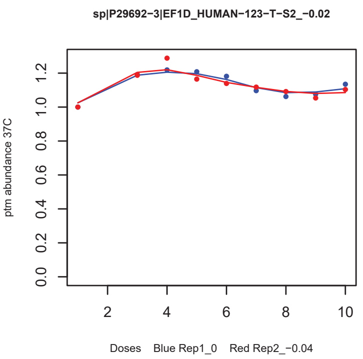
sp|P29692−3|EF1D_HUMAN−123−T−S2_−0.02
ptm abundance 37C
Doses Blue Rep1_0 Red Rep2_−0.04

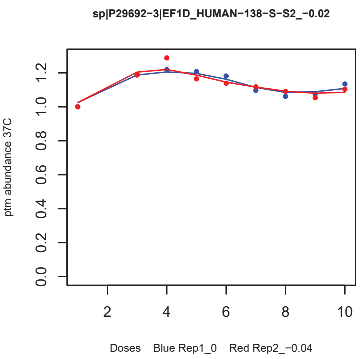
sp|P29692−3|EF1D_HUMAN−138−S−S2_−0.02
ptm abundance 37C
Doses Blue Rep1_0 Red Rep2_−0.04

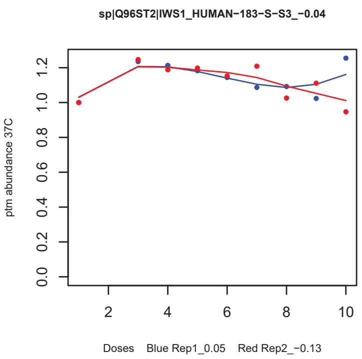
sp|Q96ST2|IWS1_HUMAN−183−S−S3_−0.04
ptm abundance 37C
Doses Blue Rep1_0.05 Red Rep2_−0.13

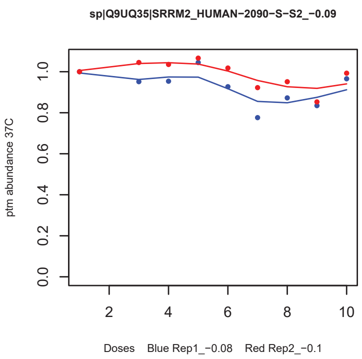
sp|Q9UQ35|SRRM2_HUMAN−2090−S−S2_−0.09
ptm abundance 37C
Doses Blue Rep1_−0.08 Red Rep2_−0.1

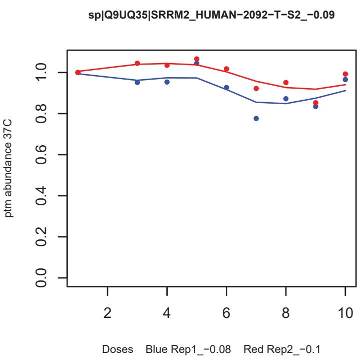
sp|Q9UQ35|SRRM2_HUMAN−2092−T−S2_−0.09
ptm abundance 37C
Doses Blue Rep1_−0.08 Red Rep2_−0.1

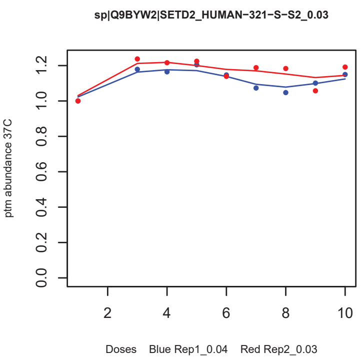
sp|Q9BYW2|SETD2_HUMAN−321−S−S2_0.03
ptm abundance 37C
Doses Blue Rep1_0.04 Red Rep2_0.03

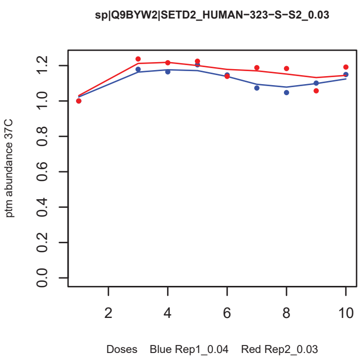
sp|Q9BYW2|SETD2_HUMAN−323−S−S2_0.03
ptm abundance 37C
Doses Blue Rep1_0.04 Red Rep2_0.03

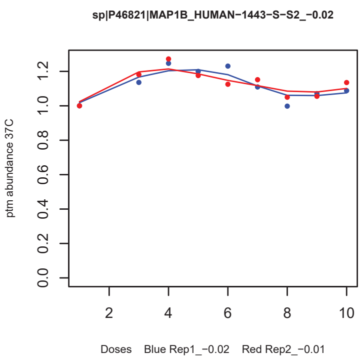
sp|P46821|MAP1B_HUMAN−1443−S−S2_−0.02
ptm abundance 37C
Doses Blue Rep1_−0.02 Red Rep2_−0.01

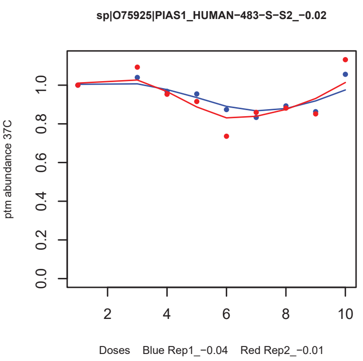
sp|O75925|PIAS1_HUMAN−483−S−S2_−0.02
ptm abundance 37C
Doses Blue Rep1_−0.04 Red Rep2_−0.01

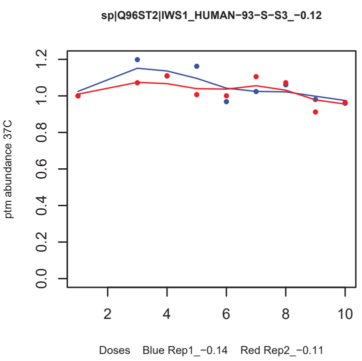
sp|Q96ST2|IWS1_HUMAN−93−S−S3_−0.12
ptm abundance 37C
Doses Blue Rep1_−0.14 Red Rep2_−0.11

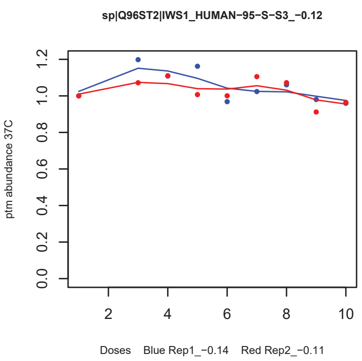
sp|Q96ST2|IWS1_HUMAN−95−S−S3_−0.12
ptm abundance 37C
Doses Blue Rep1_−0.14 Red Rep2_−0.11

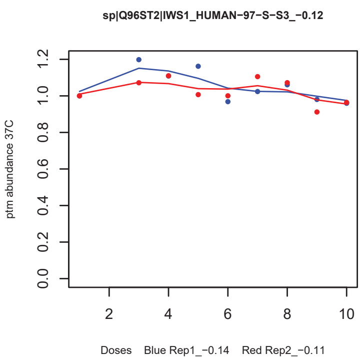
sp|Q96ST2|IWS1_HUMAN−97−S−S3_−0.12
ptm abundance 37C
Doses Blue Rep1_−0.14 Red Rep2_−0.11

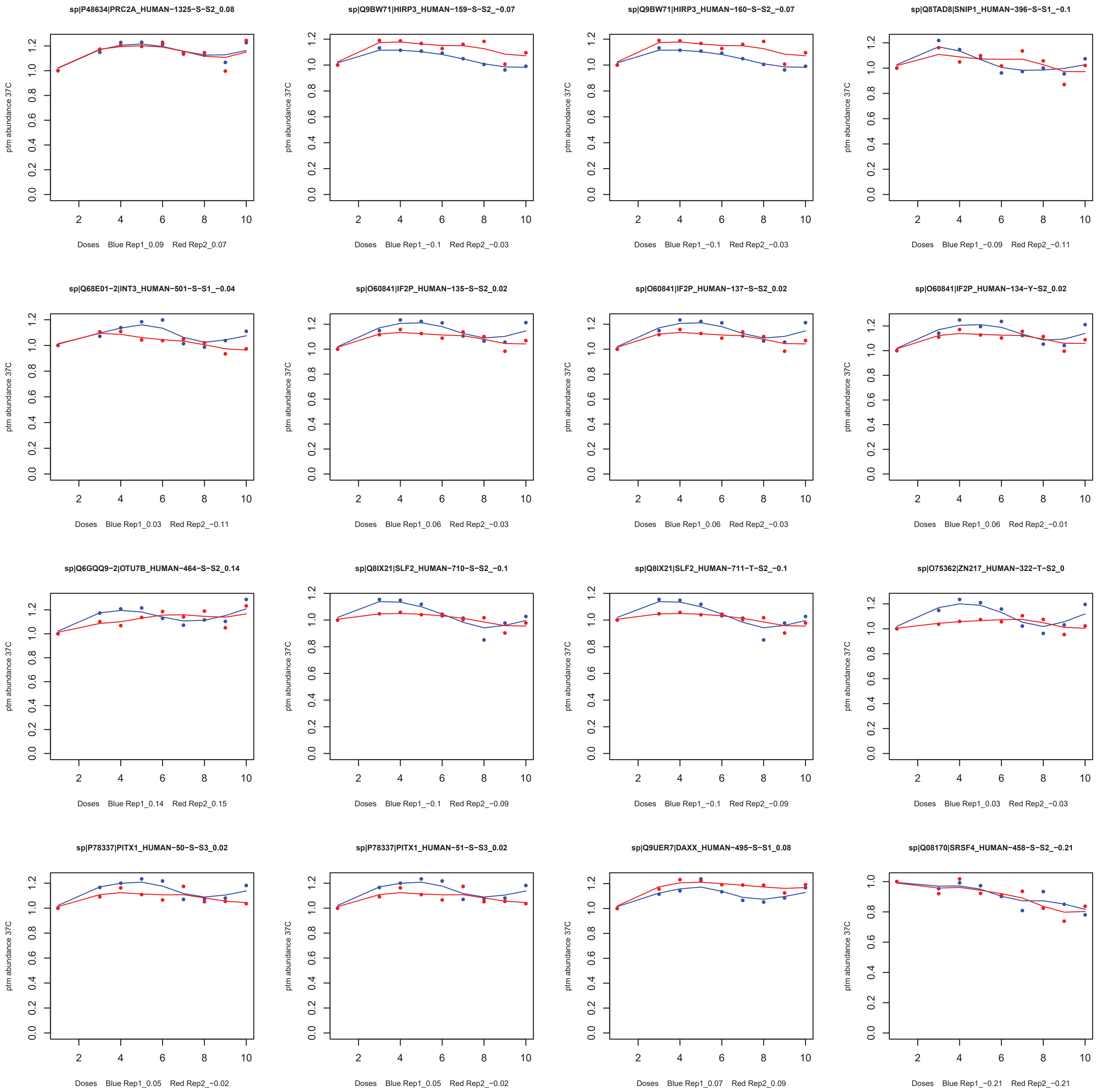
sp|P48634|PRC2A_HUMAN−1325−S−S2_0.08
ptm abundance 37C
Doses Blue Rep1_0.09 Red Rep2_0.07
sp|Q9BW71|HIRP3_HUMAN−159−S−S2_−0.07
ptm abundance 37C
Doses Blue Rep1_−0.1 Red Rep2_−0.03
sp|Q9BW71|HIRP3_HUMAN−160−S−S2_−0.07
ptm abundance 37C
Doses Blue Rep1_−0.1 Red Rep2_−0.03
sp|Q8TAD8|SNIP1_HUMAN−396−S−S1_−0.1
ptm abundance 37C
Doses Blue Rep1_−0.09 Red Rep2_−0.11
sp|Q68E01−2|INT3_HUMAN−501−S−S1_−0.04
ptm abundance 37C
Doses Blue Rep1_0.03 Red Rep2_−0.11
sp|O60841|IF2P_HUMAN−135−S−S2_0.02
ptm abundance 37C
Doses Blue Rep1_0.06 Red Rep2_−0.03
sp|O60841|IF2P_HUMAN−137−S−S2_0.02
ptm abundance 37C
Doses Blue Rep1_0.06 Red Rep2_−0.03
sp|O60841|IF2P_HUMAN−134−Y−S2_0.02
ptm abundance 37C
Doses Blue Rep1_0.06 Red Rep2_−0.01
sp|Q6GQQ9−2|OTU7B_HUMAN−464−S−S2_0.14
ptm abundance 37C
Doses Blue Rep1_0.14 Red Rep2_0.15
sp|Q8IX21|SLF2_HUMAN−710−S−S2_−0.1
ptm abundance 37C
Doses Blue Rep1_−0.1 Red Rep2_−0.09
sp|Q8IX21|SLF2_HUMAN−711−T−S2_−0.1
ptm abundance 37C
Doses Blue Rep1_−0.1 Red Rep2_−0.09
sp|O75362|ZN217_HUMAN−322−T−S2_0
ptm abundance 37C
Doses Blue Rep1_0.03 Red Rep2_−0.03
sp|P78337|PITX1_HUMAN−50−S−S3_0.02
ptm abundance 37C
Doses Blue Rep1_0.05 Red Rep2_−0.02
sp|P78337|PITX1_HUMAN−51−S−S3_0.02
ptm abundance 37C
Doses Blue Rep1_0.05 Red Rep2_−0.02
sp|Q9UER7|DAXX_HUMAN−495−S−S1_0.08
ptm abundance 37C
Doses Blue Rep1_0.07 Red Rep2_0.09
sp|Q08170|SRSF4_HUMAN−458−S−S2_−0.21
ptm abundance 37C
Doses Blue Rep1_−0.21 Red Rep2_−0.21

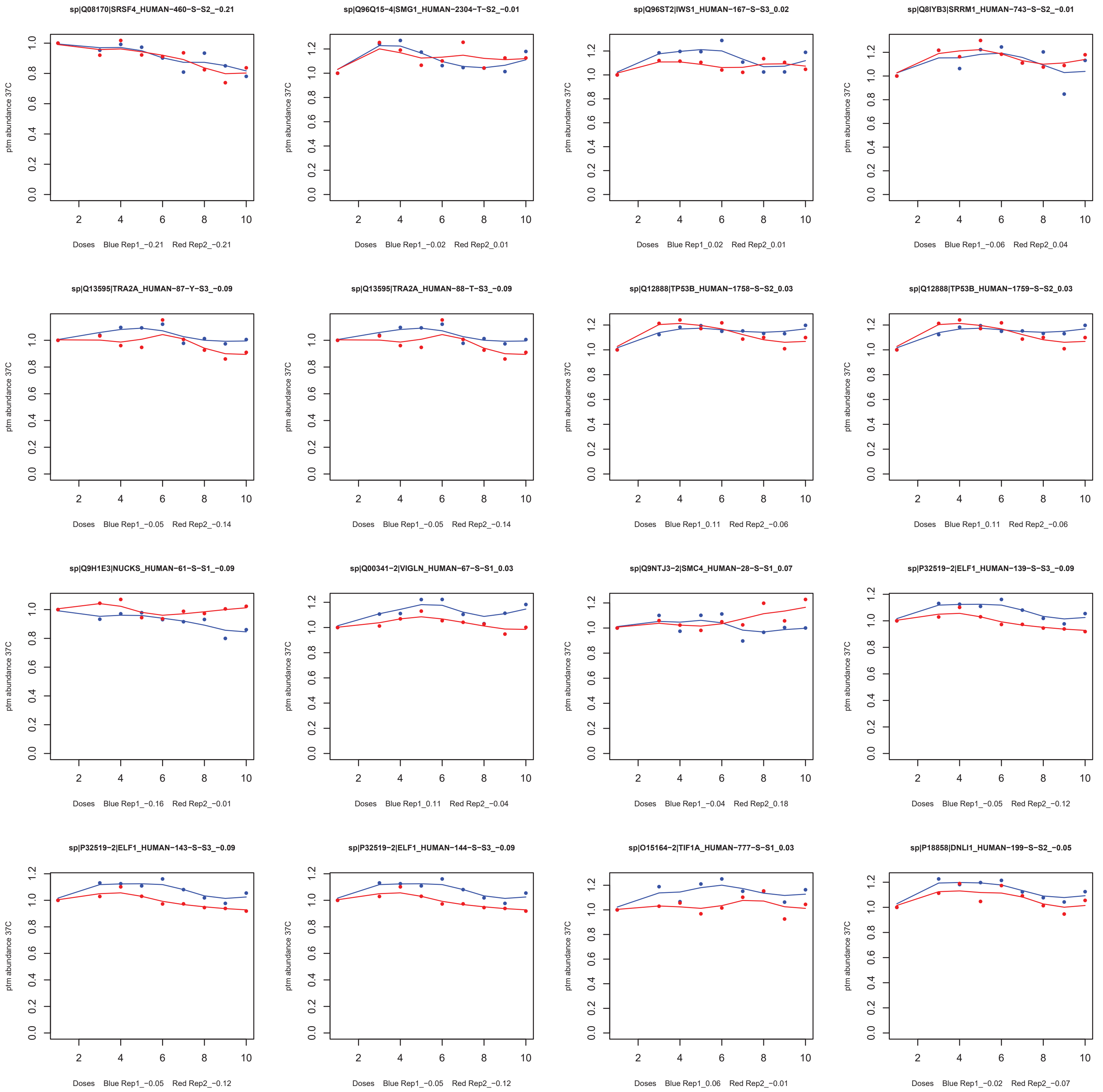

sp|Q08170|SRSF4_HUMAN−460−S−S2_−0.21
Doses Blue Rep1_−0.21 Red Rep2_−0.21
sp|Q96Q15−4|SMG1_HUMAN−2304−T−S2_−0.01
Doses Blue Rep1_−0.02 Red Rep2_0.01
sp|Q96ST2|IWS1_HUMAN−167−S−S3_0.02
Doses Blue Rep1_0.02 Red Rep2_0.01
sp|Q8IYB3|SRRM1_HUMAN−743−S−S2_−0.01
Doses Blue Rep1_−0.06 Red Rep2_0.04
sp|Q13595|TRA2A_HUMAN−87−Y−S3_−0.09
Doses Blue Rep1_−0.05 Red Rep2_−0.14
sp|Q13595|TRA2A_HUMAN−88−T−S3_−0.09
Doses Blue Rep1_−0.05 Red Rep2_−0.14
sp|Q12888|TP53B_HUMAN−1758−S−S2_0.03
Doses Blue Rep1_0.11 Red Rep2_−0.06
sp|Q12888|TP53B_HUMAN−1759−S−S2_0.03
Doses Blue Rep1_0.11 Red Rep2_−0.06
sp|Q9H1E3|NUCKS_HUMAN−61−S−S1_−0.09
Doses Blue Rep1_−0.16 Red Rep2_−0.01
sp|Q00341−2|VIGLN_HUMAN−67−S−S1_0.03
Doses Blue Rep1_0.11 Red Rep2_−0.04
sp|Q9NTJ3−2|SMC4_HUMAN−28−S−S1_0.07
Doses Blue Rep1_−0.04 Red Rep2_0.18
sp|P32519−2|ELF1_HUMAN−139−S−S3_−0.09
Doses Blue Rep1_−0.05 Red Rep2_−0.12
sp|P32519−2|ELF1_HUMAN−143−S−S3_−0.09
Doses Blue Rep1_−0.05 Red Rep2_−0.12
sp|P32519−2|ELF1_HUMAN−144−S−S3_−0.09
Doses Blue Rep1_−0.05 Red Rep2_−0.12
sp|O15164−2|TIF1A_HUMAN−777−S−S1_0.03
Doses Blue Rep1_0.06 Red Rep2_−0.01
sp|P18858|DNLI1_HUMAN−199−S−S2_−0.05
Doses Blue Rep1_−0.02 Red Rep2_−0.07
ptm abundance 37C

sp|Q5T8D3−4|ACBD5_HUMAN−78−S−S2_0.11

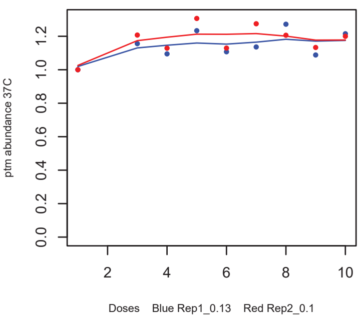

sp|Q5T8D3−4|ACBD5_HUMAN−82−S−S2_0.11

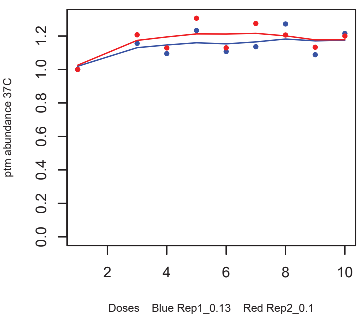

sp|Q96F63|CCD97_HUMAN−257−S−S2_0.07

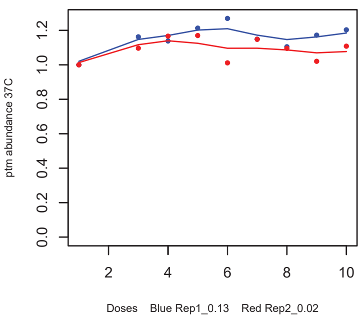

sp|Q96F63|CCD97_HUMAN−264−S−S2_0.07

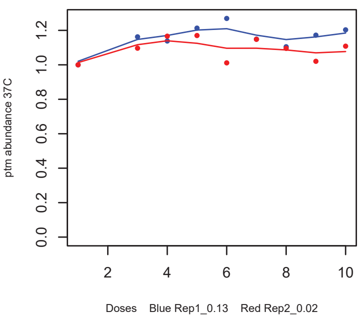

sp|P31323|KAP3_HUMAN−83−S−S1_0.07

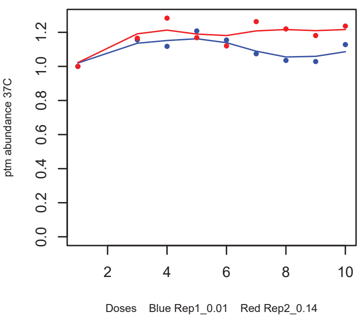

sp|P31323|KAP3_HUMAN−85−S−S1_0.07

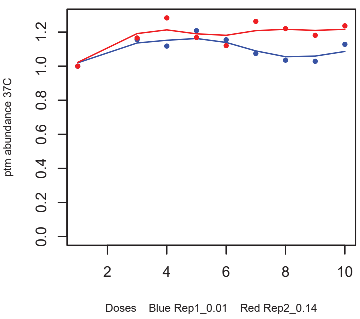

sp|O15541|R113A_HUMAN−43−S−S3_0.08

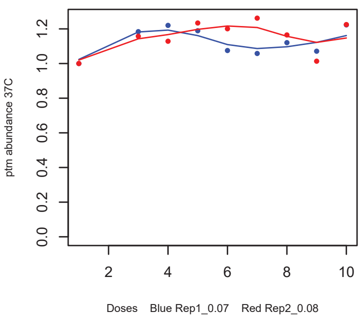

sp|O15541|R113A_HUMAN−45−S−S3_0.08

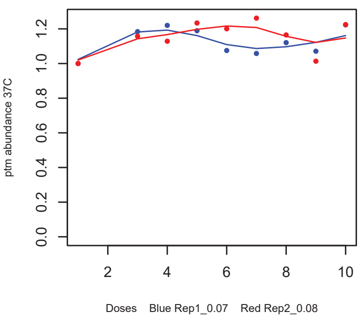

sp|O15541|R113A_HUMAN−46−S−S3_0.08

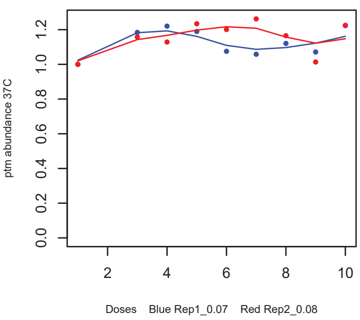

sp|Q68DK2−4|ZFY26_HUMAN−619−S−S2_−0.14

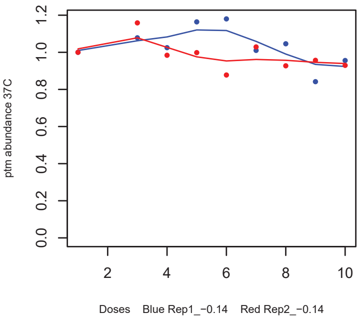

sp|Q96ST2|IWS1_HUMAN−415−S−S3_0

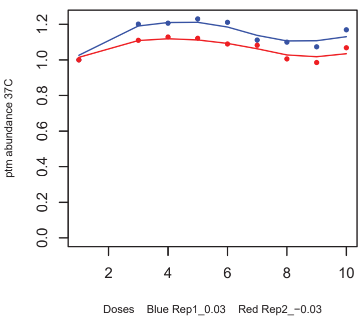

sp|Q5JTV8−3|TOIP1_HUMAN−157−S−S3_−0.01

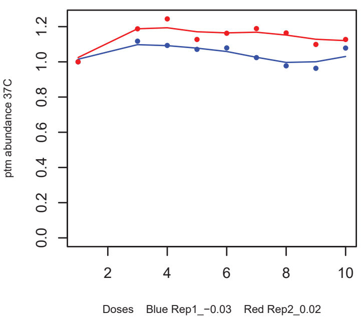

sp|Q9UHI6|DDX20_HUMAN−678−S−S2_−0.08

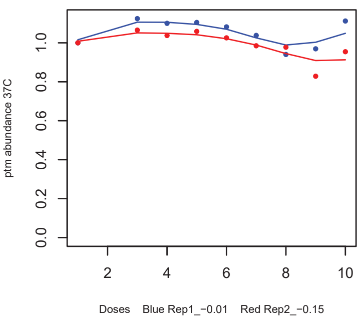

sp|P55884|EIF3B_HUMAN−154−S−S2_0.06

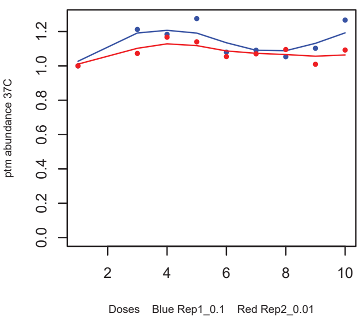

sp|P55884|EIF3B_HUMAN−164−S−S2_0.06

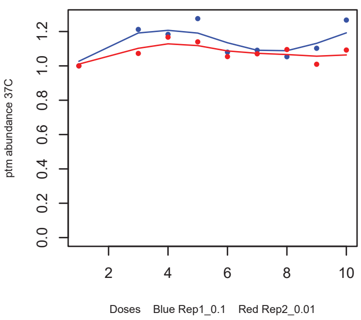

sp|Q8IXQ4|GPAM1_HUMAN−105−S−S1_0.01

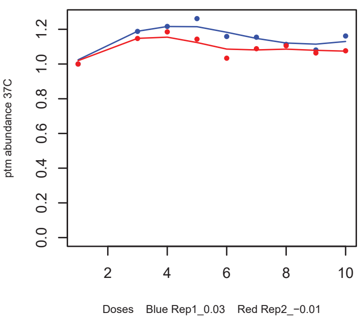

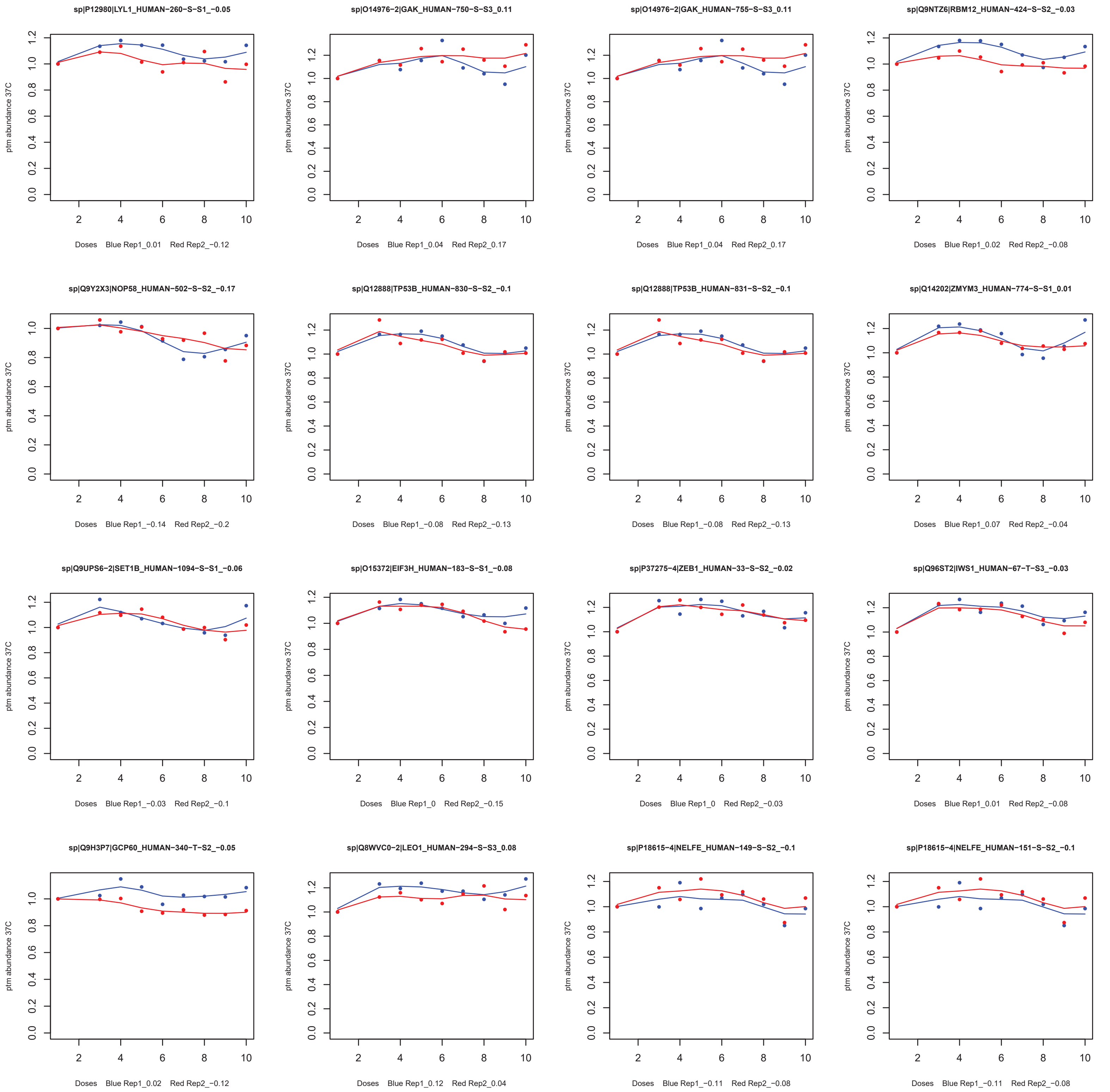

sp|P12980|LYL1_HUMAN−260−S−S1_−0.05
Doses Blue Rep1_0.01 Red Rep2_−0.12
sp|O14976−2|GAK_HUMAN−750−S−S3_0.11
Doses Blue Rep1_0.04 Red Rep2_0.17
sp|O14976−2|GAK_HUMAN−755−S−S3_0.11
Doses Blue Rep1_0.04 Red Rep2_0.17
sp|Q9NTZ6|RBM12_HUMAN−424−S−S2_−0.03
Doses Blue Rep1_0.02 Red Rep2_−0.08
sp|Q9Y2X3|NOP58_HUMAN−502−S−S2_−0.17
Doses Blue Rep1_−0.14 Red Rep2_−0.2
sp|Q12888|TP53B_HUMAN−830−S−S2_−0.1
Doses Blue Rep1_−0.08 Red Rep2_−0.13
sp|Q12888|TP53B_HUMAN−831−S−S2_−0.1
Doses Blue Rep1_−0.08 Red Rep2_−0.13
sp|Q14202|ZMYM3_HUMAN−774−S−S1_0.01
Doses Blue Rep1_0.07 Red Rep2_−0.04
sp|Q9UPS6−2|SET1B_HUMAN−1094−S−S1_−0.06
Doses Blue Rep1_−0.03 Red Rep2_−0.1
sp|O15372|EIF3H_HUMAN−183−S−S1_−0.08
Doses Blue Rep1_0 Red Rep2_−0.15
sp|P37275−4|ZEB1_HUMAN−33−S−S2_−0.02
Doses Blue Rep1_0 Red Rep2_−0.03
sp|Q96ST2|IWS1_HUMAN−67−T−S3_−0.03
Doses Blue Rep1_0.01 Red Rep2_−0.08
sp|Q9H3P7|GCP60_HUMAN−340−T−S2_−0.05
Doses Blue Rep1_0.02 Red Rep2_−0.12
sp|Q8WVC0−2|LEO1_HUMAN−294−S−S3_0.08
Doses Blue Rep1_0.12 Red Rep2_0.04
sp|P18615−4|NELFE_HUMAN−149−S−S2_−0.1
Doses Blue Rep1_−0.11 Red Rep2_−0.08
sp|P18615−4|NELFE_HUMAN−151−S−S2_−0.1
Doses Blue Rep1_−0.11 Red Rep2_−0.08
ptm abundance 37C

sp|Q9H6Y2|WDR55_HUMAN−21−S−S1_0.07

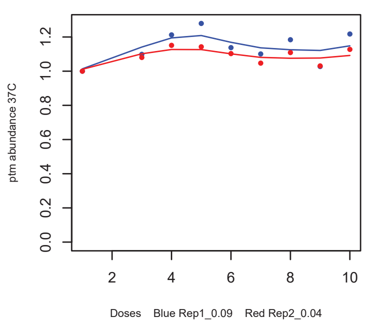

sp|Q8WU17|RN139_HUMAN−625−S−S3_−0.04

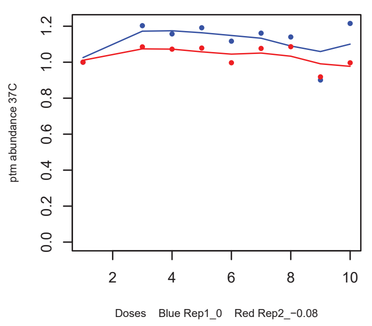

sp|Q8WU17|RN139_HUMAN−634−S−S3_−0.04

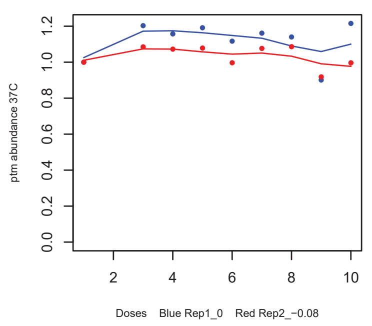

sp|Q8WU17|RN139_HUMAN−635−T−S3_−0.04

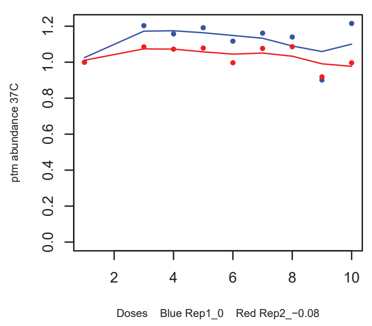

sp|P37275−4|ZEB1_HUMAN−29−T−S2_−0.01

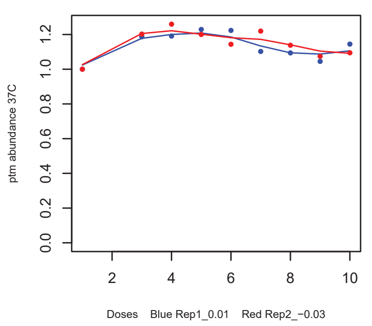

sp|P37275−4|ZEB1_HUMAN−31−S−S2_−0.01

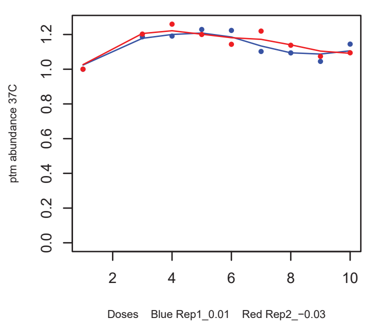

sp|Q6BDS2|URFB1_HUMAN−918−S−S2_−0.05

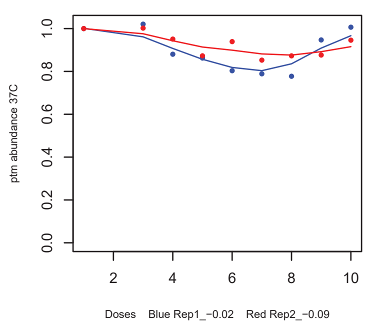

sp|O75064|DEN4B_HUMAN−736−S−S1_−0.08

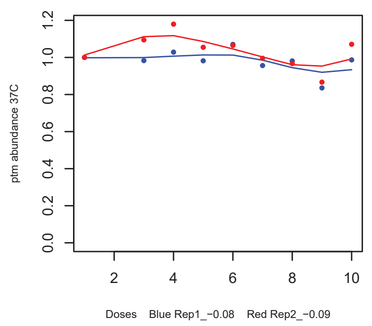

sp|O43719|HTSF1_HUMAN−553−S−S3_0.02

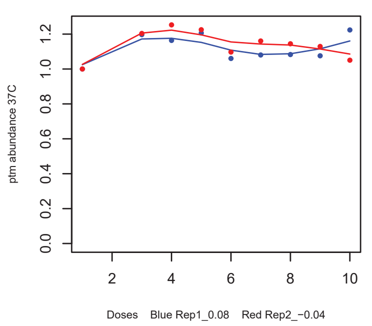

sp|Q5VT52−2|RPRD2_HUMAN−891−S−S2_−0.07

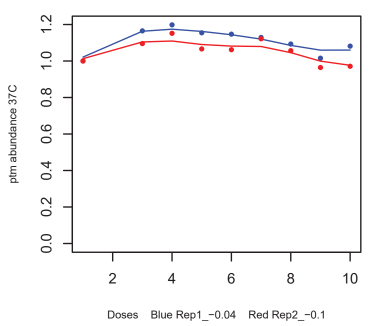

sp|Q9UKV3|ACINU_HUMAN−898−S−S1_0.13

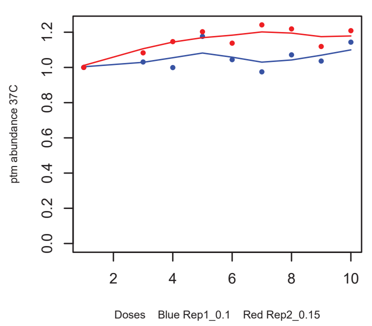

sp|Q96L91−4|EP400_HUMAN−974−T−S2_−0.05

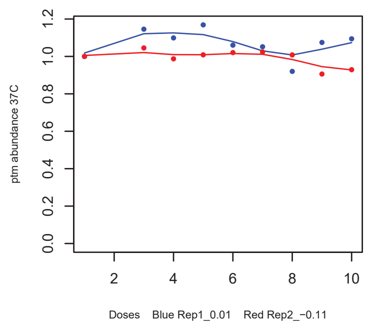

sp|Q96L91−4|EP400_HUMAN−975−S−S2_−0.05

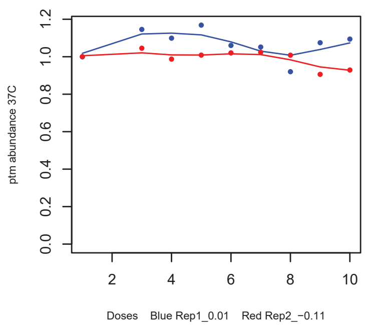

sp|Q5JSZ5|PRC2B_HUMAN−388−S−S1_0.07

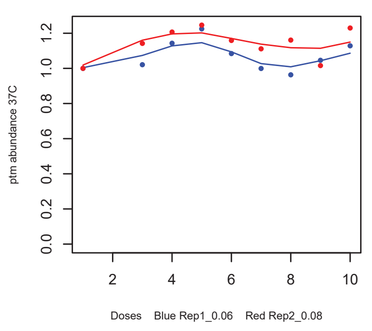

sp|Q9H4L7|SMRCD_HUMAN−54−T−S2_0.02

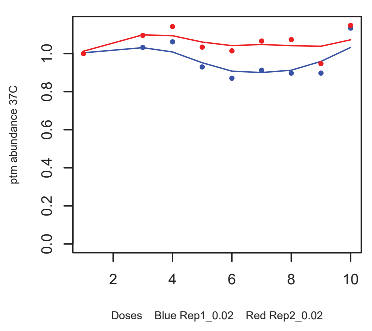

sp|O43896|KIF1C_HUMAN−674−S−S2_−0.08

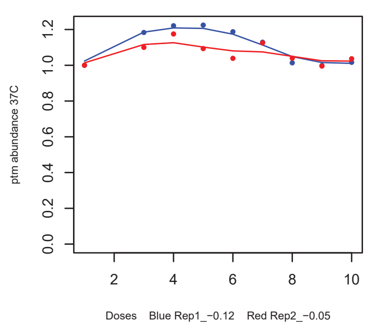

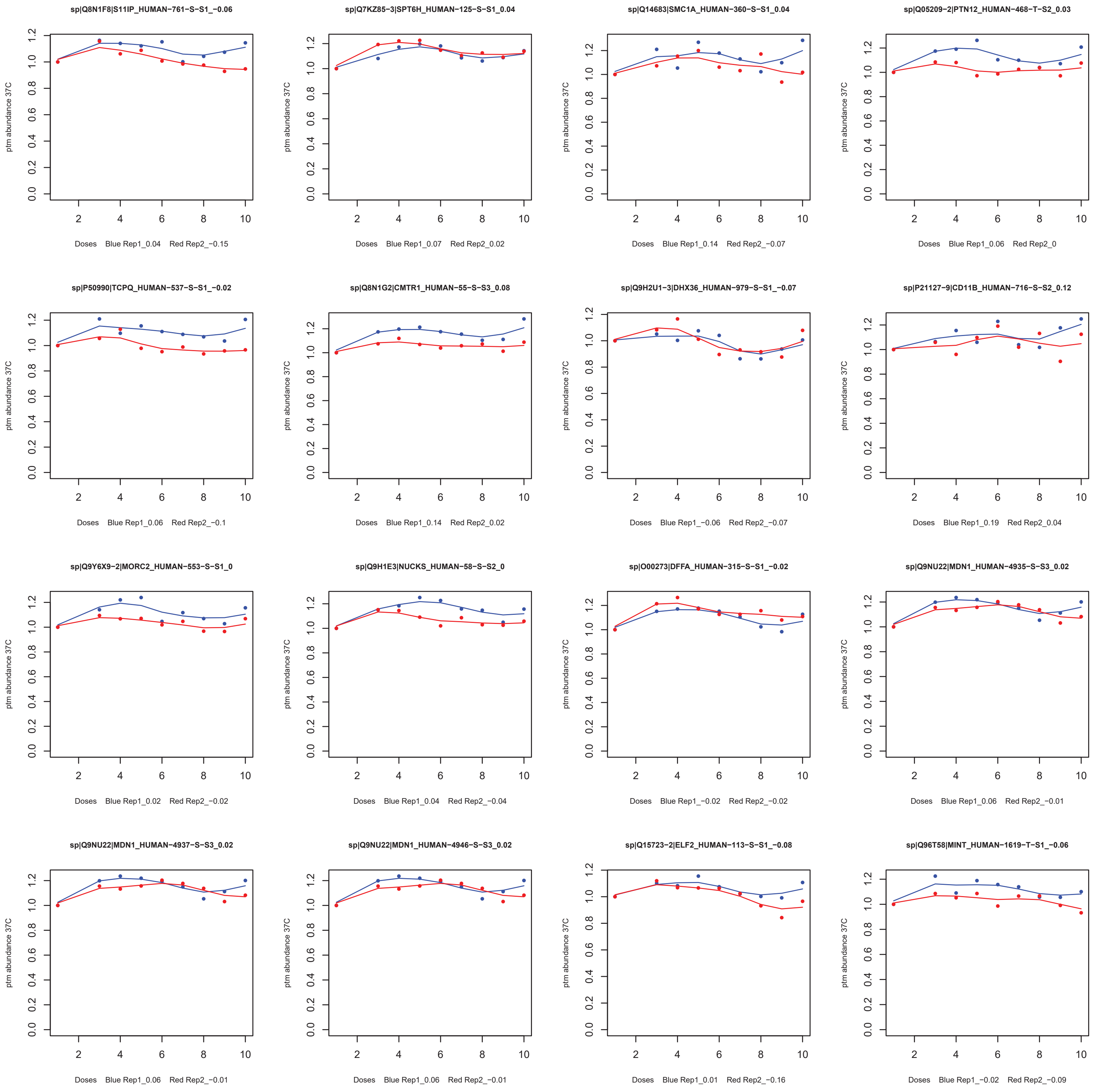
sp|Q8N1F8|S11IP_HUMAN−761−S−S1_−0.06
ptm abundance 37C
Doses Blue Rep1_0.04 Red Rep2_−0.15
sp|Q7KZ85−3|SPT6H_HUMAN−125−S−S1_0.04
ptm abundance 37C
Doses Blue Rep1_0.07 Red Rep2_0.02
sp|Q14683|SMC1A_HUMAN−360−S−S1_0.04
ptm abundance 37C
Doses Blue Rep1_0.14 Red Rep2_−0.07
sp|Q05209−2|PTN12_HUMAN−468−T−S2_0.03
ptm abundance 37C
Doses Blue Rep1_0.06 Red Rep2_0
sp|P50990|TCPQ_HUMAN−537−S−S1_−0.02
ptm abundance 37C
Doses Blue Rep1_0.06 Red Rep2_−0.1
sp|Q8N1G2|CMTR1_HUMAN−55−S−S3_0.08
ptm abundance 37C
Doses Blue Rep1_0.14 Red Rep2_0.02
sp|Q9H2U1−3|DHX36_HUMAN−979−S−S1_−0.07
ptm abundance 37C
Doses Blue Rep1_−0.06 Red Rep2_−0.07
sp|P21127−9|CD11B_HUMAN−716−S−S2_0.12
ptm abundance 37C
Doses Blue Rep1_0.19 Red Rep2_0.04
sp|Q9Y6X9−2|MORC2_HUMAN−553−S−S1_0
ptm abundance 37C
Doses Blue Rep1_0.02 Red Rep2_−0.02
sp|Q9H1E3|NUCKS_HUMAN−58−S−S2_0
ptm abundance 37C
Doses Blue Rep1_0.04 Red Rep2_−0.04
sp|O00273|DFFA_HUMAN−315−S−S1_−0.02
ptm abundance 37C
Doses Blue Rep1_−0.02 Red Rep2_−0.02
sp|Q9NU22|MDN1_HUMAN−4935−S−S3_0.02
ptm abundance 37C
Doses Blue Rep1_0.06 Red Rep2_−0.01
sp|Q9NU22|MDN1_HUMAN−4937−S−S3_0.02
ptm abundance 37C
Doses Blue Rep1_0.06 Red Rep2_−0.01
sp|Q9NU22|MDN1_HUMAN−4946−S−S3_0.02
ptm abundance 37C
Doses Blue Rep1_0.06 Red Rep2_−0.01
sp|Q15723−2|ELF2_HUMAN−113−S−S1_−0.08
ptm abundance 37C
Doses Blue Rep1_0.01 Red Rep2_−0.16
sp|Q96T58|MINT_HUMAN−1619−T−S1_−0.06
ptm abundance 37C
Doses Blue Rep1_−0.02 Red Rep2_−0.09

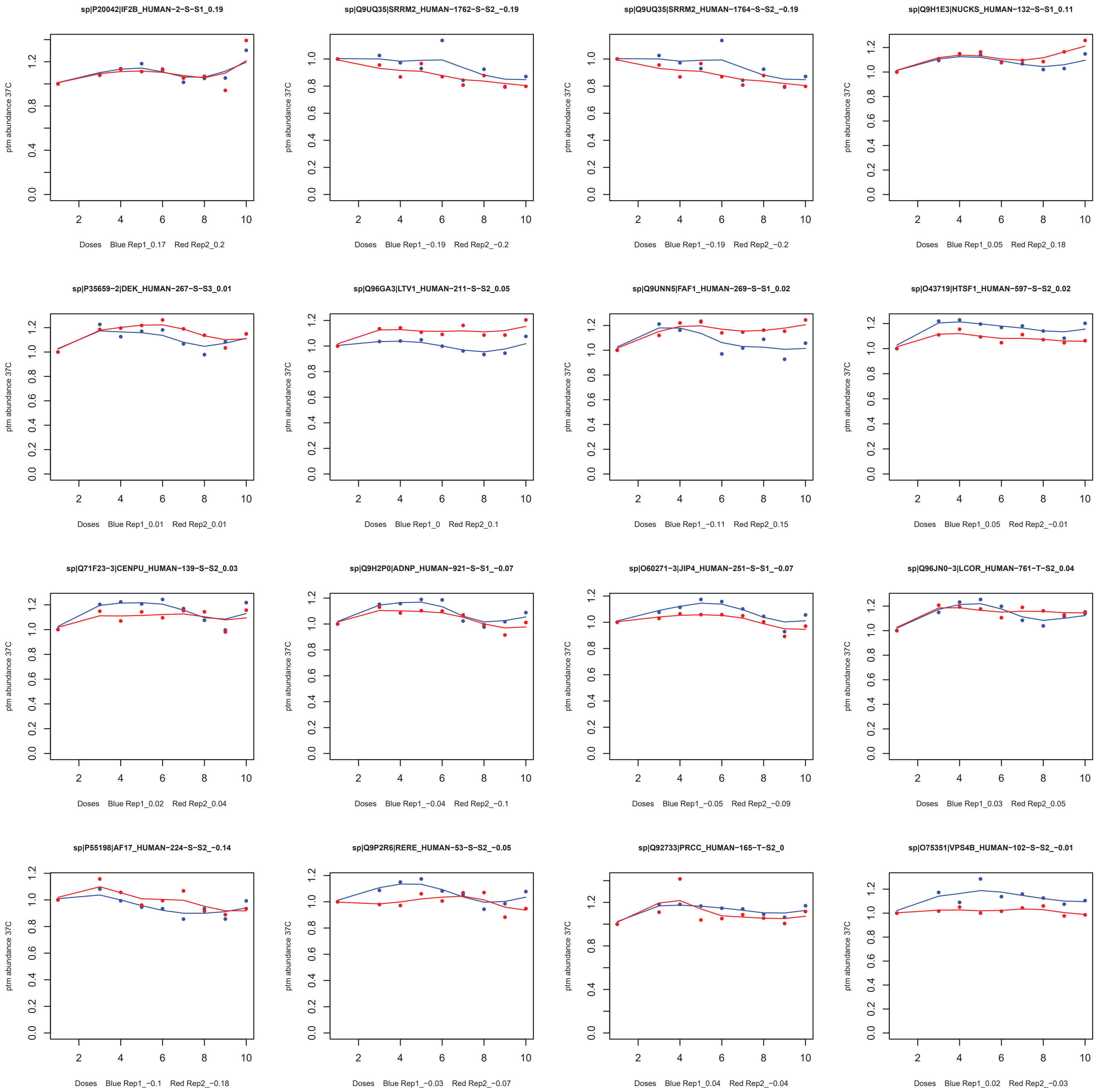
sp|P20042|IF2B_HUMAN−2−S−S1_0.19
ptm abundance 37C
Doses Blue Rep1_0.17 Red Rep2_0.2
sp|Q9UQ35|SRRM2_HUMAN−1762−S−S2_−0.19
ptm abundance 37C
Doses Blue Rep1_−0.19 Red Rep2_−0.2
sp|Q9UQ35|SRRM2_HUMAN−1764−S−S2_−0.19
ptm abundance 37C
Doses Blue Rep1_−0.19 Red Rep2_−0.2
sp|Q9H1E3|NUCKS_HUMAN−132−S−S1_0.11
ptm abundance 37C
Doses Blue Rep1_0.05 Red Rep2_0.18
sp|P35659−2|DEK_HUMAN−267−S−S3_0.01
ptm abundance 37C
Doses Blue Rep1_0.01 Red Rep2_0.01
sp|Q96GA3|LTV1_HUMAN−211−S−S2_0.05
ptm abundance 37C
Doses Blue Rep1_0 Red Rep2_0.1
sp|Q9UNN5|FAF1_HUMAN−269−S−S1_0.02
ptm abundance 37C
Doses Blue Rep1_−0.11 Red Rep2_0.15
sp|O43719|HTSF1_HUMAN−597−S−S2_0.02
ptm abundance 37C
Doses Blue Rep1_0.05 Red Rep2_−0.01
sp|Q71F23−3|CENPU_HUMAN−139−S−S2_0.03
ptm abundance 37C
Doses Blue Rep1_0.02 Red Rep2_0.04
sp|Q9H2P0|ADNP_HUMAN−921−S−S1_−0.07
ptm abundance 37C
Doses Blue Rep1_−0.04 Red Rep2_−0.1
sp|O60271−3|JIP4_HUMAN−251−S−S1_−0.07
ptm abundance 37C
Doses Blue Rep1_−0.05 Red Rep2_−0.09
sp|Q96JN0−3|LCOR_HUMAN−761−T−S2_0.04
ptm abundance 37C
Doses Blue Rep1_0.03 Red Rep2_0.05
sp|P55198|AF17_HUMAN−224−S−S2_−0.14
ptm abundance 37C
Doses Blue Rep1_−0.1 Red Rep2_−0.18
sp|Q9P2R6|RERE_HUMAN−53−S−S2_−0.05
ptm abundance 37C
Doses Blue Rep1_−0.03 Red Rep2_−0.07
sp|Q92733|PRCC_HUMAN−165−T−S2_0
ptm abundance 37C
Doses Blue Rep1_0.04 Red Rep2_−0.04
sp|O75351|VPS4B_HUMAN−102−S−S2_−0.01
ptm abundance 37C
Doses Blue Rep1_0.02 Red Rep2_−0.03

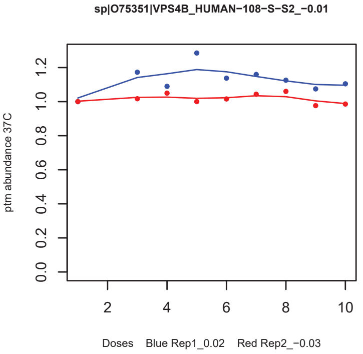
sp|O75351|VPS4B_HUMAN−108−S−S2_−0.01
ptm abundance 37C
Doses Blue Rep1_0.02 Red Rep2_−0.03

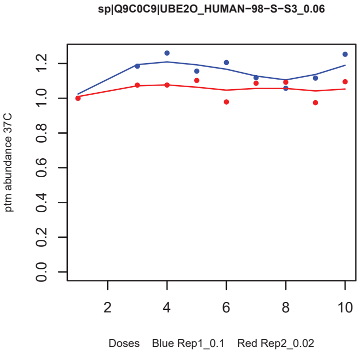
sp|Q9C0C9|UBE2O_HUMAN−98−S−S3_0.06
ptm abundance 37C
Doses Blue Rep1_0.1 Red Rep2_0.02

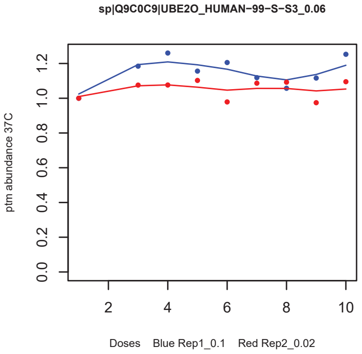
sp|Q9C0C9|UBE2O_HUMAN−99−S−S3_0.06
ptm abundance 37C
Doses Blue Rep1_0.1 Red Rep2_0.02

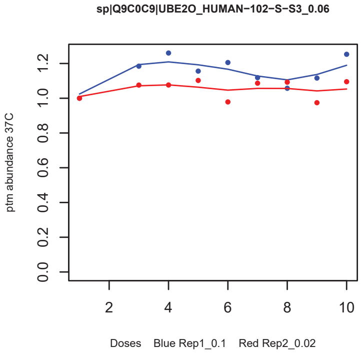
sp|Q9C0C9|UBE2O_HUMAN−102−S−S3_0.06
ptm abundance 37C
Doses Blue Rep1_0.1 Red Rep2_0.02

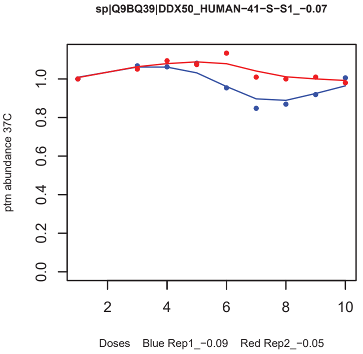
sp|Q9BQ39|DDX50_HUMAN−41−S−S1_−0.07
ptm abundance 37C
Doses Blue Rep1_−0.09 Red Rep2_−0.05

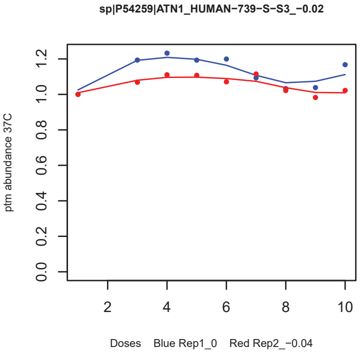
sp|P54259|ATN1_HUMAN−739−S−S3_−0.02
ptm abundance 37C
Doses Blue Rep1_0 Red Rep2_−0.04

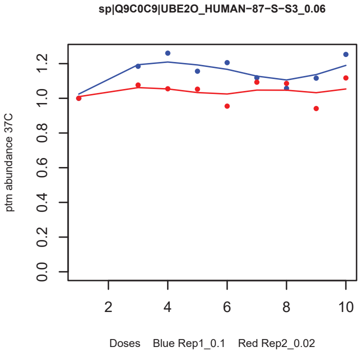
sp|Q9C0C9|UBE2O_HUMAN−87−S−S3_0.06
ptm abundance 37C
Doses Blue Rep1_0.1 Red Rep2_0.02

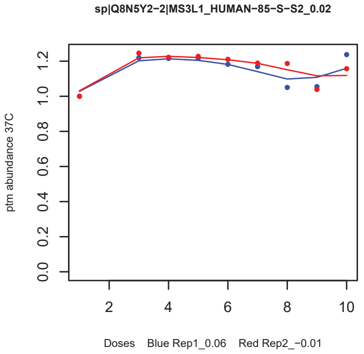
sp|Q8N5Y2−2|MS3L1_HUMAN−85−S−S2_0.02
ptm abundance 37C
Doses Blue Rep1_0.06 Red Rep2_−0.01

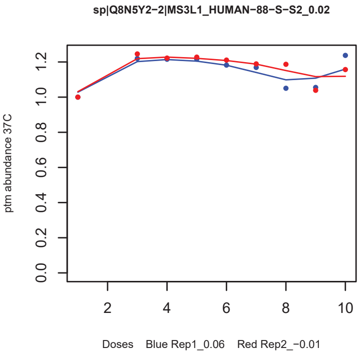
sp|Q8N5Y2−2|MS3L1_HUMAN−88−S−S2_0.02
ptm abundance 37C
Doses Blue Rep1_0.06 Red Rep2_−0.01

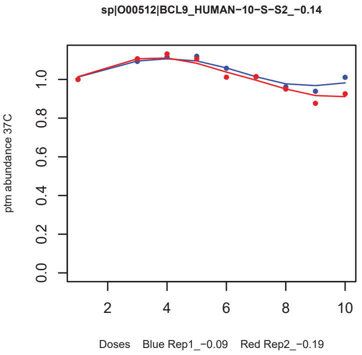
sp|O00512|BCL9_HUMAN−10−S−S2_−0.14
ptm abundance 37C
Doses Blue Rep1_−0.09 Red Rep2_−0.19

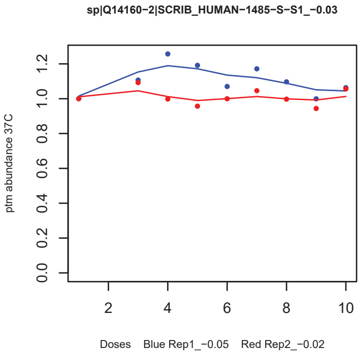
sp|Q14160−2|SCRIB_HUMAN−1485−S−S1_−0.03
ptm abundance 37C
Doses Blue Rep1_−0.05 Red Rep2_−0.02

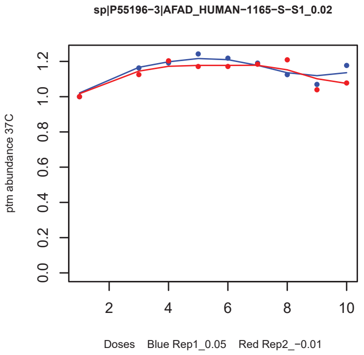
sp|P55196−3|AFAD_HUMAN−1165−S−S1_0.02
ptm abundance 37C
Doses Blue Rep1_0.05 Red Rep2_−0.01

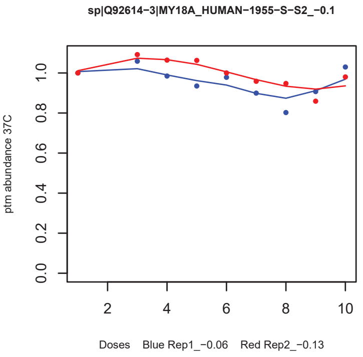
sp|Q92614−3|MY18A_HUMAN−1955−S−S2_−0.1
ptm abundance 37C
Doses Blue Rep1_−0.06 Red Rep2_−0.13

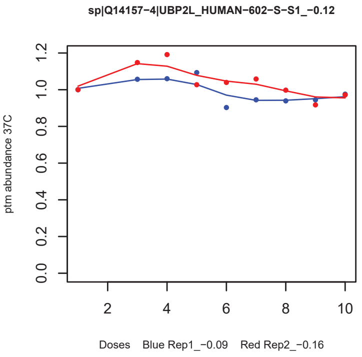
sp|Q14157−4|UBP2L_HUMAN−602−S−S1_−0.12
ptm abundance 37C
Doses Blue Rep1_−0.09 Red Rep2_−0.16

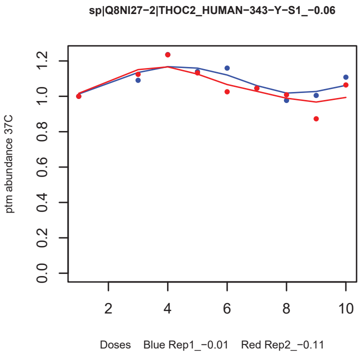
sp|Q8NI27−2|THOC2_HUMAN−343−Y−S1_−0.06
ptm abundance 37C
Doses Blue Rep1_−0.01 Red Rep2_−0.11

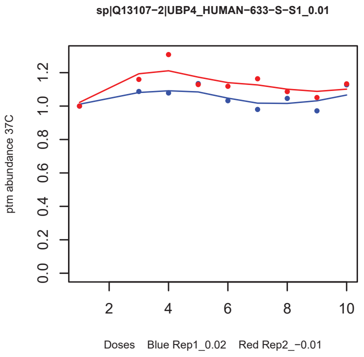
sp|Q13107−2|UBP4_HUMAN−633−S−S1_0.01
ptm abundance 37C
Doses Blue Rep1_0.02 Red Rep2_−0.01

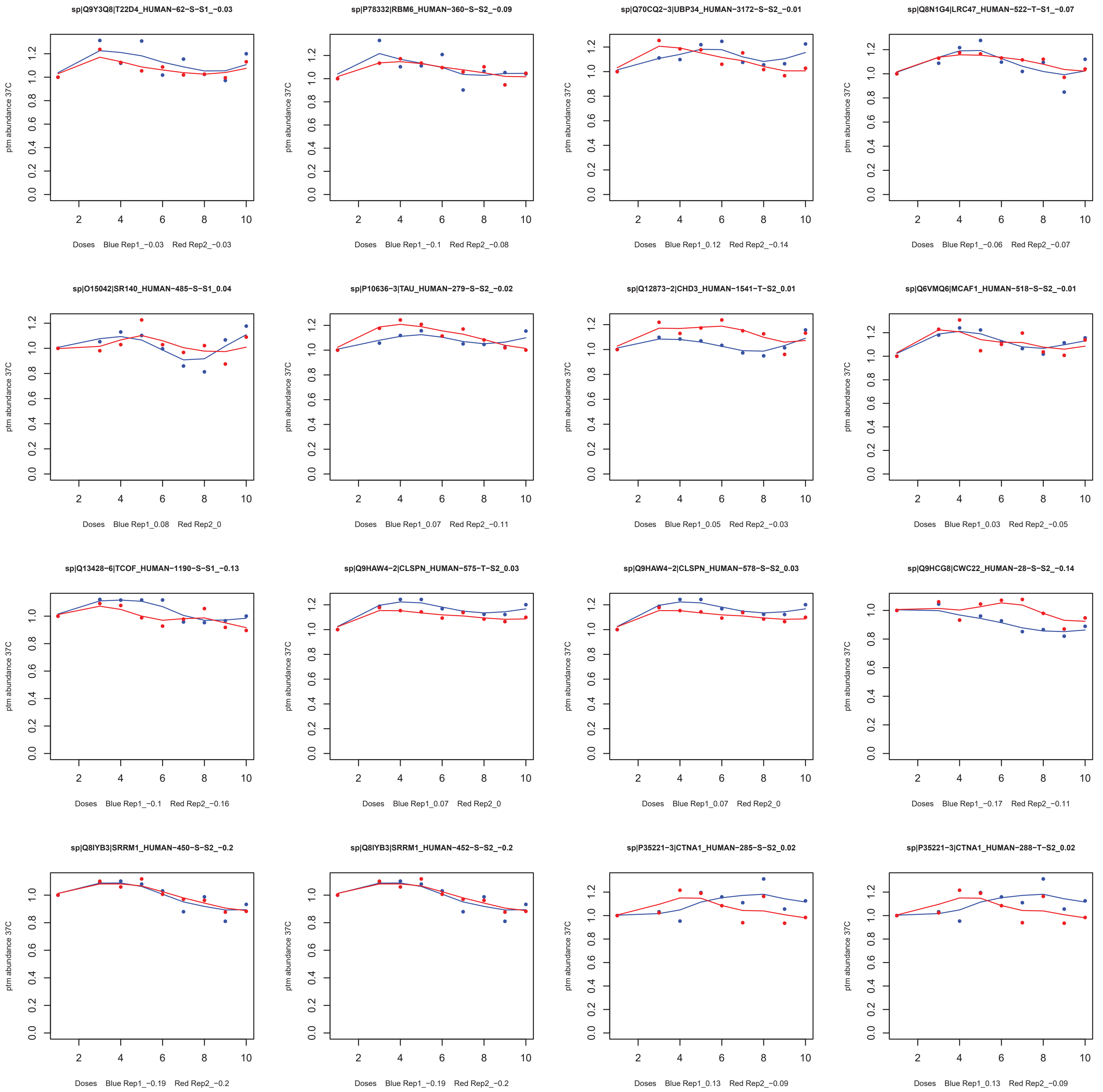
sp|Q9Y3Q8|T22D4_HUMAN−62−S−S1_−0.03
Doses Blue Rep1_−0.03 Red Rep2_−0.03
sp|P78332|RBM6_HUMAN−360−S−S2_−0.09
Doses Blue Rep1_−0.1 Red Rep2_−0.08
sp|Q70CQ2−3|UBP34_HUMAN−3172−S−S2_−0.01
Doses Blue Rep1_0.12 Red Rep2_−0.14
sp|Q8N1G4|LRC47_HUMAN−522−T−S1_−0.07
Doses Blue Rep1_−0.06 Red Rep2_−0.07
sp|O15042|SR140_HUMAN−485−S−S1_0.04
Doses Blue Rep1_0.08 Red Rep2_0
sp|P10636−3|TAU_HUMAN−279−S−S2_−0.02
Doses Blue Rep1_0.07 Red Rep2_−0.11
sp|Q12873−2|CHD3_HUMAN−1541−T−S2_0.01
Doses Blue Rep1_0.05 Red Rep2_−0.03
sp|Q6VMQ6|MCAF1_HUMAN−518−S−S2_−0.01
Doses Blue Rep1_0.03 Red Rep2_−0.05
sp|Q13428−6|TCOF_HUMAN−1190−S−S1_−0.13
Doses Blue Rep1_−0.1 Red Rep2_−0.16
sp|Q9HAW4−2|CLSPN_HUMAN−575−T−S2_0.03
Doses Blue Rep1_0.07 Red Rep2_0
sp|Q9HAW4−2|CLSPN_HUMAN−578−S−S2_0.03
Doses Blue Rep1_0.07 Red Rep2_0
sp|Q9HCG8|CWC22_HUMAN−28−S−S2_−0.14
Doses Blue Rep1_−0.17 Red Rep2_−0.11
sp|Q8IYB3|SRRM1_HUMAN−450−S−S2_−0.2
Doses Blue Rep1_−0.19 Red Rep2_−0.2
sp|Q8IYB3|SRRM1_HUMAN−452−S−S2_−0.2
Doses Blue Rep1_−0.19 Red Rep2_−0.2
sp|P35221−3|CTNA1_HUMAN−285−S−S2_0.02
Doses Blue Rep1_0.13 Red Rep2_−0.09
sp|P35221−3|CTNA1_HUMAN−288−T−S2_0.02
Doses Blue Rep1_0.13 Red Rep2_−0.09
ptm abundance 37C

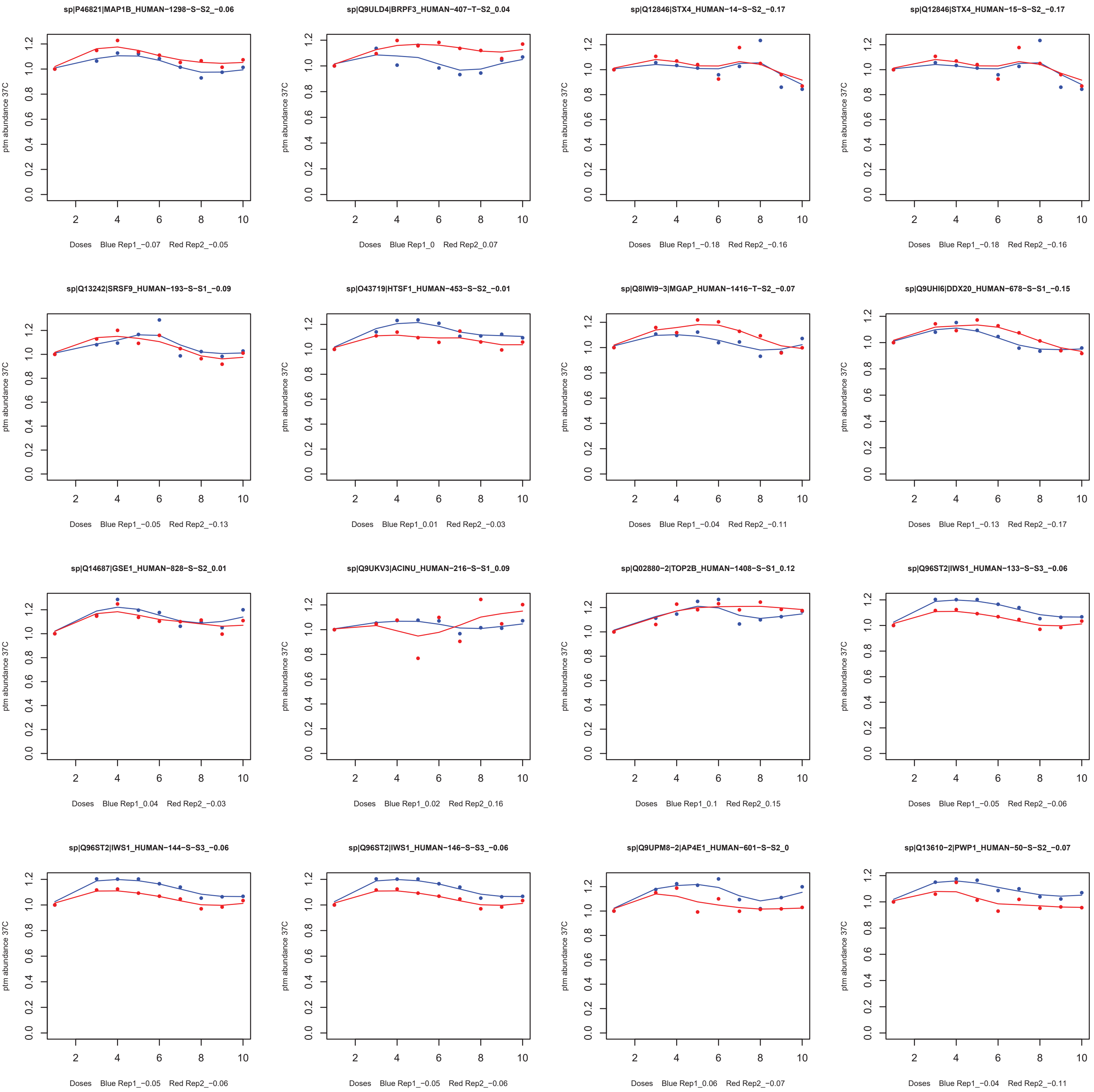
sp|P46821|MAP1B_HUMAN−1298−S−S2_−0.06
Doses Blue Rep1_−0.07 Red Rep2_−0.05
sp|Q9ULD4|BRPF3_HUMAN−407−T−S2_0.04
Doses Blue Rep1_0 Red Rep2_0.07
sp|Q12846|STX4_HUMAN−14−S−S2_−0.17
Doses Blue Rep1_−0.18 Red Rep2_−0.16
sp|Q12846|STX4_HUMAN−15−S−S2_−0.17
Doses Blue Rep1_−0.18 Red Rep2_−0.16
sp|Q13242|SRSF9_HUMAN−193−S−S1_−0.09
Doses Blue Rep1_−0.05 Red Rep2_−0.13
sp|O43719|HTSF1_HUMAN−453−S−S2_−0.01
Doses Blue Rep1_0.01 Red Rep2_−0.03
sp|Q8IWI9−3|MGAP_HUMAN−1416−T−S2_−0.07
Doses Blue Rep1_−0.04 Red Rep2_−0.11
sp|Q9UHI6|DDX20_HUMAN−678−S−S1_−0.15
Doses Blue Rep1_−0.13 Red Rep2_−0.17
sp|Q14687|GSE1_HUMAN−828−S−S2_0.01
Doses Blue Rep1_0.04 Red Rep2_−0.03
sp|Q9UKV3|ACINU_HUMAN−216−S−S1_0.09
Doses Blue Rep1_0.02 Red Rep2_0.16
sp|Q02880−2|TOP2B_HUMAN−1408−S−S1_0.12
Doses Blue Rep1_0.1 Red Rep2_0.15
sp|Q96ST2|IWS1_HUMAN−133−S−S3_−0.06
Doses Blue Rep1_−0.05 Red Rep2_−0.06
sp|Q96ST2|IWS1_HUMAN−144−S−S3_−0.06
Doses Blue Rep1_−0.05 Red Rep2_−0.06
sp|Q96ST2|IWS1_HUMAN−146−S−S3_−0.06
Doses Blue Rep1_−0.05 Red Rep2_−0.06
sp|Q9UPM8−2|AP4E1_HUMAN−601−S−S2_0
Doses Blue Rep1_0.06 Red Rep2_−0.07
sp|Q13610−2|PWP1_HUMAN−50−S−S2_−0.07
Doses Blue Rep1_−0.04 Red Rep2_−0.11
ptm abundance 37C

sp|Q9NTI5|PDS5B_HUMAN−1166−S−S1_−0.12

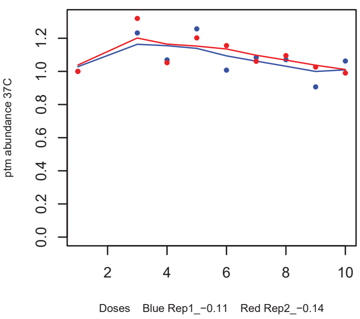

sp|Q96JM3|CHAP1_HUMAN−445−S−S1_−0.06

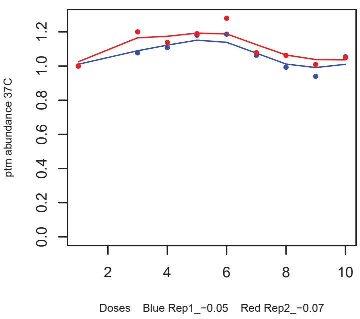

sp|Q99590−2|SCAFB_HUMAN−461−S−S1_−0.14

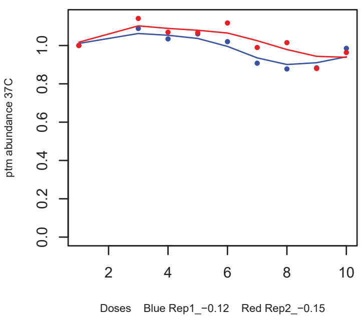

sp|Q9UER7|DAXX_HUMAN−737−S−S2_0.05

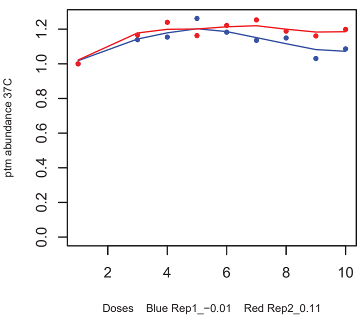

sp|Q9UER7|DAXX_HUMAN−739−S−S2_0.05

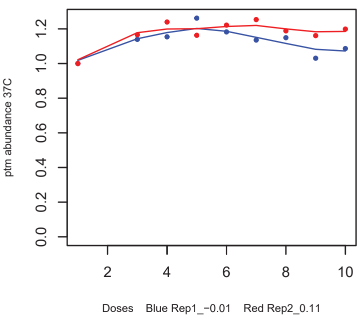

sp|Q92974−3|ARHG2_HUMAN−913−S−S2_0.09

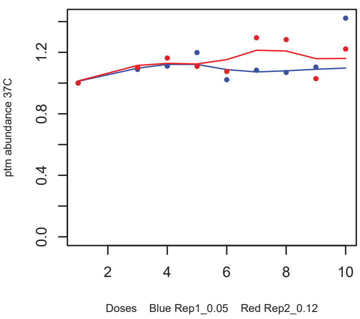

sp|Q9BRD0|BUD13_HUMAN−364−S−S3_−0.02

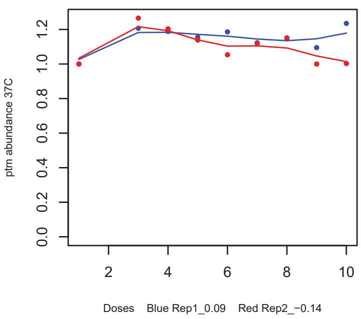

sp|Q8TAQ2−2|SMRC2_HUMAN−304−S−S3_−0.09

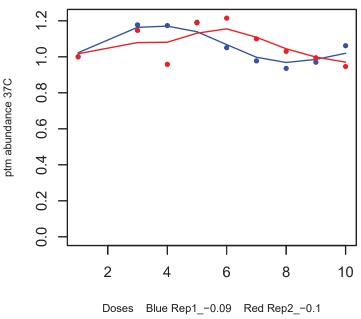

sp|Q5XUX1−3|FBXW9_HUMAN−22−S−S2_−0.07

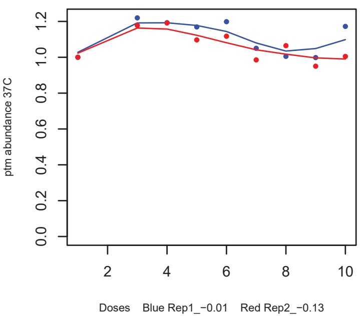

sp|O75475|PSIP1_HUMAN−134−T−S1_−0.04

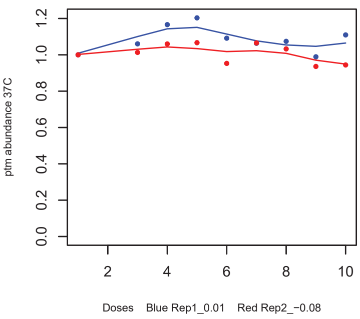

sp|P35269|T2FA_HUMAN−307−S−S3_0.12

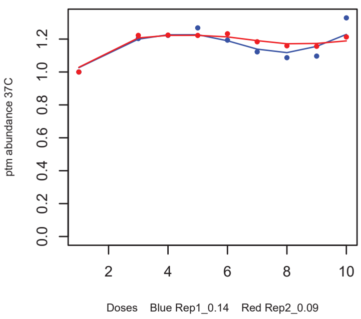

sp|P35269|T2FA_HUMAN−308−S−S3_0.12

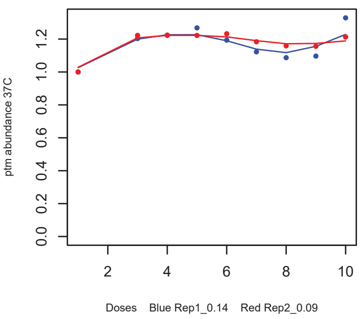

sp|P35269|T2FA_HUMAN−311−S−S3_0.12

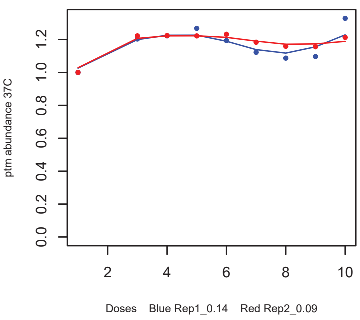

sp|P00519−2|ABL1_HUMAN−827−S−S2_−0.13

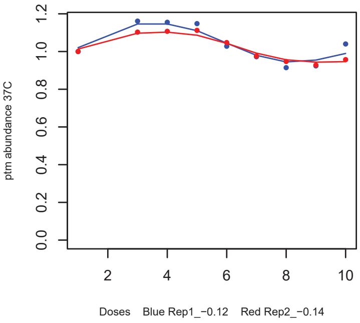

sp|Q8N163−2|CCAR2_HUMAN−675−S−S2_0.03

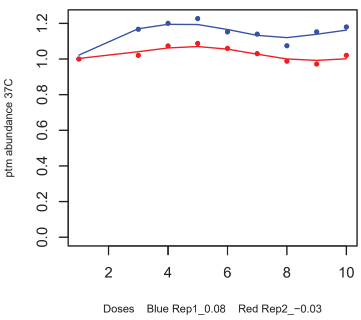

sp|Q12894|IFRD2_HUMAN−93−S−S3_0.01

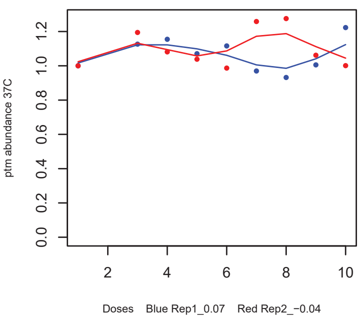

sp|Q12894|IFRD2_HUMAN-95-S-S3_0.01

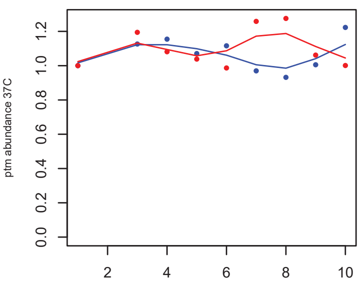

Doses Blue Rep1_0.07 Red Rep2_-0.04

sp|Q12894|IFRD2_HUMAN-96-S-S3_0.01

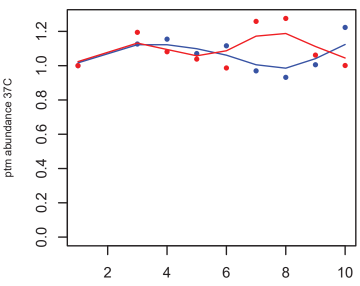

Doses Blue Rep1_0.07 Red Rep2_-0.04

sp|Q8IX07|FOG1_HUMAN-61-S-S2_-0.05

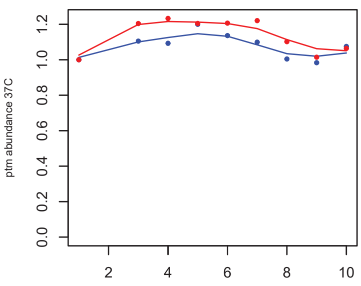

Doses Blue Rep1_-0.02 Red Rep2_-0.08

sp|Q5VT06|CE350_HUMAN-1828-S-S2_-0.08

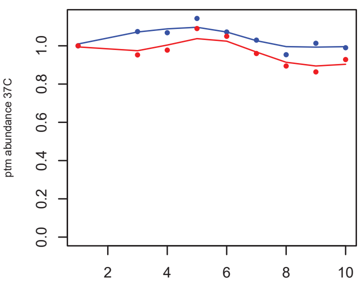

Doses Blue Rep1_-0.06 Red Rep2_-0.1

sp|Q8IWW6-3|RHG12_HUMAN-231-T-S2_-0.15

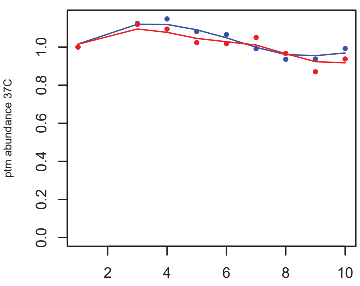

Doses Blue Rep1_-0.12 Red Rep2_-0.17

sp|Q9Y6K1|DNM3A_HUMAN-251-T-S3_0.13

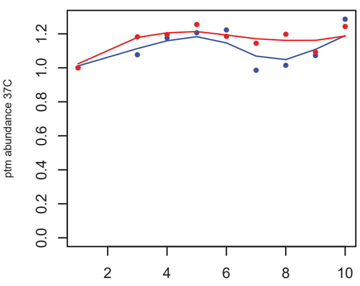

Doses Blue Rep1_0.16 Red Rep2_0.11

sp|Q9Y6K1|DNM3A_HUMAN-255-S-S3_0.13

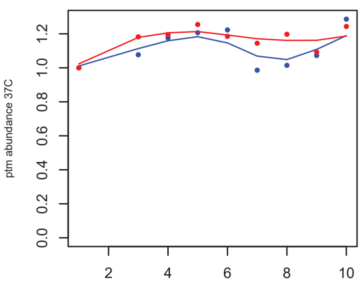

Doses Blue Rep1_0.16 Red Rep2_0.11

sp|O43314-2|VIP2_HUMAN-493-S-S2_-0.03

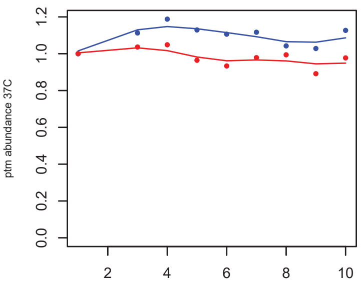

Doses Blue Rep1_0.02 Red Rep2_-0.09

sp|Q9Y4B4|ARIP4_HUMAN-1155-S-S1_-0.05

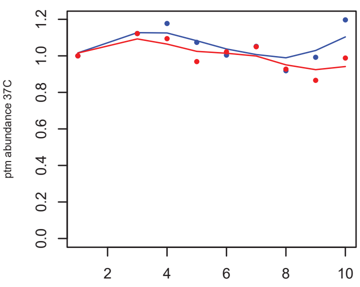

Doses Blue Rep1_0.04 Red Rep2_-0.14

sp|Q641Q2-2|WAC2A_HUMAN-1158-S-S2_0.09

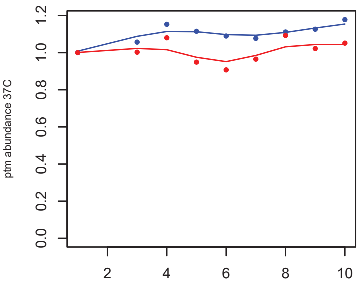

Doses Blue Rep1_0.13 Red Rep2_0.04

sp|Q641Q2-2|WAC2A_HUMAN-1159-S-S2_0.09

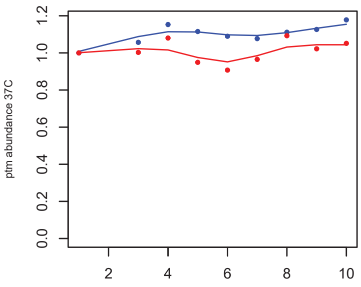

Doses Blue Rep1_0.13 Red Rep2_0.04

sp|Q9Y2K6|UBP20_HUMAN-132-S-S1_0.03

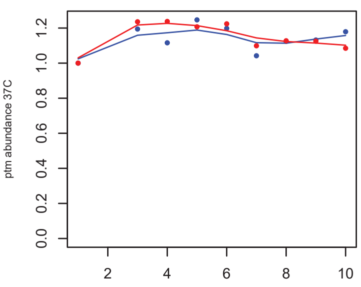

Doses Blue Rep1_0.08 Red Rep2_-0.03

sp|P18858|DNLI1_HUMAN-918-T-S2_0.02

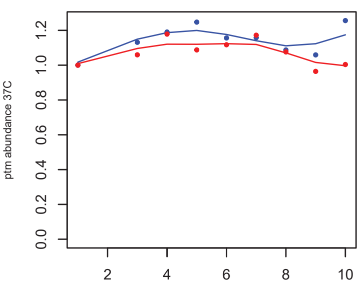

Doses Blue Rep1_0.11 Red Rep2_-0.07

sp|Q00839-2|HNRPU_HUMAN-4-S-S2_-0.09

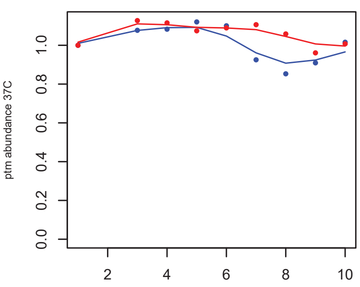

Doses Blue Rep1_-0.1 Red Rep2_-0.08

sp|Q9Y383-3|LC7L2_HUMAN-381-S-S2_0.09

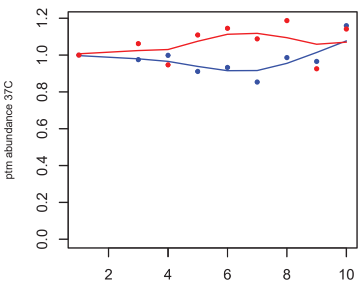

Doses Blue Rep1_0.11 Red Rep2_0.07

sp|Q15649-2|ZNHI3_HUMAN-80-S-S1_0.01

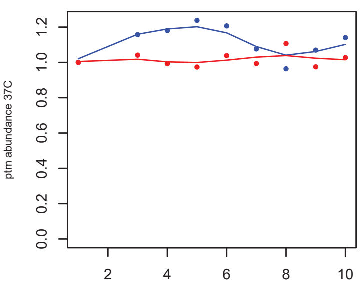

Doses Blue Rep1_0.01 Red Rep2_0

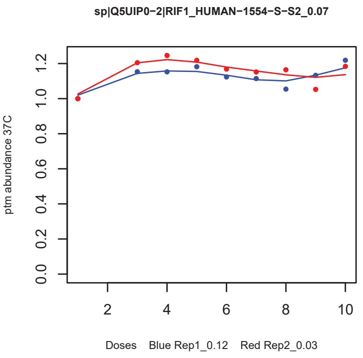
sp|Q5UIP0−2|RIF1_HUMAN−1554−S−S2_0.07
ptm abundance 37C
Doses Blue Rep1_0.12 Red Rep2_0.03

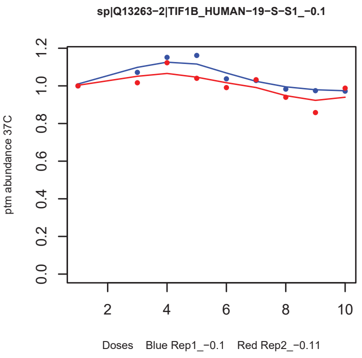
sp|Q13263−2|TIF1B_HUMAN−19−S−S1_−0.1
ptm abundance 37C
Doses Blue Rep1_−0.1 Red Rep2_−0.11

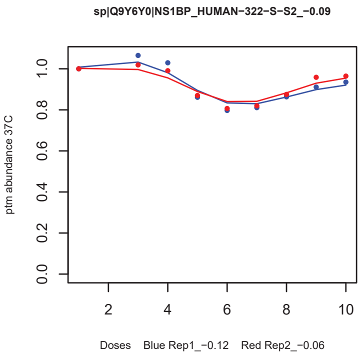
sp|Q9Y6Y0|NS1BP_HUMAN−322−S−S2_−0.09
ptm abundance 37C
Doses Blue Rep1_−0.12 Red Rep2_−0.06

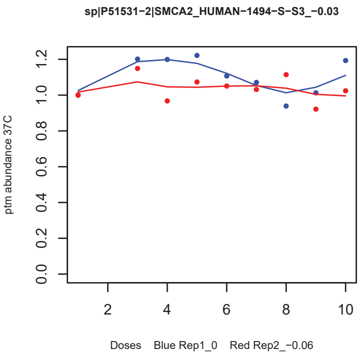
sp|P51531−2|SMCA2_HUMAN−1494−S−S3_−0.03
ptm abundance 37C
Doses Blue Rep1_0 Red Rep2_−0.06

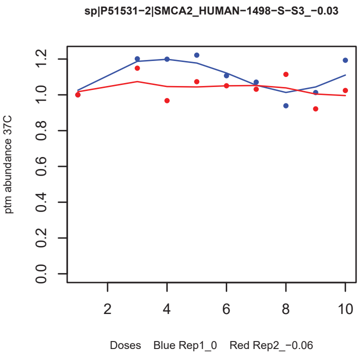
sp|P51531−2|SMCA2_HUMAN−1498−S−S3_−0.03
ptm abundance 37C
Doses Blue Rep1_0 Red Rep2_−0.06

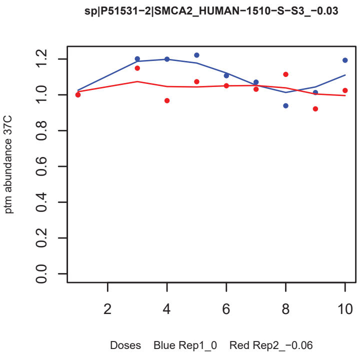
sp|P51531−2|SMCA2_HUMAN−1510−S−S3_−0.03
ptm abundance 37C
Doses Blue Rep1_0 Red Rep2_−0.06

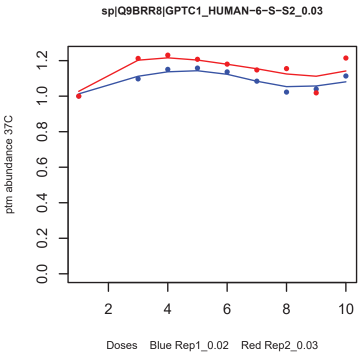
sp|Q9BRR8|GPTC1_HUMAN−6−S−S2_0.03
ptm abundance 37C
Doses Blue Rep1_0.02 Red Rep2_0.03

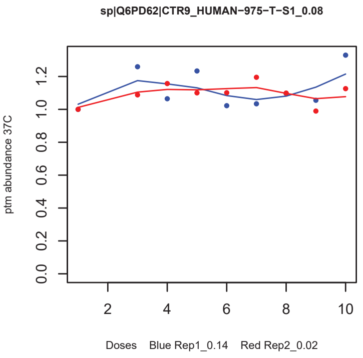
sp|Q6PD62|CTR9_HUMAN−975−T−S1_0.08
ptm abundance 37C
Doses Blue Rep1_0.14 Red Rep2_0.02

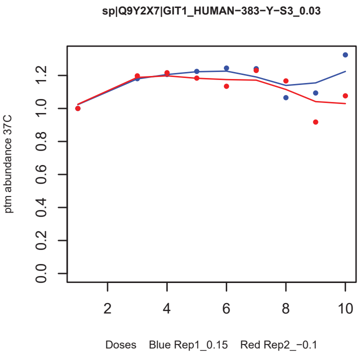
sp|Q9Y2X7|GIT1_HUMAN−383−Y−S3_0.03
ptm abundance 37C
Doses Blue Rep1_0.15 Red Rep2_−0.1

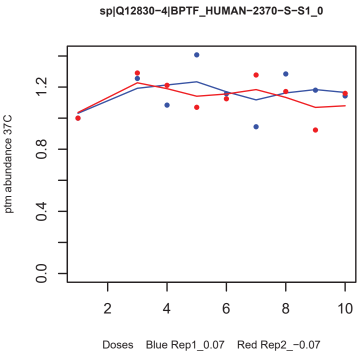
sp|Q12830−4|BPTF_HUMAN−2370−S−S1_0
ptm abundance 37C
Doses Blue Rep1_0.07 Red Rep2_−0.07

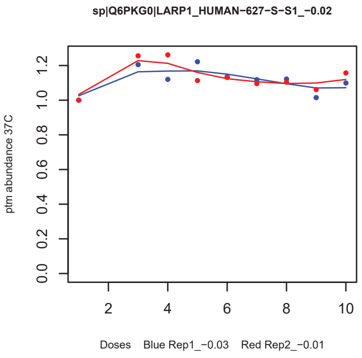
sp|Q6PKG0|LARP1_HUMAN−627−S−S1_−0.02
ptm abundance 37C
Doses Blue Rep1_−0.03 Red Rep2_−0.01

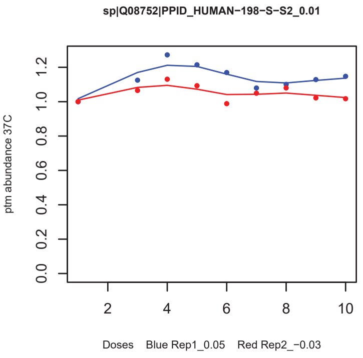
sp|Q08752|PPID_HUMAN−198−S−S2_0.01
ptm abundance 37C
Doses Blue Rep1_0.05 Red Rep2_−0.03

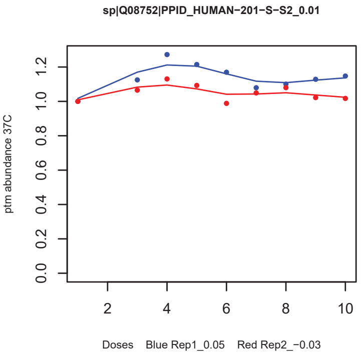
sp|Q08752|PPID_HUMAN−201−S−S2_0.01
ptm abundance 37C
Doses Blue Rep1_0.05 Red Rep2_−0.03

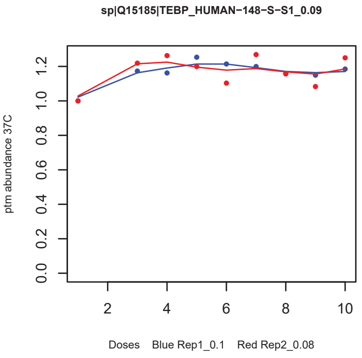
sp|Q15185|TEBP_HUMAN−148−S−S1_0.09
ptm abundance 37C
Doses Blue Rep1_0.1 Red Rep2_0.08

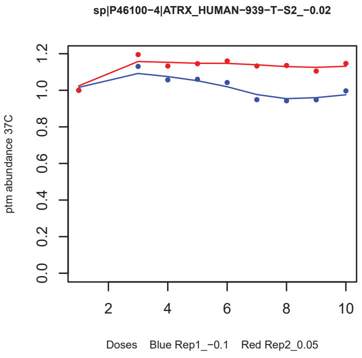
sp|P46100−4|ATRX_HUMAN−939−T−S2_−0.02
ptm abundance 37C
Doses Blue Rep1_−0.1 Red Rep2_0.05

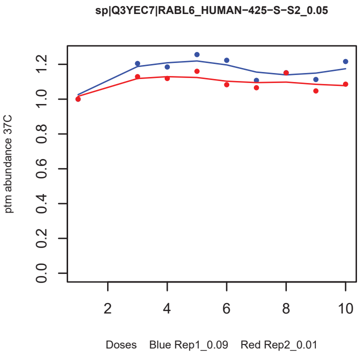
sp|Q3YEC7|RABL6_HUMAN−425−S−S2_0.05
ptm abundance 37C
Doses Blue Rep1_0.09 Red Rep2_0.01

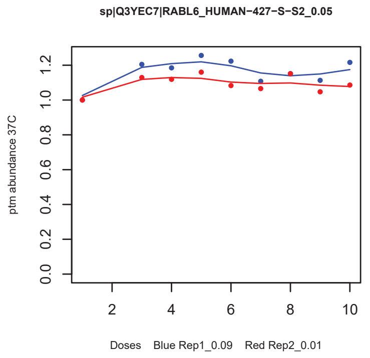
sp|Q3YEC7|RABL6_HUMAN−427−S−S2_0.05
ptm abundance 37C
Doses Blue Rep1_0.09 Red Rep2_0.01
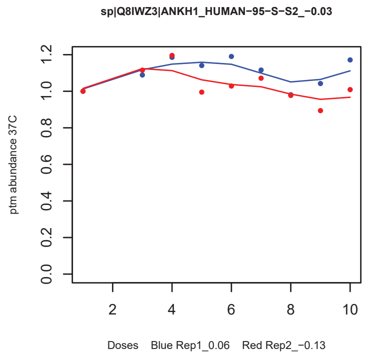
sp|Q8IWZ3|ANKH1_HUMAN−95−S−S2_−0.03
ptm abundance 37C
Doses Blue Rep1_0.06 Red Rep2_−0.13
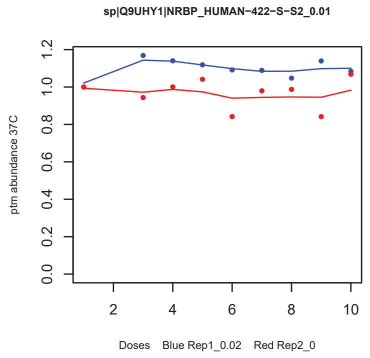
sp|Q9UHY1|NRBP_HUMAN−422−S−S2_0.01
ptm abundance 37C
Doses Blue Rep1_0.02 Red Rep2_0
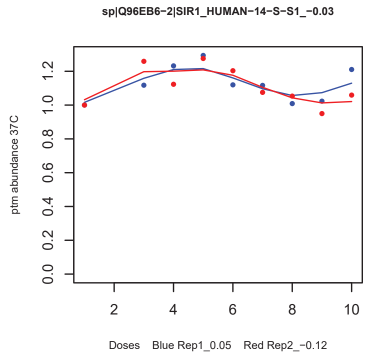
sp|Q96EB6−2|SIR1_HUMAN−14−S−S1_−0.03
ptm abundance 37C
Doses Blue Rep1_0.05 Red Rep2_−0.12
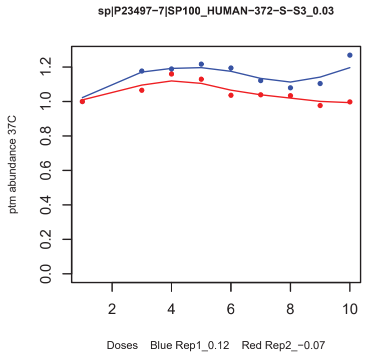
sp|P23497−7|SP100_HUMAN−372−S−S3_0.03
ptm abundance 37C
Doses Blue Rep1_0.12 Red Rep2_−0.07
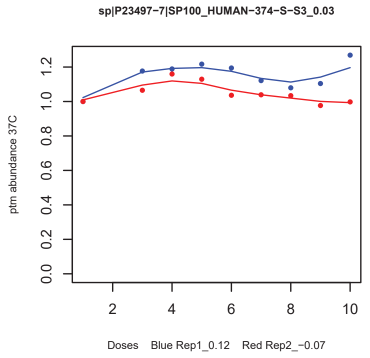
sp|P23497−7|SP100_HUMAN−374−S−S3_0.03
ptm abundance 37C
Doses Blue Rep1_0.12 Red Rep2_−0.07
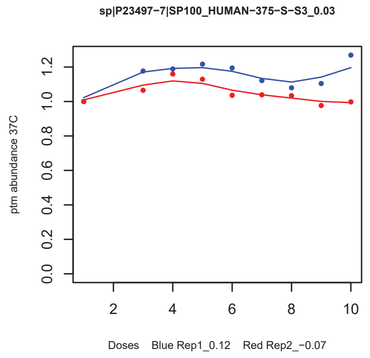
sp|P23497−7|SP100_HUMAN−375−S−S3_0.03
ptm abundance 37C
Doses Blue Rep1_0.12 Red Rep2_−0.07
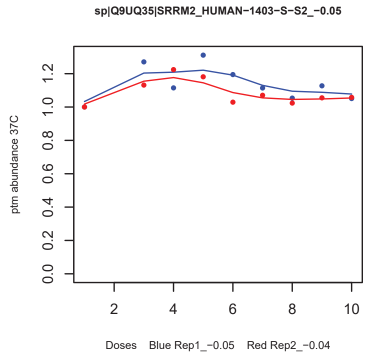
sp|Q9UQ35|SRRM2_HUMAN−1403−S−S2_−0.05
ptm abundance 37C
Doses Blue Rep1_−0.05 Red Rep2_−0.04
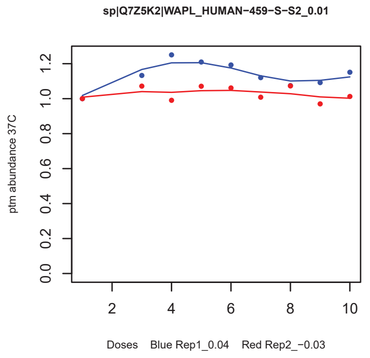
sp|Q7Z5K2|WAPL_HUMAN−459−S−S2_0.01
ptm abundance 37C
Doses Blue Rep1_0.04 Red Rep2_−0.03
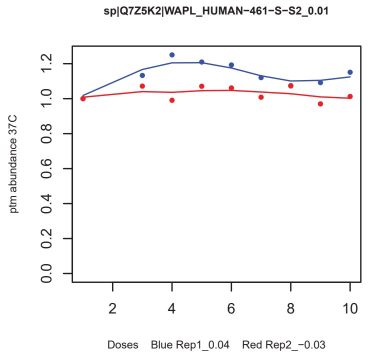
sp|Q7Z5K2|WAPL_HUMAN−461−S−S2_0.01
ptm abundance 37C
Doses Blue Rep1_0.04 Red Rep2_−0.03
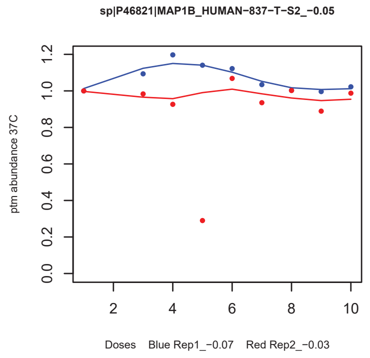
sp|P46821|MAP1B_HUMAN−837−T−S2_−0.05
ptm abundance 37C
Doses Blue Rep1_−0.07 Red Rep2_−0.03
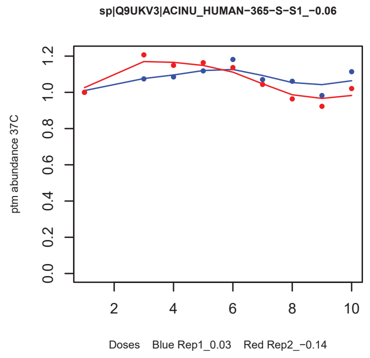
sp|Q9UKV3|ACINU_HUMAN−365−S−S1_−0.06
ptm abundance 37C
Doses Blue Rep1_0.03 Red Rep2_−0.14
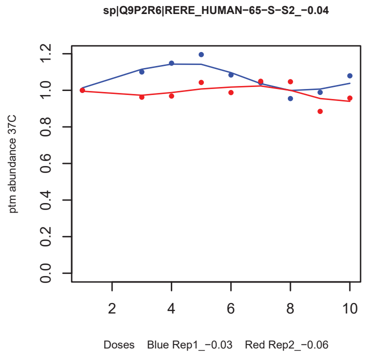
sp|Q9P2R6|RERE_HUMAN−65−S−S2_−0.04
ptm abundance 37C
Doses Blue Rep1_−0.03 Red Rep2_−0.06
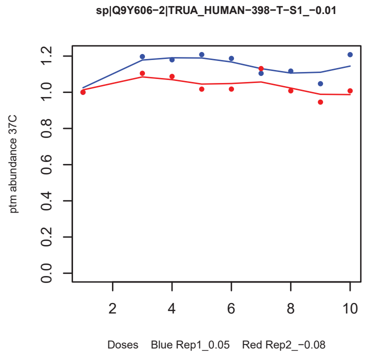
sp|Q9Y606−2|TRUA_HUMAN−398−T−S1_−0.01
ptm abundance 37C
Doses Blue Rep1_0.05 Red Rep2_−0.08
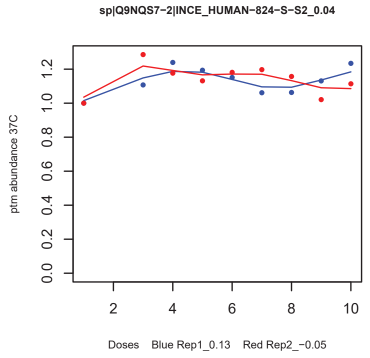
sp|Q9NQS7−2|INCE_HUMAN−824−S−S2_0.04
ptm abundance 37C
Doses Blue Rep1_0.13 Red Rep2_−0.05
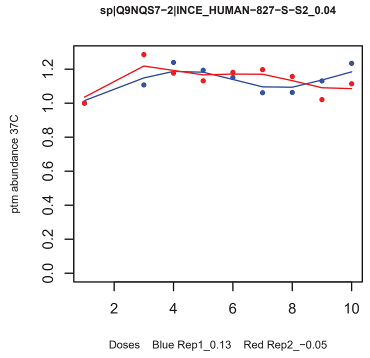
sp|Q9NQS7−2|INCE_HUMAN−827−S−S2_0.04
ptm abundance 37C
Doses Blue Rep1_0.13 Red Rep2_−0.05

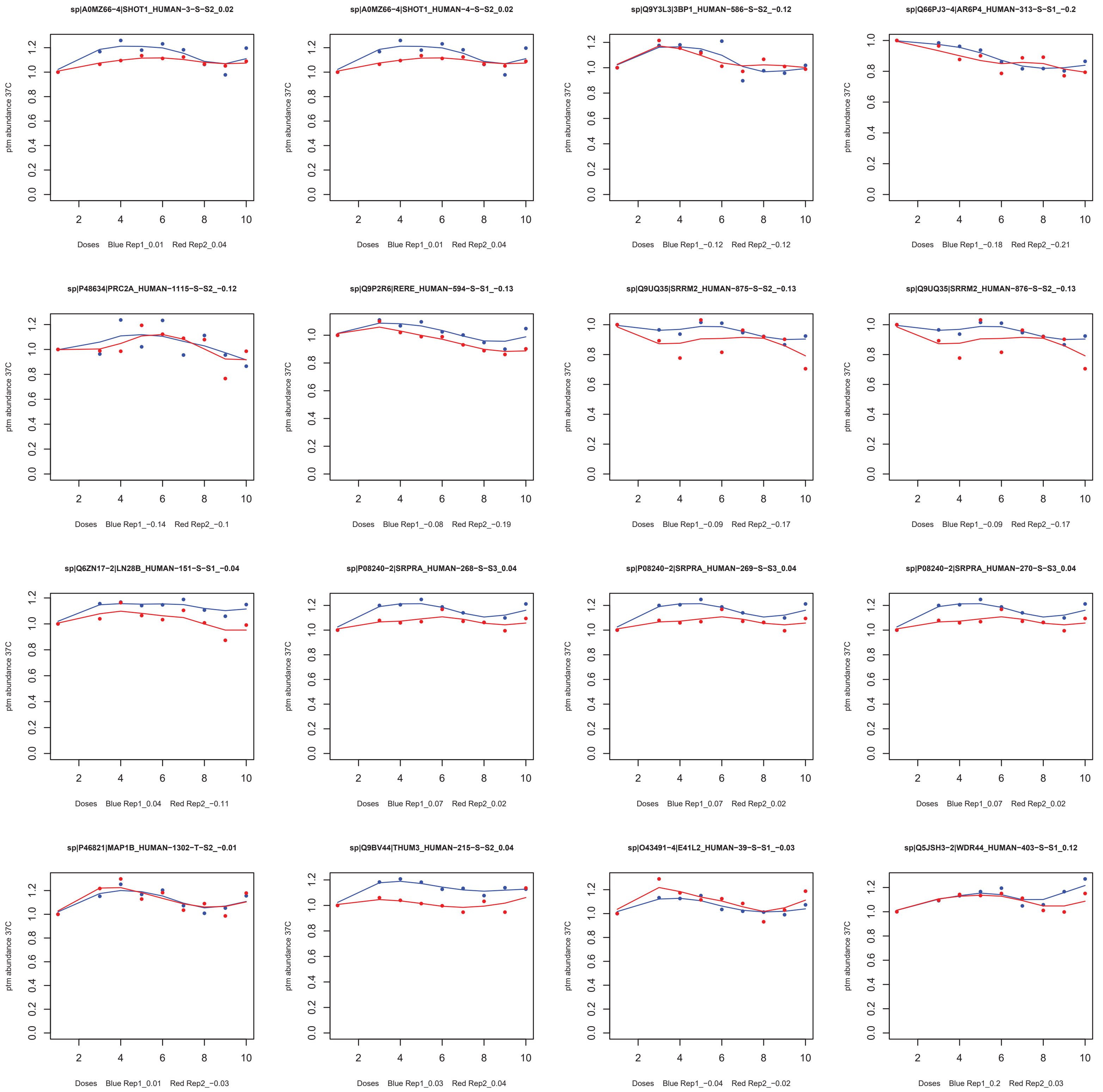
sp|A0MZ66−4|SHOT1_HUMAN−3−S−S2_0.02
ptm abundance 37C
Doses Blue Rep1_0.01 Red Rep2_0.04
sp|A0MZ66−4|SHOT1_HUMAN−4−S−S2_0.02
ptm abundance 37C
Doses Blue Rep1_0.01 Red Rep2_0.04
sp|Q9Y3L3|3BP1_HUMAN−586−S−S2_−0.12
ptm abundance 37C
Doses Blue Rep1_−0.12 Red Rep2_−0.12
sp|Q66PJ3−4|AR6P4_HUMAN−313−S−S1_−0.2
ptm abundance 37C
Doses Blue Rep1_−0.18 Red Rep2_−0.21
sp|P48634|PRC2A_HUMAN−1115−S−S2_−0.12
ptm abundance 37C
Doses Blue Rep1_−0.14 Red Rep2_−0.1
sp|Q9P2R6|RERE_HUMAN−594−S−S1_−0.13
ptm abundance 37C
Doses Blue Rep1_−0.08 Red Rep2_−0.19
sp|Q9UQ35|SRRM2_HUMAN−875−S−S2_−0.13
ptm abundance 37C
Doses Blue Rep1_−0.09 Red Rep2_−0.17
sp|Q9UQ35|SRRM2_HUMAN−876−S−S2_−0.13
ptm abundance 37C
Doses Blue Rep1_−0.09 Red Rep2_−0.17
sp|Q6ZN17−2|LN28B_HUMAN−151−S−S1_−0.04
ptm abundance 37C
Doses Blue Rep1_0.04 Red Rep2_−0.11
sp|P08240−2|SRPRA_HUMAN−268−S−S3_0.04
ptm abundance 37C
Doses Blue Rep1_0.07 Red Rep2_0.02
sp|P08240−2|SRPRA_HUMAN−269−S−S3_0.04
ptm abundance 37C
Doses Blue Rep1_0.07 Red Rep2_0.02
sp|P08240−2|SRPRA_HUMAN−270−S−S3_0.04
ptm abundance 37C
Doses Blue Rep1_0.07 Red Rep2_0.02
sp|P46821|MAP1B_HUMAN−1302−T−S2_−0.01
ptm abundance 37C
Doses Blue Rep1_0.01 Red Rep2_−0.03
sp|Q9BV44|THUM3_HUMAN−215−S−S2_0.04
ptm abundance 37C
Doses Blue Rep1_0.03 Red Rep2_0.04
sp|O43491−4|E41L2_HUMAN−39−S−S1_−0.03
ptm abundance 37C
Doses Blue Rep1_−0.04 Red Rep2_−0.02
sp|Q5JSH3−2|WDR44_HUMAN−403−S−S1_0.12
ptm abundance 37C
Doses Blue Rep1_0.2 Red Rep2_0.03

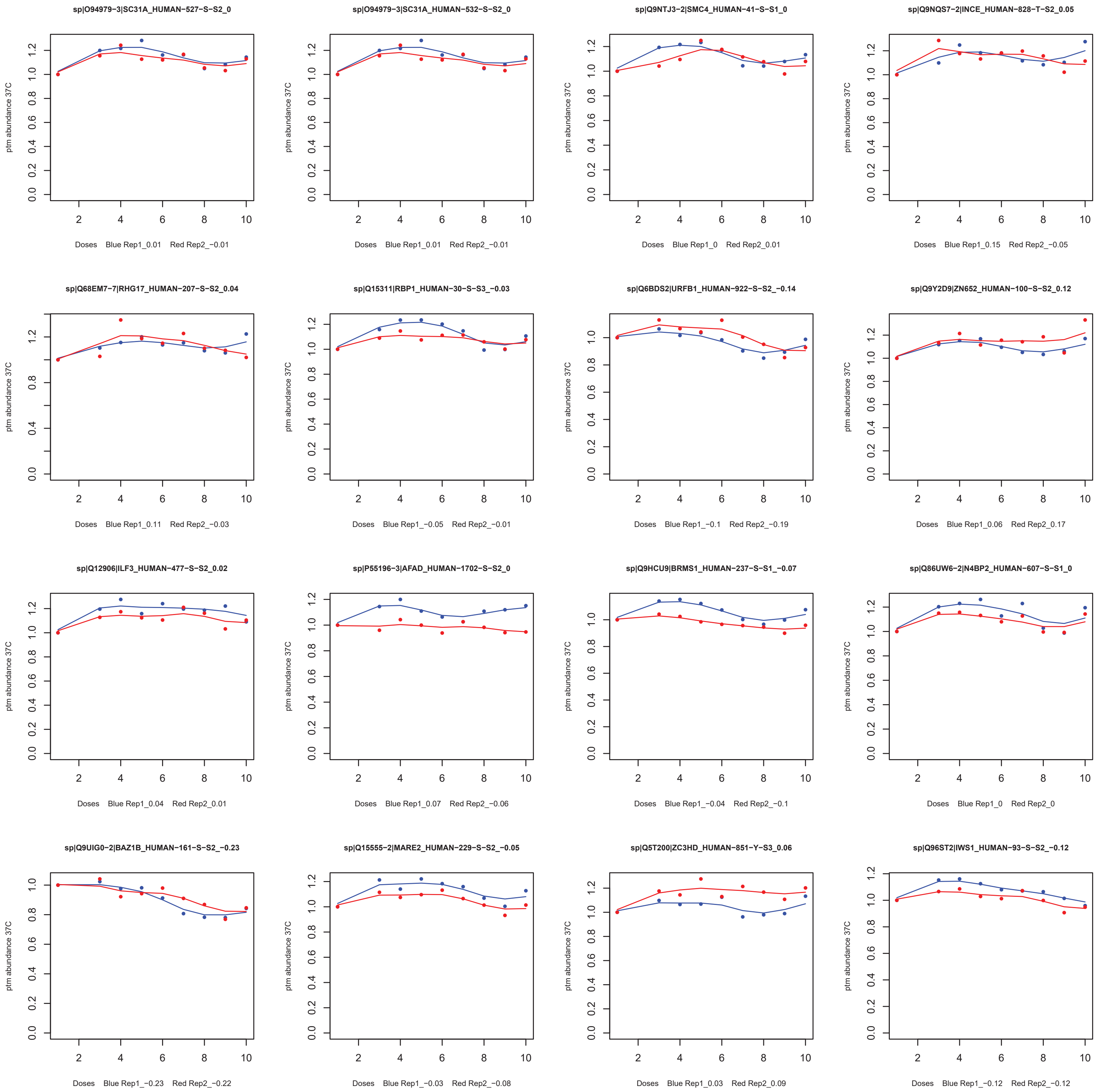

sp|O94979−3|SC31A_HUMAN−527−S−S2_0
ptm abundance 37C
Doses Blue Rep1_0.01 Red Rep2_−0.01
sp|O94979−3|SC31A_HUMAN−532−S−S2_0
ptm abundance 37C
Doses Blue Rep1_0.01 Red Rep2_−0.01
sp|Q9NTJ3−2|SMC4_HUMAN−41−S−S1_0
ptm abundance 37C
Doses Blue Rep1_0 Red Rep2_0.01
sp|Q9NQS7−2|INCE_HUMAN−828−T−S2_0.05
ptm abundance 37C
Doses Blue Rep1_0.15 Red Rep2_−0.05
sp|Q68EM7−7|RHG17_HUMAN−207−S−S2_0.04
ptm abundance 37C
Doses Blue Rep1_0.11 Red Rep2_−0.03
sp|Q15311|RBP1_HUMAN−30−S−S3_−0.03
ptm abundance 37C
Doses Blue Rep1_−0.05 Red Rep2_−0.01
sp|Q6BDS2|URFB1_HUMAN−922−S−S2_−0.14
ptm abundance 37C
Doses Blue Rep1_−0.1 Red Rep2_−0.19
sp|Q9Y2D9|ZN652_HUMAN−100−S−S2_0.12
ptm abundance 37C
Doses Blue Rep1_0.06 Red Rep2_0.17
sp|Q12906|ILF3_HUMAN−477−S−S2_0.02
ptm abundance 37C
Doses Blue Rep1_0.04 Red Rep2_0.01
sp|P55196−3|AFAD_HUMAN−1702−S−S2_0
ptm abundance 37C
Doses Blue Rep1_0.07 Red Rep2_−0.06
sp|Q9HCU9|BRMS1_HUMAN−237−S−S1_−0.07
ptm abundance 37C
Doses Blue Rep1_−0.04 Red Rep2_−0.1
sp|Q86UW6−2|N4BP2_HUMAN−607−S−S1_0
ptm abundance 37C
Doses Blue Rep1_0 Red Rep2_0
sp|Q9UIG0−2|BAZ1B_HUMAN−161−S−S2_−0.23
ptm abundance 37C
Doses Blue Rep1_−0.23 Red Rep2_−0.22
sp|Q15555−2|MARE2_HUMAN−229−S−S2_−0.05
ptm abundance 37C
Doses Blue Rep1_−0.03 Red Rep2_−0.08
sp|Q5T200|ZC3HD_HUMAN−851−Y−S3_0.06
ptm abundance 37C
Doses Blue Rep1_0.03 Red Rep2_0.09
sp|Q96ST2|IWS1_HUMAN−93−S−S2_−0.12
ptm abundance 37C
Doses Blue Rep1_−0.12 Red Rep2_−0.12

sp|P25205|MCM3_HUMAN−722−T−S2_−0.02

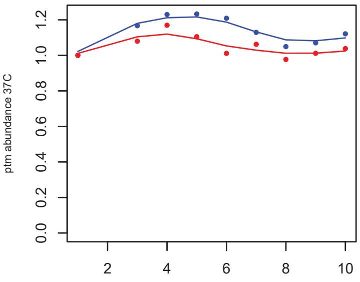

Doses Blue Rep1_0 Red Rep2_−0.04

sp|Q9BRD0|BUD13_HUMAN−185−S−S3_−0.07

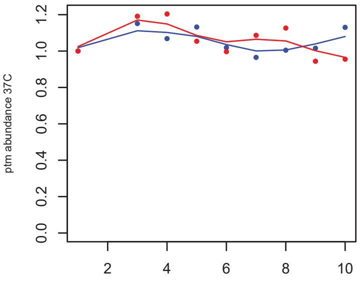

Doses Blue Rep1_0.02 Red Rep2_−0.17

sp|Q9BRD0|BUD13_HUMAN−187−T−S3_−0.07

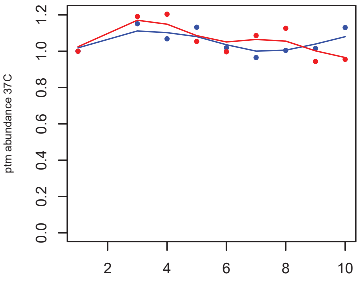

Doses Blue Rep1_0.02 Red Rep2_−0.17

sp|Q9BRD0|BUD13_HUMAN−188−S−S3_−0.07

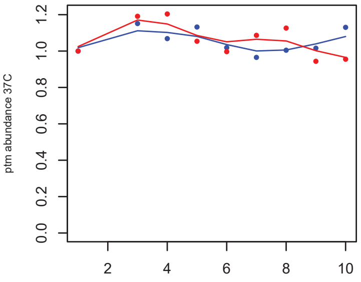

Doses Blue Rep1_0.02 Red Rep2_−0.17

sp|O43164−2|PJA2_HUMAN−428−S−S3_0.04

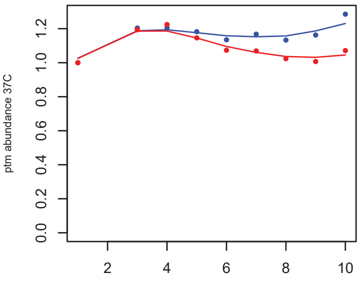

Doses Blue Rep1_0.15 Red Rep2_−0.08

sp|O43164−2|PJA2_HUMAN−429−S−S3_0.04

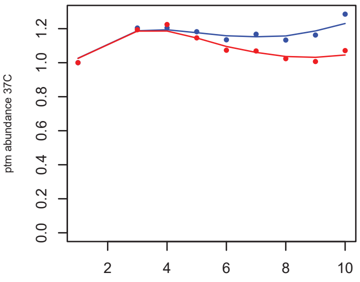

Doses Blue Rep1_0.15 Red Rep2_−0.08

sp|O43164−2|PJA2_HUMAN−432−S−S3_0.04

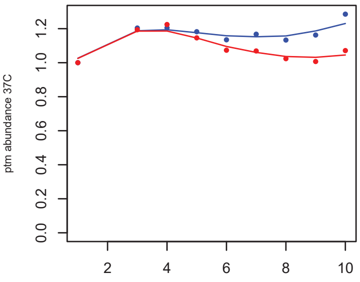

Doses Blue Rep1_0.15 Red Rep2_−0.08

sp|Q9UPT8|ZC3H4_HUMAN−1106−T−S2_0.02

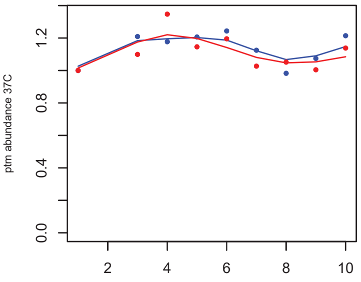

Doses Blue Rep1_0.05 Red Rep2_−0.01

sp|Q9HAS0|NJMU_HUMAN−19−S−S2_0.04

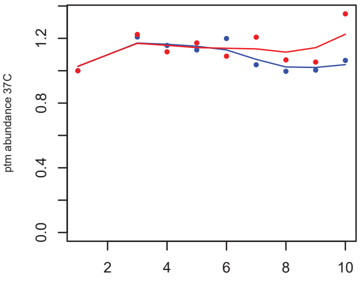

Doses Blue Rep1_−0.08 Red Rep2_0.16

sp|Q9H3Q1−2|BORG4_HUMAN−71−S−S2_−0.2

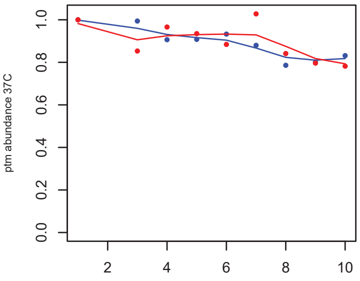

Doses Blue Rep1_−0.2 Red Rep2_−0.19

sp|O60678|ANM3_HUMAN−25−S−S2_0.02

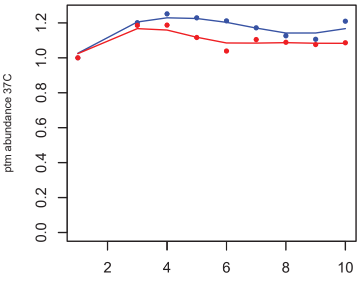

Doses Blue Rep1_0.06 Red Rep2_−0.02

sp|O60678|ANM3_HUMAN−27−S−S2_0.02

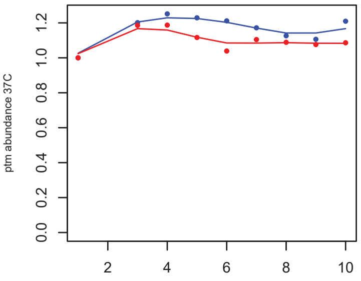

Doses Blue Rep1_0.06 Red Rep2_−0.02

sp|O43719|HTSF1_HUMAN−597−S−S3_0.01

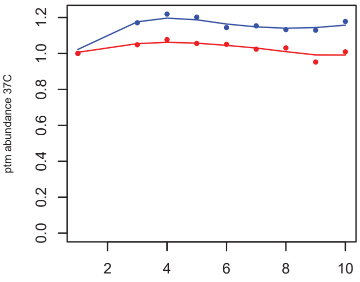

Doses Blue Rep1_0.07 Red Rep2_−0.05

sp|O43719|HTSF1_HUMAN−600−S−S3_0.01

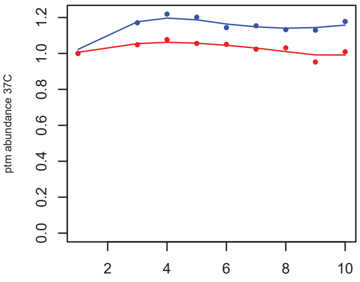

Doses Blue Rep1_0.07 Red Rep2_−0.05

sp|O43719|HTSF1_HUMAN−607−S−S3_0.01

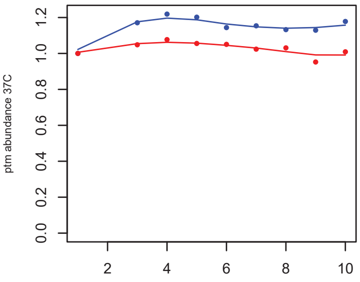

Doses Blue Rep1_0.07 Red Rep2_−0.05

sp|P31323|KAP3_HUMAN−83−S−S2_0.01

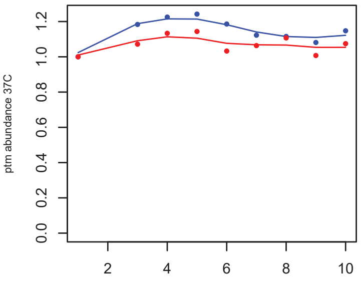

Doses Blue Rep1_0.02 Red Rep2_0

sp|P31323|KAP3_HUMAN−85−S−S2_0.01

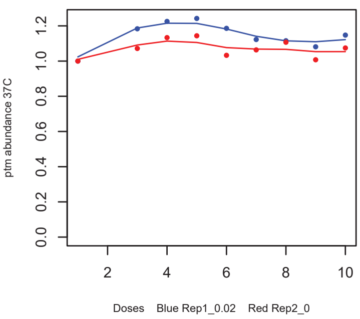

sp|Q3T8J9−2|GON4L_HUMAN−1339−S−S1_−0.02

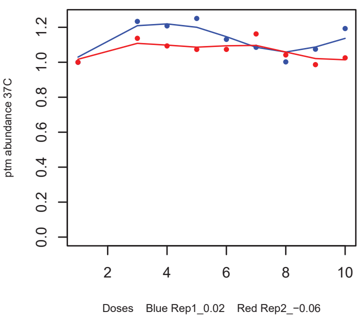

sp|P28715|ERCC5_HUMAN−384−S−S1_0.01

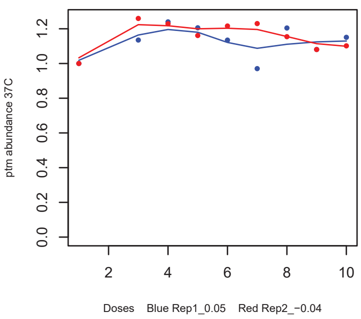

sp|P37275−4|ZEB1_HUMAN−996−S−S3_−0.01

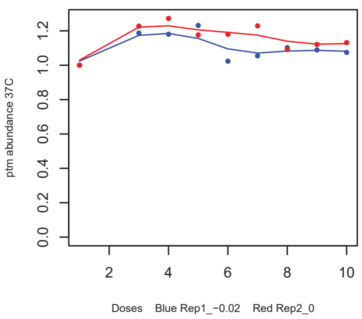

sp|P37275−4|ZEB1_HUMAN−1002−S−S3_−0.01

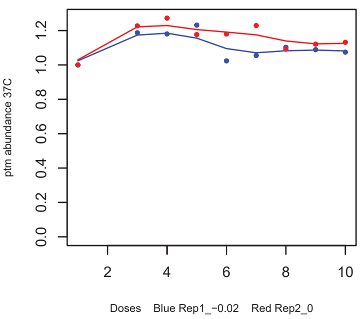

sp|Q14160−2|SCRIB_HUMAN−1219−S−S2_−0.01

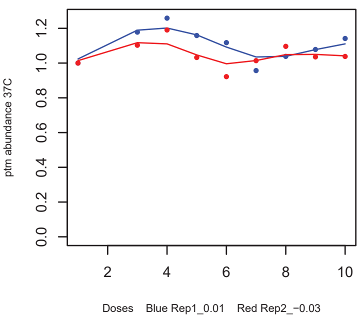

sp|Q9UKL0|RCOR1_HUMAN−260−S−S1_0.11

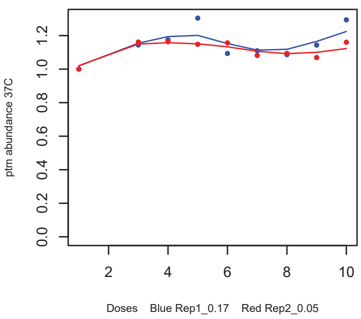

sp|Q8TDR0−2|MIPT3_HUMAN−357−S−S2_0.06

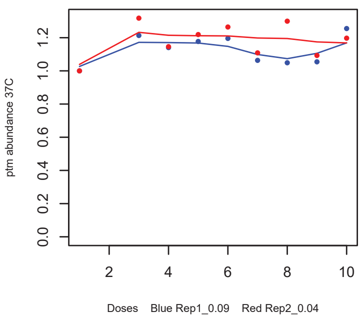

sp|Q8TDR0−2|MIPT3_HUMAN−359−S−S2_0.06

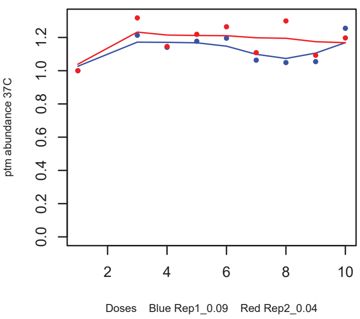

sp|Q8IZ69−2|TRM2A_HUMAN−2−S−S1_−0.11

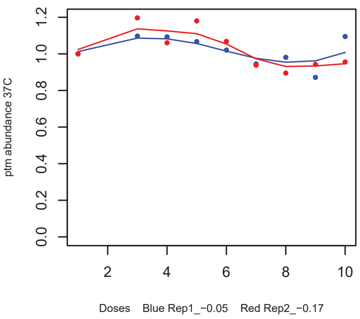

sp|Q9UQ35|SRRM2_HUMAN−1101−S−S3_−0.08

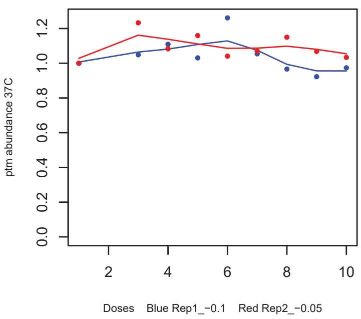

sp|Q9Y2W2|WBP11_HUMAN−250−S−S3_0.07

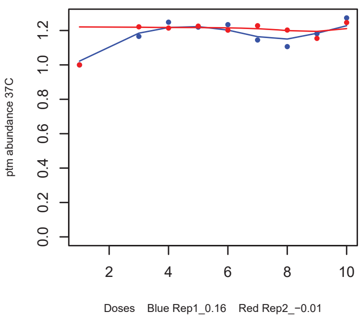

sp|P98175−2|RBM10_HUMAN−731−Y−S3_0.05

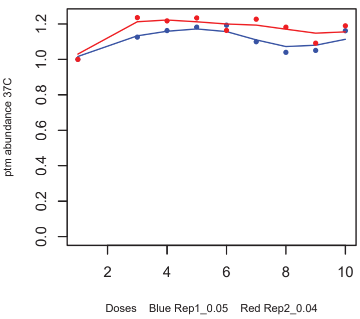

sp|P98175−2|RBM10_HUMAN−732−S−S3_0.05

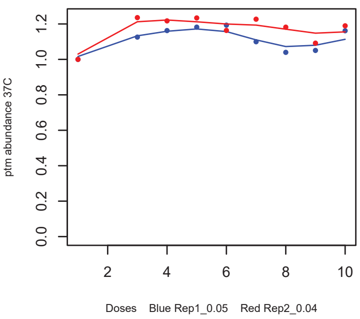

sp|Q9BRR8|GPTC1_HUMAN−8−S−S2_0.03

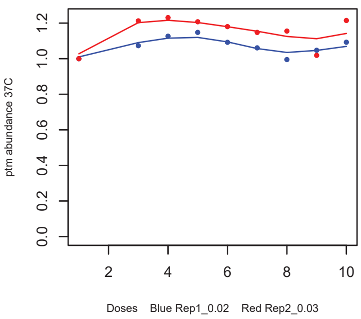

sp|P98175−2|RBM10_HUMAN−735−S−S3_0.05

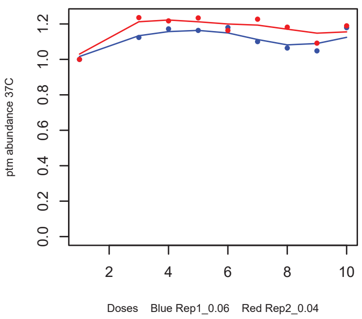

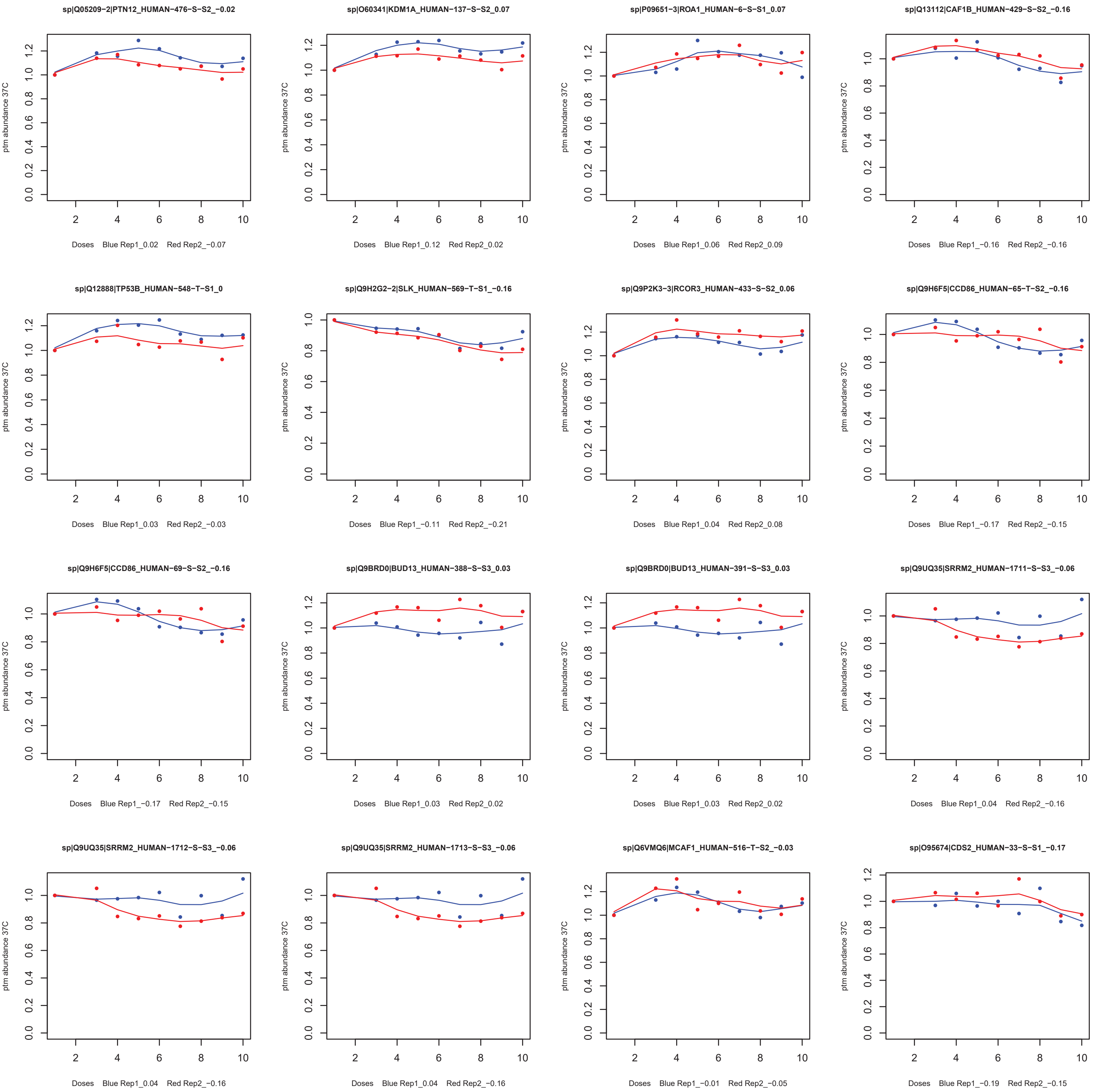

sp|Q05209−2|PTN12_HUMAN−476−S−S2_−0.02
ptm abundance 37C
Doses Blue Rep1_0.02 Red Rep2_−0.07
sp|O60341|KDM1A_HUMAN−137−S−S2_0.07
ptm abundance 37C
Doses Blue Rep1_0.12 Red Rep2_0.02
sp|P09651−3|ROA1_HUMAN−6−S−S1_0.07
ptm abundance 37C
Doses Blue Rep1_0.06 Red Rep2_0.09
sp|Q13112|CAF1B_HUMAN−429−S−S2_−0.16
ptm abundance 37C
Doses Blue Rep1_−0.16 Red Rep2_−0.16
sp|Q12888|TP53B_HUMAN−548−T−S1_0
ptm abundance 37C
Doses Blue Rep1_0.03 Red Rep2_−0.03
sp|Q9H2G2−2|SLK_HUMAN−569−T−S1_−0.16
ptm abundance 37C
Doses Blue Rep1_−0.11 Red Rep2_−0.21
sp|Q9P2K3−3|RCOR3_HUMAN−433−S−S2_0.06
ptm abundance 37C
Doses Blue Rep1_0.04 Red Rep2_0.08
sp|Q9H6F5|CCD86_HUMAN−65−T−S2_−0.16
ptm abundance 37C
Doses Blue Rep1_−0.17 Red Rep2_−0.15
sp|Q9H6F5|CCD86_HUMAN−69−S−S2_−0.16
ptm abundance 37C
Doses Blue Rep1_−0.17 Red Rep2_−0.15
sp|Q9BRD0|BUD13_HUMAN−388−S−S3_0.03
ptm abundance 37C
Doses Blue Rep1_0.03 Red Rep2_0.02
sp|Q9BRD0|BUD13_HUMAN−391−S−S3_0.03
ptm abundance 37C
Doses Blue Rep1_0.03 Red Rep2_0.02
sp|Q9UQ35|SRRM2_HUMAN−1711−S−S3_−0.06
ptm abundance 37C
Doses Blue Rep1_0.04 Red Rep2_−0.16
sp|Q9UQ35|SRRM2_HUMAN−1712−S−S3_−0.06
ptm abundance 37C
Doses Blue Rep1_0.04 Red Rep2_−0.16
sp|Q9UQ35|SRRM2_HUMAN−1713−S−S3_−0.06
ptm abundance 37C
Doses Blue Rep1_0.04 Red Rep2_−0.16
sp|Q6VMQ6|MCAF1_HUMAN−516−T−S2_−0.03
ptm abundance 37C
Doses Blue Rep1_−0.01 Red Rep2_−0.05
sp|O95674|CDS2_HUMAN−33−S−S1_−0.17
ptm abundance 37C
Doses Blue Rep1_−0.19 Red Rep2_−0.15

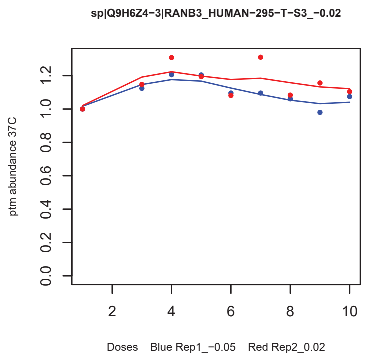
sp|Q9H6Z4−3|RANB3_HUMAN−295−T−S3_−0.02
ptm abundance 37C
Doses Blue Rep1_−0.05 Red Rep2_0.02

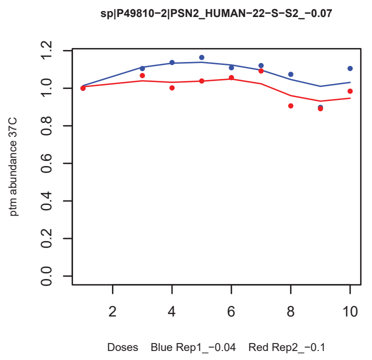
sp|P49810−2|PSN2_HUMAN−22−S−S2_−0.07
ptm abundance 37C
Doses Blue Rep1_−0.04 Red Rep2_−0.1

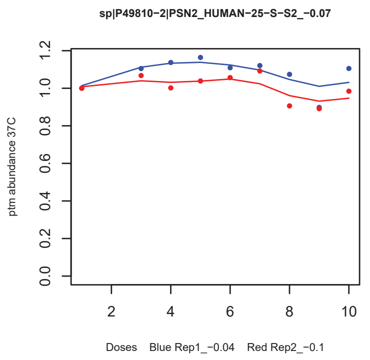
sp|P49810−2|PSN2_HUMAN−25−S−S2_−0.07
ptm abundance 37C
Doses Blue Rep1_−0.04 Red Rep2_−0.1

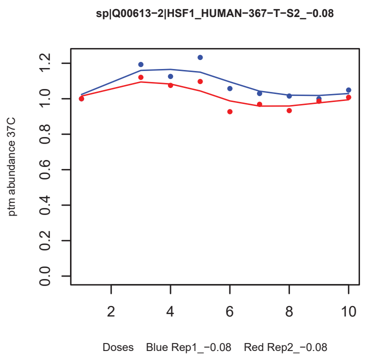
sp|Q00613−2|HSF1_HUMAN−367−T−S2_−0.08
ptm abundance 37C
Doses Blue Rep1_−0.08 Red Rep2_−0.08

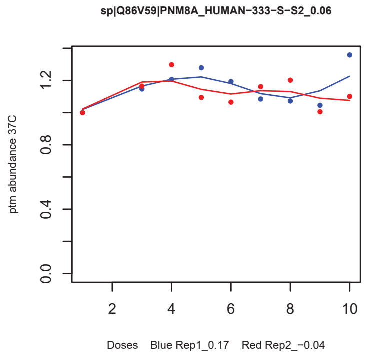
sp|Q86V59|PNM8A_HUMAN−333−S−S2_0.06
ptm abundance 37C
Doses Blue Rep1_0.17 Red Rep2_−0.04

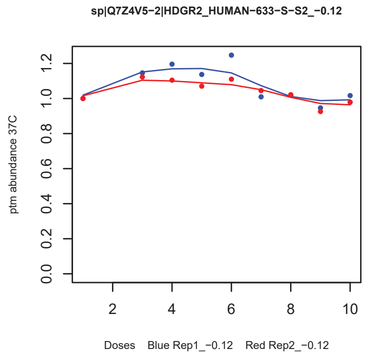
sp|Q7Z4V5−2|HDGR2_HUMAN−633−S−S2_−0.12
ptm abundance 37C
Doses Blue Rep1_−0.12 Red Rep2_−0.12

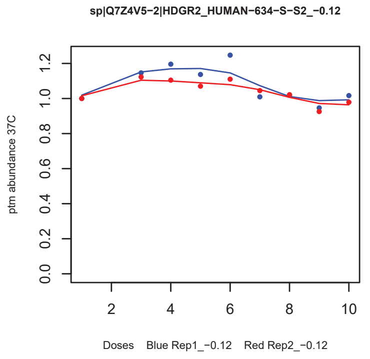
sp|Q7Z4V5−2|HDGR2_HUMAN−634−S−S2_−0.12
ptm abundance 37C
Doses Blue Rep1_−0.12 Red Rep2_−0.12

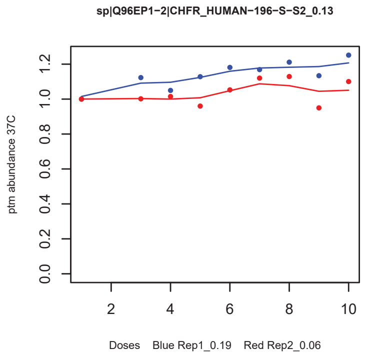
sp|Q96EP1−2|CHFR_HUMAN−196−S−S2_0.13
ptm abundance 37C
Doses Blue Rep1_0.19 Red Rep2_0.06

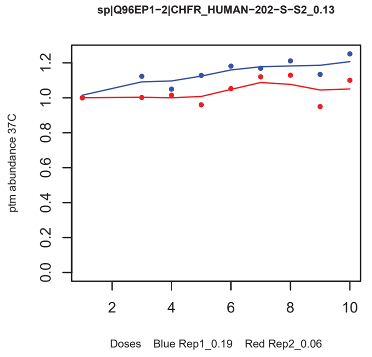
sp|Q96EP1−2|CHFR_HUMAN−202−S−S2_0.13
ptm abundance 37C
Doses Blue Rep1_0.19 Red Rep2_0.06

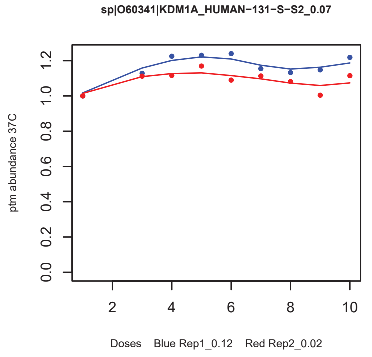
sp|O60341|KDM1A_HUMAN−131−S−S2_0.07
ptm abundance 37C
Doses Blue Rep1_0.12 Red Rep2_0.02

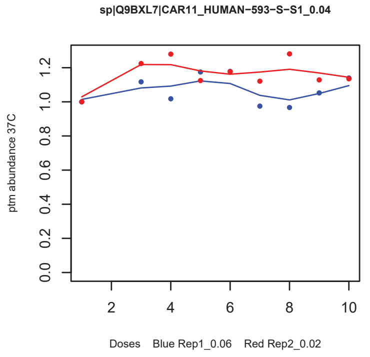
sp|Q9BXL7|CAR11_HUMAN−593−S−S1_0.04
ptm abundance 37C
Doses Blue Rep1_0.06 Red Rep2_0.02

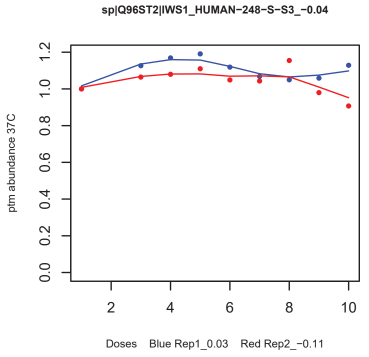
sp|Q96ST2|IWS1_HUMAN−248−S−S3_−0.04
ptm abundance 37C
Doses Blue Rep1_0.03 Red Rep2_−0.11

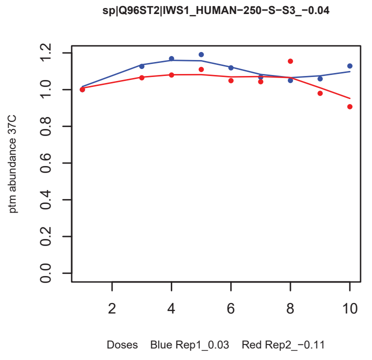
sp|Q96ST2|IWS1_HUMAN−250−S−S3_−0.04
ptm abundance 37C
Doses Blue Rep1_0.03 Red Rep2_−0.11

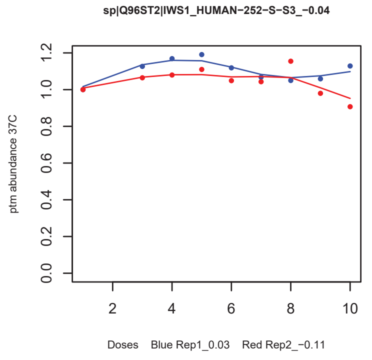
sp|Q96ST2|IWS1_HUMAN−252−S−S3_−0.04
ptm abundance 37C
Doses Blue Rep1_0.03 Red Rep2_−0.11

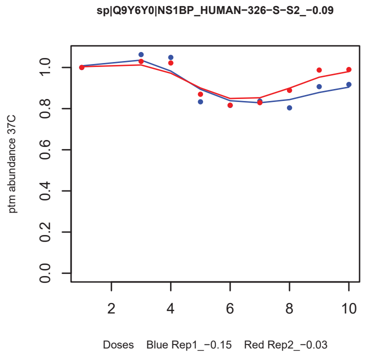
sp|Q9Y6Y0|NS1BP_HUMAN−326−S−S2_−0.09
ptm abundance 37C
Doses Blue Rep1_−0.15 Red Rep2_−0.03

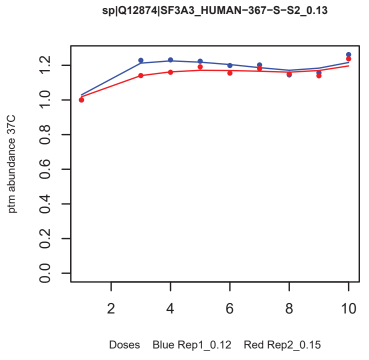
sp|Q12874|SF3A3_HUMAN−367−S−S2_0.13
ptm abundance 37C
Doses Blue Rep1_0.12 Red Rep2_0.15

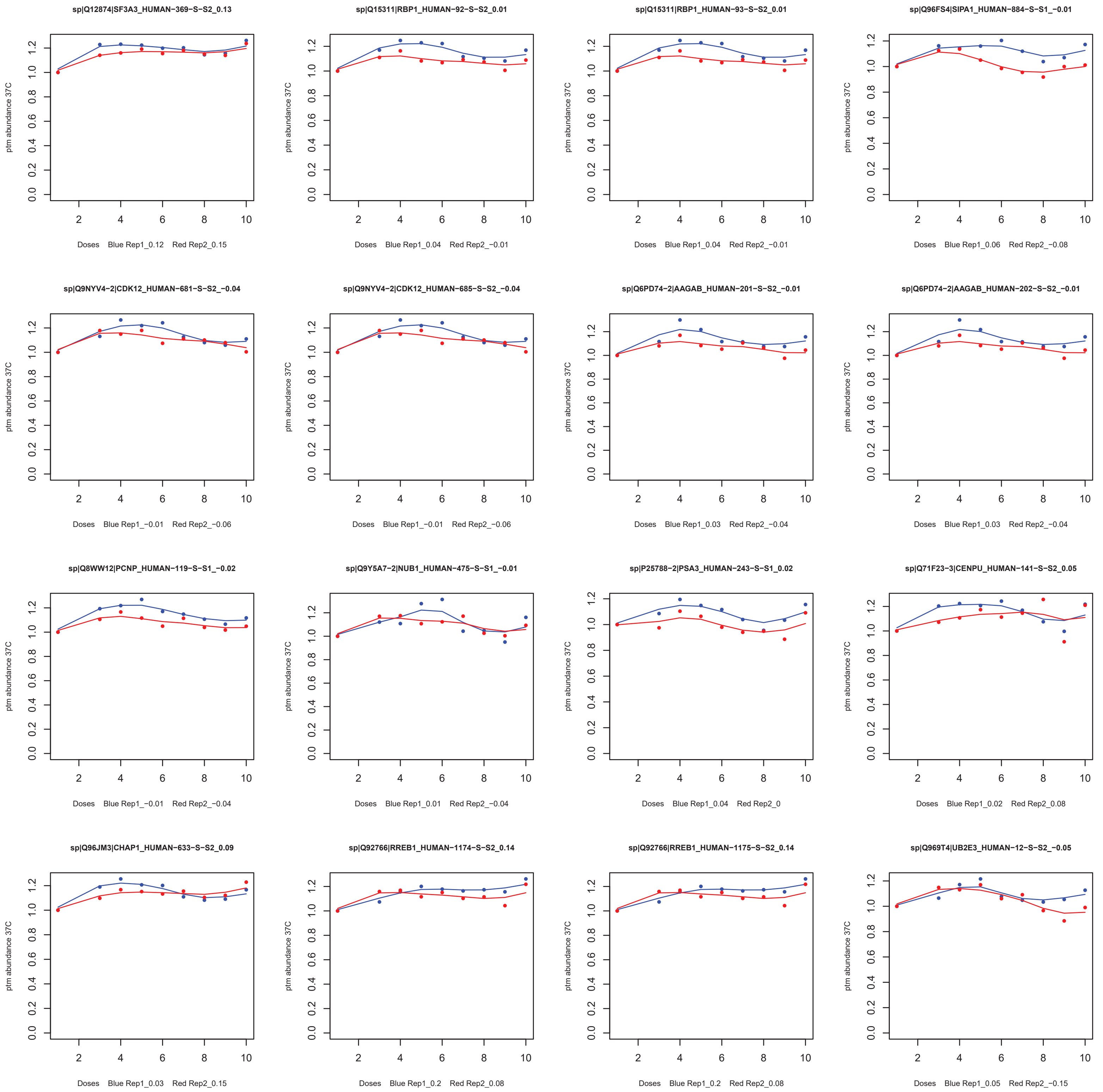
sp|Q12874|SF3A3_HUMAN−369−S−S2_0.13
ptm abundance 37C
Doses Blue Rep1_0.12 Red Rep2_0.15
sp|Q15311|RBP1_HUMAN−92−S−S2_0.01
ptm abundance 37C
Doses Blue Rep1_0.04 Red Rep2_−0.01
sp|Q15311|RBP1_HUMAN−93−S−S2_0.01
ptm abundance 37C
Doses Blue Rep1_0.04 Red Rep2_−0.01
sp|Q96FS4|SIPA1_HUMAN−884−S−S1_−0.01
ptm abundance 37C
Doses Blue Rep1_0.06 Red Rep2_−0.08
sp|Q9NYV4−2|CDK12_HUMAN−681−S−S2_−0.04
ptm abundance 37C
Doses Blue Rep1_−0.01 Red Rep2_−0.06
sp|Q9NYV4−2|CDK12_HUMAN−685−S−S2_−0.04
ptm abundance 37C
Doses Blue Rep1_−0.01 Red Rep2_−0.06
sp|Q6PD74−2|AAGAB_HUMAN−201−S−S2_−0.01
ptm abundance 37C
Doses Blue Rep1_0.03 Red Rep2_−0.04
sp|Q6PD74−2|AAGAB_HUMAN−202−S−S2_−0.01
ptm abundance 37C
Doses Blue Rep1_0.03 Red Rep2_−0.04
sp|Q8WW12|PCNP_HUMAN−119−S−S1_−0.02
ptm abundance 37C
Doses Blue Rep1_−0.01 Red Rep2_−0.04
sp|Q9Y5A7−2|NUB1_HUMAN−475−S−S1_−0.01
ptm abundance 37C
Doses Blue Rep1_0.01 Red Rep2_−0.04
sp|P25788−2|PSA3_HUMAN−243−S−S1_0.02
ptm abundance 37C
Doses Blue Rep1_0.04 Red Rep2_0
sp|Q71F23−3|CENPU_HUMAN−141−S−S2_0.05
ptm abundance 37C
Doses Blue Rep1_0.02 Red Rep2_0.08
sp|Q96JM3|CHAP1_HUMAN−633−S−S2_0.09
ptm abundance 37C
Doses Blue Rep1_0.03 Red Rep2_0.15
sp|Q92766|RREB1_HUMAN−1174−S−S2_0.14
ptm abundance 37C
Doses Blue Rep1_0.2 Red Rep2_0.08
sp|Q92766|RREB1_HUMAN−1175−S−S2_0.14
ptm abundance 37C
Doses Blue Rep1_0.2 Red Rep2_0.08
sp|Q969T4|UB2E3_HUMAN−12−S−S2_−0.05
ptm abundance 37C
Doses Blue Rep1_0.05 Red Rep2_−0.15

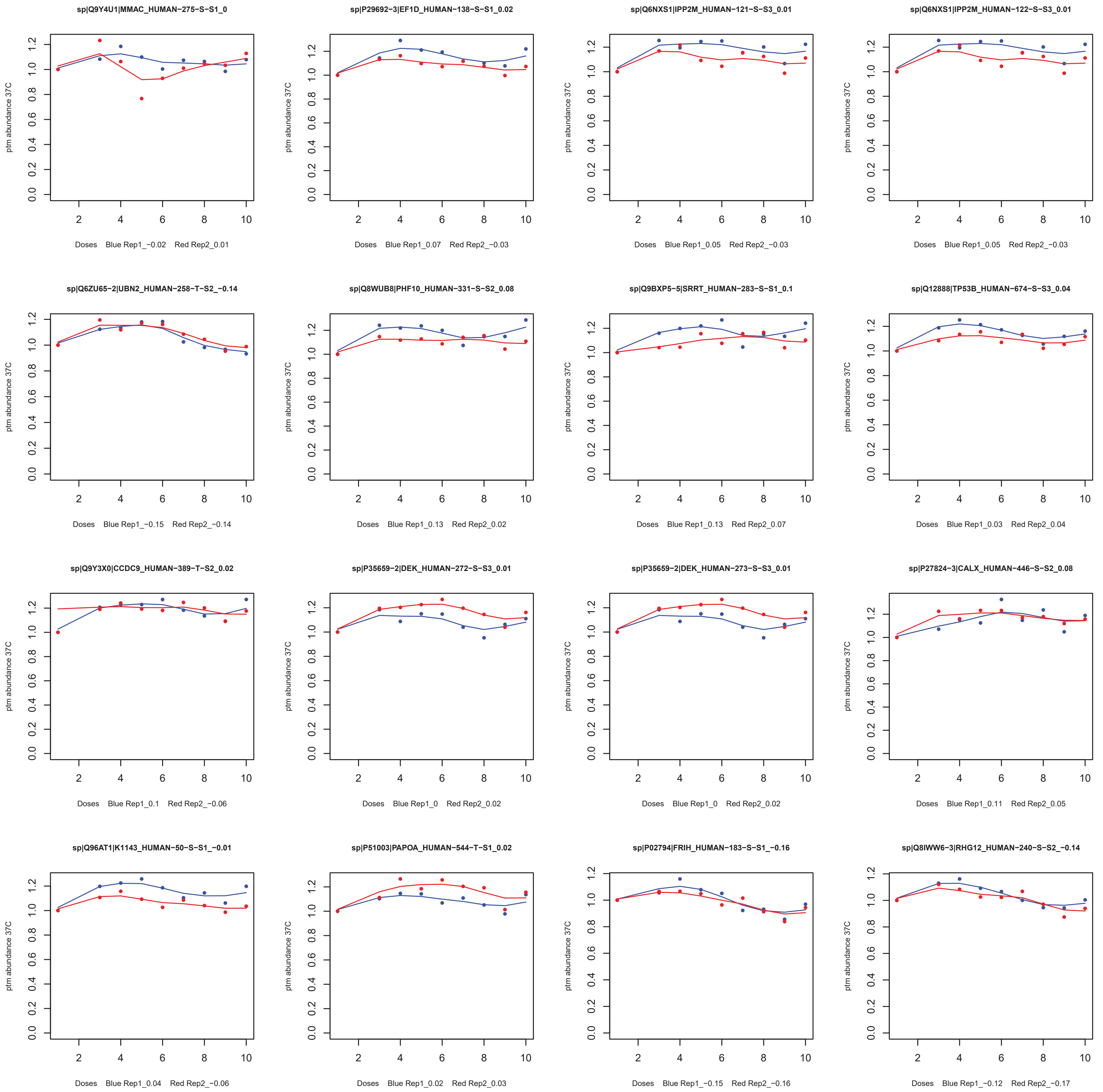
sp|Q9Y4U1|MMAC_HUMAN−275−S−S1_0
ptm abundance 37C
Doses Blue Rep1_−0.02 Red Rep2_0.01
sp|P29692−3|EF1D_HUMAN−138−S−S1_0.02
ptm abundance 37C
Doses Blue Rep1_0.07 Red Rep2_−0.03
sp|Q6NXS1|IPP2M_HUMAN−121−S−S3_0.01
ptm abundance 37C
Doses Blue Rep1_0.05 Red Rep2_−0.03
sp|Q6NXS1|IPP2M_HUMAN−122−S−S3_0.01
ptm abundance 37C
Doses Blue Rep1_0.05 Red Rep2_−0.03
sp|Q6ZU65−2|UBN2_HUMAN−258−T−S2_−0.14
ptm abundance 37C
Doses Blue Rep1_−0.15 Red Rep2_−0.14
sp|Q8WUB8|PHF10_HUMAN−331−S−S2_0.08
ptm abundance 37C
Doses Blue Rep1_0.13 Red Rep2_0.02
sp|Q9BXP5−5|SRRT_HUMAN−283−S−S1_0.1
ptm abundance 37C
Doses Blue Rep1_0.13 Red Rep2_0.07
sp|Q12888|TP53B_HUMAN−674−S−S3_0.04
ptm abundance 37C
Doses Blue Rep1_0.03 Red Rep2_0.04
sp|Q9Y3X0|CCDC9_HUMAN−389−T−S2_0.02
ptm abundance 37C
Doses Blue Rep1_0.1 Red Rep2_−0.06
sp|P35659−2|DEK_HUMAN−272−S−S3_0.01
ptm abundance 37C
Doses Blue Rep1_0 Red Rep2_0.02
sp|P35659−2|DEK_HUMAN−273−S−S3_0.01
ptm abundance 37C
Doses Blue Rep1_0 Red Rep2_0.02
sp|P27824−3|CALX_HUMAN−446−S−S2_0.08
ptm abundance 37C
Doses Blue Rep1_0.11 Red Rep2_0.05
sp|Q96AT1|K1143_HUMAN−50−S−S1_−0.01
ptm abundance 37C
Doses Blue Rep1_0.04 Red Rep2_−0.06
sp|P51003|PAPOA_HUMAN−544−T−S1_0.02
ptm abundance 37C
Doses Blue Rep1_0.02 Red Rep2_0.03
sp|P02794|FRIH_HUMAN−183−S−S1_−0.16
ptm abundance 37C
Doses Blue Rep1_−0.15 Red Rep2_−0.16
sp|Q8IWW6−3|RHG12_HUMAN−240−S−S2_−0.14
ptm abundance 37C
Doses Blue Rep1_−0.12 Red Rep2_−0.17

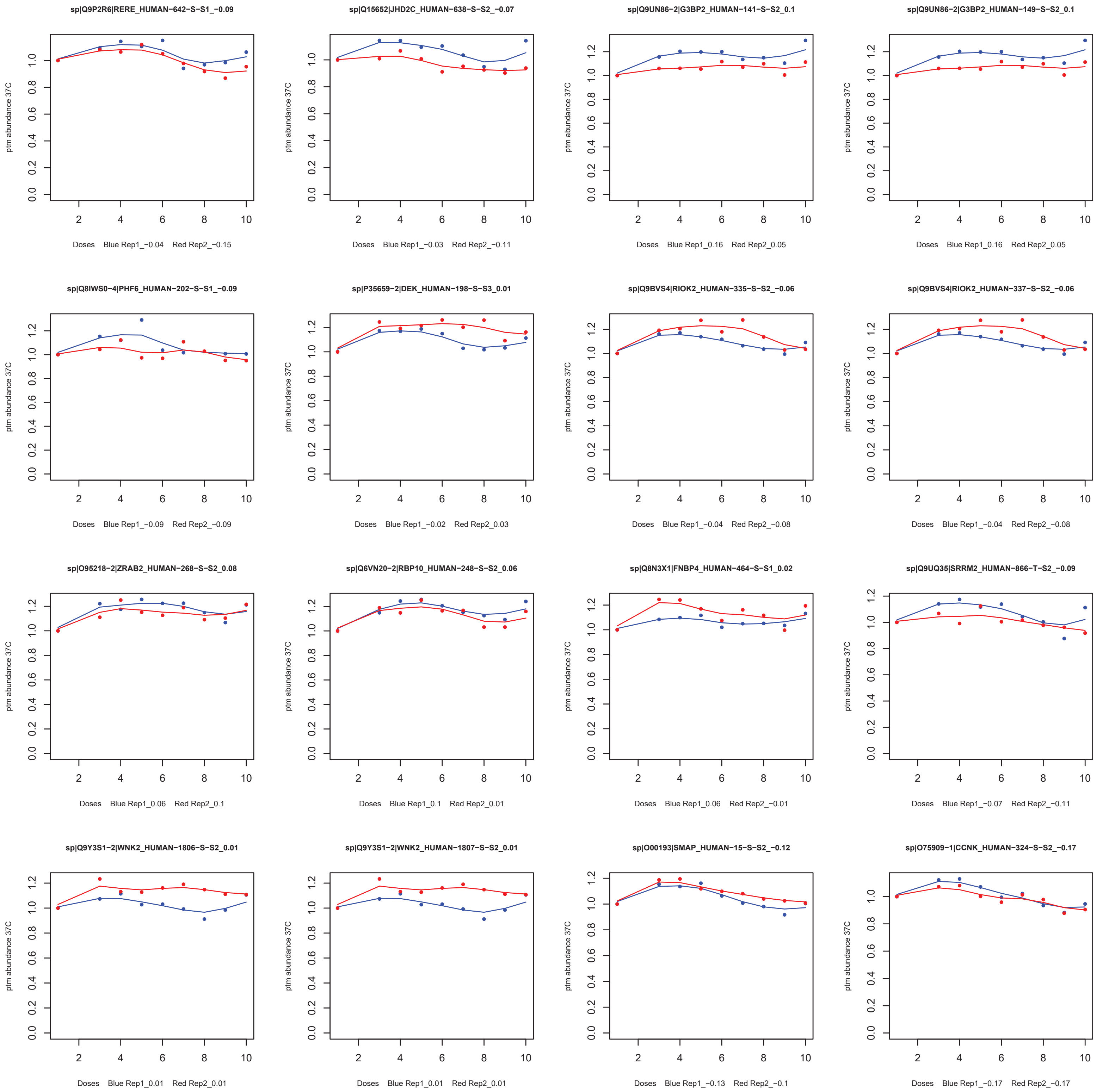
sp|Q9P2R6|RERE_HUMAN−642−S−S1_−0.09
Doses Blue Rep1_−0.04 Red Rep2_−0.15
sp|Q15652|JHD2C_HUMAN−638−S−S2_−0.07
Doses Blue Rep1_−0.03 Red Rep2_−0.11
sp|Q9UN86−2|G3BP2_HUMAN−141−S−S2_0.1
Doses Blue Rep1_0.16 Red Rep2_0.05
sp|Q9UN86−2|G3BP2_HUMAN−149−S−S2_0.1
Doses Blue Rep1_0.16 Red Rep2_0.05
sp|Q8IWS0−4|PHF6_HUMAN−202−S−S1_−0.09
Doses Blue Rep1_−0.09 Red Rep2_−0.09
sp|P35659−2|DEK_HUMAN−198−S−S3_0.01
Doses Blue Rep1_−0.02 Red Rep2_0.03
sp|Q9BVS4|RIOK2_HUMAN−335−S−S2_−0.06
Doses Blue Rep1_−0.04 Red Rep2_−0.08
sp|Q9BVS4|RIOK2_HUMAN−337−S−S2_−0.06
Doses Blue Rep1_−0.04 Red Rep2_−0.08
sp|O95218−2|ZRAB2_HUMAN−268−S−S2_0.08
Doses Blue Rep1_0.06 Red Rep2_0.1
sp|Q6VN20−2|RBP10_HUMAN−248−S−S2_0.06
Doses Blue Rep1_0.1 Red Rep2_0.01
sp|Q8N3X1|FNBP4_HUMAN−464−S−S1_0.02
Doses Blue Rep1_0.06 Red Rep2_−0.01
sp|Q9UQ35|SRRM2_HUMAN−866−T−S2_−0.09
Doses Blue Rep1_−0.07 Red Rep2_−0.11
sp|Q9Y3S1−2|WNK2_HUMAN−1806−S−S2_0.01
Doses Blue Rep1_0.01 Red Rep2_0.01
sp|Q9Y3S1−2|WNK2_HUMAN−1807−S−S2_0.01
Doses Blue Rep1_0.01 Red Rep2_0.01
sp|O00193|SMAP_HUMAN−15−S−S2_−0.12
Doses Blue Rep1_−0.13 Red Rep2_−0.1
sp|O75909−1|CCNK_HUMAN−324−S−S2_−0.17
Doses Blue Rep1_−0.17 Red Rep2_−0.17
ptm abundance 37C

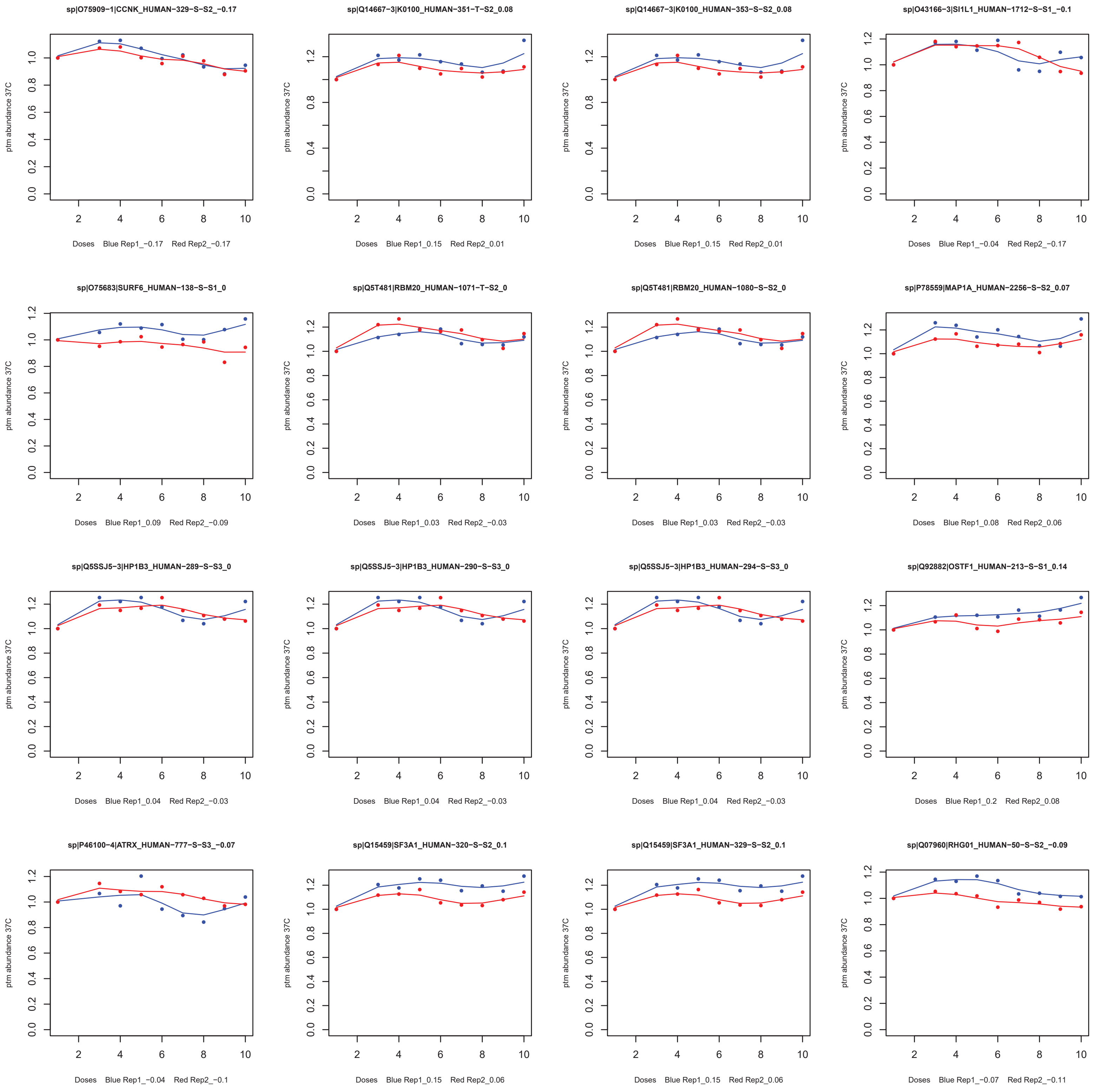
sp|O75909−1|CCNK_HUMAN−329−S−S2_−0.17
Doses Blue Rep1_−0.17 Red Rep2_−0.17
sp|Q14667−3|K0100_HUMAN−351−T−S2_0.08
Doses Blue Rep1_0.15 Red Rep2_0.01
sp|Q14667−3|K0100_HUMAN−353−S−S2_0.08
Doses Blue Rep1_0.15 Red Rep2_0.01
sp|O43166−3|SI1L1_HUMAN−1712−S−S1_−0.1
Doses Blue Rep1_−0.04 Red Rep2_−0.17
sp|O75683|SURF6_HUMAN−138−S−S1_0
Doses Blue Rep1_0.09 Red Rep2_−0.09
sp|Q5T481|RBM20_HUMAN−1071−T−S2_0
Doses Blue Rep1_0.03 Red Rep2_−0.03
sp|Q5T481|RBM20_HUMAN−1080−S−S2_0
Doses Blue Rep1_0.03 Red Rep2_−0.03
sp|P78559|MAP1A_HUMAN−2256−S−S2_0.07
Doses Blue Rep1_0.08 Red Rep2_0.06
sp|Q5SSJ5−3|HP1B3_HUMAN−289−S−S3_0
Doses Blue Rep1_0.04 Red Rep2_−0.03
sp|Q5SSJ5−3|HP1B3_HUMAN−290−S−S3_0
Doses Blue Rep1_0.04 Red Rep2_−0.03
sp|Q5SSJ5−3|HP1B3_HUMAN−294−S−S3_0
Doses Blue Rep1_0.04 Red Rep2_−0.03
sp|Q92882|OSTF1_HUMAN−213−S−S1_0.14
Doses Blue Rep1_0.2 Red Rep2_0.08
sp|P46100−4|ATRX_HUMAN−777−S−S3_−0.07
Doses Blue Rep1_−0.04 Red Rep2_−0.1
sp|Q15459|SF3A1_HUMAN−320−S−S2_0.1
Doses Blue Rep1_0.15 Red Rep2_0.06
sp|Q15459|SF3A1_HUMAN−329−S−S2_0.1
Doses Blue Rep1_0.15 Red Rep2_0.06
sp|Q07960|RHG01_HUMAN−50−S−S2_−0.09
Doses Blue Rep1_−0.07 Red Rep2_−0.11
ptm abundance 37C

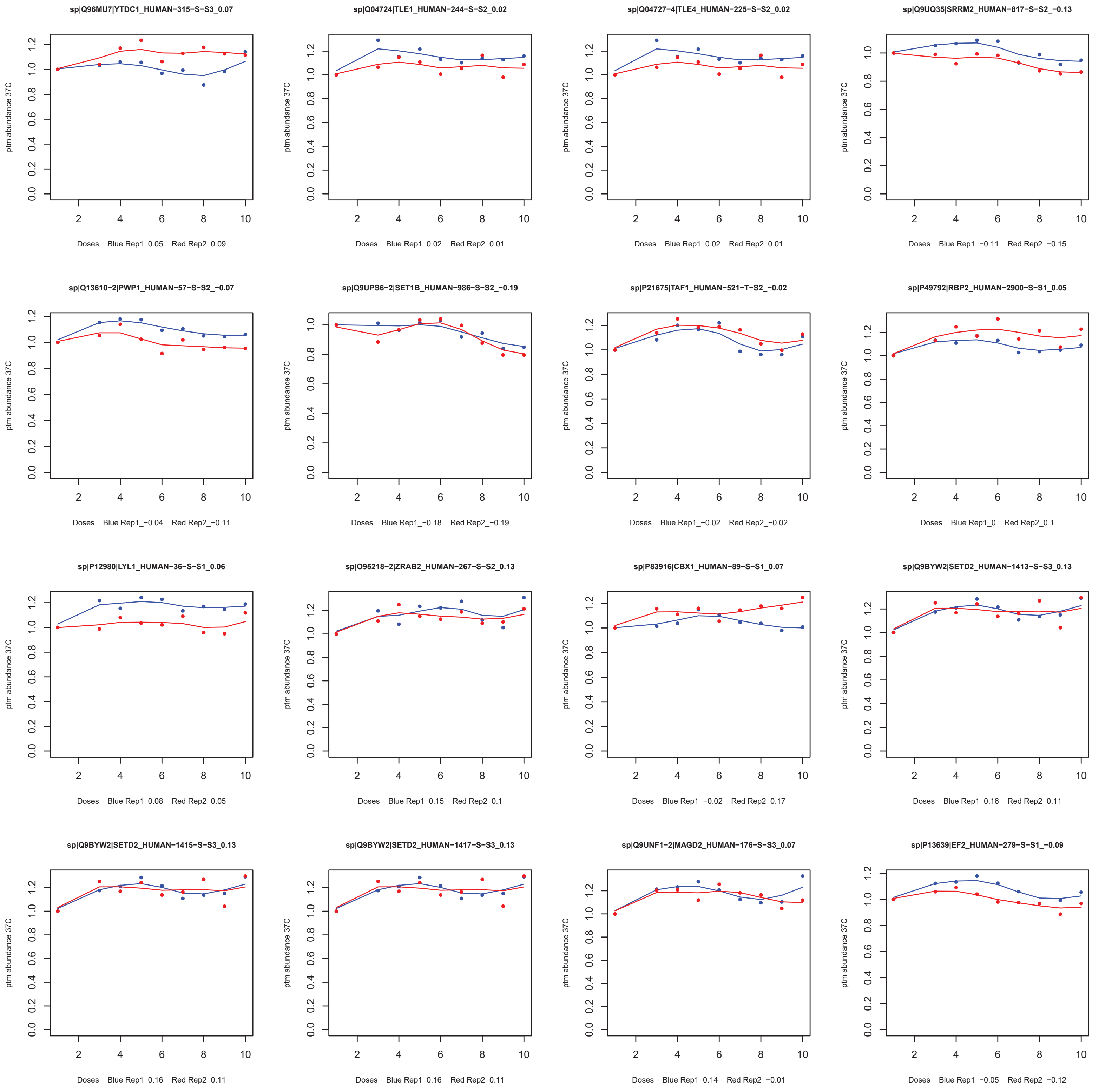

sp|Q96MU7|YTDC1_HUMAN−315−S−S3_0.07
ptm abundance 37C
Doses Blue Rep1_0.05 Red Rep2_0.09
sp|Q04724|TLE1_HUMAN−244−S−S2_0.02
ptm abundance 37C
Doses Blue Rep1_0.02 Red Rep2_0.01
sp|Q04727−4|TLE4_HUMAN−225−S−S2_0.02
ptm abundance 37C
Doses Blue Rep1_0.02 Red Rep2_0.01
sp|Q9UQ35|SRRM2_HUMAN−817−S−S2_−0.13
ptm abundance 37C
Doses Blue Rep1_−0.11 Red Rep2_−0.15
sp|Q13610−2|PWP1_HUMAN−57−S−S2_−0.07
ptm abundance 37C
Doses Blue Rep1_−0.04 Red Rep2_−0.11
sp|Q9UPS6−2|SET1B_HUMAN−986−S−S2_−0.19
ptm abundance 37C
Doses Blue Rep1_−0.18 Red Rep2_−0.19
sp|P21675|TAF1_HUMAN−521−T−S2_−0.02
ptm abundance 37C
Doses Blue Rep1_−0.02 Red Rep2_−0.02
sp|P49792|RBP2_HUMAN−2900−S−S1_0.05
ptm abundance 37C
Doses Blue Rep1_0 Red Rep2_0.1
sp|P12980|LYL1_HUMAN−36−S−S1_0.06
ptm abundance 37C
Doses Blue Rep1_0.08 Red Rep2_0.05
sp|O95218−2|ZRAB2_HUMAN−267−S−S2_0.13
ptm abundance 37C
Doses Blue Rep1_0.15 Red Rep2_0.1
sp|P83916|CBX1_HUMAN−89−S−S1_0.07
ptm abundance 37C
Doses Blue Rep1_−0.02 Red Rep2_0.17
sp|Q9BYW2|SETD2_HUMAN−1413−S−S3_0.13
ptm abundance 37C
Doses Blue Rep1_0.16 Red Rep2_0.11
sp|Q9BYW2|SETD2_HUMAN−1415−S−S3_0.13
ptm abundance 37C
Doses Blue Rep1_0.16 Red Rep2_0.11
sp|Q9BYW2|SETD2_HUMAN−1417−S−S3_0.13
ptm abundance 37C
Doses Blue Rep1_0.16 Red Rep2_0.11
sp|Q9UNF1−2|MAGD2_HUMAN−176−S−S3_0.07
ptm abundance 37C
Doses Blue Rep1_0.14 Red Rep2_−0.01
sp|P13639|EF2_HUMAN−279−S−S1_−0.09
ptm abundance 37C
Doses Blue Rep1_−0.05 Red Rep2_−0.12

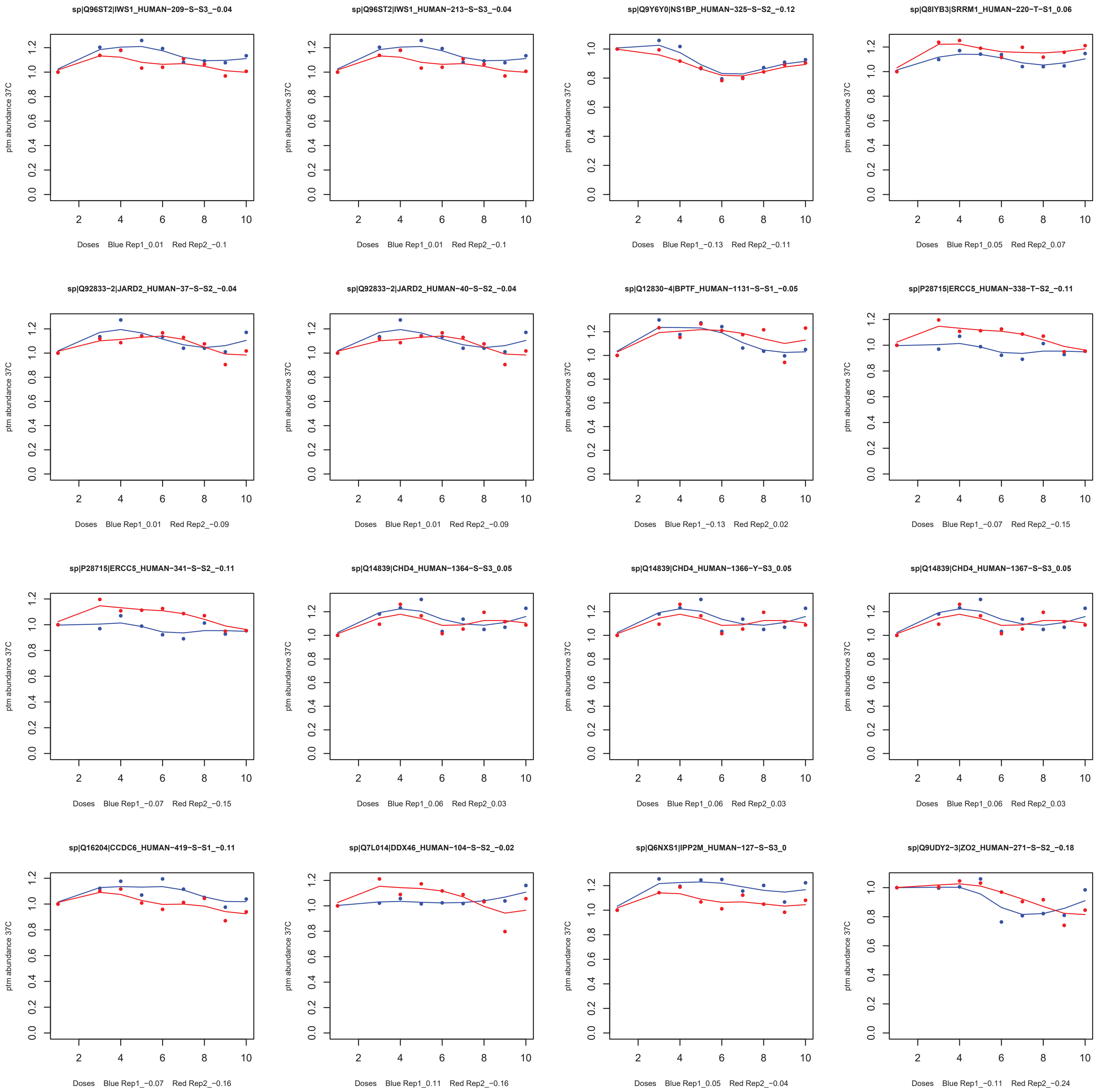

sp|Q96ST2|IWS1_HUMAN−209−S−S3_−0.04
Doses Blue Rep1_0.01 Red Rep2_−0.1
sp|Q96ST2|IWS1_HUMAN−213−S−S3_−0.04
Doses Blue Rep1_0.01 Red Rep2_−0.1
sp|Q9Y6Y0|NS1BP_HUMAN−325−S−S2_−0.12
Doses Blue Rep1_−0.13 Red Rep2_−0.11
sp|Q8IYB3|SRRM1_HUMAN−220−T−S1_0.06
Doses Blue Rep1_0.05 Red Rep2_0.07
sp|Q92833−2|JARD2_HUMAN−37−S−S2_−0.04
Doses Blue Rep1_0.01 Red Rep2_−0.09
sp|Q92833−2|JARD2_HUMAN−40−S−S2_−0.04
Doses Blue Rep1_0.01 Red Rep2_−0.09
sp|Q12830−4|BPTF_HUMAN−1131−S−S1_−0.05
Doses Blue Rep1_−0.13 Red Rep2_0.02
sp|P28715|ERCC5_HUMAN−338−T−S2_−0.11
Doses Blue Rep1_−0.07 Red Rep2_−0.15
sp|P28715|ERCC5_HUMAN−341−S−S2_−0.11
Doses Blue Rep1_−0.07 Red Rep2_−0.15
sp|Q14839|CHD4_HUMAN−1364−S−S3_0.05
Doses Blue Rep1_0.06 Red Rep2_0.03
sp|Q14839|CHD4_HUMAN−1366−Y−S3_0.05
Doses Blue Rep1_0.06 Red Rep2_0.03
sp|Q14839|CHD4_HUMAN−1367−S−S3_0.05
Doses Blue Rep1_0.06 Red Rep2_0.03
sp|Q16204|CCDC6_HUMAN−419−S−S1_−0.11
Doses Blue Rep1_−0.07 Red Rep2_−0.16
sp|Q7L014|DDX46_HUMAN−104−S−S2_−0.02
Doses Blue Rep1_0.11 Red Rep2_−0.16
sp|Q6NXS1|IPP2M_HUMAN−127−S−S3_0
Doses Blue Rep1_0.05 Red Rep2_−0.04
sp|Q9UDY2−3|ZO2_HUMAN−271−S−S2_−0.18
Doses Blue Rep1_−0.11 Red Rep2_−0.24
ptm abundance 37C

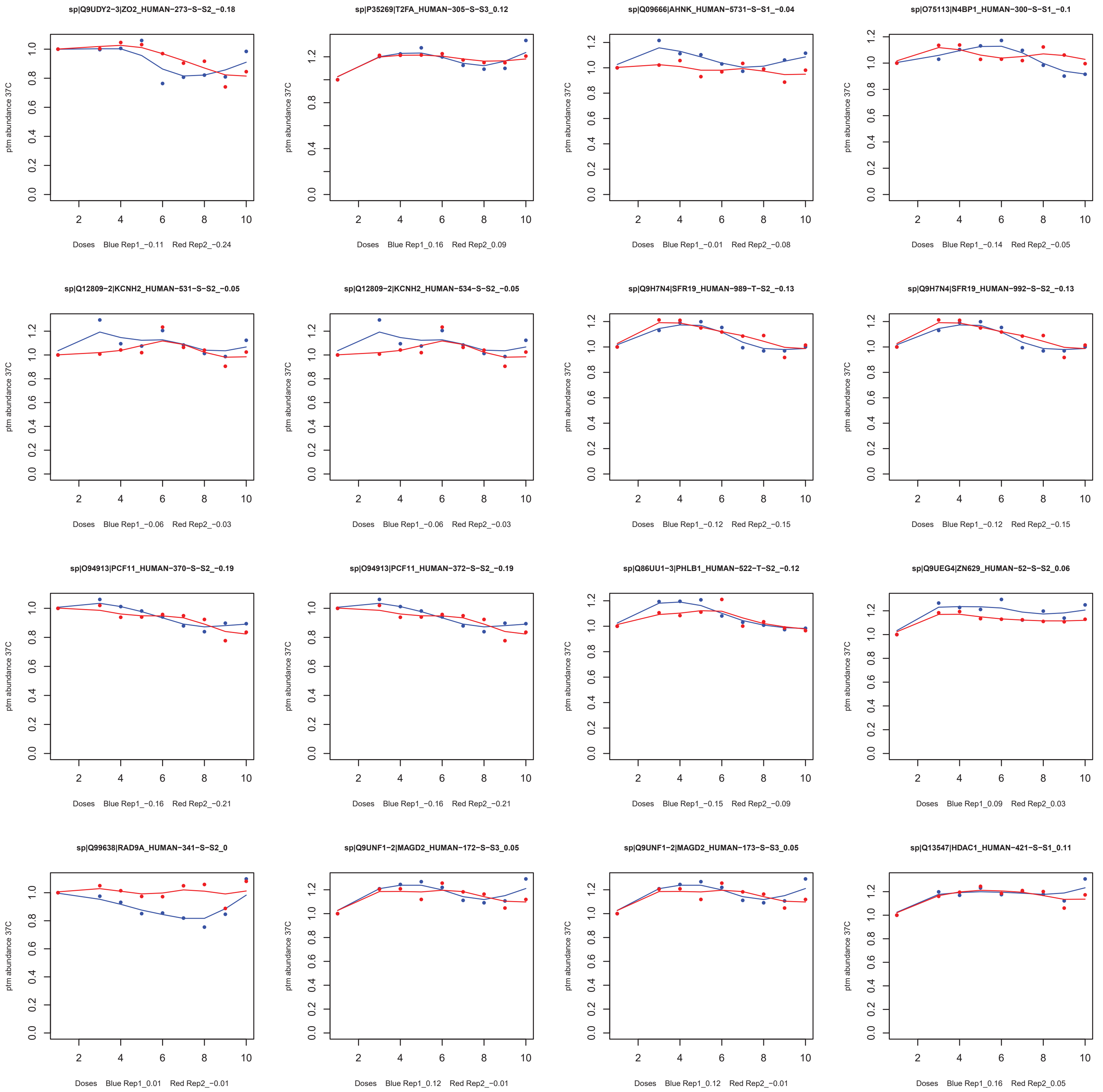
sp|Q9UDY2−3|ZO2_HUMAN−273−S−S2_−0.18
Doses Blue Rep1_−0.11 Red Rep2_−0.24
sp|P35269|T2FA_HUMAN−305−S−S3_0.12
Doses Blue Rep1_0.16 Red Rep2_0.09
sp|Q09666|AHNK_HUMAN−5731−S−S1_−0.04
Doses Blue Rep1_−0.01 Red Rep2_−0.08
sp|O75113|N4BP1_HUMAN−300−S−S1_−0.1
Doses Blue Rep1_−0.14 Red Rep2_−0.05
sp|Q12809−2|KCNH2_HUMAN−531−S−S2_−0.05
Doses Blue Rep1_−0.06 Red Rep2_−0.03
sp|Q12809−2|KCNH2_HUMAN−534−S−S2_−0.05
Doses Blue Rep1_−0.06 Red Rep2_−0.03
sp|Q9H7N4|SFR19_HUMAN−989−T−S2_−0.13
Doses Blue Rep1_−0.12 Red Rep2_−0.15
sp|Q9H7N4|SFR19_HUMAN−992−S−S2_−0.13
Doses Blue Rep1_−0.12 Red Rep2_−0.15
sp|O94913|PCF11_HUMAN−370−S−S2_−0.19
Doses Blue Rep1_−0.16 Red Rep2_−0.21
sp|O94913|PCF11_HUMAN−372−S−S2_−0.19
Doses Blue Rep1_−0.16 Red Rep2_−0.21
sp|Q86UU1−3|PHLB1_HUMAN−522−T−S2_−0.12
Doses Blue Rep1_−0.15 Red Rep2_−0.09
sp|Q9UEG4|ZN629_HUMAN−52−S−S2_0.06
Doses Blue Rep1_0.09 Red Rep2_0.03
sp|Q99638|RAD9A_HUMAN−341−S−S2_0
Doses Blue Rep1_0.01 Red Rep2_−0.01
sp|Q9UNF1−2|MAGD2_HUMAN−172−S−S3_0.05
Doses Blue Rep1_0.12 Red Rep2_−0.01
sp|Q9UNF1−2|MAGD2_HUMAN−173−S−S3_0.05
Doses Blue Rep1_0.12 Red Rep2_−0.01
sp|Q13547|HDAC1_HUMAN−421−S−S1_0.11
Doses Blue Rep1_0.16 Red Rep2_0.05
ptm abundance 37C

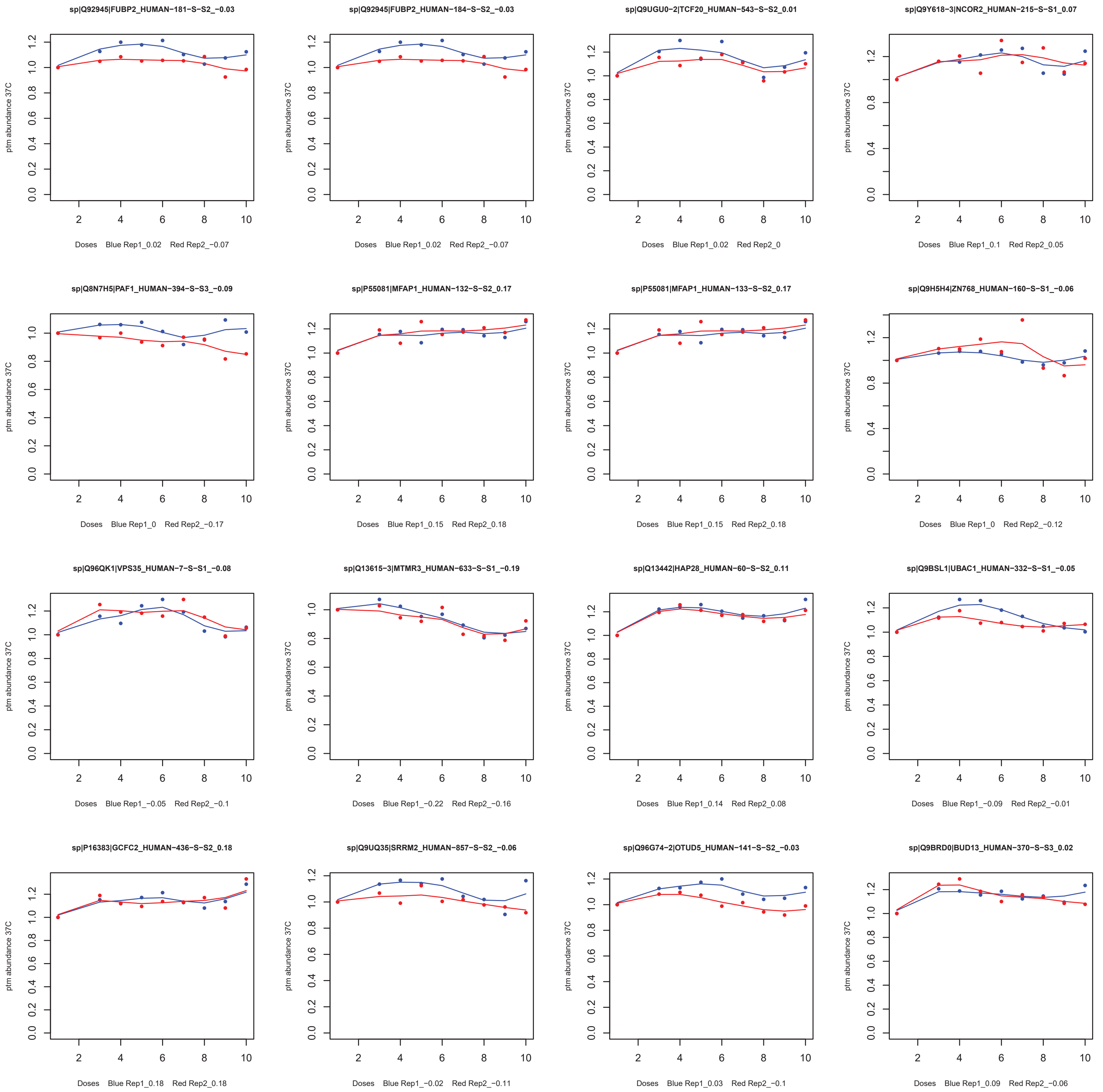
sp|Q92945|FUBP2_HUMAN−181−S−S2_−0.03
Doses Blue Rep1_0.02 Red Rep2_−0.07
sp|Q92945|FUBP2_HUMAN−184−S−S2_−0.03
Doses Blue Rep1_0.02 Red Rep2_−0.07
sp|Q9UGU0−2|TCF20_HUMAN−543−S−S2_0.01
Doses Blue Rep1_0.02 Red Rep2_0
sp|Q9Y618−3|NCOR2_HUMAN−215−S−S1_0.07
Doses Blue Rep1_0.1 Red Rep2_0.05
sp|Q8N7H5|PAF1_HUMAN−394−S−S3_−0.09
Doses Blue Rep1_0 Red Rep2_−0.17
sp|P55081|MFAP1_HUMAN−132−S−S2_0.17
Doses Blue Rep1_0.15 Red Rep2_0.18
sp|P55081|MFAP1_HUMAN−133−S−S2_0.17
Doses Blue Rep1_0.15 Red Rep2_0.18
sp|Q9H5H4|ZN768_HUMAN−160−S−S1_−0.06
Doses Blue Rep1_0 Red Rep2_−0.12
sp|Q96QK1|VPS35_HUMAN−7−S−S1_−0.08
Doses Blue Rep1_−0.05 Red Rep2_−0.1
sp|Q13615−3|MTMR3_HUMAN−633−S−S1_−0.19
Doses Blue Rep1_−0.22 Red Rep2_−0.16
sp|Q13442|HAP28_HUMAN−60−S−S2_0.11
Doses Blue Rep1_0.14 Red Rep2_0.08
sp|Q9BSL1|UBAC1_HUMAN−332−S−S1_−0.05
Doses Blue Rep1_−0.09 Red Rep2_−0.01
sp|P16383|GCFC2_HUMAN−436−S−S2_0.18
Doses Blue Rep1_0.18 Red Rep2_0.18
sp|Q9UQ35|SRRM2_HUMAN−857−S−S2_−0.06
Doses Blue Rep1_−0.02 Red Rep2_−0.11
sp|Q96G74−2|OTUD5_HUMAN−141−S−S2_−0.03
Doses Blue Rep1_0.03 Red Rep2_−0.1
sp|Q9BRD0|BUD13_HUMAN−370−S−S3_0.02
Doses Blue Rep1_0.09 Red Rep2_−0.06
ptm abundance 37C

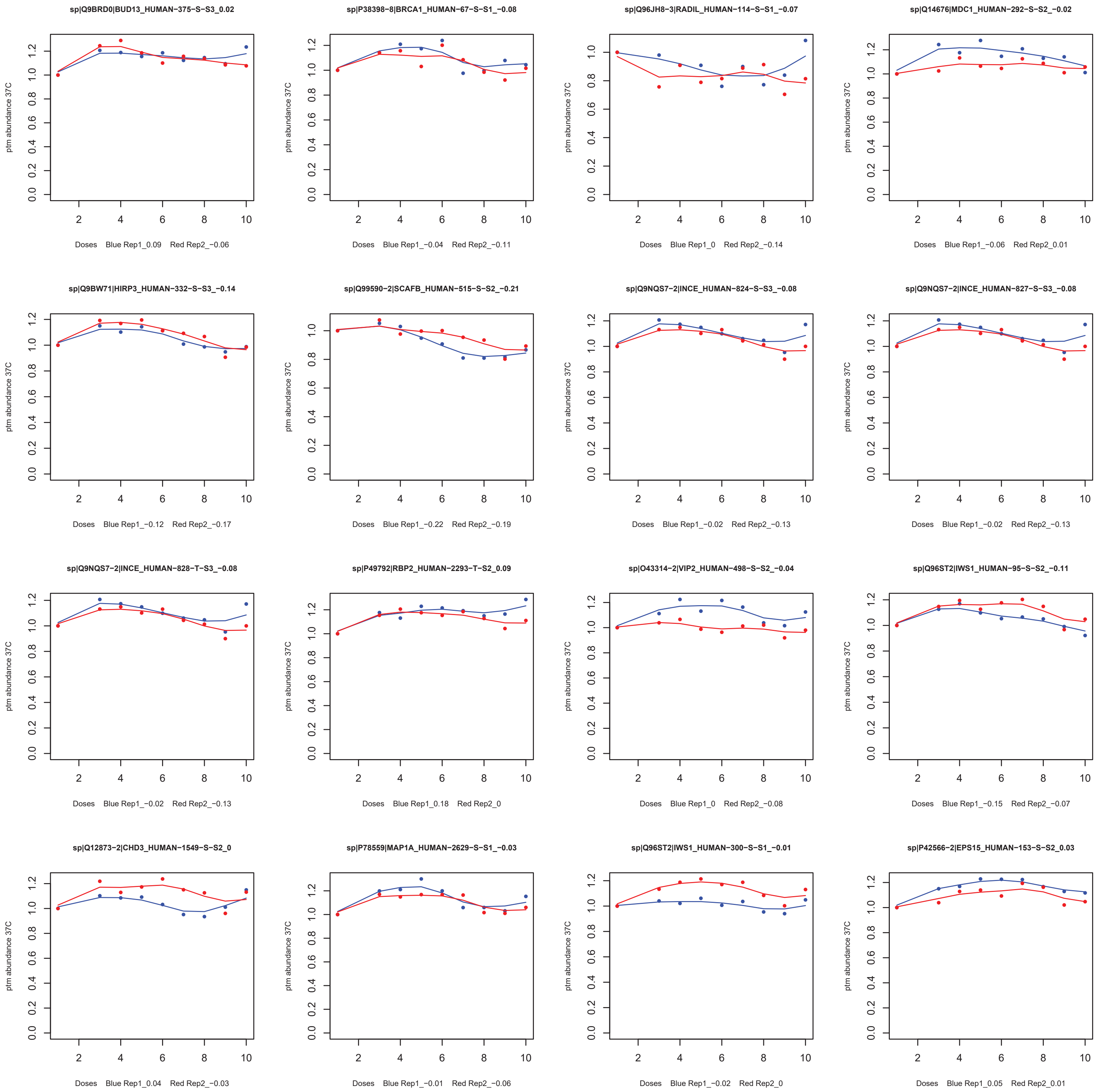

sp|Q9BRD0|BUD13_HUMAN−375−S−S3_0.02
ptm abundance 37C
Doses Blue Rep1_0.09 Red Rep2_−0.06
sp|P38398−8|BRCA1_HUMAN−67−S−S1_−0.08
ptm abundance 37C
Doses Blue Rep1_−0.04 Red Rep2_−0.11
sp|Q96JH8−3|RADIL_HUMAN−114−S−S1_−0.07
ptm abundance 37C
Doses Blue Rep1_0 Red Rep2_−0.14
sp|Q14676|MDC1_HUMAN−292−S−S2_−0.02
ptm abundance 37C
Doses Blue Rep1_−0.06 Red Rep2_0.01
sp|Q9BW71|HIRP3_HUMAN−332−S−S3_−0.14
ptm abundance 37C
Doses Blue Rep1_−0.12 Red Rep2_−0.17
sp|Q99590−2|SCAFB_HUMAN−515−S−S2_−0.21
ptm abundance 37C
Doses Blue Rep1_−0.22 Red Rep2_−0.19
sp|Q9NQS7−2|INCE_HUMAN−824−S−S3_−0.08
ptm abundance 37C
Doses Blue Rep1_−0.02 Red Rep2_−0.13
sp|Q9NQS7−2|INCE_HUMAN−827−S−S3_−0.08
ptm abundance 37C
Doses Blue Rep1_−0.02 Red Rep2_−0.13
sp|Q9NQS7−2|INCE_HUMAN−828−T−S3_−0.08
ptm abundance 37C
Doses Blue Rep1_−0.02 Red Rep2_−0.13
sp|P49792|RBP2_HUMAN−2293−T−S2_0.09
ptm abundance 37C
Doses Blue Rep1_0.18 Red Rep2_0
sp|O43314−2|VIP2_HUMAN−498−S−S2_−0.04
ptm abundance 37C
Doses Blue Rep1_0 Red Rep2_−0.08
sp|Q96ST2|IWS1_HUMAN−95−S−S2_−0.11
ptm abundance 37C
Doses Blue Rep1_−0.15 Red Rep2_−0.07
sp|Q12873−2|CHD3_HUMAN−1549−S−S2_0
ptm abundance 37C
Doses Blue Rep1_0.04 Red Rep2_−0.03
sp|P78559|MAP1A_HUMAN−2629−S−S1_−0.03
ptm abundance 37C
Doses Blue Rep1_−0.01 Red Rep2_−0.06
sp|Q96ST2|IWS1_HUMAN−300−S−S1_−0.01
ptm abundance 37C
Doses Blue Rep1_−0.02 Red Rep2_0
sp|P42566−2|EPS15_HUMAN−153−S−S2_0.03
ptm abundance 37C
Doses Blue Rep1_0.05 Red Rep2_0.01

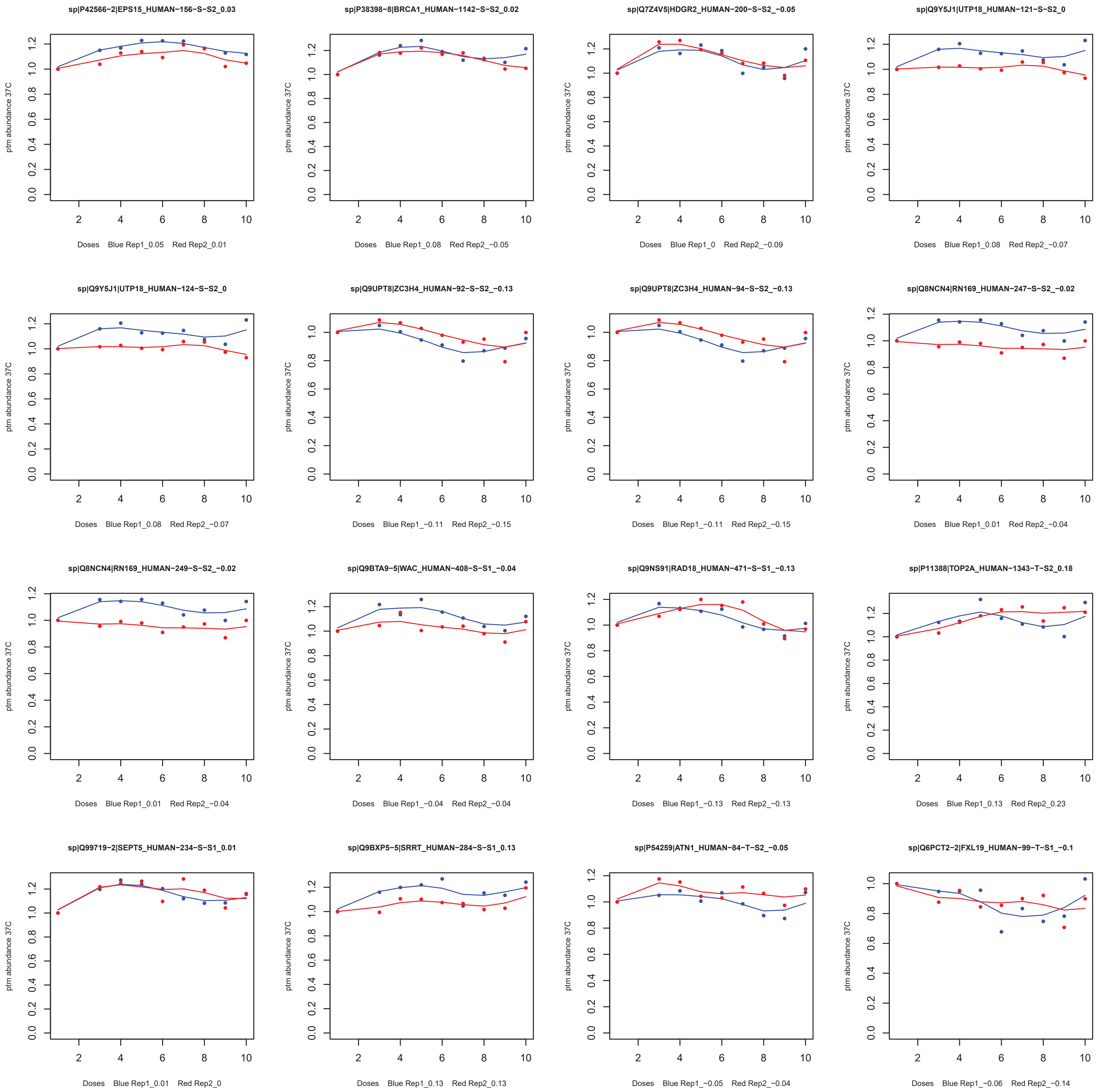
sp|P42566−2|EPS15_HUMAN−156−S−S2_0.03
ptm abundance 37C
Doses Blue Rep1_0.05 Red Rep2_0.01
sp|P38398−8|BRCA1_HUMAN−1142−S−S2_0.02
ptm abundance 37C
Doses Blue Rep1_0.08 Red Rep2_−0.05
sp|Q7Z4V5|HDGR2_HUMAN−200−S−S2_−0.05
ptm abundance 37C
Doses Blue Rep1_0 Red Rep2_−0.09
sp|Q9Y5J1|UTP18_HUMAN−121−S−S2_0
ptm abundance 37C
Doses Blue Rep1_0.08 Red Rep2_−0.07
sp|Q9Y5J1|UTP18_HUMAN−124−S−S2_0
ptm abundance 37C
Doses Blue Rep1_0.08 Red Rep2_−0.07
sp|Q9UPT8|ZC3H4_HUMAN−92−S−S2_−0.13
ptm abundance 37C
Doses Blue Rep1_−0.11 Red Rep2_−0.15
sp|Q9UPT8|ZC3H4_HUMAN−94−S−S2_−0.13
ptm abundance 37C
Doses Blue Rep1_−0.11 Red Rep2_−0.15
sp|Q8NCN4|RN169_HUMAN−247−S−S2_−0.02
ptm abundance 37C
Doses Blue Rep1_0.01 Red Rep2_−0.04
sp|Q8NCN4|RN169_HUMAN−249−S−S2_−0.02
ptm abundance 37C
Doses Blue Rep1_0.01 Red Rep2_−0.04
sp|Q9BTA9−5|WAC_HUMAN−408−S−S1_−0.04
ptm abundance 37C
Doses Blue Rep1_−0.04 Red Rep2_−0.04
sp|Q9NS91|RAD18_HUMAN−471−S−S1_−0.13
ptm abundance 37C
Doses Blue Rep1_−0.13 Red Rep2_−0.13
sp|P11388|TOP2A_HUMAN−1343−T−S2_0.18
ptm abundance 37C
Doses Blue Rep1_0.13 Red Rep2_0.23
sp|Q99719−2|SEPT5_HUMAN−234−S−S1_0.01
ptm abundance 37C
Doses Blue Rep1_0.01 Red Rep2_0
sp|Q9BXP5−5|SRRT_HUMAN−284−S−S1_0.13
ptm abundance 37C
Doses Blue Rep1_0.13 Red Rep2_0.13
sp|P54259|ATN1_HUMAN−84−T−S2_−0.05
ptm abundance 37C
Doses Blue Rep1_−0.05 Red Rep2_−0.04
sp|Q6PCT2−2|FXL19_HUMAN−99−T−S1_−0.1
ptm abundance 37C
Doses Blue Rep1_−0.06 Red Rep2_−0.14

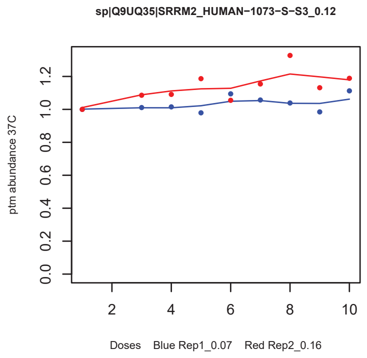
sp|Q9UQ35|SRRM2_HUMAN−1073−S−S3_0.12
ptm abundance 37C
Doses Blue Rep1_0.07 Red Rep2_0.16

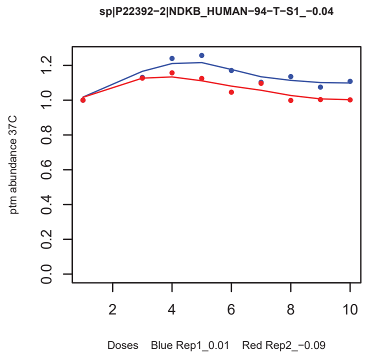
sp|P22392−2|NDKB_HUMAN−94−T−S1_−0.04
ptm abundance 37C
Doses Blue Rep1_0.01 Red Rep2_−0.09

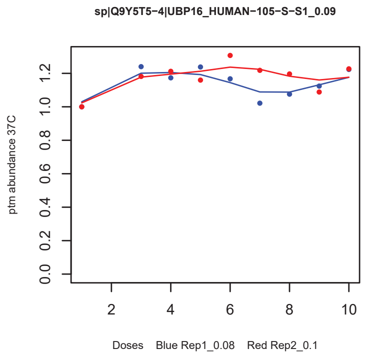
sp|Q9Y5T5−4|UBP16_HUMAN−105−S−S1_0.09
ptm abundance 37C
Doses Blue Rep1_0.08 Red Rep2_0.1

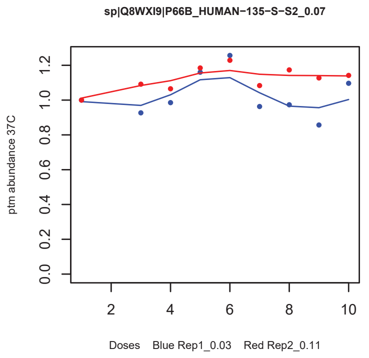
sp|Q8WXI9|P66B_HUMAN−135−S−S2_0.07
ptm abundance 37C
Doses Blue Rep1_0.03 Red Rep2_0.11

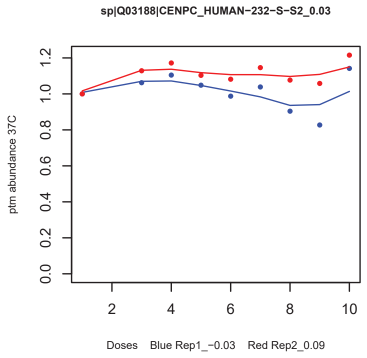
sp|Q03188|CENPC_HUMAN−232−S−S2_0.03
ptm abundance 37C
Doses Blue Rep1_−0.03 Red Rep2_0.09

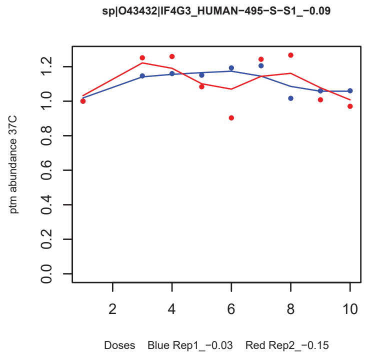
sp|O43432|IF4G3_HUMAN−495−S−S1_−0.09
ptm abundance 37C
Doses Blue Rep1_−0.03 Red Rep2_−0.15

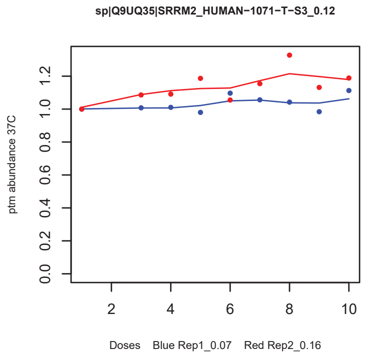
sp|Q9UQ35|SRRM2_HUMAN−1071−T−S3_0.12
ptm abundance 37C
Doses Blue Rep1_0.07 Red Rep2_0.16

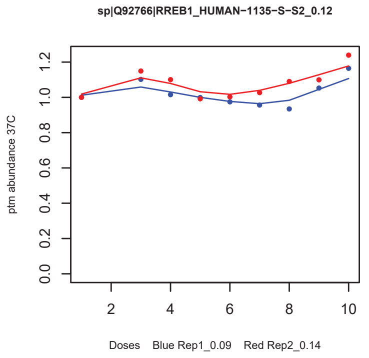
sp|Q92766|RREB1_HUMAN−1135−S−S2_0.12
ptm abundance 37C
Doses Blue Rep1_0.09 Red Rep2_0.14

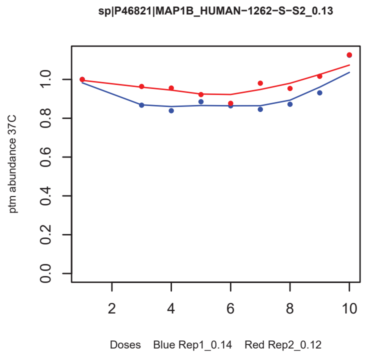
sp|P46821|MAP1B_HUMAN−1262−S−S2_0.13
ptm abundance 37C
Doses Blue Rep1_0.14 Red Rep2_0.12

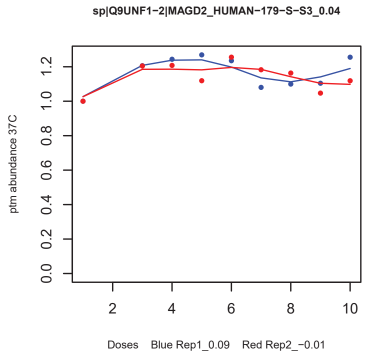
sp|Q9UNF1−2|MAGD2_HUMAN−179−S−S3_0.04
ptm abundance 37C
Doses Blue Rep1_0.09 Red Rep2_−0.01

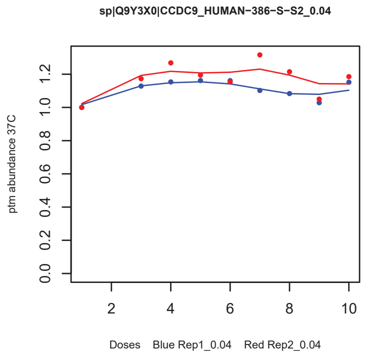
sp|Q9Y3X0|CCDC9_HUMAN−386−S−S2_0.04
ptm abundance 37C
Doses Blue Rep1_0.04 Red Rep2_0.04

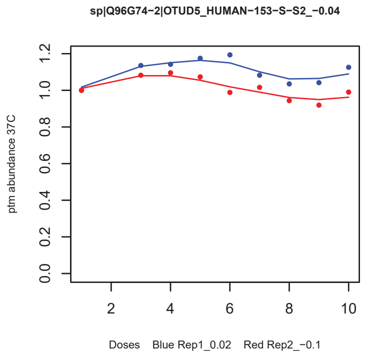
sp|Q96G74−2|OTUD5_HUMAN−153−S−S2_−0.04
ptm abundance 37C
Doses Blue Rep1_0.02 Red Rep2_−0.1

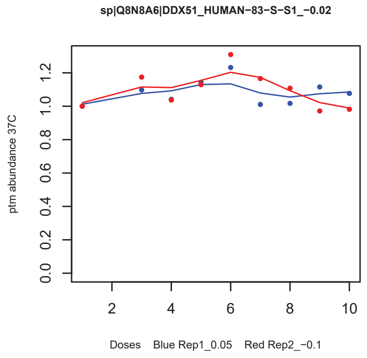
sp|Q8N8A6|DDX51_HUMAN−83−S−S1_−0.02
ptm abundance 37C
Doses Blue Rep1_0.05 Red Rep2_−0.1

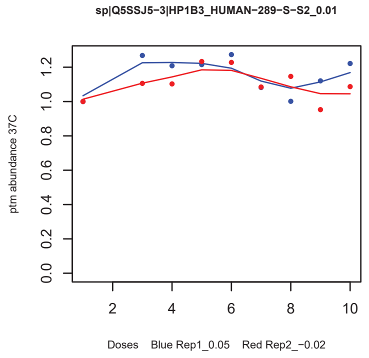
sp|Q5SSJ5−3|HP1B3_HUMAN−289−S−S2_0.01
ptm abundance 37C
Doses Blue Rep1_0.05 Red Rep2_−0.02

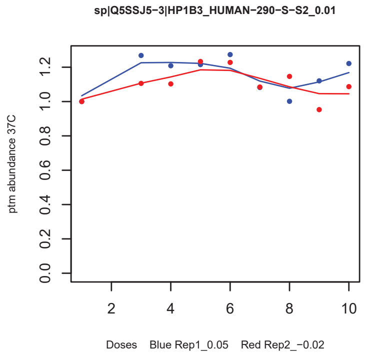
sp|Q5SSJ5−3|HP1B3_HUMAN−290−S−S2_0.01
ptm abundance 37C
Doses Blue Rep1_0.05 Red Rep2_−0.02

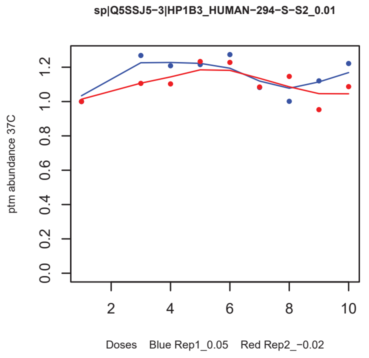
sp|Q5SSJ5−3|HP1B3_HUMAN−294−S−S2_0.01
ptm abundance 37C
Doses Blue Rep1_0.05 Red Rep2_−0.02

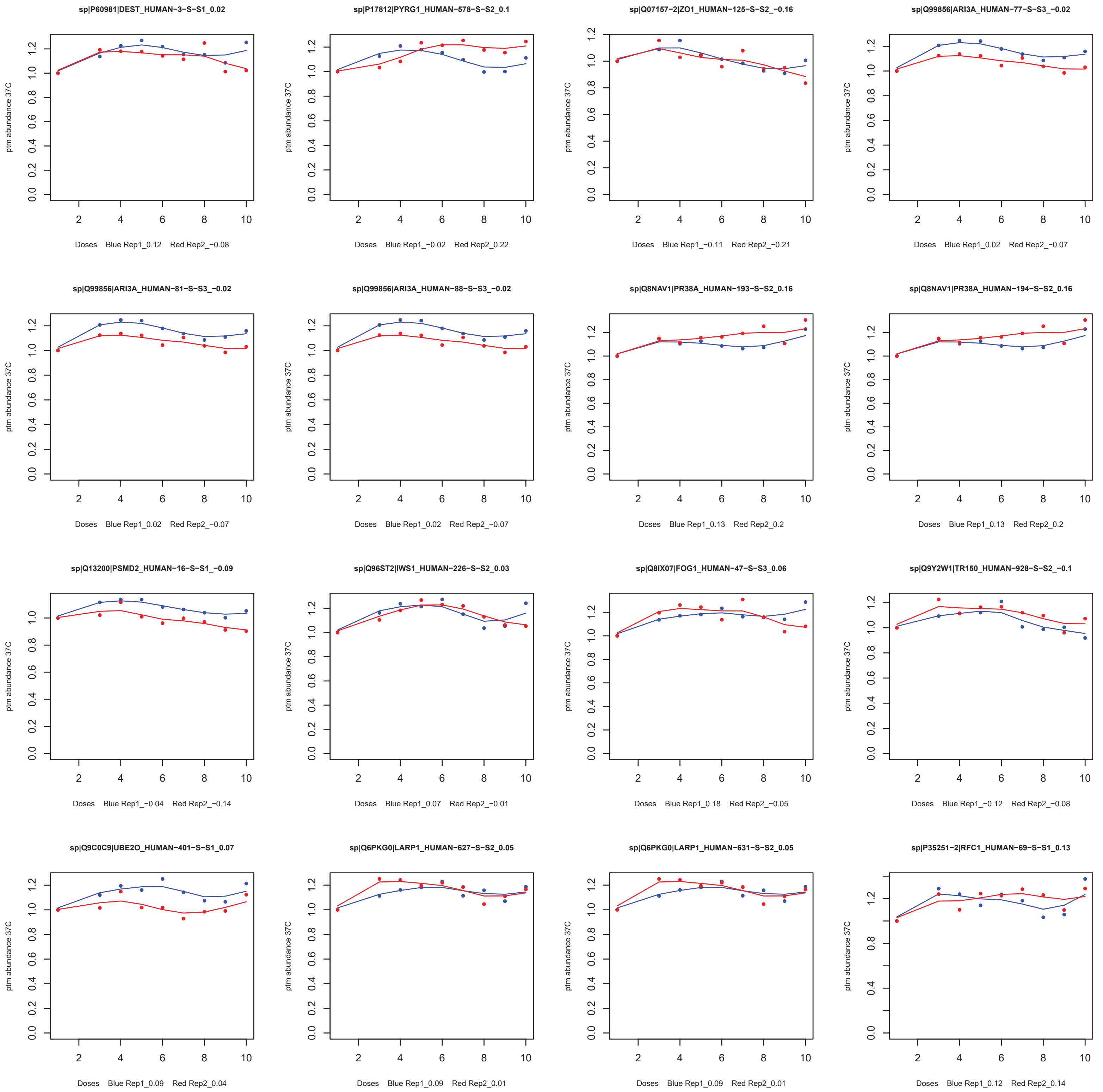
sp|P60981|DEST_HUMAN−3−S−S1_0.02
Doses Blue Rep1_0.12 Red Rep2_−0.08
sp|P17812|PYRG1_HUMAN−578−S−S2_0.1
Doses Blue Rep1_−0.02 Red Rep2_0.22
sp|Q07157−2|ZO1_HUMAN−125−S−S2_−0.16
Doses Blue Rep1_−0.11 Red Rep2_−0.21
sp|Q99856|ARI3A_HUMAN−77−S−S3_−0.02
Doses Blue Rep1_0.02 Red Rep2_−0.07
sp|Q99856|ARI3A_HUMAN−81−S−S3_−0.02
Doses Blue Rep1_0.02 Red Rep2_−0.07
sp|Q99856|ARI3A_HUMAN−88−S−S3_−0.02
Doses Blue Rep1_0.02 Red Rep2_−0.07
sp|Q8NAV1|PR38A_HUMAN−193−S−S2_0.16
Doses Blue Rep1_0.13 Red Rep2_0.2
sp|Q8NAV1|PR38A_HUMAN−194−S−S2_0.16
Doses Blue Rep1_0.13 Red Rep2_0.2
sp|Q13200|PSMD2_HUMAN−16−S−S1_−0.09
Doses Blue Rep1_−0.04 Red Rep2_−0.14
sp|Q96ST2|IWS1_HUMAN−226−S−S2_0.03
Doses Blue Rep1_0.07 Red Rep2_−0.01
sp|Q8IX07|FOG1_HUMAN−47−S−S3_0.06
Doses Blue Rep1_0.18 Red Rep2_−0.05
sp|Q9Y2W1|TR150_HUMAN−928−S−S2_−0.1
Doses Blue Rep1_−0.12 Red Rep2_−0.08
sp|Q9C0C9|UBE2O_HUMAN−401−S−S1_0.07
Doses Blue Rep1_0.09 Red Rep2_0.04
sp|Q6PKG0|LARP1_HUMAN−627−S−S2_0.05
Doses Blue Rep1_0.09 Red Rep2_0.01
sp|Q6PKG0|LARP1_HUMAN−631−S−S2_0.05
Doses Blue Rep1_0.09 Red Rep2_0.01
sp|P35251−2|RFC1_HUMAN−69−S−S1_0.13
Doses Blue Rep1_0.12 Red Rep2_0.14
ptm abundance 37C

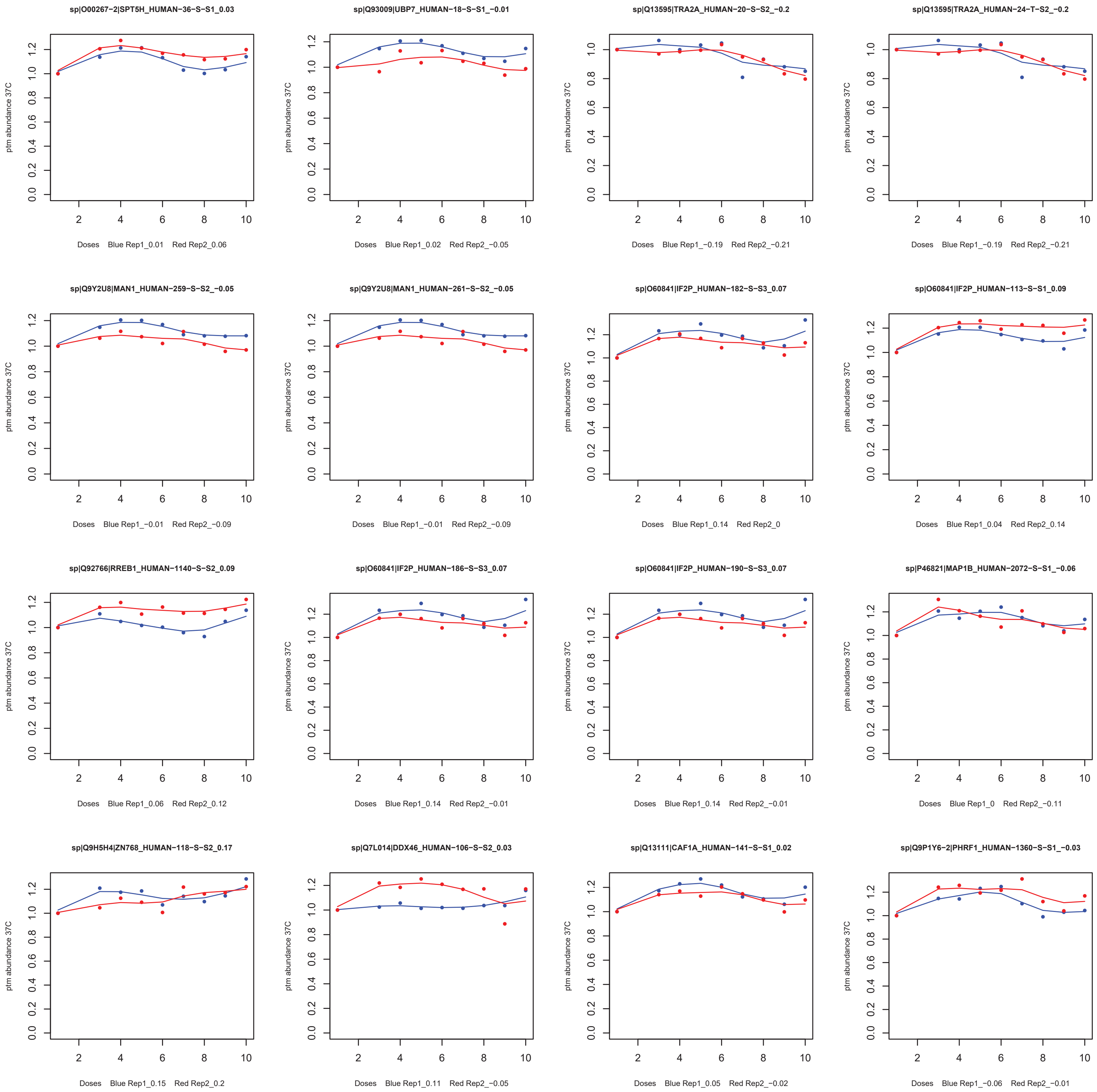
sp|O00267−2|SPT5H_HUMAN−36−S−S1_0.03
ptm abundance 37C
Doses Blue Rep1_0.01 Red Rep2_0.06
sp|Q93009|UBP7_HUMAN−18−S−S1_−0.01
ptm abundance 37C
Doses Blue Rep1_0.02 Red Rep2_−0.05
sp|Q13595|TRA2A_HUMAN−20−S−S2_−0.2
ptm abundance 37C
Doses Blue Rep1_−0.19 Red Rep2_−0.21
sp|Q13595|TRA2A_HUMAN−24−T−S2_−0.2
ptm abundance 37C
Doses Blue Rep1_−0.19 Red Rep2_−0.21
sp|Q9Y2U8|MAN1_HUMAN−259−S−S2_−0.05
ptm abundance 37C
Doses Blue Rep1_−0.01 Red Rep2_−0.09
sp|Q9Y2U8|MAN1_HUMAN−261−S−S2_−0.05
ptm abundance 37C
Doses Blue Rep1_−0.01 Red Rep2_−0.09
sp|O60841|IF2P_HUMAN−182−S−S3_0.07
ptm abundance 37C
Doses Blue Rep1_0.14 Red Rep2_0
sp|O60841|IF2P_HUMAN−113−S−S1_0.09
ptm abundance 37C
Doses Blue Rep1_0.04 Red Rep2_0.14
sp|Q92766|RREB1_HUMAN−1140−S−S2_0.09
ptm abundance 37C
Doses Blue Rep1_0.06 Red Rep2_0.12
sp|O60841|IF2P_HUMAN−186−S−S3_0.07
ptm abundance 37C
Doses Blue Rep1_0.14 Red Rep2_−0.01
sp|O60841|IF2P_HUMAN−190−S−S3_0.07
ptm abundance 37C
Doses Blue Rep1_0.14 Red Rep2_−0.01
sp|P46821|MAP1B_HUMAN−2072−S−S1_−0.06
ptm abundance 37C
Doses Blue Rep1_0 Red Rep2_−0.11
sp|Q9H5H4|ZN768_HUMAN−118−S−S2_0.17
ptm abundance 37C
Doses Blue Rep1_0.15 Red Rep2_0.2
sp|Q7L014|DDX46_HUMAN−106−S−S2_0.03
ptm abundance 37C
Doses Blue Rep1_0.11 Red Rep2_−0.05
sp|Q13111|CAF1A_HUMAN−141−S−S1_0.02
ptm abundance 37C
Doses Blue Rep1_0.05 Red Rep2_−0.02
sp|Q9P1Y6−2|PHRF1_HUMAN−1360−S−S1_−0.03
ptm abundance 37C
Doses Blue Rep1_−0.06 Red Rep2_−0.01

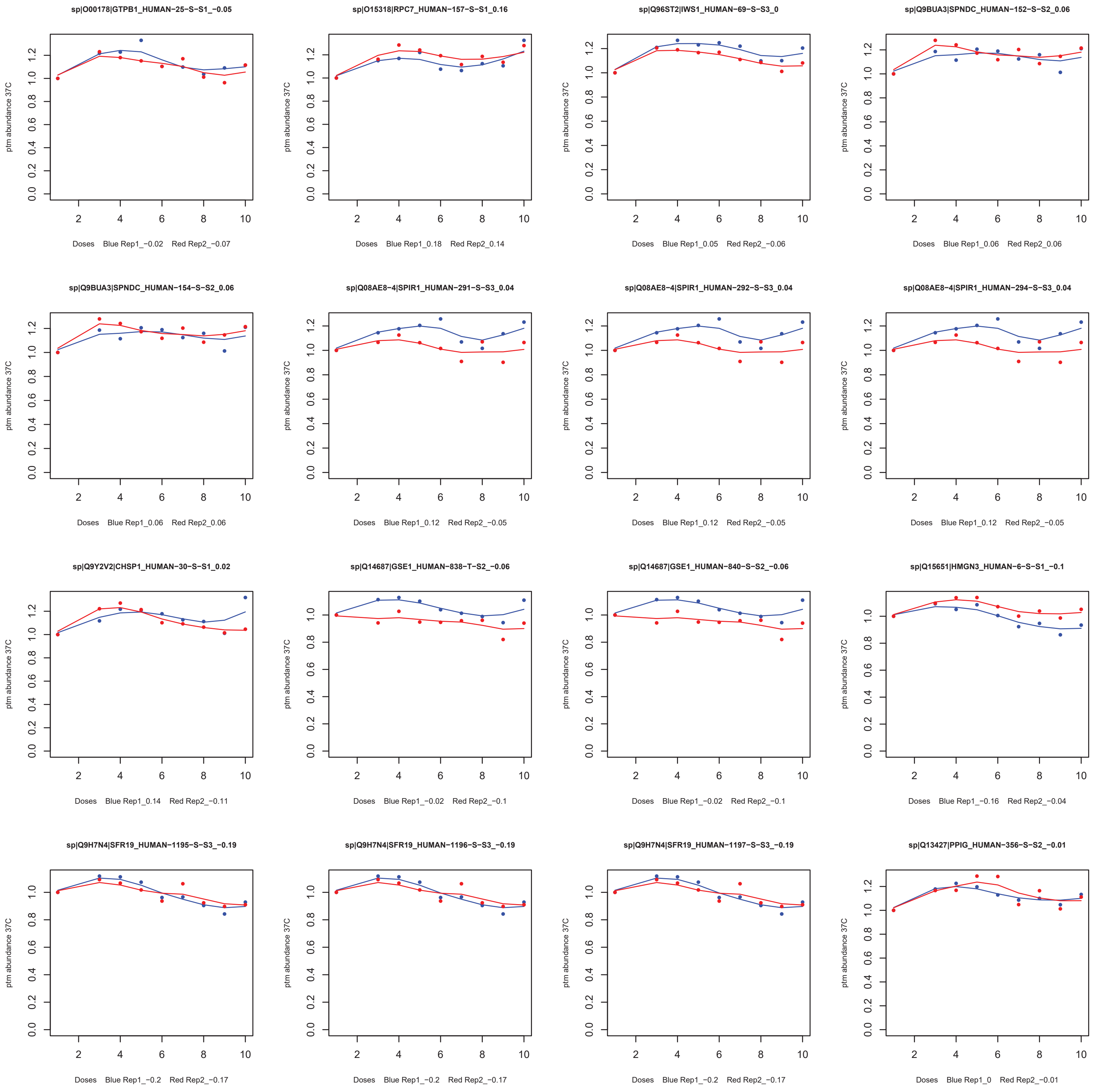
sp|O00178|GTPB1_HUMAN−25−S−S1_−0.05
Doses Blue Rep1_−0.02 Red Rep2_−0.07
sp|O15318|RPC7_HUMAN−157−S−S1_0.16
Doses Blue Rep1_0.18 Red Rep2_0.14
sp|Q96ST2|IWS1_HUMAN−69−S−S3_0
Doses Blue Rep1_0.05 Red Rep2_−0.06
sp|Q9BUA3|SPNDC_HUMAN−152−S−S2_0.06
Doses Blue Rep1_0.06 Red Rep2_0.06
sp|Q9BUA3|SPNDC_HUMAN−154−S−S2_0.06
Doses Blue Rep1_0.06 Red Rep2_0.06
sp|Q08AE8−4|SPIR1_HUMAN−291−S−S3_0.04
Doses Blue Rep1_0.12 Red Rep2_−0.05
sp|Q08AE8−4|SPIR1_HUMAN−292−S−S3_0.04
Doses Blue Rep1_0.12 Red Rep2_−0.05
sp|Q08AE8−4|SPIR1_HUMAN−294−S−S3_0.04
Doses Blue Rep1_0.12 Red Rep2_−0.05
sp|Q9Y2V2|CHSP1_HUMAN−30−S−S1_0.02
Doses Blue Rep1_0.14 Red Rep2_−0.11
sp|Q14687|GSE1_HUMAN−838−T−S2_−0.06
Doses Blue Rep1_−0.02 Red Rep2_−0.1
sp|Q14687|GSE1_HUMAN−840−S−S2_−0.06
Doses Blue Rep1_−0.02 Red Rep2_−0.1
sp|Q15651|HMGN3_HUMAN−6−S−S1_−0.1
Doses Blue Rep1_−0.16 Red Rep2_−0.04
sp|Q9H7N4|SFR19_HUMAN−1195−S−S3_−0.19
Doses Blue Rep1_−0.2 Red Rep2_−0.17
sp|Q9H7N4|SFR19_HUMAN−1196−S−S3_−0.19
Doses Blue Rep1_−0.2 Red Rep2_−0.17
sp|Q9H7N4|SFR19_HUMAN−1197−S−S3_−0.19
Doses Blue Rep1_−0.2 Red Rep2_−0.17
sp|Q13427|PPIG_HUMAN−356−S−S2_−0.01
Doses Blue Rep1_0 Red Rep2_−0.01
ptm abundance 37C

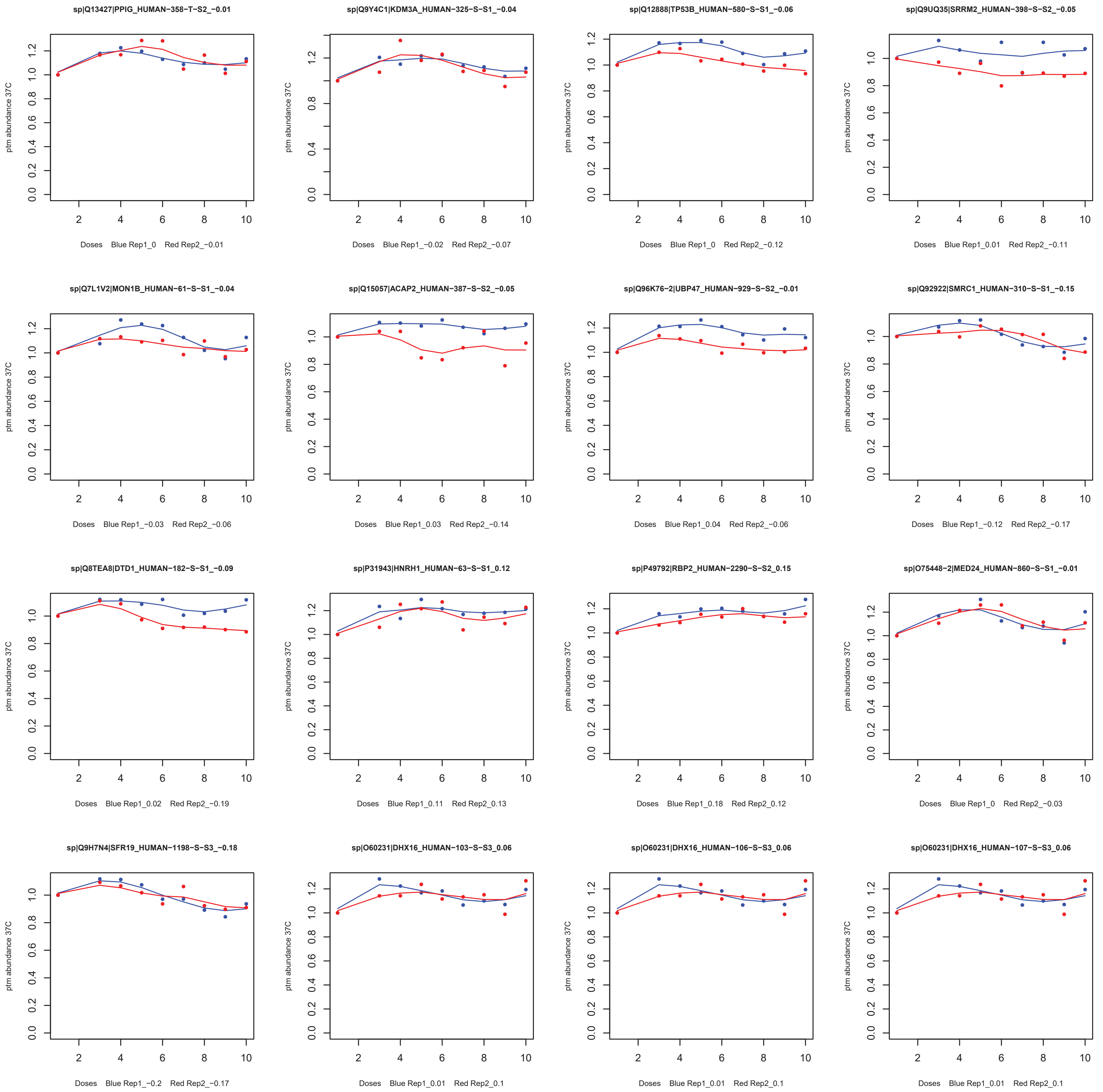
sp|Q13427|PPIG_HUMAN−358−T−S2_−0.01
ptm abundance 37C
Doses Blue Rep1_0 Red Rep2_−0.01
sp|Q9Y4C1|KDM3A_HUMAN−325−S−S1_−0.04
ptm abundance 37C
Doses Blue Rep1_−0.02 Red Rep2_−0.07
sp|Q12888|TP53B_HUMAN−580−S−S1_−0.06
ptm abundance 37C
Doses Blue Rep1_0 Red Rep2_−0.12
sp|Q9UQ35|SRRM2_HUMAN−398−S−S2_−0.05
ptm abundance 37C
Doses Blue Rep1_0.01 Red Rep2_−0.11
sp|Q7L1V2|MON1B_HUMAN−61−S−S1_−0.04
ptm abundance 37C
Doses Blue Rep1_−0.03 Red Rep2_−0.06
sp|Q15057|ACAP2_HUMAN−387−S−S2_−0.05
ptm abundance 37C
Doses Blue Rep1_0.03 Red Rep2_−0.14
sp|Q96K76−2|UBP47_HUMAN−929−S−S2_−0.01
ptm abundance 37C
Doses Blue Rep1_0.04 Red Rep2_−0.06
sp|Q92922|SMRC1_HUMAN−310−S−S1_−0.15
ptm abundance 37C
Doses Blue Rep1_−0.12 Red Rep2_−0.17
sp|Q8TEA8|DTD1_HUMAN−182−S−S1_−0.09
ptm abundance 37C
Doses Blue Rep1_0.02 Red Rep2_−0.19
sp|P31943|HNRH1_HUMAN−63−S−S1_0.12
ptm abundance 37C
Doses Blue Rep1_0.11 Red Rep2_0.13
sp|P49792|RBP2_HUMAN−2290−S−S2_0.15
ptm abundance 37C
Doses Blue Rep1_0.18 Red Rep2_0.12
sp|O75448−2|MED24_HUMAN−860−S−S1_−0.01
ptm abundance 37C
Doses Blue Rep1_0 Red Rep2_−0.03
sp|Q9H7N4|SFR19_HUMAN−1198−S−S3_−0.18
ptm abundance 37C
Doses Blue Rep1_−0.2 Red Rep2_−0.17
sp|O60231|DHX16_HUMAN−103−S−S3_0.06
ptm abundance 37C
Doses Blue Rep1_0.01 Red Rep2_0.1
sp|O60231|DHX16_HUMAN−106−S−S3_0.06
ptm abundance 37C
Doses Blue Rep1_0.01 Red Rep2_0.1
sp|O60231|DHX16_HUMAN−107−S−S3_0.06
ptm abundance 37C
Doses Blue Rep1_0.01 Red Rep2_0.1

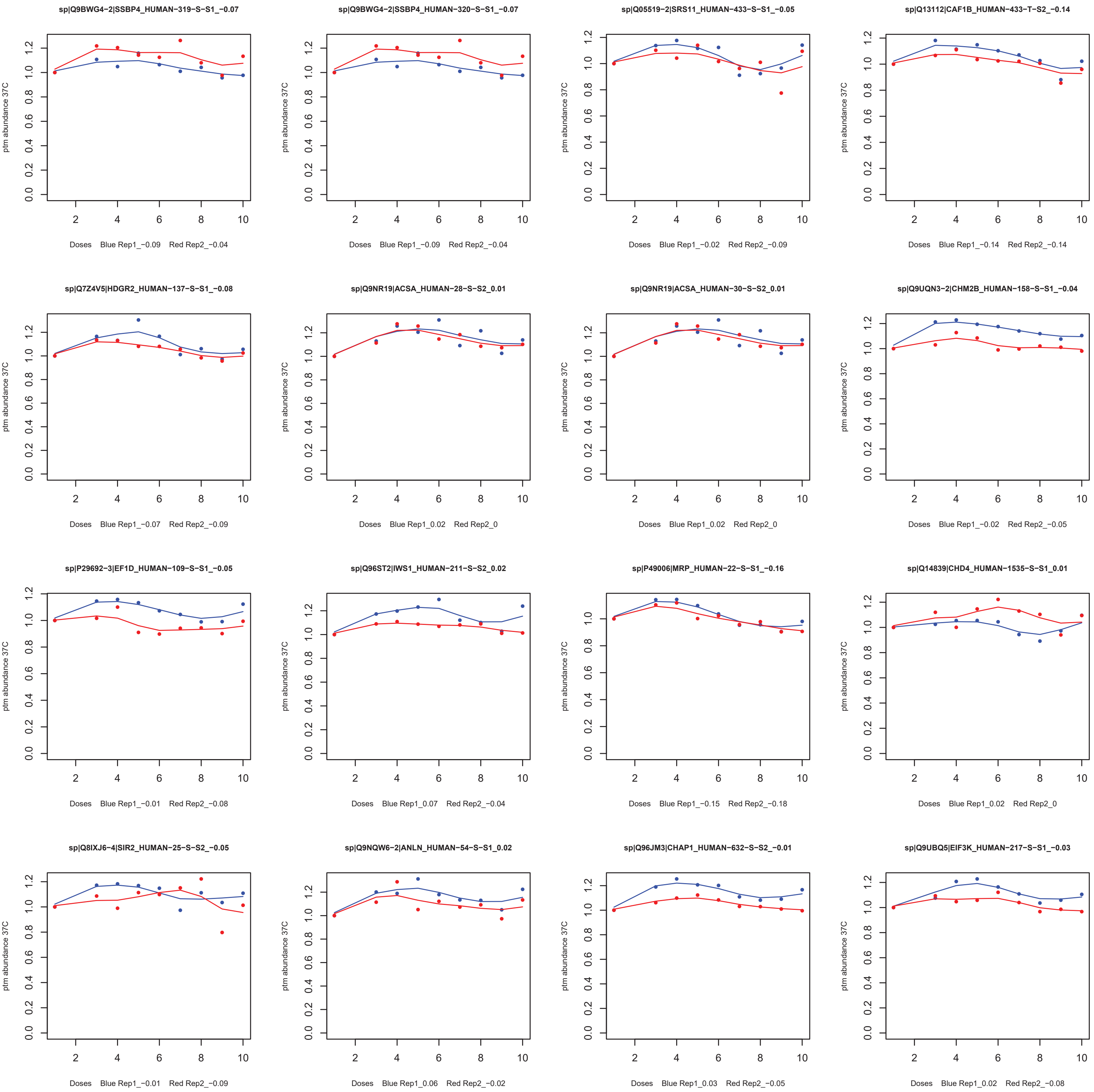
sp|Q9BWG4−2|SSBP4_HUMAN−319−S−S1_−0.07
ptm abundance 37C
Doses Blue Rep1_−0.09 Red Rep2_−0.04
sp|Q9BWG4−2|SSBP4_HUMAN−320−S−S1_−0.07
ptm abundance 37C
Doses Blue Rep1_−0.09 Red Rep2_−0.04
sp|Q05519−2|SRS11_HUMAN−433−S−S1_−0.05
ptm abundance 37C
Doses Blue Rep1_−0.02 Red Rep2_−0.09
sp|Q13112|CAF1B_HUMAN−433−T−S2_−0.14
ptm abundance 37C
Doses Blue Rep1_−0.14 Red Rep2_−0.14
sp|Q7Z4V5|HDGR2_HUMAN−137−S−S1_−0.08
ptm abundance 37C
Doses Blue Rep1_−0.07 Red Rep2_−0.09
sp|Q9NR19|ACSA_HUMAN−28−S−S2_0.01
ptm abundance 37C
Doses Blue Rep1_0.02 Red Rep2_0
sp|Q9NR19|ACSA_HUMAN−30−S−S2_0.01
ptm abundance 37C
Doses Blue Rep1_0.02 Red Rep2_0
sp|Q9UQN3−2|CHM2B_HUMAN−158−S−S1_−0.04
ptm abundance 37C
Doses Blue Rep1_−0.02 Red Rep2_−0.05
sp|P29692−3|EF1D_HUMAN−109−S−S1_−0.05
ptm abundance 37C
Doses Blue Rep1_−0.01 Red Rep2_−0.08
sp|Q96ST2|IWS1_HUMAN−211−S−S2_0.02
ptm abundance 37C
Doses Blue Rep1_0.07 Red Rep2_−0.04
sp|P49006|MRP_HUMAN−22−S−S1_−0.16
ptm abundance 37C
Doses Blue Rep1_−0.15 Red Rep2_−0.18
sp|Q14839|CHD4_HUMAN−1535−S−S1_0.01
ptm abundance 37C
Doses Blue Rep1_0.02 Red Rep2_0
sp|Q8IXJ6−4|SIR2_HUMAN−25−S−S2_−0.05
ptm abundance 37C
Doses Blue Rep1_−0.01 Red Rep2_−0.09
sp|Q9NQW6−2|ANLN_HUMAN−54−S−S1_0.02
ptm abundance 37C
Doses Blue Rep1_0.06 Red Rep2_−0.02
sp|Q96JM3|CHAP1_HUMAN−632−S−S2_−0.01
ptm abundance 37C
Doses Blue Rep1_0.03 Red Rep2_−0.05
sp|Q9UBQ5|EIF3K_HUMAN−217−S−S1_−0.03
ptm abundance 37C
Doses Blue Rep1_0.02 Red Rep2_−0.08

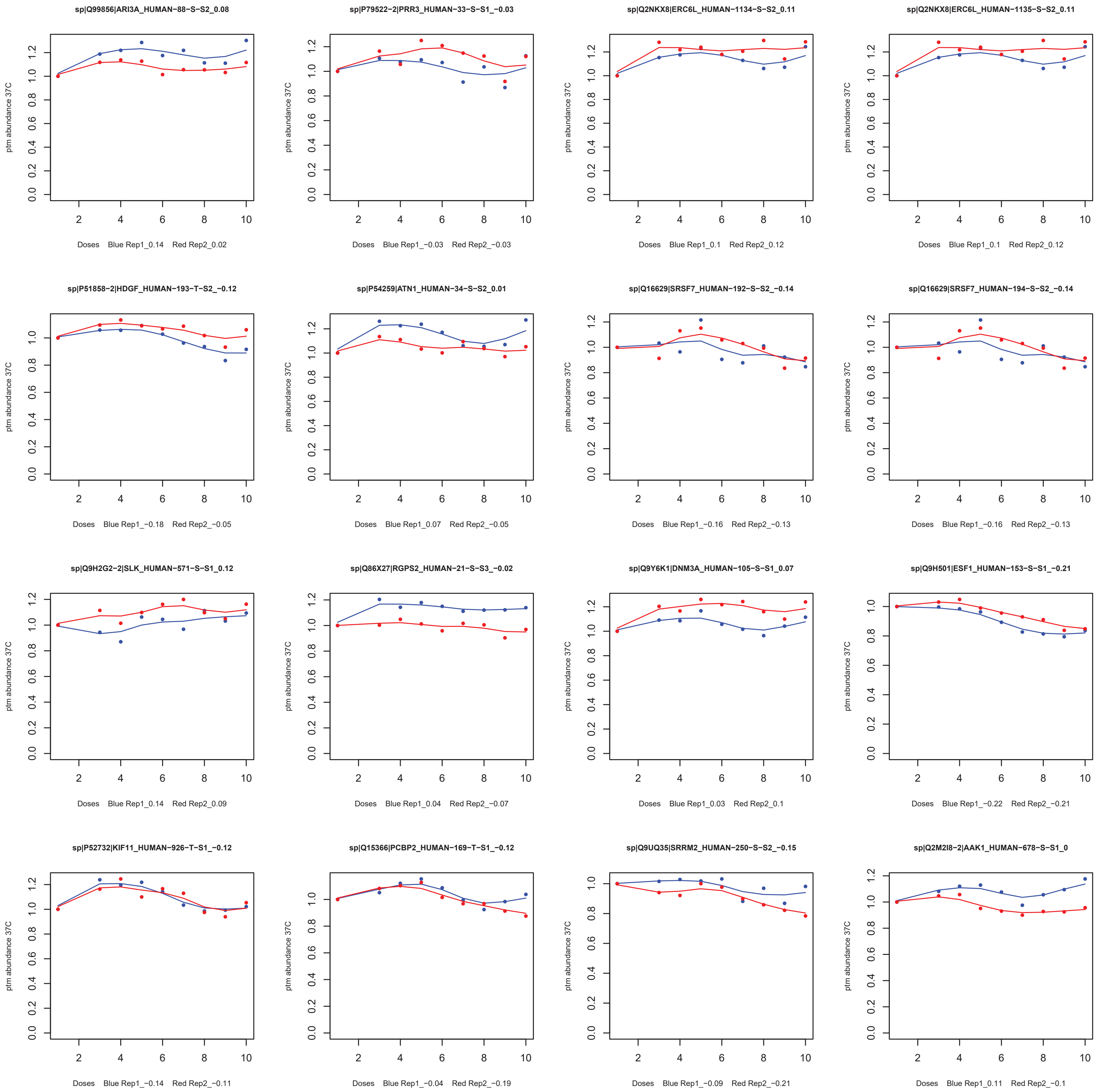
sp|Q99856|ARI3A_HUMAN−88−S−S2_0.08
ptm abundance 37C
Doses Blue Rep1_0.14 Red Rep2_0.02
sp|P79522−2|PRR3_HUMAN−33−S−S1_−0.03
ptm abundance 37C
Doses Blue Rep1_−0.03 Red Rep2_−0.03
sp|Q2NKX8|ERC6L_HUMAN−1134−S−S2_0.11
ptm abundance 37C
Doses Blue Rep1_0.1 Red Rep2_0.12
sp|Q2NKX8|ERC6L_HUMAN−1135−S−S2_0.11
ptm abundance 37C
Doses Blue Rep1_0.1 Red Rep2_0.12
sp|P51858−2|HDGF_HUMAN−193−T−S2_−0.12
ptm abundance 37C
Doses Blue Rep1_−0.18 Red Rep2_−0.05
sp|P54259|ATN1_HUMAN−34−S−S2_0.01
ptm abundance 37C
Doses Blue Rep1_0.07 Red Rep2_−0.05
sp|Q16629|SRSF7_HUMAN−192−S−S2_−0.14
ptm abundance 37C
Doses Blue Rep1_−0.16 Red Rep2_−0.13
sp|Q16629|SRSF7_HUMAN−194−S−S2_−0.14
ptm abundance 37C
Doses Blue Rep1_−0.16 Red Rep2_−0.13
sp|Q9H2G2−2|SLK_HUMAN−571−S−S1_0.12
ptm abundance 37C
Doses Blue Rep1_0.14 Red Rep2_0.09
sp|Q86X27|RGPS2_HUMAN−21−S−S3_−0.02
ptm abundance 37C
Doses Blue Rep1_0.04 Red Rep2_−0.07
sp|Q9Y6K1|DNM3A_HUMAN−105−S−S1_0.07
ptm abundance 37C
Doses Blue Rep1_0.03 Red Rep2_0.1
sp|Q9H501|ESF1_HUMAN−153−S−S1_−0.21
ptm abundance 37C
Doses Blue Rep1_−0.22 Red Rep2_−0.21
sp|P52732|KIF11_HUMAN−926−T−S1_−0.12
ptm abundance 37C
Doses Blue Rep1_−0.14 Red Rep2_−0.11
sp|Q15366|PCBP2_HUMAN−169−T−S1_−0.12
ptm abundance 37C
Doses Blue Rep1_−0.04 Red Rep2_−0.19
sp|Q9UQ35|SRRM2_HUMAN−250−S−S2_−0.15
ptm abundance 37C
Doses Blue Rep1_−0.09 Red Rep2_−0.21
sp|Q2M2I8−2|AAK1_HUMAN−678−S−S1_0
ptm abundance 37C
Doses Blue Rep1_0.11 Red Rep2_−0.1

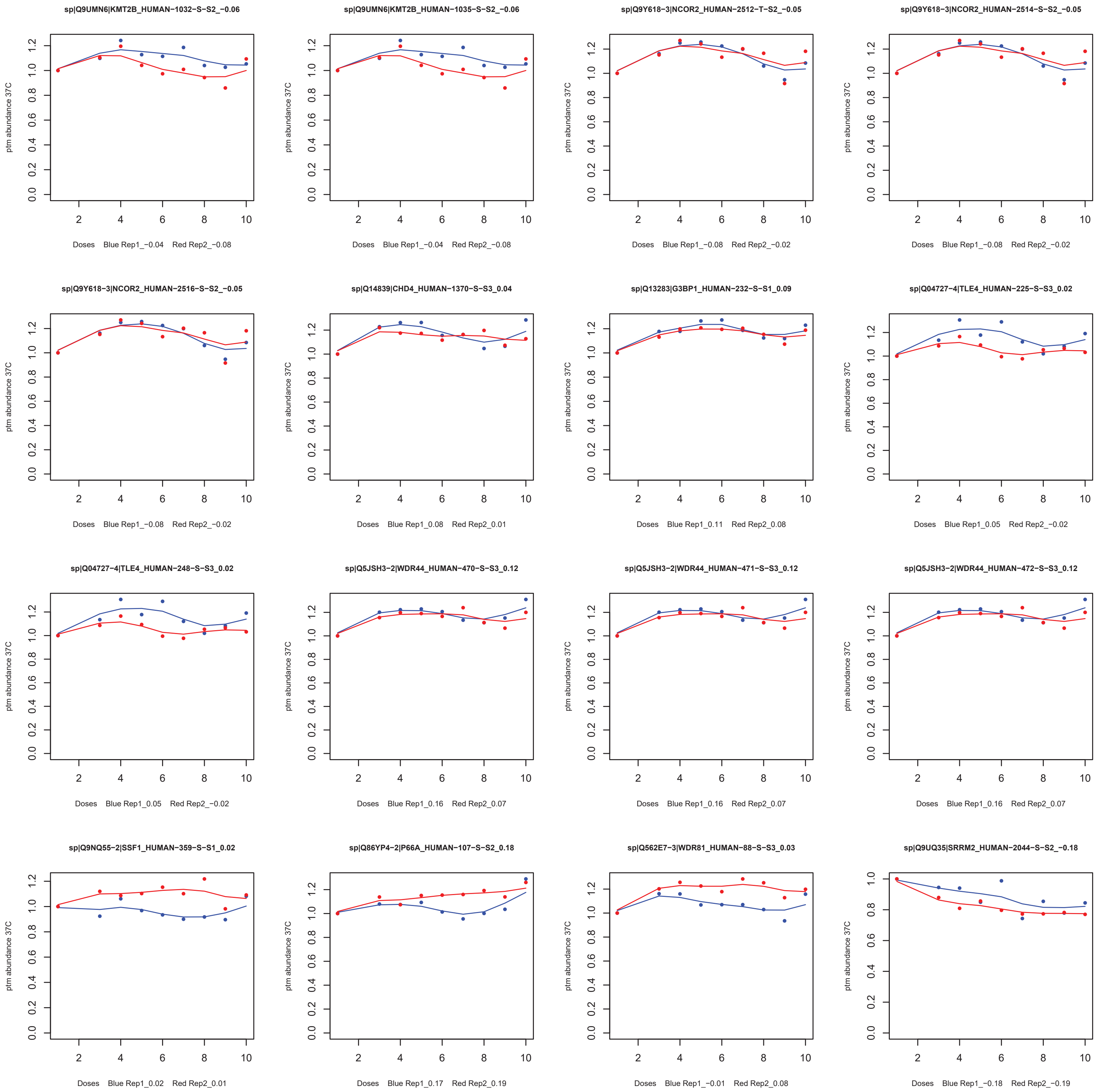
sp|Q9UMN6|KMT2B_HUMAN−1032−S−S2_−0.06
Doses Blue Rep1_−0.04 Red Rep2_−0.08
sp|Q9UMN6|KMT2B_HUMAN−1035−S−S2_−0.06
Doses Blue Rep1_−0.04 Red Rep2_−0.08
sp|Q9Y618−3|NCOR2_HUMAN−2512−T−S2_−0.05
Doses Blue Rep1_−0.08 Red Rep2_−0.02
sp|Q9Y618−3|NCOR2_HUMAN−2514−S−S2_−0.05
Doses Blue Rep1_−0.08 Red Rep2_−0.02
sp|Q9Y618−3|NCOR2_HUMAN−2516−S−S2_−0.05
Doses Blue Rep1_−0.08 Red Rep2_−0.02
sp|Q14839|CHD4_HUMAN−1370−S−S3_0.04
Doses Blue Rep1_0.08 Red Rep2_0.01
sp|Q13283|G3BP1_HUMAN−232−S−S1_0.09
Doses Blue Rep1_0.11 Red Rep2_0.08
sp|Q04727−4|TLE4_HUMAN−225−S−S3_0.02
Doses Blue Rep1_0.05 Red Rep2_−0.02
sp|Q04727−4|TLE4_HUMAN−248−S−S3_0.02
Doses Blue Rep1_0.05 Red Rep2_−0.02
sp|Q5JSH3−2|WDR44_HUMAN−470−S−S3_0.12
Doses Blue Rep1_0.16 Red Rep2_0.07
sp|Q5JSH3−2|WDR44_HUMAN−471−S−S3_0.12
Doses Blue Rep1_0.16 Red Rep2_0.07
sp|Q5JSH3−2|WDR44_HUMAN−472−S−S3_0.12
Doses Blue Rep1_0.16 Red Rep2_0.07
sp|Q9NQ55−2|SSF1_HUMAN−359−S−S1_0.02
Doses Blue Rep1_0.02 Red Rep2_0.01
sp|Q86YP4−2|P66A_HUMAN−107−S−S2_0.18
Doses Blue Rep1_0.17 Red Rep2_0.19
sp|Q562E7−3|WDR81_HUMAN−88−S−S3_0.03
Doses Blue Rep1_−0.01 Red Rep2_0.08
sp|Q9UQ35|SRRM2_HUMAN−2044−S−S2_−0.18
Doses Blue Rep1_−0.18 Red Rep2_−0.19
ptm abundance 37C

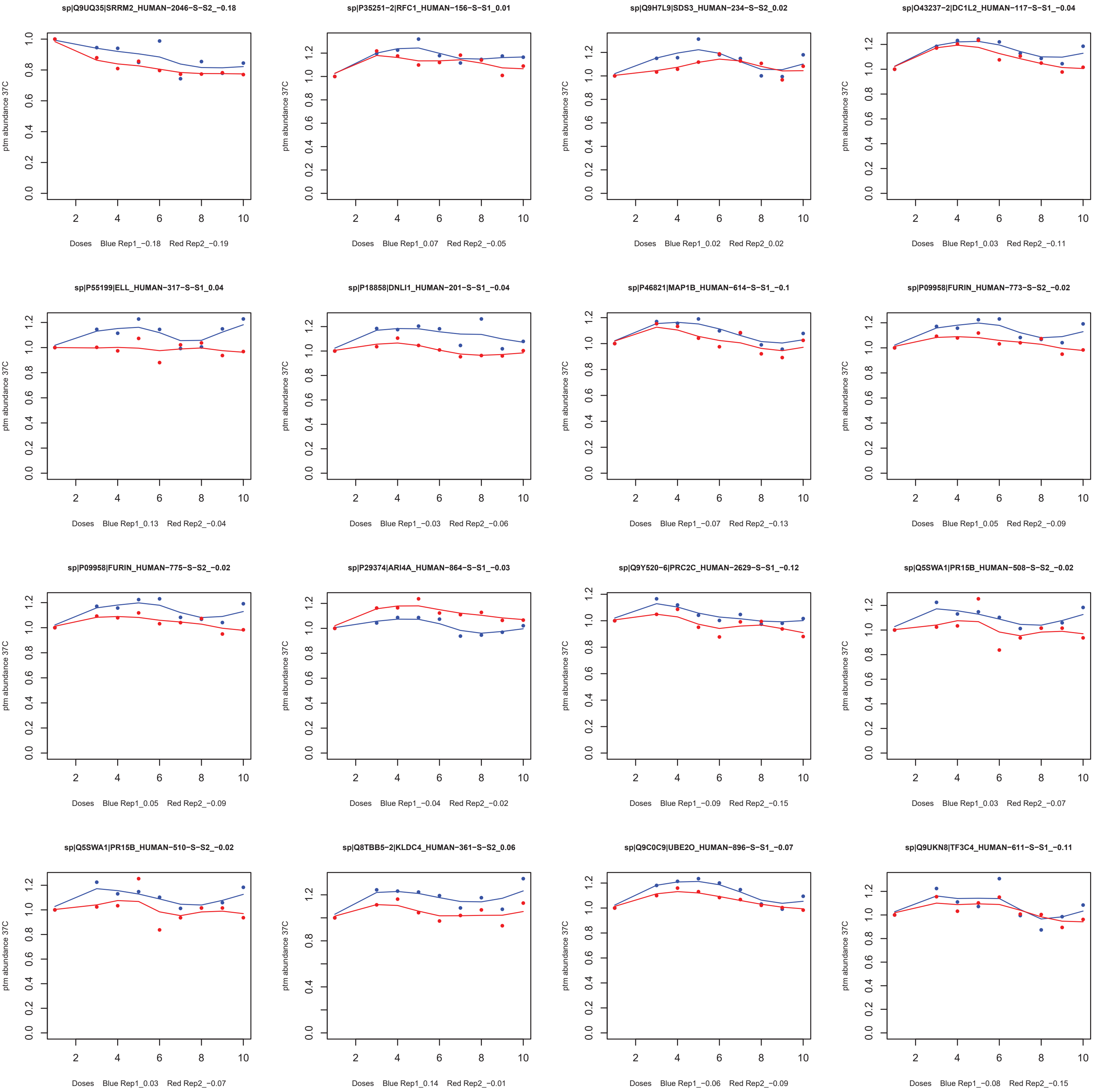
sp|Q9UQ35|SRRM2_HUMAN−2046−S−S2_−0.18
Doses Blue Rep1_−0.18 Red Rep2_−0.19
sp|P35251−2|RFC1_HUMAN−156−S−S1_0.01
Doses Blue Rep1_0.07 Red Rep2_−0.05
sp|Q9H7L9|SDS3_HUMAN−234−S−S2_0.02
Doses Blue Rep1_0.02 Red Rep2_0.02
sp|O43237−2|DC1L2_HUMAN−117−S−S1_−0.04
Doses Blue Rep1_0.03 Red Rep2_−0.11
sp|P55199|ELL_HUMAN−317−S−S1_0.04
Doses Blue Rep1_0.13 Red Rep2_−0.04
sp|P18858|DNLI1_HUMAN−201−S−S1_−0.04
Doses Blue Rep1_−0.03 Red Rep2_−0.06
sp|P46821|MAP1B_HUMAN−614−S−S1_−0.1
Doses Blue Rep1_−0.07 Red Rep2_−0.13
sp|P09958|FURIN_HUMAN−773−S−S2_−0.02
Doses Blue Rep1_0.05 Red Rep2_−0.09
sp|P09958|FURIN_HUMAN−775−S−S2_−0.02
Doses Blue Rep1_0.05 Red Rep2_−0.09
sp|P29374|ARI4A_HUMAN−864−S−S1_−0.03
Doses Blue Rep1_−0.04 Red Rep2_−0.02
sp|Q9Y520−6|PRC2C_HUMAN−2629−S−S1_−0.12
Doses Blue Rep1_−0.09 Red Rep2_−0.15
sp|Q5SWA1|PR15B_HUMAN−508−S−S2_−0.02
Doses Blue Rep1_0.03 Red Rep2_−0.07
sp|Q5SWA1|PR15B_HUMAN−510−S−S2_−0.02
Doses Blue Rep1_0.03 Red Rep2_−0.07
sp|Q8TBB5−2|KLDC4_HUMAN−361−S−S2_0.06
Doses Blue Rep1_0.14 Red Rep2_−0.01
sp|Q9C0C9|UBE2O_HUMAN−896−S−S1_−0.07
Doses Blue Rep1_−0.06 Red Rep2_−0.09
sp|Q9UKN8|TF3C4_HUMAN−611−S−S1_−0.11
Doses Blue Rep1_−0.08 Red Rep2_−0.15
ptm abundance 37C

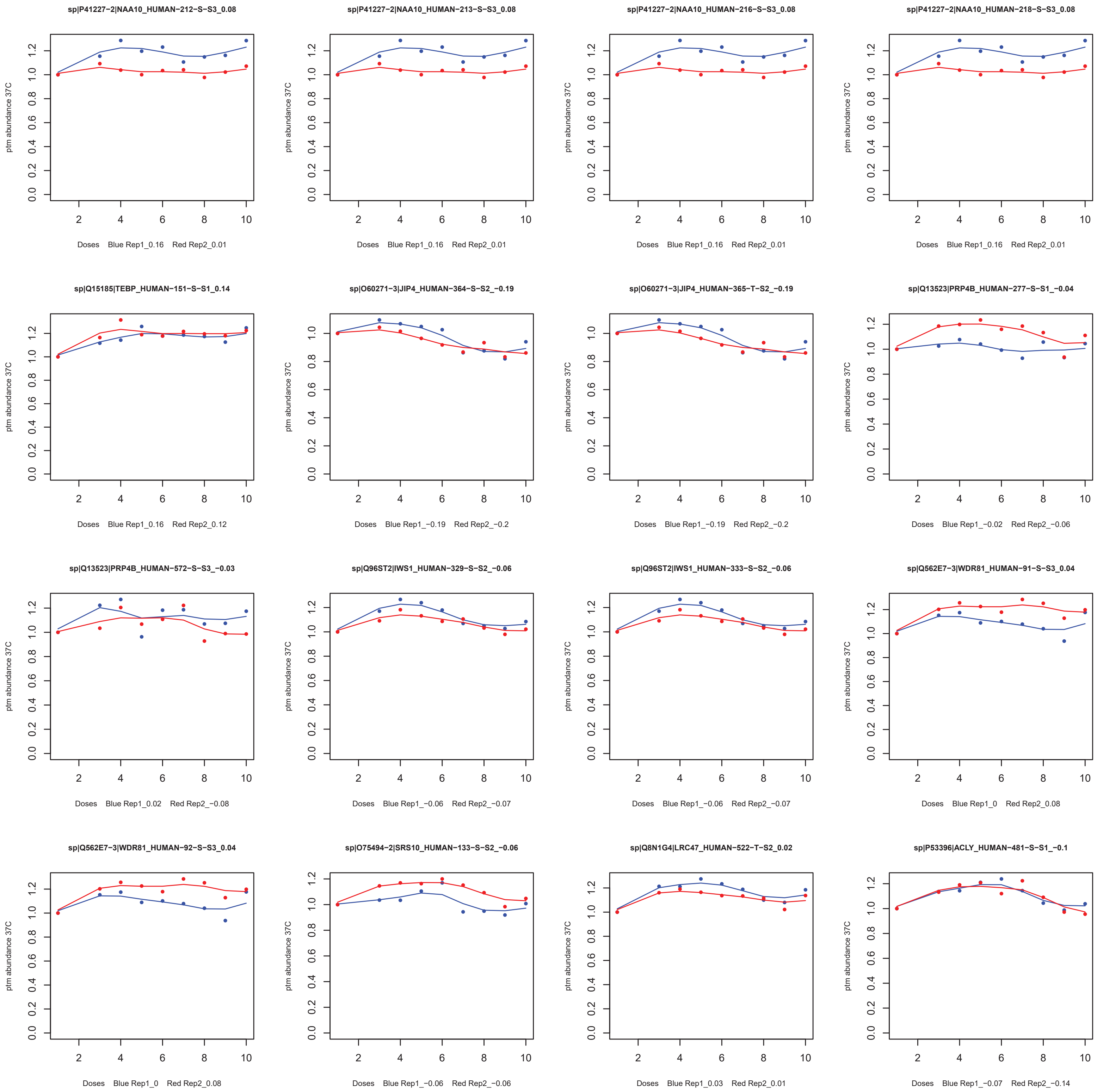
sp|P41227−2|NAA10_HUMAN−212−S−S3_0.08
Doses Blue Rep1_0.16 Red Rep2_0.01
sp|P41227−2|NAA10_HUMAN−213−S−S3_0.08
Doses Blue Rep1_0.16 Red Rep2_0.01
sp|P41227−2|NAA10_HUMAN−216−S−S3_0.08
Doses Blue Rep1_0.16 Red Rep2_0.01
sp|P41227−2|NAA10_HUMAN−218−S−S3_0.08
Doses Blue Rep1_0.16 Red Rep2_0.01
sp|Q15185|TEBP_HUMAN−151−S−S1_0.14
Doses Blue Rep1_0.16 Red Rep2_0.12
sp|O60271−3|JIP4_HUMAN−364−S−S2_−0.19
Doses Blue Rep1_−0.19 Red Rep2_−0.2
sp|O60271−3|JIP4_HUMAN−365−T−S2_−0.19
Doses Blue Rep1_−0.19 Red Rep2_−0.2
sp|Q13523|PRP4B_HUMAN−277−S−S1_−0.04
Doses Blue Rep1_−0.02 Red Rep2_−0.06
sp|Q13523|PRP4B_HUMAN−572−S−S3_−0.03
Doses Blue Rep1_0.02 Red Rep2_−0.08
sp|Q96ST2|IWS1_HUMAN−329−S−S2_−0.06
Doses Blue Rep1_−0.06 Red Rep2_−0.07
sp|Q96ST2|IWS1_HUMAN−333−S−S2_−0.06
Doses Blue Rep1_−0.06 Red Rep2_−0.07
sp|Q562E7−3|WDR81_HUMAN−91−S−S3_0.04
Doses Blue Rep1_0 Red Rep2_0.08
sp|Q562E7−3|WDR81_HUMAN−92−S−S3_0.04
Doses Blue Rep1_0 Red Rep2_0.08
sp|O75494−2|SRS10_HUMAN−133−S−S2_−0.06
Doses Blue Rep1_−0.06 Red Rep2_−0.06
sp|Q8N1G4|LRC47_HUMAN−522−T−S2_0.02
Doses Blue Rep1_0.03 Red Rep2_0.01
sp|P53396|ACLY_HUMAN−481−S−S1_−0.1
Doses Blue Rep1_−0.07 Red Rep2_−0.14
ptm abundance 37C

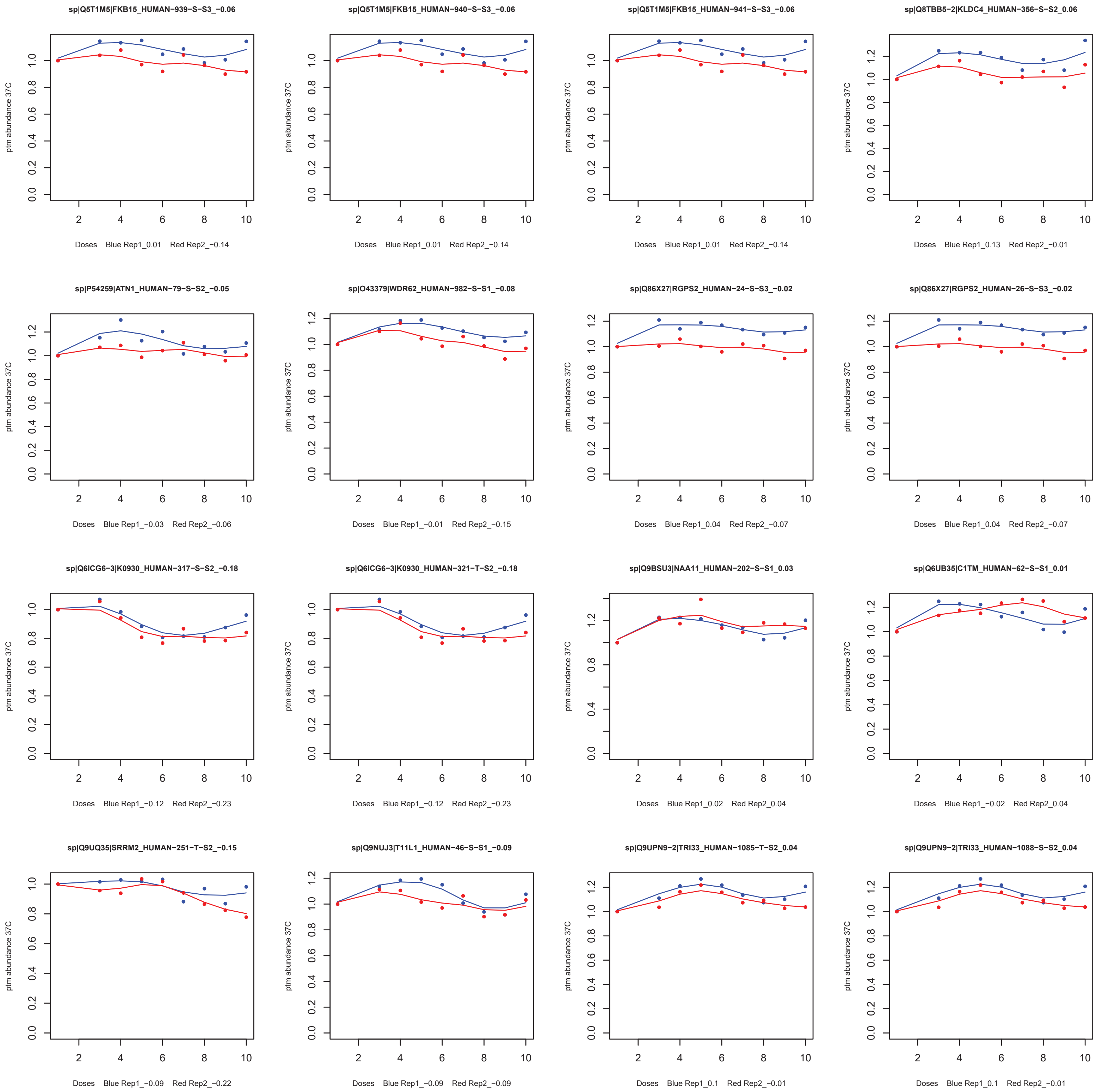
sp|Q5T1M5|FKB15_HUMAN−939−S−S3_−0.06
ptm abundance 37C
Doses Blue Rep1_0.01 Red Rep2_−0.14
sp|Q5T1M5|FKB15_HUMAN−940−S−S3_−0.06
ptm abundance 37C
Doses Blue Rep1_0.01 Red Rep2_−0.14
sp|Q5T1M5|FKB15_HUMAN−941−S−S3_−0.06
ptm abundance 37C
Doses Blue Rep1_0.01 Red Rep2_−0.14
sp|Q8TBB5−2|KLDC4_HUMAN−356−S−S2_0.06
ptm abundance 37C
Doses Blue Rep1_0.13 Red Rep2_−0.01
sp|P54259|ATN1_HUMAN−79−S−S2_−0.05
ptm abundance 37C
Doses Blue Rep1_−0.03 Red Rep2_−0.06
sp|O43379|WDR62_HUMAN−982−S−S1_−0.08
ptm abundance 37C
Doses Blue Rep1_−0.01 Red Rep2_−0.15
sp|Q86X27|RGPS2_HUMAN−24−S−S3_−0.02
ptm abundance 37C
Doses Blue Rep1_0.04 Red Rep2_−0.07
sp|Q86X27|RGPS2_HUMAN−26−S−S3_−0.02
ptm abundance 37C
Doses Blue Rep1_0.04 Red Rep2_−0.07
sp|Q6ICG6−3|K0930_HUMAN−317−S−S2_−0.18
ptm abundance 37C
Doses Blue Rep1_−0.12 Red Rep2_−0.23
sp|Q6ICG6−3|K0930_HUMAN−321−T−S2_−0.18
ptm abundance 37C
Doses Blue Rep1_−0.12 Red Rep2_−0.23
sp|Q9BSU3|NAA11_HUMAN−202−S−S1_0.03
ptm abundance 37C
Doses Blue Rep1_0.02 Red Rep2_0.04
sp|Q6UB35|C1TM_HUMAN−62−S−S1_0.01
ptm abundance 37C
Doses Blue Rep1_−0.02 Red Rep2_0.04
sp|Q9UQ35|SRRM2_HUMAN−251−T−S2_−0.15
ptm abundance 37C
Doses Blue Rep1_−0.09 Red Rep2_−0.22
sp|Q9NUJ3|T11L1_HUMAN−46−S−S1_−0.09
ptm abundance 37C
Doses Blue Rep1_−0.09 Red Rep2_−0.09
sp|Q9UPN9−2|TRI33_HUMAN−1085−T−S2_0.04
ptm abundance 37C
Doses Blue Rep1_0.1 Red Rep2_−0.01
sp|Q9UPN9−2|TRI33_HUMAN−1088−S−S2_0.04
ptm abundance 37C
Doses Blue Rep1_0.1 Red Rep2_−0.01

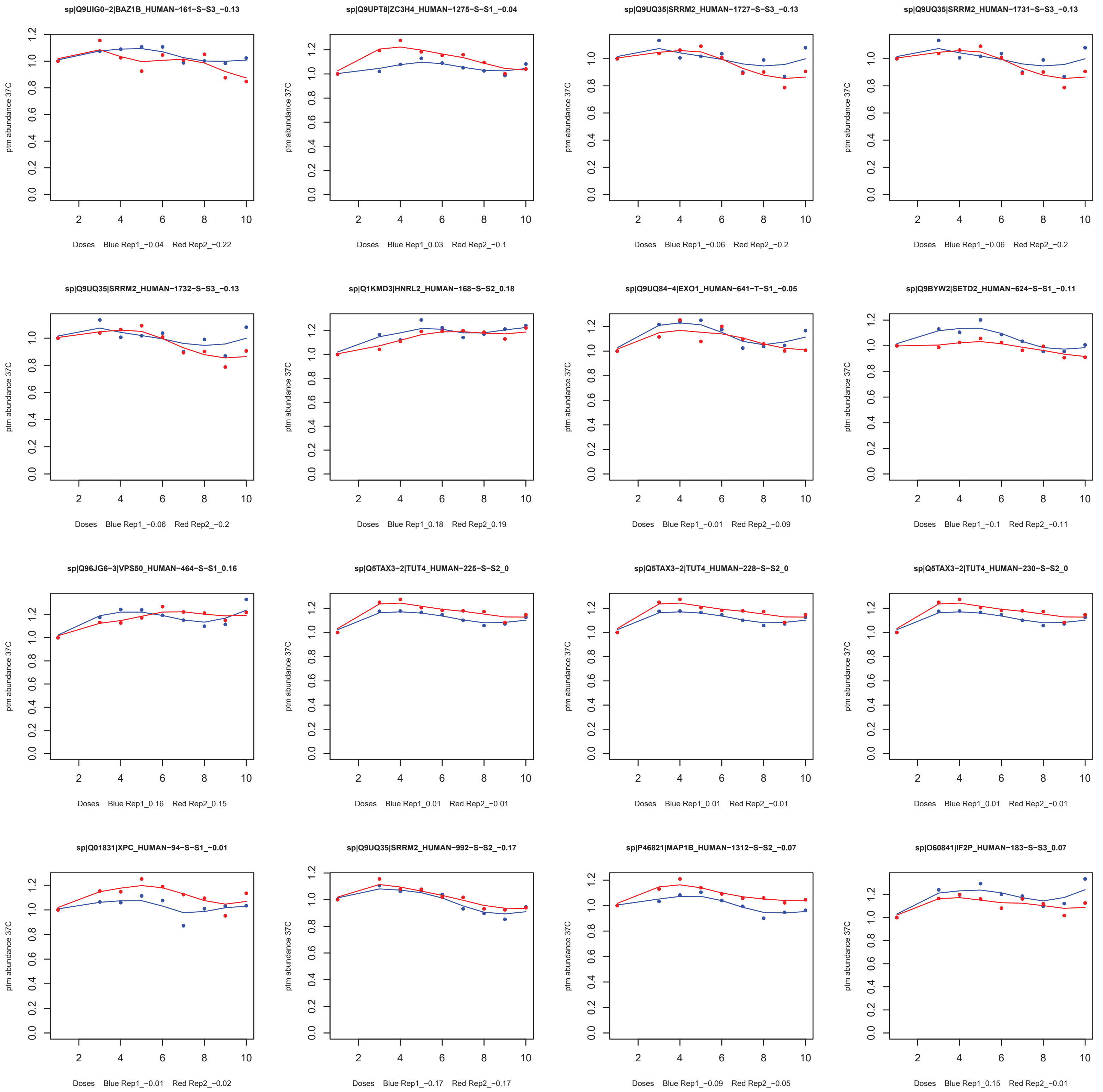
sp|Q9UIG0−2|BAZ1B_HUMAN−161−S−S3_−0.13
ptm abundance 37C
Doses Blue Rep1_−0.04 Red Rep2_−0.22
sp|Q9UPT8|ZC3H4_HUMAN−1275−S−S1_−0.04
ptm abundance 37C
Doses Blue Rep1_0.03 Red Rep2_−0.1
sp|Q9UQ35|SRRM2_HUMAN−1727−S−S3_−0.13
ptm abundance 37C
Doses Blue Rep1_−0.06 Red Rep2_−0.2
sp|Q9UQ35|SRRM2_HUMAN−1731−S−S3_−0.13
ptm abundance 37C
Doses Blue Rep1_−0.06 Red Rep2_−0.2
sp|Q9UQ35|SRRM2_HUMAN−1732−S−S3_−0.13
ptm abundance 37C
Doses Blue Rep1_−0.06 Red Rep2_−0.2
sp|Q1KMD3|HNRL2_HUMAN−168−S−S2_0.18
ptm abundance 37C
Doses Blue Rep1_0.18 Red Rep2_0.19
sp|Q9UQ84−4|EXO1_HUMAN−641−T−S1_−0.05
ptm abundance 37C
Doses Blue Rep1_−0.01 Red Rep2_−0.09
sp|Q9BYW2|SETD2_HUMAN−624−S−S1_−0.11
ptm abundance 37C
Doses Blue Rep1_−0.1 Red Rep2_−0.11
sp|Q96JG6−3|VPS50_HUMAN−464−S−S1_0.16
ptm abundance 37C
Doses Blue Rep1_0.16 Red Rep2_0.15
sp|Q5TAX3−2|TUT4_HUMAN−225−S−S2_0
ptm abundance 37C
Doses Blue Rep1_0.01 Red Rep2_−0.01
sp|Q5TAX3−2|TUT4_HUMAN−228−S−S2_0
ptm abundance 37C
Doses Blue Rep1_0.01 Red Rep2_−0.01
sp|Q5TAX3−2|TUT4_HUMAN−230−S−S2_0
ptm abundance 37C
Doses Blue Rep1_0.01 Red Rep2_−0.01
sp|Q01831|XPC_HUMAN−94−S−S1_−0.01
ptm abundance 37C
Doses Blue Rep1_−0.01 Red Rep2_−0.02
sp|Q9UQ35|SRRM2_HUMAN−992−S−S2_−0.17
ptm abundance 37C
Doses Blue Rep1_−0.17 Red Rep2_−0.17
sp|P46821|MAP1B_HUMAN−1312−S−S2_−0.07
ptm abundance 37C
Doses Blue Rep1_−0.09 Red Rep2_−0.05
sp|O60841|IF2P_HUMAN−183−S−S3_0.07
ptm abundance 37C
Doses Blue Rep1_0.15 Red Rep2_−0.01

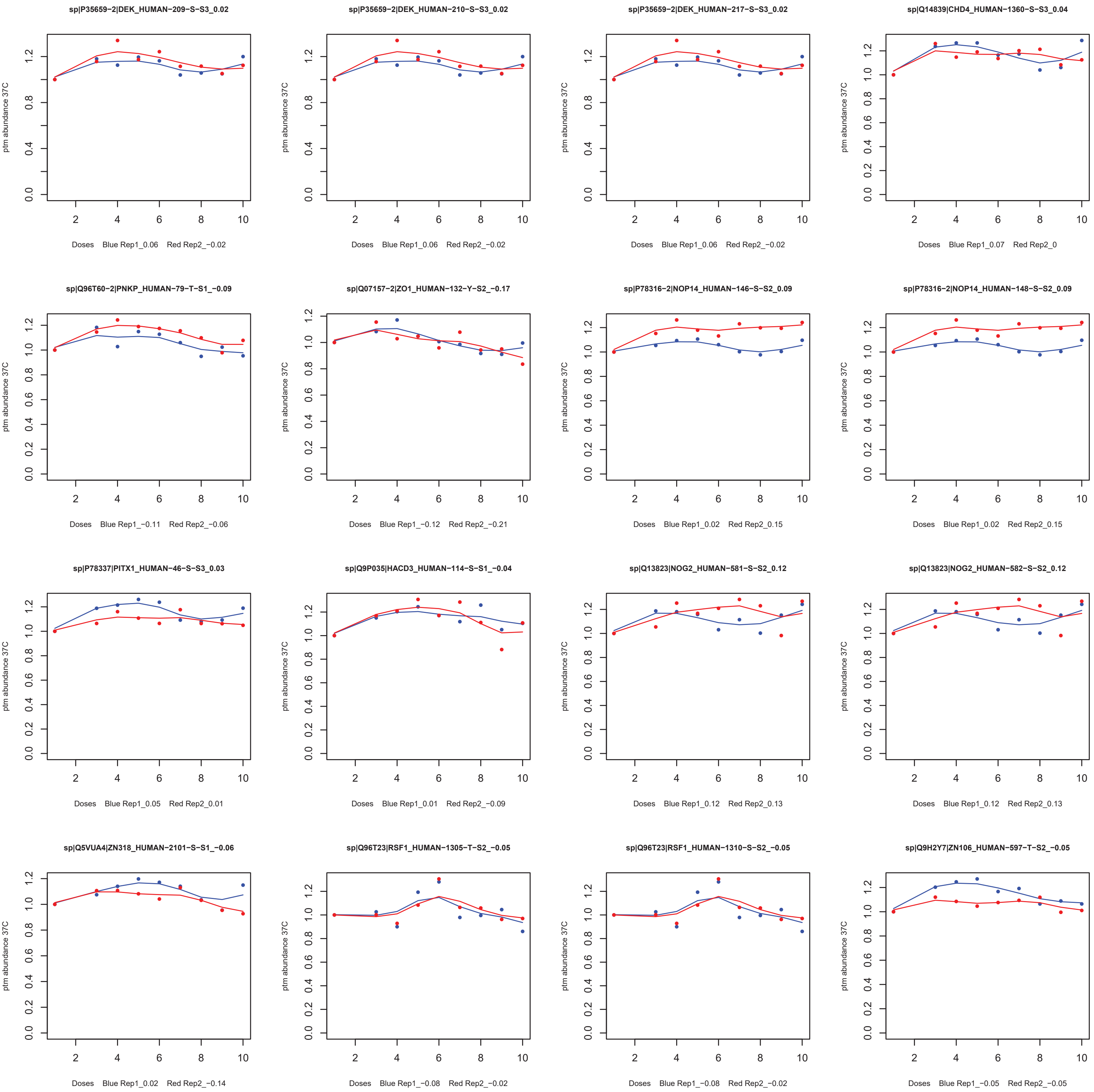
sp|P35659−2|DEK_HUMAN−209−S−S3_0.02
Doses Blue Rep1_0.06 Red Rep2_−0.02
sp|P35659−2|DEK_HUMAN−210−S−S3_0.02
Doses Blue Rep1_0.06 Red Rep2_−0.02
sp|P35659−2|DEK_HUMAN−217−S−S3_0.02
Doses Blue Rep1_0.06 Red Rep2_−0.02
sp|Q14839|CHD4_HUMAN−1360−S−S3_0.04
Doses Blue Rep1_0.07 Red Rep2_0
sp|Q96T60−2|PNKP_HUMAN−79−T−S1_−0.09
Doses Blue Rep1_−0.11 Red Rep2_−0.06
sp|Q07157−2|ZO1_HUMAN−132−Y−S2_−0.17
Doses Blue Rep1_−0.12 Red Rep2_−0.21
sp|P78316−2|NOP14_HUMAN−146−S−S2_0.09
Doses Blue Rep1_0.02 Red Rep2_0.15
sp|P78316−2|NOP14_HUMAN−148−S−S2_0.09
Doses Blue Rep1_0.02 Red Rep2_0.15
sp|P78337|PITX1_HUMAN−46−S−S3_0.03
Doses Blue Rep1_0.05 Red Rep2_0.01
sp|Q9P035|HACD3_HUMAN−114−S−S1_−0.04
Doses Blue Rep1_0.01 Red Rep2_−0.09
sp|Q13823|NOG2_HUMAN−581−S−S2_0.12
Doses Blue Rep1_0.12 Red Rep2_0.13
sp|Q13823|NOG2_HUMAN−582−S−S2_0.12
Doses Blue Rep1_0.12 Red Rep2_0.13
sp|Q5VUA4|ZN318_HUMAN−2101−S−S1_−0.06
Doses Blue Rep1_0.02 Red Rep2_−0.14
sp|Q96T23|RSF1_HUMAN−1305−T−S2_−0.05
Doses Blue Rep1_−0.08 Red Rep2_−0.02
sp|Q96T23|RSF1_HUMAN−1310−S−S2_−0.05
Doses Blue Rep1_−0.08 Red Rep2_−0.02
sp|Q9H2Y7|ZN106_HUMAN−597−T−S2_−0.05
Doses Blue Rep1_−0.05 Red Rep2_−0.05
ptm abundance 37C

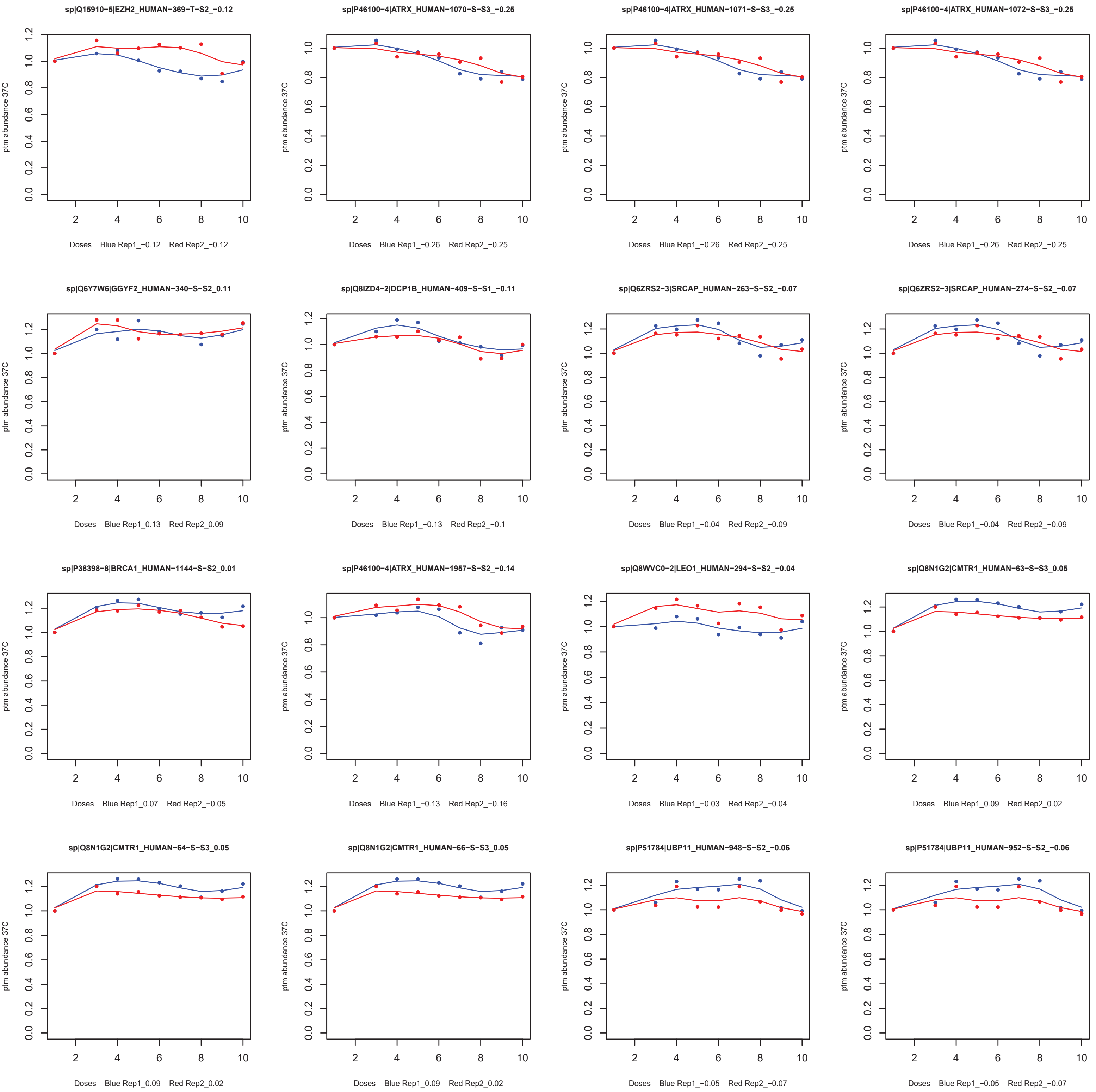
sp|Q15910−5|EZH2_HUMAN−369−T−S2_−0.12
ptm abundance 37C
Doses Blue Rep1_−0.12 Red Rep2_−0.12
sp|P46100−4|ATRX_HUMAN−1070−S−S3_−0.25
Doses Blue Rep1_−0.26 Red Rep2_−0.25
sp|P46100−4|ATRX_HUMAN−1071−S−S3_−0.25
Doses Blue Rep1_−0.26 Red Rep2_−0.25
sp|P46100−4|ATRX_HUMAN−1072−S−S3_−0.25
Doses Blue Rep1_−0.26 Red Rep2_−0.25
sp|Q6Y7W6|GGYF2_HUMAN−340−S−S2_0.11
Doses Blue Rep1_0.13 Red Rep2_0.09
sp|Q8IZD4−2|DCP1B_HUMAN−409−S−S1_−0.11
Doses Blue Rep1_−0.13 Red Rep2_−0.1
sp|Q6ZRS2−3|SRCAP_HUMAN−263−S−S2_−0.07
Doses Blue Rep1_−0.04 Red Rep2_−0.09
sp|Q6ZRS2−3|SRCAP_HUMAN−274−S−S2_−0.07
Doses Blue Rep1_−0.04 Red Rep2_−0.09
sp|P38398−8|BRCA1_HUMAN−1144−S−S2_0.01
Doses Blue Rep1_0.07 Red Rep2_−0.05
sp|P46100−4|ATRX_HUMAN−1957−S−S2_−0.14
Doses Blue Rep1_−0.13 Red Rep2_−0.16
sp|Q8WVC0−2|LEO1_HUMAN−294−S−S2_−0.04
Doses Blue Rep1_−0.03 Red Rep2_−0.04
sp|Q8N1G2|CMTR1_HUMAN−63−S−S3_0.05
Doses Blue Rep1_0.09 Red Rep2_0.02
sp|Q8N1G2|CMTR1_HUMAN−64−S−S3_0.05
Doses Blue Rep1_0.09 Red Rep2_0.02
sp|Q8N1G2|CMTR1_HUMAN−66−S−S3_0.05
Doses Blue Rep1_0.09 Red Rep2_0.02
sp|P51784|UBP11_HUMAN−948−S−S2_−0.06
Doses Blue Rep1_−0.05 Red Rep2_−0.07
sp|P51784|UBP11_HUMAN−952−S−S2_−0.06
Doses Blue Rep1_−0.05 Red Rep2_−0.07

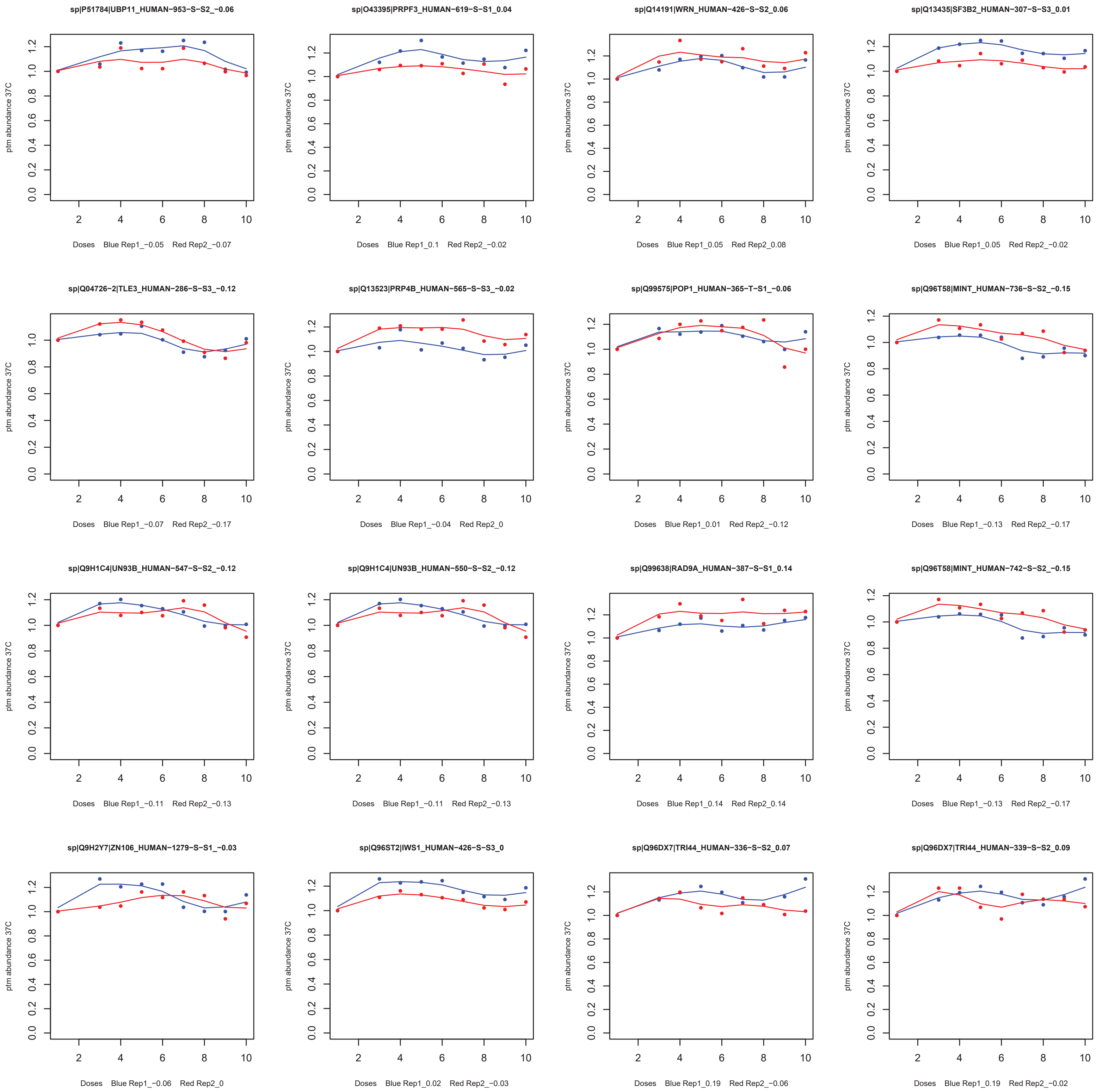
sp|P51784|UBP11_HUMAN−953−S−S2_−0.06
ptm abundance 37C
Doses Blue Rep1_−0.05 Red Rep2_−0.07
sp|O43395|PRPF3_HUMAN−619−S−S1_0.04
ptm abundance 37C
Doses Blue Rep1_0.1 Red Rep2_−0.02
sp|Q14191|WRN_HUMAN−426−S−S2_0.06
ptm abundance 37C
Doses Blue Rep1_0.05 Red Rep2_0.08
sp|Q13435|SF3B2_HUMAN−307−S−S3_0.01
ptm abundance 37C
Doses Blue Rep1_0.05 Red Rep2_−0.02
sp|Q04726−2|TLE3_HUMAN−286−S−S3_−0.12
ptm abundance 37C
Doses Blue Rep1_−0.07 Red Rep2_−0.17
sp|Q13523|PRP4B_HUMAN−565−S−S3_−0.02
ptm abundance 37C
Doses Blue Rep1_−0.04 Red Rep2_0
sp|Q99575|POP1_HUMAN−365−T−S1_−0.06
ptm abundance 37C
Doses Blue Rep1_0.01 Red Rep2_−0.12
sp|Q96T58|MINT_HUMAN−736−S−S2_−0.15
ptm abundance 37C
Doses Blue Rep1_−0.13 Red Rep2_−0.17
sp|Q9H1C4|UN93B_HUMAN−547−S−S2_−0.12
ptm abundance 37C
Doses Blue Rep1_−0.11 Red Rep2_−0.13
sp|Q9H1C4|UN93B_HUMAN−550−S−S2_−0.12
ptm abundance 37C
Doses Blue Rep1_−0.11 Red Rep2_−0.13
sp|Q99638|RAD9A_HUMAN−387−S−S1_0.14
ptm abundance 37C
Doses Blue Rep1_0.14 Red Rep2_0.14
sp|Q96T58|MINT_HUMAN−742−S−S2_−0.15
ptm abundance 37C
Doses Blue Rep1_−0.13 Red Rep2_−0.17
sp|Q9H2Y7|ZN106_HUMAN−1279−S−S1_−0.03
ptm abundance 37C
Doses Blue Rep1_−0.06 Red Rep2_0
sp|Q96ST2|IWS1_HUMAN−426−S−S3_0
ptm abundance 37C
Doses Blue Rep1_0.02 Red Rep2_−0.03
sp|Q96DX7|TRI44_HUMAN−336−S−S2_0.07
ptm abundance 37C
Doses Blue Rep1_0.19 Red Rep2_−0.06
sp|Q96DX7|TRI44_HUMAN−339−S−S2_0.09
ptm abundance 37C
Doses Blue Rep1_0.19 Red Rep2_−0.02

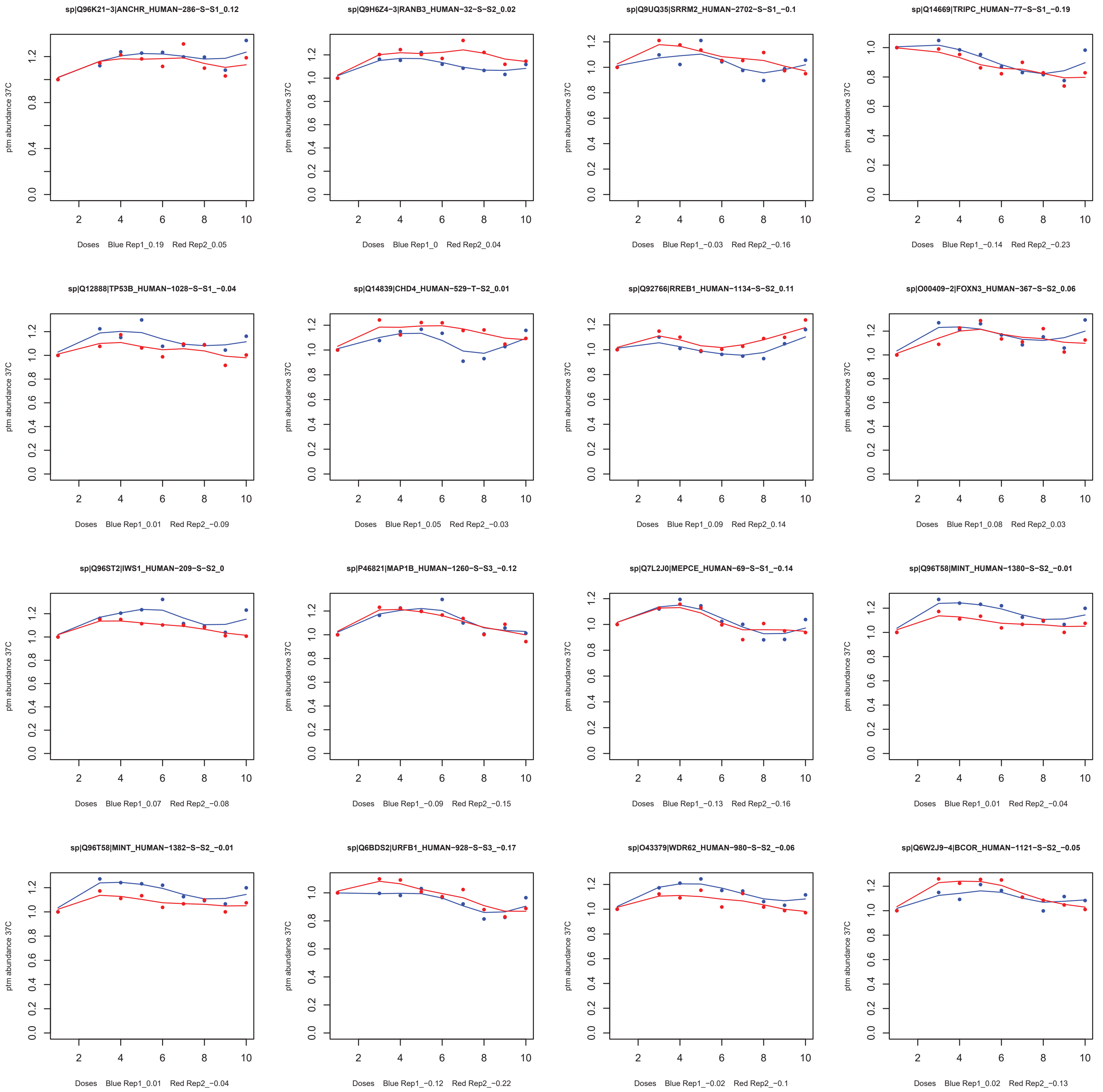
sp|Q96K21−3|ANCHR_HUMAN−286−S−S1_0.12
Doses Blue Rep1_0.19 Red Rep2_0.05
sp|Q9H6Z4−3|RANB3_HUMAN−32−S−S2_0.02
Doses Blue Rep1_0 Red Rep2_0.04
sp|Q9UQ35|SRRM2_HUMAN−2702−S−S1_−0.1
Doses Blue Rep1_−0.03 Red Rep2_−0.16
sp|Q14669|TRIPC_HUMAN−77−S−S1_−0.19
Doses Blue Rep1_−0.14 Red Rep2_−0.23
sp|Q12888|TP53B_HUMAN−1028−S−S1_−0.04
Doses Blue Rep1_0.01 Red Rep2_−0.09
sp|Q14839|CHD4_HUMAN−529−T−S2_0.01
Doses Blue Rep1_0.05 Red Rep2_−0.03
sp|Q92766|RREB1_HUMAN−1134−S−S2_0.11
Doses Blue Rep1_0.09 Red Rep2_0.14
sp|O00409−2|FOXN3_HUMAN−367−S−S2_0.06
Doses Blue Rep1_0.08 Red Rep2_0.03
sp|Q96ST2|IWS1_HUMAN−209−S−S2_0
Doses Blue Rep1_0.07 Red Rep2_−0.08
sp|P46821|MAP1B_HUMAN−1260−S−S3_−0.12
Doses Blue Rep1_−0.09 Red Rep2_−0.15
sp|Q7L2J0|MEPCE_HUMAN−69−S−S1_−0.14
Doses Blue Rep1_−0.13 Red Rep2_−0.16
sp|Q96T58|MINT_HUMAN−1380−S−S2_−0.01
Doses Blue Rep1_0.01 Red Rep2_−0.04
sp|Q96T58|MINT_HUMAN−1382−S−S2_−0.01
Doses Blue Rep1_0.01 Red Rep2_−0.04
sp|Q6BDS2|URFB1_HUMAN−928−S−S3_−0.17
Doses Blue Rep1_−0.12 Red Rep2_−0.22
sp|O43379|WDR62_HUMAN−980−S−S2_−0.06
Doses Blue Rep1_−0.02 Red Rep2_−0.1
sp|Q6W2J9−4|BCOR_HUMAN−1121−S−S2_−0.05
Doses Blue Rep1_0.02 Red Rep2_−0.13
ptm abundance 37C

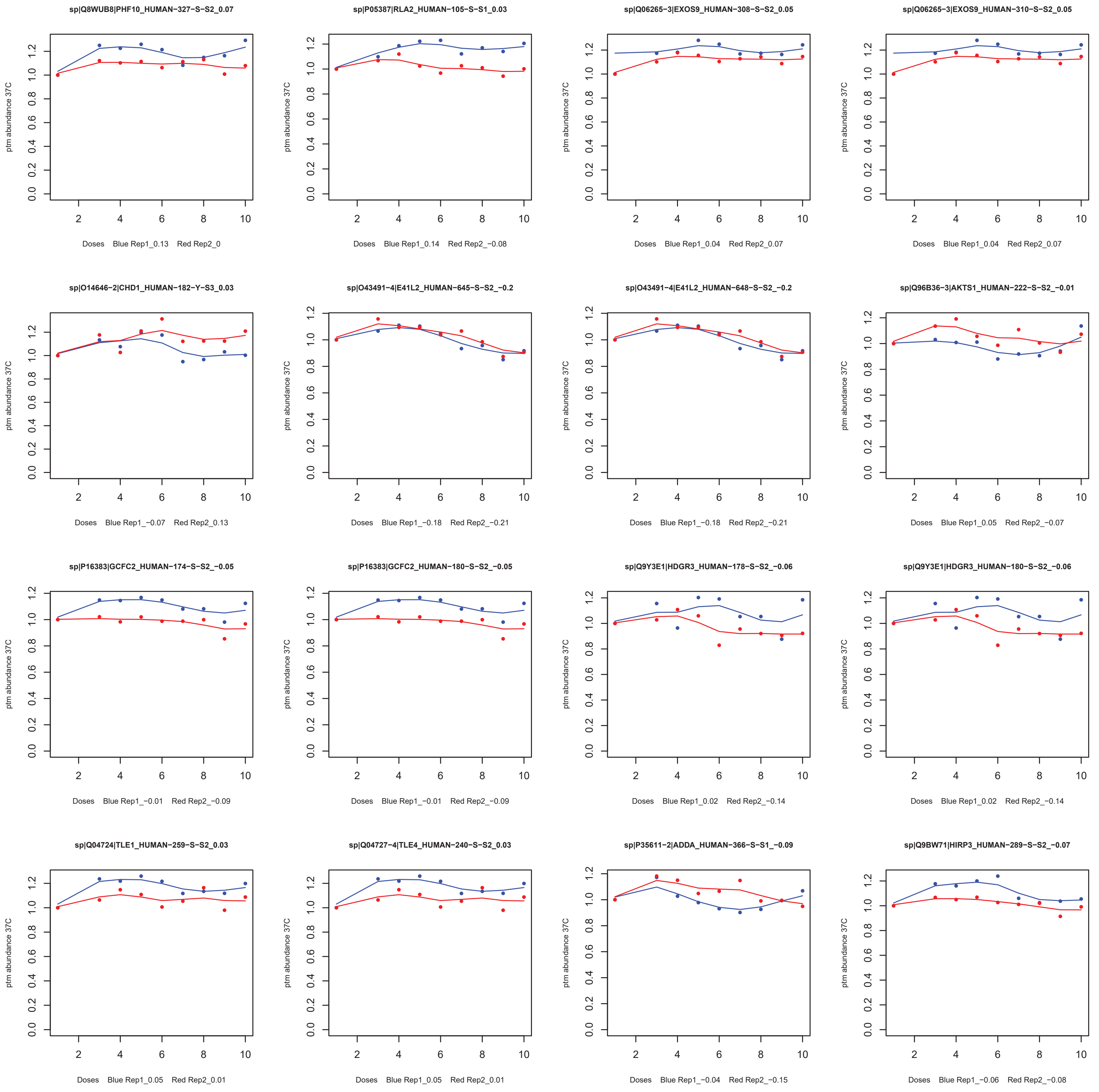
sp|Q8WUB8|PHF10_HUMAN−327−S−S2_0.07
ptm abundance 37C
Doses Blue Rep1_0.13 Red Rep2_0
sp|P05387|RLA2_HUMAN−105−S−S1_0.03
ptm abundance 37C
Doses Blue Rep1_0.14 Red Rep2_−0.08
sp|Q06265−3|EXOS9_HUMAN−308−S−S2_0.05
ptm abundance 37C
Doses Blue Rep1_0.04 Red Rep2_0.07
sp|Q06265−3|EXOS9_HUMAN−310−S−S2_0.05
ptm abundance 37C
Doses Blue Rep1_0.04 Red Rep2_0.07
sp|O14646−2|CHD1_HUMAN−182−Y−S3_0.03
ptm abundance 37C
Doses Blue Rep1_−0.07 Red Rep2_0.13
sp|O43491−4|E41L2_HUMAN−645−S−S2_−0.2
ptm abundance 37C
Doses Blue Rep1_−0.18 Red Rep2_−0.21
sp|O43491−4|E41L2_HUMAN−648−S−S2_−0.2
ptm abundance 37C
Doses Blue Rep1_−0.18 Red Rep2_−0.21
sp|Q96B36−3|AKTS1_HUMAN−222−S−S2_−0.01
ptm abundance 37C
Doses Blue Rep1_0.05 Red Rep2_−0.07
sp|P16383|GCFC2_HUMAN−174−S−S2_−0.05
ptm abundance 37C
Doses Blue Rep1_−0.01 Red Rep2_−0.09
sp|P16383|GCFC2_HUMAN−180−S−S2_−0.05
ptm abundance 37C
Doses Blue Rep1_−0.01 Red Rep2_−0.09
sp|Q9Y3E1|HDGR3_HUMAN−178−S−S2_−0.06
ptm abundance 37C
Doses Blue Rep1_0.02 Red Rep2_−0.14
sp|Q9Y3E1|HDGR3_HUMAN−180−S−S2_−0.06
ptm abundance 37C
Doses Blue Rep1_0.02 Red Rep2_−0.14
sp|Q04724|TLE1_HUMAN−259−S−S2_0.03
ptm abundance 37C
Doses Blue Rep1_0.05 Red Rep2_0.01
sp|Q04727−4|TLE4_HUMAN−240−S−S2_0.03
ptm abundance 37C
Doses Blue Rep1_0.05 Red Rep2_0.01
sp|P35611−2|ADDA_HUMAN−366−S−S1_−0.09
ptm abundance 37C
Doses Blue Rep1_−0.04 Red Rep2_−0.15
sp|Q9BW71|HIRP3_HUMAN−289−S−S2_−0.07
ptm abundance 37C
Doses Blue Rep1_−0.06 Red Rep2_−0.08

sp|Q9BW71|HIRP3_HUMAN−291−S−S2_−0.07

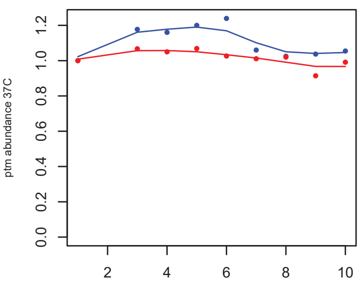

Doses Blue Rep1_−0.06 Red Rep2_−0.08

sp|Q1KMD3|HNRL2_HUMAN−161−S−S1_0.2

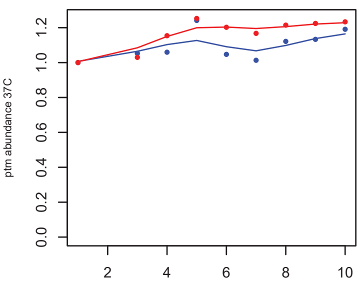

Doses Blue Rep1_0.16 Red Rep2_0.23

sp|Q96ST2|IWS1_HUMAN−211−S−S3_−0.04

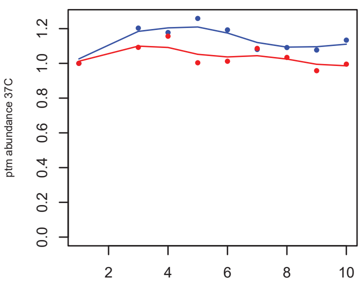

Doses Blue Rep1_0.01 Red Rep2_−0.09

sp|Q9Y520−6|PRC2C_HUMAN−1480−T−S2_0.02

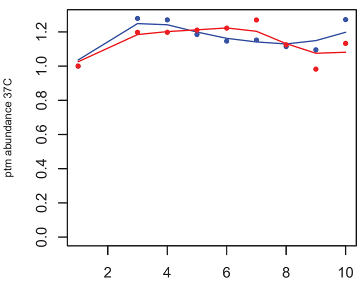

Doses Blue Rep1_0.07 Red Rep2_−0.03

sp|Q8NFC6|BD1L1_HUMAN−2986−S−S1_0.08

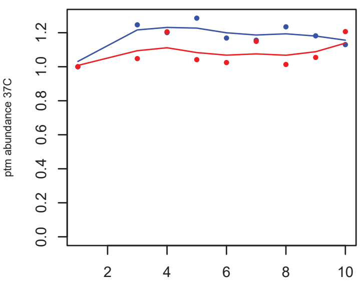

Doses Blue Rep1_0.04 Red Rep2_0.11

sp|Q13905−2|RPGF1_HUMAN−178−S−S2_−0.06

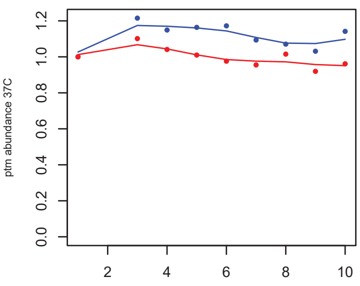

Doses Blue Rep1_0 Red Rep2_−0.11

sp|Q9H0G5|NSRP1_HUMAN−248−S−S1_0.06

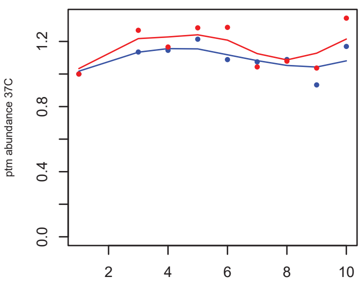

Doses Blue Rep1_0.01 Red Rep2_0.11

sp|Q9C0C9|UBE2O_HUMAN−87−S−S2_−0.02

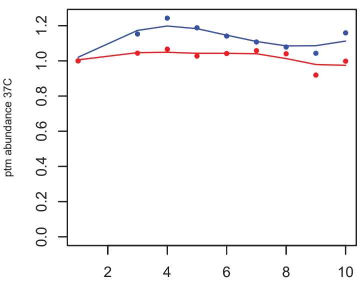

Doses Blue Rep1_0.02 Red Rep2_−0.06

sp|Q9C0C9|UBE2O_HUMAN−89−S−S2_−0.02

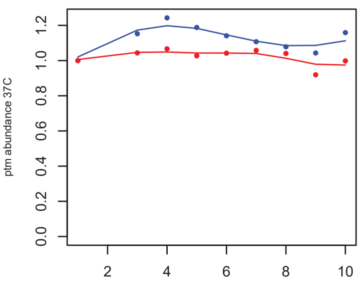

Doses Blue Rep1_0.02 Red Rep2_−0.06

sp|Q7Z4V5|HDGR2_HUMAN−232−S−S3_0.1

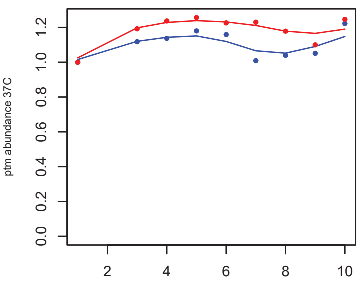

Doses Blue Rep1_0.1 Red Rep2_0.1

sp|Q7Z4V5|HDGR2_HUMAN−234−S−S3_0.1

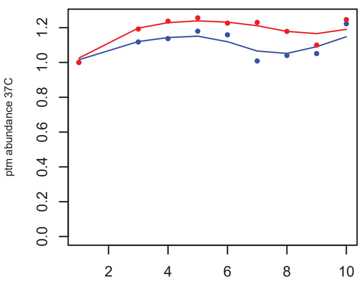

Doses Blue Rep1_0.1 Red Rep2_0.1

sp|P23588−2|IF4B_HUMAN−459−S−S1_0.03

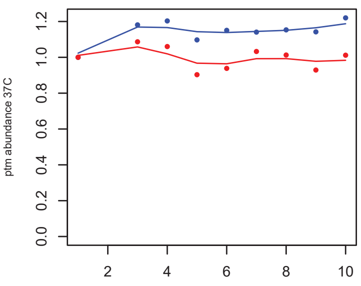

Doses Blue Rep1_0.11 Red Rep2_−0.06

sp|Q9UQ35|SRRM2_HUMAN−1102−S−S3_−0.05

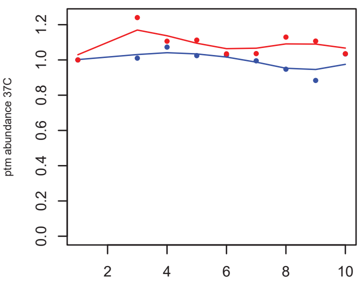

Doses Blue Rep1_−0.05 Red Rep2_−0.04

sp|Q9UQ35|SRRM2_HUMAN−876−S−S1_0.02

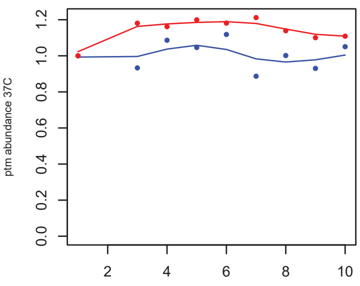

Doses Blue Rep1_0.01 Red Rep2_0.02

sp|Q14C86−3|GAPD1_HUMAN−787−S−S2_−0.16

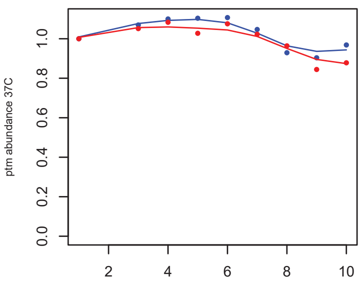

Doses Blue Rep1_−0.12 Red Rep2_−0.2

sp|O75821|EIF3G_HUMAN−41−T−S2_0.08

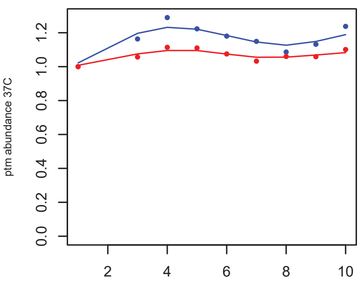

Doses Blue Rep1_0.1 Red Rep2_0.05

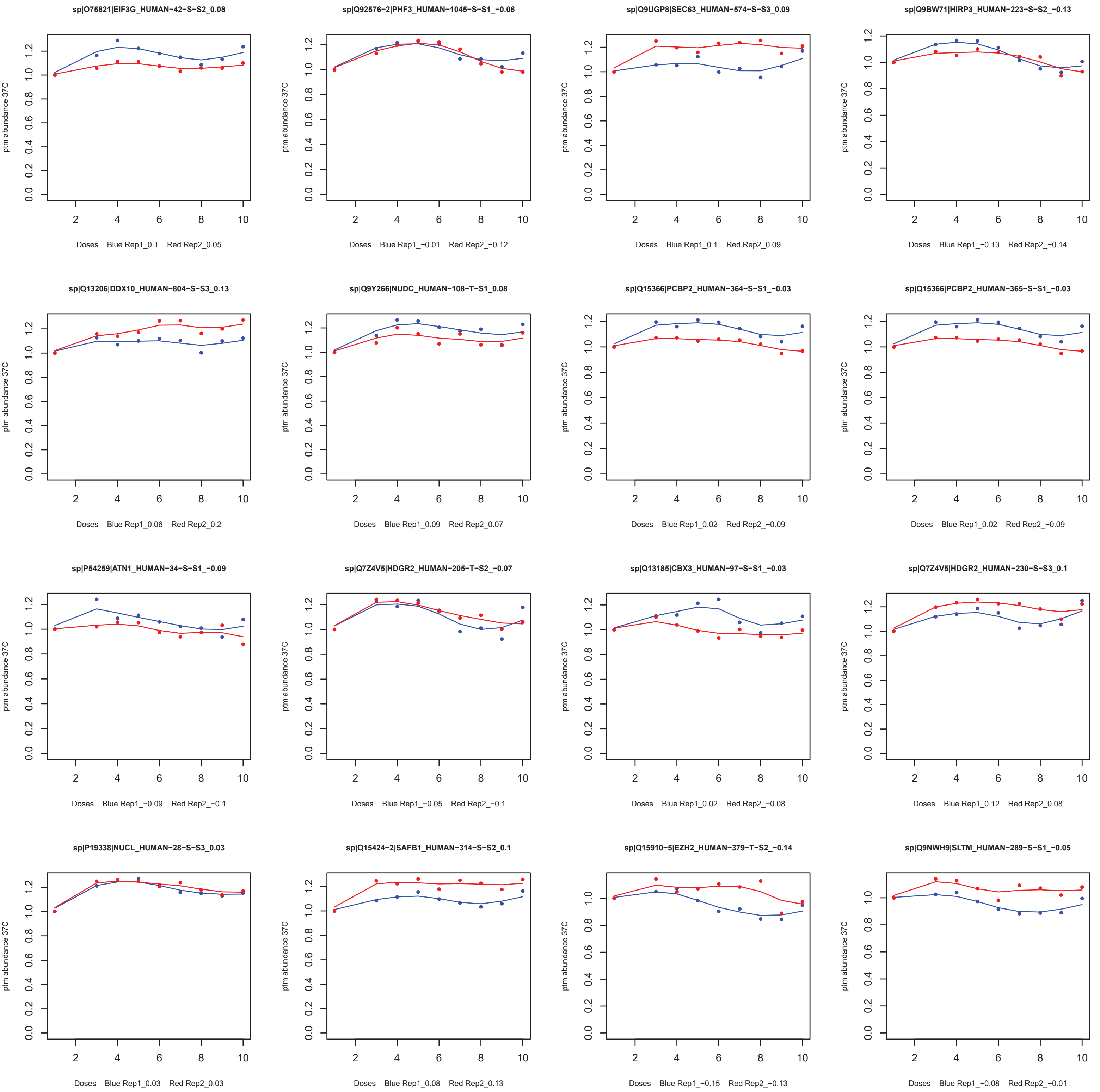
sp|O75821|EIF3G_HUMAN−42−S−S2_0.08
Doses Blue Rep1_0.1 Red Rep2_0.05
sp|Q92576−2|PHF3_HUMAN−1045−S−S1_−0.06
Doses Blue Rep1_−0.01 Red Rep2_−0.12
sp|Q9UGP8|SEC63_HUMAN−574−S−S3_0.09
Doses Blue Rep1_0.1 Red Rep2_0.09
sp|Q9BW71|HIRP3_HUMAN−223−S−S2_−0.13
Doses Blue Rep1_−0.13 Red Rep2_−0.14
sp|Q13206|DDX10_HUMAN−804−S−S3_0.13
Doses Blue Rep1_0.06 Red Rep2_0.2
sp|Q9Y266|NUDC_HUMAN−108−T−S1_0.08
Doses Blue Rep1_0.09 Red Rep2_0.07
sp|Q15366|PCBP2_HUMAN−364−S−S1_−0.03
Doses Blue Rep1_0.02 Red Rep2_−0.09
sp|Q15366|PCBP2_HUMAN−365−S−S1_−0.03
Doses Blue Rep1_0.02 Red Rep2_−0.09
sp|P54259|ATN1_HUMAN−34−S−S1_−0.09
Doses Blue Rep1_−0.09 Red Rep2_−0.1
sp|Q7Z4V5|HDGR2_HUMAN−205−T−S2_−0.07
Doses Blue Rep1_−0.05 Red Rep2_−0.1
sp|Q13185|CBX3_HUMAN−97−S−S1_−0.03
Doses Blue Rep1_0.02 Red Rep2_−0.08
sp|Q7Z4V5|HDGR2_HUMAN−230−S−S3_0.1
Doses Blue Rep1_0.12 Red Rep2_0.08
sp|P19338|NUCL_HUMAN−28−S−S3_0.03
Doses Blue Rep1_0.03 Red Rep2_0.03
sp|Q15424−2|SAFB1_HUMAN−314−S−S2_0.1
Doses Blue Rep1_0.08 Red Rep2_0.13
sp|Q15910−5|EZH2_HUMAN−379−T−S2_−0.14
Doses Blue Rep1_−0.15 Red Rep2_−0.13
sp|Q9NWH9|SLTM_HUMAN−289−S−S1_−0.05
Doses Blue Rep1_−0.08 Red Rep2_−0.01
ptm abundance 37C

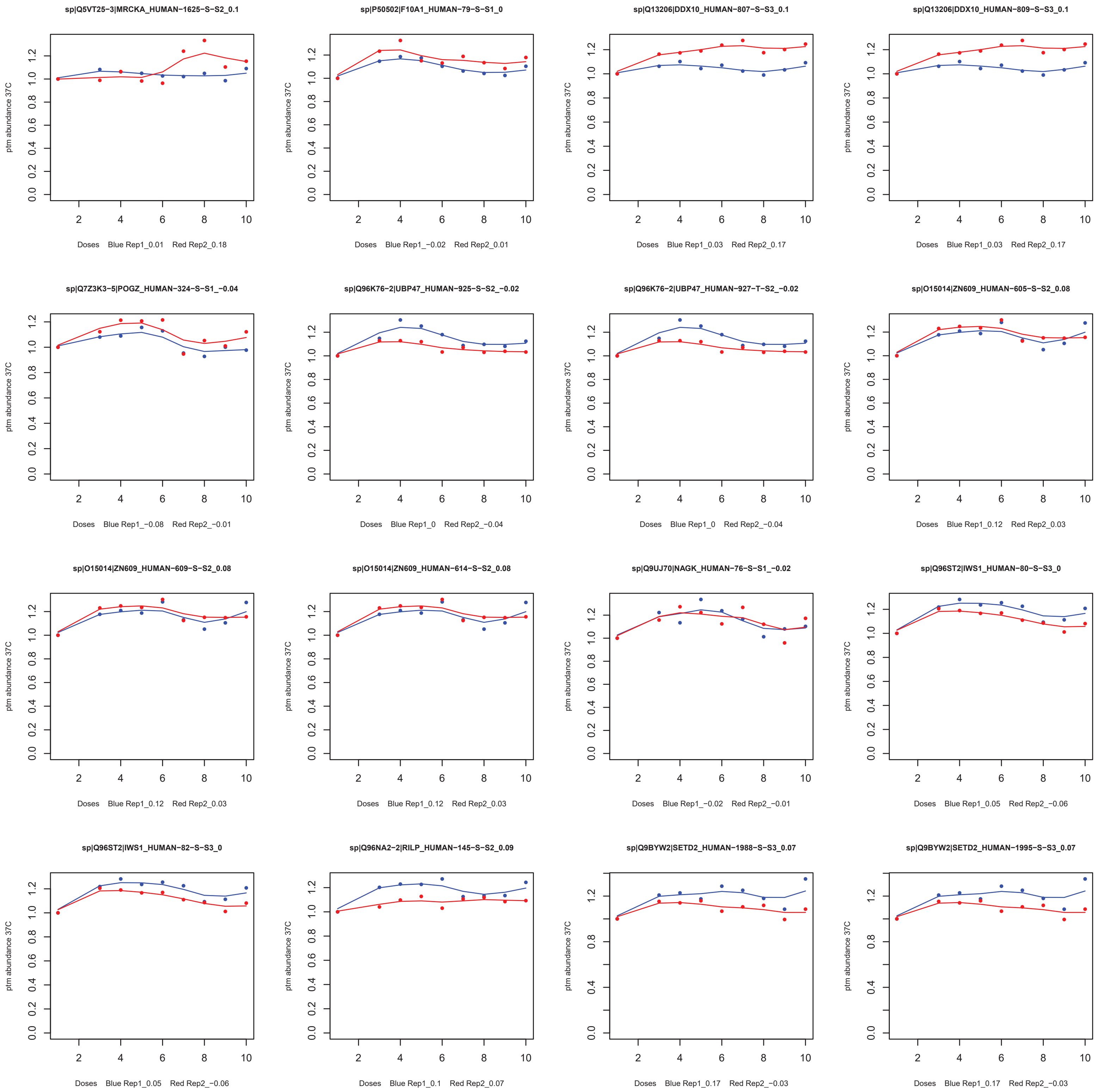
sp|Q5VT25−3|MRCKA_HUMAN−1625−S−S2_0.1
ptm abundance 37C
Doses Blue Rep1_0.01 Red Rep2_0.18
sp|P50502|F10A1_HUMAN−79−S−S1_0
ptm abundance 37C
Doses Blue Rep1_−0.02 Red Rep2_0.01
sp|Q13206|DDX10_HUMAN−807−S−S3_0.1
ptm abundance 37C
Doses Blue Rep1_0.03 Red Rep2_0.17
sp|Q13206|DDX10_HUMAN−809−S−S3_0.1
ptm abundance 37C
Doses Blue Rep1_0.03 Red Rep2_0.17
sp|Q7Z3K3−5|POGZ_HUMAN−324−S−S1_−0.04
ptm abundance 37C
Doses Blue Rep1_−0.08 Red Rep2_−0.01
sp|Q96K76−2|UBP47_HUMAN−925−S−S2_−0.02
ptm abundance 37C
Doses Blue Rep1_0 Red Rep2_−0.04
sp|Q96K76−2|UBP47_HUMAN−927−T−S2_−0.02
ptm abundance 37C
Doses Blue Rep1_0 Red Rep2_−0.04
sp|O15014|ZN609_HUMAN−605−S−S2_0.08
ptm abundance 37C
Doses Blue Rep1_0.12 Red Rep2_0.03
sp|O15014|ZN609_HUMAN−609−S−S2_0.08
ptm abundance 37C
Doses Blue Rep1_0.12 Red Rep2_0.03
sp|O15014|ZN609_HUMAN−614−S−S2_0.08
ptm abundance 37C
Doses Blue Rep1_0.12 Red Rep2_0.03
sp|Q9UJ70|NAGK_HUMAN−76−S−S1_−0.02
ptm abundance 37C
Doses Blue Rep1_−0.02 Red Rep2_−0.01
sp|Q96ST2|IWS1_HUMAN−80−S−S3_0
ptm abundance 37C
Doses Blue Rep1_0.05 Red Rep2_−0.06
sp|Q96ST2|IWS1_HUMAN−82−S−S3_0
ptm abundance 37C
Doses Blue Rep1_0.05 Red Rep2_−0.06
sp|Q96NA2−2|RILP_HUMAN−145−S−S2_0.09
ptm abundance 37C
Doses Blue Rep1_0.1 Red Rep2_0.07
sp|Q9BYW2|SETD2_HUMAN−1988−S−S3_0.07
ptm abundance 37C
Doses Blue Rep1_0.17 Red Rep2_−0.03
sp|Q9BYW2|SETD2_HUMAN−1995−S−S3_0.07
ptm abundance 37C
Doses Blue Rep1_0.17 Red Rep2_−0.03

sp|Q8IX07|FOG1_HUMAN−44−S−S2_0.04

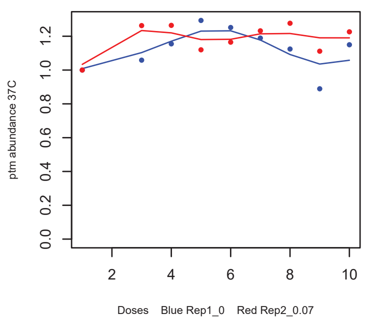

sp|Q99590−2|SCAFB_HUMAN−98−S−S2_−0.18

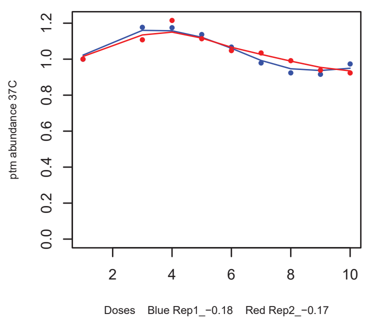

sp|Q5VT52−2|RPRD2_HUMAN−888−S−S2_0.02

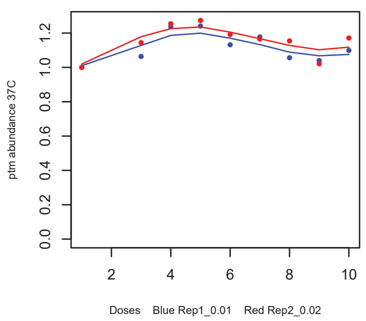

sp|Q8IXQ4|GPAM1_HUMAN−140−S−S2_0.07

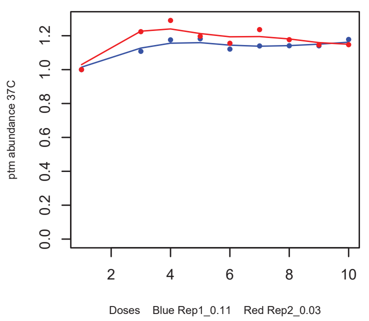

sp|Q8IXQ4|GPAM1_HUMAN−141−S−S2_0.07

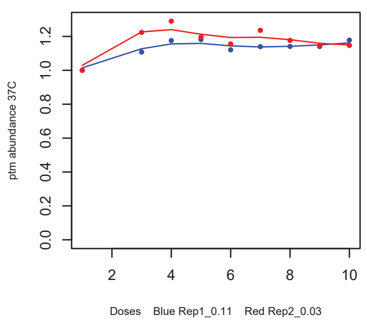

sp|Q9BW85|CCD94_HUMAN−213−S−S2_0.13

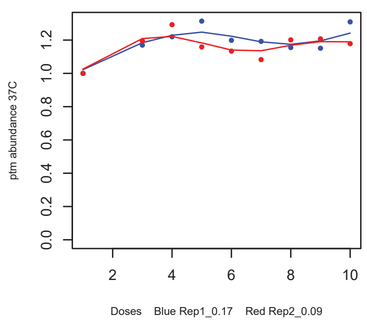

sp|Q9H0G5|NSRP1_HUMAN−248−S−S2_0.02

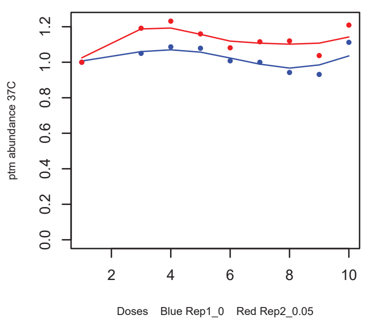

sp|Q6ZU65−2|UBN2_HUMAN−252−T−S2_−0.14

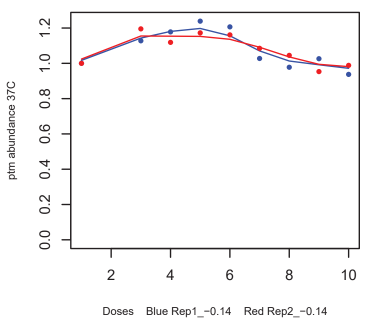

sp|Q03111|ENL_HUMAN−475−S−S1_−0.1

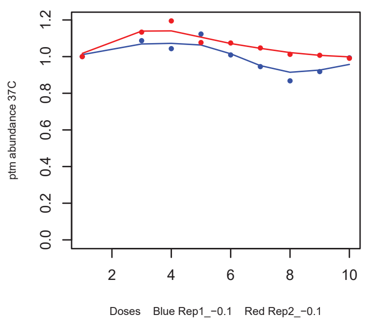

sp|Q15648|MED1_HUMAN−1482−S−S2_0.01

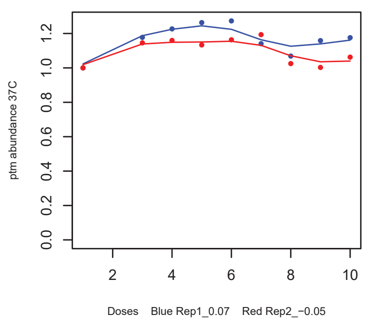

sp|Q96EV8−3|DTBP1_HUMAN−235−S−S2_0.05

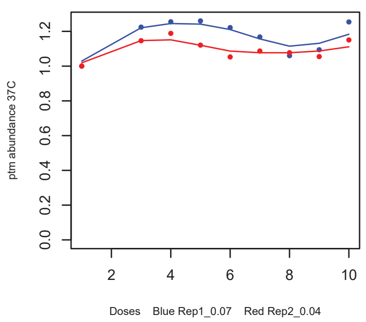

sp|Q96EV8−3|DTBP1_HUMAN−240−S−S2_0.05

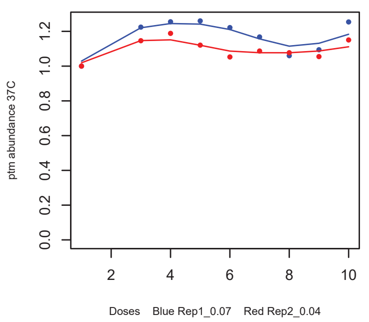

sp|Q8N1G4|LRC47_HUMAN−518−S−S2_0.04

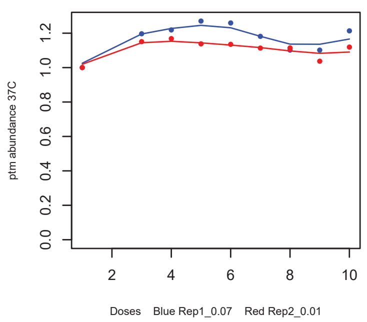

sp|Q9HAP2|MLXIP_HUMAN−33−S−S3_0.08

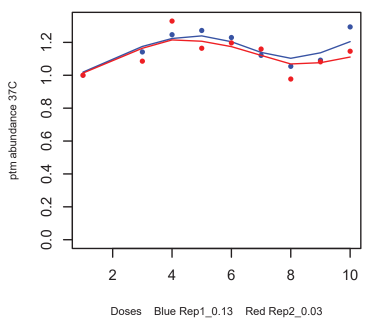

sp|Q9HAP2|MLXIP_HUMAN−35−T−S3_0.08

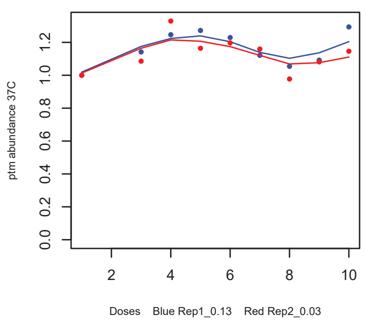

sp|Q9HAP2|MLXIP_HUMAN−39−S−S3_0.08

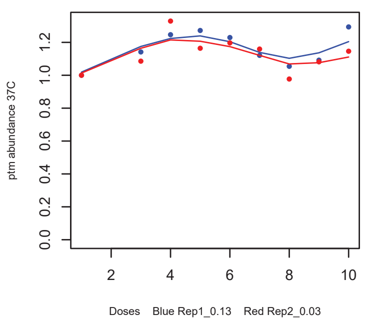

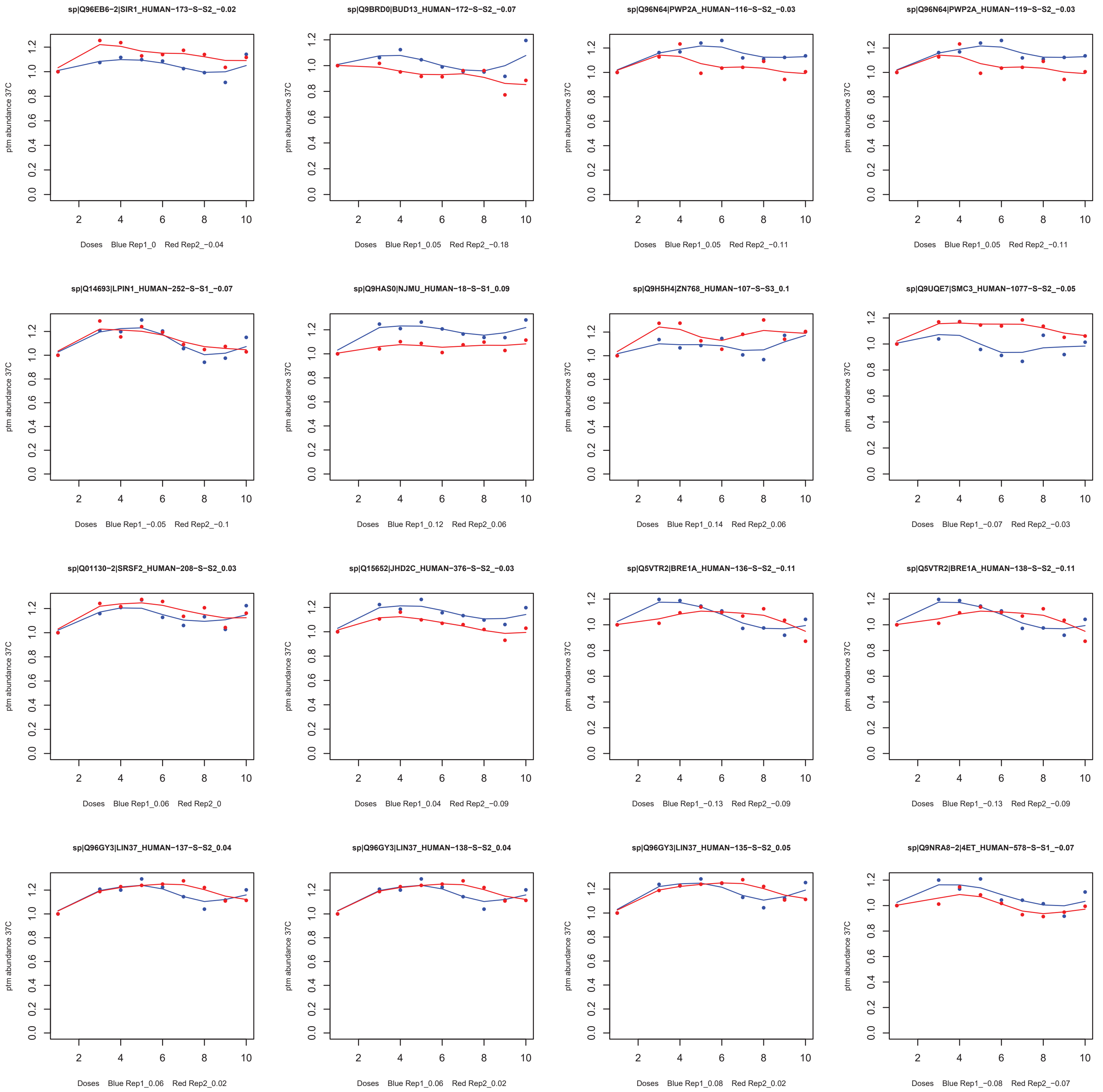
sp|Q96EB6−2|SIR1_HUMAN−173−S−S2_−0.02
ptm abundance 37C
Doses Blue Rep1_0 Red Rep2_−0.04
sp|Q9BRD0|BUD13_HUMAN−172−S−S2_−0.07
Doses Blue Rep1_0.05 Red Rep2_−0.18
sp|Q96N64|PWP2A_HUMAN−116−S−S2_−0.03
Doses Blue Rep1_0.05 Red Rep2_−0.11
sp|Q96N64|PWP2A_HUMAN−119−S−S2_−0.03
Doses Blue Rep1_0.05 Red Rep2_−0.11
sp|Q14693|LPIN1_HUMAN−252−S−S1_−0.07
Doses Blue Rep1_−0.05 Red Rep2_−0.1
sp|Q9HAS0|NJMU_HUMAN−18−S−S1_0.09
Doses Blue Rep1_0.12 Red Rep2_0.06
sp|Q9H5H4|ZN768_HUMAN−107−S−S3_0.1
Doses Blue Rep1_0.14 Red Rep2_0.06
sp|Q9UQE7|SMC3_HUMAN−1077−S−S2_−0.05
Doses Blue Rep1_−0.07 Red Rep2_−0.03
sp|Q01130−2|SRSF2_HUMAN−208−S−S2_0.03
Doses Blue Rep1_0.06 Red Rep2_0
sp|Q15652|JHD2C_HUMAN−376−S−S2_−0.03
Doses Blue Rep1_0.04 Red Rep2_−0.09
sp|Q5VTR2|BRE1A_HUMAN−136−S−S2_−0.11
Doses Blue Rep1_−0.13 Red Rep2_−0.09
sp|Q5VTR2|BRE1A_HUMAN−138−S−S2_−0.11
Doses Blue Rep1_−0.13 Red Rep2_−0.09
sp|Q96GY3|LIN37_HUMAN−137−S−S2_0.04
Doses Blue Rep1_0.06 Red Rep2_0.02
sp|Q96GY3|LIN37_HUMAN−138−S−S2_0.04
Doses Blue Rep1_0.06 Red Rep2_0.02
sp|Q96GY3|LIN37_HUMAN−135−S−S2_0.05
Doses Blue Rep1_0.08 Red Rep2_0.02
sp|Q9NRA8−2|4ET_HUMAN−578−S−S1_−0.07
Doses Blue Rep1_−0.08 Red Rep2_−0.07

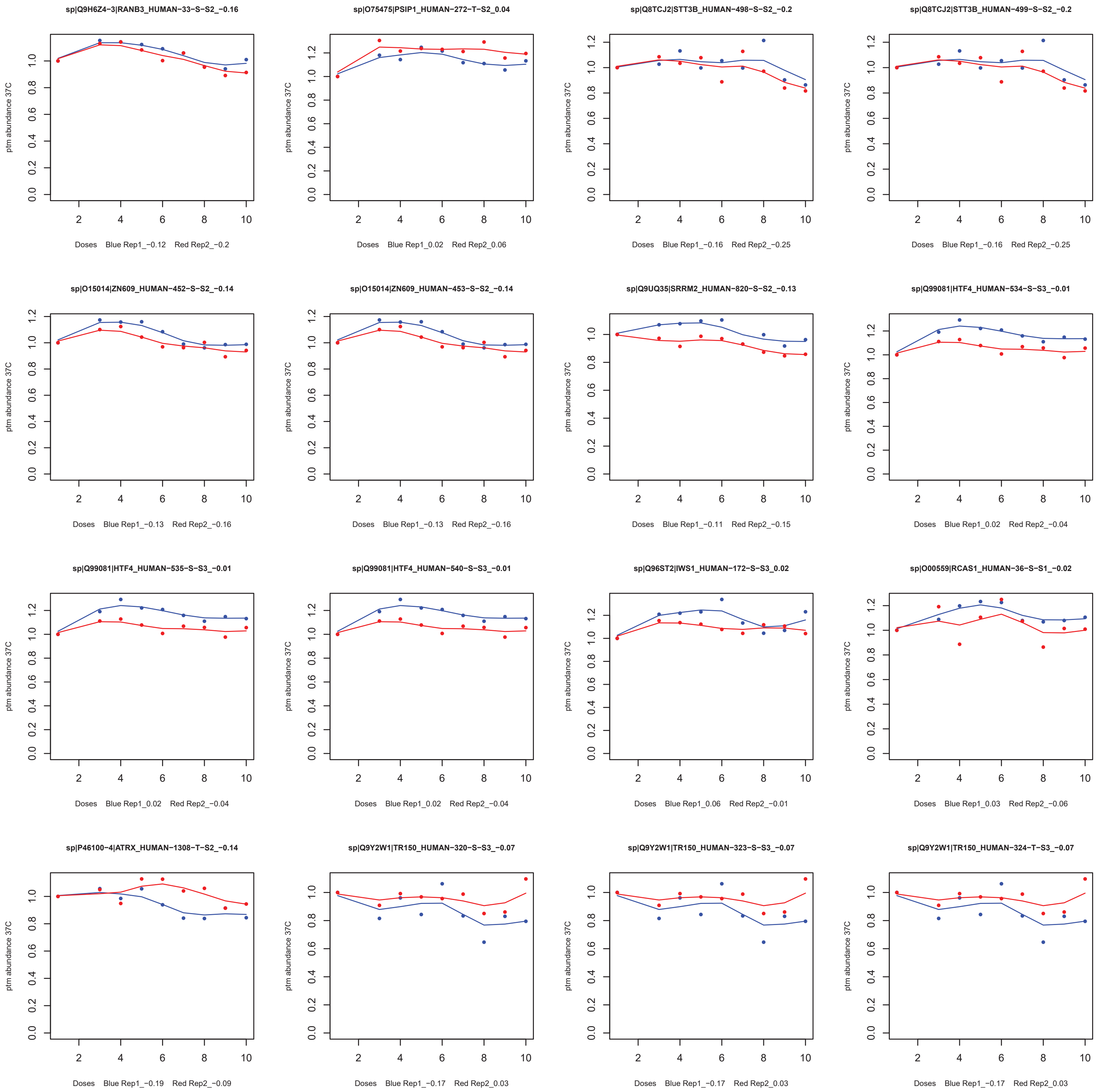
sp|Q9H6Z4-3|RANB3_HUMAN-33-S-S2_-0.16
ptm abundance 37C
Doses Blue Rep1_-0.12 Red Rep2_-0.2
sp|O75475|PSIP1_HUMAN-272-T-S2_0.04
ptm abundance 37C
Doses Blue Rep1_0.02 Red Rep2_0.06
sp|Q8TCJ2|STT3B_HUMAN-498-S-S2_-0.2
ptm abundance 37C
Doses Blue Rep1_-0.16 Red Rep2_-0.25
sp|Q8TCJ2|STT3B_HUMAN-499-S-S2_-0.2
ptm abundance 37C
Doses Blue Rep1_-0.16 Red Rep2_-0.25
sp|O15014|ZN609_HUMAN-452-S-S2_-0.14
ptm abundance 37C
Doses Blue Rep1_-0.13 Red Rep2_-0.16
sp|O15014|ZN609_HUMAN-453-S-S2_-0.14
ptm abundance 37C
Doses Blue Rep1_-0.13 Red Rep2_-0.16
sp|Q9UQ35|SRRM2_HUMAN-820-S-S2_-0.13
ptm abundance 37C
Doses Blue Rep1_-0.11 Red Rep2_-0.15
sp|Q99081|HTF4_HUMAN-534-S-S3_-0.01
ptm abundance 37C
Doses Blue Rep1_0.02 Red Rep2_-0.04
sp|Q99081|HTF4_HUMAN-535-S-S3_-0.01
ptm abundance 37C
Doses Blue Rep1_0.02 Red Rep2_-0.04
sp|Q99081|HTF4_HUMAN-540-S-S3_-0.01
ptm abundance 37C
Doses Blue Rep1_0.02 Red Rep2_-0.04
sp|Q96ST2|IWS1_HUMAN-172-S-S3_0.02
ptm abundance 37C
Doses Blue Rep1_0.06 Red Rep2_-0.01
sp|O00559|RCAS1_HUMAN-36-S-S1_-0.02
ptm abundance 37C
Doses Blue Rep1_0.03 Red Rep2_-0.06
sp|P46100-4|ATRX_HUMAN-1308-T-S2_-0.14
ptm abundance 37C
Doses Blue Rep1_-0.19 Red Rep2_-0.09
sp|Q9Y2W1|TR150_HUMAN-320-S-S3_-0.07
ptm abundance 37C
Doses Blue Rep1_-0.17 Red Rep2_0.03
sp|Q9Y2W1|TR150_HUMAN-323-S-S3_-0.07
ptm abundance 37C
Doses Blue Rep1_-0.17 Red Rep2_0.03
sp|Q9Y2W1|TR150_HUMAN-324-T-S3_-0.07
ptm abundance 37C
Doses Blue Rep1_-0.17 Red Rep2_0.03

sp|Q6QNY0|BL1S3_HUMAN−25−T−S2_−0.17

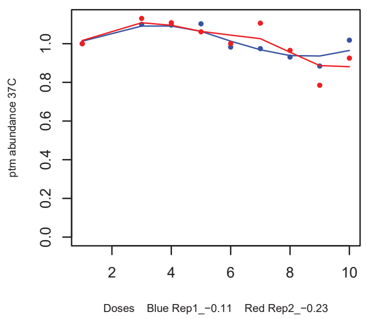

sp|Q86UV5−6|UBP48_HUMAN−11−S−S3_0.01

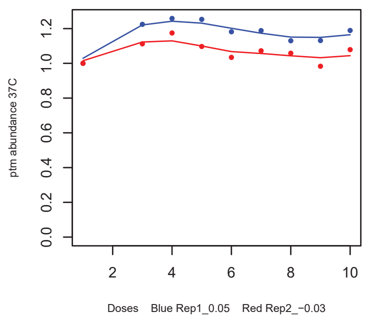

sp|Q86UV5−6|UBP48_HUMAN−12−S−S3_0.01

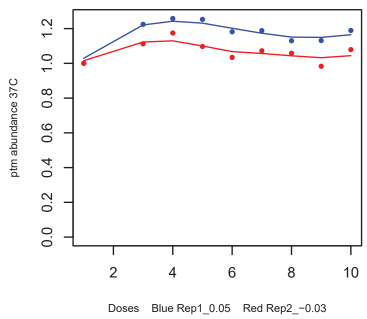

sp|Q86UV5−6|UBP48_HUMAN−13−S−S3_0.01

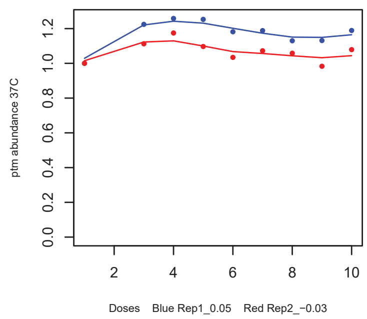

sp|P25205|MCM3_HUMAN−708−Y−S3_−0.09

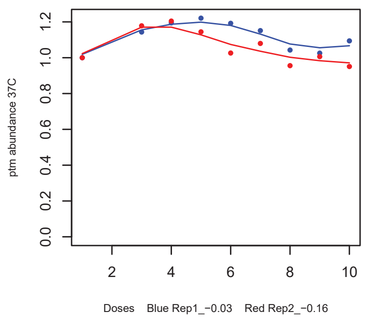

sp|Q99590−2|SCAFB_HUMAN−517−S−S1_0.18

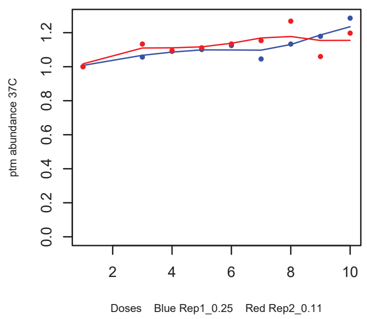

sp|Q969R5|LMBL2_HUMAN−688−S−S2_0.03

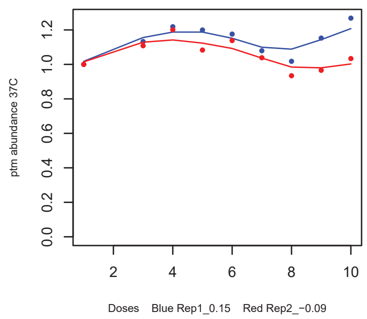

sp|Q969R5|LMBL2_HUMAN−689−S−S2_0.03

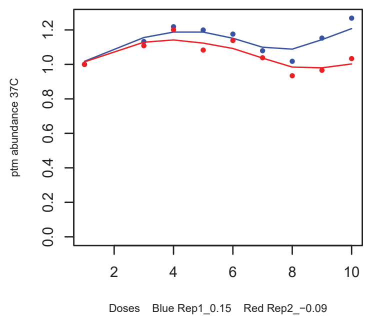

sp|Q6IBW4−2|CNDH2_HUMAN−262−S−S1_−0.17

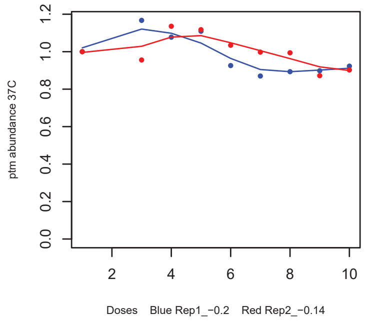

sp|Q9H1E3|NUCKS_HUMAN−132−S−S2_0.14

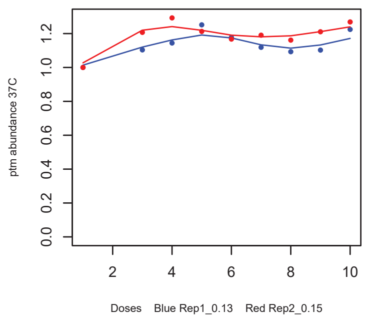

sp|Q96MY1−2|NOL4L_HUMAN−137−S−S2_0.02

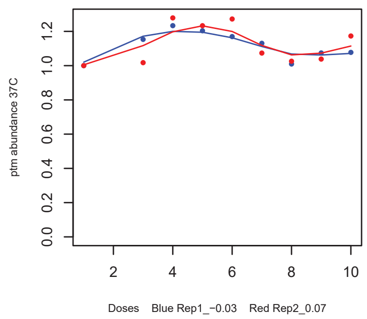

sp|P67809|YBOX1_HUMAN−313−S−S1_−0.02

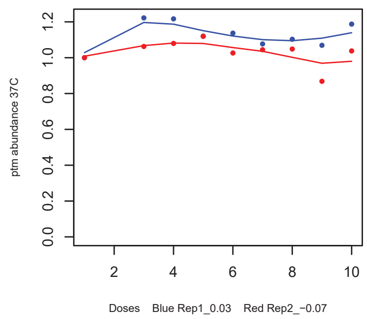

sp|Q14498|RBM39_HUMAN−136−S−S1_0.02

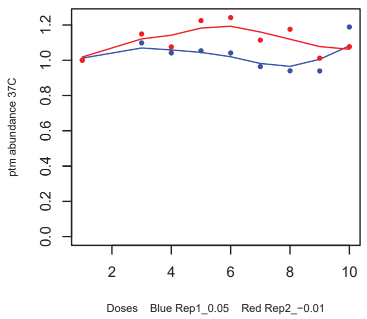

sp|Q8N1G4|LRC47_HUMAN−520−S−S2_0.02

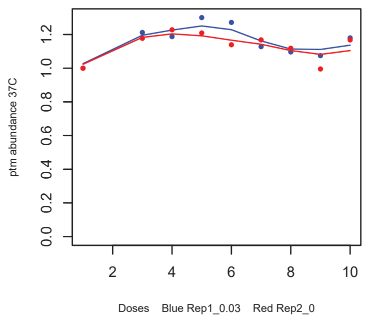

sp|P50502|F10A1_HUMAN−79−S−S2_0.12

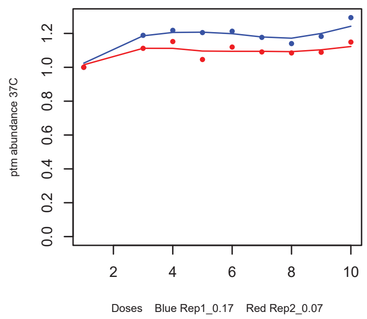

sp|Q4G0J3|LARP7_HUMAN−338−T−S1_0.07

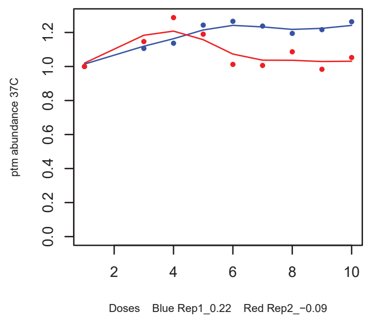

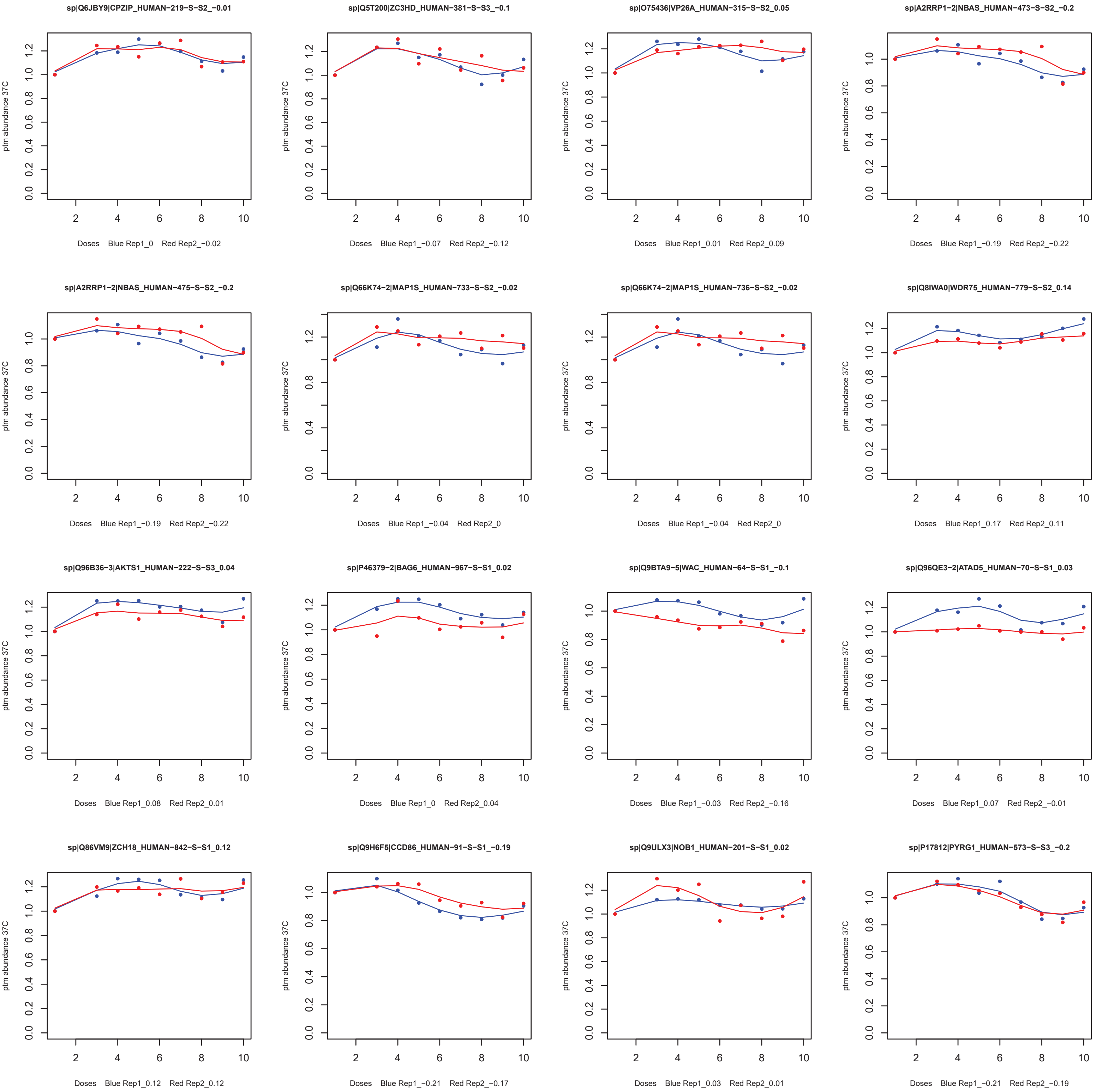
sp|Q6JBY9|CPZIP_HUMAN−219−S−S2_−0.01
Doses Blue Rep1_0 Red Rep2_−0.02
sp|Q5T200|ZC3HD_HUMAN−381−S−S3_−0.1
Doses Blue Rep1_−0.07 Red Rep2_−0.12
sp|O75436|VP26A_HUMAN−315−S−S2_0.05
Doses Blue Rep1_0.01 Red Rep2_0.09
sp|A2RRP1−2|NBAS_HUMAN−473−S−S2_−0.2
Doses Blue Rep1_−0.19 Red Rep2_−0.22
sp|A2RRP1−2|NBAS_HUMAN−475−S−S2_−0.2
Doses Blue Rep1_−0.19 Red Rep2_−0.22
sp|Q66K74−2|MAP1S_HUMAN−733−S−S2_−0.02
Doses Blue Rep1_−0.04 Red Rep2_0
sp|Q66K74−2|MAP1S_HUMAN−736−S−S2_−0.02
Doses Blue Rep1_−0.04 Red Rep2_0
sp|Q8IWA0|WDR75_HUMAN−779−S−S2_0.14
Doses Blue Rep1_0.17 Red Rep2_0.11
sp|Q96B36−3|AKTS1_HUMAN−222−S−S3_0.04
Doses Blue Rep1_0.08 Red Rep2_0.01
sp|P46379−2|BAG6_HUMAN−967−S−S1_0.02
Doses Blue Rep1_0 Red Rep2_0.04
sp|Q9BTA9−5|WAC_HUMAN−64−S−S1_−0.1
Doses Blue Rep1_−0.03 Red Rep2_−0.16
sp|Q96QE3−2|ATAD5_HUMAN−70−S−S1_0.03
Doses Blue Rep1_0.07 Red Rep2_−0.01
sp|Q86VM9|ZCH18_HUMAN−842−S−S1_0.12
Doses Blue Rep1_0.12 Red Rep2_0.12
sp|Q9H6F5|CCD86_HUMAN−91−S−S1_−0.19
Doses Blue Rep1_−0.21 Red Rep2_−0.17
sp|Q9ULX3|NOB1_HUMAN−201−S−S1_0.02
Doses Blue Rep1_0.03 Red Rep2_0.01
sp|P17812|PYRG1_HUMAN−573−S−S3_−0.2
Doses Blue Rep1_−0.21 Red Rep2_−0.19
ptm abundance 37C

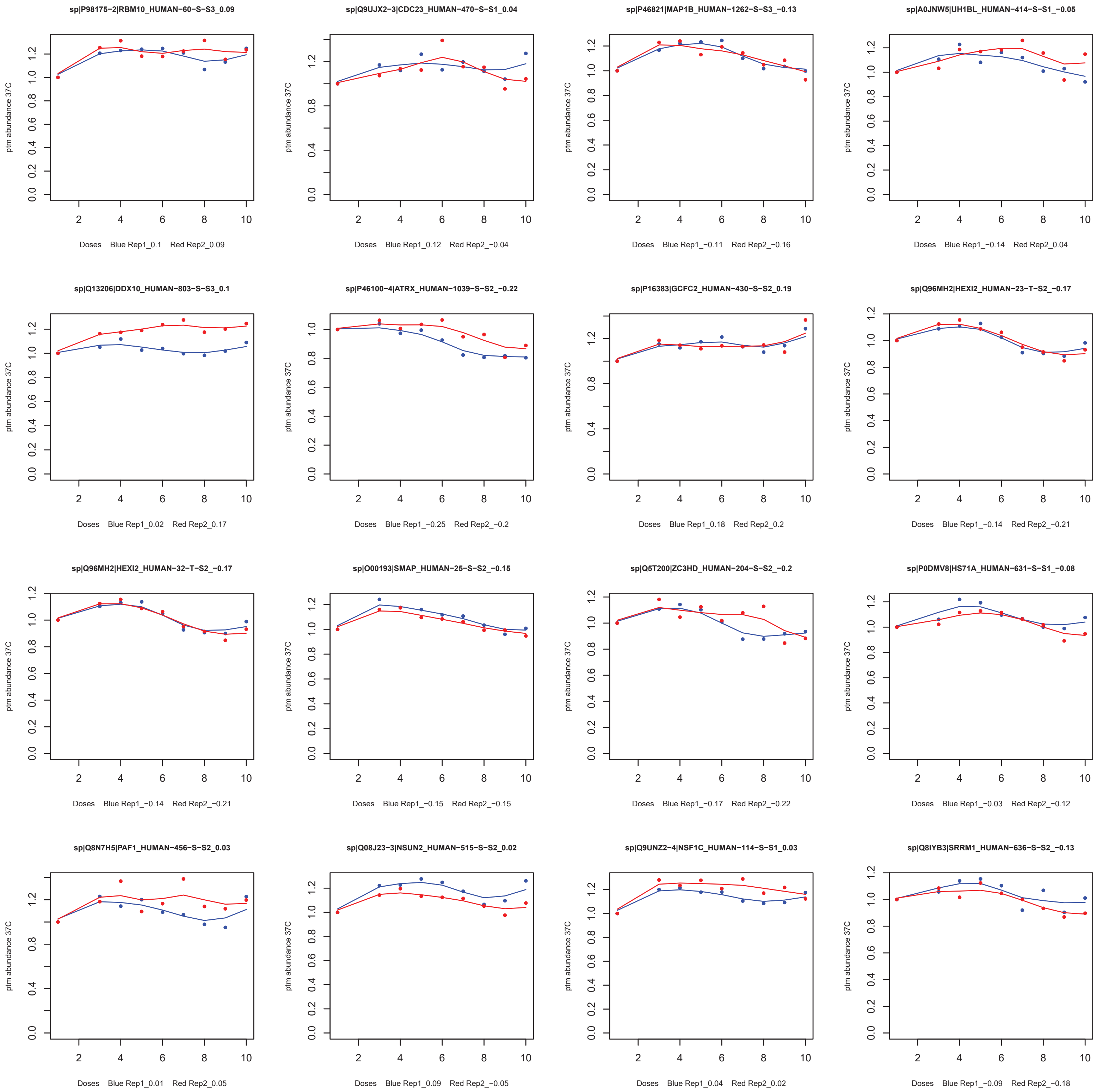
sp|P98175−2|RBM10_HUMAN−60−S−S3_0.09
Doses Blue Rep1_0.1 Red Rep2_0.09
sp|Q9UJX2−3|CDC23_HUMAN−470−S−S1_0.04
Doses Blue Rep1_0.12 Red Rep2_−0.04
sp|P46821|MAP1B_HUMAN−1262−S−S3_−0.13
Doses Blue Rep1_−0.11 Red Rep2_−0.16
sp|A0JNW5|UH1BL_HUMAN−414−S−S1_−0.05
Doses Blue Rep1_−0.14 Red Rep2_0.04
sp|Q13206|DDX10_HUMAN−803−S−S3_0.1
Doses Blue Rep1_0.02 Red Rep2_0.17
sp|P46100−4|ATRX_HUMAN−1039−S−S2_−0.22
Doses Blue Rep1_−0.25 Red Rep2_−0.2
sp|P16383|GCFC2_HUMAN−430−S−S2_0.19
Doses Blue Rep1_0.18 Red Rep2_0.2
sp|Q96MH2|HEXI2_HUMAN−23−T−S2_−0.17
Doses Blue Rep1_−0.14 Red Rep2_−0.21
sp|Q96MH2|HEXI2_HUMAN−32−T−S2_−0.17
Doses Blue Rep1_−0.14 Red Rep2_−0.21
sp|O00193|SMAP_HUMAN−25−S−S2_−0.15
Doses Blue Rep1_−0.15 Red Rep2_−0.15
sp|Q5T200|ZC3HD_HUMAN−204−S−S2_−0.2
Doses Blue Rep1_−0.17 Red Rep2_−0.22
sp|P0DMV8|HS71A_HUMAN−631−S−S1_−0.08
Doses Blue Rep1_−0.03 Red Rep2_−0.12
sp|Q8N7H5|PAF1_HUMAN−456−S−S2_0.03
Doses Blue Rep1_0.01 Red Rep2_0.05
sp|Q08J23−3|NSUN2_HUMAN−515−S−S2_0.02
Doses Blue Rep1_0.09 Red Rep2_−0.05
sp|Q9UNZ2−4|NSF1C_HUMAN−114−S−S1_0.03
Doses Blue Rep1_0.04 Red Rep2_0.02
sp|Q8IYB3|SRRM1_HUMAN−636−S−S2_−0.13
Doses Blue Rep1_−0.09 Red Rep2_−0.18
ptm abundance 37C

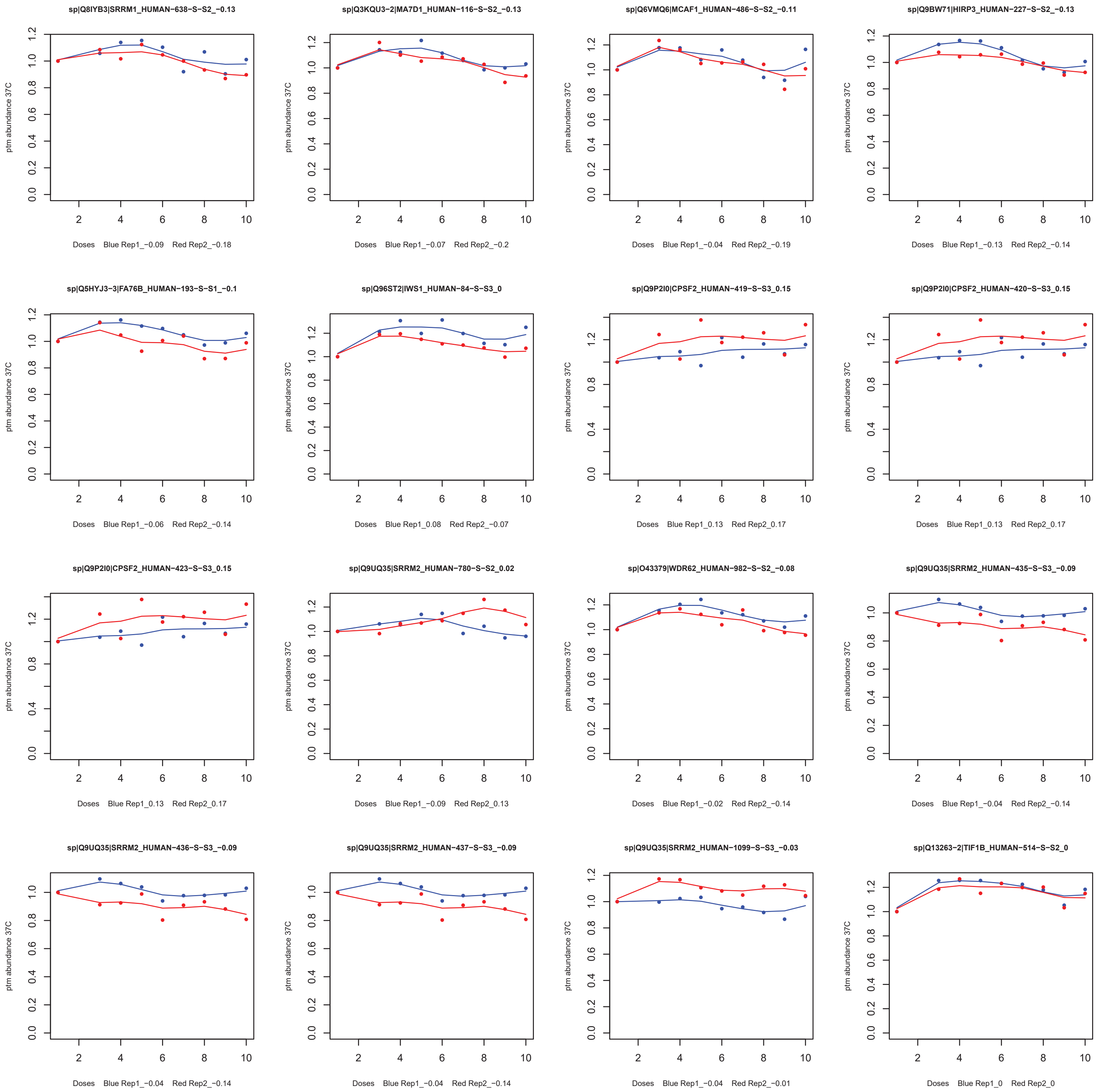
sp|Q8IYB3|SRRM1_HUMAN−638−S−S2_−0.13
Doses Blue Rep1_−0.09 Red Rep2_−0.18
sp|Q3KQU3−2|MA7D1_HUMAN−116−S−S2_−0.13
Doses Blue Rep1_−0.07 Red Rep2_−0.2
sp|Q6VMQ6|MCAF1_HUMAN−486−S−S2_−0.11
Doses Blue Rep1_−0.04 Red Rep2_−0.19
sp|Q9BW71|HIRP3_HUMAN−227−S−S2_−0.13
Doses Blue Rep1_−0.13 Red Rep2_−0.14
sp|Q5HYJ3−3|FA76B_HUMAN−193−S−S1_−0.1
Doses Blue Rep1_−0.06 Red Rep2_−0.14
sp|Q96ST2|IWS1_HUMAN−84−S−S3_0
Doses Blue Rep1_0.08 Red Rep2_−0.07
sp|Q9P2I0|CPSF2_HUMAN−419−S−S3_0.15
Doses Blue Rep1_0.13 Red Rep2_0.17
sp|Q9P2I0|CPSF2_HUMAN−420−S−S3_0.15
Doses Blue Rep1_0.13 Red Rep2_0.17
sp|Q9P2I0|CPSF2_HUMAN−423−S−S3_0.15
Doses Blue Rep1_0.13 Red Rep2_0.17
sp|Q9UQ35|SRRM2_HUMAN−780−S−S2_0.02
Doses Blue Rep1_−0.09 Red Rep2_0.13
sp|O43379|WDR62_HUMAN−982−S−S2_−0.08
Doses Blue Rep1_−0.02 Red Rep2_−0.14
sp|Q9UQ35|SRRM2_HUMAN−435−S−S3_−0.09
Doses Blue Rep1_−0.04 Red Rep2_−0.14
sp|Q9UQ35|SRRM2_HUMAN−436−S−S3_−0.09
Doses Blue Rep1_−0.04 Red Rep2_−0.14
sp|Q9UQ35|SRRM2_HUMAN−437−S−S3_−0.09
Doses Blue Rep1_−0.04 Red Rep2_−0.14
sp|Q9UQ35|SRRM2_HUMAN−1099−S−S3_−0.03
Doses Blue Rep1_−0.04 Red Rep2_−0.01
sp|Q13263−2|TIF1B_HUMAN−514−S−S2_0
Doses Blue Rep1_0 Red Rep2_0
ptm abundance 37C

sp|O15119−3|TBX3_HUMAN−371−S−S2_0

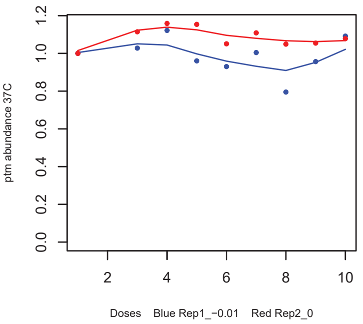

sp|O15119−3|TBX3_HUMAN−375−S−S2_0

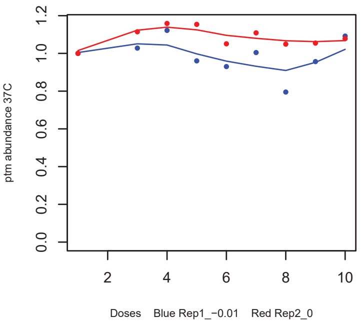

sp|Q147X3|NAA30_HUMAN−55−S−S1_0

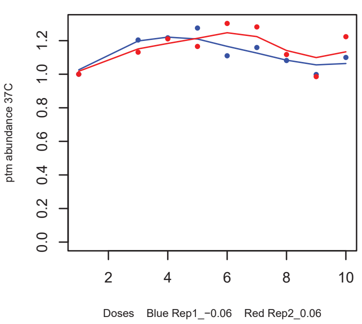

sp|Q9BVI0|PHF20_HUMAN−159−S−S1_−0.05

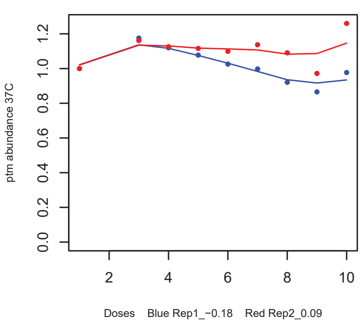

sp|Q14669|TRIPC_HUMAN−312−S−S1_−0.09

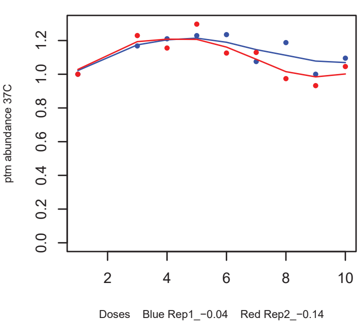

sp|P46013|KI67_HUMAN−2828−S−S1_0.09

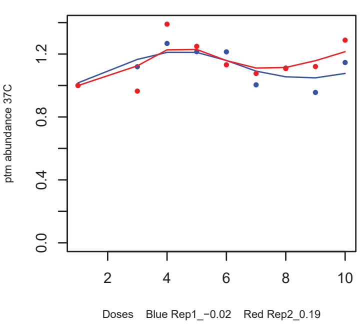

sp|P78559|MAP1A_HUMAN−1675−S−S1_0

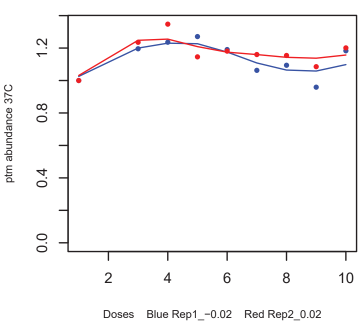

sp|Q1KMD3|HNRL2_HUMAN−165−T−S2_0.2

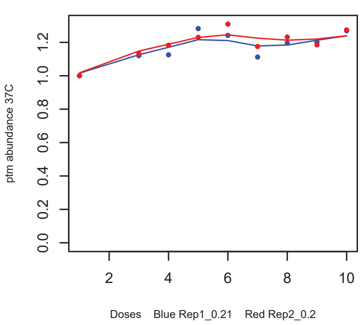

sp|Q96JM3|CHAP1_HUMAN−284−S−S2_−0.11

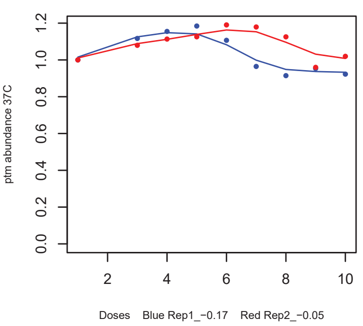

sp|Q96JM3|CHAP1_HUMAN−286−S−S2_−0.11

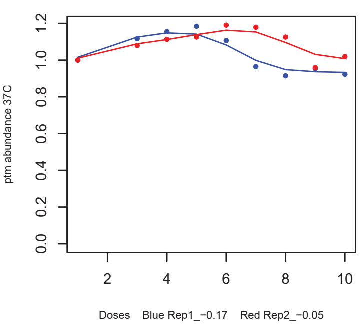

sp|Q9ULW0|TPX2_HUMAN−185−S−S2_−0.24

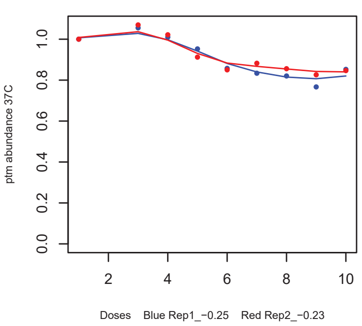

sp|Q9ULW0|TPX2_HUMAN−186−S−S2_−0.24

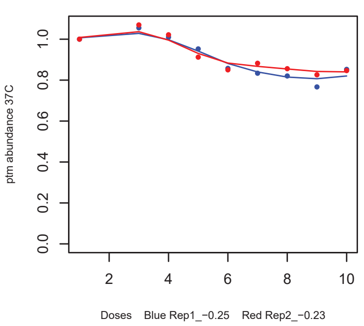

sp|Q92628|K0232_HUMAN−1117−S−S2_0.01

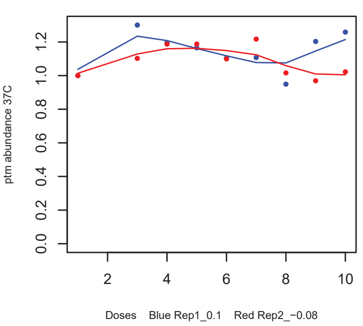

sp|Q92628|K0232_HUMAN−1122−S−S2_0.01

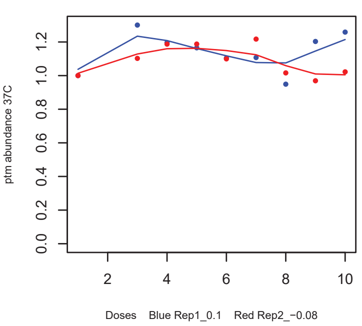

sp|P00519−2|ABL1_HUMAN−828−S−S2_−0.13

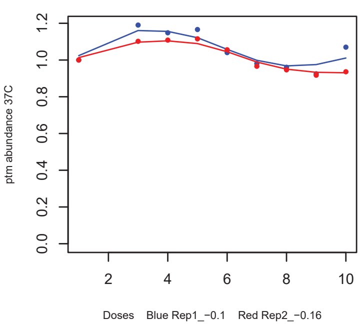

sp|Q96B36−3|AKTS1_HUMAN−231−S−S3_0.06

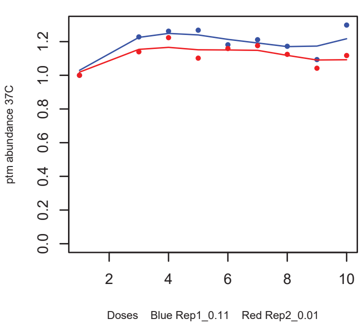

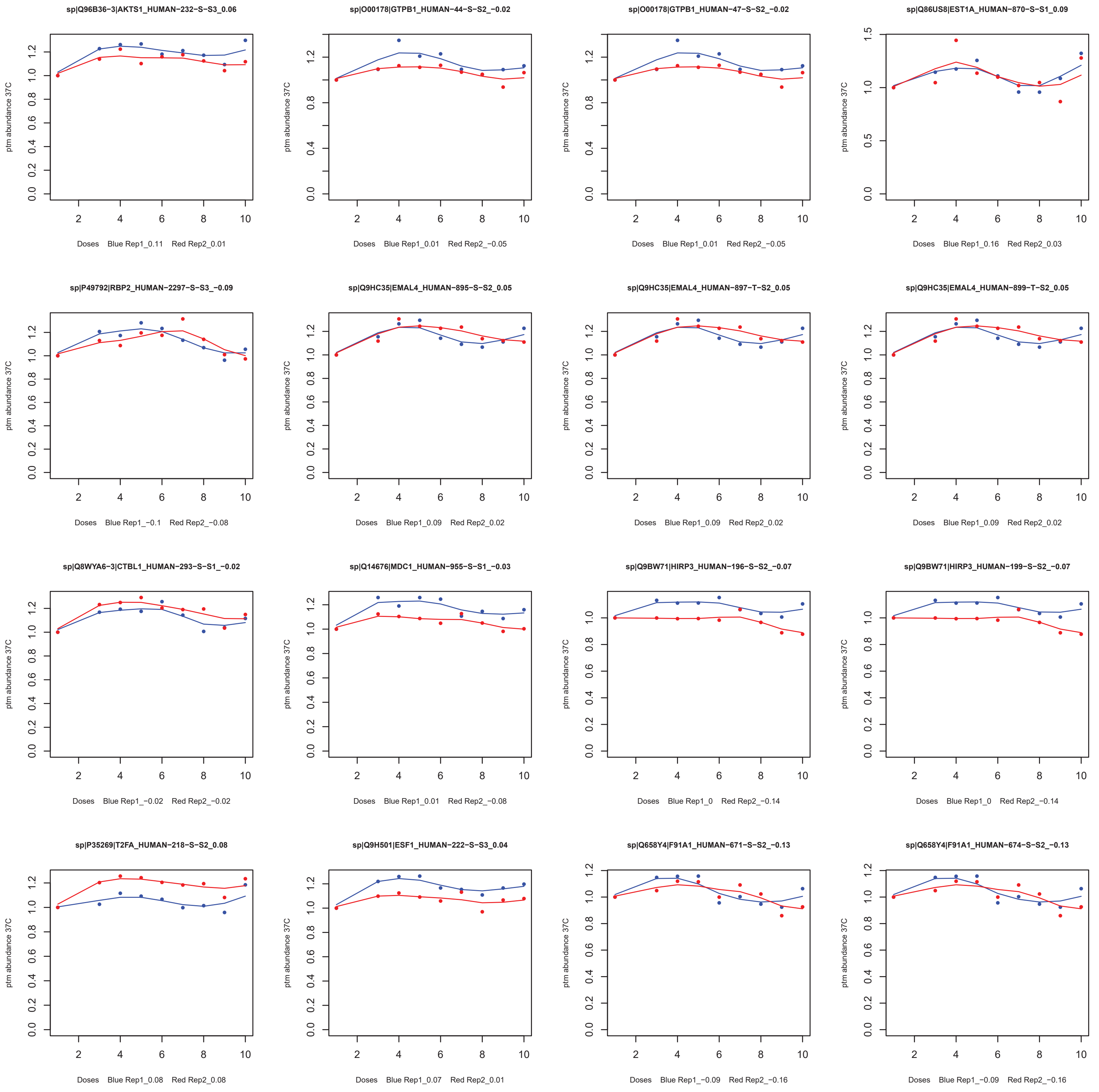
sp|Q96B36−3|AKTS1_HUMAN−232−S−S3_0.06
Doses Blue Rep1_0.11 Red Rep2_0.01
sp|O00178|GTPB1_HUMAN−44−S−S2_−0.02
Doses Blue Rep1_0.01 Red Rep2_−0.05
sp|O00178|GTPB1_HUMAN−47−S−S2_−0.02
Doses Blue Rep1_0.01 Red Rep2_−0.05
sp|Q86US8|EST1A_HUMAN−870−S−S1_0.09
Doses Blue Rep1_0.16 Red Rep2_0.03
sp|P49792|RBP2_HUMAN−2297−S−S3_−0.09
Doses Blue Rep1_−0.1 Red Rep2_−0.08
sp|Q9HC35|EMAL4_HUMAN−895−S−S2_0.05
Doses Blue Rep1_0.09 Red Rep2_0.02
sp|Q9HC35|EMAL4_HUMAN−897−T−S2_0.05
Doses Blue Rep1_0.09 Red Rep2_0.02
sp|Q9HC35|EMAL4_HUMAN−899−T−S2_0.05
Doses Blue Rep1_0.09 Red Rep2_0.02
sp|Q8WYA6−3|CTBL1_HUMAN−293−S−S1_−0.02
Doses Blue Rep1_−0.02 Red Rep2_−0.02
sp|Q14676|MDC1_HUMAN−955−S−S1_−0.03
Doses Blue Rep1_0.01 Red Rep2_−0.08
sp|Q9BW71|HIRP3_HUMAN−196−S−S2_−0.07
Doses Blue Rep1_0 Red Rep2_−0.14
sp|Q9BW71|HIRP3_HUMAN−199−S−S2_−0.07
Doses Blue Rep1_0 Red Rep2_−0.14
sp|P35269|T2FA_HUMAN−218−S−S2_0.08
Doses Blue Rep1_0.08 Red Rep2_0.08
sp|Q9H501|ESF1_HUMAN−222−S−S3_0.04
Doses Blue Rep1_0.07 Red Rep2_0.01
sp|Q658Y4|F91A1_HUMAN−671−S−S2_−0.13
Doses Blue Rep1_−0.09 Red Rep2_−0.16
sp|Q658Y4|F91A1_HUMAN−674−S−S2_−0.13
Doses Blue Rep1_−0.09 Red Rep2_−0.16
ptm abundance 37C

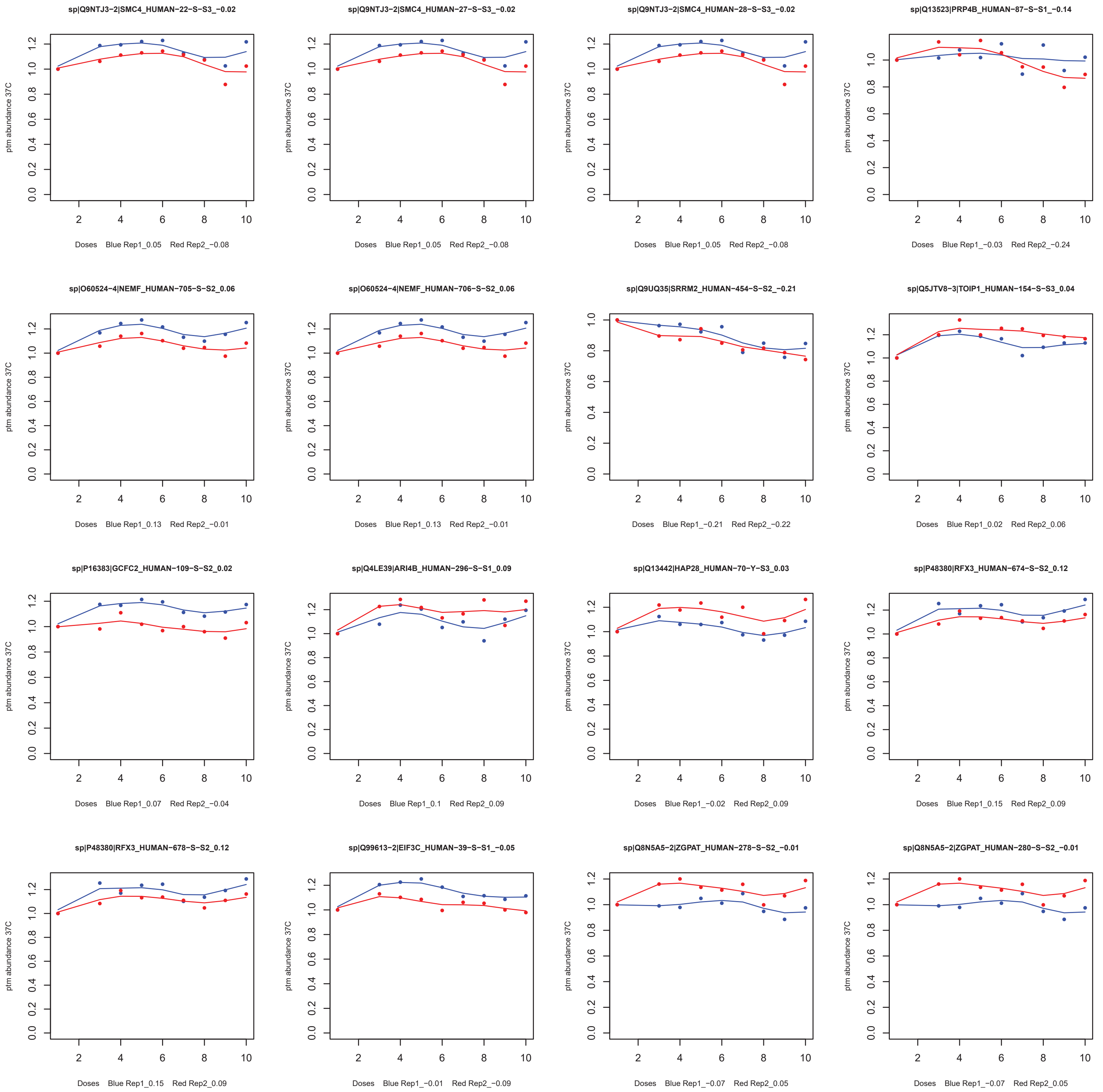

sp|Q9NTJ3−2|SMC4_HUMAN−22−S−S3_−0.02
Doses Blue Rep1_0.05 Red Rep2_−0.08
sp|Q9NTJ3−2|SMC4_HUMAN−27−S−S3_−0.02
Doses Blue Rep1_0.05 Red Rep2_−0.08
sp|Q9NTJ3−2|SMC4_HUMAN−28−S−S3_−0.02
Doses Blue Rep1_0.05 Red Rep2_−0.08
sp|Q13523|PRP4B_HUMAN−87−S−S1_−0.14
Doses Blue Rep1_−0.03 Red Rep2_−0.24
sp|O60524−4|NEMF_HUMAN−705−S−S2_0.06
Doses Blue Rep1_0.13 Red Rep2_−0.01
sp|O60524−4|NEMF_HUMAN−706−S−S2_0.06
Doses Blue Rep1_0.13 Red Rep2_−0.01
sp|Q9UQ35|SRRM2_HUMAN−454−S−S2_−0.21
Doses Blue Rep1_−0.21 Red Rep2_−0.22
sp|Q5JTV8−3|TOIP1_HUMAN−154−S−S3_0.04
Doses Blue Rep1_0.02 Red Rep2_0.06
sp|P16383|GCFC2_HUMAN−109−S−S2_0.02
Doses Blue Rep1_0.07 Red Rep2_−0.04
sp|Q4LE39|ARI4B_HUMAN−296−S−S1_0.09
Doses Blue Rep1_0.1 Red Rep2_0.09
sp|Q13442|HAP28_HUMAN−70−Y−S3_0.03
Doses Blue Rep1_−0.02 Red Rep2_0.09
sp|P48380|RFX3_HUMAN−674−S−S2_0.12
Doses Blue Rep1_0.15 Red Rep2_0.09
sp|P48380|RFX3_HUMAN−678−S−S2_0.12
Doses Blue Rep1_0.15 Red Rep2_0.09
sp|Q99613−2|EIF3C_HUMAN−39−S−S1_−0.05
Doses Blue Rep1_−0.01 Red Rep2_−0.09
sp|Q8N5A5−2|ZGPAT_HUMAN−278−S−S2_−0.01
Doses Blue Rep1_−0.07 Red Rep2_0.05
sp|Q8N5A5−2|ZGPAT_HUMAN−280−S−S2_−0.01
Doses Blue Rep1_−0.07 Red Rep2_0.05
ptm abundance 37C

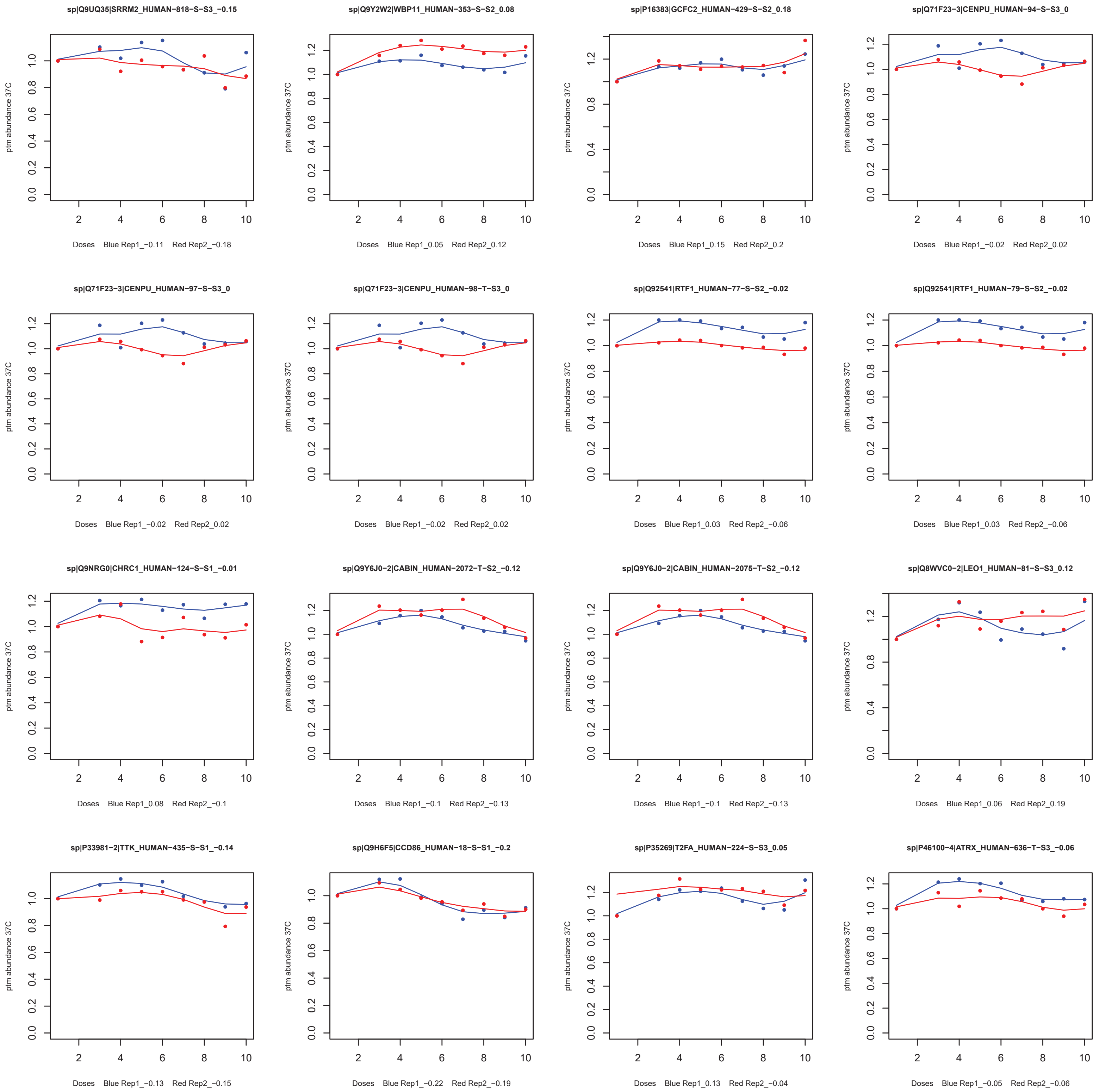

sp|Q9UQ35|SRRM2_HUMAN−818−S−S3_−0.15
ptm abundance 37C
Doses Blue Rep1_−0.11 Red Rep2_−0.18
sp|Q9Y2W2|WBP11_HUMAN−353−S−S2_0.08
ptm abundance 37C
Doses Blue Rep1_0.05 Red Rep2_0.12
sp|P16383|GCFC2_HUMAN−429−S−S2_0.18
ptm abundance 37C
Doses Blue Rep1_0.15 Red Rep2_0.2
sp|Q71F23−3|CENPU_HUMAN−94−S−S3_0
ptm abundance 37C
Doses Blue Rep1_−0.02 Red Rep2_0.02
sp|Q71F23−3|CENPU_HUMAN−97−S−S3_0
ptm abundance 37C
Doses Blue Rep1_−0.02 Red Rep2_0.02
sp|Q71F23−3|CENPU_HUMAN−98−T−S3_0
ptm abundance 37C
Doses Blue Rep1_−0.02 Red Rep2_0.02
sp|Q92541|RTF1_HUMAN−77−S−S2_−0.02
ptm abundance 37C
Doses Blue Rep1_0.03 Red Rep2_−0.06
sp|Q92541|RTF1_HUMAN−79−S−S2_−0.02
ptm abundance 37C
Doses Blue Rep1_0.03 Red Rep2_−0.06
sp|Q9NRG0|CHRC1_HUMAN−124−S−S1_−0.01
ptm abundance 37C
Doses Blue Rep1_0.08 Red Rep2_−0.1
sp|Q9Y6J0−2|CABIN_HUMAN−2072−T−S2_−0.12
ptm abundance 37C
Doses Blue Rep1_−0.1 Red Rep2_−0.13
sp|Q9Y6J0−2|CABIN_HUMAN−2075−T−S2_−0.12
ptm abundance 37C
Doses Blue Rep1_−0.1 Red Rep2_−0.13
sp|Q8WVC0−2|LEO1_HUMAN−81−S−S3_0.12
ptm abundance 37C
Doses Blue Rep1_0.06 Red Rep2_0.19
sp|P33981−2|TTK_HUMAN−435−S−S1_−0.14
ptm abundance 37C
Doses Blue Rep1_−0.13 Red Rep2_−0.15
sp|Q9H6F5|CCD86_HUMAN−18−S−S1_−0.2
ptm abundance 37C
Doses Blue Rep1_−0.22 Red Rep2_−0.19
sp|P35269|T2FA_HUMAN−224−S−S3_0.05
ptm abundance 37C
Doses Blue Rep1_0.13 Red Rep2_−0.04
sp|P46100−4|ATRX_HUMAN−636−T−S3_−0.06
ptm abundance 37C
Doses Blue Rep1_−0.05 Red Rep2_−0.06

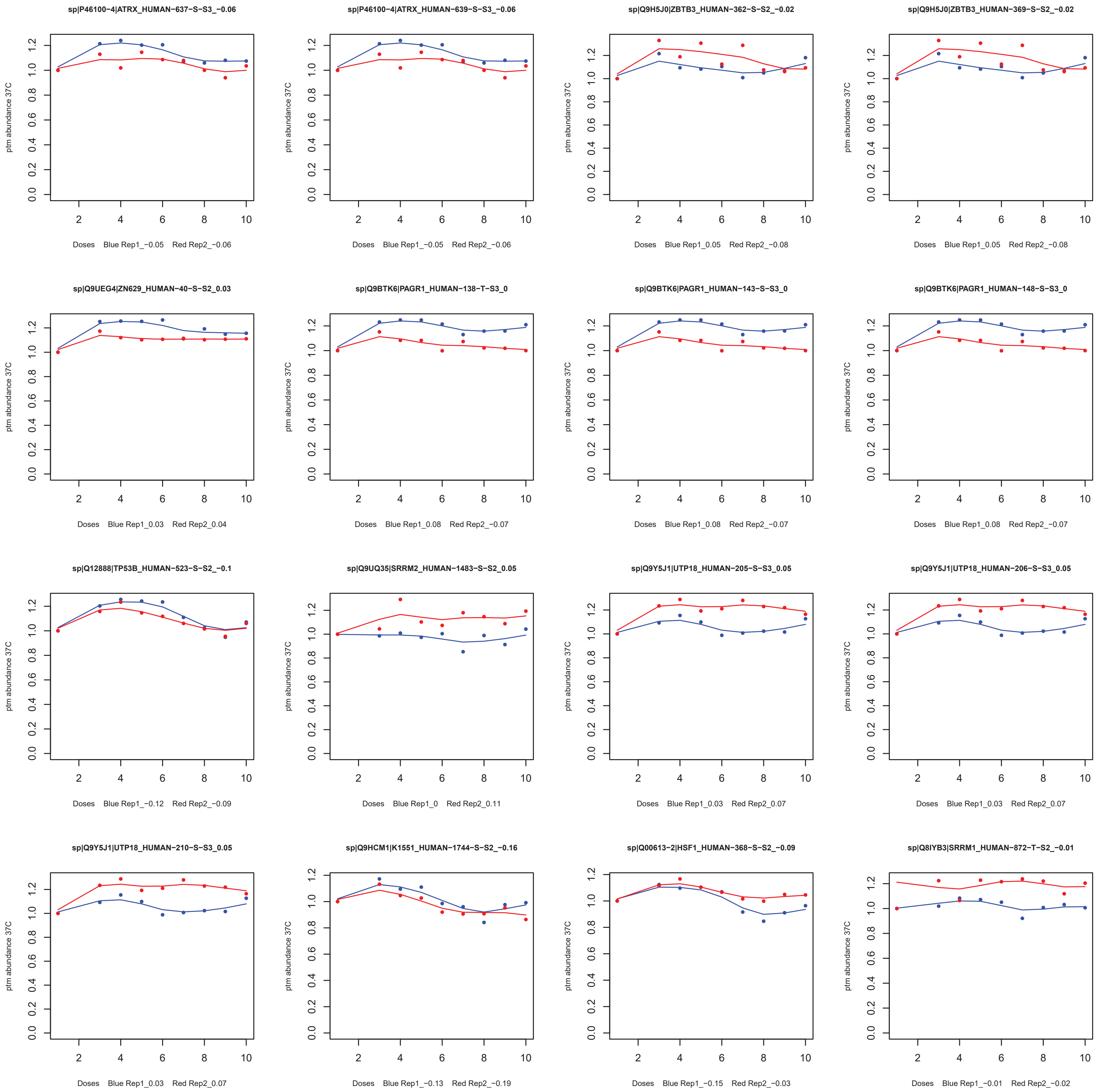

sp|P46100−4|ATRX_HUMAN−637−S−S3_−0.06
Doses Blue Rep1_−0.05 Red Rep2_−0.06
sp|P46100−4|ATRX_HUMAN−639−S−S3_−0.06
Doses Blue Rep1_−0.05 Red Rep2_−0.06
sp|Q9H5J0|ZBTB3_HUMAN−362−S−S2_−0.02
Doses Blue Rep1_0.05 Red Rep2_−0.08
sp|Q9H5J0|ZBTB3_HUMAN−369−S−S2_−0.02
Doses Blue Rep1_0.05 Red Rep2_−0.08
sp|Q9UEG4|ZN629_HUMAN−40−S−S2_0.03
Doses Blue Rep1_0.03 Red Rep2_0.04
sp|Q9BTK6|PAGR1_HUMAN−138−T−S3_0
Doses Blue Rep1_0.08 Red Rep2_−0.07
sp|Q9BTK6|PAGR1_HUMAN−143−S−S3_0
Doses Blue Rep1_0.08 Red Rep2_−0.07
sp|Q9BTK6|PAGR1_HUMAN−148−S−S3_0
Doses Blue Rep1_0.08 Red Rep2_−0.07
sp|Q12888|TP53B_HUMAN−523−S−S2_−0.1
Doses Blue Rep1_−0.12 Red Rep2_−0.09
sp|Q9UQ35|SRRM2_HUMAN−1483−S−S2_0.05
Doses Blue Rep1_0 Red Rep2_0.11
sp|Q9Y5J1|UTP18_HUMAN−205−S−S3_0.05
Doses Blue Rep1_0.03 Red Rep2_0.07
sp|Q9Y5J1|UTP18_HUMAN−206−S−S3_0.05
Doses Blue Rep1_0.03 Red Rep2_0.07
sp|Q9Y5J1|UTP18_HUMAN−210−S−S3_0.05
Doses Blue Rep1_0.03 Red Rep2_0.07
sp|Q9HCM1|K1551_HUMAN−1744−S−S2_−0.16
Doses Blue Rep1_−0.13 Red Rep2_−0.19
sp|Q00613−2|HSF1_HUMAN−368−S−S2_−0.09
Doses Blue Rep1_−0.15 Red Rep2_−0.03
sp|Q8IYB3|SRRM1_HUMAN−872−T−S2_−0.01
Doses Blue Rep1_−0.01 Red Rep2_−0.02
ptm abundance 37C

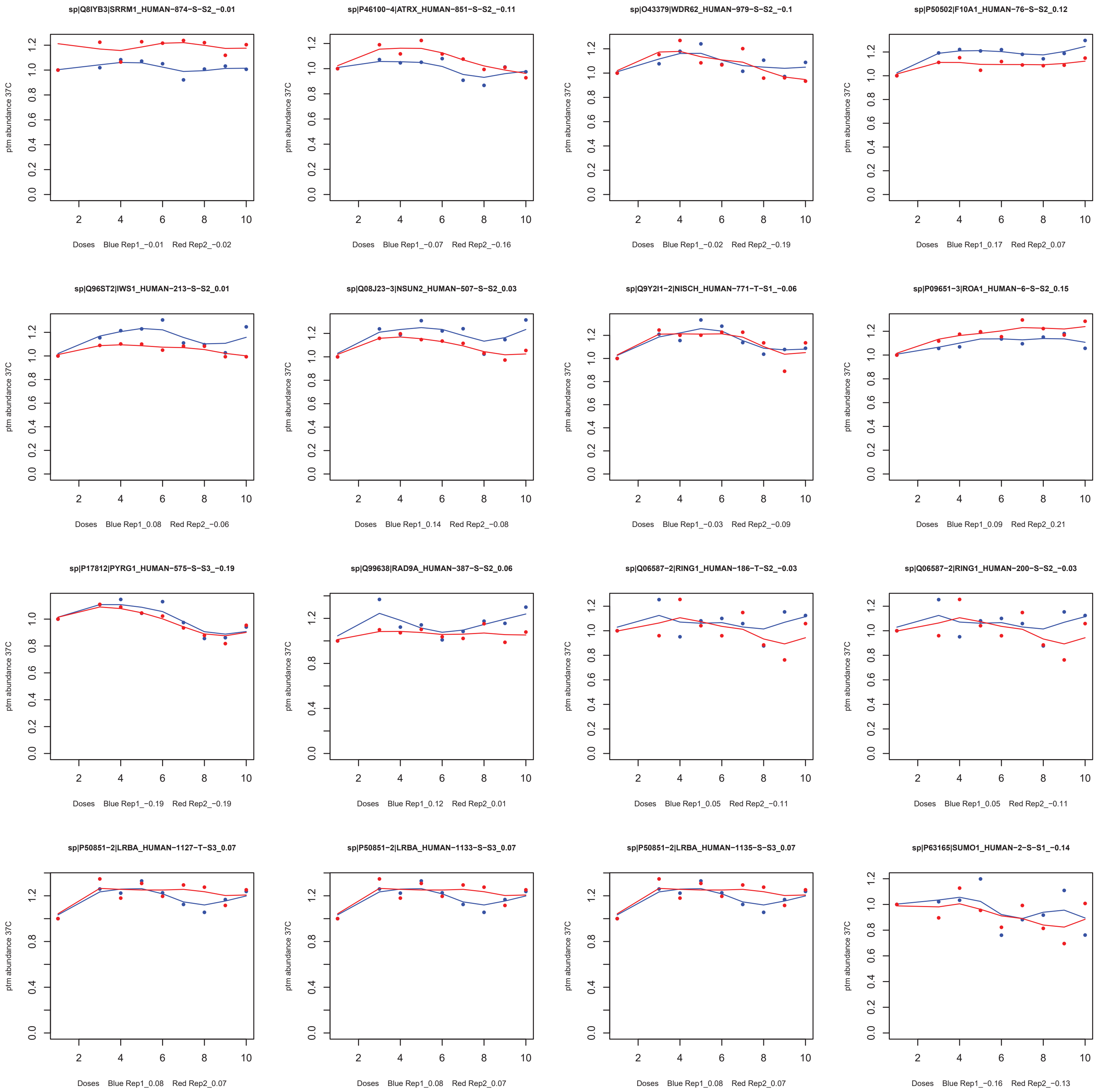

sp|Q8IYB3|SRRM1_HUMAN−874−S−S2_−0.01
Doses Blue Rep1_−0.01 Red Rep2_−0.02
sp|P46100−4|ATRX_HUMAN−851−S−S2_−0.11
Doses Blue Rep1_−0.07 Red Rep2_−0.16
sp|O43379|WDR62_HUMAN−979−S−S2_−0.1
Doses Blue Rep1_−0.02 Red Rep2_−0.19
sp|P50502|F10A1_HUMAN−76−S−S2_0.12
Doses Blue Rep1_0.17 Red Rep2_0.07
sp|Q96ST2|IWS1_HUMAN−213−S−S2_0.01
Doses Blue Rep1_0.08 Red Rep2_−0.06
sp|Q08J23−3|NSUN2_HUMAN−507−S−S2_0.03
Doses Blue Rep1_0.14 Red Rep2_−0.08
sp|Q9Y2I1−2|NISCH_HUMAN−771−T−S1_−0.06
Doses Blue Rep1_−0.03 Red Rep2_−0.09
sp|P09651−3|ROA1_HUMAN−6−S−S2_0.15
Doses Blue Rep1_0.09 Red Rep2_0.21
sp|P17812|PYRG1_HUMAN−575−S−S3_−0.19
Doses Blue Rep1_−0.19 Red Rep2_−0.19
sp|Q99638|RAD9A_HUMAN−387−S−S2_0.06
Doses Blue Rep1_0.12 Red Rep2_0.01
sp|Q06587−2|RING1_HUMAN−186−T−S2_−0.03
Doses Blue Rep1_0.05 Red Rep2_−0.11
sp|Q06587−2|RING1_HUMAN−200−S−S2_−0.03
Doses Blue Rep1_0.05 Red Rep2_−0.11
sp|P50851−2|LRBA_HUMAN−1127−T−S3_0.07
Doses Blue Rep1_0.08 Red Rep2_0.07
sp|P50851−2|LRBA_HUMAN−1133−S−S3_0.07
Doses Blue Rep1_0.08 Red Rep2_0.07
sp|P50851−2|LRBA_HUMAN−1135−S−S3_0.07
Doses Blue Rep1_0.08 Red Rep2_0.07
sp|P63165|SUMO1_HUMAN−2−S−S1_−0.14
Doses Blue Rep1_−0.16 Red Rep2_−0.13
ptm abundance 37C

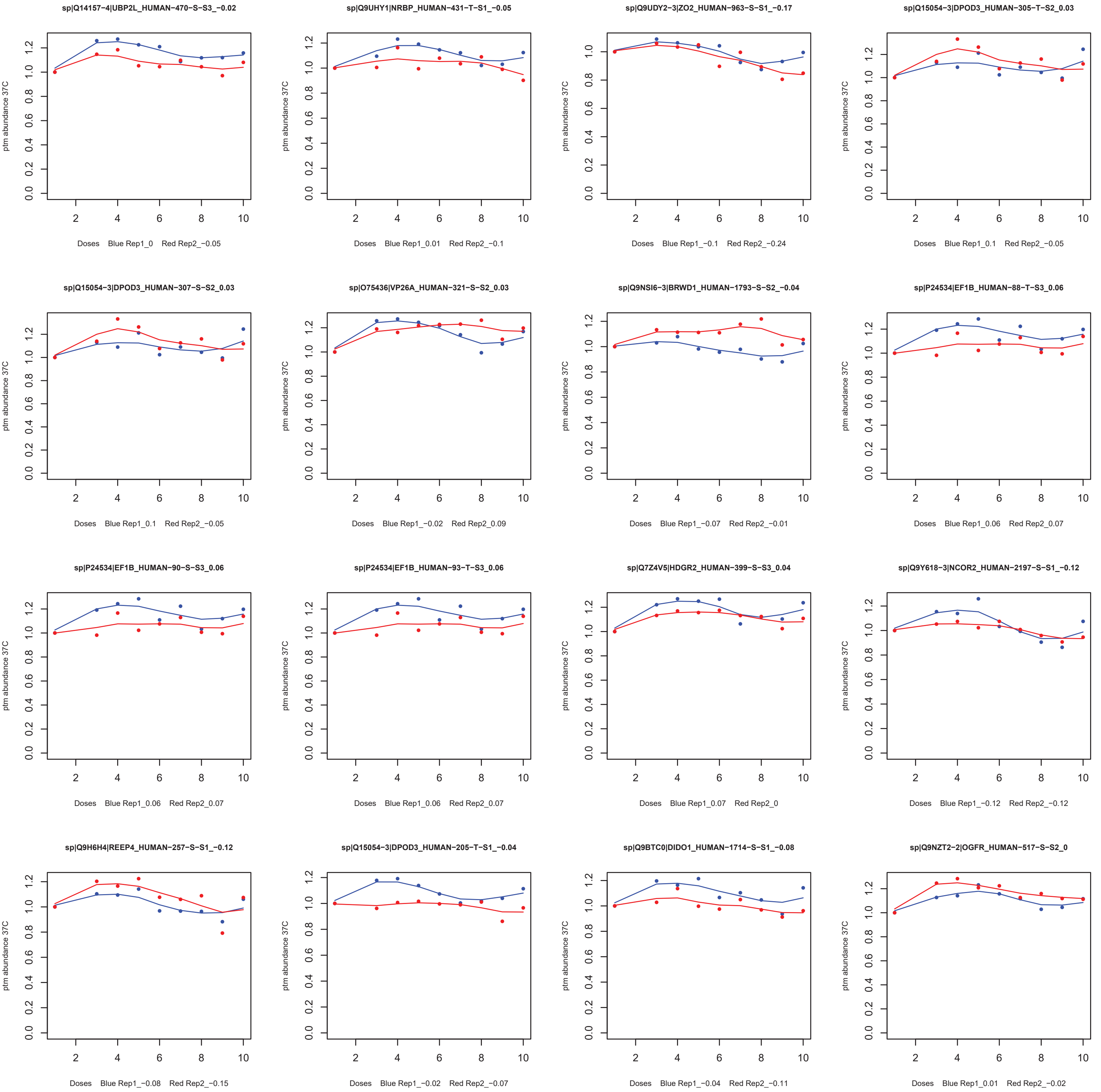

sp|Q14157−4|UBP2L_HUMAN−470−S−S3_−0.02
Doses Blue Rep1_0 Red Rep2_−0.05
sp|Q9UHY1|NRBP_HUMAN−431−T−S1_−0.05
Doses Blue Rep1_0.01 Red Rep2_−0.1
sp|Q9UDY2−3|ZO2_HUMAN−963−S−S1_−0.17
Doses Blue Rep1_−0.1 Red Rep2_−0.24
sp|Q15054−3|DPOD3_HUMAN−305−T−S2_0.03
Doses Blue Rep1_0.1 Red Rep2_−0.05
sp|Q15054−3|DPOD3_HUMAN−307−S−S2_0.03
Doses Blue Rep1_0.1 Red Rep2_−0.05
sp|O75436|VP26A_HUMAN−321−S−S2_0.03
Doses Blue Rep1_−0.02 Red Rep2_0.09
sp|Q9NSI6−3|BRWD1_HUMAN−1793−S−S2_−0.04
Doses Blue Rep1_−0.07 Red Rep2_−0.01
sp|P24534|EF1B_HUMAN−88−T−S3_0.06
Doses Blue Rep1_0.06 Red Rep2_0.07
sp|P24534|EF1B_HUMAN−90−S−S3_0.06
Doses Blue Rep1_0.06 Red Rep2_0.07
sp|P24534|EF1B_HUMAN−93−T−S3_0.06
Doses Blue Rep1_0.06 Red Rep2_0.07
sp|Q7Z4V5|HDGR2_HUMAN−399−S−S3_0.04
Doses Blue Rep1_0.07 Red Rep2_0
sp|Q9Y618−3|NCOR2_HUMAN−2197−S−S1_−0.12
Doses Blue Rep1_−0.12 Red Rep2_−0.12
sp|Q9H6H4|REEP4_HUMAN−257−S−S1_−0.12
Doses Blue Rep1_−0.08 Red Rep2_−0.15
sp|Q15054−3|DPOD3_HUMAN−205−T−S1_−0.04
Doses Blue Rep1_−0.02 Red Rep2_−0.07
sp|Q9BTC0|DIDO1_HUMAN−1714−S−S1_−0.08
Doses Blue Rep1_−0.04 Red Rep2_−0.11
sp|Q9NZT2−2|OGFR_HUMAN−517−S−S2_0
Doses Blue Rep1_0.01 Red Rep2_−0.02
ptm abundance 37C

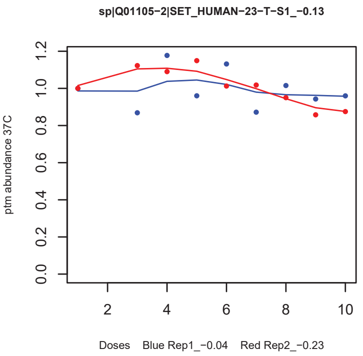
sp|Q01105−2|SET_HUMAN−23−T−S1_−0.13
ptm abundance 37C
0.0 0.2 0.4 0.6 0.8 1.0 1.2
2 4 6 8 10
Doses Blue Rep1_−0.04 Red Rep2_−0.23

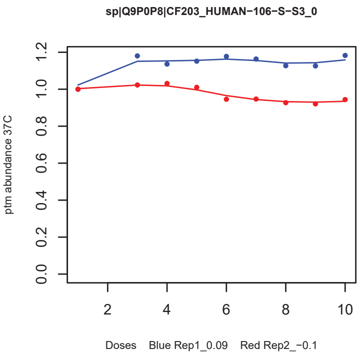
sp|Q9P0P8|CF203_HUMAN−106−S−S3_0
ptm abundance 37C
0.0 0.2 0.4 0.6 0.8 1.0 1.2
2 4 6 8 10
Doses Blue Rep1_0.09 Red Rep2_−0.1

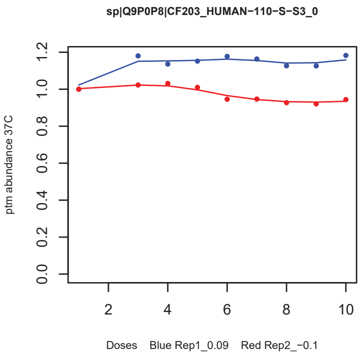
sp|Q9P0P8|CF203_HUMAN−110−S−S3_0
ptm abundance 37C
0.0 0.2 0.4 0.6 0.8 1.0 1.2
2 4 6 8 10
Doses Blue Rep1_0.09 Red Rep2_−0.1

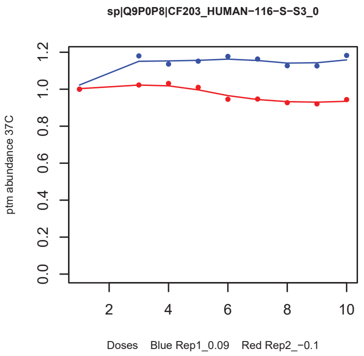
sp|Q9P0P8|CF203_HUMAN−116−S−S3_0
ptm abundance 37C
0.0 0.2 0.4 0.6 0.8 1.0 1.2
2 4 6 8 10
Doses Blue Rep1_0.09 Red Rep2_−0.1

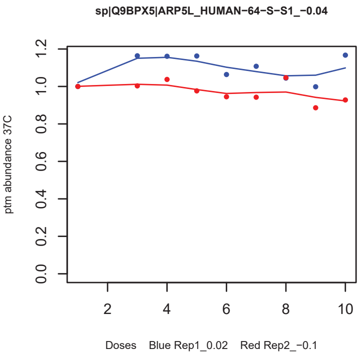
sp|Q9BPX5|ARP5L_HUMAN−64−S−S1_−0.04
ptm abundance 37C
0.0 0.2 0.4 0.6 0.8 1.0 1.2
2 4 6 8 10
Doses Blue Rep1_0.02 Red Rep2_−0.1

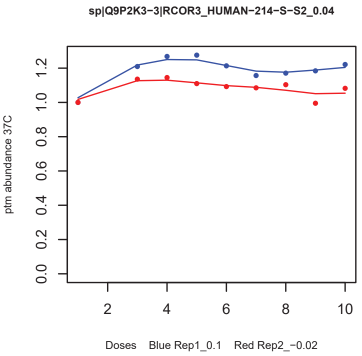
sp|Q9P2K3−3|RCOR3_HUMAN−214−S−S2_0.04
ptm abundance 37C
0.0 0.2 0.4 0.6 0.8 1.0 1.2
2 4 6 8 10
Doses Blue Rep1_0.1 Red Rep2_−0.02

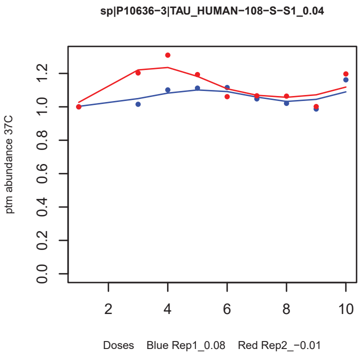
sp|P10636−3|TAU_HUMAN−108−S−S1_0.04
ptm abundance 37C
0.0 0.2 0.4 0.6 0.8 1.0 1.2
2 4 6 8 10
Doses Blue Rep1_0.08 Red Rep2_−0.01

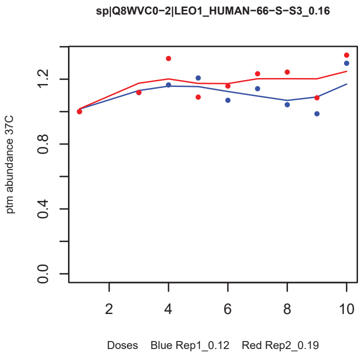
sp|Q8WVC0−2|LEO1_HUMAN−66−S−S3_0.16
ptm abundance 37C
0.0 0.4 0.8 1.2
2 4 6 8 10
Doses Blue Rep1_0.12 Red Rep2_0.19

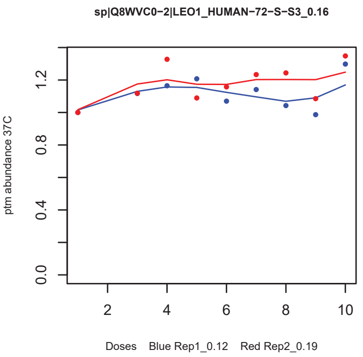
sp|Q8WVC0−2|LEO1_HUMAN−72−S−S3_0.16
ptm abundance 37C
0.0 0.4 0.8 1.2
2 4 6 8 10
Doses Blue Rep1_0.12 Red Rep2_0.19

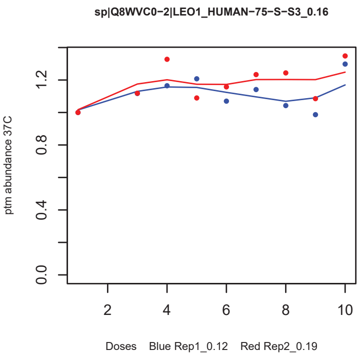
sp|Q8WVC0−2|LEO1_HUMAN−75−S−S3_0.16
ptm abundance 37C
0.0 0.4 0.8 1.2
2 4 6 8 10
Doses Blue Rep1_0.12 Red Rep2_0.19

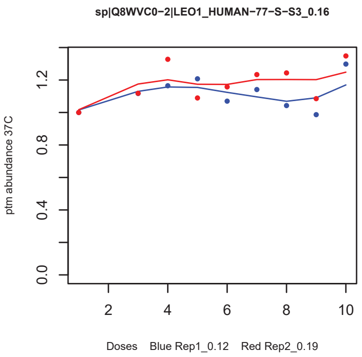
sp|Q8WVC0−2|LEO1_HUMAN−77−S−S3_0.16
ptm abundance 37C
0.0 0.4 0.8 1.2
2 4 6 8 10
Doses Blue Rep1_0.12 Red Rep2_0.19

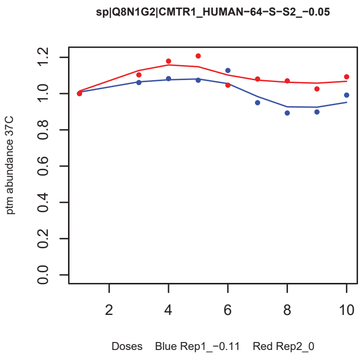
sp|Q8N1G2|CMTR1_HUMAN−64−S−S2_−0.05
ptm abundance 37C
0.0 0.2 0.4 0.6 0.8 1.0 1.2
2 4 6 8 10
Doses Blue Rep1_−0.11 Red Rep2_0

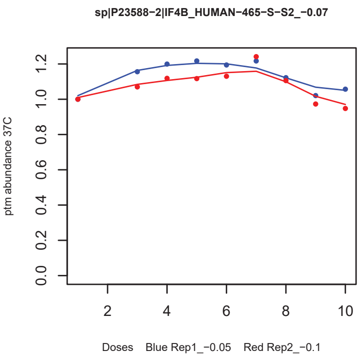
sp|P23588−2|IF4B_HUMAN−465−S−S2_−0.07
ptm abundance 37C
0.0 0.2 0.4 0.6 0.8 1.0 1.2
2 4 6 8 10
Doses Blue Rep1_−0.05 Red Rep2_−0.1

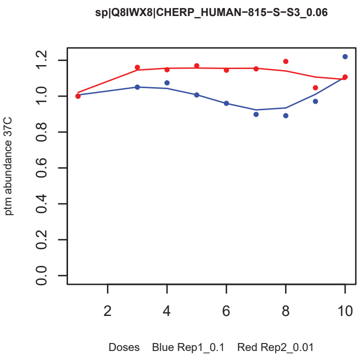
sp|Q8IWX8|CHERP_HUMAN−815−S−S3_0.06
ptm abundance 37C
0.0 0.2 0.4 0.6 0.8 1.0 1.2
2 4 6 8 10
Doses Blue Rep1_0.1 Red Rep2_0.01

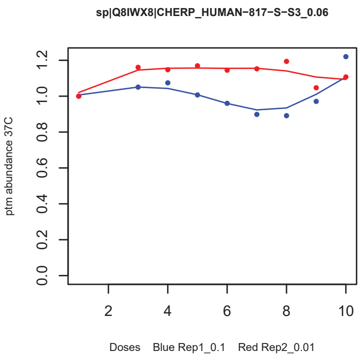
sp|Q8IWX8|CHERP_HUMAN−817−S−S3_0.06
ptm abundance 37C
0.0 0.2 0.4 0.6 0.8 1.0 1.2
2 4 6 8 10
Doses Blue Rep1_0.1 Red Rep2_0.01

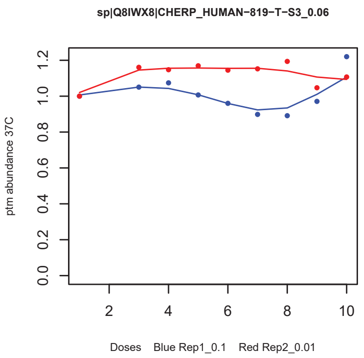
sp|Q8IWX8|CHERP_HUMAN−819−T−S3_0.06
ptm abundance 37C
0.0 0.2 0.4 0.6 0.8 1.0 1.2
2 4 6 8 10
Doses Blue Rep1_0.1 Red Rep2_0.01

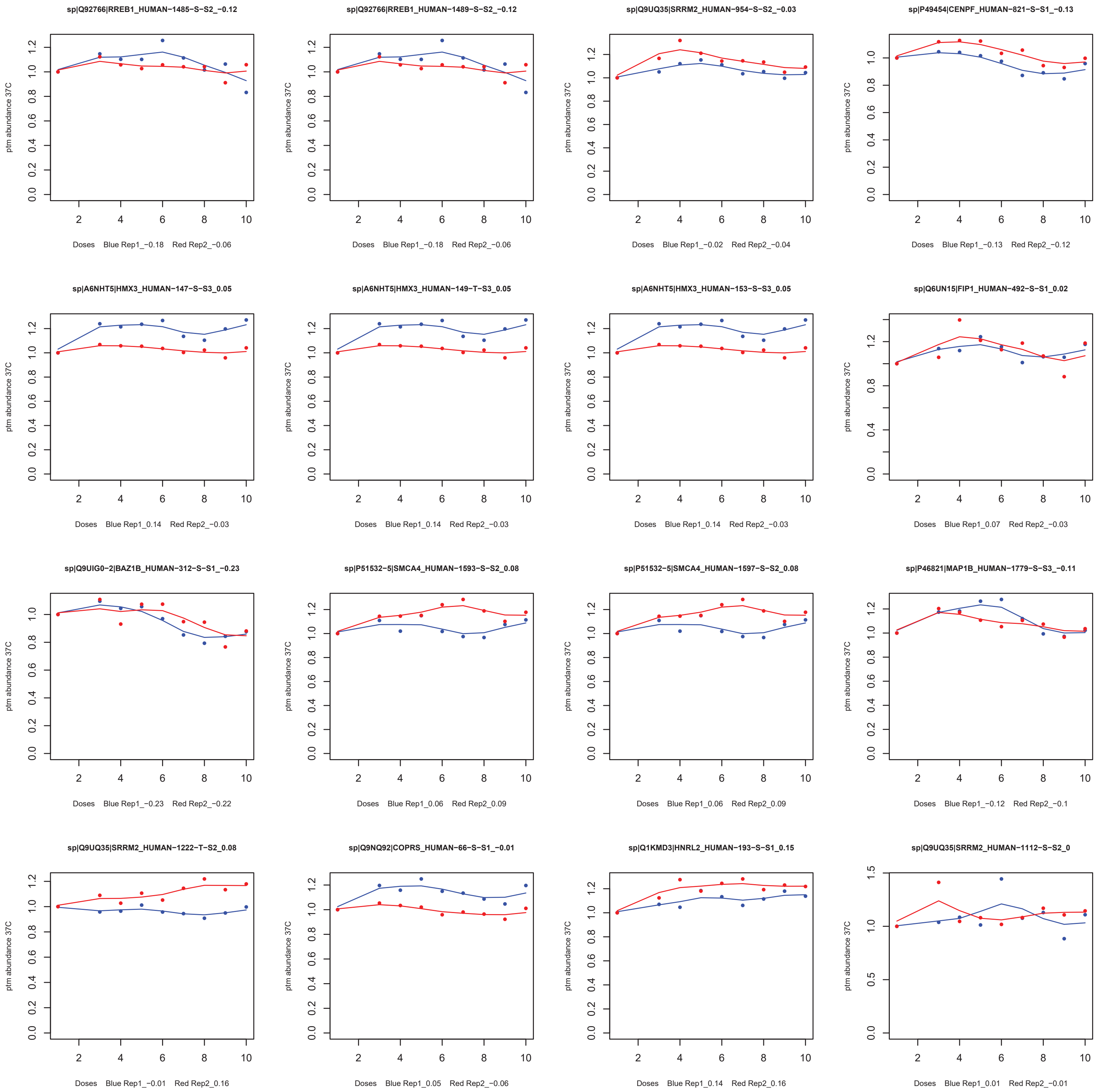
sp|Q92766|RREB1_HUMAN−1485−S−S2_−0.12
Doses Blue Rep1_−0.18 Red Rep2_−0.06
sp|Q92766|RREB1_HUMAN−1489−S−S2_−0.12
Doses Blue Rep1_−0.18 Red Rep2_−0.06
sp|Q9UQ35|SRRM2_HUMAN−954−S−S2_−0.03
Doses Blue Rep1_−0.02 Red Rep2_−0.04
sp|P49454|CENPF_HUMAN−821−S−S1_−0.13
Doses Blue Rep1_−0.13 Red Rep2_−0.12
sp|A6NHT5|HMX3_HUMAN−147−S−S3_0.05
Doses Blue Rep1_0.14 Red Rep2_−0.03
sp|A6NHT5|HMX3_HUMAN−149−T−S3_0.05
Doses Blue Rep1_0.14 Red Rep2_−0.03
sp|A6NHT5|HMX3_HUMAN−153−S−S3_0.05
Doses Blue Rep1_0.14 Red Rep2_−0.03
sp|Q6UN15|FIP1_HUMAN−492−S−S1_0.02
Doses Blue Rep1_0.07 Red Rep2_−0.03
sp|Q9UIG0−2|BAZ1B_HUMAN−312−S−S1_−0.23
Doses Blue Rep1_−0.23 Red Rep2_−0.22
sp|P51532−5|SMCA4_HUMAN−1593−S−S2_0.08
Doses Blue Rep1_0.06 Red Rep2_0.09
sp|P51532−5|SMCA4_HUMAN−1597−S−S2_0.08
Doses Blue Rep1_0.06 Red Rep2_0.09
sp|P46821|MAP1B_HUMAN−1779−S−S3_−0.11
Doses Blue Rep1_−0.12 Red Rep2_−0.1
sp|Q9UQ35|SRRM2_HUMAN−1222−T−S2_0.08
Doses Blue Rep1_−0.01 Red Rep2_0.16
sp|Q9NQ92|COPRS_HUMAN−66−S−S1_−0.01
Doses Blue Rep1_0.05 Red Rep2_−0.06
sp|Q1KMD3|HNRL2_HUMAN−193−S−S1_0.15
Doses Blue Rep1_0.14 Red Rep2_0.16
sp|Q9UQ35|SRRM2_HUMAN−1112−S−S2_0
Doses Blue Rep1_0.01 Red Rep2_−0.01
ptm abundance 37C

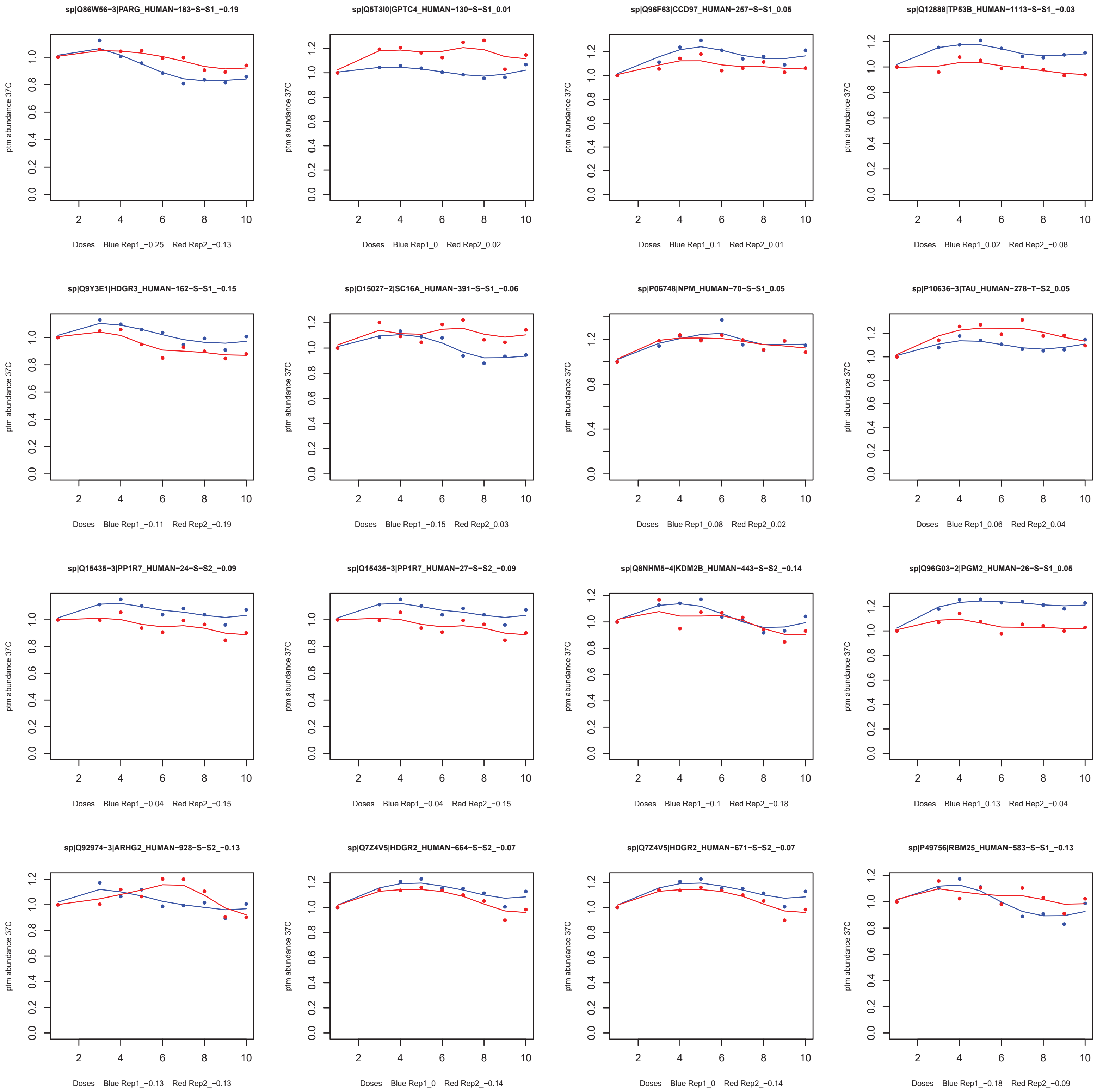

sp|Q86W56−3|PARG_HUMAN−183−S−S1_−0.19
Doses Blue Rep1_−0.25 Red Rep2_−0.13
sp|Q5T3I0|GPTC4_HUMAN−130−S−S1_0.01
Doses Blue Rep1_0 Red Rep2_0.02
sp|Q96F63|CCD97_HUMAN−257−S−S1_0.05
Doses Blue Rep1_0.1 Red Rep2_0.01
sp|Q12888|TP53B_HUMAN−1113−S−S1_−0.03
Doses Blue Rep1_0.02 Red Rep2_−0.08
sp|Q9Y3E1|HDGR3_HUMAN−162−S−S1_−0.15
Doses Blue Rep1_−0.11 Red Rep2_−0.19
sp|O15027−2|SC16A_HUMAN−391−S−S1_−0.06
Doses Blue Rep1_−0.15 Red Rep2_0.03
sp|P06748|NPM_HUMAN−70−S−S1_0.05
Doses Blue Rep1_0.08 Red Rep2_0.02
sp|P10636−3|TAU_HUMAN−278−T−S2_0.05
Doses Blue Rep1_0.06 Red Rep2_0.04
sp|Q15435−3|PP1R7_HUMAN−24−S−S2_−0.09
Doses Blue Rep1_−0.04 Red Rep2_−0.15
sp|Q15435−3|PP1R7_HUMAN−27−S−S2_−0.09
Doses Blue Rep1_−0.04 Red Rep2_−0.15
sp|Q8NHM5−4|KDM2B_HUMAN−443−S−S2_−0.14
Doses Blue Rep1_−0.1 Red Rep2_−0.18
sp|Q96G03−2|PGM2_HUMAN−26−S−S1_0.05
Doses Blue Rep1_0.13 Red Rep2_−0.04
sp|Q92974−3|ARHG2_HUMAN−928−S−S2_−0.13
Doses Blue Rep1_−0.13 Red Rep2_−0.13
sp|Q7Z4V5|HDGR2_HUMAN−664−S−S2_−0.07
Doses Blue Rep1_0 Red Rep2_−0.14
sp|Q7Z4V5|HDGR2_HUMAN−671−S−S2_−0.07
Doses Blue Rep1_0 Red Rep2_−0.14
sp|P49756|RBM25_HUMAN−583−S−S1_−0.13
Doses Blue Rep1_−0.18 Red Rep2_−0.09
ptm abundance 37C

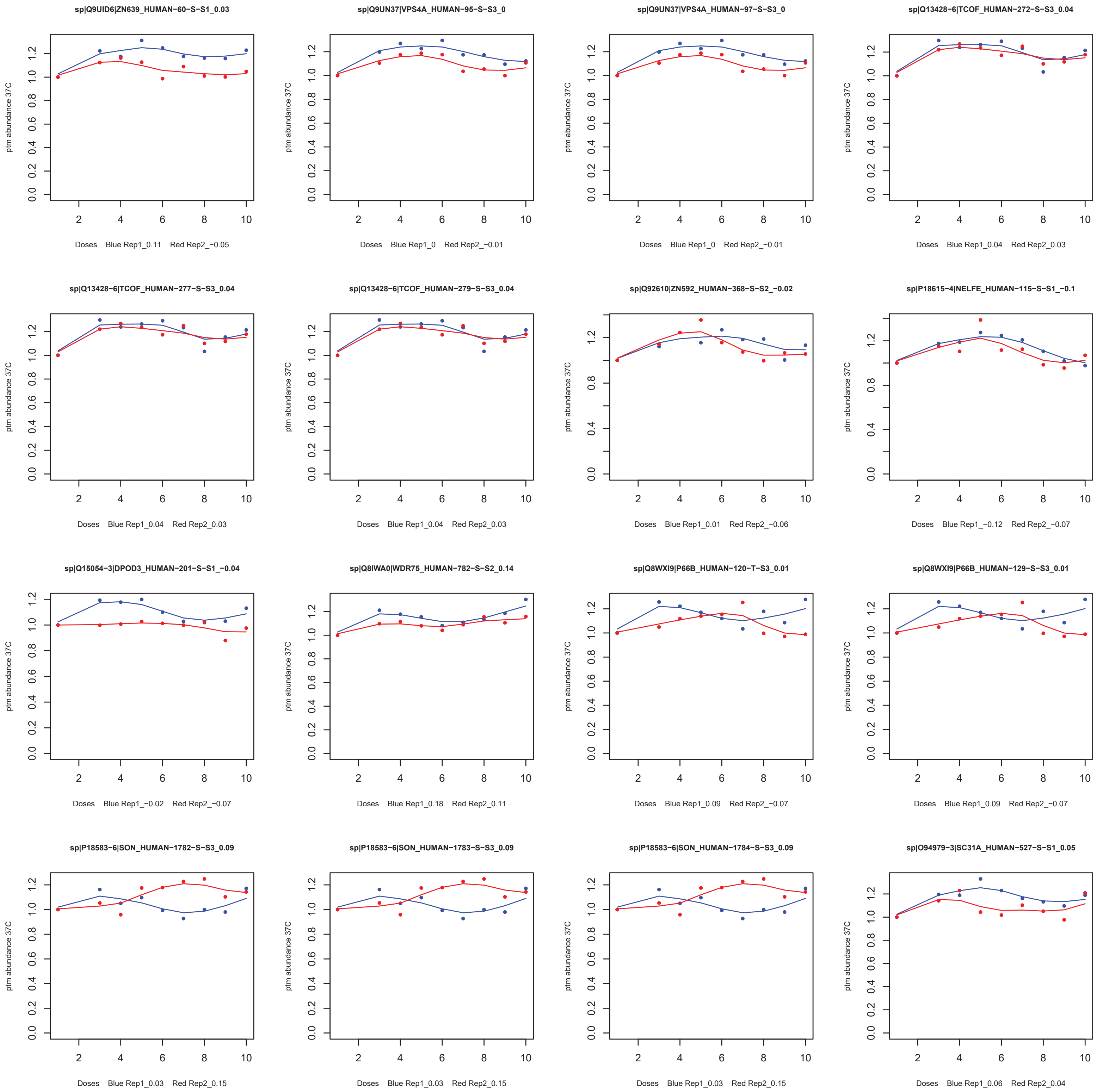
sp|Q9UID6|ZN639_HUMAN−60−S−S1_0.03
ptm abundance 37C
Doses Blue Rep1_0.11 Red Rep2_−0.05
sp|Q9UN37|VPS4A_HUMAN−95−S−S3_0
ptm abundance 37C
Doses Blue Rep1_0 Red Rep2_−0.01
sp|Q9UN37|VPS4A_HUMAN−97−S−S3_0
ptm abundance 37C
Doses Blue Rep1_0 Red Rep2_−0.01
sp|Q13428−6|TCOF_HUMAN−272−S−S3_0.04
ptm abundance 37C
Doses Blue Rep1_0.04 Red Rep2_0.03
sp|Q13428−6|TCOF_HUMAN−277−S−S3_0.04
ptm abundance 37C
Doses Blue Rep1_0.04 Red Rep2_0.03
sp|Q13428−6|TCOF_HUMAN−279−S−S3_0.04
ptm abundance 37C
Doses Blue Rep1_0.04 Red Rep2_0.03
sp|Q92610|ZN592_HUMAN−368−S−S2_−0.02
ptm abundance 37C
Doses Blue Rep1_0.01 Red Rep2_−0.06
sp|P18615−4|NELFE_HUMAN−115−S−S1_−0.1
ptm abundance 37C
Doses Blue Rep1_−0.12 Red Rep2_−0.07
sp|Q15054−3|DPOD3_HUMAN−201−S−S1_−0.04
ptm abundance 37C
Doses Blue Rep1_−0.02 Red Rep2_−0.07
sp|Q8IWA0|WDR75_HUMAN−782−S−S2_0.14
ptm abundance 37C
Doses Blue Rep1_0.18 Red Rep2_0.11
sp|Q8WXI9|P66B_HUMAN−120−T−S3_0.01
ptm abundance 37C
Doses Blue Rep1_0.09 Red Rep2_−0.07
sp|Q8WXI9|P66B_HUMAN−129−S−S3_0.01
ptm abundance 37C
Doses Blue Rep1_0.09 Red Rep2_−0.07
sp|P18583−6|SON_HUMAN−1782−S−S3_0.09
ptm abundance 37C
Doses Blue Rep1_0.03 Red Rep2_0.15
sp|P18583−6|SON_HUMAN−1783−S−S3_0.09
ptm abundance 37C
Doses Blue Rep1_0.03 Red Rep2_0.15
sp|P18583−6|SON_HUMAN−1784−S−S3_0.09
ptm abundance 37C
Doses Blue Rep1_0.03 Red Rep2_0.15
sp|O94979−3|SC31A_HUMAN−527−S−S1_0.05
ptm abundance 37C
Doses Blue Rep1_0.06 Red Rep2_0.04

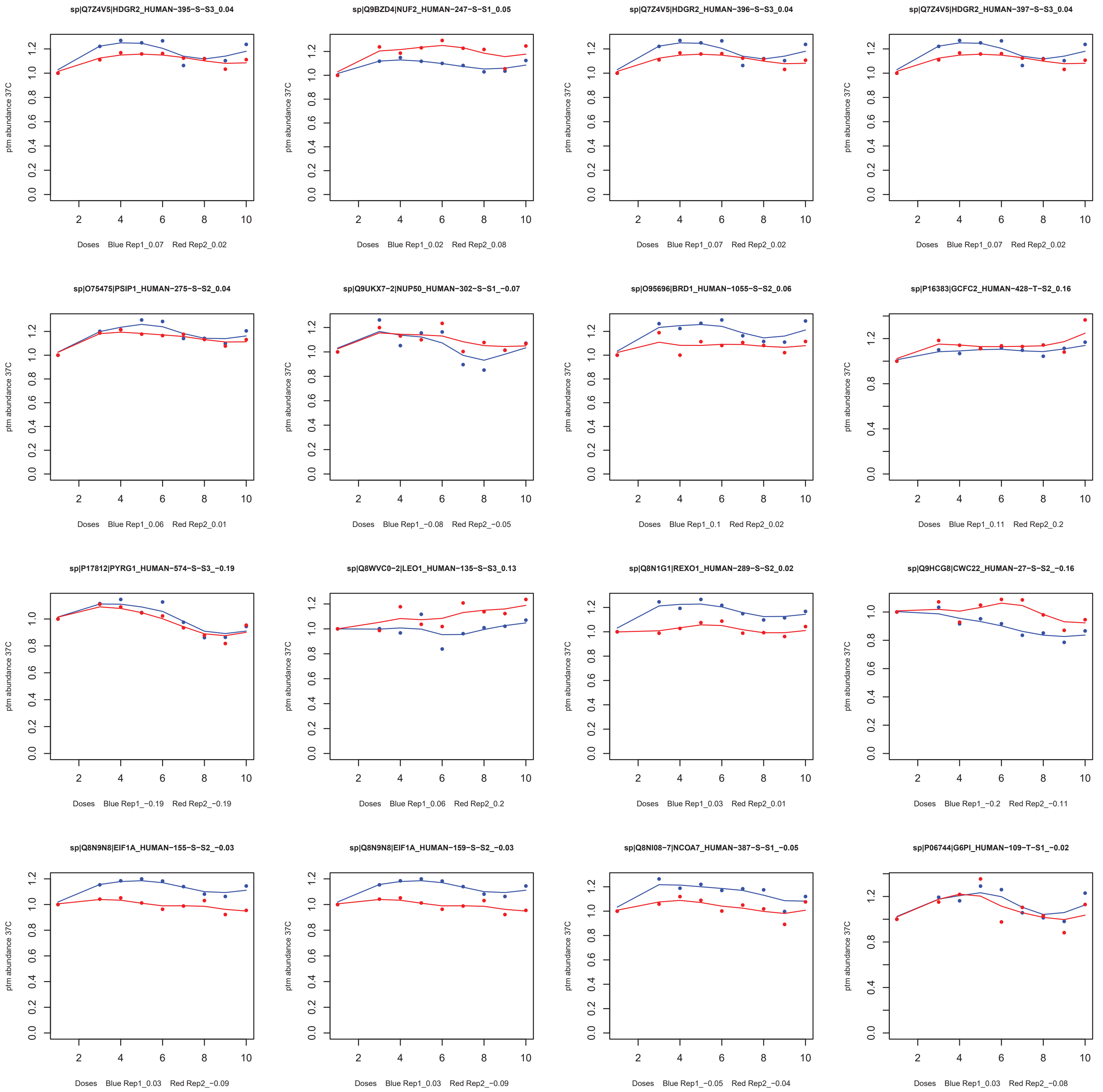

sp|Q7Z4V5|HDGR2_HUMAN−395−S−S3_0.04
ptm abundance 37C
Doses Blue Rep1_0.07 Red Rep2_0.02
sp|Q9BZD4|NUF2_HUMAN−247−S−S1_0.05
ptm abundance 37C
Doses Blue Rep1_0.02 Red Rep2_0.08
sp|Q7Z4V5|HDGR2_HUMAN−396−S−S3_0.04
ptm abundance 37C
Doses Blue Rep1_0.07 Red Rep2_0.02
sp|Q7Z4V5|HDGR2_HUMAN−397−S−S3_0.04
ptm abundance 37C
Doses Blue Rep1_0.07 Red Rep2_0.02
sp|O75475|PSIP1_HUMAN−275−S−S2_0.04
ptm abundance 37C
Doses Blue Rep1_0.06 Red Rep2_0.01
sp|Q9UKX7−2|NUP50_HUMAN−302−S−S1_−0.07
ptm abundance 37C
Doses Blue Rep1_−0.08 Red Rep2_−0.05
sp|O95696|BRD1_HUMAN−1055−S−S2_0.06
ptm abundance 37C
Doses Blue Rep1_0.1 Red Rep2_0.02
sp|P16383|GCFC2_HUMAN−428−T−S2_0.16
ptm abundance 37C
Doses Blue Rep1_0.11 Red Rep2_0.2
sp|P17812|PYRG1_HUMAN−574−S−S3_−0.19
ptm abundance 37C
Doses Blue Rep1_−0.19 Red Rep2_−0.19
sp|Q8WVC0−2|LEO1_HUMAN−135−S−S3_0.13
ptm abundance 37C
Doses Blue Rep1_0.06 Red Rep2_0.2
sp|Q8N1G1|REXO1_HUMAN−289−S−S2_0.02
ptm abundance 37C
Doses Blue Rep1_0.03 Red Rep2_0.01
sp|Q9HCG8|CWC22_HUMAN−27−S−S2_−0.16
ptm abundance 37C
Doses Blue Rep1_−0.2 Red Rep2_−0.11
sp|Q8N9N8|EIF1A_HUMAN−155−S−S2_−0.03
ptm abundance 37C
Doses Blue Rep1_0.03 Red Rep2_−0.09
sp|Q8N9N8|EIF1A_HUMAN−159−S−S2_−0.03
ptm abundance 37C
Doses Blue Rep1_0.03 Red Rep2_−0.09
sp|Q8NI08−7|NCOA7_HUMAN−387−S−S1_−0.05
ptm abundance 37C
Doses Blue Rep1_−0.05 Red Rep2_−0.04
sp|P06744|G6PI_HUMAN−109−T−S1_−0.02
ptm abundance 37C
Doses Blue Rep1_0.03 Red Rep2_−0.08

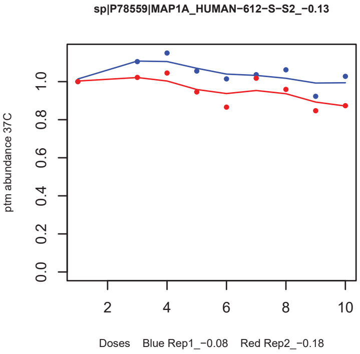
sp|P78559|MAP1A_HUMAN−612−S−S2_−0.13
ptm abundance 37C
Doses Blue Rep1_−0.08 Red Rep2_−0.18

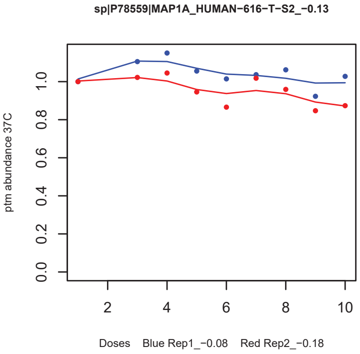
sp|P78559|MAP1A_HUMAN−616−T−S2_−0.13
ptm abundance 37C
Doses Blue Rep1_−0.08 Red Rep2_−0.18

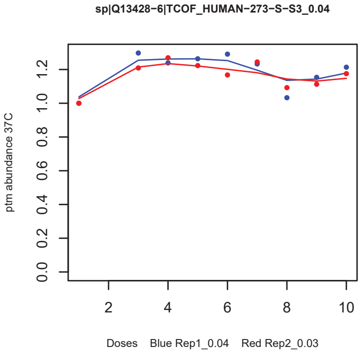
sp|Q13428−6|TCOF_HUMAN−273−S−S3_0.04
ptm abundance 37C
Doses Blue Rep1_0.04 Red Rep2_0.03

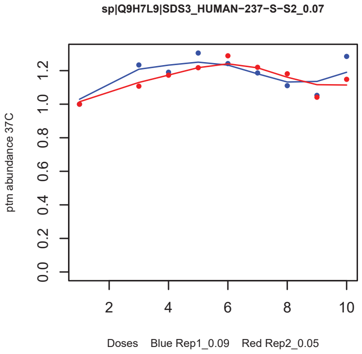
sp|Q9H7L9|SDS3_HUMAN−237−S−S2_0.07
ptm abundance 37C
Doses Blue Rep1_0.09 Red Rep2_0.05

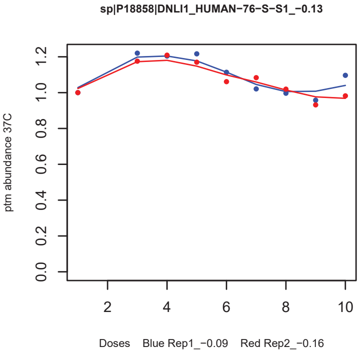
sp|P18858|DNLI1_HUMAN−76−S−S1_−0.13
ptm abundance 37C
Doses Blue Rep1_−0.09 Red Rep2_−0.16

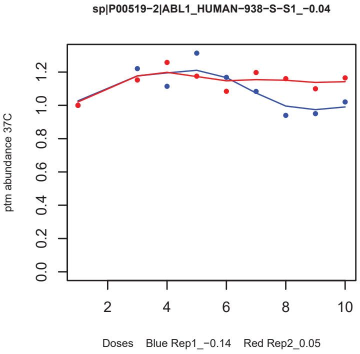
sp|P00519−2|ABL1_HUMAN−938−S−S1_−0.04
ptm abundance 37C
Doses Blue Rep1_−0.14 Red Rep2_0.05

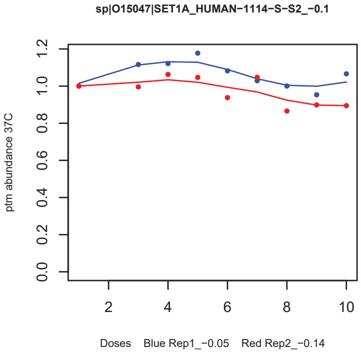
sp|O15047|SET1A_HUMAN−1114−S−S2_−0.1
ptm abundance 37C
Doses Blue Rep1_−0.05 Red Rep2_−0.14

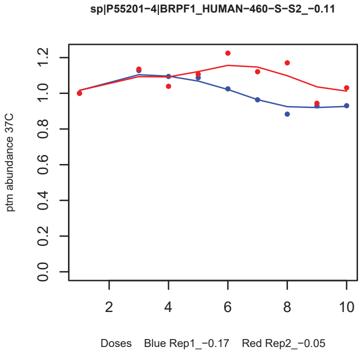
sp|P55201−4|BRPF1_HUMAN−460−S−S2_−0.11
ptm abundance 37C
Doses Blue Rep1_−0.17 Red Rep2_−0.05

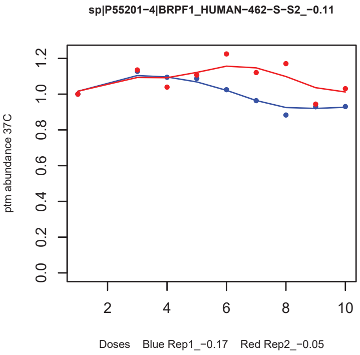
sp|P55201−4|BRPF1_HUMAN−462−S−S2_−0.11
ptm abundance 37C
Doses Blue Rep1_−0.17 Red Rep2_−0.05

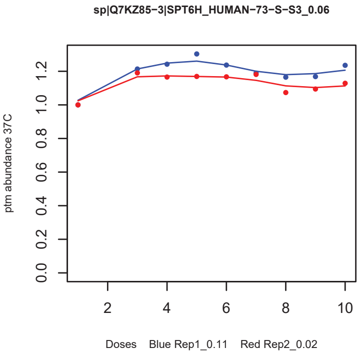
sp|Q7KZ85−3|SPT6H_HUMAN−73−S−S3_0.06
ptm abundance 37C
Doses Blue Rep1_0.11 Red Rep2_0.02

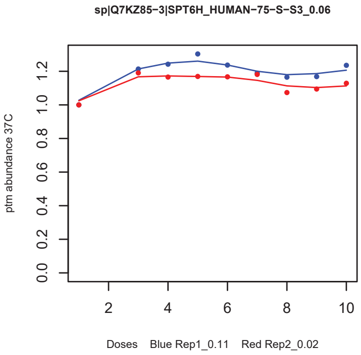
sp|Q7KZ85−3|SPT6H_HUMAN−75−S−S3_0.06
ptm abundance 37C
Doses Blue Rep1_0.11 Red Rep2_0.02

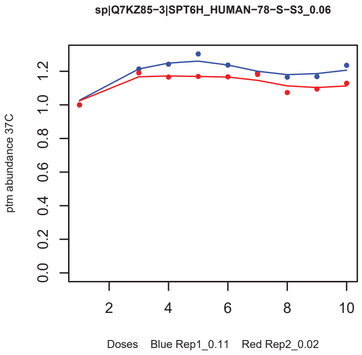
sp|Q7KZ85−3|SPT6H_HUMAN−78−S−S3_0.06
ptm abundance 37C
Doses Blue Rep1_0.11 Red Rep2_0.02

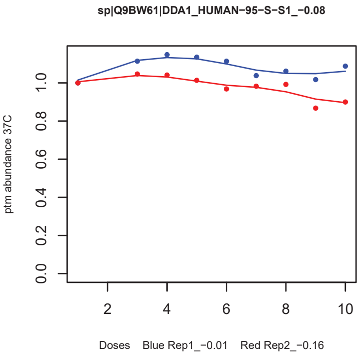
sp|Q9BW61|DDA1_HUMAN−95−S−S1_−0.08
ptm abundance 37C
Doses Blue Rep1_−0.01 Red Rep2_−0.16

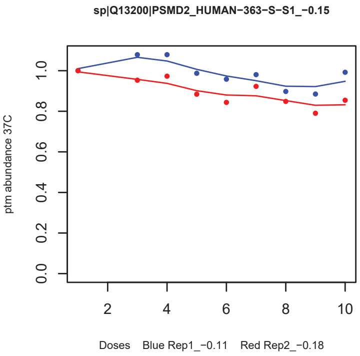
sp|Q13200|PSMD2_HUMAN−363−S−S1_−0.15
ptm abundance 37C
Doses Blue Rep1_−0.11 Red Rep2_−0.18

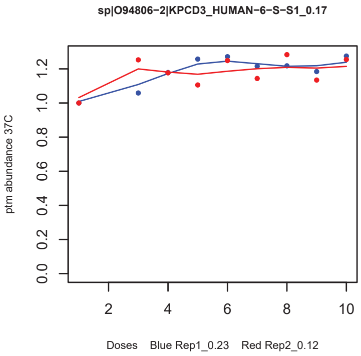
sp|O94806−2|KPCD3_HUMAN−6−S−S1_0.17
ptm abundance 37C
Doses Blue Rep1_0.23 Red Rep2_0.12

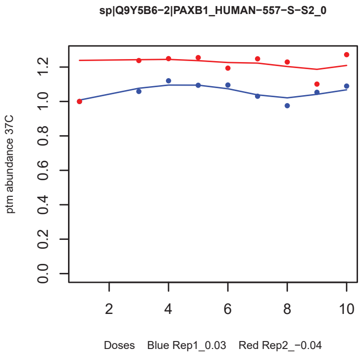
sp|Q9Y5B6−2|PAXB1_HUMAN−557−S−S2_0
ptm abundance 37C
Doses Blue Rep1_0.03 Red Rep2_−0.04

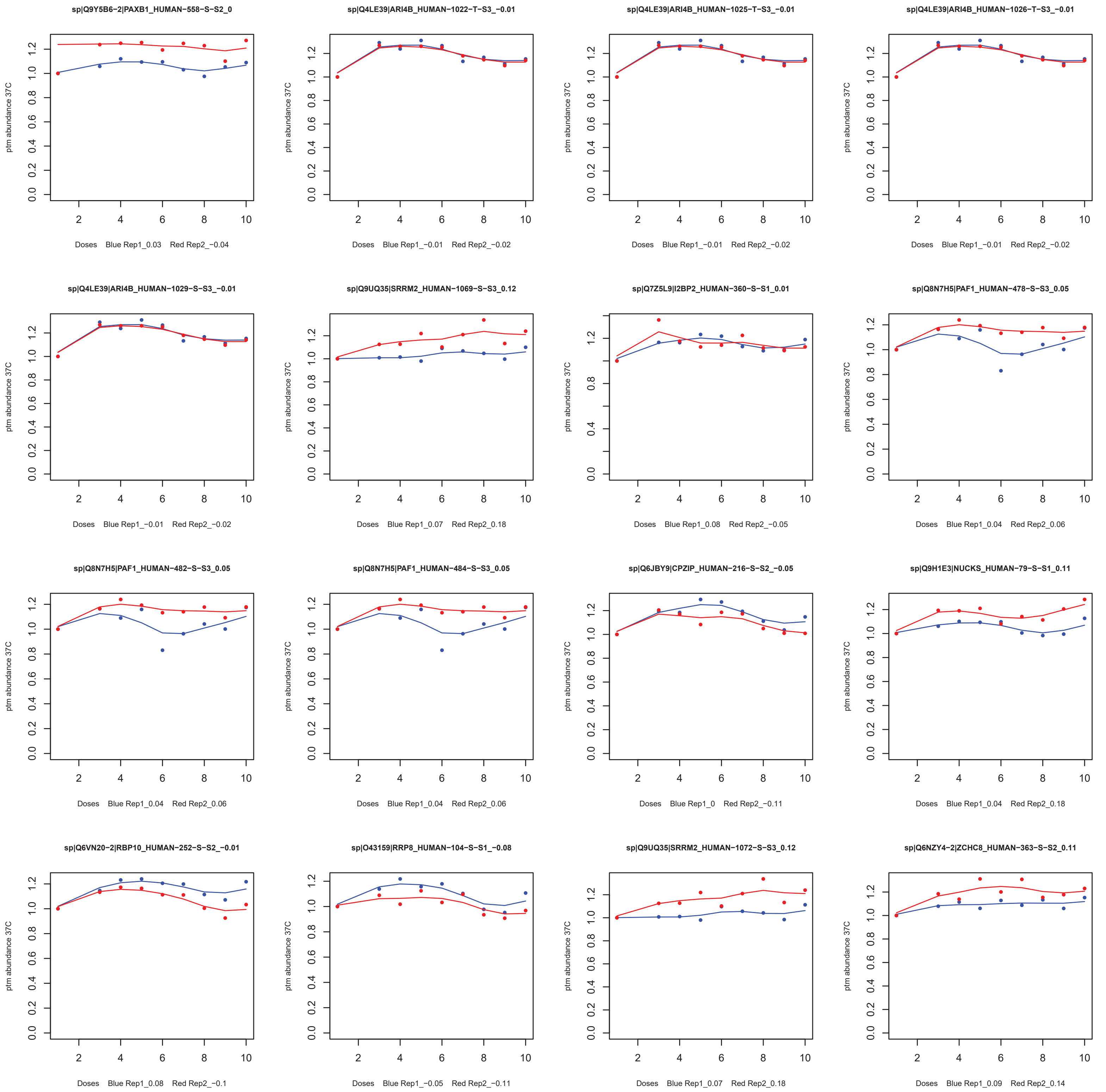
sp|Q9Y5B6−2|PAXB1_HUMAN−558−S−S2_0
ptm abundance 37C
Doses Blue Rep1_0.03 Red Rep2_−0.04
sp|Q4LE39|ARI4B_HUMAN−1022−T−S3_−0.01
ptm abundance 37C
Doses Blue Rep1_−0.01 Red Rep2_−0.02
sp|Q4LE39|ARI4B_HUMAN−1025−T−S3_−0.01
ptm abundance 37C
Doses Blue Rep1_−0.01 Red Rep2_−0.02
sp|Q4LE39|ARI4B_HUMAN−1026−T−S3_−0.01
ptm abundance 37C
Doses Blue Rep1_−0.01 Red Rep2_−0.02
sp|Q4LE39|ARI4B_HUMAN−1029−S−S3_−0.01
ptm abundance 37C
Doses Blue Rep1_−0.01 Red Rep2_−0.02
sp|Q9UQ35|SRRM2_HUMAN−1069−S−S3_0.12
ptm abundance 37C
Doses Blue Rep1_0.07 Red Rep2_0.18
sp|Q7Z5L9|I2BP2_HUMAN−360−S−S1_0.01
ptm abundance 37C
Doses Blue Rep1_0.08 Red Rep2_−0.05
sp|Q8N7H5|PAF1_HUMAN−478−S−S3_0.05
ptm abundance 37C
Doses Blue Rep1_0.04 Red Rep2_0.06
sp|Q8N7H5|PAF1_HUMAN−482−S−S3_0.05
ptm abundance 37C
Doses Blue Rep1_0.04 Red Rep2_0.06
sp|Q8N7H5|PAF1_HUMAN−484−S−S3_0.05
ptm abundance 37C
Doses Blue Rep1_0.04 Red Rep2_0.06
sp|Q6JBY9|CPZIP_HUMAN−216−S−S2_−0.05
ptm abundance 37C
Doses Blue Rep1_0 Red Rep2_−0.11
sp|Q9H1E3|NUCKS_HUMAN−79−S−S1_0.11
ptm abundance 37C
Doses Blue Rep1_0.04 Red Rep2_0.18
sp|Q6VN20−2|RBP10_HUMAN−252−S−S2_−0.01
ptm abundance 37C
Doses Blue Rep1_0.08 Red Rep2_−0.1
sp|O43159|RRP8_HUMAN−104−S−S1_−0.08
ptm abundance 37C
Doses Blue Rep1_−0.05 Red Rep2_−0.11
sp|Q9UQ35|SRRM2_HUMAN−1072−S−S3_0.12
ptm abundance 37C
Doses Blue Rep1_0.07 Red Rep2_0.18
sp|Q6NZY4−2|ZCHC8_HUMAN−363−S−S2_0.11
ptm abundance 37C
Doses Blue Rep1_0.09 Red Rep2_0.14

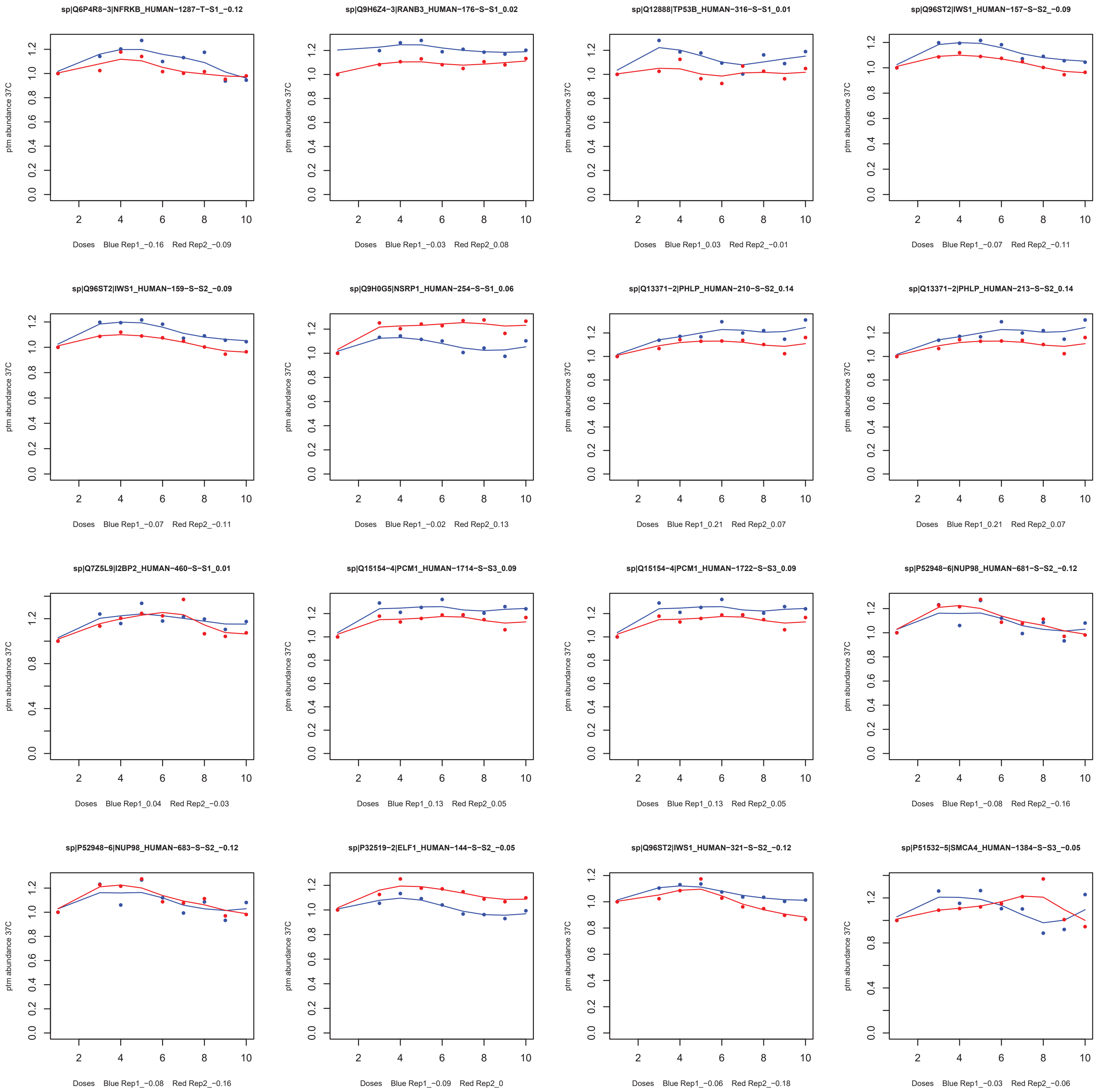

sp|Q6P4R8−3|NFRKB_HUMAN−1287−T−S1_−0.12
ptm abundance 37C
Doses Blue Rep1_−0.16 Red Rep2_−0.09
sp|Q9H6Z4−3|RANB3_HUMAN−176−S−S1_0.02
ptm abundance 37C
Doses Blue Rep1_−0.03 Red Rep2_0.08
sp|Q12888|TP53B_HUMAN−316−S−S1_0.01
ptm abundance 37C
Doses Blue Rep1_0.03 Red Rep2_−0.01
sp|Q96ST2|IWS1_HUMAN−157−S−S2_−0.09
ptm abundance 37C
Doses Blue Rep1_−0.07 Red Rep2_−0.11
sp|Q96ST2|IWS1_HUMAN−159−S−S2_−0.09
ptm abundance 37C
Doses Blue Rep1_−0.07 Red Rep2_−0.11
sp|Q9H0G5|NSRP1_HUMAN−254−S−S1_0.06
ptm abundance 37C
Doses Blue Rep1_−0.02 Red Rep2_0.13
sp|Q13371−2|PHLP_HUMAN−210−S−S2_0.14
ptm abundance 37C
Doses Blue Rep1_0.21 Red Rep2_0.07
sp|Q13371−2|PHLP_HUMAN−213−S−S2_0.14
ptm abundance 37C
Doses Blue Rep1_0.21 Red Rep2_0.07
sp|Q7Z5L9|I2BP2_HUMAN−460−S−S1_0.01
ptm abundance 37C
Doses Blue Rep1_0.04 Red Rep2_−0.03
sp|Q15154−4|PCM1_HUMAN−1714−S−S3_0.09
ptm abundance 37C
Doses Blue Rep1_0.13 Red Rep2_0.05
sp|Q15154−4|PCM1_HUMAN−1722−S−S3_0.09
ptm abundance 37C
Doses Blue Rep1_0.13 Red Rep2_0.05
sp|P52948−6|NUP98_HUMAN−681−S−S2_−0.12
ptm abundance 37C
Doses Blue Rep1_−0.08 Red Rep2_−0.16
sp|P52948−6|NUP98_HUMAN−683−S−S2_−0.12
ptm abundance 37C
Doses Blue Rep1_−0.08 Red Rep2_−0.16
sp|P32519−2|ELF1_HUMAN−144−S−S2_−0.05
ptm abundance 37C
Doses Blue Rep1_−0.09 Red Rep2_0
sp|Q96ST2|IWS1_HUMAN−321−S−S2_−0.12
ptm abundance 37C
Doses Blue Rep1_−0.06 Red Rep2_−0.18
sp|P51532−5|SMCA4_HUMAN−1384−S−S3_−0.05
ptm abundance 37C
Doses Blue Rep1_−0.03 Red Rep2_−0.06

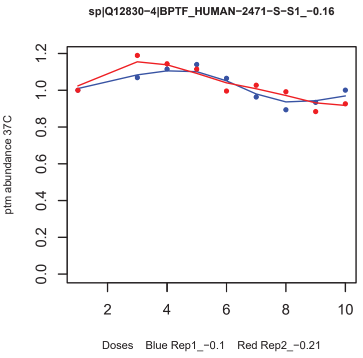
sp|Q12830−4|BPTF_HUMAN−2471−S−S1_−0.16
ptm abundance 37C
Doses Blue Rep1_−0.1 Red Rep2_−0.21

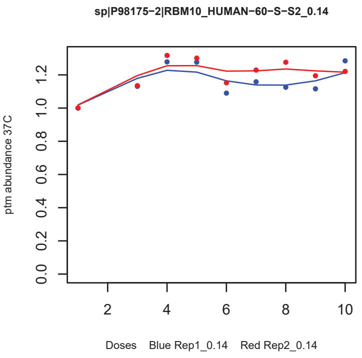
sp|P98175−2|RBM10_HUMAN−60−S−S2_0.14
ptm abundance 37C
Doses Blue Rep1_0.14 Red Rep2_0.14

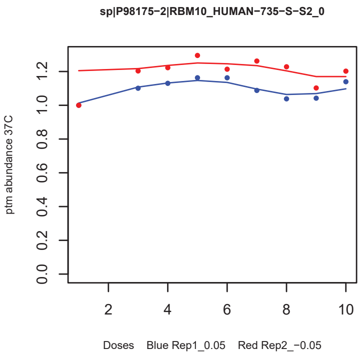
sp|P98175−2|RBM10_HUMAN−735−S−S2_0
ptm abundance 37C
Doses Blue Rep1_0.05 Red Rep2_−0.05

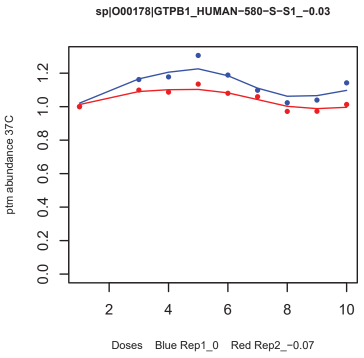
sp|O00178|GTPB1_HUMAN−580−S−S1_−0.03
ptm abundance 37C
Doses Blue Rep1_0 Red Rep2_−0.07

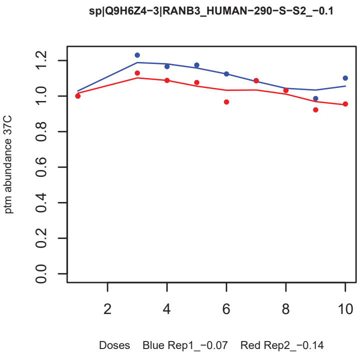
sp|Q9H6Z4−3|RANB3_HUMAN−290−S−S2_−0.1
ptm abundance 37C
Doses Blue Rep1_−0.07 Red Rep2_−0.14

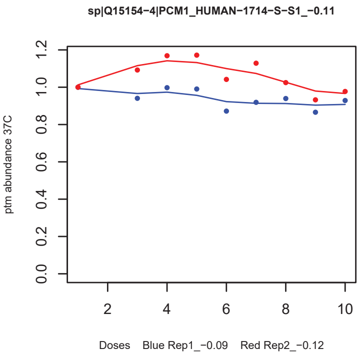
sp|Q15154−4|PCM1_HUMAN−1714−S−S1_−0.11
ptm abundance 37C
Doses Blue Rep1_−0.09 Red Rep2_−0.12

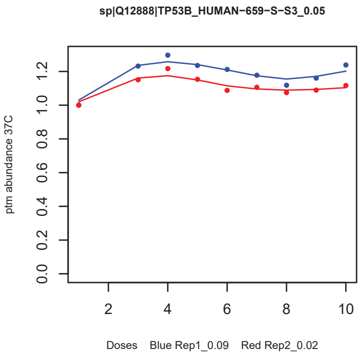
sp|Q12888|TP53B_HUMAN−659−S−S3_0.05
ptm abundance 37C
Doses Blue Rep1_0.09 Red Rep2_0.02

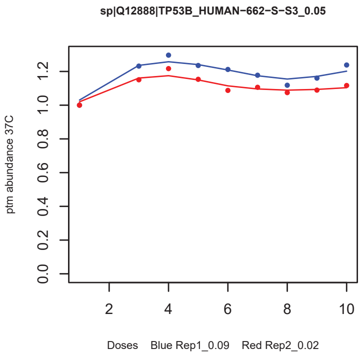
sp|Q12888|TP53B_HUMAN−662−S−S3_0.05
ptm abundance 37C
Doses Blue Rep1_0.09 Red Rep2_0.02

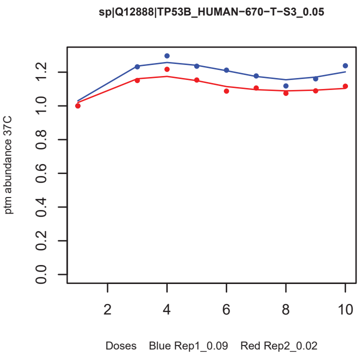
sp|Q12888|TP53B_HUMAN−670−T−S3_0.05
ptm abundance 37C
Doses Blue Rep1_0.09 Red Rep2_0.02

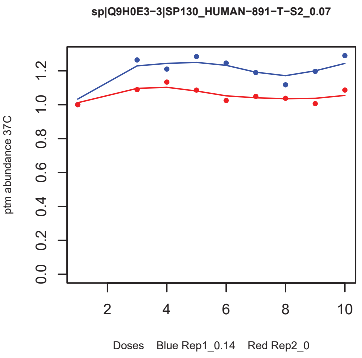
sp|Q9H0E3−3|SP130_HUMAN−891−T−S2_0.07
ptm abundance 37C
Doses Blue Rep1_0.14 Red Rep2_0

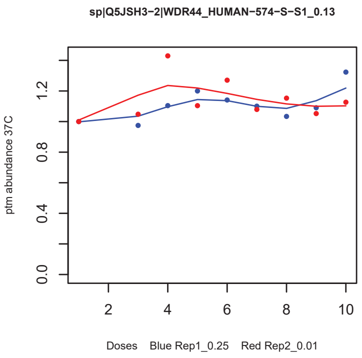
sp|Q5JSH3−2|WDR44_HUMAN−574−S−S1_0.13
ptm abundance 37C
Doses Blue Rep1_0.25 Red Rep2_0.01

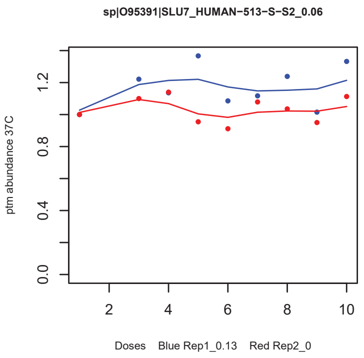
sp|O95391|SLU7_HUMAN−513−S−S2_0.06
ptm abundance 37C
Doses Blue Rep1_0.13 Red Rep2_0

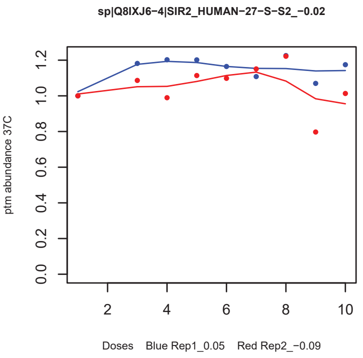
sp|Q8IXJ6−4|SIR2_HUMAN−27−S−S2_−0.02
ptm abundance 37C
Doses Blue Rep1_0.05 Red Rep2_−0.09

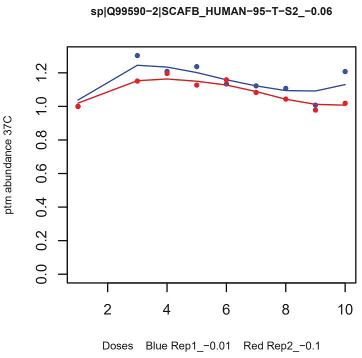
sp|Q99590−2|SCAFB_HUMAN−95−T−S2_−0.06
ptm abundance 37C
Doses Blue Rep1_−0.01 Red Rep2_−0.1

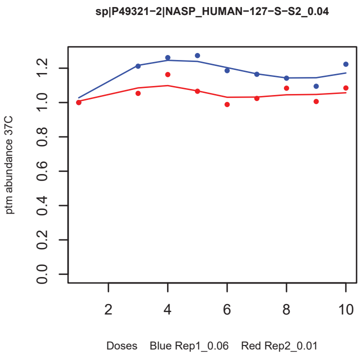
sp|P49321−2|NASP_HUMAN−127−S−S2_0.04
ptm abundance 37C
Doses Blue Rep1_0.06 Red Rep2_0.01

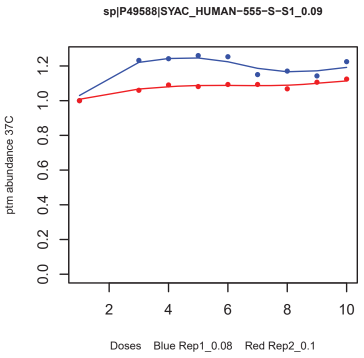
sp|P49588|SYAC_HUMAN−555−S−S1_0.09
ptm abundance 37C
Doses Blue Rep1_0.08 Red Rep2_0.1

sp|P23588-2|IF4B_HUMAN-558-S-S1_-0.05

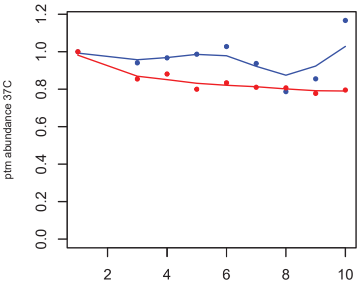

Doses Blue Rep1_0.07 Red Rep2_-0.17

sp|Q8WZ73-2|RFFL_HUMAN-226-S-S2_0.06

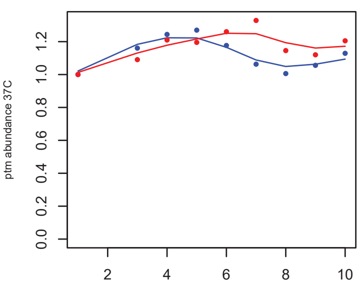

Doses Blue Rep1_-0.01 Red Rep2_0.12

sp|Q8WZ73-2|RFFL_HUMAN-229-S-S2_0.06

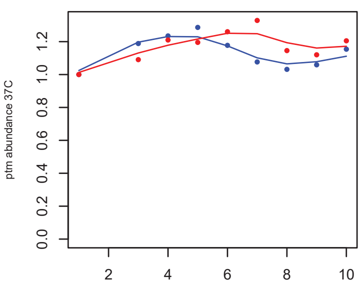

Doses Blue Rep1_0 Red Rep2_0.12

sp|Q9UN37|VPS4A_HUMAN-99-S-S3_0

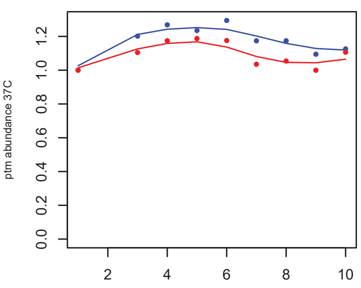

Doses Blue Rep1_0 Red Rep2_-0.01

sp|Q13435|SF3B2_HUMAN-309-S-S1_0.09

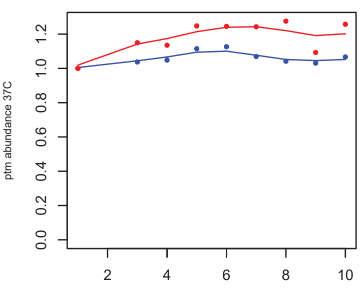

Doses Blue Rep1_0.03 Red Rep2_0.15

sp|O14795|UN13B_HUMAN-254-S-S1_-0.17

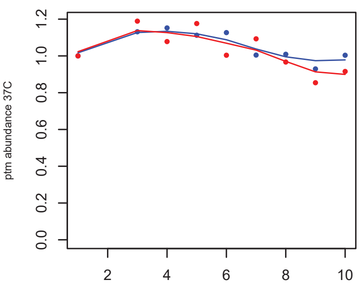

Doses Blue Rep1_-0.12 Red Rep2_-0.23

sp|Q9NVM9-2|INT13_HUMAN-522-S-S2_-0.01

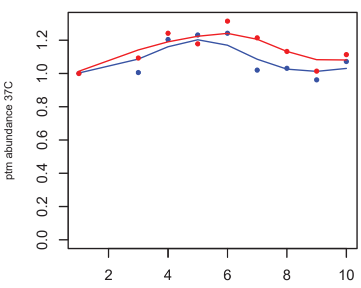

Doses Blue Rep1_-0.02 Red Rep2_0

sp|Q9NVM9-2|INT13_HUMAN-525-S-S2_-0.01

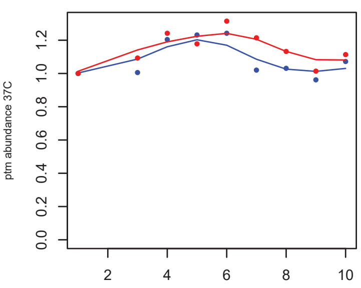

Doses Blue Rep1_-0.02 Red Rep2_0

sp|Q6VMQ6|MCAF1_HUMAN-477-S-S2_-0.2

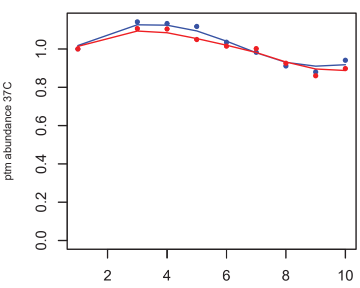

Doses Blue Rep1_-0.19 Red Rep2_-0.21

sp|Q9P2K3-2|RCOR3_HUMAN-434-S-S1_-0.07

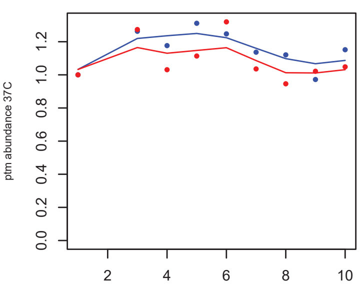

Doses Blue Rep1_-0.05 Red Rep2_-0.08

sp|Q12874|SF3A3_HUMAN-365-S-S3_0.13

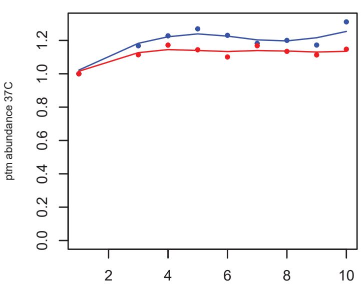

Doses Blue Rep1_0.19 Red Rep2_0.08

sp|Q12874|SF3A3_HUMAN-367-S-S3_0.13

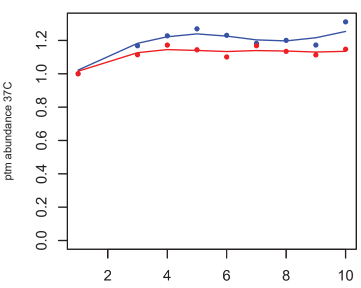

Doses Blue Rep1_0.19 Red Rep2_0.08

sp|Q12874|SF3A3_HUMAN-369-S-S3_0.13

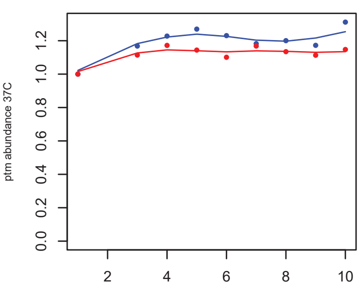

Doses Blue Rep1_0.19 Red Rep2_0.08

sp|P22059|OSBP1_HUMAN-198-S-S2_-0.12

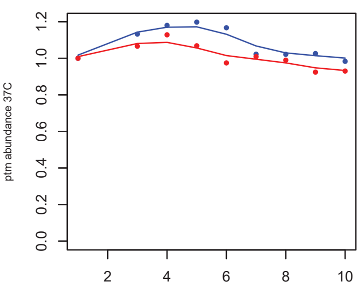

Doses Blue Rep1_-0.1 Red Rep2_-0.14

sp|Q13428-6|TCOF_HUMAN-270-S-S3_0.03

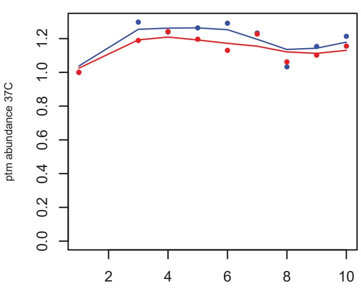

Doses Blue Rep1_0.04 Red Rep2_0.03

sp|Q15648|MED1_HUMAN-1465-S-S2_0.04

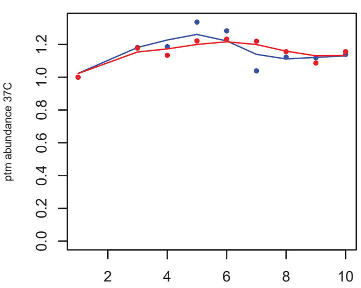

Doses Blue Rep1_0.03 Red Rep2_0.06

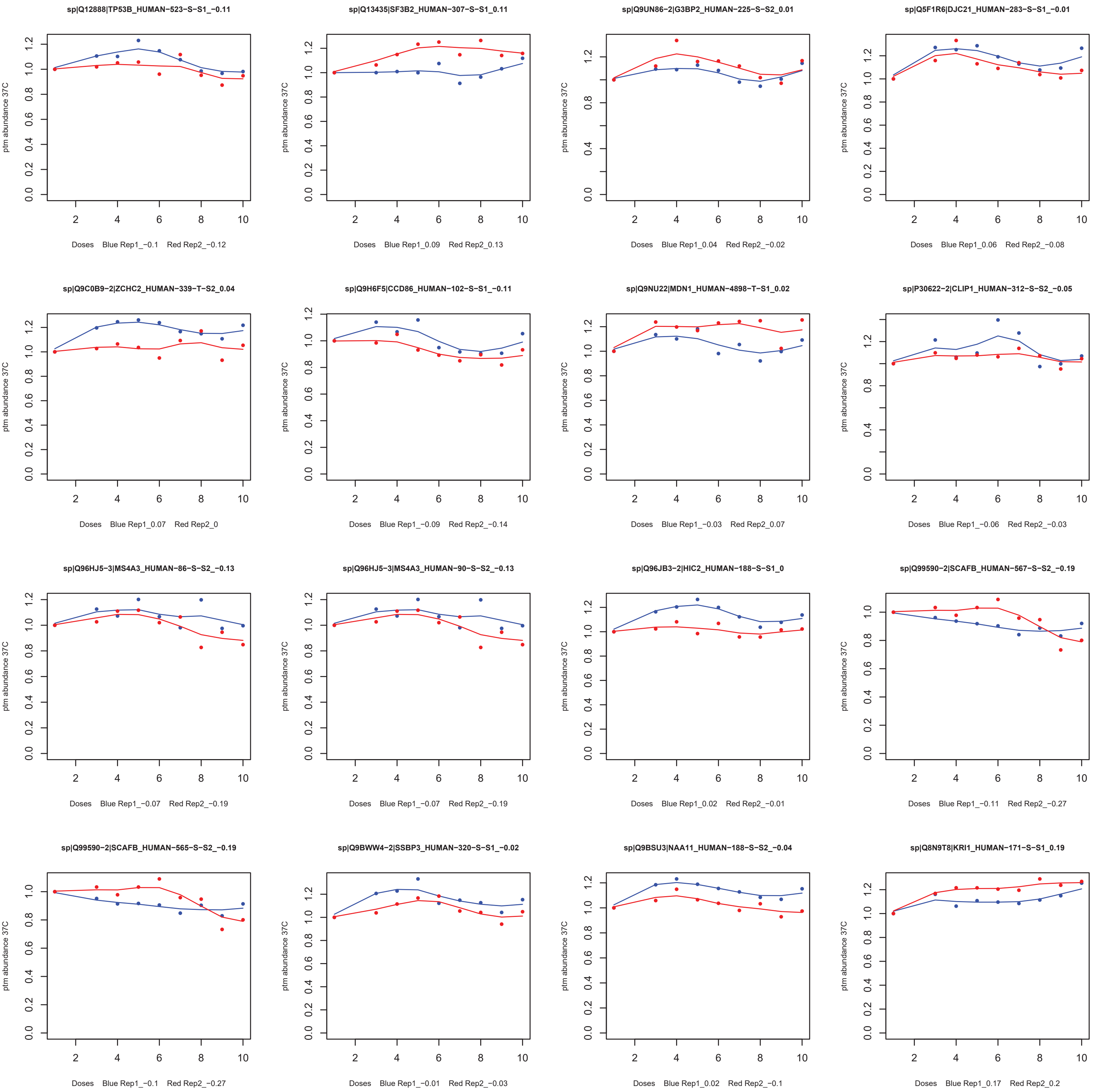
sp|Q12888|TP53B_HUMAN−523−S−S1_−0.11
Doses Blue Rep1_−0.1 Red Rep2_−0.12
sp|Q13435|SF3B2_HUMAN−307−S−S1_0.11
Doses Blue Rep1_0.09 Red Rep2_0.13
sp|Q9UN86−2|G3BP2_HUMAN−225−S−S2_0.01
Doses Blue Rep1_0.04 Red Rep2_−0.02
sp|Q5F1R6|DJC21_HUMAN−283−S−S1_−0.01
Doses Blue Rep1_0.06 Red Rep2_−0.08
sp|Q9C0B9−2|ZCHC2_HUMAN−339−T−S2_0.04
Doses Blue Rep1_0.07 Red Rep2_0
sp|Q9H6F5|CCD86_HUMAN−102−S−S1_−0.11
Doses Blue Rep1_−0.09 Red Rep2_−0.14
sp|Q9NU22|MDN1_HUMAN−4898−T−S1_0.02
Doses Blue Rep1_−0.03 Red Rep2_0.07
sp|P30622−2|CLIP1_HUMAN−312−S−S2_−0.05
Doses Blue Rep1_−0.06 Red Rep2_−0.03
sp|Q96HJ5−3|MS4A3_HUMAN−86−S−S2_−0.13
Doses Blue Rep1_−0.07 Red Rep2_−0.19
sp|Q96HJ5−3|MS4A3_HUMAN−90−S−S2_−0.13
Doses Blue Rep1_−0.07 Red Rep2_−0.19
sp|Q96JB3−2|HIC2_HUMAN−188−S−S1_0
Doses Blue Rep1_0.02 Red Rep2_−0.01
sp|Q99590−2|SCAFB_HUMAN−567−S−S2_−0.19
Doses Blue Rep1_−0.11 Red Rep2_−0.27
sp|Q99590−2|SCAFB_HUMAN−565−S−S2_−0.19
Doses Blue Rep1_−0.1 Red Rep2_−0.27
sp|Q9BWW4−2|SSBP3_HUMAN−320−S−S1_−0.02
Doses Blue Rep1_−0.01 Red Rep2_−0.03
sp|Q9BSU3|NAA11_HUMAN−188−S−S2_−0.04
Doses Blue Rep1_0.02 Red Rep2_−0.1
sp|Q8N9T8|KRI1_HUMAN−171−S−S1_0.19
Doses Blue Rep1_0.17 Red Rep2_0.2
ptm abundance 37C

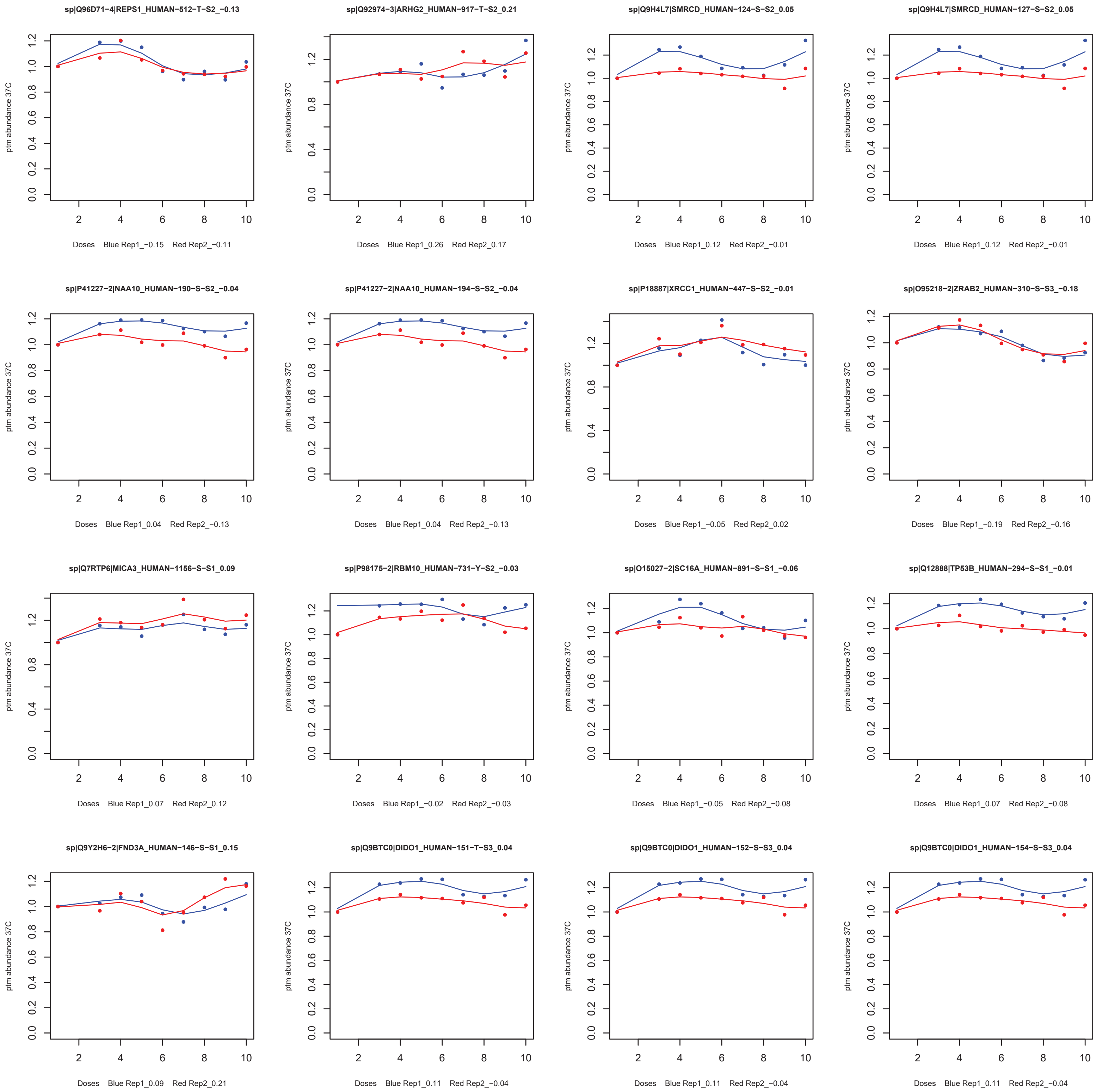

sp|Q96D71−4|REPS1_HUMAN−512−T−S2_−0.13
Doses Blue Rep1_−0.15 Red Rep2_−0.11
sp|Q92974−3|ARHG2_HUMAN−917−T−S2_0.21
Doses Blue Rep1_0.26 Red Rep2_0.17
sp|Q9H4L7|SMRCD_HUMAN−124−S−S2_0.05
Doses Blue Rep1_0.12 Red Rep2_−0.01
sp|Q9H4L7|SMRCD_HUMAN−127−S−S2_0.05
Doses Blue Rep1_0.12 Red Rep2_−0.01
sp|P41227−2|NAA10_HUMAN−190−S−S2_−0.04
Doses Blue Rep1_0.04 Red Rep2_−0.13
sp|P41227−2|NAA10_HUMAN−194−S−S2_−0.04
Doses Blue Rep1_0.04 Red Rep2_−0.13
sp|P18887|XRCC1_HUMAN−447−S−S2_−0.01
Doses Blue Rep1_−0.05 Red Rep2_0.02
sp|O95218−2|ZRAB2_HUMAN−310−S−S3_−0.18
Doses Blue Rep1_−0.19 Red Rep2_−0.16
sp|Q7RTP6|MICA3_HUMAN−1156−S−S1_0.09
Doses Blue Rep1_0.07 Red Rep2_0.12
sp|P98175−2|RBM10_HUMAN−731−Y−S2_−0.03
Doses Blue Rep1_−0.02 Red Rep2_−0.03
sp|O15027−2|SC16A_HUMAN−891−S−S1_−0.06
Doses Blue Rep1_−0.05 Red Rep2_−0.08
sp|Q12888|TP53B_HUMAN−294−S−S1_−0.01
Doses Blue Rep1_0.07 Red Rep2_−0.08
sp|Q9Y2H6−2|FND3A_HUMAN−146−S−S1_0.15
Doses Blue Rep1_0.09 Red Rep2_0.21
sp|Q9BTC0|DIDO1_HUMAN−151−T−S3_0.04
Doses Blue Rep1_0.11 Red Rep2_−0.04
sp|Q9BTC0|DIDO1_HUMAN−152−S−S3_0.04
Doses Blue Rep1_0.11 Red Rep2_−0.04
sp|Q9BTC0|DIDO1_HUMAN−154−S−S3_0.04
Doses Blue Rep1_0.11 Red Rep2_−0.04
ptm abundance 37C

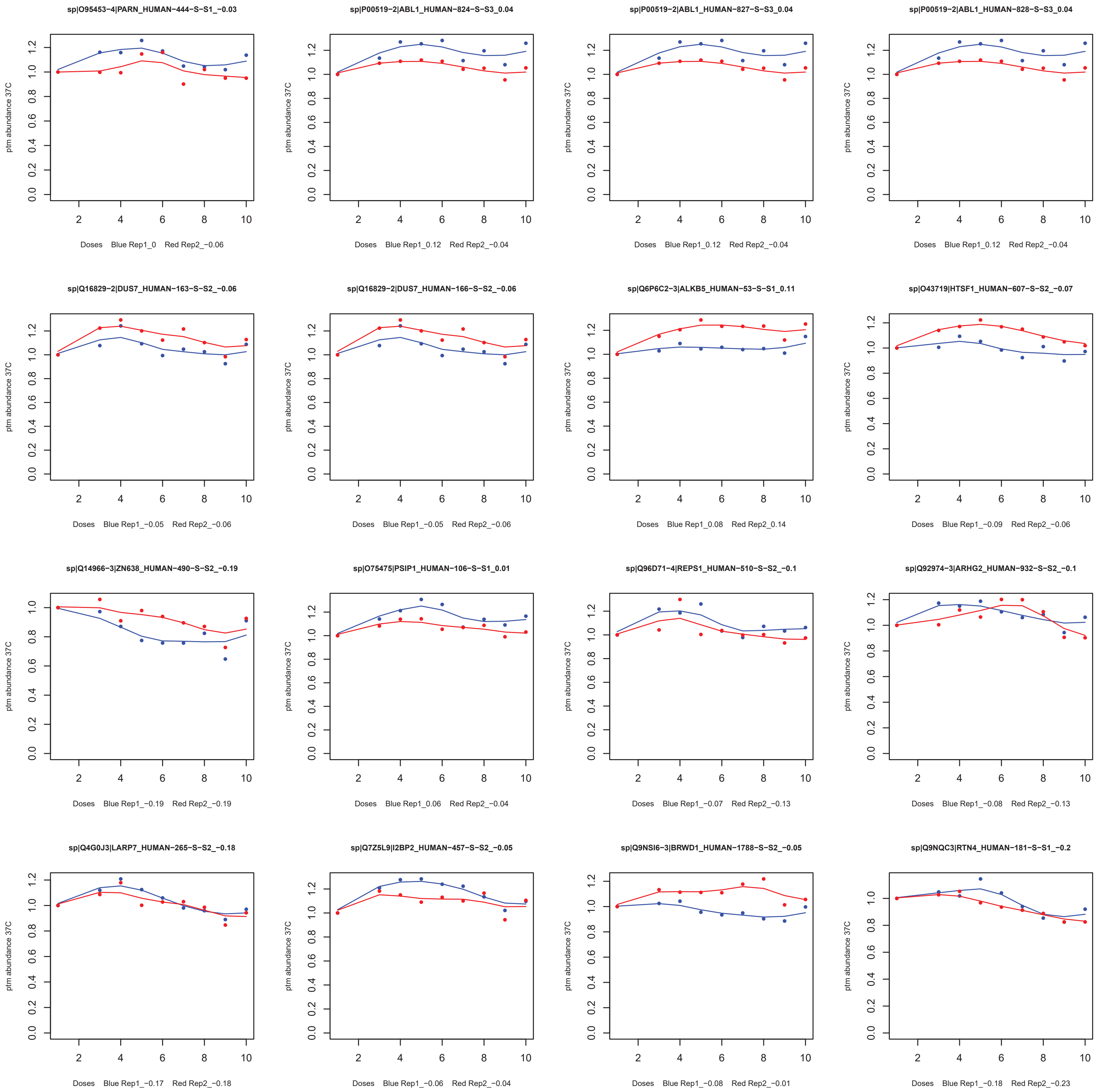
sp|O95453−4|PARN_HUMAN−444−S−S1_−0.03
ptm abundance 37C
Doses Blue Rep1_0 Red Rep2_−0.06
sp|P00519−2|ABL1_HUMAN−824−S−S3_0.04
ptm abundance 37C
Doses Blue Rep1_0.12 Red Rep2_−0.04
sp|P00519−2|ABL1_HUMAN−827−S−S3_0.04
ptm abundance 37C
Doses Blue Rep1_0.12 Red Rep2_−0.04
sp|P00519−2|ABL1_HUMAN−828−S−S3_0.04
ptm abundance 37C
Doses Blue Rep1_0.12 Red Rep2_−0.04
sp|Q16829−2|DUS7_HUMAN−163−S−S2_−0.06
ptm abundance 37C
Doses Blue Rep1_−0.05 Red Rep2_−0.06
sp|Q16829−2|DUS7_HUMAN−166−S−S2_−0.06
ptm abundance 37C
Doses Blue Rep1_−0.05 Red Rep2_−0.06
sp|Q6P6C2−3|ALKB5_HUMAN−53−S−S1_0.11
ptm abundance 37C
Doses Blue Rep1_0.08 Red Rep2_0.14
sp|O43719|HTSF1_HUMAN−607−S−S2_−0.07
ptm abundance 37C
Doses Blue Rep1_−0.09 Red Rep2_−0.06
sp|Q14966−3|ZN638_HUMAN−490−S−S2_−0.19
ptm abundance 37C
Doses Blue Rep1_−0.19 Red Rep2_−0.19
sp|O75475|PSIP1_HUMAN−106−S−S1_0.01
ptm abundance 37C
Doses Blue Rep1_0.06 Red Rep2_−0.04
sp|Q96D71−4|REPS1_HUMAN−510−S−S2_−0.1
ptm abundance 37C
Doses Blue Rep1_−0.07 Red Rep2_−0.13
sp|Q92974−3|ARHG2_HUMAN−932−S−S2_−0.1
ptm abundance 37C
Doses Blue Rep1_−0.08 Red Rep2_−0.13
sp|Q4G0J3|LARP7_HUMAN−265−S−S2_−0.18
ptm abundance 37C
Doses Blue Rep1_−0.17 Red Rep2_−0.18
sp|Q7Z5L9|I2BP2_HUMAN−457−S−S2_−0.05
ptm abundance 37C
Doses Blue Rep1_−0.06 Red Rep2_−0.04
sp|Q9NSI6−3|BRWD1_HUMAN−1788−S−S2_−0.05
ptm abundance 37C
Doses Blue Rep1_−0.08 Red Rep2_−0.01
sp|Q9NQC3|RTN4_HUMAN−181−S−S1_−0.2
ptm abundance 37C
Doses Blue Rep1_−0.18 Red Rep2_−0.23

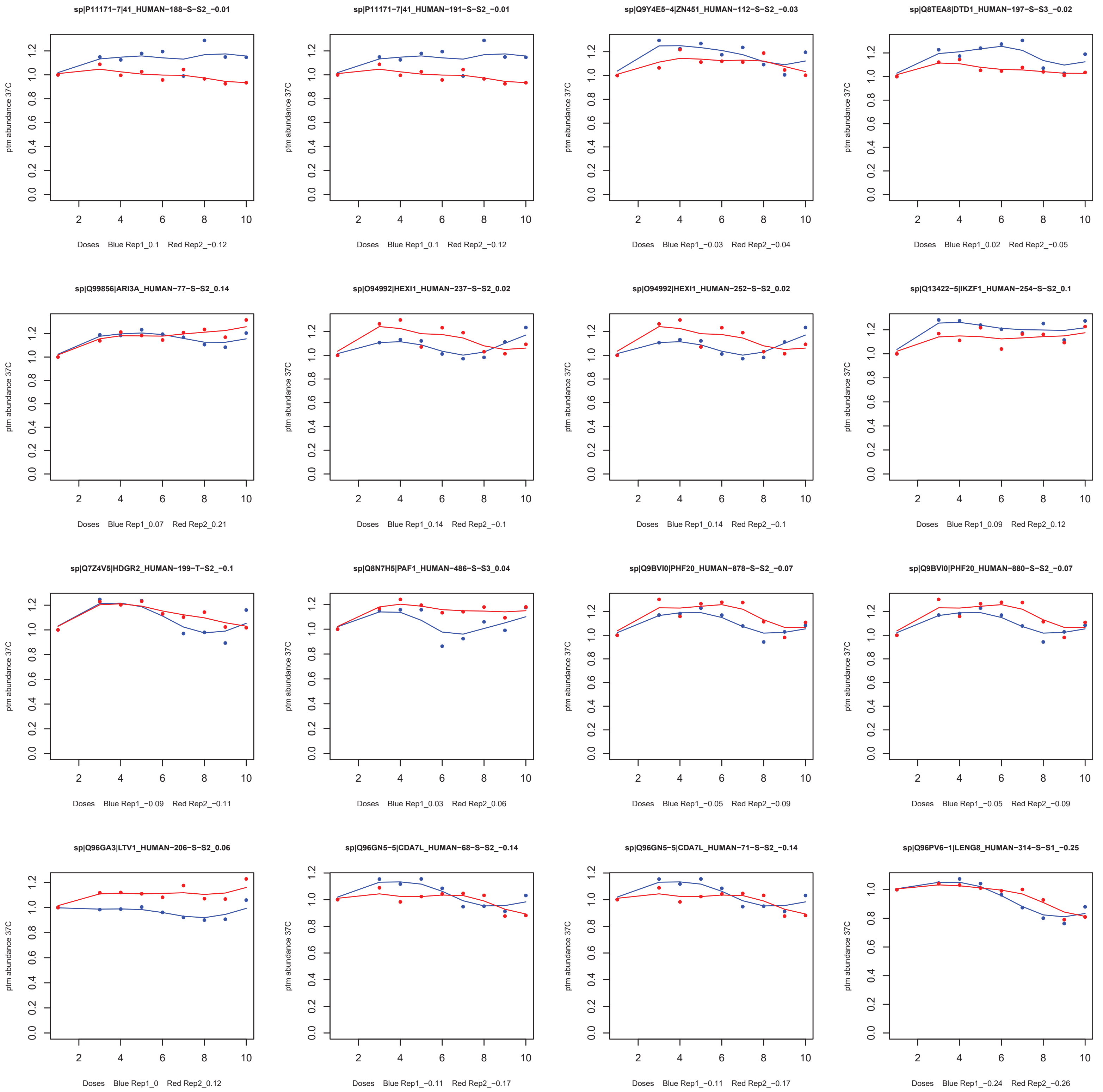
sp|P11171−7|41_HUMAN−188−S−S2_−0.01
Doses Blue Rep1_0.1 Red Rep2_−0.12
sp|P11171−7|41_HUMAN−191−S−S2_−0.01
Doses Blue Rep1_0.1 Red Rep2_−0.12
sp|Q9Y4E5−4|ZN451_HUMAN−112−S−S2_−0.03
Doses Blue Rep1_−0.03 Red Rep2_−0.04
sp|Q8TEA8|DTD1_HUMAN−197−S−S3_−0.02
Doses Blue Rep1_0.02 Red Rep2_−0.05
sp|Q99856|ARI3A_HUMAN−77−S−S2_0.14
Doses Blue Rep1_0.07 Red Rep2_0.21
sp|O94992|HEXI1_HUMAN−237−S−S2_0.02
Doses Blue Rep1_0.14 Red Rep2_−0.1
sp|O94992|HEXI1_HUMAN−252−S−S2_0.02
Doses Blue Rep1_0.14 Red Rep2_−0.1
sp|Q13422−5|IKZF1_HUMAN−254−S−S2_0.1
Doses Blue Rep1_0.09 Red Rep2_0.12
sp|Q7Z4V5|HDGR2_HUMAN−199−T−S2_−0.1
Doses Blue Rep1_−0.09 Red Rep2_−0.11
sp|Q8N7H5|PAF1_HUMAN−486−S−S3_0.04
Doses Blue Rep1_0.03 Red Rep2_0.06
sp|Q9BVI0|PHF20_HUMAN−878−S−S2_−0.07
Doses Blue Rep1_−0.05 Red Rep2_−0.09
sp|Q9BVI0|PHF20_HUMAN−880−S−S2_−0.07
Doses Blue Rep1_−0.05 Red Rep2_−0.09
sp|Q96GA3|LTV1_HUMAN−206−S−S2_0.06
Doses Blue Rep1_0 Red Rep2_0.12
sp|Q96GN5−5|CDA7L_HUMAN−68−S−S2_−0.14
Doses Blue Rep1_−0.11 Red Rep2_−0.17
sp|Q96GN5−5|CDA7L_HUMAN−71−S−S2_−0.14
Doses Blue Rep1_−0.11 Red Rep2_−0.17
sp|Q96PV6−1|LENG8_HUMAN−314−S−S1_−0.25
Doses Blue Rep1_−0.24 Red Rep2_−0.26
ptm abundance 37C

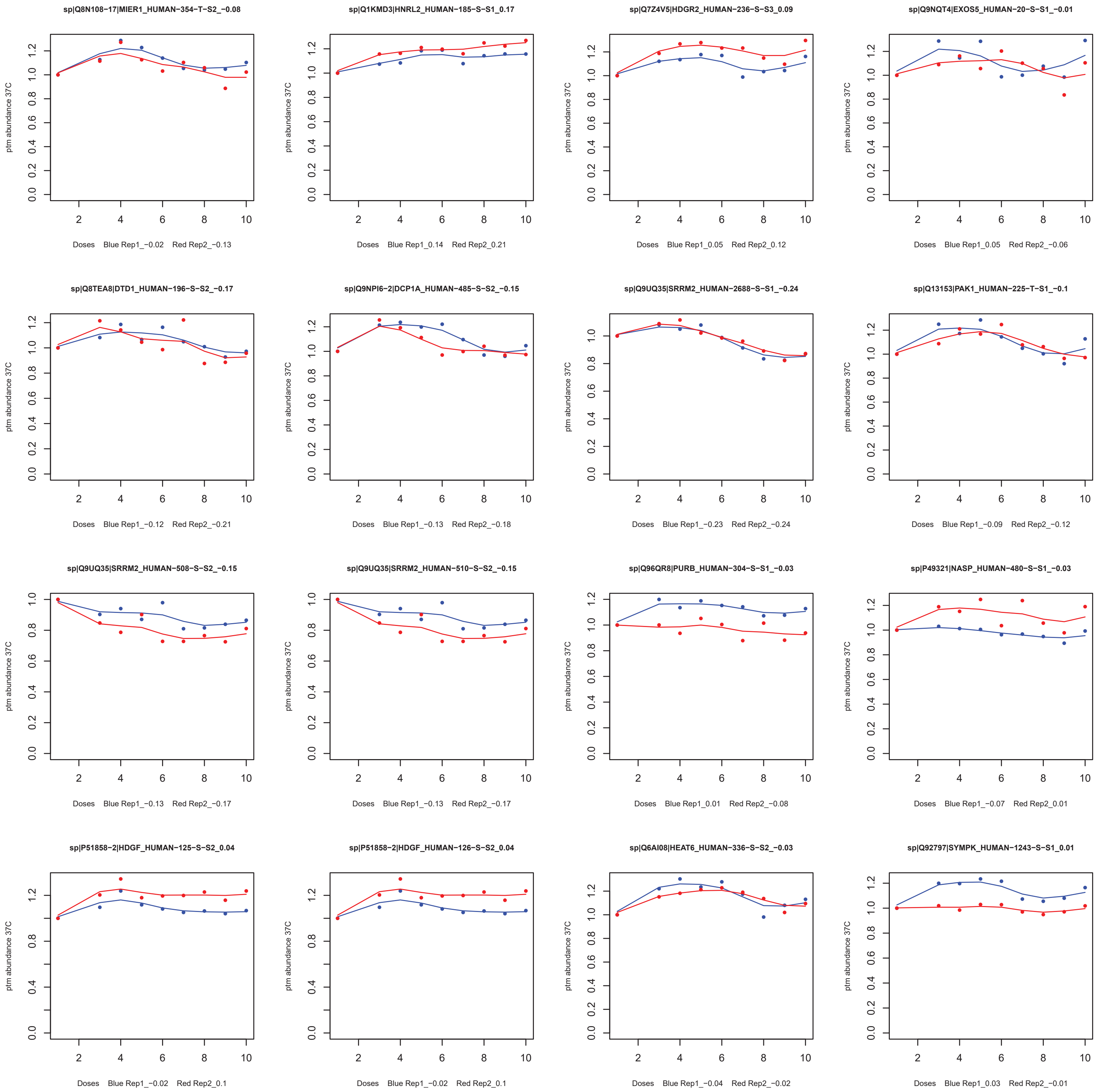
sp|Q8N108−17|MIER1_HUMAN−354−T−S2_−0.08
ptm abundance 37C
Doses Blue Rep1_−0.02 Red Rep2_−0.13
sp|Q1KMD3|HNRL2_HUMAN−185−S−S1_0.17
ptm abundance 37C
Doses Blue Rep1_0.14 Red Rep2_0.21
sp|Q7Z4V5|HDGR2_HUMAN−236−S−S3_0.09
ptm abundance 37C
Doses Blue Rep1_0.05 Red Rep2_0.12
sp|Q9NQT4|EXOS5_HUMAN−20−S−S1_−0.01
ptm abundance 37C
Doses Blue Rep1_0.05 Red Rep2_−0.06
sp|Q8TEA8|DTD1_HUMAN−196−S−S2_−0.17
ptm abundance 37C
Doses Blue Rep1_−0.12 Red Rep2_−0.21
sp|Q9NPI6−2|DCP1A_HUMAN−485−S−S2_−0.15
ptm abundance 37C
Doses Blue Rep1_−0.13 Red Rep2_−0.18
sp|Q9UQ35|SRRM2_HUMAN−2688−S−S1_−0.24
ptm abundance 37C
Doses Blue Rep1_−0.23 Red Rep2_−0.24
sp|Q13153|PAK1_HUMAN−225−T−S1_−0.1
ptm abundance 37C
Doses Blue Rep1_−0.09 Red Rep2_−0.12
sp|Q9UQ35|SRRM2_HUMAN−508−S−S2_−0.15
ptm abundance 37C
Doses Blue Rep1_−0.13 Red Rep2_−0.17
sp|Q9UQ35|SRRM2_HUMAN−510−S−S2_−0.15
ptm abundance 37C
Doses Blue Rep1_−0.13 Red Rep2_−0.17
sp|Q96QR8|PURB_HUMAN−304−S−S1_−0.03
ptm abundance 37C
Doses Blue Rep1_0.01 Red Rep2_−0.08
sp|P49321|NASP_HUMAN−480−S−S1_−0.03
ptm abundance 37C
Doses Blue Rep1_−0.07 Red Rep2_0.01
sp|P51858−2|HDGF_HUMAN−125−S−S2_0.04
ptm abundance 37C
Doses Blue Rep1_−0.02 Red Rep2_0.1
sp|P51858−2|HDGF_HUMAN−126−S−S2_0.04
ptm abundance 37C
Doses Blue Rep1_−0.02 Red Rep2_0.1
sp|Q6AI08|HEAT6_HUMAN−336−S−S2_−0.03
ptm abundance 37C
Doses Blue Rep1_−0.04 Red Rep2_−0.02
sp|Q92797|SYMPK_HUMAN−1243−S−S1_0.01
ptm abundance 37C
Doses Blue Rep1_0.03 Red Rep2_−0.01

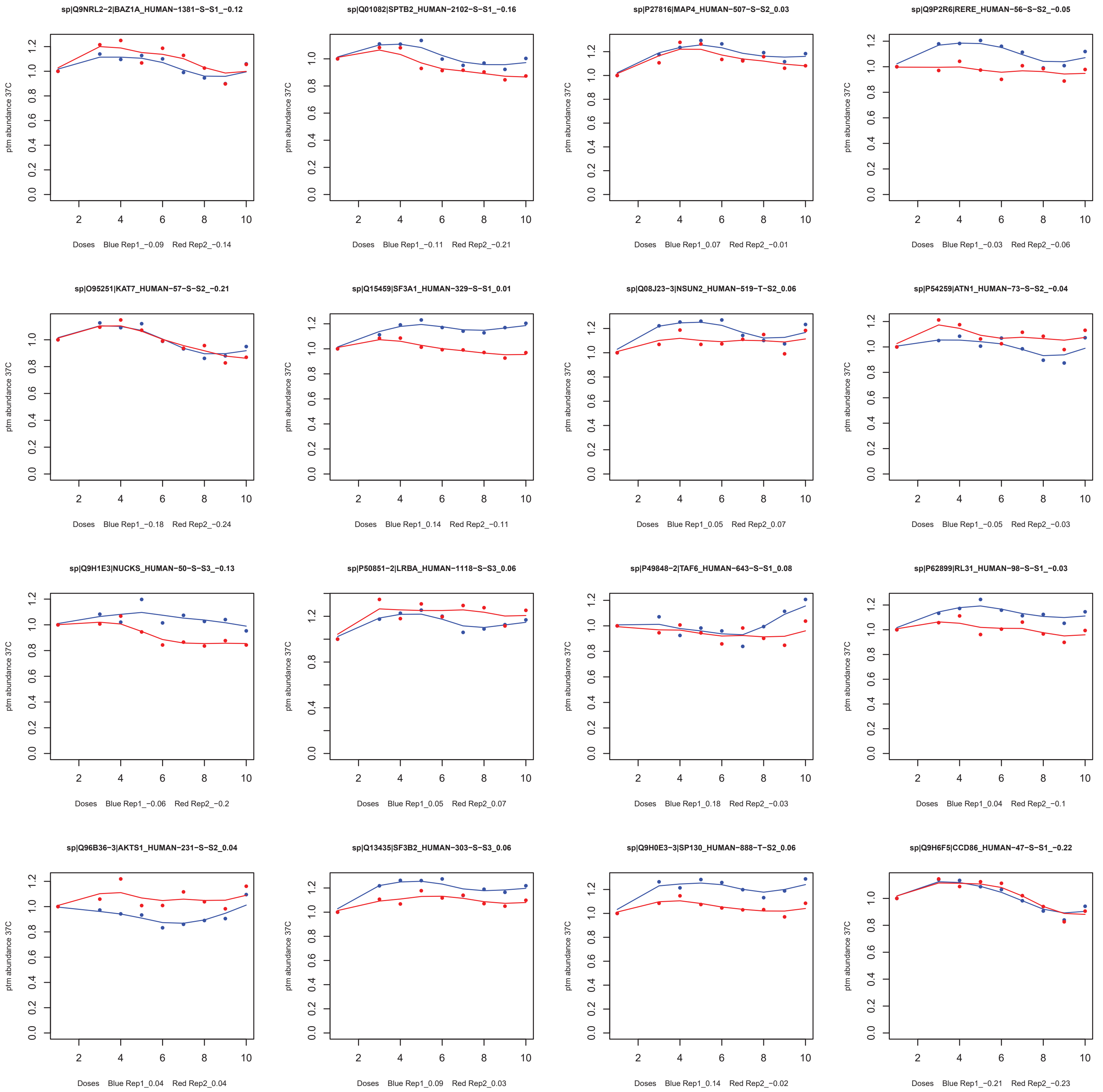
sp|Q9NRL2−2|BAZ1A_HUMAN−1381−S−S1_−0.12
ptm abundance 37C
Doses Blue Rep1_−0.09 Red Rep2_−0.14
sp|Q01082|SPTB2_HUMAN−2102−S−S1_−0.16
ptm abundance 37C
Doses Blue Rep1_−0.11 Red Rep2_−0.21
sp|P27816|MAP4_HUMAN−507−S−S2_0.03
ptm abundance 37C
Doses Blue Rep1_0.07 Red Rep2_−0.01
sp|Q9P2R6|RERE_HUMAN−56−S−S2_−0.05
ptm abundance 37C
Doses Blue Rep1_−0.03 Red Rep2_−0.06
sp|O95251|KAT7_HUMAN−57−S−S2_−0.21
ptm abundance 37C
Doses Blue Rep1_−0.18 Red Rep2_−0.24
sp|Q15459|SF3A1_HUMAN−329−S−S1_0.01
ptm abundance 37C
Doses Blue Rep1_0.14 Red Rep2_−0.11
sp|Q08J23−3|NSUN2_HUMAN−519−T−S2_0.06
ptm abundance 37C
Doses Blue Rep1_0.05 Red Rep2_0.07
sp|P54259|ATN1_HUMAN−73−S−S2_−0.04
ptm abundance 37C
Doses Blue Rep1_−0.05 Red Rep2_−0.03
sp|Q9H1E3|NUCKS_HUMAN−50−S−S3_−0.13
ptm abundance 37C
Doses Blue Rep1_−0.06 Red Rep2_−0.2
sp|P50851−2|LRBA_HUMAN−1118−S−S3_0.06
ptm abundance 37C
Doses Blue Rep1_0.05 Red Rep2_0.07
sp|P49848−2|TAF6_HUMAN−643−S−S1_0.08
ptm abundance 37C
Doses Blue Rep1_0.18 Red Rep2_−0.03
sp|P62899|RL31_HUMAN−98−S−S1_−0.03
ptm abundance 37C
Doses Blue Rep1_0.04 Red Rep2_−0.1
sp|Q96B36−3|AKTS1_HUMAN−231−S−S2_0.04
ptm abundance 37C
Doses Blue Rep1_0.04 Red Rep2_0.04
sp|Q13435|SF3B2_HUMAN−303−S−S3_0.06
ptm abundance 37C
Doses Blue Rep1_0.09 Red Rep2_0.03
sp|Q9H0E3−3|SP130_HUMAN−888−T−S2_0.06
ptm abundance 37C
Doses Blue Rep1_0.14 Red Rep2_−0.02
sp|Q9H6F5|CCD86_HUMAN−47−S−S1_−0.22
ptm abundance 37C
Doses Blue Rep1_−0.21 Red Rep2_−0.23

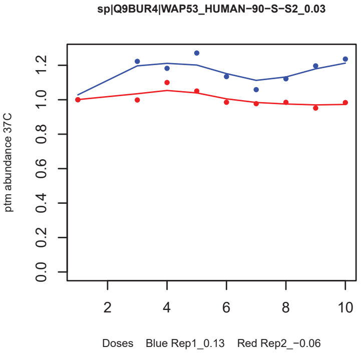
sp|Q9BUR4|WAP53_HUMAN−90−S−S2_0.03
ptm abundance 37C
Doses Blue Rep1_0.13 Red Rep2_−0.06

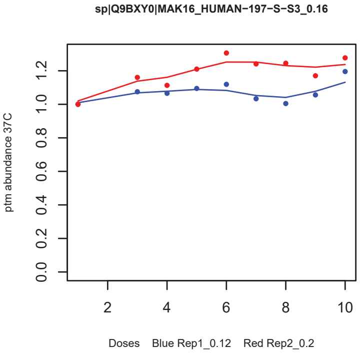
sp|Q9BXY0|MAK16_HUMAN−197−S−S3_0.16
ptm abundance 37C
Doses Blue Rep1_0.12 Red Rep2_0.2

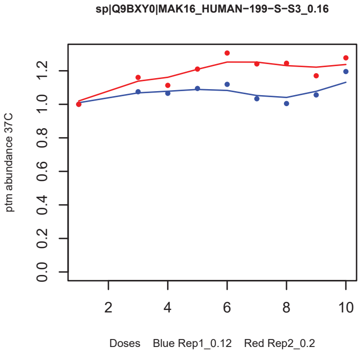
sp|Q9BXY0|MAK16_HUMAN−199−S−S3_0.16
ptm abundance 37C
Doses Blue Rep1_0.12 Red Rep2_0.2

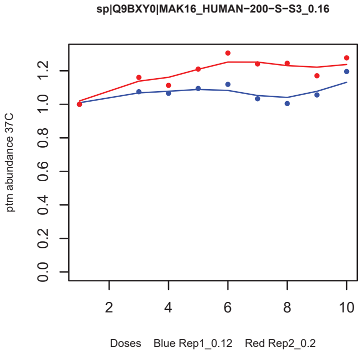
sp|Q9BXY0|MAK16_HUMAN−200−S−S3_0.16
ptm abundance 37C
Doses Blue Rep1_0.12 Red Rep2_0.2

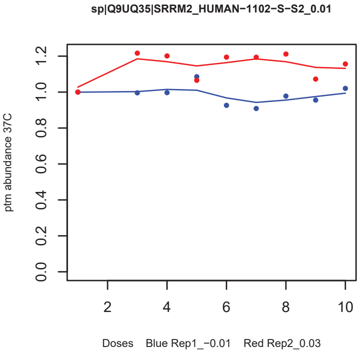
sp|Q9UQ35|SRRM2_HUMAN−1102−S−S2_0.01
ptm abundance 37C
Doses Blue Rep1_−0.01 Red Rep2_0.03

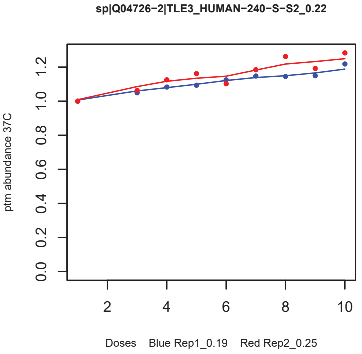
sp|Q04726−2|TLE3_HUMAN−240−S−S2_0.22
ptm abundance 37C
Doses Blue Rep1_0.19 Red Rep2_0.25

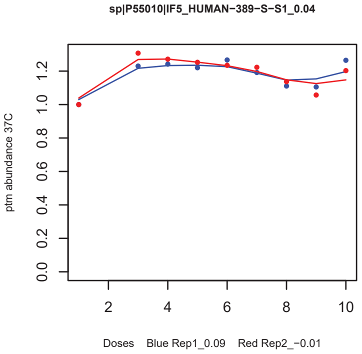
sp|P55010|IF5_HUMAN−389−S−S1_0.04
ptm abundance 37C
Doses Blue Rep1_0.09 Red Rep2_−0.01

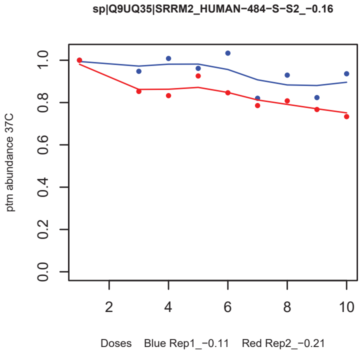
sp|Q9UQ35|SRRM2_HUMAN−484−S−S2_−0.16
ptm abundance 37C
Doses Blue Rep1_−0.11 Red Rep2_−0.21

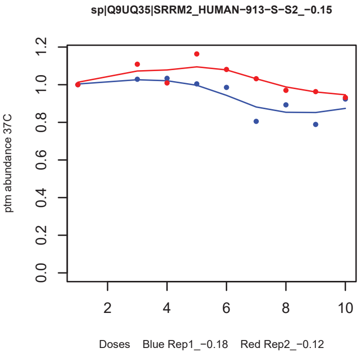
sp|Q9UQ35|SRRM2_HUMAN−913−S−S2_−0.15
ptm abundance 37C
Doses Blue Rep1_−0.18 Red Rep2_−0.12

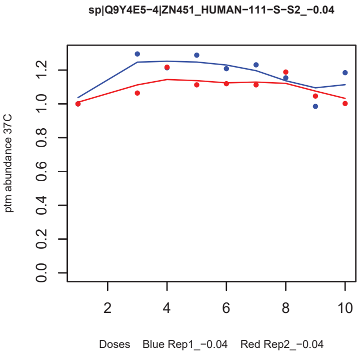
sp|Q9Y4E5−4|ZN451_HUMAN−111−S−S2_−0.04
ptm abundance 37C
Doses Blue Rep1_−0.04 Red Rep2_−0.04

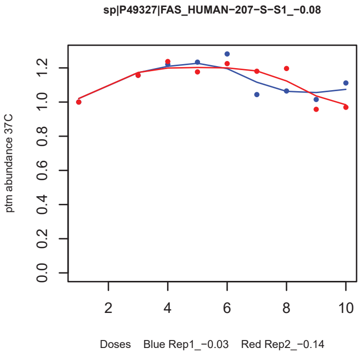
sp|P49327|FAS_HUMAN−207−S−S1_−0.08
ptm abundance 37C
Doses Blue Rep1_−0.03 Red Rep2_−0.14

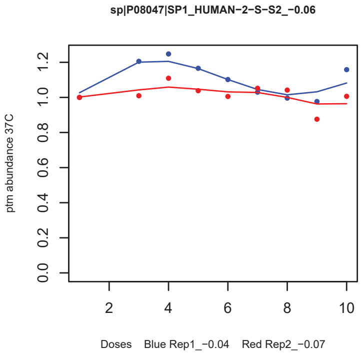
sp|P08047|SP1_HUMAN−2−S−S2_−0.06
ptm abundance 37C
Doses Blue Rep1_−0.04 Red Rep2_−0.07

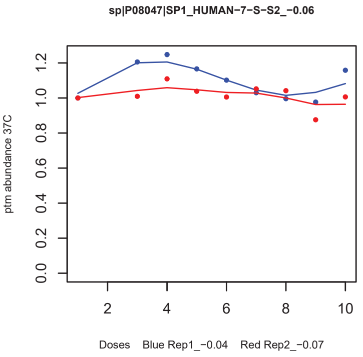
sp|P08047|SP1_HUMAN−7−S−S2_−0.06
ptm abundance 37C
Doses Blue Rep1_−0.04 Red Rep2_−0.07

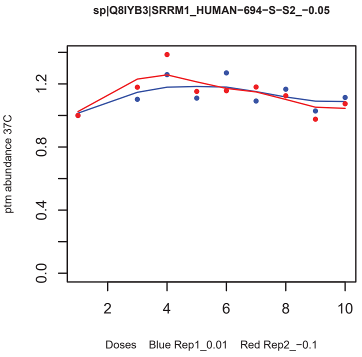
sp|Q8IYB3|SRRM1_HUMAN−694−S−S2_−0.05
ptm abundance 37C
Doses Blue Rep1_0.01 Red Rep2_−0.1

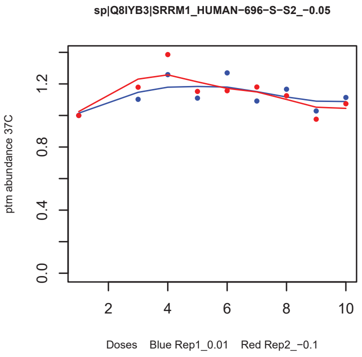
sp|Q8IYB3|SRRM1_HUMAN−696−S−S2_−0.05
ptm abundance 37C
Doses Blue Rep1_0.01 Red Rep2_−0.1

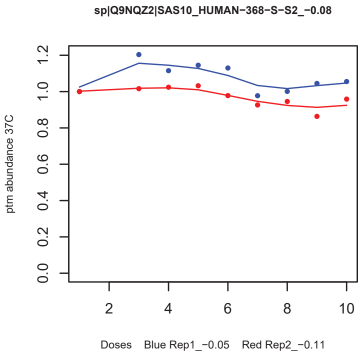
sp|Q9NQZ2|SAS10_HUMAN−368−S−S2_−0.08
ptm abundance 37C
Doses Blue Rep1_−0.05 Red Rep2_−0.11

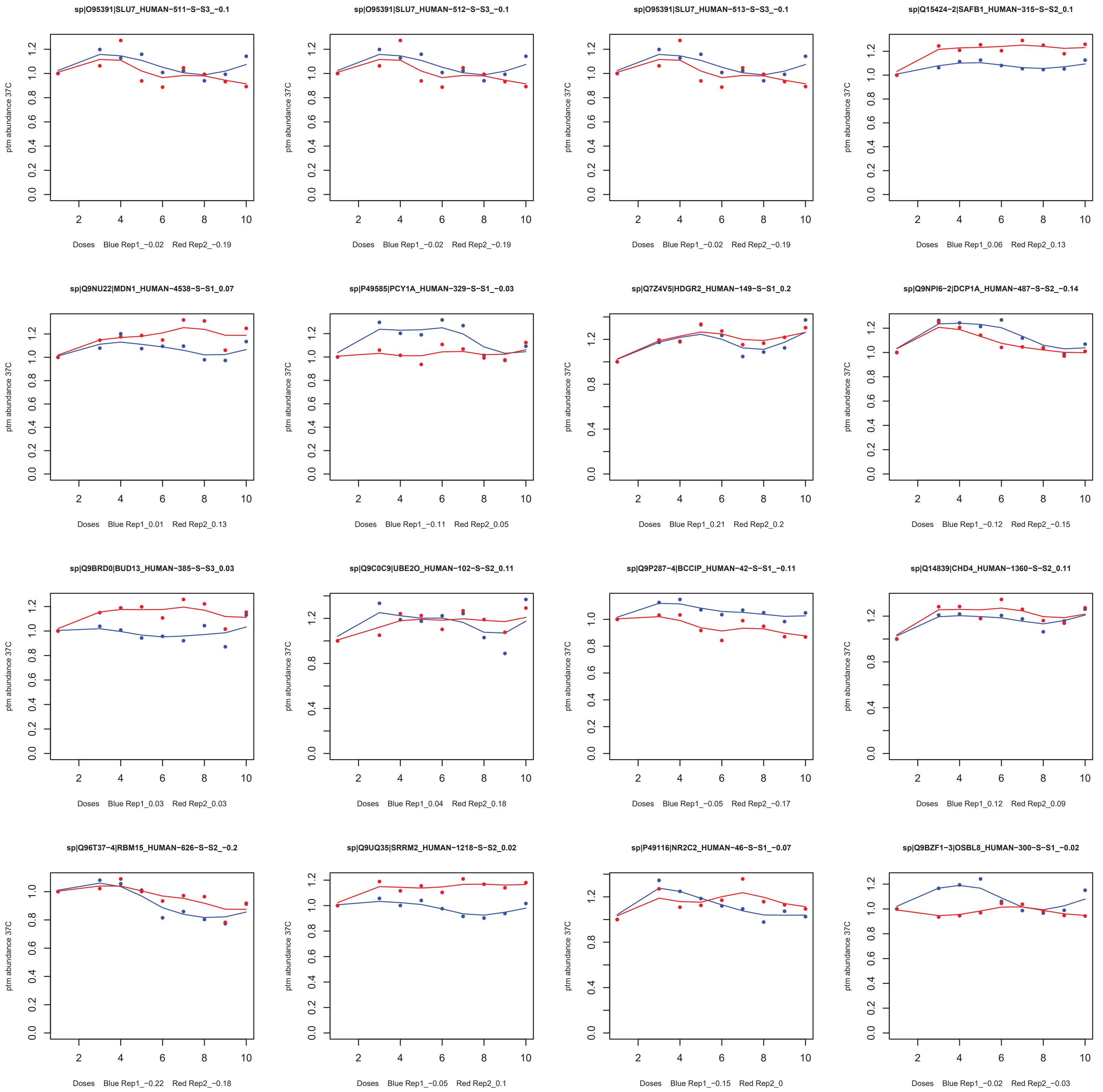

sp|O95391|SLU7_HUMAN−511−S−S3_−0.1
Doses Blue Rep1_−0.02 Red Rep2_−0.19
sp|O95391|SLU7_HUMAN−512−S−S3_−0.1
Doses Blue Rep1_−0.02 Red Rep2_−0.19
sp|O95391|SLU7_HUMAN−513−S−S3_−0.1
Doses Blue Rep1_−0.02 Red Rep2_−0.19
sp|Q15424−2|SAFB1_HUMAN−315−S−S2_0.1
Doses Blue Rep1_0.06 Red Rep2_0.13
sp|Q9NU22|MDN1_HUMAN−4538−S−S1_0.07
Doses Blue Rep1_0.01 Red Rep2_0.13
sp|P49585|PCY1A_HUMAN−329−S−S1_−0.03
Doses Blue Rep1_−0.11 Red Rep2_0.05
sp|Q7Z4V5|HDGR2_HUMAN−149−S−S1_0.2
Doses Blue Rep1_0.21 Red Rep2_0.2
sp|Q9NPI6−2|DCP1A_HUMAN−487−S−S2_−0.14
Doses Blue Rep1_−0.12 Red Rep2_−0.15
sp|Q9BRD0|BUD13_HUMAN−385−S−S3_0.03
Doses Blue Rep1_0.03 Red Rep2_0.03
sp|Q9C0C9|UBE2O_HUMAN−102−S−S2_0.11
Doses Blue Rep1_0.04 Red Rep2_0.18
sp|Q9P287−4|BCCIP_HUMAN−42−S−S1_−0.11
Doses Blue Rep1_−0.05 Red Rep2_−0.17
sp|Q14839|CHD4_HUMAN−1360−S−S2_0.11
Doses Blue Rep1_0.12 Red Rep2_0.09
sp|Q96T37−4|RBM15_HUMAN−626−S−S2_−0.2
Doses Blue Rep1_−0.22 Red Rep2_−0.18
sp|Q9UQ35|SRRM2_HUMAN−1218−S−S2_0.02
Doses Blue Rep1_−0.05 Red Rep2_0.1
sp|P49116|NR2C2_HUMAN−46−S−S1_−0.07
Doses Blue Rep1_−0.15 Red Rep2_0
sp|Q9BZF1−3|OSBL8_HUMAN−300−S−S1_−0.02
Doses Blue Rep1_−0.02 Red Rep2_−0.03
ptm abundance 37C

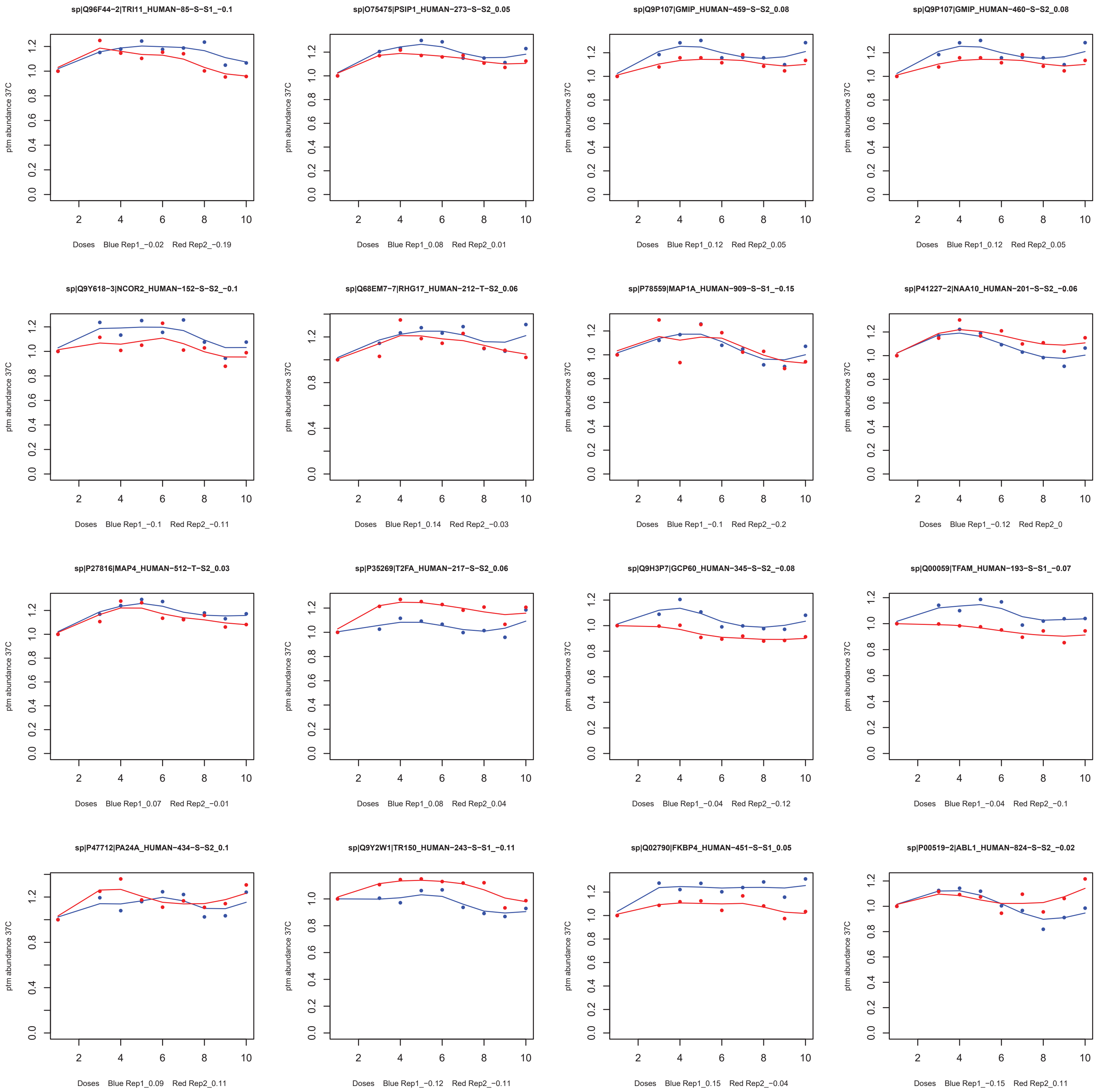
sp|Q96F44−2|TRI11_HUMAN−85−S−S1_−0.1
Doses Blue Rep1_−0.02 Red Rep2_−0.19
sp|O75475|PSIP1_HUMAN−273−S−S2_0.05
Doses Blue Rep1_0.08 Red Rep2_0.01
sp|Q9P107|GMIP_HUMAN−459−S−S2_0.08
Doses Blue Rep1_0.12 Red Rep2_0.05
sp|Q9P107|GMIP_HUMAN−460−S−S2_0.08
Doses Blue Rep1_0.12 Red Rep2_0.05
sp|Q9Y618−3|NCOR2_HUMAN−152−S−S2_−0.1
Doses Blue Rep1_−0.1 Red Rep2_−0.11
sp|Q68EM7−7|RHG17_HUMAN−212−T−S2_0.06
Doses Blue Rep1_0.14 Red Rep2_−0.03
sp|P78559|MAP1A_HUMAN−909−S−S1_−0.15
Doses Blue Rep1_−0.1 Red Rep2_−0.2
sp|P41227−2|NAA10_HUMAN−201−S−S2_−0.06
Doses Blue Rep1_−0.12 Red Rep2_0
sp|P27816|MAP4_HUMAN−512−T−S2_0.03
Doses Blue Rep1_0.07 Red Rep2_−0.01
sp|P35269|T2FA_HUMAN−217−S−S2_0.06
Doses Blue Rep1_0.08 Red Rep2_0.04
sp|Q9H3P7|GCP60_HUMAN−345−S−S2_−0.08
Doses Blue Rep1_−0.04 Red Rep2_−0.12
sp|Q00059|TFAM_HUMAN−193−S−S1_−0.07
Doses Blue Rep1_−0.04 Red Rep2_−0.1
sp|P47712|PA24A_HUMAN−434−S−S2_0.1
Doses Blue Rep1_0.09 Red Rep2_0.11
sp|Q9Y2W1|TR150_HUMAN−243−S−S1_−0.11
Doses Blue Rep1_−0.12 Red Rep2_−0.11
sp|Q02790|FKBP4_HUMAN−451−S−S1_0.05
Doses Blue Rep1_0.15 Red Rep2_−0.04
sp|P00519−2|ABL1_HUMAN−824−S−S2_−0.02
Doses Blue Rep1_−0.15 Red Rep2_0.11
ptm abundance 37C

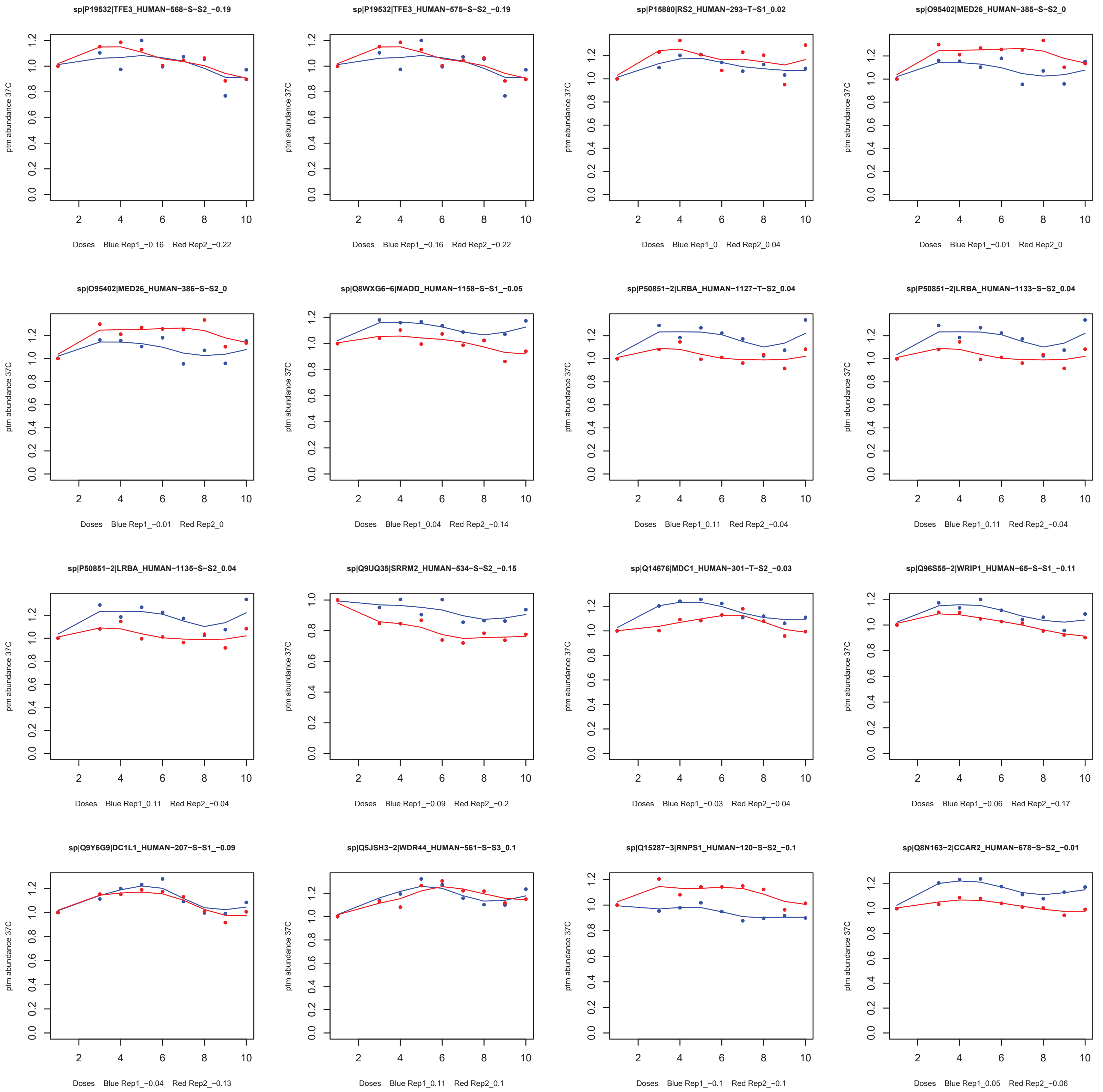
sp|P19532|TFE3_HUMAN−568−S−S2_−0.19
Doses Blue Rep1_−0.16 Red Rep2_−0.22
sp|P19532|TFE3_HUMAN−575−S−S2_−0.19
Doses Blue Rep1_−0.16 Red Rep2_−0.22
sp|P15880|RS2_HUMAN−293−T−S1_0.02
Doses Blue Rep1_0 Red Rep2_0.04
sp|O95402|MED26_HUMAN−385−S−S2_0
Doses Blue Rep1_−0.01 Red Rep2_0
sp|O95402|MED26_HUMAN−386−S−S2_0
Doses Blue Rep1_−0.01 Red Rep2_0
sp|Q8WXG6−6|MADD_HUMAN−1158−S−S1_−0.05
Doses Blue Rep1_0.04 Red Rep2_−0.14
sp|P50851−2|LRBA_HUMAN−1127−T−S2_0.04
Doses Blue Rep1_0.11 Red Rep2_−0.04
sp|P50851−2|LRBA_HUMAN−1133−S−S2_0.04
Doses Blue Rep1_0.11 Red Rep2_−0.04
sp|P50851−2|LRBA_HUMAN−1135−S−S2_0.04
Doses Blue Rep1_0.11 Red Rep2_−0.04
sp|Q9UQ35|SRRM2_HUMAN−534−S−S2_−0.15
Doses Blue Rep1_−0.09 Red Rep2_−0.2
sp|Q14676|MDC1_HUMAN−301−T−S2_−0.03
Doses Blue Rep1_−0.03 Red Rep2_−0.04
sp|Q96S55−2|WRIP1_HUMAN−65−S−S1_−0.11
Doses Blue Rep1_−0.06 Red Rep2_−0.17
sp|Q9Y6G9|DC1L1_HUMAN−207−S−S1_−0.09
Doses Blue Rep1_−0.04 Red Rep2_−0.13
sp|Q5JSH3−2|WDR44_HUMAN−561−S−S3_0.1
Doses Blue Rep1_0.11 Red Rep2_0.1
sp|Q15287−3|RNPS1_HUMAN−120−S−S2_−0.1
Doses Blue Rep1_−0.1 Red Rep2_−0.1
sp|Q8N163−2|CCAR2_HUMAN−678−S−S2_−0.01
Doses Blue Rep1_0.05 Red Rep2_−0.06
ptm abundance 37C

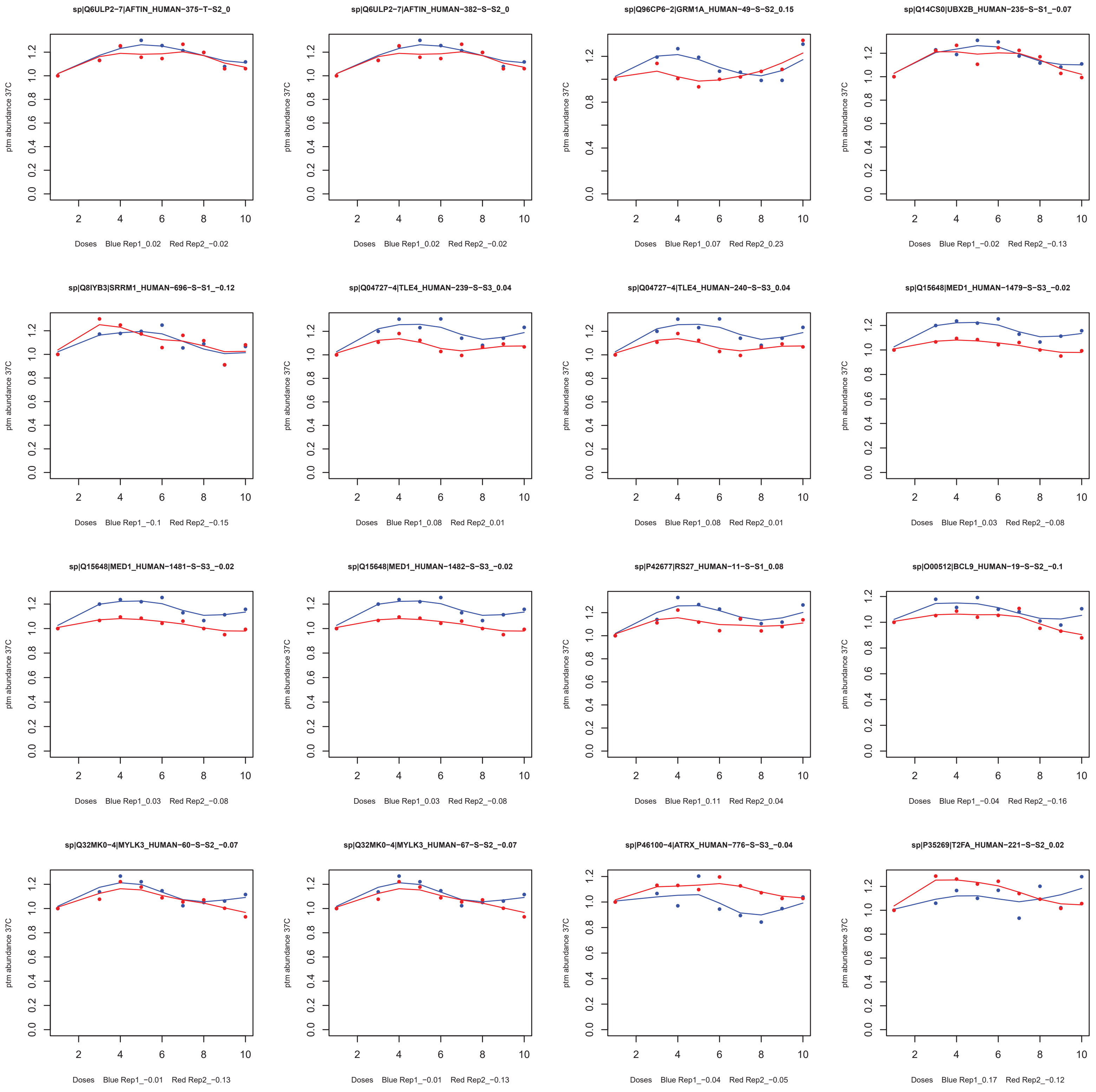
sp|Q6ULP2−7|AFTIN_HUMAN−375−T−S2_0
Doses Blue Rep1_0.02 Red Rep2_−0.02
sp|Q6ULP2−7|AFTIN_HUMAN−382−S−S2_0
Doses Blue Rep1_0.02 Red Rep2_−0.02
sp|Q96CP6−2|GRM1A_HUMAN−49−S−S2_0.15
Doses Blue Rep1_0.07 Red Rep2_0.23
sp|Q14CS0|UBX2B_HUMAN−235−S−S1_−0.07
Doses Blue Rep1_−0.02 Red Rep2_−0.13
sp|Q8IYB3|SRRM1_HUMAN−696−S−S1_−0.12
Doses Blue Rep1_−0.1 Red Rep2_−0.15
sp|Q04727−4|TLE4_HUMAN−239−S−S3_0.04
Doses Blue Rep1_0.08 Red Rep2_0.01
sp|Q04727−4|TLE4_HUMAN−240−S−S3_0.04
Doses Blue Rep1_0.08 Red Rep2_0.01
sp|Q15648|MED1_HUMAN−1479−S−S3_−0.02
Doses Blue Rep1_0.03 Red Rep2_−0.08
sp|Q15648|MED1_HUMAN−1481−S−S3_−0.02
Doses Blue Rep1_0.03 Red Rep2_−0.08
sp|Q15648|MED1_HUMAN−1482−S−S3_−0.02
Doses Blue Rep1_0.03 Red Rep2_−0.08
sp|P42677|RS27_HUMAN−11−S−S1_0.08
Doses Blue Rep1_0.11 Red Rep2_0.04
sp|O00512|BCL9_HUMAN−19−S−S2_−0.1
Doses Blue Rep1_−0.04 Red Rep2_−0.16
sp|Q32MK0−4|MYLK3_HUMAN−60−S−S2_−0.07
Doses Blue Rep1_−0.01 Red Rep2_−0.13
sp|Q32MK0−4|MYLK3_HUMAN−67−S−S2_−0.07
Doses Blue Rep1_−0.01 Red Rep2_−0.13
sp|P46100−4|ATRX_HUMAN−776−S−S3_−0.04
Doses Blue Rep1_−0.04 Red Rep2_−0.05
sp|P35269|T2FA_HUMAN−221−S−S2_0.02
Doses Blue Rep1_0.17 Red Rep2_−0.12
ptm abundance 37C

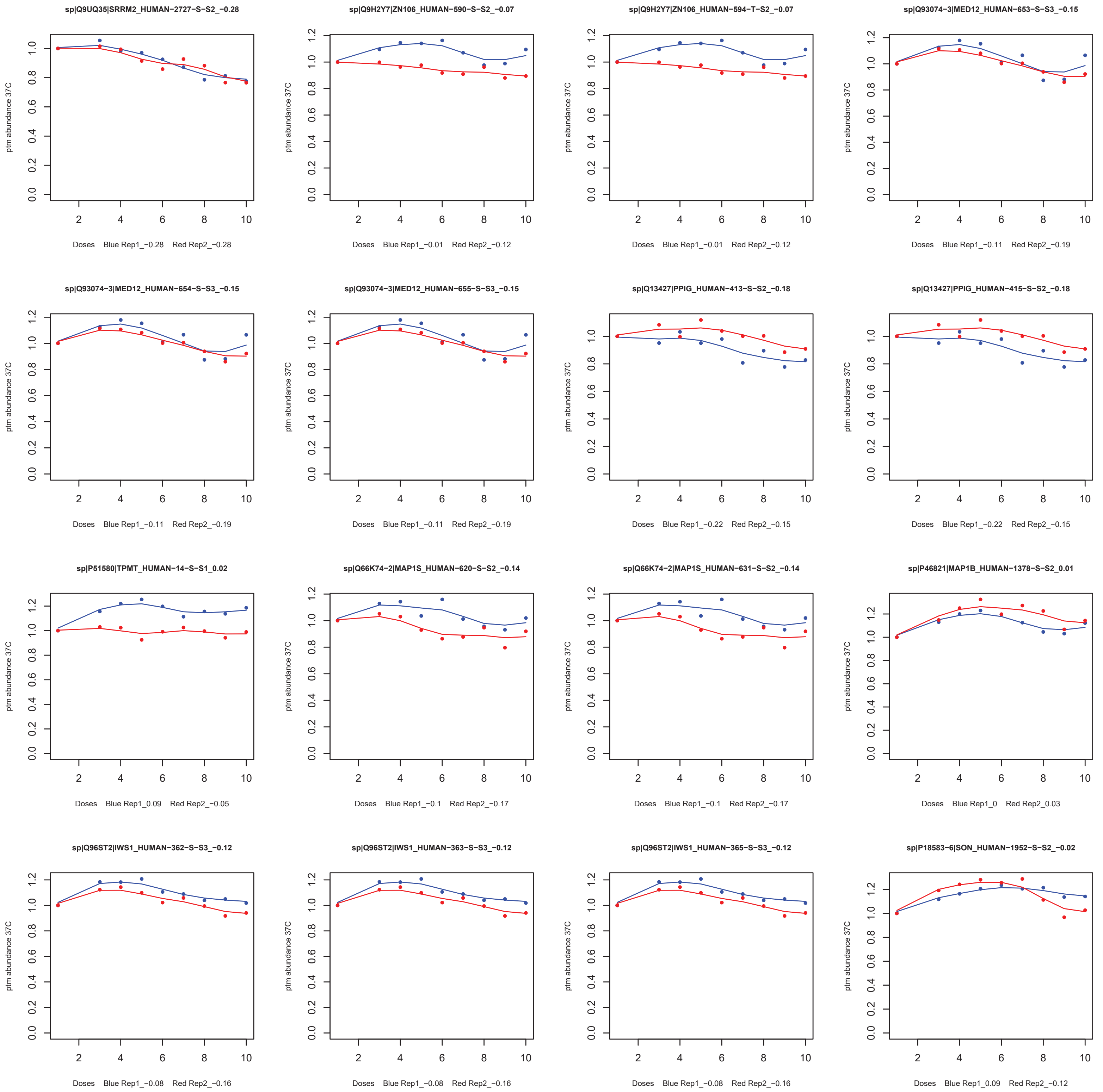
sp|Q9UQ35|SRRM2_HUMAN−2727−S−S2_−0.28
ptm abundance 37C
Doses Blue Rep1_−0.28 Red Rep2_−0.28
sp|Q9H2Y7|ZN106_HUMAN−590−S−S2_−0.07
ptm abundance 37C
Doses Blue Rep1_−0.01 Red Rep2_−0.12
sp|Q9H2Y7|ZN106_HUMAN−594−T−S2_−0.07
ptm abundance 37C
Doses Blue Rep1_−0.01 Red Rep2_−0.12
sp|Q93074−3|MED12_HUMAN−653−S−S3_−0.15
ptm abundance 37C
Doses Blue Rep1_−0.11 Red Rep2_−0.19
sp|Q93074−3|MED12_HUMAN−654−S−S3_−0.15
ptm abundance 37C
Doses Blue Rep1_−0.11 Red Rep2_−0.19
sp|Q93074−3|MED12_HUMAN−655−S−S3_−0.15
ptm abundance 37C
Doses Blue Rep1_−0.11 Red Rep2_−0.19
sp|Q13427|PPIG_HUMAN−413−S−S2_−0.18
ptm abundance 37C
Doses Blue Rep1_−0.22 Red Rep2_−0.15
sp|Q13427|PPIG_HUMAN−415−S−S2_−0.18
ptm abundance 37C
Doses Blue Rep1_−0.22 Red Rep2_−0.15
sp|P51580|TPMT_HUMAN−14−S−S1_0.02
ptm abundance 37C
Doses Blue Rep1_0.09 Red Rep2_−0.05
sp|Q66K74−2|MAP1S_HUMAN−620−S−S2_−0.14
ptm abundance 37C
Doses Blue Rep1_−0.1 Red Rep2_−0.17
sp|Q66K74−2|MAP1S_HUMAN−631−S−S2_−0.14
ptm abundance 37C
Doses Blue Rep1_−0.1 Red Rep2_−0.17
sp|P46821|MAP1B_HUMAN−1378−S−S2_0.01
ptm abundance 37C
Doses Blue Rep1_0 Red Rep2_0.03
sp|Q96ST2|IWS1_HUMAN−362−S−S3_−0.12
ptm abundance 37C
Doses Blue Rep1_−0.08 Red Rep2_−0.16
sp|Q96ST2|IWS1_HUMAN−363−S−S3_−0.12
ptm abundance 37C
Doses Blue Rep1_−0.08 Red Rep2_−0.16
sp|Q96ST2|IWS1_HUMAN−365−S−S3_−0.12
ptm abundance 37C
Doses Blue Rep1_−0.08 Red Rep2_−0.16
sp|P18583−6|SON_HUMAN−1952−S−S2_−0.02
ptm abundance 37C
Doses Blue Rep1_0.09 Red Rep2_−0.12

sp|Q13905−2|RPGF1_HUMAN−177−T−S2_−0.07

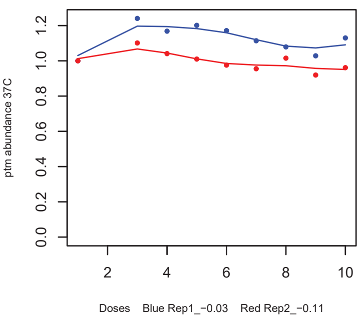

sp|Q8NB78|KDM1B_HUMAN−247−S−S1_−0.2

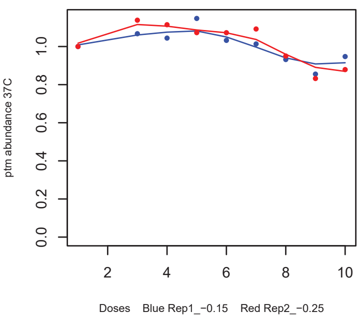

sp|O15042|SR140_HUMAN−788−S−S2_−0.02

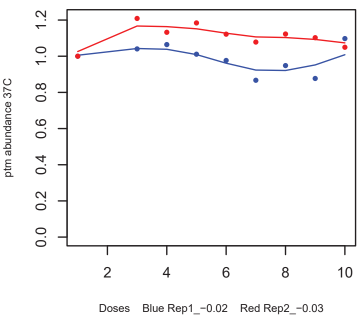

sp|O15042|SR140_HUMAN−800−S−S2_−0.02

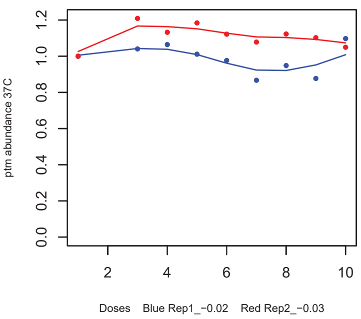

sp|P33316−2|DUT_HUMAN−11−S−S1_−0.13

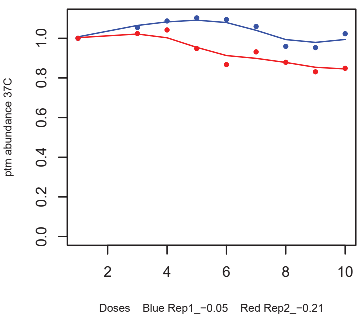

sp|P04075|ALDOA_HUMAN−39−S−S1_0.04

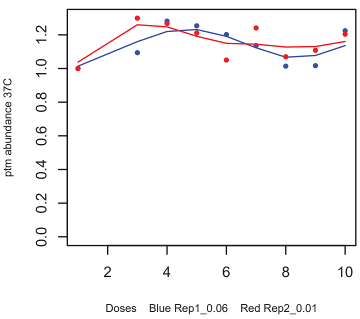

sp|O95402|MED26_HUMAN−389−S−S2_0.02

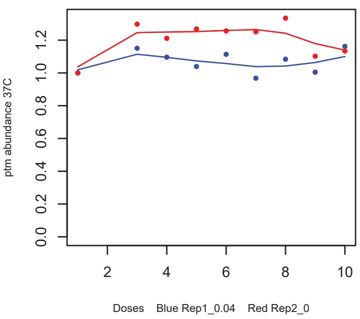

sp|Q96GA3|LTV1_HUMAN−200−S−S3_0.1

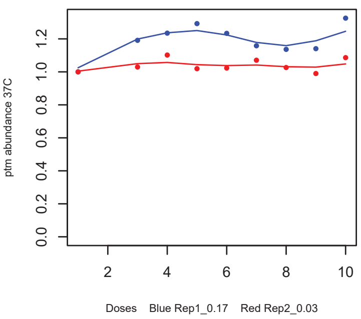

sp|Q9BUL5−3|PHF23_HUMAN−248−S−S2_0.11

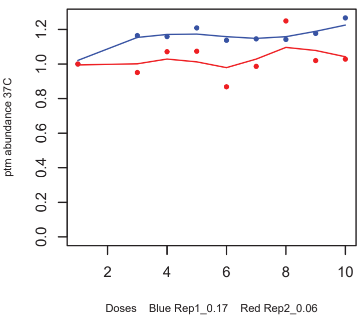

sp|Q9BUL5−3|PHF23_HUMAN−249−S−S2_0.11

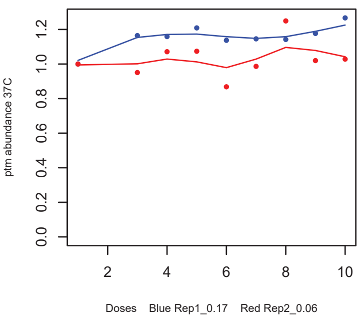

sp|Q9BUL5−3|PHF23_HUMAN−250−S−S2_0.11

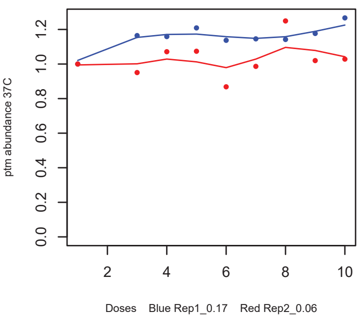

sp|P46100−4|ATRX_HUMAN−1284−S−S3_0.02

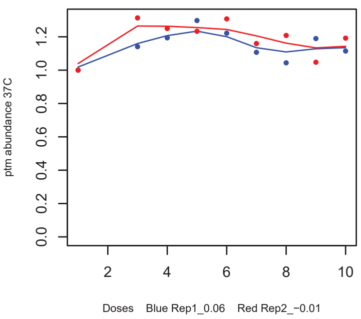

sp|P46100−4|ATRX_HUMAN−1286−S−S3_0.02

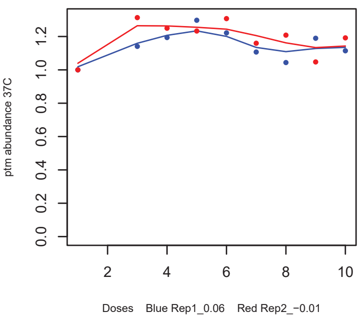

sp|P46100−4|ATRX_HUMAN−1288−S−S3_0.02

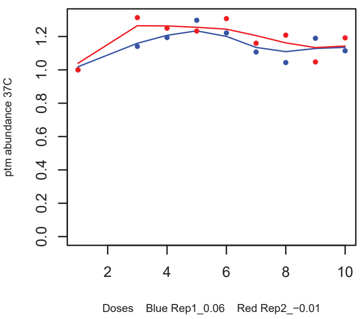

sp|Q99567|NUP88_HUMAN−35−S−S1_−0.12

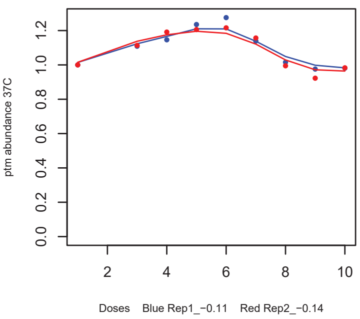

sp|P21127−9|CD11B_HUMAN−542−S−S1_−0.04

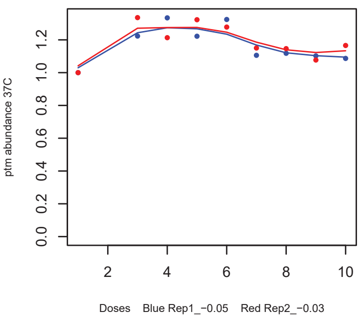

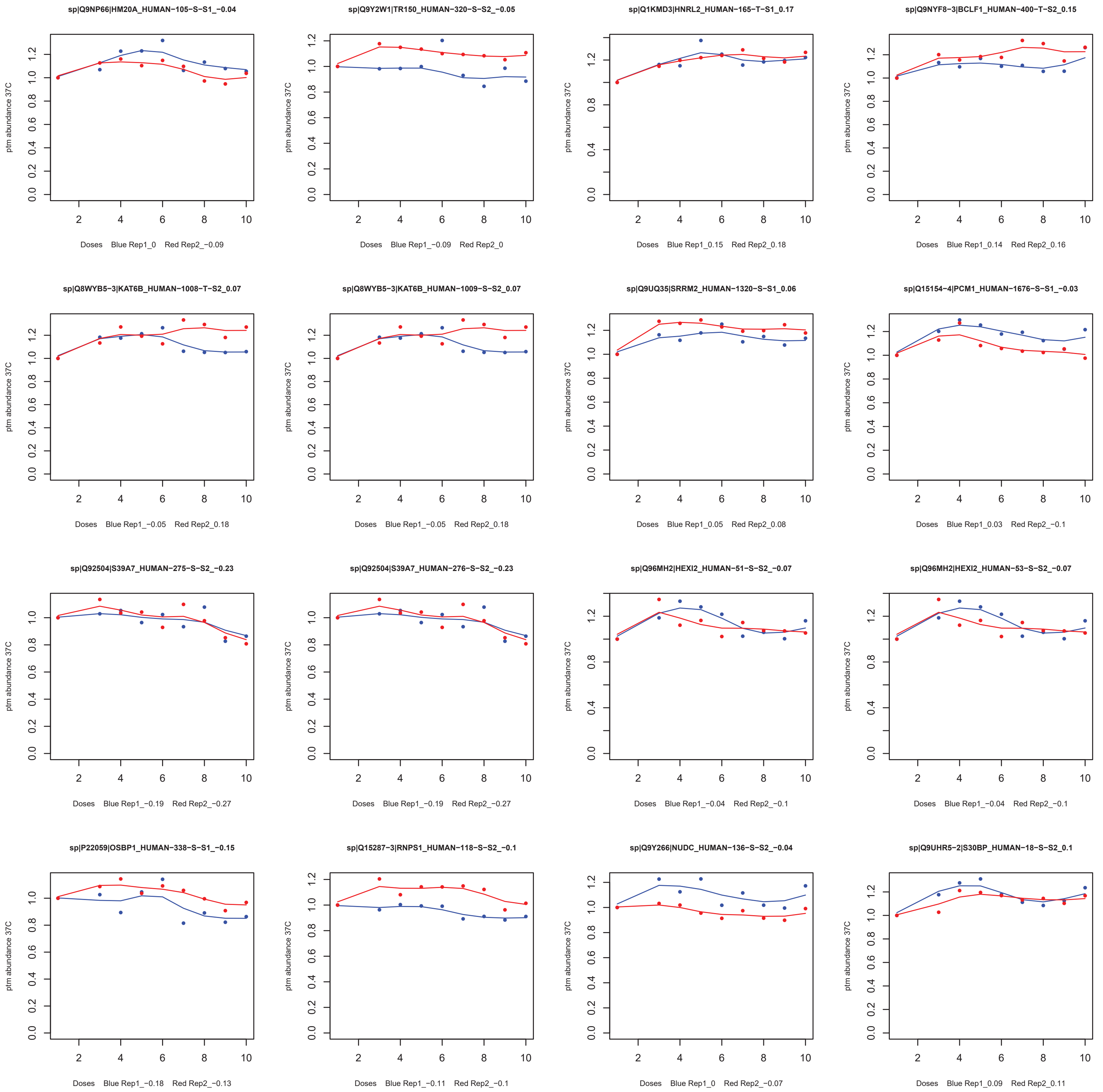

sp|Q9NP66|HM20A_HUMAN−105−S−S1_−0.04
Doses Blue Rep1_0 Red Rep2_−0.09
sp|Q9Y2W1|TR150_HUMAN−320−S−S2_−0.05
Doses Blue Rep1_−0.09 Red Rep2_0
sp|Q1KMD3|HNRL2_HUMAN−165−T−S1_0.17
Doses Blue Rep1_0.15 Red Rep2_0.18
sp|Q9NYF8−3|BCLF1_HUMAN−400−T−S2_0.15
Doses Blue Rep1_0.14 Red Rep2_0.16
sp|Q8WYB5−3|KAT6B_HUMAN−1008−T−S2_0.07
Doses Blue Rep1_−0.05 Red Rep2_0.18
sp|Q8WYB5−3|KAT6B_HUMAN−1009−S−S2_0.07
Doses Blue Rep1_−0.05 Red Rep2_0.18
sp|Q9UQ35|SRRM2_HUMAN−1320−S−S1_0.06
Doses Blue Rep1_0.05 Red Rep2_0.08
sp|Q15154−4|PCM1_HUMAN−1676−S−S1_−0.03
Doses Blue Rep1_0.03 Red Rep2_−0.1
sp|Q92504|S39A7_HUMAN−275−S−S2_−0.23
Doses Blue Rep1_−0.19 Red Rep2_−0.27
sp|Q92504|S39A7_HUMAN−276−S−S2_−0.23
Doses Blue Rep1_−0.19 Red Rep2_−0.27
sp|Q96MH2|HEXI2_HUMAN−51−S−S2_−0.07
Doses Blue Rep1_−0.04 Red Rep2_−0.1
sp|Q96MH2|HEXI2_HUMAN−53−S−S2_−0.07
Doses Blue Rep1_−0.04 Red Rep2_−0.1
sp|P22059|OSBP1_HUMAN−338−S−S1_−0.15
Doses Blue Rep1_−0.18 Red Rep2_−0.13
sp|Q15287−3|RNPS1_HUMAN−118−S−S2_−0.1
Doses Blue Rep1_−0.11 Red Rep2_−0.1
sp|Q9Y266|NUDC_HUMAN−136−S−S2_−0.04
Doses Blue Rep1_0 Red Rep2_−0.07
sp|Q9UHR5−2|S30BP_HUMAN−18−S−S2_0.1
Doses Blue Rep1_0.09 Red Rep2_0.11
ptm abundance 37C

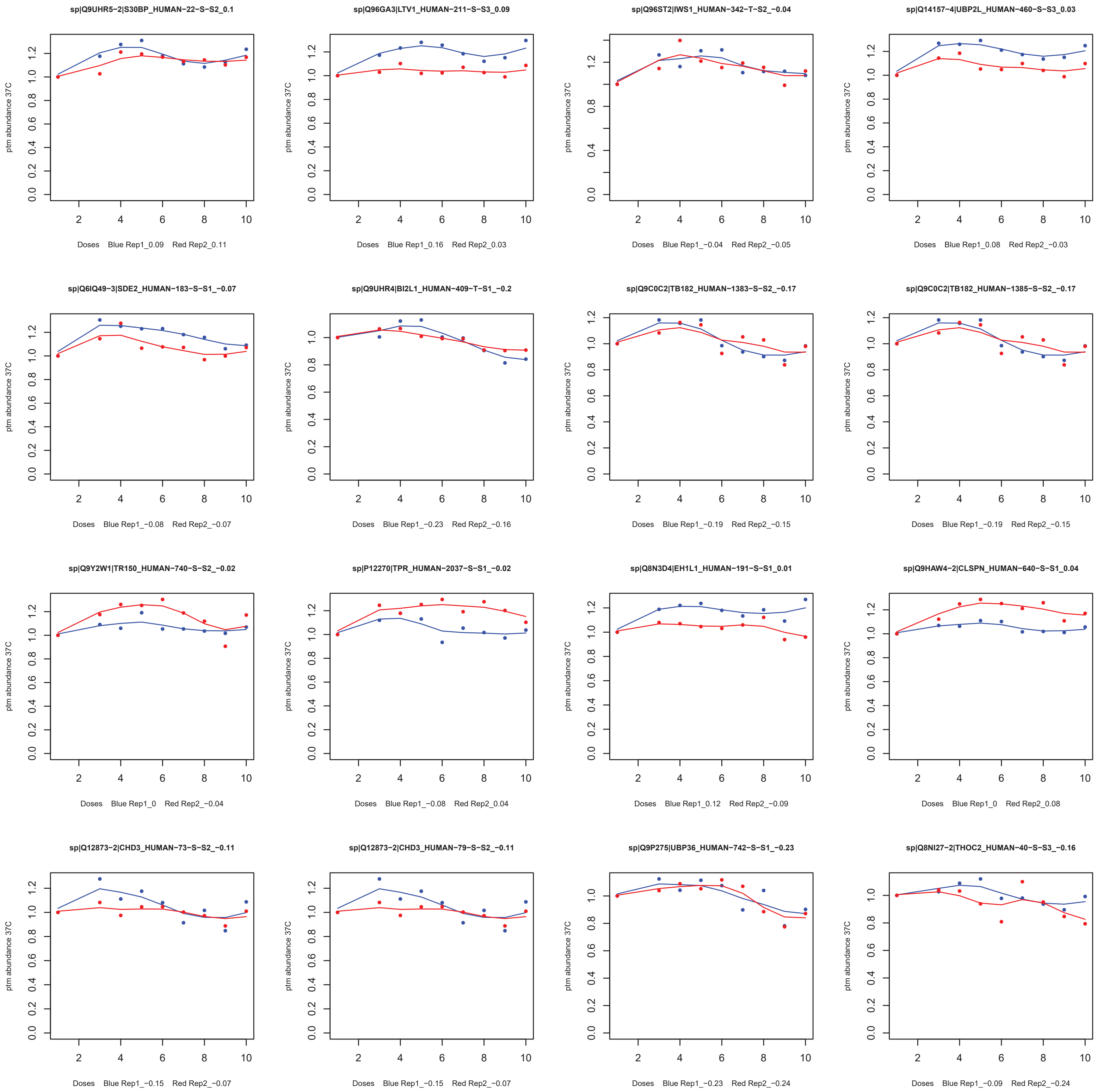
sp|Q9UHR5−2|S30BP_HUMAN−22−S−S2_0.1
Doses Blue Rep1_0.09 Red Rep2_0.11
sp|Q96GA3|LTV1_HUMAN−211−S−S3_0.09
Doses Blue Rep1_0.16 Red Rep2_0.03
sp|Q96ST2|IWS1_HUMAN−342−T−S2_−0.04
Doses Blue Rep1_−0.04 Red Rep2_−0.05
sp|Q14157−4|UBP2L_HUMAN−460−S−S3_0.03
Doses Blue Rep1_0.08 Red Rep2_−0.03
sp|Q6IQ49−3|SDE2_HUMAN−183−S−S1_−0.07
Doses Blue Rep1_−0.08 Red Rep2_−0.07
sp|Q9UHR4|BI2L1_HUMAN−409−T−S1_−0.2
Doses Blue Rep1_−0.23 Red Rep2_−0.16
sp|Q9C0C2|TB182_HUMAN−1383−S−S2_−0.17
Doses Blue Rep1_−0.19 Red Rep2_−0.15
sp|Q9C0C2|TB182_HUMAN−1385−S−S2_−0.17
Doses Blue Rep1_−0.19 Red Rep2_−0.15
sp|Q9Y2W1|TR150_HUMAN−740−S−S2_−0.02
Doses Blue Rep1_0 Red Rep2_−0.04
sp|P12270|TPR_HUMAN−2037−S−S1_−0.02
Doses Blue Rep1_−0.08 Red Rep2_0.04
sp|Q8N3D4|EH1L1_HUMAN−191−S−S1_0.01
Doses Blue Rep1_0.12 Red Rep2_−0.09
sp|Q9HAW4−2|CLSPN_HUMAN−640−S−S1_0.04
Doses Blue Rep1_0 Red Rep2_0.08
sp|Q12873−2|CHD3_HUMAN−73−S−S2_−0.11
Doses Blue Rep1_−0.15 Red Rep2_−0.07
sp|Q12873−2|CHD3_HUMAN−79−S−S2_−0.11
Doses Blue Rep1_−0.15 Red Rep2_−0.07
sp|Q9P275|UBP36_HUMAN−742−S−S1_−0.23
Doses Blue Rep1_−0.23 Red Rep2_−0.24
sp|Q8NI27−2|THOC2_HUMAN−40−S−S3_−0.16
Doses Blue Rep1_−0.09 Red Rep2_−0.24
ptm abundance 37C

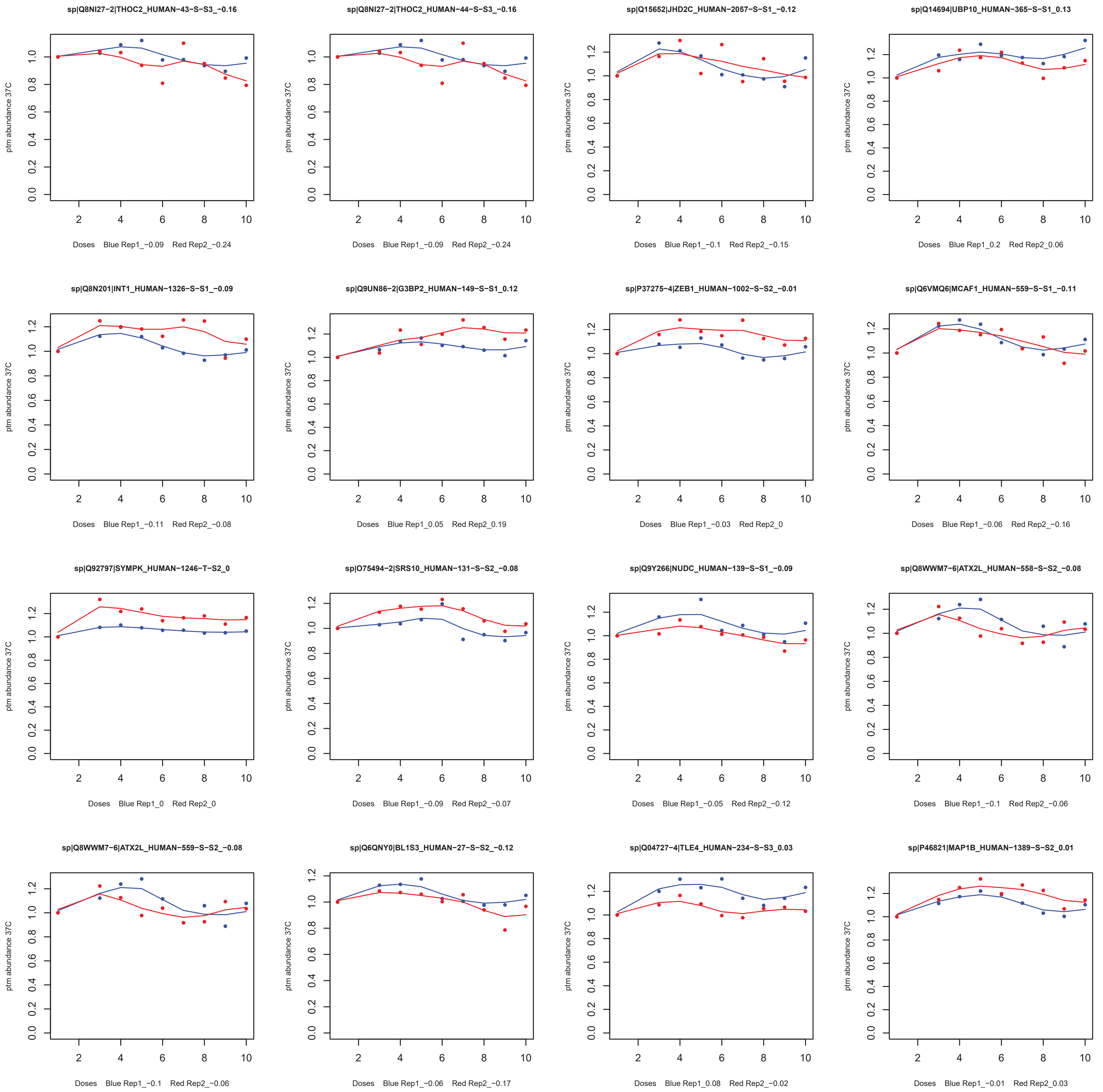
sp|Q8NI27−2|THOC2_HUMAN−43−S−S3_−0.16
Doses Blue Rep1_−0.09 Red Rep2_−0.24
sp|Q8NI27−2|THOC2_HUMAN−44−S−S3_−0.16
Doses Blue Rep1_−0.09 Red Rep2_−0.24
sp|Q15652|JHD2C_HUMAN−2057−S−S1_−0.12
Doses Blue Rep1_−0.1 Red Rep2_−0.15
sp|Q14694|UBP10_HUMAN−365−S−S1_0.13
Doses Blue Rep1_0.2 Red Rep2_0.06
sp|Q8N201|INT1_HUMAN−1326−S−S1_−0.09
Doses Blue Rep1_−0.11 Red Rep2_−0.08
sp|Q9UN86−2|G3BP2_HUMAN−149−S−S1_0.12
Doses Blue Rep1_0.05 Red Rep2_0.19
sp|P37275−4|ZEB1_HUMAN−1002−S−S2_−0.01
Doses Blue Rep1_−0.03 Red Rep2_0
sp|Q6VMQ6|MCAF1_HUMAN−559−S−S1_−0.11
Doses Blue Rep1_−0.06 Red Rep2_−0.16
sp|Q92797|SYMPK_HUMAN−1246−T−S2_0
Doses Blue Rep1_0 Red Rep2_0
sp|O75494−2|SRS10_HUMAN−131−S−S2_−0.08
Doses Blue Rep1_−0.09 Red Rep2_−0.07
sp|Q9Y266|NUDC_HUMAN−139−S−S1_−0.09
Doses Blue Rep1_−0.05 Red Rep2_−0.12
sp|Q8WWM7−6|ATX2L_HUMAN−558−S−S2_−0.08
Doses Blue Rep1_−0.1 Red Rep2_−0.06
sp|Q8WWM7−6|ATX2L_HUMAN−559−S−S2_−0.08
Doses Blue Rep1_−0.1 Red Rep2_−0.06
sp|Q6QNY0|BL1S3_HUMAN−27−S−S2_−0.12
Doses Blue Rep1_−0.06 Red Rep2_−0.17
sp|Q04727−4|TLE4_HUMAN−234−S−S3_0.03
Doses Blue Rep1_0.08 Red Rep2_−0.02
sp|P46821|MAP1B_HUMAN−1389−S−S2_0.01
Doses Blue Rep1_−0.01 Red Rep2_0.03
ptm abundance 37C

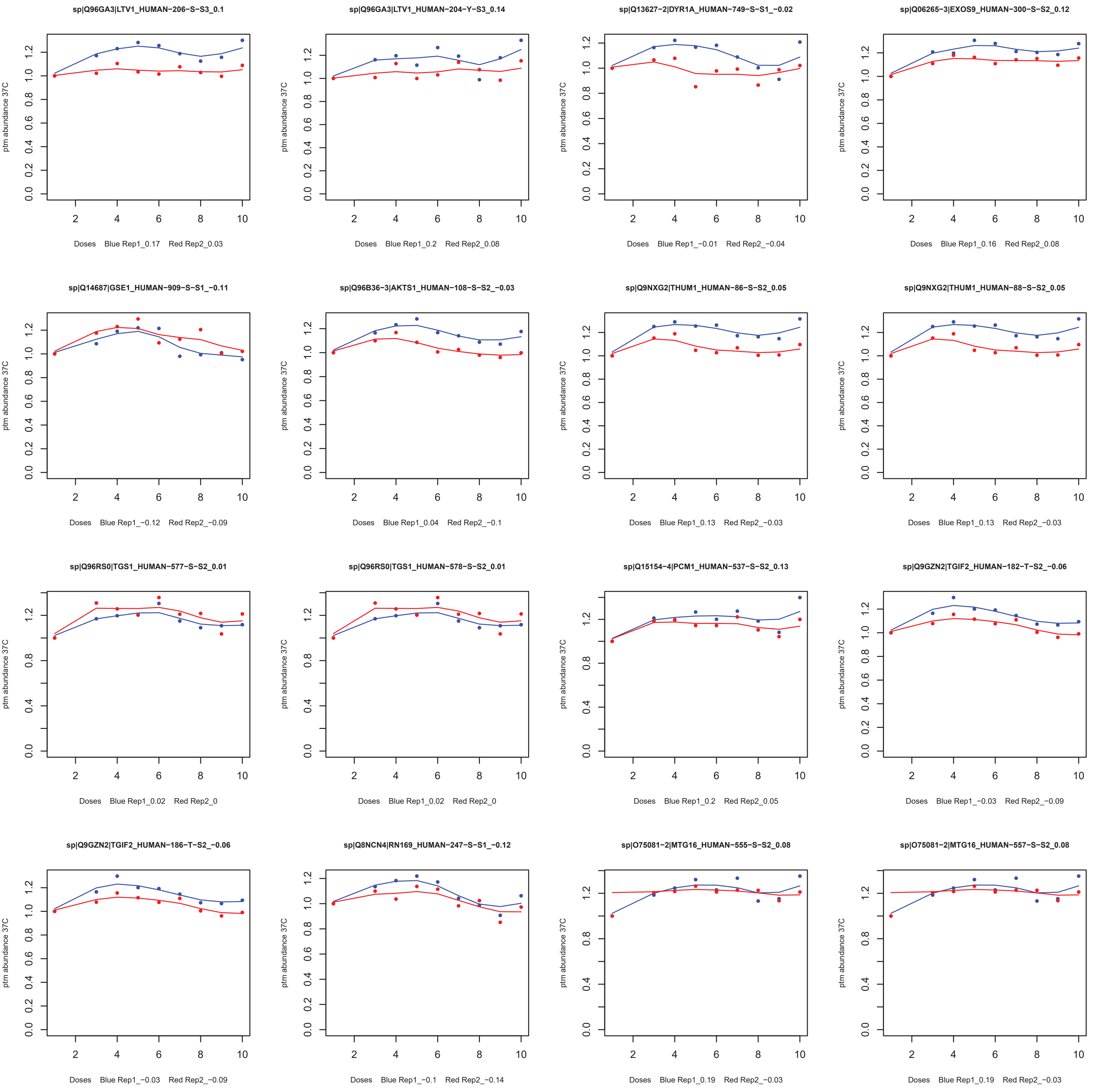
sp|Q96GA3|LTV1_HUMAN−206−S−S3_0.1
Doses Blue Rep1_0.17 Red Rep2_0.03
sp|Q96GA3|LTV1_HUMAN−204−Y−S3_0.14
Doses Blue Rep1_0.2 Red Rep2_0.08
sp|Q13627−2|DYR1A_HUMAN−749−S−S1_−0.02
Doses Blue Rep1_−0.01 Red Rep2_−0.04
sp|Q06265−3|EXOS9_HUMAN−300−S−S2_0.12
Doses Blue Rep1_0.16 Red Rep2_0.08
sp|Q14687|GSE1_HUMAN−909−S−S1_−0.11
Doses Blue Rep1_−0.12 Red Rep2_−0.09
sp|Q96B36−3|AKTS1_HUMAN−108−S−S2_−0.03
Doses Blue Rep1_0.04 Red Rep2_−0.1
sp|Q9NXG2|THUM1_HUMAN−86−S−S2_0.05
Doses Blue Rep1_0.13 Red Rep2_−0.03
sp|Q9NXG2|THUM1_HUMAN−88−S−S2_0.05
Doses Blue Rep1_0.13 Red Rep2_−0.03
sp|Q96RS0|TGS1_HUMAN−577−S−S2_0.01
Doses Blue Rep1_0.02 Red Rep2_0
sp|Q96RS0|TGS1_HUMAN−578−S−S2_0.01
Doses Blue Rep1_0.02 Red Rep2_0
sp|Q15154−4|PCM1_HUMAN−537−S−S2_0.13
Doses Blue Rep1_0.2 Red Rep2_0.05
sp|Q9GZN2|TGIF2_HUMAN−182−T−S2_−0.06
Doses Blue Rep1_−0.03 Red Rep2_−0.09
sp|Q9GZN2|TGIF2_HUMAN−186−T−S2_−0.06
Doses Blue Rep1_−0.03 Red Rep2_−0.09
sp|Q8NCN4|RN169_HUMAN−247−S−S1_−0.12
Doses Blue Rep1_−0.1 Red Rep2_−0.14
sp|O75081−2|MTG16_HUMAN−555−S−S2_0.08
Doses Blue Rep1_0.19 Red Rep2_−0.03
sp|O75081−2|MTG16_HUMAN−557−S−S2_0.08
Doses Blue Rep1_0.19 Red Rep2_−0.03
ptm abundance 37C

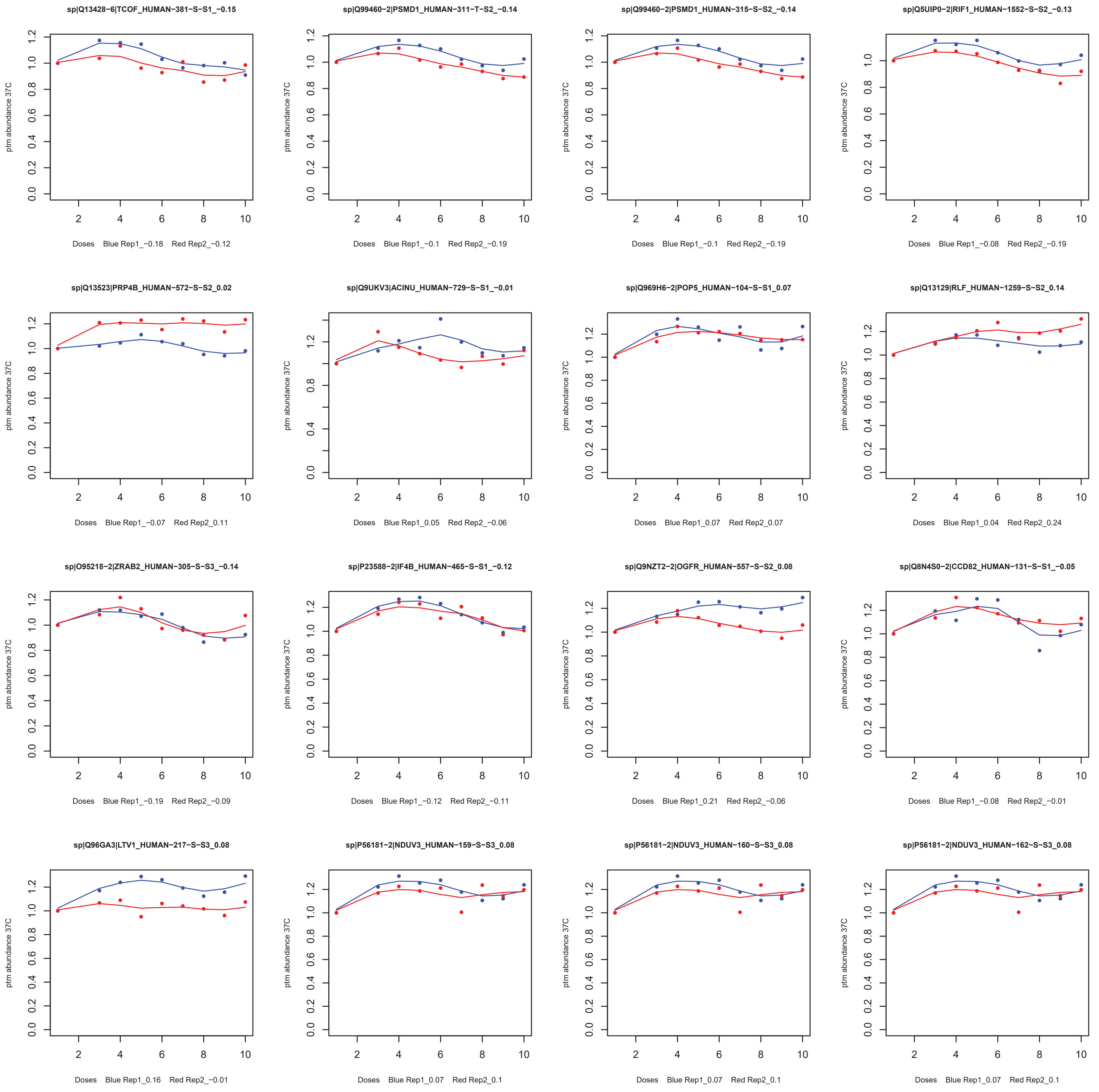
sp|Q13428−6|TCOF_HUMAN−381−S−S1_−0.15
ptm abundance 37C
Doses Blue Rep1_−0.18 Red Rep2_−0.12
sp|Q99460−2|PSMD1_HUMAN−311−T−S2_−0.14
ptm abundance 37C
Doses Blue Rep1_−0.1 Red Rep2_−0.19
sp|Q99460−2|PSMD1_HUMAN−315−S−S2_−0.14
ptm abundance 37C
Doses Blue Rep1_−0.1 Red Rep2_−0.19
sp|Q5UIP0−2|RIF1_HUMAN−1552−S−S2_−0.13
ptm abundance 37C
Doses Blue Rep1_−0.08 Red Rep2_−0.19
sp|Q13523|PRP4B_HUMAN−572−S−S2_0.02
ptm abundance 37C
Doses Blue Rep1_−0.07 Red Rep2_0.11
sp|Q9UKV3|ACINU_HUMAN−729−S−S1_−0.01
ptm abundance 37C
Doses Blue Rep1_0.05 Red Rep2_−0.06
sp|Q969H6−2|POP5_HUMAN−104−S−S1_0.07
ptm abundance 37C
Doses Blue Rep1_0.07 Red Rep2_0.07
sp|Q13129|RLF_HUMAN−1259−S−S2_0.14
ptm abundance 37C
Doses Blue Rep1_0.04 Red Rep2_0.24
sp|O95218−2|ZRAB2_HUMAN−305−S−S3_−0.14
ptm abundance 37C
Doses Blue Rep1_−0.19 Red Rep2_−0.09
sp|P23588−2|IF4B_HUMAN−465−S−S1_−0.12
ptm abundance 37C
Doses Blue Rep1_−0.12 Red Rep2_−0.11
sp|Q9NZT2−2|OGFR_HUMAN−557−S−S2_0.08
ptm abundance 37C
Doses Blue Rep1_0.21 Red Rep2_−0.06
sp|Q8N4S0−2|CCD82_HUMAN−131−S−S1_−0.05
ptm abundance 37C
Doses Blue Rep1_−0.08 Red Rep2_−0.01
sp|Q96GA3|LTV1_HUMAN−217−S−S3_0.08
ptm abundance 37C
Doses Blue Rep1_0.16 Red Rep2_−0.01
sp|P56181−2|NDUV3_HUMAN−159−S−S3_0.08
ptm abundance 37C
Doses Blue Rep1_0.07 Red Rep2_0.1
sp|P56181−2|NDUV3_HUMAN−160−S−S3_0.08
ptm abundance 37C
Doses Blue Rep1_0.07 Red Rep2_0.1
sp|P56181−2|NDUV3_HUMAN−162−S−S3_0.08
ptm abundance 37C
Doses Blue Rep1_0.07 Red Rep2_0.1

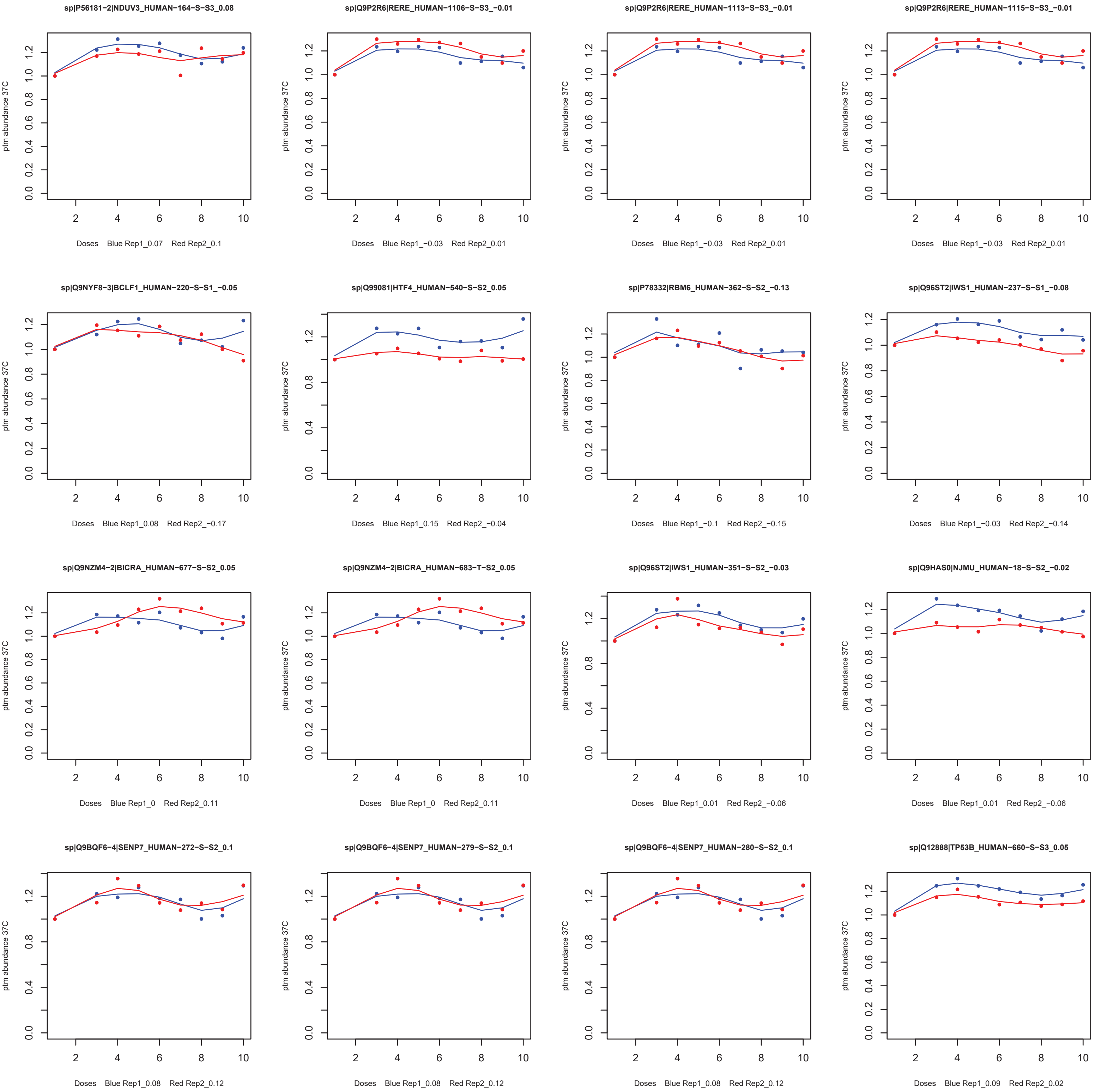
sp|P56181−2|NDUV3_HUMAN−164−S−S3_0.08
Doses Blue Rep1_0.07 Red Rep2_0.1
sp|Q9P2R6|RERE_HUMAN−1106−S−S3_−0.01
Doses Blue Rep1_−0.03 Red Rep2_0.01
sp|Q9P2R6|RERE_HUMAN−1113−S−S3_−0.01
Doses Blue Rep1_−0.03 Red Rep2_0.01
sp|Q9P2R6|RERE_HUMAN−1115−S−S3_−0.01
Doses Blue Rep1_−0.03 Red Rep2_0.01
sp|Q9NYF8−3|BCLF1_HUMAN−220−S−S1_−0.05
Doses Blue Rep1_0.08 Red Rep2_−0.17
sp|Q99081|HTF4_HUMAN−540−S−S2_0.05
Doses Blue Rep1_0.15 Red Rep2_−0.04
sp|P78332|RBM6_HUMAN−362−S−S2_−0.13
Doses Blue Rep1_−0.1 Red Rep2_−0.15
sp|Q96ST2|IWS1_HUMAN−237−S−S1_−0.08
Doses Blue Rep1_−0.03 Red Rep2_−0.14
sp|Q9NZM4−2|BICRA_HUMAN−677−S−S2_0.05
Doses Blue Rep1_0 Red Rep2_0.11
sp|Q9NZM4−2|BICRA_HUMAN−683−T−S2_0.05
Doses Blue Rep1_0 Red Rep2_0.11
sp|Q96ST2|IWS1_HUMAN−351−S−S2_−0.03
Doses Blue Rep1_0.01 Red Rep2_−0.06
sp|Q9HAS0|NJMU_HUMAN−18−S−S2_−0.02
Doses Blue Rep1_0.01 Red Rep2_−0.06
sp|Q9BQF6−4|SENP7_HUMAN−272−S−S2_0.1
Doses Blue Rep1_0.08 Red Rep2_0.12
sp|Q9BQF6−4|SENP7_HUMAN−279−S−S2_0.1
Doses Blue Rep1_0.08 Red Rep2_0.12
sp|Q9BQF6−4|SENP7_HUMAN−280−S−S2_0.1
Doses Blue Rep1_0.08 Red Rep2_0.12
sp|Q12888|TP53B_HUMAN−660−S−S3_0.05
Doses Blue Rep1_0.09 Red Rep2_0.02
ptm abundance 37C

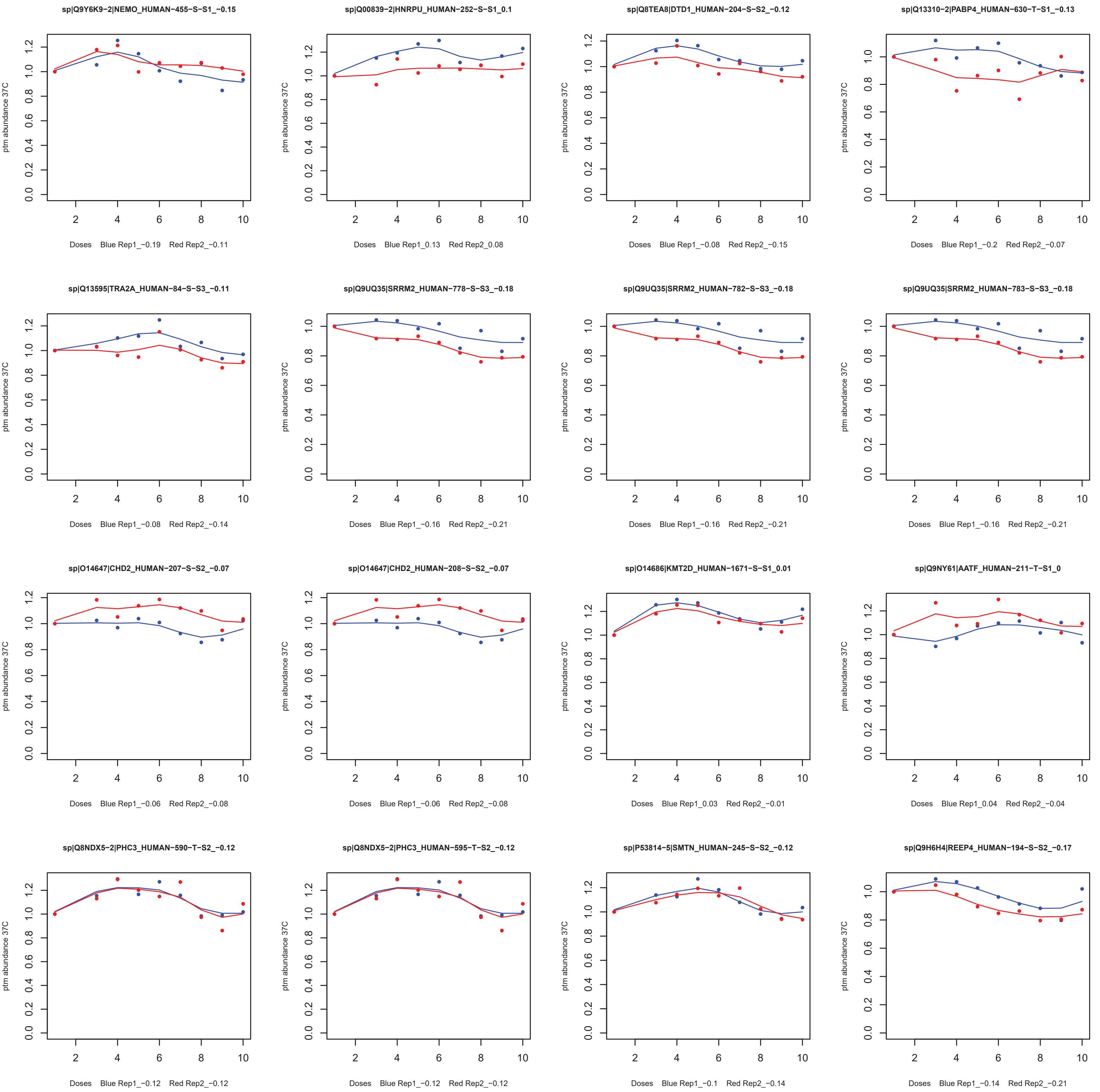
sp|Q9Y6K9−2|NEMO_HUMAN−455−S−S1_−0.15
Doses Blue Rep1_−0.19 Red Rep2_−0.11
sp|Q00839−2|HNRPU_HUMAN−252−S−S1_0.1
Doses Blue Rep1_0.13 Red Rep2_0.08
sp|Q8TEA8|DTD1_HUMAN−204−S−S2_−0.12
Doses Blue Rep1_−0.08 Red Rep2_−0.15
sp|Q13310−2|PABP4_HUMAN−630−T−S1_−0.13
Doses Blue Rep1_−0.2 Red Rep2_−0.07
sp|Q13595|TRA2A_HUMAN−84−S−S3_−0.11
Doses Blue Rep1_−0.08 Red Rep2_−0.14
sp|Q9UQ35|SRRM2_HUMAN−778−S−S3_−0.18
Doses Blue Rep1_−0.16 Red Rep2_−0.21
sp|Q9UQ35|SRRM2_HUMAN−782−S−S3_−0.18
Doses Blue Rep1_−0.16 Red Rep2_−0.21
sp|Q9UQ35|SRRM2_HUMAN−783−S−S3_−0.18
Doses Blue Rep1_−0.16 Red Rep2_−0.21
sp|O14647|CHD2_HUMAN−207−S−S2_−0.07
Doses Blue Rep1_−0.06 Red Rep2_−0.08
sp|O14647|CHD2_HUMAN−208−S−S2_−0.07
Doses Blue Rep1_−0.06 Red Rep2_−0.08
sp|O14686|KMT2D_HUMAN−1671−S−S1_0.01
Doses Blue Rep1_0.03 Red Rep2_−0.01
sp|Q9NY61|AATF_HUMAN−211−T−S1_0
Doses Blue Rep1_0.04 Red Rep2_−0.04
sp|Q8NDX5−2|PHC3_HUMAN−590−T−S2_−0.12
Doses Blue Rep1_−0.12 Red Rep2_−0.12
sp|Q8NDX5−2|PHC3_HUMAN−595−T−S2_−0.12
Doses Blue Rep1_−0.12 Red Rep2_−0.12
sp|P53814−5|SMTN_HUMAN−245−S−S2_−0.12
Doses Blue Rep1_−0.1 Red Rep2_−0.14
sp|Q9H6H4|REEP4_HUMAN−194−S−S2_−0.17
Doses Blue Rep1_−0.14 Red Rep2_−0.21
ptm abundance 37C

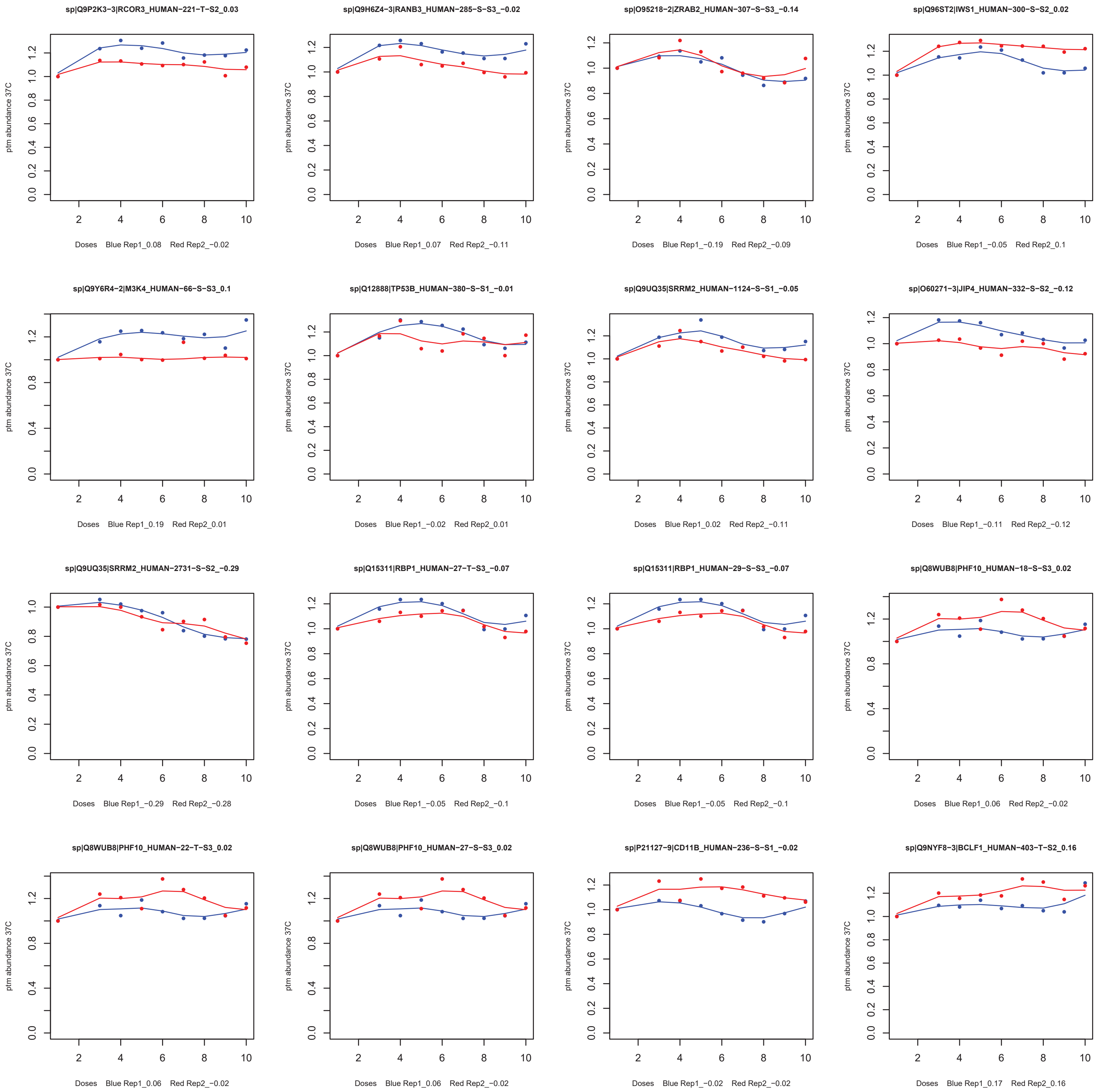
sp|Q9P2K3−3|RCOR3_HUMAN−221−T−S2_0.03
Doses Blue Rep1_0.08 Red Rep2_−0.02
sp|Q9H6Z4−3|RANB3_HUMAN−285−S−S3_−0.02
Doses Blue Rep1_0.07 Red Rep2_−0.11
sp|O95218−2|ZRAB2_HUMAN−307−S−S3_−0.14
Doses Blue Rep1_−0.19 Red Rep2_−0.09
sp|Q96ST2|IWS1_HUMAN−300−S−S2_0.02
Doses Blue Rep1_−0.05 Red Rep2_0.1
sp|Q9Y6R4−2|M3K4_HUMAN−66−S−S3_0.1
Doses Blue Rep1_0.19 Red Rep2_0.01
sp|Q12888|TP53B_HUMAN−380−S−S1_−0.01
Doses Blue Rep1_−0.02 Red Rep2_0.01
sp|Q9UQ35|SRRM2_HUMAN−1124−S−S1_−0.05
Doses Blue Rep1_0.02 Red Rep2_−0.11
sp|O60271−3|JIP4_HUMAN−332−S−S2_−0.12
Doses Blue Rep1_−0.11 Red Rep2_−0.12
sp|Q9UQ35|SRRM2_HUMAN−2731−S−S2_−0.29
Doses Blue Rep1_−0.29 Red Rep2_−0.28
sp|Q15311|RBP1_HUMAN−27−T−S3_−0.07
Doses Blue Rep1_−0.05 Red Rep2_−0.1
sp|Q15311|RBP1_HUMAN−29−S−S3_−0.07
Doses Blue Rep1_−0.05 Red Rep2_−0.1
sp|Q8WUB8|PHF10_HUMAN−18−S−S3_0.02
Doses Blue Rep1_0.06 Red Rep2_−0.02
sp|Q8WUB8|PHF10_HUMAN−22−T−S3_0.02
Doses Blue Rep1_0.06 Red Rep2_−0.02
sp|Q8WUB8|PHF10_HUMAN−27−S−S3_0.02
Doses Blue Rep1_0.06 Red Rep2_−0.02
sp|P21127−9|CD11B_HUMAN−236−S−S1_−0.02
Doses Blue Rep1_−0.02 Red Rep2_−0.02
sp|Q9NYF8−3|BCLF1_HUMAN−403−T−S2_0.16
Doses Blue Rep1_0.17 Red Rep2_0.16
ptm abundance 37C

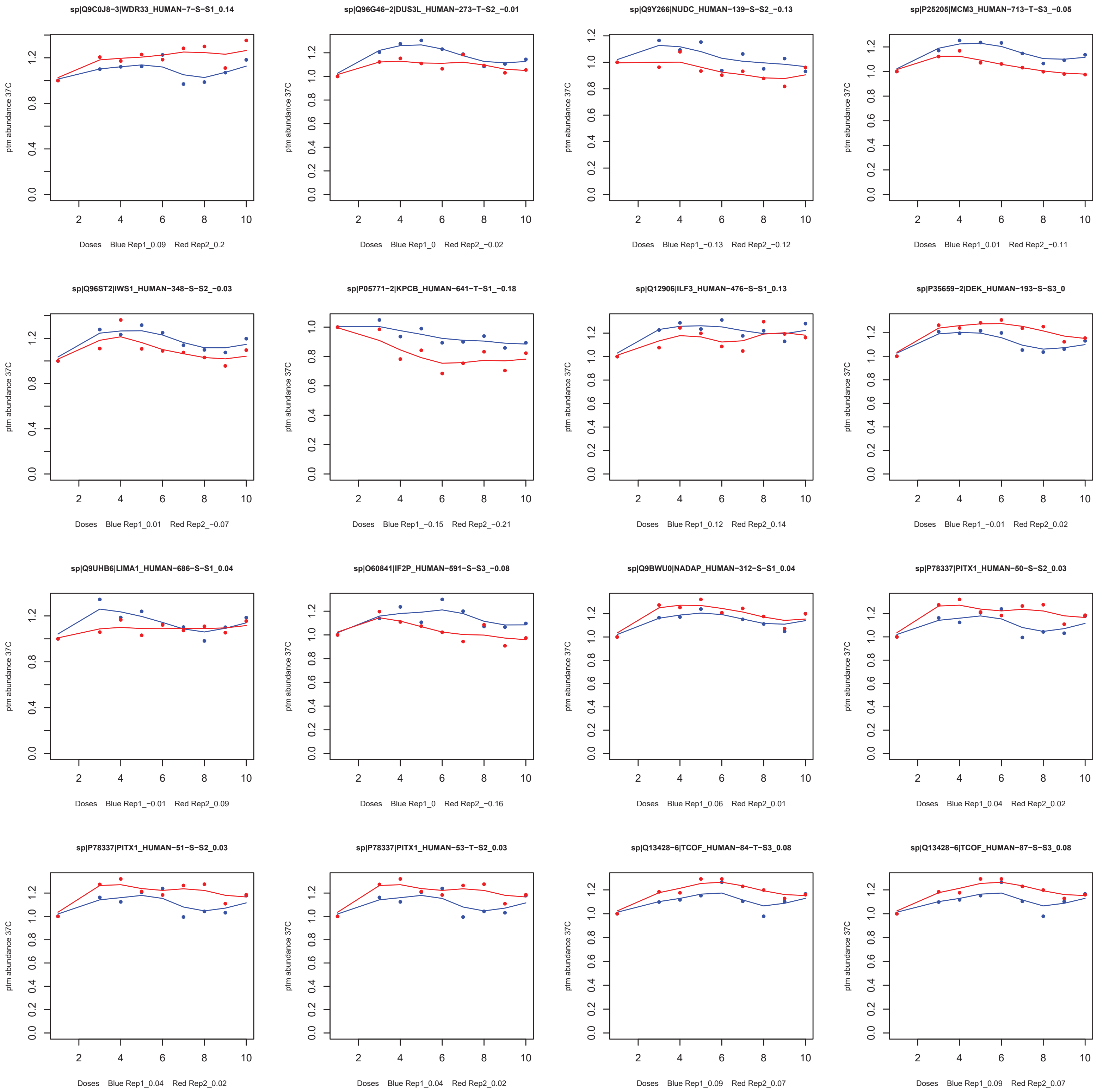
sp|Q9C0J8−3|WDR33_HUMAN−7−S−S1_0.14
Doses Blue Rep1_0.09 Red Rep2_0.2
sp|Q96G46−2|DUS3L_HUMAN−273−T−S2_−0.01
Doses Blue Rep1_0 Red Rep2_−0.02
sp|Q9Y266|NUDC_HUMAN−139−S−S2_−0.13
Doses Blue Rep1_−0.13 Red Rep2_−0.12
sp|P25205|MCM3_HUMAN−713−T−S3_−0.05
Doses Blue Rep1_0.01 Red Rep2_−0.11
sp|Q96ST2|IWS1_HUMAN−348−S−S2_−0.03
Doses Blue Rep1_0.01 Red Rep2_−0.07
sp|P05771−2|KPCB_HUMAN−641−T−S1_−0.18
Doses Blue Rep1_−0.15 Red Rep2_−0.21
sp|Q12906|ILF3_HUMAN−476−S−S1_0.13
Doses Blue Rep1_0.12 Red Rep2_0.14
sp|P35659−2|DEK_HUMAN−193−S−S3_0
Doses Blue Rep1_−0.01 Red Rep2_0.02
sp|Q9UHB6|LIMA1_HUMAN−686−S−S1_0.04
Doses Blue Rep1_−0.01 Red Rep2_0.09
sp|O60841|IF2P_HUMAN−591−S−S3_−0.08
Doses Blue Rep1_0 Red Rep2_−0.16
sp|Q9BWU0|NADAP_HUMAN−312−S−S1_0.04
Doses Blue Rep1_0.06 Red Rep2_0.01
sp|P78337|PITX1_HUMAN−50−S−S2_0.03
Doses Blue Rep1_0.04 Red Rep2_0.02
sp|P78337|PITX1_HUMAN−51−S−S2_0.03
Doses Blue Rep1_0.04 Red Rep2_0.02
sp|P78337|PITX1_HUMAN−53−T−S2_0.03
Doses Blue Rep1_0.04 Red Rep2_0.02
sp|Q13428−6|TCOF_HUMAN−84−T−S3_0.08
Doses Blue Rep1_0.09 Red Rep2_0.07
sp|Q13428−6|TCOF_HUMAN−87−S−S3_0.08
Doses Blue Rep1_0.09 Red Rep2_0.07
ptm abundance 37C

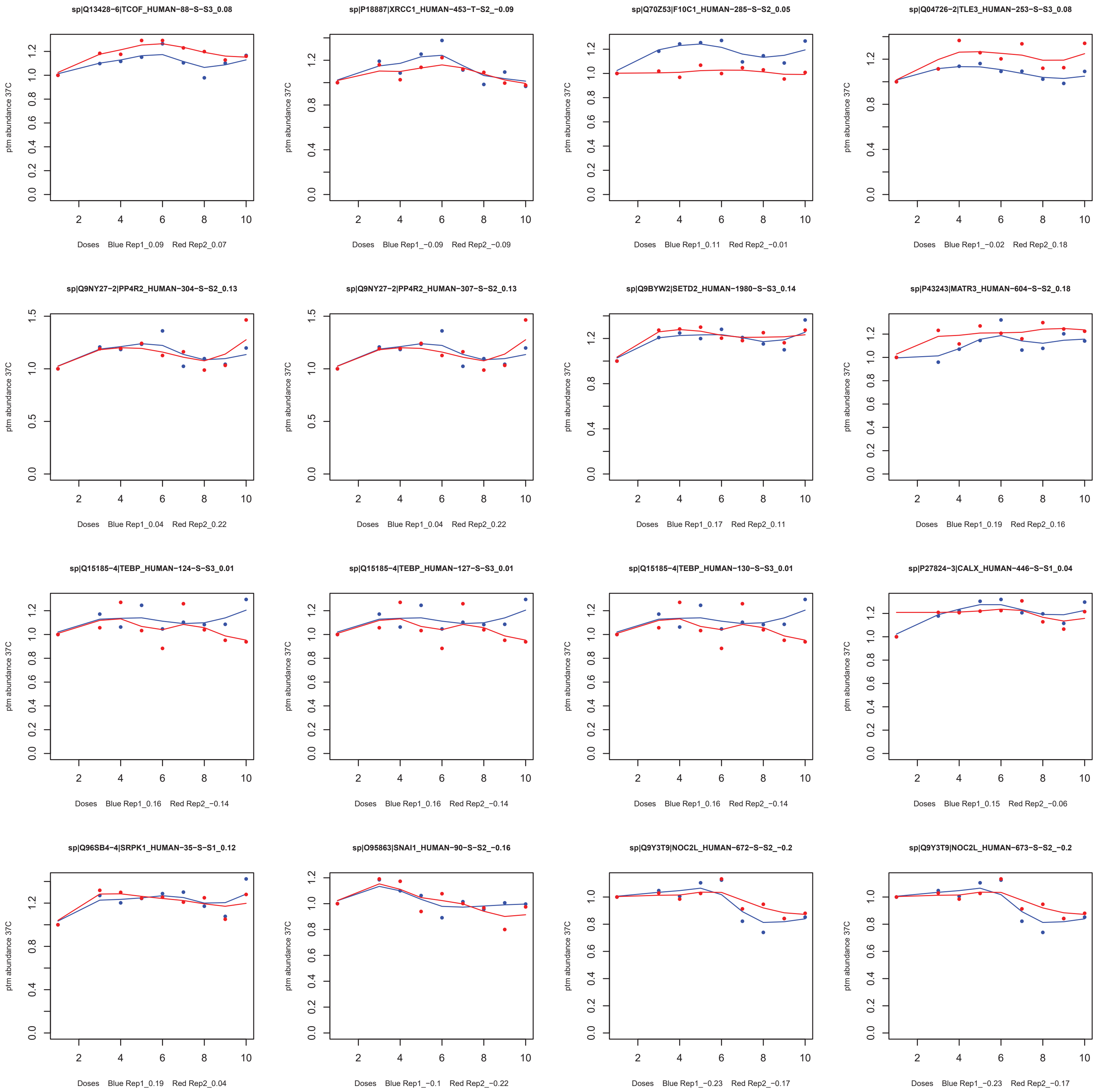
sp|Q13428−6|TCOF_HUMAN−88−S−S3_0.08
Doses Blue Rep1_0.09 Red Rep2_0.07
sp|P18887|XRCC1_HUMAN−453−T−S2_−0.09
Doses Blue Rep1_−0.09 Red Rep2_−0.09
sp|Q70Z53|F10C1_HUMAN−285−S−S2_0.05
Doses Blue Rep1_0.11 Red Rep2_−0.01
sp|Q04726−2|TLE3_HUMAN−253−S−S3_0.08
Doses Blue Rep1_−0.02 Red Rep2_0.18
sp|Q9NY27−2|PP4R2_HUMAN−304−S−S2_0.13
Doses Blue Rep1_0.04 Red Rep2_0.22
sp|Q9NY27−2|PP4R2_HUMAN−307−S−S2_0.13
Doses Blue Rep1_0.04 Red Rep2_0.22
sp|Q9BYW2|SETD2_HUMAN−1980−S−S3_0.14
Doses Blue Rep1_0.17 Red Rep2_0.11
sp|P43243|MATR3_HUMAN−604−S−S2_0.18
Doses Blue Rep1_0.19 Red Rep2_0.16
sp|Q15185−4|TEBP_HUMAN−124−S−S3_0.01
Doses Blue Rep1_0.16 Red Rep2_−0.14
sp|Q15185−4|TEBP_HUMAN−127−S−S3_0.01
Doses Blue Rep1_0.16 Red Rep2_−0.14
sp|Q15185−4|TEBP_HUMAN−130−S−S3_0.01
Doses Blue Rep1_0.16 Red Rep2_−0.14
sp|P27824−3|CALX_HUMAN−446−S−S1_0.04
Doses Blue Rep1_0.15 Red Rep2_−0.06
sp|Q96SB4−4|SRPK1_HUMAN−35−S−S1_0.12
Doses Blue Rep1_0.19 Red Rep2_0.04
sp|O95863|SNAI1_HUMAN−90−S−S2_−0.16
Doses Blue Rep1_−0.1 Red Rep2_−0.22
sp|Q9Y3T9|NOC2L_HUMAN−672−S−S2_−0.2
Doses Blue Rep1_−0.23 Red Rep2_−0.17
sp|Q9Y3T9|NOC2L_HUMAN−673−S−S2_−0.2
Doses Blue Rep1_−0.23 Red Rep2_−0.17
ptm abundance 37C

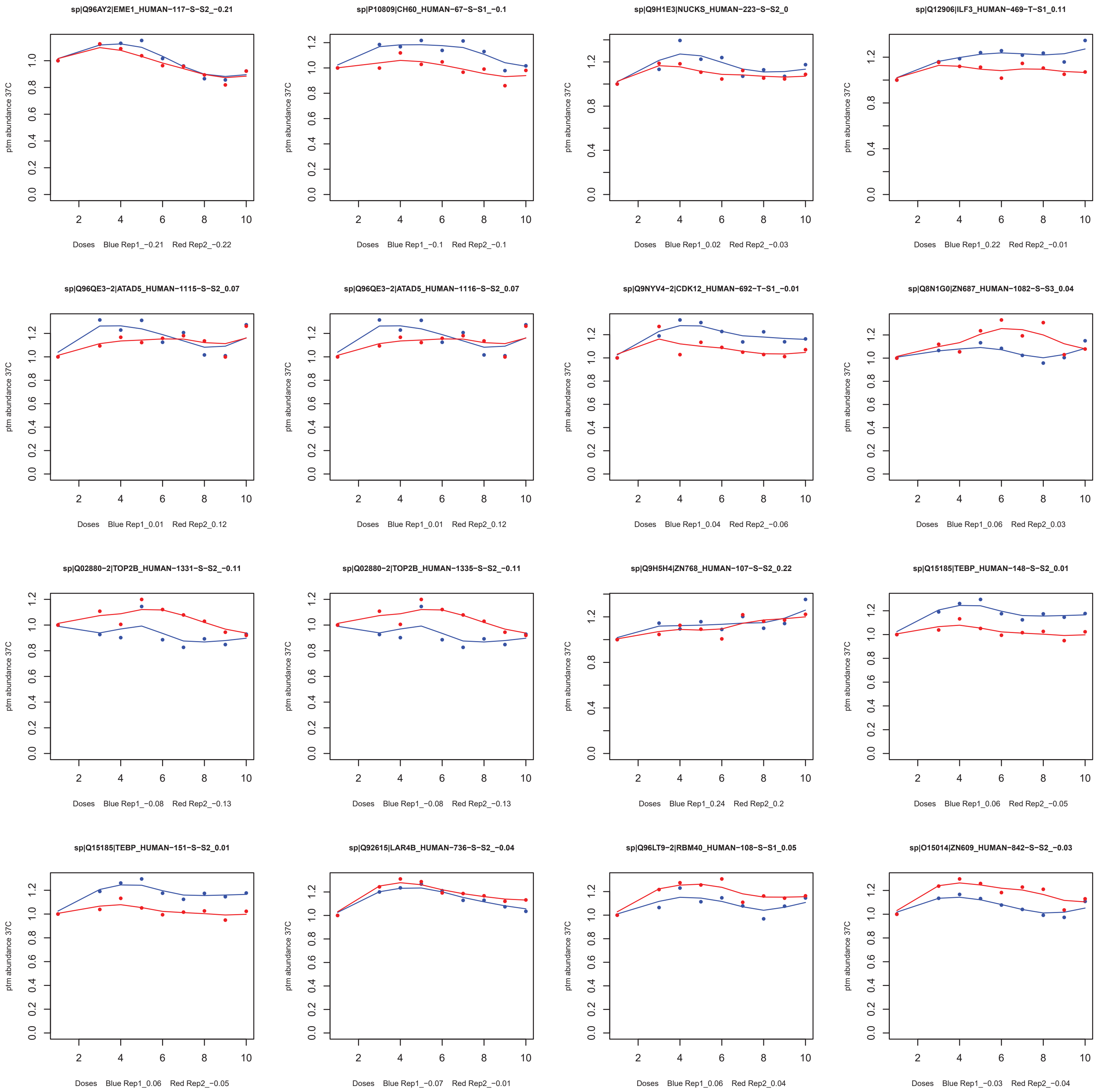

sp|Q96AY2|EME1_HUMAN−117−S−S2_−0.21
ptm abundance 37C
Doses Blue Rep1_−0.21 Red Rep2_−0.22
sp|P10809|CH60_HUMAN−67−S−S1_−0.1
ptm abundance 37C
Doses Blue Rep1_−0.1 Red Rep2_−0.1
sp|Q9H1E3|NUCKS_HUMAN−223−S−S2_0
ptm abundance 37C
Doses Blue Rep1_0.02 Red Rep2_−0.03
sp|Q12906|ILF3_HUMAN−469−T−S1_0.11
ptm abundance 37C
Doses Blue Rep1_0.22 Red Rep2_−0.01
sp|Q96QE3−2|ATAD5_HUMAN−1115−S−S2_0.07
ptm abundance 37C
Doses Blue Rep1_0.01 Red Rep2_0.12
sp|Q96QE3−2|ATAD5_HUMAN−1116−S−S2_0.07
ptm abundance 37C
Doses Blue Rep1_0.01 Red Rep2_0.12
sp|Q9NYV4−2|CDK12_HUMAN−692−T−S1_−0.01
ptm abundance 37C
Doses Blue Rep1_0.04 Red Rep2_−0.06
sp|Q8N1G0|ZN687_HUMAN−1082−S−S3_0.04
ptm abundance 37C
Doses Blue Rep1_0.06 Red Rep2_0.03
sp|Q02880−2|TOP2B_HUMAN−1331−S−S2_−0.11
ptm abundance 37C
Doses Blue Rep1_−0.08 Red Rep2_−0.13
sp|Q02880−2|TOP2B_HUMAN−1335−S−S2_−0.11
ptm abundance 37C
Doses Blue Rep1_−0.08 Red Rep2_−0.13
sp|Q9H5H4|ZN768_HUMAN−107−S−S2_0.22
ptm abundance 37C
Doses Blue Rep1_0.24 Red Rep2_0.2
sp|Q15185|TEBP_HUMAN−148−S−S2_0.01
ptm abundance 37C
Doses Blue Rep1_0.06 Red Rep2_−0.05
sp|Q15185|TEBP_HUMAN−151−S−S2_0.01
ptm abundance 37C
Doses Blue Rep1_0.06 Red Rep2_−0.05
sp|Q92615|LAR4B_HUMAN−736−S−S2_−0.04
ptm abundance 37C
Doses Blue Rep1_−0.07 Red Rep2_−0.01
sp|Q96LT9−2|RBM40_HUMAN−108−S−S1_0.05
ptm abundance 37C
Doses Blue Rep1_0.06 Red Rep2_0.04
sp|O15014|ZN609_HUMAN−842−S−S2_−0.03
ptm abundance 37C
Doses Blue Rep1_−0.03 Red Rep2_−0.04

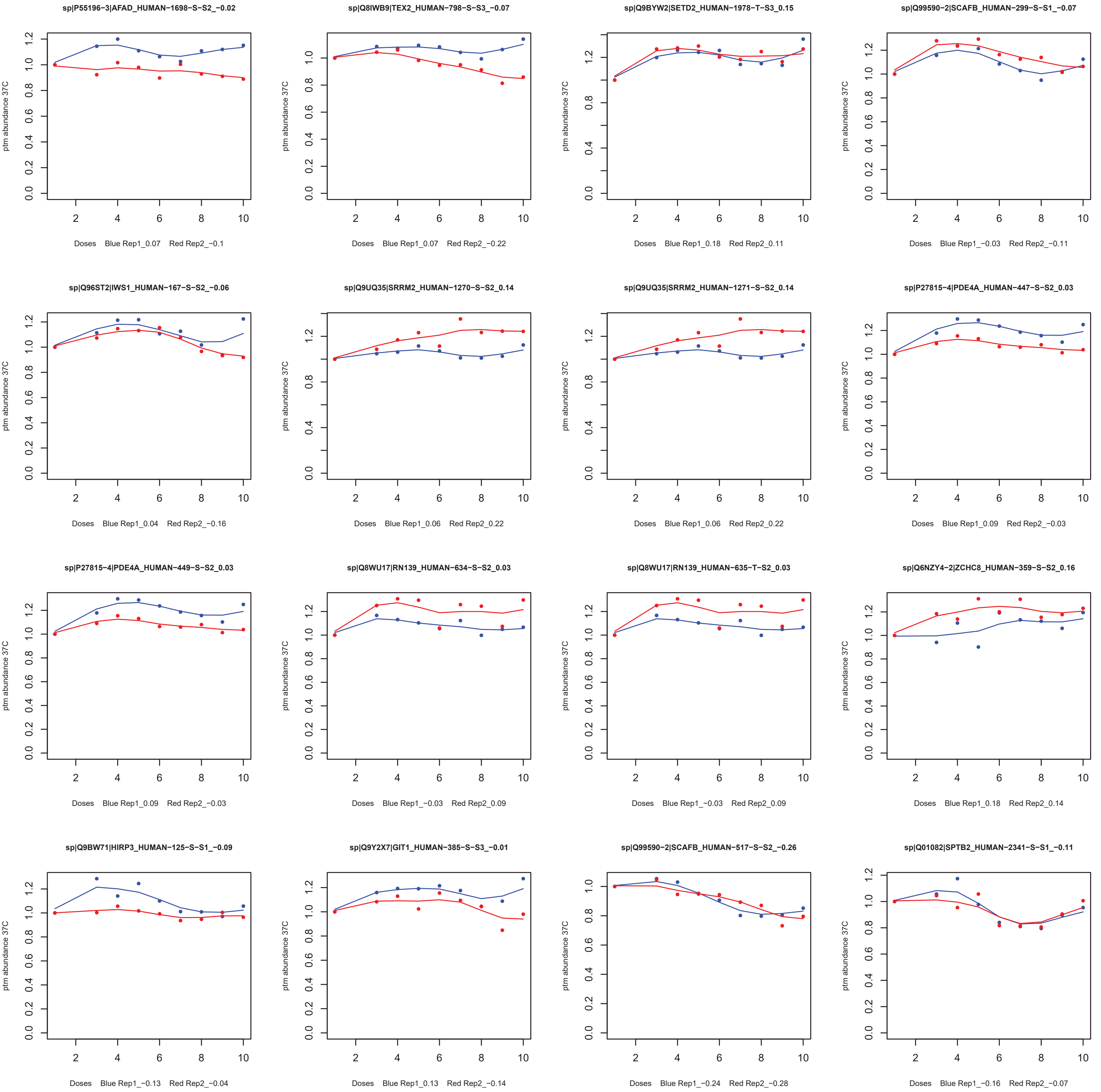
sp|P55196−3|AFAD_HUMAN−1698−S−S2_−0.02
Doses Blue Rep1_0.07 Red Rep2_−0.1
sp|Q8IWB9|TEX2_HUMAN−798−S−S3_−0.07
Doses Blue Rep1_0.07 Red Rep2_−0.22
sp|Q9BYW2|SETD2_HUMAN−1978−T−S3_0.15
Doses Blue Rep1_0.18 Red Rep2_0.11
sp|Q99590−2|SCAFB_HUMAN−299−S−S1_−0.07
Doses Blue Rep1_−0.03 Red Rep2_−0.11
sp|Q96ST2|IWS1_HUMAN−167−S−S2_−0.06
Doses Blue Rep1_0.04 Red Rep2_−0.16
sp|Q9UQ35|SRRM2_HUMAN−1270−S−S2_0.14
Doses Blue Rep1_0.06 Red Rep2_0.22
sp|Q9UQ35|SRRM2_HUMAN−1271−S−S2_0.14
Doses Blue Rep1_0.06 Red Rep2_0.22
sp|P27815−4|PDE4A_HUMAN−447−S−S2_0.03
Doses Blue Rep1_0.09 Red Rep2_−0.03
sp|P27815−4|PDE4A_HUMAN−449−S−S2_0.03
Doses Blue Rep1_0.09 Red Rep2_−0.03
sp|Q8WU17|RN139_HUMAN−634−S−S2_0.03
Doses Blue Rep1_−0.03 Red Rep2_0.09
sp|Q8WU17|RN139_HUMAN−635−T−S2_0.03
Doses Blue Rep1_−0.03 Red Rep2_0.09
sp|Q6NZY4−2|ZCHC8_HUMAN−359−S−S2_0.16
Doses Blue Rep1_0.18 Red Rep2_0.14
sp|Q9BW71|HIRP3_HUMAN−125−S−S1_−0.09
Doses Blue Rep1_−0.13 Red Rep2_−0.04
sp|Q9Y2X7|GIT1_HUMAN−385−S−S3_−0.01
Doses Blue Rep1_0.13 Red Rep2_−0.14
sp|Q99590−2|SCAFB_HUMAN−517−S−S2_−0.26
Doses Blue Rep1_−0.24 Red Rep2_−0.28
sp|Q01082|SPTB2_HUMAN−2341−S−S1_−0.11
Doses Blue Rep1_−0.16 Red Rep2_−0.07
ptm abundance 37C

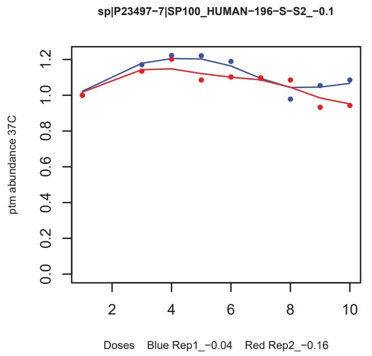
sp|P23497−7|SP100_HUMAN−196−S−S2_−0.1
ptm abundance 37C
Doses Blue Rep1_−0.04 Red Rep2_−0.16
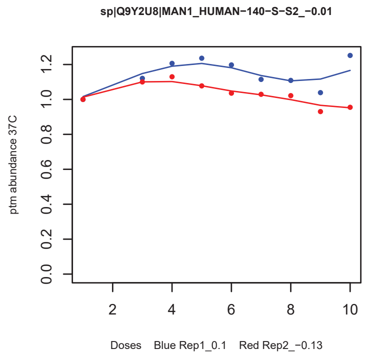
sp|Q9Y2U8|MAN1_HUMAN−140−S−S2_−0.01
ptm abundance 37C
Doses Blue Rep1_0.1 Red Rep2_−0.13
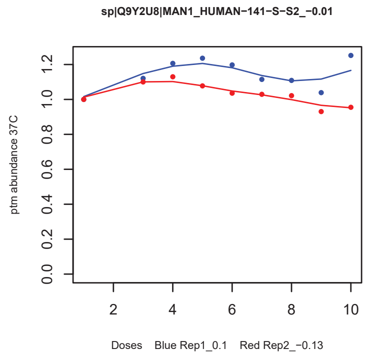
sp|Q9Y2U8|MAN1_HUMAN−141−S−S2_−0.01
ptm abundance 37C
Doses Blue Rep1_0.1 Red Rep2_−0.13
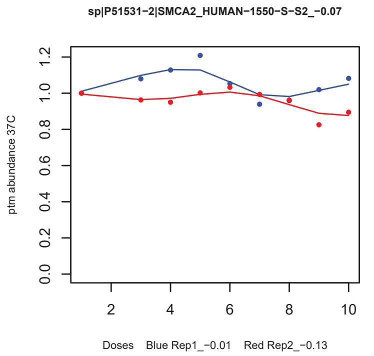
sp|P51531−2|SMCA2_HUMAN−1550−S−S2_−0.07
ptm abundance 37C
Doses Blue Rep1_−0.01 Red Rep2_−0.13
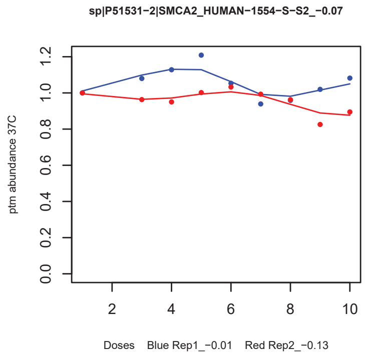
sp|P51531−2|SMCA2_HUMAN−1554−S−S2_−0.07
ptm abundance 37C
Doses Blue Rep1_−0.01 Red Rep2_−0.13
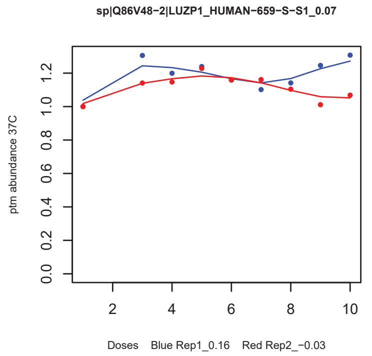
sp|Q86V48−2|LUZP1_HUMAN−659−S−S1_0.07
ptm abundance 37C
Doses Blue Rep1_0.16 Red Rep2_−0.03
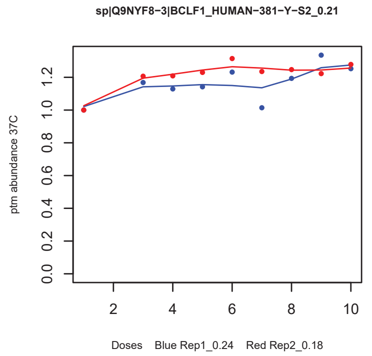
sp|Q9NYF8−3|BCLF1_HUMAN−381−Y−S2_0.21
ptm abundance 37C
Doses Blue Rep1_0.24 Red Rep2_0.18
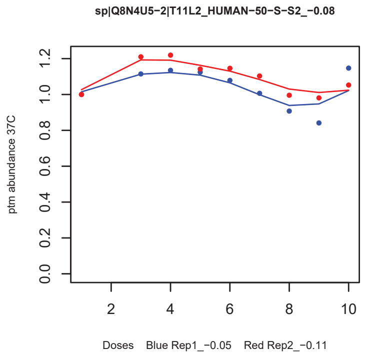
sp|Q8N4U5−2|T11L2_HUMAN−50−S−S2_−0.08
ptm abundance 37C
Doses Blue Rep1_−0.05 Red Rep2_−0.11
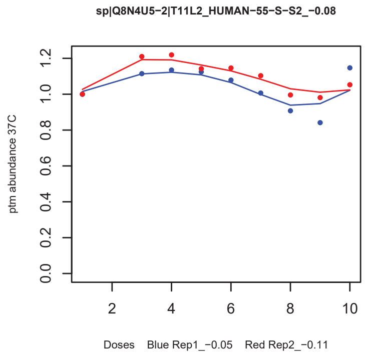
sp|Q8N4U5−2|T11L2_HUMAN−55−S−S2_−0.08
ptm abundance 37C
Doses Blue Rep1_−0.05 Red Rep2_−0.11
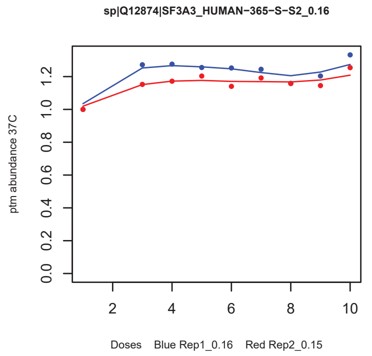
sp|Q12874|SF3A3_HUMAN−365−S−S2_0.16
ptm abundance 37C
Doses Blue Rep1_0.16 Red Rep2_0.15
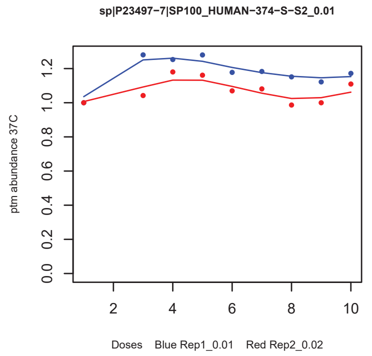
sp|P23497−7|SP100_HUMAN−374−S−S2_0.01
ptm abundance 37C
Doses Blue Rep1_0.01 Red Rep2_0.02
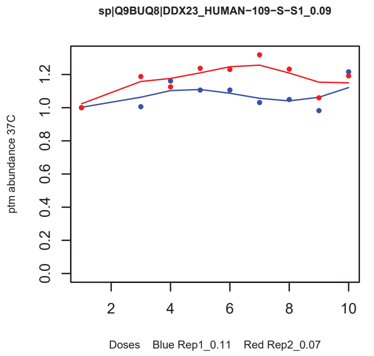
sp|Q9BUQ8|DDX23_HUMAN−109−S−S1_0.09
ptm abundance 37C
Doses Blue Rep1_0.11 Red Rep2_0.07
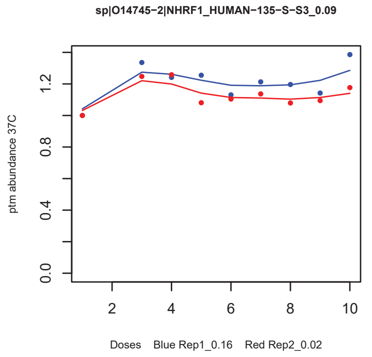
sp|O14745−2|NHRF1_HUMAN−135−S−S3_0.09
ptm abundance 37C
Doses Blue Rep1_0.16 Red Rep2_0.02
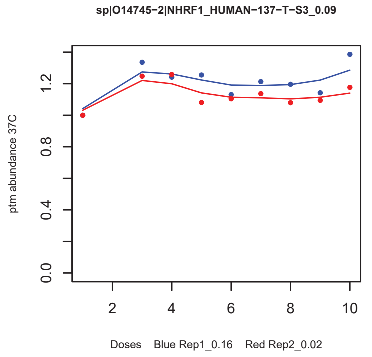
sp|O14745−2|NHRF1_HUMAN−137−T−S3_0.09
ptm abundance 37C
Doses Blue Rep1_0.16 Red Rep2_0.02
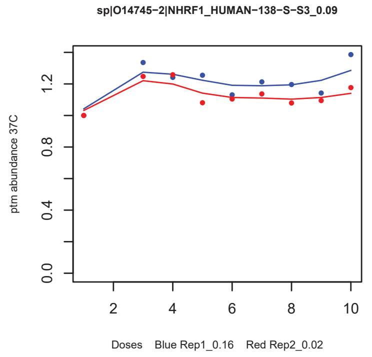
sp|O14745−2|NHRF1_HUMAN−138−S−S3_0.09
ptm abundance 37C
Doses Blue Rep1_0.16 Red Rep2_0.02
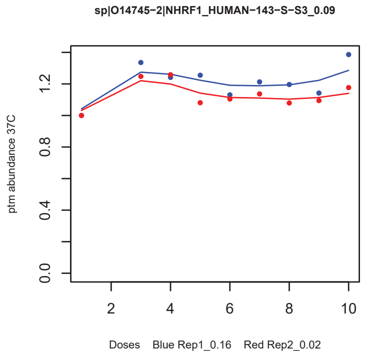
sp|O14745−2|NHRF1_HUMAN−143−S−S3_0.09
ptm abundance 37C
Doses Blue Rep1_0.16 Red Rep2_0.02

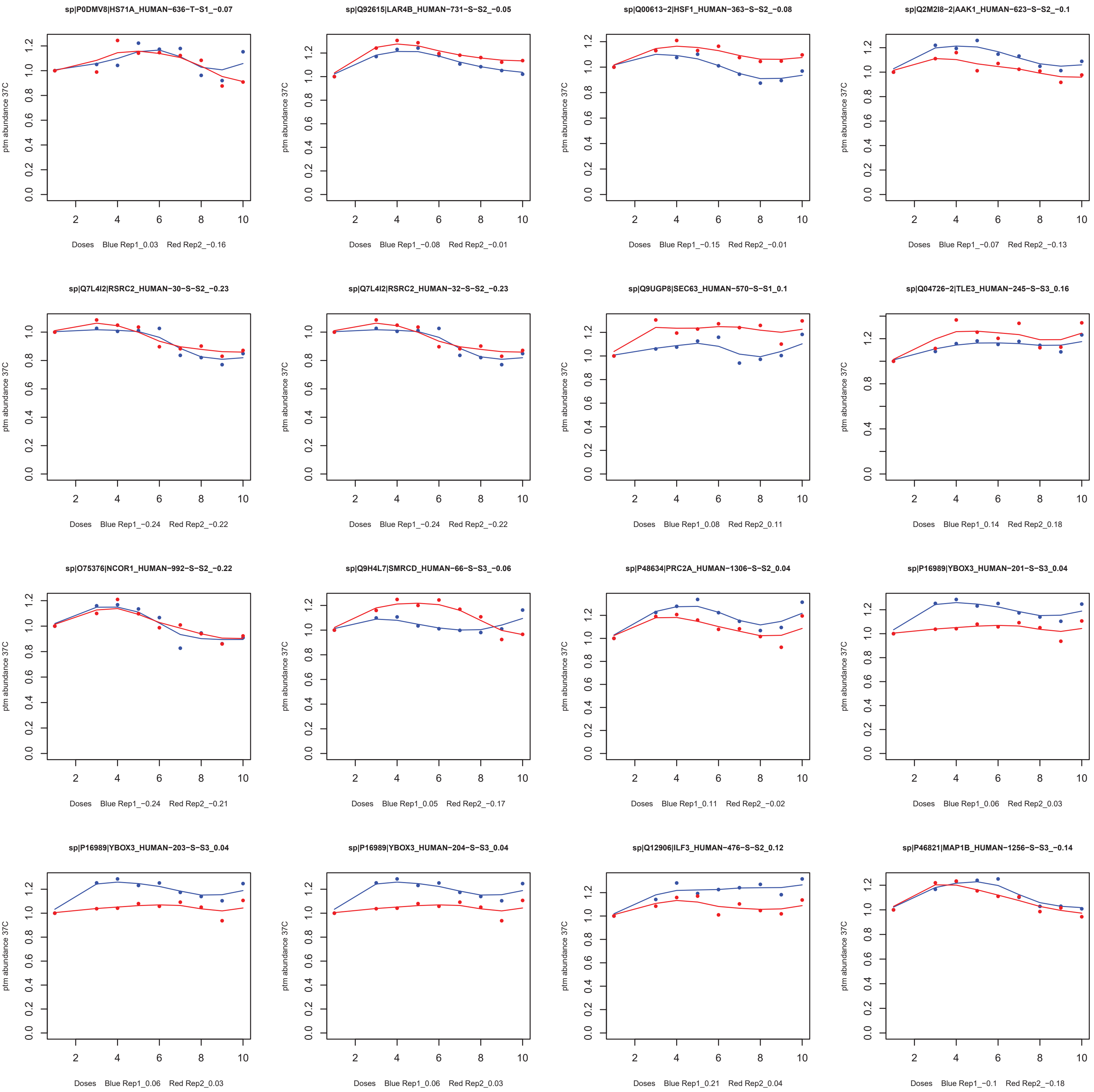
sp|P0DMV8|HS71A_HUMAN−636−T−S1_−0.07
Doses Blue Rep1_0.03 Red Rep2_−0.16
sp|Q92615|LAR4B_HUMAN−731−S−S2_−0.05
Doses Blue Rep1_−0.08 Red Rep2_−0.01
sp|Q00613−2|HSF1_HUMAN−363−S−S2_−0.08
Doses Blue Rep1_−0.15 Red Rep2_−0.01
sp|Q2M2I8−2|AAK1_HUMAN−623−S−S2_−0.1
Doses Blue Rep1_−0.07 Red Rep2_−0.13
sp|Q7L4I2|RSRC2_HUMAN−30−S−S2_−0.23
Doses Blue Rep1_−0.24 Red Rep2_−0.22
sp|Q7L4I2|RSRC2_HUMAN−32−S−S2_−0.23
Doses Blue Rep1_−0.24 Red Rep2_−0.22
sp|Q9UGP8|SEC63_HUMAN−570−S−S1_0.1
Doses Blue Rep1_0.08 Red Rep2_0.11
sp|Q04726−2|TLE3_HUMAN−245−S−S3_0.16
Doses Blue Rep1_0.14 Red Rep2_0.18
sp|O75376|NCOR1_HUMAN−992−S−S2_−0.22
Doses Blue Rep1_−0.24 Red Rep2_−0.21
sp|Q9H4L7|SMRCD_HUMAN−66−S−S3_−0.06
Doses Blue Rep1_0.05 Red Rep2_−0.17
sp|P48634|PRC2A_HUMAN−1306−S−S2_0.04
Doses Blue Rep1_0.11 Red Rep2_−0.02
sp|P16989|YBOX3_HUMAN−201−S−S3_0.04
Doses Blue Rep1_0.06 Red Rep2_0.03
sp|P16989|YBOX3_HUMAN−203−S−S3_0.04
Doses Blue Rep1_0.06 Red Rep2_0.03
sp|P16989|YBOX3_HUMAN−204−S−S3_0.04
Doses Blue Rep1_0.06 Red Rep2_0.03
sp|Q12906|ILF3_HUMAN−476−S−S2_0.12
Doses Blue Rep1_0.21 Red Rep2_0.04
sp|P46821|MAP1B_HUMAN−1256−S−S3_−0.14
Doses Blue Rep1_−0.1 Red Rep2_−0.18
ptm abundance 37C

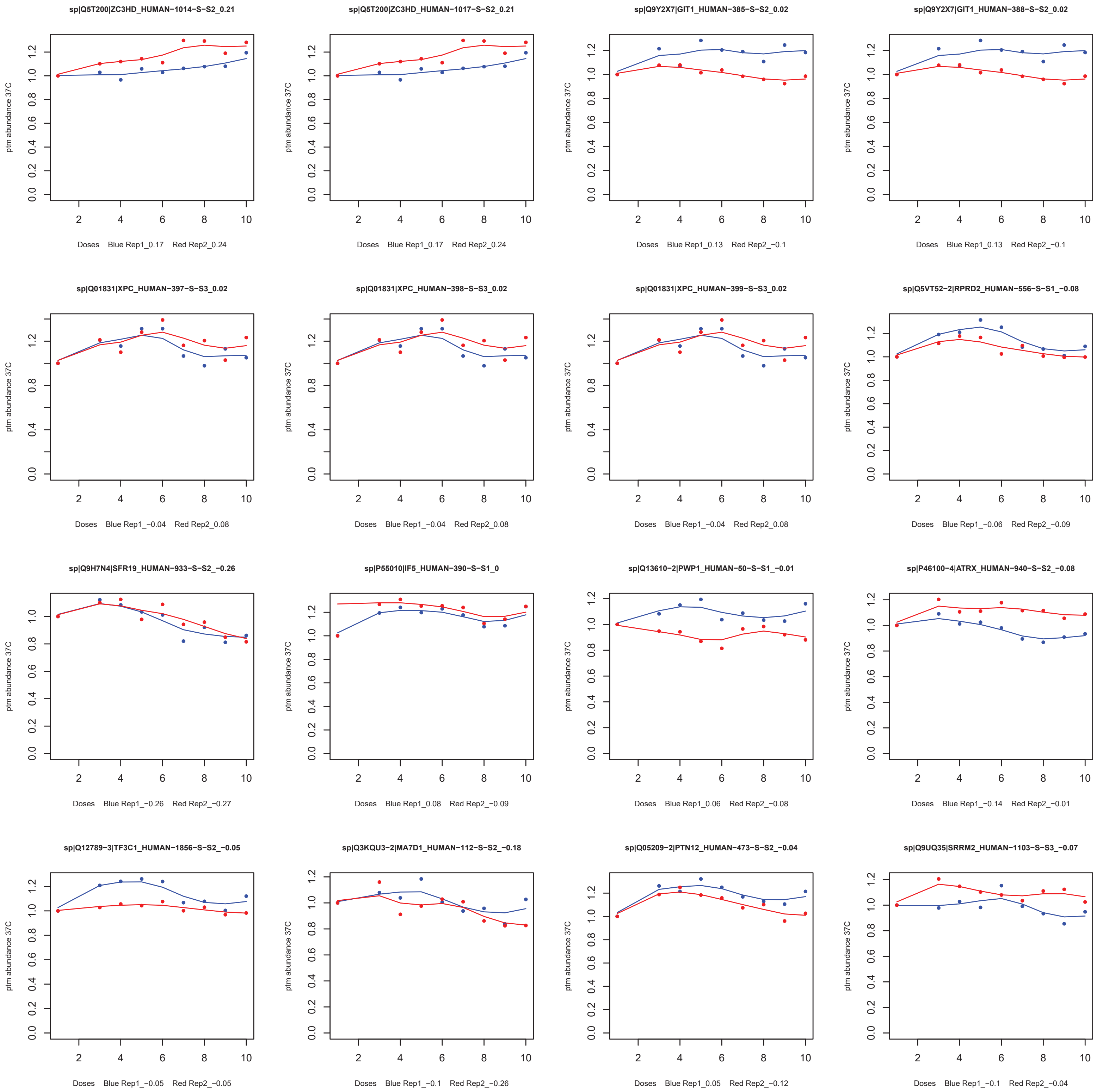
sp|Q5T200|ZC3HD_HUMAN−1014−S−S2_0.21
ptm abundance 37C
Doses Blue Rep1_0.17 Red Rep2_0.24
sp|Q5T200|ZC3HD_HUMAN−1017−S−S2_0.21
ptm abundance 37C
Doses Blue Rep1_0.17 Red Rep2_0.24
sp|Q9Y2X7|GIT1_HUMAN−385−S−S2_0.02
ptm abundance 37C
Doses Blue Rep1_0.13 Red Rep2_−0.1
sp|Q9Y2X7|GIT1_HUMAN−388−S−S2_0.02
ptm abundance 37C
Doses Blue Rep1_0.13 Red Rep2_−0.1
sp|Q01831|XPC_HUMAN−397−S−S3_0.02
ptm abundance 37C
Doses Blue Rep1_−0.04 Red Rep2_0.08
sp|Q01831|XPC_HUMAN−398−S−S3_0.02
ptm abundance 37C
Doses Blue Rep1_−0.04 Red Rep2_0.08
sp|Q01831|XPC_HUMAN−399−S−S3_0.02
ptm abundance 37C
Doses Blue Rep1_−0.04 Red Rep2_0.08
sp|Q5VT52−2|RPRD2_HUMAN−556−S−S1_−0.08
ptm abundance 37C
Doses Blue Rep1_−0.06 Red Rep2_−0.09
sp|Q9H7N4|SFR19_HUMAN−933−S−S2_−0.26
ptm abundance 37C
Doses Blue Rep1_−0.26 Red Rep2_−0.27
sp|P55010|IF5_HUMAN−390−S−S1_0
ptm abundance 37C
Doses Blue Rep1_0.08 Red Rep2_−0.09
sp|Q13610−2|PWP1_HUMAN−50−S−S1_−0.01
ptm abundance 37C
Doses Blue Rep1_0.06 Red Rep2_−0.08
sp|P46100−4|ATRX_HUMAN−940−S−S2_−0.08
ptm abundance 37C
Doses Blue Rep1_−0.14 Red Rep2_−0.01
sp|Q12789−3|TF3C1_HUMAN−1856−S−S2_−0.05
ptm abundance 37C
Doses Blue Rep1_−0.05 Red Rep2_−0.05
sp|Q3KQU3−2|MA7D1_HUMAN−112−S−S2_−0.18
ptm abundance 37C
Doses Blue Rep1_−0.1 Red Rep2_−0.26
sp|Q05209−2|PTN12_HUMAN−473−S−S2_−0.04
ptm abundance 37C
Doses Blue Rep1_0.05 Red Rep2_−0.12
sp|Q9UQ35|SRRM2_HUMAN−1103−S−S3_−0.07
ptm abundance 37C
Doses Blue Rep1_−0.1 Red Rep2_−0.04

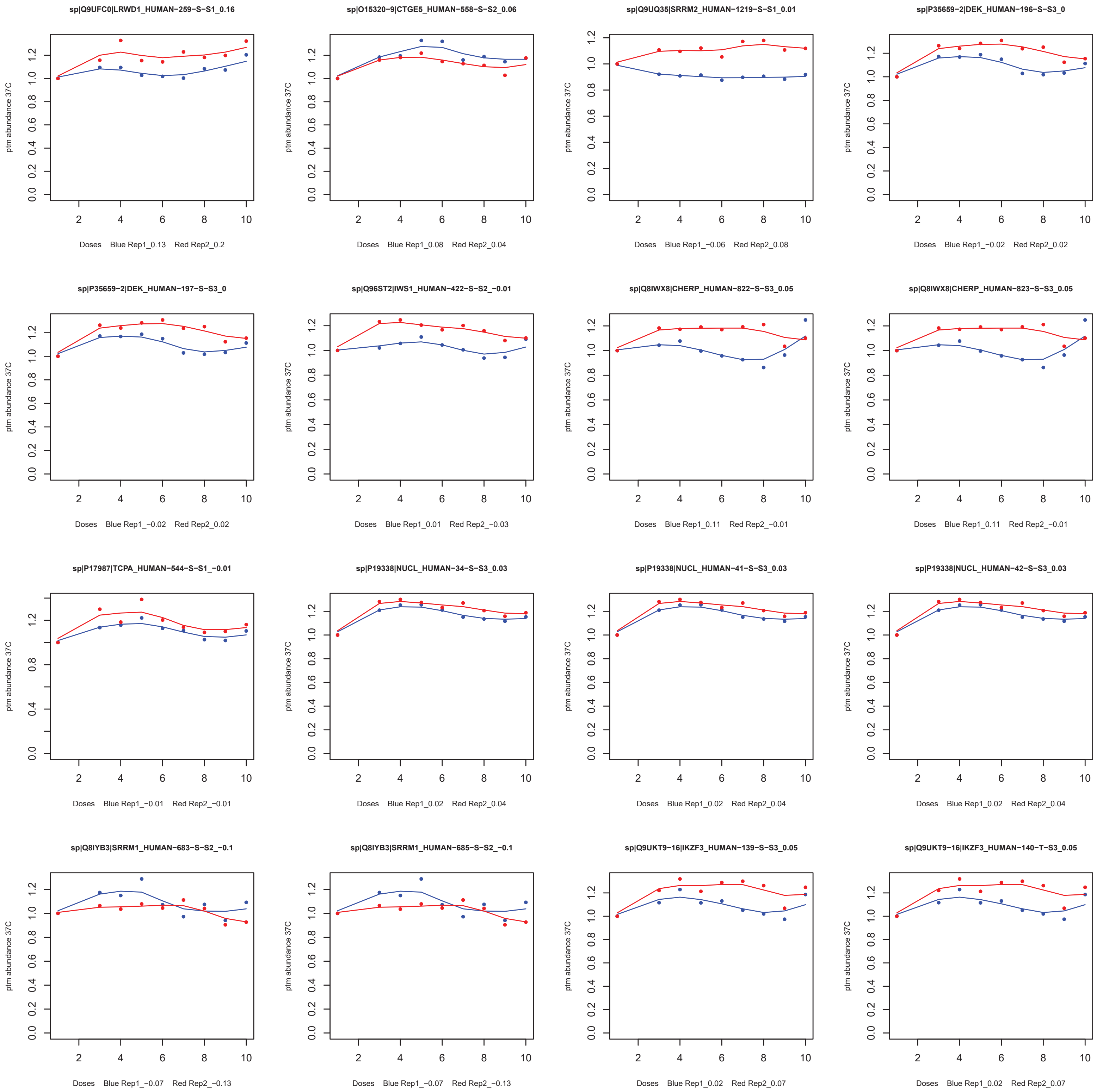
sp|Q9UFC0|LRWD1_HUMAN−259−S−S1_0.16
ptm abundance 37C
Doses Blue Rep1_0.13 Red Rep2_0.2
sp|O15320−9|CTGE5_HUMAN−558−S−S2_0.06
ptm abundance 37C
Doses Blue Rep1_0.08 Red Rep2_0.04
sp|Q9UQ35|SRRM2_HUMAN−1219−S−S1_0.01
ptm abundance 37C
Doses Blue Rep1_−0.06 Red Rep2_0.08
sp|P35659−2|DEK_HUMAN−196−S−S3_0
ptm abundance 37C
Doses Blue Rep1_−0.02 Red Rep2_0.02
sp|P35659−2|DEK_HUMAN−197−S−S3_0
ptm abundance 37C
Doses Blue Rep1_−0.02 Red Rep2_0.02
sp|Q96ST2|IWS1_HUMAN−422−S−S2_−0.01
ptm abundance 37C
Doses Blue Rep1_0.01 Red Rep2_−0.03
sp|Q8IWX8|CHERP_HUMAN−822−S−S3_0.05
ptm abundance 37C
Doses Blue Rep1_0.11 Red Rep2_−0.01
sp|Q8IWX8|CHERP_HUMAN−823−S−S3_0.05
ptm abundance 37C
Doses Blue Rep1_0.11 Red Rep2_−0.01
sp|P17987|TCPA_HUMAN−544−S−S1_−0.01
ptm abundance 37C
Doses Blue Rep1_−0.01 Red Rep2_−0.01
sp|P19338|NUCL_HUMAN−34−S−S3_0.03
ptm abundance 37C
Doses Blue Rep1_0.02 Red Rep2_0.04
sp|P19338|NUCL_HUMAN−41−S−S3_0.03
ptm abundance 37C
Doses Blue Rep1_0.02 Red Rep2_0.04
sp|P19338|NUCL_HUMAN−42−S−S3_0.03
ptm abundance 37C
Doses Blue Rep1_0.02 Red Rep2_0.04
sp|Q8IYB3|SRRM1_HUMAN−683−S−S2_−0.1
ptm abundance 37C
Doses Blue Rep1_−0.07 Red Rep2_−0.13
sp|Q8IYB3|SRRM1_HUMAN−685−S−S2_−0.1
ptm abundance 37C
Doses Blue Rep1_−0.07 Red Rep2_−0.13
sp|Q9UKT9−16|IKZF3_HUMAN−139−S−S3_0.05
ptm abundance 37C
Doses Blue Rep1_0.02 Red Rep2_0.07
sp|Q9UKT9−16|IKZF3_HUMAN−140−T−S3_0.05
ptm abundance 37C
Doses Blue Rep1_0.02 Red Rep2_0.07

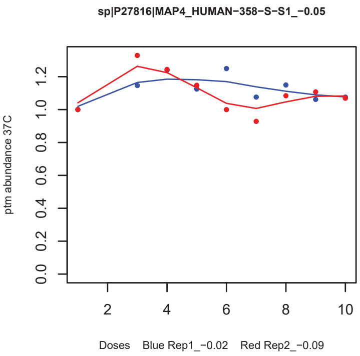
sp|P27816|MAP4_HUMAN−358−S−S1_−0.05
ptm abundance 37C
Doses Blue Rep1_−0.02 Red Rep2_−0.09

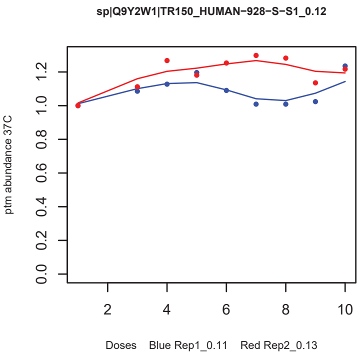
sp|Q9Y2W1|TR150_HUMAN−928−S−S1_0.12
ptm abundance 37C
Doses Blue Rep1_0.11 Red Rep2_0.13

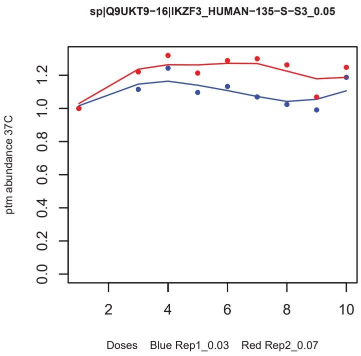
sp|Q9UKT9−16|IKZF3_HUMAN−135−S−S3_0.05
ptm abundance 37C
Doses Blue Rep1_0.03 Red Rep2_0.07

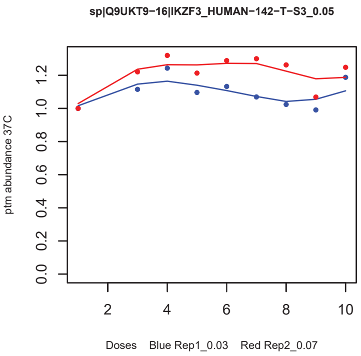
sp|Q9UKT9−16|IKZF3_HUMAN−142−T−S3_0.05
ptm abundance 37C
Doses Blue Rep1_0.03 Red Rep2_0.07

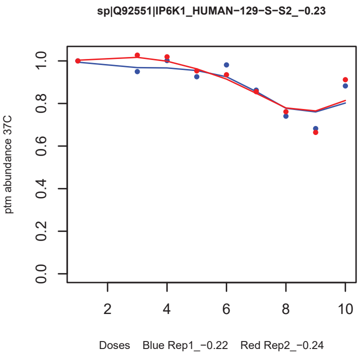
sp|Q92551|IP6K1_HUMAN−129−S−S2_−0.23
ptm abundance 37C
Doses Blue Rep1_−0.22 Red Rep2_−0.24

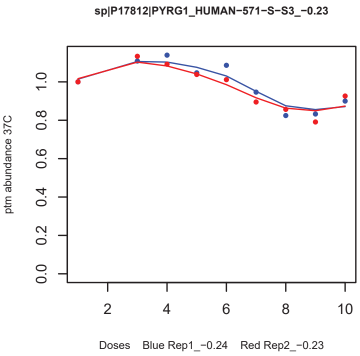
sp|P17812|PYRG1_HUMAN−571−S−S3_−0.23
ptm abundance 37C
Doses Blue Rep1_−0.24 Red Rep2_−0.23

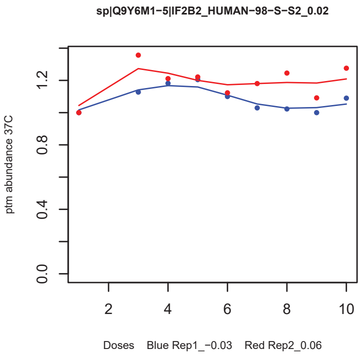
sp|Q9Y6M1−5|IF2B2_HUMAN−98−S−S2_0.02
ptm abundance 37C
Doses Blue Rep1_−0.03 Red Rep2_0.06

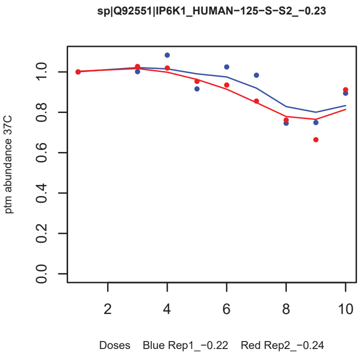
sp|Q92551|IP6K1_HUMAN−125−S−S2_−0.23
ptm abundance 37C
Doses Blue Rep1_−0.22 Red Rep2_−0.24

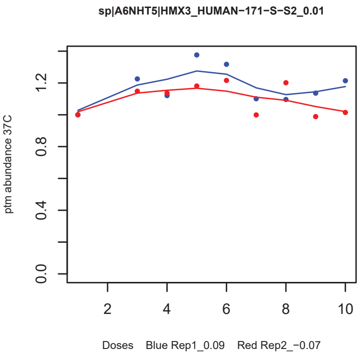
sp|A6NHT5|HMX3_HUMAN−171−S−S2_0.01
ptm abundance 37C
Doses Blue Rep1_0.09 Red Rep2_−0.07

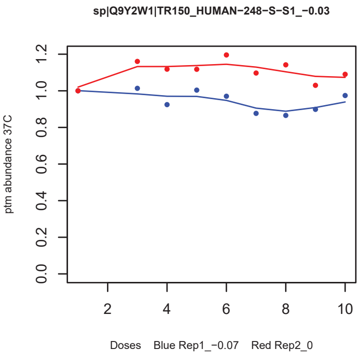
sp|Q9Y2W1|TR150_HUMAN−248−S−S1_−0.03
ptm abundance 37C
Doses Blue Rep1_−0.07 Red Rep2_0

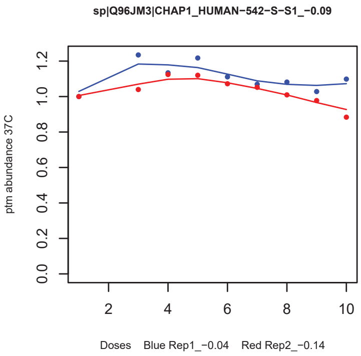
sp|Q96JM3|CHAP1_HUMAN−542−S−S1_−0.09
ptm abundance 37C
Doses Blue Rep1_−0.04 Red Rep2_−0.14

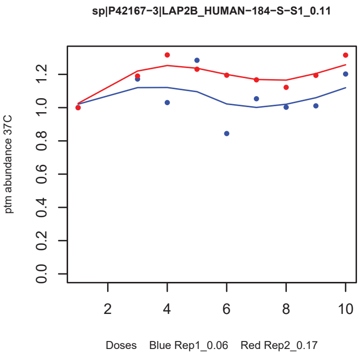
sp|P42167−3|LAP2B_HUMAN−184−S−S1_0.11
ptm abundance 37C
Doses Blue Rep1_0.06 Red Rep2_0.17

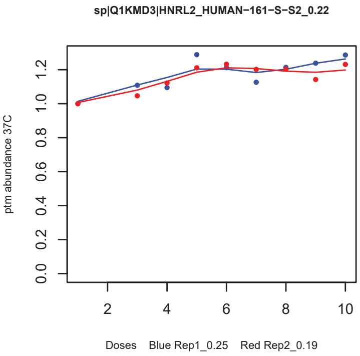
sp|Q1KMD3|HNRL2_HUMAN−161−S−S2_0.22
ptm abundance 37C
Doses Blue Rep1_0.25 Red Rep2_0.19

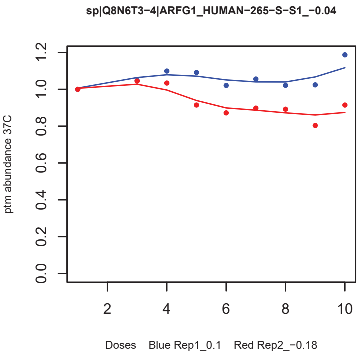
sp|Q8N6T3−4|ARFG1_HUMAN−265−S−S1_−0.04
ptm abundance 37C
Doses Blue Rep1_0.1 Red Rep2_−0.18

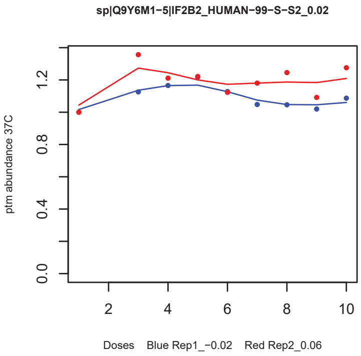
sp|Q9Y6M1−5|IF2B2_HUMAN−99−S−S2_0.02
ptm abundance 37C
Doses Blue Rep1_−0.02 Red Rep2_0.06

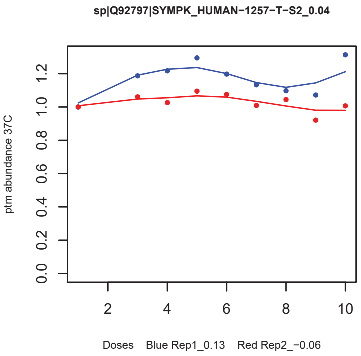
sp|Q92797|SYMPK_HUMAN−1257−T−S2_0.04
ptm abundance 37C
Doses Blue Rep1_0.13 Red Rep2_−0.06

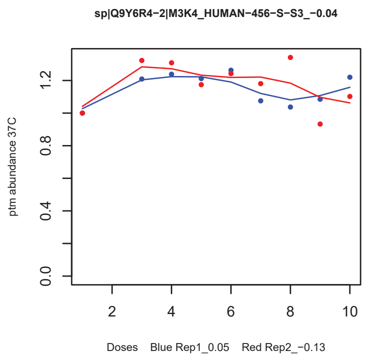
sp|Q9Y6R4−2|M3K4_HUMAN−456−S−S3_−0.04
ptm abundance 37C
Doses Blue Rep1_0.05 Red Rep2_−0.13

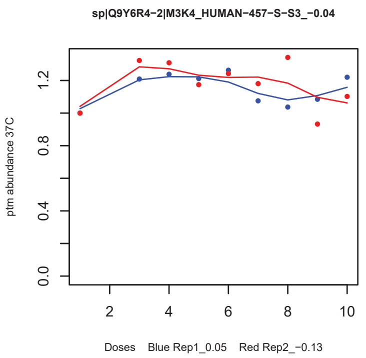
sp|Q9Y6R4−2|M3K4_HUMAN−457−S−S3_−0.04
ptm abundance 37C
Doses Blue Rep1_0.05 Red Rep2_−0.13

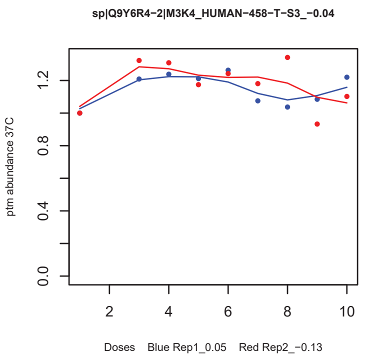
sp|Q9Y6R4−2|M3K4_HUMAN−458−T−S3_−0.04
ptm abundance 37C
Doses Blue Rep1_0.05 Red Rep2_−0.13

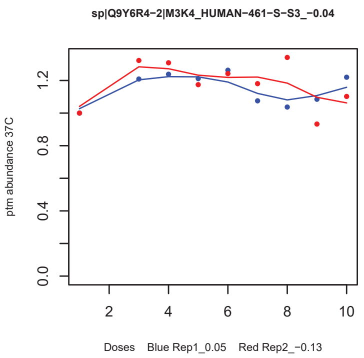
sp|Q9Y6R4−2|M3K4_HUMAN−461−S−S3_−0.04
ptm abundance 37C
Doses Blue Rep1_0.05 Red Rep2_−0.13

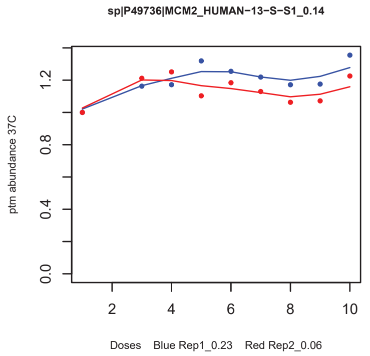
sp|P49736|MCM2_HUMAN−13−S−S1_0.14
ptm abundance 37C
Doses Blue Rep1_0.23 Red Rep2_0.06

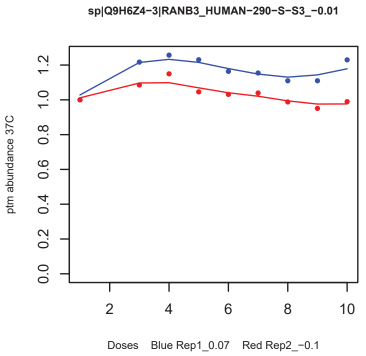
sp|Q9H6Z4−3|RANB3_HUMAN−290−S−S3_−0.01
ptm abundance 37C
Doses Blue Rep1_0.07 Red Rep2_−0.1

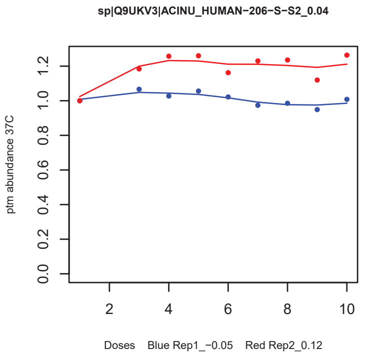
sp|Q9UKV3|ACINU_HUMAN−206−S−S2_0.04
ptm abundance 37C
Doses Blue Rep1_−0.05 Red Rep2_0.12

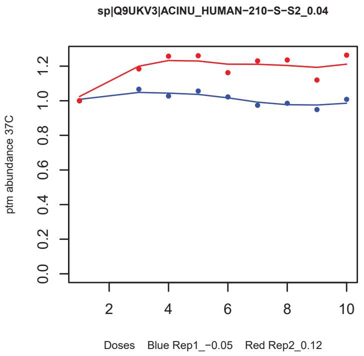
sp|Q9UKV3|ACINU_HUMAN−210−S−S2_0.04
ptm abundance 37C
Doses Blue Rep1_−0.05 Red Rep2_0.12

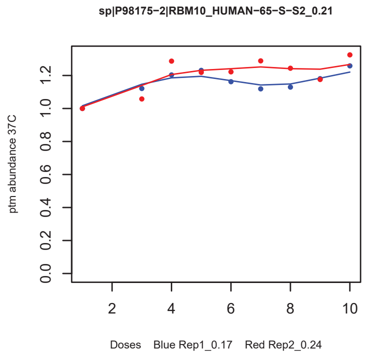
sp|P98175−2|RBM10_HUMAN−65−S−S2_0.21
ptm abundance 37C
Doses Blue Rep1_0.17 Red Rep2_0.24

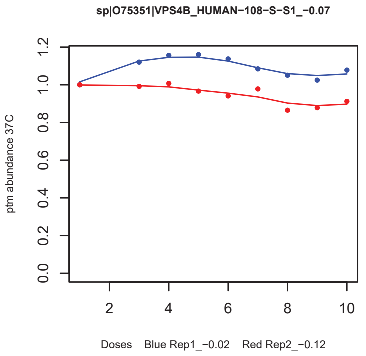
sp|O75351|VPS4B_HUMAN−108−S−S1_−0.07
ptm abundance 37C
Doses Blue Rep1_−0.02 Red Rep2_−0.12

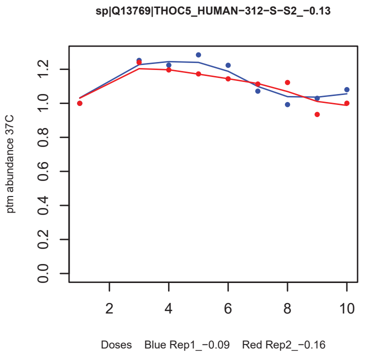
sp|Q13769|THOC5_HUMAN−312−S−S2_−0.13
ptm abundance 37C
Doses Blue Rep1_−0.09 Red Rep2_−0.16

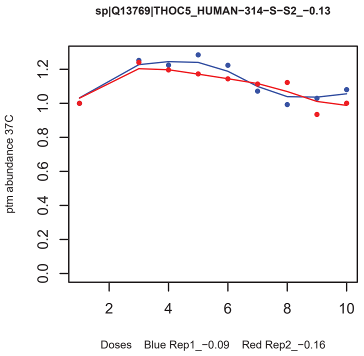
sp|Q13769|THOC5_HUMAN−314−S−S2_−0.13
ptm abundance 37C
Doses Blue Rep1_−0.09 Red Rep2_−0.16

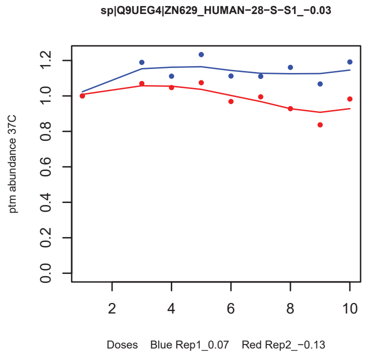
sp|Q9UEG4|ZN629_HUMAN−28−S−S1_−0.03
ptm abundance 37C
Doses Blue Rep1_0.07 Red Rep2_−0.13

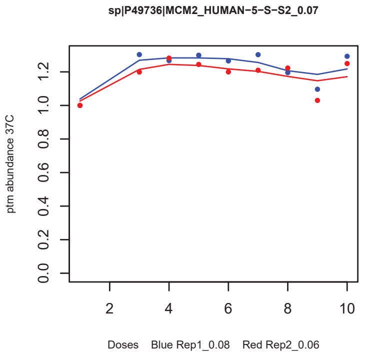
sp|P49736|MCM2_HUMAN−5−S−S2_0.07
ptm abundance 37C
Doses Blue Rep1_0.08 Red Rep2_0.06

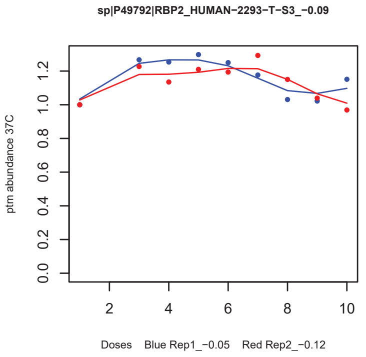
sp|P49792|RBP2_HUMAN−2293−T−S3_−0.09
ptm abundance 37C
Doses Blue Rep1_−0.05 Red Rep2_−0.12

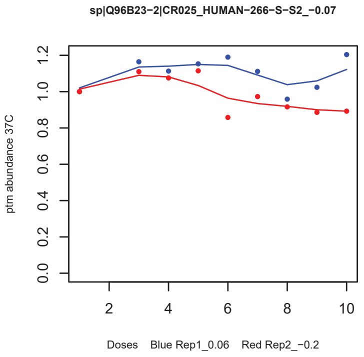
sp|Q96B23−2|CR025_HUMAN−266−S−S2_−0.07
ptm abundance 37C
Doses Blue Rep1_0.06 Red Rep2_−0.2

sp|Q96B23−2|CR025_HUMAN−267−S−S2_−0.07

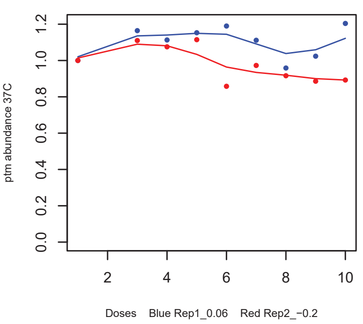

sp|Q96B23−2|CR025_HUMAN−269−S−S2_−0.07

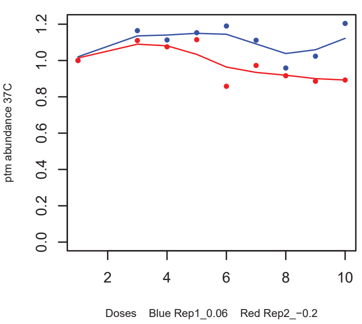

sp|Q5T890|ER6L2_HUMAN−939−S−S2_−0.05

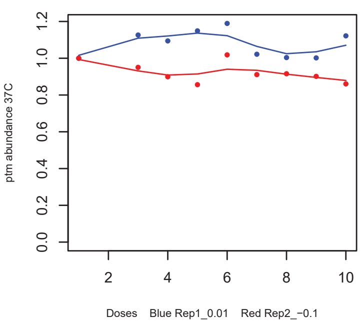

sp|Q86V59|PNM8A_HUMAN−343−S−S3_−0.03

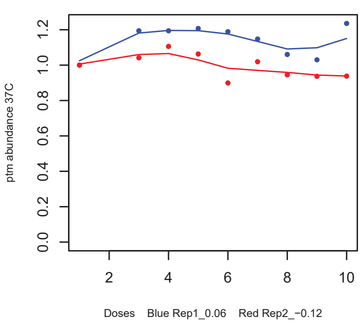

sp|Q8N163−2|CCAR2_HUMAN−686−S−S2_−0.24

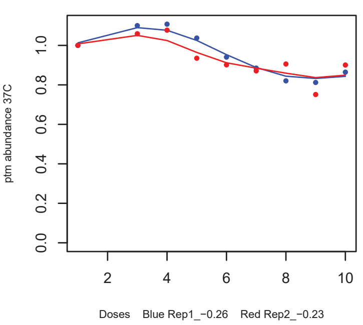

sp|Q9P258|RCC2_HUMAN−50−S−S2_0.15

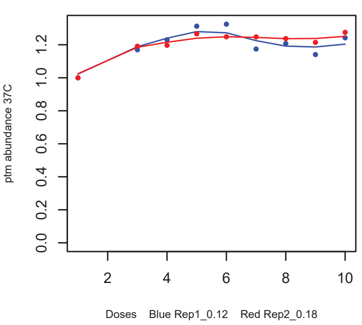

sp|Q9P258|RCC2_HUMAN−51−S−S2_0.15

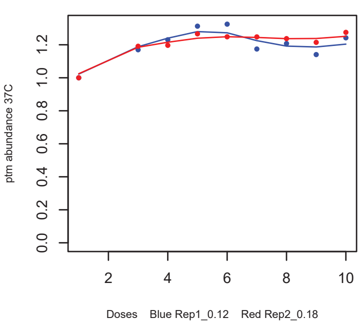

sp|Q0VDF9|HSP7E_HUMAN−76−S−S1_−0.11

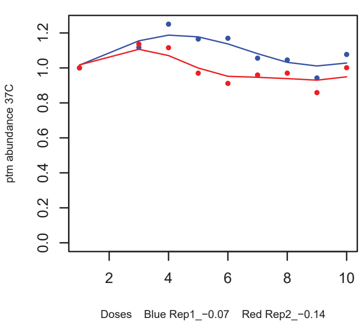

sp|Q13769|THOC5_HUMAN−307−S−S2_−0.1

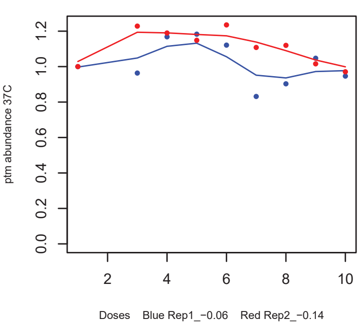

sp|O15357|SHIP2_HUMAN−132−S−S1_−0.14

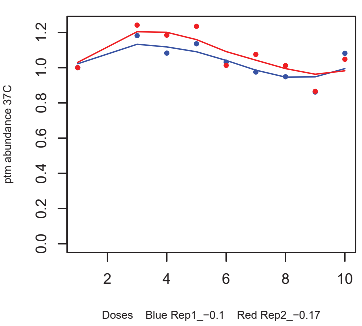

sp|Q9Y606−2|TRUA_HUMAN−398−T−S2_−0.02

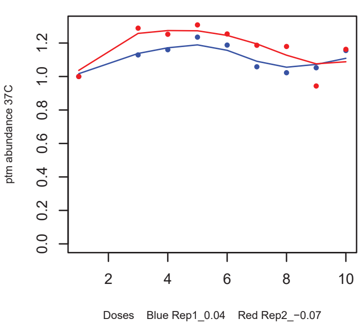

sp|Q9BZF1−3|OSBL8_HUMAN−766−S−S2_0

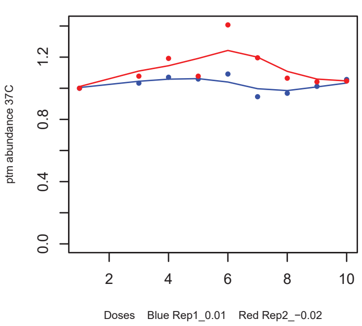

sp|P46100−4|ATRX_HUMAN−1038−S−S2_−0.24

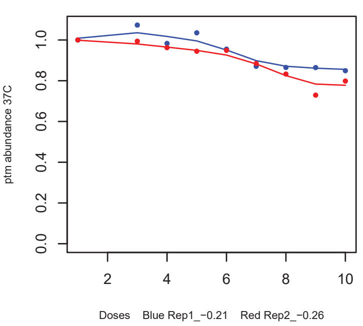

sp|Q6PD62|CTR9_HUMAN−1085−S−S1_0.16

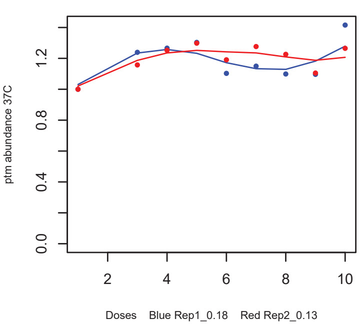

sp|O95218−2|ZRAB2_HUMAN−303−T−S3_−0.1

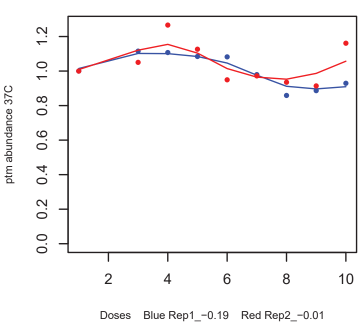

sp|Q14684−2|RRP1B_HUMAN−434−S−S3_−0.07

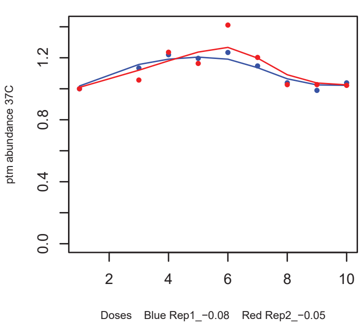

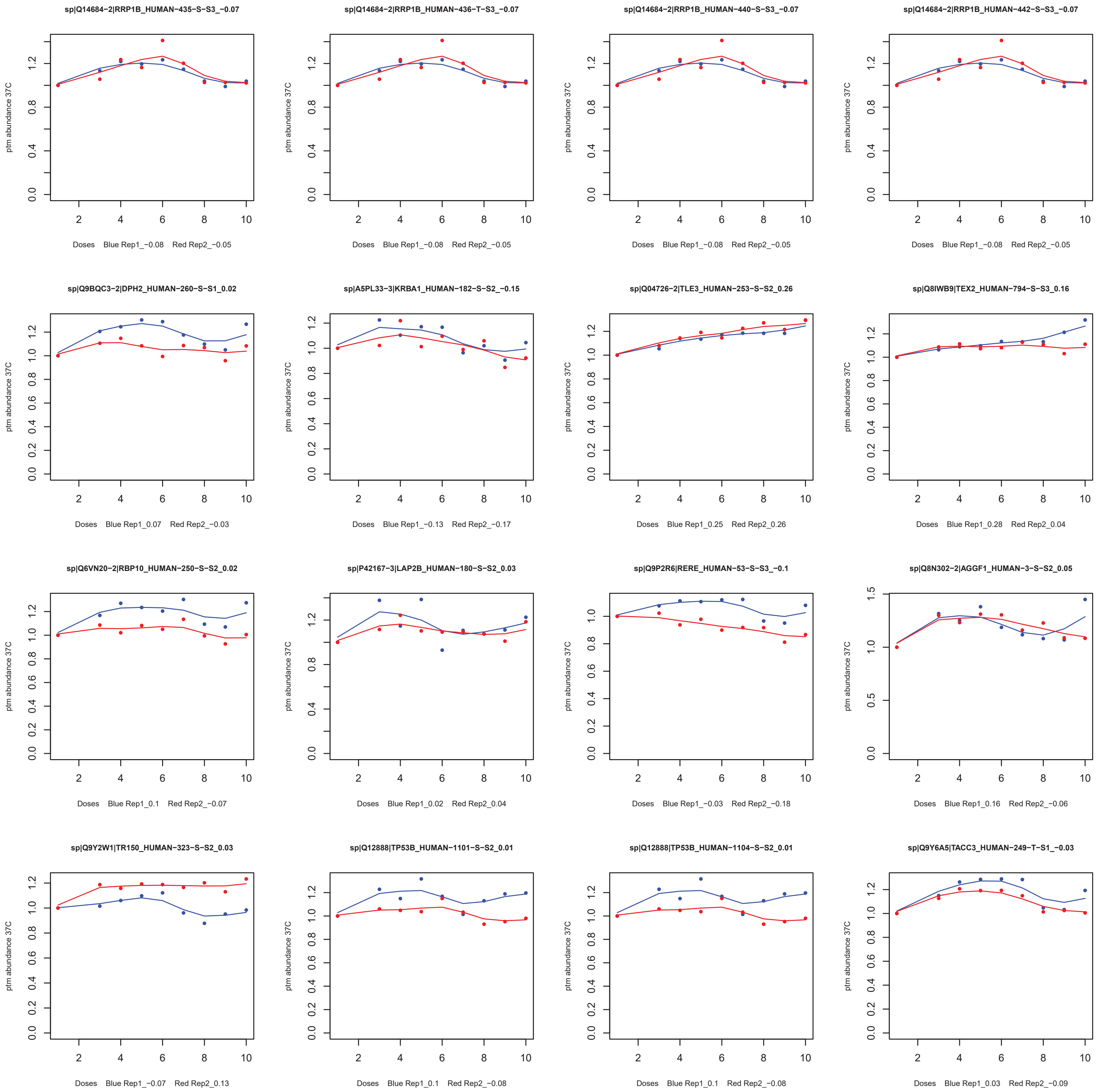
sp|Q14684−2|RRP1B_HUMAN−435−S−S3_−0.07
Doses Blue Rep1_−0.08 Red Rep2_−0.05
sp|Q14684−2|RRP1B_HUMAN−436−T−S3_−0.07
Doses Blue Rep1_−0.08 Red Rep2_−0.05
sp|Q14684−2|RRP1B_HUMAN−440−S−S3_−0.07
Doses Blue Rep1_−0.08 Red Rep2_−0.05
sp|Q14684−2|RRP1B_HUMAN−442−S−S3_−0.07
Doses Blue Rep1_−0.08 Red Rep2_−0.05
sp|Q9BQC3−2|DPH2_HUMAN−260−S−S1_0.02
Doses Blue Rep1_0.07 Red Rep2_−0.03
sp|A5PL33−3|KRBA1_HUMAN−182−S−S2_−0.15
Doses Blue Rep1_−0.13 Red Rep2_−0.17
sp|Q04726−2|TLE3_HUMAN−253−S−S2_0.26
Doses Blue Rep1_0.25 Red Rep2_0.26
sp|Q8IWB9|TEX2_HUMAN−794−S−S3_0.16
Doses Blue Rep1_0.28 Red Rep2_0.04
sp|Q6VN20−2|RBP10_HUMAN−250−S−S2_0.02
Doses Blue Rep1_0.1 Red Rep2_−0.07
sp|P42167−3|LAP2B_HUMAN−180−S−S2_0.03
Doses Blue Rep1_0.02 Red Rep2_0.04
sp|Q9P2R6|RERE_HUMAN−53−S−S3_−0.1
Doses Blue Rep1_−0.03 Red Rep2_−0.18
sp|Q8N302−2|AGGF1_HUMAN−3−S−S2_0.05
Doses Blue Rep1_0.16 Red Rep2_−0.06
sp|Q9Y2W1|TR150_HUMAN−323−S−S2_0.03
Doses Blue Rep1_−0.07 Red Rep2_0.13
sp|Q12888|TP53B_HUMAN−1101−S−S2_0.01
Doses Blue Rep1_0.1 Red Rep2_−0.08
sp|Q12888|TP53B_HUMAN−1104−S−S2_0.01
Doses Blue Rep1_0.1 Red Rep2_−0.08
sp|Q9Y6A5|TACC3_HUMAN−249−T−S1_−0.03
Doses Blue Rep1_0.03 Red Rep2_−0.09
ptm abundance 37C

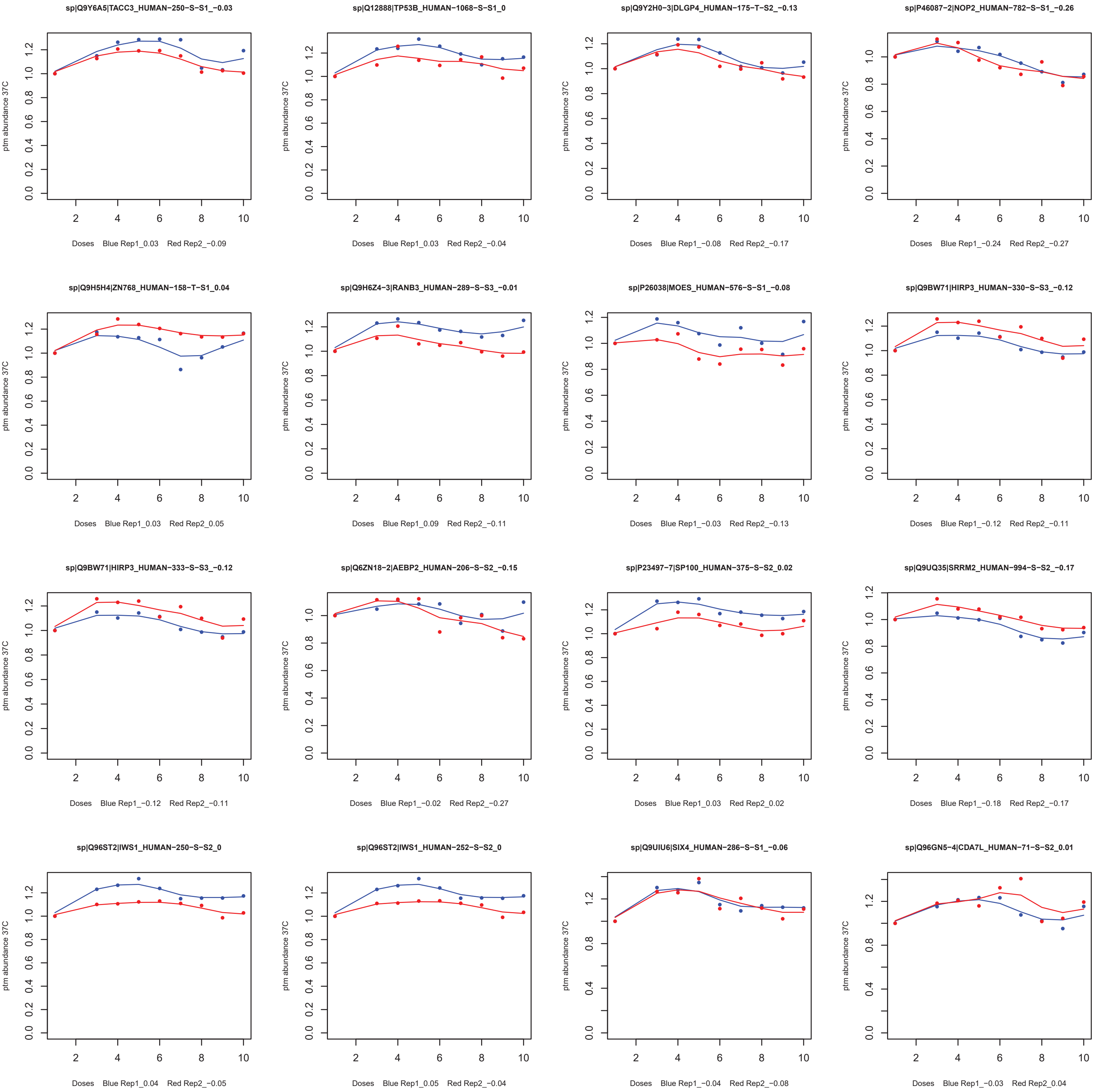
sp|Q9Y6A5|TACC3_HUMAN−250−S−S1_−0.03
Doses Blue Rep1_0.03 Red Rep2_−0.09
sp|Q12888|TP53B_HUMAN−1068−S−S1_0
Doses Blue Rep1_0.03 Red Rep2_−0.04
sp|Q9Y2H0−3|DLGP4_HUMAN−175−T−S2_−0.13
Doses Blue Rep1_−0.08 Red Rep2_−0.17
sp|P46087−2|NOP2_HUMAN−782−S−S1_−0.26
Doses Blue Rep1_−0.24 Red Rep2_−0.27
sp|Q9H5H4|ZN768_HUMAN−158−T−S1_0.04
Doses Blue Rep1_0.03 Red Rep2_0.05
sp|Q9H6Z4−3|RANB3_HUMAN−289−S−S3_−0.01
Doses Blue Rep1_0.09 Red Rep2_−0.11
sp|P26038|MOES_HUMAN−576−S−S1_−0.08
Doses Blue Rep1_−0.03 Red Rep2_−0.13
sp|Q9BW71|HIRP3_HUMAN−330−S−S3_−0.12
Doses Blue Rep1_−0.12 Red Rep2_−0.11
sp|Q9BW71|HIRP3_HUMAN−333−S−S3_−0.12
Doses Blue Rep1_−0.12 Red Rep2_−0.11
sp|Q6ZN18−2|AEBP2_HUMAN−206−S−S2_−0.15
Doses Blue Rep1_−0.02 Red Rep2_−0.27
sp|P23497−7|SP100_HUMAN−375−S−S2_0.02
Doses Blue Rep1_0.03 Red Rep2_0.02
sp|Q9UQ35|SRRM2_HUMAN−994−S−S2_−0.17
Doses Blue Rep1_−0.18 Red Rep2_−0.17
sp|Q96ST2|IWS1_HUMAN−250−S−S2_0
Doses Blue Rep1_0.04 Red Rep2_−0.05
sp|Q96ST2|IWS1_HUMAN−252−S−S2_0
Doses Blue Rep1_0.05 Red Rep2_−0.04
sp|Q9UIU6|SIX4_HUMAN−286−S−S1_−0.06
Doses Blue Rep1_−0.04 Red Rep2_−0.08
sp|Q96GN5−4|CDA7L_HUMAN−71−S−S2_0.01
Doses Blue Rep1_−0.03 Red Rep2_0.04
ptm abundance 37C

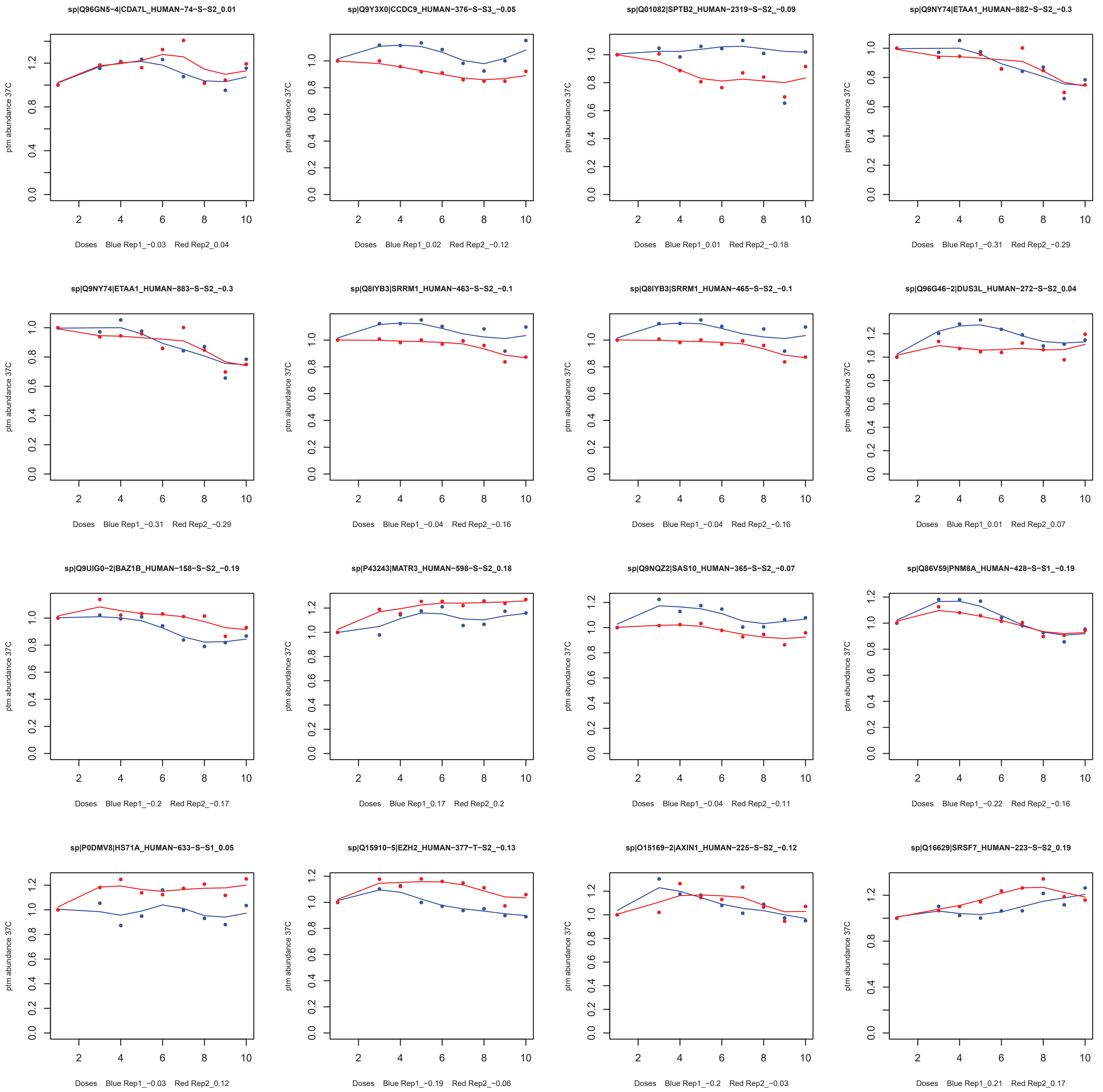
sp|Q96GN5−4|CDA7L_HUMAN−74−S−S2_0.01
Doses Blue Rep1_−0.03 Red Rep2_0.04
sp|Q9Y3X0|CCDC9_HUMAN−376−S−S3_−0.05
Doses Blue Rep1_0.02 Red Rep2_−0.12
sp|Q01082|SPTB2_HUMAN−2319−S−S2_−0.09
Doses Blue Rep1_0.01 Red Rep2_−0.18
sp|Q9NY74|ETAA1_HUMAN−882−S−S2_−0.3
Doses Blue Rep1_−0.31 Red Rep2_−0.29
sp|Q9NY74|ETAA1_HUMAN−883−S−S2_−0.3
Doses Blue Rep1_−0.31 Red Rep2_−0.29
sp|Q8IYB3|SRRM1_HUMAN−463−S−S2_−0.1
Doses Blue Rep1_−0.04 Red Rep2_−0.16
sp|Q8IYB3|SRRM1_HUMAN−465−S−S2_−0.1
Doses Blue Rep1_−0.04 Red Rep2_−0.16
sp|Q96G46−2|DUS3L_HUMAN−272−S−S2_0.04
Doses Blue Rep1_0.01 Red Rep2_0.07
sp|Q9UIG0−2|BAZ1B_HUMAN−158−S−S2_−0.19
Doses Blue Rep1_−0.2 Red Rep2_−0.17
sp|P43243|MATR3_HUMAN−598−S−S2_0.18
Doses Blue Rep1_0.17 Red Rep2_0.2
sp|Q9NQZ2|SAS10_HUMAN−365−S−S2_−0.07
Doses Blue Rep1_−0.04 Red Rep2_−0.11
sp|Q86V59|PNM8A_HUMAN−428−S−S1_−0.19
Doses Blue Rep1_−0.22 Red Rep2_−0.16
sp|P0DMV8|HS71A_HUMAN−633−S−S1_0.05
Doses Blue Rep1_−0.03 Red Rep2_0.12
sp|Q15910−5|EZH2_HUMAN−377−T−S2_−0.13
Doses Blue Rep1_−0.19 Red Rep2_−0.06
sp|O15169−2|AXIN1_HUMAN−225−S−S2_−0.12
Doses Blue Rep1_−0.2 Red Rep2_−0.03
sp|Q16629|SRSF7_HUMAN−223−S−S2_0.19
Doses Blue Rep1_0.21 Red Rep2_0.17
ptm abundance 37C

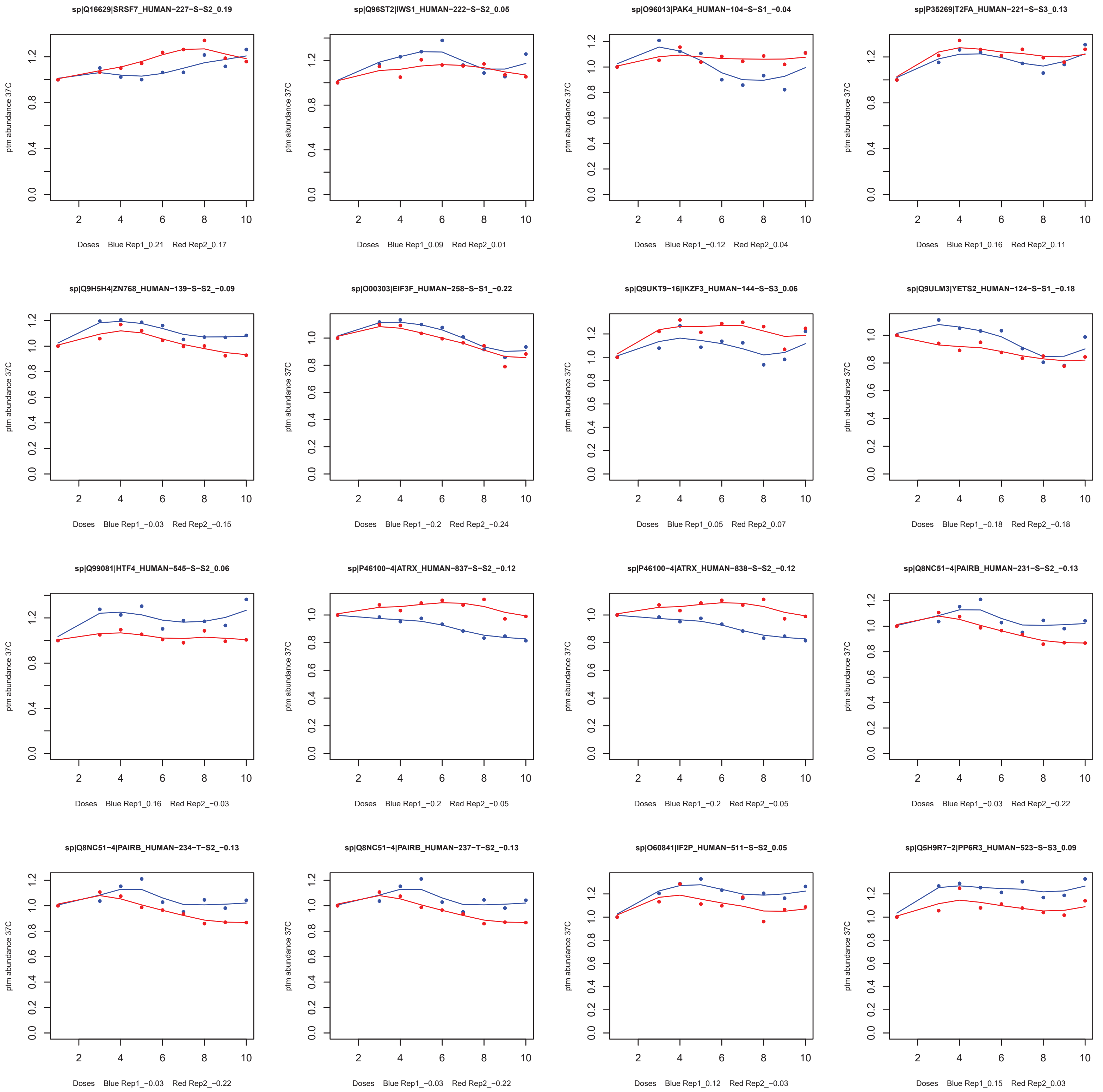
sp|Q16629|SRSF7_HUMAN−227−S−S2_0.19
ptm abundance 37C
Doses Blue Rep1_0.21 Red Rep2_0.17
sp|Q96ST2|IWS1_HUMAN−222−S−S2_0.05
ptm abundance 37C
Doses Blue Rep1_0.09 Red Rep2_0.01
sp|O96013|PAK4_HUMAN−104−S−S1_−0.04
ptm abundance 37C
Doses Blue Rep1_−0.12 Red Rep2_0.04
sp|P35269|T2FA_HUMAN−221−S−S3_0.13
ptm abundance 37C
Doses Blue Rep1_0.16 Red Rep2_0.11
sp|Q9H5H4|ZN768_HUMAN−139−S−S2_−0.09
ptm abundance 37C
Doses Blue Rep1_−0.03 Red Rep2_−0.15
sp|O00303|EIF3F_HUMAN−258−S−S1_−0.22
ptm abundance 37C
Doses Blue Rep1_−0.2 Red Rep2_−0.24
sp|Q9UKT9−16|IKZF3_HUMAN−144−S−S3_0.06
ptm abundance 37C
Doses Blue Rep1_0.05 Red Rep2_0.07
sp|Q9ULM3|YETS2_HUMAN−124−S−S1_−0.18
ptm abundance 37C
Doses Blue Rep1_−0.18 Red Rep2_−0.18
sp|Q99081|HTF4_HUMAN−545−S−S2_0.06
ptm abundance 37C
Doses Blue Rep1_0.16 Red Rep2_−0.03
sp|P46100−4|ATRX_HUMAN−837−S−S2_−0.12
ptm abundance 37C
Doses Blue Rep1_−0.2 Red Rep2_−0.05
sp|P46100−4|ATRX_HUMAN−838−S−S2_−0.12
ptm abundance 37C
Doses Blue Rep1_−0.2 Red Rep2_−0.05
sp|Q8NC51−4|PAIRB_HUMAN−231−S−S2_−0.13
ptm abundance 37C
Doses Blue Rep1_−0.03 Red Rep2_−0.22
sp|Q8NC51−4|PAIRB_HUMAN−234−T−S2_−0.13
ptm abundance 37C
Doses Blue Rep1_−0.03 Red Rep2_−0.22
sp|Q8NC51−4|PAIRB_HUMAN−237−T−S2_−0.13
ptm abundance 37C
Doses Blue Rep1_−0.03 Red Rep2_−0.22
sp|O60841|IF2P_HUMAN−511−S−S2_0.05
ptm abundance 37C
Doses Blue Rep1_0.12 Red Rep2_−0.03
sp|Q5H9R7−2|PP6R3_HUMAN−523−S−S3_0.09
ptm abundance 37C
Doses Blue Rep1_0.15 Red Rep2_0.03

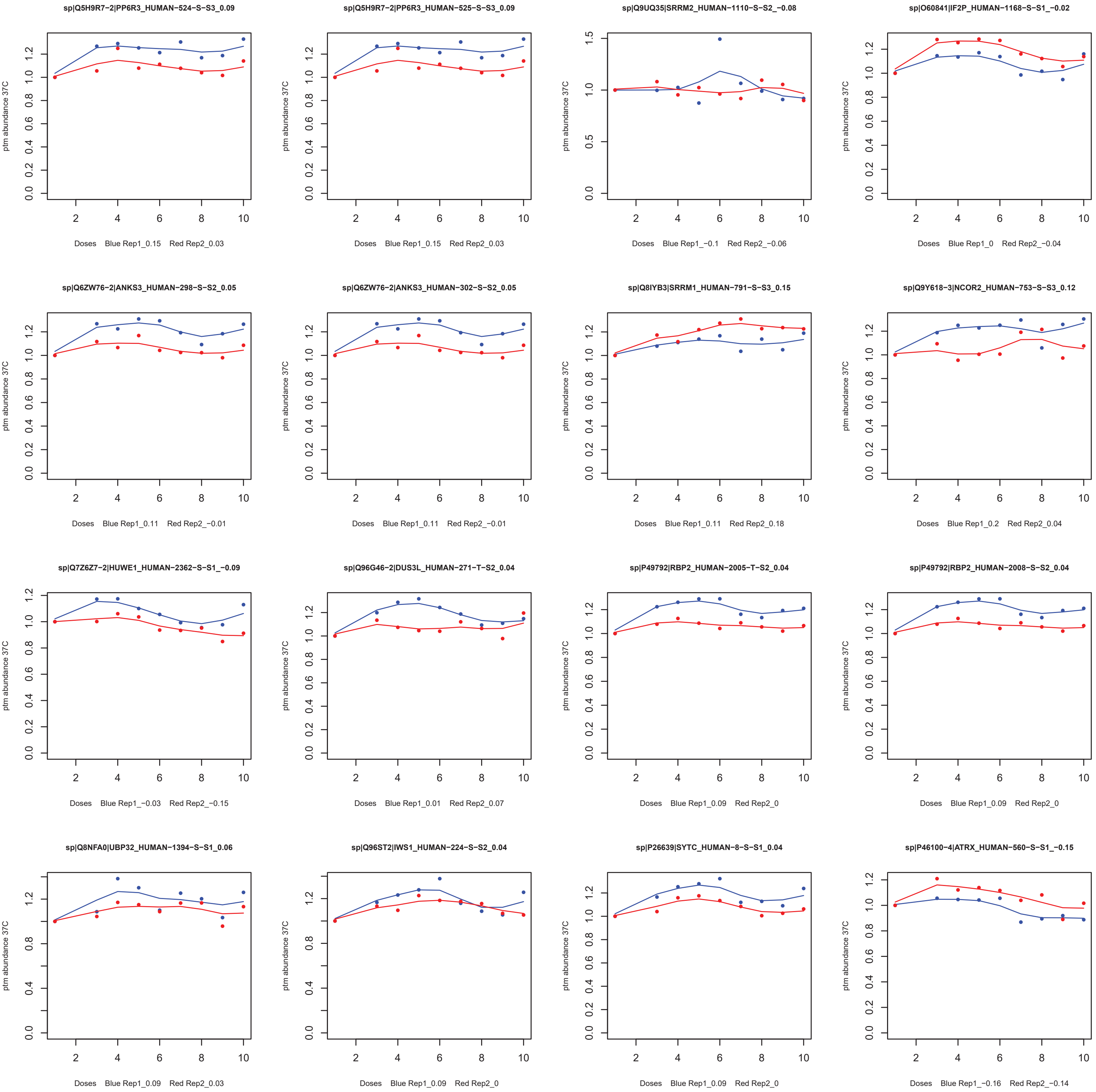
sp|Q5H9R7−2|PP6R3_HUMAN−524−S−S3_0.09
Doses Blue Rep1_0.15 Red Rep2_0.03
sp|Q5H9R7−2|PP6R3_HUMAN−525−S−S3_0.09
Doses Blue Rep1_0.15 Red Rep2_0.03
sp|Q9UQ35|SRRM2_HUMAN−1110−S−S2_−0.08
Doses Blue Rep1_−0.1 Red Rep2_−0.06
sp|O60841|IF2P_HUMAN−1168−S−S1_−0.02
Doses Blue Rep1_0 Red Rep2_−0.04
sp|Q6ZW76−2|ANKS3_HUMAN−298−S−S2_0.05
Doses Blue Rep1_0.11 Red Rep2_−0.01
sp|Q6ZW76−2|ANKS3_HUMAN−302−S−S2_0.05
Doses Blue Rep1_0.11 Red Rep2_−0.01
sp|Q8IYB3|SRRM1_HUMAN−791−S−S3_0.15
Doses Blue Rep1_0.11 Red Rep2_0.18
sp|Q9Y618−3|NCOR2_HUMAN−753−S−S3_0.12
Doses Blue Rep1_0.2 Red Rep2_0.04
sp|Q7Z6Z7−2|HUWE1_HUMAN−2362−S−S1_−0.09
Doses Blue Rep1_−0.03 Red Rep2_−0.15
sp|Q96G46−2|DUS3L_HUMAN−271−T−S2_0.04
Doses Blue Rep1_0.01 Red Rep2_0.07
sp|P49792|RBP2_HUMAN−2005−T−S2_0.04
Doses Blue Rep1_0.09 Red Rep2_0
sp|P49792|RBP2_HUMAN−2008−S−S2_0.04
Doses Blue Rep1_0.09 Red Rep2_0
sp|Q8NFA0|UBP32_HUMAN−1394−S−S1_0.06
Doses Blue Rep1_0.09 Red Rep2_0.03
sp|Q96ST2|IWS1_HUMAN−224−S−S2_0.04
Doses Blue Rep1_0.09 Red Rep2_0
sp|P26639|SYTC_HUMAN−8−S−S1_0.04
Doses Blue Rep1_0.09 Red Rep2_0
sp|P46100−4|ATRX_HUMAN−560−S−S1_−0.15
Doses Blue Rep1_−0.16 Red Rep2_−0.14
ptm abundance 37C

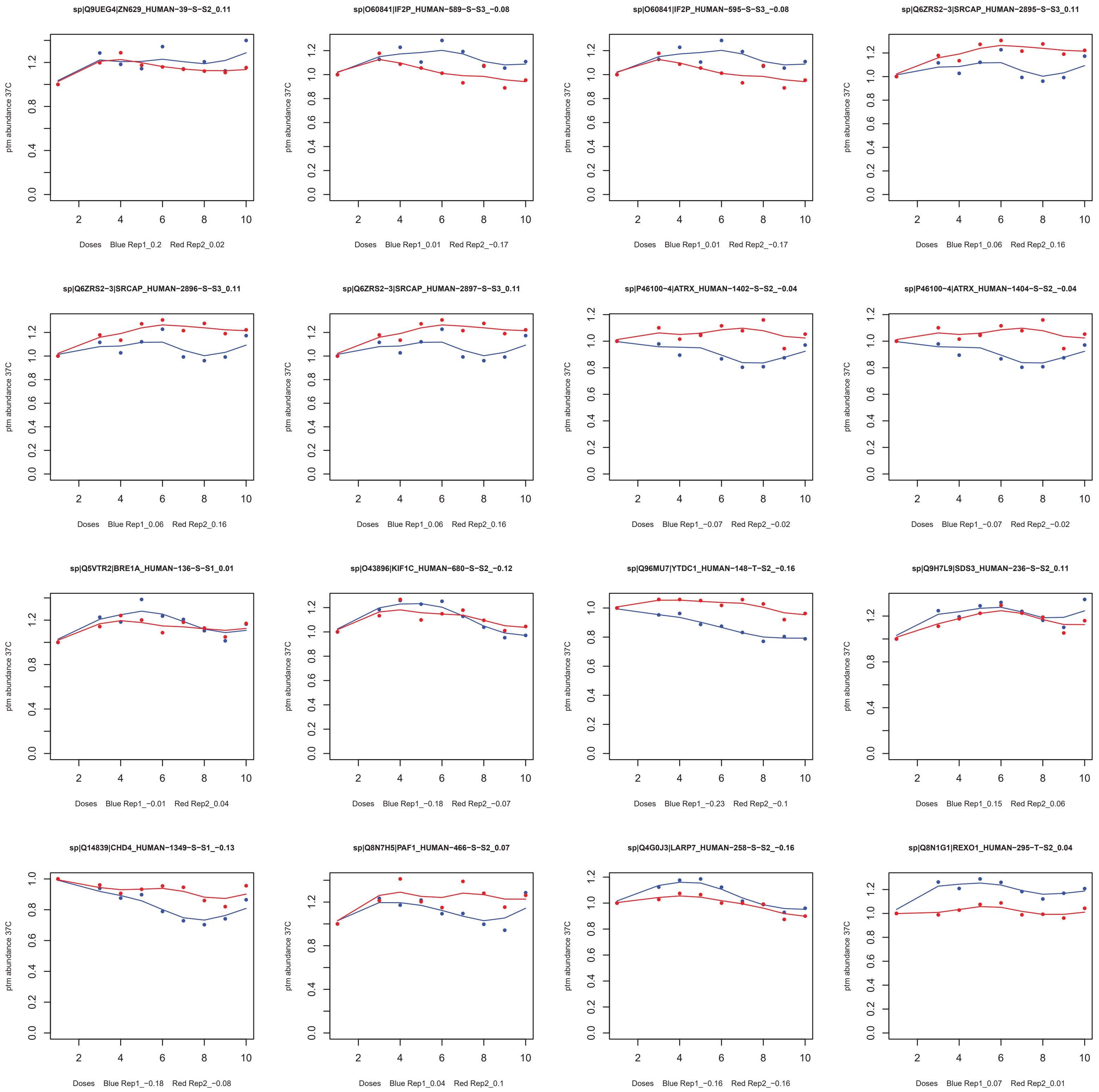
sp|Q9UEG4|ZN629_HUMAN−39−S−S2_0.11
Doses Blue Rep1_0.2 Red Rep2_0.02
sp|O60841|IF2P_HUMAN−589−S−S3_−0.08
Doses Blue Rep1_0.01 Red Rep2_−0.17
sp|O60841|IF2P_HUMAN−595−S−S3_−0.08
Doses Blue Rep1_0.01 Red Rep2_−0.17
sp|Q6ZRS2−3|SRCAP_HUMAN−2895−S−S3_0.11
Doses Blue Rep1_0.06 Red Rep2_0.16
sp|Q6ZRS2−3|SRCAP_HUMAN−2896−S−S3_0.11
Doses Blue Rep1_0.06 Red Rep2_0.16
sp|Q6ZRS2−3|SRCAP_HUMAN−2897−S−S3_0.11
Doses Blue Rep1_0.06 Red Rep2_0.16
sp|P46100−4|ATRX_HUMAN−1402−S−S2_−0.04
Doses Blue Rep1_−0.07 Red Rep2_−0.02
sp|P46100−4|ATRX_HUMAN−1404−S−S2_−0.04
Doses Blue Rep1_−0.07 Red Rep2_−0.02
sp|Q5VTR2|BRE1A_HUMAN−136−S−S1_0.01
Doses Blue Rep1_−0.01 Red Rep2_0.04
sp|O43896|KIF1C_HUMAN−680−S−S2_−0.12
Doses Blue Rep1_−0.18 Red Rep2_−0.07
sp|Q96MU7|YTDC1_HUMAN−148−T−S2_−0.16
Doses Blue Rep1_−0.23 Red Rep2_−0.1
sp|Q9H7L9|SDS3_HUMAN−236−S−S2_0.11
Doses Blue Rep1_0.15 Red Rep2_0.06
sp|Q14839|CHD4_HUMAN−1349−S−S1_−0.13
Doses Blue Rep1_−0.18 Red Rep2_−0.08
sp|Q8N7H5|PAF1_HUMAN−466−S−S2_0.07
Doses Blue Rep1_0.04 Red Rep2_0.1
sp|Q4G0J3|LARP7_HUMAN−258−S−S2_−0.16
Doses Blue Rep1_−0.16 Red Rep2_−0.16
sp|Q8N1G1|REXO1_HUMAN−295−T−S2_0.04
Doses Blue Rep1_0.07 Red Rep2_0.01
ptm abundance 37C

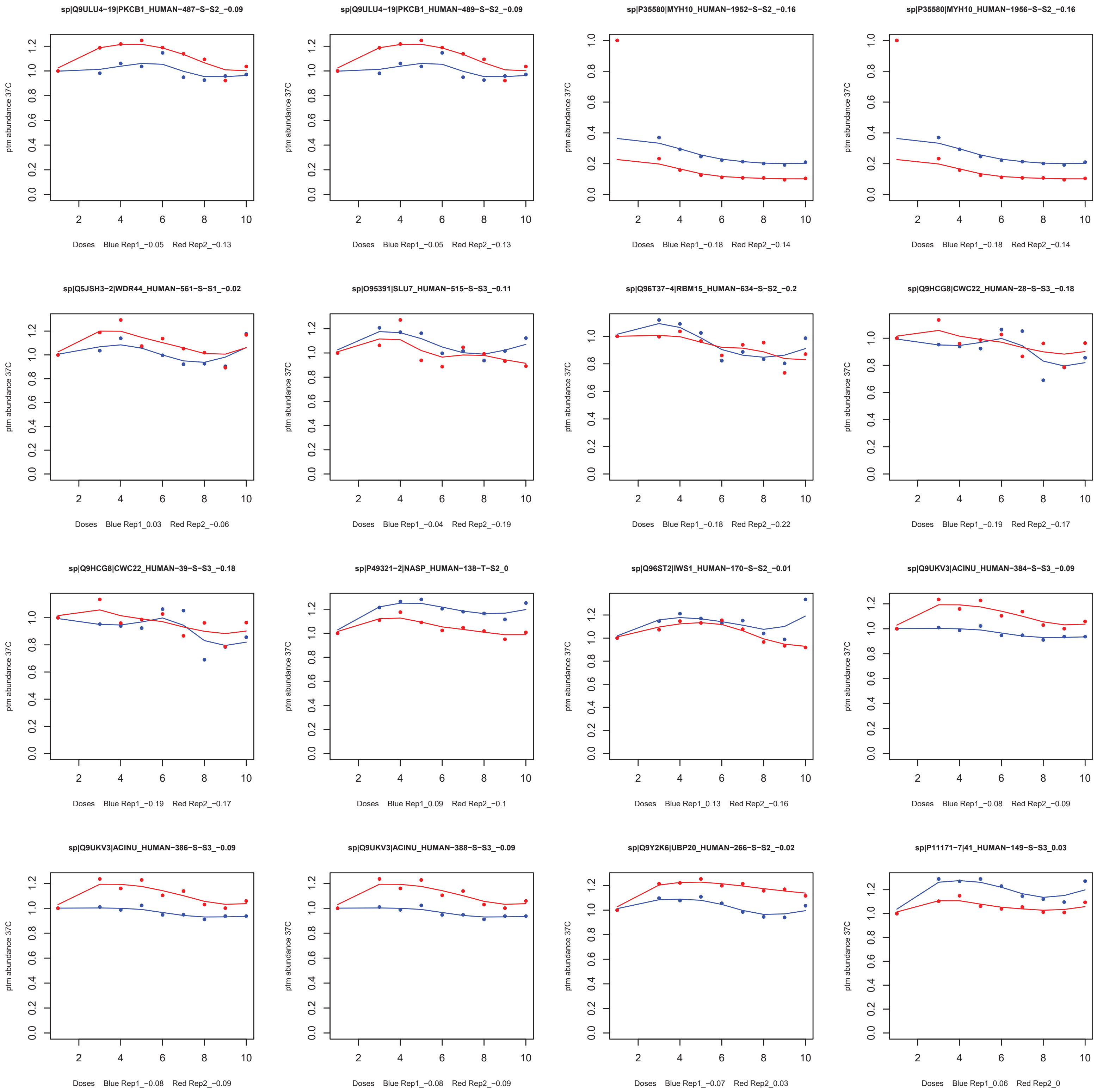
sp|Q9ULU4−19|PKCB1_HUMAN−487−S−S2_−0.09
ptm abundance 37C
Doses Blue Rep1_−0.05 Red Rep2_−0.13
sp|Q9ULU4−19|PKCB1_HUMAN−489−S−S2_−0.09
ptm abundance 37C
Doses Blue Rep1_−0.05 Red Rep2_−0.13
sp|P35580|MYH10_HUMAN−1952−S−S2_−0.16
ptm abundance 37C
Doses Blue Rep1_−0.18 Red Rep2_−0.14
sp|P35580|MYH10_HUMAN−1956−S−S2_−0.16
ptm abundance 37C
Doses Blue Rep1_−0.18 Red Rep2_−0.14
sp|Q5JSH3−2|WDR44_HUMAN−561−S−S1_−0.02
ptm abundance 37C
Doses Blue Rep1_0.03 Red Rep2_−0.06
sp|O95391|SLU7_HUMAN−515−S−S3_−0.11
ptm abundance 37C
Doses Blue Rep1_−0.04 Red Rep2_−0.19
sp|Q96T37−4|RBM15_HUMAN−634−S−S2_−0.2
ptm abundance 37C
Doses Blue Rep1_−0.18 Red Rep2_−0.22
sp|Q9HCG8|CWC22_HUMAN−28−S−S3_−0.18
ptm abundance 37C
Doses Blue Rep1_−0.19 Red Rep2_−0.17
sp|Q9HCG8|CWC22_HUMAN−39−S−S3_−0.18
ptm abundance 37C
Doses Blue Rep1_−0.19 Red Rep2_−0.17
sp|P49321−2|NASP_HUMAN−138−T−S2_0
ptm abundance 37C
Doses Blue Rep1_0.09 Red Rep2_−0.1
sp|Q96ST2|IWS1_HUMAN−170−S−S2_−0.01
ptm abundance 37C
Doses Blue Rep1_0.13 Red Rep2_−0.16
sp|Q9UKV3|ACINU_HUMAN−384−S−S3_−0.09
ptm abundance 37C
Doses Blue Rep1_−0.08 Red Rep2_−0.09
sp|Q9UKV3|ACINU_HUMAN−386−S−S3_−0.09
ptm abundance 37C
Doses Blue Rep1_−0.08 Red Rep2_−0.09
sp|Q9UKV3|ACINU_HUMAN−388−S−S3_−0.09
ptm abundance 37C
Doses Blue Rep1_−0.08 Red Rep2_−0.09
sp|Q9Y2K6|UBP20_HUMAN−266−S−S2_−0.02
ptm abundance 37C
Doses Blue Rep1_−0.07 Red Rep2_0.03
sp|P11171−7|41_HUMAN−149−S−S3_0.03
ptm abundance 37C
Doses Blue Rep1_0.06 Red Rep2_0

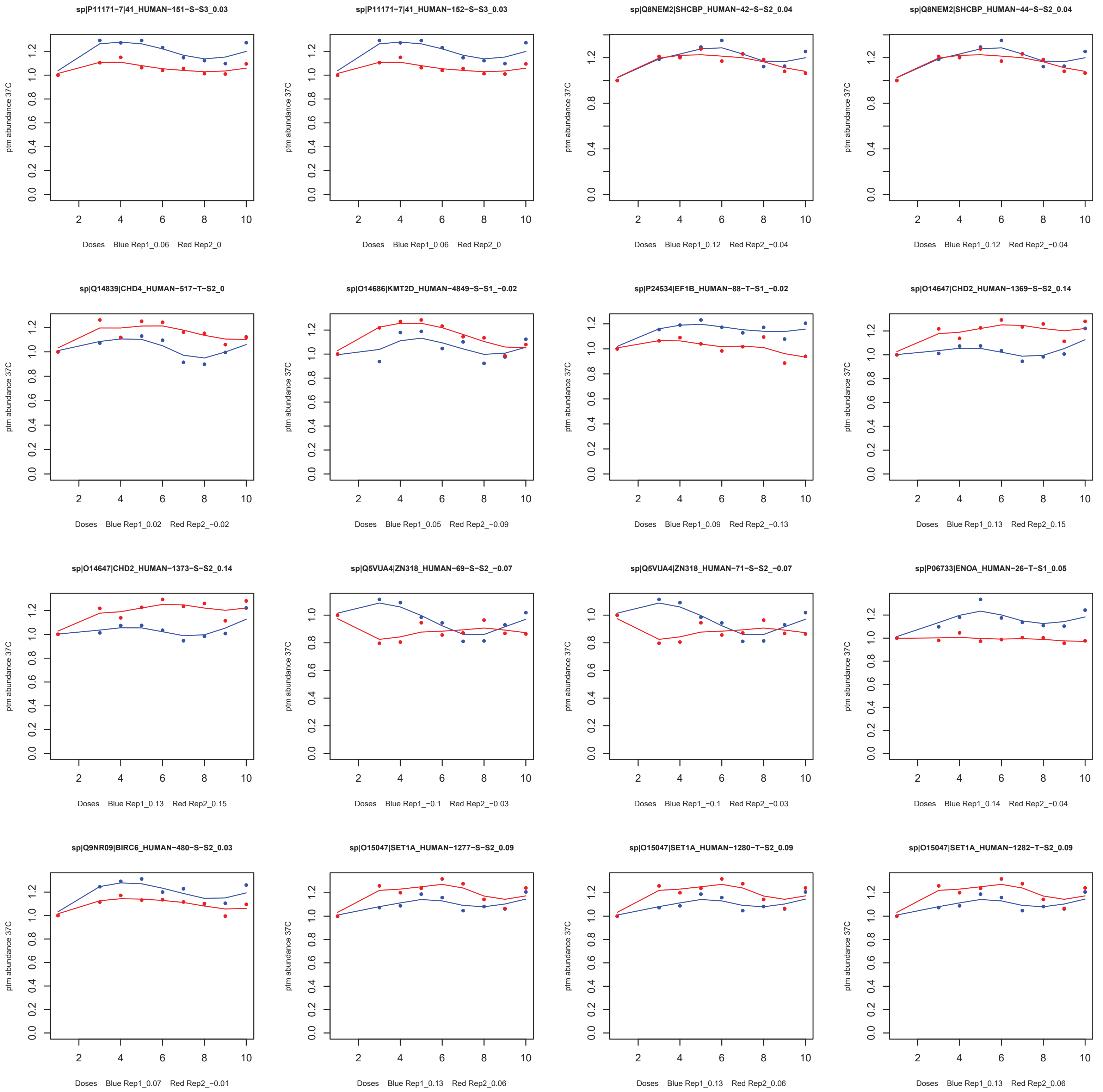
sp|P11171−7|41_HUMAN−151−S−S3_0.03
ptm abundance 37C
Doses Blue Rep1_0.06 Red Rep2_0
sp|P11171−7|41_HUMAN−152−S−S3_0.03
ptm abundance 37C
Doses Blue Rep1_0.06 Red Rep2_0
sp|Q8NEM2|SHCBP_HUMAN−42−S−S2_0.04
ptm abundance 37C
Doses Blue Rep1_0.12 Red Rep2_−0.04
sp|Q8NEM2|SHCBP_HUMAN−44−S−S2_0.04
ptm abundance 37C
Doses Blue Rep1_0.12 Red Rep2_−0.04
sp|Q14839|CHD4_HUMAN−517−T−S2_0
ptm abundance 37C
Doses Blue Rep1_0.02 Red Rep2_−0.02
sp|O14686|KMT2D_HUMAN−4849−S−S1_−0.02
ptm abundance 37C
Doses Blue Rep1_0.05 Red Rep2_−0.09
sp|P24534|EF1B_HUMAN−88−T−S1_−0.02
ptm abundance 37C
Doses Blue Rep1_0.09 Red Rep2_−0.13
sp|O14647|CHD2_HUMAN−1369−S−S2_0.14
ptm abundance 37C
Doses Blue Rep1_0.13 Red Rep2_0.15
sp|O14647|CHD2_HUMAN−1373−S−S2_0.14
ptm abundance 37C
Doses Blue Rep1_0.13 Red Rep2_0.15
sp|Q5VUA4|ZN318_HUMAN−69−S−S2_−0.07
ptm abundance 37C
Doses Blue Rep1_−0.1 Red Rep2_−0.03
sp|Q5VUA4|ZN318_HUMAN−71−S−S2_−0.07
ptm abundance 37C
Doses Blue Rep1_−0.1 Red Rep2_−0.03
sp|P06733|ENOA_HUMAN−26−T−S1_0.05
ptm abundance 37C
Doses Blue Rep1_0.14 Red Rep2_−0.04
sp|Q9NR09|BIRC6_HUMAN−480−S−S2_0.03
ptm abundance 37C
Doses Blue Rep1_0.07 Red Rep2_−0.01
sp|O15047|SET1A_HUMAN−1277−S−S2_0.09
ptm abundance 37C
Doses Blue Rep1_0.13 Red Rep2_0.06
sp|O15047|SET1A_HUMAN−1280−T−S2_0.09
ptm abundance 37C
Doses Blue Rep1_0.13 Red Rep2_0.06
sp|O15047|SET1A_HUMAN−1282−T−S2_0.09
ptm abundance 37C
Doses Blue Rep1_0.13 Red Rep2_0.06

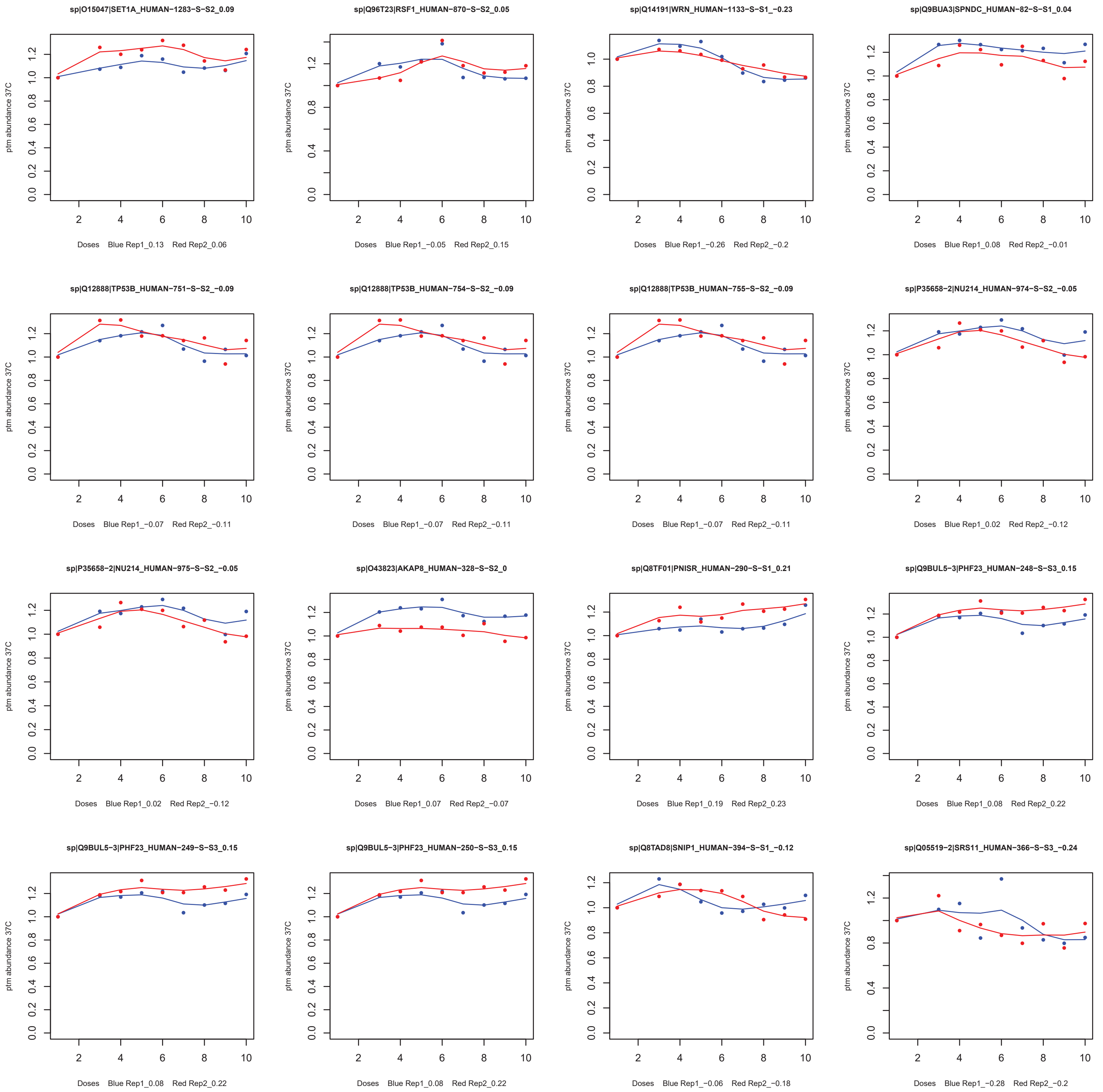
sp|O15047|SET1A_HUMAN−1283−S−S2_0.09
Doses Blue Rep1_0.13 Red Rep2_0.06
sp|Q96T23|RSF1_HUMAN−870−S−S2_0.05
Doses Blue Rep1_−0.05 Red Rep2_0.15
sp|Q14191|WRN_HUMAN−1133−S−S1_−0.23
Doses Blue Rep1_−0.26 Red Rep2_−0.2
sp|Q9BUA3|SPNDC_HUMAN−82−S−S1_0.04
Doses Blue Rep1_0.08 Red Rep2_−0.01
sp|Q12888|TP53B_HUMAN−751−S−S2_−0.09
Doses Blue Rep1_−0.07 Red Rep2_−0.11
sp|Q12888|TP53B_HUMAN−754−S−S2_−0.09
Doses Blue Rep1_−0.07 Red Rep2_−0.11
sp|Q12888|TP53B_HUMAN−755−S−S2_−0.09
Doses Blue Rep1_−0.07 Red Rep2_−0.11
sp|P35658−2|NU214_HUMAN−974−S−S2_−0.05
Doses Blue Rep1_0.02 Red Rep2_−0.12
sp|P35658−2|NU214_HUMAN−975−S−S2_−0.05
Doses Blue Rep1_0.02 Red Rep2_−0.12
sp|O43823|AKAP8_HUMAN−328−S−S2_0
Doses Blue Rep1_0.07 Red Rep2_−0.07
sp|Q8TF01|PNISR_HUMAN−290−S−S1_0.21
Doses Blue Rep1_0.19 Red Rep2_0.23
sp|Q9BUL5−3|PHF23_HUMAN−248−S−S3_0.15
Doses Blue Rep1_0.08 Red Rep2_0.22
sp|Q9BUL5−3|PHF23_HUMAN−249−S−S3_0.15
Doses Blue Rep1_0.08 Red Rep2_0.22
sp|Q9BUL5−3|PHF23_HUMAN−250−S−S3_0.15
Doses Blue Rep1_0.08 Red Rep2_0.22
sp|Q8TAD8|SNIP1_HUMAN−394−S−S1_−0.12
Doses Blue Rep1_−0.06 Red Rep2_−0.18
sp|Q05519−2|SRS11_HUMAN−366−S−S3_−0.24
Doses Blue Rep1_−0.28 Red Rep2_−0.2
ptm abundance 37C

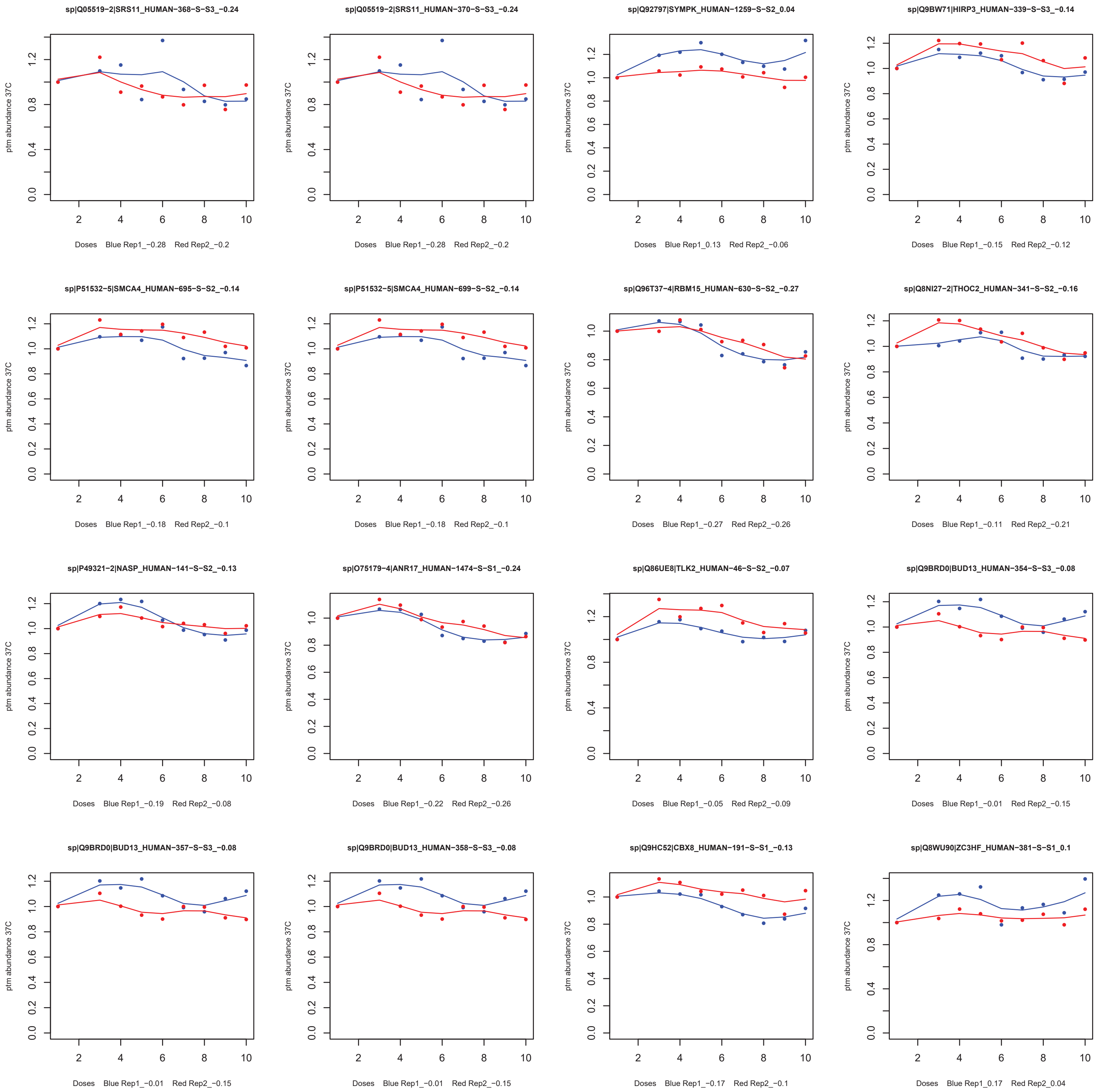
sp|Q05519−2|SRS11_HUMAN−368−S−S3_−0.24
ptm abundance 37C
Doses Blue Rep1_−0.28 Red Rep2_−0.2
sp|Q05519−2|SRS11_HUMAN−370−S−S3_−0.24
ptm abundance 37C
Doses Blue Rep1_−0.28 Red Rep2_−0.2
sp|Q92797|SYMPK_HUMAN−1259−S−S2_0.04
ptm abundance 37C
Doses Blue Rep1_0.13 Red Rep2_−0.06
sp|Q9BW71|HIRP3_HUMAN−339−S−S3_−0.14
ptm abundance 37C
Doses Blue Rep1_−0.15 Red Rep2_−0.12
sp|P51532−5|SMCA4_HUMAN−695−S−S2_−0.14
ptm abundance 37C
Doses Blue Rep1_−0.18 Red Rep2_−0.1
sp|P51532−5|SMCA4_HUMAN−699−S−S2_−0.14
ptm abundance 37C
Doses Blue Rep1_−0.18 Red Rep2_−0.1
sp|Q96T37−4|RBM15_HUMAN−630−S−S2_−0.27
ptm abundance 37C
Doses Blue Rep1_−0.27 Red Rep2_−0.26
sp|Q8NI27−2|THOC2_HUMAN−341−S−S2_−0.16
ptm abundance 37C
Doses Blue Rep1_−0.11 Red Rep2_−0.21
sp|P49321−2|NASP_HUMAN−141−S−S2_−0.13
ptm abundance 37C
Doses Blue Rep1_−0.19 Red Rep2_−0.08
sp|O75179−4|ANR17_HUMAN−1474−S−S1_−0.24
ptm abundance 37C
Doses Blue Rep1_−0.22 Red Rep2_−0.26
sp|Q86UE8|TLK2_HUMAN−46−S−S2_−0.07
ptm abundance 37C
Doses Blue Rep1_−0.05 Red Rep2_−0.09
sp|Q9BRD0|BUD13_HUMAN−354−S−S3_−0.08
ptm abundance 37C
Doses Blue Rep1_−0.01 Red Rep2_−0.15
sp|Q9BRD0|BUD13_HUMAN−357−S−S3_−0.08
ptm abundance 37C
Doses Blue Rep1_−0.01 Red Rep2_−0.15
sp|Q9BRD0|BUD13_HUMAN−358−S−S3_−0.08
ptm abundance 37C
Doses Blue Rep1_−0.01 Red Rep2_−0.15
sp|Q9HC52|CBX8_HUMAN−191−S−S1_−0.13
ptm abundance 37C
Doses Blue Rep1_−0.17 Red Rep2_−0.1
sp|Q8WU90|ZC3HF_HUMAN−381−S−S1_0.1
ptm abundance 37C
Doses Blue Rep1_0.17 Red Rep2_0.04

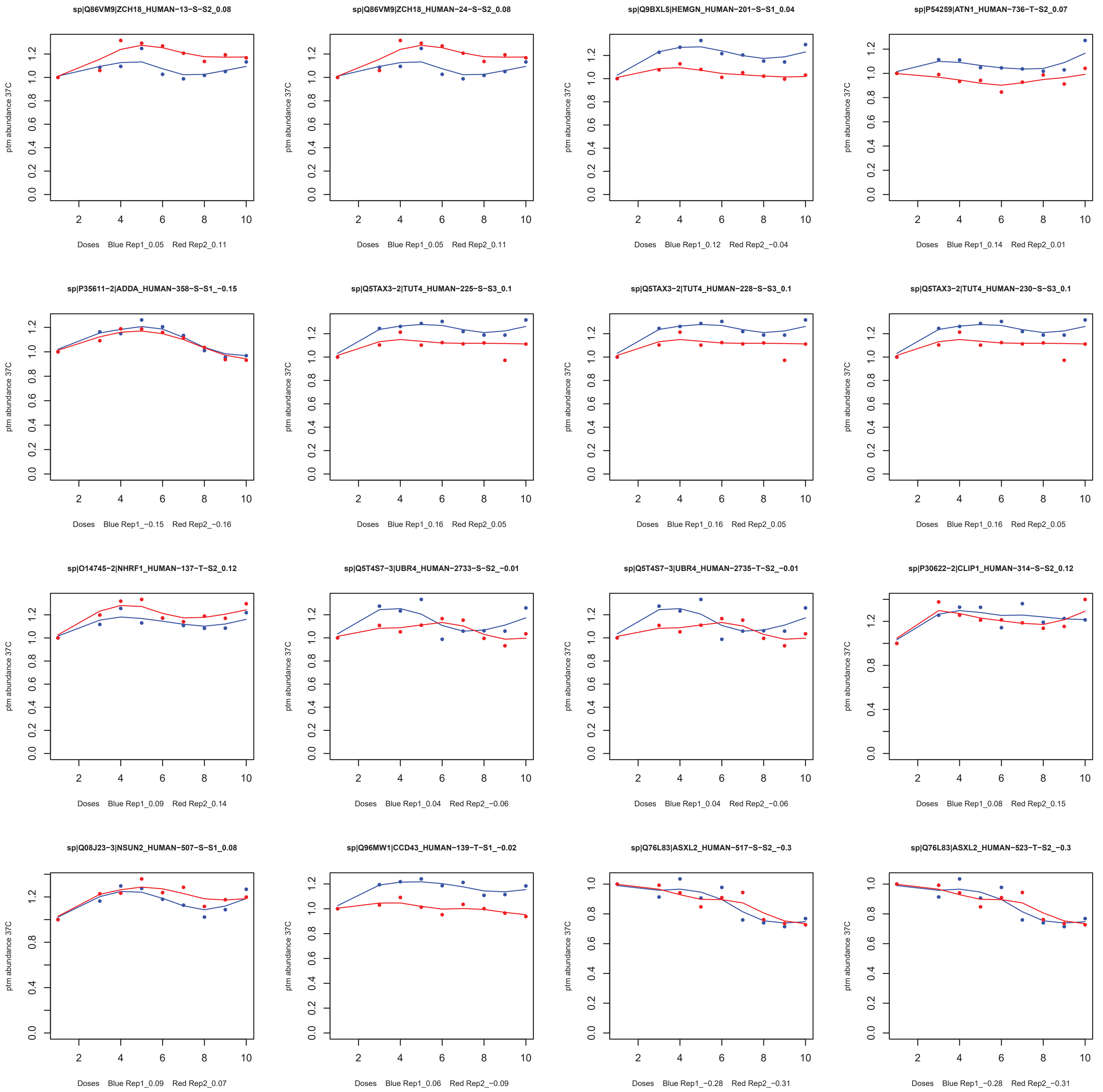
sp|Q86VM9|ZCH18_HUMAN−13−S−S2_0.08
Doses Blue Rep1_0.05 Red Rep2_0.11
sp|Q86VM9|ZCH18_HUMAN−24−S−S2_0.08
Doses Blue Rep1_0.05 Red Rep2_0.11
sp|Q9BXL5|HEMGN_HUMAN−201−S−S1_0.04
Doses Blue Rep1_0.12 Red Rep2_−0.04
sp|P54259|ATN1_HUMAN−736−T−S2_0.07
Doses Blue Rep1_0.14 Red Rep2_0.01
sp|P35611−2|ADDA_HUMAN−358−S−S1_−0.15
Doses Blue Rep1_−0.15 Red Rep2_−0.16
sp|Q5TAX3−2|TUT4_HUMAN−225−S−S3_0.1
Doses Blue Rep1_0.16 Red Rep2_0.05
sp|Q5TAX3−2|TUT4_HUMAN−228−S−S3_0.1
Doses Blue Rep1_0.16 Red Rep2_0.05
sp|Q5TAX3−2|TUT4_HUMAN−230−S−S3_0.1
Doses Blue Rep1_0.16 Red Rep2_0.05
sp|O14745−2|NHRF1_HUMAN−137−T−S2_0.12
Doses Blue Rep1_0.09 Red Rep2_0.14
sp|Q5T4S7−3|UBR4_HUMAN−2733−S−S2_−0.01
Doses Blue Rep1_0.04 Red Rep2_−0.06
sp|Q5T4S7−3|UBR4_HUMAN−2735−T−S2_−0.01
Doses Blue Rep1_0.04 Red Rep2_−0.06
sp|P30622−2|CLIP1_HUMAN−314−S−S2_0.12
Doses Blue Rep1_0.08 Red Rep2_0.15
sp|Q08J23−3|NSUN2_HUMAN−507−S−S1_0.08
Doses Blue Rep1_0.09 Red Rep2_0.07
sp|Q96MW1|CCD43_HUMAN−139−T−S1_−0.02
Doses Blue Rep1_0.06 Red Rep2_−0.09
sp|Q76L83|ASXL2_HUMAN−517−S−S2_−0.3
Doses Blue Rep1_−0.28 Red Rep2_−0.31
sp|Q76L83|ASXL2_HUMAN−523−T−S2_−0.3
Doses Blue Rep1_−0.28 Red Rep2_−0.31
ptm abundance 37C

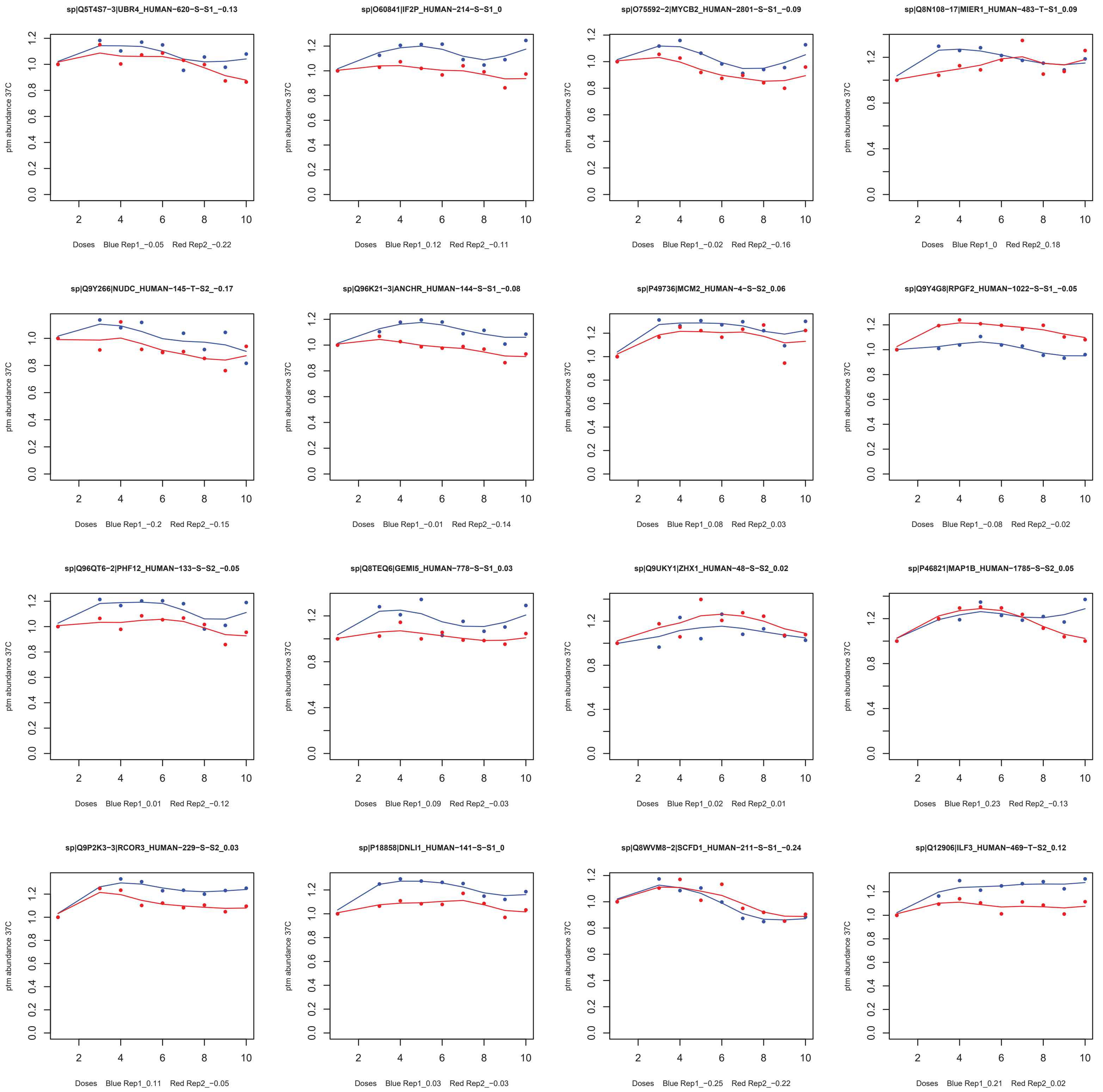
sp|Q5T4S7−3|UBR4_HUMAN−620−S−S1_−0.13
Doses Blue Rep1_−0.05 Red Rep2_−0.22
sp|O60841|IF2P_HUMAN−214−S−S1_0
Doses Blue Rep1_0.12 Red Rep2_−0.11
sp|O75592−2|MYCB2_HUMAN−2801−S−S1_−0.09
Doses Blue Rep1_−0.02 Red Rep2_−0.16
sp|Q8N108−17|MIER1_HUMAN−483−T−S1_0.09
Doses Blue Rep1_0 Red Rep2_0.18
sp|Q9Y266|NUDC_HUMAN−145−T−S2_−0.17
Doses Blue Rep1_−0.2 Red Rep2_−0.15
sp|Q96K21−3|ANCHR_HUMAN−144−S−S1_−0.08
Doses Blue Rep1_−0.01 Red Rep2_−0.14
sp|P49736|MCM2_HUMAN−4−S−S2_0.06
Doses Blue Rep1_0.08 Red Rep2_0.03
sp|Q9Y4G8|RPGF2_HUMAN−1022−S−S1_−0.05
Doses Blue Rep1_−0.08 Red Rep2_−0.02
sp|Q96QT6−2|PHF12_HUMAN−133−S−S2_−0.05
Doses Blue Rep1_0.01 Red Rep2_−0.12
sp|Q8TEQ6|GEMI5_HUMAN−778−S−S1_0.03
Doses Blue Rep1_0.09 Red Rep2_−0.03
sp|Q9UKY1|ZHX1_HUMAN−48−S−S2_0.02
Doses Blue Rep1_0.02 Red Rep2_0.01
sp|P46821|MAP1B_HUMAN−1785−S−S2_0.05
Doses Blue Rep1_0.23 Red Rep2_−0.13
sp|Q9P2K3−3|RCOR3_HUMAN−229−S−S2_0.03
Doses Blue Rep1_0.11 Red Rep2_−0.05
sp|P18858|DNLI1_HUMAN−141−S−S1_0
Doses Blue Rep1_0.03 Red Rep2_−0.03
sp|Q8WVM8−2|SCFD1_HUMAN−211−S−S1_−0.24
Doses Blue Rep1_−0.25 Red Rep2_−0.22
sp|Q12906|ILF3_HUMAN−469−T−S2_0.12
Doses Blue Rep1_0.21 Red Rep2_0.02
ptm abundance 37C

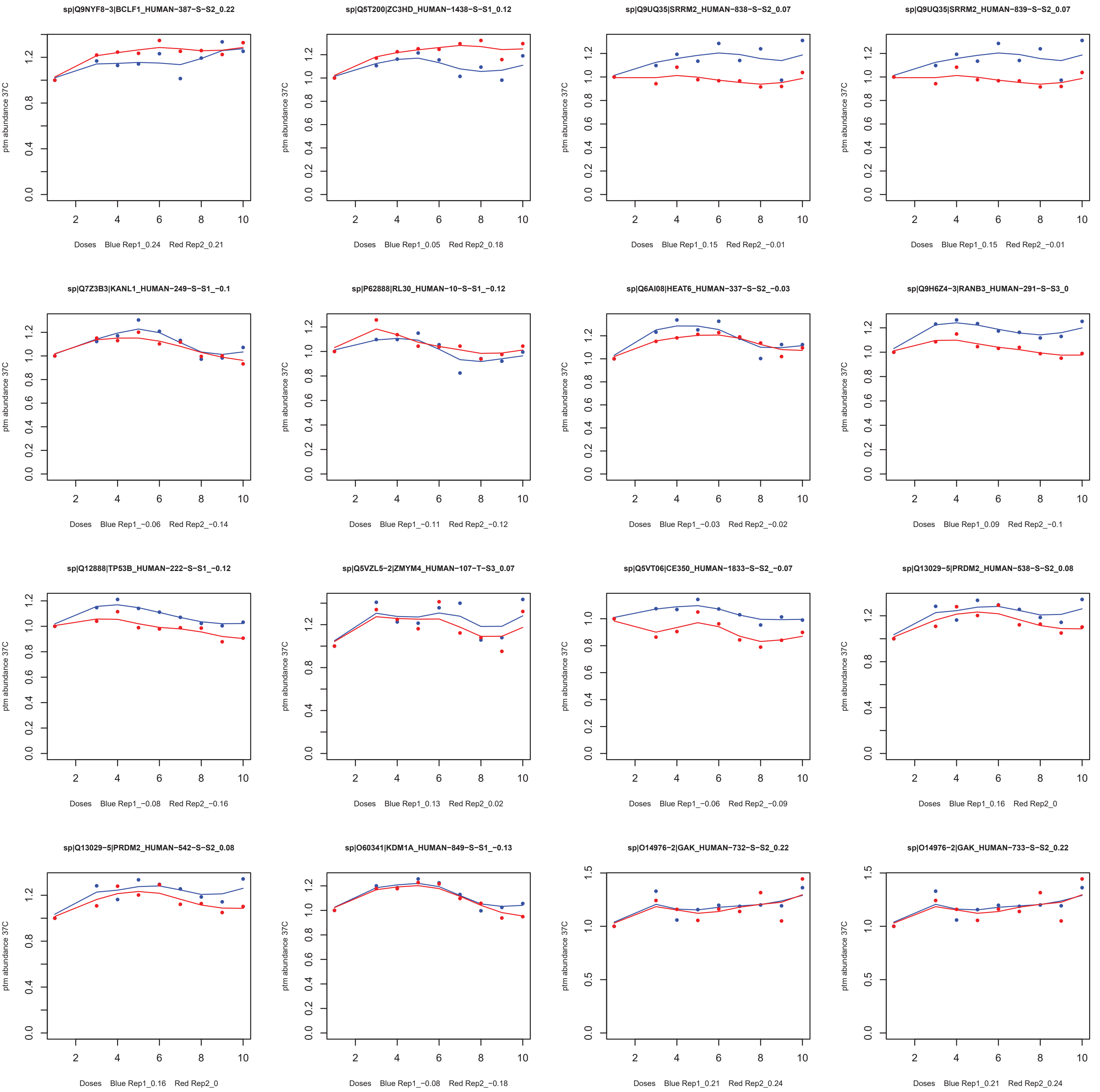
sp|Q9NYF8-3|BCLF1_HUMAN-387-S-S2_0.22
Doses Blue Rep1_0.24 Red Rep2_0.21
sp|Q5T200|ZC3HD_HUMAN-1438-S-S1_0.12
Doses Blue Rep1_0.05 Red Rep2_0.18
sp|Q9UQ35|SRRM2_HUMAN-838-S-S2_0.07
Doses Blue Rep1_0.15 Red Rep2_-0.01
sp|Q9UQ35|SRRM2_HUMAN-839-S-S2_0.07
Doses Blue Rep1_0.15 Red Rep2_-0.01
sp|Q7Z3B3|KANL1_HUMAN-249-S-S1_-0.1
Doses Blue Rep1_-0.06 Red Rep2_-0.14
sp|P62888|RL30_HUMAN-10-S-S1_-0.12
Doses Blue Rep1_-0.11 Red Rep2_-0.12
sp|Q6AI08|HEAT6_HUMAN-337-S-S2_-0.03
Doses Blue Rep1_-0.03 Red Rep2_-0.02
sp|Q9H6Z4-3|RANB3_HUMAN-291-S-S3_0
Doses Blue Rep1_0.09 Red Rep2_-0.1
sp|Q12888|TP53B_HUMAN-222-S-S1_-0.12
Doses Blue Rep1_-0.08 Red Rep2_-0.16
sp|Q5VZL5-2|ZMYM4_HUMAN-107-T-S3_0.07
Doses Blue Rep1_0.13 Red Rep2_0.02
sp|Q5VT06|CE350_HUMAN-1833-S-S2_-0.07
Doses Blue Rep1_-0.06 Red Rep2_-0.09
sp|Q13029-5|PRDM2_HUMAN-538-S-S2_0.08
Doses Blue Rep1_0.16 Red Rep2_0
sp|Q13029-5|PRDM2_HUMAN-542-S-S2_0.08
Doses Blue Rep1_0.16 Red Rep2_0
sp|O60341|KDM1A_HUMAN-849-S-S1_-0.13
Doses Blue Rep1_-0.08 Red Rep2_-0.18
sp|O14976-2|GAK_HUMAN-732-S-S2_0.22
Doses Blue Rep1_0.21 Red Rep2_0.24
sp|O14976-2|GAK_HUMAN-733-S-S2_0.22
Doses Blue Rep1_0.21 Red Rep2_0.24
ptm abundance 37C

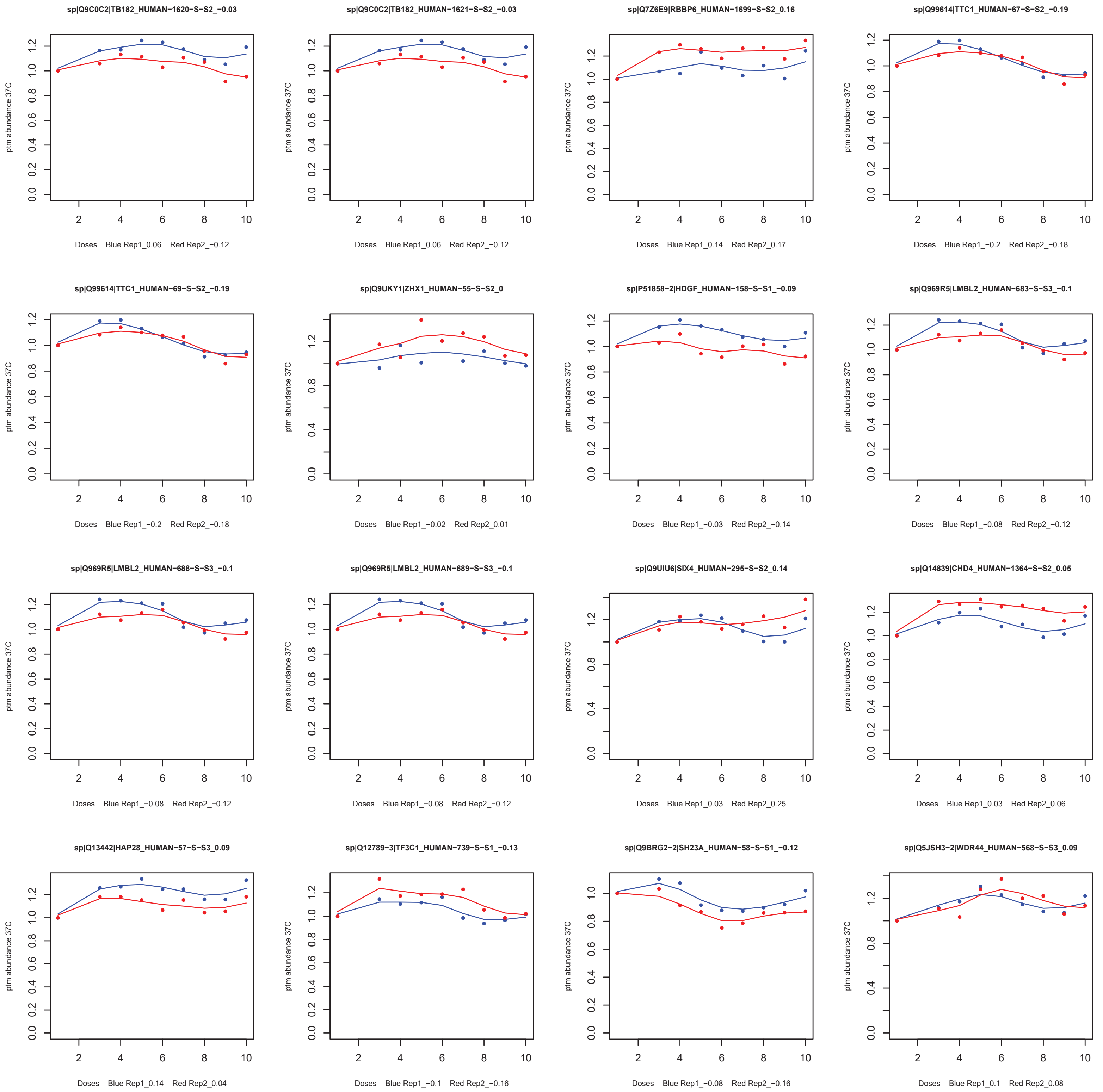
sp|Q9C0C2|TB182_HUMAN−1620−S−S2_−0.03
Doses Blue Rep1_0.06 Red Rep2_−0.12
sp|Q9C0C2|TB182_HUMAN−1621−S−S2_−0.03
Doses Blue Rep1_0.06 Red Rep2_−0.12
sp|Q7Z6E9|RBBP6_HUMAN−1699−S−S2_0.16
Doses Blue Rep1_0.14 Red Rep2_0.17
sp|Q99614|TTC1_HUMAN−67−S−S2_−0.19
Doses Blue Rep1_−0.2 Red Rep2_−0.18
sp|Q99614|TTC1_HUMAN−69−S−S2_−0.19
Doses Blue Rep1_−0.2 Red Rep2_−0.18
sp|Q9UKY1|ZHX1_HUMAN−55−S−S2_0
Doses Blue Rep1_−0.02 Red Rep2_0.01
sp|P51858−2|HDGF_HUMAN−158−S−S1_−0.09
Doses Blue Rep1_−0.03 Red Rep2_−0.14
sp|Q969R5|LMBL2_HUMAN−683−S−S3_−0.1
Doses Blue Rep1_−0.08 Red Rep2_−0.12
sp|Q969R5|LMBL2_HUMAN−688−S−S3_−0.1
Doses Blue Rep1_−0.08 Red Rep2_−0.12
sp|Q969R5|LMBL2_HUMAN−689−S−S3_−0.1
Doses Blue Rep1_−0.08 Red Rep2_−0.12
sp|Q9UIU6|SIX4_HUMAN−295−S−S2_0.14
Doses Blue Rep1_0.03 Red Rep2_0.25
sp|Q14839|CHD4_HUMAN−1364−S−S2_0.05
Doses Blue Rep1_0.03 Red Rep2_0.06
sp|Q13442|HAP28_HUMAN−57−S−S3_0.09
Doses Blue Rep1_0.14 Red Rep2_0.04
sp|Q12789−3|TF3C1_HUMAN−739−S−S1_−0.13
Doses Blue Rep1_−0.1 Red Rep2_−0.16
sp|Q9BRG2−2|SH23A_HUMAN−58−S−S1_−0.12
Doses Blue Rep1_−0.08 Red Rep2_−0.16
sp|Q5JSH3−2|WDR44_HUMAN−568−S−S3_0.09
Doses Blue Rep1_0.1 Red Rep2_0.08
ptm abundance 37C

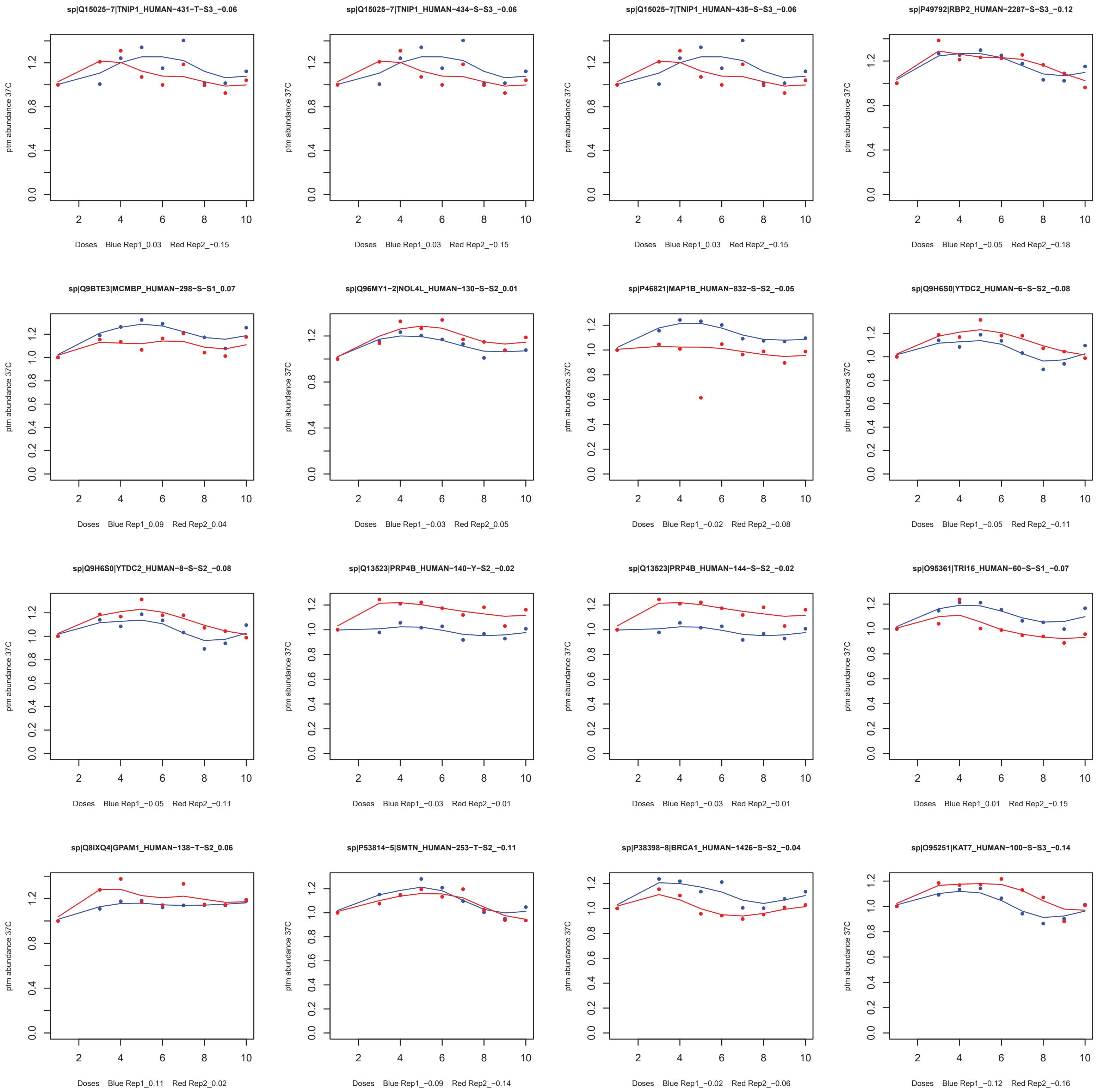
sp|Q15025−7|TNIP1_HUMAN−431−T−S3_−0.06
Doses Blue Rep1_0.03 Red Rep2_−0.15
sp|Q15025−7|TNIP1_HUMAN−434−S−S3_−0.06
Doses Blue Rep1_0.03 Red Rep2_−0.15
sp|Q15025−7|TNIP1_HUMAN−435−S−S3_−0.06
Doses Blue Rep1_0.03 Red Rep2_−0.15
sp|P49792|RBP2_HUMAN−2287−S−S3_−0.12
Doses Blue Rep1_−0.05 Red Rep2_−0.18
sp|Q9BTE3|MCMBP_HUMAN−298−S−S1_0.07
Doses Blue Rep1_0.09 Red Rep2_0.04
sp|Q96MY1−2|NOL4L_HUMAN−130−S−S2_0.01
Doses Blue Rep1_−0.03 Red Rep2_0.05
sp|P46821|MAP1B_HUMAN−832−S−S2_−0.05
Doses Blue Rep1_−0.02 Red Rep2_−0.08
sp|Q9H6S0|YTDC2_HUMAN−6−S−S2_−0.08
Doses Blue Rep1_−0.05 Red Rep2_−0.11
sp|Q9H6S0|YTDC2_HUMAN−8−S−S2_−0.08
Doses Blue Rep1_−0.05 Red Rep2_−0.11
sp|Q13523|PRP4B_HUMAN−140−Y−S2_−0.02
Doses Blue Rep1_−0.03 Red Rep2_−0.01
sp|Q13523|PRP4B_HUMAN−144−S−S2_−0.02
Doses Blue Rep1_−0.03 Red Rep2_−0.01
sp|O95361|TRI16_HUMAN−60−S−S1_−0.07
Doses Blue Rep1_0.01 Red Rep2_−0.15
sp|Q8IXQ4|GPAM1_HUMAN−138−T−S2_0.06
Doses Blue Rep1_0.11 Red Rep2_0.02
sp|P53814−5|SMTN_HUMAN−253−T−S2_−0.11
Doses Blue Rep1_−0.09 Red Rep2_−0.14
sp|P38398−8|BRCA1_HUMAN−1426−S−S2_−0.04
Doses Blue Rep1_−0.02 Red Rep2_−0.06
sp|O95251|KAT7_HUMAN−100−S−S3_−0.14
Doses Blue Rep1_−0.12 Red Rep2_−0.16
ptm abundance 37C

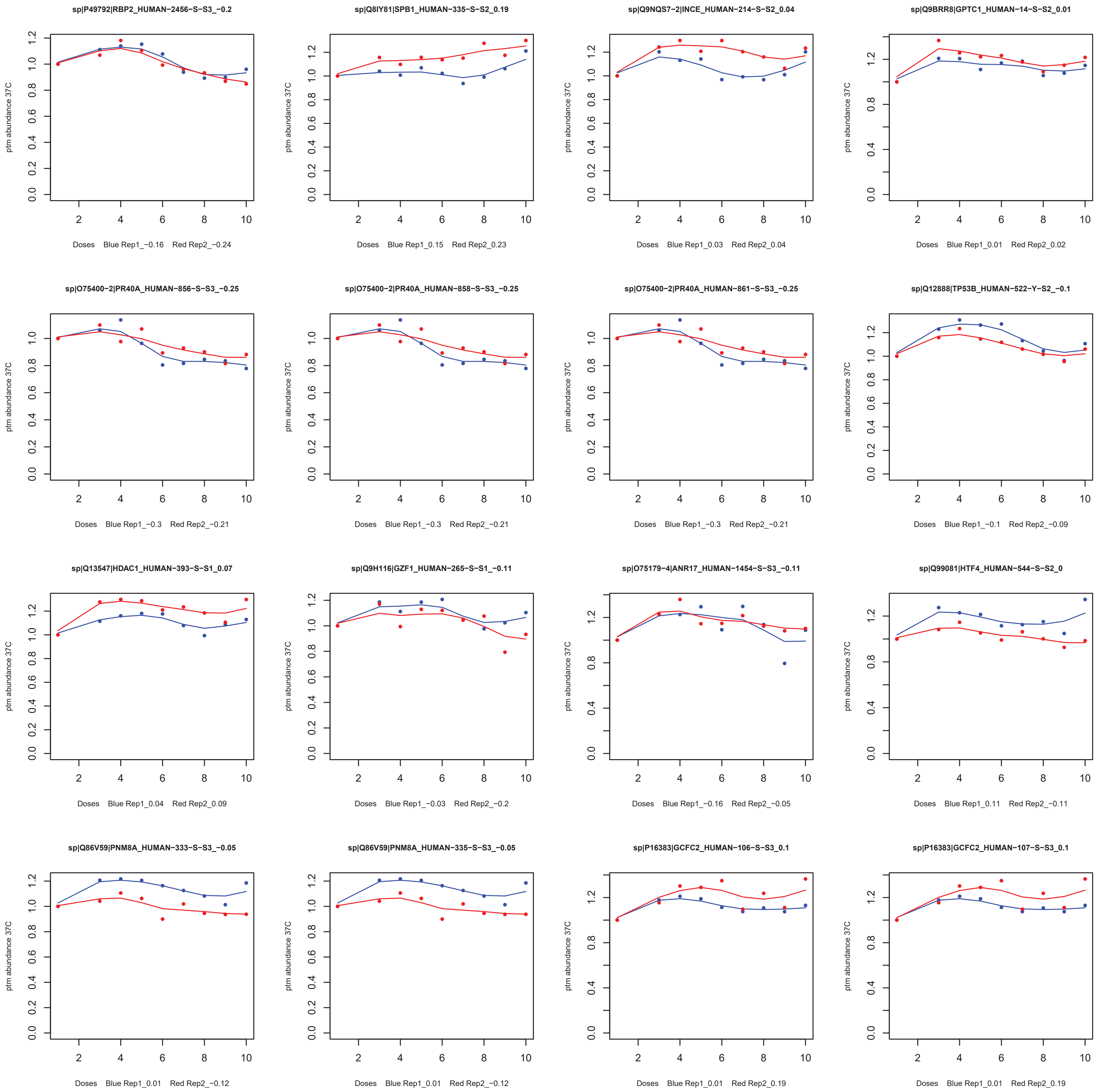

sp|P49792|RBP2_HUMAN−2456−S−S3_−0.2
Doses Blue Rep1_−0.16 Red Rep2_−0.24
sp|Q8IY81|SPB1_HUMAN−335−S−S2_0.19
Doses Blue Rep1_0.15 Red Rep2_0.23
sp|Q9NQS7−2|INCE_HUMAN−214−S−S2_0.04
Doses Blue Rep1_0.03 Red Rep2_0.04
sp|Q9BRR8|GPTC1_HUMAN−14−S−S2_0.01
Doses Blue Rep1_0.01 Red Rep2_0.02
sp|O75400−2|PR40A_HUMAN−856−S−S3_−0.25
Doses Blue Rep1_−0.3 Red Rep2_−0.21
sp|O75400−2|PR40A_HUMAN−858−S−S3_−0.25
Doses Blue Rep1_−0.3 Red Rep2_−0.21
sp|O75400−2|PR40A_HUMAN−861−S−S3_−0.25
Doses Blue Rep1_−0.3 Red Rep2_−0.21
sp|Q12888|TP53B_HUMAN−522−Y−S2_−0.1
Doses Blue Rep1_−0.1 Red Rep2_−0.09
sp|Q13547|HDAC1_HUMAN−393−S−S1_0.07
Doses Blue Rep1_0.04 Red Rep2_0.09
sp|Q9H116|GZF1_HUMAN−265−S−S1_−0.11
Doses Blue Rep1_−0.03 Red Rep2_−0.2
sp|O75179−4|ANR17_HUMAN−1454−S−S3_−0.11
Doses Blue Rep1_−0.16 Red Rep2_−0.05
sp|Q99081|HTF4_HUMAN−544−S−S2_0
Doses Blue Rep1_0.11 Red Rep2_−0.11
sp|Q86V59|PNM8A_HUMAN−333−S−S3_−0.05
Doses Blue Rep1_0.01 Red Rep2_−0.12
sp|Q86V59|PNM8A_HUMAN−335−S−S3_−0.05
Doses Blue Rep1_0.01 Red Rep2_−0.12
sp|P16383|GCFC2_HUMAN−106−S−S3_0.1
Doses Blue Rep1_0.01 Red Rep2_0.19
sp|P16383|GCFC2_HUMAN−107−S−S3_0.1
Doses Blue Rep1_0.01 Red Rep2_0.19
ptm abundance 37C

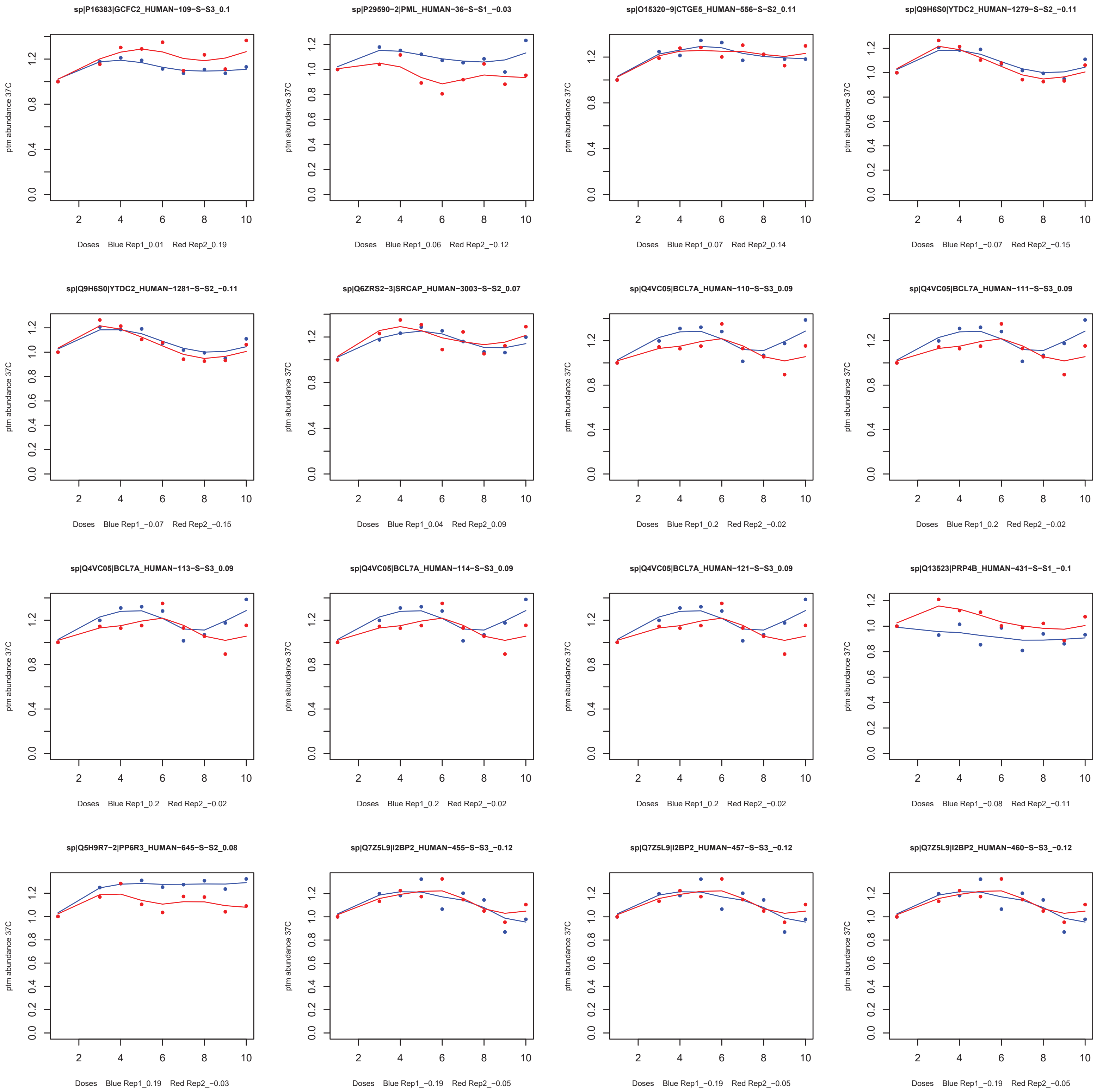
sp|P16383|GCFC2_HUMAN−109−S−S3_0.1
ptm abundance 37C
Doses Blue Rep1_0.01 Red Rep2_0.19
sp|P29590−2|PML_HUMAN−36−S−S1_−0.03
ptm abundance 37C
Doses Blue Rep1_0.06 Red Rep2_−0.12
sp|O15320−9|CTGE5_HUMAN−556−S−S2_0.11
ptm abundance 37C
Doses Blue Rep1_0.07 Red Rep2_0.14
sp|Q9H6S0|YTDC2_HUMAN−1279−S−S2_−0.11
ptm abundance 37C
Doses Blue Rep1_−0.07 Red Rep2_−0.15
sp|Q9H6S0|YTDC2_HUMAN−1281−S−S2_−0.11
ptm abundance 37C
Doses Blue Rep1_−0.07 Red Rep2_−0.15
sp|Q6ZRS2−3|SRCAP_HUMAN−3003−S−S2_0.07
ptm abundance 37C
Doses Blue Rep1_0.04 Red Rep2_0.09
sp|Q4VC05|BCL7A_HUMAN−110−S−S3_0.09
ptm abundance 37C
Doses Blue Rep1_0.2 Red Rep2_−0.02
sp|Q4VC05|BCL7A_HUMAN−111−S−S3_0.09
ptm abundance 37C
Doses Blue Rep1_0.2 Red Rep2_−0.02
sp|Q4VC05|BCL7A_HUMAN−113−S−S3_0.09
ptm abundance 37C
Doses Blue Rep1_0.2 Red Rep2_−0.02
sp|Q4VC05|BCL7A_HUMAN−114−S−S3_0.09
ptm abundance 37C
Doses Blue Rep1_0.2 Red Rep2_−0.02
sp|Q4VC05|BCL7A_HUMAN−121−S−S3_0.09
ptm abundance 37C
Doses Blue Rep1_0.2 Red Rep2_−0.02
sp|Q13523|PRP4B_HUMAN−431−S−S1_−0.1
ptm abundance 37C
Doses Blue Rep1_−0.08 Red Rep2_−0.11
sp|Q5H9R7−2|PP6R3_HUMAN−645−S−S2_0.08
ptm abundance 37C
Doses Blue Rep1_0.19 Red Rep2_−0.03
sp|Q7Z5L9|I2BP2_HUMAN−455−S−S3_−0.12
ptm abundance 37C
Doses Blue Rep1_−0.19 Red Rep2_−0.05
sp|Q7Z5L9|I2BP2_HUMAN−457−S−S3_−0.12
ptm abundance 37C
Doses Blue Rep1_−0.19 Red Rep2_−0.05
sp|Q7Z5L9|I2BP2_HUMAN−460−S−S3_−0.12
ptm abundance 37C
Doses Blue Rep1_−0.19 Red Rep2_−0.05

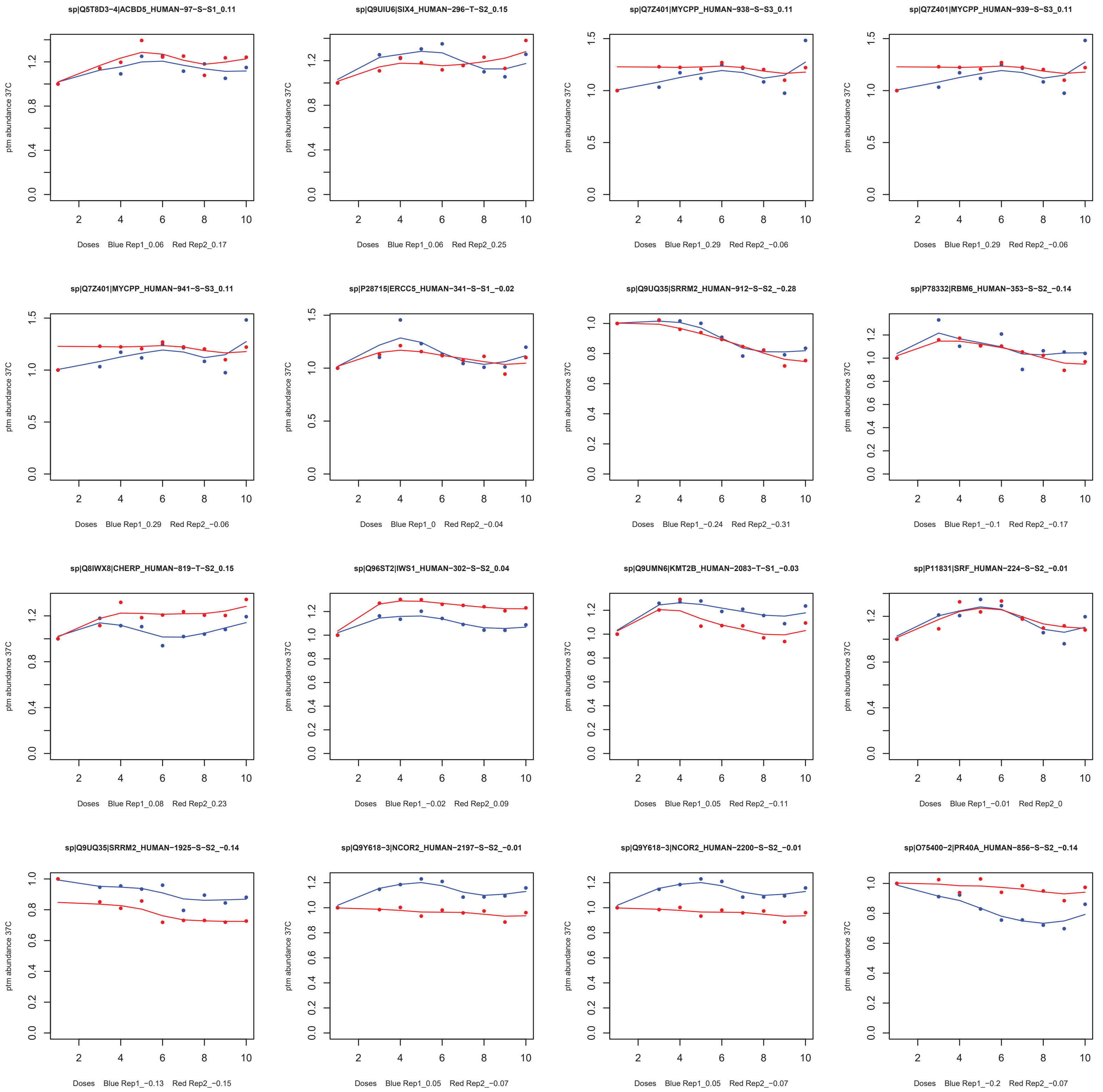

sp|Q5T8D3−4|ACBD5_HUMAN−97−S−S1_0.11
ptm abundance 37C
Doses Blue Rep1_0.06 Red Rep2_0.17
sp|Q9UIU6|SIX4_HUMAN−296−T−S2_0.15
ptm abundance 37C
Doses Blue Rep1_0.06 Red Rep2_0.25
sp|Q7Z401|MYCPP_HUMAN−938−S−S3_0.11
ptm abundance 37C
Doses Blue Rep1_0.29 Red Rep2_−0.06
sp|Q7Z401|MYCPP_HUMAN−939−S−S3_0.11
ptm abundance 37C
Doses Blue Rep1_0.29 Red Rep2_−0.06
sp|Q7Z401|MYCPP_HUMAN−941−S−S3_0.11
ptm abundance 37C
Doses Blue Rep1_0.29 Red Rep2_−0.06
sp|P28715|ERCC5_HUMAN−341−S−S1_−0.02
ptm abundance 37C
Doses Blue Rep1_0 Red Rep2_−0.04
sp|Q9UQ35|SRRM2_HUMAN−912−S−S2_−0.28
ptm abundance 37C
Doses Blue Rep1_−0.24 Red Rep2_−0.31
sp|P78332|RBM6_HUMAN−353−S−S2_−0.14
ptm abundance 37C
Doses Blue Rep1_−0.1 Red Rep2_−0.17
sp|Q8IWX8|CHERP_HUMAN−819−T−S2_0.15
ptm abundance 37C
Doses Blue Rep1_0.08 Red Rep2_0.23
sp|Q96ST2|IWS1_HUMAN−302−S−S2_0.04
ptm abundance 37C
Doses Blue Rep1_−0.02 Red Rep2_0.09
sp|Q9UMN6|KMT2B_HUMAN−2083−T−S1_−0.03
ptm abundance 37C
Doses Blue Rep1_0.05 Red Rep2_−0.11
sp|P11831|SRF_HUMAN−224−S−S2_−0.01
ptm abundance 37C
Doses Blue Rep1_−0.01 Red Rep2_0
sp|Q9UQ35|SRRM2_HUMAN−1925−S−S2_−0.14
ptm abundance 37C
Doses Blue Rep1_−0.13 Red Rep2_−0.15
sp|Q9Y618−3|NCOR2_HUMAN−2197−S−S2_−0.01
ptm abundance 37C
Doses Blue Rep1_0.05 Red Rep2_−0.07
sp|Q9Y618−3|NCOR2_HUMAN−2200−S−S2_−0.01
ptm abundance 37C
Doses Blue Rep1_0.05 Red Rep2_−0.07
sp|O75400−2|PR40A_HUMAN−856−S−S2_−0.14
ptm abundance 37C
Doses Blue Rep1_−0.2 Red Rep2_−0.07

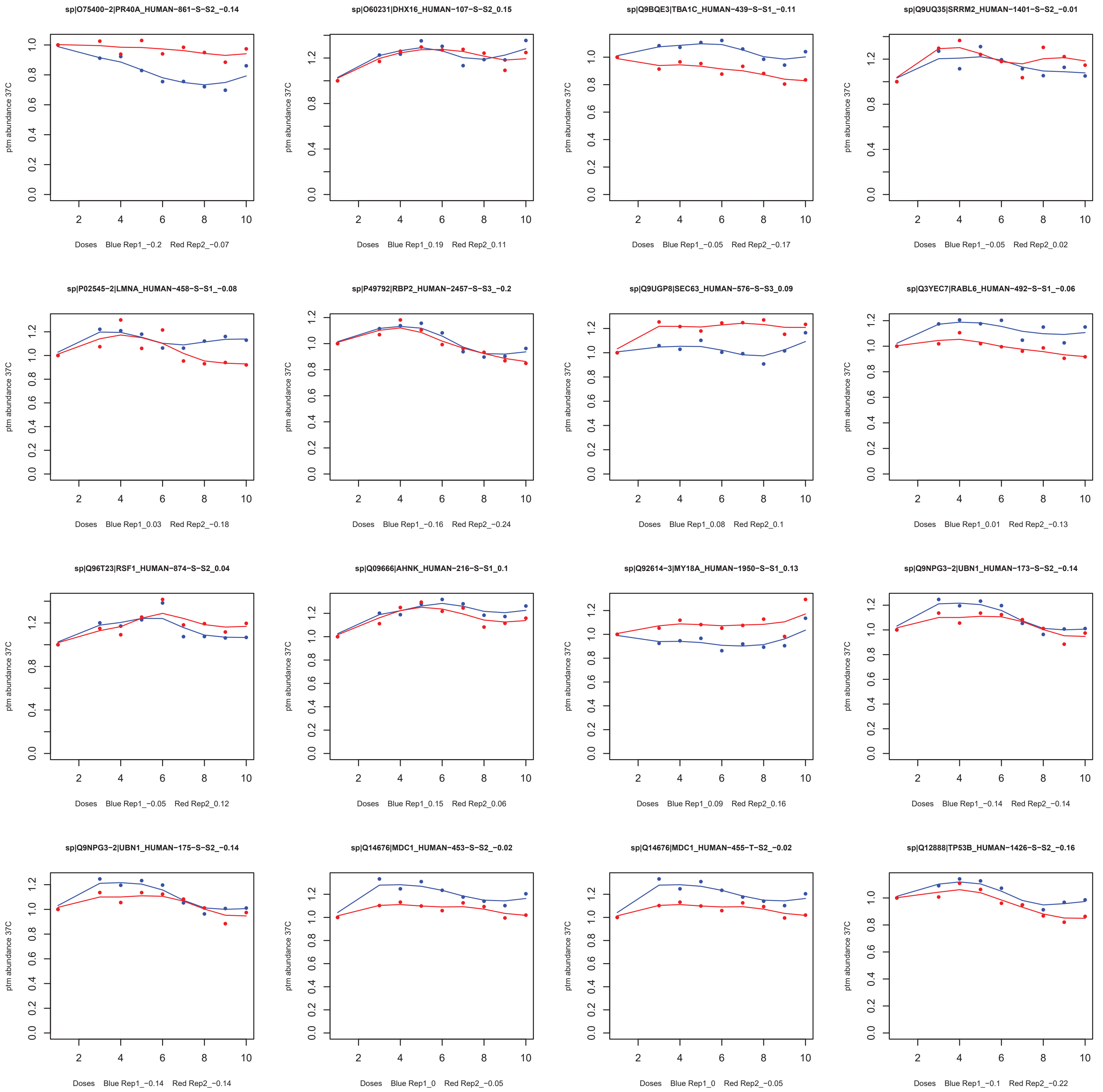

sp|O75400−2|PR40A_HUMAN−861−S−S2_−0.14
ptm abundance 37C
Doses Blue Rep1_−0.2 Red Rep2_−0.07
sp|O60231|DHX16_HUMAN−107−S−S2_0.15
ptm abundance 37C
Doses Blue Rep1_0.19 Red Rep2_0.11
sp|Q9BQE3|TBA1C_HUMAN−439−S−S1_−0.11
ptm abundance 37C
Doses Blue Rep1_−0.05 Red Rep2_−0.17
sp|Q9UQ35|SRRM2_HUMAN−1401−S−S2_−0.01
ptm abundance 37C
Doses Blue Rep1_−0.05 Red Rep2_0.02
sp|P02545−2|LMNA_HUMAN−458−S−S1_−0.08
ptm abundance 37C
Doses Blue Rep1_0.03 Red Rep2_−0.18
sp|P49792|RBP2_HUMAN−2457−S−S3_−0.2
ptm abundance 37C
Doses Blue Rep1_−0.16 Red Rep2_−0.24
sp|Q9UGP8|SEC63_HUMAN−576−S−S3_0.09
ptm abundance 37C
Doses Blue Rep1_0.08 Red Rep2_0.1
sp|Q3YEC7|RABL6_HUMAN−492−S−S1_−0.06
ptm abundance 37C
Doses Blue Rep1_0.01 Red Rep2_−0.13
sp|Q96T23|RSF1_HUMAN−874−S−S2_0.04
ptm abundance 37C
Doses Blue Rep1_−0.05 Red Rep2_0.12
sp|Q09666|AHNK_HUMAN−216−S−S1_0.1
ptm abundance 37C
Doses Blue Rep1_0.15 Red Rep2_0.06
sp|Q92614−3|MY18A_HUMAN−1950−S−S1_0.13
ptm abundance 37C
Doses Blue Rep1_0.09 Red Rep2_0.16
sp|Q9NPG3−2|UBN1_HUMAN−173−S−S2_−0.14
ptm abundance 37C
Doses Blue Rep1_−0.14 Red Rep2_−0.14
sp|Q9NPG3−2|UBN1_HUMAN−175−S−S2_−0.14
ptm abundance 37C
Doses Blue Rep1_−0.14 Red Rep2_−0.14
sp|Q14676|MDC1_HUMAN−453−S−S2_−0.02
ptm abundance 37C
Doses Blue Rep1_0 Red Rep2_−0.05
sp|Q14676|MDC1_HUMAN−455−T−S2_−0.02
ptm abundance 37C
Doses Blue Rep1_0 Red Rep2_−0.05
sp|Q12888|TP53B_HUMAN−1426−S−S2_−0.16
ptm abundance 37C
Doses Blue Rep1_−0.1 Red Rep2_−0.22

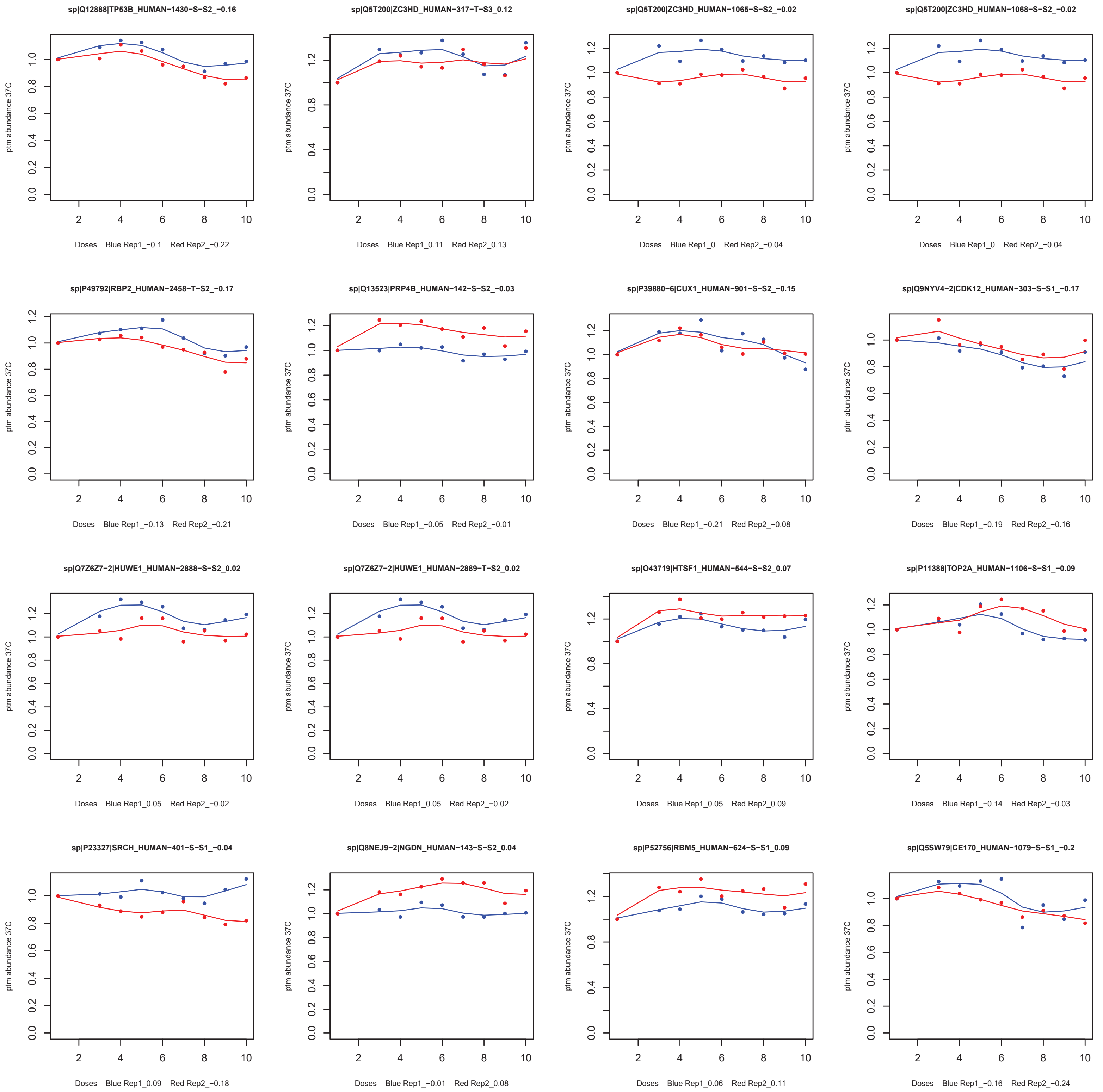
sp|Q12888|TP53B_HUMAN−1430−S−S2_−0.16
ptm abundance 37C
Doses Blue Rep1_−0.1 Red Rep2_−0.22
sp|Q5T200|ZC3HD_HUMAN−317−T−S3_0.12
ptm abundance 37C
Doses Blue Rep1_0.11 Red Rep2_0.13
sp|Q5T200|ZC3HD_HUMAN−1065−S−S2_−0.02
ptm abundance 37C
Doses Blue Rep1_0 Red Rep2_−0.04
sp|Q5T200|ZC3HD_HUMAN−1068−S−S2_−0.02
ptm abundance 37C
Doses Blue Rep1_0 Red Rep2_−0.04
sp|P49792|RBP2_HUMAN−2458−T−S2_−0.17
ptm abundance 37C
Doses Blue Rep1_−0.13 Red Rep2_−0.21
sp|Q13523|PRP4B_HUMAN−142−S−S2_−0.03
ptm abundance 37C
Doses Blue Rep1_−0.05 Red Rep2_−0.01
sp|P39880−6|CUX1_HUMAN−901−S−S2_−0.15
ptm abundance 37C
Doses Blue Rep1_−0.21 Red Rep2_−0.08
sp|Q9NYV4−2|CDK12_HUMAN−303−S−S1_−0.17
ptm abundance 37C
Doses Blue Rep1_−0.19 Red Rep2_−0.16
sp|Q7Z6Z7−2|HUWE1_HUMAN−2888−S−S2_0.02
ptm abundance 37C
Doses Blue Rep1_0.05 Red Rep2_−0.02
sp|Q7Z6Z7−2|HUWE1_HUMAN−2889−T−S2_0.02
ptm abundance 37C
Doses Blue Rep1_0.05 Red Rep2_−0.02
sp|O43719|HTSF1_HUMAN−544−S−S2_0.07
ptm abundance 37C
Doses Blue Rep1_0.05 Red Rep2_0.09
sp|P11388|TOP2A_HUMAN−1106−S−S1_−0.09
ptm abundance 37C
Doses Blue Rep1_−0.14 Red Rep2_−0.03
sp|P23327|SRCH_HUMAN−401−S−S1_−0.04
ptm abundance 37C
Doses Blue Rep1_0.09 Red Rep2_−0.18
sp|Q8NEJ9−2|NGDN_HUMAN−143−S−S2_0.04
ptm abundance 37C
Doses Blue Rep1_−0.01 Red Rep2_0.08
sp|P52756|RBM5_HUMAN−624−S−S1_0.09
ptm abundance 37C
Doses Blue Rep1_0.06 Red Rep2_0.11
sp|Q5SW79|CE170_HUMAN−1079−S−S1_−0.2
ptm abundance 37C
Doses Blue Rep1_−0.16 Red Rep2_−0.24

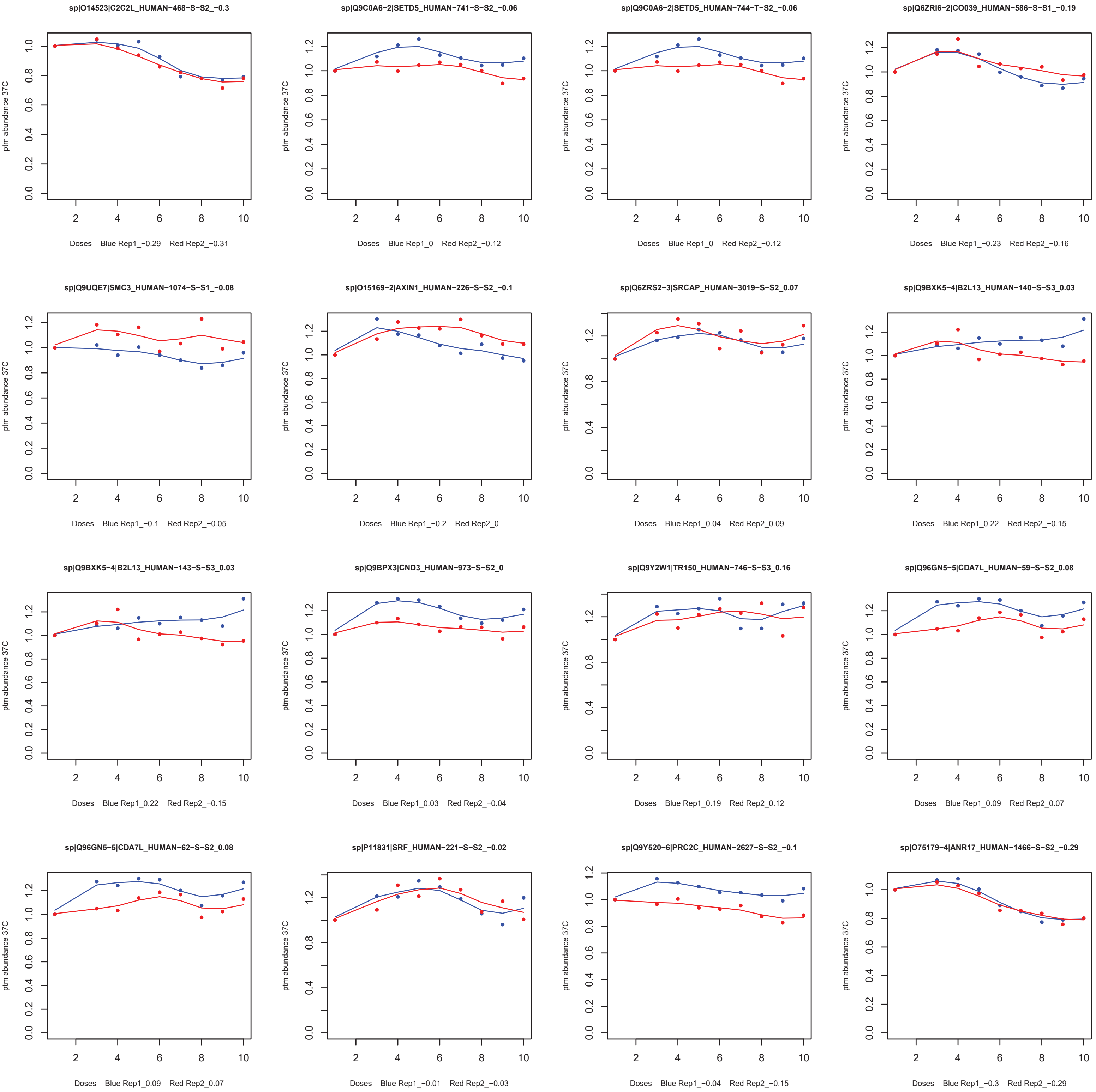
sp|O14523|C2C2L_HUMAN−468−S−S2_−0.3
Doses Blue Rep1_−0.29 Red Rep2_−0.31
sp|Q9C0A6−2|SETD5_HUMAN−741−S−S2_−0.06
Doses Blue Rep1_0 Red Rep2_−0.12
sp|Q9C0A6−2|SETD5_HUMAN−744−T−S2_−0.06
Doses Blue Rep1_0 Red Rep2_−0.12
sp|Q6ZRI6−2|CO039_HUMAN−586−S−S1_−0.19
Doses Blue Rep1_−0.23 Red Rep2_−0.16
sp|Q9UQE7|SMC3_HUMAN−1074−S−S1_−0.08
Doses Blue Rep1_−0.1 Red Rep2_−0.05
sp|O15169−2|AXIN1_HUMAN−226−S−S2_−0.1
Doses Blue Rep1_−0.2 Red Rep2_0
sp|Q6ZRS2−3|SRCAP_HUMAN−3019−S−S2_0.07
Doses Blue Rep1_0.04 Red Rep2_0.09
sp|Q9BXK5−4|B2L13_HUMAN−140−S−S3_0.03
Doses Blue Rep1_0.22 Red Rep2_−0.15
sp|Q9BXK5−4|B2L13_HUMAN−143−S−S3_0.03
Doses Blue Rep1_0.22 Red Rep2_−0.15
sp|Q9BPX3|CND3_HUMAN−973−S−S2_0
Doses Blue Rep1_0.03 Red Rep2_−0.04
sp|Q9Y2W1|TR150_HUMAN−746−S−S3_0.16
Doses Blue Rep1_0.19 Red Rep2_0.12
sp|Q96GN5−5|CDA7L_HUMAN−59−S−S2_0.08
Doses Blue Rep1_0.09 Red Rep2_0.07
sp|Q96GN5−5|CDA7L_HUMAN−62−S−S2_0.08
Doses Blue Rep1_0.09 Red Rep2_0.07
sp|P11831|SRF_HUMAN−221−S−S2_−0.02
Doses Blue Rep1_−0.01 Red Rep2_−0.03
sp|Q9Y520−6|PRC2C_HUMAN−2627−S−S2_−0.1
Doses Blue Rep1_−0.04 Red Rep2_−0.15
sp|O75179−4|ANR17_HUMAN−1466−S−S2_−0.29
Doses Blue Rep1_−0.3 Red Rep2_−0.29
ptm abundance 37C

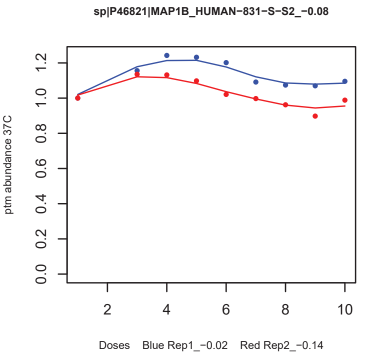
sp|P46821|MAP1B_HUMAN−831−S−S2_−0.08
ptm abundance 37C
Doses Blue Rep1_−0.02 Red Rep2_−0.14

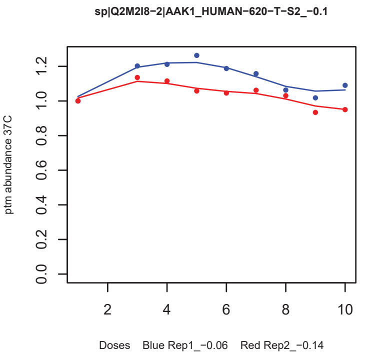
sp|Q2M2I8−2|AAK1_HUMAN−620−T−S2_−0.1
ptm abundance 37C
Doses Blue Rep1_−0.06 Red Rep2_−0.14

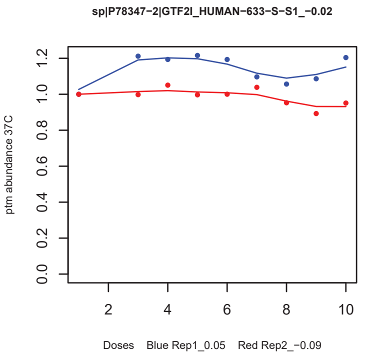
sp|P78347−2|GTF2I_HUMAN−633−S−S1_−0.02
ptm abundance 37C
Doses Blue Rep1_0.05 Red Rep2_−0.09

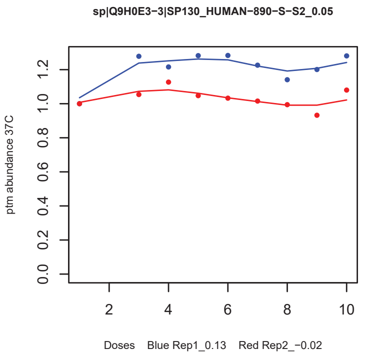
sp|Q9H0E3−3|SP130_HUMAN−890−S−S2_0.05
ptm abundance 37C
Doses Blue Rep1_0.13 Red Rep2_−0.02

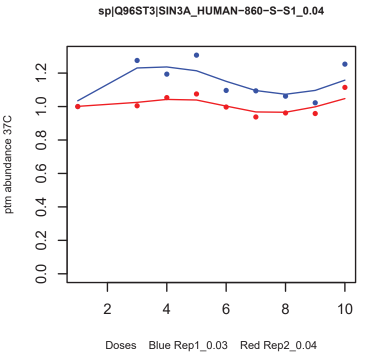
sp|Q96ST3|SIN3A_HUMAN−860−S−S1_0.04
ptm abundance 37C
Doses Blue Rep1_0.03 Red Rep2_0.04

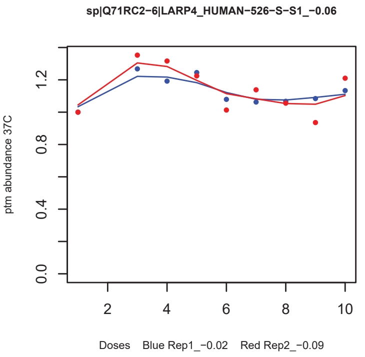
sp|Q71RC2−6|LARP4_HUMAN−526−S−S1_−0.06
ptm abundance 37C
Doses Blue Rep1_−0.02 Red Rep2_−0.09

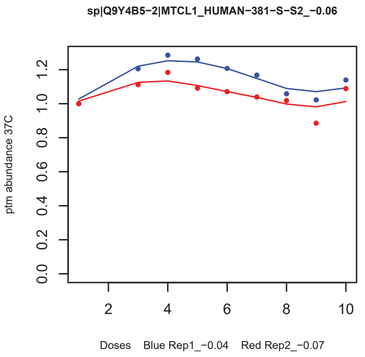
sp|Q9Y4B5−2|MTCL1_HUMAN−381−S−S2_−0.06
ptm abundance 37C
Doses Blue Rep1_−0.04 Red Rep2_−0.07

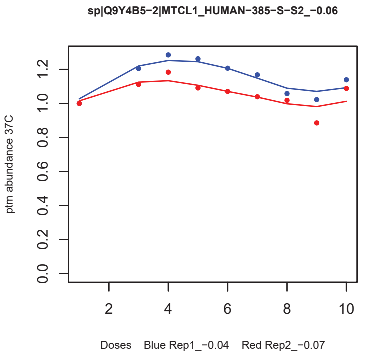
sp|Q9Y4B5−2|MTCL1_HUMAN−385−S−S2_−0.06
ptm abundance 37C
Doses Blue Rep1_−0.04 Red Rep2_−0.07

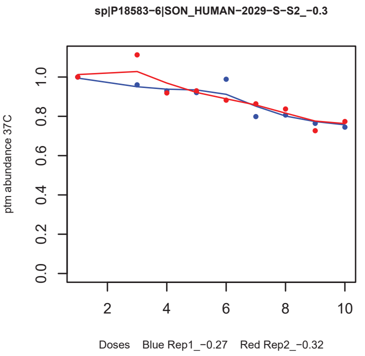
sp|P18583−6|SON_HUMAN−2029−S−S2_−0.3
ptm abundance 37C
Doses Blue Rep1_−0.27 Red Rep2_−0.32

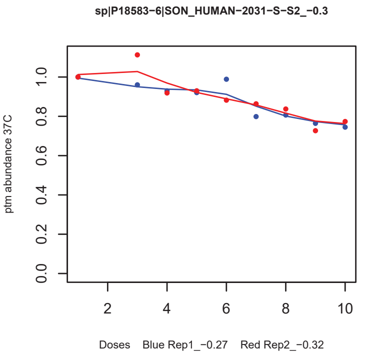
sp|P18583−6|SON_HUMAN−2031−S−S2_−0.3
ptm abundance 37C
Doses Blue Rep1_−0.27 Red Rep2_−0.32

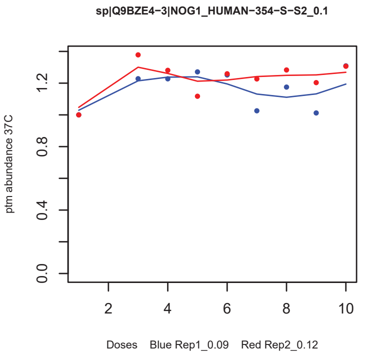
sp|Q9BZE4−3|NOG1_HUMAN−354−S−S2_0.1
ptm abundance 37C
Doses Blue Rep1_0.09 Red Rep2_0.12

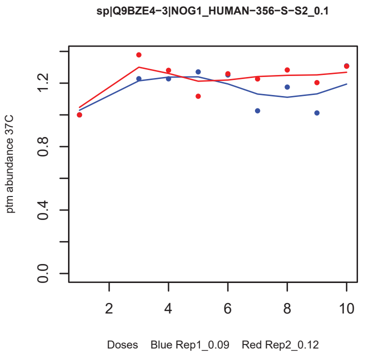
sp|Q9BZE4−3|NOG1_HUMAN−356−S−S2_0.1
ptm abundance 37C
Doses Blue Rep1_0.09 Red Rep2_0.12

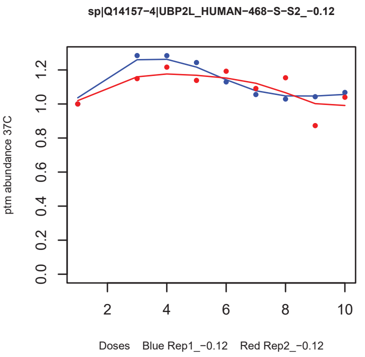
sp|Q14157−4|UBP2L_HUMAN−468−S−S2_−0.12
ptm abundance 37C
Doses Blue Rep1_−0.12 Red Rep2_−0.12

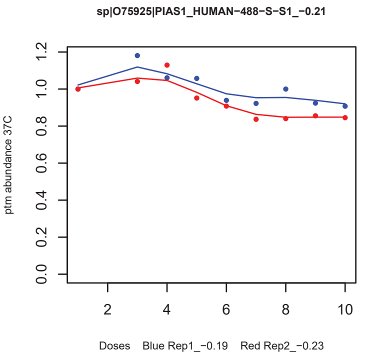
sp|O75925|PIAS1_HUMAN−488−S−S1_−0.21
ptm abundance 37C
Doses Blue Rep1_−0.19 Red Rep2_−0.23

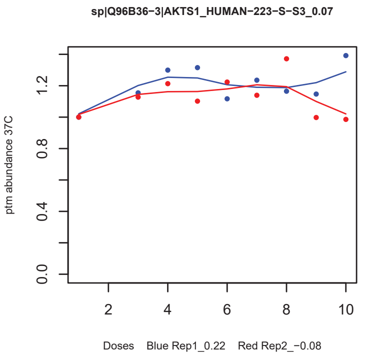
sp|Q96B36−3|AKTS1_HUMAN−223−S−S3_0.07
ptm abundance 37C
Doses Blue Rep1_0.22 Red Rep2_−0.08

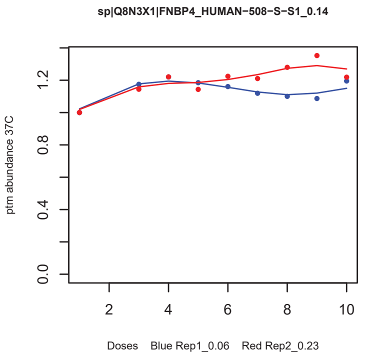
sp|Q8N3X1|FNBP4_HUMAN−508−S−S1_0.14
ptm abundance 37C
Doses Blue Rep1_0.06 Red Rep2_0.23

sp|Q8N302-2|AGGF1_HUMAN-11-S-S2_0.05

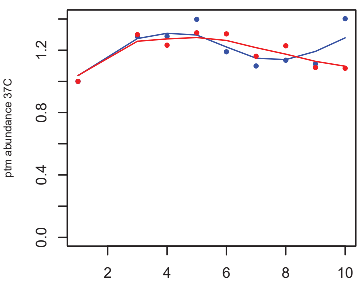

Doses Blue Rep1_0.15 Red Rep2_-0.06

sp|O14523|C2C2L_HUMAN-466-S-S2_-0.3

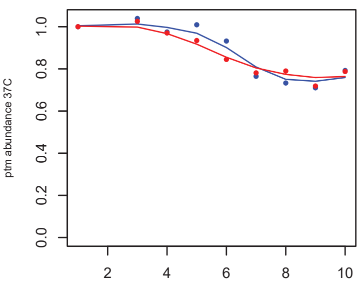

Doses Blue Rep1_-0.31 Red Rep2_-0.3

sp|Q13610-2|PWP1_HUMAN-55-T-S2_-0.02

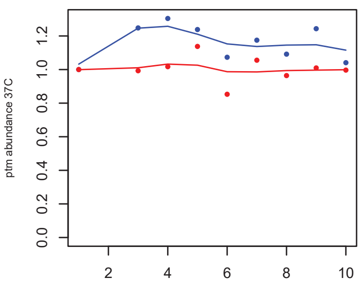

Doses Blue Rep1_-0.03 Red Rep2_-0.01

sp|O43823|AKAP8_HUMAN-339-S-S2_0

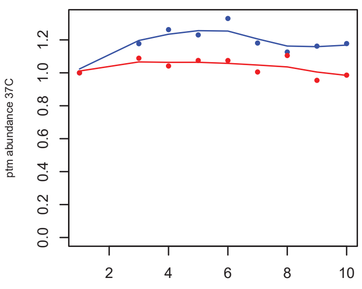

Doses Blue Rep1_0.07 Red Rep2_-0.07

sp|Q9UEG4|ZN629_HUMAN-28-S-S2_0.18

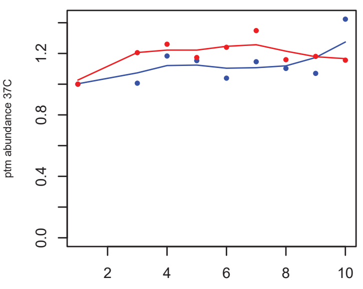

Doses Blue Rep1_0.3 Red Rep2_0.06

sp|Q9UEG4|ZN629_HUMAN-34-S-S2_0.18

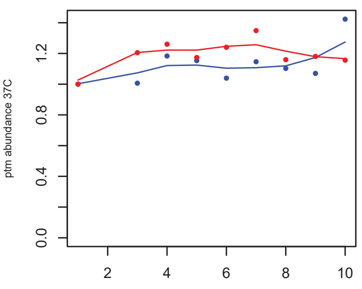

Doses Blue Rep1_0.3 Red Rep2_0.06

sp|Q15059-2|BRD3_HUMAN-263-S-S1_0.01

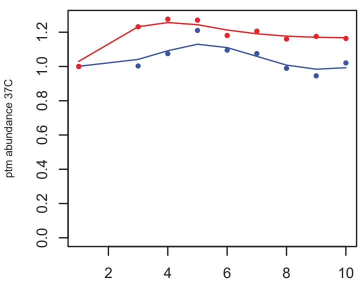

Doses Blue Rep1_-0.04 Red Rep2_0.05

sp|Q3YEC7|RABL6_HUMAN-596-S-S2_0.02

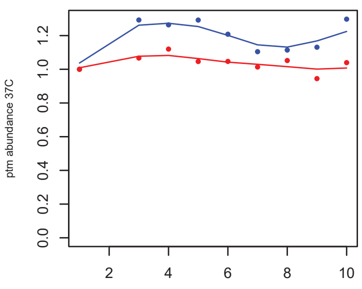

Doses Blue Rep1_0.09 Red Rep2_-0.04

sp|Q3YEC7|RABL6_HUMAN-599-T-S2_0.02

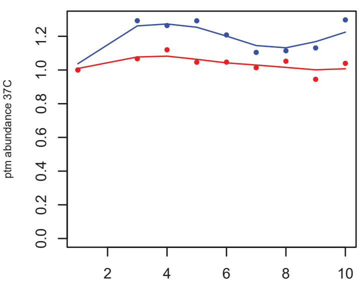

Doses Blue Rep1_0.09 Red Rep2_-0.04

sp|Q9UQ35|SRRM2_HUMAN-486-S-S2_-0.16

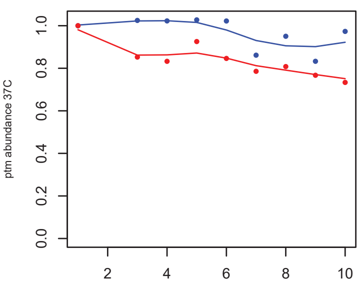

Doses Blue Rep1_-0.11 Red Rep2_-0.21

sp|Q8NFC6|BD1L1_HUMAN-3029-S-S1_-0.08

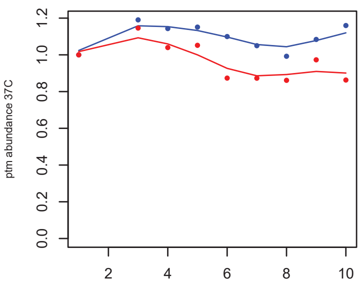

Doses Blue Rep1_0.04 Red Rep2_-0.19

sp|Q8N3X1|FNBP4_HUMAN-966-S-S3_0.09

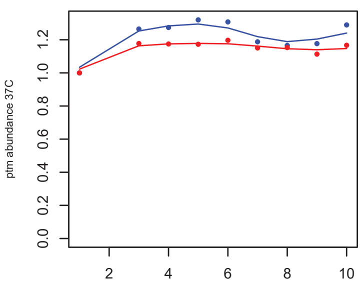

Doses Blue Rep1_0.12 Red Rep2_0.07

sp|Q99613-2|EIF3C_HUMAN-156-S-S1_-0.07

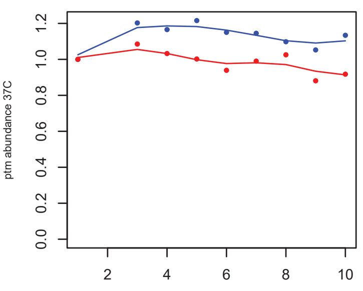

Doses Blue Rep1_0 Red Rep2_-0.15

sp|Q9Y467|SALL2_HUMAN-802-S-S2_-0.04

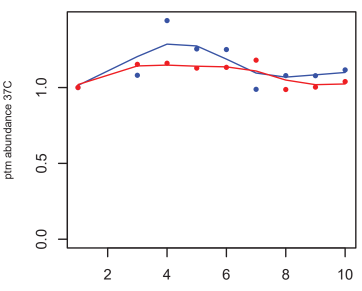

Doses Blue Rep1_-0.01 Red Rep2_-0.07

sp|Q9Y467|SALL2_HUMAN-806-S-S2_-0.04

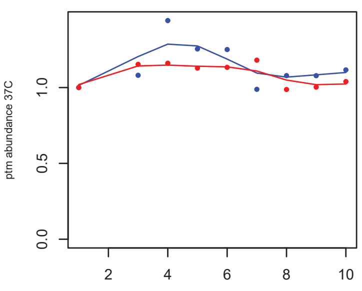

Doses Blue Rep1_-0.01 Red Rep2_-0.07

sp|Q02078-8|MEF2A_HUMAN-30-S-S2_-0.04

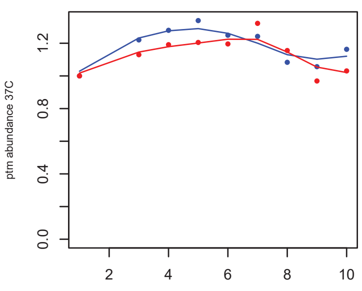

Doses Blue Rep1_-0.01 Red Rep2_-0.08

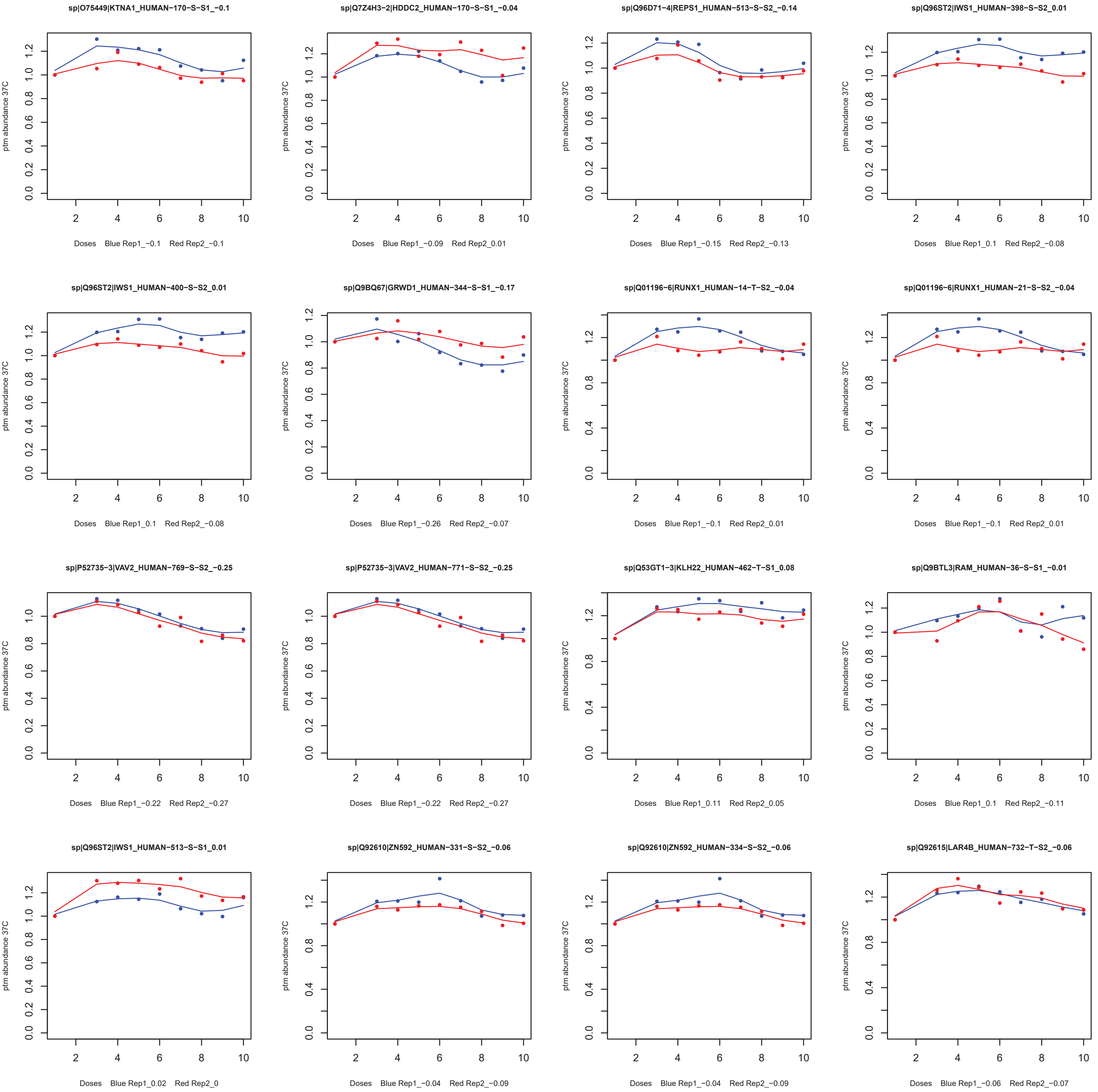
sp|O75449|KTNA1_HUMAN−170−S−S1_−0.1
Doses Blue Rep1_−0.1 Red Rep2_−0.1
sp|Q7Z4H3−2|HDDC2_HUMAN−170−S−S1_−0.04
Doses Blue Rep1_−0.09 Red Rep2_0.01
sp|Q96D71−4|REPS1_HUMAN−513−S−S2_−0.14
Doses Blue Rep1_−0.15 Red Rep2_−0.13
sp|Q96ST2|IWS1_HUMAN−398−S−S2_0.01
Doses Blue Rep1_0.1 Red Rep2_−0.08
sp|Q96ST2|IWS1_HUMAN−400−S−S2_0.01
Doses Blue Rep1_0.1 Red Rep2_−0.08
sp|Q9BQ67|GRWD1_HUMAN−344−S−S1_−0.17
Doses Blue Rep1_−0.26 Red Rep2_−0.07
sp|Q01196−6|RUNX1_HUMAN−14−T−S2_−0.04
Doses Blue Rep1_−0.1 Red Rep2_0.01
sp|Q01196−6|RUNX1_HUMAN−21−S−S2_−0.04
Doses Blue Rep1_−0.1 Red Rep2_0.01
sp|P52735−3|VAV2_HUMAN−769−S−S2_−0.25
Doses Blue Rep1_−0.22 Red Rep2_−0.27
sp|P52735−3|VAV2_HUMAN−771−S−S2_−0.25
Doses Blue Rep1_−0.22 Red Rep2_−0.27
sp|Q53GT1−3|KLH22_HUMAN−462−T−S1_0.08
Doses Blue Rep1_0.11 Red Rep2_0.05
sp|Q9BTL3|RAM_HUMAN−36−S−S1_−0.01
Doses Blue Rep1_0.1 Red Rep2_−0.11
sp|Q96ST2|IWS1_HUMAN−513−S−S1_0.01
Doses Blue Rep1_0.02 Red Rep2_0
sp|Q92610|ZN592_HUMAN−331−S−S2_−0.06
Doses Blue Rep1_−0.04 Red Rep2_−0.09
sp|Q92610|ZN592_HUMAN−334−S−S2_−0.06
Doses Blue Rep1_−0.04 Red Rep2_−0.09
sp|Q92615|LAR4B_HUMAN−732−T−S2_−0.06
Doses Blue Rep1_−0.06 Red Rep2_−0.07
ptm abundance 37C

sp|O95218−2|ZRAB2_HUMAN−266−S−S3_−0.02

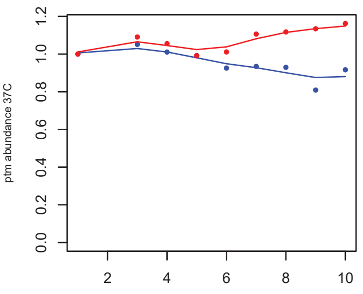

Doses Blue Rep1_−0.17 Red Rep2_0.14

sp|O95218−2|ZRAB2_HUMAN−267−S−S3_−0.02

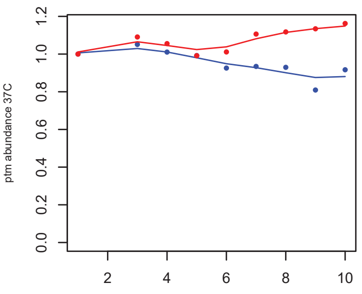

Doses Blue Rep1_−0.17 Red Rep2_0.14

sp|O95218−2|ZRAB2_HUMAN−268−S−S3_−0.02

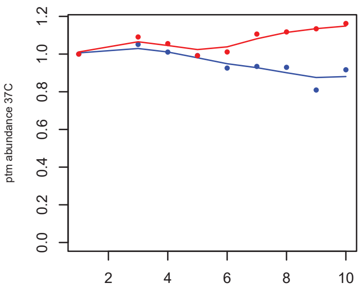

Doses Blue Rep1_−0.17 Red Rep2_0.14

sp|Q9UGU0−2|TCF20_HUMAN−574−S−S1_0.12

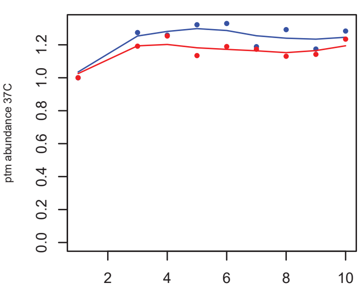

Doses Blue Rep1_0.13 Red Rep2_0.11

sp|Q9BZF1−3|OSBL8_HUMAN−757−S−S2_0

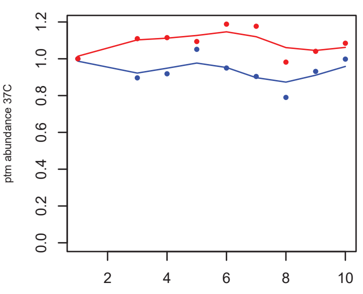

Doses Blue Rep1_0 Red Rep2_0

sp|O75376|NCOR1_HUMAN−172−S−S2_−0.03

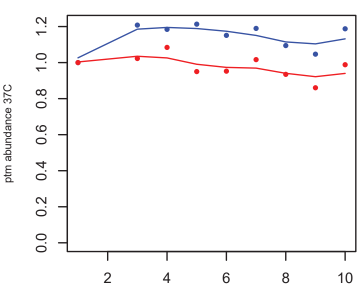

Doses Blue Rep1_0.03 Red Rep2_−0.1

sp|Q12789−3|TF3C1_HUMAN−1845−S−S2_−0.05

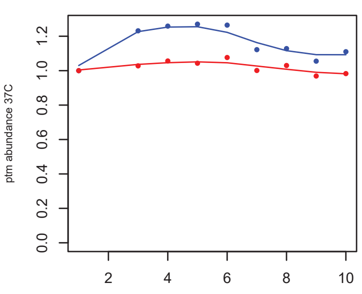

Doses Blue Rep1_−0.04 Red Rep2_−0.05

sp|Q96ST2|IWS1_HUMAN−438−S−S1_−0.03

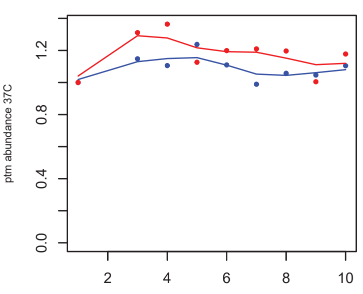

Doses Blue Rep1_0.01 Red Rep2_−0.06

sp|Q5H9R7−2|PP6R3_HUMAN−642−S−S3_0.08

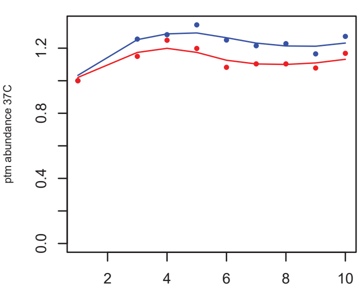

Doses Blue Rep1_0.11 Red Rep2_0.04

sp|Q5H9R7−2|PP6R3_HUMAN−643−T−S3_0.08

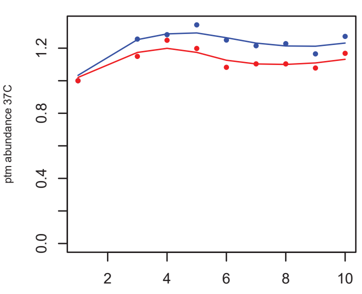

Doses Blue Rep1_0.11 Red Rep2_0.04

sp|Q5H9R7−2|PP6R3_HUMAN−645−S−S3_0.08

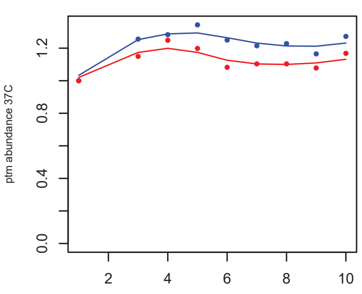

Doses Blue Rep1_0.11 Red Rep2_0.04

sp|Q9NTI5|PDS5B_HUMAN−1283−S−S1_−0.17

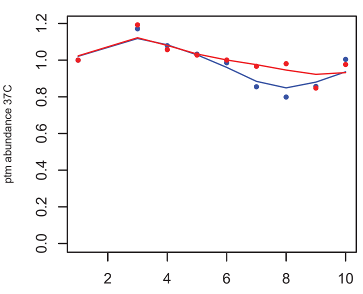

Doses Blue Rep1_−0.17 Red Rep2_−0.18

sp|Q15424−2|SAFB1_HUMAN−322−T−S2_0.06

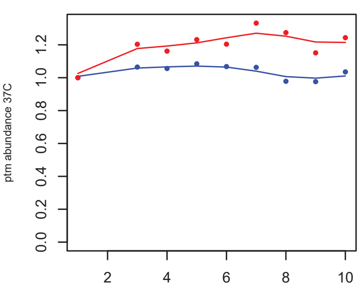

Doses Blue Rep1_−0.03 Red Rep2_0.14

sp|Q86W56−3|PARG_HUMAN−91−T−S2_0.12

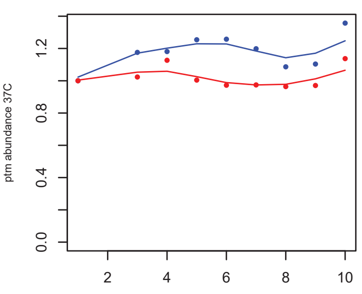

Doses Blue Rep1_0.19 Red Rep2_0.05

sp|Q8NI27−2|THOC2_HUMAN−337−S−S2_−0.2

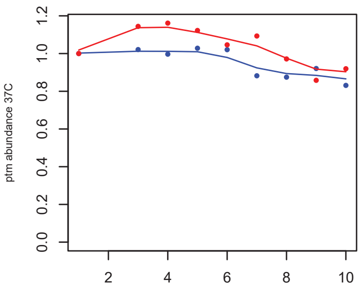

Doses Blue Rep1_−0.18 Red Rep2_−0.22

sp|Q14157−4|UBP2L_HUMAN−463−S−S3_0.05

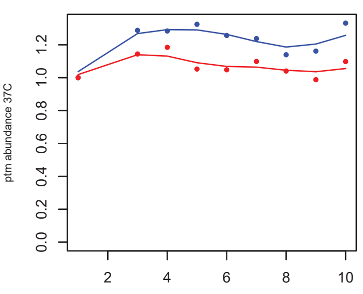

Doses Blue Rep1_0.13 Red Rep2_−0.03

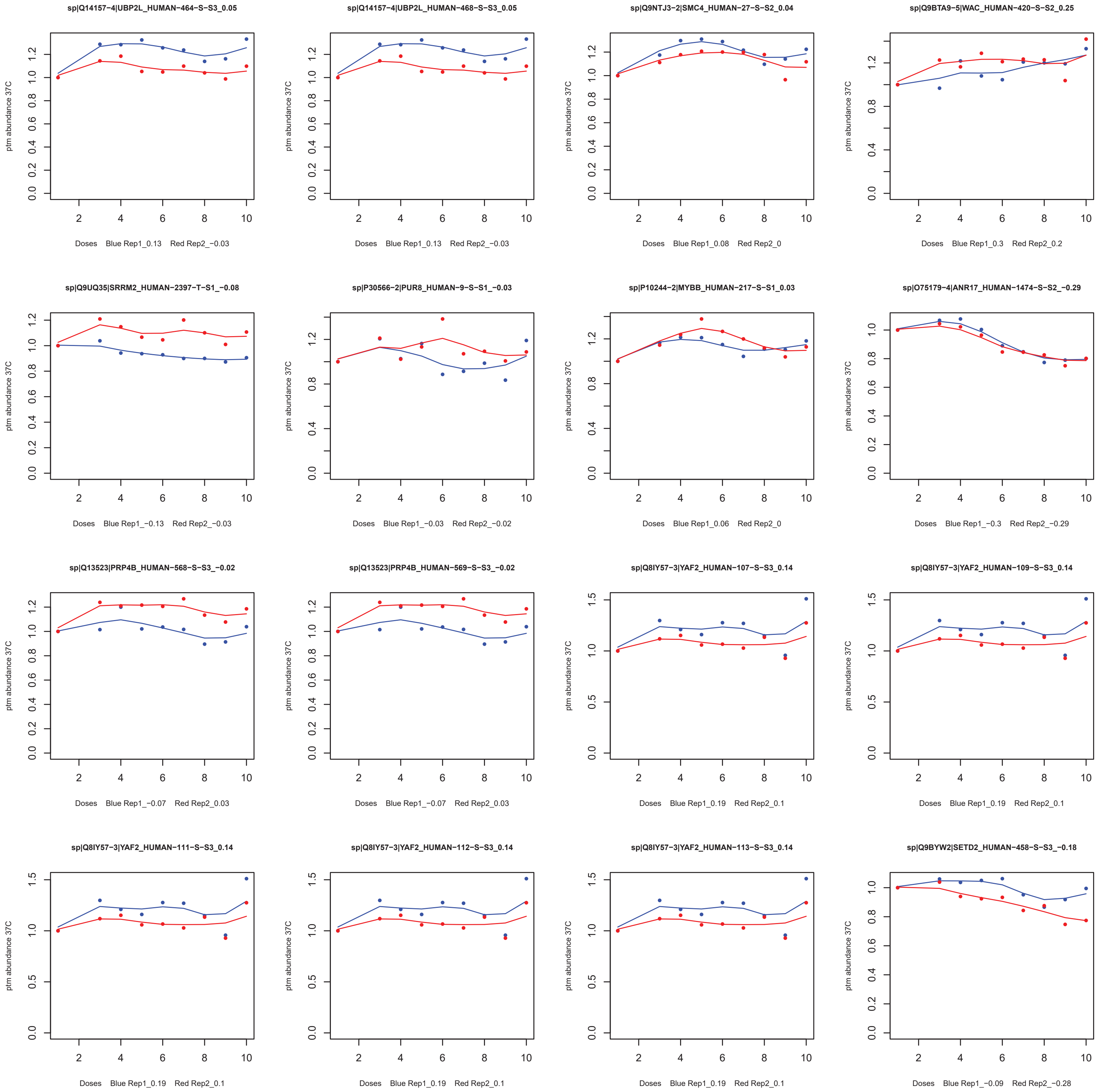
sp|Q14157−4|UBP2L_HUMAN−464−S−S3_0.05
ptm abundance 37C
Doses Blue Rep1_0.13 Red Rep2_−0.03
sp|Q14157−4|UBP2L_HUMAN−468−S−S3_0.05
ptm abundance 37C
Doses Blue Rep1_0.13 Red Rep2_−0.03
sp|Q9NTJ3−2|SMC4_HUMAN−27−S−S2_0.04
ptm abundance 37C
Doses Blue Rep1_0.08 Red Rep2_0
sp|Q9BTA9−5|WAC_HUMAN−420−S−S2_0.25
ptm abundance 37C
Doses Blue Rep1_0.3 Red Rep2_0.2
sp|Q9UQ35|SRRM2_HUMAN−2397−T−S1_−0.08
ptm abundance 37C
Doses Blue Rep1_−0.13 Red Rep2_−0.03
sp|P30566−2|PUR8_HUMAN−9−S−S1_−0.03
ptm abundance 37C
Doses Blue Rep1_−0.03 Red Rep2_−0.02
sp|P10244−2|MYBB_HUMAN−217−S−S1_0.03
ptm abundance 37C
Doses Blue Rep1_0.06 Red Rep2_0
sp|O75179−4|ANR17_HUMAN−1474−S−S2_−0.29
ptm abundance 37C
Doses Blue Rep1_−0.3 Red Rep2_−0.29
sp|Q13523|PRP4B_HUMAN−568−S−S3_−0.02
ptm abundance 37C
Doses Blue Rep1_−0.07 Red Rep2_0.03
sp|Q13523|PRP4B_HUMAN−569−S−S3_−0.02
ptm abundance 37C
Doses Blue Rep1_−0.07 Red Rep2_0.03
sp|Q8IY57−3|YAF2_HUMAN−107−S−S3_0.14
ptm abundance 37C
Doses Blue Rep1_0.19 Red Rep2_0.1
sp|Q8IY57−3|YAF2_HUMAN−109−S−S3_0.14
ptm abundance 37C
Doses Blue Rep1_0.19 Red Rep2_0.1
sp|Q8IY57−3|YAF2_HUMAN−111−S−S3_0.14
ptm abundance 37C
Doses Blue Rep1_0.19 Red Rep2_0.1
sp|Q8IY57−3|YAF2_HUMAN−112−S−S3_0.14
ptm abundance 37C
Doses Blue Rep1_0.19 Red Rep2_0.1
sp|Q8IY57−3|YAF2_HUMAN−113−S−S3_0.14
ptm abundance 37C
Doses Blue Rep1_0.19 Red Rep2_0.1
sp|Q9BYW2|SETD2_HUMAN−458−S−S3_−0.18
ptm abundance 37C
Doses Blue Rep1_−0.09 Red Rep2_−0.28

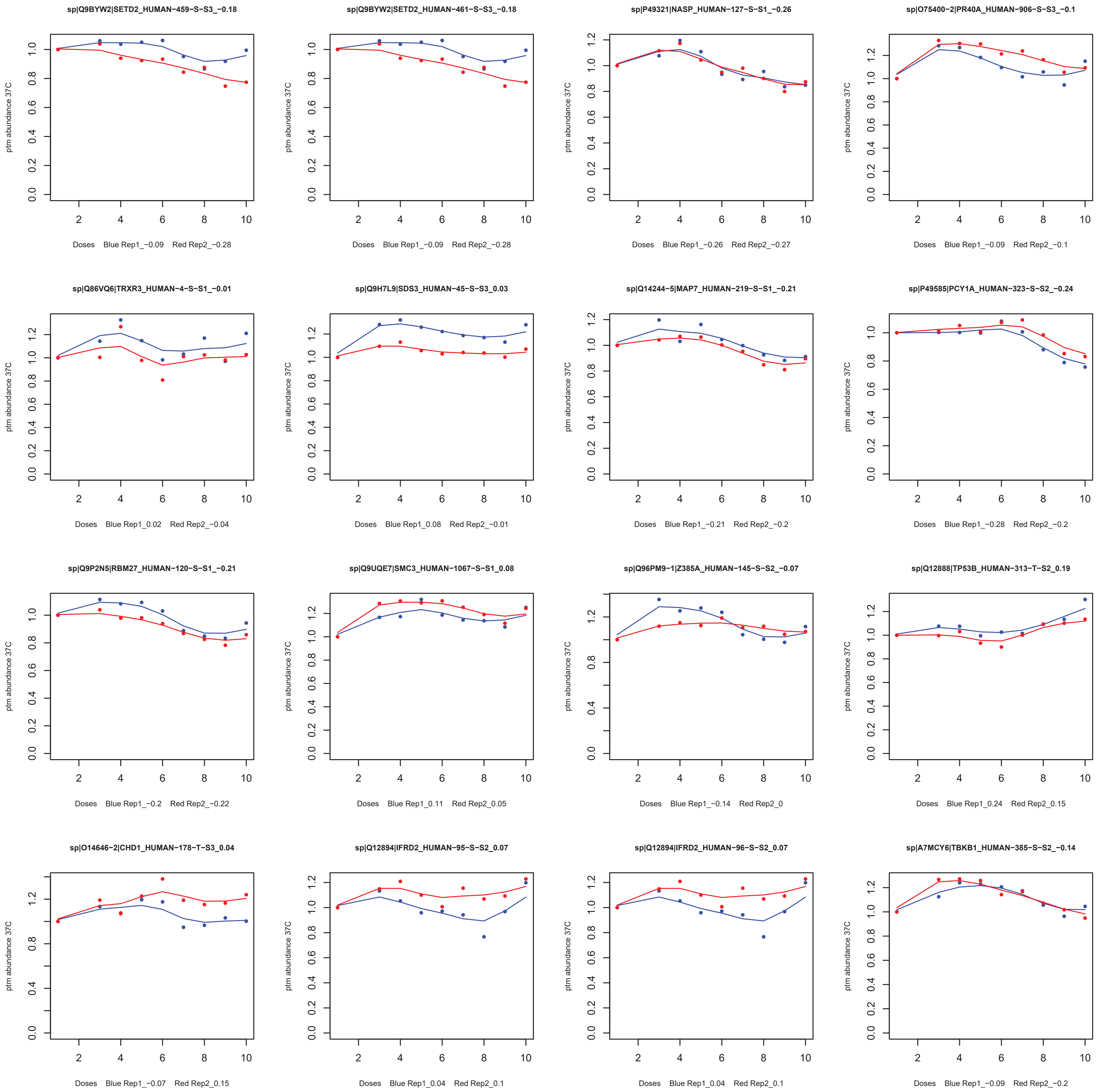

sp|Q9BYW2|SETD2_HUMAN−459−S−S3_−0.18
ptm abundance 37C
Doses Blue Rep1_−0.09 Red Rep2_−0.28
sp|Q9BYW2|SETD2_HUMAN−461−S−S3_−0.18
ptm abundance 37C
Doses Blue Rep1_−0.09 Red Rep2_−0.28
sp|P49321|NASP_HUMAN−127−S−S1_−0.26
ptm abundance 37C
Doses Blue Rep1_−0.26 Red Rep2_−0.27
sp|O75400−2|PR40A_HUMAN−906−S−S3_−0.1
ptm abundance 37C
Doses Blue Rep1_−0.09 Red Rep2_−0.1
sp|Q86VQ6|TRXR3_HUMAN−4−S−S1_−0.01
ptm abundance 37C
Doses Blue Rep1_0.02 Red Rep2_−0.04
sp|Q9H7L9|SDS3_HUMAN−45−S−S3_0.03
ptm abundance 37C
Doses Blue Rep1_0.08 Red Rep2_−0.01
sp|Q14244−5|MAP7_HUMAN−219−S−S1_−0.21
ptm abundance 37C
Doses Blue Rep1_−0.21 Red Rep2_−0.2
sp|P49585|PCY1A_HUMAN−323−S−S2_−0.24
ptm abundance 37C
Doses Blue Rep1_−0.28 Red Rep2_−0.2
sp|Q9P2N5|RBM27_HUMAN−120−S−S1_−0.21
ptm abundance 37C
Doses Blue Rep1_−0.2 Red Rep2_−0.22
sp|Q9UQE7|SMC3_HUMAN−1067−S−S1_0.08
ptm abundance 37C
Doses Blue Rep1_0.11 Red Rep2_0.05
sp|Q96PM9−1|Z385A_HUMAN−145−S−S2_−0.07
ptm abundance 37C
Doses Blue Rep1_−0.14 Red Rep2_0
sp|Q12888|TP53B_HUMAN−313−T−S2_0.19
ptm abundance 37C
Doses Blue Rep1_0.24 Red Rep2_0.15
sp|O14646−2|CHD1_HUMAN−178−T−S3_0.04
ptm abundance 37C
Doses Blue Rep1_−0.07 Red Rep2_0.15
sp|Q12894|IFRD2_HUMAN−95−S−S2_0.07
ptm abundance 37C
Doses Blue Rep1_0.04 Red Rep2_0.1
sp|Q12894|IFRD2_HUMAN−96−S−S2_0.07
ptm abundance 37C
Doses Blue Rep1_0.04 Red Rep2_0.1
sp|A7MCY6|TBKB1_HUMAN−385−S−S2_−0.14
ptm abundance 37C
Doses Blue Rep1_−0.09 Red Rep2_−0.2

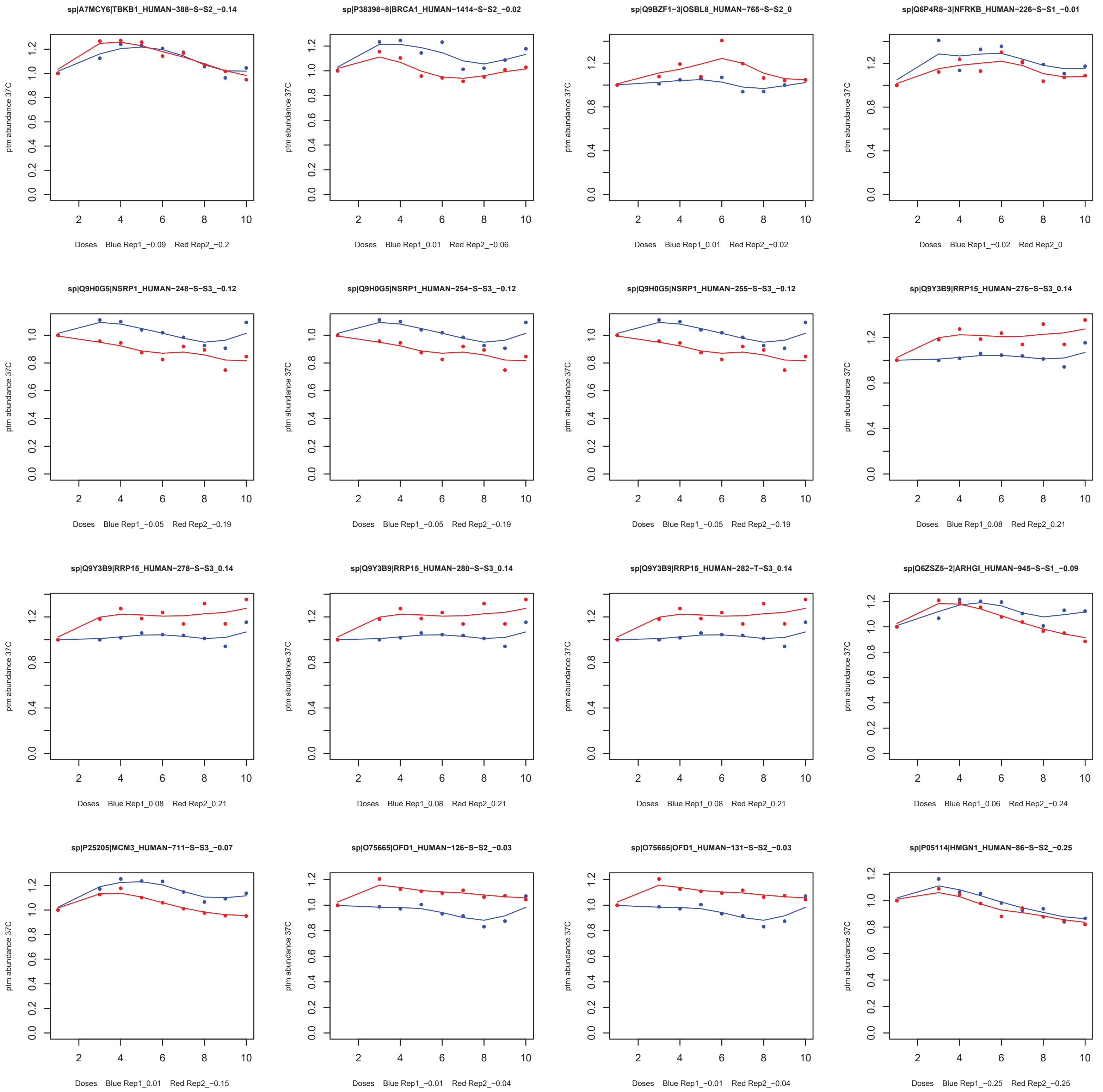
sp|A7MCY6|TBKB1_HUMAN−388−S−S2_−0.14
ptm abundance 37C
Doses Blue Rep1_−0.09 Red Rep2_−0.2
sp|P38398−8|BRCA1_HUMAN−1414−S−S2_−0.02
ptm abundance 37C
Doses Blue Rep1_0.01 Red Rep2_−0.06
sp|Q9BZF1−3|OSBL8_HUMAN−765−S−S2_0
ptm abundance 37C
Doses Blue Rep1_0.01 Red Rep2_−0.02
sp|Q6P4R8−3|NFRKB_HUMAN−226−S−S1_−0.01
ptm abundance 37C
Doses Blue Rep1_−0.02 Red Rep2_0
sp|Q9H0G5|NSRP1_HUMAN−248−S−S3_−0.12
ptm abundance 37C
Doses Blue Rep1_−0.05 Red Rep2_−0.19
sp|Q9H0G5|NSRP1_HUMAN−254−S−S3_−0.12
ptm abundance 37C
Doses Blue Rep1_−0.05 Red Rep2_−0.19
sp|Q9H0G5|NSRP1_HUMAN−255−S−S3_−0.12
ptm abundance 37C
Doses Blue Rep1_−0.05 Red Rep2_−0.19
sp|Q9Y3B9|RRP15_HUMAN−276−S−S3_0.14
ptm abundance 37C
Doses Blue Rep1_0.08 Red Rep2_0.21
sp|Q9Y3B9|RRP15_HUMAN−278−S−S3_0.14
ptm abundance 37C
Doses Blue Rep1_0.08 Red Rep2_0.21
sp|Q9Y3B9|RRP15_HUMAN−280−S−S3_0.14
ptm abundance 37C
Doses Blue Rep1_0.08 Red Rep2_0.21
sp|Q9Y3B9|RRP15_HUMAN−282−T−S3_0.14
ptm abundance 37C
Doses Blue Rep1_0.08 Red Rep2_0.21
sp|Q6ZSZ5−2|ARHGI_HUMAN−945−S−S1_−0.09
ptm abundance 37C
Doses Blue Rep1_0.06 Red Rep2_−0.24
sp|P25205|MCM3_HUMAN−711−S−S3_−0.07
ptm abundance 37C
Doses Blue Rep1_0.01 Red Rep2_−0.15
sp|O75665|OFD1_HUMAN−126−S−S2_−0.03
ptm abundance 37C
Doses Blue Rep1_−0.01 Red Rep2_−0.04
sp|O75665|OFD1_HUMAN−131−S−S2_−0.03
ptm abundance 37C
Doses Blue Rep1_−0.01 Red Rep2_−0.04
sp|P05114|HMGN1_HUMAN−86−S−S2_−0.25
ptm abundance 37C
Doses Blue Rep1_−0.25 Red Rep2_−0.25

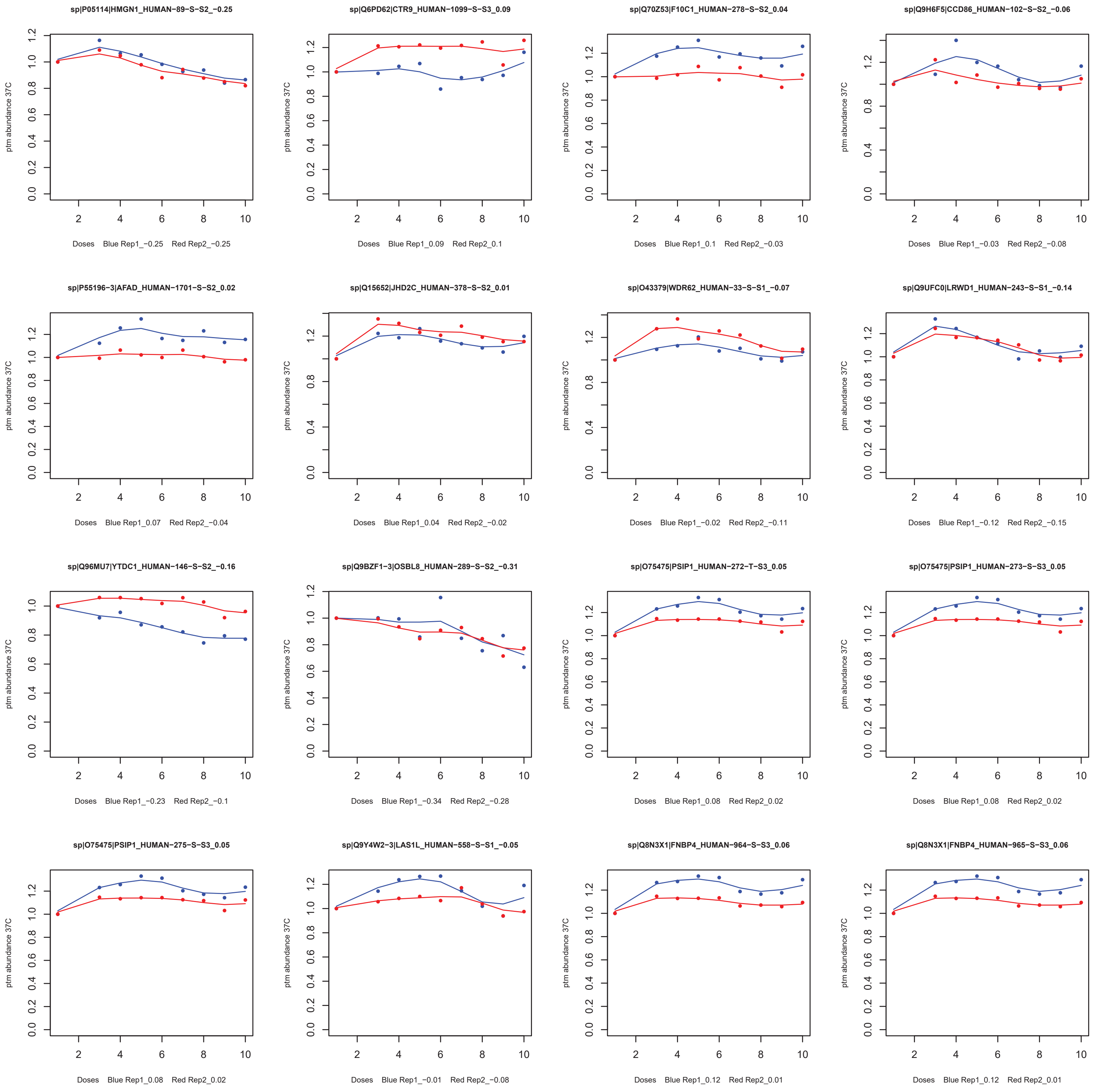

sp|P05114|HMGN1_HUMAN−89−S−S2_−0.25
Doses Blue Rep1_−0.25 Red Rep2_−0.25
sp|Q6PD62|CTR9_HUMAN−1099−S−S3_0.09
Doses Blue Rep1_0.09 Red Rep2_0.1
sp|Q70Z53|F10C1_HUMAN−278−S−S2_0.04
Doses Blue Rep1_0.1 Red Rep2_−0.03
sp|Q9H6F5|CCD86_HUMAN−102−S−S2_−0.06
Doses Blue Rep1_−0.03 Red Rep2_−0.08
sp|P55196−3|AFAD_HUMAN−1701−S−S2_0.02
Doses Blue Rep1_0.07 Red Rep2_−0.04
sp|Q15652|JHD2C_HUMAN−378−S−S2_0.01
Doses Blue Rep1_0.04 Red Rep2_−0.02
sp|O43379|WDR62_HUMAN−33−S−S1_−0.07
Doses Blue Rep1_−0.02 Red Rep2_−0.11
sp|Q9UFC0|LRWD1_HUMAN−243−S−S1_−0.14
Doses Blue Rep1_−0.12 Red Rep2_−0.15
sp|Q96MU7|YTDC1_HUMAN−146−S−S2_−0.16
Doses Blue Rep1_−0.23 Red Rep2_−0.1
sp|Q9BZF1−3|OSBL8_HUMAN−289−S−S2_−0.31
Doses Blue Rep1_−0.34 Red Rep2_−0.28
sp|O75475|PSIP1_HUMAN−272−T−S3_0.05
Doses Blue Rep1_0.08 Red Rep2_0.02
sp|O75475|PSIP1_HUMAN−273−S−S3_0.05
Doses Blue Rep1_0.08 Red Rep2_0.02
sp|O75475|PSIP1_HUMAN−275−S−S3_0.05
Doses Blue Rep1_0.08 Red Rep2_0.02
sp|Q9Y4W2−3|LAS1L_HUMAN−558−S−S1_−0.05
Doses Blue Rep1_−0.01 Red Rep2_−0.08
sp|Q8N3X1|FNBP4_HUMAN−964−S−S3_0.06
Doses Blue Rep1_0.12 Red Rep2_0.01
sp|Q8N3X1|FNBP4_HUMAN−965−S−S3_0.06
Doses Blue Rep1_0.12 Red Rep2_0.01
ptm abundance 37C

sp|Q92833−2|JARD2_HUMAN−40−S−S1_0.15

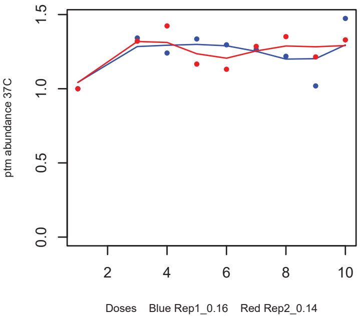

sp|Q8IYH5−4|ZZZ3_HUMAN−131−S−S2_0.03

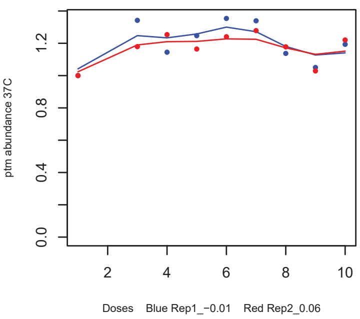

sp|P25205|MCM3_HUMAN−674−T−S1_−0.04

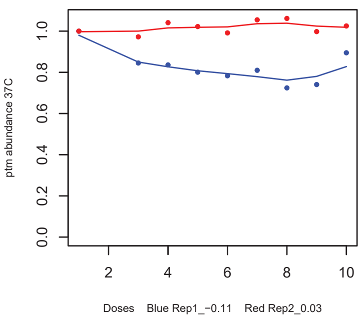

sp|Q6ZW49−1|PAXI1_HUMAN−201−S−S2_−0.01

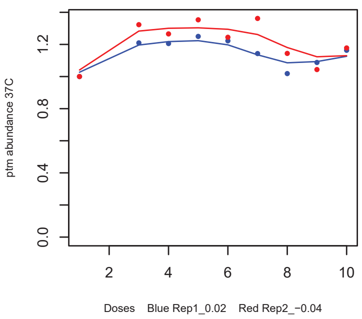

sp|Q9H2G2−2|SLK_HUMAN−565−S−S2_−0.09

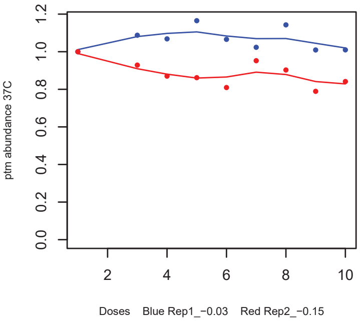

sp|Q9BW71|HIRP3_HUMAN−358−T−S3_0.01

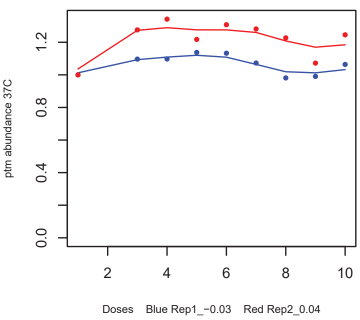

sp|Q9BW71|HIRP3_HUMAN−359−S−S3_0.01

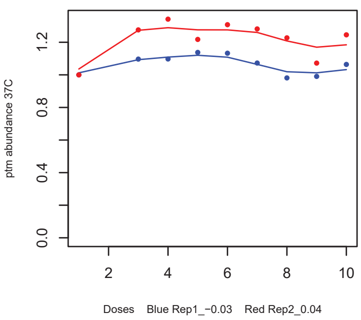

sp|Q86WC4|OSTM1_HUMAN−333−S−S1_−0.08

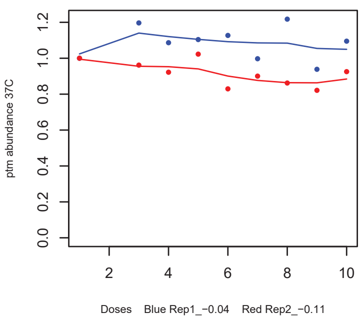

sp|Q9UQ35|SRRM2_HUMAN−2729−S−S2_−0.3

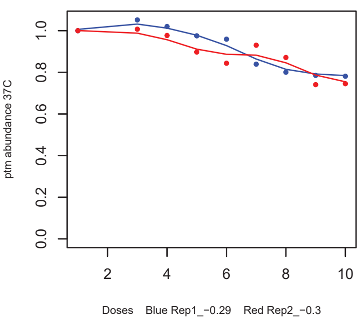

sp|Q8N1G2|CMTR1_HUMAN−53−S−S1_0.13

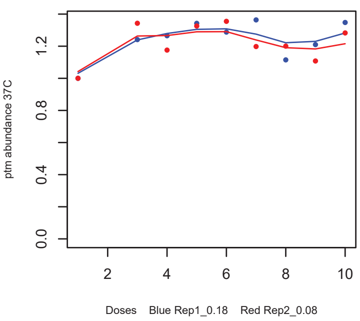

sp|Q86U70|LDB1_HUMAN−302−S−S1_−0.17

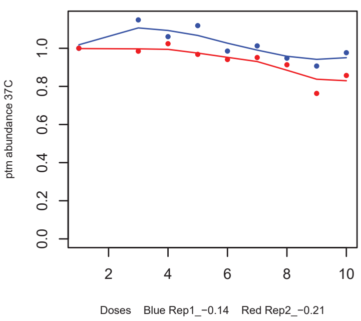

sp|O15541|R113A_HUMAN−84−S−S2_0.13

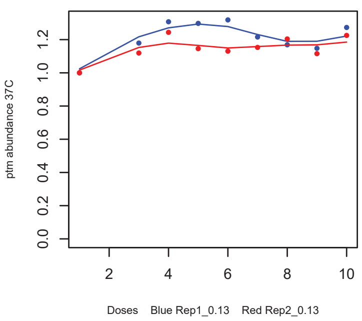

sp|O15541|R113A_HUMAN−85−S−S2_0.13

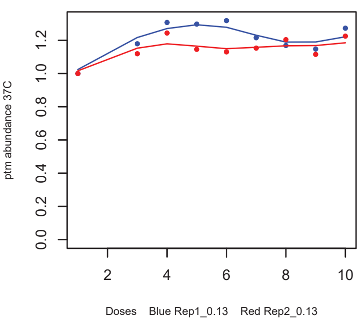

sp|O15541|R113A_HUMAN−95−S−S2_0.13

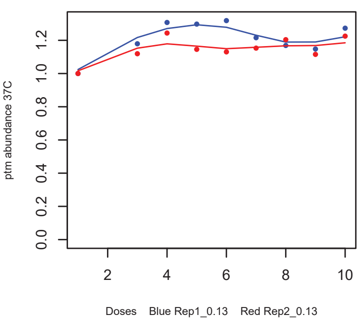

sp|P35659−2|DEK_HUMAN−272−S−S2_0.03

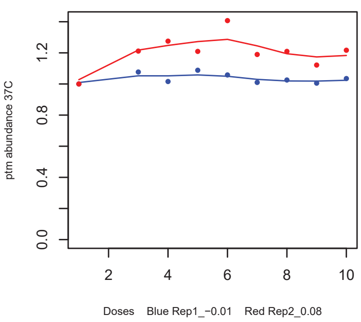

sp|P35659−2|DEK_HUMAN−273−S−S2_0.03

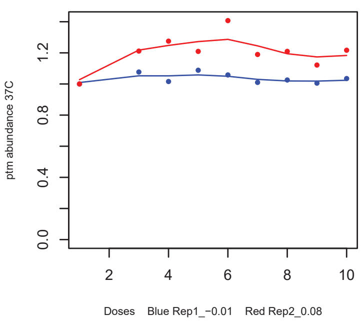

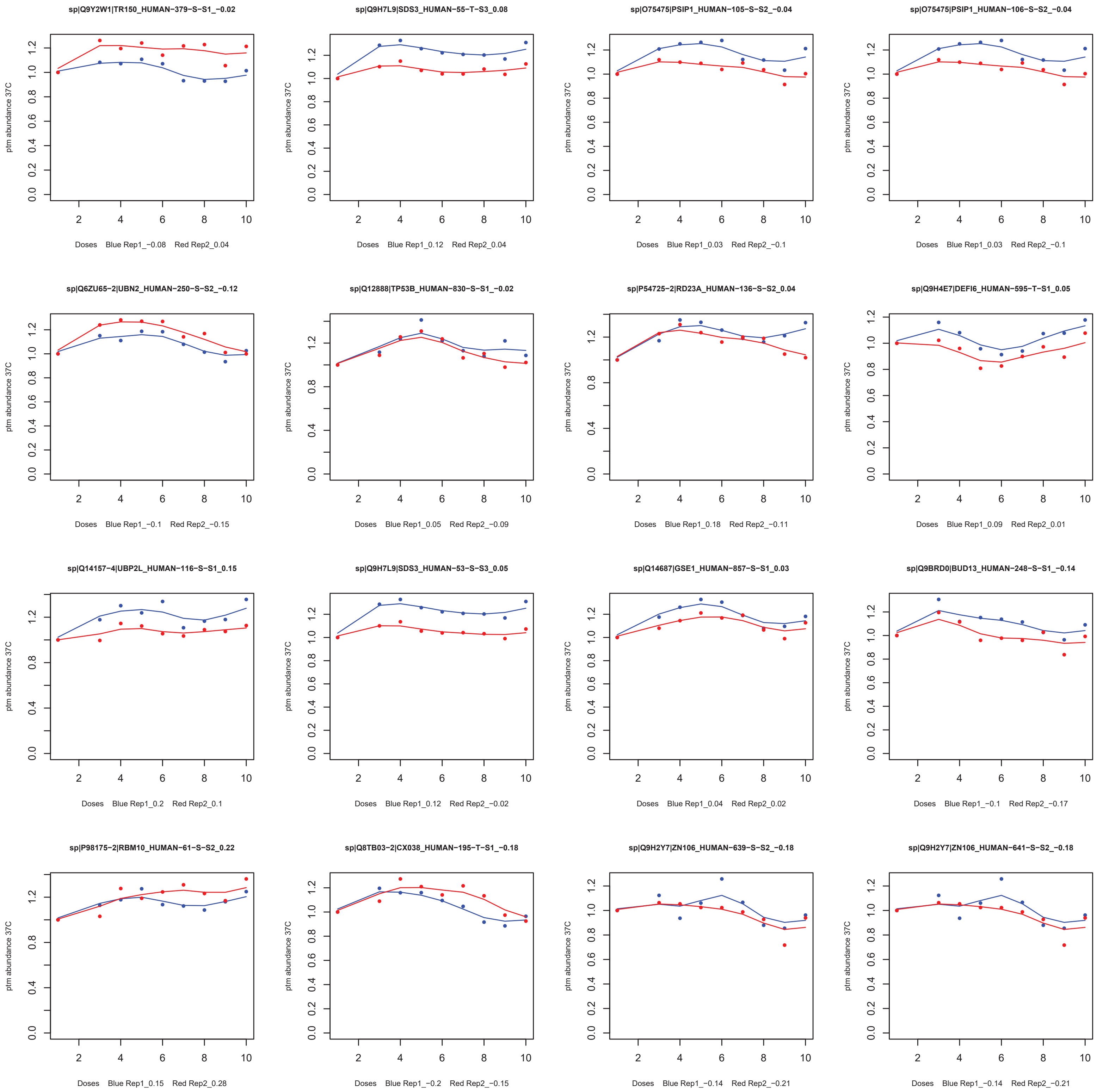
sp|Q9Y2W1|TR150_HUMAN−379−S−S1_−0.02
Doses Blue Rep1_−0.08 Red Rep2_0.04
sp|Q9H7L9|SDS3_HUMAN−55−T−S3_0.08
Doses Blue Rep1_0.12 Red Rep2_0.04
sp|O75475|PSIP1_HUMAN−105−S−S2_−0.04
Doses Blue Rep1_0.03 Red Rep2_−0.1
sp|O75475|PSIP1_HUMAN−106−S−S2_−0.04
Doses Blue Rep1_0.03 Red Rep2_−0.1
sp|Q6ZU65−2|UBN2_HUMAN−250−S−S2_−0.12
Doses Blue Rep1_−0.1 Red Rep2_−0.15
sp|Q12888|TP53B_HUMAN−830−S−S1_−0.02
Doses Blue Rep1_0.05 Red Rep2_−0.09
sp|P54725−2|RD23A_HUMAN−136−S−S2_0.04
Doses Blue Rep1_0.18 Red Rep2_−0.11
sp|Q9H4E7|DEFI6_HUMAN−595−T−S1_0.05
Doses Blue Rep1_0.09 Red Rep2_0.01
sp|Q14157−4|UBP2L_HUMAN−116−S−S1_0.15
Doses Blue Rep1_0.2 Red Rep2_0.1
sp|Q9H7L9|SDS3_HUMAN−53−S−S3_0.05
Doses Blue Rep1_0.12 Red Rep2_−0.02
sp|Q14687|GSE1_HUMAN−857−S−S1_0.03
Doses Blue Rep1_0.04 Red Rep2_0.02
sp|Q9BRD0|BUD13_HUMAN−248−S−S1_−0.14
Doses Blue Rep1_−0.1 Red Rep2_−0.17
sp|P98175−2|RBM10_HUMAN−61−S−S2_0.22
Doses Blue Rep1_0.15 Red Rep2_0.28
sp|Q8TB03−2|CX038_HUMAN−195−T−S1_−0.18
Doses Blue Rep1_−0.2 Red Rep2_−0.15
sp|Q9H2Y7|ZN106_HUMAN−639−S−S2_−0.18
Doses Blue Rep1_−0.14 Red Rep2_−0.21
sp|Q9H2Y7|ZN106_HUMAN−641−S−S2_−0.18
Doses Blue Rep1_−0.14 Red Rep2_−0.21
ptm abundance 37C

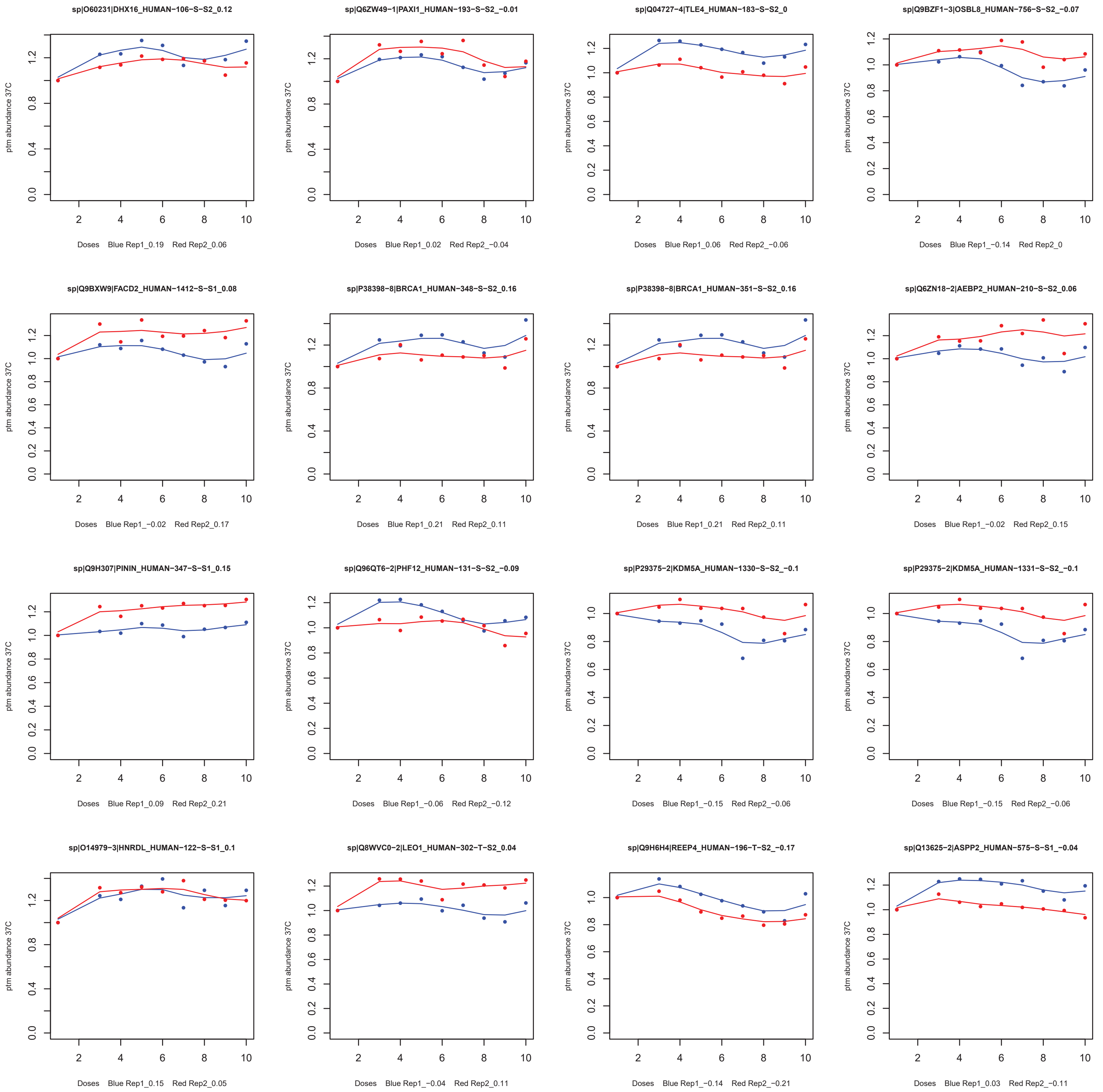

sp|O60231|DHX16_HUMAN−106−S−S2_0.12
ptm abundance 37C
Doses Blue Rep1_0.19 Red Rep2_0.06
sp|Q6ZW49−1|PAXI1_HUMAN−193−S−S2_−0.01
ptm abundance 37C
Doses Blue Rep1_0.02 Red Rep2_−0.04
sp|Q04727−4|TLE4_HUMAN−183−S−S2_0
ptm abundance 37C
Doses Blue Rep1_0.06 Red Rep2_−0.06
sp|Q9BZF1−3|OSBL8_HUMAN−756−S−S2_−0.07
ptm abundance 37C
Doses Blue Rep1_−0.14 Red Rep2_0
sp|Q9BXW9|FACD2_HUMAN−1412−S−S1_0.08
ptm abundance 37C
Doses Blue Rep1_−0.02 Red Rep2_0.17
sp|P38398−8|BRCA1_HUMAN−348−S−S2_0.16
ptm abundance 37C
Doses Blue Rep1_0.21 Red Rep2_0.11
sp|P38398−8|BRCA1_HUMAN−351−S−S2_0.16
ptm abundance 37C
Doses Blue Rep1_0.21 Red Rep2_0.11
sp|Q6ZN18−2|AEBP2_HUMAN−210−S−S2_0.06
ptm abundance 37C
Doses Blue Rep1_−0.02 Red Rep2_0.15
sp|Q9H307|PININ_HUMAN−347−S−S1_0.15
ptm abundance 37C
Doses Blue Rep1_0.09 Red Rep2_0.21
sp|Q96QT6−2|PHF12_HUMAN−131−S−S2_−0.09
ptm abundance 37C
Doses Blue Rep1_−0.06 Red Rep2_−0.12
sp|P29375−2|KDM5A_HUMAN−1330−S−S2_−0.1
ptm abundance 37C
Doses Blue Rep1_−0.15 Red Rep2_−0.06
sp|P29375−2|KDM5A_HUMAN−1331−S−S2_−0.1
ptm abundance 37C
Doses Blue Rep1_−0.15 Red Rep2_−0.06
sp|O14979−3|HNRDL_HUMAN−122−S−S1_0.1
ptm abundance 37C
Doses Blue Rep1_0.15 Red Rep2_0.05
sp|Q8WVC0−2|LEO1_HUMAN−302−T−S2_0.04
ptm abundance 37C
Doses Blue Rep1_−0.04 Red Rep2_0.11
sp|Q9H6H4|REEP4_HUMAN−196−T−S2_−0.17
ptm abundance 37C
Doses Blue Rep1_−0.14 Red Rep2_−0.21
sp|Q13625−2|ASPP2_HUMAN−575−S−S1_−0.04
ptm abundance 37C
Doses Blue Rep1_0.03 Red Rep2_−0.11

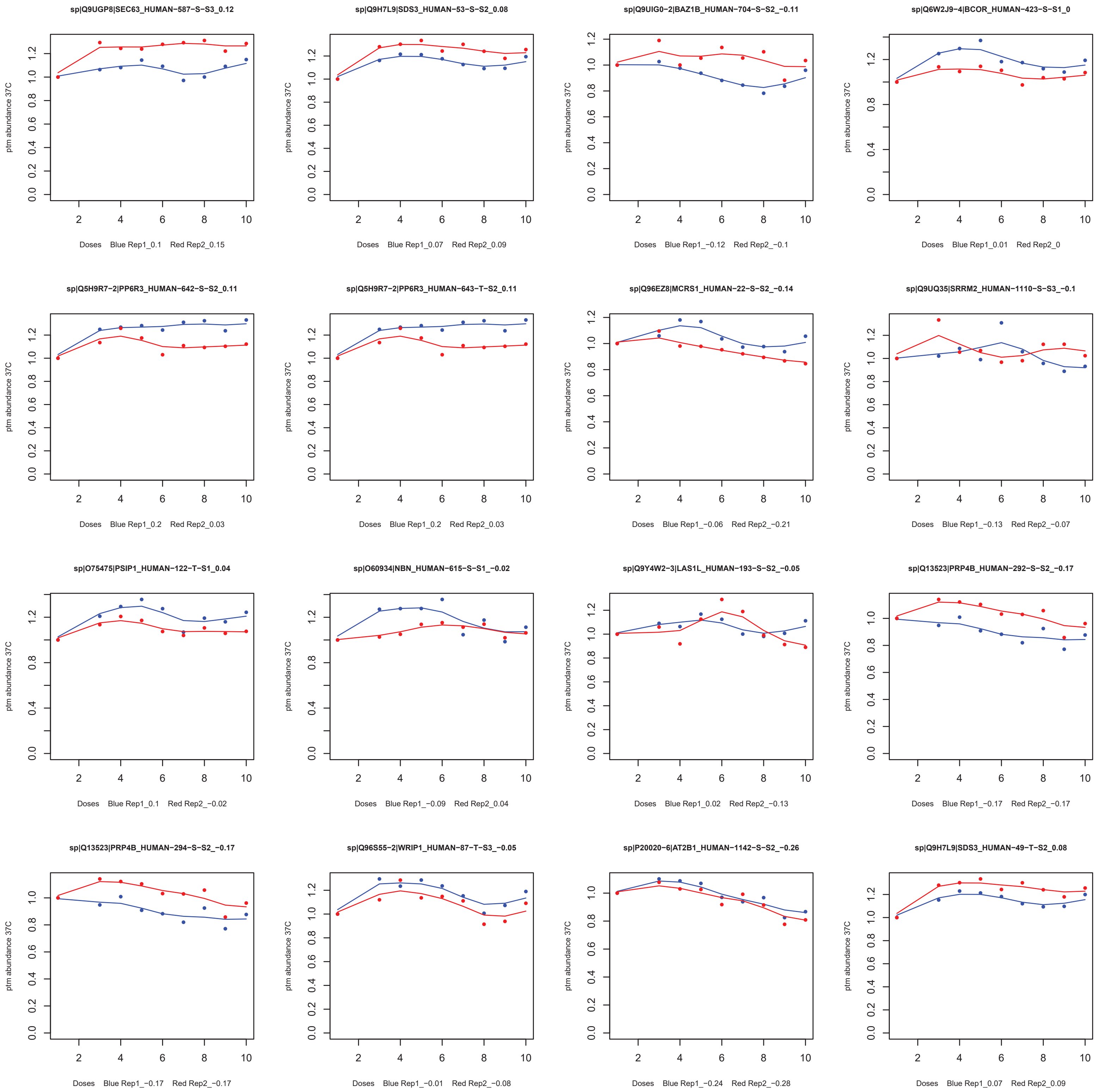
sp|Q9UGP8|SEC63_HUMAN−587−S−S3_0.12
Doses Blue Rep1_0.1 Red Rep2_0.15
sp|Q9H7L9|SDS3_HUMAN−53−S−S2_0.08
Doses Blue Rep1_0.07 Red Rep2_0.09
sp|Q9UIG0−2|BAZ1B_HUMAN−704−S−S2_−0.11
Doses Blue Rep1_−0.12 Red Rep2_−0.1
sp|Q6W2J9−4|BCOR_HUMAN−423−S−S1_0
Doses Blue Rep1_0.01 Red Rep2_0
sp|Q5H9R7−2|PP6R3_HUMAN−642−S−S2_0.11
Doses Blue Rep1_0.2 Red Rep2_0.03
sp|Q5H9R7−2|PP6R3_HUMAN−643−T−S2_0.11
Doses Blue Rep1_0.2 Red Rep2_0.03
sp|Q96EZ8|MCRS1_HUMAN−22−S−S2_−0.14
Doses Blue Rep1_−0.06 Red Rep2_−0.21
sp|Q9UQ35|SRRM2_HUMAN−1110−S−S3_−0.1
Doses Blue Rep1_−0.13 Red Rep2_−0.07
sp|O75475|PSIP1_HUMAN−122−T−S1_0.04
Doses Blue Rep1_0.1 Red Rep2_−0.02
sp|O60934|NBN_HUMAN−615−S−S1_−0.02
Doses Blue Rep1_−0.09 Red Rep2_0.04
sp|Q9Y4W2−3|LAS1L_HUMAN−193−S−S2_−0.05
Doses Blue Rep1_0.02 Red Rep2_−0.13
sp|Q13523|PRP4B_HUMAN−292−S−S2_−0.17
Doses Blue Rep1_−0.17 Red Rep2_−0.17
sp|Q13523|PRP4B_HUMAN−294−S−S2_−0.17
Doses Blue Rep1_−0.17 Red Rep2_−0.17
sp|Q96S55−2|WRIP1_HUMAN−87−T−S3_−0.05
Doses Blue Rep1_−0.01 Red Rep2_−0.08
sp|P20020−6|AT2B1_HUMAN−1142−S−S2_−0.26
Doses Blue Rep1_−0.24 Red Rep2_−0.28
sp|Q9H7L9|SDS3_HUMAN−49−T−S2_0.08
Doses Blue Rep1_0.07 Red Rep2_0.09
ptm abundance 37C

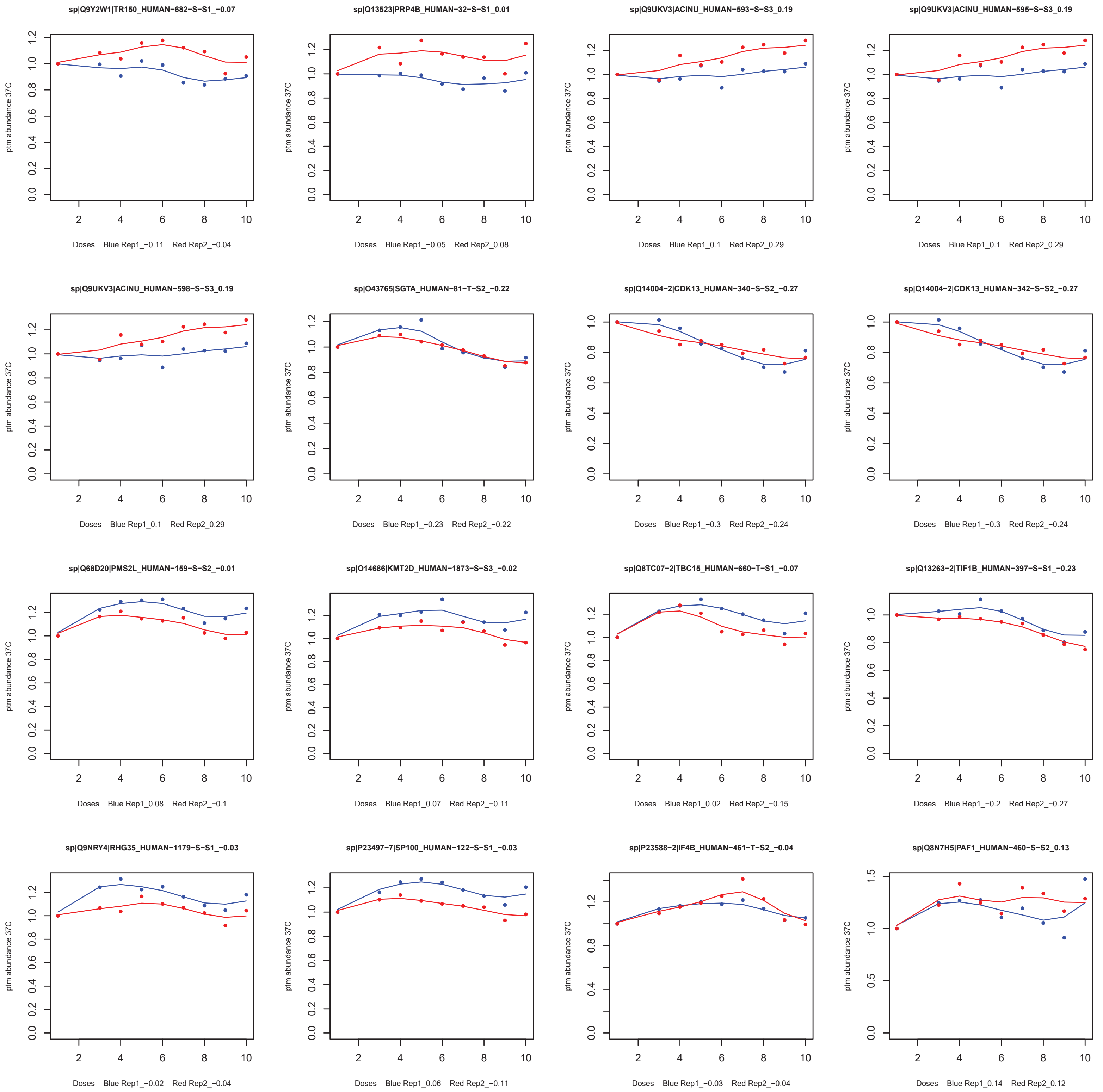
sp|Q9Y2W1|TR150_HUMAN−682−S−S1_−0.07
Doses Blue Rep1_−0.11 Red Rep2_−0.04
sp|Q13523|PRP4B_HUMAN−32−S−S1_0.01
Doses Blue Rep1_−0.05 Red Rep2_0.08
sp|Q9UKV3|ACINU_HUMAN−593−S−S3_0.19
Doses Blue Rep1_0.1 Red Rep2_0.29
sp|Q9UKV3|ACINU_HUMAN−595−S−S3_0.19
Doses Blue Rep1_0.1 Red Rep2_0.29
sp|Q9UKV3|ACINU_HUMAN−598−S−S3_0.19
Doses Blue Rep1_0.1 Red Rep2_0.29
sp|O43765|SGTA_HUMAN−81−T−S2_−0.22
Doses Blue Rep1_−0.23 Red Rep2_−0.22
sp|Q14004−2|CDK13_HUMAN−340−S−S2_−0.27
Doses Blue Rep1_−0.3 Red Rep2_−0.24
sp|Q14004−2|CDK13_HUMAN−342−S−S2_−0.27
Doses Blue Rep1_−0.3 Red Rep2_−0.24
sp|Q68D20|PMS2L_HUMAN−159−S−S2_−0.01
Doses Blue Rep1_0.08 Red Rep2_−0.1
sp|O14686|KMT2D_HUMAN−1873−S−S3_−0.02
Doses Blue Rep1_0.07 Red Rep2_−0.11
sp|Q8TC07−2|TBC15_HUMAN−660−T−S1_−0.07
Doses Blue Rep1_0.02 Red Rep2_−0.15
sp|Q13263−2|TIF1B_HUMAN−397−S−S1_−0.23
Doses Blue Rep1_−0.2 Red Rep2_−0.27
sp|Q9NRY4|RHG35_HUMAN−1179−S−S1_−0.03
Doses Blue Rep1_−0.02 Red Rep2_−0.04
sp|P23497−7|SP100_HUMAN−122−S−S1_−0.03
Doses Blue Rep1_0.06 Red Rep2_−0.11
sp|P23588−2|IF4B_HUMAN−461−T−S2_−0.04
Doses Blue Rep1_−0.03 Red Rep2_−0.04
sp|Q8N7H5|PAF1_HUMAN−460−S−S2_0.13
Doses Blue Rep1_0.14 Red Rep2_0.12
ptm abundance 37C

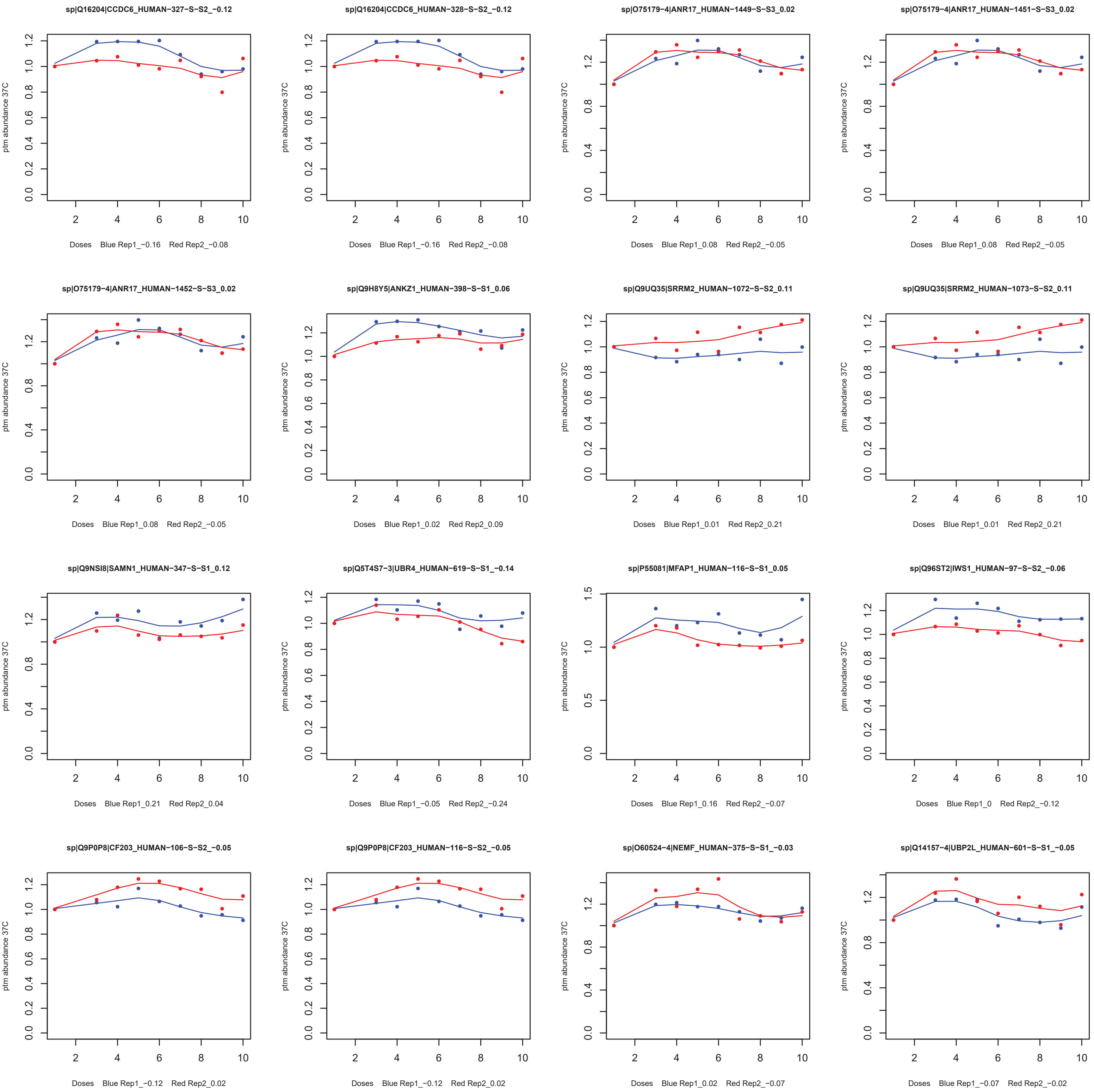
sp|Q16204|CCDC6_HUMAN−327−S−S2_−0.12
ptm abundance 37C
Doses Blue Rep1_−0.16 Red Rep2_−0.08
sp|Q16204|CCDC6_HUMAN−328−S−S2_−0.12
Doses Blue Rep1_−0.16 Red Rep2_−0.08
sp|O75179−4|ANR17_HUMAN−1449−S−S3_0.02
Doses Blue Rep1_0.08 Red Rep2_−0.05
sp|O75179−4|ANR17_HUMAN−1451−S−S3_0.02
Doses Blue Rep1_0.08 Red Rep2_−0.05
sp|O75179−4|ANR17_HUMAN−1452−S−S3_0.02
Doses Blue Rep1_0.08 Red Rep2_−0.05
sp|Q9H8Y5|ANKZ1_HUMAN−398−S−S1_0.06
Doses Blue Rep1_0.02 Red Rep2_0.09
sp|Q9UQ35|SRRM2_HUMAN−1072−S−S2_0.11
Doses Blue Rep1_0.01 Red Rep2_0.21
sp|Q9UQ35|SRRM2_HUMAN−1073−S−S2_0.11
Doses Blue Rep1_0.01 Red Rep2_0.21
sp|Q9NSI8|SAMN1_HUMAN−347−S−S1_0.12
Doses Blue Rep1_0.21 Red Rep2_0.04
sp|Q5T4S7−3|UBR4_HUMAN−619−S−S1_−0.14
Doses Blue Rep1_−0.05 Red Rep2_−0.24
sp|P55081|MFAP1_HUMAN−116−S−S1_0.05
Doses Blue Rep1_0.16 Red Rep2_−0.07
sp|Q96ST2|IWS1_HUMAN−97−S−S2_−0.06
Doses Blue Rep1_0 Red Rep2_−0.12
sp|Q9P0P8|CF203_HUMAN−106−S−S2_−0.05
Doses Blue Rep1_−0.12 Red Rep2_0.02
sp|Q9P0P8|CF203_HUMAN−116−S−S2_−0.05
Doses Blue Rep1_−0.12 Red Rep2_0.02
sp|O60524−4|NEMF_HUMAN−375−S−S1_−0.03
Doses Blue Rep1_0.02 Red Rep2_−0.07
sp|Q14157−4|UBP2L_HUMAN−601−S−S1_−0.05
Doses Blue Rep1_−0.07 Red Rep2_−0.02

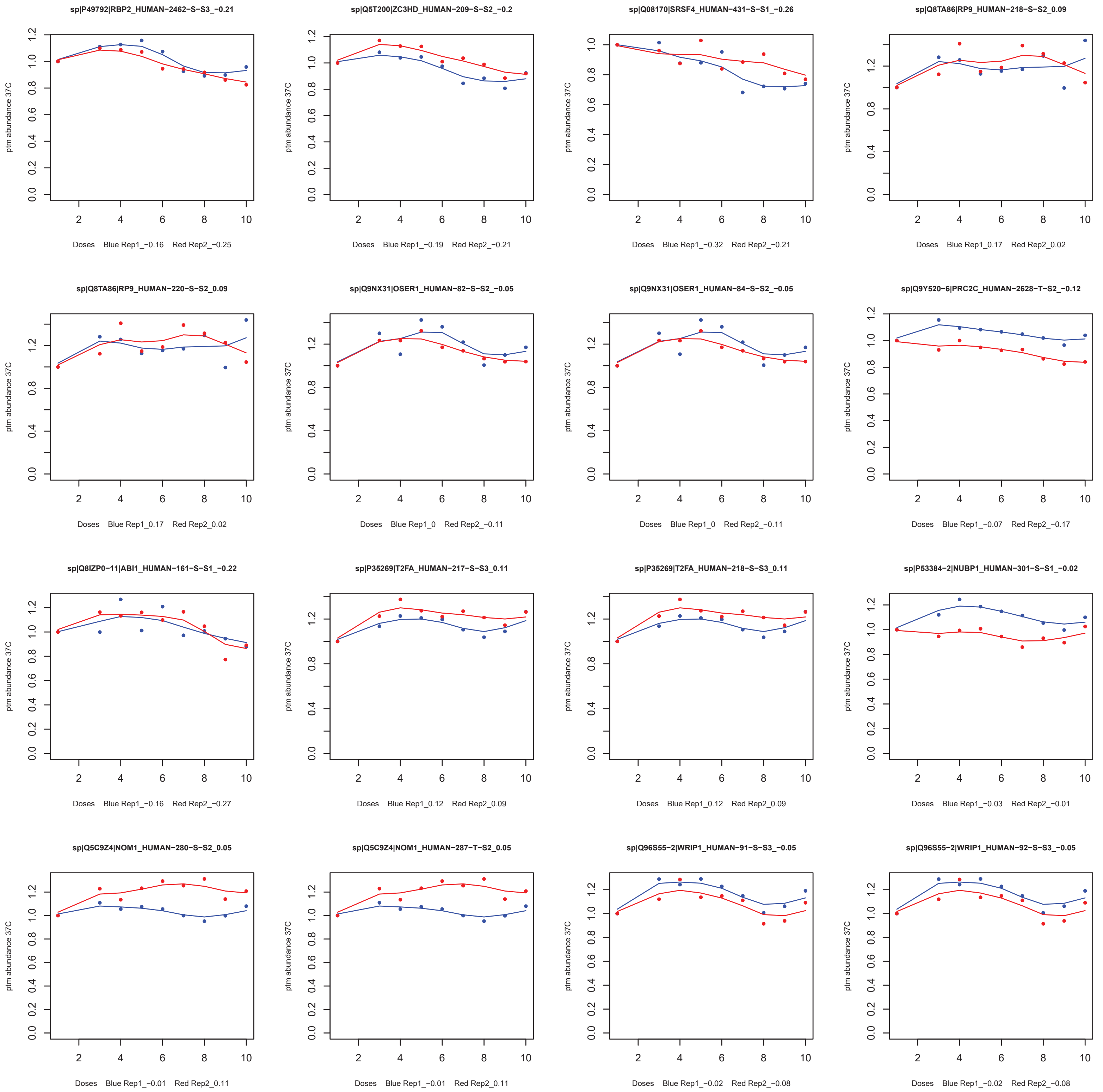
sp|P49792|RBP2_HUMAN−2462−S−S3_−0.21
Doses Blue Rep1_−0.16 Red Rep2_−0.25
sp|Q5T200|ZC3HD_HUMAN−209−S−S2_−0.2
Doses Blue Rep1_−0.19 Red Rep2_−0.21
sp|Q08170|SRSF4_HUMAN−431−S−S1_−0.26
Doses Blue Rep1_−0.32 Red Rep2_−0.21
sp|Q8TA86|RP9_HUMAN−218−S−S2_0.09
Doses Blue Rep1_0.17 Red Rep2_0.02
sp|Q8TA86|RP9_HUMAN−220−S−S2_0.09
Doses Blue Rep1_0.17 Red Rep2_0.02
sp|Q9NX31|OSER1_HUMAN−82−S−S2_−0.05
Doses Blue Rep1_0 Red Rep2_−0.11
sp|Q9NX31|OSER1_HUMAN−84−S−S2_−0.05
Doses Blue Rep1_0 Red Rep2_−0.11
sp|Q9Y520−6|PRC2C_HUMAN−2628−T−S2_−0.12
Doses Blue Rep1_−0.07 Red Rep2_−0.17
sp|Q8IZP0−11|ABI1_HUMAN−161−S−S1_−0.22
Doses Blue Rep1_−0.16 Red Rep2_−0.27
sp|P35269|T2FA_HUMAN−217−S−S3_0.11
Doses Blue Rep1_0.12 Red Rep2_0.09
sp|P35269|T2FA_HUMAN−218−S−S3_0.11
Doses Blue Rep1_0.12 Red Rep2_0.09
sp|P53384−2|NUBP1_HUMAN−301−S−S1_−0.02
Doses Blue Rep1_−0.03 Red Rep2_−0.01
sp|Q5C9Z4|NOM1_HUMAN−280−S−S2_0.05
Doses Blue Rep1_−0.01 Red Rep2_0.11
sp|Q5C9Z4|NOM1_HUMAN−287−T−S2_0.05
Doses Blue Rep1_−0.01 Red Rep2_0.11
sp|Q96S55−2|WRIP1_HUMAN−91−S−S3_−0.05
Doses Blue Rep1_−0.02 Red Rep2_−0.08
sp|Q96S55−2|WRIP1_HUMAN−92−S−S3_−0.05
Doses Blue Rep1_−0.02 Red Rep2_−0.08
ptm abundance 37C

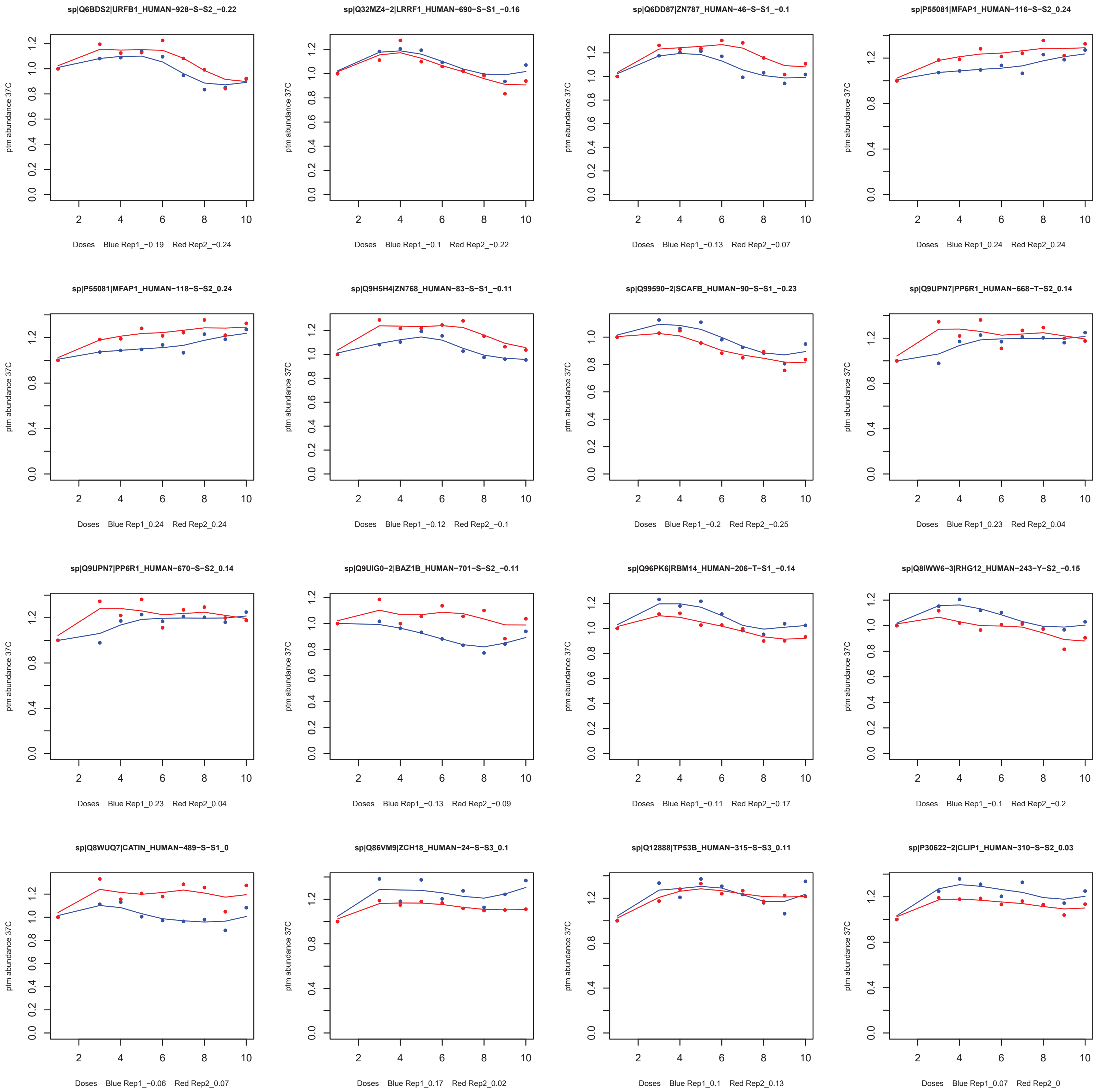

sp|Q6BDS2|URFB1_HUMAN−928−S−S2_−0.22
Doses Blue Rep1_−0.19 Red Rep2_−0.24
sp|Q32MZ4−2|LRRF1_HUMAN−690−S−S1_−0.16
Doses Blue Rep1_−0.1 Red Rep2_−0.22
sp|Q6DD87|ZN787_HUMAN−46−S−S1_−0.1
Doses Blue Rep1_−0.13 Red Rep2_−0.07
sp|P55081|MFAP1_HUMAN−116−S−S2_0.24
Doses Blue Rep1_0.24 Red Rep2_0.24
sp|P55081|MFAP1_HUMAN−118−S−S2_0.24
Doses Blue Rep1_0.24 Red Rep2_0.24
sp|Q9H5H4|ZN768_HUMAN−83−S−S1_−0.11
Doses Blue Rep1_−0.12 Red Rep2_−0.1
sp|Q99590−2|SCAFB_HUMAN−90−S−S1_−0.23
Doses Blue Rep1_−0.2 Red Rep2_−0.25
sp|Q9UPN7|PP6R1_HUMAN−668−T−S2_0.14
Doses Blue Rep1_0.23 Red Rep2_0.04
sp|Q9UPN7|PP6R1_HUMAN−670−S−S2_0.14
Doses Blue Rep1_0.23 Red Rep2_0.04
sp|Q9UIG0−2|BAZ1B_HUMAN−701−S−S2_−0.11
Doses Blue Rep1_−0.13 Red Rep2_−0.09
sp|Q96PK6|RBM14_HUMAN−206−T−S1_−0.14
Doses Blue Rep1_−0.11 Red Rep2_−0.17
sp|Q8IWW6−3|RHG12_HUMAN−243−Y−S2_−0.15
Doses Blue Rep1_−0.1 Red Rep2_−0.2
sp|Q8WUQ7|CATIN_HUMAN−489−S−S1_0
Doses Blue Rep1_−0.06 Red Rep2_0.07
sp|Q86VM9|ZCH18_HUMAN−24−S−S3_0.1
Doses Blue Rep1_0.17 Red Rep2_0.02
sp|Q12888|TP53B_HUMAN−315−S−S3_0.11
Doses Blue Rep1_0.1 Red Rep2_0.13
sp|P30622−2|CLIP1_HUMAN−310−S−S2_0.03
Doses Blue Rep1_0.07 Red Rep2_0
ptm abundance 37C

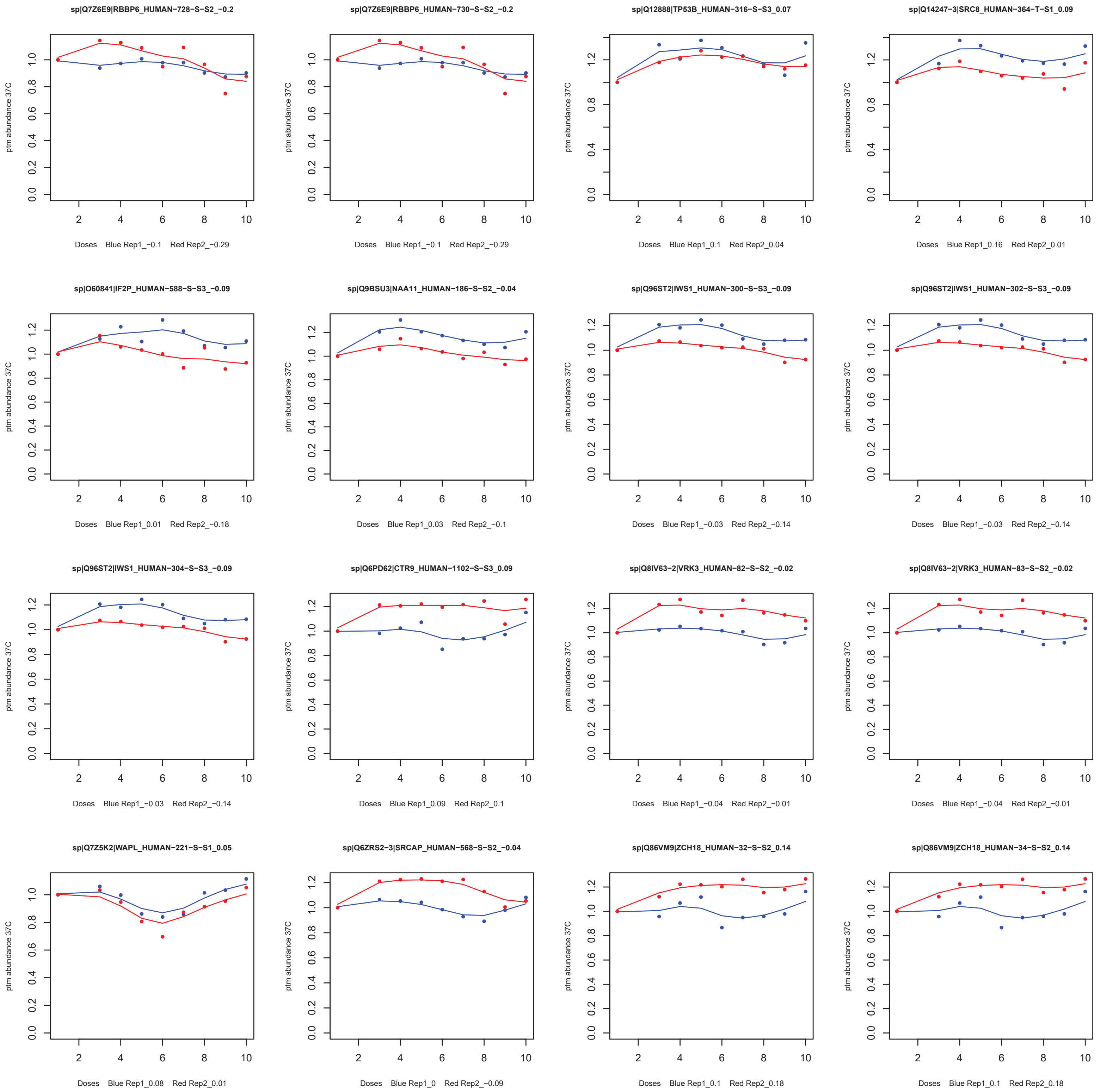
sp|Q7Z6E9|RBBP6_HUMAN−728−S−S2_−0.2
ptm abundance 37C
Doses Blue Rep1_−0.1 Red Rep2_−0.29
sp|Q7Z6E9|RBBP6_HUMAN−730−S−S2_−0.2
ptm abundance 37C
Doses Blue Rep1_−0.1 Red Rep2_−0.29
sp|Q12888|TP53B_HUMAN−316−S−S3_0.07
ptm abundance 37C
Doses Blue Rep1_0.1 Red Rep2_0.04
sp|Q14247−3|SRC8_HUMAN−364−T−S1_0.09
ptm abundance 37C
Doses Blue Rep1_0.16 Red Rep2_0.01
sp|O60841|IF2P_HUMAN−588−S−S3_−0.09
ptm abundance 37C
Doses Blue Rep1_0.01 Red Rep2_−0.18
sp|Q9BSU3|NAA11_HUMAN−186−S−S2_−0.04
ptm abundance 37C
Doses Blue Rep1_0.03 Red Rep2_−0.1
sp|Q96ST2|IWS1_HUMAN−300−S−S3_−0.09
ptm abundance 37C
Doses Blue Rep1_−0.03 Red Rep2_−0.14
sp|Q96ST2|IWS1_HUMAN−302−S−S3_−0.09
ptm abundance 37C
Doses Blue Rep1_−0.03 Red Rep2_−0.14
sp|Q96ST2|IWS1_HUMAN−304−S−S3_−0.09
ptm abundance 37C
Doses Blue Rep1_−0.03 Red Rep2_−0.14
sp|Q6PD62|CTR9_HUMAN−1102−S−S3_0.09
ptm abundance 37C
Doses Blue Rep1_0.09 Red Rep2_0.1
sp|Q8IV63−2|VRK3_HUMAN−82−S−S2_−0.02
ptm abundance 37C
Doses Blue Rep1_−0.04 Red Rep2_−0.01
sp|Q8IV63−2|VRK3_HUMAN−83−S−S2_−0.02
ptm abundance 37C
Doses Blue Rep1_−0.04 Red Rep2_−0.01
sp|Q7Z5K2|WAPL_HUMAN−221−S−S1_0.05
ptm abundance 37C
Doses Blue Rep1_0.08 Red Rep2_0.01
sp|Q6ZRS2−3|SRCAP_HUMAN−568−S−S2_−0.04
ptm abundance 37C
Doses Blue Rep1_0 Red Rep2_−0.09
sp|Q86VM9|ZCH18_HUMAN−32−S−S2_0.14
ptm abundance 37C
Doses Blue Rep1_0.1 Red Rep2_0.18
sp|Q86VM9|ZCH18_HUMAN−34−S−S2_0.14
ptm abundance 37C
Doses Blue Rep1_0.1 Red Rep2_0.18

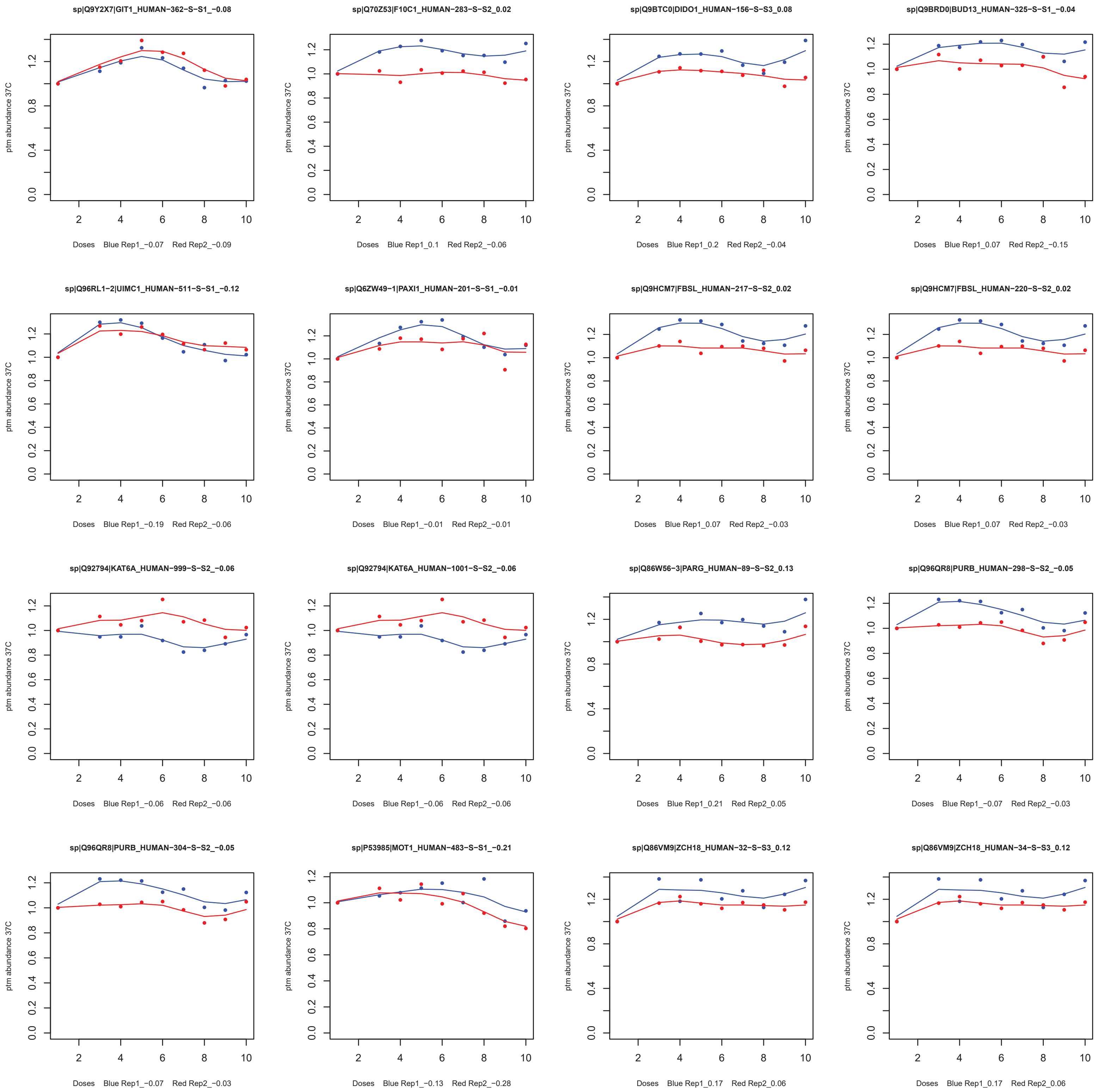
sp|Q9Y2X7|GIT1_HUMAN−362−S−S1_−0.08
Doses Blue Rep1_−0.07 Red Rep2_−0.09
sp|Q70Z53|F10C1_HUMAN−283−S−S2_0.02
Doses Blue Rep1_0.1 Red Rep2_−0.06
sp|Q9BTC0|DIDO1_HUMAN−156−S−S3_0.08
Doses Blue Rep1_0.2 Red Rep2_−0.04
sp|Q9BRD0|BUD13_HUMAN−325−S−S1_−0.04
Doses Blue Rep1_0.07 Red Rep2_−0.15
sp|Q96RL1−2|UIMC1_HUMAN−511−S−S1_−0.12
Doses Blue Rep1_−0.19 Red Rep2_−0.06
sp|Q6ZW49−1|PAXI1_HUMAN−201−S−S1_−0.01
Doses Blue Rep1_−0.01 Red Rep2_−0.01
sp|Q9HCM7|FBSL_HUMAN−217−S−S2_0.02
Doses Blue Rep1_0.07 Red Rep2_−0.03
sp|Q9HCM7|FBSL_HUMAN−220−S−S2_0.02
Doses Blue Rep1_0.07 Red Rep2_−0.03
sp|Q92794|KAT6A_HUMAN−999−S−S2_−0.06
Doses Blue Rep1_−0.06 Red Rep2_−0.06
sp|Q92794|KAT6A_HUMAN−1001−S−S2_−0.06
Doses Blue Rep1_−0.06 Red Rep2_−0.06
sp|Q86W56−3|PARG_HUMAN−89−S−S2_0.13
Doses Blue Rep1_0.21 Red Rep2_0.05
sp|Q96QR8|PURB_HUMAN−298−S−S2_−0.05
Doses Blue Rep1_−0.07 Red Rep2_−0.03
sp|Q96QR8|PURB_HUMAN−304−S−S2_−0.05
Doses Blue Rep1_−0.07 Red Rep2_−0.03
sp|P53985|MOT1_HUMAN−483−S−S1_−0.21
Doses Blue Rep1_−0.13 Red Rep2_−0.28
sp|Q86VM9|ZCH18_HUMAN−32−S−S3_0.12
Doses Blue Rep1_0.17 Red Rep2_0.06
sp|Q86VM9|ZCH18_HUMAN−34−S−S3_0.12
Doses Blue Rep1_0.17 Red Rep2_0.06
ptm abundance 37C

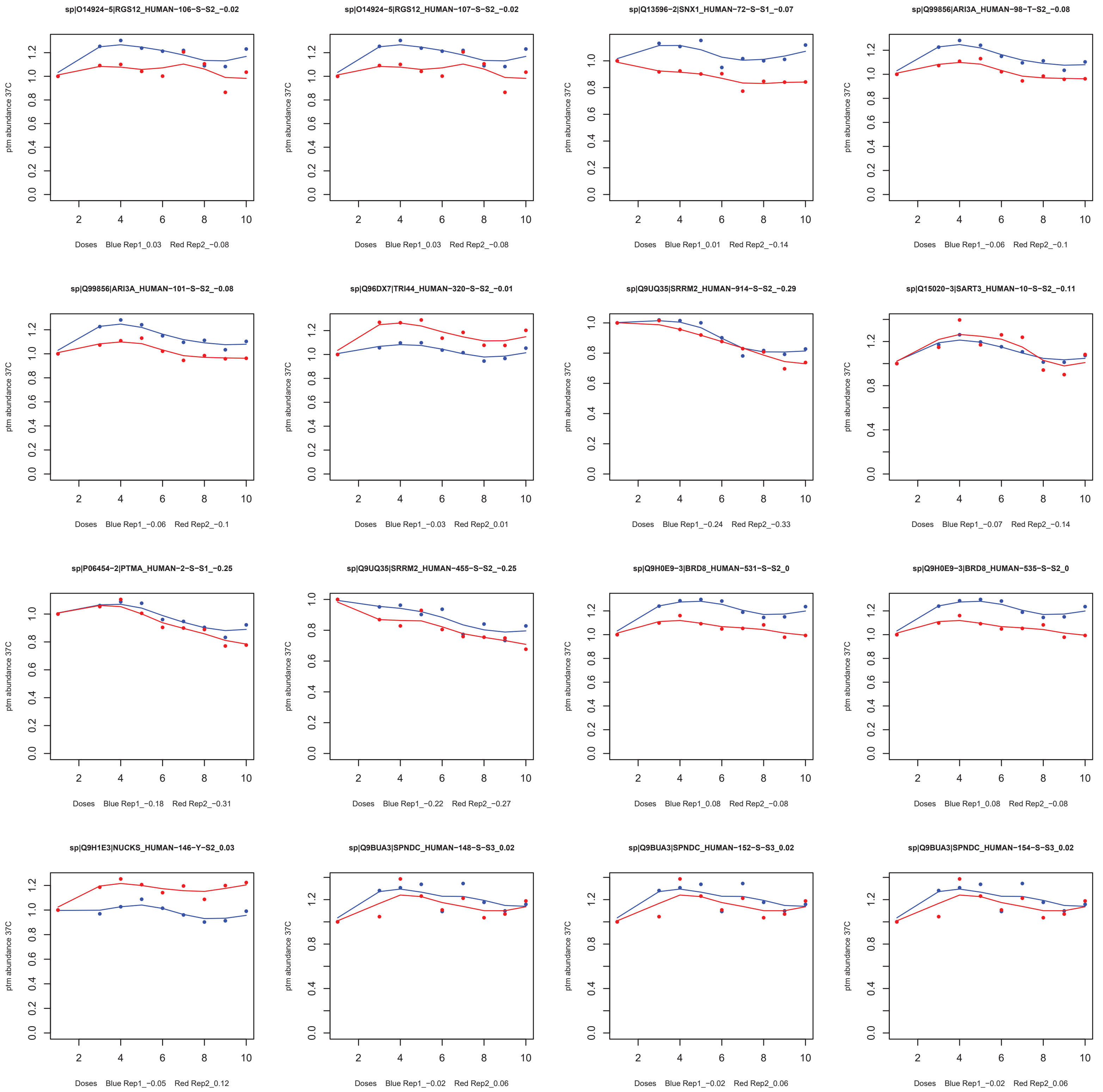
sp|O14924−5|RGS12_HUMAN−106−S−S2_−0.02
ptm abundance 37C
Doses Blue Rep1_0.03 Red Rep2_−0.08
sp|O14924−5|RGS12_HUMAN−107−S−S2_−0.02
ptm abundance 37C
Doses Blue Rep1_0.03 Red Rep2_−0.08
sp|Q13596−2|SNX1_HUMAN−72−S−S1_−0.07
ptm abundance 37C
Doses Blue Rep1_0.01 Red Rep2_−0.14
sp|Q99856|ARI3A_HUMAN−98−T−S2_−0.08
ptm abundance 37C
Doses Blue Rep1_−0.06 Red Rep2_−0.1
sp|Q99856|ARI3A_HUMAN−101−S−S2_−0.08
ptm abundance 37C
Doses Blue Rep1_−0.06 Red Rep2_−0.1
sp|Q96DX7|TRI44_HUMAN−320−S−S2_−0.01
ptm abundance 37C
Doses Blue Rep1_−0.03 Red Rep2_0.01
sp|Q9UQ35|SRRM2_HUMAN−914−S−S2_−0.29
ptm abundance 37C
Doses Blue Rep1_−0.24 Red Rep2_−0.33
sp|Q15020−3|SART3_HUMAN−10−S−S2_−0.11
ptm abundance 37C
Doses Blue Rep1_−0.07 Red Rep2_−0.14
sp|P06454−2|PTMA_HUMAN−2−S−S1_−0.25
ptm abundance 37C
Doses Blue Rep1_−0.18 Red Rep2_−0.31
sp|Q9UQ35|SRRM2_HUMAN−455−S−S2_−0.25
ptm abundance 37C
Doses Blue Rep1_−0.22 Red Rep2_−0.27
sp|Q9H0E9−3|BRD8_HUMAN−531−S−S2_0
ptm abundance 37C
Doses Blue Rep1_0.08 Red Rep2_−0.08
sp|Q9H0E9−3|BRD8_HUMAN−535−S−S2_0
ptm abundance 37C
Doses Blue Rep1_0.08 Red Rep2_−0.08
sp|Q9H1E3|NUCKS_HUMAN−146−Y−S2_0.03
ptm abundance 37C
Doses Blue Rep1_−0.05 Red Rep2_0.12
sp|Q9BUA3|SPNDC_HUMAN−148−S−S3_0.02
ptm abundance 37C
Doses Blue Rep1_−0.02 Red Rep2_0.06
sp|Q9BUA3|SPNDC_HUMAN−152−S−S3_0.02
ptm abundance 37C
Doses Blue Rep1_−0.02 Red Rep2_0.06
sp|Q9BUA3|SPNDC_HUMAN−154−S−S3_0.02
ptm abundance 37C
Doses Blue Rep1_−0.02 Red Rep2_0.06

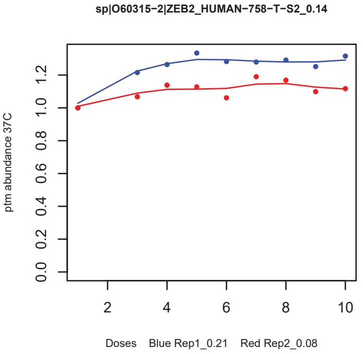
sp|O60315−2|ZEB2_HUMAN−758−T−S2_0.14
ptm abundance 37C
Doses Blue Rep1_0.21 Red Rep2_0.08
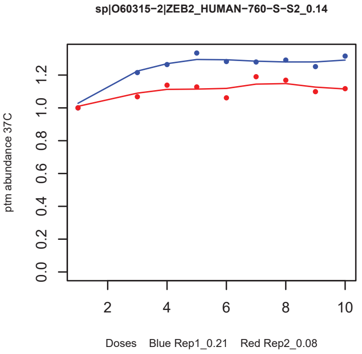
sp|O60315−2|ZEB2_HUMAN−760−S−S2_0.14
ptm abundance 37C
Doses Blue Rep1_0.21 Red Rep2_0.08
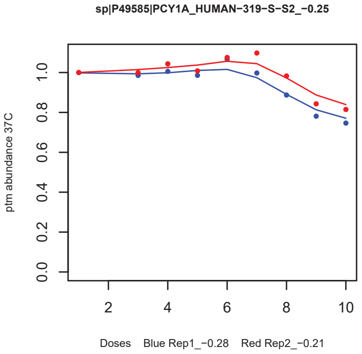
sp|P49585|PCY1A_HUMAN−319−S−S2_−0.25
ptm abundance 37C
Doses Blue Rep1_−0.28 Red Rep2_−0.21
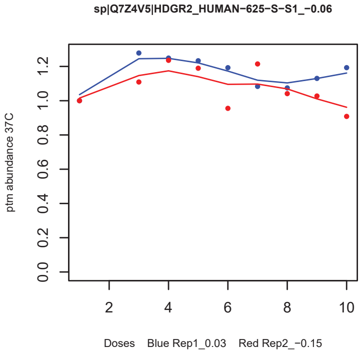
sp|Q7Z4V5|HDGR2_HUMAN−625−S−S1_−0.06
ptm abundance 37C
Doses Blue Rep1_0.03 Red Rep2_−0.15
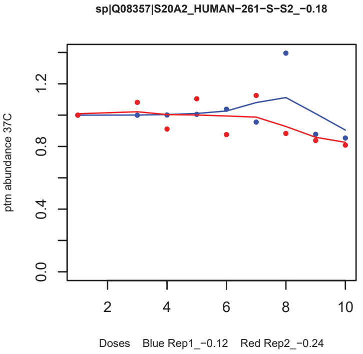
sp|Q08357|S20A2_HUMAN−261−S−S2_−0.18
ptm abundance 37C
Doses Blue Rep1_−0.12 Red Rep2_−0.24
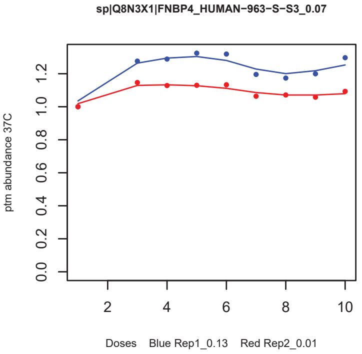
sp|Q8N3X1|FNBP4_HUMAN−963−S−S3_0.07
ptm abundance 37C
Doses Blue Rep1_0.13 Red Rep2_0.01
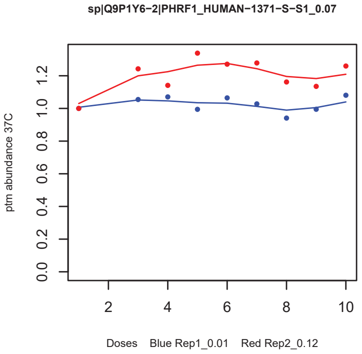
sp|Q9P1Y6−2|PHRF1_HUMAN−1371−S−S1_0.07
ptm abundance 37C
Doses Blue Rep1_0.01 Red Rep2_0.12
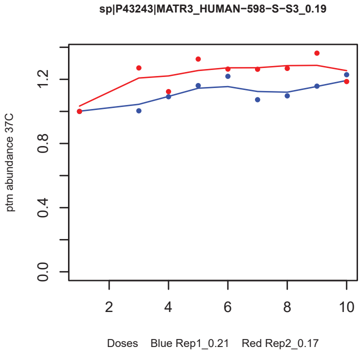
sp|P43243|MATR3_HUMAN−598−S−S3_0.19
ptm abundance 37C
Doses Blue Rep1_0.21 Red Rep2_0.17
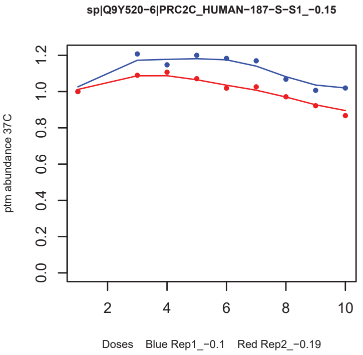
sp|Q9Y520−6|PRC2C_HUMAN−187−S−S1_−0.15
ptm abundance 37C
Doses Blue Rep1_−0.1 Red Rep2_−0.19
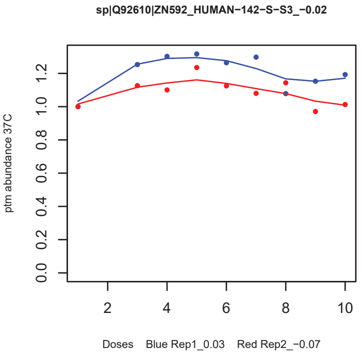
sp|Q92610|ZN592_HUMAN−142−S−S3_−0.02
ptm abundance 37C
Doses Blue Rep1_0.03 Red Rep2_−0.07
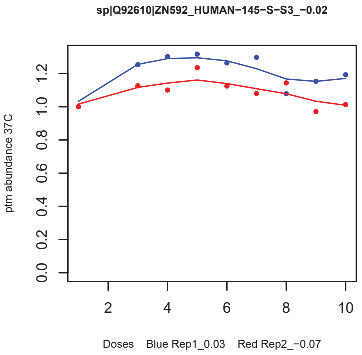
sp|Q92610|ZN592_HUMAN−145−S−S3_−0.02
ptm abundance 37C
Doses Blue Rep1_0.03 Red Rep2_−0.07
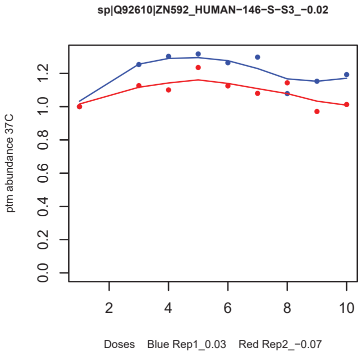
sp|Q92610|ZN592_HUMAN−146−S−S3_−0.02
ptm abundance 37C
Doses Blue Rep1_0.03 Red Rep2_−0.07
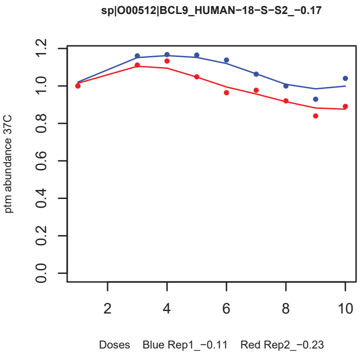
sp|O00512|BCL9_HUMAN−18−S−S2_−0.17
ptm abundance 37C
Doses Blue Rep1_−0.11 Red Rep2_−0.23
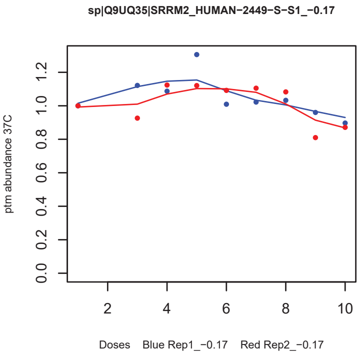
sp|Q9UQ35|SRRM2_HUMAN−2449−S−S1_−0.17
ptm abundance 37C
Doses Blue Rep1_−0.17 Red Rep2_−0.17
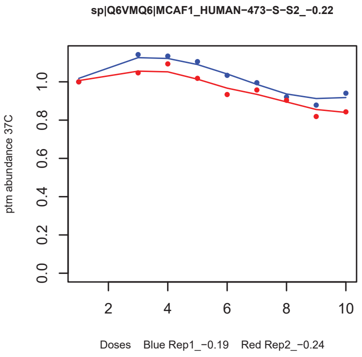
sp|Q6VMQ6|MCAF1_HUMAN−473−S−S2_−0.22
ptm abundance 37C
Doses Blue Rep1_−0.19 Red Rep2_−0.24
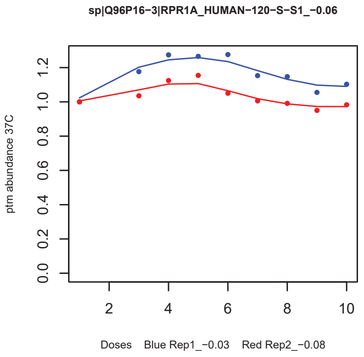
sp|Q96P16−3|RPR1A_HUMAN−120−S−S1_−0.06
ptm abundance 37C
Doses Blue Rep1_−0.03 Red Rep2_−0.08

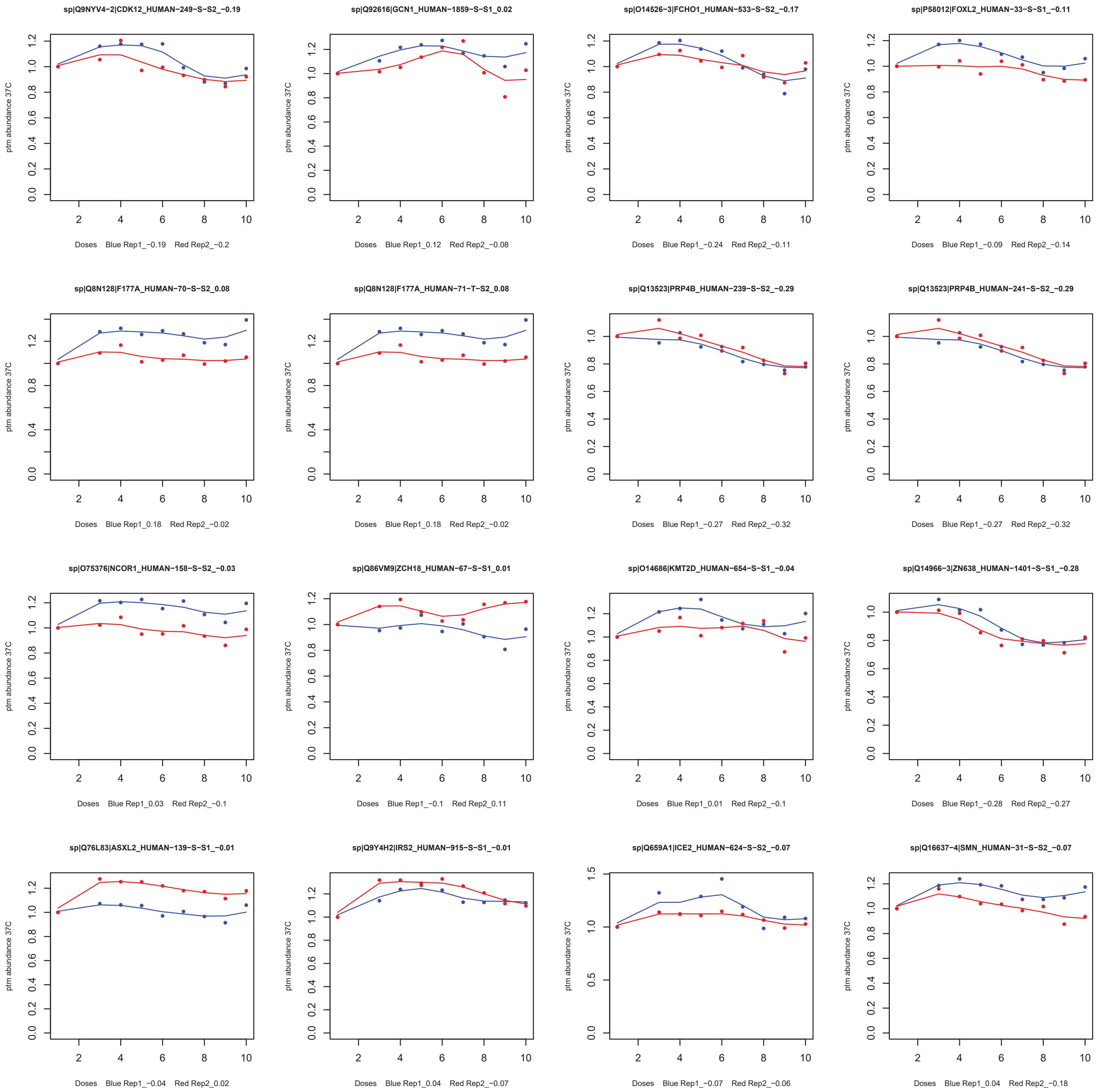
sp|Q9NYV4−2|CDK12_HUMAN−249−S−S2_−0.19
ptm abundance 37C
Doses Blue Rep1_−0.19 Red Rep2_−0.2
sp|Q92616|GCN1_HUMAN−1859−S−S1_0.02
ptm abundance 37C
Doses Blue Rep1_0.12 Red Rep2_−0.08
sp|O14526−3|FCHO1_HUMAN−533−S−S2_−0.17
ptm abundance 37C
Doses Blue Rep1_−0.24 Red Rep2_−0.11
sp|P58012|FOXL2_HUMAN−33−S−S1_−0.11
ptm abundance 37C
Doses Blue Rep1_−0.09 Red Rep2_−0.14
sp|Q8N128|F177A_HUMAN−70−S−S2_0.08
ptm abundance 37C
Doses Blue Rep1_0.18 Red Rep2_−0.02
sp|Q8N128|F177A_HUMAN−71−T−S2_0.08
ptm abundance 37C
Doses Blue Rep1_0.18 Red Rep2_−0.02
sp|Q13523|PRP4B_HUMAN−239−S−S2_−0.29
ptm abundance 37C
Doses Blue Rep1_−0.27 Red Rep2_−0.32
sp|Q13523|PRP4B_HUMAN−241−S−S2_−0.29
ptm abundance 37C
Doses Blue Rep1_−0.27 Red Rep2_−0.32
sp|O75376|NCOR1_HUMAN−158−S−S2_−0.03
ptm abundance 37C
Doses Blue Rep1_0.03 Red Rep2_−0.1
sp|Q86VM9|ZCH18_HUMAN−67−S−S1_0.01
ptm abundance 37C
Doses Blue Rep1_−0.1 Red Rep2_0.11
sp|O14686|KMT2D_HUMAN−654−S−S1_−0.04
ptm abundance 37C
Doses Blue Rep1_0.01 Red Rep2_−0.1
sp|Q14966−3|ZN638_HUMAN−1401−S−S1_−0.28
ptm abundance 37C
Doses Blue Rep1_−0.28 Red Rep2_−0.27
sp|Q76L83|ASXL2_HUMAN−139−S−S1_−0.01
ptm abundance 37C
Doses Blue Rep1_−0.04 Red Rep2_0.02
sp|Q9Y4H2|IRS2_HUMAN−915−S−S1_−0.01
ptm abundance 37C
Doses Blue Rep1_0.04 Red Rep2_−0.07
sp|Q659A1|ICE2_HUMAN−624−S−S2_−0.07
ptm abundance 37C
Doses Blue Rep1_−0.07 Red Rep2_−0.06
sp|Q16637−4|SMN_HUMAN−31−S−S2_−0.07
ptm abundance 37C
Doses Blue Rep1_0.04 Red Rep2_−0.18

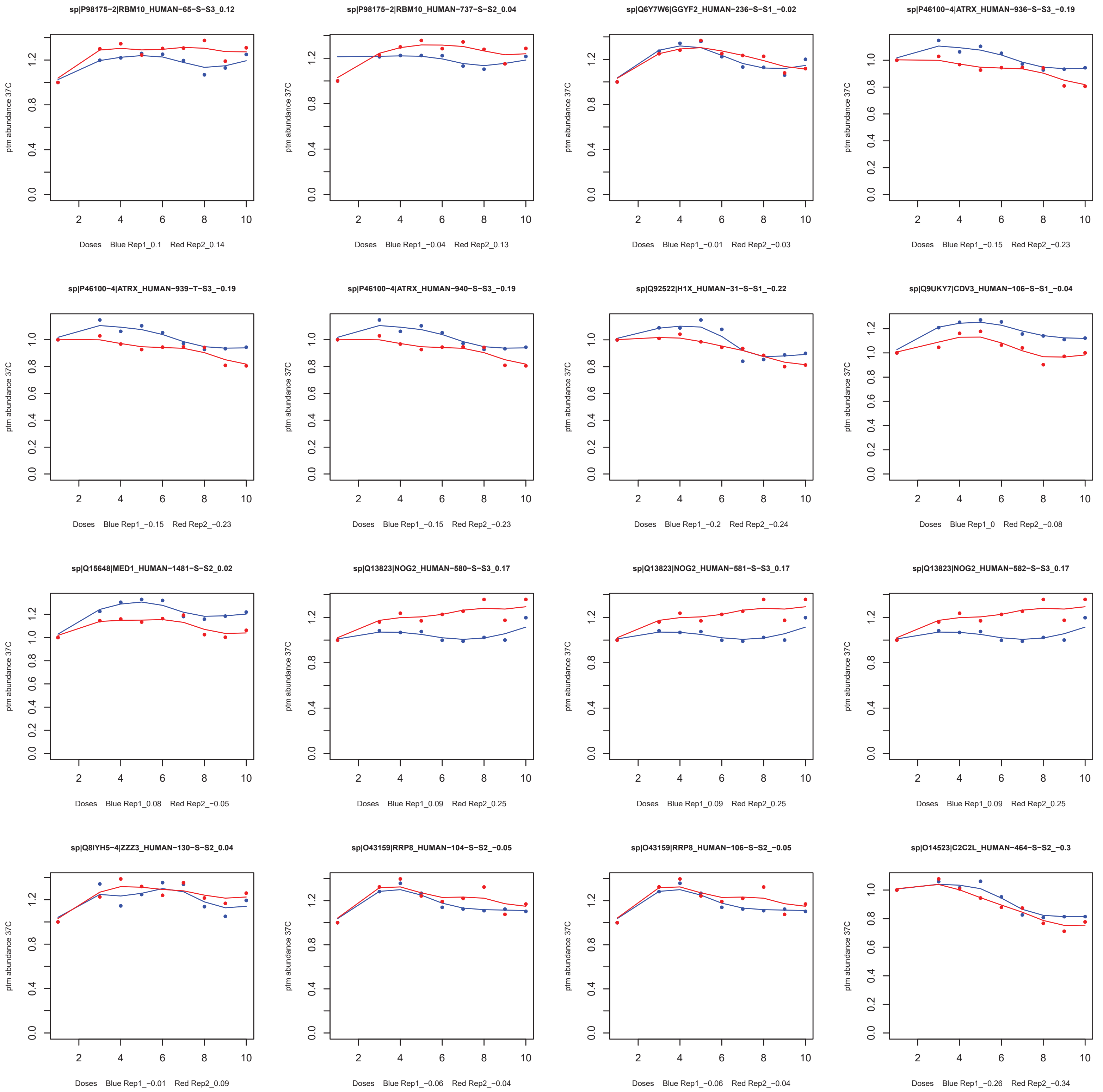

sp|P98175−2|RBM10_HUMAN−65−S−S3_0.12
Doses Blue Rep1_0.1 Red Rep2_0.14
sp|P98175−2|RBM10_HUMAN−737−S−S2_0.04
Doses Blue Rep1_−0.04 Red Rep2_0.13
sp|Q6Y7W6|GGYF2_HUMAN−236−S−S1_−0.02
Doses Blue Rep1_−0.01 Red Rep2_−0.03
sp|P46100−4|ATRX_HUMAN−936−S−S3_−0.19
Doses Blue Rep1_−0.15 Red Rep2_−0.23
sp|P46100−4|ATRX_HUMAN−939−T−S3_−0.19
Doses Blue Rep1_−0.15 Red Rep2_−0.23
sp|P46100−4|ATRX_HUMAN−940−S−S3_−0.19
Doses Blue Rep1_−0.15 Red Rep2_−0.23
sp|Q92522|H1X_HUMAN−31−S−S1_−0.22
Doses Blue Rep1_−0.2 Red Rep2_−0.24
sp|Q9UKY7|CDV3_HUMAN−106−S−S1_−0.04
Doses Blue Rep1_0 Red Rep2_−0.08
sp|Q15648|MED1_HUMAN−1481−S−S2_0.02
Doses Blue Rep1_0.08 Red Rep2_−0.05
sp|Q13823|NOG2_HUMAN−580−S−S3_0.17
Doses Blue Rep1_0.09 Red Rep2_0.25
sp|Q13823|NOG2_HUMAN−581−S−S3_0.17
Doses Blue Rep1_0.09 Red Rep2_0.25
sp|Q13823|NOG2_HUMAN−582−S−S3_0.17
Doses Blue Rep1_0.09 Red Rep2_0.25
sp|Q8IYH5−4|ZZZ3_HUMAN−130−S−S2_0.04
Doses Blue Rep1_−0.01 Red Rep2_0.09
sp|O43159|RRP8_HUMAN−104−S−S2_−0.05
Doses Blue Rep1_−0.06 Red Rep2_−0.04
sp|O43159|RRP8_HUMAN−106−S−S2_−0.05
Doses Blue Rep1_−0.06 Red Rep2_−0.04
sp|O14523|C2C2L_HUMAN−464−S−S2_−0.3
Doses Blue Rep1_−0.26 Red Rep2_−0.34
ptm abundance 37C

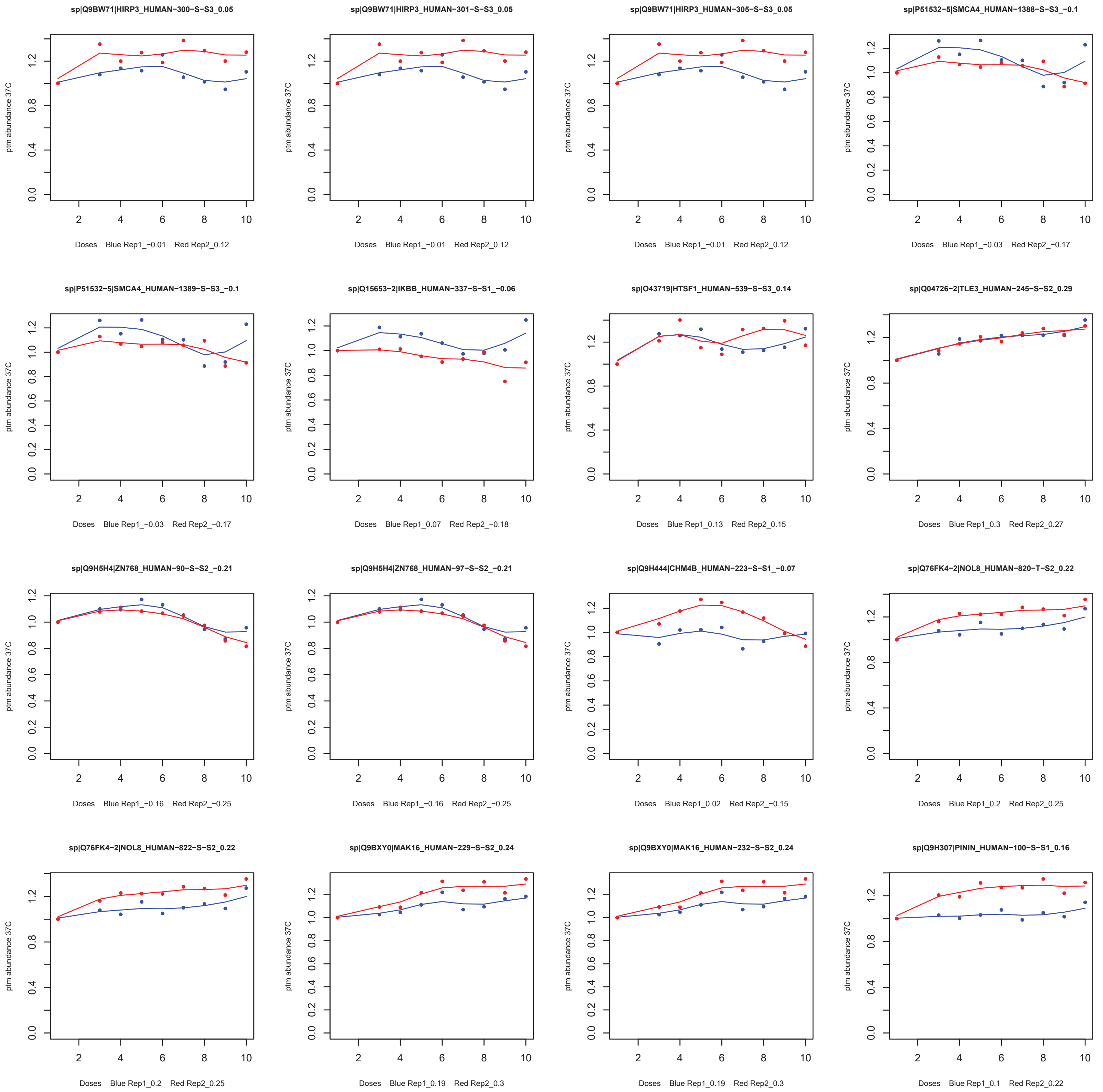

sp|Q9BW71|HIRP3_HUMAN−300−S−S3_0.05
Doses Blue Rep1_−0.01 Red Rep2_0.12
sp|Q9BW71|HIRP3_HUMAN−301−S−S3_0.05
Doses Blue Rep1_−0.01 Red Rep2_0.12
sp|Q9BW71|HIRP3_HUMAN−305−S−S3_0.05
Doses Blue Rep1_−0.01 Red Rep2_0.12
sp|P51532−5|SMCA4_HUMAN−1388−S−S3_−0.1
Doses Blue Rep1_−0.03 Red Rep2_−0.17
sp|P51532−5|SMCA4_HUMAN−1389−S−S3_−0.1
Doses Blue Rep1_−0.03 Red Rep2_−0.17
sp|Q15653−2|IKBB_HUMAN−337−S−S1_−0.06
Doses Blue Rep1_0.07 Red Rep2_−0.18
sp|O43719|HTSF1_HUMAN−539−S−S3_0.14
Doses Blue Rep1_0.13 Red Rep2_0.15
sp|Q04726−2|TLE3_HUMAN−245−S−S2_0.29
Doses Blue Rep1_0.3 Red Rep2_0.27
sp|Q9H5H4|ZN768_HUMAN−90−S−S2_−0.21
Doses Blue Rep1_−0.16 Red Rep2_−0.25
sp|Q9H5H4|ZN768_HUMAN−97−S−S2_−0.21
Doses Blue Rep1_−0.16 Red Rep2_−0.25
sp|Q9H444|CHM4B_HUMAN−223−S−S1_−0.07
Doses Blue Rep1_0.02 Red Rep2_−0.15
sp|Q76FK4−2|NOL8_HUMAN−820−T−S2_0.22
Doses Blue Rep1_0.2 Red Rep2_0.25
sp|Q76FK4−2|NOL8_HUMAN−822−S−S2_0.22
Doses Blue Rep1_0.2 Red Rep2_0.25
sp|Q9BXY0|MAK16_HUMAN−229−S−S2_0.24
Doses Blue Rep1_0.19 Red Rep2_0.3
sp|Q9BXY0|MAK16_HUMAN−232−S−S2_0.24
Doses Blue Rep1_0.19 Red Rep2_0.3
sp|Q9H307|PININ_HUMAN−100−S−S1_0.16
Doses Blue Rep1_0.1 Red Rep2_0.22
ptm abundance 37C

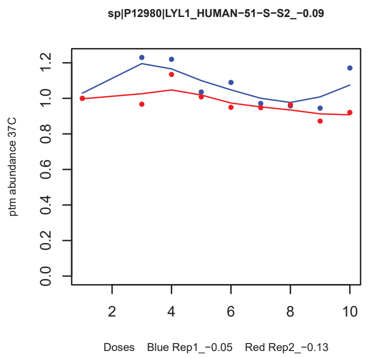
sp|P12980|LYL1_HUMAN−51−S−S2_−0.09
ptm abundance 37C
Doses Blue Rep1_−0.05 Red Rep2_−0.13

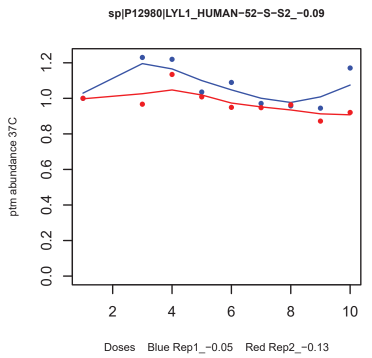
sp|P12980|LYL1_HUMAN−52−S−S2_−0.09
ptm abundance 37C
Doses Blue Rep1_−0.05 Red Rep2_−0.13

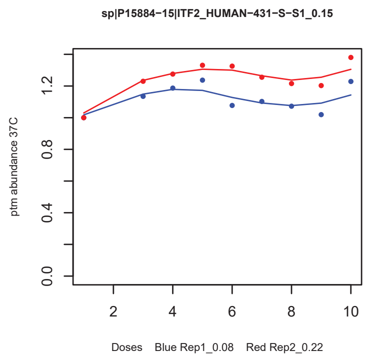
sp|P15884−15|ITF2_HUMAN−431−S−S1_0.15
ptm abundance 37C
Doses Blue Rep1_0.08 Red Rep2_0.22

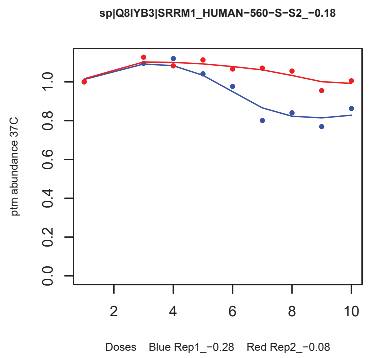
sp|Q8IYB3|SRRM1_HUMAN−560−S−S2_−0.18
ptm abundance 37C
Doses Blue Rep1_−0.28 Red Rep2_−0.08

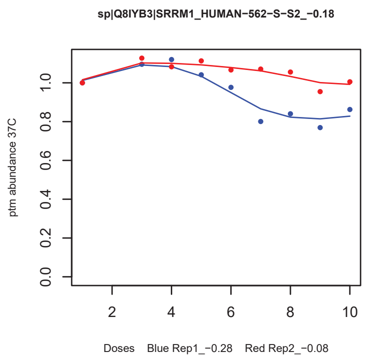
sp|Q8IYB3|SRRM1_HUMAN−562−S−S2_−0.18
ptm abundance 37C
Doses Blue Rep1_−0.28 Red Rep2_−0.08

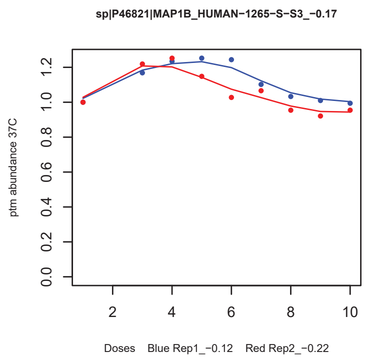
sp|P46821|MAP1B_HUMAN−1265−S−S3_−0.17
ptm abundance 37C
Doses Blue Rep1_−0.12 Red Rep2_−0.22

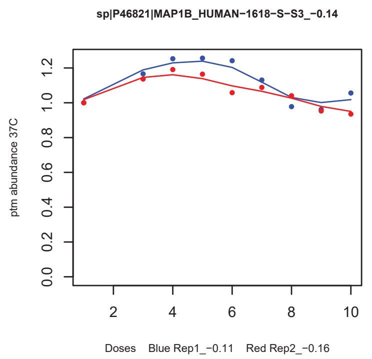
sp|P46821|MAP1B_HUMAN−1618−S−S3_−0.14
ptm abundance 37C
Doses Blue Rep1_−0.11 Red Rep2_−0.16

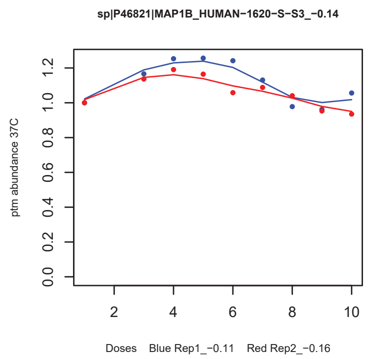
sp|P46821|MAP1B_HUMAN−1620−S−S3_−0.14
ptm abundance 37C
Doses Blue Rep1_−0.11 Red Rep2_−0.16

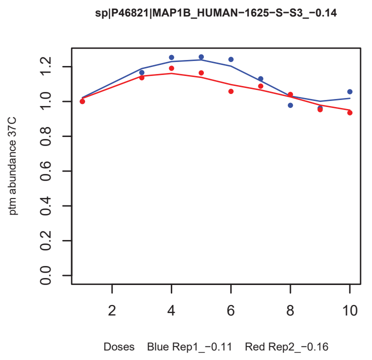
sp|P46821|MAP1B_HUMAN−1625−S−S3_−0.14
ptm abundance 37C
Doses Blue Rep1_−0.11 Red Rep2_−0.16

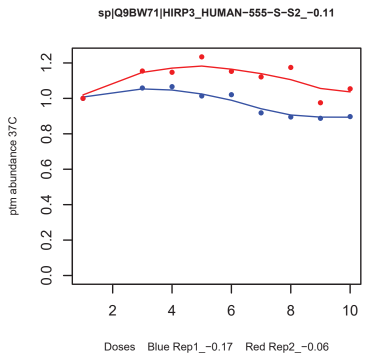
sp|Q9BW71|HIRP3_HUMAN−555−S−S2_−0.11
ptm abundance 37C
Doses Blue Rep1_−0.17 Red Rep2_−0.06

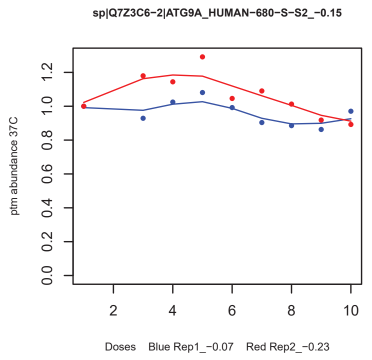
sp|Q7Z3C6−2|ATG9A_HUMAN−680−S−S2_−0.15
ptm abundance 37C
Doses Blue Rep1_−0.07 Red Rep2_−0.23

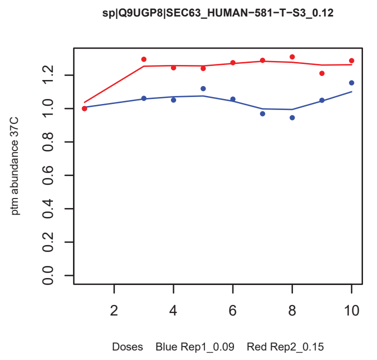
sp|Q9UGP8|SEC63_HUMAN−581−T−S3_0.12
ptm abundance 37C
Doses Blue Rep1_0.09 Red Rep2_0.15

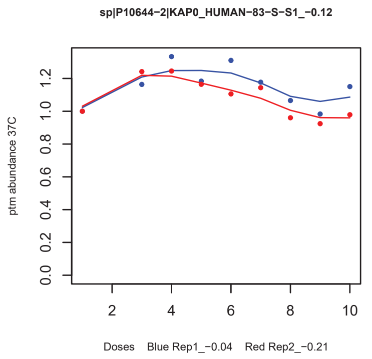
sp|P10644−2|KAP0_HUMAN−83−S−S1_−0.12
ptm abundance 37C
Doses Blue Rep1_−0.04 Red Rep2_−0.21

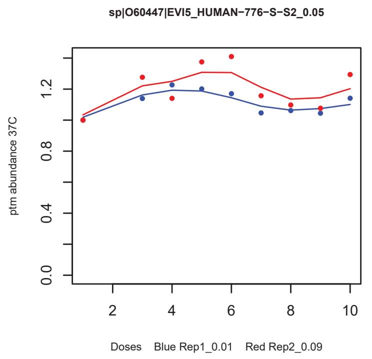
sp|O60447|EVI5_HUMAN−776−S−S2_0.05
ptm abundance 37C
Doses Blue Rep1_0.01 Red Rep2_0.09

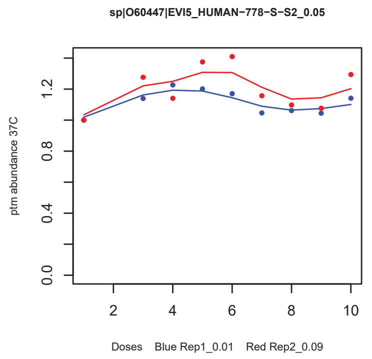
sp|O60447|EVI5_HUMAN−778−S−S2_0.05
ptm abundance 37C
Doses Blue Rep1_0.01 Red Rep2_0.09

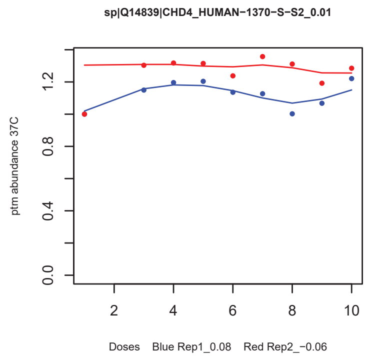
sp|Q14839|CHD4_HUMAN−1370−S−S2_0.01
ptm abundance 37C
Doses Blue Rep1_0.08 Red Rep2_−0.06

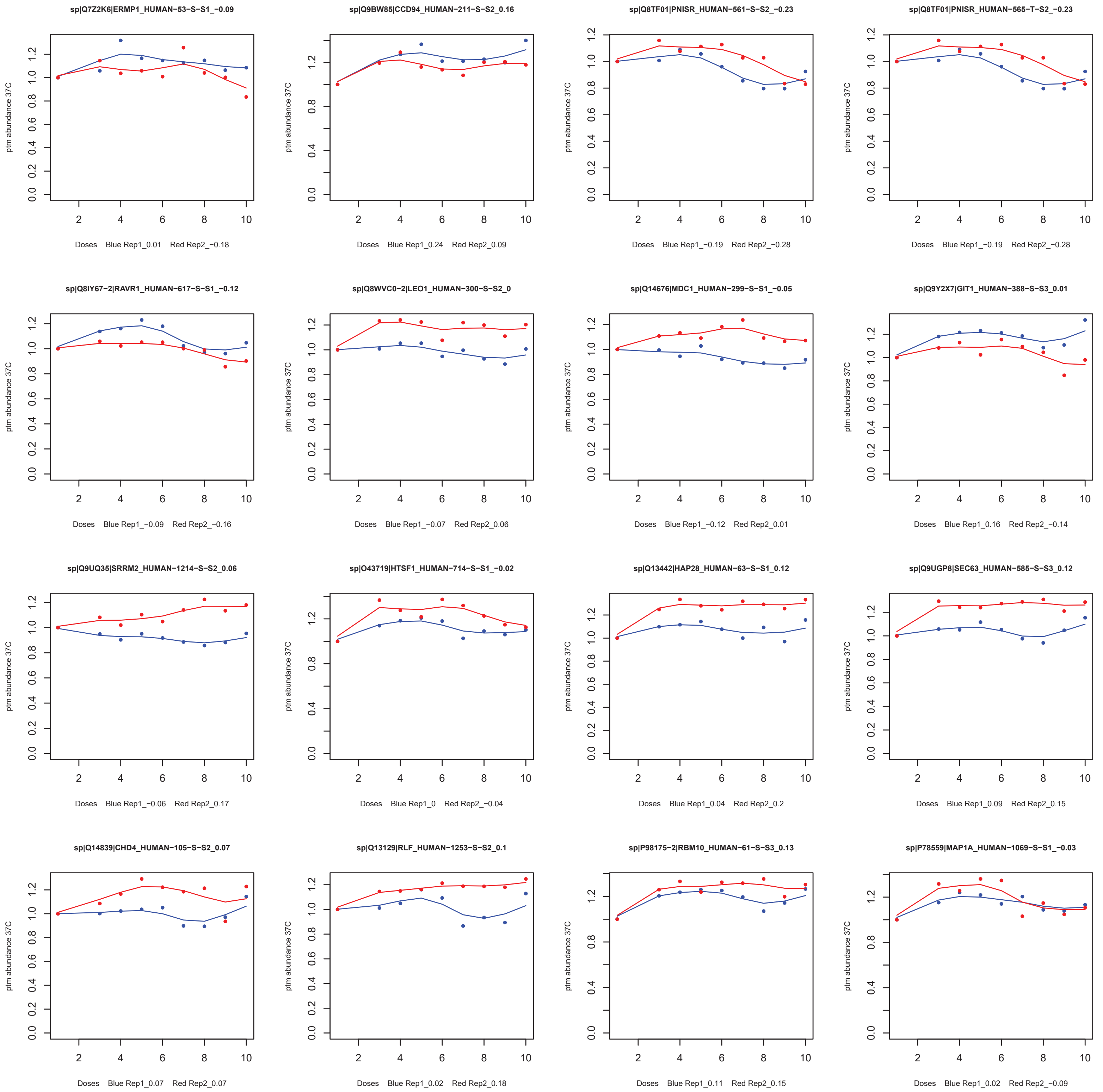

sp|Q7Z2K6|ERMP1_HUMAN−53−S−S1_−0.09
ptm abundance 37C
Doses Blue Rep1_0.01 Red Rep2_−0.18
sp|Q9BW85|CCD94_HUMAN−211−S−S2_0.16
ptm abundance 37C
Doses Blue Rep1_0.24 Red Rep2_0.09
sp|Q8TF01|PNISR_HUMAN−561−S−S2_−0.23
ptm abundance 37C
Doses Blue Rep1_−0.19 Red Rep2_−0.28
sp|Q8TF01|PNISR_HUMAN−565−T−S2_−0.23
ptm abundance 37C
Doses Blue Rep1_−0.19 Red Rep2_−0.28
sp|Q8IY67−2|RAVR1_HUMAN−617−S−S1_−0.12
ptm abundance 37C
Doses Blue Rep1_−0.09 Red Rep2_−0.16
sp|Q8WVC0−2|LEO1_HUMAN−300−S−S2_0
ptm abundance 37C
Doses Blue Rep1_−0.07 Red Rep2_0.06
sp|Q14676|MDC1_HUMAN−299−S−S1_−0.05
ptm abundance 37C
Doses Blue Rep1_−0.12 Red Rep2_0.01
sp|Q9Y2X7|GIT1_HUMAN−388−S−S3_0.01
ptm abundance 37C
Doses Blue Rep1_0.16 Red Rep2_−0.14
sp|Q9UQ35|SRRM2_HUMAN−1214−S−S2_0.06
ptm abundance 37C
Doses Blue Rep1_−0.06 Red Rep2_0.17
sp|O43719|HTSF1_HUMAN−714−S−S1_−0.02
ptm abundance 37C
Doses Blue Rep1_0 Red Rep2_−0.04
sp|Q13442|HAP28_HUMAN−63−S−S1_0.12
ptm abundance 37C
Doses Blue Rep1_0.04 Red Rep2_0.2
sp|Q9UGP8|SEC63_HUMAN−585−S−S3_0.12
ptm abundance 37C
Doses Blue Rep1_0.09 Red Rep2_0.15
sp|Q14839|CHD4_HUMAN−105−S−S2_0.07
ptm abundance 37C
Doses Blue Rep1_0.07 Red Rep2_0.07
sp|Q13129|RLF_HUMAN−1253−S−S2_0.1
ptm abundance 37C
Doses Blue Rep1_0.02 Red Rep2_0.18
sp|P98175−2|RBM10_HUMAN−61−S−S3_0.13
ptm abundance 37C
Doses Blue Rep1_0.11 Red Rep2_0.15
sp|P78559|MAP1A_HUMAN−1069−S−S1_−0.03
ptm abundance 37C
Doses Blue Rep1_0.02 Red Rep2_−0.09

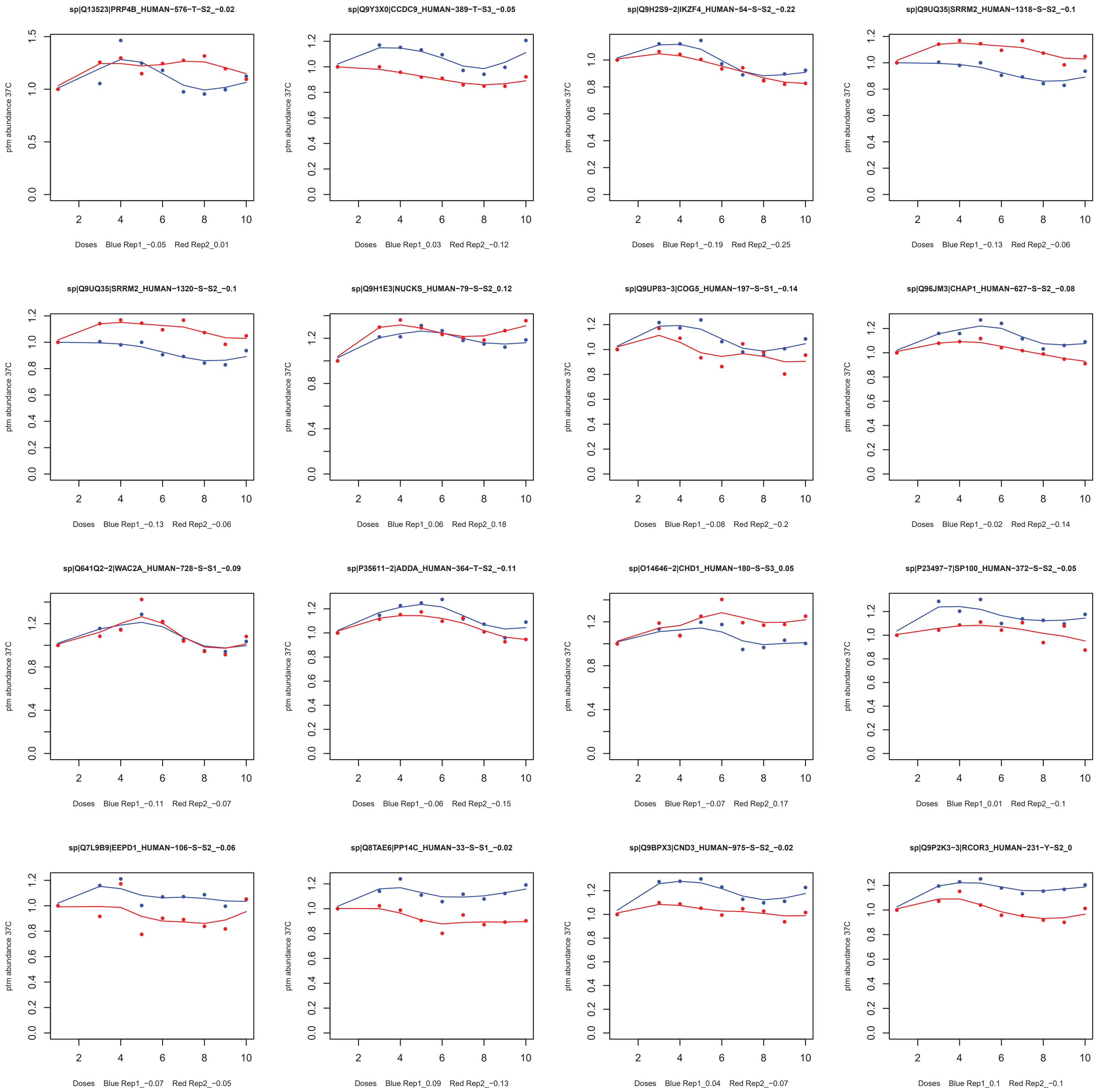
sp|Q13523|PRP4B_HUMAN−576−T−S2_−0.02
Doses Blue Rep1_−0.05 Red Rep2_0.01
sp|Q9Y3X0|CCDC9_HUMAN−389−T−S3_−0.05
Doses Blue Rep1_0.03 Red Rep2_−0.12
sp|Q9H2S9−2|IKZF4_HUMAN−54−S−S2_−0.22
Doses Blue Rep1_−0.19 Red Rep2_−0.25
sp|Q9UQ35|SRRM2_HUMAN−1318−S−S2_−0.1
Doses Blue Rep1_−0.13 Red Rep2_−0.06
sp|Q9UQ35|SRRM2_HUMAN−1320−S−S2_−0.1
Doses Blue Rep1_−0.13 Red Rep2_−0.06
sp|Q9H1E3|NUCKS_HUMAN−79−S−S2_0.12
Doses Blue Rep1_0.06 Red Rep2_0.18
sp|Q9UP83−3|COG5_HUMAN−197−S−S1_−0.14
Doses Blue Rep1_−0.08 Red Rep2_−0.2
sp|Q96JM3|CHAP1_HUMAN−627−S−S2_−0.08
Doses Blue Rep1_−0.02 Red Rep2_−0.14
sp|Q641Q2−2|WAC2A_HUMAN−728−S−S1_−0.09
Doses Blue Rep1_−0.11 Red Rep2_−0.07
sp|P35611−2|ADDA_HUMAN−364−T−S2_−0.11
Doses Blue Rep1_−0.06 Red Rep2_−0.15
sp|O14646−2|CHD1_HUMAN−180−S−S3_0.05
Doses Blue Rep1_−0.07 Red Rep2_0.17
sp|P23497−7|SP100_HUMAN−372−S−S2_−0.05
Doses Blue Rep1_0.01 Red Rep2_−0.1
sp|Q7L9B9|EEPD1_HUMAN−106−S−S2_−0.06
Doses Blue Rep1_−0.07 Red Rep2_−0.05
sp|Q8TAE6|PP14C_HUMAN−33−S−S1_−0.02
Doses Blue Rep1_0.09 Red Rep2_−0.13
sp|Q9BPX3|CND3_HUMAN−975−S−S2_−0.02
Doses Blue Rep1_0.04 Red Rep2_−0.07
sp|Q9P2K3−3|RCOR3_HUMAN−231−Y−S2_0
Doses Blue Rep1_0.1 Red Rep2_−0.1
ptm abundance 37C

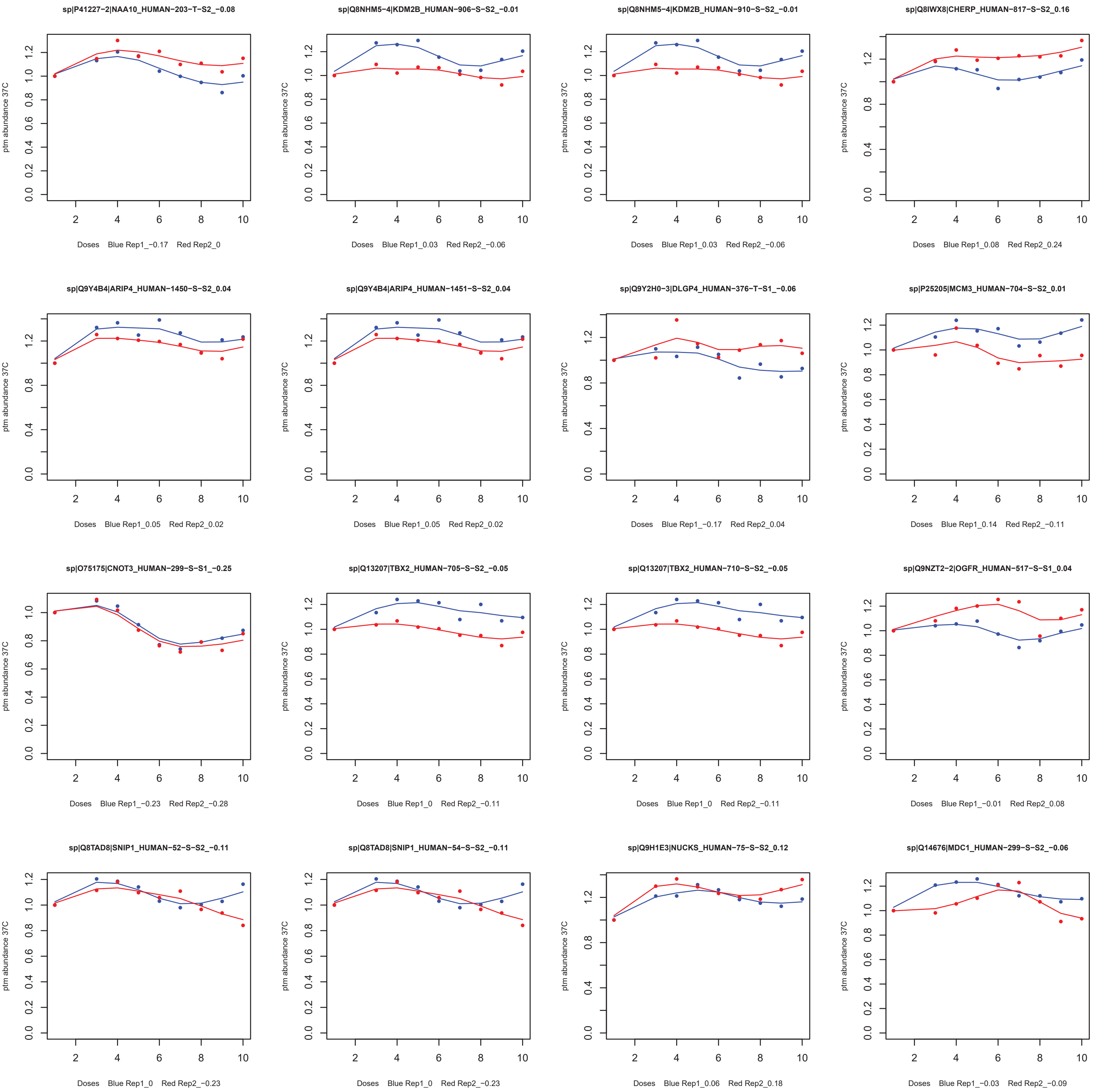
sp|P41227−2|NAA10_HUMAN−203−T−S2_−0.08
ptm abundance 37C
Doses Blue Rep1_−0.17 Red Rep2_0
sp|Q8NHM5−4|KDM2B_HUMAN−906−S−S2_−0.01
ptm abundance 37C
Doses Blue Rep1_0.03 Red Rep2_−0.06
sp|Q8NHM5−4|KDM2B_HUMAN−910−S−S2_−0.01
ptm abundance 37C
Doses Blue Rep1_0.03 Red Rep2_−0.06
sp|Q8IWX8|CHERP_HUMAN−817−S−S2_0.16
ptm abundance 37C
Doses Blue Rep1_0.08 Red Rep2_0.24
sp|Q9Y4B4|ARIP4_HUMAN−1450−S−S2_0.04
ptm abundance 37C
Doses Blue Rep1_0.05 Red Rep2_0.02
sp|Q9Y4B4|ARIP4_HUMAN−1451−S−S2_0.04
ptm abundance 37C
Doses Blue Rep1_0.05 Red Rep2_0.02
sp|Q9Y2H0−3|DLGP4_HUMAN−376−T−S1_−0.06
ptm abundance 37C
Doses Blue Rep1_−0.17 Red Rep2_0.04
sp|P25205|MCM3_HUMAN−704−S−S2_0.01
ptm abundance 37C
Doses Blue Rep1_0.14 Red Rep2_−0.11
sp|O75175|CNOT3_HUMAN−299−S−S1_−0.25
ptm abundance 37C
Doses Blue Rep1_−0.23 Red Rep2_−0.28
sp|Q13207|TBX2_HUMAN−705−S−S2_−0.05
ptm abundance 37C
Doses Blue Rep1_0 Red Rep2_−0.11
sp|Q13207|TBX2_HUMAN−710−S−S2_−0.05
ptm abundance 37C
Doses Blue Rep1_0 Red Rep2_−0.11
sp|Q9NZT2−2|OGFR_HUMAN−517−S−S1_0.04
ptm abundance 37C
Doses Blue Rep1_−0.01 Red Rep2_0.08
sp|Q8TAD8|SNIP1_HUMAN−52−S−S2_−0.11
ptm abundance 37C
Doses Blue Rep1_0 Red Rep2_−0.23
sp|Q8TAD8|SNIP1_HUMAN−54−S−S2_−0.11
ptm abundance 37C
Doses Blue Rep1_0 Red Rep2_−0.23
sp|Q9H1E3|NUCKS_HUMAN−75−S−S2_0.12
ptm abundance 37C
Doses Blue Rep1_0.06 Red Rep2_0.18
sp|Q14676|MDC1_HUMAN−299−S−S2_−0.06
ptm abundance 37C
Doses Blue Rep1_−0.03 Red Rep2_−0.09

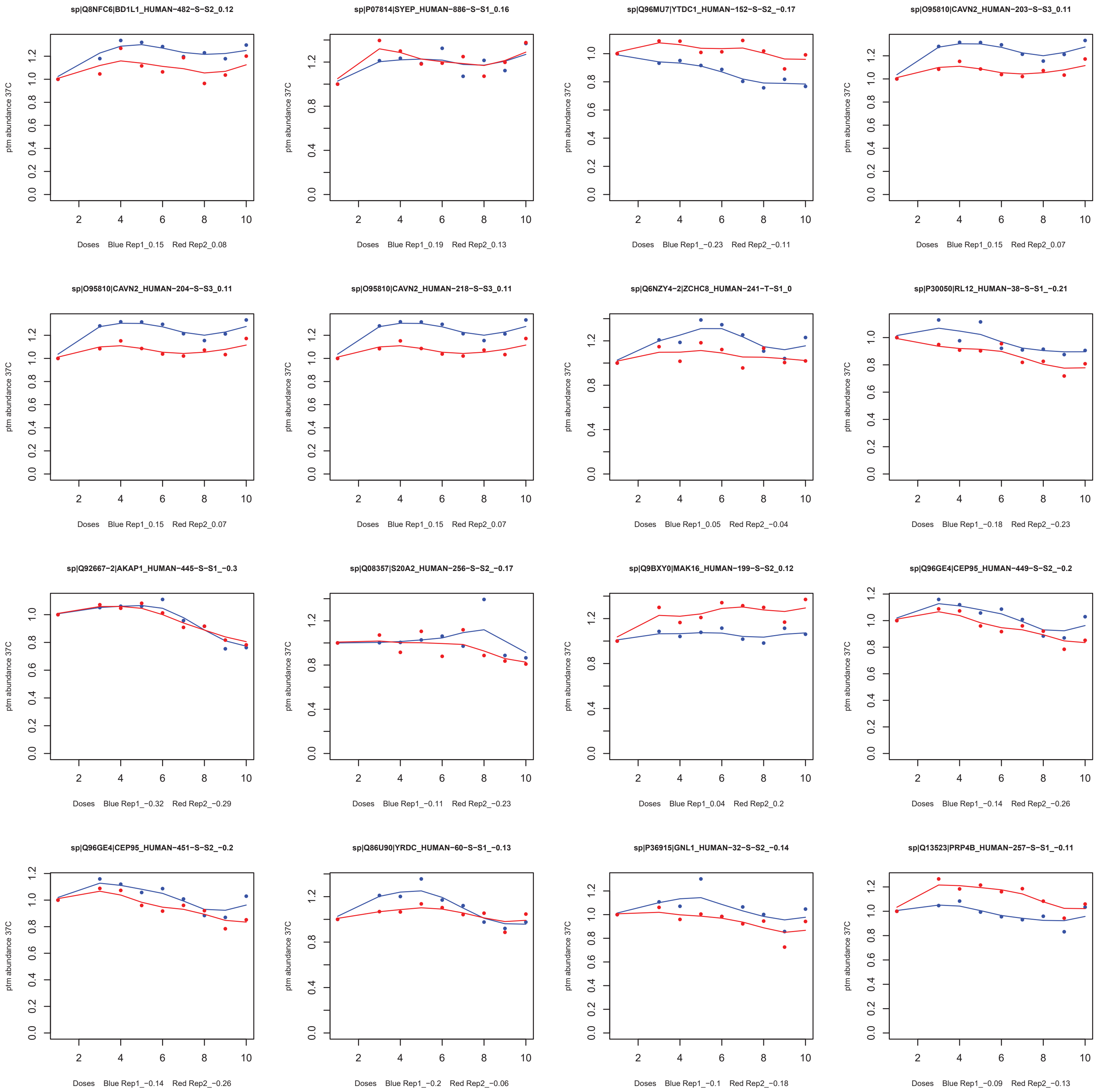

sp|Q8NFC6|BD1L1_HUMAN−482−S−S2_0.12
Doses Blue Rep1_0.15 Red Rep2_0.08
sp|P07814|SYEP_HUMAN−886−S−S1_0.16
Doses Blue Rep1_0.19 Red Rep2_0.13
sp|Q96MU7|YTDC1_HUMAN−152−S−S2_−0.17
Doses Blue Rep1_−0.23 Red Rep2_−0.11
sp|O95810|CAVN2_HUMAN−203−S−S3_0.11
Doses Blue Rep1_0.15 Red Rep2_0.07
sp|O95810|CAVN2_HUMAN−204−S−S3_0.11
Doses Blue Rep1_0.15 Red Rep2_0.07
sp|O95810|CAVN2_HUMAN−218−S−S3_0.11
Doses Blue Rep1_0.15 Red Rep2_0.07
sp|Q6NZY4−2|ZCHC8_HUMAN−241−T−S1_0
Doses Blue Rep1_0.05 Red Rep2_−0.04
sp|P30050|RL12_HUMAN−38−S−S1_−0.21
Doses Blue Rep1_−0.18 Red Rep2_−0.23
sp|Q92667−2|AKAP1_HUMAN−445−S−S1_−0.3
Doses Blue Rep1_−0.32 Red Rep2_−0.29
sp|Q08357|S20A2_HUMAN−256−S−S2_−0.17
Doses Blue Rep1_−0.11 Red Rep2_−0.23
sp|Q9BXY0|MAK16_HUMAN−199−S−S2_0.12
Doses Blue Rep1_0.04 Red Rep2_0.2
sp|Q96GE4|CEP95_HUMAN−449−S−S2_−0.2
Doses Blue Rep1_−0.14 Red Rep2_−0.26
sp|Q96GE4|CEP95_HUMAN−451−S−S2_−0.2
Doses Blue Rep1_−0.14 Red Rep2_−0.26
sp|Q86U90|YRDC_HUMAN−60−S−S1_−0.13
Doses Blue Rep1_−0.2 Red Rep2_−0.06
sp|P36915|GNL1_HUMAN−32−S−S2_−0.14
Doses Blue Rep1_−0.1 Red Rep2_−0.18
sp|Q13523|PRP4B_HUMAN−257−S−S1_−0.11
Doses Blue Rep1_−0.09 Red Rep2_−0.13
ptm abundance 37C

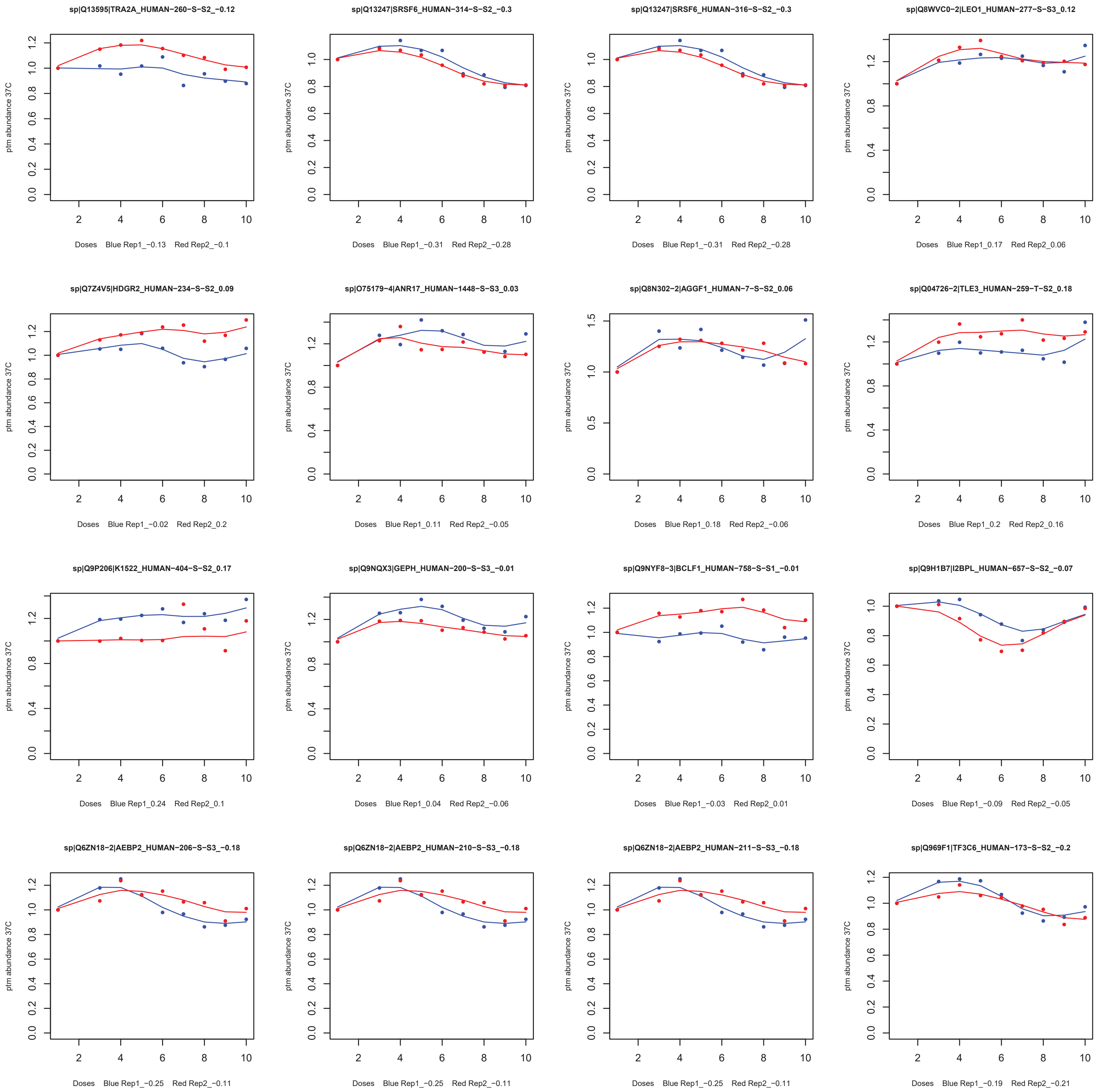

sp|Q13595|TRA2A_HUMAN−260−S−S2_−0.12
Doses Blue Rep1_−0.13 Red Rep2_−0.1
sp|Q13247|SRSF6_HUMAN−314−S−S2_−0.3
Doses Blue Rep1_−0.31 Red Rep2_−0.28
sp|Q13247|SRSF6_HUMAN−316−S−S2_−0.3
Doses Blue Rep1_−0.31 Red Rep2_−0.28
sp|Q8WVC0−2|LEO1_HUMAN−277−S−S3_0.12
Doses Blue Rep1_0.17 Red Rep2_0.06
sp|Q7Z4V5|HDGR2_HUMAN−234−S−S2_0.09
Doses Blue Rep1_−0.02 Red Rep2_0.2
sp|O75179−4|ANR17_HUMAN−1448−S−S3_0.03
Doses Blue Rep1_0.11 Red Rep2_−0.05
sp|Q8N302−2|AGGF1_HUMAN−7−S−S2_0.06
Doses Blue Rep1_0.18 Red Rep2_−0.06
sp|Q04726−2|TLE3_HUMAN−259−T−S2_0.18
Doses Blue Rep1_0.2 Red Rep2_0.16
sp|Q9P206|K1522_HUMAN−404−S−S2_0.17
Doses Blue Rep1_0.24 Red Rep2_0.1
sp|Q9NQX3|GEPH_HUMAN−200−S−S3_−0.01
Doses Blue Rep1_0.04 Red Rep2_−0.06
sp|Q9NYF8−3|BCLF1_HUMAN−758−S−S1_−0.01
Doses Blue Rep1_−0.03 Red Rep2_0.01
sp|Q9H1B7|I2BPL_HUMAN−657−S−S2_−0.07
Doses Blue Rep1_−0.09 Red Rep2_−0.05
sp|Q6ZN18−2|AEBP2_HUMAN−206−S−S3_−0.18
Doses Blue Rep1_−0.25 Red Rep2_−0.11
sp|Q6ZN18−2|AEBP2_HUMAN−210−S−S3_−0.18
Doses Blue Rep1_−0.25 Red Rep2_−0.11
sp|Q6ZN18−2|AEBP2_HUMAN−211−S−S3_−0.18
Doses Blue Rep1_−0.25 Red Rep2_−0.11
sp|Q969F1|TF3C6_HUMAN−173−S−S2_−0.2
Doses Blue Rep1_−0.19 Red Rep2_−0.21
ptm abundance 37C

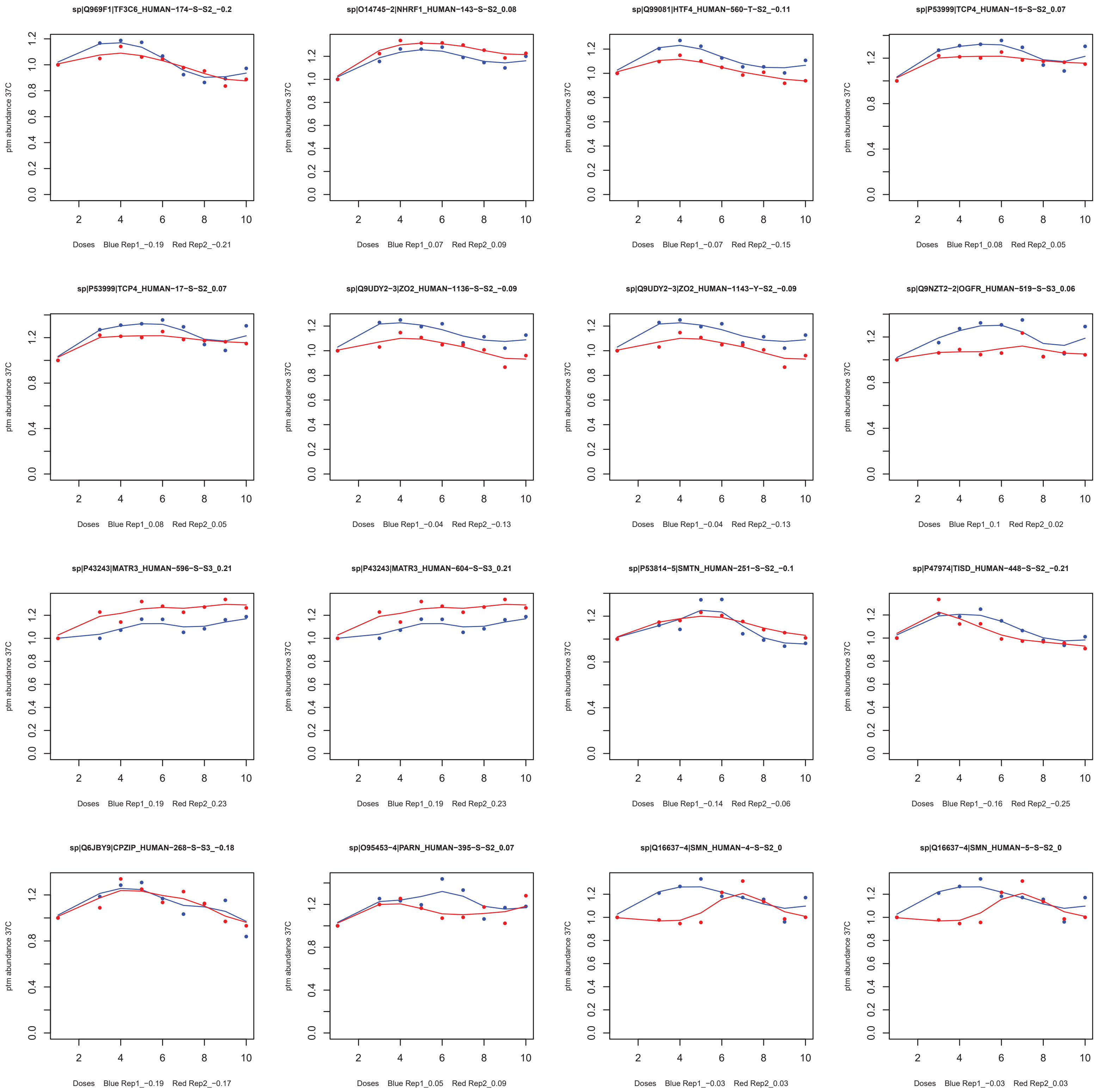
sp|Q969F1|TF3C6_HUMAN−174−S−S2_−0.2
ptm abundance 37C
Doses Blue Rep1_−0.19 Red Rep2_−0.21
sp|O14745−2|NHRF1_HUMAN−143−S−S2_0.08
ptm abundance 37C
Doses Blue Rep1_0.07 Red Rep2_0.09
sp|Q99081|HTF4_HUMAN−560−T−S2_−0.11
ptm abundance 37C
Doses Blue Rep1_−0.07 Red Rep2_−0.15
sp|P53999|TCP4_HUMAN−15−S−S2_0.07
ptm abundance 37C
Doses Blue Rep1_0.08 Red Rep2_0.05
sp|P53999|TCP4_HUMAN−17−S−S2_0.07
ptm abundance 37C
Doses Blue Rep1_0.08 Red Rep2_0.05
sp|Q9UDY2−3|ZO2_HUMAN−1136−S−S2_−0.09
ptm abundance 37C
Doses Blue Rep1_−0.04 Red Rep2_−0.13
sp|Q9UDY2−3|ZO2_HUMAN−1143−Y−S2_−0.09
ptm abundance 37C
Doses Blue Rep1_−0.04 Red Rep2_−0.13
sp|Q9NZT2−2|OGFR_HUMAN−519−S−S3_0.06
ptm abundance 37C
Doses Blue Rep1_0.1 Red Rep2_0.02
sp|P43243|MATR3_HUMAN−596−S−S3_0.21
ptm abundance 37C
Doses Blue Rep1_0.19 Red Rep2_0.23
sp|P43243|MATR3_HUMAN−604−S−S3_0.21
ptm abundance 37C
Doses Blue Rep1_0.19 Red Rep2_0.23
sp|P53814−5|SMTN_HUMAN−251−S−S2_−0.1
ptm abundance 37C
Doses Blue Rep1_−0.14 Red Rep2_−0.06
sp|P47974|TISD_HUMAN−448−S−S2_−0.21
ptm abundance 37C
Doses Blue Rep1_−0.16 Red Rep2_−0.25
sp|Q6JBY9|CPZIP_HUMAN−268−S−S3_−0.18
ptm abundance 37C
Doses Blue Rep1_−0.19 Red Rep2_−0.17
sp|O95453−4|PARN_HUMAN−395−S−S2_0.07
ptm abundance 37C
Doses Blue Rep1_0.05 Red Rep2_0.09
sp|Q16637−4|SMN_HUMAN−4−S−S2_0
ptm abundance 37C
Doses Blue Rep1_−0.03 Red Rep2_0.03
sp|Q16637−4|SMN_HUMAN−5−S−S2_0
ptm abundance 37C
Doses Blue Rep1_−0.03 Red Rep2_0.03

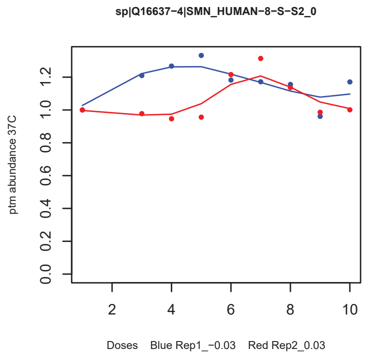
sp|Q16637−4|SMN_HUMAN−8−S−S2_0
ptm abundance 37C
Doses Blue Rep1_−0.03 Red Rep2_0.03

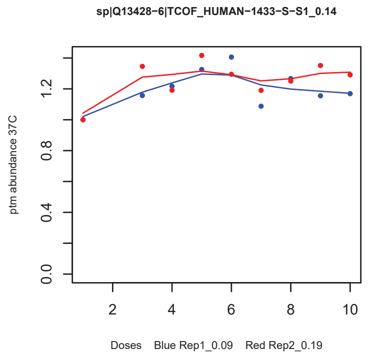
sp|Q13428−6|TCOF_HUMAN−1433−S−S1_0.14
ptm abundance 37C
Doses Blue Rep1_0.09 Red Rep2_0.19

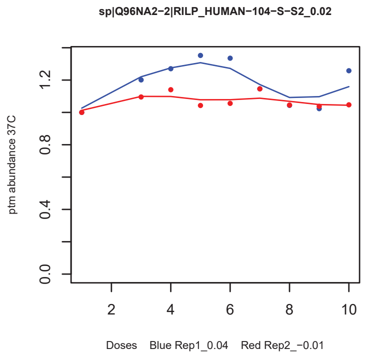
sp|Q96NA2−2|RILP_HUMAN−104−S−S2_0.02
ptm abundance 37C
Doses Blue Rep1_0.04 Red Rep2_−0.01

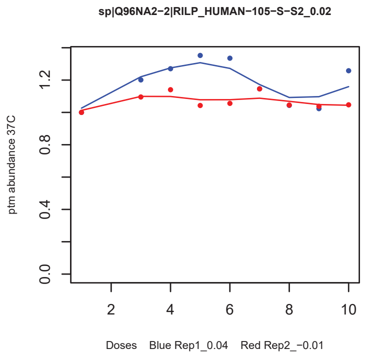
sp|Q96NA2−2|RILP_HUMAN−105−S−S2_0.02
ptm abundance 37C
Doses Blue Rep1_0.04 Red Rep2_−0.01

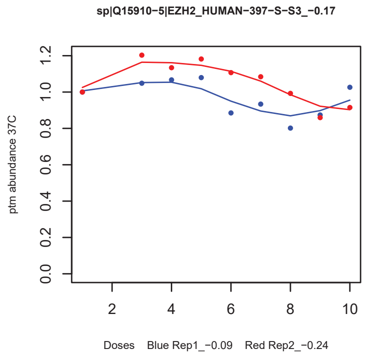
sp|Q15910−5|EZH2_HUMAN−397−S−S3_−0.17
ptm abundance 37C
Doses Blue Rep1_−0.09 Red Rep2_−0.24

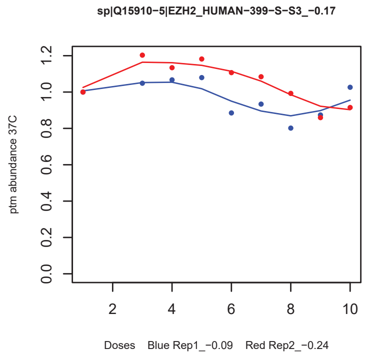
sp|Q15910−5|EZH2_HUMAN−399−S−S3_−0.17
ptm abundance 37C
Doses Blue Rep1_−0.09 Red Rep2_−0.24

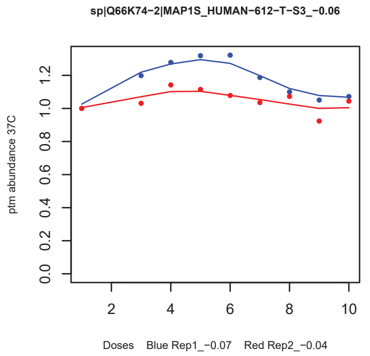
sp|Q66K74−2|MAP1S_HUMAN−612−T−S3_−0.06
ptm abundance 37C
Doses Blue Rep1_−0.07 Red Rep2_−0.04

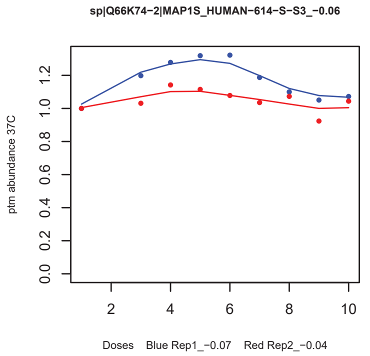
sp|Q66K74−2|MAP1S_HUMAN−614−S−S3_−0.06
ptm abundance 37C
Doses Blue Rep1_−0.07 Red Rep2_−0.04

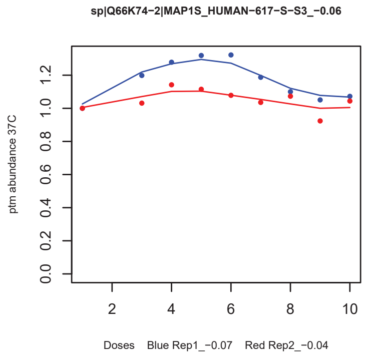
sp|Q66K74−2|MAP1S_HUMAN−617−S−S3_−0.06
ptm abundance 37C
Doses Blue Rep1_−0.07 Red Rep2_−0.04

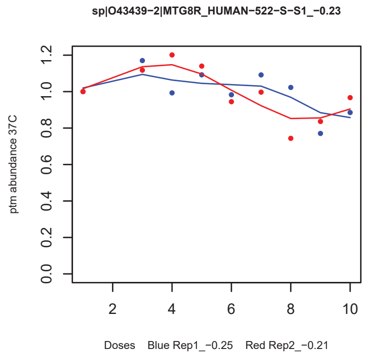
sp|O43439−2|MTG8R_HUMAN−522−S−S1_−0.23
ptm abundance 37C
Doses Blue Rep1_−0.25 Red Rep2_−0.21

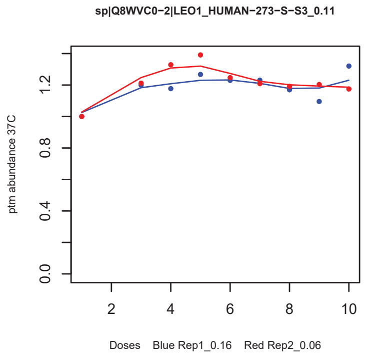
sp|Q8WVC0−2|LEO1_HUMAN−273−S−S3_0.11
ptm abundance 37C
Doses Blue Rep1_0.16 Red Rep2_0.06

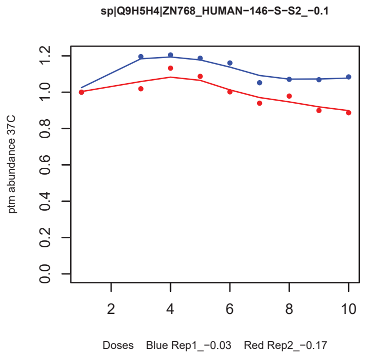
sp|Q9H5H4|ZN768_HUMAN−146−S−S2_−0.1
ptm abundance 37C
Doses Blue Rep1_−0.03 Red Rep2_−0.17

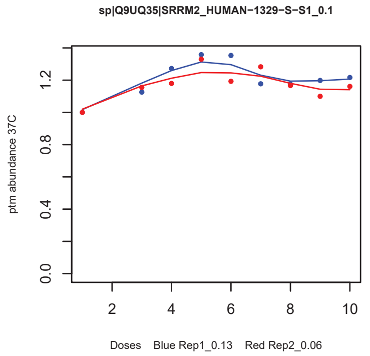
sp|Q9UQ35|SRRM2_HUMAN−1329−S−S1_0.1
ptm abundance 37C
Doses Blue Rep1_0.13 Red Rep2_0.06

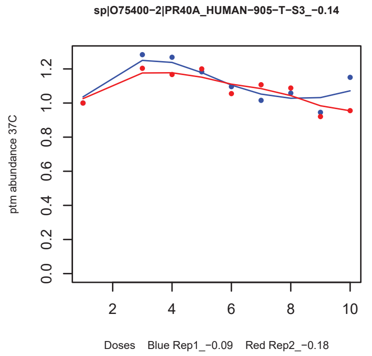
sp|O75400−2|PR40A_HUMAN−905−T−S3_−0.14
ptm abundance 37C
Doses Blue Rep1_−0.09 Red Rep2_−0.18

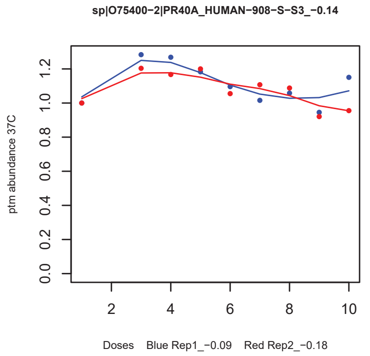
sp|O75400−2|PR40A_HUMAN−908−S−S3_−0.14
ptm abundance 37C
Doses Blue Rep1_−0.09 Red Rep2_−0.18

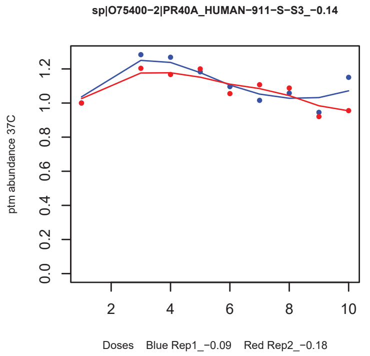
sp|O75400−2|PR40A_HUMAN−911−S−S3_−0.14
ptm abundance 37C
Doses Blue Rep1_−0.09 Red Rep2_−0.18

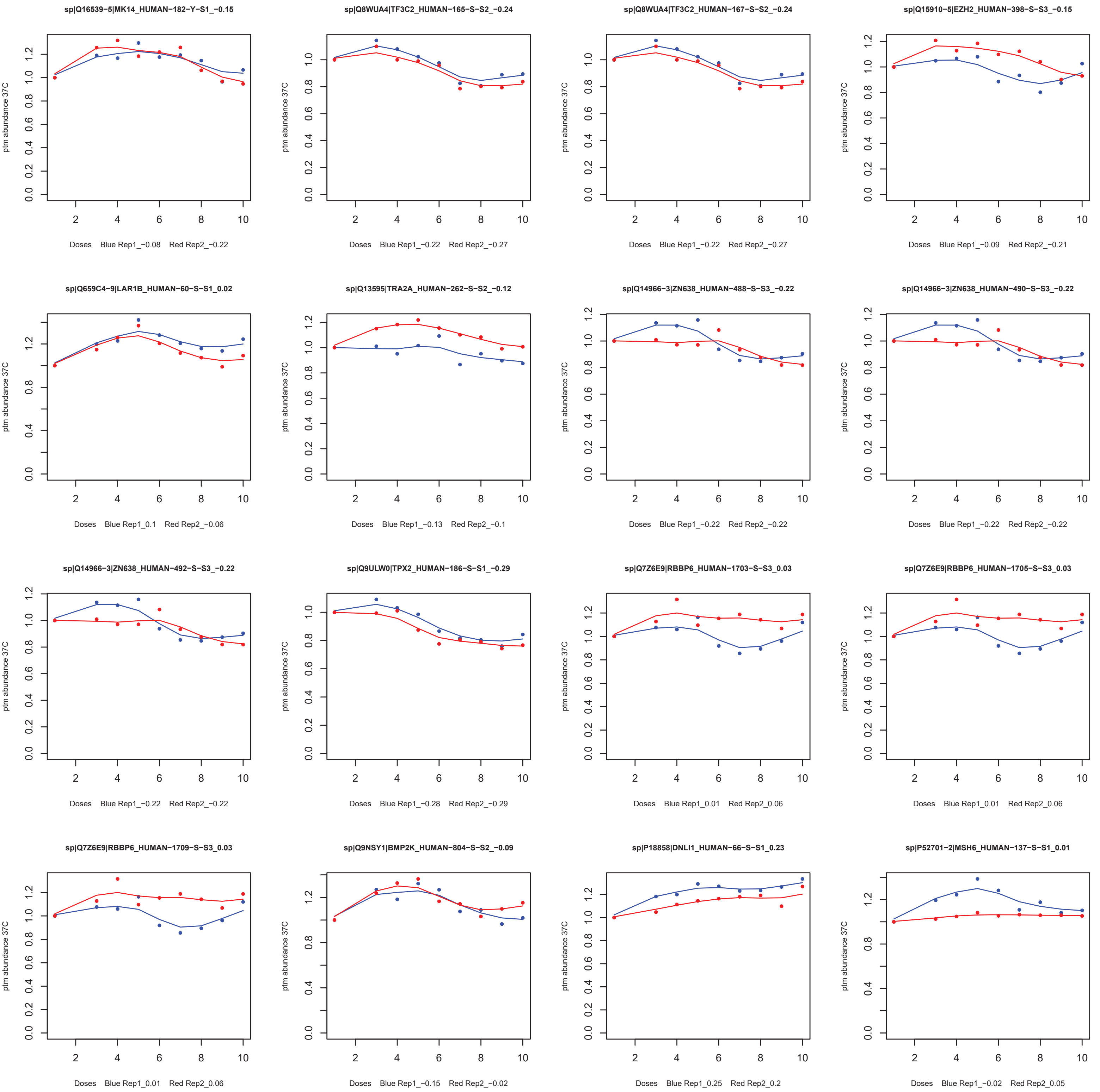
sp|Q16539−5|MK14_HUMAN−182−Y−S1_−0.15
ptm abundance 37C
Doses Blue Rep1_−0.08 Red Rep2_−0.22
sp|Q8WUA4|TF3C2_HUMAN−165−S−S2_−0.24
ptm abundance 37C
Doses Blue Rep1_−0.22 Red Rep2_−0.27
sp|Q8WUA4|TF3C2_HUMAN−167−S−S2_−0.24
ptm abundance 37C
Doses Blue Rep1_−0.22 Red Rep2_−0.27
sp|Q15910−5|EZH2_HUMAN−398−S−S3_−0.15
ptm abundance 37C
Doses Blue Rep1_−0.09 Red Rep2_−0.21
sp|Q659C4−9|LAR1B_HUMAN−60−S−S1_0.02
ptm abundance 37C
Doses Blue Rep1_0.1 Red Rep2_−0.06
sp|Q13595|TRA2A_HUMAN−262−S−S2_−0.12
ptm abundance 37C
Doses Blue Rep1_−0.13 Red Rep2_−0.1
sp|Q14966−3|ZN638_HUMAN−488−S−S3_−0.22
ptm abundance 37C
Doses Blue Rep1_−0.22 Red Rep2_−0.22
sp|Q14966−3|ZN638_HUMAN−490−S−S3_−0.22
ptm abundance 37C
Doses Blue Rep1_−0.22 Red Rep2_−0.22
sp|Q14966−3|ZN638_HUMAN−492−S−S3_−0.22
ptm abundance 37C
Doses Blue Rep1_−0.22 Red Rep2_−0.22
sp|Q9ULW0|TPX2_HUMAN−186−S−S1_−0.29
ptm abundance 37C
Doses Blue Rep1_−0.28 Red Rep2_−0.29
sp|Q7Z6E9|RBBP6_HUMAN−1703−S−S3_0.03
ptm abundance 37C
Doses Blue Rep1_0.01 Red Rep2_0.06
sp|Q7Z6E9|RBBP6_HUMAN−1705−S−S3_0.03
ptm abundance 37C
Doses Blue Rep1_0.01 Red Rep2_0.06
sp|Q7Z6E9|RBBP6_HUMAN−1709−S−S3_0.03
ptm abundance 37C
Doses Blue Rep1_0.01 Red Rep2_0.06
sp|Q9NSY1|BMP2K_HUMAN−804−S−S2_−0.09
ptm abundance 37C
Doses Blue Rep1_−0.15 Red Rep2_−0.02
sp|P18858|DNLI1_HUMAN−66−S−S1_0.23
ptm abundance 37C
Doses Blue Rep1_0.25 Red Rep2_0.2
sp|P52701−2|MSH6_HUMAN−137−S−S1_0.01
ptm abundance 37C
Doses Blue Rep1_−0.02 Red Rep2_0.05

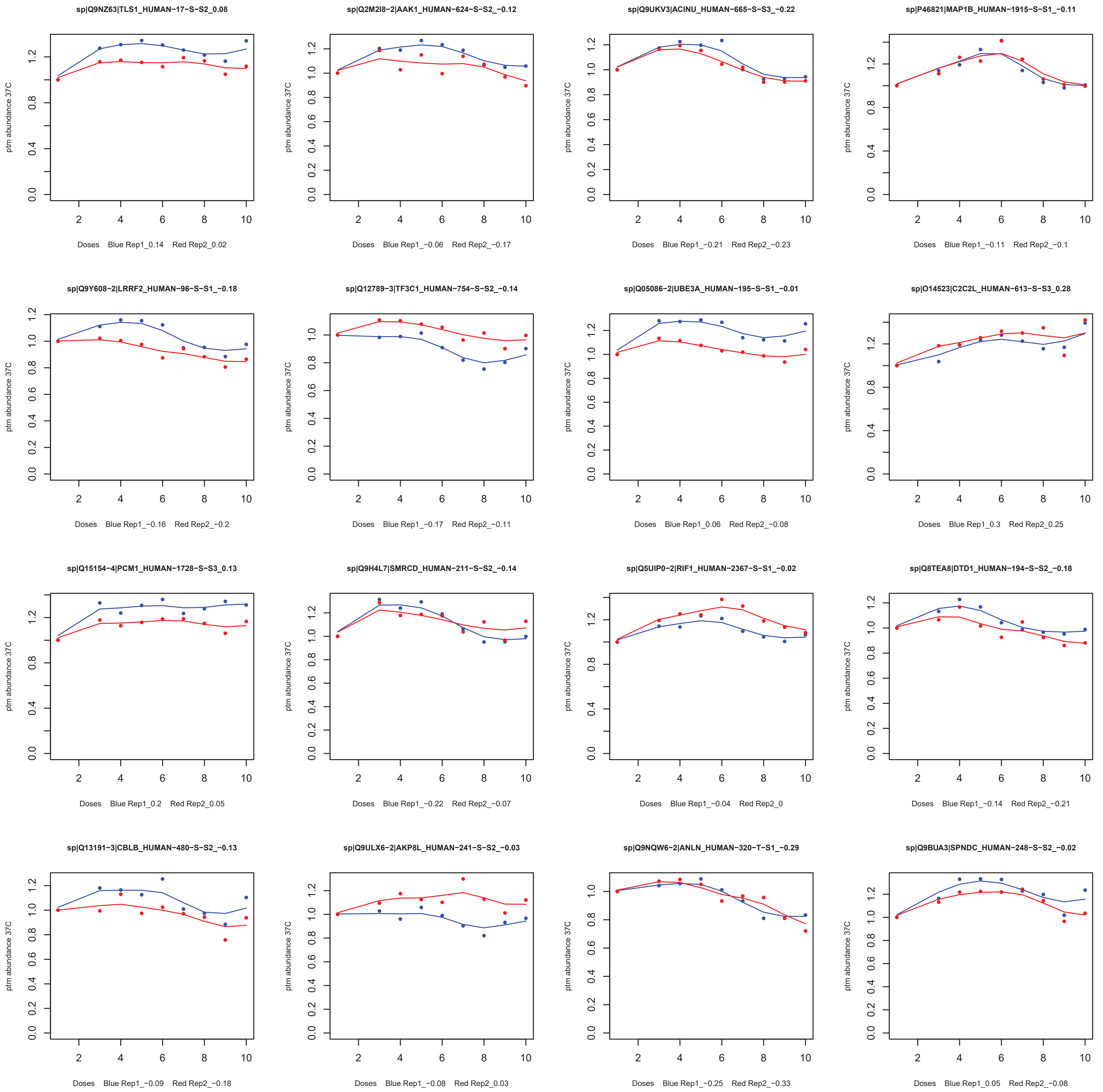
sp|Q9NZ63|TLS1_HUMAN−17−S−S2_0.08
Doses Blue Rep1_0.14 Red Rep2_0.02
sp|Q2M2I8−2|AAK1_HUMAN−624−S−S2_−0.12
Doses Blue Rep1_−0.06 Red Rep2_−0.17
sp|Q9UKV3|ACINU_HUMAN−665−S−S3_−0.22
Doses Blue Rep1_−0.21 Red Rep2_−0.23
sp|P46821|MAP1B_HUMAN−1915−S−S1_−0.11
Doses Blue Rep1_−0.11 Red Rep2_−0.1
sp|Q9Y608−2|LRRF2_HUMAN−96−S−S1_−0.18
Doses Blue Rep1_−0.16 Red Rep2_−0.2
sp|Q12789−3|TF3C1_HUMAN−754−S−S2_−0.14
Doses Blue Rep1_−0.17 Red Rep2_−0.11
sp|Q05086−2|UBE3A_HUMAN−195−S−S1_−0.01
Doses Blue Rep1_0.06 Red Rep2_−0.08
sp|O14523|C2C2L_HUMAN−613−S−S3_0.28
Doses Blue Rep1_0.3 Red Rep2_0.25
sp|Q15154−4|PCM1_HUMAN−1728−S−S3_0.13
Doses Blue Rep1_0.2 Red Rep2_0.05
sp|Q9H4L7|SMRCD_HUMAN−211−S−S2_−0.14
Doses Blue Rep1_−0.22 Red Rep2_−0.07
sp|Q5UIP0−2|RIF1_HUMAN−2367−S−S1_−0.02
Doses Blue Rep1_−0.04 Red Rep2_0
sp|Q8TEA8|DTD1_HUMAN−194−S−S2_−0.18
Doses Blue Rep1_−0.14 Red Rep2_−0.21
sp|Q13191−3|CBLB_HUMAN−480−S−S2_−0.13
Doses Blue Rep1_−0.09 Red Rep2_−0.18
sp|Q9ULX6−2|AKP8L_HUMAN−241−S−S2_−0.03
Doses Blue Rep1_−0.08 Red Rep2_0.03
sp|Q9NQW6−2|ANLN_HUMAN−320−T−S1_−0.29
Doses Blue Rep1_−0.25 Red Rep2_−0.33
sp|Q9BUA3|SPNDC_HUMAN−248−S−S2_−0.02
Doses Blue Rep1_0.05 Red Rep2_−0.08
ptm abundance 37C

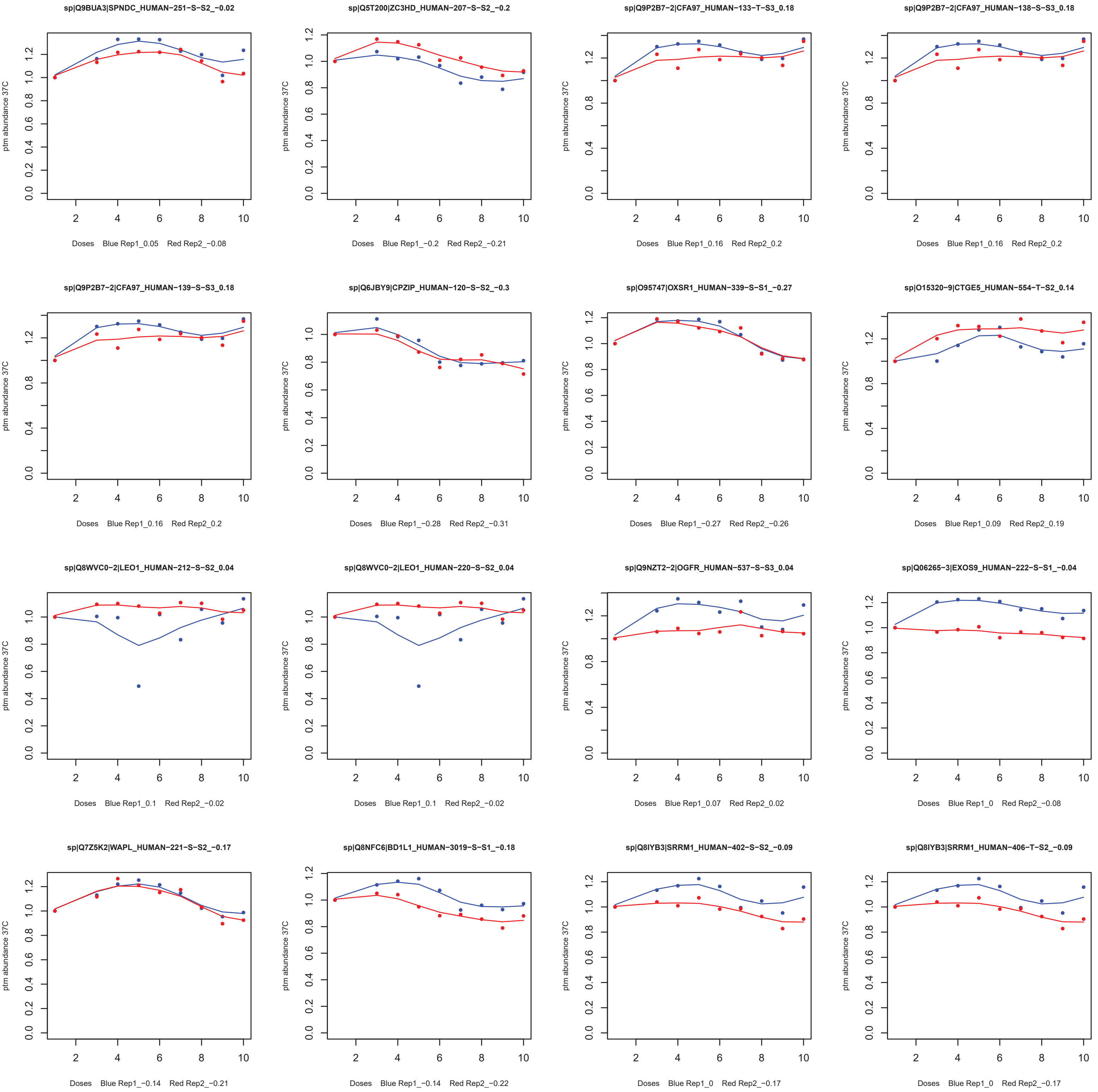
sp|Q9BUA3|SPNDC_HUMAN−251−S−S2_−0.02
ptm abundance 37C
Doses Blue Rep1_0.05 Red Rep2_−0.08
sp|Q5T200|ZC3HD_HUMAN−207−S−S2_−0.2
ptm abundance 37C
Doses Blue Rep1_−0.2 Red Rep2_−0.21
sp|Q9P2B7−2|CFA97_HUMAN−133−T−S3_0.18
ptm abundance 37C
Doses Blue Rep1_0.16 Red Rep2_0.2
sp|Q9P2B7−2|CFA97_HUMAN−138−S−S3_0.18
ptm abundance 37C
Doses Blue Rep1_0.16 Red Rep2_0.2
sp|Q9P2B7−2|CFA97_HUMAN−139−S−S3_0.18
ptm abundance 37C
Doses Blue Rep1_0.16 Red Rep2_0.2
sp|Q6JBY9|CPZIP_HUMAN−120−S−S2_−0.3
ptm abundance 37C
Doses Blue Rep1_−0.28 Red Rep2_−0.31
sp|O95747|OXSR1_HUMAN−339−S−S1_−0.27
ptm abundance 37C
Doses Blue Rep1_−0.27 Red Rep2_−0.26
sp|O15320−9|CTGE5_HUMAN−554−T−S2_0.14
ptm abundance 37C
Doses Blue Rep1_0.09 Red Rep2_0.19
sp|Q8WVC0−2|LEO1_HUMAN−212−S−S2_0.04
ptm abundance 37C
Doses Blue Rep1_0.1 Red Rep2_−0.02
sp|Q8WVC0−2|LEO1_HUMAN−220−S−S2_0.04
ptm abundance 37C
Doses Blue Rep1_0.1 Red Rep2_−0.02
sp|Q9NZT2−2|OGFR_HUMAN−537−S−S3_0.04
ptm abundance 37C
Doses Blue Rep1_0.07 Red Rep2_0.02
sp|Q06265−3|EXOS9_HUMAN−222−S−S1_−0.04
ptm abundance 37C
Doses Blue Rep1_0 Red Rep2_−0.08
sp|Q7Z5K2|WAPL_HUMAN−221−S−S2_−0.17
ptm abundance 37C
Doses Blue Rep1_−0.14 Red Rep2_−0.21
sp|Q8NFC6|BD1L1_HUMAN−3019−S−S1_−0.18
ptm abundance 37C
Doses Blue Rep1_−0.14 Red Rep2_−0.22
sp|Q8IYB3|SRRM1_HUMAN−402−S−S2_−0.09
ptm abundance 37C
Doses Blue Rep1_0 Red Rep2_−0.17
sp|Q8IYB3|SRRM1_HUMAN−406−T−S2_−0.09
ptm abundance 37C
Doses Blue Rep1_0 Red Rep2_−0.17

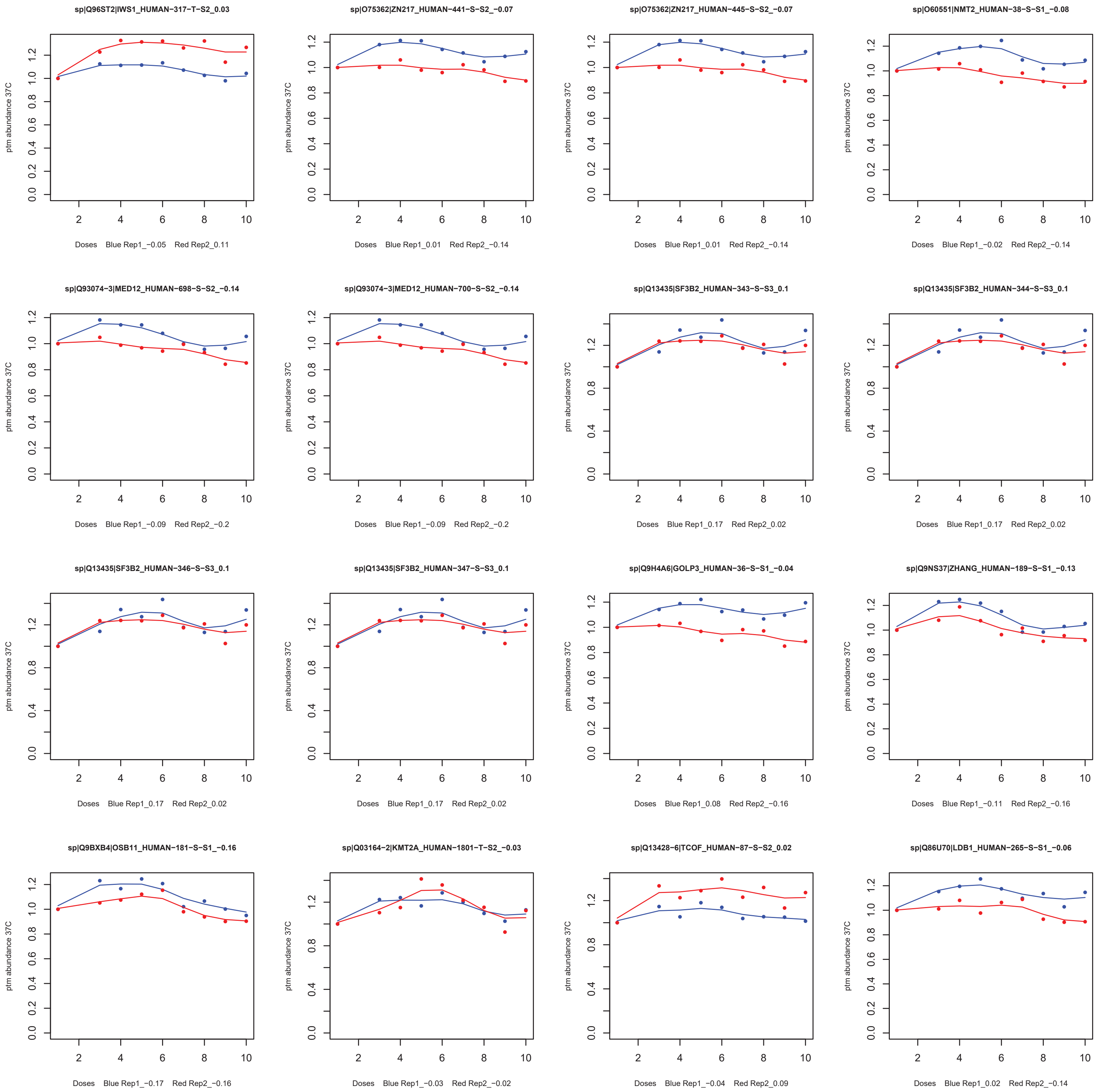
sp|Q96ST2|IWS1_HUMAN−317−T−S2_0.03
Doses Blue Rep1_−0.05 Red Rep2_0.11
sp|O75362|ZN217_HUMAN−441−S−S2_−0.07
Doses Blue Rep1_0.01 Red Rep2_−0.14
sp|O75362|ZN217_HUMAN−445−S−S2_−0.07
Doses Blue Rep1_0.01 Red Rep2_−0.14
sp|O60551|NMT2_HUMAN−38−S−S1_−0.08
Doses Blue Rep1_−0.02 Red Rep2_−0.14
sp|Q93074−3|MED12_HUMAN−698−S−S2_−0.14
Doses Blue Rep1_−0.09 Red Rep2_−0.2
sp|Q93074−3|MED12_HUMAN−700−S−S2_−0.14
Doses Blue Rep1_−0.09 Red Rep2_−0.2
sp|Q13435|SF3B2_HUMAN−343−S−S3_0.1
Doses Blue Rep1_0.17 Red Rep2_0.02
sp|Q13435|SF3B2_HUMAN−344−S−S3_0.1
Doses Blue Rep1_0.17 Red Rep2_0.02
sp|Q13435|SF3B2_HUMAN−346−S−S3_0.1
Doses Blue Rep1_0.17 Red Rep2_0.02
sp|Q13435|SF3B2_HUMAN−347−S−S3_0.1
Doses Blue Rep1_0.17 Red Rep2_0.02
sp|Q9H4A6|GOLP3_HUMAN−36−S−S1_−0.04
Doses Blue Rep1_0.08 Red Rep2_−0.16
sp|Q9NS37|ZHANG_HUMAN−189−S−S1_−0.13
Doses Blue Rep1_−0.11 Red Rep2_−0.16
sp|Q9BXB4|OSB11_HUMAN−181−S−S1_−0.16
Doses Blue Rep1_−0.17 Red Rep2_−0.16
sp|Q03164−2|KMT2A_HUMAN−1801−T−S2_−0.03
Doses Blue Rep1_−0.03 Red Rep2_−0.02
sp|Q13428−6|TCOF_HUMAN−87−S−S2_0.02
Doses Blue Rep1_−0.04 Red Rep2_0.09
sp|Q86U70|LDB1_HUMAN−265−S−S1_−0.06
Doses Blue Rep1_0.02 Red Rep2_−0.14
ptm abundance 37C

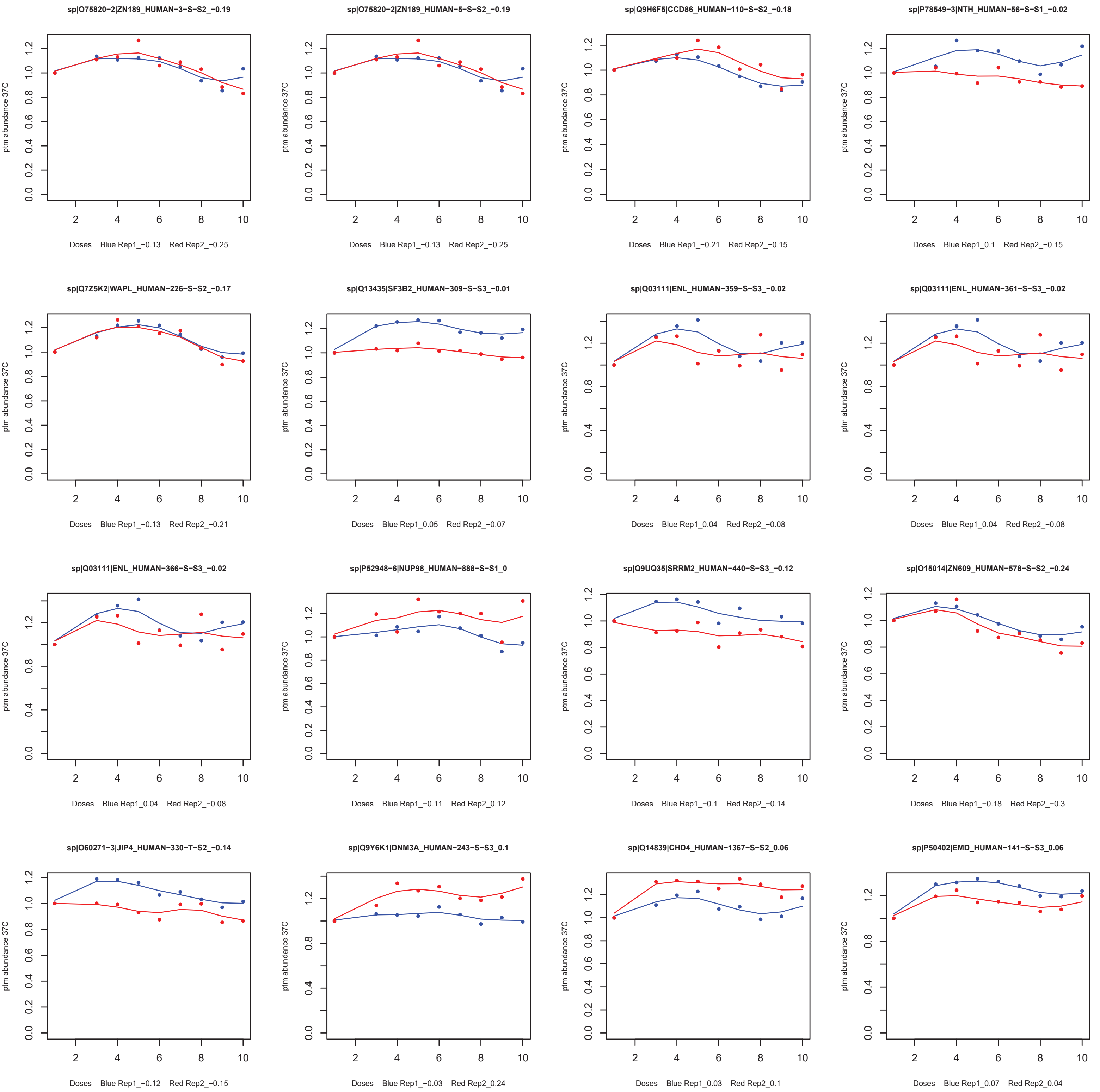
sp|O75820−2|ZN189_HUMAN−3−S−S2_−0.19
ptm abundance 37C
Doses Blue Rep1_−0.13 Red Rep2_−0.25
sp|O75820−2|ZN189_HUMAN−5−S−S2_−0.19
ptm abundance 37C
Doses Blue Rep1_−0.13 Red Rep2_−0.25
sp|Q9H6F5|CCD86_HUMAN−110−S−S2_−0.18
ptm abundance 37C
Doses Blue Rep1_−0.21 Red Rep2_−0.15
sp|P78549−3|NTH_HUMAN−56−S−S1_−0.02
ptm abundance 37C
Doses Blue Rep1_0.1 Red Rep2_−0.15
sp|Q7Z5K2|WAPL_HUMAN−226−S−S2_−0.17
ptm abundance 37C
Doses Blue Rep1_−0.13 Red Rep2_−0.21
sp|Q13435|SF3B2_HUMAN−309−S−S3_−0.01
ptm abundance 37C
Doses Blue Rep1_0.05 Red Rep2_−0.07
sp|Q03111|ENL_HUMAN−359−S−S3_−0.02
ptm abundance 37C
Doses Blue Rep1_0.04 Red Rep2_−0.08
sp|Q03111|ENL_HUMAN−361−S−S3_−0.02
ptm abundance 37C
Doses Blue Rep1_0.04 Red Rep2_−0.08
sp|Q03111|ENL_HUMAN−366−S−S3_−0.02
ptm abundance 37C
Doses Blue Rep1_0.04 Red Rep2_−0.08
sp|P52948−6|NUP98_HUMAN−888−S−S1_0
ptm abundance 37C
Doses Blue Rep1_−0.11 Red Rep2_0.12
sp|Q9UQ35|SRRM2_HUMAN−440−S−S3_−0.12
ptm abundance 37C
Doses Blue Rep1_−0.1 Red Rep2_−0.14
sp|O15014|ZN609_HUMAN−578−S−S2_−0.24
ptm abundance 37C
Doses Blue Rep1_−0.18 Red Rep2_−0.3
sp|O60271−3|JIP4_HUMAN−330−T−S2_−0.14
ptm abundance 37C
Doses Blue Rep1_−0.12 Red Rep2_−0.15
sp|Q9Y6K1|DNM3A_HUMAN−243−S−S3_0.1
ptm abundance 37C
Doses Blue Rep1_−0.03 Red Rep2_0.24
sp|Q14839|CHD4_HUMAN−1367−S−S2_0.06
ptm abundance 37C
Doses Blue Rep1_0.03 Red Rep2_0.1
sp|P50402|EMD_HUMAN−141−S−S3_0.06
ptm abundance 37C
Doses Blue Rep1_0.07 Red Rep2_0.04

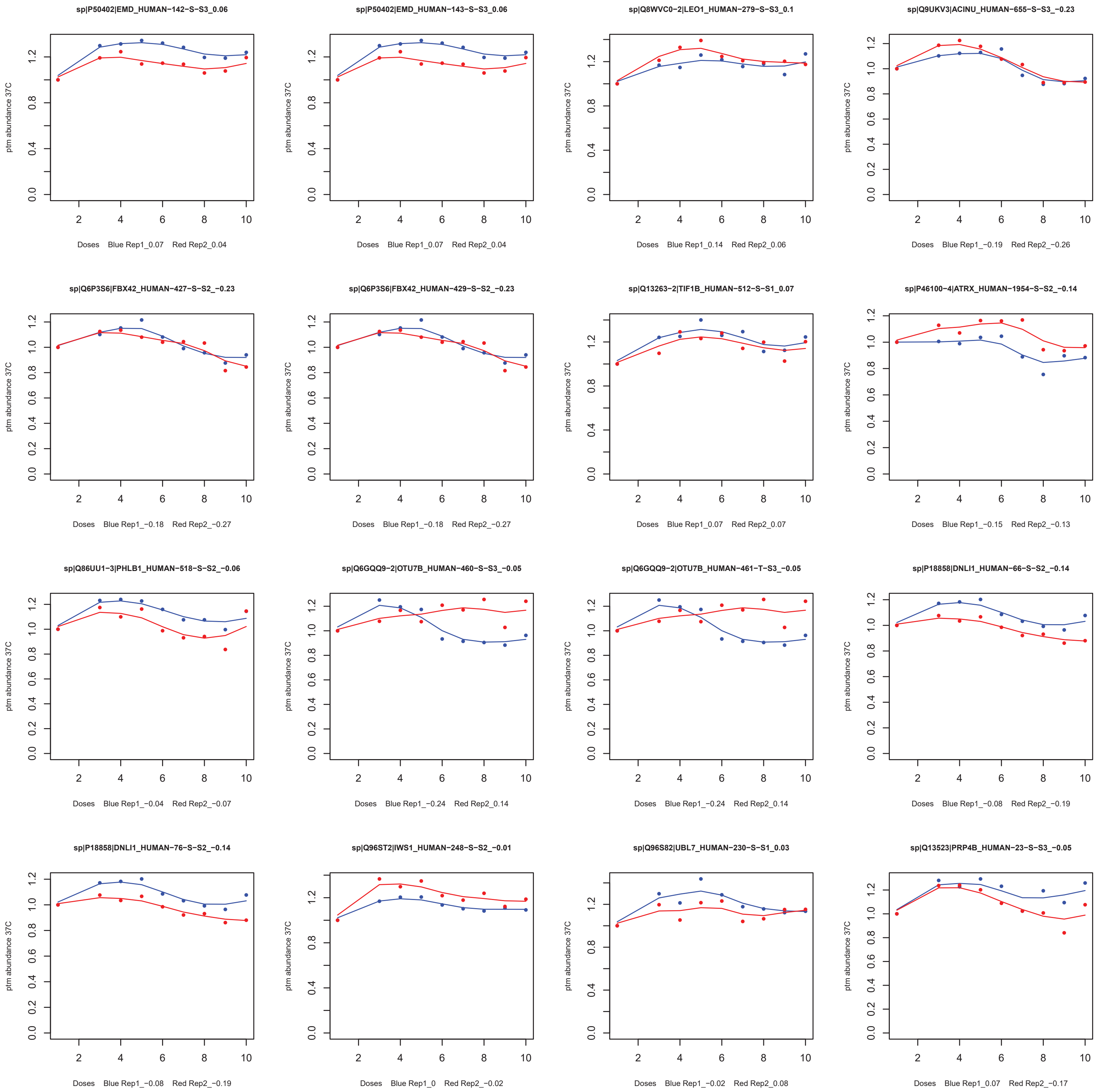
sp|P50402|EMD_HUMAN−142−S−S3_0.06
Doses Blue Rep1_0.07 Red Rep2_0.04
sp|P50402|EMD_HUMAN−143−S−S3_0.06
Doses Blue Rep1_0.07 Red Rep2_0.04
sp|Q8WVC0−2|LEO1_HUMAN−279−S−S3_0.1
Doses Blue Rep1_0.14 Red Rep2_0.06
sp|Q9UKV3|ACINU_HUMAN−655−S−S3_−0.23
Doses Blue Rep1_−0.19 Red Rep2_−0.26
sp|Q6P3S6|FBX42_HUMAN−427−S−S2_−0.23
Doses Blue Rep1_−0.18 Red Rep2_−0.27
sp|Q6P3S6|FBX42_HUMAN−429−S−S2_−0.23
Doses Blue Rep1_−0.18 Red Rep2_−0.27
sp|Q13263−2|TIF1B_HUMAN−512−S−S1_0.07
Doses Blue Rep1_0.07 Red Rep2_0.07
sp|P46100−4|ATRX_HUMAN−1954−S−S2_−0.14
Doses Blue Rep1_−0.15 Red Rep2_−0.13
sp|Q86UU1−3|PHLB1_HUMAN−518−S−S2_−0.06
Doses Blue Rep1_−0.04 Red Rep2_−0.07
sp|Q6GQQ9−2|OTU7B_HUMAN−460−S−S3_−0.05
Doses Blue Rep1_−0.24 Red Rep2_0.14
sp|Q6GQQ9−2|OTU7B_HUMAN−461−T−S3_−0.05
Doses Blue Rep1_−0.24 Red Rep2_0.14
sp|P18858|DNLI1_HUMAN−66−S−S2_−0.14
Doses Blue Rep1_−0.08 Red Rep2_−0.19
sp|P18858|DNLI1_HUMAN−76−S−S2_−0.14
Doses Blue Rep1_−0.08 Red Rep2_−0.19
sp|Q96ST2|IWS1_HUMAN−248−S−S2_−0.01
Doses Blue Rep1_0 Red Rep2_−0.02
sp|Q96S82|UBL7_HUMAN−230−S−S1_0.03
Doses Blue Rep1_−0.02 Red Rep2_0.08
sp|Q13523|PRP4B_HUMAN−23−S−S3_−0.05
Doses Blue Rep1_0.07 Red Rep2_−0.17
ptm abundance 37C

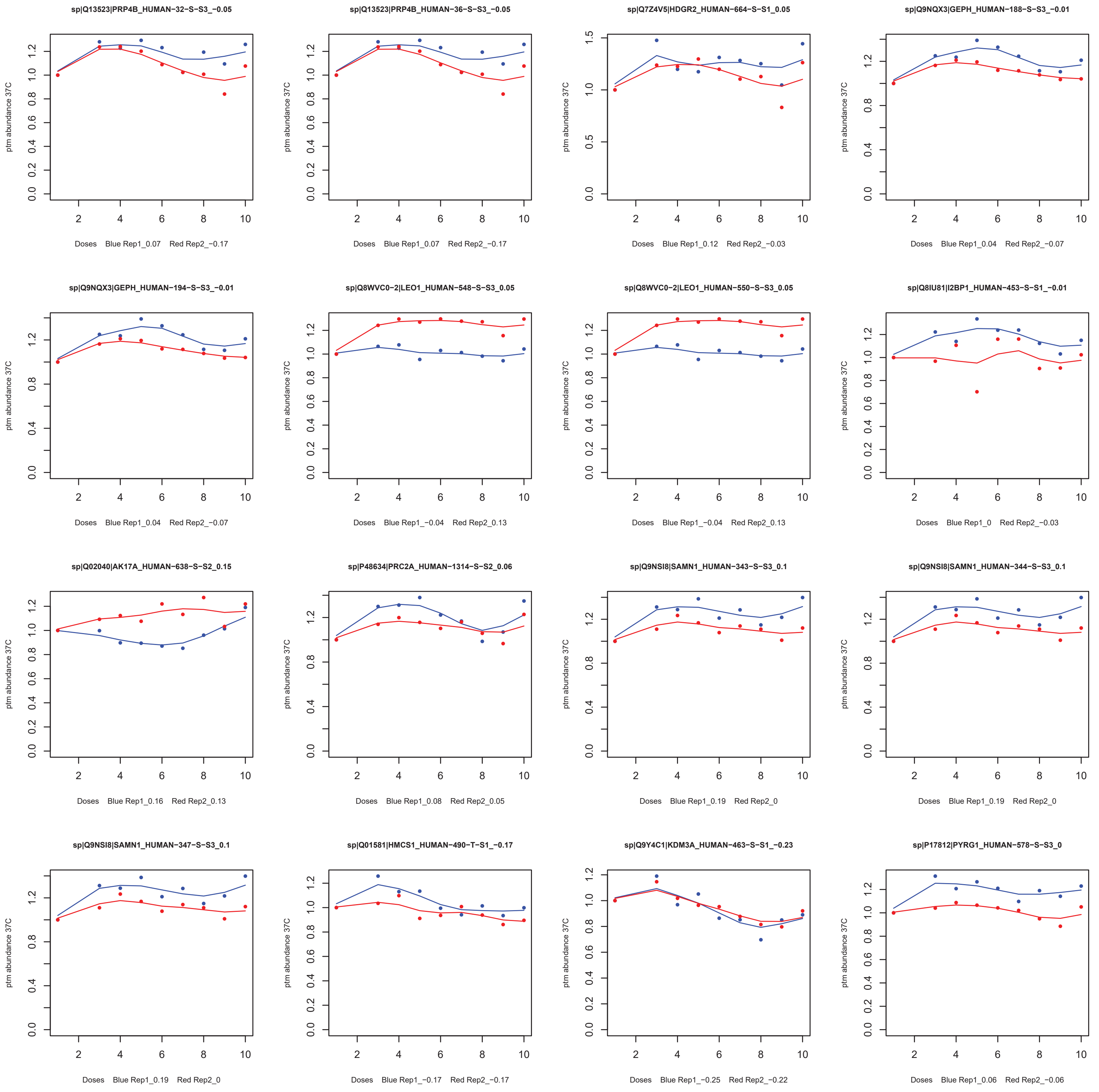

sp|Q13523|PRP4B_HUMAN−32−S−S3_−0.05
ptm abundance 37C
Doses Blue Rep1_0.07 Red Rep2_−0.17
sp|Q13523|PRP4B_HUMAN−36−S−S3_−0.05
ptm abundance 37C
Doses Blue Rep1_0.07 Red Rep2_−0.17
sp|Q7Z4V5|HDGR2_HUMAN−664−S−S1_0.05
ptm abundance 37C
Doses Blue Rep1_0.12 Red Rep2_−0.03
sp|Q9NQX3|GEPH_HUMAN−188−S−S3_−0.01
ptm abundance 37C
Doses Blue Rep1_0.04 Red Rep2_−0.07
sp|Q9NQX3|GEPH_HUMAN−194−S−S3_−0.01
ptm abundance 37C
Doses Blue Rep1_0.04 Red Rep2_−0.07
sp|Q8WVC0−2|LEO1_HUMAN−548−S−S3_0.05
ptm abundance 37C
Doses Blue Rep1_−0.04 Red Rep2_0.13
sp|Q8WVC0−2|LEO1_HUMAN−550−S−S3_0.05
ptm abundance 37C
Doses Blue Rep1_−0.04 Red Rep2_0.13
sp|Q8IU81|I2BP1_HUMAN−453−S−S1_−0.01
ptm abundance 37C
Doses Blue Rep1_0 Red Rep2_−0.03
sp|Q02040|AK17A_HUMAN−638−S−S2_0.15
ptm abundance 37C
Doses Blue Rep1_0.16 Red Rep2_0.13
sp|P48634|PRC2A_HUMAN−1314−S−S2_0.06
ptm abundance 37C
Doses Blue Rep1_0.08 Red Rep2_0.05
sp|Q9NSI8|SAMN1_HUMAN−343−S−S3_0.1
ptm abundance 37C
Doses Blue Rep1_0.19 Red Rep2_0
sp|Q9NSI8|SAMN1_HUMAN−344−S−S3_0.1
ptm abundance 37C
Doses Blue Rep1_0.19 Red Rep2_0
sp|Q9NSI8|SAMN1_HUMAN−347−S−S3_0.1
ptm abundance 37C
Doses Blue Rep1_0.19 Red Rep2_0
sp|Q01581|HMCS1_HUMAN−490−T−S1_−0.17
ptm abundance 37C
Doses Blue Rep1_−0.17 Red Rep2_−0.17
sp|Q9Y4C1|KDM3A_HUMAN−463−S−S1_−0.23
ptm abundance 37C
Doses Blue Rep1_−0.25 Red Rep2_−0.22
sp|P17812|PYRG1_HUMAN−578−S−S3_0
ptm abundance 37C
Doses Blue Rep1_0.06 Red Rep2_−0.06

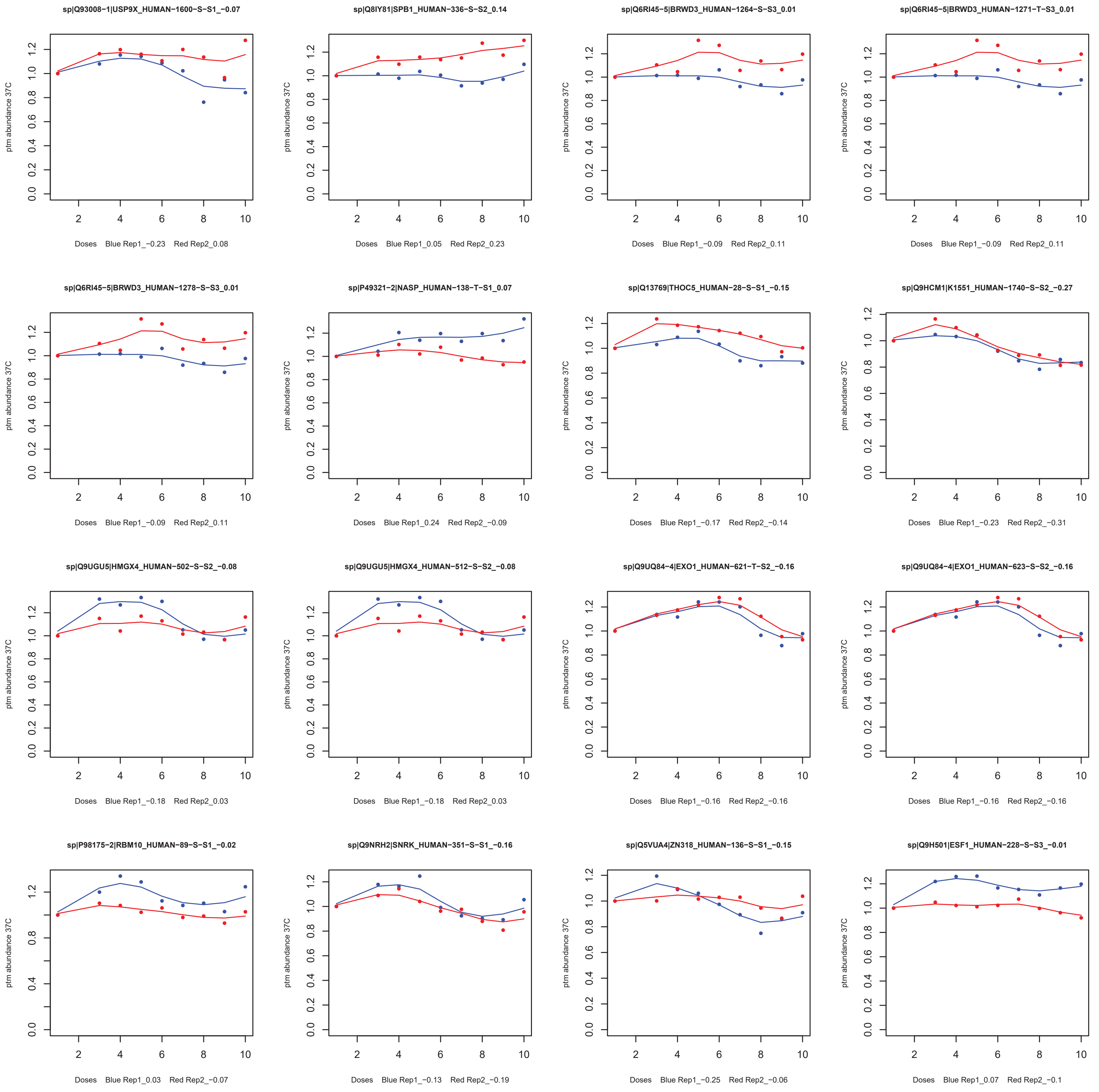
sp|Q93008−1|USP9X_HUMAN−1600−S−S1_−0.07
Doses Blue Rep1_−0.23 Red Rep2_0.08
sp|Q8IY81|SPB1_HUMAN−336−S−S2_0.14
Doses Blue Rep1_0.05 Red Rep2_0.23
sp|Q6RI45−5|BRWD3_HUMAN−1264−S−S3_0.01
Doses Blue Rep1_−0.09 Red Rep2_0.11
sp|Q6RI45−5|BRWD3_HUMAN−1271−T−S3_0.01
Doses Blue Rep1_−0.09 Red Rep2_0.11
sp|Q6RI45−5|BRWD3_HUMAN−1278−S−S3_0.01
Doses Blue Rep1_−0.09 Red Rep2_0.11
sp|P49321−2|NASP_HUMAN−138−T−S1_0.07
Doses Blue Rep1_0.24 Red Rep2_−0.09
sp|Q13769|THOC5_HUMAN−28−S−S1_−0.15
Doses Blue Rep1_−0.17 Red Rep2_−0.14
sp|Q9HCM1|K1551_HUMAN−1740−S−S2_−0.27
Doses Blue Rep1_−0.23 Red Rep2_−0.31
sp|Q9UGU5|HMGX4_HUMAN−502−S−S2_−0.08
Doses Blue Rep1_−0.18 Red Rep2_0.03
sp|Q9UGU5|HMGX4_HUMAN−512−S−S2_−0.08
Doses Blue Rep1_−0.18 Red Rep2_0.03
sp|Q9UQ84−4|EXO1_HUMAN−621−T−S2_−0.16
Doses Blue Rep1_−0.16 Red Rep2_−0.16
sp|Q9UQ84−4|EXO1_HUMAN−623−S−S2_−0.16
Doses Blue Rep1_−0.16 Red Rep2_−0.16
sp|P98175−2|RBM10_HUMAN−89−S−S1_−0.02
Doses Blue Rep1_0.03 Red Rep2_−0.07
sp|Q9NRH2|SNRK_HUMAN−351−S−S1_−0.16
Doses Blue Rep1_−0.13 Red Rep2_−0.19
sp|Q5VUA4|ZN318_HUMAN−136−S−S1_−0.15
Doses Blue Rep1_−0.25 Red Rep2_−0.06
sp|Q9H501|ESF1_HUMAN−228−S−S3_−0.01
Doses Blue Rep1_0.07 Red Rep2_−0.1
ptm abundance 37C

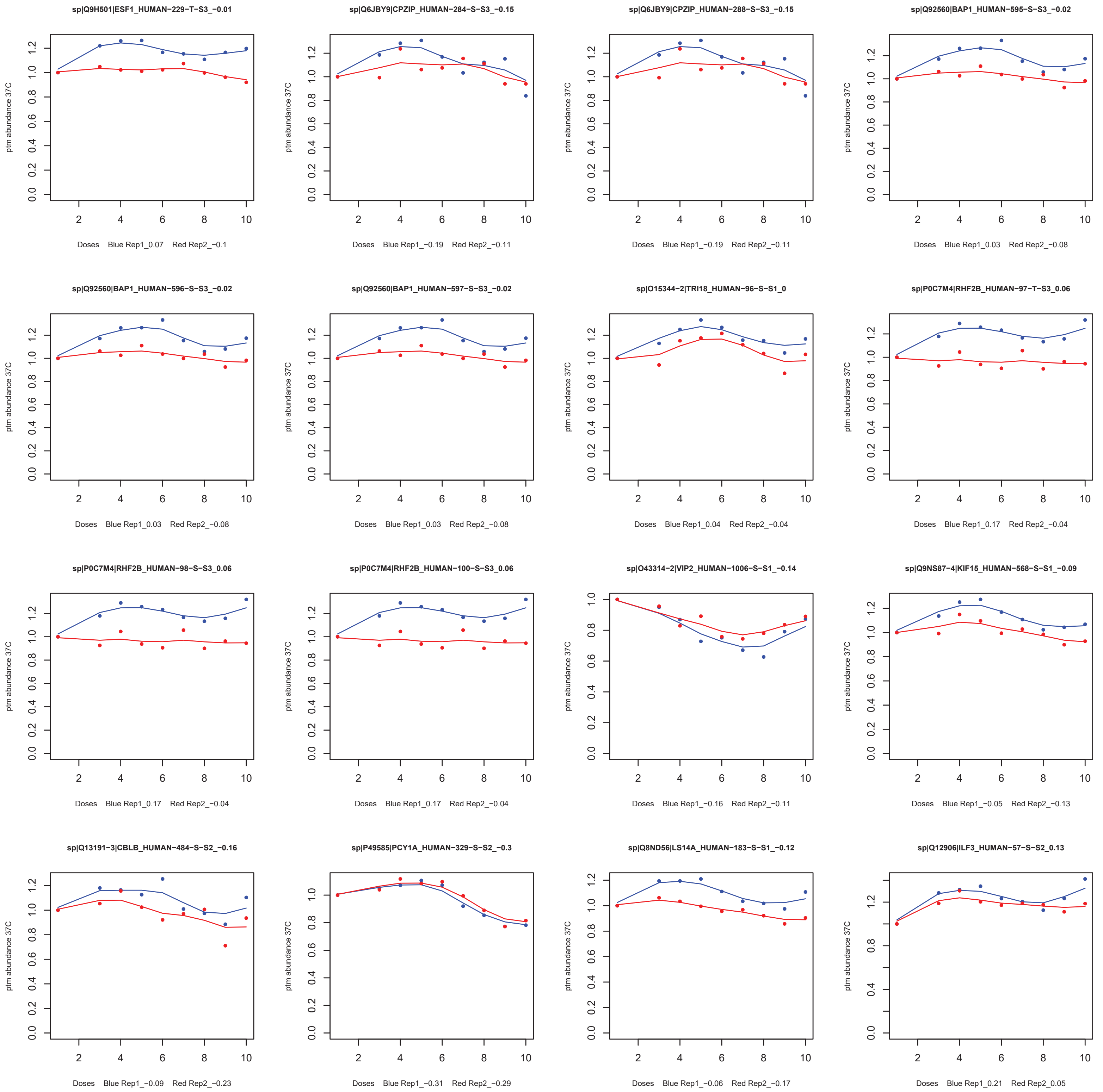
sp|Q9H501|ESF1_HUMAN−229−T−S3_−0.01
Doses Blue Rep1_0.07 Red Rep2_−0.1
sp|Q6JBY9|CPZIP_HUMAN−284−S−S3_−0.15
Doses Blue Rep1_−0.19 Red Rep2_−0.11
sp|Q6JBY9|CPZIP_HUMAN−288−S−S3_−0.15
Doses Blue Rep1_−0.19 Red Rep2_−0.11
sp|Q92560|BAP1_HUMAN−595−S−S3_−0.02
Doses Blue Rep1_0.03 Red Rep2_−0.08
sp|Q92560|BAP1_HUMAN−596−S−S3_−0.02
Doses Blue Rep1_0.03 Red Rep2_−0.08
sp|Q92560|BAP1_HUMAN−597−S−S3_−0.02
Doses Blue Rep1_0.03 Red Rep2_−0.08
sp|O15344−2|TRI18_HUMAN−96−S−S1_0
Doses Blue Rep1_0.04 Red Rep2_−0.04
sp|P0C7M4|RHF2B_HUMAN−97−T−S3_0.06
Doses Blue Rep1_0.17 Red Rep2_−0.04
sp|P0C7M4|RHF2B_HUMAN−98−S−S3_0.06
Doses Blue Rep1_0.17 Red Rep2_−0.04
sp|P0C7M4|RHF2B_HUMAN−100−S−S3_0.06
Doses Blue Rep1_0.17 Red Rep2_−0.04
sp|O43314−2|VIP2_HUMAN−1006−S−S1_−0.14
Doses Blue Rep1_−0.16 Red Rep2_−0.11
sp|Q9NS87−4|KIF15_HUMAN−568−S−S1_−0.09
Doses Blue Rep1_−0.05 Red Rep2_−0.13
sp|Q13191−3|CBLB_HUMAN−484−S−S2_−0.16
Doses Blue Rep1_−0.09 Red Rep2_−0.23
sp|P49585|PCY1A_HUMAN−329−S−S2_−0.3
Doses Blue Rep1_−0.31 Red Rep2_−0.29
sp|Q8ND56|LS14A_HUMAN−183−S−S1_−0.12
Doses Blue Rep1_−0.06 Red Rep2_−0.17
sp|Q12906|ILF3_HUMAN−57−S−S2_0.13
Doses Blue Rep1_0.21 Red Rep2_0.05
ptm abundance 37C

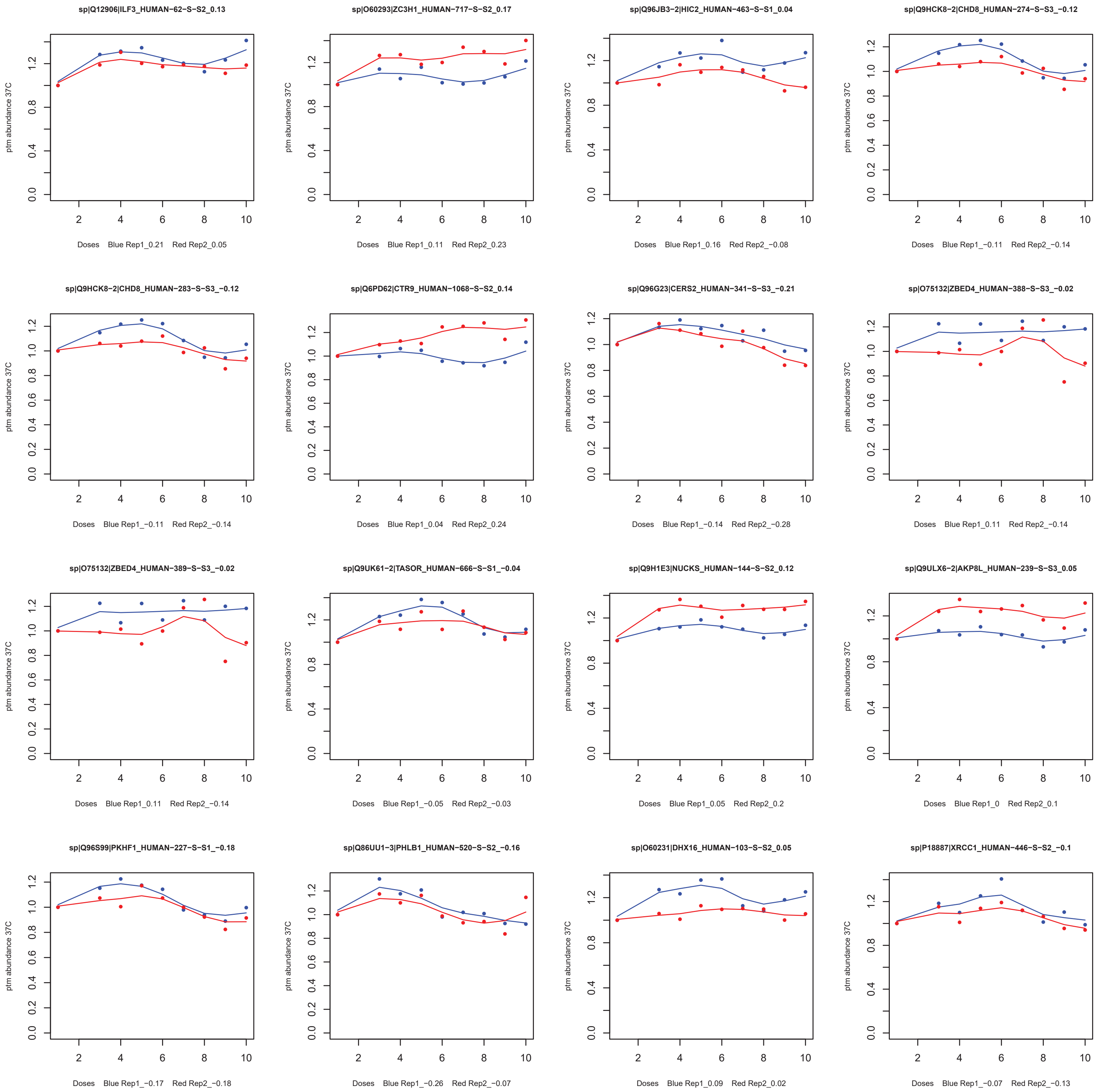
sp|Q12906|ILF3_HUMAN−62−S−S2_0.13
Doses Blue Rep1_0.21 Red Rep2_0.05
sp|O60293|ZC3H1_HUMAN−717−S−S2_0.17
Doses Blue Rep1_0.11 Red Rep2_0.23
sp|Q96JB3−2|HIC2_HUMAN−463−S−S1_0.04
Doses Blue Rep1_0.16 Red Rep2_−0.08
sp|Q9HCK8−2|CHD8_HUMAN−274−S−S3_−0.12
Doses Blue Rep1_−0.11 Red Rep2_−0.14
sp|Q9HCK8−2|CHD8_HUMAN−283−S−S3_−0.12
Doses Blue Rep1_−0.11 Red Rep2_−0.14
sp|Q6PD62|CTR9_HUMAN−1068−S−S2_0.14
Doses Blue Rep1_0.04 Red Rep2_0.24
sp|Q96G23|CERS2_HUMAN−341−S−S3_−0.21
Doses Blue Rep1_−0.14 Red Rep2_−0.28
sp|O75132|ZBED4_HUMAN−388−S−S3_−0.02
Doses Blue Rep1_0.11 Red Rep2_−0.14
sp|O75132|ZBED4_HUMAN−389−S−S3_−0.02
Doses Blue Rep1_0.11 Red Rep2_−0.14
sp|Q9UK61−2|TASOR_HUMAN−666−S−S1_−0.04
Doses Blue Rep1_−0.05 Red Rep2_−0.03
sp|Q9H1E3|NUCKS_HUMAN−144−S−S2_0.12
Doses Blue Rep1_0.05 Red Rep2_0.2
sp|Q9ULX6−2|AKP8L_HUMAN−239−S−S3_0.05
Doses Blue Rep1_0 Red Rep2_0.1
sp|Q96S99|PKHF1_HUMAN−227−S−S1_−0.18
Doses Blue Rep1_−0.17 Red Rep2_−0.18
sp|Q86UU1−3|PHLB1_HUMAN−520−S−S2_−0.16
Doses Blue Rep1_−0.26 Red Rep2_−0.07
sp|O60231|DHX16_HUMAN−103−S−S2_0.05
Doses Blue Rep1_0.09 Red Rep2_0.02
sp|P18887|XRCC1_HUMAN−446−S−S2_−0.1
Doses Blue Rep1_−0.07 Red Rep2_−0.13
ptm abundance 37C

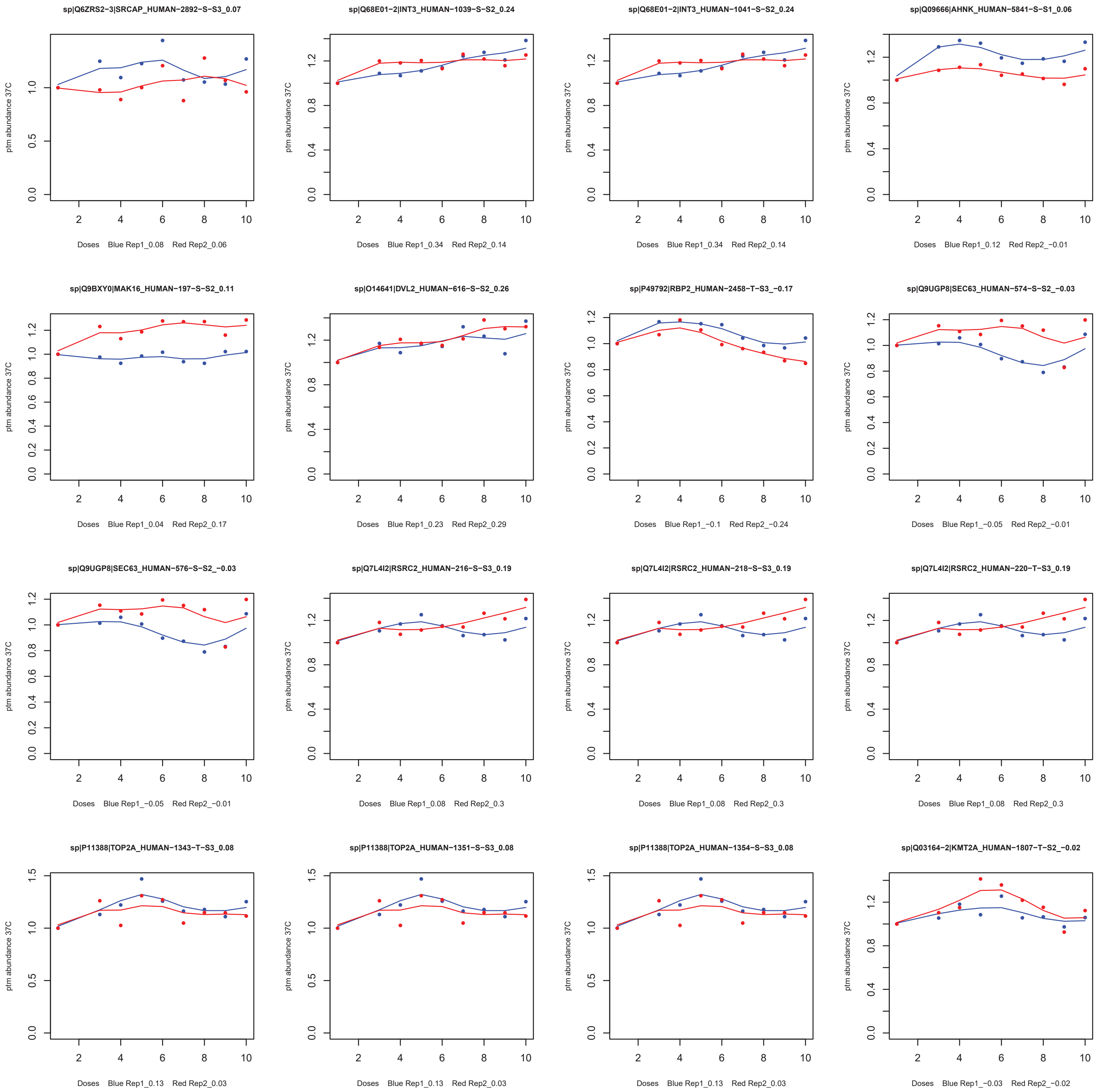
sp|Q6ZRS2−3|SRCAP_HUMAN−2892−S−S3_0.07
ptm abundance 37C
Doses Blue Rep1_0.08 Red Rep2_0.06
sp|Q68E01−2|INT3_HUMAN−1039−S−S2_0.24
ptm abundance 37C
Doses Blue Rep1_0.34 Red Rep2_0.14
sp|Q68E01−2|INT3_HUMAN−1041−S−S2_0.24
ptm abundance 37C
Doses Blue Rep1_0.34 Red Rep2_0.14
sp|Q09666|AHNK_HUMAN−5841−S−S1_0.06
ptm abundance 37C
Doses Blue Rep1_0.12 Red Rep2_−0.01
sp|Q9BXY0|MAK16_HUMAN−197−S−S2_0.11
ptm abundance 37C
Doses Blue Rep1_0.04 Red Rep2_0.17
sp|O14641|DVL2_HUMAN−616−S−S2_0.26
ptm abundance 37C
Doses Blue Rep1_0.23 Red Rep2_0.29
sp|P49792|RBP2_HUMAN−2458−T−S3_−0.17
ptm abundance 37C
Doses Blue Rep1_−0.1 Red Rep2_−0.24
sp|Q9UGP8|SEC63_HUMAN−574−S−S2_−0.03
ptm abundance 37C
Doses Blue Rep1_−0.05 Red Rep2_−0.01
sp|Q9UGP8|SEC63_HUMAN−576−S−S2_−0.03
ptm abundance 37C
Doses Blue Rep1_−0.05 Red Rep2_−0.01
sp|Q7L4I2|RSRC2_HUMAN−216−S−S3_0.19
ptm abundance 37C
Doses Blue Rep1_0.08 Red Rep2_0.3
sp|Q7L4I2|RSRC2_HUMAN−218−S−S3_0.19
ptm abundance 37C
Doses Blue Rep1_0.08 Red Rep2_0.3
sp|Q7L4I2|RSRC2_HUMAN−220−T−S3_0.19
ptm abundance 37C
Doses Blue Rep1_0.08 Red Rep2_0.3
sp|P11388|TOP2A_HUMAN−1343−T−S3_0.08
ptm abundance 37C
Doses Blue Rep1_0.13 Red Rep2_0.03
sp|P11388|TOP2A_HUMAN−1351−S−S3_0.08
ptm abundance 37C
Doses Blue Rep1_0.13 Red Rep2_0.03
sp|P11388|TOP2A_HUMAN−1354−S−S3_0.08
ptm abundance 37C
Doses Blue Rep1_0.13 Red Rep2_0.03
sp|Q03164−2|KMT2A_HUMAN−1807−T−S2_−0.02
ptm abundance 37C
Doses Blue Rep1_−0.03 Red Rep2_−0.02

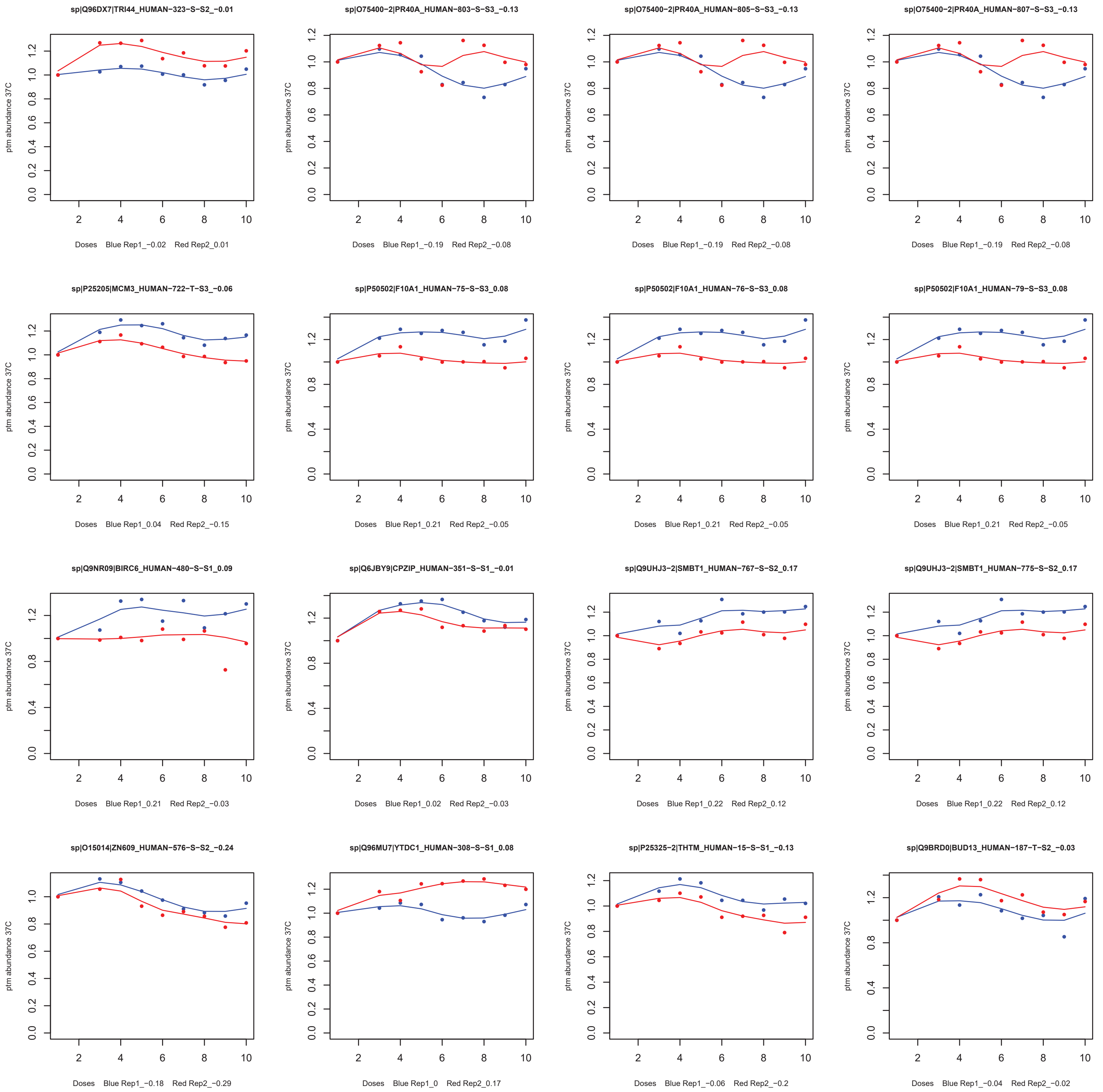
sp|Q96DX7|TRI44_HUMAN−323−S−S2_−0.01
Doses Blue Rep1_−0.02 Red Rep2_0.01
sp|O75400−2|PR40A_HUMAN−803−S−S3_−0.13
Doses Blue Rep1_−0.19 Red Rep2_−0.08
sp|O75400−2|PR40A_HUMAN−805−S−S3_−0.13
Doses Blue Rep1_−0.19 Red Rep2_−0.08
sp|O75400−2|PR40A_HUMAN−807−S−S3_−0.13
Doses Blue Rep1_−0.19 Red Rep2_−0.08
sp|P25205|MCM3_HUMAN−722−T−S3_−0.06
Doses Blue Rep1_0.04 Red Rep2_−0.15
sp|P50502|F10A1_HUMAN−75−S−S3_0.08
Doses Blue Rep1_0.21 Red Rep2_−0.05
sp|P50502|F10A1_HUMAN−76−S−S3_0.08
Doses Blue Rep1_0.21 Red Rep2_−0.05
sp|P50502|F10A1_HUMAN−79−S−S3_0.08
Doses Blue Rep1_0.21 Red Rep2_−0.05
sp|Q9NR09|BIRC6_HUMAN−480−S−S1_0.09
Doses Blue Rep1_0.21 Red Rep2_−0.03
sp|Q6JBY9|CPZIP_HUMAN−351−S−S1_−0.01
Doses Blue Rep1_0.02 Red Rep2_−0.03
sp|Q9UHJ3−2|SMBT1_HUMAN−767−S−S2_0.17
Doses Blue Rep1_0.22 Red Rep2_0.12
sp|Q9UHJ3−2|SMBT1_HUMAN−775−S−S2_0.17
Doses Blue Rep1_0.22 Red Rep2_0.12
sp|O15014|ZN609_HUMAN−576−S−S2_−0.24
Doses Blue Rep1_−0.18 Red Rep2_−0.29
sp|Q96MU7|YTDC1_HUMAN−308−S−S1_0.08
Doses Blue Rep1_0 Red Rep2_0.17
sp|P25325−2|THTM_HUMAN−15−S−S1_−0.13
Doses Blue Rep1_−0.06 Red Rep2_−0.2
sp|Q9BRD0|BUD13_HUMAN−187−T−S2_−0.03
Doses Blue Rep1_−0.04 Red Rep2_−0.02
ptm abundance 37C

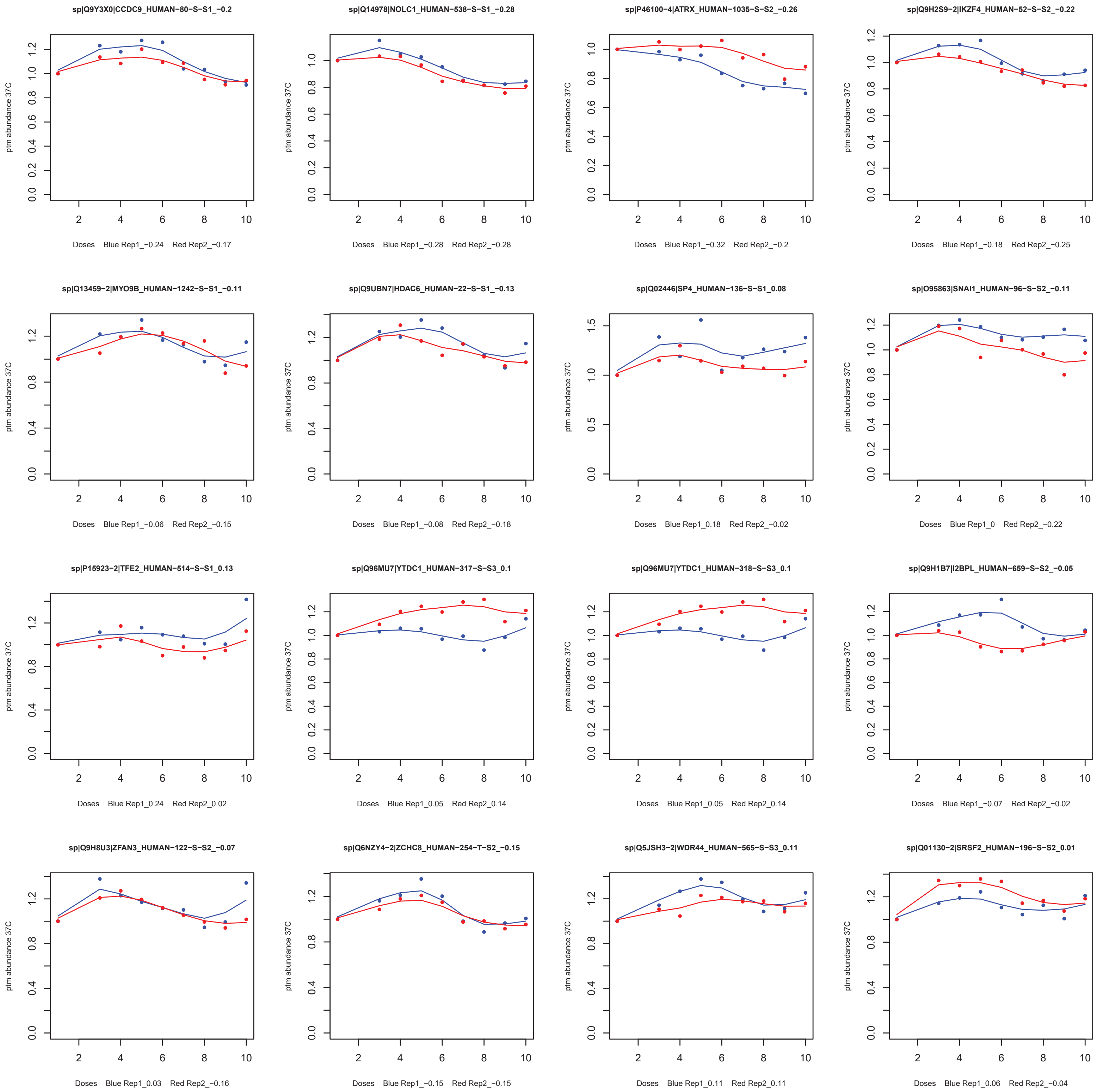
sp|Q9Y3X0|CCDC9_HUMAN−80−S−S1_−0.2
Doses Blue Rep1_−0.24 Red Rep2_−0.17
sp|Q14978|NOLC1_HUMAN−538−S−S1_−0.28
Doses Blue Rep1_−0.28 Red Rep2_−0.28
sp|P46100−4|ATRX_HUMAN−1035−S−S2_−0.26
Doses Blue Rep1_−0.32 Red Rep2_−0.2
sp|Q9H2S9−2|IKZF4_HUMAN−52−S−S2_−0.22
Doses Blue Rep1_−0.18 Red Rep2_−0.25
sp|Q13459−2|MYO9B_HUMAN−1242−S−S1_−0.11
Doses Blue Rep1_−0.06 Red Rep2_−0.15
sp|Q9UBN7|HDAC6_HUMAN−22−S−S1_−0.13
Doses Blue Rep1_−0.08 Red Rep2_−0.18
sp|Q02446|SP4_HUMAN−136−S−S1_0.08
Doses Blue Rep1_0.18 Red Rep2_−0.02
sp|O95863|SNAI1_HUMAN−96−S−S2_−0.11
Doses Blue Rep1_0 Red Rep2_−0.22
sp|P15923−2|TFE2_HUMAN−514−S−S1_0.13
Doses Blue Rep1_0.24 Red Rep2_0.02
sp|Q96MU7|YTDC1_HUMAN−317−S−S3_0.1
Doses Blue Rep1_0.05 Red Rep2_0.14
sp|Q96MU7|YTDC1_HUMAN−318−S−S3_0.1
Doses Blue Rep1_0.05 Red Rep2_0.14
sp|Q9H1B7|I2BPL_HUMAN−659−S−S2_−0.05
Doses Blue Rep1_−0.07 Red Rep2_−0.02
sp|Q9H8U3|ZFAN3_HUMAN−122−S−S2_−0.07
Doses Blue Rep1_0.03 Red Rep2_−0.16
sp|Q6NZY4−2|ZCHC8_HUMAN−254−T−S2_−0.15
Doses Blue Rep1_−0.15 Red Rep2_−0.15
sp|Q5JSH3−2|WDR44_HUMAN−565−S−S3_0.11
Doses Blue Rep1_0.11 Red Rep2_0.11
sp|Q01130−2|SRSF2_HUMAN−196−S−S2_0.01
Doses Blue Rep1_0.06 Red Rep2_−0.04
ptm abundance 37C

sp|P49736|MCM2_HUMAN−13−S−S2_0.11

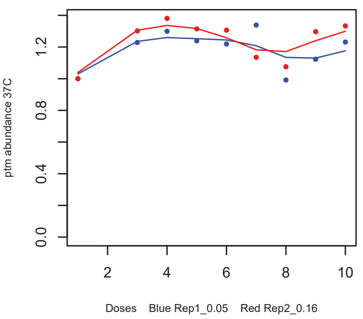

sp|P49915−2|GUAA_HUMAN−233−S−S1_−0.05

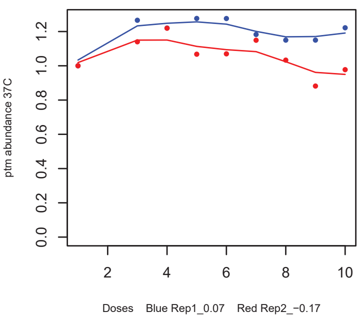

sp|O75351|VPS4B_HUMAN−93−S−S1_−0.06

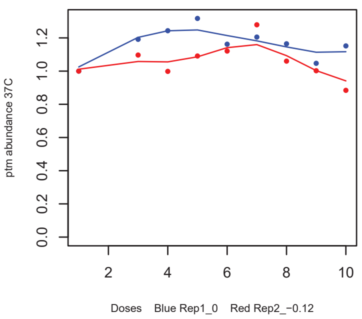

sp|Q9UGU0−2|TCF20_HUMAN−1762−T−S2_−0.21

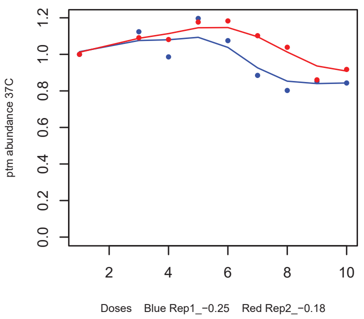

sp|Q9UGU0−2|TCF20_HUMAN−1764−T−S2_−0.21

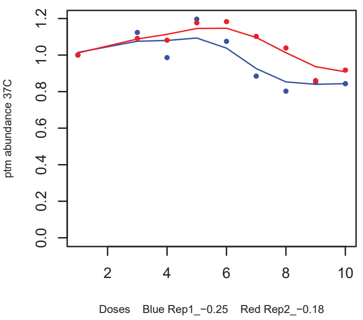

sp|P49585|PCY1A_HUMAN−331−S−S2_−0.3

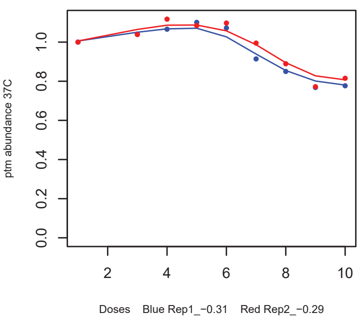

sp|Q9H6Z4−3|RANB3_HUMAN−287−S−S2_−0.08

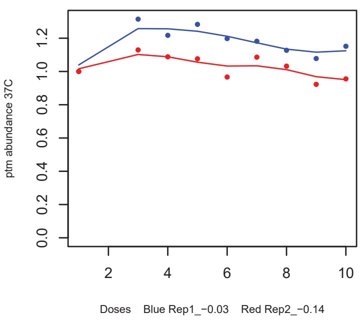

sp|Q96RT1−7|ERBIN_HUMAN−602−S−S2_−0.22

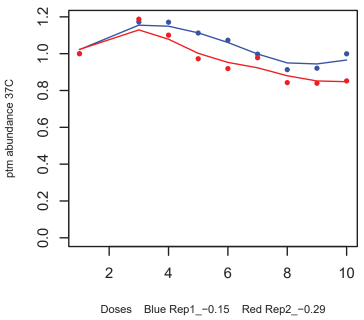

sp|Q96RT1−7|ERBIN_HUMAN−603−S−S2_−0.22

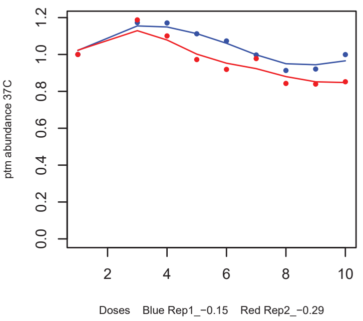

sp|P67809|YBOX1_HUMAN−165−S−S2_−0.13

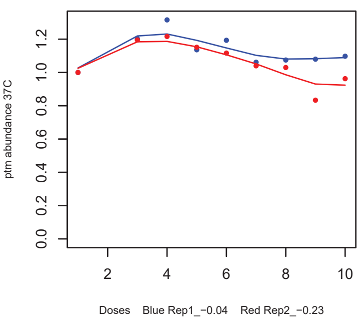

sp|O00193|SMAP_HUMAN−24−S−S2_−0.04

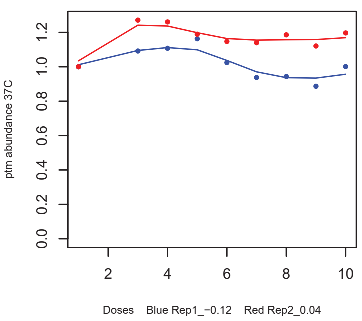

sp|P49450−2|CENPA_HUMAN−17−S−S2_−0.1

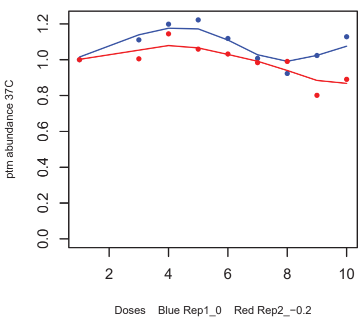

sp|Q86VM9|ZCH18_HUMAN−83−S−S1_0.01

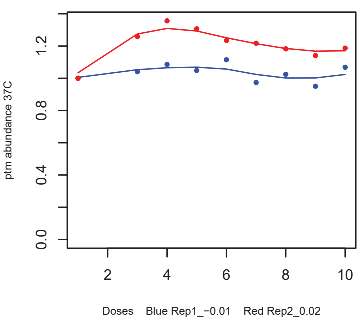

sp|Q8WVK2|SNR27_HUMAN−59−S−S3_−0.26

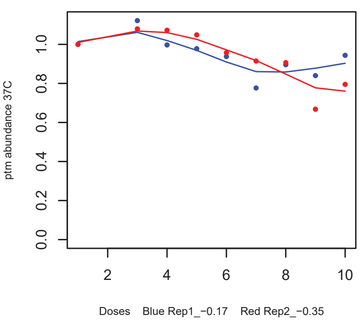

sp|Q8WVK2|SNR27_HUMAN−61−S−S3_−0.26

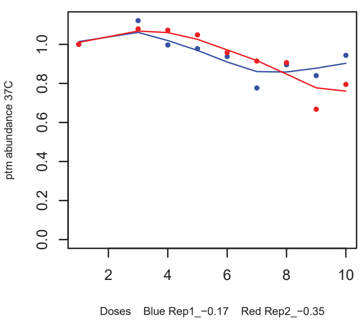

sp|Q8WVK2|SNR27_HUMAN−63−S−S3_−0.26

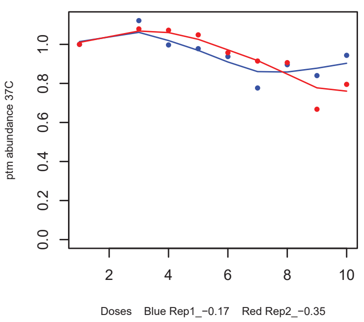

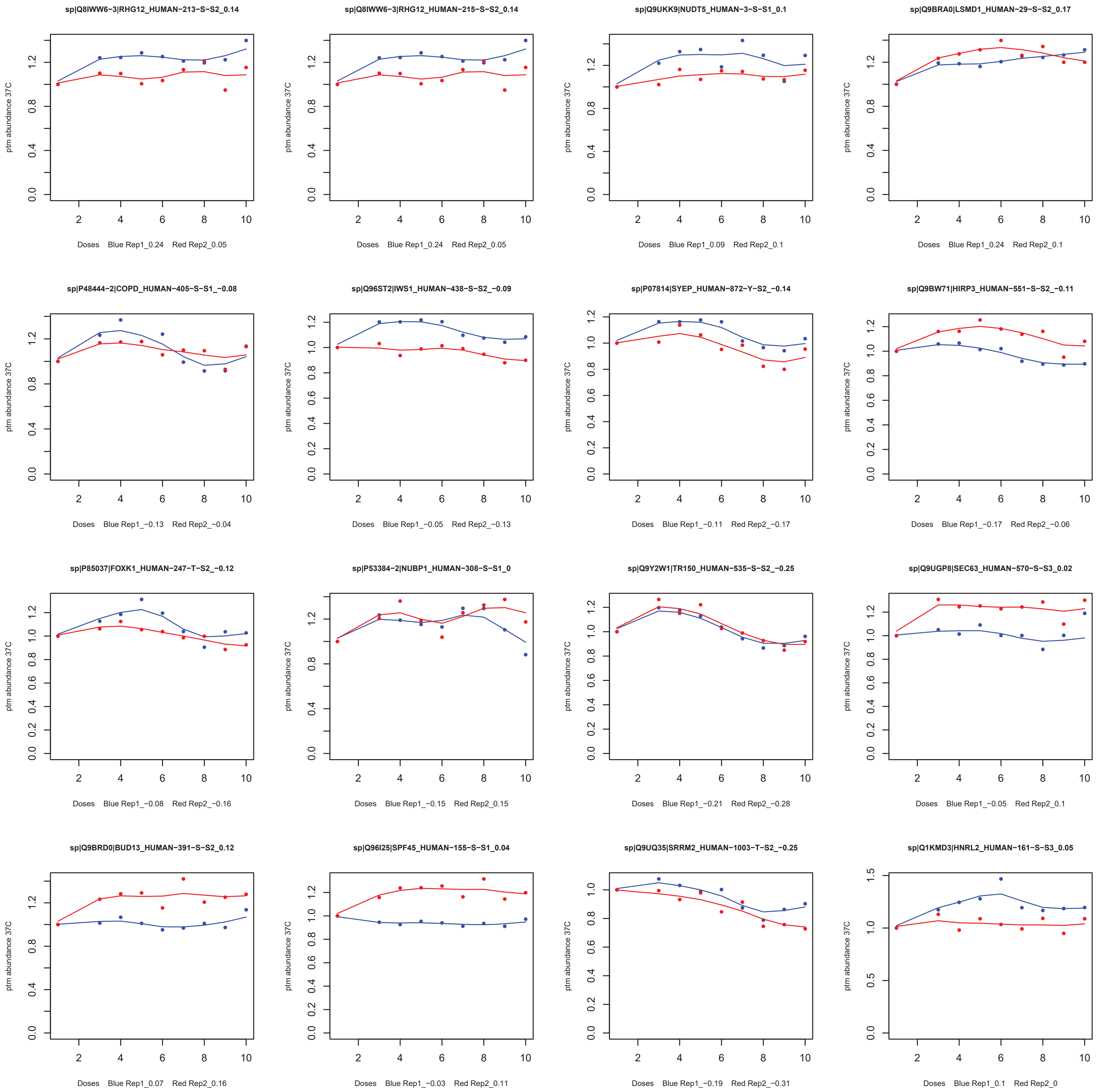

sp|Q8IWW6−3|RHG12_HUMAN−213−S−S2_0.14
Doses Blue Rep1_0.24 Red Rep2_0.05
sp|Q8IWW6−3|RHG12_HUMAN−215−S−S2_0.14
Doses Blue Rep1_0.24 Red Rep2_0.05
sp|Q9UKK9|NUDT5_HUMAN−3−S−S1_0.1
Doses Blue Rep1_0.09 Red Rep2_0.1
sp|Q9BRA0|LSMD1_HUMAN−29−S−S2_0.17
Doses Blue Rep1_0.24 Red Rep2_0.1
sp|P48444−2|COPD_HUMAN−405−S−S1_−0.08
Doses Blue Rep1_−0.13 Red Rep2_−0.04
sp|Q96ST2|IWS1_HUMAN−438−S−S2_−0.09
Doses Blue Rep1_−0.05 Red Rep2_−0.13
sp|P07814|SYEP_HUMAN−872−Y−S2_−0.14
Doses Blue Rep1_−0.11 Red Rep2_−0.17
sp|Q9BW71|HIRP3_HUMAN−551−S−S2_−0.11
Doses Blue Rep1_−0.17 Red Rep2_−0.06
sp|P85037|FOXK1_HUMAN−247−T−S2_−0.12
Doses Blue Rep1_−0.08 Red Rep2_−0.16
sp|P53384−2|NUBP1_HUMAN−308−S−S1_0
Doses Blue Rep1_−0.15 Red Rep2_0.15
sp|Q9Y2W1|TR150_HUMAN−535−S−S2_−0.25
Doses Blue Rep1_−0.21 Red Rep2_−0.28
sp|Q9UGP8|SEC63_HUMAN−570−S−S3_0.02
Doses Blue Rep1_−0.05 Red Rep2_0.1
sp|Q9BRD0|BUD13_HUMAN−391−S−S2_0.12
Doses Blue Rep1_0.07 Red Rep2_0.16
sp|Q96I25|SPF45_HUMAN−155−S−S1_0.04
Doses Blue Rep1_−0.03 Red Rep2_0.11
sp|Q9UQ35|SRRM2_HUMAN−1003−T−S2_−0.25
Doses Blue Rep1_−0.19 Red Rep2_−0.31
sp|Q1KMD3|HNRL2_HUMAN−161−S−S3_0.05
Doses Blue Rep1_0.1 Red Rep2_0
ptm abundance 37C

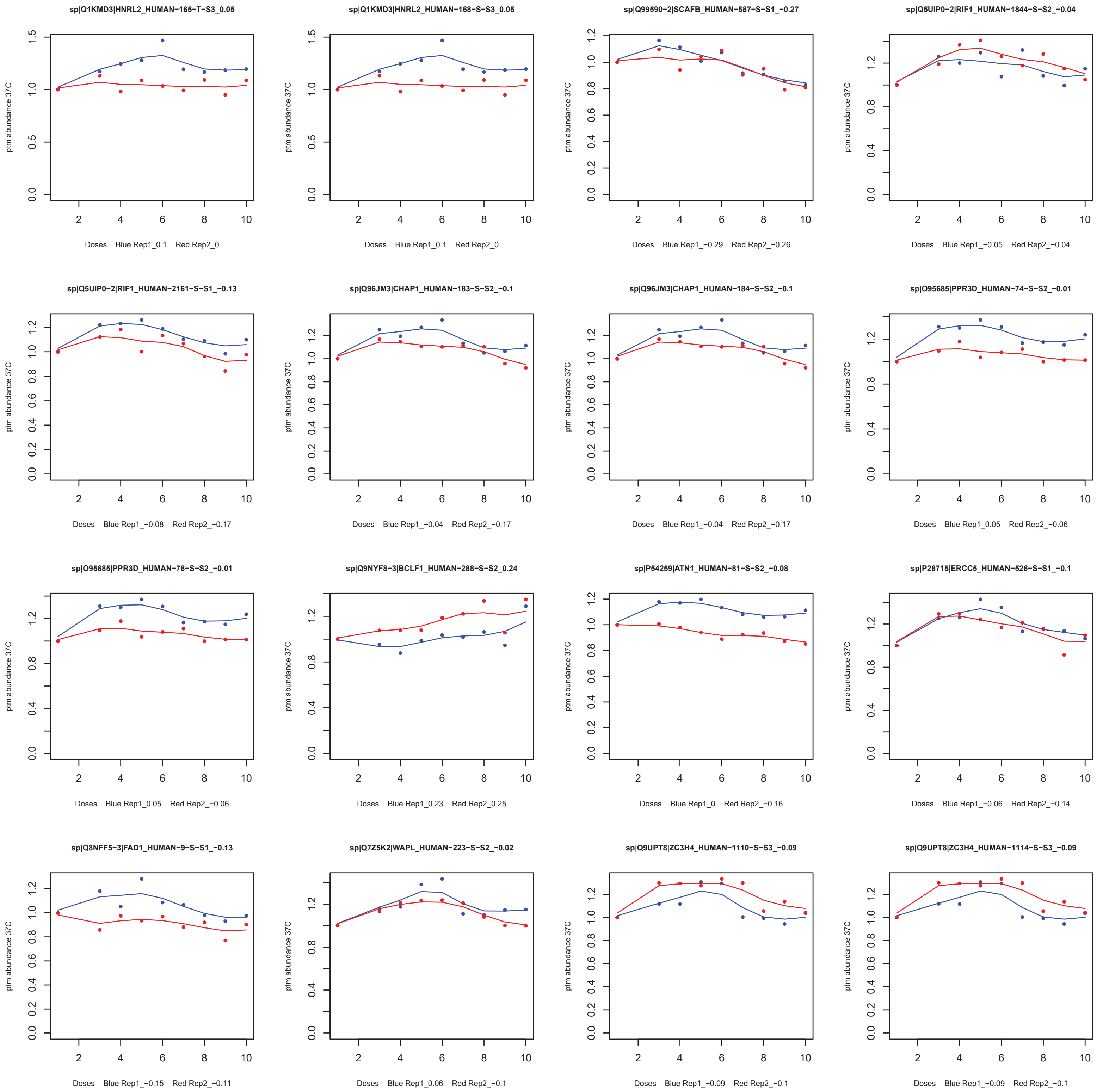
sp|Q1KMD3|HNRL2_HUMAN−165−T−S3_0.05
Doses Blue Rep1_0.1 Red Rep2_0
sp|Q1KMD3|HNRL2_HUMAN−168−S−S3_0.05
Doses Blue Rep1_0.1 Red Rep2_0
sp|Q99590−2|SCAFB_HUMAN−587−S−S1_−0.27
Doses Blue Rep1_−0.29 Red Rep2_−0.26
sp|Q5UIP0−2|RIF1_HUMAN−1844−S−S2_−0.04
Doses Blue Rep1_−0.05 Red Rep2_−0.04
sp|Q5UIP0−2|RIF1_HUMAN−2161−S−S1_−0.13
Doses Blue Rep1_−0.08 Red Rep2_−0.17
sp|Q96JM3|CHAP1_HUMAN−183−S−S2_−0.1
Doses Blue Rep1_−0.04 Red Rep2_−0.17
sp|Q96JM3|CHAP1_HUMAN−184−S−S2_−0.1
Doses Blue Rep1_−0.04 Red Rep2_−0.17
sp|O95685|PPR3D_HUMAN−74−S−S2_−0.01
Doses Blue Rep1_0.05 Red Rep2_−0.06
sp|O95685|PPR3D_HUMAN−78−S−S2_−0.01
Doses Blue Rep1_0.05 Red Rep2_−0.06
sp|Q9NYF8−3|BCLF1_HUMAN−288−S−S2_0.24
Doses Blue Rep1_0.23 Red Rep2_0.25
sp|P54259|ATN1_HUMAN−81−S−S2_−0.08
Doses Blue Rep1_0 Red Rep2_−0.16
sp|P28715|ERCC5_HUMAN−526−S−S1_−0.1
Doses Blue Rep1_−0.06 Red Rep2_−0.14
sp|Q8NFF5−3|FAD1_HUMAN−9−S−S1_−0.13
Doses Blue Rep1_−0.15 Red Rep2_−0.11
sp|Q7Z5K2|WAPL_HUMAN−223−S−S2_−0.02
Doses Blue Rep1_0.06 Red Rep2_−0.1
sp|Q9UPT8|ZC3H4_HUMAN−1110−S−S3_−0.09
Doses Blue Rep1_−0.09 Red Rep2_−0.1
sp|Q9UPT8|ZC3H4_HUMAN−1114−S−S3_−0.09
Doses Blue Rep1_−0.09 Red Rep2_−0.1
ptm abundance 37C

sp|Q9H869−7|YYAP1_HUMAN−395−S−S1_−0.17

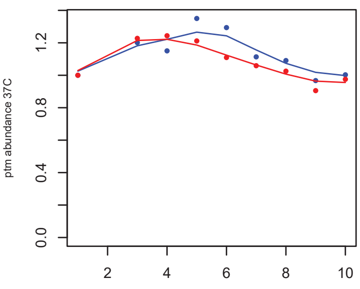

Doses Blue Rep1_−0.13 Red Rep2_−0.21

sp|Q8N163−2|CCAR2_HUMAN−687−S−S2_−0.06

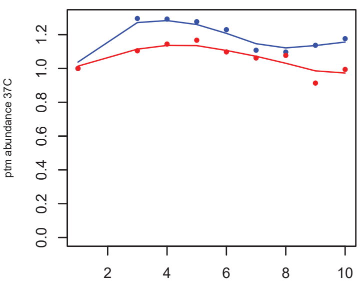

Doses Blue Rep1_0 Red Rep2_−0.11

sp|Q12789−3|TF3C1_HUMAN−748−S−S2_−0.16

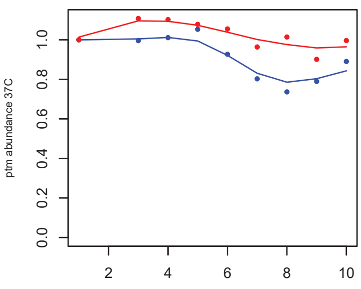

Doses Blue Rep1_−0.2 Red Rep2_−0.11

sp|Q9BV44|THUM3_HUMAN−220−T−S2_0.04

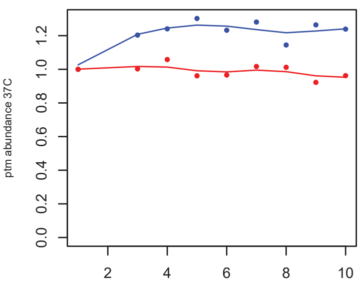

Doses Blue Rep1_0.15 Red Rep2_−0.07

sp|Q8TEH3|DEN1A_HUMAN−523−S−S1_−0.06

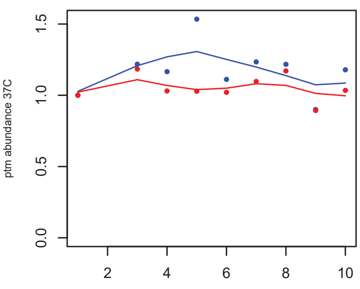

Doses Blue Rep1_−0.04 Red Rep2_−0.09

sp|O75528−2|TADA3_HUMAN−298−S−S1_−0.02

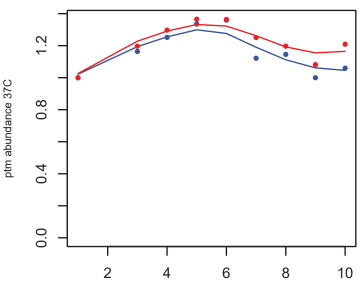

Doses Blue Rep1_−0.08 Red Rep2_0.05

sp|Q99856|ARI3A_HUMAN−81−S−S2_0.22

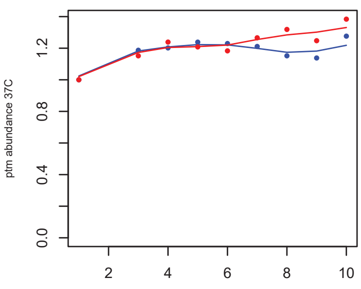

Doses Blue Rep1_0.14 Red Rep2_0.29

sp|P25205|MCM3_HUMAN−668−S−S3_0.11

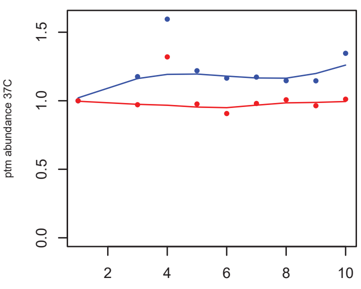

Doses Blue Rep1_0.21 Red Rep2_0.01

sp|P25205|MCM3_HUMAN−672−S−S3_0.11

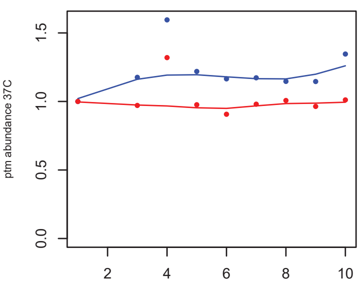

Doses Blue Rep1_0.21 Red Rep2_0.01

sp|P25205|MCM3_HUMAN−674−T−S3_0.11

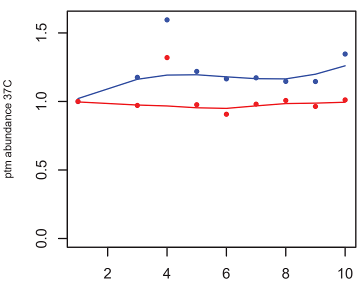

Doses Blue Rep1_0.21 Red Rep2_0.01

sp|Q8NE71|ABCF1_HUMAN−20−S−S2_−0.09

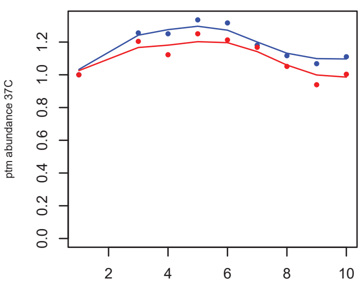

Doses Blue Rep1_−0.05 Red Rep2_−0.14

sp|Q8NE71|ABCF1_HUMAN−21−T−S2_−0.09

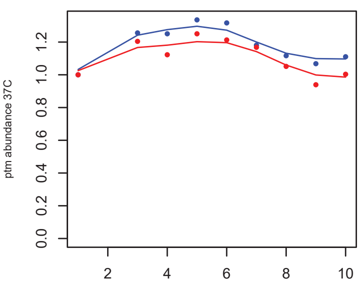

Doses Blue Rep1_−0.05 Red Rep2_−0.14

sp|Q7Z6Z7−2|HUWE1_HUMAN−1905−T−S2_−0.14

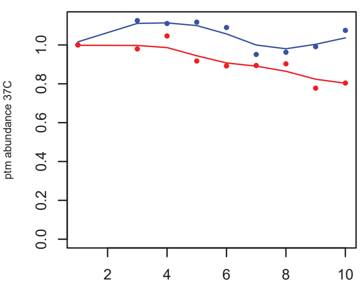

Doses Blue Rep1_−0.03 Red Rep2_−0.24

sp|P52948−6|NUP98_HUMAN−932−S−S2_−0.04

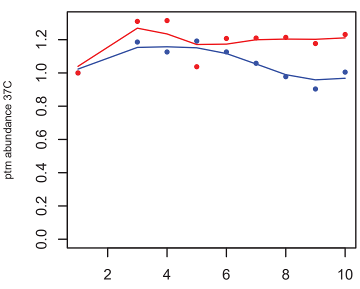

Doses Blue Rep1_−0.15 Red Rep2_0.07

sp|P52948−6|NUP98_HUMAN−934−S−S2_−0.04

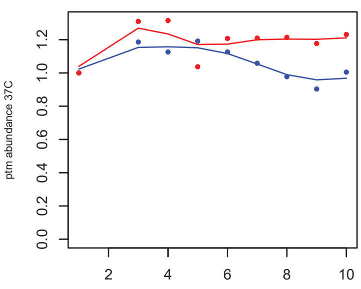

Doses Blue Rep1_−0.15 Red Rep2_0.07

sp|Q71F23−3|CENPU_HUMAN−110−T−S2_0.01

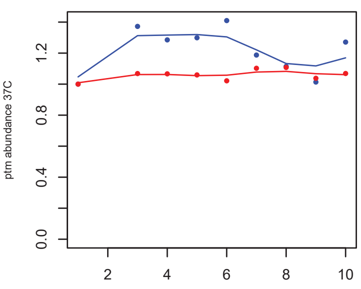

Doses Blue Rep1_−0.01 Red Rep2_0.03

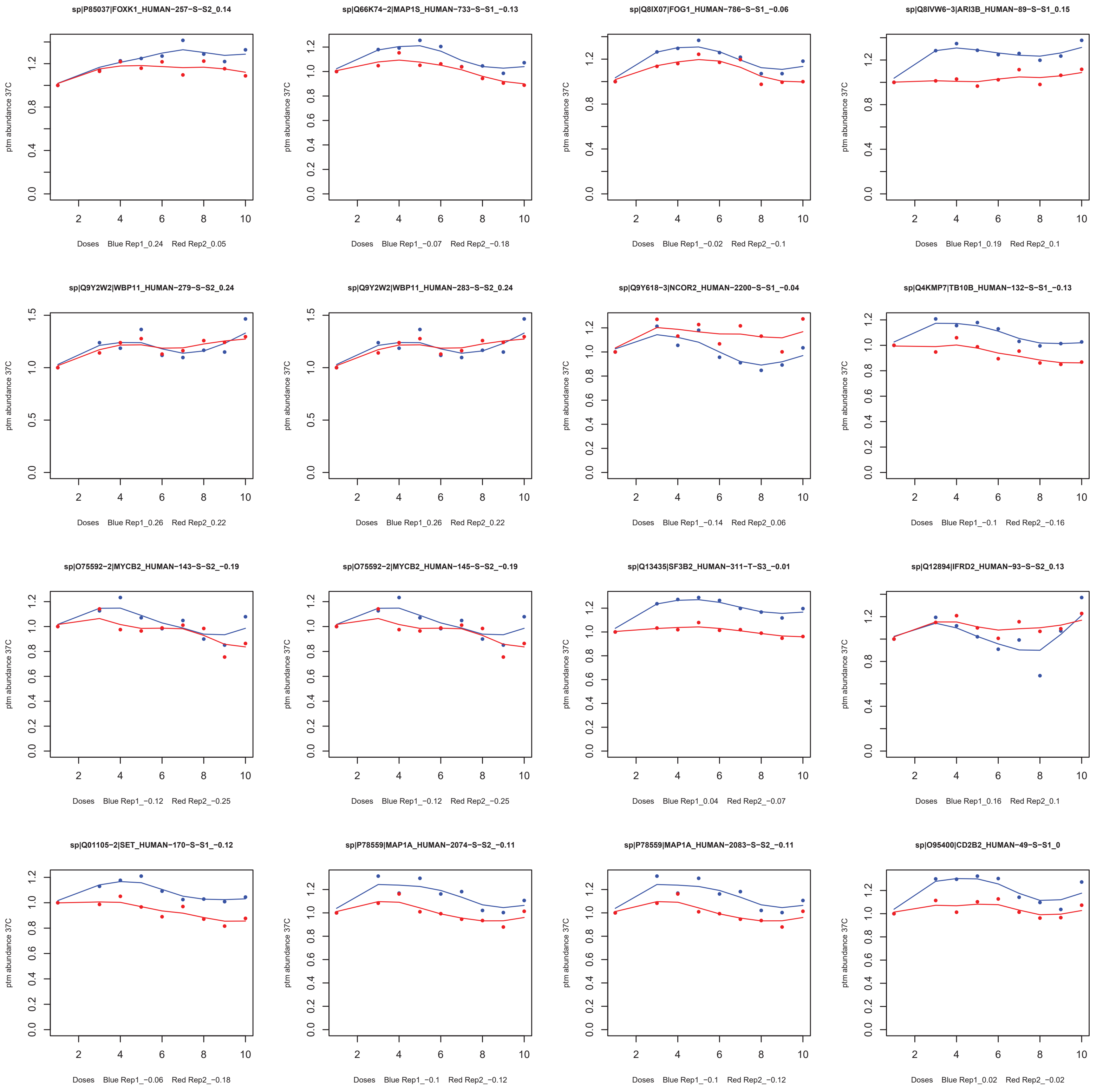
sp|P85037|FOXK1_HUMAN−257−S−S2_0.14
Doses Blue Rep1_0.24 Red Rep2_0.05
sp|Q66K74−2|MAP1S_HUMAN−733−S−S1_−0.13
Doses Blue Rep1_−0.07 Red Rep2_−0.18
sp|Q8IX07|FOG1_HUMAN−786−S−S1_−0.06
Doses Blue Rep1_−0.02 Red Rep2_−0.1
sp|Q8IVW6−3|ARI3B_HUMAN−89−S−S1_0.15
Doses Blue Rep1_0.19 Red Rep2_0.1
sp|Q9Y2W2|WBP11_HUMAN−279−S−S2_0.24
Doses Blue Rep1_0.26 Red Rep2_0.22
sp|Q9Y2W2|WBP11_HUMAN−283−S−S2_0.24
Doses Blue Rep1_0.26 Red Rep2_0.22
sp|Q9Y618−3|NCOR2_HUMAN−2200−S−S1_−0.04
Doses Blue Rep1_−0.14 Red Rep2_0.06
sp|Q4KMP7|TB10B_HUMAN−132−S−S1_−0.13
Doses Blue Rep1_−0.1 Red Rep2_−0.16
sp|O75592−2|MYCB2_HUMAN−143−S−S2_−0.19
Doses Blue Rep1_−0.12 Red Rep2_−0.25
sp|O75592−2|MYCB2_HUMAN−145−S−S2_−0.19
Doses Blue Rep1_−0.12 Red Rep2_−0.25
sp|Q13435|SF3B2_HUMAN−311−T−S3_−0.01
Doses Blue Rep1_0.04 Red Rep2_−0.07
sp|Q12894|IFRD2_HUMAN−93−S−S2_0.13
Doses Blue Rep1_0.16 Red Rep2_0.1
sp|Q01105−2|SET_HUMAN−170−S−S1_−0.12
Doses Blue Rep1_−0.06 Red Rep2_−0.18
sp|P78559|MAP1A_HUMAN−2074−S−S2_−0.11
Doses Blue Rep1_−0.1 Red Rep2_−0.12
sp|P78559|MAP1A_HUMAN−2083−S−S2_−0.11
Doses Blue Rep1_−0.1 Red Rep2_−0.12
sp|O95400|CD2B2_HUMAN−49−S−S1_0
Doses Blue Rep1_0.02 Red Rep2_−0.02
ptm abundance 37C

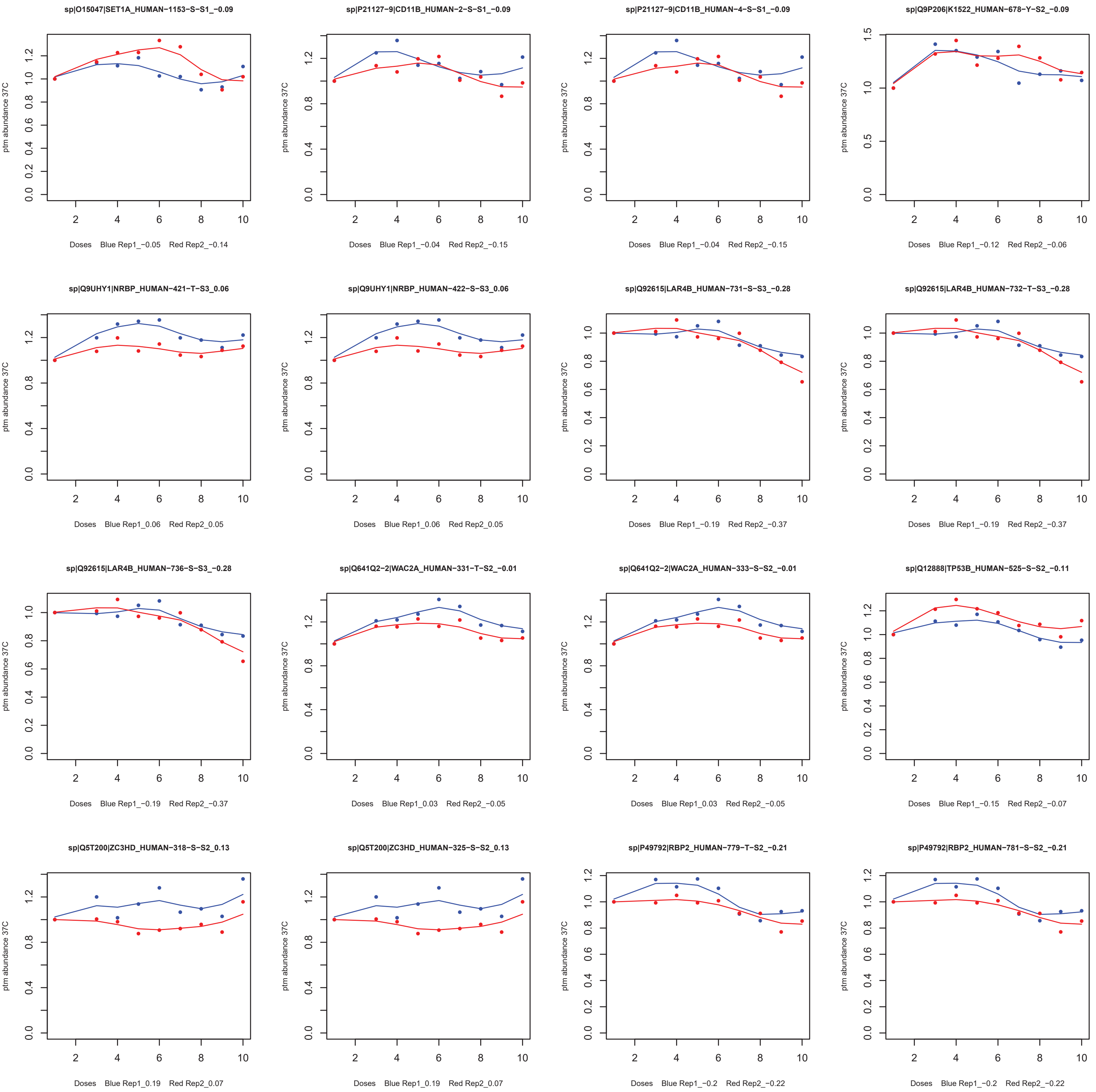
sp|O15047|SET1A_HUMAN−1153−S−S1_−0.09
ptm abundance 37C
Doses Blue Rep1_−0.05 Red Rep2_−0.14
sp|P21127−9|CD11B_HUMAN−2−S−S1_−0.09
ptm abundance 37C
Doses Blue Rep1_−0.04 Red Rep2_−0.15
sp|P21127−9|CD11B_HUMAN−4−S−S1_−0.09
ptm abundance 37C
Doses Blue Rep1_−0.04 Red Rep2_−0.15
sp|Q9P206|K1522_HUMAN−678−Y−S2_−0.09
ptm abundance 37C
Doses Blue Rep1_−0.12 Red Rep2_−0.06
sp|Q9UHY1|NRBP_HUMAN−421−T−S3_0.06
ptm abundance 37C
Doses Blue Rep1_0.06 Red Rep2_0.05
sp|Q9UHY1|NRBP_HUMAN−422−S−S3_0.06
ptm abundance 37C
Doses Blue Rep1_0.06 Red Rep2_0.05
sp|Q92615|LAR4B_HUMAN−731−S−S3_−0.28
ptm abundance 37C
Doses Blue Rep1_−0.19 Red Rep2_−0.37
sp|Q92615|LAR4B_HUMAN−732−T−S3_−0.28
ptm abundance 37C
Doses Blue Rep1_−0.19 Red Rep2_−0.37
sp|Q92615|LAR4B_HUMAN−736−S−S3_−0.28
ptm abundance 37C
Doses Blue Rep1_−0.19 Red Rep2_−0.37
sp|Q641Q2−2|WAC2A_HUMAN−331−T−S2_−0.01
ptm abundance 37C
Doses Blue Rep1_0.03 Red Rep2_−0.05
sp|Q641Q2−2|WAC2A_HUMAN−333−S−S2_−0.01
ptm abundance 37C
Doses Blue Rep1_0.03 Red Rep2_−0.05
sp|Q12888|TP53B_HUMAN−525−S−S2_−0.11
ptm abundance 37C
Doses Blue Rep1_−0.15 Red Rep2_−0.07
sp|Q5T200|ZC3HD_HUMAN−318−S−S2_0.13
ptm abundance 37C
Doses Blue Rep1_0.19 Red Rep2_0.07
sp|Q5T200|ZC3HD_HUMAN−325−S−S2_0.13
ptm abundance 37C
Doses Blue Rep1_0.19 Red Rep2_0.07
sp|P49792|RBP2_HUMAN−779−T−S2_−0.21
ptm abundance 37C
Doses Blue Rep1_−0.2 Red Rep2_−0.22
sp|P49792|RBP2_HUMAN−781−S−S2_−0.21
ptm abundance 37C
Doses Blue Rep1_−0.2 Red Rep2_−0.22

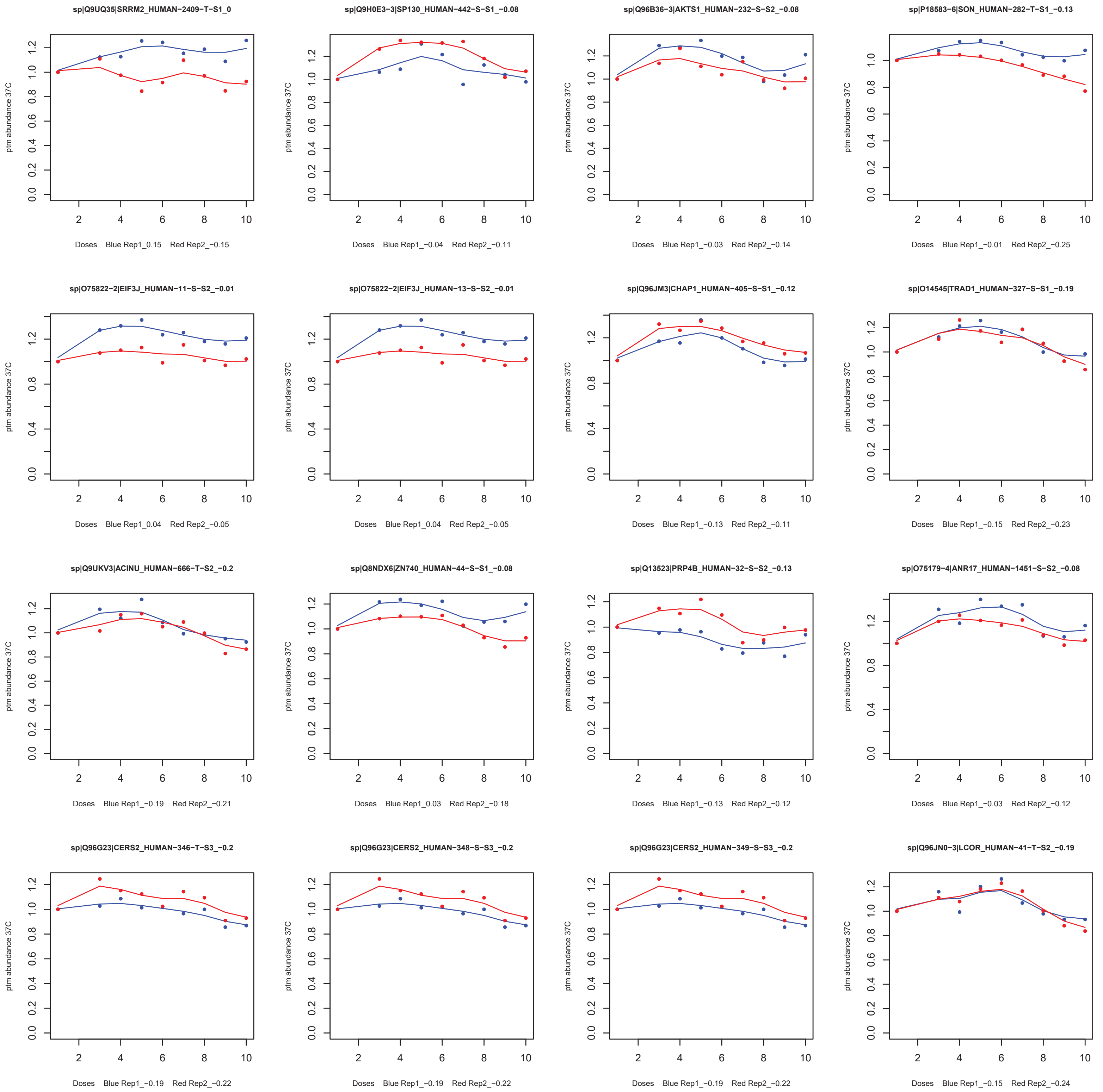

sp|Q9UQ35|SRRM2_HUMAN−2409−T−S1_0
Doses Blue Rep1_0.15 Red Rep2_−0.15
sp|Q9H0E3−3|SP130_HUMAN−442−S−S1_−0.08
Doses Blue Rep1_−0.04 Red Rep2_−0.11
sp|Q96B36−3|AKTS1_HUMAN−232−S−S2_−0.08
Doses Blue Rep1_−0.03 Red Rep2_−0.14
sp|P18583−6|SON_HUMAN−282−T−S1_−0.13
Doses Blue Rep1_−0.01 Red Rep2_−0.25
sp|O75822−2|EIF3J_HUMAN−11−S−S2_−0.01
Doses Blue Rep1_0.04 Red Rep2_−0.05
sp|O75822−2|EIF3J_HUMAN−13−S−S2_−0.01
Doses Blue Rep1_0.04 Red Rep2_−0.05
sp|Q96JM3|CHAP1_HUMAN−405−S−S1_−0.12
Doses Blue Rep1_−0.13 Red Rep2_−0.11
sp|O14545|TRAD1_HUMAN−327−S−S1_−0.19
Doses Blue Rep1_−0.15 Red Rep2_−0.23
sp|Q9UKV3|ACINU_HUMAN−666−T−S2_−0.2
Doses Blue Rep1_−0.19 Red Rep2_−0.21
sp|Q8NDX6|ZN740_HUMAN−44−S−S1_−0.08
Doses Blue Rep1_0.03 Red Rep2_−0.18
sp|Q13523|PRP4B_HUMAN−32−S−S2_−0.13
Doses Blue Rep1_−0.13 Red Rep2_−0.12
sp|O75179−4|ANR17_HUMAN−1451−S−S2_−0.08
Doses Blue Rep1_−0.03 Red Rep2_−0.12
sp|Q96G23|CERS2_HUMAN−346−T−S3_−0.2
Doses Blue Rep1_−0.19 Red Rep2_−0.22
sp|Q96G23|CERS2_HUMAN−348−S−S3_−0.2
Doses Blue Rep1_−0.19 Red Rep2_−0.22
sp|Q96G23|CERS2_HUMAN−349−S−S3_−0.2
Doses Blue Rep1_−0.19 Red Rep2_−0.22
sp|Q96JN0−3|LCOR_HUMAN−41−T−S2_−0.19
Doses Blue Rep1_−0.15 Red Rep2_−0.24
ptm abundance 37C

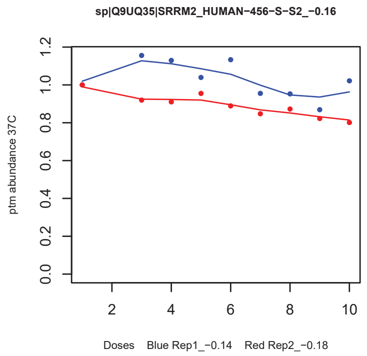
sp|Q9UQ35|SRRM2_HUMAN−456−S−S2_−0.16
ptm abundance 37C
Doses Blue Rep1_−0.14 Red Rep2_−0.18

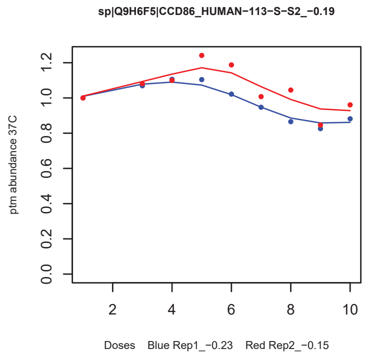
sp|Q9H6F5|CCD86_HUMAN−113−S−S2_−0.19
ptm abundance 37C
Doses Blue Rep1_−0.23 Red Rep2_−0.15

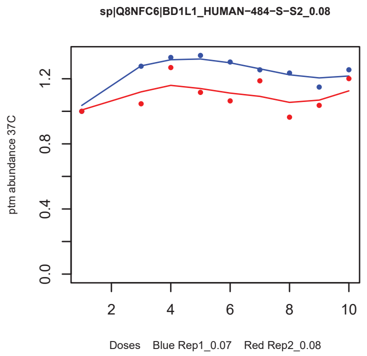
sp|Q8NFC6|BD1L1_HUMAN−484−S−S2_0.08
ptm abundance 37C
Doses Blue Rep1_0.07 Red Rep2_0.08

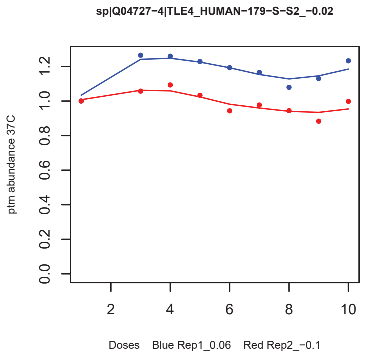
sp|Q04727−4|TLE4_HUMAN−179−S−S2_−0.02
ptm abundance 37C
Doses Blue Rep1_0.06 Red Rep2_−0.1

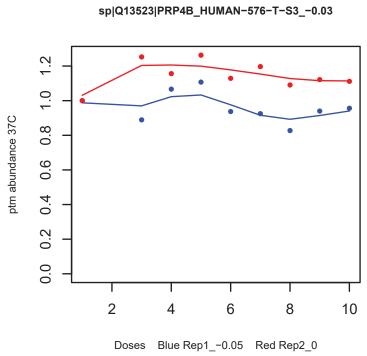
sp|Q13523|PRP4B_HUMAN−576−T−S3_−0.03
ptm abundance 37C
Doses Blue Rep1_−0.05 Red Rep2_0

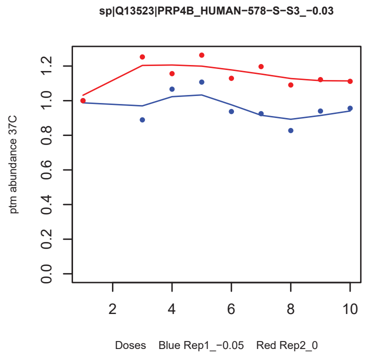
sp|Q13523|PRP4B_HUMAN−578−S−S3_−0.03
ptm abundance 37C
Doses Blue Rep1_−0.05 Red Rep2_0

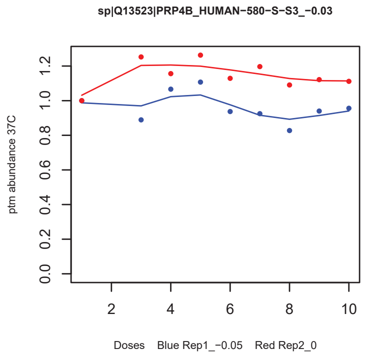
sp|Q13523|PRP4B_HUMAN−580−S−S3_−0.03
ptm abundance 37C
Doses Blue Rep1_−0.05 Red Rep2_0

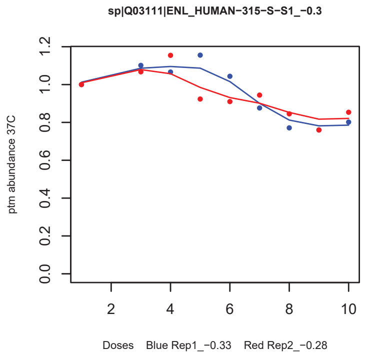
sp|Q03111|ENL_HUMAN−315−S−S1_−0.3
ptm abundance 37C
Doses Blue Rep1_−0.33 Red Rep2_−0.28

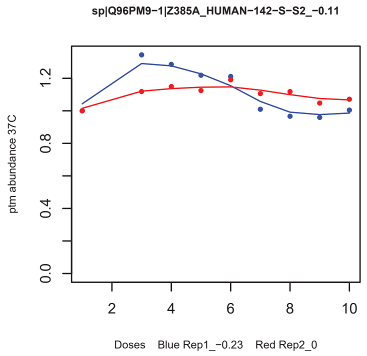
sp|Q96PM9−1|Z385A_HUMAN−142−S−S2_−0.11
ptm abundance 37C
Doses Blue Rep1_−0.23 Red Rep2_0

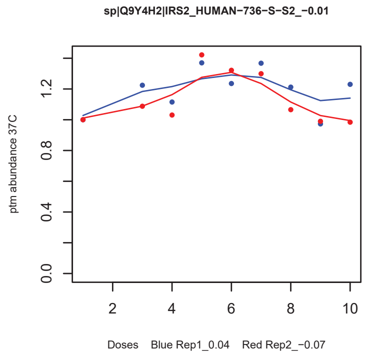
sp|Q9Y4H2|IRS2_HUMAN−736−S−S2_−0.01
ptm abundance 37C
Doses Blue Rep1_0.04 Red Rep2_−0.07

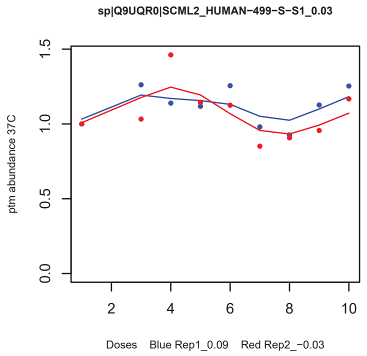
sp|Q9UQR0|SCML2_HUMAN−499−S−S1_0.03
ptm abundance 37C
Doses Blue Rep1_0.09 Red Rep2_−0.03

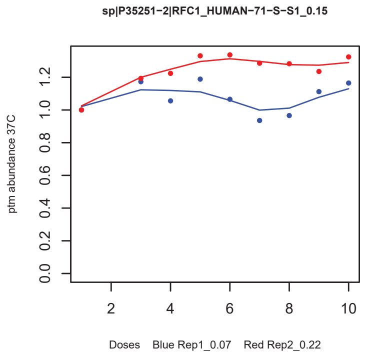
sp|P35251−2|RFC1_HUMAN−71−S−S1_0.15
ptm abundance 37C
Doses Blue Rep1_0.07 Red Rep2_0.22

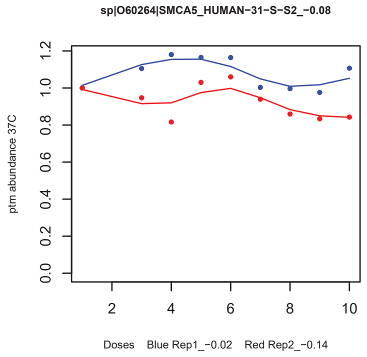
sp|O60264|SMCA5_HUMAN−31−S−S2_−0.08
ptm abundance 37C
Doses Blue Rep1_−0.02 Red Rep2_−0.14

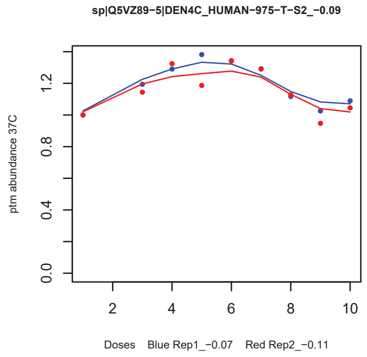
sp|Q5VZ89−5|DEN4C_HUMAN−975−T−S2_−0.09
ptm abundance 37C
Doses Blue Rep1_−0.07 Red Rep2_−0.11

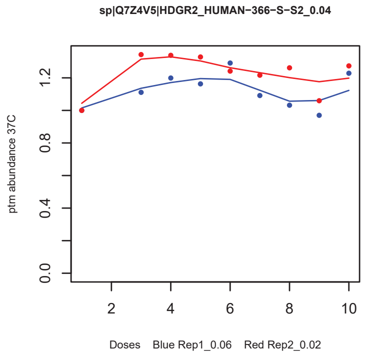
sp|Q7Z4V5|HDGR2_HUMAN−366−S−S2_0.04
ptm abundance 37C
Doses Blue Rep1_0.06 Red Rep2_0.02

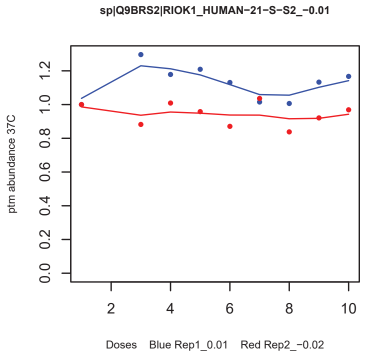
sp|Q9BRS2|RIOK1_HUMAN−21−S−S2_−0.01
ptm abundance 37C
Doses Blue Rep1_0.01 Red Rep2_−0.02

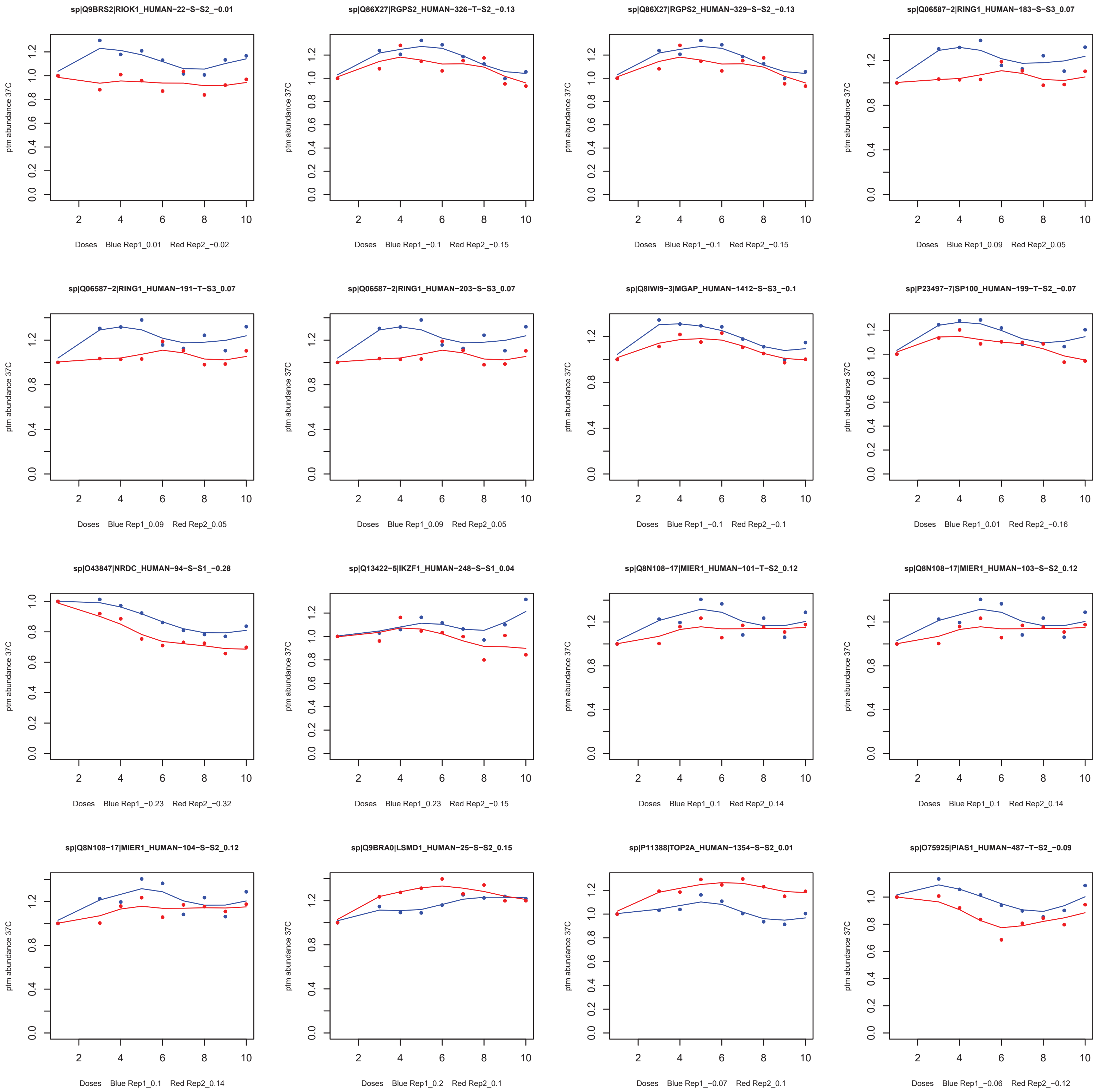
sp|Q9BRS2|RIOK1_HUMAN−22−S−S2_−0.01
ptm abundance 37C
Doses Blue Rep1_0.01 Red Rep2_−0.02
sp|Q86X27|RGPS2_HUMAN−326−T−S2_−0.13
ptm abundance 37C
Doses Blue Rep1_−0.1 Red Rep2_−0.15
sp|Q86X27|RGPS2_HUMAN−329−S−S2_−0.13
ptm abundance 37C
Doses Blue Rep1_−0.1 Red Rep2_−0.15
sp|Q06587−2|RING1_HUMAN−183−S−S3_0.07
ptm abundance 37C
Doses Blue Rep1_0.09 Red Rep2_0.05
sp|Q06587−2|RING1_HUMAN−191−T−S3_0.07
ptm abundance 37C
Doses Blue Rep1_0.09 Red Rep2_0.05
sp|Q06587−2|RING1_HUMAN−203−S−S3_0.07
ptm abundance 37C
Doses Blue Rep1_0.09 Red Rep2_0.05
sp|Q8IWI9−3|MGAP_HUMAN−1412−S−S3_−0.1
ptm abundance 37C
Doses Blue Rep1_−0.1 Red Rep2_−0.1
sp|P23497−7|SP100_HUMAN−199−T−S2_−0.07
ptm abundance 37C
Doses Blue Rep1_0.01 Red Rep2_−0.16
sp|O43847|NRDC_HUMAN−94−S−S1_−0.28
ptm abundance 37C
Doses Blue Rep1_−0.23 Red Rep2_−0.32
sp|Q13422−5|IKZF1_HUMAN−248−S−S1_0.04
ptm abundance 37C
Doses Blue Rep1_0.23 Red Rep2_−0.15
sp|Q8N108−17|MIER1_HUMAN−101−T−S2_0.12
ptm abundance 37C
Doses Blue Rep1_0.1 Red Rep2_0.14
sp|Q8N108−17|MIER1_HUMAN−103−S−S2_0.12
ptm abundance 37C
Doses Blue Rep1_0.1 Red Rep2_0.14
sp|Q8N108−17|MIER1_HUMAN−104−S−S2_0.12
ptm abundance 37C
Doses Blue Rep1_0.1 Red Rep2_0.14
sp|Q9BRA0|LSMD1_HUMAN−25−S−S2_0.15
ptm abundance 37C
Doses Blue Rep1_0.2 Red Rep2_0.1
sp|P11388|TOP2A_HUMAN−1354−S−S2_0.01
ptm abundance 37C
Doses Blue Rep1_−0.07 Red Rep2_0.1
sp|O75925|PIAS1_HUMAN−487−T−S2_−0.09
ptm abundance 37C
Doses Blue Rep1_−0.06 Red Rep2_−0.12

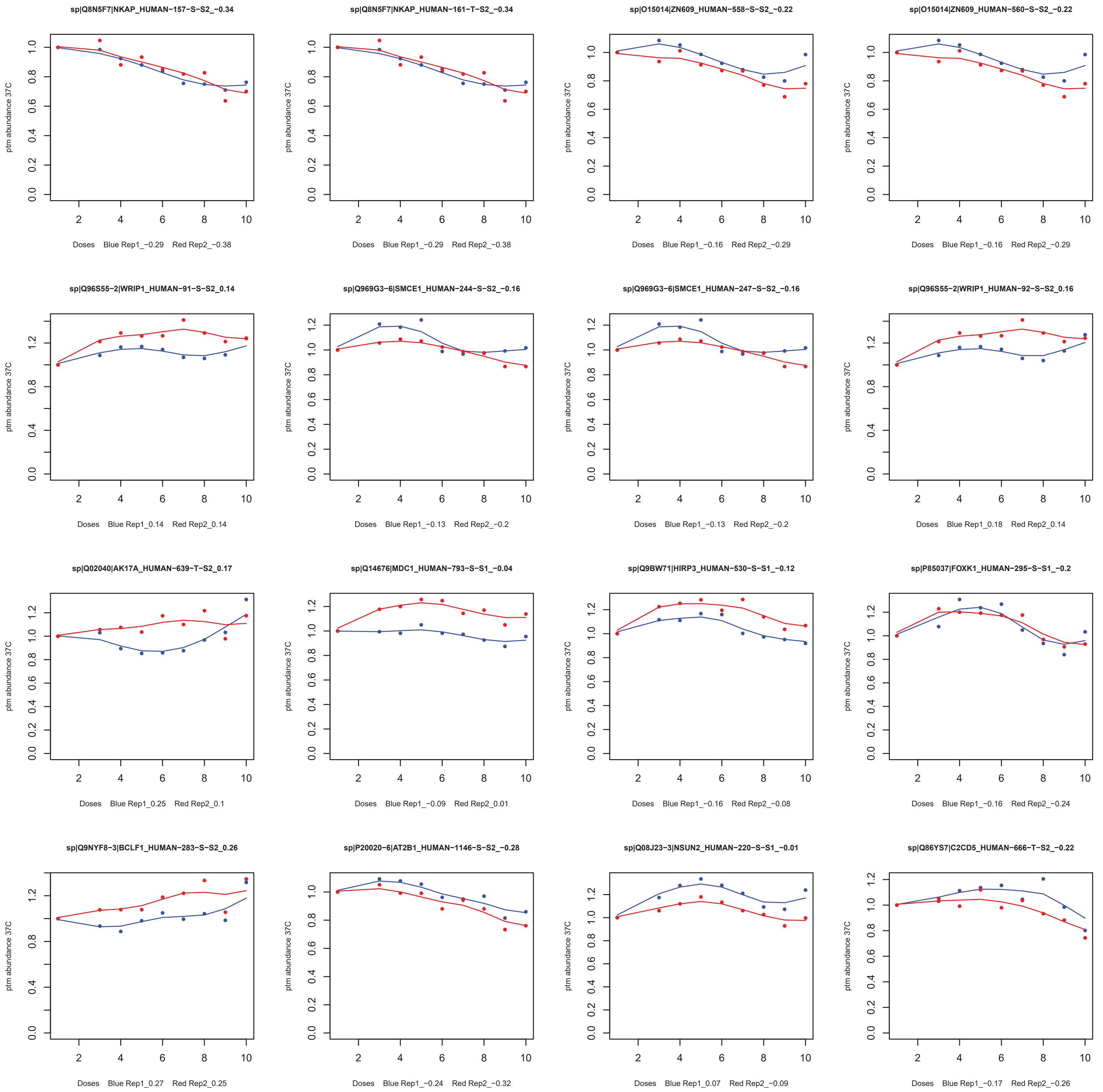
sp|Q8N5F7|NKAP_HUMAN−157−S−S2_−0.34
Doses Blue Rep1_−0.29 Red Rep2_−0.38
sp|Q8N5F7|NKAP_HUMAN−161−T−S2_−0.34
Doses Blue Rep1_−0.29 Red Rep2_−0.38
sp|O15014|ZN609_HUMAN−558−S−S2_−0.22
Doses Blue Rep1_−0.16 Red Rep2_−0.29
sp|O15014|ZN609_HUMAN−560−S−S2_−0.22
Doses Blue Rep1_−0.16 Red Rep2_−0.29
sp|Q96S55−2|WRIP1_HUMAN−91−S−S2_0.14
Doses Blue Rep1_0.14 Red Rep2_0.14
sp|Q969G3−6|SMCE1_HUMAN−244−S−S2_−0.16
Doses Blue Rep1_−0.13 Red Rep2_−0.2
sp|Q969G3−6|SMCE1_HUMAN−247−S−S2_−0.16
Doses Blue Rep1_−0.13 Red Rep2_−0.2
sp|Q96S55−2|WRIP1_HUMAN−92−S−S2_0.16
Doses Blue Rep1_0.18 Red Rep2_0.14
sp|Q02040|AK17A_HUMAN−639−T−S2_0.17
Doses Blue Rep1_0.25 Red Rep2_0.1
sp|Q14676|MDC1_HUMAN−793−S−S1_−0.04
Doses Blue Rep1_−0.09 Red Rep2_0.01
sp|Q9BW71|HIRP3_HUMAN−530−S−S1_−0.12
Doses Blue Rep1_−0.16 Red Rep2_−0.08
sp|P85037|FOXK1_HUMAN−295−S−S1_−0.2
Doses Blue Rep1_−0.16 Red Rep2_−0.24
sp|Q9NYF8−3|BCLF1_HUMAN−283−S−S2_0.26
Doses Blue Rep1_0.27 Red Rep2_0.25
sp|P20020−6|AT2B1_HUMAN−1146−S−S2_−0.28
Doses Blue Rep1_−0.24 Red Rep2_−0.32
sp|Q08J23−3|NSUN2_HUMAN−220−S−S1_−0.01
Doses Blue Rep1_0.07 Red Rep2_−0.09
sp|Q86YS7|C2CD5_HUMAN−666−T−S2_−0.22
Doses Blue Rep1_−0.17 Red Rep2_−0.26
ptm abundance 37C

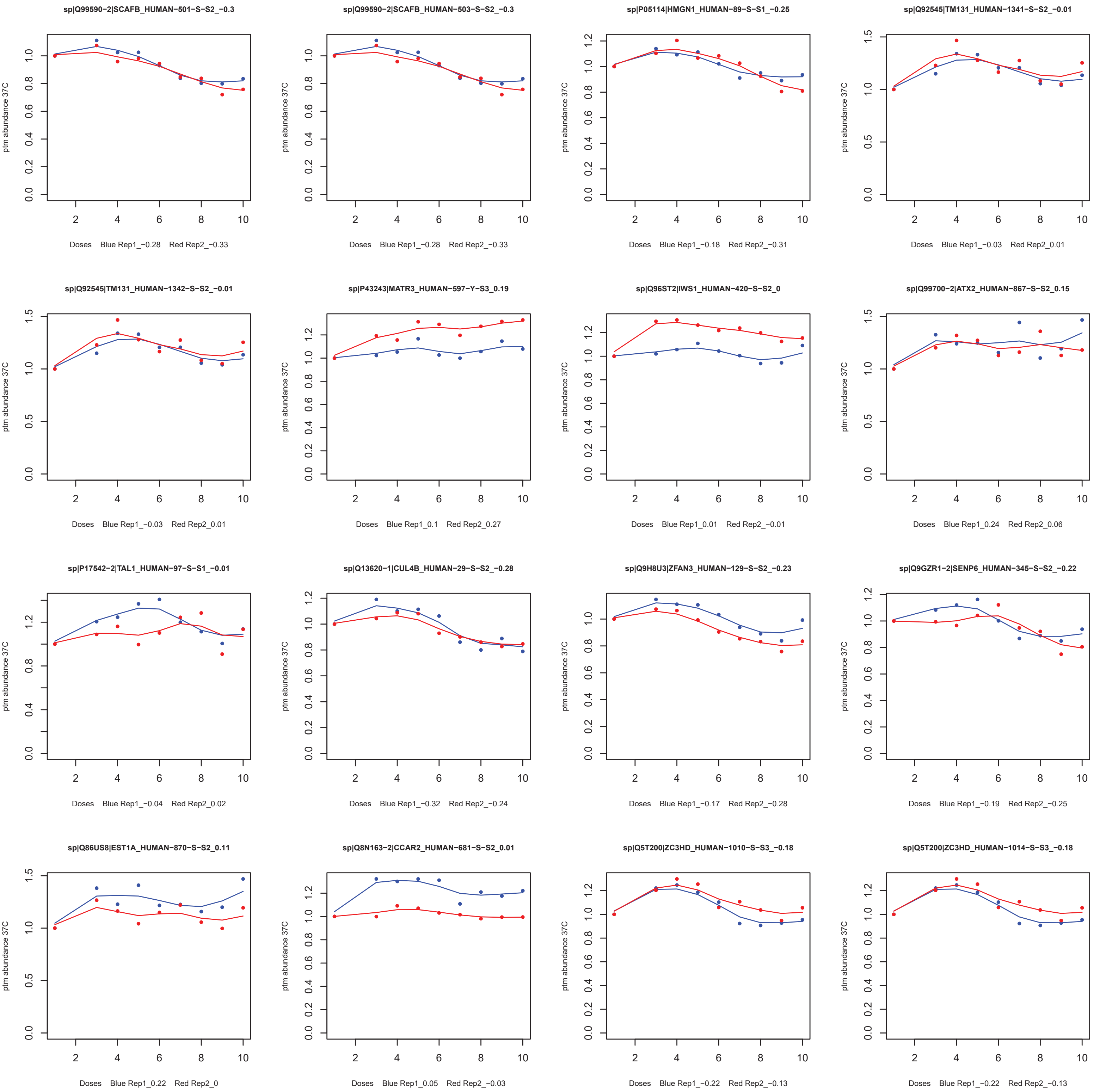
sp|Q99590−2|SCAFB_HUMAN−501−S−S2_−0.3
Doses Blue Rep1_−0.28 Red Rep2_−0.33
sp|Q99590−2|SCAFB_HUMAN−503−S−S2_−0.3
Doses Blue Rep1_−0.28 Red Rep2_−0.33
sp|P05114|HMGN1_HUMAN−89−S−S1_−0.25
Doses Blue Rep1_−0.18 Red Rep2_−0.31
sp|Q92545|TM131_HUMAN−1341−S−S2_−0.01
Doses Blue Rep1_−0.03 Red Rep2_0.01
sp|Q92545|TM131_HUMAN−1342−S−S2_−0.01
Doses Blue Rep1_−0.03 Red Rep2_0.01
sp|P43243|MATR3_HUMAN−597−Y−S3_0.19
Doses Blue Rep1_0.1 Red Rep2_0.27
sp|Q96ST2|IWS1_HUMAN−420−S−S2_0
Doses Blue Rep1_0.01 Red Rep2_−0.01
sp|Q99700−2|ATX2_HUMAN−867−S−S2_0.15
Doses Blue Rep1_0.24 Red Rep2_0.06
sp|P17542−2|TAL1_HUMAN−97−S−S1_−0.01
Doses Blue Rep1_−0.04 Red Rep2_0.02
sp|Q13620−1|CUL4B_HUMAN−29−S−S2_−0.28
Doses Blue Rep1_−0.32 Red Rep2_−0.24
sp|Q9H8U3|ZFAN3_HUMAN−129−S−S2_−0.23
Doses Blue Rep1_−0.17 Red Rep2_−0.28
sp|Q9GZR1−2|SENP6_HUMAN−345−S−S2_−0.22
Doses Blue Rep1_−0.19 Red Rep2_−0.25
sp|Q86US8|EST1A_HUMAN−870−S−S2_0.11
Doses Blue Rep1_0.22 Red Rep2_0
sp|Q8N163−2|CCAR2_HUMAN−681−S−S2_0.01
Doses Blue Rep1_0.05 Red Rep2_−0.03
sp|Q5T200|ZC3HD_HUMAN−1010−S−S3_−0.18
Doses Blue Rep1_−0.22 Red Rep2_−0.13
sp|Q5T200|ZC3HD_HUMAN−1014−S−S3_−0.18
Doses Blue Rep1_−0.22 Red Rep2_−0.13
ptm abundance 37C

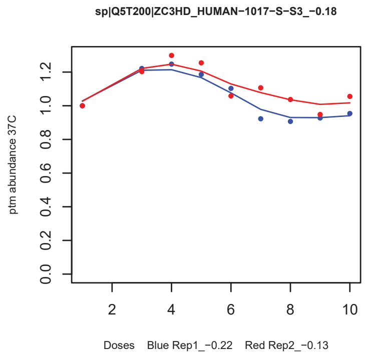
sp|Q5T200|ZC3HD_HUMAN−1017−S−S3_−0.18
ptm abundance 37C
Doses Blue Rep1_−0.22 Red Rep2_−0.13

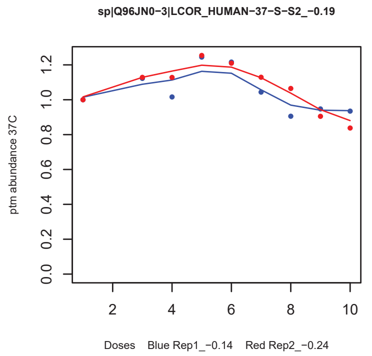
sp|Q96JN0−3|LCOR_HUMAN−37−S−S2_−0.19
ptm abundance 37C
Doses Blue Rep1_−0.14 Red Rep2_−0.24

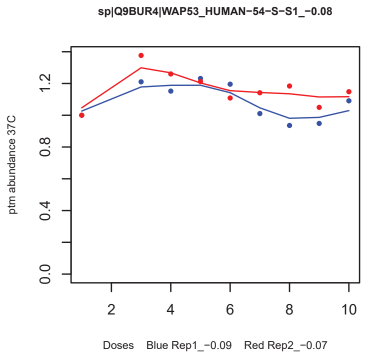
sp|Q9BUR4|WAP53_HUMAN−54−S−S1_−0.08
ptm abundance 37C
Doses Blue Rep1_−0.09 Red Rep2_−0.07

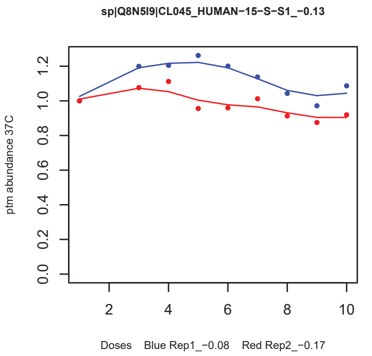
sp|Q8N5I9|CL045_HUMAN−15−S−S1_−0.13
ptm abundance 37C
Doses Blue Rep1_−0.08 Red Rep2_−0.17

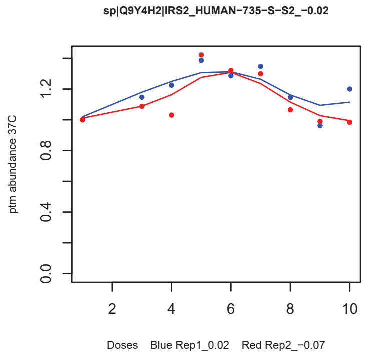
sp|Q9Y4H2|IRS2_HUMAN−735−S−S2_−0.02
ptm abundance 37C
Doses Blue Rep1_0.02 Red Rep2_−0.07

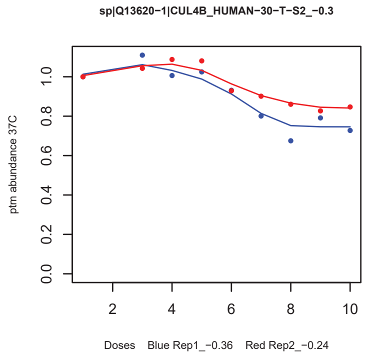
sp|Q13620−1|CUL4B_HUMAN−30−T−S2_−0.3
ptm abundance 37C
Doses Blue Rep1_−0.36 Red Rep2_−0.24

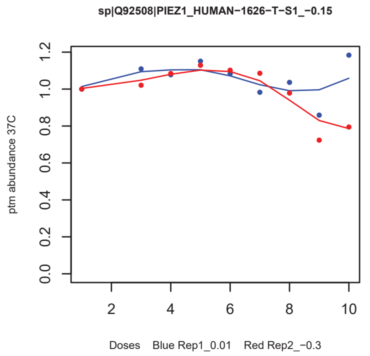
sp|Q92508|PIEZ1_HUMAN−1626−T−S1_−0.15
ptm abundance 37C
Doses Blue Rep1_0.01 Red Rep2_−0.3

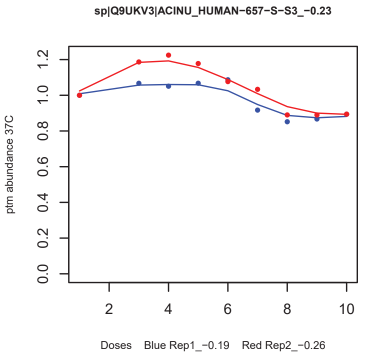
sp|Q9UKV3|ACINU_HUMAN−657−S−S3_−0.23
ptm abundance 37C
Doses Blue Rep1_−0.19 Red Rep2_−0.26

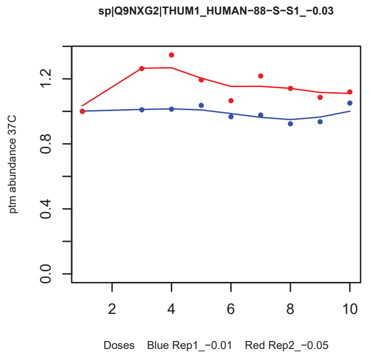
sp|Q9NXG2|THUM1_HUMAN−88−S−S1_−0.03
ptm abundance 37C
Doses Blue Rep1_−0.01 Red Rep2_−0.05

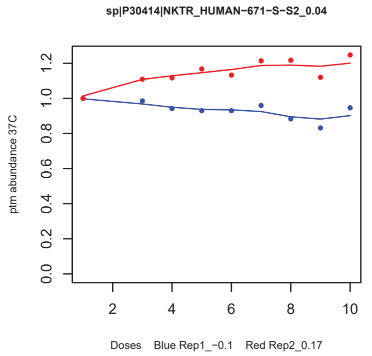
sp|P30414|NKTR_HUMAN−671−S−S2_0.04
ptm abundance 37C
Doses Blue Rep1_−0.1 Red Rep2_0.17

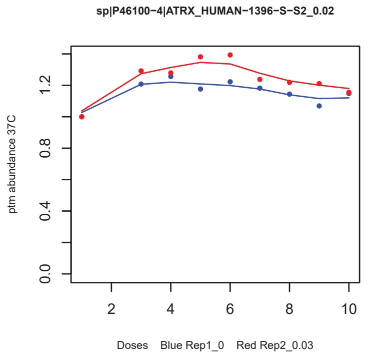
sp|P46100−4|ATRX_HUMAN−1396−S−S2_0.02
ptm abundance 37C
Doses Blue Rep1_0 Red Rep2_0.03

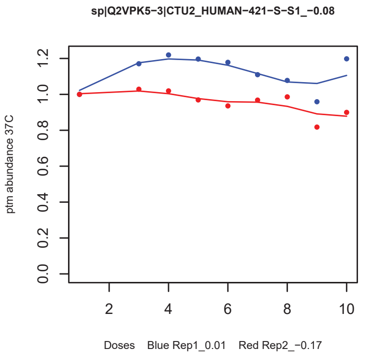
sp|Q2VPK5−3|CTU2_HUMAN−421−S−S1_−0.08
ptm abundance 37C
Doses Blue Rep1_0.01 Red Rep2_−0.17

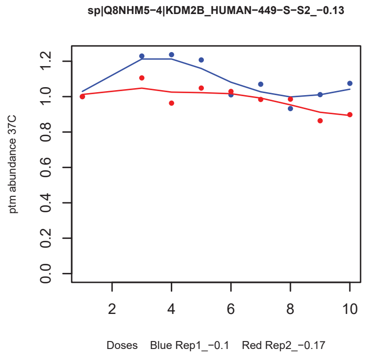
sp|Q8NHM5−4|KDM2B_HUMAN−449−S−S2_−0.13
ptm abundance 37C
Doses Blue Rep1_−0.1 Red Rep2_−0.17

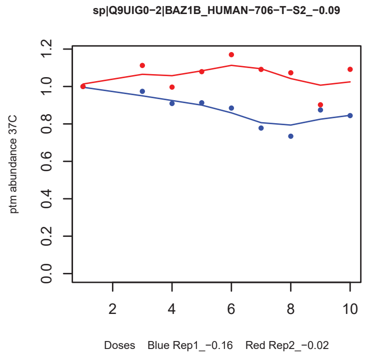
sp|Q9UIG0−2|BAZ1B_HUMAN−706−T−S2_−0.09
ptm abundance 37C
Doses Blue Rep1_−0.16 Red Rep2_−0.02

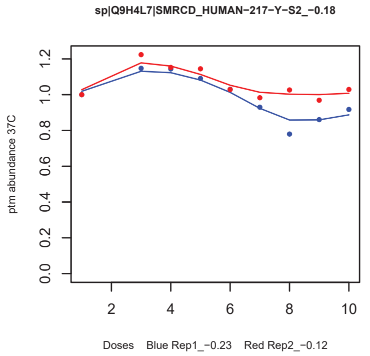
sp|Q9H4L7|SMRCD_HUMAN−217−Y−S2_−0.18
ptm abundance 37C
Doses Blue Rep1_−0.23 Red Rep2_−0.12

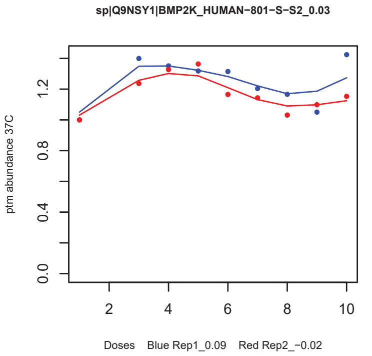
sp|Q9NSY1|BMP2K_HUMAN−801−S−S2_0.03
ptm abundance 37C
Doses Blue Rep1_0.09 Red Rep2_−0.02

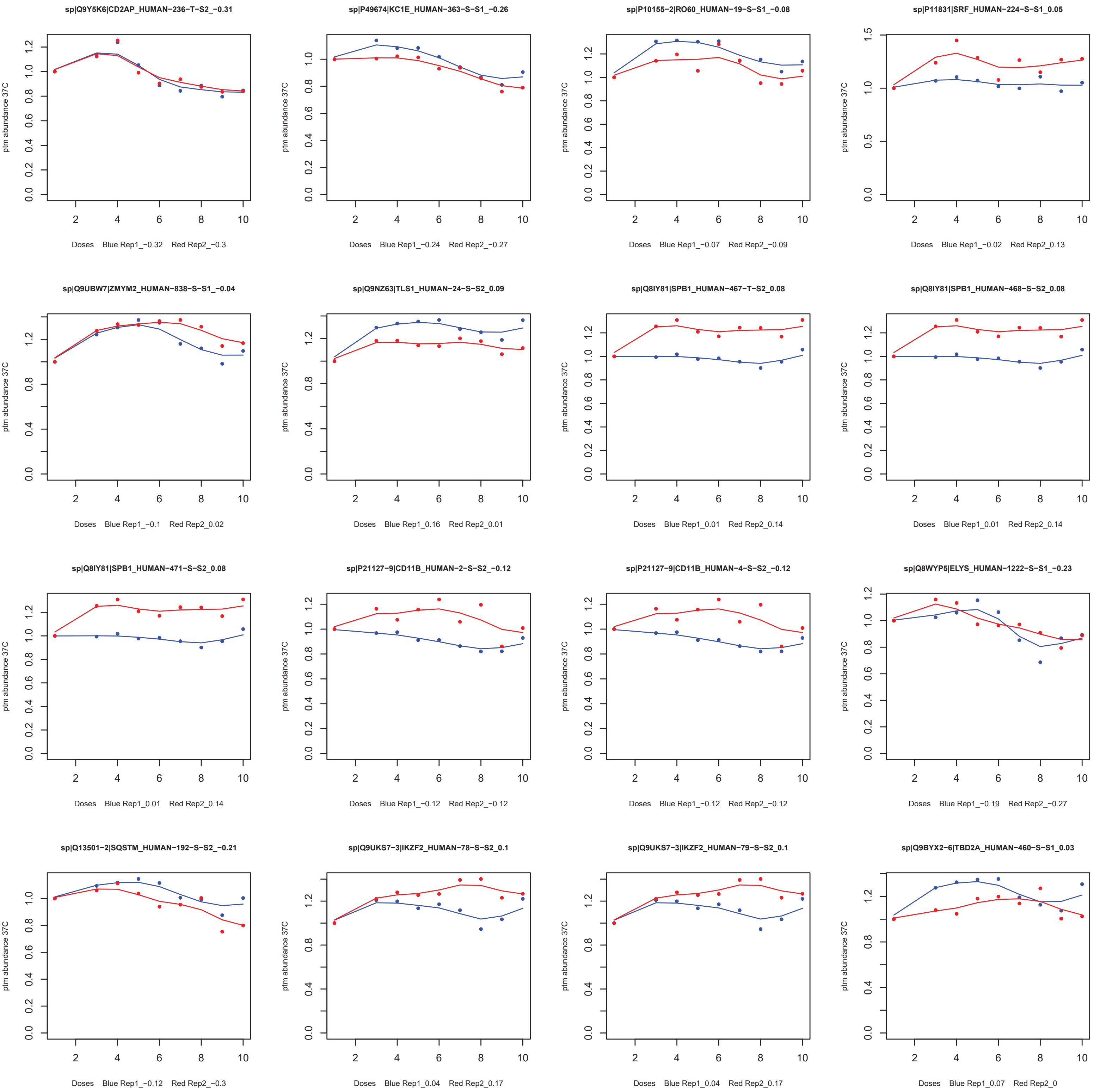
sp|Q9Y5K6|CD2AP_HUMAN−236−T−S2_−0.31
Doses Blue Rep1_−0.32 Red Rep2_−0.3
sp|P49674|KC1E_HUMAN−363−S−S1_−0.26
Doses Blue Rep1_−0.24 Red Rep2_−0.27
sp|P10155−2|RO60_HUMAN−19−S−S1_−0.08
Doses Blue Rep1_−0.07 Red Rep2_−0.09
sp|P11831|SRF_HUMAN−224−S−S1_0.05
Doses Blue Rep1_−0.02 Red Rep2_0.13
sp|Q9UBW7|ZMYM2_HUMAN−838−S−S1_−0.04
Doses Blue Rep1_−0.1 Red Rep2_0.02
sp|Q9NZ63|TLS1_HUMAN−24−S−S2_0.09
Doses Blue Rep1_0.16 Red Rep2_0.01
sp|Q8IY81|SPB1_HUMAN−467−T−S2_0.08
Doses Blue Rep1_0.01 Red Rep2_0.14
sp|Q8IY81|SPB1_HUMAN−468−S−S2_0.08
Doses Blue Rep1_0.01 Red Rep2_0.14
sp|Q8IY81|SPB1_HUMAN−471−S−S2_0.08
Doses Blue Rep1_0.01 Red Rep2_0.14
sp|P21127−9|CD11B_HUMAN−2−S−S2_−0.12
Doses Blue Rep1_−0.12 Red Rep2_−0.12
sp|P21127−9|CD11B_HUMAN−4−S−S2_−0.12
Doses Blue Rep1_−0.12 Red Rep2_−0.12
sp|Q8WYP5|ELYS_HUMAN−1222−S−S1_−0.23
Doses Blue Rep1_−0.19 Red Rep2_−0.27
sp|Q13501−2|SQSTM_HUMAN−192−S−S2_−0.21
Doses Blue Rep1_−0.12 Red Rep2_−0.3
sp|Q9UKS7−3|IKZF2_HUMAN−78−S−S2_0.1
Doses Blue Rep1_0.04 Red Rep2_0.17
sp|Q9UKS7−3|IKZF2_HUMAN−79−S−S2_0.1
Doses Blue Rep1_0.04 Red Rep2_0.17
sp|Q9BYX2−6|TBD2A_HUMAN−460−S−S1_0.03
Doses Blue Rep1_0.07 Red Rep2_0
ptm abundance 37C

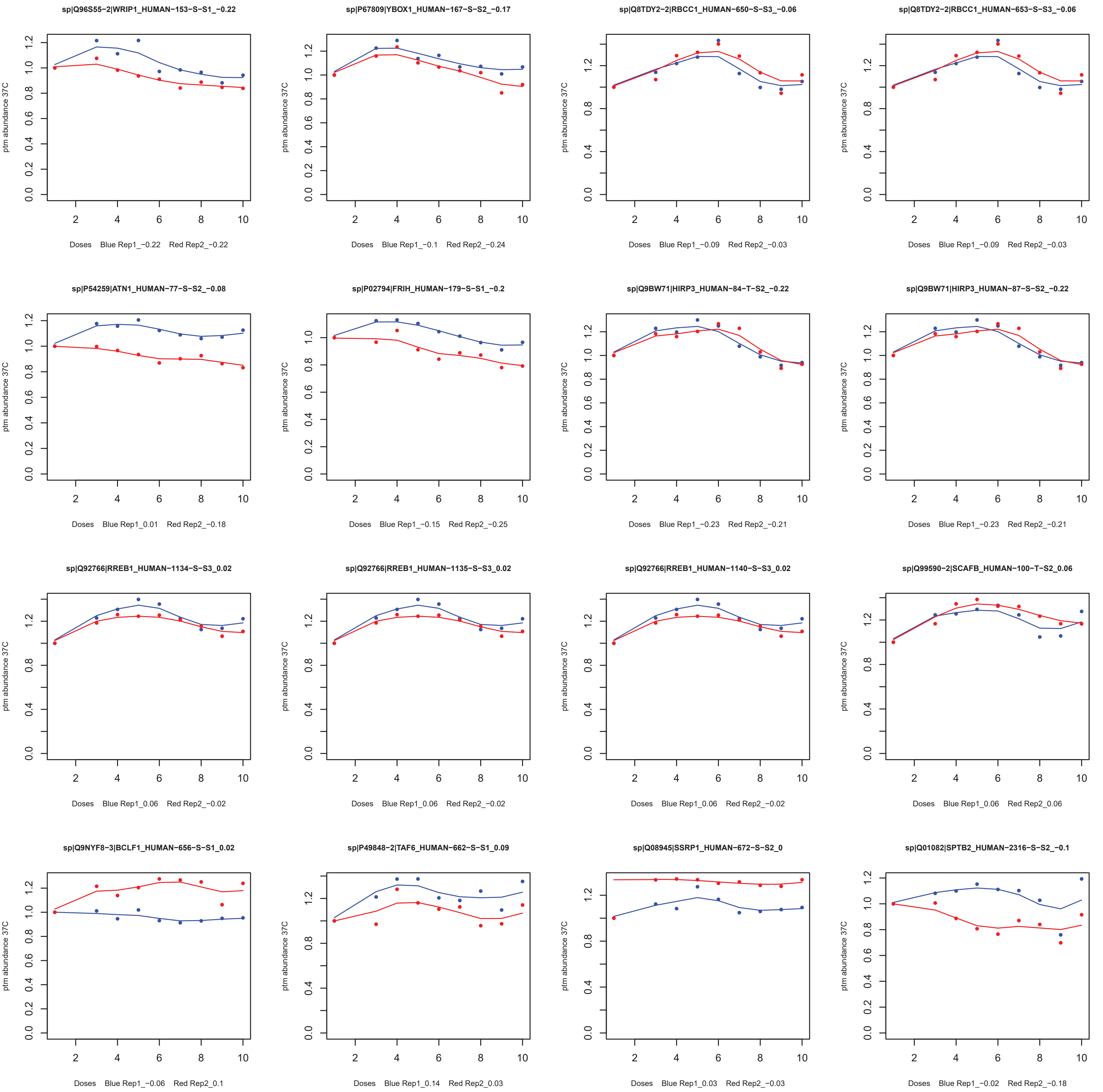

sp|Q96S55−2|WRIP1_HUMAN−153−S−S1_−0.22
Doses Blue Rep1_−0.22 Red Rep2_−0.22
sp|P67809|YBOX1_HUMAN−167−S−S2_−0.17
Doses Blue Rep1_−0.1 Red Rep2_−0.24
sp|Q8TDY2−2|RBCC1_HUMAN−650−S−S3_−0.06
Doses Blue Rep1_−0.09 Red Rep2_−0.03
sp|Q8TDY2−2|RBCC1_HUMAN−653−S−S3_−0.06
Doses Blue Rep1_−0.09 Red Rep2_−0.03
sp|P54259|ATN1_HUMAN−77−S−S2_−0.08
Doses Blue Rep1_0.01 Red Rep2_−0.18
sp|P02794|FRIH_HUMAN−179−S−S1_−0.2
Doses Blue Rep1_−0.15 Red Rep2_−0.25
sp|Q9BW71|HIRP3_HUMAN−84−T−S2_−0.22
Doses Blue Rep1_−0.23 Red Rep2_−0.21
sp|Q9BW71|HIRP3_HUMAN−87−S−S2_−0.22
Doses Blue Rep1_−0.23 Red Rep2_−0.21
sp|Q92766|RREB1_HUMAN−1134−S−S3_0.02
Doses Blue Rep1_0.06 Red Rep2_−0.02
sp|Q92766|RREB1_HUMAN−1135−S−S3_0.02
Doses Blue Rep1_0.06 Red Rep2_−0.02
sp|Q92766|RREB1_HUMAN−1140−S−S3_0.02
Doses Blue Rep1_0.06 Red Rep2_−0.02
sp|Q99590−2|SCAFB_HUMAN−100−T−S2_0.06
Doses Blue Rep1_0.06 Red Rep2_0.06
sp|Q9NYF8−3|BCLF1_HUMAN−656−S−S1_0.02
Doses Blue Rep1_−0.06 Red Rep2_0.1
sp|P49848−2|TAF6_HUMAN−662−S−S1_0.09
Doses Blue Rep1_0.14 Red Rep2_0.03
sp|Q08945|SSRP1_HUMAN−672−S−S2_0
Doses Blue Rep1_0.03 Red Rep2_−0.03
sp|Q01082|SPTB2_HUMAN−2316−S−S2_−0.1
Doses Blue Rep1_−0.02 Red Rep2_−0.18
ptm abundance 37C

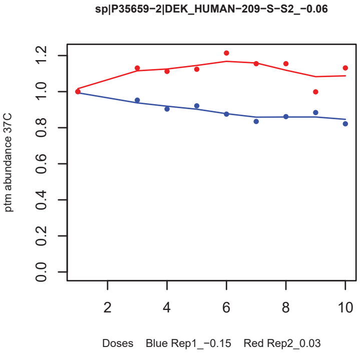
sp|P35659−2|DEK_HUMAN−209−S−S2_−0.06
ptm abundance 37C
Doses Blue Rep1_−0.15 Red Rep2_0.03

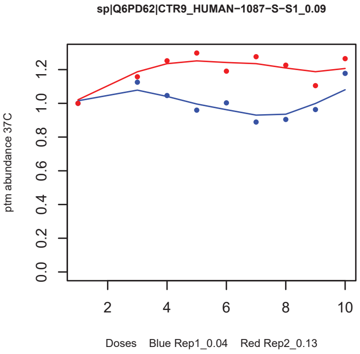
sp|Q6PD62|CTR9_HUMAN−1087−S−S1_0.09
ptm abundance 37C
Doses Blue Rep1_0.04 Red Rep2_0.13

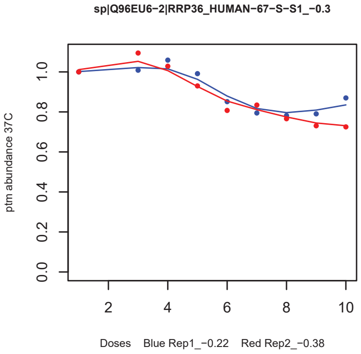
sp|Q96EU6−2|RRP36_HUMAN−67−S−S1_−0.3
ptm abundance 37C
Doses Blue Rep1_−0.22 Red Rep2_−0.38

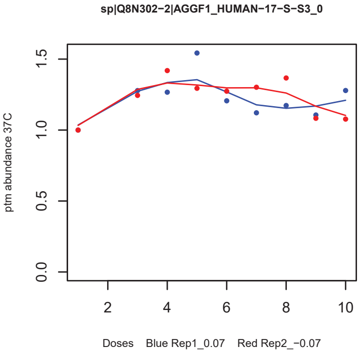
sp|Q8N302−2|AGGF1_HUMAN−17−S−S3_0
ptm abundance 37C
Doses Blue Rep1_0.07 Red Rep2_−0.07

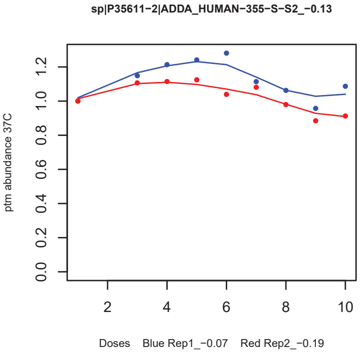
sp|P35611−2|ADDA_HUMAN−355−S−S2_−0.13
ptm abundance 37C
Doses Blue Rep1_−0.07 Red Rep2_−0.19

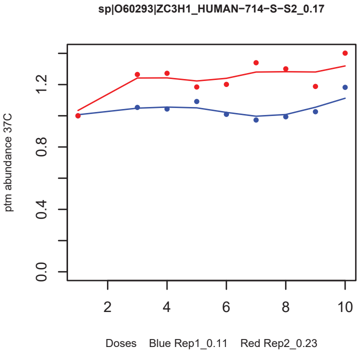
sp|O60293|ZC3H1_HUMAN−714−S−S2_0.17
ptm abundance 37C
Doses Blue Rep1_0.11 Red Rep2_0.23

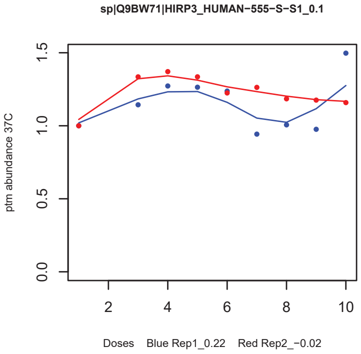
sp|Q9BW71|HIRP3_HUMAN−555−S−S1_0.1
ptm abundance 37C
Doses Blue Rep1_0.22 Red Rep2_−0.02

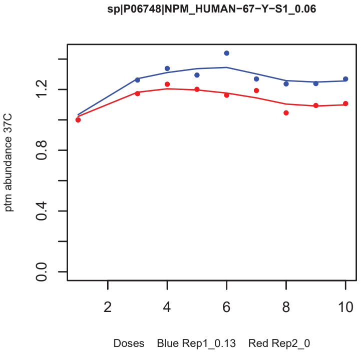
sp|P06748|NPM_HUMAN−67−Y−S1_0.06
ptm abundance 37C
Doses Blue Rep1_0.13 Red Rep2_0

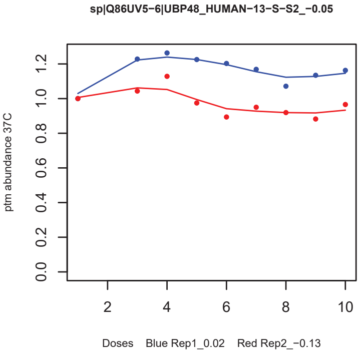
sp|Q86UV5−6|UBP48_HUMAN−13−S−S2_−0.05
ptm abundance 37C
Doses Blue Rep1_0.02 Red Rep2_−0.13

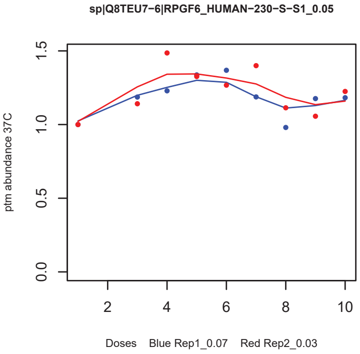
sp|Q8TEU7−6|RPGF6_HUMAN−230−S−S1_0.05
ptm abundance 37C
Doses Blue Rep1_0.07 Red Rep2_0.03

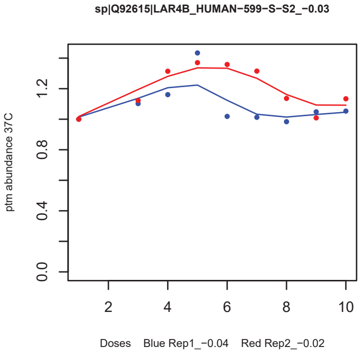
sp|Q92615|LAR4B_HUMAN−599−S−S2_−0.03
ptm abundance 37C
Doses Blue Rep1_−0.04 Red Rep2_−0.02

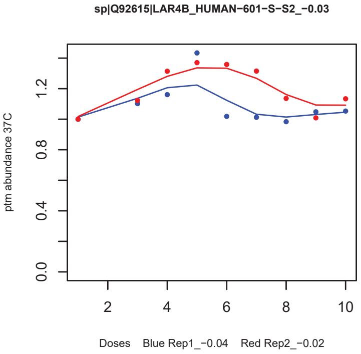
sp|Q92615|LAR4B_HUMAN−601−S−S2_−0.03
ptm abundance 37C
Doses Blue Rep1_−0.04 Red Rep2_−0.02

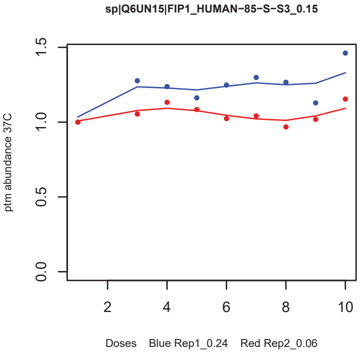
sp|Q6UN15|FIP1_HUMAN−85−S−S3_0.15
ptm abundance 37C
Doses Blue Rep1_0.24 Red Rep2_0.06

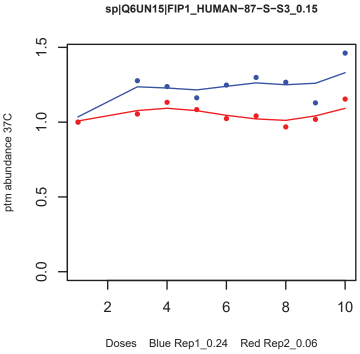
sp|Q6UN15|FIP1_HUMAN−87−S−S3_0.15
ptm abundance 37C
Doses Blue Rep1_0.24 Red Rep2_0.06

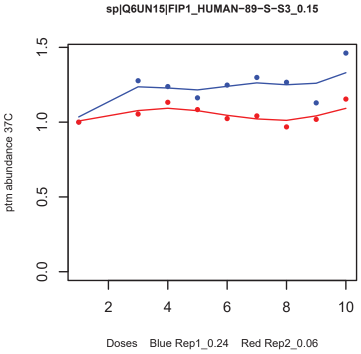
sp|Q6UN15|FIP1_HUMAN−89−S−S3_0.15
ptm abundance 37C
Doses Blue Rep1_0.24 Red Rep2_0.06

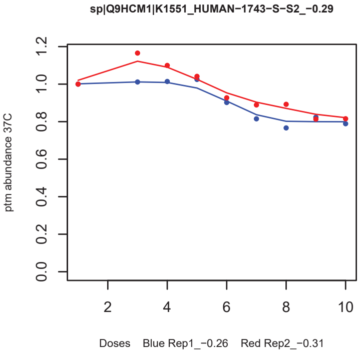
sp|Q9HCM1|K1551_HUMAN−1743−S−S2_−0.29
ptm abundance 37C
Doses Blue Rep1_−0.26 Red Rep2_−0.31

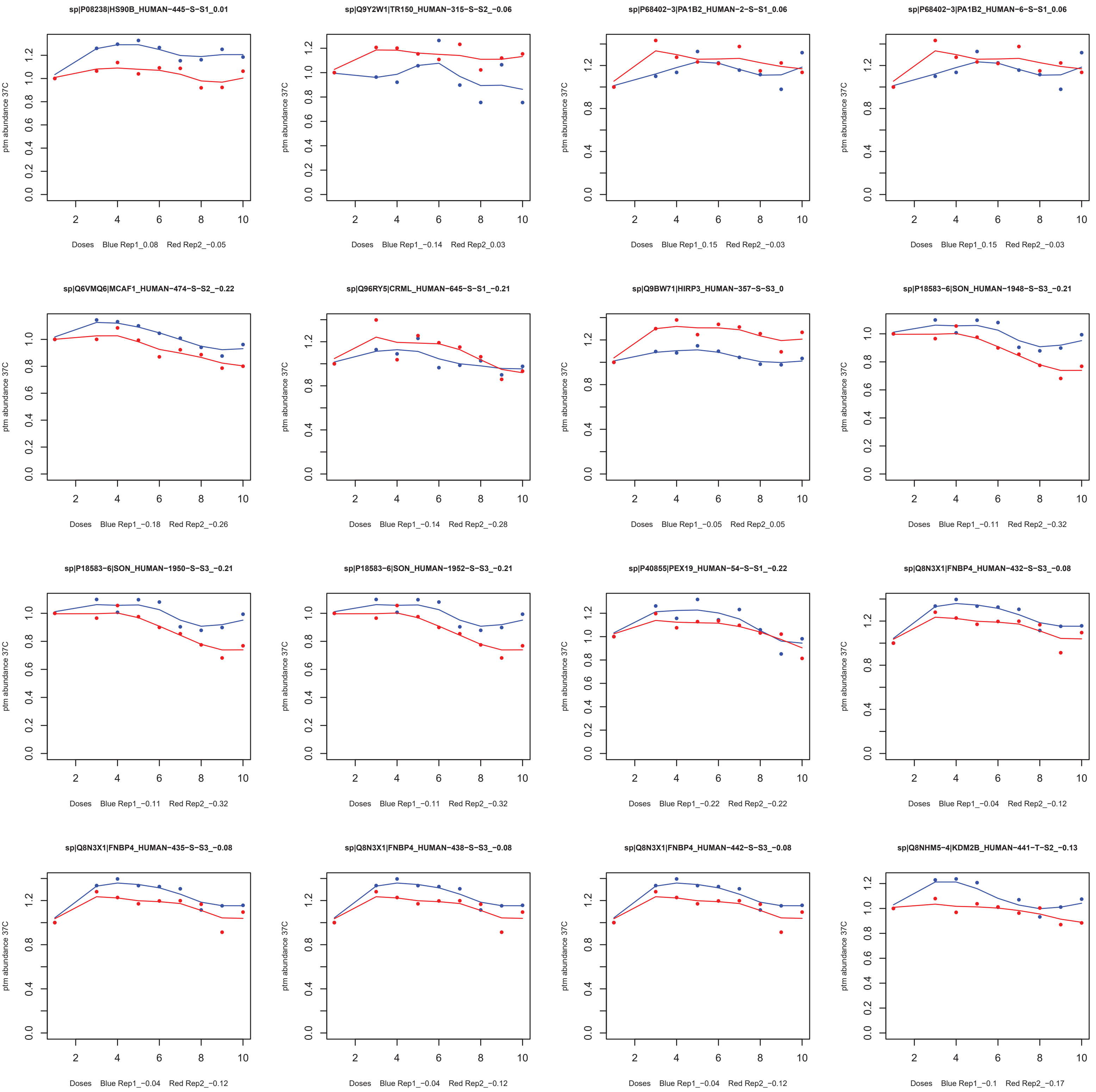
sp|P08238|HS90B_HUMAN-445-S-S1_0.01
ptm abundance 37C
Doses Blue Rep1_0.08 Red Rep2_-0.05
sp|Q9Y2W1|TR150_HUMAN-315-S-S2_-0.06
ptm abundance 37C
Doses Blue Rep1_-0.14 Red Rep2_0.03
sp|P68402-3|PA1B2_HUMAN-2-S-S1_0.06
ptm abundance 37C
Doses Blue Rep1_0.15 Red Rep2_-0.03
sp|P68402-3|PA1B2_HUMAN-6-S-S1_0.06
ptm abundance 37C
Doses Blue Rep1_0.15 Red Rep2_-0.03
sp|Q6VMQ6|MCAF1_HUMAN-474-S-S2_-0.22
ptm abundance 37C
Doses Blue Rep1_-0.18 Red Rep2_-0.26
sp|Q96RY5|CRML_HUMAN-645-S-S1_-0.21
ptm abundance 37C
Doses Blue Rep1_-0.14 Red Rep2_-0.28
sp|Q9BW71|HIRP3_HUMAN-357-S-S3_0
ptm abundance 37C
Doses Blue Rep1_-0.05 Red Rep2_0.05
sp|P18583-6|SON_HUMAN-1948-S-S3_-0.21
ptm abundance 37C
Doses Blue Rep1_-0.11 Red Rep2_-0.32
sp|P18583-6|SON_HUMAN-1950-S-S3_-0.21
ptm abundance 37C
Doses Blue Rep1_-0.11 Red Rep2_-0.32
sp|P18583-6|SON_HUMAN-1952-S-S3_-0.21
ptm abundance 37C
Doses Blue Rep1_-0.11 Red Rep2_-0.32
sp|P40855|PEX19_HUMAN-54-S-S1_-0.22
ptm abundance 37C
Doses Blue Rep1_-0.22 Red Rep2_-0.22
sp|Q8N3X1|FNBP4_HUMAN-432-S-S3_-0.08
ptm abundance 37C
Doses Blue Rep1_-0.04 Red Rep2_-0.12
sp|Q8N3X1|FNBP4_HUMAN-435-S-S3_-0.08
ptm abundance 37C
Doses Blue Rep1_-0.04 Red Rep2_-0.12
sp|Q8N3X1|FNBP4_HUMAN-438-S-S3_-0.08
ptm abundance 37C
Doses Blue Rep1_-0.04 Red Rep2_-0.12
sp|Q8N3X1|FNBP4_HUMAN-442-S-S3_-0.08
ptm abundance 37C
Doses Blue Rep1_-0.04 Red Rep2_-0.12
sp|Q8NHM5-4|KDM2B_HUMAN-441-T-S2_-0.13
ptm abundance 37C
Doses Blue Rep1_-0.1 Red Rep2_-0.17

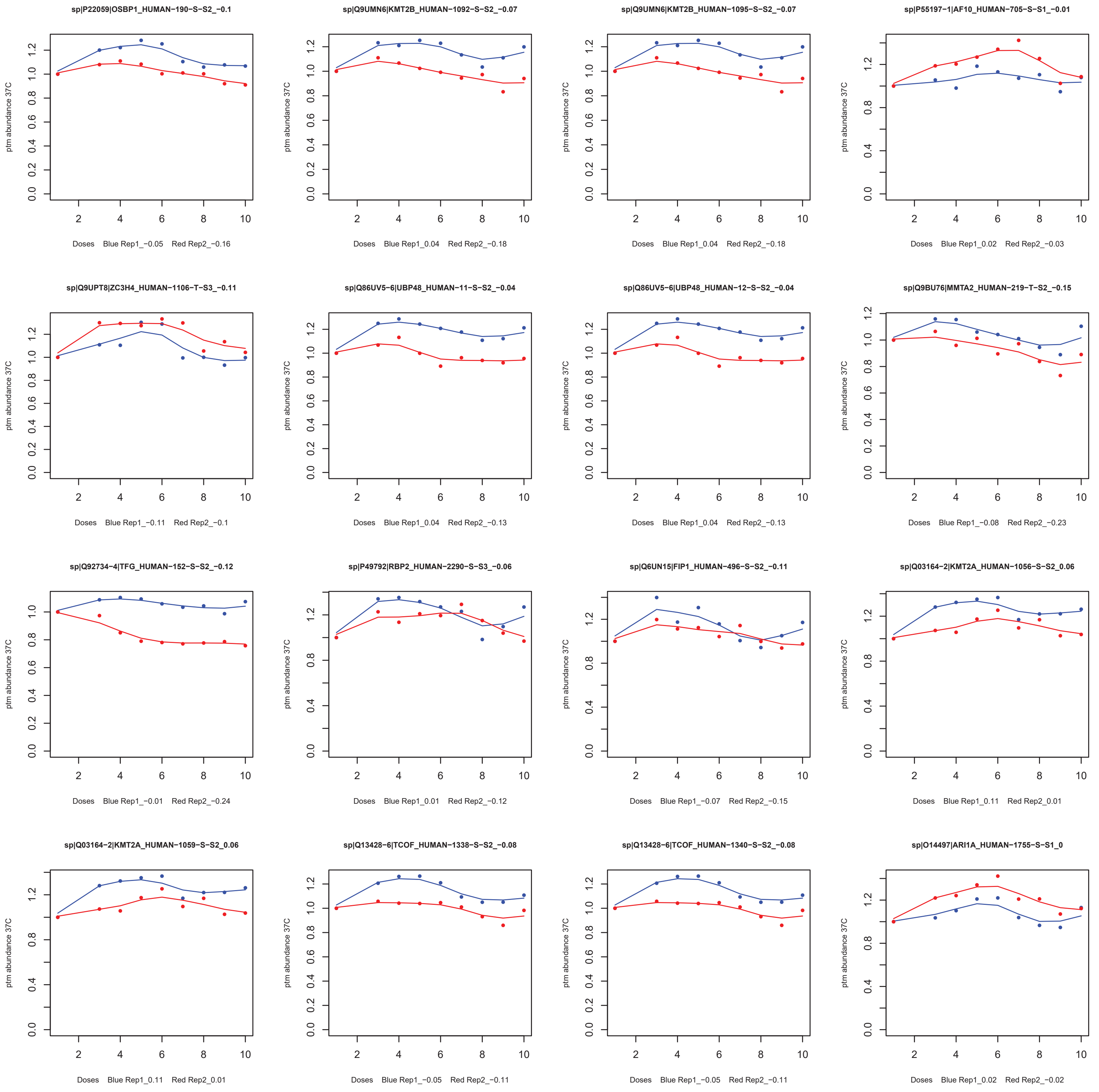
sp|P22059|OSBP1_HUMAN−190−S−S2_−0.1
ptm abundance 37C
Doses Blue Rep1_−0.05 Red Rep2_−0.16
sp|Q9UMN6|KMT2B_HUMAN−1092−S−S2_−0.07
ptm abundance 37C
Doses Blue Rep1_0.04 Red Rep2_−0.18
sp|Q9UMN6|KMT2B_HUMAN−1095−S−S2_−0.07
ptm abundance 37C
Doses Blue Rep1_0.04 Red Rep2_−0.18
sp|P55197−1|AF10_HUMAN−705−S−S1_−0.01
ptm abundance 37C
Doses Blue Rep1_0.02 Red Rep2_−0.03
sp|Q9UPT8|ZC3H4_HUMAN−1106−T−S3_−0.11
ptm abundance 37C
Doses Blue Rep1_−0.11 Red Rep2_−0.1
sp|Q86UV5−6|UBP48_HUMAN−11−S−S2_−0.04
ptm abundance 37C
Doses Blue Rep1_0.04 Red Rep2_−0.13
sp|Q86UV5−6|UBP48_HUMAN−12−S−S2_−0.04
ptm abundance 37C
Doses Blue Rep1_0.04 Red Rep2_−0.13
sp|Q9BU76|MMTA2_HUMAN−219−T−S2_−0.15
ptm abundance 37C
Doses Blue Rep1_−0.08 Red Rep2_−0.23
sp|Q92734−4|TFG_HUMAN−152−S−S2_−0.12
ptm abundance 37C
Doses Blue Rep1_−0.01 Red Rep2_−0.24
sp|P49792|RBP2_HUMAN−2290−S−S3_−0.06
ptm abundance 37C
Doses Blue Rep1_0.01 Red Rep2_−0.12
sp|Q6UN15|FIP1_HUMAN−496−S−S2_−0.11
ptm abundance 37C
Doses Blue Rep1_−0.07 Red Rep2_−0.15
sp|Q03164−2|KMT2A_HUMAN−1056−S−S2_0.06
ptm abundance 37C
Doses Blue Rep1_0.11 Red Rep2_0.01
sp|Q03164−2|KMT2A_HUMAN−1059−S−S2_0.06
ptm abundance 37C
Doses Blue Rep1_0.11 Red Rep2_0.01
sp|Q13428−6|TCOF_HUMAN−1338−S−S2_−0.08
ptm abundance 37C
Doses Blue Rep1_−0.05 Red Rep2_−0.11
sp|Q13428−6|TCOF_HUMAN−1340−S−S2_−0.08
ptm abundance 37C
Doses Blue Rep1_−0.05 Red Rep2_−0.11
sp|O14497|ARI1A_HUMAN−1755−S−S1_0
ptm abundance 37C
Doses Blue Rep1_0.02 Red Rep2_−0.02

sp|P30622−2|CLIP1_HUMAN−200−S−S2_−0.26

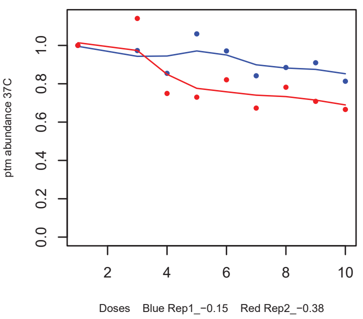

sp|P30622−2|CLIP1_HUMAN−204−S−S2_−0.26

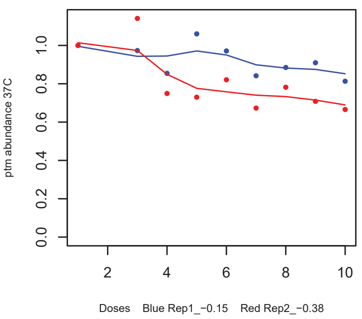

sp|Q6IN85−4|P4R3A_HUMAN−529−S−S2_−0.25

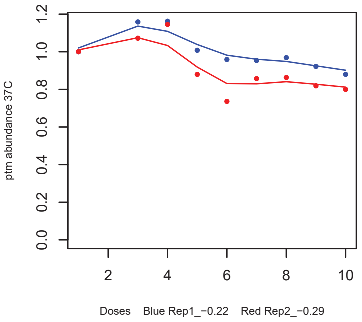

sp|Q68D20|PMS2L_HUMAN−161−S−S2_−0.06

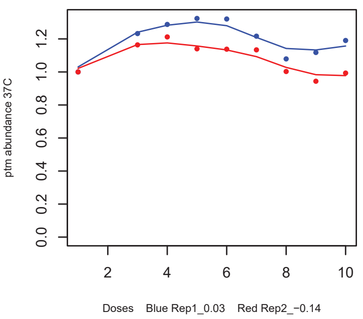

sp|O95696|BRD1_HUMAN−1052−S−S2_0.07

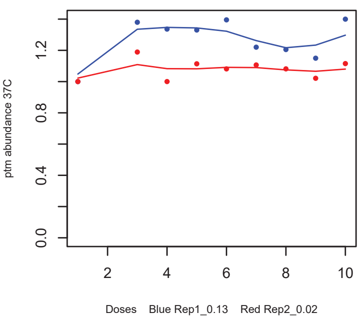

sp|Q9NYF8−3|BCLF1_HUMAN−510−S−S1_0.13

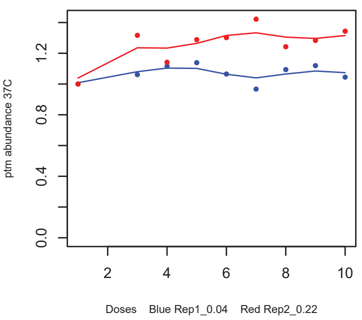

sp|Q5VUA4|ZN318_HUMAN−1971−S−S1_−0.16

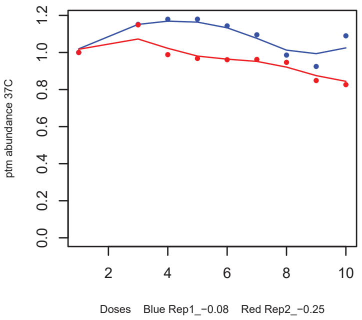

sp|P49450−2|CENPA_HUMAN−19−S−S2_−0.1

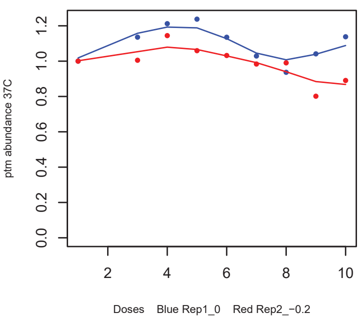

sp|Q9Y5K6|CD2AP_HUMAN−231−T−S2_−0.3

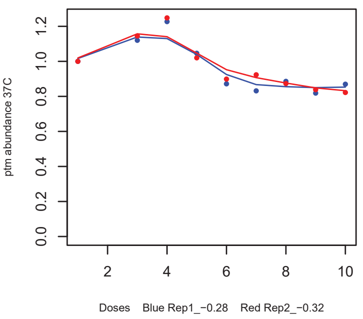

sp|Q9BSE4|HERP2_HUMAN−93−S−S2_−0.26

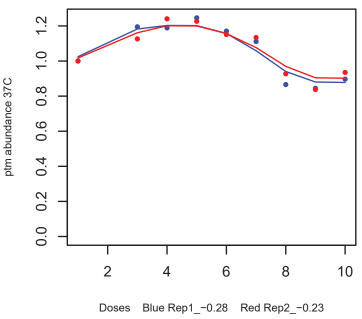

sp|Q9NYV4−2|CDK12_HUMAN−238−S−S2_−0.2

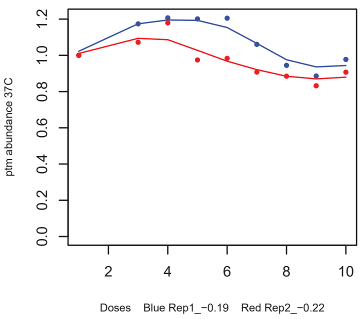

sp|Q96T58|MINT_HUMAN−2120−S−S1_−0.08

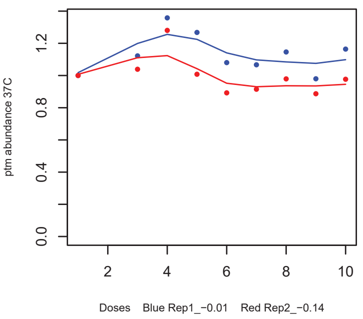

sp|Q15032−2|R3HD1_HUMAN−355−S−S2_0.06

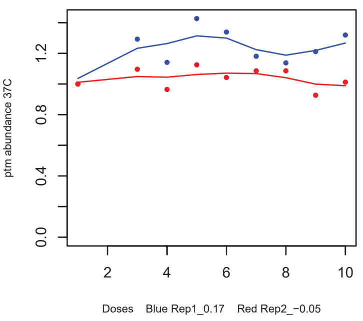

sp|Q15032−2|R3HD1_HUMAN−360−S−S2_0.06

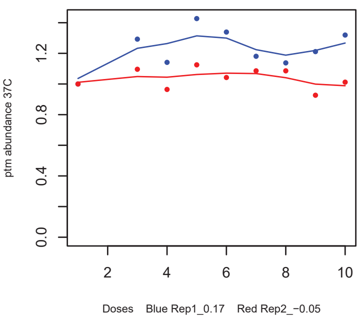

sp|P43243|MATR3_HUMAN−596−S−S2_0.23

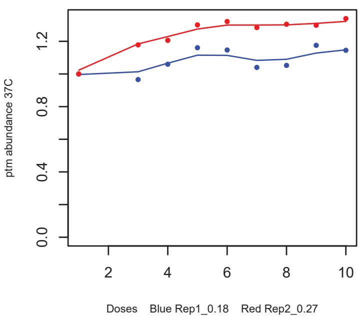

sp|Q9Y2W2|WBP11_HUMAN−353−S−S3_0.16

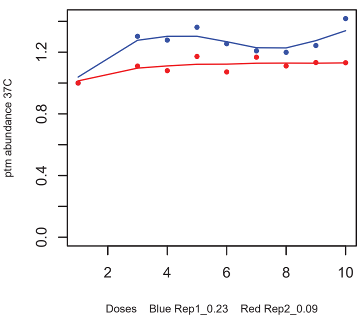

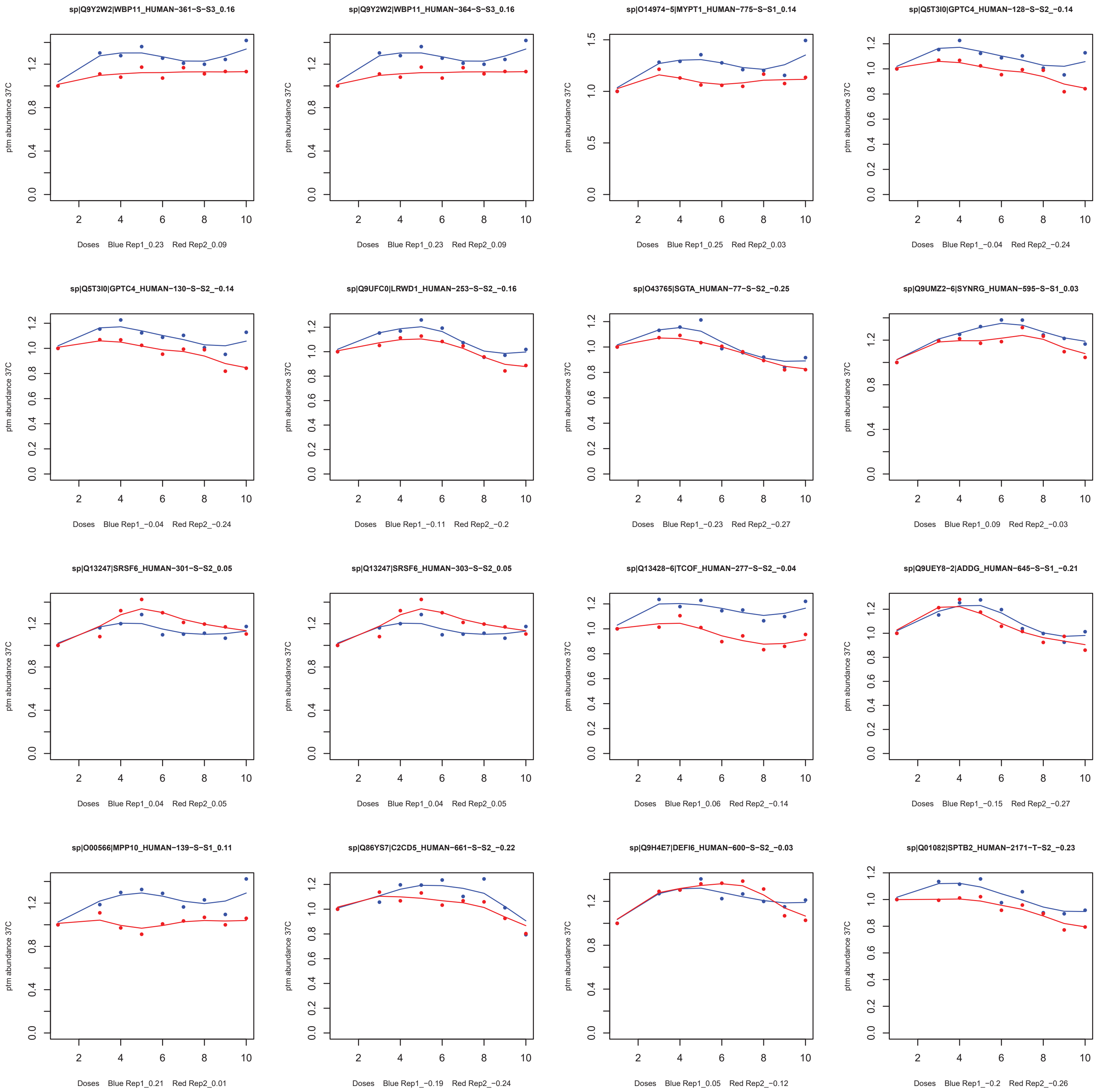

sp|Q9Y2W2|WBP11_HUMAN−361−S−S3_0.16
ptm abundance 37C
Doses Blue Rep1_0.23 Red Rep2_0.09
sp|Q9Y2W2|WBP11_HUMAN−364−S−S3_0.16
ptm abundance 37C
Doses Blue Rep1_0.23 Red Rep2_0.09
sp|O14974−5|MYPT1_HUMAN−775−S−S1_0.14
ptm abundance 37C
Doses Blue Rep1_0.25 Red Rep2_0.03
sp|Q5T3I0|GPTC4_HUMAN−128−S−S2_−0.14
ptm abundance 37C
Doses Blue Rep1_−0.04 Red Rep2_−0.24
sp|Q5T3I0|GPTC4_HUMAN−130−S−S2_−0.14
ptm abundance 37C
Doses Blue Rep1_−0.04 Red Rep2_−0.24
sp|Q9UFC0|LRWD1_HUMAN−253−S−S2_−0.16
ptm abundance 37C
Doses Blue Rep1_−0.11 Red Rep2_−0.2
sp|O43765|SGTA_HUMAN−77−S−S2_−0.25
ptm abundance 37C
Doses Blue Rep1_−0.23 Red Rep2_−0.27
sp|Q9UMZ2−6|SYNRG_HUMAN−595−S−S1_0.03
ptm abundance 37C
Doses Blue Rep1_0.09 Red Rep2_−0.03
sp|Q13247|SRSF6_HUMAN−301−S−S2_0.05
ptm abundance 37C
Doses Blue Rep1_0.04 Red Rep2_0.05
sp|Q13247|SRSF6_HUMAN−303−S−S2_0.05
ptm abundance 37C
Doses Blue Rep1_0.04 Red Rep2_0.05
sp|Q13428−6|TCOF_HUMAN−277−S−S2_−0.04
ptm abundance 37C
Doses Blue Rep1_0.06 Red Rep2_−0.14
sp|Q9UEY8−2|ADDG_HUMAN−645−S−S1_−0.21
ptm abundance 37C
Doses Blue Rep1_−0.15 Red Rep2_−0.27
sp|O00566|MPP10_HUMAN−139−S−S1_0.11
ptm abundance 37C
Doses Blue Rep1_0.21 Red Rep2_0.01
sp|Q86YS7|C2CD5_HUMAN−661−S−S2_−0.22
ptm abundance 37C
Doses Blue Rep1_−0.19 Red Rep2_−0.24
sp|Q9H4E7|DEFI6_HUMAN−600−S−S2_−0.03
ptm abundance 37C
Doses Blue Rep1_0.05 Red Rep2_−0.12
sp|Q01082|SPTB2_HUMAN−2171−T−S2_−0.23
ptm abundance 37C
Doses Blue Rep1_−0.2 Red Rep2_−0.26

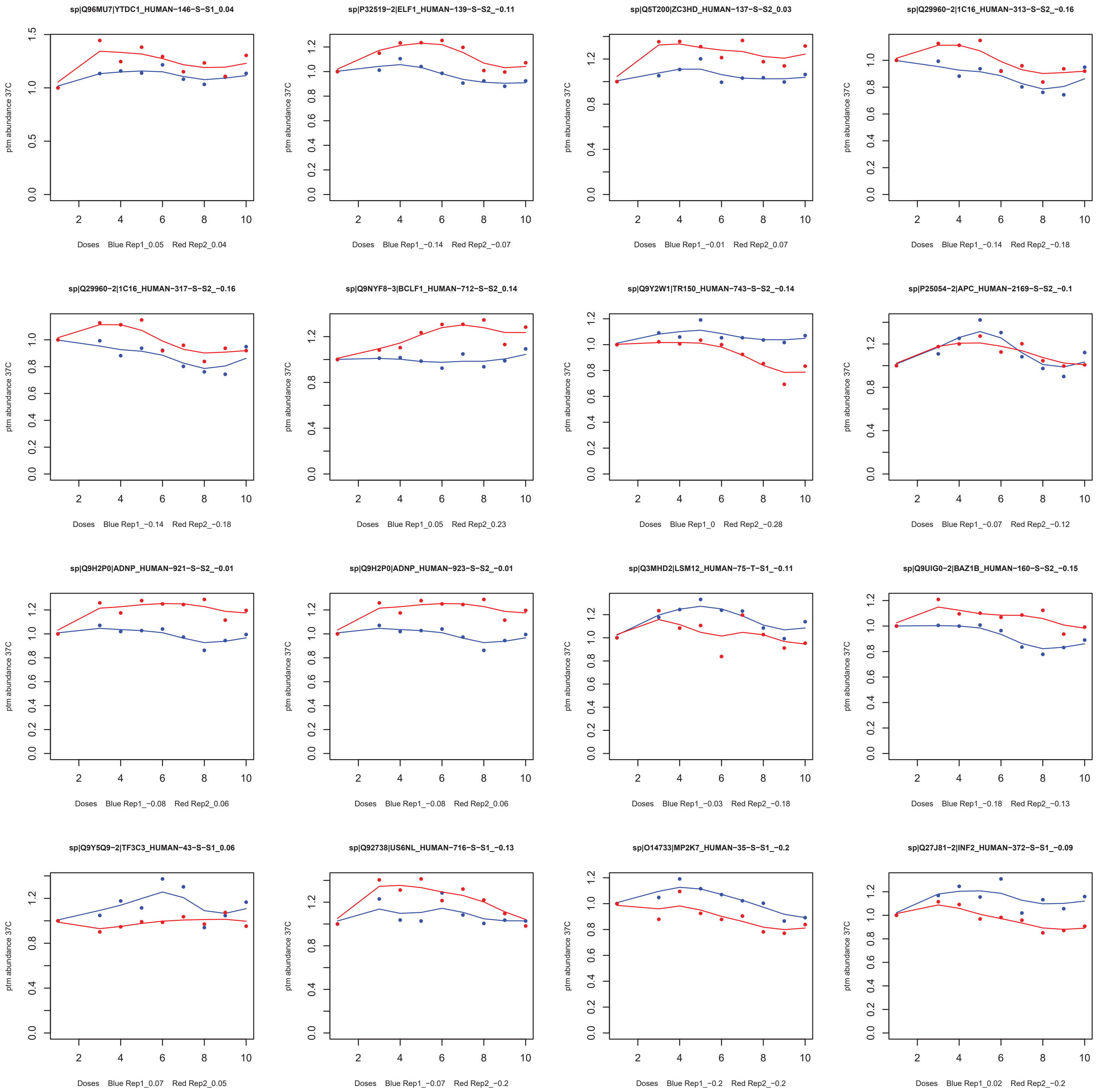
sp|Q96MU7|YTDC1_HUMAN−146−S−S1_0.04
Doses Blue Rep1_0.05 Red Rep2_0.04
sp|P32519−2|ELF1_HUMAN−139−S−S2_−0.11
Doses Blue Rep1_−0.14 Red Rep2_−0.07
sp|Q5T200|ZC3HD_HUMAN−137−S−S2_0.03
Doses Blue Rep1_−0.01 Red Rep2_0.07
sp|Q29960−2|1C16_HUMAN−313−S−S2_−0.16
Doses Blue Rep1_−0.14 Red Rep2_−0.18
sp|Q29960−2|1C16_HUMAN−317−S−S2_−0.16
Doses Blue Rep1_−0.14 Red Rep2_−0.18
sp|Q9NYF8−3|BCLF1_HUMAN−712−S−S2_0.14
Doses Blue Rep1_0.05 Red Rep2_0.23
sp|Q9Y2W1|TR150_HUMAN−743−S−S2_−0.14
Doses Blue Rep1_0 Red Rep2_−0.28
sp|P25054−2|APC_HUMAN−2169−S−S2_−0.1
Doses Blue Rep1_−0.07 Red Rep2_−0.12
sp|Q9H2P0|ADNP_HUMAN−921−S−S2_−0.01
Doses Blue Rep1_−0.08 Red Rep2_0.06
sp|Q9H2P0|ADNP_HUMAN−923−S−S2_−0.01
Doses Blue Rep1_−0.08 Red Rep2_0.06
sp|Q3MHD2|LSM12_HUMAN−75−T−S1_−0.11
Doses Blue Rep1_−0.03 Red Rep2_−0.18
sp|Q9UIG0−2|BAZ1B_HUMAN−160−S−S2_−0.15
Doses Blue Rep1_−0.18 Red Rep2_−0.13
sp|Q9Y5Q9−2|TF3C3_HUMAN−43−S−S1_0.06
Doses Blue Rep1_0.07 Red Rep2_0.05
sp|Q92738|US6NL_HUMAN−716−S−S1_−0.13
Doses Blue Rep1_−0.07 Red Rep2_−0.2
sp|O14733|MP2K7_HUMAN−35−S−S1_−0.2
Doses Blue Rep1_−0.2 Red Rep2_−0.2
sp|Q27J81−2|INF2_HUMAN−372−S−S1_−0.09
Doses Blue Rep1_0.02 Red Rep2_−0.2
ptm abundance 37C

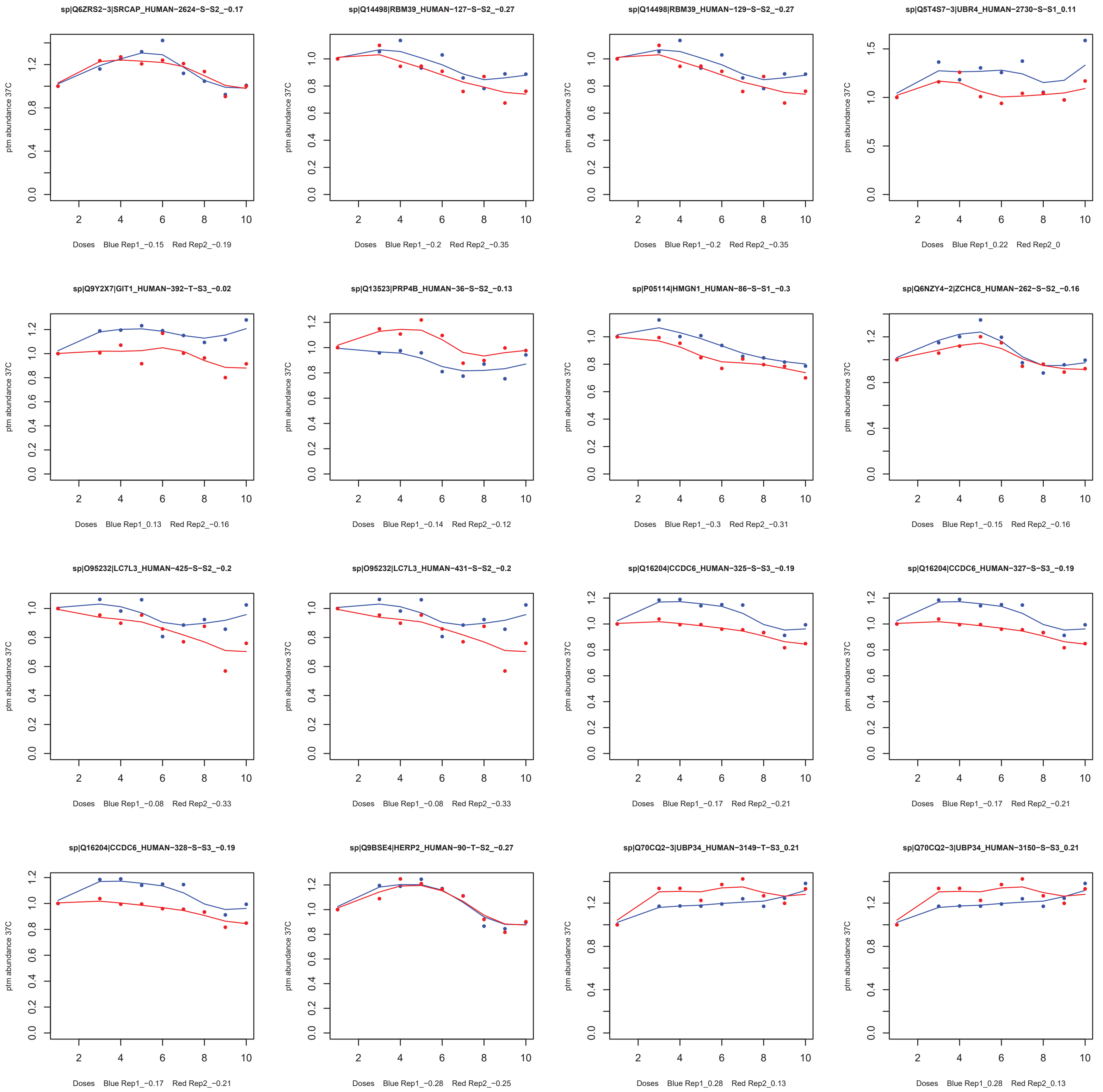

sp|Q6ZRS2−3|SRCAP_HUMAN−2624−S−S2_−0.17
ptm abundance 37C
Doses Blue Rep1_−0.15 Red Rep2_−0.19
sp|Q14498|RBM39_HUMAN−127−S−S2_−0.27
ptm abundance 37C
Doses Blue Rep1_−0.2 Red Rep2_−0.35
sp|Q14498|RBM39_HUMAN−129−S−S2_−0.27
ptm abundance 37C
Doses Blue Rep1_−0.2 Red Rep2_−0.35
sp|Q5T4S7−3|UBR4_HUMAN−2730−S−S1_0.11
ptm abundance 37C
Doses Blue Rep1_0.22 Red Rep2_0
sp|Q9Y2X7|GIT1_HUMAN−392−T−S3_−0.02
ptm abundance 37C
Doses Blue Rep1_0.13 Red Rep2_−0.16
sp|Q13523|PRP4B_HUMAN−36−S−S2_−0.13
ptm abundance 37C
Doses Blue Rep1_−0.14 Red Rep2_−0.12
sp|P05114|HMGN1_HUMAN−86−S−S1_−0.3
ptm abundance 37C
Doses Blue Rep1_−0.3 Red Rep2_−0.31
sp|Q6NZY4−2|ZCHC8_HUMAN−262−S−S2_−0.16
ptm abundance 37C
Doses Blue Rep1_−0.15 Red Rep2_−0.16
sp|O95232|LC7L3_HUMAN−425−S−S2_−0.2
ptm abundance 37C
Doses Blue Rep1_−0.08 Red Rep2_−0.33
sp|O95232|LC7L3_HUMAN−431−S−S2_−0.2
ptm abundance 37C
Doses Blue Rep1_−0.08 Red Rep2_−0.33
sp|Q16204|CCDC6_HUMAN−325−S−S3_−0.19
ptm abundance 37C
Doses Blue Rep1_−0.17 Red Rep2_−0.21
sp|Q16204|CCDC6_HUMAN−327−S−S3_−0.19
ptm abundance 37C
Doses Blue Rep1_−0.17 Red Rep2_−0.21
sp|Q16204|CCDC6_HUMAN−328−S−S3_−0.19
ptm abundance 37C
Doses Blue Rep1_−0.17 Red Rep2_−0.21
sp|Q9BSE4|HERP2_HUMAN−90−T−S2_−0.27
ptm abundance 37C
Doses Blue Rep1_−0.28 Red Rep2_−0.25
sp|Q70CQ2−3|UBP34_HUMAN−3149−T−S3_0.21
ptm abundance 37C
Doses Blue Rep1_0.28 Red Rep2_0.13
sp|Q70CQ2−3|UBP34_HUMAN−3150−S−S3_0.21
ptm abundance 37C
Doses Blue Rep1_0.28 Red Rep2_0.13

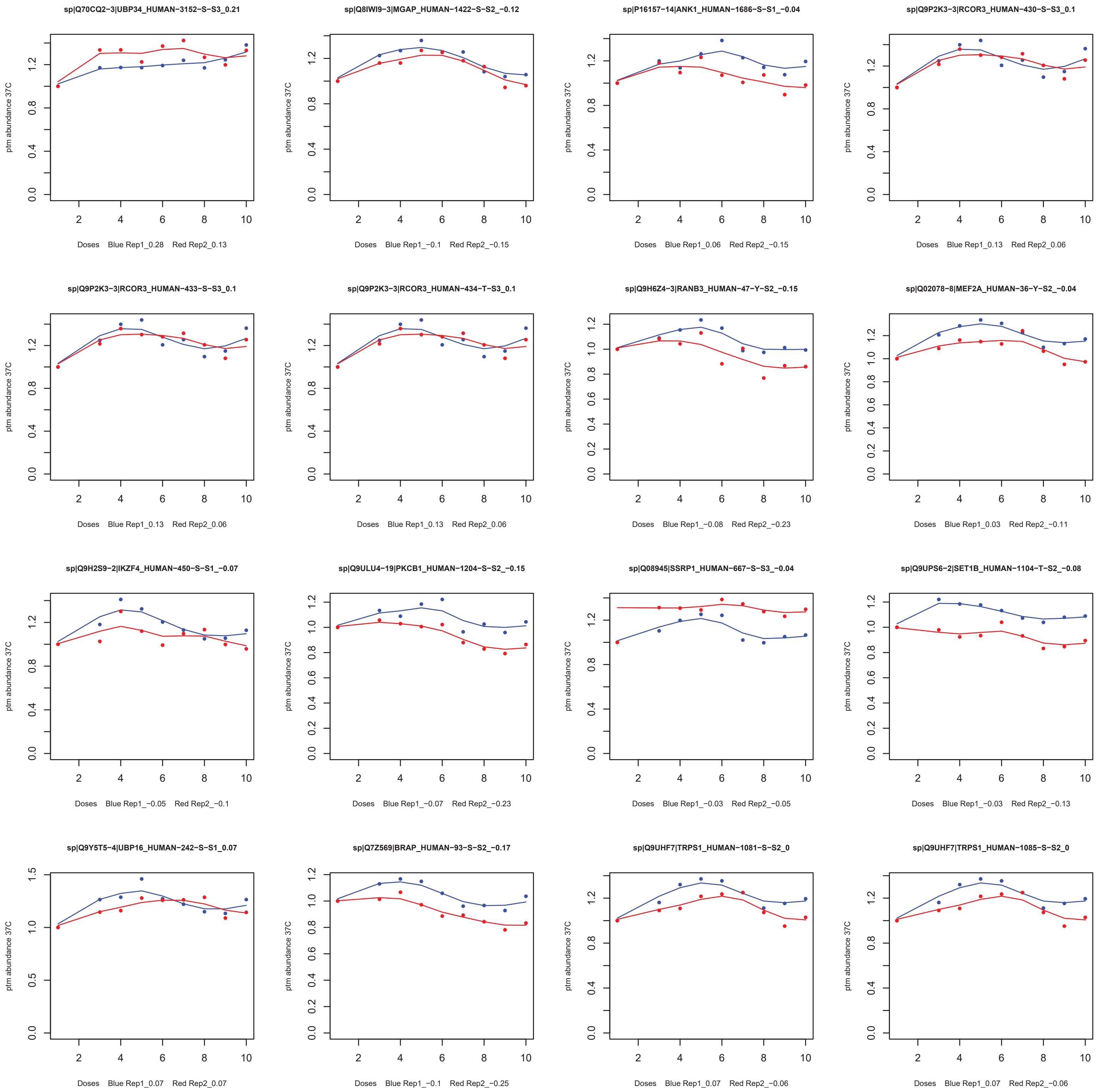
sp|Q70CQ2−3|UBP34_HUMAN−3152−S−S3_0.21
Doses Blue Rep1_0.28 Red Rep2_0.13
sp|Q8IWI9−3|MGAP_HUMAN−1422−S−S2_−0.12
Doses Blue Rep1_−0.1 Red Rep2_−0.15
sp|P16157−14|ANK1_HUMAN−1686−S−S1_−0.04
Doses Blue Rep1_0.06 Red Rep2_−0.15
sp|Q9P2K3−3|RCOR3_HUMAN−430−S−S3_0.1
Doses Blue Rep1_0.13 Red Rep2_0.06
sp|Q9P2K3−3|RCOR3_HUMAN−433−S−S3_0.1
Doses Blue Rep1_0.13 Red Rep2_0.06
sp|Q9P2K3−3|RCOR3_HUMAN−434−T−S3_0.1
Doses Blue Rep1_0.13 Red Rep2_0.06
sp|Q9H6Z4−3|RANB3_HUMAN−47−Y−S2_−0.15
Doses Blue Rep1_−0.08 Red Rep2_−0.23
sp|Q02078−8|MEF2A_HUMAN−36−Y−S2_−0.04
Doses Blue Rep1_0.03 Red Rep2_−0.11
sp|Q9H2S9−2|IKZF4_HUMAN−450−S−S1_−0.07
Doses Blue Rep1_−0.05 Red Rep2_−0.1
sp|Q9ULU4−19|PKCB1_HUMAN−1204−S−S2_−0.15
Doses Blue Rep1_−0.07 Red Rep2_−0.23
sp|Q08945|SSRP1_HUMAN−667−S−S3_−0.04
Doses Blue Rep1_−0.03 Red Rep2_−0.05
sp|Q9UPS6−2|SET1B_HUMAN−1104−T−S2_−0.08
Doses Blue Rep1_−0.03 Red Rep2_−0.13
sp|Q9Y5T5−4|UBP16_HUMAN−242−S−S1_0.07
Doses Blue Rep1_0.07 Red Rep2_0.07
sp|Q7Z569|BRAP_HUMAN−93−S−S2_−0.17
Doses Blue Rep1_−0.1 Red Rep2_−0.25
sp|Q9UHF7|TRPS1_HUMAN−1081−S−S2_0
Doses Blue Rep1_0.07 Red Rep2_−0.06
sp|Q9UHF7|TRPS1_HUMAN−1085−S−S2_0
Doses Blue Rep1_0.07 Red Rep2_−0.06
ptm abundance 37C

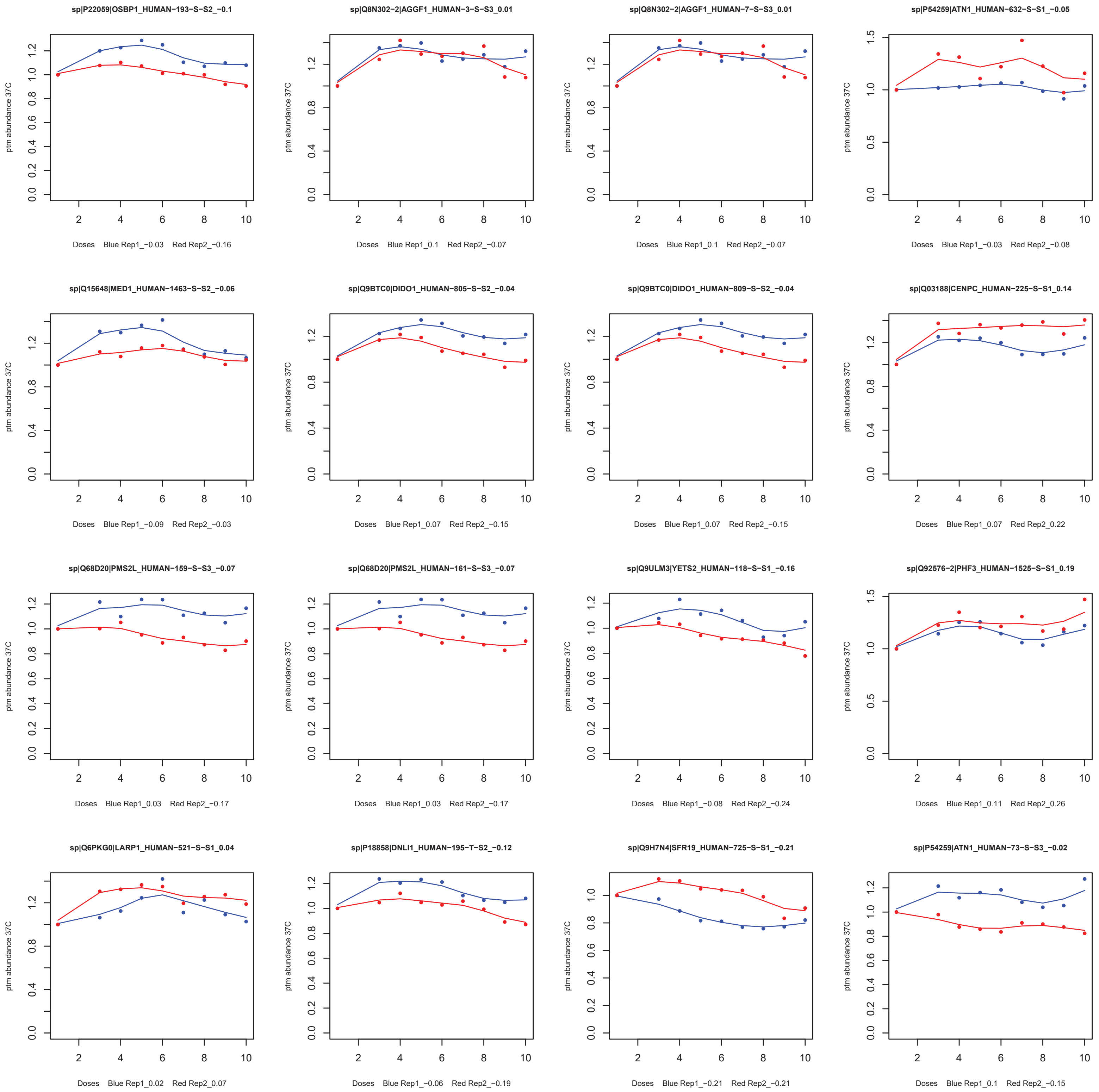

sp|P22059|OSBP1_HUMAN−193−S−S2_−0.1
ptm abundance 37C
Doses Blue Rep1_−0.03 Red Rep2_−0.16
sp|Q8N302−2|AGGF1_HUMAN−3−S−S3_0.01
ptm abundance 37C
Doses Blue Rep1_0.1 Red Rep2_−0.07
sp|Q8N302−2|AGGF1_HUMAN−7−S−S3_0.01
ptm abundance 37C
Doses Blue Rep1_0.1 Red Rep2_−0.07
sp|P54259|ATN1_HUMAN−632−S−S1_−0.05
ptm abundance 37C
Doses Blue Rep1_−0.03 Red Rep2_−0.08
sp|Q15648|MED1_HUMAN−1463−S−S2_−0.06
ptm abundance 37C
Doses Blue Rep1_−0.09 Red Rep2_−0.03
sp|Q9BTC0|DIDO1_HUMAN−805−S−S2_−0.04
ptm abundance 37C
Doses Blue Rep1_0.07 Red Rep2_−0.15
sp|Q9BTC0|DIDO1_HUMAN−809−S−S2_−0.04
ptm abundance 37C
Doses Blue Rep1_0.07 Red Rep2_−0.15
sp|Q03188|CENPC_HUMAN−225−S−S1_0.14
ptm abundance 37C
Doses Blue Rep1_0.07 Red Rep2_0.22
sp|Q68D20|PMS2L_HUMAN−159−S−S3_−0.07
ptm abundance 37C
Doses Blue Rep1_0.03 Red Rep2_−0.17
sp|Q68D20|PMS2L_HUMAN−161−S−S3_−0.07
ptm abundance 37C
Doses Blue Rep1_0.03 Red Rep2_−0.17
sp|Q9ULM3|YETS2_HUMAN−118−S−S1_−0.16
ptm abundance 37C
Doses Blue Rep1_−0.08 Red Rep2_−0.24
sp|Q92576−2|PHF3_HUMAN−1525−S−S1_0.19
ptm abundance 37C
Doses Blue Rep1_0.11 Red Rep2_0.26
sp|Q6PKG0|LARP1_HUMAN−521−S−S1_0.04
ptm abundance 37C
Doses Blue Rep1_0.02 Red Rep2_0.07
sp|P18858|DNLI1_HUMAN−195−T−S2_−0.12
ptm abundance 37C
Doses Blue Rep1_−0.06 Red Rep2_−0.19
sp|Q9H7N4|SFR19_HUMAN−725−S−S1_−0.21
ptm abundance 37C
Doses Blue Rep1_−0.21 Red Rep2_−0.21
sp|P54259|ATN1_HUMAN−73−S−S3_−0.02
ptm abundance 37C
Doses Blue Rep1_0.1 Red Rep2_−0.15

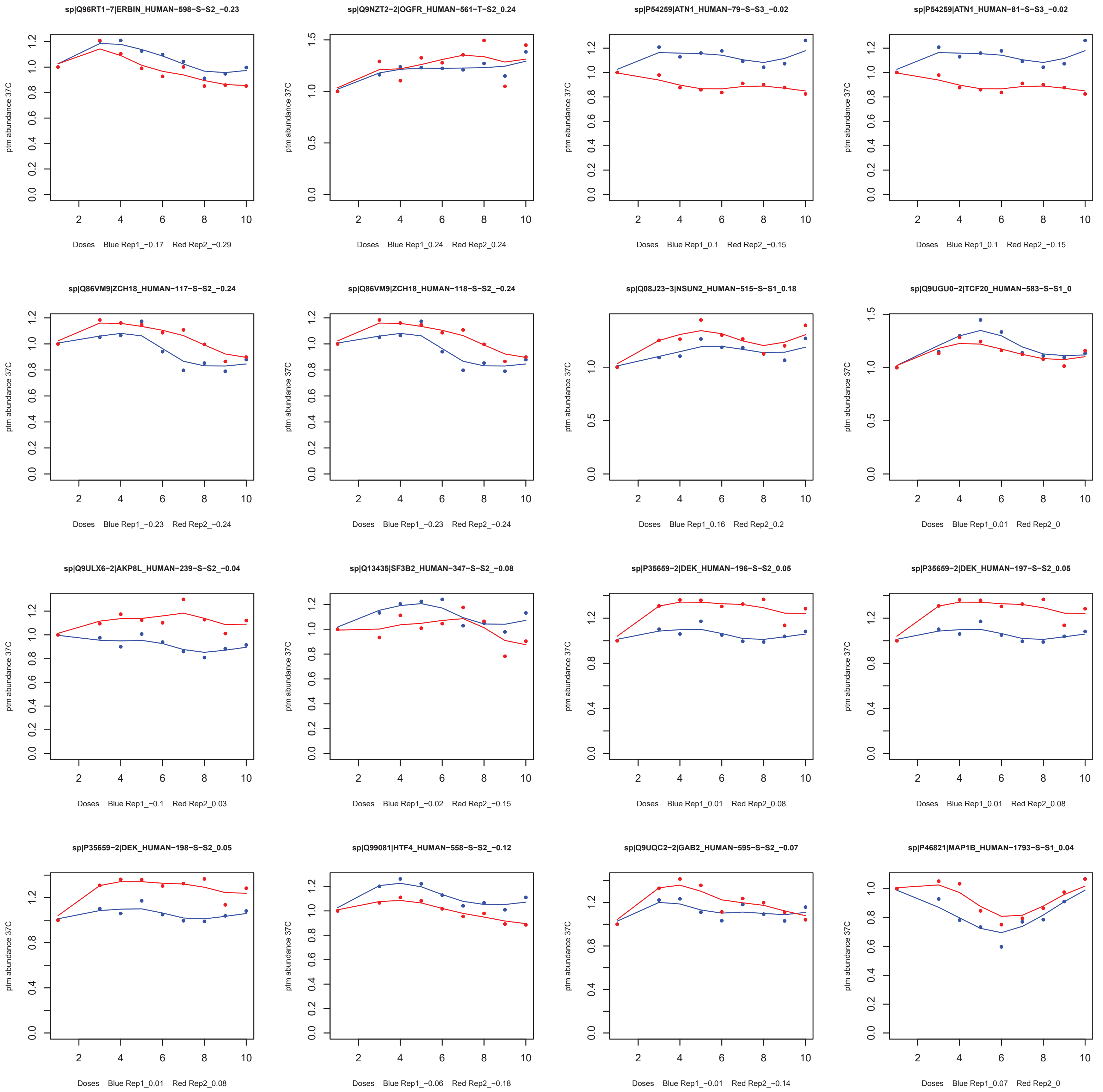
sp|Q96RT1−7|ERBIN_HUMAN−598−S−S2_−0.23
Doses Blue Rep1_−0.17 Red Rep2_−0.29
sp|Q9NZT2−2|OGFR_HUMAN−561−T−S2_0.24
Doses Blue Rep1_0.24 Red Rep2_0.24
sp|P54259|ATN1_HUMAN−79−S−S3_−0.02
Doses Blue Rep1_0.1 Red Rep2_−0.15
sp|P54259|ATN1_HUMAN−81−S−S3_−0.02
Doses Blue Rep1_0.1 Red Rep2_−0.15
sp|Q86VM9|ZCH18_HUMAN−117−S−S2_−0.24
Doses Blue Rep1_−0.23 Red Rep2_−0.24
sp|Q86VM9|ZCH18_HUMAN−118−S−S2_−0.24
Doses Blue Rep1_−0.23 Red Rep2_−0.24
sp|Q08J23−3|NSUN2_HUMAN−515−S−S1_0.18
Doses Blue Rep1_0.16 Red Rep2_0.2
sp|Q9UGU0−2|TCF20_HUMAN−583−S−S1_0
Doses Blue Rep1_0.01 Red Rep2_0
sp|Q9ULX6−2|AKP8L_HUMAN−239−S−S2_−0.04
Doses Blue Rep1_−0.1 Red Rep2_0.03
sp|Q13435|SF3B2_HUMAN−347−S−S2_−0.08
Doses Blue Rep1_−0.02 Red Rep2_−0.15
sp|P35659−2|DEK_HUMAN−196−S−S2_0.05
Doses Blue Rep1_0.01 Red Rep2_0.08
sp|P35659−2|DEK_HUMAN−197−S−S2_0.05
Doses Blue Rep1_0.01 Red Rep2_0.08
sp|P35659−2|DEK_HUMAN−198−S−S2_0.05
Doses Blue Rep1_0.01 Red Rep2_0.08
sp|Q99081|HTF4_HUMAN−558−S−S2_−0.12
Doses Blue Rep1_−0.06 Red Rep2_−0.18
sp|Q9UQC2−2|GAB2_HUMAN−595−S−S2_−0.07
Doses Blue Rep1_−0.01 Red Rep2_−0.14
sp|P46821|MAP1B_HUMAN−1793−S−S1_0.04
Doses Blue Rep1_0.07 Red Rep2_0
ptm abundance 37C

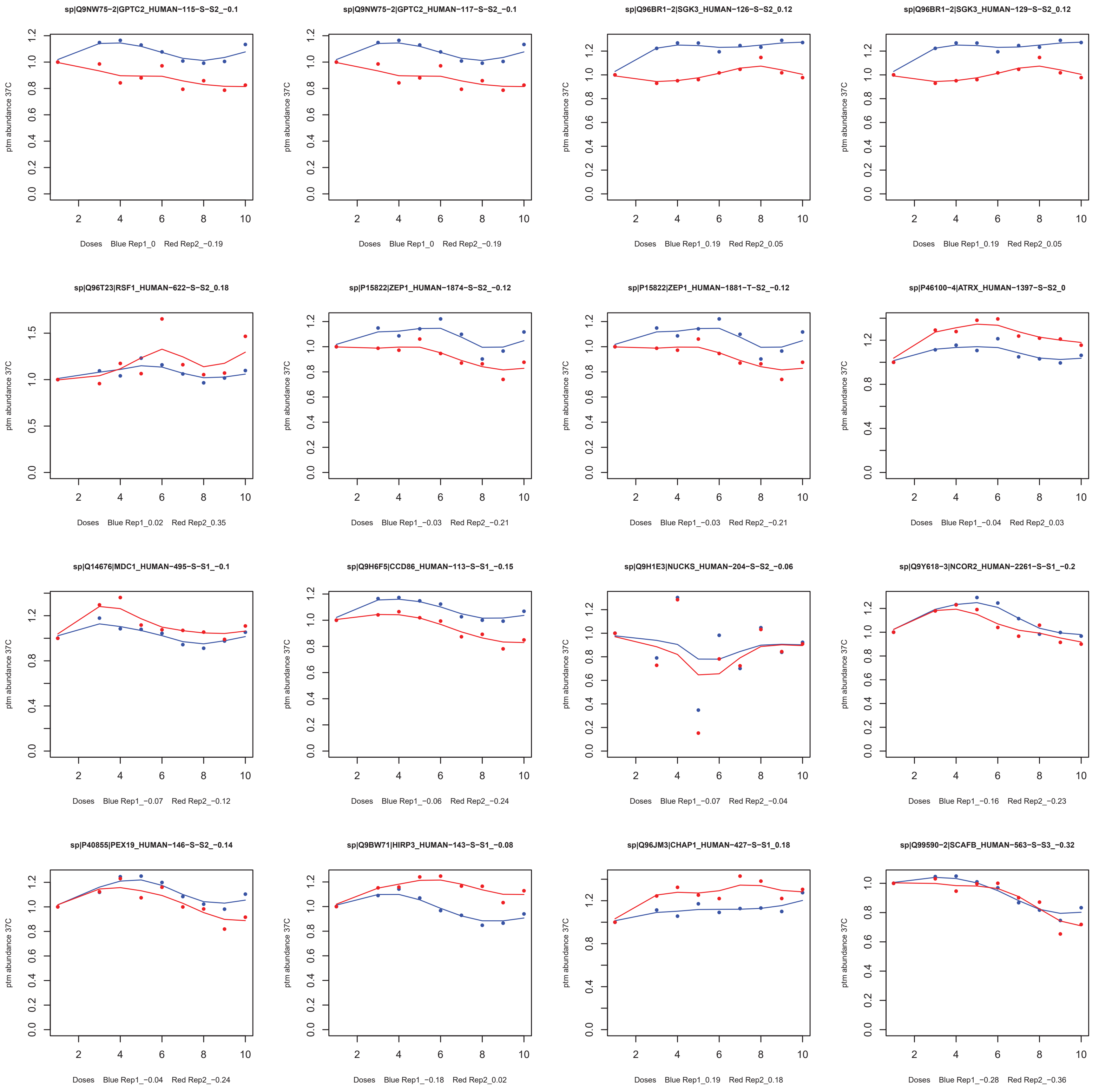

sp|Q9NW75−2|GPTC2_HUMAN−115−S−S2_−0.1
Doses Blue Rep1_0 Red Rep2_−0.19
sp|Q9NW75−2|GPTC2_HUMAN−117−S−S2_−0.1
Doses Blue Rep1_0 Red Rep2_−0.19
sp|Q96BR1−2|SGK3_HUMAN−126−S−S2_0.12
Doses Blue Rep1_0.19 Red Rep2_0.05
sp|Q96BR1−2|SGK3_HUMAN−129−S−S2_0.12
Doses Blue Rep1_0.19 Red Rep2_0.05
sp|Q96T23|RSF1_HUMAN−622−S−S2_0.18
Doses Blue Rep1_0.02 Red Rep2_0.35
sp|P15822|ZEP1_HUMAN−1874−S−S2_−0.12
Doses Blue Rep1_−0.03 Red Rep2_−0.21
sp|P15822|ZEP1_HUMAN−1881−T−S2_−0.12
Doses Blue Rep1_−0.03 Red Rep2_−0.21
sp|P46100−4|ATRX_HUMAN−1397−S−S2_0
Doses Blue Rep1_−0.04 Red Rep2_0.03
sp|Q14676|MDC1_HUMAN−495−S−S1_−0.1
Doses Blue Rep1_−0.07 Red Rep2_−0.12
sp|Q9H6F5|CCD86_HUMAN−113−S−S1_−0.15
Doses Blue Rep1_−0.06 Red Rep2_−0.24
sp|Q9H1E3|NUCKS_HUMAN−204−S−S2_−0.06
Doses Blue Rep1_−0.07 Red Rep2_−0.04
sp|Q9Y618−3|NCOR2_HUMAN−2261−S−S1_−0.2
Doses Blue Rep1_−0.16 Red Rep2_−0.23
sp|P40855|PEX19_HUMAN−146−S−S2_−0.14
Doses Blue Rep1_−0.04 Red Rep2_−0.24
sp|Q9BW71|HIRP3_HUMAN−143−S−S1_−0.08
Doses Blue Rep1_−0.18 Red Rep2_0.02
sp|Q96JM3|CHAP1_HUMAN−427−S−S1_0.18
Doses Blue Rep1_0.19 Red Rep2_0.18
sp|Q99590−2|SCAFB_HUMAN−563−S−S3_−0.32
Doses Blue Rep1_−0.28 Red Rep2_−0.36
ptm abundance 37C

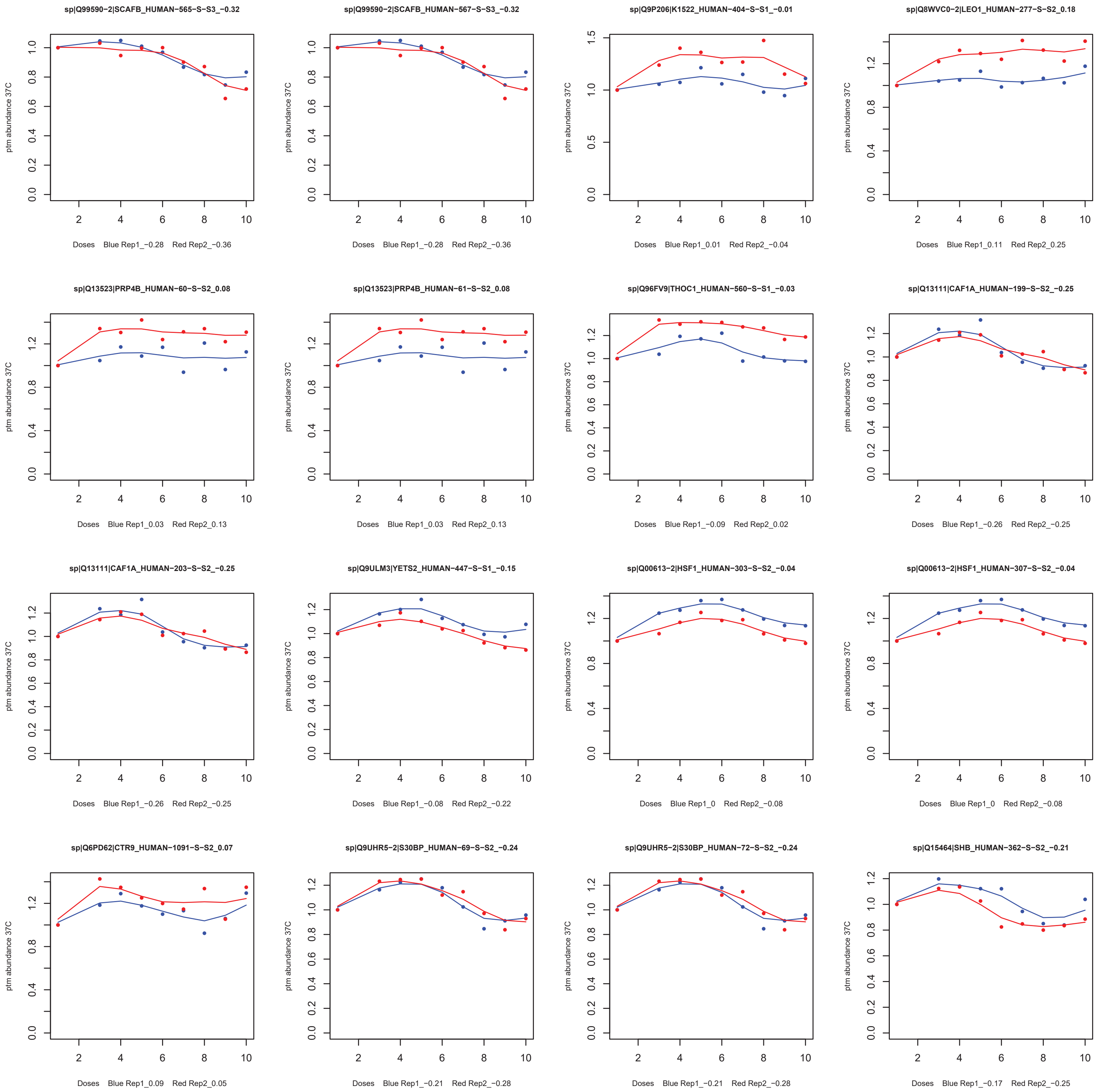
sp|Q99590−2|SCAFB_HUMAN−565−S−S3_−0.32
Doses Blue Rep1_−0.28 Red Rep2_−0.36
sp|Q99590−2|SCAFB_HUMAN−567−S−S3_−0.32
Doses Blue Rep1_−0.28 Red Rep2_−0.36
sp|Q9P206|K1522_HUMAN−404−S−S1_−0.01
Doses Blue Rep1_0.01 Red Rep2_−0.04
sp|Q8WVC0−2|LEO1_HUMAN−277−S−S2_0.18
Doses Blue Rep1_0.11 Red Rep2_0.25
sp|Q13523|PRP4B_HUMAN−60−S−S2_0.08
Doses Blue Rep1_0.03 Red Rep2_0.13
sp|Q13523|PRP4B_HUMAN−61−S−S2_0.08
Doses Blue Rep1_0.03 Red Rep2_0.13
sp|Q96FV9|THOC1_HUMAN−560−S−S1_−0.03
Doses Blue Rep1_−0.09 Red Rep2_0.02
sp|Q13111|CAF1A_HUMAN−199−S−S2_−0.25
Doses Blue Rep1_−0.26 Red Rep2_−0.25
sp|Q13111|CAF1A_HUMAN−203−S−S2_−0.25
Doses Blue Rep1_−0.26 Red Rep2_−0.25
sp|Q9ULM3|YETS2_HUMAN−447−S−S1_−0.15
Doses Blue Rep1_−0.08 Red Rep2_−0.22
sp|Q00613−2|HSF1_HUMAN−303−S−S2_−0.04
Doses Blue Rep1_0 Red Rep2_−0.08
sp|Q00613−2|HSF1_HUMAN−307−S−S2_−0.04
Doses Blue Rep1_0 Red Rep2_−0.08
sp|Q6PD62|CTR9_HUMAN−1091−S−S2_0.07
Doses Blue Rep1_0.09 Red Rep2_0.05
sp|Q9UHR5−2|S30BP_HUMAN−69−S−S2_−0.24
Doses Blue Rep1_−0.21 Red Rep2_−0.28
sp|Q9UHR5−2|S30BP_HUMAN−72−S−S2_−0.24
Doses Blue Rep1_−0.21 Red Rep2_−0.28
sp|Q15464|SHB_HUMAN−362−S−S2_−0.21
Doses Blue Rep1_−0.17 Red Rep2_−0.25
ptm abundance 37C

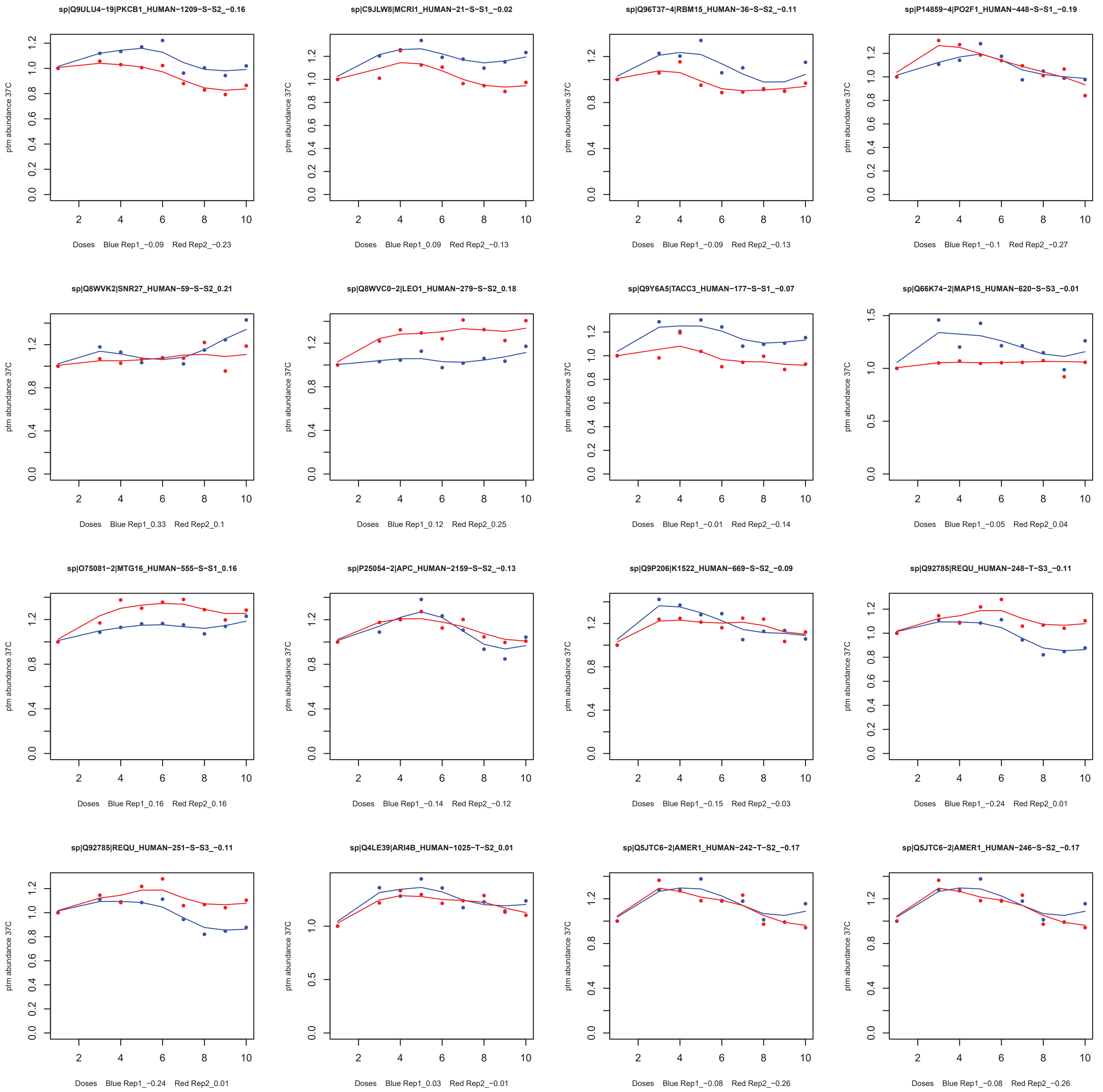

sp|Q9ULU4−19|PKCB1_HUMAN−1209−S−S2_−0.16
Doses Blue Rep1_−0.09 Red Rep2_−0.23
sp|C9JLW8|MCRI1_HUMAN−21−S−S1_−0.02
Doses Blue Rep1_0.09 Red Rep2_−0.13
sp|Q96T37−4|RBM15_HUMAN−36−S−S2_−0.11
Doses Blue Rep1_−0.09 Red Rep2_−0.13
sp|P14859−4|PO2F1_HUMAN−448−S−S1_−0.19
Doses Blue Rep1_−0.1 Red Rep2_−0.27
sp|Q8WVK2|SNR27_HUMAN−59−S−S2_0.21
Doses Blue Rep1_0.33 Red Rep2_0.1
sp|Q8WVC0−2|LEO1_HUMAN−279−S−S2_0.18
Doses Blue Rep1_0.12 Red Rep2_0.25
sp|Q9Y6A5|TACC3_HUMAN−177−S−S1_−0.07
Doses Blue Rep1_−0.01 Red Rep2_−0.14
sp|Q66K74−2|MAP1S_HUMAN−620−S−S3_−0.01
Doses Blue Rep1_−0.05 Red Rep2_0.04
sp|O75081−2|MTG16_HUMAN−555−S−S1_0.16
Doses Blue Rep1_0.16 Red Rep2_0.16
sp|P25054−2|APC_HUMAN−2159−S−S2_−0.13
Doses Blue Rep1_−0.14 Red Rep2_−0.12
sp|Q9P206|K1522_HUMAN−669−S−S2_−0.09
Doses Blue Rep1_−0.15 Red Rep2_−0.03
sp|Q92785|REQU_HUMAN−248−T−S3_−0.11
Doses Blue Rep1_−0.24 Red Rep2_0.01
sp|Q92785|REQU_HUMAN−251−S−S3_−0.11
Doses Blue Rep1_−0.24 Red Rep2_0.01
sp|Q4LE39|ARI4B_HUMAN−1025−T−S2_0.01
Doses Blue Rep1_0.03 Red Rep2_−0.01
sp|Q5JTC6−2|AMER1_HUMAN−242−T−S2_−0.17
Doses Blue Rep1_−0.08 Red Rep2_−0.26
sp|Q5JTC6−2|AMER1_HUMAN−246−S−S2_−0.17
Doses Blue Rep1_−0.08 Red Rep2_−0.26
ptm abundance 37C

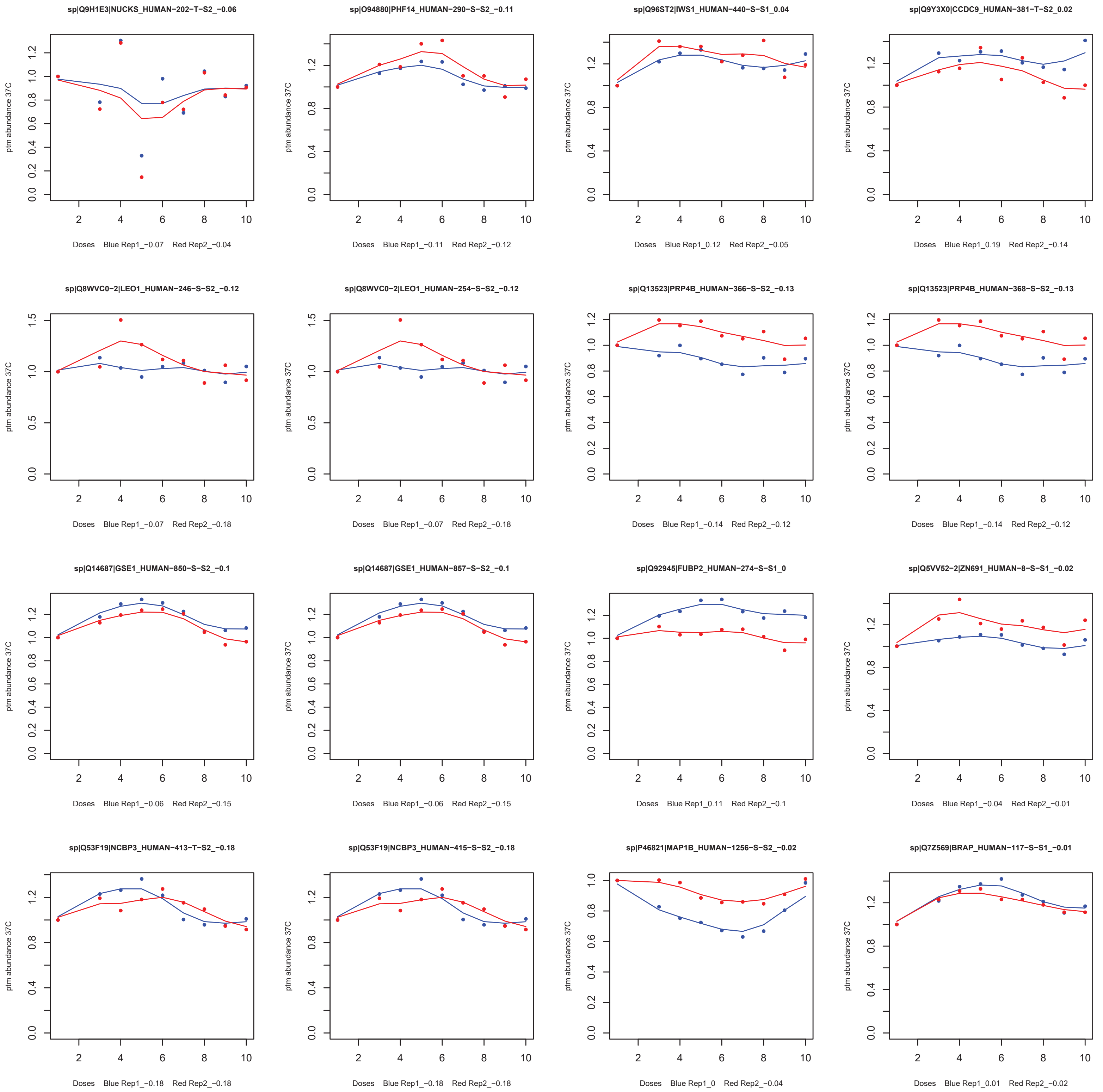
sp|Q9H1E3|NUCKS_HUMAN−202−T−S2_−0.06
Doses Blue Rep1_−0.07 Red Rep2_−0.04
sp|O94880|PHF14_HUMAN−290−S−S2_−0.11
Doses Blue Rep1_−0.11 Red Rep2_−0.12
sp|Q96ST2|IWS1_HUMAN−440−S−S1_0.04
Doses Blue Rep1_0.12 Red Rep2_−0.05
sp|Q9Y3X0|CCDC9_HUMAN−381−T−S2_0.02
Doses Blue Rep1_0.19 Red Rep2_−0.14
sp|Q8WVC0−2|LEO1_HUMAN−246−S−S2_−0.12
Doses Blue Rep1_−0.07 Red Rep2_−0.18
sp|Q8WVC0−2|LEO1_HUMAN−254−S−S2_−0.12
Doses Blue Rep1_−0.07 Red Rep2_−0.18
sp|Q13523|PRP4B_HUMAN−366−S−S2_−0.13
Doses Blue Rep1_−0.14 Red Rep2_−0.12
sp|Q13523|PRP4B_HUMAN−368−S−S2_−0.13
Doses Blue Rep1_−0.14 Red Rep2_−0.12
sp|Q14687|GSE1_HUMAN−850−S−S2_−0.1
Doses Blue Rep1_−0.06 Red Rep2_−0.15
sp|Q14687|GSE1_HUMAN−857−S−S2_−0.1
Doses Blue Rep1_−0.06 Red Rep2_−0.15
sp|Q92945|FUBP2_HUMAN−274−S−S1_0
Doses Blue Rep1_0.11 Red Rep2_−0.1
sp|Q5VV52−2|ZN691_HUMAN−8−S−S1_−0.02
Doses Blue Rep1_−0.04 Red Rep2_−0.01
sp|Q53F19|NCBP3_HUMAN−413−T−S2_−0.18
Doses Blue Rep1_−0.18 Red Rep2_−0.18
sp|Q53F19|NCBP3_HUMAN−415−S−S2_−0.18
Doses Blue Rep1_−0.18 Red Rep2_−0.18
sp|P46821|MAP1B_HUMAN−1256−S−S2_−0.02
Doses Blue Rep1_0 Red Rep2_−0.04
sp|Q7Z569|BRAP_HUMAN−117−S−S1_−0.01
Doses Blue Rep1_0.01 Red Rep2_−0.02
ptm abundance 37C

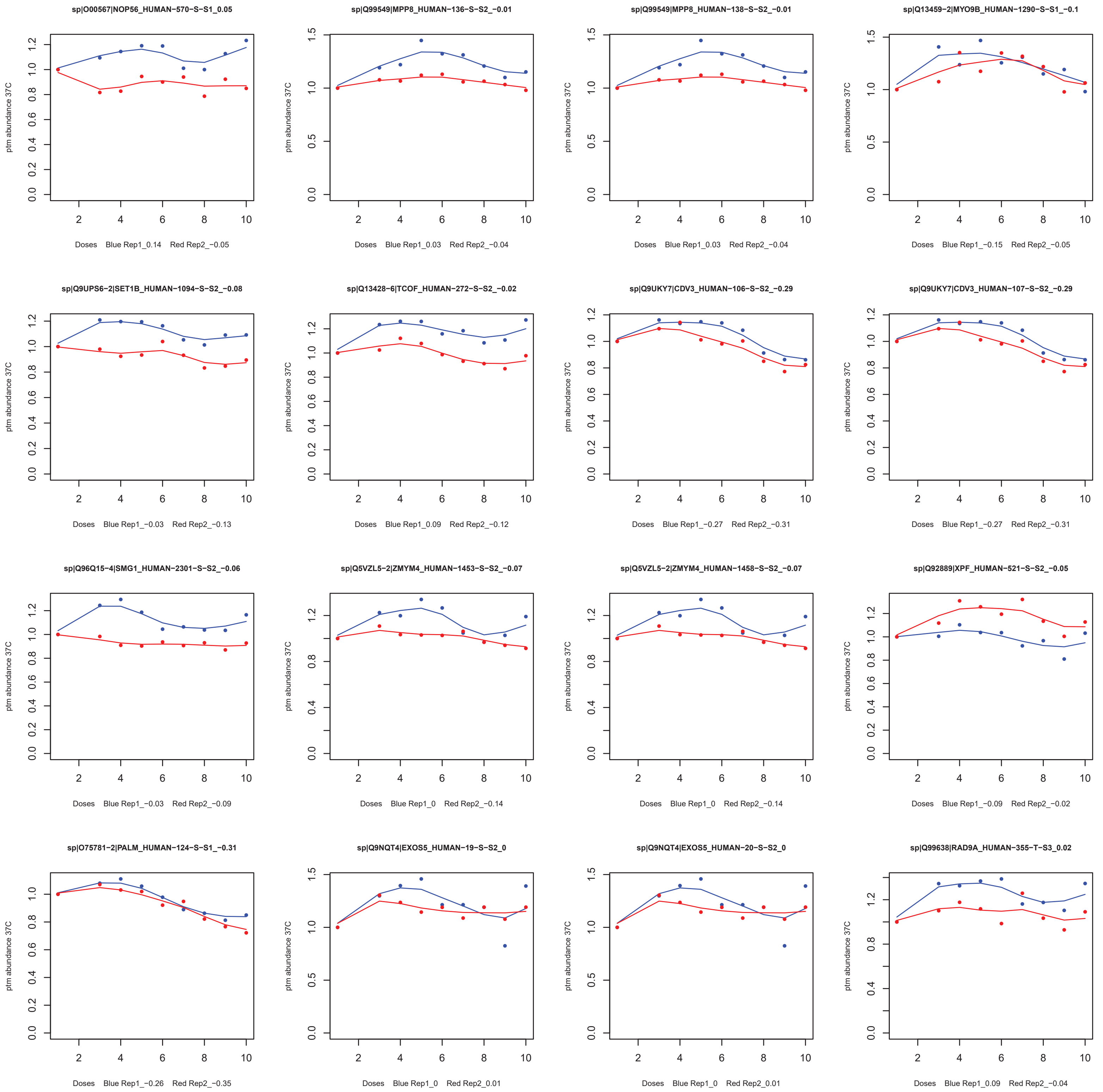
sp|O00567|NOP56_HUMAN−570−S−S1_0.05
Doses Blue Rep1_0.14 Red Rep2_−0.05
sp|Q99549|MPP8_HUMAN−136−S−S2_−0.01
Doses Blue Rep1_0.03 Red Rep2_−0.04
sp|Q99549|MPP8_HUMAN−138−S−S2_−0.01
Doses Blue Rep1_0.03 Red Rep2_−0.04
sp|Q13459−2|MYO9B_HUMAN−1290−S−S1_−0.1
Doses Blue Rep1_−0.15 Red Rep2_−0.05
sp|Q9UPS6−2|SET1B_HUMAN−1094−S−S2_−0.08
Doses Blue Rep1_−0.03 Red Rep2_−0.13
sp|Q13428−6|TCOF_HUMAN−272−S−S2_−0.02
Doses Blue Rep1_0.09 Red Rep2_−0.12
sp|Q9UKY7|CDV3_HUMAN−106−S−S2_−0.29
Doses Blue Rep1_−0.27 Red Rep2_−0.31
sp|Q9UKY7|CDV3_HUMAN−107−S−S2_−0.29
Doses Blue Rep1_−0.27 Red Rep2_−0.31
sp|Q96Q15−4|SMG1_HUMAN−2301−S−S2_−0.06
Doses Blue Rep1_−0.03 Red Rep2_−0.09
sp|Q5VZL5−2|ZMYM4_HUMAN−1453−S−S2_−0.07
Doses Blue Rep1_0 Red Rep2_−0.14
sp|Q5VZL5−2|ZMYM4_HUMAN−1458−S−S2_−0.07
Doses Blue Rep1_0 Red Rep2_−0.14
sp|Q92889|XPF_HUMAN−521−S−S2_−0.05
Doses Blue Rep1_−0.09 Red Rep2_−0.02
sp|O75781−2|PALM_HUMAN−124−S−S1_−0.31
Doses Blue Rep1_−0.26 Red Rep2_−0.35
sp|Q9NQT4|EXOS5_HUMAN−19−S−S2_0
Doses Blue Rep1_0 Red Rep2_0.01
sp|Q9NQT4|EXOS5_HUMAN−20−S−S2_0
Doses Blue Rep1_0 Red Rep2_0.01
sp|Q99638|RAD9A_HUMAN−355−T−S3_0.02
Doses Blue Rep1_0.09 Red Rep2_−0.04
ptm abundance 37C

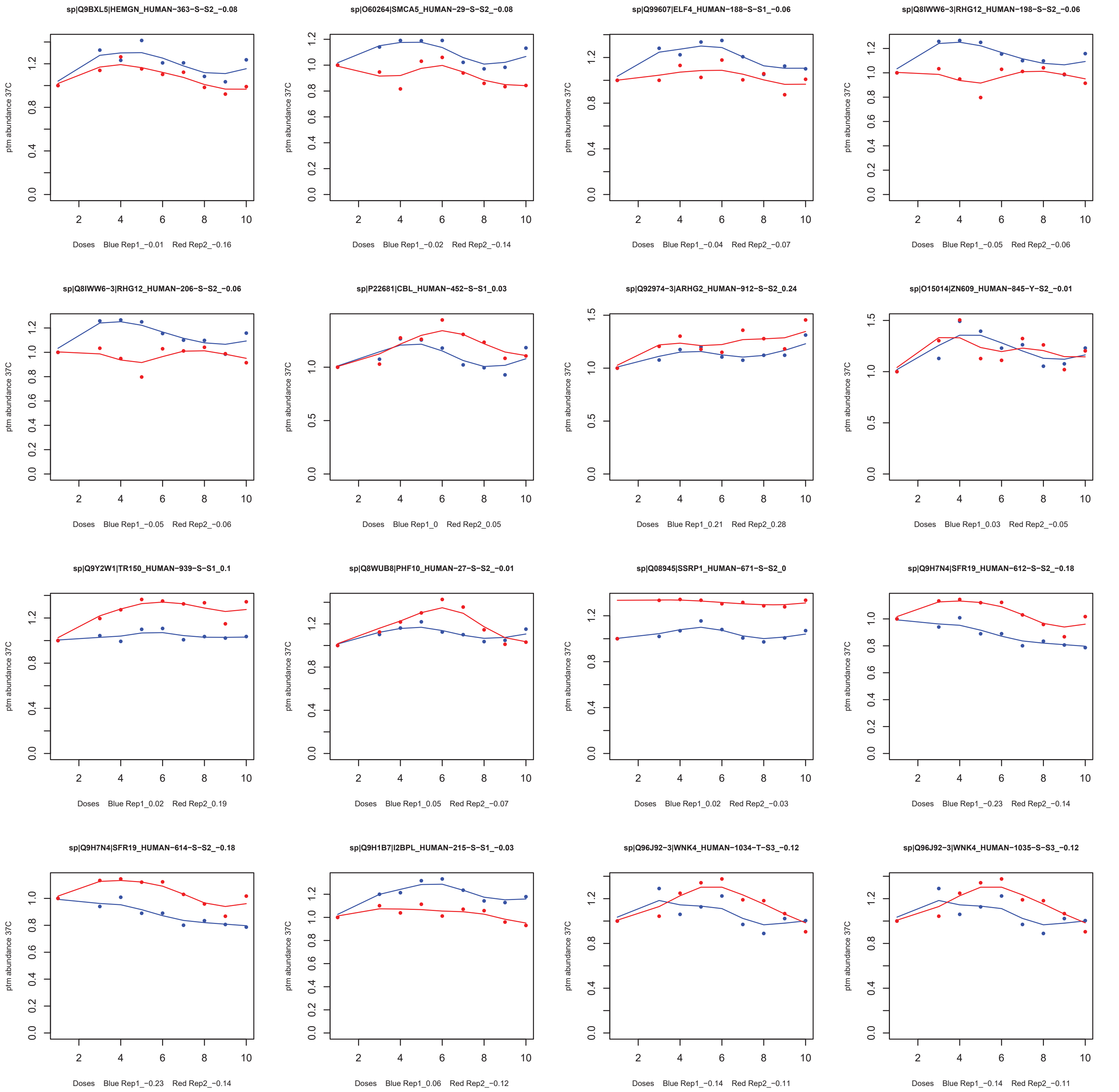
sp|Q9BXL5|HEMGN_HUMAN−363−S−S2_−0.08
Doses Blue Rep1_−0.01 Red Rep2_−0.16
sp|O60264|SMCA5_HUMAN−29−S−S2_−0.08
Doses Blue Rep1_−0.02 Red Rep2_−0.14
sp|Q99607|ELF4_HUMAN−188−S−S1_−0.06
Doses Blue Rep1_−0.04 Red Rep2_−0.07
sp|Q8IWW6−3|RHG12_HUMAN−198−S−S2_−0.06
Doses Blue Rep1_−0.05 Red Rep2_−0.06
sp|Q8IWW6−3|RHG12_HUMAN−206−S−S2_−0.06
Doses Blue Rep1_−0.05 Red Rep2_−0.06
sp|P22681|CBL_HUMAN−452−S−S1_0.03
Doses Blue Rep1_0 Red Rep2_0.05
sp|Q92974−3|ARHG2_HUMAN−912−S−S2_0.24
Doses Blue Rep1_0.21 Red Rep2_0.28
sp|O15014|ZN609_HUMAN−845−Y−S2_−0.01
Doses Blue Rep1_0.03 Red Rep2_−0.05
sp|Q9Y2W1|TR150_HUMAN−939−S−S1_0.1
Doses Blue Rep1_0.02 Red Rep2_0.19
sp|Q8WUB8|PHF10_HUMAN−27−S−S2_−0.01
Doses Blue Rep1_0.05 Red Rep2_−0.07
sp|Q08945|SSRP1_HUMAN−671−S−S2_0
Doses Blue Rep1_0.02 Red Rep2_−0.03
sp|Q9H7N4|SFR19_HUMAN−612−S−S2_−0.18
Doses Blue Rep1_−0.23 Red Rep2_−0.14
sp|Q9H7N4|SFR19_HUMAN−614−S−S2_−0.18
Doses Blue Rep1_−0.23 Red Rep2_−0.14
sp|Q9H1B7|I2BPL_HUMAN−215−S−S1_−0.03
Doses Blue Rep1_0.06 Red Rep2_−0.12
sp|Q96J92−3|WNK4_HUMAN−1034−T−S3_−0.12
Doses Blue Rep1_−0.14 Red Rep2_−0.11
sp|Q96J92−3|WNK4_HUMAN−1035−S−S3_−0.12
Doses Blue Rep1_−0.14 Red Rep2_−0.11
ptm abundance 37C

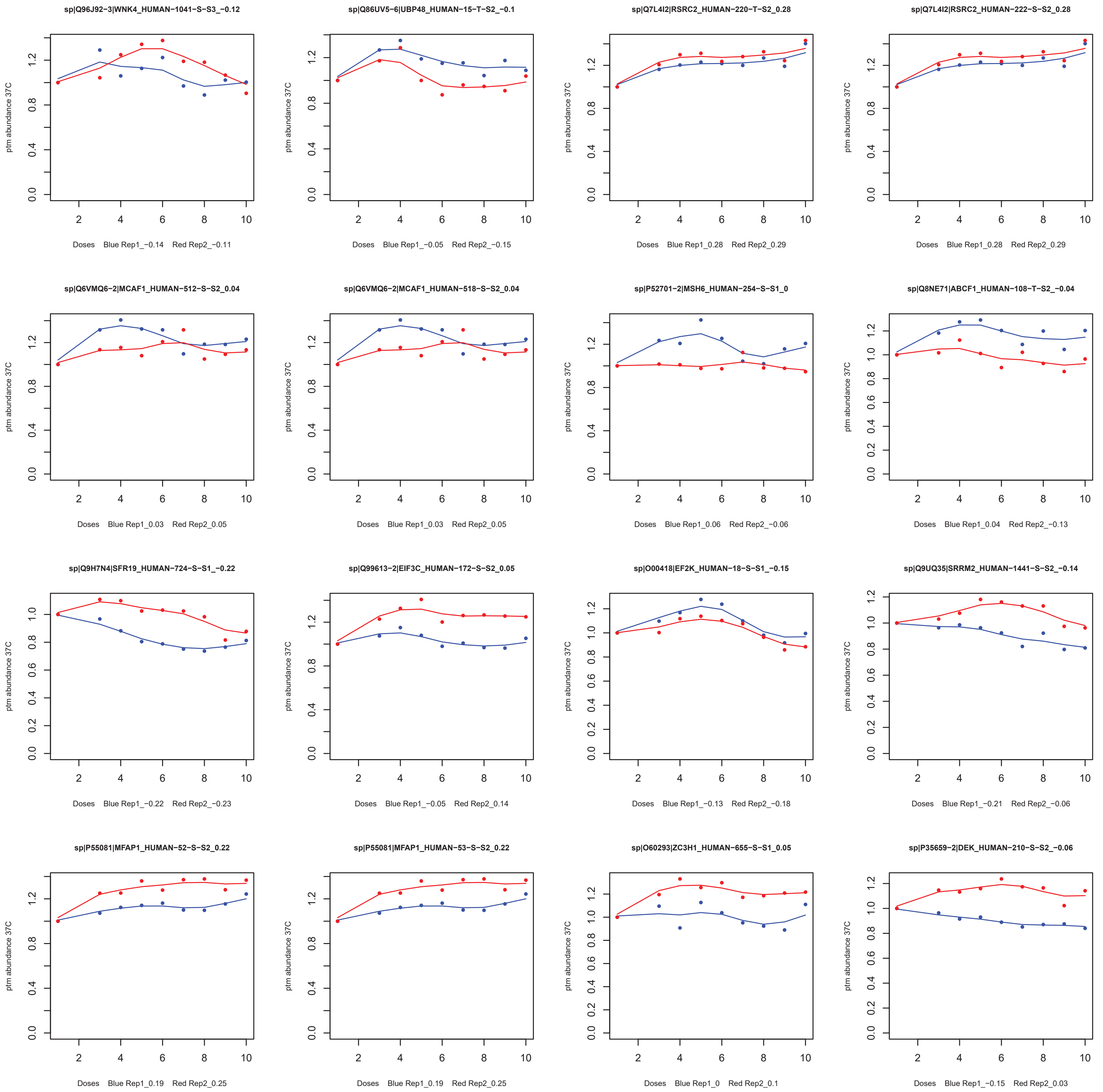
sp|Q96J92−3|WNK4_HUMAN−1041−S−S3_−0.12
Doses Blue Rep1_−0.14 Red Rep2_−0.11
sp|Q86UV5−6|UBP48_HUMAN−15−T−S2_−0.1
Doses Blue Rep1_−0.05 Red Rep2_−0.15
sp|Q7L4I2|RSRC2_HUMAN−220−T−S2_0.28
Doses Blue Rep1_0.28 Red Rep2_0.29
sp|Q7L4I2|RSRC2_HUMAN−222−S−S2_0.28
Doses Blue Rep1_0.28 Red Rep2_0.29
sp|Q6VMQ6−2|MCAF1_HUMAN−512−S−S2_0.04
Doses Blue Rep1_0.03 Red Rep2_0.05
sp|Q6VMQ6−2|MCAF1_HUMAN−518−S−S2_0.04
Doses Blue Rep1_0.03 Red Rep2_0.05
sp|P52701−2|MSH6_HUMAN−254−S−S1_0
Doses Blue Rep1_0.06 Red Rep2_−0.06
sp|Q8NE71|ABCF1_HUMAN−108−T−S2_−0.04
Doses Blue Rep1_0.04 Red Rep2_−0.13
sp|Q9H7N4|SFR19_HUMAN−724−S−S1_−0.22
Doses Blue Rep1_−0.22 Red Rep2_−0.23
sp|Q99613−2|EIF3C_HUMAN−172−S−S2_0.05
Doses Blue Rep1_−0.05 Red Rep2_0.14
sp|O00418|EF2K_HUMAN−18−S−S1_−0.15
Doses Blue Rep1_−0.13 Red Rep2_−0.18
sp|Q9UQ35|SRRM2_HUMAN−1441−S−S2_−0.14
Doses Blue Rep1_−0.21 Red Rep2_−0.06
sp|P55081|MFAP1_HUMAN−52−S−S2_0.22
Doses Blue Rep1_0.19 Red Rep2_0.25
sp|P55081|MFAP1_HUMAN−53−S−S2_0.22
Doses Blue Rep1_0.19 Red Rep2_0.25
sp|O60293|ZC3H1_HUMAN−655−S−S1_0.05
Doses Blue Rep1_0 Red Rep2_0.1
sp|P35659−2|DEK_HUMAN−210−S−S2_−0.06
Doses Blue Rep1_−0.15 Red Rep2_0.03
ptm abundance 37C

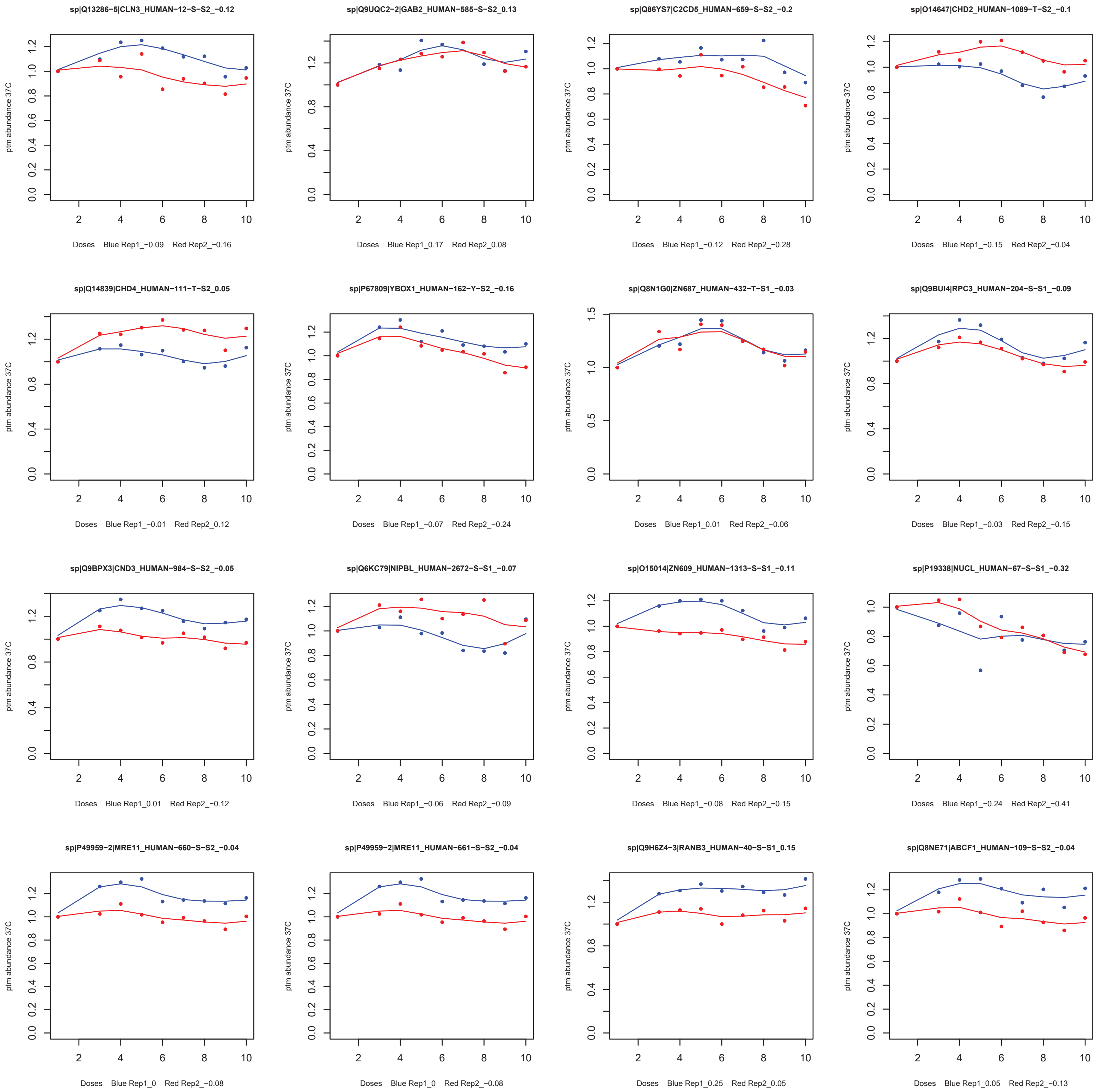
sp|Q13286−5|CLN3_HUMAN−12−S−S2_−0.12
Doses Blue Rep1_−0.09 Red Rep2_−0.16
sp|Q9UQC2−2|GAB2_HUMAN−585−S−S2_0.13
Doses Blue Rep1_0.17 Red Rep2_0.08
sp|Q86YS7|C2CD5_HUMAN−659−S−S2_−0.2
Doses Blue Rep1_−0.12 Red Rep2_−0.28
sp|O14647|CHD2_HUMAN−1089−T−S2_−0.1
Doses Blue Rep1_−0.15 Red Rep2_−0.04
sp|Q14839|CHD4_HUMAN−111−T−S2_0.05
Doses Blue Rep1_−0.01 Red Rep2_0.12
sp|P67809|YBOX1_HUMAN−162−Y−S2_−0.16
Doses Blue Rep1_−0.07 Red Rep2_−0.24
sp|Q8N1G0|ZN687_HUMAN−432−T−S1_−0.03
Doses Blue Rep1_0.01 Red Rep2_−0.06
sp|Q9BUI4|RPC3_HUMAN−204−S−S1_−0.09
Doses Blue Rep1_−0.03 Red Rep2_−0.15
sp|Q9BPX3|CND3_HUMAN−984−S−S2_−0.05
Doses Blue Rep1_0.01 Red Rep2_−0.12
sp|Q6KC79|NIPBL_HUMAN−2672−S−S1_−0.07
Doses Blue Rep1_−0.06 Red Rep2_−0.09
sp|O15014|ZN609_HUMAN−1313−S−S1_−0.11
Doses Blue Rep1_−0.08 Red Rep2_−0.15
sp|P19338|NUCL_HUMAN−67−S−S1_−0.32
Doses Blue Rep1_−0.24 Red Rep2_−0.41
sp|P49959−2|MRE11_HUMAN−660−S−S2_−0.04
Doses Blue Rep1_0 Red Rep2_−0.08
sp|P49959−2|MRE11_HUMAN−661−S−S2_−0.04
Doses Blue Rep1_0 Red Rep2_−0.08
sp|Q9H6Z4−3|RANB3_HUMAN−40−S−S1_0.15
Doses Blue Rep1_0.25 Red Rep2_0.05
sp|Q8NE71|ABCF1_HUMAN−109−S−S2_−0.04
Doses Blue Rep1_0.05 Red Rep2_−0.13
ptm abundance 37C

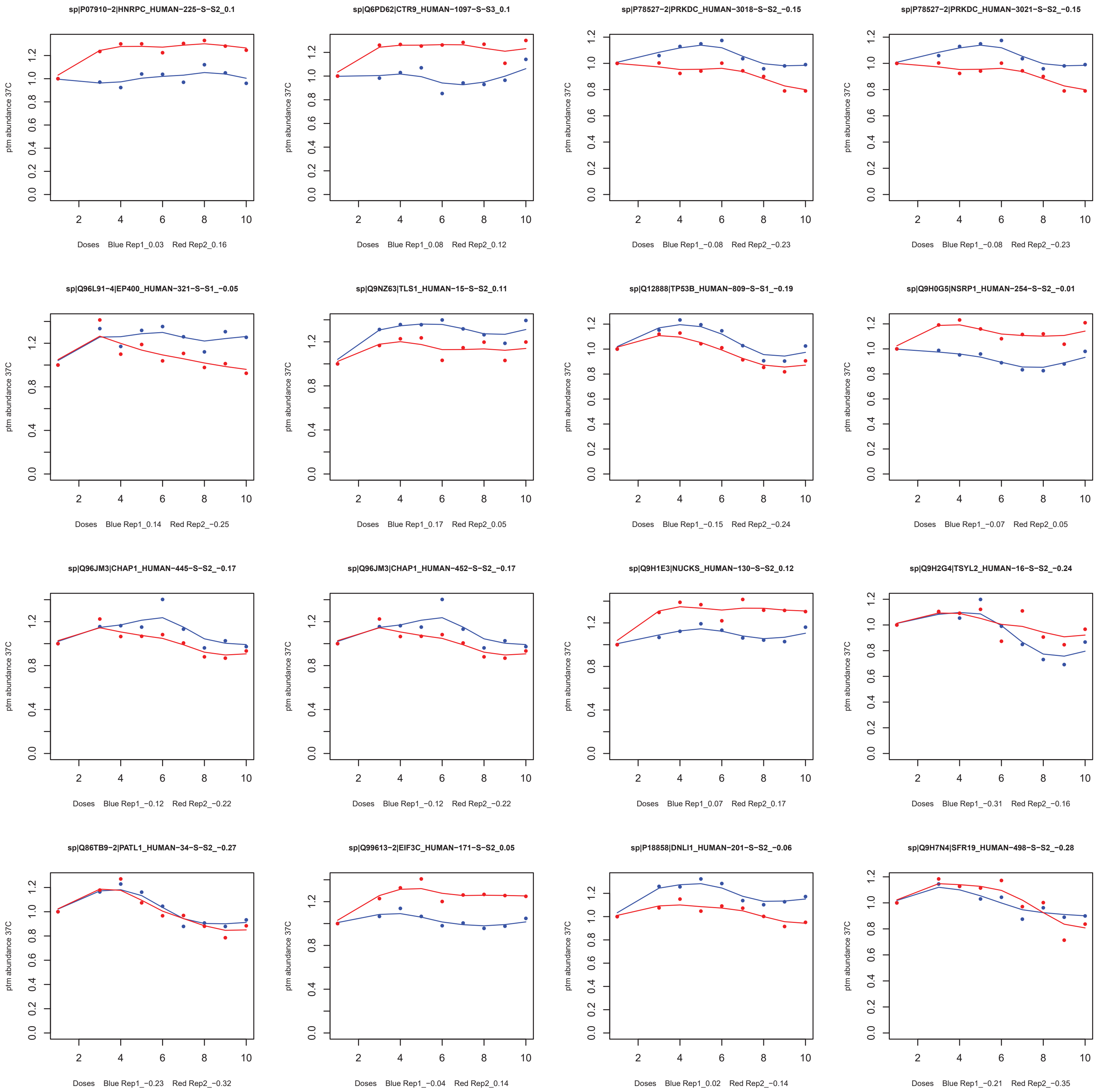

sp|P07910−2|HNRPC_HUMAN−225−S−S2_0.1
ptm abundance 37C
Doses Blue Rep1_0.03 Red Rep2_0.16
sp|Q6PD62|CTR9_HUMAN−1097−S−S3_0.1
ptm abundance 37C
Doses Blue Rep1_0.08 Red Rep2_0.12
sp|P78527−2|PRKDC_HUMAN−3018−S−S2_−0.15
ptm abundance 37C
Doses Blue Rep1_−0.08 Red Rep2_−0.23
sp|P78527−2|PRKDC_HUMAN−3021−S−S2_−0.15
ptm abundance 37C
Doses Blue Rep1_−0.08 Red Rep2_−0.23
sp|Q96L91−4|EP400_HUMAN−321−S−S1_−0.05
ptm abundance 37C
Doses Blue Rep1_0.14 Red Rep2_−0.25
sp|Q9NZ63|TLS1_HUMAN−15−S−S2_0.11
ptm abundance 37C
Doses Blue Rep1_0.17 Red Rep2_0.05
sp|Q12888|TP53B_HUMAN−809−S−S1_−0.19
ptm abundance 37C
Doses Blue Rep1_−0.15 Red Rep2_−0.24
sp|Q9H0G5|NSRP1_HUMAN−254−S−S2_−0.01
ptm abundance 37C
Doses Blue Rep1_−0.07 Red Rep2_0.05
sp|Q96JM3|CHAP1_HUMAN−445−S−S2_−0.17
ptm abundance 37C
Doses Blue Rep1_−0.12 Red Rep2_−0.22
sp|Q96JM3|CHAP1_HUMAN−452−S−S2_−0.17
ptm abundance 37C
Doses Blue Rep1_−0.12 Red Rep2_−0.22
sp|Q9H1E3|NUCKS_HUMAN−130−S−S2_0.12
ptm abundance 37C
Doses Blue Rep1_0.07 Red Rep2_0.17
sp|Q9H2G4|TSYL2_HUMAN−16−S−S2_−0.24
ptm abundance 37C
Doses Blue Rep1_−0.31 Red Rep2_−0.16
sp|Q86TB9−2|PATL1_HUMAN−34−S−S2_−0.27
ptm abundance 37C
Doses Blue Rep1_−0.23 Red Rep2_−0.32
sp|Q99613−2|EIF3C_HUMAN−171−S−S2_0.05
ptm abundance 37C
Doses Blue Rep1_−0.04 Red Rep2_0.14
sp|P18858|DNLI1_HUMAN−201−S−S2_−0.06
ptm abundance 37C
Doses Blue Rep1_0.02 Red Rep2_−0.14
sp|Q9H7N4|SFR19_HUMAN−498−S−S2_−0.28
ptm abundance 37C
Doses Blue Rep1_−0.21 Red Rep2_−0.35

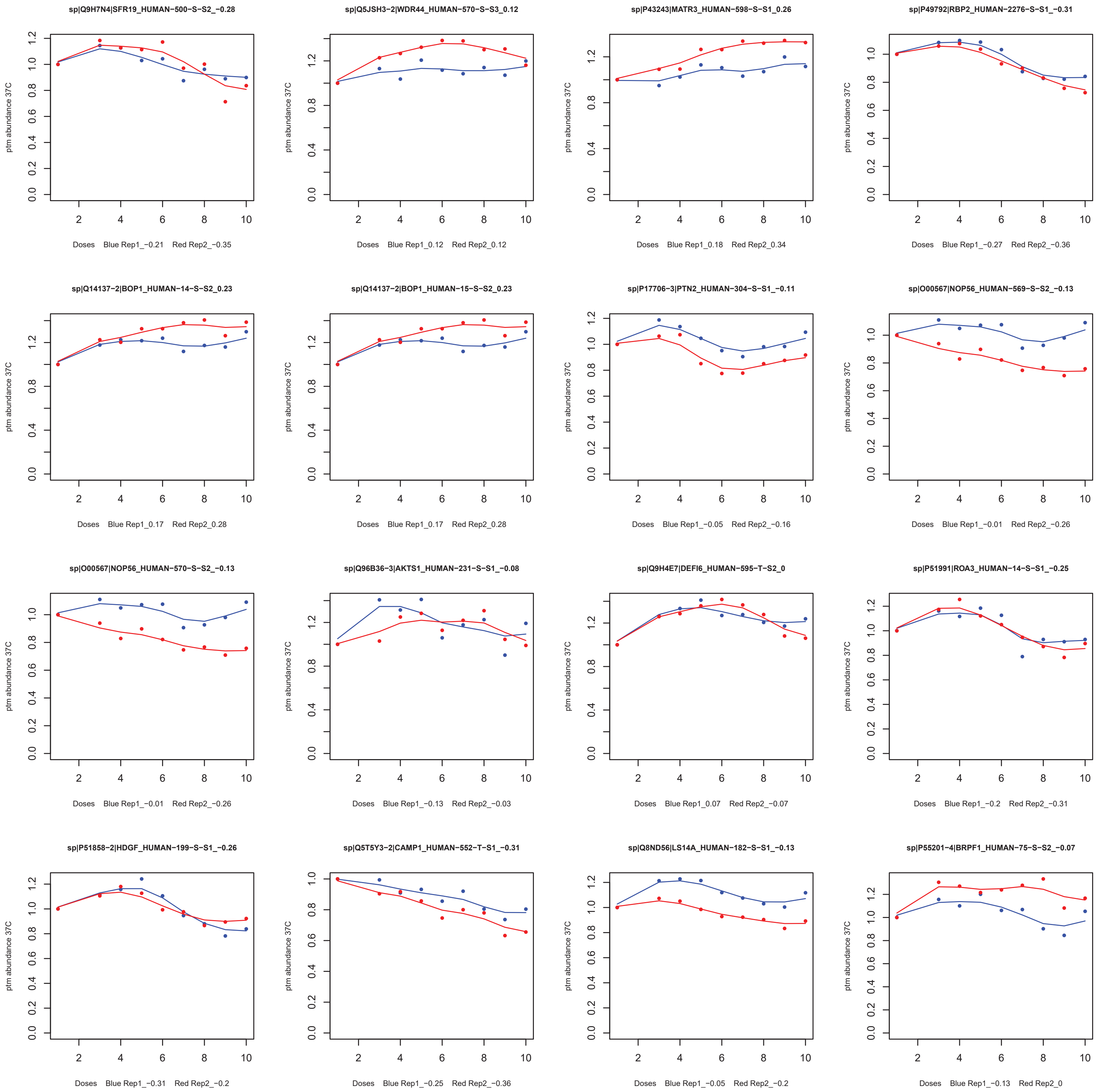

sp|Q9H7N4|SFR19_HUMAN−500−S−S2_−0.28
Doses Blue Rep1_−0.21 Red Rep2_−0.35
sp|Q5JSH3−2|WDR44_HUMAN−570−S−S3_0.12
Doses Blue Rep1_0.12 Red Rep2_0.12
sp|P43243|MATR3_HUMAN−598−S−S1_0.26
Doses Blue Rep1_0.18 Red Rep2_0.34
sp|P49792|RBP2_HUMAN−2276−S−S1_−0.31
Doses Blue Rep1_−0.27 Red Rep2_−0.36
sp|Q14137−2|BOP1_HUMAN−14−S−S2_0.23
Doses Blue Rep1_0.17 Red Rep2_0.28
sp|Q14137−2|BOP1_HUMAN−15−S−S2_0.23
Doses Blue Rep1_0.17 Red Rep2_0.28
sp|P17706−3|PTN2_HUMAN−304−S−S1_−0.11
Doses Blue Rep1_−0.05 Red Rep2_−0.16
sp|O00567|NOP56_HUMAN−569−S−S2_−0.13
Doses Blue Rep1_−0.01 Red Rep2_−0.26
sp|O00567|NOP56_HUMAN−570−S−S2_−0.13
Doses Blue Rep1_−0.01 Red Rep2_−0.26
sp|Q96B36−3|AKTS1_HUMAN−231−S−S1_−0.08
Doses Blue Rep1_−0.13 Red Rep2_−0.03
sp|Q9H4E7|DEFI6_HUMAN−595−T−S2_0
Doses Blue Rep1_0.07 Red Rep2_−0.07
sp|P51991|ROA3_HUMAN−14−S−S1_−0.25
Doses Blue Rep1_−0.2 Red Rep2_−0.31
sp|P51858−2|HDGF_HUMAN−199−S−S1_−0.26
Doses Blue Rep1_−0.31 Red Rep2_−0.2
sp|Q5T5Y3−2|CAMP1_HUMAN−552−T−S1_−0.31
Doses Blue Rep1_−0.25 Red Rep2_−0.36
sp|Q8ND56|LS14A_HUMAN−182−S−S1_−0.13
Doses Blue Rep1_−0.05 Red Rep2_−0.2
sp|P55201−4|BRPF1_HUMAN−75−S−S2_−0.07
Doses Blue Rep1_−0.13 Red Rep2_0
ptm abundance 37C

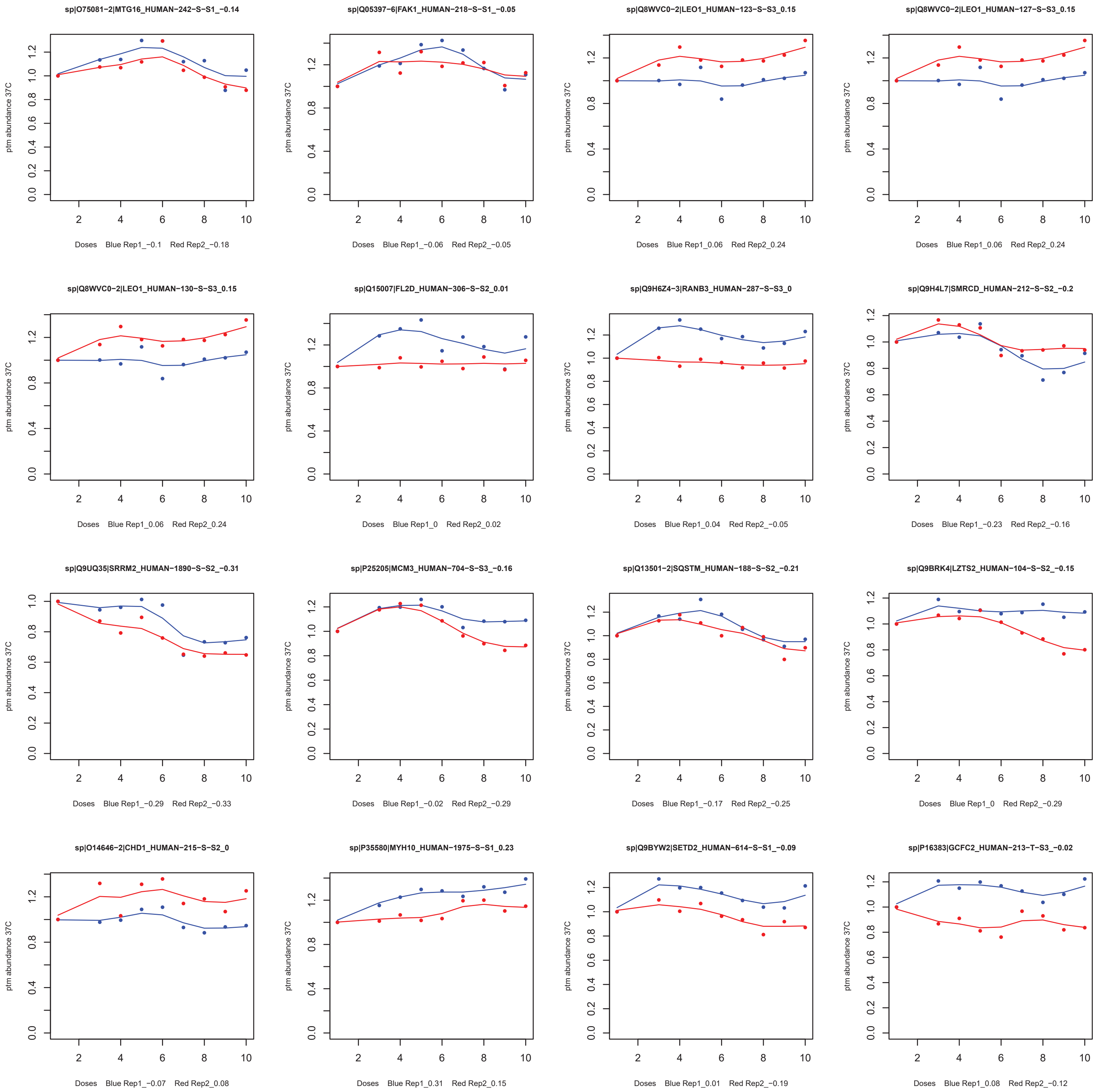

sp|O75081−2|MTG16_HUMAN−242−S−S1_−0.14
Doses Blue Rep1_−0.1 Red Rep2_−0.18
sp|Q05397−6|FAK1_HUMAN−218−S−S1_−0.05
Doses Blue Rep1_−0.06 Red Rep2_−0.05
sp|Q8WVC0−2|LEO1_HUMAN−123−S−S3_0.15
Doses Blue Rep1_0.06 Red Rep2_0.24
sp|Q8WVC0−2|LEO1_HUMAN−127−S−S3_0.15
Doses Blue Rep1_0.06 Red Rep2_0.24
sp|Q8WVC0−2|LEO1_HUMAN−130−S−S3_0.15
Doses Blue Rep1_0.06 Red Rep2_0.24
sp|Q15007|FL2D_HUMAN−306−S−S2_0.01
Doses Blue Rep1_0 Red Rep2_0.02
sp|Q9H6Z4−3|RANB3_HUMAN−287−S−S3_0
Doses Blue Rep1_0.04 Red Rep2_−0.05
sp|Q9H4L7|SMRCD_HUMAN−212−S−S2_−0.2
Doses Blue Rep1_−0.23 Red Rep2_−0.16
sp|Q9UQ35|SRRM2_HUMAN−1890−S−S2_−0.31
Doses Blue Rep1_−0.29 Red Rep2_−0.33
sp|P25205|MCM3_HUMAN−704−S−S3_−0.16
Doses Blue Rep1_−0.02 Red Rep2_−0.29
sp|Q13501−2|SQSTM_HUMAN−188−S−S2_−0.21
Doses Blue Rep1_−0.17 Red Rep2_−0.25
sp|Q9BRK4|LZTS2_HUMAN−104−S−S2_−0.15
Doses Blue Rep1_0 Red Rep2_−0.29
sp|O14646−2|CHD1_HUMAN−215−S−S2_0
Doses Blue Rep1_−0.07 Red Rep2_0.08
sp|P35580|MYH10_HUMAN−1975−S−S1_0.23
Doses Blue Rep1_0.31 Red Rep2_0.15
sp|Q9BYW2|SETD2_HUMAN−614−S−S1_−0.09
Doses Blue Rep1_0.01 Red Rep2_−0.19
sp|P16383|GCFC2_HUMAN−213−T−S3_−0.02
Doses Blue Rep1_0.08 Red Rep2_−0.12
ptm abundance 37C

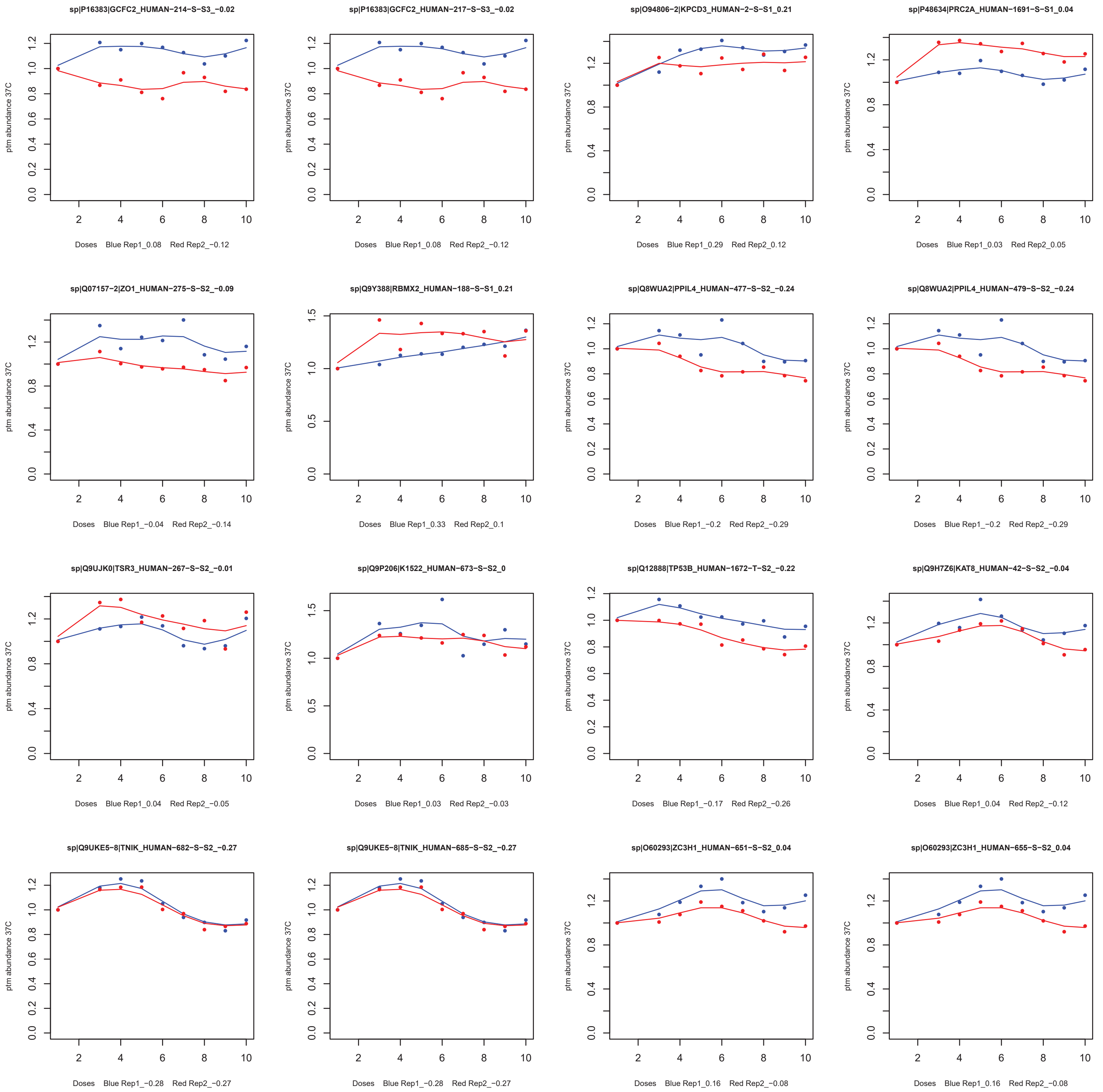

sp|P16383|GCFC2_HUMAN−214−S−S3_−0.02
Doses Blue Rep1_0.08 Red Rep2_−0.12
sp|P16383|GCFC2_HUMAN−217−S−S3_−0.02
Doses Blue Rep1_0.08 Red Rep2_−0.12
sp|O94806−2|KPCD3_HUMAN−2−S−S1_0.21
Doses Blue Rep1_0.29 Red Rep2_0.12
sp|P48634|PRC2A_HUMAN−1691−S−S1_0.04
Doses Blue Rep1_0.03 Red Rep2_0.05
sp|Q07157−2|ZO1_HUMAN−275−S−S2_−0.09
Doses Blue Rep1_−0.04 Red Rep2_−0.14
sp|Q9Y388|RBMX2_HUMAN−188−S−S1_0.21
Doses Blue Rep1_0.33 Red Rep2_0.1
sp|Q8WUA2|PPIL4_HUMAN−477−S−S2_−0.24
Doses Blue Rep1_−0.2 Red Rep2_−0.29
sp|Q8WUA2|PPIL4_HUMAN−479−S−S2_−0.24
Doses Blue Rep1_−0.2 Red Rep2_−0.29
sp|Q9UJK0|TSR3_HUMAN−267−S−S2_−0.01
Doses Blue Rep1_0.04 Red Rep2_−0.05
sp|Q9P206|K1522_HUMAN−673−S−S2_0
Doses Blue Rep1_0.03 Red Rep2_−0.03
sp|Q12888|TP53B_HUMAN−1672−T−S2_−0.22
Doses Blue Rep1_−0.17 Red Rep2_−0.26
sp|Q9H7Z6|KAT8_HUMAN−42−S−S2_−0.04
Doses Blue Rep1_0.04 Red Rep2_−0.12
sp|Q9UKE5−8|TNIK_HUMAN−682−S−S2_−0.27
Doses Blue Rep1_−0.28 Red Rep2_−0.27
sp|Q9UKE5−8|TNIK_HUMAN−685−S−S2_−0.27
Doses Blue Rep1_−0.28 Red Rep2_−0.27
sp|O60293|ZC3H1_HUMAN−651−S−S2_0.04
Doses Blue Rep1_0.16 Red Rep2_−0.08
sp|O60293|ZC3H1_HUMAN−655−S−S2_0.04
Doses Blue Rep1_0.16 Red Rep2_−0.08
ptm abundance 37C

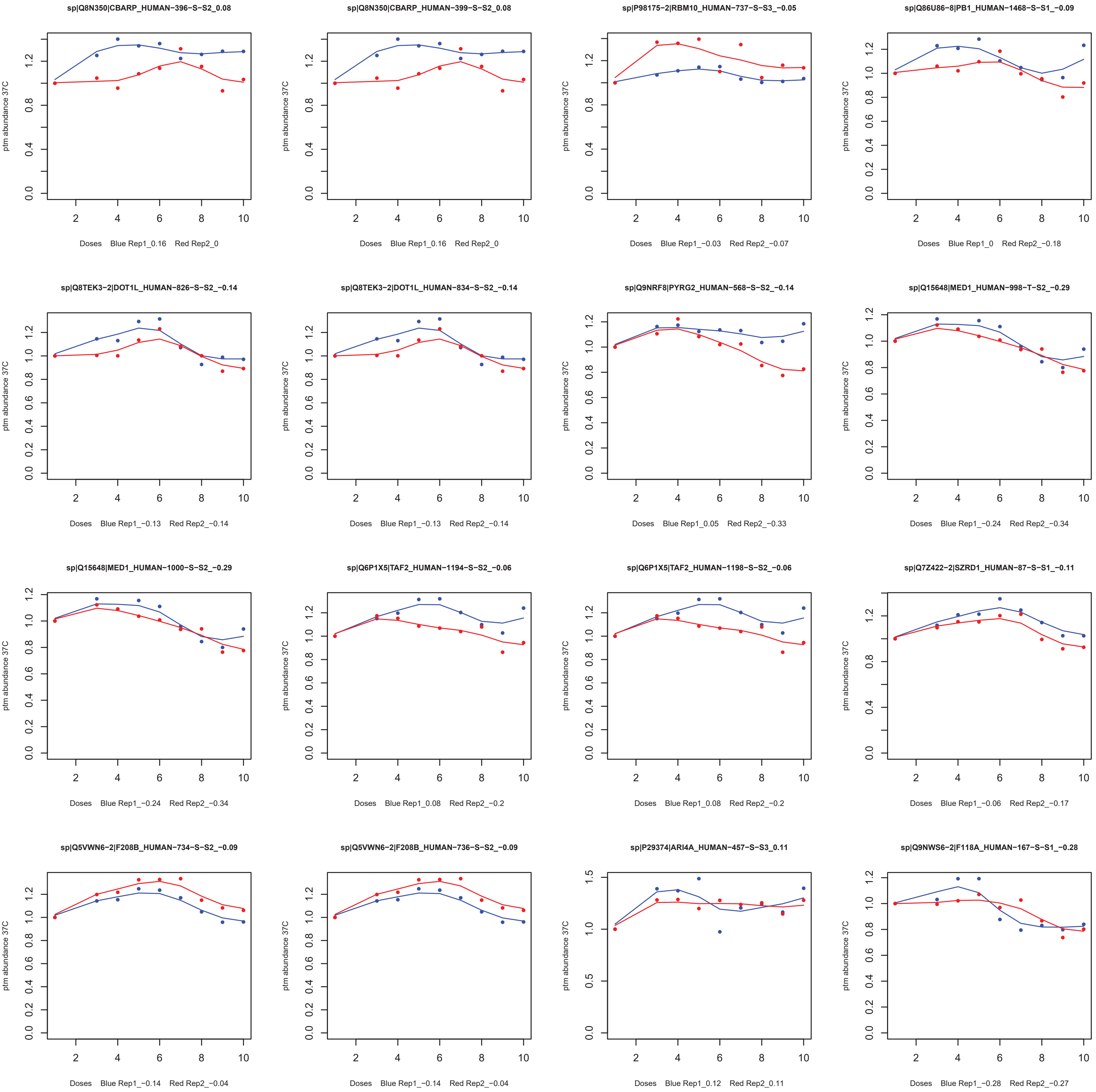
sp|Q8N350|CBARP_HUMAN−396−S−S2_0.08
Doses Blue Rep1_0.16 Red Rep2_0
sp|Q8N350|CBARP_HUMAN−399−S−S2_0.08
Doses Blue Rep1_0.16 Red Rep2_0
sp|P98175−2|RBM10_HUMAN−737−S−S3_−0.05
Doses Blue Rep1_−0.03 Red Rep2_−0.07
sp|Q86U86−8|PB1_HUMAN−1468−S−S1_−0.09
Doses Blue Rep1_0 Red Rep2_−0.18
sp|Q8TEK3−2|DOT1L_HUMAN−826−S−S2_−0.14
Doses Blue Rep1_−0.13 Red Rep2_−0.14
sp|Q8TEK3−2|DOT1L_HUMAN−834−S−S2_−0.14
Doses Blue Rep1_−0.13 Red Rep2_−0.14
sp|Q9NRF8|PYRG2_HUMAN−568−S−S2_−0.14
Doses Blue Rep1_0.05 Red Rep2_−0.33
sp|Q15648|MED1_HUMAN−998−T−S2_−0.29
Doses Blue Rep1_−0.24 Red Rep2_−0.34
sp|Q15648|MED1_HUMAN−1000−S−S2_−0.29
Doses Blue Rep1_−0.24 Red Rep2_−0.34
sp|Q6P1X5|TAF2_HUMAN−1194−S−S2_−0.06
Doses Blue Rep1_0.08 Red Rep2_−0.2
sp|Q6P1X5|TAF2_HUMAN−1198−S−S2_−0.06
Doses Blue Rep1_0.08 Red Rep2_−0.2
sp|Q7Z422−2|SZRD1_HUMAN−87−S−S1_−0.11
Doses Blue Rep1_−0.06 Red Rep2_−0.17
sp|Q5VWN6−2|F208B_HUMAN−734−S−S2_−0.09
Doses Blue Rep1_−0.14 Red Rep2_−0.04
sp|Q5VWN6−2|F208B_HUMAN−736−S−S2_−0.09
Doses Blue Rep1_−0.14 Red Rep2_−0.04
sp|P29374|ARI4A_HUMAN−457−S−S3_0.11
Doses Blue Rep1_0.12 Red Rep2_0.11
sp|Q9NWS6−2|F118A_HUMAN−167−S−S1_−0.28
Doses Blue Rep1_−0.28 Red Rep2_−0.27
ptm abundance 37C

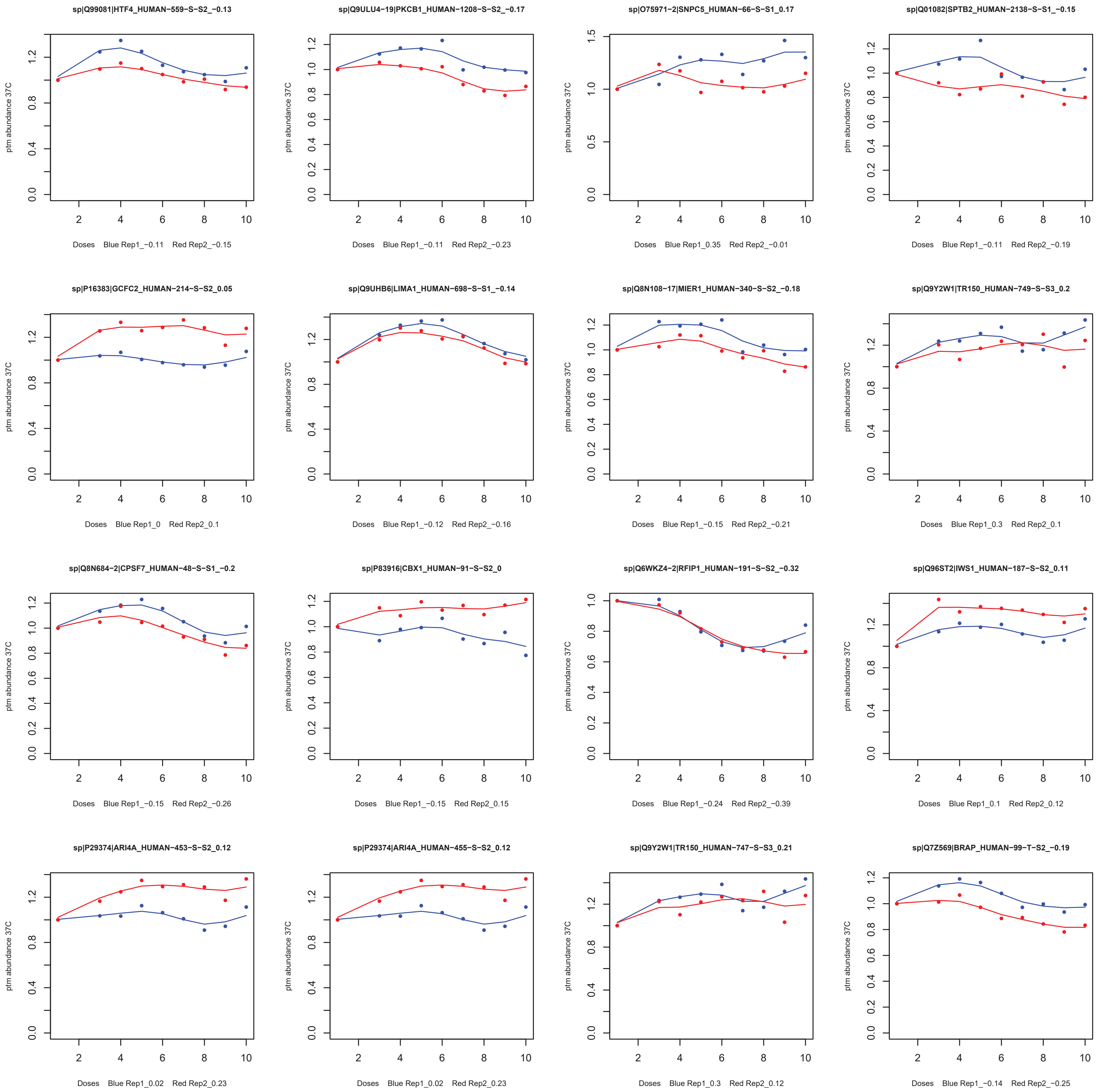
sp|Q99081|HTF4_HUMAN−559−S−S2_−0.13
ptm abundance 37C
Doses Blue Rep1_−0.11 Red Rep2_−0.15
sp|Q9ULU4−19|PKCB1_HUMAN−1208−S−S2_−0.17
ptm abundance 37C
Doses Blue Rep1_−0.11 Red Rep2_−0.23
sp|O75971−2|SNPC5_HUMAN−66−S−S1_0.17
ptm abundance 37C
Doses Blue Rep1_0.35 Red Rep2_−0.01
sp|Q01082|SPTB2_HUMAN−2138−S−S1_−0.15
ptm abundance 37C
Doses Blue Rep1_−0.11 Red Rep2_−0.19
sp|P16383|GCFC2_HUMAN−214−S−S2_0.05
ptm abundance 37C
Doses Blue Rep1_0 Red Rep2_0.1
sp|Q9UHB6|LIMA1_HUMAN−698−S−S1_−0.14
ptm abundance 37C
Doses Blue Rep1_−0.12 Red Rep2_−0.16
sp|Q8N108−17|MIER1_HUMAN−340−S−S2_−0.18
ptm abundance 37C
Doses Blue Rep1_−0.15 Red Rep2_−0.21
sp|Q9Y2W1|TR150_HUMAN−749−S−S3_0.2
ptm abundance 37C
Doses Blue Rep1_0.3 Red Rep2_0.1
sp|Q8N684−2|CPSF7_HUMAN−48−S−S1_−0.2
ptm abundance 37C
Doses Blue Rep1_−0.15 Red Rep2_−0.26
sp|P83916|CBX1_HUMAN−91−S−S2_0
ptm abundance 37C
Doses Blue Rep1_−0.15 Red Rep2_0.15
sp|Q6WKZ4−2|RFIP1_HUMAN−191−S−S2_−0.32
ptm abundance 37C
Doses Blue Rep1_−0.24 Red Rep2_−0.39
sp|Q96ST2|IWS1_HUMAN−187−S−S2_0.11
ptm abundance 37C
Doses Blue Rep1_0.1 Red Rep2_0.12
sp|P29374|ARI4A_HUMAN−453−S−S2_0.12
ptm abundance 37C
Doses Blue Rep1_0.02 Red Rep2_0.23
sp|P29374|ARI4A_HUMAN−455−S−S2_0.12
ptm abundance 37C
Doses Blue Rep1_0.02 Red Rep2_0.23
sp|Q9Y2W1|TR150_HUMAN−747−S−S3_0.21
ptm abundance 37C
Doses Blue Rep1_0.3 Red Rep2_0.12
sp|Q7Z569|BRAP_HUMAN−99−T−S2_−0.19
ptm abundance 37C
Doses Blue Rep1_−0.14 Red Rep2_−0.25

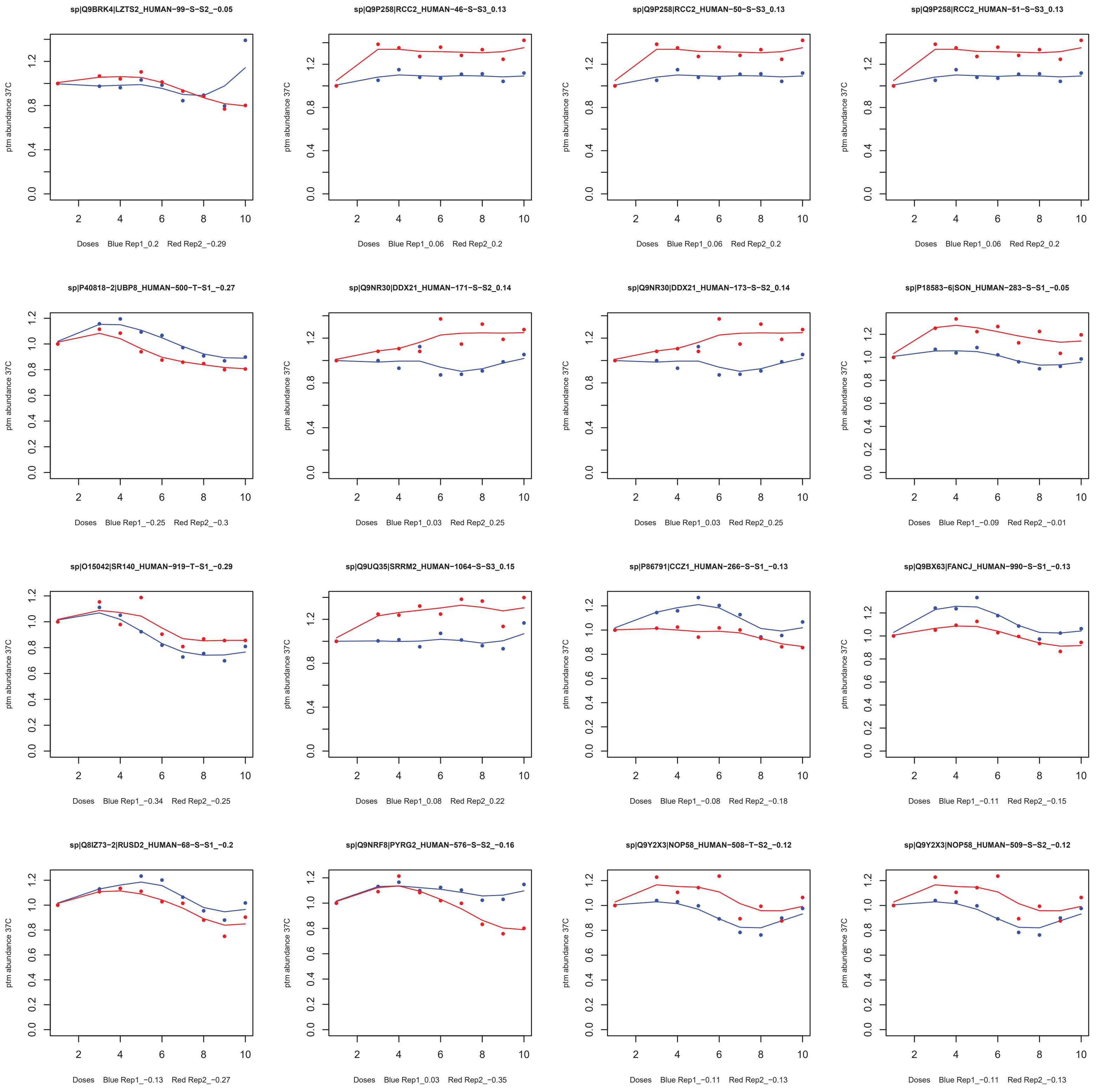
sp|Q9BRK4|LZTS2_HUMAN−99−S−S2_−0.05
Doses Blue Rep1_0.2 Red Rep2_−0.29
sp|Q9P258|RCC2_HUMAN−46−S−S3_0.13
Doses Blue Rep1_0.06 Red Rep2_0.2
sp|Q9P258|RCC2_HUMAN−50−S−S3_0.13
Doses Blue Rep1_0.06 Red Rep2_0.2
sp|Q9P258|RCC2_HUMAN−51−S−S3_0.13
Doses Blue Rep1_0.06 Red Rep2_0.2
sp|P40818−2|UBP8_HUMAN−500−T−S1_−0.27
Doses Blue Rep1_−0.25 Red Rep2_−0.3
sp|Q9NR30|DDX21_HUMAN−171−S−S2_0.14
Doses Blue Rep1_0.03 Red Rep2_0.25
sp|Q9NR30|DDX21_HUMAN−173−S−S2_0.14
Doses Blue Rep1_0.03 Red Rep2_0.25
sp|P18583−6|SON_HUMAN−283−S−S1_−0.05
Doses Blue Rep1_−0.09 Red Rep2_−0.01
sp|O15042|SR140_HUMAN−919−T−S1_−0.29
Doses Blue Rep1_−0.34 Red Rep2_−0.25
sp|Q9UQ35|SRRM2_HUMAN−1064−S−S3_0.15
Doses Blue Rep1_0.08 Red Rep2_0.22
sp|P86791|CCZ1_HUMAN−266−S−S1_−0.13
Doses Blue Rep1_−0.08 Red Rep2_−0.18
sp|Q9BX63|FANCJ_HUMAN−990−S−S1_−0.13
Doses Blue Rep1_−0.11 Red Rep2_−0.15
sp|Q8IZ73−2|RUSD2_HUMAN−68−S−S1_−0.2
Doses Blue Rep1_−0.13 Red Rep2_−0.27
sp|Q9NRF8|PYRG2_HUMAN−576−S−S2_−0.16
Doses Blue Rep1_0.03 Red Rep2_−0.35
sp|Q9Y2X3|NOP58_HUMAN−508−T−S2_−0.12
Doses Blue Rep1_−0.11 Red Rep2_−0.13
sp|Q9Y2X3|NOP58_HUMAN−509−S−S2_−0.12
Doses Blue Rep1_−0.11 Red Rep2_−0.13
ptm abundance 37C

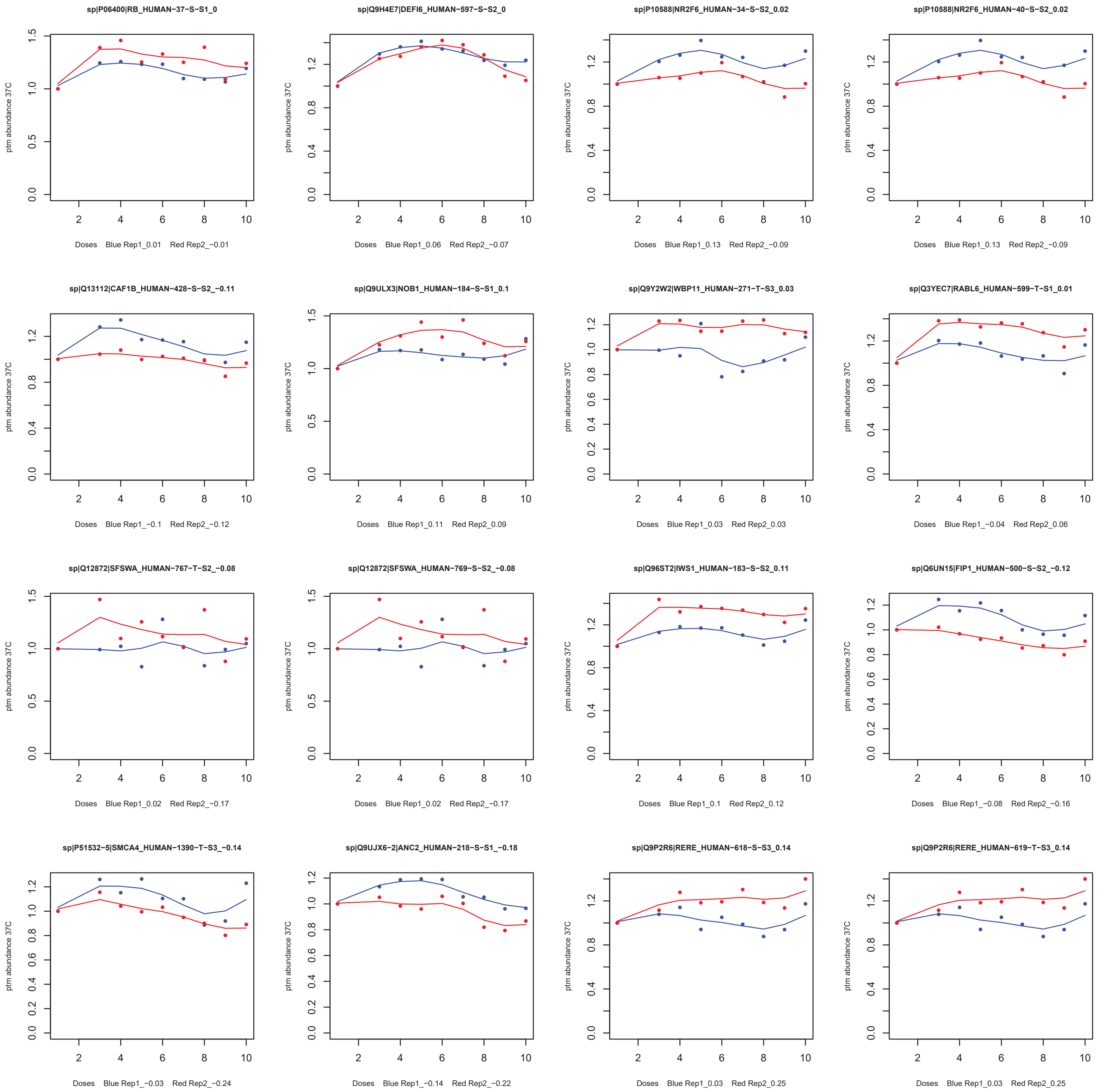
sp|P06400|RB_HUMAN−37−S−S1_0
ptm abundance 37C
Doses Blue Rep1_0.01 Red Rep2_−0.01
sp|Q9H4E7|DEFI6_HUMAN−597−S−S2_0
ptm abundance 37C
Doses Blue Rep1_0.06 Red Rep2_−0.07
sp|P10588|NR2F6_HUMAN−34−S−S2_0.02
ptm abundance 37C
Doses Blue Rep1_0.13 Red Rep2_−0.09
sp|P10588|NR2F6_HUMAN−40−S−S2_0.02
ptm abundance 37C
Doses Blue Rep1_0.13 Red Rep2_−0.09
sp|Q13112|CAF1B_HUMAN−428−S−S2_−0.11
ptm abundance 37C
Doses Blue Rep1_−0.1 Red Rep2_−0.12
sp|Q9ULX3|NOB1_HUMAN−184−S−S1_0.1
ptm abundance 37C
Doses Blue Rep1_0.11 Red Rep2_0.09
sp|Q9Y2W2|WBP11_HUMAN−271−T−S3_0.03
ptm abundance 37C
Doses Blue Rep1_0.03 Red Rep2_0.03
sp|Q3YEC7|RABL6_HUMAN−599−T−S1_0.01
ptm abundance 37C
Doses Blue Rep1_−0.04 Red Rep2_0.06
sp|Q12872|SFSWA_HUMAN−767−T−S2_−0.08
ptm abundance 37C
Doses Blue Rep1_0.02 Red Rep2_−0.17
sp|Q12872|SFSWA_HUMAN−769−S−S2_−0.08
ptm abundance 37C
Doses Blue Rep1_0.02 Red Rep2_−0.17
sp|Q96ST2|IWS1_HUMAN−183−S−S2_0.11
ptm abundance 37C
Doses Blue Rep1_0.1 Red Rep2_0.12
sp|Q6UN15|FIP1_HUMAN−500−S−S2_−0.12
ptm abundance 37C
Doses Blue Rep1_−0.08 Red Rep2_−0.16
sp|P51532−5|SMCA4_HUMAN−1390−T−S3_−0.14
ptm abundance 37C
Doses Blue Rep1_−0.03 Red Rep2_−0.24
sp|Q9UJX6−2|ANC2_HUMAN−218−S−S1_−0.18
ptm abundance 37C
Doses Blue Rep1_−0.14 Red Rep2_−0.22
sp|Q9P2R6|RERE_HUMAN−618−S−S3_0.14
ptm abundance 37C
Doses Blue Rep1_0.03 Red Rep2_0.25
sp|Q9P2R6|RERE_HUMAN−619−T−S3_0.14
ptm abundance 37C
Doses Blue Rep1_0.03 Red Rep2_0.25

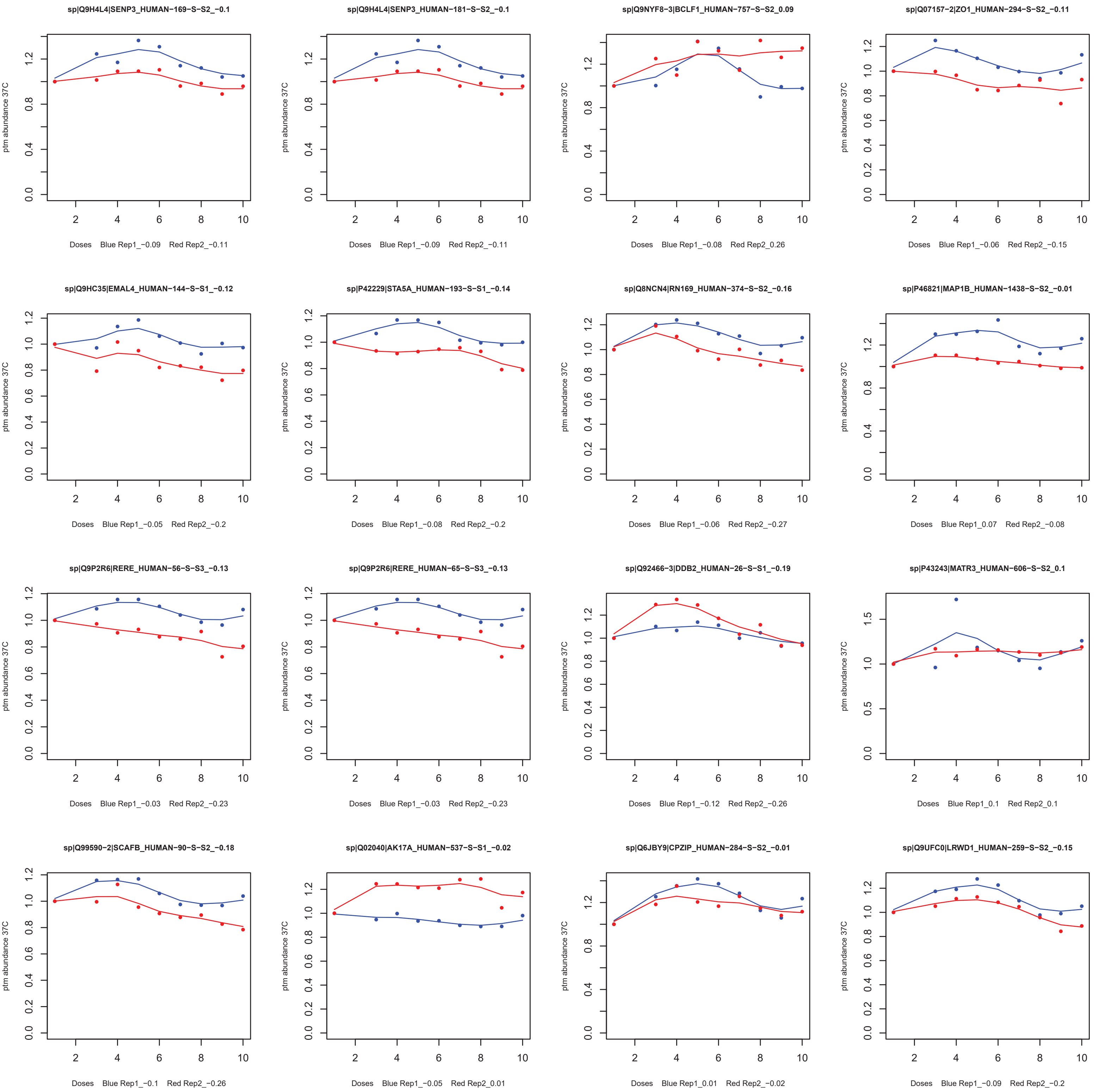

sp|Q9H4L4|SENP3_HUMAN−169−S−S2_−0.1
Doses Blue Rep1_−0.09 Red Rep2_−0.11
sp|Q9H4L4|SENP3_HUMAN−181−S−S2_−0.1
Doses Blue Rep1_−0.09 Red Rep2_−0.11
sp|Q9NYF8−3|BCLF1_HUMAN−757−S−S2_0.09
Doses Blue Rep1_−0.08 Red Rep2_0.26
sp|Q07157−2|ZO1_HUMAN−294−S−S2_−0.11
Doses Blue Rep1_−0.06 Red Rep2_−0.15
sp|Q9HC35|EMAL4_HUMAN−144−S−S1_−0.12
Doses Blue Rep1_−0.05 Red Rep2_−0.2
sp|P42229|STA5A_HUMAN−193−S−S1_−0.14
Doses Blue Rep1_−0.08 Red Rep2_−0.2
sp|Q8NCN4|RN169_HUMAN−374−S−S2_−0.16
Doses Blue Rep1_−0.06 Red Rep2_−0.27
sp|P46821|MAP1B_HUMAN−1438−S−S2_−0.01
Doses Blue Rep1_0.07 Red Rep2_−0.08
sp|Q9P2R6|RERE_HUMAN−56−S−S3_−0.13
Doses Blue Rep1_−0.03 Red Rep2_−0.23
sp|Q9P2R6|RERE_HUMAN−65−S−S3_−0.13
Doses Blue Rep1_−0.03 Red Rep2_−0.23
sp|Q92466−3|DDB2_HUMAN−26−S−S1_−0.19
Doses Blue Rep1_−0.12 Red Rep2_−0.26
sp|P43243|MATR3_HUMAN−606−S−S2_0.1
Doses Blue Rep1_0.1 Red Rep2_0.1
sp|Q99590−2|SCAFB_HUMAN−90−S−S2_−0.18
Doses Blue Rep1_−0.1 Red Rep2_−0.26
sp|Q02040|AK17A_HUMAN−537−S−S1_−0.02
Doses Blue Rep1_−0.05 Red Rep2_0.01
sp|Q6JBY9|CPZIP_HUMAN−284−S−S2_−0.01
Doses Blue Rep1_0.01 Red Rep2_−0.02
sp|Q9UFC0|LRWD1_HUMAN−259−S−S2_−0.15
Doses Blue Rep1_−0.09 Red Rep2_−0.2
ptm abundance 37C

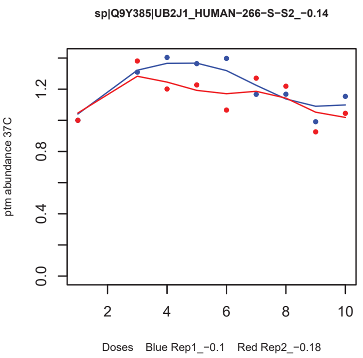
sp|Q9Y385|UB2J1_HUMAN−266−S−S2_−0.14
ptm abundance 37C
Doses Blue Rep1_−0.1 Red Rep2_−0.18

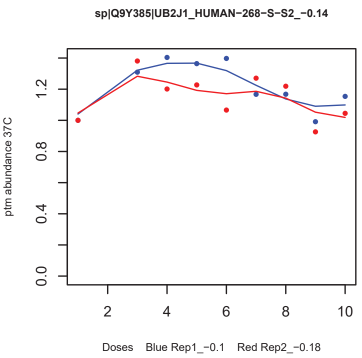
sp|Q9Y385|UB2J1_HUMAN−268−S−S2_−0.14
ptm abundance 37C
Doses Blue Rep1_−0.1 Red Rep2_−0.18

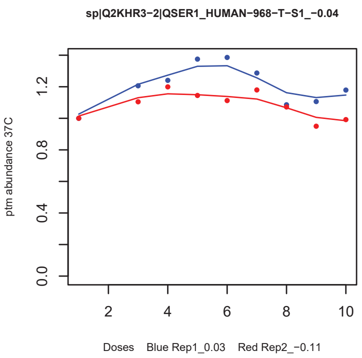
sp|Q2KHR3−2|QSER1_HUMAN−968−T−S1_−0.04
ptm abundance 37C
Doses Blue Rep1_0.03 Red Rep2_−0.11

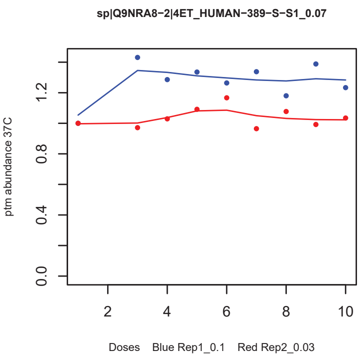
sp|Q9NRA8−2|4ET_HUMAN−389−S−S1_0.07
ptm abundance 37C
Doses Blue Rep1_0.1 Red Rep2_0.03

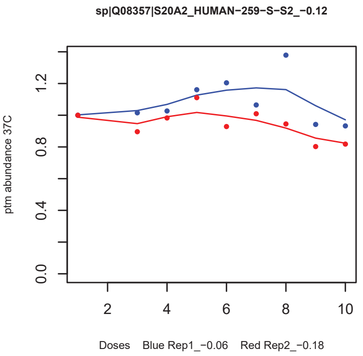
sp|Q08357|S20A2_HUMAN−259−S−S2_−0.12
ptm abundance 37C
Doses Blue Rep1_−0.06 Red Rep2_−0.18

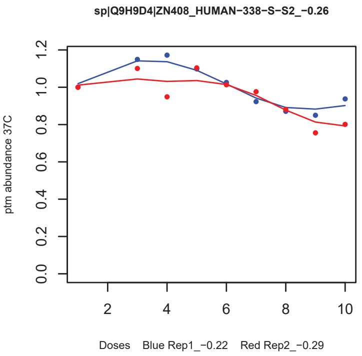
sp|Q9H9D4|ZN408_HUMAN−338−S−S2_−0.26
ptm abundance 37C
Doses Blue Rep1_−0.22 Red Rep2_−0.29

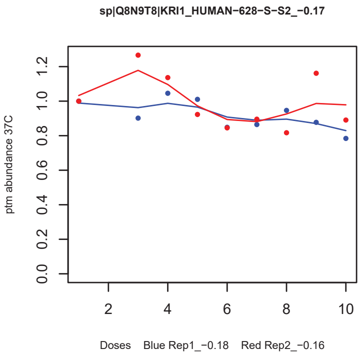
sp|Q8N9T8|KRI1_HUMAN−628−S−S2_−0.17
ptm abundance 37C
Doses Blue Rep1_−0.18 Red Rep2_−0.16

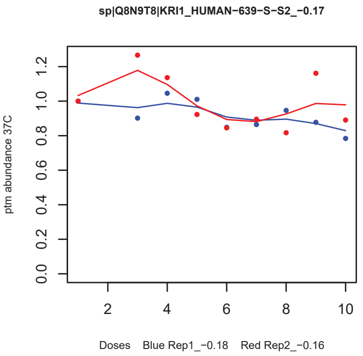
sp|Q8N9T8|KRI1_HUMAN−639−S−S2_−0.17
ptm abundance 37C
Doses Blue Rep1_−0.18 Red Rep2_−0.16

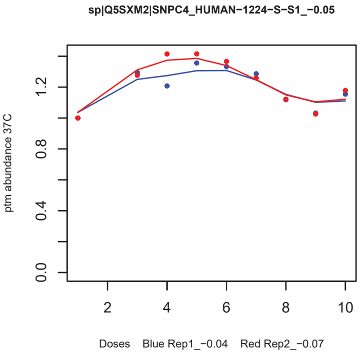
sp|Q5SXM2|SNPC4_HUMAN−1224−S−S1_−0.05
ptm abundance 37C
Doses Blue Rep1_−0.04 Red Rep2_−0.07

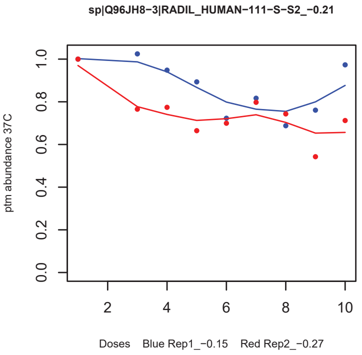
sp|Q96JH8−3|RADIL_HUMAN−111−S−S2_−0.21
ptm abundance 37C
Doses Blue Rep1_−0.15 Red Rep2_−0.27

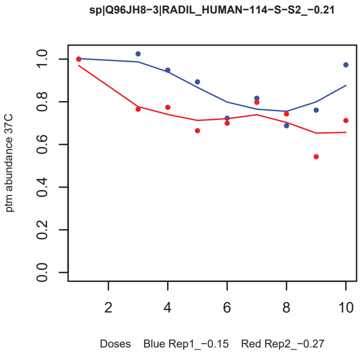
sp|Q96JH8−3|RADIL_HUMAN−114−S−S2_−0.21
ptm abundance 37C
Doses Blue Rep1_−0.15 Red Rep2_−0.27

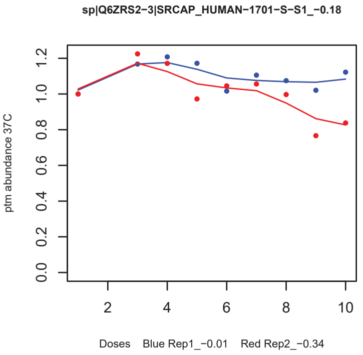
sp|Q6ZRS2−3|SRCAP_HUMAN−1701−S−S1_−0.18
ptm abundance 37C
Doses Blue Rep1_−0.01 Red Rep2_−0.34

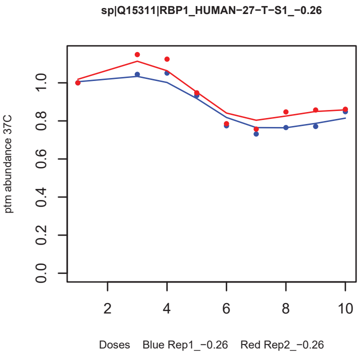
sp|Q15311|RBP1_HUMAN−27−T−S1_−0.26
ptm abundance 37C
Doses Blue Rep1_−0.26 Red Rep2_−0.26

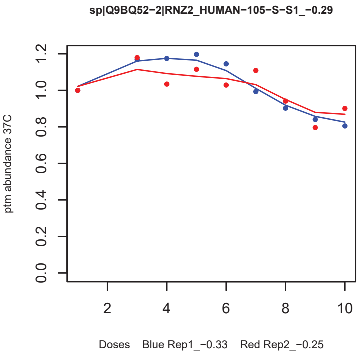
sp|Q9BQ52−2|RNZ2_HUMAN−105−S−S1_−0.29
ptm abundance 37C
Doses Blue Rep1_−0.33 Red Rep2_−0.25

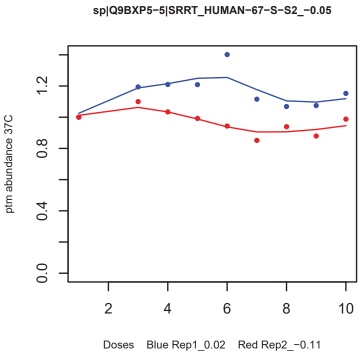
sp|Q9BXP5−5|SRRT_HUMAN−67−S−S2_−0.05
ptm abundance 37C
Doses Blue Rep1_0.02 Red Rep2_−0.11

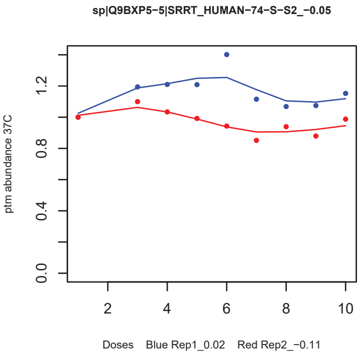
sp|Q9BXP5−5|SRRT_HUMAN−74−S−S2_−0.05
ptm abundance 37C
Doses Blue Rep1_0.02 Red Rep2_−0.11

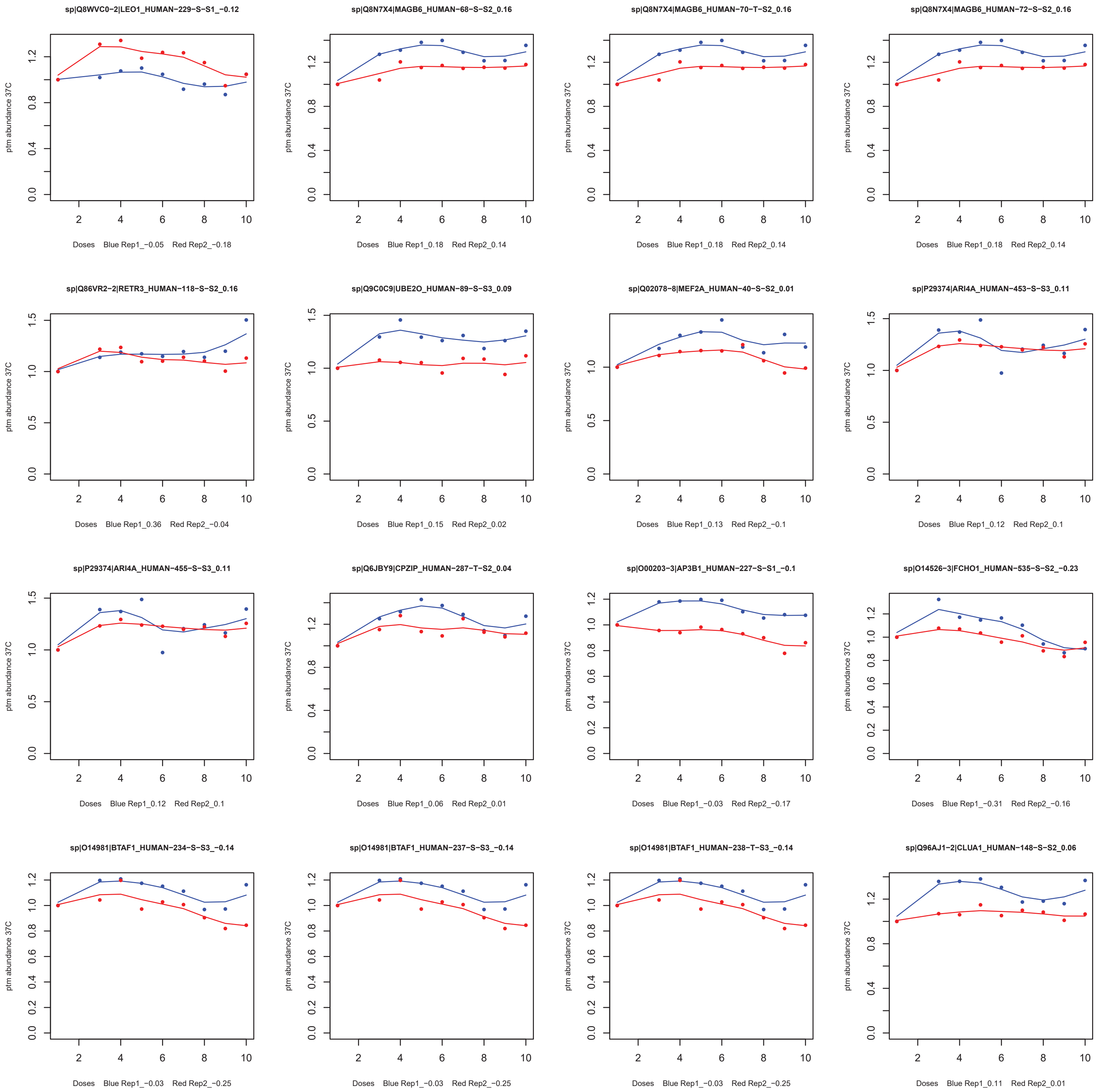

sp|Q8WVC0−2|LEO1_HUMAN−229−S−S1_−0.12
ptm abundance 37C
Doses Blue Rep1_−0.05 Red Rep2_−0.18
sp|Q8N7X4|MAGB6_HUMAN−68−S−S2_0.16
ptm abundance 37C
Doses Blue Rep1_0.18 Red Rep2_0.14
sp|Q8N7X4|MAGB6_HUMAN−70−T−S2_0.16
ptm abundance 37C
Doses Blue Rep1_0.18 Red Rep2_0.14
sp|Q8N7X4|MAGB6_HUMAN−72−S−S2_0.16
ptm abundance 37C
Doses Blue Rep1_0.18 Red Rep2_0.14
sp|Q86VR2−2|RETR3_HUMAN−118−S−S2_0.16
ptm abundance 37C
Doses Blue Rep1_0.36 Red Rep2_−0.04
sp|Q9C0C9|UBE2O_HUMAN−89−S−S3_0.09
ptm abundance 37C
Doses Blue Rep1_0.15 Red Rep2_0.02
sp|Q02078−8|MEF2A_HUMAN−40−S−S2_0.01
ptm abundance 37C
Doses Blue Rep1_0.13 Red Rep2_−0.1
sp|P29374|ARI4A_HUMAN−453−S−S3_0.11
ptm abundance 37C
Doses Blue Rep1_0.12 Red Rep2_0.1
sp|P29374|ARI4A_HUMAN−455−S−S3_0.11
ptm abundance 37C
Doses Blue Rep1_0.12 Red Rep2_0.1
sp|Q6JBY9|CPZIP_HUMAN−287−T−S2_0.04
ptm abundance 37C
Doses Blue Rep1_0.06 Red Rep2_0.01
sp|O00203−3|AP3B1_HUMAN−227−S−S1_−0.1
ptm abundance 37C
Doses Blue Rep1_−0.03 Red Rep2_−0.17
sp|O14526−3|FCHO1_HUMAN−535−S−S2_−0.23
ptm abundance 37C
Doses Blue Rep1_−0.31 Red Rep2_−0.16
sp|O14981|BTAF1_HUMAN−234−S−S3_−0.14
ptm abundance 37C
Doses Blue Rep1_−0.03 Red Rep2_−0.25
sp|O14981|BTAF1_HUMAN−237−S−S3_−0.14
ptm abundance 37C
Doses Blue Rep1_−0.03 Red Rep2_−0.25
sp|O14981|BTAF1_HUMAN−238−T−S3_−0.14
ptm abundance 37C
Doses Blue Rep1_−0.03 Red Rep2_−0.25
sp|Q96AJ1−2|CLUA1_HUMAN−148−S−S2_0.06
ptm abundance 37C
Doses Blue Rep1_0.11 Red Rep2_0.01

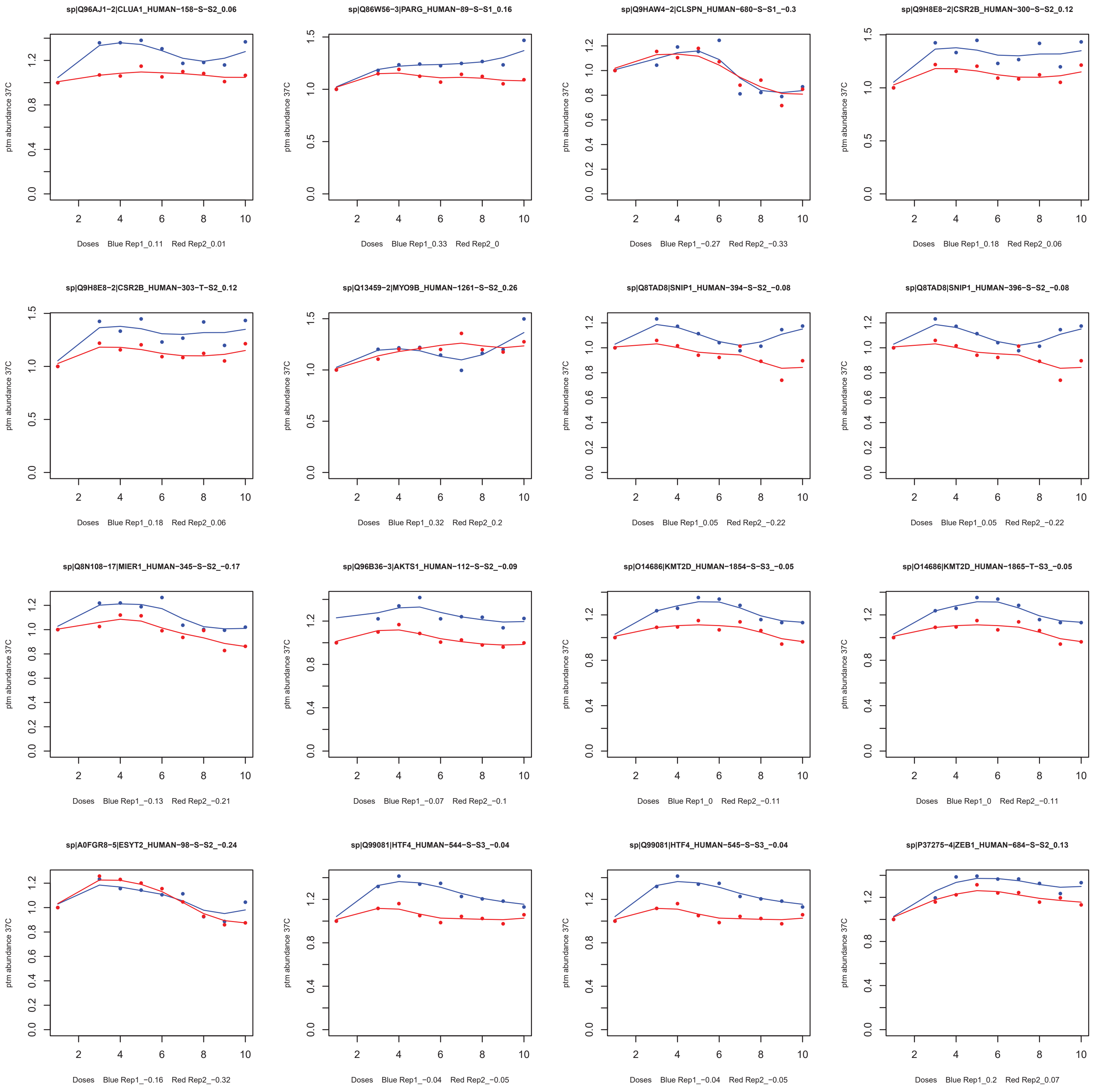
sp|Q96AJ1-2|CLUA1_HUMAN-158-S-S2_0.06
Doses Blue Rep1_0.11 Red Rep2_0.01
sp|Q86W56-3|PARG_HUMAN-89-S-S1_0.16
Doses Blue Rep1_0.33 Red Rep2_0
sp|Q9HAW4-2|CLSPN_HUMAN-680-S-S1_-0.3
Doses Blue Rep1_-0.27 Red Rep2_-0.33
sp|Q9H8E8-2|CSR2B_HUMAN-300-S-S2_0.12
Doses Blue Rep1_0.18 Red Rep2_0.06
sp|Q9H8E8-2|CSR2B_HUMAN-303-T-S2_0.12
Doses Blue Rep1_0.18 Red Rep2_0.06
sp|Q13459-2|MYO9B_HUMAN-1261-S-S2_0.26
Doses Blue Rep1_0.32 Red Rep2_0.2
sp|Q8TAD8|SNIP1_HUMAN-394-S-S2_-0.08
Doses Blue Rep1_0.05 Red Rep2_-0.22
sp|Q8TAD8|SNIP1_HUMAN-396-S-S2_-0.08
Doses Blue Rep1_0.05 Red Rep2_-0.22
sp|Q8N108-17|MIER1_HUMAN-345-S-S2_-0.17
Doses Blue Rep1_-0.13 Red Rep2_-0.21
sp|Q96B36-3|AKTS1_HUMAN-112-S-S2_-0.09
Doses Blue Rep1_-0.07 Red Rep2_-0.1
sp|O14686|KMT2D_HUMAN-1854-S-S3_-0.05
Doses Blue Rep1_0 Red Rep2_-0.11
sp|O14686|KMT2D_HUMAN-1865-T-S3_-0.05
Doses Blue Rep1_0 Red Rep2_-0.11
sp|A0FGR8-5|ESYT2_HUMAN-98-S-S2_-0.24
Doses Blue Rep1_-0.16 Red Rep2_-0.32
sp|Q99081|HTF4_HUMAN-544-S-S3_-0.04
Doses Blue Rep1_-0.04 Red Rep2_-0.05
sp|Q99081|HTF4_HUMAN-545-S-S3_-0.04
Doses Blue Rep1_-0.04 Red Rep2_-0.05
sp|P37275-4|ZEB1_HUMAN-684-S-S2_0.13
Doses Blue Rep1_0.2 Red Rep2_0.07
ptm abundance 37C

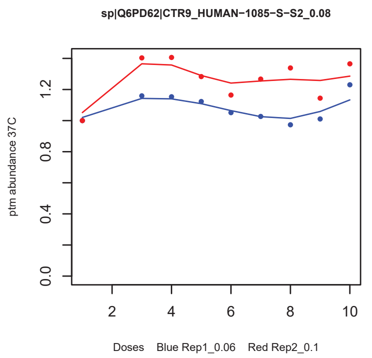
sp|Q6PD62|CTR9_HUMAN−1085−S−S2_0.08
ptm abundance 37C
Doses Blue Rep1_0.06 Red Rep2_0.1
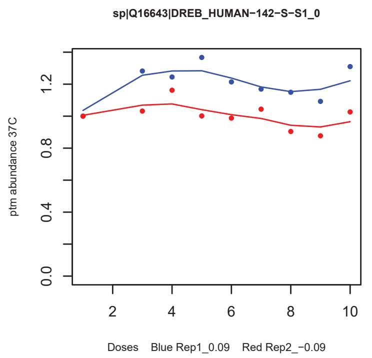
sp|Q16643|DREB_HUMAN−142−S−S1_0
ptm abundance 37C
Doses Blue Rep1_0.09 Red Rep2_−0.09
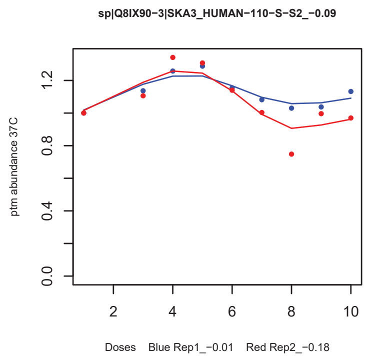
sp|Q8IX90−3|SKA3_HUMAN−110−S−S2_−0.09
ptm abundance 37C
Doses Blue Rep1_−0.01 Red Rep2_−0.18
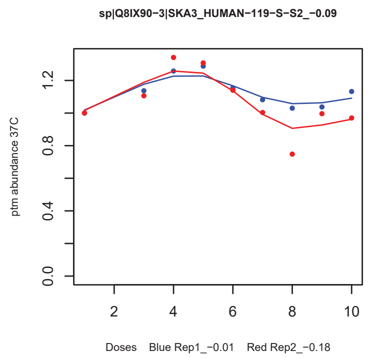
sp|Q8IX90−3|SKA3_HUMAN−119−S−S2_−0.09
ptm abundance 37C
Doses Blue Rep1_−0.01 Red Rep2_−0.18
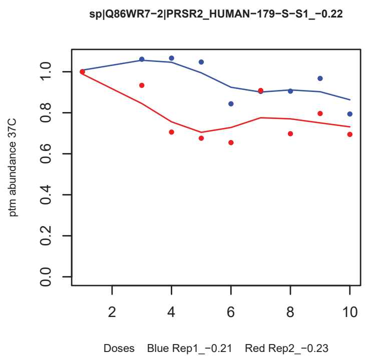
sp|Q86WR7−2|PRSR2_HUMAN−179−S−S1_−0.22
ptm abundance 37C
Doses Blue Rep1_−0.21 Red Rep2_−0.23
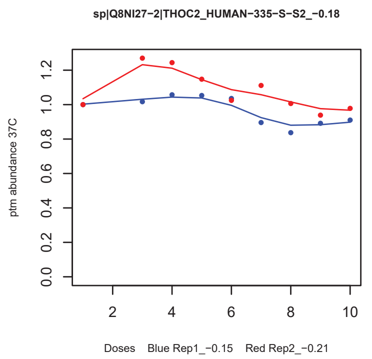
sp|Q8NI27−2|THOC2_HUMAN−335−S−S2_−0.18
ptm abundance 37C
Doses Blue Rep1_−0.15 Red Rep2_−0.21
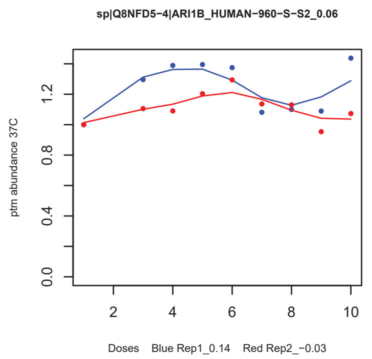
sp|Q8NFD5−4|ARI1B_HUMAN−960−S−S2_0.06
ptm abundance 37C
Doses Blue Rep1_0.14 Red Rep2_−0.03
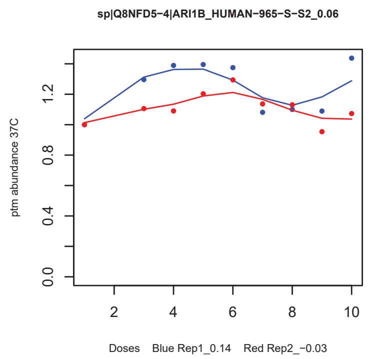
sp|Q8NFD5−4|ARI1B_HUMAN−965−S−S2_0.06
ptm abundance 37C
Doses Blue Rep1_0.14 Red Rep2_−0.03
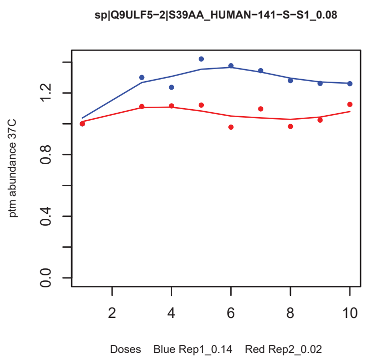
sp|Q9ULF5−2|S39AA_HUMAN−141−S−S1_0.08
ptm abundance 37C
Doses Blue Rep1_0.14 Red Rep2_0.02
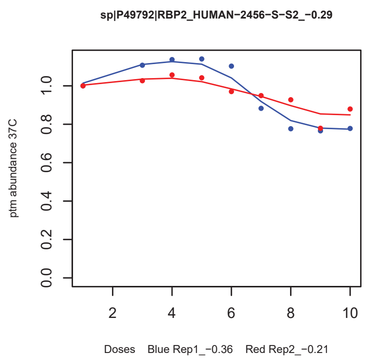
sp|P49792|RBP2_HUMAN−2456−S−S2_−0.29
ptm abundance 37C
Doses Blue Rep1_−0.36 Red Rep2_−0.21
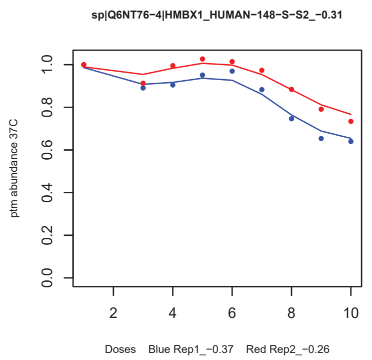
sp|Q6NT76−4|HMBX1_HUMAN−148−S−S2_−0.31
ptm abundance 37C
Doses Blue Rep1_−0.37 Red Rep2_−0.26
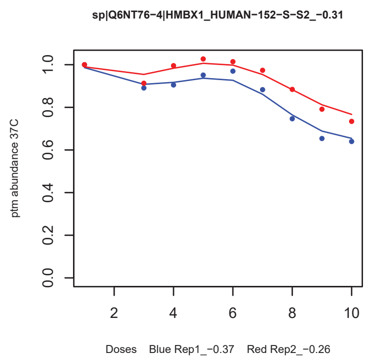
sp|Q6NT76−4|HMBX1_HUMAN−152−S−S2_−0.31
ptm abundance 37C
Doses Blue Rep1_−0.37 Red Rep2_−0.26
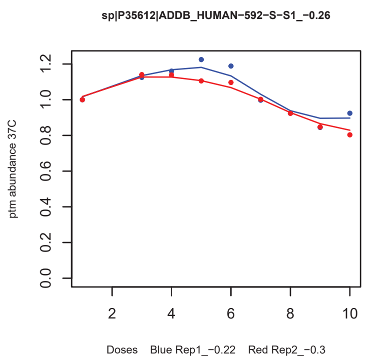
sp|P35612|ADDB_HUMAN−592−S−S1_−0.26
ptm abundance 37C
Doses Blue Rep1_−0.22 Red Rep2_−0.3
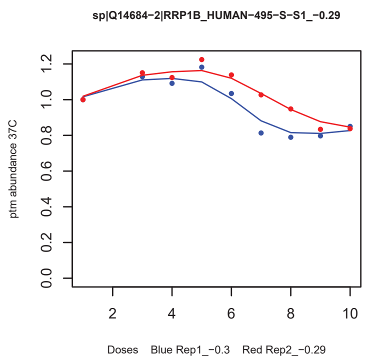
sp|Q14684−2|RRP1B_HUMAN−495−S−S1_−0.29
ptm abundance 37C
Doses Blue Rep1_−0.3 Red Rep2_−0.29
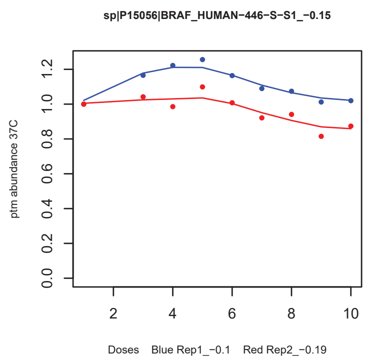
sp|P15056|BRAF_HUMAN−446−S−S1_−0.15
ptm abundance 37C
Doses Blue Rep1_−0.1 Red Rep2_−0.19
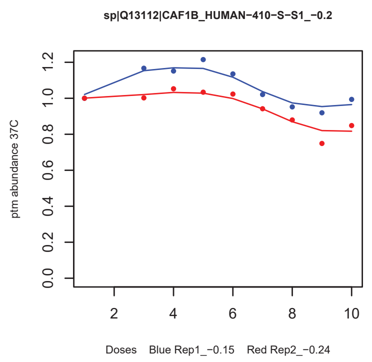
sp|Q13112|CAF1B_HUMAN−410−S−S1_−0.2
ptm abundance 37C
Doses Blue Rep1_−0.15 Red Rep2_−0.24

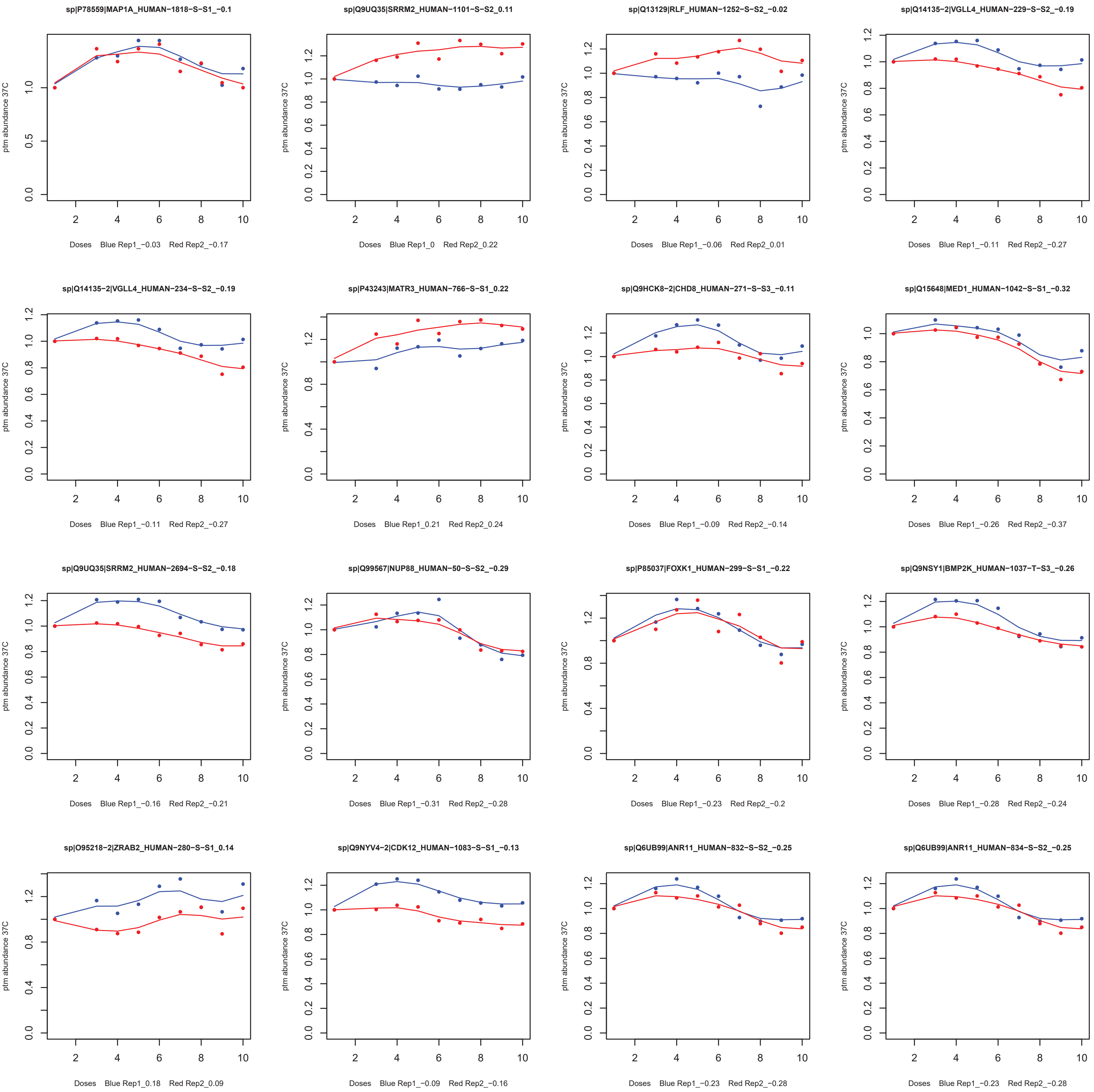
sp|P78559|MAP1A_HUMAN−1818−S−S1_−0.1
Doses Blue Rep1_−0.03 Red Rep2_−0.17
sp|Q9UQ35|SRRM2_HUMAN−1101−S−S2_0.11
Doses Blue Rep1_0 Red Rep2_0.22
sp|Q13129|RLF_HUMAN−1252−S−S2_−0.02
Doses Blue Rep1_−0.06 Red Rep2_0.01
sp|Q14135−2|VGLL4_HUMAN−229−S−S2_−0.19
Doses Blue Rep1_−0.11 Red Rep2_−0.27
sp|Q14135−2|VGLL4_HUMAN−234−S−S2_−0.19
Doses Blue Rep1_−0.11 Red Rep2_−0.27
sp|P43243|MATR3_HUMAN−766−S−S1_0.22
Doses Blue Rep1_0.21 Red Rep2_0.24
sp|Q9HCK8−2|CHD8_HUMAN−271−S−S3_−0.11
Doses Blue Rep1_−0.09 Red Rep2_−0.14
sp|Q15648|MED1_HUMAN−1042−S−S1_−0.32
Doses Blue Rep1_−0.26 Red Rep2_−0.37
sp|Q9UQ35|SRRM2_HUMAN−2694−S−S2_−0.18
Doses Blue Rep1_−0.16 Red Rep2_−0.21
sp|Q99567|NUP88_HUMAN−50−S−S2_−0.29
Doses Blue Rep1_−0.31 Red Rep2_−0.28
sp|P85037|FOXK1_HUMAN−299−S−S1_−0.22
Doses Blue Rep1_−0.23 Red Rep2_−0.2
sp|Q9NSY1|BMP2K_HUMAN−1037−T−S3_−0.26
Doses Blue Rep1_−0.28 Red Rep2_−0.24
sp|O95218−2|ZRAB2_HUMAN−280−S−S1_0.14
Doses Blue Rep1_0.18 Red Rep2_0.09
sp|Q9NYV4−2|CDK12_HUMAN−1083−S−S1_−0.13
Doses Blue Rep1_−0.09 Red Rep2_−0.16
sp|Q6UB99|ANR11_HUMAN−832−S−S2_−0.25
Doses Blue Rep1_−0.23 Red Rep2_−0.28
sp|Q6UB99|ANR11_HUMAN−834−S−S2_−0.25
Doses Blue Rep1_−0.23 Red Rep2_−0.28
ptm abundance 37C

sp|Q9ULH1|ASAP1_HUMAN−717−S−S2_−0.26

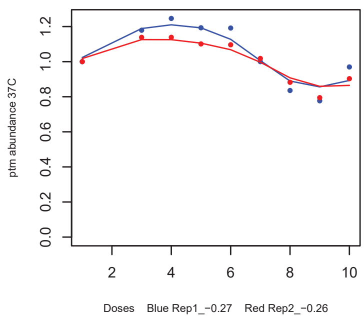

sp|Q9ULH1|ASAP1_HUMAN−726−S−S2_−0.26

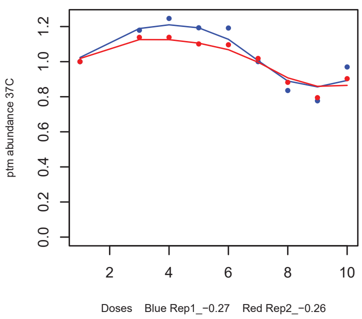

sp|Q9UQ35|SRRM2_HUMAN−2067−S−S3_−0.21

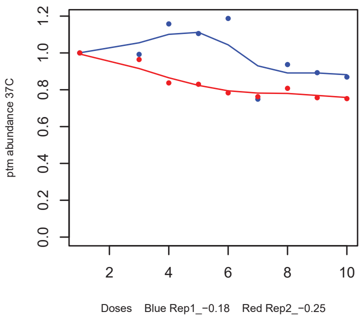

sp|Q9UQ35|SRRM2_HUMAN−2069−T−S3_−0.21

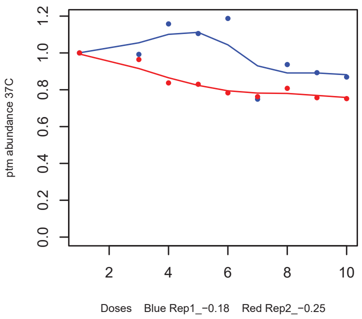

sp|Q9UQ35|SRRM2_HUMAN−2071−S−S3_−0.21

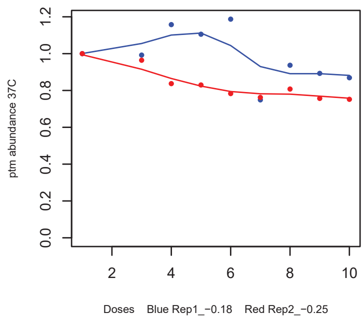

sp|O75362|ZN217_HUMAN−327−S−S3_0.09

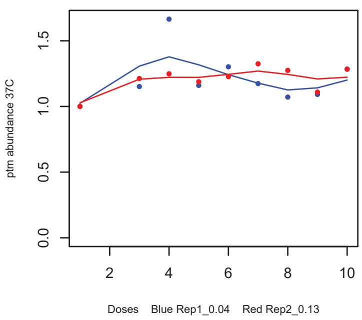

sp|Q9Y3X0|CCDC9_HUMAN−381−T−S3_−0.04

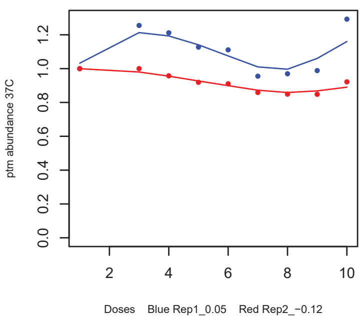

sp|Q9BTK6|PAGR1_HUMAN−138−T−S2_−0.02

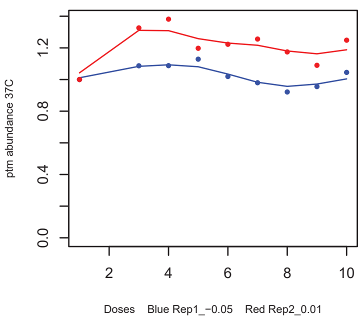

sp|Q9BTK6|PAGR1_HUMAN−148−S−S2_−0.02

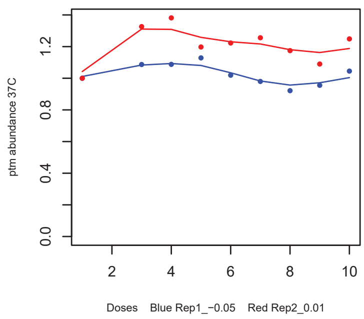

sp|Q5JSZ5|PRC2B_HUMAN−980−S−S1_−0.01

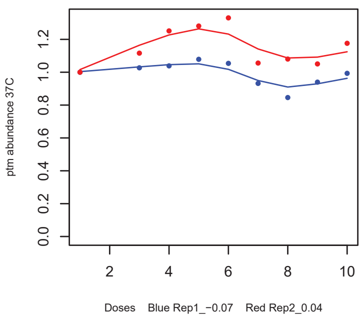

sp|Q12872|SFSWA_HUMAN−870−S−S2_−0.33

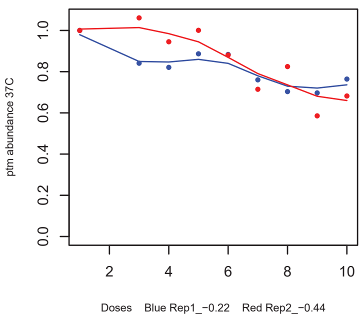

sp|Q12872|SFSWA_HUMAN−866−S−S2_−0.33

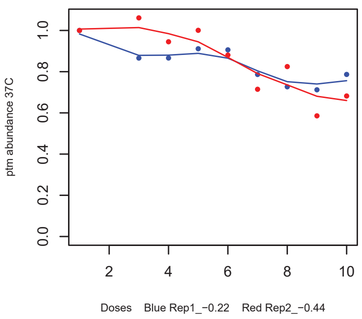

sp|Q9NSY1|BMP2K_HUMAN−1029−S−S3_−0.26

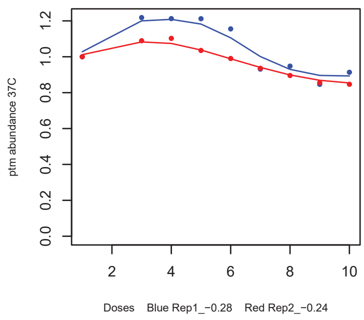

sp|Q9NSY1|BMP2K_HUMAN−1031−S−S3_−0.26

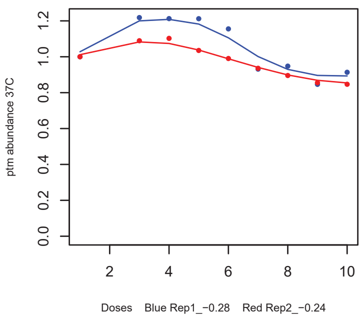

sp|Q9NSY1|BMP2K_HUMAN−1032−S−S3_−0.26

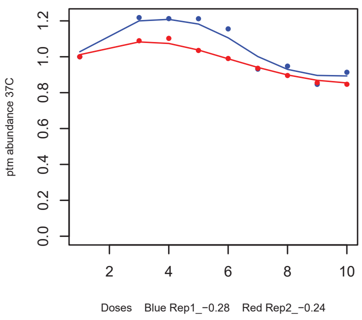

sp|P18583−6|SON_HUMAN−1829−S−S3_−0.19

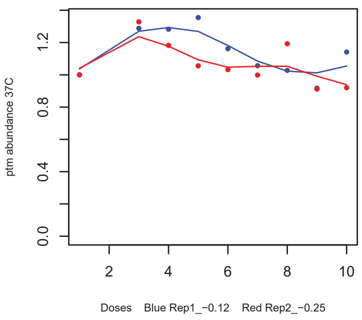

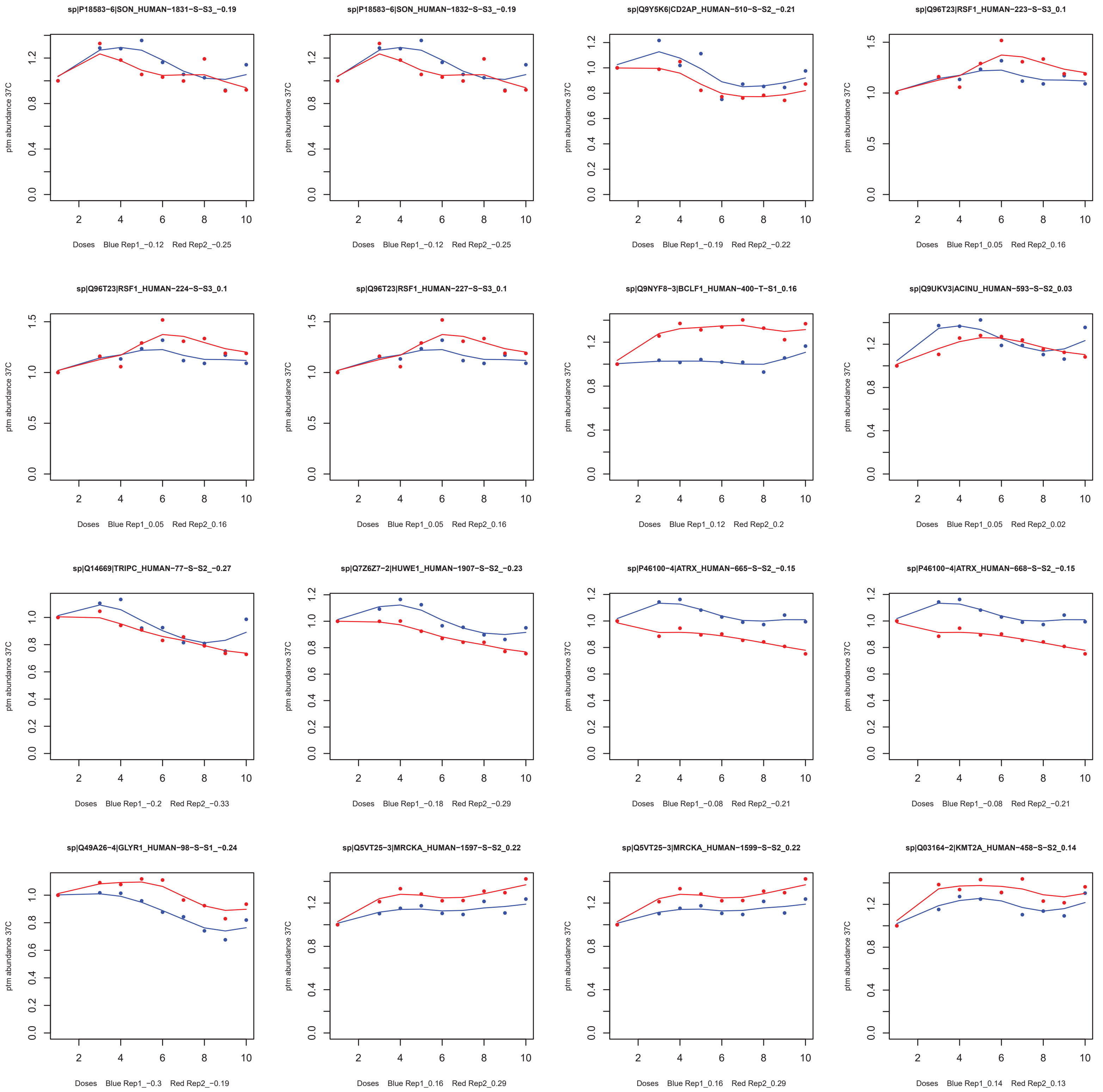
sp|P18583−6|SON_HUMAN−1831−S−S3_−0.19
ptm abundance 37C
Doses Blue Rep1_−0.12 Red Rep2_−0.25
sp|P18583−6|SON_HUMAN−1832−S−S3_−0.19
Doses Blue Rep1_−0.12 Red Rep2_−0.25
sp|Q9Y5K6|CD2AP_HUMAN−510−S−S2_−0.21
Doses Blue Rep1_−0.19 Red Rep2_−0.22
sp|Q96T23|RSF1_HUMAN−223−S−S3_0.1
Doses Blue Rep1_0.05 Red Rep2_0.16
sp|Q96T23|RSF1_HUMAN−224−S−S3_0.1
Doses Blue Rep1_0.05 Red Rep2_0.16
sp|Q96T23|RSF1_HUMAN−227−S−S3_0.1
Doses Blue Rep1_0.05 Red Rep2_0.16
sp|Q9NYF8−3|BCLF1_HUMAN−400−T−S1_0.16
Doses Blue Rep1_0.12 Red Rep2_0.2
sp|Q9UKV3|ACINU_HUMAN−593−S−S2_0.03
Doses Blue Rep1_0.05 Red Rep2_0.02
sp|Q14669|TRIPC_HUMAN−77−S−S2_−0.27
Doses Blue Rep1_−0.2 Red Rep2_−0.33
sp|Q7Z6Z7−2|HUWE1_HUMAN−1907−S−S2_−0.23
Doses Blue Rep1_−0.18 Red Rep2_−0.29
sp|P46100−4|ATRX_HUMAN−665−S−S2_−0.15
Doses Blue Rep1_−0.08 Red Rep2_−0.21
sp|P46100−4|ATRX_HUMAN−668−S−S2_−0.15
Doses Blue Rep1_−0.08 Red Rep2_−0.21
sp|Q49A26−4|GLYR1_HUMAN−98−S−S1_−0.24
Doses Blue Rep1_−0.3 Red Rep2_−0.19
sp|Q5VT25−3|MRCKA_HUMAN−1597−S−S2_0.22
Doses Blue Rep1_0.16 Red Rep2_0.29
sp|Q5VT25−3|MRCKA_HUMAN−1599−S−S2_0.22
Doses Blue Rep1_0.16 Red Rep2_0.29
sp|Q03164−2|KMT2A_HUMAN−458−S−S2_0.14
Doses Blue Rep1_0.14 Red Rep2_0.13

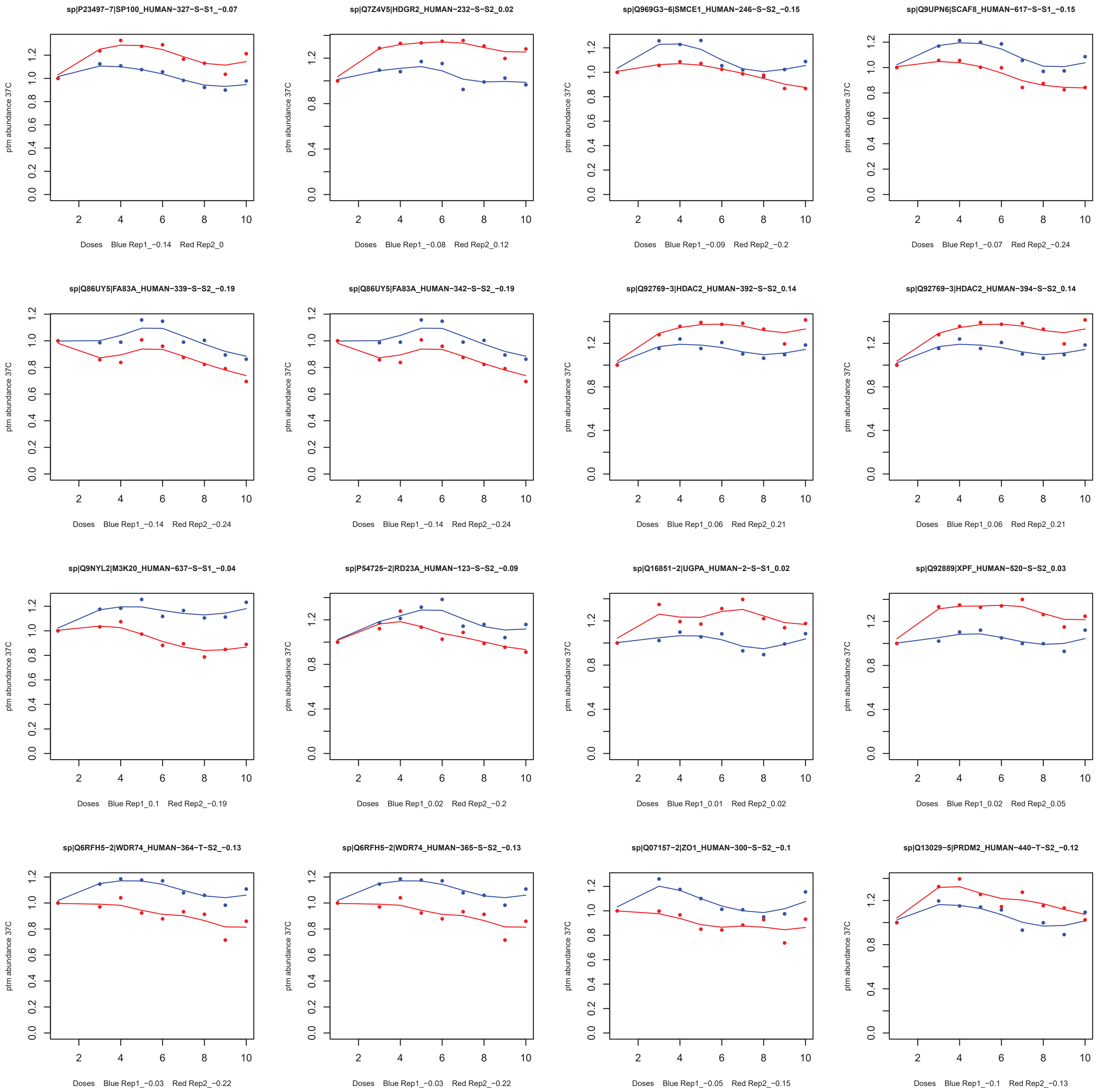
sp|P23497−7|SP100_HUMAN−327−S−S1_−0.07
Doses Blue Rep1_−0.14 Red Rep2_0
sp|Q7Z4V5|HDGR2_HUMAN−232−S−S2_0.02
Doses Blue Rep1_−0.08 Red Rep2_0.12
sp|Q969G3−6|SMCE1_HUMAN−246−S−S2_−0.15
Doses Blue Rep1_−0.09 Red Rep2_−0.2
sp|Q9UPN6|SCAF8_HUMAN−617−S−S1_−0.15
Doses Blue Rep1_−0.07 Red Rep2_−0.24
sp|Q86UY5|FA83A_HUMAN−339−S−S2_−0.19
Doses Blue Rep1_−0.14 Red Rep2_−0.24
sp|Q86UY5|FA83A_HUMAN−342−S−S2_−0.19
Doses Blue Rep1_−0.14 Red Rep2_−0.24
sp|Q92769−3|HDAC2_HUMAN−392−S−S2_0.14
Doses Blue Rep1_0.06 Red Rep2_0.21
sp|Q92769−3|HDAC2_HUMAN−394−S−S2_0.14
Doses Blue Rep1_0.06 Red Rep2_0.21
sp|Q9NYL2|M3K20_HUMAN−637−S−S1_−0.04
Doses Blue Rep1_0.1 Red Rep2_−0.19
sp|P54725−2|RD23A_HUMAN−123−S−S2_−0.09
Doses Blue Rep1_0.02 Red Rep2_−0.2
sp|Q16851−2|UGPA_HUMAN−2−S−S1_0.02
Doses Blue Rep1_0.01 Red Rep2_0.02
sp|Q92889|XPF_HUMAN−520−S−S2_0.03
Doses Blue Rep1_0.02 Red Rep2_0.05
sp|Q6RFH5−2|WDR74_HUMAN−364−T−S2_−0.13
Doses Blue Rep1_−0.03 Red Rep2_−0.22
sp|Q6RFH5−2|WDR74_HUMAN−365−S−S2_−0.13
Doses Blue Rep1_−0.03 Red Rep2_−0.22
sp|Q07157−2|ZO1_HUMAN−300−S−S2_−0.1
Doses Blue Rep1_−0.05 Red Rep2_−0.15
sp|Q13029−5|PRDM2_HUMAN−440−T−S2_−0.12
Doses Blue Rep1_−0.1 Red Rep2_−0.13
ptm abundance 37C

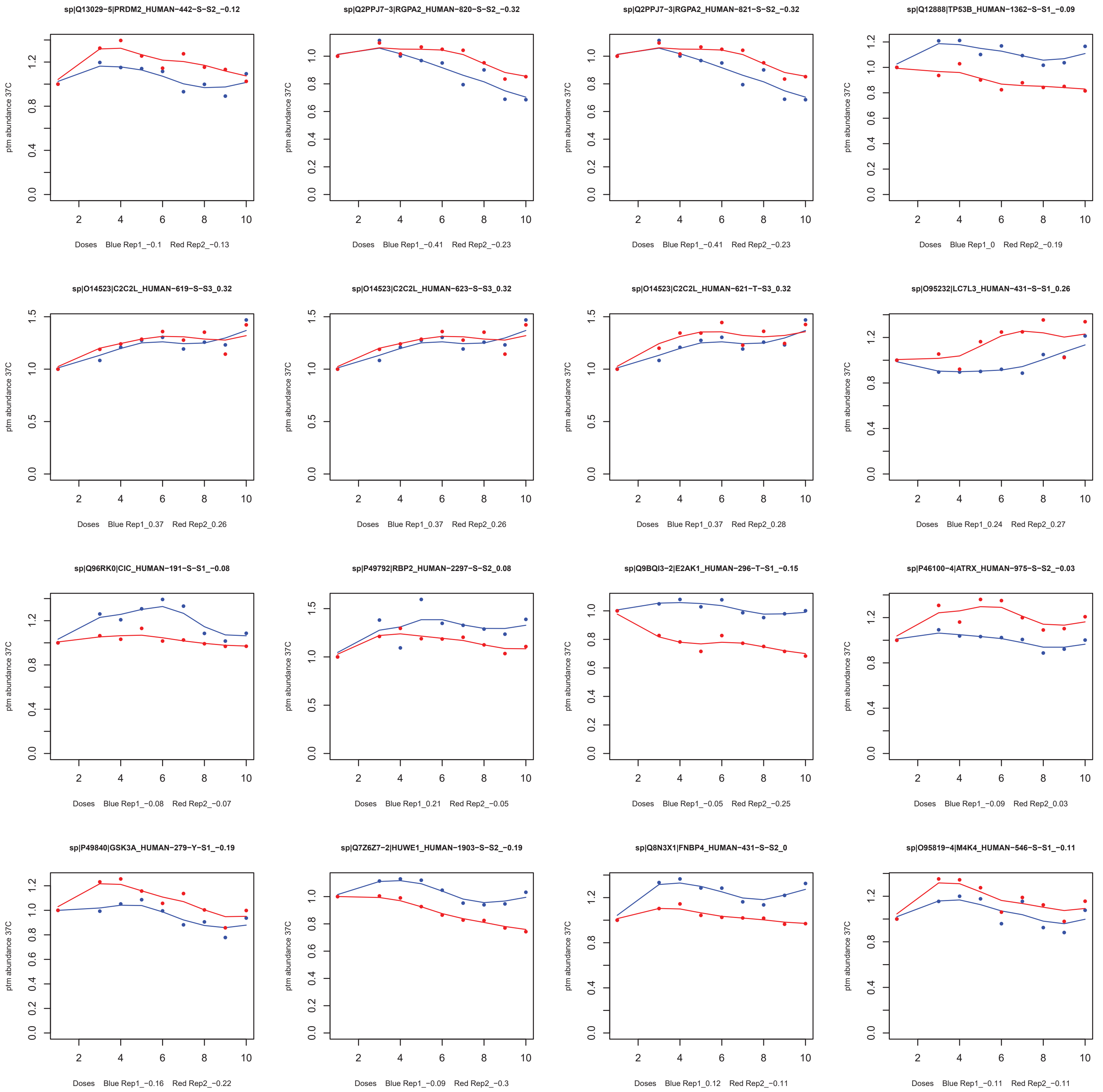
sp|Q13029-5|PRDM2_HUMAN-442-S-S2_-0.12
Doses Blue Rep1_-0.1 Red Rep2_-0.13
sp|Q2PPJ7-3|RGPA2_HUMAN-820-S-S2_-0.32
Doses Blue Rep1_-0.41 Red Rep2_-0.23
sp|Q2PPJ7-3|RGPA2_HUMAN-821-S-S2_-0.32
Doses Blue Rep1_-0.41 Red Rep2_-0.23
sp|Q12888|TP53B_HUMAN-1362-S-S1_-0.09
Doses Blue Rep1_0 Red Rep2_-0.19
sp|O14523|C2C2L_HUMAN-619-S-S3_0.32
Doses Blue Rep1_0.37 Red Rep2_0.26
sp|O14523|C2C2L_HUMAN-623-S-S3_0.32
Doses Blue Rep1_0.37 Red Rep2_0.26
sp|O14523|C2C2L_HUMAN-621-T-S3_0.32
Doses Blue Rep1_0.37 Red Rep2_0.28
sp|O95232|LC7L3_HUMAN-431-S-S1_0.26
Doses Blue Rep1_0.24 Red Rep2_0.27
sp|Q96RK0|CIC_HUMAN-191-S-S1_-0.08
Doses Blue Rep1_-0.08 Red Rep2_-0.07
sp|P49792|RBP2_HUMAN-2297-S-S2_0.08
Doses Blue Rep1_0.21 Red Rep2_-0.05
sp|Q9BQI3-2|E2AK1_HUMAN-296-T-S1_-0.15
Doses Blue Rep1_-0.05 Red Rep2_-0.25
sp|P46100-4|ATRX_HUMAN-975-S-S2_-0.03
Doses Blue Rep1_-0.09 Red Rep2_0.03
sp|P49840|GSK3A_HUMAN-279-Y-S1_-0.19
Doses Blue Rep1_-0.16 Red Rep2_-0.22
sp|Q7Z6Z7-2|HUWE1_HUMAN-1903-S-S2_-0.19
Doses Blue Rep1_-0.09 Red Rep2_-0.3
sp|Q8N3X1|FNBP4_HUMAN-431-S-S2_0
Doses Blue Rep1_0.12 Red Rep2_-0.11
sp|O95819-4|M4K4_HUMAN-546-S-S1_-0.11
Doses Blue Rep1_-0.11 Red Rep2_-0.11
ptm abundance 37C

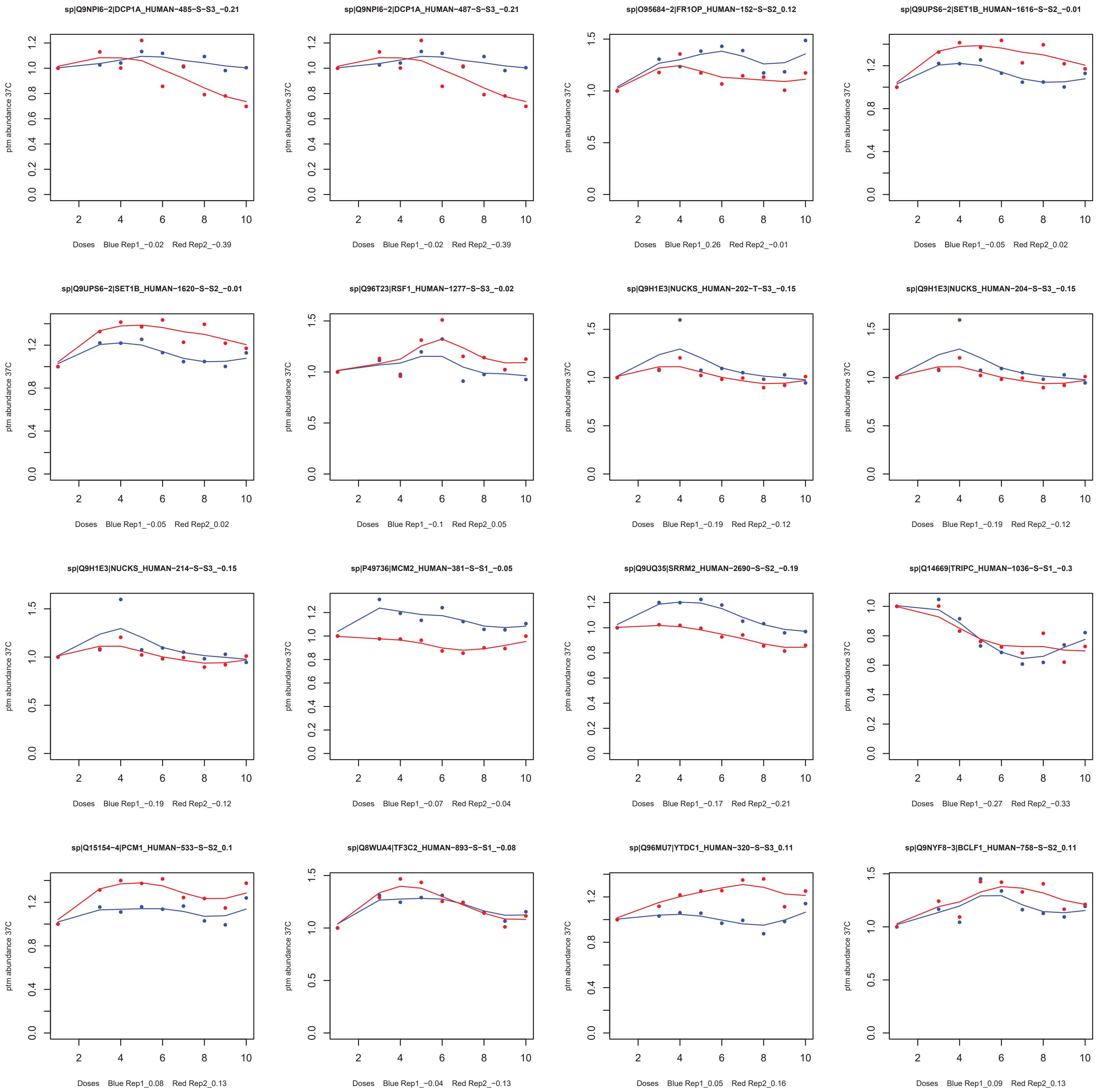
sp|Q9NPI6−2|DCP1A_HUMAN−485−S−S3_−0.21
ptm abundance 37C
Doses Blue Rep1_−0.02 Red Rep2_−0.39
sp|Q9NPI6−2|DCP1A_HUMAN−487−S−S3_−0.21
ptm abundance 37C
Doses Blue Rep1_−0.02 Red Rep2_−0.39
sp|O95684−2|FR1OP_HUMAN−152−S−S2_0.12
ptm abundance 37C
Doses Blue Rep1_0.26 Red Rep2_−0.01
sp|Q9UPS6−2|SET1B_HUMAN−1616−S−S2_−0.01
ptm abundance 37C
Doses Blue Rep1_−0.05 Red Rep2_0.02
sp|Q9UPS6−2|SET1B_HUMAN−1620−S−S2_−0.01
ptm abundance 37C
Doses Blue Rep1_−0.05 Red Rep2_0.02
sp|Q96T23|RSF1_HUMAN−1277−S−S3_−0.02
ptm abundance 37C
Doses Blue Rep1_−0.1 Red Rep2_0.05
sp|Q9H1E3|NUCKS_HUMAN−202−T−S3_−0.15
ptm abundance 37C
Doses Blue Rep1_−0.19 Red Rep2_−0.12
sp|Q9H1E3|NUCKS_HUMAN−204−S−S3_−0.15
ptm abundance 37C
Doses Blue Rep1_−0.19 Red Rep2_−0.12
sp|Q9H1E3|NUCKS_HUMAN−214−S−S3_−0.15
ptm abundance 37C
Doses Blue Rep1_−0.19 Red Rep2_−0.12
sp|P49736|MCM2_HUMAN−381−S−S1_−0.05
ptm abundance 37C
Doses Blue Rep1_−0.07 Red Rep2_−0.04
sp|Q9UQ35|SRRM2_HUMAN−2690−S−S2_−0.19
ptm abundance 37C
Doses Blue Rep1_−0.17 Red Rep2_−0.21
sp|Q14669|TRIPC_HUMAN−1036−S−S1_−0.3
ptm abundance 37C
Doses Blue Rep1_−0.27 Red Rep2_−0.33
sp|Q15154−4|PCM1_HUMAN−533−S−S2_0.1
ptm abundance 37C
Doses Blue Rep1_0.08 Red Rep2_0.13
sp|Q8WUA4|TF3C2_HUMAN−893−S−S1_−0.08
ptm abundance 37C
Doses Blue Rep1_−0.04 Red Rep2_−0.13
sp|Q96MU7|YTDC1_HUMAN−320−S−S3_0.11
ptm abundance 37C
Doses Blue Rep1_0.05 Red Rep2_0.16
sp|Q9NYF8−3|BCLF1_HUMAN−758−S−S2_0.11
ptm abundance 37C
Doses Blue Rep1_0.09 Red Rep2_0.13

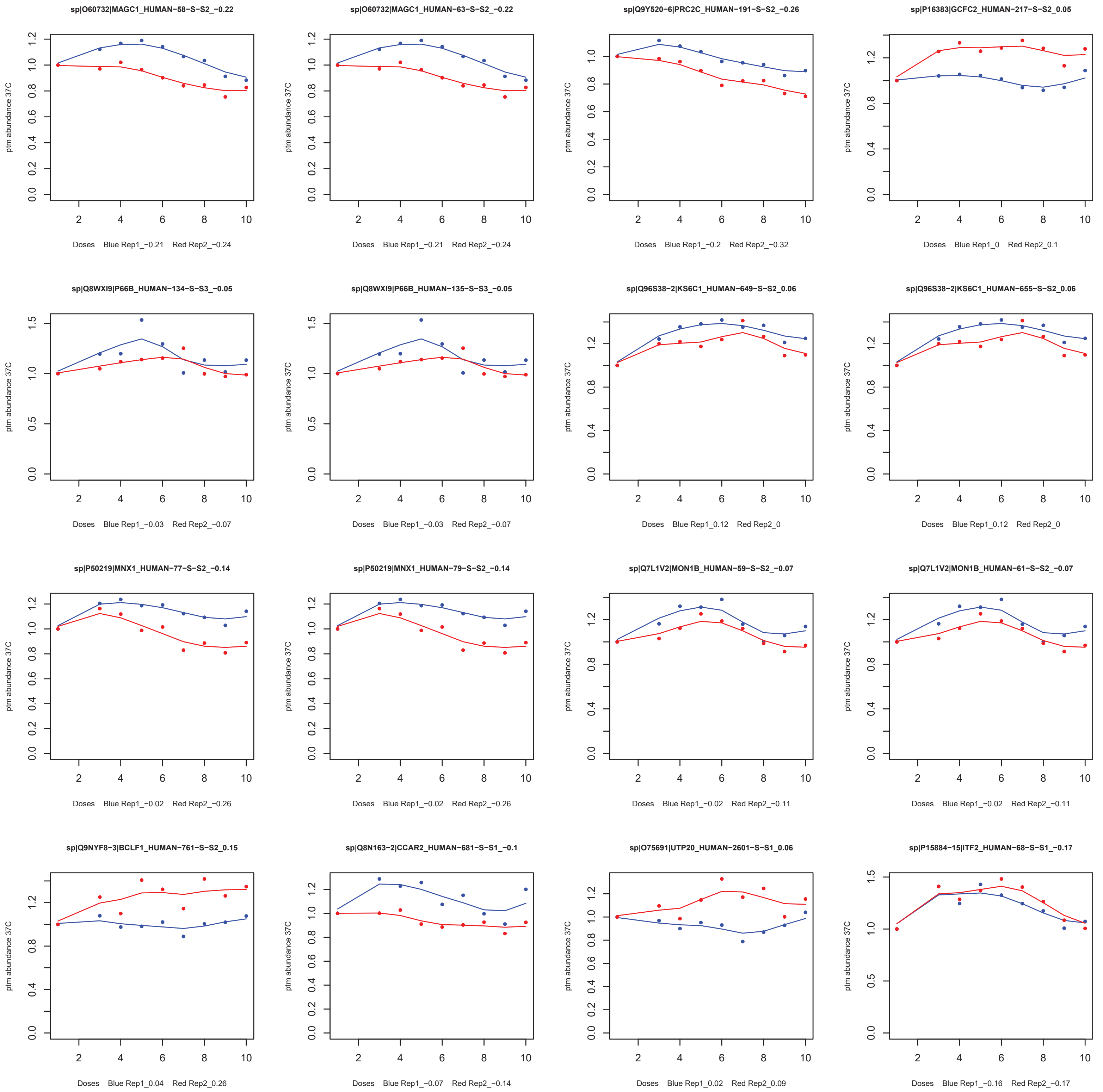
sp|O60732|MAGC1_HUMAN−58−S−S2_−0.22
ptm abundance 37C
Doses Blue Rep1_−0.21 Red Rep2_−0.24
sp|O60732|MAGC1_HUMAN−63−S−S2_−0.22
ptm abundance 37C
Doses Blue Rep1_−0.21 Red Rep2_−0.24
sp|Q9Y520−6|PRC2C_HUMAN−191−S−S2_−0.26
ptm abundance 37C
Doses Blue Rep1_−0.2 Red Rep2_−0.32
sp|P16383|GCFC2_HUMAN−217−S−S2_0.05
ptm abundance 37C
Doses Blue Rep1_0 Red Rep2_0.1
sp|Q8WXI9|P66B_HUMAN−134−S−S3_−0.05
ptm abundance 37C
Doses Blue Rep1_−0.03 Red Rep2_−0.07
sp|Q8WXI9|P66B_HUMAN−135−S−S3_−0.05
ptm abundance 37C
Doses Blue Rep1_−0.03 Red Rep2_−0.07
sp|Q96S38−2|KS6C1_HUMAN−649−S−S2_0.06
ptm abundance 37C
Doses Blue Rep1_0.12 Red Rep2_0
sp|Q96S38−2|KS6C1_HUMAN−655−S−S2_0.06
ptm abundance 37C
Doses Blue Rep1_0.12 Red Rep2_0
sp|P50219|MNX1_HUMAN−77−S−S2_−0.14
ptm abundance 37C
Doses Blue Rep1_−0.02 Red Rep2_−0.26
sp|P50219|MNX1_HUMAN−79−S−S2_−0.14
ptm abundance 37C
Doses Blue Rep1_−0.02 Red Rep2_−0.26
sp|Q7L1V2|MON1B_HUMAN−59−S−S2_−0.07
ptm abundance 37C
Doses Blue Rep1_−0.02 Red Rep2_−0.11
sp|Q7L1V2|MON1B_HUMAN−61−S−S2_−0.07
ptm abundance 37C
Doses Blue Rep1_−0.02 Red Rep2_−0.11
sp|Q9NYF8−3|BCLF1_HUMAN−761−S−S2_0.15
ptm abundance 37C
Doses Blue Rep1_0.04 Red Rep2_0.26
sp|Q8N163−2|CCAR2_HUMAN−681−S−S1_−0.1
ptm abundance 37C
Doses Blue Rep1_−0.07 Red Rep2_−0.14
sp|O75691|UTP20_HUMAN−2601−S−S1_0.06
ptm abundance 37C
Doses Blue Rep1_0.02 Red Rep2_0.09
sp|P15884−15|ITF2_HUMAN−68−S−S1_−0.17
ptm abundance 37C
Doses Blue Rep1_−0.16 Red Rep2_−0.17

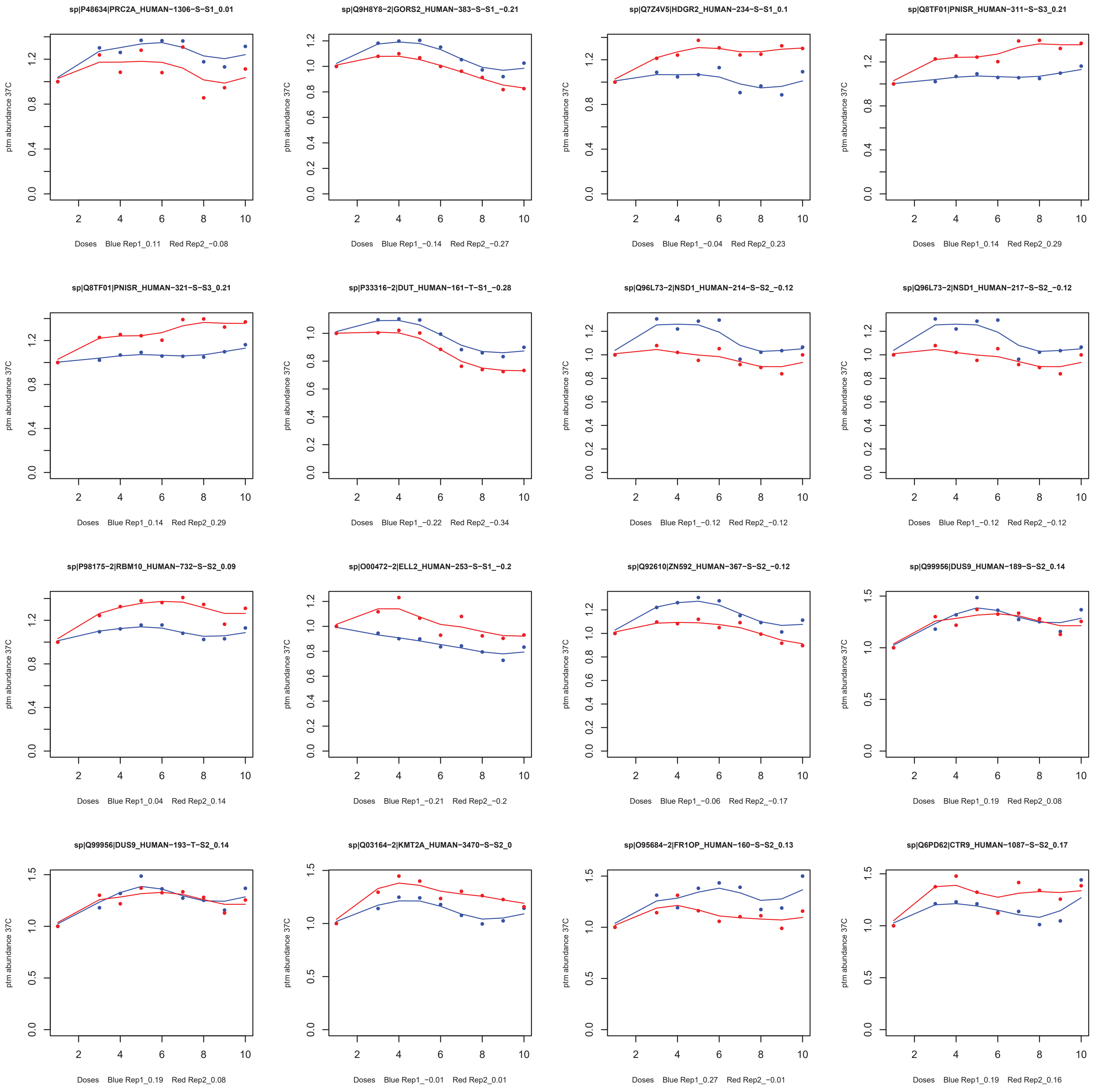
sp|P48634|PRC2A_HUMAN−1306−S−S1_0.01
Doses Blue Rep1_0.11 Red Rep2_−0.08
sp|Q9H8Y8−2|GORS2_HUMAN−383−S−S1_−0.21
Doses Blue Rep1_−0.14 Red Rep2_−0.27
sp|Q7Z4V5|HDGR2_HUMAN−234−S−S1_0.1
Doses Blue Rep1_−0.04 Red Rep2_0.23
sp|Q8TF01|PNISR_HUMAN−311−S−S3_0.21
Doses Blue Rep1_0.14 Red Rep2_0.29
sp|Q8TF01|PNISR_HUMAN−321−S−S3_0.21
Doses Blue Rep1_0.14 Red Rep2_0.29
sp|P33316−2|DUT_HUMAN−161−T−S1_−0.28
Doses Blue Rep1_−0.22 Red Rep2_−0.34
sp|Q96L73−2|NSD1_HUMAN−214−S−S2_−0.12
Doses Blue Rep1_−0.12 Red Rep2_−0.12
sp|Q96L73−2|NSD1_HUMAN−217−S−S2_−0.12
Doses Blue Rep1_−0.12 Red Rep2_−0.12
sp|P98175−2|RBM10_HUMAN−732−S−S2_0.09
Doses Blue Rep1_0.04 Red Rep2_0.14
sp|O00472−2|ELL2_HUMAN−253−S−S1_−0.2
Doses Blue Rep1_−0.21 Red Rep2_−0.2
sp|Q92610|ZN592_HUMAN−367−S−S2_−0.12
Doses Blue Rep1_−0.06 Red Rep2_−0.17
sp|Q99956|DUS9_HUMAN−189−S−S2_0.14
Doses Blue Rep1_0.19 Red Rep2_0.08
sp|Q99956|DUS9_HUMAN−193−T−S2_0.14
Doses Blue Rep1_0.19 Red Rep2_0.08
sp|Q03164−2|KMT2A_HUMAN−3470−S−S2_0
Doses Blue Rep1_−0.01 Red Rep2_0.01
sp|O95684−2|FR1OP_HUMAN−160−S−S2_0.13
Doses Blue Rep1_0.27 Red Rep2_−0.01
sp|Q6PD62|CTR9_HUMAN−1087−S−S2_0.17
Doses Blue Rep1_0.19 Red Rep2_0.16
ptm abundance 37C

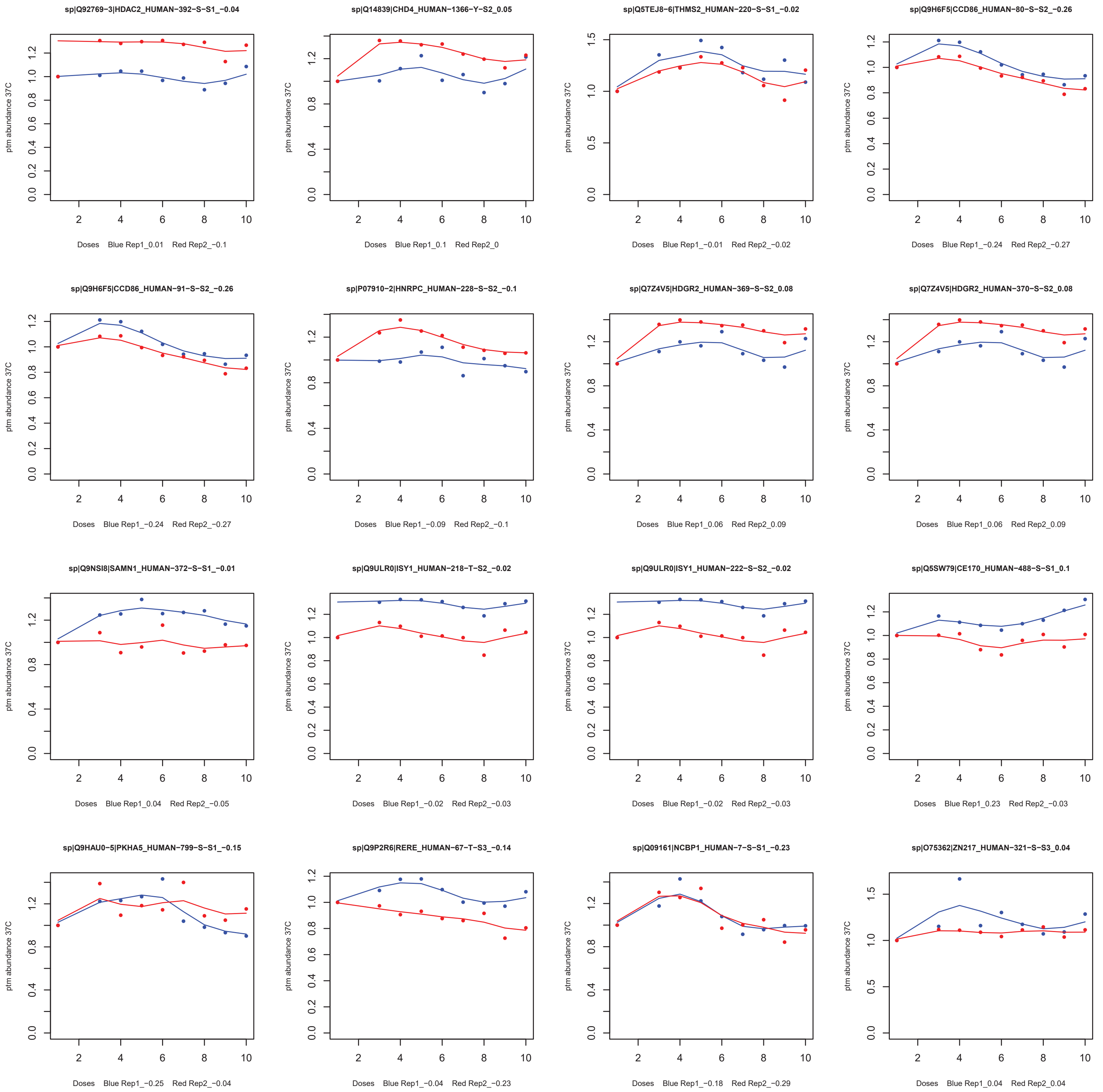

sp|Q92769−3|HDAC2_HUMAN−392−S−S1_−0.04
Doses Blue Rep1_0.01 Red Rep2_−0.1
sp|Q14839|CHD4_HUMAN−1366−Y−S2_0.05
Doses Blue Rep1_0.1 Red Rep2_0
sp|Q5TEJ8−6|THMS2_HUMAN−220−S−S1_−0.02
Doses Blue Rep1_−0.01 Red Rep2_−0.02
sp|Q9H6F5|CCD86_HUMAN−80−S−S2_−0.26
Doses Blue Rep1_−0.24 Red Rep2_−0.27
sp|Q9H6F5|CCD86_HUMAN−91−S−S2_−0.26
Doses Blue Rep1_−0.24 Red Rep2_−0.27
sp|P07910−2|HNRPC_HUMAN−228−S−S2_−0.1
Doses Blue Rep1_−0.09 Red Rep2_−0.1
sp|Q7Z4V5|HDGR2_HUMAN−369−S−S2_0.08
Doses Blue Rep1_0.06 Red Rep2_0.09
sp|Q7Z4V5|HDGR2_HUMAN−370−S−S2_0.08
Doses Blue Rep1_0.06 Red Rep2_0.09
sp|Q9NSI8|SAMN1_HUMAN−372−S−S1_−0.01
Doses Blue Rep1_0.04 Red Rep2_−0.05
sp|Q9ULR0|ISY1_HUMAN−218−T−S2_−0.02
Doses Blue Rep1_−0.02 Red Rep2_−0.03
sp|Q9ULR0|ISY1_HUMAN−222−S−S2_−0.02
Doses Blue Rep1_−0.02 Red Rep2_−0.03
sp|Q5SW79|CE170_HUMAN−488−S−S1_0.1
Doses Blue Rep1_0.23 Red Rep2_−0.03
sp|Q9HAU0−5|PKHA5_HUMAN−799−S−S1_−0.15
Doses Blue Rep1_−0.25 Red Rep2_−0.04
sp|Q9P2R6|RERE_HUMAN−67−T−S3_−0.14
Doses Blue Rep1_−0.04 Red Rep2_−0.23
sp|Q09161|NCBP1_HUMAN−7−S−S1_−0.23
Doses Blue Rep1_−0.18 Red Rep2_−0.29
sp|O75362|ZN217_HUMAN−321−S−S3_0.04
Doses Blue Rep1_0.04 Red Rep2_0.04
ptm abundance 37C

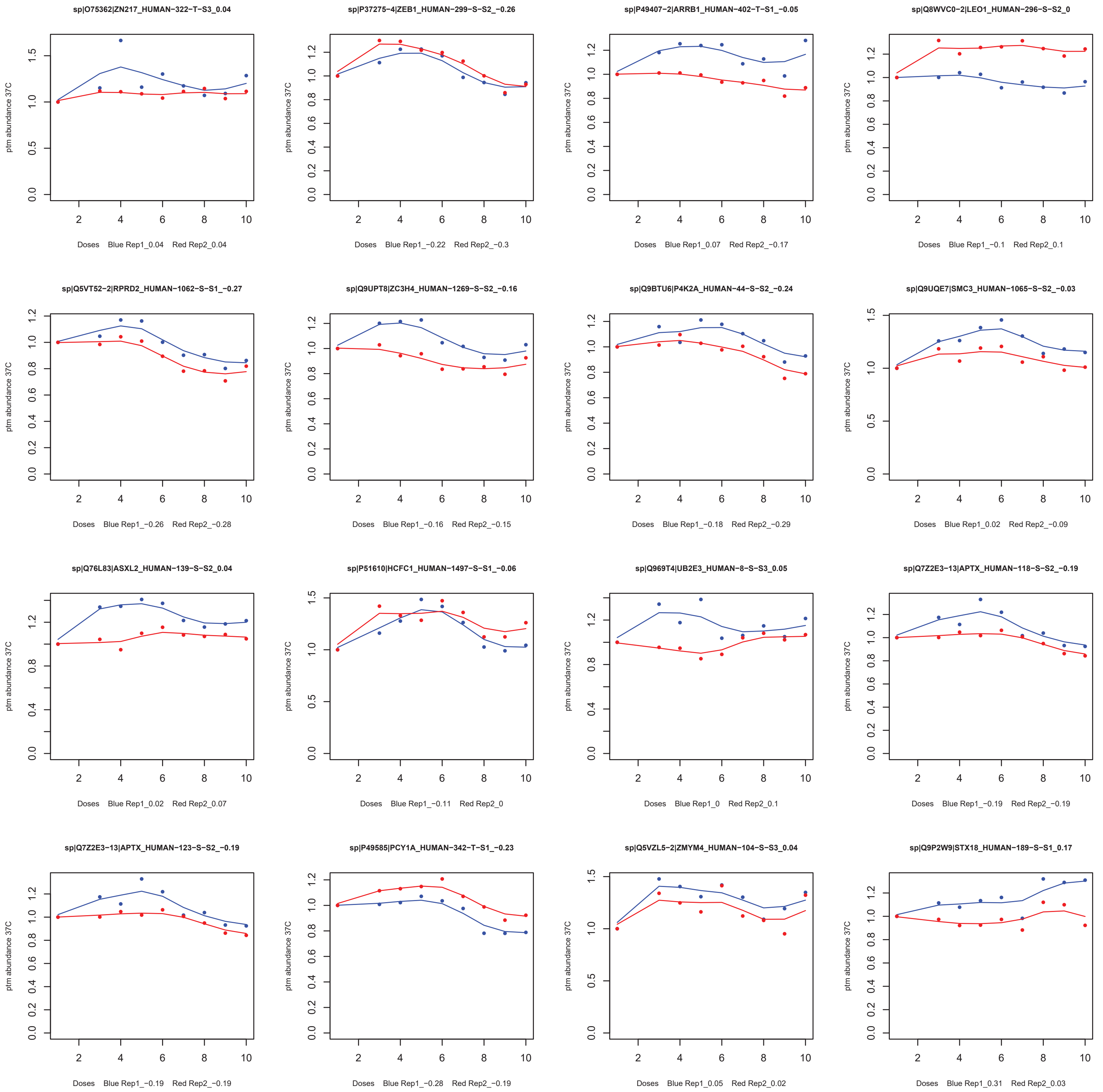
sp|O75362|ZN217_HUMAN−322−T−S3_0.04
ptm abundance 37C
Doses Blue Rep1_0.04 Red Rep2_0.04
sp|P37275−4|ZEB1_HUMAN−299−S−S2_−0.26
ptm abundance 37C
Doses Blue Rep1_−0.22 Red Rep2_−0.3
sp|P49407−2|ARRB1_HUMAN−402−T−S1_−0.05
ptm abundance 37C
Doses Blue Rep1_0.07 Red Rep2_−0.17
sp|Q8WVC0−2|LEO1_HUMAN−296−S−S2_0
ptm abundance 37C
Doses Blue Rep1_−0.1 Red Rep2_0.1
sp|Q5VT52−2|RPRD2_HUMAN−1062−S−S1_−0.27
ptm abundance 37C
Doses Blue Rep1_−0.26 Red Rep2_−0.28
sp|Q9UPT8|ZC3H4_HUMAN−1269−S−S2_−0.16
ptm abundance 37C
Doses Blue Rep1_−0.16 Red Rep2_−0.15
sp|Q9BTU6|P4K2A_HUMAN−44−S−S2_−0.24
ptm abundance 37C
Doses Blue Rep1_−0.18 Red Rep2_−0.29
sp|Q9UQE7|SMC3_HUMAN−1065−S−S2_−0.03
ptm abundance 37C
Doses Blue Rep1_0.02 Red Rep2_−0.09
sp|Q76L83|ASXL2_HUMAN−139−S−S2_0.04
ptm abundance 37C
Doses Blue Rep1_0.02 Red Rep2_0.07
sp|P51610|HCFC1_HUMAN−1497−S−S1_−0.06
ptm abundance 37C
Doses Blue Rep1_−0.11 Red Rep2_0
sp|Q969T4|UB2E3_HUMAN−8−S−S3_0.05
ptm abundance 37C
Doses Blue Rep1_0 Red Rep2_0.1
sp|Q7Z2E3−13|APTX_HUMAN−118−S−S2_−0.19
ptm abundance 37C
Doses Blue Rep1_−0.19 Red Rep2_−0.19
sp|Q7Z2E3−13|APTX_HUMAN−123−S−S2_−0.19
ptm abundance 37C
Doses Blue Rep1_−0.19 Red Rep2_−0.19
sp|P49585|PCY1A_HUMAN−342−T−S1_−0.23
ptm abundance 37C
Doses Blue Rep1_−0.28 Red Rep2_−0.19
sp|Q5VZL5−2|ZMYM4_HUMAN−104−S−S3_0.04
ptm abundance 37C
Doses Blue Rep1_0.05 Red Rep2_0.02
sp|Q9P2W9|STX18_HUMAN−189−S−S1_0.17
ptm abundance 37C
Doses Blue Rep1_0.31 Red Rep2_0.03

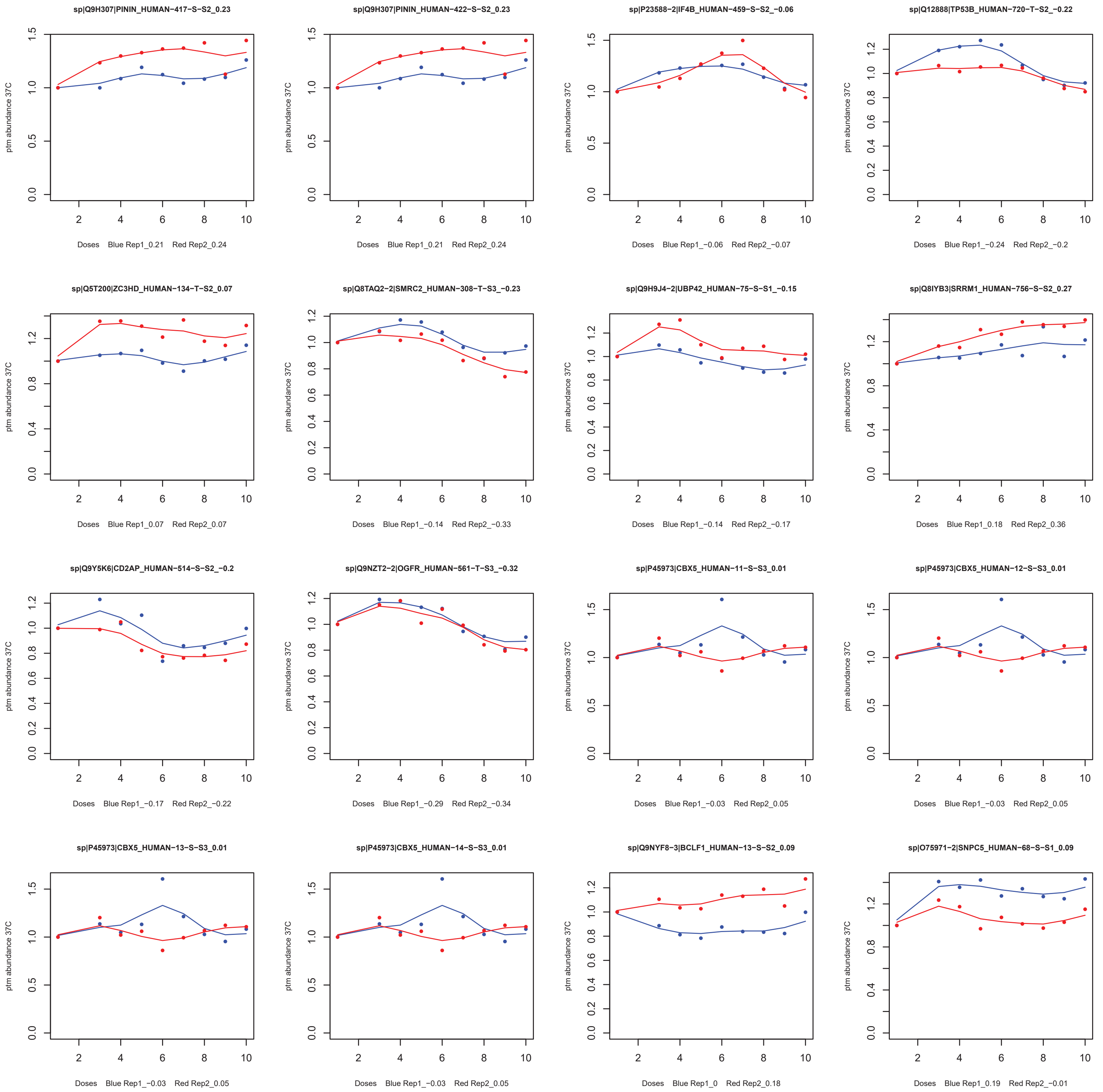

sp|Q9H307|PININ_HUMAN−417−S−S2_0.23
ptm abundance 37C
Doses Blue Rep1_0.21 Red Rep2_0.24
sp|Q9H307|PININ_HUMAN−422−S−S2_0.23
ptm abundance 37C
Doses Blue Rep1_0.21 Red Rep2_0.24
sp|P23588−2|IF4B_HUMAN−459−S−S2_−0.06
ptm abundance 37C
Doses Blue Rep1_−0.06 Red Rep2_−0.07
sp|Q12888|TP53B_HUMAN−720−T−S2_−0.22
ptm abundance 37C
Doses Blue Rep1_−0.24 Red Rep2_−0.2
sp|Q5T200|ZC3HD_HUMAN−134−T−S2_0.07
ptm abundance 37C
Doses Blue Rep1_0.07 Red Rep2_0.07
sp|Q8TAQ2−2|SMRC2_HUMAN−308−T−S3_−0.23
ptm abundance 37C
Doses Blue Rep1_−0.14 Red Rep2_−0.33
sp|Q9H9J4−2|UBP42_HUMAN−75−S−S1_−0.15
ptm abundance 37C
Doses Blue Rep1_−0.14 Red Rep2_−0.17
sp|Q8IYB3|SRRM1_HUMAN−756−S−S2_0.27
ptm abundance 37C
Doses Blue Rep1_0.18 Red Rep2_0.36
sp|Q9Y5K6|CD2AP_HUMAN−514−S−S2_−0.2
ptm abundance 37C
Doses Blue Rep1_−0.17 Red Rep2_−0.22
sp|Q9NZT2−2|OGFR_HUMAN−561−T−S3_−0.32
ptm abundance 37C
Doses Blue Rep1_−0.29 Red Rep2_−0.34
sp|P45973|CBX5_HUMAN−11−S−S3_0.01
ptm abundance 37C
Doses Blue Rep1_−0.03 Red Rep2_0.05
sp|P45973|CBX5_HUMAN−12−S−S3_0.01
ptm abundance 37C
Doses Blue Rep1_−0.03 Red Rep2_0.05
sp|P45973|CBX5_HUMAN−13−S−S3_0.01
ptm abundance 37C
Doses Blue Rep1_−0.03 Red Rep2_0.05
sp|P45973|CBX5_HUMAN−14−S−S3_0.01
ptm abundance 37C
Doses Blue Rep1_−0.03 Red Rep2_0.05
sp|Q9NYF8−3|BCLF1_HUMAN−13−S−S2_0.09
ptm abundance 37C
Doses Blue Rep1_0 Red Rep2_0.18
sp|O75971−2|SNPC5_HUMAN−68−S−S1_0.09
ptm abundance 37C
Doses Blue Rep1_0.19 Red Rep2_−0.01

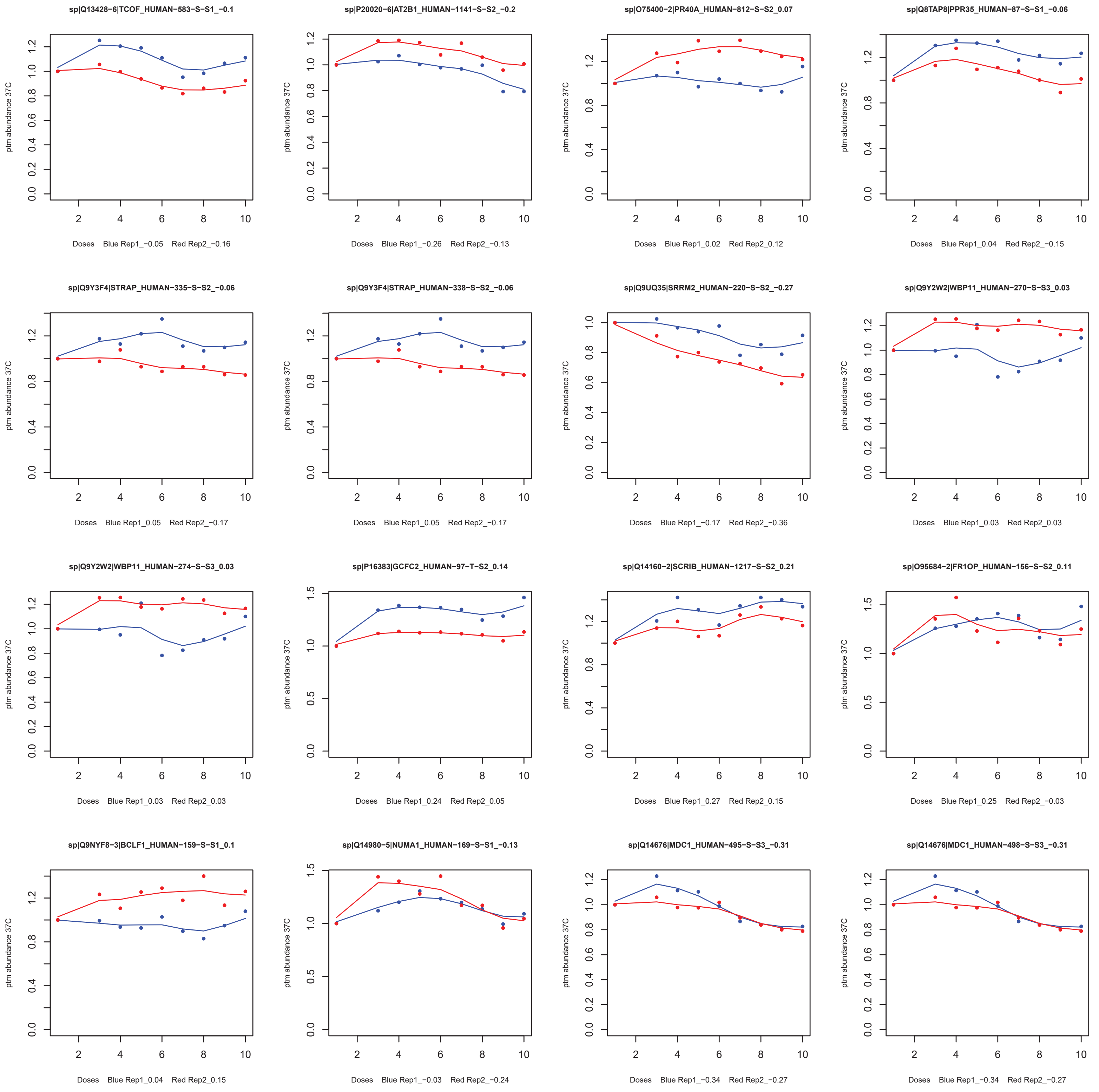
sp|Q13428−6|TCOF_HUMAN−583−S−S1_−0.1
Doses Blue Rep1_−0.05 Red Rep2_−0.16
sp|P20020−6|AT2B1_HUMAN−1141−S−S2_−0.2
Doses Blue Rep1_−0.26 Red Rep2_−0.13
sp|O75400−2|PR40A_HUMAN−812−S−S2_0.07
Doses Blue Rep1_0.02 Red Rep2_0.12
sp|Q8TAP8|PPR35_HUMAN−87−S−S1_−0.06
Doses Blue Rep1_0.04 Red Rep2_−0.15
sp|Q9Y3F4|STRAP_HUMAN−335−S−S2_−0.06
Doses Blue Rep1_0.05 Red Rep2_−0.17
sp|Q9Y3F4|STRAP_HUMAN−338−S−S2_−0.06
Doses Blue Rep1_0.05 Red Rep2_−0.17
sp|Q9UQ35|SRRM2_HUMAN−220−S−S2_−0.27
Doses Blue Rep1_−0.17 Red Rep2_−0.36
sp|Q9Y2W2|WBP11_HUMAN−270−S−S3_0.03
Doses Blue Rep1_0.03 Red Rep2_0.03
sp|Q9Y2W2|WBP11_HUMAN−274−S−S3_0.03
Doses Blue Rep1_0.03 Red Rep2_0.03
sp|P16383|GCFC2_HUMAN−97−T−S2_0.14
Doses Blue Rep1_0.24 Red Rep2_0.05
sp|Q14160−2|SCRIB_HUMAN−1217−S−S2_0.21
Doses Blue Rep1_0.27 Red Rep2_0.15
sp|O95684−2|FR1OP_HUMAN−156−S−S2_0.11
Doses Blue Rep1_0.25 Red Rep2_−0.03
sp|Q9NYF8−3|BCLF1_HUMAN−159−S−S1_0.1
Doses Blue Rep1_0.04 Red Rep2_0.15
sp|Q14980−5|NUMA1_HUMAN−169−S−S1_−0.13
Doses Blue Rep1_−0.03 Red Rep2_−0.24
sp|Q14676|MDC1_HUMAN−495−S−S3_−0.31
Doses Blue Rep1_−0.34 Red Rep2_−0.27
sp|Q14676|MDC1_HUMAN−498−S−S3_−0.31
Doses Blue Rep1_−0.34 Red Rep2_−0.27
ptm abundance 37C

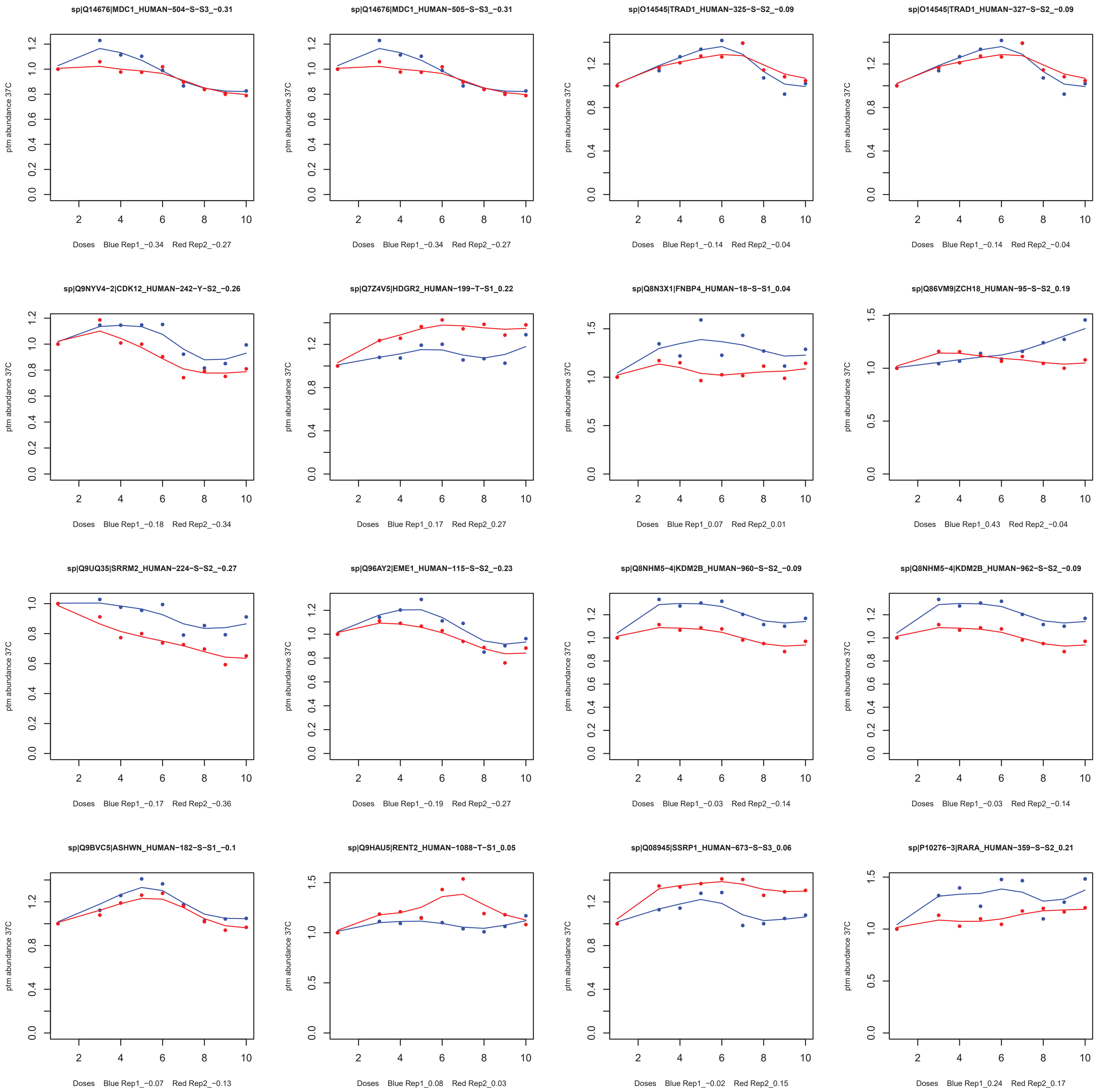
sp|Q14676|MDC1_HUMAN−504−S−S3_−0.31
ptm abundance 37C
Doses Blue Rep1_−0.34 Red Rep2_−0.27
sp|Q14676|MDC1_HUMAN−505−S−S3_−0.31
ptm abundance 37C
Doses Blue Rep1_−0.34 Red Rep2_−0.27
sp|O14545|TRAD1_HUMAN−325−S−S2_−0.09
ptm abundance 37C
Doses Blue Rep1_−0.14 Red Rep2_−0.04
sp|O14545|TRAD1_HUMAN−327−S−S2_−0.09
ptm abundance 37C
Doses Blue Rep1_−0.14 Red Rep2_−0.04
sp|Q9NYV4−2|CDK12_HUMAN−242−Y−S2_−0.26
ptm abundance 37C
Doses Blue Rep1_−0.18 Red Rep2_−0.34
sp|Q7Z4V5|HDGR2_HUMAN−199−T−S1_0.22
ptm abundance 37C
Doses Blue Rep1_0.17 Red Rep2_0.27
sp|Q8N3X1|FNBP4_HUMAN−18−S−S1_0.04
ptm abundance 37C
Doses Blue Rep1_0.07 Red Rep2_0.01
sp|Q86VM9|ZCH18_HUMAN−95−S−S2_0.19
ptm abundance 37C
Doses Blue Rep1_0.43 Red Rep2_−0.04
sp|Q9UQ35|SRRM2_HUMAN−224−S−S2_−0.27
ptm abundance 37C
Doses Blue Rep1_−0.17 Red Rep2_−0.36
sp|Q96AY2|EME1_HUMAN−115−S−S2_−0.23
ptm abundance 37C
Doses Blue Rep1_−0.19 Red Rep2_−0.27
sp|Q8NHM5−4|KDM2B_HUMAN−960−S−S2_−0.09
ptm abundance 37C
Doses Blue Rep1_−0.03 Red Rep2_−0.14
sp|Q8NHM5−4|KDM2B_HUMAN−962−S−S2_−0.09
ptm abundance 37C
Doses Blue Rep1_−0.03 Red Rep2_−0.14
sp|Q9BVC5|ASHWN_HUMAN−182−S−S1_−0.1
ptm abundance 37C
Doses Blue Rep1_−0.07 Red Rep2_−0.13
sp|Q9HAU5|RENT2_HUMAN−1088−T−S1_0.05
ptm abundance 37C
Doses Blue Rep1_0.08 Red Rep2_0.03
sp|Q08945|SSRP1_HUMAN−673−S−S3_0.06
ptm abundance 37C
Doses Blue Rep1_−0.02 Red Rep2_0.15
sp|P10276−3|RARA_HUMAN−359−S−S2_0.21
ptm abundance 37C
Doses Blue Rep1_0.24 Red Rep2_0.17

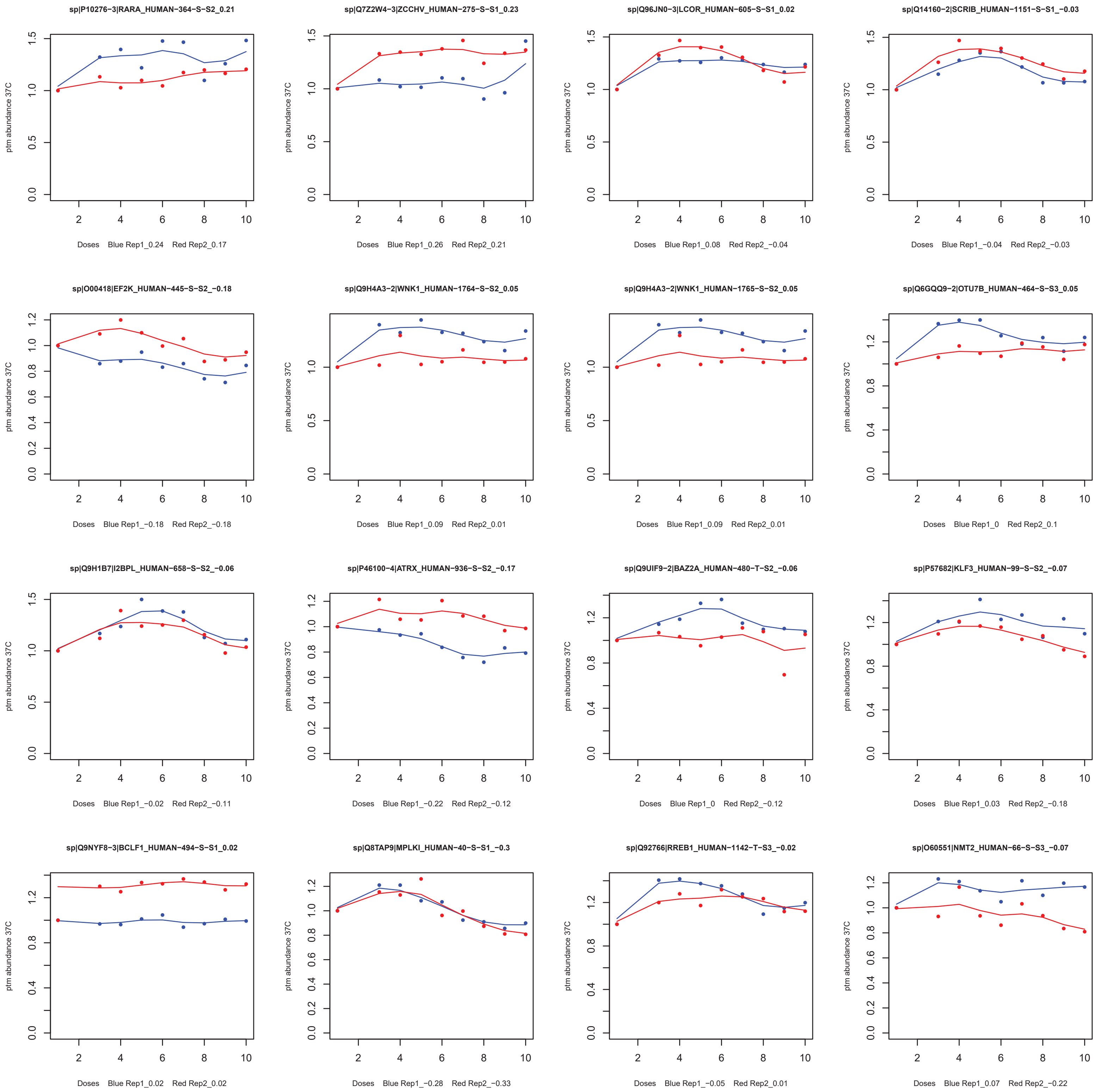

sp|P10276−3|RARA_HUMAN−364−S−S2_0.21
Doses Blue Rep1_0.24 Red Rep2_0.17
sp|Q7Z2W4−3|ZCCHV_HUMAN−275−S−S1_0.23
Doses Blue Rep1_0.26 Red Rep2_0.21
sp|Q96JN0−3|LCOR_HUMAN−605−S−S1_0.02
Doses Blue Rep1_0.08 Red Rep2_−0.04
sp|Q14160−2|SCRIB_HUMAN−1151−S−S1_−0.03
Doses Blue Rep1_−0.04 Red Rep2_−0.03
sp|O00418|EF2K_HUMAN−445−S−S2_−0.18
Doses Blue Rep1_−0.18 Red Rep2_−0.18
sp|Q9H4A3−2|WNK1_HUMAN−1764−S−S2_0.05
Doses Blue Rep1_0.09 Red Rep2_0.01
sp|Q9H4A3−2|WNK1_HUMAN−1765−S−S2_0.05
Doses Blue Rep1_0.09 Red Rep2_0.01
sp|Q6GQQ9−2|OTU7B_HUMAN−464−S−S3_0.05
Doses Blue Rep1_0 Red Rep2_0.1
sp|Q9H1B7|I2BPL_HUMAN−658−S−S2_−0.06
Doses Blue Rep1_−0.02 Red Rep2_−0.11
sp|P46100−4|ATRX_HUMAN−936−S−S2_−0.17
Doses Blue Rep1_−0.22 Red Rep2_−0.12
sp|Q9UIF9−2|BAZ2A_HUMAN−480−T−S2_−0.06
Doses Blue Rep1_0 Red Rep2_−0.12
sp|P57682|KLF3_HUMAN−99−S−S2_−0.07
Doses Blue Rep1_0.03 Red Rep2_−0.18
sp|Q9NYF8−3|BCLF1_HUMAN−494−S−S1_0.02
Doses Blue Rep1_0.02 Red Rep2_0.02
sp|Q8TAP9|MPLKI_HUMAN−40−S−S1_−0.3
Doses Blue Rep1_−0.28 Red Rep2_−0.33
sp|Q92766|RREB1_HUMAN−1142−T−S3_−0.02
Doses Blue Rep1_−0.05 Red Rep2_0.01
sp|O60551|NMT2_HUMAN−66−S−S3_−0.07
Doses Blue Rep1_0.07 Red Rep2_−0.22
ptm abundance 37C

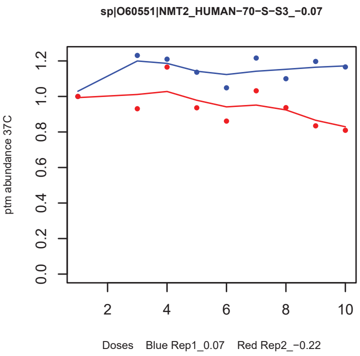
sp|O60551|NMT2_HUMAN−70−S−S3_−0.07
ptm abundance 37C
Doses Blue Rep1_0.07 Red Rep2_−0.22

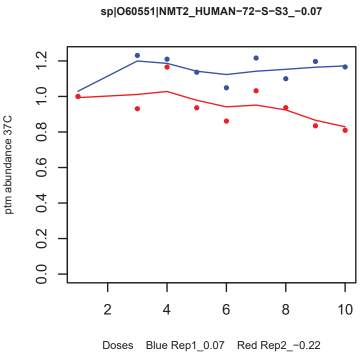
sp|O60551|NMT2_HUMAN−72−S−S3_−0.07
ptm abundance 37C
Doses Blue Rep1_0.07 Red Rep2_−0.22

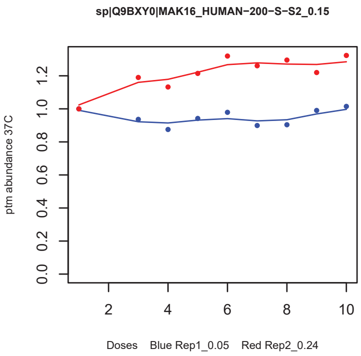
sp|Q9BXY0|MAK16_HUMAN−200−S−S2_0.15
ptm abundance 37C
Doses Blue Rep1_0.05 Red Rep2_0.24

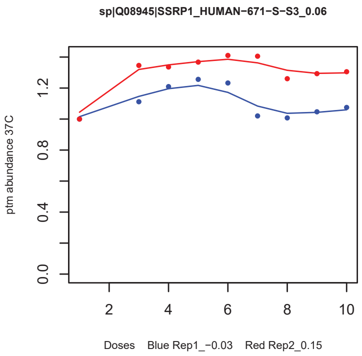
sp|Q08945|SSRP1_HUMAN−671−S−S3_0.06
ptm abundance 37C
Doses Blue Rep1_−0.03 Red Rep2_0.15

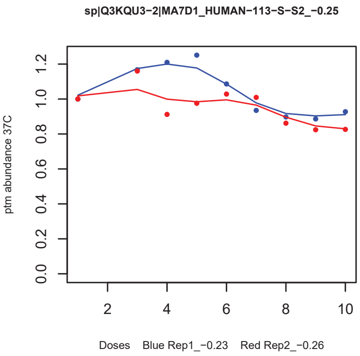
sp|Q3KQU3−2|MA7D1_HUMAN−113−S−S2_−0.25
ptm abundance 37C
Doses Blue Rep1_−0.23 Red Rep2_−0.26

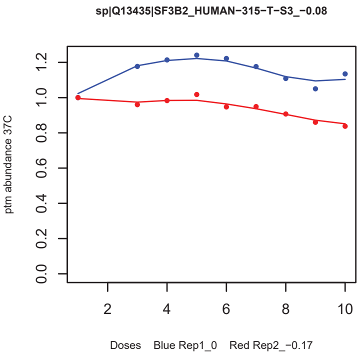
sp|Q13435|SF3B2_HUMAN−315−T−S3_−0.08
ptm abundance 37C
Doses Blue Rep1_0 Red Rep2_−0.17

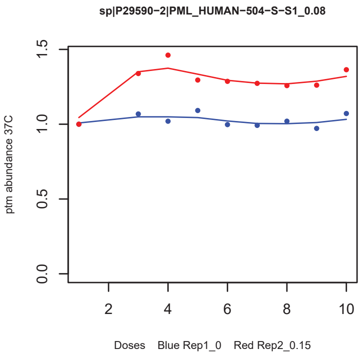
sp|P29590−2|PML_HUMAN−504−S−S1_0.08
ptm abundance 37C
Doses Blue Rep1_0 Red Rep2_0.15

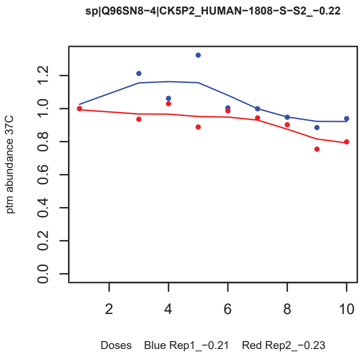
sp|Q96SN8−4|CK5P2_HUMAN−1808−S−S2_−0.22
ptm abundance 37C
Doses Blue Rep1_−0.21 Red Rep2_−0.23

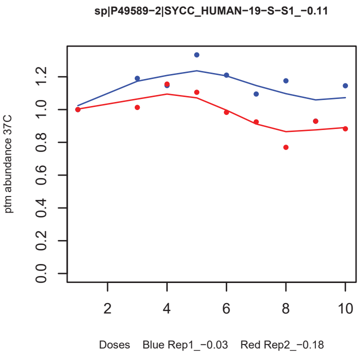
sp|P49589−2|SYCC_HUMAN−19−S−S1_−0.11
ptm abundance 37C
Doses Blue Rep1_−0.03 Red Rep2_−0.18

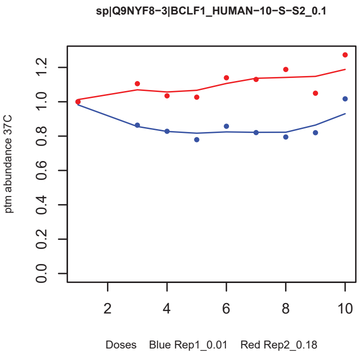
sp|Q9NYF8−3|BCLF1_HUMAN−10−S−S2_0.1
ptm abundance 37C
Doses Blue Rep1_0.01 Red Rep2_0.18

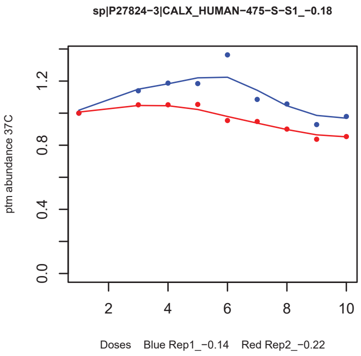
sp|P27824−3|CALX_HUMAN−475−S−S1_−0.18
ptm abundance 37C
Doses Blue Rep1_−0.14 Red Rep2_−0.22

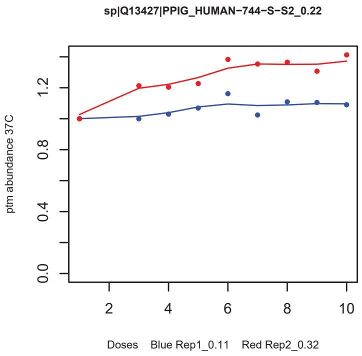
sp|Q13427|PPIG_HUMAN−744−S−S2_0.22
ptm abundance 37C
Doses Blue Rep1_0.11 Red Rep2_0.32

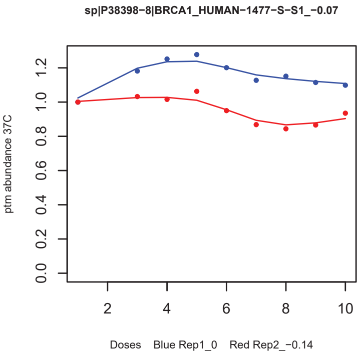
sp|P38398−8|BRCA1_HUMAN−1477−S−S1_−0.07
ptm abundance 37C
Doses Blue Rep1_0 Red Rep2_−0.14

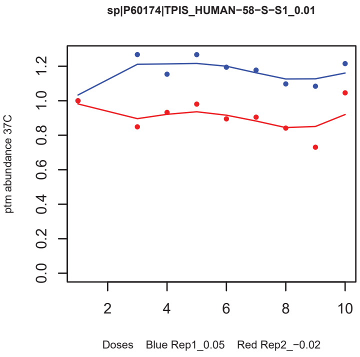
sp|P60174|TPIS_HUMAN−58−S−S1_0.01
ptm abundance 37C
Doses Blue Rep1_0.05 Red Rep2_−0.02

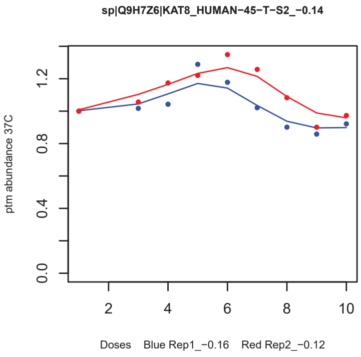
sp|Q9H7Z6|KAT8_HUMAN−45−T−S2_−0.14
ptm abundance 37C
Doses Blue Rep1_−0.16 Red Rep2_−0.12

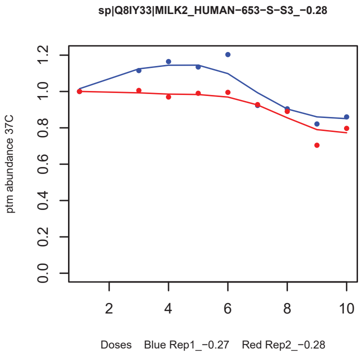
sp|Q8IY33|MILK2_HUMAN−653−S−S3_−0.28
ptm abundance 37C
Doses Blue Rep1_−0.27 Red Rep2_−0.28

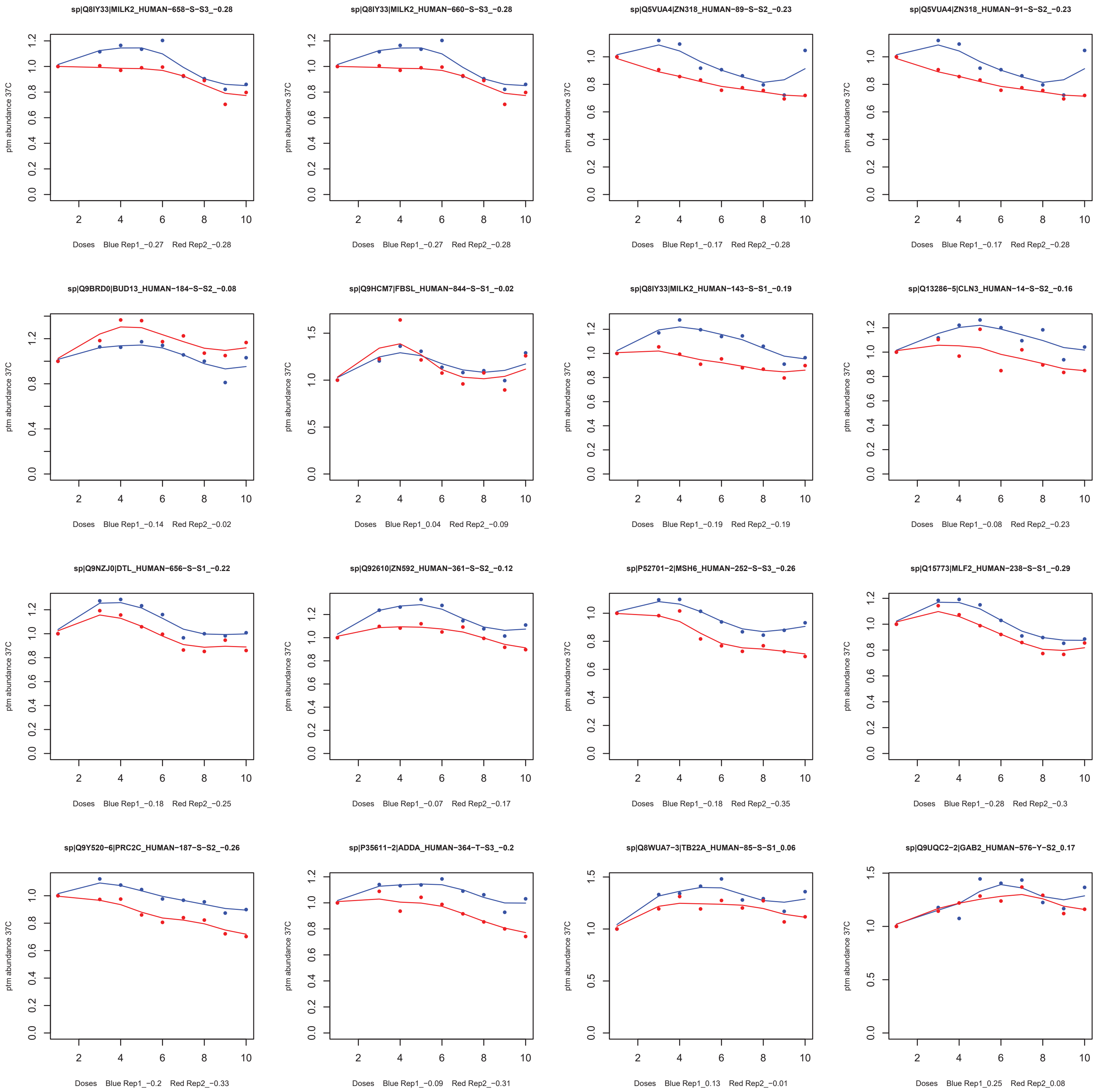
sp|Q8IY33|MILK2_HUMAN−658−S−S3_−0.28
Doses Blue Rep1_−0.27 Red Rep2_−0.28
sp|Q8IY33|MILK2_HUMAN−660−S−S3_−0.28
Doses Blue Rep1_−0.27 Red Rep2_−0.28
sp|Q5VUA4|ZN318_HUMAN−89−S−S2_−0.23
Doses Blue Rep1_−0.17 Red Rep2_−0.28
sp|Q5VUA4|ZN318_HUMAN−91−S−S2_−0.23
Doses Blue Rep1_−0.17 Red Rep2_−0.28
sp|Q9BRD0|BUD13_HUMAN−184−S−S2_−0.08
Doses Blue Rep1_−0.14 Red Rep2_−0.02
sp|Q9HCM7|FBSL_HUMAN−844−S−S1_−0.02
Doses Blue Rep1_0.04 Red Rep2_−0.09
sp|Q8IY33|MILK2_HUMAN−143−S−S1_−0.19
Doses Blue Rep1_−0.19 Red Rep2_−0.19
sp|Q13286−5|CLN3_HUMAN−14−S−S2_−0.16
Doses Blue Rep1_−0.08 Red Rep2_−0.23
sp|Q9NZJ0|DTL_HUMAN−656−S−S1_−0.22
Doses Blue Rep1_−0.18 Red Rep2_−0.25
sp|Q92610|ZN592_HUMAN−361−S−S2_−0.12
Doses Blue Rep1_−0.07 Red Rep2_−0.17
sp|P52701−2|MSH6_HUMAN−252−S−S3_−0.26
Doses Blue Rep1_−0.18 Red Rep2_−0.35
sp|Q15773|MLF2_HUMAN−238−S−S1_−0.29
Doses Blue Rep1_−0.28 Red Rep2_−0.3
sp|Q9Y520−6|PRC2C_HUMAN−187−S−S2_−0.26
Doses Blue Rep1_−0.2 Red Rep2_−0.33
sp|P35611−2|ADDA_HUMAN−364−T−S3_−0.2
Doses Blue Rep1_−0.09 Red Rep2_−0.31
sp|Q8WUA7−3|TB22A_HUMAN−85−S−S1_0.06
Doses Blue Rep1_0.13 Red Rep2_−0.01
sp|Q9UQC2−2|GAB2_HUMAN−576−Y−S2_0.17
Doses Blue Rep1_0.25 Red Rep2_0.08
ptm abundance 37C

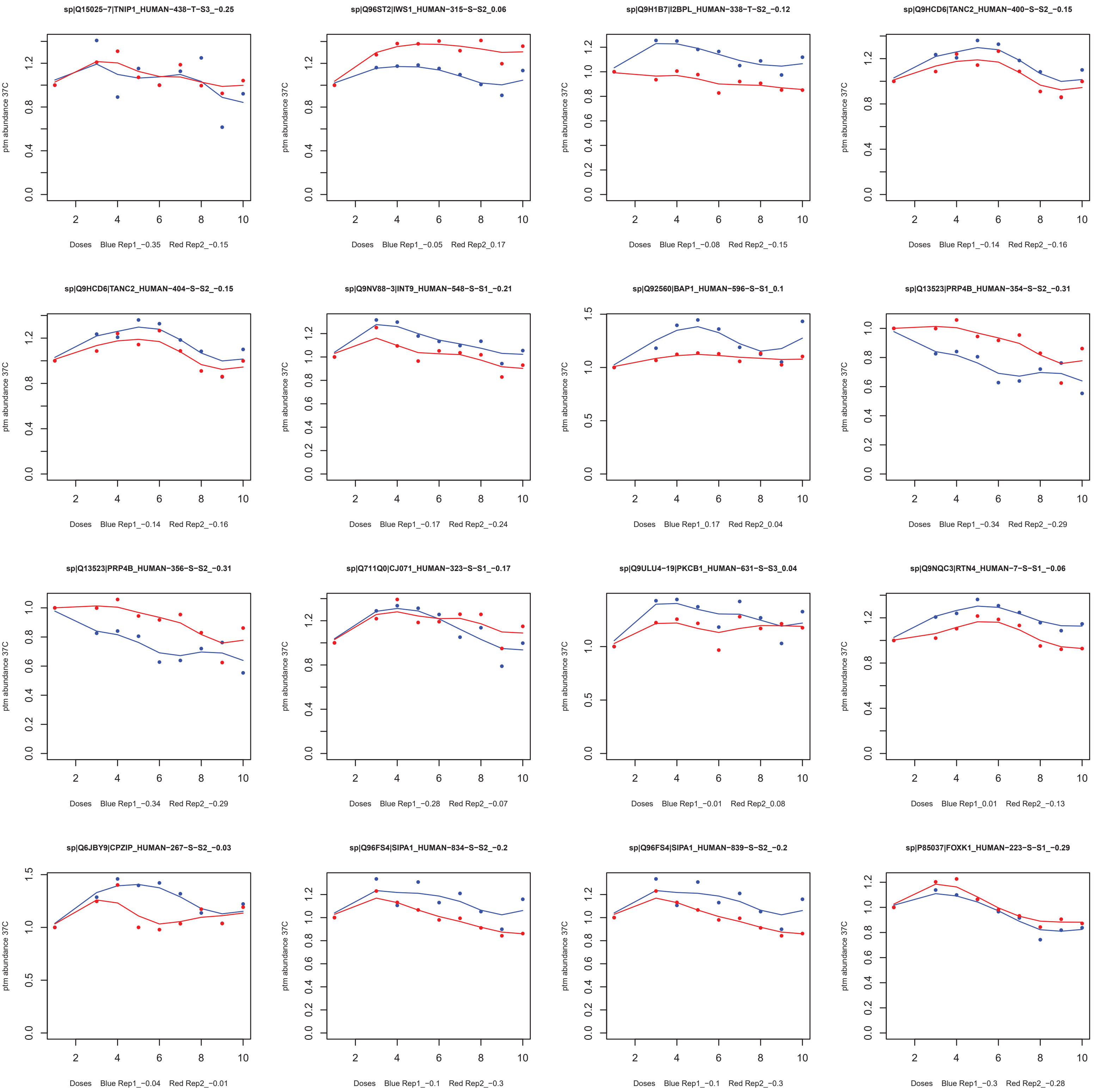
sp|Q15025−7|TNIP1_HUMAN−438−T−S3_−0.25
Doses Blue Rep1_−0.35 Red Rep2_−0.15
sp|Q96ST2|IWS1_HUMAN−315−S−S2_0.06
Doses Blue Rep1_−0.05 Red Rep2_0.17
sp|Q9H1B7|I2BPL_HUMAN−338−T−S2_−0.12
Doses Blue Rep1_−0.08 Red Rep2_−0.15
sp|Q9HCD6|TANC2_HUMAN−400−S−S2_−0.15
Doses Blue Rep1_−0.14 Red Rep2_−0.16
sp|Q9HCD6|TANC2_HUMAN−404−S−S2_−0.15
Doses Blue Rep1_−0.14 Red Rep2_−0.16
sp|Q9NV88−3|INT9_HUMAN−548−S−S1_−0.21
Doses Blue Rep1_−0.17 Red Rep2_−0.24
sp|Q92560|BAP1_HUMAN−596−S−S1_0.1
Doses Blue Rep1_0.17 Red Rep2_0.04
sp|Q13523|PRP4B_HUMAN−354−S−S2_−0.31
Doses Blue Rep1_−0.34 Red Rep2_−0.29
sp|Q13523|PRP4B_HUMAN−356−S−S2_−0.31
Doses Blue Rep1_−0.34 Red Rep2_−0.29
sp|Q711Q0|CJ071_HUMAN−323−S−S1_−0.17
Doses Blue Rep1_−0.28 Red Rep2_−0.07
sp|Q9ULU4−19|PKCB1_HUMAN−631−S−S3_0.04
Doses Blue Rep1_−0.01 Red Rep2_0.08
sp|Q9NQC3|RTN4_HUMAN−7−S−S1_−0.06
Doses Blue Rep1_0.01 Red Rep2_−0.13
sp|Q6JBY9|CPZIP_HUMAN−267−S−S2_−0.03
Doses Blue Rep1_−0.04 Red Rep2_−0.01
sp|Q96FS4|SIPA1_HUMAN−834−S−S2_−0.2
Doses Blue Rep1_−0.1 Red Rep2_−0.3
sp|Q96FS4|SIPA1_HUMAN−839−S−S2_−0.2
Doses Blue Rep1_−0.1 Red Rep2_−0.3
sp|P85037|FOXK1_HUMAN−223−S−S1_−0.29
Doses Blue Rep1_−0.3 Red Rep2_−0.28
ptm abundance 37C

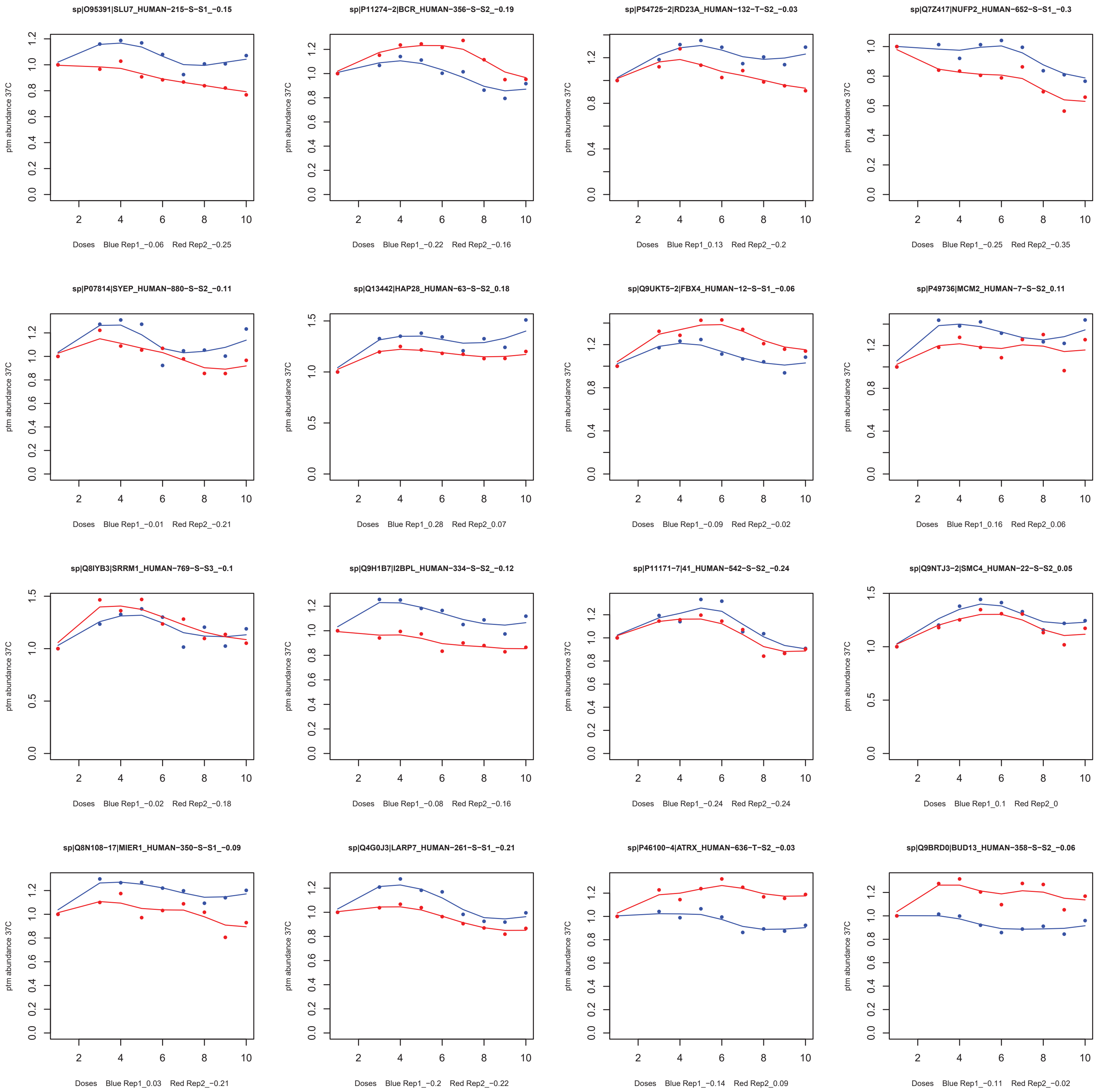
sp|O95391|SLU7_HUMAN−215−S−S1_−0.15
Doses Blue Rep1_−0.06 Red Rep2_−0.25
sp|P11274−2|BCR_HUMAN−356−S−S2_−0.19
Doses Blue Rep1_−0.22 Red Rep2_−0.16
sp|P54725−2|RD23A_HUMAN−132−T−S2_−0.03
Doses Blue Rep1_0.13 Red Rep2_−0.2
sp|Q7Z417|NUFP2_HUMAN−652−S−S1_−0.3
Doses Blue Rep1_−0.25 Red Rep2_−0.35
sp|P07814|SYEP_HUMAN−880−S−S2_−0.11
Doses Blue Rep1_−0.01 Red Rep2_−0.21
sp|Q13442|HAP28_HUMAN−63−S−S2_0.18
Doses Blue Rep1_0.28 Red Rep2_0.07
sp|Q9UKT5−2|FBX4_HUMAN−12−S−S1_−0.06
Doses Blue Rep1_−0.09 Red Rep2_−0.02
sp|P49736|MCM2_HUMAN−7−S−S2_0.11
Doses Blue Rep1_0.16 Red Rep2_0.06
sp|Q8IYB3|SRRM1_HUMAN−769−S−S3_−0.1
Doses Blue Rep1_−0.02 Red Rep2_−0.18
sp|Q9H1B7|I2BPL_HUMAN−334−S−S2_−0.12
Doses Blue Rep1_−0.08 Red Rep2_−0.16
sp|P11171−7|41_HUMAN−542−S−S2_−0.24
Doses Blue Rep1_−0.24 Red Rep2_−0.24
sp|Q9NTJ3−2|SMC4_HUMAN−22−S−S2_0.05
Doses Blue Rep1_0.1 Red Rep2_0
sp|Q8N108−17|MIER1_HUMAN−350−S−S1_−0.09
Doses Blue Rep1_0.03 Red Rep2_−0.21
sp|Q4G0J3|LARP7_HUMAN−261−S−S1_−0.21
Doses Blue Rep1_−0.2 Red Rep2_−0.22
sp|P46100−4|ATRX_HUMAN−636−T−S2_−0.03
Doses Blue Rep1_−0.14 Red Rep2_0.09
sp|Q9BRD0|BUD13_HUMAN−358−S−S2_−0.06
Doses Blue Rep1_−0.11 Red Rep2_−0.02
ptm abundance 37C

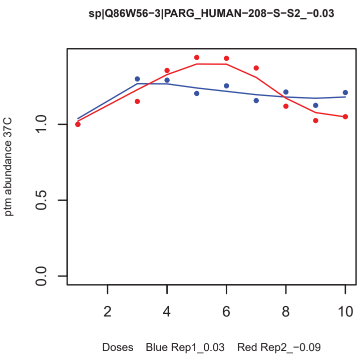
sp|Q86W56−3|PARG_HUMAN−208−S−S2_−0.03
ptm abundance 37C
Doses Blue Rep1_0.03 Red Rep2_−0.09

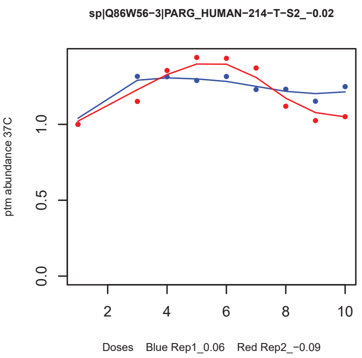
sp|Q86W56−3|PARG_HUMAN−214−T−S2_−0.02
ptm abundance 37C
Doses Blue Rep1_0.06 Red Rep2_−0.09

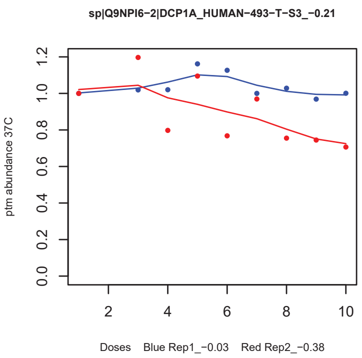
sp|Q9NPI6−2|DCP1A_HUMAN−493−T−S3_−0.21
ptm abundance 37C
Doses Blue Rep1_−0.03 Red Rep2_−0.38

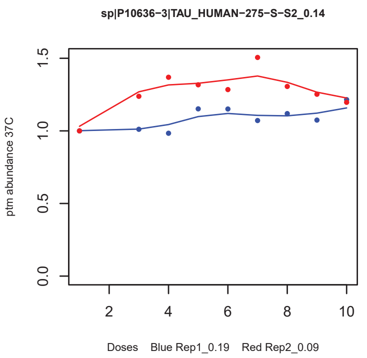
sp|P10636−3|TAU_HUMAN−275−S−S2_0.14
ptm abundance 37C
Doses Blue Rep1_0.19 Red Rep2_0.09

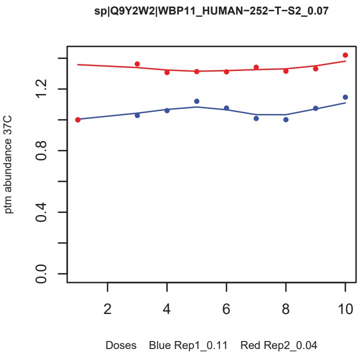
sp|Q9Y2W2|WBP11_HUMAN−252−T−S2_0.07
ptm abundance 37C
Doses Blue Rep1_0.11 Red Rep2_0.04

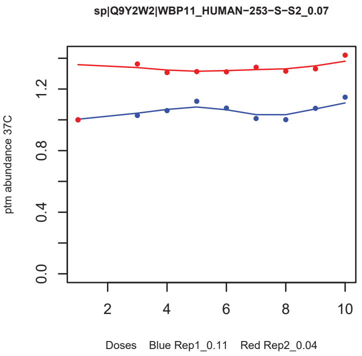
sp|Q9Y2W2|WBP11_HUMAN−253−S−S2_0.07
ptm abundance 37C
Doses Blue Rep1_0.11 Red Rep2_0.04

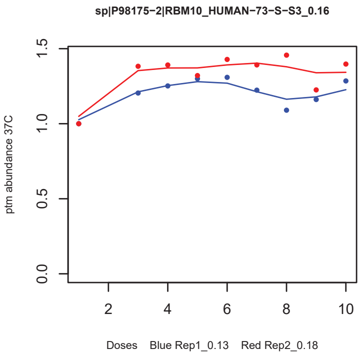
sp|P98175−2|RBM10_HUMAN−73−S−S3_0.16
ptm abundance 37C
Doses Blue Rep1_0.13 Red Rep2_0.18

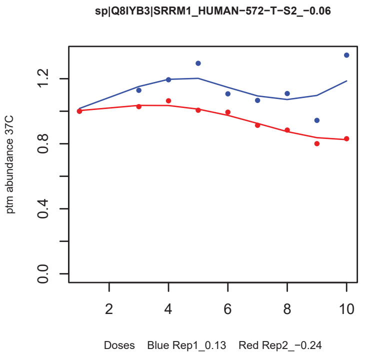
sp|Q8IYB3|SRRM1_HUMAN−572−T−S2_−0.06
ptm abundance 37C
Doses Blue Rep1_0.13 Red Rep2_−0.24

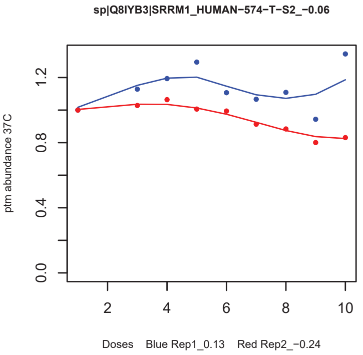
sp|Q8IYB3|SRRM1_HUMAN−574−T−S2_−0.06
ptm abundance 37C
Doses Blue Rep1_0.13 Red Rep2_−0.24

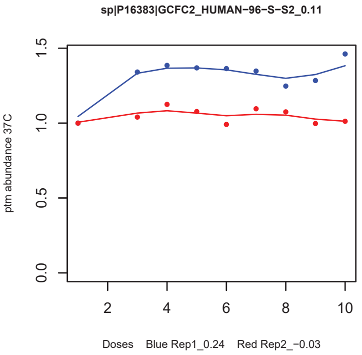
sp|P16383|GCFC2_HUMAN−96−S−S2_0.11
ptm abundance 37C
Doses Blue Rep1_0.24 Red Rep2_−0.03

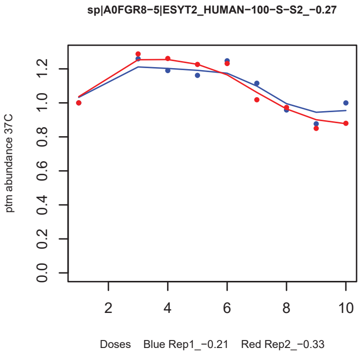
sp|A0FGR8−5|ESYT2_HUMAN−100−S−S2_−0.27
ptm abundance 37C
Doses Blue Rep1_−0.21 Red Rep2_−0.33

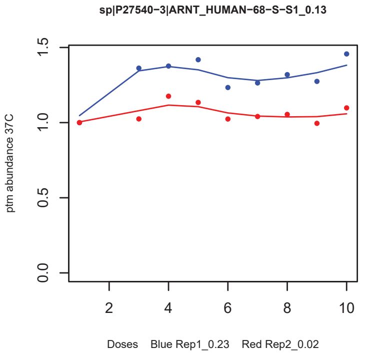
sp|P27540−3|ARNT_HUMAN−68−S−S1_0.13
ptm abundance 37C
Doses Blue Rep1_0.23 Red Rep2_0.02

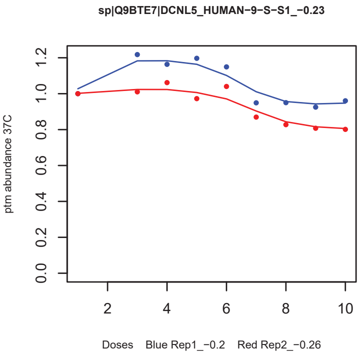
sp|Q9BTE7|DCNL5_HUMAN−9−S−S1_−0.23
ptm abundance 37C
Doses Blue Rep1_−0.2 Red Rep2_−0.26

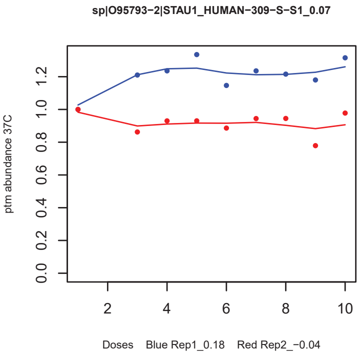
sp|O95793−2|STAU1_HUMAN−309−S−S1_0.07
ptm abundance 37C
Doses Blue Rep1_0.18 Red Rep2_−0.04

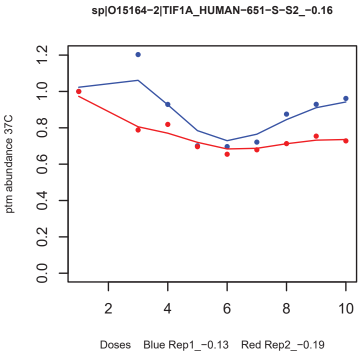
sp|O15164−2|TIF1A_HUMAN−651−S−S2_−0.16
ptm abundance 37C
Doses Blue Rep1_−0.13 Red Rep2_−0.19

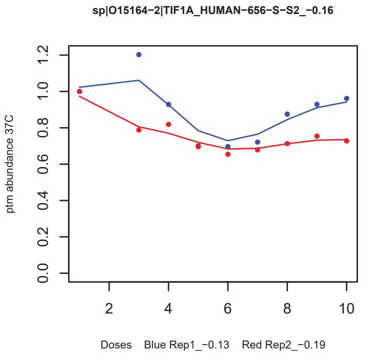
sp|O15164−2|TIF1A_HUMAN−656−S−S2_−0.16
ptm abundance 37C
Doses Blue Rep1_−0.13 Red Rep2_−0.19

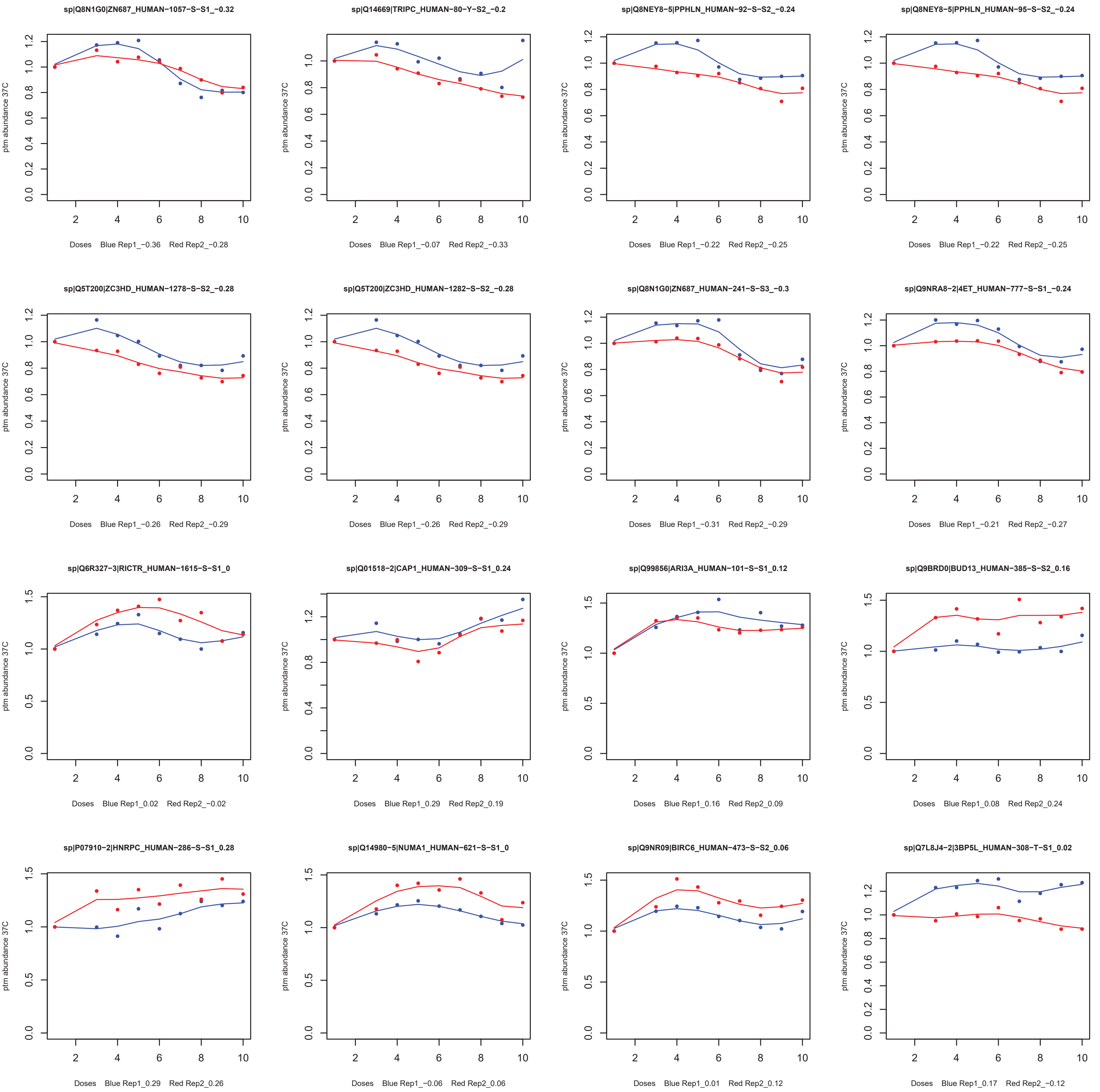

sp|Q8N1G0|ZN687_HUMAN−1057−S−S1_−0.32
ptm abundance 37C
Doses Blue Rep1_−0.36 Red Rep2_−0.28
sp|Q14669|TRIPC_HUMAN−80−Y−S2_−0.2
ptm abundance 37C
Doses Blue Rep1_−0.07 Red Rep2_−0.33
sp|Q8NEY8−5|PPHLN_HUMAN−92−S−S2_−0.24
ptm abundance 37C
Doses Blue Rep1_−0.22 Red Rep2_−0.25
sp|Q8NEY8−5|PPHLN_HUMAN−95−S−S2_−0.24
ptm abundance 37C
Doses Blue Rep1_−0.22 Red Rep2_−0.25
sp|Q5T200|ZC3HD_HUMAN−1278−S−S2_−0.28
ptm abundance 37C
Doses Blue Rep1_−0.26 Red Rep2_−0.29
sp|Q5T200|ZC3HD_HUMAN−1282−S−S2_−0.28
ptm abundance 37C
Doses Blue Rep1_−0.26 Red Rep2_−0.29
sp|Q8N1G0|ZN687_HUMAN−241−S−S3_−0.3
ptm abundance 37C
Doses Blue Rep1_−0.31 Red Rep2_−0.29
sp|Q9NRA8−2|4ET_HUMAN−777−S−S1_−0.24
ptm abundance 37C
Doses Blue Rep1_−0.21 Red Rep2_−0.27
sp|Q6R327−3|RICTR_HUMAN−1615−S−S1_0
ptm abundance 37C
Doses Blue Rep1_0.02 Red Rep2_−0.02
sp|Q01518−2|CAP1_HUMAN−309−S−S1_0.24
ptm abundance 37C
Doses Blue Rep1_0.29 Red Rep2_0.19
sp|Q99856|ARI3A_HUMAN−101−S−S1_0.12
ptm abundance 37C
Doses Blue Rep1_0.16 Red Rep2_0.09
sp|Q9BRD0|BUD13_HUMAN−385−S−S2_0.16
ptm abundance 37C
Doses Blue Rep1_0.08 Red Rep2_0.24
sp|P07910−2|HNRPC_HUMAN−286−S−S1_0.28
ptm abundance 37C
Doses Blue Rep1_0.29 Red Rep2_0.26
sp|Q14980−5|NUMA1_HUMAN−621−S−S1_0
ptm abundance 37C
Doses Blue Rep1_−0.06 Red Rep2_0.06
sp|Q9NR09|BIRC6_HUMAN−473−S−S2_0.06
ptm abundance 37C
Doses Blue Rep1_0.01 Red Rep2_0.12
sp|Q7L8J4−2|3BP5L_HUMAN−308−T−S1_0.02
ptm abundance 37C
Doses Blue Rep1_0.17 Red Rep2_−0.12

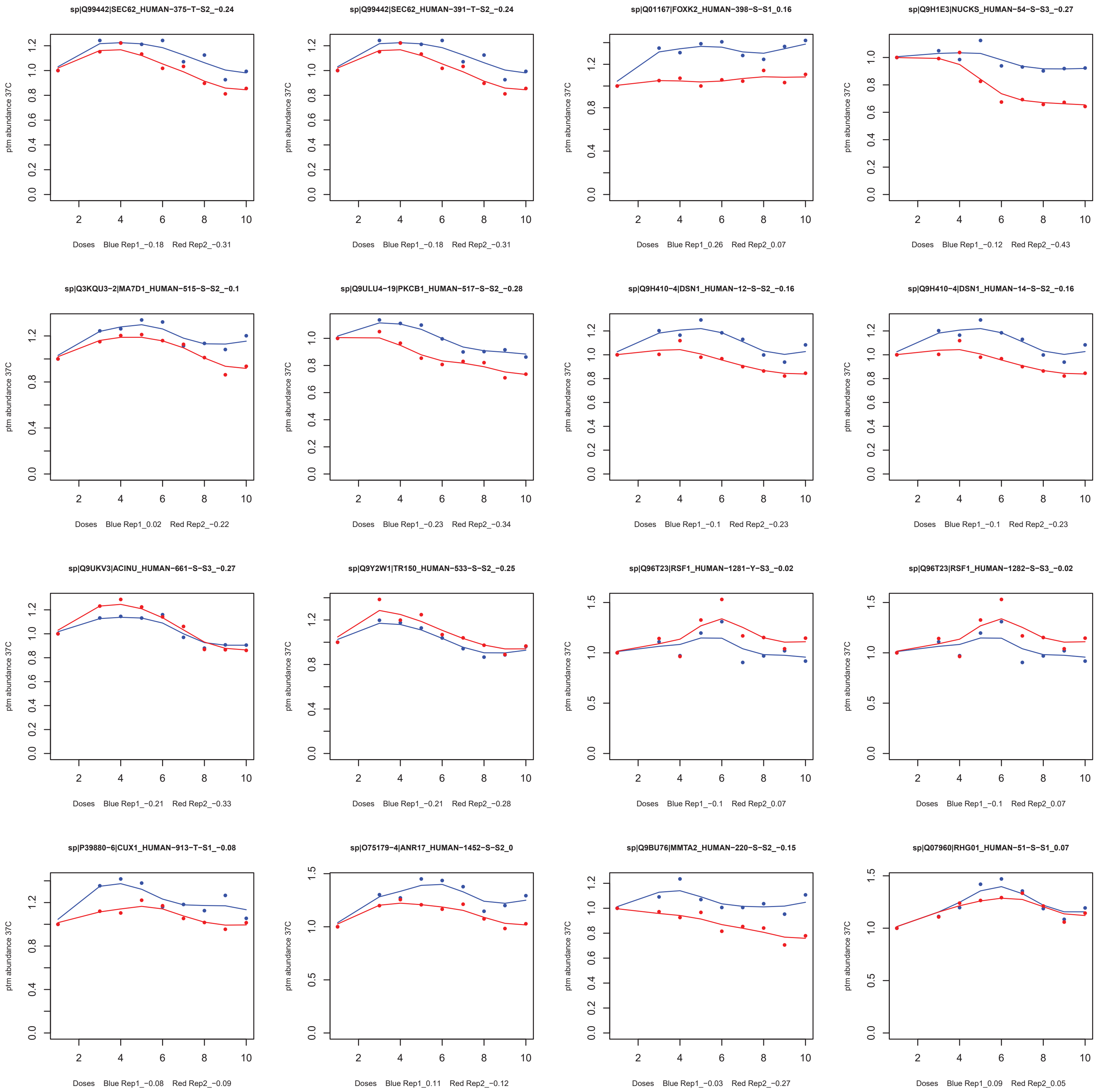
sp|Q99442|SEC62_HUMAN−375−T−S2_−0.24
ptm abundance 37C
Doses Blue Rep1_−0.18 Red Rep2_−0.31
sp|Q99442|SEC62_HUMAN−391−T−S2_−0.24
ptm abundance 37C
Doses Blue Rep1_−0.18 Red Rep2_−0.31
sp|Q01167|FOXK2_HUMAN−398−S−S1_0.16
ptm abundance 37C
Doses Blue Rep1_0.26 Red Rep2_0.07
sp|Q9H1E3|NUCKS_HUMAN−54−S−S3_−0.27
ptm abundance 37C
Doses Blue Rep1_−0.12 Red Rep2_−0.43
sp|Q3KQU3−2|MA7D1_HUMAN−515−S−S2_−0.1
ptm abundance 37C
Doses Blue Rep1_0.02 Red Rep2_−0.22
sp|Q9ULU4−19|PKCB1_HUMAN−517−S−S2_−0.28
ptm abundance 37C
Doses Blue Rep1_−0.23 Red Rep2_−0.34
sp|Q9H410−4|DSN1_HUMAN−12−S−S2_−0.16
ptm abundance 37C
Doses Blue Rep1_−0.1 Red Rep2_−0.23
sp|Q9H410−4|DSN1_HUMAN−14−S−S2_−0.16
ptm abundance 37C
Doses Blue Rep1_−0.1 Red Rep2_−0.23
sp|Q9UKV3|ACINU_HUMAN−661−S−S3_−0.27
ptm abundance 37C
Doses Blue Rep1_−0.21 Red Rep2_−0.33
sp|Q9Y2W1|TR150_HUMAN−533−S−S2_−0.25
ptm abundance 37C
Doses Blue Rep1_−0.21 Red Rep2_−0.28
sp|Q96T23|RSF1_HUMAN−1281−Y−S3_−0.02
ptm abundance 37C
Doses Blue Rep1_−0.1 Red Rep2_0.07
sp|Q96T23|RSF1_HUMAN−1282−S−S3_−0.02
ptm abundance 37C
Doses Blue Rep1_−0.1 Red Rep2_0.07
sp|P39880−6|CUX1_HUMAN−913−T−S1_−0.08
ptm abundance 37C
Doses Blue Rep1_−0.08 Red Rep2_−0.09
sp|O75179−4|ANR17_HUMAN−1452−S−S2_0
ptm abundance 37C
Doses Blue Rep1_0.11 Red Rep2_−0.12
sp|Q9BU76|MMTA2_HUMAN−220−S−S2_−0.15
ptm abundance 37C
Doses Blue Rep1_−0.03 Red Rep2_−0.27
sp|Q07960|RHG01_HUMAN−51−S−S1_0.07
ptm abundance 37C
Doses Blue Rep1_0.09 Red Rep2_0.05

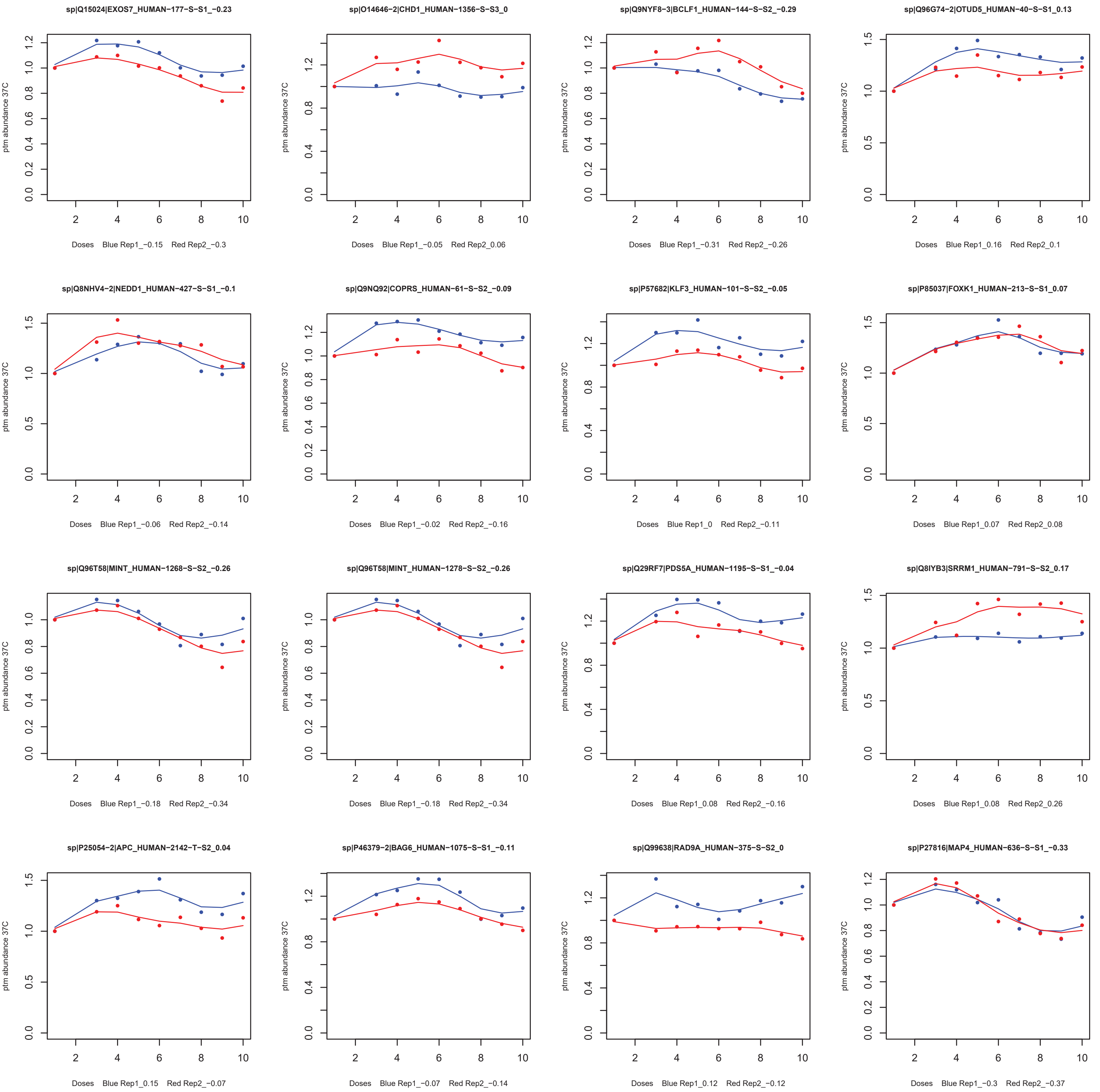
sp|Q15024|EXOS7_HUMAN−177−S−S1_−0.23
Doses Blue Rep1_−0.15 Red Rep2_−0.3
sp|O14646−2|CHD1_HUMAN−1356−S−S3_0
Doses Blue Rep1_−0.05 Red Rep2_0.06
sp|Q9NYF8−3|BCLF1_HUMAN−144−S−S2_−0.29
Doses Blue Rep1_−0.31 Red Rep2_−0.26
sp|Q96G74−2|OTUD5_HUMAN−40−S−S1_0.13
Doses Blue Rep1_0.16 Red Rep2_0.1
sp|Q8NHV4−2|NEDD1_HUMAN−427−S−S1_−0.1
Doses Blue Rep1_−0.06 Red Rep2_−0.14
sp|Q9NQ92|COPRS_HUMAN−61−S−S2_−0.09
Doses Blue Rep1_−0.02 Red Rep2_−0.16
sp|P57682|KLF3_HUMAN−101−S−S2_−0.05
Doses Blue Rep1_0 Red Rep2_−0.11
sp|P85037|FOXK1_HUMAN−213−S−S1_0.07
Doses Blue Rep1_0.07 Red Rep2_0.08
sp|Q96T58|MINT_HUMAN−1268−S−S2_−0.26
Doses Blue Rep1_−0.18 Red Rep2_−0.34
sp|Q96T58|MINT_HUMAN−1278−S−S2_−0.26
Doses Blue Rep1_−0.18 Red Rep2_−0.34
sp|Q29RF7|PDS5A_HUMAN−1195−S−S1_−0.04
Doses Blue Rep1_0.08 Red Rep2_−0.16
sp|Q8IYB3|SRRM1_HUMAN−791−S−S2_0.17
Doses Blue Rep1_0.08 Red Rep2_0.26
sp|P25054−2|APC_HUMAN−2142−T−S2_0.04
Doses Blue Rep1_0.15 Red Rep2_−0.07
sp|P46379−2|BAG6_HUMAN−1075−S−S1_−0.11
Doses Blue Rep1_−0.07 Red Rep2_−0.14
sp|Q99638|RAD9A_HUMAN−375−S−S2_0
Doses Blue Rep1_0.12 Red Rep2_−0.12
sp|P27816|MAP4_HUMAN−636−S−S1_−0.33
Doses Blue Rep1_−0.3 Red Rep2_−0.37
ptm abundance 37C

sp|Q96RK0|CIC_HUMAN−1397−S−S2_−0.32

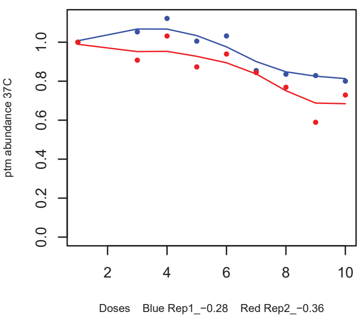

sp|Q9BRD0|BUD13_HUMAN−271−S−S2_−0.3

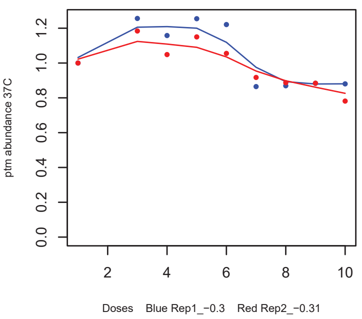

sp|Q9BRD0|BUD13_HUMAN−280−Y−S2_−0.3

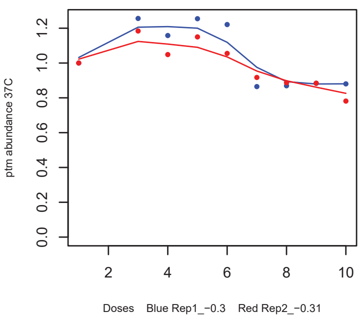

sp|Q5VTL8|PR38B_HUMAN−527−S−S1_−0.2

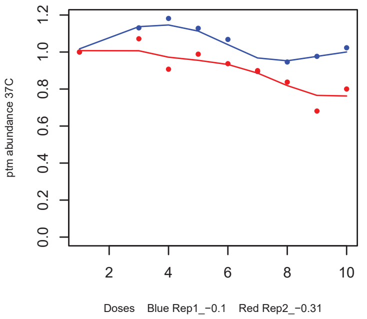

sp|Q9BU76|MMTA2_HUMAN−217−S−S2_−0.15

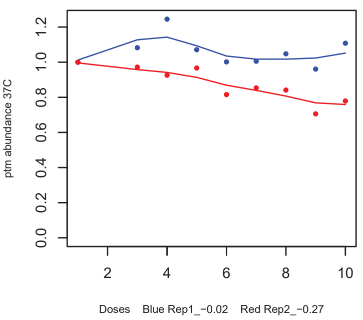

sp|Q5VV41|ARHGG_HUMAN−239−S−S2_0.22

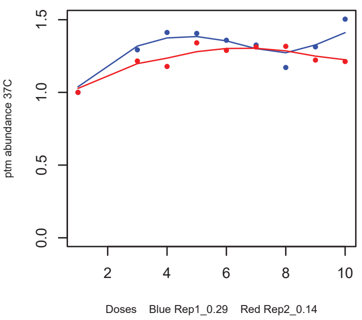

sp|Q7LBC6|KDM3B_HUMAN−311−S−S2_−0.09

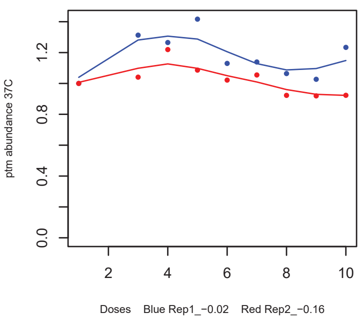

sp|O00429−7|DNM1L_HUMAN−413−S−S1_−0.2

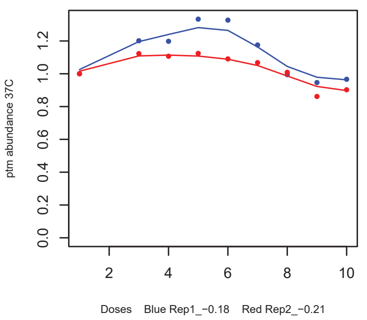

sp|Q3KQU3−2|MA7D1_HUMAN−511−S−S2_−0.09

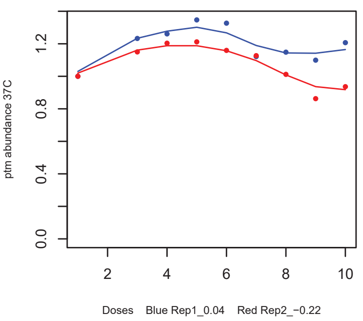

sp|Q86U90|YRDC_HUMAN−37−S−S1_−0.06

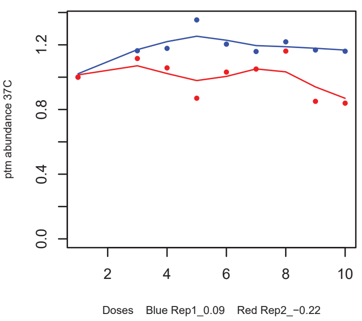

sp|P46100−4|ATRX_HUMAN−639−S−S1_−0.09

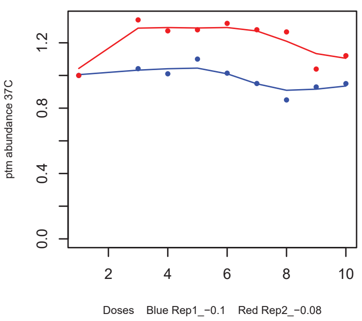

sp|Q99590−2|SCAFB_HUMAN−101−S−S2_−0.05

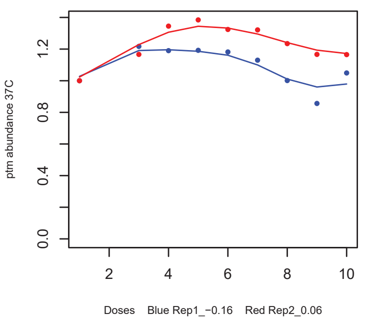

sp|Q8TAP8|PPR35_HUMAN−84−T−S2_−0.12

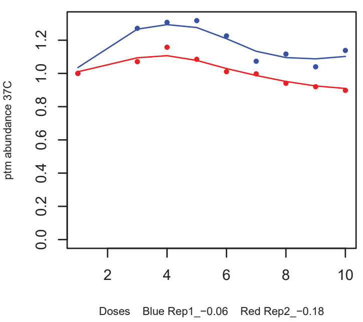

sp|Q8TAP8|PPR35_HUMAN−87−S−S2_−0.12

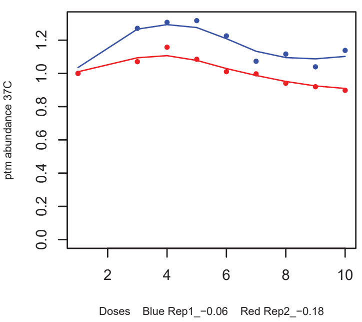

sp|O43683−2|BUB1_HUMAN−655−S−S1_−0.22

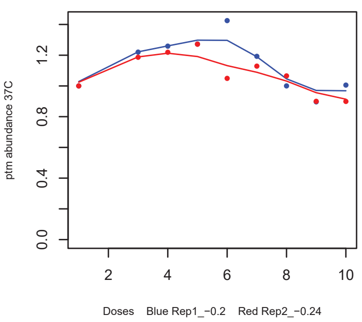

sp|Q03164−2|KMT2A_HUMAN−2353−S−S2_0.3

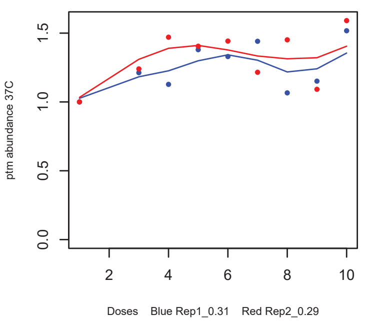

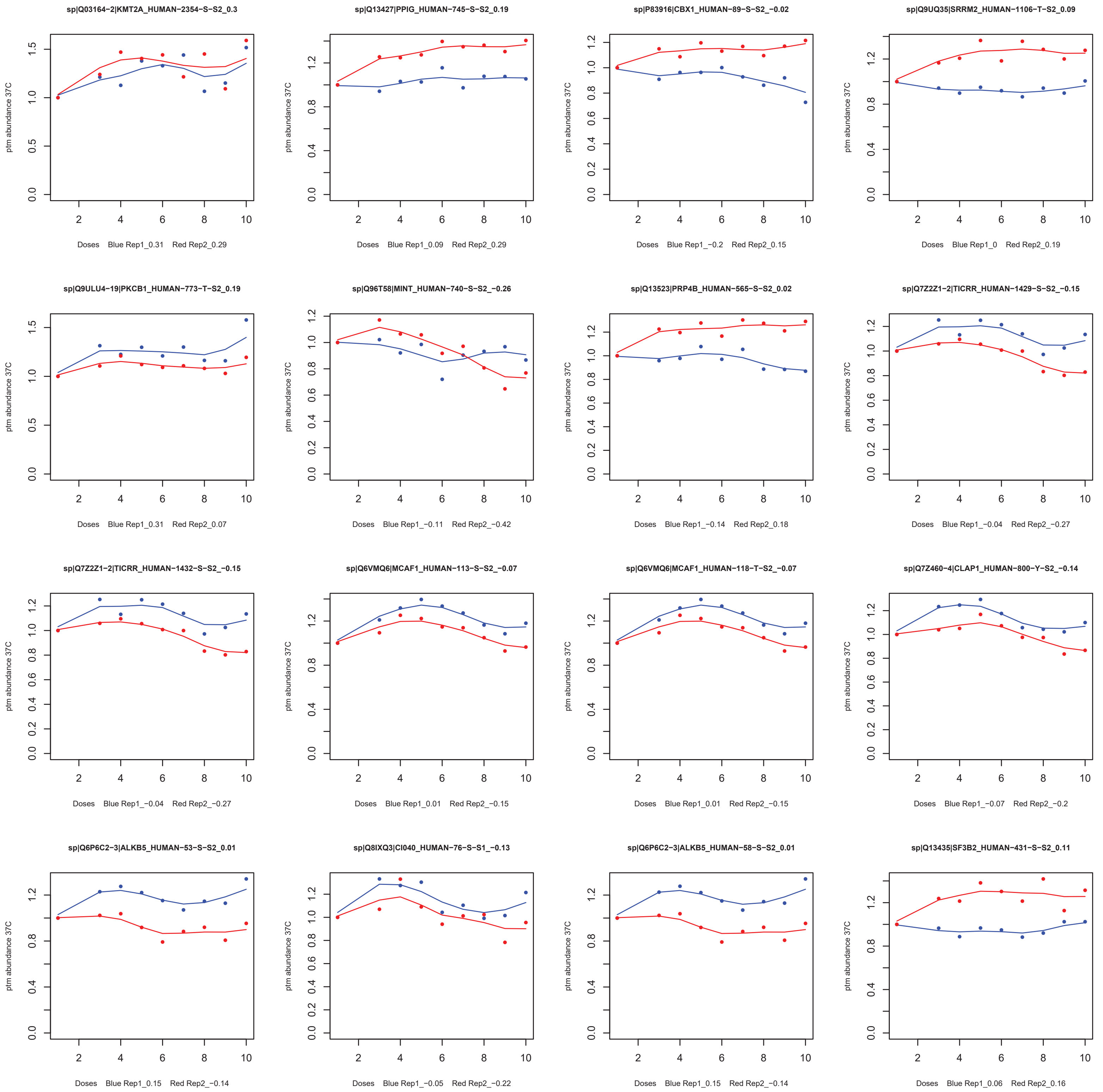
sp|Q03164−2|KMT2A_HUMAN−2354−S−S2_0.3
Doses Blue Rep1_0.31 Red Rep2_0.29
sp|Q13427|PPIG_HUMAN−745−S−S2_0.19
Doses Blue Rep1_0.09 Red Rep2_0.29
sp|P83916|CBX1_HUMAN−89−S−S2_−0.02
Doses Blue Rep1_−0.2 Red Rep2_0.15
sp|Q9UQ35|SRRM2_HUMAN−1106−T−S2_0.09
Doses Blue Rep1_0 Red Rep2_0.19
sp|Q9ULU4−19|PKCB1_HUMAN−773−T−S2_0.19
Doses Blue Rep1_0.31 Red Rep2_0.07
sp|Q96T58|MINT_HUMAN−740−S−S2_−0.26
Doses Blue Rep1_−0.11 Red Rep2_−0.42
sp|Q13523|PRP4B_HUMAN−565−S−S2_0.02
Doses Blue Rep1_−0.14 Red Rep2_0.18
sp|Q7Z2Z1−2|TICRR_HUMAN−1429−S−S2_−0.15
Doses Blue Rep1_−0.04 Red Rep2_−0.27
sp|Q7Z2Z1−2|TICRR_HUMAN−1432−S−S2_−0.15
Doses Blue Rep1_−0.04 Red Rep2_−0.27
sp|Q6VMQ6|MCAF1_HUMAN−113−S−S2_−0.07
Doses Blue Rep1_0.01 Red Rep2_−0.15
sp|Q6VMQ6|MCAF1_HUMAN−118−T−S2_−0.07
Doses Blue Rep1_0.01 Red Rep2_−0.15
sp|Q7Z460−4|CLAP1_HUMAN−800−Y−S2_−0.14
Doses Blue Rep1_−0.07 Red Rep2_−0.2
sp|Q6P6C2−3|ALKB5_HUMAN−53−S−S2_0.01
Doses Blue Rep1_0.15 Red Rep2_−0.14
sp|Q8IXQ3|CI040_HUMAN−76−S−S1_−0.13
Doses Blue Rep1_−0.05 Red Rep2_−0.22
sp|Q6P6C2−3|ALKB5_HUMAN−58−S−S2_0.01
Doses Blue Rep1_0.15 Red Rep2_−0.14
sp|Q13435|SF3B2_HUMAN−431−S−S2_0.11
Doses Blue Rep1_0.06 Red Rep2_0.16
ptm abundance 37C

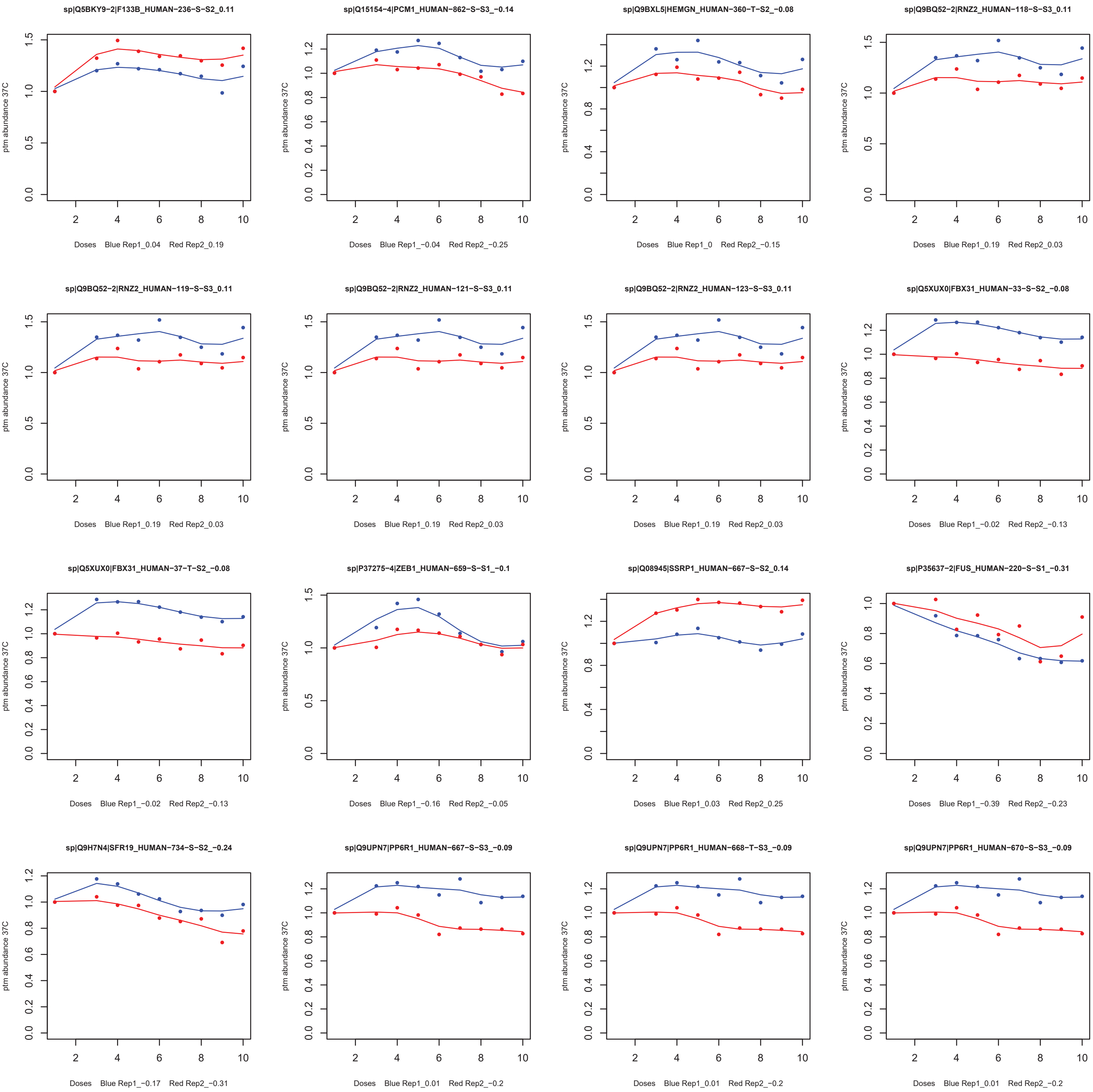
sp|Q5BKY9−2|F133B_HUMAN−236−S−S2_0.11
Doses Blue Rep1_0.04 Red Rep2_0.19
sp|Q15154−4|PCM1_HUMAN−862−S−S3_−0.14
Doses Blue Rep1_−0.04 Red Rep2_−0.25
sp|Q9BXL5|HEMGN_HUMAN−360−T−S2_−0.08
Doses Blue Rep1_0 Red Rep2_−0.15
sp|Q9BQ52−2|RNZ2_HUMAN−118−S−S3_0.11
Doses Blue Rep1_0.19 Red Rep2_0.03
sp|Q9BQ52−2|RNZ2_HUMAN−119−S−S3_0.11
Doses Blue Rep1_0.19 Red Rep2_0.03
sp|Q9BQ52−2|RNZ2_HUMAN−121−S−S3_0.11
Doses Blue Rep1_0.19 Red Rep2_0.03
sp|Q9BQ52−2|RNZ2_HUMAN−123−S−S3_0.11
Doses Blue Rep1_0.19 Red Rep2_0.03
sp|Q5XUX0|FBX31_HUMAN−33−S−S2_−0.08
Doses Blue Rep1_−0.02 Red Rep2_−0.13
sp|Q5XUX0|FBX31_HUMAN−37−T−S2_−0.08
Doses Blue Rep1_−0.02 Red Rep2_−0.13
sp|P37275−4|ZEB1_HUMAN−659−S−S1_−0.1
Doses Blue Rep1_−0.16 Red Rep2_−0.05
sp|Q08945|SSRP1_HUMAN−667−S−S2_0.14
Doses Blue Rep1_0.03 Red Rep2_0.25
sp|P35637−2|FUS_HUMAN−220−S−S1_−0.31
Doses Blue Rep1_−0.39 Red Rep2_−0.23
sp|Q9H7N4|SFR19_HUMAN−734−S−S2_−0.24
Doses Blue Rep1_−0.17 Red Rep2_−0.31
sp|Q9UPN7|PP6R1_HUMAN−667−S−S3_−0.09
Doses Blue Rep1_0.01 Red Rep2_−0.2
sp|Q9UPN7|PP6R1_HUMAN−668−T−S3_−0.09
Doses Blue Rep1_0.01 Red Rep2_−0.2
sp|Q9UPN7|PP6R1_HUMAN−670−S−S3_−0.09
Doses Blue Rep1_0.01 Red Rep2_−0.2
ptm abundance 37C

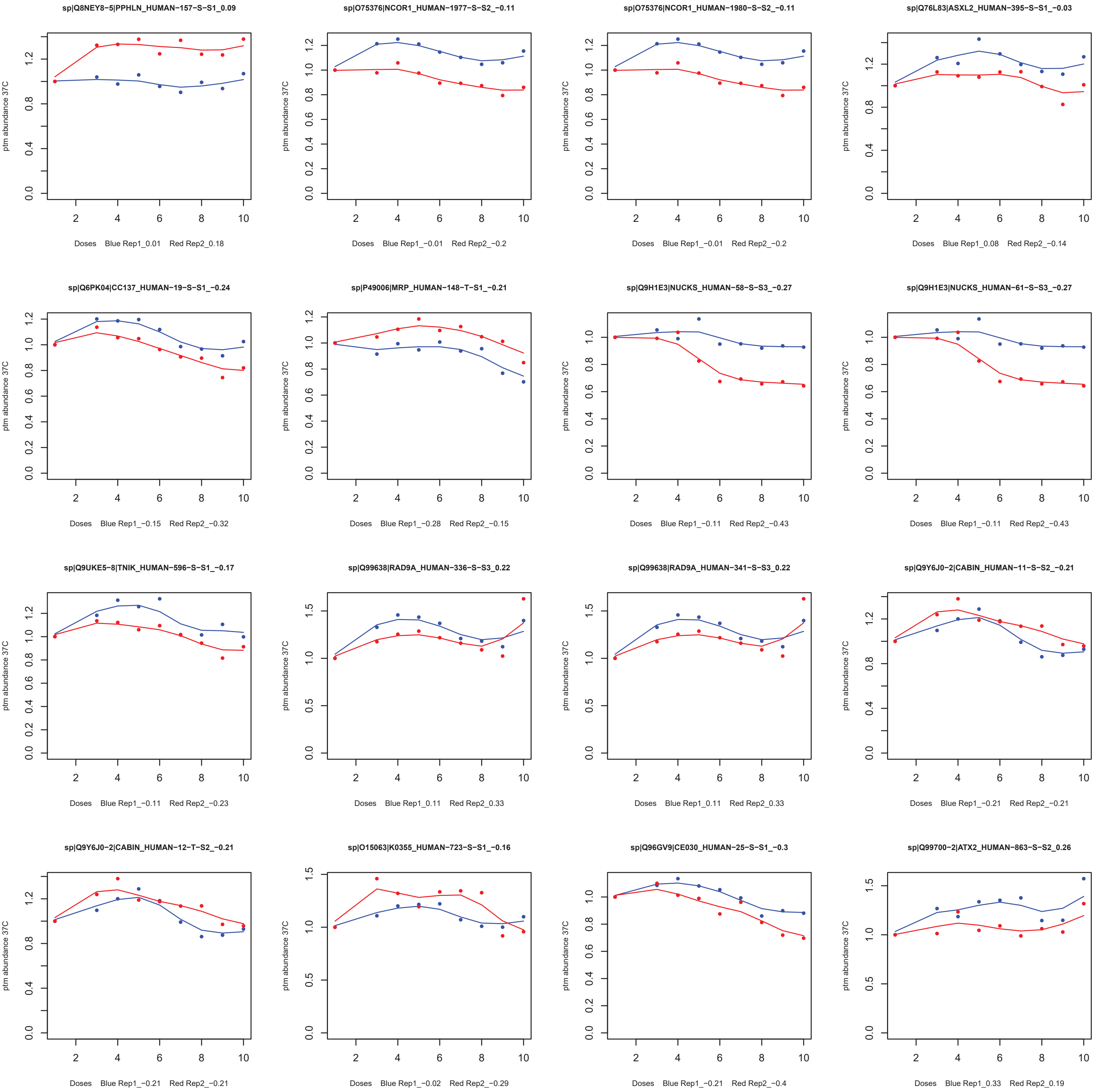
sp|Q8NEY8−5|PPHLN_HUMAN−157−S−S1_0.09
ptm abundance 37C
Doses Blue Rep1_0.01 Red Rep2_0.18
sp|O75376|NCOR1_HUMAN−1977−S−S2_−0.11
ptm abundance 37C
Doses Blue Rep1_−0.01 Red Rep2_−0.2
sp|O75376|NCOR1_HUMAN−1980−S−S2_−0.11
ptm abundance 37C
Doses Blue Rep1_−0.01 Red Rep2_−0.2
sp|Q76L83|ASXL2_HUMAN−395−S−S1_−0.03
ptm abundance 37C
Doses Blue Rep1_0.08 Red Rep2_−0.14
sp|Q6PK04|CC137_HUMAN−19−S−S1_−0.24
ptm abundance 37C
Doses Blue Rep1_−0.15 Red Rep2_−0.32
sp|P49006|MRP_HUMAN−148−T−S1_−0.21
ptm abundance 37C
Doses Blue Rep1_−0.28 Red Rep2_−0.15
sp|Q9H1E3|NUCKS_HUMAN−58−S−S3_−0.27
ptm abundance 37C
Doses Blue Rep1_−0.11 Red Rep2_−0.43
sp|Q9H1E3|NUCKS_HUMAN−61−S−S3_−0.27
ptm abundance 37C
Doses Blue Rep1_−0.11 Red Rep2_−0.43
sp|Q9UKE5−8|TNIK_HUMAN−596−S−S1_−0.17
ptm abundance 37C
Doses Blue Rep1_−0.11 Red Rep2_−0.23
sp|Q99638|RAD9A_HUMAN−336−S−S3_0.22
ptm abundance 37C
Doses Blue Rep1_0.11 Red Rep2_0.33
sp|Q99638|RAD9A_HUMAN−341−S−S3_0.22
ptm abundance 37C
Doses Blue Rep1_0.11 Red Rep2_0.33
sp|Q9Y6J0−2|CABIN_HUMAN−11−S−S2_−0.21
ptm abundance 37C
Doses Blue Rep1_−0.21 Red Rep2_−0.21
sp|Q9Y6J0−2|CABIN_HUMAN−12−T−S2_−0.21
ptm abundance 37C
Doses Blue Rep1_−0.21 Red Rep2_−0.21
sp|O15063|K0355_HUMAN−723−S−S1_−0.16
ptm abundance 37C
Doses Blue Rep1_−0.02 Red Rep2_−0.29
sp|Q96GV9|CE030_HUMAN−25−S−S1_−0.3
ptm abundance 37C
Doses Blue Rep1_−0.21 Red Rep2_−0.4
sp|Q99700−2|ATX2_HUMAN−863−S−S2_0.26
ptm abundance 37C
Doses Blue Rep1_0.33 Red Rep2_0.19

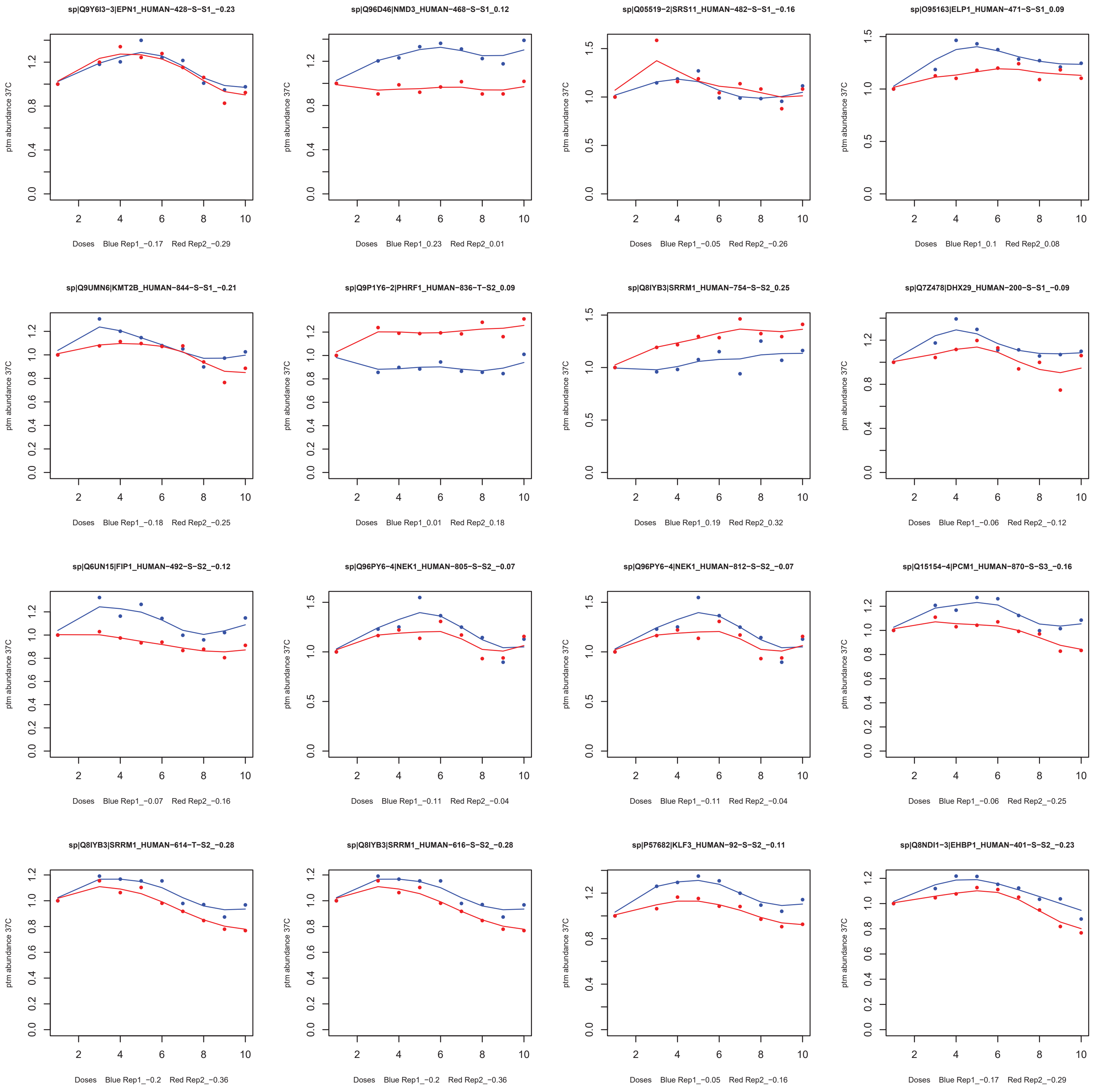

sp|Q9Y6I3−3|EPN1_HUMAN−428−S−S1_−0.23
Doses Blue Rep1_−0.17 Red Rep2_−0.29
sp|Q96D46|NMD3_HUMAN−468−S−S1_0.12
Doses Blue Rep1_0.23 Red Rep2_0.01
sp|Q05519−2|SRS11_HUMAN−482−S−S1_−0.16
Doses Blue Rep1_−0.05 Red Rep2_−0.26
sp|O95163|ELP1_HUMAN−471−S−S1_0.09
Doses Blue Rep1_0.1 Red Rep2_0.08
sp|Q9UMN6|KMT2B_HUMAN−844−S−S1_−0.21
Doses Blue Rep1_−0.18 Red Rep2_−0.25
sp|Q9P1Y6−2|PHRF1_HUMAN−836−T−S2_0.09
Doses Blue Rep1_0.01 Red Rep2_0.18
sp|Q8IYB3|SRRM1_HUMAN−754−S−S2_0.25
Doses Blue Rep1_0.19 Red Rep2_0.32
sp|Q7Z478|DHX29_HUMAN−200−S−S1_−0.09
Doses Blue Rep1_−0.06 Red Rep2_−0.12
sp|Q6UN15|FIP1_HUMAN−492−S−S2_−0.12
Doses Blue Rep1_−0.07 Red Rep2_−0.16
sp|Q96PY6−4|NEK1_HUMAN−805−S−S2_−0.07
Doses Blue Rep1_−0.11 Red Rep2_−0.04
sp|Q96PY6−4|NEK1_HUMAN−812−S−S2_−0.07
Doses Blue Rep1_−0.11 Red Rep2_−0.04
sp|Q15154−4|PCM1_HUMAN−870−S−S3_−0.16
Doses Blue Rep1_−0.06 Red Rep2_−0.25
sp|Q8IYB3|SRRM1_HUMAN−614−T−S2_−0.28
Doses Blue Rep1_−0.2 Red Rep2_−0.36
sp|Q8IYB3|SRRM1_HUMAN−616−S−S2_−0.28
Doses Blue Rep1_−0.2 Red Rep2_−0.36
sp|P57682|KLF3_HUMAN−92−S−S2_−0.11
Doses Blue Rep1_−0.05 Red Rep2_−0.16
sp|Q8NDI1−3|EHBP1_HUMAN−401−S−S2_−0.23
Doses Blue Rep1_−0.17 Red Rep2_−0.29
ptm abundance 37C

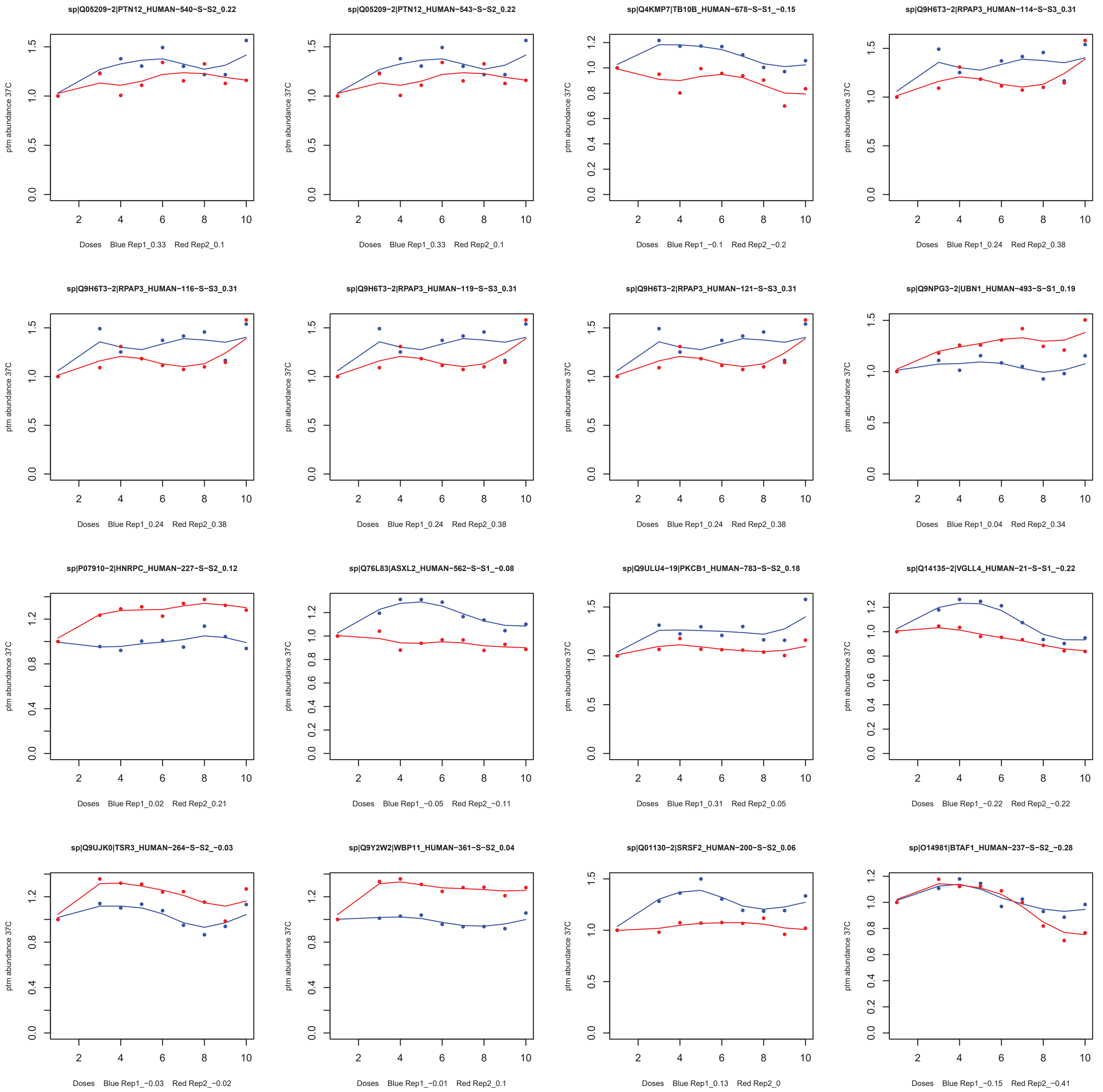
sp|Q05209−2|PTN12_HUMAN−540−S−S2_0.22
Doses Blue Rep1_0.33 Red Rep2_0.1
sp|Q05209−2|PTN12_HUMAN−543−S−S2_0.22
Doses Blue Rep1_0.33 Red Rep2_0.1
sp|Q4KMP7|TB10B_HUMAN−678−S−S1_−0.15
Doses Blue Rep1_−0.1 Red Rep2_−0.2
sp|Q9H6T3−2|RPAP3_HUMAN−114−S−S3_0.31
Doses Blue Rep1_0.24 Red Rep2_0.38
sp|Q9H6T3−2|RPAP3_HUMAN−116−S−S3_0.31
Doses Blue Rep1_0.24 Red Rep2_0.38
sp|Q9H6T3−2|RPAP3_HUMAN−119−S−S3_0.31
Doses Blue Rep1_0.24 Red Rep2_0.38
sp|Q9H6T3−2|RPAP3_HUMAN−121−S−S3_0.31
Doses Blue Rep1_0.24 Red Rep2_0.38
sp|Q9NPG3−2|UBN1_HUMAN−493−S−S1_0.19
Doses Blue Rep1_0.04 Red Rep2_0.34
sp|P07910−2|HNRPC_HUMAN−227−S−S2_0.12
Doses Blue Rep1_0.02 Red Rep2_0.21
sp|Q76L83|ASXL2_HUMAN−562−S−S1_−0.08
Doses Blue Rep1_−0.05 Red Rep2_−0.11
sp|Q9ULU4−19|PKCB1_HUMAN−783−S−S2_0.18
Doses Blue Rep1_0.31 Red Rep2_0.05
sp|Q14135−2|VGLL4_HUMAN−21−S−S1_−0.22
Doses Blue Rep1_−0.22 Red Rep2_−0.22
sp|Q9UJK0|TSR3_HUMAN−264−S−S2_−0.03
Doses Blue Rep1_−0.03 Red Rep2_−0.02
sp|Q9Y2W2|WBP11_HUMAN−361−S−S2_0.04
Doses Blue Rep1_−0.01 Red Rep2_0.1
sp|Q01130−2|SRSF2_HUMAN−200−S−S2_0.06
Doses Blue Rep1_0.13 Red Rep2_0
sp|O14981|BTAF1_HUMAN−237−S−S2_−0.28
Doses Blue Rep1_−0.15 Red Rep2_−0.41
ptm abundance 37C

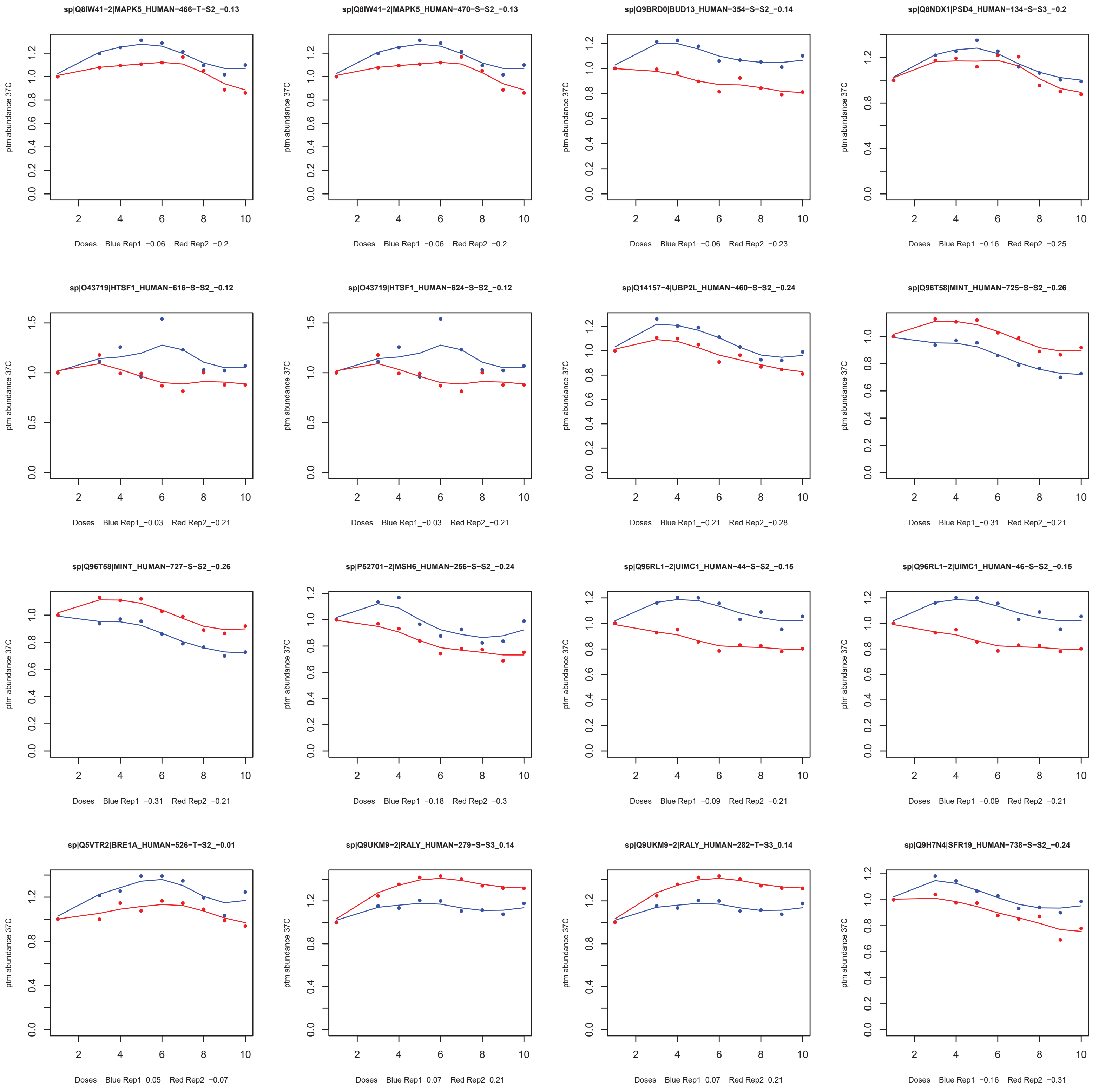
sp|Q8IW41−2|MAPK5_HUMAN−466−T−S2_−0.13
Doses Blue Rep1_−0.06 Red Rep2_−0.2
sp|Q8IW41−2|MAPK5_HUMAN−470−S−S2_−0.13
Doses Blue Rep1_−0.06 Red Rep2_−0.2
sp|Q9BRD0|BUD13_HUMAN−354−S−S2_−0.14
Doses Blue Rep1_−0.06 Red Rep2_−0.23
sp|Q8NDX1|PSD4_HUMAN−134−S−S3_−0.2
Doses Blue Rep1_−0.16 Red Rep2_−0.25
sp|O43719|HTSF1_HUMAN−616−S−S2_−0.12
Doses Blue Rep1_−0.03 Red Rep2_−0.21
sp|O43719|HTSF1_HUMAN−624−S−S2_−0.12
Doses Blue Rep1_−0.03 Red Rep2_−0.21
sp|Q14157−4|UBP2L_HUMAN−460−S−S2_−0.24
Doses Blue Rep1_−0.21 Red Rep2_−0.28
sp|Q96T58|MINT_HUMAN−725−S−S2_−0.26
Doses Blue Rep1_−0.31 Red Rep2_−0.21
sp|Q96T58|MINT_HUMAN−727−S−S2_−0.26
Doses Blue Rep1_−0.31 Red Rep2_−0.21
sp|P52701−2|MSH6_HUMAN−256−S−S2_−0.24
Doses Blue Rep1_−0.18 Red Rep2_−0.3
sp|Q96RL1−2|UIMC1_HUMAN−44−S−S2_−0.15
Doses Blue Rep1_−0.09 Red Rep2_−0.21
sp|Q96RL1−2|UIMC1_HUMAN−46−S−S2_−0.15
Doses Blue Rep1_−0.09 Red Rep2_−0.21
sp|Q5VTR2|BRE1A_HUMAN−526−T−S2_−0.01
Doses Blue Rep1_0.05 Red Rep2_−0.07
sp|Q9UKM9−2|RALY_HUMAN−279−S−S3_0.14
Doses Blue Rep1_0.07 Red Rep2_0.21
sp|Q9UKM9−2|RALY_HUMAN−282−T−S3_0.14
Doses Blue Rep1_0.07 Red Rep2_0.21
sp|Q9H7N4|SFR19_HUMAN−738−S−S2_−0.24
Doses Blue Rep1_−0.16 Red Rep2_−0.31
ptm abundance 37C

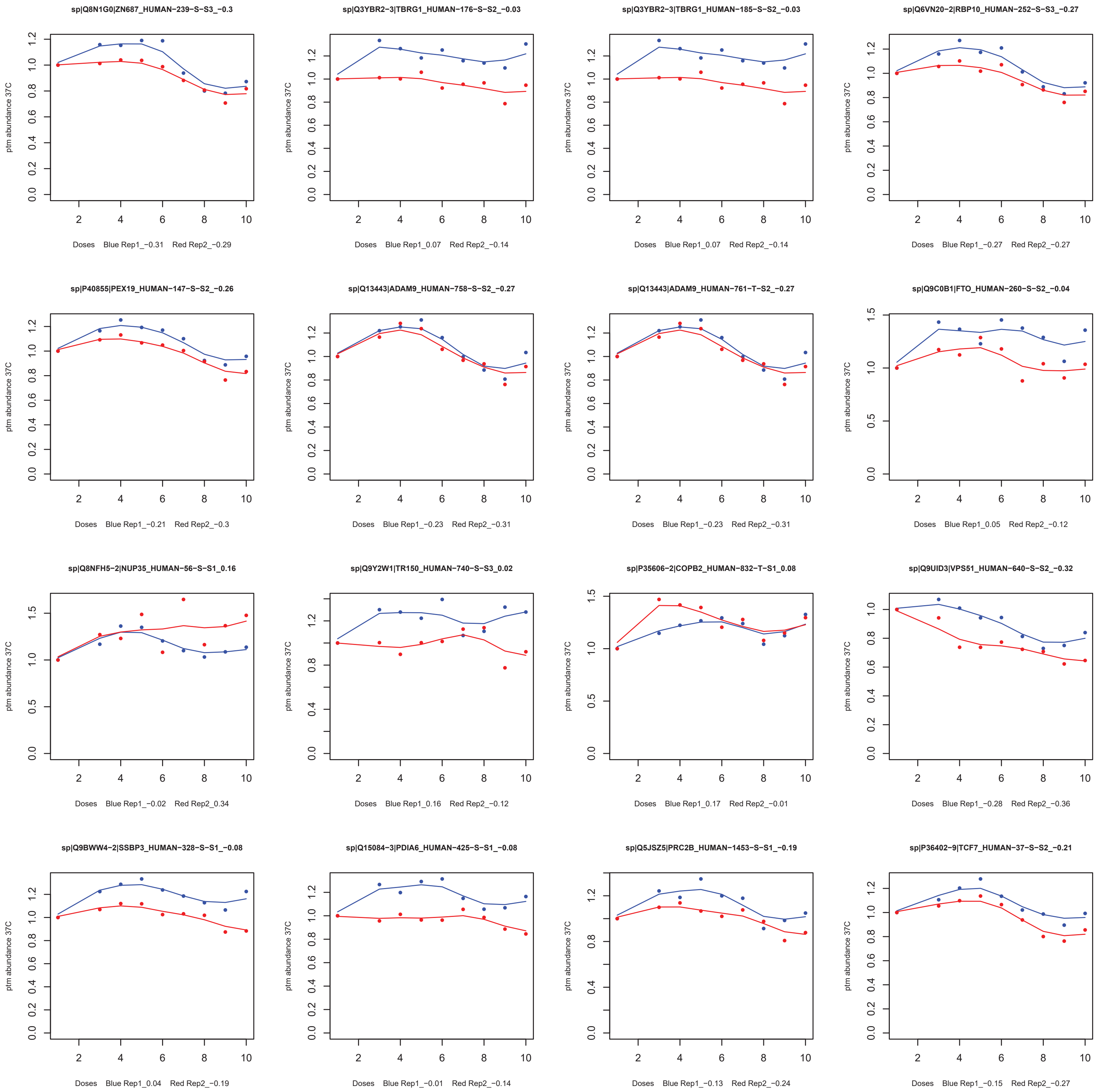
sp|Q8N1G0|ZN687_HUMAN−239−S−S3_−0.3
ptm abundance 37C
Doses Blue Rep1_−0.31 Red Rep2_−0.29
sp|Q3YBR2−3|TBRG1_HUMAN−176−S−S2_−0.03
ptm abundance 37C
Doses Blue Rep1_0.07 Red Rep2_−0.14
sp|Q3YBR2−3|TBRG1_HUMAN−185−S−S2_−0.03
ptm abundance 37C
Doses Blue Rep1_0.07 Red Rep2_−0.14
sp|Q6VN20−2|RBP10_HUMAN−252−S−S3_−0.27
ptm abundance 37C
Doses Blue Rep1_−0.27 Red Rep2_−0.27
sp|P40855|PEX19_HUMAN−147−S−S2_−0.26
ptm abundance 37C
Doses Blue Rep1_−0.21 Red Rep2_−0.3
sp|Q13443|ADAM9_HUMAN−758−S−S2_−0.27
ptm abundance 37C
Doses Blue Rep1_−0.23 Red Rep2_−0.31
sp|Q13443|ADAM9_HUMAN−761−T−S2_−0.27
ptm abundance 37C
Doses Blue Rep1_−0.23 Red Rep2_−0.31
sp|Q9C0B1|FTO_HUMAN−260−S−S2_−0.04
ptm abundance 37C
Doses Blue Rep1_0.05 Red Rep2_−0.12
sp|Q8NFH5−2|NUP35_HUMAN−56−S−S1_0.16
ptm abundance 37C
Doses Blue Rep1_−0.02 Red Rep2_0.34
sp|Q9Y2W1|TR150_HUMAN−740−S−S3_0.02
ptm abundance 37C
Doses Blue Rep1_0.16 Red Rep2_−0.12
sp|P35606−2|COPB2_HUMAN−832−T−S1_0.08
ptm abundance 37C
Doses Blue Rep1_0.17 Red Rep2_−0.01
sp|Q9UID3|VPS51_HUMAN−640−S−S2_−0.32
ptm abundance 37C
Doses Blue Rep1_−0.28 Red Rep2_−0.36
sp|Q9BWW4−2|SSBP3_HUMAN−328−S−S1_−0.08
ptm abundance 37C
Doses Blue Rep1_0.04 Red Rep2_−0.19
sp|Q15084−3|PDIA6_HUMAN−425−S−S1_−0.08
ptm abundance 37C
Doses Blue Rep1_−0.01 Red Rep2_−0.14
sp|Q5JSZ5|PRC2B_HUMAN−1453−S−S1_−0.19
ptm abundance 37C
Doses Blue Rep1_−0.13 Red Rep2_−0.24
sp|P36402−9|TCF7_HUMAN−37−S−S2_−0.21
ptm abundance 37C
Doses Blue Rep1_−0.15 Red Rep2_−0.27

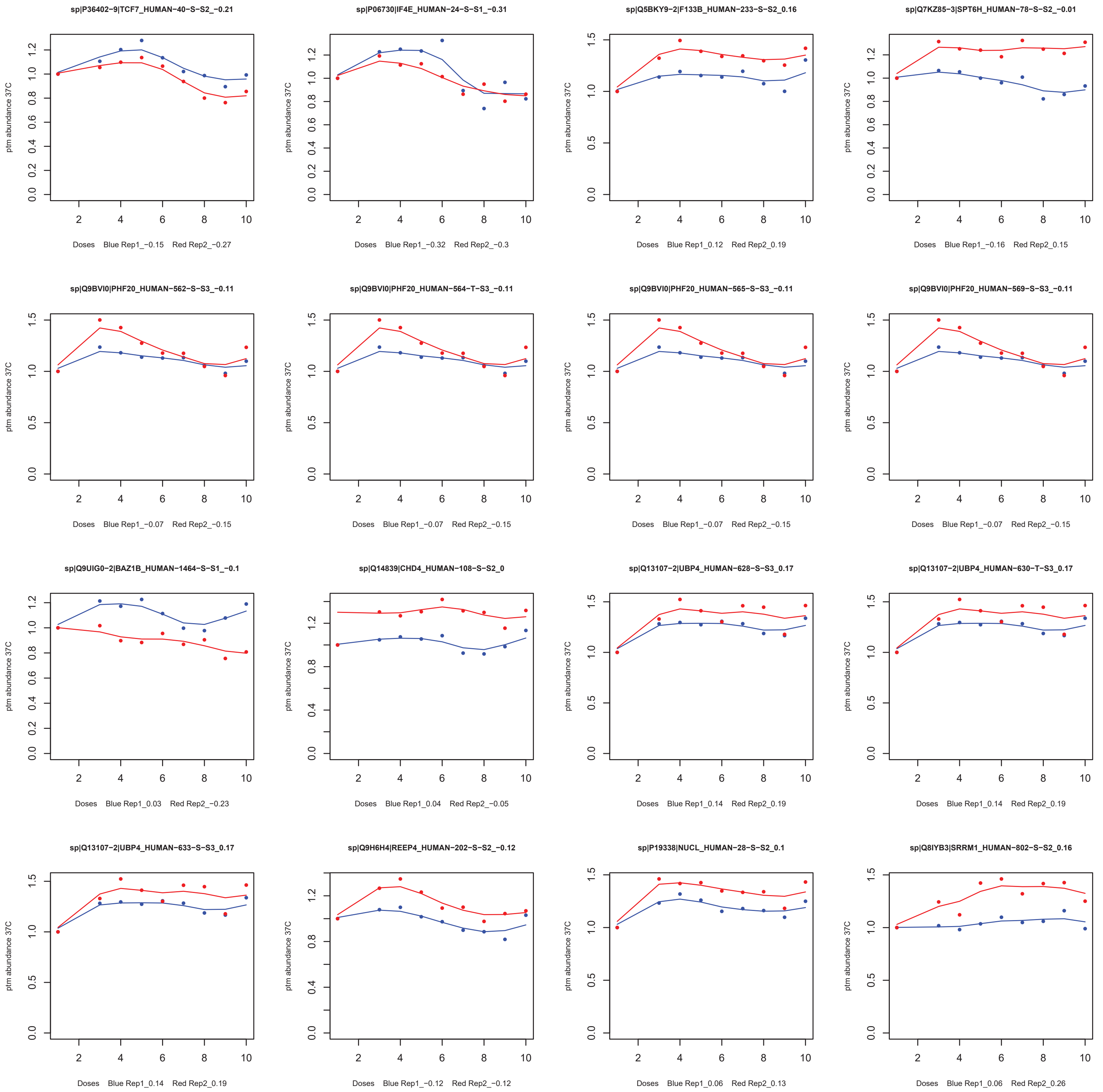

sp|P36402-9|TCF7_HUMAN-40-S-S2_-0.21
ptm abundance 37C
Doses Blue Rep1_-0.15 Red Rep2_-0.27
sp|P06730|IF4E_HUMAN-24-S-S1_-0.31
ptm abundance 37C
Doses Blue Rep1_-0.32 Red Rep2_-0.3
sp|Q5BKY9-2|F133B_HUMAN-233-S-S2_0.16
ptm abundance 37C
Doses Blue Rep1_0.12 Red Rep2_0.19
sp|Q7KZ85-3|SPT6H_HUMAN-78-S-S2_-0.01
ptm abundance 37C
Doses Blue Rep1_-0.16 Red Rep2_0.15
sp|Q9BVI0|PHF20_HUMAN-562-S-S3_-0.11
ptm abundance 37C
Doses Blue Rep1_-0.07 Red Rep2_-0.15
sp|Q9BVI0|PHF20_HUMAN-564-T-S3_-0.11
ptm abundance 37C
Doses Blue Rep1_-0.07 Red Rep2_-0.15
sp|Q9BVI0|PHF20_HUMAN-565-S-S3_-0.11
ptm abundance 37C
Doses Blue Rep1_-0.07 Red Rep2_-0.15
sp|Q9BVI0|PHF20_HUMAN-569-S-S3_-0.11
ptm abundance 37C
Doses Blue Rep1_-0.07 Red Rep2_-0.15
sp|Q9UIG0-2|BAZ1B_HUMAN-1464-S-S1_-0.1
ptm abundance 37C
Doses Blue Rep1_0.03 Red Rep2_-0.23
sp|Q14839|CHD4_HUMAN-108-S-S2_0
ptm abundance 37C
Doses Blue Rep1_0.04 Red Rep2_-0.05
sp|Q13107-2|UBP4_HUMAN-628-S-S3_0.17
ptm abundance 37C
Doses Blue Rep1_0.14 Red Rep2_0.19
sp|Q13107-2|UBP4_HUMAN-630-T-S3_0.17
ptm abundance 37C
Doses Blue Rep1_0.14 Red Rep2_0.19
sp|Q13107-2|UBP4_HUMAN-633-S-S3_0.17
ptm abundance 37C
Doses Blue Rep1_0.14 Red Rep2_0.19
sp|Q9H6H4|REEP4_HUMAN-202-S-S2_-0.12
ptm abundance 37C
Doses Blue Rep1_-0.12 Red Rep2_-0.12
sp|P19338|NUCL_HUMAN-28-S-S2_0.1
ptm abundance 37C
Doses Blue Rep1_0.06 Red Rep2_0.13
sp|Q8IYB3|SRRM1_HUMAN-802-S-S2_0.16
ptm abundance 37C
Doses Blue Rep1_0.06 Red Rep2_0.26

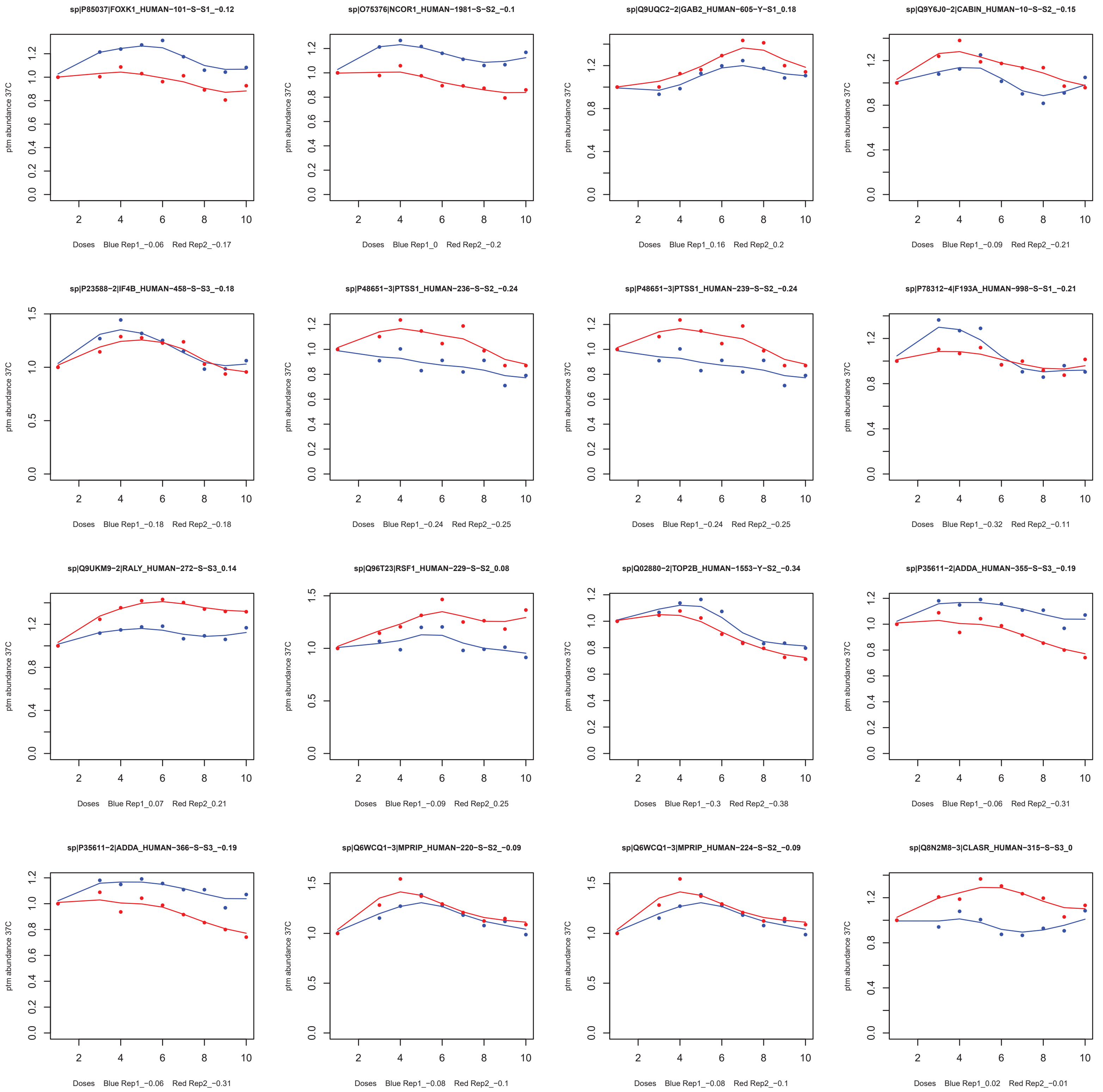
sp|P85037|FOXK1_HUMAN−101−S−S1_−0.12
ptm abundance 37C
Doses Blue Rep1_−0.06 Red Rep2_−0.17
sp|O75376|NCOR1_HUMAN−1981−S−S2_−0.1
ptm abundance 37C
Doses Blue Rep1_0 Red Rep2_−0.2
sp|Q9UQC2−2|GAB2_HUMAN−605−Y−S1_0.18
ptm abundance 37C
Doses Blue Rep1_0.16 Red Rep2_0.2
sp|Q9Y6J0−2|CABIN_HUMAN−10−S−S2_−0.15
ptm abundance 37C
Doses Blue Rep1_−0.09 Red Rep2_−0.21
sp|P23588−2|IF4B_HUMAN−458−S−S3_−0.18
ptm abundance 37C
Doses Blue Rep1_−0.18 Red Rep2_−0.18
sp|P48651−3|PTSS1_HUMAN−236−S−S2_−0.24
ptm abundance 37C
Doses Blue Rep1_−0.24 Red Rep2_−0.25
sp|P48651−3|PTSS1_HUMAN−239−S−S2_−0.24
ptm abundance 37C
Doses Blue Rep1_−0.24 Red Rep2_−0.25
sp|P78312−4|F193A_HUMAN−998−S−S1_−0.21
ptm abundance 37C
Doses Blue Rep1_−0.32 Red Rep2_−0.11
sp|Q9UKM9−2|RALY_HUMAN−272−S−S3_0.14
ptm abundance 37C
Doses Blue Rep1_0.07 Red Rep2_0.21
sp|Q96T23|RSF1_HUMAN−229−S−S2_0.08
ptm abundance 37C
Doses Blue Rep1_−0.09 Red Rep2_0.25
sp|Q02880−2|TOP2B_HUMAN−1553−Y−S2_−0.34
ptm abundance 37C
Doses Blue Rep1_−0.3 Red Rep2_−0.38
sp|P35611−2|ADDA_HUMAN−355−S−S3_−0.19
ptm abundance 37C
Doses Blue Rep1_−0.06 Red Rep2_−0.31
sp|P35611−2|ADDA_HUMAN−366−S−S3_−0.19
ptm abundance 37C
Doses Blue Rep1_−0.06 Red Rep2_−0.31
sp|Q6WCQ1−3|MPRIP_HUMAN−220−S−S2_−0.09
ptm abundance 37C
Doses Blue Rep1_−0.08 Red Rep2_−0.1
sp|Q6WCQ1−3|MPRIP_HUMAN−224−S−S2_−0.09
ptm abundance 37C
Doses Blue Rep1_−0.08 Red Rep2_−0.1
sp|Q8N2M8−3|CLASR_HUMAN−315−S−S3_0
ptm abundance 37C
Doses Blue Rep1_0.02 Red Rep2_−0.01

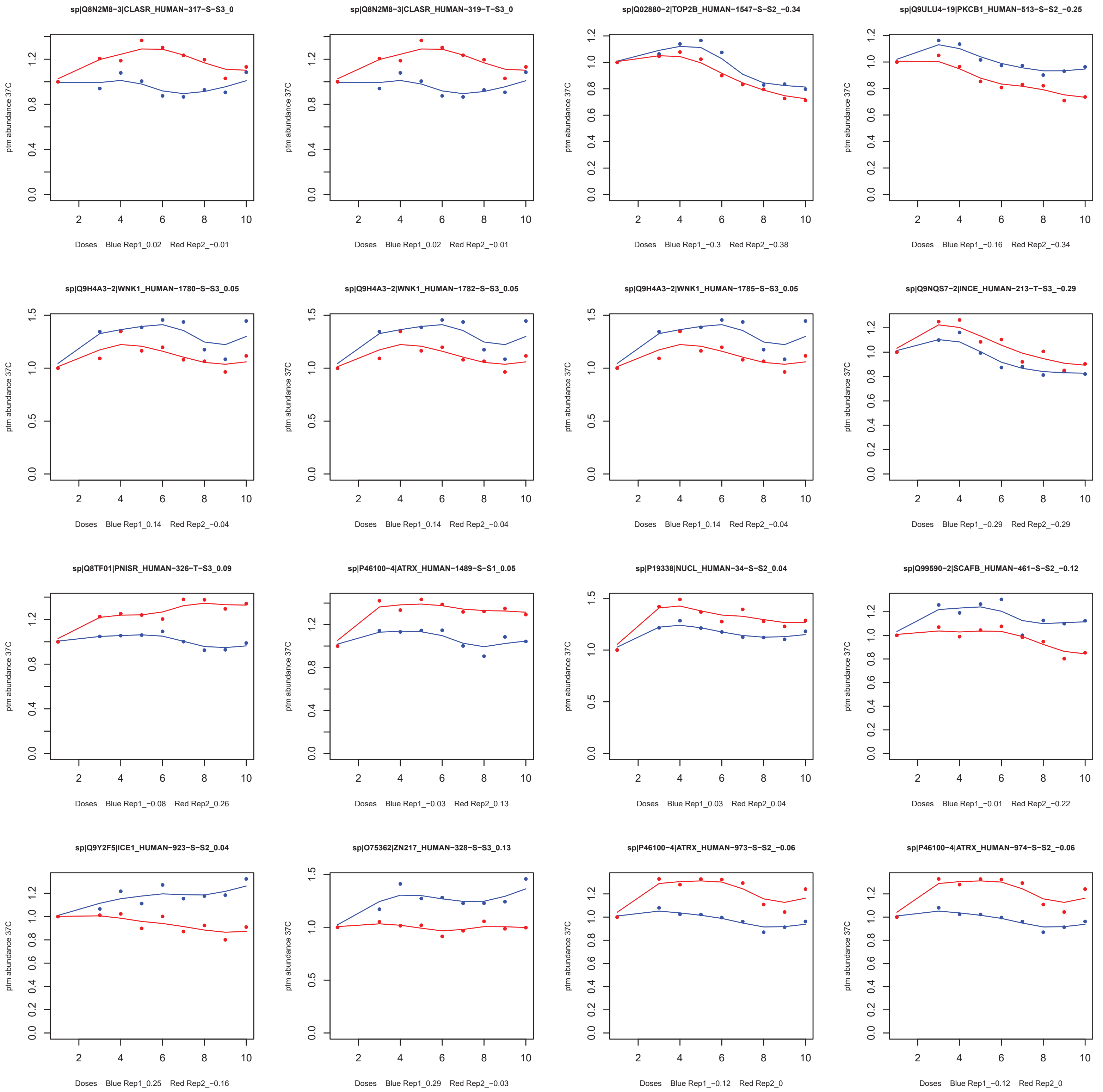

sp|Q8N2M8−3|CLASR_HUMAN−317−S−S3_0
Doses Blue Rep1_0.02 Red Rep2_−0.01
sp|Q8N2M8−3|CLASR_HUMAN−319−T−S3_0
Doses Blue Rep1_0.02 Red Rep2_−0.01
sp|Q02880−2|TOP2B_HUMAN−1547−S−S2_−0.34
Doses Blue Rep1_−0.3 Red Rep2_−0.38
sp|Q9ULU4−19|PKCB1_HUMAN−513−S−S2_−0.25
Doses Blue Rep1_−0.16 Red Rep2_−0.34
sp|Q9H4A3−2|WNK1_HUMAN−1780−S−S3_0.05
Doses Blue Rep1_0.14 Red Rep2_−0.04
sp|Q9H4A3−2|WNK1_HUMAN−1782−S−S3_0.05
Doses Blue Rep1_0.14 Red Rep2_−0.04
sp|Q9H4A3−2|WNK1_HUMAN−1785−S−S3_0.05
Doses Blue Rep1_0.14 Red Rep2_−0.04
sp|Q9NQS7−2|INCE_HUMAN−213−T−S3_−0.29
Doses Blue Rep1_−0.29 Red Rep2_−0.29
sp|Q8TF01|PNISR_HUMAN−326−T−S3_0.09
Doses Blue Rep1_−0.08 Red Rep2_0.26
sp|P46100−4|ATRX_HUMAN−1489−S−S1_0.05
Doses Blue Rep1_−0.03 Red Rep2_0.13
sp|P19338|NUCL_HUMAN−34−S−S2_0.04
Doses Blue Rep1_0.03 Red Rep2_0.04
sp|Q99590−2|SCAFB_HUMAN−461−S−S2_−0.12
Doses Blue Rep1_−0.01 Red Rep2_−0.22
sp|Q9Y2F5|ICE1_HUMAN−923−S−S2_0.04
Doses Blue Rep1_0.25 Red Rep2_−0.16
sp|O75362|ZN217_HUMAN−328−S−S3_0.13
Doses Blue Rep1_0.29 Red Rep2_−0.03
sp|P46100−4|ATRX_HUMAN−973−S−S2_−0.06
Doses Blue Rep1_−0.12 Red Rep2_0
sp|P46100−4|ATRX_HUMAN−974−S−S2_−0.06
Doses Blue Rep1_−0.12 Red Rep2_0
ptm abundance 37C

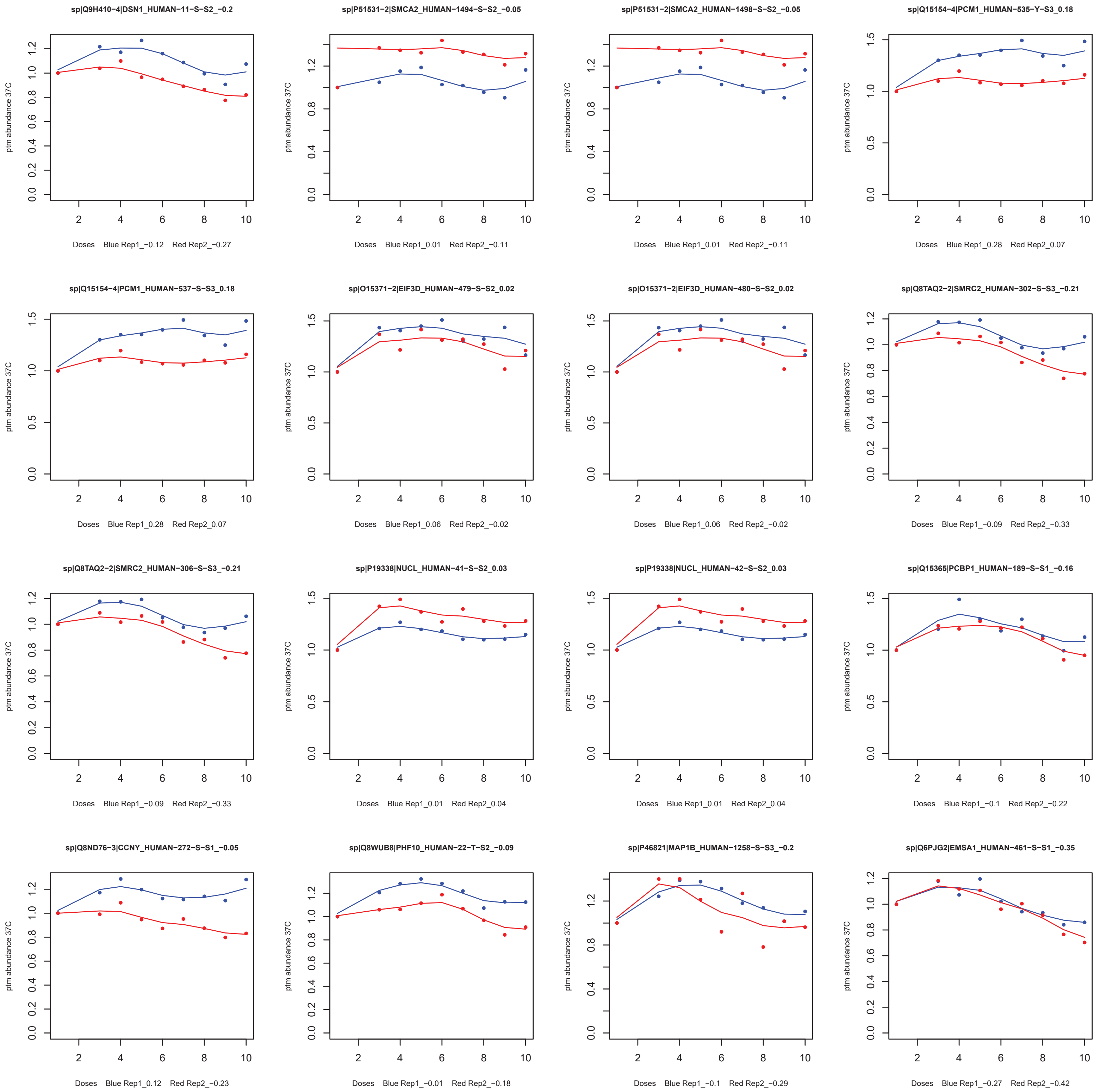

sp|Q9H410−4|DSN1_HUMAN−11−S−S2_−0.2
Doses Blue Rep1_−0.12 Red Rep2_−0.27
sp|P51531−2|SMCA2_HUMAN−1494−S−S2_−0.05
Doses Blue Rep1_0.01 Red Rep2_−0.11
sp|P51531−2|SMCA2_HUMAN−1498−S−S2_−0.05
Doses Blue Rep1_0.01 Red Rep2_−0.11
sp|Q15154−4|PCM1_HUMAN−535−Y−S3_0.18
Doses Blue Rep1_0.28 Red Rep2_0.07
sp|Q15154−4|PCM1_HUMAN−537−S−S3_0.18
Doses Blue Rep1_0.28 Red Rep2_0.07
sp|O15371−2|EIF3D_HUMAN−479−S−S2_0.02
Doses Blue Rep1_0.06 Red Rep2_−0.02
sp|O15371−2|EIF3D_HUMAN−480−S−S2_0.02
Doses Blue Rep1_0.06 Red Rep2_−0.02
sp|Q8TAQ2−2|SMRC2_HUMAN−302−S−S3_−0.21
Doses Blue Rep1_−0.09 Red Rep2_−0.33
sp|Q8TAQ2−2|SMRC2_HUMAN−306−S−S3_−0.21
Doses Blue Rep1_−0.09 Red Rep2_−0.33
sp|P19338|NUCL_HUMAN−41−S−S2_0.03
Doses Blue Rep1_0.01 Red Rep2_0.04
sp|P19338|NUCL_HUMAN−42−S−S2_0.03
Doses Blue Rep1_0.01 Red Rep2_0.04
sp|Q15365|PCBP1_HUMAN−189−S−S1_−0.16
Doses Blue Rep1_−0.1 Red Rep2_−0.22
sp|Q8ND76−3|CCNY_HUMAN−272−S−S1_−0.05
Doses Blue Rep1_0.12 Red Rep2_−0.23
sp|Q8WUB8|PHF10_HUMAN−22−T−S2_−0.09
Doses Blue Rep1_−0.01 Red Rep2_−0.18
sp|P46821|MAP1B_HUMAN−1258−S−S3_−0.2
Doses Blue Rep1_−0.1 Red Rep2_−0.29
sp|Q6PJG2|EMSA1_HUMAN−461−S−S1_−0.35
Doses Blue Rep1_−0.27 Red Rep2_−0.42
ptm abundance 37C

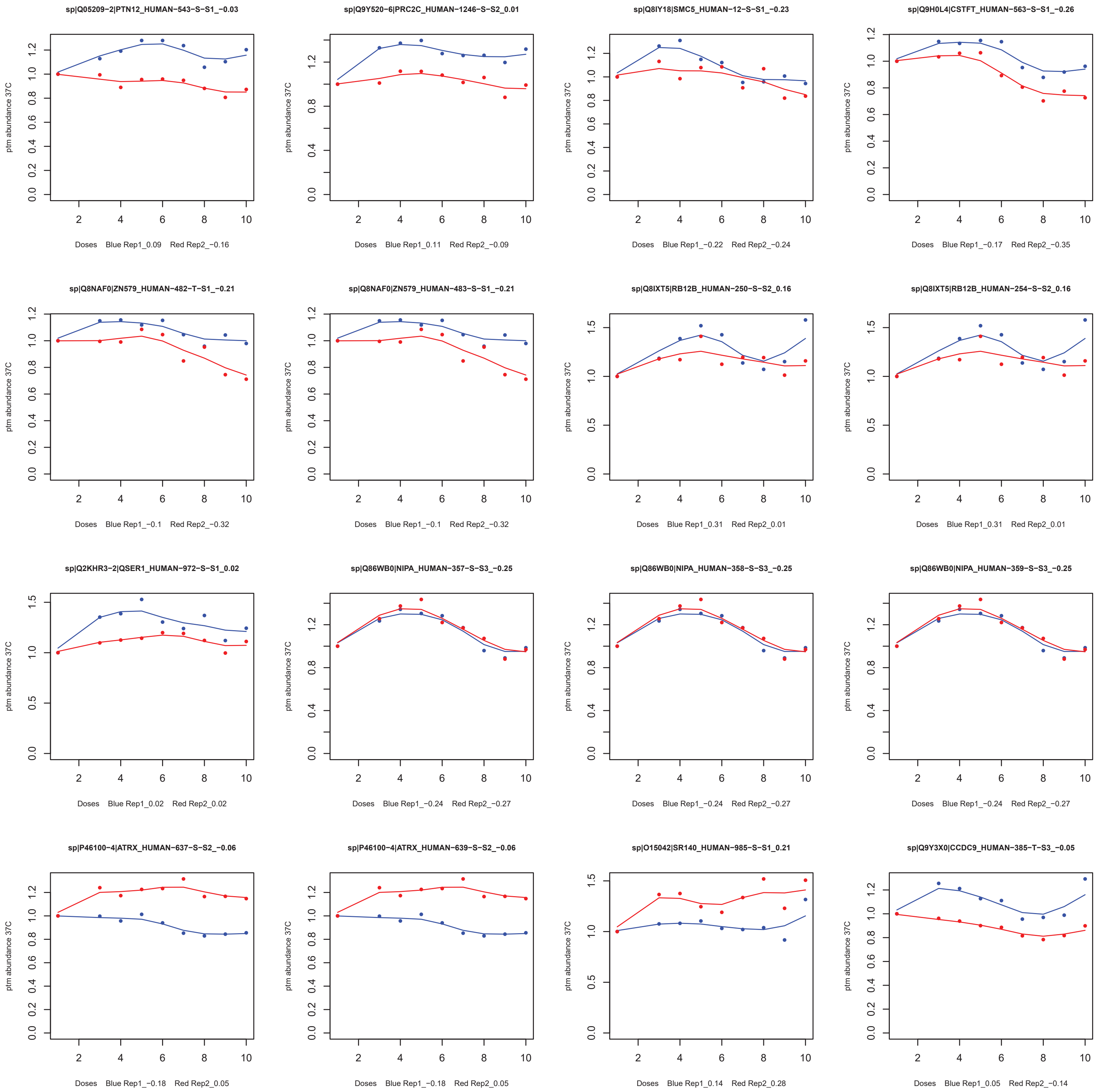
sp|Q05209−2|PTN12_HUMAN−543−S−S1_−0.03
Doses Blue Rep1_0.09 Red Rep2_−0.16
sp|Q9Y520−6|PRC2C_HUMAN−1246−S−S2_0.01
Doses Blue Rep1_0.11 Red Rep2_−0.09
sp|Q8IY18|SMC5_HUMAN−12−S−S1_−0.23
Doses Blue Rep1_−0.22 Red Rep2_−0.24
sp|Q9H0L4|CSTFT_HUMAN−563−S−S1_−0.26
Doses Blue Rep1_−0.17 Red Rep2_−0.35
sp|Q8NAF0|ZN579_HUMAN−482−T−S1_−0.21
Doses Blue Rep1_−0.1 Red Rep2_−0.32
sp|Q8NAF0|ZN579_HUMAN−483−S−S1_−0.21
Doses Blue Rep1_−0.1 Red Rep2_−0.32
sp|Q8IXT5|RB12B_HUMAN−250−S−S2_0.16
Doses Blue Rep1_0.31 Red Rep2_0.01
sp|Q8IXT5|RB12B_HUMAN−254−S−S2_0.16
Doses Blue Rep1_0.31 Red Rep2_0.01
sp|Q2KHR3−2|QSER1_HUMAN−972−S−S1_0.02
Doses Blue Rep1_0.02 Red Rep2_0.02
sp|Q86WB0|NIPA_HUMAN−357−S−S3_−0.25
Doses Blue Rep1_−0.24 Red Rep2_−0.27
sp|Q86WB0|NIPA_HUMAN−358−S−S3_−0.25
Doses Blue Rep1_−0.24 Red Rep2_−0.27
sp|Q86WB0|NIPA_HUMAN−359−S−S3_−0.25
Doses Blue Rep1_−0.24 Red Rep2_−0.27
sp|P46100−4|ATRX_HUMAN−637−S−S2_−0.06
Doses Blue Rep1_−0.18 Red Rep2_0.05
sp|P46100−4|ATRX_HUMAN−639−S−S2_−0.06
Doses Blue Rep1_−0.18 Red Rep2_0.05
sp|O15042|SR140_HUMAN−985−S−S1_0.21
Doses Blue Rep1_0.14 Red Rep2_0.28
sp|Q9Y3X0|CCDC9_HUMAN−385−T−S3_−0.05
Doses Blue Rep1_0.05 Red Rep2_−0.14
ptm abundance 37C

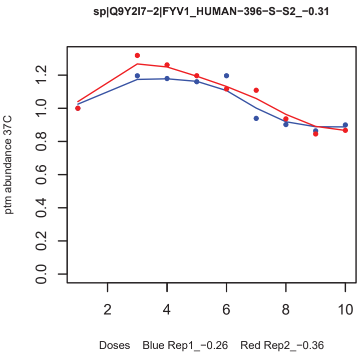
sp|Q9Y2I7−2|FYV1_HUMAN−396−S−S2_−0.31
ptm abundance 37C
Doses Blue Rep1_−0.26 Red Rep2_−0.36

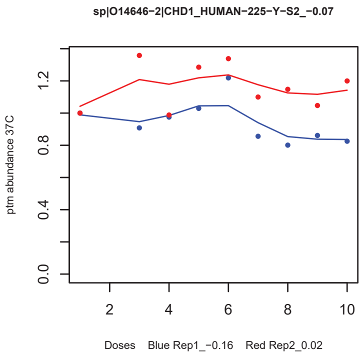
sp|O14646−2|CHD1_HUMAN−225−Y−S2_−0.07
ptm abundance 37C
Doses Blue Rep1_−0.16 Red Rep2_0.02

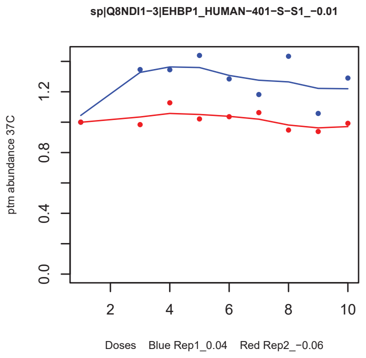
sp|Q8NDI1−3|EHBP1_HUMAN−401−S−S1_−0.01
ptm abundance 37C
Doses Blue Rep1_0.04 Red Rep2_−0.06

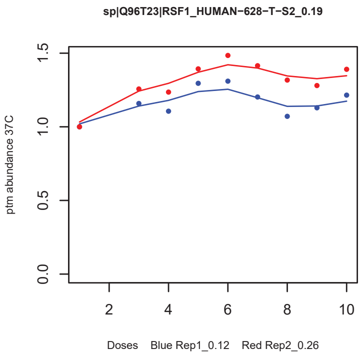
sp|Q96T23|RSF1_HUMAN−628−T−S2_0.19
ptm abundance 37C
Doses Blue Rep1_0.12 Red Rep2_0.26

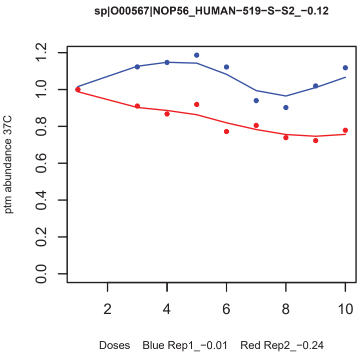
sp|O00567|NOP56_HUMAN−519−S−S2_−0.12
ptm abundance 37C
Doses Blue Rep1_−0.01 Red Rep2_−0.24

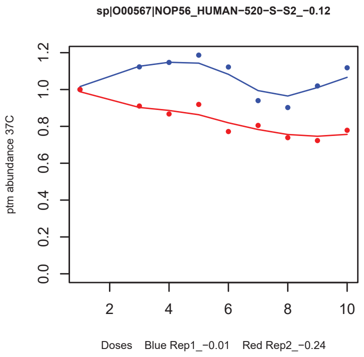
sp|O00567|NOP56_HUMAN−520−S−S2_−0.12
ptm abundance 37C
Doses Blue Rep1_−0.01 Red Rep2_−0.24

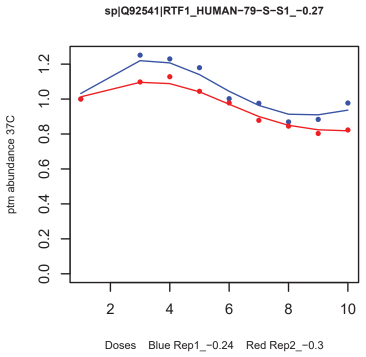
sp|Q92541|RTF1_HUMAN−79−S−S1_−0.27
ptm abundance 37C
Doses Blue Rep1_−0.24 Red Rep2_−0.3

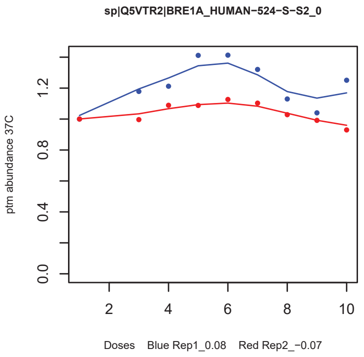
sp|Q5VTR2|BRE1A_HUMAN−524−S−S2_0
ptm abundance 37C
Doses Blue Rep1_0.08 Red Rep2_−0.07

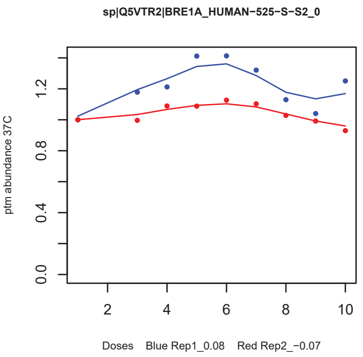
sp|Q5VTR2|BRE1A_HUMAN−525−S−S2_0
ptm abundance 37C
Doses Blue Rep1_0.08 Red Rep2_−0.07

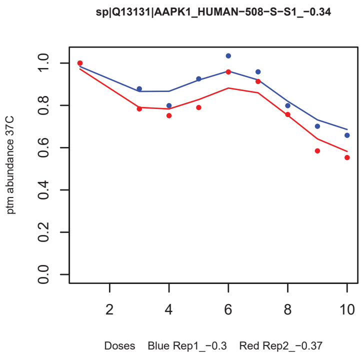
sp|Q13131|AAPK1_HUMAN−508−S−S1_−0.34
ptm abundance 37C
Doses Blue Rep1_−0.3 Red Rep2_−0.37

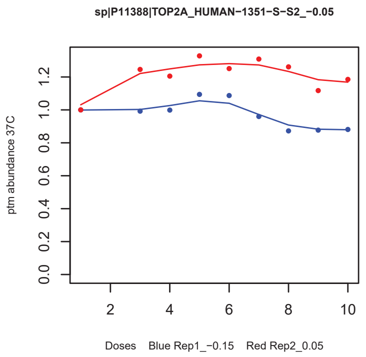
sp|P11388|TOP2A_HUMAN−1351−S−S2_−0.05
ptm abundance 37C
Doses Blue Rep1_−0.15 Red Rep2_0.05

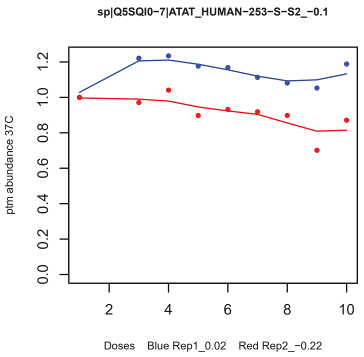
sp|Q5SQI0−7|ATAT_HUMAN−253−S−S2_−0.1
ptm abundance 37C
Doses Blue Rep1_0.02 Red Rep2_−0.22

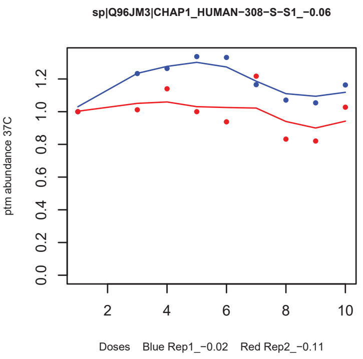
sp|Q96JM3|CHAP1_HUMAN−308−S−S1_−0.06
ptm abundance 37C
Doses Blue Rep1_−0.02 Red Rep2_−0.11

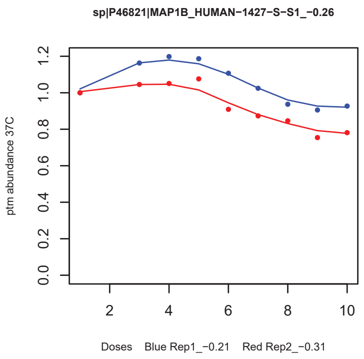
sp|P46821|MAP1B_HUMAN−1427−S−S1_−0.26
ptm abundance 37C
Doses Blue Rep1_−0.21 Red Rep2_−0.31

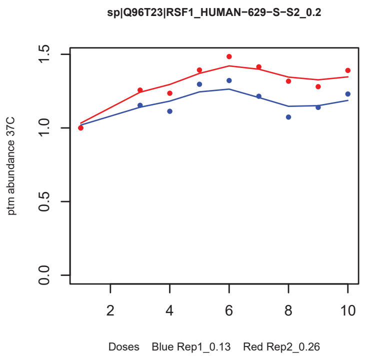
sp|Q96T23|RSF1_HUMAN−629−S−S2_0.2
ptm abundance 37C
Doses Blue Rep1_0.13 Red Rep2_0.26

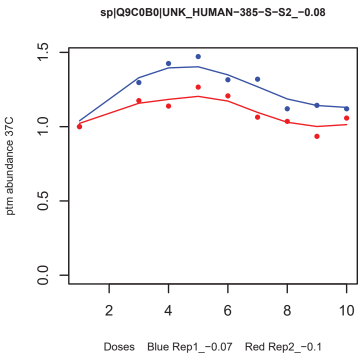
sp|Q9C0B0|UNK_HUMAN−385−S−S2_−0.08
ptm abundance 37C
Doses Blue Rep1_−0.07 Red Rep2_−0.1

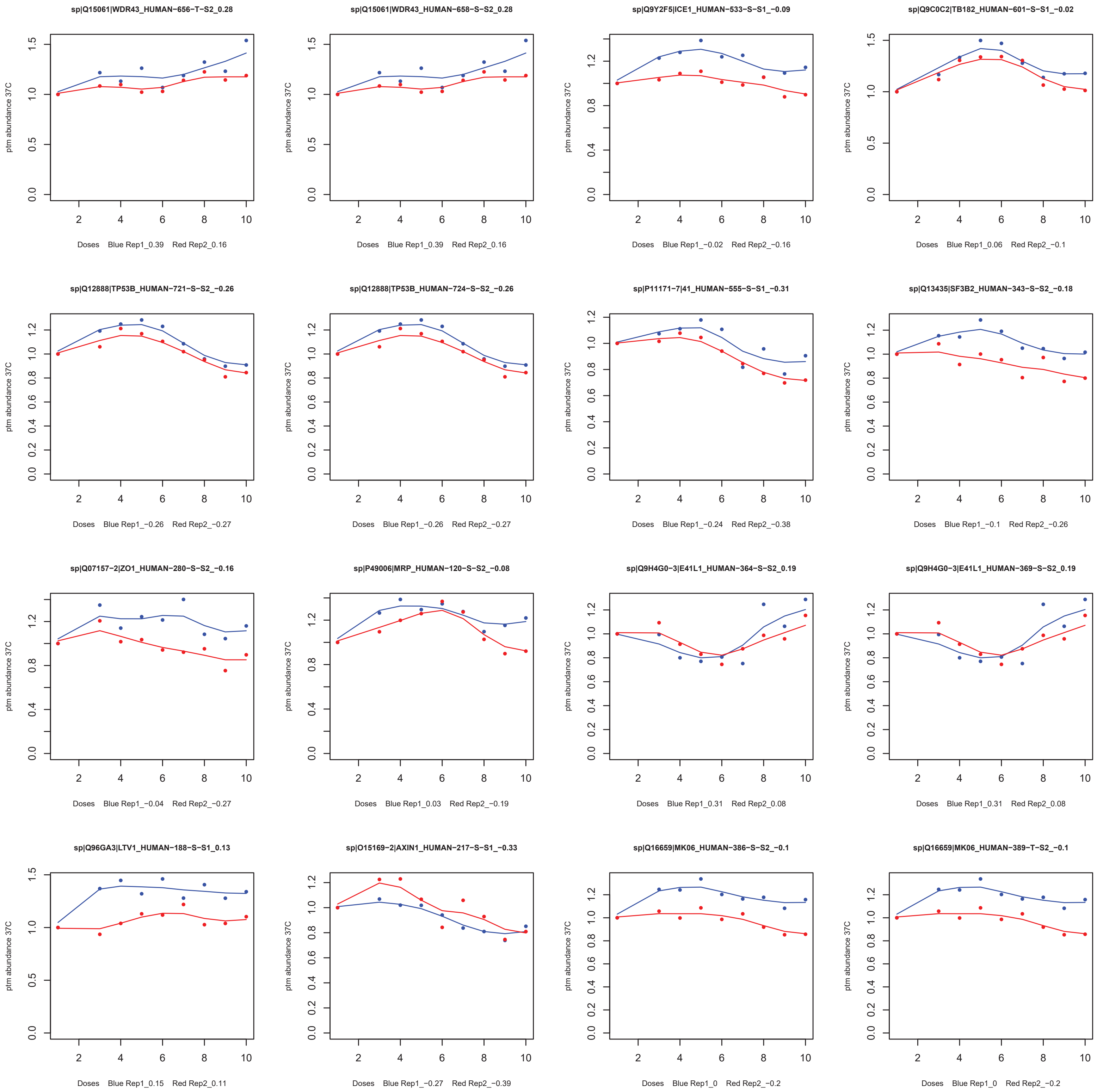
sp|Q15061|WDR43_HUMAN−656−T−S2_0.28
ptm abundance 37C
Doses Blue Rep1_0.39 Red Rep2_0.16
sp|Q15061|WDR43_HUMAN−658−S−S2_0.28
ptm abundance 37C
Doses Blue Rep1_0.39 Red Rep2_0.16
sp|Q9Y2F5|ICE1_HUMAN−533−S−S1_−0.09
ptm abundance 37C
Doses Blue Rep1_−0.02 Red Rep2_−0.16
sp|Q9C0C2|TB182_HUMAN−601−S−S1_−0.02
ptm abundance 37C
Doses Blue Rep1_0.06 Red Rep2_−0.1
sp|Q12888|TP53B_HUMAN−721−S−S2_−0.26
ptm abundance 37C
Doses Blue Rep1_−0.26 Red Rep2_−0.27
sp|Q12888|TP53B_HUMAN−724−S−S2_−0.26
ptm abundance 37C
Doses Blue Rep1_−0.26 Red Rep2_−0.27
sp|P11171−7|41_HUMAN−555−S−S1_−0.31
ptm abundance 37C
Doses Blue Rep1_−0.24 Red Rep2_−0.38
sp|Q13435|SF3B2_HUMAN−343−S−S2_−0.18
ptm abundance 37C
Doses Blue Rep1_−0.1 Red Rep2_−0.26
sp|Q07157−2|ZO1_HUMAN−280−S−S2_−0.16
ptm abundance 37C
Doses Blue Rep1_−0.04 Red Rep2_−0.27
sp|P49006|MRP_HUMAN−120−S−S2_−0.08
ptm abundance 37C
Doses Blue Rep1_0.03 Red Rep2_−0.19
sp|Q9H4G0−3|E41L1_HUMAN−364−S−S2_0.19
ptm abundance 37C
Doses Blue Rep1_0.31 Red Rep2_0.08
sp|Q9H4G0−3|E41L1_HUMAN−369−S−S2_0.19
ptm abundance 37C
Doses Blue Rep1_0.31 Red Rep2_0.08
sp|Q96GA3|LTV1_HUMAN−188−S−S1_0.13
ptm abundance 37C
Doses Blue Rep1_0.15 Red Rep2_0.11
sp|O15169−2|AXIN1_HUMAN−217−S−S1_−0.33
ptm abundance 37C
Doses Blue Rep1_−0.27 Red Rep2_−0.39
sp|Q16659|MK06_HUMAN−386−S−S2_−0.1
ptm abundance 37C
Doses Blue Rep1_0 Red Rep2_−0.2
sp|Q16659|MK06_HUMAN−389−T−S2_−0.1
ptm abundance 37C
Doses Blue Rep1_0 Red Rep2_−0.2

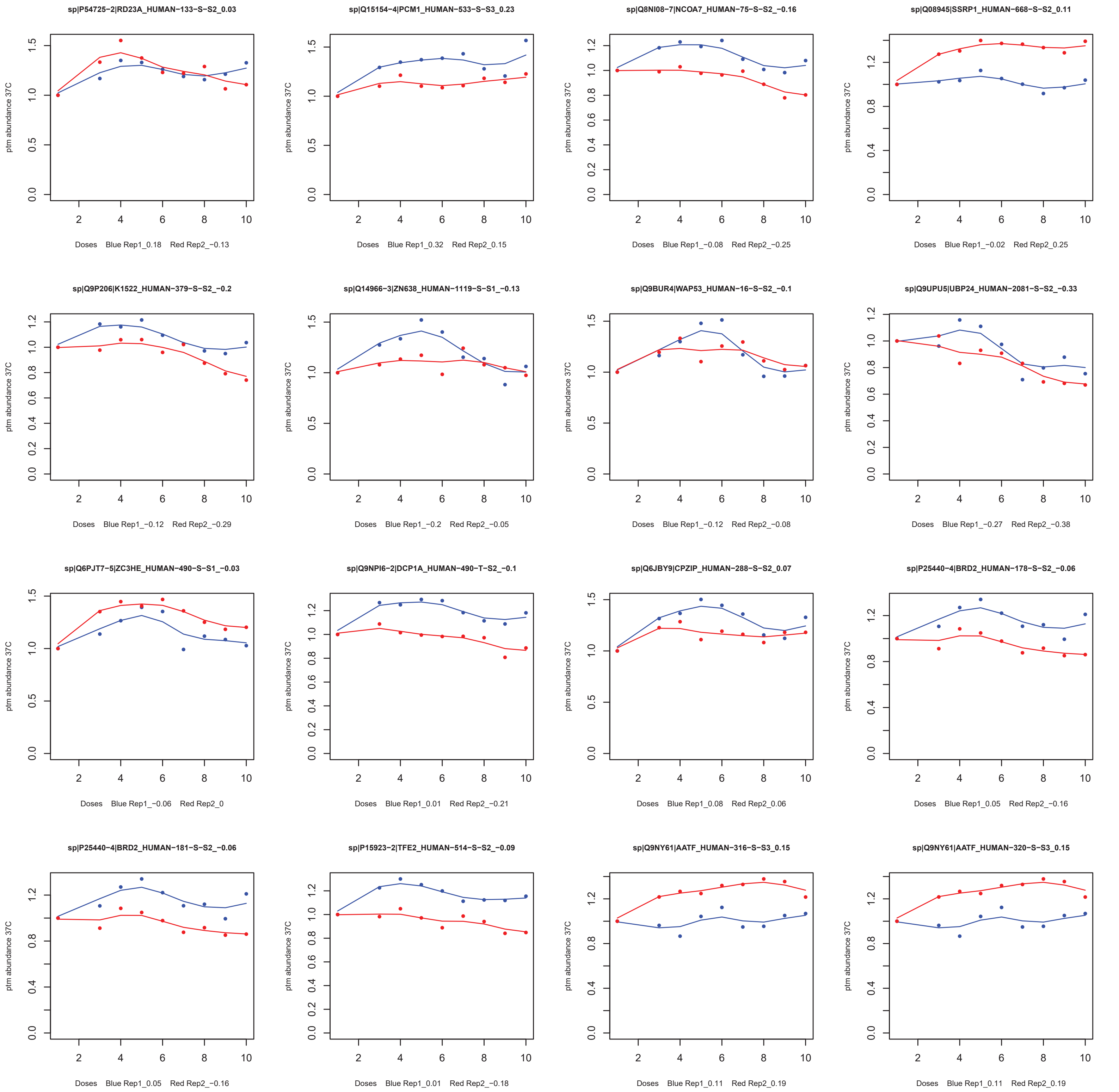
sp|P54725−2|RD23A_HUMAN−133−S−S2_0.03
Doses Blue Rep1_0.18 Red Rep2_−0.13
sp|Q15154−4|PCM1_HUMAN−533−S−S3_0.23
Doses Blue Rep1_0.32 Red Rep2_0.15
sp|Q8NI08−7|NCOA7_HUMAN−75−S−S2_−0.16
Doses Blue Rep1_−0.08 Red Rep2_−0.25
sp|Q08945|SSRP1_HUMAN−668−S−S2_0.11
Doses Blue Rep1_−0.02 Red Rep2_0.25
sp|Q9P206|K1522_HUMAN−379−S−S2_−0.2
Doses Blue Rep1_−0.12 Red Rep2_−0.29
sp|Q14966−3|ZN638_HUMAN−1119−S−S1_−0.13
Doses Blue Rep1_−0.2 Red Rep2_−0.05
sp|Q9BUR4|WAP53_HUMAN−16−S−S2_−0.1
Doses Blue Rep1_−0.12 Red Rep2_−0.08
sp|Q9UPU5|UBP24_HUMAN−2081−S−S2_−0.33
Doses Blue Rep1_−0.27 Red Rep2_−0.38
sp|Q6PJT7−5|ZC3HE_HUMAN−490−S−S1_−0.03
Doses Blue Rep1_−0.06 Red Rep2_0
sp|Q9NPI6−2|DCP1A_HUMAN−490−T−S2_−0.1
Doses Blue Rep1_0.01 Red Rep2_−0.21
sp|Q6JBY9|CPZIP_HUMAN−288−S−S2_0.07
Doses Blue Rep1_0.08 Red Rep2_0.06
sp|P25440−4|BRD2_HUMAN−178−S−S2_−0.06
Doses Blue Rep1_0.05 Red Rep2_−0.16
sp|P25440−4|BRD2_HUMAN−181−S−S2_−0.06
Doses Blue Rep1_0.05 Red Rep2_−0.16
sp|P15923−2|TFE2_HUMAN−514−S−S2_−0.09
Doses Blue Rep1_0.01 Red Rep2_−0.18
sp|Q9NY61|AATF_HUMAN−316−S−S3_0.15
Doses Blue Rep1_0.11 Red Rep2_0.19
sp|Q9NY61|AATF_HUMAN−320−S−S3_0.15
Doses Blue Rep1_0.11 Red Rep2_0.19
ptm abundance 37C

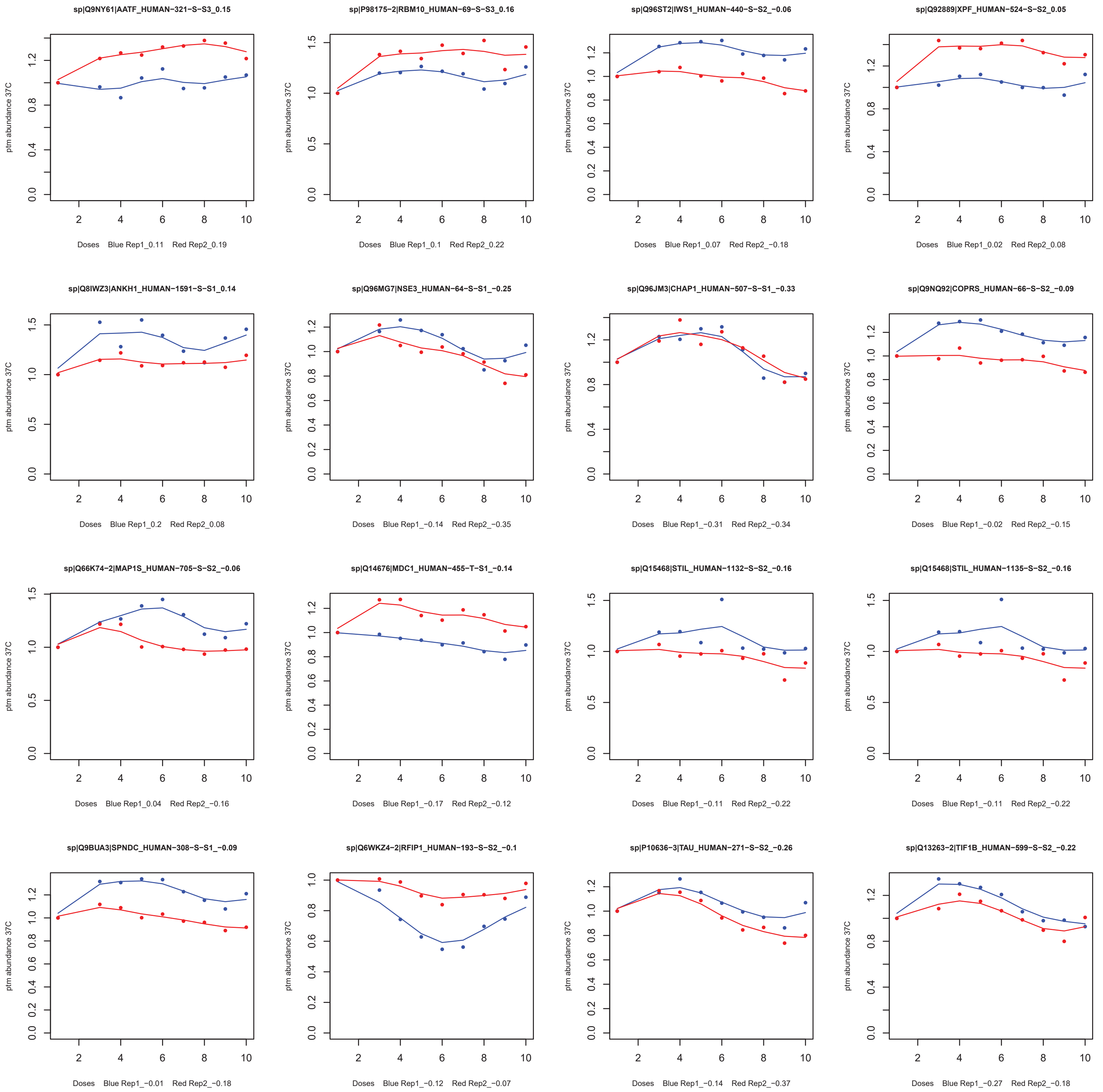

sp|Q9NY61|AATF_HUMAN−321−S−S3_0.15
Doses Blue Rep1_0.11 Red Rep2_0.19
sp|P98175−2|RBM10_HUMAN−69−S−S3_0.16
Doses Blue Rep1_0.1 Red Rep2_0.22
sp|Q96ST2|IWS1_HUMAN−440−S−S2_−0.06
Doses Blue Rep1_0.07 Red Rep2_−0.18
sp|Q92889|XPF_HUMAN−524−S−S2_0.05
Doses Blue Rep1_0.02 Red Rep2_0.08
sp|Q8IWZ3|ANKH1_HUMAN−1591−S−S1_0.14
Doses Blue Rep1_0.2 Red Rep2_0.08
sp|Q96MG7|NSE3_HUMAN−64−S−S1_−0.25
Doses Blue Rep1_−0.14 Red Rep2_−0.35
sp|Q96JM3|CHAP1_HUMAN−507−S−S1_−0.33
Doses Blue Rep1_−0.31 Red Rep2_−0.34
sp|Q9NQ92|COPRS_HUMAN−66−S−S2_−0.09
Doses Blue Rep1_−0.02 Red Rep2_−0.15
sp|Q66K74−2|MAP1S_HUMAN−705−S−S2_−0.06
Doses Blue Rep1_0.04 Red Rep2_−0.16
sp|Q14676|MDC1_HUMAN−455−T−S1_−0.14
Doses Blue Rep1_−0.17 Red Rep2_−0.12
sp|Q15468|STIL_HUMAN−1132−S−S2_−0.16
Doses Blue Rep1_−0.11 Red Rep2_−0.22
sp|Q15468|STIL_HUMAN−1135−S−S2_−0.16
Doses Blue Rep1_−0.11 Red Rep2_−0.22
sp|Q9BUA3|SPNDC_HUMAN−308−S−S1_−0.09
Doses Blue Rep1_−0.01 Red Rep2_−0.18
sp|Q6WKZ4−2|RFIP1_HUMAN−193−S−S2_−0.1
Doses Blue Rep1_−0.12 Red Rep2_−0.07
sp|P10636−3|TAU_HUMAN−271−S−S2_−0.26
Doses Blue Rep1_−0.14 Red Rep2_−0.37
sp|Q13263−2|TIF1B_HUMAN−599−S−S2_−0.22
Doses Blue Rep1_−0.27 Red Rep2_−0.18
ptm abundance 37C

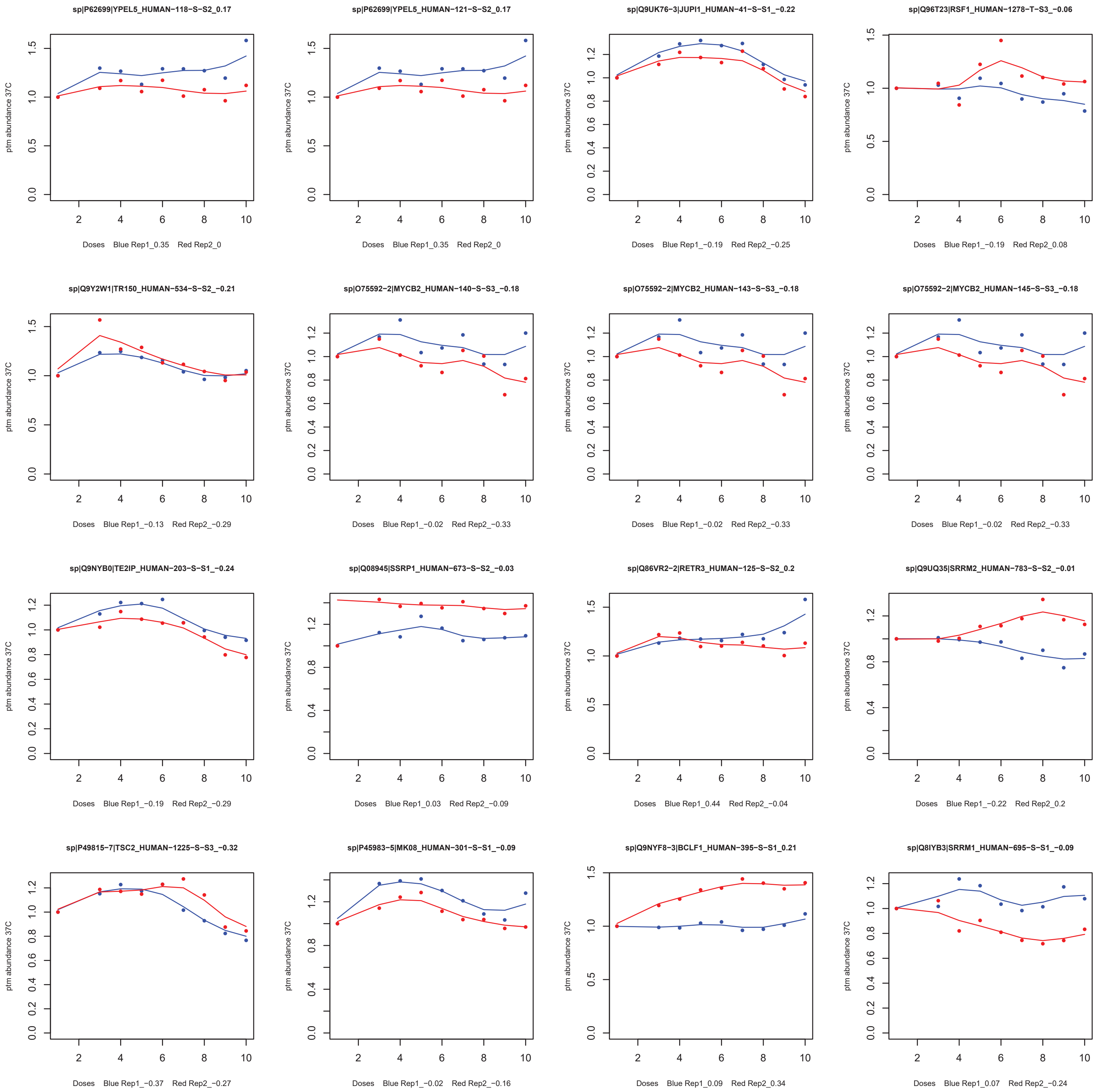

sp|P62699|YPEL5_HUMAN−118−S−S2_0.17
ptm abundance 37C
Doses Blue Rep1_0.35 Red Rep2_0
sp|P62699|YPEL5_HUMAN−121−S−S2_0.17
ptm abundance 37C
Doses Blue Rep1_0.35 Red Rep2_0
sp|Q9UK76−3|JUPI1_HUMAN−41−S−S1_−0.22
ptm abundance 37C
Doses Blue Rep1_−0.19 Red Rep2_−0.25
sp|Q96T23|RSF1_HUMAN−1278−T−S3_−0.06
ptm abundance 37C
Doses Blue Rep1_−0.19 Red Rep2_0.08
sp|Q9Y2W1|TR150_HUMAN−534−S−S2_−0.21
ptm abundance 37C
Doses Blue Rep1_−0.13 Red Rep2_−0.29
sp|O75592−2|MYCB2_HUMAN−140−S−S3_−0.18
ptm abundance 37C
Doses Blue Rep1_−0.02 Red Rep2_−0.33
sp|O75592−2|MYCB2_HUMAN−143−S−S3_−0.18
ptm abundance 37C
Doses Blue Rep1_−0.02 Red Rep2_−0.33
sp|O75592−2|MYCB2_HUMAN−145−S−S3_−0.18
ptm abundance 37C
Doses Blue Rep1_−0.02 Red Rep2_−0.33
sp|Q9NYB0|TE2IP_HUMAN−203−S−S1_−0.24
ptm abundance 37C
Doses Blue Rep1_−0.19 Red Rep2_−0.29
sp|Q08945|SSRP1_HUMAN−673−S−S2_−0.03
ptm abundance 37C
Doses Blue Rep1_0.03 Red Rep2_−0.09
sp|Q86VR2−2|RETR3_HUMAN−125−S−S2_0.2
ptm abundance 37C
Doses Blue Rep1_0.44 Red Rep2_−0.04
sp|Q9UQ35|SRRM2_HUMAN−783−S−S2_−0.01
ptm abundance 37C
Doses Blue Rep1_−0.22 Red Rep2_0.2
sp|P49815−7|TSC2_HUMAN−1225−S−S3_−0.32
ptm abundance 37C
Doses Blue Rep1_−0.37 Red Rep2_−0.27
sp|P45983−5|MK08_HUMAN−301−S−S1_−0.09
ptm abundance 37C
Doses Blue Rep1_−0.02 Red Rep2_−0.16
sp|Q9NYF8−3|BCLF1_HUMAN−395−S−S1_0.21
ptm abundance 37C
Doses Blue Rep1_0.09 Red Rep2_0.34
sp|Q8IYB3|SRRM1_HUMAN−695−S−S1_−0.09
ptm abundance 37C
Doses Blue Rep1_0.07 Red Rep2_−0.24

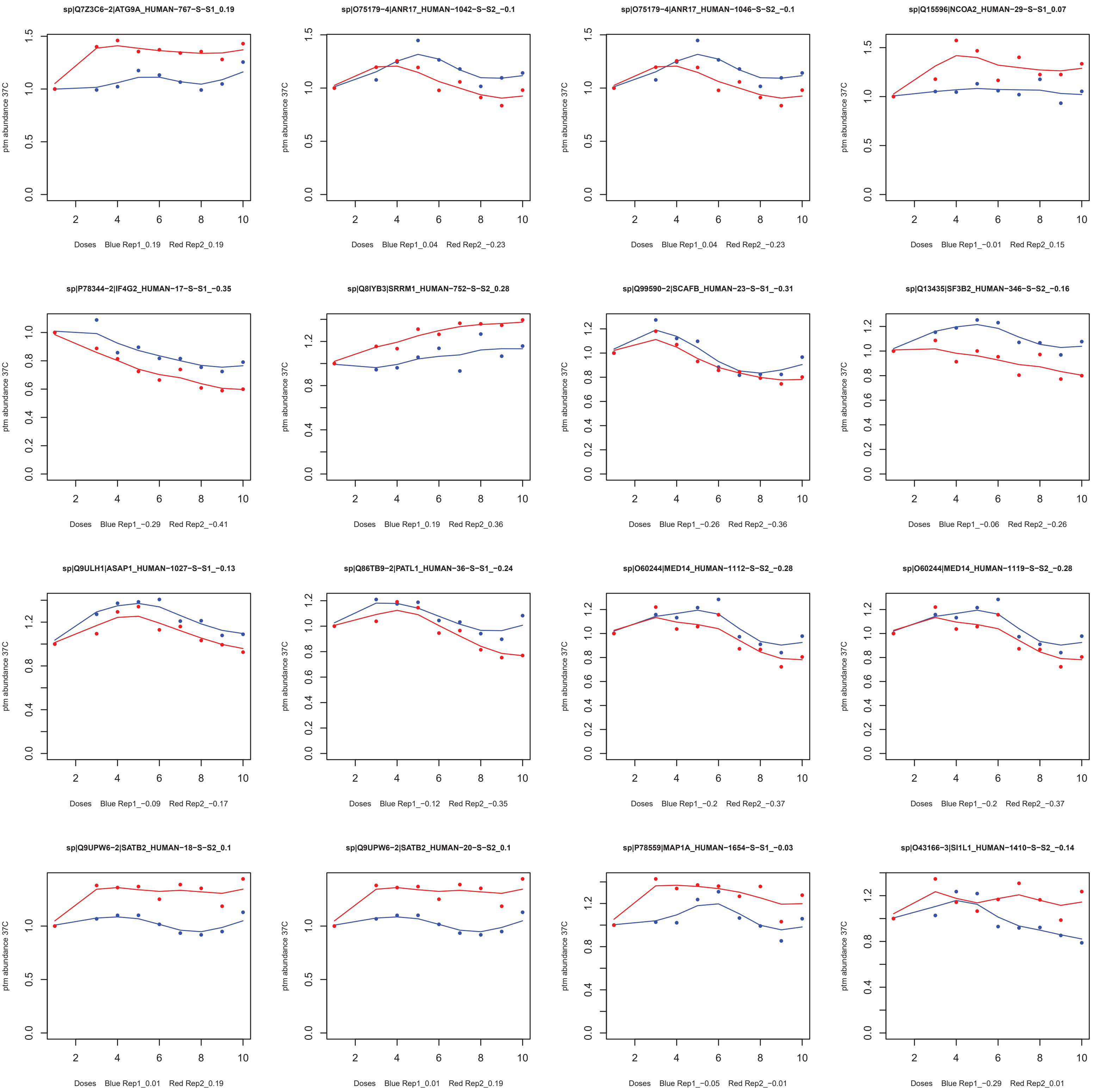
sp|Q7Z3C6−2|ATG9A_HUMAN−767−S−S1_0.19
Doses Blue Rep1_0.19 Red Rep2_0.19
sp|O75179−4|ANR17_HUMAN−1042−S−S2_−0.1
Doses Blue Rep1_0.04 Red Rep2_−0.23
sp|O75179−4|ANR17_HUMAN−1046−S−S2_−0.1
Doses Blue Rep1_0.04 Red Rep2_−0.23
sp|Q15596|NCOA2_HUMAN−29−S−S1_0.07
Doses Blue Rep1_−0.01 Red Rep2_0.15
sp|P78344−2|IF4G2_HUMAN−17−S−S1_−0.35
Doses Blue Rep1_−0.29 Red Rep2_−0.41
sp|Q8IYB3|SRRM1_HUMAN−752−S−S2_0.28
Doses Blue Rep1_0.19 Red Rep2_0.36
sp|Q99590−2|SCAFB_HUMAN−23−S−S1_−0.31
Doses Blue Rep1_−0.26 Red Rep2_−0.36
sp|Q13435|SF3B2_HUMAN−346−S−S2_−0.16
Doses Blue Rep1_−0.06 Red Rep2_−0.26
sp|Q9ULH1|ASAP1_HUMAN−1027−S−S1_−0.13
Doses Blue Rep1_−0.09 Red Rep2_−0.17
sp|Q86TB9−2|PATL1_HUMAN−36−S−S1_−0.24
Doses Blue Rep1_−0.12 Red Rep2_−0.35
sp|O60244|MED14_HUMAN−1112−S−S2_−0.28
Doses Blue Rep1_−0.2 Red Rep2_−0.37
sp|O60244|MED14_HUMAN−1119−S−S2_−0.28
Doses Blue Rep1_−0.2 Red Rep2_−0.37
sp|Q9UPW6−2|SATB2_HUMAN−18−S−S2_0.1
Doses Blue Rep1_0.01 Red Rep2_0.19
sp|Q9UPW6−2|SATB2_HUMAN−20−S−S2_0.1
Doses Blue Rep1_0.01 Red Rep2_0.19
sp|P78559|MAP1A_HUMAN−1654−S−S1_−0.03
Doses Blue Rep1_−0.05 Red Rep2_−0.01
sp|O43166−3|SI1L1_HUMAN−1410−S−S2_−0.14
Doses Blue Rep1_−0.29 Red Rep2_0.01
ptm abundance 37C

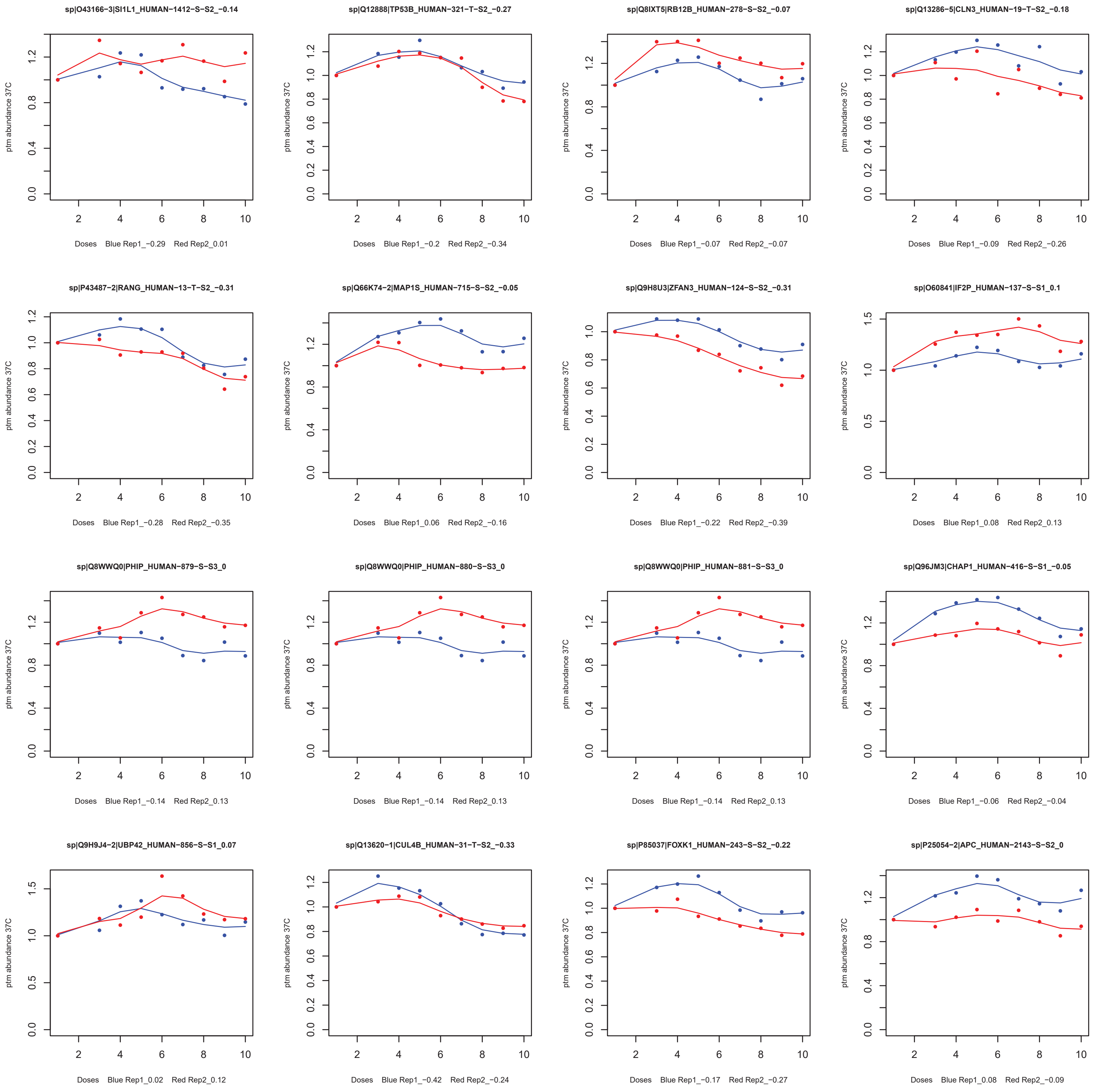

sp|O43166−3|SI1L1_HUMAN−1412−S−S2_−0.14
ptm abundance 37C
Doses Blue Rep1_−0.29 Red Rep2_0.01
sp|Q12888|TP53B_HUMAN−321−T−S2_−0.27
ptm abundance 37C
Doses Blue Rep1_−0.2 Red Rep2_−0.34
sp|Q8IXT5|RB12B_HUMAN−278−S−S2_−0.07
ptm abundance 37C
Doses Blue Rep1_−0.07 Red Rep2_−0.07
sp|Q13286−5|CLN3_HUMAN−19−T−S2_−0.18
ptm abundance 37C
Doses Blue Rep1_−0.09 Red Rep2_−0.26
sp|P43487−2|RANG_HUMAN−13−T−S2_−0.31
ptm abundance 37C
Doses Blue Rep1_−0.28 Red Rep2_−0.35
sp|Q66K74−2|MAP1S_HUMAN−715−S−S2_−0.05
ptm abundance 37C
Doses Blue Rep1_0.06 Red Rep2_−0.16
sp|Q9H8U3|ZFAN3_HUMAN−124−S−S2_−0.31
ptm abundance 37C
Doses Blue Rep1_−0.22 Red Rep2_−0.39
sp|O60841|IF2P_HUMAN−137−S−S1_0.1
ptm abundance 37C
Doses Blue Rep1_0.08 Red Rep2_0.13
sp|Q8WWQ0|PHIP_HUMAN−879−S−S3_0
ptm abundance 37C
Doses Blue Rep1_−0.14 Red Rep2_0.13
sp|Q8WWQ0|PHIP_HUMAN−880−S−S3_0
ptm abundance 37C
Doses Blue Rep1_−0.14 Red Rep2_0.13
sp|Q8WWQ0|PHIP_HUMAN−881−S−S3_0
ptm abundance 37C
Doses Blue Rep1_−0.14 Red Rep2_0.13
sp|Q96JM3|CHAP1_HUMAN−416−S−S1_−0.05
ptm abundance 37C
Doses Blue Rep1_−0.06 Red Rep2_−0.04
sp|Q9H9J4−2|UBP42_HUMAN−856−S−S1_0.07
ptm abundance 37C
Doses Blue Rep1_0.02 Red Rep2_0.12
sp|Q13620−1|CUL4B_HUMAN−31−T−S2_−0.33
ptm abundance 37C
Doses Blue Rep1_−0.42 Red Rep2_−0.24
sp|P85037|FOXK1_HUMAN−243−S−S2_−0.22
ptm abundance 37C
Doses Blue Rep1_−0.17 Red Rep2_−0.27
sp|P25054−2|APC_HUMAN−2143−S−S2_0
ptm abundance 37C
Doses Blue Rep1_0.08 Red Rep2_−0.09

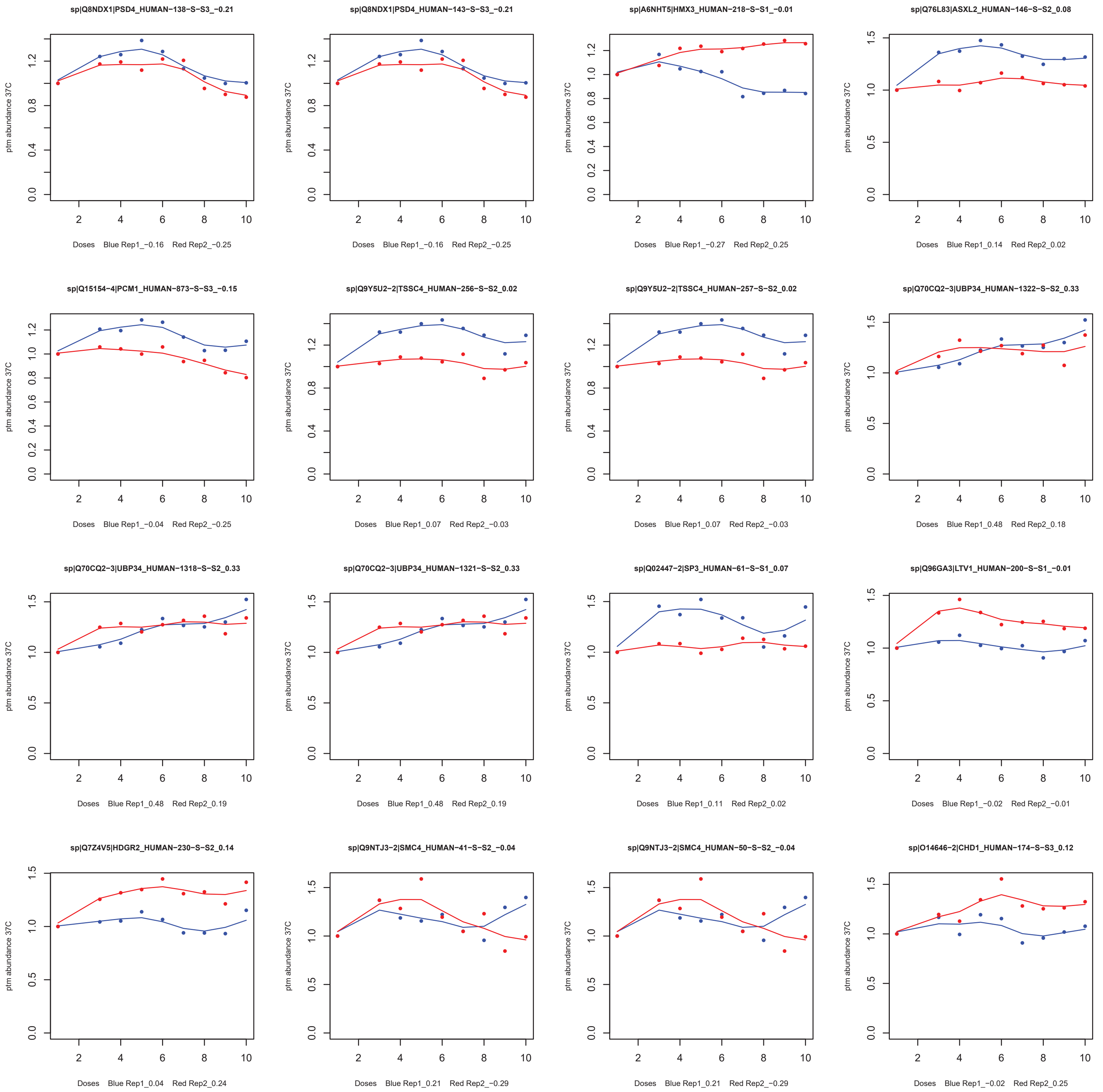

sp|Q8NDX1|PSD4_HUMAN−138−S−S3_−0.21
Doses Blue Rep1_−0.16 Red Rep2_−0.25
sp|Q8NDX1|PSD4_HUMAN−143−S−S3_−0.21
Doses Blue Rep1_−0.16 Red Rep2_−0.25
sp|A6NHT5|HMX3_HUMAN−218−S−S1_−0.01
Doses Blue Rep1_−0.27 Red Rep2_0.25
sp|Q76L83|ASXL2_HUMAN−146−S−S2_0.08
Doses Blue Rep1_0.14 Red Rep2_0.02
sp|Q15154−4|PCM1_HUMAN−873−S−S3_−0.15
Doses Blue Rep1_−0.04 Red Rep2_−0.25
sp|Q9Y5U2−2|TSSC4_HUMAN−256−S−S2_0.02
Doses Blue Rep1_0.07 Red Rep2_−0.03
sp|Q9Y5U2−2|TSSC4_HUMAN−257−S−S2_0.02
Doses Blue Rep1_0.07 Red Rep2_−0.03
sp|Q70CQ2−3|UBP34_HUMAN−1322−S−S2_0.33
Doses Blue Rep1_0.48 Red Rep2_0.18
sp|Q70CQ2−3|UBP34_HUMAN−1318−S−S2_0.33
Doses Blue Rep1_0.48 Red Rep2_0.19
sp|Q70CQ2−3|UBP34_HUMAN−1321−S−S2_0.33
Doses Blue Rep1_0.48 Red Rep2_0.19
sp|Q02447−2|SP3_HUMAN−61−S−S1_0.07
Doses Blue Rep1_0.11 Red Rep2_0.02
sp|Q96GA3|LTV1_HUMAN−200−S−S1_−0.01
Doses Blue Rep1_−0.02 Red Rep2_−0.01
sp|Q7Z4V5|HDGR2_HUMAN−230−S−S2_0.14
Doses Blue Rep1_0.04 Red Rep2_0.24
sp|Q9NTJ3−2|SMC4_HUMAN−41−S−S2_−0.04
Doses Blue Rep1_0.21 Red Rep2_−0.29
sp|Q9NTJ3−2|SMC4_HUMAN−50−S−S2_−0.04
Doses Blue Rep1_0.21 Red Rep2_−0.29
sp|O14646−2|CHD1_HUMAN−174−S−S3_0.12
Doses Blue Rep1_−0.02 Red Rep2_0.25
ptm abundance 37C

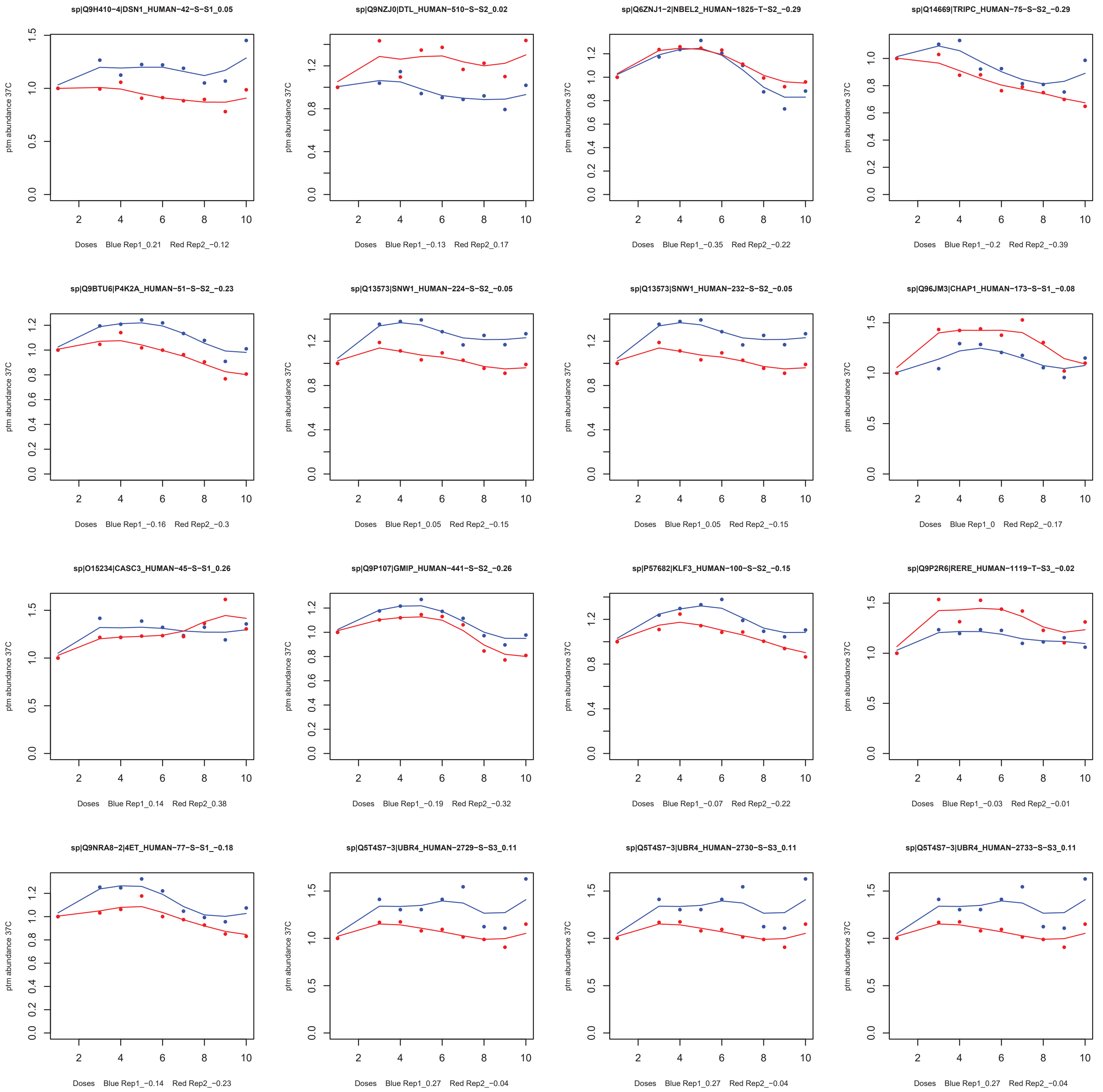
sp|Q9H4I0-4|DSN1_HUMAN-42-S-S1_0.05
Doses Blue Rep1_0.21 Red Rep2_-0.12
sp|Q9NZJ0|DTL_HUMAN-510-S-S2_0.02
Doses Blue Rep1_-0.13 Red Rep2_0.17
sp|Q6ZNJ1-2|NBEL2_HUMAN-1825-T-S2_-0.29
Doses Blue Rep1_-0.35 Red Rep2_-0.22
sp|Q14669|TRIPC_HUMAN-75-S-S2_-0.29
Doses Blue Rep1_-0.2 Red Rep2_-0.39
sp|Q9BTU6|P4K2A_HUMAN-51-S-S2_-0.23
Doses Blue Rep1_-0.16 Red Rep2_-0.3
sp|Q13573|SNW1_HUMAN-224-S-S2_-0.05
Doses Blue Rep1_0.05 Red Rep2_-0.15
sp|Q13573|SNW1_HUMAN-232-S-S2_-0.05
Doses Blue Rep1_0.05 Red Rep2_-0.15
sp|Q96JM3|CHAP1_HUMAN-173-S-S1_-0.08
Doses Blue Rep1_0 Red Rep2_-0.17
sp|O15234|CASC3_HUMAN-45-S-S1_0.26
Doses Blue Rep1_0.14 Red Rep2_0.38
sp|Q9P107|GMIP_HUMAN-441-S-S2_-0.26
Doses Blue Rep1_-0.19 Red Rep2_-0.32
sp|P57682|KLF3_HUMAN-100-S-S2_-0.15
Doses Blue Rep1_-0.07 Red Rep2_-0.22
sp|Q9P2R6|RERE_HUMAN-1119-T-S3_-0.02
Doses Blue Rep1_-0.03 Red Rep2_-0.01
sp|Q9NRA8-2|4ET_HUMAN-77-S-S1_-0.18
Doses Blue Rep1_-0.14 Red Rep2_-0.23
sp|Q5T4S7-3|UBR4_HUMAN-2729-S-S3_0.11
Doses Blue Rep1_0.27 Red Rep2_-0.04
sp|Q5T4S7-3|UBR4_HUMAN-2730-S-S3_0.11
Doses Blue Rep1_0.27 Red Rep2_-0.04
sp|Q5T4S7-3|UBR4_HUMAN-2733-S-S3_0.11
Doses Blue Rep1_0.27 Red Rep2_-0.04
ptm abundance 37C

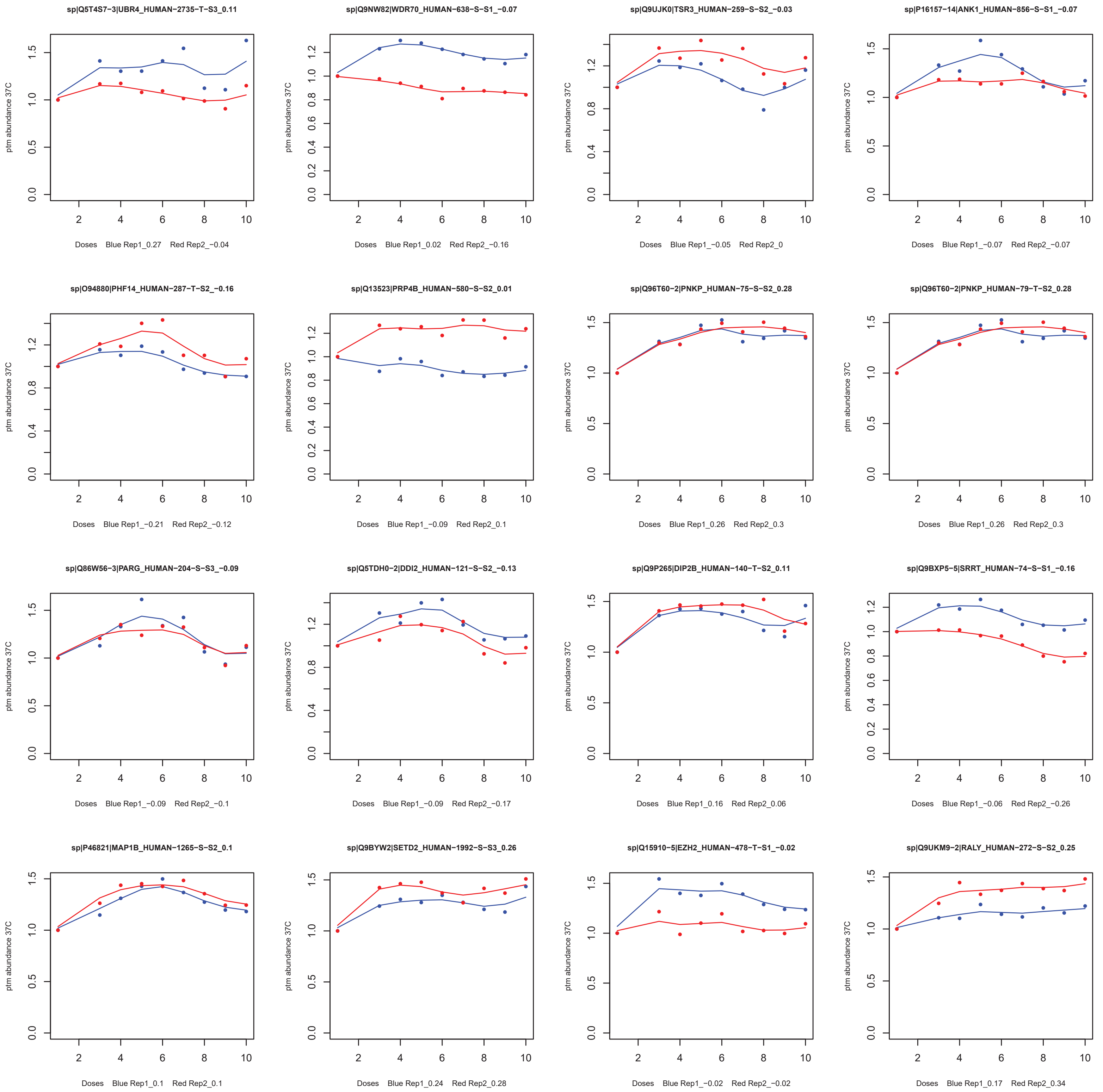
sp|Q5T4S7−3|UBR4_HUMAN−2735−T−S3_0.11
Doses Blue Rep1_0.27 Red Rep2_−0.04
sp|Q9NW82|WDR70_HUMAN−638−S−S1_−0.07
Doses Blue Rep1_0.02 Red Rep2_−0.16
sp|Q9UJK0|TSR3_HUMAN−259−S−S2_−0.03
Doses Blue Rep1_−0.05 Red Rep2_0
sp|P16157−14|ANK1_HUMAN−856−S−S1_−0.07
Doses Blue Rep1_−0.07 Red Rep2_−0.07
sp|O94880|PHF14_HUMAN−287−T−S2_−0.16
Doses Blue Rep1_−0.21 Red Rep2_−0.12
sp|Q13523|PRP4B_HUMAN−580−S−S2_0.01
Doses Blue Rep1_−0.09 Red Rep2_0.1
sp|Q96T60−2|PNKP_HUMAN−75−S−S2_0.28
Doses Blue Rep1_0.26 Red Rep2_0.3
sp|Q96T60−2|PNKP_HUMAN−79−T−S2_0.28
Doses Blue Rep1_0.26 Red Rep2_0.3
sp|Q86W56−3|PARG_HUMAN−204−S−S3_−0.09
Doses Blue Rep1_−0.09 Red Rep2_−0.1
sp|Q5TDH0−2|DDI2_HUMAN−121−S−S2_−0.13
Doses Blue Rep1_−0.09 Red Rep2_−0.17
sp|Q9P265|DIP2B_HUMAN−140−T−S2_0.11
Doses Blue Rep1_0.16 Red Rep2_0.06
sp|Q9BXP5−5|SRRT_HUMAN−74−S−S1_−0.16
Doses Blue Rep1_−0.06 Red Rep2_−0.26
sp|P46821|MAP1B_HUMAN−1265−S−S2_0.1
Doses Blue Rep1_0.1 Red Rep2_0.1
sp|Q9BYW2|SETD2_HUMAN−1992−S−S3_0.26
Doses Blue Rep1_0.24 Red Rep2_0.28
sp|Q15910−5|EZH2_HUMAN−478−T−S1_−0.02
Doses Blue Rep1_−0.02 Red Rep2_−0.02
sp|Q9UKM9−2|RALY_HUMAN−272−S−S2_0.25
Doses Blue Rep1_0.17 Red Rep2_0.34
ptm abundance 37C

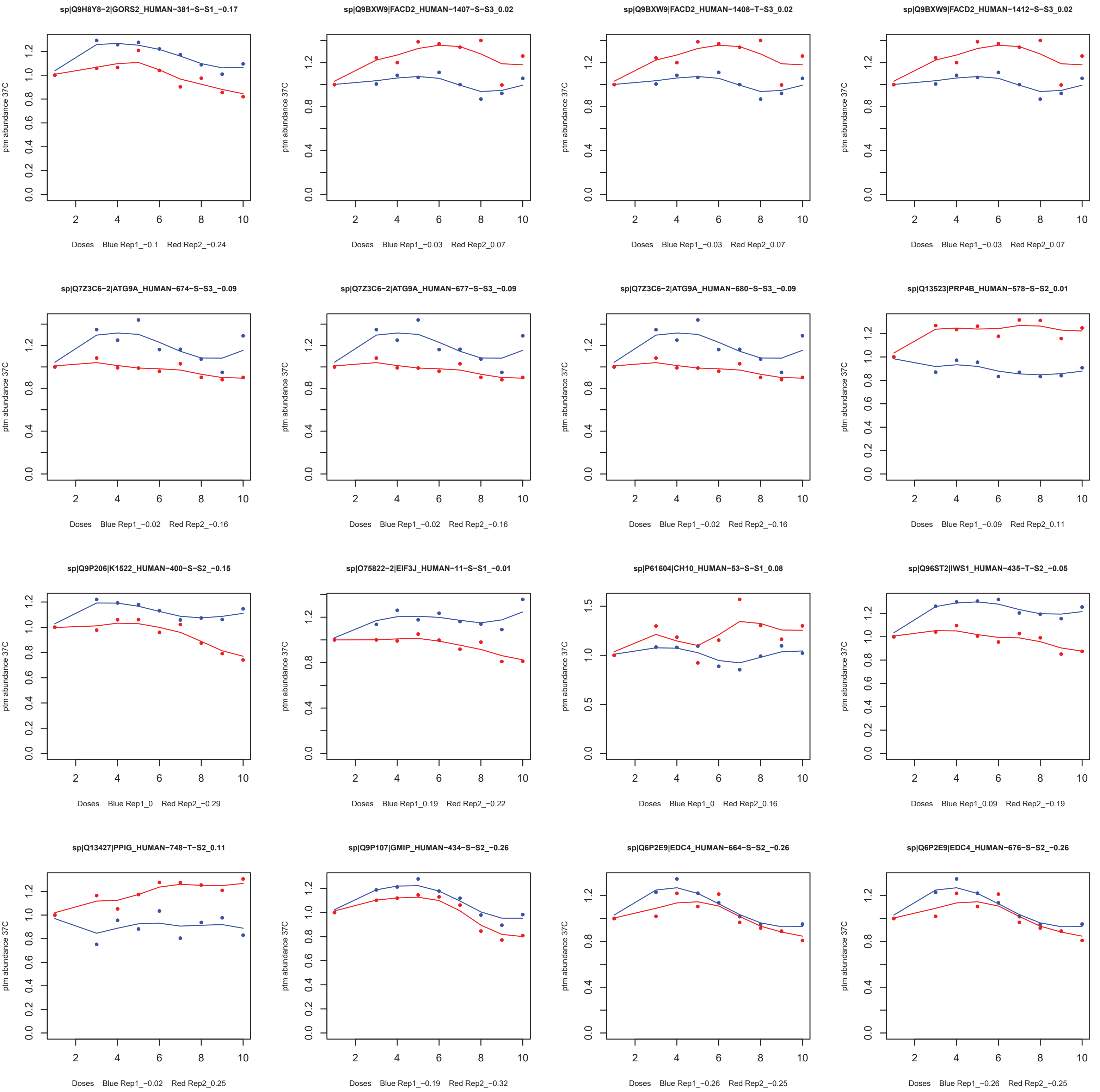

sp|Q9H8Y8−2|GORS2_HUMAN−381−S−S1_−0.17
Doses Blue Rep1_−0.1 Red Rep2_−0.24
sp|Q9BXW9|FACD2_HUMAN−1407−S−S3_0.02
Doses Blue Rep1_−0.03 Red Rep2_0.07
sp|Q9BXW9|FACD2_HUMAN−1408−T−S3_0.02
Doses Blue Rep1_−0.03 Red Rep2_0.07
sp|Q9BXW9|FACD2_HUMAN−1412−S−S3_0.02
Doses Blue Rep1_−0.03 Red Rep2_0.07
sp|Q7Z3C6−2|ATG9A_HUMAN−674−S−S3_−0.09
Doses Blue Rep1_−0.02 Red Rep2_−0.16
sp|Q7Z3C6−2|ATG9A_HUMAN−677−S−S3_−0.09
Doses Blue Rep1_−0.02 Red Rep2_−0.16
sp|Q7Z3C6−2|ATG9A_HUMAN−680−S−S3_−0.09
Doses Blue Rep1_−0.02 Red Rep2_−0.16
sp|Q13523|PRP4B_HUMAN−578−S−S2_0.01
Doses Blue Rep1_−0.09 Red Rep2_0.11
sp|Q9P206|K1522_HUMAN−400−S−S2_−0.15
Doses Blue Rep1_0 Red Rep2_−0.29
sp|O75822−2|EIF3J_HUMAN−11−S−S1_−0.01
Doses Blue Rep1_0.19 Red Rep2_−0.22
sp|P61604|CH10_HUMAN−53−S−S1_0.08
Doses Blue Rep1_0 Red Rep2_0.16
sp|Q96ST2|IWS1_HUMAN−435−T−S2_−0.05
Doses Blue Rep1_0.09 Red Rep2_−0.19
sp|Q13427|PPIG_HUMAN−748−T−S2_0.11
Doses Blue Rep1_−0.02 Red Rep2_0.25
sp|Q9P107|GMIP_HUMAN−434−S−S2_−0.26
Doses Blue Rep1_−0.19 Red Rep2_−0.32
sp|Q6P2E9|EDC4_HUMAN−664−S−S2_−0.26
Doses Blue Rep1_−0.26 Red Rep2_−0.25
sp|Q6P2E9|EDC4_HUMAN−676−S−S2_−0.26
Doses Blue Rep1_−0.26 Red Rep2_−0.25
ptm abundance 37C

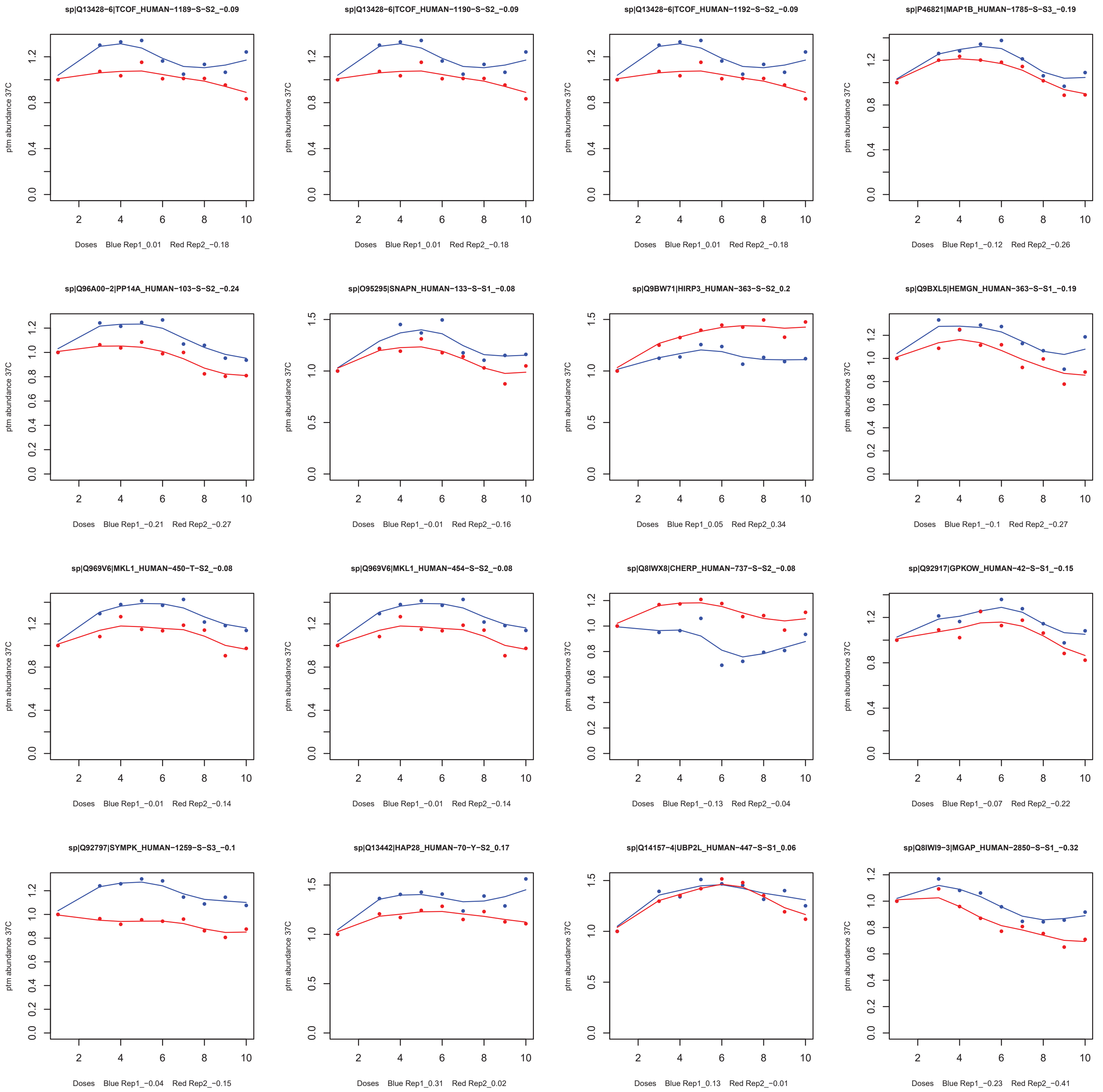

sp|Q13428−6|TCOF_HUMAN−1189−S−S2_−0.09
Doses Blue Rep1_0.01 Red Rep2_−0.18
sp|Q13428−6|TCOF_HUMAN−1190−S−S2_−0.09
Doses Blue Rep1_0.01 Red Rep2_−0.18
sp|Q13428−6|TCOF_HUMAN−1192−S−S2_−0.09
Doses Blue Rep1_0.01 Red Rep2_−0.18
sp|P46821|MAP1B_HUMAN−1785−S−S3_−0.19
Doses Blue Rep1_−0.12 Red Rep2_−0.26
sp|Q96A00−2|PP14A_HUMAN−103−S−S2_−0.24
Doses Blue Rep1_−0.21 Red Rep2_−0.27
sp|O95295|SNAPN_HUMAN−133−S−S1_−0.08
Doses Blue Rep1_−0.01 Red Rep2_−0.16
sp|Q9BW71|HIRP3_HUMAN−363−S−S2_0.2
Doses Blue Rep1_0.05 Red Rep2_0.34
sp|Q9BXL5|HEMGN_HUMAN−363−S−S1_−0.19
Doses Blue Rep1_−0.1 Red Rep2_−0.27
sp|Q969V6|MKL1_HUMAN−450−T−S2_−0.08
Doses Blue Rep1_−0.01 Red Rep2_−0.14
sp|Q969V6|MKL1_HUMAN−454−S−S2_−0.08
Doses Blue Rep1_−0.01 Red Rep2_−0.14
sp|Q8IWX8|CHERP_HUMAN−737−S−S2_−0.08
Doses Blue Rep1_−0.13 Red Rep2_−0.04
sp|Q92917|GPKOW_HUMAN−42−S−S1_−0.15
Doses Blue Rep1_−0.07 Red Rep2_−0.22
sp|Q92797|SYMPK_HUMAN−1259−S−S3_−0.1
Doses Blue Rep1_−0.04 Red Rep2_−0.15
sp|Q13442|HAP28_HUMAN−70−Y−S2_0.17
Doses Blue Rep1_0.31 Red Rep2_0.02
sp|Q14157−4|UBP2L_HUMAN−447−S−S1_0.06
Doses Blue Rep1_0.13 Red Rep2_−0.01
sp|Q8IWI9−3|MGAP_HUMAN−2850−S−S1_−0.32
Doses Blue Rep1_−0.23 Red Rep2_−0.41
ptm abundance 37C

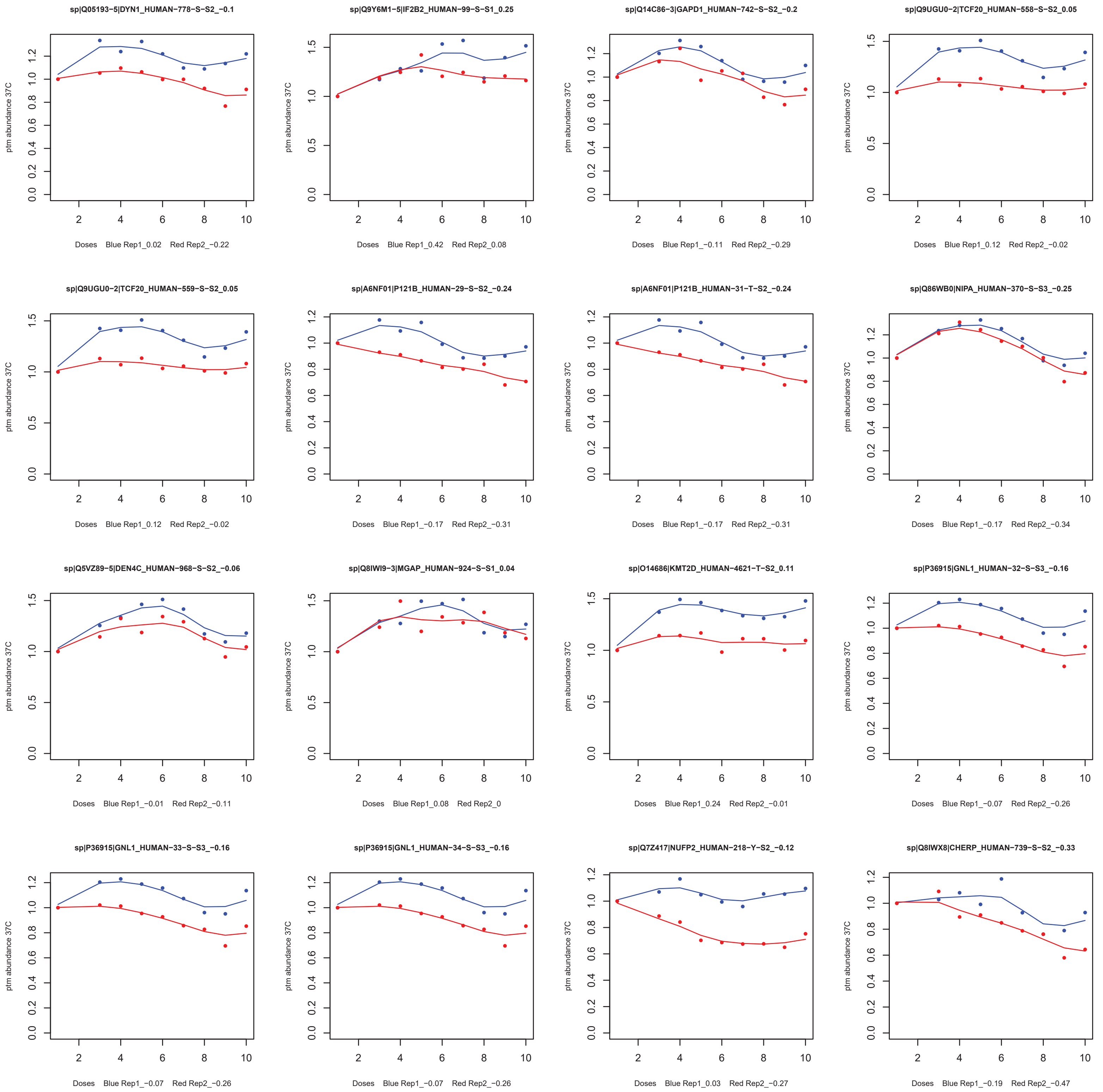

sp|Q05193−5|DYN1_HUMAN−778−S−S2_−0.1
ptm abundance 37C
Doses Blue Rep1_0.02 Red Rep2_−0.22
sp|Q9Y6M1−5|IF2B2_HUMAN−99−S−S1_0.25
ptm abundance 37C
Doses Blue Rep1_0.42 Red Rep2_0.08
sp|Q14C86−3|GAPD1_HUMAN−742−S−S2_−0.2
ptm abundance 37C
Doses Blue Rep1_−0.11 Red Rep2_−0.29
sp|Q9UGU0−2|TCF20_HUMAN−558−S−S2_0.05
ptm abundance 37C
Doses Blue Rep1_0.12 Red Rep2_−0.02
sp|Q9UGU0−2|TCF20_HUMAN−559−S−S2_0.05
ptm abundance 37C
Doses Blue Rep1_0.12 Red Rep2_−0.02
sp|A6NF01|P121B_HUMAN−29−S−S2_−0.24
ptm abundance 37C
Doses Blue Rep1_−0.17 Red Rep2_−0.31
sp|A6NF01|P121B_HUMAN−31−T−S2_−0.24
ptm abundance 37C
Doses Blue Rep1_−0.17 Red Rep2_−0.31
sp|Q86WB0|NIPA_HUMAN−370−S−S3_−0.25
ptm abundance 37C
Doses Blue Rep1_−0.17 Red Rep2_−0.34
sp|Q5VZ89−5|DEN4C_HUMAN−968−S−S2_−0.06
ptm abundance 37C
Doses Blue Rep1_−0.01 Red Rep2_−0.11
sp|Q8IWI9−3|MGAP_HUMAN−924−S−S1_0.04
ptm abundance 37C
Doses Blue Rep1_0.08 Red Rep2_0
sp|O14686|KMT2D_HUMAN−4621−T−S2_0.11
ptm abundance 37C
Doses Blue Rep1_0.24 Red Rep2_−0.01
sp|P36915|GNL1_HUMAN−32−S−S3_−0.16
ptm abundance 37C
Doses Blue Rep1_−0.07 Red Rep2_−0.26
sp|P36915|GNL1_HUMAN−33−S−S3_−0.16
ptm abundance 37C
Doses Blue Rep1_−0.07 Red Rep2_−0.26
sp|P36915|GNL1_HUMAN−34−S−S3_−0.16
ptm abundance 37C
Doses Blue Rep1_−0.07 Red Rep2_−0.26
sp|Q7Z417|NUFP2_HUMAN−218−Y−S2_−0.12
ptm abundance 37C
Doses Blue Rep1_0.03 Red Rep2_−0.27
sp|Q8IWX8|CHERP_HUMAN−739−S−S2_−0.33
ptm abundance 37C
Doses Blue Rep1_−0.19 Red Rep2_−0.47

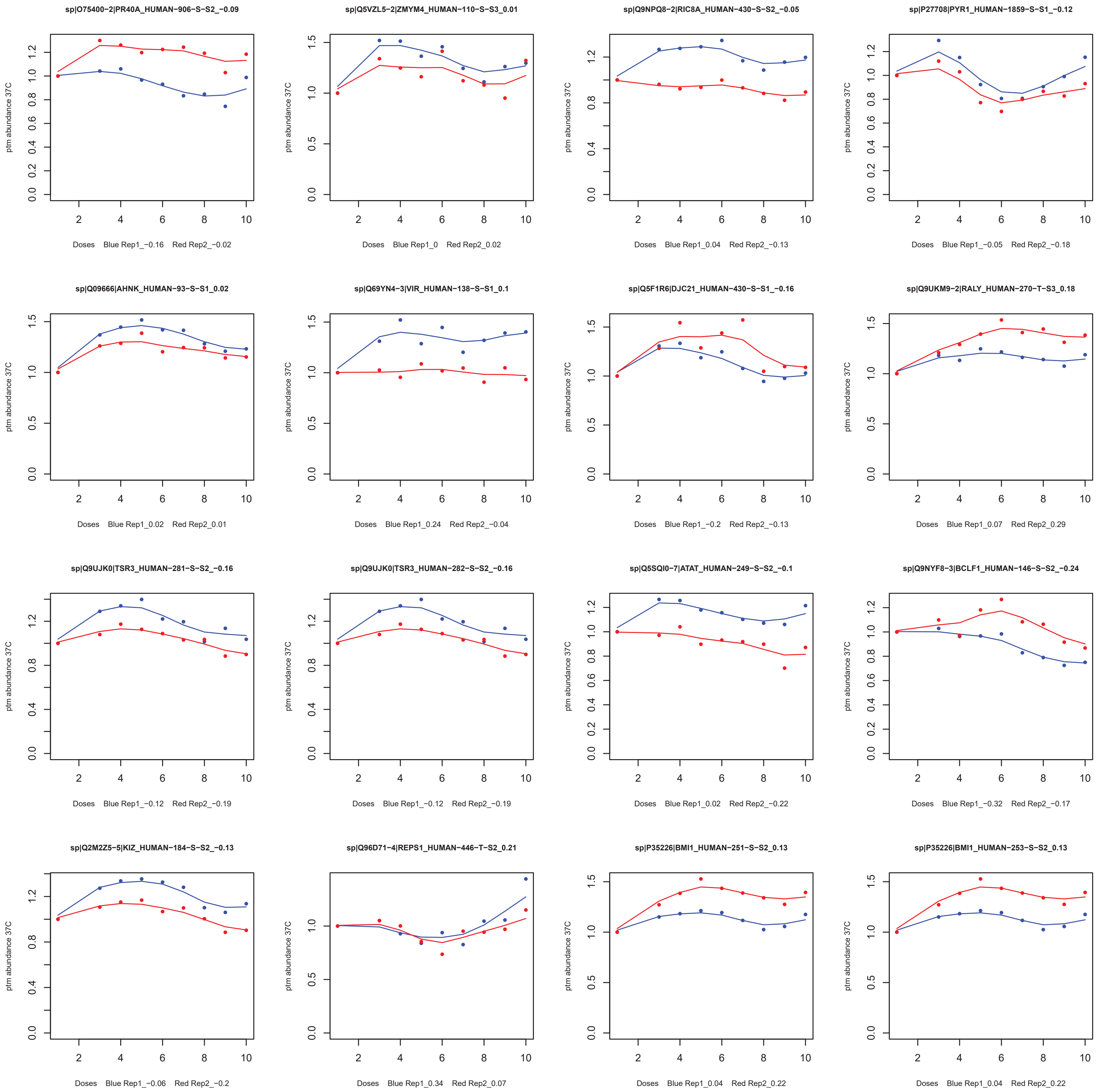
sp|O75400−2|PR40A_HUMAN−906−S−S2_−0.09
Doses Blue Rep1_−0.16 Red Rep2_−0.02
sp|Q5VZL5−2|ZMYM4_HUMAN−110−S−S3_0.01
Doses Blue Rep1_0 Red Rep2_0.02
sp|Q9NPQ8−2|RIC8A_HUMAN−430−S−S2_−0.05
Doses Blue Rep1_0.04 Red Rep2_−0.13
sp|P27708|PYR1_HUMAN−1859−S−S1_−0.12
Doses Blue Rep1_−0.05 Red Rep2_−0.18
sp|Q09666|AHNK_HUMAN−93−S−S1_0.02
Doses Blue Rep1_0.02 Red Rep2_0.01
sp|Q69YN4−3|VIR_HUMAN−138−S−S1_0.1
Doses Blue Rep1_0.24 Red Rep2_−0.04
sp|Q5F1R6|DJC21_HUMAN−430−S−S1_−0.16
Doses Blue Rep1_−0.2 Red Rep2_−0.13
sp|Q9UKM9−2|RALY_HUMAN−270−T−S3_0.18
Doses Blue Rep1_0.07 Red Rep2_0.29
sp|Q9UJK0|TSR3_HUMAN−281−S−S2_−0.16
Doses Blue Rep1_−0.12 Red Rep2_−0.19
sp|Q9UJK0|TSR3_HUMAN−282−S−S2_−0.16
Doses Blue Rep1_−0.12 Red Rep2_−0.19
sp|Q5SQI0−7|ATAT_HUMAN−249−S−S2_−0.1
Doses Blue Rep1_0.02 Red Rep2_−0.22
sp|Q9NYF8−3|BCLF1_HUMAN−146−S−S2_−0.24
Doses Blue Rep1_−0.32 Red Rep2_−0.17
sp|Q2M2Z5−5|KIZ_HUMAN−184−S−S2_−0.13
Doses Blue Rep1_−0.06 Red Rep2_−0.2
sp|Q96D71−4|REPS1_HUMAN−446−T−S2_0.21
Doses Blue Rep1_0.34 Red Rep2_0.07
sp|P35226|BMI1_HUMAN−251−S−S2_0.13
Doses Blue Rep1_0.04 Red Rep2_0.22
sp|P35226|BMI1_HUMAN−253−S−S2_0.13
Doses Blue Rep1_0.04 Red Rep2_0.22
ptm abundance 37C

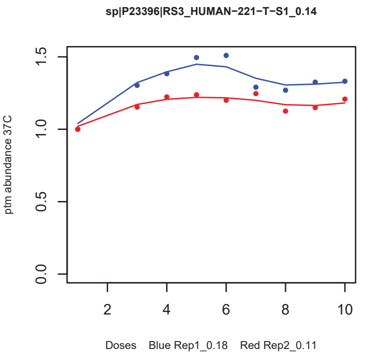
sp|P23396|RS3_HUMAN−221−T−S1_0.14
ptm abundance 37C
Doses Blue Rep1_0.18 Red Rep2_0.11
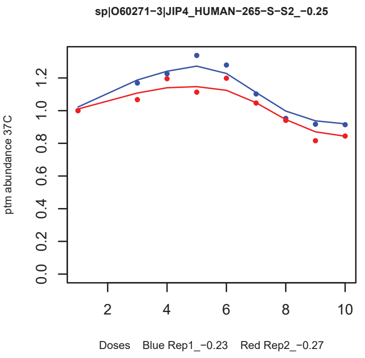
sp|O60271−3|JIP4_HUMAN−265−S−S2_−0.25
ptm abundance 37C
Doses Blue Rep1_−0.23 Red Rep2_−0.27
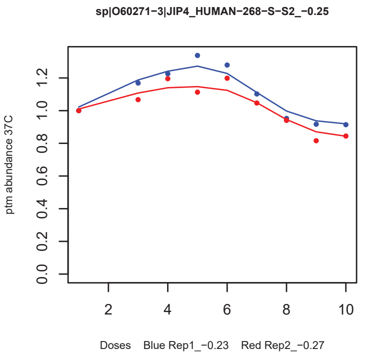
sp|O60271−3|JIP4_HUMAN−268−S−S2_−0.25
ptm abundance 37C
Doses Blue Rep1_−0.23 Red Rep2_−0.27
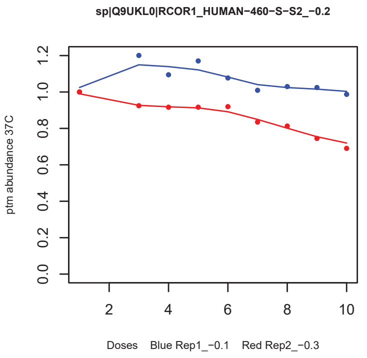
sp|Q9UKL0|RCOR1_HUMAN−460−S−S2_−0.2
ptm abundance 37C
Doses Blue Rep1_−0.1 Red Rep2_−0.3
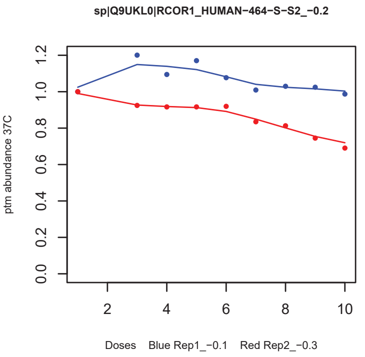
sp|Q9UKL0|RCOR1_HUMAN−464−S−S2_−0.2
ptm abundance 37C
Doses Blue Rep1_−0.1 Red Rep2_−0.3
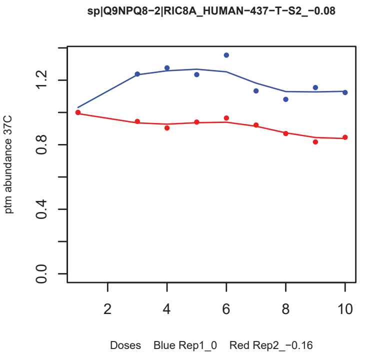
sp|Q9NPQ8−2|RIC8A_HUMAN−437−T−S2_−0.08
ptm abundance 37C
Doses Blue Rep1_0 Red Rep2_−0.16
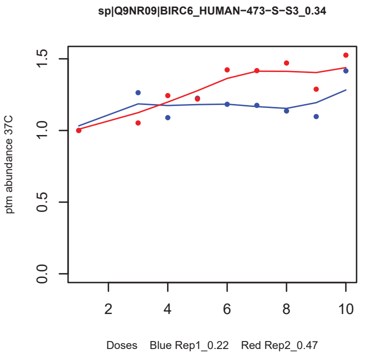
sp|Q9NR09|BIRC6_HUMAN−473−S−S3_0.34
ptm abundance 37C
Doses Blue Rep1_0.22 Red Rep2_0.47
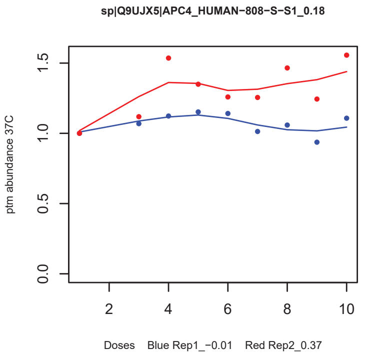
sp|Q9UJX5|APC4_HUMAN−808−S−S1_0.18
ptm abundance 37C
Doses Blue Rep1_−0.01 Red Rep2_0.37
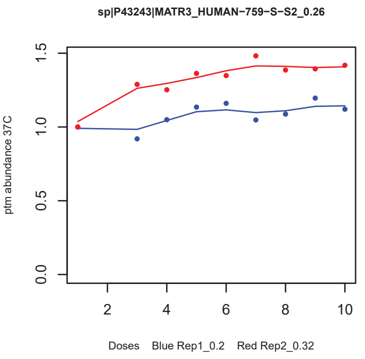
sp|P43243|MATR3_HUMAN−759−S−S2_0.26
ptm abundance 37C
Doses Blue Rep1_0.2 Red Rep2_0.32
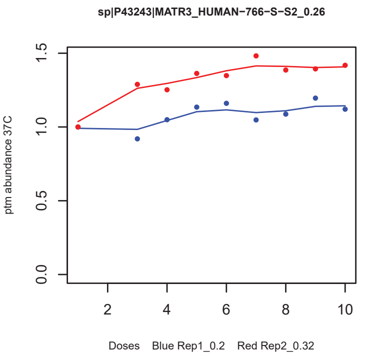
sp|P43243|MATR3_HUMAN−766−S−S2_0.26
ptm abundance 37C
Doses Blue Rep1_0.2 Red Rep2_0.32
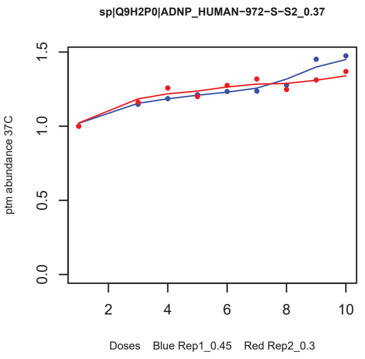
sp|Q9H2P0|ADNP_HUMAN−972−S−S2_0.37
ptm abundance 37C
Doses Blue Rep1_0.45 Red Rep2_0.3
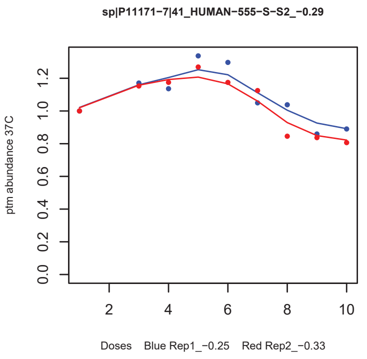
sp|P11171−7|41_HUMAN−555−S−S2_−0.29
ptm abundance 37C
Doses Blue Rep1_−0.25 Red Rep2_−0.33
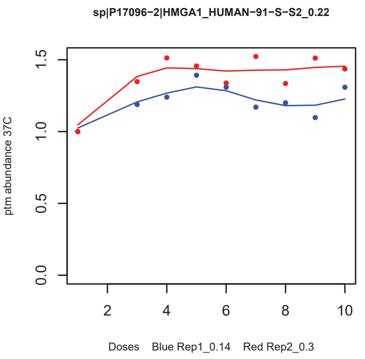
sp|P17096−2|HMGA1_HUMAN−91−S−S2_0.22
ptm abundance 37C
Doses Blue Rep1_0.14 Red Rep2_0.3
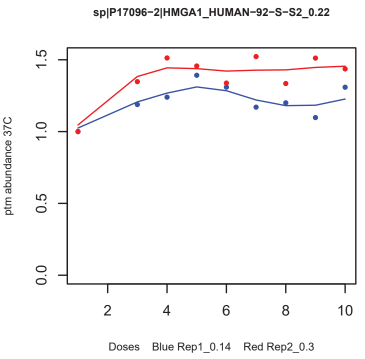
sp|P17096−2|HMGA1_HUMAN−92−S−S2_0.22
ptm abundance 37C
Doses Blue Rep1_0.14 Red Rep2_0.3
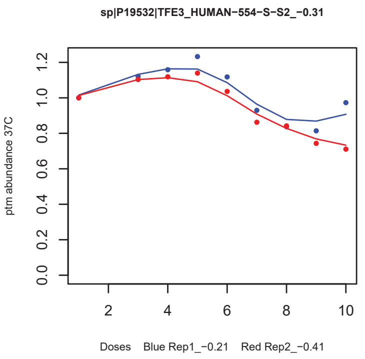
sp|P19532|TFE3_HUMAN−554−S−S2_−0.31
ptm abundance 37C
Doses Blue Rep1_−0.21 Red Rep2_−0.41
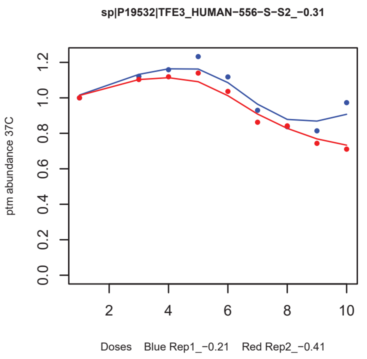
sp|P19532|TFE3_HUMAN−556−S−S2_−0.31
ptm abundance 37C
Doses Blue Rep1_−0.21 Red Rep2_−0.41

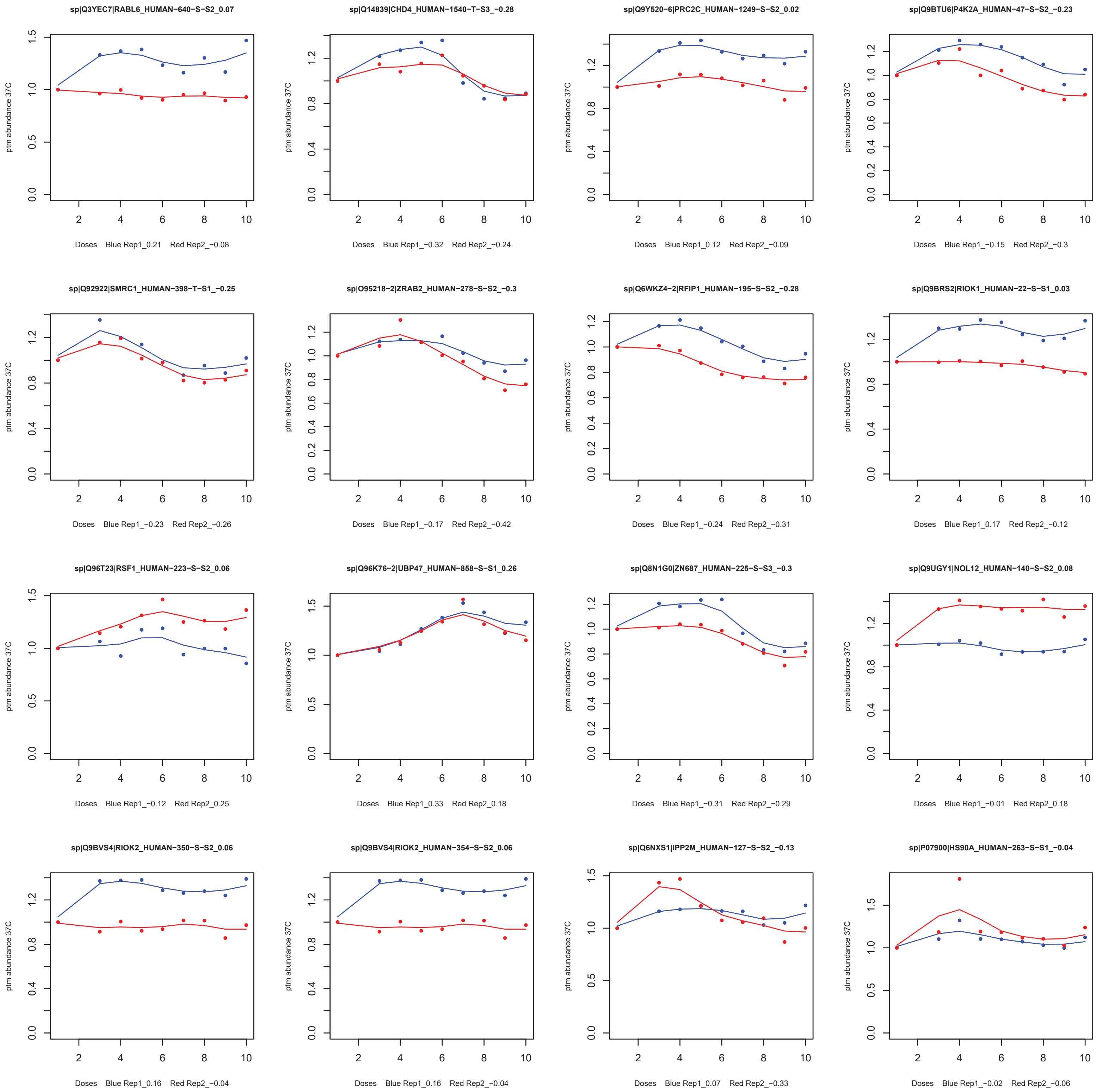

sp|Q3YEC7|RABL6_HUMAN−640−S−S2_0.07
Doses Blue Rep1_0.21 Red Rep2_−0.08
sp|Q14839|CHD4_HUMAN−1540−T−S3_−0.28
Doses Blue Rep1_−0.32 Red Rep2_−0.24
sp|Q9Y520−6|PRC2C_HUMAN−1249−S−S2_0.02
Doses Blue Rep1_0.12 Red Rep2_−0.09
sp|Q9BTU6|P4K2A_HUMAN−47−S−S2_−0.23
Doses Blue Rep1_−0.15 Red Rep2_−0.3
sp|Q92922|SMRC1_HUMAN−398−T−S1_−0.25
Doses Blue Rep1_−0.23 Red Rep2_−0.26
sp|O95218−2|ZRAB2_HUMAN−278−S−S2_−0.3
Doses Blue Rep1_−0.17 Red Rep2_−0.42
sp|Q6WKZ4−2|RFIP1_HUMAN−195−S−S2_−0.28
Doses Blue Rep1_−0.24 Red Rep2_−0.31
sp|Q9BRS2|RIOK1_HUMAN−22−S−S1_0.03
Doses Blue Rep1_0.17 Red Rep2_−0.12
sp|Q96T23|RSF1_HUMAN−223−S−S2_0.06
Doses Blue Rep1_−0.12 Red Rep2_0.25
sp|Q96K76−2|UBP47_HUMAN−858−S−S1_0.26
Doses Blue Rep1_0.33 Red Rep2_0.18
sp|Q8N1G0|ZN687_HUMAN−225−S−S3_−0.3
Doses Blue Rep1_−0.31 Red Rep2_−0.29
sp|Q9UGY1|NOL12_HUMAN−140−S−S2_0.08
Doses Blue Rep1_−0.01 Red Rep2_0.18
sp|Q9BVS4|RIOK2_HUMAN−350−S−S2_0.06
Doses Blue Rep1_0.16 Red Rep2_−0.04
sp|Q9BVS4|RIOK2_HUMAN−354−S−S2_0.06
Doses Blue Rep1_0.16 Red Rep2_−0.04
sp|Q6NXS1|IPP2M_HUMAN−127−S−S2_−0.13
Doses Blue Rep1_0.07 Red Rep2_−0.33
sp|P07900|HS90A_HUMAN−263−S−S1_−0.04
Doses Blue Rep1_−0.02 Red Rep2_−0.06
ptm abundance 37C

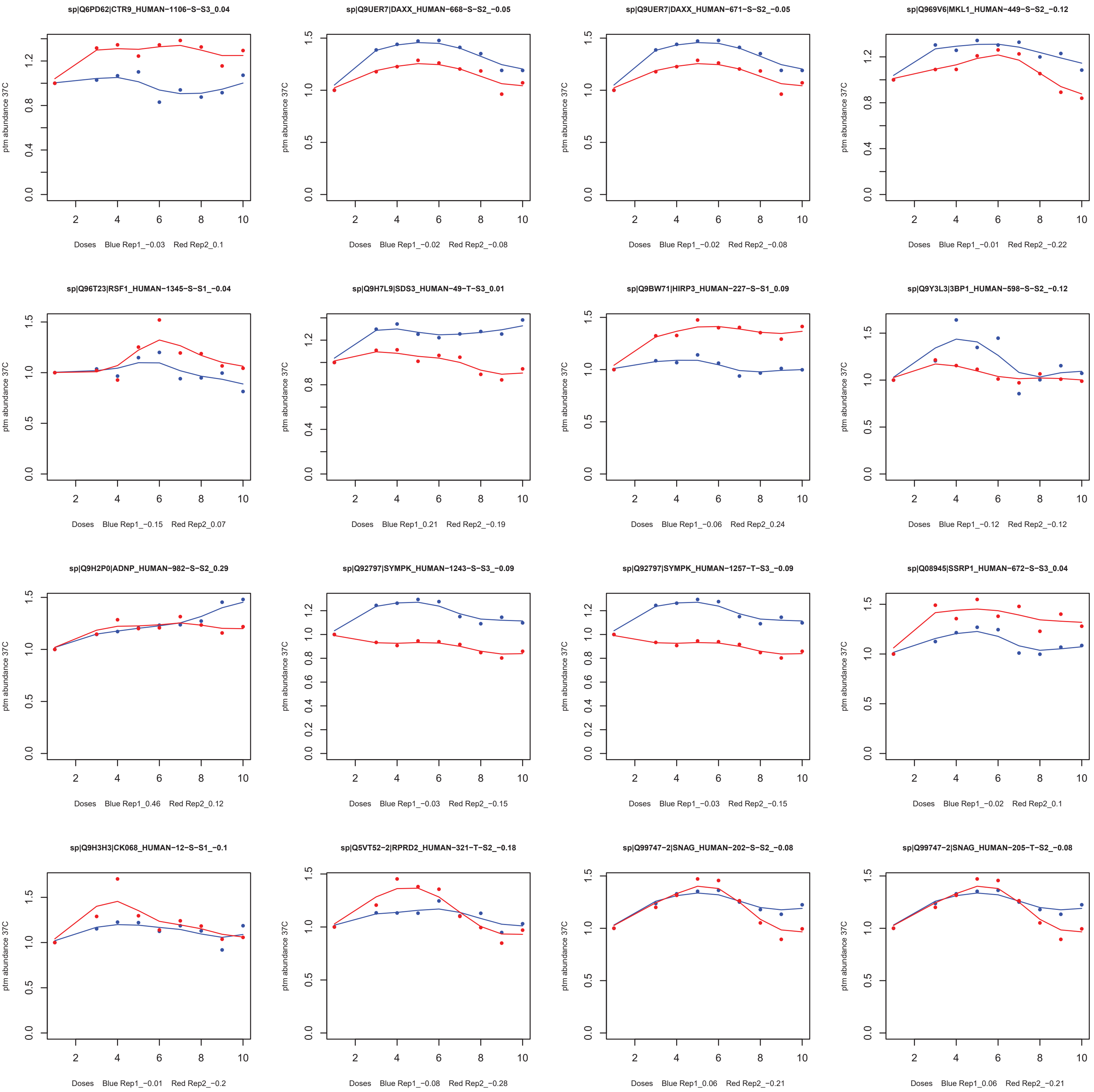

sp|Q6PD62|CTR9_HUMAN−1106−S−S3_0.04
Doses Blue Rep1_−0.03 Red Rep2_0.1
sp|Q9UER7|DAXX_HUMAN−668−S−S2_−0.05
Doses Blue Rep1_−0.02 Red Rep2_−0.08
sp|Q9UER7|DAXX_HUMAN−671−S−S2_−0.05
Doses Blue Rep1_−0.02 Red Rep2_−0.08
sp|Q969V6|MKL1_HUMAN−449−S−S2_−0.12
Doses Blue Rep1_−0.01 Red Rep2_−0.22
sp|Q96T23|RSF1_HUMAN−1345−S−S1_−0.04
Doses Blue Rep1_−0.15 Red Rep2_0.07
sp|Q9H7L9|SDS3_HUMAN−49−T−S3_0.01
Doses Blue Rep1_0.21 Red Rep2_−0.19
sp|Q9BW71|HIRP3_HUMAN−227−S−S1_0.09
Doses Blue Rep1_−0.06 Red Rep2_0.24
sp|Q9Y3L3|3BP1_HUMAN−598−S−S2_−0.12
Doses Blue Rep1_−0.12 Red Rep2_−0.12
sp|Q9H2P0|ADNP_HUMAN−982−S−S2_0.29
Doses Blue Rep1_0.46 Red Rep2_0.12
sp|Q92797|SYMPK_HUMAN−1243−S−S3_−0.09
Doses Blue Rep1_−0.03 Red Rep2_−0.15
sp|Q92797|SYMPK_HUMAN−1257−T−S3_−0.09
Doses Blue Rep1_−0.03 Red Rep2_−0.15
sp|Q08945|SSRP1_HUMAN−672−S−S3_0.04
Doses Blue Rep1_−0.02 Red Rep2_0.1
sp|Q9H3H3|CK068_HUMAN−12−S−S1_−0.1
Doses Blue Rep1_−0.01 Red Rep2_−0.2
sp|Q5VT52−2|RPRD2_HUMAN−321−T−S2_−0.18
Doses Blue Rep1_−0.08 Red Rep2_−0.28
sp|Q99747−2|SNAG_HUMAN−202−S−S2_−0.08
Doses Blue Rep1_0.06 Red Rep2_−0.21
sp|Q99747−2|SNAG_HUMAN−205−T−S2_−0.08
Doses Blue Rep1_0.06 Red Rep2_−0.21
ptm abundance 37C

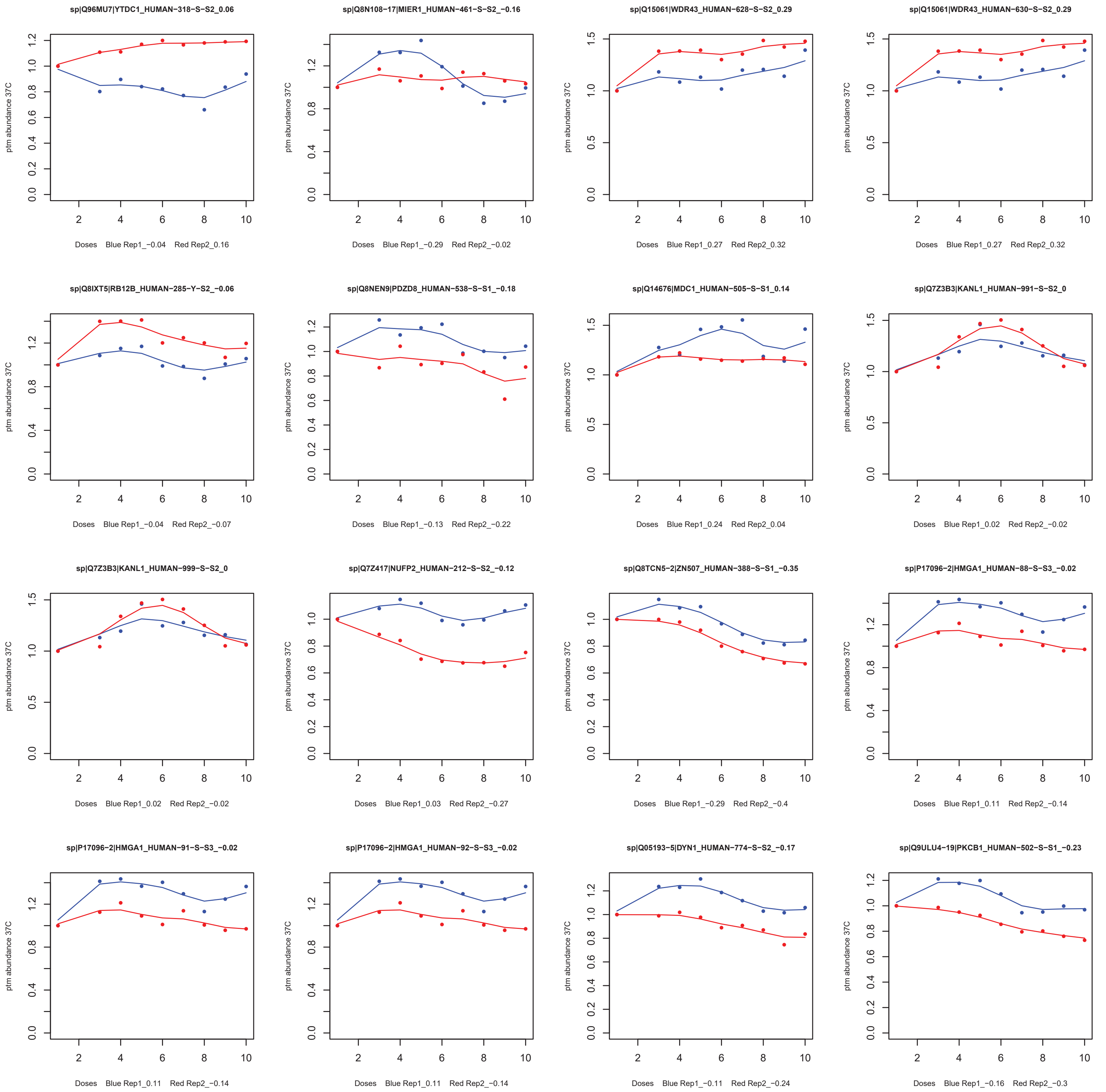
sp|Q96MU7|YTDC1_HUMAN−318−S−S2_0.06
ptm abundance 37C
Doses Blue Rep1_−0.04 Red Rep2_0.16
sp|Q8N108−17|MIER1_HUMAN−461−S−S2_−0.16
ptm abundance 37C
Doses Blue Rep1_−0.29 Red Rep2_−0.02
sp|Q15061|WDR43_HUMAN−628−S−S2_0.29
ptm abundance 37C
Doses Blue Rep1_0.27 Red Rep2_0.32
sp|Q15061|WDR43_HUMAN−630−S−S2_0.29
ptm abundance 37C
Doses Blue Rep1_0.27 Red Rep2_0.32
sp|Q8IXT5|RB12B_HUMAN−285−Y−S2_−0.06
ptm abundance 37C
Doses Blue Rep1_−0.04 Red Rep2_−0.07
sp|Q8NEN9|PDZD8_HUMAN−538−S−S1_−0.18
ptm abundance 37C
Doses Blue Rep1_−0.13 Red Rep2_−0.22
sp|Q14676|MDC1_HUMAN−505−S−S1_0.14
ptm abundance 37C
Doses Blue Rep1_0.24 Red Rep2_0.04
sp|Q7Z3B3|KANL1_HUMAN−991−S−S2_0
ptm abundance 37C
Doses Blue Rep1_0.02 Red Rep2_−0.02
sp|Q7Z3B3|KANL1_HUMAN−999−S−S2_0
ptm abundance 37C
Doses Blue Rep1_0.02 Red Rep2_−0.02
sp|Q7Z417|NUFP2_HUMAN−212−S−S2_−0.12
ptm abundance 37C
Doses Blue Rep1_0.03 Red Rep2_−0.27
sp|Q8TCN5−2|ZN507_HUMAN−388−S−S1_−0.35
ptm abundance 37C
Doses Blue Rep1_−0.29 Red Rep2_−0.4
sp|P17096−2|HMGA1_HUMAN−88−S−S3_−0.02
ptm abundance 37C
Doses Blue Rep1_0.11 Red Rep2_−0.14
sp|P17096−2|HMGA1_HUMAN−91−S−S3_−0.02
ptm abundance 37C
Doses Blue Rep1_0.11 Red Rep2_−0.14
sp|P17096−2|HMGA1_HUMAN−92−S−S3_−0.02
ptm abundance 37C
Doses Blue Rep1_0.11 Red Rep2_−0.14
sp|Q05193−5|DYN1_HUMAN−774−S−S2_−0.17
ptm abundance 37C
Doses Blue Rep1_−0.11 Red Rep2_−0.24
sp|Q9ULU4−19|PKCB1_HUMAN−502−S−S1_−0.23
ptm abundance 37C
Doses Blue Rep1_−0.16 Red Rep2_−0.3

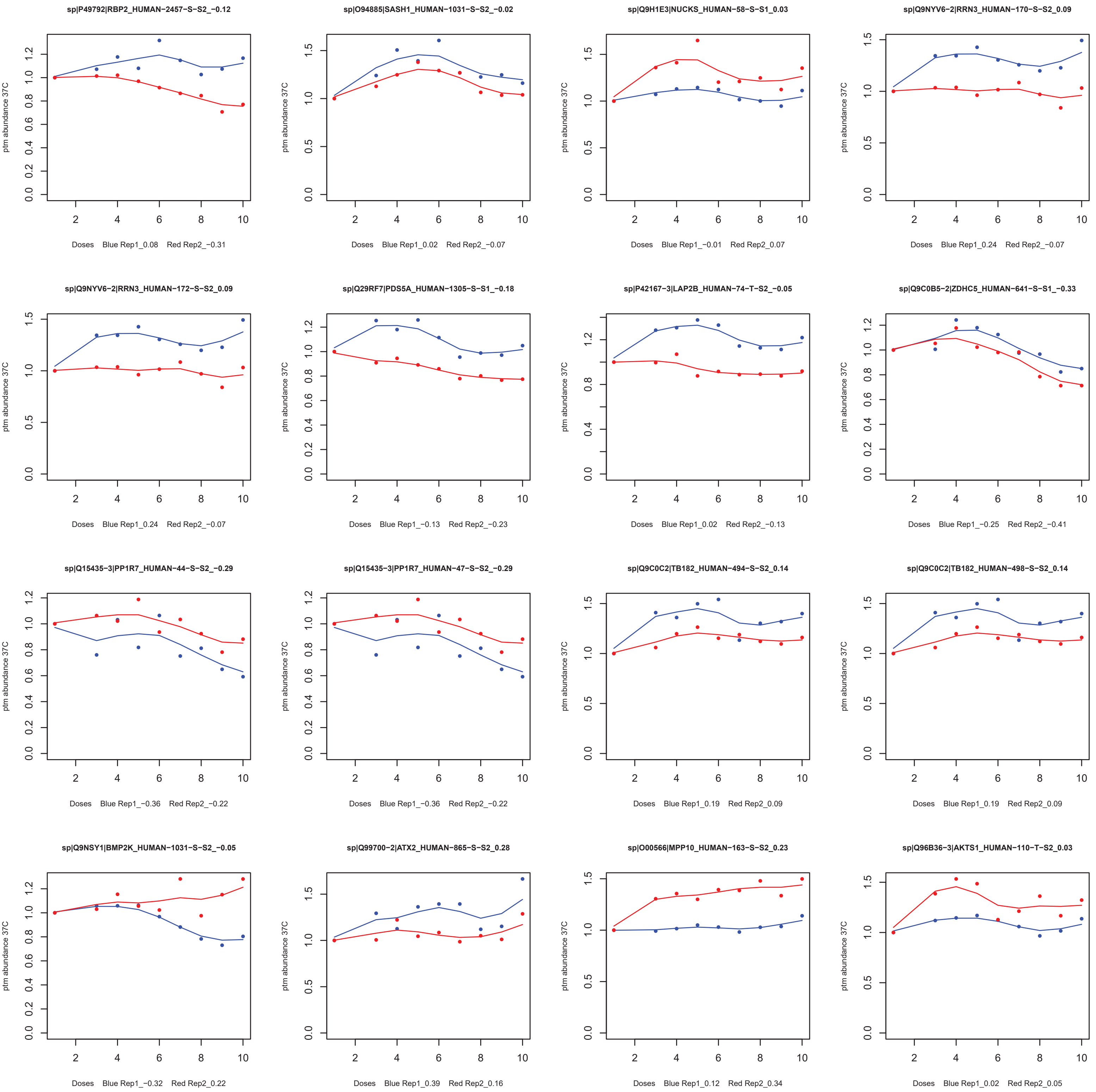
sp|P49792|RBP2_HUMAN−2457−S−S2_−0.12
ptm abundance 37C
Doses Blue Rep1_0.08 Red Rep2_−0.31
sp|O94885|SASH1_HUMAN−1031−S−S2_−0.02
ptm abundance 37C
Doses Blue Rep1_0.02 Red Rep2_−0.07
sp|Q9H1E3|NUCKS_HUMAN−58−S−S1_0.03
ptm abundance 37C
Doses Blue Rep1_−0.01 Red Rep2_0.07
sp|Q9NYV6−2|RRN3_HUMAN−170−S−S2_0.09
ptm abundance 37C
Doses Blue Rep1_0.24 Red Rep2_−0.07
sp|Q9NYV6−2|RRN3_HUMAN−172−S−S2_0.09
ptm abundance 37C
Doses Blue Rep1_0.24 Red Rep2_−0.07
sp|Q29RF7|PDS5A_HUMAN−1305−S−S1_−0.18
ptm abundance 37C
Doses Blue Rep1_−0.13 Red Rep2_−0.23
sp|P42167−3|LAP2B_HUMAN−74−T−S2_−0.05
ptm abundance 37C
Doses Blue Rep1_0.02 Red Rep2_−0.13
sp|Q9C0B5−2|ZDHC5_HUMAN−641−S−S1_−0.33
ptm abundance 37C
Doses Blue Rep1_−0.25 Red Rep2_−0.41
sp|Q15435−3|PP1R7_HUMAN−44−S−S2_−0.29
ptm abundance 37C
Doses Blue Rep1_−0.36 Red Rep2_−0.22
sp|Q15435−3|PP1R7_HUMAN−47−S−S2_−0.29
ptm abundance 37C
Doses Blue Rep1_−0.36 Red Rep2_−0.22
sp|Q9C0C2|TB182_HUMAN−494−S−S2_0.14
ptm abundance 37C
Doses Blue Rep1_0.19 Red Rep2_0.09
sp|Q9C0C2|TB182_HUMAN−498−S−S2_0.14
ptm abundance 37C
Doses Blue Rep1_0.19 Red Rep2_0.09
sp|Q9NSY1|BMP2K_HUMAN−1031−S−S2_−0.05
ptm abundance 37C
Doses Blue Rep1_−0.32 Red Rep2_0.22
sp|Q99700−2|ATX2_HUMAN−865−S−S2_0.28
ptm abundance 37C
Doses Blue Rep1_0.39 Red Rep2_0.16
sp|O00566|MPP10_HUMAN−163−S−S2_0.23
ptm abundance 37C
Doses Blue Rep1_0.12 Red Rep2_0.34
sp|Q96B36−3|AKTS1_HUMAN−110−T−S2_0.03
ptm abundance 37C
Doses Blue Rep1_0.02 Red Rep2_0.05

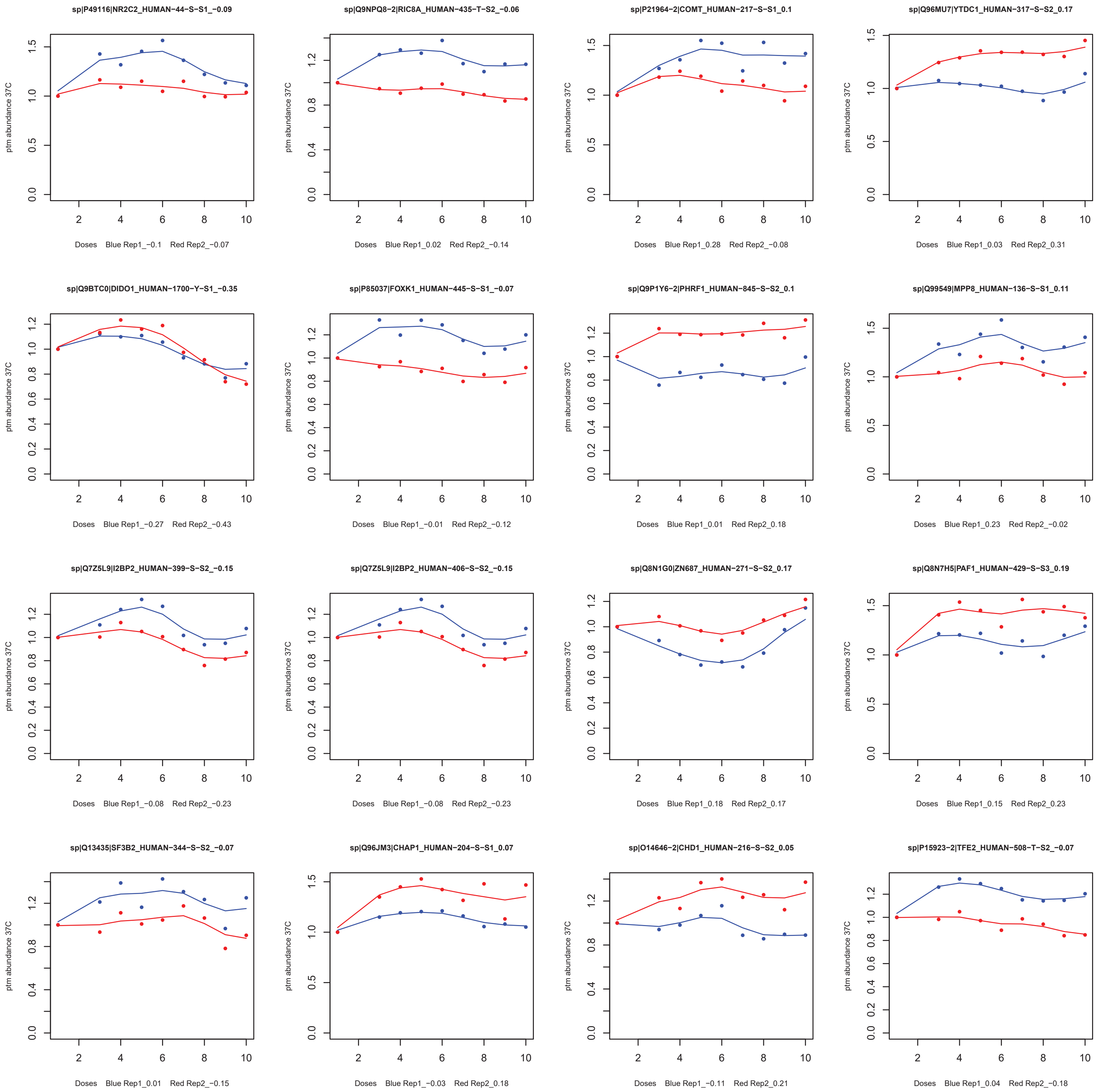
sp|P49116|NR2C2_HUMAN−44−S−S1_−0.09
Doses Blue Rep1_−0.1 Red Rep2_−0.07
sp|Q9NPQ8−2|RIC8A_HUMAN−435−T−S2_−0.06
Doses Blue Rep1_0.02 Red Rep2_−0.14
sp|P21964−2|COMT_HUMAN−217−S−S1_0.1
Doses Blue Rep1_0.28 Red Rep2_−0.08
sp|Q96MU7|YTDC1_HUMAN−317−S−S2_0.17
Doses Blue Rep1_0.03 Red Rep2_0.31
sp|Q9BTC0|DIDO1_HUMAN−1700−Y−S1_−0.35
Doses Blue Rep1_−0.27 Red Rep2_−0.43
sp|P85037|FOXK1_HUMAN−445−S−S1_−0.07
Doses Blue Rep1_−0.01 Red Rep2_−0.12
sp|Q9P1Y6−2|PHRF1_HUMAN−845−S−S2_0.1
Doses Blue Rep1_0.01 Red Rep2_0.18
sp|Q99549|MPP8_HUMAN−136−S−S1_0.11
Doses Blue Rep1_0.23 Red Rep2_−0.02
sp|Q7Z5L9|I2BP2_HUMAN−399−S−S2_−0.15
Doses Blue Rep1_−0.08 Red Rep2_−0.23
sp|Q7Z5L9|I2BP2_HUMAN−406−S−S2_−0.15
Doses Blue Rep1_−0.08 Red Rep2_−0.23
sp|Q8N1G0|ZN687_HUMAN−271−S−S2_0.17
Doses Blue Rep1_0.18 Red Rep2_0.17
sp|Q8N7H5|PAF1_HUMAN−429−S−S3_0.19
Doses Blue Rep1_0.15 Red Rep2_0.23
sp|Q13435|SF3B2_HUMAN−344−S−S2_−0.07
Doses Blue Rep1_0.01 Red Rep2_−0.15
sp|Q96JM3|CHAP1_HUMAN−204−S−S1_0.07
Doses Blue Rep1_−0.03 Red Rep2_0.18
sp|O14646−2|CHD1_HUMAN−216−S−S2_0.05
Doses Blue Rep1_−0.11 Red Rep2_0.21
sp|P15923−2|TFE2_HUMAN−508−T−S2_−0.07
Doses Blue Rep1_0.04 Red Rep2_−0.18
ptm abundance 37C

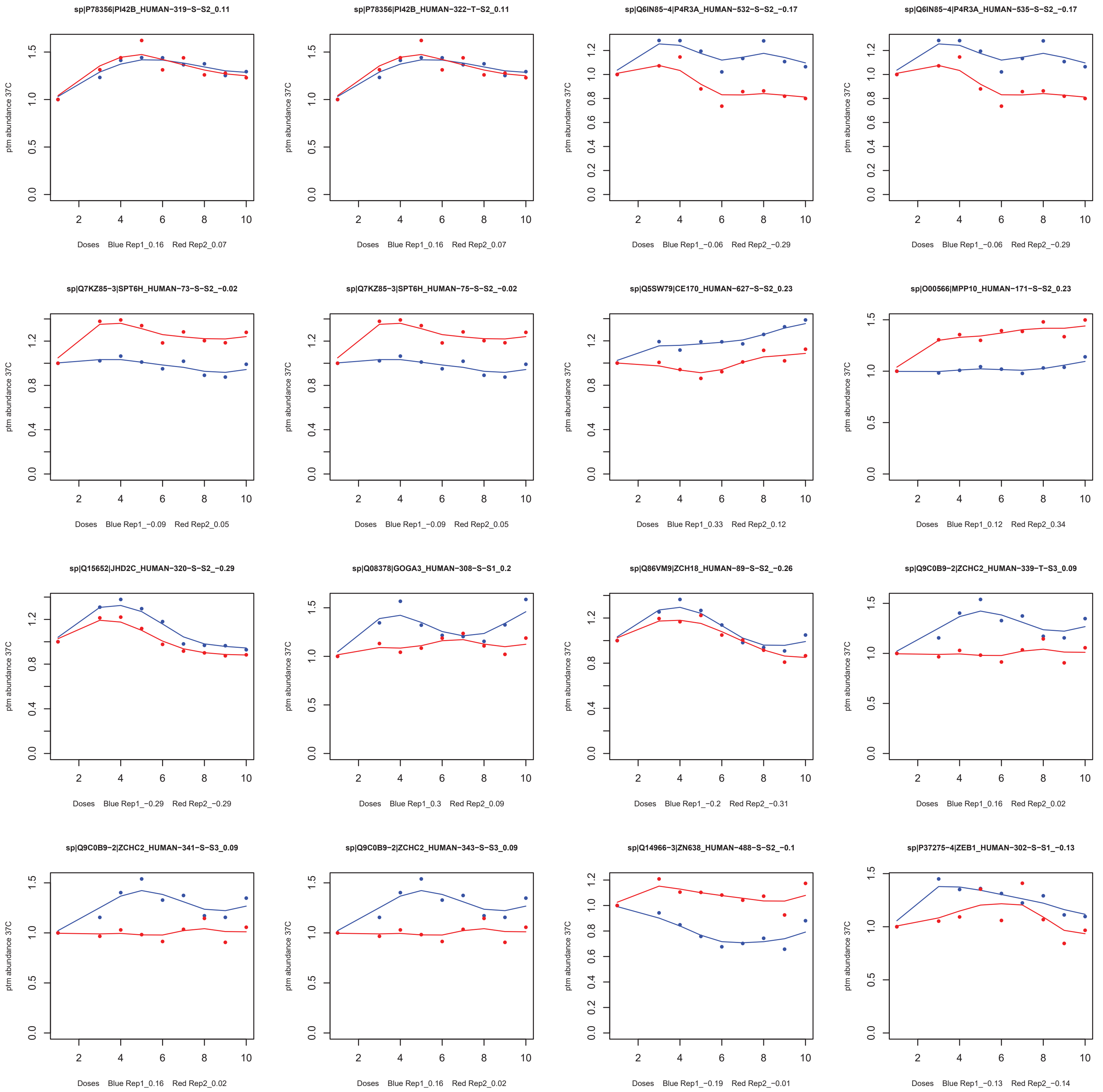

sp|P78356|PI42B_HUMAN−319−S−S2_0.11
Doses Blue Rep1_0.16 Red Rep2_0.07
sp|P78356|PI42B_HUMAN−322−T−S2_0.11
Doses Blue Rep1_0.16 Red Rep2_0.07
sp|Q6IN85−4|P4R3A_HUMAN−532−S−S2_−0.17
Doses Blue Rep1_−0.06 Red Rep2_−0.29
sp|Q6IN85−4|P4R3A_HUMAN−535−S−S2_−0.17
Doses Blue Rep1_−0.06 Red Rep2_−0.29
sp|Q7KZ85−3|SPT6H_HUMAN−73−S−S2_−0.02
Doses Blue Rep1_−0.09 Red Rep2_0.05
sp|Q7KZ85−3|SPT6H_HUMAN−75−S−S2_−0.02
Doses Blue Rep1_−0.09 Red Rep2_0.05
sp|Q5SW79|CE170_HUMAN−627−S−S2_0.23
Doses Blue Rep1_0.33 Red Rep2_0.12
sp|O00566|MPP10_HUMAN−171−S−S2_0.23
Doses Blue Rep1_0.12 Red Rep2_0.34
sp|Q15652|JHD2C_HUMAN−320−S−S2_−0.29
Doses Blue Rep1_−0.29 Red Rep2_−0.29
sp|Q08378|GOGA3_HUMAN−308−S−S1_0.2
Doses Blue Rep1_0.3 Red Rep2_0.09
sp|Q86VM9|ZCH18_HUMAN−89−S−S2_−0.26
Doses Blue Rep1_−0.2 Red Rep2_−0.31
sp|Q9C0B9−2|ZCHC2_HUMAN−339−T−S3_0.09
Doses Blue Rep1_0.16 Red Rep2_0.02
sp|Q9C0B9−2|ZCHC2_HUMAN−341−S−S3_0.09
Doses Blue Rep1_0.16 Red Rep2_0.02
sp|Q9C0B9−2|ZCHC2_HUMAN−343−S−S3_0.09
Doses Blue Rep1_0.16 Red Rep2_0.02
sp|Q14966−3|ZN638_HUMAN−488−S−S2_−0.1
Doses Blue Rep1_−0.19 Red Rep2_−0.01
sp|P37275−4|ZEB1_HUMAN−302−S−S1_−0.13
Doses Blue Rep1_−0.13 Red Rep2_−0.14
ptm abundance 37C

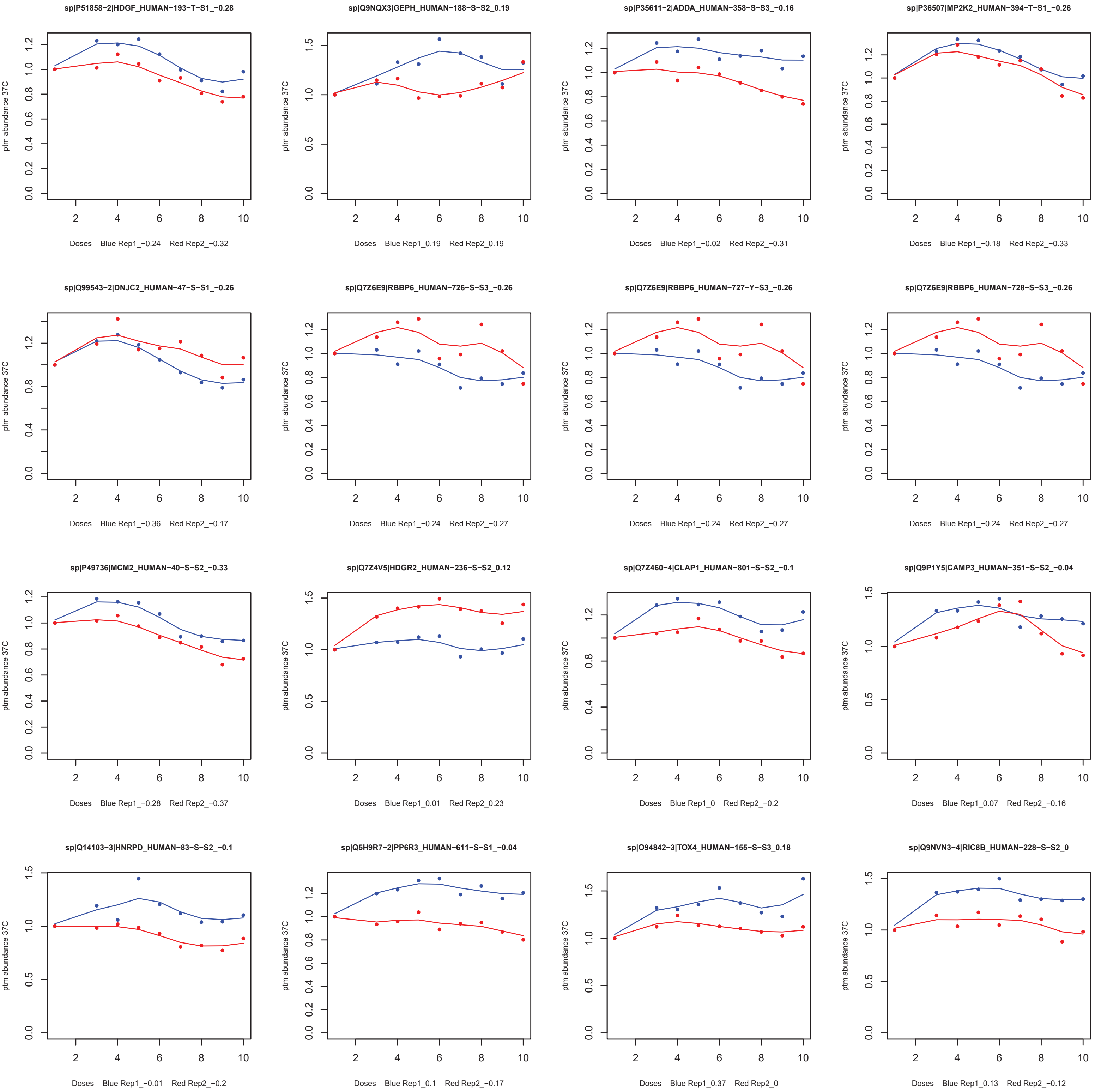

sp|P51858−2|HDGF_HUMAN−193−T−S1_−0.28
ptm abundance 37C
Doses Blue Rep1_−0.24 Red Rep2_−0.32
sp|Q9NQX3|GEPH_HUMAN−188−S−S2_0.19
ptm abundance 37C
Doses Blue Rep1_0.19 Red Rep2_0.19
sp|P35611−2|ADDA_HUMAN−358−S−S3_−0.16
ptm abundance 37C
Doses Blue Rep1_−0.02 Red Rep2_−0.31
sp|P36507|MP2K2_HUMAN−394−T−S1_−0.26
ptm abundance 37C
Doses Blue Rep1_−0.18 Red Rep2_−0.33
sp|Q99543−2|DNJC2_HUMAN−47−S−S1_−0.26
ptm abundance 37C
Doses Blue Rep1_−0.36 Red Rep2_−0.17
sp|Q7Z6E9|RBBP6_HUMAN−726−S−S3_−0.26
ptm abundance 37C
Doses Blue Rep1_−0.24 Red Rep2_−0.27
sp|Q7Z6E9|RBBP6_HUMAN−727−Y−S3_−0.26
ptm abundance 37C
Doses Blue Rep1_−0.24 Red Rep2_−0.27
sp|Q7Z6E9|RBBP6_HUMAN−728−S−S3_−0.26
ptm abundance 37C
Doses Blue Rep1_−0.24 Red Rep2_−0.27
sp|P49736|MCM2_HUMAN−40−S−S2_−0.33
ptm abundance 37C
Doses Blue Rep1_−0.28 Red Rep2_−0.37
sp|Q7Z4V5|HDGR2_HUMAN−236−S−S2_0.12
ptm abundance 37C
Doses Blue Rep1_0.01 Red Rep2_0.23
sp|Q7Z460−4|CLAP1_HUMAN−801−S−S2_−0.1
ptm abundance 37C
Doses Blue Rep1_0 Red Rep2_−0.2
sp|Q9P1Y5|CAMP3_HUMAN−351−S−S2_−0.04
ptm abundance 37C
Doses Blue Rep1_0.07 Red Rep2_−0.16
sp|Q14103−3|HNRPD_HUMAN−83−S−S2_−0.1
ptm abundance 37C
Doses Blue Rep1_−0.01 Red Rep2_−0.2
sp|Q5H9R7−2|PP6R3_HUMAN−611−S−S1_−0.04
ptm abundance 37C
Doses Blue Rep1_0.1 Red Rep2_−0.17
sp|O94842−3|TOX4_HUMAN−155−S−S3_0.18
ptm abundance 37C
Doses Blue Rep1_0.37 Red Rep2_0
sp|Q9NVN3−4|RIC8B_HUMAN−228−S−S2_0
ptm abundance 37C
Doses Blue Rep1_0.13 Red Rep2_−0.12

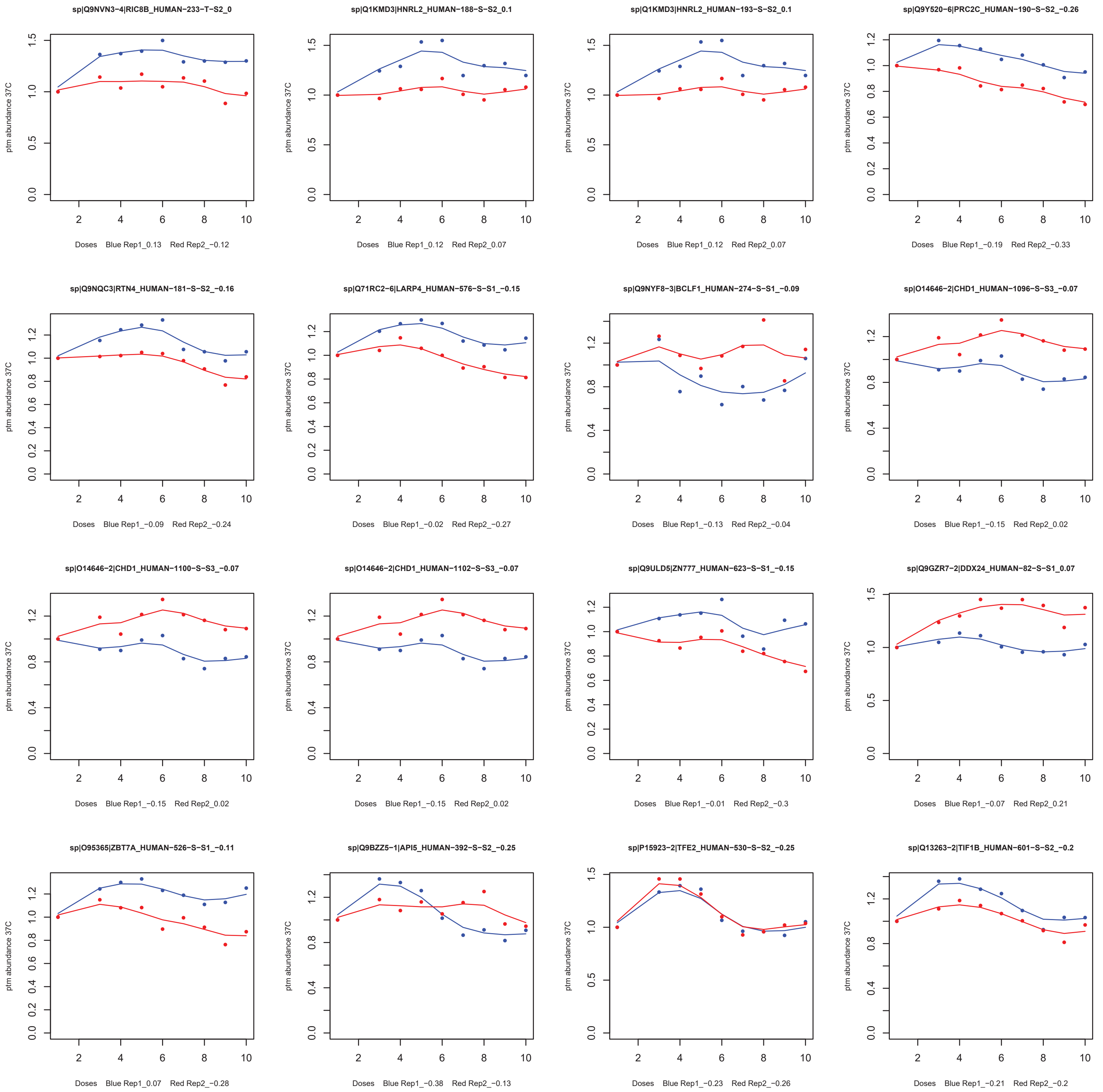
sp|Q9NVN3−4|RIC8B_HUMAN−233−T−S2_0
ptm abundance 37C
Doses Blue Rep1_0.13 Red Rep2_−0.12
sp|Q1KMD3|HNRL2_HUMAN−188−S−S2_0.1
ptm abundance 37C
Doses Blue Rep1_0.12 Red Rep2_0.07
sp|Q1KMD3|HNRL2_HUMAN−193−S−S2_0.1
ptm abundance 37C
Doses Blue Rep1_0.12 Red Rep2_0.07
sp|Q9Y520−6|PRC2C_HUMAN−190−S−S2_−0.26
ptm abundance 37C
Doses Blue Rep1_−0.19 Red Rep2_−0.33
sp|Q9NQC3|RTN4_HUMAN−181−S−S2_−0.16
ptm abundance 37C
Doses Blue Rep1_−0.09 Red Rep2_−0.24
sp|Q71RC2−6|LARP4_HUMAN−576−S−S1_−0.15
ptm abundance 37C
Doses Blue Rep1_−0.02 Red Rep2_−0.27
sp|Q9NYF8−3|BCLF1_HUMAN−274−S−S1_−0.09
ptm abundance 37C
Doses Blue Rep1_−0.13 Red Rep2_−0.04
sp|O14646−2|CHD1_HUMAN−1096−S−S3_−0.07
ptm abundance 37C
Doses Blue Rep1_−0.15 Red Rep2_0.02
sp|O14646−2|CHD1_HUMAN−1100−S−S3_−0.07
ptm abundance 37C
Doses Blue Rep1_−0.15 Red Rep2_0.02
sp|O14646−2|CHD1_HUMAN−1102−S−S3_−0.07
ptm abundance 37C
Doses Blue Rep1_−0.15 Red Rep2_0.02
sp|Q9ULD5|ZN777_HUMAN−623−S−S1_−0.15
ptm abundance 37C
Doses Blue Rep1_−0.01 Red Rep2_−0.3
sp|Q9GZR7−2|DDX24_HUMAN−82−S−S1_0.07
ptm abundance 37C
Doses Blue Rep1_−0.07 Red Rep2_0.21
sp|O95365|ZBT7A_HUMAN−526−S−S1_−0.11
ptm abundance 37C
Doses Blue Rep1_0.07 Red Rep2_−0.28
sp|Q9BZZ5−1|API5_HUMAN−392−S−S2_−0.25
ptm abundance 37C
Doses Blue Rep1_−0.38 Red Rep2_−0.13
sp|P15923−2|TFE2_HUMAN−530−S−S2_−0.25
ptm abundance 37C
Doses Blue Rep1_−0.23 Red Rep2_−0.26
sp|Q13263−2|TIF1B_HUMAN−601−S−S2_−0.2
ptm abundance 37C
Doses Blue Rep1_−0.21 Red Rep2_−0.2

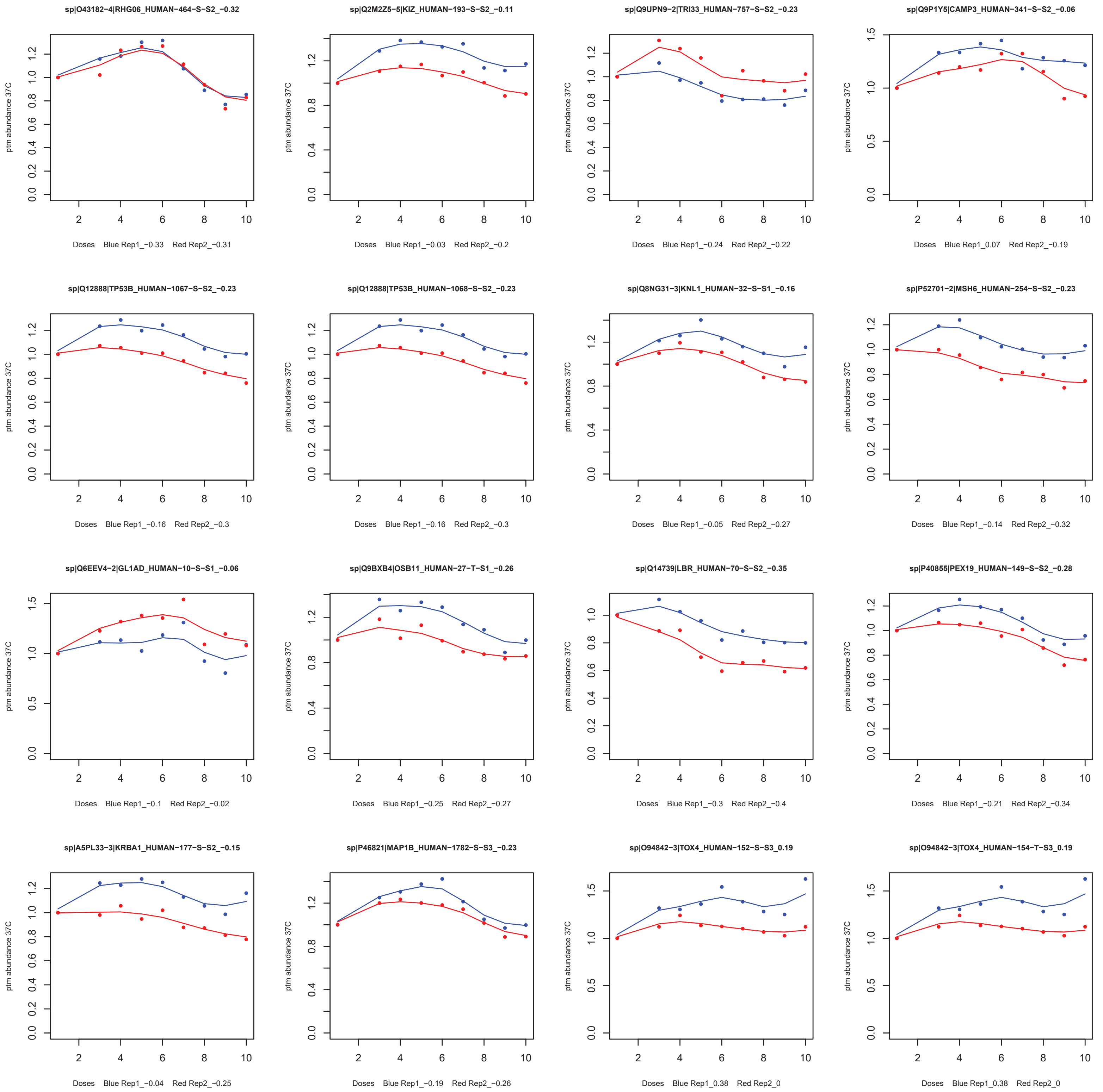
sp|O43182−4|RHG06_HUMAN−464−S−S2_−0.32
ptm abundance 37C
Doses Blue Rep1_−0.33 Red Rep2_−0.31
sp|Q2M2Z5−5|KIZ_HUMAN−193−S−S2_−0.11
ptm abundance 37C
Doses Blue Rep1_−0.03 Red Rep2_−0.2
sp|Q9UPN9−2|TRI33_HUMAN−757−S−S2_−0.23
ptm abundance 37C
Doses Blue Rep1_−0.24 Red Rep2_−0.22
sp|Q9P1Y5|CAMP3_HUMAN−341−S−S2_−0.06
ptm abundance 37C
Doses Blue Rep1_0.07 Red Rep2_−0.19
sp|Q12888|TP53B_HUMAN−1067−S−S2_−0.23
ptm abundance 37C
Doses Blue Rep1_−0.16 Red Rep2_−0.3
sp|Q12888|TP53B_HUMAN−1068−S−S2_−0.23
ptm abundance 37C
Doses Blue Rep1_−0.16 Red Rep2_−0.3
sp|Q8NG31−3|KNL1_HUMAN−32−S−S1_−0.16
ptm abundance 37C
Doses Blue Rep1_−0.05 Red Rep2_−0.27
sp|P52701−2|MSH6_HUMAN−254−S−S2_−0.23
ptm abundance 37C
Doses Blue Rep1_−0.14 Red Rep2_−0.32
sp|Q6EEV4−2|GL1AD_HUMAN−10−S−S1_−0.06
ptm abundance 37C
Doses Blue Rep1_−0.1 Red Rep2_−0.02
sp|Q9BXB4|OSB11_HUMAN−27−T−S1_−0.26
ptm abundance 37C
Doses Blue Rep1_−0.25 Red Rep2_−0.27
sp|Q14739|LBR_HUMAN−70−S−S2_−0.35
ptm abundance 37C
Doses Blue Rep1_−0.3 Red Rep2_−0.4
sp|P40855|PEX19_HUMAN−149−S−S2_−0.28
ptm abundance 37C
Doses Blue Rep1_−0.21 Red Rep2_−0.34
sp|A5PL33−3|KRBA1_HUMAN−177−S−S2_−0.15
ptm abundance 37C
Doses Blue Rep1_−0.04 Red Rep2_−0.25
sp|P46821|MAP1B_HUMAN−1782−S−S3_−0.23
ptm abundance 37C
Doses Blue Rep1_−0.19 Red Rep2_−0.26
sp|O94842−3|TOX4_HUMAN−152−S−S3_0.19
ptm abundance 37C
Doses Blue Rep1_0.38 Red Rep2_0
sp|O94842−3|TOX4_HUMAN−154−T−S3_0.19
ptm abundance 37C
Doses Blue Rep1_0.38 Red Rep2_0

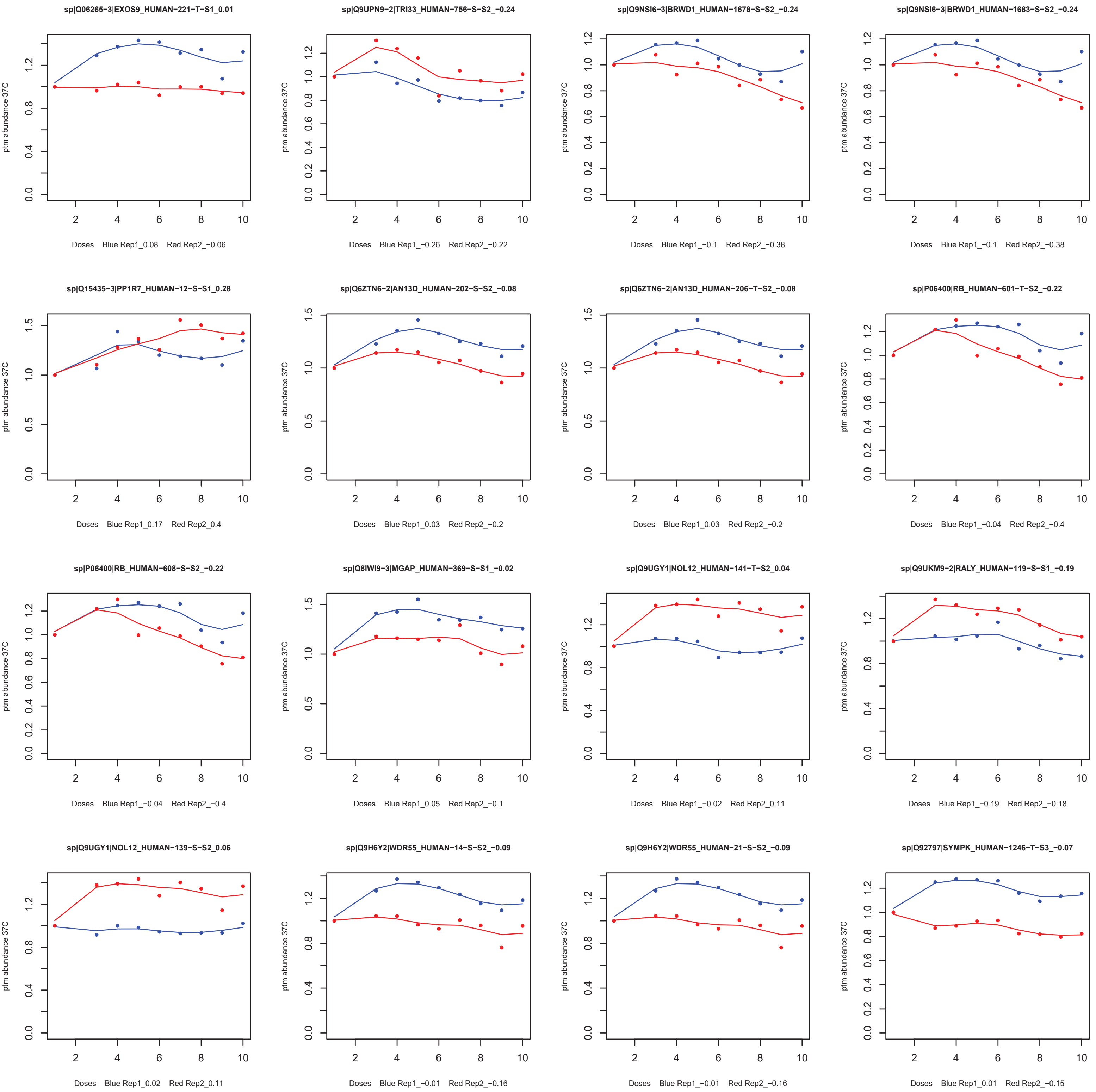

sp|Q06265−3|EXOS9_HUMAN−221−T−S1_0.01
Doses Blue Rep1_0.08 Red Rep2_−0.06
sp|Q9UPN9−2|TRI33_HUMAN−756−S−S2_−0.24
Doses Blue Rep1_−0.26 Red Rep2_−0.22
sp|Q9NSI6−3|BRWD1_HUMAN−1678−S−S2_−0.24
Doses Blue Rep1_−0.1 Red Rep2_−0.38
sp|Q9NSI6−3|BRWD1_HUMAN−1683−S−S2_−0.24
Doses Blue Rep1_−0.1 Red Rep2_−0.38
sp|Q15435−3|PP1R7_HUMAN−12−S−S1_0.28
Doses Blue Rep1_0.17 Red Rep2_0.4
sp|Q6ZTN6−2|AN13D_HUMAN−202−S−S2_−0.08
Doses Blue Rep1_0.03 Red Rep2_−0.2
sp|Q6ZTN6−2|AN13D_HUMAN−206−T−S2_−0.08
Doses Blue Rep1_0.03 Red Rep2_−0.2
sp|P06400|RB_HUMAN−601−T−S2_−0.22
Doses Blue Rep1_−0.04 Red Rep2_−0.4
sp|P06400|RB_HUMAN−608−S−S2_−0.22
Doses Blue Rep1_−0.04 Red Rep2_−0.4
sp|Q8IWI9−3|MGAP_HUMAN−369−S−S1_−0.02
Doses Blue Rep1_0.05 Red Rep2_−0.1
sp|Q9UGY1|NOL12_HUMAN−141−T−S2_0.04
Doses Blue Rep1_−0.02 Red Rep2_0.11
sp|Q9UKM9−2|RALY_HUMAN−119−S−S1_−0.19
Doses Blue Rep1_−0.19 Red Rep2_−0.18
sp|Q9UGY1|NOL12_HUMAN−139−S−S2_0.06
Doses Blue Rep1_0.02 Red Rep2_0.11
sp|Q9H6Y2|WDR55_HUMAN−14−S−S2_−0.09
Doses Blue Rep1_−0.01 Red Rep2_−0.16
sp|Q9H6Y2|WDR55_HUMAN−21−S−S2_−0.09
Doses Blue Rep1_−0.01 Red Rep2_−0.16
sp|Q92797|SYMPK_HUMAN−1246−T−S3_−0.07
Doses Blue Rep1_0.01 Red Rep2_−0.15
ptm abundance 37C

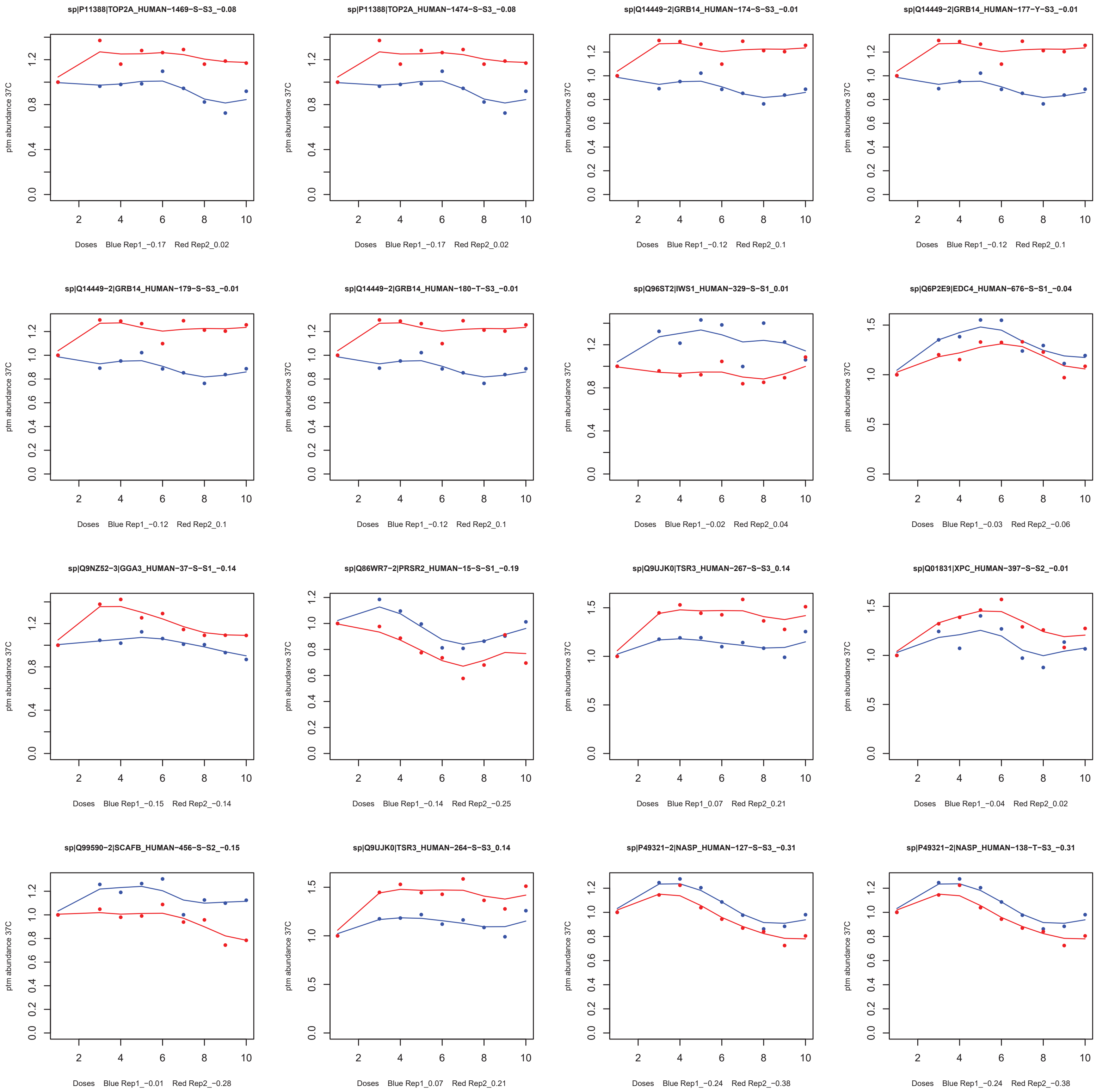
sp|P11388|TOP2A_HUMAN−1469−S−S3_−0.08
Doses Blue Rep1_−0.17 Red Rep2_0.02
sp|P11388|TOP2A_HUMAN−1474−S−S3_−0.08
Doses Blue Rep1_−0.17 Red Rep2_0.02
sp|Q14449−2|GRB14_HUMAN−174−S−S3_−0.01
Doses Blue Rep1_−0.12 Red Rep2_0.1
sp|Q14449−2|GRB14_HUMAN−177−Y−S3_−0.01
Doses Blue Rep1_−0.12 Red Rep2_0.1
sp|Q14449−2|GRB14_HUMAN−179−S−S3_−0.01
Doses Blue Rep1_−0.12 Red Rep2_0.1
sp|Q14449−2|GRB14_HUMAN−180−T−S3_−0.01
Doses Blue Rep1_−0.12 Red Rep2_0.1
sp|Q96ST2|IWS1_HUMAN−329−S−S1_0.01
Doses Blue Rep1_−0.02 Red Rep2_0.04
sp|Q6P2E9|EDC4_HUMAN−676−S−S1_−0.04
Doses Blue Rep1_−0.03 Red Rep2_−0.06
sp|Q9NZ52−3|GGA3_HUMAN−37−S−S1_−0.14
Doses Blue Rep1_−0.15 Red Rep2_−0.14
sp|Q86WR7−2|PRSR2_HUMAN−15−S−S1_−0.19
Doses Blue Rep1_−0.14 Red Rep2_−0.25
sp|Q9UJK0|TSR3_HUMAN−267−S−S3_0.14
Doses Blue Rep1_0.07 Red Rep2_0.21
sp|Q01831|XPC_HUMAN−397−S−S2_−0.01
Doses Blue Rep1_−0.04 Red Rep2_0.02
sp|Q99590−2|SCAFB_HUMAN−456−S−S2_−0.15
Doses Blue Rep1_−0.01 Red Rep2_−0.28
sp|Q9UJK0|TSR3_HUMAN−264−S−S3_0.14
Doses Blue Rep1_0.07 Red Rep2_0.21
sp|P49321−2|NASP_HUMAN−127−S−S3_−0.31
Doses Blue Rep1_−0.24 Red Rep2_−0.38
sp|P49321−2|NASP_HUMAN−138−T−S3_−0.31
Doses Blue Rep1_−0.24 Red Rep2_−0.38
ptm abundance 37C

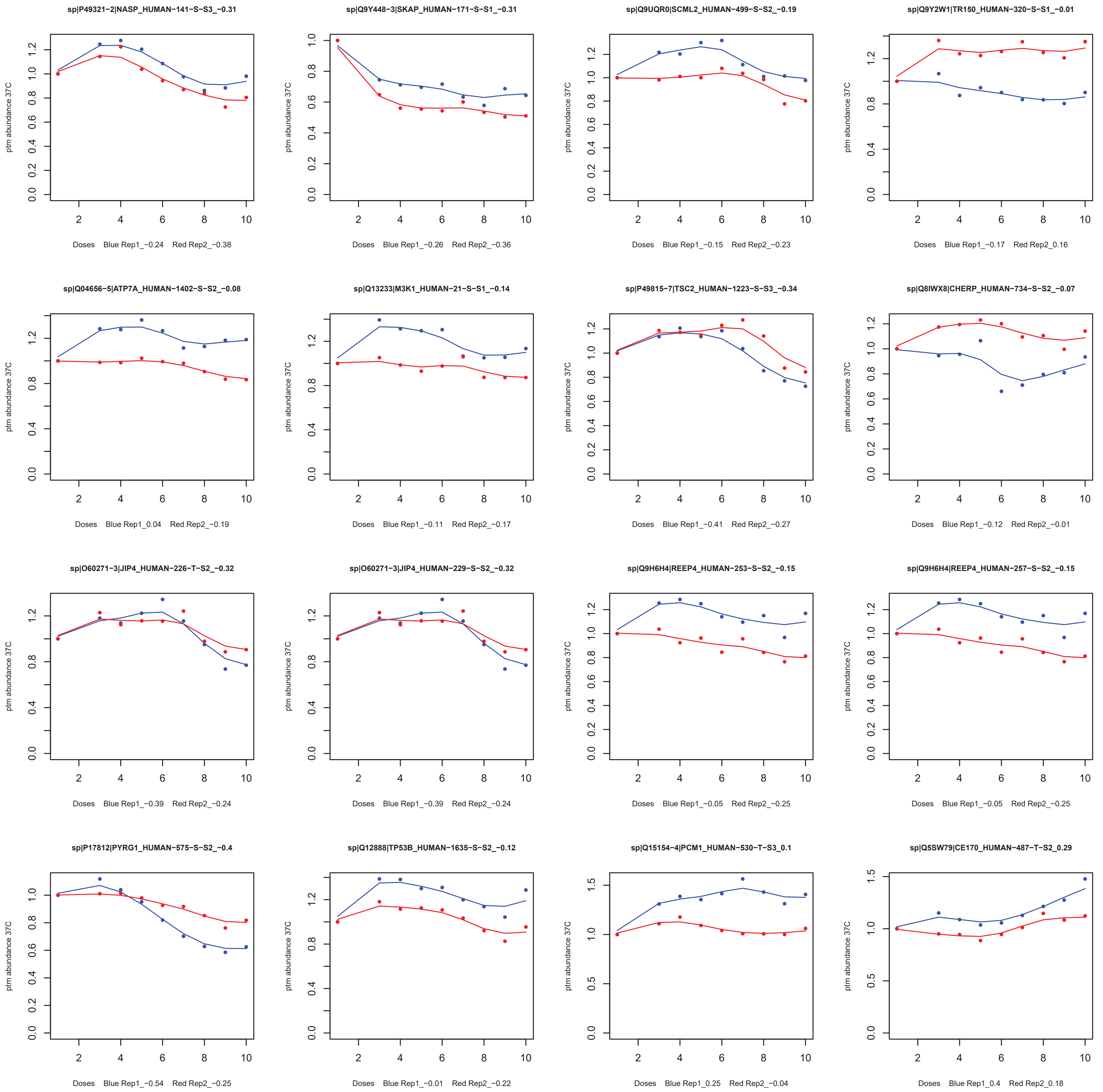

sp|P49321-2|NASP_HUMAN-141-S-S3_-0.31
ptm abundance 37C
Doses Blue Rep1_-0.24 Red Rep2_-0.38
sp|Q9Y448-3|SKAP_HUMAN-171-S-S1_-0.31
ptm abundance 37C
Doses Blue Rep1_-0.26 Red Rep2_-0.36
sp|Q9UQR0|SCML2_HUMAN-499-S-S2_-0.19
ptm abundance 37C
Doses Blue Rep1_-0.15 Red Rep2_-0.23
sp|Q9Y2W1|TR150_HUMAN-320-S-S1_-0.01
ptm abundance 37C
Doses Blue Rep1_-0.17 Red Rep2_0.16
sp|Q04656-5|ATP7A_HUMAN-1402-S-S2_-0.08
ptm abundance 37C
Doses Blue Rep1_0.04 Red Rep2_-0.19
sp|Q13233|M3K1_HUMAN-21-S-S1_-0.14
ptm abundance 37C
Doses Blue Rep1_-0.11 Red Rep2_-0.17
sp|P49815-7|TSC2_HUMAN-1223-S-S3_-0.34
ptm abundance 37C
Doses Blue Rep1_-0.41 Red Rep2_-0.27
sp|Q8IWX8|CHERP_HUMAN-734-S-S2_-0.07
ptm abundance 37C
Doses Blue Rep1_-0.12 Red Rep2_-0.01
sp|O60271-3|JIP4_HUMAN-226-T-S2_-0.32
ptm abundance 37C
Doses Blue Rep1_-0.39 Red Rep2_-0.24
sp|O60271-3|JIP4_HUMAN-229-S-S2_-0.32
ptm abundance 37C
Doses Blue Rep1_-0.39 Red Rep2_-0.24
sp|Q9H6H4|REEP4_HUMAN-253-S-S2_-0.15
ptm abundance 37C
Doses Blue Rep1_-0.05 Red Rep2_-0.25
sp|Q9H6H4|REEP4_HUMAN-257-S-S2_-0.15
ptm abundance 37C
Doses Blue Rep1_-0.05 Red Rep2_-0.25
sp|P17812|PYRG1_HUMAN-575-S-S2_-0.4
ptm abundance 37C
Doses Blue Rep1_-0.54 Red Rep2_-0.25
sp|Q12888|TP53B_HUMAN-1635-S-S2_-0.12
ptm abundance 37C
Doses Blue Rep1_-0.01 Red Rep2_-0.22
sp|Q15154-4|PCM1_HUMAN-530-T-S3_0.1
ptm abundance 37C
Doses Blue Rep1_0.25 Red Rep2_-0.04
sp|Q5SW79|CE170_HUMAN-487-T-S2_0.29
ptm abundance 37C
Doses Blue Rep1_0.4 Red Rep2_0.18

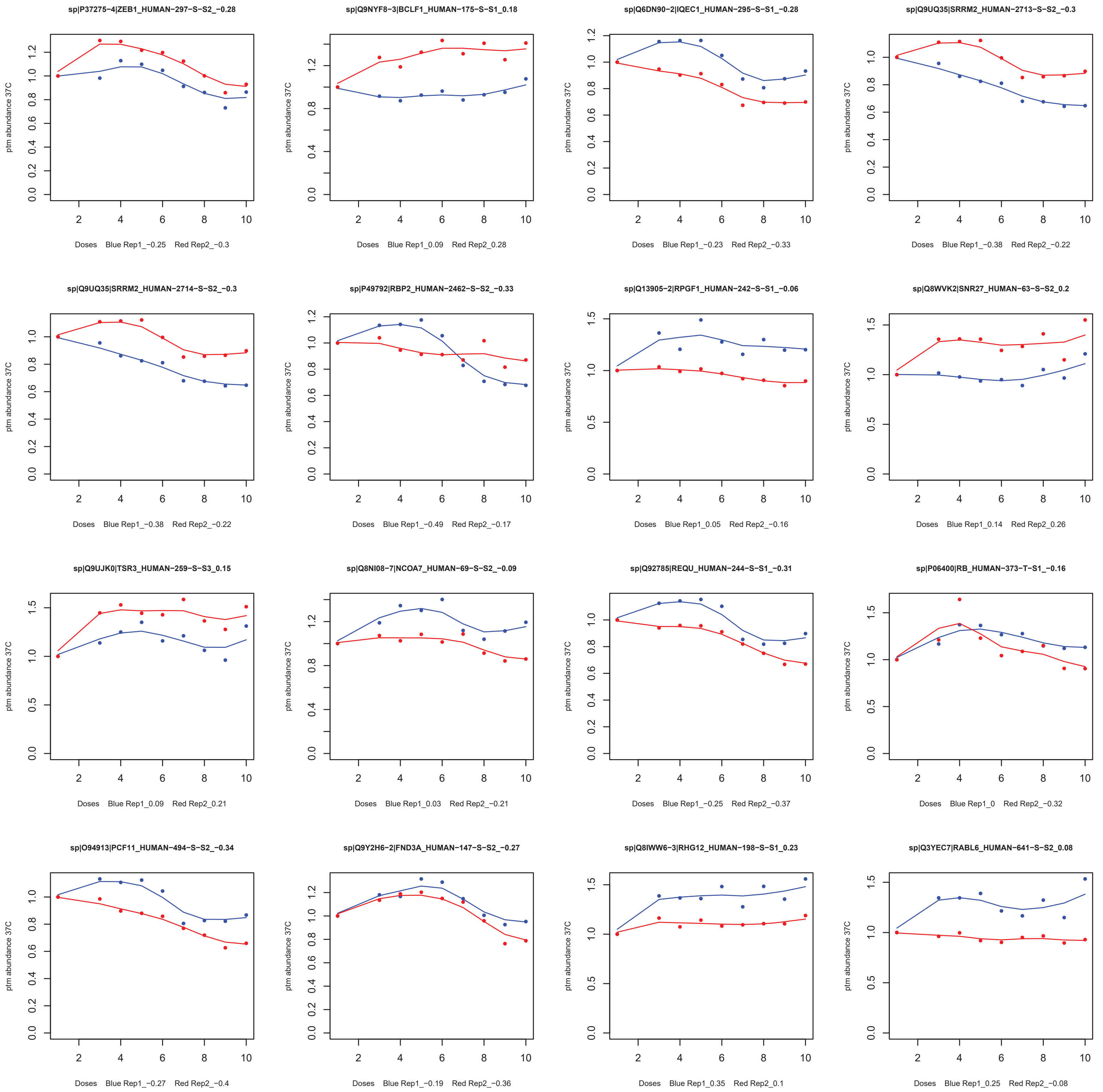
sp|P37275−4|ZEB1_HUMAN−297−S−S2_−0.28
Doses Blue Rep1_−0.25 Red Rep2_−0.3
sp|Q9NYF8−3|BCLF1_HUMAN−175−S−S1_0.18
Doses Blue Rep1_0.09 Red Rep2_0.28
sp|Q6DN90−2|IQEC1_HUMAN−295−S−S1_−0.28
Doses Blue Rep1_−0.23 Red Rep2_−0.33
sp|Q9UQ35|SRRM2_HUMAN−2713−S−S2_−0.3
Doses Blue Rep1_−0.38 Red Rep2_−0.22
sp|Q9UQ35|SRRM2_HUMAN−2714−S−S2_−0.3
Doses Blue Rep1_−0.38 Red Rep2_−0.22
sp|P49792|RBP2_HUMAN−2462−S−S2_−0.33
Doses Blue Rep1_−0.49 Red Rep2_−0.17
sp|Q13905−2|RPGF1_HUMAN−242−S−S1_−0.06
Doses Blue Rep1_0.05 Red Rep2_−0.16
sp|Q8WVK2|SNR27_HUMAN−63−S−S2_0.2
Doses Blue Rep1_0.14 Red Rep2_0.26
sp|Q9UJK0|TSR3_HUMAN−259−S−S3_0.15
Doses Blue Rep1_0.09 Red Rep2_0.21
sp|Q8NI08−7|NCOA7_HUMAN−69−S−S2_−0.09
Doses Blue Rep1_0.03 Red Rep2_−0.21
sp|Q92785|REQU_HUMAN−244−S−S1_−0.31
Doses Blue Rep1_−0.25 Red Rep2_−0.37
sp|P06400|RB_HUMAN−373−T−S1_−0.16
Doses Blue Rep1_0 Red Rep2_−0.32
sp|O94913|PCF11_HUMAN−494−S−S2_−0.34
Doses Blue Rep1_−0.27 Red Rep2_−0.4
sp|Q9Y2H6−2|FND3A_HUMAN−147−S−S2_−0.27
Doses Blue Rep1_−0.19 Red Rep2_−0.36
sp|Q8IWW6−3|RHG12_HUMAN−198−S−S1_0.23
Doses Blue Rep1_0.35 Red Rep2_0.1
sp|Q3YEC7|RABL6_HUMAN−641−S−S2_0.08
Doses Blue Rep1_0.25 Red Rep2_−0.08
ptm abundance 37C

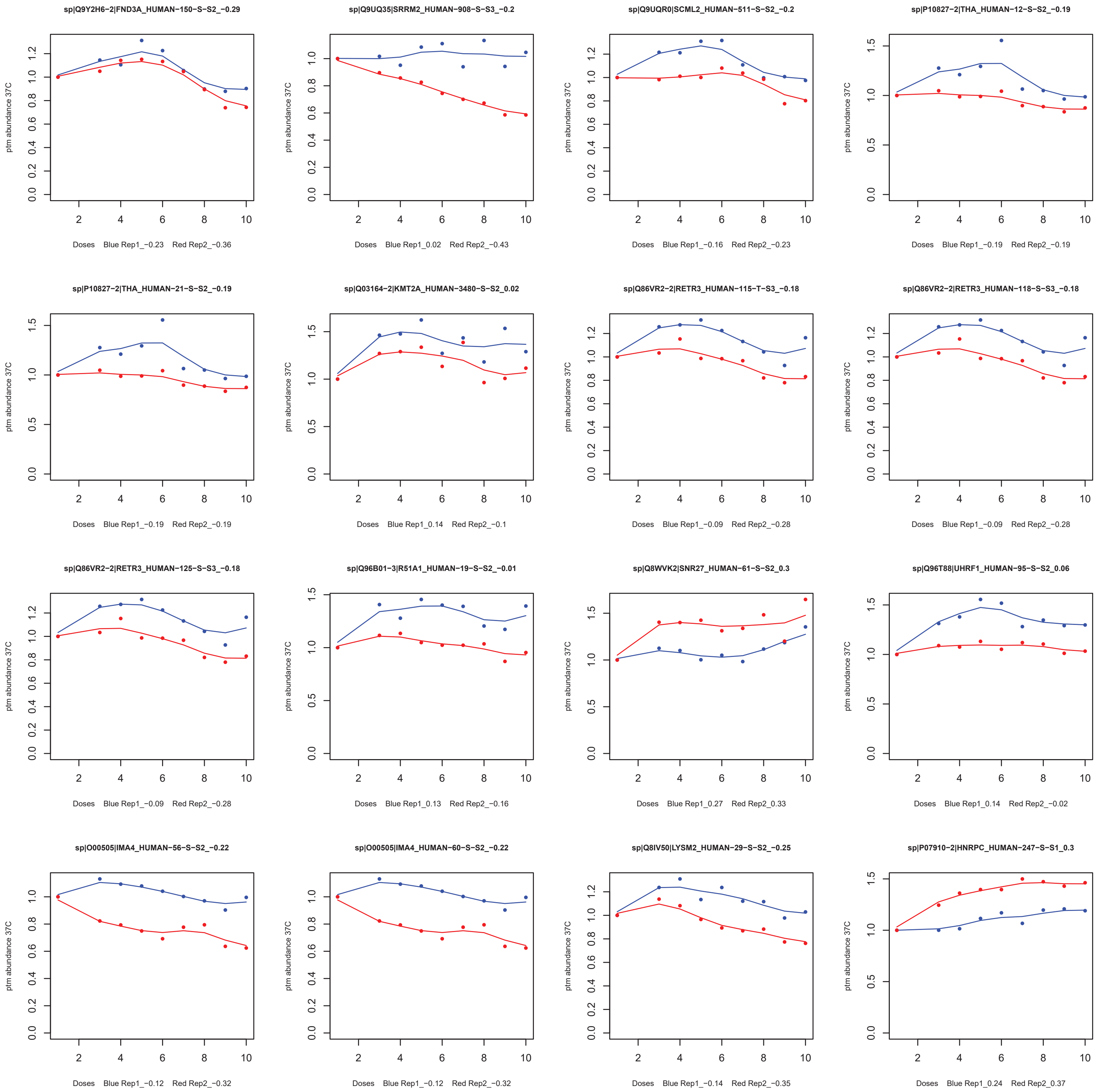
sp|Q9Y2H6−2|FND3A_HUMAN−150−S−S2_−0.29
ptm abundance 37C
Doses Blue Rep1_−0.23 Red Rep2_−0.36
sp|Q9UQ35|SRRM2_HUMAN−908−S−S3_−0.2
ptm abundance 37C
Doses Blue Rep1_0.02 Red Rep2_−0.43
sp|Q9UQR0|SCML2_HUMAN−511−S−S2_−0.2
ptm abundance 37C
Doses Blue Rep1_−0.16 Red Rep2_−0.23
sp|P10827−2|THA_HUMAN−12−S−S2_−0.19
ptm abundance 37C
Doses Blue Rep1_−0.19 Red Rep2_−0.19
sp|P10827−2|THA_HUMAN−21−S−S2_−0.19
ptm abundance 37C
Doses Blue Rep1_−0.19 Red Rep2_−0.19
sp|Q03164−2|KMT2A_HUMAN−3480−S−S2_0.02
ptm abundance 37C
Doses Blue Rep1_0.14 Red Rep2_−0.1
sp|Q86VR2−2|RETR3_HUMAN−115−T−S3_−0.18
ptm abundance 37C
Doses Blue Rep1_−0.09 Red Rep2_−0.28
sp|Q86VR2−2|RETR3_HUMAN−118−S−S3_−0.18
ptm abundance 37C
Doses Blue Rep1_−0.09 Red Rep2_−0.28
sp|Q86VR2−2|RETR3_HUMAN−125−S−S3_−0.18
ptm abundance 37C
Doses Blue Rep1_−0.09 Red Rep2_−0.28
sp|Q96B01−3|R51A1_HUMAN−19−S−S2_−0.01
ptm abundance 37C
Doses Blue Rep1_0.13 Red Rep2_−0.16
sp|Q8WVK2|SNR27_HUMAN−61−S−S2_0.3
ptm abundance 37C
Doses Blue Rep1_0.27 Red Rep2_0.33
sp|Q96T88|UHRF1_HUMAN−95−S−S2_0.06
ptm abundance 37C
Doses Blue Rep1_0.14 Red Rep2_−0.02
sp|O00505|IMA4_HUMAN−56−S−S2_−0.22
ptm abundance 37C
Doses Blue Rep1_−0.12 Red Rep2_−0.32
sp|O00505|IMA4_HUMAN−60−S−S2_−0.22
ptm abundance 37C
Doses Blue Rep1_−0.12 Red Rep2_−0.32
sp|Q8IV50|LYSM2_HUMAN−29−S−S2_−0.25
ptm abundance 37C
Doses Blue Rep1_−0.14 Red Rep2_−0.35
sp|P07910−2|HNRPC_HUMAN−247−S−S1_0.3
ptm abundance 37C
Doses Blue Rep1_0.24 Red Rep2_0.37

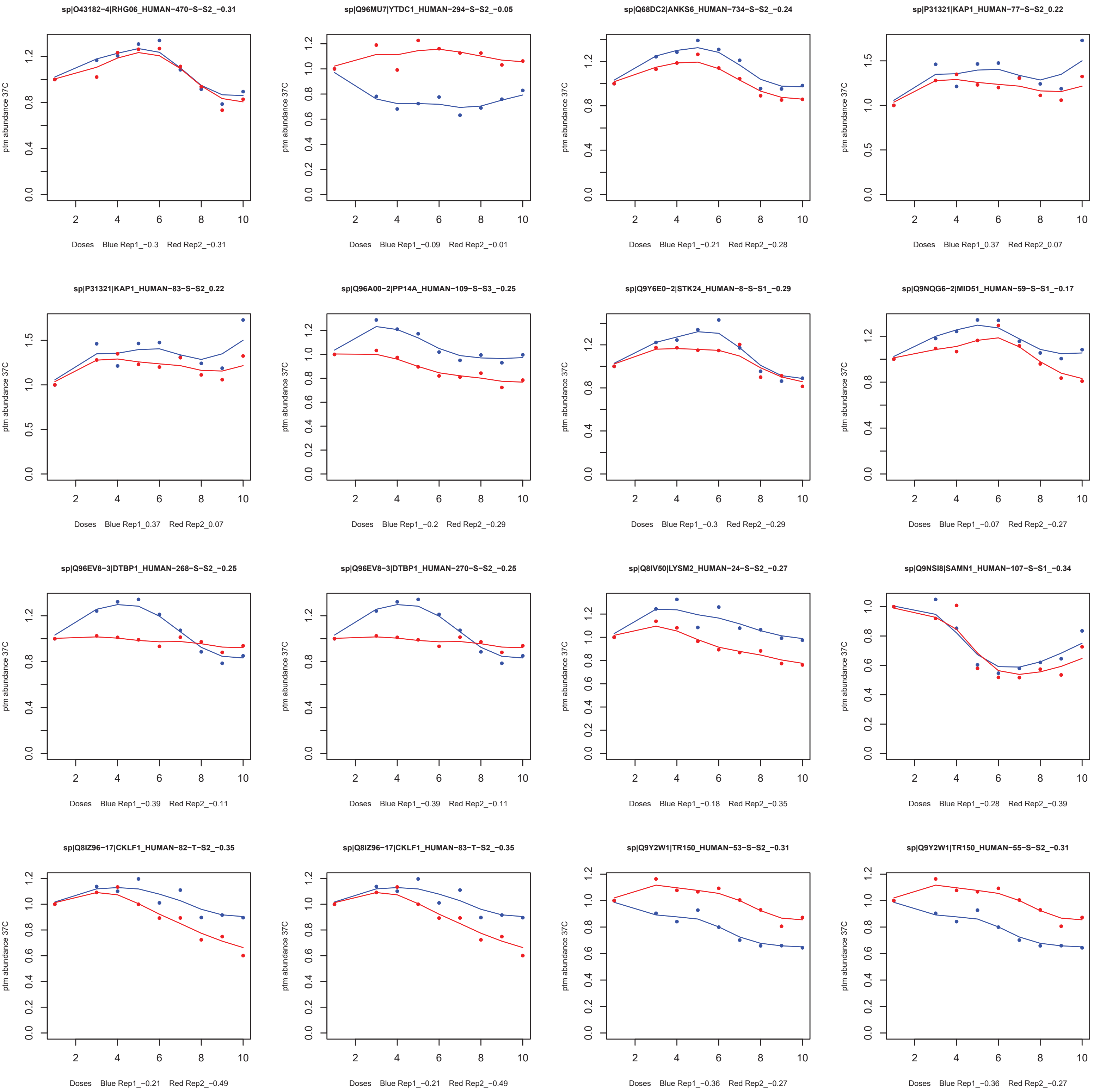
sp|O43182−4|RHG06_HUMAN−470−S−S2_−0.31
ptm abundance 37C
Doses Blue Rep1_−0.3 Red Rep2_−0.31
sp|Q96MU7|YTDC1_HUMAN−294−S−S2_−0.05
ptm abundance 37C
Doses Blue Rep1_−0.09 Red Rep2_−0.01
sp|Q68DC2|ANKS6_HUMAN−734−S−S2_−0.24
ptm abundance 37C
Doses Blue Rep1_−0.21 Red Rep2_−0.28
sp|P31321|KAP1_HUMAN−77−S−S2_0.22
ptm abundance 37C
Doses Blue Rep1_0.37 Red Rep2_0.07
sp|P31321|KAP1_HUMAN−83−S−S2_0.22
ptm abundance 37C
Doses Blue Rep1_0.37 Red Rep2_0.07
sp|Q96A00−2|PP14A_HUMAN−109−S−S3_−0.25
ptm abundance 37C
Doses Blue Rep1_−0.2 Red Rep2_−0.29
sp|Q9Y6E0−2|STK24_HUMAN−8−S−S1_−0.29
ptm abundance 37C
Doses Blue Rep1_−0.3 Red Rep2_−0.29
sp|Q9NQG6−2|MID51_HUMAN−59−S−S1_−0.17
ptm abundance 37C
Doses Blue Rep1_−0.07 Red Rep2_−0.27
sp|Q96EV8−3|DTBP1_HUMAN−268−S−S2_−0.25
ptm abundance 37C
Doses Blue Rep1_−0.39 Red Rep2_−0.11
sp|Q96EV8−3|DTBP1_HUMAN−270−S−S2_−0.25
ptm abundance 37C
Doses Blue Rep1_−0.39 Red Rep2_−0.11
sp|Q8IV50|LYSM2_HUMAN−24−S−S2_−0.27
ptm abundance 37C
Doses Blue Rep1_−0.18 Red Rep2_−0.35
sp|Q9NSI8|SAMN1_HUMAN−107−S−S1_−0.34
ptm abundance 37C
Doses Blue Rep1_−0.28 Red Rep2_−0.39
sp|Q8IZ96−17|CKLF1_HUMAN−82−T−S2_−0.35
ptm abundance 37C
Doses Blue Rep1_−0.21 Red Rep2_−0.49
sp|Q8IZ96−17|CKLF1_HUMAN−83−T−S2_−0.35
ptm abundance 37C
Doses Blue Rep1_−0.21 Red Rep2_−0.49
sp|Q9Y2W1|TR150_HUMAN−53−S−S2_−0.31
ptm abundance 37C
Doses Blue Rep1_−0.36 Red Rep2_−0.27
sp|Q9Y2W1|TR150_HUMAN−55−S−S2_−0.31
ptm abundance 37C
Doses Blue Rep1_−0.36 Red Rep2_−0.27

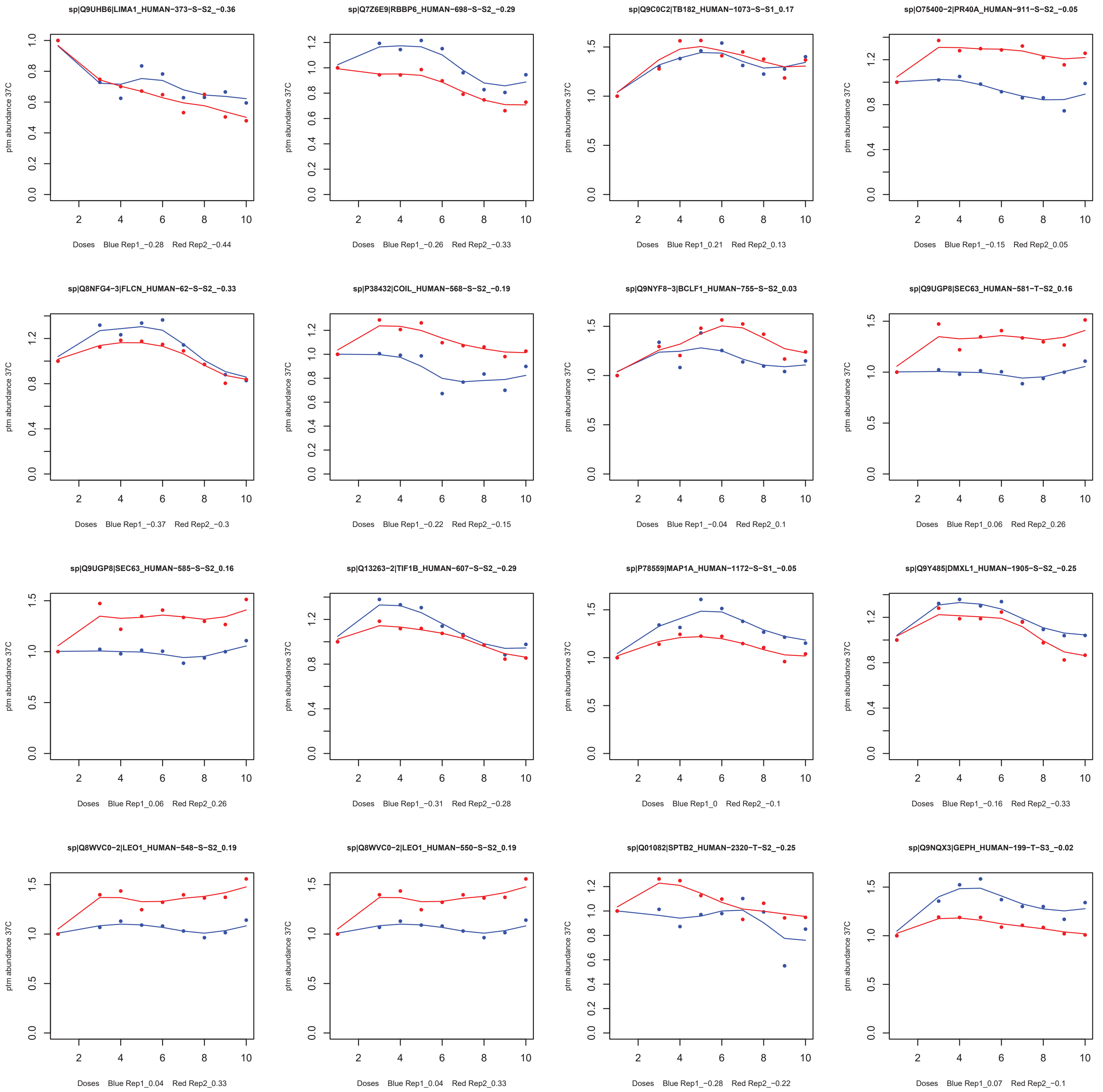
sp|Q9UHB6|LIMA1_HUMAN−373−S−S2_−0.36
ptm abundance 37C
Doses Blue Rep1_−0.28 Red Rep2_−0.44
sp|Q7Z6E9|RBBP6_HUMAN−698−S−S2_−0.29
ptm abundance 37C
Doses Blue Rep1_−0.26 Red Rep2_−0.33
sp|Q9C0C2|TB182_HUMAN−1073−S−S1_0.17
ptm abundance 37C
Doses Blue Rep1_0.21 Red Rep2_0.13
sp|O75400−2|PR40A_HUMAN−911−S−S2_−0.05
ptm abundance 37C
Doses Blue Rep1_−0.15 Red Rep2_0.05
sp|Q8NFG4−3|FLCN_HUMAN−62−S−S2_−0.33
ptm abundance 37C
Doses Blue Rep1_−0.37 Red Rep2_−0.3
sp|P38432|COIL_HUMAN−568−S−S2_−0.19
ptm abundance 37C
Doses Blue Rep1_−0.22 Red Rep2_−0.15
sp|Q9NYF8−3|BCLF1_HUMAN−755−S−S2_0.03
ptm abundance 37C
Doses Blue Rep1_−0.04 Red Rep2_0.1
sp|Q9UGP8|SEC63_HUMAN−581−T−S2_0.16
ptm abundance 37C
Doses Blue Rep1_0.06 Red Rep2_0.26
sp|Q9UGP8|SEC63_HUMAN−585−S−S2_0.16
ptm abundance 37C
Doses Blue Rep1_0.06 Red Rep2_0.26
sp|Q13263−2|TIF1B_HUMAN−607−S−S2_−0.29
ptm abundance 37C
Doses Blue Rep1_−0.31 Red Rep2_−0.28
sp|P78559|MAP1A_HUMAN−1172−S−S1_−0.05
ptm abundance 37C
Doses Blue Rep1_0 Red Rep2_−0.1
sp|Q9Y485|DMXL1_HUMAN−1905−S−S2_−0.25
ptm abundance 37C
Doses Blue Rep1_−0.16 Red Rep2_−0.33
sp|Q8WVC0−2|LEO1_HUMAN−548−S−S2_0.19
ptm abundance 37C
Doses Blue Rep1_0.04 Red Rep2_0.33
sp|Q8WVC0−2|LEO1_HUMAN−550−S−S2_0.19
ptm abundance 37C
Doses Blue Rep1_0.04 Red Rep2_0.33
sp|Q01082|SPTB2_HUMAN−2320−T−S2_−0.25
ptm abundance 37C
Doses Blue Rep1_−0.28 Red Rep2_−0.22
sp|Q9NQX3|GEPH_HUMAN−199−T−S3_−0.02
ptm abundance 37C
Doses Blue Rep1_0.07 Red Rep2_−0.1

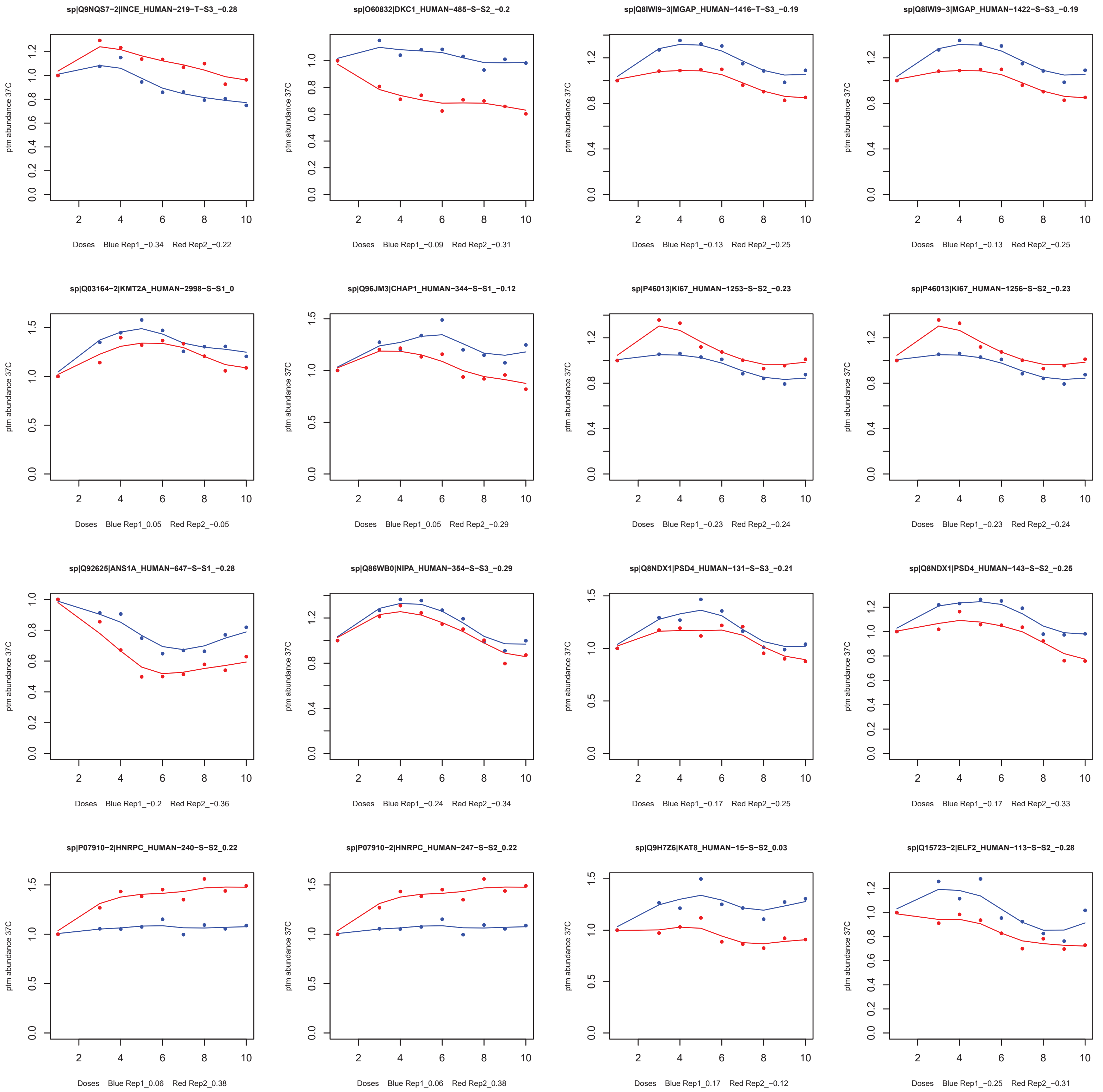
sp|Q9NQS7−2|INCE_HUMAN−219−T−S3_−0.28
Doses Blue Rep1_−0.34 Red Rep2_−0.22
sp|O60832|DKC1_HUMAN−485−S−S2_−0.2
Doses Blue Rep1_−0.09 Red Rep2_−0.31
sp|Q8IWI9−3|MGAP_HUMAN−1416−T−S3_−0.19
Doses Blue Rep1_−0.13 Red Rep2_−0.25
sp|Q8IWI9−3|MGAP_HUMAN−1422−S−S3_−0.19
Doses Blue Rep1_−0.13 Red Rep2_−0.25
sp|Q03164−2|KMT2A_HUMAN−2998−S−S1_0
Doses Blue Rep1_0.05 Red Rep2_−0.05
sp|Q96JM3|CHAP1_HUMAN−344−S−S1_−0.12
Doses Blue Rep1_0.05 Red Rep2_−0.29
sp|P46013|KI67_HUMAN−1253−S−S2_−0.23
Doses Blue Rep1_−0.23 Red Rep2_−0.24
sp|P46013|KI67_HUMAN−1256−S−S2_−0.23
Doses Blue Rep1_−0.23 Red Rep2_−0.24
sp|Q92625|ANS1A_HUMAN−647−S−S1_−0.28
Doses Blue Rep1_−0.2 Red Rep2_−0.36
sp|Q86WB0|NIPA_HUMAN−354−S−S3_−0.29
Doses Blue Rep1_−0.24 Red Rep2_−0.34
sp|Q8NDX1|PSD4_HUMAN−131−S−S3_−0.21
Doses Blue Rep1_−0.17 Red Rep2_−0.25
sp|Q8NDX1|PSD4_HUMAN−143−S−S2_−0.25
Doses Blue Rep1_−0.17 Red Rep2_−0.33
sp|P07910−2|HNRPC_HUMAN−240−S−S2_0.22
Doses Blue Rep1_0.06 Red Rep2_0.38
sp|P07910−2|HNRPC_HUMAN−247−S−S2_0.22
Doses Blue Rep1_0.06 Red Rep2_0.38
sp|Q9H7Z6|KAT8_HUMAN−15−S−S2_0.03
Doses Blue Rep1_0.17 Red Rep2_−0.12
sp|Q15723−2|ELF2_HUMAN−113−S−S2_−0.28
Doses Blue Rep1_−0.25 Red Rep2_−0.31
ptm abundance 37C

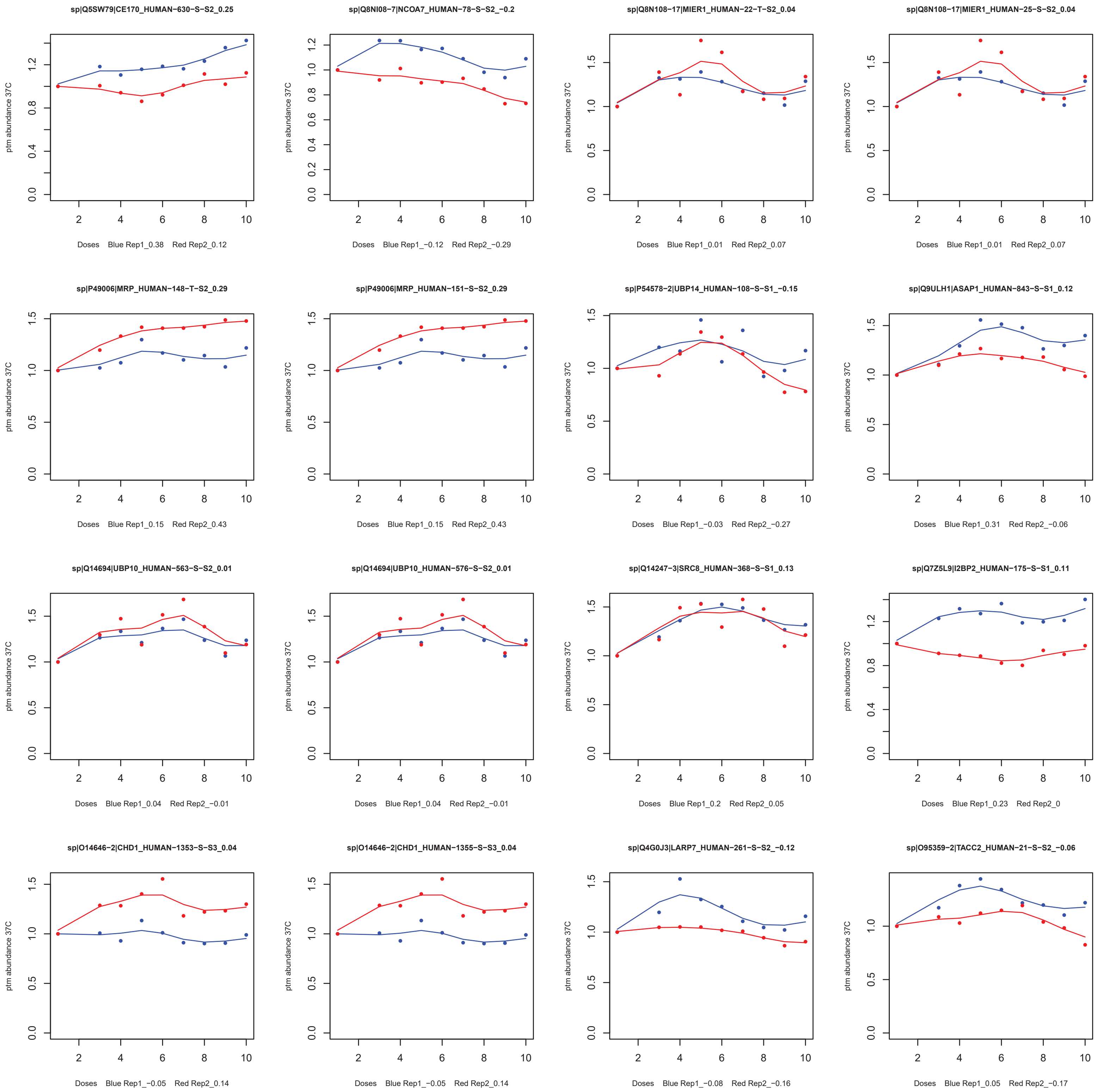
sp|Q5SW79|CE170_HUMAN−630−S−S2_0.25
ptm abundance 37C
Doses Blue Rep1_0.38 Red Rep2_0.12
sp|Q8NI08−7|NCOA7_HUMAN−78−S−S2_−0.2
ptm abundance 37C
Doses Blue Rep1_−0.12 Red Rep2_−0.29
sp|Q8N108−17|MIER1_HUMAN−22−T−S2_0.04
ptm abundance 37C
Doses Blue Rep1_0.01 Red Rep2_0.07
sp|Q8N108−17|MIER1_HUMAN−25−S−S2_0.04
ptm abundance 37C
Doses Blue Rep1_0.01 Red Rep2_0.07
sp|P49006|MRP_HUMAN−148−T−S2_0.29
ptm abundance 37C
Doses Blue Rep1_0.15 Red Rep2_0.43
sp|P49006|MRP_HUMAN−151−S−S2_0.29
ptm abundance 37C
Doses Blue Rep1_0.15 Red Rep2_0.43
sp|P54578−2|UBP14_HUMAN−108−S−S1_−0.15
ptm abundance 37C
Doses Blue Rep1_−0.03 Red Rep2_−0.27
sp|Q9ULH1|ASAP1_HUMAN−843−S−S1_0.12
ptm abundance 37C
Doses Blue Rep1_0.31 Red Rep2_−0.06
sp|Q14694|UBP10_HUMAN−563−S−S2_0.01
ptm abundance 37C
Doses Blue Rep1_0.04 Red Rep2_−0.01
sp|Q14694|UBP10_HUMAN−576−S−S2_0.01
ptm abundance 37C
Doses Blue Rep1_0.04 Red Rep2_−0.01
sp|Q14247−3|SRC8_HUMAN−368−S−S1_0.13
ptm abundance 37C
Doses Blue Rep1_0.2 Red Rep2_0.05
sp|Q7Z5L9|I2BP2_HUMAN−175−S−S1_0.11
ptm abundance 37C
Doses Blue Rep1_0.23 Red Rep2_0
sp|O14646−2|CHD1_HUMAN−1353−S−S3_0.04
ptm abundance 37C
Doses Blue Rep1_−0.05 Red Rep2_0.14
sp|O14646−2|CHD1_HUMAN−1355−S−S3_0.04
ptm abundance 37C
Doses Blue Rep1_−0.05 Red Rep2_0.14
sp|Q4G0J3|LARP7_HUMAN−261−S−S2_−0.12
ptm abundance 37C
Doses Blue Rep1_−0.08 Red Rep2_−0.16
sp|O95359−2|TACC2_HUMAN−21−S−S2_−0.06
ptm abundance 37C
Doses Blue Rep1_0.05 Red Rep2_−0.17

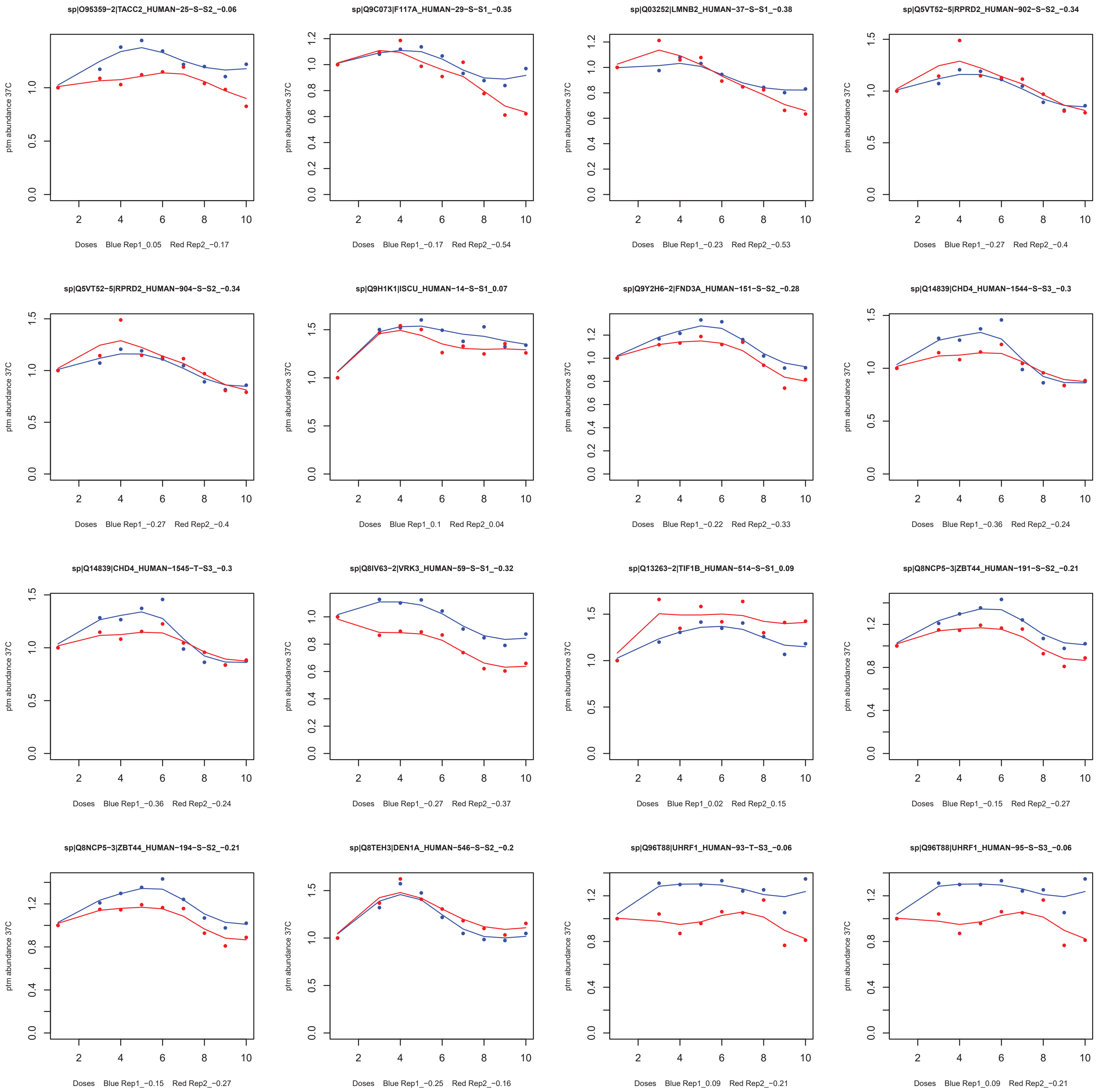
sp|O95359−2|TACC2_HUMAN−25−S−S2_−0.06
ptm abundance 37C
Doses Blue Rep1_0.05 Red Rep2_−0.17
sp|Q9C073|F117A_HUMAN−29−S−S1_−0.35
ptm abundance 37C
Doses Blue Rep1_−0.17 Red Rep2_−0.54
sp|Q03252|LMNB2_HUMAN−37−S−S1_−0.38
ptm abundance 37C
Doses Blue Rep1_−0.23 Red Rep2_−0.53
sp|Q5VT52−5|RPRD2_HUMAN−902−S−S2_−0.34
ptm abundance 37C
Doses Blue Rep1_−0.27 Red Rep2_−0.4
sp|Q5VT52−5|RPRD2_HUMAN−904−S−S2_−0.34
ptm abundance 37C
Doses Blue Rep1_−0.27 Red Rep2_−0.4
sp|Q9H1K1|ISCU_HUMAN−14−S−S1_0.07
ptm abundance 37C
Doses Blue Rep1_0.1 Red Rep2_0.04
sp|Q9Y2H6−2|FND3A_HUMAN−151−S−S2_−0.28
ptm abundance 37C
Doses Blue Rep1_−0.22 Red Rep2_−0.33
sp|Q14839|CHD4_HUMAN−1544−S−S3_−0.3
ptm abundance 37C
Doses Blue Rep1_−0.36 Red Rep2_−0.24
sp|Q14839|CHD4_HUMAN−1545−T−S3_−0.3
ptm abundance 37C
Doses Blue Rep1_−0.36 Red Rep2_−0.24
sp|Q8IV63−2|VRK3_HUMAN−59−S−S1_−0.32
ptm abundance 37C
Doses Blue Rep1_−0.27 Red Rep2_−0.37
sp|Q13263−2|TIF1B_HUMAN−514−S−S1_0.09
ptm abundance 37C
Doses Blue Rep1_0.02 Red Rep2_0.15
sp|Q8NCP5−3|ZBT44_HUMAN−191−S−S2_−0.21
ptm abundance 37C
Doses Blue Rep1_−0.15 Red Rep2_−0.27
sp|Q8NCP5−3|ZBT44_HUMAN−194−S−S2_−0.21
ptm abundance 37C
Doses Blue Rep1_−0.15 Red Rep2_−0.27
sp|Q8TEH3|DEN1A_HUMAN−546−S−S2_−0.2
ptm abundance 37C
Doses Blue Rep1_−0.25 Red Rep2_−0.16
sp|Q96T88|UHRF1_HUMAN−93−T−S3_−0.06
ptm abundance 37C
Doses Blue Rep1_0.09 Red Rep2_−0.21
sp|Q96T88|UHRF1_HUMAN−95−S−S3_−0.06
ptm abundance 37C
Doses Blue Rep1_0.09 Red Rep2_−0.21

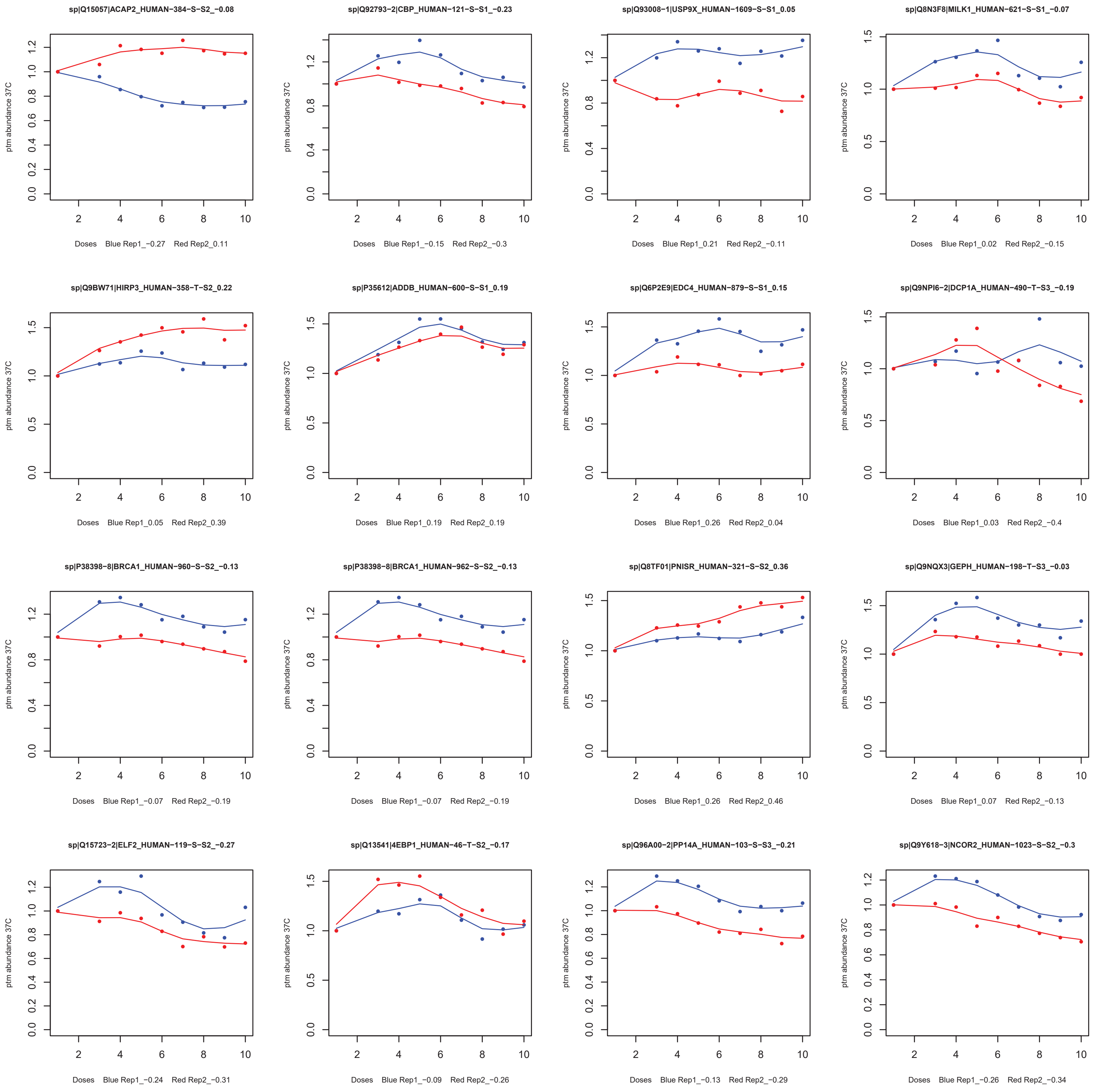
sp|Q15057|ACAP2_HUMAN−384−S−S2_−0.08
Doses Blue Rep1_−0.27 Red Rep2_0.11
sp|Q92793−2|CBP_HUMAN−121−S−S1_−0.23
Doses Blue Rep1_−0.15 Red Rep2_−0.3
sp|Q93008−1|USP9X_HUMAN−1609−S−S1_0.05
Doses Blue Rep1_0.21 Red Rep2_−0.11
sp|Q8N3F8|MILK1_HUMAN−621−S−S1_−0.07
Doses Blue Rep1_0.02 Red Rep2_−0.15
sp|Q9BW71|HIRP3_HUMAN−358−T−S2_0.22
Doses Blue Rep1_0.05 Red Rep2_0.39
sp|P35612|ADDB_HUMAN−600−S−S1_0.19
Doses Blue Rep1_0.19 Red Rep2_0.19
sp|Q6P2E9|EDC4_HUMAN−879−S−S1_0.15
Doses Blue Rep1_0.26 Red Rep2_0.04
sp|Q9NPI6−2|DCP1A_HUMAN−490−T−S3_−0.19
Doses Blue Rep1_0.03 Red Rep2_−0.4
sp|P38398−8|BRCA1_HUMAN−960−S−S2_−0.13
Doses Blue Rep1_−0.07 Red Rep2_−0.19
sp|P38398−8|BRCA1_HUMAN−962−S−S2_−0.13
Doses Blue Rep1_−0.07 Red Rep2_−0.19
sp|Q8TF01|PNISR_HUMAN−321−S−S2_0.36
Doses Blue Rep1_0.26 Red Rep2_0.46
sp|Q9NQX3|GEPH_HUMAN−198−T−S3_−0.03
Doses Blue Rep1_0.07 Red Rep2_−0.13
sp|Q15723−2|ELF2_HUMAN−119−S−S2_−0.27
Doses Blue Rep1_−0.24 Red Rep2_−0.31
sp|Q13541|4EBP1_HUMAN−46−T−S2_−0.17
Doses Blue Rep1_−0.09 Red Rep2_−0.26
sp|Q96A00−2|PP14A_HUMAN−103−S−S3_−0.21
Doses Blue Rep1_−0.13 Red Rep2_−0.29
sp|Q9Y618−3|NCOR2_HUMAN−1023−S−S2_−0.3
Doses Blue Rep1_−0.26 Red Rep2_−0.34
ptm abundance 37C

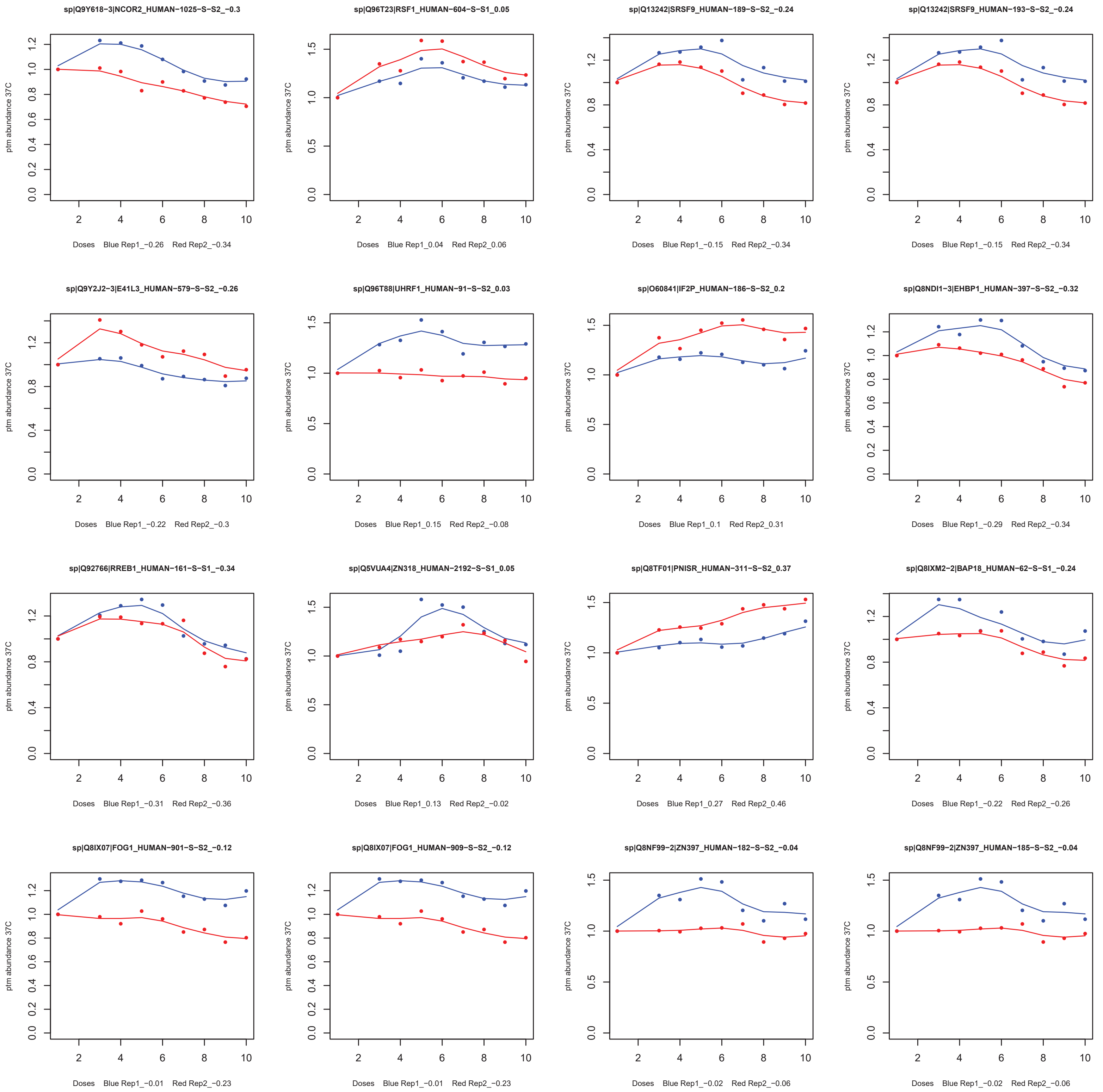

sp|Q9Y618−3|NCOR2_HUMAN−1025−S−S2_−0.3
ptm abundance 37C
Doses Blue Rep1_−0.26 Red Rep2_−0.34
sp|Q96T23|RSF1_HUMAN−604−S−S1_0.05
ptm abundance 37C
Doses Blue Rep1_0.04 Red Rep2_0.06
sp|Q13242|SRSF9_HUMAN−189−S−S2_−0.24
ptm abundance 37C
Doses Blue Rep1_−0.15 Red Rep2_−0.34
sp|Q13242|SRSF9_HUMAN−193−S−S2_−0.24
ptm abundance 37C
Doses Blue Rep1_−0.15 Red Rep2_−0.34
sp|Q9Y2J2−3|E41L3_HUMAN−579−S−S2_−0.26
ptm abundance 37C
Doses Blue Rep1_−0.22 Red Rep2_−0.3
sp|Q96T88|UHRF1_HUMAN−91−S−S2_0.03
ptm abundance 37C
Doses Blue Rep1_0.15 Red Rep2_−0.08
sp|O60841|IF2P_HUMAN−186−S−S2_0.2
ptm abundance 37C
Doses Blue Rep1_0.1 Red Rep2_0.31
sp|Q8NDI1−3|EHBP1_HUMAN−397−S−S2_−0.32
ptm abundance 37C
Doses Blue Rep1_−0.29 Red Rep2_−0.34
sp|Q92766|RREB1_HUMAN−161−S−S1_−0.34
ptm abundance 37C
Doses Blue Rep1_−0.31 Red Rep2_−0.36
sp|Q5VUA4|ZN318_HUMAN−2192−S−S1_0.05
ptm abundance 37C
Doses Blue Rep1_0.13 Red Rep2_−0.02
sp|Q8TF01|PNISR_HUMAN−311−S−S2_0.37
ptm abundance 37C
Doses Blue Rep1_0.27 Red Rep2_0.46
sp|Q8IXM2−2|BAP18_HUMAN−62−S−S1_−0.24
ptm abundance 37C
Doses Blue Rep1_−0.22 Red Rep2_−0.26
sp|Q8IX07|FOG1_HUMAN−901−S−S2_−0.12
ptm abundance 37C
Doses Blue Rep1_−0.01 Red Rep2_−0.23
sp|Q8IX07|FOG1_HUMAN−909−S−S2_−0.12
ptm abundance 37C
Doses Blue Rep1_−0.01 Red Rep2_−0.23
sp|Q8NF99−2|ZN397_HUMAN−182−S−S2_−0.04
ptm abundance 37C
Doses Blue Rep1_−0.02 Red Rep2_−0.06
sp|Q8NF99−2|ZN397_HUMAN−185−S−S2_−0.04
ptm abundance 37C
Doses Blue Rep1_−0.02 Red Rep2_−0.06

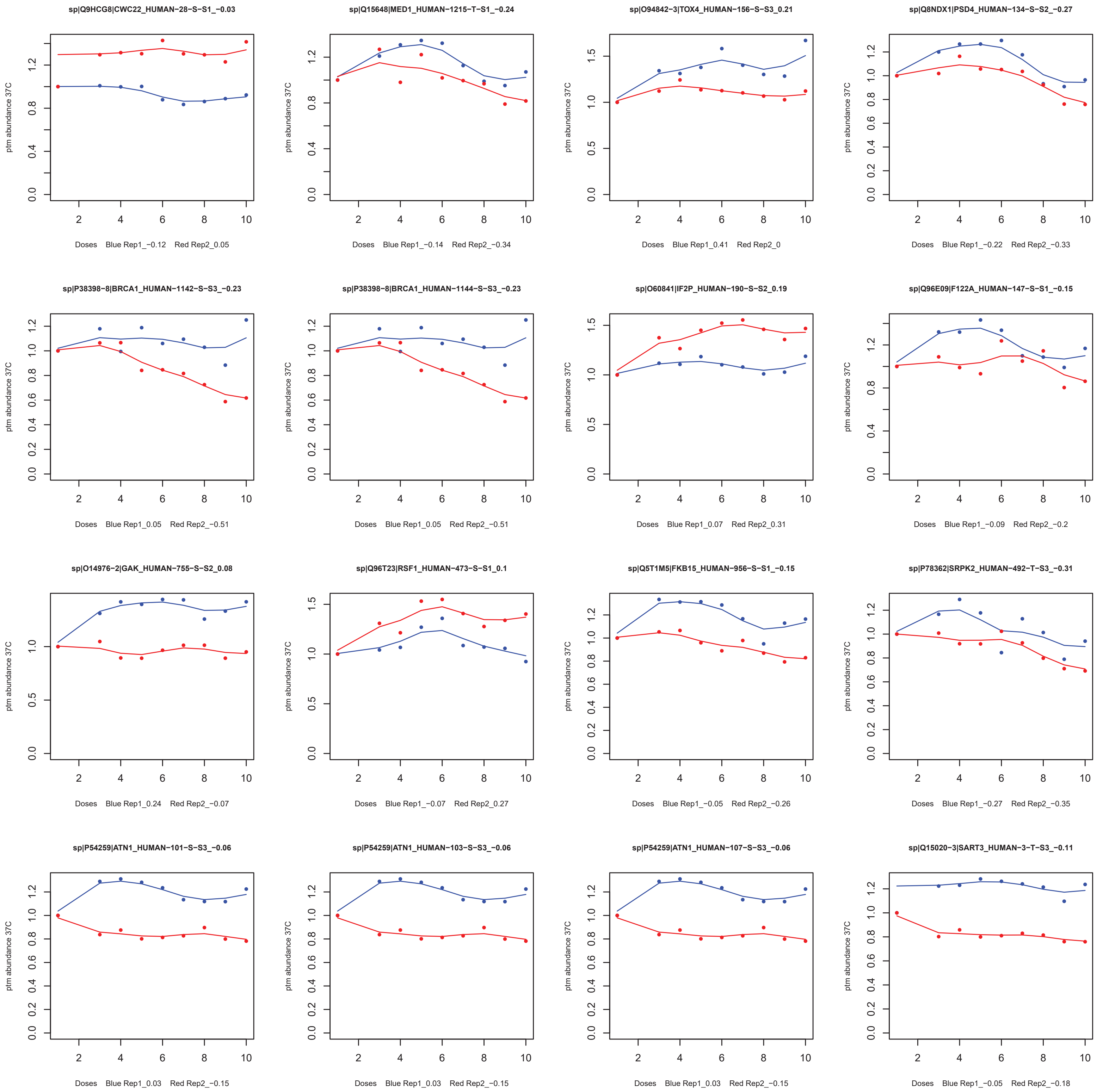
sp|Q9HCG8|CWC22_HUMAN−28−S−S1_−0.03
Doses Blue Rep1_−0.12 Red Rep2_0.05
sp|Q15648|MED1_HUMAN−1215−T−S1_−0.24
Doses Blue Rep1_−0.14 Red Rep2_−0.34
sp|O94842−3|TOX4_HUMAN−156−S−S3_0.21
Doses Blue Rep1_0.41 Red Rep2_0
sp|Q8NDX1|PSD4_HUMAN−134−S−S2_−0.27
Doses Blue Rep1_−0.22 Red Rep2_−0.33
sp|P38398−8|BRCA1_HUMAN−1142−S−S3_−0.23
Doses Blue Rep1_0.05 Red Rep2_−0.51
sp|P38398−8|BRCA1_HUMAN−1144−S−S3_−0.23
Doses Blue Rep1_0.05 Red Rep2_−0.51
sp|O60841|IF2P_HUMAN−190−S−S2_0.19
Doses Blue Rep1_0.07 Red Rep2_0.31
sp|Q96E09|F122A_HUMAN−147−S−S1_−0.15
Doses Blue Rep1_−0.09 Red Rep2_−0.2
sp|O14976−2|GAK_HUMAN−755−S−S2_0.08
Doses Blue Rep1_0.24 Red Rep2_−0.07
sp|Q96T23|RSF1_HUMAN−473−S−S1_0.1
Doses Blue Rep1_−0.07 Red Rep2_0.27
sp|Q5T1M5|FKB15_HUMAN−956−S−S1_−0.15
Doses Blue Rep1_−0.05 Red Rep2_−0.26
sp|P78362|SRPK2_HUMAN−492−T−S3_−0.31
Doses Blue Rep1_−0.27 Red Rep2_−0.35
sp|P54259|ATN1_HUMAN−101−S−S3_−0.06
Doses Blue Rep1_0.03 Red Rep2_−0.15
sp|P54259|ATN1_HUMAN−103−S−S3_−0.06
Doses Blue Rep1_0.03 Red Rep2_−0.15
sp|P54259|ATN1_HUMAN−107−S−S3_−0.06
Doses Blue Rep1_0.03 Red Rep2_−0.15
sp|Q15020−3|SART3_HUMAN−3−T−S3_−0.11
Doses Blue Rep1_−0.05 Red Rep2_−0.18
ptm abundance 37C

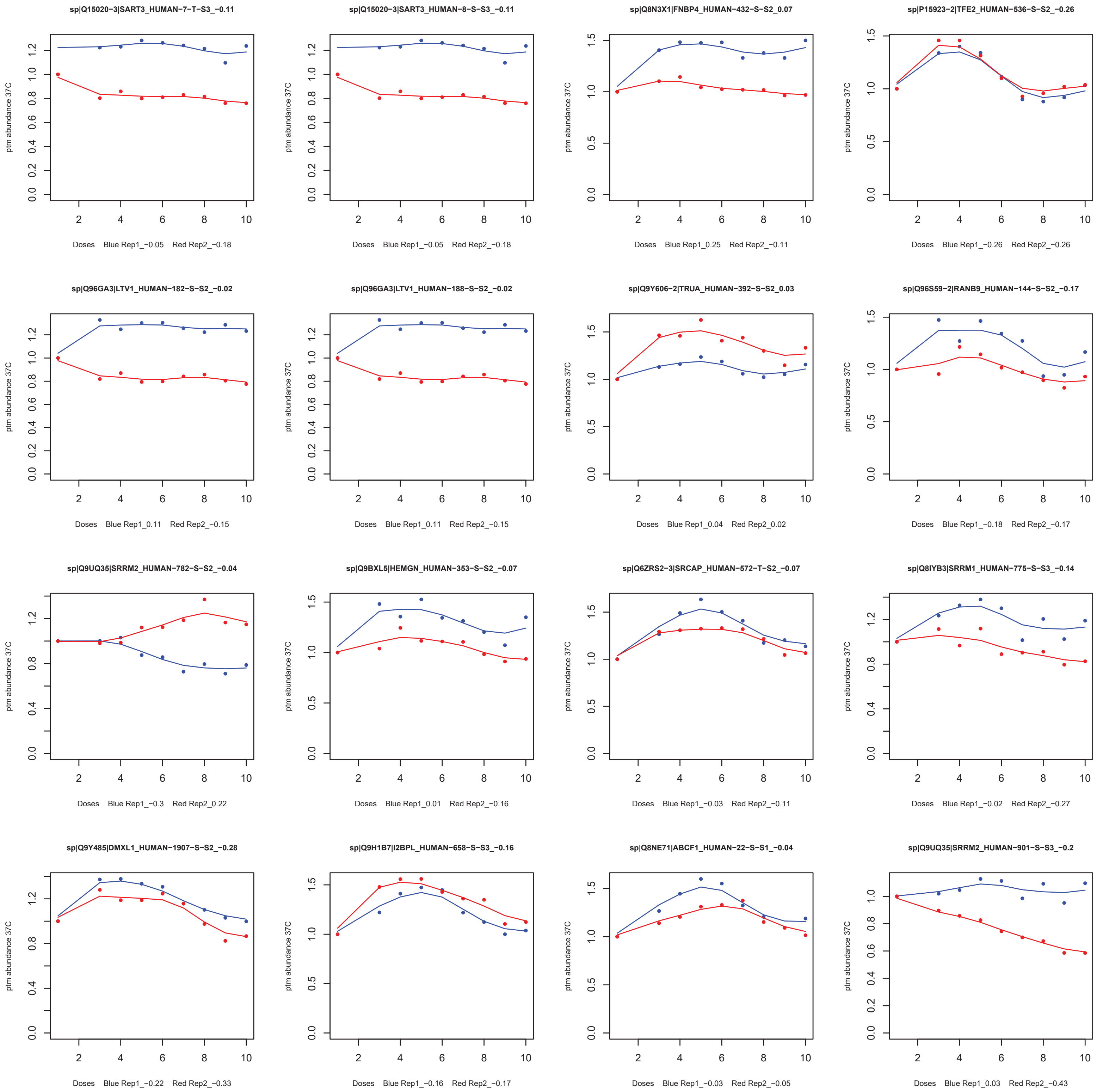
sp|Q15020−3|SART3_HUMAN−7−T−S3_−0.11
Doses Blue Rep1_−0.05 Red Rep2_−0.18
sp|Q15020−3|SART3_HUMAN−8−S−S3_−0.11
Doses Blue Rep1_−0.05 Red Rep2_−0.18
sp|Q8N3X1|FNBP4_HUMAN−432−S−S2_0.07
Doses Blue Rep1_0.25 Red Rep2_−0.11
sp|P15923−2|TFE2_HUMAN−536−S−S2_−0.26
Doses Blue Rep1_−0.26 Red Rep2_−0.26
sp|Q96GA3|LTV1_HUMAN−182−S−S2_−0.02
Doses Blue Rep1_0.11 Red Rep2_−0.15
sp|Q96GA3|LTV1_HUMAN−188−S−S2_−0.02
Doses Blue Rep1_0.11 Red Rep2_−0.15
sp|Q9Y606−2|TRUA_HUMAN−392−S−S2_0.03
Doses Blue Rep1_0.04 Red Rep2_0.02
sp|Q96S59−2|RANB9_HUMAN−144−S−S2_−0.17
Doses Blue Rep1_−0.18 Red Rep2_−0.17
sp|Q9UQ35|SRRM2_HUMAN−782−S−S2_−0.04
Doses Blue Rep1_−0.3 Red Rep2_0.22
sp|Q9BXL5|HEMGN_HUMAN−353−S−S2_−0.07
Doses Blue Rep1_0.01 Red Rep2_−0.16
sp|Q6ZRS2−3|SRCAP_HUMAN−572−T−S2_−0.07
Doses Blue Rep1_−0.03 Red Rep2_−0.11
sp|Q8IYB3|SRRM1_HUMAN−775−S−S3_−0.14
Doses Blue Rep1_−0.02 Red Rep2_−0.27
sp|Q9Y485|DMXL1_HUMAN−1907−S−S2_−0.28
Doses Blue Rep1_−0.22 Red Rep2_−0.33
sp|Q9H1B7|I2BPL_HUMAN−658−S−S3_−0.16
Doses Blue Rep1_−0.16 Red Rep2_−0.17
sp|Q8NE71|ABCF1_HUMAN−22−S−S1_−0.04
Doses Blue Rep1_−0.03 Red Rep2_−0.05
sp|Q9UQ35|SRRM2_HUMAN−901−S−S3_−0.2
Doses Blue Rep1_0.03 Red Rep2_−0.43
ptm abundance 37C

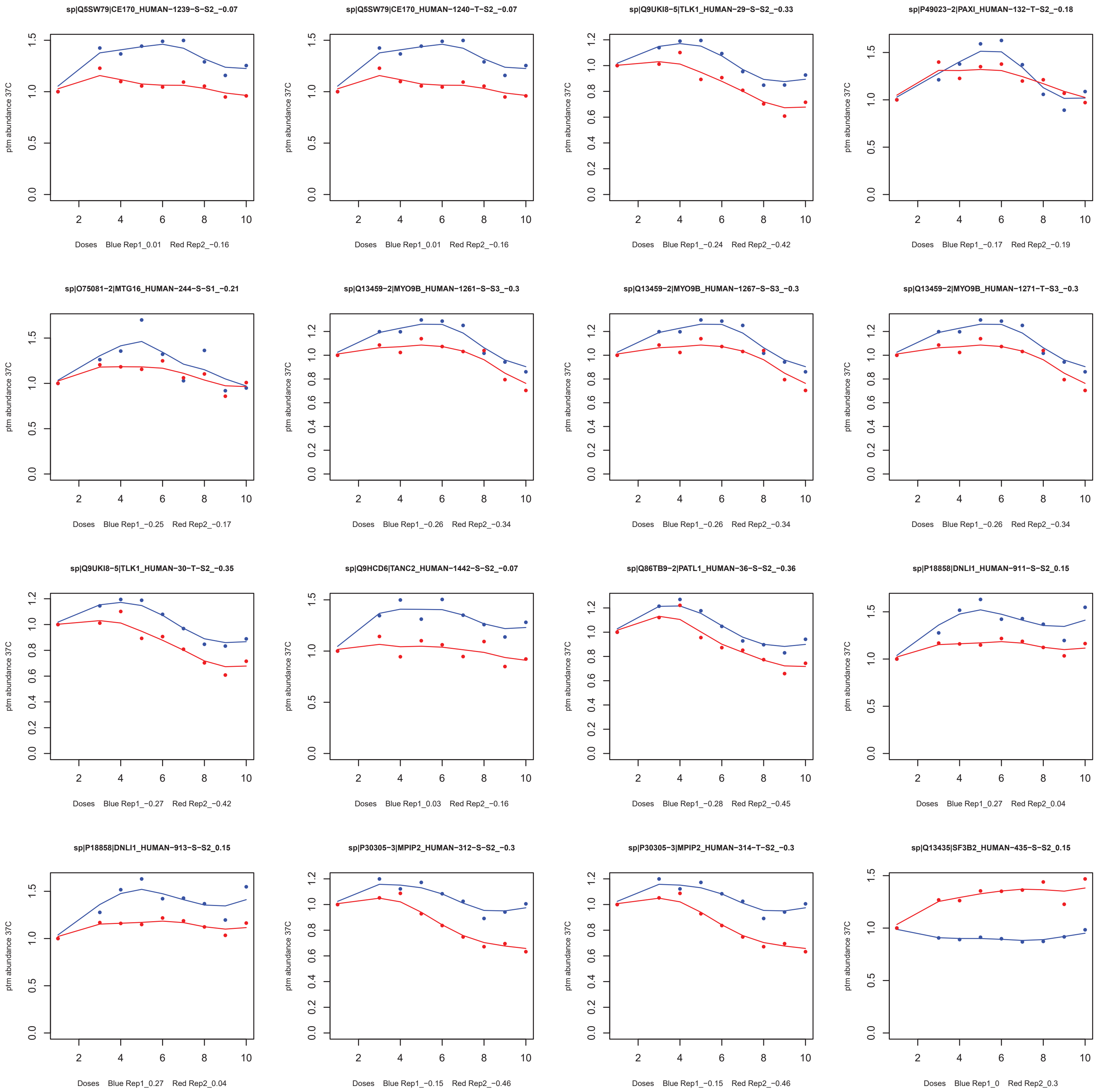
sp|Q5SW79|CE170_HUMAN−1239−S−S2_−0.07
Doses Blue Rep1_0.01 Red Rep2_−0.16
sp|Q5SW79|CE170_HUMAN−1240−T−S2_−0.07
Doses Blue Rep1_0.01 Red Rep2_−0.16
sp|Q9UKI8−5|TLK1_HUMAN−29−S−S2_−0.33
Doses Blue Rep1_−0.24 Red Rep2_−0.42
sp|P49023−2|PAXI_HUMAN−132−T−S2_−0.18
Doses Blue Rep1_−0.17 Red Rep2_−0.19
sp|O75081−2|MTG16_HUMAN−244−S−S1_−0.21
Doses Blue Rep1_−0.25 Red Rep2_−0.17
sp|Q13459−2|MYO9B_HUMAN−1261−S−S3_−0.3
Doses Blue Rep1_−0.26 Red Rep2_−0.34
sp|Q13459−2|MYO9B_HUMAN−1267−S−S3_−0.3
Doses Blue Rep1_−0.26 Red Rep2_−0.34
sp|Q13459−2|MYO9B_HUMAN−1271−T−S3_−0.3
Doses Blue Rep1_−0.26 Red Rep2_−0.34
sp|Q9UKI8−5|TLK1_HUMAN−30−T−S2_−0.35
Doses Blue Rep1_−0.27 Red Rep2_−0.42
sp|Q9HCD6|TANC2_HUMAN−1442−S−S2_−0.07
Doses Blue Rep1_0.03 Red Rep2_−0.16
sp|Q86TB9−2|PATL1_HUMAN−36−S−S2_−0.36
Doses Blue Rep1_−0.28 Red Rep2_−0.45
sp|P18858|DNLI1_HUMAN−911−S−S2_0.15
Doses Blue Rep1_0.27 Red Rep2_0.04
sp|P18858|DNLI1_HUMAN−913−S−S2_0.15
Doses Blue Rep1_0.27 Red Rep2_0.04
sp|P30305−3|MPIP2_HUMAN−312−S−S2_−0.3
Doses Blue Rep1_−0.15 Red Rep2_−0.46
sp|P30305−3|MPIP2_HUMAN−314−T−S2_−0.3
Doses Blue Rep1_−0.15 Red Rep2_−0.46
sp|Q13435|SF3B2_HUMAN−435−S−S2_0.15
Doses Blue Rep1_0 Red Rep2_0.3
ptm abundance 37C

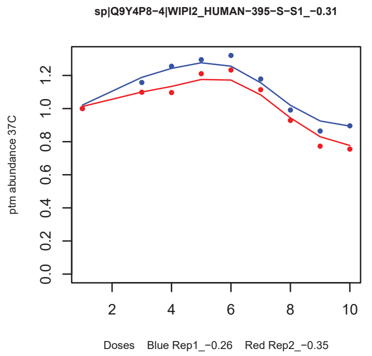
sp|Q9Y4P8−4|WIPI2_HUMAN−395−S−S1_−0.31
ptm abundance 37C
Doses Blue Rep1_−0.26 Red Rep2_−0.35

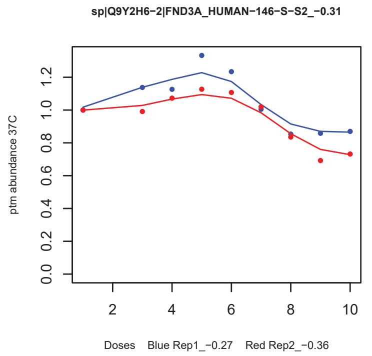
sp|Q9Y2H6−2|FND3A_HUMAN−146−S−S2_−0.31
ptm abundance 37C
Doses Blue Rep1_−0.27 Red Rep2_−0.36

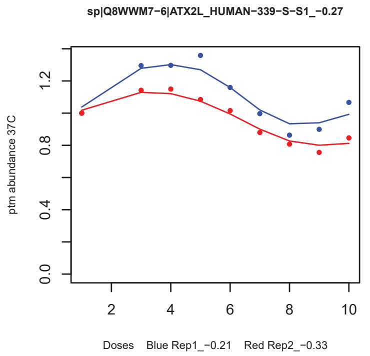
sp|Q8WWM7−6|ATX2L_HUMAN−339−S−S1_−0.27
ptm abundance 37C
Doses Blue Rep1_−0.21 Red Rep2_−0.33

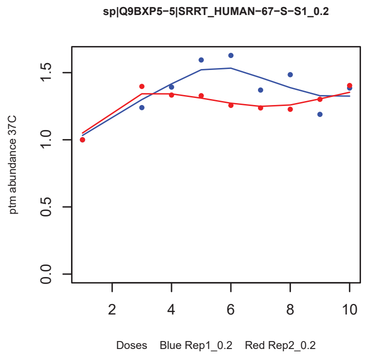
sp|Q9BXP5−5|SRRT_HUMAN−67−S−S1_0.2
ptm abundance 37C
Doses Blue Rep1_0.2 Red Rep2_0.2

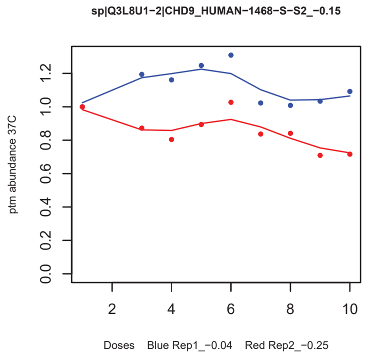
sp|Q3L8U1−2|CHD9_HUMAN−1468−S−S2_−0.15
ptm abundance 37C
Doses Blue Rep1_−0.04 Red Rep2_−0.25

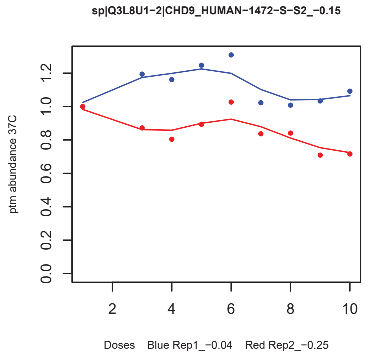
sp|Q3L8U1−2|CHD9_HUMAN−1472−S−S2_−0.15
ptm abundance 37C
Doses Blue Rep1_−0.04 Red Rep2_−0.25

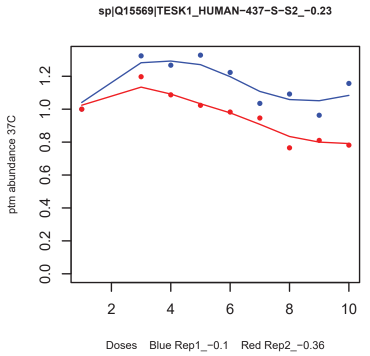
sp|Q15569|TESK1_HUMAN−437−S−S2_−0.23
ptm abundance 37C
Doses Blue Rep1_−0.1 Red Rep2_−0.36

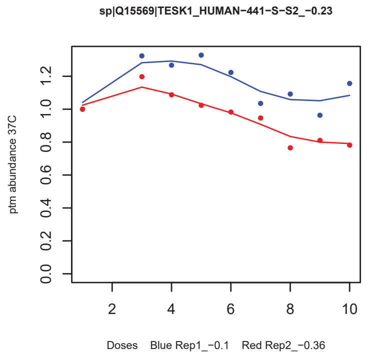
sp|Q15569|TESK1_HUMAN−441−S−S2_−0.23
ptm abundance 37C
Doses Blue Rep1_−0.1 Red Rep2_−0.36

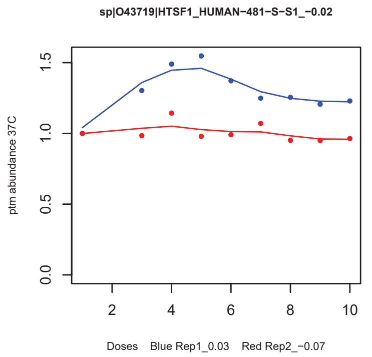
sp|O43719|HTSF1_HUMAN−481−S−S1_−0.02
ptm abundance 37C
Doses Blue Rep1_0.03 Red Rep2_−0.07

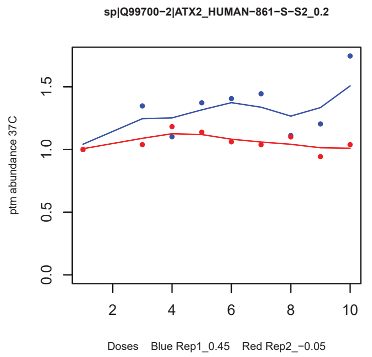
sp|Q99700−2|ATX2_HUMAN−861−S−S2_0.2
ptm abundance 37C
Doses Blue Rep1_0.45 Red Rep2_−0.05

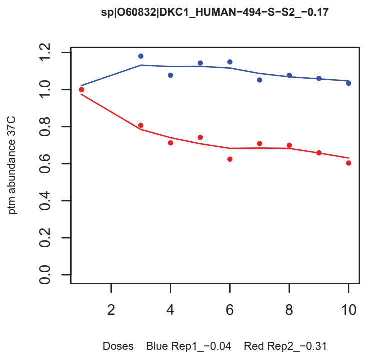
sp|O60832|DKC1_HUMAN−494−S−S2_−0.17
ptm abundance 37C
Doses Blue Rep1_−0.04 Red Rep2_−0.31

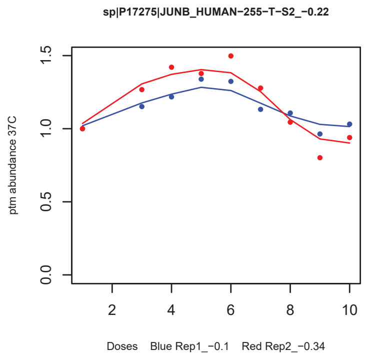
sp|P17275|JUNB_HUMAN−255−T−S2_−0.22
ptm abundance 37C
Doses Blue Rep1_−0.1 Red Rep2_−0.34

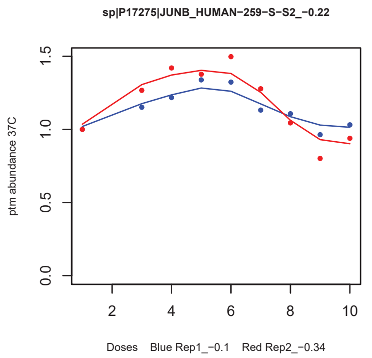
sp|P17275|JUNB_HUMAN−259−S−S2_−0.22
ptm abundance 37C
Doses Blue Rep1_−0.1 Red Rep2_−0.34

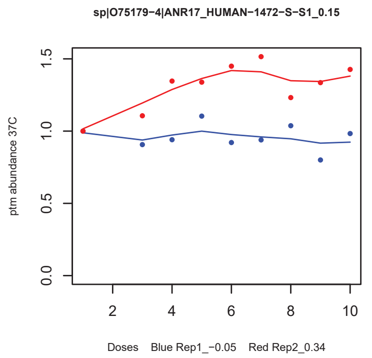
sp|O75179−4|ANR17_HUMAN−1472−S−S1_0.15
ptm abundance 37C
Doses Blue Rep1_−0.05 Red Rep2_0.34

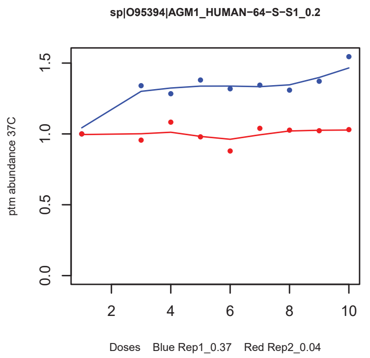
sp|O95394|AGM1_HUMAN−64−S−S1_0.2
ptm abundance 37C
Doses Blue Rep1_0.37 Red Rep2_0.04

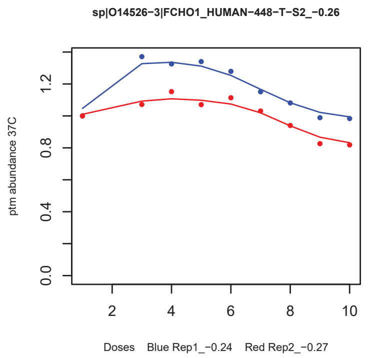
sp|O14526−3|FCHO1_HUMAN−448−T−S2_−0.26
ptm abundance 37C
Doses Blue Rep1_−0.24 Red Rep2_−0.27

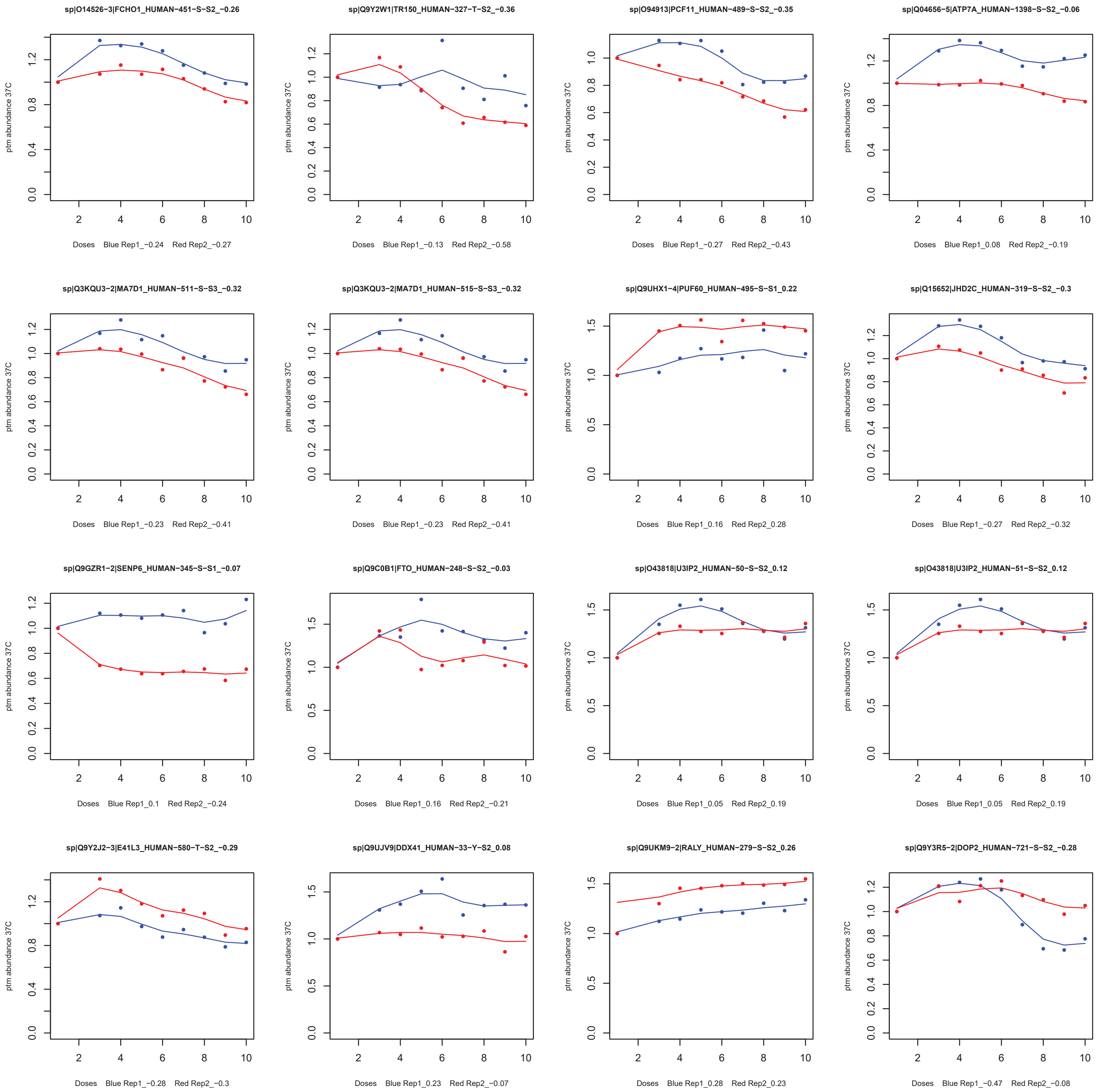
sp|O14526-3|FCHO1_HUMAN-451-S-S2_-0.26
ptm abundance 37C
Doses Blue Rep1_-0.24 Red Rep2_-0.27
sp|Q9Y2W1|TR150_HUMAN-327-T-S2_-0.36
ptm abundance 37C
Doses Blue Rep1_-0.13 Red Rep2_-0.58
sp|O94913|PCF11_HUMAN-489-S-S2_-0.35
ptm abundance 37C
Doses Blue Rep1_-0.27 Red Rep2_-0.43
sp|Q04656-5|ATP7A_HUMAN-1398-S-S2_-0.06
ptm abundance 37C
Doses Blue Rep1_0.08 Red Rep2_-0.19
sp|Q3KQU3-2|MA7D1_HUMAN-511-S-S3_-0.32
ptm abundance 37C
Doses Blue Rep1_-0.23 Red Rep2_-0.41
sp|Q3KQU3-2|MA7D1_HUMAN-515-S-S3_-0.32
ptm abundance 37C
Doses Blue Rep1_-0.23 Red Rep2_-0.41
sp|Q9UHX1-4|PUF60_HUMAN-495-S-S1_0.22
ptm abundance 37C
Doses Blue Rep1_0.16 Red Rep2_0.28
sp|Q15652|JHD2C_HUMAN-319-S-S2_-0.3
ptm abundance 37C
Doses Blue Rep1_-0.27 Red Rep2_-0.32
sp|Q9GZR1-2|SENP6_HUMAN-345-S-S1_-0.07
ptm abundance 37C
Doses Blue Rep1_0.1 Red Rep2_-0.24
sp|Q9C0B1|FTO_HUMAN-248-S-S2_-0.03
ptm abundance 37C
Doses Blue Rep1_0.16 Red Rep2_-0.21
sp|O43818|U3IP2_HUMAN-50-S-S2_0.12
ptm abundance 37C
Doses Blue Rep1_0.05 Red Rep2_0.19
sp|O43818|U3IP2_HUMAN-51-S-S2_0.12
ptm abundance 37C
Doses Blue Rep1_0.05 Red Rep2_0.19
sp|Q9Y2J2-3|E41L3_HUMAN-580-T-S2_-0.29
ptm abundance 37C
Doses Blue Rep1_-0.28 Red Rep2_-0.3
sp|Q9UJV9|DDX41_HUMAN-33-Y-S2_0.08
ptm abundance 37C
Doses Blue Rep1_0.23 Red Rep2_-0.07
sp|Q9UKM9-2|RALY_HUMAN-279-S-S2_0.26
ptm abundance 37C
Doses Blue Rep1_0.28 Red Rep2_0.23
sp|Q9Y3R5-2|DOP2_HUMAN-721-S-S2_-0.28
ptm abundance 37C
Doses Blue Rep1_-0.47 Red Rep2_-0.08

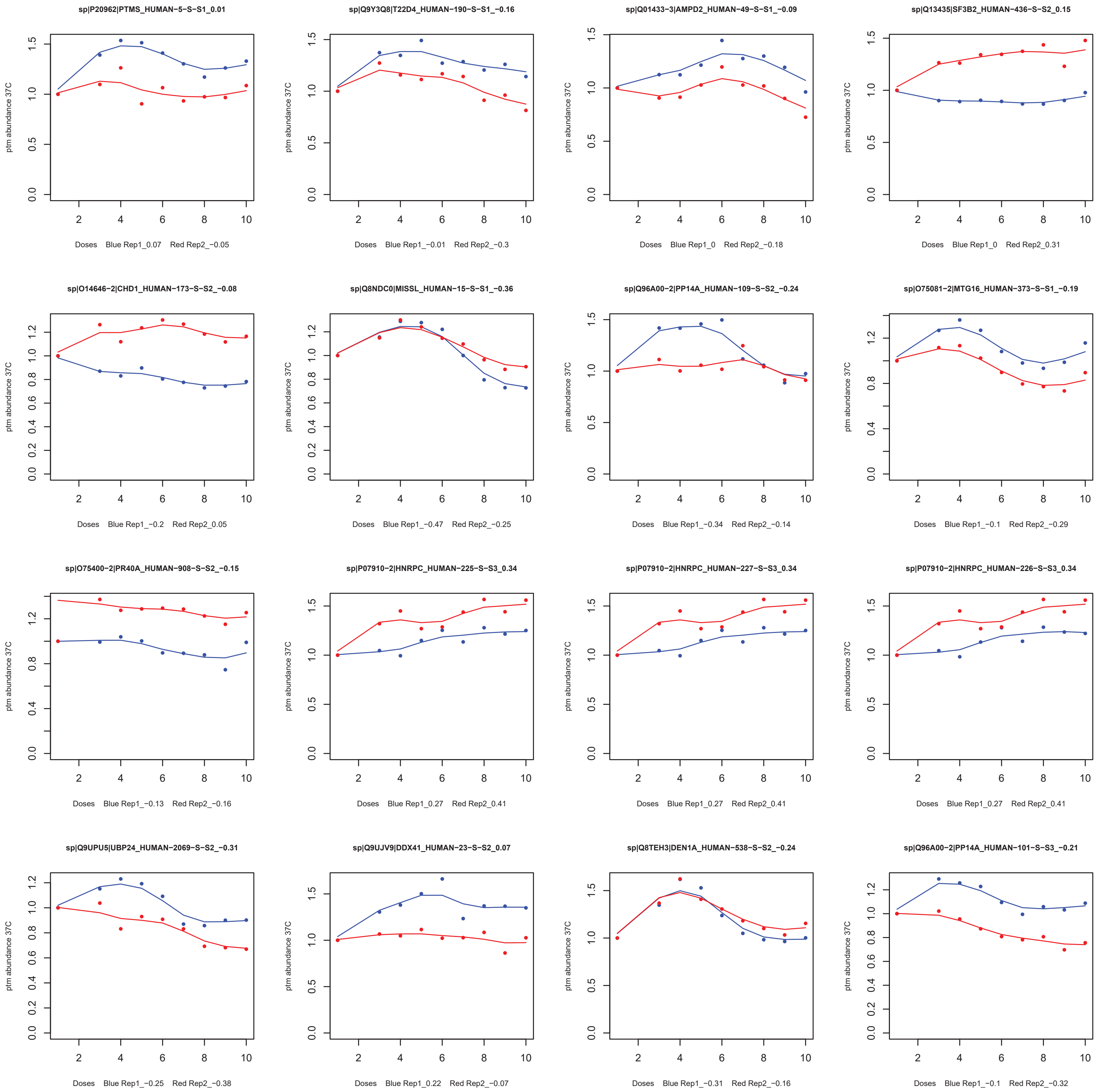
sp|P20962|PTMS_HUMAN−5−S−S1_0.01
Doses Blue Rep1_0.07 Red Rep2_−0.05
sp|Q9Y3Q8|T22D4_HUMAN−190−S−S1_−0.16
Doses Blue Rep1_−0.01 Red Rep2_−0.3
sp|Q01433−3|AMPD2_HUMAN−49−S−S1_−0.09
Doses Blue Rep1_0 Red Rep2_−0.18
sp|Q13435|SF3B2_HUMAN−436−S−S2_0.15
Doses Blue Rep1_0 Red Rep2_0.31
sp|O14646−2|CHD1_HUMAN−173−S−S2_−0.08
Doses Blue Rep1_−0.2 Red Rep2_0.05
sp|Q8NDC0|MISSL_HUMAN−15−S−S1_−0.36
Doses Blue Rep1_−0.47 Red Rep2_−0.25
sp|Q96A00−2|PP14A_HUMAN−109−S−S2_−0.24
Doses Blue Rep1_−0.34 Red Rep2_−0.14
sp|O75081−2|MTG16_HUMAN−373−S−S1_−0.19
Doses Blue Rep1_−0.1 Red Rep2_−0.29
sp|O75400−2|PR40A_HUMAN−908−S−S2_−0.15
Doses Blue Rep1_−0.13 Red Rep2_−0.16
sp|P07910−2|HNRPC_HUMAN−225−S−S3_0.34
Doses Blue Rep1_0.27 Red Rep2_0.41
sp|P07910−2|HNRPC_HUMAN−227−S−S3_0.34
Doses Blue Rep1_0.27 Red Rep2_0.41
sp|P07910−2|HNRPC_HUMAN−226−S−S3_0.34
Doses Blue Rep1_0.27 Red Rep2_0.41
sp|Q9UPU5|UBP24_HUMAN−2069−S−S2_−0.31
Doses Blue Rep1_−0.25 Red Rep2_−0.38
sp|Q9UJV9|DDX41_HUMAN−23−S−S2_0.07
Doses Blue Rep1_0.22 Red Rep2_−0.07
sp|Q8TEH3|DEN1A_HUMAN−538−S−S2_−0.24
Doses Blue Rep1_−0.31 Red Rep2_−0.16
sp|Q96A00−2|PP14A_HUMAN−101−S−S3_−0.21
Doses Blue Rep1_−0.1 Red Rep2_−0.32
ptm abundance 37C

sp|Q7Z6E9|RBBP6_HUMAN−703−S−S2_−0.31

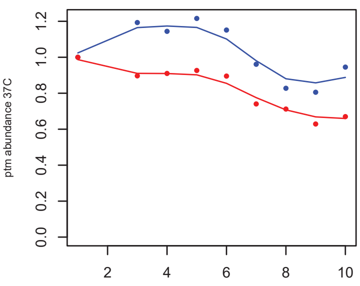

Doses Blue Rep1_−0.26 Red Rep2_−0.36

sp|Q9H9D4|ZN408_HUMAN−345−S−S2_−0.21

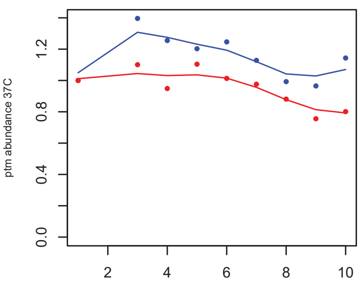

Doses Blue Rep1_−0.14 Red Rep2_−0.29

sp|Q96D71−4|REPS1_HUMAN−170−S−S3_−0.31

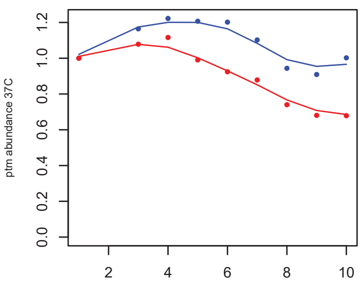

Doses Blue Rep1_−0.16 Red Rep2_−0.45

sp|Q13428−6|TCOF_HUMAN−1219−S−S1_−0.34

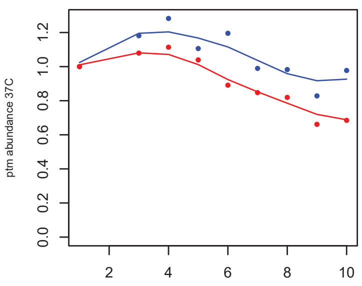

Doses Blue Rep1_−0.23 Red Rep2_−0.45

sp|Q96T23|RSF1_HUMAN−1281−Y−S2_0.04

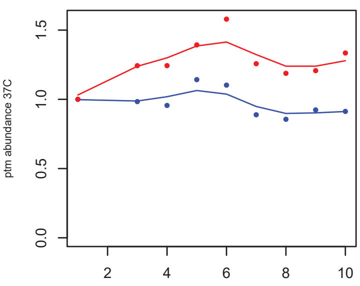

Doses Blue Rep1_−0.1 Red Rep2_0.18

sp|Q5UIP0−2|RIF1_HUMAN−2365−S−S2_−0.31

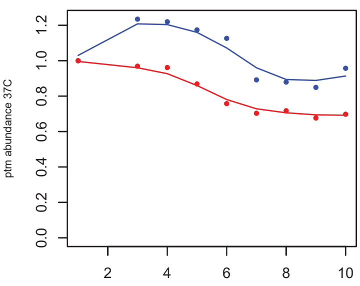

Doses Blue Rep1_−0.26 Red Rep2_−0.36

sp|Q5UIP0−2|RIF1_HUMAN−2367−S−S2_−0.31

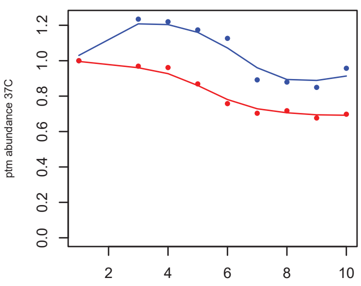

Doses Blue Rep1_−0.26 Red Rep2_−0.36

sp|Q8NI08−7|NCOA7_HUMAN−105−S−S3_−0.1

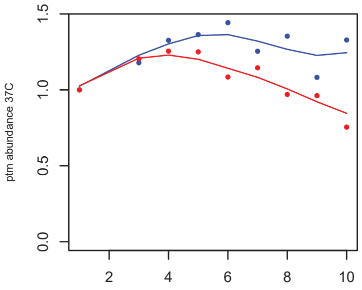

Doses Blue Rep1_0.15 Red Rep2_−0.34

sp|Q8NI08−7|NCOA7_HUMAN−106−T−S3_−0.1

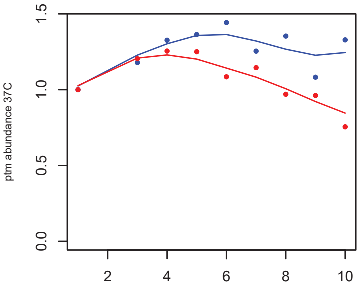

Doses Blue Rep1_0.15 Red Rep2_−0.34

sp|Q5SW79|CE170_HUMAN−488−S−S2_0.25

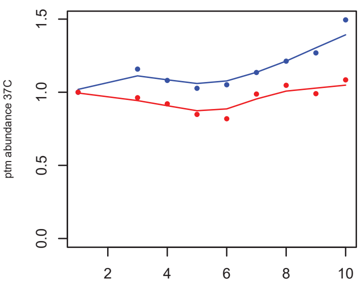

Doses Blue Rep1_0.41 Red Rep2_0.1

sp|Q9UJX2−3|CDC23_HUMAN−470−S−S2_−0.34

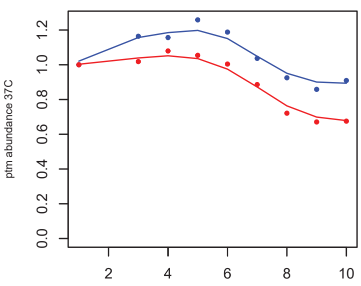

Doses Blue Rep1_−0.24 Red Rep2_−0.43

sp|Q9UJX2−3|CDC23_HUMAN−478−T−S2_−0.34

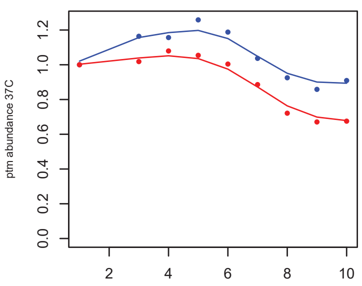

Doses Blue Rep1_−0.24 Red Rep2_−0.43

sp|Q5VTD9−2|GFI1B_HUMAN−93−S−S2_−0.03

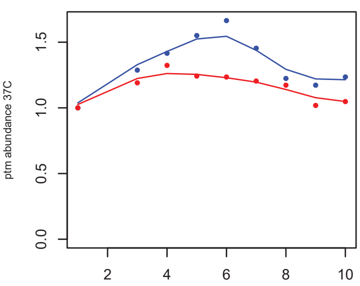

Doses Blue Rep1_0.04 Red Rep2_−0.1

sp|Q5VTD9−2|GFI1B_HUMAN−98−S−S2_−0.03

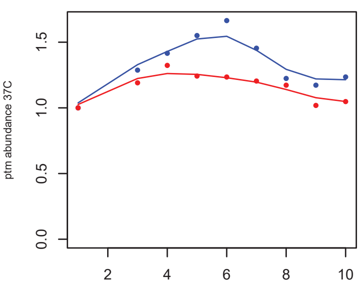

Doses Blue Rep1_0.04 Red Rep2_−0.1

sp|P46821|MAP1B_HUMAN−1260−S−S2_0.27

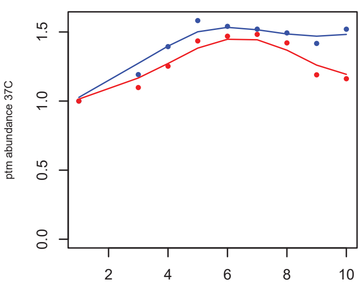

Doses Blue Rep1_0.42 Red Rep2_0.13

sp|Q00341−2|VIGLN_HUMAN−911−S−S1_0.03

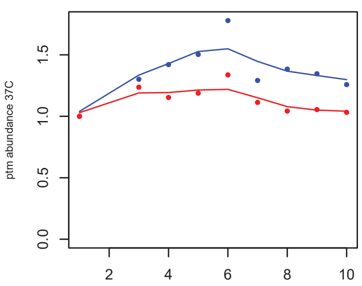

Doses Blue Rep1_0.14 Red Rep2_−0.09

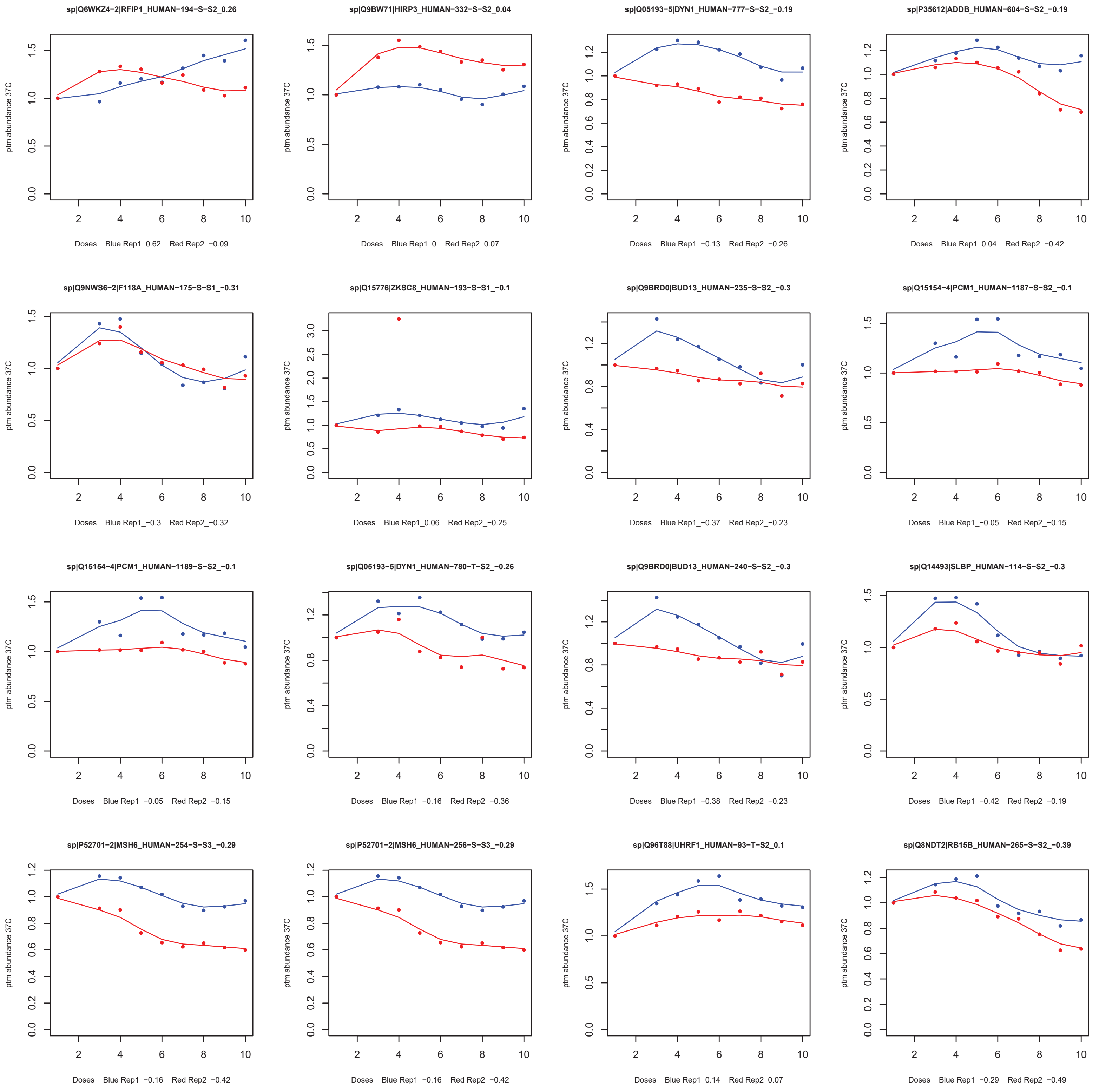

sp|Q6WKZ4−2|RFIP1_HUMAN−194−S−S2_0.26
Doses Blue Rep1_0.62 Red Rep2_−0.09
sp|Q9BW71|HIRP3_HUMAN−332−S−S2_0.04
Doses Blue Rep1_0 Red Rep2_0.07
sp|Q05193−5|DYN1_HUMAN−777−S−S2_−0.19
Doses Blue Rep1_−0.13 Red Rep2_−0.26
sp|P35612|ADDB_HUMAN−604−S−S2_−0.19
Doses Blue Rep1_0.04 Red Rep2_−0.42
sp|Q9NWS6−2|F118A_HUMAN−175−S−S1_−0.31
Doses Blue Rep1_−0.3 Red Rep2_−0.32
sp|Q15776|ZKSC8_HUMAN−193−S−S1_−0.1
Doses Blue Rep1_0.06 Red Rep2_−0.25
sp|Q9BRD0|BUD13_HUMAN−235−S−S2_−0.3
Doses Blue Rep1_−0.37 Red Rep2_−0.23
sp|Q15154−4|PCM1_HUMAN−1187−S−S2_−0.1
Doses Blue Rep1_−0.05 Red Rep2_−0.15
sp|Q15154−4|PCM1_HUMAN−1189−S−S2_−0.1
Doses Blue Rep1_−0.05 Red Rep2_−0.15
sp|Q05193−5|DYN1_HUMAN−780−T−S2_−0.26
Doses Blue Rep1_−0.16 Red Rep2_−0.36
sp|Q9BRD0|BUD13_HUMAN−240−S−S2_−0.3
Doses Blue Rep1_−0.38 Red Rep2_−0.23
sp|Q14493|SLBP_HUMAN−114−S−S2_−0.3
Doses Blue Rep1_−0.42 Red Rep2_−0.19
sp|P52701−2|MSH6_HUMAN−254−S−S3_−0.29
Doses Blue Rep1_−0.16 Red Rep2_−0.42
sp|P52701−2|MSH6_HUMAN−256−S−S3_−0.29
Doses Blue Rep1_−0.16 Red Rep2_−0.42
sp|Q96T88|UHRF1_HUMAN−93−T−S2_0.1
Doses Blue Rep1_0.14 Red Rep2_0.07
sp|Q8NDT2|RB15B_HUMAN−265−S−S2_−0.39
Doses Blue Rep1_−0.29 Red Rep2_−0.49
ptm abundance 37C

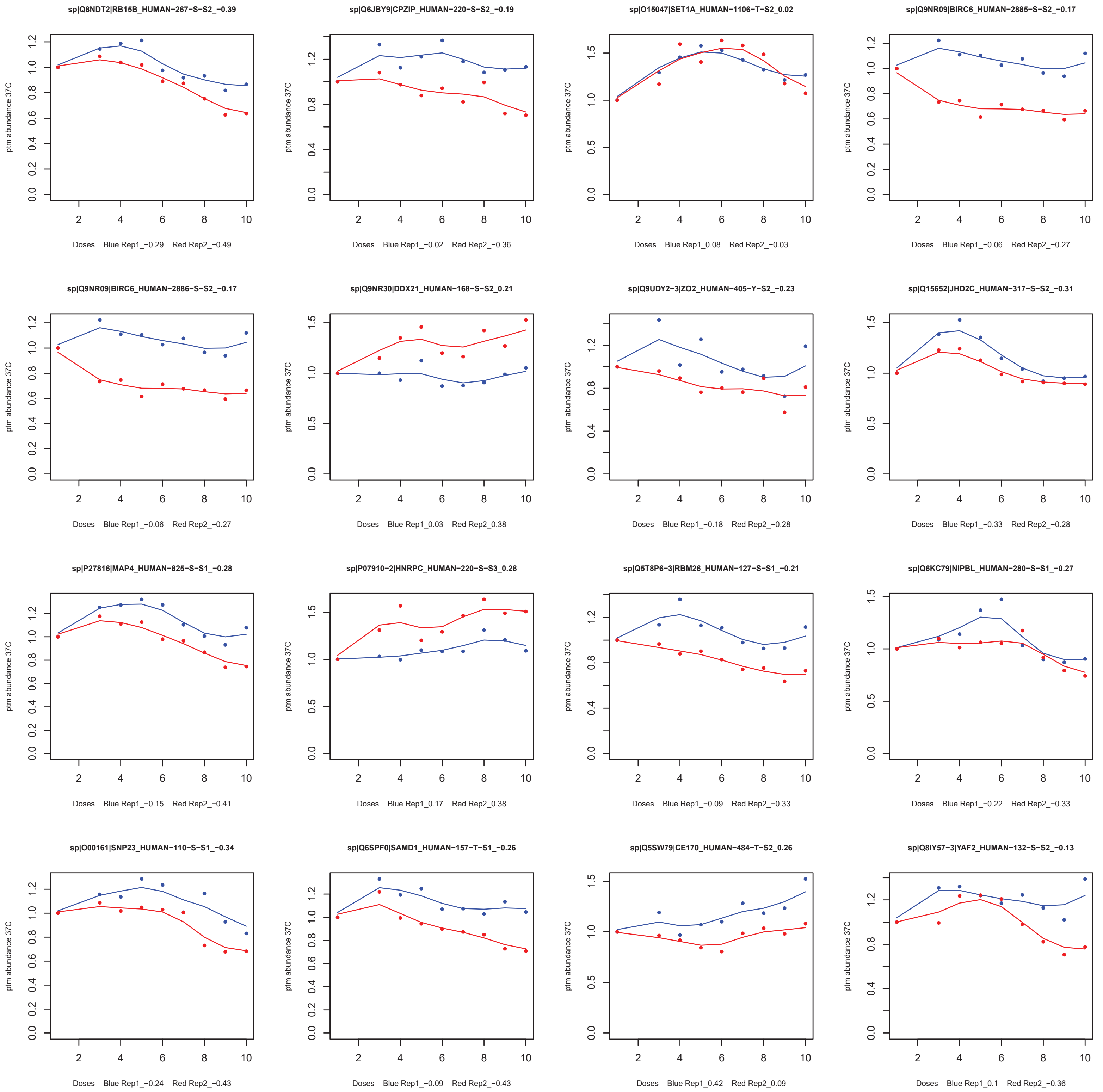

sp|Q8NDT2|RB15B_HUMAN−267−S−S2_−0.39
Doses Blue Rep1_−0.29 Red Rep2_−0.49
sp|Q6JBY9|CPZIP_HUMAN−220−S−S2_−0.19
Doses Blue Rep1_−0.02 Red Rep2_−0.36
sp|O15047|SET1A_HUMAN−1106−T−S2_0.02
Doses Blue Rep1_0.08 Red Rep2_−0.03
sp|Q9NR09|BIRC6_HUMAN−2885−S−S2_−0.17
Doses Blue Rep1_−0.06 Red Rep2_−0.27
sp|Q9NR09|BIRC6_HUMAN−2886−S−S2_−0.17
Doses Blue Rep1_−0.06 Red Rep2_−0.27
sp|Q9NR30|DDX21_HUMAN−168−S−S2_0.21
Doses Blue Rep1_0.03 Red Rep2_0.38
sp|Q9UDY2−3|ZO2_HUMAN−405−Y−S2_−0.23
Doses Blue Rep1_−0.18 Red Rep2_−0.28
sp|Q15652|JHD2C_HUMAN−317−S−S2_−0.31
Doses Blue Rep1_−0.33 Red Rep2_−0.28
sp|P27816|MAP4_HUMAN−825−S−S1_−0.28
Doses Blue Rep1_−0.15 Red Rep2_−0.41
sp|P07910−2|HNRPC_HUMAN−220−S−S3_0.28
Doses Blue Rep1_0.17 Red Rep2_0.38
sp|Q5T8P6−3|RBM26_HUMAN−127−S−S1_−0.21
Doses Blue Rep1_−0.09 Red Rep2_−0.33
sp|Q6KC79|NIPBL_HUMAN−280−S−S1_−0.27
Doses Blue Rep1_−0.22 Red Rep2_−0.33
sp|O00161|SNP23_HUMAN−110−S−S1_−0.34
Doses Blue Rep1_−0.24 Red Rep2_−0.43
sp|Q6SPF0|SAMD1_HUMAN−157−T−S1_−0.26
Doses Blue Rep1_−0.09 Red Rep2_−0.43
sp|Q5SW79|CE170_HUMAN−484−T−S2_0.26
Doses Blue Rep1_0.42 Red Rep2_0.09
sp|Q8IY57−3|YAF2_HUMAN−132−S−S2_−0.13
Doses Blue Rep1_0.1 Red Rep2_−0.36
ptm abundance 37C

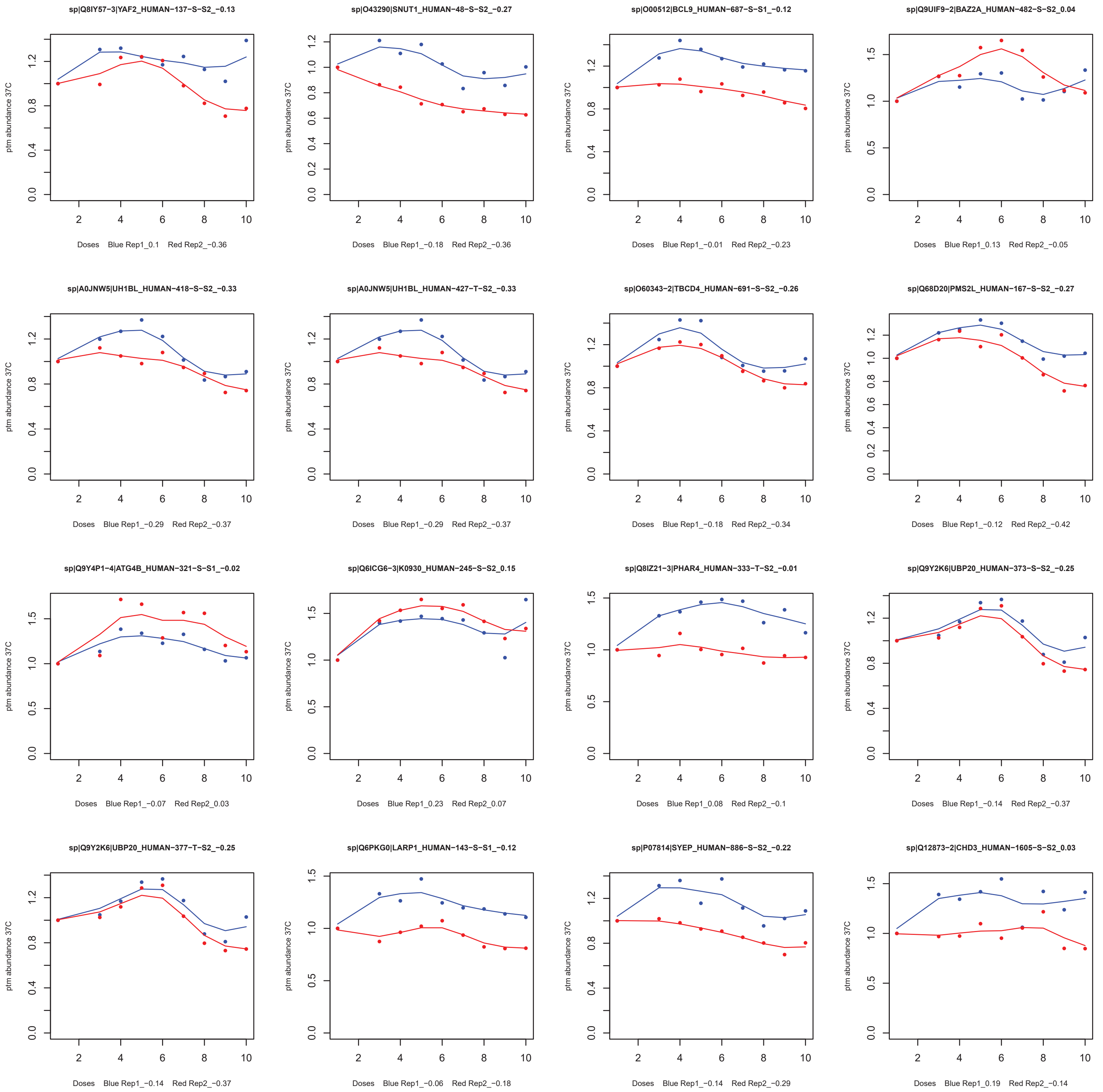

sp|Q8IY57−3|YAF2_HUMAN−137−S−S2_−0.13
ptm abundance 37C
Doses Blue Rep1_0.1 Red Rep2_−0.36
sp|O43290|SNUT1_HUMAN−48−S−S2_−0.27
ptm abundance 37C
Doses Blue Rep1_−0.18 Red Rep2_−0.36
sp|O00512|BCL9_HUMAN−687−S−S1_−0.12
ptm abundance 37C
Doses Blue Rep1_−0.01 Red Rep2_−0.23
sp|Q9UIF9−2|BAZ2A_HUMAN−482−S−S2_0.04
ptm abundance 37C
Doses Blue Rep1_0.13 Red Rep2_−0.05
sp|A0JNW5|UH1BL_HUMAN−418−S−S2_−0.33
ptm abundance 37C
Doses Blue Rep1_−0.29 Red Rep2_−0.37
sp|A0JNW5|UH1BL_HUMAN−427−T−S2_−0.33
ptm abundance 37C
Doses Blue Rep1_−0.29 Red Rep2_−0.37
sp|O60343−2|TBCD4_HUMAN−691−S−S2_−0.26
ptm abundance 37C
Doses Blue Rep1_−0.18 Red Rep2_−0.34
sp|Q68D20|PMS2L_HUMAN−167−S−S2_−0.27
ptm abundance 37C
Doses Blue Rep1_−0.12 Red Rep2_−0.42
sp|Q9Y4P1−4|ATG4B_HUMAN−321−S−S1_−0.02
ptm abundance 37C
Doses Blue Rep1_−0.07 Red Rep2_0.03
sp|Q6ICG6−3|K0930_HUMAN−245−S−S2_0.15
ptm abundance 37C
Doses Blue Rep1_0.23 Red Rep2_0.07
sp|Q8IZ21−3|PHAR4_HUMAN−333−T−S2_−0.01
ptm abundance 37C
Doses Blue Rep1_0.08 Red Rep2_−0.1
sp|Q9Y2K6|UBP20_HUMAN−373−S−S2_−0.25
ptm abundance 37C
Doses Blue Rep1_−0.14 Red Rep2_−0.37
sp|Q9Y2K6|UBP20_HUMAN−377−T−S2_−0.25
ptm abundance 37C
Doses Blue Rep1_−0.14 Red Rep2_−0.37
sp|Q6PKG0|LARP1_HUMAN−143−S−S1_−0.12
ptm abundance 37C
Doses Blue Rep1_−0.06 Red Rep2_−0.18
sp|P07814|SYEP_HUMAN−886−S−S2_−0.22
ptm abundance 37C
Doses Blue Rep1_−0.14 Red Rep2_−0.29
sp|Q12873−2|CHD3_HUMAN−1605−S−S2_0.03
ptm abundance 37C
Doses Blue Rep1_0.19 Red Rep2_−0.14

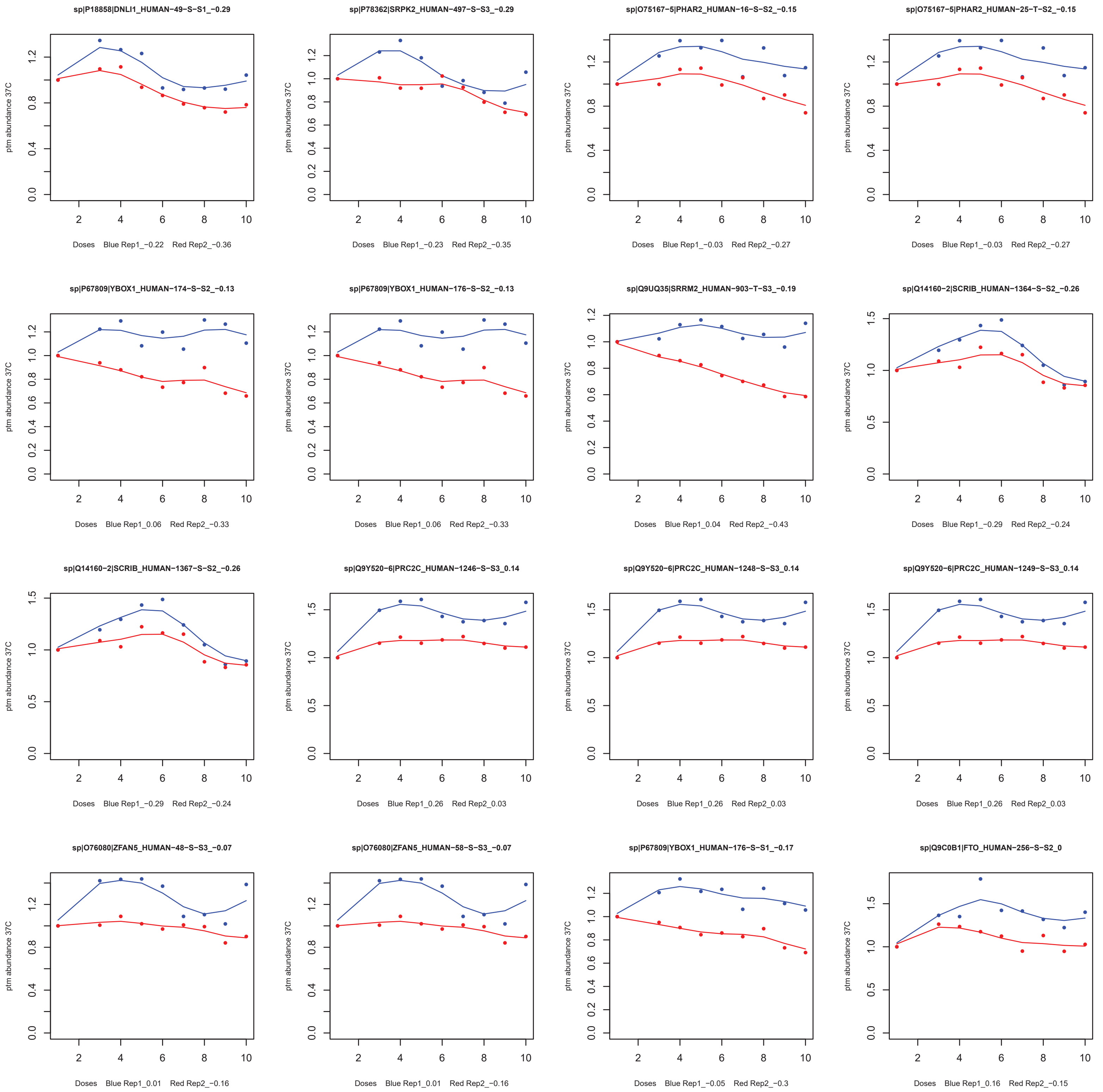
sp|P18858|DNLI1_HUMAN−49−S−S1_−0.29
Doses Blue Rep1_−0.22 Red Rep2_−0.36
sp|P78362|SRPK2_HUMAN−497−S−S3_−0.29
Doses Blue Rep1_−0.23 Red Rep2_−0.35
sp|O75167−5|PHAR2_HUMAN−16−S−S2_−0.15
Doses Blue Rep1_−0.03 Red Rep2_−0.27
sp|O75167−5|PHAR2_HUMAN−25−T−S2_−0.15
Doses Blue Rep1_−0.03 Red Rep2_−0.27
sp|P67809|YBOX1_HUMAN−174−S−S2_−0.13
Doses Blue Rep1_0.06 Red Rep2_−0.33
sp|P67809|YBOX1_HUMAN−176−S−S2_−0.13
Doses Blue Rep1_0.06 Red Rep2_−0.33
sp|Q9UQ35|SRRM2_HUMAN−903−T−S3_−0.19
Doses Blue Rep1_0.04 Red Rep2_−0.43
sp|Q14160−2|SCRIB_HUMAN−1364−S−S2_−0.26
Doses Blue Rep1_−0.29 Red Rep2_−0.24
sp|Q14160−2|SCRIB_HUMAN−1367−S−S2_−0.26
Doses Blue Rep1_−0.29 Red Rep2_−0.24
sp|Q9Y520−6|PRC2C_HUMAN−1246−S−S3_0.14
Doses Blue Rep1_0.26 Red Rep2_0.03
sp|Q9Y520−6|PRC2C_HUMAN−1248−S−S3_0.14
Doses Blue Rep1_0.26 Red Rep2_0.03
sp|Q9Y520−6|PRC2C_HUMAN−1249−S−S3_0.14
Doses Blue Rep1_0.26 Red Rep2_0.03
sp|O76080|ZFAN5_HUMAN−48−S−S3_−0.07
Doses Blue Rep1_0.01 Red Rep2_−0.16
sp|O76080|ZFAN5_HUMAN−58−S−S3_−0.07
Doses Blue Rep1_0.01 Red Rep2_−0.16
sp|P67809|YBOX1_HUMAN−176−S−S1_−0.17
Doses Blue Rep1_−0.05 Red Rep2_−0.3
sp|Q9C0B1|FTO_HUMAN−256−S−S2_0
Doses Blue Rep1_0.16 Red Rep2_−0.15
ptm abundance 37C

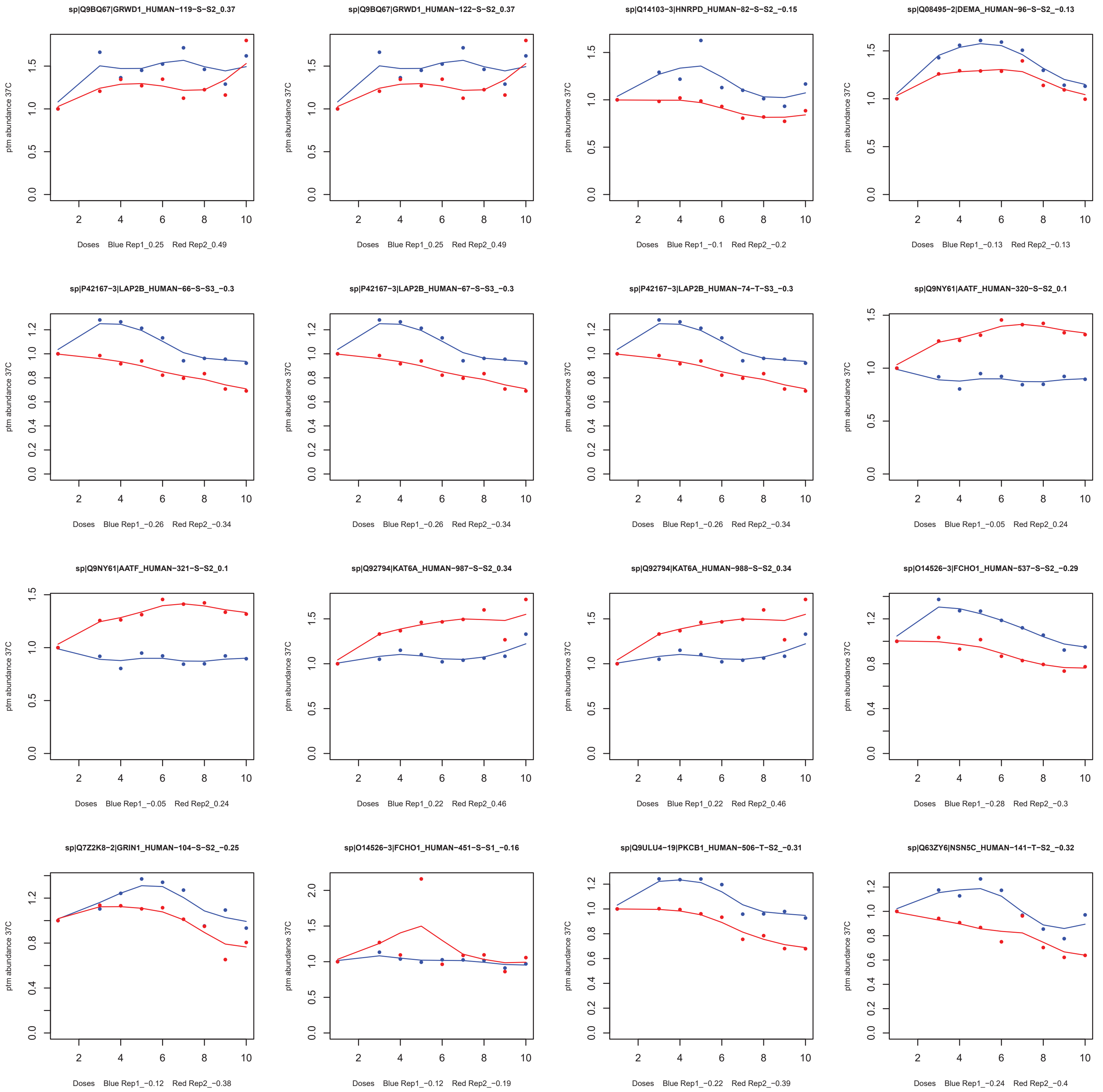
sp|Q9BQ67|GRWD1_HUMAN−119−S−S2_0.37
Doses Blue Rep1_0.25 Red Rep2_0.49
sp|Q9BQ67|GRWD1_HUMAN−122−S−S2_0.37
Doses Blue Rep1_0.25 Red Rep2_0.49
sp|Q14103−3|HNRPD_HUMAN−82−S−S2_−0.15
Doses Blue Rep1_−0.1 Red Rep2_−0.2
sp|Q08495−2|DEMA_HUMAN−96−S−S2_−0.13
Doses Blue Rep1_−0.13 Red Rep2_−0.13
sp|P42167−3|LAP2B_HUMAN−66−S−S3_−0.3
Doses Blue Rep1_−0.26 Red Rep2_−0.34
sp|P42167−3|LAP2B_HUMAN−67−S−S3_−0.3
Doses Blue Rep1_−0.26 Red Rep2_−0.34
sp|P42167−3|LAP2B_HUMAN−74−T−S3_−0.3
Doses Blue Rep1_−0.26 Red Rep2_−0.34
sp|Q9NY61|AATF_HUMAN−320−S−S2_0.1
Doses Blue Rep1_−0.05 Red Rep2_0.24
sp|Q9NY61|AATF_HUMAN−321−S−S2_0.1
Doses Blue Rep1_−0.05 Red Rep2_0.24
sp|Q92794|KAT6A_HUMAN−987−S−S2_0.34
Doses Blue Rep1_0.22 Red Rep2_0.46
sp|Q92794|KAT6A_HUMAN−988−S−S2_0.34
Doses Blue Rep1_0.22 Red Rep2_0.46
sp|O14526−3|FCHO1_HUMAN−537−S−S2_−0.29
Doses Blue Rep1_−0.28 Red Rep2_−0.3
sp|Q7Z2K8−2|GRIN1_HUMAN−104−S−S2_−0.25
Doses Blue Rep1_−0.12 Red Rep2_−0.38
sp|O14526−3|FCHO1_HUMAN−451−S−S1_−0.16
Doses Blue Rep1_−0.12 Red Rep2_−0.19
sp|Q9ULU4−19|PKCB1_HUMAN−506−T−S2_−0.31
Doses Blue Rep1_−0.22 Red Rep2_−0.39
sp|Q63ZY6|NSN5C_HUMAN−141−T−S2_−0.32
Doses Blue Rep1_−0.24 Red Rep2_−0.4
ptm abundance 37C

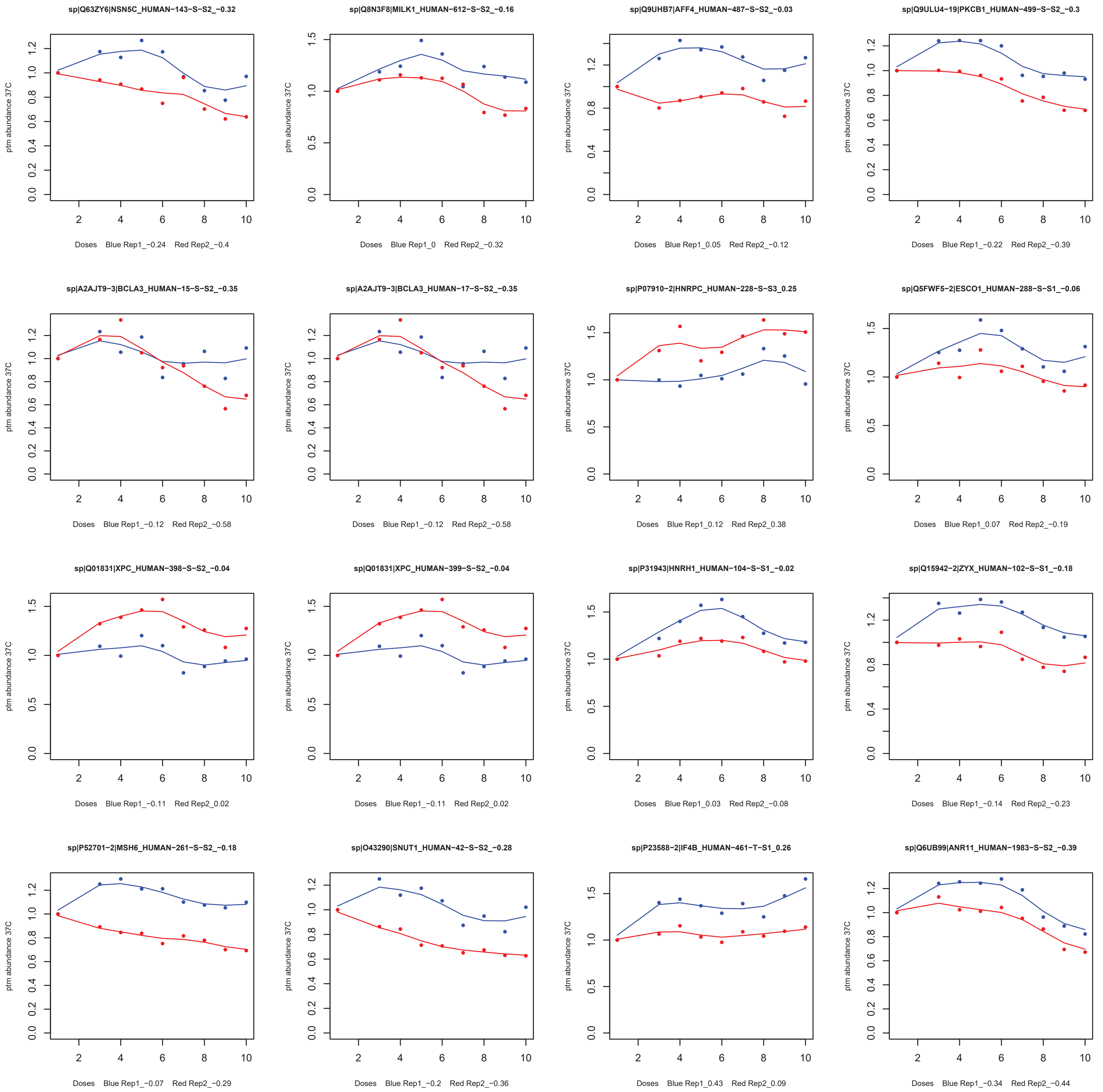

sp|Q63ZY6|NSN5C_HUMAN−143−S−S2_−0.32
Doses Blue Rep1_−0.24 Red Rep2_−0.4
sp|Q8N3F8|MILK1_HUMAN−612−S−S2_−0.16
Doses Blue Rep1_0 Red Rep2_−0.32
sp|Q9UHB7|AFF4_HUMAN−487−S−S2_−0.03
Doses Blue Rep1_0.05 Red Rep2_−0.12
sp|Q9ULU4−19|PKCB1_HUMAN−499−S−S2_−0.3
Doses Blue Rep1_−0.22 Red Rep2_−0.39
sp|A2AJT9−3|BCLA3_HUMAN−15−S−S2_−0.35
Doses Blue Rep1_−0.12 Red Rep2_−0.58
sp|A2AJT9−3|BCLA3_HUMAN−17−S−S2_−0.35
Doses Blue Rep1_−0.12 Red Rep2_−0.58
sp|P07910−2|HNRPC_HUMAN−228−S−S3_0.25
Doses Blue Rep1_0.12 Red Rep2_0.38
sp|Q5FWF5−2|ESCO1_HUMAN−288−S−S1_−0.06
Doses Blue Rep1_0.07 Red Rep2_−0.19
sp|Q01831|XPC_HUMAN−398−S−S2_−0.04
Doses Blue Rep1_−0.11 Red Rep2_0.02
sp|Q01831|XPC_HUMAN−399−S−S2_−0.04
Doses Blue Rep1_−0.11 Red Rep2_0.02
sp|P31943|HNRH1_HUMAN−104−S−S1_−0.02
Doses Blue Rep1_0.03 Red Rep2_−0.08
sp|Q15942−2|ZYX_HUMAN−102−S−S1_−0.18
Doses Blue Rep1_−0.14 Red Rep2_−0.23
sp|P52701−2|MSH6_HUMAN−261−S−S2_−0.18
Doses Blue Rep1_−0.07 Red Rep2_−0.29
sp|O43290|SNUT1_HUMAN−42−S−S2_−0.28
Doses Blue Rep1_−0.2 Red Rep2_−0.36
sp|P23588−2|IF4B_HUMAN−461−T−S1_0.26
Doses Blue Rep1_0.43 Red Rep2_0.09
sp|Q6UB99|ANR11_HUMAN−1983−S−S2_−0.39
Doses Blue Rep1_−0.34 Red Rep2_−0.44
ptm abundance 37C

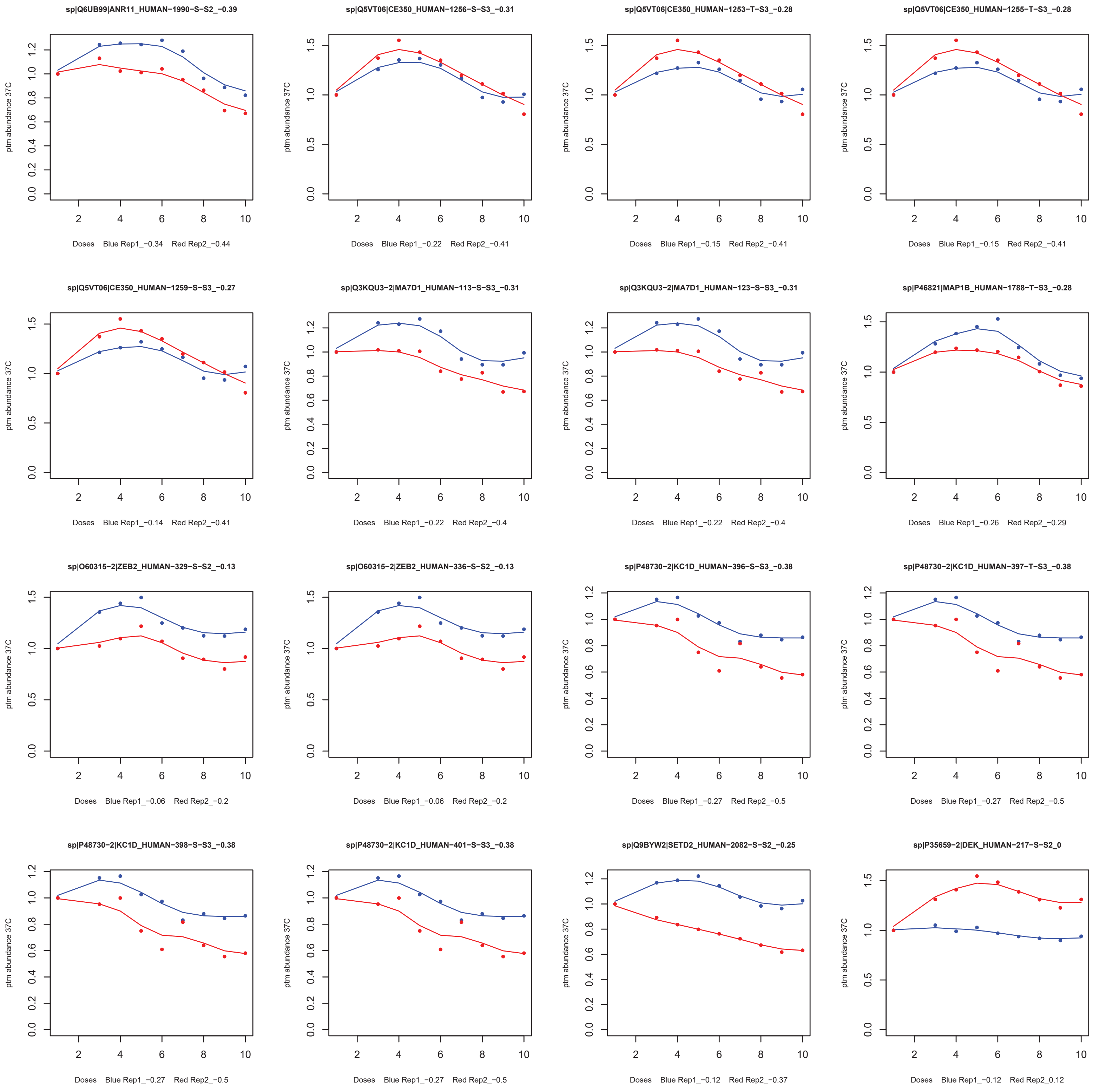

sp|Q6UB99|ANR11_HUMAN−1990−S−S2_−0.39
ptm abundance 37C
Doses Blue Rep1_−0.34 Red Rep2_−0.44
sp|Q5VT06|CE350_HUMAN−1256−S−S3_−0.31
Doses Blue Rep1_−0.22 Red Rep2_−0.41
sp|Q5VT06|CE350_HUMAN−1253−T−S3_−0.28
Doses Blue Rep1_−0.15 Red Rep2_−0.41
sp|Q5VT06|CE350_HUMAN−1255−T−S3_−0.28
Doses Blue Rep1_−0.15 Red Rep2_−0.41
sp|Q5VT06|CE350_HUMAN−1259−S−S3_−0.27
Doses Blue Rep1_−0.14 Red Rep2_−0.41
sp|Q3KQU3−2|MA7D1_HUMAN−113−S−S3_−0.31
Doses Blue Rep1_−0.22 Red Rep2_−0.4
sp|Q3KQU3−2|MA7D1_HUMAN−123−S−S3_−0.31
Doses Blue Rep1_−0.22 Red Rep2_−0.4
sp|P46821|MAP1B_HUMAN−1788−T−S3_−0.28
Doses Blue Rep1_−0.26 Red Rep2_−0.29
sp|O60315−2|ZEB2_HUMAN−329−S−S2_−0.13
Doses Blue Rep1_−0.06 Red Rep2_−0.2
sp|O60315−2|ZEB2_HUMAN−336−S−S2_−0.13
Doses Blue Rep1_−0.06 Red Rep2_−0.2
sp|P48730−2|KC1D_HUMAN−396−S−S3_−0.38
Doses Blue Rep1_−0.27 Red Rep2_−0.5
sp|P48730−2|KC1D_HUMAN−397−T−S3_−0.38
Doses Blue Rep1_−0.27 Red Rep2_−0.5
sp|P48730−2|KC1D_HUMAN−398−S−S3_−0.38
Doses Blue Rep1_−0.27 Red Rep2_−0.5
sp|P48730−2|KC1D_HUMAN−401−S−S3_−0.38
Doses Blue Rep1_−0.27 Red Rep2_−0.5
sp|Q9BYW2|SETD2_HUMAN−2082−S−S2_−0.25
Doses Blue Rep1_−0.12 Red Rep2_−0.37
sp|P35659−2|DEK_HUMAN−217−S−S2_0
Doses Blue Rep1_−0.12 Red Rep2_0.12

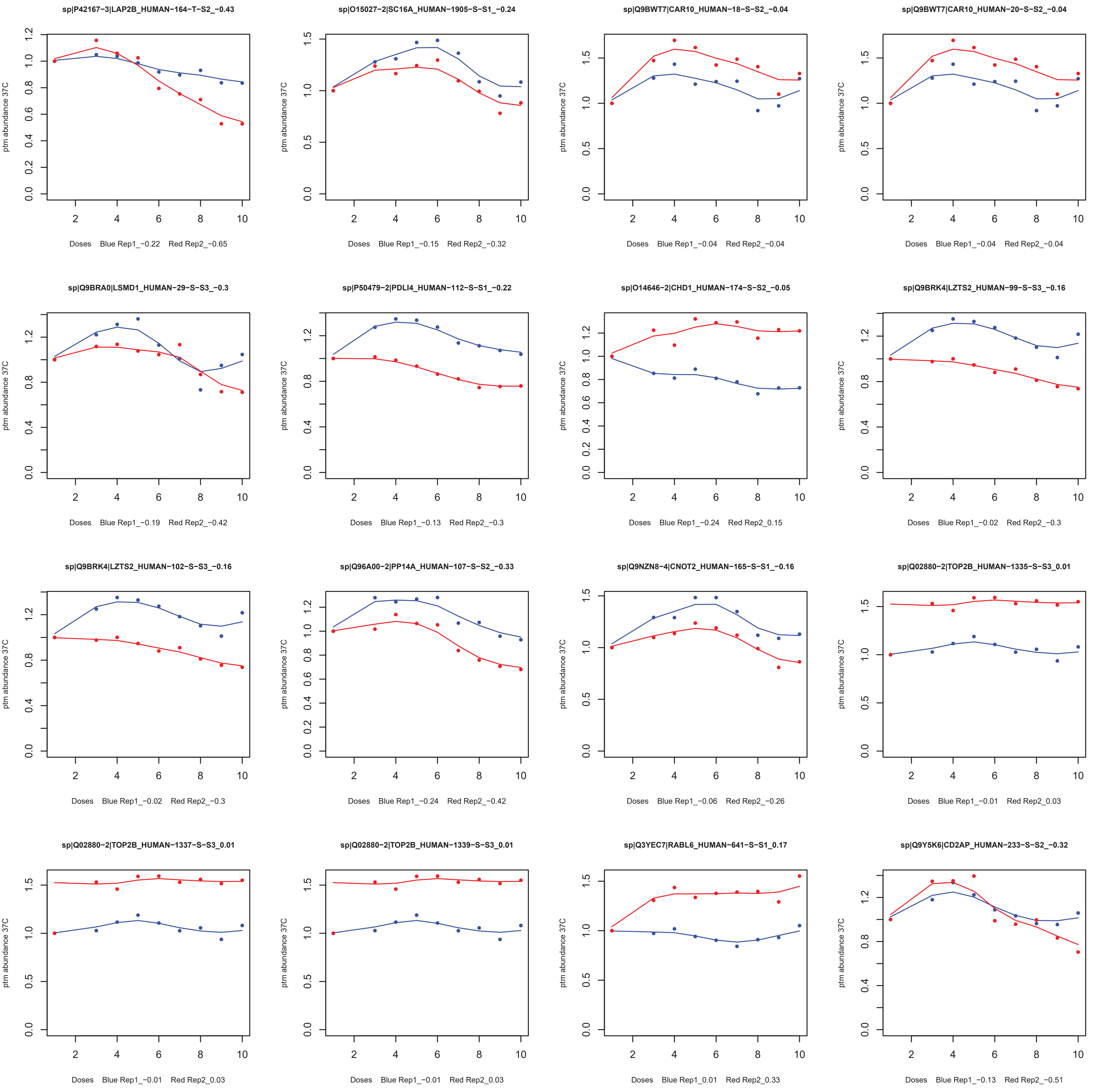
sp|P42167−3|LAP2B_HUMAN−164−T−S2_−0.43
Doses Blue Rep1_−0.22 Red Rep2_−0.65
sp|O15027−2|SC16A_HUMAN−1905−S−S1_−0.24
Doses Blue Rep1_−0.15 Red Rep2_−0.32
sp|Q9BWT7|CAR10_HUMAN−18−S−S2_−0.04
Doses Blue Rep1_−0.04 Red Rep2_−0.04
sp|Q9BWT7|CAR10_HUMAN−20−S−S2_−0.04
Doses Blue Rep1_−0.04 Red Rep2_−0.04
sp|Q9BRA0|LSMD1_HUMAN−29−S−S3_−0.3
Doses Blue Rep1_−0.19 Red Rep2_−0.42
sp|P50479−2|PDLI4_HUMAN−112−S−S1_−0.22
Doses Blue Rep1_−0.13 Red Rep2_−0.3
sp|O14646−2|CHD1_HUMAN−174−S−S2_−0.05
Doses Blue Rep1_−0.24 Red Rep2_0.15
sp|Q9BRK4|LZTS2_HUMAN−99−S−S3_−0.16
Doses Blue Rep1_−0.02 Red Rep2_−0.3
sp|Q9BRK4|LZTS2_HUMAN−102−S−S3_−0.16
Doses Blue Rep1_−0.02 Red Rep2_−0.3
sp|Q96A00−2|PP14A_HUMAN−107−S−S2_−0.33
Doses Blue Rep1_−0.24 Red Rep2_−0.42
sp|Q9NZN8−4|CNOT2_HUMAN−165−S−S1_−0.16
Doses Blue Rep1_−0.06 Red Rep2_−0.26
sp|Q02880−2|TOP2B_HUMAN−1335−S−S3_0.01
Doses Blue Rep1_−0.01 Red Rep2_0.03
sp|Q02880−2|TOP2B_HUMAN−1337−S−S3_0.01
Doses Blue Rep1_−0.01 Red Rep2_0.03
sp|Q02880−2|TOP2B_HUMAN−1339−S−S3_0.01
Doses Blue Rep1_−0.01 Red Rep2_0.03
sp|Q3YEC7|RABL6_HUMAN−641−S−S1_0.17
Doses Blue Rep1_0.01 Red Rep2_0.33
sp|Q9Y5K6|CD2AP_HUMAN−233−S−S2_−0.32
Doses Blue Rep1_−0.13 Red Rep2_−0.51
ptm abundance 37C

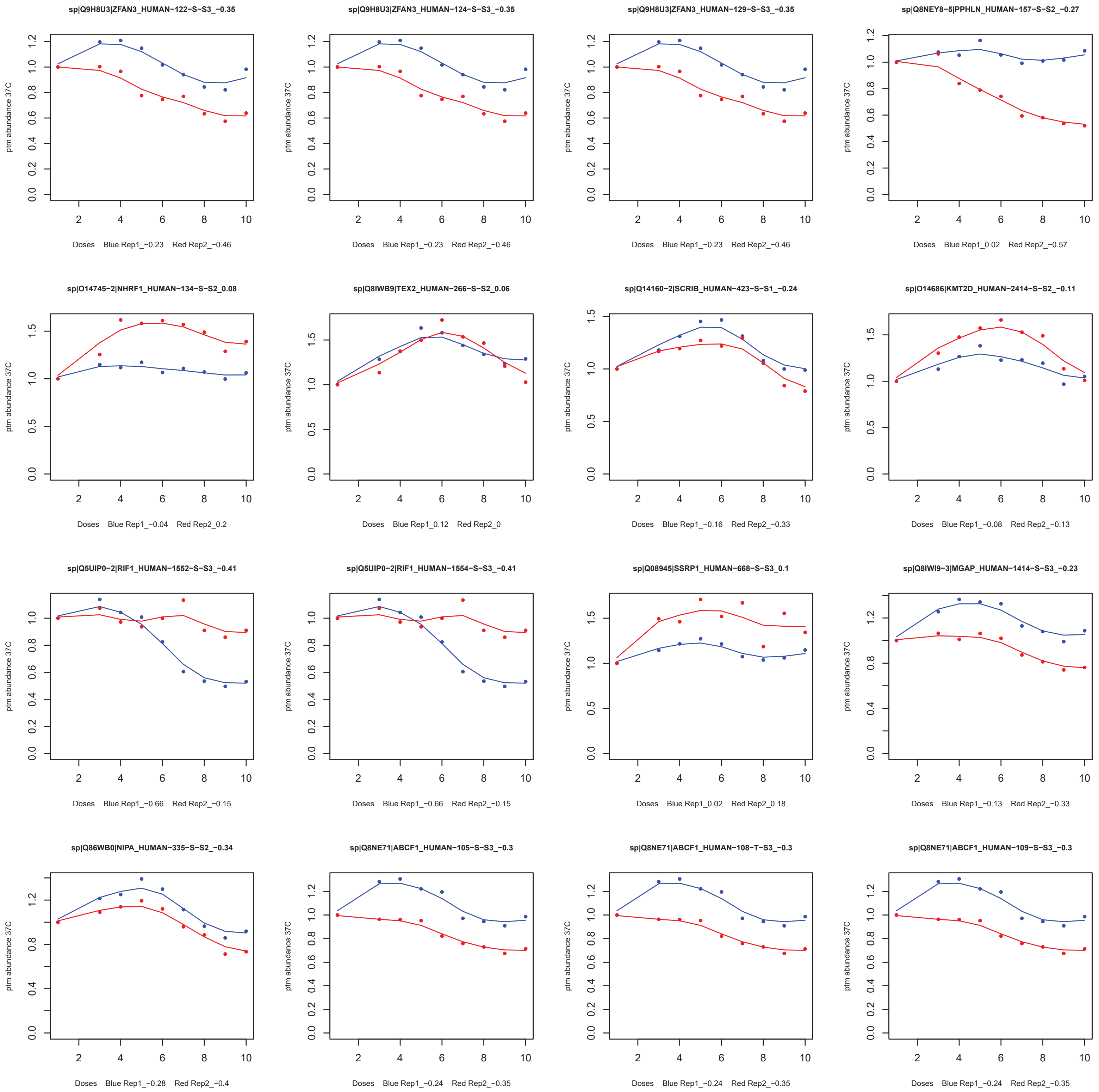
sp|Q9H8U3|ZFAN3_HUMAN−122−S−S3_−0.35
Doses Blue Rep1_−0.23 Red Rep2_−0.46
sp|Q9H8U3|ZFAN3_HUMAN−124−S−S3_−0.35
Doses Blue Rep1_−0.23 Red Rep2_−0.46
sp|Q9H8U3|ZFAN3_HUMAN−129−S−S3_−0.35
Doses Blue Rep1_−0.23 Red Rep2_−0.46
sp|Q8NEY8−5|PPHLN_HUMAN−157−S−S2_−0.27
Doses Blue Rep1_0.02 Red Rep2_−0.57
sp|O14745−2|NHRF1_HUMAN−134−S−S2_0.08
Doses Blue Rep1_−0.04 Red Rep2_0.2
sp|Q8IWB9|TEX2_HUMAN−266−S−S2_0.06
Doses Blue Rep1_0.12 Red Rep2_0
sp|Q14160−2|SCRIB_HUMAN−423−S−S1_−0.24
Doses Blue Rep1_−0.16 Red Rep2_−0.33
sp|O14686|KMT2D_HUMAN−2414−S−S2_−0.11
Doses Blue Rep1_−0.08 Red Rep2_−0.13
sp|Q5UIP0−2|RIF1_HUMAN−1552−S−S3_−0.41
Doses Blue Rep1_−0.66 Red Rep2_−0.15
sp|Q5UIP0−2|RIF1_HUMAN−1554−S−S3_−0.41
Doses Blue Rep1_−0.66 Red Rep2_−0.15
sp|Q08945|SSRP1_HUMAN−668−S−S3_0.1
Doses Blue Rep1_0.02 Red Rep2_0.18
sp|Q8IWI9−3|MGAP_HUMAN−1414−S−S3_−0.23
Doses Blue Rep1_−0.13 Red Rep2_−0.33
sp|Q86WB0|NIPA_HUMAN−335−S−S2_−0.34
Doses Blue Rep1_−0.28 Red Rep2_−0.4
sp|Q8NE71|ABCF1_HUMAN−105−S−S3_−0.3
Doses Blue Rep1_−0.24 Red Rep2_−0.35
sp|Q8NE71|ABCF1_HUMAN−108−T−S3_−0.3
Doses Blue Rep1_−0.24 Red Rep2_−0.35
sp|Q8NE71|ABCF1_HUMAN−109−S−S3_−0.3
Doses Blue Rep1_−0.24 Red Rep2_−0.35
ptm abundance 37C

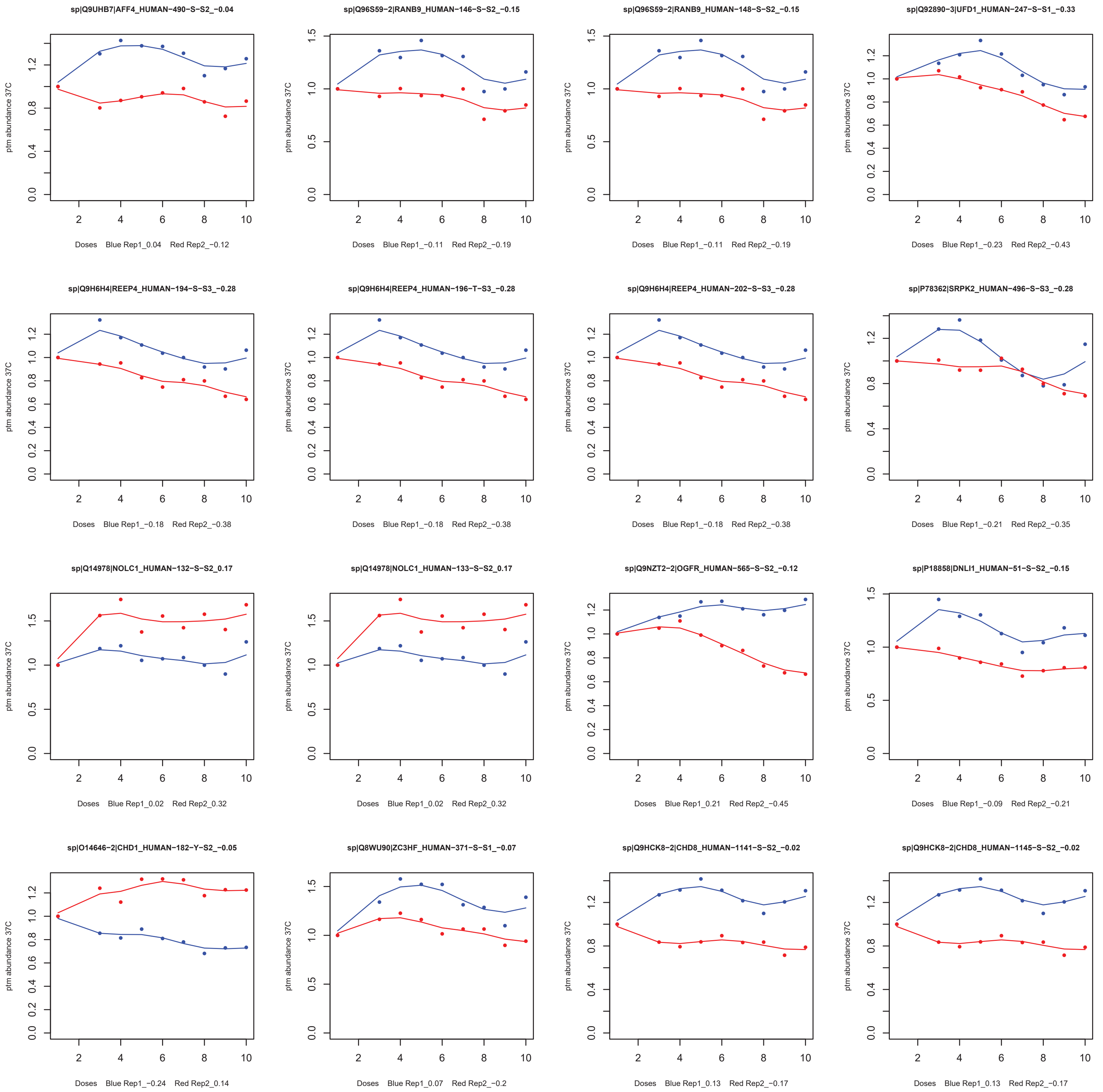
sp|Q9UHB7|AFF4_HUMAN−490−S−S2_−0.04
ptm abundance 37C
Doses Blue Rep1_0.04 Red Rep2_−0.12
sp|Q96S59−2|RANB9_HUMAN−146−S−S2_−0.15
ptm abundance 37C
Doses Blue Rep1_−0.11 Red Rep2_−0.19
sp|Q96S59−2|RANB9_HUMAN−148−S−S2_−0.15
ptm abundance 37C
Doses Blue Rep1_−0.11 Red Rep2_−0.19
sp|Q92890−3|UFD1_HUMAN−247−S−S1_−0.33
ptm abundance 37C
Doses Blue Rep1_−0.23 Red Rep2_−0.43
sp|Q9H6H4|REEP4_HUMAN−194−S−S3_−0.28
ptm abundance 37C
Doses Blue Rep1_−0.18 Red Rep2_−0.38
sp|Q9H6H4|REEP4_HUMAN−196−T−S3_−0.28
ptm abundance 37C
Doses Blue Rep1_−0.18 Red Rep2_−0.38
sp|Q9H6H4|REEP4_HUMAN−202−S−S3_−0.28
ptm abundance 37C
Doses Blue Rep1_−0.18 Red Rep2_−0.38
sp|P78362|SRPK2_HUMAN−496−S−S3_−0.28
ptm abundance 37C
Doses Blue Rep1_−0.21 Red Rep2_−0.35
sp|Q14978|NOLC1_HUMAN−132−S−S2_0.17
ptm abundance 37C
Doses Blue Rep1_0.02 Red Rep2_0.32
sp|Q14978|NOLC1_HUMAN−133−S−S2_0.17
ptm abundance 37C
Doses Blue Rep1_0.02 Red Rep2_0.32
sp|Q9NZT2−2|OGFR_HUMAN−565−S−S2_−0.12
ptm abundance 37C
Doses Blue Rep1_0.21 Red Rep2_−0.45
sp|P18858|DNLI1_HUMAN−51−S−S2_−0.15
ptm abundance 37C
Doses Blue Rep1_−0.09 Red Rep2_−0.21
sp|O14646−2|CHD1_HUMAN−182−Y−S2_−0.05
ptm abundance 37C
Doses Blue Rep1_−0.24 Red Rep2_0.14
sp|Q8WU90|ZC3HF_HUMAN−371−S−S1_−0.07
ptm abundance 37C
Doses Blue Rep1_0.07 Red Rep2_−0.2
sp|Q9HCK8−2|CHD8_HUMAN−1141−S−S2_−0.02
ptm abundance 37C
Doses Blue Rep1_0.13 Red Rep2_−0.17
sp|Q9HCK8−2|CHD8_HUMAN−1145−S−S2_−0.02
ptm abundance 37C
Doses Blue Rep1_0.13 Red Rep2_−0.17

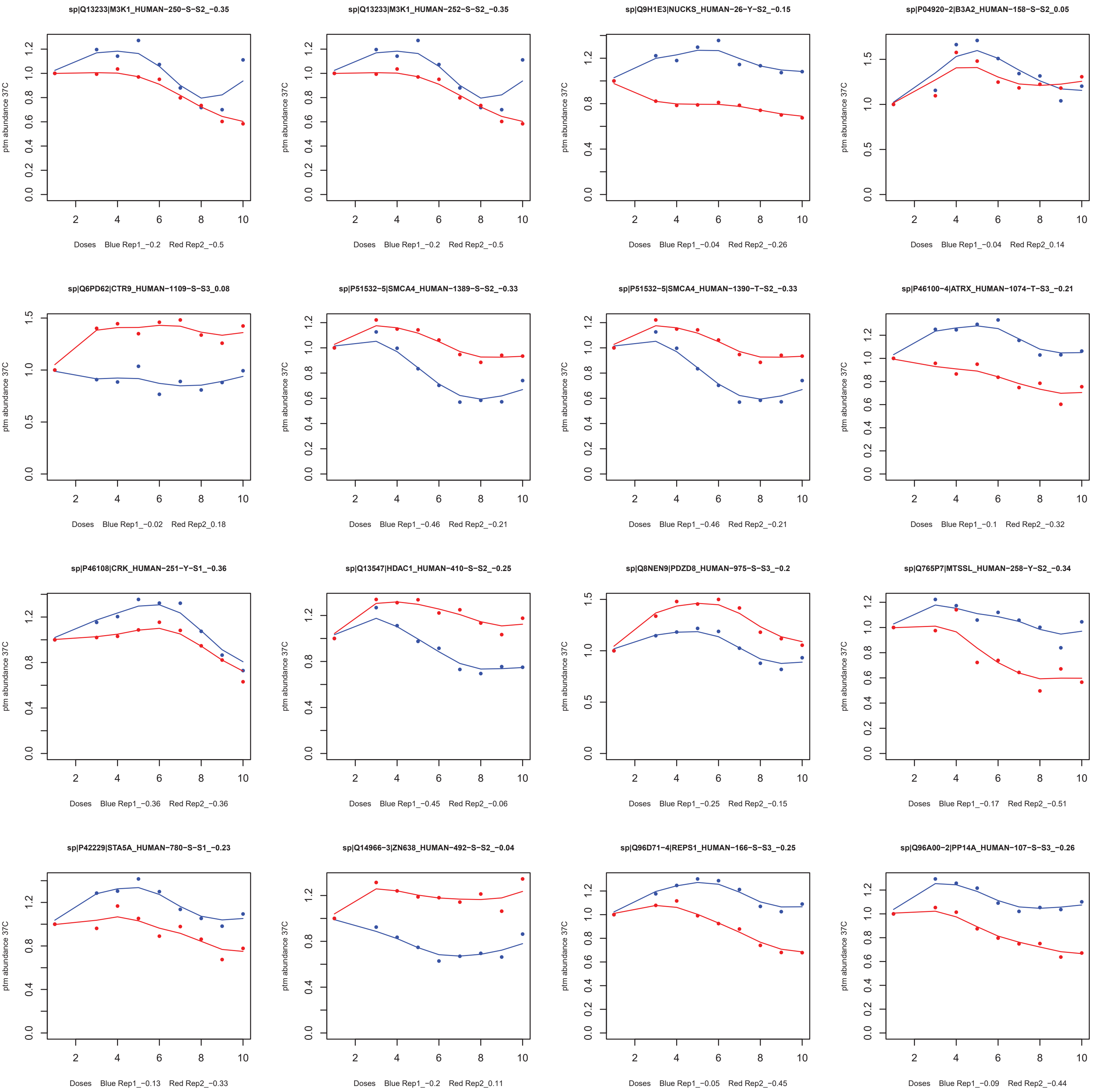

sp|Q13233|M3K1_HUMAN−250−S−S2_−0.35
ptm abundance 37C
Doses Blue Rep1_−0.2 Red Rep2_−0.5
sp|Q13233|M3K1_HUMAN−252−S−S2_−0.35
ptm abundance 37C
Doses Blue Rep1_−0.2 Red Rep2_−0.5
sp|Q9H1E3|NUCKS_HUMAN−26−Y−S2_−0.15
ptm abundance 37C
Doses Blue Rep1_−0.04 Red Rep2_−0.26
sp|P04920−2|B3A2_HUMAN−158−S−S2_0.05
ptm abundance 37C
Doses Blue Rep1_−0.04 Red Rep2_0.14
sp|Q6PD62|CTR9_HUMAN−1109−S−S3_0.08
ptm abundance 37C
Doses Blue Rep1_−0.02 Red Rep2_0.18
sp|P51532−5|SMCA4_HUMAN−1389−S−S2_−0.33
ptm abundance 37C
Doses Blue Rep1_−0.46 Red Rep2_−0.21
sp|P51532−5|SMCA4_HUMAN−1390−T−S2_−0.33
ptm abundance 37C
Doses Blue Rep1_−0.46 Red Rep2_−0.21
sp|P46100−4|ATRX_HUMAN−1074−T−S3_−0.21
ptm abundance 37C
Doses Blue Rep1_−0.1 Red Rep2_−0.32
sp|P46108|CRK_HUMAN−251−Y−S1_−0.36
ptm abundance 37C
Doses Blue Rep1_−0.36 Red Rep2_−0.36
sp|Q13547|HDAC1_HUMAN−410−S−S2_−0.25
ptm abundance 37C
Doses Blue Rep1_−0.45 Red Rep2_−0.06
sp|Q8NEN9|PDZD8_HUMAN−975−S−S3_−0.2
ptm abundance 37C
Doses Blue Rep1_−0.25 Red Rep2_−0.15
sp|Q765P7|MTSSL_HUMAN−258−Y−S2_−0.34
ptm abundance 37C
Doses Blue Rep1_−0.17 Red Rep2_−0.51
sp|P42229|STA5A_HUMAN−780−S−S1_−0.23
ptm abundance 37C
Doses Blue Rep1_−0.13 Red Rep2_−0.33
sp|Q14966−3|ZN638_HUMAN−492−S−S2_−0.04
ptm abundance 37C
Doses Blue Rep1_−0.2 Red Rep2_0.11
sp|Q96D71−4|REPS1_HUMAN−166−S−S3_−0.25
ptm abundance 37C
Doses Blue Rep1_−0.05 Red Rep2_−0.45
sp|Q96A00−2|PP14A_HUMAN−107−S−S3_−0.26
ptm abundance 37C
Doses Blue Rep1_−0.09 Red Rep2_−0.44

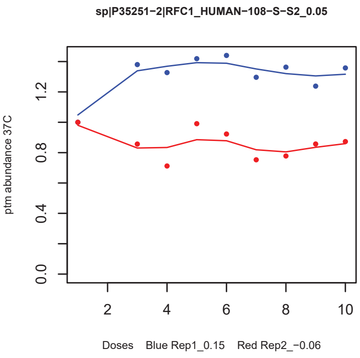
sp|P35251−2|RFC1_HUMAN−108−S−S2_0.05
ptm abundance 37C
Doses Blue Rep1_0.15 Red Rep2_−0.06

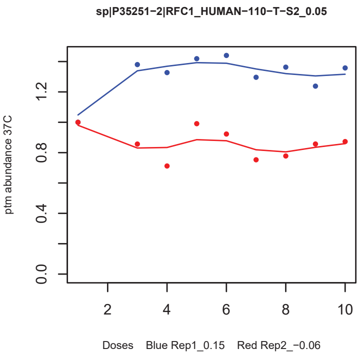
sp|P35251−2|RFC1_HUMAN−110−T−S2_0.05
ptm abundance 37C
Doses Blue Rep1_0.15 Red Rep2_−0.06

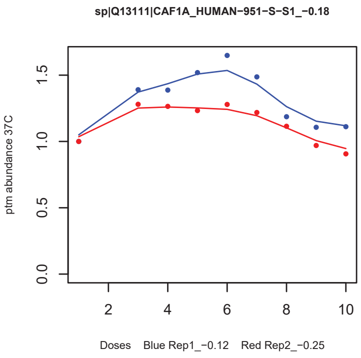
sp|Q13111|CAF1A_HUMAN−951−S−S1_−0.18
ptm abundance 37C
Doses Blue Rep1_−0.12 Red Rep2_−0.25

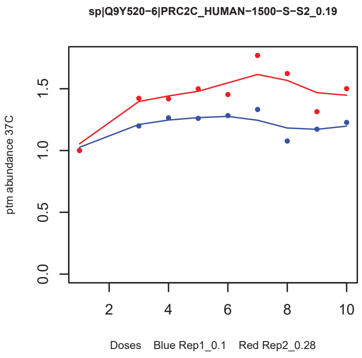
sp|Q9Y520−6|PRC2C_HUMAN−1500−S−S2_0.19
ptm abundance 37C
Doses Blue Rep1_0.1 Red Rep2_0.28

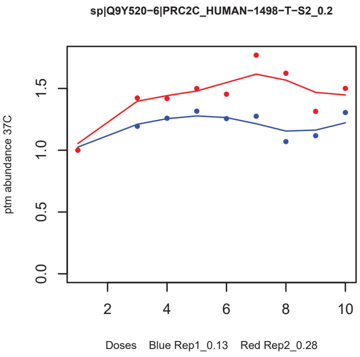
sp|Q9Y520−6|PRC2C_HUMAN−1498−T−S2_0.2
ptm abundance 37C
Doses Blue Rep1_0.13 Red Rep2_0.28

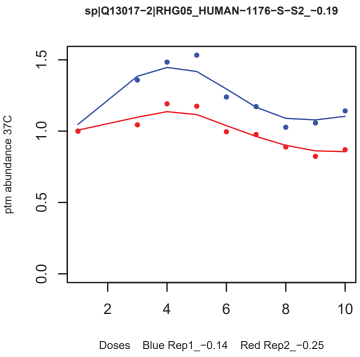
sp|Q13017−2|RHG05_HUMAN−1176−S−S2_−0.19
ptm abundance 37C
Doses Blue Rep1_−0.14 Red Rep2_−0.25

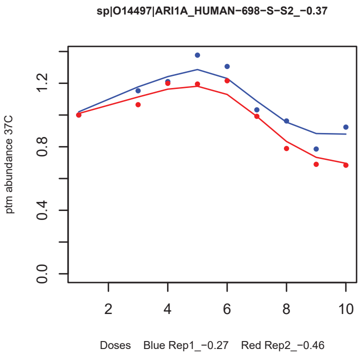
sp|O14497|ARI1A_HUMAN−698−S−S2_−0.37
ptm abundance 37C
Doses Blue Rep1_−0.27 Red Rep2_−0.46

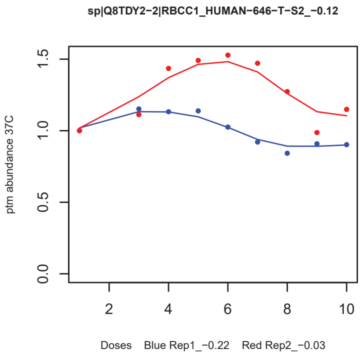
sp|Q8TDY2−2|RBCC1_HUMAN−646−T−S2_−0.12
ptm abundance 37C
Doses Blue Rep1_−0.22 Red Rep2_−0.03

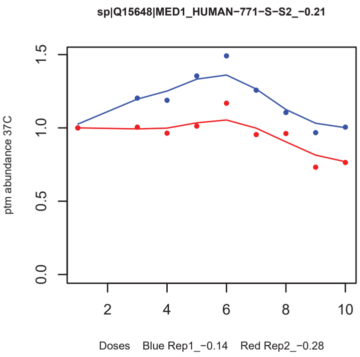
sp|Q15648|MED1_HUMAN−771−S−S2_−0.21
ptm abundance 37C
Doses Blue Rep1_−0.14 Red Rep2_−0.28

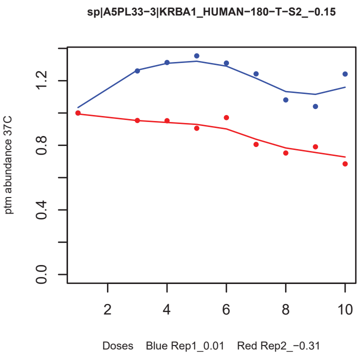
sp|A5PL33−3|KRBA1_HUMAN−180−T−S2_−0.15
ptm abundance 37C
Doses Blue Rep1_0.01 Red Rep2_−0.31

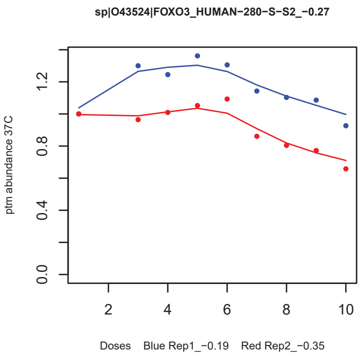
sp|O43524|FOXO3_HUMAN−280−S−S2_−0.27
ptm abundance 37C
Doses Blue Rep1_−0.19 Red Rep2_−0.35

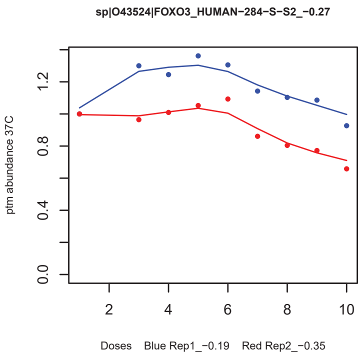
sp|O43524|FOXO3_HUMAN−284−S−S2_−0.27
ptm abundance 37C
Doses Blue Rep1_−0.19 Red Rep2_−0.35

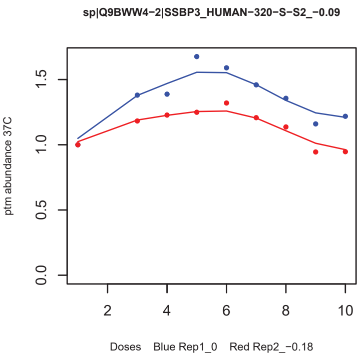
sp|Q9BWW4−2|SSBP3_HUMAN−320−S−S2_−0.09
ptm abundance 37C
Doses Blue Rep1_0 Red Rep2_−0.18

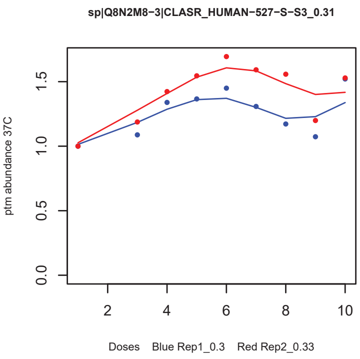
sp|Q8N2M8−3|CLASR_HUMAN−527−S−S3_0.31
ptm abundance 37C
Doses Blue Rep1_0.3 Red Rep2_0.33

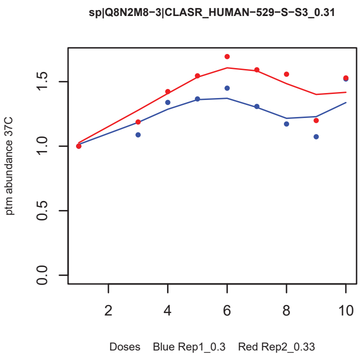
sp|Q8N2M8−3|CLASR_HUMAN−529−S−S3_0.31
ptm abundance 37C
Doses Blue Rep1_0.3 Red Rep2_0.33

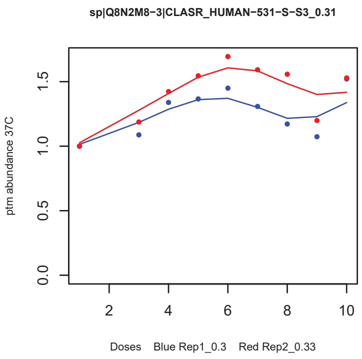
sp|Q8N2M8−3|CLASR_HUMAN−531−S−S3_0.31
ptm abundance 37C
Doses Blue Rep1_0.3 Red Rep2_0.33

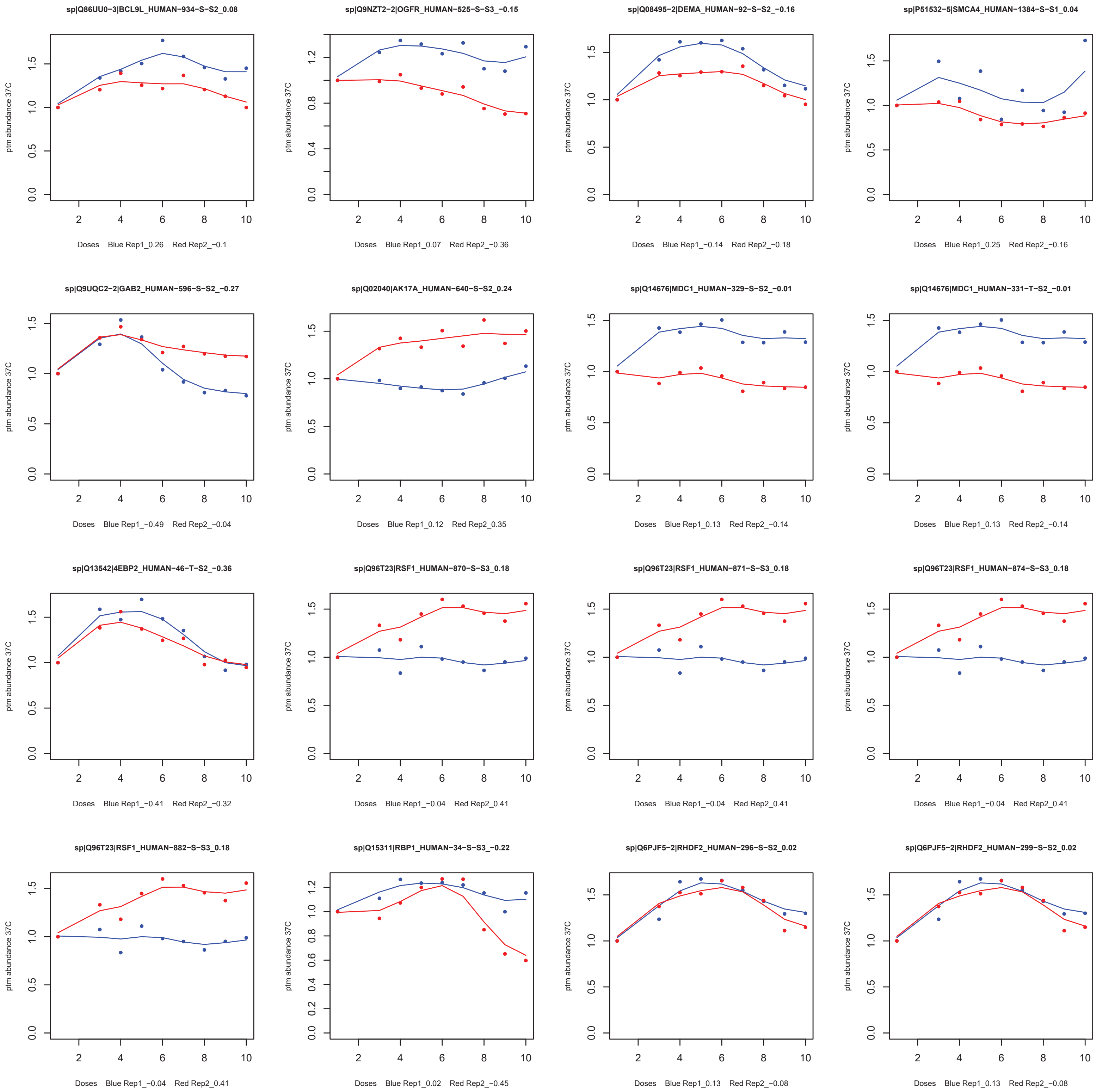
sp|Q86UU0-3|BCL9L_HUMAN-934-S-S2_0.08
ptm abundance 37C
Doses Blue Rep1_0.26 Red Rep2_-0.1
sp|Q9NZT2-2|OGFR_HUMAN-525-S-S3_-0.15
ptm abundance 37C
Doses Blue Rep1_0.07 Red Rep2_-0.36
sp|Q08495-2|DEMA_HUMAN-92-S-S2_-0.16
ptm abundance 37C
Doses Blue Rep1_-0.14 Red Rep2_-0.18
sp|P51532-5|SMCA4_HUMAN-1384-S-S1_0.04
ptm abundance 37C
Doses Blue Rep1_0.25 Red Rep2_-0.16
sp|Q9UQC2-2|GAB2_HUMAN-596-S-S2_-0.27
ptm abundance 37C
Doses Blue Rep1_-0.49 Red Rep2_-0.04
sp|Q02040|AK17A_HUMAN-640-S-S2_0.24
ptm abundance 37C
Doses Blue Rep1_0.12 Red Rep2_0.35
sp|Q14676|MDC1_HUMAN-329-S-S2_-0.01
ptm abundance 37C
Doses Blue Rep1_0.13 Red Rep2_-0.14
sp|Q14676|MDC1_HUMAN-331-T-S2_-0.01
ptm abundance 37C
Doses Blue Rep1_0.13 Red Rep2_-0.14
sp|Q13542|4EBP2_HUMAN-46-T-S2_-0.36
ptm abundance 37C
Doses Blue Rep1_-0.41 Red Rep2_-0.32
sp|Q96T23|RSF1_HUMAN-870-S-S3_0.18
ptm abundance 37C
Doses Blue Rep1_-0.04 Red Rep2_0.41
sp|Q96T23|RSF1_HUMAN-871-S-S3_0.18
ptm abundance 37C
Doses Blue Rep1_-0.04 Red Rep2_0.41
sp|Q96T23|RSF1_HUMAN-874-S-S3_0.18
ptm abundance 37C
Doses Blue Rep1_-0.04 Red Rep2_0.41
sp|Q96T23|RSF1_HUMAN-882-S-S3_0.18
ptm abundance 37C
Doses Blue Rep1_-0.04 Red Rep2_0.41
sp|Q15311|RBP1_HUMAN-34-S-S3_-0.22
ptm abundance 37C
Doses Blue Rep1_0.02 Red Rep2_-0.45
sp|Q6PJF5-2|RHDF2_HUMAN-296-S-S2_0.02
ptm abundance 37C
Doses Blue Rep1_0.13 Red Rep2_-0.08
sp|Q6PJF5-2|RHDF2_HUMAN-299-S-S2_0.02
ptm abundance 37C
Doses Blue Rep1_0.13 Red Rep2_-0.08

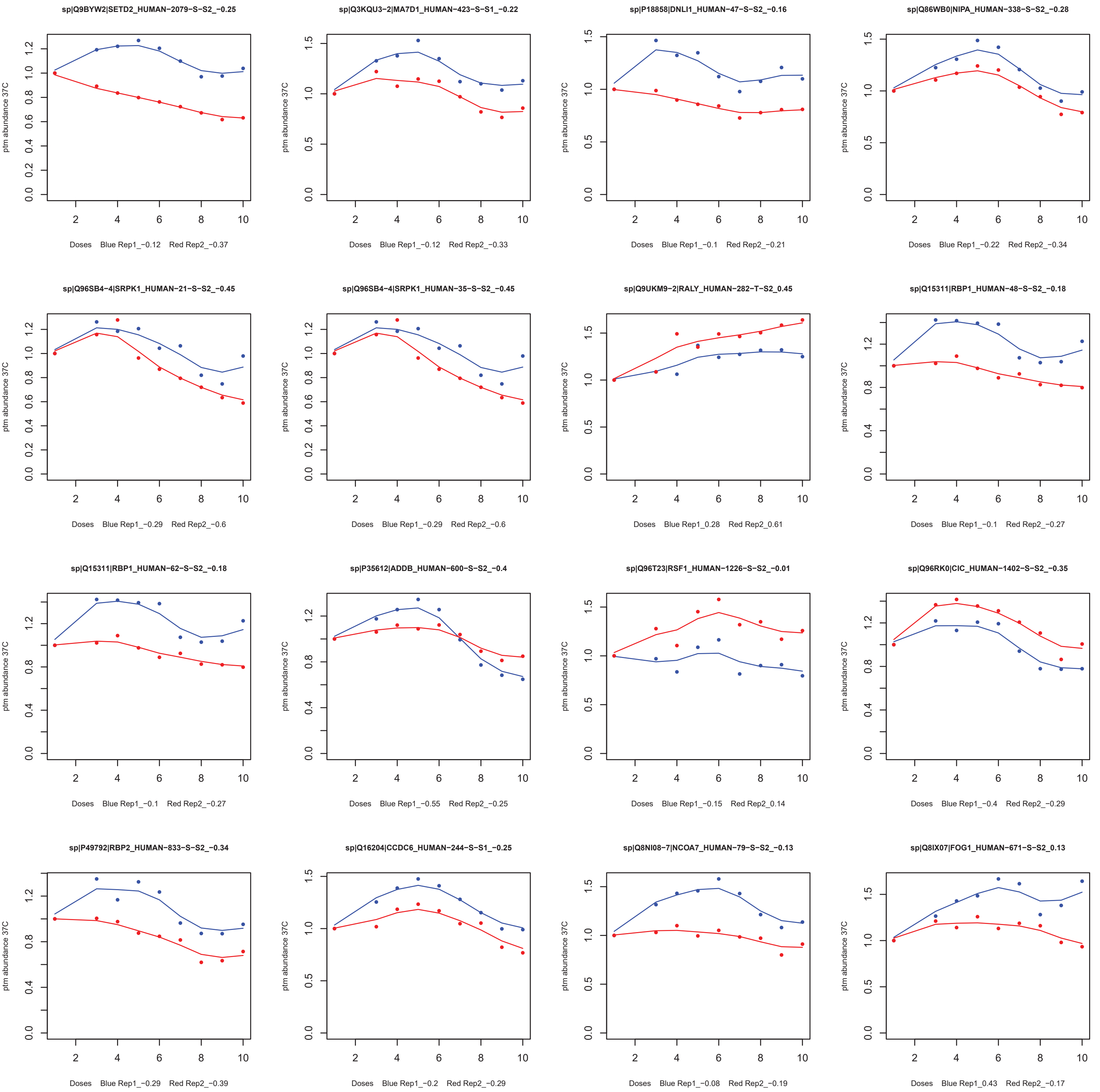
sp|Q9BYW2|SETD2_HUMAN−2079−S−S2_−0.25
Doses Blue Rep1_−0.12 Red Rep2_−0.37
sp|Q3KQU3−2|MA7D1_HUMAN−423−S−S1_−0.22
Doses Blue Rep1_−0.12 Red Rep2_−0.33
sp|P18858|DNLI1_HUMAN−47−S−S2_−0.16
Doses Blue Rep1_−0.1 Red Rep2_−0.21
sp|Q86WB0|NIPA_HUMAN−338−S−S2_−0.28
Doses Blue Rep1_−0.22 Red Rep2_−0.34
sp|Q96SB4−4|SRPK1_HUMAN−21−S−S2_−0.45
Doses Blue Rep1_−0.29 Red Rep2_−0.6
sp|Q96SB4−4|SRPK1_HUMAN−35−S−S2_−0.45
Doses Blue Rep1_−0.29 Red Rep2_−0.6
sp|Q9UKM9−2|RALY_HUMAN−282−T−S2_0.45
Doses Blue Rep1_0.28 Red Rep2_0.61
sp|Q15311|RBP1_HUMAN−48−S−S2_−0.18
Doses Blue Rep1_−0.1 Red Rep2_−0.27
sp|Q15311|RBP1_HUMAN−62−S−S2_−0.18
Doses Blue Rep1_−0.1 Red Rep2_−0.27
sp|P35612|ADDB_HUMAN−600−S−S2_−0.4
Doses Blue Rep1_−0.55 Red Rep2_−0.25
sp|Q96T23|RSF1_HUMAN−1226−S−S2_−0.01
Doses Blue Rep1_−0.15 Red Rep2_0.14
sp|Q96RK0|CIC_HUMAN−1402−S−S2_−0.35
Doses Blue Rep1_−0.4 Red Rep2_−0.29
sp|P49792|RBP2_HUMAN−833−S−S2_−0.34
Doses Blue Rep1_−0.29 Red Rep2_−0.39
sp|Q16204|CCDC6_HUMAN−244−S−S1_−0.25
Doses Blue Rep1_−0.2 Red Rep2_−0.29
sp|Q8NI08−7|NCOA7_HUMAN−79−S−S2_−0.13
Doses Blue Rep1_−0.08 Red Rep2_−0.19
sp|Q8IX07|FOG1_HUMAN−671−S−S2_0.13
Doses Blue Rep1_0.43 Red Rep2_−0.17
ptm abundance 37C

sp|Q8IX07|FOG1_HUMAN−672−S−S2_0.13

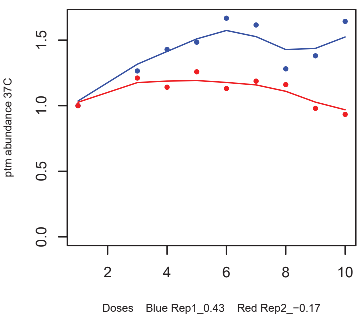

sp|Q8N9T8|KRI1_HUMAN−91−T−S3_0.14

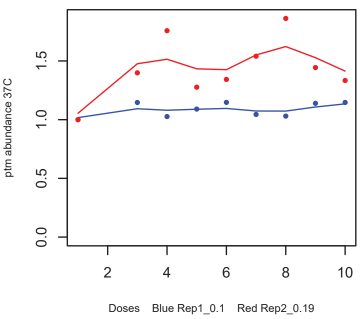

sp|Q8N9T8|KRI1_HUMAN−93−S−S3_0.14

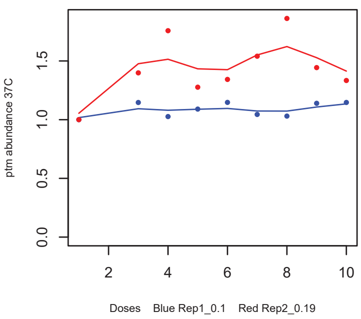

sp|Q8N9T8|KRI1_HUMAN−94−S−S3_0.14

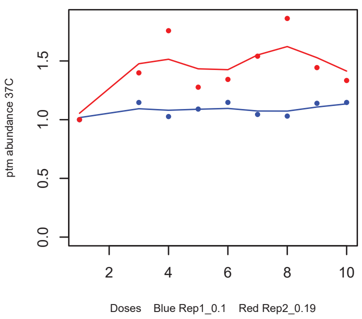

sp|Q8N9T8|KRI1_HUMAN−95−S−S3_0.14

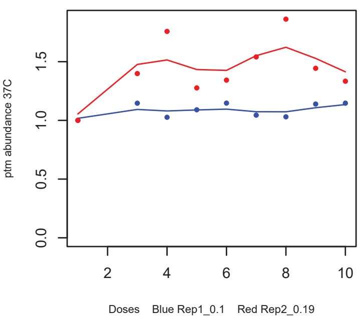

sp|Q8N9T8|KRI1_HUMAN−97−S−S3_0.14

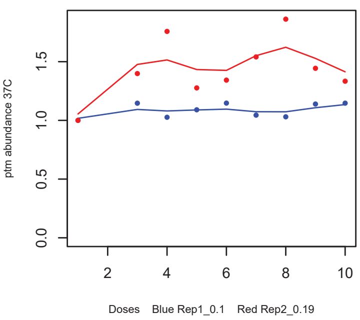

sp|O43149−3|ZZEF1_HUMAN−985−S−S1_−0.31

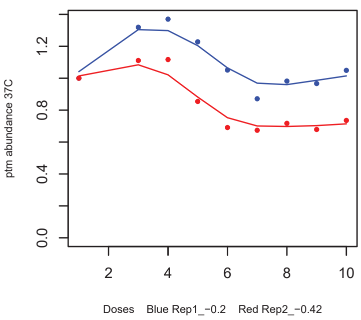

sp|Q6IN85−4|P4R3A_HUMAN−529−S−S3_−0.26

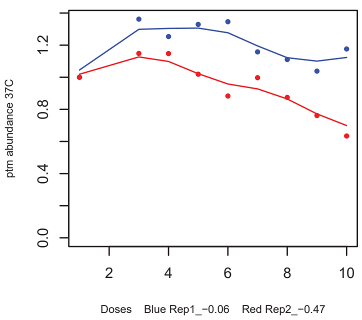

sp|Q6IN85−4|P4R3A_HUMAN−532−S−S3_−0.26

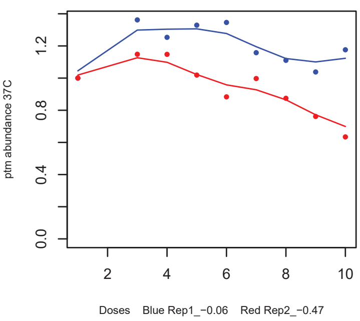

sp|Q6IN85−4|P4R3A_HUMAN−535−S−S3_−0.26

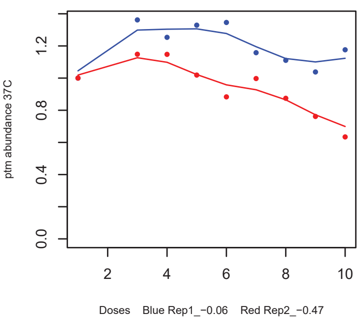

sp|Q6IN85−4|P4R3A_HUMAN−538−S−S3_−0.26

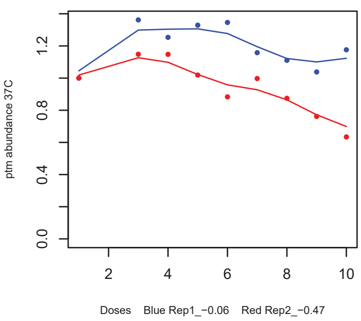

sp|Q6IN85−4|P4R3A_HUMAN−541−S−S3_−0.26

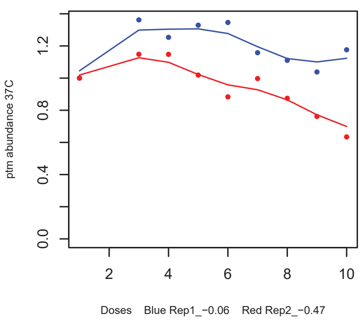

sp|Q9NZT2−2|OGFR_HUMAN−557−S−S3_−0.39

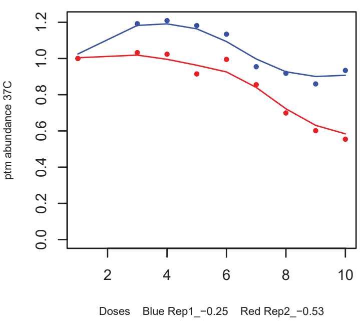

sp|Q9NZT2−2|OGFR_HUMAN−559−S−S3_−0.39

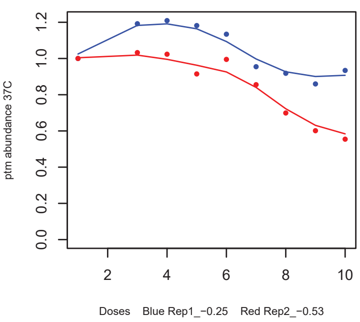

sp|Q12888|TP53B_HUMAN−316−S−S2_−0.32

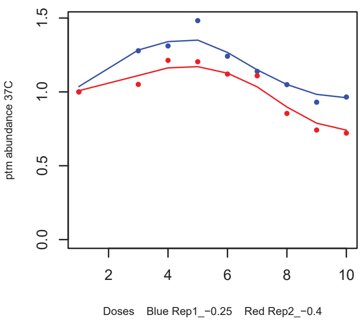

sp|P85037|FOXK1_HUMAN−213−S−S2_−0.38

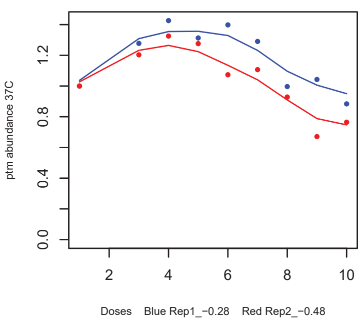

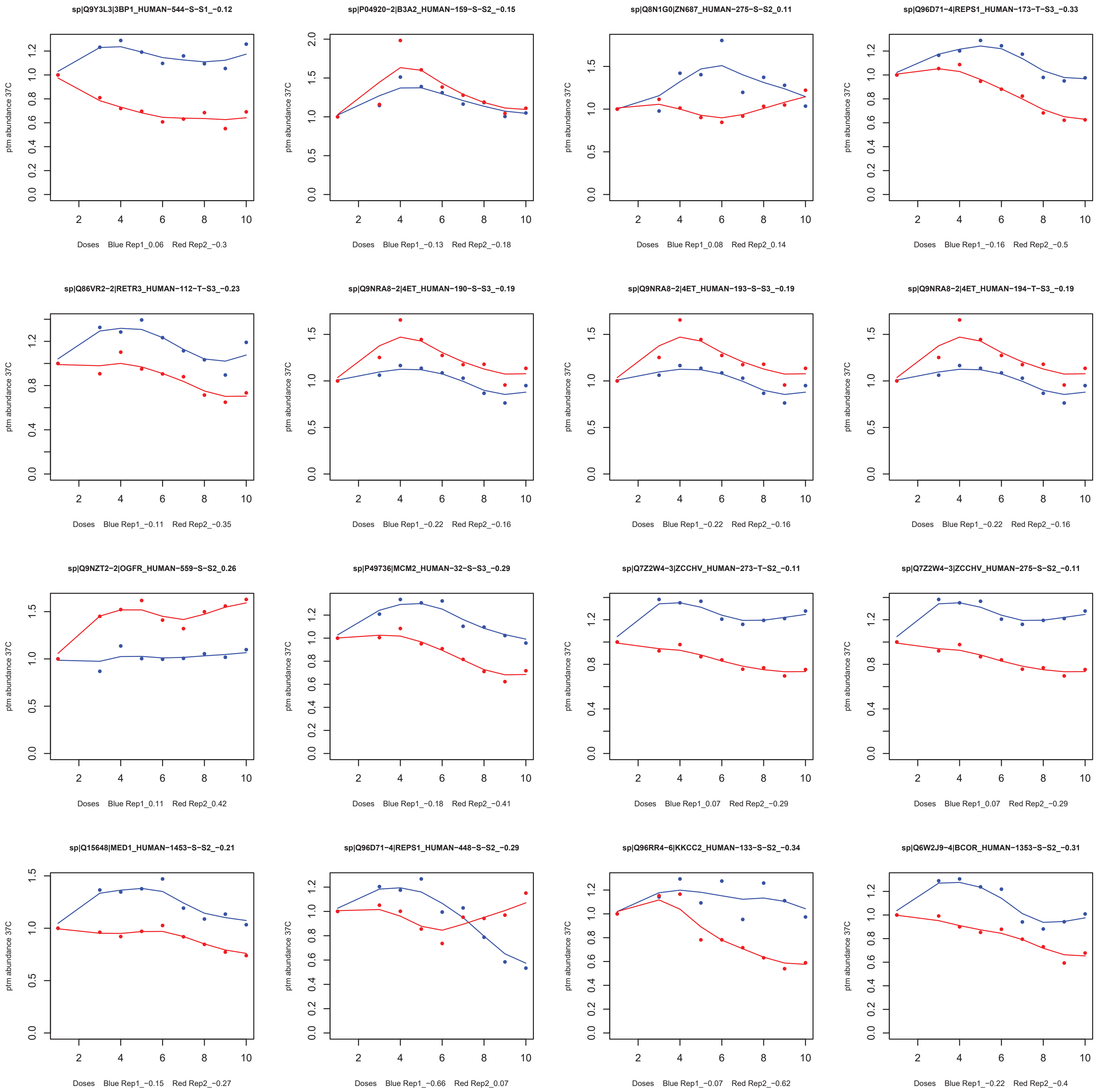
sp|Q9Y3L3|3BP1_HUMAN−544−S−S1_−0.12
Doses Blue Rep1_0.06 Red Rep2_−0.3
sp|P04920−2|B3A2_HUMAN−159−S−S2_−0.15
Doses Blue Rep1_−0.13 Red Rep2_−0.18
sp|Q8N1G0|ZN687_HUMAN−275−S−S2_0.11
Doses Blue Rep1_0.08 Red Rep2_0.14
sp|Q96D71−4|REPS1_HUMAN−173−T−S3_−0.33
Doses Blue Rep1_−0.16 Red Rep2_−0.5
sp|Q86VR2−2|RETR3_HUMAN−112−T−S3_−0.23
Doses Blue Rep1_−0.11 Red Rep2_−0.35
sp|Q9NRA8−2|4ET_HUMAN−190−S−S3_−0.19
Doses Blue Rep1_−0.22 Red Rep2_−0.16
sp|Q9NRA8−2|4ET_HUMAN−193−S−S3_−0.19
Doses Blue Rep1_−0.22 Red Rep2_−0.16
sp|Q9NRA8−2|4ET_HUMAN−194−T−S3_−0.19
Doses Blue Rep1_−0.22 Red Rep2_−0.16
sp|Q9NZT2−2|OGFR_HUMAN−559−S−S2_0.26
Doses Blue Rep1_0.11 Red Rep2_0.42
sp|P49736|MCM2_HUMAN−32−S−S3_−0.29
Doses Blue Rep1_−0.18 Red Rep2_−0.41
sp|Q7Z2W4−3|ZCCHV_HUMAN−273−T−S2_−0.11
Doses Blue Rep1_0.07 Red Rep2_−0.29
sp|Q7Z2W4−3|ZCCHV_HUMAN−275−S−S2_−0.11
Doses Blue Rep1_0.07 Red Rep2_−0.29
sp|Q15648|MED1_HUMAN−1453−S−S2_−0.21
Doses Blue Rep1_−0.15 Red Rep2_−0.27
sp|Q96D71−4|REPS1_HUMAN−448−S−S2_−0.29
Doses Blue Rep1_−0.66 Red Rep2_0.07
sp|Q96RR4−6|KKCC2_HUMAN−133−S−S2_−0.34
Doses Blue Rep1_−0.07 Red Rep2_−0.62
sp|Q6W2J9−4|BCOR_HUMAN−1353−S−S2_−0.31
Doses Blue Rep1_−0.22 Red Rep2_−0.4
ptm abundance 37C

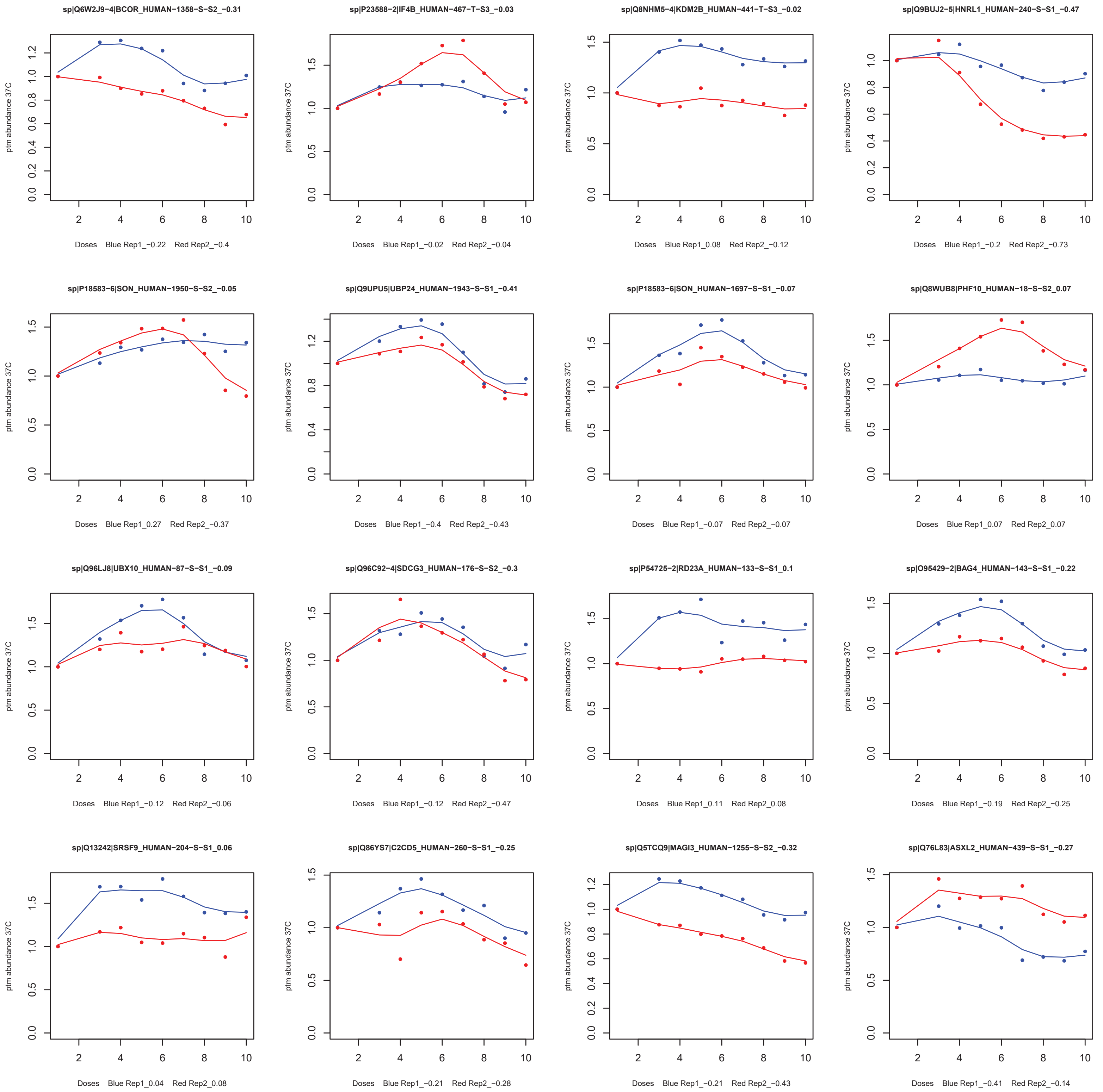

sp|Q6W2J9−4|BCOR_HUMAN−1358−S−S2_−0.31
ptm abundance 37C
Doses Blue Rep1_−0.22 Red Rep2_−0.4
sp|P23588−2|IF4B_HUMAN−467−T−S3_−0.03
ptm abundance 37C
Doses Blue Rep1_−0.02 Red Rep2_−0.04
sp|Q8NHM5−4|KDM2B_HUMAN−441−T−S3_−0.02
ptm abundance 37C
Doses Blue Rep1_0.08 Red Rep2_−0.12
sp|Q9BUJ2−5|HNRL1_HUMAN−240−S−S1_−0.47
ptm abundance 37C
Doses Blue Rep1_−0.2 Red Rep2_−0.73
sp|P18583−6|SON_HUMAN−1950−S−S2_−0.05
ptm abundance 37C
Doses Blue Rep1_0.27 Red Rep2_−0.37
sp|Q9UPU5|UBP24_HUMAN−1943−S−S1_−0.41
ptm abundance 37C
Doses Blue Rep1_−0.4 Red Rep2_−0.43
sp|P18583−6|SON_HUMAN−1697−S−S1_−0.07
ptm abundance 37C
Doses Blue Rep1_−0.07 Red Rep2_−0.07
sp|Q8WUB8|PHF10_HUMAN−18−S−S2_0.07
ptm abundance 37C
Doses Blue Rep1_0.07 Red Rep2_0.07
sp|Q96LJ8|UBX10_HUMAN−87−S−S1_−0.09
ptm abundance 37C
Doses Blue Rep1_−0.12 Red Rep2_−0.06
sp|Q96C92−4|SDCG3_HUMAN−176−S−S2_−0.3
ptm abundance 37C
Doses Blue Rep1_−0.12 Red Rep2_−0.47
sp|P54725−2|RD23A_HUMAN−133−S−S1_0.1
ptm abundance 37C
Doses Blue Rep1_0.11 Red Rep2_0.08
sp|O95429−2|BAG4_HUMAN−143−S−S1_−0.22
ptm abundance 37C
Doses Blue Rep1_−0.19 Red Rep2_−0.25
sp|Q13242|SRSF9_HUMAN−204−S−S1_0.06
ptm abundance 37C
Doses Blue Rep1_0.04 Red Rep2_0.08
sp|Q86YS7|C2CD5_HUMAN−260−S−S1_−0.25
ptm abundance 37C
Doses Blue Rep1_−0.21 Red Rep2_−0.28
sp|Q5TCQ9|MAGI3_HUMAN−1255−S−S2_−0.32
ptm abundance 37C
Doses Blue Rep1_−0.21 Red Rep2_−0.43
sp|Q76L83|ASXL2_HUMAN−439−S−S1_−0.27
ptm abundance 37C
Doses Blue Rep1_−0.41 Red Rep2_−0.14

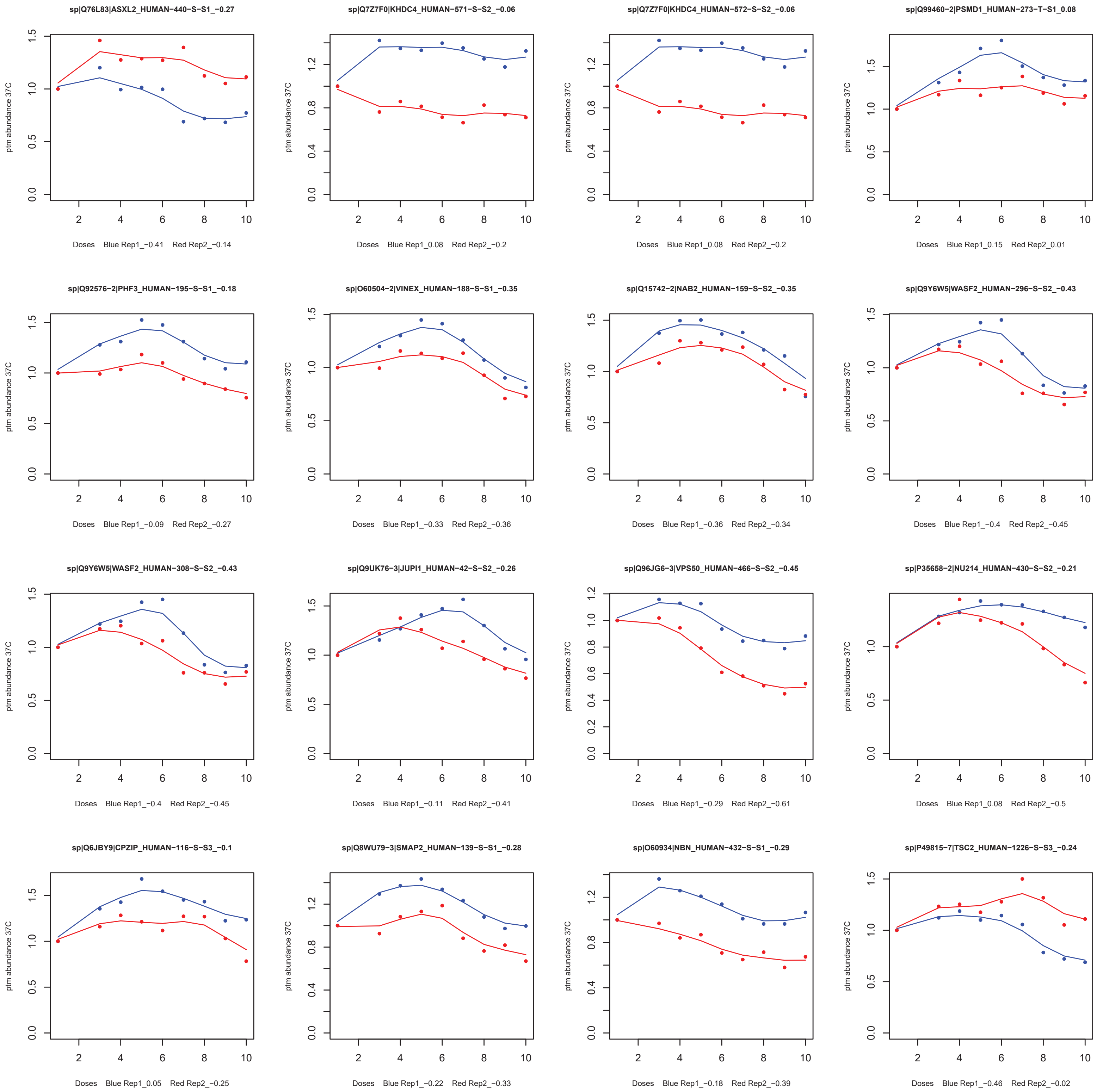
sp|Q76L83|ASXL2_HUMAN−440−S−S1_−0.27
Doses Blue Rep1_−0.41 Red Rep2_−0.14
sp|Q7Z7F0|KHDC4_HUMAN−571−S−S2_−0.06
Doses Blue Rep1_0.08 Red Rep2_−0.2
sp|Q7Z7F0|KHDC4_HUMAN−572−S−S2_−0.06
Doses Blue Rep1_0.08 Red Rep2_−0.2
sp|Q99460−2|PSMD1_HUMAN−273−T−S1_0.08
Doses Blue Rep1_0.15 Red Rep2_0.01
sp|Q92576−2|PHF3_HUMAN−195−S−S1_−0.18
Doses Blue Rep1_−0.09 Red Rep2_−0.27
sp|O60504−2|VINEX_HUMAN−188−S−S1_−0.35
Doses Blue Rep1_−0.33 Red Rep2_−0.36
sp|Q15742−2|NAB2_HUMAN−159−S−S2_−0.35
Doses Blue Rep1_−0.36 Red Rep2_−0.34
sp|Q9Y6W5|WASF2_HUMAN−296−S−S2_−0.43
Doses Blue Rep1_−0.4 Red Rep2_−0.45
sp|Q9Y6W5|WASF2_HUMAN−308−S−S2_−0.43
Doses Blue Rep1_−0.4 Red Rep2_−0.45
sp|Q9UK76−3|JUPI1_HUMAN−42−S−S2_−0.26
Doses Blue Rep1_−0.11 Red Rep2_−0.41
sp|Q96JG6−3|VPS50_HUMAN−466−S−S2_−0.45
Doses Blue Rep1_−0.29 Red Rep2_−0.61
sp|P35658−2|NU214_HUMAN−430−S−S2_−0.21
Doses Blue Rep1_0.08 Red Rep2_−0.5
sp|Q6JBY9|CPZIP_HUMAN−116−S−S3_−0.1
Doses Blue Rep1_0.05 Red Rep2_−0.25
sp|Q8WU79−3|SMAP2_HUMAN−139−S−S1_−0.28
Doses Blue Rep1_−0.22 Red Rep2_−0.33
sp|O60934|NBN_HUMAN−432−S−S1_−0.29
Doses Blue Rep1_−0.18 Red Rep2_−0.39
sp|P49815−7|TSC2_HUMAN−1226−S−S3_−0.24
Doses Blue Rep1_−0.46 Red Rep2_−0.02
ptm abundance 37C

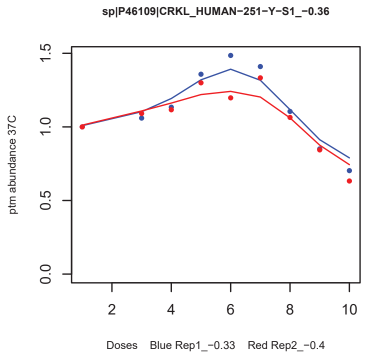
sp|P46109|CRKL_HUMAN−251−Y−S1_−0.36
ptm abundance 37C
Doses Blue Rep1_−0.33 Red Rep2_−0.4

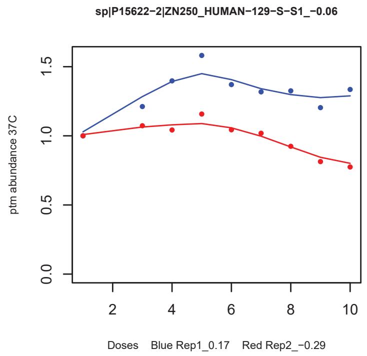
sp|P15622−2|ZN250_HUMAN−129−S−S1_−0.06
ptm abundance 37C
Doses Blue Rep1_0.17 Red Rep2_−0.29

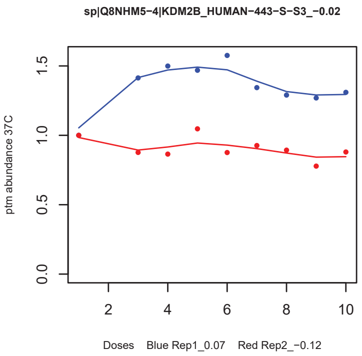
sp|Q8NHM5−4|KDM2B_HUMAN−443−S−S3_−0.02
ptm abundance 37C
Doses Blue Rep1_0.07 Red Rep2_−0.12

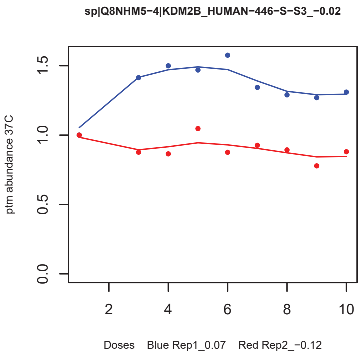
sp|Q8NHM5−4|KDM2B_HUMAN−446−S−S3_−0.02
ptm abundance 37C
Doses Blue Rep1_0.07 Red Rep2_−0.12

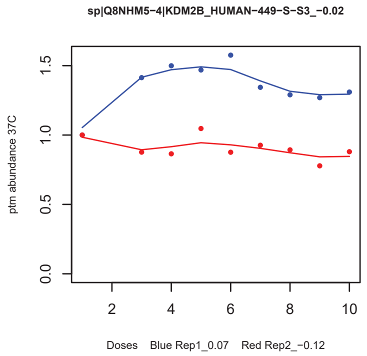
sp|Q8NHM5−4|KDM2B_HUMAN−449−S−S3_−0.02
ptm abundance 37C
Doses Blue Rep1_0.07 Red Rep2_−0.12

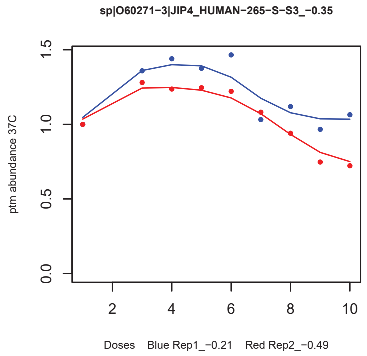
sp|O60271−3|JIP4_HUMAN−265−S−S3_−0.35
ptm abundance 37C
Doses Blue Rep1_−0.21 Red Rep2_−0.49

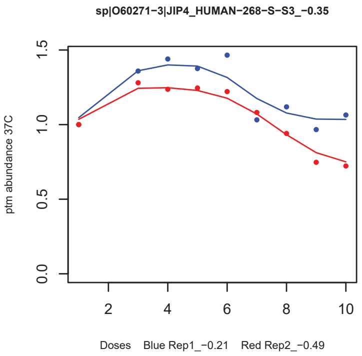
sp|O60271−3|JIP4_HUMAN−268−S−S3_−0.35
ptm abundance 37C
Doses Blue Rep1_−0.21 Red Rep2_−0.49

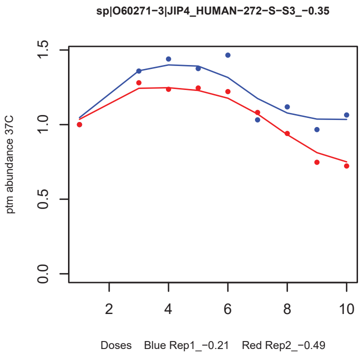
sp|O60271−3|JIP4_HUMAN−272−S−S3_−0.35
ptm abundance 37C
Doses Blue Rep1_−0.21 Red Rep2_−0.49

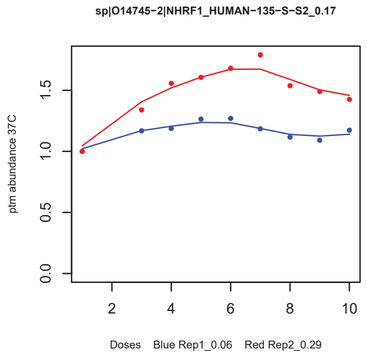
sp|O14745−2|NHRF1_HUMAN−135−S−S2_0.17
ptm abundance 37C
Doses Blue Rep1_0.06 Red Rep2_0.29

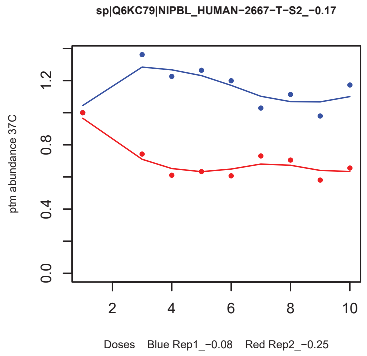
sp|Q6KC79|NIPBL_HUMAN−2667−T−S2_−0.17
ptm abundance 37C
Doses Blue Rep1_−0.08 Red Rep2_−0.25

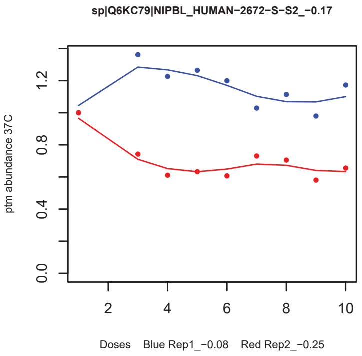
sp|Q6KC79|NIPBL_HUMAN−2672−S−S2_−0.17
ptm abundance 37C
Doses Blue Rep1_−0.08 Red Rep2_−0.25

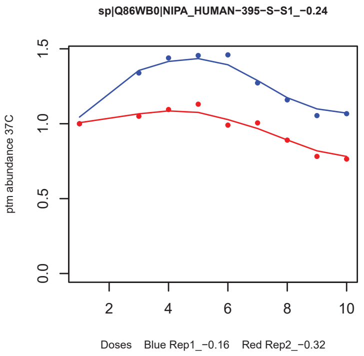
sp|Q86WB0|NIPA_HUMAN−395−S−S1_−0.24
ptm abundance 37C
Doses Blue Rep1_−0.16 Red Rep2_−0.32

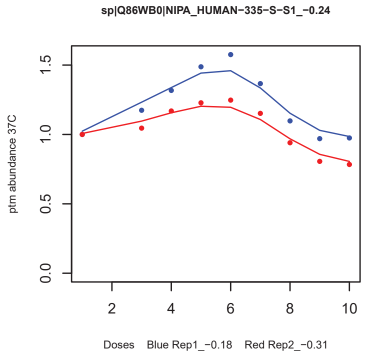
sp|Q86WB0|NIPA_HUMAN−335−S−S1_−0.24
ptm abundance 37C
Doses Blue Rep1_−0.18 Red Rep2_−0.31

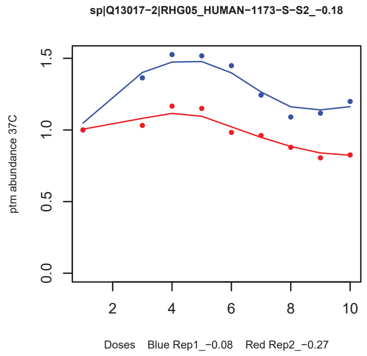
sp|Q13017−2|RHG05_HUMAN−1173−S−S2_−0.18
ptm abundance 37C
Doses Blue Rep1_−0.08 Red Rep2_−0.27

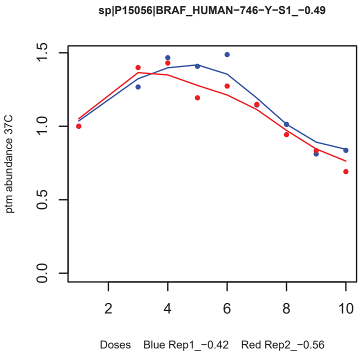
sp|P15056|BRAF_HUMAN−746−Y−S1_−0.49
ptm abundance 37C
Doses Blue Rep1_−0.42 Red Rep2_−0.56

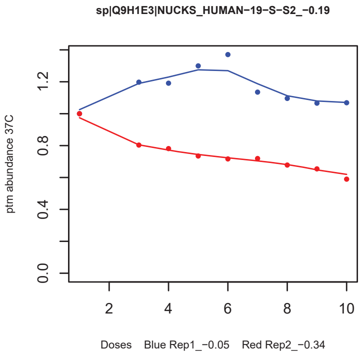
sp|Q9H1E3|NUCKS_HUMAN−19−S−S2_−0.19
ptm abundance 37C
Doses Blue Rep1_−0.05 Red Rep2_−0.34

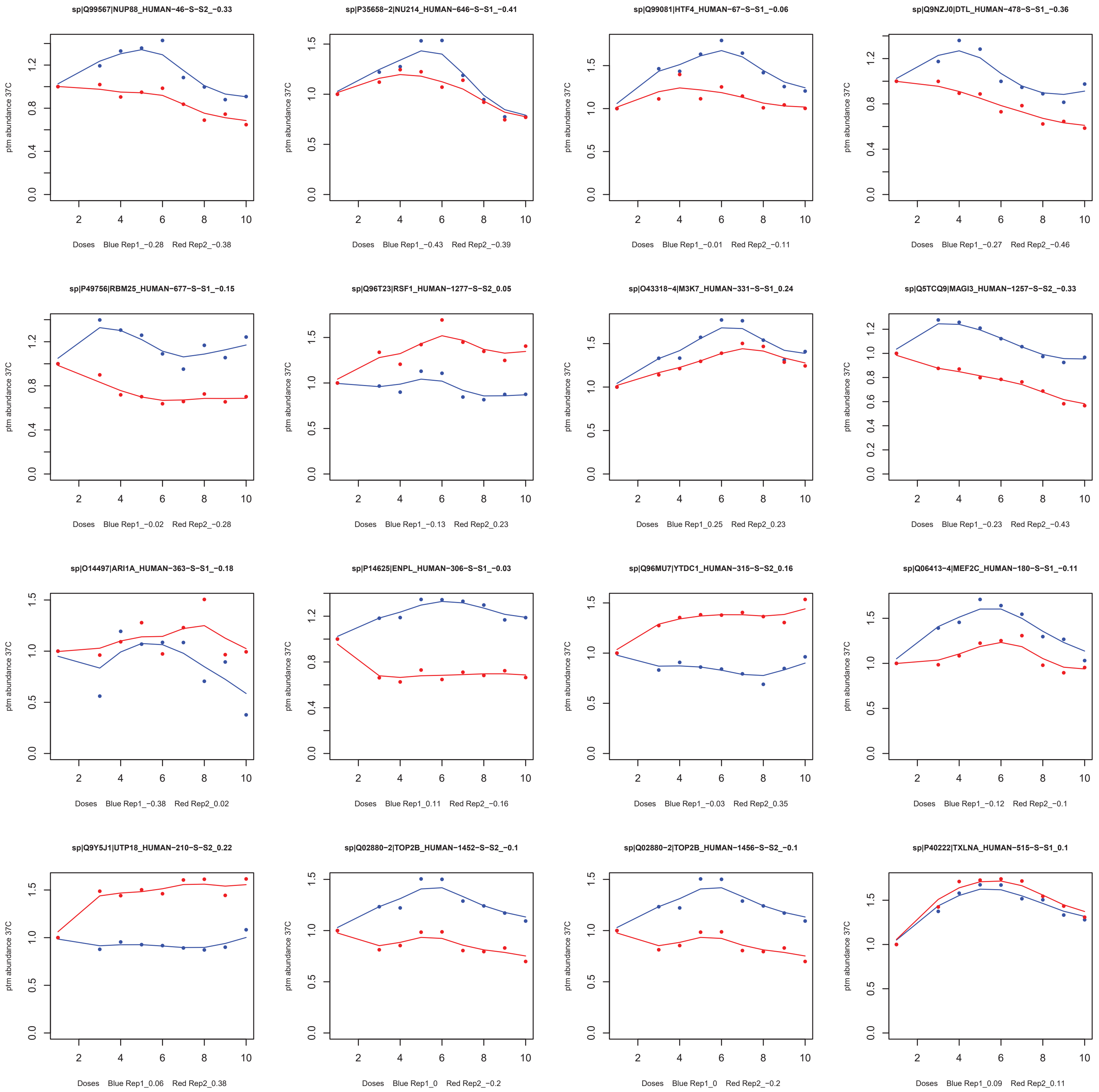

sp|Q99567|NUP88_HUMAN−46−S−S2_−0.33
Doses Blue Rep1_−0.28 Red Rep2_−0.38
sp|P35658−2|NU214_HUMAN−646−S−S1_−0.41
Doses Blue Rep1_−0.43 Red Rep2_−0.39
sp|Q99081|HTF4_HUMAN−67−S−S1_−0.06
Doses Blue Rep1_−0.01 Red Rep2_−0.11
sp|Q9NZJ0|DTL_HUMAN−478−S−S1_−0.36
Doses Blue Rep1_−0.27 Red Rep2_−0.46
sp|P49756|RBM25_HUMAN−677−S−S1_−0.15
Doses Blue Rep1_−0.02 Red Rep2_−0.28
sp|Q96T23|RSF1_HUMAN−1277−S−S2_0.05
Doses Blue Rep1_−0.13 Red Rep2_0.23
sp|O43318−4|M3K7_HUMAN−331−S−S1_0.24
Doses Blue Rep1_0.25 Red Rep2_0.23
sp|Q5TCQ9|MAGI3_HUMAN−1257−S−S2_−0.33
Doses Blue Rep1_−0.23 Red Rep2_−0.43
sp|O14497|ARI1A_HUMAN−363−S−S1_−0.18
Doses Blue Rep1_−0.38 Red Rep2_0.02
sp|P14625|ENPL_HUMAN−306−S−S1_−0.03
Doses Blue Rep1_0.11 Red Rep2_−0.16
sp|Q96MU7|YTDC1_HUMAN−315−S−S2_0.16
Doses Blue Rep1_−0.03 Red Rep2_0.35
sp|Q06413−4|MEF2C_HUMAN−180−S−S1_−0.11
Doses Blue Rep1_−0.12 Red Rep2_−0.1
sp|Q9Y5J1|UTP18_HUMAN−210−S−S2_0.22
Doses Blue Rep1_0.06 Red Rep2_0.38
sp|Q02880−2|TOP2B_HUMAN−1452−S−S2_−0.1
Doses Blue Rep1_0 Red Rep2_−0.2
sp|Q02880−2|TOP2B_HUMAN−1456−S−S2_−0.1
Doses Blue Rep1_0 Red Rep2_−0.2
sp|P40222|TXLNA_HUMAN−515−S−S1_0.1
Doses Blue Rep1_0.09 Red Rep2_0.11
ptm abundance 37C

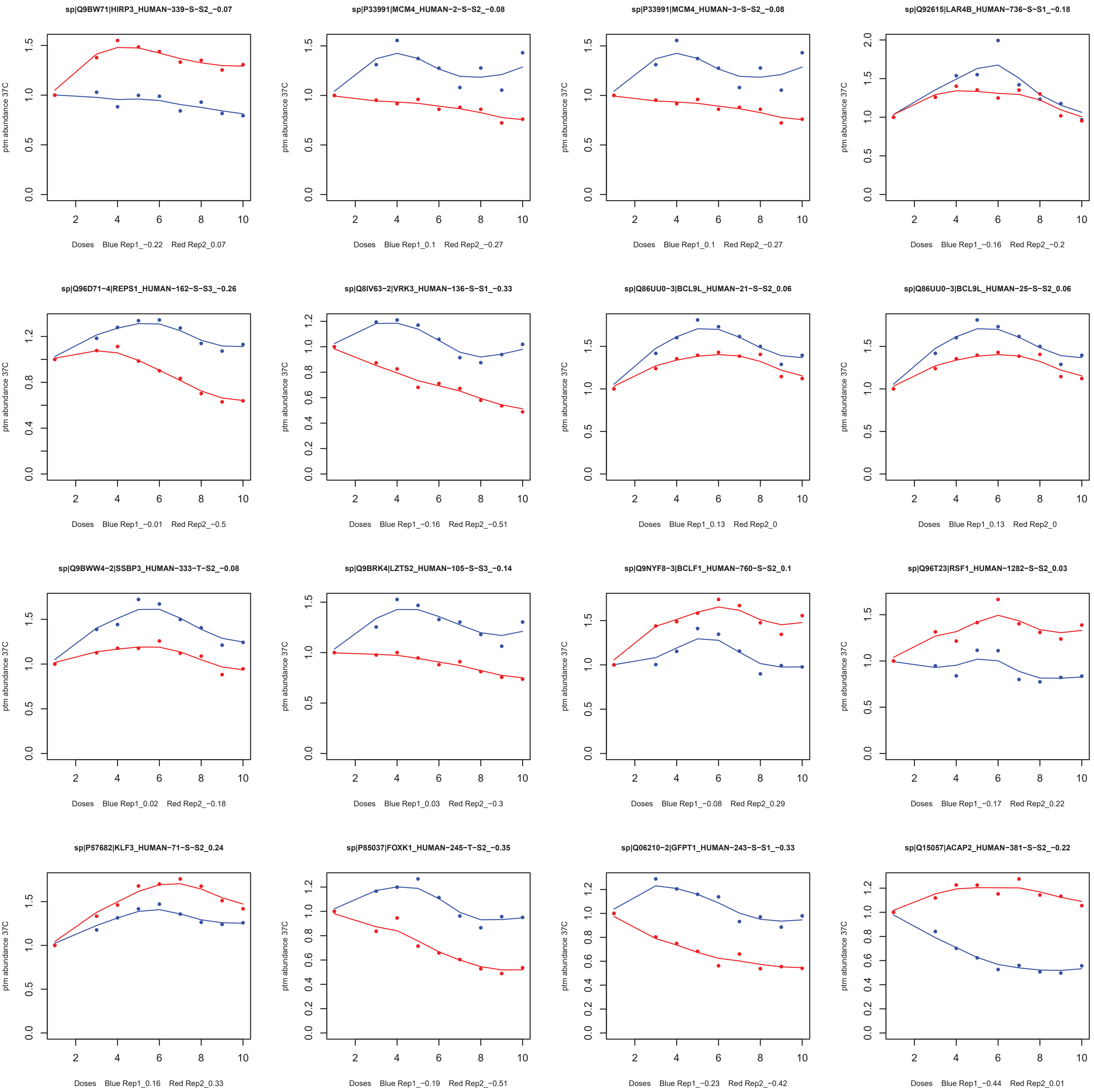
sp|Q9BW71|HIRP3_HUMAN−339−S−S2_−0.07
ptm abundance 37C
Doses Blue Rep1_−0.22 Red Rep2_0.07
sp|P33991|MCM4_HUMAN−2−S−S2_−0.08
ptm abundance 37C
Doses Blue Rep1_0.1 Red Rep2_−0.27
sp|P33991|MCM4_HUMAN−3−S−S2_−0.08
ptm abundance 37C
Doses Blue Rep1_0.1 Red Rep2_−0.27
sp|Q92615|LAR4B_HUMAN−736−S−S1_−0.18
ptm abundance 37C
Doses Blue Rep1_−0.16 Red Rep2_−0.2
sp|Q96D71−4|REPS1_HUMAN−162−S−S3_−0.26
ptm abundance 37C
Doses Blue Rep1_−0.01 Red Rep2_−0.5
sp|Q8IV63−2|VRK3_HUMAN−136−S−S1_−0.33
ptm abundance 37C
Doses Blue Rep1_−0.16 Red Rep2_−0.51
sp|Q86UU0−3|BCL9L_HUMAN−21−S−S2_0.06
ptm abundance 37C
Doses Blue Rep1_0.13 Red Rep2_0
sp|Q86UU0−3|BCL9L_HUMAN−25−S−S2_0.06
ptm abundance 37C
Doses Blue Rep1_0.13 Red Rep2_0
sp|Q9BWW4−2|SSBP3_HUMAN−333−T−S2_−0.08
ptm abundance 37C
Doses Blue Rep1_0.02 Red Rep2_−0.18
sp|Q9BRK4|LZTS2_HUMAN−105−S−S3_−0.14
ptm abundance 37C
Doses Blue Rep1_0.03 Red Rep2_−0.3
sp|Q9NYF8−3|BCLF1_HUMAN−760−S−S2_0.1
ptm abundance 37C
Doses Blue Rep1_−0.08 Red Rep2_0.29
sp|Q96T23|RSF1_HUMAN−1282−S−S2_0.03
ptm abundance 37C
Doses Blue Rep1_−0.17 Red Rep2_0.22
sp|P57682|KLF3_HUMAN−71−S−S2_0.24
ptm abundance 37C
Doses Blue Rep1_0.16 Red Rep2_0.33
sp|P85037|FOXK1_HUMAN−245−T−S2_−0.35
ptm abundance 37C
Doses Blue Rep1_−0.19 Red Rep2_−0.51
sp|Q06210−2|GFPT1_HUMAN−243−S−S1_−0.33
ptm abundance 37C
Doses Blue Rep1_−0.23 Red Rep2_−0.42
sp|Q15057|ACAP2_HUMAN−381−S−S2_−0.22
ptm abundance 37C
Doses Blue Rep1_−0.44 Red Rep2_0.01

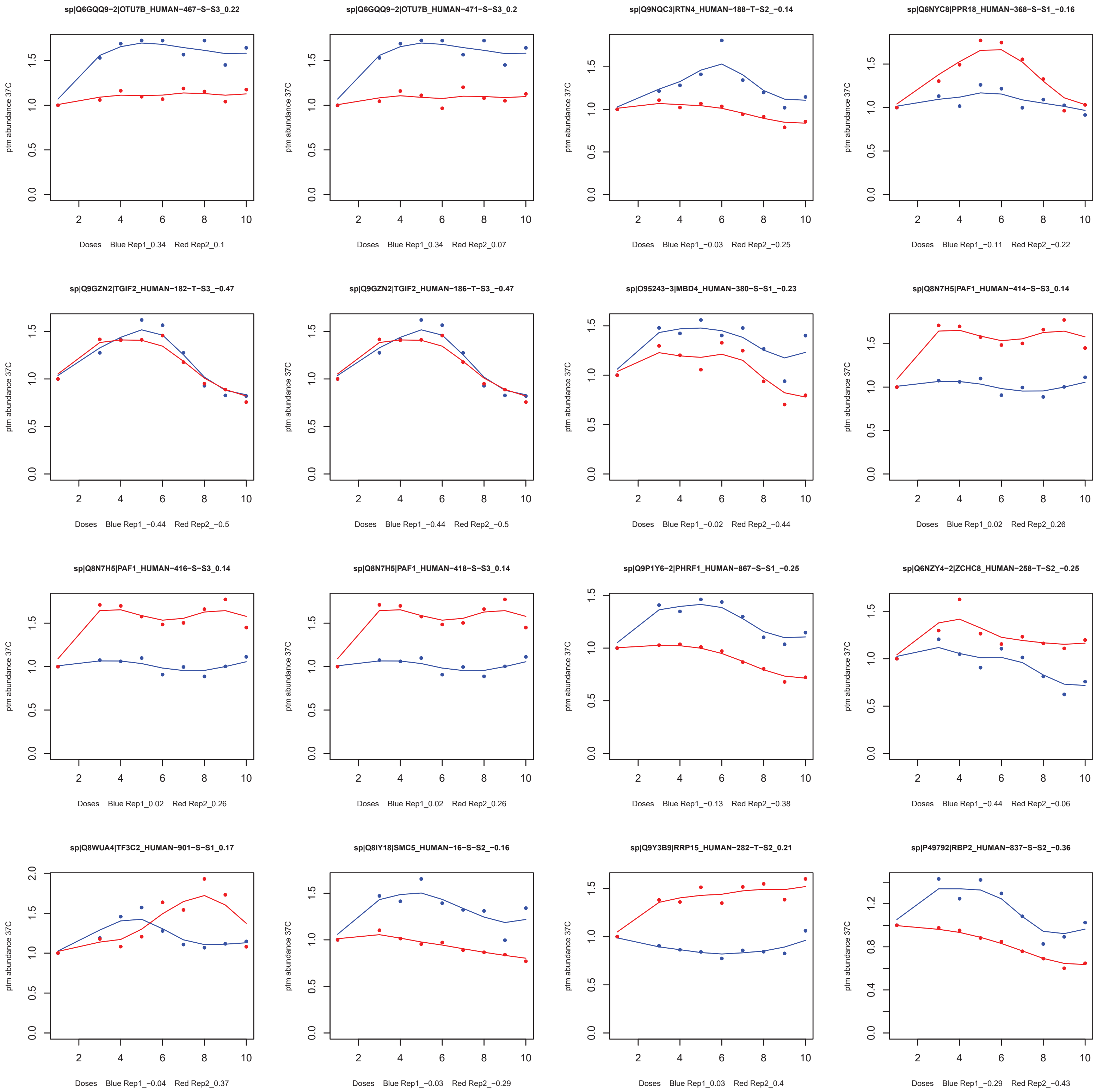
sp|Q6GQQ9−2|OTU7B_HUMAN−467−S−S3_0.22
ptm abundance 37C
Doses Blue Rep1_0.34 Red Rep2_0.1
sp|Q6GQQ9−2|OTU7B_HUMAN−471−S−S3_0.2
ptm abundance 37C
Doses Blue Rep1_0.34 Red Rep2_0.07
sp|Q9NQC3|RTN4_HUMAN−188−T−S2_−0.14
ptm abundance 37C
Doses Blue Rep1_−0.03 Red Rep2_−0.25
sp|Q6NYC8|PPR18_HUMAN−368−S−S1_−0.16
ptm abundance 37C
Doses Blue Rep1_−0.11 Red Rep2_−0.22
sp|Q9GZN2|TGIF2_HUMAN−182−T−S3_−0.47
ptm abundance 37C
Doses Blue Rep1_−0.44 Red Rep2_−0.5
sp|Q9GZN2|TGIF2_HUMAN−186−T−S3_−0.47
ptm abundance 37C
Doses Blue Rep1_−0.44 Red Rep2_−0.5
sp|O95243−3|MBD4_HUMAN−380−S−S1_−0.23
ptm abundance 37C
Doses Blue Rep1_−0.02 Red Rep2_−0.44
sp|Q8N7H5|PAF1_HUMAN−414−S−S3_0.14
ptm abundance 37C
Doses Blue Rep1_0.02 Red Rep2_0.26
sp|Q8N7H5|PAF1_HUMAN−416−S−S3_0.14
ptm abundance 37C
Doses Blue Rep1_0.02 Red Rep2_0.26
sp|Q8N7H5|PAF1_HUMAN−418−S−S3_0.14
ptm abundance 37C
Doses Blue Rep1_0.02 Red Rep2_0.26
sp|Q9P1Y6−2|PHRF1_HUMAN−867−S−S1_−0.25
ptm abundance 37C
Doses Blue Rep1_−0.13 Red Rep2_−0.38
sp|Q6NZY4−2|ZCHC8_HUMAN−258−T−S2_−0.25
ptm abundance 37C
Doses Blue Rep1_−0.44 Red Rep2_−0.06
sp|Q8WUA4|TF3C2_HUMAN−901−S−S1_0.17
ptm abundance 37C
Doses Blue Rep1_−0.04 Red Rep2_0.37
sp|Q8IY18|SMC5_HUMAN−16−S−S2_−0.16
ptm abundance 37C
Doses Blue Rep1_−0.03 Red Rep2_−0.29
sp|Q9Y3B9|RRP15_HUMAN−282−T−S2_0.21
ptm abundance 37C
Doses Blue Rep1_0.03 Red Rep2_0.4
sp|P49792|RBP2_HUMAN−837−S−S2_−0.36
ptm abundance 37C
Doses Blue Rep1_−0.29 Red Rep2_−0.43

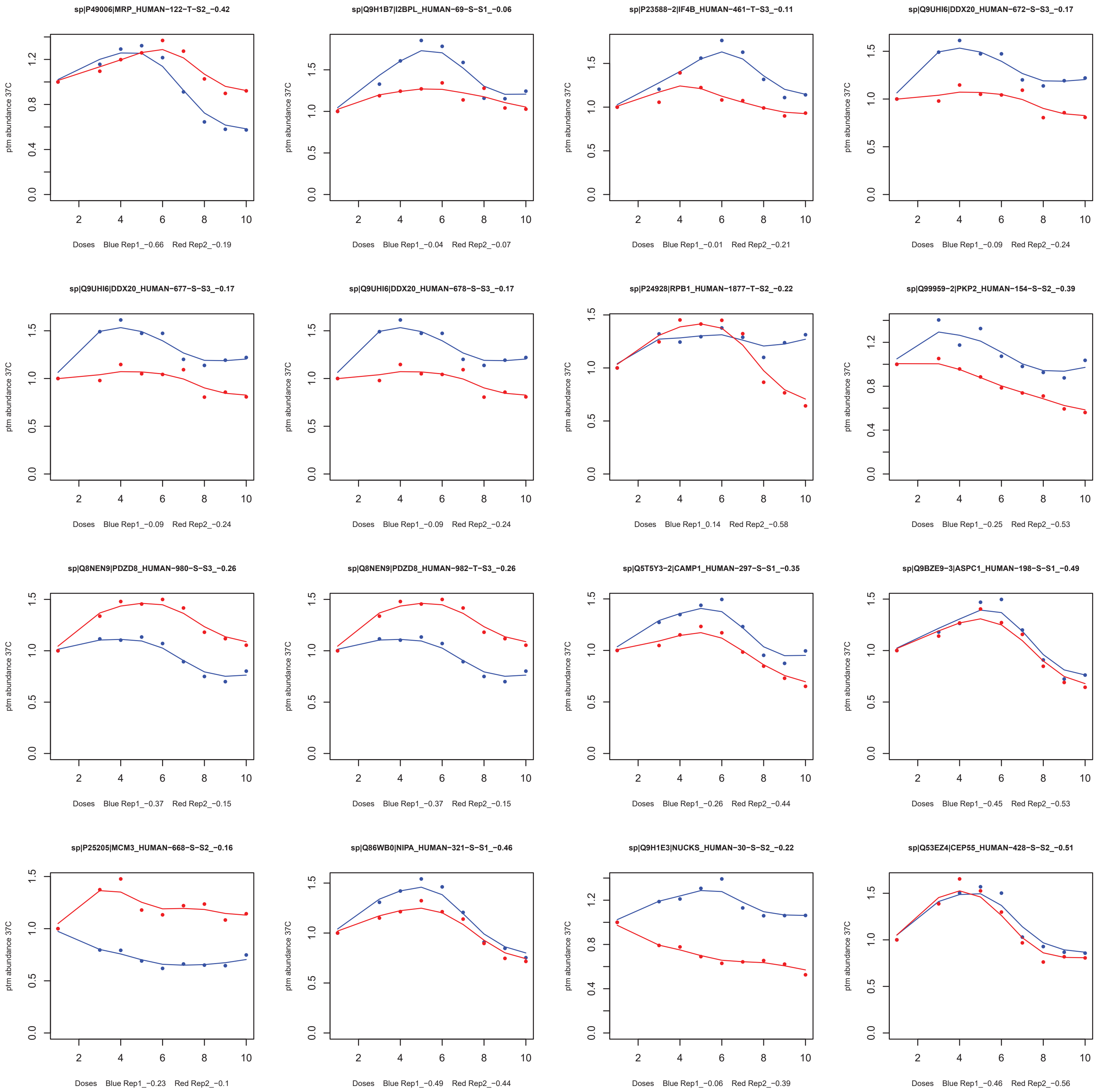
sp|P49006|MRP_HUMAN−122−T−S2_−0.42
Doses Blue Rep1_−0.66 Red Rep2_−0.19
sp|Q9H1B7|I2BPL_HUMAN−69−S−S1_−0.06
Doses Blue Rep1_−0.04 Red Rep2_−0.07
sp|P23588−2|IF4B_HUMAN−461−T−S3_−0.11
Doses Blue Rep1_−0.01 Red Rep2_−0.21
sp|Q9UHI6|DDX20_HUMAN−672−S−S3_−0.17
Doses Blue Rep1_−0.09 Red Rep2_−0.24
sp|Q9UHI6|DDX20_HUMAN−677−S−S3_−0.17
Doses Blue Rep1_−0.09 Red Rep2_−0.24
sp|Q9UHI6|DDX20_HUMAN−678−S−S3_−0.17
Doses Blue Rep1_−0.09 Red Rep2_−0.24
sp|P24928|RPB1_HUMAN−1877−T−S2_−0.22
Doses Blue Rep1_0.14 Red Rep2_−0.58
sp|Q99959−2|PKP2_HUMAN−154−S−S2_−0.39
Doses Blue Rep1_−0.25 Red Rep2_−0.53
sp|Q8NEN9|PDZD8_HUMAN−980−S−S3_−0.26
Doses Blue Rep1_−0.37 Red Rep2_−0.15
sp|Q8NEN9|PDZD8_HUMAN−982−T−S3_−0.26
Doses Blue Rep1_−0.37 Red Rep2_−0.15
sp|Q5T5Y3−2|CAMP1_HUMAN−297−S−S1_−0.35
Doses Blue Rep1_−0.26 Red Rep2_−0.44
sp|Q9BZE9−3|ASPC1_HUMAN−198−S−S1_−0.49
Doses Blue Rep1_−0.45 Red Rep2_−0.53
sp|P25205|MCM3_HUMAN−668−S−S2_−0.16
Doses Blue Rep1_−0.23 Red Rep2_−0.1
sp|Q86WB0|NIPA_HUMAN−321−S−S1_−0.46
Doses Blue Rep1_−0.49 Red Rep2_−0.44
sp|Q9H1E3|NUCKS_HUMAN−30−S−S2_−0.22
Doses Blue Rep1_−0.06 Red Rep2_−0.39
sp|Q53EZ4|CEP55_HUMAN−428−S−S2_−0.51
Doses Blue Rep1_−0.46 Red Rep2_−0.56
ptm abundance 37C

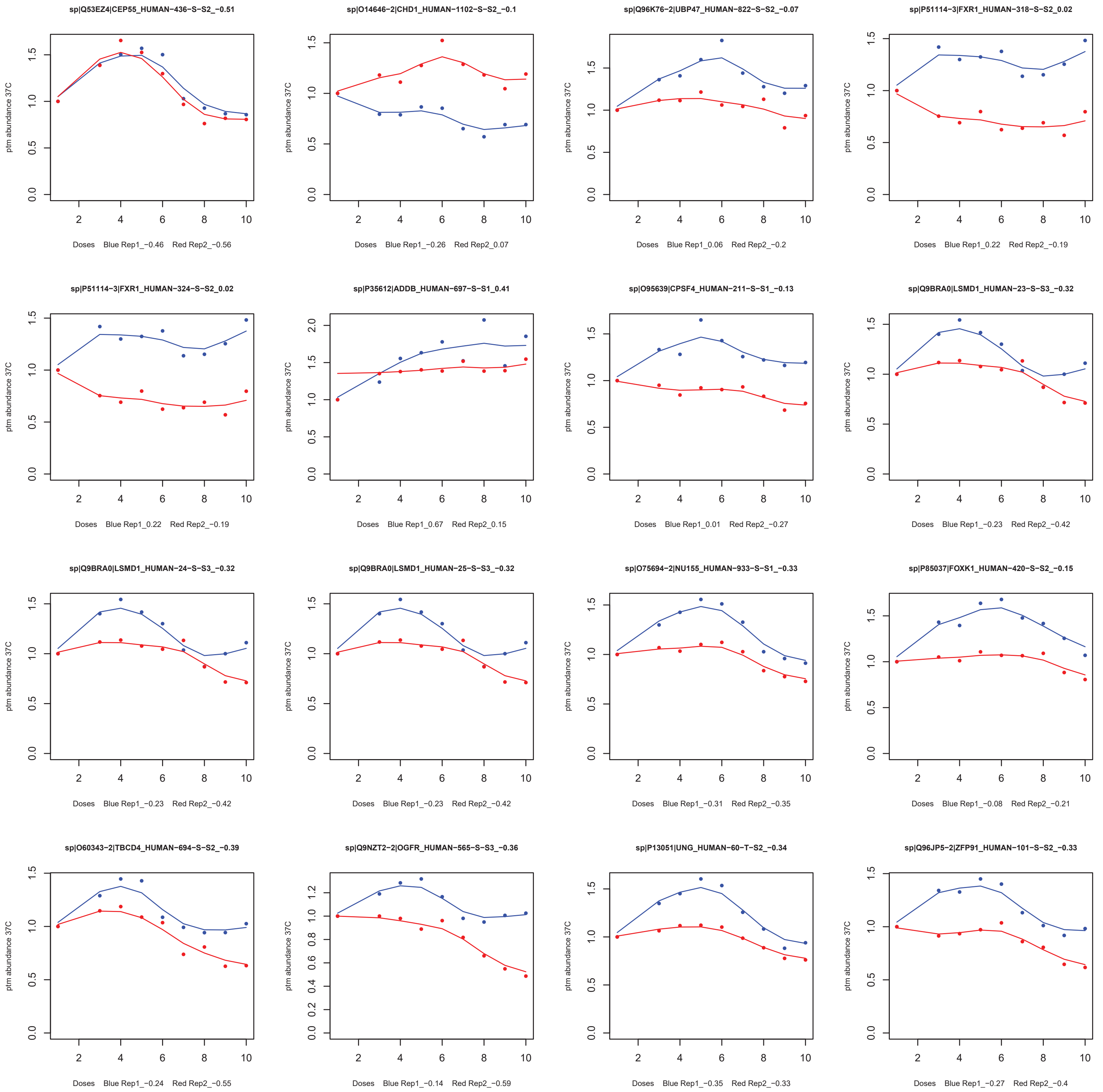
sp|Q53EZ4|CEP55_HUMAN−436−S−S2_−0.51
Doses Blue Rep1_−0.46 Red Rep2_−0.56
sp|O14646−2|CHD1_HUMAN−1102−S−S2_−0.1
Doses Blue Rep1_−0.26 Red Rep2_0.07
sp|Q96K76−2|UBP47_HUMAN−822−S−S2_−0.07
Doses Blue Rep1_0.06 Red Rep2_−0.2
sp|P51114−3|FXR1_HUMAN−318−S−S2_0.02
Doses Blue Rep1_0.22 Red Rep2_−0.19
sp|P51114−3|FXR1_HUMAN−324−S−S2_0.02
Doses Blue Rep1_0.22 Red Rep2_−0.19
sp|P35612|ADDB_HUMAN−697−S−S1_0.41
Doses Blue Rep1_0.67 Red Rep2_0.15
sp|O95639|CPSF4_HUMAN−211−S−S1_−0.13
Doses Blue Rep1_0.01 Red Rep2_−0.27
sp|Q9BRA0|LSMD1_HUMAN−23−S−S3_−0.32
Doses Blue Rep1_−0.23 Red Rep2_−0.42
sp|Q9BRA0|LSMD1_HUMAN−24−S−S3_−0.32
Doses Blue Rep1_−0.23 Red Rep2_−0.42
sp|Q9BRA0|LSMD1_HUMAN−25−S−S3_−0.32
Doses Blue Rep1_−0.23 Red Rep2_−0.42
sp|O75694−2|NU155_HUMAN−933−S−S1_−0.33
Doses Blue Rep1_−0.31 Red Rep2_−0.35
sp|P85037|FOXK1_HUMAN−420−S−S2_−0.15
Doses Blue Rep1_−0.08 Red Rep2_−0.21
sp|O60343−2|TBCD4_HUMAN−694−S−S2_−0.39
Doses Blue Rep1_−0.24 Red Rep2_−0.55
sp|Q9NZT2−2|OGFR_HUMAN−565−S−S3_−0.36
Doses Blue Rep1_−0.14 Red Rep2_−0.59
sp|P13051|UNG_HUMAN−60−T−S2_−0.34
Doses Blue Rep1_−0.35 Red Rep2_−0.33
sp|Q96JP5−2|ZFP91_HUMAN−101−S−S2_−0.33
Doses Blue Rep1_−0.27 Red Rep2_−0.4
ptm abundance 37C

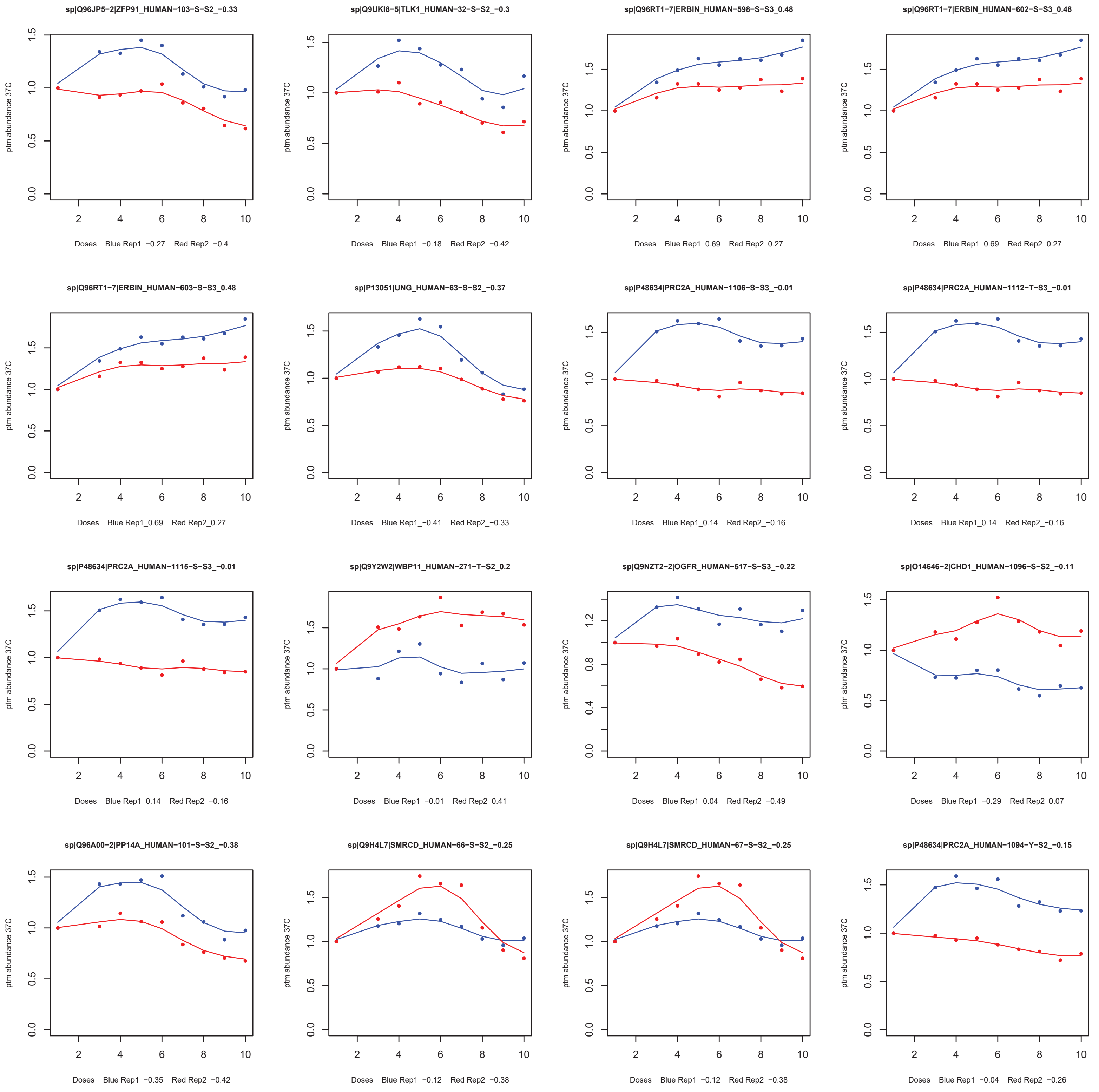
sp|Q96JP5−2|ZFP91_HUMAN−103−S−S2_−0.33
Doses Blue Rep1_−0.27 Red Rep2_−0.4
sp|Q9UKI8−5|TLK1_HUMAN−32−S−S2_−0.3
Doses Blue Rep1_−0.18 Red Rep2_−0.42
sp|Q96RT1−7|ERBIN_HUMAN−598−S−S3_0.48
Doses Blue Rep1_0.69 Red Rep2_0.27
sp|Q96RT1−7|ERBIN_HUMAN−602−S−S3_0.48
Doses Blue Rep1_0.69 Red Rep2_0.27
sp|Q96RT1−7|ERBIN_HUMAN−603−S−S3_0.48
Doses Blue Rep1_0.69 Red Rep2_0.27
sp|P13051|UNG_HUMAN−63−S−S2_−0.37
Doses Blue Rep1_−0.41 Red Rep2_−0.33
sp|P48634|PRC2A_HUMAN−1106−S−S3_−0.01
Doses Blue Rep1_0.14 Red Rep2_−0.16
sp|P48634|PRC2A_HUMAN−1112−T−S3_−0.01
Doses Blue Rep1_0.14 Red Rep2_−0.16
sp|P48634|PRC2A_HUMAN−1115−S−S3_−0.01
Doses Blue Rep1_0.14 Red Rep2_−0.16
sp|Q9Y2W2|WBP11_HUMAN−271−T−S2_0.2
Doses Blue Rep1_−0.01 Red Rep2_0.41
sp|Q9NZT2−2|OGFR_HUMAN−517−S−S3_−0.22
Doses Blue Rep1_0.04 Red Rep2_−0.49
sp|O14646−2|CHD1_HUMAN−1096−S−S2_−0.11
Doses Blue Rep1_−0.29 Red Rep2_0.07
sp|Q96A00−2|PP14A_HUMAN−101−S−S2_−0.38
Doses Blue Rep1_−0.35 Red Rep2_−0.42
sp|Q9H4L7|SMRCD_HUMAN−66−S−S2_−0.25
Doses Blue Rep1_−0.12 Red Rep2_−0.38
sp|Q9H4L7|SMRCD_HUMAN−67−S−S2_−0.25
Doses Blue Rep1_−0.12 Red Rep2_−0.38
sp|P48634|PRC2A_HUMAN−1094−Y−S2_−0.15
Doses Blue Rep1_−0.04 Red Rep2_−0.26
ptm abundance 37C

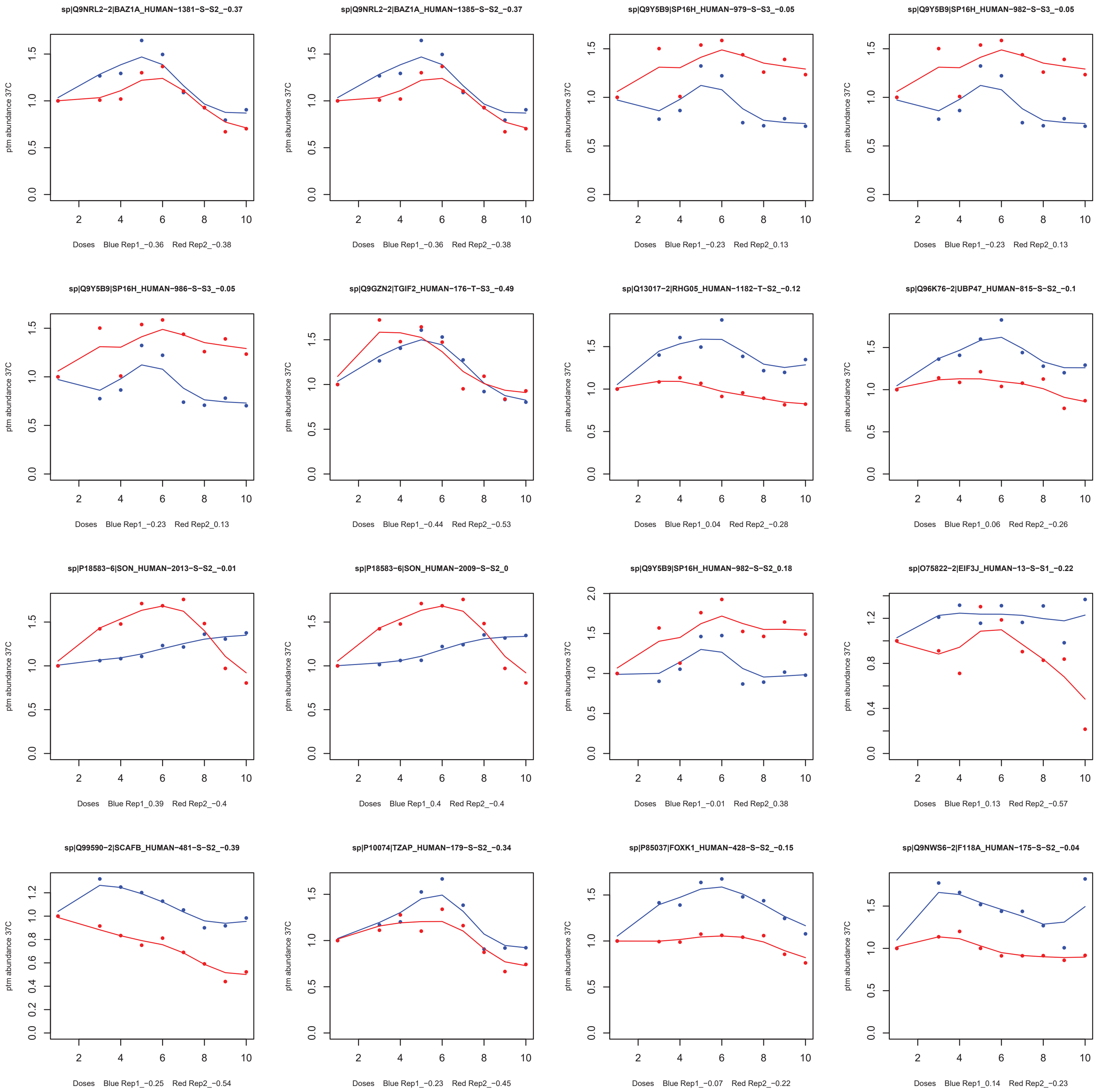
sp|Q9NRL2−2|BAZ1A_HUMAN−1381−S−S2_−0.37
ptm abundance 37C
Doses Blue Rep1_−0.36 Red Rep2_−0.38
sp|Q9NRL2−2|BAZ1A_HUMAN−1385−S−S2_−0.37
Doses Blue Rep1_−0.36 Red Rep2_−0.38
sp|Q9Y5B9|SP16H_HUMAN−979−S−S3_−0.05
Doses Blue Rep1_−0.23 Red Rep2_0.13
sp|Q9Y5B9|SP16H_HUMAN−982−S−S3_−0.05
Doses Blue Rep1_−0.23 Red Rep2_0.13
sp|Q9Y5B9|SP16H_HUMAN−986−S−S3_−0.05
Doses Blue Rep1_−0.23 Red Rep2_0.13
sp|Q9GZN2|TGIF2_HUMAN−176−T−S3_−0.49
Doses Blue Rep1_−0.44 Red Rep2_−0.53
sp|Q13017−2|RHG05_HUMAN−1182−T−S2_−0.12
Doses Blue Rep1_0.04 Red Rep2_−0.28
sp|Q96K76−2|UBP47_HUMAN−815−S−S2_−0.1
Doses Blue Rep1_0.06 Red Rep2_−0.26
sp|P18583−6|SON_HUMAN−2013−S−S2_−0.01
Doses Blue Rep1_0.39 Red Rep2_−0.4
sp|P18583−6|SON_HUMAN−2009−S−S2_0
Doses Blue Rep1_0.4 Red Rep2_−0.4
sp|Q9Y5B9|SP16H_HUMAN−982−S−S2_0.18
Doses Blue Rep1_−0.01 Red Rep2_0.38
sp|O75822−2|EIF3J_HUMAN−13−S−S1_−0.22
Doses Blue Rep1_0.13 Red Rep2_−0.57
sp|Q99590−2|SCAFB_HUMAN−481−S−S2_−0.39
Doses Blue Rep1_−0.25 Red Rep2_−0.54
sp|P10074|TZAP_HUMAN−179−S−S2_−0.34
Doses Blue Rep1_−0.23 Red Rep2_−0.45
sp|P85037|FOXK1_HUMAN−428−S−S2_−0.15
Doses Blue Rep1_−0.07 Red Rep2_−0.22
sp|Q9NWS6−2|F118A_HUMAN−175−S−S2_−0.04
Doses Blue Rep1_0.14 Red Rep2_−0.23

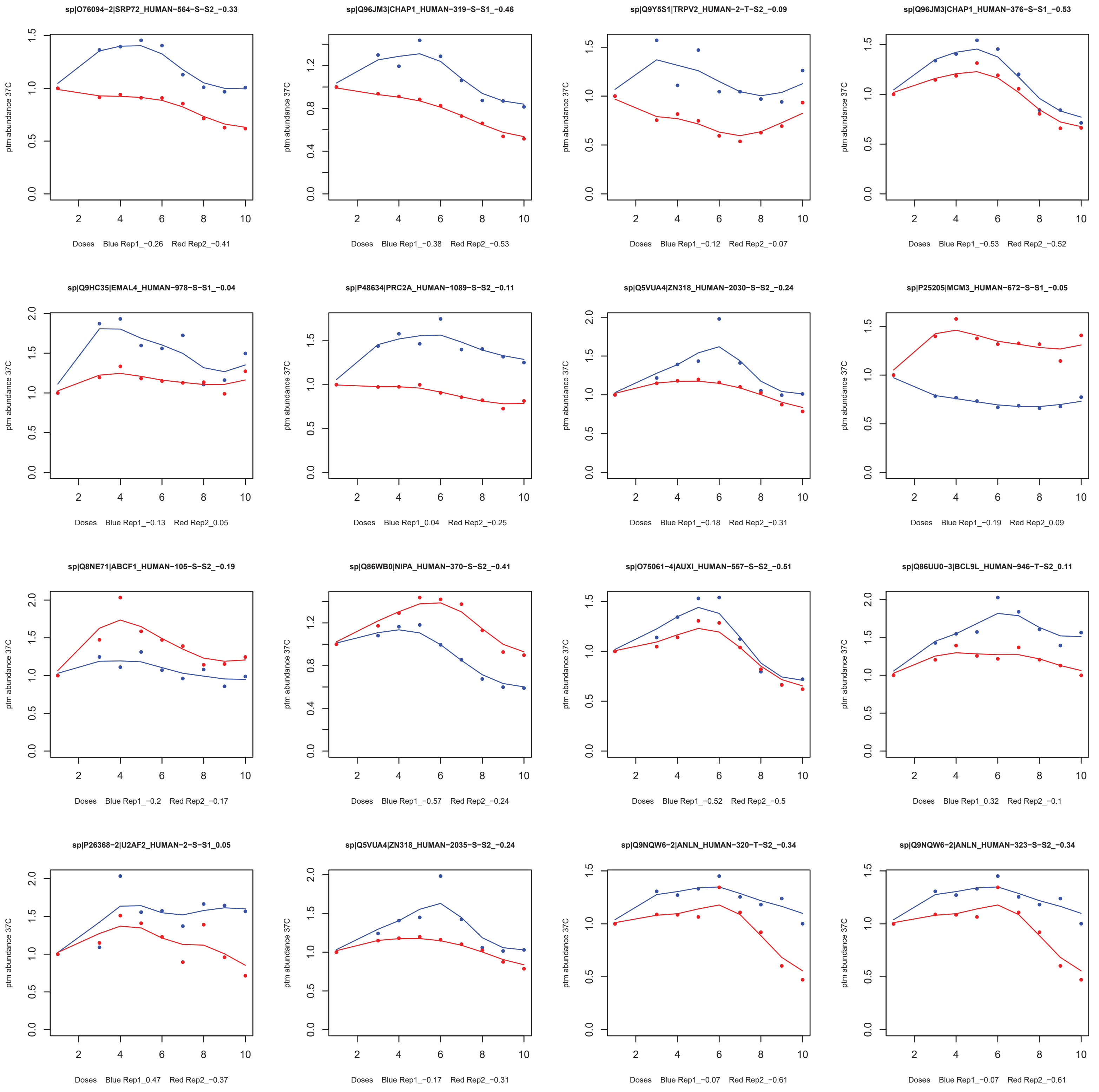

sp|O76094−2|SRP72_HUMAN−564−S−S2_−0.33
ptm abundance 37C
Doses Blue Rep1_−0.26 Red Rep2_−0.41
sp|Q96JM3|CHAP1_HUMAN−319−S−S1_−0.46
ptm abundance 37C
Doses Blue Rep1_−0.38 Red Rep2_−0.53
sp|Q9Y5S1|TRPV2_HUMAN−2−T−S2_−0.09
ptm abundance 37C
Doses Blue Rep1_−0.12 Red Rep2_−0.07
sp|Q96JM3|CHAP1_HUMAN−376−S−S1_−0.53
ptm abundance 37C
Doses Blue Rep1_−0.53 Red Rep2_−0.52
sp|Q9HC35|EMAL4_HUMAN−978−S−S1_−0.04
ptm abundance 37C
Doses Blue Rep1_−0.13 Red Rep2_0.05
sp|P48634|PRC2A_HUMAN−1089−S−S2_−0.11
ptm abundance 37C
Doses Blue Rep1_0.04 Red Rep2_−0.25
sp|Q5VUA4|ZN318_HUMAN−2030−S−S2_−0.24
ptm abundance 37C
Doses Blue Rep1_−0.18 Red Rep2_−0.31
sp|P25205|MCM3_HUMAN−672−S−S1_−0.05
ptm abundance 37C
Doses Blue Rep1_−0.19 Red Rep2_0.09
sp|Q8NE71|ABCF1_HUMAN−105−S−S2_−0.19
ptm abundance 37C
Doses Blue Rep1_−0.2 Red Rep2_−0.17
sp|Q86WB0|NIPA_HUMAN−370−S−S2_−0.41
ptm abundance 37C
Doses Blue Rep1_−0.57 Red Rep2_−0.24
sp|O75061−4|AUXI_HUMAN−557−S−S2_−0.51
ptm abundance 37C
Doses Blue Rep1_−0.52 Red Rep2_−0.5
sp|Q86UU0−3|BCL9L_HUMAN−946−T−S2_0.11
ptm abundance 37C
Doses Blue Rep1_0.32 Red Rep2_−0.1
sp|P26368−2|U2AF2_HUMAN−2−S−S1_0.05
ptm abundance 37C
Doses Blue Rep1_0.47 Red Rep2_−0.37
sp|Q5VUA4|ZN318_HUMAN−2035−S−S2_−0.24
ptm abundance 37C
Doses Blue Rep1_−0.17 Red Rep2_−0.31
sp|Q9NQW6−2|ANLN_HUMAN−320−T−S2_−0.34
ptm abundance 37C
Doses Blue Rep1_−0.07 Red Rep2_−0.61
sp|Q9NQW6−2|ANLN_HUMAN−323−S−S2_−0.34
ptm abundance 37C
Doses Blue Rep1_−0.07 Red Rep2_−0.61

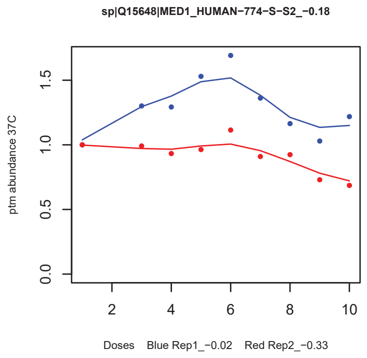
sp|Q15648|MED1_HUMAN−774−S−S2_−0.18
ptm abundance 37C
Doses Blue Rep1_−0.02 Red Rep2_−0.33

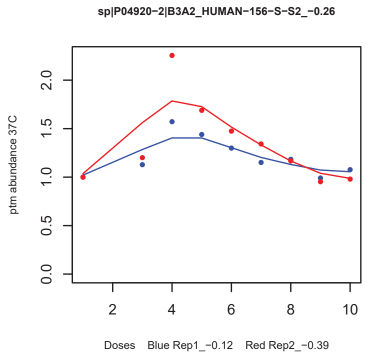
sp|P04920−2|B3A2_HUMAN−156−S−S2_−0.26
ptm abundance 37C
Doses Blue Rep1_−0.12 Red Rep2_−0.39

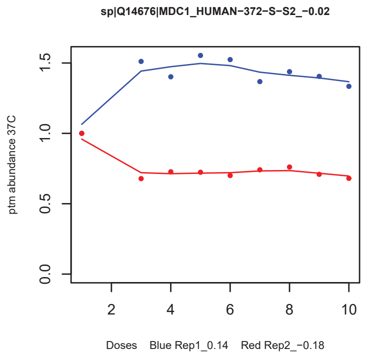
sp|Q14676|MDC1_HUMAN−372−S−S2_−0.02
ptm abundance 37C
Doses Blue Rep1_0.14 Red Rep2_−0.18

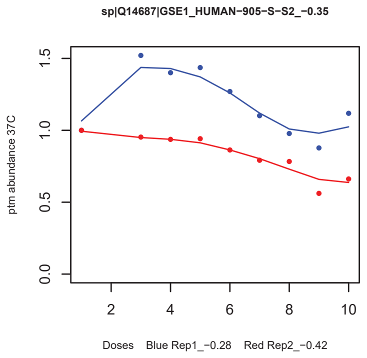
sp|Q14687|GSE1_HUMAN−905−S−S2_−0.35
ptm abundance 37C
Doses Blue Rep1_−0.28 Red Rep2_−0.42

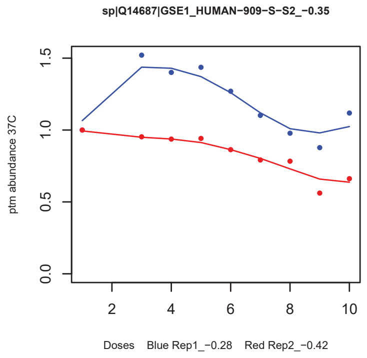
sp|Q14687|GSE1_HUMAN−909−S−S2_−0.35
ptm abundance 37C
Doses Blue Rep1_−0.28 Red Rep2_−0.42

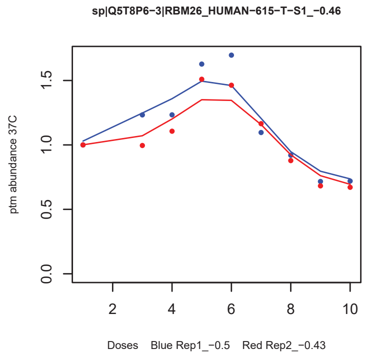
sp|Q5T8P6−3|RBM26_HUMAN−615−T−S1_−0.46
ptm abundance 37C
Doses Blue Rep1_−0.5 Red Rep2_−0.43

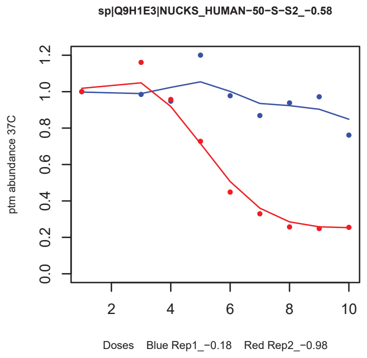
sp|Q9H1E3|NUCKS_HUMAN−50−S−S2_−0.58
ptm abundance 37C
Doses Blue Rep1_−0.18 Red Rep2_−0.98

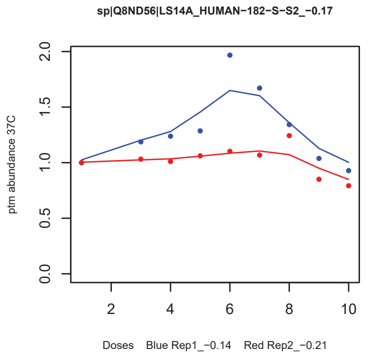
sp|Q8ND56|LS14A_HUMAN−182−S−S2_−0.17
ptm abundance 37C
Doses Blue Rep1_−0.14 Red Rep2_−0.21

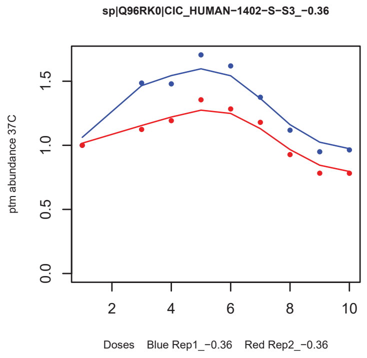
sp|Q96RK0|CIC_HUMAN−1402−S−S3_−0.36
ptm abundance 37C
Doses Blue Rep1_−0.36 Red Rep2_−0.36

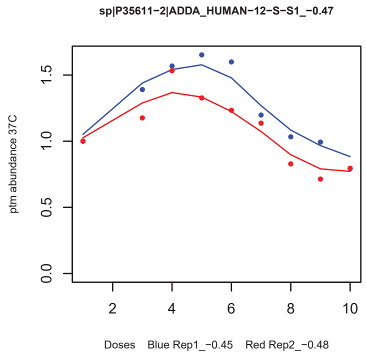
sp|P35611−2|ADDA_HUMAN−12−S−S1_−0.47
ptm abundance 37C
Doses Blue Rep1_−0.45 Red Rep2_−0.48

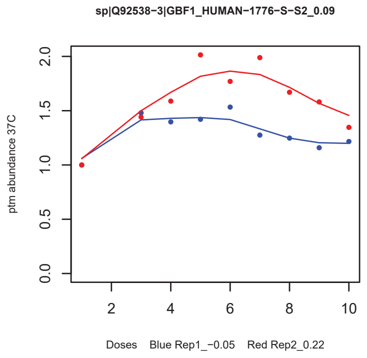
sp|Q92538−3|GBF1_HUMAN−1776−S−S2_0.09
ptm abundance 37C
Doses Blue Rep1_−0.05 Red Rep2_0.22

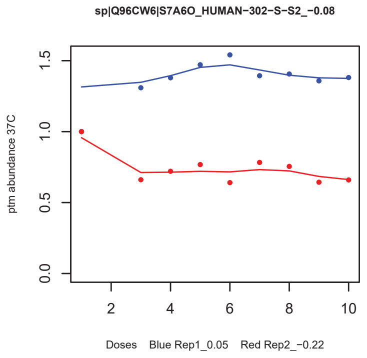
sp|Q96CW6|S7A6O_HUMAN−302−S−S2_−0.08
ptm abundance 37C
Doses Blue Rep1_0.05 Red Rep2_−0.22

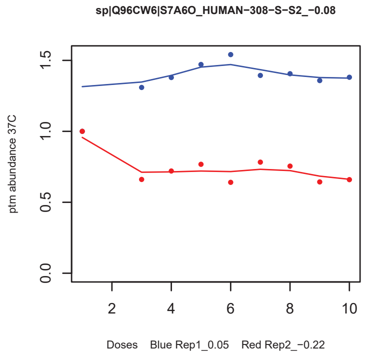
sp|Q96CW6|S7A6O_HUMAN−308−S−S2_−0.08
ptm abundance 37C
Doses Blue Rep1_0.05 Red Rep2_−0.22

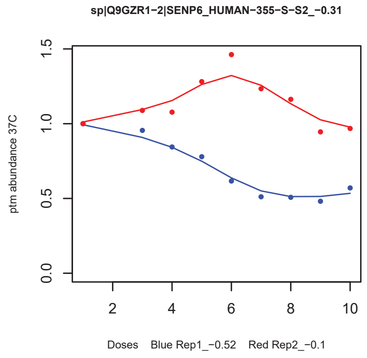
sp|Q9GZR1−2|SENP6_HUMAN−355−S−S2_−0.31
ptm abundance 37C
Doses Blue Rep1_−0.52 Red Rep2_−0.1

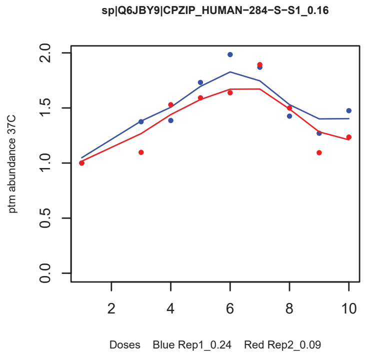
sp|Q6JBY9|CPZIP_HUMAN−284−S−S1_0.16
ptm abundance 37C
Doses Blue Rep1_0.24 Red Rep2_0.09

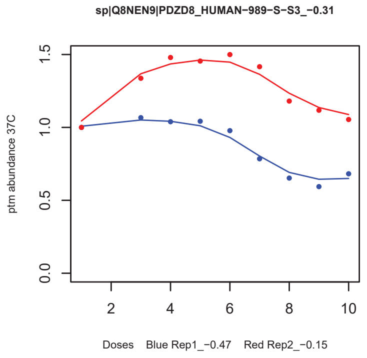
sp|Q8NEN9|PDZD8_HUMAN−989−S−S3_−0.31
ptm abundance 37C
Doses Blue Rep1_−0.47 Red Rep2_−0.15

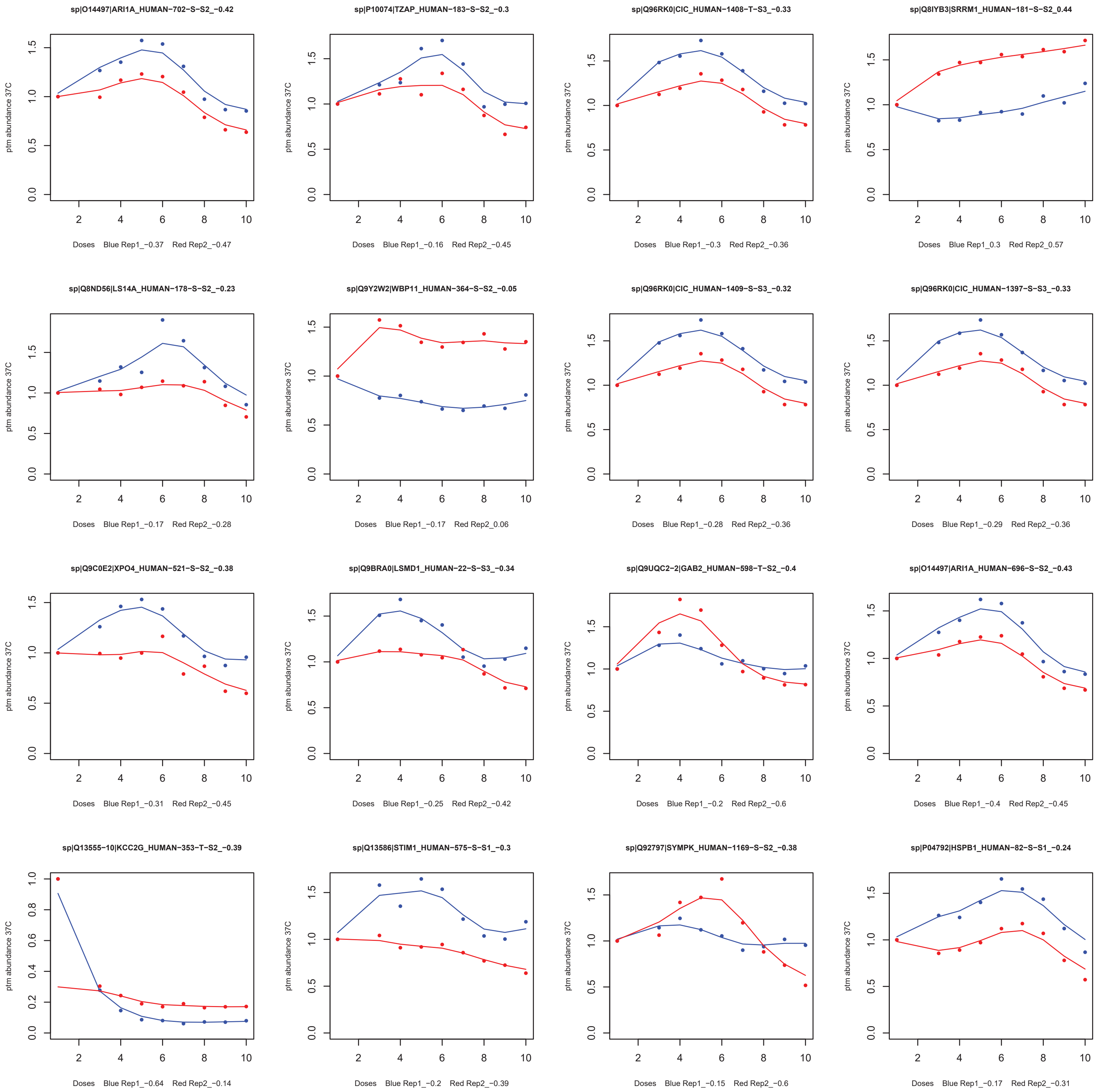
sp|O14497|ARI1A_HUMAN−702−S−S2_−0.42
Doses Blue Rep1_−0.37 Red Rep2_−0.47
sp|P10074|TZAP_HUMAN−183−S−S2_−0.3
Doses Blue Rep1_−0.16 Red Rep2_−0.45
sp|Q96RK0|CIC_HUMAN−1408−T−S3_−0.33
Doses Blue Rep1_−0.3 Red Rep2_−0.36
sp|Q8IYB3|SRRM1_HUMAN−181−S−S2_0.44
Doses Blue Rep1_0.3 Red Rep2_0.57
sp|Q8ND56|LS14A_HUMAN−178−S−S2_−0.23
Doses Blue Rep1_−0.17 Red Rep2_−0.28
sp|Q9Y2W2|WBP11_HUMAN−364−S−S2_−0.05
Doses Blue Rep1_−0.17 Red Rep2_0.06
sp|Q96RK0|CIC_HUMAN−1409−S−S3_−0.32
Doses Blue Rep1_−0.28 Red Rep2_−0.36
sp|Q96RK0|CIC_HUMAN−1397−S−S3_−0.33
Doses Blue Rep1_−0.29 Red Rep2_−0.36
sp|Q9C0E2|XPO4_HUMAN−521−S−S2_−0.38
Doses Blue Rep1_−0.31 Red Rep2_−0.45
sp|Q9BRA0|LSMD1_HUMAN−22−S−S3_−0.34
Doses Blue Rep1_−0.25 Red Rep2_−0.42
sp|Q9UQC2−2|GAB2_HUMAN−598−T−S2_−0.4
Doses Blue Rep1_−0.2 Red Rep2_−0.6
sp|O14497|ARI1A_HUMAN−696−S−S2_−0.43
Doses Blue Rep1_−0.4 Red Rep2_−0.45
sp|Q13555−10|KCC2G_HUMAN−353−T−S2_−0.39
Doses Blue Rep1_−0.64 Red Rep2_−0.14
sp|Q13586|STIM1_HUMAN−575−S−S1_−0.3
Doses Blue Rep1_−0.2 Red Rep2_−0.39
sp|Q92797|SYMPK_HUMAN−1169−S−S2_−0.38
Doses Blue Rep1_−0.15 Red Rep2_−0.6
sp|P04792|HSPB1_HUMAN−82−S−S1_−0.24
Doses Blue Rep1_−0.17 Red Rep2_−0.31
ptm abundance 37C

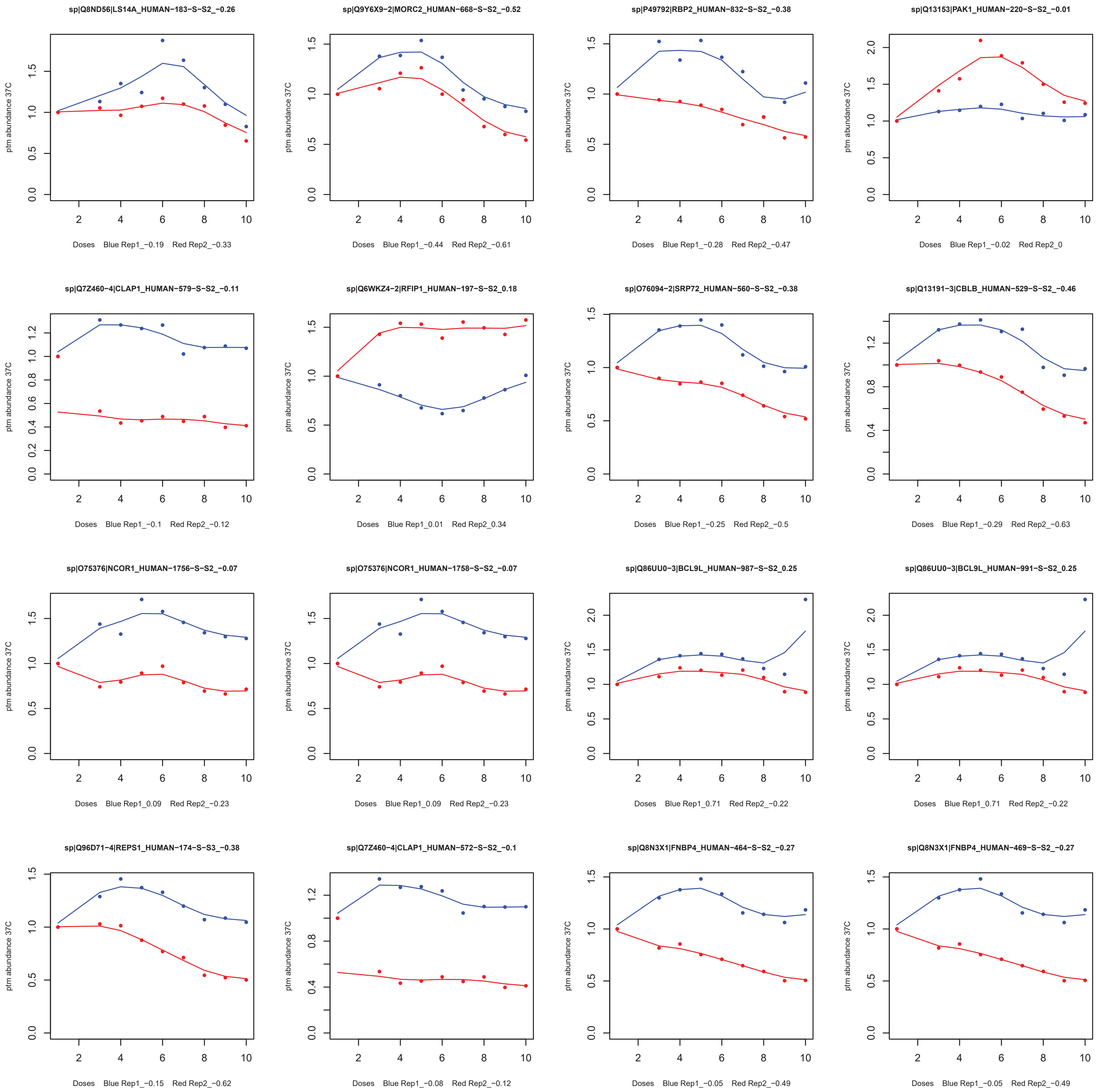
sp|Q8ND56|LS14A_HUMAN−183−S−S2_−0.26
Doses Blue Rep1_−0.19 Red Rep2_−0.33
sp|Q9Y6X9−2|MORC2_HUMAN−668−S−S2_−0.52
Doses Blue Rep1_−0.44 Red Rep2_−0.61
sp|P49792|RBP2_HUMAN−832−S−S2_−0.38
Doses Blue Rep1_−0.28 Red Rep2_−0.47
sp|Q13153|PAK1_HUMAN−220−S−S2_−0.01
Doses Blue Rep1_−0.02 Red Rep2_0
sp|Q7Z460−4|CLAP1_HUMAN−579−S−S2_−0.11
Doses Blue Rep1_−0.1 Red Rep2_−0.12
sp|Q6WKZ4−2|RFIP1_HUMAN−197−S−S2_0.18
Doses Blue Rep1_0.01 Red Rep2_0.34
sp|O76094−2|SRP72_HUMAN−560−S−S2_−0.38
Doses Blue Rep1_−0.25 Red Rep2_−0.5
sp|Q13191−3|CBLB_HUMAN−529−S−S2_−0.46
Doses Blue Rep1_−0.29 Red Rep2_−0.63
sp|O75376|NCOR1_HUMAN−1756−S−S2_−0.07
Doses Blue Rep1_0.09 Red Rep2_−0.23
sp|O75376|NCOR1_HUMAN−1758−S−S2_−0.07
Doses Blue Rep1_0.09 Red Rep2_−0.23
sp|Q86UU0−3|BCL9L_HUMAN−987−S−S2_0.25
Doses Blue Rep1_0.71 Red Rep2_−0.22
sp|Q86UU0−3|BCL9L_HUMAN−991−S−S2_0.25
Doses Blue Rep1_0.71 Red Rep2_−0.22
sp|Q96D71−4|REPS1_HUMAN−174−S−S3_−0.38
Doses Blue Rep1_−0.15 Red Rep2_−0.62
sp|Q7Z460−4|CLAP1_HUMAN−572−S−S2_−0.1
Doses Blue Rep1_−0.08 Red Rep2_−0.12
sp|Q8N3X1|FNBP4_HUMAN−464−S−S2_−0.27
Doses Blue Rep1_−0.05 Red Rep2_−0.49
sp|Q8N3X1|FNBP4_HUMAN−469−S−S2_−0.27
Doses Blue Rep1_−0.05 Red Rep2_−0.49
ptm abundance 37C

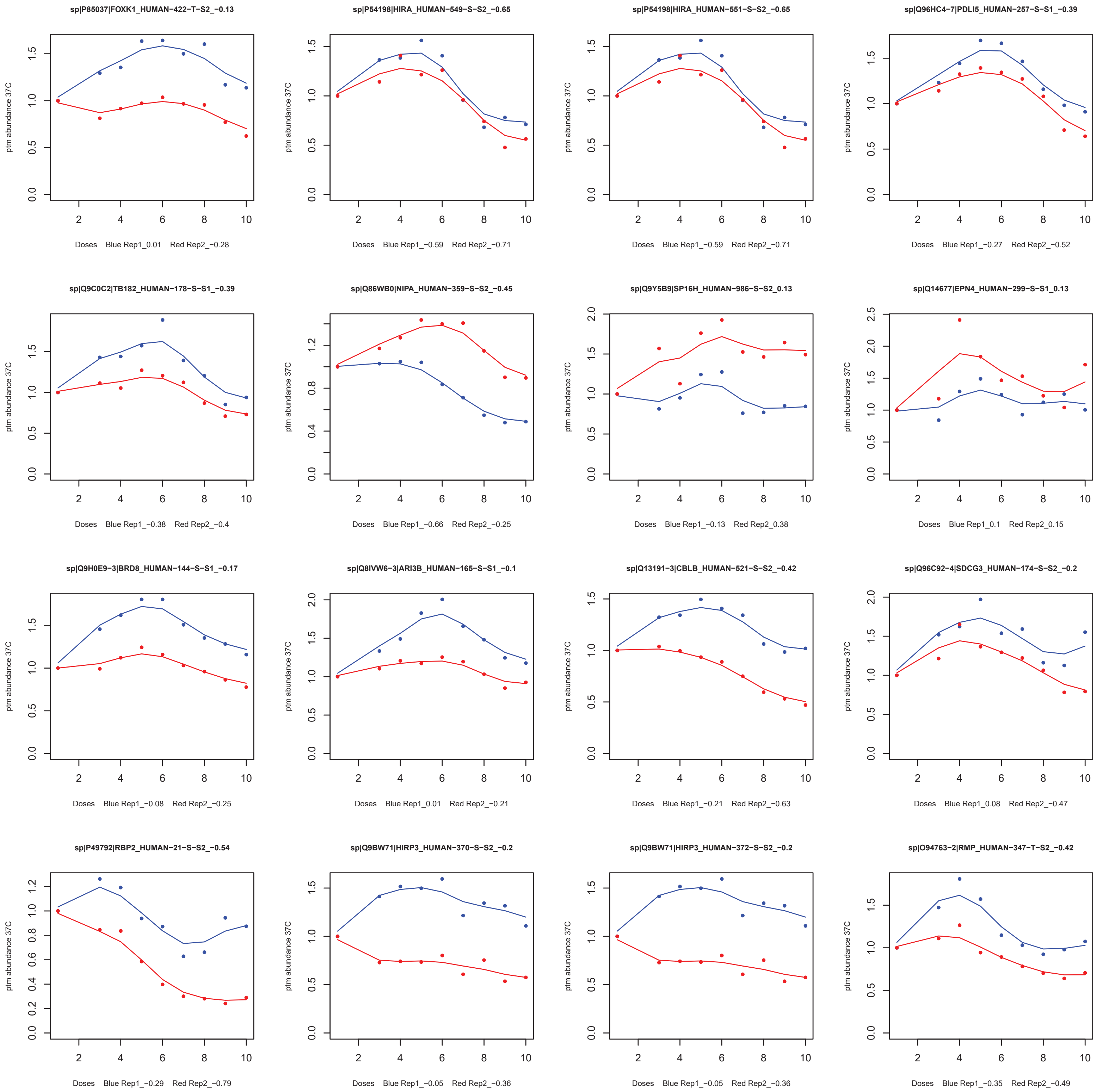
sp|P85037|FOXK1_HUMAN−422−T−S2_−0.13
ptm abundance 37C
Doses Blue Rep1_0.01 Red Rep2_−0.28
sp|P54198|HIRA_HUMAN−549−S−S2_−0.65
ptm abundance 37C
Doses Blue Rep1_−0.59 Red Rep2_−0.71
sp|P54198|HIRA_HUMAN−551−S−S2_−0.65
ptm abundance 37C
Doses Blue Rep1_−0.59 Red Rep2_−0.71
sp|Q96HC4−7|PDLI5_HUMAN−257−S−S1_−0.39
ptm abundance 37C
Doses Blue Rep1_−0.27 Red Rep2_−0.52
sp|Q9C0C2|TB182_HUMAN−178−S−S1_−0.39
ptm abundance 37C
Doses Blue Rep1_−0.38 Red Rep2_−0.4
sp|Q86WB0|NIPA_HUMAN−359−S−S2_−0.45
ptm abundance 37C
Doses Blue Rep1_−0.66 Red Rep2_−0.25
sp|Q9Y5B9|SP16H_HUMAN−986−S−S2_0.13
ptm abundance 37C
Doses Blue Rep1_−0.13 Red Rep2_0.38
sp|Q14677|EPN4_HUMAN−299−S−S1_0.13
ptm abundance 37C
Doses Blue Rep1_0.1 Red Rep2_0.15
sp|Q9H0E9−3|BRD8_HUMAN−144−S−S1_−0.17
ptm abundance 37C
Doses Blue Rep1_−0.08 Red Rep2_−0.25
sp|Q8IVW6−3|ARI3B_HUMAN−165−S−S1_−0.1
ptm abundance 37C
Doses Blue Rep1_0.01 Red Rep2_−0.21
sp|Q13191−3|CBLB_HUMAN−521−S−S2_−0.42
ptm abundance 37C
Doses Blue Rep1_−0.21 Red Rep2_−0.63
sp|Q96C92−4|SDCG3_HUMAN−174−S−S2_−0.2
ptm abundance 37C
Doses Blue Rep1_0.08 Red Rep2_−0.47
sp|P49792|RBP2_HUMAN−21−S−S2_−0.54
ptm abundance 37C
Doses Blue Rep1_−0.29 Red Rep2_−0.79
sp|Q9BW71|HIRP3_HUMAN−370−S−S2_−0.2
ptm abundance 37C
Doses Blue Rep1_−0.05 Red Rep2_−0.36
sp|Q9BW71|HIRP3_HUMAN−372−S−S2_−0.2
ptm abundance 37C
Doses Blue Rep1_−0.05 Red Rep2_−0.36
sp|O94763−2|RMP_HUMAN−347−T−S2_−0.42
ptm abundance 37C
Doses Blue Rep1_−0.35 Red Rep2_−0.49

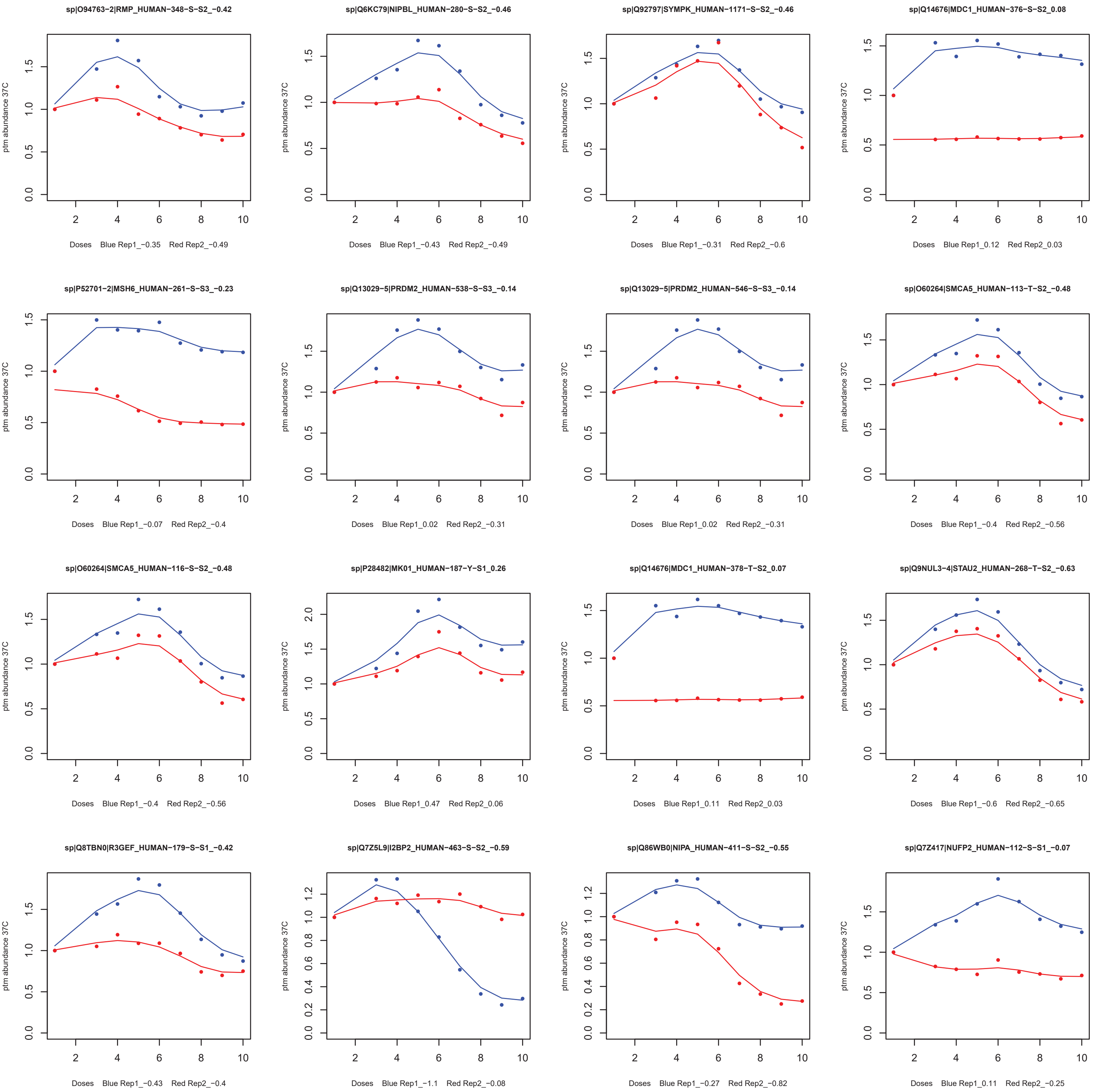
sp|O94763-2|RMP_HUMAN-348-S-S2_-0.42
Doses Blue Rep1_-0.35 Red Rep2_-0.49
sp|Q6KC79|NIPBL_HUMAN-280-S-S2_-0.46
Doses Blue Rep1_-0.43 Red Rep2_-0.49
sp|Q92797|SYMPK_HUMAN-1171-S-S2_-0.46
Doses Blue Rep1_-0.31 Red Rep2_-0.6
sp|Q14676|MDC1_HUMAN-376-S-S2_0.08
Doses Blue Rep1_0.12 Red Rep2_0.03
sp|P52701-2|MSH6_HUMAN-261-S-S3_-0.23
Doses Blue Rep1_-0.07 Red Rep2_-0.4
sp|Q13029-5|PRDM2_HUMAN-538-S-S3_-0.14
Doses Blue Rep1_0.02 Red Rep2_-0.31
sp|Q13029-5|PRDM2_HUMAN-546-S-S3_-0.14
Doses Blue Rep1_0.02 Red Rep2_-0.31
sp|O60264|SMCA5_HUMAN-113-T-S2_-0.48
Doses Blue Rep1_-0.4 Red Rep2_-0.56
sp|O60264|SMCA5_HUMAN-116-S-S2_-0.48
Doses Blue Rep1_-0.4 Red Rep2_-0.56
sp|P28482|MK01_HUMAN-187-Y-S1_0.26
Doses Blue Rep1_0.47 Red Rep2_0.06
sp|Q14676|MDC1_HUMAN-378-T-S2_0.07
Doses Blue Rep1_0.11 Red Rep2_0.03
sp|Q9NUL3-4|STAU2_HUMAN-268-T-S2_-0.63
Doses Blue Rep1_-0.6 Red Rep2_-0.65
sp|Q8TBN0|R3GEF_HUMAN-179-S-S1_-0.42
Doses Blue Rep1_-0.43 Red Rep2_-0.4
sp|Q7Z5L9|I2BP2_HUMAN-463-S-S2_-0.59
Doses Blue Rep1_-1.1 Red Rep2_-0.08
sp|Q86WB0|NIPA_HUMAN-411-S-S2_-0.55
Doses Blue Rep1_-0.27 Red Rep2_-0.82
sp|Q7Z417|NUFP2_HUMAN-112-S-S1_-0.07
Doses Blue Rep1_0.11 Red Rep2_-0.25
ptm abundance 37C

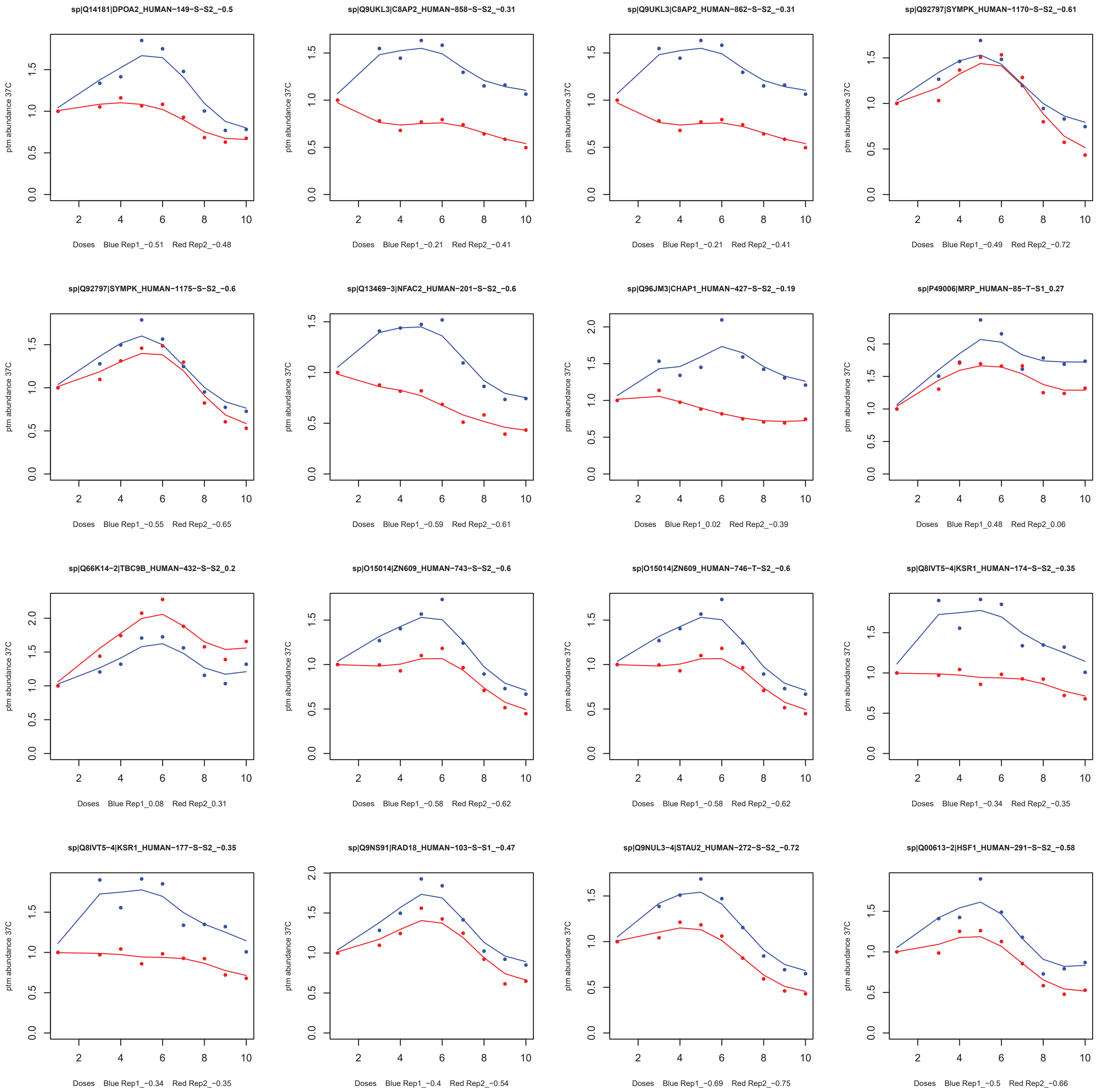
sp|Q14181|DPOA2_HUMAN−149−S−S2_−0.5
Doses Blue Rep1_−0.51 Red Rep2_−0.48
sp|Q9UKL3|C8AP2_HUMAN−858−S−S2_−0.31
Doses Blue Rep1_−0.21 Red Rep2_−0.41
sp|Q9UKL3|C8AP2_HUMAN−862−S−S2_−0.31
Doses Blue Rep1_−0.21 Red Rep2_−0.41
sp|Q92797|SYMPK_HUMAN−1170−S−S2_−0.61
Doses Blue Rep1_−0.49 Red Rep2_−0.72
sp|Q92797|SYMPK_HUMAN−1175−S−S2_−0.6
Doses Blue Rep1_−0.55 Red Rep2_−0.65
sp|Q13469−3|NFAC2_HUMAN−201−S−S2_−0.6
Doses Blue Rep1_−0.59 Red Rep2_−0.61
sp|Q96JM3|CHAP1_HUMAN−427−S−S2_−0.19
Doses Blue Rep1_0.02 Red Rep2_−0.39
sp|P49006|MRP_HUMAN−85−T−S1_0.27
Doses Blue Rep1_0.48 Red Rep2_0.06
sp|Q66K14−2|TBC9B_HUMAN−432−S−S2_0.2
Doses Blue Rep1_0.08 Red Rep2_0.31
sp|O15014|ZN609_HUMAN−743−S−S2_−0.6
Doses Blue Rep1_−0.58 Red Rep2_−0.62
sp|O15014|ZN609_HUMAN−746−T−S2_−0.6
Doses Blue Rep1_−0.58 Red Rep2_−0.62
sp|Q8IVT5−4|KSR1_HUMAN−174−S−S2_−0.35
Doses Blue Rep1_−0.34 Red Rep2_−0.35
sp|Q8IVT5−4|KSR1_HUMAN−177−S−S2_−0.35
Doses Blue Rep1_−0.34 Red Rep2_−0.35
sp|Q9NS91|RAD18_HUMAN−103−S−S1_−0.47
Doses Blue Rep1_−0.4 Red Rep2_−0.54
sp|Q9NUL3−4|STAU2_HUMAN−272−S−S2_−0.72
Doses Blue Rep1_−0.69 Red Rep2_−0.75
sp|Q00613−2|HSF1_HUMAN−291−S−S2_−0.58
Doses Blue Rep1_−0.5 Red Rep2_−0.66
ptm abundance 37C

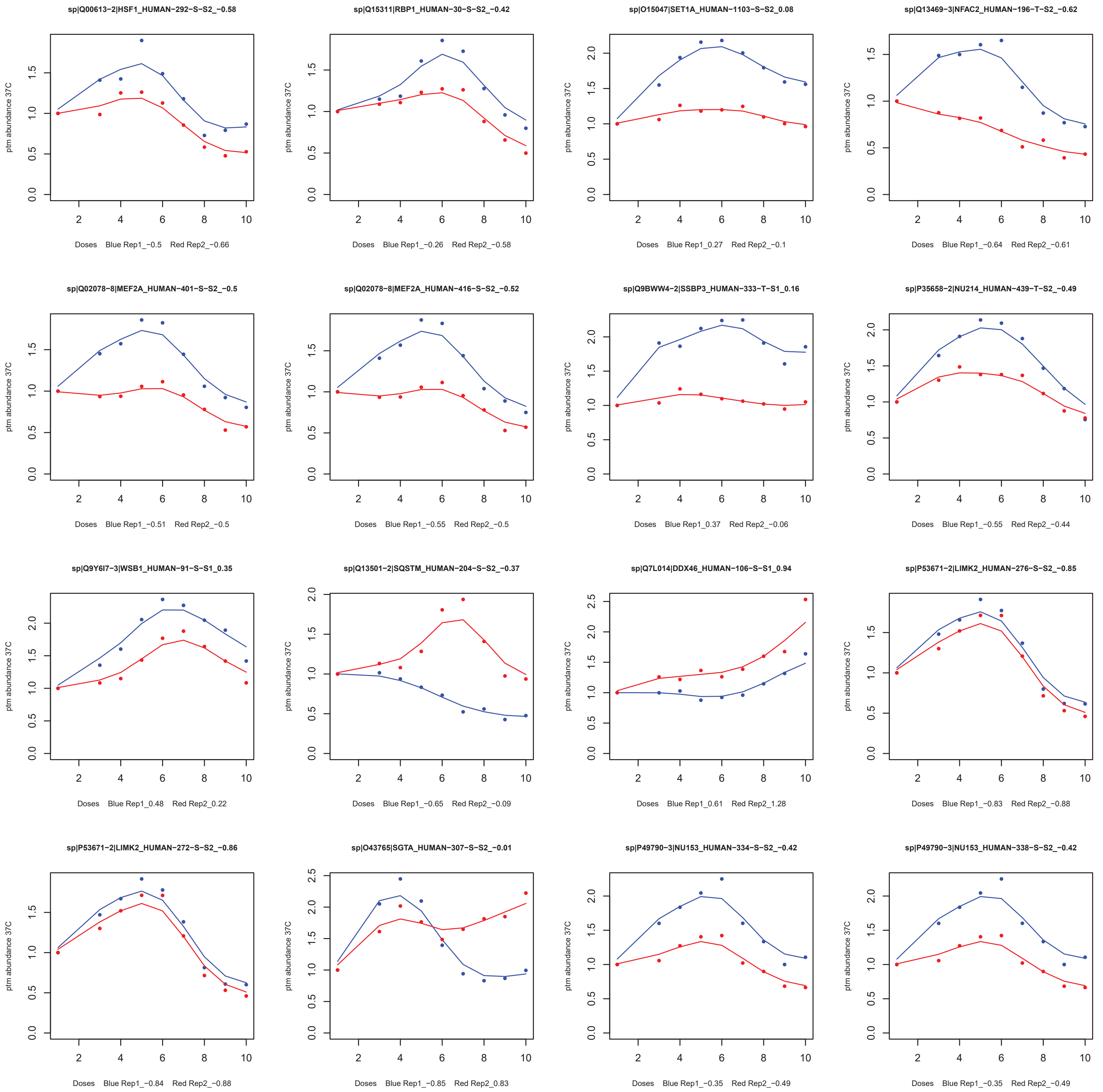

sp|Q00613−2|HSF1_HUMAN−292−S−S2_−0.58
Doses Blue Rep1_−0.5 Red Rep2_−0.66
sp|Q15311|RBP1_HUMAN−30−S−S2_−0.42
Doses Blue Rep1_−0.26 Red Rep2_−0.58
sp|O15047|SET1A_HUMAN−1103−S−S2_0.08
Doses Blue Rep1_0.27 Red Rep2_−0.1
sp|Q13469−3|NFAC2_HUMAN−196−T−S2_−0.62
Doses Blue Rep1_−0.64 Red Rep2_−0.61
sp|Q02078−8|MEF2A_HUMAN−401−S−S2_−0.5
Doses Blue Rep1_−0.51 Red Rep2_−0.5
sp|Q02078−8|MEF2A_HUMAN−416−S−S2_−0.52
Doses Blue Rep1_−0.55 Red Rep2_−0.5
sp|Q9BWW4−2|SSBP3_HUMAN−333−T−S1_0.16
Doses Blue Rep1_0.37 Red Rep2_−0.06
sp|P35658−2|NU214_HUMAN−439−T−S2_−0.49
Doses Blue Rep1_−0.55 Red Rep2_−0.44
sp|Q9Y6I7−3|WSB1_HUMAN−91−S−S1_0.35
Doses Blue Rep1_0.48 Red Rep2_0.22
sp|Q13501−2|SQSTM_HUMAN−204−S−S2_−0.37
Doses Blue Rep1_−0.65 Red Rep2_−0.09
sp|Q7L014|DDX46_HUMAN−106−S−S1_0.94
Doses Blue Rep1_0.61 Red Rep2_1.28
sp|P53671−2|LIMK2_HUMAN−276−S−S2_−0.85
Doses Blue Rep1_−0.83 Red Rep2_−0.88
sp|P53671−2|LIMK2_HUMAN−272−S−S2_−0.86
Doses Blue Rep1_−0.84 Red Rep2_−0.88
sp|O43765|SGTA_HUMAN−307−S−S2_−0.01
Doses Blue Rep1_−0.85 Red Rep2_0.83
sp|P49790−3|NU153_HUMAN−334−S−S2_−0.42
Doses Blue Rep1_−0.35 Red Rep2_−0.49
sp|P49790−3|NU153_HUMAN−338−S−S2_−0.42
Doses Blue Rep1_−0.35 Red Rep2_−0.49
ptm abundance 37C

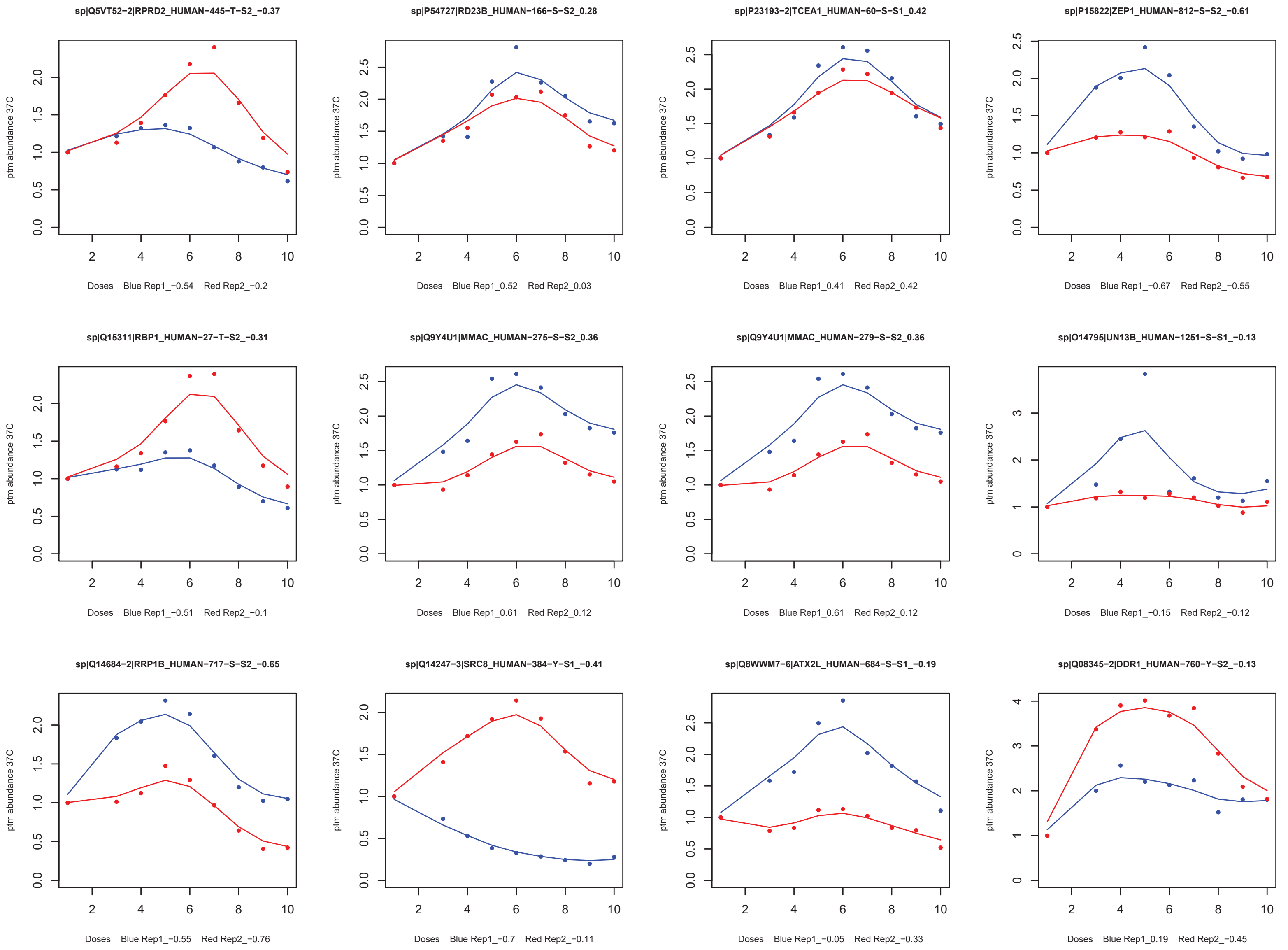
sp|Q5VT52−2|RPRD2_HUMAN−445−T−S2_−0.37
ptm abundance 37C
Doses Blue Rep1_−0.54 Red Rep2_−0.2
sp|P54727|RD23B_HUMAN−166−S−S2_0.28
ptm abundance 37C
Doses Blue Rep1_0.52 Red Rep2_0.03
sp|P23193−2|TCEA1_HUMAN−60−S−S1_0.42
ptm abundance 37C
Doses Blue Rep1_0.41 Red Rep2_0.42
sp|P15822|ZEP1_HUMAN−812−S−S2_−0.61
ptm abundance 37C
Doses Blue Rep1_−0.67 Red Rep2_−0.55
sp|Q15311|RBP1_HUMAN−27−T−S2_−0.31
ptm abundance 37C
Doses Blue Rep1_−0.51 Red Rep2_−0.1
sp|Q9Y4U1|MMAC_HUMAN−275−S−S2_0.36
ptm abundance 37C
Doses Blue Rep1_0.61 Red Rep2_0.12
sp|Q9Y4U1|MMAC_HUMAN−279−S−S2_0.36
ptm abundance 37C
Doses Blue Rep1_0.61 Red Rep2_0.12
sp|O14795|UN13B_HUMAN−1251−S−S1_−0.13
ptm abundance 37C
Doses Blue Rep1_−0.15 Red Rep2_−0.12
sp|Q14684−2|RRP1B_HUMAN−717−S−S2_−0.65
ptm abundance 37C
Doses Blue Rep1_−0.55 Red Rep2_−0.76
sp|Q14247−3|SRC8_HUMAN−384−Y−S1_−0.41
ptm abundance 37C
Doses Blue Rep1_−0.7 Red Rep2_−0.11
sp|Q8WWM7−6|ATX2L_HUMAN−684−S−S1_−0.19
ptm abundance 37C
Doses Blue Rep1_−0.05 Red Rep2_−0.33
sp|Q08345−2|DDR1_HUMAN−760−Y−S2_−0.13
ptm abundance 37C
Doses Blue Rep1_0.19 Red Rep2_−0.45
